# Ferroptosis-Related Long Noncoding RNAs Have Excellent Predictive Ability for Multiomic Characteristics of Bladder Cancer

**DOI:** 10.1155/2022/9316847

**Published:** 2022-08-29

**Authors:** Jingchao Liu, Jingyi Cui, Shuangyi Zhao, Meng Wu, Jiawen Wang, Yaoguang Zhang, Bin Jin, Jianye Wang

**Affiliations:** ^1^Department of Urology, Beijing Hospital, National Center of Gerontology, Institute of Geriatric Medicine, Chinese Academy of Medical Sciences, No. 1 DaHua Road, Dong Dan, Beijing 100730, China; ^2^Graduate School of Peking Union Medical College, Chinese Academy of Medical Sciences, 9 DongDan SanTiao, Beijing 100730, China; ^3^The Key Laboratory of Geriatrics, Beijing Institute of Geriatrics, Institute of Geriatric Medicine, Chinese Academy of Medical Sciences, Beijing Hospital, National Center of Gerontology of National Health Commission, Beijing 100730, China

## Abstract

**Background:**

The role of ferroptosis-related long non-coding RNAs (lncRNAs) in bladder cancer remains elusive. This study is aimed at examining the prognostic role of ferroptosis-related lncRNAs in bladder cancer.

**Materials and Methods:**

The transcriptomic matrix and clinical information of patients with bladder cancer were obtained from The Cancer Genome Atlas (TCGA) database. A ferroptosis-related lncRNA signature was developed via the least absolute shrinkage and selection operator (LASSO) analysis using data from the training cohort, and the signature was further validated using data from the test cohort. The role of AC006160.1, the most significant lncRNA in the risk signature, was examined in various cell lines including SV-HUC-1, BIU-87, HT-1376, T24, RT4, RT-112, 5637, and UMUC3. The pcDNA3.1-AC006160.1 plasmid was constructed and transfected into the bladder cancer cell lines T24 and BIU-87. In addition, cell proliferation, colony formation, transwell, and wound healing assays were performed to examine the biological function of AC006160.1 in T24 and BIU-87 cell lines.

**Results:**

Two clusters were identified through consensus clustering based on prognostic ferroptosis-related lncRNAs. A 5-lncRNA risk signature was successfully constructed using data from the training cohort and validated using data from the test cohort. The risk signature had excellent ability to predict survival outcomes, clinical stages, pathological grades, expression of immune checkpoints, and immunotherapeutic responses in bladder cancer samples. Furthermore, AC006160.1 expression was found to be lower in the cancer cell lines BIU-87, T24, RT4, RT-112, and 5637 than in the normal control cell line SV-HUC-1. Cell proliferation, colony formation, transwell migration, and wound healing assays validated that overexpression of AC006160.1 significantly inhibited the proliferation and invasion abilities of both T24 and BIU-87 cells. Drug sensitivity analysis revealed that patients with high expression of AC006160.1 were sensitive to metformin and methotrexate, and the results were further validated via in vitro drug experiments.

**Conclusions:**

Ferroptosis-related lncRNAs play a vital role in predicting the multiomic characteristics of bladder cancer. The lncRNA AC006160.1 serves as a protective factor for the development of bladder cancer.

## 1. Introduction

Bladder cancer, one of the most malignant tumours, originates from the transitional epithelium of the urinary tract and leads to >2,00,000 deaths annually worldwide [1]. Non-muscle-invasive bladder cancer accounts for 50% of bladder cancer cases, whereas muscle-invasive bladder cancer accounts for 30% of cases [1, 2]. Although bladder cancer can be treated via transurethral tumour resection or radical cystectomy, a considerable proportion of patients develops tumour recurrence or metastasis [3]. During the past decade, limited progress has been made in the development of relative treatment modalities for bladder cancer owing to unclear mechanisms underlying cancer development. At present, cisplatin-based chemotherapy is recommended as the first-line treatment for advanced bladder cancer, and immunotherapy is recommended as the second-line treatment [4, 5]. However, only a limited number of patients can benefit from chemotherapy and immunotherapy. Moreover, patients with the same clinical stage or grade often have distinct survival outcomes after receiving similar treatment strategies. These discrepancies indicate that other biological mechanisms may be involved in the development of bladder cancer, which remain elusive at present. Recent developments in large-scale gene expression and sequencing technology have helped clinicians to explore valuable tools for improving the diagnosis and treatment of bladder cancer [6].

Ferroptosis is a novel cell death mechanism, which is different from previously known programmed cell death mechanisms including autophagy and apoptosis [7, 8]. It is typically characterised by iron dependence and accumulation of reactive oxygen species in cells [8, 9]. The unique morphological characteristics of ferroptosis include the loss of mitochondrial cristae, shrinkage of cell mitochondria, and enhanced density of the mitochondrial membrane [10]. These characteristics confirm that ferroptosis is a novel cell death mechanism, which may offer promising directions for research into cell death and cancer treatment. Several studies have demonstrated that ferroptosis plays a vital regulatory role in various malignant cancers including colorectal cancer, non-small-cell lung cancer, hepatocellular carcinoma, breast cancer, and acute myeloid leukaemia [9, 11, 12]. Identification of drugs interfering with ferroptosis in cancer cells may help to develop novel treatment strategies for cancer in the future, especially for patients who are resistant to chemotherapy or immunotherapy [13–15]. Eling et al. reported that pancreatic cancer cells can be inhibited by inducing ferroptosis using the combination of cotylenin A and phenylethyl isothiocyanate [16]. A recent study reported that sorafenib plays a therapeutic role by inducing ferroptosis in hepatocellular carcinoma [17]. Previous high-quality studies have validated that several mRNAs participate in the regulation of ferroptosis in various malignancies, and their results have been experimentally validated and widely recognised in recent studies [15, 18–20]. In addition, studies employing cutting-edge technology have reported a vital role of lncRNAs in various biological activities including differentiation, apoptosis, metastasis, cell cycle, and proliferation in multiple cancers [21, 22]. Coexpression analysis can help to screen for lncRNAs that are coexpressed with known ferroptosis mRNAs and further identify ferroptosis-related lncRNAs in malignancies [23, 24]. Studies have shown that lncRNAs participate in multiple ferroptosis-related regulatory activities in various cancers. The competing endogenous RNA LINC00336 inhibits ferroptosis in cancer cells, thereby promoting the development and deterioration of lung cancer [25]. In addition, LINC00618 enhances vincristine-induced ferroptosis and is a potential prognostic factor for predicting the survival of patients with leukaemia [26]. Furthermore, studies have also discovered that lncRNAs regulate tumour progression by affecting the immune microenvironment [22, 27]. However, the role of ferroptosis-related lncRNAs in the development of bladder cancer remains elusive. This study is aimed at examining the prognostic role of ferroptosis-related lncRNAs in bladder cancer; in addition, a risk signature based on prognostic lncRNAs was established to guide the currently available diagnostic and treatment strategies of bladder cancer from a genetic perspective.

## 2. Materials and Methods

### 2.1. Extraction and Processing of Sequencing Data

The mRNA and lncRNA sequencing data of 411 bladder cancer samples were extracted from The Cancer Genome Atlas (TCGA) database (https://portal.gdc.cancer.gov). Samples without clinical information were excluded, and a total of 403 bladder cancer samples with both transcriptomic and clinical information were eventually included for further analysis. The 403 bladder cancer samples were randomly divided into the training (203 samples) and validation (200 samples) cohorts for the construction and validation of an lncRNA signature, respectively.

### 2.2. Identification of Ferroptosis-Related lncRNAs and Prognostic lncRNAs

High-quality studies concerning ferroptosis (impact factor of >10 points in the last 4 years) were comprehensively reviewed, and a total of 60 ferroptosis-related mRNAs were identified, which were listed in Supplementary Table 1 [18–20, 28]. Pearson's correlation analysis (|*R*| > 0.5 and *p* < 0.001) of ferroptosis-related mRNAs and lncRNAs was performed to identify ferroptosis-related lncRNAs. Subsequently, univariate Cox regression analysis was performed to identify ferroptosis-related lncRNAs associated with prognosis (overall survival) (*p* < 0.01).

### 2.3. Distinct Ferroptosis Mediation Patterns in Bladder Cancer

Based on the expression of prognostic ferroptosis-related lncRNAs, distinct ferroptosis mediation patterns were identified via consensus clustering using the “ConsensusClusterPlus” package of R software. The Euclidean distance was calculated to assess similarity between samples, and the *K*-means algorithm was used for clustering. Differences in survival between distinct patterns were evaluated via Kaplan–Meier survival analysis using the “survival” and “survminer” packages. The potential relationship between distinct ferroptosis mediation patterns and various clinical parameters including age, sex, clinical stage, and tumour grade were investigated using the “pheatmap” package.

### 2.4. Construction and Validation of Ferroptosis-Related lncRNA Signature

Based on the prognostic ferroptosis-related lncRNAs identified via univariate Cox regression analysis, an lncRNA risk signature was developed in the training cohort via the least absolute shrinkage and selection operator (LASSO) analysis using the “glmnet” package. Each ferroptosis mediation pattern was quantified using the following formula: risk score = *Σ* (ferroptosis − related lncRNA expression∗corresponding regression coefficient). All cancer samples in both training and validation cohorts were divided into the high- or low-risk groups based on the corresponding median risk score. Kaplan–Meier analysis was performed to compare the survival of patients in the high- and low-risk groups in both training and validation cohorts. In addition, receiver operating characteristic (ROC) analysis was performed to examine the performance of risk scores in predicting survival outcomes at 1–5 years in both training and validation cohorts.

### 2.5. Independent Prognostic Analysis and Stratified Survival Analysis of the Ferroptosis-Related lncRNA Risk Signature

Univariate and multivariate Cox regression analyses were performed to examine the independent prognostic role of the ferroptosis-related lncRNA risk signature in the training cohort and verify its role in the validation cohort. Furthermore, stratified survival analysis was performed to investigate the predictive value of the risk signature in different subgroups, including old (age, >65 years) or young (age, ≤65 years) group, male or female group, stage I–II or stage III–IV group, T1–T2 or T3–T4 group, and N0 or N1–3 stage group. Differences in risk scores between these groups were analysed using Student's t-test.

### 2.6. Expression of Immune Checkpoints, Infiltration Analysis of Immune Cells, and Gene Set Enrichment Analysis between Distinct Ferroptosis Mediation Patterns

Differences in the expression of classic immune checkpoints including PD-1, PD-L1 and CTLA-4 between distinct ferroptosis mediation patterns were investigated using the “limma,” “ggplot2” and “ggpubr” packages in R. The CIBERSORT algorithm (https://cibersort.stanford.edu/) was used to analyse the infiltration levels of 22 types of immune cells in bladder cancer [29]. The ESTIMATE algorithm was used to evaluate the immune cell microenvironment scores of each bladder cancer sample [30]. Differences in the immune infiltration levels of 22 immune cell types between distinct ferroptosis mediation patterns were further examined. Gene set enrichment analysis (GSEA) (version 4.0.4) was performed based on the whole transcriptomic profiles of bladder cancer samples to investigate potential biological activities of distinct ferroptosis mediation patterns.

### 2.7. Guidance of the lncRNA Risk Signature for Immunotherapy

Differences in the expression of immune checkpoints between the high- and low-risk groups were examined using the “limma” and “ggpubr” packages in R. The standard symbol names of PD-L1, PD-1 and CTLA-4 were derived from the NCBI website (https://www.ncbi.nlm.nih.gov/gene), which were CD274, PDCD1, and CTLA-4, respectively. Additionally, correlation analysis was performed to examine the relationship between risk scores and infiltration levels of immune cells, and the Tumour Immune Dysfunction and Exclusion (TIDE) algorithm was used to evaluate the response of patients to immunotherapy [31].

### 2.8. Cell Lines and Cell Culture

The biological functions of the most significant lncRNA, AC006160.1, were examined via in vitro experiments. Human cell lines SV-HUC-1 (CL-0222), BIU-87 (CL-0035), HT-1376 (CL-0672), T24 (CL-0227), RT4 (CL-0431), RT-112 (CL-0682), 5637 (CL-0002), and UMUC3 (CL-0463) were purchased from Procell Life Science & Technology Co., Ltd. (Wuhan, China). BIU-87, T24, RT4, RT-112, and 5637 cells were cultured in the Roswell Park Memorial Institute (RPMI)-1640 (Macgene, China) medium supplemented with 10% foetal bovine serum (FBS) (Gibco, MA, USA). HT-1376 and UMUC3 cells were cultured in MEM (Macgene, China) supplemented with 10% FBS (Gibco, MA, USA). SV-HUC-1 cells were cultured in Ham's F-12K medium (Macgene, China) supplemented with 10% FBS (Gibco, MA, USA). All cell lines were cultured at 37°C in a humidified incubator containing 5% CO_2_.

### 2.9. Vector Construction

The lncRNA AC006160.1 was cloned into the pcDNA3.1 vector at the NheI and KpnI sites to produce the pcDNA3.1-AC006160.1 plasmid. The primers used for plasmid construction were as follows: forward, 5′-CTAGCTAGCCACGTGACAGGACCGAGC-3′; reverse, 5′-GGGGTACCTCATCTTCCGATTTAAAATTTTTTTCCC-3′. Empty pcDNA3.1 vector was used as the negative control. The pcDNA3.1-AC006160.1 and negative control plasmids were transfected into bladder cancer cells using the Lipo8000 Transfection Reagent (Beyotime Biotechnology, China) according to the manufacturer's instructions and cultured in 6-well plates.

### 2.10. RNA Extraction and Reverse Transcription Polymerase Chain Reaction

The TRIzol reagent (Invitrogen, USA) was used to isolate total RNA from bladder cancer cells, and first-strand complementary deoxyribonucleic acid (cDNA) was synthesised using the Evo M-MLV RT Premix (Accurate Biology, China). Quantitative polymerase chain reaction (qPCR) was performed using the Taq Pro Universal SYBR qPCR Master Mix (Vazyme, China). All reactions were performed on the iQ5 Real-Time PCR Thermal Cycler (Bio-Rad, USA). The specific qPCR primers used for detecting lncRNA AC006160.1 were as follows: forward, 5′-ATGCCTGGAGAGACTTTGGC-3′; reverse, 5′-GCCTGTCTTGTTCCCGCTAT-3′; GAPDH forward, 5′-GGTATCGTGGAAGGACTCATGAC-3′; reverse, 5′-ATGCCAGTGAGCTTCCCGTTCAG-3′.

### 2.11. Cell Proliferation Assay

Cell viability was measured using the CCK-8 kit (Meilunbio, China). According to bioinformatic analysis and experimental results, AC006160.1 was found to be significantly downregulated in bladder cancer and served as a protective factor for cancer development. Therefore, cell lines with low expression of AC006160.1 were randomly selected to validate the biological functions of AC006160.1. BIU-87 and T24 cells were selected as ideal experimental cells. Briefly, BIU-87 or T24 cells were transfected with the pcDNA3.1-AC006160.1 or negative control plasmid for 24 h, and the transfected cells were seeded in a 96-well plate at a density of 3 × 10^3^ cells/well. The cells were cultured for 1, 2, 3, 4, or 5 days, and 10 *μ*L of the CCK-8 reagent was added to each well. After 2 h of incubation, absorbance was measured at 450 nm using a multimode microplate reader (BioTek, USA). For drug sensitivity analysis, BIU-87 cells were transfected with the pcDNA3.1-AC006160.1 or negative control plasmid for 24 h, and the transfected cells were seeded in a 96-well plate at a density of 3 × 10^3^ cells/well and incubated for 24 h. Thereafter, the cells were cultured with or without 1-mM metformin (MET) for 72 h, and absorbance was measured as mentioned above.

### 2.12. Colony Formation Assay

BIU-87 or T24 cells were transfected with the pcDNA3.1-AC006160.1 or negative control plasmid for 24 h, and approximately 2.0 × 10^3^ transfected cells were seeded in 6-well plates and incubated at 37°C in RPMI-1640 medium supplemented with 10% FBS. After 14 days, cell colonies were washed with phosphate-buffered saline (PBS), fixed in methanol for 15 min and stained with crystal violet for 30 min. Subsequently, photographs were captured for evaluating colony formation. For drug sensitivity analysis, BIU-87 cells were transfected with the pcDNA3.1-AC006160.1 or negative control plasmid for 24 h, and approximately 2.0 × 10^3^ transfected cells were seeded in 6-well plates and incubated for 24 h at 37°C in RPMI-1640 medium supplemented with 10% FBS. Thereafter, the cells were cultured with or without 1-mM MET, and subsequent analysis was performed as mentioned above. The number of colonies formed was evaluated using the ImageJ software.

### 2.13. Wound Healing Assay

Transfected BIU-87 and T24 cells were seeded in a 12-well plate and cultured to complete confluence. A clear wound was created by scratching the cell monolayer with a 200 *μ*L pipette tip. Thereafter, the cells were cultured in a serum-free medium. At 0 h and 48 h, images were captured with the aid of an optical microscope, and the wound area was measured using the ImagePro software.

### 2.14. Transwell Migration Assay

Transfected BIU-87 and T24 cells in 200 *μ*L of serum-free medium were added to the upper transwell chamber, whereas 600 *μ*L of the medium containing 10% FBS was added to the lower chamber. After 48 h of incubation, cells in the upper chamber were removed with a cotton swab, whereas those in the bottom chamber were fixed with ethanol, stained with 0.1% crystal violet for 30 min, and photographed. Thereafter, the number of migrated cells was evaluated using the ImageJ software.

### 2.15. Drug Sensitivity Analysis

All bladder cancer samples were divided into the high- and low-expression groups based on the median expression of the target lncRNA AC006160.1. Data from the Genomics of Drug Sensitivity in Cancer (GDSC) database were used to perform drug sensitivity analysis in both groups [32]. To validate the results of drug sensitivity analysis, BIU-87 cells were transfected with the pcDNA3.1-AC006160.1 or negative control plasmid for 24 h, and the transfected cells were seeded in a 96-well plate at a density of 3 × 10^3^ cells/per well and incubated for 24 h. Subsequently, the cells were cultured with or without 1-mM MET for 72 h, and absorbance was measured as mentioned in the section Cell proliferation assay. Furthermore, colony formation assay was performed as mentioned earlier to validate the results of drug sensitivity analysis. BIU-87 cells were transfected with the pcDNA3.1-AC006160.1 or negative control plasmid for 24 h, and approximately 2.0 × 10^3^ transfected cells were seeded in 6-well plates and incubated at 37°C in RPMI-1640 medium supplemented with 10% FBS for 24 h. Thereafter, the cells were cultured with or without 1-mM MET, and subsequent analysis was performed as mentioned above.

### 2.16. Statistical Analysis

The chi-squared test was used to compare categorical variables, whereas the Student's *t*-test or Mann–Whitney *U* test was used to compare continuous variables. One-way ANOVA was used to compare data among ≥3 groups, and Kaplan–Meier analysis with the log-rank test was used to compare survival. All statistical analyses were performed using either the R or the SPSS Statistics software.

## 3. Results

### 3.1. Identification of Ferroptosis-Related lncRNAs in Bladder Cancer Samples

**Figure 1:**
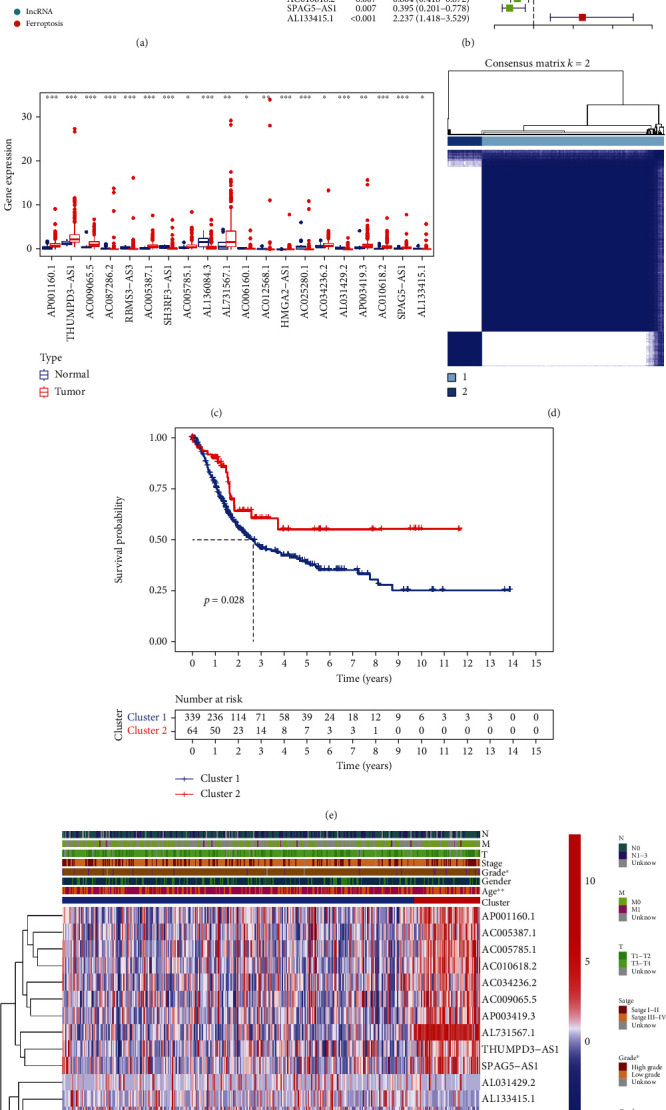
Ferroptosis-related lncRNAs associated with the prognosis of bladder cancer. (a) Coexpression network of ferroptosis-related mRNAs and lncRNAs. (b) The 20 prognostic ferroptosis-related lncRNAs identified via univariate Cox regression analysis. (c) The 20 prognostic ferroptosis-related lncRNAs were differentially expressed between tumour and healthy tissues. (d) Consensus clustering when *k* was 2. (e) Cluster 1 had significantly poorer survival outcomes than cluster 2. (f) Heatmap demonstrating the relationship between the two clusters and various clinical parameters including the age, sex, clinical stage, and tumour grade of patients with bladder cancer.

The transcriptomic data of 411 bladder cancer samples and 19 normal bladder samples were used to screen for ferroptosis-related lncRNAs. The mRNAs and lncRNAs were separated based on annotations in the GENCODE database [33]. Pearson's correlation analysis (|*R*| > 0.5 and *p* < 0.001) were conducted between these ferroptosis-related mRNAs and lncRNAs to identify ferroptosis-related lncRNAs. A total of 263 ferroptosis-related lncRNAs were identified via Pearson's correlation analysis (Supplementary Tables 2 and 3). The coexpression network of ferroptosis-related mRNAs and lncRNAs is demonstrated in Figure 1(a). Only 403 samples with available clinical information were selected for analysing the relationship between the ferroptosis-related lncRNAs and prognosis via univariate Cox regression analysis (Supplementary Table 4). A total of 20 ferroptosis-related lncRNAs were identified to be significantly associated with the prognosis of bladder cancer, including AP001160.1, THUMPD3-AS1, AC009065.5, AC087286.2, RBMS3-AS3, AC005387.1, SH3RF3-AS1, AC005785.1, AL136084.3, AL731567.1, AC006160.1, AC012568.1, HMGA2-AS1, AC025280.1, AC034236.2, AL031429.2, AP003419.3, AC010618.2, SPAG5-AS1, and AL133415.1 (Figure 1(b)). The expression of these 20 prognostic lncRNAs was found to be different between tumour and healthy bladder tissues (*p* < 0.05) (Figure 1(c)). These results verified the important role of ferroptosis-related lncRNAs in the development of bladder cancer.

### 3.2. Distinct Ferroptosis Mediation Patterns Based on Prognostic Ferroptosis-Related lncRNAs in Bladder Cancer

Based on the 20 prognostic ferroptosis-related lncRNAs, two ferroptosis mediation patterns were identified via consensus clustering using the “ConsensusClusterPlus” package in R (Figure 1(d)). The results of cluster analysis for all bladder cancer samples are shown in Supplementary Table 5. Kaplan–Meier survival analysis (Figure 1(e)) revealed that survival outcomes were significantly poorer in cluster 1 than in cluster 2 (*p* < 0.05). Detailed clinical characteristics of bladder cancer samples are mentioned in Supplementary Table 6, and the relationship between the two ferroptosis mediation patterns and various clinical parameters including age, sex, grade, and tumour stage is demonstrated in Figure 1(f). Significant differences in the tumour grade and age of patients were observed between clusters 1 and 2.

### 3.3. Construction and Validation of a Ferroptosis-Related-lncRNA Risk Signature

**Figure 2:**
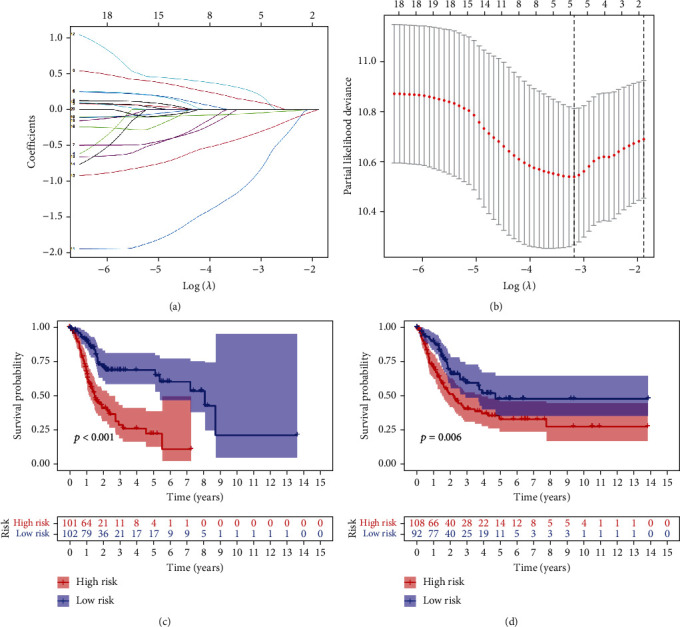
Construction and validation of the ferroptosis-related lncRNA signature for predicting prognosis in the training and validation cohorts. (a) LASSO coefficients of the five ferroptosis-related lncRNAs used to construct the signature. (b) Ten-fold cross-validation for tuning parameter selection in the LASSO model. (c) Kaplan–Meier survival curves of the high- and low-risk groups in the training cohort. (d) Kaplan–Meier survival curves of the high- and low-risk groups in the validation cohort.

**Figure 3:**
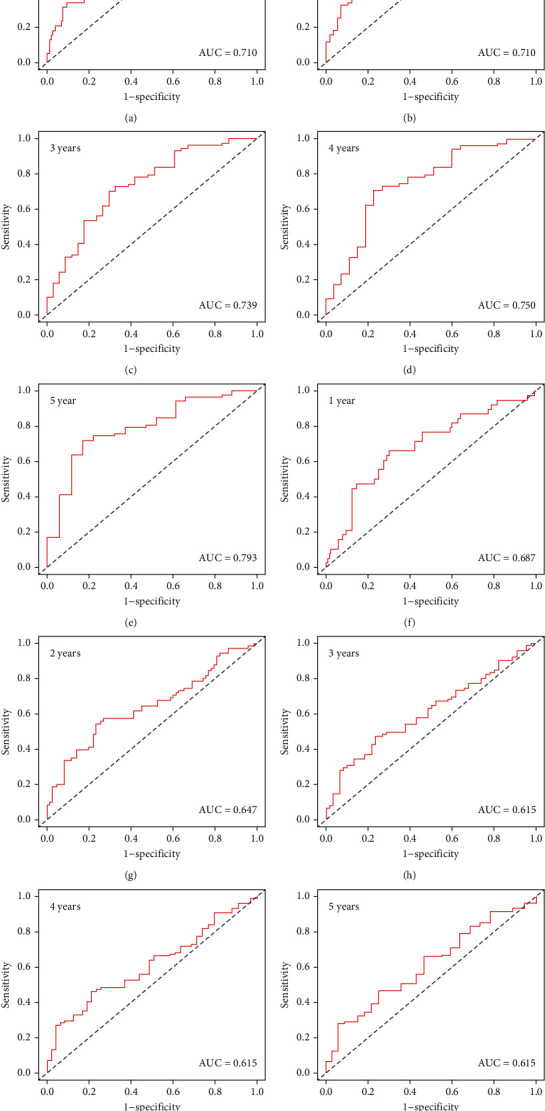
ROC curve analysis of the relationship between the ferroptosis-related lncRNA signature and survival in both training and validation cohorts. (a–e) ROC curves for predicting survival at 1–5 years in the training cohort. (f–J) ROC curves for predicting survival at 1–5 years in the validation cohort.

The 403 bladder cancer samples were randomly divided into the training (203 samples) and validation (200 samples) cohorts to investigate the prognostic role of ferroptosis-related lncRNAs. LASSO regression analysis was performed to examine the 20 ferroptosis-related lncRNAs associated with prognosis, and a risk signature including 5 lncRNAs was successfully constructed in the training cohort. The five targeted lncRNAs are listed in Table 1.  Figure 2(a) demonstrates the LASSO coefficients of the five lncRNAs, and Figure 2(b) demonstrates the 10-fold cross-validation of the LASSO model, indicating that the selection of these five lncRNAs is optimal for constructing the signature. All samples were further divided into the high- and low-risk groups according to the median risk score. The risk scores and grouping of the training and validation cohorts are shown in Supplementary Tables 7 and 8, respectively. Survival outcomes were significantly poorer in the high-risk group than in the low-risk group in both training (*p* < 0.001) (Figure 2(c)) and validation (*p* = 0.006) (Figure 2(d)) cohorts. To verify the predictive role of the risk signature, ROC analysis was performed to compare survival outcomes at 1–5 years in the training and validation cohorts. The area under the ROC curve (AUC) for predicting 1-, 2-, 3-, 4-, and 5-year survival in the training cohort was 0.710, 0.710, 0.739, 0.750, and 0.793, respectively. These results demonstrated promising performance of the risk signature in predicting the survival of patients with bladder cancer (Figures 3(a)–3(e)). As shown in Figures 3(f)–3(j), the risk signature showed excellent predictive performance in the validation cohort. These results indicate that the identified ferroptosis-related lncRNAs can be used to predict survival outcomes in bladder cancer.

### 3.4. Independent Predictive Role of the Ferroptosis-Related lncRNA Signature

**Figure 4:**
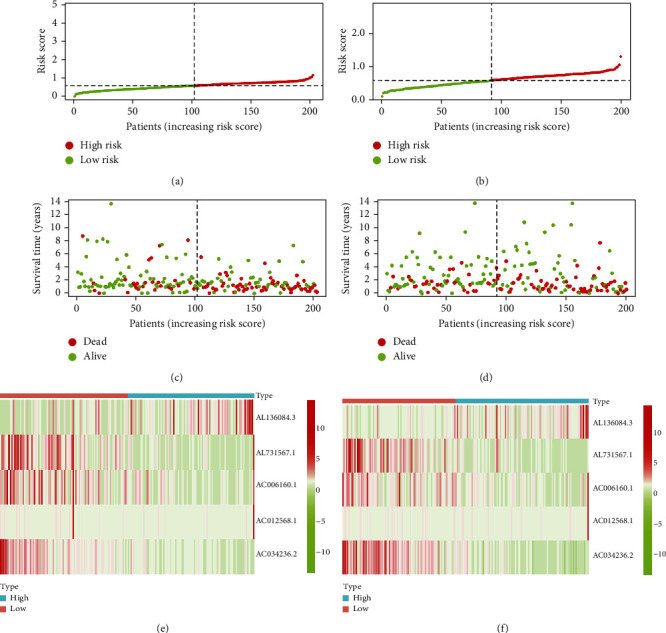
Distribution of risk scores and survival outcomes in both training and validation cohorts. (a) Distribution of risk scores in the training cohort. (b) Distribution of risk scores in the validation cohort. (c) Survival outcomes in the training cohort. (d) Survival outcomes in the validation cohort. (e) Heatmap demonstrating the expression of the five target lncRNAs in the training cohort. (f) Heatmap demonstrating the expression of the five target lncRNAs in the validation cohort.

**Figure 5:**
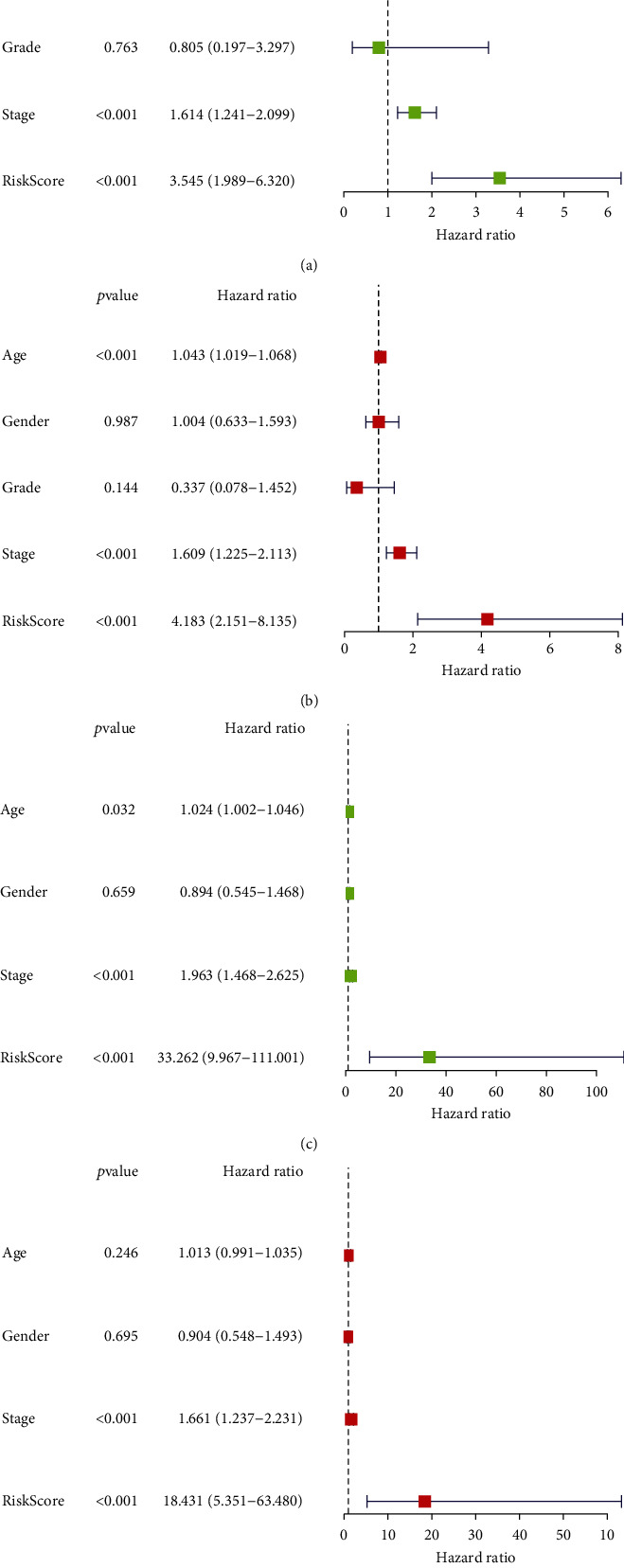
The lncRNA risk signature was identified as an independent prognostic factor in both training and validation cohorts. (a) Univariate Cox regression analysis in the validation cohort. (b) Multivariate Cox regression analysis in the validation cohort. (c) Univariate Cox regression analysis in the training cohort. (d) Multivariate Cox regression analysis in the training cohort.

The distribution of risk scores in the training cohort is demonstrated in Figure 4(a), and the corresponding survival status is demonstrated in Figure 4(c). In both training and validation cohorts, the death rate is significantly higher in the high-risk group than in the low-risk group (Figures 4(b) and 4(d)). Heatmaps (Figures 4(e) and 4(f)) are plotted to demonstrate the expression of the five target lncRNAs in the high- and low-risk groups. Univariate and multivariate cox regression analyses are performed in both training and validation cohorts to investigate the independent predictive role of the lncRNA signature. As shown in Figures 5(a) and 5(b), the risk score (*p* < 0.001), stage (*p* < 0.001), and age (*p* < 0.001) are identified as independent risk factors for survival in the validation cohort. As shown in Figures 5(c) and 5(d), the risk score (*p* < 0.001) and stage (*p* < 0.001) are identified as independent risk factors for overall survival in the training cohort. These results demonstrate that the lncRNA risk signature established in this study can independently predict survival outcomes.

### 3.5. Prognostic Role of the lncRNA Signature in Different Subgroups

**Figure 6:**
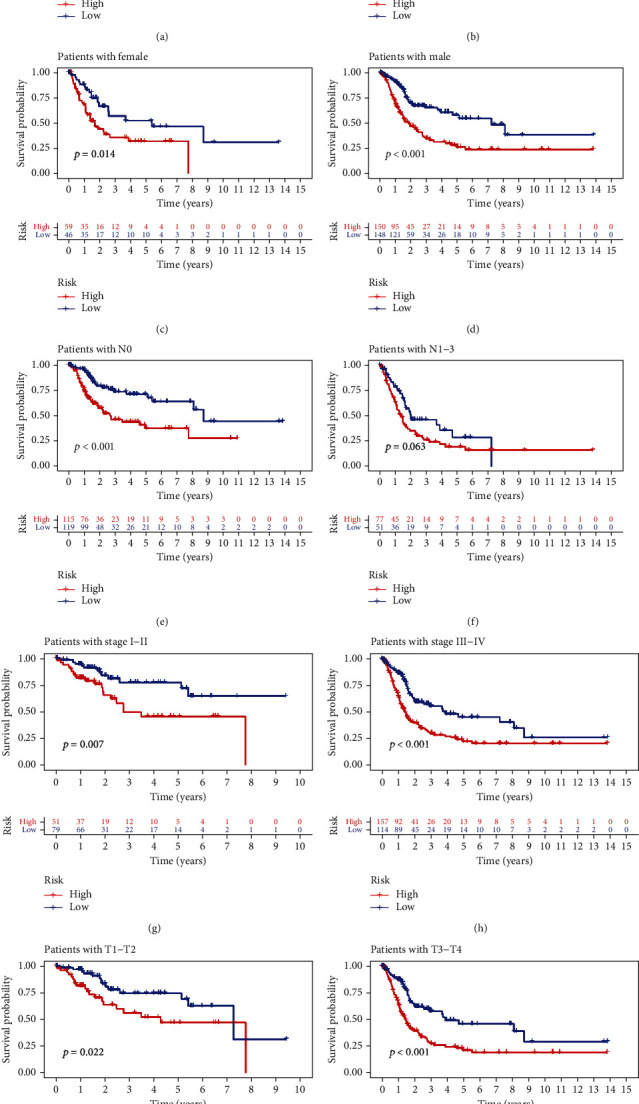
The lncRNA signature showed excellent performance in predicting survival in different subgroups. (a, b) Age (age, >65 or ≤65 years). (c, d) Sex (female or male). (e, f) Lymphatic invasion (no or yes). (g, h) Tumour stage (I–II or III–IV). (i, j) Clinical T stage (T1–T2 or T3–T4).

**Figure 7:**
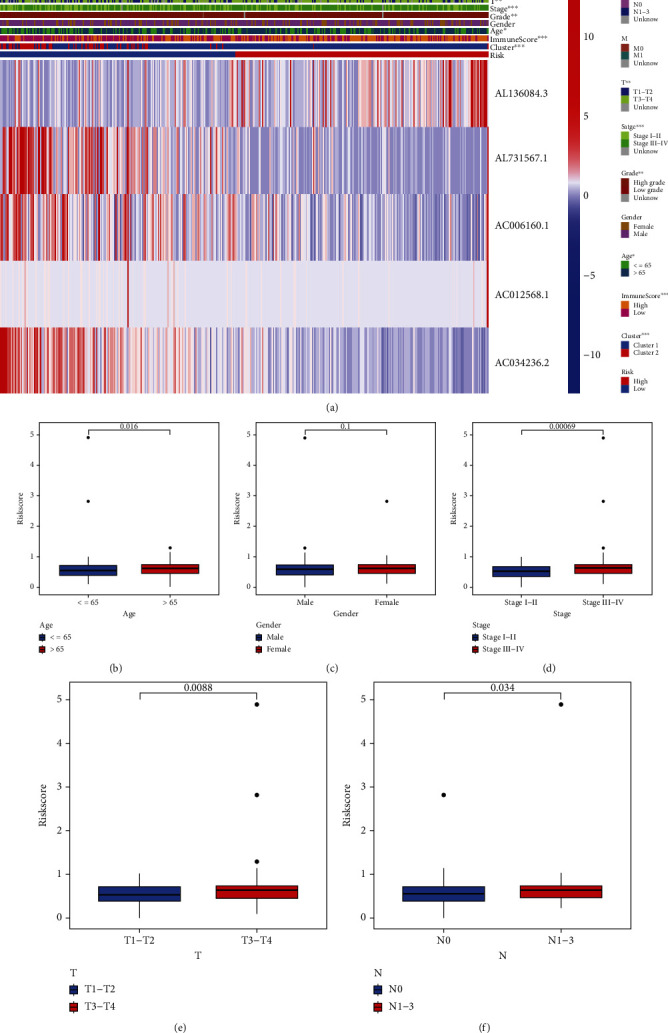
Correlation between the lncRNA risk signature and clinicopathological features. (a) Heatmap demonstrating the correlation between risk scores and clinicopathological features. (b) Differences in risk scores between patients aged >65 years and those aged ≤65 years. (c) Differences in risk scores between female and male patients. (d) Differences in risk scores between patients with stage I–II disease and those with stage III–IV disease. (e) Differences in risk scores between patients with T1–T2-stage disease and those with T3–T4-stage disease. (f) Differences in risk scores between patients with and without lymphatic invasion (^∗^*p* < 0.05;  ^∗∗^*p* < 0.01;  ^∗∗∗^*p* < 0.001).

An excellent prognostic tool in clinical practice should have consistent predictive performance in different subgroups. As shown in Figure 6, the 5-lncRNA risk signature was significantly associated with prognosis in the old (age > 65 years) (*p* < 0.001), male (*p* < 0.001), female (*p* = 0.014), stage I–II (*p* = 0.007), stage III–IV (*p* < 0.001), T1–T2 (*p* = 0.022), T3–T4 (*p* < 0.001), and N0 stage (*p* < 0.001) groups. However, the lncRNA risk signature was not associated with prognosis among patients aged ≤65 years, suggesting that the prognosis of younger patients may be influenced by various other mechanisms underlying the development of bladder cancer. Furthermore, the subgroup analysis verified that the lncRNA risk signature can serve as a biomarker to predict survival outcomes in bladder cancer. As shown in Figure 7(a), the risk signature was significantly correlated with the ferroptosis mediation patterns (*p* < 0.001), immune scores (*p* < 0.001), clinical stages (*p* < 0.001), and tumour grades (*p* < 0.01). In addition, elderly patients, patients with advanced-stage disease, and those with advanced T- or N-grade disease had higher risk scores (Figures 7(b)–7(f)). These results indicate that the lncRNA risk signature reflects various clinical characteristics from a genetic or molecular perspective, which should be further investigated in future studies.

### 3.6. Infiltration Abundance of Immune Checkpoints and Immune Cells in Clusters 1 and 2

**Figure 8:**
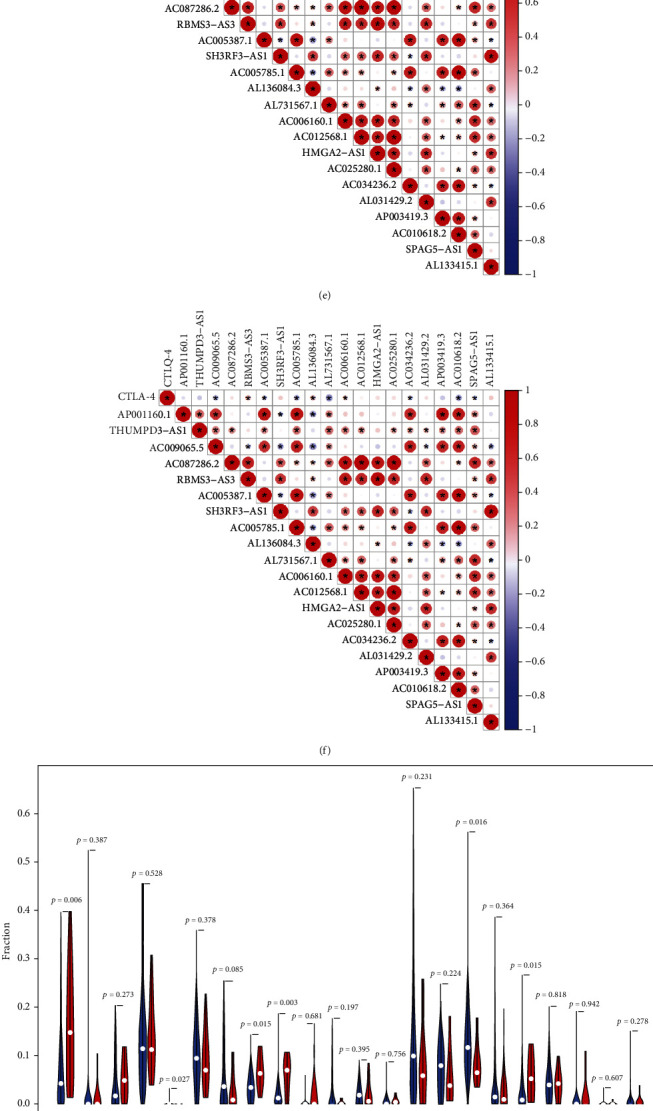
Correlation of ferroptosis-related lncRNAs with immune checkpoints and immune microenvironment in bladder cancer. (a–c) Differences in the expression of PD-1, PD-L1, and CTLA-4 between clusters 1 and 2. (d–f) Correlation between the expression of PD-1, PD-L1, and CTLA-4 and the 20 prognostic lncRNAs. (g) Infiltration of 22 types of immune cells in clusters 1 and 2. (h–l) The two clusters had significantly different infiltration levels of various immune cells including naive B cells, activated dendritic cells, M2 macrophages, follicular helper T cells, and Tregs (^∗^*p* < 0.05;  ^∗∗^*p* < 0.01;  ^∗∗∗^*p* < 0.001).

The expression of PD-1 (*p* < 0.001), PD-L1 (*p* < 0.001), and CTLA-4 (*p* < 0.001) was significantly higher in cluster 1 than in cluster 2 (Figures 8(a)–8(c)). Higher expression of these immune checkpoints was associated with poorer survival outcomes in cluster 1. As shown in Figure 8(d), PD-1 expression was positively correlated with AL136084.3 and negatively correlated with AC005785.1, AL731567.1 and AC010618.2. As shown in Figure 8(e), PD-L1 expression was positively correlated with AC006160.1 and negatively correlated with AC009065.5, AC005387.1, AL731567.1, AC034236.2, AP003419.3, and AC010618.2. As shown in Figure 8(f), CTLA-4 expression was significantly correlated with various ferroptosis-related lncRNAs. Furthermore, based on the cellular biomarkers of immune cells shown in Supplementary Table 9, the infiltration levels of 22 types of immune cells were calculated using the CIBERSORT algorithm (Supplementary Table 10). The ESTIMATE scores of each sample are shown in Supplementary Table 11, and the relationship between the infiltration of 22 types of immune cells and ferroptosis mediation patterns is demonstrated in Figure 8(g). As shown in box plots in Figures 8(h)–8(l), cluster 1 had lower infiltration levels of naive B cells (*p* = 0.006), activated dendritic cells (*p* = 0.015), follicular helper T cells (*p* = 0.015), and Tregs (*p* = 0.003) and higher infiltration levels of M2 macrophages. These results indicate that poorer survival outcomes observed in cluster 1 may be associated with the tumour immune microenvironment of bladder cancer. Furthermore, GSEA was performed to investigate mechanisms underlying poorer survival outcomes in cluster 1. The results revealed that the toll-like receptor signalling pathway, T-cell receptor signalling pathway, regulation of the actin cytoskeleton, cytokine and cytokine–receptor interaction, and chemokine signalling pathway were significantly enriched in cluster 1 (*p* < 0.01) (Supplementary Figure 1). These results indicate that ferroptosis-related lncRNAs play a regulatory role through these pathways during the development of bladder cancer.

### 3.7. Guidance of Immunotherapy for Bladder Cancer Based on the lncRNA Signature

**Figure 9:**
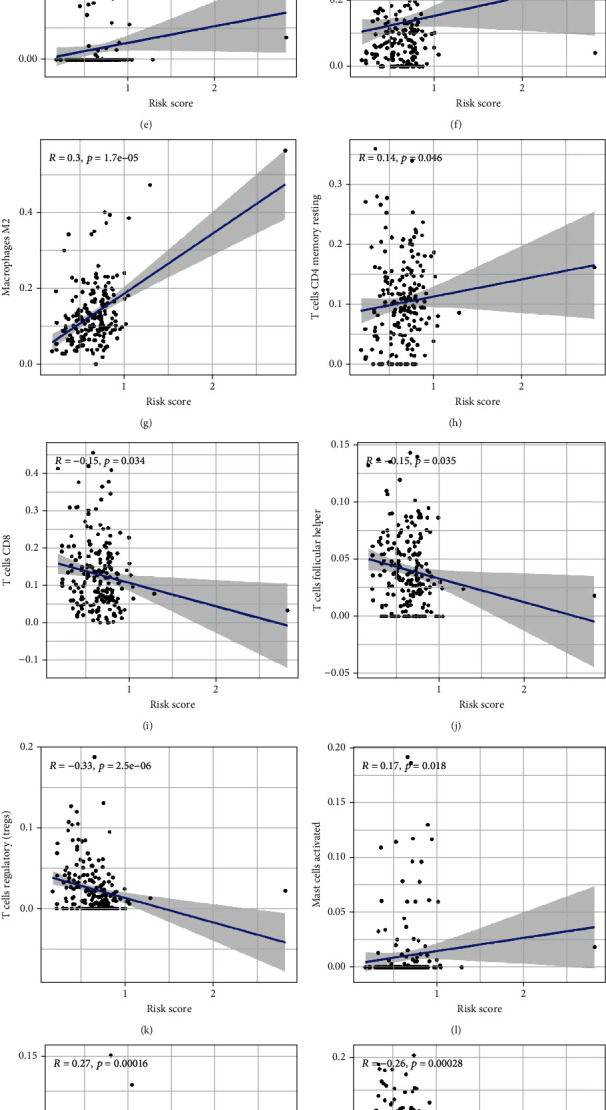
The lncRNA risk signature plays a vital role in predicting the response of patients to immunotherapy. (a–c) The high-risk group had significantly higher expression of various immune checkpoints including PD-1, PD-L1, and CTLA-4. (d–n) Risk scores were significantly correlated with the infiltration of 11 types of immune cells, including naive B cells, eosinophils, M0 macrophages, M2 macrophages, CD4 memory resting T cells, CD8 T cells, follicular helper T cells, Tregs, activated mast cells, neutrophils, and plasma cells. (o, p) As evaluated using the TIDE algorithm, high risk scores indicated a response to immunotherapy.

The expression of PD-1 (*p* < 0.001), PD-L1 (*p* < 0.001), and CTLA-4 (*p* < 0.001) (Figures 9(a)–9(c)) was higher in the high-risk group, which indicated the potential role of the lncRNA signature in immunotherapy. Therefore, the correlation between risk scores and immune cell infiltration was further investigated. As shown in Figures 9(d)–9(n), risk scores were significantly correlated with the infiltration levels of naive B cells, eosinophils, M0 macrophages, M2 macrophages, CD4 memory resting T cells, CD8 T cells, follicular helper T cells, Tregs, activated mast cells, neutrophils, and plasma cells. Furthermore, the response of patients to immunotherapy was evaluated using the TIDE algorithm (Supplementary Table 12). As shown in Figures 9(o) and 9(p), patients with high risk scores posed a better response to immunotherapy (*p* < 0.001). With the rapid development of sequencing technology, the lncRNA risk signature established in this study can be used to guide immunotherapy for bladder cancer in the future.

### 3.8. lncRNA AC006160.1 Inhibited the Proliferation and Migration of Bladder Cancer Cells

**Figure 10:**
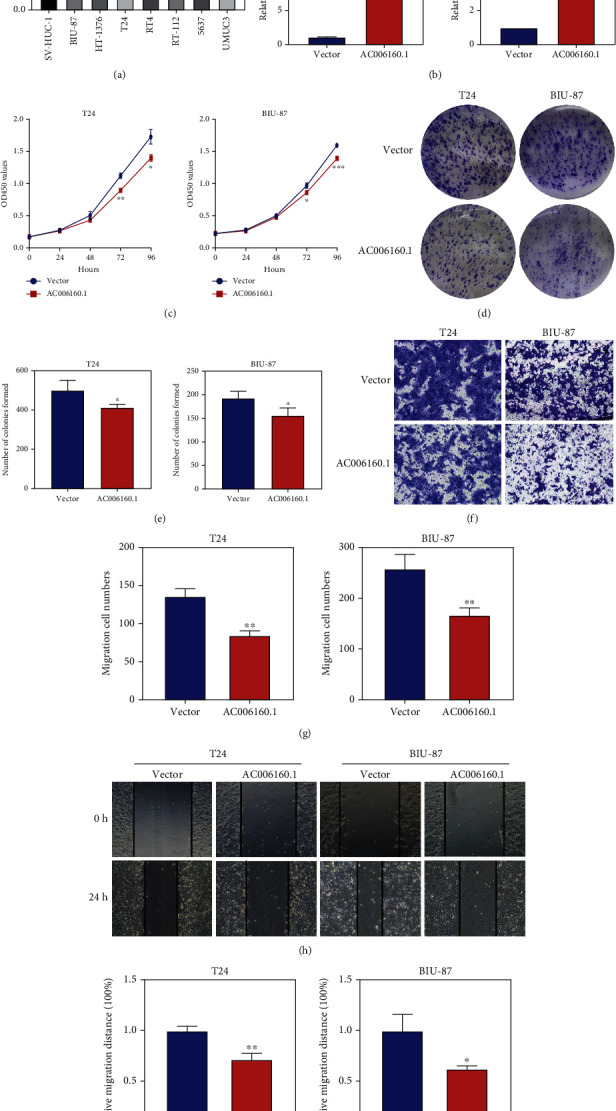
The lncRNA AC006160.1 suppresses the viability of bladder cancer cells. (a) Expression of AC006160.1 in bladder cancer cell lines and SV-HUC-1 cells was measured via qRT-PCR and normalised to GAPDH expression. (b) T24 and BIU-87 cells were transfected with the pcDNA3.1-AC006160.1 or negative control plasmid for 24 h, and the relative expression of AC006160.1 was measured via qRT-PCR and normalised to GAPDH expression. (c) T24 and BIU-87 cells were transfected with the pcDNA3.1-AC006160.1 or negative control plasmid, and cell proliferation was examined via the CCK8 assay. (d, e) Colony formation assay was performed to detect the proliferation of cells transfected with the indicated vectors. (f, g) The migration ability of T24 and BIU-87 cells transfected with the pcDNA3.1-AC006160.1 or negative control plasmid was measured via transwell assay. (h, i) The migration ability of cells transfected with the pcDNA3.1-AC006160.1 or negative control plasmid was detected via wound healing assay. All experiments were performed in triplicate. All data are expressed as the mean ± standard deviation (SD) (^∗^*p* < 0.05,  ^∗∗^*p* < 0.01, and^∗∗∗^*p* < 0.001 versus the control group).

Because the lncRNA AC006160.1 had the highest correlation with the risk signature, it was selected for further analysis. The qRT-PCR indicated that AC006160.1 expression was lower in the bladder cancer cell lines BIU-87, T24, RT4, RT-112, and 5637 than in the normal human uroepithelial cell line SV-HUC-1 (Figure 10(a)). Furthermore, CCK-8 assay was performed to examine the influence of AC006160.1 on the viability of bladder cancer cells. AC006160.1 expression was lower in T24 and BIU-87 cells than in the other bladder cancer cell lines; therefore, these two cell lines were used for examining the biological functions of AC006160.1. A plasmid overexpressing AC006160.1 was constructed, and the results of qPCR verified that transfection with this plasmid increased AC006160.1 expression by approximately 15- and 5-fold in T24 and BIU-87 cells, respectively (Figure 10(b)). CCK8 assay revealed that AC006160.1 overexpression significantly suppressed the proliferation of T24 and BIU-87 cells (Figure 10(c)). Furthermore, the influence of AC006160.1 on colony formation was examined. As shown in Figure 10(d), AC006160.1 overexpression decreased the number of colonies of both T24 and BIU-87 cells compared with the control group (Figure 10(e)). Transwell (Figures 10(f) and 10(g)) and wound healing (Figures 10(h) and 10(I)) assays revealed that AC006160.1 overexpression significantly inhibited the migration of T24 and BIU-87 cells. Altogether, these results indicate that the ferroptosis-related lncRNA AC006160.1 suppresses the viability of bladder cancer cells, which is consistent with the results of bioinformatic analysis.

### 3.9. Drug Sensitivity Analysis

**Figure 11:**
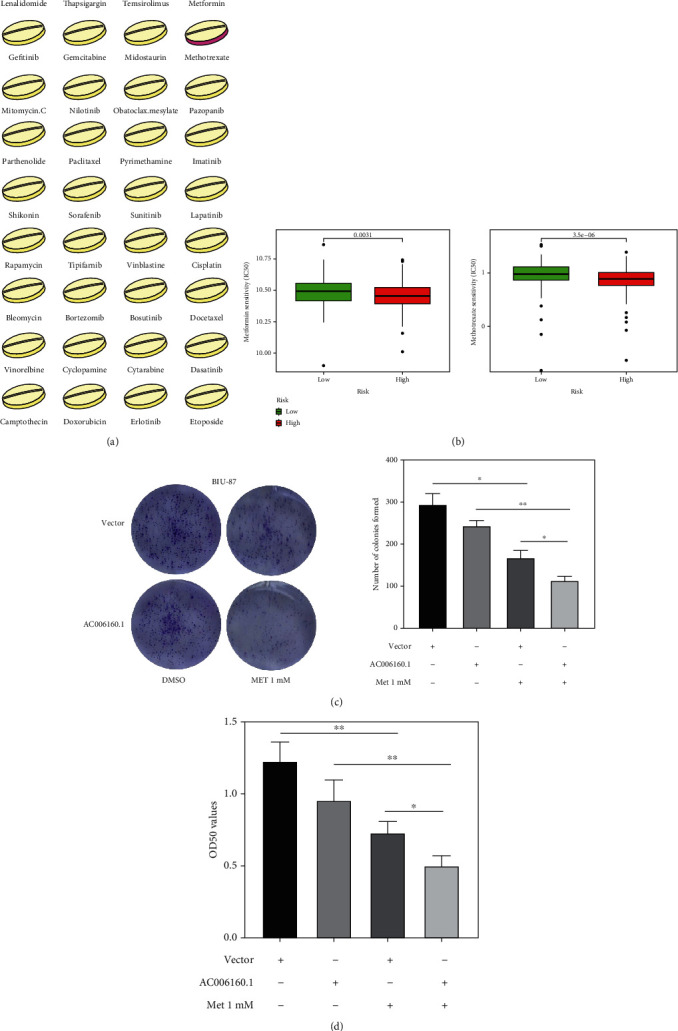
Drug sensitivity analysis based on IC50 values. (a) Drug sensitivity analysis of patients with bladder cancer with high or low AC006160.1 expression. The red colour indicates sensitive drugs for samples with high AC006160.1 expression. (b) Compared with patients with low AC006160.1 expression, those with high AC006160.1 expression were more sensitive metformin and methotrexate (*p* < 0.01). (c) Crystal violet staining of BIU-87 cells transfected with the pcDNA3.1-AC006160.1 or negative control plasmid and subsequently treated with 1-mM metformin for 14 days. (d) CCK-8 assay was performed to examine the proliferation of BIU-87 cells transfected with the pcDNA3.1-AC006160.1 or negative control plasmid and subsequently treated with 1-mM metformin for 72 h. All experiments were performed in triplicate. All data are expressed as the mean ± standard deviation (SD) (^∗^*p* < 0.05,  ^∗∗^*p* < 0.01,  ^∗∗∗^*p* < 0.001 versus the control group).

As shown in Figure 11(a), drug sensitivity analysis based on IC50 values revealed that patients with low expression of AC006160.1 were sensitive to most anticancer drugs. However, compared with patients with low expression of AC006160.1, those with high expression of AC006160.1 were more sensitive to MET and methotrexate (*p* < 0.01) (Figure 11(b)). Furthermore, in vitro drug sensitivity analysis was performed to examine whether AC006160.1 could enhance MET sensitivity in BIU-87 cells. AC006160.1 overexpression combined with MET treatment decreased the number of BIU-87 colonies (Figure 11(c)). In addition, CCK8 assay revealed that AC006160.1 overexpression combined with MET treatment significantly suppressed the proliferation of BIU-87 cells (Figure 11(d)). These results indicate that AC006160.1 may enhance drug sensitivity in the treatment of bladder cancer.

## 4. Discussion

Bladder cancer is one of the most malignant tumours. According to recent statistical data, >500,000 new cases of bladder cancer are reported annually worldwide [34]. Bladder cancer is classified as non-muscle-invasive and muscle-invasive bladder cancer, and its metastasis depends on its clinical characteristics. The currently available diagnostic strategies for bladder cancer include urine cytology and cystoscopy, which are invasive and can lead to many complications in patients [35]. Transurethral resection of tumours combined with intravesical perfusion therapy is recommended for non-muscle-invasive cancer, whereas radical cystectomy is recommended for muscle-invasive cancer. In addition, cisplatin-based chemotherapy is recommended for patients with advanced cancer with metastasis [36]. For chemotherapy-resistant tumours, immune checkpoint inhibitor (ICI) therapy is recommended in clinical settings [37]. However, owing to the irregular expression of various immune checkpoints and heterogeneity of genetic mutations, chemotherapy or ICI therapy has limited benefits in only a small number of patients. Moreover, because mechanisms underlying the development of bladder cancer remain unclear, limited progress has been achieved in the development of relative treatment strategies for bladder cancer during the past decade. Furthermore, bladder cancer has a high genetic mutation burden, and cancers with a high mutation burden such as lung cancer and melanoma may benefit the most from ICI therapy if robust biomarkers are discovered for clinical application [38–40]. However, the rapid development of next-generation sequencing and large-scale gene expression tools may help to improve the treatment of cancer in the future. The next-generation sequencing can provide vital genetic information for improving the diagnosis of bladder cancer and distinguishing treatment responses. Whole-transcriptome matrix information can also help to discover distinct molecular subtypes of bladder cancer with different mechanisms and molecular characteristics [6, 41]. In addition, establishing valuable risk signatures for predicting survival outcomes and response to ICI therapy can greatly improve therapeutic efficacy among patients with bladder cancer. Identifying patients who are sensitive to ICIs or chemotherapeutic agents can significantly improve the guidance of targeted therapy and increase the therapeutic benefits, especially among patients with advanced cancer. In addition, it can help to avoid unnecessary ICI or chemotherapy toxicity and potential delay in radical cystectomy for patients with treatment resistance. In a previous study, we reported that ferroptosis-related genes identified using sequencing data played a vital role in predicting the clinical information of bladder cancer samples [42]. In this study, we investigated the potential role of ferroptosis-related lncRNAs in bladder cancer and successfully constructed a risk signature based on five ferroptosis-related lncRNAs to predict survival outcomes, the tumour microenvironment, and the response of patients with bladder cancer to ICI therapy. In addition, the lncRNA AC006160.1 was identified as a protective factor for the development of bladder cancer.

Univariate cox regression analysis was used to construct a risk signature based on 20 prognostic ferroptosis-related lncRNAs, which were differentially expressed between tumour and healthy bladder tissues (Figure 1(c)). Significant differences in expression indicated the potential role of these lncRNAs in the development of bladder cancer. These prognostic lncRNAs were further used to divide samples into two groups with different survival outcomes. However, differences in only the tumour grade and age of patients were observed between clusters 1 and 2, which indicated that more accurate scoring methods are required for prognostic analysis. Therefore, a prognostic risk signature was constructed based on LASSO regression analysis, and the risk scores were found to have a significant relationship with various clinical parameters, including survival outcomes, immune microenvironment, ferroptosis clusters, age, clinical stages and tumour grades (Figure 7). The predictive ability of the lncRNA-based risk signature was validated in different clinical groups via subgroup analysis. The risk signature can be used as a novel tool for the treatment of bladder cancer in the future, especially considering the wide development and popularisation of whole-transcriptome sequencing technology.

The risk signature comprised five ferroptosis-related lncRNAs, namely, AC006160.1, AL136084.3, AL731567.1, AC012568.1, and AC034236.2. AL136084.3, has been identified as a risk factor for tumour invasiveness and resistance [43, 44]. To the best of our knowledge, no study has reported on the other four lncRNAs to date. In this study, AC006160.1 was found to have the highest coefficient in the risk signature and was hence analysed as the most important lncRNA. AC006160.1 overexpression significantly suppressed the viability of bladder cancer cells, indicating that AC006160.1 plays a protective role by inhibiting the proliferation and invasion abilities of tumour cells. Therefore, examining the underlying regulatory mechanisms of AC006160.1 may help to identify novel therapeutic targets for bladder cancer. Previous studies have demonstrated the vital role of lncRNAs in tumour development and treatment. For example, lncRNAs can influence tumour invasiveness and resistance through the miR-302a-3p/AKT axis, miR-124-3p/MCP-1 pathway, or miR-132-3p/USP22 pathway [45, 46]. In addition, studies have reported the involvement of the WNT signalling pathway and CeRNAs in regulatory mechanisms of lncRNAs in malignant tumours [47, 48]. Moreover, studies employing whole-transcriptome sequencing have broadened the horizon for cancer treatment.

In this study, the independent prognostic role of the ferroptosis-related-lncRNA risk signature was validated in both training and validation cohorts. The ability of the risk signature to predict survival outcomes was better than that of various clinicopathological parameters including age, sex, tumour stages, and grades. However, in the training cohort, 6 samples had a low grade, of which 5 were included in the low-risk group, Therefore, “grade” was excluded from multivariate Cox regression analysis to avoid statistical bias. The risk scores were used to evaluate bladder cancer samples at the transcriptomic (lncRNA) level and were theoretically more accurate than the currently used clinical parameters. Elderly patients, patients with advanced-stage disease, and those with higher T or N grades had higher risk scores, indicating that the risk signature can reflect various clinical characteristics from a genetic or molecular perspective. Therefore, the lncRNA risk signature has valuable application prospects for the treatment of bladder cancer.

Furthermore, the relationship between the immune microenvironment and the two ferroptosis mediation patterns was examined. Cluster 1 had lower infiltration of naive B cells, activated dendritic cells, and follicular helper T cells, which are responsible for activation of the immune system [5]. However, cluster 1 had higher infiltration of M2 macrophages, which are responsible for immune suppression [49]. These results were consistent with the significantly poorer survival outcomes of cluster 1. Bladder, skin and lung cancers are classical malignancies with a high mutation burden [50, 51]. Patients with such malignancies can potentially benefit from immunotherapy, which is closely associated with the tumour immune microenvironment. Therefore, this study mainly focused on the relationship among the risk signature, expression of immune checkpoints, and immune microenvironment of bladder cancer. Both cluster 1 (Figure 8) and high-risk group (Figure 9) had significantly higher expression of PD-1, PD-L1, and CTLA-4. Higher expression of these immune checkpoints in cluster 1 or the high-risk group might have led to poorer survival outcomes. Furthermore, PD-1 expression was positively correlated with AL136084.3 and negatively correlated with AC005785.1, AL731567.1, and AC010618.2. Similarly, various ferroptosis-related lncRNAs were significantly correlated with the expression of PD-L1 and CTLA-4. Cluster analysis revealed that the prognostic lncRNAs were significantly associated with the infiltration of various immune cells. These results indicate that ferroptosis-related lncRNAs not only regulate the malignant phenotype of bladder cancer through genetic alterations but also play a vital role in remodelling the tumour immune microenvironment of bladder cancer. Furthermore, the toll-like receptor signalling pathway, T-cell receptor signalling pathway, regulation of the actin cytoskeleton, cytokine and cytokine–receptor interaction, and chemokine signalling pathway were significantly enriched in cluster 1, indicating that ferroptosis-related lncRNAs may play a regulatory role in the development of bladder cancer through these biological pathways. Investigating the role of lncRNAs in these biological pathways may provide novel insights into the treatment of bladder cancer. The risk signature established in this study can be used to examine ferroptosis mediation patterns. The risk scores were significantly correlated with the infiltration of naive B cells, eosinophils, M0 macrophages, M2 macrophages, CD4 memory resting T cells, CD8 T cells, follicular helper T cells, Tregs, activated mast cells, neutrophils, and plasma cells, which indicated that the risk signature can be used to guide immunotherapy. In addition, the risk signature can be used to predict the response of patients to immunotherapy. Previous studies have demonstrated that malignant and immune cells coexist in the tumour microenvironment and react with each other to promote tumour growth and progression [52, 53]. However, the role of immune cells in tumour development remains elusive. The ferroptosis-related lncRNAs identified in this study may help to examine the relationship between immune cells and tumour progression. In conclusion, a novel lncRNA-based risk signature was constructed to predict survival outcomes, clinical stages, tumour grades, immune cell infiltration, immune checkpoint expression, and immunotherapeutic responses in bladder cancer samples. In addition, the lncRNA AC006160.1 was identified as a protective factor for the progression of bladder cancer in vitro.

However, this study has several limitations. First, all analysed lncRNA-sequencing data were extracted from public databases; therefore, large-scale sequencing studies in multicentre institutions are required to validate the lncRNA-based risk signature established in this study. Second, the TIDE algorithm was used to evaluate the potential response to immunotherapy; however, prospective clinical trials concerning different immunotherapeutic strategies should be conducted for bladder cancer with varying risk factors. In addition, the regulatory role of AC006160.1 in the progression of bladder cancer and the underlying mechanisms should be investigated in future studies.

## 5. Conclusion

Ferroptosis-related lncRNAs can accurately predict survival outcomes, clinical stages, tumour grades, immune cell infiltration, immune checkpoint expression, and immunotherapeutic responses in bladder cancer. The lncRNA AC006160.1 may serve as a protective factor for the progression of bladder cancer.

## Figures and Tables

**Table 1:** The five ferroptosis-related lncRNAs included in the risk signature.

lncRNA	Coefficient
AL136084.3	0.12
AL731567.1	-0.05
AC006160.1	-0.98
AC012568.1	0.25
AC034236.2	-0.35

## Data Availability

The data used to support the findings of this study are included within the article and the supplementary information files.

## Abbreviations

TCGA: The Cancer Genome Atlas
lncRNA: Long noncoding RNA
PD-1: Programmed cell death 1
ssGSEA: Single-sample gene set enrichment analysis
PD-L1: Programmed cell death-ligand 1
DEGs: Differentially expressed genes
CTLA-4: Cytotoxic T lymphocyte antigen 4
LASSO: Least absolute shrinkage and selection operator
ROC: Receiver operating characteristic
GSVA: Gene set variation analysis
TMB: Tumour mutation burden
TIDE: Tumour Immune Dysfunction and Exclusion
GDSC: Genomics of Drug Sensitivity in Cancer
MET: Metformin
qPCR: Quantitative polymerase chain reaction
cDNA: Complementary deoxyribonucleic acid.

## Supplementary Materials

Supplementary Materials Supplementary Table 1: expression matrix of 60 ferroptosis-related genes. Supplementary Table 2: results for coexpression analysis of ferroptosis-related lncRNAs and mRNAs. Supplementary Table 3: expression of 263 ferroptosis-related lncRNAs. Supplementary Table 4: expression of lncRNAs and clinical information of 403 bladder cancer samples. Supplementary Table 5: cluster analysis based on 20 prognostic lncRNAs. Supplementary Table 6: detailed clinical information of bladder cancer samples. Supplementary Table 7: data of the high- and low-risk groups of the training cohort. Supplementary Table 8: data of the high- and low-risk groups of the validation cohort. Supplementary Table 9: markers of different immune cells. Supplementary Table 10: infiltration of 22 types of immune cells analysed using the CIBERSORT algorithm. Supplementary Table 11: immune scores evaluated using the ESTIMATE algorithm. Supplementary Table 12: immunotherapeutic response evaluated using the Tumour Immune Dysfunction and Exclusion (TIDE) algorithm. Supplementary Figure 1: gene set enrichment analysis. (A–F) Most significantly enriched biological pathways in cluster 1. (G–I) Most significantly enriched biological pathways in cluster 2.
