# Supplementary material for: Ferroptosis-Related Long Noncoding RNAs Have Excellent Predictive Ability for Multiomic Characteristics of Bladder Cancer
Source: Oxid Med Cell Longev. 2022 Aug 29;2022:9316847. doi: 10.1155/2022/9316847 (PMC9444476; doi:10.1155/2022/9316847)
Supplement: Supplementary Materials — Supplementary Table 1: expression matrix of 60 ferroptosis-related genes. Supplementary Table 2: results for coexpression analysis of ferroptosis-related lncRNAs and mRNAs. Supplementary Table 3: expression of 263 ferroptosis-related lncRNAs. Supplementary Table 4: expression of lncRNAs and clinical information of 403 bladder cancer samples. Supplementary Table 5: cluster analysis based on 20 prognostic lncRNAs. Supplementary Table 6: detailed clinical information of bladder cancer samples. Supplementary Table 7: data of the high- and low-risk groups of the training cohort. Supplementary Table 8: data of the high- and low-risk groups of the validation cohort. Supplementary Table 9: markers of different immune cells. Supplementary Table 10: infiltration of 22 types of immune cells analysed using the CIBERSORT algorithm. Supplementary Table 11: immune scores evaluated using the ESTIMATE algorithm. Supplementary Table 12: immunotherapeutic response evaluated using the Tumour Immune Dysfunction and Exclusion (TIDE) algorithm. Supplementary Figure 1: gene set enrichment analysis. (A–F) Most significantly enriched biological pathways in cluster 1. (G–I) Most significantly enriched biological pathways in cluster 2. [file 9316847.f1.zip › s-table 1.pdf]

ID TCGA-GD-A3OP-11A-11R-A220-07 TCGA-BT-A20Q-11A-11R-A14Y-07 TCGA-GD-A2C5-11A-11R-A180-07 TCGA-BT-A20N-11A-11R-A14Y-07 TCGA-BT-A2LB-11A-11R-A18C-07 TCGA-GC-A3BM-11A-11R-A22U-07 TCGA-CU-A0YR-11A-13R-A10U-07 TCGA-GC-A3WC-11A-11R-A22U-07 TCGA-CU-A0YN-11A-11R-A10U-07 TCGA-BT-A20R-11A-11R-A16R-07 TCGA-BT-A20U-11A-11R-A14Y-07 TCGA-BT-A20W-11A-11R-A14Y-07 TCGA-GD-A3OQ-11A-21R-A220-07 TCGA-K4-A5RI-11A-11R-A28M-07 TCGA-BL-A13J-11A-13R-A10U-07 TCGA-K4-A54R-11A-11R-A26T-07 TCGA-K4-A3WV-11A-21R-A22U-07 TCGA-GC-A6I3-11A-11R-A31N-07 TCGA-BT-A2LA-11A-11R-A18C-07 TCGA-CF-A47X-01A-31R-A23W-07 TCGA-ZF-AA51-01A-21R-A39I-07 TCGA-BL-A0C8-01A-11R-A10U-07 TCGA-BT-A42C-01A-11R-A23N-07 TCGA-G2-A3IB-01A-11R-A20F-07 TCGA-2F-A9KP-01A-11R-A38B-07 TCGA-DK-A3IT-01A-31R-A20F-07 TCGA-FD-A43P-01A-31R-A23W-07 TCGA-DK-A3IV-01A-22R-A21D-07 TCGA-2F-A9KW-01A-11R-A38B-07 TCGA-GU-A762-01A-11R-A33J-07 TCGA-XF-AAME-01A-12R-A42T-07 TCGA-XF-AAN0-01A-11R-A42T-07 TCGA-CF-A47W-01A-11R-A23W-07 TCGA-GC-A3I6-01A-11R-A20F-07 TCGA-FD-A6TA-01A-12R-A33J-07 TCGA-DK-A1A7-01A-11R-A13Y-07 TCGA-FD-A3SR-01A-11R-A22U-07 TCGA-GU-AATP-01A-11R-A39I-07 TCGA-DK-AA6X-01A-12R-A42T-07 TCGA-XF-A9T3-01A-11R-A42T-07 TCGA-PQ-A6FN-01A-11R-A31N-07 TCGA-DK-A3WW-01A-22R-A23N-07 TCGA-XF-AAMY-01A-11R-A42T-07 TCGA-K4-A3WS-01A-11R-A22U-07 TCGA-UY-A9PA-01A-11R-A38B-07 TCGA-G2-AA3F-01A-12R-A42T-07 TCGA-FJ-A871-01A-11R-A352-07 TCGA-ZF-A9RN-01A-11R-A42T-07 TCGA-GC-A3RB-01A-12R-A220-07 TCGA-UY-A9PF-01A-11R-A38B-07 TCGA-YC-A89H-01A-11R-A36F-07 TCGA-XF-AAMQ-01A-11R-A42T-07 TCGA-CF-A1HR-01A-11R-A13Y-07 TCGA-E7-A3X6-01A-12R-A22U-07 TCGA-DK-A6AW-01A-11R-A30C-07 TCGA-GC-A3BM-01A-11R-A22U-07 TCGA-GV-A6ZA-01A-12R-A33J-07 TCGA-G2-A2EJ-01A-11R-A180-07 TCGA-FD-A43N-01A-11R-A23W-07 TCGA-UY-A8OD-01A-11R-A36F-07 TCGA-2F-A9KT-01A-11R-A38B-07 TCGA-BT-A20Q-01A-11R-A14Y-07 TCGA-CU-A0YR-01A-12R-A10U-07 TCGA-SY-A9G5-01A-11R-A38B-07 TCGA-4Z-AA84-01A-11R-A39I-07 TCGA-4Z-AA7Q-01A-11R-A39I-07 TCGA-FD-A5BT-01A-11R-A26T-07 TCGA-CF-A27C-01A-11R-A16R-07 TCGA-E7-A6MF-01A-12R-A32O-07 TCGA-FT-A61P-01A-11R-A30C-07 TCGA-DK-AA77-01A-11R-A39I-07 TCGA-ZF-AA5N-01A-11R-A42T-07 TCGA-DK-AA6U-01A-11R-A39I-07 TCGA-BT-A20O-01A-21R-A14Y-07 TCGA-GD-A3OS-01A-12R-A220-07 TCGA-XF-A9SZ-01A-11R-A39I-07 TCGA-4Z-AA82-01A-11R-A39I-07 TCGA-DK-AA76-01A-11R-A39I-07 TCGA-DK-A3IL-01A-11R-A20F-07 TCGA-E7-A678-01A-11R-A30C-07 TCGA-ZF-A9R1-01A-11R-A39I-07 TCGA-XF-A9SH-01A-11R-A39I-07 TCGA-FD-A5BU-01A-31R-A26T-07 TCGA-C4-A0F7-01A-11R-A084-07 TCGA-C4-A0F0-01A-12R-A10U-07 TCGA-FD-A3N5-01A-11R-A21D-07 TCGA-ZF-AA5H-01A-11R-A39I-07 TCGA-DK-A3WX-01A-22R-A22U-07 TCGA-YF-AA3L-01A-11R-A38B-07 TCGA-BT-A2LA-01A-11R-A18C-07 TCGA-4Z-AA87-01A-11R-A39I-07 TCGA-BT-A2LB-01A-11R-A18C-07 TCGA-XF-A8HB-01A-11R-A36F-07 TCGA-DK-A2I6-01A-12R-A18C-07 TCGA-ZF-AA4X-01A-11R-A38B-07 TCGA-BT-A20P-01A-11R-A14Y-07 TCGA-BT-A20W-01A-21R-A14Y-07 TCGA-FD-A5BX-01A-11R-A26T-07 TCGA-BL-A13J-01A-11R-A10U-07 TCGA-CF-A8HX-01A-11R-A36F-07 TCGA-PQ-A6FI-01A-11R-A31N-07 TCGA-FJ-A3Z7-01A-12R-A23N-07 TCGA-E7-A7PW-01A-11R-A352-07 TCGA-4Z-AA7S-01A-

11R-A39I-07 TCGA-CF-A3MH-01A-11R-A20F-07 TCGA-DK-A6B2-01A-11R-A30C-07

TCGA-4Z-AA86-01A-11R-A39I-07 TCGA-BL-A0C8-01B-04R-A277-07 TCGA-5N-A9KI-01A-31R-A42T-07 TCGA-GU-AATO-01A-11R-A39I-07 TCGA-CF-A9FH-01A-11R-A38B-07 TCGA-FD-A6TG-01A-11R-A32O-07 TCGA-4Z-AA7O-01A-31R-A39I-07

TCGA-GV-A3QI-01A-11R-A220-07 TCGA-DK-A6AV-01A-12R-A30C-07 TCGA-DK-A1A3-01A-11R-A13Y-07 TCGA-GD-A6C6-01A-21R-A31N-07 TCGA-GC-A3RD-01A-12R-A22U-07 TCGA-BT-A20R-01A-12R-A16R-07 TCGA-XF-A9T0-01A-11R-A39I-07

TCGA-YF-AA3M-01A-11R-A42T-07 TCGA-4Z-AA7Y-01A-11R-A39I-07 TCGA-FD-A62O-01A-11R-A30C-07 TCGA-BL-A3JM-01A-12R-A21D-07 TCGA-FJ-A3ZF-01A-11R-A23N-07 TCGA-CU-A0YO-01A-11R-A10U-07 TCGA-CF-A5UA-01A-11R-A28M-07 TCGA-K4-AAQO-01A-11R-A38B-07 TCGA-XF-AAMJ-01A-11R-A42T-07 TCGA-GU-A763-01A-11R-A32O-07 TCGA-G2-A2EC-01A-11R-A180-07 TCGA-FD-A3B7-01A-31R-A20F-07 TCGA-FD-A5C0-01A-11R-A28M-07 TCGA-FD-A5BS-01A-21R-A26T-07 TCGA-CU-A0YN-01A-21R-A10U-07 TCGA-FD-A5BZ-01A-11R-A28M-07 TCGA-UY-A9PE-01A-11R-A38B-07 TCGA-GV-A40E-01A-12R-A23N-07 TCGA-BT-A2LD-01A-12R-A20F-07 TCGA-UY-A9PD-01A-11R-A38B-07 TCGA-G2-AA3B-01A-11R-A39I-07 TCGA-K4-A54R-01A-11R-A26T-07 TCGA-DK-A1AA-01A-11R-A13Y-07 TCGA-CU-A5W6-01A-11R-A28M-07 TCGA-DK-AA6R-01A-11R-A42T-07 TCGA-BT-A3PH-01A-11R-A220-07 TCGA-BL-A13I-01B-04R-A277-07 TCGA-CF-A3MF-01A-12R-A21D-07 TCGA-CF-A47T-01A-11R-A23W-07 TCGA-YC-A8S6-01A-31R-A38B-07 TCGA-DK-A3IS-01A-21R-A21D-07 TCGA-XF-A9SJ-01A-11R-A39I-07 TCGA-DK-AA6T-01A-11R-A39I-07

TCGA-BT-A20N-01A-11R-A14Y-07 TCGA-C4-A0F6-01A-11R-A10U-07 TCGA-CF-A7I0-01A-22R-A352-07 TCGA-FD-A3B6-01A-21R-A20F-07 TCGA-GC-A3YS-01A-11R-A23N-07 TCGA-XF-A9T2-01A-11R-A42T-07 TCGA-UY-A78O-01A-12R-A33J-07

TCGA-GC-A3OO-01A-11R-A22U-07 TCGA-E7-A6ME-01A-22R-A32O-07 TCGA-XF-AAN5-01A-11R-A42T-07 TCGA-FD-A3SN-01A-12R-A22U-07 TCGA-ZF-A9R2-01A-11R-A39I-07 TCGA-ZF-AA4T-01A-11R-A38B-07 TCGA-E7-A4IJ-01A-31R-A26T-07

TCGA-FD-A6TE-01A-12R-A33J-07 TCGA-H4-A2HO-01A-11R-A180-07 TCGA-FD-A6TB-01A-12R-A33J-07 TCGA-XF-AAN3-01A-11R-A42T-07 TCGA-XF-AAMH-01A-11R-A42T-07 TCGA-FD-A3B8-01A-31R-A20F-07 TCGA-XF-A9SY-01A-21R-A42T-07

TCGA-CF-A9FF-01A-11R-A38B-07 TCGA-XF-A9ST-01A-11R-A42T-07 TCGA-DK-A6B5-01A-11R-A31N-07 TCGA-GC-A3RC-01A-11R-A22U-07 TCGA-GV-A3QK-01B-11R-A23N-07 TCGA-GU-A764-01A-11R-A352-07 TCGA-GU-A42R-01A-11R-A23N-07 TCGA-DK-A6B6-01A-11R-A30C-07 TCGA-R3-A69X-01A-22R-A30C-07 TCGA-5N-A9KM-01A-11R-A42T-07 TCGA-FD-A6TF-01A-52R-A32O-07 TCGA-XF-AAMR-01A-31R-A42T-07 TCGA-XF-A8HF-01A-11R-A36F-07 TCGA-ZF-AA4U-01A-11R-A38B-07

TCGA-DK-A3IQ-01A-31R-A32Y-07 TCGA-E7-A4XJ-01A-11R-A26T-07 TCGA-XF-A9T6-01A-11R-A42T-07 TCGA-4Z-AA7M-01A-11R-A39I-07 TCGA-ZF-AA56-01A-31R-A39I-07 TCGA-XF-A8HH-01A-11R-A38B-07 TCGA-C4-A0EZ-01A-21R-A24X-07

TCGA-FD-A62P-01A-32R-A30C-07 TCGA-CF-A47V-01A-11R-A23W-07 TCGA-XF-A9SI-01A-11R-A39I-07 TCGA-GU-A42Q-01A-11R-A23W-07 TCGA-BT-A42F-01A-11R-A23W-07 TCGA-ZF-A9RF-01A-11R-A38B-07 TCGA-KQ-A41N-01A-11R-A33J-07 TCGA-GD-A3OQ-01A-32R-A220-07 TCGA-BT-A20U-01A-11R-A14Y-07 TCGA-XF-A9SW-01A-11R-A42T-07 TCGA-FD-A3B3-01A-12R-A206-07 TCGA-ZF-A9RC-01A-

11R-A38B-07 TCGA-E7-A6MD-01A-41R-A352-07 TCGA-E5-A4TZ-01A-11R-A31N-07
TCGA-ZF-A9R4-01A-11R-A38B-07 TCGA-GV-A3QH-01A-11R-A220-07 TCGA-BT-
A0YX-01A-11R-A10U-07 TCGA-FT-A3EE-01A-11R-A206-07 TCGA-ZF-A9RL-01A-11R-
A38B-07 TCGA-ZF-A9R9-01A-11R-A38B-07 TCGA-ZF-A9RE-01A-11R-A38B-07 TCGA-
DK-A6B1-01A-12R-A30C-07 TCGA-CF-A5U8-01A-11R-A28M-07 TCGA-XF-AAMW-
01A-11R-A42T-07 TCGA-BT-A3PJ-01A-21R-A220-07 TCGA-ZF-A9R7-01A-11R-
A38B-07 TCGA-FD-A3SO-01A-11R-A22U-07 TCGA-E7-A97P-01A-11R-A38B-07 TCGA-
GU-A767-01A-11R-A32O-07 TCGA-4Z-AA81-01A-11R-A39I-07 TCGA-2F-A9KQ-
01A-11R-A38B-07 TCGA-SY-A9G0-01A-12R-A38B-07 TCGA-GV-A40G-01A-11R-
A23N-07 TCGA-FD-A43Y-01A-21R-A26T-07 TCGA-S5-AA26-01A-11R-A38B-07 TCGA-
BT-A42E-01A-11R-A23W-07 TCGA-FD-A43X-01A-11R-A23W-07 TCGA-ZF-AA4R-
01A-11R-A38B-07 TCGA-BT-A20T-01A-11R-A14Y-07 TCGA-E7-A7DU-01A-11R-
A32O-07 TCGA-DK-A3WY-01A-11R-A22U-07 TCGA-G2-A2EF-01A-12R-A18C-07
TCGA-BT-A20V-01A-11R-A14Y-07 TCGA-K4-A3WV-01A-11R-A22U-07 TCGA-GU-
AATQ-01A-11R-A39I-07 TCGA-G2-A3VY-01A-11R-A22U-07 TCGA-XF-AAN8-01A-
11R-A42T-07 TCGA-CU-A3QU-01A-11R-A22U-07 TCGA-GD-A3OP-01A-21R-A220-
07 TCGA-KQ-A41P-01A-12R-A33J-07 TCGA-UY-A9PH-01A-11R-A38B-07 TCGA-FD-
A5BY-01A-31R-A28M-07 TCGA-K4-A3WU-01B-11R-A23N-07 TCGA-FD-A3SJ-01A-12R-
A22U-07 TCGA-FD-A3B5-01A-11R-A20F-07 TCGA-GC-A6I3-01A-11R-A31N-07 TCGA-
4Z-AA80-01A-11R-A39I-07 TCGA-G2-A3IE-01A-11R-A20F-07 TCGA-ZF-A9R0-01A-
11R-A38B-07 TCGA-FD-A43S-01A-21R-A23W-07 TCGA-XF-A8HG-01A-11R-A36F-07
TCGA-K4-A83P-01A-11R-A352-07 TCGA-GD-A2C5-01A-12R-A180-07 TCGA-XF-
AAMX-01A-11R-A42T-07 TCGA-DK-A1AE-01A-11R-A13Y-07 TCGA-FJ-A3ZE-01A-11R-
A23N-07 TCGA-DK-A2I1-01A-11R-A180-07 TCGA-DK-A6B0-01A-11R-A31N-07 TCGA-
FD-A5BR-01A-11R-A26T-07 TCGA-KQ-A41Q-01A-11R-A33J-07 TCGA-DK-A1AF-
01A-11R-A13Y-07 TCGA-LT-A5Z6-01A-11R-A28M-07 TCGA-GC-A4ZW-01A-11R-
A26T-07 TCGA-K4-A4AB-01B-12R-A28M-07 TCGA-DK-AA74-01A-11R-A39I-07 TCGA-
G2-A2EO-01A-11R-A180-07 TCGA-DK-A1AG-01A-11R-A13Y-07 TCGA-GV-A3JZ-01A-
11R-A21D-07 TCGA-XF-A9SV-01A-21R-A42T-07 TCGA-UY-A78K-01A-11R-A33J-07
TCGA-FD-A6TH-01A-11R-A32O-07 TCGA-DK-AA6W-01A-12R-A39I-07 TCGA-CF-
A3MG-01A-11R-A20F-07 TCGA-DK-A3X1-01A-12R-A22U-07 TCGA-DK-A1A6-01A-
11R-A13Y-07 TCGA-XF-AAMG-01A-11R-A42T-07 TCGA-E7-A541-01A-11R-A26T-07
TCGA-XF-A8HE-01A-11R-A36F-07 TCGA-DK-AA6Q-01A-11R-A39I-07 TCGA-UY-
A78N-01A-12R-A33J-07 TCGA-DK-AA75-01A-11R-A39I-07 TCGA-ZF-AA5P-01A-
11R-A39I-07 TCGA-GC-A3WC-01A-31R-A22U-07 TCGA-K4-A4AC-01A-21R-A26T-07
TCGA-FD-A3SL-01A-21R-A22U-07 TCGA-K4-A5RH-01A-11R-A30C-07 TCGA-FD-
A5C1-01A-11R-A28M-07 TCGA-BL-A13J-01A-11R-A277-07 TCGA-XF-AAN7-01A-
11R-A42T-07 TCGA-2F-A9KO-01A-11R-A38B-07 TCGA-DK-A3IM-01A-11R-A20F-07
TCGA-FD-A3SS-01A-12R-A22U-07 TCGA-4Z-AA7N-01A-11R-A39I-07 TCGA-HQ-
A2OE-01A-11R-A206-07 TCGA-UY-A8OC-01A-11R-A36F-07 TCGA-GV-A3JV-01A-
11R-A220-07 TCGA-XF-A9T8-01A-11R-A39I-07 TCGA-KQ-A41S-01A-12R-A33J-07
TCGA-CU-A3YL-01A-11R-A22U-07 TCGA-E7-A7XN-01A-11R-A352-07 TCGA-DK-
A2HX-01A-12R-A18C-07 TCGA-CF-A8HY-01A-11R-A36F-07 TCGA-4Z-AA7W-01A-
11R-A39I-07 TCGA-FD-A43U-01A-11R-A23W-07 TCGA-BL-A0C8-01A-11R-A277-07

TCGA-BT-A0S7-01A-11R-A10U-07 TCGA-ZF-A9R3-01A-11R-A38B-07 TCGA-4Z-AA89-01A-11R-A39I-07 TCGA-GU-A42P-01A-11R-A23W-07 TCGA-FD-A3NA-01A-11R-A21D-07 TCGA-XF-A9SL-01A-11R-A39I-07 TCGA-XF-AAN2-01A-11R-A42T-07 TCGA-ZF-AA54-01A-11R-A39I-07 TCGA-GU-A766-01A-11R-A32O-07 TCGA-FD-A6TD-01A-51R-A33J-07 TCGA-FD-A3B4-01A-12R-A206-07 TCGA-DK-A1AB-01A-11R-A13Y-07 TCGA-BT-A20X-01A-11R-A16R-07 TCGA-GV-A3JW-01A-11R-A20F-07 TCGA-UY-A9PB-01A-11R-A38B-07 TCGA-FD-A6TI-01A-11R-A32O-07 TCGA-XF-AAML-01A-11R-A42T-07 TCGA-ZF-AA58-01A-12R-A42T-07 TCGA-FD-A3SP-01A-31R-A22U-07 TCGA-E7-A97Q-01A-11R-A38B-07 TCGA-YC-A9TC-01A-22R-A39I-07

TCGA-GV-A3QG-01A-11R-A220-07 TCGA-K4-A6FZ-01A-11R-A31N-07 TCGA-KQ-A41R-01A-21R-A352-07 TCGA-DK-AA6M-01A-11R-A39I-07 TCGA-ZF-AA52-01A-12R-A39I-07 TCGA-K4-A5RJ-01A-11R-A28M-07 TCGA-HQ-A5ND-01A-11R-A26T-07

TCGA-BT-A3PK-01A-21R-A220-07 TCGA-FD-A3N6-01A-11R-A21D-07 TCGA-UY-A78P-01A-12R-A36F-07 TCGA-CF-A9FL-01A-11R-A38B-07 TCGA-CF-A9FM-01A-11R-A38B-07 TCGA-ZF-A9RD-01A-11R-A42T-07 TCGA-G2-AA3D-01A-11R-A39I-07

TCGA-DK-A2I2-01A-11R-A180-07 TCGA-FD-A62S-01A-11R-A30C-07 TCGA-MV-A51V-01A-11R-A26T-07 TCGA-E5-A2PC-01A-11R-A206-07 TCGA-DK-A3X2-01A-11R-A22U-07 TCGA-GD-A76B-01A-11R-A32O-07 TCGA-GC-A6I1-01A-12R-A31N-07

TCGA-BL-A13J-01B-04R-A277-07 TCGA-XF-A9SK-01A-11R-A42T-07 TCGA-DK-A1AD-01A-11R-A13Y-07 TCGA-K4-A5RI-01A-11R-A28M-07 TCGA-FD-A3SQ-01A-21R-A22U-07 TCGA-GV-A3QF-01A-31R-A22U-07 TCGA-E7-A7DV-01A-11R-A33J-07

TCGA-XF-AAN1-01A-31R-A42T-07 TCGA-DK-AA6P-01A-11R-A39I-07 TCGA-CF-A47Y-01A-11R-A23W-07 TCGA-CF-A3MI-01A-11R-A20F-07 TCGA-G2-A2EL-01A-12R-A18C-07 TCGA-XF-AAMZ-01A-11R-A42T-07 TCGA-DK-AA6L-01A-11R-A39I-07

TCGA-XF-A9T4-01A-11R-A39I-07 TCGA-K4-A6MB-01A-11R-A31N-07 TCGA-UY-A8OB-01A-12R-A42T-07 TCGA-ZF-A9R5-01A-12R-A42T-07 TCGA-CU-A3KJ-01A-11R-A21D-07 TCGA-DK-AA6S-01A-21R-A39I-07 TCGA-E7-A85H-01A-11R-A352-07

TCGA-G2-AA3C-01A-21R-A39I-07 TCGA-E7-A8O8-01A-11R-A36F-07 TCGA-XF-A9SM-01A-11R-A42T-07 TCGA-DK-A3IN-01A-11R-A20F-07 TCGA-XF-A8HC-01A-11R-A36F-07 TCGA-ZF-AA4W-01A-12R-A38B-07 TCGA-LC-A66R-01A-41R-A30C-07

TCGA-E7-A8O7-01A-11R-A36F-07 TCGA-LT-A8JT-01A-11R-A36F-07 TCGA-FJ-A3Z9-01A-11R-A26T-07 TCGA-DK-A1AC-01A-11R-A13Y-07 TCGA-C4-A0F1-01A-11R-A034-07 TCGA-G2-A2ES-01A-11R-A180-07 TCGA-XF-AAN4-01A-11R-A42T-07

TCGA-4Z-AA83-01A-11R-A39I-07 TCGA-E7-A5KE-01A-11R-A28M-07 TCGA-ZF-AA4V-01A-11R-A38B-07 TCGA-ZF-AA53-01A-11R-A39I-07 TCGA-ZF-A9RM-01A-11R-A38B-07 TCGA-BL-A13I-01A-11R-A13Y-07 TCGA-CU-A72E-01A-12R-A33J-07

TCGA-2F-A9KR-01A-11R-A38B-07 TCGA-E5-A4U1-01A-11R-A31N-07 TCGA-E7-A3Y1-01A-11R-A22U-07 TCGA-FD-A6TC-01A-21R-A33J-07 TCGA-FD-A5BV-01A-11R-A26T-07 TCGA-DK-A2I4-01A-11R-A21D-07 TCGA-UY-A78M-01A-21R-A352-07

TCGA-4Z-AA7R-01A-11R-A39I-07 TCGA-E7-A519-01A-11R-A26T-07 TCGA-DK-A3IU-01A-11R-A20F-07 TCGA-FD-A3SM-01A-11R-A22U-07 TCGA-BL-A5ZZ-01A-31R-A30C-07 TCGA-FD-A62N-01A-11R-A30C-07 TCGA-BT-A20J-01A-11R-A14Y-07

TCGA-DK-AA71-01A-31R-A39I-07 TCGA-KQ-A41O-01A-12R-A352-07 TCGA-BL-A13I-01A-11R-A277-07 TCGA-E7-A677-01A-11R-A30C-07 TCGA-DK-A1A5-01A-

11R-A13Y-07 TCGA-HQ-A5NE-01A-12R-A28M-07 TCGA-XF-AAMT-01A-11R-A42T-07 TCGA-XF-A9SU-01A-31R-A39I-07 TCGA-CF-A47S-01A-11R-A23W-07 TCGA-FD-A6TK-01A-42R-A33J-07 TCGA-E7-A5KF-01A-11R-A28M-07 TCGA-UY-A78L-01A-12R-A33J-07 TCGA-S5-A6DX-01A-11R-A31N-07 TCGA-XF-A8HI-01A-11R-A38B-07 TCGA-XF-A9T5-01A-11R-A42T-07 TCGA-XF-A9SX-01A-21R-A39I-07 TCGA-G2-A2EK-01A-22R-A18C-07 TCGA-XF-A8HD-01A-11R-A36F-07 TCGA-GV-A3JX-01A-11R-A20F-07 TCGA-H4-A2HQ-01A-11R-A180-07 TCGA-XF-A9SP-01A-11R-A39I-07 TCGA-DK-A3IK-01A-32R-A21D-07

ACSL4 4.960116 10.98861 8.213418 12.28393 4.811481 5.333087 11.77768 7.586674 8.272641 17.00878 3.400133 16.87811 10.22609 14.27767 9.235378 9.982893 6.638522 5.456251 5.031157 12.71382 13.01401 18.2123 4.559561 3.83911 6.468616 2.906341 10.70202 2.087376 7.720133 8.668112 11.65725 2.490481 2.017734 10.62341 12.01361 4.938973 3.876739 4.429899 6.580069 8.033393 3.424778 12.93926 5.767193 9.413828 9.286295 55.35082 6.047114 2.949549 6.043039 11.57897 6.029808 11.66079 4.794883 5.124241 3.630641 3.330513 8.957516 7.569492 8.771184 5.536179 5.195983 7.150073 9.544303 4.113171 24.83982 2.107757 3.34917 3.047493 5.708343 10.86928 5.361841 1.076694 13.71839 23.77464 17.36885 2.873053 4.886517 17.76937 1.867361 2.088469 10.34927 4.331252 6.944055 7.81119 36.16264 5.532518 9.699426 18.68221 2.02062 3.430436 16.03506 5.055126 5.275055 2.537992 9.061539 4.889263 18.56804 9.618053 3.206815 1.521326 5.170866 9.417269 3.39669 2.959667 3.97646 10.83539 13.91591 30.05306 4.592735 3.58121 5.06339 2.969982 1.672947 9.684157 7.14224 4.378202 0.6586598 14.33506 12.21696 6.579091 4.380215 9.091901 6.926032 5.661194 19.08189 8.966618 4.102148 7.262082 11.1549 1.463981 8.460273 18.43551 3.951471 8.54354 7.524024 6.399994 8.546802 6.181612 2.149279 14.075 9.579713 3.222954 6.01816 3.988159 5.074138 1.528914 60.67102 2.601214 3.188822 20.57368 4.472761 4.161826 4.305995 2.052264 4.504408 7.572114 10.34118 7.106386 4.964626 3.358784 11.39789 13.63348 11.0482 2.734716 6.127025 10.17291 14.15508 6.823749 8.40105 5.940685 4.605507 1.818372 18.41144 17.79934 4.858169 3.966594 2.798168 7.091415 9.001186 5.50204 4.872102 12.59805 6.432248 9.471192 5.626288 8.714423 2.854046 4.117645 6.9764 3.450418 3.520358 4.502571 12.12546 17.51685 9.703191 3.03749 6.150296 10.24036 4.215242 3.37182 28.42428 0.7089679 7.016755 4.777935 8.321673 8.103277 6.396857 16.85463 0.9416815 13.77856 14.07154 4.205971 8.885502 3.269672 6.500353 7.637025 16.16815 4.2054 21.22638 12.57735 2.931629 3.394742 12.85789 2.69034 2.041832 3.38626 10.06445 55.67371 6.427101 2.051893 5.313722 4.239918 11.66449 3.525059 4.297386 8.52716 19.84059 5.384767 1.578953 4.818495 13.44312 27.94619 13.97009 11.76976 11.09753 10.92867 5.384848 13.38866 11.03126 3.377141 4.662244 8.241445 7.245044 4.970115 5.550845 18.86433 15.84903 4.253041 3.733075 6.711921 1.079974 28.33547 5.045654 6.691819 16.98949 53.99914 6.453393 4.649267 10.55015 7.534595 5.26905 9.527796 11.79179 4.549953 3.202199 2.948883 3.718643 2.988134 15.44509 6.186886 18.4253 7.327284 5.254164 7.179378 4.008384 5.452296 14.89082 8.997861 9.515807 15.65108 17.87232 8.017118 7.243288 14.71831 8.323884 4.045292 1.722292 6.176822 5.738076 12.65792 13.80365 5.212307 6.67663 10.40331 10.16084 6.571708 2.425574 2.335477 4.33871 2 37.22965 3.423256 4.277146 5.132139 3.195455 23.62338 5.180939 5.120912 12.71989 6.845247 8.186667

19.07054 9.760922 4.993902 18.34126 11.87654 6.794344 10.67175 22.46773 11.00604
9.271781 12.5088 12.75671 13.52153 13.01073 8.341501 5.081764 31.98621 12.41863
9.70267 5.871287 8.09222 3.862878 11.29172 9.817475 7.963357 10.68437 8.654767
6.206307 4.875766 3.804255 29.50747 9.295138 10.97671 6.929899 16.29993 9.303006
6.612388 61.13144 21.38617 7.647198 16.64213 2.976447 6.353155 6.704796 5.987592
4.644409 15.40141 5.686798 7.410599 5.281574 3.506723 7.43694 3.21019 13.5659
2.514741 10.01718 13.87072 3.760469 2.976299 8.66146 11.73794 4.609998 4.036456
6.678636 3.712086 12.75206 15.79961 11.07469 7.936249 6.247334 7.955548 24.27219
27.11573 1.613901 5.620152 4.535313 1.903799 3.983836 8.902938 18.4361 1.915851
10.67005 1.494034 6.148792 5.193863 24.12486 12.85911 6.410139 22.4115 2.548996
35.50455 25.32985 11.94686 9.782833 15.76296 4.488407 5.604954 12.05002 3.369367
11.16728 2.616354 6.624486 4.680151 7.629252 8.47472 27.82772 10.03064 5.271889
3.221737 8.394265
AKR1C1 1.759884 1.498762 7.850994 1.246685 1.90982 8.135607 3.019859 2.274693
2.982943 3.266205 44.85443 7.43323 17.67098 9.152227 2.617984 17.62552 8.84323
10.04717 17.16188 5.858784 2.394624 1.24667 913.76633 138.869 0.832956 0.4359824
1.214463 5.477764 0.2561038 0.2836844 4.515036 0.7770971 6.622012 1.39239
2.721463 2.117994 1.209112 1.034233 4.285298 0.8383302 4.002478 0.336755
1.043993 2.033449 2.59037 1.816023 2.089364 0.1011073 38.39534 0.7509624
10.84232 4.568401 2.611431 1.218859 0.795965 0.5618794 4.125233 1.054408
4.386039 0.9323422 1.687744 11.80419 2.943404 0.4001823 0.9503476
0.6061148 0.7077161 1.777223 0.7228348 0.599593 1.50487 915.34114
1.254508 100.7188 1.934207 0.747385 0.1761461 4.086143 1.054162 3.400347
52.99395 108.3985 59.63451 1.363621 2.158246 2.831058 0.121241 4.004263 3.18048
0.1278299 0.3857079 2.786886 8.100766 0.3797551 10.04552 12.78897
1.584737 0.3062329 0.7937153 1.626084 2.131646 0.1670486 3.39575
16.15213 4.335203 3.600847 0.4411281 5.291261 2.374187 0.3462519 5.789146
0.9552308 1.204522 17.77638 0.7404635 0.195083 10.12903 7.29657 6.604586
1.097003 0.8592462 14.25145 3.183614 0.4224174 0.5040465 1.212663
4.924772 6.407078 13.22631 12.10224 8.716625 0.3966203 0.4227928 0.6217028
0.5616301 2.550133 4.096254 0.5325827 0.1311127 3.767588 21.04679
3.202837 3.944599 12.61434 1.789203 0.1554693 90.34944 1.441164 4.279428
25.87157 1.082091 0.6758185 0.6346392 42.20111 8.28393 2.67281 0.04254656
3.643377 0.4397728 27.26632 0.7876952 1.18677 111.93463 0.0779673
9.375251 15.10068 1.392072 7.210547 23.95851 3.213249 193.9345 0.8261638
0.5101258 0.2594341 6.824339 0.1156798 1.510937 0.9271978 4.061909
0.8351534 14.73789 1.744285 24.11779 51.55641 3.1953 6.179065 3.376727
1.259278 0.5184627 0.7403631 3.986226 2.1225 25.53754 1.109055 0.08989661
0.5979242 2.73452 1.236352 220.4968 1.66474 0.376562 3.562013 18.63607
146.9516 1.514405 0.6120789 1.175485 9.923728 0.336651 0.6918766 0.7533029
132.7894 0.6394731 8.793435 13.27021 8.700767 1.098898 4.973984 2.574923
0.7369221 6.537607 1.038209 0.9899506 10.02555 0.01439534 1.720127
2.017282 0.51552 36.7122 5.85828 0.5081694 2.059056 2.881657 2.300224
2.508101 0.9034048 0.1054396 0.7868216 4.873625 209.9243 2.762561

7.626717 0.3730743 2.686204 2.660965 2.084143 2.003192 0.7200587 1.367622
0.9482096 4.052309 6.133955 22.88246 2.48053 7.455676 1.348677 2.272528
2.027925 2.338775 228.2562 0.3108905 0.5532582 4.208861 9.150163 1.784947
2.148995 0.9910542 0.05937302 10.78154 6.251847 18.00977 1.177843 0.2068246
20.13986 1.263868 0.8273573 2.606472 6.087991 12.92574 0.4090951 0.6898815
2.171283 0.8720971 216.1935 1.114848 2.585933 2.69345 309.514 19.4654
0.8719524 0.2059262 0.4487691 1.252235 10.50458 69.65566 1.491619
0.8517113 11.15524 5.826659 3.555254 43.74963 0.6174162 1.916598 2.051062
0.6263844 0.5275127 2.525654 0.1979602 0.9109434 1.742225 0.9804977
8.452406 4.079189 0.5411588 21.05134 1.764754 0.2575358 0.9597055
0.411542 1.837038 0.64822 4.1463 2.351811 32.80307 0.2474463 22.09306
2.130768 0.1526046 0.2709952 0.8250985 14.0309 1.366782 11.53854
1.672476 1.188202 24.23182 9.846029 10.90516 0.08124092 64.18157 12.11075
0.8591775 20.28395 30.09545 2.522185 0.2218034 0.2670905 0.5373328
100.4185 0.1435087 1.947038 3.235112 6.389469 17.88608 3.496147 296.9034
11.40439 124.714 18.71487 2.040912 8.353701 3.012441 1.059942 0.1360854
21.91545 18.66878 2.414791 1.166605 0.01577188 0.3185656 0.3097812
0.9894036 13.51779 1.471029 0.5330335 0.2586497 0.4990617 1.742768
6.116951 0.1102741 3.644368 1.849576 27.70903 1.675661 1.441011 14.96304
1.676172 3.231329 2.407546 0.6681867 5.465334 20.8798 167.1281 32.37336
3.083739 14.29117 2.379047 0.8124785 0.6088977 0.1463774 0.9522656
1.298704 3.477508 0.1919699 6.604544 0.8541984 0.2884595 0.1403476
13.93426 5.553025 123.9554 4.572904 1.059713 5.853689 1.491509 1.096239 3.639147
0.203354 5.117093 0.6342295 4.184168 2.996883 39.56465 0.07684483 3.073496
0.3591394 1.494323 1.69433 17.12126 1.409126
AKR1C2 21.25216 1.076168 42.3998 2.440934 10.84006 31.04872 7.591472 6.311096
1.104816 1.268539 153.1206 11.42221 25.61105 41.24916 0.7622255 53.96567 51.3228
61.35 87.41302 8.860229 17.82413 34.54554 46.32242 179.1609 14.56791 2.587079
0.8447412 9.644022 3.601489 0.3340337 6.61966 3.796337 45.98468 11.60651
101.4359 27.95428 0.4389668 2.677387 32.06235 0.6781493 14.99808 0.1653429
17.11172 11.66702 39.10929 89.5995 1.458609 0.07363876 20.25431 16.12194
1.870002 20.53728 33.04063 14.8324 1.467228 11.93012 37.96789 3.676805 143.6973
0.555266 0.8948362 126.2268 47.53326 0.4739021 0.4876601 24.63802
0.440447 65.17801 5.696378 0.6872869 35.04746 0.8586943 9.599676 36.64836
3.012229 14.15878 0.2927058 18.43061 15.20769 37.40792 13.55136 97.30727
167.6977 7.385531 1.179799 6.776768 0.1079633 3.423855 39.80154 0.1495164
1.854722 26.13778 89.80798 9.072298 70.15658 184.5015 35.45713 0.5702092
4.488343 32.39759 4.072057 0.0770547 27.50044 24.32866 41.65557 8.135759
0.2894689 26.90903 2.417176 4.733224 80.67011 4.054178 27.09115 38.2001
3.158736 0.2640521 129.9478 37.14348 23.43422 15.4164 4.966006 180.7962
51.34656 0.6323399 0.510224 15.89706 28.12318 3.099742 42.24292 66.84841 98.2367
0.4260976 0.2923047 0.6486544 3.70091 35.90312 35.86042 0.5821313
0.07642888 85.16939 53.87307 81.76274 32.94669 320.7106 1.180678 17.26692
65.95867 21.3925 32.40906 8.203271 13.1433 0.4703554 4.232266 67.4652

7.283369 32.92343 0.03364371 19.53729 1.878872 30.20256 29.30856 1.703513
14.73148 0.1631982 46.09915 15.8651 8.224899 3.155571 1.787776 3.216643
320.6098 2.031835 0.5167707 0.2605049 114.2426 0.1333994 15.97446 1.87189
104.527 0.9195772 20.04054 114.4508 175.6013 165.9127 5.527301 52.37099
122.8688 46.00339 0.4263947 9.020244 0.4211378 12.22131 52.79256 6.318299
0.1269389 5.964781 29.94825 0.8676523 260.5739 27.46385 0.1622446
17.35186 23.16648 136.4883 0.798344 0.5811078 4.938311 14.90487 3.242086
6.379931 0.2681422 172.0237 5.245796 107.6167 9.838175 65.69118 0.6836342
36.91562 9.79545 0.256544 28.80908 17.48384 4.03592 144.8986 0.01897187
44.81561 36.16147 0.7673089 164.4278 53.80803 8.728919 22.28317 19.28171
12.63127 17.61826 0.5444511 0.3521607 0.9920756 20.29161 311.9862
12.98458 5.494822 0.1816556 19.62485 141.5827 42.22422 17.30385 1.348431
2.831168 1.640743 86.82822 93.10152 34.44238 72.66265 26.13262 54.15444 17.75143
44.55731 22.95778 325.2933 3.991543 13.91173 26.10091 9.577762 1.930883 6.537656
30.47009 0.06402169 77.82166 11.45708 20.44973 27.18934 0.3713067 15.25637
0.4767176 0.5462288 1.518801 159.488 12.38058 1.680041 0.7671433
14.20399 11.51851 211.7313 3.189471 2.388496 12.50912 181.0467 23.89469 49.6085
0.1572097 0.3985795 4.04795 7.081137 60.86553 1.633734 1.069904 31.37179
36.3711 3.529674 59.43327 5.548514 16.04938 34.06127 1.465791 2.532581 10.50568
0.272577 10.70569 32.50877 0.9463433 108.3278 75.01653 0.5188935 38.35184
1.078632 0.2365591 0.8371682 0.7610334 1.402173 0.444067 6.488946
3.471523 46.25231 0.09544792 43.94953 5.287341 0.02914787 0.07692445 5.39435
120.3362 1.409527 15.01499 10.00584 0.6458971 2.096372 122.4598 11.33809
0.1800703 205.0591 17.84539 7.339892 27.07525 56.70591 71.34982 3.68029
1.670662 2.339524 25.13945 0.1279426 15.15208 20.37472 5.386918 58.11837
101.6833 463.443 56.42249 328.8092 2.092611 24.41468 60.25359 57.71174 24.66637
0.1727067 53.42777 22.35561 14.48538 2.217484 0.01247162 0.5917474
0.5179762 2.832991 11.44404 0.8097361 35.18325 0.2143672 4.684265
46.25998 3.70523 0.4359963 46.25708 24.72884 37.11223 0.4049892 16.55116
155.0841 0.3786953 95.49815 2.080149 0.602526 36.64318 14.38517 63.6798
283.6357 14.45687 73.016 25.59493 0.7446391 4.418343 0.1062107 78.30328
3.825329 40.90199 0.05622233 665.8594 0.334529 0.3229727 0.2273546
10.21902 50.32352 49.23946 27.32801 0.4474377 7.866836 1.979299 12.96407
58.86757 0.1813763 90.07992 0.1918803 44.42554 31.33157 148.3456 0.09645251
29.57721 0.1883352 1.597297 1.943284 43.39198 6.825741
AKR1C3 14.12386 1.029042 37.50349 1.389557 5.10104 21.83634 7.079105 6.446482
2.246266 2.781758 118.787 15.15265 28.70463 32.72244 1.306624 34.42963 44.22276
148.5001 86.35903 1.023797 25.48418 153.4159 113.3178 274.5675 28.20842 4.972324
16.99701 35.4357 21.95259 0.8207596 8.272302 4.219902 28.04661 9.855862
33.62481 21.81549 13.43707 6.336499 78.42977 12.41263 12.05277 1.822718 23.3503
25.75325 29.63019 104.5914 3.746077 0.1857678 11.96283 23.99515 1.250835
6.777231 22.93876 7.100218 3.036115 5.551937 43.79634 3.751267 158.3983 17.5073
1.053451 179.1381 56.39555 0.2474574 0.9241369 11.37507 1.427016 11.54187
9.069248 1.055364 20.34006 13.33703 25.44864 117.6484 2.318878 15.16641 0.519855

7.557657 14.12362 12.15522 15.59301 151.4671 113.0777 7.304441 0.7323427
7.097647 0.3082184 2.792783 31.91528 0.9079858 48.08107 25.86862 65.29327
19.03793 64.43274 261.6349 32.00004 2.653526 3.139705 32.32119 4.033867 3.203306
55.08667 19.92326 22.3084 9.050491 0.3150058 105.6139 20.23368 5.729381
89.34742 0.9128105 6.934757 45.88834 1.599821 4.918491 20.60904 8.975249
39.25875 0.73325 22.42034 101.8482 72.83992 3.672666 5.880031 14.00622 42.7086
10.50894 37.09381 84.77579 89.78815 1.873806 1.362132 56.88724 3.05756 246.5682
79.55096 1.496058 0.1369205 102.4368 153.5392 17.9847 20.74832 163.7865
3.651591 0.8160658 48.67633 11.73047 10.42405 17.90907 2.865993 3.80443
4.115072 31.0329 4.152943 55.29075 0.6559952 19.60407 2.108815 30.89814
14.43103 9.65576 319.70493 0.7115781 17.34755 58.75972 14.88874 40.9273
89.90058 52.02004 358.3402 4.172288 1.148079 1.389648 128.8657 0.1514365 21.0211
2.42406 334.90958 3.993046 5.940397 17.82517 85.95738 277.7501 6.26841 129.7444
43.64476 6.968218 1.718721 10.96023 16.14459 13.0154 838.76944 4.270265 0.7056867
2.16943 35.72239 1.935323 426.81 3.412984 1.713413 15.79305 13.29305 147.4313
2.728612 0.8804573 17.11337 6.973984 4.345888 7.474684 3.518699 175.3862
4.573078 128.9349 52.46897 19.71305 57.84062 24.90878 8.739191 0.3337028
40.20876 13.73792 10.85529 80.49146 0.1116737 44.47732 9.06891 444.64506
82.97833 28.57513 5.939026 15.88332 14.43954 8.093437 11.48632 1.53196 0.4930179
2.576309 15.56118 533.2047 35.8642 8.901225 43.65139 30.04773 146.6719 26.81195
23.75987 4.397311 2.403139 2.80805 967.01272 119.547 30.45798 33.29567 36.63527
19.58899 20.21536 66.93905 24.75239 335.3733 1.128087 12.39559 18.26265 20.74312
11.58582 6.823155 18.33094 0.4030196 50.34836 4.411254 25.50549 25.60012
1.234936 17.27627 6.11745 3.985577 2.54958 92.35001 14.17384 0.3046097
19.76106 13.68707 5.078651 412.2015 1.838584 14.84324 4.616052 347.404 51.60599
45.60523 1.380765 0.468284 3.673307 9.708843 54.04173 5.5922 1.335582 15.15324
87.45314 1.949715 46.6881 1.97401 13.24734 15.63689 7.599765 6.424321 8.128368
2.148836 12.62123 165.6379 1.597614 125.4242 34.77225 7.330445 57.89614 7.202291
1.2259642 2.922551 2.118643 9.287628 0.5134451 12.95342 4.930576 66.42511
0.9129788 94.81929 27.72681 0.3174091 0.1859709 11.90241 170.479
3.829572 20.56752 17.88254 4.485731 39.85032 109.4782 24.99405 0.6803694
162.3882 52.75454 5.827073 29.18196 31.63926 30.19104 8.448482 1.107717 28.21873
117.324 0.8022215 47.42969 5.66718 1.599507 216.5349 209.8893 570.1092
95.25638 525.4427 17.20833 31.58738 34.63182 31.04917 8.078531 0.33235 77.46288
22.64435 16.52407 11.15442 0.02447048 3.108601 1.302157 2.118737 10.13859
12.25383 10.03617 1.815166 15.01007 104.0164 3.530399 0.5307054 42.60984
18.34525 23.51014 3.298869 12.36901 39.01497 1.565682 45.83362 4.387085 0.3891671
15.21443 64.29236 125.2536 112.485 7.122605 54.0407 10.18004 2.688504 3.007823
1.626759 13.50889 23.65321 28.73994 0.3516241 109.494 0.7320031 0.6782597
0.5500528 6.13942 29.49746 69.14733 53.94573 0.7511282 6.131309 7.387861
7.124812 61.91886 0.3784515 46.69855 1.922404 93.7229 920.92885 22.60313
0.4400034 67.15078 25.93525 25.11465 0.5556494 93.41797 31.48567
ALOX15 1.087433 0.01788304 1.398909 0.009297051 0.6247088 0.1282463
0.04978184 0.06824558 0.04612481 0.04912758 2.984754 1.763795 0.9145375

0.1434206 0.01596744 0.1684767 0.786186 0.1086775 0.7245125
0.8093389 0.7571885 0.8823705 2.906827 0.08996382 4.199314 0.2476814
5.42802 1.727237 1.00791 0 1.806469 0.2429217 1.327052 0.5955701
0.3667027 1.320883 3.789121 2.187304 4.825516 0.1800513 0.2305056
0.05140826 0.5397942 1.615077 0.2201316 0.2637823 0.01638151
0.04833535 0.1824635 0.4579346 0.1278161 3.996182 0.8849871
0.7388404 0.01372541 0.5648284 0.5135111 0.04485871 1.284451
0.2398435 0.3524141 0.1905892 0.2624241 0.1881424 0.7184812
0.6593798 1.406642 2.11926 0.312648 0.8434068 0.5349911 0 4.805999
0.08034706 0.1815778 0.4118842 2.477783 0.4390546 1.511696 0.1657187
1.062872 0.4738098 0.006913049 2.264417 0 0.3962487 0.1190539
0.04917252 1.38349 0.08708224 3.998518 1.343377 4.340894 0.1329688
4.638781 0.4357278 9.05481 0.0145822 0.925565 1.185461 0.1252193
0.6611205 2.292384 7.628886 0.8562961 1.178066 0.1197498 0.4452775
3.568384 0.8125518 0.9370409 1.004999 0.1071756 3.113561 0.8933929
0.4857513 0.04255803 1.117075 5.367934 0.4459932 0.2966381 0.2974674
0.5920693 0.02895755 4.16462 0.4209471 1.703684 1.549061 1.321224
0.1409366 0.3951749 0.03902566 3.687393 0.02240878 0.2639626
6.474025 2.877018 0.4223234 0.3729788 0.05931795 2.518553 3.250438
0.979513 0.05031548 0.6173562 0.7148456 0.03978337 0.03809931
0.148942 0.1923036 0.8973811 9.170058 2.438697 0.05436371 12.09156 1.74609
0.01656241 1.097426 0.05016661 3.665312 0.5438731 3.836871 0.8094792
0.05356041 5.630876 1.604358 2.225534 1.042316 2.722362 0.05322744 0.8415702
0.1282762 0.0444583 0 0.7745581 2.379166 3.346505 0.02359066
1.475622 0.6964573 3.260256 1.012976 0.7911586 0.09796329 0.129305
0.7991262 3.9625 7.015893 0.02607568 1.451521 1.033093 0.9898269
0.9972009 2.681159 0.07776602 0.1781882 0.3198147 0.02690616
0.03553148 0.02193048 0.02505761 0.09495011 0.1435908 0.8482261
0.1521351 0.01129234 0.5561413 7.00079 2.307037 0.04603964 1.221849
0.7534083 0.4689308 3.825889 0.1957798 19.7366 10.53492 0.1192767
0.06149442 0.01804195 1.127899 0.06041126 0.8239292 0.8656225
0.02988682 1.441196 1.055625 1.436103 0.1904431 0.9865412 0.1377842
0.8989745 2.759111 1.150372 0.386672 0.8070264 0.0749685 1.538089
25.20031 5.598524 9.518494 0.03931027 15.34805 3.509218 3.294166 0.8484285
0.4438611 4.888998 3.524524 0.06638786 0.7778227 7.047988 1.396516
4.873712 0.3639086 5.019735 1.738541 3.487548 0.9962641 0.04599612
0.5621695 1.176635 0.3617171 1.807631 5.602897 0.3591271 1.197801
2.227211 0.6924491 0.04332358 0.3747085 1.675925 2.227291 3.015382
1.726593 0.8718949 0.5390739 0.1870167 1.154158 0.976363 2.860393 1.14249
0.44281 1.44635 2.806641 4.026581 3.252431 5.806985 0.01393936 2.538252
0.01303453 0.05063641 0.569807 0 0.1851855 0.4037802 0.8508256
1.126501 1.817202 0.01358841 0.1403613 0.226413 0.2725435 0.3251532
0.1471983 0.2550916 1.073464 0.07667801 1.304963 0.5740048 11.50259
1.770823 0.5889854 2.543583 0.7619798 0.126112 1.032908 0.01427284

0.02795881 0.7861885 0.09160761 0.2428631 0.1662923 9.732594
0.1409637 0.1770175 1.41437 0.02295866 0.03786902 3.616242 0.02313389
0.2111851 0.5241844 1.342091 0.09150149 3.084196 0.05614114 0.2787639
0.09583408 0.07558442 0.706498 1.586023 0.9678828 0.6186621 5.748801
0.06331822 0.3892296 0.2190277 0.05229946 6.254652 5.669511 0.03994785
0.7644109 0.4266888 0.1297487 0.5585298 0.1804307 0.09162654
0.01912146 1.099103 2.241333 0.2022312 0.6408948 0.02180039 4.189163
1.175373 0.08221234 3.953048 0.02455855 0.04556811 1.590169 0.4126633
3.880774 0.03066336 0.2049362 0.228361 0.305362 2.91434 0.02999453
0.1907875 0.2166284 2.378274 0.07645118 4.512833 0.03261353 0.7867854
0.1509174 0.05123761 4.152763 0.9931959 0 4.497438 0.02310689
3.358885 3.194559 1.672877 0.3676544 0.3580522 0.2602683 0.7768249
0.06630324 6.816773 0.1025542 1.065588 0.162773 0.068738 0.09274752
0.02276422 3.596214 3.398254 0 0.03031295 1.766425 0.1593725 0.1125838
0.4233542 0.1475664 0.005330715 1.239522 0.305379 1.214563 0.9319539
0.3048703 0.3228807 2.297391 0.009759499 2.037312 0.8524979 0.04936192
1.495581
ALOX5 51.84653 1.297446 32.86214 2.893212 4.435117 35.40695 9.910434 19.63718
1.901224 1.465722 5.689229 8.163178 37.36783 27.45754 2.032779 53.49287 99.7538
12.66442 61.39815 24.63549 15.42976 93.76021 15.52517 0.7216248 8.613235
6.544718 24.80171 32.89963 49.73688 6.801287 14.74707 41.9957 67.95493 8.623115
20.15281 2.786767 118.6727 21.1463 29.98659 6.791682 2.011563 4.655031 51.72473
30.17537 73.80472 61.58355 1.624543 3.257903 16.02212 54.06606 4.193667 32.69106
30.20856 10.38791 42.61282 23.25894 8.352301 0.2538161 15.95032 54.8169
6.279116 14.75521 8.446821 15.8169 26.51747 4.654503 5.294441 98.3263 31.12929
7.327005 45.99767 5.779676 15.52405 14.79161 2.993792 24.65127 6.336271 32.62498
39.9574 101.0174 51.62704 54.5609 4.681168 2.996943 0.7015801 0.9417103
8.88203 5.70908 33.70335 2.433335 40.88516 70.79958 8.265523 6.727643 68.9303
10.10601 20.44567 4.471396 45.23959 21.42538 0.8203326 14.47024 70.48478
68.84704 43.73445 20.93077 7.422549 66.17122 54.6358 41.08978 5.066728 28.56374
66.87655 14.01246 27.236 28.83927 16.35747 15.93087 5.214023 9.008567 23.31858
35.39954 9.744229 1.204301 2.168195 54.55023 22.05116 21.96382 11.87581 51.78272
49.98981 7.025028 47.72994 26.933 4.440799 23.35606 18.82502 7.738282 3.481438
63.82351 27.40663 4.034526 64.49803 32.51564 5.040629 34.21533 10.02962 45.55957
26.84806 20.88407 30.39895 33.07276 8.308744 17.16587 28.62734 58.60893 11.20012
3.977757 2.254767 35.32477 47.6143 57.58223 3.669435 5.235512 16.63419 35.61615
85.24733 76.60962 122.6004 32.63187 19.99052 2.151765 12.16278 4.279098 48.38432
3.698623 25.2078 2.588844 59.32779 6.274518 50.37002 62.75375 29.42576 2.157553
9.57883 31.02651 22.31716 14.23952 15.08709 20.95717 19.42041 12.543 33.29114
35.15468 6.737016 42.8354 50.79926 21.22743 1.134912 6.774659 7.477702 0.7427273
20.21606 4.925264 23.10505 4.864148 28.16072 70.85842 4.115294 31.5171 33.28056
9.029208 14.68979 35.0654 20.13853 9.188795 28.9581 136.2162 9.764852 2.572671
15.14324 4.785401 5.38184 34.736 37.65959 95.20416 23.70033 21.95556 8.509172
20.91632 4.140504 26.68378 11.23689 47.04376 35.7386 33.25506 1.854082 10.43872

1.2211235.00815415.717159.73097749.7631850.3778725.6024320.073116.098213
19.3578488.6236292.78693716.369813.03579257.4095851.0549855.1966842.91091
19.6921345.5936818.582471.73070627.2431718.9925 96.030364.6648 24.91237
62.7255 48.3921 10.271437.6241117.65043310.3442639.3087916.878137.258966
34.7314715.921276.16633783.4710214.7291 36.099675.23994 39.9548911.14105
3.84109515.130987.20521512.352834.9618032.86224112.9915615.8653910.89998
39.834761.15475412.935570.5246977 9.2929173 7.109141.096332.405753
21.225148.71532132.603494.0279033.0429418.4776811.6024647.84435416.90287
83.8784 1.864647102.801768.9849632.4026214.1094113.4049522.6878716.26873
4.46516415.887792.42443323.979691.39043613.925836.44647314.6095829.729
8.74749414.6333914.38739133.111214.7023648.117238.13054 8.06651620.15715
15.341050.8383638 4.5565740.4866808 18.4479980.64892101.60333.696475
56.5879712.3920911.2451129.5629642.544465.9529131 3.543 5.89986536.5758
31.0474125.0223 22.7708327.1744 16.2498515.3300543.5458413.3457942.98705
17.758643.30257827.314989.45358712.8534923.6310511.6393755.463152.520997
15.8449619.8572 13.6139317.0894 10.5464323.182758.1314860.9580557
3.675715106.481383.2983347.7013319.877131.04667 2.5916739.26013435.62292
10.261219.1472017.27812 76.7747912.1779517.090577.7241918.9667645.38798
8.70837737.3925924.7697347.4000625.5199439.087 12.7724340.127424.987061
16.8013315.9549225.6353433.7946713.3172629.4444614.0438744.715147.030628
27.7865397.225755.541022155.559 13.7833 3.40597854.474931 1.2454622.02159
57.2102323.7492511.4246125.8550119.4927915.45383
ALOX12 0.6395769 0.5701107 0.3077474 0.3750237 116.06330.483065
0.2683631 0.4795348 0.4401369 0.2753733 25.390141.04147 0.557641
0.6734627 0.5505959 0.3379732 0.6671762 0.7179465 0.3079658
2.54506614.358530.3827203 3.97244414.7561 12.726851.9850066.265623
0.6766097 0.2375934 0.1334886 0.6644609 0.5069454 1.268528
5.0820129.0374492.3840922.6578441.4741120.5694973 0.4100021 0.7592213
6.8157185.2679462.0201191.5533880.5186733 0.3694775 0.3144754
6.9895849.5478590.4751923 0.4077432 1.6465370.8262 0.4107759
0.3149862 9.2785730.6479202 0.6199216 0.5599261 0.6987724
0.3141321 22.518720.4308748 15.470879.5000286.0506060.4222821
0.7970446 23.412494.0550320.3800415 1.5164650.5607653 1.31788
0.2533597 0.3793117 1.3846960.5017183 0.2803281 16.613696.347481
0.2428763 3.2877890.07694936 0.5585753 0.3042988 1.26547 2.097354
1.07378 2.7528540.4616286 0.2377628 11.276971.0462560.4148627
0.7757450.09487351 0.6021833 1.2072131.2220373.69491 4.3350141.565235
0.7664373 2.1475790.8663658 2.8035751.51989 1.5424310.9652787
2.0387151.2318910.4533077 1.2612070.2136578 1.0429436.80425 0.1926372
0.3675465 0.5723336 0.2408445 0.4727542 0.7374561 2.445516
5.5470160.3813975 0.9800984 0.3650372 0.4112382 0.1779961.358394
0.8738042 0.2915882 0.5301453.7780442.3560376.0948640.4172124
1.0677380.4503927 0.9879229 0.3199972 10.794642.1963060.2111437
3.15779 0.3470299 1.1459880.5362075.4550440.5043018 0.3311259 5.97242

0.2825666 7.14583 0.3879251 1.52481 0.5711822 1.500676 0.3823208
4.413211 0.9324411 0.5885276 9.366106 0.2777119 2.876081 3.993507
0.4940938 0.8003468 0.3272625 0.1271741 4.563736 2.669976 1.019197
4.922071 0.5026898 0.2839446 1.41897 0.4349983 6.468414 1.596146
0.8350977 0.178461 0.4169789 1.379158 0.1239447 3.1034 0.4071633
5.447904 0.1463166 4.306395 2.949048 0.4303752 0.3975359 4.608269
1.686126 0.4638946 3.137334 3.759677 0.3831152 0.1676768 0.8492895
5.894654 0.1814649 1.087346 3.628962 3.558434 0.8111831 0.5541476
0.9539389 5.023293 3.483672 4.578899 0.3760644 2.789224 0.6896866
0.6811804 0.5867984 4.149491 2.288537 0.3458775 0.4896277 8.560397
0.3791719 4.513477 0.8710661 5.53303 0.7335145 1.249325 0.4290107
0.5816158 0.6858925 4.409761 1.369677 2.27654 110.75497 0.4255844
2.512198 7.6852 0.7687667 0.4219993 0.7447324 0.5197842 1.453592
11.79895 0.1740599 2.233656 2.79672 2.752816 13.55003 3.271124 4.368219
0.8985921 0.2889314 7.113395 0.691035 0.3963984 0.3689646 13.51257
0.7653742 0.1686195 1.198081 0.5237168 3.623534 0.4673046 0.7267125
0.2825191 0.7758876 0.1832144 0.1300212 9.107041 1.039393 0.8396362
0.4289784 0.2279663 0.8455901 0.08111682 7.637584 0.3268675
0.5460161 0.7249639 0.6242108 1.7227 1.696786 2.524343 3.636438
1.248484 0.4262483 0.3427467 0.4791437 0.5106419 0.5214566
0.4075485 16.71713 10.69091 0.369038 1.576161 5.372096 0.2121787 0.3774589
6.745902 0.2364267 4.395507 9.660173 0.2046909 8.852674 2.13519 0.3855473
0.4251632 9.502355 0.94839 7.986711 2.031124 0.4406693 0.4318418
1.040975 0.1764354 9.779118 0.5892519 0.2221491 6.213344 4.158071
15.20217 2.384529 0.2894006 1.080499 0.3211491 0.5349971 0.5050667
0.4289586 0.4991246 0.3133885 2.600961 0.8453535 3.334066 0.5009291
0.6265406 1.371717 17.68154 4.820174 6.018608 0.9578775 1.792303 0.6166699
0.6965797 0.4162805 0.427506 0.9475109 7.212226 0.5847445 0.2339148
2.601443 2.557966 1.549636 8.308151 2.961265 0.3099891 0.1119658 0.1479494
9.591505 1.038742 5.284126 0.1645296 3.173445 2.049426 0.96765 210.59507
0.08521641 0.9063555 3.742009 0.3281465 4.302667 0.1396496 2.233342
0.4232118 0.5960162 0.7149242 1.892934 0.8137319 0.8392392
1.081013 0.7958405 2.850643 1.655062 19.61545 0.6006832 6.411582 0.7204895
0.286417 4.46896 0.5270101 0.428458 1.627439 1.936999 5.608314 0.9815459
0.4585105 0.1693336 0.4831954 1.027461 6.026092 0.3396804 0.7322998
2.506347 0.4174029 4.680863 0.1184853 13.95524 0.3093501 0.1406541
0.8381831 0.2114854 0.6967943 0.5092503 0.2203513 0.7335042
0.1872841 8.665644 0.3434861 8.174214 1.107988 0.6457976 0.1729988
0.9604556 0.374629 0.4767144 4.27035 0.4863195 8.129615
ATP5MC3 27.93578 16.92886 19.41802 16.78046 26.5559 916.8222 18.58818 17.80342
20.12786 18.72471 40.21982 22.79707 20.57113 24.09398 13.6404 18.42571 26.51808
25.54815 32.20178 32.94322 29.0867 34.80068 58.98607 31.34161 41.64764 58.9401
27.54136 10.33585 22.76429 40.44986 19.16204 21.88881 48.38375 20.103 26.92771
15.24338 11.03383 24.38722 42.52722 19.06632 41.00777 33.17769 27.90378 23.22618

	23.086	26.00746	35.95514	26.96811	29.79512	23.37626	45.89214	20.68162	40.26561
	27.49056	47.56545	27.96161	45.75416	33.61856	22.03137	16.56088	23.61236	34.65505
	49.83504	19.74585	29.58979	20.84827	19.27667	36.95137	26.0121	16.87543	16.01529
	36.50451	29.66098	39.24661	13.45579	25.40917	27.88374	30.16582	20.48296	45.61108
	25.36413	18.13319	28.83887	66.70526	41.35844	20.49937	11.12414	22.18061	16.36697
	15.56465	28.78978	38.40637	54.88445	36.1814	16.67542	25.7981	23.83667	43.1256
	16.88387	9.467865	40.11665	9.965923	26.39562	25.83872	33.32223	24.38724	18.15689
	12.61301	30.35683	16.27975	35.56642	15.46586	29.72504	31.48938	22.1097	11.49321
	31.34523	22.29128	19.53706	36.59932	44.47919	45.35531	21.35185	28.68044	29.36373
	28.94725	42.43075	18.14463	22.41726	61.07173	20.91884	15.64534	33.444	19.30613
	12.17226	15.20904	40.36093	25.8213	16.88621	31.24572	29.63175	23.85954	29.70555
	44.74151	20.54922	14.80513	11.76459	2.891565	41.84674	33.39672	32.66553	21.60499
	45.89489	39.77921	48.81874	20.36236	21.09618	24.28978	38.11407	28.83795	14.99317
	32.34195	25.64387	31.45328	24.1524	25.97895	37.89465	17.23569	28.05661	24.23833
	23.36988	31.08824	20.11862	21.88339	33.72756	32.75632	25.51035	18.31681	13.09863
	23.93679	25.82248	25.32965	26.08272	44.09503	16.73229	31.41288	33.49197	38.87943
	13.29329	25.1316	31.97465	15.48155	37.86503	13.15378	19.64729	28.35574	19.54497
	26.07167	44.75323	55.60313	22.4091	24.30887	30.55386	43.23227	18.08336	20.96199
	21.5204	27.36366	15.63567	30.20538	22.7589	22.76749	26.15985	29.91029	12.21188
	36.14408	20.54928	33.98787	28.61523	14.70377	13.38913	26.3116	28.53318	37.99944
	19.56668	22.21617	19.21761	36.20117	21.2762	21.38226	23.79182	23.22528	18.02069
	27.76459	27.02782	28.67311	22.94288	8.667508	44.93048	36.36154	23.0941	15.00453
	18.7758	19.09359	31.28998	29.87088	33.31716	18.41055	19.98174	26.80258	34.60463
	28.01096	21.71921	15.8173	29.38776	13.17081	22.19199	17.68643	39.36489	47.26612
	30.263	20.68656	40.25313	15.95299	22.08404	25.04332	14.34812	26.08441	23.6244
	23.49679	45.40155	23.53338	20.20479	17.89288	35.94532	22.53583	25.93421	33.04043
	22.11563	31.57729	22.9636	17.57637	45.4921	40.97627	26.04435	39.72425	22.57974
	38.00203	17.61864	14.69129	21.53363	31.44478	10.77359	19.99576	16.63093	25.8539
	18.70732	16.67539	12.90614	21.94381	20.60087	34.56565	17.62885	17.52195	30.68265
	9.005634	40.8459	34.6938	18.63613	25.05902	30.75593	25.00775	34.20885	19.82802
	29.99344	22.63729	33.5963	32.66214	28.44485	20.12113	23.11147	30.43606	39.26941
	18.82148	28.86357	49.12012	23.5526	22.48866	16.53291	11.30926	76.29125	21.03262
	21.08479	14.07847	28.51017	15.98891	61.83714	32.31429	24.6172	31.79223	34.74859
	30.61871	30.87772	46.72607	25.54885	35.10178	20.43328	13.08964	37.69195	26.79104
	29.11525	35.17226	7.297798	26.18524	26.16159	48.61861	22.17943	55.88994	35.41278
	45.43483	40.26495	16.63274	19.32903	15.47872	39.2408	22.67609	21.29838	31.98979
	21.44595	34.78132	23.12778	16.21405	33.44576	41.60883	27.90282	31.14714	28.62432
	31.04676	31.92927	23.2232	26.56005	23.45841	36.49883	18.60164	39.6308	50.32848
	19.09858	26.74072	29.17675	23.01331	21.74865	23.37825	36.09411	32.93076	50.30879
	22.75412	41.42691	17.79392	10.88774	18.96723	34.41126	53.60479	15.97091	13.22663
	18.95964	27.5426	15.70913	27.94799	38.59427	12.32566	8.46613	19.42333	24.90723
	19.85341	20.07961	32.90719	28.78908	22.06663	39.19487	25.19387	19.57384	32.29192
	51.37989	25.84178	26.51984	26.09198	26.48676	33.68079	26.52477	12.96114	
CARS1	6.178098	5.746536	4.57026	4.474178	4.134522	3.946517	4.387239	5.045644	

3.480569	4.889474	6.118454	4.368242	4.700362	5.479447	4.849383	4.535335	8.668991
5.659037	6.696551	4.556138	5.095224	5.261203	5.124183	7.3804	6.523973	4.526452
6.150875	9.50289	17.27468	12.94872	5.132152	4.862312	6.695558	5.605076	4.666191
3.302784	6.786734	8.866297	6.157996	7.307268	9.852477	5.830994	6.924922	4.916971
7.626457	7.44709	11.15587	7.568744	20.32434	3.377009	7.775844	6.196307	6.223937
4.835298	4.957475	3.936729	7.523929	8.020617	4.596929	3.373291	5.250322	8.405015
5.157182	6.842724	8.701811	6.821924	7.796033	3.160107	8.649268	6.614337	8.958582
17.95976	6.798935	8.776757	10.01585	6.788863	6.437274	5.585134	4.134686	8.332048
5.037538	3.831003	6.353668	5.146501	4.243223	5.912976	9.155386	6.429764	3.553194
10.02593	9.789819	8.476724	6.369455	6.650294	6.137657	7.435604	8.169519	8.832634
4.637732	6.708006	4.311969	10.59144	7.190747	3.306591	3.280107	9.833976	6.342056
4.394012	10.05595	6.286307	3.892963	6.946157	3.213776	4.541319	6.025497	6.724185
11.2532	5.707332	4.083593	8.722454	10.6258	6.093431	4.577269	9.340996	8.161783
6.616913	3.286604	4.118506	6.979105	5.424346	5.45365	4.52484	5.646844	9.271791
6.881489	5.03916	3.473436	9.441948	9.762227	5.981573	5.511077	7.91239	5.828292
5.601341	6.207698	6.086196	8.068419	2.063084	9.063785	6.621265	5.618979	5.7098
5.739163	4.67251	3.689042	3.202406	8.934873	5.759434	6.244051	5.871439	5.737091
7.885562	6.29621	5.960058	4.465254	4.824609	7.028233	7.03367	7.294535	7.01815
7.668463	10.23386	5.126458	15.39659	3.350005	7.564844	4.464468	10.75488	5.579798
7.598086	3.061187	4.935789	5.415137	3.862818	6.518867	4.241222	4.376169	7.484871
5.836671	5.91016	6.108803	5.845193	6.782364	7.241004	5.299284	5.328058	4.983715
8.239959	4.455179	4.181799	6.934505	4.075837	7.213226	5.905175	5.146688	5.634829
5.941298	4.971157	6.233127	6.956091	7.657227	8.039213	7.188184	3.384755	5.966316
7.244984	5.656775	4.404787	7.598366	8.878438	3.193083	4.142711	7.799882	5.561086
9.129977	6.482478	5.786377	5.224259	5.626893	3.124744	5.67255	5.801291	5.737337
3.992818	4.400406	7.07622	8.741185	4.532832	9.367726	5.132782	5.475135	4.749485
9.333158	4.63963	7.432935	4.679379	6.681206	6.831289	6.681812	4.504525	7.353953
5.561033	3.983887	5.0556	6.172985	3.215939	6.237436	6.544506	3.563315	5.370981
3.873163	5.938287	7.564935	3.810006	3.201046	5.84192	6.963206	14.21859	5.812309
5.836787	14.67992	7.79112	12.44577	5.083801	9.651413	7.53065	8.086844	4.251878
8.035769	5.817258	11.20227	5.462849	8.968896	6.140617	6.801212	7.600666	5.858195
10.37875	9.079102	4.83002	8.675426	7.446683	3.907418	4.401826	8.530981	4.843512
5.400071	4.728351	2.98026	6.120013	6.26771	5.810207	4.650476	5.52596	9.951764
4.961525	4.638755	3.851337	5.772678	4.848804	3.586655	6.631862	6.218581	4.566852
5.214696	7.075105	12.53545	18.04805	14.81862	5.985356	4.92291	8.188759	5.315058
11.88176	10.18293	6.680776	5.301201	13.52483	5.203288	4.600762	16.89385	6.931294
8.650096	4.687217	8.667318	4.980523	11.83744	4.026605	4.655322	5.787608	9.392099
5.887553	8.416678	5.383964	6.070324	7.250427	6.983625	4.120186	6.41674	6.454721
5.42988	9.289493	5.507986	7.956435	4.590522	5.028674	6.162647	5.676312	6.424963
6.086194	6.557523	4.506805	3.695058	9.966434	5.979748	5.647146	7.621902	5.083811
6.910221	6.137527	6.120854	5.385589	5.776618	8.137477	4.237517	8.779735	4.576371
4.474734	7.451355	9.669754	9.35888	3.353385	7.075765	16.04008	4.802196	4.138303
5.943238	5.410035	2.961886	7.093617	8.679779	4.5041	10.49319	4.938993	3.214516
5.279558	5.110067	5.923167	5.873023	5.852496	9.534244	10.079	5.896919	6.120145

6.76797 7.471547 5.104677 5.16493 7.905969 2.724741 7.551849 5.87787 5.187142
6.684957 10.61285 7.664222 6.930429 9.321461 5.583383 7.323474 5.709499 6.109883
4.450806 11.43345 4.421435 11.10302 10.30962 7.891034 6.062582 5.167091
CBS 0.02836492 0.05382304 0.04038689 0.02798157 0.004608339 0.03047258
0.05351061 0.0410801 0.02699337 0.03412166 0.02073705 0.02602226
0.03102665 0.05630304 0.05206242 0.1162033 0.1063077 0 0.08964624
0 0 0.1702368 0.006867154 0.004750288 0.01777607 0.02823687
0.1017008 0.004536222 0.1025832 0.01210819 0.1432233 0 0.05376624
0 0.02207349 0.09024426 0.01187939 0.1923374 0.219541 0.3010583
0.2854528 0.02292219 0.2707721 0.1475866 0.06370537 1.884807
0.04930387 0.5541949 0.01562665 0.005342083 0.006106214 0.09061243
0.1713407 0.008686356 0.1239292 0.02602008 0.6064729 0.01350124
0.5271606 0.04953963 0.1599423 0.1593392 0.1209642 0.06417579
2.855519 0.003985045 0.02559176 0.06906224 0.06081194 0.01311847
0.5568527 0.1002408 0.4800127 0.3077742 0.1295406 1.716739
0.1030218 0.007465729 0.05219059 0.428109 0.009996727 0.8858719
0.04161276 0.2723887 0.008475413 0.01863437 0.04052425 0.03288795
0.07411516 0.7696401 0.2741269 0.02610045 0.4830595 0.447282 0 0
0.4550751 0.09509161 1.005946 0.04524527 0 0.3615331 1.98946
0.06669128 0.01738007 0.1884165 0.1850125 0.1368984 0.1347609 0
0.003916995 0.04394968 0.03136088 0.1791187 0.1648834 0.2882774
0.252618 1.163101 0.04400684 0.8650488 1.960054 0.009947725 0.5231151
0.008300408 0.02964609 0.01005504 0.07657737 0.06415939 1.525241
0.0407042 0.0508277 0.06117529 0.015038 0.08992571 0.01151384
0.216754 0.1560186 0.011005 0.07549407 0.004959188 0 0.6188713
0.00627248 0.8581362 0.09553922 0.1649915 0.04989044 0.02866712
1.526733 0.0137805 0 0.5502738 0.5886031 1.456996 0.08912398
0.03778287 0.02492416 0.05909238 0.1509878 0.009974307 0 0.2010278
0.02662634 0.2567294 0.03008858 0.04060813 0.1414584 0.08043806
0.0355119 0.1572333 0.05580134 0.1654613 0.03221287 0.1688654
0.03492446 4.126214 0.01211167 0.04733428 0.01736547 0.04471777
0.01444711 0.04366742 0.06660617 0.03603632 0.2086867 0.01406521
0.6307041 0.01395171 0.002616022 0.1978111 0.2220947 1.212868
0.009094852 0.1270796 0.005572724 0.05586433 0.06085115 0.04723842
0.1425867 0.02200157 0.09112832 0.1258766 0.002182672 0.02337846
0.0572356 0.05664476 0.5538418 0.03429437 0.01826048 0.2338313
0.09193576 0.04060356 0.07507197 0.01877834 0.1496492 0.2230949
0.009411465 0.3058065 0.04112919 0.002262556 0.01438419 0.01515178
0.02928899 0.68854 0.01124389 0 0.3142702 0.6499842 0.06878177
0.01325544 0.03208933 0.01511546 0.08675995 0.0363906 0.03232713
0.0118196 0 0.2172721 0.4890199 0.4135353 0.03763292 0.0739457
0.0095698 0.07541054 0.01273544 0.1595961 0.0244 0.2855711
0.8922491 0 0.003981349 1.401976 0.2047679 0.1035239 0.0309397
0.01034796 0.2355481 0.5322053 0.003844208 0.1899054 0.3994944

1.008842 0.04398664 0.141679 0.08394656 0.1377585 0.02580304 0.5480682
0.003216176 0 0.07205196 0.03436738 0.3858455 0.01244921 0.08408694
0.6029038 1.585422 0.0243215 0.01238396 0.5373141 0.3823883
0.02476345 0.3516944 0.1151618 0.4803622 0.06789302 0.1233898
0.2799661 0.006992277 0.3363276 0.07846075 0.02540029 0.8323538
0.09426587 0.5767103 0.1056754 0.02032343 0.1822828 0.02328769
0.05112174 0.2370409 0.008736424 0.3189983 0.009061315 0.3691889
0.1322246 0.007132084 0.1269289 0.0758953 0.1816192 0.1898007
0.2708177 0.01554987 0.05016417 0.3491933 0.02663597 0.01523907
0.06801579 0.01963461 0.02208465 0.008354964 0.1571938 0.006763436
0.2864668 0.05499696 0.01593769 0.1281637 0.1381985 0.46947
0.1038416 1.546292 0.01513355 0.01421308 0.3294485 0.01835963
0.01586767 0.293685 0.1239436 0.03365067 0.2843605 0.01277096
0.3495236 0.03419087 0.02342143 0.1619293 0.002887432 0.2112933
0.02929837 0.01412627 0.730793 0.01572689 0.1770083 1.639963 0.03057658
0.01627116 0.05013573 0.230649 0.01225648 0.04316276 0.01901148
0.2414869 0.1841999 0.02276009 0.0306195 0.1814555 0.07075101
0.02624328 0.08395975 0.004106361 0.5877751 0.06040204 0.07666687
0.8933385 0.01153604 0.0738877 0.01735617 0.2165592 0.06390794
0.2933947 0.3030596 0.2247397 0.00817118 0.2262622 0.007059455 0
0.01401188 0.02671882 0 0.003156232 0.177739 0.1449456 0.4548406
0.01738635 0.002376428 0.02312826 0.09527468 0.01271884 0.3364058
0.1818459 0.0363979 1.024985 0.6387609 0.009353332 0.210569 0.1932391
0.5344465 0.1628344 0.1865105 0.02004374 0.00701496 0 0.2204813
0.01072731 0.02878006 0.02151406 0.09768714 0.2960896 0.02941398
0.01413114 0.1350101 0.04438248 0.00336322 0.2525112 0 0
0.4454968 0.2330782 0.2154231 0.1238049 0.02178044
CD44 53.4031833.5995619.0043151.4089345.9661138.8635133.2901935.30471
36.4251650.4027769.1280937.1164234.8513844.7424929.1627448.4113824.39567
22.7552530.798791.49809620.362252.33564820.7849691.7990923.8350211.10215
14.0131410.614975.54736297.6586 44.1814368.2984613.7791 56.2164721.99621
4.8153289.4785255.37408927.6824660.7084780.8159261.438155.2597832.9371
52.447988.7516737.5928214.0042527.313865.05724237.3907424.3940515.10323
49.368492.1834538.095114.554222101.28977.7416931 2.1197 44.548933.97896
14.0268128.7582 283.062550.6217 14.6063828.3561181.0338915.1674521.65024
73.2830512.3386 58.1506 17.9842128.89098257.66511.17122619.1946822.69999
28.0785613.02648132.93 58.5856993.2052994.43205135.4156114.536612.52965
1.8583152.47051618.7897435.0985647.016763.7284447.9927558.54222 37.75031
54.8664139.48327134.78425.2989457.34290422.3951 36.9090820.6902269.08562
3.4761329.92525513.0685411.8829636.5666315.4293 21.8168942.693676.300939
2.48720222.6020310.764 18.3285323.3489744.8267513.0873618.685450.6521243
42.714085.80574 18.8126937.132557.4272 16.8634498.4618745.9514642.50095
93.9739325.8898 11.5764 81.134694.3166058.3354631.30412 28.4574 82.55681
17.98827281.989728.4274159.3626914.0883323.5229234.4910523.5131962.60508

56.33244 28.26583 5.773128 14.59423 97.23793 56.37806 161.3008 4.363415 42.50039
4.930921 116.9602 77.01042 21.7134 30.94263 3.85926 6.748303 3.592091 30.03856
53.5594 96.4639 71.48389 292.5285 10.14491 0.7722541 97.50648 215.0791
7.879962 38.54475 9.455795 6.278673 26.23909 11.39078 29.25278 26.37046 4.793036
37.48643 41.40031 26.39658 60.07215 29.66911 69.02249 8.25081 4.940926 128.9032
25.33747 20.95508 62.22958 66.54376 81.46682 1.501296 114.892 25.01869 29.51851
75.27934 24.30202 20.44442 58.16592 2.284868 1.110704 81.47778 92.18216 11.85729
55.31296 41.50055 3.676895 18.77956 213.5071 105.5203 10.8543 91.5023 78.12917
5.673501 87.87356 40.24785 22.44588 11.11807 137.7652 23.18923 87.56063 26.98372
24.39234 26.24898 11.04821 48.12708 87.25959 27.107 18.16981 53.94113 1.704502
43.02293 1.867762 22.96157 6.68128 27.79788 48.48853 23.17725 9.434881 87.16247
9.851141 5.843024 25.0393 5.351826 25.32341 4.717727 28.2151 6.620379 14.92527
36.60836 10.11644 14.90918 17.72091 31.53076 4.520133 46.21627 5.205801 1.370198
25.87009 44.61817 13.53465 53.44294 10.35824 21.63459 12.17976 4.990683 9.183023
22.6941 21.11383 5.403029 10.70893 14.4153 58.03201 91.3666 2.032686 3.687792
33.34242 58.2283 94.03814 12.74097 42.02468 59.38644 50.12742 31.45542 10.58506
89.7105 5.128779 34.80378 62.94362 152.1855 12.77654 151.3034 11.90191 19.61829
54.36049 15.97309 15.39487 34.12854 36.38303 4.985621 14.21884 4.484325 29.47559
1.296543 48.42459 20.00814 46.31293 103.1116 73.89842 50.68707 92.37398 61.77086
106.7302 5.3156 70.47077 3.94826 1.958131 86.48548 62.46727 4.473373 2.951964
28.02598 57.37894 3.250686 54.47414 15.01357 59.11563 51.37972 29.95668 102.0709
54.99749 32.67428 56.69556 308.3262 3.921823 560.1262 58.27905 2.120246 69.45111
28.26273 5.829404 71.53378 52.45865 17.52144 37.29891 45.70933 24.12285 6.76288
274.9004 35.12325 1.511909 38.49015 7.509798 5.022007 18.42887 45.10058 145.965
9.87414 60.2867 5.235167 105.5833 34.39871 8.007126 90.08928 1.500016 66.48883
44.43777 9.869577 98.73082 57.95337 42.89691 10.17615 14.25018 10.23812 167.2753
59.02018 44.87626 59.39782 30.00506 84.2727 90.23829 8.656781 28.91817 84.21923
26.08635 10.34526 26.44999 48.31064 8.482116 89.60363 10.67613 3.664626 10.29987
34.58063 60.32964 82.67114 74.60609 38.74341 29.85418 14.94562 28.8668 124.9904
50.28587 70.05246 73.69219 27.49793 23.62888 127.9477 20.45648 70.68199 35.37859
39.05998 22.34896 22.96285 14.53549 24.07283 14.90289 14.37087 18.83181 5.583012
CHAC1 1.323807 1.03527 1.831098 0.7073709 0.2507245 0.1339728 0.2470224
1.061076 0.3051678 0.1250133 1.20345 0.2451576 0.299612 0.7322952
0.3433387 0.1973885 0.1809221 0.9955728 0.2796191 0.8227263
2.764476 2.382553 1.207658 5.427398 0.4982223 2.243892 0.9755518 4.322755
6.149 2.155963 1.898126 6.785122 0.9961871 2.093322 6.392914 0.7204308
6.149819 16.43177 1.15083 4.392709 1.405911 1.16209 0.721138 3.789647 0.9802827
6.932397 1.707021 3.620515 10.17289 2.184242 3.68461 3.859792 12.21116 3.284304
2.440495 2.745536 1.806243 5.757748 2.448997 1.096784 2.595313 6.882757 7.050513
1.506178 8.467291 4.467666 1.543052 0.7401036 0.327164 1.319324 6.824737
7.195421 4.376968 3.382825 1.48833 9.228113 0.7780263 2.991004 0.5972604
1.00048 1.615185 0.5877728 2.858603 1.081467 1.047998 1.863818 3.481249
0.9759956 1.166386 3.374579 4.895377 1.134467 1.772533 1.034987 1.56523
3.076193 4.958973 5.113323 0.7054217 1.459937 1.190922 15.31817 2.519039

0.2722649 0.7122645 1.393174 2.662062 0.5464383 20.72058 1.343988
3.073962 1.116726 1.447721 6.496837 3.011165 3.259059 5.560998 1.038695 1.532694
1.856513 1.945678 0.8747038 2.759852 8.457197 2.228798 2.365075 0.4141072
0.5053877 0.8082795 0.5015479 0.7374313 1.387819 0.6446171 12.9171
6.435146 1.554828 2.333408 6.631563 6.851051 5.690604 1.973656 1.360434 2.516398
7.989453 4.106237 6.027953 1.118651 1.155321 0.6962208 2.196243 2.599368
4.085251 0.5845921 1.605703 0.5224449 0.6455733 2.629899 0.8204202
2.427084 1.12919 0.5687026 3.216119 0.5926302 1.171366 1.364184 6.256795
1.134587 1.038835 3.747075 3.17922 0.5439941 8.436407 1.60144 2.306795
2.317576 9.248387 0.9904307 1.492505 2.748503 2.718634 0.8574758 1.348251
2.068141 1.814787 20.89463 1.660343 2.646851 1.48363 0.8884787 1.554316
6.172493 4.986606 3.088882 10.48737 1.543531 2.713964 2.188899 5.574085 0.235081
3.319046 3.326051 2.647425 0.8996362 1.387577 1.719515 0.3424283 0.8657658
3.641222 2.78479 1.551578 3.211912 2.276048 2.728447 0.6687278 1.54203
4.715254 3.087797 0.8658736 4.407598 4.20772 1.501952 0.5195955 1.963729
1.421541 6.067998 1.474715 1.107244 4.518605 1.537196 0.6993307 0.3825459
2.932333 1.117075 3.256966 1.741047 1.062033 1.165604 1.081952 20.61448 2.136956
2.125039 0.1137858 0.8756576 0.4760004 1.606252 1.596287 1.045928
2.796154 1.055608 5.095078 1.207776 6.708148 1.448119 2.008756 0.8773704
0.5459381 2.098914 2.423821 4.183014 1.504025 1.954401 2.949297 2.057762
1.191281 2.081561 2.257231 0.9237511 2.131554 0.6575063 1.200282 22.38439
1.699833 5.508543 0.4104972 2.560092 3.436883 8.940889 1.672683 2.225483
11.81412 3.192682 5.797464 12.74618 3.493534 2.687634 5.093016 2.260348 4.947688
1.487123 1.891239 1.805612 8.197452 0.5903444 1.056822 5.143337 2.33753
4.117637 4.027069 0.7075489 0.910265 1.96564 2.957547 3.033482 1.394333 6.6584
7.179841 1.528616 1.141452 3.149047 1.431438 0.5632599 4.375641 1.217752
2.251016 6.122762 6.071168 2.445449 28.47095 15.88971 1.643683 1.549657 1.56794
0.6541801 1.344838 2.124335 2.049549 4.286411 1.699353 1.297473 4.023256
14.23429 2.181387 2.48389 1.794783 4.510125 2.132108 0.5306246 1.247024
4.459492 2.708748 5.263833 0.8341982 4.588524 2.123804 2.374337 0.9330529
2.654567 5.736086 0.312749 1.730273 1.659439 5.726505 0.5057444 2.911249
0.8431051 0.593194 3.398346 6.345033 1.826489 2.914992 2.441034 0.5017457
2.110741 5.062623 2.026592 1.29607 1.396905 2.516049 2.139354 0.3876231
7.843714 0.8089584 3.86015 0.938287 0.6143852 1.831354 1.49782 1.962409
1.699209 2.40183 3.371376 1.293286 1.062203 16.11591 0.2067848 2.233119
0.6020303 5.099046 1.904533 1.598379 12.86342 2.709604 3.611749 0.4466506
0.686363 1.776192 1.121863 2.867146 2.383083 1.680246 2.283083 2.672089 5.536041
1.4268 2.620524 11.18947 1.260133 1.995597 1.295398 1.052459 2.145487 2.569604
0.4598356 2.47791 2.234608 3.305141 2.123493 2.777411 0.5311909 1.189998
6.745207 1.175519 2.802778 6.94272 0.7401529 2.138886 6.155762 1.378254
2.880946 4.069703
CISD1 6.156846 9.034133 4.934281 12.45062 5.555 4.93786 10.4052 9.415912
11.44913 10.91425 8.176336 8.662028 7.464259 8.380541 11.74766 4.106667 4.553219
12.56003 7.487059 6.338228 6.172091 6.784086 7.274703 6.160504 4.503686 9.349381

3.228143	11.23782	12.44394	9.325297	8.439247	5.854159	5.077857	8.228604	4.307946
2.414256	4.740314	4.942215	13.91891	10.86921	15.06219	10.20848	3.759913	8.010453
6.42581	2.577145	8.718001	4.245178	5.811418	4.973938	5.634646	6.975026	7.279242
5.157999	8.67541	4.655855	4.60807	10.28327	3.327862	7.766053	5.31857	7.478478
3.354841	4.832977	12.88178	4.93145	8.779238	4.938714	6.765952	6.87061	7.16301
5.347955	4.191111	8.459075	5.312414	6.852562	9.330148	5.816339	3.842886	7.542479
9.600658	5.003746	11.86073	14.27763	8.59592	20.43861	8.303171	6.815629	7.697737
7.00099	3.023478	4.305785	17.98138	4.971811	4.701567	4.647044	3.500067	9.781622
4.411093	2.993735	7.230567	2.330812	2.995905	5.046375	6.168632	5.573788	9.125621
7.923725	10.38194	10.55823	5.619447	8.652097	5.863345	6.38169	4.800029	8.991051
16.25133	2.158297	5.827317	2.727765	7.495203	6.48983	3.414906	6.77713	2.140645
5.46919	9.363595	6.197298	6.005624	6.92544	8.130957	8.119626	7.71520	321.77316
8.641933	8.255473	4.989574	5.789851	5.753671	5.182829	3.196102	2.770299	6.153085
7.770871	6.322643	6.834578	8.34245	1.14241	6.141605	6.131741	6.521547	7.857777
4.674241	13.30605	4.833564	6.502257	16.67476	14.23519	6.634635	7.288981	8.93797
6.870478	18.97302	13.84078	5.127755	3.007586	3.723184	4.256347	3.626987	9.005404
8.77936	13.76251	10.67645	9.492515	4.064708	10.91844	10.37944	4.651761	4.650575
9.03487	7.213022	5.03511	910.11189	9.143012	11.92973	13.28968	4.653066	4.109157
7.450134	4.65846	12.9955	3.520011	8.604902	5.55892	11.99221	8.067553	2.969533
8.751724	8.533391	9.197505	8.844583	6.108978	4.78513	8.65141	317.17465	8.637705
6.577477	7.942021	6.305297	3.428398	2.49043	9.992909	5.444471	5.985677	6.586925
9.486066	4.588218	4.816066	7.180275	10.24297	3.917654	9.9473	12.14344	6.77747
3.356143	6.861231	10.91059	6.325903	7.342652	5.87712	11.80811	4.888726	8.056507
6.371542	4.722216	7.61511	11.97807	10.54255	6.346657	14.67491	1.972088	8.009057
3.26749	4.580266	7.955605	7.455154	12.60529	5.788799	5.60728	5.10976	5.333508
8.026387	4.607702	7.632105	13.19854	5.169715	5.281741	6.652191	4.108875	9.948766
4.049823	6.385807	6.875952	6.545288	4.5541	6.091075	4.421482	7.835725	10.53435
12.20773	13.13307	6.028065	3.539982	5.337311	4.726822	8.834888	6.176491	10.87705
4.9343	0.621178	6.232802	6.369216	8.675927	7.002485	8.897102	3.305846	10.61593
4.998563	11.85825	9.108336	13.10742	15.02248	5.587886	6.090136	5.506833	10.47935
5.556711	5.570772	6.218801	9.171659	5.078364	10.12645	5.865338	4.491051	8.837671
7.532432	6.039202	18.80163	5.362971	8.175277	6.17621	9.346442	5.734055	3.108284
7.985877	10.92925	8.288913	9.489076	8.714363	12.43204	11.1677	10.92107	13.35507
3.984501	8.836173	11.83795	2.402719	7.782301	8.127939	3.314312	13.25785	3.733197
6.296084	2.608375	8.678018	12.08881	12.4033	14.26675	5.503593	8.83047	15.61434
3.890056	5.595423	16.84229	4.844544	7.749859	6.867331	3.140251	4.4961	9.194483
3.18761	11.31204	4.778012	12.7368	4.576977	10.45131	9.715524	2.627342	15.74267
7.225027	5.265692	3.639618	3.243612	14.36897	5.019025	3.575712	4.826395	5.85951
30.13833	4.196039	4.139475	6.073055	2.704598	15.02467	6.791066	10.0918	8.662834
5.018733	13.20492	11.20161	4.780789	4.80033	2.869288	4.141634	6.035897	13.02963
22.02168	5.402707	6.237289	7.45832	10.76113	4.883164	11.2395	9.793269	4.638056
7.607101	4.085906	6.785046	7.586354	5.477051	5.343113	4.29495	4.198864	6.947101
5.462571	8.135255	9.05351	14.79992	6.922242	3.115806	3.6061	5.051816	5.722741
10.1475	5.97877	8.040286	4.001649	6.592681	4.115014	6.974757	8.720951	5.807555

	6.014588	9.866406	7.392768	5.21518	3.566537	6.346321	6.629035	3.282191	
CS	21.60147	19.32141	18.4214	18.53252	27.41812	17.88364	22.56331	20.61815	21.72095
	20.81688	29.86763	25.01788	21.9995	18.60363	21.20385	25.17214	20.31951	13.68611
	18.37803	20.83079	18.4805	34.10062	23.17894	29.46105	59.47258	34.93793	27.60195
	25.03927	36.71791	21.6453	22.72247	16.75959	20.25359	26.29273	19.04945	9.457478
	22.25309	14.6048	33.18196	20.74255	14.52358	17.31472	20.99613	21.42217	21.25734
	32.45198	29.75403	52.7738	36.21118	21.51187	29.62774	31.77211	23.67637	29.28866
	14.80383	32.76292	24.84649	24.33835	19.24191	14.57933	23.85993	22.89224	51.28035
	18.48683	16.77962	25.20505	10.49031	16.93381	25.31224	20.19401	29.10376	19.20734
	34.27182	25.11621	18.18079	16.97866	42.11995	12.93071	19.75287	22.81274	38.89211
	12.32161	20.83403	43.62526	17.61719	32.54485	20.44655	41.67006	14.03022	27.39117
	32.43733	27.56287	36.14435	19.04344	23.28029	22.20175	33.83029	20.10091	17.36451
	26.43234	29.25962	26.60054	30.13461	34.15073	23.78878	22.43801	20.0465	25.07344
	17.0357	15.52298	14.63144	22.95586	22.10115	32.9087	33.7671	23.8198	16.13411
	25.31227	27.59427	26.35794	35.55886	34.93009	18.37694	62.01906	26.34613	27.20672
	72.51455	20.73481	12.87127	20.89387	19.43322	24.27006	21.71665	15.78135	24.62554
	19.90808	23.07598	24.68626	20.64923	17.09987	18.50092	32.44637	24.31655	25.44228
	29.62665	15.43911	21.77719	8.69057	16.49986	23.62938	18.9165	18.08517	45.92867
	33.71226	27.04459	13.5985	19.50907	31.44038	24.84686	24.58477	22.67919	27.76033
	24.96362	24.10867	39.80288	24.15152	27.65845	17.39599	13.96174	22.99025	25.66782
	22.97555	25.92134	44.18172	14.5023	31.50189	19.72709	24.54109	26.2991	22.14173
	25.84946	27.17447	22.82481	15.31733	27.60267	26.91794	13.7421	15.80896	14.07264
	25.02283	24.99896	23.37413	23.99168	22.49523	28.59275	24.29795	15.86433	30.91936
	34.54071	26.29763	21.02425	14.38485	26.62481	30.78758	17.36472	26.73487	17.72057
	22.06166	23.42774	38.44228	28.84061	28.55804	25.74866	19.98828	13.33444	34.9016
	24.78282	20.22761	19.09512	23.50541	15.22155	20.01793	20.53851	19.88977	27.38035
	19.9432	17.67364	22.87782	28.1544	16.55619	20.66748	15.81916	24.45802	20.46041
	18.61223	25.71486	32.80196	41.55348	22.53984	31.28568	39.09238	11.05173	29.79744
	19.77333	27.14377	11.77415	24.92923	27.46945	29.58588	31.70087	15.14192	43.73807
	17.08984	12.40831	15.16464	23.83853	32.69277	22.2998	31.6448	39.90368	18.1256
	15.07293	21.1765	21.54666	23.06764	25.08198	27.96418	33.01687	14.49202	15.18041
	23.20101	20.03583	23.54833	25.86402	28.20686	16.8692	35.67959	22.64986	12.32931
	36.74559	27.37985	18.5429	36.15077	27.53479	27.72837	20.40217	24.70495	33.12362
	23.42833	14.30779	26.25649	23.8949	9.901506	27.78444	27.73014	24.63608	14.93371
	21.36329	42.63367	16.38648	20.86692	22.65105	16.93076	21.37394	23.86453	18.48698
	16.62416	14.31856	11.42021	22.52401	41.95717	26.00492	26.89955	20.63347	26.46489
	18.83889	31.98858	30.61021	25.48617	25.72014	19.67826	27.03867	44.89539	36.7879
	21.86923	28.34513	23.02191	14.98449	20.66456	14.27128	37.67026	17.52166	23.14075
	16.33186	25.13857	11.73155	35.33901	31.18709	27.89832	21.65959	20.62359	17.6917
	24.97052	25.72002	33.60531	23.42295	30.26673	18.02009	16.40463	35.16981	18.79288
	18.64573	15.56368	14.8157	24.21497	32.69228	17.68638	25.4019	19.43431	23.14939
	33.14623	16.16317	7.403442	35.71793	55.01529	22.33593	22.28172	18.27554	20.13978
	24.53356	18.80829	15.12756	61.0157	21.1748	18.37105	25.14163	24.93962	20.99554
	22.77026	32.55207	22.18586	27.30619	19.46311	47.1447	24.37264	35.53669	23.33215

17.48578 23.01401 23.97533 14.9206 32.43399 23.84125 21.11651 32.50407 9.345402
20.33382 20.7151 19.25556 24.01106 22.02094 36.72372 11.2181 16.84295 17.50863
22.86419 21.56813 38.36948 43.1506 17.1505 16.84 31.66113 26.00489 19.51388
24.52624 24.27941 12.74072 23.69265 28.44464 21.72217 7.415938 26.12039 36.77458
16.04689 22.28948 19.29868 31.44057 19.7978 12.27196 12.74907
DPP4 0.5438997 0.5408039 0.9506662 0.7644852 1.070709 0.4082438
2.044334 0.8162547 0.8463244 0.9116935 0.1517063 2.785062 0.6755418
2.398494 1.383515 0.6010876 0.4088355 0.988517 0.3151116 0.03724012
0.4831916 0.4920111 0.94274 0.5534536 0.1163979 0.608242 1.014565
1.103117 1.464209 0.9145065 3.406779 0.7195168 0.01734296 2.957683
1.121412 0.1608677 1.951366 3.683531 0.9227821 0.9584762 0.3083974
1.191701 0.7054454 1.420884 1.472949 0.3983563 0.2449149 1.060508
0.3931643 0.2533037 0.5956173 0.2004216 0.3165351 0.2353588
0.2844878 0.07441878 0.1855285 9.794809 1.47914 0.3595414 0.7583926
0.8980051 1.026645 2.664544 7.056342 0.5128846 4.5171 0.04009837
0.2775096 1.532869 0.1181594 0.1462487 0.4395901 2.377669 0.4250309
1.445338 0.5357685 0.04551427 0.07070579 0.04504742 0.1760613
0.2341746 1.531531 0.3338767 0.4305811 5.194887 12.36132 1.002495
0.4929138 0.5128852 0.3897615 1.060797 0.08054527 0.1195974
0.2017541 0.06499986 0.6517478 13.15516 1.245652 0.04816157 4.698521
0.6260488 0.1024192 0.1376774 0.6525565 0.4679756 5.650969
0.4002133 1.46452 0.298833 0.03714616 0.6654619 0.2731276 9.123539
0.3264751 1.115849 0.05784289 0.5416553 3.148616 0.5000929 0.3614688
0.1033221 0.5120901 0.1967888 0.08568186 0.3360139 0.03734787
0.5601554 3.954332 0.04063277 0.9605887 1.4255 0.7212006 6.007637
3.002713 2.014335 0.3774429 3.473832 51.30276 0.3829555 0.04642541
3.568672 0.1161354 1.359181 11.19817 0.3308157 0.6083068 0.04746757
0.01320229 4.23793 0.04854442 12.70457 0.3314564 0.2216659 0.3353937
0.3443474 11.6006 2.869378 0.2301214 0.1486409 1.372206 0.1714763
0.2705426 0.6228173 0.03057214 0.2300599 0.07769285 0.9111469
1.491415 1.539325 0.6853078 0.4690767 4.649418 3.812871 0.08871433
0.1989155 0.2658164 0.3847599 0.2470237 0.9314469 0.02740134
0.08604937 0.7970799 0.8610183 0.951317 0.4134936 0.3236566
0.00945062 3.717745 0.07412402 1.518754 0.1797192 0.5051756 0.7651668
4.31466 2.997041 0.05246105 1.37136 0.2587111 1.132662 5.551314 0.09217984
0.156721 0.1646957 2.059133 3.292152 0.572469 0.9756744 0.247386 0.09777334
0.6553756 0.4358337 10.6835 0.005088033 0.7222575 0.1090047
0.2964442 0.02251603 3.524305 3.160245 0.2663242 1.182358 1.895803
0.4201835 0.7464069 0.1079004 0.9390076 2.749806 3.748023 0.05387388
0.5250247 0.01706487 4.589619 0.3380612 0.3138681 1.569249 1.5335
0.04805744 0.1861943 0.18396 0.07647543 6.478228 0.8394705 0.7618463
0.7289595 0.6414367 2.094936 11.38197 0.274215 7.600483 0.2319324
0.05161259 0.07665322 0.08630779 1.592805 0.03271105 3.191115 0.2534247
0.6926619 0.2620854 0.04244859 1.743651 0.03724463 0.7485709

0.222906 3.474187 0.1947603 0.8234153 0.4095722 2.995693 1.375073
0.1181898 3.696438 0.2529856 0.4746573 1.442536 1.244559 0.2353559
0.2936028 0.7279324 1.259467 1.10231 2.277823 0.1456157 0.1073088
0.007664904 0.8915401 0.7159579 1.705116 0.5492134 1.41506 1.204397
2.22124 1.081259 0.5558276 3.56123 0.3212229 2.354254 25.38814 0.3601404
0.5150859 0.8482302 4.267645 0.09002342 1.117755 1.904008 0.1449341
0.7503638 1.066758 0.6061248 0.3535602 0.3815691 0.03159954
3.649182 0.9397543 1.705035 16.05516 1.299732 7.888864 5.723159 1.802548
1.551947 2.968761 0.0789344 10.65461 0.286862 0.09301674 1.372991 5.051394
0.8528761 6.579345 22.03124 1.588564 0.1487747 0.3606573 1.289814
0.9647059 4.193545 1.358975 0.8047249 0.9386122 0.3247001 0.1667537
0.6187437 0.107354 1.811155 2.561775 0.9675 0.0847528 0.2754275
0.3018376 2.17181 1.534452 1.703561 0.2519262 0.4155226 1.265719
0.1079299 24.54011 0.09658507 0.08581172 0.04701608 0.1657354
0.1600024 0.237899 0.516529 0.7263047 0.3144727 0.5730033 0.09732495
4.262399 1.324283 0.2276012 6.402509 0.1001002 2.247887 2.261219 0.69264
0.2191235 3.298994 0.1539072 0.19557 0.1350816 0.5674567 0.8865359
0.7529829 1.152291 0.9865634 0.02137971 7.577714 37.75291 0.02875603
0.4004241 0.5215578 0.09399981 0.04467962 0.08615497 11.81473
1.364443 13.47336 0.7946618 0.5338592 0.0760292 12.73687 1.935793 5.57828
2.01693 0.3867508 0.2389595 0.01900719 0.5876638 7.166216 5.211513
1.611591 3.344551 0.696238 0.052147 14.00146 0.00765773 9.771426 0.1366538
0.2004799 0.356547 1.335894 0.3730706 1.087702 0.236824 0.1263218
1.480782 0.4688381
FANCD2 0.9136971 0.1453184 1.962751 0.1775384 1.140326 1.231364 0.6299102
0.6682528 1.870938 0.2180317 1.041387 2.815348 1.335529 0.9019508
0.1557025 1.046752 1.261051 2.938067 0.9831993 1.414681 4.419617 3.533
4.863258 2.416312 7.064734 7.62985 10.41114 4.036155 5.561108 3.496328 1.896281
3.476834 2.158811 4.821015 3.006661 2.789186 1.857056 8.299314 3.205382 4.60878
2.830136 2.046955 4.500269 3.080114 1.788799 8.440654 2.651245 6.705234 12.47644
2.284642 3.296444 5.700776 4.17293 3.733644 1.826358 2.945915 12.18567 1.525532
3.776479 1.771278 3.787627 1.182202 4.507005 1.953874 7.281172 1.962503 1.308874
1.650199 1.620275 2.342962 3.445284 2.148489 8.599213 5.428004 4.629821 2.300295
2.218034 5.360737 1.95203 1.464468 3.775963 1.662586 1.769536 9.770711 3.380964
1.953762 2.891947 3.405293 1.514436 6.399034 13.84983 3.70925 4.065172 10.05193
4.141418 2.418068 7.056077 0.856131 4.431599 2.77999 3.194341 4.978179 3.061697
4.877099 1.888394 2.226767 3.495346 6.256235 2.816633 1.499231 3.566094 10.04291
1.923267 11.08818 5.708492 4.881231 8.464151 3.687387 2.241154 6.043287 4.372139
3.177319 3.231146 4.701164 9.390564 2.150113 1.838451 4.044821 2.838508 2.811086
1.039526 3.625127 3.3277 1.579672 2.685877 1.469211 8.579739 4.12041 3.376465
3.530799 3.151301 3.830235 1.459818 1.635463 4.520335 6.30957 11.14109 3.570681
1.147163 2.139366 4.571204 3.047909 6.482157 12.34093 2.787279 3.861581 5.497875
2.191647 3.826872 2.894966 6.367689 4.061659 4.318745 4.407956 3.274867 5.214832
3.164837 3.471212 3.804677 3.60921 1.37264 2.166658 2.8159 2.667158 2.75455

7.019724 7.322326 2.851519 3.79421 2.105313 3.278857 1.945332 4.169852 1.117129
2.649687 1.632548 2.869054 2.375948 0.7395035 2.059033 3.383681 14.66842
2.880354 3.533989 5.606123 5.420819 2.025294 5.094326 4.962038 2.586244 7.958968
2.419419 4.416257 5.074862 1.679248 4.182821 6.948315 4.424988 5.796065 7.356137
6.50159 2.31734 3.017363 3.975907 0.7613668 4.319076 3.620965 1.650414
3.389675 3.885157 4.089459 4.653775 3.460988 2.165494 3.542745 3.724268 2.235177
4.856098 3.924584 5.042633 3.033027 1.709966 2.677093 1.983287 2.360949 7.812077
4.695389 2.593096 5.586761 3.10958 6.930811 0.3833244 4.019066 2.609028
7.981564 6.390217 5.617769 4.965144 10.49257 3.353709 3.531164 6.165408 5.235426
1.809271 1.42093 6.727654 3.079878 2.947101 12.89394 4.488074 6.425109 2.009155
1.469665 2.455799 5.695253 2.508007 4.684566 6.442581 0.8284006 2.165103
2.550102 1.952862 5.431437 3.231791 5.29204 2.776084 8.440037 5.513155 3.03179
7.40247 8.749058 3.309548 4.38542 2.335075 5.51339 3.212443 3.092684 3.335251
2.854452 3.592514 5.082798 1.769337 6.907254 4.988418 2.881114 5.387125 2.699692
3.258037 5.140038 2.558984 5.176342 2.870625 5.229924 3.337013 2.712118 2.078068
1.449985 3.479952 2.32575 5.622382 3.816459 2.20892 2.622921 9.82232 3.409357
1.833838 3.250407 6.387694 3.730534 2.351006 2.937026 0.9135471 0.9495613
17.65802 4.75055 1.108346 6.428795 4.934593 3.391573 5.233185 3.839635 6.670023
2.043431 3.248563 2.699069 3.177773 3.929883 5.137993 5.726423 3.292807 2.591224
1.852326 1.592397 4.045534 5.634266 1.386883 3.219223 3.191827 2.603867 7.530375
4.03384 1.61768 7.989121 1.94416 8.570306 2.233488 0.955387 3.856828 5.226655
3.815075 3.341219 1.623879 0.8411053 9.272081 5.883744 2.820768 2.154497
5.784476 3.831626 1.883181 3.204545 2.437367 10.76306 2.39205 4.241363 5.288665
15.85733 4.72347 1.142517 8.561366 2.288431 2.318674 3.42617 10.48492 1.746917
4.615392 0.9991238 1.464196 10.95963 3.359179 2.46546 7.48857 4.853793
1.988376 2.933114 5.069505 1.985708 2.366894 6.4997 2.672413 2.662078 6.983378
0.9432118 2.292202 1.785646 2.190052 1.962235 2.783995 2.587859 6.522898 5.84119
3.696405 3.798782 2.730004 2.097646 3.763573 1.278274 3.35495 2.859191 3.366881
1.650379 2.473516 3.6988 2.156871 1.550102 2.313756 3.861085 3.385589 2.088674
1.595592
GCLC 14.72601 6.484436 19.61861 5.216565 6.81573 18.5836 7.314234 8.550623 5.64611
8.759833 11.97872 16.74926 23.88515 20.63935 6.608697 42.38698 15.71755 2.464708
14.90294 22.2787 3.283278 5.903005 40.3269 22.71299 17.48207 3.14491 3.280459
16.71175 2.717374 8.254965 8.741293 2.916501 6.421651 7.953555 10.56954 12.87849
6.005772 5.786923 7.62829 5.896234 15.37899 4.133204 3.820421 6.423166 12.72616
29.05931 2.36545 5.707473 4.781139 36.88105 8.649184 6.97951 816.71767 5.804247
26.91595 13.07247 29.98827 5.223487 5.71115 2.85722 4.350329 10.98524 13.07426
1.734469 5.890921 4.489512 0.6507955 29.4507 317.09481 2.940048 17.58061
2.881222 11.39203 36.30115 2.786672 1.225948 3.576098 75.45788 2.614311 15.80071
3.24105 926.25191 27.26633 5.539073 3.323221 4.077464 4.364471 5.638996 5.308068
3.896264 11.25803 5.152209 10.94875 10.80355 32.39308 17.85505 19.15893 2.299188
3.12425 4.822598 4.151587 20.78293 26.39476 8.813347 6.384723 6.892477 1.951683
9.60208 5.280704 2.28009 14.62992 3.343726 19.7256 437.04301 8.864894 2.165686
5.317915 54.60713 3.261804 6.520754 4.33234 37.99481 6.860141 3.990048 28.25799

	14.59056	14.22832	9.571772	11.44747	15.49465	7.484812	5.274178	5.732429	2.528228
	3.145905	7.857712	7.613381	5.987278	2.650355	13.80638	11.27141	4.752431	8.245103
	3.690899	5.798394	4.148845	49.78137	1.953856	13.75793	5.480703	10.68472	4.277586
	1.691691	21.25399	12.78799	20.46353	3.648422	8.274538	5.543929	19.04284	7.231567
	18.9103	33.35962	3.315251	3.272133	23.9918	10.53649	4.452203	5.053015	6.900904
	15.85297	2.292312	10.69945	6.16731	20.67741	6.689605	16.67103	3.015024	11.13777
	3.76943	8.695188	17.14434	12.09907	26.65215	3.082112	9.978858	11.25112	16.71351
	2.2214	7.016117	9.910579	8.351604	38.14297	8.774543	2.953647	2.129229	5.338407
	4.063748	103.1481	3.367381	3.378887	6.350613	5.344991	59.76931	3.523638	6.025749
	1.967173	10.18277	7.571767	40.98525	24.57445	56.86075	4.057208	30.89152	4.653564
	15.39896	92.6241	16.86078	4.345005	11.24813	5.109501	9.252946	3.12092	6.155182
	1.914364	9.294529	11.82308	11.88378	26.40565	14.36303	3.91897	6.987312	7.00648
	5.70252	9.154536	3.343177	5.593555	5.7492	22.83763	30.45684	16.22887	1.877607
	73.88027	19.59491	28.81266	13.45829	9.872978	9.397055	51.6428	4.338286	4.245916
	55.44762	26.56234	8.645804	5.916896	23.71687	6.588058	8.919398	10.58514	118.367
	15.54133	5.347718	6.637448	20.48855	6.378484	9.492824	14.67808	6.265191	10.59142
	2.583356	7.822149	6.902033	7.353906	6.693141	9.751838	2.748202	9.115647	29.71831
	5.707263	3.012079	11.67952	17.75447	6.93383	81.10992	9.266742	7.426464	16.39644
	59.5728	7.535419	8.01341	3.357993	3.161985	4.336953	2.252476	25.85891	6.932395
	2.471591	10.39701	4.290806	3.435098	4.39042	3.276119	5.291587	7.217317	5.233325
	2.896833	2.198884	2.451902	1.754685	9.373607	4.280356	24.27558	14.61315	24.15702
	17.56043	3.62016	8.549469	2.870421	2.186151	7.895428	6.12825	4.589608	3.539552
	76.0487	4.025603	7.298936	20.49472	1.936929	2.461013	7.733208	28.70809	2.697379
	8.720344	15.02684	2.936948	2.97091	5.845297	21.36487	5.21149	45.54846	9.654053
	7.809999	7.903951	35.17083	6.000354	2.722755	3.585787	11.58778	12.88372	2.548799
	45.23597	2.434284	7.719673	3.392626	11.00601	42.12829	5.92056	28.31874	3.030277
	5.766129	101.0773	11.97008	5.922134	3.773999	2.989768	11.29267	4.228139	6.690675
	4.76769	17.29422	6.853266	3.098331	9.042706	3.854207	9.14197	5.115862	7.187271
	121.4102	4.584488	2.992815	17.03244	16.56784	10.59656	10.34187	2.669485	7.495842
	5.616739	4.073685	10.38642	2.405545	2.742879	28.81703	30.90052	8.029872	13.21746
	11.01562	14.84139	3.616408	6.561537	3.121123	2.960213	30.00493	1.012487	2.759011
	110.6522	2.923765	2.704616	5.35641	3.783335	10.38075	33.2169	4.465509	6.886457
	13.2001	4.646962	2.009329	8.748834	2.444306	7.281276	1.946543	1.84733	10.40955
	14.30154	1.883108	6.774583	1.964703	9.346407	10.63999	4.365137	8.50327	
GCLM	3.846409	1.561156	2.752664	3.112165	3.369549	3.344709	2.05457	2.815984	
	2.583018	10.23888	37.78194	6.585943	3.676523	5.792181	3.003463	7.494936	3.250226
	3.743485	3.133612	5.419227	18.95232	8.108848	5.740517	44.50218	13.05324	11.12571
	5.784945	7.996713	14.65101	5.002736	8.72115	9.92252	1.208499	3.898693	6.770982
	4.290589	2.439229	3.595763	4.905404	6.096906	5.243307	1.495949	13.15638	5.024521
	8.582284	18.76087	2.122823	5.720077	15.76662	17.05554	8.562579	4.08418	14.66561
	3.737572	8.344319	6.396129	12.80233	3.206116	7.439166	2.507586	3.64702	10.73296
	59.26176	7.935743	5.740269	16.45502	1.76229	5.858205	2.272129	4.549644	11.31054
	1.351587	6.77205	94.25751	4.06232	3.33464	16.90137	9.436113	4.047494	1.025175
	4.501659	16.01487	55.47825	4.456702	11.2853	3.968826	9.031286	11.73536	2.281898

3.776318 6.113305 6.200009 5.409125 13.30516 13.39369 6.314119 7.504651 2.307884
8.468682 1.654943 1.533543 1.919509 3.347719 9.296273 4.476418 7.05922 20.07757
13.58577 10.05012 4.436769 5.183732 7.389708 9.333792 8.814584 18.09781 3.978844
0.7840309 5.92734 7.131729 2.623636 7.940315 10.0137 10.73282 4.679158
6.213515 5.581216 1.714283 4.46721 4.666132 1.292847 5.040112 21.30212 9.496851
4.123596 14.92022 7.222579 4.27655 11.38456 4.084943 12.26908 6.447185 4.793112
2.74343 20.71008 7.5978 5.197408 121.7804 1.710856 2.680187 4.767744 3.673182
3.246771 1.296319 10.4038 6.168218 9.908302 8.994201 8.496639 4.221918 3.13452
8.515852 4.712247 20.71893 8.962741 2.382741 10.00674 4.154907 4.448061 6.576327
5.367242 82.80057 1.127884 7.327916 14.49767 4.57488 3.219412 20.52925 13.0751
7.248265 5.889235 2.423819 10.99422 7.376796 7.497881 5.94593 4.877986 10.83988
3.751243 2.882296 3.946837 6.126348 5.623369 40.16962 14.57159 2.10363 4.537021
2.967847 3.476816 125.9255 10.63644 12.61344 7.560597 10.08665 67.63833 1.958725
5.886436 5.326541 6.861422 4.138142 4.73482 1.959547 62.00532 8.150179 6.065846
9.579046 37.43365 5.13463 1.972658 13.83283 9.860929 4.448328 17.42248 4.958691
7.661571 4.345313 5.806671 4.107239 45.31423 60.16216 3.47967 5.417685 2.466558
5.95374 8.656801 1.767387 2.926401 4.181018 8.110967 9.496147 32.9588 9.02006
4.5427 23.43016 5.470746 7.382601 4.888905 28.08647 7.085366 6.351047 5.459202
3.793407 14.2047 4.864387 11.6803 2.130558 6.508209 11.93696 5.964043 10.57911
72.439 2.759156 5.358411 1.414677 4.483439 2.320687 5.129221 6.590459 4.759396
15.7325 3.183468 2.949513 2.012432 6.189922 4.450024 9.88222 5.642209 3.329969
6.808026 5.221335 33.71107 5.727568 10.90032 5.35737 61.03395 6.03667 5.100244
3.231368 138.7649 7.016762 8.508317 7.101584 7.926626 10.88143 2.997801 18.066
8.437441 7.718478 4.024341 12.33752 7.859928 12.20333 5.053477 3.066785 7.703278
2.954287 3.826321 2.893184 3.804418 2.997189 11.28644 2.768461 4.601204 1.995561
3.265965 20.79655 3.00975 6.188204 10.85089 2.142243 12.15048 4.202571 15.10281
3.836014 7.574515 7.077599 2.303314 3.352567 33.74779 12.10576 5.700246 12.55678
4.054798 5.783437 6.539889 8.051125 4.395527 6.381901 10.12258 3.912223 5.91006
5.020742 6.305807 5.436613 15.90844 6.789641 7.790648 5.672659 8.542421 59.86126
8.9296 7.416766 3.474829 12.76285 4.2781 6.690739 89.58958 4.430387 53.11068
22.9044 3.225297 9.006028 1.453932 2.863879 5.23841 8.852991 26.78632 25.04904
4.547989 9.794148 1.876575 20.94371 4.444299 6.193408 22.57995 1.379599 4.911077
18.43741 11.44209 2.015654 3.900905 7.980699 4.283194 2.660016 8.541078 10.57251
2.6812 4.142566 7.851794 6.721834 7.775221 4.282838 14.96577 76.74751 13.82351
5.820409 3.775883 1.027121 6.996037 7.857757 5.790229 8.456984 4.822358 0.5445452
2.624797 21.48846 9.709766 6.515628 17.895 20.74884 2.410916 67.20877 13.06474
17.14052 7.1026 5.865365 4.089314 2.303981 8.962395 2.844971 4.466468 8.486775
3.048656 5.338952 6.280321 4.46766 4.459137 7.96334 4.794504 8.454544 5.175192
GLS2 0.2181105 0.02940056 0.2564607 0.007642396 0.1510369 0.2940704
0.1052276 0.09536912 0.01895784 0.02485171 0.08835457 0.1949421
0.3195937 0.1076434 0.006562806 0.1442624 0.6912209 0.02061588
0.1627882 0.377694 0.2049454 0.2665741 0.1913085 0.06227569
0.2670274 0.5367636 0.2045381 0.3233644 0.3602285 0.02480263
0.06318981 0.08665679 0.1258694 0.2259566 0.1145465 0.09729367

0.09246906 0.06447067 0.2859705 0 0.01184257 0.03286796 0.2815938
0.1595216 0.04175848 0.07709701 0.01346599 0.1305506 0.2414463
0.7791288 0.3001939 0.06060805 0.05743266 0.09015267 0.1184674
0.1563467 0.3259394 0.01106248 0.1766147 0.01739621 0.1310515
0.1671134 0.05247216 0.09279449 0.1760468 0.03918259 0 0.2667689
0.9598315 0.0752419 0.08795507 0.08845206 0.2548803 0.01801287
0.1293598 0.06155974 0.2505151 0.2936244 0.1509292 0.2111482
0.8245598 0.2301490 0.07387497 1.0749380 0.07803847 0.01747592
0.1122805 0.1490585 0.4226845 0.1645662 0.1594218 1.826784
0.2314663 0.3609818 0.2096655 0.3267083 0.005993458 0.3878773
0.1324018 0.01102856 0.05790932 0.1434486 0.2498041 0.1261316
0.06081728 0.02362491 0.4014508 0.09601623 0.07072272 0.06418915
0.1736998 0.06607564 1.861397 0.09699492 0.03936751 0.02332247
0.2183975 0.02704332 0.1222056 0.1009008 0.2879964 0.7300424
0.1734276 0.0712537 0.1208353 0.0050196 0.3855141 0.1487777
0.09829972 0.1374336 0.01604001 0.2513615 0.02763082 0.05188735
0.3614338 0.2739355 0.2434627 0.05378902 0.2925646 0.07861938
0.09751594 0.1130682 0.1189115 0.01619622 0.5695949 0.2207444
0.0469778 0.0851714 0.06774777 0.1247797 0.04319774 0.06391691
0.4660347 0.3407846 0.1125747 0.01815293 0.07645004 0.4742386
0.2724207 0.1336091 0.5490520 0.02181676 0.3668994 0.3389870 0.2903823
0.2506084 0.1318166 0.01058083 0.1896016 0.05367341 0.04519118
0.0487277 0.03953222 0.09300187 0.1295595 0.4168040 0.1454406
0.2134304 0.09618074 0.0946999 0.2276887 0.3092578 0.1986361
0.09242761 0.1083310 0.1879194 0.2057684 0.04286965 0.06897019
0.2310821 0.2173904 0.0596162 0.3470829 0.1643799 0.07934057
0.05439212 0.1216462 0.1585562 0.04957527 0.09784026 0.005575089
0.07153639 0.05363554 0.08337241 0.01547095 0.1680740 0.1067788
0.5984816 0.2507276 0.09685177 0.3122189 0.4620199 0.215409
0.1149540 0.10788 0.1002488 0.1852023 0.008424977 0.07044683 0.1335739
0.05959138 0.03199792 0.1728080 0.1228384 0.6074083 0.1199328
0.0615250 0.04383364 0.2317029 0.05663096 0.2188036 0.3588265
0.0851922 0.07063411 0.05810757 0.1479021 1.1408170 0.3561662 0.11126
0.2312639 0.04039246 0.4195032 0.4907807 0.25044 0.02615356
0.2848941 0.2107675 0.1760007 0.04001975 0.1109144 0.4307751
0.3134839 0.1696482 0.03549137 0.8591834 0.1424527 0.2547349
0.2015884 0.1774157 0.1625966 0.06817423 0.1531752 0.4759573
0.3521693 0.03125763 0.7309150 0.5239140 0.0632455 0.008903252 0.2926183
0.2685984 0.04863834 0.1734080 0.1607622 0.0267932 0.5281156
0.1081819 0.05073504 0.00779217 0.04024248 0.2898623 0.1112223
0.2767889 0.8206835 0.1576164 0.4493404 0.2319725 0.0401047
0.07381496 0.02142939 0.08324866 0.4941308 0.03089535 0.2029692
0.09741038 0.08511202 0.1194853 0.2153446 0.01117 0.2653747
0.0524945 0.03733954 0.06929576 0.01578268 0.4263724 0.05843796

0.01260623 0.1088257 0.2322932 0.2177231 0.1775193 0.133781
0.07863159 0.2126549 0.03819313 0.0915669 0.02933153 0.05056216
0.07755187 0.1129554 0.0257599 0.02216695 0.1551549 0.01931259
0.0447731 0.09751221 0.01415442 0.0400233 0.1754866 0.2234451
0.09565667 0.1902136 0.0782433 0.0376082 0.05200579 0.07911316
0.09374338 0.07615195 0.1104572 0.04708849 0.1854704 0.465047
0.01919075 0.1242107 0.004731734 0.03506366 0.3780965 0.04299139
0.3466667 0.2534266 0.005473012 0.3770183 0.2066909 0.4113893
0.04349603 0.2679299 0.125532 0.003929572 0.5919406 0.2205725
0.0699953 0.009324437 0.5376131 0.383687 0.3800325 0.05222128
0.2104283 0.06729236 0.5565198 0.06695903 0.1758913 0.1529494
0.02520601 0.1105534 0.02844215 0.1860972 0.1570922 0.03698432
0.03485153 0.1295081 0.2678079 0.08798256 0.1041172 0 0.00765392
0.03648755 0.1403951 0.2379224 0.1015022 0.006786497 0.3378979
0.04748603 0.08956969 0.4169118 0.07806496 0.1875852 0.1681871
0.06112763 0.2175343 0.05450283 0.1145707 0.02299144 0.02275165
0.1338032 0.1742215 0.1252525 0.03742545 0.4072950 0.1322001
0.3554224 0.3737694 0.1582129 0.05764352 0.09695354 0.06264129
0.08491215 0.02629184 0.7456615 0.04254725 0.04628343 0.181877
0.07576599 0.09367609 0.2957906 0.03610143 0.1946493 0.3003315
0.1159333 0.06345308
GPX4 72.63173 60.52713 141.9534 56.20937 39.89953 88.05886 79.80672 86.66387
58.58346 58.11359 60.97241 85.25107 66.30892 132.8349 58.06824 83.49856 91.37856
198.2109 143.3653 89.12809 323.4019 256.1448 143.6868 33.60854 87.81756 65.675
63.66465 108.2506 103.9663 194.3561 103.6826 131.9253 176.5988 82.37655 154.6625
86.5412 93.62991 130.3644 102.586 112.3944 198.067 79.32548 117.1208 80.23329
75.81212 96.45756 161.4144 278.7279 80.57667 71.98206 157.5274 104.7793 103.385
85.63025 242.3537 110.184 168.3212 44.98533 98.66302 211.0823 181.984 147.9511
83.43034 106.5174 204.7293 154.2857 217.079 69.62033 58.86524 72.59762 88.34953
166.7995 113.7912 83.24865 61.90774 130.5654 64.59489 120.0065 93.44525 99.64761
113.7238 51.12546 111.1448 55.04937 71.84284 86.04863 118.4695 48.03828 306.061
118.0217 110.4628 68.59332 163.9999 90.53704 115.8537 177.655 60.86109 171.2001
91.45678 169.8216 29.31519 107.8458 212.263 101.0828 156.8043 85.06116 58.68716
84.17868 116.8686 213.0351 90.98534 173.2818 93.05854 61.38507 179.7121 72.71518
481.648 115.5574 95.06919 134.1456 140.7081 108.5857 114.3414 118.1909 6.064911
92.03695 268.6449 89.09699 79.26152 110.6502 99.30664 54.30688 122.4271 209.4002
64.47224 75.61324 117.2573 61.07738 110.7524 191.7507 153.5019 145.2182 76.43016
193.8496 47.91242 113.076 62.84843 86.12815 128.0025 114.1874 113.0963 62.49568
146.2181 130.6626 36.86153 79.85416 14.07367 99.64131 85.27785 255.5672 75.15917
103.0895 92.93848 131.2188 118.1196 179.0955 82.81852 116.4191 51.02011 134.222
119.8214 148.0636 61.99689 54.85142 179.0246 117.5406 191.1167 58.1289 51.47929
89.71005 77.09026 82.13742 83.38149 112.3559 112.4248 123.2815 113.5828 113.3513
95.18023 118.0758 121.6163 130.5638 80.44593 110.4588 125.7928 182.156 199.356
194.9943 96.37936 105.4972 145.6033 189.7228 105.1852 93.5847 76.03003 79.98158

	115.9784	145.1772	50.58047	59.51671	86.28527	76.06705	84.72977	59.53035	127.6787
	49.40324	119.5554	104.3031	58.63022	93.18686	137.1744	31.82886	60.54204	164.2479
	108.8144	73.97604	97.06239	145.33	87.52744	103.2578	152.5975	156.6121	54.41928
	85.81015	127.7768	123.2813	78.46641	105.9036	97.33943	129.885	57.05348	167.6297
	131.7951	61.08388	163.2036	125.5744	131.7287	94.35588	79.15877	49.43523	224.2621
	88.48068	112.5271	109.096	204.6117	107.7607	141.3267	137.9485	122.7591	80.85942
	68.66345	120.1283	105.2544	135.6674	129.5182	63.68961	119.1737	174.2079	179.2804
	202.6938	204.8878	143.4382	76.57137	95.1502	170.8639	100.6401	81.5866	77.74069
	82.37259	152.5229	202.7014	257.0314	92.41916	76.94892	142.9461	208.6446	82.06883
	66.94742	112.5606	61.45653	79.72345	123.3094	70.18279	97.7539	69.15665	82.03237
	91.17661	105.889	48.7408	156.119	141.3649	69.09286	117.2692	115.1023	32.24103
	77.60079	135.5584	250.0104	211.8624	215.7217	94.9564	142.0425	87.62091	101.647
	78.73058	102.4058	51.91958	97.03715	74.5253	175.2312	37.11688	28.57656	86.12542
	41.07551	172.7674	178.5105	150.2762	103.7372	119.092	68.64765	162.6927	130.781
	79.96093	124.499	330.1532	201.2896	125.3071	61.85725	109.5692	37.47269	97.00846
	93.6462	78.374	80.11835	143.0003	96.049	129.6108	139.4883	65.48086	68.93187
	58.11866	124.9705	29.02738	290.1454	73.19667	104.2084	152.7983	131.8545	119.3065
	128.293	85.56966	93.97891	61.84884	99.51809	262.939	220.5719	71.64175	54.45525
	42.67506	67.27856	41.98241	96.08988	119.4307	111.2151	185.3277	127.5559	133.218
	181.8945	172.2929	243.4286	90.21225	76.20319	124.0103	109.4541	33.3031	47.8092
	59.94405	120.2058	181.1794	93.38343	98.35314	66.14396	268.3415	114.1551	128.808
	258.1382	122.3367	129.8944	117.8043	71.67444	140.1798	142.5298	253.1638	164.8872
	112.1057	70.597	144.9892	134.2791	107.8972	114.6351	139.4168	129.1605	79.81186
	109.8551	58.53515	216.5715	169.2598	101.6306	120.6392	148.2284	231.2446	69.05625
	83.34071	353.3291	80.9206	136.2585	97.05023	86.75655	170.6198	69.33381	
GSS	34.45089	10.49932	36.39709	9.072625	26.1632	26.63437	15.07659	15.89412	13.47891
	8.834706	36.13954	26.23737	34.87466	27.60958	7.62132	28.81896	36.37169	55.80314
	39.83796	40.50094	52.66016	27.62179	62.52194	39.02805	63.06527	51.17386	41.80774
	337.794	44.66582	52.73254	33.92906	43.98917	51.11913	61.03625	36.29164	46.10003
	34.65502	39.77278	81.96531	45.99898	99.86621	37.48131	39.27013	43.91516	39.31678
	36.07648	54.6215	45.71327	43.64183	28.9332	106.0389	29.83138	39.00566	39.59354
	47.36547	70.98413	48.29375	49.47226	32.88199	33.89495	36.75058	47.16658	40.1965
	41.79832	18.9037	25.53703	44.86858	36.65406	34.83929	46.54892	49.98103	63.67837
	85.48695	38.34349	19.42388	25.13378	26.07186	36.24394	61.56758	45.3457	112.1806
	41.80499	38.94659	45.65235	53.27989	46.90262	60.43952	43.60209	48.3272	15.84151
	32.37058	33.19176	83.71999	50.53495	348.9884	88.20025	38.10251	33.47066	33.33126
	64.12715	58.43701	29.04196	45.47454	57.90239	53.8511	48.30058	33.4077	13.96618
	71.52513	99.68645	59.2327	38.09041	38.18458	67.97676	57.02118	34.12121	84.78242
	50.17701	27.52076	34.20206	54.0585	66.81452	45.87613	86.42407	26.02828	24.82725
	46.29842	43.39258	36.84671	58.87744	35.74788	28.29488	69.23538	33.89317	33.87318
	55.86636	67.99119	50.22051	50.00648	29.03057	62.60886	36.59954	35.67757	48.1318
	55.09949	47.04553	19.81665	40.09235	64.3411	31.33368	66.67264	55.8424	14.87275
	43.40944	76.48224	59.81165	67.99441	49.55685	48.02271	67.83825	57.68358	56.41343
	38.4029	32.82133	47.16775	72.88665	65.80548	98.41473	44.86388	81.44895	32.07104

47.06004 32.4404 39.91784 44.96107 44.18377 66.99194 81.55603 43.00285 35.20834
42.01049 61.48837 47.79753 62.18241 34.11229 38.72487 88.53317 82.7802 25.58477
67.06233 95.97736 68.42265 44.22778 69.27113 43.88643 36.00288 53.24299 37.15722
58.8744 60.02791 67.14517 31.32645 33.02172 38.84439 25.597 34.95921 59.95258
22.66674 54.84975 32.91138 35.41202 42.70991 42.00849 50.95866 79.81628 38.58344
63.1767 38.57617 33.96209 42.35449 33.48504 17.45051 49.86687 40.33094 41.3132
42.8727 29.15342 88.47368 32.97331 37.05075 37.03091 48.17361 37.7215 29.07414
42.97269 30.61001 58.48721 88.0155 44.60899 68.51424 45.13442 14.49864 65.94586
35.0007 45.45959 44.72196 60.74583 52.90699 50.81984 34.81867 68.87899 48.55797
50.65571 37.63363 37.29409 62.65843 23.7515 45.3764 84.44955 42.83044 50.38136
40.93451 42.70847 51.03553 135.3198 23.24165 37.98984 57.94024 36.17812 59.11983
70.51128 44.40141 27.62782 51.80024 54.49829 34.07317 48.37778 73.11374 41.29731
60.56428 54.31355 57.70529 35.46545 39.95538 25.81008 66.63929 38.5189 46.41392
78.79052 27.85256 35.80498 65.42209 30.5745 24.45149 45.42479 40.30344 60.14736
18.40654 45.52149 46.14665 41.08363 45.31229 34.28134 31.35291 44.65884 25.19457
59.6921 58.19127 36.19738 26.91654 73.29534 73.21435 44.45679 46.99694 72.09941
23.54616 46.59473 44.67926 40.71292 50.40475 24.71266 29.20708 34.02053 47.9228
50.26426 54.88848 50.5192 51.39604 32.28881 54.36483 59.01985 29.30363 39.35947
60.13151 50.07521 47.2842 44.58725 64.04776 41.66848 30.70724 31.2251 50.58478
48.96306 49.71974 48.52349 70.49599 52.01314 71.88043 55.29778 63.68071 32.84415
60.63318 18.62032 64.7335 31.32587 61.75709 46.55206 54.07076 74.47864 70.31215
37.51516 32.60253 30.15471 39.44404 72.22976 85.20969 53.8867 32.26798 52.38242
44.8947 57.90045 26.56798 53.4786 49.10712 32.67744 68.04461 46.59427 68.35469
38.5005 38.25761 39.99701 39.04362 48.77127 35.97479 36.34124 28.98218 19.85774
61.38932 42.75837 31.13943 44.31834 51.18553 49.97839 37.95838 46.54556 132.7819
42.75087 105.1458 54.93582 35.24953 62.58902 46.55284 51.43454 38.10412 50.16118
24.45958 34.72979 27.06619 55.00118 47.96265 40.39421 39.7682 51.88858 50.12941
40.58143 62.90744 53.78597 41.70124 46.33044 57.86159 62.97959 36.73974 63.50029
52.64337 48.6954 68.57734 83.55099 64.19637 46.55926 67.18508
HMGCR 7.192403 2.198903 8.921458 3.080697 24.40136 8.491814 3.13168 3.472471
2.930495 3.255811 15.8677 10.41541 7.76272 4.467245 2.606153 9.354471 10.55905
5.311324 6.552833 5.102346 4.572008 9.810184 13.69897 12.37032 16.5302 13.85242
13.55248 7.800951 12.53689 4.511536 8.847605 7.629034 5.059897 10.29092 7.226427
2.712317 7.873279 6.056831 6.859428 4.676012 3.515262 6.238268 10.40458 7.344148
6.580213 12.87116 3.185157 13.62257 14.44206 19.09686 11.88432 5.905957 7.538575
6.028924 5.487779 11.44003 8.565778 17.86689 10.59092 4.914559 9.655581 9.360039
15.01412 4.233168 7.083312 7.566683 2.938005 12.39912 8.813595 6.195009 15.48603
8.688678 18.28249 6.721017 7.160507 4.556957 19.01754 25.76326 12.40609 2.473355
10.83149 5.768178 8.996311 14.83632 9.425288 12.14906 5.405169 9.782351 2.464517
5.573567 11.89544 6.318876 8.376834 10.01385 10.92837 9.121133 13.19821 5.590744
14.27877 4.146253 12.6757 5.71347 4.222835 20.8469 3.207089 6.486641 5.72188
10.25877 6.752537 6.131087 13.12143 6.260962 8.311058 7.360302 11.81748 14.2018
2.956401 8.036469 8.540878 12.66559 9.888311 37.09622 12.30055 18.24156 10.8482
6.869097 5.040184 17.15155 4.996117 6.11922 4.126237 5.950164 8.502072 2.927217

15.54801 8.233559 6.905372 7.095495 12.06346 16.50435 6.261064 7.337732 8.576932
14.189 4.996417 25.44919 9.6746 2.876204 4.422872 9.970867 11.91098 3.338744
8.436153 27.19344 10.20156 11.16487 6.984941 5.42999 7.521723 8.113565 9.439815
11.18622 12.59572 9.719803 12.02829 8.754934 9.29651 15.83348 12.7533 5.208678
5.496771 7.648064 5.224455 6.238183 13.98532 19.29966 5.998597 17.53672 11.50453
4.885671 12.89086 10.51113 8.384653 4.049179 3.4025 7.548184 5.175307 10.26358
3.119153 5.399622 4.873085 13.07332 9.255168 9.909417 11.88953 12.39456 3.750942
8.355706 9.41905 7.996642 9.373651 3.956084 14.2873 12.44154 4.228767 8.772245
3.673149 4.538352 34.30359 8.523089 10.62379 12.36236 14.28632 15.00452 3.222007
16.39252 8.904274 4.685521 8.695294 5.90679 4.596542 15.01188 9.654377 7.348668
6.15483 12.14133 8.639699 8.662454 9.487767 8.042318 7.260851 6.260587 5.359067
9.223958 8.206591 4.103956 9.657496 14.98931 12.52859 7.561421 9.687067 3.121925
17.36366 10.28295 3.789248 14.62032 5.541482 8.411671 9.334058 14.68019 5.364362
42.85358 3.41255 8.977595 3.746453 10.70738 6.316438 9.398308 21.93977 13.46243
22.80202 7.194009 6.495773 6.280645 10.02751 8.883739 13.18931 10.80995 5.481702
3.13218 15.3273 6.745817 7.583308 10.60931 6.677821 9.96369 14.66971 11.02834
3.458181 16.34136 9.714044 4.30686 27.2678 6.857146 13.36626 5.788369 4.61063
10.87424 3.69519 7.269138 4.053228 4.960906 22.41199 6.402028 12.91932 10.98795
7.219352 13.85249 29.11418 5.317645 15.20625 7.815685 6.359696 12.18259 7.010573
12.87711 5.458925 4.237507 4.650975 16.38196 12.86951 12.39866 9.537673 23.05228
14.4627 4.609678 11.63631 7.911145 6.082418 8.891051 14.93821 11.69914 9.007023
20.37879 4.426526 12.03967 12.64446 2.983329 5.096393 5.226201 10.28219 10.5588
8.765589 6.912812 7.166591 3.93722 4.458591 7.90124 6.49464 23.70963 5.410384
15.96692 7.836015 7.703944 13.7909 6.267594 7.69214 9.381111 11.63639 16.00368
6.548176 6.77542 21.08825 3.935349 8.982017 5.551476 8.632684 9.213877 5.767982
12.34591 8.186843 3.410349 4.771423 30.95385 17.06446 3.321803 9.742483 11.5967
9.460461 5.904345 20.33244 4.595712 16.30833 7.662511 3.925067 7.481758 8.281707
11.72731 12.39509 6.890911 9.03889 9.225248 13.36582 13.4531 14.6325 23.91013
3.909262 7.73609 10.90381 4.022515 7.162505 26.5266 4.280828 7.309441 20.93847
6.125649 3.655852 6.057551 5.121647 7.415379 6.728135 7.699673 1.894449 4.381015
7.387723 8.244976 3.018881 5.851707 10.19415 7.337759 6.986037 12.08048 12.48358
6.951734 5.024063 5.221805 4.189873 9.506447 12.82494 8.011388 8.489327 10.30423
5.751089 5.357336 9.849082 5.299912 7.152697 17.16016 4.333465 3.440391
HSPB1 1434.036 783.5554 1104.879 1072.014 1789.28 457.6705 422.236 467.8432
525.9412 472.3811 3258.007 382.7236 479.3908 607.4941 594.7912 336.5129 705.1401
1038.882 973.3752 96.09551 650.273 882.8615 544.7468 1215.488 793.2019 1330.753
601.5128 552.9036 1097.296 190.0909 742.3525 1252.695 2004.219 1029.718 1773.027
462.3852 522.3724 730.7176 755.8202 300.8835 3554.295 1757.672 1288.523 779.3173
432.9044 307.0594 345.0188 111.4392 567.5933 839.1728 1356.433 458.9871 1145.705
1272.213 214.7064 872.8236 1098.131 926.9037 2098.836 953.2431 612.639 1430.593
510.2984 1513.751 354.9918 1557.096 848.3927 1089.007 661.048 155.5841 885.6873
585.8622 229.9087 201.5578 490.606 2754.138 1263.067 292.6267 2367.43 1404.88
841.6342 1509.544 1423.313 318.4142 176.7487 1262.035 251.0551 112.7839 410.6334
215.5785 735.3477 731.2829 785.5654 223.1518 834.3084 807.4517 753.0007 734.2995

1120.586 1465.836 800.5164 53.12638 1663.053 1338.485 1105.655 536.0437 420.8
84.20361 634.2671 881.0777 933.9856 1099.282 1546.36 111.0558 569.317 880.88
2300.459 1109.473 456.9808 1127.409 298.1579 598.4723 729.286 99.67459 224.169
995.6219 2282.522 753.7535 994.0932 2038.063 782.3003 130.9227 1711.606 459.8243
404.8065 953.3684 1614.147 459.9169 604.7114 1116.544 993.0264 1095.865 961.4734
3071.348 1169.034 2289.632 32.55032 886.6783 1149.725 579.4389 1083.092 501.6683
132.3547 829.5399 793.9532 1039.6 144.6495 514.7164 210.794 680.9988 627.4029
485.5995 420.6708 990.1248 959.52 640.4084 220.4466 296.4404 517.6847 869.1418
573.8659 834.0601 392.1709 274.2909 1553.537 109.5714 1730.824 438.3225 484.1603
871.5853 394.0003 553.4825 691.8841 574.1048 454.7822 827.9196 1748.352 1666.207
910.4421 1553.107 668.5931 722.1856 1557.433 1249.416 147.2043 2047.966 1559.238
293.2627 1366.129 462.3106 440.8097 338.4693 448.2189 489.8616 976.0465 724.5487
803.3497 1352.178 1005.118 285.7675 93.84322 2450.558 450.915 350.9232 1293.307
1265.875 173.5019 1681.457 838.9306 213.0444 1483.043 305.2129 902.6286 647.6652
62.94604 909.0871 564.0454 2342.968 815.0247 1362.502 1831.785 1543.154 912.8983
2766.653 2364.714 213.908 126.6593 416.9689 1138.458 841.3779 560.6982 272.1284
437.485 334.3509 1230.888 1361.617 1388.898 623.2352 547.1373 1954.308 1405.287
165.3532 835.6979 953.4295 987.131 1081.611 848.0796 1527.533 914.7939 880.5468
893.9728 827.5888 992.1844 753.4222 1196.791 579.0201 408.8767 69.64072 1255.55
1101.967 2491.832 939.2032 226.9356 1089.248 479.5276 562.1376 171.4522 638.5816
560.0144 700.3541 172.4864 956.6124 1936.289 1738.198 76.5931 688.4984 893.9761
628.4452 1032.929 555.3775 312.8474 2706.423 205.3188 175.2031 1072.159 596.6346
553.6379 456.3571 383.463 838.3922 720.8565 2839.276 939.3775 814.3485 885.0267
382.9552 1199.073 1491.921 1763.466 268.1869 364.0102 915.128 1664.448 782.4396
968.7037 542.6285 501.1764 222.7495 743.2485 1492.06 959.3693 1341.924 1833.114
493.6735 248.5506 1360.065 401.0417 389.728 294.8906 571.1959 135.6037 734.5865
585.9206 3297.222 683.8067 635.1289 323.2803 1241.93 1290.256 937.2972 1516.377
1108.544 892.138 1668.604 1723.941 773.7396 373.1045 217.0702 1910.203 863.635
598.5679 1875.154 131.7319 1154.257 1409.731 695.4703 1325.422 751.0082 826.6637
591.469 404.0022 1012.382 1477.814 35.92347 558.9167 1544.413 344.6833 1065.54
581.8385 902.2858 814.3906 329.1274 858.1474 768.8302 1037.185 249.5703 608.5334
466.6066 1825.593 960.9348 370.5374 491.2077 1035.019 207.5512 935.4529 1371.628
548.1953 823.8532 322.7278 406.3292 1936.124 346.4213 244.0065 4818.262 1628.638
852.194 1603.457 657.9834 571.5746 193.6454 903.5509 121.9484 2175.984 431.4131
822.7432 304.9741 462.2382 278.5793 1941.678 295.1768 82.64261 380.2431 321.414
436.3141 303.4477 1233.276 738.0418 295.5959 1302.162 922.6696 5145.286 1031.412
1649.749 429.8337 985.2521 964.8825 256.0848 474.6194 1368.771 1079.701
CRYAB 19.04249 123.3227 10.33165 659.6879 44.23355 21.0528 71.94639 61.53021
48.11005 80.92704 43.88623 23.52221 22.55859 25.08111 81.9149 12.2786 11.8273
13.84384 11.43434 0.5864417 2.185982 2.369175 1.07516 9.195711 1.192766
8.311332 1.380325 0.2942324 9.03841 5.192933 27.36448 1.717556 1.242649
1.503451 3.743945 2.491191 6.84179 5.377396 2.61886 229.10609 7.176596 2.249425
4.577111 18.47857 0.2089805 0.9010061 2.591476 0.4028507 0.3495137
2.688385 2.765638 1.042295 3.196481 1.269319 2.101996 0.8406355 0.5980129

3.814959 2.064691 19.01852 4.264578 3.763439 1.360731 4.458148 0.7673461
2.130244 18.81555 0.2096354 1.274247 15.85171 0.9153567 0.6553649
0.9656591 3.72055 44.85947 10.1245 3.612451 0.3005682 6.488934 0.3532637
5.172426 2.150529 15.50445 4.011856 3.156264 23.14088 1.602872 3.592126 1.996772
0.7304389 1.560133 8.836247 0.8588078 0.956536 0.3816623 1.14466
1.657632 14.07005 10.67963 2.739572 5.990892 3.009539 0.8454482 1.193321
0.5331177 10.1269 4.546519 0.8380046 7.286334 6.853414 0.5957447
10.38512 1.683441 0.5129967 0.9077826 4.359914 0.9390446 2.106905
14.72728 2.376817 2.180502 0.964149 2.053207 5.467428 0.5437252 9.782986
1.623705 54.99322 8.873787 1.360825 8.634287 3.6232 16.21868 11.20056 3.096732
11.56723 5.670589 1.778387 6.680742 5.889839 0.8993166 3.566975 1.365523
4.311484 6.012736 1.697531 0.7364776 1.73832 1.453093 9.328847 0.8916098
5.181029 4.39362 0.8190546 1.373226 4.893729 0.5822428 4.931992 42.6355
2.044996 19.50533 1.498764 1.563288 4.079591 0.6813889 0.5201866 0.8722188
0.8195986 2.281742 19.2891 6.549714 5.434681 58.09917 2.760756 2.275065
1.187295 1.43033 1.568203 0.2961588 13.15238 0.6979639 0.2130956
10.47168 2.102945 29.31754 32.57262 5.742404 0.3432562 20.42044 3.181763
1.135425 2.187165 3.790395 5.834666 13.48008 11.89515 1.664166 16.48986 4.214257
1.168734 0.1827372 3.195882 0.9714932 11.74421 88.03239 9.079778 9.561322
5.215909 24.44413 0.2001601 0.4161524 2.361216 1.753725 0.8400118
10.70036 22.53615 1.199859 0.6839826 0.8854196 1.75095 0.3860689
3.646482 5.480941 22.4899 0.226338 1.055129 30.35043 0.8764715 3.71782
1.264973 23.75122 0.8113922 8.91714 4.871958 1.048415 15.57761 0.6812991
0.8330775 1.31538 3.409833 0.3419476 51.16087 0.3317667 3.657666
2.777261 2.022778 8.172501 23.3012 12.42662 13.4667 1.674119 0.732807 0.4459419
6.127085 42.40656 1.859298 16.66442 4.914645 1.48748 1.560071 0.3562438
7.804452 0.725573 5.149406 0.9913684 6.598459 0.379253 0.5343394 8.538641
26.79458 5.044856 0.532162 1.526849 3.104666 1.955961 10.02908 5.111926 0.4118759
1.378005 0.9784438 2.079324 1.297643 1.863042 4.60204 4.886247 1.771618
70.76587 9.181723 4.965473 24.5085 13.23482 4.047826 4.271338 1.096367 7.661486
5.647998 15.42488 20.11335 1.432377 7.516048 77.94663 22.98595 15.46103 3.702106
0.6893191 14.8538 1.268183 3.389542 8.897799 0.9808697 11.27095 1.538541
0.7390671 1.522014 4.934169 31.06856 0.6021592 1.809518 16.39941 5.412352
25.34911 6.069285 9.44457 0.8416319 5.869287 1.930033 0.07166438 5.409283
12.18776 3.433055 1.58975 19.84972 1.335167 1.313536 2.597303 24.56826 7.432539
1.933422 10.77004 8.880657 2.556495 2.456967 0.4894275 4.544447 0.4469447
6.52276 4.504556 1.294222 0.5822608 3.023264 2.48574 4.003878 5.673564
12.40408 2.854245 4.247981 106.2585 0.7812839 4.451568 1.116204 0.7791924
1.594226 1.399926 5.038549 1.845975 1.547288 0.8469147 3.115083 42.80894
0.255626 26.06205 6.550423 1.234639 2.451199 0.5820062 2.30589 2.138304
1.206054 25.20564 63.93089 0.5799972 2.16267 0.2659009 1.636849 7.995088
3.463037 61.38418 6.170174 0.8612456 1.144434 15.33895 0.4883135 2.223052
5.628839 3.066841 3.016131 1.742416 4.259477 1.032458 4.736113 4.530967 1.137262
2.573178 9.336579 2.751525 42.57704 7.224612 2.451873 1.093873 0.7295859

0.3638276 20.32076 4.098046 4.48451 9.250963 21.28873 0.8311227 6.913807
0.2907792 1.887316 9.656081 0.707101 1.089786 4.901116 8.664329 1.401559
0.9874903 1.265693 22.27134 3.761899
LPCAT3 5.465083 1.513794 5.901832 2.069624 2.13776 5.407339 3.381463 2.433702
2.182063 2.267852 4.705613 4.447378 6.62037 3.656754 2.122362 6.078843 6.604561
0.7648703 5.358745 6.981615 3.11332 18.49992 6.749265 3.984234 6.646264 9.34353
7.422594 10.45312 1.86266 3.590965 3.500787 2.800103 0.838373 7.322942 5.854254
2.667195 3.808884 1.46278 9.256962 1.88084 0.4981849 1.79829 7.822477
5.096121 8.850428 6.452742 0.8536508 1.586884 8.832865 10.16573 2.679139
7.380611 9.244697 4.66159 1.35467 11.92927 4.050668 3.619533 2.984669 1.814283
4.291904 14.52685 7.327215 4.781927 2.6428 6.23838 0.1732719 5.285226
6.858295 4.27211 6.802664 2.635665 5.249375 4.825412 1.034875 2.346872 6.510193
15.75629 4.254834 2.131068 15.02242 5.973513 3.740221 6.554629 4.047292 4.481234
2.527129 5.211415 0.9628297 3.549069 3.558878 10.94909 6.243889 2.425838
7.664059 4.050184 5.160127 0.6408269 6.49148 0.8679582 3.479563 0.9330532
1.645772 8.228077 6.878565 13.0053 6.277043 9.820261 4.060181 3.195493 3.465339
5.495163 3.976406 6.390429 10.53474 11.52852 0.08175945 10.96658 5.384038
4.021653 6.816453 7.476773 14.64779 5.310791 6.185081 8.476841 1.205377 5.375131
5.104734 1.612237 9.63942 3.971243 7.006206 1.751603 5.407294 5.816394 2.744888
1.845266 3.813692 1.680872 2.165497 6.495201 5.804461 10.91984 4.897067 9.699532
3.883548 0.4300128 1.198976 2.948923 5.445347 1.911859 1.257834 20.80215 11.8968
4.90837 6.809165 2.796174 4.047814 6.408052 11.99776 8.415484 6.959771 15.03146
6.600776 2.820616 7.533437 9.20986 16.54778 12.28785 10.10826 2.446749 6.911102
5.964907 1.730457 4.531034 9.688826 6.832094 9.389954 4.363166 4.307451 11.40838
11.79796 5.250783 2.871583 5.108661 2.177607 3.436399 2.900485 1.287492 1.903696
5.36726 7.02297 5.850475 1.468641 9.852468 1.739932 4.647271 7.046683 2.903108
2.229433 1.798055 4.122173 5.667616 1.34262 6.860741 2.180046 8.294246 5.048414
9.473618 7.956817 5.584729 5.59933 7.864239 1.954472 6.408136 7.349682 3.289199
3.204512 3.840863 2.389492 5.945061 2.126598 2.766176 12.12743 11.42538 3.691193
4.637175 3.40255 2.07507 2.183786 3.183939 3.204098 3.665935 1.973186 2.877296
3.121723 11.48832 3.810003 6.931969 14.04235 1.146961 5.848096 9.713313 4.266262
4.225111 6.89034 6.953503 9.397224 4.35229 0.5660795 12.77044 8.211183
3.745593 1.181979 11.10165 3.705102 9.217585 11.29047 3.571114 5.14125 2.606508
2.495341 7.854253 7.146989 5.417975 12.68445 6.080077 10.00946 0.6788461
3.333867 3.341164 6.948159 13.87444 3.723583 4.981039 2.76641 11.88628 2.232108
8.345128 2.791257 4.476114 5.618131 5.667513 6.594336 1.511363 4.617338 5.963502
6.055472 2.712734 3.385233 1.787503 3.667454 2.743777 7.999158 2.545311 2.805321
4.729724 20.96797 1.72783 4.34477 2.358868 3.587418 1.886997 6.657789 5.672489
0.496787 0.7788928 0.7402924 11.70598 6.535345 12.06121 3.32197 15.94394
11.12128 2.190061 6.128138 4.992147 1.77654 3.969251 2.501858 5.917742 7.162148
3.486617 2.595258 11.57557 4.910658 1.405896 2.829505 4.809646 1.208299 3.117372
7.243101 0.9325872 2.326969 1.578138 14.39856 8.617346 4.130959 3.003978
4.044309 1.03265 6.746972 2.758286 3.839735 2.865509 6.606922 5.622679 11.09465
7.780933 24.52927 2.53738 4.515735 5.401396 6.873674 8.856549 6.838966 3.951792

2.693118 6.116119 12.33774 1.200043 0.4494576 5.374425 6.111601 7.756377
4.337082 5.170848 2.300031 4.103541 5.536617 3.176622 3.737947 1.170802 1.264174
3.365113 5.336651 5.283315 1.409971 3.039123 5.735356 8.480762 3.86642 4.717885
3.618314 2.626147 3.274523 3.119129 11.43663 2.734452 4.954435 10.01196 2.472041
3.95567 4.156818 0.8999374 1.187334 5.584804 4.660797 6.743388 1.880259
4.039221 0.080599 5.533236 3.130987 5.311069 2.868379 5.384625 4.387082 1.753019
2.88131 7.21205 8.3503 5.94286 6.616095 2.177659 2.793834 4.581562 7.285909
5.302425 0.2491721 4.385844 3.968765 1.067273 7.158779 3.881095 11.11417
10.81475 2.4131 3.399186
MT1G 57.33072 0.1623303 0.4567757 0.5485508 1.563609 0.1225404
0.8714952 0.6194879 0.06978171 1.097717 1.726185 2.15268 0.08020832
3.198088 6.667321 0.6372165 0.217197 4.173663 0.7672749 3.099363 1.760223
891.4242 0.6834741 10.20789 0.2144505 1.073046 1.526678 0.3488717
90.23166 11.6402 2.541927 0.7280929 0.6718015 2.521155 1.331473 0.5371908
1.961596 10.99534 1.731739 2.24728 6.309823 14.8291 4.617206 3.437852 0.3458437
0.4256783 23.24684 2.099761 3.453955 0.290011 2.486206 1.807041 0.2466371
0.6287532 1.713114 2.458932 1.94713 2.422825 1.356729 0.4162179 4.036808
0.5190124 1.931442 7.104577 1.536408 3.894122 41.84515 0.08926788 0.2606336
3.798269 1.214073 0.2093028 1.237435 2.420073 3.131661 84.46312 19.93579
0.1013249 0.4999981 0.1880355 1.175855 0.0977484 3.451363 51.35365
7.975296 7.025159 6.156086 6.720127 0.2438524 39.82486 1.080527 4.551387
0.2913815 2.662505 0.2526466 0.6752859 1.84308 12.11163 3.500692
0.1754482 14.39089 0.6148782 2.124074 0.05746883 0.06739496 1.446491
9.283053 196.9464 3.339867 1.77887 0.7442604 5.192929 0.6080427 0.276699
0.2295177 0.7762899 8.78863 3.014613 3.384478 12.28023 4.08545 0.2100164
0.5191192 7.866945 3.880507 0.2729341 1.025451 1.548038 1.683975 0.833502
0.2069501 4.450253 1.605553 98.19712 2.864874 2.786954 4.537468 12.84495
1.811621 1.929442 2.232431 14.39371 0.7945476 12.70281 1.550028 0.4777098
2.166762 0.02882001 0.2645211 0.41562 0.2634223 1.848446 0.3529066
1.057452 0.9855924 0.1243125 27.46257 18.12243 14.91366 0.6016502
2.950801 5.254598 4.45693 2.079794 0.9528452 1.302679 3.078716 2.021677
0.4381523 6.683757 2.656163 36.67879 5.448098 6.911901 0.3159965 0.06812771
5.369732 4.844924 0.628491 17.9797 0.2091479 0.5028582 5.298321 0.7706794
3.699846 0.6872149 2.824484 0.2524702 0.8521115 0.152323 26.8254
1.028815 2.002395 1.565027 6.428806 11.18753 0.1251321 2.472891 1.658731
12.09352 6.852121 0.3078192 1.32317 2.919091 2.18655 310.14793 1.484788
1.582507 1.50764 0.8619541 0.7130012 0.7912821 0.2415115 0.1359251
0.8039528 18.20016 1.426345 0.09022623 1.628099 9.744483 0.5639751
10.25458 5.94793 21.15924 0.7121433 0.3603153 0.4674275 1.486188
26.93338 0.4397625 6.365239 0.5014704 0.4859549 1.034824 0.3412452
3.502408 6.549948 0.5215594 2.197867 2.736444 0.2979398 4.594217 0.7359942
0.3119152 2.160567 12.89996 10.26586 18.34335 4.055223 10.6062 10.6989
1.309873 0.780101 0.5043684 1.007793 0.06241892 2.765536 2.669921 0.2782591
1.316801 11.22188 8.234026 0.3979911 4.775121 1.124132 1.054679 0.1334091

18.53238 2.327997 31.80511 19.98295 0.8611091 1.668837 0.3942415 1.318752
6.231729 6.837288 0.1257491 3.949763 4.21627 8.030659 2.88076 8 19.20444
0.3125953 2.011328 6.5013 1.984766 1.166949 4.396996 1.195501 5.413085
17.86865 0.738904 5.458673 5.393178 27.29016 2.451818 0.439812 0.3762625
6.722386 0.2335862 4.558387 3.401707 0.3006182 1.684734 0.9262206
0.1935933 6.125081 89.18525 234.6298 5.409548 0.5635825 0.2110428
10.95245 7.108877 0.7230069 2.849584 8.065068 1.72578 2 18.64053 2.910435
4.160198 30.08782 1.757255 14.35967 2.925285 1.076795 19.79832 11.34374 0.7633915
12.52089 6.396512 8.487594 0.3456059 3.737653 6.532465 23.80615 1.552765
4.509074 11.9306 25.24804 1.054163 2.010833 2.701942 0.4675967 27.48401
4.113961 1.491141 0.8947016 3.753551 6.189915 17.40576 0.5871303 4.149853
2.082132 2.748453 1.003831 0.1940691 27.20738 0.5590515 2.328251 0.1207711
0.8494749 1.642482 0.4766544 16.21726 0.932836 3.038671 30.02072 0.2068185
2.555774 8.22013 4.439577 19.27515 13.34165 11.99775 8.745736 0.2891195 107.819
0.7697076 0.3351832 0.3203756 0.971562 8.750731 46.42955 29.49737
1.369927 1.744128 0.2570183 3.021434 17.5362 5.569505 5.768096 0.9030805
2.570963 2.586136 0.1150801 6.010215 4.134449 12.26263 16.1407 12.45038
7.701235 7.516229 2.634955 10.42529 13.53054 4.614924 0.94305 0.1586784
0.4906014 0.3095558 0.8735461 1.128409 6.423754 8.992449 0.4956185
2.612988 0.1022869 3.523763 5.731537 0.3347346 26.83743 7.413443 0.2721931
1.284558 2.656392 1.65823 3.808634 1.02914
NCOA4 51.35263 52.70312 37.42868 51.65648 36.03317 44.61768 59.65698 48.84219 55.0628
54.2181 46.60822 56.32257 59.85391 43.04052 60.16291 53.80176 56.52613 18.2782
37.82093 45.43879 19.41128 71.43266 63.345 52.63649 39.91374 36.05413 38.36472
31.87707 49.66869 35.19104 40.78125 36.47477 20.93505 57.30477 42.13284 38.81365
80.27264 17.04625 47.82742 29.12464 18.56886 24.78256 41.07057 38.61636 38.46476
33.13708 16.0995 20.23175 44.20634 27.59513 29.72903 54.8349 40.23736 30.34709
24.58624 32.24965 15.28078 55.96594 18.39766 19.07348 33.44291 55.69406 54.24633
29.46731 32.65436 22.91222 10.79594 43.935 59.95616 24.58524 76.85806 25.54069
20.79404 57.88947 24.23807 12.2866 50.19006 70.13748 16.22123 42.52579 52.98077
38.51584 48.5063 55.73221 86.53913 76.00743 33.95023 38.72826 14.83537 11.21221
14.41748 39.22176 57.13664 47.86177 47.63576 20.01699 46.47766 22.57979 75.79373
10.7854 20.97793 10.36052 39.23379 63.65388 24.6308 51.91527 49.13924 81.45062
18.25719 26.21414 53.91737 53.77939 57.44282 36.31838 33.95782 61.47325 6.641389
54.32572 27.00595 37.3306 18.24187 70.26351 32.88523 30.5833 20.30361 74.88011
21.87159 50.68884 26.32612 40.85845 72.61517 31.50104 47.634 22.14143 43.58702
36.9205 20.4403 17.89188 22.60167 29.73415 36.12931 15.72751 61.04921 37.87949
29.95804 86.28407 88.46957 6.226436 34.00815 38.04161 35.88295 21.89428 33.98384
58.36206 35.06597 35.61146 42.21588 25.67925 33.4187 27.02558 42.02229 44.5918
38.39419 44.3495 36.59142 27.05983 40.52236 61.51269 34.01884 43.10643 38.57232
21.64426 44.28654 41.6916 23.45135 67.88136 67.8675 2 33.97948 43.55519 25.74512
50.93942 56.66466 61.12495 61.97903 16.25648 59.13051 19.46442 20.94763 24.58081
15.41808 25.97547 20.58135 26.80131 31.37752 29.04612 40.11217 19.86959 34.61235
32.97266 38.378 22.70416 12.25919 59.55509 48.25117 36.16229 41.31963 31.51854

56.19778 57.22629 34.29547 30.99752 43.35659 36.24671 119.1511 20.09597 58.05765
26.33169 31.96296 38.76236 64.25293 19.95774 64.1169 47.83852 28.99315 34.49354
51.63475 37.47212 23.22738 46.10825 28.82238 36.77007 22.43926 43.93361 36.28999
24.04002 42.55694 47.42869 31.86778 36.5621 49.2467 54.15569 30.07411 30.9897
41.03852 24.94795 36.91487 38.37551 43.18953 40.74497 39.37227 24.4475 56.41464
31.62317 30.15956 21.7935 47.14702 29.47873 41.51297 44.42237 41.45833 77.48641
37.79324 34.91051 34.51875 27.92025 53.88925 23.01503 41.44552 34.94456 19.36064
20.74772 28.70188 33.45687 56.36509 32.80436 32.26994 22.10511 86.84942 21.19995
44.02254 25.61556 18.73311 36.90711 36.67637 31.86403 13.93047 33.2263 32.45169
31.77459 41.29025 31.03367 41.85557 131.0956 19.91674 33.3164 35.83883 25.69631
42.70821 51.11229 22.20895 36.06558 28.75367 22.5026 27.25523 51.27222 19.65934
15.16971 20.88608 14.24664 123.9845 68.0701 48.44249 41.69505 21.69111 39.03634
32.56987 59.55272 53.31935 26.01533 20.86362 42.31409 56.37925 45.42785 53.78066
39.09464 40.74919 41.82893 36.67113 29.94101 20.09361 23.60116 37.86236 40.62688
15.48935 19.57098 13.71383 45.06378 48.89005 35.01052 49.26982 30.99307 33.90319
48.69616 35.59443 32.26979 25.21439 39.43661 13.10552 27.22432 51.25467 27.02734
37.82106 96.77659 29.48466 45.69703 25.65531 48.55242 30.50405 20.93186 35.89023
41.07709 19.54573 19.4861 19.22982 39.39011 17.5118 45.92973 34.62808 49.6788
47.12141 38.57831 23.65346 45.33121 24.93661 19.66764 35.54446 35.25443 18.06775
32.53402 31.3478 56.86363 54.72462 52.55199 77.06292 32.15186 74.29921 45.56184
53.71208 39.54935 27.11427 37.8104 45.61847 47.94267 42.01018 24.58691 20.63588
23.63528 33.50841 19.68751 46.28395 25.39202 13.48164 6.761084 37.83365 43.16448
30.34987 24.63516 34.20121 38.88767 13.37427 70.81185 47.03606 49.74865 44.84736
27.39856 12.68336 35.24955 38.48465 62.88704 29.17752 19.05655 44.75039 30.53038
19.67777 48.50399 20.53223 21.17015 32.60253 24.24527 28.04579
PTGS2 17.15012 5.609083 23.66669 225.8246 9.116356 115.1912 17.43137 50.58396
13.40937 213.1877 10.90199 89.1625 30.42634 38.2145 35.48068 135.0019 7.097488
51.03211 100.6436 5.928557 102.3929 1.398326 30.43022 1.838443 5.067202 5.942295
1.42889 7.679634 5.482896 0.08186986 6.782408 2.777504 1.018907 5.575376
25.32995 1.327429 0.3863147 0.9570743 1.420995 13.63862 6.533713 29.20068
4.613969 15.14156 92.4777 0.7952686 0.2081355 1.949439 2.366211 47.48662
11.74527 30.42316 12.69864 9.005735 3.58235 6.523578 1.020447 2.602462 11.92211
10.69605 32.60625 3.468127 4.49961 16.49164 0.629307 1.31389 3.140607 7.293571
1.026409 16.74335 10.196 21.86776 3.412261 1.684639 7.200047 0.9869701
39.31264 2.822058 1.79113 0.3211841 1.321822 1.600076 4.1267 20.09891
6.369237 9.559762 40.10513 1.242464 0.6030938 2.719178 0.2936265 75.83248
2.909688 43.47983 12.85849 0.5698635 8.400709 3.556325 159.3784 1.040569
1.144115 7.887572 1.275998 0.9529966 6.126814 1.930279 23.70417 2.180712
11.98318 1.525424 0.2774606 0.6659206 17.33308 28.91768 6.691907 1.524881
1.530517 46.61262 5.820006 7.155246 116.3525 24.80787 55.57148 0.1603527
2.672705 28.81374 1.021753 12.45441 7.863344 0.3007445 4.880156 4.317598
17.96538 7.383286 7.043678 13.30661 1.499859 151.2449 0.7567 3.276526 8.729812
15.76219 22.0944 4.664605 40.86823 2.476267 11.93204 0.3692068 6.032761
62.85178 1.589256 11.55993 0.168967 101.5607 2.576256 8.2237 0.7097686

22.23488 4.132247 0.3554064 12.81878 3.773312 119.1059 28.53154 10.65663 10.7452
2.442274 13.48776 2.615276 27.75812 3.715218 7.47426 43.14997 3.557408 10.47462
0.3636538 8.523153 5.456127 5.979886 10.04989 7.963616 1.053217 6.083428
15.31581 22.58756 23.61255 0.2174312 1.194909 8.493757 4.802553 16.27693
2.250351 23.59851 1.204772 0.1220119 15.56001 8.374991 2.003337 1.010014
1.788713 0.607061 20.52323 78.47145 8.334273 8.059433 5.332892 54.18427 5.790434
2.607543 1.82923 9.946021 3.515749 105.8067 1.170545 1.234707 16.13266 1.412111
3.476167 11.94489 5.210176 66.19794 1.377698 16.49272 1.849451 2.283191 20.602
17.66493 0.456337 0.5167444 11.24464 4.809964 2.602137 15.11138 8.034486
4.191201 1.868573 13.81548 22.0378 5.16612 0.5882409 10.73865 7.409317
3.675945 19.6686 5.798143 20.87657 15.92463 10.34304 1.803507 5.832959 1.256267
11.38326 0.6141963 3.651177 16.16615 1.239434 35.01697 11.2282 0.2772557
1.190905 3.937874 1.048146 7.977149 15.06745 10.41802 62.26643 0.4035315
0.5520208 29.27669 15.17698 2.622509 2.37313 50.61905 1.106309 0.2382522
1.568765 0.3816601 4.3227 8.05446 1.451389 19.6315 30.40324 4.752011
1.888584 1.447936 4.036819 39.47714 6.461923 7.028735 9.21625 1.267341 20.9038
257.7688 10.92654 9.306822 0.8619699 1.366904 26.59876 31.91411 9.998935
6.510783 4.35067 26.14344 3.178174 3.320106 5.031897 5.322988 7.009526 14.43795
2.704219 2.835577 15.54901 6.098171 2.547761 103.2008 5.625979 5.174869 1.549106
4.027957 19.34127 3.87914 5.342191 2.636439 4.296003 7.973564 45.92909 9.638522
6.051569 10.47102 11.50988 0.653865 18.3435 51.60227 0.3997031 3.274909
7.735077 0.2331616 35.62575 16.96621 2.55819 13.84909 6.778049 43.99074
12.18654 2.382799 3.503066 2.735047 9.886225 5.93104 8.937823 4.830776 5.806805
174.8809 15.83072 2.906869 7.353421 29.07381 38.2516 1.859169 0.7774013
0.2906192 8.595397 4.008547 0.1042215 2.0279 18.22925 120.7276 3.819626
102.2661 0.3154174 1.633641 76.23226 0.161419 0.1683837 0.488216 2.266613
41.39415 0.735625 1.995908 41.61976 18.86479 6.949182 30.56793 7.816042 2.166672
10.7765 12.00959 40.86404 7.085197 0.1560763 1.584087 13.48107 1.537588
0.7437321 1.417016 0.5568931 19.21459 27.70665 22.97574 34.38583 5.498423
9.659172 5.577416 4.85765 6.144752 38.06109 3.359737 16.17242 0.9654639
0.3478337 2.094995 18.9519 54.38456 92.5665 10.38018 14.86773 10.4391
19.75748 21.48286 0.5651129 0.9509454 30.87079 0.3939185 7.484872
0.7629689 26.53482 7.934667 33.10713 7.079562 8.630435
RPL8 696.8171 356.9255 690.0825 400.6832 662.1621 826.222 626.4418 648.59
342.8766 426.7062 832.1414 560.2809 542.9038 1450.326 355.1333 670.3833 1245.225
1356.594 1341.274 1722.613 774.1522 599.9259 455.5511 681.6851 1219.712 1160.094
478.4628 300.9628 946.7741 877.5299 782.0549 461.0934 1550.503 673.2604 429.8995
632.8725 408.3231 576.0853 348.0299 880.5687 3128.059 1177.765 578.351 715.2283
548.6939 400.2748 1041.695 1440.205 737.3155 700.7816 1737.302 925.3112 617.1721
617.5957 1574.815 1092.593 1215.901 516.0718 588.6005 1563.553 641.4549 562.0431
879.0453 605.2912 2105.261 604.5299 1788.045 1376.137 940.1541 827.1534 1134.714
2390.114 793.6048 469.5209 1419.551 554.3275 689.5901 573.2552 435.9544 1990.01
552.0762 1549.758 578.0331 1223.014 2362.929 648.9438 824.9994 577.5501 1335.848
1370.346 1363.679 639.6641 2050.223 720.0232 691.5893 875.8319 1222.9 1493.193

1153.074 756.9506 1147.76 296.3633 1211.816 683.028 1670.046 1137.773 694.0064
137.1261 430.2549 441.9996 768.1079 530.487 941.7339 486.7956 787.2064 752.6057
3299.566 973.139 415.9421 2844.213 1092.232 447.1377 1773.047 501.1082 892.3658
833.8353 1645.823 848.0283 631.2696 2250.73 881.0075 841.9925 577.83 1141.207
709.5204 583.6453 1074.785 652.3944 609.6111 907.1516 1301.871 810.4201 1032.884
552.7728 713.1403 887.4085 116.8404 481.437 1874.783 744.2899 943.7335 801.1362
1216.91 1254.591 1203.91 1135.471 805.7584 719.2326 1063.083 1282.432 572.3965
715.8447 1218.21 1057.916 732.9809 588.9836 749.5708 944.5181 808.4712 624.4961
831.2171 2192.247 431.0324 820.5112 1252.167 1430.126 239.0546 552.0014 642.1
468.3064 357.3962 519.1621 441.7574 474.4633 851.3766 381.7242 1867.982 1533.962
482.1988 955.5418 1147.296 329.8381 752.6278 666.6985 737.851 1084.212 655.9524
935.792 1091.464 1896.478 840.0324 1743.909 604.3535 804.7531 543.6532 569.1211
704.8532 691.0182 742.5109 891.3707 828.5391 692.6533 1452.158 683.6824 675.7831
807.2819 624.4038 1272.769 768.6958 371.5468 618.8214 704.7096 739.7032 1578.554
1280.944 685.0413 1715.439 552.6149 884.6233 1038.323 1279.588 1207.856 411.7336
502.8966 1233.861 1149.769 623.4434 1131.224 562.4897 1156.252 873.5557 778.8251
618.1545 718.1978 1140.777 518.2172 732.6596 787.4629 636.6875 580.93 1658.625
586.8418 1634.957 1036.274 1564.416 2319.004 891.8914 506.2248 644.8114 1192.057
810.1138 1784.481 1519.185 2003.392 1174.479 515.813 420.5608 1423.457 845.159
1203.558 1016.655 1161.69 898.5215 851.1226 594.5415 1066.256 1424.885 859.3886
541.6703 773.5203 624.422 643.9252 792.8273 843.8795 1583.658 1110.602 567.8358
953.2322 1176.833 489.2289 560.4045 761.3479 722.7495 1075.707 1006.446 457.0188
590.8453 1083.462 575.3103 1285.026 1382.057 650.5098 603.9895 646.5526 700.5318
345.6971 2488.097 1042.017 1053.787 414.4101 1508.598 665.7848 1162.91 669.6882
562.2384 747.338 920.6523 1133.562 1131.297 1195.421 739.0606 555.6849 1116.344
656.5976 874.065 759.04 953.5803 1369.023 407.9057 1058.228 1299.21 737.1396
736.8212 1420.582 2131.824 1044.412 335.4096 818.636 677.6112 1382.333 930.3107
755.7497 779.6877 752.2859 643.3082 930.6432 778.7109 529.1397 569.9831 1309.567
834.157 1595.584 248.4964 420.7757 970.1246 999.5286 910.2442 368.9598 1059.98
1029.585 672.1831 1150.365 1236.378 597.3392 983.3159 643.1572 463.093 579.6392
1318.493 1156.821 660.8489 443.6319 997.1381 1072.003 1933.452 766.2886 710.4055
799.4141 1683.313 870.2827 542.1304 1084.166 1445.229 1009.128 1567.42 1313.95
479.5427 676.9301 779.636 668.6851 1204.805 552.7004 566.0107 1037.694 931.3674
2245.731 1167.304 617.0438 1023.436 725.248 1887.043 306.1997 2352.128 915.6064
380.7124 652.2981 987.0378 713.2087 1256.244 1467.716 284.9533 862.6413 331.6967
513.5672 708.5783 1299.658 1415.289 866.3147 788.7642 477.3239 1018.372 761.7048
1221.195 1068.497 1328.633 590.9135 568.2105 1034.368 1002.531 1061.541
SAT1 95.54275 10.49087 118.5322 55.09748 87.33178 79.89287 39.85922 146.0806
17.52574 75.39627 85.32306 65.03606 58.9642 165.0005 27.70967 156.3642 86.06023
212.8905 101.2824 15.3369 183.7383 125.594 85.43426 58.15416 48.84533 43.3781
91.75562 206.2374 137.5161 160.3194 178.8256 83.93721 57.06771 105.6143 117.0202
24.99292 60.42789 113.9878 105.5168 86.79401 76.70119 365.7573 101.0846 86.89943
162.5036 39.30026 28.67008 35.78412 28.41788 100.4822 57.07093 92.87591 77.37206
93.42887 89.83281 33.39495 47.1317 298.1115 133.94 83.33265 71.21142 128.29

72.65136 369.0433 28.44944 66.10153 117.492 39.67337 39.83061 119.8214 75.03511
116.5703 55.41592 211.3619 44.10929 221.559 46.61127 34.87352 94.83384 69.54225
36.94236 47.89593 92.22516 54.99391 83.5659 89.61718 72.98775 167.5843 201.4857
12.05309 61.63197 113.4267 76.37391 109.831 282.744 62.70705 51.45133 101.0442
56.83082 75.18708 85.54302 300.0079 98.39456 150.8039 65.19612 77.87058 156.8438
88.33474 195.6899 206.1842 106.1739 63.62919 72.69494 37.42581 77.44385 87.4305
47.09064 31.81043 130.9945 56.40937 97.76691 38.43674 58.55768 93.39658 18.78094
84.26757 137.39 50.8647 106.2864 58.72746 70.41935 69.55929 190.1325 258.0463
64.2454 51.7962 81.9366 252.0522 47.7046 36.19926 67.50442 207.0581 49.76714
268.9182 143.9101 159.2047 162.2306 43.10022 91.61522 266.5425 22.99693 135.1997
37.59538 44.44853 34.71614 75.04845 65.34604 132.0476 623.0472 35.59503 65.75616
91.06835 292.6569 83.99095 53.97762 66.7907 78.36239 133.3493 269.1795 129.9057
188.4705 293.7485 138.0482 147.0792 89.75161 21.28309 120.8236 67.7734 150.3453
158.2038 76.8135 76.71851 38.83436 146.2378 164.6116 74.79828 111.8093 61.31409
59.8519 113.1218 160.0661 39.90881 118.5187 46.19249 61.83407 108.3563 163.3379
147.3923 43.69415 269.6772 106.8848 24.33411 135.5932 120.1469 158.4735 173.407
52.28039 91.99862 56.69338 58.58923 64.64368 143.7666 56.35048 60.28684 155.5497
116.7299 412.7462 71.1281 124.0068 206.6296 173.8087 195.2674 296.9126 52.37314
99.39298 44.87021 58.81437 136.7389 84.15549 27.72722 250.448 33.64367 76.83486
77.8999 80.8374 135.0176 122.0492 57.63831 468.286 91.91751 41.20612 405.2799
305.8749 45.16247 104.0238 166.2681 90.37878 148.3744 37.48567 136.1937 94.05268
15.11009 45.27721 25.20255 134.3454 19.26541 179.0227 40.86818 52.87327 69.06203
48.60243 72.39266 80.91912 72.59826 68.6695 127.0313 68.75402 220.9727 76.76308
288.2758 229.7922 73.9495 90.52914 45.08097 323.2489 72.19788 64.44272 20.75577
54.4915 114.2728 97.00735 80.38011 239.689 61.87043 32.44995 38.82385 102.9429
135.0529 171.3542 79.69224 244.934 314.2426 68.16727 186.6312 143.8293 69.31069
49.56025 109.7494 234.0494 131.7655 70.71214 494.3296 133.7786 91.27512 228.3712
191.8232 64.59568 300.2891 195.2908 132.5272 26.07685 192.4434 77.0078 213.2817
54.94882 90.65625 93.94597 134.1137 510.712 489.1956 76.55087 100.0154 134.2052
20.97204 252.2887 256.287 75.06709 224.0514 98.89488 44.29746 50.83297 116.0575
199.2578 69.97636 315.6232 167.541 195.2793 33.74789 293.5276 51.79662 189.8328
40.57557 198.9532 236.5138 168.6848 233.2703 138.3252 272.9878 27.52588 34.77924
247.3462 222.6364 71.90626 157.5092 54.94285 70.18171 78.14737 167.3937 179.8102
64.13151 33.22027 67.4297 84.77591 52.04698 52.61707 46.3523 133.1934 55.58512
423.3968 107.3271 229.0166 113.7681 36.25741 78.40892 71.91933 251.7961 68.32158
239.0075 176.0309 342.0382 307.7768 122.553 65.19539 91.80282 47.90862 373.2904
123.0897 127.8306 97.61653 215.9251 163.3635 61.68155 160.2401 43.42776 70.89872
43.45201 38.78424 102.1445 60.76172 143.704 45.09325 73.70888 126.702 87.23082
37.16782 210.2079 299.6344 106.3489 133.6319 19.51401 94.41818 392.1546 81.02507
426.0807 84.85413 89.99649 345.669 117.1265 58.87227 178.0432 143.6954 71.20937
147.3183 168.4242 31.1743 171.4862 100.8573 49.90029 78.70005 92.94002
SLC7A11 0.2655803 0.1861875 1.864753 0.2723395 0.7360069 2.858641
0.3011907 0.3107074 0.103099 0.3694444 13.14861 1.208372 0.6907521
3.653247 0.2507741 13.05897 0.1954338 0.8025139 1.293849 0.6486389

0.1433937 0.4790986 1.431756 12.16893 0.1834336 0.1549611 0.4960019
0.7212974 0.3737643 0.408914 4.195463 0.1860268 0.4935744 1.356155
0.7661664 0.1604053 0.505663 0.4521058 0.5386436 0.9531797
0.8872466 0.0443507 0.3846817 0.3424463 3.714219 1.412997 2.942789
0.6847949 2.34184 0.4998894 0.4425077 0.951088 0.6822111 1.723449
2.392983 1.409649 1.383335 0.9230019 0.4731011 0.09709592 0.2191407
3.18385 1.474364 0.1301458 1.172774 0.6757125 0.04887281 0.5622627
0.09007555 0.4453403 2.346012 0.2332821 0.8150703 74.72534 0.3916249
0.4545987 0.301626 2.744544 0.1209603 0.2339477 0.5251655 5.058442
19.01836 0.5384351 1.335736 1.520843 1.469115 0.3702273 0.3650201
0.4507631 0.2329709 0.1085126 0.4845319 0.4361594 0.5588174
7.55402 0.8934557 0.4039989 0.09287355 0.1614415 0.7402413
1.208718 0.5402989 1.269141 0.3441137 0.1888054 0.3550106 1.287627
0.591561 0.03036421 1.176772 0.1073175 0.4097748 6.029161 0.6038374
0.1569482 0.5181887 0.9461955 0.07353522 0.1469103 1.700346
2.878918 0.5366432 6.632753 0.3587628 1.240395 0.2090469 0.1454546
0.1533035 3.184907 0.329003 2.554945 0.2475811 0.984634 0.6905994 0.37992
0.06664676 1.829252 3.064556 0.3244929 0.02732513 0.2149206 1.963862
0.9078671 0.7625902 0.7181745 259.6162 0.06723175 0.6467899
0.736869 0.7689321 0.1159162 0.0224527 1.789829 0.4459063 0.3310811
1.030731 3.411423 14.2483 0.2175485 0.3022638 0.4583656 9.891385
0.3024483 0.9552797 0.2571385 0.4357663 0.2248054 0.1476407
0.7451584 25.50655 4.984276 1.823599 0.9561359 1.025883 3.989127 0.05112894
2.158471 0.4826087 0.4640703 1.191297 1.027837 0.3905214 0.3664853
0.6865163 0.5086522 0.2487683 0.4196384 0.1159561 0.2191271
1.448512 0.5066743 16.3002 0.26078 0.1627149 0.2148666 0.8095637
0.9614662 46.762 1.042952 0.3006809 1.811969 0.795481 46.05265 0.2013483
1.184991 0.0923663 1.537 1.808307 0.3879383 1.943597 37.56876 0.4295938
0.3646514 0.06251577 19.75694 0.3128749 0.5355574 3.309732 3.139191
0.1146977 0.1833596 0.6411085 0.4495262 0.1081162 0.6767496
0.2362581 0.3780682 7.49558 0.6124219 0.2726644 0.3669036
0.1714998 0.4096574 0.3752863 0.224532 0.3915866 0.5646743
1.601131 50.64336 0.372342 0.1872954 0.1817935 0.4487117 0.8512342
0.1908898 0.2188828 0.34356 0.3850879 4.979698 0.1624688 0.8202116
0.08151328 0.1736883 0.5746873 0.05581785 0.4952057 0.1464423
0.1298254 143.1583 0.4923406 0.1719614 0.2359784 0.5083787
0.06142538 1.027391 0.1867313 0.6673987 2.538892 0.2369973 1.63764
3.982276 1.409566 0.2058359 0.2622833 0.7621028 1.055523 1.036501
0.2875312 2.089267 1.392095 1.490911 3.080028 23.81872 0.3074105 0.2388398
0.2315046 25.73547 1.259419 0.3274823 0.3599707 0.2166866 0.1745825
0.9550555 1.990675 0.813212 0.5020571 0.765225 0.1380994 0.6474254
0.2708054 0.971186 0.2184281 1.24319 1.707592 0.5266709 0.2132637
0.1894333 0.1957934 1.159398 0.6042659 1.62341 1.020204 0.2639404
6.79118 0.4825668 0.5539627 1.480966 3.418093 1.129853 1.695907 0.9580549

1.050064 1.281047 0.2183481 1.179008 0.7568094 0.6380935 0.3341269
0.2146154 48.49784 0.6555892 4.841663 0.4131952 0.7039989 0.1172233
0.07076369 6.426254 1.519763 32.72248 1.682508 0.1662865 0.7409219
14.67435 0.4678391 0.5728916 1.068855 0.4509239 113.0495 0.7770526
0.451824 0.9164189 0.543716 0.0484042 0.1962517 66.51289 0.5916006
0.07114286 0.5044516 0.2140166 1.295175 0.6761677 0.4223554
0.09848307 0.07412977 0.1880532 0.4813871 0.5629228 0.7078395
0.4617919 2.54235 0.3533138 0.7598675 2.280734 0.1627378 0.7375685
0.3511851 0.08149753 0.8151172 0.6658629 0.6290114 0.2184641
0.3210839 0.9718509 0.5831865 16.5819 0.3383774 0.7625098
0.3664076 0.2766326 0.7051216 0.6208299 60.52709 13.92441 2.075907
0.4640751 0.4988891 0.1384019 0.6101878 0.4805402 0.1489112
1.788726 0.4540745 0.3581684 13.8314 4.874164 0.2267949 1.478284
1.903812 0.2443104 97.89138 0.4693887 0.2354816 2.535623 0.5354642
0.2002147 0.2229044 0.1335768 0.3817861 0.03653461 0.4428484
1.583044 1.95177 0.4088936 0.09931679 0.630327 3.027479 0.5270902
0.2463863 0.1379179

FDFT1 32.983 14.5456 24.46213 10.88365 17.43355 30.26851 17.2231 21.96252
11.66047 10.89299 29.73495 26.6573 25.45828 22.00152 14.68538 20.01867 42.30134
44.80768 38.5597 40.1808 23.43846 42.71708 62.00262 61.10298 29.01312 43.77096
40.63276 35.33335 38.13859 16.1347 14.63418 26.8521 45.66984 41.66625 43.4178
6.33848 20.66478 31.43141 42.56942 13.66701 11.71324 39.84062 36.54487 21.15334
34.29406 45.62647 12.3131 51.7494 38.79837 19.38006 21.79373 19.39989 47.66428
28.30568 20.25308 48.83288 49.7782 19.491 33.07589 31.49053 21.19937 32.71265
19.83608 22.32295 38.60639 21.29299 11.303 32.11443 31.31411 21.86616 52.89252
52.45647 29.07344 18.22482 16.1698 58.93223 46.49521 43.09521 47.72585 27.95048
40.46968 35.71847 12.62098 35.66842 13.0518 28.53279 7.495533 11.47636 30.20028
36.20078 60.41283 26.75244 26.45662 34.06184 74.31996 32.34424 31.08437 18.6118
28.84586 20.26997 17.54957 34.05588 30.34362 91.95344 37.48375 40.03009 13.26476
30.81979 78.02638 61.89665 73.15065 53.00538 23.85066 65.98615 38.0158 38.63542
35.32109 16.38961 34.93565 21.17312 120.9769 158.1568 55.69727 46.21605 24.51627
33.48715 34.8354 86.33223 22.22396 33.90483 25.18582 6.553427 23.84255 24.3194
19.41307 19.37617 40.916 10.88185 93.71622 39.97998 46.98581 33.88142 42.0741
79.5212 15.43702 136.4279 10.36756 12.07066 34.01433 42.43087 65.68905 27.93094
114.134 114.913 48.34565 41.47783 5.834371 17.16176 25.40826 70.92116 68.3024
53.09883 19.60735 61.22105 103.9599 38.59341 35.86409 51.88853 83.28852 14.29969
27.83057 30.32607 11.9794 11.23451 63.87927 28.3669 30.48068 16.71813 67.14131
7.867833 46.9158 66.35797 36.52539 27.43208 29.48561 25.14783 44.79014 35.59917
9.937478 28.73076 17.15339 41.16646 39.35361 28.54555 10.91532 15.37661 19.35073
16.15388 26.68495 5.700739 16.40499 17.8363 41.06364 58.14589 20.25047 9.687371
39.50659 19.80952 13.65525 23.14858 28.63472 42.82607 38.21278 33.04062 50.95166
31.61698 68.61749 21.68499 9.912383 8.184509 27.62708 17.03783 20.36032 29.04177
17.5865 76.27249 16.60077 54.91169 11.68926 45.15439 12.10412 24.57531 23.12069
28.59817 33.68032 23.40551 10.05662 53.13465 18.44564 70.29838 24.83752 8.032073

39.42692 34.51203 9.282712 41.25688 18.54879 24.64082 17.55606 19.07964 29.69217
49.80704 18.07273 12.59051 33.54385 28.61317 43.3656 18.52164 29.30126 46.03403
37.50162 37.56122 45.96537 21.70249 64.23309 48.48736 72.64795 41.84772 22.61792
22.21174 66.2396 27.08899 61.74978 63.35212 54.43612 32.4086 22.32958 77.94504
29.6603 77.24445 35.20148 24.09408 79.91305 39.9858 33.24148 40.46743 17.39729
38.72923 6.275532 30.75872 12.38885 21.69904 28.43987 29.46353 36.22233 13.4005
46.57547 47.47034 94.40629 15.96071 81.15938 33.6885 36.5751 16.93479 12.29562
92.93421 33.58203 12.05211 31.03816 47.42483 31.40739 57.1927 34.97602 37.28889
32.36669 43.30727 24.61062 10.67053 9.905036 33.34069 12.40855 30.75887 20.0809
74.42555 10.04921 48.57595 83.02741 7.838413 12.20598 11.67929 35.31312 35.37148
29.81691 41.31973 11.87326 28.10454 19.80109 21.47498 14.5049 31.47011 17.11784
59.22611 38.52268 15.2766 56.74171 17.98952 11.41604 38.23401 98.3144 40.17197
59.49895 21.38722 29.09641 13.40206 10.95246 58.79253 25.46826 41.73413 13.95757
41.32994 36.97603 17.26943 33.46119 36.69342 163.3975 11.28557 17.60622 18.28107
17.8808 16.46386 21.30229 10.31122 68.49525 26.14469 7.761198 10.63225 22.64487
67.83012 17.17391 15.5362 57.54558 17.51321 36.58466 73.9026 33.43796 62.94194
13.36095 20.75944 28.47524 15.39412 28.21633 98.70389 16.3186 25.30877 56.38486
14.82824 15.82849 10.03383 29.85902 13.47064 48.64014 32.67964 24.2564 25.34665
25.38553 21.56774 12.64099 30.91929 160.0093 33.70772 11.38795 47.41226 52.6417
19.79379 12.42931 68.66406 33.64223 25.67334 118.0885 29.38502 49.59656 44.5488
26.23141 31.79786 16.12305 72.08174 25.90565 51.05318 12.28348 24.11909
TFRC 15.84653 4.090674 14.91384 14.31285 16.18525 13.33367 7.581368 4.910072
7.657674 16.2988 9.331485 13.37941 12.36761 21.81026 4.810258 25.27718 6.399775
20.05361 12.80807 16.03359 28.35984 43.70081 26.38746 38.27833 53.59478 56.21155
89.14243 29.69387 65.79055 11.11332 24.89005 17.59205 2.467235 36.55241 18.22292
7.259943 22.40458 12.90052 19.26716 8.146542 8.959902 52.71778 18.46313 13.6062
20.64962 54.30525 6.459807 52.4246 330.5585 27.77112 26.05888 33.94105 47.66088
10.81205 8.743582 24.19868 41.31005 59.91215 25.79709 19.48224 41.85664 43.48684
107.9043 9.107566 34.73946 23.86064 7.083901 12.58884 13.04765 5.719774 13.20172
25.76147 27.67361 35.4467 33.32918 29.20885 8.964106 160.4375 35.63868 3.386641
14.21494 6.074418 27.28246 52.93599 43.40361 10.85083 14.36895 14.86059 11.57406
61.20525 90.57945 22.363 31.78792 24.79029 38.02886 45.99635 19.88691 20.00429
18.0545 20.63246 11.86849 8.58818 11.88288 29.37108 15.96433 9.697375 12.8568
100.597 28.51078 36.38557 10.59926 15.10353 18.71849 209.7984 33.62593 35.95619
24.10479 49.0517 43.96913 12.46366 33.28726 41.01784 55.4714 54.24477 20.89241
17.76707 66.62008 19.24436 23.48511 4.844099 10.3252 24.31335 39.50715 10.35339
18.8893 18.47944 15.006 21.31886 46.85822 48.42933 19.4558 9 48.20981 11.31902
69.28753 9.013902 46.94686 52.34383 11.24737 8.60831 18.14879 15.22249 8.544913
6.190113 314.1283 64.97995 18.04517 16.04788 23.11228 7.832511 18.06209 16.03473
35.63078 38.60232 14.06766 31.87498 23.34903 7.111884 20.90309 12.71069 26.06643
55.9962 18.84733 17.0539 23.99053 9.003466 25.60944 20.27494 9.631832 15.58191
18.06897 21.44233 22.0921 28.53707 34.8984 33.02108 22.89167 33.20913 24.00519
7.853157 13.47456 12.76366 23.19955 85.31338 22.14432 9.325866 14.62484 8.600748
13.89738 98.49397 67.33007 33.46361 8.717056 55.02099 87.57225 8.114596 33.73273

	10.32829	10.68448	88.8223	21.23167	91.62816	180.8661	38.04909	21.90881	15.37861
	192.3481	18.53756	7.192816	13.01824	25.52331	12.57009	84.9702	17.42184	17.10819
	8.15542	45.16461	27.27697	176.5651	14.87392	41.90586	22.7738	9.615317	11.06931
	31.09589	5.910552	19.61212	14.18493	90.02192	107.9166	85.23439	15.57251	14.72239
	36.73754	24.95604	31.67297	14.29927	26.21606	18.67556	8.823149	10.63781	13.91122
	37.43355	9.956655	12.19948	6.197279	53.008	38.77682	14.25872	17.26551	71.71065
	133.3074	19.17678	4.527357	25.47428	36.22084	17.54759	20.41543	21.97589	10.80871
	10.81229	31.12625	16.53635	42.35698	31.44313	32.69919	82.74876	20.67826	22.83358
	34.88777	99.35095	17.60344	59.12873	64.22265	91.95146	16.30577	9.414611	15.78909
	27.47698	36.47308	41.33962	30.94111	7.198322	39.38609	6.110035	23.24163	43.7342
	36.1501	17.91075	37.12389	9.92895	80.76246	94.16605	89.0989	24.17139	18.34091
	165.1027	12.9234	5.890723	16.83938	98.5808	38.56043	38.23501	9.726645	33.55706
	43.22377	6.872439	13.38709	17.99983	11.43944	15.04663	16.02168	8.392268	12.1429
	22.89729	11.49865	42.40793	25.68561	13.21843	12.95389	8.727952	25.04362	31.40283
	19.41146	9.025486	11.39336	11.80608	51.12595	62.01338	16.63151	42.27987	24.81955
	13.42693	8.495898	16.00562	24.91537	26.92296	29.70202	17.39696	67.28615	64.93419
	34.11282	18.23847	83.88519	15.29163	27.69753	56.86718	10.68119	32.96791	18.95863
	23.03959	34.32393	8.75349	5.906679	77.30032	101.0934	65.43321	13.66562	36.28534
	24.75661	14.94975	19.3069	6.59974	13.82389	14.95133	8.847733	21.48603	46.52751
	106.3647	13.6284	13.06512	22.84702	36.08526	18.73186	30.30307	21.72612	74.67004
	10.18794	19.74777	21.11675	13.68004	8.893724	32.62263	17.88132	10.76732	22.20514
	8.878484	5.791758	60.40317	9.796853	56.05904	15.1666	37.2328	3.279412	27.0726
	22.08495	34.46324	13.48136	20.01015	32.77717	36.39524	27.99171	42.44765	22.89177
	23.60412	12.90432	23.165	11.25132	9.122342	6.558284	39.92884	18.28107	18.39541
	13.47633	7.50463	19.2316	6.821599	21.24966	101.5113	7.943506	11.26688	
TP53	20.39278	9.844058	15.51889	6.737886	11.94159	14.35437	14.44742	12.00494	
	8.616339	6.029938	17.61166	25.57923	22.71907	15.76869	7.374133	9.901868	33.68871
	13.27405	25.16368	23.03626	4.512041	15.99264	35.95674	13.25945	40.52105	17.78868
	43.93486	22.49272	49.31297	25.6978	16.71046	14.10363	52.91267	26.9719	24.43232
	15.9847	18.32033	4.853508	12.64603	19.28272	13.8299	12.21656	40.04899	6.853962
	25.04204	25.96106	31.12094	13.20353	6.51219	18.25463	23.16079	26.56757	13.36041
	25.17968	8.967972	1.66982	30.26088	6.69998	30.76424	10.16386	10.52945	17.36195
	14.86243	16.2257	40.44279	17.53366	9.48631	10.41005	25.64839	22.42511	4.054236
	27.98415	29.78836	25.93765	63.62722	16.90392	30.01178	29.74616	33.15536	37.62859
	20.86609	22.01574	29.37915	36.2439	1.578132	23.16018	6.905828	10.95759	19.19036
	42.29305	39.56423	32.25639	21.16684	6.064369	24.49867	18.95198	70.4635	17.11975
	24.18655	13.87405	12.55441	6.945499	16.05925	26.68665	23.58224	41.43265	26.76924
	9.602472	23.02684	14.60015	24.22385	18.91205	23.31912	15.30898	25.03061	11.96977
	14.15182	28.47088	24.6867	9.546361	43.42667	23.25639	20.08026	37.91597	38.25591
	21.88927	29.55268	17.72318	9.407247	32.08959	14.4504	21.33054	26.90682	21.34701
	3.106657	19.79967	6.763164	10.84813	38.91165	39.4329	4.080564	19.06897	38.54213
	3.100423	7.588377	11.16598	18.42449	13.17988	42.12108	22.78466	2.196988	17.97453
	25.44952	2.471686	26.14736	23.84156	18.48247	21.76485	29.10113	21.00846	26.24835
	25.06352	27.39446	3.993904	27.85054	19.266	4.310856	2.650369	17.11532	14.34186

	13.54417	13.98876	10.51799	4.193778	22.46688	18.30413	2.635251	11.09873	8.053738
	43.37684	15.14409	3.866982	18.31614	8.811121	6.631874	12.90565	20.03429	33.33876
	10.40513	26.65217	17.85699	27.03385	19.85126	4.454953	35.18395	16.50195	25.66066
	24.88668	2.212485	9.876698	26.28121	7.572306	25.24672	59.68582	14.61547	17.72536
	22.65519	17.44821	29.12143	28.32965	45.14376	16.214	35.98455	2.11088	11.29591
	16.6654	30.76076	25.95494	3.456874	31.71772	11.68886	7.178589	22.33077	31.93411
	22.44187	22.06023	21.34657	24.04698	11.80586	19.79876	19.07001	16.60044	4.679766
	22.45214	17.60289	35.28451	26.615	43.38429	37.42206	1.372734	39.9797	9.272678
	5.242606	19.50213	29.87247	6.356133	13.78769	19.91946	29.94726	11.104	17.29088
	5.182373	19.47744	15.18487	22.96402	13.98717	21.58158	22.54854	22.0041	56.05191
	20.86995	4.97592	42.92359	16.10939	35.03649	19.60349	5.770554	34.57425	5.611501
	8.20546	18.03945	36.97344	5.135434	21.18447	17.17931	31.10974	4.872865	12.19632
	20.40621	5.709915	12.24186	12.49723	12.7121	18.015	7.352348	48.1577	20.20978
	22.57775	26.009276	6.217937	42.56873	13.47707	14.85953	46.51391	30.19596	30.42252
	33.7234	20.04002	15.29713	20.26004	25.94098	23.71817	6.030141	24.70956	24.98453
	11.4498	29.32673	18.63424	18.92418	9.531279	52.16088	13.98404	32.51968	35.84711
	5.987848	14.53453	32.9294	4.65357	6.073971	13.66378	14.82601	4.634367	2.440982
	40.80478	32.38761	13.73897	82.7041	44.16857	37.2093	5.180732	31.211	25.72253
	15.91163	13.60724	31.41616	17.11194	8.427406	11.81319	21.42747	14.93301	6.242168
	17.60764	38.48713	13.10348	11.8206	19.97245	18.25589	11.86422	16.77009	25.32214
	35.64201	12.29923	18.59192	5.120103	31.01045	23.26207	13.65127	14.56997	12.12158
	18.1423	30.39312	30.35385	18.49692	7.153129	32.75788	9.365413	26.77573	7.071648
	1.415943	40.276	10.91308	16.60845	3.466581	16.77316	29.16093	49.67901	38.85743
	20.50776	1.225683	6.453341	49.20448	31.28434	31.95475	65.85378	16.61298	20.16361
	12.47608	22.9611	29.99961	29.23226	12.30929	31.8797	30.44517	16.91309	17.08932
	17.72131	27.86547	25.15594	19.69535	18.94774	25.42315	10.08135	26.74488	30.53117
	23.6028	8.233436	9.37329	23.62482	28.82755	17.041	35.61721	13.89763	29.18848
	23.8779	16.12114	22.4366	36.1448	22.20734	43.65902	26.52223	13.48824	32.54106
	8.676359	19.34964	35.39261	14.15452	29.05274	27.12385	5.464324	32.11473	
EMC2	8.889375	6.826373	10.30645	8.713222	6.873099	7.681058	8.05446	8.798333	
	6.125048	8.488588	8.776028	9.247351	8.301116	9.560513	8.208319	5.877526	8.198125
	5.19312	6.161797	14.49312	7.174371	8.785819	13.89018	8.645002	9.230844	11.59524
	7.612286	9.338237	9.994682	6.160831	10.8555	8.05477	6.589413	11.87343	5.916092
	6.598027	9.231862	7.54733	8.961522	10.46886	10.51878	10.66038	8.261521	10.6855
	6.806807	10.78691	5.289662	14.62134	11.25852	6.356695	13.60038	8.081609	6.667505
	9.078549	5.356699	10.79286	7.870964	11.70383	17.44661	11.08224	8.422901	8.487772
	15.55472	11.1996	13.91955	6.0078559	4.744031	10.59776	8.013397	7.728496	11.72812
	13.43778	11.8253	8.634255	11.701039	9.000706	12.37039	6.860399	5.047147	9.130735
	12.51737	9.840118	9.951784	17.51962	12.92966	7.978321	10.13264	8.952291	7.074201
	6.085283	8.695122	7.980537	18.9668	6.517187	11.11997	8.438841	14.18815	14.2113
	14.00233	3.074172	11.28546	4.834786	6.384249	9.239319	7.691332	11.70249	10.91896
	14.28941	10.22138	5.645714	9.891321	5.572589	9.648919	10.1108	7.99002	11.49344
	2.177547	15.59095	10.0455	17.32574	10.40616	8.087777	10.83029	9.943927	7.431343
	7.200522	9.241348	8.30366	8.668583	5.605964	6.832654	8.129234	11.15752	9.340756

7.056967 9.070356 8.830521 12.33473 5.584709 10.15555 13.70299 8.608137 8.855992
9.541011 18.74348 7.249035 9.679439 1.154782 4.862262 7.098297 10.60726 11.9223
5.91204 13.98376 11.39245 6.990729 11.38206 8.718988 8.626428 7.560409 8.233899
7.897452 18.85236 9.954741 6.561229 13.72169 10.03186 15.62375 6.784929 8.25292
10.14347 13.1379 10.13087 27.9044 7.302352 12.2165 7.134272 4.640603 6.377527
7.509122 9.832329 8.421502 9.877183 10.23067 10.04181 9.519408 15.66456 15.31197
6.422951 4.600206 10.42074 6.258333 11.76352 8.229806 10.89687 11.49017 4.47775
11.20331 8.636942 12.42379 8.611952 7.52248 8.014957 7.359575 12.31551 8.536382
5.676929 14.50124 6.935637 6.331345 10.26554 9.938861 9.275854 12.15992 12.18704
15.09715 11.24119 7.654076 13.24095 17.3719 6.901533 6.825657 13.1709 9.073419
10.5273 7.883936 11.6826 9.374657 9.512277 6.137917 13.10456 6.271244 8.252819
12.11081 5.908323 7.753493 7.485025 15.80499 10.69846 14.63349 11.79033 6.865791
12.67124 11.3689 9.079981 8.535828 10.71267 14.66782 9.838082 11.04121 4.844702
18.69384 7.427569 7.389158 5.991626 20.64604 15.18891 8.478427 8.617404 18.61853
13.59016 14.03241 8.738292 16.17858 8.509514 7.002707 8.501258 7.661141 11.95438
6.669654 5.807985 8.36968 10.73758 12.1548 11.04706 6.015574 26.04125 9.397508
7.062397 13.30464 9.702595 7.098427 8.12819 9.232075 8.080236 7.158799 9.061221
12.96267 21.53491 6.930949 6.993984 9.601868 14.71893 7.252394 12.3165 6.329983
10.29832 10.11998 11.43117 12.7351 10.29462 7.981025 10.76043 4.743351 8.590839
7.624601 4.390538 6.061055 6.697193 11.32127 8.05631 11.7868 9.530694 10.40858
7.42747 8.30019 11.04049 9.9264 11.29677 9.376913 10.03927 12.04324 13.25553
7.119532 8.439407 16.3617 10.72074 11.33204 7.315413 11.02501 8.164274 7.643922
10.47999 9.126312 10.24782 11.59795 7.328787 12.86214 7.506234 21.72998 11.79851
8.441828 9.750821 9.310955 9.627717 11.88291 10.94462 7.171095 8.080464 15.74414
13.50146 12.45577 17.75536 8.368852 9.424189 11.16303 10.63197 9.139952 25.93851
7.41281 6.991431 4.987676 3.415996 9.024354 8.131237 7.315534 6.846389 10.6102
6.920379 8.902329 15.94226 8.028895 8.892505 5.107101 4.693274 13.42574 6.275638
8.021804 16.39806 12.37657 7.442112 10.70569 6.937893 11.59732 14.30892 12.78777
10.66976 5.981361 12.23573 6.732822 11.93685 9.80686 9.579747 11.35376 10.66207
4.807604 5.255988 7.378259 14.55277 8.450061 8.215818 8.618147 1.871752 8.289354
9.329907 8.230598 7.365871 10.92353 9.820839 5.709653 1.849 8.207609 12.52214
11.2547 9.735212 7.996358 7.152789 11.67644 7.004999 6.901208 4.388323 8.426513
8.629908 9.062582 12.97892 8.338282 9.013996 15.67308 11.11002 9.468643
AIFM2 2.462462 5.713963 1.831328 4.831816 4.256915 2.783111 4.389062 5.288569
3.878083 4.45413 5.332898 4.205272 3.619918 4.522203 6.345613 3.224861 4.776581
4.372426 4.479417 6.20901 6.512997 7.614022 7.232834 2.106133 5.798464 6.42947
6.25569 7.847067 5.950364 9.700726 3.286787 2.129892 14.32566 2.748446 3.850511
2.058796 2.829716 5.926958 6.754765 10.15523 4.125669 1.310134 3.45444 6.672808
2.766974 20.11299 8.864836 5.991776 10.36676 1.849632 1.105518 4.5699 8.413931
2.031816 17.1645 1.860666 8.557583 4.282115 5.923097 2.716904 5.156236 5.689595
9.88759 3.831395 7.025166 2.159188 4.43525 3.31821 5.904649 5.244232 4.654735
0.7642509 13.77133 13.61091 5.804425 4.482799 3.86916 3.763918 3.103688
6.135412 4.474079 4.083161 3.986924 1.491291 1.06964 3.9319 4.354751 2.328842
4.754765 2.097413 5.799809 5.023325 14.70548 4.38393 2.328842 16.66845 7.268232

5.15228 2.177531 6.56009 4.07036 2.884599 4.784978 2.627226 3.500994 5.659
3.918311 5.408372 6.031799 7.997249 6.967412 5.674791 1.568951 19.0002 8.626468
1.19278 18.10727 3.954728 3.932791 8.785721 4.149822 4.009563 1.873218 11.41888
6.174432 2.148215 6.480749 3.702403 4.109257 5.928375 6.293807 2.13429 2.448353
10.71775 2.92523 5.477622 3.195815 5.708252 3.877894 5.92451 4.027457 5.541459
3.050426 4.140092 5.341973 4.081698 7.674387 2.411957 3.893551 4.170364 1.923726
10.11109 3.645587 3.310329 1.851748 2.32859 12.37728 6.230895 18.01618 5.566875
3.48931 6.277496 2.542277 8.643565 1.883755 8.537521 9.069114 4.886867 2.704855
4.124373 7.821517 7.568073 5.635602 10.23874 5.509554 1.746312 13.50249 1.407446
4.331585 4.206853 4.410281 3.48867 10.57723 6.58059 6.667252 5.128275 10.59118
2.498275 2.392329 1.802068 14.2698 6.321154 4.503126 1.520773 1.196725 2.131104
3.209327 6.438845 8.906731 1.588249 8.787077 5.080366 1.139355 3.911318 4.12647
2.602533 4.944475 8.629053 1.873213 4.577963 3.341108 2.303182 0.9105185
4.104555 6.594201 7.109613 7.785831 7.928838 1.949914 7.726186 4.764916 4.917738
2.945953 8.99262 6.929256 2.704186 4.568455 5.240626 10.33694 4.455237 3.617614
6.1624 2.877293 4.541152 5.14787 4.947581 3.528469 2.478687 4.565983 5.212092
4.326737 4.221532 12.63403 1.944725 4.859698 9.280007 3.301089 3.220759 6.160378
1.760345 3.882934 8.845819 4.108918 5.093496 4.849245 4.120743 5.217667 5.647446
3.540895 8.908036 2.888139 8.676137 5.93281 4.666745 2.684675 3.388939 4.710563
7.651395 3.485195 4.867917 2.210714 5.079733 6.228091 1.965998 13.11245 7.729815
2.707071 5.372192 3.571799 2.755452 9.286081 1.982223 0.8439259 18.62788
10.24134 1.41312 5.371987 4.552007 3.331174 4.341455 11.70155 2.261574 1.851273
2.018101 3.779978 1.230684 3.861554 3.083395 10.04588 3.900369 16.0597 3.425121
3.489181 4.977484 4.921326 4.729181 2.422272 7.553335 7.844068 7.282351 2.238615
7.340339 5.389091 2.547953 8.817733 3.924794 7.048552 6.448005 7.191168 7.079515
4.308845 2.298065 3.152138 6.515917 6.717664 1.718054 6.20018 5.528422 4.887998
8.075048 22.13678 4.502695 2.510641 3.022369 5.907942 5.158479 42.55922 3.779857
4.675703 2.107195 5.134849 2.701027 5.340734 5.138009 4.735156 2.236056 3.343669
4.733319 8.104639 6.843803 3.387691 5.02707 2.666176 6.207607 3.09331 7.479057
4.56053 6.750171 6.778301 10.84102 7.407991 6.900532 5.105722 5.586956 8.191772
3.236097 1.880205 5.443292 7.000358 4.828833 1.474367 3.383979 3.918767 17.09483
5.905339 4.390046 6.128591 3.510184 3.08193 7.553271 6.422984 2.796778 5.959923
3.992899 1.455717 1.663441 5.23168 2.694596 2.519296 4.420118 4.389195 8.158666
19.89756 5.982641 2.68967 21.23475 3.685195 2.433814 5.274558 8.584528 5.681863
9.256083 14.10345 3.311729 8.337265 8.012115 8.85787 8.093505 3.401083 5.06164
17.5556 9.286172 2.792739 2.810245 4.257578 6.00013 9.044623 5.434891 10.02078
2.81009 6.446419 4.79568 11.76874 14.35944 5.584128 6.453414 4.401093 2.855355
4.777426 2.731212
PHKG2 3.69127 1.317353 3.109196 1.445595 2.51127 2.808932 1.940783 2.945228
1.481084 0.9113833 3.069677 2.338234 2.529542 3.621098 1.483872 5.966745
4.276751 10.10332 3.820828 5.898931 3.993429 3.107681 9.017251 5.142905 3.61987
3.697572 5.11764 18.16878 8.237593 5.334398 3.492205 8.186422 7.177347 4.589752
4.229009 6.859216 6.583601 8.852882 6.623594 5.835925 7.134942 4.435629 6.381048
6.104096 3.68074 4.206507 5.903759 4.166317 4.323365 4.620132 3.36891 2.939607

	5.18021	4.147414	8.689078	5.6148	7.301826	3.479399	3.813343	6.721856	7.831911
	3.599933	2.241002	8.344696	6.019258	5.524467	8.897544	3.541916	2.25901	4.273858
	4.465466	4.18104	6.378482	3.142324	3.552278	7.522976	3.333667	4.928607	8.326088
	6.678526	5.769841	4.642366	3.892232	10.88973	2.99864	3.369906	3.326856	3.692878
	9.008679	9.600273	4.277682	4.421562	7.121335	3.774453	6.43757	8.363509	4.798015
	5.010716	4.940344	10.42958	4.601999	3.923134	8.863675	4.792896	5.515275	4.342011
	3.862941	3.053862	11.9641	9.776394	6.463765	3.590975	5.02451	8.212059	6.105495
	1.944601	9.292563	4.467229	4.454202	5.984493	4.284582	3.035594	2.894433	5.52464
	6.053122	3.156183	7.042216	3.069816	5.32483	9.862257	2.813444	2.865977	4.446671
	5.886797	5.834736	4.027648	6.702368	6.019447	5.67752	5.620046	7.890856	5.58208
	2.941971	6.249707	3.352385	3.795229	2.533876	6.707014	5.518929	4.080648	4.772348
	7.346209	7.793092	4.851407	3.870525	5.332822	6.864922	7.774309	4.906514	5.675775
	3.941966	8.834639	4.174868	5.433358	3.157615	7.332968	8.399312	3.561963	3.702326
	5.429071	3.648395	6.09196	2.604339	3.313736	7.223701	10.95201	10.27345	3.636021
	3.895897	8.193563	4.227557	4.513124	3.762064	6.557726	6.152224	4.813636	4.536787
	3.918053	2.732945	6.38229	5.326791	5.155038	3.835608	4.699025	7.244512	5.230294
	9.841091	5.034382	3.108695	4.940966	6.804151	9.776919	2.802943	3.059259	4.132229
	3.192596	5.602065	3.914334	5.878937	4.001978	4.352007	2.568585	2.596153	5.864927
	6.411235	3.919912	6.891409	7.888336	3.08968	3.069105	14.47679	4.658323	3.288027
	6.491521	3.471771	4.376736	2.820656	15.69018	6.360953	7.746515	6.152381	7.31497
	6.589864	4.839051	5.998716	4.894824	6.373872	6.571787	9.089995	4.674268	5.197069
	2.494833	8.522975	3.635299	4.431047	6.711477	8.597002	5.177262	4.890299	3.26833
	13.15791	8.930887	11.96507	7.052694	6.79165	3.154612	4.072377	6.702518	9.928893
	4.171821	7.168673	4.161424	6.790038	4.417295	7.911078	2.576078	4.370574	5.238726
	6.504765	6.559614	8.988629	6.292897	6.238184	4.010818	7.485082	2.960679	8.509586
	4.207546	8.555957	5.233941	7.412323	5.997603	4.499828	7.628591	3.657614	9.985685
	4.791802	4.434777	4.802462	3.188704	3.790527	6.002637	3.340952	2.35691	4.484265
	4.275639	6.560957	3.431913	2.394671	4.185676	3.529287	2.7107	4.787569	9.095085
	3.46117	1.975496	5.310989	8.238092	14.12336	1.989601	2.28987	6.931099	5.705378
	6.244285	5.307143	4.086111	3.025202	3.461558	5.122977	4.624609	4.035748	3.079118
	2.510086	7.770922	4.707452	5.973186	5.886317	5.375566	4.990659	6.997188	6.02747
	3.624253	4.626189	6.347211	5.902561	6.872377	4.224708	7.519014	3.85491	2.64976
	5.17051	5.204332	5.160102	6.217602	6.420191	7.267226	5.875914	7.112491	5.972726
	2.500195	6.011944	4.041384	4.035681	10.58326	2.974511	4.464214	5.18728	5.848324
	5.874866	7.387817	3.534699	5.03253	3.977462	4.787137	5.416768	9.146799	2.532579
	8.683308	4.649861	7.108979	2.697204	6.093619	5.506672	7.937128	3.95415	9.778052
	3.124166	20.71946	4.674037	3.132799	3.812045	3.871899	4.259352	3.525869	1.673107
	3.391509	3.118886	5.408059	8.554155	13.88924	3.095097	5.1189	4.632515	2.849885
	6.51824	27.5855	7.603422	5.163105	9.468338	3.146152	6.404823	4.434261	9.192368
	3.452485	5.069283	3.962576	4.405669	4.492438	4.481364	5.483692	4.214115	7.221182
	2.627192	3.59213	4.265218	6.778475	6.15302	4.163688	3.672805	5.119757	8.980799
	5.185207	8.61368	11.45554	3.034453	5.733766	4.415263	7.389167	4.331524	14.57659
HSBP1	10.97623	4.47367	14.69955	4.777807	9.194516	9.219478	6.508892	7.67043	
	6.236397	4.679612	10.83224	12.18828	9.814467	10.77246	5.144291	8.43182	911.92507

17.19358	13.8075	17.25333	10.70852	9.465514	31.99004	13.22923	17.64784	21.19336
13.56242	8.756535	16.9817	14.60228	11.71673	10.27848	16.85614	15.55998	18.06138
10.38343	9.273732	16.24417	18.8855	13.64133	16.76887	17.44528	24.35107	10.97777
14.32997	11.55113	17.78161	16.28678	17.57911	6.766924	13.49251	16.68443	15.18386
15.43622	12.1598	19.00533	17.21103	11.25083	8.683197	10.9336	15.25723	13.82444
11.14254	17.57587	5.162508	7.472536	10.64003	18.50903	11.59056	7.913838	17.55626
23.11121	15.01849	8.700359	19.66355	15.33528	15.92951	11.94357	15.538	14.57497
20.09138	12.86507	20.01395	17.52884	18.28074	8.945	6.801432	10.74494	13.24147
37.41046	19.31529	14.35536	16.07981	13.96243	13.56953	23.40333	14.96943	14.74804
11.60952	12.44255	11.341	6.117796	14.42035	23.76585	22.71263	14.01542	8.175674
6.801576	14.94404	13.92145	20.78459	16.4312	18.28916	6.796089	17.16262	14.09547
13.14834	10.98812	16.58731	13.00066	10.00526	19.45664	7.542737	15.28653	14.22857
11.91072	33.0811	11.86677	13.31463	17.97654	13.97592	9.991146	6.773191	8.332682
6.32446	7.55054	21.93705	12.32709	11.18028	20.12117	20.70158	14.485	15.84477
18.44903	9.667726	16.21988	9.106371	4.352502	15.49635	14.81833	25.38491	8.25157
10.53667	18.88703	11.4539	13.92887	10.33457	18.78539	10.51047	17.00965	13.79781
12.05969	16.96365	14.09428	34.28742	7.477322	10.85708	8.460796	12.92607	9.597743
10.05287	15.81139	7.165623	9.403966	12.93844	15.7552	16.50871	7.577109	16.98039
12.67183	21.66616	10.03454	15.701	16.0461	13.85308	18.37483	16.12856	23.31373
7.471817	9.733078	13.24103	12.28638	12.11459	9.825747	56.22252	11.41465	11.08848
8.209315	14.08398	9.769333	9.81465	19.41442	14.66077	12.51515	10.38907	12.17676
8.914575	17.45717	10.05473	8.699752	8.828302	12.46336	13.55293	23.47919	10.93149
7.433794	15.80563	17.45321	13.90752	10.06977	20.31813	4.646059	24.16129	21.23481
6.95855	18.87345	8.302665	16.24776	20.08304	14.52444	17.10059	13.38571	15.64416
16.68163	19.01991	8.711285	15.11331	22.93787	16.15382	9.084834	17.62336	8.952419
9.68826	10.14646	9.170784	17.19186	21.24923	14.6812	13.14758	8.596497	24.80498
11.0875	9.319366	12.69715	13.0074	8.529766	10.34254	15.33741	17.39667	11.97816
16.75329	11.27756	18.09567	11.46403	16.38711	13.16499	6.357942	51.70173	19.68028
16.81817	18.87707	17.57439	10.73137	24.48377	13.37523	8.702783	11.2345	23.04327
17.45598	15.27831	7.700495	10.12679	6.976159	14.6124	12.65704	21.86717	8.363465
11.59553	8.04775	12.58827	14.61284	20.39216	11.92048	9.635059	13.77938	12.35656
7.028868	8.432616	9.218726	13.93233	11.65924	11.53134	8.333816	8.512853	17.15154
9.408068	22.91616	18.83671	10.6745	9.698158	16.28409	13.75481	17.21854	8.207541
10.70088	7.500485	13.93307	18.85751	14.55204	11.75555	12.02091	13.99052	16.27063
26.67188	13.70813	14.77566	12.91045	17.17687	12.98245	16.72664	18.29594	12.66241
11.55036	8.718384	10.54587	14.50968	7.2945	23.48846	10.75867	8.826924	17.23039
16.17411	15.96244	20.08623	30.35574	14.89564	10.15628	8.130174	19.50429	8.013264
17.28104	16.91512	9.204119	18.36664	15.47896	14.31902	11.02217	13.07183	16.12031
18.12262	9.344027	9.610333	12.37627	30.22099	19.70005	9.003057	14.45395	17.35906
9.461195	13.72586	16.21834	36.5767	21.93001	14.99647	10.79133	12.7362	8.551562
11.98878	6.758521	16.98508	12.25662	16.86872	12.71009	8.858529	8.655981	23.95335
9.99368	14.87163	15.19848	9.900051	13.23565	16.44863	15.56602	14.44353	24.52561
20.40244	11.77758	10.07017	20.59252	9.556468	18.24311	15.57685	14.69012	7.731694
10.76358	14.05747	6.704897	8.718405	21.00871	12.25447	6.682995	11.38152	11.20248

	12.68975	13.43455	18.00426	14.15028	15.56629	15.89272	9.859138	20.58742	17.98749
	12.57775	7.306877	12.49187	10.67617	7.111601	29.36664	9.702403	10.95002	
ACO1	5.064191	12.5575	5.765622	11.61583	4.217917	5.772213	11.83975	10.54072	
	15.93988	13.42405	6.247259	9.285971	7.17615	7.127861	12.87066	5.936741	5.46432
	4.76477	7.164231	2.649467	5.579227	10.63906	6.612276	2.195254	3.776124	4.976166
	7.098235	9.295958	15.65091	3.94789	7.66675	12.62615	3.523129	5.97318	6.704728
	4.462488	8.456154	3.380599	5.440548	7.138944	6.432656	4.313398	2.72677	5.624109
	4.51283	6.993688	6.805187	5.730751	5.867075	4.894296	3.976394	4.821899	10.48622
	5.334103	4.254699	13.52176	3.107372	4.472839	3.806961	4.389326	3.791927	4.883273
	9.491036	3.19413	10.64543	6.120808	5.579237	3.447241	7.198616	10.03043	3.886882
	5.838593	3.198727	10.55764	4.192419	6.974419	8.134171	3.141675	5.732937	3.912531
	2.789535	5.14295	4.450993	6.283014	5.941733	7.877848	7.049992	6.594892	4.414882
	5.519197	2.08925	3.079658	5.391366	11.07644	10.35834	4.185023	4.24326	7.453886
	7.285671	2.606759	3.346863	3.034918	3.423813	3.574211	2.236835	5.377159	6.565215
	12.92416	9.446694	6.477912	2.026539	4.79055	5.742402	13.90689	2.316443	9.364799
	2.943371	4.872605	13.40724	1.644423	6.46609	3.368562	2.964721	9.164717	10.18274
	4.979406	5.95659	8.487442	12.22799	8.255099	5.126956	4.66842	7.728892	9.581632
	6.150834	5.191766	6.771668	6.605541	5.89945	3.354169	5.062327	10.33363	3.40295
	5.568385	5.308667	3.120052	6.833867	0.7295601	1.855674	6.073388	2.892643	
	25.87926	2.990608	5.784142	5.634794	3.492433	5.442579	5.240709	4.571496	2.191982
	11.63841	11.06133	6.708475	6.234181	3.530266	7.493905	5.278132	11.71863	12.05426
	8.177961	7.496432	3.954929	8.192779	13.4162	2.446829	7.09457	6.988579	4.610358
	7.102889	4.852566	7.843046	7.227665	11.97029	9.366209	11.039	10.10179	3.384734
	5.933717	7.852847	2.237968	5.800995	3.744861	7.194268	4.191641	2.154865	3.017375
	1.880708	13.35035	2.483706	4.41683	19.98334	3.899179	5.846224	6.429845	8.836768
	6.112272	4.455102	4.863896	4.264469	8.276751	3.627529	8.668792	9.285947	7.030144
	4.30134	1.024929	10.23296	1.833187	6.575814	21.39357	5.417813	9.711757	5.410628
	2.966916	6.749774	3.799792	8.336976	8.989209	3.923074	1.805775	3.924369	2.369169
	6.427281	5.393275	0.3145637	6.184124	21.27845	2.465346	5.115902	5.882102	5.65556
	10.68018	15.62917	9.84476	3.491796	6.254895	7.876207	8.474414	6.906767	4.895578
	3.833579	5.745435	2.744358	7.832325	7.317223	7.104231	5.820759	9.260129	1.909464
	5.829857	3.473391	4.551797	7.153667	4.686274	3.441343	8.307476	7.522473	5.733823
	9.108422	6.051195	4.018145	3.561835	15.14822	2.381775	35.02737	7.082191	4.807822
	4.52522	2.377633	1.537559	7.483781	4.448572	8.942088	5.675401	3.67436	2.474869
	8.106009	5.145229	5.300788	8.698686	6.129811	2.734933	9.016713	5.757617	8.190796
	4.558093	9.346442	5.763254	1.934851	4.953045	3.497258	7.143541	9.91849	2.569209
	1.652284	8.784844	2.386284	3.240625	5.320113	15.27081	8.899134	10.24334	6.236307
	7.755872	6.306908	8.415542	7.391483	4.247548	2.534555	6.446933	5.495198	6.133096
	3.90623	6.101257	7.596858	8.660841	8.403637	8.619397	11.15147	4.028179	4.283961
	12.21248	8.225798	2.358743	3.941315	10.74642	77.60173	13.97306	6.579078	3.522853
	5.437276	2.484663	8.396301	4.34423	5.839543	11.46859	8.78543	9.177769	4.431878
	3.548551	2.740463	4.298762	6.109391	6.818008	7.203093	4.31518	4.625966	5.573271
	32.6201	4.234937	5.764029	3.728637	1.653361	6.355119	4.195568	7.364679	5.015796
	3.0964	3.976081	7.150753	4.586051	4.641379	3.818026	9.8161	1.535095	6.547605

9.88712 15.4988 2.510302 3.863502 9.587437 2.864836 2.359554 13.62236 6.0737
7.045871 10.49965 3.851472 4.791097 20.78079 6.405449 10.73653 5.74953 2.866685
5.680233 1.751036 3.267329 6.811496 5.331467 8.392504 3.723357 7.91823 4.473685
5.709407 5.150871 12.31604 7.363741 4.174478 3.661922 1.08691 4.967704 3.807134
7.78541 6.333403 5.855235 4.787763 4.268522 7.546519 4.891796 13.97379 1.913516
6.262302 6.77871 8.065469 7.696873 9.440927 9.87788 7.038049 8.661138 2.68237
FTH1 182.9878 170.1769 171.9744 202.2144 138.6144 218.1167 206.1632 203.5041
146.7736 193.4966 94.81727 241.6173 176.1719 387.3947 223.437 203.8308 246.261
280.8251 331.1323 188.211 362.5273 97.91921 239.2771 502.6508 185.8747 304.2154
233.9467 401.708 194.9383 412.6117 282.2379 133.0934 124.6319 277.9787 307.5629
70.62339 185.2325 198.5567 393.8 386.4808 301.7656 103.7415 211.092 210.4132
219.5881 269.2271 167.4071 188.5131 165.4666 87.8962 197.141 239.0287 1324.186
202.8058 147.0225 160.5194 140.9119 279.5746 288.7539 283.1827 218.3962 786.977
529.8144 178.1739 227.6345 190.8562 336.2954 441.101 223.6677 157.3123 269.6902
132.0258 291.5646 627.6201 173.7353 329.536 132.9779 496.8538 153.6222 160.9648
272.3537 144.0766 270.4095 99.9444 105.4096 109.9524 322.8673 172.0282 252.171
124.3495 200.7661 260.7826 539.9887 140.5226 302.5536 662.6986 117.571 156.7015
220.9666 79.48108 149.4562 93.32215 133.0495 302.4545 347.5986 213.4617 354.4317
29.55338 211.6307 336.1751 263.8039 200.8622 173.3071 201.5804 175.2899 205.3076
143.6306 283.1819 271.213 220.3203 291.5206 595.0107 214.5372 113.3607 130.3926
191.1547 154.5958 204.2523 282.6117 131.399 261.4741 248.9586 207.6447 376.0428
181.9285 308.0344 152.3852 170.7706 218.3185 339.5809 151.4329 552.4354 226.8598
841.7732 144.2139 148.8431 179.5017 12.30884 126.7117 208.849 242.6874 290
70.28894 370.7072 159.7064 83.84204 200.8887 186.9743 143.028 216.5496 252.7581
326.4586 328.64 227.9045 137.9983 391.1864 100.1984 158.2435 179.8043 665.5989
787.9804 249.5463 323.9436 402.8866 150.721 139.3933 171.2786 211.7635 183.7581
236.7751 153.036 207.137 467.6402 613.2727 226.5441 502.2649 161.289 124.8165
243.5129 58.1763 305.304 153.8155 403.4146 196.0847 135.385 211.7581 98.62936
221.1773 476.7747 140.3206 210.9923 86.62533 333.2466 326.5925 214.9066 276.3136
138.5543 169.325 248.3654 129.7756 288.1201 313.1439 76.66393 161.3901 186.1421
163.3613 245.7276 126.6723 128.071 617.5138 172.9431 99.90251 119.8079 266.7577
89.59424 356.6118 178.0772 202.5022 470.5852 253.7103 184.5622 195.2939 215.1579
388.4413 113.8645 193.4865 159.8789 489.5475 477.1257 788.3541 270.9921 583.978
379.9109 204.8456 232.2769 173.532 530.7176 134.6966 208.4618 169.811 259.3407
221.544 163.814 160.6543 498.425 160.1368 471.5235 278.242 282.7654 211.3854
164.9851 246.1342 189.7197 283.0783 294.9106 216.0415 206.1269 191.4794 199.2308
468.5915 379.1441 136.7576 175.6786 220.1274 176.3282 183.3555 128.9793 393.2176
111.4015 151.7123 211.9113 681.7226 111.4406 650.2907 205.1045 121.819 223.1487
360.7475 397.5014 167.2891 242.4206 339.2136 72.49043 250.3292 320.6329 223.7668
157.1715 166.6763 195.8307 239.5346 112.5984 168.1212 245.7878 110.1672 149.4711
188.3025 334.2107 170.2235 684.9776 52.42023 95.44116 520.5866 145.4038 192.0653
156.9313 226.7521 180.9121 328.9762 314.3013 329.6006 185.661 143.5011 74.18832
124.1819 578.0543 363.9152 481.9106 399.6398 449.6475 144.199 403.4271 255.7434
225.6358 29.83085 197.7765 248.2429 377.3808 146.8094 170.1322 99.6345 383.0922

112.0336 280.4804 360.781 233.7177 471.0309 352.6342 158.5776 416.4046 315.0011
173.8318 495.6885 79.33358 235.1413 191.9888 523 456.155 450.0122 402.0074
158.0713 93.44322 81.51154 83.20707 151.2538 221.0886 579.5427 227.6466 172.0479
128.6767 90.39393 183.7608 250.0074 200.3408 598.4258 44.41633 332.7168 193.7827
311.0661 65.52343 222.4344 282.14 223.8289 105.1856 256.0228 125.5452 153.3064
243.7466 617.2753 235.5895 106.525 381.8947 115.0892 499.7941 195.7654 97.76965
96.99235 55.44251 327.6081 155.8991 398.0353 312.8314 208.2701 75.41565 287.2461
966.0722 343.9932 450.5736 221.7927 463.7705 38.71597 179.0816 219.1988 230.1988
232.1597 328.9189 314.571 335.7252 233.6422 221.1323 156.7641 206.7793 190.7335
178.1664 605.1661 133.7346 122.1089 266.0713 115.5753 319.8207 147.5917
STEAP3 7.546051 1.134213 10.77233 0.8532663 3.43432 10.05781 3.794139 3.208111
1.158695 1.266084 16.51521 7.902013 11.11941 7.258427 0.7506026 9.936429
14.68089 7.098603 12.96011 28.09077 20.01569 22.52605 23.10276 13.42555 18.35516
39.61675 12.48752 3.451425 7.351717 13.04896 5.830071 33.05077 21.01887 15.16064
10.73139 9.273077 1.970279 11.00846 9.713316 32.1539 14.99044 14.38889 27.25702
14.6622 17.1393 23.88579 10.04347 65.26418 52.44344 23.4736 23.17534 21.65386
17.51074 35.58875 3.973665 16.53564 49.6318 7.320315 13.05878 4.816199 26.09552
10.40983 23.2751 2.57747 15.346 11.70736 3.48593 16.92118 13.63537 6.044397
7.898993 23.34126 19.31049 6.948991 38.01761 1.838411 24.25728 24.09298 32.00339
15.4072 21.76004 9.256315 12.69966 6.41121 9.455417 44.03583 21.68495 12.03457
7.436642 4.121438 7.759652 31.71967 17.48849 11.96143 3.248999 23.59927 13.0808
15.02083 17.6786 10.87418 11.41231 9.308085 12.43511 12.16426 15.06119 40.17212
18.04215 14.57613 2.313977 5.164223 18.63914 19.58185 10.48281 13.309 7.257327
6.131022 21.28661 9.475107 4.700876 10.7101 7.288978 19.63601 21.28078 2.707058
24.30427 22.00663 8.315376 9.633879 8.832134 24.59193 12.13484 6.850373 15.13493
7.992458 3.592367 11.18863 9.258278 28.59429 0.8642052 11.34555 10.36818
6.922011 19.65904 2.524807 12.87367 1.161682 5.343286 4.221607 16.55431 21.1477
11.9161 20.63983 1.165642 15.80267 40.91332 7.893857 22.85045 20.39748 7.346132
24.36703 8.671455 25.94362 5.653875 45.00486 16.3139 39.62007 20.0809 3.479881
15.04528 11.47804 21.53975 4.430242 5.536235 11.14797 20.71519 11.0099 44.40266
19.07231 6.147832 28.85215 18.04313 14.78624 25.54123 16.5296 5.197482 35.71608
8.36777 27.62257 2.089653 17.83 28.79437 7.746561 23.21064 8.123107 55.64298
7.672762 18.2226 18.85174 23.85351 19.20284 17.08446 0.3947265 43.31146 30.1713
3.244714 1.230187 20.40079 12.86549 14.04984 18.3961 10.78736 20.52603 16.05318
19.91056 3.72166 18.90485 4.780865 17.42422 22.17006 33.7667 42.51495 34.10954
9.565494 17.27311 6.088029 13.4011 11.0722 12.8693 17.79114 17.25285 12.42161
19.3665 13.18828 11.32889 14.00928 4.755856 1.479581 11.79326 32.31075 26.79055
21.03067 2.280556 4.074806 29.726 10.66549 19.15027 25.83307 15.4221 7.548594
13.09077 12.21174 21.2965 27.80617 32.38577 10.34596 26.40353 10.03169 13.38373
19.74522 28.60758 3.615992 10.75988 21.37528 10.49527 22.91132 11.22942 11.23501
5.936724 16.21324 7.928073 9.920926 19.70544 6.49948 14.93498 10.08977 2.35302
5.300719 17.25191 8.717834 8.721103 6.836093 31.67876 13.67984 15.20036 8.817745
18.4814 5.995739 21.68821 23.63119 13.20406 8.179481 8.590925 13.78297 1.73874
12.48215 17.41238 7.49734 7.067891 18.83952 11.19366 21.68662 13.93549 5.367914

27.40231 17.01951 19.03402 16.76755 12.54711 10.62621 16.8026 5.45602 10.9815
19.5048 4.464191 17.88513 21.09827 17.88521 5.079992 3.024969 23.58235 12.33795
19.37254 9.440242 29.01233 14.05349 3.986229 16.66775 6.304179 3.100142 4.088494
7.961947 2.302579 24.16534 16.54118 25.11159 12.85197 17.73763 15.11335 8.31399
12.90896 17.81907 9.279302 24.6158 8.35277 17.20432 37.77944 14.42597 4.467103
49.4944 4.577089 52.29302 47.9754 12.80274 20.54687 28.76407 31.78743 38.66512
4.876948 20.02834 23.12571 36.07442 14.47426 6.055949 2.505067 14.81926 48.06659
22.91194 25.17647 4.245223 26.555 9.024475 17.14627 16.35472 30.83066 6.584944
7.208216 20.20009 11.92452 15.95754 1.898117 26.88372 24.5646 20.18256 18.2052
19.4072 16.08019 3.64986 13.47029 9.333485 8.280145 24.08643 15.27262 9.24146
35.42726 26.65281 31.22781 21.67684 5.601997 5.91625 19.08986 21.07104 11.66192
18.45577 4.591635 9.574729 7.791778 10.58834 14.53422 19.86883 22.78437 10.48523
12.35249 8.000804 23.11291 8.168582 11.38473 21.88301 7.080105 35.07002 13.66018
28.20219 23.25305 9.575456 7.794534 31.18315 3.611964 6.117345 24.38236 14.17509
9.748354
NFS1 5.00186 2.829462 6.838556 2.364081 2.748775 3.592912 3.492185 3.555577
1.933072 1.992362 3.312472 3.77312 4.066838 2.674423 2.67074 2.510944 4.759296
2.754035 4.342396 5.106914 5.807509 6.204847 11.25044 4.984616 9.665224 5.785995
5.556421 8.360004 12.00992 5.568448 4.858822 3.913447 5.208792 5.785909 5.801986
4.448946 6.503683 4.220081 8.560786 4.281648 8.124501 2.992091 4.059154 4.503047
4.224491 5.107662 4.119255 7.01554 6.377436 5.620648 4.704971 4.924567 5.013984
5.83846 4.110605 9.14192 4.495818 5.044341 4.05397 4.161541 3.257356 6.573094
7.362339 6.195991 4.329306 3.97288 3.768357 4.164983 5.487554 4.082388 6.242682
4.586317 12.27672 3.070823 3.513642 5.425067 2.976209 6.702849 6.719624 5.529628
12.43993 4.740165 3.445425 6.339359 6.82169 6.458388 4.074892 8.697038 6.231411
4.177979 6.200506 4.62146 9.727971 7.396584 6.322456 6.461721 4.38917 2.587363
5.296777 7.998418 6.855949 3.735814 4.890112 7.380366 4.265327 3.968433 4.371312
5.527399 6.577218 8.108069 4.390409 3.730653 5.208334 9.500701 6.324691 5.872432
3.31467 7.448501 4.501888 6.825507 5.27721 6.566747 7.151271 12.5365 33.88372
5.210395 4.230384 7.13918 3.298283 3.800975 4.394482 5.912787 6.528758 3.368251
4.76009 7.689277 4.594714 7.825649 5.220939 5.173095 6.521345 5.094593 4.776556
6.521572 6.527974 4.879496 3.338356 6.275672 4.628972 3.441077 5.865545 5.779362
5.963527 5.999073 8.288382 7.432903 7.01299 4.295337 3.57891 7.318379 7.820968
8.093727 4.484165 5.552046 7.511892 8.816275 7.644591 10.52118 5.484177 10.35665
3.431329 2.854546 3.595264 5.321794 3.99815 6.83228 9.094904 13.55706 5.144831
3.280627 6.877638 7.217675 5.286591 7.997157 5.654732 5.995511 7.657442 8.270818
3.350662 5.788917 9.336393 13.30851 4.31327 9.2772 4.428875 5.982625 4.779697
5.074316 5.223118 8.398895 6.134552 4.809675 2.684973 6.062478 3.839878 5.689724
6.238995 17.48116 5.710836 5.814522 5.612241 5.140434 5.639754 5.436497 7.196212
4.779107 8.004479 4.800383 3.428552 4.322689 6.206017 5.816906 3.541342 3.511326
4.167173 5.873206 4.631404 9.489567 3.714844 5.803638 3.414637 6.947209 4.300321
5.551731 4.533831 4.250721 9.815432 8.761255 5.627597 3.448644 5.111037 2.060272
5.980384 5.59696 6.814537 6.112657 6.053892 5.515459 6.719368 5.009368 4.767518
8.26567 4.331129 5.548375 3.189085 5.824574 3.692697 5.645042 12.19895 5.532121

6.253331 4.252919 4.890034 7.090255 15.1834 2.72169 7.262591 5.425957 3.811399
4.4739 4.668815 5.294973 3.830675 6.528152 4.70144 4.899216 6.22168 5.096032
2.866818 7.954358 7.650897 6.511696 4.51091 5.275651 3.359006 7.418665 4.949869
6.233187 7.734977 3.606041 3.338396 7.82573 4.925272 3.06535 8.105499 7.522015
5.270758 3.860402 11.28038 3.597351 4.510345 4.458213 6.144238 6.706339 3.630892
5.32377 4.888841 6.161338 3.562317 6.31743 7.376423 6.125897 5.999515 4.680472
6.634033 3.570627 5.327406 5.359324 5.51061 4.435431 3.729432 4.181253 4.474875
6.336467 8.315077 5.76827 5.218148 4.193625 4.799437 8.29074 5.215863 3.969715
4.603147 4.388919 4.679305 5.581417 6.924862 4.134951 4.054274 3.97447 4.981296
4.492342 6.455249 5.111919 7.704012 7.377066 4.95324 7.335238 4.414766 8.049742
5.574712 5.989489 4.778009 8.589555 6.043455 7.016512 6.018724 5.132697 4.835246
6.521269 7.063954 3.374132 3.339494 2.633762 11.13038 10.65331 5.441684 2.890258
4.59341 6.124657 5.985742 4.741426 6.939243 3.144858 3.857717 7.437435 4.638074
7.149696 4.499986 3.779301 4.409573 5.565827 2.773472 4.70519 2.83807 4.261762
3.451305 7.740099 7.313615 4.332075 2.593825 7.333798 3.992203 4.125306 5.441531
8.945239 5.573474 5.636214 5.236764 3.871073 6.668979 5.008283 2.771092 4.436509
4.237149 3.146201 3.268083 5.552721 8.178169 4.697924 2.748638 4.17306 4.843367
5.136858 4.904651 4.803659 4.477817 3.780365 4.976169 5.209016 4.476968 4.966917
6.253946 4.813492 7.048826 6.763939 7.864213 9.772107 4.646192 7.017162
ACSL3 15.55295 9.105855 11.41072 9.148827 15.5778 10.41234 12.41506 10.93837
8.660065 10.08321 12.17903 13.7179 12.72504 9.120836 9.29409 18.4927 15.62764
6.827271 10.85736 10.89843 7.328474 13.81804 20.04766 13.70229 14.42876 19.09802
12.44838 9.651514 7.269665 7.799542 10.01137 11.58297 6.275342 14.28354 22.42033
6.869216 4.890815 7.76418 8.45257 7.393111 4.717736 13.52737 14.04157 7.661233
18.36698 5.346701 4.397857 5.694393 9.030768 13.82459 18.5378 19.50972 13.75654
14.68213 5.25291 18.7249 9.727705 24.23992 12.49534 14.35116 11.00675 12.39285
19.97802 8.690124 8.86414 18.06321 15.00899 10.69689 15.18263 9.88586 18.31486
13.66684 7.228761 15.74934 8.281053 13.57337 24.02515 14.85935 6.02553 9.45891
20.91665 9.447437 11.35972 12.70287 25.46431 7.14936 15.69701 14.3442 5.434262
5.480524 8.257605 12.89908 13.94526 14.13077 11.43714 18.97243 10.39129 8.916297
12.06101 2.647475 11.61707 3.691817 5.702272 31.16533 10.52814 7.880171 13.28057
22.44818 4.289062 10.28346 35.10198 8.476214 17.22088 18.2976 12.06632 13.41352
3.358232 10.06133 5.797823 6.381587 76.91961 37.69976 24.46815 22.67456 7.937838
15.61014 10.45337 10.60735 9.879542 6.441622 19.01568 19.3944 11.30138 6.68499
9.809782 13.96114 5.285356 15.48783 23.49541 9.500945 13.29103 8.407204 14.52542
15.3561 8.590454 33.2735 20.63605 3.764713 4.311255 7.969399 19.30956 13.15362
7.407981 25.16925 23.81683 11.49655 11.66245 5.634884 9.245956 17.72541 11.37177
12.64171 25.37471 12.13506 13.49809 9.155572 7.191999 16.6893 14.41848 16.38906
14.81858 10.14662 11.79148 12.16843 7.144292 4.103895 11.2273 9.208734 8.471932
7.245822 23.24477 12.73269 37.23138 24.97011 4.667981 13.20168 10.49448 15.96682
7.132805 8.481381 11.44923 11.57417 16.88995 12.44708 7.197672 11.18436 8.866403
14.07036 22.80088 35.45165 12.62948 8.94759 14.94868 31.47507 9.262966 10.97958
6.483131 16.7566 10.87685 9.689408 8.643744 17.56496 15.0658 28.43937 7.797863
16.6393 10.28563 7.433694 8.466564 13.18637 8.660062 32.42521 23.19419 3.209376

15.04241 12.09346 12.3878 11.81282 17.24178 6.025751 7.447551 9.582027 9.313016
9.93049 7.948696 6.306395 12.82308 9.912234 14.76023 13.72811 11.81868 7.258939
5.271944 12.52885 11.05351 7.569166 4.39103 16.8996 10.4034 10.41068 5.052708
26.02058 9.694863 10.76311 8.427271 15.41506 7.106934 13.97224 20.40463 13.8139
45.40308 8.261646 11.64701 9.740493 13.51136 12.17701 12.75932 8.327873 15.20814
8.824983 15.36247 11.05625 12.97371 15.62419 7.676492 27.9388 12.34634 23.5747
8.609783 28.12253 5.649065 9.015663 23.06039 16.36736 6.679359 7.577944 8.73818
8.338158 8.398709 6.363857 8.133907 14.84993 18.10264 12.25775 12.46522 11.09347
8.125458 10.35365 20.61284 5.868118 23.68272 7.347893 8.107565 6.216954 21.21364
9.286993 7.580621 9.70503 4.460828 24.56299 16.66023 14.95599 18.58648 11.72396
26.71004 10.11735 10.28886 12.4381 10.98419 8.126142 13.99408 16.90136 7.085247
15.72109 9.680025 13.31214 10.90581 12.68609 8.981208 8.552872 13.35123 8.435767
19.05908 4.627371 11.83074 8.808328 13.06207 15.52229 9.193314 12.55253 20.61556
16.77654 23.17628 9.198195 15.83628 11.03657 15.11399 10.04392 42.81206 17.86579
11.0285 15.75506 15.69748 5.753336 7.46202 13.92894 11.08567 25.06088 9.349864
8.392901 9.706816 5.719944 4.410571 6.738217 18.41769 5.16884 12.86456 13.71374
13.43253 11.96715 14.12696 6.27691 18.5785 12.45977 4.134187 7.639363 17.1567
6.214672 19.95842 8.860541 14.91889 17.14037 15.30886 8.409923 10.97642 20.4003
10.34291 14.95999 17.82975 11.3637 10.90345 19.04224 12.50726 9.749367 16.98622
4.932123 5.072504 12.15817 9.151471 13.39334 7.991755 7.321489 2.152252 16.19692
14.69892 11.18211 6.597072 8.266249 15.97258 5.437584 14.07768 22.52368 18.64128
12.78332 9.298573 5.851971 13.7679 22.29872 23.34832 24.12123 7.19291 13.66216
13.75393 8.67535 6.36597 10.87265 20.17207 34.69011 10.4371 8.102347
ACACA 5.039078 7.679429 8.162917 9.235287 3.906619 5.731718 6.976979 7.398922
10.07659 9.512419 6.555759 6.547156 8.720759 2.640227 10.16824 4.458385 6.869699
3.180825 3.820954 5.006894 4.405101 3.954958 9.49588 9.224822 7.40172 7.879387
16.65933 8.83988 4.204789 3.636774 5.19424 13.76689 3.121422 7.94095 11.33871
5.601106 8.755135 6.670222 6.883541 5.716831 4.638977 3.189803 7.05194 5.551676
5.115782 10.36186 3.519462 7.731934 8.824062 3.112914 5.94889 8.987202 8.56266
14.04913 2.932652 9.305819 6.140574 6.452849 5.850961 3.317234 6.68606 9.098743
16.43685 7.363446 9.890718 6.197244 1.521978 7.210811 16.79218 5.435552 8.996699
6.142478 11.66167 6.166244 6.9342 4.203575 7.175732 10.32294 8.50435 2.851322
9.715698 6.092367 4.869941 11.15142 6.097172 8.033999 4.984743 9.96819 5.203866
5.313974 7.868175 6.434206 3.931231 7.724703 10.09803 5.548215 7.420543 3.136707
5.573126 3.390475 5.804621 3.040357 4.522991 9.696871 6.248333 4.374036 3.710269
9.994624 5.30321 7.787361 10.28246 9.568911 4.856676 8.315237 4.303016 5.077394
2.257059 9.449107 3.301567 4.932933 14.25638 10.30293 6.544273 14.88813 7.843192
5.30845 3.171935 7.452787 10.85307 3.550636 6.180819 6.907436 8.998223 1.973251
6.778367 5.872016 4.924195 6.190316 5.922962 7.719919 4.515747 5.997599 7.318867
9.195372 5.819343 9.983275 13.24764 1.927538 2.34025 5.216817 6.741176 4.381628
3.38508 10.44404 7.953075 5.791311 4.795 5.303909 3.206479 3.567788 9.115247
8.770404 6.514599 4.249951 11.77832 6.465097 7.435432 16.6406 3.868526 8.991832
7.581357 8.235344 6.456912 3.471304 5.625226 15.7946 10.15241 7.096236 11.29003
3.805432 6.556974 7.857159 13.19953 6.246525 7.511856 9.302513 3.419866 5.873599

4.210172 5.719576 6.102072 6.295642 8.742704 7.104587 6.061456 7.285812 2.911861
7.11135 10.65292 5.628406 8.04405 3.7661 10.56671 16.16465 3.254588 5.591917
6.480539 7.505128 15.37276 5.986294 12.20035 7.4854 12.8813 9.039992 2.371341
11.70517 5.562762 4.238025 8.94993 5.730491 4.479844 8.846564 7.975873 6.03839
5.243938 5.396611 5.5675 9.975409 4.724464 5.257769 4.375664 4.992783 5.166202
4.31484 5.621461 4.168241 5.557819 11.12644 13.40752 11.00881 13.1426 1.692197
10.11203 6.318019 4.888183 3.988054 6.383578 7.631916 13.11821 14.27111 4.450981
11.10786 5.064259 5.028811 2.965026 11.62437 5.987498 5.114354 3.070332 9.655126
7.220718 5.304968 3.33017 6.214064 8.63601 6.282168 5.649734 13.10447 5.660935
2.618878 2.02158 7.62799 14.77693 5.523563 4.062028 10.42934 8.782908 10.14135
4.02486 4.828447 7.177761 4.436607 12.90899 6.652583 4.842612 9.681018 4.958268
7.847445 3.901241 2.97177 2.889069 4.140874 10.82277 3.927565 6.011095 8.934288
5.69226 6.789648 26.11102 3.530994 7.538316 5.493438 7.077083 11.68539 5.379348
4.380154 5.277753 4.486645 3.701944 9.151164 15.06019 8.606772 5.360029 10.38819
6.914953 3.47212 8.556745 4.853619 3.70649 5.925135 4.223492 5.384635 5.163141
10.31288 3.233858 6.807082 9.200456 2.691556 6.077514 4.775598 6.60015 3.631634
5.476964 2.752353 3.852683 4.370694 8.386634 14.67091 5.190383 6.193124 5.854534
3.749239 5.632816 4.17566 7.25048 12.10021 5.410904 6.211176 13.55124 20.33754
11.9032 5.519583 15.30114 7.780177 10.28542 9.451098 7.335239 10.97201 5.046333
7.071455 10.66068 3.084731 2.621066 12.89065 8.574503 5.604064 4.847438 6.506882
4.502909 4.34222 8.004587 4.585483 5.536785 5.655017 2.865786 4.740856 6.212212
4.761287 5.720047 4.59599 9.572613 6.230695 7.990451 5.284774 6.965555 9.529419
5.28507 4.013497 7.045544 4.589774 3.515021 18.41299 4.382367 6.061051 10.55561
3.699035 2.386996 6.212255 3.724067 7.764983 4.496953 9.277336 1.541902 4.361383
8.812961 5.691611 2.974888 5.37128 9.693081 4.783041 10.52258 10.05537 6.695306
6.013922 6.216378 4.934386 3.746517 3.195873 10.77185 4.037646 4.340211 5.842556
5.070669 3.337477 7.040476 3.041713 10.03251 13.17382 5.491689 4.158637
PEBP1 152.3155 142.7287 170.0688 127.9932 147.6118 153.2831 187.2191 159.666
110.8912 117.7261 156.0587 421.7965 195.6007 178.1574 148.1124 155.3349 333.1951
302.2175 257.6436 232.5168 479.6643 551.956 321.8227 131.9843 536.435 355.2635
265.4738 163.3218 578.0921 176.5418 137.6702 80.38531 295.3513 103.9834 407.268
184.9868 210.7501 319.6677 240.4891 169.7224 194.2869 149.2581 370.6285 214.5385
158.9327 350.2541 241.2832 411.9413 387.404 424.7521 168.1564 120.6809 240.2337
173.8896 367.274 178.0492 240.0972 52.04635 381.5085 162.3951 183.3123 132.4988
387.4955 146.3793 167.6405 141.4719 99.16783 205.2346 154.3058 150.9247 196.6133
161.3866 676.8987 135.0745 91.1472 369.1455 185.1782 181.5167 375.0337 260.8358
210.8688 578.6444 106.912 221.1814 225.0968 148.0335 101.1848 104.2436 302.4737
156.3503 674.4118 322.6563 159.0006 164.4471 152.7589 255.3662 542.9607 135.6395
194.1982 223.6965 94.14367 636.8134 1203.757 424.8295 368.7129 329.0629 137.6409
218.294 157.8016 188.3499 272.2882 449.0295 202.345 209.4729 550.7296 418.6601
197.7279 192.9668 198.4823 429.5485 204.3182 181.6069 298.129 459.3384 745.1209
217.1845 148.2474 396.046 492.4555 255.7759 236.704 77.59769 420.6435 112.6622
88.68964 163.0072 599.2177 75.9176 185.1998 403.4207 380.7901 336.3146 202.2637
538.6949 117.8598 662.6798 51.12584 324.7473 274.7378 171.811 286.9755 115.0798

235.211 143.7808 153.6527 555.3076 139.4997 66.43429 337.433 552.8041 119.3941
604.5825 143.4071 272.0796 283.9895 395.8141 303.1977 253.1298 1112.882 160.0704
75.34048 135.5398 106.6248 89.77603 350.7528 661.6617 165.8386 44.88825 550.4482
91.43068 112.7548 329.1834 147.0235 270.6578 250.0944 186.7856 271.9845 146.7084
116.6667 356.67 143.335 440.7289 117.2636 386.6633 249.4827 243.5013 174.2007
283.9631 101.8361 163.9603 119.151 472.6106 137.9976 142.4251 183.8721 60.00536
429.7962 206.5077 113.3776 176.3748 296.6749 106.4039 184.5426 384.0505 396.6694
112.4078 826.8251 309.8865 81.12401 96.7197 101.7787 73.10615 136.8581 518.8675
145.072 683.2055 147.1215 477.0502 108.37 199.3249 155.9314 69.6597 181.9496
305.6708 232.3329 130.3602 168.8215 183.329 111.0062 121.2528 352.049 123.4379
1089.197 251.4159 256.6098 233.9848 144.7648 435.322 180.3858 151.8496 382.5851
587.9031 571.7328 189.8971 221.646 163.5867 425.1146 160.9606 401.8392 177.8912
167.7707 420.4706 200.5607 222.5881 240.3971 219.3818 303.8102 522.7734 156.0853
138.4428 333.7101 218.7846 595.9012 488.1823 757.6664 223.5375 195.1231 321.6801
209.2676 511.8458 123.6323 25.39553 130.1503 190.2207 151.4048 731.3746 178.5715
108.0643 103.9359 214.9863 164.3107 60.99397 147.3218 237.8346 209.3122 112.4351
174.1325 180.7275 489.4435 115.5204 476.8236 115.34 137.9772 149.4416 115.4624
151.2426 301.7418 142.0022 242.0692 436.1092 192.5107 220.7284 158.0598 405.4536
308.3913 256.4669 187.8076 104.3071 138.2129 149.265 99.80067 86.86556 119.7783
362.6745 129.0508 210.048 325.0278 103.1333 138.4758 430.8923 209.9203 441.4045
115.776 340.4788 150.9585 162.1673 277.1168 146.1533 111.3971 92.27566 112.7481
232.7069 286.7002 83.1075 291.1971 32.24222 137.0516 242.827 140.1369 158.5846
332.1693 101.4521 100.4614 290.5917 248.578 201.6254 184.8569 288.487 295.901
366.3612 212.2592 184.7187 566.4865 197.2613 378.8465 84.11027 89.7009 211.3312
169.2767 182.7132 89.47511 236.0321 287.2401 70.41123 277.2775 164.0039 134.5529
353.3694 98.70277 70.57905 257.7238 234.4253 344.1745 1062.407 62.11932 134.5786
147.2072 90.24393 386.9473 221.8335 180.0795 232.6009 117.5861 120.305 222.9523
363.6963 203.7378 279.5198 368.051 104.8305 440.4189 316.5742 191.8593 136.9728
235.0167 110.3099 158.9205 229.8707 283.5874 190.0664 63.46122 174.7048 331.5467
123.1423 155.3241 344.39 303.5174 116.7241 262.0275 124.7076 453.6151 172.3507
122.2447 152.2957 232.0758 118.2997 280.5258 107.1206 313.1443 444.4856
ZEB1 1.990745 31.02104 2.348566 33.6155 2.049865 4.890106 20.3097 16.71274
26.48579 32.7233 3.38708 11.65387 9.764088 5.241533 32.5145 2.547024 2.308278
1.648191 1.495901 0.6535802 2.667225 3.42784 0.6823966 0.4471971
1.071478 3.659521 1.867699 0.9199253 3.7422 1.101881 6.328889 1.293252
0.2912434 1.171862 2.920774 1.143937 4.943987 2.064455 2.08003 4.127296
0.7426878 0.8878937 2.39793 7.178546 0.4897787 0.9942233 12.17109
0.4157495 0.5604226 2.212801 0.6922096 1.617575 1.304468 0.9040599
0.8993174 1.154467 0.5755443 0.4077859 1.608297 4.977403 1.046735
3.720089 2.442213 2.481785 0.8319112 1.169237 1.382442 0.5534093 0.6327521
2.871343 0.8079201 0.2177717 0.6332336 8.904964 4.184123 1.809482
2.927824 0.5329806 2.244693 0.1989046 1.482236 1.169591 2.260706 2.559934
1.818511 0.8137814 1.897458 2.526337 0.4942239 2.238916 0.2746741
1.798168 0.2643033 0.4355427 0.5160027 0.7235206 3.668707 2.197836

2.413942 1.011616 0.4012559 3.102824 2.650151 0.8171868 0.5044883 2.77264
3.074412 7.913536 1.978823 0.8802812 0.4517187 5.080568 0.9981741
0.9185219 1.014919 2.735412 0.6782815 0.8269196 4.501033 1.053059
0.2978751 0.7543941 1.310595 1.629564 0.8512286 3.407732 0.4109172
8.078542 6.801903 0.1075631 4.376256 3.635915 2.741725 3.002678 2.71207
5.459351 1.307036 0.9086778 0.6978339 1.1088 0.2885528 1.322703 0.96612
1.002398 0.9950437 0.9492067 41.06123 0.5247574 0.5249647 6.360427
0.523565 0.9435241 2.061249 0.7854483 1.592589 2.303913 1.223163 2.500918
2.53683 0.5607867 5.458949 1.793199 1.394314 1.693219 0.1327767 0.4460041
0.7258387 0.9833762 1.94256 3.761039 2.441023 0.4350898 8.932423
2.428733 2.739854 0.2894443 0.678422 1.002622 1.129036 1.765037 1.328331
2.052334 4.763742 1.292747 2.710173 14.95034 0.9396065 0.3611929 12.53129
0.7131884 0.2627356 0.8325674 1.828936 3.040699 2.371729 1.639094
1.655649 2.917069 0.9268442 0.6753141 2.309898 0.9101129 1.109586
1.826724 8.920496 3.045505 3.514571 3.734307 0.1629526 0.3646206 0.2988011
0.9654164 1.345415 0.3182062 4.079688 0.4045542 1.408379 0.4328773
2.613562 1.022397 1.154782 1.775873 2.272216 1.282285 0.3998823 1.42508
11.53708 1.089789 1.286044 0.6031424 0.8017063 0.6759178 2.72904
3.760277 0.4860842 5.345642 3.425189 0.2504598 0.7246541 0.6585283
2.166276 4.462983 0.5912228 2.751796 1.168954 1.252045 0.8781399 4.025754
4.498001 0.6670673 0.5840763 0.3377295 0.3698998 1.580576 2.417537
0.2029517 4.925521 2.641535 1.510923 3.532416 0.3584718 2.943443 0.2976307
1.803383 1.625346 6.220001 0.7778985 0.289399 2.941955 2.258987 1.968558
0.8565464 1.540107 1.831221 0.8531724 1.444603 0.7846259 0.3107384
1.488873 0.7330103 1.706348 1.187831 1.294828 1.162445 1.152595 0.4172242
8.314077 2.887347 0.4580394 8.36552 3.009692 1.41729 6.024745 8.933449
2.714605 0.27289 1.048755 5.419793 0.5467768 2.310074 2.772838 1.740865
3.963891 2.187104 0.6850424 6.422979 0.3944603 0.6065133 1.1015
5.692322 1.206092 0.8647075 0.2653283 0.7905066 1.414149 6.135633
0.6734753 7.613483 1.395119 1.160815 2.516944 5.117777 1.618322 0.822761
2.037088 1.062777 0.3447275 3.245762 6.584752 1.983155 0.5211918 4.562395
1.003525 0.8427888 0.8137857 2.598588 2.518602 1.166818 3.386646 0.9650417
1.232329 1.540767 0.6973988 0.8773734 0.418945 1.683055 3.955194 1.12051
0.2992189 0.3944224 0.8307506 0.6812224 8.408953 2.41675 1.376782
1.707468 5.675753 0.7187447 2.024169 1.017926 2.45662 0.4984424 0.7789457
0.7377958 1.005658 0.3885335 1.345578 3.119003 0.3382549 0.6686162
0.4571348 3.981654 0.8117096 2.322865 0.5972971 1.707454 1.717572
0.4386929 0.3541088 1.705981 1.13536 0.5000082 0.4001375 1.508237
1.009629 1.093748 14.50861 1.569379 0.4952182 0.8894442 0.873951 0.3092496
9.820616 1.534342 1.169012 0.4053415 2.584312 2.775563 1.499756 3.550832
0.5787691 1.341289 0.4072463 4.540089 2.67111 6.83919 1.848391 1.030206
0.4339959 0.2559033 11.95309 0.7888007 3.064985 2.192595 3.3988
1.929238 0.3077774 3.46972 0.2815845 0.9776916 1.259794 1.374646
1.353199 1.988146 1.419664 1.43063 1.098869 0.9761714 4.312394 1.545419

SQLE	3.195175	1.194853	12.00429	1.198255	17.32864	6.751465	1.675736	1.456984	
	2.157884	1.488085	21.22756	13.71209	4.017403	4.723518	0.5618387		10.87931
	8.665175	13.09475	5.526365	21.99832	15.0628	26.40518	13.88814	54.55692	25.82092
	74.33608	19.60175	11.2906	17.66601	9.113667	13.11601	7.16858	4.665401	14.56237
	11.60979	8.890789	15.89332	22.20813	11.52659	8.186164	19.21733	6.905805	15.73488
	19.61121	8.127261	25.35344	4.688987	37.50393	34.84077	49.9688	34.28038	9.85839
	11.71803	10.33773	1.237219	14.29167	22.36805	27.90437	31.8168	55.48828	17.30326
	9.157565	82.7256	13.77571	20.26074	10.67881	6.944321	11.53951	2.714303	17.87676
	37.43745	36.81639	35.90481	13.53212	21.03216	11.41658	41.79287	22.93637	16.43916
	3.157947	66.04664	14.34722	13.65458	32.97371	32.81179	32.31736	22.87192	9.914687
	5.326305	20.48978	28.06563	14.77009	11.10545	26.38075	19.3324	15.11746	22.88649
	14.96629	27.22203	11.02919	16.48195	11.75767	12.97049	15.90405	7.365698	17.95513
	20.74406	23.56696	10.29797	8.669857	20.00501	18.45244	21.26384	21.90247	31.8819
	27.82742	1.256304	15.88473	11.36339	25.66708	16.71806	49.19025	36.92034	23.87501
	24.21617	6.133884	8.444831	81.00341	13.67416	8.574109	7.867787	11.15514	16.11906
	7.946887	52.38808	15.46509	17.68963	16.46688	24.60233	33.75468	20.38256	12.98206
	8.265826	24.09472	20.05565	92.47306	10.62462	2.71129	8.714961	21.27337	24.66186
	8.648033	10.58049	81.46432	27.12174	10.5579	9.079921	22.09562	8.49869	16.24665
	16.51472	15.2866	29.79961	27.49029	20.10982	21.59494	19.88757	25.51398	10.77824
	11.15783	7.203161	21.4112	8.202496	29.31188	29.03287	28.59821	12.29887	23.42392
	9.741473	6.106103	12.34756	19.47981	22.267	22.87148	14.9363	9.052453	27.06387
	29.17812	6.004037	8.538313	17.30441	9.607057	50.51148	17.62228	17.11186	37.95357
	3.492362	23.09393	24.41058	16.57763	18.77802	23.71388	21.21449	15.18287	6.185141
	9.01824	12.86256	15.81209	53.63201	14.58433	21.97592	46.61435	37.15501	29.54714
	16.80738	15.97486	9.661525	3.924474	12.35054	5.089369	4.70861	23.38229	15.88707
	21.70211	9.966697	10.96108	23.33066	31.82469	22.0095	13.67206	28.56361	6.734984
	18.87292	22.78121	24.16233	6.036546	16.67497	41.32915	12.56025	39.62716	35.74091
	4.001931	22.74055	16.91692	14.14782	30.85116	10.6299	28.99844	29.57143	20.54593
	10.15429	102.8525	10.20145	27.67875	3.808972	33.81387	12.129	22.9789	28.54701
	30.02444	92.64375	24.04631	6.110273	17.59837	19.57888	9.197121	9.628001	16.85758
	15.50815	9.685975	51.69682	7.538455	53.10148	21.48803	18.20309	14.94806	36.9378
	26.37664	10.75114	53.22805	15.96488	10.66765	137.4323	31.3866	13.39193	14.53691
	6.121353	18.42107	7.610672	21.66537	7.374312	10.02968	32.43474	13.88839	30.42037
	13.34003	33.2106	55.27341	69.7567	11.82966	38.98899	32.79402	5.091246	7.779698
	13.31918	8.965703	12.25416	9.264257	10.07919	35.35302	17.48731	13.92948	11.74707
	43.01288	31.44922	8.103387	16.48672	10.5803	10.70876	20.68838	26.55398	20.25029
	45.28899	35.66554	15.02869	14.61063	54.5829	6.871139	7.182128	39.50464	11.52864
	34.3207	17.5462	24.31735	14.41232	9.28582	2.530303	36.08892	31.67218	45.77686
	11.89236	29.48928	4.139158	10.77122	18.11733	12.40278	33.28944	19.97424	25.21617
	16.55609	16.84094	20.31051	28.76139	11.50126	23.73571	16.5915	43.64723	28.67083
	56.69811	28.64933	15.44471	2.760481	13.92023	24.0777	38.15265	5.204513	9.219031
	29.03055	34.83706	3.886211	57.48573	7.408135	22.93434	10.64996	9.945497	10.03515
	14.74108	16.69111	22.29984	5.414102	8.821429	7.981484	28.25461	45.54635	68.10987
	71.17176	5.355885	8.352638	14.27879	17.13714	14.85112	28.45886	10.68386	13.61289

40.22794 12.45356 1.674074 7.032934 26.20964 17.44177 32.88574 27.52147 0.5012865
11.69749 15.79222 27.19398 7.988413 21.16184 14.83705 20.53973 8.632533 20.34044
40.43406 14.77426 11.22936 16.62367 6.261156 37.85107 11.82803 27.55575 15.35949
13.3703 8.027975 8.898114 25.68727 7.848106 13.49498 55.72035 11.51411 25.16202
FADS2 1.466414 6.525749 1.054839 5.137712 0.8182502 2.427664 4.463565 3.449012
8.401526 4.168565 1.891434 11.32526 6.657505 2.184998 2.748221 1.126947 1.146038
1.736933 1.127322 0.2719863 1.570773 1.701978 44.68735 11.31135 5.156556
30.83641 9.639203 1.408482 4.586327 4.868043 4.249722 0.9480622 0.8729634
3.410384 6.899031 1.914711 18.48826 21.31017 4.652891 4.571973 1.441047 0.597808
3.015686 5.393925 0.4103745 11.43907 5.780586 55.70717 3.47741 1.058674 2.24922
1.762946 3.496705 3.471372 0.5529774 1.377219 5.450346 0.3019081 6.007248
11.25058 1.792593 1.747536 3.220967 2.418627 4.316822 0.6548478 11.78367
0.1578745 0.3210841 3.191177 15.08163 0.3749076 46.83574 17.75145
14.94994 3.192844 12.31288 16.6585 26.88521 0.1304617 29.38448 0.5851074
4.942876 6.094443 1.165827 1.685885 1.55552 1.998802 1.16939 31.20772 38.1942
2.567261 0.699801 15.34816 0.7256009 4.324325 9.138544 12.04255 21.1031
1.334638 0.4846094 0.9034125 12.51604 2.126545 0.9015741 1.398569
3.226718 1.033229 11.71606 0.9916148 13.2976 1.662719 0.6617552 5.901839
0.8794778 74.23404 0.4094901 6.101381 13.85526 8.291182 39.09816 66.50119
1.605613 3.094488 2.461968 1.873028 0.6485071 6.002051 6.847602 0.2346937
7.639096 4.906626 0.9070097 14.32056 3.587411 13.05469 4.279854 1.254712
52.47307 19.67419 0.8365935 9.824932 1.418063 44.20873 1.212497 1.096683
3.224055 0.2274026 1.249514 4.701451 6.644917 7.373643 2.850057 41.48623
2.843149 1.426927 1.793039 2.731478 5.776904 6.646199 2.146123 11.80871 1.114336
3.624934 6.492926 29.40021 4.141289 33.0356 0.3616167 5.284004 0.7338988
31.26865 9.716045 3.548431 18.09829 5.118383 5.700956 1.955491 5.709878 2.010808
0.7326625 1.121433 60.8764 1.01014 6.456571 3.189918 6.177386 1.590675
5.947495 0.6372118 6.906056 13.69997 9.689215 1.864045 37.53527 5.491943
0.8205197 23.7548 36.05502 1.118822 11.18612 0.7181797 7.781939 31.54796
4.015753 1.967394 10.80666 5.713562 0.4649926 0.2078736 20.89637 1.743508
3.278386 12.48872 2.731407 13.74398 1.391607 0.1554778 1.227687 1.427312
5.898922 2.607334 1.818627 17.44117 0.286031 20.14642 4.12009 1.439839 5.611347
2.344099 1.637691 0.5690258 15.53592 4.168996 2.062539 4.746563 3.03079
46.92518 59.56638 28.0837 3.781089 3.043116 1.871476 1.653584 2.4494 3.572083
8.35701 27.31195 31.40895 6.316738 1.337885 41.60684 0.1923544 1.823207
3.456118 0.6496074 13.61156 7.243353 1.251579 18.24282 21.08541 4.547907
1.954244 1.464909 1.287499 6.28576 38.05898 3.005527 4.214163 8.764011 17.34649
2.113529 69.96632 4.099127 16.71579 16.02601 26.02621 4.450002 4.731207 22.68315
11.72826 1.863729 3.499305 14.27696 1.685783 16.74188 4.818022 7.988045 0.7423413
3.868206 9.849952 2.008804 14.10788 2.030572 3.39455 7.066153 8.946101 7.804459
0.853808 6.407984 2.107429 1.648907 1.207773 5.145552 0.3734294 2.16046
3.700325 3.271527 9.563892 1.442213 3.729277 0.8283795 1.0288 10.76823
13.79797 4.704856 1.972706 2.621796 1.275747 3.015503 3.492771 3.019673 1.042034
33.75536 3.094685 0.9682805 54.24052 4.649667 7.692748 6.682565 26.51113 15.7939

0.5802508 0.6464829 2.265552 9.25312 40.73801 16.3849 2.506951 1.976975
1.550374 8.596437 0.3577296 1.899152 7.275186 3.067244 5.150627 3.989539
43.79703 14.6209 13.23628 3.13461 7.595332 10.14552 0.5622235 3.858847
7.097696 70.68264 4.468774 1.778506 1.647048 0.2891671 0.348444 33.17376
36.03224 1.211975 3.507191 13.15848 1.240893 0.1446995 0.842991 3.645038
1.905458 47.39898 0.6663004 3.704493 6.898171 7.47316 0.1226957 2.169515
0.556308 0.2832997 14.00093 19.47182 1.500222 23.15744 3.168252 1.795379
4.974785 1.077328 2.061966 8.888827 4.531665 6.439752 4.193044 8.170871 0.5342519
1.58791 0.9728245 7.916865 6.812608 43.20694 0.6965425 4.921784 22.85296
8.833294 5.376955 9.412673 33.63042 1.498134 5.839963 1.373339 5.35832 1.83426
3.661115 3.897473 0.4027275 19.42777 6.910694 1.613913 23.7917 5.729507
5.382409 3.052007 1.471219 1.456785 2.092424 18.87429 2.501414 4.095931
NFE2L2 52.85166 20.96834 49.65975 30.50386 50.50414 58.13445 31.41114 42.25759 26.1769
49.18778 36.16068 46.93883 49.34291 37.53556 32.99916 47.98307 42.1908 14.6734
43.06331 16.96958 21.14955 17.51378 44.42078 30.68002 32.07544 33.03835 24.10601
16.65535 11.40174 15.09265 30.66341 18.12795 17.32768 56.00907 43.08664 10.18587
13.25695 16.70473 26.46983 16.2475 15.28042 23.06648 17.70629 23.35116 42.08566
84.84397 9.338857 16.77454 25.70681 35.1297 16.19977 29.76139 21.88637 57.91373
16.71855 40.83301 27.42218 27.5747 18.82425 19.1972 19.39983 39.67029 22.85491
25.91739 27.16643 37.75731 4.854821 37.90694 34.04496 16.15467 14.87514 14.60639
54.24576 24.4188 12.76131 19.20312 21.91436 55.08555 12.89927 26.90803 28.17592
25.00113 45.80065 30.41062 16.40101 26.66717 13.101 18.81277 8.010391 9.009064
20.2417 27.94554 32.52442 17.10879 23.41668 11.24791 19.26782 11.42774 26.11493
13.08012 34.38498 17.97431 21.90694 23.24489 17.35316 17.77851 13.8235 21.51548
18.69646 8.22896 20.72577 16.83947 38.16157 62.47842 15.1054 15.10513 1.950466
19.95601 20.59671 27.6514 29.91573 29.2647 51.07917 14.9931 12.49668 41.22465
13.44373 24.49959 33.76689 18.49628 53.85178 15.35008 40.27297 12.49636 20.90536
20.21596 15.15272 15.45371 19.34216 22.39034 22.59719 29.46936 36.23143 28.07299
15.23316 11.353 39.01979 4.92384 14.96107 27.18317 26.8516 8.672582 9.348652
41.18208 17.49951 11.10267 19.00026 24.787 15.20168 19.69778 26.049 28.28351
33.7937 21.15221 26.96973 43.0179 39.44357 16.77292 15.48812 30.28356 52.63108
5.265187 25.59813 12.63571 18.08993 6.392314 58.1583 27.1906 12.40209 14.50914
41.35669 31.76881 30.97868 40.53585 10.77044 35.04495 17.96087 38.64485 16.05102
11.28761 9.898801 20.42489 44.47925 12.10345 8.042095 20.40356 15.77493 18.75735
63.30231 20.59498 14.01273 13.27499 31.16904 79.93075 20.51319 29.96568 27.29965
40.16092 23.44014 27.62371 13.63684 40.72046 18.8662 32.65135 23.34875 50.19302
16.41518 24.60509 20.65612 25.39691 26.02853 24.95607 26.61961 14.03485 17.01535
13.10731 28.71583 39.34653 32.11524 20.90692 23.65932 17.23855 24.35495 43.36155
18.82829 13.36462 20.09872 18.50993 36.96533 17.25391 38.40105 20.99837 26.7735
30.71663 12.55723 19.38771 51.64806 18.66756 16.46774 21.15839 13.52905 23.3319
20.20708 13.78327 20.77094 12.22227 26.01797 31.15582 22.73596 72.29568 32.36435
20.36816 25.49849 24.33735 46.93346 28.92273 15.63859 16.81508 39.18201 9.822932
21.36868 25.36892 20.90004 16.43262 16.85724 11.89254 28.51308 24.0354 27.76302
17.20765 10.76241 25.16677 32.77692 67.52683 10.49506 8.906471 24.30542 37.03081

23.15737 19.53602 18.58935 18.06474 28.14132 11.68322 52.26299 17.79841 13.36834
29.41229 21.16925 17.08161 19.74343 39.26908 17.64691 19.15973 39.21291 16.64581
8.479217 13.77745 10.77815 20.59316 19.85621 24.02102 31.33521 15.49126 42.02649
19.3479 37.75124 17.31223 17.59221 13.96504 39.90775 92.84492 41.39068 15.53745
14.0133 26.58542 29.02492 17.18831 16.96142 10.65504 14.12231 17.61293 30.11204
6.063926 19.34257 14.0383 17.5703 28.86312 26.70935 39.71631 33.96831 18.96224
55.42229 24.27662 28.79954 26.56462 19.28818 20.30086 41.68333 17.66436 18.54274
17.4214 34.19325 39.94815 36.05349 28.90991 47.43524 40.04909 11.12312 20.40153
47.60472 19.42457 12.33482 8.057565 22.95977 41.36365 24.36658 20.55504 13.41789
37.47297 34.00311 22.66735 19.86667 9.680156 18.07376 23.23667 17.52893 25.10121
19.48447 16.09391 46.1038 27.2101 23.76065 19.94792 28.66548 43.16632 28.71789
21.36282 19.1578 15.52701 9.988063 25.73586 27.56047 26.91785 56.09611 8.509325
18.83837 20.744 11.1874 20.66746 12.05539 37.68432 1.873684 11.60546 26.47298
22.26417 11.9751 17.829 20.0738 14.62978 21.90844 53.85774 28.41935 22.95957
14.52863 11.14025 28.19153 18.31874 38.53399 10.74692 5.279994 35.33165 18.4671
14.28964 35.92437 23.44218 22.74767 33.01469 23.89158 14.8019
KEAP1 26.74695 19.06658 32.79622 13.13513 15.90217 15.60875 20.17886 20.33389
18.08322 11.02373 37.03437 20.26992 19.27767 24.20616 19.10526 13.54572 27.37959
26.42146 23.43617 29.68976 53.81394 20.96875 27.78766 39.16509 48.11024 17.86236
27.72095 34.69101 21.84742 27.95309 22.05009 35.30671 38.99373 33.10417 29.76431
14.77453 25.21719 19.96245 20.31381 25.9922 22.52505 16.52279 20.93275 19.72825
21.12672 31.64681 28.21494 34.31662 33.69337 14.35211 27.94035 37.78683 26.74158
38.39327 26.60915 31.51269 39.04638 27.43386 14.52949 20.10518 23.84623 29.42243
20.95263 31.79535 46.60145 40.8406 34.36109 35.14433 42.27817 24.23782 29.26438
17.59958 28.71162 17.71733 21.32066 29.66711 27.27083 33.4135 17.13301 36.96464
34.33676 37.31839 52.62012 20.3209 20.93509 29.02711 21.00802 12.44656 40.276
30.7496 19.42688 18.29786 53.9714 28.86209 34.78565 29.34674 36.98057 27.25395
20.71276 26.55515 23.06332 16.15097 27.6399 31.1888 30.74077 24.88929 18.76325
9.867272 27.48653 16.81091 31.23112 36.2162 34.29031 78.36056 32.02324 22.61994
52.20521 25.17764 17.06148 27.21333 24.76504 24.08723 22.82924 40.38036 16.61776
21.64387 33.01689 19.43536 23.01527 34.25144 25.76558 13.30729 33.8025 25.20001
16.31838 20.72095 18.41851 14.76324 28.0365 24.57093 24.62507 18.83962 25.99626
32.73148 29.29307 27.36813 23.98071 8.839107 25.42525 26.05775 37.02987 25.79661
13.71031 28.8332 17.96572 24.53569 27.96187 15.90895 27.31094 34.93162 30.96635
37.24555 19.14478 20.56476 37.79196 36.70139 18.92184 21.02845 11.72561 19.39312
34.78371 27.79214 18.08933 34.67606 21.01532 22.70848 31.02713 29.17668 21.54902
32.15786 20.9631 36.809 18.84792 12.11157 43.42873 22.55847 19.11583 33.18454
18.29041 21.41472 28.48979 29.71419 46.94328 19.76358 35.21731 38.09433 29.27013
34.99409 42.96872 17.49176 52.71191 25.6707 24.96038 36.05712 16.09874 20.79522
20.34132 37.29362 40.67783 23.41825 25.09138 32.09408 22.98485 19.15004 13.98162
34.08323 36.22509 29.94546 17.49493 23.29386 23.91182 19.32283 21.21487 47.61735
17.96115 22.33501 17.21316 63.54558 23.37971 27.32903 19.41398 37.88665 20.73804
32.28588 35.01629 22.85102 30.82533 28.81788 25.95701 48.5551 37.22691 18.58107
38.31948 26.62963 33.97967 26.59794 24.78063 16.60626 15.42067 26.91061 24.98448

	19.81059	18.66767	21.83275	27.76722	15.01141	25.1965	34.35551	44.15542	65.7019
	88.75865	24.06217	38.13013	23.7163	23.11968	21.529	40.02539	46.88956	29.8969
	26.96753	27.69648	34.41994	20.98115	20.97969	24.46717	17.88825	26.36019	27.39582
	21.42275	19.16653	24.67981	37.30853	28.32719	71.18103	31.88888	22.90748	17.82921
	61.59362	24.21683	22.62099	47.69344	21.49424	20.76482	36.20534	23.45805	19.67089
	15.48277	13.39696	26.75055	20.34185	30.108	51.31971	21.36807	39.44565	31.65567
	24.56156	35.97381	30.21659	35.59829	22.03561	24.14335	24.88147	28.89844	18.71622
	23.94623	22.38971	27.42264	27.19893	39.02017	36.31245	14.11013	37.64072	26.94086
	45.03813	23.21306	23.34616	42.0869	22.08329	20.07992	12.67622	52.46535	16.30533
	16.82296	21.55442	37.87177	22.17405	20.09983	24.66837	18.40278	34.14031	32.54211
	21.06364	31.9065	33.56457	57.63213	16.34319	25.69738	31.84351	29.92671	34.26808
	22.97522	24.53783	9.845584	28.39163	18.64664	44.92683	28.46757	20.1131	20.30043
	30.67506	38.61449	21.64631	15.42549	22.99458	39.21426	26.49583	20.53001	36.40196
	31.75556	25.24643	17.61371	33.15784	35.22028	19.2049	31.1438	21.16326	23.13939
	41.49703	32.96373	41.16444	26.46441	23.34851	17.49232	20.5115	26.0347	20.82373
	24.89993	21.7958	29.49343	33.90144	19.75646	68.6082	56.86394	49.25831	46.81564
	75.94887	25.12309	52.12308	16.13608	24.39559	32.19244	22.16166	28.54624	18.04464
	19.18137	15.62072	32.11449	18.3851	30.32079	28.15582	76.21214	27.70995	23.83806
	23.52919	25.8188	30.80677	28.74994	23.70865	32.65646	21.35245	42.42036	23.82408
	22.23495	34.55218	27.11047	31.13672	18.30987	22.93848	30.71184	20.45274	
NQO1	12.69518	25.9378	57.0724	22.3981	20.67674	7.463423	20.68931	20.35372	26.2566
	49.89394	342.6533	47.11986	15.70921	36.4186	28.94715	11.43377	10.32682	23.8131
	16.20918	94.61723	1.873975	75.04279	116.2769	207.46	65.73272	86.10914	34.75389
	128.4761	31.67698	88.3021	35.04352	12.27446	21.41071	36.78838	96.73791	82.10402
	13.25392	31.75125	69.81443	81.00668	53.35783	12.22554	60.46389	17.28164	7.723206
	148.1747	30.62891	59.59266	95.8237	35.36013	1.445321	30.61638	53.34619	62.44781
	213.5625	17.47876	121.648	41.14521	34.30487	9.954811	29.65952	62.17834	166.9682
	65.22455	20.3655	29.66034	11.88624	113.3423	2.402891	11.63386	49.43832	57.48753
	92.34124	380.9183	16.5297	38.15949	36.5306	95.64988	18.95096	68.1643	102.264
	66.41724	216.496	2.438357	31.44366	42.22436	13.75744	17.94657	29.23334	2.698191
	48.30923	24.27117	119.3574	22.96758	94.66483	196.2889	55.27867	12.10437	40.08666
	21.58056	23.78432	13.48046	104.6681	79.17638	78.34094	69.09616	8.042327	50.36689
	133.6626	43.01041	129.6525	106.4212	7.287543	51.1522	79.37722	11.9521	97.23873
	29.59322	40.23113	53.41245	68.63252	115.6551	30.27132	20.77627	45.31768	12.8331
	83.00412	28.37306	31.63749	60.22431	49.75535	23.73442	101.0004	20.42949	16.54382
	44.56024	7.413708	53.45674	45.99923	56.98473	41.8638	55.79427	41.66247	79.23523
	38.50841	59.75043	206.0367	9.997107	73.25001	75.98412	39.09581	41.0304	3.046548
	63.97031	3.200959	41.20908	46.45649	45.06444	23.22202	126.1704	118.0984	96.19001
	176.0359	68.86635	40.89583	41.14948	105.1722	39.22046	231.0062	1.998332	282.607
	4.996957	13.60092	41.51266	171.2932	6.877675	80.46717	19.3168	51.02173	14.14576
	48.9648	153.909	143.1566	60.27822	42.48515	33.7964	36.50265	55.28119	11.51119
	20.39789	115.2101	5.698955	197.4181	30.13085	6.54874	42.49192	12.70164	13.59474
	208.2123	14.89471	0.5045944	68.21639	88.19144	266.8026	17.77153	6.154514	
	63.27689	66.43364	28.99869	10.5294	1.61141	333.7798	0.9984895	70.1138	

11.53733 70.44337 69.76411 111.5281 39.06923 23.74303 35.80673 26.68137 40.70658
76.70445 24.5661 16.91613 9.094653 55.88374 278.4007 34.30538 33.05341 29.08981
48.10209 32.87129 142.4389 6.162248 2.980125 80.99509 34.40054 434.8557 159.0465
7.916053 121.0203 40.26848 83.22233 82.72969 114.643 18.76632 133.7397 22.72343
46.47098 13.61758 71.26903 24.1384 20.69887 20.50099 118.846 147.0754 64.35918
398.7555 39.03135 36.10204 82.54089 22.69149 71.22342 71.18066 95.89036 10.55772
62.20854 34.10381 61.33856 33.10092 15.65098 70.99306 83.08448 75.98023 2.162995
101.6953 66.68896 15.28089 7.513544 1.932906 4.021026 302.4845 5.349843 123.9117
25.53913 287.2151 24.2862 268.7634 3.787229 41.68125 40.55265 31.02114 55.80002
22.30879 34.93785 8.274352 17.74701 35.36641 3.176456 44.92862 33.56541 30.48049
23.19129 17.66551 36.00871 32.08506 8.184115 81.1274 34.48553 62.82527 73.685
16.35163 72.7186 24.38537 49.5769 47.52466 48.58616 19.51461 25.60416 20.39433
72.15962 220.9445 18.1296 2.241122 115.2918 22.97224 13.97832 28.06414 494.0188
39.17049 15.40808 41.41176 14.84609 43.78762 24.44808 92.79955 27.29187 87.1029
28.46401 10.85032 32.81354 321.8808 113.8456 35.84627 13.9052 95.0713 577.6567
29.36317 62.97643 32.41459 28.0615 98.61321 7.610558 340.0427 22.47319 167.3098
144.941 51.66026 153.6243 105.3759 73.96179 5.062783 131.871 63.41221 16.34431
57.79596 11.28675 4.051837 37.93382 28.93095 60.55021 406.2614 48.43049 66.61351
28.33087 47.72104 73.64226 24.85908 39.26608 46.71356 177.6253 33.59075 16.23397
10.80684 19.62284 32.44557 26.08491 46.81585 29.45017 52.51749 400.5793 162.097
47.12309 29.89505 71.45645 85.08992 58.27463 18.75756 33.39351 110.9547 49.5251
4.232031 355.747 36.56452 15.73157 18.9318 91.09867 18.99606 278.7336 5.608757
79.35919 20.22637 18.56897 60.02654 29.7376 54.88895 153.9372 16.90952 45.31071
100.2829 52.66694 34.42219 49.89489 25.99819 44.71368 46.48066 9.088176 32.76415
NOX1 1.182646 0.1083165 0.6028031 0.1001096 0.4946174 0.508768
0.3350285 4.014178 0.05173614 0.1068174 0.5898179 0.5486238
0.6600774 0.3776912 0.2149196 0.1795242 0.43248 3.004335 0.5396019
1.032622 1.031594 1.004942 1.007313 0.4078824 2.058967 2.068446 2.468425
28.64663 0.1769521 0.4710989 0.3473547 3.78174 0.5289884 0.7322575
0.9674106 0.5862581 0.7889227 3.471905 2.990757 0.2625421 0.3781263
0.3459742 0.2794445 1.086936 0.5242129 1.664778 0.5585822 0.446117
0.5570779 0.7167137 0.253962 1.419132 1.044895 0.4428496 0.4156699
0.5042484 0.516197 0.2234031 0.798416 0.5222188 0.3614054 0.5045099
0.4964164 6.310669 0.6044163 0.4277187 0.3727784 0.4831373
0.369291 0.3432043 0.666084 0.1448319 0.8274896 0.1966292 0.4833715
2.217562 0.2496839 0.6510594 0.5107401 0.8699149 1.55579 0.6957181
0.1581828 0.2287149 0.5458045 0.3722306 0.3319361 0.3824054
7.114168 1.054904 1.704163 0.4520419 0.571649 0.2474056 8.100209 0.8296599
0.6396192 0.4514314 0.2931289 0.7544482 0.3431063 0.4522236
2.284778 0.5027671 1.085941 3.156002 0.4384139 1.063341 0.6996181 3.20385
0.9038919 0.8255032 4.099299 0.4630406 1.145773 0.4604329 0.3723365
0.5851723 0.4575693 0.750376 0.6608623 0.2491297 1.122929 0.5512387
2.932691 4.658621 2.523271 0.726886 0.4384972 0.7530467 0.3955155
0.2626401 0.8070213 7.495236 0.2317109 0.08162951 1.03165 0.4798533

0.2466085 1.477062 3.635142 0.5748235 0.2468515 1.837012 0.3977967
0.5548725 0.2141915 0.5811864 0.7583147 0.4930249 0.8533161
0.5216503 0.5708615 0.1777054 0.305573 1.1152 0.4681485 0.550791
0.2194517 0.6066461 0.4291303 5.903572 0.3393674 0.2963766
0.4844155 0.9311362 1.148974 0.7434398 1.256071 0.4776228 0.7844565
0.4521997 0.1728719 0.1834025 0.4685595 0.1313257 0.8774709
0.1746399 1.438661 0.6674446 1.953784 8.48962 0.2755307 0.4131528
0.5675307 0.4038267 0.5812117 0.7206481 0.3299166 0.5194882
0.4162127 4.779985 1.700144 0.8240576 0.927095 0.5196509 0.5863247
0.1991843 0.07515341 0.4796695 0.2698177 0.4929489 0.7262315
0.5896697 0.6655082 0.1367941 2.708021 0.3680847 0.6185982
1.278105 0.8575373 0.5950372 0.2238207 1.108523 11.1932 0.3042192
37.81718 0.6421795 0.7035506 0.3035528 1.067842 0.1544942 0.7859038
1.331561 0.4726704 0.238369 0.2310337 1.592974 0.2665872 0.6757916
0.205326 0.8517372 1.164004 1.70183 0.7228545 0.412298 0.3935357
0.7495721 0.677528 0.4637254 3.502724 0.1455055 22.82167 5.908537
2.955936 0.5281627 0.8347667 14.75792 0.3269787 0.2859429 0.8119118
0.6133511 4.287143 0.4380423 0.7720831 0.3794744 0.5267001
0.6150524 0.8458351 0.4143219 0.308274 1.304663 0.8188207 6.101626
1.180318 0.2672809 3.353031 0.8012978 0.3969745 1.428667 0.5547878
0.6809879 1.07515 0.4342602 0.5189451 0.274196 0.4174605 0.8483962
1.511944 0.7336394 0.461253 0.7973656 0.6208503 0.4841169 3.668004
2.246832 1.912957 0.6668183 0.4409116 0.4109412 0.3508861 0.2981822
0.2528415 0.5058823 2.149843 0.4962233 0.6786369 0.277165 1.236355
0.5121149 0.6171531 0.4063318 0.4891199 1.450735 0.4134824
1.808309 1.511849 0.639887 3.394132 0.8201475 0.1909828 7.78999 0.7510419
0.491598 0.9116574 0.5449711 0.477057 0.4514597 0.5268505 2.6328
0.7230017 0.4217942 0.1391358 0.3387354 0.5270425 0.2504818
1.301901 0.7725511 0.2330112 0.9839314 0.4592841 0.1914352
0.7881766 4.624989 1.13307 0.7983244 0.2590807 0.6907319 0.2579828
0.1526034 0.8652668 1.080781 0.477066 0.8641339 0.6101498 0.4261274
0.2296538 1.621446 0.3411419 1.002575 1.455885 0.3853464 0.6173321
1.446049 0.7297456 2.397762 0.8930882 0.3453187 0.4310985 0.9309874
0.9241619 0.5587273 2.198576 0.4460141 0.7853144 0.7277366
0.4979551 1.03367 0.4297212 0.7622948 0.1430076 5.934971 0.4221709
0.433361 1.775728 0.5029706 0.1559015 2.936638 0.3330709 0.4422623
2.001291 0.4677443 0.318997 0.33465 0.1682733 1.052736 0.2867757
0.4482733 9.400629 1.279019 0.4000411 0.2115468 0.217711 0.2231812
1.125936 1.996022 0.9385237 0.7343748 0.9508621 0.5055628 1.865996
0.434888 0.3262682 0.2731937 0.5258166 0.1542007 0.3388431
0.5821663 0.4087496 1.402318 0 2.810156 0.9729095 0.6177983
2.323557 0.9003299 0.6554539 0.208077 0.2881747 0.5155368 0.3031391
0.6046376 0.3817221 0.5624146 0.5960977 0.3678123 5.039383
0.6039214 0.5125409 1.909134

ABCC1 8.246676 9.362119 10.22997 9.383435 9.955496 8.805318 8.225994 10.39636
10.52253 7.696244 27.34551 14.16693 12.42038 10.30068 12.89766 9.132385 6.227132
10.19936 9.064045 12.92822 6.357053 3.524564 15.05981 63.91131 10.58451 6.716024
9.249516 15.99893 6.718422 6.361775 13.07975 4.665149 5.064315 27.26769 9.953089
4.720897 3.988109 5.231771 15.06825 12.17698 6.817056 5.92168 5.537102 8.332311
16.87748 10.31005 14.13145 21.93781 14.99894 4.986658 12.86865 15.59874 13.41678
13.88237 9.544713 18.50027 10.73463 11.37129 8.901433 8.745964 14.27927 15.45047
18.28814 8.556064 12.23786 4.269683 3.232589 6.220027 9.648317 10.75314 12.77181
3.661073 11.54795 62.78797 12.95384 9.254481 8.996904 10.72773 13.11876 5.11233
9.083846 6.818193 29.08961 5.428135 8.401242 14.50846 8.680312 9.867448 5.794511
12.79816 6.292537 11.20842 15.15051 5.307436 7.551604 13.14754 10.31612 3.436969
5.720992 20.21708 11.18972 7.127716 7.581652 11.65309 9.310736 5.99465 9.871329
5.325127 8.978222 3.69005 7.512784 9.470112 8.777488 20.68785 8.555783 4.882172
3.611329 10.88083 6.504821 7.382035 6.802955 13.94663 9.28791 10.21686 11.8963
9.359026 6.497905 7.216512 13.64341 6.554017 7.676959 13.24926 3.661598 7.512144
18.07114 7.310601 5.206358 8.259157 4.41364 5.064618 6.737406 5.117636 13.81936
4.041612 9.036913 6.603984 84.06481 2.531025 6.856014 11.68263 9.792568 7.291058
11.20979 10.50123 6.298565 5.87715 8.621127 8.673376 8.61726 7.294264 7.141029
16.47815 19.42826 10.56344 16.15928 12.09088 10.68594 6.593468 5.448706 14.34341
35.31517 4.449557 12.77283 9.962646 8.154118 5.858309 18.03075 6.75549 3.570264
7.934392 14.28314 8.591976 15.64062 11.63356 10.48942 17.22573 3.056195 10.1238
6.599411 13.44891 7.557385 9.0658 74.08836 4.604324 1.030905 7.683667 8.459424
7.048994 107.4617 11.19277 7.524637 15.89993 15.20268 63.73495 6.073124 7.867125
6.508812 7.410224 7.105723 7.01356 15.63661 37.34527 11.32982 13.90204 4.168489
21.67637 7.632327 5.440373 11.73981 5.549628 13.06206 19.95861 14.36984 10.78328
6.977056 9.95052 7.925441 8.809919 14.28501 8.937747 7.20664 13.66067 11.11811
13.46389 8.012263 9.601114 8.294186 19.64686 30.77758 60.59544 10.66599 8.289287
8.129371 9.822628 9.600618 5.199444 25.41875 8.094663 15.48856 14.13483 9.38031
17.5082 6.142813 5.899754 7.641595 3.612263 16.07975 6.880145 12.77306 71.39374
7.485149 5.322419 7.287474 7.380511 6.569718 13.27889 4.415049 172.6338 18.08807
6.5706 10.176 15.92436 15.26966 7.483339 9.983548 2.14944 13.51264 12.03643
8.747668 6.304385 14.80548 8.8071 16.09306 58.37864 3.884401 10.07101 8.087677
130.8499 10.42638 4.99022 6.662755 6.928786 6.297286 21.96192 10.58594 20.20383
4.112662 11.26746 11.00177 9.647897 9.082738 11.93273 6.202782 11.10474 14.80254
5.347548 4.271192 3.611096 6.680034 4.825503 8.314534 11.2668 10.73323 14.01515
11.57864 7.874332 5.879372 6.352897 8.460551 6.927347 10.85659 13.01888 15.5049
13.35992 8.424924 10.44716 12.90842 6.283052 10.44377 7.232742 32.05268 9.615975
8.999872 9.889118 5.82235 6.496217 7.53966 23.04948 6.211443 53.07124 9.467229
6.632132 7.400111 22.82305 9.780949 12.84546 9.171953 5.91535 44.00652 6.453222
8.000091 5.822264 9.153103 9.974028 7.627614 39.7988 12.87613 7.570616 8.427256
14.8683 17.23934 6.052902 2.383015 12.82167 12.6164 8.887522 14.83302 9.005846
5.863766 13.68454 7.616871 17.7507 6.192093 21.36823 6.481028 10.63198 15.43268
6.08296 11.49399 15.43702 14.57328 10.5702 5.775091 6.547607 8.84712 14.41019
8.772482 8.569061 7.716203 11.36777 4.403713 4.634237 56.68673 17.99561 16.6198

10.800196.3578893.6419354.35666813.079145.25202517.926985.4506076.239513
13.9635313.730166.62683813.833866.9166576.947286139.651416.007347.180568
10.4733713.9562 7.0047816.9681018.6093515.5270355.7915323.05426511.00014
12.2153445.9787217.8486154.7123866.22588814.963536.8858996.753258

SLC1A5 87.0214522.96472108.203224.8656338.19862109.730630.1948639.21068
12.8013443.6800182.8788 51.4225469.3457875.2346922.57646121.600996.96061
141.3274207.730768.13146145.370980.8751181.471639.838973 91.08072101.4607
44.7296871.7049 85.99398102.861860.0041498.4161563.416792.898181 74.3484
59.113568 0.2446 51.9559430.3927270.4804294.1799 56.5922873.92725107.8177
95.6640698.88803721.908 32.99982133.707948.599191.21187171.9146128.0268
90.9599944.84234104.2488127.492961.6158 72.6771343.58584102.2912129.8569
68.4293874.26143148.5164116.034550.73563127.1706117.058346.30102109.9183
101.761977.20316121.975737.3971 90.82824151.304289.25986120.228976.92129
82.557698 4.95046117.172846.46698118.920753.156755 3.8588340.70157332.4062
20.9489 68.46526194.2196230.4175122.469622.2797173.459496 0.9136681.85324
59.8023687.9939872.4010474.1120187.6153250.8500895.53013141.219174.28816
31.6962273.2857280.8234157.99576121.958 89.2811842.87239312.013587.98961
135.6648121.918658.2394383.91954102.472162.90632117.8796532.41049.463191
90.92341158.154590.3133374.5888873.64465126.253839.03171295.8408106.3723
41.55794130.218380.3680264.63893112.2258113.372 44.475728 8.276645 9.33387
80.124 57.84292104.337731.2646635.10168109.595275.8519 102.414 91.76955
27.427 132.4116109.068157.4390335.4142 135.9684414.925284.0924473.94928
42.7688271.07883152.492479.50164105.416450.1910365.9699428.38391133.5454
124.9437433.544649.9760287.0347364.690616 6.11629123.0035114.766275.00406
62.9541299.7563967.6381382.1423985.53516142.287950.59639129.0989124.6889
35.4726856.8811180.0680597.47039144.186978.5566735.76225145.221344.02343
49.09278189.642995.1345365.5272314.8135759.40647101.329845.1372856.18589
84.8896171.22035163.713162.5911556.28276115.427887.9336789.6827330.11585
79.408446.76613754.4552674.23611125.331748.7371837.0238344.949889 3.94715
83.5754668.2765752.4838885.31848122.5172121.9059125.313 64.8421385.57163
104.8073110.651624.8603391.74149176.954769.45578172.5152101.428444.12548
142.934769.92379216.958 82.84077214.166163.5584929.7897756.9391786.74445
65.2681 218.168871.9039698.5007350.3688134.65411106.1361100.894585.00136
70.3751378.1074645.8501385.9577290.4482750.8203820.2395948.78382177.7766
72.38681270.362787.8198263.5131469.6100664.25401243.878331.54961107.3674
98.9055 107.260913.16063128.9227106.4535140.419338.8567118.171234 5.49183
94.4517892.7702248.363764 0.135 58.0029335.2022548.111 107.143 35.46649
84.9849 46.87522110.266861.3447223.3159287.7359557.7632148.38746106.0957
49.02826118.315997.1509789.1737152.4970736.9832653.7762544.2813 16.25313
70.7904651.4195862.33086152.789790.36177105.980867.8889579.0033650.92897
80.1315886.9339 38.6771335.8063646.2938433.9453635.5559750.8362678.36514
118.473421.42209115.857584.67685126.482697.4556 44.8241451.54281109.5326
59.3068447.7199252.38698144.755735.21883109.155 55.39567144.522936.19025
25.2767 128.867731.30924153.899255.9323689.7161684.06023178.632348.85126

	60.95347	70.72472	46.81147	89.32326	103.883	128.0329	296.8546	42.96411	67.7336
	170.3726	36.43752	45.54836	29.07343	84.98434	67.93034	101.5876	57.6523	80.65791
	2.513635	56.84215	49.7779	49.71917	90.19718	73.72941	26.61255	34.41221	45.0834
	31.77891	50.06778	121.4254	83.30064	75.86598	66.55409	76.13	104.788	49.32393
	72.41342	53.74951	111.9431	101.4379	46.83613	122.4235	28.78829	84.70197	59.87847
	67.54157	122.4344	64.72893	111.6681	81.33251	48.41787	43.61964	109.3943	100.0043
	126.5604	64.89674	133.92	77.11726	88.25632	66.07241	29.43696	207.6998	43.17293
	64.24004	51.32198	52.90253	77.14089	163.2296	137.2844	128.4992	87.71619	
GOT1	34.54236	9.994966	9.278257	11.63268	31.18833	11.0711	13.05285	14.14548	
	13.89373	8.00276	38.59396	16.69293	15.5354	12.98619	12.01869	15.78254	18.50936
	17.79723	21.83396	17.25539	12.13732	15.40938	19.68602	22.58743	18.70304	28.99526
	18.57099	13.931	27.97975	24.73628	13.63245	20.08821	17.97625	23.9567	24.40896
	11.37541	10.51734	13.04516	37.68008	18.35228	25.32928	18.05605	18.87781	9.107312
	14.49338	19.27044	22.89705	21.28425	25.61693	20.08482	30.93227	14.29353	33.23889
	13.07471	33.76885	10.90222	17.91843	20.54886	14.88251	11.80816	16.36678	15.00582
	20.9359	8.63427	17.85077	16.47764	11.62684	13.6581	24.78385	7.583757	20.62272
	15.73657	15.38986	21.2019	14.52865	23.60944	25.78014	17.57719	10.84636	19.54824
	21.81033	15.60587	18.69747	73.90543	15.19712	11.32024	20.5878	11.05607	11.75124
	23.23486	19.01833	20.14693	146.9113	30.03556	12.73308	21.237	17.1078	33.27618
	11.74883	10.8479	27.95923	8.956827	12.28632	19.71935	20.8663	14.92908	10.58757
	7.644784	12.62193	24.06963	18.37842	21.48811	14.36074	31.27296	17.82455	19.69547
	22.54092	12.8294	9.478497	23.4864	25.02269	27.91369	9.391461	24.2827	12.23337
	22.64129	21.55793	18.5003	9.912316	21.94468	11.2909	16.66707	15.72817	12.09961
	14.31953	10.79472	11.30935	21.51977	10.99918	23.72651	14.36457	10.89497	13.70253
	10.84593	21.26788	32.88689	9.76283	6.366797	21.96158	17.45038	18.58928	22.97827
	17.69893	18.58393	13.18208	13.89477	18.21911	14.89735	19.93692	17.90803	36.57249
	15.52851	17.98292	37.00547	14.15117	16.19461	18.25943	18.03996	11.44185	20.67994
	17.45206	18.22816	10.8008	24.06469	16.13225	37.76324	30.16338	16.2245	16.49862
	12.71437	15.77345	16.55893	20.23138	11.63833	11.81071	26.74786	13.03734	12.05912
	8.737013	18.6162	25.8966	12.28721	20.08031	16.18708	19.35288	24.53277	12.88489
	12.84658	37.08024	22.05818	18.94003	7.104986	24.49048	25.43314	17.30499	19.283
	8.108451	22.38889	19.76439	12.42146	11.38576	34.64307	26.85433	24.20325	7.449342
	34.91367	12.60066	17.02908	17.34317	16.53183	14.92623	17.71354	21.49171	16.12387
	24.25853	16.27662	10.59377	14.21548	17.24436	23.27951	18.69243	12.46906	11.54282
	15.70341	16.12408	16.84232	22.3241	28.50106	36.11333	14.0824	9.949324	5.975728
	12.55144	12.17213	34.38724	25.79468	18.09306	14.28649	25.51051	14.12712	12.68393
	24.02898	16.6921	11.88244	14.45618	14.95775	14.4444	15.12776	11.48292	31.28236
	17.95997	19.38403	23.13508	16.06356	12.75628	17.3723	23.06384	21.9329	13.41551
	9.597335	30.84406	13.58065	9.769068	9.357659	16.84692	29.62575	19.79649	26.66138
	12.17747	24.733	21.6162	14.7267	33.63948	41.21298	28.49781	15.07864	11.66787
	22.14801	12.31262	16.09043	16.7301	24.57678	11.02658	22.9767	10.82476	14.50227
	11.54347	11.63488	20.54542	15.63776	12.50322	17.47092	17.13515	13.00492	31.55461
	10.04218	14.11618	16.1625	10.82708	16.87785	18.27837	25.47403	22.49512	13.21919
	24.3005	15.25838	12.68479	53.22475	29.11691	18.32701	14.26191	22.10226	17.83609

	20.42972	19.05857	50.08648	11.10983	24.34973	12.4591	10.91961	83.32026	10.36768
	16.76467	21.64047	17.05124	11.41636	23.65154	18.18535	12.30948	16.94742	23.4178
	11.76987	22.61805	26.30932	32.8803	13.25674	17.3861	7.997042	6.830258	54.64596
	13.20091	30.19963	6.4077	15.04407	10.32711	22.96872	16.34691	12.0178	9.492209
	21.7681	28.91473	11.11303	16.27969	23.03403	19.53287	13.1495	15.30237	20.7255
	17.28965	21.28153	16.63107	9.057356	31.5786	13.91923	17.51022	16.94773	13.45598
	12.38956	19.76529	27.59587	8.856635	14.60967	17.89686	21.32425	25.92216	47.01277
	12.15491	12.14798	22.84077	19.99372	25.3668	16.95225	18.6256	24.01003	19.78504
	39.49537	28.95695	14.70119	15.11572	12.13163	27.47562	10.7026	11.82239	8.647995
	19.9246	13.73112	12.14995	23.96502	18.3488	7.580428	15.63488	18.05955	8.420735
	14.86963	14.58288	18.79848	8.757374	15.81765	27.04335	18.04766	23.41528	18.11514
	44.87362	13.70924	8.765168	11.86183	15.39657	18.29936	12.10102	10.58245	
G6PD	13.1657	19.4734	13.10659	11.84315	17.57111	10.90896	14.64632	15.55192	
	10.60413	12.86387	147.5965	19.16659	11.76282	24.45396	17.66588	12.7628	9.467807
	18.88168	13.70208	12.35654	11.89844	16.58382	119.9878	141.783	24.02378	16.8574
	28.1984	19.91877	14.89427	29.22005	20.16211	13.04022	20.04425	28.36512	19.29765
	8.542333	14.92079	10.59703	28.98225	19.67795	34.38358	8.392947	6.00203	23.90009
	22.38644	17.20459	32.15863	20.28548	28.46397	4.632826	12.98181	29.95292	47.82792
	26.46646	10.14667	17.47685	19.85358	17.57542	15.39334	18.34123	19.8219	82.45566
	37.4155	30.41772	20.60621	39.02172	17.92643	17.73956	11.70591	29.44396	30.85306
	10.84887	29.46742	282.1759	31.15915	14.96935	44.00916	16.12483	35.59459	15.10266
	35.25102	37.93054	108.9046	10.40121	21.29063	30.13488	22.77761	28.17049	17.47409
	22.62152	17.66824	11.18734	24.26618	23.24757	24.92521	151.4243	13.89264	17.3177
	12.70016	25.26	25.06125	3.739998	36.85786	49.76035	32.34377	11.13052	21.46175
	6.564946	29.49596	73.73113	32.34263	44.09806	12.01659	20.04264	33.24572	17.59995
	10.41557	29.44307	17.81899	12.40916	40.10449	69.2525	11.49129	15.42185	8.197561
	14.32493	15.70871	14.51024	21.22009	25.42258	17.93865	20.71089	25.01534	32.75144
	23.09861	15.86295	15.90377	48.00637	51.50833	16.43184	12.35558	16.43271	20.94398
	25.09193	35.05019	15.04918	90.23914	11.4806	18.91134	12.81654	21.09367	35.82821
	24.29536	22.78696	28.48142	23.33373	33.55752	32.33795	16.78205	18.24295	19.4037
	13.52691	45.93886	25.79052	19.8043	18.76332	19.10318	31.06508	6.243286	33.19114
	289.2443	21.53858	34.7999	37.90604	21.64542	13.34221	48.17043	26.54922	9.869153
	22.73968	12.04792	20.27857	50.80667	30.96415	25.2926	25.91647	14.2086	28.42331
	12.75066	6.737796	47.41984	16.12985	180.7822	22.26238	28.81864	24.21686	10.53114
	23.38484	193.9435	35.31799	29.52248	10.17073	20.77379	253.1838	11.63539	15.15525
	10.62851	41.24863	20.96025	10.15673	15.71124	275.7129	16.21058	29.93174	11.71345
	68.36602	18.97992	16.48715	19.89425	30.48801	17.09751	17.87498	19.98209	28.69632
	17.3558	13.89378	16.03671	44.00519	144.4612	17.19481	23.24494	18.98997	28.50451
	31.74933	24.7821	18.41385	18.8522	24.89785	12.06985	537.6305	22.79045	13.30365
	22.29764	9.843274	69.94901	23.7394	37.83438	10.64906	11.90337	32.16219	25.72083
	12.29556	11.41679	12.81416	22.15613	15.65101	24.22609	31.80513	9.066022	185.9256
	22.52942	14.70917	17.75046	11.23703	14.99642	16.02221	15.12048	65.60283	23.13717
	24.52258	21.80296	25.27723	14.99938	23.85903	29.73839	21.2126	914.71357	77.76466
	16.68175	33.82898	15.64194	40.54356	17.3283	246.8427	35.39332	25.4542	21.46549

	135.6308	41.15539	11.40588	25.5433	36.63773	7.286825	39.85188	37.48974	18.89134
	36.93593	12.00619	17.05811	24.70506	7.308335	42.66181	15.34662	23.06085	24.69868
	13.03239	23.68373	22.46997	15.82406	10.14704	10.15736	21.45963	23.59224	16.78387
	32.47955	15.91751	14.09872	10.03395	14.82103	30.69136	22.14599	12.66529	15.52576
	18.3842	30.94865	12.7636	20.41727	16.85657	11.45921	19.37294	227.411	13.01751
	17.66992	8.840484	24.5549	19.20437	204.3683	33.10226	21.59549	40.207	130.6616
	11.62653	19.79263	75.47295	18.36221	10.51817	16.84328	16.30244	434.6139	21.81521
	11.79733	19.49241	6.152068	47.69142	15.85453	341.7643	24.1713	78.8053	116.7495
	20.48746	18.44802	21.71535	10.68116	19.21139	36.24064	58.21488	14.4353	22.12932
	37.78294	12.54628	15.31238	27.42278	36.4947	292.837	11.60829	41.20516	19.12474
	25.67261	10.16472	18.03516	13.46968	16.80881	10.00545	7.646275	11.31416	20.4235
	19.90428	19.91475	16.01311	13.52996	30.89878	15.13455	266.1554	186.0867	27.80736
	17.17672	10.22736	29.17315	17.13009	20.25233	15.21274	75.32457	18.25946	16.54068
	382.597	19.13594	32.07225	15.3001	81.90347	21.35677	235.7109	14.45609	37.21619
	27.31682	26.41204	20.40616	14.28804	18.06747	10.64684	46.2013	13.53864	16.26948
	48.00848	24.64764	12.45607	13.4623	29.91236	22.50709	12.73008	12.62346	
PGD	99.7516	10.74426	38.29501	15.75956	234.1249	22.61224	17.60522	23.93571	18.73942
	13.86574	755.1429	78.44439	30.56996	54.6973	13.61578	29.29348	34.53005	71.68885
	46.3314	43.57887	77.1719	42.14279	95.55775	353.1119	124.1938	83.20345	92.00552
	95.47754	50.66236	62.06785	43.58839	69.41465	55.01042	60.58354	228.6168	37.33893
	50.49219	28.30351	62.74815	52.53825	50.21399	27.4874	102.4809	53.25813	43.67744
	120.9128	39.43119	141.5042	103.7339	52.48261	44.8875	53.6734	78.86638	73.65221
	55.95213	53.07619	47.71442	19.16718	85.23689	21.23566	72.90807	98.80222	74.48033
	46.68321	37.5502	96.49966	33.45837	88.1242	27.33606	47.52738	96.80402	44.38688
	74.75363	404.4008	54.87196	103.7123	45.50659	188.165	98.66646	51.08529	104.1125
	112.2286	154.7194	66.05137	42.25332	71.14217	41.53706	27.58996	43.96495	68.72201
	184.2304	43.98898	102.5745	122.6673	67.15095	134.788	72.25789	45.45392	50.01931
	24.68165	11.85101	50.18326	84.27099	127.8735	53.31268	82.07574	37.45342	14.77051
	84.4136	99.89195	58.93286	152.268	45.69545	32.20014	149.0782	79.53176	110.1485
	108.8623	105.121	106.7473	119.4253	112.8198	20.30785	66.90917	62.15321	79.5981
	55.49751	35.32798	73.85378	46.18186	41.10277	50.852	163.4142	66.40378	60.05513
	44.98062	71.41744	38.44153	54.43095	60.98623	38.26092	107.021	42.0819	125.1423
	29.32081	98.29311	148.6521	8.592074	51.99453	56.31048	57.30039	28.8361	66.70136
	153.9116	75.88829	42.55429	54.77542	66.08395	66.09598	35.66846	110.1299	57.49219
	133.0476	87.45797	87.32426	118.4838	96.63921	68.12423	45.70611	65.53356	469.5214
	88.86434	63.4337	43.63939	37.62513	92.80276	207.0262	37.33557	79.70529	60.4339
	115.6889	158.6267	77.23645	67.72027	80.58115	38.97024	54.89984	63.62561	33.65519
	45.3252	79.02741	75.48751	371.6516	53.33632	84.64089	91.46347	21.92765	48.95171
	643.6544	90.79727	77.20793	61.2499	100.4852	660.1203	36.08429	56.47935	56.01747
	156.6045	82.26748	52.38275	42.10858	817.1173	52.4057	104.3068	24.15967	136.6518
	57.91239	30.83084	23.79571	35.00396	34.5226	52.87983	67.27175	97.15962	41.93214
	44.28015	29.39151	123.9623	74.12026	49.33276	27.02917	34.15658	50.52315	57.08009
	74.77579	35.33092	57.65068	100.6279	98.03408	350.6507	72.23913	36.99459	137.769
	38.95818	116.9154	91.59045	130.1851	58.18954	168.4158	42.28126	128.6015	61.63344

68.30774 47.38019 50.21691 58.25731 118.9596 86.48317 74.70709 504.565 46.35789
76.85937 66.45905 57.30191 115.2076 66.59063 142.36 184.3776 73.57649 40.07539
80.53989 48.01279 94.25939 178.7303 126.2559 68.11439 67.43621 57.61733 109.4953
101.491 47.09089 80.38825 79.12412 276.9637 23.76371 191.6051 40.04842 573.5233
39.79463 61.65571 72.98687 41.47166 28.23494 86.35575 58.54386 52.10316 79.86389
41.20457 52.58794 45.33484 16.79084 83.89767 90.46829 65.09048 31.83335 49.08153
66.64967 21.54273 48.60561 35.40129 42.9474 21.93426 62.44653 97.77086 87.58537
29.14515 36.17921 29.28026 39.58966 65.10909 34.77122 77.31544 21.49376 87.62577
49.352 60.33281 97.86537 45.32694 53.38907 18.71703 161.9095 47.03492 28.55126
26.53437 45.71253 84.77918 142.2284 99.72361 63.6857 81.21858 89.05972 31.16488
56.58109 171.1555 174.86 26.19375 43.29401 43.23604 342.9144 185.7369 53.35534
62.70354 21.90536 40.88116 85.35684 434.5103 50.4062 127.5629 130.2721 20.98727
191.5563 20.16176 43.98257 133.2701 83.91539 87.7983 36.04282 208.6198 73.4976
36.70525 19.8514 101.1843 254.401 287.9811 33.70707 51.4796 476.00664 48.22415
26.78182 54.91076 57.2162 37.95726 37.87779 247.7161 35.92003 93.59929 28.80279
28.74538 98.82263 27.21504 71.45106 82.62015 484.3594 363.9323 159.6395 71.17944
44.39904 80.75695 28.59015 74.8258 172.0565 80.98996 39.89987 43.23726 808.1785
53.20391 71.52269 62.00545 266.6498 23.46472 269.5744 30.89731 95.54226 50.28497
51.30231 89.0507 22.40946 40.99007 64.64919 43.60746 45.77788 61.24301 67.11408
49.99313 25.90593 46.61739 74.80089 57.30566 62.28414 65.46278
IREB2 3.969498 2.946236 5.126363 3.113171 3.746238 3.555101 4.231842 3.663268
3.322477 2.946333 4.109916 4.93749 6.240528 3.578958 3.616502 3.098072 4.395366
1.880265 2.775262 4.250495 2.149607 6.571157 4.125592 4.849041 7.164847 5.738176
5.656544 7.725611 7.834012 2.250711 3.794861 6.156477 2.164135 6.652962 4.567466
2.210208 7.790182 3.357271 7.593362 2.736643 1.71545 3.604093 3.737625 3.395715
4.34465 7.982575 2.542329 3.947918 6.489392 2.734117 4.760665 4.664901 4.35288
3.732968 2.680659 7.51831 4.177125 6.089026 3.584154 3.179899 9.830027 7.626767
9.517793 4.063329 1.917267 3.923993 1.168608 5.912263 6.841853 2.640341 5.959559
3.471917 6.614851 5.78912 4.866988 3.963203 2.867498 7.923332 4.231815 2.166427
6.554012 2.910471 2.94858 3.168606 2.993865 4.796559 2.38815 10.19404 3.173398
6.672457 8.873263 2.732773 5.314137 3.097139 5.028694 3.177036 4.754836 2.305958
4.822213 2.98137 3.857192 1.773621 1.904009 6.154526 2.928182 3.712821 4.028752
18.09896 4.496309 2.734277 6.28236 5.906339 5.25271 8.860151 5.410849 5.616356
0.8088414 6.744875 5.135042 4.043839 3.570207 4.466617 5.393892 7.982986
4.566198 5.148349 4.293422 5.237843 3.383755 1.746261 4.709649 3.654237 6.221462
2.092991 6.187511 4.262646 3.912093 3.168679 4.846035 3.927124 2.146259 3.821642
5.727335 5.70596 2.397817 3.660961 17.35705 1.178164 2.366312 3.946198 6.472202
1.839556 2.467193 5.16934 10.82679 4.009872 4.137341 3.734347 4.977943 2.620319
3.24337 5.119828 4.477029 3.130707 5.559901 5.002887 3.406891 5.207136 7.095748
5.473554 4.73072 2.930565 4.72896 2.812713 2.70038 4.562968 3.340528 4.15038
4.514771 2.594081 7.835062 4.193144 4.120022 4.032299 3.483504 7.692427 2.224406
3.697335 3.837629 2.709103 3.119302 5.026972 4.687177 4.562129 5.368362 4.651864
2.374453 5.704155 4.91363 5.675187 3.147494 3.202533 5.989888 7.948462 3.23887
3.74692 4.578132 6.54489 7.298935 3.420884 5.248316 5.423314 3.899335 4.870304

	2.18791	7.852499	8.074884	2.776256	3.825845	3.215383	3.986584	5.834647	3.936108
	2.872877	3.158616	7.235008	4.18775	6.280372	3.378721	3.751444	2.664661	5.337821
	5.54698	4.518312	2.801529	4.399702	3.505772	4.02926	6.613623	4.507819	6.352815
	2.476627	7.357358	5.854635	2.699209	3.835233	2.894098	5.519661	6.155722	5.138985
	2.080187	5.866272	3.025274	2.302903	2.279098	3.413805	4.161146	5.519538	2.566191
	7.976888	7.108161	2.743151	2.929757	2.43339	4.284849	6.679917	5.553312	4.785402
	2.098477	1.849218	4.06244	4.440949	5.820645	5.097276	4.050166	2.633615	4.743154
	4.898437	3.029115	8.008615	5.624421	4.831938	6.486269	5.797641	5.604779	2.636311
	3.187724	5.614206	3.58309	4.269429	3.63382	2.04372	9.294291	5.268578	7.522826
	4.454585	5.309323	4.132395	6.267559	1.964744	3.972002	4.396386	4.913352	5.963503
	4.463518	8.645015	2.11849	2.299495	2.829876	12.82412	8.063332	5.040855	4.604232
	4.989693	3.237461	2.920948	5.824878	4.457873	2.624578	2.112022	4.025598	4.759311
	2.379922	6.29181	2.474346	4.040623	3.325747	2.245601	3.633271	3.419497	4.294107
	5.197131	3.961475	2.503651	2.08685	2.471233	5.340879	4.44855	6.27422	5.587381
	4.473136	4.425445	3.753189	2.897533	5.551791	2.868581	4.020978	2.440157	4.221367
	9.297577	2.887155	2.479044	14.96647	3.5619	3.526683	3.985562	4.693678	3.22884
	2.248254	5.832159	8.320863	2.13579	1.970645	5.977337	7.4877	4.867612	5.05241
	6.460414	3.011958	5.604375	3.740719	4.280144	5.414094	2.214388	2.283703	3.860568
	4.156164	4.108635	2.392853	2.303834	5.200209	4.202596	3.513695	5.618377	2.402309
	3.837938	3.803241	3.483629	6.703342	2.896977	2.668292	6.352474	4.680337	3.675682
	6.145805	3.484006	2.597848	5.908052	3.215068	7.481065	2.665838	6.62167	0.7400922
	3.708365	4.736114	3.948094	1.815591	4.094618	4.669835	3.786343	8.546706	4.191961
	10.53467	3.745216	3.884267	2.152445	2.522272	2.777445	4.926921	2.649507	1.469588
	5.841931	2.595239	2.02524	5.182167	3.535493	3.946347	4.888336	2.203587	3.463517
HMOX1	13.23041	4.568704	8.917098	5.526739	53.34476	4.413748	7.237295	11.59125	8.10614
	29.90515	11.31624	14.98918	5.152676	21.65871	5.265816	11.00888	2.5545	16.87292
	19.69574	3.054736	191.157	244.6017	14.11256	32.71128	17.66374	32.74591	24.40475
	12.47951	332.3931	19.1237	29.69519	66.65066	1.018611	8.299921	43.31785	9.716341
	26.96616	87.7576	20.3787	9.577804	6.382347	7.130722	51.5858	28.29091	11.50416
	52.86685	84.74536	15.14862	9.536579	21.20819	12.63778	11.82362	69.53013	10.26136
	5.94458	3.736202	3.854883	11.5683	36.89532	43.21264	19.03759	19.21569	261.9894
	19.03468	26.99746	15.59583	31.55588	1.315356	1.399799	13.23299	14.52912	4.677766
	32.81617	59.60066	26.04863	58.84863	7.59541	13.09219	3.170466	1.53634	3.403642
	3.673186	14.49103	5.991769	4.639395	18.45528	10.32708	8.931746	3.602652	17.33198
	107.7285	40.43143	8.576485	4.516848	15.30236	113.6574	22.33024	55.82965	10.49309
	4.17647	5.570194	120.488	14.8421	9.731468	3.558902	43.15951	54.43404	64.83329
	18.09467	20.74191	3.315683	25.9982	5.423084	21.68311	9.519497	13.96353	18.9141
	17.7424	20.87619	123.3887	9.772535	15.64363	38.00547	11.57564	48.75682	6.497079
	1.892794	9.584719	41.62761	1.196877	26.89777	45.63901	8.078965	44.18184	16.29258
	24.18493	114.27	16.15478	22.26477	56.88136	58.08626	40.57724	2.808172	358.3399
	6.609248	14.94085	27.50684	1.393067	1.878961	15.65704	1.750391	55.51473	15.02738
	7.089983	8.941303	1.909269	19.73832	13.03176	7.260267	10.15988	13.2103	17.49992
	7.569294	45.98212	1.422051	39.90536	43.15049	18.09422	155.4302	54.13403	13.04099
	14.84471	26.36582	15.70096	25.22716	255.6277	18.89852	35.40994	10.70011	19.36811

5.617317 36.31422 47.80658 21.60256 14.02064 16.06914 45.4609 1.899431 12.43032
15.99629 7.967625 52.58509 23.54082 27.87886 3.247031 12.4316 2.200868 32.22737
2.902493 15.59763 22.01562 11.36563 25.84179 12.21675 19.82827 11.70489 14.56235
7.750935 6.30527 4.325247 19.94353 9.350501 11.29732 10.41692 104.7716 5.438376
46.1337 1.331822 40.83545 18.91602 8.360866 13.87749 14.0033 7.534911 7.952654
12.66261 25.58218 84.00691 20.00062 4.62536 13.90198 2.15383 11.79721 10.6201
3.922242 24.94613 13.58729 3.515709 9.785064 9.630786 8.056851 561.5242 28.44698
7.161567 42.42976 39.04272 20.74033 15.79562 85.17261 21.70577 47.6765 37.30105
16.63391 14.54909 17.87906 10.58916 126.8885 21.80412 15.45683 4.982823 2.114462
47.6654 5.647172 12.00293 25.05089 18.87897 118.6745 5.472022 6.361457 34.79883
18.04096 4.863506 293.3715 5.339297 43.90129 36.849 11.25682 8.382183 37.35389
15.12743 96.83869 14.05447 10.78526 32.0069 12.50862 29.26207 23.11391 16.51812
14.30393 29.89316 21.21621 19.56591 8.321976 23.4774 103.5439 10.49828 8.366002
47.38723 63.18393 10.08762 25.42915 22.39944 14.08806 4.678871 9.185177 11.87863
1.630047 14.68396 63.97666 196.3599 6.962095 12.11934 3.970763 39.13392 33.54651
32.29857 13.85998 54.54421 30.54203 19.55786 10.43763 10.92221 6.386731 5.938956
33.58552 8.653536 23.42842 25.84191 28.81341 22.24982 14.25154 68.44386 11.75045
59.10237 39.07311 21.02072 68.63186 3.909712 20.14737 7.506502 14.9004 3.728863
9.300063 20.63548 16.45824 9.809277 15.21416 16.88513 5.597342 8.348861 12.27717
19.63877 4.660645 27.5866 17.18386 35.1663 37.42536 68.28442 42.02396 6.745632
35.6219 1.02442 20.02873 2.567214 24.11349 29.88943 7.150978 20.28305 12.25862
1.68612 4.661808 59.1216 60.63135 94.96019 3.997437 32.19454 46.40239 15.92486
7.662909 15.28714 6.048628 3.097187 7.747681 370.0904 25.35761 22.37092 19.68946
59.92645 3.072464 21.34467 79.13212 18.97703 77.84641 5.303955 6.97341 4.280587
1.704668 12.89478 4.354775 43.19488 12.92496 30.97158 12.62836 31.91412 37.36867
50.33983 40.01964 32.09124 26.32109 2.127808 41.15544 35.30502 7.627638 13.42613
11.31381 59.14193 6.646027 40.42617 2.878811 51.86745 252.5217 4.858744 25.52926
59.32941 4.570651 54.66671 21.38022 5.252847 17.80588 28.22203
ACSF2 15.89456 0.9975879 10.54575 0.8079661 3.59276 13.85635 3.979716
5.213604 1.160119 1.098433 2.218727 7.654615 15.63056 8.30449 1.457981 19.73645
17.90795 13.03799 18.8836 10.2425 13.88628 54.49203 33.43843 1.189993 28.18894
18.40129 6.123038 19.59437 18.41581 7.788563 3.57629 10.35916 9.024263 7.892214
24.59045 7.39483 13.27683 15.24888 19.47238 8.369987 2.749357 3.598955 19.74341
12.51265 18.39489 28.00811 6.855305 5.769763 7.128906 17.17203 10.73982 15.58384
27.17299 4.019866 135.7754 10.02448 17.77141 2.649909 19.17938 79.9701 15.47105
20.92792 13.83944 10.91073 9.212669 8.228912 14.66651 23.67304 9.737604 14.91505
14.29368 13.05209 15.28446 4.971037 7.134234 12.11931 5.076782 54.97098 16.52788
10.50949 14.02706 18.10967 3.64935 0.7938955 1.09135 4.345734 10.25658
3.371855 34.04921 1.356287 17.05189 17.55706 26.20021 7.431314 33.27145 13.51687
20.15179 3.570635 5.925129 24.11392 1.042533 25.03001 17.00543 35.27983 22.01061
25.46482 3.4371 67.47538 16.91717 63.12158 22.68785 10.66481 11.70039 11.49901
10.11093 11.16829 14.54839 12.07651 21.33028 29.71984 24.21185 20.73562 25.73103
2.769204 14.73064 8.007855 25.74515 16.02664 5.34574 7.608722 19.43541 5.611526
13.61812 4.750197 5.168876 19.83127 17.65701 5.098255 12.27243 20.15003 23.07665

15.93335	14.61177	16.89989	3.501709	19.78268	2.984343	32.39827	7.780032	9.764955
13.39378	34.12857	4.23375	9.011233	15.11612	25.18229	8.013773	4.754793	0.8321842
31.13594	16.06356	9.14714	12.63121	8.089816	5.507818	12.10442	12.2001	32.79688
49.77114	16.95766	7.688039	19.35105	3.408274	3.50637	36.67695	4.988824	15.93889
2.503829	22.72683	12.77264	8.307107	14.1595	18.58274	79.87793	18.12697	11.1368
19.29662	8.721261	4.945662	26.40755	13.16021	8.700652	7.23606	14.41733	0.8610969
17.07248	26.35933	5.683799	1.4277	7.233981	4.00216	437.65037	10.06433	7.925471
6.841476	2.037886	19.52675	4.873624	3.404099	3.07855	239.23023	1.462484	11.2818
32.5334	16.95963	2.838468	49.78633	17.31528	4.375272	2.632669	24.46591	9.631057
5.958119	18.97388	8.142992	16.62779	8.290044	44.29557	2.164994	19.43232	5.776178
16.65618	8.477982	8.647605	17.83004	4.932566	11.2475	9.571485	5.986399	7.998269
31.64128	5.020552	47.84851	31.22196	20.15958	12.51098	12.18917	20.66991	14.64678
3.864512	14.58764	24.57282	13.54634	19.50156	15.49414	21.21457	5.386658	15.34066
21.26724	0.4342206	52.97592	7.374891	12.98539	14.71092	14.94464	7.274104	
26.59782	5.922199	13.06787	8.967465	4.51426	9.318037	9.460313	19.4696	835.63261
20.60348	5.70647	21.6216	12.47443	10.42553	8.44739	830.77308	0.8053339	
3.217444	10.70492	26.65756	6.403542	1.196766	8.021494	17.65002	6.637829	8.711979
5.42699	4.693474	7.276584	2.146784	16.76188	8.969126	24.42169	2.68896	343.70743
3.081708	17.79085	47.72191	2.26234	16.44193	15.07803	14.0389	18.45723	55.19418
6.615462	60.28466	15.41319	38.82575	11.52559	10.34404	10.75688	5.950521	6.737708
9.425049	4.252014	3.83895	0.9685516	13.81428	7.587612	88.35034	22.55795	
7.054105	7.941678	22.46535	0.9373116	23.5362	8.768176	18.14811	8.693466	
27.99219	16.19577	5.866806	2.224691	0.5153382	6.794666	8.732519	16.20172	
4.407323	35.23309	7.047391	4.934299	27.8276	13.96372	1.579082	18.72909	4.460813
7.243947	26.87615	10.47319	6.328073	17.66955	61.33557	12.72989	18.95008	12.41964
9.115642	8.492484	0.8013549	33.99976	19.27901	11.89543	12.17273	8.003536	
3.478489	2.38324	6.641253	7.644878	4.271813	10.30399	7.232931	11.28717	96.31914
2.562662	3.896172	18.77708	16.55103	20.94219	11.38206	1.113931	0.4811367	
1.730672	17.1344	23.7828	7.726677	5.604094	26.5971	4.660736	1.78039	6.482304
21.81251	2.851195	26.71155	23.39385	14.44397	14.62122	11.68479	11.35418	5.942923
10.46923	2.956588	7.247252	4.437895	30.71348	23.06681	3.80836	29.32735	12.07273
8.014499	6.015941	18.8086	23.271	4.466791	18.09694	22.4817	21.70748	10.03542
3.151583	15.85912	19.68823	19.2984	21.27385	19.71861	18.7121	10.28917	
