# Supplementary material for: Ferroptosis-Related Long Noncoding RNAs Have Excellent Predictive Ability for Multiomic Characteristics of Bladder Cancer
Source: Oxid Med Cell Longev. 2022 Aug 29;2022:9316847. doi: 10.1155/2022/9316847 (PMC9444476; doi:10.1155/2022/9316847)
Supplement: Supplementary Materials — Supplementary Table 1: expression matrix of 60 ferroptosis-related genes. Supplementary Table 2: results for coexpression analysis of ferroptosis-related lncRNAs and mRNAs. Supplementary Table 3: expression of 263 ferroptosis-related lncRNAs. Supplementary Table 4: expression of lncRNAs and clinical information of 403 bladder cancer samples. Supplementary Table 5: cluster analysis based on 20 prognostic lncRNAs. Supplementary Table 6: detailed clinical information of bladder cancer samples. Supplementary Table 7: data of the high- and low-risk groups of the training cohort. Supplementary Table 8: data of the high- and low-risk groups of the validation cohort. Supplementary Table 9: markers of different immune cells. Supplementary Table 10: infiltration of 22 types of immune cells analysed using the CIBERSORT algorithm. Supplementary Table 11: immune scores evaluated using the ESTIMATE algorithm. Supplementary Table 12: immunotherapeutic response evaluated using the Tumour Immune Dysfunction and Exclusion (TIDE) algorithm. Supplementary Figure 1: gene set enrichment analysis. (A–F) Most significantly enriched biological pathways in cluster 1. (G–I) Most significantly enriched biological pathways in cluster 2. [file 9316847.f1.zip › s-table 10.pdf]

Mixture B cells naive B cells memory Plasma cells T cells CD8 T cells CD4 naive T cells CD4 memory resting T cells CD4 memory activated T cells follicular helper T cells regulatory (Tregs) T cells gamma delta NK cells resting NK cells activated Monocytes Macrophages M0 Macrophages M1 Macrophages M2 Dendritic cells resting Dendritic cells activated Mast cells resting Mast cells activated Eosinophils Neutrophils P-value Correlation RMSE

TCGA-GD-A3OP-11A-11R-A220-07 0.0161710399295584 0 0.00537396706861971 0.151435975414936 0 0.207791864906784 0.0424037706147541 0.00256726265543052 0 0.0152455255508599 0 0 0.0305622820785503 0 0.209513195286685 0.10163686256871 0.112317950177314 0.0369129801438529 0.0680673236039456 0 0 0 0.098 0.110584177400225 1.06015381218083

TCGA-BT-A20Q-11A-11R-A14Y-07 0 0.00112359416624234 0 0.0511764430618364 0 0.274533720322075 0 0.00185270052966052 0.0549333089157158 0 0 0.0324523892311536 0.079222545285068 0 0.00451774244108637 0.1653353686305 0.0579338916289317 0 0.27691829578773 0 0 0 0.108 0.106039306055898 1.04413904997505

TCGA-GD-A2C5-11A-11R-A180-07 0.117569924637317 0.0125344904904807 0.0689341839673839 0.100618630232976 0 0.347405573147848 0.00276143549746051 0.0201920725234019 0.0670463187129218 0 0 0.0391472481987654 0.0126078312200423 0.0122206594141602 0.00432506605287033 0.0298138595525777 0.0898200810268384 0.0243524597917993 0.0453733640624686 0 0 0.00527680147068799 0.056 0.152644217285323 1.03864281518733

TCGA-BT-A20N-11A-11R-A14Y-07 0.222882605247587 0 0 0.00709156436349284 0 0.128843119558491 0 0.0277021387144975 0 0.00203963273732235 0 0 0.0828399138992844 0 0.0245373648377983 0.219179806004026 0.0386967940867684 0.00696484308331167 0.169196119590046 0 0 0.0700260978773745 0.051 0.159304616609996 1.01694459412326

TCGA-BT-A2LB-11A-11R-A18C-07 0.182529746323259 0 0.0104706469225918 0.187927783364529 0 0.155549589504467 0.0701518773962926 0.0295975798744461 0.00681613364356333 0 0 0.00627374664297745 0.00786955040823954 0 0.031566127518989 0.155524428940592 0 0.0674163746574254 0.0187019727445265 0.0318649922597459 0.0144811587689853 0.0232582910293696 0.054 0.15583819354421 1.02266860962948

TCGA-GC-A3BM-11A-11R-A22U-07 0.0260266404412066 0 0.00340034802322837 0.329903569520475 0 0.0162066863269801 0 0.0183142986727882 0.0346714713904119 0 0 0.018112964401798 0.0209149408175491 0 0.00326747644452134 0.0674699132632192 0.0125619559119332 0.00157136265456602 0 0.419427427454565 0.0278241396939299 0.000326804982828444 0.3 0.0428430832818963 1.14669568560233

TCGA-CU-A0YR-11A-13R-A10U-07 0.233085807882693 0 0.0720123306514228
0.15533325040894 0 0.119563457664068 0 0.0795079097478596
0.0168458811136265 0 0 0.0846715490151731 7.35843931576815e-05
0.0114363645848368 0.0304247454843112 0.0784760796240044 0 0
0.115790129841156 0.00277890958875149 0 0 0.001 0.407668427475824
0.915658141230852
TCGA-GC-A3WC-11A-11R-A22U-07 0.259845379786475 0 0.044827316927685
0.0465744532729527 0 0.369992706971842 0.0436672802720383
0.0339083403534718 0 0 0.00624122330389125 0 0.00567058007875526
0.0121707557928318 0.0176975144032251 0.06688009513927
0.00707081821962057 0.0177164435317254 0.0677370919462158 0 0 0
0.004 0.33874837403063 0.951986938341134
TCGA-CU-A0YN-11A-11R-A10U-07 0.157202336883721 0 0 0.0392990419009631
0 0.200773613841764 0 0.00913295266485445 0.00272884581720867 0 0
0.00779230549699647 0.0283189373188256 0 0.0116051370624927
0.342976787531223 0 0 0.200170041481951 0 0 0 0.077
0.132237534309087 1.028858090735
TCGA-BT-A20R-11A-11R-A16R-07 0.0351696331480143 0 0.00556411880943247
0.0200446407384881 0 0.111473752231 0.0146862901913712 0 0 0 0
0.0151085288728137 0.0205027541640384 0 0.0385313686711263
0.180961814599367 0.055599718912872 0 0.4553785358892
0.0303949626994959 0 0.0165838810727809 0.00900000000000001
0.268117958526293 0.993349036448483
TCGA-BT-A20U-11A-11R-A14Y-07 0.124050245301558 0.0395870167316055
0.0324769724774433 0.257594497208784 0 0.0567976244038228
0.077407492272353 0.0444157852097337 0.00960408130911379
0.0193908141763004 0 0.0282050148353142 0 0.00707119045589169
0.0248141903743139 0.0979228312358288 0.087224452934047
0.0100523022086796 0.0668253285044646 0 0.0165601603607462 0
0.00900000000000001 0.264444278280809 0.976840402829615
TCGA-BT-A20W-11A-11R-A14Y-07 0.0433196104966659 0 0 0.26075611398042
0 0.182799872677161 0.0427669806551867 0.0606542653045228
0.0400938633124288 0.0111119461173972 0 0.0460224296014332
0.0206858909886105 0 0.0543994065748336 0.0840994261021153
0.0452047008466712 0.0110993671063094 0.0969861262362435 0 0 0
0.00900000000000001 0.26712663069298 0.985307839210536
TCGA-GD-A3OQ-11A-21R-A220-07 0.0594024880430081 0
0.00361167596186517 0.261375665361622 0 0.21293698292291 0
0.00283651730134346 0.0180815962787415 0 0 0 0.0154742489355055 0
0.0143288603143166 0.148688291942217 0.117213538191496 0
0.0710132769537658 0.0471034986042476 0.0239025647612085
0.00403079442775225 0.269 0.0486019879066777 1.06861715135711
TCGA-K4-A5RI-11A-11R-A28M-07 0.166170846226971 0 0.107343894548853
0.117820389948988 0 0.127163886823652 0.0689532822466676 0

0.0672160889661851	0	0	0.015283048142667	0.007099743181023
0.0480967842723029	0.0224682949284274	0.101887005969196	0
0.0327768789946541	0.100056776507774	0	0	0.0176630792426397	0.001
0.4211344284011 0.906224409561297
TCGA-BL-A13J-11A-13R-A10U-07	0.0264076156717557	0	0	0.0148899120984716
0	0.252856635488767	0	0.0112336716171822	0	0	0
0.0508313820014833	0.054781667431391	0	0	0.29692530456142	0
0.000724053905414176	0.208614110642845	0	0.0344370683532519
0.0482985782280178	0.077	0.130964508175198	1.02859714021494
TCGA-K4-A54R-11A-11R-A26T-07	0	0	0.00358268062696957 0.29075972282624
0	0.1822564478526 0.117607656141537	0	0	0	0.0658374806156768	0
0.0112075073119485	0	0.0066091367504714	0.0631033289741803
0.0292436104300093	0.0694154189099168	0.0366438284349518
0.101317184185118	0.0184912132834851	0.00392478365689514 0.246
0.0551353934835833	1.08481638022406
TCGA-K4-A3WV-11A-21R-A22U-070.0464081630535691	0	0.0260952981275007
0.368220077158517	0	0.141897925915944	0	0.0441046619244792	0
0.0572235179595928	0	0	0	0	0.0240064901224109	0.169354545406431
0.0254707841992688	0.050513260065774	0.00997544866999592
0.0367298273965165	0	0	0.148	0.086174686446791	1.07592750935238
TCGA-GC-A6I3-11A-11R-A31N-07	0.105197240810493	0.0629588989219491
0.140598387679153	0.147429197313266	0	0.0580430530825952
0.0903892857423274	0.111996945502676	0.0444263121694792	0
0.0387322016866541	0	0.0041331070565139	0.0466044591955552
0.00214710654281249 0.0549415847600468	0	0.0623934556088334
0.0146892954882258	0.0038880561085688	0	0.0114314123308517	0.001
0.405362507859555	0.91609667626772
TCGA-BT-A2LA-11A-11R-A18C-07	0	0	0	0.300452260512681	0
0.241412965601117	0.0337056408862016	0.010504868941463	0
0.0587763192929615	0	0	0.00703441945217866 0	0.00989975831106781
0.140625133864275	0.0431752093440213	0.0244755835063617	0
0.119259185576332	0.0106786547113393	0	0.148	0.0863575081622781
1.07815711432939
TCGA-CF-A47X-01A-31R-A23W-07 0.00138970372746311 0.0797047034168756
0.0398054967235299	0.124598107029748	0	0.032378562025286	0
0.0780716731463025	0.0797041974198762	0	0	0	0	0.117639774333916
0	0.132044381905473	0.0870867879185809	0.211209435220117	0
0.0163671771328323	0	0	0.532	0.0102333400225524	1.08198402822789
TCGA-ZF-AA51-01A-21R-A39I-07	0.0053972920251132	0	0.03303382665734
0.147214182048647	0	0.0949677831162563	0.0638505743299221
0.0712041766734538	0.118290227454479	0	0	0.00863863777723926 0
0.206916021919355	0.105831420234611	0.120912772055827
0.00451768187937414 0.00766325368391598 0.00676234603279873 0	0
0.0047998041166659 0.202	0.0667750236451522	1.0709346745211

TCGA-BL-A0C8-01A-11R-A10U-07 0.0116416433330551 0 0 0.0371500753925435
0 0.154565656828757 0 0 0.0106743439454047 0 0.0438318182437906
0 0.075478948631912 0.0338686220358198 0 0.406175389743727 0
0.164779014931874 0.0558443207145858 0 0 0.0059901661985294 0.655
-0.00359784870562845 1.09034623494709
TCGA-BT-A42C-01A-11R-A23N-07 0.183133871403364 0 0.0261364187051448
0.122022230487201 0 0.045767018611142 0 0.0590527425852587
0.0900585557625549 0 0.0318826564196788 0 0.00106787355548618 0
0.022818717835961 0 0.0549491266507819 0.198509142530627 0
0.126731827941387 0.0207170652532402 0.0171527522581718 0.093
0.114099390214757 1.0481105958476
TCGA-G2-A3IB-01A-11R-A20F-07 0 0.00628661448522449 0.00206906710811909
0.109947654651065 0 0.211917713091393 0.0287188565104067 0 0 0
0.0158979396197178 0.0422147421317694 0 0.326006618263731
0.0288293276503043 0.0979961614437186 0.0273282822866204
0.0861102597547225 0.0159649834487121 0 0.000711779554495908 0
0.191 0.0705486277036442 1.07717467223314
TCGA-2F-A9KP-01A-11R-A38B-07 0.177333426428699 0.0768975316380486
0.00598571147924275 0.0677457019872861 0 0.0649972631975928
0.058204491389895 0.0147806696963626 0.0123455841789722 0
0.0608253203959842 0 0.0305283319057437 0 0 0.115568454792881 0
0.233429806230622 0.0134702538372178 0.0187924603020678 0
0.0490949925393842 0.507 0.0136014491329872 1.08286922736964
TCGA-DK-A3IT-01A-31R-A20F-07 0.243520986842863 0 0.203530076328052
0.0629865447847656 0 0.127227237490724 0.0077659012785053 0 0 0
0.0131282173340819 0 0.0223162091096812 0.0420553227868293
0.0391917238974463 0.121150757649198 0 0.0276578146943057
0.0894692078035469 0 0 0 0.001 0.502691042818589
0.86419165833498
TCGA-FD-A43P-01A-31R-A23W-07 0.0609219179601933 0 0.0338228646884176
0.270967353848787 0 0 0.161329759219094 0.0453432468207776
0.0603541880770927 0.0312842457830405 0 0.0233543743743531 0
0.113822491102937 0.131478580477297 0.0479657372459824
0.00160380057143241 0.000517597199791512 0.017233842630803 0 0 0
0.017 0.225541355940883 1.00362630483067
TCGA-DK-A3IV-01A-22R-A21D-07 0.0876909725124662 0.0179586448209897 0
0.0523138699563902 0 0.26589846104355 0 0.0750835515383951
0.103387914665202 0.00585663611371529 0 0.04723860236588 0
0.0631918234933871 0.119855490983476 0.0517159640849493
0.0173893899797973 0.0618719330406195 0.0305467454011829 0 0 0
0.00600000000000001 0.307502860429485 0.966196019004904
TCGA-2F-A9KW-01A-11R-A38B-07 0.108790850881825 0 0.0127797071454817
0.0110983544276664 0 0.033843311021465 0.0088404747454937 0
0.0407278515139437 0 0.00568965691744846 0 0 0.33132356909697

0.0273027916088977 0.177736347731173 0 0.121853654793615
0.00994302073963182 0 0.0515149700285031 0.0585554393478851 0.069
0.13918146993471 1.05128363439994
TCGA-GU-A762-01A-11R-A33J-07 0.0304872899414805 0 0.00832054783338106
0.253559664376634 0 0.0245594819537569 0.185590750540385
0.0274221644139415 0.0312151488793496 0 0.0886852352666279 0
0.0112878111054619 0.0199904636392502 0.107031216916187
0.152718991471752 0.0114127528953466 0.0144318049915809
0.0332866757748642 0 0 0 0.012 0.243362575181321
0.990927506278605
TCGA-XF-AAME-01A-12R-A42T-07 0.130500566708846 0 0.0654557636139875
0.0603872938160191 0 0.170318157935557 0.0384516952975555 0
0.0424365909545036 0.0328648614587157 0 0.00317171037762998 0
0.11055863956844 0.0196065045884966 0.174383569541002
0.0263537222036117 0.0178492104329475 0.0824615168566191 0
0.0058792998695273 0.0193208967765406 0.005 0.320043483203073
0.949450887019236
TCGA-XF-AAN0-01A-11R-A42T-07 0.128633395296673 0 0.130073239916684
0.13143507236881 0 0.135303945367606 0.031694013992183
0.063865907092987 0.00125538138233193 0 0 0.0187816233386472
0.00562501061301293 0.0216526352183584 0.102540552874722
0.142794437939584 0.00213148414308559 0 0.044266260675064 0 0
0.0399470397802496 0.002 0.381665678160227 0.925771491353068
TCGA-CF-A47W-01A-11R-A23W-07 0 0.0450338920438011 0.0321584009570568
0.160531832741624 0 0.126369062072679 0 0 0 0
0.0348357236680683 0.0821781694463277 0.0445170081496632 0
0.0106858352974641 0.266006400098519 0.0188529881319048
0.108394446554959 0.0704362408379335 0 0 0 0.756 -
0.0131111859165917 1.08542514098362
TCGA-GC-A3I6-01A-11R-A20F-07 0 0 0.00477375862907049 0.037628354656605
0 0.145118876843663 0.0455145757433405 0.0596945318924321 0 0
0.0347727821080551 0.051911559690233 0 0.101935861256437
0.15055849194538 0.132720853316173 0 0.114369878978787
0.121000474939824 0 0 0 0.003 0.346163306359681
0.944193878368476
TCGA-FD-A6TA-01A-12R-A33J-07 0.173056931360728 0 0.174109504482321
0.0825325286777698 0 0.146184301054919 0.00168361312766473
0.0311560544138222 0.017138828502029 0 0 0.0222973329688529
0.0129999744185416 0.109386453806299 0.09073832950098
0.103712298268284 0.00172140013779095 0 0.0322873544740775 0 0
0.000995094805920327 0.001 0.423322735531076 0.907605945758877
TCGA-DK-A1A7-01A-11R-A13Y-07 0.243656484999258 0 0.101258085556724
0.124254333457885 0 0.139019958970379 0.00733076724307894
0.0700996847478418 0.0579680077844109 0 0 0.0440536445051596 0

0.03394947614825 0.0110442863046474 0.049540786443165
0.0146879050920566 0.0339181446876239 0.0692184340595195 0 0 0
0.002 0.366496803695222 0.938502671642693
TCGA-FD-A3SR-01A-11R-A22U-07 0.0134701097760006 0 0.00155493473436257
0.148671649039694 0 0.227734517531001 0 0.0265236320988672
0.0762153199971839 0 0 0.015118031460941 0.00691443398466327
0.0726084739641248 0.181083184194999 0.114577372130209
0.0142224451593336 0.00252149920192385 0.0987843967266959 0 0 0
0.045 0.167582453351507 1.03399160332496
TCGA-GU-AATP-01A-11R-A39I-07 0.0969813605238136 0 0.0156833004326444
0.0815720783110424 0 0.0978263774235833 0.0250529360901695
0.0479453897186216 0.0312297452040049 0 0.00535511083978896 0 0
0.257288046526212 0.0359143833632505 0.177544396237622 0
0.0875521733069777 0.0395963395361403 0 0 0.00045836248612844 0.043
0.170400920385561 1.01921825064235
TCGA-DK-AA6X-01A-12R-A42T-07 0.0392953228036602 0.0399476230722913 0
0.0793743140714271 0 0.129075652310704 0.0105468432111726
0.0125798656691551 0.0574490525012339 0 0 0.0141879107353793
0.0142452087098291 0.101737829097426 0.063110911013402
0.0872335549344836 0.192292639235504 0.00363771170985223
0.146312195202087 0 0 0.00897336572239275 0.168 0.0795187036273998
1.03968756433039
TCGA-XF-A9T3-01A-11R-A42T-07 0.198949996821258 0 0.0222305079087967
0.0580555926915162 0 0.116601829870854 0.0430296619989984
0.0285873524125109 0.0106172588458553 0 0.00572327338061762
0.0169348153852475 0 0.231878118935767 0.0893421679745023
0.129904399206571 0 0 0.0481450245675048 0 0 0 0.001
0.404850692723611 0.914105330179824
TCGA-PQ-A6FN-01A-11R-A31N-07 0.12146115575533 0 0.152223934256415
0.160373940073024 0 0 0.0522929355923437 0.0224791014633237
0.0174452051852569 0 0.00181094742205492 0.000369342800624573 0
0.270690143530386 0.0883202691249627 0.0721476835950608
0.0341582677697214 0 0.00535709785740451 0 0 0.000869975574091666
0.001 0.405537953163875 0.916298563333604
TCGA-DK-A3WW-01A-22R-A23N-07 0.0100731264746326 0
0.00562513732294626 0.300709063757813 0 0 0.206970195285306
0.0481914712021261 0.00978560374508177 0 0.080887023913695 0 0
0.0196289304718316 0.143278265421311 0.112208231297159
0.0307602471073564 0.0101112684613247 0.0149743837864374 0 0
0.00679705175297993 0.003 0.361206106284279 0.93998409879981
TCGA-XF-AAMY-01A-11R-A42T-07 0 0.0238962645593531 0.0152724251846546
0.0519899195285269 0 0.00359473758655223 0.0259181222732299
0.00527381159966325 0.0151875739996221 0 0.0252251946318173 0
0.0440513693702314 0.329223228848764 0.0338774229205239

0.168541131897382 0 0.0373442131268981 0.0584545315640155 0
0.151222680978152 0.0109273719306137 0.088 0.122308616435112
1.05254950268281
TCGA-K4-A3WS-01A-11R-A22U-07 0.205051841084455 0 0.0669123812509965
0.115643064290729 0 0.0123227629109693 0.0498462055879264
0.0140006606736832 0.0803254530609563 0 0.00922462048565805
0.00749785937389328 0.00893428491792908 0.0407698913730648
0.180204755081702 0.108723703641659 0.0347127012576242
0.0154161526766696 0.0504136623320838 0 0 0 0.001
0.458642253849657 0.888043936512029
TCGA-UY-A9PA-01A-11R-A38B-07 0 0 0.00114834099518966 0.280989531325739
0 0.127212288136977 0.186655943526155 0.0652391146113237 0 0
0.0167739554248755 0.00728373851567843 0 0.0375621082778196
0.0838569050571417 0.0568951664296842 0.0512295012485657
0.0430357829232697 0.0421176235275804 0 0 0 0.212
0.0641455439850891 1.07770766762199
TCGA-G2-AA3F-01A-12R-A42T-07 0 0.0368676955452207 0.0124958769990686
0.0732571190382511 0 0.0393307488107054 0.0296441379981884
0.0141591207452423 0.0525819022971267 0 0.109330508169912 0 0
0.229220034257088 0.112782486912438 0.230595649086081
0.0149092621187741 0.00166541968328517 0.0431600383386186 0 0 0
0.433 0.0232882043768149 1.0863177627745
TCGA-FJ-A871-01A-11R-A352-07 0.0433125035969954 0 0.0166378193116718
0.13793350791707 0.0315301940324615 0.0689965285997988
0.15918788688964 0 0 0 0.0611366962831632 0 0
0.277468539276536 0.019324700793209 0.133119921069288 0 0
0.051351702230167 0 0 0 0.029 0.188540634663855
1.01070521492029
TCGA-ZF-A9RN-01A-11R-A42T-07 0.00166345301225786 0 0 0.377432278550714
0 0.124267705838101 0.138352009629974 0.0537422872400869
0.0236252845790008 0 0.00501620554478301 0.0151972420589414
0.00961275200784374 0.0518070750025915 0.104374957616989
0.0595881664428489 0 0 0.0353205824758679 0 0 0 0.029
0.19003000289545 1.03700445431686
TCGA-GC-A3RB-01A-12R-A220-07 0 0.0298309586976022 0.0124921849898195
0.146421078246531 0 0.118179306131185 0.00280456533793635
0.0479813919011304 0.0155928365682782 0 0 0.0451420838903312
0.00957854960034666 0.070128065881309 0.0688279591250027
0.177608803867394 0.190610604212982 0.0480331896578804 0
0.0167684218922713 0 0 0.131 0.0941903489044093 1.04580003125988
TCGA-UY-A9PF-01A-11R-A38B-07 0.149518950446085 0 0 0.0759287521677998
0 0.143671775063409 0 0.0870987128666718 0.0224719972654087 0 0
0.0637075011655699 0 0.043167134032307 0.0796244663280451
0.0804745300076261 0.0416023832842691 0.087617096333181

0.125116701039628	0	0	0	0.0659999999999999	0.141701645604687
1.02613316814404
TCGA-YC-A89H-01A-11R-A36F-07	0.135994458092926	0	0.104098936129744
0.0826064617579451	0	0	0.0603835804884046	0.0124089132661363
0.00365183393116442	0	0.00568252592596527	0.0280339812328621	0
0.312316624914136	0.0638727932670749	0.165274054672649
0.0201446771167514	0	0	0.00333754512496159	0.00076273646149721
0.00143087761778202	0.002	0.371830063167697	0.934199412868502
TCGA-XF-AAMQ-01A-11R-A42T-07	0.1077725300374	0	0.11734198217391
0.209359990175502	0	0.104726155755685	0.078824019301748
0.0728283962726226	0.00672154305272124	0	0	0.0102340973299325	0	0
0.0680919007593749	0.07647893357015	0.0623669617856937
0.0481263236860346	0.0349619820193921	0	0.00216518407983295	0	0.003
0.365042709489951	0.933739038774483
TCGA-CF-A1HR-01A-11R-A13Y-07	0.18451690788203	0	0.113147517658059
0.201104330955395	0	0.108735054102346	0.000837874386855903
0.000522199216917256	0.0063354222982768	0	0	0.0500400729478171
0.0188346220240006	0.00631917156356521	0.0329427120933275
0.0956883786315735	0.00512690144026155	0.0818876100066676
0.0931216307863319	0	0	0.000839594006574329	0.003
0.358199869043706	0.938272290282405
TCGA-E7-A3X6-01A-12R-A22U-07	0	0	0.0138373700984168	0.186477971376287
0	0	0.106092107376719	0.0919625099520689	0.032553382346659
0.0348224005007441	0	0.0334905489759074	0	0.0450492270110634
0.151253675678108	0.0926694985832654	0.0958682494830136
0.0287367579489709	0.081078480744647	0	0	0.00610781992412853
0.00800000000000001	0.278085447659009	0.973276088351462
TCGA-DK-A6AW-01A-11R-A30C-07	0	0.0328729400673636	0.0187901601122496
0.145829139961308	0	0.168051280718547	0	0.0449558692984589
0.105656248986128	0	0	0.127037695683556	0.0235489923046821
0.0632450815895281	0	0.116037318284854	0	0.101545070022141
0.0508065427953633	0	0	0.00162366017581956	0.414	0.0258496639886369
1.09198337759785
TCGA-GC-A3BM-01A-11R-A22U-07	0.00614319999572321	0.0311580053069122	0
0.161328098567715	0	0.171962078608246	0.0157890957971537
0.173314566277772	0	0	0	0.0126099059173669	0.0124537283644208	0
0.210570680379448	0.0624810368474187	0.118202446743458
0.00702073233088711	0.0169664248634786	0	0	0	0.138
0.0928484004616576	1.08122105627811
TCGA-GV-A6ZA-01A-12R-A33J-07	0.0621701877832359	0	0.0445589351617393
0.175677547302729	0	0.116632353346289	0.0106653701804778
0.134933415077724	0.0414939988546639	0	0.0907338460648134
0.00944528328197921	0.0109840843422896	0.0691225416860968
0.0803864002579543	0.0361272038490675	0.0436293214338728

0.0263554264006952 0.0470840849763725 0 0 0 0.034
0.183307192693106 1.0194840053243
TCGA-G2-A2EJ-01A-11R-A180-07 0 0.0131786543121497 0 0 0
0.11147987803132 0.0350471836650633 0.0134223378978362
0.00624682839362809 0.014669748740802 0 0.0294969440046966 0
0.183926942766109 0.0969232669871921 0.0630412735227461 0
0.225356629806191 0 0.19147908345929 0.0115726566724944
0.00415857174048162 0.00800000000000001 0.27945916910368
0.991406436698123
TCGA-FD-A43N-01A-11R-A23W-07 0.272053698561545 0 0.122613103964411
0.0833711392330829 0 0.0616135779777936 0 0 0.00468467293443956 0
0 0 0 0.167517522083644 0.0519854981568687 0.0998877741165685 0
0.0482472047410849 0 0.0784394886674372 0.00904642161874873
0.000539897944376435 0.001 0.454487074275394 0.891420702360249
TCGA-UY-A8OD-01A-11R-A36F-07 0.0892971862356406 0 0 0.109394951899862
0 0.118106498432263 0.00310347387986181 0.038494766095259
0.0389252462707759 0.0180359904761067 0 0.0446441199452343 0
0.335731303506116 0.065476581087374 0.0590734576336307 0 0
0.0797164245378758 0 0 0 0.03 0.187365321133551 1.01794531717358
TCGA-2F-A9KT-01A-11R-A38B-07 0 0.00229342506290232 0.0107505337276358
0.0769493292946229 0 0.155066485419491 0 0.0643634219534842 0 0
0.0107962685743406 0.0306896290342949 0 0.190179122294205
0.0696916725698648 0.222768585912985 0.00185982353845567 0
0.0106796736336676 0 0.0198743279193887 0.134037701064662 0.272
0.0481126448248799 1.07924154638933
TCGA-BT-A20Q-01A-11R-A14Y-07 0.0415355689355311 0 0.146829329044847
0.126349925170309 0 0.108657403159758 0.0648997092861466
0.0651794119865891 0.0426858178470813 0 0 0.0192708349814967
0.0124096509679609 0.00235585375428545 0.0915692096740419
0.100117132409657 0.117530069903514 0.0151755794089325
0.0454345034698482 0 0 0 0.005 0.319523919720256
0.952190184618955
TCGA-CU-A0YR-01A-12R-A10U-07 0.0511592954308121 0 0.0188987564625971
0.0968696601349672 0 0.0637661906161476 0.0176888295588179
0.0245347990932656 0.0106177911373516 0 0 0.0506609287993893 0
0.340671325213313 0.063432153754242 0.180634707587005
0.029871866174909 0.0436111781074433 0 0.00138508126970137 0
0.00619743666003779 0.026 0.197409482333865 1.02116329312458
TCGA-SY-A9G5-01A-11R-A38B-07 0.0335882768632162 0 0.0366094562118352
0.160394836032529 0 0.0819265189836537 0.0208894056579993
0.14295756912219 0 0.0552191300044169 0 0.0285473299365037 0
0.0444002086751204 0 0.170520018166007 3.40673398303895e-05
0.175966961138189 0.0489462218685101 0 0 0 0.005
0.318389746198725 0.953670521696448

TCGA-4Z-AA84-01A-11R-A39I-07 0.144123428754011 0 0.141059788850891
0.059515754386499 0 0.0866654209809726 0 0.0628000568650171
0.0122685217232801 0 0 0.0164838707478213 0 0.319593665009613
0.0407925995234716 0.0859305578362278 0 0.0166805790067085 0
0.0130313449191707 0 0.00105441139631565 0.00600000000000001
0.306432085441291 0.969590977683466
TCGA-4Z-AA7Q-01A-11R-A39I-07 0.0135445056171615 0 0.00350076278534536
0.281984735342048 0 0.108404500431796 0.112040463156609
0.045027729002279 0.0411121825817431 0 0 0.00515541120413514
0.00800983913154007 0.0490498838895282 0.160661979745543
0.0923660248107237 0 0.0339641426910897 0.0451778396104581 0 0 0
0.092 0.116910843104188 1.06158362889789
TCGA-FD-A5BT-01A-11R-A26T-07 0.0422906003736378 0 0.0096005858881896
0.227414927301096 0 0.0381451577237718 0.092141163628757
0.086243503877066 0.0241943571386264 0 0 0.0241772060081945
0.0153390646782913 0.0571681863674134 0.105869081185474
0.239666297790728 0.0105857568516374 0 0.0271641111871168 0 0 0
0.002 0.370279464753864 0.930032185072182
TCGA-CF-A27C-01A-11R-A16R-07 0 0 0.0368525557442483 0.292498361490768
0 0.159570254523678 0 0.0469047242975024 0.100980629007985 0 0
0.0670367053797429 0.0196008954000181 0 0 0.025417793885231
0.0989909988021423 0.111117306989839 0.0323791380592228 0 0
0.0086506364196221 0.682 -0.00613292622952986 1.12270684725801
TCGA-E7-A6MF-01A-12R-A32O-07 0.00913791332612782 0.0278800822727926 0
0.139415724239787 0 0.257768689698112 0 0.0835784395960124
0.0294023973828928 0 0 0.029043051664292 0.0956472839136986 0
0.024681413261091 0.15628033515544 0.0676941328732223
0.0395178060933245 0.0276155116066041 0 0 0.0123372189166022 0.2
0.0671742387499026 1.06269477284589
TCGA-FT-A61P-01A-11R-A30C-07 0.0307100853003706 0 0.013845878900749
0.0716884311969858 0 0.109274226234258 0.119701747482732
0.0683439213964616 0 0 0.015138998621118 0.0191022722141759
0.0092940702886026 0.144847910005006 0.0878538336217863
0.185202920578176 0.0493708823175642 0.00765815899050609
0.0679666628515069 0 0 0 0.014 0.236754475104165
0.983553075544359
TCGA-DK-AA77-01A-11R-A39I-07 0.00951339990079729 0 0.00808825254001723
0.264075377743358 0 0.107680969379688 0.119616997305593
0.0902090301578543 0 0 0.000646568033976061 0
0.00722944445911641 0 0.0755594435996054 0.0858526354922623
0.0515448054762459 0.13889101736536 0.0369310476128855 0 0
0.00416101093324143 0.05 0.161905059590252 1.03088040969506
TCGA-ZF-AA5N-01A-11R-A42T-07 0.177720221907872 0 0.0443890393406911
0.00263997628099646 0 0.0320023055994034 0.146382252793828

0.00763880351873747 0.0329865879196922 0 0 0.05298452218421 0
0.13450832955766 0.0351604938159572 0.0983552070684111 0
0.195969957542116 0.00779914180002545 0.0120673593586037 0
0.019395801311796 0.039 0.177163662536372 1.01614721955254
TCGA-DK-AA6U-01A-11R-A39I-07 0.149159922841938 0 0.0246106500183742
0.196175340631367 0 0.0105727621259092 0 0 0.112364460835911 0
0 0.0218095746002094 0.0677524349776161 0 0.06241260143946
0.134907054716616 0.0705248730713322 0.0820443987473368
0.0238627208231244 0 0 0.0438032051708049 0.267 0.0497304118699965
1.06078549946048
TCGA-BT-A20O-01A-21R-A14Y-07 0.0294366480035695 0 0.00592196665255138
0.339257923087528 0 0.120769621586501 0.108618046865276
0.0420472893884823 0 0 0 0.0131481414069627 0.00894591683704305 0
0.0804101876289454 0.157715198289585 0.0108449265899136 0
0.0764039163921771 0 0 0.00648021727146494 0.088 0.122239445913869
1.04908262440589
TCGA-GD-A3OS-01A-12R-A220-07 0.0604900142165343 0 0 0.0618288427527674
0 0.183655973413756 0.000214550147035357 0.0496675668060003
0.0510239264790949 0 0.0086645579961823 0 0 0.359454454274919
0.011026162756719 0.199279938044368 0 0.0146940131126231 0 0 0
0 0.069 0.139004207604632 1.05403908339785
TCGA-XF-A9SZ-01A-11R-A39I-07 0 0.0203853318413098 0 0.0261867529941745
0 0.0511444000183604 0.00635820227491373 0.00597463926081103
0.00964212751624727 0 0.0723408114400103 0 0 0.488104538844369
0.0118200286365673 0.165010313550313 0 0 0 0.103863712343436 0
0.039169141279488 0.122 0.0985748189718502 1.10805821833239
TCGA-4Z-AA82-01A-11R-A39I-07 0 0.0501544540207217 0.00527034361905301 0
0 0.179015280464736 0 0.065118059949842 0.00200700922739444 0 0
0.0732767065482584 0.0101448843400152 0.030568233451697 0
0.0361551280943274 0.245066902114761 0.219562606805835
0.0767241781585408 0 0 0.00693621320481921 0.014 0.236703088972684
0.987987475390132
TCGA-DK-AA76-01A-11R-A39I-07 0 0.080185176973679 0 0.0779843095187232
0 0.210159346179281 0 0 0.111624224477626 0 0
0.00257678528400193 0.0231228316118143 0.182050584279645 0
0.145915773288521 0 0.079640884248777 0 0.0867400841379313 0 0
0.488 0.0171761376656654 1.08203950090241
TCGA-DK-A3IL-01A-11R-A20F-07 0.405135380038017 0.0064697049638907
0.0233983314684501 0.0619732276684585 0 0.0517006616999406 0
0.0889379498527562 0.00631137539994779 0 0 0.0258672649398822 0
0.149036741200886 0.0531581593505245 0.0361460746915981 0
0.043962936208848 0.0479021925168 0 0 0 0.189 0.0713505267864752
1.09473677122163
TCGA-E7-A678-01A-11R-A30C-07 0 0 0.182351722529855 0.138722657810515

0 0.359201173118697 0 0.00938467261473742 0.050636642437211
0.0151886544957579 0 0.068865189394611 0 0.0332672069697264
0.0174412259400122 0.0329883396076033 0 0.0425852912419932
0.0493672238392806 0 0 0 0.014 0.238383618691549
1.01161586014888
TCGA-ZF-A9R1-01A-11R-A39I-07 0.237781046333257 0.00127960519361933
0.0263091734634373 0.196788352950018 0 0.0825448155361695 0
0.0340030953782759 0.0435739057441624 0 0 0.0531575266521375
0.00952406154924274 0.0886971315836295 0.0255462141723269
0.0666554306392917 0.0343814801587565 0.0306264301377756
0.0691317305078998 0 0 0 0.077 0.132105365125351
1.03969423019399
TCGA-XF-A9SH-01A-11R-A39I-07 0.0011818812617028 0.0502689607902392
0.0480277702192685 0.0446948438225239 0 0.188067737843504 0
0.00221112253849258 0.0538680907296188 0 0 0.0373823561542528 0
0.129790341923305 0.0096487198178598 0.219733603002314
0.112984390753654 0.0309308082830531 0.057731469086308 0
0.008530142542497 0.00494776123140684 0.045 0.16685246809814
1.01173022931196
TCGA-FD-A5BU-01A-31R-A26T-07 0.119414215190647 0 0.119693453600567
0.014623201040583 0 0.157481353705465 0.0248982767938614
0.040081939358403 0.0145364159035374 0 0.0167979199052611
0.0234284146740174 0.00780831444297888 0.132485237728658
0.100780511755048 0.0482302437135031 0.112530961801111 0
0.0650829428131901 0 0 0.00212659757316829 0.003 0.36123533465105
0.933241921097294
TCGA-C4-A0F7-01A-11R-A084-07 0 0.00885529584290128 0 0.155305670632912
0 0.225523407488574 0 0 0 0 0.0207708044709052 0
0.0650161067429791 0.153033376944491 0.0578150977751654
0.0708013361347883 0.0492855290621911 0.108007460695006
0.0449081477331412 0.00628882980265077 0 0.0343889366742951 0.479
0.0179059165236307 1.08006295296729
TCGA-C4-A0F0-01A-12R-A10U-07 0.00683940410878107 0 0.00351894243397081
0.1582585517499 0 0.186373258107263 0.0659697370354189 0 0 0
0.00460493632369749 0 0 0.102244325932784 0.101092260271183
0.172197208788847 0.113903279009207 0.00883632153460983
0.0491165375009207 0.00657430678979116 0 0.0204709304136264 0.01
0.252583409494098 0.980244952346411
TCGA-FD-A3N5-01A-11R-A21D-07 0.00865475519389695 0 0.00900861911044108
0.110482690096845 0 0.0939387219074469 0.100640976913316
0.038508101373415 0 0 0.00261667358419169 0.0634998669152737 0
0.360056752124427 0.0368660352708413 0.090797994153168
0.0538857350488566 0.00654138029287407 0 0.0109383055496169 0
0.0135633924653894 0.019 0.219371807205625 1.00957426458858

TCGA-ZF-AA5H-01A-11R-A39I-07 0.0456012235579887 0 0.000519195541425012
0.101871275734849 0 0.158479736399072 0.075151020468482
0.0112375415275983 0 0 0.048355711455929 0.0286618349391716 0
0.264625616177689 0.117414265004351 0.143526716360308
0.00256948574936809 0.00198637708376935 0 0 0 0 0.05
0.161807550402984 1.03196452972035
TCGA-DK-A3WX-01A-22R-A22U-07 0.0293389989917264 0 0.0148808264565563
0.0810733890472177 0 0.219975758327534 0.031152713373456
0.0435835196922254 0 0 0.0417126734863358 0.0135998067566625 0
0.125787958273537 0.116158482297398 0.0466061912180927
0.00740076201144866 0 0 0.0778462408459275 0 0.150882679221881
0.033 0.183782238708487 1.01909237429933
TCGA-YF-AA3L-01A-11R-A38B-07 0 0.0972511001034129 0.000973107742470952
0.0185723472748683 0 0.175512817584756 0 0.0206563479803397
0.0816161954586574 0 0.0638696953990543 0 0.027160512035202
0.00172011771694638 0.0238165758344066 0.203234215693309
0.256100953103886 0 0.0295160140726908 0 0 0 0.404
0.0270881659211194 1.06473101116154
TCGA-BT-A2LA-01A-11R-A18C-07 0.163627770810987 0 0 0.196341786050382
0 0.0478301260715379 0 0.105040233668537 0 0 0
0.0522041392308615 0.0255602434376126 0.250268280012737 0
0.0424455163262071 0 0.0123228933385841 0 0.0966123463664721
0.00774666468608188 0 0.852 -0.0205793148455593 1.10972003079132
TCGA-4Z-AA87-01A-11R-A39I-07 0 0.0373488006058858 0.0162529558933627
0.212064010358719 0 0 0.094230779417615 0.0405724470888005
0.0468835735066916 0 0.0319870992621258 0 0.00753048178113474
0.11311457040828 0.0921863577857625 0.0903505236025175
0.217195894524589 0 0 0 0.000282505764515594 0 0.093
0.114522583706275 1.03638046323434
TCGA-BT-A2LB-01A-11R-A18C-07 0.0352900280502239 0.00390350933381304
0.033033450892688 0.148388337130015 0 0.0432262494627057
0.136590196289918 0.0361258909939496 0 0 0.037680410292203 0
0.000619630467191522 0.0690198817858309 0.124987133780192
0.123197722353393 0.0203679926229427 0.113200497209367
0.0154921104327913 0 0 0.0588769589027751 0.045 0.16703972869181
1.01952064372692
TCGA-XF-A8HB-01A-11R-A36F-07 0 0.0796197868229479 0 0 0
0.19348938693971 0 0.0860703367879571 0 0 0.00666330066138225
0.0214276256339402 0.0369173223106726 0 0.00209639316984026
0.266075844608921 0.0846370327111765 0.184543006792821
0.0384599635606304 0 0 0 0.404 0.0270549594287334
1.0670846910291
TCGA-DK-A2I6-01A-12R-A18C-07 0 0.0492874441021313 0.00389350310992312
0.138401175734078 0 0.26143000819897 0.0527055585050532

0.00584945271043376 0 0 0.0541103594817203 0 0.0193506544827501
0.0741549503827014 0.0258932759248172 0.0755606307441108
0.0127057141670004 0.210808245617215 0 0.0155627779658261 0
0.000286248873268761 0.109 0.104643949767859 1.05429965342296
TCGA-ZF-AA4X-01A-11R-A38B-07 0.0544344965143941 0.0513011824009197 0
0.0717569181890369 0 0.00800129210775991 0.000410163097923529
0.0515195248392046 0 0 0.0794859340057897 0 0.0102768495715489
0.12559048930199 0 0.0789251289645932 0 0.412797737521433
0.0527085597773698 0 0.00279172370803594 0 0.805 -
0.0171284038755522 1.12967697497874
TCGA-BT-A20P-01A-11R-A14Y-07 0 0.0540990632432298 0.0268060676686123
0.156356670924528 0 0.156739348916407 0 0.0261465712732071 0 0
0 0.0779942967816545 0.0114509012447368 0.0826771249719651 0
0.199890932548953 0.000104877384017294 0.203434192655116 0
0.00429995238757379 0 0 0.388 0.0286014225536384 1.08180460615253
TCGA-BT-A20W-01A-21R-A14Y-07 0.150294794536117 0 0.00438079550935992
0.247573577252495 0 0.100494915937252 0.0154261292257204
0.0860927497249945 0.0985933628702006 0 0 0.025098211875094
0.0269789823123881 0.0229862955942421 0.0595550008693183
0.0814765585008338 0.0111761287350144 0.0203542340963925
0.0461060738601096 0 0.00341218910046779 0 0.108 0.106754248806746
1.05930109315519
TCGA-FD-A5BX-01A-11R-A26T-07 0 0.00900967966921827 0 0.0689571633599578
0 0.105341377371789 0.00512009097198749 0 0 0.0154534129504062 0
0.0184692561916681 0.0146243248435296 0.319718881275999
0.0540585799678201 0.229822815875267 0.122081669875021 0
0.0373427476473361 0 0 0 0 0.65178899821256 0.774492986085764
TCGA-BL-A13J-01A-11R-A10U-07 0.0294025304772864 0 0.0117135214353761
0.131744586531937 0 0 0.0896280306205653 0.0297775773173645
0.0197320875161285 0 0.0388474032568929 0 0 0.243361255353356
0.118498871000622 0.17381266607062 0 0.0506723711344959
0.0606882121919556 0 0 0.00212088709339808 0.666 -
0.00444174452763401 1.09605638030204
TCGA-CF-A8HX-01A-11R-A36F-07 0 0.110567821014039 0.0344779270947906 0
0.0293957538489678 0.224366131510723 0 0 0.00637744938321581 0
0.0357243654604726 0.00397155429579291 0.00883667567954643 0 0
0.160148175911747 0.163751090069315 0.218394921280618 0 0 0
0.0039881344507722 0.596 0.00454834057821427 1.08164709514866
TCGA-PQ-A6FI-01A-11R-A31N-07 0.0191165672537319 0 0.0051320879346423
0.151907802597491 0 0.117911184522454 0.10583551155648
0.0675934402122755 0 0 0.0289192685911823 0.0474361669947617
0.0405308722630461 0.0681633332319101 0.0190207750995319
0.0556730513240015 0 0.218584794012097 0.053872676833215 0 0
0.000302467573179275 0.096 0.112486185053192 1.04612771331176

TCGA-FJ-A3Z7-01A-12R-A23N-07 0.173380026380347 0 0.0307618671347847
0.200517679851919 0 0.173177250051633 0.0454452460483521
0.0359985484860265 0 0 0.0328227126123157 0 0.00674187344187702
0.0749891874532296 0.13159369414148 0.0606884750530038 0
0.00844082196033229 0 0.0254426173847003 0 0 0.0639999999999999
0.145171446081897 1.04200782968357
TCGA-E7-A7PW-01A-11R-A352-07 0 0.174211045741378 0.014844104601043
0.219864620943098 0 0.127271404449226 0 0.0469978204274994
0.0245164286860204 0 0 0.0564866321745944 0.0297827031441031
0.116698209942922 0.0723865507351738 0.0183602068268399
0.0200530068407704 0.0489469865196978 0 0.025319229391719
0.00426104957591404 0 0.085 0.123793838175378 1.04612291519021
TCGA-4Z-AA7S-01A-11R-A39I-07 0.03855234422224 0.114372796128428
0.00693141132596463 0.0267737899633859 0.00131838459646738
0.103640849357864 0 0.136831556934005 0.000370224830922236 0 0
0 0 0 0.00611923798275547 0.1625781258574 0.0505895754859053
0.163117675094227 0.171761127625297 0 0 0.017042900595138 0.501
0.0144329897882992 1.06639229328205
TCGA-CF-A3MH-01A-11R-A20F-07 0 0.120632445350537 0.0191521521692921
0.0815308791014867 0.151073862078352 0.0246992076073522 0
0.0377236466986727 0 0.0495169966300013 0 0 0.00539498242181975 0
0.00784536842053568 0.0845949001016767 0.175990878729547
0.175559918067765 0 0.0470916650354311 0.00540454539646605
0.0137885521910653 0.382 0.0296738784297058 1.06822944542458
TCGA-DK-A6B2-01A-11R-A30C-07 0.154012391751101 0 0.122973732937242
0.0857114480712387 0 0.0889384391913207 0.0207743891459745
0.0216983896357379 0.0370747401343437 0 0 0.0166727363185281
0.00437726992965323 0.0808841066216041 0.117000306914258
0.137889201828028 0.0148032390722645 0.0282763311601191
0.0675720728127717 0 0 0.00134120447581506 0.001 0.476189094778874
0.879042752105645
TCGA-4Z-AA86-01A-11R-A39I-07 0.0147914660178928 0 0.00320101729074147
0.111356420252201 0 0.226536492901984 0.0739285460281792
0.00153256889681364 0.000627673290209266 0 0.0911794252235477 0 0
0.222312307423709 0.0751811425251511 0.10815057066468 0 0 0
0.07120236948489 0 0 0.0600000000000001 0.147490202557972
1.0352743853833
TCGA-BL-A0C8-01B-04R-A277-07 0 0 0.00761135033039761 0 0
0.169936108909454 0 0.0421504845638924 0 0 0.0201178623777716 0
0.00794119669387085 0 0 0.316449238576246 0 0.201830811467233
0.0242082648549524 0 0.209754682226183 0 0.962 -0.03398846428801
1.10191289538818
TCGA-5N-A9KI-01A-31R-A42T-07 0.0120887921453537 0.0793553819595273
0.0168877349835271 0.0408679972564542 0 0.117688206102285 0

0.0456325315061539 0.0244312428716828 0 0 0.0245058454771323 0
0.106820481972378 0.0636832070068712 0.254379908236916
0.0125680612558488 0.0839492624053475 0.0531802586292581 0
0.000133529185401668 0.0638275590058623 0.239 0.0571943752940214
1.05636431178817
TCGA-GU-AATO-01A-11R-A39I-07 0 0.0240602075571968 0.118205651746503
0.148538805898586 0 0.0652612351326635 0.0973857359243625
0.0796288722809943 0.0279046448531572 0 0 0.0219809402218897
0.0115024069680977 0.0801163279973794 0.0906966141261359
0.136568527855602 0.00472776893168366 0 0.0523206625337926 0 0
0.0411015979719565 0.011 0.245250260557601 0.982170861585115
TCGA-CF-A9FH-01A-11R-A38B-07 0 0 0.000834780889498668
0.0687148927178948 0.108801026678444 0 0 0.0573323204180079
0.0144064147570407 0 0 0.0524678094842327 0.0245412095785612 0
0.022748732232834 0.0809635657143131 0 0.507772332467009
0.0508089260774053 0 0.0106079889847588 0 0.0649999999999999
0.14381644572871 1.08716223480948
TCGA-FD-A6TG-01A-11R-A32O-07 0.0801460607936454 0 0.103251485701707
0.122988633230392 0 0.0138126165480958 0 0.0898941475881283
0.0109687335932024 0.00521550366076802 0 0.0517930207206097
0.0138618113273198 0.0709888335583269 0.0867846876815792
0.156364903564402 0.0653408520695525 0 0.128588709962271 0 0 0
0.00600000000000001 0.309338048225933 0.95423127076135
TCGA-4Z-AA7O-01A-31R-A39I-07 0.0616145240360123 0 0.0482276376750649
0.308747348583891 0 0.113736109221792 0 0.109642496488819 0 0
0 0.0836581215931919 0.018906333753568 0 0.0405237387731914
0.0602475812025492 0.0266348437712117 0.0879874847947161
0.0288559338058044 0 0 0.0112178463001882 0.032 0.185028822369125
1.03047345425241
TCGA-GV-A3QI-01A-11R-A220-07 0.0714839954203526 0 0 0
0.00241979105652253 0.304679161295846 0 0 0 0 0.0959974182060377
0 0.00868592608607481 0.140158517496073 0.0025282584856308
0.190946447417746 0.0232994007635391 0.0551429798723175
0.10465810389986 0 0 0 0.618 0.00159893977257481
1.07600120174014
TCGA-DK-A6AV-01A-12R-A30C-07 0.22289606245504 0 0.0141573160499918
0.2089392545675 0 0.0815469045122903 0 0.119357912635073
0.00998098942501931 0 0 0 0.022876466644473 0.072092683558906
0.0573958858624926 0.0651657969311956 0 0.0915256216193723 0
0.0252646648417337 0.00880044089691289 0 0.00700000000000001
0.294139641935754 0.969503697715047
TCGA-DK-A1A3-01A-11R-A13Y-07 0.149955783494793 0 0.0757088148512233
0.0859930511545209 0 0.118271908040294 0.0362663730538043
0.0541375100741668 0.0227051792925028 0 0 0.0339329606273004

0.0248600007948882 0.0598253262411009 0.0394784980225931
0.115157278651112 0.0381441445777295 0.0606621720215786
0.0849009991023916 0 0 0 0.004 0.339140648789375
0.941634869329982
TCGA-GD-A6C6-01A-21R-A31N-07 0 0.0314024517543714 0.0105500547340415
0.173430418654082 0 0 0 0.0495039637901207 0.054745549816253 0
0 0.0751860672462185 0.011209079340862 0.135437990937555 0
0.213484938491741 0 0.153552953628704 0.0889754281773412 0 0
0.002521103428709 0.18 0.0758009279802776 1.05378360893041
TCGA-GC-A3RD-01A-12R-A22U-07 0 0.0200926372759582 0 0.0282928047446684
0 0.0551897830923574 0 0.0245121751718842 0.00850794825456906 0
0.0687440839783988 0 0 0.428317404954192 0.00197901306169818
0.0951816588419451 0 0 0 0.234677135275272 0.0241028110687221
0.0104025442803344 0.077 0.132572561352213 1.08748139387643
TCGA-BT-A20R-01A-12R-A16R-07 0.0738385139210928 0 0.00834340660839761
0.0184321364857336 0 0.0579633257229553 0 0 0.00125247381127205 0
0 0.0307340411222581 0 0.652823928183147 0.0276975696375755
0.0822726586882569 0 0 0.0466419458193109 0 0 0 0.001
0.529154784749289 0.857186841679081
TCGA-XF-A9T0-01A-11R-A39I-07 0.0446479270628773 0 0.189452412215983
0.15318453099271 0 0.0470239444260752 0.0874177312806296 0 0 0
0.0360062942245469 0 0.0108095567115972 0.167199579388385
0.0796358117108129 0.127023368680668 0.0183597558747621 0 0
0.0327099259346334 0 0.00652916149631916 0.003 0.352295450162787
0.940572961396877
TCGA-YF-AA3M-01A-11R-A42T-07 0 0.0237448234213465 0.028990561332716
0.111701221802447 0 0 0.0367484717309138 0.0348654033687184
0.0307488179147593 0 0 0.0273808972684568 0 0.147862747916365 0
0.117170435694 0.0573230608318354 0.136467413719665 0.196683246170561
0 0.0187300231554616 0.0315828756727539 0.218 0.0621637582057291
1.04951650489496
TCGA-4Z-AA7Y-01A-11R-A39I-07 0 0.0111773074592915 0 0.0886112207787856
0 0.20087936275717 0 0.0585837148341559 0 0 0
0.123286657062362 0.0473000587839545 0.074548281365475 0
0.122075356334264 0.055905164570396 0.169842951623915
0.0411766899559673 0 0 0.00661323447426267 0.631 -
0.000465625989855082 1.09139579537453
TCGA-FD-A62O-01A-11R-A30C-07 0.0897400792497386 0 0.124112472086995
0.0751597128496099 0 0.0921280446501402 0 0.0149848326936606
0.0309001486615856 0 0 0 0.0119166316543429 0.165552301353567 0
0.114181798199362 0 0.095673355854945 0 0.108288193013057 0
0.0773624297329964 0.124 0.0973550613562641 1.05003377578996
TCGA-BL-A3JM-01A-12R-A21D-07 0.0263448541315035 0 0.0113763182670943
0.0700834424031177 0 0.0315156424283183 0.021089838813406

0.0536762258554395 0.00257617003248005 0.032799206574072 0
0.0194752576905122 0 0.459223253641276 0.128042936234188
0.0854592225927529 0.00568935835351723 0 0.052648272982323 0 0 0
0 0.663728531959323 0.757963577095113
TCGA-FJ-A3ZF-01A-11R-A23N-07 0 0.102668386880652 0.0352977336193645
0.201523577880513 0 0 0 0.0576301982159454 0.067953702109823 0
0 0.0990297015319789 0.0178130414379462 0.0147847558512673
0.00826668994929836 0.192930072669425 0 0.132705721353663
0.0693964185001239 0 0 0 0.151 0.0832620201337696
1.05174913302457
TCGA-CU-A0YO-01A-11R-A10U-07 0.00762504588020465 0.0270989703114995
0.02425912279698 0.100994010393388 0 0.154511837887107
0.0399233966853753 0.0297510364828894 0.0510816394724039 0 0
0.0410763544708403 0 0.0423912391084022 0.0613766752998848
0.0889999275179629 0.0538901140750182 0.141284926467551
0.121336550642414 0 0.00366986163309716 0.0107292908749805 0.069
0.13934531597893 1.02433410581946
TCGA-CF-A5UA-01A-11R-A28M-07 0 0.0818374843966048 0.0121508158954502
0.205884548013692 0 0.0578053912804885 0 0.0756104132218403
0.038211993432033 0 0.00667617626129881 0.00495401922241635 0
0.115872617388841 0 0.159569049068235 0.190104623720209
0.0231644455045125 0.0281584225943793 0 0 0 0.089
0.121238332225877 1.03453675946911
TCGA-K4-AAQO-01A-11R-A38B-07 0.237015245677014 0 0.0665893545771775
0.0918241012851361 0 0.0566355779157287 0.0115238012339253
0.0202729787561416 0.0511277366733376 0 0 0.0404870251159324
0.0165521413483128 0.0250139663743199 0.0624494375275065
0.156678246058185 0.0273818058067983 0.0126290656217102
0.123819516028775 0 0 0 0.001 0.438601937283686
0.897852443434941
TCGA-XF-AAMJ-01A-11R-A42T-07 0.156829135133872 0 0.153166101124879
0.0816010570490158 0 0.0154179534395639 0.0220816305417352
0.0328124082437623 0.0295841929200627 0 0.00297403228336201
0.020880427298853 0 0.222224735756658 0.0908679682757979
0.0900579366847055 0.000865003795568243 0 0.072373057877131 0 0
0.00826435957503293 0.002 0.388673010041457 0.92432745379107
TCGA-GU-A763-01A-11R-A32O-07 0 0.0866170437059448 0 0.178023133547064
0 0.110265929256667 0 0.202166384753784 0 0.0426210373591343 0
0.0668115443107151 0 0 0.0324579993041592 0.0851556671151795
0.107091360443402 0.064139167543058 0.024650732660892 0 0 0
0.201 0.0670293538874386 1.07527243949689
TCGA-G2-A2EC-01A-11R-A180-07 0.0484600546624105 0 0.101812550000665
0.0529793888678929 0 0.339389310040926 0.00853305090982729
0.031995520896277 0.130074604414068 0 0 0.0440311380281512

0.00329725794488122 0 0.0115545313645994 0.0608781611792901
0.0202873321263703 0.0580442157767612 0.0886628837878789 0 0 0
0.018 0.225437678527374 1.00507941252037
TCGA-FD-A3B7-01A-31R-A20F-07 0.0238217064565235 0.00126186126279843
0.00640211389223643 0.309612196688941 0 0.0118808588878625
0.154855181369946 0 0 0 0.033711854881314 0 0
0.167658511451302 0.106860164671754 0.123235009239311
0.0113175434205719 0.00259629569107886 0 0.0278466098660335 0
0.0189400922203273 0.051 0.160370940524079 1.02818736800981
TCGA-FD-A5C0-01A-11R-A28M-07 0.0493543045287493 0 0 0.17685272721891
0 0.216816174061758 0 0.0157498114279913 0 0 0 0
0.0445482717305989 0.0749180223833975 0.0367252189050054
0.203946376240701 0 0.033255230248735 0.0223595073258348
0.110046977873195 0 0.0154273780551232 0.099 0.108649126526769
1.04242732683426
TCGA-FD-A5BS-01A-21R-A26T-07 0.000312285870756893 0.0319089793710592
0.0616660555383875 0.26203144147194 0 0.0897239568436186
0.0553599553025992 0.0281611143466454 0.0463879194437011 0
0.0446754842789627 0.0405730335895658 0.0132580038387031
0.111817769205946 0.119753907110209 0.0770987804901535 0 0
0.01510079263811 0 0 0.00217052065964077 0.04 0.175535731598643
1.02805835600513
TCGA-CU-A0YN-01A-21R-A10U-07 0.0212745744794416 0 0.00252986076112043
0.142570011796881 0 0.268600771034345 0.00526347604147159 0 0 0
0.0399925849296431 0 0 0.229634793041709 0.0169801013461576
0.0799772660970072 0.0399139404654695 0.0311245219059082 0
0.0958830273437439 0 0.0262550707571013 0.111 0.103609622597282
1.05474594518033
TCGA-FD-A5BZ-01A-11R-A28M-07 0.110614015416635 0 0.00934249140006939
0.00614476826217387 0 0.0639117045160342 0 0.0328492368836819
0.0220304556264652 0.0077842807958316 0 0.0166444819731673 0
0.423824793486204 0.0876882541342692 0.114395768036805 0 0
0.104769749468663 0 0 0 0.039 0.177672073307968
1.03655234206671
TCGA-UY-A9PE-01A-11R-A38B-07 0.0970072573609013 0 0.180439714610145
0.0212653193413354 0 0 0 0.0647849947537562 0.0127454321878715
0.00867338236864563 0 0.0354158336342644 0 0.341279852814504
0.120160656477713 0.0681661026978935 0 0 0.0500614537529706 0 0
0 0.00600000000000001 0.310991964339574 0.972848539533355
TCGA-GV-A40E-01A-12R-A23N-07 0 0.0022526859527009 0.00199205086118947
0.148096610057725 0 0.0826944787688251 0.111482055872839
0.0112653143271141 0 0 0.0606643400601248 0 0.00789584121743692
0.153377713448076 0.0117082525023284 0.126901374704366 0
0.185148476758108 0 0.0744550058831963 0 0.0220657995859693 0.07

0.137148664652421 1.03371127065185
TCGA-BT-A2LD-01A-12R-A20F-07 0.0161853784873138 0 0.0888576352575037
0.130510314295147 0 0.0950848341891508 0.0486215956722398
0.0862553322201806 0.0180806985072457 0 0 0.0151868965417704 0
0.0811090755227755 0.141351337229824 0.227975117942312 0
0.0044208382451354 0.0438675708730614 0 0 0.00249337501633984 0.044
0.169092780924129 1.01918562754968
TCGA-UY-A9PD-01A-11R-A38B-07 0.0490784913147695 0 0.156665742107126
0.0284616461673388 0 0 0 0.0289833554323312 0.0347325384261749 0
0.0142611653947982 0 0 0.49908976085771 0.0691024578886472
0.100360437906077 0 0 0.0192644045050281 0 0 0 0.017
0.226390539210551 1.03481611922627
TCGA-G2-AA3B-01A-11R-A39I-07 0.048986545652094 0 0.014776428357493
0.140170236593226 0 0 0.279353989481614 0.14177510389533
0.0785280306366513 0 0.0834612216502705 0 0 0.00590681202457452
0.0385745063808786 0.0483394319571998 0.0130241588350416
0.0944606427334621 0.0123871637549173 0 0 0.000255728047247118
0.129 0.0946780250309238 1.06203771653727
TCGA-K4-A54R-01A-11R-A26T-07 0 0 0.00498165451516556 0.236476024227253
0 0.0736065712568648 0.134243201814511 0.0091156370607345 0 0
0.176770920648523 0 0 0.0655969217602795 0.146900249168659
0.104158692417094 0.00495893731074113 0.00723329902927789
0.0359578907908972 0 0 0 0.001 0.439649228735418
0.899960307078292
TCGA-DK-A1AA-01A-11R-A13Y-07 0 0.0323575069095716 0 0 0
0.225063822162384 0 0.0207688311015946 0.0277273328530175
0.014317492982542 0 0.0615979652911754 0.0186578258908554 0
0.0306902881335191 0.117488362907607 0.223576516696554
0.10160149032748 0.116554626952528 0 0 0.00959793779117156 0.337
0.0364746394947159 1.05908628084943
TCGA-CU-A5W6-01A-11R-A28M-07 0.0559171205320436 0 0.135509793509881
0.0538200837302194 0 0.13329659062892 0 0.0386910738173794
0.00942185466605268 0 0 0.0647149896340168 0.0101267870829245
0.0869295538992819 0.0196439150337914 0.0714115163480708
0.0145707292142156 0.195963477978708 0.0692530529627383 0 0
0.0407294609617563 0.00900000000000001 0.265249857998391
0.976255592084798
TCGA-DK-AA6R-01A-11R-A42T-07 0 0.0054560914021689 0 0 0
0.0415681874590793 0.091215256380088 0.139401595938301
0.00978109834975106 0 0.138168724357143 0.0204345366732852
0.00328054624722083 0.107712550736588 0.159216291664172
0.0189137920651684 0.0842071272717484 0.178790669081495 0 0
0.00185353237379035 0 0.039 0.176759449490255 1.03895075741679
TCGA-BT-A3PH-01A-11R-A220-07 0 0.0530516984700821 0 0.0636699396706547

0 0.144987361339984 0 0.0602644340886274 0 0 0
0.00886919011647096 0 0.102580714588921 0 0.145409714127176 0
0.187458382680421 0.198625372618282 0 0.00388246347114559
0.0312007288282352 0.469 0.0187902261089963 1.07065015746568
TCGA-BL-A13I-01B-04R-A277-07 0.0156775601111426 0 0.00137635450970935
0.00124213574624336 0 0.186618101744243 0.00525644867161043
0.00751058856515946 0 0 0.000920176202565849 0.00168518663414294
0.0312170702708959 0 0.0454618276796871 0.649833180648861 0 0 0
0.042595320629547 0.0106060485861919 0 0.001 0.433641774879654
0.904229431005186
TCGA-CF-A3MF-01A-12R-A21D-07 0 0.00179218729853774 0.075737302098782
0.244515878628718 0.0155477522948802 0 0 0.105542717121009
0.0193282851860172 0 0.0289644557136446 0 0.0219876332135315 0 0
0.242792908575616 0.0473061967212926 0.159120077362185 0
0.0346493859901204 0 0.00271521979566598 0.24 0.057009083221792
1.06182109038431
TCGA-CF-A47T-01A-11R-A23W-07 0.0578110913496275 0.0302964339412014 0
0.134931572044935 0 0.120652752912957 0 0.116461042139685
0.010900455116166 0 0.011989119976966 0.013164467351764
0.0600553917872409 0 0 0.159254358280367 0.171557978480462
0.107141490342079 0.00167196955976614 0.000965704621903958 0
0.00314617209488056 0.118 0.101645363271633 1.03961476600228
TCGA-YC-A8S6-01A-31R-A38B-07 0 0.0107065813900202 0 0.0240867295812396
0 0.183244138923671 0.0495153784542469 0 0.0945028430176809 0 0
0 0.0737021099001622 0 0.00373372731267506 0.100857888514719
0.0591127529571861 0.165727470408681 0.094473812295817
0.0960586880834813 0.00974956759243713 0.0345283115679832
0.00800000000000001 0.279049431081609 0.967415917063418
TCGA-DK-A3IS-01A-21R-A21D-07 0 0.00447673225781637 0 0.286211233138383
0 0.0692751999668415 0.0794531120192574 0.158313456691568
0.00364624990038983 0 0 0.0479795670696481 0.00790873411056472 0
0.0744667221495415 0.081421496793542 0.0808710644254939
0.0314540043654807 0.0699317483557463 0 0 0.00459067875572665 0.486
0.0173443411704608 1.09863705974776
TCGA-XF-A9SJ-01A-11R-A39I-07 0.0957961539018802 0 0.0671382410511746
0.232532246043005 0 0.093969958615876 0.077970467296661
0.0194186255566076 0.0147461124644083 0 0.0590329974268572
0.00758583882023102 0.0143730645268105 0.0506840947144037
0.106185615867768 0.131192111879974 0.00658635931433568 0
0.0227881125200067 0 0 0 0.00700000000000001 0.296443710026154
0.965365909353848
TCGA-DK-AA6T-01A-11R-A39I-07 0.0658843904073441 0.52460740990776 0
0.11948262312424 0 0 0.0256196010638407 0.0550017526747557
0.0089321046785311 0 0 0.0175698405573666 0.00683400302869054 0

0.00971418603062558 0.0613697038513511 0 0.0908956780633646
0.0140887066121298 0 0 0 0.001 0.406281673919073
0.933090465466346
TCGA-BT-A20N-01A-11R-A14Y-07 0.193737498867763 0 0.147922853623842
0.0674010377892181 0 0.0939467527441384 0.0161358989180631
0.0609892985293672 0 0 0.00321234282463845 0 0.00785941519045957
0.0605410601956608 0.151853979419203 0.0756957177095093
0.0950047856281855 0.0106486963471528 0 0 0 0.0150506622127989
0.00700000000000001 0.289360451426643 0.978679056717898
TCGA-C4-A0F6-01A-11R-A10U-07 0.0516675646198845 0 0.0596567772413303
0.184239562179064 0 0.0359236328775946 0 0 0
0.000604792953455357 0 0.0154378124974537 0 0.374487449775243
0.0374108576627804 0.188541872102521 0.00592853471715888
0.0102297935034794 0.0358713498700347 0 0 0 0.045
0.167321570109142 1.03829055095842
TCGA-CF-A7I0-01A-22R-A352-07 0.0936367815145695 0 0.0156639959883999
0.29199145763736 0 0.112519582871321 0.072523002065842
0.00289533912892501 0.0044203609557819 0 0 0.0482075641105047
0.0172316647854098 0 0.0291131647127271 0.102008772065411
0.0942167170420617 0.0128143959363442 0.102757201185342 0 0 0
0.269 0.0486661246225707 1.06731965882433
TCGA-FD-A3B6-01A-21R-A20F-07 0.0154742150913355 0 0.00687179267653065
0.191632233812612 0 0.118252949937472 0.127026010253372
0.050600662231904 0 0 0.0593324843847542 0 0.00209456323473358
0.139581318768085 0.0799208179957094 0.162952799606835
0.0109690515755885 0 0.0249080401235899 0 0 0.0103830603074779
0.01 0.247902685136259 0.985436437000611
TCGA-GC-A3YS-01A-11R-A23N-07 0 0.0125753816882938 0.0085018982047519
0.0207051334722131 0 0.277071449893358 0 0.0188790930891463
0.0324189561528496 0 0.017887599208668 0.0567998885323203 0
0.251413086839412 0.0149140912844206 0.137533621441011
0.0796106027784826 0.039386768598842 0.0314925298976353 0 0
0.000809898918595697 0.01 0.254361736108894 0.98566872301234
TCGA-XF-A9T2-01A-11R-A42T-07 0.00428723849872891 0 0.00010249360260405
0.0297896744041025 0 0.0503718513830767 0 0.0317222063695006
0.0329705865827046 0.00358641743703169 0 0.090554670416926 0
0.395057041424615 0.00341405652160635 0.148364262828774
0.0078631396331405 0 0.20116301169121 0 0 0.000753349205979316
0 0.658283602790049 0.767844977595874
TCGA-UY-A78O-01A-12R-A33J-07 0.183069609992503 0 0.134323394613298
0.0159777215521987 0.00872707439650404 0.0201501668794032 0
0.106594554504795 0 0.00194894210968962 0 0.0293678842449158 0
0.23329137786425 0.0195713434441609 0.0548305607122875
0.0459439278666329 0.0955588411818659 0.0506446006374957 0 0 0

0.002 0.375129403931258 0.930711554845125
TCGA-GC-A3OO-01A-11R-A22U-07 0.00626893890867077 0.0413081253836242 0.00121798592090915 0.0209860322926458 0 0.0581706030369897 0 0.0280970182174807 0.000152354748812191 0.000228235552831033 0 0.0192101353581911 0 0.385530999149769 0.031219528778269 0.214109764339526 0.0180143237357241 0.00985615041792521 0.145241075742774 0 0.0203887284158584 0 0.001 0.444277359273502 0.898221195174602
TCGA-E7-A6ME-01A-22R-A32O-07 0.0556170465286807 0 0.00562251459127475 0.329743568060098 0 0.115656617451954 0.0434169614535476 0.134196233530559 0.0325562731072524 0 0 0.0315105122964085 0.00123740313234338 0.0395975373584538 0.0634396100074066 0.0716817236756541 0 0.0517306192972239 0.0202602728218324 0 0 0.0037331066873109 0.0639999999999999 0.144784484384859 1.05676532844846
TCGA-XF-AAN5-01A-11R-A42T-07 0.0140363350485814 0 0.00369464905355514 0.169215872431089 0 0.131707739022012 0.125472155110611 0.0503368535065232 0 0 0.0583699879201668 0.0210977930224916 0 0.0971022785282187 0.0960713599260969 0.121132618949115 0.0273212165303131 0 0.0336698341120684 0.0153245668733914 0.0202863867664376 0.0151603531993288 0.017 0.22689500280068 0.994251456381801
TCGA-FD-A3SN-01A-12R-A22U-07 0 0 0.00604872421133705 0.239233006125664 0 0.0556150145564749 0.104888317767513 0.0392543506627105 0 0 0.0154699542502512 0.0467028935749633 0.00039595981891703 0.153368305243541 0.0570974798836598 0.177974752008757 0 0.0565762667193009 0.034537057168873 0 0 0.0128379180080368 0.091 0.119924083566529 1.04212715075613
TCGA-ZF-A9R2-01A-11R-A39I-07 0 0.0713725615835627 0.0287146718602557 0.0888685976705026 0 0.181287628477392 0 0.21880114543594 0 0 0.00471764139482917 0 0.0148487718047355 0.00267538888900694 0 0.132652272718611 0.0678290431318374 0.153537501971779 0 0 0.0236005681490455 0.0110942069125026 0.468 0.0190707388028368 1.08437531163875
TCGA-ZF-AA4T-01A-11R-A38B-07 0.0183996087627392 0.00453401389438615 0.00972785295199035 0.0576235323160128 0.004482626752061 0 0 0.0484964198092066 0.0496040955051279 0 0.0354496569105422 0 0 0.285360467297626 0.00630779138030045 0.00339637492582957 0 0.269324821668838 0 0.202079862532982 0 0.00521287529235762 0.261 0.0508855389583957 1.10609921696588
TCGA-E7-A4IJ-01A-31R-A26T-07 0.0124109304330777 0 0 0.261211491454528 0 0.0812151583837534 0.0302777293176443 0.199826756211053 0.0072387651268103 0.0910020391798968 0.0450191577906342 0.0256308144381688 0 0.0128599778260139 0.0343625897659717

0.0720139085816878 0.00955476082995946 0.0943749612922467
0.0230009593685539 0 0 0 0.14 0.0902640340939357 1.09226588750315
TCGA-FD-A6TE-01A-12R-A33J-07 0.0574501575614613 0 0.101365584708385
0.134457254669328 0 0.195125211786055 0.0601106616009861
0.136954807030388 0.000416037746203272 0 0.00477507637177741
0.0172958123319749 0.00282667271092782 0.0380874215301226
0.135974996552766 0.0282079620587054 0.0200776829181632
0.0339096159437469 0.0329650444790084 0 0 0 0.024
0.201703675329096 1.01742697205063
TCGA-H4-A2HO-01A-11R-A180-07 0.0916255458868729 0 0.0106496841759894
0.154131572590207 0 0.142230981481999 0.030259633370879
0.00742783516491888 0.109999620749888 0 0.0347048103959783 0
0.00403010860964109 0.223516100699123 0.00908882573818977
0.0630075483826 0.00472927579118513 0.0915138579008078 0
0.023084599061721 0 0 0.49 0.0169341801634733 1.08714365486894
TCGA-FD-A6TB-01A-12R-A33J-07 0.289941220650043 0 0.0563385815074002
0.135687955720223 0 0.0491771515028828 0.0940699331176379 0
0.0203583805392449 0 0 0.050651912688951 0 0.0789093257333341
0.0783169210844041 0.0905068898354419 0 0 0.0529068449576035 0 0
0.00313488266283332 0.001 0.450797419729163 0.891997524875581
TCGA-XF-AAN3-01A-11R-A42T-07 0 0.0247322360429259 0.00171016806420311
0.0345478796626047 0 0.196034969258914 0.00900539969699089
0.0364732214751905 0.0525677624288018 0 0.00726450531192211 0
0.00856670855514467 0 0.0185951655949 0.195661102845654
0.223909980723698 0.0788283708409273 0.0939768820140705 0 0
0.0181256474840521 0.036 0.179931751471055 1.00536195684618
TCGA-XF-AAMH-01A-11R-A42T-07 0.0496034857284206 0 0.0111437258417654
0.18240718102792 0 0 0.0542224839087071 0.00377491234580382
0.0102691117548945 0 0 0.0311883119086009 0 0.330219177688427
0.0179286136111572 0.196648808184569 0 0.0497613570292761
0.0628328309704581 0 0 0 0.151 0.0842810098606029
1.06649856475588
TCGA-FD-A3B8-01A-31R-A20F-07 0.0730837734559938 0 0 0.140391085404043
0 0.179740651617425 0.0616411065095197 0 0 0 0.051240405575785
0 0 0.0989106469739022 0.160138341974708 0.0973299459860337
0.0334464204731789 0 0.0851537444022633 0 0 0.0189238776271471
0.001 0.494118625175623 0.87052058632576
TCGA-XF-A9SY-01A-21R-A42T-07 0.028530474710956 0 0.00416587282739804
0.11692616190857 0 0.126985019956849 0.101533863667482
0.0203140823723412 0 0 0.0411337565742011 0.0242693854531186 0
0.166664878931124 0.134995684074162 0.123697697383179
0.00847664936205306 0 0 0.102306472778564 0 0 0.119
0.100085848086731 1.0557911554026
TCGA-CF-A9FF-01A-11R-A38B-07 0.398270165139288 0 0.048766241435248

0.106615531009188 0 0.0585312076143044 0.0054323044578606
0.0611928939355292 0.0683393144573459 0 0 0.0331187663517612
0.00172513051474549 0.0453587176782046 0.0362695101629632
0.0377208773252709 0 0.0386040018944466 0.0600553380238444 0 0 0
0.018 0.222639257661924 1.02525836296329
TCGA-XF-A9ST-01A-11R-A42T-07 0.241360863854057 0 0.0277511200674898
0.0138013227307504 0 0.0463946300439091 0 0.0785674650829753
0.0135621521005241 0 0.0464225251053355 0 0 0.381044740561655
0.0170824606024782 0.102026617995569 0 0.00897071923442437 0
0.0230153826208325 0 0 0.069 0.139132120025252 1.05510826601205
TCGA-DK-A6B5-01A-11R-A31N-07 0 0.00933671397417835 0.0140459265427774
0.11319184355306 0 0.0383354586274257 0.00582381951269325
0.166059496416764 0 0 0 0 0.0146426243275828 0.131489422745409
0.0800813378311038 0.113273004441732 0.158177666715517
0.0530469172428767 0.0938744628839689 0 0.00695858884649984
0.0016627163384113 0.188 0.0717628434444516 1.04986762109492
TCGA-GC-A3RC-01A-11R-A22U-07 0.009115163939196 0 0.00261530371717348
0.105100457382147 0 0.236790035189725 0.00296419225959653
0.0317162517949981 0 0 0.0199639333126383 0.0266392086222962 0
0.272721594376506 0.0435532520433273 0.182623202752417
0.0252588087960729 0.0334022699680519 0.0075363258458543 0 0 0
0.00900000000000001 0.267873709097601 0.984131192971179
TCGA-GV-A3QK-01B-11R-A23N-07 0.0135967043427012 0.0153803931163482 0
0.198712483353986 0 0.0679885849737908 0.124194826030695
0.112519885854326 0.030528818436536 0 0 0.011971322320734 0
0.095814344267091 0.0134961202006079 0.0688275053132597
0.0226401882079084 0.179810642810525 0.0445181807714907 0 0 0
0.325 0.0385613768444099 1.07554071378142
TCGA-GU-A764-01A-11R-A352-07 0.00183944149926074 0.0118023119619436
0.0655504501160404 0.0760245853870451 0 0.145478361560879
0.105941414585204 0.0351567050126197 0 0 0 0.082692888082051
0.051596533164339 0.0837064171892502 0.146239982600055
0.114294669164614 0.0360382636489627 0 0.0436379760277344 0 0 0
0.085 0.124110880134376 1.03614473980352
TCGA-GU-A42R-01A-11R-A23N-07 0 0.0125718954907094 0.0220202669236585
0.208856966147893 0 0.00622706678644599 0 0.0316520659966964 0 0
0 0.0976032335350997 0.0618961146693304 0.0307088191680695
0.00269394713126835 0.147440572263156 0.0122649353905649
0.169517586759884 0.177870953749066 0 0.0186755759881582 0 0.811
-0.0175191563342318 1.08744744477793
TCGA-DK-A6B6-01A-11R-A30C-07 0.0577182375115701 0 0.115805752767506
0.0388142157648666 0 0.0476185450854563 0.106545560790573
0.0735259843596358 0.0962491513720483 0.166067277598212
0.0123452519376177 0 0 0.0355461319602463 0.0586575416907263

0.0615960191146867 0.00909561850304266 0.0658369604755857
0.0534780516104756 0 0 0.00109969945775081 0.00600000000000001
0.300545684054179 0.962751894818796
TCGA-R3-A69X-01A-22R-A30C-07 0.118462997017569 0 0 0.10515900273275
0 0.253276556238673 0.00371023469616977 0.0330396886038533
0.0212768169923944 0 0 0.0277915527782858 0.0663800508841829 0
0.0477352590646898 0.191647461820679 0.0791250006579416 0
0.0507812969102815 0 0 0.00161408160253029 0.02 0.216295227124502
0.993965003663696
TCGA-5N-A9KM-01A-11R-A42T-07 0.0320812957914823 0 0.0512787414656062
0.304357086898024 0 0.140488403283243 0.0448230790750967
0.00447565702029136 0 0 0 0.0316104869610717 0.0870532983141656 0
0.0457130790516808 0.184291660755065 0.0106752178905436
0.00564484382177184 0.0575071496719588 0 0 0 0.02 0.216674712129586
0.999025139290282
TCGA-FD-A6TF-01A-52R-A32O-07 0.169410004432717 0 0 0.134419194822818
0 0.0977020528006073 0 0.0289318887955914 0.00777678984833729 0 0
0.0197937172158061 0.0175106250033378 0.0893140237358631
0.0439495547772149 0.237505423339458 0.0108152498821535 0
0.142871475346096 0 0 0 0.00900000000000001 0.266095985970367
0.972402996359766
TCGA-XF-AAMR-01A-31R-A42T-07 0.0222397448623321 0 0.00161887745642671
0.0185205034571419 0 0.20856392358993 0 0 0.00617306315537673 0
0 0.0520891274064426 0.00975236429180345 0 0.00262028334930107
0.349530323207813 0.130892937389708 0.0032603204086183
0.176594163670293 0 0.00142932400795558 0.016715043746857 0.039
0.177708263608295 1.01022043097516
TCGA-XF-A8HF-01A-11R-A36F-07 0.0426830750930816 0.00682974263351278
0.00229004570637092 0.120123334356596 0 0.128368689410817
0.00149339667502385 0 0.0573118537358767 0 0.0458633489657754 0
0.0268929359916587 0.00842147986100501 0.0990213426519213
0.336170389580678 0.00946344440044274 0.0222196312510841 0
0.0709664089668826 0.0188202770281036 0.00306060369116928 0.088
0.12254799822696 1.03700912980929
TCGA-ZF-AA4U-01A-11R-A38B-07 0 0.0033280706357282 0 0.133581585320525
0 0.0938748064512492 0.0512109434651284 0.111861439165397
0.0273664822415307 0.00274118893316429 0 0 0.0544795820285916 0
0.166738636472744 0.0981866694654954 0.182030146799564
0.0141275698277758 0.0588161148228708 0 0.00165676437023576 0 0.491
0.0164743910491768 1.08426617781169
TCGA-DK-A3IQ-01A-31R-A32Y-07 0.0455725633073349 0 0.00517562665402284
0.0178819665895008 0 0.318943812220295 0 0 0 0
0.0186319796658049 0.00587487178084963 0.0221468045475721
0.150502005194665 0 0.271712675666635 0 0.013980734312047

0.129576960061273	0	0	0	0.08 0.126597183196584	1.0340669221909
TCGA-E7-A4XJ-01A-11R-A26T-07	0	0.128709265866293	0.0780597417536753
0.0470335958081245	0	0.0670714212656498	0	0.0252850410570389
0.0966600599958468	0	0	0.0568165132989711	0.0213241957712403	0	0
0.151877276106328	0.17463865931568	0.105110045595717
0.0235406849069105	0.0238734992585245	0	0	0.616
0.00179060968749018 1.07418818973159
TCGA-XF-A9T6-01A-11R-A42T-07	0	0.00174783367802747 0.000519820719049411
0.146115537816407	0	0	0.066450512711825	0.0365935095813117
0.0165529001687864	0	0	0.0467963738415719	0	0.13138344100725
0.0433200389924573	0.245014342032128	0.0945011904599345
0.130517763721066	0.0361591031158584	0	0	0.0043276321543267	0.075
0.134104770437004	1.03353432681014
TCGA-4Z-AA7M-01A-11R-A39I-07	0.0685437215245769	0	0.0174597564367415
0.0991017821343319	0.101905172014006	0	0	0	0.0273314377798319	0
0.0707254429139865	0	0	0.377052222305156	0.0757418710217582
0.136185065630882	0.0212014582720245	0	0	0.00475206996670468 0	0
0.073	0.135042606071249	1.05276158132762
TCGA-ZF-AA56-01A-31R-A39I-07	0.0603744706510284	0.0453468792563601
0.0642576878160042	0.0643642934855118	0	0.214819914823033
0.00315554230493931 0.0401700275149797	0.0102108482340096	0
0.0183593097409263	0	0.0389969866688419	0	0.0342248360404173
0.195917290277412	0.0318183204689809	0.0741865941297632
0.0322368541218532	0.0191875233327566	0.00153397001567929
0.050838651117504	0.00700000000000001 0.297830229272014
0.958156233134331
TCGA-XF-A8HH-01A-11R-A38B-07 0.062907348531618	0	0.00858074205554028
0.0523035784757566	0	0.0611369691952931	0	0.0515744913068937
0.0638239452783269	0	0	0.0136754020369054	0.00602447868971975
0.222137390002912	0.112739406730976	0.182740432215925
0.0579477285913457	0	0.101450355419651	0	0.00295773146913699 0
0.18 0.07577838670292	1.05691844991376
TCGA-C4-A0EZ-01A-21R-A24X-07	0.00300470325076792 0.0140371431375049
0.0310366142118034	0.105392742988325	0	0.165341624292435	0
0.0749411476614618	0.0309514923940337	0	0.0923778236413877	0
0.0287262223378279	0.327376286751431	0.0243317732056246
0.0539426624068515	0.0180310692264946	0.000990917526253938
0.0295177769677985	0	0	0	0.52 0.0121617328140329	1.09742475271305
TCGA-FD-A62P-01A-32R-A30C-07	0.0157074055526101	0.00968116895129312 0
0.183871518595773	0	0	0.129720433398125	0.0276512394934949
0.00423583730849243 0	0.0212299234807121	0	0	0.327381015888073
0.0632894310841222	0.153766514128843	0.010322359448269	0
0.041012171020992	0	0	0.0121309816492007	0.001	0.412409106120456
0.911811973549055

TCGA-CF-A47V-01A-11R-A23W-07 0.00735658611937163 0.020996685788213
0.0111888707645932 0.303215615454687 0 0.0131090274930749
0.0317840429883018 0.0497954011188366 0.0212379156209014 0 0
0.0269851907207004 0.0358504723129341 0 0.04508468197639
0.103633631512522 0.16125011062442 0.0852205955108533
0.0832911719941999 0 0 0 0.315 0.0405659207272405
1.06443515384129
TCGA-XF-A9SI-01A-11R-A39I-07 0.0829259030596642 0 0.0255054960287308
0.243642573828474 0 0.200759281573043 0.117514459527578
0.0400526697725562 0.0296900087520261 0 0.0150622015051571 0
0.00599704279344995 0.0416027011810311 0.112687641349682
0.0585516455002284 0 0.00157641131642617 0.0226423600210583 0 0
0.00178960379089539 0.0580000000000001 0.150110376483196
1.04322395506186
TCGA-GU-A42Q-01A-11R-A23W-07 0 0.0245648666542446
0.00632573400522842 0.0754212654648779 0 0.108032405789867 0
0.00992323786187078 0 0 0.0729514741187105 0.00368397168986439 0
0.555946142117949 0.044683337351281 0 0.0866734367544224 0 0
0.00155710734885171 0.0102370208428323 0 0.04 0.176264547131413
1.0621869402125
TCGA-BT-A42F-01A-11R-A23W-07 0.121624055594178 0 0.066251252132676
0.151612103031598 0 0.0328392323765572 0.0754941661122007
0.054721297371304 0.0146023965065223 0 0.0164858750995448
0.0158961626422196 0 0.19953214757088 0.0457900776205757
0.0934162733597532 0.00116348463557641 0.00416816643798615 0
0.0963500243371928 0 0.010053285171236 0.00600000000000001
0.31382859877998 0.95546985161045
TCGA-ZF-A9RF-01A-11R-A38B-07 0.0442417114472998 0 0.0120732865909377
0.419350516886372 0 0.016002537750015 0.0813029512742301
0.0455142907902503 0.00786942868741242 0 0 0.0222648762007476
0.0106156689430022 0.0232870172822219 0.0752082305788006
0.222194359850857 0 0 0 0.0200751237178535 0 0
0.00700000000000001 0.286492534090336 0.976510310453281
TCGA-KQ-A41N-01A-11R-A33J-07 0 0.14566586761128 0.0407387423096138
0.0607289444765281 0 0.105621340656407 0 0.0524550602140212
0.016550273073392 0 0.0423488046643834 0 0.00786936417783416
0.132492351709356 0 0.156268545101386 0.0302063283744862
0.132597231689864 0.0564474181325177 0.0200097278089297 0 0 0.587
0.00578387588248575 1.07144444414924
TCGA-GD-A3OQ-01A-32R-A220-07 0.0601134337260243 0
0.00752956924101878 0.0639086775475019 0 0.0544098127349446
0.0577909840859784 0.0979820649436131 0 0 0 0.0435446051717546
0.00490949349542242 0.122449337104909 0.0643855591151327
0.110121123553602 0.0321747497011834 0.128189768104928 0

0.0923772980289223 0.0356434461422961 0.0244700773027679 0.25
0.05331590857423 1.06155594331323
TCGA-BT-A20U-01A-11R-A14Y-07 0 0.0125890949032657 0.0114669472570751
0.0885255555127249 0 0.104705200004398 0.019240290092413
0.0880822807506585 0.0237426292928669 0 0.0288638485938165 0 0
0.265653941368576 0.0787271417951455 0.190440123862965
0.0296048830895389 0.011941949675564 0.0353178737575208 0 0
0.0110982400434706 0.099 0.109889782562313 1.04997012942505
TCGA-XF-A9SW-01A-11R-A42T-07 0.0744708349770409 0 0.0246632070393389
0.0471818573332564 0 0.132213902391867 0 0.010921794537093
0.0152362688433199 0 0 0.0366997971163356 0.0213319673919158 0
0.00713795911603237 0.392899599311254 0.0100502584886727
0.0852028539358488 0.129017657937984 0 0 0.0129720415800398 0.034
0.183432704583206 1.00530074741319
TCGA-FD-A3B3-01A-12R-A206-07 0.047931579183059 0 0.117246210014488
0.141683357877414 0 0.113297337822247 0.0778833027342085
0.0688666680318992 0 0 0.0182617900958325 0.0229410929531859 0
0.104645001965416 0.127863199264196 0.0913858529319515
0.0104197850709076 0.00485882168698233 0.0461027457861444 0 0
0.006613254582069 0.00800000000000001 0.279695438279191
0.971729613088184
TCGA-ZF-A9RC-01A-11R-A38B-07 0.0518325489925538 0.0172097466515485
0.0142193787955266 0.0594583182486734 0.00700703560457055
0.010533802885288 0.0741214248287895 0.00613051860694672 0 0
0.0228187795381272 0 0 0.308011121265027 0.0445514782681671
0.159231873131732 0 0.021289666686383 0.173836512759242 0 0
0.0297477937374253 0.113 0.102667958567794 1.05100207368416
TCGA-E7-A6MD-01A-41R-A352-07 0.193542228805424 0 0.0353971795084681
0.0989954102586061 0 0.270565101284655 0.0135295477619337
0.0357974264691081 0.0405933033480082 0 0 0.00286562750799557
0.0117063106815425 0.0421094873923884 0.0467161730938392
0.109275569824134 0.0393360839069519 0.015850043463918
0.0437205066930277 0 0 0 0.002 0.366891754645319
0.932588128273122
TCGA-E5-A4TZ-01A-11R-A31N-07 0.115686910975564 0 0.0120847358709167
0.060918080311484 0 0 0 0.113335439422196 0.00637372015769519 0
0.0264373541193579 0 0 0.4234262705993 0 0.0900441147404159 0
0.101258305719728 0.0400837935561748 0 0 0.0103512745271667 0.092
0.117143021841954 1.06321670743144
TCGA-ZF-A9R4-01A-11R-A38B-07 0.0103622038386706 0.00545182924595608
0.00429595319598099 0.15658033878047 0 0.115817863710958
0.145038838207606 0.0669840594685332 0 0.0351939755332422
0.0068175248668355 0.0716334738215571 0 0.0337638169271008
0.102761232444508 0.151374922026233 0.0204102173188227

0.00355945320644641 0.0699542974070802 0 0 0 0.183
0.0733683630598175 1.06442298665072
TCGA-GV-A3QH-01A-11R-A220-070 0.0703826661611657 0 0.0923519305571021
0 0.0732139341782893 0 0.0430926344912474 0 0 0.0380428737478429
0.00387797852588223 0.0586638598244743 0.174314279470421
0.0123271627367657 0.0939428290994841 0 0.339789851207325 0 0 0
0 0.913 -0.0256253539553341 1.12385594638652
TCGA-BT-A0YX-01A-11R-A10U-07 0.0260336532538001 0 0.0023094259276814
0.209227498700948 0 0.189182108872239 0.09233301146504
0.0674829935368836 0 0 0 0.0423551090092259 0.0210761454966616
0.0163523200209291 0.0680039173394561 0.109065118062013
0.0353283203957652 0.0354881258699288 0.0633765524260467 0 0
0.0223856996233814 0.117 0.101865092468168 1.05088180221819
TCGA-FT-A3EE-01A-11R-A206-07 0.134099208511083 0.0740693246091337 0
0.0604520561063425 0 0.0723590824912061 0.00846360576072274
0.0553707869540927 0.00576109230905995 0 0 0 0.000131622519144196
0.0283304258950404 0 0.139164158374755 0.081035429653376
0.23497258867 0.0666460862701526 0 0 0.0391445318758909 0.01
0.247324056753708 0.982501519741543
TCGA-ZF-A9RL-01A-11R-A38B-07 0 0.0361806230898982 0.0133482008008876
0.264730839293621 0.176550687753564 0 0 0.0241284750902131 0 0
0 0.0330159522639798 0.00173707241366554 0 0.0733886613300779
0.0442242145748333 0.289874294122713 0.0428209792665465 0 0 0 0
0.498 0.0148709204347542 1.09449872030642
TCGA-ZF-A9R9-01A-11R-A38B-07 0.120530934056474 0 0.0824654367607793
0.068807261654992 0 0.188853823077179 0.00532980826904063 0
0.060836912258179 0.00393234857313528 0 0.0172343537171443
0.00890094499509866 0.12584576421467 0.107278624517608
0.161374068817329 0.00772293304324358 0 0.0330983683741215
0.00549322445365217 0 0.00229519321735408 0.00800000000000001
0.272150821102644 0.974120312198069
TCGA-ZF-A9RE-01A-11R-A38B-07 0 0.0791669598261818 0.000374283434408326
0.153042784542035 0 0.048762824079616 0.000738553969309312
0.0782923561966989 0 0 0.00327216828412577 0 0.0252693163046806
0.0744265428927394 0 0.0283436667827316 0 0.230691270656144 0
0.14330085362725 0 0.134318419404079 0.186 0.0722438555681358
1.06565545929404
TCGA-DK-A6B1-01A-12R-A30C-07 0 0 0.000324617463155279
0.125129521358232 0 0.269811536660703 0 0.00263113501069095
0.0381145567327475 0 0 0 0.111205067203462 0.0978441588313964 0
0.070233402620604 0.0188968752156587 0.150136305105363
0.109090955823503 0 0.00658186797448385 0 0.758 -
0.0133044302370913 1.08852321287692
TCGA-CF-A5U8-01A-11R-A28M-07 0.0701577188633161 0 0.154678596833972

0.341165314171519	0	0.0246251408467047	0	0	0.000825931509250583
0	0	0.0924370312145227	0	0.0572960000691329	0.031229701874464
0.0771666335840229	0.031044180758486	0.0154247381116489
0.0429258785570852	0	0	0.0610231336058758	0.432	0.0233746411256258
1.10026406409179
TCGA-XF-AAMW-01A-11R-A42T-07	0.00606503774050054 0.00683206193798897
0.00323298907612101 0.00801269717343052 0	0.228256346153868
0.0403627665784117	0.0180476274823576	0.0154098650746147	0
0.0137931417605766	0	0.0106009638986835	0.156653983052218
0.116962087961085	0.17196781787824	0.112490729612647
0.0507871593926042	0.0178031891064877	0	0.00798630505121975
0.0147352310689453	0.078	0.129784956631477	1.03653221599
TCGA-BT-A3PJ-01A-21R-A220-07	0	0	0.00302753365367569 0.101964588552273
0	0.170478110354753	0.0878655325703382	0.0586699353056396
0.0218734206263679	0	0.0419030067147062	0.0207459612220135	0
0.130880665745765	0.159859476691534	0.130126216017201
0.00586052186413753 0.00315412073488382 0	0.0597613629433638	0
0.00382954700334765 0.01 0.251840074730535	0.991280265200112
TCGA-ZF-A9R7-01A-11R-A38B-07	0.0559871877167392	0	0.0108354182446695
0.412794662220841	0	0.0236404940478176	0.0972349358883105
0.131909593837548	0.0211443445491942	0	0	0.058146741973397
0.0130080379447273	0.0191745696784286	0.072444213880831
0.0339528290375397	0.00948550849322134 0	0.0402414624867346	0	0	0
0.016	0.234382551092817	1.01877346225433
TCGA-FD-A3SO-01A-11R-A22U-07 0.0125868774122404	0	0	0.0663145731215792
0	0.150107560882271	0.0552123750968776	0.042084588626759
0.0173336872508821	0	0.0332300618824084	0.00781183379382808
0.0723175265937837	0.149427489128918	0.161892677995869
0.148307650925245	0.0106593936414701	0	0.0588439107360073	0	0
0.0138697929118607	0.182	0.0741583386430002	1.06068677840634
TCGA-E7-A97P-01A-11R-A38B-07	0.015088585842809	0	0.00742863515333915
0.345695489158097	0	0.0141450391452071	0.178292118477199
0.0719491221430862	0	0	0.0626343213437947	0.0273843262100585	0
0.0276009187404442	0.132559260258471	0.0447628598999519
0.0213205314531241	0.011104985780138	0.0400338063942803	0	0	0
0.00700000000000001 0.299725810624909	0.977324655110583
TCGA-GU-A767-01A-11R-A32O-07 0.0278844539557779	0.0188338562382144
0.0228369727007982	0.0666315529293246	0	0.17944819752467	0
0.00270377852784214 0.0126494048456097	0	0	0.102580943367297
0.126356379367956	0	0.0069356882692485	0.15529014317047	0
0.0803970680954158	0.197451561007375	0	0	0	0.417
0.0254549541189306	1.06479001997511
TCGA-4Z-AA81-01A-11R-A39I-07	0.0698091819477797	0	0.00969886468358162
0.329647404446366	0	0	0.173022743326105	0.086915028243876	0	0

0.0053526058596114	0.0471866799864153	0.00671977980823695 0
0.00214121614596473 0.0777019327330279	0	0.146808369328537
0.0449961934904985	0	0	0	0.028	0.194727652252807
1.01794462801714
TCGA-2F-A9KQ-01A-11R-A38B-07 0.0121713079505105	0	0	0.0940421937780537
0	0.184665645578625	0	0.0252665043417402	0	0	0
0.034969107080993	0.101514129821747	0.0109356532433011	0
0.142641369910736	0.0581063579006541	0.2091860997355 0.117030490879465
0	0	0.00947113977867387 0.884	-0.0229835286145535 1.08566052483913
TCGA-SY-A9G0-01A-12R-A38B-07 0.361944695894241	0	0.122009768442446
0.0483326995265074	0	0.101396486003332	0	0.0154151720140231
0.017013503423235	0	0	0.0322077546952751	0.000808069406157021
0.0366548196803077	0.00886159504261473 0.0882356251049905
0.0218767078294705	0.0193154531022614	0.125927649835139	0	0	0
0.001	0.512858924516022	0.858269364077328
TCGA-GV-A40G-01A-11R-A23N-07 0.00722639157669276 0	0.00905414001966797
0.0727711112624766	0	0.313244491324418	0	0.0749614955857758	0	0
0	0.108972082626221	0.0732471778307394	0.142831272242074	0
0.0112403448065928	0	0.0322975906853779	0.11681171523154	0	0
0.0373421868084235	0.544	0.00913628583759365 1.09426883410761
TCGA-FD-A43Y-01A-21R-A26T-07	0	0.000940895782338825
0.00266444811954557 0.0932010605906526	0	0.0665468758447833
0.043022524159891	0.0584064347670343	0.0142646380247959	0
0.0144086271492968	0.0359938183016165	0.0222147852639567
0.169404380170572	0.154530532112581	0.126006556888283
0.111428127585919	0.0177516186599797	0.0692146765787543	0	0	0
0.016	0.233000118508828	0.993590863282044
TCGA-S5-AA26-01A-11R-A38B-07	0	0.0936558349025282	0.00511488064452972
0.0140695244872902	0	0.16178252715326	0	0.0576375964097662
0.000959346305388458	0	0	0.113713893527906	0.000203438004115936
0.0101613271908412	0.00243777664293423 0.348007877002462
0.0748938102561141	0.0327030908084484	0.0625961921533259	0
0.0220628845110891	0	0.918	-0.0261777449453058 1.0894555041456
TCGA-BT-A42E-01A-11R-A23W-07 0	0	0.000981845305259755
0.146312495239997	0	0.0881607250133265	0.0882857641740089
0.101168525160354	0.0270329897767394	0	0	0.0741701241145155	0
0.101865684907131	0.135518948557033	0.130258182629622
0.0802979385945192	0.0187982358784293	0	0.00714854064906407 0	0
0.013	0.239745144005041	0.99526640414524
TCGA-FD-A43X-01A-11R-A23W-07 0	0.0669579347540415	0.0805522427819781
0.144334509637632	0	0.111217159286261	0	0.0599036940247682
0.0126296805354488	0	0.0586458334621698	0.0168211920991423
0.0299112065761399	0.125122325285502	0.00835683206192923
0.186383513890069	0	0.0135367165700025	0.0856271590349162	0	0	0

0.613 0.00232984721014079 1.07739727501876
TCGA-ZF-AA4R-01A-11R-A38B-07 0 0.00489169119137966 0.0113199461327588 0.0914128023205124 0 0.0479237268367144 0 0.0778454800251935 0.0326538285013145 0 0 0.0436878829506553 0 0.426996861328644 0.128830665837245 0.0287960114593915 0 0.0656856326995526 0.039955470716639 0 0 0 0.026 0.196679398930135 1.03509002174728
TCGA-BT-A20T-01A-11R-A14Y-07 0 0.0114563211274339 0.00176026015299688 0.167271098843362 0 0.209320258630803 0.0429046407238938 0.0596811453917008 0.0339894834155805 0.011390723190335 0 0.0457604675664606 0 0.0641046409115296 0.0780455834885556 0.12172953386848 0.0740616996387651 0.0268023671655672 0.051721775884536 0 0 0 0.073 0.135590133378562 1.03738211837399
TCGA-E7-A7DU-01A-11R-A32O-07 0 0.0231091167040665 0.0124827644414083 0.0636029606786888 0 0.214641494800368 0 0.0260251978218936 0.0258306546952891 0 0 0.0477487837851013 0.013377825799083 0 0 0.266387053216941 0 0.209031923177474 0.0977622248796867 0 0 0 0.734 -0.0109853405780439 1.08700606622217
TCGA-DK-A3WY-01A-11R-A22U-07 0.328006076992583 0.123544837181621 0.0770181424683671 0.0926595263053487 0 0.0914299463872285 0.0242425005279959 0.0684652081962647 0.0472332695958276 0 0 0 0 0.0632744772544489 0.0424406721748534 0.0274417852753806 0 0 0.0142435576400803 0 0 0 0 0.5541301467033 0.832420082384616
TCGA-G2-A2EF-01A-12R-A18C-07 0.0262153659077732 0 0.00984337486100311 0.177286147122574 0 0.0611314114947085 0.253776919231877 0.00796289277588524 0 0 0.0743747734712879 0 0.00381023533902546 0.0584261207460595 0.12992704925739 0.122478924043635 0.0723777073528996 0 0.00238907839588219 0 0 0 0.021 0.213135479653348 1.00120525298813
TCGA-BT-A20V-01A-11R-A14Y-07 0 0.0280375324788369 0 0.13234531284404 0 0.136405411331552 0.121636175922828 0.0202462810468682 0.00206907369136876 0 0 0.096223092078911 0.0725363393202904 0.115189082391973 0.10243305025264 0.0082454118900865 0.0609589747510471 0.036067371114991 0.062303742759094 0 0.00530314812547302 0 0.0659999999999999 0.14154627120804 1.03022726672475
TCGA-K4-A3WV-01A-11R-A22U-070 0.0415383400676105 0.00845221591324395 0.0603017952801523 0 0.0166524181738833 0 0.101224942160796 0 0 0 0.0174850150209982 0 0.440719486224237 0.0844473220632843 0.0172499851233311 0 0.0670807973377074 0 0.136433315998817 0 0.00841436663593849 0.543 0.00933816652984847 1.13244947011902
TCGA-GU-AATQ-01A-11R-A39I-07 0 0.00941291974373621 0 0.0095213723263311 0 0.195548119193417 0 0.0652982708582484 0.0141455378511378 0 0

0.0509171587797644 0 0.272498363884358 0.187122991108244
0.122038399940373 0.0361272699966061 0 0.037369596317785 0 0 0
0.014 0.237060460594699 1.00580335283027
TCGA-G2-A3VY-01A-11R-A22U-07 0.00951794464991323 0.167058365237519
0.049071581896975 0.29141267808555 0 0.100504460935743 0 0 0
0 0.039535919075117 0 0.0301367467424492 0 0 0.0652225651156991
0 0.126114849769199 0 0.114279627394392 0.00714526109744273 0
0.049 0.16375588338684 1.02875130157959
TCGA-XF-AAN8-01A-11R-A42T-07 0.0471034069690091 0 0.0134764146081577
0.0643481654631143 0 0.0801414560855584 0.0533501440609684 0
0.00570380693145042 0 0 0.00795085458269626 0.0196418414149242
0.0362516083700341 0.0192254826540305 0.38422286913634 0
0.00883944056189642 0.130045508774818 0 0 0.129699000387002 0.002
0.374042377041912 0.927915261408329
TCGA-CU-A3QU-01A-11R-A22U-07 0 0.0411815407696527 0.0125845277180829
0 0 0.0849775107587241 0 0.0154956483237396 0.0141125496759276
0.00250907395583336 0.0618620645608922 0 0.0814360552216648
0.368617473814889 0 0.0633371559819007 0.0081677101620963
0.0760796229625197 0 0.119864597648607 0.0497744684454704 0 0.527
0.0115293966659843 1.10651075493327
TCGA-GD-A3OP-01A-21R-A220-07 0.113865007657705 0 0.00827573652081258
0.0961224965177494 0 0.187131830580342 0.0056834100617646
0.0694267270006539 0.000885394754958548 0 0 0.0455775991194987
0.00517241335464076 0 0.110426409796198 0.17172155511628
0.0485554210038858 0 0.13715599851551 0 0 0 0.372
0.0310982573408205 1.06831015563716
TCGA-KQ-A41P-01A-12R-A33J-07 0.109485398315528 0 0.00604030148093613
0.0467484163850116 0 0.0863307923390937 0 0.00886302751466248
0.0210524720199683 0 0 0.0351342941432291 0 0.408608307069725
0.046201504092548 0.155501564886883 0 0.0133007656753446
0.0627331560770692 0 0 0 0.001 0.475767080930746
0.879488807299803
TCGA-UY-A9PH-01A-11R-A38B-07 0.0170179601594497 0 0.0603685493733888
0.19026514623823 0 0.0584145565512485 0.128968517881642
0.0240699333966672 0 0 0 0.0643754517312833 0.0197645566836676
0.0175102281393734 0.102800390324982 0.191862406230035
0.020332123964554 0.0151475780799673 0.0832250669972605 0 0
0.00587753424825128 0.024 0.200377226311932 1.00072620004207
TCGA-FD-A5BY-01A-31R-A28M-07 0.0102679051975039 0 0.00105088110552312
0.0402673371336988 0 0.155765647343549 0 0.00968462636548803
0.0126052496694809 0.0207810436318621 0 0.0522923196238752 0
0.250275371383332 0.0599559144694456 0.136586492003656
0.0870460223447511 0.000246197171428213 0.152955460926716 0 0
0.0102195316296908 0.004 0.328512960240664 0.949459144602158

TCGA-K4-A3WU-01B-11R-A23N-070.2472477916529 0.00903313782420876
0.034179302983399 0.0600943828888249 0 0.279582327614838
0.0296410576128468 0 0.0333267869184048 0 0 0.0150337007210601 0
0.143598228798391 0.00884319091103838 0.0646351359262315
0.0155532102180346 0.0132390450882408 0.0402061378103231 0 0
0.0057865630312589 0.002 0.3892071478471 0.923172241053484
TCGA-FD-A3SJ-01A-12R-A22U-07 0.147890828833343 0 0.0198667098863846
0.0282173170495127 0 0.126892760002442 0 0.0229087002385143
0.0415506393199304 0 0.0230473924250433 0 0.00858598979883754
0.14269107588292 0.100658190784903 0.145766685956905
0.0627795673162976 0 0.129144142504968 0 0 0 0.039
0.177367323663421 1.00762116477979
TCGA-FD-A3B5-01A-11R-A20F-07 0 0 0.000985907902747607
0.0608781860222994 0 0.216528127836356 0 0.0829411924366764 0 0
0 0.00858384420970045 0 0.317019102014832 0.0512272116451399
0.139634572197807 0 0.105445048852114 0 0.00623843310722866 0
0.010518373775098 0.181 0.0751983366571232 1.07995855696276
TCGA-GC-A6I3-01A-11R-A31N-07 0.0297852148837573 0 0.163441760096724
0.205818507216286 0 0.0435437005856399 0.035909307548058
0.0545689438678572 0.0432711067843588 0 0 0 0.000656474281520955
0.161940179451281 0.0661369786051319 0.118635542428932
0.00475377652868426 0.0359890160151307 0.0355494917066378 0 0 0
0.00600000000000001 0.305373382458424 0.961586503729881
TCGA-4Z-AA80-01A-11R-A39I-07 0.159146802637004 0 0.0860740965347625
0.185939441497357 0 0 0.0204377117865861 0.0872701800178221
0.0526728556046762 0 0.0528678631743773 0.00479239071258078 0
0.0219371455443343 0.111197066688889 0.0959433118786274
0.0178447560843473 0.0825824800089207 0.0122041521890478 0 0
0.00908974564066719 0.018 0.223177377022173 1.00393549267051
TCGA-G2-A3IE-01A-11R-A20F-07 0 0.104043938940034 0 0.112671185235688
0 0.0905651857191425 0.00018185468545321 0.0183625210294412
0.0154660592906514 0 0.0337757050683142 0 0.0162787069477985 0 0
0.286308859304118 0 0.248604487120496 0 0.0549508506382862
0.01695043390331 0.00184021211726758 0.0620000000000001
0.146248130782698 1.02804220924969
TCGA-ZF-A9R0-01A-11R-A38B-07 0.0208008693495458 0.0122894164237175
0.00494530360142392 0.0992495018479295 0 0.0417372534440783
0.0877797866289688 0.0504629630099433 0.0304841155267587 0
0.0413094500483999 0 0 0.298396086491141 0.169569313383229
0.0928811382099187 0.0279277240958946 0 0.0221670779390509 0 0 0
0.521 0.0120482069286639 1.10408276876115
TCGA-FD-A43S-01A-21R-A23W-07 0.107240717346205 0.067523201854426
0.152231924563787 0.257775222487344 0 0.0139465432871759
0.0230117051486761 0.0338293153978315 0.0829468641503352 0 0

0.0153075393627292 0.0151607741974868 0.0240009775010134
0.0123426949432113 0.140114736171677 0 0.0077196406541246
0.0449358133001113 0 0 0.00191232963386547 0.001 0.452269949074037
0.891854534723194
TCGA-XF-A8HG-01A-11R-A36F-07 0 0.0821067271690722 0 0.0495301757786578
0.00893019667322864 0.0403220761254332 0 0 0 0 0.0427964595260919
0 0.0566891634651112 0.0274452935729259 0.00252269367305229
0.314973930035303 0.234470354391503 0.0907686574581317
0.0440119821536976 0 0 0.00543228997779127 0.417 0.0254584938852177
1.07578710878139
TCGA-K4-A83P-01A-11R-A352-07 0.1784851546363 0.076698981911561
0.091341843791383 0.113008711324281 0 0.117557624813726
0.0386082436090834 0.0535862558615476 0.0350741274669887 0 0
0.0289362179974565 0.00401831779819567 0.0998693321137055
0.0647782031404498 0.0649722217384323 0.016025918873832 0 0
0.011618075351912 0 0.00542076957114481 0.001 0.469178810210674
0.882296979085098
TCGA-GD-A2C5-01A-12R-A180-07 0 0.011338666302063 0.0220420018261532
0.0197466709839499 0 0.211366426141325 0 0 0.0287982694704061 0
0.0174841560578382 0.00890989060450265 0.0190965784095687
0.0632876352634505 0.0343781779171795 0.173209245390388
0.116313678027931 0.0961715083548621 0.114261600862248 0
0.0433597627202229 0.0202357316679122 0.449 0.021019503604447
1.06007960890652
TCGA-XF-AAMX-01A-11R-A42T-07 0 0.0331961762330058 0.0279619908321771
0.0820869461473342 0 0.0600555566659141 0.0444561588750512
0.0976187593355221 0.0341678109157203 0 0 0 0 0.159211706724198
0.083063838820354 0.329214348446967 0 0 0.0489667070037565 0 0
0 0.108 0.10629952668236 1.04557561244924
TCGA-DK-A1AE-01A-11R-A13Y-07 0 0 0.00847929845631611 0.0578332745936931
0 0.241343037490523 0.0853101684653216 0.124097156903109 0 0
0.0563289726540424 0.0594289813393181 0.0114086975056661
0.0482480943988628 0.07411497496056 0.0340008502832766
0.10915083644804 0.0195329684251407 0.0707226880761308 0 0 0
0.129 0.0948868525614826 1.0541205836607
TCGA-FJ-A3ZE-01A-11R-A23N-07 0 0.140930171829901 0.0398081560037267
0.218236515080957 0 0.134100096777998 0 0 0.00385158672272714 0
0 0.0152128827001637 0 0.132528605298197 0 0.00422938052320141 0
0.141722130670204 0.159165710814516 0 0.00489936757657619
0.00531539600183309 0.978 -0.0401760377531169 1.10099245899296
TCGA-DK-A2I1-01A-11R-A180-07 0.0871409092987724 0 0.0765445109939239
0.148652919131163 0 0.0131960424039809 0.0274472569345475
0.00403564399089421 0.0681488568181337 0 0 0.0519082113581424
0.00645239520295658 0.112631930130426 0.139555725262431

0.138037187159762 0.0552548209156388 0 0.0694788140621549 0 0
0.00151477633707213 0.01 0.249923503057524 0.982947086822528
TCGA-DK-A6B0-01A-11R-A31N-07 0 0 0.000702122836965793
0.0545644555769394 0 0.19819516171659 0 0.0248847418754196
0.00454717616029685 0 0 0.0860647373584125 0.119942544949656
0.0072833943929308 0.0180879342421781 0.353987187624968
0.00402175544288325 0.0163459607128155 0.108649840318513 0
0.00272298679143073 0 0.616 0.00169277859350709 1.08244512128822
TCGA-FD-A5BR-01A-11R-A26T-07 0.160544796320959 0 0.127765426757026
0.0980626214672746 0 0.144130791418672 0.0772128068010202
0.0370660301569486 0.0850087820275803 0 0 0 0 0.101813699411065
0.010758486118368 0.0836888061974334 0.0191548067670973
0.0118484687966852 0.0339181988852183 0 0 0.00902627887465274 0.002
0.36827016215691 0.933047196337424
TCGA-KQ-A41Q-01A-11R-A33J-07 0 0.090356576470643 0.0158548881674383
0.0706258665917344 0.13295176323265 0 0 0 0.0265484075701104 0
0.0708645500355179 0 0.0219163690631694 0.159531637700854 0
0.0430451210850173 0 0.214585074168639 0 0.128137012734011
0.0255827331802145 0 0.152 0.0827495342121363 1.05599547146725
TCGA-DK-A1AF-01A-11R-A13Y-07 0.0272438546522405 0 7.25536983170227e-05
0.127782540941858 0 0.146099058625549 0.0463301777749251
0.0300184324755236 0.00875194137268645 0 0 0.023609678580558
0.00654780550962029 0.294527444614801 0.0879948203607392
0.105637358598075 0.00962125689761906 0 0 0.0596619846527807
0.00561946764943305 0.0204816235952735 0.04 0.176396506449071
1.02518636710603
TCGA-LT-A5Z6-01A-11R-A28M-07 0.198523219386558 0 0.118642329150974
0.203390723181968 0 0.0244867980715207 0.0103164757307316
0.0652295910414511 0.0318657778662042 0 0 0.0532644556214775 0
0.13720301058973 0.0223989411854384 0.0875365485541674 0
0.00221679619244698 0.0449253334273327 0 0 0 0.005
0.321791939861731 0.958756235637802
TCGA-GC-A4ZW-01A-11R-A26T-070.103990038541383 0 0.082413012518308
0.147846895140326 0 0.0871064792272887 0.0873304227192971
0.0731499873109978 0 0 0 0.0512258342091455 0 0.115102121010047
0.0346414688467929 0.105785253267845 0.0314321824100786
0.00713185105702018 0.0728444537414707 0 0 0 0.081
0.126307826654051 1.03003775030917
TCGA-K4-A4AB-01B-12R-A28M-07 0.00742419509477152 0.0337159606276679 0
0.0368659713088256 0 0.279342011077756 0.0315570788954968
0.0363763012718007 0.0143430301431879 0 0.00894713963629165
0.030382463525717 0.00281371692123341 0.0223698275429606
0.0116860785916102 0.169375683218309 0.143799920363565
0.0936740413398964 0.058466768137791 0 0.0100609993812555

0.0087988129218642 0.092 0.11468662857572 1.0314946536434
TCGA-DK-AA74-01A-11R-A39I-07 0.020685725539581 0 0.00369758558459336
0.134552886454451 0 0.0482283338151196 0.0346823315943148
0.0702210400215459 0.0258480070281321 0 0 0.0328191046057502 0
0.110222275440245 0.152798311083357 0.229282454811646
0.0593352890381405 0 0.058301386117653 0 0 0.0193252688654699
0.001 0.448216142810057 0.893122140370972
TCGA-G2-A2EO-01A-11R-A180-07 0.0244427846548335 0 0.0306097644826706
0.236501241806341 0 0.0677258410587996 0.188672805994334
0.0258722169023354 0 0 0 0.0747221732770168 0.0156264573956511
0.0947080327801811 0.0468592191875529 0.0956528571292724
0.00682098147768272 0.00483806167159524 0.0869475621817331 0 0 0
0.07 0.137735247192702 1.03175910120687
TCGA-DK-A1AG-01A-11R-A13Y-07 0.0136186318573801 0 0.0134734310551139
0.0954889707185795 0 0.378960037192946 0.00844206165375943 0
0.0137794348383858 0.0508588668748689 0 0.0773575672370164
0.0285885661944606 0.053849582264492 0 0.0704713577203582
0.00884334724102975 0.0603281307829792 0.0319036199479704 0 0
0.09403639442066 0.212 0.0639866726342442 1.08048687222996
TCGA-GV-A3JZ-01A-11R-A21D-07 0.115707867235263 0 0.0506018130435264
0.0894075674269419 0 0.0856616208789598 0.0463669188631996
0.0286790909752186 0.01077828056015 0 0 0.024750124361672 0
0.197098808888937 0.0725197098777792 0.19599041248685
0.0627683986211184 0 0.0169129103736008 0 0 0.00275647640678376
0.014 0.236953745273579 0.987878169394803
TCGA-XF-A9SV-01A-21R-A42T-07 0.0418537757074063 0 0.0122309755374412
0.130290784937896 0 0 0.00435062092307954 0.0314415550161897
0.0207452353746197 0.0126596708291203 0 0.0118707906269426 0
0.470060504248398 0.0431439332377085 0.159907041354963 0 0
0.0614451122062356 0 0 0 0.12 0.0994683021095089 1.08731035974305
TCGA-UY-A78K-01A-11R-A33J-07 0.0942092560796199 0.0871515928865196
0.115860260593919 0.15119546192484 0 0.0862503970474441
0.0431469635108124 0.0428438947463524 0.0840987301226741 0
0.0206801992612736 0 0.00090530314665733 0.0824883461604419
0.0589607381313953 0.106158765381451 0.0124657294346739 0
0.0135843615719259 0 0 0 0.001 0.456423135780128
0.888939739837943
TCGA-FD-A6TH-01A-11R-A32O-07 0.137121641095432 0 0.00687706418077607
0.0395335936891959 0 0.128526974967673 0 0.0945030131897234 0
0.00192963272902223 0 0.0401916155622047 0 0.257430407565866
0.0984885809634826 0.165443183577931 0 0 0.0262951579376552 0 0
0.00365913454103735 0.00700000000000001 0.281702965362302
0.973153406203796
TCGA-DK-AA6W-01A-12R-A39I-07 0.00885947217327909 0.00217432663906866 0

0.049736792314345 0 0.124178510780952 0.000455548337659914 0
0.038277448317134 0 0 0.0283358774178207 0 0.134830619509625
0.08028236724862 0.130388492476559 0.386378636818705 0 0
0.00299407258402552 0.0131078353822069 0 0.01 0.254231942720585
0.988985263043365
TCGA-CF-A3MG-01A-11R-A20F-07 0.0419091227403121 0 0 0.083018170536238
0 0.268977542513643 0 0 0 0 0.00731951916609748
0.025949390404264 0.0455359608255914 0.0937190134094433 0
0.00872989490758278 0.112907742371528 0.0638469369768556
0.248086706148445 0 0 0 0.951 -0.0323626949059256
1.0916180218911
TCGA-DK-A3X1-01A-12R-A22U-07 0.0109949941416261 0 0.0347222001421827
0.092251655135621 0 0.126383702863676 0.0798987009247831
0.0208893791212885 0.0144116033496342 0 0.049610148081075 0 0
0.185804188297097 0.10124277104919 0.180220525533701
0.0206795828311928 0.0326388413907216 0 0.0502517071382114 0 0
0.179 0.0765782490676639 1.05701535988244
TCGA-DK-A1A6-01A-11R-A13Y-07 0 0 0.00119817266760093 0.309951227004348
0 0 0.119555482041633 0.0705329558476011 0.00517098097470729
0.000601488313429278 0.0114957560964011 0 0.000705962087066073 0
0 0.167401711356309 0 0.266153264600579 0 0.0341714467388633 0
0.0130615522714621 0.013 0.239478479171603 0.99755804266245
TCGA-XF-AAMG-01A-11R-A42T-07 0 0.105681015896671 0
0.00209521254390393 0 0.310281379150714 0 0 0.0139119177043433 0
0.0465805473986484 0 0.0487847657334957 0.0898665971998319
0.0301619962114717 0.280766103804125 0.00585404602580226
0.0179206617837989 0 0.0408986910489079 0 0.00719706549828633 0.123
0.0979824390422519 1.04296082097602
TCGA-E7-A541-01A-11R-A26T-07 0 0.00874396191334495 0.00702886663322309
0.390336918160219 0 0.0600336571754315 0.110763746714926
0.0346867459770732 0.0286272751671063 0 0.00439505680671435
0.0172959952741974 0.00750773818867544 0.0137507460305083
0.0511725471736782 0.0996892317531277 0.0950719474113314
0.0137677178611129 0.0374847243049072 0 0.013859774176135
0.00578334927828751 0.054 0.154478441034567 1.0363899400148
TCGA-XF-A8HE-01A-11R-A36F-07 0.0703393659764322 0 0.0272001968450791
0.205860821901383 0 0.112032028370466 0.0972040847180655
0.0645167938615209 0.00631670853174384 0 0.0363682677145243
0.0166590204072548 0 0.0614181756849936 0.108065123691498
0.0486849442625854 0.000253091671061009 0.0158162474366153
0.0448954146746913 0 0 0.0843697142520861 0.004 0.3298036527799
0.949941045441551
TCGA-DK-AA6Q-01A-11R-A39I-07 0 0 0 0.364376277595903 0 0
0.147652552408711 0.0818958250767007 0.0147236492691955 0

0.0330962399568951	0.00281605992416084	0.0269295448808852
0.00633211513450307	0.121981389287129	0.0383552984495976
0.0925027613338529	0	0.069338286682467	0	0	0	0.001
0.445497767808178	0.895075919743814
TCGA-UY-A78N-01A-12R-A33J-07	0.0256434858584567	0	0.0111527073721664
0.234629703330982	0	0.0824573468442609	0	0.10988468620774
0.0108696595716271	0	0	0	0.0795890684580227	0
0.00120555623495605	0.140662256190391	0.145519599584695
0.0704063651560516	0	0.00634513233128009	0.0733696580067459
0.00826477485262439	0.269	0.0487367930010818	1.06157921737827
TCGA-DK-AA75-01A-11R-A39I-07	0.036239494459172	0.0659463523045637
0.0024372391833141	0.114029807249458	0	0.12343179896742
0.00384158264202525	0.00467114655298522	0	0.0652028894145628	0
0.00513861916864345	0.03213354661541	0.216514993260195
0.0610999086839054	0	0.0596500889696051	0	0.185376862596678	0	0
0.0242856699320625	0.752	-0.0128029548916783	1.08277081561603
TCGA-ZF-AA5P-01A-11R-A39I-07	0.339730576975613	0	0.130831593552732
0.0911906249276967	0	0.0678397675347733	0	0.0349934549171448	0	0
0	0.0341159289852248	0.000357413470146726	0.00802368143484823
0.131767420293155	0.0679218566810082	0	0.0408996595198289
0.0467739328385918	0.00555408886923729	0	0	0.001	0.46724438444393
0.889878593192511
TCGA-GC-A3WC-01A-31R-A22U-07	0.0304981800748854	0.0352076411591586
0.0186920370541212	0.153247943333066	0	0.0551629846588243
0.134787452210284	0.045868074247734	0.0257856764622163	0
0.0157404687226636	0	0	0.0979646167970223	0.137739540159327
0.0994267486344674	0.101645589621575	0	0.047491610319511	0	0
0.000741436545143547	0.013	0.239602339779822	0.984243000327912
TCGA-K4-A4AC-01A-21R-A26T-07	0	0.00117105718168928	0.00372299074938368
0.0662653577196265	0	0.211825935216086	0.0837408060952862	0	0	0
0.0596488593779012	0	0	0.217850891590656	0.108166033789842
0.230158871540629	0.0104133304447551	0	5.49997279096069e-06
0.00557698840237154	0	0.00145337791898139	0.00700000000000001
0.282590567643249	0.974582391320884
TCGA-FD-A3SL-01A-21R-A22U-07	0.0594739567495805	0	0	0.0615866314041503
0	0.107854245937546	0	0.0320007077992091	0	0	0
0.0140738082607098	0	0.210298554986089	0.143632420745376
0.226987897071315	0.0377649363820301	0	0.106326840663994	0	0	0
0.023	0.210022711645478	1.00636923574873
TCGA-K4-A5RH-01A-11R-A30C-07	0.00336041806819357	0	0.00065177568384047
0.455073135670724	0	0	0.176800388518419	0.0222182120420812	0	0
0.0211097136663114	0	0.0105726547433923	0.00283748940052396
0.0841779871367476	0.212886455622616	0	0	0.0103117694471502	0	0
0	0.003	0.347151940197146	0.948846899936617

TCGA-FD-A5C1-01A-11R-A28M-07 0.0135530367216392 0 0.0279529371027837
0.178840458512686 0 0.0980961939670946 0.118174839214844
0.0452542445598644 0 0 0.00721289920282522 0 0.0104166951921255
0.0993435934194658 0.0914148285560138 0.183033647235489
0.0169990934910749 0.0822732030604328 0.0204066061418193 0 0
0.00702772362184196 0.014 0.238850125063535 0.986702429552899
TCGA-BL-A13J-01A-11R-A277-07 0.0151477806602386 0 0.0177217554864054
0.0527217181267573 0.0268685396977006 0.0758919131334837
0.0318942702175826 0 0 0 0.0362781273136002 0 0
0.285253520960208 0.0835961143307608 0.214267142480957 0
0.0621149580024807 0.0486535083132468 0 0.0495906512765778 0 0.742
-0.0118084365201026 1.10634016954082
TCGA-XF-AAN7-01A-11R-A42T-07 0.0598855235054323 0 0.0089996655067708
0.07615277784047 0 0.264280300455734 0.0313740186737895
0.018331921691293 0 0 0 0.0307060553261284 0.0102098010202324
0.147391535013019 0.0781996196897572 0.211185451940239 0 0
0.063283329337135 0 0 0 0.111 0.104039265359765
1.04252565517048
TCGA-2F-A9KO-01A-11R-A38B-07 0.0886103210383544 0 0.0277664656432932
0.231473838303256 0 0.162239400896409 0.0790896627922393
0.0541408757329817 0.00876779193753011 0 0 0.0500904572234571 0
0.0366511794825546 0.0712604955574296 0.0950490814075375
0.0265573315210901 0.0128450952683994 0.0554580031954685 0 0 0
0.02 0.218401231278178 1.0023370781563
TCGA-DK-A3IM-01A-11R-A20F-07 0 0.00549914784433122 0 0.06537061322541
0 0.0938279984611342 0 0.0834810958006167 0 0 0 0 0
0.587051464339163 0.0512564897337823 0.0369746787183865 0
0.0110731982175946 0 0.0444963812865442 0 0.0209689323730368
0.00900000000000001 0.26164820664828 1.02528107325229
TCGA-FD-A3SS-01A-12R-A22U-07 0.0710397353817644 0 0.0207503775793619
0.0328842893576862 0 0.161393777232844 0 0.0441631952280776 0 0
0.0526917001480591 0 0 0.140993129664676 0.0689092046959887
0.184699908417301 0 0.173237433239959 0 0.00924615401919671
0.0399910950350855 0 0.054 0.15559418307181 1.02585999334866
TCGA-4Z-AA7N-01A-11R-A39I-07 0.287502462288083 0.103572957841695
0.0132106488536323 0.0688980599527966 0 0.139225758173428
0.0299744926772078 0.090020437316485 0.0730463744328025 0 0 0 0
0.106015902316802 0.0215213813773542 0.0349426841671254
0.0094352130854934 0.0101030576216506 0.0125305698954442 0 0 0
0.001 0.527689915527628 0.848660417525967
TCGA-HQ-A2OE-01A-11R-A206-07 0.056189089907513 0 0.0674568779547452
0.128526528152822 0 0.0738890616527057 0.0449033355262208
0.0274412376673265 0.0660797310193025 0 0.041921396665283 0
0.0186273466079445 0 0.0237830835235215 0.137507868475446

0.141982575922404 0.113807888152108 0 0.0380168381866002
0.00707735912604824 0.0127897814600095 0.0600000000000001
0.148014769831717 1.01647031204465
TCGA-UY-A8OC-01A-11R-A36F-07 0 0.00935478648084123 0.048336340800824
0.0311693096450483 0 0.0144627488063646 0 0.0738034235722235
0.0209564067642036 0 0 0.0105807059777927 0 0.586055446368855
0.020082147732611 0.130606450886814 0.0493909010854674 0
0.00249850677971365 0 0.00270282509924077 0 0.001 0.514888177496883
0.864257930681193
TCGA-GV-A3JV-01A-11R-A220-07 0.142810791727047 0 0.108475304213524
0.177632099196776 0 0.116824740843819 0.077129420646037
0.0564101382822776 0.041606467752932 0.0102261110961149 0
0.00566752212334096 0 0.042740272943147 0.0721572427412554
0.0699579021567897 0.038014883143268 0 0.0403471031336717 0 0 0
0.002 0.365975156224132 0.93378888937237
TCGA-XF-A9T8-01A-11R-A39I-07 0.00159480121435555 0 0.00118769908450269
0.214475152229217 0 0.123262315953386 0.0881628896867491
0.035969321289942 0 0 0.038017377669349 0.0208423642235258 0
0.116243550656403 0.0878904386367648 0.125785252884507
0.00104876264930962 0.0477476675708383 0.0244000227006364 0
0.0109085298576936 0.0624638536928194 0.002 0.381150683735652
0.925825261540797
TCGA-KQ-A41S-01A-12R-A33J-07 0.0484447228229647 0 0 0.0765004949113299
0 0.228543395985878 0.00619276789886672 0.0207767643457485 0 0 0
0.046462657729866 0.0361020704493546 0.0451419794892575
0.0319132321682615 0.301427831671817 0.0125110714034951
0.0316494206404597 0.0861618912544506 0 0.0122781674307065
0.0158935317975437 0.384 0.0293526731967848 1.06529810326713
TCGA-CU-A3YL-01A-11R-A22U-07 0.0232909045312016 0 0.0184532615123717
0.266261902017832 0 0.152601854763041 0.0215123157585969
0.0949789281530026 0.0343535680757239 0 0.00277992497376303
0.0102917685292058 0.0159035511780629 0.0298313825488802
0.118974201082928 0.0743395604102125 0.0870786666788007
0.00631023703933324 0.0430379727470443 0 0 0 0.429
0.0238642537647033 1.0952441572452
TCGA-E7-A7XN-01A-11R-A352-07 0 0 0.002573430303655 0.375983973633355
0 0 0.177919105173303 0.0260993700299365 0.0239091258144145 0
0.0885092349114321 0 0.0110472210963805 0 0.143668104498923
0.0595657209767236 0.0574926582670603 0.0123871313539714
0.0208449239408451 0 0 0 0.005 0.321752789566299
0.96666664322098
TCGA-DK-A2HX-01A-12R-A18C-07 0.0499047745295398 0 0.00116851434280966
0.0626978976710802 0 0.159199435068349 0.00523619620357835
0.0084228566336835 0.0564253642139501 0 0 0.0597368172267124

0.0187041308222999 0.0329208782268595 0.00437068135175412
0.19955735084688 0 0.127871548757345 0.213152751167403 0 0
0.000630802937754911 0.172 0.0787060717559628 1.04618045444636
TCGA-CF-A8HY-01A-11R-A36F-07 0 0.0698973995445063 0.0114269790876023
0.138999615114962 0.0374592007607726 0.118389728097281 0 0 0 0
0.0844124541344378 0 0.0297143043304606 0 0 0.012312010913844
0.326956061879648 0.122666448639312 0.0108611086961325
0.0369046888010403 0 0 0.415 0.0256922146659004 1.07453911168229
TCGA-4Z-AA7W-01A-11R-A39I-07 0.00629578108126326 0 0.0642411968187228
0.408241618665218 0 0 0.117506460931486 0.0866510012252646
0.00441553127175518 0 0.0340207667552044 0.0472515120240147
0.00281863483801775 0.00399617312543365 0.0686787214086493
0.0947526601763476 0.014947868945052 0.0281999412114094
0.0179821315221615 0 0 0 0.027 0.19571333737562
1.03002738800476
TCGA-FD-A43U-01A-11R-A23W-070.0177370742839148 0.0228966330286977
0.00778950252214549 0.0894221841619609 0 0.0803561345055106
0.0194552820562096 0.00663153364496667 0.0526127579193234 0 0
0.0295005160069616 0.026518245620243 0.451298381535877
0.0209924839487347 0.122783694110111 0.00857342346532758 0
0.0434321531900156 0 0 0 0.108 0.107254960198694
1.07535381118262
TCGA-BL-A0C8-01A-11R-A277-07 0 0 0.00616694551207068 0 0
0.252537209379734 0 0.00221828970234429 0 0 0.000384813844851874
0.0261033731350265 0.0455536945249692 0.34820890835389
0.00307046321230971 0.304442978366271 0 0 0 0.00260073091864005
0.00260067275196683 0.00611192029792635 0.443 0.0217519840356921
1.12078242019822
TCGA-BT-A0S7-01A-11R-A10U-07 0.123089855985377 0 0 0.0948216969961413
0 0.169401719898495 0.0217086278315434 0.046804188538164
0.0102624602628427 0 0.0338320256695685 0 0 0.137156102757276
0.0292427759207364 0.228302256804196 0.06095769683875
0.00192252312395045 0.0102253861657185 0.0158841780311051 0
0.0163885051761354 0.195 0.0688146412018932 1.05454902486636
TCGA-ZF-A9R3-01A-11R-A38B-07 0.00209173505224068 0.0489546007714609
0.0448825780107038 0.0657926078530279 0 0.187115178359535 0
0.0476404049329328 0.101486538444132 0 0 0.0369540922975885
0.0146900737606202 0 0 0.0518915042495869 0.187334781426237
0.167590305165892 0.0435755996760413 0 0 0 0.242
0.0562039391518445 1.06113868707636
TCGA-4Z-AA89-01A-11R-A39I-07 0 0.0559949040582185 3.05851048200118e-05
0.166834558557353 0 0.030539008446046 0 0.233113358680042 0 0
0 0.021427127697059 0 0 0.191168093917517 0.163332495198596
0.0239635900804124 0.0791466788317781 0.0344495994281576 0 0 0

0.527 0.0114306246407388 1.11432839244762
TCGA-GU-A42P-01A-11R-A23W-07 0.106889722961147 0 0
0.0496226447890697 0 0.219005170150425 0 0.0514670881639057 0 0
0 0 0.0118532787486615 0.20995471840793 0 0 0
0.193167739254988 0.158039637523874 0 0 0 0.774 -
0.0150561210916989 1.09263740750959
TCGA-FD-A3NA-01A-11R-A21D-07 0.00141923472877266 0 0.00151090969108338
0.219817773011654 0 0 0.197914216042579 0.00324872112779971
0.000333915694875562 0 0.0363220125740398 0.011644643700822
0.0127202017656196 0.0935031033545967 0.0767274084925804
0.140353904779114 0.077282033465312 0.0324024302510279
0.08164238706611 0 0 0.0131571042540136 0.112 0.102891608407427
1.03645974218702
TCGA-XF-A9SL-01A-11R-A39I-07 0.24334253614812 0 0.0549198320927034
0.121578847531563 0 0.0680468392392257 0.0381658309187789
0.00999999803053961 0.0609407203561304 0 0 0.0291654438942461
0.0346128585374388 0.0701014856969171 0.00664461780277101
0.135194530291317 0 0.0113560894935696 0.115930369966679 0 0 0
0.001 0.489889036935463 0.873013460852481
TCGA-XF-AAN2-01A-11R-A42T-07 0 0 0.00263825209375859 0.185450402251481
0 0.123097637386763 0.0840189794305939 0.0894881306235176
0.00515932117044066 0 0.0897930636395442 0.0177884586976507
0.00369580602578458 0.0906846212892321 0.103278966745679
0.100185944918732 0 0.0733477071062369 0.029393274609704 0 0
0.00197943401088192 0.00700000000000001 0.292327411027543
0.972193694667138
TCGA-ZF-AA54-01A-11R-A39I-07 0.00335498255837337 0.00298271513016248
0.00014294957251928 0.0777075383568868 0 0.0858623089652564
0.0127094716817271 0.0242086781603198 0.012895511411667 0
0.00580745113988882 0.00977586243708165 0 0.214882489052238
0.0443867383046656 0.471182727486782 0.00365831608200373 0
0.0304422596604273 0 0 0 0 0.646403979799457 0.77505671215926
TCGA-GU-A766-01A-11R-A32O-07 0.00912086202237311 0 0.00178513703827758
0.15391398522338 0 0.0331124405362998 0.244575335392162
0.0389296616121798 0 0 0.0636167445286816 0.0313109311397944
0.0485137616245816 0.0355871832921906 0.18107398934383
0.0493207092655254 0 0.100987560888769 0.00521207005222562 0 0
0.00293962803972914 0.001 0.40622339614452 0.918838550416273
TCGA-FD-A6TD-01A-51R-A33J-07 0.0257066742041936 0.00474267460040124
4.64324936457516e-05 0.13489546076465 0 0.11200814960764
0.0633358128988623 0.0194191647955895 0 0 0 0.0593474404258644 0
0.127744685702296 0.0873170930357523 0.273901522454919
0.0319391427021232 0.0146974502160598 0.0448982960980042 0 0 0
0.114 0.102526320727606 1.04569936402779

TCGA-FD-A3B4-01A-12R-A206-07 0.175059085313567 0 0.0673948100215729
0.0859267075815995 0 0.148649694583252 0.0343873227242518
0.038589207214548 0 0 0.0293786308329598 0 0 0.130680726522112
0.114649734417394 0.0899203875062504 0 0.00577572236190924
0.0756741829586314 0 0 0.00391378796195256 0.001 0.398932336609818
0.91663752205652
TCGA-DK-A1AB-01A-11R-A13Y-07 0.0174836463225686 0 0.0266542427556828
0.05558682200466 0 0.164358922411665 0.0494586484085168
0.0244264460281627 0.0287692153753785 0 0.0133852402575833 0 0
0.159060428539539 0.149891620534387 0.106813815983811
0.0316891591348471 0.0533702232422536 0.106180640311998 0 0
0.0128709286889468 0.00800000000000001 0.278969230518001
0.971387657860232
TCGA-BT-A20X-01A-11R-A16R-07 0.014005324054687 0 0.0799051181863245
0.163022090029837 0 0.0620694356922011 0.177437463235658
0.02371687191202 0.00252352951936988 0 0.0538957892041152 0
0.0169064101101655 0.111017815020854 0.123590667658653
0.0944374340651193 0.00588984111458346 0.047166356445816
0.0232083317839232 0 0 0.00120752196667278 0.028 0.19508170364304
1.00652872468021
TCGA-GV-A3JW-01A-11R-A20F-07 0.046729374613845 0.06008218399645
0.0391445216111418 0.0881189860994572 0.0858875186480095 0 0 0
0.0273869026776384 0 0.0686090480554433 0 0 0.129964833011553 0
0.225487513801387 0.0485031319631384 0.0665948291101525
0.113491156411784 0 0 0 0.889 -0.0234218863496268
1.07990119672814
TCGA-UY-A9PB-01A-11R-A38B-07 0.0113592247696829 0 0.0633083449977444
0.151591398709577 0 0.154884148083523 0.107471806122804
0.0293547638277413 0.0191615993010297 0 0.0519682254194363
0.00167060860808633 0 0.139953386728245 0.0971789409787279
0.130573585635754 0.00603412876731969 0 0.0354898380503292 0 0 0
0.069 0.13933606945215 1.02938161524599
TCGA-FD-A6TI-01A-11R-A32O-07 0.0851317724914003 0 0.0183094447072988
0.176969489880328 0 0.22974433154289 0.0159295807633832
0.0530938003269462 0 0 0 0.0320709071774477 0.0131848287542175
0.00707839802018418 0.0396936806824127 0.142981783474697
0.0157634032894682 0.0440325118797199 0.0681433302621134
0.0529065782687921 0 0.00496615847870177 0.625 0.000141964151333826
1.08805048356378
TCGA-XF-AAML-01A-11R-A42T-07 0.0959642698701727 0 0.0331864321870794
0.217346815621337 0 0.0641211523065909 0.134604445953926
0.0631061988755938 0.00967298986474902 0 0.0264376835586326 0
0.0365354401043565 0.0659356477556513 0.025460016038346
0.0499075912440767 0.00841241645247837 0.0485311608094958 0

0.0782281842121122 0.0310041632389386 0.0115453919064632 0.129
0.0949808642014405 1.04387785234642
TCGA-ZF-AA58-01A-12R-A42T-07 0.0220042359812803 0 0.00752763078740693
0.110231868121781 0 0.0931375315680373 0.09171959691792
0.0185767784540895 0.0227506934316648 0 0.0391913738417753 0 0
0.248742711369698 0.114389949255 0.213721083257677 0 0 0
0.0155375403642904 0 0.00246900664937915 0.005 0.315913145538855
0.958948276710458
TCGA-FD-A3SP-01A-31R-A22U-07 0.032680372510291 0 0.00616894839087575
0.068284932505125 0 0.206930911469734 0.0143782356091887 0
0.00913543882885658 0 0.0321750982790244 0 0 0.323462640777092
0.0664274453781545 0.164938350663032 0.0458293707582609 0 0
0.0256525275052302 0 0.00393572732513471 0.00800000000000001
0.270453722020786 0.983857042760248
TCGA-E7-A97Q-01A-11R-A38B-07 0.0845601846296101 0 0.070391937669637
0.224814256696144 0 0.000302752484951954 0.0822501851263067
0.0137973988217704 0.0243091295125063 0 0 0.0517133349969803
0.00832136522554811 0.00423005893482189 0.150639225408939
0.147112920735787 0.0107321826675898 0.00870591374033178
0.118119153349075 0 0 0 0.213 0.0637282904117561
1.06538894885734
TCGA-YC-A9TC-01A-22R-A39I-07 0.0113023759529123 0.0226172515320768 0
0.12106461987648 0 0.0647652715629177 0 0.0205389792713462
0.0359216478197321 0 0 0.136093503559977 0 0.121367538593368
0.00687565554366045 0.215942482318225 0 0.0321240651481803 0
0.20558138769552 0.00571204548379671 9.31756418058219e-05 0.177
0.076834457189849 1.08418068347786
TCGA-GV-A3QG-01A-11R-A220-070.225015558970244 0 0.0572439534176294
0.141298958205614 0 0.0788357543564481 0.0389748859918939
0.0472692134464129 0.0262799442050525 0.0025624477530815 0
0.0252442781426937 0.00372922816428819 0.168316548680639
0.0823102251963761 0.0712055724917497 0.00122048523384697 0
0.0304929457440296 0 0 0 0.001 0.464507134179127
0.884783753439238
TCGA-K4-A6FZ-01A-11R-A31N-07 0.0484099941499532 0 0.00552924580089079
0.119978803190689 0 0.200706668787976 0.0016930142583759
0.0270132601687681 0.00507280459395356 0 0 0 0.00745798360018893
0.175038335785447 0.0744733504339305 0.10114926281492 0
0.0715524032414285 0 0.11718336770435 0 0.0447415054691273 0.039
0.177828581270213 1.01788295355808
TCGA-KQ-A41R-01A-21R-A352-07 0 0.0284164235163872 0.00170412990688372
0.0454183129756648 0 0.158717621118517 0 0.0434749722241603
0.0714120174327945 0 0 0.0533441667077324 0 0.099726410676555
0.0654536297820611 0.187851569481975 0 0.0980406758222641

0.139711534806294 0 0 0.00672853554871064 0.682 -
0.00618162399525279 1.08212996871803
TCGA-DK-AA6M-01A-11R-A39I-07 0.00637545354701382 0 0.00181683998253958
0.0404551774904096 0 0.207594014829394 0.0022072661878258
0.00031797120443626 0.015216755859354 0 0 0.0852109601108595
0.0311725578705678 0 0.0683679682470904 0.187608571361666
0.23770102607132 0 0.109128349953675 0 0 0.00682708728384727
0.00600000000000001 0.305811936289931 0.956130958812757
TCGA-ZF-AA52-01A-12R-A39I-07 0.0262103043891675 0 0 0.0826972366205894
0 0.074542249485677 0.0133022831838572 0.00884343765400242
0.00538287196437646 0 0 0.0492001277335648 0 0.35259410531087
0.0375075500199528 0.248622287691583 0.03427604920562 0 0
0.0484092315570076 0 0.0184122651837321 0.123 0.0980816643668297
1.07871386903655
TCGA-K4-A5RJ-01A-11R-A28M-07 0.00284613303000989 0 0.00107810057057314
0.308544959980704 0 0 0.0911849581550494 0.0359226417002355
0.0411381831079189 0.00944988398956532 0 4.39688282835635e-05
0.00349402941167129 0.0260186346698056 0.0868306985977373
0.299010802201456 0.078791103817196 0 0.0132880318109138 0 0
0.00235787012887976 0.001 0.44500168358996 0.894735469680047
TCGA-HQ-A5ND-01A-11R-A26T-07 0 0.00733508354767459
0.00263727188388333 0.212565352940419 0 0.146705745794757
0.126625579523775 0 0 0 0.0462965160794033 0.0154753067010517 0
0.068450186550055 0.204929187247926 0.0392859907998078 0
0.0694387345971751 0.058601821555951 0 0 0.00165322277812172 0.045
0.168272722698472 1.04711383696706
TCGA-BT-A3PK-01A-21R-A220-07 0.0197131620958949 0 0.00239199004756767
0.0344233576876605 0 0.0771140453545964 0.0652078498846654
0.0281041873495302 0.012178742425468 0 0.0242018768864376 0 0
0.210793444251677 0.0935840103463461 0.0918966262611042 0
0.165678650306577 0 0.116490526411013 0 0.0582215306914619 0.016
0.231516761246473 1.00256180998055
TCGA-FD-A3N6-01A-11R-A21D-07 0.00107839746247286 0 0.00906648569423226
0.192194008312256 0 0.0908550098671097 0.0914983614122454
0.0750915985168387 0 0 0.010096599864147 0.016265168788953 0
0.15744978959602 0.248111093833362 0.078494377137188
0.0106145709345565 0 0.0191845385806194 0 0 0 0.005
0.319513555288838 0.970910479612664
TCGA-UY-A78P-01A-12R-A36F-07 0.0126941052100467 0.0037955787058521
0.00275586490412633 0.117457729944152 0 0.0749383205448524
0.0655228801671655 0.0753159440240419 0 0 0.0160536756896474 0 0
0.308406317744092 0.099814173281485 0.12041376731195
0.0269897112115444 0 0.0621347064060814 0 0 0.0137072248549639
0.004 0.337271276482939 0.948058214843384

TCGA-CF-A9FL-01A-11R-A38B-07 0.0192997344797778 0.0285639165134095 0.0921055788435034 0.100485247460586 0 0.0824368670952333 0.000835653441059969 0.0660814763917072 0.00501572009088124 0 0 0 0.0127348902897677 0.189910909659756 0.0195411975693612 0.133161873878502 0.0364799532426507 0.00725131596339912 0.199954451014964 0 0.00482821337750679 0.00131300068793484 0.269 0.048752807679282 1.05964662914432
TCGA-CF-A9FM-01A-11R-A38B-07 0 0.000845128087006627 0 0.0885062982738656 0 0.140945143071682 0 0.00819279517293481 0.0866226754043835 0 0 0.0880054212884888 0.0151231692001807 0.0382129014514672 0.0456640146559173 0.0961277816291431 0.0959766801038148 0.190904083667938 0 0.0653656386685268 0 0.0395082693246508 0.296 0.0438933292831223 1.07842965617484
TCGA-ZF-A9RD-01A-11R-A42T-07 0.0958052679384131 0 0 0.133320418173456 0 0.151549520450322 0.125527903922498 0.0427033756958312 0 0 0 0.0317271946120178 0.00707460439808559 0.0777766344346835 0.119976191226032 0.106890215363053 0.0135828003964809 0 0.0940658733891274 0 0 0 0.023 0.208315699221771 0.998838709653336
TCGA-G2-AA3D-01A-11R-A39I-07 0.181412981244331 0.0782182979264901 0.0496556745379739 0.167080263462487 0 0.0226930611212386 0.0194091767996297 0.0346382668131809 0.118609867385606 0 0 0.0307745481272871 0.0094763423552757 0.010657049087203 0 0.0944092821604025 0 0.130132026963068 0.048365542573932 0 0 0.00446761944189392 0.614 0.00207891485811782 1.09963082109203
TCGA-DK-A2I2-01A-11R-A180-07 0 0.0451561269823483 0 0.0563217410602656 0 0.160556899763707 0.00135852008763346 0 0.00217986825366021 0 0.0231761089889094 0 0 0.186122857313277 0.0419247395498943 0.121667643452379 0.125562470517054 0 0 0.186009807840835 7.77340137206686e-05 0.0498854821763163 0.00700000000000001 0.298149158438592 0.965766106890962
TCGA-FD-A62S-01A-11R-A30C-07 0.0147159189999524 0.0209596774154108 0.0146410250519319 0.0506570995268274 0 0.243059602783663 0.000924878614859304 0 0.0235630089327851 0 0 0.00942317330112375 0 0.230691063198602 0.0716313279125659 0.129885631393174 0.0960742970778481 0.026764039517841 0.0670092562734154 0 0 0 0.098 0.11050684481381 1.04207565719221
TCGA-MV-A51V-01A-11R-A26T-07 0.111184245416099 0 0.0360320661338172 0 0 0.354059176327971 0 0.118392322412473 0 0 0 0.0469108389070822 0.0101468448736093 0 0.0411900729743908 0.114617704659627 0 0.080644537371267 0 0.0868221909236636 0 0 0.52 0.0121379993083649 1.091633026405
TCGA-E5-A2PC-01A-11R-A206-07 0 0 0.00607828577034511 0.1635608013805 0

0.129748742474792 0.0801382775814932 0.11521011259859
0.0395235633968507 0 0.041048366795968 0.0460987224999875
0.0463821485725867 0.0329088566359674 0.12429339357022
0.0599488321701698 0.0704741452737826 0 0.0445857512787474 0 0 0
0.091 0.119758223659396 1.04999035151699
TCGA-DK-A3X2-01A-11R-A22U-07 0 0.0355928926832122 0 0.199376022602945
0 0 0 0.0543349189153206 0 0 0 0 0.0140798414658213
0.234556369114168 0 0.23354714117974 0 0.180821501683315
0.0394972856196954 0 0.00142545369093732 0.00676857304484523 0.512
0.0131236673062452 1.09479774971268
TCGA-GD-A76B-01A-11R-A32O-07 0.0120318857189907 0.00725007169812712
0.0773275286385538 0.141173829049938 0 0.040034132030634
0.104465632709077 0.0375866001370214 0.186977360372467 0
0.00315998762561511 0.0268147105745088 0.0154076148329007
0.0151059687584741 0.11604157944506 0.0628106556223951
0.132178795929405 0.00894142529299296 0 0.0095259804086317
0.000447424266709387 0.00271881688849815 0.024 0.201742583895673
1.00745973390922
TCGA-GC-A6I1-01A-12R-A31N-07 0 0.0112431188881438 0.0423725513026475
0.179083246759767 0 0.0868811587774048 0.119109780544112
0.0177156755057317 0.0295231243730202 0 0.0427321666532698
0.000533341480527794 0 0.232109231537922 0.0726415156607599
0.137586231780028 0.000768123351914855 0 0 0.0249069846367489 0
0.00279374874800171 0.078 0.129775680089387 1.03759374080212
TCGA-BL-A13J-01B-04R-A277-07 0.0491633854994836 0 0.00818347498617891 0
0 0.1997625156113 0.047821433976379 0.0444525568242327 0 0
0.0536691813192423 0 0.00105782648933744 0.0255689369561346
0.047106697298456 0.294714624892984 0 0.097373178457889
0.0428296106828805 0 0.088296577005502 0 0.412 0.0265782623670891
1.06237711572967
TCGA-XF-A9SK-01A-11R-A42T-07 0.0116041427008177 0 0.0444592332114346
0.0486260363730255 0 0.204467117579888 0 0.0195164096566587
0.0843150405352793 0 0 0.0351475093602545 0.0675023432607608
0.00203390685934985 0.0798016286761234 0.227028964990339
0.0282566193231972 0.084604021735207 0.0608785842206695 0 0
0.00175844151699565 0.029 0.191809657990306 1.00313835981617
TCGA-DK-A1AD-01A-11R-A13Y-07 0.126742508248002 0 0.00979596152647738
0.250981069486271 0 0 0.0854726922849955 0.0270621882734167
0.0596056948795291 0 0 0 0.0250251840478754 0.0117823017861983
0.211674850676198 0.123299734601728 0.02452089312229
0.0326870807125426 0 0.000284028960492144 0.0110658113939848 0
0.012 0.244262892116011 1.0020676523125
TCGA-K4-A5RI-01A-11R-A28M-07 0 0.010161195477403 0.00116253823262102
0.00927824493764494 0 0.0864194667256608 0 0.0493581230363072

0.0161993693849185 0 0.0675978791524715 0.08622942311144
0.0473148209319456 0.0040757647378716 0.00214094220315635
0.109792469523798 0.230610511941027 0.0592314158209756
0.20062369888235 0 0.019804135900409 0 0.02 0.215550086675961
0.993331419237244
TCGA-FD-A3SQ-01A-21R-A22U-07 0.276695207858854 0 0.121031168701053
0.0326144253852774 0 0.0642989395853015 0.00265652932439543
0.0314033218002162 0.0247716246315716 0 0.023440067976125
0.0157834157288709 0 0.280722952993185 0.0192893577704351
0.0379590865843012 0.0143441090336417 0 0.0549897926267716 0 0 0
0.001 0.444085854111817 0.898045439100313
TCGA-GV-A3QF-01A-31R-A22U-07 0 0.112521978201234 0 0
0.14602937668418 0 0.0149612381741357 0 0 0 0.111037638044835
0 0.0545581070666118 0.0881611534731943 0 0.000598711859482137 0
0.231892985758529 0 0.0199537898282423 0.00667778925615605
0.213607231653401 0.332 0.0373944516748654 1.08827866699357
TCGA-E7-A7DV-01A-11R-A33J-07 0 0.00276777853360682 0 0.0352071713032614
0 0.122886277673379 0.02171421908605 0 0 0 0.0201474071469011
0.00504034428320894 0.0865064310352606 0.211221807678431
0.08505169072116 0.399789101524811 0 0 0 0.00880833015752012 0
0.000859440856410013 0.003 0.357029159325839 0.944547157822491
TCGA-XF-AAN1-01A-31R-A42T-07 0.00627341802278851 0.0357500078495027
0.00051733080556038 0.159009517117592 0 0.189118988108042 0
0.0737381557810324 0.00849549125867437 0 0 0 0.0438020065803535
0.0148598884152512 0.0262625491322998 0 0.166220022945116
0.206699835819556 0 0.0692527881642303 0 0 0.317
0.0398930926899248 1.07854446605925
TCGA-DK-AA6P-01A-11R-A39I-07 0.0550671151825919 0.0199504074021327 0
0.109505073830135 0 0.11621580438419 0 0.0250502581025802
0.0837356909116579 0 0.037470593242783 0.0147383888628869
0.00920178659271075 0.169694393432443 0.019965446464337
0.0688497989230549 0.0460476499007033 0.0622229444365816
0.162284648331212 0 0 0 0.466 0.0192013250121626
1.06835037863344
TCGA-CF-A47Y-01A-11R-A23W-07 0.0256438402791207 0 0 0.192602064388806
0 0.341169738222231 0 0.0228984225508161 0 0.0350365343360978 0
0 0.0255658530899675 0 0.0378489161555739 0.181124706270006
0.0563244173836111 0 0.0736361309764128 0 0.00814937634735665 0
0.379 0.0299571966084143 1.08900328951218
TCGA-CF-A3MI-01A-11R-A20F-07 0 0.046173911688538 0.00287608844469034
0.182924811287812 0.0344605435827265 0.0284045516460488
0.0925610459438852 0 0.00659495421338856 0 0 0.0274969601507018
0.0102694104020329 0.144755308796981 0.00843962672981655
0.24386796556552 0.0227605291574099 0.0728119177986172

0.0756023745918315 0 0 0 0.327 0.0377804740491692
1.06267990484763
TCGA-G2-A2EL-01A-12R-A18C-07 0 0.0355675093789098 0 0.142736428533291
0 0 0.0948190817374493 0.107848175199017 0.00323344274402676 0 0
0.0842921254367612 0 0.238160920891692 0.145998426735824
0.00231435306293608 0.0875263840279574 0 0.0575031522521348 0 0 0
0.955 -0.0327719785115601 1.11276543774858
TCGA-XF-AAMZ-01A-11R-A42T-07 0.396794479261848 0 0.0259785117839057
0.0625188449836842 0 0.153196363704993 0.011522736974074 0
0.119428119479818 0 0 0 0.0243487914596571 0.0263791732008488
0.0339877934760531 0.127650086047611 0 0.00237048918120557 0
0.00637481509323484 0 0.00944979535306693 0.001 0.415186608055685
0.916708420252789
TCGA-DK-AA6L-01A-11R-A39I-07 0.045798876395867 0 0.0158406288249164
0.0706269219399079 0 0.0935195851203428 0.0178069159609752
0.0510450212106721 0.0385549067629306 0 0 0.033054112741826
0.00791514918973439 0.23174920013063 0.0483025482034054
0.120707931435891 0.188051824838714 0 0.0370263772441875 0 0 0
0.00900000000000001 0.256988171203703 0.979913091648923
TCGA-XF-A9T4-01A-11R-A39I-07 0.00496293430916424 0 0.0219328583429961
0.120495519836275 0 0.19946068212603 0.0180994331718642
0.0464679403730501 0 0 0 0.0786036673774714 0.00155708562556429
0.113097577483964 0.127807173013796 0.10147152366512
0.0661771323494635 0.00972291544312474 0 0.0597417170768993
0.0168954630633806 0.0135063767418363 0.021 0.213773025012669
1.00473464500989
TCGA-K4-A6MB-01A-11R-A31N-07 0 0 0 0.0354161671546602 0
0.0930529406503348 0 0.0341011569012169 0.0183664854208638 0
0.0185839668678767 0 0 0.499908667182833 0.0206658435551831
0.148530225960669 0.00322442056148957 0.0561174571775344
0.065888503458273 0 0.00145933108638781 0.00468483402267721 0.136
0.093100888196416 1.10157787683249
TCGA-UY-A8OB-01A-12R-A42T-07 0 0 0.00233009733966097 0.268171096165401
0 0.106773636704107 0.140055631169242 0 0 0 0.0928029202584979
0.04339156123734 0.0154510718824813 0.105566362793959
0.0425200617863646 0.100820200009653 0 0.0563788726774693
0.0257384879758239 0 0 0 0.054 0.154196089136888
1.03974417729584
TCGA-ZF-A9R5-01A-12R-A42T-07 0 0.0752847178579395 0.0106292974078931
0.147853289376068 0 0.342575498240878 0.0310479108532436
0.0713692581079537 0.0296055998853544 0 0.0271411615010605 0
0.0152207636658438 0.0152856702031496 0 0.094737291557871 0
0.0547526252304878 0.0844969161122572 0 0 0 0.092
0.117251137866713 1.05634438185091

TCGA-CU-A3KJ-01A-11R-A21D-07 0 0 0.00364934445657117 0.121764932037473 0 0.211510583079277 0.0787996714875754 0.0437122594660287 0 0 0.0435850583807298 0 0.00747635267319519 0.160595503715669 0.0726240270082276 0.0780479328608001 0.0360504211772761 0.0449866899185003 0 0.0776398663724659 0 0.0195573573662097 0.326 0.0380337739852264 1.0749135643651
TCGA-DK-AA6S-01A-21R-A39I-07 0 0 0.00314695812841578 0.132153896771709 0 0.21121402645281 0.0780667643125569 0.079854910805596 0.0407975000898261 0 0 0 0 0.1048304240237 0.163954078270587 0.115439381299035 0.0395916204948061 0 0.0309504393509574 0 0 0 0.002 0.368889292706882 0.934560152857049
TCGA-E7-A85H-01A-11R-A352-07 0.107797255613552 0 0.0465023846398196 0.156466269034332 0 0 0.0693786926783103 0.0550507718874571 0.083601284080758 0 0 0.0535131880225655 0.00803825807026904 0.0878463048115969 0.0954083969193548 0.0741057411057878 0.109814699422001 0 0.0524767537141966 0 0 0 0.0590000000000001 0.149514531057772 1.02025271983011
TCGA-G2-AA3C-01A-21R-A39I-07 0 0 0 0.0475464738016453 0 0.0857176508151562 0 0 0 0 0 0.056886458338822 0.185974952110096 0 0.0105815262769532 0.552568884676448 0 0 0.0607240539808791 0 0 0 0.315 0.040284661364522 1.09611136544605
TCGA-E7-A8O8-01A-11R-A36F-07 0 0.0175640745464063 0.0105901873144623 0.10422925823951 0 0.144327523888715 0.0190722762227423 0.0704855901385989 0.00282010354228553 0.0029166945670559 0 0.128492237035349 0 0.147669538606324 0.0181746564470008 0.107268797508889 0.0673926353230017 0.0591640353724358 0.0998323912472231 0 0 0 0.202 0.0667879673732891 1.0603071215882
TCGA-XF-A9SM-01A-11R-A42T-07 0.0122766360970192 0 0.00179044714030276 0.21852508099734 0 0.101598507263432 0.189760586654828 0.0343088199267592 0.00874649104217056 0 0.0283901921978194 0 0.00502478750996432 0.0421967518876813 0.0777375468784473 0.214956272996552 0.0158903040187999 0 0.0430295683383189 0 0 0.0057680070505651 0.01 0.250596171041503 0.982611891542382
TCGA-DK-A3IN-01A-11R-A20F-07 0.0693906282425389 0 0.0393062192415176 0.146808521485649 0 0.224411005336046 0.00356451187136251 0 0.0105192054504327 0 0 0.0192177193734268 0.00726876782126498 0.0105659908672198 0.0337177901322481 0.371095346628847 0.0156587570457999 0.00554902865895598 0 0.0242756300178641 0 0.0186508778268257 0.039 0.177491651097042 1.0149803065189
TCGA-XF-A8HC-01A-11R-A36F-07 0 0.0737420451295053 0 0.0987529502224941 0 0.250309323029495 0 0.0442053327045476 0 0.0682703524436909 0 0 0 0.242353953417966 0 0.0263336884157225 0 0.017904124228539

0.153418941935108	0	0	0.0247092884729316	0.777	-0.0153564454547325	1.09924812489439
TCGA-ZF-AA4W-01A-12R-A38B-07	0	0.0246550380853206	0.00121085890440907	0.056762640717182	0	0.0658141242503892	0.0855605953548976	0.0555470327599422	0	0	0	0.140352814338952	0	0.159145281180265	0.15051805692645	0	0	0.145261898892521	0.0625539226219354	0	0	0.0526177359677358	0.0610000000000001	0.147127709899095	1.05176790911622
TCGA-LC-A66R-01A-41R-A30C-07	0	0.011184776438077	0.0346555961169076	0.185427361760791	0	0.131511241509937	0.157187450824587	0.0290059395168814	0.002645381321594	0	0.100894717526372	0	0.0037789109217044	0.0446332821005381	0.0784378749714176	0.136002988406971	0	0.0560268244114586	0.0234389352072892	0	0	0.00516871896547418	0.152	0.0828542517922052	1.05799814962304
TCGA-E7-A8O7-01A-11R-A36F-07	0.0536369730098912	0	0.0725580233062383	0.111052814959301	0	0.0412975490757189	0	0.048873925298213	0.106938019317115	0.00507758574354449	0	0.00312784048584535	0.00634696424129055	0.0117780158543612	0.00625341140885496	0.0648646146361298	0.196665183119915	0.12462362479271	0	0.109029575127372	0	0.0378758796234984	0.029	0.191680903603164	1.00213379212416
TCGA-LT-A8JT-01A-11R-A36F-07	0.0204928333868346	0.0483304239016585	0	0.150938036900983	0	0.335719060228447	0	0.0962432597259365	0.0075325455408854	0	0	0.0116255816594392	0.0308050523946558	0.043635200982862	0.0620494559518705	0.048959368192055	0.0312154818823678	0.0699288943970736	0.0425248048549307	0	0	0	0.124	0.096870894953078	1.06569367010145
TCGA-FJ-A3Z9-01A-11R-A26T-07	0.00479517984015402	0	0.0165256438022077	0.027284186964531	0.174799463523682	0.0498207600593638	0	0	0.0282434546202708	0	0.00838901481767924	0.0630445904156763	0.014900507831712	0.291867702161876	0	0.0378845189143922	0.104907620328266	0.116697668922017	0.0604800457463203	0	0	0.000359642051851964	0.303	0.0422884593682692	1.07698215542346
TCGA-DK-A1AC-01A-11R-A13Y-07	0.0873575477994603	0	0.0890148879039316	0.186561006805962	0	0.157221099503566	0.0766118355826607	0.0572255295630474	0.0221828117866809	0	0.00138986687607767	0	0.00964335644892154	0.100084439235256	0.0504793330924479	0.0999062295961289	0.00685934229936496	0	0	0.0367249164563672	0.018737797050127	0	0.00600000000000001	0.304286772669759	0.960520105042583
TCGA-C4-A0F1-01A-11R-A034-07	0.0316304692829907	0	0.0577751115618316	0.0274233796762805	0	0.180493456374924	0	0.0256660733270195	0.00905567747830635	0	0	0.0499915482124999	0	0.0952608414170548	0.148871574444207	0.101447862455519	0.0848039077241476

0.0158805999168765 0 0.129716165651671 0.0200489050172816
0.0219344274593903 0.017 0.230079004767614 0.995334296882413
TCGA-G2-A2ES-01A-11R-A180-07 0 0 0.00203000829522398 0.0870230897850075
0 0.12605738205904 0.148635684265757 0.0213412873678636
0.00689795661423626 0 0.0397011807416891 0.00708876106791175 0
0.187881188246769 0.090177790000045 0.160739151868573 0
0.078062095594293 0.00809603255300648 0 0.0333555083630311
0.00291288317755226 0.00800000000000001 0.269241308449389
0.976383001062354
TCGA-XF-AAN4-01A-11R-A42T-07 0.0304669527378248 0 0 0.0727130481954705
0 0.0767444130978995 0.0575213367688324 0.0188774454880135
0.00531430458547175 0.00979178134521152 0 0.0386297783014668
0.0744494413830589 0 0.145919393990174 0.202176482294564
0.0275268269000519 0.0230443772510037 0.189745108458795 0
0.0270793092021622 0 0.001 0.481120571024135 0.878380726153302
TCGA-4Z-AA83-01A-11R-A39I-07 0 0 0 0.0408023320531115 0
0.0882655613013809 0 0.0200180518942865 0.0811241178880778 0 0
0.0859754446341042 0 0.205963921484573 0.043867464138128
0.119901693478764 0.0566977701760806 0.169660095881535 0
0.0405763400925178 0 0.0471472069774408 0.151 0.0843271484858587
1.06559015791448
TCGA-E7-A5KE-01A-11R-A28M-07 0.0444026540474572 0 0 0.044673387975595
0.0754911382503901 0 0 0 0.0279588662891904 0 0.29733879180387
0 0.0416315537236424 0.0405735652134584 0.0132688657653883 0 0
0.244043856644529 0 0.164198849120955 0.00541250283792762
0.00100596832759584 0.784 -0.0156352478711498 1.13969343771357
TCGA-ZF-AA4V-01A-11R-A38B-07 0.00253945500324118 0 0.0159082297955375
0.255320394476736 0 0.0655793030178285 0.0949774006546438
0.0539780228562253 0.0227543623766036 0.0597199857269916 0
0.0482626444887443 0 0.116351125929361 0.0976741364410677
0.0912711650622764 0.0326503192474099 0.00579903189971066
0.0372144230236221 0 0 0 0.013 0.241666237466689
0.995545182499662
TCGA-ZF-AA53-01A-11R-A39I-07 0.0133542332612322 0 0.00179023642919068
0.13233502050669 0 0.0947329335535367 0.0478882449715115
0.0457511211727325 0.00104473478908089 0 0 0.0598758410370503
0.0155467332169108 0.0848184848397187 0.172537143712603
0.199488035040164 0.0944663186385847 0 0.0151515860355029 0
0.0109802951754327 0.0102390376200582 0.00900000000000001
0.264691022846101 0.982415636111023
TCGA-ZF-A9RM-01A-11R-A38B-07 0.0292099467298504 0 0.000541344929193205
0.211477365660208 0 0.257818620516419 0 0.033889561780514 0 0
0 0.132863303504413 0.0303266199101802 0.0688045964447717
0.0231009021862478 0.0584440328462538 0.046162941278137

0.0455183217197438 0.0618424424940678 0 0 0 0.69 -
0.00713492031936894 1.11726375440396
TCGA-BL-A13I-01A-11R-A13Y-07 0.00786879310788694 0 0 0.042191592626282
0 0.167042581845197 0.00685190087522757 0.0446831052355492
0.0359405641258586 0 0 0.00941177238065108 0.0319257234327599
0.0305242520614252 0.0240023068832996 0.523033141084997 0 0
0.0744824591841984 0.00204180715666758 0 0 0 0.604050459326932
0.80992869220873
TCGA-CU-A72E-01A-12R-A33J-07 0 0.0168008348266425 0 0.0318951503824108
0 0.145030849875088 0 0.0226886334655166 0.00511381236932669 0 0
0.0253837942270811 0 0.427364579565722 0.0308084839932663
0.193242535504175 0.0112140695860449 0 0.0675649615121047 0
0.0228922946926212 0 0.019 0.219288554396397 1.02260656111584
TCGA-2F-A9KR-01A-11R-A38B-07 0.0626886707547808 0 0.0487918290265976
0.114322877498256 0 0.128151552926457 0.0151267035023001
0.167686062997816 0 0 0.000344302959184559 0.0750313180030195
0.0211881620791825 0.0571801198084198 0.000802257212939917
0.045975923524175 0.0343553135668916 0.111705206298455
0.0734229716468822 0 0.0239075323946952 0.0193191957999477 0.315
0.040628775245854 1.06711764797578
TCGA-E5-A4U1-01A-11R-A31N-07 0.0632550714685323 0 0 0.096121285143487
0 0.225980342299661 0 0.035761052939729 0 0 0.169951104329564
0 0.0180133846754785 0.0581350349654754 0 0 0.131293958287019
0.0447336646266275 0.156755101264426 0 0 0 0.79 -
0.0160048617191371 1.09460558209142
TCGA-E7-A3Y1-01A-11R-A22U-07 0 0.0350493003371257 0.017369710315597
0.141696103953152 0 0.202264314298799 0 0.116065122467859
0.0299752247690156 0.00851835676470503 0 0.0932901505679879
0.00343789151118945 0 0.0320455225623084 0.147973281287107
0.0486195687041113 0.00405291549877907 0.119642536962263 0 0 0
0.324 0.0388644109238926 1.08116279731836
TCGA-FD-A6TC-01A-21R-A33J-07 0 0.0177045205965637 0.0305329791352131
0.00479253153866801 0 0.104195889621153 0 0 0.00395311875279318 0
0.0256166988332493 0 0 0.455286739542758 0.00197079781024959
0.229558630181341 0 0.0406493564608724 0 0.0769808232062445 0
0.00875791432089368 0.092 0.115309375718445 1.09314707054948
TCGA-FD-A5BV-01A-11R-A26T-07 0.0968393506027499 0 0 0.140108558041207
0 0.173756965340289 0 0 0 0 0 0.0631957301360439
0.019566032163189 0 0.0408178585427503 0.340792816350162 0 0
0.098964923669349 0 0.0205547883316748 0.00540297682258492 0.376
0.0305836780676271 1.06860400749261
TCGA-DK-A2I4-01A-11R-A21D-07 0.0814053048652701 0 0.0405564625316941
0.171130775873856 0 0.15597815489304 0.0961819001896014
0.0313292780675219 0 0 0 0.043647533855059 0.0122657287311368

0.0452259312578433	0.0755295859197793	0.169310513685413
0.0168002920325417	0	0.0538382660087039	0	0	0.00680027208853908
0.004	0.338398264791188	0.942939155589552
TCGA-UY-A78M-01A-21R-A352-07 0.0494537146526077	0	0.00967325887647745
0.0204076560675953	0	0.0728971822767014	0	0	0.045096465460013	0
0	0.0447318661715473	0	0.532344515046802	0.0508526179619869
0.0622775208406551	0	0.0418133995704139	0.0690372638193225	0	0
0.00141453925587691 0.198	0.0678563649444664	1.11196949536091
TCGA-4Z-AA7R-01A-11R-A39I-07	0.0920996437568527	0	0.0423699206588811
0.0833386967701807	0	0.0814376528434184	0	0.0396823233340784
0.0377433111003432	0	0.0200659503346905	0	0	0.16903928979301
0.0108859767876897	0.34145797498852	0	0.0151373198554991	0
0.0603291066396717	0	0.00641283313716492 0.045	0.167179181718321
1.02276484735391
TCGA-E7-A519-01A-11R-A26T-07	0	0.00912617582936204 0.0916947079807078
0.101477076391897	0	0.160268612931676	0.00477878495410194 0
0.126160060140705	0	0.0957790936754652	0	0.063221311985843
0.175674144911178	0	0.0613540087350772	0.000911493142954354
0.0938059403180863	0.0147105631828785	0	0	0.00103802582006743
0.00900000000000001 0.26240912927687	0.978609411964877
TCGA-DK-A3IU-01A-11R-A20F-07	0.148450367246654	0	0.0164064713135927
0.185725911676814	0	0.147078176857928	0.105802712054633
0.00463457420052443 0.0382456951169632	0	0	0.00659334196539446 0
0.107127553421016	0.100135768923335	0.0890046880628012	0	0
0.0354077088989265	0	0	0.0153870302614186	0.001	0.483449166503294
0.87598531141132
TCGA-FD-A3SM-01A-11R-A22U-07 0	0.00706504276836195 0.0241609312089867
0.0180935192423316	0	0.147975521042953	0	0.0450195272152951	0
0.00271584565418242 0	0.0196244438480297	0	0.518524174906169
0.0634540688990839	0.0610791483928689	0	0.0152828231361899
0.0770049536855474	0	0	0	0.175	0.0780457175084432
1.10288724033057
TCGA-BL-A5ZZ-01A-31R-A30C-07	0.00407331360239903 0	0.000845530171464626
0.0122443241090006	0	0.117795115073974	0.0353304112519708
0.00373408765213526 0	3.64758384109012e-05	0.0337115009410939
0.0191890089775023	0	0.452219283767792	0.00174168586409873
0.233708918022843	0	0.0196050341623689	0	0.0610215332319822	0
0.00474377733296307 0.001	0.440710704852047	0.906820812713582
TCGA-FD-A62N-01A-11R-A30C-07 0.0437112757608249	0	0.0497170173184656
0.240785723897982	0	0.104529679393681	0.0918913984887927
0.0203018733287642	0.0208592749558748	0	0	0.010407084213576
0.00633262840985112 0.0561081303025047	0.139917028028636
0.113046602165191	0.0422114898539635	0	0.0565673507602677	0	0
0.00361344312162479 0.04 0.175936019085028	1.0169997802254

TCGA-BT-A20J-01A-11R-A14Y-07 0.0337870027743535 0 0 0.196715715085887
0 0.170430887258596 0.0196416708100716 0.0523249998010867
0.0183303898709537 0 0 0.0385013249427453 0 0.1256742369756
0.147360252036138 0.125919620780541 0.0256326599176895 0
0.0450061354369801 0 0 0.000675104309358272 0.12 0.0993109002544971
1.05979472637657
TCGA-DK-AA71-01A-31R-A39I-07 0.00976622993144734 0 0.0101170128133029
0.265764964351522 0 0 0.117224665811421 0.04983406844335 0 0
0 0.036572051326745 0.00895846842713489 0.163033435467254
0.115250928607612 0.152677002733573 0 0.00665124408008568
0.0616950196678695 0 0.00245490833868299 0 0.652 -
0.00339105497283978 1.09306187009822
TCGA-KQ-A41O-01A-12R-A352-07 0 0 0.0592345248695506 0.617910694040137
0 0 0 0 0 0 0.0472727231653827 0.00786322604552785
0.00688611789136776 0 0 0.141116139580796 0 0.119716574407238 0
0 0 0 0.432 0.0236357067964657 1.13865103120796
TCGA-BL-A13I-01A-11R-A277-07 0.0108627106644693 0 0 0.0549477507391666
0 0.131331310598263 0.0122808243839176 0.00211132215736146
0.0296854384377377 0 0.018824691973884 0 0.0245717725837851
0.0931464523271626 0.0273660138574892 0.513254931618954
0.00402010329956028 0 0.062183505143191 0.0106228398271322 0
0.00479033238792571 0.001 0.514709625314418 0.858242942608196
TCGA-E7-A677-01A-11R-A30C-07 0 0.0388844363475488 0.000316370303426455
0.162347791277481 0 0 0.0417837349063442 0.0206025921591807
0.152103773082343 0 0 0.013295640829284 0.0172135376645868
0.064236340068549 0.0410335650892492 0.00311346572294158
0.174952400625058 0.136810394503113 0.133305957420895 0 0 0
0.152 0.0831407047173899 1.04599812384309
TCGA-DK-A1A5-01A-11R-A13Y-07 0 0.0233844985945651 0 0.150871941806356
0 0.0381306477521582 0.0715834052149643 0.0864988894244792
0.00251843573308823 0 0 0.00480602149767609 0 0.273856063925052
0.0412342979578441 0.0806526861467712 0.113128433882004
0.085206783196778 0.0281278948682636 0 0 0 0.039
0.178051708230217 1.01757758507698
TCGA-HQ-A5NE-01A-12R-A28M-07 0.00682269679884298 0
0.00347652040359356 0.0447750392339343 0 0.118021850040928
0.00916220748094409 0.0853771759749588 0 0 0.0890964842721342 0 0
0.243381380227714 0.0067485354954355 0.0331510610932818 0
0.190651271725577 0 0.168918751390504 0.000417025862153186 0
0.075 0.133769413622754 1.04889875619341
TCGA-XF-AAMT-01A-11R-A42T-07 0.00278762800662785 0.0249123749483024
0.00614853284282479 0.0291996392607976 0 0.0881204654456576 0 0
0.0346553288774439 0 0 0.0125134581588934 0 0.337285957960386
0.00905304102180179 0.342142336005159 0 0.00943693907187828

0.0901293653665266 0 0 0.0136149330337009 0.004 0.32907405594569
0.963325114166686
TCGA-XF-A9SU-01A-31R-A39I-07 0.232175807633593 0 0.181726989253318
0.104176330066787 0 0.00280246352145362 0.0216734699551 0
0.0266510383104051 0.0192768141797633 0 7.08096058937193e-05
0.00854009410116047 0.0765222670996604 0.149149509198168
0.118958281517901 0 0 0.0540041804782198 0 0
0.00427194507857713 0.001 0.448866477631434 0.897592856681285
TCGA-CF-A47S-01A-11R-A23W-07 0 0.0102334164687135 0.031142241476521
0.11844746204936 0 0.108845064832647 0 0.0275802062986399
0.151249509151104 0 0 0.0403337354893641 0.031030323032101
0.0320669434162043 0.109661553475088 0 0.0940518715041378
0.179494806719419 0 0.0584401520032657 0.00742271408343469 0 0.087
0.123223653798689 1.05349971830474
TCGA-FD-A6TK-01A-42R-A33J-07 0.026943360808309 0 0.029806609836151
0.172652692457422 0 0.084279856624323 0.177146640103895
0.035298431626289 0.0041758470618738 0 0.00873807753661083
0.00306823719859881 0.00298544106720792 0.040125558016896
0.1150461019971 0.176671660025393 0.0255148928289508 0.0209933137814902
0.0728864359275403 0 0 0.00366684310194977 0.023 0.206309323330908
0.99717167752463
TCGA-E7-A5KF-01A-11R-A28M-07 0 0.0408807879506569 0.0114016469924738
0.0487599068650172 0 0.238715421761961 0 0 0.0857048038498363 0
0.073412998676592 0 0.00748395644857985 0.0225810595113857 0
0.138490557362439 0 0.237614974107441 0 0.0711196857541815
0.0116524153764721 0.012181785342964 0.731 -0.0105954566701421
1.09633601974269
TCGA-UY-A78L-01A-12R-A33J-07 0 0.00676279522813653 0.010409886220192
0.0479526301730612 0 0.125435913278646 0 0.0445924642106138
0.0157719960178247 0 0 0.0340458365069959 0 0.300103803236259
0.0417495551371301 0.179949960833527 0.0355340236380224
0.0466286360741627 0.0132382438282853 0 0 0.0978242556171434 0.158
0.0813774787080326 1.07251499497156
TCGA-S5-A6DX-01A-11R-A31N-07 0.156656160739179 0 0.16020466413609
0.168033097758523 0 0 0.0901786344134724 0.00970385999199624
0.0264358775474213 0 0 0.0240005540360726 0.0107003112430889
0.0602978097894422 0.110811412700591 0.0759689726914139
0.0307014349890238 0 0.0716769288905434 0 0 0.00463028107314317
0.005 0.322598277624526 0.956423584585339
TCGA-XF-A8HI-01A-11R-A38B-07 0 0.036051570839607 0.0156028087089168
0.0745121348817361 0 0.27528327485288 0.0151948153385283
0.0159446086253825 0.0941155126969497 0 0.0180857747307224 0 0
0.0541731807202232 0 0.102697837439848 0.0310625710148516
0.138527640558859 0 0.11936117333051 0 0.00938709626098479 0.183

0.073649213282177	1.06064180432259
TCGA-XF-A9T5-01A-11R-A42T-07	0.113315265102751	0	0.0878623607032397	0.140446527587899	0	0.103186560393084	0.0690672032171883	0.0445963558642811	0.0265473663032053	0	0.00672454839375686	0	0	0.150419847584349	0.0920585454693315	0.092850856262386	0.0480123210000317	0	0.0219514090709869	0	0	0.0029608330475103	0.002	0.385482669996303	0.922584462646793
TCGA-XF-A9SX-01A-21R-A39I-07	0.0561496868759974	0	0.00233917128103756	0.130740843934083	0	0.155167340885767	0.0779604919492951	0.0418996850328389	0.0503844394005139	0	0	0.0577385695777963	0	0.0630807745055708	0.0791227908636856	0.189781599395182	0.00671085461212039	0.0164100081312912	0.0702959283253913	0	0	0.00221781522943071	0.00600000000000001	0.301266331513215	0.960106294986755
TCGA-G2-A2EK-01A-22R-A18C-07	0.0890229091422189	0.107269506896099	0.013283272365564	0.0642843295309573	0	0.252441751758382	0.00602773844485814	0	0.104491172757864	0	0	0.0494293586777466	0.00779139071409915	0.0562130156581977	0	0.0452798102245656	0.0497102202776093	0.0749350876662455	0.0798204358855926	0	0	0	0.01	0.247373273757645	0.985825307489333
TCGA-XF-A8HD-01A-11R-A36F-07	0.206566392253794	0	0.0506419743591235	0.205925695508715	0	0.00888104191263834	0.0807967154781553	0.0533284034192047	0.00544220234189783	0	0.00511750299044132	0.072355616419944	0.0121062834051938	0.00920776286709741	0.0345596908323622	0.053389404028466	0	0.130984104967535	0.0661508134907214	0	0	0.00454639572471018	0.001	0.474563689195831	0.879608851466445
TCGA-GV-A3JX-01A-11R-A20F-07	0.096788121038545	0	0.0164563727144362	0.14666004459527	0	0.134557616930852	0.0364148338358011	0.0892566375925563	0.0171753919568117	0.0119071062678309	0.0291851642681237	0	0	0.0566530489711308	0.0965110454575792	0.205435379958797	0.0206247757466795	0.0173871655173352	0.024987295148252	0	0	0	0.012	0.243971292557035	0.985595192396582
TCGA-H4-A2HQ-01A-11R-A180-07	0	0.00300843532719338	0.00800103100146493	0.0869180678679968	0	0.216603447770348	0.0304170324029233	0.119461991495812	0	0	0.0393095522977675	0	0.0101685361451769	0	0.15146752751598	0.104574868082463	0.0825116093379901	0.142487952521078	0.00506994823380556	0	0	0	0.244	0.0554571000930441	1.08958854184822
TCGA-XF-A9SP-01A-11R-A39I-07	0.205852921291759	0	0.119894529331831	0.137894465312317	0	0.0251047740649511	0	0.0176871913301762	0.0137428182666243	0.00242498823131804	0	0.0467572677976099	0.00427432441431933	0.0154269224339194	0.0536011040655501

0.25830391478523 0 0.0158858540248175 0.0831489246495778 0 0 0
0.001 0.413895841112972 0.910469310356232
TCGA-DK-A3IK-01A-32R-A21D-07 0 0.189685954103041 0.0764486230251883
0.12397310586603 0 0.0530960303469752 0 0.0584678835759138 0
0.0168676966969602 0 0.0364585222661032 0.00327463514770142
0.18398653312977 0.114759097704471 0.0879761462573835 0
0.0169861655162569 0.0380196063642061 0 0 0 0.016
0.234644269147471 0.994790728287916
