# Supplementary material for: Ferroptosis-Related Long Noncoding RNAs Have Excellent Predictive Ability for Multiomic Characteristics of Bladder Cancer
Source: Oxid Med Cell Longev. 2022 Aug 29;2022:9316847. doi: 10.1155/2022/9316847 (PMC9444476; doi:10.1155/2022/9316847)
Supplement: Supplementary Materials — Supplementary Table 1: expression matrix of 60 ferroptosis-related genes. Supplementary Table 2: results for coexpression analysis of ferroptosis-related lncRNAs and mRNAs. Supplementary Table 3: expression of 263 ferroptosis-related lncRNAs. Supplementary Table 4: expression of lncRNAs and clinical information of 403 bladder cancer samples. Supplementary Table 5: cluster analysis based on 20 prognostic lncRNAs. Supplementary Table 6: detailed clinical information of bladder cancer samples. Supplementary Table 7: data of the high- and low-risk groups of the training cohort. Supplementary Table 8: data of the high- and low-risk groups of the validation cohort. Supplementary Table 9: markers of different immune cells. Supplementary Table 10: infiltration of 22 types of immune cells analysed using the CIBERSORT algorithm. Supplementary Table 11: immune scores evaluated using the ESTIMATE algorithm. Supplementary Table 12: immunotherapeutic response evaluated using the Tumour Immune Dysfunction and Exclusion (TIDE) algorithm. Supplementary Figure 1: gene set enrichment analysis. (A–F) Most significantly enriched biological pathways in cluster 1. (G–I) Most significantly enriched biological pathways in cluster 2. [file 9316847.f1.zip › s-table 11.pdf]

ID	StromalScore	ImmuneScore	ESTIMATEScore
TCGA-CF-A47X-01A-31R-A23W-07	-1772.23869809142	-602.429051032574	-2374.66774912399
TCGA-ZF-AA51-01A-21R-A39I-07	64.3441392254353	1450.54662334637	1514.89076257181
TCGA-BL-A0C8-01A-11R-A10U-07	-625.200675069726	-211.674358804004	-836.875033873729
TCGA-BT-A42C-01A-11R-A23N-07	-1729.01589157274	-438.730301824338	-2167.74619339708
TCGA-G2-A3IB-01A-11R-A20F-07	-1436.34728994	-218.627253281216	-1654.97454322122
TCGA-2F-A9KP-01A-11R-A38B-07	-1630.63052327507	-674.193999823542	-2304.82452309861
TCGA-DK-A3IT-01A-31R-A20F-07	-53.3954836772599	529.93283129294	476.537347615681
TCGA-FD-A43P-01A-31R-A23W-07	-257.918314092308	1960.04947408487	1702.13115999257
TCGA-DK-A3IV-01A-22R-A21D-07	-1488.33800517209	1268.08811578202	-220.249889390063
TCGA-2F-A9KW-01A-11R-A38B-07	-18.8384464968865	357.681108801005	338.842662304119
TCGA-GU-A762-01A-11R-A33J-07	436.953392960058	2383.71665033574	2820.6700432958
TCGA-XF-AAME-01A-12R-A42T-07	1564.83999742588	2374.88671678768	3939.72671421356
TCGA-XF-AAN0-01A-11R-A42T-07	-433.471711504356	926.142039176469	492.670327672113
TCGA-CF-A47W-01A-11R-A23W-07	-1628.41563670245	-364.794427590086	-1993.21006429253
TCGA-GC-A3I6-01A-11R-A20F-07	-483.413782656876	834.807380461024	351.393597804149
TCGA-FD-A6TA-01A-12R-A33J-07	538.860345099576	1265.02252638968	1803.88287148925
TCGA-DK-A1A7-01A-11R-A13Y-07	-1073.92465083149	58.2864381036851	-1015.6382127278
TCGA-FD-A3SR-01A-11R-A22U-07	361.358864769324	993.789629045046	1355.14849381437
TCGA-GU-AATP-01A-11R-A39I-07	528.365798725223	759.807122468201	1288.17292119342
TCGA-DK-AA6X-01A-12R-A42T-07	-559.782348043752	878.575438424505	318.793090380753
TCGA-XF-A9T3-01A-11R-A42T-07	517.884018403117	1031.17078641073	1549.05480481385
TCGA-PQ-A6FN-01A-11R-A31N-07	-516.939276659981	705.863785487601	

188.92450882762
TCGA-DK-A3WW-01A-22R-A23N-07 -432.686228255479 1973.5087331237 1540.82250486822
TCGA-XF-AAMY-01A-11R-A42T-07 186.511652334003 211.246976159031 397.758628493034
TCGA-K4-A3WS-01A-11R-A22U-07 1127.38075925364 1241.7727865668 2369.15354582044
TCGA-UY-A9PA-01A-11R-A38B-07 -1625.69938043669 831.079793038577 -794.61958739811
TCGA-G2-AA3F-01A-12R-A42T-07 -1119.97605350379 -443.460647735005 -1563.4367012388
TCGA-FJ-A871-01A-11R-A352-07 -842.415544805501 -485.807087926978 -1328.22263273248
TCGA-ZF-A9RN-01A-11R-A42T-07 -1127.82177767135 1053.21311558385 -74.6086620875008
TCGA-GC-A3RB-01A-12R-A220-07 -1314.19503076671 -180.545891778136 -1494.74092254485
TCGA-UY-A9PF-01A-11R-A38B-07 -445.634966376598 475.539642043842 29.904675667244
TCGA-YC-A89H-01A-11R-A36F-07 -646.889902122956 366.617780491821 -280.272121631135
TCGA-XF-AAMQ-01A-11R-A42T-07 -687.052164908733 1151.38113551108 464.328970602352
TCGA-CF-A1HR-01A-11R-A13Y-07 -800.926656101546 394.753596135759 -406.173059965786
TCGA-E7-A3X6-01A-12R-A22U-07 -640.818854292803 1095.32963835949 454.510784066687
TCGA-DK-A6AW-01A-11R-A30C-07 -1108.43371356432 -292.640683425998 -1401.07439699032
TCGA-GC-A3BM-01A-11R-A22U-07 -1498.32829552124 -25.2754104678103 -1523.60370598905
TCGA-GV-A6ZA-01A-12R-A33J-07 -1345.99152456011 400.943369803567 -945.048154756548
TCGA-G2-A2EJ-01A-11R-A180-07 -1304.13732027335 409.712642913094 -894.424677360258
TCGA-FD-A43N-01A-11R-A23W-07 -40.2218988865042 362.428193085891 322.206294199386
TCGA-UY-A8OD-01A-11R-A36F-07 556.062493748461 982.297243423872 1538.35973717233
TCGA-2F-A9KT-01A-11R-A38B-07 -936.774492675418 -92.6058276869062 -1029.38032036232
TCGA-BT-A20Q-01A-11R-A14Y-07 415.792071119799 1057.2686425733 1473.0607136931
TCGA-CU-A0YR-01A-12R-A10U-07 132.007546226705 1301.81023028688

1433.81777651358
TCGA-SY-A9G5-01A-11R-A38B-07 782.544867388454 1708.13074419519
2490.67561158365
TCGA-4Z-AA84-01A-11R-A39I-07 -1002.54691088598 -335.878454891326 -
1338.4253657773
TCGA-4Z-AA7Q-01A-11R-A39I-07 -495.605887182969 1804.00124132829
1308.39535414532
TCGA-FD-A5BT-01A-11R-A26T-07 1480.22858209892 2385.29578281764
3865.52436491656
TCGA-CF-A27C-01A-11R-A16R-07 -2154.7783267956 -413.555572104406 -
2568.3338989
TCGA-E7-A6MF-01A-12R-A32O-07 -1829.58243603786 -280.732073207146 -
2110.31450924501
TCGA-FT-A61P-01A-11R-A30C-07 665.471639835075 1202.08893064583
1867.5605704809
TCGA-DK-AA77-01A-11R-A39I-07 -1416.70372058611 505.000899082875 -
911.702821503238
TCGA-ZF-AA5N-01A-11R-A42T-07 -1527.70055102854 -157.085023827182 -
1684.78557485572
TCGA-DK-AA6U-01A-11R-A39I-07 -1854.50461137816 -396.647392254239 -
2251.15200363239
TCGA-BT-A20O-01A-21R-A14Y-07 1047.31838300991 3086.7190490448
4134.03743205471
TCGA-GD-A3OS-01A-12R-A220-07 -395.648328054756 -49.7914093022977 -
445.439737357054
TCGA-XF-A9SZ-01A-11R-A39I-07 627.973212500302 845.836871465156
1473.81008396546
TCGA-4Z-AA82-01A-11R-A39I-07 -176.62895426241 334.78769025177
158.158735989359
TCGA-DK-AA76-01A-11R-A39I-07 -1808.12941570949 -1096.84214558378 -
2904.97156129327
TCGA-DK-A3IL-01A-11R-A20F-07 -771.938911922325 -257.122150814334 -
1029.06106273666
TCGA-E7-A678-01A-11R-A30C-07 -1881.91492363438 18.3914334381903 -
1863.52349019619
TCGA-ZF-A9R1-01A-11R-A39I-07 -1205.23281809858 -139.801117845579 -
1345.03393594416
TCGA-XF-A9SH-01A-11R-A39I-07 -874.429295631732 142.129309401516 -
732.299986230216
TCGA-FD-A5BU-01A-31R-A26T-07 488.458718747592 1313.44550513484
1801.90422388243
TCGA-C4-A0F7-01A-11R-A084-07 -943.004137572052 -441.07233417505 -
1384.0764717471
TCGA-C4-A0F0-01A-12R-A10U-07 -175.336608722605 855.420613210572

680.084004487967
TCGA-FD-A3N5-01A-11R-A21D-07 -900.477298493645 367.209423791031 -
533.267874702615
TCGA-ZF-AA5H-01A-11R-A39I-07 -186.552659792117 1291.88632751138
1105.33366771927
TCGA-DK-A3WX-01A-22R-A22U-07 118.433107187052 1445.59972516426
1564.03283235132
TCGA-YF-AA3L-01A-11R-A38B-07 -1289.55461266317 -118.319795296147 -
1407.87440795932
TCGA-BT-A2LA-01A-11R-A18C-07 -1390.28208103529 -1428.58349447607 -
2818.86557551136
TCGA-4Z-AA87-01A-11R-A39I-07 -806.492710775063 704.33225437673 -
102.160456398333
TCGA-BT-A2LB-01A-11R-A18C-07 231.692807708646 1020.59184216397
1252.28464987262
TCGA-XF-A8HB-01A-11R-A36F-07 -1906.58662517618 -385.181903710974 -
2291.76852888715
TCGA-DK-A2I6-01A-12R-A18C-07 -936.359882672186 -228.128482391235 -
1164.48836506342
TCGA-ZF-AA4X-01A-11R-A38B-07 -2432.85542937504 -358.01958120171 -
2790.87501057675
TCGA-BT-A20P-01A-11R-A14Y-07 -1490.13142918067 -373.796045650345 -
1863.92747483101
TCGA-BT-A20W-01A-21R-A14Y-07 -601.658466636404 609.619273929175
7.96080729277082
TCGA-FD-A5BX-01A-11R-A26T-07 820.859564215631 1854.84338592508
2675.70295014072
TCGA-BL-A13J-01A-11R-A10U-07 -99.876947942544 -92.4877997214408 -
192.364747663985
TCGA-CF-A8HX-01A-11R-A36F-07 -1588.31639735985 -473.644692004299 -
2061.96108936415
TCGA-PQ-A6FI-01A-11R-A31N-07 -1190.29295404381 474.266540076472 -
716.026413967338
TCGA-FJ-A3Z7-01A-12R-A23N-07 131.687557044745 363.626546499494
495.314103544238
TCGA-E7-A7PW-01A-11R-A352-07 -1856.24600504095 -128.784586516948 -
1985.0305915579
TCGA-4Z-AA7S-01A-11R-A39I-07 -1368.7235608233 -597.177547679784 -
1965.90110850309
TCGA-CF-A3MH-01A-11R-A20F-07 -1948.40377192882 -105.080405193428 -
2053.48417712225
TCGA-DK-A6B2-01A-11R-A30C-07 627.697874126739 1292.89255090424
1920.59042503098
TCGA-4Z-AA86-01A-11R-A39I-07 649.033347932914 1865.05391631594

2514.08726424885
TCGA-BL-A0C8-01B-04R-A277-07 -903.814619824186 -841.675248087716 -
1745.4898679119
TCGA-5N-A9KI-01A-31R-A42T-07 -7.61895828693348 747.177131807088
739.558173520154
TCGA-GU-AATO-01A-11R-A39I-07 -87.0765586366839 1160.9561159361
1073.87955729941
TCGA-CF-A9FH-01A-11R-A38B-07 -1919.51909940743 -249.268545211843 -
2168.78764461927
TCGA-FD-A6TG-01A-11R-A32O-07 538.620550088055 853.169437982637
1391.78998807069
TCGA-4Z-AA7O-01A-31R-A39I-07 -613.506660434985 655.126392392588
41.6197319576032
TCGA-GV-A3QI-01A-11R-A220-07 -1848.14536149155 -1152.82072409293 -
3000.96608558448
TCGA-DK-A6AV-01A-12R-A30C-07 -982.624237339837 332.311850279071 -
650.312387060766
TCGA-DK-A1A3-01A-11R-A13Y-07 234.876844970719 675.634192571103
910.511037541822
TCGA-GD-A6C6-01A-21R-A31N-07 -1022.44361443498 171.224473007284 -
851.219141427692
TCGA-GC-A3RD-01A-12R-A22U-07 -1158.04707845745 -101.318992024398 -
1259.36607048185
TCGA-BT-A20R-01A-12R-A16R-07 856.174396533834 598.027717692392
1454.20211422623
TCGA-XF-A9T0-01A-11R-A39I-07 -198.49375700566 646.955169259925
448.461412254264
TCGA-YF-AA3M-01A-11R-A42T-07 -1497.93343888491 -109.463571953721 -
1607.39701083863
TCGA-4Z-AA7Y-01A-11R-A39I-07 -1716.5571124156 -470.628695568016 -
2187.18580798361
TCGA-FD-A62O-01A-11R-A30C-07 -861.528937774776 -416.111606643086 -
1277.64054441786
TCGA-BL-A3JM-01A-12R-A21D-07 -569.674823135513 -173.372752284253 -
743.047575419767
TCGA-FJ-A3ZF-01A-11R-A23N-07 -1833.72550512973 -1010.51234510936 -
2844.23785023908
TCGA-CU-A0YO-01A-11R-A10U-07 -332.658429454169 880.150664640368
547.492235186198
TCGA-CF-A5UA-01A-11R-A28M-07 -1544.77020941995 -217.346377306724 -
1762.11658672668
TCGA-K4-AAQO-01A-11R-A38B-07 452.013034635691 845.118753760633
1297.13178839632
TCGA-XF-AAMJ-01A-11R-A42T-07 1198.45816053696 622.59367210835

1821.05183264531
TCGA-GU-A763-01A-11R-A32O-07 -1940.23038134644 -41.6755553991329 -
1981.90593674557
TCGA-G2-A2EC-01A-11R-A180-07 361.143434656282 971.885925806413
1333.02936046269
TCGA-FD-A3B7-01A-31R-A20F-07 1295.6682256484 2136.02755235085
3431.69577799925
TCGA-FD-A5C0-01A-11R-A28M-07 -428.897133366416 212.367709616357 -
216.529423750059
TCGA-FD-A5BS-01A-21R-A26T-07 1668.8212482347 3164.37053676924
4833.19178500395
TCGA-CU-A0YN-01A-21R-A10U-07 -111.821500959118 916.942435966247
805.12093500713
TCGA-FD-A5BZ-01A-11R-A28M-07 1018.37986315376 157.720133847225
1176.09999700098
TCGA-UY-A9PE-01A-11R-A38B-07 -515.070402271291 -246.463799383914 -
761.534201655205
TCGA-GV-A40E-01A-12R-A23N-07 -584.006482953346 413.859054461905 -
170.14742849144
TCGA-BT-A2LD-01A-12R-A20F-07 -602.038937200312 222.210429842253 -
379.828507358059
TCGA-UY-A9PD-01A-11R-A38B-07 -253.961053032468 -35.5836300214698 -
289.544683053938
TCGA-G2-AA3B-01A-11R-A39I-07 -1707.76953223638 306.223249188435 -
1401.54628304795
TCGA-K4-A54R-01A-11R-A26T-07 250.009467474398 1384.52457993991
1634.53404741431
TCGA-DK-A1AA-01A-11R-A13Y-07 -1422.60657077233 70.9970010821334 -
1351.60956969019
TCGA-CU-A5W6-01A-11R-A28M-07 -942.174125293519 162.680335552978 -
779.493789740541
TCGA-DK-AA6R-01A-11R-A42T-07 -896.952919698162 204.253299429789 -
692.699620268373
TCGA-BT-A3PH-01A-11R-A220-07 -992.095740309142 -376.76183276733 -
1368.85757307647
TCGA-BL-A13I-01B-04R-A277-07 1142.45976337523 2074.86217943835
3217.32194281358
TCGA-CF-A3MF-01A-12R-A21D-07 -1290.97814361958 -264.898707226835 -
1555.87685084641
TCGA-CF-A47T-01A-11R-A23W-07 -1292.57828525093 -517.354105585169 -
1809.9323908361
TCGA-YC-A8S6-01A-31R-A38B-07 607.974779097187 1324.76171792077
1932.73649701796
TCGA-DK-A3IS-01A-21R-A21D-07 -1814.67839483102 -171.590681531205 -

1986.26907636223
TCGA-XF-A9SJ-01A-11R-A39I-07 217.375664309722 1411.41137534361 1628.78703965333
TCGA-DK-AA6T-01A-11R-A39I-07 -123.947939194131 2167.22856111362 2043.28062191949
TCGA-BT-A20N-01A-11R-A14Y-07 -1019.45064211244 -670.939395641637 -1690.39003775408
TCGA-C4-A0F6-01A-11R-A10U-07 -373.917212243134 113.835096354957 -260.082115888178
TCGA-CF-A7I0-01A-22R-A352-07 -471.129485076529 101.192747905829 -369.9367371707
TCGA-FD-A3B6-01A-21R-A20F-07 -180.940081292244 1718.70246422347 1537.76238293123
TCGA-GC-A3YS-01A-11R-A23N-07 486.693931652269 936.46362806001 1423.15755971228
TCGA-XF-A9T2-01A-11R-A42T-07 -164.244668498083 -72.2636552382903 -236.508323736373
TCGA-UY-A78O-01A-12R-A33J-07 -869.453721166465 -27.2651679011085 -896.718889067574
TCGA-GC-A3OO-01A-11R-A22U-07 -24.0139122263739 415.472522403669 391.458610177295
TCGA-E7-A6ME-01A-22R-A32O-07 -1589.43467695471 211.81862685483 -1377.61605009988
TCGA-XF-AAN5-01A-11R-A42T-07 -563.182557959682 1453.0727814455 889.89022348582
TCGA-FD-A3SN-01A-12R-A22U-07 -291.831287098834 319.256204416089 27.4249173172552
TCGA-ZF-A9R2-01A-11R-A39I-07 -2418.60351026673 -747.304096141287 -3165.90760640802
TCGA-ZF-AA4T-01A-11R-A38B-07 -1758.12203716159 -371.221484529669 -2129.34352169126
TCGA-E7-A4IJ-01A-31R-A26T-07 -1334.51874542549 539.823778542714 -794.694966882781
TCGA-FD-A6TE-01A-12R-A33J-07 -1905.55464847937 -88.8401841536066 -1994.39483263298
TCGA-H4-A2HO-01A-11R-A180-07 -793.78652913317 550.632754266554 -243.153774866615
TCGA-FD-A6TB-01A-12R-A33J-07 489.24396114357 1965.66752953793 2454.9114906815
TCGA-XF-AAN3-01A-11R-A42T-07 283.191439533779 739.142662585179 1022.33410211896
TCGA-XF-AAMH-01A-11R-A42T-07 -863.885897384193 -588.474104093356 -1452.36000147755
TCGA-FD-A3B8-01A-31R-A20F-07 1656.25973688676 2689.67969567099

4345.93943255775
TCGA-XF-A9SY-01A-21R-A42T-07	568.580293659178	2093.00884452631
2661.58913818549
TCGA-CF-A9FF-01A-11R-A38B-07	-625.082733799333	464.870711648536	-
160.212022150797
TCGA-XF-A9ST-01A-11R-A42T-07	-1452.33494512298	-910.760543273716	-
2363.0954883967
TCGA-DK-A6B5-01A-11R-A31N-07	-931.125244359989	784.560181600577	-
146.565062759412
TCGA-GC-A3RC-01A-11R-A22U-07	-398.153421909789	1419.57250031378
1021.41907840399
TCGA-GV-A3QK-01B-11R-A23N-07	-1237.47014813954	907.964349640562	-
329.505798498975
TCGA-GU-A764-01A-11R-A352-07	294.825535772183	1445.39113058881
1740.21666636099
TCGA-GU-A42R-01A-11R-A23N-07	-1597.99363018847	-648.915837689319	-
2246.90946787779
TCGA-DK-A6B6-01A-11R-A30C-07	-1443.43262376977	788.351227643504	-
655.081396126269
TCGA-R3-A69X-01A-22R-A30C-07	497.743965948862	1287.80872062057
1785.55268656944
TCGA-5N-A9KM-01A-11R-A42T-07	-65.2996265573967	881.091235166846
815.791608609449
TCGA-FD-A6TF-01A-52R-A32O-07	461.656722401288	443.298894908113
904.955617309401
TCGA-XF-AAMR-01A-31R-A42T-07	575.856505686584	581.173489801797
1157.02999548838
TCGA-XF-A8HF-01A-11R-A36F-07	-174.945662703635	487.877590338229
312.931927634594
TCGA-ZF-AA4U-01A-11R-A38B-07	-2233.30650191097	-270.486562086867	-
2503.79306399784
TCGA-DK-A3IQ-01A-31R-A32Y-07	1736.9871898156	399.57919062322
2136.56638043882
TCGA-E7-A4XJ-01A-11R-A26T-07	-1361.02968958196	-464.279747306729	-
1825.30943688869
TCGA-XF-A9T6-01A-11R-A42T-07	-1195.75635162973	310.790277204735	-
884.966074424999
TCGA-4Z-AA7M-01A-11R-A39I-07	-1044.07469020777	-147.900415420209	-
1191.97510562798
TCGA-ZF-AA56-01A-31R-A39I-07	-169.837972957191	1033.42470508847
863.586732131281
TCGA-XF-A8HH-01A-11R-A38B-07	229.908000882765	313.769589913473
543.677590796238
TCGA-C4-A0EZ-01A-21R-A24X-07	-1288.90661706792	-1110.19414368343	-

2399.10076075136
TCGA-FD-A62P-01A-32R-A30C-07 161.154103258849 1162.68173969056 1323.83584294941
TCGA-CF-A47V-01A-11R-A23W-07 -830.477753871231 397.55309455007 -432.924659321161
TCGA-XF-A9SI-01A-11R-A39I-07 727.199080083299 2808.41826800697 3535.61734809026
TCGA-GU-A42Q-01A-11R-A23W-07 -1423.69837115476 -512.006765460406 -1935.70513661516
TCGA-BT-A42F-01A-11R-A23W-07 -202.524157314375 2064.31467805958 1861.79052074521
TCGA-ZF-A9RF-01A-11R-A38B-07 -738.401296655835 1894.27495336705 1155.87365671121
TCGA-KQ-A41N-01A-11R-A33J-07 -1236.93792798338 -731.981114305755 -1968.91904228914
TCGA-GD-A3OQ-01A-32R-A220-07 -1178.23996742716 -99.3533508635437 -1277.5933182907
TCGA-BT-A20U-01A-11R-A14Y-07 -334.644992683887 200.369184370325 -134.275808313562
TCGA-XF-A9SW-01A-11R-A42T-07 1486.6365612092 1153.95579223805 2640.59235344725
TCGA-FD-A3B3-01A-12R-A206-07 730.911610889806 1978.75443131461 2709.66604220442
TCGA-ZF-A9RC-01A-11R-A38B-07 4.13544028675025 -232.294228456223 -228.158788169473
TCGA-E7-A6MD-01A-41R-A352-07 66.1610586144643 1097.07833990619 1163.23939852065
TCGA-E5-A4TZ-01A-11R-A31N-07 -1973.42384559385 -262.474176725722 -2235.89802231958
TCGA-ZF-A9R4-01A-11R-A38B-07 -1581.33267957473 186.128644960002 -1395.20403461473
TCGA-GV-A3QH-01A-11R-A220-07 -2213.64027408464 -725.978767242824 -2939.61904132746
TCGA-BT-A0YX-01A-11R-A10U-07 -517.228708371959 956.506000649337 439.277292277379
TCGA-FT-A3EE-01A-11R-A206-07 -1058.68108746608 -231.710699642173 -1290.39178710825
TCGA-ZF-A9RL-01A-11R-A38B-07 -2185.41115589709 -893.943014636792 -3079.35417053388
TCGA-ZF-A9R9-01A-11R-A38B-07 754.640811127372 548.224852835442 1302.86566396281
TCGA-ZF-A9RE-01A-11R-A38B-07 -1996.40247644546 -326.808620305714 -2323.21109675117
TCGA-DK-A6B1-01A-12R-A30C-07 -1211.78740896666 -389.904518587565 -

1601.69192755423
TCGA-CF-A5U8-01A-11R-A28M-07 -1750.59511208749 -455.534907505999 -2206.13001959349
TCGA-XF-AAMW-01A-11R-A42T-07 -433.066221180463 814.910711458103 381.844490277641
TCGA-BT-A3PJ-01A-21R-A220-07 -627.122844600103 1041.80740196493 414.684557364831
TCGA-ZF-A9R7-01A-11R-A38B-07 -950.979577016314 2017.5198974305 1066.54032041418
TCGA-FD-A3SO-01A-11R-A22U-07 393.336387488797 1017.33852978541 1410.67491727421
TCGA-E7-A97P-01A-11R-A38B-07 127.32569362289 1733.07872608373 1860.40441970662
TCGA-GU-A767-01A-11R-A32O-07 -357.043029549337 -98.4177505040322 -455.460780053369
TCGA-4Z-AA81-01A-11R-A39I-07 -1264.40528741215 811.527374162421 -452.877913249733
TCGA-2F-A9KQ-01A-11R-A38B-07 -1210.75210430486 -567.434583695289 -1778.18668800015
TCGA-SY-A9G0-01A-12R-A38B-07 1111.70720788801 705.648509389613 1817.35571727762
TCGA-GV-A40G-01A-11R-A23N-07 -1813.09368629993 -652.233408321241 -2465.32709462117
TCGA-FD-A43Y-01A-21R-A26T-07 77.8547350270306 805.939846248049 883.79458127508
TCGA-S5-AA26-01A-11R-A38B-07 -1876.11556238347 -584.273507501117 -2460.38906988459
TCGA-BT-A42E-01A-11R-A23W-07 -496.34046658957 1699.1688433453 1202.82837675573
TCGA-FD-A43X-01A-11R-A23W-07 -1790.5637959364 -499.391270154646 -2289.95506609105
TCGA-ZF-AA4R-01A-11R-A38B-07 167.080833005086 -112.660018954096 54.4208140509901
TCGA-BT-A20T-01A-11R-A14Y-07 -43.5489793141607 782.71288247368 739.163903159519
TCGA-E7-A7DU-01A-11R-A32O-07 -1532.31573398478 -177.180169060665 -1709.49590304545
TCGA-DK-A3WY-01A-11R-A22U-07 877.070572766707 3046.43663439239 3923.5072071591
TCGA-G2-A2EF-01A-12R-A18C-07 -664.77684773964 1658.84309640537 994.066248665731
TCGA-BT-A20V-01A-11R-A14Y-07 -1647.91122358842 475.648345572327 -1172.26287801609
TCGA-K4-A3WV-01A-11R-A22U-07 -1350.80144022128 -827.785022119772 -

2178.58646234105
TCGA-GU-AATQ-01A-11R-A39I-07 -1069.43577823296 159.550819738715 -
909.884958494244
TCGA-G2-A3VY-01A-11R-A22U-07 -2049.17595147835 -988.817753449597 -
3037.99370492795
TCGA-XF-AAN8-01A-11R-A42T-07 2133.42545753471 2204.50551055213
4337.93096808684
TCGA-CU-A3QU-01A-11R-A22U-07 -2026.62150968583 -382.080452689975 -
2408.70196237581
TCGA-GD-A3OP-01A-21R-A220-07 18.6098669609692 343.478466657246
362.088333618215
TCGA-KQ-A41P-01A-12R-A33J-07 -76.0761802569038 541.42076544462
465.344585187716
TCGA-UY-A9PH-01A-11R-A38B-07 237.8776800423 2027.42427611512
2265.30195615742
TCGA-FD-A5BY-01A-31R-A28M-07 -180.540968255667 437.070438985763
256.529470730097
TCGA-K4-A3WU-01B-11R-A23N-07184.201182198523 1561.5199324101
1745.72111460862
TCGA-FD-A3SJ-01A-12R-A22U-07 -205.679743527125 12.9152543578159 -
192.764489169309
TCGA-FD-A3B5-01A-11R-A20F-07 -766.638014061358 -247.387340107271 -
1014.02535416863
TCGA-GC-A6I3-01A-11R-A31N-07 -204.899418715885 1659.14233478994
1454.24291607405
TCGA-4Z-AA80-01A-11R-A39I-07 -1708.05124654491 -462.830235248841 -
2170.88148179375
TCGA-G2-A3IE-01A-11R-A20F-07 -1340.96313298937 -122.958523718651 -
1463.92165670802
TCGA-ZF-A9R0-01A-11R-A38B-07 -607.693984943553 394.330448394794 -
213.36353654876
TCGA-FD-A43S-01A-21R-A23W-07 814.736949704908 2279.04329522962
3093.78024493453
TCGA-XF-A8HG-01A-11R-A36F-07 -1634.78198714386 -475.927531332647 -
2110.70951847651
TCGA-K4-A83P-01A-11R-A352-07 1137.63409297628 2526.22654573794
3663.86063871422
TCGA-GD-A2C5-01A-12R-A180-07 -187.87193619422 51.2987186878716 -
136.573217506348
TCGA-XF-AAMX-01A-11R-A42T-07 -375.471416658761 -239.550048645797 -
615.021465304558
TCGA-DK-A1AE-01A-11R-A13Y-07 -1228.79321888643 -360.855149512982 -
1589.64836839942
TCGA-FJ-A3ZE-01A-11R-A23N-07 -2032.58769776254 -1008.63136943982 -

3041.21906720236
TCGA-DK-A2I1-01A-11R-A180-07 808.297604644903 1245.28264352501
2053.58024816991
TCGA-DK-A6B0-01A-11R-A31N-07 -1727.93319948494 -211.279921538154 -
1939.21312102309
TCGA-FD-A5BR-01A-11R-A26T-07 -136.148837856608 1052.95805430287
916.809216446266
TCGA-KQ-A41Q-01A-11R-A33J-07 -1406.93874253132 -404.835818999476 -
1811.77456153079
TCGA-DK-A1AF-01A-11R-A13Y-07 1233.75697832623 1749.28436048849
2983.04133881472
TCGA-LT-A5Z6-01A-11R-A28M-07 -1795.20718002432 74.9059896058723 -
1720.30119041844
TCGA-GC-A4ZW-01A-11R-A26T-07 -1658.71878958017 -542.020986439372 -
2200.73977601954
TCGA-K4-A4AB-01B-12R-A28M-07 162.191502421529 553.239262383828
715.430764805357
TCGA-DK-AA74-01A-11R-A39I-07 1432.21182713722 2384.66188894924
3816.87371608646
TCGA-G2-A2EO-01A-11R-A180-07 481.806384842526 1423.97030880791
1905.77669365044
TCGA-DK-A1AG-01A-11R-A13Y-07 -1748.76475523567 194.117419692186 -
1554.64733554348
TCGA-GV-A3JZ-01A-11R-A21D-07 -360.685738450838 124.840672904817 -
235.845065546021
TCGA-XF-A9SV-01A-21R-A42T-07 174.011494206248 -57.1817035516831
116.829790654565
TCGA-UY-A78K-01A-11R-A33J-07 -519.052299967316 1803.75858577481
1284.70628580749
TCGA-FD-A6TH-01A-11R-A32O-07 32.6496987390192 157.024345303011
189.67404404203
TCGA-DK-AA6W-01A-12R-A39I-07 -1173.55681199002 -63.586955616615 -
1237.14376760663
TCGA-CF-A3MG-01A-11R-A20F-07 -2140.71393130213 -594.644819164725 -
2735.35875046685
TCGA-DK-A3X1-01A-12R-A22U-07 -523.919944123092 119.594396716374 -
404.325547406718
TCGA-DK-A1A6-01A-11R-A13Y-07 -459.520824821251 976.249269605633
516.728444784382
TCGA-XF-AAMG-01A-11R-A42T-07 -177.348278351586 -12.6414663308286 -
189.989744682415
TCGA-E7-A541-01A-11R-A26T-07 -812.952266735791 1269.41313334994
456.460866614154
TCGA-XF-A8HE-01A-11R-A36F-07 -256.606839648727 1334.74874129075

1078.14190164202
TCGA-DK-AA6Q-01A-11R-A39I-07 -1509.46478970951 1086.97023901499 -
422.49455069452
TCGA-UY-A78N-01A-12R-A33J-07 -1172.83468229566 -869.392270788331 -
2042.22695308399
TCGA-DK-AA75-01A-11R-A39I-07 -1882.35491414549 -727.72111841558 -
2610.07603256107
TCGA-ZF-AA5P-01A-11R-A39I-07 732.775059977352 949.890897980438
1682.66595795779
TCGA-GC-A3WC-01A-31R-A22U-07 -167.17239431638 1757.86732560138
1590.694931285
TCGA-K4-A4AC-01A-21R-A26T-07 -523.945764548176 966.501407401279
442.555642853104
TCGA-FD-A3SL-01A-21R-A22U-07 1129.88984900382 753.399900106675
1883.28974911049
TCGA-K4-A5RH-01A-11R-A30C-07 891.464277888764 2493.92868210705
3385.39295999582
TCGA-FD-A5C1-01A-11R-A28M-07 701.105619756801 1805.82659109374
2506.93221085054
TCGA-BL-A13J-01A-11R-A277-07 344.380747623484 -42.5649875159941
301.81576010749
TCGA-XF-AAN7-01A-11R-A42T-07 -154.731999344926 63.1241284988687 -
91.607870846057
TCGA-2F-A9KO-01A-11R-A38B-07 -114.753831166899 1490.74693725784
1375.99310609094
TCGA-DK-A3IM-01A-11R-A20F-07 -1737.41573368933 -639.715983535885 -
2377.13171722522
TCGA-FD-A3SS-01A-12R-A22U-07 -958.72325079688 -406.527065853809 -
1365.25031665069
TCGA-4Z-AA7N-01A-11R-A39I-07 826.302726508366 2591.45437520603
3417.75710171439
TCGA-HQ-A2OE-01A-11R-A206-07 -1745.33654133416 -390.552031005961 -
2135.88857234012
TCGA-UY-A8OC-01A-11R-A36F-07 350.970042512313 328.515162659298
679.485205171611
TCGA-GV-A3JV-01A-11R-A220-07 -177.653164313987 632.429044599162
454.775880285175
TCGA-XF-A9T8-01A-11R-A39I-07 369.017844327711 1995.76792593932
2364.78577026703
TCGA-KQ-A41S-01A-12R-A33J-07 353.971710952567 303.416326172519
657.388037125085
TCGA-CU-A3YL-01A-11R-A22U-07 -961.922583244866 -109.762621998064 -
1071.68520524293
TCGA-E7-A7XN-01A-11R-A352-07 -895.821398563133 1911.59250164216

1015.77110307902
TCGA-DK-A2HX-01A-12R-A18C-07 974.057304470224 293.222654677199
1267.27995914742
TCGA-CF-A8HY-01A-11R-A36F-07 -1612.82895106668 -88.5171415211215 -
1701.3460925878
TCGA-4Z-AA7W-01A-11R-A39I-07 -58.4117668942496 2475.46711419258
2417.05534729833
TCGA-FD-A43U-01A-11R-A23W-07 1033.45329488401 2301.74441945265
3335.19771433666
TCGA-BL-A0C8-01A-11R-A277-07 -449.592246309703 -122.304480982962 -
571.896727292665
TCGA-BT-A0S7-01A-11R-A10U-07 -464.777068420294 -67.8764696476256 -
532.653538067919
TCGA-ZF-A9R3-01A-11R-A38B-07 -1182.91022840462 253.290712694302 -
929.619515710321
TCGA-4Z-AA89-01A-11R-A39I-07 -2158.294363701 -57.7930899753459 -
2216.08745367635
TCGA-GU-A42P-01A-11R-A23W-07 -1909.34791982124 -959.417681808155 -
2868.7656016294
TCGA-FD-A3NA-01A-11R-A21D-07 -321.773283396217 698.017697206538
376.244413810321
TCGA-XF-A9SL-01A-11R-A39I-07 1105.16162020127 2044.32635681624
3149.48797701752
TCGA-XF-AAN2-01A-11R-A42T-07 -901.798831236067 948.311875314414
46.5130440783471
TCGA-ZF-AA54-01A-11R-A39I-07 873.727549304878 1762.36268053392
2636.0902298388
TCGA-GU-A766-01A-11R-A32O-07 242.011714770769 2421.33416777284
2663.34588254361
TCGA-FD-A6TD-01A-51R-A33J-07 286.947985167223 1631.53527030588
1918.4832554731
TCGA-FD-A3B4-01A-12R-A206-07 147.647213485489 1364.43974608364
1512.08695956913
TCGA-DK-A1AB-01A-11R-A13Y-07 904.627106195868 979.728693227383
1884.35579942325
TCGA-BT-A20X-01A-11R-A16R-07 -223.947970053036 459.802028745757
235.854058692721
TCGA-GV-A3JW-01A-11R-A20F-07 -1546.02268516892 -981.624590791572 -
2527.64727596049
TCGA-UY-A9PB-01A-11R-A38B-07 763.072803490882 1939.60988445854
2702.68268794942
TCGA-FD-A6TI-01A-11R-A32O-07 -693.920231777971 -21.6333481950401 -
715.553579973011
TCGA-XF-AAML-01A-11R-A42T-07 -1949.33197322945 245.007207086883 -

1704.32476614256
TCGA-ZF-AA58-01A-12R-A42T-07 832.421028511303 2408.90926962996 3241.33029814126
TCGA-FD-A3SP-01A-31R-A22U-07 1689.58127151147 1927.84872828072 3617.42999979219
TCGA-E7-A97Q-01A-11R-A38B-07 -152.857801951363 308.461824408523 155.60402245716
TCGA-YC-A9TC-01A-22R-A39I-07 -931.067500488275 -161.159956289126 -1092.2274567774
TCGA-GV-A3QG-01A-11R-A220-07 1357.86136405346 2695.53252943873 4053.39389349219
TCGA-K4-A6FZ-01A-11R-A31N-07 -909.570806340159 921.416710885304 11.845904545145
TCGA-KQ-A41R-01A-21R-A352-07 -1303.46774622012 -347.870294916384 -1651.33804113651
TCGA-DK-AA6M-01A-11R-A39I-07 -11.3999136635055 1639.37948641968 1627.97957275618
TCGA-ZF-AA52-01A-12R-A39I-07 846.292363366363 1078.71789570361 1925.01025906998
TCGA-K4-A5RJ-01A-11R-A28M-07 478.80077242076 2801.215369322 3280.01614174276
TCGA-HQ-A5ND-01A-11R-A26T-07 -1677.70079274035 -151.747050054836 -1829.44784279519
TCGA-BT-A3PK-01A-21R-A220-07 306.16329258565 1002.16901400892 1308.33230659457
TCGA-FD-A3N6-01A-11R-A21D-07 -1046.85996799916 323.828665629366 -723.031302369793
TCGA-UY-A78P-01A-12R-A36F-07 201.816430034873 1991.65980853899 2193.47623857386
TCGA-CF-A9FL-01A-11R-A38B-07 -352.97179372608 24.1416699230391 -328.830123803041
TCGA-CF-A9FM-01A-11R-A38B-07 -1518.77480299465 227.322721063996 -1291.45208193066
TCGA-ZF-A9RD-01A-11R-A42T-07 -193.121943129963 1848.44094266946 1655.31899953949
TCGA-G2-AA3D-01A-11R-A39I-07 -2081.87527012191 -342.514907955678 -2424.39017807759
TCGA-DK-A2I2-01A-11R-A180-07 248.762378948983 1599.57107133301 1848.33345028199
TCGA-FD-A62S-01A-11R-A30C-07 1228.01053955981 1430.42634501072 2658.43688457054
TCGA-MV-A51V-01A-11R-A26T-07 -1224.20316892502 -552.797718192875 -1777.0008871179
TCGA-E5-A2PC-01A-11R-A206-07 -1523.62718741926 452.595072385873 -

1071.03211503339
TCGA-DK-A3X2-01A-11R-A22U-07 -1794.67618515797 -937.914934425342 -
2732.59111958331
TCGA-GD-A76B-01A-11R-A32O-07 -718.44362776124 1396.59807991946
678.154452158218
TCGA-GC-A6I1-01A-12R-A31N-07 -166.131682816432 2365.04898254227
2198.91729972584
TCGA-BL-A13J-01B-04R-A277-07 -202.208910011313 96.3841148638609 -
105.824795147453
TCGA-XF-A9SK-01A-11R-A42T-07 745.382465182645 1242.28337236767
1987.66583755031
TCGA-DK-A1AD-01A-11R-A13Y-07 -331.0121845321 310.168349833909 -
20.8438346981912
TCGA-K4-A5RI-01A-11R-A28M-07 -566.691747384443 228.556556143882 -
338.135191240561
TCGA-FD-A3SQ-01A-21R-A22U-07 803.541658254063 891.756762782574
1695.29842103664
TCGA-GV-A3QF-01A-31R-A22U-07 -1693.48975167865 -428.639944514986 -
2122.12969619363
TCGA-E7-A7DV-01A-11R-A33J-07 595.294169019933 2109.71002106544
2705.00419008537
TCGA-XF-AAN1-01A-31R-A42T-07 -1453.78713063721 -126.442477107213 -
1580.22960774442
TCGA-DK-AA6P-01A-11R-A39I-07 -1605.05876552268 -782.593791136675 -
2387.65255665935
TCGA-CF-A47Y-01A-11R-A23W-07 -1624.68248148741 -376.218007364147 -
2000.90048885155
TCGA-CF-A3MI-01A-11R-A20F-07 -1024.21735865767 347.571424696541 -
676.645933961132
TCGA-G2-A2EL-01A-12R-A18C-07 -2040.28584853238 -1568.14746083726 -
3608.43330936964
TCGA-XF-AAMZ-01A-11R-A42T-07 -973.872086697145 314.327715836028 -
659.544370861117
TCGA-DK-AA6L-01A-11R-A39I-07 -923.02319102306 831.704845498504 -
91.3183455245564
TCGA-XF-A9T4-01A-11R-A39I-07 -718.018002739846 635.46679586265 -
82.5512068771961
TCGA-K4-A6MB-01A-11R-A31N-07 -433.891083441411 -192.608514448187 -
626.499597889598
TCGA-UY-A8OB-01A-12R-A42T-07 -1272.65995314996 580.526628831692 -
692.133324318265
TCGA-ZF-A9R5-01A-12R-A42T-07 -1723.28754754897 -75.5974073801236 -
1798.88495492909
TCGA-CU-A3KJ-01A-11R-A21D-07 -1045.76657443499 436.616766778907 -

609.14980765608
TCGA-DK-AA6S-01A-21R-A39I-07 778.818925387025 1141.69148126021 1920.51040664724
TCGA-E7-A85H-01A-11R-A352-07 -534.715271573857 608.701823472203 73.986551898346
TCGA-G2-AA3C-01A-21R-A39I-07 -17.8576771025931 2029.08106918844 2011.22339208585
TCGA-E7-A8O8-01A-11R-A36F-07 -1417.88701186195 -204.305748354888 -1622.19276021684
TCGA-XF-A9SM-01A-11R-A42T-07 666.28377128677 2459.52995822561 3125.81372951238
TCGA-DK-A3IN-01A-11R-A20F-07 481.799794022937 1027.67921867083 1509.47901269377
TCGA-XF-A8HC-01A-11R-A36F-07 -2017.84906652496 -932.863744983032 -2950.71281150799
TCGA-ZF-AA4W-01A-12R-A38B-07 -1278.47459226969 6.70580173231864 -1271.76879053737
TCGA-LC-A66R-01A-41R-A30C-07 -267.877519335175 1537.19868726588 1269.32116793071
TCGA-E7-A8O7-01A-11R-A36F-07 -1312.76049886002 651.163964501938 -661.59653435808
TCGA-LT-A8JT-01A-11R-A36F-07 -1481.12956791891 173.191911923928 -1307.93765599498
TCGA-FJ-A3Z9-01A-11R-A26T-07 -1931.44448339208 -721.075810155327 -2652.52029354741
TCGA-DK-A1AC-01A-11R-A13Y-07 8.80091179662419 953.785396065404 962.586307862028
TCGA-C4-A0F1-01A-11R-A034-07 -368.222923983684 321.679364037092 -46.5435599465918
TCGA-G2-A2ES-01A-11R-A180-07 -27.312057183619 862.008122716481 834.696065532862
TCGA-XF-AAN4-01A-11R-A42T-07 1629.95268748855 2066.68853911871 3696.64122660726
TCGA-4Z-AA83-01A-11R-A39I-07 -833.78110129157 469.588299768067 -364.192801523502
TCGA-E7-A5KE-01A-11R-A28M-07 -2171.9519261966 -953.389035873166 -3125.34096206977
TCGA-ZF-AA4V-01A-11R-A38B-07 -449.691856880621 859.659773780432 409.967916899811
TCGA-ZF-AA53-01A-11R-A39I-07 656.076765225657 1689.96893904514 2346.04570427079
TCGA-ZF-A9RM-01A-11R-A38B-07 -2485.34263522628 -853.289509848077 -3338.63214507436
TCGA-BL-A13I-01A-11R-A13Y-07 745.797096263816 2226.70862514418

2972.50572140799
TCGA-CU-A72E-01A-12R-A33J-07 -148.207798363489 294.732762849119 146.52496448563
TCGA-2F-A9KR-01A-11R-A38B-07 -1435.12042202624 -210.880182178551 -1646.00060420479
TCGA-E5-A4U1-01A-11R-A31N-07 -2037.85348988987 -936.04058380524 -2973.89407369511
TCGA-E7-A3Y1-01A-11R-A22U-07 -1269.25135970556 -107.626011827154 -1376.87737153271
TCGA-FD-A6TC-01A-21R-A33J-07 296.619442099809 140.138506476349 436.757948576158
TCGA-FD-A5BV-01A-11R-A26T-07 -1058.56180205714 -452.073388791714 -1510.63519084886
TCGA-DK-A2I4-01A-11R-A21D-07 1199.40745230096 2758.49038399081 3957.89783629177
TCGA-UY-A78M-01A-21R-A352-07 -300.033173084078 -233.22784561422 -533.261018698298
TCGA-4Z-AA7R-01A-11R-A39I-07 -948.698466618074 -654.90810916688 -1603.60657578495
TCGA-E7-A519-01A-11R-A26T-07 -781.430640349037 1313.26313642851 531.83249607947
TCGA-DK-A3IU-01A-11R-A20F-07 917.344709126541 2730.25526296488 3647.59997209142
TCGA-FD-A3SM-01A-11R-A22U-07 154.036706669144 -40.5038960108056 113.532810658339
TCGA-BL-A5ZZ-01A-31R-A30C-07 935.830069132174 1253.64892432889 2189.47899346107
TCGA-FD-A62N-01A-11R-A30C-07 1360.71434562603 3006.37526757426 4367.0896132003
TCGA-BT-A20J-01A-11R-A14Y-07 -325.973402452671 1766.75835208646 1440.78494963379
TCGA-DK-AA71-01A-31R-A39I-07 -1247.75247983422 -220.522373868609 -1468.27485370283
TCGA-KQ-A41O-01A-12R-A352-07 -2502.81683610988 -1426.77319398445 -3929.59003009433
TCGA-BL-A13I-01A-11R-A277-07 777.129374010517 2073.58085361043 2850.71022762095
TCGA-E7-A677-01A-11R-A30C-07 -1077.47670300041 167.589549739366 -909.887153261041
TCGA-DK-A1A5-01A-11R-A13Y-07 334.520173901917 384.891273091952 719.411446993869
TCGA-HQ-A5NE-01A-12R-A28M-07 -436.961017941409 354.578787699562 -82.3822302418473
TCGA-XF-AAMT-01A-11R-A42T-07 849.711835524479 1037.36319251755

1887.07502804203
TCGA-XF-A9SU-01A-31R-A39I-07 455.149957590174 961.231276715512 1416.38123430569
TCGA-CF-A47S-01A-11R-A23W-07 -1481.40518224087 267.163316288429 -1214.24186595244
TCGA-FD-A6TK-01A-42R-A33J-07 904.790348518546 1598.65569786854 2503.44604638708
TCGA-E7-A5KF-01A-11R-A28M-07 -2385.07344194345 -526.199074894634 -2911.27251683808
TCGA-UY-A78L-01A-12R-A33J-07 -233.36122727095 226.736692537261 -6.62453473368876
TCGA-S5-A6DX-01A-11R-A31N-07 362.704896270794 1887.87861577723 2250.58351204802
TCGA-XF-A8HI-01A-11R-A38B-07 -1526.667462905 28.5389557869321 -1498.12850711807
TCGA-XF-A9T5-01A-11R-A42T-07 109.353541738119 2144.473106133 2253.82664787111
TCGA-XF-A9SX-01A-21R-A39I-07 977.305618013402 1911.62828751059 2888.93390552399
TCGA-G2-A2EK-01A-22R-A18C-07 -896.109348200544 344.419676264481 -551.689671936063
TCGA-XF-A8HD-01A-11R-A36F-07 -255.093238502541 1428.60975449535 1173.5165159928
TCGA-GV-A3JX-01A-11R-A20F-07 -1270.51880290438 627.91602032096 -642.602782583424
TCGA-H4-A2HQ-01A-11R-A180-07 -1727.80728318017 -333.128808351955 -2060.93609153213
TCGA-XF-A9SP-01A-11R-A39I-07 941.019599083924 849.81682449322 1790.83642357714
TCGA-DK-A3IK-01A-32R-A21D-07 -394.154105026893 -11.2664414147934 -405.420546441686
