# Supplementary material for: Ferroptosis-Related Long Noncoding RNAs Have Excellent Predictive Ability for Multiomic Characteristics of Bladder Cancer
Source: Oxid Med Cell Longev. 2022 Aug 29;2022:9316847. doi: 10.1155/2022/9316847 (PMC9444476; doi:10.1155/2022/9316847)
Supplement: Supplementary Materials — Supplementary Table 1: expression matrix of 60 ferroptosis-related genes. Supplementary Table 2: results for coexpression analysis of ferroptosis-related lncRNAs and mRNAs. Supplementary Table 3: expression of 263 ferroptosis-related lncRNAs. Supplementary Table 4: expression of lncRNAs and clinical information of 403 bladder cancer samples. Supplementary Table 5: cluster analysis based on 20 prognostic lncRNAs. Supplementary Table 6: detailed clinical information of bladder cancer samples. Supplementary Table 7: data of the high- and low-risk groups of the training cohort. Supplementary Table 8: data of the high- and low-risk groups of the validation cohort. Supplementary Table 9: markers of different immune cells. Supplementary Table 10: infiltration of 22 types of immune cells analysed using the CIBERSORT algorithm. Supplementary Table 11: immune scores evaluated using the ESTIMATE algorithm. Supplementary Table 12: immunotherapeutic response evaluated using the Tumour Immune Dysfunction and Exclusion (TIDE) algorithm. Supplementary Figure 1: gene set enrichment analysis. (A–F) Most significantly enriched biological pathways in cluster 1. (G–I) Most significantly enriched biological pathways in cluster 2. [file 9316847.f1.zip › s-table 12.pdf]

| id | Overall Response |
| --- | --- |
| TCGA-2F-A9KL | Responder |
| TCGA-2F-A9KO | Responder |
| TCGA-2F-A9KP | Responder |
| TCGA-2F-A9KQ | Responder |
| TCGA-2F-A9KR | Non responder |
| TCGA-4Z-AA7M | Responder |
| TCGA-4Z-AA7N | Responder |
| TCGA-4Z-AA7Q | Responder |
| TCGA-4z-AA7S | Non responder |
| TCGA-4Z-AA7W | Responder |
| TCGA-4Z-AA7Y | Non responder |
| TCGA-4Z-AA80 | Non responder |
| TCGA-4Z-AA81 | Responder |
| TCGA-4Z-AA82 | Responder |
| TCGA-4Z-AA83 | Responder |
| TCGA-4Z-AA84 | Responder |
| TCGA-4Z-AA86 | Responder |
| TCGA-4Z-AA89 | Non responder |
| TCGA-5N-A9KI | Responder |
| TCGA-5N-A9KN | Responder |
| TCGA-BL-A0C8 | Non responder |
| TCGA-BL-A13I | Responder |
| TCGA-BL-A13J | Responder |
| TCGA-BL-A3JM | Non responder |
| TCGA-BL-A5ZZ | Responder |
| TCGA-BT-A0S7 | Responder |
| TCGA-BT-A0YX | Responder |
| TCGA-BT-A20J | Non responder |
| TCGA-BT-A20O | Responder |
| TCGA-BT-A20P | Responder |
| TCGA-BT-A20R | Responder |
| TCGA-BT-A20T | Responder |
| TCGA-BT-A20U | Responder |
| TCGA-BT-A20V | Non responder |
| TCGA-BT-A20W | Non responder |
| TCGA-BT-A2LB | Responder |
| TCGA-BT-A2ON | Responder |
| TCGA-BT-A3PH | Responder |
| TCGA-BT-A3PJ | Responder |
| TCGA-BT-A3PK | Responder |
| TCGA-BT-A42C | Non responder |
| TCGA-BT-A42E | Responder |
| TCGA-BT-A42F | Non responder |

| | |
|---|---|
| TCGA-BT-Z20X | Non responder |
| TCGA-C4_A0F6 | Responder |
| TCGA-C4-A0F0 | Non responder |
| TCGA-C4-A0F1 | Responder |
| TCGA-CF-A1HR | Non responder |
| TCGA-CF-A1HS | Responder |
| TCGA-CF-A27C | Non responder |
| TCGA-CF-A3MH | Responder |
| TCGA-CF-A3MI | Responder |
| TCGA-CF-A47S | Non responder |
| TCGA-CF-A47T | Responder |
| TCGA-CF-A47V | Non responder |
| TCGA-CF-A47W | Responder |
| TCGA-CF-A47Y | Responder |
| TCGA-CF-A5U8 | Non responder |
| TCGA-CF-A5UA | Responder |
| TCGA-CF-A710 | Responder |
| TCGA-CF-A8HX | Non responder |
| TCGA-CF-A8HY | Responder |
| TCGA-CF-A9FF | Responder |
| TCGA-CF-A9FL | Responder |
| TCGA-CF-A9FM | Non responder |
| TCGA-CU-A0YN | Non responder |
| TCGA-CU-A0YO | Responder |
| TCGA-CU-A0YR | Non responder |
| TCGA-CU-A3KJ | Non responder |
| TCGA-CU-A3QU | Non responder |
| TCGA-CU-A3YL | Non responder |
| TCGA-CU-A5W6 | Non responder |
| TCGA-CU-A72E | Responder |
| TCGA-CV-A3JW | Non responder |
| TCGA-DK-A1A3 | Non responder |
| TCGA-DK-A1A6 | Non responder |
| TCGA-DK-A1A7 | Non responder |
| TCGA-DK-A1AA | Responder |
| TCGA-DK-A1AB | Responder |
| TCGA-DK-A1AC | Non responder |
| TCGA-Dk-A1AD | Responder |
| TCGA-DK-A1AE | Responder |
| TCGA-DK-A1AF | Responder |
| TCGA-DK-A2HX | Responder |
| TCGA-DK-A2I1 | Responder |
| TCGA-DK-A2I2 | Responder |
| TCGA-DK-A2I4 | Responder |

| | |
|---|---|
| TCGA-DK-A2I6 | Responder |
| TCGA-DK-A3IK | Responder |
| TCGA-DK-A3IM | Responder |
| TCGA-DK-A3IN | Responder |
| TCGA-DK-A3IQ | Responder |
| TCGA-DK-A3IS | Non responder |
| TCGA-DK-A3IT | Responder |
| TCGA-DK-A3IU | Responder |
| TCGA-DK-A3IV | Non responder |
| TCGA-DK-A3WH | Responder |
| TCGA-DK-A3WW | Responder |
| TCGA-DK-A3WX | Responder |
| TCGA-DK-A3X1 | Responder |
| TCGA-DK-A3X2 | Responder |
| TCGA-DK-A6AV | Non responder |
| TCGA-Dk-A6AW | Responder |
| TCGA-DK-A6B0 | Responder |
| TCGA-DK-A6B2 | Responder |
| TCGA-DK-A6B5 | Non responder |
| TCGA-DK-A6B6 | Non responder |
| TCGA-DK-A6BI | Non responder |
| TCGA-DK-AA6L | Responder |
| TCGA-DK-AA6P | Non responder |
| TCGA-DK-AA6Q | Responder |
| TCGA-DK-AA6R | Responder |
| TCGA-DK-AA6S | Responder |
| TCGA-DK-AA6T | Responder |
| TCGA-DK-AA6U | Non responder |
| TCGA-DK-AA6W | Non responder |
| TCGA-DK-AA71 | Responder |
| TCGA-DK-AA74 | Responder |
| TCGA-DK-AA75 | Responder |
| TCGA-DK-AA76 | Non responder |
| TCGA-Dk-AA77 | Non responder |
| TCGA-E5-A2PC | Responder |
| TCGA-E5-A4TZ | Non responder |
| TCGA-E5-A4U1 | Non responder |
| TCGA-E7-A3X6 | Responder |
| TCGA-E7-A3Y1 | Responder |
| TCGA-E7-A4IJ | Non responder |
| TCGA-E7-A4XJ | Responder |
| TCGA-E7-A519 | Responder |
| TCGA-E7-A541 | Responder |
| TCGA-E7-A5KE | Non responder |

| | |
|---|---|
| TCGA-E7-A5KF | Non responder |
| TCGA-E7-A677 | Responder |
| TCGA-E7-A678 | Responder |
| TCGA-E7-A6MD | Responder |
| TCGA-E7-A6ME | Non responder |
| TCGA-E7-A6MF | Non responder |
| TCGA-E7-A763 | Responder |
| TCGA-E7-A7DU | Responder |
| TCGA-E7-A7DV | Responder |
| TCGA-E7-A7PW | Non responder |
| TCGA-E7-A7XN | Responder |
| TCGA-E7-A807 | Non responder |
| TCGA-E7-A85H | Non responder |
| TCGA-E7-A97P | Responder |
| TCGA-FD-A3B3 | Responder |
| TCGA-FD-A3B4 | Responder |
| TCGA-FD-A3B5 | Responder |
| TCGA-FD-A3B6 | Responder |
| TCGA-FD-A3B7 | Responder |
| TCGA-FD-A3B8 | Responder |
| TCGA-FD-A3N5 | Responder |
| TCGA-FD-A3N6 | Non responder |
| TCGA-FD-A3NA | Responder |
| TCGA-FD-A3SI | Responder |
| TCGA-FD-A3SM | Responder |
| TCGA-FD-A3SN | Responder |
| TCGA-FD-A3SO | Responder |
| TCGA-FD-A3SP | Responder |
| TCGA-FD-A3SQ | Responder |
| TCGA-FD-A3SR | Responder |
| TCGA-FD-A3SS | Responder |
| TCGA-FD-A43N | Responder |
| TCGA-FD-A43S | Responder |
| TCGA-FD-A43U | Responder |
| TCGA-FD-A43X | Responder |
| TCGA-FD-A43Y | Responder |
| TCGA-FD-A5BS | Responder |
| TCGA-FD-A5BT | Responder |
| TCGA-FD-A5BU | Responder |
| TCGA-FD-A5BX | Responder |
| TCGA-FD-A5BY | Responder |
| TCGA-FD-A5BZ | Responder |
| TCGA-FD-A5CI | Responder |
| TCGA-FD-A62O | Responder |

| | |
|---|---|
| TCGA-FD-A62P | Responder |
| TCGA-FD-A62S | Responder |
| TCGA-FD-A6TA | Responder |
| TCGA-FD-A6TC | Responder |
| TCGA-FD-A6TD | Responder |
| TCGA-FD-A6TE | Non responder |
| TCGA-FD-A6TF | Responder |
| TCGA-FD-A6TG | Responder |
| TCGA-FD-A6TH | Responder |
| TCGA-FD-A6TI | Responder |
| TCGA-FD-A6Tk | Responder |
| TCGA-FD-A9RC | Responder |
| TCGA-FJ-A3JZ | Responder |
| TCGA-FJ-A3Z9 | Responder |
| TCGA-FJ-A3ZE | Non responder |
| TCGA-FJ-A3ZF | Non responder |
| TCGA-FJ-A871 | Responder |
| TCGA-FT-A3EE | Responder |
| TCGA-FT-A6IP | Responder |
| TCGA-G2-A2EC | Responder |
| TCGA-G2-A2EJ | Responder |
| TCGA-G2-A2EK | Non responder |
| TCGA-G2-A2EO | Non responder |
| TCGA-G2-A2ES | Non responder |
| TCGA-G2-A3IB | Responder |
| TCGA-G2-A3IE | Responder |
| TCGA-G2-A3VY | Non responder |
| TCGA-G2-AA3B | Non responder |
| TCGA-G2-AA3C | Responder |
| TCGA-G2-AA3D | Non responder |
| TCGA-G2-AA3F | Non responder |
| TCGA-G4-A0EZ | Responder |
| TCGA-GC-A3BM | Non responder |
| TCGA-GC-A3I6 | Responder |
| TCGA-GC-A3OO | Responder |
| TCGA-GC-A3RB | Responder |
| TCGA-GC-A3RC | Responder |
| TCGA-GC-A3RD | Responder |
| TCGA-GC-A3WC | Responder |
| TCGA-GC-A3YS | Responder |
| TCGA-GC-A4ZW | Non responder |
| TCGA-GC-A6I1 | Responder |
| TCGA-GC-A6I3 | Responder |
| TCGA-GD-A2C5 | Responder |

| | |
|---|---|
| TCGA-GD-A3OP | Responder |
| TCGA-GD-A3OQ | Responder |
| TCGA-GD-A3OS | Responder |
| TCGA-GD-A6C6 | Responder |
| TCGA-GU-A42P | Non responder |
| TCGA-GU-A42Q | Responder |
| TCGA-GU-A42R | Non responder |
| TCGA-GU-A762 | Responder |
| TCGA-GU-A763 | Responder |
| TCGA-GU-A764 | Responder |
| TCGA-GU-A766 | Responder |
| TCGA-GU-A767 | Responder |
| TCGA-GU-AATO | Responder |
| TCGA-GU-AATP | Responder |
| TCGA-GU-AATQ | Responder |
| TCGA-GV-A3JV | Responder |
| TCGA-Gv-A3JW | Non responder |
| TCGA-GV-A3JX | Non responder |
| TCGA-GV-A3JZ | Responder |
| TCGA-Gv-A3OK | Responder |
| TCGA-Gv-A3QF | Non responder |
| TCGA-GV-A3QI | Non responder |
| TCGA-GV-A3QU | Non responder |
| TCGA-GV-A40E | Responder |
| TCGA-GV-A6ZA | Non responder |
| TCGA-H4-A2HO | Non responder |
| TCGA-HQ-A20F | Non responder |
| TCGA-HQ-A2OE | Non responder |
| TCGA-HQ-A41R | Responder |
| TCGA-HQ-A5NE | Responder |
| TCGA-K4-A3WS | Responder |
| TCGA-K4-A3WU | Responder |
| TCGA-k4-A3WV | Non responder |
| TCGA-K4-A4AB | Responder |
| TCGA-K4-A4AC | Responder |
| TCGA-K4-A54R | Responder |
| TCGA-K4-A5RH | Responder |
| TCGA-K4-A5RI | Responder |
| TCGA-K4-A5RJ | Responder |
| TCGA-K4-A6FZ | Non responder |
| TCGA-K4-A6MB | Responder |
| TCGA-K4-A83P | Responder |
| TCGA-K4-AAQO | Responder |
| TCGA-KQ-A41N | Responder |

| | |
|---|---|
| TCGA-KQ-A41P | Responder |
| TCGA-KQ-A41Q | Non responder |
| TCGA-KQ-A41S | Responder |
| TCGA-KQ-A4IO | Responder |
| TCGA-LC-A66R | Responder |
| TCGA-LT-A5Z6 | Non responder |
| TCGA-LT-A8JT | Responder |
| TCGA-MV-A51V | Non responder |
| TCGA-PQ-A6FI | Non responder |
| TCGA-PQ-A6FN | Non responder |
| TCGA-R3-A69X | Responder |
| TCGA-S5-A6DX | Responder |
| TCGA-S5-AA26 | Responder |
| TCGA-SY-A9G0 | Responder |
| TCGA-SY-A9G5 | Responder |
| TCGA-UY-A78K | Responder |
| TCGA-UY-A78L | Responder |
| TCGA-UY-A78M | Responder |
| TCGA-UY-A78N | Responder |
| TCGA-UY-A78O | Responder |
| TCGA-UY-A78P | Responder |
| TCGA-UY-A8OB | Responder |
| TCGA-UY-A8OC | Responder |
| TCGA-UY-A8OD | Responder |
| TCGA-UY-A9PA | Responder |
| TCGA-UY-A9PB | Responder |
| TCGA-UY-A9PD | Responder |
| TCGA-UY-A9PE | Responder |
| TCGA-UY-A9PF | Responder |
| TCGA-UY-A9PH | Responder |
| TCGA-XF-A8HB | Non responder |
| TCGA-XF-A8HC | Non responder |
| TCGA-XF-A8HD | Responder |
| TCGA-XF-A8HE | Responder |
| TCGA-XF-A8HF | Responder |
| TCGA-XF-A8HG | Responder |
| TCGA-XF-A8HH | Responder |
| TCGA-XF-A8HL | Non responder |
| TCGA-XF-A9R0 | Responder |
| TCGA-XF-A9SH | Responder |
| TCGA-XF-A9SJ | Responder |
| TCGA-XF-A9SK | Responder |
| TCGA-XF-A9SL | Responder |
| TCGA-XF-A9SP | Responder |

| | |
|---|---|
| TCGA-XF-A9ST | Responder |
| TCGA-XF-A9SU | Responder |
| TCGA-XF-A9SV | Responder |
| TCGA-XF-A9SW | Responder |
| TCGA-XF-A9SY | Responder |
| TCGA-XF-A9SZ | Responder |
| TCGA-XF-A9T0 | Non responder |
| TCGA-XF-A9T2 | Responder |
| TCGA-XF-A9T3 | Responder |
| TCGA-XF-A9T4 | Responder |
| TCGA-XF-A9T5 | Responder |
| TCGA-XF-A9T8 | Responder |
| TCGA-XF-AAME | Responder |
| TCGA-XF-AAMH | Responder |
| TCGA-XF-AAMJ | Responder |
| TCGA-XF-AAML | Non responder |
| TCGA-XF-AAMQ | Responder |
| TCGA-XF-AAMR | Responder |
| TCGA-XF-AAMT | Responder |
| TCGA-XF-AAMW | Responder |
| TCGA-XF-AAMY | Responder |
| TCGA-XF-AAN0 | Non responder |
| TCGA-XF-AAN3 | Responder |
| TCGA-XF-AAN4 | Responder |
| TCGA-xF-AAN5 | Non responder |
| TCGA-XF-AAN7 | Responder |
| TCGA-XF-AANI | Responder |
| TCGA-YC-A89H | Responder |
| TCGA-YC-A8S6 | Responder |
| TCGA-YC-A9TC | Responder |
| TCGA-YF-AA3L | Responder |
| TCGA-YF-AA3M | Responder |
| TCGA-ZF-A9R1 | Non responder |
| TCGA-ZF-A9R2 | Non responder |
| TCGA-ZF-A9R3 | Non responder |
| TCGA-ZF-A9R4 | Non responder |
| TCGA-ZF-A9R5 | Non responder |
| TCGA-ZF-A9R7 | Responder |
| TCGA-ZF-A9R9 | Responder |
| TCGA-ZF-A9RD | Responder |
| TCGA-ZF-A9RE | Non responder |
| TCGA-ZF-A9RM | Non responder |
| TCGA-ZF-AA4N | Non responder |
| TCGA-ZF-AA4R | Responder |

| | |
|---|---|
| TCGA-ZF-AA4T | Non responder |
| TCGA-ZF-AA4V | Non responder |
| TCGA-ZF-AA4X | Non responder |
| TCGA-ZF-AA51 | Non responder |
| TCGA-ZF-AA52 | Responder |
| TCGA-ZF-AA53 | Responder |
| TCGA-ZF-AA54 | Responder |
| TCGA-ZF-AA56 | Responder |
| TCGA-ZF-AA58 | Responder |
| TCGA-ZF-AA5H | Responder |
| TCGA-ZF-AA5N | Responder |
| TCGA-ZF-AA5P | Responder |
