# Supplementary material for: Ferroptosis-Related Long Noncoding RNAs Have Excellent Predictive Ability for Multiomic Characteristics of Bladder Cancer
Source: Oxid Med Cell Longev. 2022 Aug 29;2022:9316847. doi: 10.1155/2022/9316847 (PMC9444476; doi:10.1155/2022/9316847)
Supplement: Supplementary Materials — Supplementary Table 1: expression matrix of 60 ferroptosis-related genes. Supplementary Table 2: results for coexpression analysis of ferroptosis-related lncRNAs and mRNAs. Supplementary Table 3: expression of 263 ferroptosis-related lncRNAs. Supplementary Table 4: expression of lncRNAs and clinical information of 403 bladder cancer samples. Supplementary Table 5: cluster analysis based on 20 prognostic lncRNAs. Supplementary Table 6: detailed clinical information of bladder cancer samples. Supplementary Table 7: data of the high- and low-risk groups of the training cohort. Supplementary Table 8: data of the high- and low-risk groups of the validation cohort. Supplementary Table 9: markers of different immune cells. Supplementary Table 10: infiltration of 22 types of immune cells analysed using the CIBERSORT algorithm. Supplementary Table 11: immune scores evaluated using the ESTIMATE algorithm. Supplementary Table 12: immunotherapeutic response evaluated using the Tumour Immune Dysfunction and Exclusion (TIDE) algorithm. Supplementary Figure 1: gene set enrichment analysis. (A–F) Most significantly enriched biological pathways in cluster 1. (G–I) Most significantly enriched biological pathways in cluster 2. [file 9316847.f1.zip › s-table 2.pdf]

Ferroptosis	lncRNA	cor	pvalue	Regulation
IREB2	AC092802.3	0.514996679937929	3.23676273223084e-29	postive
IREB2	AC009095.1	0.566070129117645	3.40344560752245e-36	postive
IREB2	TXNDC12-AS1	0.525928121492263	1.30323221493306e-30	postive
IREB2	AL450998.3	0.551351674465339	4.64550462574244e-34	postive
CRYAB	AC079313.2	0.570135866137488	8.37653473930232e-37	postive
RPL8	ZFAS1	0.581461107724884	1.51946023312867e-38	postive
CRYAB	AC027449.1	0.56148244949498	1.61765851746639e-35	postive
ZEB1	AL035530.2	0.511438828651977	8.98220511589738e-29	postive
SLC7A11	AC004846.2	0.506495920085327	3.63606996998749e-28	postive
ZEB1	AC018752.1	0.572166450223707	4.12855832148133e-37	postive
IREB2	AC018752.1	0.616131728121857	2.53040163994096e-44	postive
IREB2	MIRLET7A1HG	0.507133188503864	3.04016814905431e-28	postive
CRYAB	MIR100HG	0.546844940996223	1.99457377066346e-33	postive
ZEB1	MIR100HG	0.587030001601883	1.99599628714535e-39	postive
IREB2	AC073487.1	0.529282489063559	4.74986722263781e-31	postive
PHKG2	AP001160.1	0.584683297075321	4.71742641781347e-39	postive
IREB2	SSBP3-AS1	0.520356042783259	6.7991893127737e-30	postive
CRYAB	AL139220.2	0.505840727367863	4.36900954631568e-28	postive
ZEB1	AL137779.1	0.582314506282273	1.11609161580677e-38	postive
SLC7A11	AC069277.1	0.602300003075678	6.21433786200243e-42	postive
ZEB1	AC069277.1	0.510679165511573	1.1152073788788e-28	postive
ZEB1	AP001189.3	0.514284158439067	3.97467853498819e-29	postive
IREB2	AC011405.1	0.509243796878104	1.67599418487964e-28	postive
SLC7A11	AL356124.1	0.504714613345113	5.98484036868526e-28	postive
ZEB1	AL356124.1	0.563588512098145	7.93272619087793e-36	postive
ZEB1	FO680682.1	0.514866932130793	3.36022176014369e-29	postive
IREB2	FO680682.1	0.511483470121567	8.86856468482854e-29	postive
FANCD2	THUMPD3-AS1	0.508503679936615	2.06618224593252e-28	postive
RPL8	GAS5	0.521632556286845	4.66948055583239e-30	postive
IREB2	AC073529.1	0.520049601734285	7.4392581684651e-30	postive
SLC7A11	OSMR-AS1	0.543755820614797	5.3469718253479e-33	postive
ZEB1	OSMR-AS1	0.521357888290094	5.06337724218617e-30	postive
SLC7A11	AL162724.2	0.667817898621479	2.09259648843551e-54	postive
ZEB1	AL162724.2	0.692114835576307	6.98065037612683e-60	postive
IREB2	AL162727.2	0.501743066803539	1.36540999177793e-27	postive
CRYAB	AC053503.3	0.588298547149006	1.25015565026792e-39	postive
PHKG2	AC009065.5	0.52741171992004	8.35125606529916e-31	postive
ZEB1	BNC2-AS1	0.508749406195601	1.92759076246913e-28	postive
IREB2	AC078778.1	0.544430527673557	4.31468425827825e-33	postive
ZEB1	AC087286.2	0.596829448158394	5.09217819887294e-41	postive
IREB2	AC087286.2	0.517127139287246	1.74650067336383e-29	postive
IREB2	AL031670.1	0.51720932656162	1.70527481876534e-29	postive
ZEB1	AC022893.1	0.556804786832552	7.73329487146157e-35	postive

IREB2	AC022154.1	0.520118307160932	7.29076092400476e-30	postive
MT1G	AC011472.4	0.858987947396005	5.64170781263485e-121	postive
IREB2	AP001107.1	0.519829589110702	7.93519635689315e-30	postive
CRYAB	MIR497HG	0.520235557909693	7.04407850034961e-30	postive
SLC7A11	MAP3K20-AS1	0.598997827228621	2.2228742582285e-41	postive
ZEB1	MAP3K20-AS1	0.607854017459874	7.04255910190464e-43	postive
SLC7A11	ENTPD1-AS1	0.599268628715474	2.00338399186721e-41	postive
ZEB1	ENTPD1-AS1	0.687166114325665	1.00699425650768e-58	postive
IREB2	ENTPD1-AS1	0.573923476155975	2.2294503963298e-37	postive
ZEB1	ARAP1-AS2	0.515151162232154	3.09560483395037e-29	postive
IREB2	ARAP1-AS2	0.516780318031138	1.93159650078062e-29	postive
PHKG2	AL589765.6	0.534541911202692	9.53812077711172e-32	postive
SLC7A11	RBMS3-AS3	0.727703763217616	5.7730778619658e-69	postive
ZEB1	RBMS3-AS3	0.693544100956199	3.19702114332052e-60	postive
PHKG2	AL390719.2	0.515367315279742	2.90825658039358e-29	postive
IREB2	AP001619.1	0.524864363249469	1.79066104105623e-30	postive
IREB2	AC092171.1	0.526854521728254	9.87302901789391e-31	postive
ZEB1	AC129507.1	0.513058434884181	5.65253287418949e-29	postive
PHKG2	AC005387.1	0.526038708825187	1.26080616023409e-30	postive
IREB2	AP005899.1	0.50262974368818	1.06842540706056e-27	postive
RPL8	EPB41L4A-AS1	0.50516541398807	5.27718841122082e-28	postive
IREB2	AL157871.5	0.541134724634932	1.22466685121887e-32	postive
IREB2	AC108449.2	0.654639400471509	1.20236379682901e-51	postive
ZEB1	SH3RF3-AS1	0.647770784414144	2.91156588302921e-50	postive
SLC7A11	AP003171.1	0.62294175397505	1.52121745907451e-45	postive
ZEB1	AP003171.1	0.610774539959886	2.20259798910587e-43	postive
PHKG2	AC005785.1	0.541415667739465	1.12096468707455e-32	postive
SLC7A11	LSAMP-AS1	0.644561413171571	1.25508836688321e-49	postive
ZEB1	LSAMP-AS1	0.561750119559223	1.47801262473437e-35	postive
ZEB1	CARMN	0.511128103506276	9.81406977607493e-29	postive
PHKG2	AC020765.2	0.576991749252603	7.53364540011367e-38	postive
SLC7A11	GRK5-IT1	0.596927844704096	4.904868073402e-41	postive
ZEB1	GRK5-IT1	0.645767802346476	7.26181722264635e-50	postive
IREB2	AC024270.4	0.505339767832745	5.02625019720257e-28	postive
PHKG2	AC010422.2	0.52139577367788	5.0071534427329e-30	postive
CRYAB	AC002398.2	0.603680937347446	3.63078593763855e-42	postive
CRYAB	HAND2-AS1	0.581163658265402	1.69160221896282e-38	postive
ZEB1	HAND2-AS1	0.522338505439175	3.79073467315356e-30	postive
PHKG2	AC109460.3	0.555430704258449	1.21878734489862e-34	postive
IREB2	AC008750.5	0.527447928017279	8.26081706680097e-31	postive
IREB2	AC012557.1	0.535820811389007	6.42789068803632e-32	postive
ZEB1	AL136084.3	0.528489316875444	6.03633194923843e-31	postive
ZEB1	AC058791.1	0.566037297930631	3.44192252256772e-36	postive
IREB2	AC058791.1	0.507803463835571	2.51744568080328e-28	postive

IREB2	AC005070.3	0.516018408997787	2.40905414977216e-29	postive
CRYAB	AC036108.3	0.504929563939587	5.63643536152825e-28	postive
ZEB1	AC036108.3	0.636979043009626	3.69831322972052e-48	postive
IREB2	AL731566.2	0.560890196928382	1.97476028504693e-35	postive
IREB2	AC024075.1	0.590374527184449	5.78754193544695e-40	postive
ALOX5	AL731567.1	0.63524428713245	7.91414507321002e-48	postive
SLC7A11	AC006160.1	0.502287024993809	1.17477000291339e-27	postive
ZEB1	AC006160.1	0.543874853742903	5.14858561316547e-33	postive
CRYAB	GATA6-AS1	0.510587386878249	1.14470338947339e-28	postive
CRYAB	MIR1-1HG-AS1	0.587941517212129	1.42640785339066e-39	postive
IREB2	AP001001.1	0.510468119969883	1.18418830997237e-28	postive
ZEB1	AC090825.1	0.628302271424087	1.58375645109789e-46	postive
IREB2	AC025031.2	0.509814783826894	1.42559374183938e-28	postive
IREB2	CRTC3-AS1	0.522726012074527	3.38011499152252e-30	postive
IREB2	DCUN1D2-AS	0.531320592247346	2.55827959525874e-31	postive
IREB2	AC006059.1	0.514386358019161	3.85941880134442e-29	postive
SLC7A11	AP003900.1	0.740328812298131	1.52856566004516e-72	postive
ZEB1	AP003900.1	0.638291164747833	2.07342177214655e-48	postive
IREB2	AC004837.2	0.520050611081341	7.43705511083487e-30	postive
ZEB1	AC087854.1	0.647372687125115	3.49343694930371e-50	postive
SLC7A11	AC040904.1	0.601188520687089	9.55925543884065e-42	postive
ZEB1	AC040904.1	0.616629294703422	2.06532728098601e-44	postive
IREB2	AC008434.1	0.525126332263424	1.65605897372659e-30	postive
IREB2	AP001469.2	0.534240677717029	1.04647025903879e-31	postive
SLC7A11	AC011510.1	0.656902911911327	4.13012048748929e-52	postive
ZEB1	AC011510.1	0.714089493829776	2.51164527220307e-65	postive
ZEB1	AC012568.1	0.513658334110688	4.7584711591164e-29	postive
SLC7A11	MKNK1-AS1	0.56068149561942	2.11835737589524e-35	postive
ZEB1	MKNK1-AS1	0.596580751769157	5.59779090459247e-41	postive
IREB2	MKNK1-AS1	0.511743280224186	8.23483865051672e-29	postive
SLC7A11	C1QTNF7-AS1	0.708454138827872	6.9886911756454e-64	postive
ZEB1	C1QTNF7-AS1	0.680699151286549	3.04269441366441e-57	postive
CRYAB	ADAMTS9-AS1	0.543156158385372	6.46737962031974e-33	postive
ZEB1	ADAMTS9-AS1	0.594323176871942	1.3170191795135e-40	postive
IREB2	AL513327.1	0.528445027057914	6.1175478880072e-31	postive
CRYAB	AF001548.1	0.51361815323986	4.81371644858716e-29	postive
ZEB1	AF001548.1	0.528936386765605	5.27394815552647e-31	postive
CRYAB	LINC02489	0.57238084607086	3.83028835277619e-37	postive
CRYAB	MBNL1-AS1	0.629978579634274	7.73549084964145e-47	postive
ZEB1	MBNL1-AS1	0.51056487453286	1.15205542684059e-28	postive
IREB2	AC004918.5	0.538359774379022	2.92164520723038e-32	postive
SLC7A11	HMGA2-AS1	0.665456305246385	6.69159575630218e-54	postive
ZEB1	HMGA2-AS1	0.731492043925333	5.12178399952815e-70	postive
SLC7A11	AL445649.1	0.55931553581628	3.34959362038961e-35	postive

ZEB1	AL445649.1	0.560451787123989	2.28837733855075e-35	postive
SLC7A11	AL355102.1	0.719932581557199	7.32537662987304e-67	postive
ZEB1	AL355102.1	0.685424726933044	2.54337354448034e-58	postive
CRYAB	AC005180.1	0.599809682788969	1.62714190454447e-41	postive
PHKG2	LINC00685	0.508448210482053	2.09880485433035e-28	postive
PHKG2	AL513320.1	0.509186849381521	1.70323394620711e-28	postive
ZEB1	AC079313.1	0.630659723032694	5.77417851174903e-47	postive
SLC7A11	GNG12-AS1	0.641780095565673	4.38997264811042e-49	postive
ZEB1	GNG12-AS1	0.704943831854402	5.33109629011516e-63	postive
PHKG2	AC145285.6	0.544307414143491	4.4870758771485e-33	postive
IREB2	AC008870.4	0.568413970434603	1.5203870570839e-36	postive
IREB2	AC139887.2	0.586445298936709	2.47466549161178e-39	postive
IREB2	AC244093.5	0.569136426196899	1.18445109933809e-36	postive
ZEB1	AC004637.1	0.51510370845891	3.13830540807446e-29	postive
IREB2	AC008635.2	0.505723381369255	4.5149014500813e-28	postive
PHKG2	AC139530.1	0.505880968451147	4.32005996078104e-28	postive
IREB2	AC020978.1	0.509619405765947	1.50680729357446e-28	postive
PHKG2	AC127024.6	0.506399805036631	3.7354442186672e-28	postive
SLC7A11	STARD13-AS	0.664617148001256	1.00880668419282e-53	postive
ZEB1	STARD13-AS	0.679228528946074	6.52461355061268e-57	postive
IREB2	AC015849.4	0.500961940448071	1.69373019265911e-27	postive
PHKG2	AC093249.2	0.530666345893534	3.12185186752346e-31	postive
IREB2	DHDDS-AS1	0.508212414486642	2.24325675551828e-28	postive
ZEB1	MSC-AS1	0.501276544591963	1.55304035749656e-27	postive
IREB2	FMR1-IT1	0.50376386793703	7.79909249629906e-28	postive
ZEB1	AC025280.1	0.559004228390466	3.71718850156092e-35	postive
IREB2	AC024267.6	0.560842595849766	2.00663941565375e-35	postive
IREB2	AC010976.1	0.518494781409538	1.1726415370045e-29	postive
IREB2	UBL7-AS1	0.604830920490462	2.31616108282458e-42	postive
IREB2	Z98885.30.555312732362391	1.26720538547407e-34	postive	
ZEB1	AC026356.1	0.544170969711357	4.6861041944446e-33	postive
GSS AL121906.1	0.612653493134758	1.03605093677095e-43	postive	
ZEB1	LINC-PINT	0.55699376996821	7.26308289840581e-35	postive
SLC7A11	AC023825.2	0.651418065079583	5.41553441878165e-51	postive
ZEB1	AC023825.2	0.661300769508514	5.04360575932237e-53	postive
CRYAB	AC005180.2	0.617975527823259	1.19003164369308e-44	postive
IREB2	AC023024.1	0.506154770198499	4.00109517491808e-28	postive
ZEB1	AC104984.6	0.521248927162184	5.22858379482635e-30	postive
IREB2	AC104984.6	0.510697440208495	1.10942453587123e-28	postive
PHKG2	MIR762HG	0.549955570311959	7.31299483360482e-34	postive
IREB2	AC011442.1	0.530293628615577	3.49611667825448e-31	postive
IREB2	AL021368.2	0.500814790912538	1.76378727021579e-27	postive
ZEB1	COL4A2-AS1	0.515471066118748	2.82235744746922e-29	postive
IREB2	AC138393.3	0.556369572424924	8.93384344146052e-35	postive

IREB2	AC007619.1	0.500786398545708	1.77763055276272e-27	postive
IREB2	AL354993.2	0.531649214392237	2.31444367371985e-31	postive
PHKG2	AC034236.2	0.516604715826636	2.03257944198433e-29	postive
ZEB1	AC019183.1	0.515507823054798	2.79253070417476e-29	postive
ZEB1	AL031429.2	0.635008511860966	8.77296960790041e-48	postive
ZEB1	AC131097.3	0.52340265430146	2.76588408178455e-30	postive
CRYAB	LINC01081	0.573237919374404	2.83662542659264e-37	postive
ZEB1	LINC01081	0.518284387367797	1.24689722569084e-29	postive
IREB2	AL513327.2	0.517754808882734	1.45501456049435e-29	postive
PHKG2	AP003419.3	0.525149956671868	1.64442416170682e-30	postive
IREB2	OPA1-AS1	0.541208310621667	1.19662128483757e-32	postive
IREB2	AC007365.1	0.513708689734032	4.69012060473802e-29	postive
IREB2	LINC00630	0.621841420827797	2.40720316183439e-45	postive
ZEB1	AL161719.1	0.508572729946119	2.02627211765257e-28	postive
IREB2	AP001432.1	0.524257421463637	2.14549828309505e-30	postive
SLC7A11	AC009716.1	0.591441574955165	3.88717898530262e-40	postive
ZEB1	AC009716.1	0.597389517681149	4.11324556175895e-41	postive
IREB2	AC068790.3	0.501028613136517	1.66289970127895e-27	postive
SLC7A11	AC037487.2	0.548060363667903	1.34936395475255e-33	postive
ZEB1	AC037487.2	0.601870791440043	7.34015503979304e-42	postive
IREB2	AC037487.2	0.514641696979534	3.58569025188727e-29	postive
ZEB1	AL117336.1	0.512161948689094	7.30653261764982e-29	postive
IREB2	LINC00641	0.555965924975813	1.02113665993015e-34	postive
NOX1	AC015908.2	0.52095110682794	5.70782341828083e-30	postive
IREB2	ITFG1-AS1	0.509925679746219	1.38143799913588e-28	postive
IREB2	AC138932.6	0.505595403209815	4.67950342315483e-28	postive
IREB2	AC127024.4	0.532935708061411	1.56202457410381e-31	postive
IREB2	AL359220.1	0.504003687647556	7.29578743340945e-28	postive
CRYAB	ACTA2-AS1	0.5463225673272	2.35820401155613e-33	postive
ZEB1	ACTA2-AS1	0.609404991837854	3.80458191198202e-43	postive
IREB2	AC138956.2	0.577655479910574	5.9486903998723e-38	postive
IREB2	AC024075.3	0.551224535365107	4.84191508399778e-34	postive
IREB2	AL354726.1	0.543469569454151	5.85555176841237e-33	postive
ZEB1	AC067852.3	0.535557870301662	6.97215168340689e-32	postive
IREB2	AC067852.3	0.562962840837623	9.8082102067088e-36	postive
MT1G	HID1-AS1	0.549201432123882	9.33594689979105e-34	postive
PHKG2	AC008764.6	0.519115188696343	9.78197197937109e-30	postive
ZEB1	AC104170.1	0.521579086716174	4.74370807224964e-30	postive
IREB2	AC068790.2	0.516038963778074	2.39475855340771e-29	postive
ZEB1	PRR34	0.539544074004835	2.0178977781524e-32	postive
SLC7A11	AL139383.1	0.629859260929303	8.14145504945276e-47	postive
ZEB1	AL139383.1	0.605890890225655	1.52796465848392e-42	postive
ZEB1	AC090559.1	0.623784192183903	1.06917532781007e-45	postive
IREB2	AC022973.4	0.521850243635384	4.3789557516321e-30	postive

ZEB1	AC136604.2	0.517898797581333	1.3952504518927e-29	postive
ZEB1	AL353804.1	0.558676598912649	4.14721555893249e-35	postive
IREB2	AL353804.1	0.566649114766526	2.79080148552109e-36	postive
KEAP1	AC114271.1	0.54049728807003	1.49647512750693e-32	postive
IREB2	WASHC5-AS1	0.536160916563647	5.78582521530069e-32	postive
IREB2	PABPC4-AS1	0.535402586100125	7.31472998129075e-32	postive
IREB2	LINC01409	0.524208189856718	2.17715776322503e-30	postive
PHKG2	H1FX-AS1	0.532925235920849	1.56704261540879e-31	postive
SLC7A11	AC079921.2	0.607798526760218	7.1989819089003e-43	postive
ZEB1	AC079921.2	0.69528051837826	1.23034638618192e-60	postive
ZEB1	AL355073.2	0.551196058611977	4.88702149589548e-34	postive
SLC7A11	KLF7-IT1	0.536060481977981	5.9685256275904e-32	postive
ZEB1	KLF7-IT1	0.605464409788658	1.80668256778447e-42	postive
IREB2	KLF7-IT1	0.50386461795635	7.5836024011975e-28	postive
ZEB1	AL133371.2	0.613788602862051	6.55287473439159e-44	postive
IREB2	NCBP2-AS1	0.520470994418214	6.57340037106462e-30	postive
ZEB1	AC108463.2	0.683576011587883	6.75328567025664e-58	postive
ZEB1	SDCBP2-AS1	0.501236398930216	1.57032947283381e-27	postive
IREB2	SDCBP2-AS1	0.536340861873325	5.47221736063827e-32	postive
SLC7A11	ANKRD44-IT1	0.681784164190439	1.7281499996435e-57	postive
ZEB1	ANKRD44-IT1	0.678113803446489	1.15981128370357e-56	postive
IREB2	MCM3AP-AS1	0.519892392063794	7.79038842264731e-30	postive
IREB2	AP000766.1	0.508621915192748	1.99830918837018e-28	postive
CRYAB	AP003071.4	0.616208119693328	2.45277792832381e-44	postive
IREB2	AC007622.2	0.567615311610663	2.00224931090339e-36	postive
IREB2	AC004884.2	0.504788495335934	5.86275198573319e-28	postive
ACO1	AC061992.2	0.604683209093976	2.45408784520264e-42	postive
IREB2	AC234775.3	0.532477837952718	1.79699045915963e-31	postive
IREB2	AC078795.1	0.562754321660871	1.0525993552824e-35	postive
IREB2	AC007390.1	0.506980069842827	3.17387703581955e-28	postive
IREB2	AL139021.3	0.511022934776216	1.01125234891255e-28	postive
IREB2	LAMTOR5-AS1	0.528911647371952	5.31352748878549e-31	postive
ZEB1	AC087286.1	0.57732226047949	6.69810308743551e-38	postive
IREB2	AC087286.1	0.519454930990708	8.85603754345297e-30	postive
PHKG2	AC012645.4	0.517158475513143	1.73066720790664e-29	postive
ZEB1	AC110609.1	0.533932321271251	1.15053850622045e-31	postive
SLC7A11	AC069549.1	0.626541115020574	3.3465470770133e-46	postive
ZEB1	AC069549.1	0.653439342591407	2.11086268178966e-51	postive
IREB2	AL353804.2	0.517983994798565	1.36103808782426e-29	postive
ZEB1	MAGI2-AS3	0.509714779124155	1.46660741220596e-28	postive
NCOA4	AL731577.2	0.509514741927382	1.55217713437601e-28	postive
IREB2	AL731577.2	0.510596560037345	1.14172095229732e-28	postive
PHKG2	AC010618.2	0.515829168784463	2.54469571510175e-29	postive
IREB2	AC008115.3	0.512449958818533	6.72878175482729e-29	postive

IREB2	AC087294.1	0.508776658853081	1.91279785104834e-28	postive
SLC7A11	AC138207.4	0.661648063287971	4.26546538129047e-53	postive
ZEB1	AC138207.4	0.597205635522151	4.41210905289498e-41	postive
IREB2	PRC1-AS1	0.505044912767734	5.45781443889601e-28	postive
ZEB1	AC005740.4	0.558018871986049	5.16463547413856e-35	postive
ZEB1	FENDRR	0.806205084035172	2.87859558593042e-95	postive
SLC7A11	AC234772.2	0.668865331422596	1.24532979701626e-54	postive
ZEB1	AC234772.2	0.659951471656457	9.6508068627277e-53	postive
IREB2	AL590729.1	0.553034224770499	2.68103740755203e-34	postive
SLC7A11	RASA3-IT1	0.599370529197694	1.92647424900658e-41	postive
ZEB1	RASA3-IT1	0.647468383023282	3.3438214760693e-50	postive
CRYAB	LINC00702	0.576440250603025	9.16357437530289e-38	postive
IREB2	AL133243.3	0.512879836387131	5.94944188302448e-29	postive
ZEB1	AL606534.1	0.551211160052863	4.86304967292644e-34	postive
SLC7A11	WWTR1-IT1	0.705509240796594	3.85093314176244e-63	postive
ZEB1	WWTR1-IT1	0.695016086538526	1.42355514380615e-60	postive
SLC7A11	AC093423.2	0.613193283147909	8.33438360726458e-44	postive
ZEB1	AC093423.2	0.656406252853428	5.22555686169219e-52	postive
SLC7A11	AL133445.2	0.579139364780764	3.50129133943076e-38	postive
ZEB1	AL133445.2	0.646545432408819	5.096842802925e-50	postive
IREB2	AL157838.1	0.53325010107564	1.4185557816989e-31	postive
IREB2	AC091057.1	0.543382308312758	6.01990039053772e-33	postive
CRYAB	AP003071.3	0.595750923266028	7.67255802450159e-41	postive
IREB2	AC073130.2	0.50383523621139	7.64583073637303e-28	postive
ZEB1	AL132642.1	0.59295649250819	2.20350135035416e-40	postive
IREB2	AC087276.3	0.510295594745831	1.2436980745184e-28	postive
IREB2	AC068790.6	0.500172220598666	2.10479726716322e-27	postive
IREB2	GTF3C2-AS1	0.523590744146862	2.61570192852627e-30	postive
IREB2	AC106820.5	0.515710042407315	2.6339258068279e-29	postive
MT1G	AC063919.1	0.768927168780757	1.78867974973691e-81	postive
GCLM	AL596223.1	0.506581063744777	3.55022425276854e-28	postive
SLC7A11	AL596223.1	0.764012482685731	7.53975585391193e-80	postive
ZEB1	AL596223.1	0.520253327116891	7.00742127665805e-30	postive
ABCC1	AL596223.1	0.538299902937231	2.9767070269694e-32	postive
IREB2	AL117329.1	0.528620172630013	5.80255447733718e-31	postive
IREB2	AC015849.3	0.552790963247116	2.90336775642944e-34	postive
IREB2	AC022272.1	0.525940959674666	1.29823539986951e-30	postive
IREB2	AP001020.3	0.506883358864918	3.26130606456745e-28	postive
SLC7A11	LINC02595	0.537709302443676	3.57796803865416e-32	postive
IREB2	AL139353.2	0.518199692311897	1.27808483557673e-29	postive
ZEB1	GSN-AS1	0.588396282712777	1.20578982494491e-39	postive
IREB2	GSN-AS1	0.532214918900198	1.94737576041731e-31	postive
IREB2	FTX	0.52906857495567	5.06735657275784e-31	postive
IREB2	AC005519.1	0.500834178060936	1.75439591903669e-27	postive

MT1G	AC016924.1	0.603579513027879	3.77729861822847e-42	postive
ZEB1	AC099811.5	0.531013593143075	2.80895022415922e-31	postive
IREB2	AC099811.5	0.519433758336862	8.91111956289359e-30	postive
IREB2	SPAG5-AS1	0.505214495389893	5.20532221185612e-28	postive
ZEB1	TGFB2-AS1	0.500481150617343	1.93341251399962e-27	postive
IREB2	CDC42-IT1	0.50308505610628	9.41727304042659e-28	postive
SLC7A11	SBF2-AS1	0.54413482937673	4.74026854924565e-33	postive
SLC7A11	AL133415.1	0.500551107110959	1.89656012778592e-27	postive
ZEB1	AL133415.1	0.608396431806035	5.68032837002867e-43	postive
PHKG2	AC106782.5	0.600534096603427	1.23084055173433e-41	postive
ZEB1	AC093010.2	0.566763976290889	2.68293423139715e-36	postive
IREB2	AC105339.2	0.500224127339372	2.07498668805981e-27	postive
ZEB1	SOCAR	0.601285719048737	9.20656712028132e-42	postive
ZEB1	RNF216-IT1	0.568419244299246	1.51762178279357e-36	postive
IREB2	AC087301.1	0.507770346460521	2.54104751661927e-28	postive
ACO1	LINC00092	0.586041790801261	2.86967308222369e-39	postive
ZEB1	AC087286.4	0.508034059391568	2.3590063672731e-28	postive
IREB2	AC087286.4	0.507797698163623	2.52153908233963e-28	postive
PHKG2	AC092119.2	0.517162844736837	1.72847084336693e-29	postive
AKR1C1	LINC01697	0.603068253345738	4.60990502411418e-42	postive
GCLM	LINC01697	0.592681295926722	2.44338985181513e-40	postive
SLC7A11	LINC01697	0.551672662903578	4.1840190862199e-34	postive
ABCC1	LINC01697	0.502436442943172	1.12717798102453e-27	postive
IREB2	RORA-AS1	0.54460714743691	4.07875617290343e-33	postive
