# Supplementary material for: Ferroptosis-Related Long Noncoding RNAs Have Excellent Predictive Ability for Multiomic Characteristics of Bladder Cancer
Source: Oxid Med Cell Longev. 2022 Aug 29;2022:9316847. doi: 10.1155/2022/9316847 (PMC9444476; doi:10.1155/2022/9316847)
Supplement: Supplementary Materials — Supplementary Table 1: expression matrix of 60 ferroptosis-related genes. Supplementary Table 2: results for coexpression analysis of ferroptosis-related lncRNAs and mRNAs. Supplementary Table 3: expression of 263 ferroptosis-related lncRNAs. Supplementary Table 4: expression of lncRNAs and clinical information of 403 bladder cancer samples. Supplementary Table 5: cluster analysis based on 20 prognostic lncRNAs. Supplementary Table 6: detailed clinical information of bladder cancer samples. Supplementary Table 7: data of the high- and low-risk groups of the training cohort. Supplementary Table 8: data of the high- and low-risk groups of the validation cohort. Supplementary Table 9: markers of different immune cells. Supplementary Table 10: infiltration of 22 types of immune cells analysed using the CIBERSORT algorithm. Supplementary Table 11: immune scores evaluated using the ESTIMATE algorithm. Supplementary Table 12: immunotherapeutic response evaluated using the Tumour Immune Dysfunction and Exclusion (TIDE) algorithm. Supplementary Figure 1: gene set enrichment analysis. (A–F) Most significantly enriched biological pathways in cluster 1. (G–I) Most significantly enriched biological pathways in cluster 2. [file 9316847.f1.zip › s-table 3.pdf]

ID	TCGA-GD-A3OP-11A-11R-A220-07	TCGA-BT-A20Q-11A-11R-A14Y-07	TCGA-GD-A2C5-11A-11R-A180-07	TCGA-BT-A20N-11A-11R-A14Y-07	TCGA-BT-A2LB-11A-11R-A18C-07	TCGA-GC-A3BM-11A-11R-A22U-07	TCGA-CU-A0YR-11A-13R-A10U-07	TCGA-GC-A3WC-11A-11R-A22U-07	TCGA-CU-A0YN-11A-11R-A10U-07	TCGA-BT-A20R-11A-11R-A16R-07	TCGA-BT-A20U-11A-11R-A14Y-07	TCGA-BT-A20W-11A-11R-A14Y-07	TCGA-GD-A3OQ-11A-21R-A220-07	TCGA-K4-A5RI-11A-11R-A28M-07	TCGA-BL-A13J-11A-13R-A10U-07	TCGA-K4-A54R-11A-11R-A26T-07	TCGA-K4-A3WV-11A-21R-A22U-07	TCGA-GC-A6I3-11A-11R-A31N-07	TCGA-BT-A2LA-11A-11R-A18C-07	TCGA-CF-A47X-01A-31R-A23W-07	TCGA-ZF-AA51-01A-21R-A39I-07	TCGA-BL-A0C8-01A-11R-A10U-07	TCGA-BT-A42C-01A-11R-A23N-07	TCGA-G2-A3IB-01A-11R-A20F-07	TCGA-2F-A9KP-01A-11R-A38B-07	TCGA-DK-A3IT-01A-31R-A20F-07	TCGA-FD-A43P-01A-31R-A23W-07	TCGA-DK-A3IV-01A-22R-A21D-07	TCGA-2F-A9KW-01A-11R-A38B-07	TCGA-GU-A762-01A-11R-A33J-07	TCGA-XF-AAME-01A-12R-A42T-07	TCGA-XF-AAN0-01A-11R-A42T-07	TCGA-CF-A47W-01A-11R-A23W-07	TCGA-GC-A3I6-01A-11R-A20F-07	TCGA-FD-A6TA-01A-12R-A33J-07	TCGA-DK-A1A7-01A-11R-A13Y-07	TCGA-FD-A3SR-01A-11R-A22U-07	TCGA-GU-AATP-01A-11R-A39I-07	TCGA-DK-AA6X-01A-12R-A42T-07	TCGA-XF-A9T3-01A-11R-A42T-07	TCGA-PQ-A6FN-01A-11R-A31N-07	TCGA-DK-A3WW-01A-22R-A23N-07	TCGA-XF-AAMY-01A-11R-A42T-07	TCGA-K4-A3WS-01A-11R-A22U-07	TCGA-UY-A9PA-01A-11R-A38B-07	TCGA-G2-AA3F-01A-12R-A42T-07	TCGA-FJ-A871-01A-11R-A352-07	TCGA-ZF-A9RN-01A-11R-A42T-07	TCGA-GC-A3RB-01A-12R-A220-07	TCGA-UY-A9PF-01A-11R-A38B-07	TCGA-YC-A89H-01A-11R-A36F-07	TCGA-XF-AAMQ-01A-11R-A42T-07	TCGA-CF-A1HR-01A-11R-A13Y-07	TCGA-E7-A3X6-01A-12R-A22U-07	TCGA-DK-A6AW-01A-11R-A30C-07	TCGA-GC-A3BM-01A-11R-A22U-07	TCGA-GV-A6ZA-01A-12R-A33J-07	TCGA-G2-A2EJ-01A-11R-A180-07	TCGA-FD-A43N-01A-11R-A23W-07	TCGA-UY-A8OD-01A-11R-A36F-07	TCGA-2F-A9KT-01A-11R-A38B-07	TCGA-BT-A20Q-01A-11R-A14Y-07	TCGA-CU-A0YR-01A-12R-A10U-07	TCGA-SY-A9G5-01A-11R-A38B-07	TCGA-4Z-AA84-01A-11R-A39I-07	TCGA-4Z-AA7Q-01A-11R-A39I-07	TCGA-FD-A5BT-01A-11R-A26T-07	TCGA-CF-A27C-01A-11R-A16R-07	TCGA-E7-A6MF-01A-12R-A32O-07	TCGA-FT-A61P-01A-11R-A30C-07	TCGA-DK-AA77-01A-11R-A39I-07	TCGA-ZF-AA5N-01A-11R-A42T-07	TCGA-DK-AA6U-01A-11R-A39I-07	TCGA-BT-A20O-01A-21R-A14Y-07	TCGA-GD-A3OS-01A-12R-A220-07	TCGA-XF-A9SZ-01A-11R-A39I-07	TCGA-4Z-AA82-01A-11R-A39I-07	TCGA-DK-AA76-01A-11R-A39I-07	TCGA-DK-A3IL-01A-11R-A20F-07	TCGA-E7-A678-01A-11R-A30C-07	TCGA-ZF-A9R1-01A-11R-A39I-07	TCGA-XF-A9SH-01A-11R-A39I-07	TCGA-FD-A5BU-01A-31R-A26T-07	TCGA-C4-A0F7-01A-11R-A084-07	TCGA-C4-A0F0-01A-12R-A10U-07	TCGA-FD-A3N5-01A-11R-A21D-07	TCGA-ZF-AA5H-01A-11R-A39I-07	TCGA-DK-A3WX-01A-22R-A22U-07	TCGA-YF-AA3L-01A-11R-A38B-07	TCGA-BT-A2LA-01A-11R-A18C-07	TCGA-4Z-AA87-01A-11R-A39I-07	TCGA-BT-A2LB-01A-11R-A18C-07	TCGA-XF-A8HB-01A-11R-A36F-07	TCGA-DK-A2I6-01A-12R-A18C-07	TCGA-ZF-AA4X-01A-11R-A38B-07	TCGA-BT-A20P-01A-11R-A14Y-07	TCGA-BT-A20W-01A-21R-A14Y-07	TCGA-FD-A5BX-01A-11R-A26T-07	TCGA-BL-A13J-01A-11R-A10U-07	TCGA-CF-A8HX-01A-11R-A36F-07	TCGA-PQ-A6FI-01A-11R-A31N-07	TCGA-FJ-A3Z7-01A-12R-A23N-07	TCGA-E7-A7PW-01A-11R-A352-07	TCGA-4Z-AA7S-01A-

11R-A39I-07 TCGA-CF-A3MH-01A-11R-A20F-07 TCGA-DK-A6B2-01A-11R-A30C-07

TCGA-4Z-AA86-01A-11R-A39I-07 TCGA-BL-A0C8-01B-04R-A277-07 TCGA-5N-A9KI-01A-31R-A42T-07 TCGA-GU-AATO-01A-11R-A39I-07 TCGA-CF-A9FH-01A-11R-A38B-07TCGA-FD-A6TG-01A-11R-A32O-07 TCGA-4Z-AA7O-01A-31R-A39I-07

TCGA-GV-A3QI-01A-11R-A220-07 TCGA-DK-A6AV-01A-12R-A30C-07 TCGA-DK-A1A3-01A-11R-A13Y-07 TCGA-GD-A6C6-01A-21R-A31N-07TCGA-GC-A3RD-01A-12R-A22U-07 TCGA-BT-A20R-01A-12R-A16R-07 TCGA-XF-A9T0-01A-11R-A39I-07

TCGA-YF-AA3M-01A-11R-A42T-07 TCGA-4Z-AA7Y-01A-11R-A39I-07 TCGA-FD-A62O-01A-11R-A30C-07 TCGA-BL-A3JM-01A-12R-A21D-07 TCGA-FJ-A3ZF-01A-11R-A23N-07TCGA-CU-A0YO-01A-11R-A10U-07TCGA-CF-A5UA-01A-11R-A28M-07TCGA-K4-AAQO-01A-11R-A38B-07 TCGA-XF-AAMJ-01A-11R-A42T-07 TCGA-GU-A763-01A-11R-A32O-07 TCGA-G2-A2EC-01A-11R-A180-07 TCGA-FD-A3B7-01A-31R-A20F-07 TCGA-FD-A5C0-01A-11R-A28M-07TCGA-FD-A5BS-01A-21R-A26T-07 TCGA-CU-A0YN-01A-21R-A10U-07 TCGA-FD-A5BZ-01A-11R-A28M-07TCGA-UY-A9PE-01A-11R-A38B-07 TCGA-GV-A40E-01A-12R-A23N-07 TCGA-BT-A2LD-01A-12R-A20F-07 TCGA-UY-A9PD-01A-11R-A38B-07 TCGA-G2-AA3B-01A-11R-A39I-07 TCGA-K4-A54R-01A-11R-A26T-07 TCGA-DK-A1AA-01A-11R-A13Y-07 TCGA-CU-A5W6-01A-11R-A28M-07 TCGA-DK-AA6R-01A-11R-A42T-07 TCGA-BT-A3PH-01A-11R-A220-07 TCGA-BL-A13I-01B-04R-A277-07 TCGA-CF-A3MF-01A-12R-A21D-07 TCGA-CF-A47T-01A-11R-A23W-07 TCGA-YC-A8S6-01A-31R-A38B-07 TCGA-DK-A3IS-01A-21R-A21D-07 TCGA-XF-A9SJ-01A-11R-A39I-07 TCGA-DK-AA6T-01A-11R-A39I-07

TCGA-BT-A20N-01A-11R-A14Y-07 TCGA-C4-A0F6-01A-11R-A10U-07 TCGA-CF-A7I0-01A-22R-A352-07 TCGA-FD-A3B6-01A-21R-A20F-07 TCGA-GC-A3YS-01A-11R-A23N-07 TCGA-XF-A9T2-01A-11R-A42T-07 TCGA-UY-A78O-01A-12R-A33J-07

TCGA-GC-A3OO-01A-11R-A22U-07 TCGA-E7-A6ME-01A-22R-A32O-07 TCGA-XF-AAN5-01A-11R-A42T-07 TCGA-FD-A3SN-01A-12R-A22U-07 TCGA-ZF-A9R2-01A-11R-A39I-07 TCGA-ZF-AA4T-01A-11R-A38B-07 TCGA-E7-A4IJ-01A-31R-A26T-07

TCGA-FD-A6TE-01A-12R-A33J-07 TCGA-H4-A2HO-01A-11R-A180-07 TCGA-FD-A6TB-01A-12R-A33J-07 TCGA-XF-AAN3-01A-11R-A42T-07 TCGA-XF-AAMH-01A-11R-A42T-07TCGA-FD-A3B8-01A-31R-A20F-07 TCGA-XF-A9SY-01A-21R-A42T-07

TCGA-CF-A9FF-01A-11R-A38B-07 TCGA-XF-A9ST-01A-11R-A42T-07 TCGA-DK-A6B5-01A-11R-A31N-07 TCGA-GC-A3RC-01A-11R-A22U-07 TCGA-GV-A3QK-01B-11R-A23N-07 TCGA-GU-A764-01A-11R-A352-07 TCGA-GU-A42R-01A-11R-A23N-07 TCGA-DK-A6B6-01A-11R-A30C-07 TCGA-R3-A69X-01A-22R-A30C-07 TCGA-5N-A9KM-01A-11R-A42T-07 TCGA-FD-A6TF-01A-52R-A32O-07 TCGA-XF-AAMR-01A-31R-A42T-07TCGA-XF-A8HF-01A-11R-A36F-07 TCGA-ZF-AA4U-01A-11R-A38B-07

TCGA-DK-A3IQ-01A-31R-A32Y-07 TCGA-E7-A4XJ-01A-11R-A26T-07 TCGA-XF-A9T6-01A-11R-A42T-07 TCGA-4Z-AA7M-01A-11R-A39I-07 TCGA-ZF-AA56-01A-31R-A39I-07 TCGA-XF-A8HH-01A-11R-A38B-07 TCGA-C4-A0EZ-01A-21R-A24X-07

TCGA-FD-A62P-01A-32R-A30C-07 TCGA-CF-A47V-01A-11R-A23W-07 TCGA-XF-A9SI-01A-11R-A39I-07 TCGA-GU-A42Q-01A-11R-A23W-07 TCGA-BT-A42F-01A-11R-A23W-07 TCGA-ZF-A9RF-01A-11R-A38B-07 TCGA-KQ-A41N-01A-11R-A33J-07 TCGA-GD-A3OQ-01A-32R-A220-07 TCGA-BT-A20U-01A-11R-A14Y-07 TCGA-XF-A9SW-01A-11R-A42T-07 TCGA-FD-A3B3-01A-12R-A206-07 TCGA-ZF-A9RC-01A-

11R-A38B-07 TCGA-E7-A6MD-01A-41R-A352-07 TCGA-E5-A4TZ-01A-11R-A31N-07

TCGA-ZF-A9R4-01A-11R-A38B-07 TCGA-GV-A3QH-01A-11R-A220-07 TCGA-BT-A0YX-01A-11R-A10U-07 TCGA-FT-A3EE-01A-11R-A206-07 TCGA-ZF-A9RL-01A-11R-A38B-07 TCGA-ZF-A9R9-01A-11R-A38B-07 TCGA-ZF-A9RE-01A-11R-A38B-07 TCGA-DK-A6B1-01A-12R-A30C-07 TCGA-CF-A5U8-01A-11R-A28M-07 TCGA-XF-AAMW-01A-11R-A42T-07 TCGA-BT-A3PJ-01A-21R-A220-07 TCGA-ZF-A9R7-01A-11R-A38B-07 TCGA-FD-A3SO-01A-11R-A22U-07 TCGA-E7-A97P-01A-11R-A38B-07 TCGA-GU-A767-01A-11R-A32O-07 TCGA-4Z-AA81-01A-11R-A39I-07 TCGA-2F-A9KQ-01A-11R-A38B-07 TCGA-SY-A9G0-01A-12R-A38B-07 TCGA-GV-A40G-01A-11R-A23N-07 TCGA-FD-A43Y-01A-21R-A26T-07 TCGA-S5-AA26-01A-11R-A38B-07 TCGA-BT-A42E-01A-11R-A23W-07 TCGA-FD-A43X-01A-11R-A23W-07 TCGA-ZF-AA4R-01A-11R-A38B-07 TCGA-BT-A20T-01A-11R-A14Y-07 TCGA-E7-A7DU-01A-11R-A32O-07 TCGA-DK-A3WY-01A-11R-A22U-07 TCGA-G2-A2EF-01A-12R-A18C-07

TCGA-BT-A20V-01A-11R-A14Y-07 TCGA-K4-A3WV-01A-11R-A22U-07 TCGA-GU-AATQ-01A-11R-A39I-07 TCGA-G2-A3VY-01A-11R-A22U-07 TCGA-XF-AAN8-01A-11R-A42T-07 TCGA-CU-A3QU-01A-11R-A22U-07 TCGA-GD-A3OP-01A-21R-A220-07 TCGA-KQ-A41P-01A-12R-A33J-07 TCGA-UY-A9PH-01A-11R-A38B-07 TCGA-FD-A5BY-01A-31R-A28M-07 TCGA-K4-A3WU-01B-11R-A23N-07 TCGA-FD-A3SJ-01A-12R-A22U-07 TCGA-FD-A3B5-01A-11R-A20F-07 TCGA-GC-A6I3-01A-11R-A31N-07 TCGA-4Z-AA80-01A-11R-A39I-07 TCGA-G2-A3IE-01A-11R-A20F-07 TCGA-ZF-A9R0-01A-11R-A38B-07 TCGA-FD-A43S-01A-21R-A23W-07 TCGA-XF-A8HG-01A-11R-A36F-07

TCGA-K4-A83P-01A-11R-A352-07 TCGA-GD-A2C5-01A-12R-A180-07 TCGA-XF-AAMX-01A-11R-A42T-07 TCGA-DK-A1AE-01A-11R-A13Y-07 TCGA-FJ-A3ZE-01A-11R-A23N-07 TCGA-DK-A2I1-01A-11R-A180-07 TCGA-DK-A6B0-01A-11R-A31N-07 TCGA-FD-A5BR-01A-11R-A26T-07 TCGA-KQ-A41Q-01A-11R-A33J-07 TCGA-DK-A1AF-01A-11R-A13Y-07 TCGA-LT-A5Z6-01A-11R-A28M-07 TCGA-GC-A4ZW-01A-11R-A26T-07 TCGA-K4-A4AB-01B-12R-A28M-07 TCGA-DK-AA74-01A-11R-A39I-07 TCGA-G2-A2EO-01A-11R-A180-07 TCGA-DK-A1AG-01A-11R-A13Y-07 TCGA-GV-A3JZ-01A-11R-A21D-07 TCGA-XF-A9SV-01A-21R-A42T-07 TCGA-UY-A78K-01A-11R-A33J-07

TCGA-FD-A6TH-01A-11R-A32O-07 TCGA-DK-AA6W-01A-12R-A39I-07 TCGA-CF-A3MG-01A-11R-A20F-07 TCGA-DK-A3X1-01A-12R-A22U-07 TCGA-DK-A1A6-01A-11R-A13Y-07 TCGA-XF-AAMG-01A-11R-A42T-07 TCGA-E7-A541-01A-11R-A26T-07

TCGA-XF-A8HE-01A-11R-A36F-07 TCGA-DK-AA6Q-01A-11R-A39I-07 TCGA-UY-A78N-01A-12R-A33J-07 TCGA-DK-AA75-01A-11R-A39I-07 TCGA-ZF-AA5P-01A-11R-A39I-07 TCGA-GC-A3WC-01A-31R-A22U-07 TCGA-K4-A4AC-01A-21R-A26T-07

TCGA-FD-A3SL-01A-21R-A22U-07 TCGA-K4-A5RH-01A-11R-A30C-07 TCGA-FD-A5C1-01A-11R-A28M-07 TCGA-BL-A13J-01A-11R-A277-07 TCGA-XF-AAN7-01A-11R-A42T-07 TCGA-2F-A9KO-01A-11R-A38B-07 TCGA-DK-A3IM-01A-11R-A20F-07

TCGA-FD-A3SS-01A-12R-A22U-07 TCGA-4Z-AA7N-01A-11R-A39I-07 TCGA-HQ-A2OE-01A-11R-A206-07 TCGA-UY-A8OC-01A-11R-A36F-07 TCGA-GV-A3JV-01A-11R-A220-07 TCGA-XF-A9T8-01A-11R-A39I-07 TCGA-KQ-A41S-01A-12R-A33J-07

TCGA-CU-A3YL-01A-11R-A22U-07 TCGA-E7-A7XN-01A-11R-A352-07 TCGA-DK-A2HX-01A-12R-A18C-07 TCGA-CF-A8HY-01A-11R-A36F-07 TCGA-4Z-AA7W-01A-11R-A39I-07 TCGA-FD-A43U-01A-11R-A23W-07 TCGA-BL-A0C8-01A-11R-A277-07

TCGA-BT-A0S7-01A-11R-A10U-07 TCGA-ZF-A9R3-01A-11R-A38B-07 TCGA-4Z-AA89-01A-11R-A39I-07 TCGA-GU-A42P-01A-11R-A23W-07 TCGA-FD-A3NA-01A-11R-A21D-07 TCGA-XF-A9SL-01A-11R-A39I-07 TCGA-XF-AAN2-01A-11R-A42T-07 TCGA-ZF-AA54-01A-11R-A39I-07 TCGA-GU-A766-01A-11R-A32O-07 TCGA-FD-A6TD-01A-51R-A33J-07 TCGA-FD-A3B4-01A-12R-A206-07 TCGA-DK-A1AB-01A-11R-A13Y-07 TCGA-BT-A20X-01A-11R-A16R-07 TCGA-GV-A3JW-01A-11R-A20F-07 TCGA-UY-A9PB-01A-11R-A38B-07 TCGA-FD-A6TI-01A-11R-A32O-07 TCGA-XF-AAML-01A-11R-A42T-07 TCGA-ZF-AA58-01A-12R-A42T-07 TCGA-FD-A3SP-01A-31R-A22U-07 TCGA-E7-A97Q-01A-11R-A38B-07 TCGA-YC-A9TC-01A-22R-A39I-07

TCGA-GV-A3QG-01A-11R-A220-07 TCGA-K4-A6FZ-01A-11R-A31N-07 TCGA-KQ-A41R-01A-21R-A352-07 TCGA-DK-AA6M-01A-11R-A39I-07 TCGA-ZF-AA52-01A-12R-A39I-07 TCGA-K4-A5RJ-01A-11R-A28M-07 TCGA-HQ-A5ND-01A-11R-A26T-07

TCGA-BT-A3PK-01A-21R-A220-07 TCGA-FD-A3N6-01A-11R-A21D-07 TCGA-UY-A78P-01A-12R-A36F-07 TCGA-CF-A9FL-01A-11R-A38B-07 TCGA-CF-A9FM-01A-11R-A38B-07 TCGA-ZF-A9RD-01A-11R-A42T-07 TCGA-G2-AA3D-01A-11R-A39I-07

TCGA-DK-A2I2-01A-11R-A180-07 TCGA-FD-A62S-01A-11R-A30C-07 TCGA-MV-A51V-01A-11R-A26T-07 TCGA-E5-A2PC-01A-11R-A206-07 TCGA-DK-A3X2-01A-11R-A22U-07 TCGA-GD-A76B-01A-11R-A32O-07 TCGA-GC-A6I1-01A-12R-A31N-07

TCGA-BL-A13J-01B-04R-A277-07 TCGA-XF-A9SK-01A-11R-A42T-07 TCGA-DK-A1AD-01A-11R-A13Y-07 TCGA-K4-A5RI-01A-11R-A28M-07 TCGA-FD-A3SQ-01A-21R-A22U-07 TCGA-GV-A3QF-01A-31R-A22U-07 TCGA-E7-A7DV-01A-11R-A33J-07

TCGA-XF-AAN1-01A-31R-A42T-07 TCGA-DK-AA6P-01A-11R-A39I-07 TCGA-CF-A47Y-01A-11R-A23W-07 TCGA-CF-A3MI-01A-11R-A20F-07 TCGA-G2-A2EL-01A-12R-A18C-07 TCGA-XF-AAMZ-01A-11R-A42T-07 TCGA-DK-AA6L-01A-11R-A39I-07

TCGA-XF-A9T4-01A-11R-A39I-07 TCGA-K4-A6MB-01A-11R-A31N-07 TCGA-UY-A8OB-01A-12R-A42T-07 TCGA-ZF-A9R5-01A-12R-A42T-07 TCGA-CU-A3KJ-01A-11R-A21D-07 TCGA-DK-AA6S-01A-21R-A39I-07 TCGA-E7-A85H-01A-11R-A352-07

TCGA-G2-AA3C-01A-21R-A39I-07 TCGA-E7-A8O8-01A-11R-A36F-07 TCGA-XF-A9SM-01A-11R-A42T-07 TCGA-DK-A3IN-01A-11R-A20F-07 TCGA-XF-A8HC-01A-11R-A36F-07 TCGA-ZF-AA4W-01A-12R-A38B-07 TCGA-LC-A66R-01A-41R-A30C-07

TCGA-E7-A8O7-01A-11R-A36F-07 TCGA-LT-A8JT-01A-11R-A36F-07 TCGA-FJ-A3Z9-01A-11R-A26T-07 TCGA-DK-A1AC-01A-11R-A13Y-07 TCGA-C4-A0F1-01A-11R-A034-07 TCGA-G2-A2ES-01A-11R-A180-07 TCGA-XF-AAN4-01A-11R-A42T-07

TCGA-4Z-AA83-01A-11R-A39I-07 TCGA-E7-A5KE-01A-11R-A28M-07 TCGA-ZF-AA4V-01A-11R-A38B-07 TCGA-ZF-AA53-01A-11R-A39I-07 TCGA-ZF-A9RM-01A-11R-A38B-07 TCGA-BL-A13I-01A-11R-A13Y-07 TCGA-CU-A72E-01A-12R-A33J-07

TCGA-2F-A9KR-01A-11R-A38B-07 TCGA-E5-A4U1-01A-11R-A31N-07 TCGA-E7-A3Y1-01A-11R-A22U-07 TCGA-FD-A6TC-01A-21R-A33J-07 TCGA-FD-A5BV-01A-11R-A26T-07 TCGA-DK-A2I4-01A-11R-A21D-07 TCGA-UY-A78M-01A-21R-A352-07

TCGA-4Z-AA7R-01A-11R-A39I-07 TCGA-E7-A519-01A-11R-A26T-07 TCGA-DK-A3IU-01A-11R-A20F-07 TCGA-FD-A3SM-01A-11R-A22U-07 TCGA-BL-A5ZZ-01A-31R-A30C-07 TCGA-FD-A62N-01A-11R-A30C-07 TCGA-BT-A20J-01A-11R-A14Y-07

TCGA-DK-AA71-01A-31R-A39I-07 TCGA-KQ-A41O-01A-12R-A352-07 TCGA-BL-A13I-01A-11R-A277-07 TCGA-E7-A677-01A-11R-A30C-07 TCGA-DK-A1A5-01A-

11R-A13Y-07TCGA-HQ-A5NE-01A-12R-A28M-07 TCGA-XF-AAMT-01A-11R-A42T-07 TCGA-XF-A9SU-01A-31R-A39I-07 TCGA-CF-A47S-01A-11R-A23W-07 TCGA-FD-A6TK-01A-42R-A33J-07 TCGA-E7-A5KF-01A-11R-A28M-07 TCGA-UY-A78L-01A-12R-A33J-07 TCGA-S5-A6DX-01A-11R-A31N-07 TCGA-XF-A8HI-01A-11R-A38B-07 TCGA-XF-A9T5-01A-11R-A42T-07 TCGA-XF-A9SX-01A-21R-A39I-07 TCGA-G2-A2EK-01A-22R-A18C-07 TCGA-XF-A8HD-01A-11R-A36F-07 TCGA-GV-A3JX-01A-11R-A20F-07TCGA-H4-A2HQ-01A-11R-A180-07TCGA-XF-A9SP-01A-11R-A39I-07 TCGA-DK-A3IK-01A-32R-A21D-07

AC092802.3 0.0939763 0.06439407 0.04831901 0.022318166 0.011026865 0.06481334 0.05121622 0.04914837 0.1845426 0.02721552 0.03969581 0.088951768 0.2757515 0.074845722 0.134158017 0.03370327 0.1805234 0.02006826 0.02318985 0.3238117 0.155167763 0.3077708 0.1643177 0.1591313 0.1559606 0.1621569 0.04424554 0.3147748 0.07013194 0 0.09226692 0.110028138 0.02450519 0.04124147 0.1408469 0.113651088 0.3268885 0.06275813 0.4040916 0.1080562 0.01729199 0.06856047 0.3737915 0.18033 0.02032462 0.1125736 0.09175805 0.04972805 0.1175163 0.1278257 0.08766594 0.1946938 0.05590706 0.166278174 0.1482693 0.2628799 0.2652681 0.1076862 0.1569734 0.1016047 0.1007134 0.03050143 0.01702611 0.05419774 0.0497527 0.17163798 0 0.4839541 0.6892637 0.12555983 0.08026753 0 0.08983289 0.105206328 0.4165164 0.01797735 0.04771186 1.0361150.2497640.07956366 0.04784047 0.1292512 0.03319043 0.04433833 0 0.04954273 0.05103511 0.2229678 0.3063202 0.07963676 0.02597753 0.045420608 0.08298539 0.02816472 0.3711898 0.178584076 0.1659287 0.05250827 0.1089156 0.1546617 0.05367802 0.1430958 0.1904157 0.3039607 0.1425845 0.06830974 0.06899208 5.2756240.042059578 0 0.3374140.02474426 0.1072008 0.6481213 0.1618602 0.04927087 0.1191905 0.05798076 0.1447873 0.1249074 0.02455513 0.3173731 0.2584182 0.1886821 0.2837494 0.08019924 0.08795273 0.06823156 0.130343142 0.1127758 0.2675659 0.1171045 0.07196604 0.134484275 0.1377520.09099128 0 0.07899852 0.04712419 0.04746552 0.2623920.01898512 0.1600942 0.07549096 0.03153198 0.01052781 1.6235430 0.0466363 0.04396544 0.1178927 0.0630754 0.06787535 0.093216835 0.09478065 0.2958778 0.026506089 0.05953543 0.1505352 0.09546636 0.1500692 0.1068935 0.09556754 0.07143067 0.1439923 0.07066721 0.3659271 0.1283152 0.06179863 0.1561705 0.1393271 0.01466359 0.1067250.096205284 0.3133781 0 0.39414 0.1132618 0.1142686 0.02675025 0.2212425 0.2849665 0.03984392 0.1254225 0.1214628 0.1009660.01524397 0.2503781 0.1126734 0 0 0.1451080 0.05067948 0.1466790.01782301 0.1985523 0.1291799 0.09748069 0.052645513 0.3158001 0.2767768 0.1253451 0.06712817 0.121736655 0.1174680.1177988 0.2954153 0.0655407 0.2901179 0.2199845 0.0298943 0.03193471 0.2156781 0.1119005 0.1225168 0.1013392 0.1113511 0.295242581 0.064966298 0.1376743 0.159523211 0.093443856 0.01484273 0.16142679 0.1759161 0.1854217

0.08983605 0.018286831 0.3806125 0.01181285 0.2531786 0.118628454
0.0746363 0.128921 0.1272691 0.3779301 0.16976 0.1223666 0.1181505
0.2701449 0 0.1259428 0.2783975 0.137130477 0.07637652 0.102172863
0.1251878 0.1877991 0.1381192 0.07621279 0.2552458 0.5157579
0.01905482 0.02961309 0.1155498 0.053678197 0.08825991 0.2023658
0.1358974 0.1999295 0 0 0.08475262 0.1767637 0.05071222
0.7761814 0.06725237 0.2308706 0.02600026 0.01499186 0.215074862
0.08116516 0.08936564 0.05365435 0.1075861 0.49636 0.01662761
0.1185296 0.1024 0.1175205 0.1692976 0.132874218 0.055112009
0.09782246 0.08122744 0.147624 0.2709727 0.01673117 0.1293376
0.1095158 0 0.2855968 0.2405973 0.2222748 0.08428698 0.1512934
0.2617005 0.1592084 0.01630993 0.4604933 0.05574553 0.136303805
0.3469116 0.06145375 0.1326788 0.1023942 0 0.03026713 0.7207661
0 0.2462455 0.09301956 0.1461273 0.033873731 0.1115358 0.170166
0.0513943 0.05369353 0 0.109954974 0.1410504 0.05394532 0.3950106
0.07519834 0.01089594 0.1314304 0.096449022 0.181814 0.6677708
0.1249524 0.1138081 0.068018289 0.1142474 0.04393104 0.0569524
0.173276234 0.045626628 0.06134823 0.02016058 0.04583767 0.2548853
0.114537 0.05604295 0.1154153 0.05527253 0 0.1226844 0.02897266
0.1314772 0.137981824 0.01598289 1.947942 0.1097458 0.1891068
0.08468126 0.1117769 0.3226012 0.01147557 0.2047086 0.4546871
0.204408 0.01361513 0.2407329 0.1120485 0.05643116 0.08073654
0.02363523 0.01965146 0.9688755 0.1445304 0.2568286 0.2169491 0
0.2152335 0.01661198 0.1263864 0.1338044 0 0.1017773 0.4254793
0.2997982 0.2018786 0.2027029 0.1435333 0 0.106555 0.5124965
0.5135532 0.01288773 0 0.1306015 0.06933697 0.04549066 0.2371776
0.2455104 0.3195541 0.09550303 0.178511644 0.09221628 0.07234781
0.1520827 0 0.04429458 0.065124497 0.055003323 0.03180663 0.145725242
0.07673728 0.2685671 0.5189718 0.3365528 0.1232082 0.04591008
0.154437 0 0.2338001 0.03839017 0.4463328 0.037275353 0.05792656
0.5955177 0.05106004 0.04559388 0.3433053 0.05857076 0.1179773
0.5077722 0.0507842 0.3590239
AC009095.1 0.1786605 0.02295394 0.1033429 0.023866636 0.106127335
0.2079306 0.05476968 0.1751945 0.03946929 0.009701258 0.06367495
0.209271444 0.3402502 0.016007727 0.040990319 0.3784374 0.08774926
0.04292125 0.0123994 0.2597087 0.047409586 0.3097641 0.3631512
0.2917236 0.4093724 0.2023088 0.1182884 0.3946513 0.2624923
0.046474066 0.08457304 0.188259306 0.03930809 0.2058134 0.1694465
0.157997297 0.1823836 0.2237079 0.3745111 0.1925888 0.03698347
0.1026442 0.5329677 0.3856832 0.3042868 0.2708643 0.07008883
0.05317826 0.2513396 0.08201669 0.03124945 0.09463724 0.2192153
0.08890741 0.1233217 0.3255061 0.2502995 0.1612207 0.2637871
0.0724361 0.2154021 0.09785302 0.3641481 0.1159161 0.05320462
0.163152442 0 0.6437588 0.2456953 0.033567843 0.2918445 0

0.2470259	0.150007615	0.1346603	0.05767396	0.1530665	0.1146209	
0.2199589	0.02127098	0.3410648	0.08293133	0.05323986	0.09482919	0
0.09536415	0.109152	0.3506436	0.03448139	0.09580735	0.1250095	
0.255002802	0.1267758	0.06023767	0.1428997	0.136410375	0.2883417	
0.1123028	0.3261225	0.1819316	0.05740229	0.1391127	0.1425389	
0.2762925	0.2922481	0.1899279	0.07377886	3.908598	0.044977738	
0.07362061	0.4009159	0.1323053	0.3668433	0.08943083	0.04327257	
0.0878156	0.09104294	0.1705098	0.1407572	0.0954098	0.02625881	
0.1357572	0.1554456	0.1592946	0.3742382	0.1543745	0	0.1641725
0.116155465	0.02192734	0.2341064	0.03756882	0.2462693	0	0.1031166
0.1362262	0.1710961	0.05631971	0.1847771	0.05075875	0.07014929	
0.06090701	0.1391015	0.03229146	0.1011592	0.1013242	3.06386	
0.2445137	0.016624	0.1175396	0.10315	0.01686292	0.108877	0.119621242
0.07601751	0.1054687	0.056690248	0.04774957	0.09658775	0.08507497	
0.128385	0.1524132	0.06813212	0.168050617	0.1539827	0.2456032	
0.2152237	0.194392	0.1652158	0.3188294	0.09932924	0.01568097	
0.08876759	0.144032225	0.2680966	0.1348682	0.247933	0.03028002	
0.3221549	0.01430311	0.5027593	0.2437903	0.198839	0.08382781	
0.04329669	0.1511596	0.04890486	0.1427999	0.0535515	0.2369277	
0.0180413	0.4073364	0.046544244	0.3613047	0.1568558	0.05717879	
0.2547938	0.05180348	0.1303051	0.042223607	0.06432588	0.05223177	
0.078191	0	0.06509147	0.1449439	0.1049765	0.1579558	0.3270774
0.05909462	0.5153038	0.03196841	0.06830077	0.5958256	0.1675301	
0.2246009	0.1685759	0.2381537	0.052621164	0.046315842	0.3926036	
0.077541449	0.09992714	0.01587254	0.05754228	0.2037982	0.132191	
0.1801294	0.078222404	0.5426933	0.1263245	0.1547112	0.190288629	
0.1064196	0.08271942	0.1663435	0.221321	0.1815383	0.3107843	
0.06317397	0.5898129	0	0.03673114	0.1653962	0.081469338	0.1633513
0.046826477	0.178498	0.1479792	0.05680851	0.04075028	0.1364776	
0.3171367	0.1426381	0.01583385	0.2824385	0.071753095	0.3910174	
0.4918325	0.2634037	0.3207014	0.1064515	0.05627703	0.1268861	
0.4983463	0.151846	0.2075085	0.08989806	0.08229626	0	0.04809607
0.257055582	0.1410444	0.09556597	0.1004097	0.1882647	0.246442	
0.03556253	0.1109092	0.1095047	0.2154417	0.1991481	0.157881382	
0.137516794	0.07845716	0.1158175	0.08770355	0.3380687	0.03578401	0
0.1003836	0.09749228	0.1928918	0.0321613	0.6338577	0.05633434	
0.1040081	0.2176671	0.4086108	0.01744153	0.1561405	0.1043232	
0.131184703	0.3400658	0.2135819	0.1091418	0	0.01968416	0.04855067
0.6347551	0.04856665	0.2633304	0.3183149	0.4464739	0.024149296	
0.05111757	0.2079685	0.03664008	0.1148377	0.08072959	0.192409897	
0.1206694	0.04615051	0.5218093	0	0.1398231	0.04684976	0.132609612
0.111102	0.7639229	0.1336218	0.1770244	0.084860424	0.0244348	
0.04697905	0.1015064	0.102943564	0.146376828	0.0492035	0.1832545	
0.01633932	0.0170356	0.06999071	0	0.2821096	0.1477686	0.1231889

0.1874234 0.08262089 0.1687191 0.067070556 0.01709181 4.92093
0.1369201 0.1427487 0.1207421 0.1344737 0.2979406 0 0.4378232
0.2633768 0.1502807 0.1747172 0.3805566 0.1198226 0.4023097
0.04796565 0.2148382 0.02101491 0.3676476 0.2181998 0.1569416
0.1637656 0.01967913 0.09864291 0.05329364 0.1081242 0.143088
0.01924988 0.1360484 0.1516666 0.3624159 0.3728927 0.1083834
0.09767662 0.1195131 0.09115833 0.1534552 0.5814893 0.08269142
0.04238746 0.2793257 0.1038068 0.1216172 0.2874512 0.2250379
0.6346327 0.2772078 0.119310667 0.06574293 0.0928409 0.3090055 0
0.1302615 0.097500114 0.088229313 0 0.077917946 0.08206143 0.2513006
0.8324684 0.4766288 0.0768580.08182566 0.1101014 0.02173601
0.03788205 0.1094766 0.4483728 0.079723153 0.1445397 0.3442355
0.2002098 0.02437863 0.2723826 0.1127421 0.1835095 0.8720946
0.07241025 0.2972389
TXNDC12-AS1 0 0 0 0 0 0 0 0 0 0.03289992 0 0
0.07692614 0 0 0 0.05951689 0 0.04205014 0.07339593 0.080390167
0.06565653 0.07945525 0.04122181 0.1028375 0.09801298 0.0401152
0.0393642 0.0635851 0 0 0.039902788 0.04443524 0.09971082
0.1276988 0.041216686 0.05154319 0.2275985 0.2930956 0 0 0
0.1807456 0.05449872 0.07370924 0.0510324 0 0 0.03874405 0
0.1059764 0.0962831 0.1351683 0 0 0.1003537 0.05658946
0.1562138 0.1626513 0.1228264 0.2191482 0.1106165 0.06174686
0.1965534 0.1804331 0.138324897 0 0.04280749 0.05554851 0.113838771
0.05821962 0.1338253 0.1396234 0.190770553 0 0.1955899 0.1153545
0.3887144 0.2131279 0 0.2313309 0.09374847 0.1203684 0.09647839
0 0.07186863 0.07403354 0.09513114 0 0.1805066 0.3768403
0.041180577 0 0 0.05384625 0.092521819 0.6769762 0 0 0.1121792
0.03893373 0.1887094 0.1381123 0 0 0 0.125103285 5.877326
0.3050664 0.2496699 0.1359628 0.04486874 0 0 0 0.1191238 0
0.2102729 0.1432051 0.2588509 0 0 0.0585737 0.1440577
0.3087136 0 0 0 0.078783707 0 0.0882140 0 0 0.04995711
0.06599777 0 0 0.2848342 0.1721384 0.0396496 0 0.03628734 0
0 0.03818013 4.5025420 0.2818852 0.0797225 0.07773626 0
0.1230783 0 0 0 0 0.1619333 0.2183723 0.1154060.2721204
0.1938299 0 0 0.06527529 0.1922112 0 0.07755792 0 0
0.06316044 0.1063579 0 0 0.07576642 0 0.04204081 0 0.263713
0.2910372 0.2005894 0.06889727 0 0.1705714 0 0.1220544
0.05528375 0 0.04540236 0 0.1223671 0.2631242 0.052615233
0.06126471 0 0.06463689 0.04800464 0 0.2209521 0.095462069
0.05453716 0.1771338 0.03788135 0.2434469 0 0.03276994 0.14240300
0.1584599 0.1002039 0.1139708 0.0542073 0 0.13036300 0.1269483
0.08167033 0 0.089227191 0.039267779 0.1664298 0 0 0 0
0.1063295 0.1494333 0.04072491 0.198956985 0.2300548 0.08568096
0.08744544 0.071702962 0.09022529 0 0 0 0.07695644 0.27735900

0.08164225	0.08555764	0.04152216	0	0.110514928	0.09232899	0
0.03783381	0.0716918	0.2697169	0.1381966	0.1322391	0.09352234	0
0	0.1795941	0.048667316	0.09145236	0.06671814	0.0924085	0.0453165
0	0	0.06147275	0.2331095	0	0.1919249	0	0.05581833	0
0.2174779	0.045881774	0.07358837	0	0.04864569	0	0.06428923	0
0.05373243	0.1237879	0.06088568	0	0.107084763	0	0.133036	0.0981931
0.05948588	0.1637849	0	0.05211727	0.05673856	0	0.3270773	0
0.2015254	0.03820938	0.1567659	0	0.3175617	0	0.0814646	0
0.247159585	0.05242122	0.1671511	0.03701329	0	0	0.05488337
0.2306408	0	0.1880071	0.06746892	0.1135597	0	0	0.08816055
0.06212884	0.04868121	0.1095114	0.03625114	0.1364089	0.0391276
0.1685347	0	0	0.07944089	0.149906518	0.1412928	0.3942362
0.05035023	0.03752148	0	0.08286585	0.07966012	0	0.069822591	0
0.08343202	0.1462287	0.05541161	0.05777291	0.1186799	0	0.1793849
0	0.0464190	0	0.1430445	0.181965335	0.1738906	4.0953060
0.04034202	0	0.05067124	0.1595380	0.08248842	0.06870698	0	0
0.1138750	0.2728709	0	0.04285774	0	0.05667294	0.1233303	0
0.09256319	0.06673791	0.1115094	0	0.04583527	0	0	0	0.0428623
0.04727164	0.0665575	0.08168024	0	0.04053054	0	0	0.4382238
0.09347726	0.07187439	0.05262657	0	0.08248817	0.1146864	0.1907930
0	0	0	0.1049506	0.1103086	0.08116588	0.08031932	0.04723608	0
0	0	0	0.06087407	0	0.1319505	0.03723555	0	0.0933468
0.07371344	0	0.09281715	0.0490506	0	0.1400509	0.2918518
0.06172476	0.2480260	0.1274476	0.4278565	0.05580261	0.0613913
0.1260034
AL450998.3	0.2221456	0.07941795	0.06704145	0.06193182	0.163194719
0.3447199	0.07895695	0.2576148	0.01706989	0.1174782	0.3121028
0.148102082	0.2844963	0.069231072	0.088638556	0.8261368	0.2580618
0.3898190.02145023	0.3650406	0.22554345	0.2176987	0.5471684
0.2943877	0.4721268	0.6624667	0.1637056	0.4016021	0.1946127
0.107196542	0.1950752	0.33585497	0.2493359	0.4196245	0.2117068
0.252300968	0.3286593	0.2322010.5731265	0.09995019	0.03198961
0.1395181	0.2996509	0.6672094	0.3195990.3254023	0.2667490.1073278
0.1877557	0.2601208	0.2702985	0.1882744	0.1379017	0.307609199
0.1676237	0.3967341	0.4618704	0.3486279	0.3318808	0.2976117
0.7638977	0.1974933	0.1102421	0.1503961	0.2147620.105841555
0.1618355	1.1791750.3541987	0.130658394	0.3118339	0.1365315
0.2492819	0.210847298	0.5196677	0.2328026	0.4119051	0.4626707
0.4348755	0.2207853	0.3097616	0.2869327	0.06140122	0.04101223
0.018758693	0.2108006	0.2454745	0.3882195	0.08947616	0.1933646
0.1441728	0.199563278	0.2631776	0.2692030.1098702	0.377571104
0.2494071	0.09713859	0.06044709	0.4005667	0.1390236	0.1323612
0.3346497	0.2389849	0.3407116	0.1769191	0.255266194	3.508401
0.207490259	0.08490623	0.5895269	0.2746564	0.2379815	0.2320651

0.1871475	0.1063411	0.09449936	0.1877094	0.1704511	0.2145694	
0.2952703	0.3963127	0.2689117	0.4684695	0.2712124	0.148366	
0.0677957	0.1104479	0.34160163	0.1232823	0.168746	0.2707994	
0.2662697	0.124395761	0.5478988	0.1683309	0.09866206	0.1096085	
0.04358911	0.1536669	0.1516927	0.245853	0.1851057	0.1117247	
0.05833314	0.2434513	5.432805	0.3806948	0.1150342	0.1830029	
0.1189623	0.07292964	0.2668303	0.206937726	0.1534235	0.380114	
0.1225885	0.2753465	0.3063338	0.2060446	0.2359797	0.3295824	
0.206263	0.264288819	0.2996787	0.1960981	0.2369336	0.3758498	
0.1714882	0.3808364	0.2845997	0.05425431	0.1755002	0.391548604	
0.2512203	0.1999835	0.2359002	0.1309566	0.2882633	0.1855766	
0.6650985	0.2635893	0.1474199	0.261031	0.2122187	0.2739495	
0.05640169	0.2007162	0.196862	0.1676745	0.09363119	0.5872234	
0.093938612	0.3593957	0.1973464	0.2802618	0.2938523	0.2837871	
0.4395693	0.219133085	0.4173	0.1505965	0.1739132	0.1448824	
0.05630221	0.2841771	0.2905652	0.3643391	0.5052007	0.3066907	
0.4844811	0.1659104	0.09600202	0.6982454	0.2277135	0.5018723	
0.135398	0.3237079	0.273094572	0.230355604	0.7216301	0.147556369	
0.072028369	0.1784807	0.199089531	0.2576393	0.2858534	0.3323875	
0.202980251	0.6454444	0.218534	0.3122481	0.292611694	0.06903736	0.1669497
0.2354437	0.2829926	0.1766534	0.6508257	0.2732181	0.6351107	
0.2182194	0.1800377	0.2765882	0.155030889	0.3532352	0.108009408	
0.2508927	0.2742807	0.1572406	0.2114868	0.1686415	0.465141	
0.1233778	0.1643497	0.3817204	0.260670146	0.2332542	0.6126057	
0.2356929	0.1271404	0.1956646	0.07301697	0.1724685	0.2972792	
0.1594871	0.7505894	0.1088629	0.09965739	0.04809963	0.01386723	
0.36277429	0.1876911	0.2479853	0.285369	0.2894994	0.4755222	
0.2153238	0.109638	0.06314555	0.3105845	0.2975353	0.31409435	
0.11894799	0.1809684	0.1502681	0.1365498	0.5012906	0.09285635	
0.02658559	0.08682887	0.08432796	0.5005371	0.1669115	0.4112012	
0.3703294	0.2299079	0.9951715	0.3387098	0.09051849	0.5298388	
0.09023649	0.214333955	0.4947017	0.412117	0.1321662	0.07892746	
0.05107866	0.139983	0.7255231	0.04200873	0.5634386	0.2064998	
0.3765322	0.261105407	0.05895359	0.3597733	0.1426167	0.1614133	
0.1536232	0.277381522	0.1739591	0.09979708	0.6125461	0.1391145	
0.1511786	0.2228802	0.216662019	0.2282375	0.416573	0.2054736	
0.162691	0.188747424	0.1268123	0.2234952	0.1931601	0.409598542	
0.253223319	0.1702383	0.3636401	0.1413303	0.1915588	0.1816197	
0.1814358	0.2745186	0.06390774	0.224949	0.2755968	0.11613	0.3283584
0.081219687	0.02956783	5.640462	0.1861072	0.1337629	0.195822	0.4523392
0.1898915	0.148606	0.2103912	0.578295	0.2599768	0.1259377	0.3872591
0.440483	0.3305867	0.1078711	0.2404842	0.1272409	0.9251035	
0.4167928	0.2714997	0.3069137	0.1872405	0.1279848	0.1997555	
0.151977	0.3005768	0.08325291	0.2118202	0.5247486	0.3375929	

0.2206861	0.2916619	0.1569052	0.1447255	0.3351094	0.7774435
0.4610569	0.1192094	0.4033030.3087220.3078507	0.1788320.4533966
0.4541862	0.5067126	0.3281153	0.227040495	0.1232090.1338411
0.2813481	0.08280719	0.1434012	0.337338948	0.254385885	0.1323927
0.134793472	0.1064711	0.2173677	0.4800405	0.3533742	0.2279311
0.2831072	0.2857033	0.09400507	0.2031545	0.3077557	0.3127656
0.206874562	0.1607434	0.5955072	0.2046621	0.1054340.5326676
0.08668319	0.3769846	0.6120086	0.17224	0.5034932
AC079313.2	0.1762677	10.672690.1297658	7.672038785	0.067688719	0.2237954
3.9823015.07859412.7442217.987141.3402060.409524698	1.3509111.263467062
12.823581616	0.2327495	0.07555553	0.3695687	0.01779394	0.01552912
0	0.1389161	0.05043345	0	0.02175837	0.7672910	0	0.08072004
0.022231101	0.7484348	0.033770541	0.09401611	0.02109682	0.4052779
0.06976504	0.8070080.03210354	0.08268426	1.4924380.6103473	0.02104302
0.03824216	0.6687889	0.03119081	0	0.1408148	0.05087617	0
0.1176993	0.1569574	0.05432423	0.02859892	0.042529302	0.02528208	0
0.02394641	0.04957761	0.03441378	2.8846270	0.3276593	0	0.02772453
0.0254507	0.234134349	0.02684998	0.01811442	0.04701183	0.457634325
0.04927245	0.05662956	0.03938869	0	0.1337859	0.02758862	0.1708470
0.4509362	0	0.5383951	0	0.6876239	0.04082581	0.155611883
0.1368537	0.2506244	0.02012786	0.04948303	0.2444263	0.1594638
0.15683382	0.01819315	0.02881499	0	0	0.06365986	0.5909264
0.2005744	0.07120464	0.1153264	0.1397450.08766541	0	0	0.02096606
0.088231213	0.2116638	0.430306502	0	0.02876702	1.4619730	0
0.03104947	0.05040843	0	0.02224478	0.02019957	0.08215155	0.07536619
0	0.2726466	0.01523987	0	0.4430748	0.3599335	3.4030860.300043143
0.01573358	0.03732865	0.1078274	1.3032080.165107101	0.1056993
0.3351315	0.1227670.04041122	0.0482122	0.2185263	0	0	0.06142143
0.1390210.09677999	0	0.1099209	0	0.04771304	0.1349416	0.01644744
0.07259814	0.05208185	0	0	0	0	0.04568249	0.1386097	0.02441762
1.1284750.05468072	0.09777401	0.285011465	0.0276219	0.02711204	0	0
0.02370954	0.4575409	0.3563598	0.02250321	4.2401760.029527942
0.03206133	0.3041416	0	0	0.06376735	0.2463107	0.02122036	0
0.6726033	0.1202984	0.9941374.3694670.04678777	0	1.2488110.06181939
0.02589044	0.02783592	0.044529342	0.8555179	1.4938340.1367588
0.1625092	0.09912181	0.1121977	0.040395745	0.0461559	0.02498533	0
0.2060341	7.052491652	0.4576087	0.4820741	0.1511179	0	0	0
0.09175349	0.04900802	0	0.6182139	0.02685974	0.01727982	0.04882372
0	0	0.1056397	0	0.119501613	0.7061220	0.06749163	1.8337930
0.140317787	0.03245001	0.05438516	0	0.091025513	0.0763595	0
1.4756850.01380913	0.0325649	0	0.2266469	0	0.07240917	0
0.04747087	0.023382758	0.1172098	0.134398071	0.4963026	1.183147
0.01630478	0.2631568	0	0.01978747	0.5556006	1.4542460	1.503367111
0.03869899	0.02823246	0.0782072	0.03835227	0.4773902	0.04038059

0.2341154 0.04932133 0.1089544 0 0 0.3543014 0.3990083
0.06902098 0.01941534 0 0.04571446 0.06175476 0.01500956 0 0
0.04547485 0 0.05152879 0.0519619 0.022657004 0.056384437 0.2064170
6.444047 0.8085843 0.2310857 1.499669 0.8643419 0.1165898 0.3229476
0.04615355 0.2558326 0.04850606 0.09950561 1.115595 0.1465958
0.3003568 0.03447257 0.192485 0 0.02218257 0 0.7987901 0
0.02824805 0.1625712 0.1301307 0.1045445 0.01988928 0.1142006 0
0.034655809 4.670398 0 0.1051618 0.1441997 0.06951132 0.460201161
0.6349509 0.04967173 0 0.05770089 0.1839339 0 0.253737872
0.3786668 0.02383215 0.06391864 0.04763279 0.104383045 0.2103932
0.03370899 0.2330693 0 0 0.1294521 0.03093908 0.07034396
0.09778878 0.1004412 0.2580159 0.02530285 0.1060287 0.1767841 0
0 0.04035382 0.115500724 0.2943343 0.6065396 0.05613979 0.119498
0.3682046 0.1500944 0 0.1408862 0 0.05814812 0 0 0.2730617
0.08597662 0.1154681 0.04130029 0.01813569 0 0 0.01304712
0.3096794 0.2741834 0.5648165 0 0.07647983 0.0969783 0.02933436
0.1381241 0 0 0.04000695 0 0 0.02002461 0.06860364 13.40886
0.06291948 0 0.05933378 0.1216575 0 0.02128135 0.1396228
0.1213268 0.026912 0.02335229 0.3559363 0 0.1257938 0.1332327
0.0233391 0.1030385 0.9176743 0 0.147717258 0.04881147 0.027954328
0.08832261 0.1030379 0 0.3210581 0.1418094 0.1174251 0.2962549
1.933941 0.07610848 0.4909565 0.04151252 0.019067984 0 0.09880006
0.02611946 0 0.01699507 0.05393074 0 0 0.961199 0.1244125
ZFAS1 29.96944 9.593807 21.47436 11.767156233 19.99997942 34.35693 21.30494
28.32645 9.344932 15.47908 36.47595 23.742135007 26.68545 42.906733057
12.261569415 38.19251 49.35383 69.05497 48.02004 65.22578 42.419235446
15.50832 33.10997 23.29633 84.76615 22.26258 35.95742 37.37716 32.70936
73.044717822 45.5225 13.869981293 102.0452 50.63261 44.07573
23.041965162 26.90113 25.3391 54.07285 41.76135 70.23684 81.38981 28.48286
33.1177 826.99575 36.34895 61.0663 66.4275 60.33905 48.93855 38.00987 49.81515
30.0187 38.123243649 83.57966 86.97848 36.6177 41.18721 39.80816 52.27524
54.20856 56.65249 28.9544 18.55924 41.20358 18.325736182 142.5848 49.26548
43.90373 29.20101521 75.63938 100.5923 40.23263 25.395844416 33.35554 47.4606
27.41809 80.46587 32.90781 106.6596 101.1871 51.21659 11.37258 126.164
49.526787666 16.29912 44.87254 24.69249 74.0567 27.06699 77.45772
26.288042523 68.52346 16.98044 19.87119 58.239701037 34.74755 54.99331
54.47827 49.07349 53.08738 33.30704 31.82813 72.68475 36.19888 42.27779
20.935697336 14.71408 23.212724075 50.25396 55.67377 23.67575 54.32535
40.10779 39.86885 40.56573 171.165 29.91032 17.4079 109.8066 53.44736 30.1361
103.8211 81.26922 37.74224 63.00116 119.305 36.65417 23.305892366 135.2932
65.92323 22.89075 36.09664 44.652232702 24.6103 37.51695 22.96044 67.69638
58.72877 30.78737 40.93122 36.05456 71.78938 28.47247 24.94762 39.26118 21.64467
13.74186 91.1613 44.58233 90.31737 40.97215 69.71811 52.707583054 53.17837
59.79387 23.761225703 46.12493 94.65281 96.2124 19.71443 32.00892 71.35219

20.537715639 49.79361 19.24377 27.14718 42.63502 74.77695 30.94714 48.23907
74.47645 20.87833 31.261441778 54.34932 37.42772 40.98129 18.7868 30.38649
24.50435 48.8748 25.3655 20.20149 30.45755 29.96417 27.83821 145.5101 69.56155
27.50304 72.04442 118.3553 23.87421 46.052074848 33.55553 71.94915 22.98572
26.85347 78.15448 74.39826 47.751229028 19.06138 37.5888 29.3921 52.00032
46.099386404 23.80953 35.69648 31.6244 31.82831 63.15396 25.22497 40.96325
51.23405 17.71405 63.31433 40.75174 57.27457 94.45998 25.088752736
14.607578946 72.31256 12.98199416 33.485214949 78.46409 54.972143352
36.82979 66.12598 28.02043 26.994314604 54.01897 34.3302 81.40028
15.236607611 77.96411 53.40038 48.9543 39.39389 31.40464 36.78411 27.56187
46.03304 62.1782 33.36786 25.57865 52.015430094 41.36363 23.687015844
33.4904 27.00046 35.99494 97.10858 31.73099 25.65249 44.3061 60.56266 38.31959
39.143753922 30.76006 20.5275 90.94949 112.6401 53.53087 72.26121 47.4073
15.64473 27.74447 35.28531 24.2555 21.10493 53.60131 119.3336 53.283447567
13.70023 58.54867 46.31734 49.03866 79.99489 32.28172 31.57495 75.46077 30.80066
47.71312 77.281493466 37.663345724 40.3289 56.5891 26.31801 55.92188
31.06542 15.7808 14.24257 55.42543 47.22838 20.87614 121.8891 32.3575 36.88522
103.035 48.70443 87.88028 35.66561 63.88442 26.350715869 23.50312 45.05674
28.98956 149.4241 70.37897 48.46781 16.39037 137.326 46.98068 99.95835 19.65057
37.782494806 31.9418 41.44792 65.20927 63.99379 70.09865 24.139735985
34.80029 70.78551 44.67075 73.1515 20.52024 22.79405 26.767592086 27.98809
62.57747 78.04559 28.85124 29.046781144 50.74593 178.4536 39.37712
19.766486123 12.528194561 44.47033 29.499 29.49002 48.13928 56.15837
49.47763 35.83587 79.05783 28.41019 14.74742 23.20052 72.46264 17.866412047
49.81376 40.90613 56.67195 28.40397 58.9594 26.21639 15.16609 54.85301 120.3699
50.95019 70.45966 54.06266 58.91688 102.3224 17.17165 53.71756 21.28478 33.58054
94.31704 31.72708 29.28355 89.3833 20.83606 118.5252 33.08102 19.47964 21.95707
79.23895 35.03448 25.78505 85.22218 53.45185 76.18314 71.98994 76.11435 25.36886
73.93558 20.9312 26.38155 48.33746 22.70533 28.00997 32.54046 55.4882 84.44476
86.79105 52.75997 47.948462386 25.89084 126.7569 23.34401 100.7256 50.46759
34.88006514 18.942318105 24.39076 53.548179111 160.0426 88.94621
12.79544 18.60256 32.98678 21.5357 30.33143 117.0225 52.43077 21.09049 47.99399
35.096243376 95.4209 61.7325 80.27936 87.7401 38.26797 36.82043 30.16583
45.96251 59.18061 23.27551
AC027449.1 0.254657 1.495672 0.5471208 1.399628697 0.461015058 0.3387174
2.557623 2.625594 0.7072471 3.097442 0.8989589 0.433874815 0.7020692
2.503341127 2.804462927 0.7045391 0.2858858 0.4195104 0.2423824 0
0 0.06307543 0 0.1584052 0 0.1412398 0.1541528 0.1890835 0
0.100941216 1.056228 0.038334128 0 0.2394775 0.06133935 0.039596374
0.1485508 0 0 0.878432 0 0 0 0.2617813 0 0 0 0 0 0
0.05090512 0 0 0 0 0.09640855 0 0 0 0.1769968 0 0 0
0.06294218 0 0.132887063 0.3047835 0 0.3735535 0.492135917
0.05593089 0 0 0.244361299 0.0337478 0 0.1108197 0 0.2047494
0 0.0555592 0 0.1156365 0 0 0 0.4623003 0.04569567

0.05616992 0.2080926 0.04525325 0 0.1239101 0 0 0 0 0 0
0 0 0.1359681 0.1326828 0 0 0.2379932 0.08012348 0.2402671
0.244228015 0.07995162 0 0.3879437 0.07469812 0.03642054 0
0.1144408 0 0 0.5503017 0 0 0 0 0 0 0.05588331
0.153215 1.188604 0 0.03571947 0.04237307 0.2039978 0 0.187418871 0
0.06340326 0 0.04587219 0 0 0 0 0 0 0.05492918 0 0 0
0 0 0 0 0.05911993 0 0 0.05726927 0.046173998 0.1037116
0.3146815 0 0.3137074 0.06207001 0.1109867 0 0 0 0 0 0
0.2473194 0.06067747 0.05108837 0.08262957 0 0 0 0 0 0
0.139798 0.04817596 0 0.3701797 0 0.4231799 1.383623 0.1593313 0
0.1308525 0 0 0 0 0.05885627 0 0 0 0.2250333 0 0 0
0.2268938 0 0.07795885 2.014636957 0.09444505 0 0.1143595 0 0
0 0.1041526 0 0 0 0 0 0 0 0.113172249 0 0.101051559
0.054260192 0.3102751 0 0 0.6460146 0 0.06371186 0 0.04115633
0 0.068884172 0 0 0.5419447 0.06270093 0 0.1065822 0.1029099
0 0.7397477 0 0.2514673 0.212340722 0 0 0.2544254 0.03443672
0 0.1991457 0 0 0.1327751 0.5158643 0 0.607803363 0.08785718
0 0 0 0.1734088 0.04583743 0.1181123 0 0.1766829 0 0
0.160872 0.09058567 0.05223209 0 0 0.3113525 0.2336667 0 0 0
0 0 0.1169843 0 0 0.064003955 0.04260204 0 0.8572106
0.2622436 0.05829188 0.1502053 0.2725403 0 0.1047397 0
0.06453434 0.03670729 0.03765077 0.3039241 0 0.05682426 0
0.04855477 0.047488648 0 0 0.5333734 0 0 0.05272579 0 0
0.04515403 0 0.03636514 0 1.276809 0.08469478 0.1790593 0.1403024
0 0.034826034 0 0 0 0.1964949 0.2657325 0 0 0.1357383 0
0.2902252 0.2883715 0 0 0.07652852 0.2645651 0.067077723
0.105976344 0.05343476 0 0.05323326 0.05550174 0 0 0 0.1444283
0.1337825 0.06106228 0.06729441 0 0.39332679 0 0.1967155 0
0.1550244 0.049172 0.04867925 0 0.1999061 0 0 0 0 0 0
0.06553595 0 0 0 0 0 0.2556567 0 0 0 0.1736299 0 0
0.06271583 0.08864876 0 0 0 0.03923461 0 0 1.113722 0 0 0
0 0 0 0 0 0 0.053016 0.04753362 0 0.1784912 0 0 0
0.1157427 0 0 0.1662228 0 0 0 0 0.03169081 0.03577175
0.3199041 0.1345157 0.2124468 0 0.1337525 0 0 0.4709083 0
0 0.397126 0 0 0 0 0.3538673 0.04035
AL035530.2 0.2049078 0.4056177 0.4389823 0.37308287 0.208374395
0.4710677 0.5707725 0.6429846 0.4560312 0.3428606 0.2163839
0.271533136 0.4470864 0.293751235 0.543253033 0.2449576 0.4532557
0.3063007 0.2696727 0.1985753 0.177220968 0.05263291 0.1194272
0.1652253 0.3091451 0.1571423 0.3858955 0.4260049 0.140174
0.063172334 0.3448811 0.991618021 0.133579 0.3596949 0.1279606
0.272587854 0.2375848 0.06081737 0.4699144 0.3926797 0.2010869
0.1694227 0.2173394 0.5024162 0.1477207 0.07159184 0.4573054
0.4578073 0.01552939 0.1486474 0.1380518 0.1157766 0.05417818

0.171207144	0.2394736	0.2916223	0.1701164	0.07044035	0.2200294	
0.1846174	0.07319918	0.1884335	0.08662284	0.1969564	1.458478	
0.304938688	0.1271624	0.5404802	0.512094	0.159701034	0.2566915	
0.1340997	0.09327307	0.382323354	0.3660884	0.1567928	0.3236548	
0.06491855	0.1388172	0.3469644	0.2202149	0.3945508	0.06030758	
0.6251727	0.058958639	0.1656368	0.2077189	0.1620545	0.105459	
1.244432	0.2265678	0.115542018	0.04308168	0.2524672	0.2589919	
0.166880719	0.3165699	0.1526535	0.4116351	0.1011683	0.1092379	
0.25528	0.2352722	0.08836827	0.1554471	0.2184511	0.183861004	
0.8270187	0.448348056	0.1834666	0.2043623	0.1438743	0.1714113	
0.1063681	0.07352566	0.2029256	0.1856324	0.2844504	0.5644283	
0.155629	0.07138735	0.1499348	0.2347752	0.166006	0.1649848	0.1865259
0.2876609	0.3347403	0.236835695	0.1788354	0.1149133	0.2468258	
0.1150719	0.488720403	0.09010716	0.05290647	0.1938095	0.2679444	0
0.2414878	0.1112465	0.09658959	0.334528	0.2304428	0.1260471	
0.1071236	2.342651	0.04431575	0.05649263	0.1118403	0.04673738	
0.2177572	0.1973291	0.149051378	0.1205526	0.310622	0.375663694	
0.2271714	0.262584	0.1618997	0.1963284	0.2201242	0.3009896	
0.488013165	0.1962274	0.0256807	0.07978749	0.1243471	0.03368675	
0.2373303	0.1814311	0.1492063	0.1723743	0.083907182	0.1062905	
0.07856861	0.168508	0.4115971	0.2189531	0.2527494	0.2311508	
0.138077	0.2123648	0.1367369	0.2452222	0.557709	0.06647652	0.1698447
0.2456754	0.1903061	0.6130899	0.2504804	0.147624697	0.2210051	
0.5524195	0.1295388	0.1924122	0.340347	0.2302613	0.153052461	
0.5464897	0.05916566	0.06832624	0.1626307	0.552993904	0.1116462	
0.2568509	0.2624231	0.1508455	0.150614	0.06852267	0.1303643	
0.1508673	0.1175672	0.1789261	0.3053006	0.3191673	0.5433922	
0.160938229	0.070826917	0.5336673	0.158103476	0.135831254	0.582541	
0.078217389	0.127857	0.4342452	0.2203654	0.106327943	0.215158	0.2575699
0.1401995	0.273029883	0.1084923	0.3560624	0.5961114	0.2354418	
0.07711422	0.211225	0.2146814	0.3190576	0.2914924	0.1581077	
0.2997648	0.088593207	0.3885768	0.127302734	0.4321897	0.2442519	
0.138996	0.2492639	0.03975307	0.1874282	0.1384917	0.2690374	
0.3239321	0.468164117	0.09163985	0.1470808	0.2160619	0.4540941	
0.2351373	0.200806	0.2587149	0.2102287	0.4054378	0.07692734	
0.09775896	0.2349177	0.09448582	0.1416504	0.468953594	0.09586101	
0.1082526	0.1754835	0.09241149	0.1932631	0.1329357	0.08614815	
0.05788612	0.3294568	0.18457	0.321912935	0.080111563	0.4088137	
0.03935777	0.3934118	0.4485965	0.1702446	0.3237895	0.3354438	
0.09939116	0.6664207	0.06557542	0.094238	0.3369319	0.2356309	
0.3381435	0.08099961	0.213375	0.08979479	0.1519359	0.158506444	
0.2941607	0.2009925	0.1631924	0.161245	0.1872972	0.4179688	
0.3235592	0.165042	0.1601336	0.3109933	0.1289648	0.073858971	
0.521132	0.1413461	0.1494149	0.09756204	0.109736	0.167097131	0.2323705

0.4626523 0.2279883 0.1776276 0.2850929 0.254732 0.140199603
0.3114815 0.3950445 0.3229021 0.3083069 0.06591487 0.2158929
0.1596469 0.1655735 0.181910934 0.077377397 0.1839268 0.293057
0.3442563 0.04631309 0.2021695 0.101831 0.1078516 0.1606898
0.1767538 0.1783356 0.04913423 0.0573351 0.109403044 0.1510142
1.661999 0.4254078 0.1131893 0.1333516 0.2031005 0.04263069
0.1501294 0.2314411 0.05507828 0.2507036 0.04947783 0.4792555
0.1628752 0.1777297 0.1043198 0.1975498 0.02856562 0.3520926
0.4201827 0.1599984 0.2597083 0.08024969 0.0893904 0.2294003
0.06430094 0.1250357 0.2878306 0.3328758 0.1632107 0.3221062
0.2134207 0.2291736 0.2181256 0.3898909 0.4027133 0.08939662
0.1207586 0.2622729 0.2160652 0.4218758 0.120947 0.4628808
0.5401312 0.2804035 0.353911 0.07932828 0.291922809 0.2308584
0.1577486 0.1547487 0.01626646 0.20121 0.170398531 0.149912953
0.1964968 0.211828183 0.1394329 0.3415937 0.3771922 0.1189989
0.1268604 0.456026 0.3273836 0.1329562 0.08753841 0.1581127
0.3440582 0.189643852 0.2385747 0.2924502 0.1855539 0.2485345
0.2656145 0.3150152 0.09354189 0.1453842 0.2952824 0.4545424
AC004846.2 0.2477276 0.1958618 0.2204516 0.203649678 0.221360405
0.3844173 0.03115598 0.08969419 0 0.03311163 0.1086654 0.064933646
0.1741976 0.382454894 0.104928885 0.1845222 0.1197998 0.512735
0.1269622 0.4062753 0.242722435 0.1321581 0.5797575 0.3733837
0.1034993 0.3452529 0.4239201 0.8319676 0.3199713 0.26436985
0.2886586 0.120478687 0.1118029 0.2759693 0.1606507 0.228150537
0.1296872 0.4199491 0.7374542 0.262932 0.1893439 0.3002895
0.272863 0.191973 0.2596424 0.3081648 0.4784442 0.2420056 0.2339602
0.1866223 0.2399813 0.1938054 0.1360381 0.227589238 0.2705866
0.2272487 0.7973506 0.6485287 0.2455469 0.3399463 0.5513961
0.05566414 0 0.8242428 0.1210628 0.556860073 0.1915782 0.3015807
0.1677179 0.085928494 0.3222684 0.1346865 0.6557685 0.191998163
0.6187097 0.1640405 0.0870726 0.3260131 0.3485615 0.653405
0.2910244 0.1887035 0.1211429 0.09709923 0.037010394 0.09041388
0.1490199 0.4308449 0.5001798 0.4723372 0.2133367 0.082891148
0.1947159 0.1027994 0.2709637 0.605261787 0.2649626 0.03194197
0.07950698 1.157236 0.2155135 0.07122141 0.4517533 0.05547184
0.3903183 0.1246631 0.272801372 1.51025 0.12792896 0.2093971
0.4105132 0 0.2347655 0.09538714 0.2584659 0.08991775 0.279667
0.1322663 0.2642322 0.5535979 0.2240616 0.1158391 0.05895062
0.5255697 2.433818 0.08781662 0.3477736 0.2490408 0.118936021
0.2993632 0.08878166 0.5342799 0.05253428 0.147257684 0.2262536
0.0996337 0.2919864 2.739225 0.1433336 0.2165576 0.4189998
0.03464723 0.5112919 0.578628 0.402814 0.2497679 7.407285 1.168379
0.7659877 0.3209421 0.09779562 0.1438882 0.1238703 0.170117725
0.08648582 0.1799891 0.169304658 0.4074384 0.1648332 0.4065204

0.3286458 0.2926157 0.4069513 0.078215205 0.1642383 0.1934481
0.2671221 0.4293135 0.5075123 0.5441027 0.1059448 0.1338029
0.2164108 0.351143097 0.3812699 0.2959215 0.3384907 0.1033496
0.4739465 0.3905467 0.3028203 0.2426922 0.2423796 0.286115
0.4187018 0.1105558 0.5285753 0.3046213 0.1370836 1.065967
0.4926182 0.6620434 0.185338343 0.2466358 0.9733978 0.2276849
0.6763896 0.2357492 0.2223739 0.096076368 0.2744405 1.753025
0.2859384 0.2450135 0 0.5276930.5374475 0.1198052 0.2591543
0.3529706 0.2485257 0.08183419 0.07284976 0.4920069 0.2042148
0.2555305 0.3082346 0.4064244 0.404106158 0.158081871 1.172505
0.264658844 0.170532031 0.7042752 0.196399132 0.5083153 0.1503949
0.79924600.233610154 0.6174271 2.6085280.8800815 0.469068409 0.1362088
0.7058298 0.3612965 0.6568668 0.2323550.8095170.5929572 0.1643352
0.1722164 0.1462628 0.1505382 0.389291324 0.09292312 0.372924377
0.6663523 0.1262680.1551158 0.6954294 0.09981756 0.6353381
0.4520676 0.5404293 0.2108747 0.171431718 0.4371941 0.3693111
0.1705058 0.4788852 0.2270829 0.3361412 0.4021441 0.2932619
0.1480771 0.9014129 0.1227334 0.05617753 0.5219460.2462370.438681727
0.2036702 0.05436314 0.4651079 0.1070952 0.6146778 0.2427589
0.2433519 0.1661126 2.2672670.3089627 0.511925809 0.603465863
0.7587221 0.6917748 0.1197374 0.6318821 0.2442707 0 0.171311
0.3327536 0.2743184 0.1097706 0.0338037 0.6729670.2761057
0.2122645 0.2760227 0.1785905 0.1639777 0.2797680.223875053
0.6067233 0.2803778 0.1862574 0.4048765 0.3023305 0.3314193
0.6963750.08288209 0.3311296 0.5092731 1.14290400.144243095 0.2326277
0.3105475 0.5627577 0.4654475 0.2204323 0.510781829 0.20593
0.4134836 0.5936672 0.2744691 0.1789627 0.1998802 0.226306751
0.09480136 1.1619780.9374733 0.3021035 0.517213286 3.3776630.6012955
0.2078726 0.175679751 0.305312801 0.2519067 0.53349 0.6413341
0.7849532 0.2687481 0.3068297 0.7522469 0.2017412 0.09343541
0.41580500.2291214 0.1919533 0.160244248 0.1458413 1.03041500.2002825
0.1827073 0.7984597 0.1784906 0.2676077 0.2722531 0.2490577 0
0.4896124 0.4969433 0.4584311 0.1022425 0.7552238 0.1473416
0.1725341 0.2510429 0.7129704 0.3568563 0.7365348 0.1397383
0.63809 0.9539290.3637959 0.1845209 0.4186093 0.1642557 0.4179156
0.1294144 0.5233341 1.1722510.1438602 0.1428783 0.2651438
0.2333512 0.3367043 0.2205219 0.3527954 0.3978529 0.1324131
0.4808434 0.1245285 1.0965320.4800520.4720949 0.3236813 0.203611254
0.2056899 0.1584389 0.2775461 1.9605160.1818814 0.142620134
0.326232709 0 0.398915816 0.1400431 0.3982276 1.8942140.929597
0.01873758 0.08378442 0.3288162 0.2225633 0.4654666 0.116768
0.1974650.1133772 0.1057141 0.2056109 0.1863659 0.2496221
0.28294500.3634251 0.3523171 0.1123234 0.5251840.3170357
AC018752.1 0.8625139 1.9207770.08315092 2.150777863 0.075903369 0.8922846

3.0554040.9021668 0.7621789 1.4987030.2732459 0.734758875 1.679118
0.206080064 1.319251516 0 0.1129665 0 0 0.4179293 0 0.8723394
0 0.1564828 0.7807664 0.5581030.2284230.2241466 0.3620646 0
0.5443876 0.378689077 0 0.4731424 0.1211899 0.078231671 1.0761520
0.5563125 0.2479347 0.1190293 0.1887743 0.1715328 0.2068835
0.5596177 0.2905874 0 0.1141009 0.2206154 0 0 0.2436679
0.1282787 0.190762501 0 1.1428630 0.2965030.1543608 0 0.138652
0.5248912 0.4687965 0 0.2283150.131274356 0 0.9750133 0.8434744
0.21607262 0.5525211 0.2540083 0.4416888 0.362093623 0.4000589 0
0.4378991 0.1229671 0 0 0.1097699 0.17794 0.1142331 0.1831216
0.977182195 0.4774380.4215599 0 0 0.2055672 0 0.625305068 0 0
0.2044066 0.175611803 0.2855423 0 0 0 0.07389848 0.1790907
0.2621451 0.6276935 0 0.3761678 0.31660443 6.4084820.096505633 0
0.1290327 0.8516349 0.1475832 0.5756567 0.1392704 0.1130519 0
0.5986654 0.5436233 0 0 0.1092318 0.5558815 0.2050725
0.1302125 0.5520511 0 0.1174179 0 0 0.3348706 0.4030442
0.6935291 0 0.0948215 0.3758028 0.1835545 0.09063098 0.1081264
0 0 0 0.2755019 0 0.1085251 0.1449362 7.56 0 0.1070069 0
0.07377398 0.1085448 0.2336098 0.256663053 0.4893166 0.3394455 0
0.2049059 0 0 0.3099002 0.6131673 0.2192796 0 0.3716889 0
0 0.4416284 0.2126953 0.2931815 0.1198822 0 0.9795214
0.132445722 0.2876182 0.1240189 0.0797959 0.5847277 0.2860244 0
0.1903652 0.1307710.2742654 0 0 0.6486656 0.1049318 0.1148984
0.4308816 0 0 0.2497125 0 0.2325680.2753629 0 0.2733468
0.1111512 0.5032559 0.181192567 0.2070294 0 0.6471092 0.4620765
0.418986838 0.3731955 0 0.4518866 0.3759576 0.1901929 0.1442155
0.4115545 0.1099112 0.1237182 0.1540533 0.3614330.3875376
0.1094978 0.338716812 0.074532533 0.1579467 0.299475614 0.214406777 0
0.246929006 0.4036392 0.7090829 0.07729825 0 0 0 0 0.136096398
0 0 0.1946792 0.24776 0.4382034 0.2105774 0 0.6198470.1623934
0.2364344 0.3548793 0.104881886 0.1752458 0 0.2154323 0.1360752
0.4388044 0 0 0.4437771 0.3934913 0 0.5681334 0.184746803
0.1735819 0.1266349 0.7015869 0.3440534 0 0 0.1166789
0.2212277 0.6283392 0.2428564 0.3471985 0 0 0 0.435431402
0.0698375 0.4100986 0 0 0.2440494 0.2289122 0.3059618
0.07831887 0 0.1165359 0.203253119 0 0.1683401 0 0.338723
0.8289952 0 0.1978432 0.2692327 0 1.7589670 0.2550046
0.07252362 0.1487754 0.8006286 0.3835685 0 0.1546246 0
0.093824658 0 0.1057541 1.4050680 0 0 1.6051562.9698740.6244846
0.5122397 0.2873905 0.077723221 0.1096794 0.6693355 0.3537724 0
0.1039295 0.550454204 0.64728 0.2227994 1.2795550 0.5250153 0 0
0.8045460.3206928 0 0.3560898 0.156067854 0 0 0 0.132527346
0.523451117 0 0.2081629 0.1051745 0 0.2252614 0 0.1134943
0.09511705 0.1762119 0 0.1329555 0.1810045 0 0 4.2752210

0.7657151 0.4857525 0.2885309 0 0 0.1565678 0 0 0
1.945272 0.1928214 0.1294812 0.1234999 0.2440395 0 0.4302742
0.4096542 0.2525541 0 0.1266724 0 0 0.08699801 0.1315774 0
0 0.2440654 0.3588973 0.25266 0.1550339 0.1796383 0 1.320247
0.2822214 0 0.08871264 0 0.3995532 0.2863684 0.07828368
0.1088407 0.1207121 0 0.09391352 0 0.5642403 0 0.1046861 0
0.07622538 0.717254656 0.662576439 0 0.626936871 0 0 0 0.5008994
0.1413505 0.2106812 0.4429442 0 0.04876854 0.3523447 0.4655045
0 0.1329124 0.8863228 0 0 0.8385337 0.4838058 0.2214809
0.4236664 0 0.1594413
MIRLET7A1HG 0.2476346 0.08484162 0.1074298 0.11026885 0.087169862
0.4803414 0.1686983 0.2266419 0.07294256 0.2599667 0.03922555
0.070318387 0.2724847 0.251460762 0.32195253 0.516212 0.3243359
0.01983052 0.04583026 0.2499809 0 0.2325659 0.1407216 0.2246373
0.02802054 0.02670597 0.03279103 0.4933829 0.06930111 0.085887961
0.1172235 0.195704404 0.01210744 0.1494273 0.03479459 0.01123047 0
0.02067155 0.4259248 0.1779601 0 0.0270993 0.1477453 0.01484948
0.2209222 0.2641949 0.142483 0.01637965 0.01055674 0.05052457
0.08662739 0.0349795 0.09207458 0.013692361 0.1465128 0.109375
0.01541915 0.1383336 0.04431823 0.08366749 0.01990406 0.04521014
0.01682441 0.1428151 0.06555106 0.01884496 0 0.1399671 0.6659627
0.062036181 0 0 0.1521749 0 0.1244318 0.03552876 0.09429326
0.4942677 0.01451795 0.2751738 0.01575791 0 0.01639862 0.03505045
0 0.03916465 0.0201722 0.1684849 0 0.05902 0.05133957 0.056103155
0.1288607 0.009277021 0.6895697 0.025209778 0.02049537 0 0.02152506
0 0.1166927 0.03856379 0.01881599 0.1051259 0.02348256 0.0540004
0.034087376 12.53876 0.027707541 0.02267617 0.2778473 0.01222556 0
0.1756054 0.03998566 0 0.0168255 0.04297041 0.0260131 0.01763253
0.07279271 0.07840333 0.1117186 0.07850393 0.2990805 0.03169966 0
0.03371162 0.128799008 0.05065445 0.2643961 0 0.0142227 0
0.1361201 0.07193067 0.02634999 0.2862297 0.03104395 0.0234516
0.1296417 0.03752042 0.02966205 0.01491933 0.01557922 0.02080618
4.671372 0.03012544 0.1075289 0.0434446 0 0.01558204 0.1676781 0
0.04682891 0.01624292 0.01309604 0.0441226 0 0.01572257 0.05931656
0.1232317 0.2833062 0.028233781 0.03557162 0.01745751 0.05423882
0.07396378 0.122133 0.1683495 0.09178437 0.02897974 0.01171785
0.038026228 0 0.1246239 0.08018515 0.01399 0.09238489 0.06608336
0.437243 0.506863 0.05249587 0.09295249 0.05334394 0.1263752 0.03012676
0.03298826 0.06185478 0.09951413 0.01667092 0.07169446 0 0.03338606
0 0 0.1308001 0.1595619 0.1083666 0.039016379 0.1485995
0.01608811 0.09289511 0.02211098 0 0.08036056 0 0 0.2590571
0.6689223 0.2277298 0.01477007 0.01577819 0.6571292 0.02211497
0.1902456 0.08901213 0.09431313 0.048624156 0 0.793586 0.014330308
0.015389476 0 0.070895296 0.04345802 0.1832251 0.02219295 0.072280772

0.1462625 0.07003747 0.04765319 0.019537183 0 0.02547873 0.05589395
0.2311851 0.04193724 0.03022923 0.1167508 0.2335765 0.1165611
0.03394111 0.2445329 0.03011243 0.02515724 0.05769283 0.3092618
0.02930121 0.01049869 0.01882748 0.09007926 0.3185299 0.07531633
0.02926227 0.1468041 0.026521144 0.1245919 0.1090737 0.04196484
0.7778964 0 0.1170052 0.05024915 0.03175812 0.02004458 0.6798287
0 0.01520904 0 0 0 0.01002545 0.02943565 0.1325468 0.04832346
0.3328256 0.01643063 0.01464068 0.02248598 0.08294879 0 0.043766699
0.072612153 0.01208295 0 0.1296668 0.6694064 0.04959888 0.02840119
0.02318966 0.06005796 0.5198668 0.02971838 0.07321388 0.0728774
0.0427146 0.5746672 0.01573223 0 0.01109849 0 0.013468906
0.01428341 0.01518143 0.02017031 0 0.09094493 0.05981713 1.5920380
0.2049080.1654517 0.2269083 0 0.03148985 0.1441287 0.01692848
0.1061149 0.02983901 0.128407348 0.08362767 0.01066125 0.2755278
0.03715374 0.06460118 0 0 0.0384986 0.2608749 0.1234721
0.06134174 0.15682916 0.02257878 0.0651159 0.0562777 0.095124158
0.015028701 0.03031073 0.07968699 0.04529465 0.01574161 0.01616859
0.05537902 0.3095588 0.02730887 0.07588779 0.08659354 0.02862943
0.05196784 0.061975998 0.04738065 5.6909050.05422282 0.03297644
0.06973172 0.09664613 0.02897995 0.01133962 0.1573316 0.4305797
0.1262415 0 0.1034265 0.05536056 0.1115253 0.03545781 0.2452299
0 1.1118170.05040642 0.1450206 0.0504421 0.1272904 0.0151917
0.04924555 0 0.05666541 0.01778769 0.1257144 0.5021908 0.180324
0.7435424 0.1112787 0.07736339 0.01104351 0.2948194 0.2430841
0.3432880.08914538 0 0.3584841 0.05481245 0.02247587 0.09374715
0.1906158 0.7367931 0.04044499 0 0 0.2144722 0.04508431 0
0.02188492 0.038611792 0.149467219 0.03142982 0.03599972 0.0379141 0
0.2564119 0.7010846 0.01014572 0.01512207 0.3052148 0.04016997
0.08401104 0.1011610.1737451 0.012277921 0 0.3021836 0.1177287
0.02252687 0.3392383 0.02315076 0.06358893 0.1824570.01672752
0.1487748
MIR100HG 2.13037 12.945422.28644614.429539717 2.26291652 5.672251
11.7384410.0894212.4056914.425532.8314378.283665569 6.80424 8.538814686
10.233699266 7.4906743.0245573.2067372.2085390.05846821 1.404461516
0.11182 0.1222251 0.1154987 0.5254293 1.6100320.6479419 0.2097746
0.4611148 0.989988383 4.3647390.787004975 0.1049726 0.3697651
1.6767350.418913727 1.7103620.9878166 1.2076764.4063190.4754482
0.4125342 0.7844668 3.6228590.1498316 0.2607406 13.327570.04293414
0.1702844 0.4991759 0.1979556 0.3579354 0.7240346 0.209819988
0.07877694 0.1212930.07461508 0.5578439 0.5406208 4.7539280.3732325
1.5679030.2374612 1.2526170.2610368 0.577555519 2.3808920.04468411
0.07019077 5.897686517 0.07036745 0.5073028 0.2275656 2.525853592
4.22849 1.2428950.7541561 0.03915181 1.4402080.1149296 1.66171
0.5150436 1.1572580.7491253 0.763676742 0.8094183 0.5084149

0.6768186 0.2569752 4.23647 0.2743172 1.251115336 0.01889613
0.4582786 0.1153718 0.233820071 0.4421753 4.090569 1.254288 0.4807139
0.8855358 0.9149304 0.07967119 0.202881 0.02604133 1.091532 1.33795066
0.3915945 1.494433865 1.005883 0.1605973 3.271106 0.6792103 0.03749008
0.1934953 0.8540607 0.05767289 0.2368193 3.605951 0.4764028
0.9344476 0.05691044 0.5502734 0.3145962 0.4654681 2.368087
0.07301589 5.536377 2.272351468 0.0408538 2.435305 1.710239 1.597321
2.454405093 0.4446232 2.422063 1.147596 0.8525705 0.3912118 0.7423815
0.01742643 0.4841752 0.1355638 0.4361865 0.5999761 0.241221
3.391749 0.07289029 0.1765456 2.146134 0.07260263 0.7603169 1.132599
0.234015252 0.3163106 0.9694174 5.011769963 1.233639 1.08574 0.3106737
4.428187 0.5359894 0.4823719 0.816913138 0.02868921 0.09503881
0.1202978 0.0937408 0.4432621 2.472277 0.9496134 1.148185 4.014175
0.590376292 0.5994032 0.0682043 0.2540642 0.09590728 0.1345323
0.9460311 0.1294868 0.109769 1.876145 0.9495947 2.670111 6.242848
0.5497382 0.02327997 6.066271 0.1886112 0.1344542 0.3794633
0.589686867 1.703102 0.2550502 1.352961 0.2083483 2.052603 0.2621994
0.550680796 9.941425 0.230313 0.3204985 0.610778 7.894990706 0.9775865
3.176324 1.111779 0.05876285 0.06606109 0.02295856 0.166773 0.3292703
0.08952494 3.455755 0.05928239 0.2221004 0.1616389 0.990212447
0.146698289 0.4343147 1.285791276 2.116231339 1.245017 0.125077851
0.1226741 8.365684 0.06488405 0.553810666 0.1516674 0.1647526
0.04323739 1.331476745 1.387925 0.07705912 2.600527 1.869932 0.08033011
0.9081724 0.2000938 0.07400801 11.06954 0.01596827 1.514079 0.783232045
0.5275349 0.616527781 4.57904 2.412433 0.1587637 0.478319 0.02906028
0.2080895 1.203492 2.991375 0.1677339 5.711082561 1.431923 0.5754709
0.3858383 0.2589223 1.700719 0.02883437 1.327257 0.2209169 3.831424
0.08786781 0.08039668 1.355436 1.839015 0.9827222 0.040331092 0.8853868
1.020838 0.5799412 0.6761957 1.306832 0.02319034 0.3985199 0.174553
0.4716438 0.2698485 0.182376622 0.047582539 0.9745135 0.1078924
8.598339 0.9478058 0.5967035 4.521094 1.867175 0.6115288 2.458794
2.193114 1.572164 0.0965623 0.9344607 3.667289 0.09357658 0.9261389
0.5370689 0.4137287 2.123704063 0.388795 0.05815958 3.780218 0.1054059
0.2237141 1.495554 0.2491993 0.6198339 0.4312315 0.04448 0.05406996
0.267712104 6.03183 0.0581213 1.727124 0.8852566 0.5354628 1.065504389
1.585013 0.4084291 0.2268481 1.741728 1.324262 0.02618636 1.745967553
3.1438 1.215992 0.1881006 1.405867 0.53078859 0.04097301 0.3238564
3.771075 0.395105413 0.206056014 0.6829656 0.0803364 0.5662308
0.755407 0.1662636 0.7090452 0.190534 0.6442352 1.372012 0.2165021
0.03848367 0.04191308 0.194940944 1.461663 4.75857 1.51239 0.9242161
1.15292 3.454722 0.03213774 2.700247 0.1450183 0.1585368 0.2545407
0.1356347 0.05839087 0.4967243 0.3035722 5.717697 1.165505 0.6264609
0.2023806 0.06775628 2.313654 0.4983612 4.740792 0.0275679 0.3938652
0.727742 0.0571272 0.1362882 0.8668944 0.1813201 0.1869877

0.2157391 0.8997279 0.1793859 0.3874462 12.36019 0.469708 0.02708497
0.1412272 0.6238943 0.0838461 0.8592796 0.6956325 0.5670668
0.1677113 0.2304188 1.274886 0.733566976 1.34125 0.5621721 0.2787706
0.4905078 1.972456 0.986136969 7.189058768 1.051974 0.264939628 0.14907
0.03009918 0.3619021 0.335276 1.045342 1.027533 1.88724 1.67254 0.06069858
6.919709 0.01347393 0.425802516 1.173372 0.1859945 0.2984158 2.102988
0.9907052 0.686179 0.0427381 0.1318265 3.622354 1.096061
AC073487.1 0.3918541 0 0.3238013 0.119648998 0.206904836 0.3474681
0.1830488 0.2195727 0.1978687 0.07295199 0.3192173 0.286125384
0.5117261 0.28087661 0.051373499 0.4968843 0.2199537 0.05379355
0.06216108 0.8408621 0.178256458 0.1941149 0.5285502 0.517961
0.6840931 0.3984441 0.2965036 0.5528102 0.4229791 0.232985301
0.3533203 0.235946919 0.1313737 0.4421958 0.3775443 0.365574086
0.26668 0.33645 1.227603 0.1930985 0 0.1470227 0.2671891 0.8056333
0.2724037 0.9807096 0.4567831 0.088865 0.3722798 0.2055844
0.1958259 0.308385 0.1498605 0.297142345 0.17664 0.2596105 0.9620208
0.1154624 0.3907167 0.4539238 1.025868 0.3679199 0.4107508
0.2905573 0.04445453 0.869041198 0.04689864 0.5062451 0.6569215
0.210354252 0.4303189 0.1978288 0.5847992 0.376011525 0.311577
0.1445665 0.4689411 0.2394255 0.7876467 0.6931364 0.9831563
0.2771694 0 0.1901603 0 0.2390413 0.1368011 0.7031432
0.0432158 0.4269373 0.2089006 0.304378176 0.1588891 0.1006618
0.4377934 0.615471234 0.8339562 0 0.2919514 0.7047779 0.2014397
0.6974043 0.6124979 0.5296041 0.2229512 0.146485 0.21575784 7.85637
0.338225791 0.06151287 0.6532126 0.06632771 0.1149419 0.1681266
0.3254032 0.264144 0 0.1942739 0.4586715 0.5261426 0.1974623
0.212682 0.3896424 0.4791485 1.191605 0.04299528 0.1178799 0.3200692
0.058231435 0.2748171 0.3586091 0.156951 0.1543257 0.072097808
0.5169475 0.2926858 0.4288722 0.3176368 0.2947415 0.4453145
0.8791867 0.2035607 0.1341054 0.3237692 0.0422612 0.1975407
6.399934 0.2860209 0.4583698 0.2946266 0.05745724 0.1690755
0.7277675 0.29984455 0.06351558 0.3965545 0.248676219 0.518656
0.6456225 0.1279502 0.2815855 0.2865311 0.4269527 0.306355206 0
0.4262075 0.3923514 0.6592423 0.2898928 0.4186197 0.6535732
0.1179185 0.1589331 0.257881084 0.616014 0.1448841 0.3728837
0.07590038 0.5012186 0.5019338 1.186094 0.3564685 0.2136056
0.3782235 0.2894085 0.2525995 0.1225857 0.4474306 0.1006748
0.2699485 0.2261131 0.6806908 0.1944476 0.588674 0.5361508
0.2388755 0.4967436 0.2164191 0.2939623 0.03527946 0.1612403
0.2182083 0.08399779 0.2399187 0.407898339 0.2422126 0.7894078
0.3519419 0.3220869 0.2962551 0.5896749 0.1201988 0.1926043
1.686217 0.6598956 0.2345784 0.7847454 0.29848 0.131901065 0.261216098
1.968213 0.038873331 0.45921149 0.1989319 0.144236414 0.1571828
0.4970283 0.6923235 0.392147101 0.7935223 0.2533176 0.4201183

0.264989206 0.3334413 0.4838069 0.0379054 0.5065263 0.5119276
0.369008 0.6334116 0.9956805 0.06323825 0.09207088 0.4422239
0.449267208 0.3412158 0.195626681 0.4753901 0.4769063 0.08543829
0.153218 0.3420969 1.278816 0.357539 0.1984469 0.3097347 0.251800463
0.5069641 0.6903877 0.1138366 0.9378545 0.03335414 0.1763311
0.3180546 0.516895 0.2446841 0.7565734 0 0.1237711 0 0
0.983465859 0.3263484 0.7186417 0.6831548 0.1048683 1.282989
0.1782832 0.1191458 0.3354831 0.4050222 0.09076143 0.356173235
0.295458712 0.262216 0.2177325 0.1319035 0.4842335 0.08969677
0.1155644 0.314529 0.08145858 0.4432135 0.2418481 0.6951166
0.3106589 0.3765789 1.325048 1.024231 0.1748769 0.3311713 0.07471377
0.365366343 1.084891 0.2059107 0.4377224 0.0457451 0 0.2028298
0.6250701 0 0.5905874 0.7978933 0.8393541 0.181599057 0.1281321
0.5212972 0.3214492 0.07196353 0.1214149 0.455503456 0.2268539
0.144602 0.5294188 0.05039284 0.1168276 0.2348687 0.480135369
0.2436789 0.8741759 0.4465846 0.1386663 0.273487524 0.367492
0.05887922 0.101775 0.464472021 0.122303512 0.1233343 0.2702052
0.04095641 0.1708069 0.3508797 0.07511238 2.033029 0.07407982
0.2401679 0.1879194 0.1035494 0.211457 0.067248059 0.1713705 6.96202
0.2941767 0.2683621 0.1891588 0.2247159 0.7861292 0.06152121
0.3048485 0.355484 0.2397157 0.145983 0.5050108 0.3003493 1.058858
0.1202315 0.2534197 0.1053526 2.178212 0.04557859 0.2458702
0.513122 0.3452961 0.4945199 0.2226445 0.2371477 0.5636191
0.04825206 0.3410212 1.045467 0.7686779 1.131478 0.3924203 0.1748842
0.05991471 0.3998731 0.6044552 0.6478091 0.2418216 0.05312455
0.6612643 0.1486878 0.1524238 0.9324503 0.3290489 2.284198
0.1828562 0.119626423 0.1098615 0.07757217 0.5707271 0 0.2077826
0.558617993 0.589752078 0.1278879 0.488275975 0.411393 0.5849204
3.477798 1.145961 0.3027412 0.08204221 0.379475 0.1089677 0.246885
0.3773219 0.5800767 0.066611784 0 0.819723 0.1368679 0.2444314
0.8311845 0.3768015 0.229994 0.8249081 0.3176332 0.9002855
AP001160.1 0.5225819 0 0.649338 0.041368837 0.408786773 0.3904474
0.06328941 0.6680751 0.06841344 0.3363103 0.4414797 0.065952213
0.1572712 1.193109288 0.177624714 1.811694 0.7604936 1.487937 0.2579075
0.9565937 1.109384307 0.1677889 1.27923 1.011308 1.024947 0.6762895
1.025166 1.52908 0.9099734 0.671292071 0.6596697 0.632237503 1.271835
0.4586698 0.6200487 0.800519639 0.6322632 1.589818 0.7490221
0.8011693 1.891088 0.6354166 0.8314297 2.005553 0.1130208 1.69541
1.62793 0.5223287 0.7722981 1.089911 1.32706 0.3608833 0.5526882
0.590740345 0.8550302 1.179713 3.008044 0.4191738 1.434042 0.9730583
2.202846 0.1696119 0.6311901 0.6027639 0.7685112 0.67164422
0.3567362 1.334642 0.2839146 0.378197696 0.7141607 0.8207955
0.9752295 0.260013199 1.077283 0.5664864 0.4421922 1.357621 1.633981
0.8848727 0.7389737 0.81457 0.3383689 0.3123041 0.187954745 0.25713

0.3027149 1.458678 1.613729 0.4981982 1.926073 0.968201116 0.5273873

0.4002458 1.596242 1.938846128 0.6920201 0.5190883 0.8479186 3.61217

1.47256 3.038221 1.482405 0.9014719 1.123249 0.5824455 0.660730684

1.022627 0.597704169 1.276091 0.7644131 0.2981279 1.073017 0.6588071

0.9375723 0.1826564 3.093032 1.397147 0.6831414 1.786071 1.046851

0.1176562 0.5089404 0.5338139 1.472678 0.3567766 0.3804004

0.3161842 0.845611786 1.064207 0.4734152 0.3473029 0.1867542

0.697982175 0.9447445 0.5734473 0.8402718 1.098234 0.4658622

1.407709 3.18166 0.7390051 0.4636716 0.5317327 0.2922376 0.3512572

1.15065 1.66704 1.181412 1.263159 0.5165143 0.9353298 0.9436005

0.691144953 0.4831335 1.157812 1.228288698 0.9380137 0.5022563

1.356662 0.5007015 0.8255716 0.8857175 0.688498284 0.2001776

0.8514245 0.6782806 0.4360479 0.8018474 0.8157984 0.548794

1.114394 0.1758444 0.713302448 2.672029 1.636401 0.9669385 0.577339

0.8664857 1.264395 1.589114 0.7394973 0.4923632 0.6102665 0.9506029

0.26201 1.582344 0.7425593 0.2552621 1.306692 1.56358 1.781935 0.403383452

1.722219 1.161684 0.2973297 1.692963 0.4788944 0.5646553 0.878251031

0.6968636 2.414268 0.232339 0.2073807 0.451300855 1.239432 2.32908

0.5780013 0.5264389 0.614584 1.495128 0.1662357 0.3699625 0.7662445

1.866764 0.9083858 0.5217827 0.9435422 0.410445078 0.923229126

2.594456 0.618263817 0.057735681 1.238058 0.531946408 0.6521544 1.03109

2.43535 1.254165883 3.057172 1.642218 1.095011 0.916204509 0.7378424

1.003662 0.8125629 1.034114 0.3933329 1.360908 0.7665108 0.7093804

1.005778 0.7427853 0.5160362 2.089960087 1.368521 0.51405066 0.9088523

1.429056 0.5711149 3.284472 1.419367 2.198814 2.013235 1.399712 0.8261327

0.696483372 1.262043 0.9548107 0.6769778 1.204412 1.568386 0.9754685

2.010842 3.544559 0.3007997 1.765709 0.3116466 0.4279406 0.9638789

0.861454 1.313238342 1.297608 0.4417272 1.317758 0.2900671 1.018627

0.3698503 0.8238973 0.6748732 4.107754 0.9414274 0.875715398

0.544828439 0.8612852 1.756565 0.6688857 0.9208349 2.046845 0.186464

0.4204958 0.6196176 0.3622078 0.5574625 1.545028 0.6835233

1.141778 1.077971 0.5902156 0.6348719 0.8952055 0.4391509 0.555834445

2.464962 0.6549845 0.4729476 1.803075 1.467126 5.722506 0.1178831

0.0420911 0.2882775 1.413849 0.9673602 0.544164146 0.6497611

0.9462566 0.5398305 1.368483 0.9515327 0.537322454 0.1220102

0.2799797 2.562664 0.8014766 0.2827533 2.436187 0.791728498 0.3851538

2.360411 1.389666 0.9972376 0.504313378 1.694146 0.6921579 0.8093444

0.249809716 0.507439671 0.3980017 0.672652 0.8496447 1.476419 0.6369155

0.4674641 1.253037 1.050142 0.4033295 0.9421151 0.6444429 0.5117813

0.325515766 0.5332646 2.668774 0.8476007 0.804151 0.3924119 0.3884795

1.467749 0.9146555 1.180501 0.6321042 1.18403 1.211372 1.04765 0.7269239

1.708475 0.3325618 0.7666774 0.3278325 1.535207 0.8037024 0.2040241

1.868748 1.296199 1.510333 0.8005889 0.4685383 1.558978 0.8341615

0.6131249 0.7667589 0.7731539 0.7824204 1.544664 0.2418659

0.5800371 0.671533 0.9879593 1.259893 0.5494385 0.5143012 1.049023
0.5654996 0.5691684 0.5568662 9.13404 2.143648 0.5057826 2.398939813
0.3988404 1.287394 0.9584591 2.406117 0.2463126 0.26557174 0.280373149
1.061219 0.337644432 0.8178789 1.804578 0.4809817 0.5732515 0.1332205
0.2552961 1.264331 1.130273 0.8930062 0.8776377 0.5264765 0.391529264
1.646376 1.253017 0.8518017 1.309945 0.8416228 0.6513987 0.795208
1.682759 0.9413332 1.309502
SSBP3-AS1 0.299064 0.1391933 0.1501406 0.168849177 0.041712141 0.1313433
0.3505761 0.3187152 0.2293701 0.151974 0.2359662 0.153821472
0.343881 0.202231771 0.103569288 0.145705 0.2394511 0.04337925 0.08772197
0.4429349 0.143746442 0.2054519 0.106556 0.2272698 0.1302517
0.04381449 0.2331237 0.7566657 0.08527285 0.039141649 0.1851969
0.511345709 0.0926976 0.2005806 0.04757073 0.036849979 0.3532987
0.06782852 0.6259929 0.2141082 0.03737811 0.03704983 0.1885292
0.1542953 0.2086837 0.1748988 0.06375306 0.1074914 0.1674233
0.1243378 0.08685298 0.07173531 0.04028263 0.164736214 0.02670806
0.5458069 0.219241 0.1163865 0.1999514 0.4301032 0.2503557
0.04120716 0.009200847 0.1952551 0.01792412 0.123670049 0 0.3954799
0.6704561 0.050889054 0.1995309 0.0598236 0.1178959 0.066328634
0.5129820 0.01942979 0.1461054 0.2509953 0.3255196 0.04299591
0.1464993 0.08381627 0.08071195 0.0527126 0.032877755 0.08031813
0.05515837 0.2055434 0.06098632 0.4088364 0.1123053 0.067499108
0.17938 0 0.3690848 0.179225765 0.3026269 0 0.1412582 0.3928195
0.01740444 1.110717 0.1234799 0.1478333 0.1990514 0.05168007
0.06835229 2.515525 0.037881403 0.04960413 0.2836358 0.06685853
0.1042756 0.02259628 0.1093358 0.05325159 0.009201443 0.1253304
0.1493721 0.2314269 0.132695 0.03430146 0.06982413 0.1878268
0.2760069 0.05200725 0.06337245 0.1013982 0.270008336 0.1385083
0.08544051 0.03164143 0.02333411 0.087209772 0.1488813 0.08850844
0.0864609 0.05692068 0.2122145 0.2052015 0.1358875 0.02051898
0.07570004 0.05711305 0.04259942 0.03413514 1.935346 0.115324 0.09240764
0.1306732 0.1042507 0.02556429 0.1650582 0.010074807 0.06402389
0.2043055 0.028647587 0.088475 0.03253947 0.02579483 0.3000584
0.3080792 0.1032887 0.077201745 0.1361726 0.05728246 0.06921099
0.09245472 0.1085362 0.0767218 0.2572472 0.02377245 0.1153476
0.093580171 0.1580585 0.1265711 0.256843 0.1147617 0.2077056
0.09396228 0.4931793 0.1642612 0.1148347 0.01694446 0.07293116
0.1891471 0.04942671 0.02706068 0.09471513 0.03265307 0 0.06861384
0.070561358 0.155193 0.3026473 0.03852595 0.1859814 0.08726044
0.1251107 0.021337082 0.1381511 0.4047166 0.03386801 0.08464358
0.082232554 0.1562566 0.1697548 0.1152965 0.3836945 0.1418474
0.4245672 0.05654169 0.07334394 0.1942527 0.1209412 0.4729115
0.05476332 0.07736624 0.132956675 0.03510755 0.6075893 0.054858211
0.050496718 0.0561467 0.048463582 0.2455831 0.2672032 0.394445

0.474342568 1.016981 0.1723578 0.149847 0.117528368 0.02688879 0.05573473
0.1069845 0.1118413 0.1720082 0.6860618 0.1276962 0.4075423
0.08924209 0.136118 0.2061654 0.074104808 0.01375786 0.126203066
0.2875168 0.2136547 0.09186352 0.0926665 0.118229 0.35536 0.1853488
0.1440252 0.08920387 0.166793131 0.3066128 0.08947452 0.05048894
0.1350515 0.08069059 0.03554847 0.08244001 0.442877 0.1096188
0.4671098 0.2180576 0.1330791 0 0.04050774 0.218777729 0.1315841
0.08048811 0.1449731 0.06342454 0.3544483 0.035942 0.1040861
0.01844553 0.3719735 0.2195707 0.111696264 0.059564656 0.2510988
0.1170534 0.06204758 0.2440544 0.2260366 0.06989354 0.08454564
0.0821105 0.3167938 0.2112791 0.2102039 0.1537258 0.1985562
0.2985576 0.1591659 0.008813824 0.1578066 0.1054364 0.169413578
0.2812047 0.1162328 0.06618383 0.05533344 0.009947102 0.08178124
0.3436759 0.0490849 0.07704062 0.1306953 0.603531 0.097627948
0.1722101 0.03941019 0.1388665 0.04352368 0.06527282 0.237677063
0.355708 0.03498222 0.3076368 0.1727068 0.07654567 0.2959355
0.044674885 0.06316177 0.4951347 0.1650582 0.09504774 0.049009113
0.07408662 0.07122052 0.03077687 0.052021024 0.147938776 0.02486427
0.2560256 0.02477051 0.07747823 0.08842196 0.0454281 0.14256
0.01493454 0.2005888 0.1894234 0.06262689 0.1420996 0.081343498
0.03454839 2.273137 0.05930621 0.1623059 0.04576144 0.1585601
0.1505599 0.01860407 0.04916612 0.1740455 0.082846 0.1103635
0.367649 0.0302753 0.2439616 0.03393424 0.555599 0.0424783 0.1942301
0.05972636 0.1288757 0.1172385 0.02983368 0.09138755 0.01795411
0.1024481 0.2272519 0.03891058 0.1237501 0.2107669 0.147922
0.07934141 0.352962 0.3243621 0.114749 0.1842621 0.1550927 0.5305573
0.1671476 0 0.2823063 0.08243278 0.05531171 0.2990632 0.5780748
0.3042561 0.08110062 0.325576258 0.1384259 0.03909649 0.2794288
0.01209446 0.07180988 0.07038608 0.074308987 0.06875274 0.078749387
0.03110141 0.2267698 0.1402252 0.2506883 0.1220656 0.03307952
0.1669143 0.01098398 0.04977216 0.138306 0.07308988 0.040286929 0
0.3566065 0.1839511 0.03695815 0.4129337 0.02532114 0.09273386
0.1413567 0.07318292 0.3004107
AL139220.2 0.07959482 1.906351 0.04925229 1.586512209 0.046914146 0.0201068
0.7081784 1.855792 3.477527 2.563446 0.3342557 0.167147923 0.1936521
0.339662404 3.139287542 0.05675919 0.02327401 0.163647 0.03699823
0.03408291 0.165041546 0.04814046 0.1184578 0.09268852 0.02764747
0.4838893 0.03725663 0.08466329 0.1056757 0.256801317 0.3318004
0.09167315 0.2563012 0.08773156 0.3870077 0.165206825 0.2469099
0.3003822 0.2196782 1.136551 0.1256809 0.03889236 0.2252936
0.5274637 0.01801495 0.03492329 0.113863 0.08227705 0.1401451
0.07704381 0.06734316 0.0376514 0.06607177 0.06386573 0.02920446
0.02943239 0.1825663 0.03436158 0.07553047 0.4683036 0.04641957
0.2243928 0.01207302 0.307448 0.1205368 0.101422118 0.2636326

0.06277433	0.01357636	0.539762856	0.05976263	0.05233225	0.02047483	
0.0528421	0.2764586	0.2900065	0.2142689	0.09183715	0.1953362	
0.133992	0.3703276	0.01374759	0.3647919	0.05973563	0.079091846	
0.1387642	0.2732225	0.05812645	0.1028881	0.2999943	0.07828659	
0.136880757	0.08616443	0.1414633	0.09475432	0.063315969	0.1139813	
0.0961853	0.1892155	0.09870197	0.02474061	0.0991615	0.1822792	
0.07274308	0.1664021	0.04843766	0.256837873	0.06723813	0.27587171	
0.1952663	0.1129822	0.3794295	0.01520297	0.04632817	0.04303995	
0.07860918	1.36434	0.1259101	0.2450009	0.09489691	0.1088235	
0.03938299	0.0543998	0.08626085	0.04191739	0.122267	0.397585	0.6108256
0.184849682	0.1453964	0.07545997	0.1536193	0.2832182	0.29561994	
0.06349093	0.2516315	0.3592611	0.03734463	0.2339066	0.4207158	
0.08527712	0.1043315	0.01596389	0.1070596	0.07546153	0.03359317	
0.1692994	0.01621328	0.06889431	0.01169077	0.03039868	0.1146105	
0.1082917	0.185077196	0.03360394	0.03205332	0.401746684	0.1266477	
0.04269712	0.08179705	0.5666459	0.06316413	0.1355317	0.217797908	
0.03828874	0.01565916	0.1556849	0.01895559	0.01643277	0.3523505	
0.1903867	0.2235523	1.540878	0.15690157	0.08888513	0.06387772	
0.1500151	0.02007816	0.1049662	0.3011217	0.06128146	0.09429769	
0.2684023	0.1222865	0.8564939	1.024985	0.1864609	0.03254905	
0.8255862	0.02856413	0.2691648	0.06430901	0.059153493	0.3234262	
0.245838	0.1769333	0.05396996	0.12595	0.1360848	0.100325276	0.1946061
0.5310545	0.03518197	0.05553313	1.418919171	0.237071	0.7065227	
0.09310027	0.02904636	0.03183751	0.04456815	0.07419197	0.1033157	
0.1051427	0.2539116	0.03102687	0.01596855	0.05921836	0.082868959	
0.069100295	0.0772851	0.092549533	0.14080245	0.3867868	0.031796084	
0.1637214	1.132192	0.05573901	0.051867938	0.1386924	0.5088639	
0.03419544	0.252354266	0.1014372	0.07770392	0.1504085	0.01914185	
0.2746053	0.2087873	0.07330676	0.06385222	0.3094793	0.03044474	
0.06945856	0.099938709	0.15796	0.147486719	0.09986537	0.6027524	
0.137491	0.2803409	0.1195839	0.13943	0.2668532	0.5407063	0.04974629
0.166523871	0.07823012	0.05218011	0.07076681	0.07309898	0.145584	
0.04198088	0.1502428	0.03418395	0.1384439	0.02501734	0.0447074	
0.4065409	0.4102119	0.3800425	0.038126785	0.1187037	0.02112272	
0.0856028	0.03467642	0.1162736	0.07074271	0.136578	0.0161357	
0.2708305	0.06902702	0.044492588	0.123751115	0.3663328	0.316786	
1.651594	0.1147525	0.2372776	0.792288	0.174727	0.2020171	0.3517339
0.04798261	0.0689555	0.02614804	0.1226062	0.1134032	0.1397049	
0.1329996	0.04579392	0.1432909	0.096651479	0.007687222	0.05991722	
0.7327466	0.04235382	0.09462866	0.06975176	0.04133793	0.02415279	
0.04594997	0.04287342	0.09436569	0.118095766	0.9829604	0.0301657	
0.4190958	0.08328571	0.1124139	0.23744746	0.3200552	0.1128434	
0.2471449	0.4599047	0.16225	0.1747422	0.422558094	0.5318043	
0.0908476	0.3076469	0.06969548	0.128615988	0.05670805	0.124604	

0.4374966 0.017065029 0.072794977 0.1740053 0.02501737 0.05958878
0.03953606 0.07251507 0.0745114 0.1812164 0.2057639 0.2246322
0.1491327 0.1369612 0.07458317 0.097841343 0.1218329 0.3152884
0.2788531 0.1400094 0.2226725 0.2724553 0.1325727 1.735257
0.06854614 0.1175466 0.05888325 0.02172224 0.1410138 0.04469194
0.1667279 0.05406884 0.1445505 0.1149606 0.1218904 0.04370678
0.2439030.09501642 1.3212 0.06813378 0.1884699 0.1277073 0.01355417
0.04467493 0.08570082 0.03352251 0.0231069 0.01952043 0.09183028
0.04394951 0.05943534 3.2527250.01453622 0.0321313 0.3838189
0.1756650.08489069 0.04424945 0.1834612 0.04484799 0.1896322
0.04046289 0.1838117 0.158225723 0.1998013 0.3847576 0.2480323
0.05951217 0.3454966 0.110829698 0.231575749 0.2255372 0.051666056
0.09182283 0.04463388 0 0.1048106 0.1237678 0.2441574 0.2007674
0.5693047 0.04395814 0.3334699 0.004795296 0.147576039 0.2053753
0.09415587 0.06939502 0.1616505 0.05104259 0.1723572 0.04563076
0.0409154 0.4081190.1847757
AL137779.1 0.2471868 0.1016258 0.2859614 0 0.052207326 0.1150734
0.2020718 0.2326958 0 0.1288534 0.09397105 0.042114727 0.2761764
0.318925242 0.136109756 0.2792476 0 0.2375358 0.1921389 0.04790953
0.052475051 0.0428576 0.02593239 0.05381545 0.03356383 0.2559137
0.2094830.1027806 0.04150546 0.068586101 0.3120307 0.026046728
0.05801061 0.3254335 0.04167799 0.080713143 0.06729011 0.09904399
0.06377317 0 0 0.09738104 0.1179825 0.1067229 0.09622808
0.03331163 0 0 0 0 0 0.06284924 0.04411587 0 0.07799882 0
0.03693903 0 0.1327142 0.2806143 0.04768334 0.0361027 0
0.04276705 0 0.090292211 0.12425400 0.1087786 0.03715439 0
0.1747102 0 0.124526355 0.1605133 0.04255741 0 0.04228918
0.03478006 0.09417468 0.2642540.1835842 0.03928551 0.06297671 0
0.04691258 0.02416287 0.1862919 0.1144966 0.1413918 0.1537402
0.161284863 0.08419267 0.1111229 0.1054452 0.120788085 0.09819985
0.08286782 0.05156672 0.07322547 0.07624251 0.0923857 0.2253835
0.03597798 0 0 0.054441162 3.0201850.099566747 0.1629732 0.2218759
0.08786483 0.2030193 0.04949302 0.09579203 0.07775858 0 0.03431415
0.06231853 0 0.1162577 0 0.03823425 0.2350859 0.08956186
0.07594159 0.06940288 0.2019039 0 0.07281048 0.2015373 0.1663314
0.06814547 0.254689418 0.1304389 0.04308035 0.0631256 0 0.03718537
0.05618206 0.02588146 0.2696584 0.02368673 0 0.0373225 0.02492226
4.5216230.1443406 0.07360074 0.05203923 0.07611405 0.03732926
0.1205099 0.088268095 0 0.1167375 0.156868256 0 0.1425435
0.1129977 0.1421023 0 0.1508233 0.101457786 0.17043500 0.04331258
0.02531315 0.1462946 0.3360901 0.05497102 0.0347128 0.1122879
0.091097892 0 0 0.05488466 0.06703055 0.04918283 0.09498794
0.2946052 0.2248650.1886433 0.1484550.09584543 0.09560586 0.07217345
0.03951432 0.08890979 0.04768039 0.03993785 0.04293888 0 0.03999082

0.1262655	0	0.1566762	0.3822561	0.08653649	0.311566518	0.1423975
0.1156249	0.07418168	0.1059407	0.216138452	0.2566887	0.0464771
0.03885168	0.02585885	0	0.04959663	0.1415363	0.1133975	0.08509502
0.3178796	0.0828661	0.1599321	0.07531405	0.058243458	0.025632223
0.6518269	0.034330538	0.073735887	0.03513688	0	0	0.0975433	0
0.216450142	0.2002257	0.05592864	0.142701	0.046804437	0.05889497
0.06103837	0.5356115	0.08520627	0.05023367	0.1086284	0.06992377
0.05329235	0	0.05420756	0.1464544	0.10820869	0.1808045	0.10365928
0.246962	0.1403914	0.176059	0.3608338	0.04315985	0.03052357	0.1353242
0.3154609	0.03907695	0.222374699	0.1790878	0.08710115	0	0
0.02945633	0.03114497	0.1605065	0.07608165	0.09603997	0.3758395
0.1592051	0.1457427	0.1846496	0.07097984	0.029949539	0.04803514
0.1410356	0.0952611	0.02315331	0.04196509	0	0.2104445	0.05386874
0.07948682	0	0.139800142	0	0.05789326	0.064096	0.1164891	0.1069113
0.118822	0.1360791	0.1851818	0	0.5693366	0.07119513	0.08769776
0.09976539	0.05116482	0.4130121	0.05653359	0.03861014	0.07976459	0
0.064533822	0.06843638	0	0.1449636	0	0.2614477	0.3224283
0.6022074	0.05375575	0.1227225	0.08808129	0.1976709	0.053459044
0.1508779	0.1726416	0.1622195	0.1271075	0.07148409	0.047326196
0.08904152	0.05108144	0.08250885	0.08900771	0.02579374	0.05185541
0.097852168	0.06148636	0.03676278	0.09859903	0.09796928	0.026836375	0
0.103997	0.08988147	0.045577017	0.072007299	0.05446064	0.1431772
0.1446807	0.03771153	0.1162033	0.06633464	0.03903144	0.09813412
0.1515011	0.04148973	0.02286212	0	0.029694677	0.2270158	3.60886
0.1731992	0.237001	0.2672855	0.2646071	0.0694261	0	0	0
0.09072951	0.06446159	0.09910989	0.06631252	0.04452943	0.02123621
0.02797559	0.09304095	0.1479741	0.06037831	0.34742	0.06042105
0.04356348	0	0.1573008	0.02991918	0.04525034	0.04261326	0.2409352
0.05595712	0.06171356	0.1737829	0.1599515	0	0.2910215	0.4540415
0	0	0.1525443	0.2345817	0.03435224	0.06565598	0.02692227
0.07486208	0	0.07204512	0.1291899	0.369763477	0.2183016	0.1370140	0
0	0.1835008	0	0.195312301	0.1882379	0.043121534	0.04541463
0.1192075	0	0.1076641	0.04861136	0.03622732	0.09139883	0.09623357
0.01677182	0.03029341	0.09605402	0.088241153	0.3199661	0.07620305
0.2014556	0.05396671	0.02621611	0.166384	0.07616871	0.03642541
0.1202204	0.1644987
AC069277.1	0	0	0.06799506	0	0.010344753	0.02280152	0.02402402
0.007684682	0.0173127	0	0.0279302	0.058414505	0.08457295	0	0	0
0	0.04706714	0.03263302	0.7784388	0.322331861	0.1188898	0.005138439
0	0.01330118	0	0.3268798	1.807459	0	0.027180334	0.0494625
0.01032219	0	0	0.04955035	0.063972442	0.1400004	0.02943798	0
0.1689532	0	0.1286388	0.4324919	0.2114687	0.08580311	0
0.1906101	0.2332596	0.2906509	0.1439023	0.246729	0.0207557
0.1136389	0.188490767	0	0.1946987	0.2415395	0.05556366	0.2734888

0 0.08503503 0 0.1118102 0.01694837 0 1.574423765 0 0.2491557

0 0.051534356 0.007530225 0 0.04213795 0.016449727 0.01817447

0.1011917 0 0.6284623 0.1516148 0.009330241 0.3141677 0

0.2335298 0.3078074 0.009512761 0.009295612 0.1005441 0.01845664

0.1361233 0.1167352 1.675478 0.314255615 0 0 0.1532205 0

0.1459354 0.2709309 0 0 0.1208581 1.354644 0.2590233 0.08554738

0.2786758 0.08971783 0 0.9542727 0.605019768 0 0 0.0638374

0.1206833 0.01471038 0.2942048 0.02311151 0.1517523 0.1835802

0.07408958 0.2594717 1.071182 0.02977407 0 0.004658168 0.403732

0.09028583 0.4400643 0.2000338 0.112090216 0.1202268 0.09698288

0.4778933 0.33082 0.340646043 0.006461538 0.05121759 0.1000655

0.08028772 0.0663137 0.7347342 0.2102622 0.06233751 0.06101507

0.6940474 1.220235 0.004938284 8.298722 0.007150184 0.2041733 0.01031145

0.2211999 0 0 0.09619558 0.1667205 0.007710418 0.22379802

0.1535947 0 0.1119511 0 0.1504217 0.2390822 0 0.3968125

0.02486093 0.2832152 0 0.2536442 0.3995726 0 0.1650781

0.07231112 0.009025411 0.06859831 0 0.1631289 0.03320485 0.009745456

0.1317514 1.303715 0.00891129 0.03114934 0 0 0.05998954 0.6006416

0.1565933 0.005872419 0 0.1820125 0.587068 0.210965676 0.2456466

0.006254798 0.008360246 0.130389 0.06816879 0.1886169 0.092604206 0

0.1298278 0 0.08396758 0.014275767 0.2034489 0.04604661 0.5773774

0.2459457 0 0.06879215 0.3926309 0.0561736 0.09273753 0

0.3612337 0 0 0.56549909 0.010157924 0.1937368 0.006802511

0.094968797 0.6544549 0 0.01375284 0.01932796 0.00526743 0.360268123

0.3173939 0 0.005655171 0.009274184 0.1867183 0.3265543 0.02653252

0 0.1791662 0.1004474 0.08313134 0.4963082 0 0.005370548

0.004836595 0.221559948 0.07165194 0 0.08318933 0.2457276 0.3638075

0.214495 0.05986412 1.070526 0 0.04861718 0 0.025178856 0 0.4314719

0.2111568 0.02930657 0.09338719 0 0.2385297 0.271357 0 0.01654926

0.1340709 0.7508424 0.02439191 0.1125158 0.302655793 0.4521071

0.08383756 0.05033534 0 0.008315279 0 0.2432446 0.2935338

0.03150023 0.00794125 0.36703896 0.215428463 0.1835425 0.1778065

0.007694006 0.3742545 0.1569619 0.02696375 0.003669331 0 0.02820316

0.2962499 0.1303282 0.4299598 0.005069092 0 0.01120199 0.09180605

0.01053677 0.1045943 0.006393609 0.1559457 0.05765228 0.01436209

0.1921204 0.03453681 0.01419742 0.1591012 0.149122 0.01215858

0.4101476 0.6903361 0 0.007474021 0.5131277 0.08839435 0.06296512

0.3753565 0 0.3219913 0.1518249 0.3487771 0.1499116 0.01022193

0.0513751 0.019389166 0.04264179 0.02185337 0.3321316 0.07764948

0.010635123 0.3751304 0.875787 0.008904896 0.090309736 0.185484895

0.06834453 0.4019112 0.2365121 0.1419765 0.007675131 0 0.2010836

0.2398221 0.1681096 0.6823502 0.4212968 0.1480129 0.20005352

0.3523639 0.1721505 0 0.1617551 0.05296197 0 0.05502644

0.0107657 0.1173611 0.4887673 0.2337118 0 0.05891514 0.1313966

0 0 0.3048812 0.01843584 0 0.3270109 0.4216475 0.5626972
1.3379590 0.07792189 0.2074944 0.01793249 0.2955299 0 0.09424601
0 0.008608661 0.9508196 0.2264643 0.09436121 0.009996356 0.1442382
0.8431242 0.006045254 0.07437085 0.2110111 1.0928040.2133832
0.8158555 0.9953258 0.5924358 0.1599916 0.701276764 0.009612429
0.1968301 0.6420376 0.01049813 0.1246637 0.030547978 0.051600868 0
0.145255238 0.1709771 0.2913216 0.1217172 0.00853335 0.06260944
0.8183324 0.2475095 0.1430133 0 0.09604103 0.5519531 0.104908602
0.02717164 0.007549723 0.06386869 0.06416022 0.1818130.07143191
0.07546321 0.2093105 0.2382137 0.08148751
AP001189.3 0.7278151 1.2156070.4999273 1.361168172 0.984761684 0.8823475
3.6442451.2846521.2461021.5215330.3026272 1.782533308 0.5082325
1.304224064 1.66983602 0.6607083 0.5719473 0.3059870.1515355
0.5069506 0.531118447 1.0252910.1193049 0.02475841 0.2779456
0.3237740.2409377 0.07092811 1.6421750.189322925 1.9810360.431391428 0
0.5988776 0.9970707 0.024755337 0.8358548 0.2278316 0.4694332
1.1768330.03766519 0.2090726 0.4885128 1.5057050 0.06130159
0.1142093 0 0.1396214 0.6682281 0.1909528 0.3469741 0.1623681
0.45273128 0.3588425 0.1205477 0.1019653 0.09382429 0.3174948
0.5532850.3948708 0.9301297 0.4450327 0.1574038 0.07224714
0.290779756 0.1143289 0.07713239 0.1668162 0.615358889 0.2447728 0
0.02795327 1.183988673 0.3797794 0.1566322 0.3810597 0.1945562
0.2240135 0 0.06947034 0.3378401 0.2530325 0.7533021 0.353389567
0.2374094 0.2223281 0.3142543 0.03511699 0.1734638 0 0.964612312
0.0774676 0.1635948 0 0.277849704 0.5421356 0 0.7117157 0
0.07015247 2.0401490 0.2317292 0.1811692 1.1605730.275509078
1.0514910.335916689 0 0.08166122 1.4282870.3736053 0.1138492
0.04407022 0.6439271 0 0.1262930.9461217 0.2332044 0 0.2765193
0.4573427 0.6921852 0.1648158 0.3843158 0 0.4087081 3.123029702
0 0.6622826 1.4284230.2194577 0.234345562 0.9601588 1.149537
0.2323332 0.02867893 0.2395057 0.3618608 0 0.2067657 0.4794834
0.2630935 0.1373651 0.2751785 0.5200561 0 0.1354435 2.058947
0.09337904 0.1717375 0.9609940.081217494 0.4129 0.3938472 0.057735229
0.1620991 1.1148390.1732863 0.5556941 0.1552227 0 0.311178771 0
0 0.3586760.09316482 0.5720883 0.3710931 0.6196062 0.03194004
2.0663740.461016905 0.6825961 0.1569763 0.2272528 0.3700581
0.1357627 0.2039347 0.1807153 0.08276138 1.4464590.1365968
0.3821548 1.2755520.03320422 0.03635803 3.2723170 0 0.1185271
0.505623497 0.5151506 0.1742696 0.3882185 0.8649683 0.6682729
0.0265414 0.086003845 0.06551161 0.1063891 0.2730251 0.6823495
0.729204435 0.6691913 0.7269989 0.5362248 0.04758665 0.06018392
0.0228175 0.1953461 0.3130190 0.8287169 0.1143705 0.1962095
0.03464909 0.321546835 0.212263158 0.04998007 0.189529882 0.203538231
0.2263117 0.039068645 0.3831783 2.7823060.2201397 0 0 0.07719183

0.07878149	0.602921677	0.1625718	0.2246512	0.5852337	0.03920012
0.04622115	0.1332687	0	0.04903551	2.3638090.1496329	1.504775
0.630580186	0.2218165	0.127172368	0.7726002	0.3660026	0.2777072	0
0	0.02808543	0.1245149	0.06450285	0.0359556	1.139981424	0.3570294
0.4007188	0.2775094	0	0.2439310.05731439	0.3692142	0.03500223
2.23130600	0.1098662	0.6034554	0.2265337	0.3592059	0.413358843
0.3535859	0.5839653	0.2629558	0.5539011	0.1544522	0	0.3227247
0.3221781	1.2799090.2581333	0.578849896	0	0.2663446	0.05897619
1.21475500.4262767	0.03644362	1.47121200.51117	0.06619292	0.425636
0.7205909	0.6051953	0.04589821	0.07061689	1.58342500.1213750.8881519
0.53821700.2124924	1.068822833	0.3463343	0.03346445	1.24492	0.07434458
0	0.1977825	0.4155786	0.2473095	0.1411497	0	0.2955575
0.319727782	0.6247179	0.1059010.3358393	0.3508637	0.1973225
0.174183665	1.0036320.6815175	0.2783670.2456941	0.11866700	0.360144077
1.66896300.4735679	0.1512054	1.1718690.44446974	0	0.09569004
0.2481060.083872913	0.033127782	0.5512153	0.1317406	0.09984304
0.2775940.1425617	0.1220720.1077412	0.3611818	1.67279500.648986
0.04207193	0.08591458	0.245904767	0	0.5534324	0.3585703	0.12115
0.2151936	1.3086570.4152235	0	0.1486312	0.4126641	0.1391372
0.2075941	0.1139916	0.4271097	0.04097255	0.2149392	0.5405606
0.04280455	0.03403859	0.07407396	0.8790899	0.1389870.2405025
0.2344095	0.1447360.7708211	0.04163587	0.07841887	0.1662675
0.05148743	0.05678406	0	0.7849333	0.2273763	0.4138349	1.067645
0.3572203	0.03290044	0.08421569	0.08633759	0.03160828	0.3926752
0.3220333	0.1377646	0	0.09943554	0.1485882	0.291623861	1.004322
0.09455226	0.5300235	0	0.5306504	0.794378813	0.898556593	0.3810444
0.238062664	0.1253611	0	0	0.4160696	0.5591053	0.6666717
0.4765562	0.1328201	0.07716068	0.6968415	0.11784200.216513879
0.2102915	0.2804647	0.1112183	0.3475920.2170984	0.2296406
0.02336152	0	1.17992	0.6306624
AC011405.1	0.04976971	0.01278863	0.07197096	0	0.032848937	0.03861568	0
0.009760838	0	0.03242992	0.03547604	0.042397851	0.02527573	0.044592955
0.022837463	0.05020088	0.0782222	0.02391327	0.08289885	0.09646323
0.026413912	0.2696607	0.08484686	0.02031646	0.1098158	0.09661279
0.2767949	0.05820278	0.1044612	0.025892694	0.02355963	0.104887328
0.0876009	0.1310485	0.05244772	0.108341004	0.00846781	0.07478237
0.09630284	0.1072995	0.02060507	0.03267857	0.1336226	0.1611605
0.6660156	0.1676779	0.05466929	0.02962789	0.05728585	0.09139003
0.03482081	0.03163588	0.1221342	0.024767064	0.009815398	0.1154067
0.1394526	0.05774333	0.1002048	0.02017863	0.02400195	0.05451811
0.04057651	0.03229092	0.009880859	0.034087207	0.03127233	0.07032659
0.07300661	0.009351042	0.02869396	0	0.09175249	0.073128712	0.02308463
0.02142175	0.05685329	0.08514696	0.1225486	0.1303607	0.04750545
0.06160613	0.02966221	0.1003835	0	0.01180699	0.04256928	0.007814344

0.05763317 0.3617868 0.01547737 0.060888424 0.09182189 0.03356099
0.01769234 0.121600106 0.2347925 0.03128434 0.02595669 0.1382208
0.04477379 0.03100226 0.3176582 0.0724397 0.06371372 0.05697836
0.047956259 4.458014 0 0.0410172 0.04467349 0.04422776 0.03832202
1.650478 0.048218 0 0.1623166 0.1036345 0.1019484 0.04252551
0.04388972 0.04727259 0.105851 0.1479164 0.1352459 0.05733909
0.1834073 0.162609 0.090601263 0.1221666 0.06521535 0.06279361
0.02572635 0.064100404 0.05745068 0.02168498 0.01588749 0.03137812
0.04679419 0.08483963 0.08468021 0.1018017 0.05961491 0.1349322
0.05636011 0 3.115289 0.1180652 0.1018814 0.01309727 0.02554191
0.02818516 0.01011001 0.088861494 0.02823508 0.03917411 0.015792283
0.1064133 0.08968863 0.1421967 0.0268233 0.09553043 0.07591864
0.008511655 0.08579038 0.01052585 0.1417122 0.1401582 0.05522928
0.03383496 0.06571693 0.03494616 0.04945623 0.022927579 0.09957873
0.04293764 0.07597374 0.03374059 0.0742702 0.0318755 0.3048243
0.2376956 0.03165192 0.1587938 0.008040814 0.008020715 0.1271526
0.01988998 0.02237688 0.08400164 0.2211348 0.06484131 0.051863587
0.1308439 0.02383393 0.01061892 0.03943238 0.03848259 0.02177956
0.039207635 0.0358387 0.07760148 0.03111683 0.01333162 0.036265247
0.01076726 0.01169739 0.08800396 0.2017534 0.05761725 0.02496503
0.03562194 0.03805329 0.1820426 0.08000415 0.08342318 0.05366908
0.03791018 0.058635011 0.019353406 0.3144334 0.077762996 0.046394744
0.1238058 0.053432117 0.05240526 0.04909952 0.1271199 0.065371581
0.1385806 0.04222847 0.1221113 0.10601795 0.02964545 0.01536218
0.05055115 0.05897312 0.1264284 0.1458116 0.07039385 0.1140076
0.04216769 0.05457198 0.0552896 0.018156024 0.227525 0.104356149
0.04972444 0.05888969 0.04431064 0.05675931 0.065175 0.03072877
0.136234 0.07057371 0.09834914 0.047972069 0.1051702 0.05480419
0.09108838 0.1861213 0.09637666 0.0548701 0.05049547 0.1053155
0.0241714 0.06306103 0.02003442 0.07336124 0.06196364 0.2054389
0.052764041 0.04835807 0.03549593 0.04795076 0.0291362 0 0.03962681
0.1235846 0.05423088 0.03000794 0.01008672 0.035184994 0.032835643
0.1748474 0.1935807 0.009772681 0.01793834 0.0199368 0.01712424
0.1165167 0.02715856 0.5104742 0.05375531 0.06621549 0.1757631
0.03863159 0 0.04742804 0.02915228 0.05353388 0.06642619 0.024362873
0.1291809 0.04576755 0.03648453 0.01016771 0.05483444 0.07213243
0.6946682 0.08117569 0.1235475 0.09975761 0.01243749 0.074000344
0.02847979 0.04345056 0.06124128 0.04798577 0.02698675 0.041688811
0.08964012 0.03214053 0.1522831 0.01120076 0.1038686 0.05220401
0.049254999 0.03868732 0.06476738 0.07444641 0.006164243 0.027016788
0.1089094 0.03926106 0.01131071 0.195004523 0.072491382 0.1142224
0.06005821 0.01820667 0.07593011 0.1754767 0.01669515 0.2062926
0.01646564 0.09913772 0.09397946 0.0632935 0.04700033 0.029894305
0.03809033 4.507761 0.02179545 0.01988285 0.1345412 0.09989474

0.04368302 0.0273485 0.16262 0.05643788 0.1522324 0.05678308
0.0561241 0.1001375 0.1232792 0.0481027 0.09857281 0 0.1582794
0.1063724 0.02185972 0.3801702 0.01096409 0.05495819 0 0.06777072
0.04555454 0.0643496 0.06063873 0.05633331 0.1553211 0.03280334
0.03354724 0.05441983 0.0599273 0.03809116 0.03664135 0.2429795 0
0.04723174 2.7839460 0.2439293 0.09420669 0.01044819 0.05439709
0.0325146 0.106356938 0.0305235 0.04310471 0.0724885 0.1600127
0.01979298 0.031040853 0.057349053 0.09475167 0.03255857 0.1257298
0.1300096 0.4638038 0.17884 0.02446908 0.02735315 0.0843455
0.09688051 0.08020171 0.0228728 0.04834988 0.088834371 0.2300836
0.1054836 0.09126446 0.05432951 0.03299044 0.03489636 0.006390064
0.03667028 0.01008571 0.1656045
AL356124.1 0.1028228 0 0.03965067 0 0.054291993 0.1063719 0.02801875
0.05377499 0 0.01488874 0.03257445 0 0.1044379 0 0 0.05531393
0.2962756 0.0988083 0.03805927 0.03321506 0.14552109 0.2377009
0.1258501 0.01865478 0.2326937 0.1108885 0.09076993 0.08907061
0.0863256 0.071324783 0.02163269 0.036115718 0.08043601 0 0.2022637
0.037304917 0.04665136 0.2403307 0.08842622 0.1182281 0.2270374
0.2475477 0.2044894 0.4932639 0.1667842 0.06928357 0.2796739 0
0.2279352 0.1258728 0.02397961 0.01452419 0.1835098 0.068224089
0.2974156 0.2043660.2048748 0.0530205 0.09200906 0.08337697 0
0.1001181 0.05588662 0.1185994 0.4627078 0.312992098 0.3158602
0.07748948 0.2011062 0.051517314 0.02634707 0.4844973 0.02106201
0.057555005 0.0158974 0.3540540.1044065 0.02931854 0.07233769
0.09793513 0.05234397 0.2121276 0 0 0.033283653 0.01626194
0.0335036 0.04305125 0.1058387 0.04901256 0.02131721 0.14908894
0.03891312 0.1386721 0.07310376 0.167481627 0.03404034 0.143628
0.4647570.1269157 0.03523863 0.1067497 0.09375329 0.1496584
0.05850257 0.1121102 0.113230057 6.7908820.023009445 0.1129872
0.1230589 0.08122073 0.1055630.5833188 0 0 0.05589025 0.02378956
0.04320463 0.08785652 0.04029998 0.1823059 0.2650731 0.2281748
0.1397072 0.07897398 0 0.1399774 0.463492571 0.1177833 0.0998023
0.2114116 0.09448875 0.088286436 0.02260791 0.1194682 0.3938774
0.1080439 0.2062411 0.07790059 0.1076597 0 0.0821085 0.1486753
0.1552512 0.05183484 7.1708150.2001390.05102644 0.2164687 0.03517926
0.1035196 0.08354796 0.091792689 0.07777718 0.4046631 0.108754728
0.04885488 0.04941179 0 0.04925883 0 0.4182555 0.164125192
0.02954009 0.2609534 0.03002805 0.03509855 0.07606811 0.2330069
0.09527674 0.0721978 0.07784776 0.031578494 0.1714391 0.08870796
0.1712287 0.06970712 0.03409782 0.2853669 0.0453880.0623584
0.08718932 1.0034890.1328968 0.1877999 0.1501108 0 0.12328
0.0661124 0.2215071 0.4167655 0.047621658 0.02772512 0.08753821
0.5265213 0.4344864 0 0 0.172804022 0.04936118 0.05344084
0.1714307 0.03672367 0 0.01482991 0 0.05387073 0.05378282 0

0.08596176 0.02453131 0.4323937 0.3244740.3305727 0 0.2587172
0.2088570.161517739 0.035540976 0.2259516 0.095203667 0.127800336 0 0
0.07217855 0.2028765 0 0 0.1735174 0.0387746 0.1582924
0.097346729 0.08166224 0.1692684 0.02320828 0.0443043 0.1044791
0.1506213 0.04847724 0.1108407 0.07743758 0.0187907 0.1015349
0.075019682 0.04178314 0.071865635 0.05136466 0.04866578 0.08718529
0.03127018 0.05984432 0.3809087 0.1250914 0.07290166 0.05418309
0.110121069 0.1655457 0.03019305 0.02787942 0.1230469 0.1633735
0.1295544 0.1947348 0.3692249 0.1331665 0.02895163 0.02759371
0.02526038 0 0.0492094 0.083054508 0.03330214 0.04888907 0.02201443
0.4976086 0.08728156 0.2183144 0.02431641 0.0746930.08266077
0.1944963 0.07269122 0 0.04013665 0 0.1076805 0.1235337
0.05491851 0.07075643 0.1668994 0.07481179 1.5048460.1974346
0.06079972 0.08645756 0.4433989 0 1.0321090.1873754 0.0368665
0.09148977 0.156591617 0.1186151 0.07564359 0.03350046 0.05601657 0
0.3477217 1.2873 0.07453632 0.06381142 0.3969265 0.01713034
0.018531231 0.1046017 0.1196902 0.2811617 0.1542135 0.1238975
0.032810638 0.1388955 0.01770705 0.7436301 0.1542697 0.1251773
0.1797534 0.158292492 0.08525539 0.1274358 0.02278581 0.1018812
0.111631868 0.1125019 0.1081497 0.1557841 0.189587731 0.07488259
0.03775685 0 0.05015264 0.2614492 0.2148326 0.1839558 0.05411998
0.06803511 0.08402699 0.02876429 0.06340006 0.02157808 0.020586935
0.05246237 2.2239780.2701727 0.2921061 0.0463264 0.2522419 0
0.1130025 0.1493193 0.2176516 0.1258032 0.02234519 0 0
0.06174336 0.4564065 0.1745560 0.4360012 0.0418595 0.1204309
0.2094456 0.06040399 0.1513895 0.08179093 0.08297032 0.1254859 0
0.2923152 0.3685463 0.04278521 0.03012035 0.01848205 0 0.01834195
0 0.3028 0.02478957 0.08460557 0.4553708 0.1190798 0.1820737
0.09332429 0.1038018 0 0.07492192 0.1119571 0 0.4035885 0
0.07487962 0.03673132 0.1635670.021376513 0.112839572 0.07830173
0.269060407 0 0.02754833 3.8328230.8807770.1011049 0.05023186
0.1267312 0.03335874 0.1162769 0.08400811 0.2441744 0.040784298
0.9506928 0.2113223 0.05586662 0.2244865 0.1454023 0.07690124
0.1056136 0.02525326 0.1944768 0.285112
FO680682.1 0 0.2646690.04964953 0.183461797 0 0.06659805 0 0.06733564
0 0 0 0 0 0 0 0.06926266 0 0.08248345 0.04765682 0
0.091108856 0.1488215 0.5853204 0.1401542 0.05827459 0.2221627
0.09092779 0.08922551 0.1441262 0.238162752 0.05417577 0.135669478 0
0.1695084 0.07236266 0 0.3504937 0.1719633 0.1107250 0.4264354
0.1127174 0 0.06176522 0.08353713 0.2313469 0 0.06812983
0.3512794 0.1050765 0 0.07274723 0 0.113904566 0 0.1706012 0
0 0 0 0.9106827 0.2507306 0 0.07425352 0.3408180.235152324 0
0 0 0.387051823 0 0.3033375 0.1054932 0.072068876 0.1592505
0.07388953 0 0 0 0 0 0.1062483 0 0.1822370.083353844

0.1221767 0.2936664 0.05390765 0 0.245489 0.2135429 0 0.194904
0.07717406 0 0 0 0.1438777 0 0.1271364 0.3088742 0.05346767
0 0.06246612 0 0.05615259 0 8.078196 0.115247307 0 0 0.1525537
0.3524885 0.1718627 0.5821101 0.1350069 0.5598745 0.1191547
0.1622991 0.4400466 0 0 0.1327671 0.1632654 0 0 0 0
0.178576402 0 0.04998794 0.144395 0.05915817 0.110549972 0.05661806 0
0.328802 0.1623477 0 0.1950902 0.1797448 0.07803159 0.04112565
0.06205576 0.129601 0.08654164 8.831909 0.06265219 0.06389398 0 0
0.1296245 0.2092332 0.459761643 0 0.06756114 0 0.1223496
0.1237443 0 0 0.2196738 0 0.058718081 0 0.07261312 0.3760034
0.04394949 0.2540013 0 0.04772122 0 0.09747894 0.316334129
0.08586861 0.9626742 0.09529249 0 0.08539279 0 0.170501 0
0.1091762 0.06443808 0.05546996 0.08299697 0.1879648 0.06860602
0.154368 0 0.06934136 0.1491037 0.178891792 0.06943334 0.2192261
0.1465103 0 0 0.1001649 0.054095172 0.2472351 0.1338344
0.0429322 0.09196884 0 0 0.16139 0.06745553 0.1795879 0.05678222
0 0 0.03281407 0.07387234 0.1839709 0.07193738 0.04627986 0
0.10112415 0.13351045 1.131723 0.178817323 0.256045194 0.1830173
0.294883335 0.0602534 0.08467889 0.1846196 0.075161528 0 0.04855254
0.09910483 0.162526713 0.1022553 0.1059768 0.2324864 0.480798
0.0872173 0.1257361 0.1214039 0.09252788 0 0.1882338 0.2966585
0.062625126 0 0.959874917 0.04287832 0.0812507 0 0.0783114
0.2248065 0.05299599 0 0 0 0.110312584 0.103646 0.07561389
0.279279 0.1027174 0.05114302 0 0.06966911 0.1320954 0 0.07250495
0.06910425 0.1265216 0.5343242 0 0 0 0.1224352 0.05513179
0.1607981 0.07286113 0.0683419 0.06089675 0.2338216 0.06900378 0
0 0.377530576 0.2512903 0.222571 0 0.06187428 0.4126052 0
0.4822778 0 0.8031565 0.1236112 0 0.1299119 0 0.2390283
0.09815542 0 0.04616327 0.1718417 0.056022839 0 0 0 0
0.1513113 0.1866035 1.045572 0.2799973 0 0.3058591 0.04290032
0.23204324 0 0.09991529 0.2816507 0.1103441 0 0.123253876 0
0.1330338 0.2387575 0.154538 0.08956782 0.270099 0.226525405 0.2135091
0 0 0.04252434 0.046594171 0 0.09028147 0.07802754 0 0.125021368
0.03151876 0.08286292 0.1255996 0.3273798 0 0.3455169 0.2710705
0 0.3156492 0.2881431 0 0.108078 0.10311369 0.197076 6.03375 0
0.1828838 0 0.1148548 0.0602699 0.09433252 0 0 0 0.05596014
0.1290583 0.2302678 0.3092537 0.07374197 0.09714422 0.3230813 0
0.1747179 0.0754002 0.1049049 0.0756363 0.06318865 0.1365553
0.1558399 0.1571302 0.2219595 0.1045798 0 0 0 0.1388564
0.05363114 0.2756077 0 0 0.1862451 0.1589113 0.1629153
0.4175041 0.113994 0.1402299 0.3249448 0 0 0.2243036 0
0.04211358 0.237888 0 0 0 0.160602673 0 0.06536492 0.299475931 0
0.1379812 0 0 0.08440059 0.1886971 0.1586896 0.08354189
0.1164791 0.05259638 0 0.102138069 0 0.06615308 0.06995473

0.09369872 0.04551724 0.2407343 0.1322465 0.2529718 0.2783072
0.2380065
THUMPD3-AS1 1.576213 0.8779417 1.498625 0.968327445 0.887083583 2.105401
1.118785 1.391997 0.8590657 1.07216 1.278268 1.382946957 1.958074 1.745457094
0.757912624 2.414314 1.924734 2.376312 1.145017 3.542064 3.386200387 2.168287
3.312163 0.7525887 2.624028 2.11922 8.441178 3.899972 1.909701 1.721888232
1.858639 2.657932637 1.608669 1.901195 1.468068 2.468081444 1.718257 26.57203
4.364859 2.856919 2.532109 1.620571 4.916035 3.436607 1.14822 6.890773 1.43961
2.172554 3.490892 1.432724 2.26499 2.077817 2.501465 1.468552051 1.93214
2.507498 6.403456 0.987981 3.559893 2.097034 3.686929 1.040782 0.9965008
1.837109 4.429711 3.141672525 1.581474 3.822337 1.914082 1.276808404 2.633716
1.617719 3.555415 1.858343336 2.696726 2.16935 1.150052 2.680467 3.061077
2.126074 2.533331 1.916024 0.8062763 1.897675 0.586598523 0.6000773 1.17403
1.950211 2.185928 4.397774 10.07931 1.916796704 1.67435 2.887386 5.156943
3.072800756 3.993314 1.265666 1.747475 2.949767 1.797659 27.268225 3.859737
2.040029 1.584184 1.929546 1.447323727 11.35284 2.22087529 1.965383 2.740674
2.108037 1.177326 3.635513 3.264455 1.065156 5.229083 3.734146 1.252225 4.479885
2.186256 1.136742 2.164315 2.975659 7.083187 1.409978 3.627227 2.339788 2.18453722
1.44104 1.602283 0.9447227 1.964523 1.160906835 1.873945 1.447555 6.315095
3.76375 1.171383 4.252888 3.938131 1.844779 1.168984 1.978862 1.346715 2.224396
7.073268 2.187335 1.029279 1.43066 2.530888 1.771001 2.143514 2.182310841
1.729517 2.544444 2.509702106 1.658154 1.476353 2.800909 3.279296 3.957455
2.109207 1.656130611 2.310256 3.10599 3.084366 4.243104 1.585029 2.409399
1.549305 1.527579 0.6886829 1.560938788 2.488 1.437196 11.05469 1.599653
3.584558 2.079451 4.799569 3.619033 2.214928 3.082245 1.936587 1.875467 4.564324
1.365451 1.270246 2.76275 2.763985 11.07514 1.13108077 4.23737 3.480324
2.078228 2.189562 2.484933 1.332713 1.796390755 7.081956 3.270733 1.295868
1.663573 2.3314364 1.123056 4.942393 6.323351 4.349389 6.231294 3.101034
1.124773 1.759017 5.239344 2.235356 2.361208 2.246767 2.476728 2.01821241
1.529253164 6.180739 1.736879019 0.992455656 1.073313 1.722600356 2.063835
3.021518 4.780839 2.487696842 5.361248 2.143546 2.860615 1.858632178 2.254425
2.621975 3.163575 4.609737 1.970833 4.572936 2.796739 4.397876 0.959609 1.655858
1.710252 10.005748614 4.964916 2.328652724 5.72154 10.79715 1.411726
3.560704 1.812782 7.406686 2.872058 1.977442 3.558624 1.616321072 2.330672
5.778589 1.671752 7.722834 3.064914 1.881935 2.704272 3.268177 0.8388536
7.095664 2.469582 1.116465 0.7696822 2.672976 2.595906707 3.340425 3.056101
4.225423 3.010255 3.264817 1.83739 4.131566 3.668968 8.445072 1.989375 1.394559327
2.183600755 3.603203 2.318906 2.43895 1.721338 1.141815 8.992234 3.40638
1.795769 2.843071 1.590301 3.084886 2.176102 3.011048 4.041101 2.836809 1.146238
2.299483 1.102251 2.18997966 5.141323 1.086664 1.745889 1.650556 2.757784
3.200955 3.056319 0.8672108 2.43962 3.165642 8.052476 1.347219013 1.447454
2.686249 2.96154 1.880685 0.8870863 1.190406561 0.4611109 0.9849805
11.10274 4.834516 2.107663 2.653212 1.662660371 1.15920 210.50687 2.054971
1.856366 1.15278805 5.550692 1.747216 3.19173 2.101387387 0.649563213

2.594158 1.512529 1.384548 2.530711 1.996659 1.228444 3.096198 1.230292 1.078866
2.158491 1.433821 2.195621 4.47866712 2.026193 11.60438 2.670125 3.142177
1.486225 2.004926 3.933296 2.964033 2.333522 2.500212 3.161219 2.079844 2.303717
2.513038 3.22628 1.161568 4.753487 1.087986 4.382386 1.008631 5.111413 4.7295
1.37646 3.842373 2.901773 11.64118 4.302236 0.5938964 4.392853 2.913823
2.886621 4.201163 4.305766 0.9347203 2.48003 1.7096 3.326119 9.082677
1.843498 0.662825 5.486104 2.306403 2.07652 5.652642 4.62461 4.666245 2.713091
3.15151284 1.606893 3.943349 2.670321 2.240476 0.8958517 1.303888952
1.339271753 1.132037 2.350408809 2.557767 3.542764 2.404142 3.385381 1.257535
1.137746 2.093944 2.094472 1.831552 1.578897 2.298398 2.507347169 2.69218
4.564563 1.134604 1.493953 2.429968 1.972103 2.19582 3.275431 1.553087 3.001841
GAS5 33.55621 18.27389 19.70482 17.632113649 24.550088735 42.95327
40.95593 36.22494 12.61923 25.86236 49.35915 23.517612738 33.82235
50.689282451 21.944846088 45.8301 82.36215 62.36826 48.15656 62.86093
65.574664536 21.38012 35.38781 24.21876 90.77307 23.88991 22.34179 22.75011
29.42878 48.805244534 38.92039 18.27894944 109.1449 18.69891 36.94232
29.12458907 56.61376 27.32718 38.65251 37.75381 61.8167 126.9868 36.22134
57.21328 18.85107 37.92351 54.65182 39.7141 53.01915 44.93733 46.40096 48.38808
26.36718 27.083704362 147.0672 57.69032 101.2967 21.20376 55.90761 97.32001
39.99197 21.57191 23.22544 19.57115 44.97334 11.804568956 39.97788 55.25156
66.7256 28.826086919 73.57664 82.37228 26.7576 39.764215547 59.94985
54.06583 40.62554 46.61614 27.93068 141.7024 36.00204 89.56456 15.32076 182.87
23.862591601 21.78554 48.72219 15.81175 148.0403 91.3647 56.62378
34.327328079 56.80652 15.34368 24.32996 56.35131745 56.33503 53.21951
142.1584 68.85534 51.23694 165.1535 50.23173 54.79402 45.64612 46.17209 58.47573093
20.03712 27.342748847 31.42905 51.79984 25.13418 93.71829 36.73743 18.39429
39.19505 93.54615 33.3871 15.4877 202.951 33.7164 36.12303 73.72136 51.49825
81.71981 32.0841 75.27327 49.66852 35.801347227 170.6022 40.3052 25.87994
13.78812 46.395822852 23.94448 23.55224 81.63318 48.33834 27.86248 64.80368
45.94539 27.55852 62.26306 34.57553 22.40543 56.47396 27.27287 34.03872 123.1906
42.7095 74.81945 39.49835 92.09889 76.331068861 39.04084 68.44291
27.508626495 75.65593 118.2578 64.87085 21.6842 33.59751 75.93704
48.471282736 23.65993 27.4111 41.1135 24.51652 46.67511 36.18845 62.9103
77.11894 12.84636 20.844206255 105.2977 99.53898 29.69335 8.282381 39.08325
27.61577 33.84757 21.91609 15.73125 30.60684 31.69144 24.99589 73.84308 57.78097
19.55151 138.0047 101.3703 63.42615 33.343291739 60.734 63.01536 23.01091
64.44078 37.82978 59.10649 63.204867751 45.1616 84.63333 20.17577 48.28858
59.771339879 17.03348 44.79207 33.918 28.38098 63.71158 18.85104 29.5073
55.3509 36.66019 82.89027 65.50116 43.7706 112.9754 31.544134171
21.070640572 80.54953 31.885231581 21.092525006 70.23586
54.481746692 34.58464 61.55824 29.69803 57.388962423 146.9215 47.05492
95.44994 17.35736952 52.14473 82.14614 65.0156 40.85474 27.94364 29.37129
36.37051 31.79904 61.49794 25.55328 33.84057 59.116583911 24.94533 24.9268261
63.49877 98.92469 21.19393 116.0834 34.56764 31.43111 65.80433 67.60134 116.9259

40.148873968 49.56961 23.56505 88.0489 129.2137 33.04852 182.6021 64.6737
65.24042 21.773 60.57558 14.09053 21.60257 26.13582 99.66477 74.42214607 24.6762
45.03213 21.98538 65.00132 75.6328 27.539 65.53353 21.16797 23.05232 31.00138
46.753647112 58.168613593 93.08448 103.9934 24.21342 70.58908 18.77206
17.28554 12.86118 43.29927 120.9311 57.32706 108.4979 45.5273 43.28749 71.8245
38.63555 74.07034 49.55979 86.93882 38.155395396 44.56098 30.59309 18.25067
173.5528 39.31353 38.01936 19.6869 158.9854 23.4947 112.1256 55.96188
22.007936883 43.61363 27.80636 47.21324 30.91838 32.4002 48.823097332
27.9165 35.1272 43.60168 49.8283 31.54768 17.91312 44.477991834 46.7099
69.55599 231.6455 34.94591 32.296073862 123.1953 45.54975 33.38433
30.781821114 23.745968536 55.4929 50.51466 26.79154 89.09393 60.94099
38.88089 24.41006 52.57654 17.86491 25.56417 27.78106 65.17767 23.479379081
35.7277 125.3648 29.4867 52.2475 42.7047 23.62653 32.22904 52.5188 85.87765
40.27075 121.4313 66.70784 36.57085 53.04547 34.7904 30.80804 23.15389 24.99554
147.583 23.30095 38.82259 91.33773 27.74744 263.15 23.22493 18.33125 24.97746
76.0277 28.94552 39.50934 76.51876 64.09597 46.88517 120.5659 72.02272 32.61586
62.7489 77.43817 28.93469 48.42736 37.7991 23.51986 49.26314 52.61045 128.148
262.775 33.38562 71.473707382 30.18277 109.0819 43.14848 183.1782 25.60564
31.002596681 33.079552987 34.66168 43.656699803 69.68287 152.25
11.14696 44.94996 13.53263 21.89738 35.73465 58.50118 66.6001 14.38767 72.47901
23.087394876 72.85737 86.37596 67.61748 62.45313 28.67793 32.08681 27.15388
130.288 48.84757 114.0526
AC073529.1 0.05288031 0.00988212 0.08342089 0.041100208 0.076149809
0.02237954 0.03143922 0.03771233 0.1699230 0.05429554 0.08223992
0.131047903 0.06347655 0.04824147 0.035294261 0.02327495 0.05288887
0.09239218 0.0640582 0.02795241 0.132669877 0.2083742 0.2067771
0.04709726 0.1305502 0.06843406 0.5347174 0.1099386 0.02421601
0.21341857 0.09102585 0.055721393 0.07333257 0.4810076 0.09726669
0.094182804 0.1112362 0.04815531 0.1860396 0.2155744 0.08757156
0.05050324 0.05736325 0.05534805 0.05614346 0.05182771 0.05431429
0.2060485 0.1426360 0.07061957 0.0134535 0.2240875 0.06863742
0.140346697 0.02275388 0.1910955 0.1293106 0.07436641 0.07226893
0.07796288 0.1298287 0.02106381 0.2586752 0.06653887 0.03817605
0.1843808 0.03221997 0.1304239 0.07756952 0.130064492 0.1478173
0.08494434 0.08862456 0.121089751 0.1114883 0.0662127 0.3002026
0.0740198 0.08116852 0.07326057 0.1174680 0.07140711 0.03056106
0.1592207 0.289438103 0.06842687 0.09868334 0.1207671 0.1261817
0.2016517 0.2690952 0.162061614 0.3493085 0.2204347 0.1230422
0.105709183 0.2673714 0.05640662 0.1504308 0.04984325 0.1383917
0.07785795 0.2103970 0.1399403 0.0547037 0.04402874 0.100583583 1.52398
0.096818963 0.1479105 0.06904085 0.08543999 0.1579332 1.073235
0.1304078 0.09829644 0.1254264 0.1334687 0.1939158 0.09036671
0.3504528 0.1241980 0.07435817 0.1325869 0.1045082 0.1255379
0.06073879 0.1492122 0.080011505 0.05664088 0.06719158 0.102436

0.1259032	0.061915163	0.05073566	0.03351315	0.06138349	0.1636654	
0.1518684	0.0655579	0.1006688	0.332141	0.05527928	0.13207	0.3266325
0.1114786	0.5386126	0.02807144	0.04294173	0.0607237	0.1134874	
0.116157	0.06249821	0.24032995	0.2618162	0.1135159	0.103726588	
0.3837329	0.1108878	0.1245299	0.1105445	0.1558401	0.07333051	
0.078926252	0.1408717	0.1138706	0.1263518	0.2806061	0.02133859	
0.09804448	0.2111432	0.06750963	0.1091891	0.097442209	0.0288552	
0.05806339	0.16011	0.1694698	0.06695571	0.1416287	0.1400544	
0.02623912	0.06114576	0.08661482	0.0994137	0.1208576	0.168436	
0.06147812	0.0288187	0.102002	0.1553427	0.1252616	0.180343835	
0.124439	0.3315083	0.03282211	0.02437638	0.1189462	0.2131757	
0.042415533	0.1523145	0.07495598	0.05770757	0.1648273	0.140115728	
0.07904151	0.1265445	0.1284502	0.2162489	0.2416932	0.1687976	
0.1376302	0.02940481	0.1489439	0.02060713	0.1369847	0.191806	
0.06591202	0.067963308	0.094714394	0.07394779	0.08011945	0.078871064	
0.1571691	0.066061526	0.08098995	0.04742568	0.2223078	0.084190672	
0.07788001	0.1631555	0.02775257	0.081922962	0.1030853	0.07122464	
0.2343735	0.2112797	0.3907788	0.183093	0.2039822	0.1399184	0
0.1950328	0.1376655	0.119252066	0.1640938	0.302395659	0.1008615	
0.0546068	0.1663088	0.1491222	0.1846625	0.02374496	0	0.06135101
0.2203915	0.12356442	0.05804849	0.0677579	0.152504	0.1208097	
0.126031	0.0545138	0.03901923	0.110973	0.08872004	0.2517664	0.1780332
0.04251617	0.09576199	0.213965	0.046596815	0.04203855	0.123429	0.04322833
0.1576004	0.1713892	0.1224829	0.1159609	0.2671487	0.5410523	
0.2494171	0.095159415	0.135322648	0.3602916	0.1495851	0.06041294	
0.2079217	0.1694628	0.03308093	0.1368541	0.06995388	0.5397838	
0.4153819	0.2558326	0.2764845	0.2537393	0.08032281	0.3958086	
0.02252676	0.04136709	0.1283233	0.100404569	0.1131311	0.1343902	
0.1362642	0.03928435	0.1779627	0.05573869	0.6831861	0.3345423	
0.0358007	0.08565048	0.1922157	0.08317394	0.08069273	0.06715086	
0.1340813	0.08033981	0.1112181	0.220896557	0.07792579	0.1887526	
0.06953424	0.1644475	0.06521294	0.1008487	0.101495149	0.119579	
0.1072447	0.08309423	0.07621246	0.130478806	0.1157162	0.06067618	
0.03496039	0.168412997	0.063018076	0.04942715	0.167071	0.03517198	
0.07334158	0.07533092	0.09030556	0.07590855	0.1526814	0.1060704	
0.1613789	0.1333871	0.3813436	0.109725688	0.1398088	1.845613	
0.06736775	0.2304604	0.240416	0.09648923	0.1485223	0.0792485	
0.1047174	0.08722218	0.04117195	0.06268262	0.3373115	0.2192404	
0.02598032	0.06608047	0.07072919	0.09952059	0.07913972	0.2074492	
0.0168916	0.08813038	0.04236124	0.1557149	0.1147197	0.06400568	
0.1848065	0.04143724	0.1640002	0.04353028	0.04800834	0.08449345	
0.2021984	0.1441772	0.0771791	0	0.07550338	0.2851123	0.1008674
0.03649725	0.253872	0.08299725	0.09948127	0.2256678	0.09688322	
0.0980796	0.2009993	0.267100945	0.1651044	0.01332327	0.1680415	

0.01030385 0.02039276 0.053968755 0.044315177 0.08053892 0.109021879
0.1059871 0.2627467 0 0.1340068 0.1039936 0.1902287 0.1007267
0.07486222 0.03914151 0.02945739 0.09340318 0.137289482 0.05333757
0.07410004 0.1567168 0.04198189 0.01529557 0.2534745 0.2617022
0.1912689 0.07793505 0.1119712
OSMR-AS1 0.2357364 0.3230607 0.2337556 0.575839151 0.165963091
0.3135511 0.3120086 0.2641864 0.1785544 1.1898350.3200644
0.095628208 0.1824298 0.257482901 0.185435353 0.1268153 0.08821495
1.1218750.1994433 0 0.166814168 0.09731502 0.0471069 0.4154683
0.03048481 0.02905464 0.08324132 0.03500698 0.03769791 0.342618516
0.3542578 0.201087058 0.05268894 0.7241697 0.1324911 0.024436276
0.07639647 0.1574266 0.20273 1.5876091.3012910.1326715 0.1339491
0.1938650.3496020.01512788 0.3945805 0.1603814 0.05742579 0.05496797
0.03141529 0.02854184 0.1001721 0.119172328 0.3719284 0.1338683
0.03355039 0.3820370.06026977 0.9648720.6496358 0.1311631 0.1098242
0.4467033 0.1604610.266529738 0.2821385 0.02537942 0.1975994
0.219348929 0.06903373 0 0.2069474 0.452411138 0.4790192 0.07730671
1.1113470.2304584 0.06317894 0.04276773 0.2400124 0.1111619
0.3032936 0.2859973 0.130813083 0.2023927 0.5157370.4653060.01733219
0.2568421 0.01396366 0.073244604 0.1401935 1.0193820.1117340.274268623
0.02229784 0.7150255 0.04683618 0 0.5770693 0.2657169 0.1023539
0 0.07664324 0.01468737 0.655171943 0.4077618 0.331587209 0.02467044
0.2216740.05320297 0 0.37086 0 0.01765633 0.1830522 0.2181641
0.1132033 0.1150994 0.4223706 0.1364777 0 0 0 0.068975
0.1103132 0.1100292 0.093417526 0.1102185 0.0784496 0.2140198
0.1083146 0.318072133 0.6664104 0.07825664 0 0.6086499 0.1350965
0.02551407 0 0 0.05378449 0.3570911 0.1694934 0.01131799
4.9281920 0 0.1417961 0.1152194 0.3220958 0.0547274 0.200426796
0 0.1060285 0.056991106 0.9280575 0.06473357 0.01710529 0.5001321
0.09576381 0.5479496 0.030716813 0.07739997 0 0.09834812 0.3333697
0 0.4426249 0.4181486 0.09458514 0.4589418 0.206852302 0
0.1549531 0.7103589 0.2283054 0.02233549 0.02875804 0.3419068
0.08169471 0.1856161 0.1853999 0.1450882 0.2677423 0.1311051
0.1256130.3499320.02165319 0.1269594 0.01949991 0.421121314 0.01816111
0.01433529 0.1724467 0.08538201 0.3124704 0.5239864 0.396178441
1.3095120 0.05614712 0.2405553 0.19631074 0.5439958 0.1688539
0.3352320.2818399 0 0 0.1606903 0.05149744 0.01932218 0.1202994
0.1881607 0.08473529 0 0.185151495 0.128044518 0.3206834 0.685986092
0.334858196 0.01595678 0.077130213 0 0.3986777 0.1327959 1.730025762
0.1136611 0.5968760.05184407 0.255064667 0.1872226 0.05543894
0.3344526 0.6384656 1.2090740.1644388 0.6033377 0.03630264
0.4818860 0.08867947 0.0819017 0 0.109841638 0.1794453 0.04250416
0.7081640.04096654 0.03920053 0.08317036 0.04097003 0.4934542 0
0.302962346 0.01355492 0.01977771 0.7122250.1612017 0.1070165

0.07071962 0.2004506 0 0.2616889 0.01896452 0.03615006 0.2647456
0.3354211 0.1128197 0.231217706 0.01090715 0.03202438 0.1297833
0.01051466 0 0 0.5097044 0 0 0.01820045 0.222206973 1.046724274
0.03943677 0.05821608 0.4055774 0.5340703 0.5575957 0.1699442
0.6980047 0.1796844 0.1131173 0 0.2986976 0.4077598 0.08132453
0.1563016 0 0.0175341 0.0241491 0.4344897 0.380989286 0.06215829
0.346848 0.1645815 0.2751989 0.6926019 0.1301556 0.02279013
0.0976488 0.01393305 0.06000079 0.06732651 0.279190767 0.4796293
0.1568041 0.1473381 0.8802848 0.03246321 0.752231769 0.4549117
0.3827623 0.02497993 0.5456868 0.2342752 0 0.325877146 0.1954604
0.1168661 0.2089592 0.1223502 0.536239206 0 0.3542127 0.3877714
0.186281804 0.277956909 0.4451816 0.6177026 0.1971124 0 0.1407243
0.5422442 0.1063525 0.371382 0.2889662 0.03768362 0.09344178
0.05653817 0.040455902 0.4467455 1.153299 0.3932765 0.04783542
0.6979516 0.1802498 0 0.5921708 0.04890519 0.04073454 0.1648127
0.0439111 0 0.06022926 0.3033335 0.1832368 0.1143415 0.4436551
0.1343995 0.4752751 0.3944362 0.02743913 0.3363206 0 0.9643764
0.2989196 0 0.5031531 0.5744359 0.1524715 0 0 0.02421302
0.08416714 0.3604422 0.5269421 0.02203852 0.0162382 0.1939706
0.3835117 0.03120089 0.7305036 0.330109 0.9349246 0.05655806
0.06543597 0.1466731 0.023988771 0.2974132 0.07777801 0 0.09624217
0.1666672 0.084015045 0.399138975 0.478715 0.254577246 0.02062423 0 0
0.02933622 0.08830388 0.6745313 0.4704141 0.1311082 0.03808309
0.3852017 0.05816159 1.309055387 0.1660649 0.2249405 0.4574371
0.196064 0.1309613 0.1889007 0 0.4962582 0.1637877 0.09960545
AL162724.2 0.09614603 0.988212 0.2224557 0.924754675 0.152299617 0.5967877
1.336167 0.3771233 0.169923 1.127677 0.09137769 0.08190494 0.2929687
0.482414696 0.617649573 0.1551664 0.3777777 0.8315296 0.2669092 0
0 0.1666994 0.05043345 0.05233028 0.1958253 0.06221278 0.1527764
0.3997766 0.3228802 1.06709285 0.4247873 0.709181363 0.5640967
0.3164524 0 0 0 0.1926213 0.3720792 2.653223 0.7961051
0.4419034 0.229453 0.4151104 0.09357244 0.06478464 0.3017461
0.2289428 0.2459241 0 0.134535 0.4074317 0.1715935 0.191381859
0.1516925 0.127397 0.1436784 0.3966209 0.1032413 0.7796288
0.1854696 0.07021272 0.1567729 0.1663472 0.1527042 0.175600762
1.530449 0.05434327 0.4231065 0.361290257 0 0 0.1772491 0.403632502
0.5351437 0.3310635 1.610843 0 0.2705617 0 0.07341752 0.1190119
0.4584159 0.04082581 0 0.09123583 0.2349603 0.3623014 0.1484491
0.7791087 0.2391957 0.15683382 0.05457945 0.4754473 0.06835676 0
0.3819591 0.7252279 0.2005744 0.1424093 0.6919585 0.5390166 0
0.2798807 0 0.1886946 0.529387278 3.333705 0.258183901 0.1056504 0
0.22784 0.09870823 0.3850172 0.09314842 0.3024506 0.07839152
0.3336717 0.1817961 0.08215155 0 0 0.1487163 0.09143924
0.5225411 0.0738458 0.7423629 0.314131 0.500071905 0.09440147

0.3359579 0.2156548 0.1987945 0.495321302 0.2536783 0.1675658
0.368301 0.6667851 0.1446366 0.3277895 0.2013376 0.08740552
0.04606607 0 0.217755 0 17.14767 0 0 0 0.04934233 0.2177944
2.81242 0.085832125 0 0.07567725 0.549140759 1.09638 0.9702679 0
0.1381806 0 1.613271 0.328859383 0 0 0.4211726 0.2461457
0.1422572 0.3268149 0.2939968 0.2025289 0.3275672 0 0.096184
0.3317908 0.2668499 0.1303614 0.04782551 0.8620875 0.4456276
0.08746374 0 0.2887161 0.3106678 0.5887934 0.8421798 0.230543
0.1729122 0.2781872 2.640825 0.334031 0.267176052 0.07777436 0.1227809
0.1641105 0.06094095 0.5203895 0.6170876 0.181780855 1.938548
0.8245158 0.1923586 0 0.280231456 0.2496048 0.3615556 0.3022358
0 0.4452242 0.2893674 0.06881512 0.1102681 0.08274662 0.4121426
0.4834753 0 0 0.226544361 0.099699362 0.8451176 0.200298625
0.215102903 0.6150095 0 0.06749163 1.138216 0 0.673525378 0.09735002
0.2175406 0 0.273076539 0.2290785 0 1.367179 0.6628384 0.2930841
0.5633629 1.359881 0.414573 0.3258413 0 0.2373543 0.561186194
0.2344197 0.806388425 0.5763515 0.3185397 0.3424004 0.2631568
0.33575 0.296812 0 1.431524 0.07599706 0.6178221 0.05804849 0
0.3519324 0.2301136 0.1718605 0.1211418 0.6243076 0 0.1400843
0 0 0.7086029 0.239405 0.06902098 0.116492038 0.093419 0.4114301
0.1235095 0.3602294 0.1632278 0 0.1364245 0 0.2318796 0
0.339855053 0.33830662 0.3377733 0.1246542 0.3775809 0.2079217 0
0 0.5402137 0 0.6228274 0.5538426 0.5116652 0.6790848
0.5970337 0.2677427 0 0.4505352 0.2585443 0.2566466 0.125505711
0.1330954 0.07073167 0.2349383 0 0 0.3483668 0.6831861 0 0
0.171301 0.672755 0.051983713 1.026998 0.2238362 0.4732281 0.1235997
0.06951132 0.138060348 0.432921 0.1490152 0.3209273 0.259654 0.3009828
0.1008487 0.697779148 0.5978949 0.3574823 1.470129 0.4286951
0.41753218 0 0.3033809 0.6118068 0.886384196 0.420120507 0.07061022
0.5104948 0.6330956 0.07334158 0 0.3870238 0.07590855 0.1272345
0.4714241 0.08068944 0.2667743 0.06053072 0.115500724 0.5886687
4.808992 0.4210484 0.1024268 0.2599092 0.2573046 0 0.475491 0
0.1744444 0.1176342 0.1253652 0 0 0.6062075 0.04130029
0.1632212 0.4523663 0.2877808 0.0782827 0.3378321 0.1175072
0.7625023 0.07077949 1.682556 0.2909349 0 1.325992 0.585715 0 0
0.2534804 0.05184574 0.2402954 0.2058109 2.256613 0 0.06953958 0
0.3649725 0 0.7661285 0.1570757 1.237533 0 0 0.06281229
0.410924531 0.2830361 0.1998491 0.2100519 0.1030385 0.3568734
0.239861135 0.569766567 0.8786064 1.006355809 0.5299357 0.3863922
4.778585 0.1675086 0.09453962 0.2818203 0.8887646 0.09357777
0.09785377 0.05891478 0.06226878 0.114407902 0.1777919 0 1.332092
0.7346832 0.1529557 0.5393074 0.09875554 0.7792435 0.1558701
0.2132786
AL162727.2 0.03748478 0.07705552 0 0.160238785 0.039585048 0 0.06128658

0 0 0 0.07125147 0.127730235 0.07614714 0.053737333 0 0 0 0
0.04162431 0.07265268 0.159152179 0.1299833 0.07865064 0.08160875
0.2544903 0.1940409 0.07941794 0.4286213 0.0629412 0.052003892
0.09463616 0.157994835 0.04398526 0.1480516 0.1264057 0.081598604 0
0 0.09670918 0.1293026 0.06207604 0 0.08945762 0.1618405
0.2188884 0.2020625 0 0 0 0 0.0524516 0.09530808 0.06689976
0.099486266 0.05914088 0 0.1680492 0.03865798 0.2012553 0.3039566
0.1446193 0.05474814 0.1833647 0.194563 0.0595353 0.136924138 0
0.169496 0.6048459 0 0.2305202 0 0 0.1888387 0.03477305
0.06453643 0.1141863 0.2565187 0.1054848 0 0.1144942 0.1855982
0 0 0.072802724 0.07114085 0 0.2825034 0 0.07147147 0
0.040763558 0 0.03370259 0.2132039 0 0.1489157 0 0.07819864
0.05552159 0 0.4669961 0.2050705 0.2727951 0.08531007 0.04904467
0 3.713495 0.10065904 0.08238053 0.134586 0.04441437 0.07696743
0.150108 0 0 0.2445022 0.05203589 0.09450329 0.06405741 0.08814982
0.05696647 0.1159611 0.1782486 0.237679 0 0.0526232 0.2449427 0
0.03680462 0.1746414 0.1681562 0 0 0.04945122 0.1959883
0.09572717 0.1417973 0 0.425988 0.1177442 0 0.2155192 0.2168024
0.2263917 0.1133805 1.542789 0.1641646 0.1116123 0.2367456
0.1538981 0.05660817 0 0 0.08506263 0.2950455 0.142730256
0.1068623 0 0 0 0.2558227 0.1143585 0.051285413 0.06461428
0.1268432 0 0.3454764 0.05546232 0.1528997 0.1667222 0
0.04256992 0.069072959 0 0 0.04161508 0 0.1864589 0.048015
0.2978372 0.4091974 0.2383911 0.05628136 0.04844845 0.1208184
0.1641718 0 0.1797703 0.4338312 0.06056397 0.1302298 0.05208242
0.1212886 0 0.2559294 0.2851111 0 0 0 0.1079698 0
0.1874887 0 0 0.03243809 0.07048046 0.1767506 0.2744966
0.2975676 0.1128166 0.05365837 0.02866039 0.06452141 0.08034172
0.06283138 0.04042165 0 0.176647249 0.116610393 0.1647444 0.104121479
0.055908603 0.05328352 0.064389083 0.3157583 0 0.04031251 0.13129482
0.2277251 0.12722 0.2163998 0.212930567 0.1786232 0.09256198
0.05076444 0.1938173 0 0.05491006 0 0.161631 0.08469123 0.04110168
0.148061 0.164093685 0.274182 0 0.2996055 0.1419316 0.03814081
0.1367971 0.1309 0.09257528 0.1368088 0.05315362 0 0.192697931
0.1357894 0.06604251 0.06098181 0.0448576 0.1786769 0.04722996
0.06085024 0.4038106 0.1092303 0.8865795 0.06035687 0.05525308
0.09333764 0.05381889 0.090834298 0.1456863 0 0.09630616 0.1053329
0.0636382 0 0 0 0.1205382 0.1215514 0.265000902 0.065948379
0.2194814 0.09719874 0.05888349 0.4863788 0.06006278 0 0.08424599
0 0.1079217 0 0.06649488 0.03782245 0 0.2087715 0.1714614
0.1171011 0.1612793 0.05002985 0.097862681 0.4670133 0.1103056
0.3663847 0.1225274 0.06607898 0 0.7610174 0.407591 0.1395774
0.1335714 0.1498796 0.040534136 0 0.2618034 0.1229994 0.09637648
0 0.10765212 0.2025413 0.03873137 0.291949 0 0.1173452 0 0

0.2331033	0.1672474	0.1495211	0.03714152	0.244177047	0.1640534
0.1577069	0	0.414693163	0.109195879	0.08258714	0.03618697	0.05485048
0	0.05873904	0.1005935	0.1775683	0	0.09189787	0.566256	0.0693388
0.09439728	0.135091986	0	3.648451	0.1313245	0.1198005	0	0.3511068
0.3684856	0.04119585	0.1633062	0.1360224	0.1834497	0.09775315
0.1127218	0	0.2025807	0.09661132	0.2121187	0	0.05609904
0.03052035	0	0.09162584	0.1321242	0.1103802	0.1192698	0.09074223
0.5489611	0.1292422	0.09134188	0.2121412	0.04679294	0.131767	0
0.04684239	0.04012011	0	0.5151433	0.1626698	0.04626533	0
0.6251237	0.04978219	0	0.22705	0	0.1638798	0.09795537	0
0.03678275	0	0.1091915	0	0.1192589	0.233788701	0.148091226	0
0.065391896	0.06886928	0	0	0.6857249	0.03685849	0.1098743
0.09240152	0	0.07630116	0.09187723	0.1456617	0.089209199	0
0.3466756	0	0.08183812	0.2385334	0.126157	0.1925108	0.110475
0.06076961	0.1663033
AC053503.3	3.503321	24.45825	0.6117532	22.039986422	0.628235922	1.641166
17.72386	29.66075	13.43454	16.65411	2.512886	1.80190867	5.236816	9.570762992
26.205989036	1.70683	4.051665	1.65151	0.9542003	0	0	0	0.06934599
0	0	2.65182	0	0	0.1109901	0	4.005138	0	0	0.0870244
0.5572571	0	1.169616	0.2648542	0	6.840341	0.5473223	0	1.41974
4.851603	0	0	0.3319207	0	0	0.8091825	0	0	0	0	0	0	0
0	0.1419568	11.363309	0.1275104	1.15851	0	1.258001	0	0.482902094	0
0	0	9.041288678	0	0	0	0	0	0.2276061	1.510165	0	3.348202	0
2.22088	0	3.466771	0.3929484	0	1.379942	0.3876845	0.0830274
0.3061762	0	0	0.934468177	0	0	0	0	0.1312985	1.107987
0.5515797	0.09790638	0.4077613	0.08234975	0.1205399	0.09620898	0
0	0.727907508	0	1.242510025	0.4358077	0	3.916	0	0	0
0.1039674	0	0.09175973	0	0	1.243542	0	0.1022425	0	0
1.827683	0	22.56835	0.550079095	0	0.5389324	0	4.282365	0.510800093	0
1.382418	2.532069	0.08334814	0	2.103316	0	0	0.06334085	0.09557695
0.4990218	0	0.3022826	0	0	0	0	0	0	0	0	2.393293	0	0
0	0	6.364945	0.5638949	0	0.180872661	0	0	0	0	0	4.044335
1.102488	0.09282574	9.758773	0.243605524	0.132253	0	0	0	0	1.016032	0
0	3.194866	0.3969846	4.698851	19.5154	0	0	7.528887	0	0	0
0.091841768	1.604096	0.4220592	2.25652	0	1.737729	0.6942236	0	0	0
0.06612326	0	19.458571753	2.173641	0.8699931	0.2077871	0	0	0
0	0	0	3.966873	0	0	0	0	0	0	0	0.887299474	5.449668	0	0
15.780890	0	0.4015688	0	0.07631957	0.12516008	0.7874574	0
0.9846942	0	0	0	0.7479346	0	0.1493439	0	0.2610898
0.482269385	0	0.461993368	9.113557	6.757592	0.06725722	0.9649083	0	0
4.10121	7.404726	0	6.28633987	0.5587167	0	0.5914419	0	0.4726163
0	0.9657259	0	0.5136423	0	0	1.558926	0	0.09490384	0.080088276
0	0	0	0	0	0	0	0	0.1062781	0	0.093460139	0	2.089973
0.1713996	38.21118	2.477733	1.4828	4.366682	0.5447155	0	0.2854626	0

1.289823 0.06669583 0.4104607 4.969974 0 0.309743 0 0.4411114
0.431425883 0.7320248 0.2917681 2.002849 0 0.6991392 0.4790044 0
0 0 0 0 0.071477605 19.76972 0 0.2168962 1.274622 0 0.822609576
1.131006 0.06829863 0 0.2380162 0.4828265 0 1.046668723 1.397579
0.1966153 0 0.1309902 0 0 0 8.412344 0 0.962776162 0 0 0
0.1008447 0 0 0 0 0.9723123 0 0 0.08322974 0.317626991 0
2.680688 1.042095 0.7041844 1.161469 2.034315 0 0 0.1439865 0
0.161747 0 0 0.7093071 0.2381529 0 0.5236681 0 0 0
1.393557 0.1615724 0 0 0 0.5600497 0 0 0 0 0 0 0 0
0.07074751 44.51904 0 0 0 0.1254593 0 0 0.1439861 0.6005677
0 0.09632818 0.6909352 0.141255308 0.4540371 0.2747925 0 0
2.874105 0.247356795 0.34819068 0.5033682 0 0 0 0 0.05758107
0.5849639 0.1937515 0.8147009 4.374761 0 1.782172 0 0 0 0 0
0 0.8412562 0.2224643 0.06789443 0 5.465196 0
AC009065.5 0.7554331 0.3726971 0.4544452 0.419809265 0.255283168
0.398569 0.6669606 0.5689174 0.3738306 0.2100223 0.6318115
0.283157077 0.5371093 0.953015155 0.499161696 0.5364323 1.021079
3.949106 0.4026515 1.449532 0.930147047 0.8906509 0.6974225 0.6743131
0.574421 1.58376 1.808581 1.476318 0.5581214 0.733626335 0.8582438
1.846766162 1.187826 0.8752168 1.222781 0.559116966 0.9459754 2.028027
2.221844 1.250805 1.175961 0.2976084 0.8293087 1.239401 0.3529018
3.054133 1.631153 0.4796896 0.8656528 0.9987624 1.099343 0.6914699
0.8359057 1.042575463 1.358731 1.301269 2.348116 0.2493045 0.5191564
1.02911 2.215037 0.6178719 0.8868865 1.150172 2.207666 1.79363635
1.670836 1.588376 1.108136 1.158193338 0.5110257 1.388233 3.101016 0.380567788
0.8829871 0.9624488 1.058554 0.982231 4.740628 2.705408 2.561223 1.234323
0.3841962 0.1411407 0.850974699 0.6308306 0.4282991 0.7780854
1.959528 1.20991 0.8081399 0.920091743 0.7547558 0.7742999 4.490063
2.067201795 0.7202658 0.6837863 0.3467072 3.893877 0.5747492
0.9223172 3.664414 1.099531 0.8940148 1.304688 0.632239664 1.895903
0.750577485 1.427789 2.169855 2.112402 0.9306776 2.737747 1.141733 0.7366832
5.297027 2.370023 0.2856796 1.574963 0.5329466 0.1607268 0.9114187
1.551855 2.025469 0.5570083 4.263284 0.9132238 1.540221467 2.551537
1.003074 0.4236077 1.666087 1.634560297 0.4982967 1.053271 2.392202
1.409774 1.090973 0.927176 3.037321 0.2747031 0.3329919 1.398154 0.5703106
0.4569928 0.829118 2.514399 0.7872651 0.5725377 1.256115 1.026745
1.718701 0.377661349 0.8571362 2.735192 0.575290319 0.7968331 0.6534457
2.302233 0.9771344 1.211569 0.599215 1.343625479 0.3646091 0.4345673
1.932581 0.8200168 0.5812224 1.047675 0.8399909 1.845906 0.240216
0.306246944 1.874214 0.7038705 2.633427 0.3687364 0.8116672 0.9676493
1.140443 1.869225 0.8647757 0.9300782 1.132606 1.090817 2.183652 0.8936284
0.6883553 4.488087 1.098495 1.259774 0.776718953 3.153195 2.952003 0.6705088
2.33665 1.588781 0.6699808 0.666529801 0.8703685 4.428828 0.7708083
0.7446661 0.792654691 0.3399379 3.124873 1.614803 1.027362 1.639152

0.8488109 0.2811589 0.8201843 1.19628 1.003862 1.392869 1.107883
1.726267 0.320398454 0.172337469 5.743781 0.965248612 0.585899335 1.675169
0.648818562 1.336334 0.9837441 3.57465 0.71441799 3.120763 0.6495141
2.721338 1.401792899 0.7559591 3.171192 0.920753 0.651002 1.258866 0.8410204
0.4273912 1.856695 0.682715 0.7123593 0.9100843 1.741681437 2.725966
0.40127424 1.977434 1.001125 0.7840271 5.348349 0.6858892 1.361943
2.591689 2.249537 0.7165437 1.126201428 0.9486782 1.197863 0.5161675
0.9582592.466607 1.27545 0.9565285 0.7440475 0.3081854 3.981854
1.848891 1.291682 0.8276572 1.062923 1.409553654 0.8514474 0.8620441
1.882638 0.5943785 2.077657 0.697715 2.293881 0.7572955 2.283462 1.886217
0.640869528 0.691112096 1.645439 2.311446 1.49522 1.677235 1.331494 1.247624
0.7130821 0.9014057 0.2174953 0.7832917 0.7236407 0.2286714
1.110198 1.388437 0.4952843 0.6371855 1.121344 0.5041273 1.084727934
3.869274 1.822856 0.4873291 1.851977 1.917639 1.970761 0.6441469 0.2628546
1.537725 1.076749 1.495164 0.702522747 1.106643 0.4924397 1.041102 0.5438387
0.5243139 0.593002068 0.449001 0.6244446 1.7651 0.6528443 0.5990991
0.8240777 0.897144619 0.5449385 5.055821 2.711976 0.6137825 0.442882348
1.124101 1.620921 2.362323 1.309316084 0.748214617 0.5991781 0.4521525
0.7074592 0.9681089 1.112745 0.6081803 3.14912 0.7197837 0.6852487
1.521572 1.215728 0.8180295 1.851311606 0.4625254 0.490183 2.328999
1.142791 1.082336 1.394959 0.4667843 2.822756 1.875938 1.83665 1.571256
1.418418 0.9389651 1.134891 3.837665 0.3504625 2.205818 0.5402546
3.233423 0.2460314 1.778445 1.403371 1.251472 3.025318 0.5288034 0.1828734
3.180683 0.8595268 0.2577146 2.000838 1.489973 2.310292 0.3258875
0.2265642 0.5336383 0.8942353 1.720398 1.835845 1.715594 0.3154405
2.12069 0.3210443 1.102522 4.072421 6.800279 3.41277 1.006791 0.613451622
0.6523308 5.087587 0.9682393 5.343282 0.4005721 0.565386961 0.656588139
0.4372113 0.711637322 1.027066 1.967288 0 1.23717 0.460543 1.107151
1.061439 1.529328 1.886248 0.6850947 1.467764 0.791048922 3.380586 2.095973
2.733588 0.5277724 2.69202 1.389872 1.10183 1.24679 1.151212 1.876851
BNC2-AS1 0.4497298 0.4266859 0.2401274 1.70066514 0.18266465
0.4831471 1.357469 1.682604 0.9782483 1.472732 0.4603044 0.884114067
0.4919319 1.587009094 1.269933702 0.502478 0.217487 1.329757 0.1536599 0
0.2937622 0 0 0.03765824 0 0.3581596 0.4764149 0.03596122
0.1161765 1.199856155 1.266422 0.182266238 0.04059387 0 0.1166594 0
0.4708736 0.5544612 0.1785053 8.114647 0.2291592 0.1817173
0.4953611 0.9459603 0.06733717 0.1398622 1.302866 0 0 0.08469948
0 0.02931985 0.4321912 0 0.2729047 0 0.1033948 0 0.1857379
1.122083 0.333672 0.05052691 0 0.538685 0.1098899 0.063183452 1.970839 0
0 0.207995139 0 0.4890253 0.1275531 0.058092902 1.636689 0.7742863
0.4742202 0 0.04867583 0 0.316999 0.08564404 0.4948322 0.1762759
0 0.4595898 0.5072508 0.08690719 0.1602418 0.2968237 0.04303288
0.225723442 0 0.2177282 0.09838263 0.169046876 0.2061508 2.087572
0.2165079 0.05124072 0.1778398 0.04309894 0.189259 0.1007047 0

0.3621055 0.571441408 0.1142391 0.510938702 0.4561726 0 0.4918788
0.2130991 0.5195033 0.1340641 0.2176514 0.4513007 0.09604756
0.7849514 0.295592 0.08135323 0.05257419 0 0.2961093 0.03133619
0.1062827 0.1942632 0.5651423 1.367486349 0.03396688 0.3223521
0.1939886 0.5722309 0.712892342 0.09127678 0.6632159 0.08834634 0
0.05204214 0.4717718 0.03622194 0.5031944 0.0663007 0 0.05223406
0.06975903 7.435587 0 0.05150332 0.1456612 0 0.7314093 0.05621919
0 0.1570081 0.05445933 0.087816902 0.3944918 0.4987358 0.1054294
0.5469112 0.05902452 0.1055411 0.189324654 0 0 0 0.2125594 0
0.4703692 0.307735 0.1943268 0.4321642 0.318736199 0 0 0.07681287
0 0 1.06351 0.09162437 0 0.2640125 0.2077676 0.715407 0.8474223
0.8080728 0 1.990915 0 0 0.2403774 0.04806672 0.2798423
0.1325345 0.1180982 0 0.6419759 0.08074043 0.043604753 6.825682
0.05394028 0.03460656 0.4448026 1.814957003 0.2394962 1.040739
0.271871 0 0 0 0.04952116 0 0 1.112207 0 0 0 0 1.112067184
0.07602109 0.192186843 0.20639157 0.4425769 0 0 1.63818 0.4092473
0.302929054 0.07005562 0.07827396 0.03994295 0.982565116 0 0.08542519
0.7027554 1.4608 0 0.05067634 0.1957212 0.0372922 2.032194
0.07586526 0.1024838 0.201922135 0.1686945 0.531940028 0.4838839
0.458459 0.1760002 0.2524994 0 0.08543747 0.2525209 1.618826 0
0.889201901 0.2506393 0.1219009 0.02813997 0 0.9069522 0.0435884
0.3369511 0.1597181 1.176097 0.1168888 0.1671096 0.4589363
1.808962 1.291402 0.083830719 0.6722675 0.2960759 0.2666421 0.2592305
0 0 0.3436114 0.07539106 3.504199 0.2243589 0.097827436 0
0.162047 0.1794089 1.902019 0.3491271 0 0.4285062 3.343267 0.7551096
0.7470049 3.885956 0.06136794 0.1745312 0.1074103 0.1926746 0
0.9726508 0.0744221 0.5078965 0.451585971 0.04788948 0 0.371949
0.05654009 0 1.153192 0.07023408 0.1504658 0 0 1.971109 0
2.639482 0.1610784 0.6810946 0.08894558 1.050465 0.198703679 0.1869248
0.03574508 0.2309477 1.557115 0.2165951 0.07257334 0.502140135
1.850122 0.1543522 0.5059728 0.2399446 0.713609321 0.07570222
1.164378 1.383713 3.06175326 0.050388285 0.1016259 0.03339686 0.3037281
0.105557 0 0.185675 0.05462578 0.1831225 0.5088737 0.1161325
0.03199629 0 0 0.2118107 3.08655 0.9695919 0.3685451 0.3740749
0.3703263 0 1.710879 0 0 0.04232631 0.04510805 0 0.3712262
0.1246408 0.5349739 0.1957638 0.06510693 0.05177365 0 0.5470038
0.04228062 6.096851 0 0.2751844 0.3768559 0 0.1192773 2.950471 0
0 0.0608037 0.1119286 0.04323071 0.2591871 2.259363 0 0
0.3842833 2.232472 0 1.010765 0.1507144 0.1571579 0.1161995 0
0.6780205 0 1.289975 0.5273277 0.05038628 0 0.6236946 0.215762937
2.186767634 0.2634451 0.120699996 0.1906778 0 0 0.3616306
0.2040996 0.2535066 0.6822168 1.212138 0 0.08479324 0 0.12349638
1.215465 0 0.3947212 0.8308099 0.1467612 0.03880997 0.07106707
0.05097855 1.23385 0.1151106

AC078778.1 0.02611374 0 0 0 0 0 0 0.08194284 0 0 0.04963726
0.044491572 0.07957175 0.074872122 0.047930478 0.1264318 0.1231275 0
0.02899754 0.1012268 0.110873211 0.09055274 0.4657311 0.3126896
0.1772904 0.4731244 0.2213057 0.2443079 0.04384792 0.036228461
0.1318564 0.055033474 0 0.5844602 0.04403019 0 0.1421756
0.2092675 0.6063512 0.1801571 0 0 0.06232056 0.3382381
0.05082947 0.3167249 0.03278229 0.04145466 0.05343536 0 0.03654036
0.1106605 0.09321131 0.103960516 0 0.2422116 0.1951189 0
0.1121634 0.1270506 0.2518723 0.152561 0.2554817 0 0.04147521
0.047694034 0 0.4132772 0 0.039251287 0.2007396 0 0.2888504
0.131554297 0.2906953 0.04495924 0.1193217 0.1340276 0.1469718 0
0.2791679 0.1939452 0 0.08870794 0 0.1734607 0.07657966
0.4920142 0 0.09958107 0 0.141989467 0 0 0.2227924 0.127605049
0.103742 0.04377233 0.108954 0.07735813 0.1073939 0.06506647 0.04762072
0.3420764 0.05943118 0.1025008 0.02875684 7.588541 0.105186034 0
0.1875184 0.03094123 0 0.1307157 0.1517974 0.04107354 0
0.1087523 0.06583563 0.1785021 0 0.07937127 0.2827446 0.4222009
0.3311578 0.08022753 0 0.08531954 0.0543288 0.0256399 0.1216638
0 0.1079871 0 0.1722507 0.2730701 0.06668824 0.3951319 0
0.05935283 0.08202641 0.04747954 0.07507063 0.1510352 0 0.07898642
3.761739 0 0.03887729 0.05497619 0.1608195 0 0 0.186499432
0.0592588 0.08221726 0 0.1861138 0 0.03979168 0.1876527
0.08910932 0.4780063 0.035727933 0.1350404 0.04418259 0.04575703
0.2406758 0.1159133 0.2485407 0.2032571 0 0.08896887 0.192478438
0.0522481 0.1802321 0.08697331 0.03540679 0.3377308 0 0.8299519
0.1425335 0.1328599 0 0.06750313 0.0336672 0 0.1252332
0.09392763 0.05037135 0 0.1814489 0.072566335 0.2534868 0.2334352
0 0.2317261 0.3634466 0.2133142 0 0.03760851 0 0.0783683 0
0 0.1581857 0.1964006 0.1641775 0.1365913 0.3109503 0.392968
0.1121432 0.09983116 0.1348463 0 0.4377142 0.1126388 0.3182583
0.123061134 0.135394195 0.3443072 0.036268064 0.038948674 0 0.134569776
0 0 0.1404183 0 0.2644075 0.1477128 0.1206038 0.148337873
0.3110943 0.06448321 0.035365 0.2025339 0.1061374 0.1147591
0.2216103 0.1689001 0 0.02863345 0.07735993 0.114315706 0.3183477
0.182515899 0.2087199 0.04943826 0.02657075 0.04764979 0.136787
0.03224624 0.04765385 0.03702942 0.2064118 0.033560707 0.06306503
0.1380254 0.08496584 0.09375 0 0 0.08478252 0.1205632 0.07609516
0.7058683 0 0.03849201 0 0.03749288 0.063279625 0.02537306
0.07449764 0.1006374 0.1467601 0.3546679 0.08316739 0.03705358
0.08536343 0.1679457 0.3387146 0.073845048 0.229714372 0.2446424 0
0 0.301187 0 0 0.1173798 0 0.03759178 0.2256396 0.3242652
0.1580938 0.2702622 0.07272024 0.1592645 0 0.1404438 0.1045597
0.204527826 0.1084481 0.03842214 0.1020966 0 0 0.03784726
0.3180972 0 0.3241215 0.1395786 0.5481708 0.056476132 0

0.2431801	0.04284369	0.06714058	0.07551847	0.224987234	0.0235167
0.02698217	0.1162206	0.1410466	0	0.05478199	0.103374689	0
0.2330255	0.06944247	0.2328714	0.085052851	0	0	0	0	0.076071203
0.07671234	0.1008385	0.1528461	0.1195196	0.0409205	0.07007839
0.1237028	0.06911503	0.1600514	0.1314939	0.1207620	0.062741134	0
2.6122920.09148706	0.1669178	0.1764815	0.1397704	0.07334433
0.05739809	0.2275342	0.2368997	0.03195002	0.1702491	0.2879343
0.07005503	0.3292979	0.04486945	0.08866338	0	0.3517321	0.04252394
0.1376353	0.3510708	0	0.03844812	0.0830892	0.2528619	0.3824332
0	0	0.0295576	0.4237773	0.2294884	0.2534680.06526541	0.05589926
0.05329627	0.1025355	1.0199140.03223069	0.09912834	0.2540369
0.06936143	0.05688338	0.1581742	0	0.07611116	0.06824051	0
0.0768740.03618667	0.3423068	0	0.05538775	0.065147469	0.034389203	0
0.045555201	0.04797771	0.2098921	2.5957740.6141980.05135486	0.1148157
0.2253	0.1016647	0.01771838	0.1920185	0.06765004	0.031073751
0.04828916	0.3220150.08513009	0	0.1661741	0.08788713	0.2145799
0.4232928	0.04233509	0.4344563
AC087286.2	0.04486815	0.1844662	0	0.767203879	0.047382103	0.1392505
0.5868654	0.2463872	0.3171896	1.2279150.04264292	0.305778441
0.1367187	0.096482939	0.329413106	0.1810274	0.1410370	0.1245576
0.04348153	0.047625084	0.1944826	0.02353561	0.0732624	0.06092344
0.2322611	0.04753043	0.09328122	0.1883468	0	0.2831915	0.141836273
0	0.2953555	0.03782593	0.024417764	0.09160631	0	0.05787898
0.1547713	0	0	0.3212342	0.3228636	0.1310014	0.03023283
0.02816297	0.03561332	0	0.2197053	0.03139149	0.1140809	0	0
0.1061848	0.08917791	0	0.04627243	0.02408964	0	0.04327624
0.1638297	0.2560624	0.03881434	0	0.040973511	0	0.1014408
0.06581656	0.067440848	0.03449071	0	0	0.414396035	0.1248669
0.03862407	0.2733552	0.07676127	0.03156554	0.08547066	0.1027845	0
0.07130915	0.3429368	0.130713982	0.04257672	0.06578889	0.0281790	0
0	0.146378232	0	0.02017049	0	0.054812169	0.2228095	0	0.3276049
0	0	0.02794901	0.08182105	0.03265274	0.05105679	0.2054674
0.247047396	8.6676340	0.0493035	0	0.1594880.09212768	0.2245933	0
0.2822872	0	0.03114269	0.4524703	0.03833739	0	0.06818713	0
0.1280149	0	0.1723069	0	0.03664862	0.233366889	0	0.2351705
0.5786737	0.06184718	0	0.1775748	0.2736908	0.05729126	0.02828785
0	0	0	0	0.1074875	0.03243824	0.0338730.06785651	13.79874
0.03275001	0.06679825	0.1889182	0.02302642	0	0.03645729	0
0.1527261	0.1412642	0.028473965	0.03197774	0	0	0.3224215
0.0382765	0.4106508	0.12277417	0	0.03795686	0.07861889	0.0229736
0.06638671	0.1830164	0.08730814	0	0.8152784	0.206695596	0.04488587
0	0.04981198	0.1825059	0	0	0.05941701	0	0.4280203	0
0.02899566	0.2747703	0	0	0.8069237	0.04327357	0	0.03897028
0.124682158	0.1451788	0.02864887	0.1531698	0.05687822	0.03469263

0.02617947 0.028277022 0.03230913 0.03497946 0.04488367 0.1442239
0.06538734 0.3106193 0.08436297 0.1410434 0.0704066 0 0
0.1605686 0.08576404 0 0.1923332 0 0.04838349 0 0.105720702
0.023263184 0.1478956 0.311575639 0.100381355 0 0 0.2519687
0.5311676 0.02412642 0 0 0 0.0259024 0 0.05345165 0.05539694
0.1822905 0.09666393 0.04559086 0.03286284 0 0.02418342 0.1520593
0.04919747 0.1993776 0.065471723 0.05469793 0 0.2017230.08494392
0.09130678 0 0.07834166 0.08310735 0.04093899 0 0.03546529
0.259485282 0.1083572 0.1581018 0.1459868 0 0.08020156 0 0
0.1035748 0.8280537 0.07580063 0 0.06613627 0.1117223 0.06441958
0.027181475 0.0653933 0 0 0.02101338 0.07617299 0 0 0.07333494
0 0 0.095159415 0 0.05254252 0 0.1057226 0.09703012 0.03594666
0.4013819 0.1176465 0.03264514 2.7127590.06461497 0.07959236
0.02263616 0.0928719 0.3123665 0.08551419 0.1401665 0.1447848 0
0.29284666 0.1242224 0 0.2631309 0.03666539 0 0.06502848
1.3663720.09757482 0.02784499 0 0.04485033 0.072777198 0.06846656
0.1044569 0.07361326 0.02883993 0.09731585 0.107380271 0.7273073
0.04636028 0.1497661 0 0.04681954 0 0.148013759 0.6417406
0.2001901 0.0298287 0.5112586 0.048712088 0 0 0.08157424
0.041364596 0.032676039 0.4118929 0.04331471 0.06565436 0.06845215
0.03515443 0.1204074 0 0.02968805 0.6049943 0.1129652 0
0.02824767 0 0 12.8586640 0.07169878 0.0909682 0.48030220 0
0.04886814 0 0.1097919 0 0.08994974 0.06018364 0.08082767
0.09636735 0.05077993 0 0 0.01826596 0.1182412 0.02741834 0 0
0 0.1900775 0 0 0.1640002 0.02539266 0.05600973 0 0.09677871
0.1401723 0 0.4578634 0 0.0649036 0 0.04258013 0.03117726 0
0.21990600 0 0.0326932 0.1172496 0 0.4843062 0 0.03267474 0
0.1427493 0.279837991 0.295434516 0 0 0 0.03606327 0 0.03908533
0.2867702 0.1972742 0.2488541 0 0.0152217 0.1649614 0
0.053390354 0 0.06916004 0 0 0.07137931 0.05033535 0 0 0
0.19906
AL031670.1 0.2655872 0 0.3072482 0.189220688 0.186978703 0.2747543
0.4342278 0.2083479 0.3911531 0.09614251 0.4206925 0.377081037
1.0115960.38073985 0.203113462 1.8573570.6261284 0.2977542 0.07372917
0.8150351.174609692 0.5372255 0.7894455 0.9395985 0.8114019
0.5155570.3047916 1.1733350.4459511 0.153524196 0.6425781 0.746283527
0.07791111 1.4569110.3731707 0.144535644 0.3614958 0.3547226 1.71301
0.6871015 0.4031695 1.0172361.0563751.1785250.9046734 0.805305
0.3056250.8783554 0.3170179 0.4334993 0.2787226 0.4314270.6319977
0.146850057 0.2095125 0.7918039 0.9591448 0.4793243 0.9030916
0.8614374 0.8965751 0.6788279 0.2887058 0.4595061 0.4218196
0.282956085 0 1.3009890.3895868 0.731869292 0.5444274 0.1564296
0.9792418 0.668980147 1.0676210.07620893 0.5056458 0.4543717
0.7162404 0.2529625 1.8590330.3835419 0.1406997 0.3947105 0

0.693065 0.6490384 2.390792 0.03417214 0.5696885 0.6332072 0.409158884
0.3266608 0.278588 1.195886 0.486673068 0.8792482 0 0.7387375
0.06556362 1.297034 0.3584492 0.4843219 1.095258 0.3777744 0.4343642
0.536192556 6.723886 0.208013637 0.2918414 1.629013 0.02622373 0.3635532
0.576087 0.1286534 0.1044336 0.1443623 1.106054 0.8090697 0.5673246
1.040932 0.7736029 1.266645 3.194417 0.6214777 0.3399777 0 0.6869562
0.322318394 0.2390375 0.7733559 0.2730338 0.2745682 0.513090676
0.9927202 1.00289 0.2826026 0.8651263 0.1331781 0.5030352
0.3476007 2.293709 0.3181244 0.1280074 1.203023 0.7586946 6.477613
0.03230943 0.4942471 0.279565 0.5451996 0.2673869 0.6114362
0.316129082 0.5524621 0.4877733 0.308999982 0.6624985 0.3190717
0.2360735 0.9224434 0.6419467 2.160674 0.333086873 0.3815043
0.823817 0.2326837 1.677176 0.4912021 0.4513856 0.8244215 0.2486453
0.42729 1.3050556 0.6642303 0.42007 0.6142734 0.4801351 0.7926596
0.4819443 0.4396326 0.5637423 0.2815081 0.6978384 0.4290838
0.9844259 0.2907975 0.3537979 0.3449629 0 0.286072 1.499395
0.430516868 1.038386 1.017484 0.03777732 0.6172435 0.9240998 1.265537
0.390552589 0.3824938 0.1380355 0.3542388 0.2845673 0.387046137
1.091694 0.7906664 0.3130784 1.366035 0.9077516 2.17595 0.3484987
0.2876749 1.638111 0.5218009 1.81779 0.6682562 0.6069029 1.355879853
1.17046157 1.994048 0.368860846 0.231072176 0.1258415 0.684314465
0.4039409 1.266386 0.3808297 0.503885578 1.658297 1.371107 0.6388484
0.335256897 0.2109303 0.8197754 0.7493256 1.525816 0.4497753
0.615994 0.4382517 0.548736 0.2500227 0.2426781 0.917912 0.968864057
0.8094312 0.340314397 0.9950462 0.4399562 0.3828333 0.08076961
0.309151 0.6285848 0.9693178 0.09415102 0.8397164 0.398213587
0.6413968 0.4289308 0.5580884 0.8210482 0.0263742 0.1394308
0.6826323 0.579028 0.4299547 1.121713 0.249457 0.4567258 0.330658
0.1906588 0.697210939 0.2150452 0.8839495 0.3127431 0.6841128
1.127224 0.2114615 0.1256166 0.4340902 0.8184529 0.5741441
0.438103135 0.272566918 0.3887675 0.2295576 0.5910354 1.021064
0.9929661 0.2132212 0.9119303 0.1610299 1.01953 0.7012028
1.648954 0.4019695 1.191093 1.355923 0.7086557 0.4148426 0.6665728
0.4135502 0.46225123 1.011048 0.3582046 0.6489774 0.07234427 0
0.2886914 0.4493302 0.09626216 1.373519 0.7886501 1.039804 0.311125241
0.3715002 0.9789906 1.343524 0.2560676 0.28802 0.78392413 0.7374541
0.1143415 1.649889 0.7172505 0.3926123 0.3714366 2.0735201
0.7982657 2.172466 0.500266 0.4824511 0.672794753 0 0.325904 0.4023841
0.816162429 0.805911287 0.3900977 0.726444 0.2266989 0.2701251
0.5895855 0.1781814 0.8736858 0.1757319 0.7325043 0.4086335
0.4503394 1.031103 0.42540177 0.3387705 7.539586 0.8916912 0.4951395
0.4786369 0.8292209 0.6837796 0.1459404 0.9160037 0.401561
0.2978658 0.2308669 0.6655474 0.29687 1.554936 0.9126809 0.4759196
0.4581815 2.28547 1.333497 0.7387867 0.02704948 0.1950263 0.2606886

1.0563130.5893498 0.6887656 0.3815447 1.0246950.8266849 1.547175
1.5170930.5489913 0.1382866 0.3790121 0.7227261 1.5207891.34464
0.1092664 0.3360584 0.5536410.7054337 0.2169476 1.2735510.4832093
0.9676015 0.5494432 0.331073726 0.4995087 0.5213801 0.9025848
0.04743775 0.2816579 0.30368069 0.437190091 0.3370837 1.042458259
0.44729 0.4980936 1.6500051.3495830.1740999 0.9406648 1.145696
0.1292464 0.2703046 0.4611028 0.6880282 0.447712836 0.04092669
1.1257890.8297321 0.09663994 1.00934 1.7628660.9547845 2.3156
0.4664454 1.178292
AC022893.1 0.5840824 5.7203250.2702815 4.886628254 0.127767463 0.233065
2.1895842.6313836.4885782.6496160.7295797 0.952488212 1.7501080.496415461
3.499492144 0.06733057 0.2032697 0.1042373 0.08802211 0.7034966
0.115137565 1.25863 0.3370188 0.1407860.2039364 0.5183171 0.1281674
0.3252621 0.1821375 0.046303832 0.8584318 0.083527137 0.04405962
0.2032286 0.1266194 0.476796577 0.5735396 0.1588081 0.2690908
0.69078 0.13818 0.1314877 0.5675228 0.8706128 0.09744822 0.3485848
0.09951074 0.08609814 0.1664717 0.9295225 0.2335123 0.102541
0.1042422 0.077509024 0.03949391 0.3372126 0.1246913 0.1204726
0.3180729 0.6766010.2736317 0.3168574 0.1564637 0.2309831
0.4041992 0.297170519 0.06291474 0.1603502 0.1774766 0.501673256
0.1218691 0.1179503 0.57941 0.126105302 0.7740409 0.1364739
0.1779235 0.4853544 0.4167825 0.1430532 0.6371540.1859120.2320712
0.1877826 0.105337275 0.1227275 0.09379876 0.1624520.08374051
0.2107996 0.9756544 0.158792954 0.0426301 0.04876383 0.4567904
0.173286147 0.5469474 0.1958099 0.1392548 0.2718978 0.08149863
0.5821332 0.6086435 0.2307497 0.1091916 0.4967345 0.087291472
5.4832210.207260055 0.1100264 0.1048549 0.7217179 0.1027967
0.1002412 0.07275498 0.2362336 0.1632770.1158308 0.1788080.4420304
0.1864087 0.05706278 0.3420182 0.5634244 0.2305227 0.5126967
0.07613979 1.5130330.295111467 0.05325211 0.3158578 0.1310092
0.5693286 0.085972928 0.1706199 0.1526931 0.3622474 0.1052126
0.07531372 0.2180953 0.3494616 0.09102587 0.09994611 0.1146169
0.1196865 0.06730203 1.9937540.0609045 0.09316747 0.1053981
0.1370296 0.07560517 0.08813851 0.104285185 0.1704129 0.3349502
0.063542915 0.1486708 0.2646434 0.1716458 1.1452370.1637186
0.3945673 0.114160259 0.1294470.1552926 0.2119985 0.5084098
0.9753179 0.3460242 0.2180331 0.1347529 1.1560690.069189735
0.2003359 0.3599309 0.1991637 0.1923280.3112903 0.1710086
0.2651917 0.3567554 0.4669751 0.1691295 0.4799113 2.425831
0.1096329 0.3267921.0504330.3138523 0.03370354 0.3696083 0.06376391
1.0056980.2930273 0.2919679 0.1269303 0.3871029 0.2726383
0.042068942 0.06609307 0.2797174 0.1252038 0.1519857 2.50494864
0.2960467 0.5334193 0.2295085 0.1614849 0.2759914 0.4227313
0.1134703 0.08612653 0.1867103 0.6974721 0.2587435 0.08097997

0.06991325 0.157285237 0.095176511 0.5134048 0.115886121 0.143119091
0.3380328 0.035832189 0.08200166 2.527124 0.1390889 0.058451907
0.5407054 0.1368747 0.1252424 0.323885651 0.08946261 0.1854369
0.2881515 0.09707248 0.2119609 0.1344515 0.1770259 0.3148138
0.07540837 0.08691692 0.2307065 0.219161472 0.2034412 0.297425316
0.4001493 1.35063 0.03820529 0.1141903 0.0874142 0.9942909
0.7384937 0.2484696 1.727996 0.55226228 0.2871511 0.2572662
0.240946 0.1148299 0.4872205 0.08410633 0.4131267 0.1605132
0.190463 0.585004 0.1007649 0.1844883 0.05194191 0.0419299 0.217359896
0.3202411 0.3213537 0.2143755 0.08987972 0.5595463 0.06643549
0.3137495 0.48642 0.7110364 0.1420497 0.082584107 0.058719887
0.6351294 0.4110885 2.470734 0.5714087 0.1336985 0.9933413
0.1375219 0.03035474 0.3062951 1.670267 0.3700405 0.1220784
0.3108814 0.4182489 0.1971959 0.09123264 0.2243777 0.1503433
0.17427223 0.2656658 0.09207673 1.186647 0.08182303 0.02941808
0.1511651 1.86341 0.09072891 0.2692702 0.1189308 0.4003546
0.130830804 1.419683 0.2816716 0.2464146 0.09117611 0.06635795
0.407373336 0.3719535 0.08190447 0.8076986 0.105159 0.1393109
0.288821 0.286268369 0.3684071 0.8066267 0.1886041 0.0702748
0.108706603 0.06390641 0.1316446 0.3868398 0.10000232 0.206607587
0.05515118 0.1288823 0.04883841 0.1527588 0.05230075 0.1343515
0.1054036 0.07729433 0.1994487 0.09803687 0.1196187 0.3256959
0.456079782 0.08940333 0.9700532 0.1461628 0.5022349 0.3045089
0.5191768 0.2753667 0.04126549 0.08179114 0.8856406 0.1020888
0.1142382 0.4600134 0.1343067 0.323174 0.02508972 0.2266425
0.05496206 0.2185318 0.09511271 0.2565391 0.3875187 0.1470528
0.07985379 0.1128341 0.1009952 0.152747 0.05034584 0.05083128
0.1416666 0.1979042 0.2126502 0.4724404 0.1042702 0.09377184
5.125903 0.1556233 0.09655988 0.05664211 0.04751122 0.3478781
0.2271689 0.1317742 0.3285156 0.2312203 0.2553552 0.1417303
0.222888059 0.2169757 0.3757846 0.4921927 0.09836417 0.4203242
0.16132672 0.197788789 0.07624986 0.101892699 0.2759428 0.2011983
1.762515 0.2980132 0.3076733 0.07337334 0.4833571 0.3086036
0.1019069 0.1738393 0.07565596 0.104253354 0.1465819 0.1286155
0.1156057 0.07286798 0.7831813 0.135731 0.257115 0.4426472 0.6898866
0.4210884
AC022154.1 0.07792888 0.06865473 0.1159111 0 0.011756462 0.1036526
0.1274116 0.2270679 0 0.0483604 0.1269669 0.094837298 0.3166118
0.047878752 0.102167599 0.01796663 0.1049824 0.02139608 0.01236211
0.1834071 0.094534001 0.2316244 0.3036624 0.1454231 0.2267451
0.172886 0.176899 0.2661671 0.1121584 0.108113355 0.08431868 0.269808849
0.02612658 0.02931348 0.1126246 0.024234172 0.09091754 0.06691054
0.3733847 0.1536077 0.03687224 0.04385808 0.106273 0.3044143
0.1300164 0.2250414 0.08385365 0.1060367 0.02278034 0.02725667

0.1713355 0.1698347 0.1788185 0.118186692 0.1405152 0.1032586
0.1164552 0.04592452 0.09563407 0.1263819 0.2147543 0.1138185
0.01815265 0.0385225 0.1414523 0.142329038 0.01865367 0.2768647
0.228626 0.100400661 0.08557846 0.2360558 0.4378364 0.130861906
0.1962194 0.03833367 0.1017375 0.09523015 0.2506256 0.06362102
0.4420507 0.02756064 0 0.06618079 0.021621862 0.09507734 0.09794136
0.3216219 0.03437768 0.2971709 0.08308904 0.121064703 0.2275101
0.1401318 0.2532798 0.027200024 0.1769074 0 0 0.0164895
0.02289186 0.02773886 0.2436176 0.2916651 0.07600935 0.1019613
0.073556969 1.10288 0.074737445 0.1957312 0.2797971 0.02638147
0.0457175 0.1114523 0.08628485 0.08755149 0.05446148 0.1081799
0.05613353 0.09512285 0.0785395 0.1353489 0.06887915 0.5611482
0.1714302 0.0513034 0 0.2909845 0.04632245 0.08744557 0.2723027
0.1373381 0.06138216 0.028676496 0.1174931 0.1940235 0.0568605
0.07018791 0.05024218 0 0 0.1214477 0.05333966 0.06438868
0.03361831 0.08979508 1.32368 0.1300151 0.06629601 0.04687444
0.05713323 0.05043661 0.05427477 0.099384566 0.1768407 0.2628789
0.141299377 0.1586865 0 0.05089146 0.04799958 0.03798871 0.169818
0.106619674 0.2302795 0.1318501 0.09753471 0.2622099 0.03294378
0.1210935 0.08665169 0.04690143 0.1264295 0 0.1113709 0.01920894
0.07415619 0.03018895 0.2104322 0.04278028 0.4717622 0.2228024
0.02832014 0.0835757 0.0575553 0.2224693 0.03250519 0.0533889
0.1067809 0.0429482 0.1079223 0.5994977 0.046404262 0.1620981
0.2416844 0.01900227 0.1411264 0.1377272 0.05196527 0.098225444
0.160331 0.06943291 0.06681929 0.09542632 0.064895706 0.04816934
0.1674573 0.122485 0.1863393 0.2356676 0.1898656 0.1434252
0.1702384 0.1149742 0.02386089 0.373209 0.1320542 0.01695982
0.209851619 0.10389723 0.2446393 0.015461648 0.066417738 0.06329923
0.05736922 0.171926 0.1317935 0.1795877 0.116980724 0.315619 0.138539
0.2956379 0.147557147 0.02652488 0.3298825 0.1507666 0.2110617
0.1357442 0.1630787 0.1889519 0.3600239 0 0.09765513 0.2308583
0.146203772 0.1357167 0.108933173 0.233574 0.08430524 0.1019478
0.04062772 0.1166289 0.2886887 0.08126236 0.03157245 0.3695857
0.114459673 0.2285277 0.1176848 0.2445004 0.06661185 0.01326642
0.04208083 0.1265044 0.1199287 0.07569466 0.3009228 0.07170215
0.08204875 0 0.01598381 0.202328276 0.1406202 0.2223166 0.1001077
0.1981262 0.3213011 0.08863893 0.09477916 0.1819589 0.2863916
0.1263495 0.188887861 0.097930864 0.2085899 0.1154692 0.05246387
0.2568015 0.05351458 0.04596508 0.05004085 0.03239969 0.1602597
0.09619371 0.07899392 0.1460288 0.2534775 0.2170125 0.1442811
0.06955632 0.263443 0.04457547 0.174386883 0.1849326 0.0982798
0.09793216 0.01819486 0.05887488 0 0.1808132 0.04842059 0.3178097
0 0.2225656 0.072230001 0.1019277 0.1814251 0.1095897 0.05724618
0.09658415 0.03197187 0.08020432 0.02300585 0.1362533 0.08017386

0.06970127 0.02335443 0.058760349 0.09692191 0.2649132 0.05920884
0.03309225 0.060432289 0.09744526 0.1170944 0.1012011 0.041053584
0.162151775 0.0817592 0.08597807 0.01629018 0.1188906 0.1918956
0.02987553 0.193367 0.02946483 0.08187893 0.1308018 0.07207585
0.2382999 0.200606521 0.03408082 0.662177 0.09750595 0.1185995
0.1354263 0.3873112 0.2657767 0.01223486 0.07275107 0.04039764
0.1089664 0.1306438 0.189706 0.1194623 0.1403849 0.07651423
0.08819673 0.209517 0.3498809 0.2538008 0.0391174 0.08163656 0
0.04917312 0.1239778 0.02694976 0.305695 0.03838397 0.0542557
0.05040348 0.2223544 0.07826762 0.1200638 0.04173551 0.05957685
0.2272104 0.2841311 0.2737663 0.1099236 0 0.01547142 0.1034946
0.07275085 0.3203027 0.01869676 0.2595791 0.116368 0.023790368
0.0873936 0 0.0972872 0 0.03541901 0.111093578 0.146606602
0.01695556 0.213629917 0.06136097 0.07158424 1.383275 0.1260722
0.1204136 0.09789548 0.04116383 0.02167064 0.06798262 0.05457369
0.2451424 0.119225077 0.04117286 0.4118402 0.1088769 0 0.2479492
0.1748491 0.1829576 0.09843076 0.1082887 0.5432991
AC011472.4 1.326854 3.78137 0.3488613 4.189542565 0.318454054 0.9826943
4.042917 5.724901 7.354793 4.322888 1.547651 0.924808523 1.868397 1.513072571
6.309837595 0.2433362 0.9005112 0.6375248 0.7032051 0.5552529
0.640174244 36.80831 0.6643657 0.295437 0.163786 0.3512298 0.2236157
0.3134705 0.3038098 0.125508375 0.7993961 0.508415152 0.5661647
0.5558218 0.4576091 0.393866989 0.5746384 0.664563 1.011409 1.040215
0.04993898 0 0.3598346 1.345375 0.05869717 0.3251107 0.2271392
0.09574253 0.1234128 0.1476635 0.3797667 0.1789049 0.4305565
0.120052164 0.7136654 0.4395327 0.405577 0.0932988 0.161906 1.369352
1.105263 0.2642629 0.1966845 0.4173925 0.28737 0.936298969 1.566376
0.3068015 0.5750571 0.135980528 0.1854491 0.319709 0.2964982
0.15191708 0.3916394 0.2595915 0.2755821 0.2063643 0.3818723
0.2872232 0.4605417 0.5972407 0.335487 0.05121935 0 0.08584715
0.2947771 0.1893904 0.9777685 2.472389 0.2625794 0.065586988
0.1369488 0.1084523 0.9433512 0.736782717 0.5989991 0.2527384
0.1258186 1.429314 0 0.6386718 0.6049092 0.6583751 0.3088364
0.4340103 0.033208 25.99068 0.769293376 0.1988206 0.541359 0.6074186
0.1857564 0.5434153 0.3505871 0.1897246 0.09834863 0.2511712
0.5701955 0.7729942 0.1418297 0.2749705 0.2798654 0.2007566
0.5189937 0.4632282 0.3386747 3.54693 0.062738145 0.4737377
0.4566108 0.2029176 0.5819429 0.310710715 0.2784778 0.4204502
0.3080426 0.2281464 0.5897402 0.8224779 1.199824 0.5482872
0.2600716 0.7412567 0.04553193 0.3952538 3.65448 1.496762 0.3142647
0.3174287 0.1238081 0.1821607 0.6860802 0.161525221 0.5474499
0.3323017 0.612393225 0.2149217 0.4347433 1.148772 1.560231 0.6688644
0.551995 0.082516244 0.1559428 0.3061286 0 0.4632151 0.2677102
0.2870114 0.5029681 0.6775712 0.3767134 0.222271435 0.6033538 0

0.5356564	0	0.3900068	0.4635257	0.998351 0.3840567	0.3068496
1.041377 0.5456617	2.604854 0.6163407	0.3856469	0.4700207	0.2908407
0.9744509	1.361976 0.125697898	0.6342332	0.3465871	0.463253 0.5734159
0.6062379	0.03519033	0.19004923	2.17149	1.551635 0.1206648	0.3231085
2.812587815	0.2087665	0.2268007	0.2843848	0.2839207	0.1994895
0.5142999	0	0.09222691	0.4671561	0.4524335	0.5054663	0.6828871
0.1837601	0.142109294	0.093810805	2.054273 0.083763716	0.089954778
0.9001769	0.051799771	0.2540214	1.01149	2.464728 0.105624143	1.465604
0.1705767	0.5570862	0.570995071	0.8621936	0.07446432	0.5309071
0.571715 0.1838491	0.3533926	0.6824332	0.520116 1.021987 0.3967861
0.506227 0.572044175	0.1470494	0.337226931	1.416033 0.4567249	0.06136708
0.6603039	0.1579598	0.2606626	0.8804801	1.539398 0.333706 0.54257615
0.8739195	0.9032087	0.3188815	2.453921 0.8624526	0.3039647
0.4405756	0.371266 0.3222033	0.5094543	0.3398916	0.1333501
0.6007066	0.4329625	0.43844661	0.4102064	0.4301442	0.4648587
0.1694766	0.3071742	0.09604055	0.2995227	0.2628707	2.08487
0.2444643	0.085275239	0.371379365	0.8122153	0.312778 3.268574 0.5651856
0.09663868	0.2905193	0.5647856	0.04388145	0.1736419	0.1737103
0.5884323	0.2434194	1.061123 0.251929 0.5287599	0	0.3243652
0.2817363	0.511736119	1.628047 0.08873872	0.5894989	0.3449982
0.4252741	0.5244659	19.04016 0.06557982	0.6737239	0.3223668
0.4220133	0.260871422	2.6229250.2106157	0.09895061	0.3101321
0.3924346	0.202076111	0.3258811	0.03115864	0.4697347	0.3800502
0.503477 0.8223993	0.278543204	0.3000434	0.9866802	0.400956 0.08963889
0.163696425	0.3959334	0.1268721	0.822388 0.444816835	0.087845972
0.1550261	0.02911172	0.04412615	0.690098 0.2835265	0.3237022
0.9047186	0.1995327	0.4435803	0.5567735	0.05578172	0.2657927
0.217357778	0.04615834	5.544093 1.05648	0.2891315	0.3668372	0.3631611
0.2964395	0.4639776	0.3284417	0.4377097	0.5534315	0.7470849	0
0.08089857	3.042149 0.2072585	0.5801943	0.3972716	0.2707836
0.2946363	0.1059595	0.3685561	0.2657283	0.8879871	0.1439254
0.1460007	0.7176464	0.9357559	0.07348278	0.2047962	0.2635081
0.318012 0.2927014	0	0.1936551	2.831107 0.1184065	0.5234588
0.5582934	0.05723602	0.7124416	0.4004881	0.2627525	0.2283217
0.6583849	0.5712987	0.7092288	0	0.2663191	0.08357572	0.3513699
0.6463515	0.5436686	0.112847092	0.277985227	0.2296425	0.105213031
0.387828 0.6301892	0	0.4203066	0.4151271	0.1767834	1.115028
0.5870052	0.3069141	0.2956538	0.234364 0.574136599	1.2268	0.6507523
0.2949211	1.185069 0.7995646	0.7442661	0.2168197	0.2666251
1.222199 0.4682572
AP001107.1	0.1272764	0.03270444	0.1165662	0.034004828	0.078404483
0.1975043	0.06069391	0.08320485	0.08435289	0.06911104	0.1411249
0.225883966	0.2262323	0.09883281	0.048668663	0.2909925	0.2667175
0.2242297	0.0883324	0.1901538	0.191387336	0.1747	0.367196 0.3232782

0.3456401	0.2402055	0.1572999	0.2646086	0.1157604	0.183932104	
0.1071097	0.195583685	0.1306797	0.3141855	0.2682498	0.075037398	
0.3753496	0.1806169	0.3967778	0.3292771	0.06147574	0.146246	
0.2531215	0.4045046	0.1754818	0.4573907	0.07323179	0.1262793	
0.09766499	0.3375845	0.06678562	0.1483215	0.1325056	0.147786278	
0.2259088	0.3091842	0.6339965	0.1421982	0.398618	0.1720098	
0.6240335	0.1781472	0.1470026	0.1651556	0.1010735	0.164657161	
0.06220117	0.6894122	0.1789211	0.103625085	0.1386052	0.1499304	
0.2802635	0.195917896	0.2508965	0.109564	0.1130821	0.2358924	
0.3357795	0.1515329	0.364458	0.0919017	0.1011405	0.06755561	
0.102998123	0.1459381	0.1503342	0.3264	0.09006903	0.171895	0.1649183
0.207613825	0.09633507	0.10013	0.3091723	0.207312501	0.2633493	
0.04444647	0.2765801	0.1099693	0.08178593	0.1519577	0.2707831	
0.2470007	0.1629355	0.1318339	0.18687826	1.961378	0.085444815	
0.0582742	0.3427314	0.1445217	0.1960023	0.2866944	0.07192979	
0.1167771	0.1470122	0.3091961	0.2673983	0.3171897	0.1371816	
0.306256	0.2296794	0.1664378	0.2834174	0.08960944	0.0297796	
0.2512373	0.143430366	0.1093461	0.1791294	0.249795	0.08772021	
0.054641462	0.2658534	0.1386379	0.09480144	0.1203652	0.06382245	
0.1687474	0.1166056	0.1542745	0.1880261	0.1380252	0.1121012	
0.3315053	1.515744	0.1780606	0.0947425	0.1339749	0.130637	0.05606074
0.06032689	0.293526192	0.2166172	0.333934	0.16154284	0.143625	0.1681983
0.09697084	0.1981645	0.1357226	0.1132526	0.15962401	0.1005548	
0.161507	0.09292347	0.3855812	0.180471	0.2235264	0.3213747	0.1340521
0.1385199	0.21498711	0.3289272	0.09150392	0.2001757	0.1294275	
0.4537257	0.08151544	0.4915949	0.2605116	0.155142	0.07166197	
0.1507941	0.1880214	0.06967892	0.09325209	0.1653153	0.1841297	
0.1285249	0.6632765	0.07368394	0.2402314	0.2167135	0.1086233	
0.2890766	0.1476177	0.3341821	0.140372322	0.1374758	0.2563323	
0.1697606	0.1591008	0	0.1789788	0.1695116	0.3750885	0.166434
0.2104928	0.5852296	0.08350488	0.1256972	0.1643078	0.1363968	
0.4622338	0.160123	0.09694795	0.324886684	0.230965428	0.5011075	
0.220959801	0.07118735	0.1206131	0.109313844	0.1861338	0.1778803	
0.1311745	0.157887665	0.2362621	0.2939759	0.2632916	0.261078907	
0.2274375	0.196429	0.1292748	0.2924846	0.07544046	0.4194955	
0.135014	0.4287534	0.04792699	0.1628168	0.2408908	0.216675758	
0.07758016	0.051891519	0.1960393	0.1706789	0.2320278	0.1645044	
0.1851916	0.3798173	0.2032287	0.1052792	0.3185779	0.184018932	
0.2497416	0.3737363	0.1380391	0.3109666	0.2148666	0.2138205	
0.3013089	0.4243895	0.1493831	0.8242504	0.09392931	0.1876072	
0.02641001	0.09898281	0.3148456	0.1751941	0.166419	0.2452496	
0.1490204	0.1170423	0.1351172	0.1354473	0.1502421	0.2216919	
0.2321536	0.344918795	0.083970805	0.2297794	0.06875627	0.1416199	
0.3134712	0.2719174	0.08028523	0.07548545	0.06945278	0.2137561	

0.07637156 0.4045185 0.1337739 0.2414936 0.2953608 0.3881217
0.06626784 0.3251435 0.1415601 0.173064824 0.491862 0.1482528
0.1399532 0.03466928 0.01869713 0.05380221 0.1614981 0.06919705
0.394936 0.2078681 0.4664949 0.108957266 0.04855437 0.185194 0.08700707
0.09544447 0.2530491 0.172608401 0.06686087 0.2520591 0.5369478
0.08593134 0.1272781 0.2781285 0.174944629 0.1253181 0.8675859
0.1339727 0.2364579 0.207270631 0.0580239 0.07809089 0.09641655
0.293344913 0.146761295 0.112946 0.2355005 0.07760007 0.1779952
0.1329623 0.2703992 0.2930853 0.133341 0.2275229 0.1602229
0.09319263 0.3071631 0.076448903 0.1298782 2.795904 0.1950817
0.1412404 0.1146877 0.2128846 0.3872644 0.1748463 0.2772462
0.2020605 0.2011409 0.1936157 0.2711057 0.1422679 0.1719614
0.1731299 0.2040655 0.0798446 0.563502 0.4490601 0.121121 0.2138866
0.05607706 0.06246442 0.08436887 0.1925672 0.1359131 0.04571157
0.2326077 0.2941256 0.430304 0.2889482 0.4175142 0.1855576
0.1248724 0.1515279 0.541396 0.536989 0.04581792 0.06039302 0.390609
0.1126875 0.1328467 0.1606103 0.6412614 0.2936767 0.1940162
0.192657669 0.09366954 0.1763711 0.3398548 0.06820027 0.1181057
0.191837362 0.090788974 0.1050006 0.092513607 0.1364066 0.5114991
1.449664 0.263322 0.07300409 0.116584 0.3268141 0.05161525 0.1259388
0.1949758 0.2198142 0.113588361 0.4314892 0.4822872 0.06915295
0.05789051 0.35434 0.297469 0.2396732 0.5235891 0.0859742 0.2529245
MIR497HG 0.3853849 1.10118 0.2050854 0.971984612 0.154651633 0.5561683
1.449215 1.439076 1.804934 1.001118 0.2710412 0.636905771 0.5597568
0.972363198 1.994741256 0.1243916 0.3634217 0.8221509 0.2910018
0.02240849 0.155444704 0.1737283 0.07681846 0.03775626 0.05232883
0.129672 0.1265581 0.08012181 0.1423631 0.10693148 0.7102638
0.040609032 0.1808868 0.05073781 0.129959 0.062919278 0.1258931
0.1389761 0.1093705 0.3855184 0.1212599 0.0506084 0.1471557
0.4048821 0.04500829 0.1090648 0.1403017 0.4343676 0.05914468
0.1604043 0.1024595 0.1273834 0.1238046 0.061369717 0.145928
0.07659737 0.1785322 0.03577021 0.1158711 0.362501 0.1115135
0.1125743 0.04398781 0.1333548 0.02448354 0.161889094 1.259196
0.01742606 0.03957217 0.405487659 0.04740004 0.09533576 0.08052025
0.232976428 0.3932559 0.1857816 0.04108866 0.07252562 0.1518301
0.07341311 0.2471965 0.12403 0.2572475 0.05563872 0.052394464
0.05851251 0.07157667 0.1500631 0.2380131 0.09552473 0.05752651
0.121537366 0.04375448 0.03464999 0.2575566 0.075327499 0.2985485
0.1744171 0.0964762 0.1883717 0.06735879 0.2112543 0.302197
0.1234039 0.0526249 0.2218627 0.063658751 0.3945145 0.160407333
0.2710281 0.1314508 0.2191818 0.06330481 1.014704 0.08214129
0.04849286 0.2765127 0.06419837 0.1991776 0.1053729 0.09062775
0.0644246 0.1669091 0.2052504 0.1815248 0.2604785 0.1136151
0.7932585 0.104231541 0.1135176 0.2603484 0.2506805 0.2496746

0.307738664	0.08134609	0.228364	0.3149379	0.06803221	0.1275452	
0.1401481	0.3147406	0.231231	0.05170143	0.1281657	0.06982668	
0.08548296	0.1938637	0.1575274	0.1262247	0.3326474	0.05142288	
0.3317369	0.1565709	0.027523472	0.06996302	0.2972725	0.14185104	
0.1153596	0.3889153	0.1115762	0.3212466	0.1775344	0.1058158	
0.089636009	0.04650142	0.07824527	0.06077514	0.0749841	0.3079155	
0.3720348	0.1028453	0.08118024	0.4114058	0.049710237	0.3084297	
0.04654744	0.09412676	0.07837969	0.0498421	0.2073318	0.1224838	
0.04908168	0.2745034	0.1446586	0.4034638	0.6185888	0.4332192	
0.04312429	0.2125473	0.1709768	0.03113322	0.1338906	0.053546476	
0.1496377	0.3051306	0.1315618	0.2540422	0.5065739	0.06296157	
0.102009246	0.1054546	0.1021524	0.02698627	0.1156193	1.606261617	
0.1700847	0.2173851	0.1574899	0.07660063	0.04588985	0.1237205	
0.03861671	0.04125246	0.03316757	0.2725805	0.0322988	0.6940171	
0.1056788	0.036322573	0.05195162	0.0931565	0.096343534	0.057480213	
0.4163382	0.046339358	0.05410573	0.6919557	0.174072	0.11473773	
0.249735	0.07411782	0.1067918	0.138646877	0.06427554	0.09516393	
1.064706	0.08634841	0.02349555	0.1354886	0.04360681	0.1578658	
1.758853	0.101417	0.2207235	0.213694795	0.1409447	0.123903687	0.604504
0.2225301	0.05097684	0.3445742	0.04710287	0.0713832	0.1265891	
1.10935	0.1218485	0.495286848	0.1489136	0.08147879	0.1912229	
0.07840155	0.197477	0.09711523	0.2877795	0.20165	0.1347609	0.1823003
0.08687494	0.09657066	0.4414224	0.1770616	0.107395835	0.1610153	
0.08795452	0.1881254	0.1985387	0.157025	0.05523206	0.09296186	
0.05039153	0.2106752	0.1437135	0.065388041	0.081362654	0.1805209	
0.1798759	0.9686197	0.2000204	0.1543779	0.2970223	0.1299213	
0.1289832	0.09431212	0.7658943	0.2734563	0.01166569	0.08774727	
0.2468362	0.06463644	0.1805893	0.2528647	0.07715431	0.186135227	
0.1813867	0.05670316	0.2260104	0.1196731	0.06114283	0.3574704	
0.3207881	0.3603818	0.1243675	0.08926198	0.07704638	0.045840921	
0.8879981	0.04486046	0.082197	0.1783542	0.1671745	0.125435361	
0.09023508	0.03982013	0.2572764	0.1595861	0.7680989	0.08893162	
0.147474406	0.2252766	0.5903575	0.1229793	0.4467713	0.062760238	
0.3879295	0.1297121	0.3713518	0.113693423	0.028066354	0.1358539	
0.04836546	0.1071455	0.1469886	0.04831217	0.09307913	0.03651197	
0.08159963	0.160618	0.1293719	0.03920836	0.1843966	0.106481927	
0.08258522	0.06251683	0.1890225	0.09032329	0.1510612	0.3558197	
0.1190652	0.2287109	0.05036904	0.09089995	0.1556004	0.1256263	
0.04249317	0.1447415	0.08331014	0.03641994	0.1003175	0.06527639	
0.1326548	0.1160996	0.162497	0.08478133	0.09508676	0.1021347	
0.06744239	0.09795768	1.389817	0.01328753	0.06573667	0.09160394	
0.07697329	0.08805616	0.1288452	0.05297506	0.02887353	0.7629468	
0.1815854	0.03902325	0.06183562	0.1097197	0.02677901	0.1330721	
0.09654036	0.08753719	0.3365612	0.308892	0.09063804	0.164712071	

0.1966470.1495314 0.2469741 0.4047520.2247875 0.120180293 0.162404398
0.1936959 0.053784069 0.08496626 0.2973671 0 0.1275716 0.1250521
0.06212957 0.1044989 0.4276032 0.1359731 0.1889199 0.04492688
0.082545212 0.3848301 0.07128416 0.1381979 0.3617961 0.2084529
0.05188131 0.07125204 0.07382734 0.6872565 0.1538803
MAP3K20-AS1 0.04370561 0.2395823 0.03370762 0.342524198 0.046154427
0.1431782 0.2064327 0.1447639 0.2488934 0.5315996 0.1292295
0.14892784 0.08878422 0.139234153 0.169352261 0.05486039 0.04579424
0.1959963 0.04853212 0.05647322 0.072163951 0.05893798 0.1528387
0.06872104 0.05275103 0.1508288 0.06687621 0.01514405 0.07338667
0.067371392 0.1348620.061405011 0.05128488 0.03836038 0.06550375
0.026427885 0.03304918 0.1070188 0.03758622 0.3350249 0.2814698
1.9131270.02317859 0.1537547 0.02835714 0.006544332 0.3413917
0.03854508 0.02484245 0.0118896 0.02038542 0.02469445 0.008666909
0.006444256 0.03064696 0.06434615 0.06531266 0.2353839 0.08343281
0.03150222 0.2810330.04255595 0.1187751 0.07561731 0.07712838
0.124170299 0.06509513 0.03293748 0 0.145985437 0.02239804 0.05148479
0.05371538 0.260951259 0.06757308 0.05852516 0.1849115 0.02492413
0.1298235 0.02775203 0.04449839 0 0.2006666 0.2433213 0.056589851
0.2810985 0.07595176 0.2988878 0.04498752 0.1435173 0.01208139
0.095057035 0.03859406 0.06549291 0.01381035 0.11864884 0.1639831
0.08140017 0.1620910.1007 1.11839 0.08469952 0.0974124 0.02827263
0.02762994 0.05083016 0.042781576 1.3149680.345571827 0.05336227
0.04358925 0.03452347 0.02991355 0.1312645 0.04704774 0.053467
0.3801050.03370643 0.1163082 0.05809077 0.06851921 0.05904035
0.03755709 0.05542136 0.08797566 0.05221762 0.02726948 0.08726434
0.111134379 0.05721676 0.02828114 0.1143699 0.06024471 0.075053605
0.3587605 0.05924431 0.2604319 0.08572639 0.1534125 0.04414963
0.08643846 0.05297652 0.004653444 0.06319552 0.1759752 0.01958469
5.0078380.007089212 0.02168917 0.06134109 0.0149532 0.06600264
0.02367512 0.104045764 0.1432590.05351268 0.419124815 0.2976478
0.1820247 0.02219929 0.05583422 0.0414275 0.2370435 0.079728716
0.01674165 0.05751417 0.06807277 0.03481076 0.05748144 0.05942479
0.02699872 0.08865482 0.1489040.626391376 0.1263105 0.1005493
0.2264330.1119338 0.07246765 0.2052724 0.0192925 0.0265059
0.0555907 0.1093693 0.1380836 0.2316511 0.02126856 0.06210319
0.1397362 0.09367188 0.4629201 0.06748544 0.047231188 0.03928255
0.1488351 0.04144478 0.04924844 0.03003888 0.2890132 0.532524301
0.04895644 0.1514362 0.1263043 0.07284511 0.297234677 0.4286424
0.2100082 0.06869446 0.04572157 0.01285003 0.004871824 0.07646626
0.02970378 0.008358793 0.08326659 0.008139848 0.03665656 0.04438811
0.160193389 0.276960839 0 0.276524657 0.057943976 0.0414175 0.083416489
0.02727113 0.1533052 0.09400522 0.26364465 0.06883786 0.5823439
0.01682084 0.119536292 0.1272742 0.08394027 0.1841439 0.07951234

0.07895039 0.1849545 0.09615952 0.02617425 0.2304079 0.021299
0.009590703 0.035430745 0.1420819 0.101823399 0.1407011 0.055162
0.3359994 0.06202756 0.05087455 0 0.01772367 0.08263304 0.05373882
0.081133476 0.02345547 0.04277926 0.05530164 0.01162266 0.09837777 0
0.04729914 0.1943089 0.08962208 0 0.04691564 0.06442267 0.2176552
0.3974196 0.058838222 0.06133973 0.02770755 0.07485917 0.0181946
0.04946623 0.0618641 0.1584834 0.03704028 0.07027115 0.06298825
0.130457971 0.111067539 0.0796151 0.1636981 0.1830815 0.08401427
0.2334352 0.06683454 0.1382456 0.9751786 0.272636 0.05594737
0.09475902 0.3429946 0.1809312 0.05409298 0.1036604 0.2199727
0.08357536 0.09722101 0.158477202 0.03361216 0.05001557 0.04271885
0.06349402 0.1540904 0.04222905 0.2070397 0.04224295 0.02410979
0.05191279 0.04368826 0.063014694 0.2148983 0.3843904 0.1035752
0.4806971 0.09830528 0.158059251 0.07871808 1.204242 0.04322536
0.3934409 0.06587597 0.03056221 0.185830327 0.1207948 0.06500104
0.03874111 0.06255221 0.115988769 0.02125323 0.1021552 0.1765792
0.080585667 0.091951597 0.3138438 0.5297493 0.03552958 0.07408727
0.05326778 0.2997351 0.03067213 0.4884069 0.4643118 0.04075491
0.02694866 0.04280231 0.02333501 1.21904 1.549272 0.144612 0.04138729
0.1050207 0.05848216 0.0136393 0.213478 0.03173464 0.05286539
0.01188302 0.006331993 0.03894183 0.01302762 0.1574668 0.1335047
0.08244036 0.1827863 0.02180301 0.3400381 0.1109117 0.01780528
0.1283758 0 0.3322069 0.09404573 0.1066774 0.4102138 0.1301674
0.03847626 0.006062056 0.03414101 0.03142372 0.1153008 0.1663228
0.1982223 0.05720328 0.02809864 0.1798111 0.1659075 0.03374383
0.3611617 0.04760182 0.08824346 0.01631138 0.007076915 0.03807055
0.18679613 0.1191308 0.07402304 0.07072919 0.03122584 0.07725038
0.03634498 0.409286781 0.04437697 0.059300949 0.03568824 0.03903208
1.930868 0.03384233 0.1432512 0.1067571 0.1436481 0.1606996
0.04612938 0.1190276 0.01887057 0.242699347 0.06285984 0.02245603
0.07123956 0.1802371 0.07725541 0.09806232 0.1047476 0.0500924
0.1102184 0.02693088
ENTPD1-AS1 0.09809535 0.2089381 0.1791116 0.255137545 0.053667947
0.1412179 0.1198043 0.1464905 0.2214099 0.1571018 0.07413497
0.100681359 0.159658 0.086409529 0.173541387 0.08010983 0.1263119
0.06132929 0.06299465 0.06757546 0.067742576 0.07172011 0.06571485
0.06818643 0.08665834 0.1453022 0.1464839 0.1265417 0.1448682
0.067225657 0.1104013 0.125782327 0.03744435 0.0949155 0.117572
0.034732164 0.2187788 0.07813707 0.1311147 0.1386123 0.0939465
0.06208093 0.1776942 0.1292692 0.07131463 0.1226399 0.06379829
0.1388374 0.1729164 0.2112349 0.06615065 0.07612677 0.109684
0.067439904 0.0372935 0.1644324 0.09713892 0.0865392 0.08629815
0.1111693 0.09803478 0.1104752 0.08093925 0.06952377 0.1332749
0.064756986 0.03366535 0.1108901 0.1040204 0.046187937 0.1108394

0.05847381	0.1118465	0.166711049	0.1074445	0.07528735	0.1494093	
0.1556916	0.07815796	0.04727903	0.1570319	0.09362874	0.1164579	
0.2418916	0.075749003	0.128974	0.07162839	0.1514216	0.05474415	
0.2377391	0.1102614	0.086111715	0.05769877	0.04675535	0.1193188	
0.080853199	0.1103377	0.03565935	0.07889773	0.05076627	0.08505876	
0.05006192	0.08836517	0.1066531	0.02958751	0.1175224	0.169194338	
1.362079	0.077756055	0.08311701	0.07213799	0.152639	0.03882776	0.2472893
0.08473175	0.1747396	0.03661775	0.1132055	0.08342963	0.07876792	
0.06114479	0.06645633	0.1133416	0.3023597	0.1059848	0.1198226	
0.04147941	0.139012	0.081141936	0.06266304	0.115633	0.06759869	0.1498787
0.048709758	0.1387658	0.1277074	0.04527327	0.08494485	0.05156026	
0.04835209	0.04331136	0.04942375	0.1053254	0.04272277	0.1070698	
0.2430875	2.540255	0.04658407	0.04574784	0.07713254	0.05337542	
0.04105087	0.09219085	0.137161489	0.04022957	0.06883924	0.088503848	
0.09770976	0.04770794	0.1008513	0.1002162	0.09679116	0.09014127	
0.108338797	0.1038996	0.07798608	0.07041059	0.06535592	0.03497384	
0.1462319	0.07293599	0.04481243	0.2080414	0.165514864	0.08749304	
0.1835337	0.1246492	0.07211081	0.09053558	0.0711525	0.1612056	
0.0838613	0.2059472	0.0780787	0.08096011	0.2140842	0.06901644	
0.03778588	0.1742924	0.07295161	0.0821104	0.1621895	0.096885441	
0.1453179	0.140363	0.1351606	0.05843093	0.09503921	0.08688877	
0.086402011	0.1140413	0.0884538	0.07211911	0.09370867	0.075784177	
0.1442081	0.07111055	0.1839035	0.1446572	0.104767	0.07706916	
0.08628256	0.08042433	0.1139219	0.1266562	0.1030138	0.08666391	
0.07382015	0.097467601	0.096818522	0.07272005	0.11654242	0.074036057	
0.07391983	0.079175752	0.1028752	0.1982127	0.08261683	0.089002336	
0.06222693	0.04813399	0.0818754	0.05370854	0.09011006	0.03210263	
0.1472525	0.1700878	0.1032781	0.1436962	0.06017865	0.122307	
0.05073497	0.07127507	0.1283775	0.075881977	0.09509266	0.133818769	
0.1369724	0.2058507	0.1382939	0.04528784	0.1960418	0.06567392	
0.07548617	0.07373961	0.2821257	0.113918347	0.09989827	0.08537345	
0.1788128	0.08344559	0.08168677	0.06552177	0.124707	0.09821747	
0.1446464	0.1078199	0.1141809	0.1271729	0.04708604	0.07635941	
0.063006868	0.05856584	0.08766317	0.1047583	0.06310053	0.1665372	
0.06022466	0.05869479	0.1480982	0.2546332	0.07473256	0.145382439	
0.118520734	0.1439383	0.07968	0.1837994	0.05793305	0.07574967	
0.1886838	0.08942618	0.05331415	0.2603069	0.09190922	0.1027306	
0.1001715	0.07828283	0.1250663	0.2883237	0.05722795	0.09025937	
0.08044791	0.092566473	0.0670789	0.09216346	0.09241505	0.03863212	
0.03125143	0.04110996	0.2927317	0.1901961	0.1525606	0.05053706	
0.1193217	0.088183099	0.07033562	0.08529646	0.1337942	0.05925446	
0.08373761	0.095037711	0.1192053	0.1257811	0.1814694	0.08724219	
0.07522968	0.04462843	0.06394081	0.110244	0.1300724	0.04714306	
0.1030523	0.089818744	0.07500127	0.04475159	0.07735488	0.102420958	

0.125665037 0.07290979 0.09812192 0.05361144 0.04688054 0.1055643
0.06343303 0.07651446 0.09853361 0.1216943 0.03570739 0.03935176
0.2366148 0.053951967 0.1013066 0.9234623 0.08281157 0.08813547
0.1261995 0.1850301 0.05975034 0.0623462 0.08753202 0.06861923
0.05350251 0.03082096 0.3009094 0.1521885 0.08090509 0.06599871
0.1939511 0.05783125 0.1149699 0.08468129 0.07890299 0.07366709
0.02499475 0.03132196 0.1090546 0.1630796 0.05408875 0.07131122
0.08927852 0.04146982 0.05606338 0.08724515 0.1695250.1092914
0.1277611 0.2050298 0.05336705 0.1230930.05688995 0.05608015
0.1691754 0.09574568 0.1506061 0.1216987 0.07741068 0.04478092
0.1111850.128807522 0.1217724 0.06223483 0.06541208 0.02279875
0.1027777 0.10319696 0.099610105 0.05400119 0.105150044 0.09554183
0.1082934 0.6461464 0.06383188 0.08483512 0.1403028 0.08011746
0.04601206 0.03688783 0.09704385 0.08113631 0.071724111 0.04371001
0.0819785 0.06935167 0.03870457 0.07646156 0.04640592 0.1068275
0.1027547 0.08622125 0.09831447
ARAP1-AS2 0.1461167 0 0.05634569 0.034700833 0.01714484 0.1763534
0.07963223 0.07641709 0.01434657 0.01410512 0.09258003 0.041491318
0.2143726 0.03491159 0.014899441 0.2751140.1020662 0.06240524 0
0.1258676 0.034465521 0.07037199 0.2640014 0 0.2755583 0.3361673
0.08599256 0.3290924 0.1635643 0.090094462 0.0819765 0.179628174
0.009525317 0.2885546 0.08212209 0.035341501 0.1657351 0.09757787
0.1675444 0.08400419 0.13443 0.07461965 0.1743541 0.3037467
0.1422055 0.2953668 0.07133383 0.06443199 0.09966398 0.1391226
0.1590227 0.2201561 0.05795045 0.043088898 0.03842212 0.3549515
0.09704596 0.04185829 0.1568997 0.0921535 0.2505467 0.2015536
0.05294522 0.02808933 0.03867836 0.118607532 0.02720327 0.4404665
0.2143368 0.097611754 0.09984154 0.05737468 0.2294651 0.122683037
0.1280159 0.05590326 0.04945571 0.1388773 0.3654957 0.01546344
0.2107534 0.1406741 0.03870398 0 0 0.08473327 0.06348051
0.1019635 0.01253353 0.04643295 0.05048813 0.132414519 0.0552976
0.0437912 0.1962259 0.158666804 0.04837311 0.01360686 0.1693446
0.04809436 0.0918059 0.06067877 0.0592126 0.2126725 0.1385587
0.1168306 0.080452935 4.7178760.043596843 0.05352025 0.2914553
0.1442737 0.3000211 0.1219010.01572901 0 0 0.2366435 0.0409307
0.04161625 0.07635785 0.1480379 0.2636780.04632119 0.2573553
0.01246958 0 0.09282709 0.016888393 0.08767329 0.06618468 0.05462309
0.03356837 0 0.1606351 0.08488529 0.02073039 0.3582508 0.02442328
0.1107008 0.05949613 0.08855559 0.1322379 0.04695008 0.03677003 0
8.6680860 0.01208521 0.08544819 0.01666386 0 0.1978767 0.17392299
0.3315764 0.05111534 0.21636467 0.03471268 0.09362234 0.03710836
0.05833283 0.1108004 0.1485908 0.144380808 0.08395605 0.09614073
0.04267144 0.1330050.02402151 0.1434832 0.08123596 0.01139965
0.1290634 0.179498807 0.03248319 0.01400652 0.1802407 0.03301916

0.2745772 0.02079597 0.2579949 0.147691 0.196176 0.08531686 0.01049185
0.1046563 0.08295594 0.06488234 0.09732633 0.2192148 0.01311555
0.4653359 0.045115254 0.1969943 0.02073273 0 0.1337761 0.07531954
0.1515654 0.07162272 0.09352644 0.02531408 0.081204 0.06958169 0
0.05619756 0.06105215 0.1658652 0.1868245 0.1074006 0.3908998
0.05810048 0.08689251 0.1257531 0.01739856 0.2313118 0.1050431
0.04946613 0.095635503 0.033670399 0.5886633 0.101467067 0.048429601
0.01153892 0.111551261 0.2165356 0.3043148 0.1222194 0.042649222
0.2137004 0.1102015 0.1124709 0.061482145 0.1160463 0.08017978
0.1758943 0.1469037 0.01649669 0.8561634 0.1837032 0.3325221
0.07336192 0.1691163 0.1923819 0.130297343 0.1385441 0.056736028
0.1378735 0.06915664 0.1238949 0.01481219 0.1275629 0.1002391
0.01481345 0.04604315 0.2438239 0.073027656 0.1372287 0.1001138
0.0660302 0.3011411 0.06771403 0.03068394 0.2503734 0.1499109
0.08673346 0.1508532 0 0.0837581 0 0.06992915 0.147531034
0.2129584 0.06947395 0.06256732 0.08363879 0.137813 0.02585302
0.0345549 0.1326783 0.3523959 0.06580701 0.241028781 0.114252674
0.08555443 0.04209814 0.02550327 0.1404383 0.06503508 0.02234413
0.07297624 0 0.7712498 0 0.3887982 0.04095358 0.1008149
0.3164766 0.1423361 0.05071815 0.3405301 0.173349 0.052982126
0.1348467 0.02388745 0.09521182 0.01326708 0 0.02353004 0.6921754
0.0882667 0.3224156 0.2458695 0.1217155 0.043889757 0.06193521
0.1700861 0.1065455 0.09391951 0.1056389 0.101022229 0.05117203
0.07548796 0.2077347 0.08769016 0.05929449 0.08514635 0.074980654
0.1413445 0.3863317 0.04317311 0.1286921 0.12338259 0.1598711
0.01707627 0.04427549 0.119739619 0.059117835 0.05961608 0.1097116
0.07126954 0.02476887 0.2035256 0 0.230722 0.02148477 0.1293573
0.05450076 0.03753951 0.09199077 0.019503412 0.06212649 10.31516
0.05687847 0.03459151 0.03291613 0.1737935 0.07979815 0.0178425
0.3006034 0.1325548 0.1092501 0.05292283 0.0488214 0.08710789
0.07311713 0.01394791 0.06431011 0 0.3523106 0.1454067 0.1426155
0.08928999 0 0.0358554 0.06457179 0.1375561 0.04458051 0.02798831
0.1780265 0.09188135 0.5573336 0.1284078 0.1138105 0.01014405
0.0868829 0.08283713 0.3187369 0.1761364 0.03005724 0 0.4512498
0.04312273 0.05304749 0.09833856 1.431471 0.1537871 0.07424523
0.121430001 0.1194834 0.4049574 0.1655234 0.01739904 0.04304394
0.101257168 0.096210583 0.04945372 0.07080537 0.01491413 0.1565905
3.025913 0.07778495 0.07183767 0.04758808 0.06003059 0.01580151
0.04406281 0.05968997 0.4626461 0.077275513 0.1350984 0.2502501
0.01323157 0.01772262 0.1119215 0.1912412 0.100055 0.3110139
0.05264034 0.1080424
AL589765.6 0 0 0 0 0.056962091 0 0 0.1269442 0 0 0
0.045950312 0 0 0.049501969 0 0.2967172 0.05183386 0 0.3920466
0.057254199 0.2338042 0.5658835 0 0.2929652 0.3141236 0.2571319

0.3924947 0.09057112 0.074832559 0.1361795 0.682054207 0.1898817
0.1420289 0 0.058709378 0.6607668 0.270161 0.2087439 0.09303196
0.08932618 0 0.2574554 0.3881421 0 0.2907638 0.03385712
0.4281383 0.5242797 0.06603165 0.07547681 0.06857321 0.1444011
0.17894843 0.2127563 0.07147228 0.6851551 0.0834421 0.05792046 0
0.3641826 0.1181722 0.04397636 0.0933241 0.4711856 0.246288864
0.09038026 0.2134137 0.03956187 0.283767502 0.2073212 0.1906219
0.430908 0.045289184 0.2251698 0.04643331 0.08215593 0.3691252
0.4933193 0.1027516 0.2471322 0.06676803 0.08572684 0.0229041 0
0.02559256 0.1581809 0.4403931 0.3331316 0.3342496 0.0670968
0.117315887 0.2755815 0.02424868 0.6135922 0.131788821 0.160715 0
0.1687894 0.6391557 0 0.5039985 0.6885487 0.3140373 0.1227595
0.2117229 0.059399374 1.068729 0.144846342 0.4741757 0.1936665
0.09586709 0.1107546 0.4860053 0 0 0.2198961 0.3369537
0.1699854 0.276532 0.3171146 0.0409868 0.04171642 0.4360436
0.5130231 0.04142897 0.1135856 0.1321753 0 0.02648056 0.1884791
0.03024667 0.0743518 0.347356469 0.1778983 0 0.2066242 0.2040435
0.6085802 0.06129882 0.423579 0.2942175 0.1292199 0.3899679 0
0.2175364 0.06166785 0.3149728 0.1204559 0.05677869 0.1384102
0.4072901 0.6135981 0.385228334 0.2448068 0.6368468 0.171154983
0.1537727 0.07776282 0.3698669 0.1938052 0.4601547 0 0.110698022
0.2324466 0.1368936 0.3308008 0.5247521 0.3990458 0.07333989
0.23991 0.07574851 0 0.049697302 0.1618835 0.5118886 0.5389493
0.03656767 0.2414796 0.2418242 0.1428606 0.2453445 0.03430399
0.08098775 0.5228726 0.1043131 0.3937331 0.04311307 0 0.2080915
0.3050262 0.5153447 0 0.2181649 2.376447 0 0.1367564 0.166828
0.03147259 0.203965403 0.8933563 0.08410361 0.02697925 0.1155893
0.157215462 0.1166944 1.06491 0.2119504 0.1692836 0.2854625
0.1623409 0 0.06186259 0.2321126 0 0.1356197 0.1744978
0.1232599 0 0.02796667 1.422384 0.299657445 0 0.1916848 0.231636499
0.1893208 0 3.799582 0.23616327 0.8192297 0.183067 0.6539294
1.429879824 0.06425882 0.06659742 0.07304901 0.116208 0.05480868
0.4345796 0.07629206 0.4360945 0 0.08871675 0.1331605 0.314836699
0.1972712 0.188500026 0.2155631 0.2042367 0 0.7381812 0.04709062
0.7659804 0.1968651 0.1147305 0.1705435 0.242627404 0.3907964
0.2851016 0.0219379 1.097336 0.1285562 0.2378704 0.1313434
0.1660215 0.02619669 1.458023 0.1737047 0.07950808 0.0671555
0.03872215 0.653543671 0.4978943 0 0.5196849 0.2778819 1.23625
0.08589419 0.420953 0.146937 0.9539866 0.52473 0.076266198 0.237245991
0.3789952 0.6993352 0 0.1166482 0.3889311 0 0.06061414
0.1177366 0.3494187 0 0.2870544 0 0.1674739 0.07510451
0.08224316 0 0.1160388 0.07199195 0.176028047 1.269376 0.07936377
0.5799423 0.1763145 0 0.3908815 0.1095089 0.05865153 0.1004245
0.09610327 0.5661436 0.087491713 0.04115476 0.5023064 0.08849681

0.03467096	0.03899724	0.077454622	0.09715095	0	0.5101321	0.04855703
0	0.3960468	0.071176015	0	1.002774	0.3585964	0.1603377	0.175682939
0.1180348	0.1134685	0.1961348	0.8453748	0.117848011	0.01980687	0
0.03946437	0.3703149	0.1267865	0	0.4684484	0.2141433	0.1322392
0.3168787	0.6236071	0.0679179	0.097197331	0	0.5104213	1.13384
0.229854	0.1093607	0.2165296	0.2272472	0.38532	0.4699887	0.4404005
0.06599512	0	0.2433067	0.2170557	0.5344343	0.06951088	2.106119
0.1522719	0.4439897	0	0.7107396	0.2636955	0.190124	0.1588349
0.04290672	0	1.530516	0.04649425	0.1314391	0.2136869	0.1683352
0.04740252	0.174519	0	0	0.1100874	0.582435	1.911642	1.631084	0
0.1874043	0.07163557	0.1174968	0.5717608	2.944144	0.4716396
0.1761948	0.115268448	0.2381834	1.08382	0.3142489	0	0.02860187
0.033641726	0	0.04107632	0.376390515	0.198203	0.346838	0	0.1409635
0.05303862	0.03952671	0	0.367493	0.237891	0.1322095	0.2794723
1.508350445	0.249362	0.2494297	0.04396062	0.2944085	0.7436971
0.272306	0.2216153	0.2781999	0.2186156	0.4187874
RBMS3-AS3	0.1449248	0.7944386	0.1676583	0.30976013	0.102029978
0.2998542	0.9477925	0.6821441	0.5122638	0.4616722	0.09182489
0.781904904	0.6133385	0.380895127	0.97534223	0.1559257	0.1138879
0.1392665	0.3755016	0	0	0.1256364	0	0.1577592	0	0.03125862
0.1023494	0.05021664	0	1.910061387	0.8537324	0.02545186	0.0850286
0	0.04072613	0.262899255	0.09862996	0.145173	0.1246334	0.166638
0.1600002	1.205322	0.05764398	0.2433328	0	0	0.6670902	0.03834387
0.02471276	0	0	0.02047128	0.08621666	0	0.03810872	0.03200512
0.0360954	0	0	0.1566889	0	0.03527817	0.03938503	0.04179032	0
0.573495472	0.4451928	0	0.07086286	0.145223359	0	0	0	0.283925505
0.806644	0.1663418	0	0	0.06797147	0	0.07377682	0.1195943	0
2.010255	0.328384229	0.06876175	0	0.06067908	0.07458779	1.059247	0
0.026266893	0	0	0	0	0.1919142	0.3643886	0	0	0.4221737	0
0	0.1054689	0.08245713	0.03160301	0	1.196436	0	0.2123348	0
0.08585813	0	0	0	0.03799135	0.03938758	0	0.1217905	0	0	0
0	0.1378301	0.0218791	0.0742072	0.1356356	0.1972927	0.301511543
0.02371587	0.05626701	0.08126633	0.03329457	0.124436347	0.03186498
0.1683858	0	0.03045674	0	0.1097979	0	0	0	0.1746267
0.1458804	0.02435307	16.18223	0	0	0.2034029	0.09916763	0.1459069
0.03925255	0	0.1096239	0.190119	0.061314248	0.5508727	0	0.03680568
0.2082853	0.08242249	0.07368938	0.231328166	0	0	0.04232338
0.02473503	0.1072151	0.06568287	0.1880045	0.13568	0.3566012
0.089017353	0	0	0	0.5894971	0.09611913	0.835367	0	0	0.3379475
0	0.2497506	0.2646967	0	0	0.2606377	0	0	0.04195822
0.302044053	0.1953875	0.09253631	0.04122842	0.0306196	0.2988207
0.05637342	0.456676209	0	0	0.0241625	0.0517606	0.422404349
0.1881197	0	0.1518575	0.2274145	0	0	0	0.05540385	0
0.4659296	0.04048678	0.02604657	0.07359399	0.056913265	0.82654512	0

0.167732399 0.07205187 0.1373376 0 0.03391096 0.42892 0.02597625
0.338410793 0.04891322 0.08197697 0 0.091470988 0.0575499 0.1192887
0.7850684 2.2272130.1472592 0.0353825 0.9565755 0 0.9277351 0
0.1431096 0.140983155 0.1177835 0.27011162 0.5067756 0.02286419
1.5237660 0 0 0.04407787 0.4452591 0 0.155211425 0.1166652 0
1.5521510 0.05756719 0.03043366 0.03921019 0 0.3284630 0
0.1780177 0.3007208 0.1040381 0 0.02346905 0.2756291 0.09308548
0.09049809 0 0 0 0.02631923 0.03883573 0.1566486 0.307366462
2.08226897 0.02828553 0 0.2656001 0.03482321 0.1161083 0.09972847
0.09047625 0.1054443 0.1043126 1.4609520.1713897 0.02437172
0.02499815 0.1345265 0 0.1509134 0.02598096 0.7092323 0.094589949
0.0668734 0.03553891 0.1416528 0 0.04257944 0.03500717 0.1471135
0 0 0 0.02414455 0 0.6265863 0 0.1188860.1863069 0.5238863
0.231226685 0.1087599 0.1996593 0 1.1306740.07561394 0.05067111
0.31872457 0.5407389 0.07184636 0.4817359 0.0478659 0.209787784
0.1057114 0.1016219 0.1317431 1.024330448 0.07036276 0.03547789
0.1165893 0 0 0 0.5833769 0 0.6712502 0.08882461 0.08108433
0.02233999 0 0.05803299 0.1109156 1.5020020.0423109 0 0.9467816
0.06464097 0.03392026 0.05309089 0 0 0.02955246 0 0 0.06479804
0 0.3942730.02733667 0 0.03614865 0.1573316 0.04243568 0.02952056
0.08513711 0 0.4995518 0 0.04421689 0.1249201 0.05885815 0 0
0.04245348 0 1.2979090 0.5915625 0.04742056 0.0698799 0
0.1833793 0.03356768 7.0892920.0526148 0 0 0.07039971 0.03155985
0.051616948 0.09480709 0.1004136 0.03517998 0.1553141 0.07684712 0
0.159043051 0.5150287 0.463503736 0 0.03882832 1.8007390.04208208 0
0.03539994 0.5656390 0 0.02960155 0 0 0 0 0 0.1582026 0
0.1083893 0.04961942 0 0.2741076 0

AL390719.2 7.6957730 2.4990770 1.520835896 3.7013540.0981082 1.0120810
0 2.8515021.507980305 6.4148372.946297659 0 9.417827 11.93027 7.409721
1.14941320.1786 6.847010542 5.1239363.2735248.83454 8.84038 2.698535
6.53146214.362190.6045416 1.227913581 1.969429 12.614374886 23.58804
3.1995352.0993974.457540766 4.14502320.166498.39861 4.1915284.248152
1.55628819.58324 12.219757.00797816.415780.39548 6.8109124.5124464.444191
9.8449054.04311 3.56087 6.330531862 11.431813.97551 18.337853.991525
10.4544474.18456110.070611.2927111.5899666.0215321.9060952.054902528 0
16.5342313.973523.697972331 3.4364914.13516312.979830 0.7375621
6.09532 52.35343 11.805849.81505922.8900310.447169.6931350.2622616
1.5670160 1.3950670.7918663 3.91936414.128980.1001108 17.93289
5.187751319 2.91245 22.14457115.7424212.168634679 13.141020.1760206
2.69139635.507380.9254156 12.4097 32.061914.7599561 0.600884.906951
1.486789803 6.0436783.122011006 4.91236717.720481.6352776.4685392.102576
5.9007210.7314585 19.790246.28912 1.2480756.3577141.8697233.16894
6.7755599.87284424.975742.0278797.3920392.59770812.015912151 11.53305
2.4811640.1514172 1.6749491.893462283 2.6123472.9543876.0913326.639477

0.8575616 9.171906 35.1841 4.036771 3.406929 3.101845 1.562894 2.465385
0.03430157 22.3815 13.71293 14.93832 6.836538 1.472558 4.339415 0.589258843
3.914863 9.729626 0.266564781 2.929511 7.482958 5.349018 6.532709 8.830355
1.281464 2.29875041 8.507546 5.736216 17.87445 3.103181 26.30246 0.4691301
10.4421 10.80729 0.4429514 0.63579314 12.63626 0.4918034 5.962298
0.6508823 11.3126 1.518043 13.34987 9.361721 2.251552 1.193769 2.210368
1.373192 9.920997 6.95444 1.49285 39.35396 1.623937 7.270504 3.043154186
14.19796 17.03084 1.408325 38.71868 4.222167 1.628061 0.132360528 1.253084
11.92916 4.006788 2.507479 0.393516513 0.1687635 9.81585 10.94051 14.26536
29.77183 24.20014 2.147422 1.273166 15.28641 4.98371 10.76218 6.082495 21.77953
1.59063062 0.451121936 33.49303 1.145916483 2.103228382 11.62169 6.828700321
9.266936 5.801405 19.00488 2.548409852 27.88767 1.985637 23.40034 3.15296883
1.930108 18.52177 0.2641088 0.2714817 4.115649 11.2293 13.11273 16.75345
0.101681 6.809887 9.969555 14.666461875 24.57915 1.950201793 9.862014
7.994797 1.144804 20.88582 4.583821 34.71479 20.86022 14.9331 7.470349 3.701674541
7.481257 10.20213 6.00365 16.67753 10.2255 13.49571 11.12906 4.571143 1.937998
24.12718 7.125719 7.849913 0.4108937 6.461538 16.685625457 10.07203
4.194058 15.12773 0.05620601 19.45769 5.327137 6.109111 2.566351 8.152513 6.46981
6.320821914 4.222834409 4.848605 20.18868 2.87497 9.537855 2.163355 2.023333
0.03371547 0.08731832 0.9501922 0 3.486097 0.7114222 2.748049
6.516971 1.521061 2.108888 8.003505 2.182407 3.740248223 18.0047 2.582458
2.492678 12.11184 3.067618 22.00295 2.710594 0.2283666 15.56611 15.55558
10.04702 4.444791782 2.472288 5.064096 0 0.5592668 1.930541 1.077066547
0.9997099 0.5580143 24.6196 0.8912946 1.330586 3.839402 0.633444901
1.473959 74.65244 13.40387 1.070218 3.159622949 35.48632 3.913112 11.07327
0.055320432 1.332864588 0.5398427 3.779834 5.202459 13.96091 11.37764
1.972632 15.58656 1.032314 2.335388 13.9999 3.177326 2.720019 10.236149276
1.722169 6.85445 7.016279 2.956576 9.327236 3.733654 3.28645 0.1318935
13.95341 23.51658 12.86634 11.09082 31.4578 6.922033 7.701968 2.139414 25.29736
0.7622854 43.82432 0.6351448 11.22753 18.02277 0 28.3379 0.07159814
1.906552 16.36733 3.827508 1.425655 15.36681 13.44574 26.47222 4.950716 0.8623366
2.954336 0.2755531 12.69367 34.54687 1.184992 0.1993112 20.63953 0.01992296
5.832967 14.06152 176.158 41.49353 1.999302 5.962777243 1.118762 15.57028
8.084268 33.95447 0.5568237 1.403442811 0.809975482 1.302331 0 11.16247
14.49327 0.3727974 10.65046 1.062062 2.836192 5.657838 11.94344 22.00622
1.323702 15.64227 1.26741236 15.22958 19.7474 3.325507 2.489139 15.38524
3.954921 6.933899 10.25728 5.253154 17.83674
AP001619.1 0.1324965 0 0.07664022 0.070799016 0.069960152 0.05140118
0.05415705 0.1039409 0 0.1151129 0.0629627 0.112871237 0.06728886
0 0.182393163 0.05345776 0.1561819 0.1273234 0 0.1284018
0.140637832 0.1148622 0.03475056 0.1081727 0.4947474 0.2572018
0.1754479 0.5853553 0.05561918 0.045954222 0.04181352 0.139615123
0.03886841 0.2616573 0 0.180265373 0.4959447 0.3318084 0.0854589
0.1142607 0 0.08699664 0.07905092 0.3813691 0 0.580308 0.1247487

0.1051669 0.3389021 0.2432978 0.0926997 0.1684416 0.1182345

0.219782299 0 0.04389069 0.1979998 0.03416086 0.2489802 0.1074388

0.5750803 0.2418962 0 0.1146195 0.420876 0.241991206 0 0.4867796

0.2915365 0.149365636 0 0.2341195 0.4478151 0.111247258 0.3687344

0 0 0.4533552 0.4194615 0 0.4046997 0.08200369 0 0 0

0.1257299 0.06475864 0.3328526 0 0.09473568 0.2060187 0.3241934

0 0.1786916 1.083309 0.242792157 0.9211453 0.05552329 0.2764068

0.1471881 0.2383929 0.8253398 0.6644528 0.2410605 0.03769293

0.173357 0 4.92225 0.088949264 0 0.3567883 0.2747332 0.1360274

0.6963901 0 0.1563 0 0.6897375 0.04175481 0.05660554 0

0.100679 0.3586492 0.1890153 0.3300465 0.1526477 0 0.05411206

0.206741136 0.0325231 0.270069 0.1485944 0.3196129 0.255971411

0.3932865 0.3463776 0.676729 0.0835346 0.04983005 0.1505729

0.2774585 0.1806772 0.09522382 0.09579077 0 0.1001908 1.590534

0.1450672 0.09862829 0 0.06799749 0.05002288 0.2153182 0.295707992

0.4510032 0.208578 0 0.04721546 0.09550735 0.151422 0.0952117

0.2825782 0.3031651 0.045319324 0.1141953 0 0.3482447 0.6105734

0 0.1351127 0.09207954 0 0 0.122075251 0.1325489 0.2286165

0.2206437 0.04491197 0.2636288 0.08485873 0.7018385 0.4218609

0.2106588 0.1492023 0 0.2775849 0 0.2647548 0.03971437

0.2555754 0.107037 1.4385 0.046023615 0.6430739 0.3384027 0.0565392

0.1259719 0.05122402 0.07730851 0 0.2862286 0.103295 0.1325424

0.1419653 0.193090131 0.1146581 0.3736883 0.05206299 0.2425641

0.4820772 0.1993851 0.1896649 0.1013052 0.1710467 0 0.3331328

0.2857548 0.1513863 0.07804884 0.137393304 0.2911591 0.046004457

0.049404694 0.141255 0.11379726 0.2790258 0.1960686 0.2137375

0.2320423 0.6037008 0.03747344 0.1529806 0.188160121 0 0.2453824

0.7626023 0.3425406 0.1346307 0.2426116 0.09370098 0.6070202 0

0.1452811 0.2289646 0.096669658 0.08076204 0 0.3971281 0.1567757

0.1348154 0.5439752 0.05783613 0.6135442 0.06044683 0 0.3141892

0.170281169 0.1199929 0.4668777 0.2424948 0.2378356 0.0394728 0

0.2688573 0.1529292 0.03217446 0.839403 0.106671 0 0 0.2377904

0.120401166 0.2252923 0.094497 0.1276541 0.2171853 0.4498808 0

0.1880034 0.03609326 0.2130318 0.05370559 0.046834611 0.233106128

0.349108 0.0858915 0.104067 0.1910213 0.1061512 0.04558803 0.07444556

0 0.5245189 0.09540465 0.2350378 0.1671126 0.1028447 0

0.2777777 0 0.1781469 0.08841965 0.432391042 0.6419547 0

0.06475256 0 0 0.0480076 0.5379899 0.0720351 0.3700206

0.2950822 0.3642208 0.07163751 0.05054578 0.1542317 0 0

0.09579188 0.095128831 0.05965981 0 0.5528277 0.1192743 0

0.1389771 0 0.1235917 0.4926378 0.1761695 0.09846241 0.179809719

0.07248445 0 0.06022259 0 0.048246501 0.07297969 0.09593191 0

0.3032108 0.1557176 0.08889138 0.2615194 0.0876694 0.1218109

0.1667943 0.09190881 0.2919558 0.198960979 0 4.656921 0.05802367

0.141152 0.08954365 0.6648475 0.04651704 0.03640349 0.1443086
0.3605964 0.405272 0.1295721 0.3652322 0.08886175 0.2386857
0.1422874 0.412374 0 0.9418866 0.1887892 0.3491687 0.08096691
0.05837701 0.195079 0.105395 0.04009304 0.2425499 0.05710367 0.2421479
0.1499701 0.2480968 0.3493152 0.2143421 0 0.2836231 0.33802
0.1950926 0.5270695 0.2861826 0.12574 1.381006 0.08798195 0.07215408
0.6019112 0 0.09654368 0.04328006 0.141571315 0.06500755 0.1836048
0.1447338 0.07099744 0 0 0.261727222 0 0.057784785 0.1217153 0
0.8231567 0.1731296 0 0.2427311 0 0 0.06742497 0.04059452
0.04290556 0.039415698 0.1225054 0.408462 0 0 0.2459153 0.03716033
0.06804632 0.3416817 0 0.6613067
AC092171.1 0.1966333 0.08084178 0.09099118 0.042028101 0.04153013
0.03051305 0.225043 0.03085099 0.1042555 0.06833396 0.03737627 0
0.1398053 0.056377813 0.036091077 0.03173389 0.1545226 0.07558244 0
0.0762226 0.041743101 0.1704628 0.02062882 0.06421405 0.02669952
0.2544693 0.04166014 0.5110027 0.03301696 0.054559196 0.173751
0.227917516 0.2076595 0.1812142 0.165771 0.085608097 0.214113 0.157576
0.2029223 0.2034842 0.1953788 0.05164342 0.1877066 0.05659761
0.03827399 0.2914879 0.2221621 0.2809338 0.1207086 0.240713 0
0.06666081 0.1052805 0.156562052 0.3102343 0.1563278 0.4407665
0.02027876 0.1900299 0.1275568 0.2655196 0.1723149 0 0.1360822
0.03123034 0.179565188 0 0.4001051 0.317283 0.02955575 0.06046181
0.06948966 0.09666707 0.165098022 0.237131 0.06770754 0.05989855
0.1009212 0.249003 0.2247436 0.2102101 0.09735896 0 0.100194 0
0.3918415 0.03844238 0.1234936 0.1214404 0.3936626 0.2690555
0.192449468 0.08929864 0.07071726 0.16776 0.28825523 0.2734077 0
0.04102054 0.02912487 0.1415161 0.2939657 0.4661518 0.2289595
0.08950194 0.07718185 0.06496067 5.973811 0.29041403 0.1296427
0.1058993 0.1630886 0.121124 0.2362257 0.1143016 0.1855673
0.3527098 0.1091855 0.04957344 0.2352175 0.04624061 0.149414
0.1520738 0.1122043 0.2849804 0.1208207 0 0.06424459 0.081817873
0.1158393 0.1145142 0.0441047 0.1084174 0 0.2594054 0.1028093
0.3515081 0.1239707 0.1183216 0.04469197 0.08235321 0.03575153 0
0.05686384 0.1187579 0.09912638 6.699193 0.2296415 0.1463707
0.04139641 0.1210951 0 0 0.105323882 0.1338635 0.4333603 0
0.0560566 0.2267824 0.1498133 0.08478014 0.1341965 0.05998883
0.188318945 0.3050515 0.03326896 0.2411815 0.2214984 0.2327502
0.1336773 0.05466076 0.1380675 0.0669925 0.036233491 0.1573688
0.1696407 0.960518 0.07998267 0.1760588 0.1007486 0.5728652
0.1431014 0.1750734 0.206664 0.4066324 0.025351 0.05741288 0.1257321
0.07072638 0 0.09530984 0.61483 0.136603958 0.2863088 0.05022112
0.1006893 0.04985342 0.09122366 0.4359769 0.049569281 0.05663752
0.1532964 0.1573611 0.04213712 0 0.08507998 0.1848591 0.1236237
0.2057032 0.2341418 0.2564469 0.05629497 0.1653777 0.1353836

0.1264342 0.4284717 0.02120392 0.08986676 0.185327127 0.081560169
0.3456789 0.081928256 0.175967315 0.05590171 0.168882388 0.2208491
0.2715797 0.06344 0.10330967 0.2787339 0.1334711 0.06810989
0.14892886 0.2811003 0.4369958 0.2662942 0.03389014 0.1198803
1.094555 0.1112466 0.3603427 0 0.02156064 0.0582511 0.286927868
0.1917696 0.027486458 0.176809 0.09306604 0.1400522 0.1793986
0.274664 0.1942483 0.1435311 0.02788271 0.06217024 0.126354055
0.07123082 0.3117943 0.07997283 0.3059014 0.1405924 0.1982028
0.287281 0.03026089 0.07639833 0.1328776 0.4432583 0.1159361
0.09792395 0.2258535 0.166770805 0.1528449 0.1682875 0.2525958
0.05525431 0.2336781 0.1252481 0.05580181 0.02142588 0.4426139
0.159405 0.278022194 0.069188871 0.1381596 0.2039495 0.03088842
0.02834877 0.03150703 0.08118666 0.02946185 0.08583982 0.877491
0.1132693 0.1046433 0.03968092 0.1221025 0.1095149 0.04497161
0.3685653 0.08460202 0.07873224 0.256678347 0.2722005 0.05786283
0.07687754 0.09641099 0 0.1424927 0.8782525 0 0.1708417
0.2802693 0.1965553 0.085051705 0.0900158 0.1831117 0.09678237
0.02527803 0.113729 0.112941799 0.08853896 0.06095176 0.2625381
0.1062064 0.1436297 0.08250037 0.05189327 0.1222786 0.9650597
0.2353021 0.1948327 0.042695854 0.1290859 0.08272804 0.1787483
0.326302389 0.286403533 0.1155269 0.7403191 0.1150913 0.08999684
0.1540632 0.263841 0.2794402 0.1561284 0.1205168 0.06600889
0.2000512 0.1733124 0.047243324 0 5.475772 0.1722217 0 0.2126216
0.1841795 0.0276137 0.1080502 0.2141632 0.1070296 0.1443479 0
0.0197101 0 0.07084497 0.1689308 0.1335249 0.07401266 0.3531334
0.06404003 0.06909182 0.02403201 0.06930816 0.02895097 0.03128259
0.04760051 0.1079879 0.06779639 0.1916603 0.2670782 0.1472766
0.06912081 0 0.09828814 0.1894117 0.04013145 0.2702279 0.2844381
0.09707731 0.1119637 0.08198004 0.05222833 0.06424876 0.5359648
0.0330235 0.1146216 0.1027686 0 0.05788521 0.3542257 0.2291137
0.1685836 0.08341263 0.024527633 0 0.08984421 0.274420176 0.1806332
0.4109202 0 0.01712903 0.1546784 0.08645484 0.315061 0.1913808
0.08005036 0.04819589 0.4075174 0.023398163 0.07272232 0.2424734
0.3525607 0.08585939 0.1668361 0.1544152 0.04039403 0.202831
0.03187782 0.239903
AC129507.1 0.1491021 0.1332615 0.2024892 0.290976515 0.06161333
0.4627457 0.1907827 0.5187262 0.1947716 0.07885033 0.01232239
0.287169333 0.3028883 0.37173812 0.440251097 0.1046218 0.366795
0.261643 0.07918461 0.03141179 0.055048258 0.0786786 0.07481109
0.007056798 0.008802423 0.1426209 0.05493885 0.03369396 0.02177038
0.080943011 0.6955799 0.081971926 0.1293174 0 0.1093044 0.014111843
0.1676508 0.0129876 0 0.2236188 0.02147114 0.01702605 0.07735499
0.1679343 0.06309175 0.122308 0.02441448 0.07203745 0.01326526
0.03174376 0.009071096 0.03845984 0.03470938 0.06021881 0.07159568

0.03435926	0.0581256	0	0.06264995	0.3469417	0.02501079	0.02840479	
0.0317115	0.03364816	0.01029617	0.011839981	0.3801788	0.007328252		
0.04754699	0.087696724	0	0.02290968	0.02390225	0.174176968	0.05412355	
0.05580536	0.02962146	0	0.04560695	0	0.01980087	0.08024442	
0.03090896	0.005505407	0.025181328	0.04306139	0.03168467	0.008142793		
0.02001853	0.01236043	0.0887035	0.028198959	0	0.0233144	0.08296189	
0	0.141646	0.09779781	0.04057154	0.04801013	0.0266604	0.01615267	
0.02364356	0.03774223	0.02950743	0.2883844	0.057110782	3.275322		
0.121857376	0.0997295	0.04655119	0.1152168	0	0.07139	0.03768351	
0.07137516	0.08456948	0.0179984	0.1144053	0.03323469	0	0.009851907	
0.03008185	0.09248014	0.03523263	0.07966552	0	0.1270828	0.134870531	
0.006365073	0.1208115	0.08724389	0.04467944	0.400767849	0	0.101684	
0.0496658	0.04904549	0.009752204	0.02946852	0.0135753	0.01178673	0	
0.02812071	0.04894084	0	1.452651	0.03785465	0.01930247	0.2593076	0
0.2153787	0.2212338	0	0.1176874	0.1530775	0.057596199	0.2587341	
0.09345838	0.06914763	0.02795072	0.05530318	0.0197774	0.008869412		
0.02234907	0	0.04543649	0.03319303	0.01918355	0.290871	0.08649994	
0.04551875	0.09570776	0	0.01297052	0	0.09356104	0.008789689	
0.09029054	0.05812674	0.111602	0.1297404	0.272104	0.01946684	0.05865147	
0.2799876	0.0378563	0.03108898	0.05440729	0.02500924	0.05237034		
0.1238723	0.036028995	0.08390368	0.01655714	0.04426099	0.02465387		
0.3909757	0.01512999	0.024513355	0.2334066	0.0303237	0	0.06945983	
0.321211013	0.02243966	0.01218905	0.1222705	0.01356344	0.017154		
0.01951076	0.03711919	0.02973954	0.01115848	0.1945227	0.03259859		
0.04893426	0.0395036	0.015274882	0.21511316	0.0854738	0.045017496		
0.019337914	0.04607485	0.03340677	0.01820265	0.1790714	0.0209152		
0.102178872	0.07876656	0.0146678	0	0.135023834	0	0	0.3862898
0.005586532	0	0.06647387	0.05501446	0.02096461	0.4540473	0.02132465	
0.1024240	0.009459575	0	0.045309332	0.4728067	0.1165933	0.05276924	
0.05914499	0	0	0.01183001	0.5607427	0.01024829	0.241610639	
0.07827905	0.03426461	0.01054633	0	0.05407636	0.01633611	0.1157593	
0.01995311	0.277061	0.1752309	0.01043825	0.01911118	0.2421303		
0.01861511	0.007854542	0.01259766	0.4068667	0.2664864	0.06072168		
0.03301719	0	0	0.007063786	0.0416923	0.04204273	0.009165968	0
0.03036608	0	0.02036688	0.09346159	0	0.05353201	0.08741812	
0.08490024	0.0933211	0.05601473	0.05749886	0.02616439	0	0.03610541	
0.1828596	0.02025173	0.027892	0.02595682	0.186170469	0.03589614		
0.009538246	0.1013814	0.01059508	0.01142784	0.01879107	0.3158689		
0.07048969	0.008046274	0.01155006	0.0712814	0.007010063	0.6133213		
0.01509228	0.2446255	0.05833646	0.1499789	0.018617595	0.1050838		
0.006698289	0.07212901	0.1517301	0.03382323	0.01359956	0.025662631		
0.1451283	0.04820689	0	0.957077	0.014076172	0.01418588	0.02727417	
0.1414334	0.047811967	0	0.0142828	0.01251651	0.009485963	0.03956078	
0.02031692	0.01739687	0.01023635	0.02573657	0.2145558	0.03264319	0	

0.008162637 0.046726212 0 0.9990376 0.1703366 0.05524949 0.01752452
0.06939564 0.01820763 0.1781125 0.01412127 0.01176201 0.03965775
0.04226411 0.01299624 0.01739107 0.05839127 0.01113878 0.02201057 0
0.04850946 0.01055651 0.0569463 0.01584598 0.3084729 0.009544695
0.08250714 0.01569316 0.01186732 0 0.03159373 0.007337635 0.01618495
0.02278808 0.01398291 0.05670718 0.0346923 0.1587687 0.03818144
0.05626495 0.008001228 0.08612968 0.00900919 1.205322 0.01412122 0 0
0.02834174 0.1185843 0.069267051 0.1335871 0.1167828 0 0
0.1581243 0.032345547 0.845170372 0.2270887 0.033927057 0 0.01042109
1.772093 0.1129436 0.02549755 0.03800379 0.08789067 0.1261907
0.03518851 0.08739198 0.0503821 0.007714018 0.09590176 0.01998495
0.02113343 0.2972186 0.09625582 0.02909048 0.2996392 0.009552898
0.4834443 0.04314129
AC005387.1 0.2103194 0.2594057 0.4379597 0 0.177682887 0.1305473
0.2750932 0.2639863 0.1486826 0.1096352 0 0 0.6835937
0.482414696 0 0.4073117 0.7933331 0.7275884 0 0.7337508
2.411019872 0.4375858 0.7060683 0.366312 0.628273 0.598798 0.4901576
0.8307858 0.1412601 0.875349604 0.5309841 1.241067386 1.92498
0.2215167 0.8510835 0.366266462 0.4007776 0.6741744 0.7596616
0.8705888 0.2089776 0.718093 0.301157 1.331812 0.4093794 1.020358 1.42575
0.2670999 0.2582203 0.3089606 0.9417447 0.3565028 0.3002887
0.111639418 1.592771 0.4458895 2.325795 0.1735216 1.129202 0.7503928
3.651433 0.1843084 0.2743525 0.7277689 0.6680808 1.152379997 0
0.9510073 0.7404363 0.758709539 0.6467009 0.4459578 0.8788602
0.635721191 0.8584597 0.7966215 0.3844057 0.2158911 1.242893
0.5609012 1.220566 0.6248122 0.1337046 0.2500581 0 0.119747
0.6578889 0.8453699 1.363876 0.7218213 1.203454 1.143579937 0.6208412
0.3025574 1.196243 1.952683514 1.086196 0.2820331 0.7897618 4.236676
0.5189689 1.414918 0.6903651 1.224478 0.8137175 0.3302155 0.416892482
0.4167132 0.508299556 1.848881 0.528594 0.2492 1.122806 0.3790013
0.570534 0.2646443 0.480148 0.3503553 0.3181432 1.797065 1.187018
0.1278509 0.2602536 0.4400513 1.333569 0.1938452 0.295258 0.6871616
0.350050333 0.9912154 0.4899386 0 0 1.083515349 0.3329528
0.4398601 1.93358 1.325993 0 1.051658 1.981917 0.6118386 0.5240015
0.3649302 0.6986305 0.3816929 0.577085 1.719376 0.876727 0.7969986
0.4317454 0.6352338 0.5468594 0.600824874 0.1909076 2.118963
0.160166055 0.5995826 0.9702679 1.922888 0.2418161 0.1435369
0.5133136 0.748155096 0.3625375 1.850397 0.8844625 0.4738304
0.8713256 0.2859631 0.7249694 0.7088511 0.04777022 0.542575939
2.104025 0.3628962 1.167468 0.1140662 1.046183 0.4849242 1.169772
0.3826538 0.2140102 1.199976 0.3805681 0.3796168 0.7369073
1.344834 0.6556255 1.054793 0.1359248 1.315247 0.116889523 0.8846833
0.6983162 0.5025885 3.35937 0.5854382 0.8835573 0.159058248
1.151013 2.623459 0.2103922 0.1802798 0.735607573 0.3640069 2.135438

1.1239390 0.5280495 0.7791424 0.8861876 0.2408529 0.3537767
0.7964362 1.081874 0.4935477 0.4989547 1.666109 0.396452632 0.567040122
4.3444433 0.525783891 0.18821504 1.016474 0.289019177 0.5314966 0.4979696
2.442801 0.884002058 2.470257 0.9041532 2.816886 0.318589295 0.7015529
1.3503 0.6266236 0.7974774 0.4274143 0.677796 0.9519168 0.6801588
0.3801481 0.09224526 0.8722772 1.841392199 1.128145 0.29399578
0.8405125 0.4379921 0.4280005 2.149114 0.8078984 0.5713631
1.535212 1.670111 0.8644666 1.081188675 1.47298 0.8893225 0.2737252
1.459783 0.2005039 0.9539916 0.4097019 3.107244 0.3268633 0.3553155
0.4063801 0.3720165 0.4189587 0.3019668 1.936680123 0.4495789
0.8400032 0.7024604 0.2758006 0.6427096 0.4688798 0.7162289
0.2291717 0.8115785 1.023 0.71369561 0.444027439 1.034431 0.8725796
0.3964599 0.6670821 0.3369999 0.1736749 0.06302493 0.06120964
0.6055267 0.2423061 0.895414 0.5093136 0.2176685 0.7028246
0.3527461 1.51117 0.6786788 0.6736974 0.384361241 2.445628 0.2475608
0.08222839 2.406166 0.4449067 3.292067 0.1707965 0 1.044187 1.498883
1.513699 0.500343235 1.026998 0.58757 0.5520994 0.6488985 0.7906913
0.362408414 0.2651641 0.3477021 0.7020284 0.4543945 0.2194666
1.147154 0.777072233 0.3662106 1.188629 1.39822 0.541823 0.685013733
1.104564 0.1769722 0.8412344 0.310234469 0.673943313 0.1235679
0.3248603 0.1231019 1.15513 0.593231 0.2257639 1.12914 0.4453207
0.154686 0.6354293 0.3890458 0.3177863 0.202126267 0.3863138
1.023536 1.768403 0.5377408 0.5116961 0.5628539 0.826999 0.2311415
0.7330221 0.6868746 2.573247 0.7130148 0.3794755 1.015599 1.439743
0.144551 0.5712742 0.3166564 1.510849 0.6849737 1.625817 0.3598657
0.8154538 0.8670488 0.468439 0.254568 1.386049 0.4350911 0.5125006
0.9046137 0.8401459 0.7393177 0.2268251 0.1051292 0.7653594
0.2575482 1.569055 1.095248 0.6749217 0.319351 0.7599457 0.6703624
0.8704611 3.184828 7.700197 1.042096 0.8244113 0.629228189 0.3302088
1.340654 0.428856 1.081904 0.267655 0.367287363 0.166181915 0.2562602
0.366900555 0.6182583 1.758085 0 1.502342 0.3722498 0.4931856
0.6739798 0.900686 0.8562205 0.3093026 0.3269111 0.400427657
1.477895 1.296751 0.3428179 0.3673416 0.4907328 1.368492 0.9073165
0.7438234 1.091091 0.6998203
AP005899.1 0.1905247 0 0.1259492 0.058174926 0.1437141 0.2956513
0.6230051 0.2135183 0.1443096 0.07094045 0.4138872 0.463726496
1.050523 0.117056507 0 1.317773 0.2994444 0 0.06044707 0.9495599
0.693365192 0.1415719 3.283737 0.5629353 0.5543585 0.2113405
0.7496528 1.188307 0.9140357 0.226561074 0.2748624 0.43020193
0.06387565 0.931673 0.4589176 0.08887348 2.593267 0.4907593 0.8426499
0.8449833 0 0.393164 0.4546881 1.488496 1.271484 0.953668 0.03416831
0.129622 0.2227783 0.9995784 0.1142558 0.2998817 0.3886089
0.108355906 0.1717695 0.3967105 0.5287578 0.1684181 0.7306602
0.9710965 0.2100171 0.2385167 0.6213278 0.09418186 0.3458301

0.347973568 0.09121096 1.292251 0.3194039 0.286375659 0.3766082
0.2885609 0.1338057 0.27423267 0.5807227 0.3748807 0.7876549
0.6519064 0.8042249 0.207392 0.9560473 0.2021451 0.1297722
0.2311461 0 0.6198669 1.117444 0.5470041 0.04202419 0.05189565
0.135427 0.503175172 1.452375 0.04894311 1.044954 0.798000693 1.18941
0.091246 0.5678026 0.6853447 1.119345 0.4747221 0.545975 0.5942319
0.3716634 0.07122295 0.599453241 8.718294 0.255810888 0.2990837
0.5863396 0.09674822 0.614749 1.335174 0.2109538 0.08562021 0.3550675
0.1133502 0.2058574 0.4186105 0.6400585 0.7445433 0.4630983
1.837861 1.158748 0.334478 0 0.3557072 0.453006314 0.05344789 0.09510572
0.09157401 0.3751759 0.21032945 0.4667867 1.04359 0.1390156
0.5147973 0.2456695 0.3711734 0.3704759 0.2474347 0.1825707
0.3148417 0.2465755 0.1372099 9.024025 0.1192004 0.121563 0.2865028
0.1117459 0.08220672 0.3980815 0.437365166 0.1852927 0.4284668
0.172728098 0.1551861 0.2354326 0.3317924 0.1564692 0.5108225
0.8303602 0.670292801 0.04691662 1.381518 0.4292244 3.065962 0.4429849
0.7401397 0.5447588 0 0.216371 0.601848941 0.7079425 0.1408891
0.8158544 0.3321339 1.028952 0.2789107 0.1441736 0.4951991
0.2769543 0.3677945 0.5980355 0.280725 0 0.3480747 0.06526589 0
0.4837325 0.4728012 0.226903192 0.6605102 1.876922 0.09291553
0.5520533 0.6313549 1.588093 0.274453447 0.1567943 0.2546299
0.3539539 0.5832583 0.317320914 0.2355339 0.4605846 0.2566782
0.6264117 0.4321294 1.63833 0.389615 0.3953975 0.6090395 0.8167091
0.4562205 0.6750564 0.4975711 0.38479226 0.846711495 1.256025
0.340213106 0.121786202 0 0.140259307 0.5731826 0.3759182 0.4683365
0.762668443 0.5511729 1.447205 0.1885542 0.669974547 0.1945483
0.4704667 0.2580215 1.806052 3.595309 0.9170182 0.5389529 0.8802055
0.1229891 1.014698 1.290091 0.158865209 0.4645302 1.674046558 0.5982472
0.2576424 0.4708006 0.248322 0.5702812 0.2688767 0.2483431 0.23157
0.9896382 0.489714871 0.3943883 0.863166 0.3320931 0.1954274
0.1297378 0.06858763 0.2209177 1.130945 0.4758745 5.195966 0.08765061
0.4011943 0.06777273 0.2344683 0.395730304 0.4495789 0.2329421
0.6643178 0.5098835 0.6931182 0.1300255 0.8882642 0.1482876
1.269086 0.8384588 0.577253803 0.383082497 0.2549858 0.2823052
0.3847993 0.9417629 0.6977881 0.1498371 0.1427329 0.1188187
1.528064 0.07839316 1.834721 0.7415007 0.845066 0.9095377 0.89224
0.08502748 0.5855268 0.3632682 1.030346521 1.431754 0.6407457
0.1330165 0.04448375 0 0.1183423 1.989277 0.1183812 0.6080853
0.678906 3.019977 0.35318346 0.08306605 1.457404 0.1786204 0.3848859
0.118067 1.328830853 0.3431536 0.08436889 0.6662387 0.14701 0.5112281
0.2854907 0.502811445 0.1354056 0.8500718 0.2533246 0.2157485
0.768289912 0.2382393 0.1145114 0.4948437 0.150554963 0.911805659
0.2998338 1.10357 0.07965419 0.8304856 0.1279518 0.2921651
1.418262 0.1440744 0.100091 1.279165 0.1762149 0.6168793 0.163484481

0.1666452 8.5361530.1907102 0.4639333 0.4782520.3641996 0.8791207
0 0.7114627 0.2962989 0.1998051 0.1064683 0.4638033 0.1460338
0.1470945 0.2338325 0.4004520.6659098 0.6517389 0.6648274
0.4303633 0.2328543 0.09593575 0.1202210.6062151 0.2306087
0.7972045 0.1876863 1.0611780.6469528 1.8007541.1959550.4990152
0.1360496 0.2330506 0.0555496 0.2137412 0.9449201 0.2687471
0.1033194 0.1891267 0.3253229 0.02964419 0.2060771 0.1828433
0.7536264 0.2133771.163279004 0.4006210.03771662 0.3171372 0.05833797
0.1731885 0.339509327 0.358431582 0.08290771 0.28488749 0.2500309
0.3937791 1.3527611.0195240.2141044 1.03714 0.7715697 0 0.2031425
0.1334246 0.9166331 0.485812966 0.3019848 0.7551666 0.3549174
0.2971145 0.5195994 1.6488532.3483491.8048660.1765 0.5132015
EPB41L4A-AS1 5.6409893.8137184.1593363.217713635 3.343243794 7.99087
7.3406447.1067892.6449865.2432574.5027564.639483746 4.5018249.489604881
4.421070629 6.64561214.412227.21631512.734387.5434734.333255989 3.715641
3.6232450.8531214 5.3027441.9597034.2449053.5348673.4794593.427684765
4.3764272.085553088 10.9368 3.7541242.5308545.069898915 2.5914942.10236
2.2504263.4518083.9229137.36118 3.6772863.9954371.9564034.0515975.369492
3.4722995.6899074.4663784.9503554.1204212.9317661.698094306 11.65937
5.9843074.7045232.3927725.42017 8.1789222.19513 4.1388552.7651852.068396
9.0648022.482671293 5.1265795.62095415.036052.921697261 5.8339235.273255
2.5413483.145146943 4.11157 2.0277644.7207726.04495 3.8501653.9424
2.5696132.298941.9633477.2761273.319447174 0.8865481 2.1381391.407096
9.1266898.4254697.7164235.031751722 6.9775512.4523072.8646889.044007853
5.6552577.2363755.38516 7.0633138.6039583.6186614.0937448.0235533.058369
4.3507342.398960245 4.0940242.544470291 2.5884346.9074923.0113853.682077
9.20685 2.3336134.0253797.2780342.26809 2.37770211.970354.2482734.131602
9.5814414.8679363.4251655.9037778.9882754.4774 3.389961123 13.3162
6.2144841.5443442.7892265.212279098 2.0386063.1098883.7540852.657569
2.3912613.4216623.0876171.7550114.6757065.63401 1.81176 3.3749692.156475
3.3159310.079064.4743785.76721 3.2363495.3606612.102887059 3.978917
8.3922092.090588503 4.80928410.6474110.633232.6854313.2862397.226914
2.071814112 3.3277132.82429 2.3120166.32529610.586934.1961325.9499110.2286
2.1873731.803147105 6.3873777.5100422.0891543.5360524.2728322.557904
5.3768495.3732662.1682612.8919093.62255 4.5354224.5830465.3156333.158682
9.5486558.1769513.0227613.303667044 4.7063728.4306832.6905493.771924
4.6013311.873772.410988178 3.7431834.0525542.2678065.6456056.839859889
2.7319683.6714544.4261641.90839 3.9660112.5941532.6240293.92709 4.001235
6.1780725.4698446.4649258.31031 3.484609975 1.868707123 4.2811882.312219214
2.436889463 10.290623.863730056 5.1844235.70481 2.2380436.630015438
9.1009464.64349 7.5764511.894767913 6.1293576.1446877.3635762.636636
3.8692241.4139671.8286823.4031817.9130842.8596036.0731489.051896387 3.69211
2.779033792 5.4235183.0009791.6939815.2677533.8114253.3735026.30245
10.146246.19476 5.161253308 2.5503151.2481710.1386410.856974.822646

9.880228 4.693602 4.081664 2.911664 7.914184 1.90357 3.198037 3.5832 13.52175
7.210244005 2.172606 6.240024 3.253501 2.488427 7.043496 2.354975 3.342401
2.2579443.089694 2.936189 6.498386347 6.302073654 11.77022 5.419179 4.576677
5.285588 2.809515 2.900675 2.242361 4.123597 4.551011 3.698357 6.896259 4.592758
1.370166 4.722488 3.493705 6.480396 4.829336 4.296299 3.427461896 4.572528
3.693868 4.648068 8.155638 3.652919 4.383005 4.000234 5.815973 3.302928 12.02262
4.594209 2.829692365 6.790351 4.731485 4.039043 4.149535 3.751782 4.077624499
2.755315 5.677609 4.00033 4.049691 3.687039 2.795897 4.533895137 2.74245
2.541887 11.7215 3.689661 3.264030347 6.181684 4.927279 3.799642 2.465547619
1.380132719 2.481113 4.898552 2.332458 4.201701 5.342548 4.788571 4.579483 6.24035
2.192743 2.24444 2.797035 8.541204 1.606371912 3.93769 3.56741 2.993876
3.891545 5.033175 4.194742 2.219839 2.710438 8.246499 3.454457 9.707913 3.683429
10.98482 5.784756 2.472688 2.883413 3.177087 3.641549 13.35246 2.851654 4.115106
9.535088 2.262982 8.207627 3.007871 1.779297 4.239202 9.075847 4.143163 3.623466
7.196513 8.070236 3.595775 6.899797 6.511479 4.500315 6.084976 4.85496 3.470245
3.412013 3.944333 2.851981 4.750162 7.066967 23.05226 11.65986 3.083588 6.263895653
2.072495 5.890289 1.786363 10.48688 2.826813 1.513334397 3.072907698 3.156047
1.714777332 11.16932 3.630053 1.430432 5.727801 2.137715 3.160281 3.558177
5.972971 7.733023 1.703877 4.679991 1.412034369 7.385382 5.111928 7.238871
7.491835 3.827246 2.746919 1.450797 2.65558 6.352732 5.274435
AL157871.5 0.05555905 0 0.06427426 0 0.058672023 0 0 0.04358498 0
0 0.1056072 0.047329684 0.2539429 0.079648205 0 0.1793292
0.3056243 0 0.03084729 0.2692102 0.058972899 0.09632909 0.2331482
0.1511982 0.3017596 0.3595035 0.1765671 1.010692 0.1399349 0
0.07013374 0.23417621 0.03259696 0.2560118 0.1873555 0.181415283
0.4159236 0.1669625 0.4300202 0.287474 0.04600382 0.1823991
0.198888 0.5197317 0.05407188 0.1871826 0 0.04409905 0.08526599
0.2040415 0 0.1177195 0.1983145 0.147456192 0 0.5153245
0.2905917 0.05729795 0.1193183 0.1351552 0.6430541 0.0811464
0.2264824 0.04806279 0 0.15220929 0.09309336 0.5966545 0.3259957
0.041755122 0.1281269 0.2945162 0.1365672 0.279892257 0.4123183
0.09565437 0.2538665 0.2376287 0.2736075 0.1058361 0.3818262
0.206317 0.04415013 0.2123249 0.053953238 0.1581649 0.1629293
0.4187198 0.04289148 0 0.1727774 0.120837565 0.09461803 0.09990638
0.3555065 0.203617438 0.7725177 0 0 0.0411464 0.05712228
0.03460853 0.2026337 0.2425983 0.09483343 0.07269285 0.214138644
4.403243 0 0.1221025 0.3490902 0.06582994 0.2281586 0.3893513
0.1076537 0.04369361 0.04529941 0.03856319 0.1050529 0 0.06532679
0.08443435 0.3867183 0.2906155 0.3522823 0.04267262 0 0.1815241
0 0.1091019 0.4206302 0.0934639 0.03829188 0.071556736 0.256534
0.2420744 0 0.03502811 0.1671597 0 0.05817258 0.05050825
0.3194375 0.08033485 0.08388809 0.02800832 4.128738 0.1622139
0.04135727 0.1169662 0.05702604 0.04195165 0.1805765 0.049599052 0
0.1749238 0.035258569 0.0395972 0 0.12699 0.2794723 0.1421904

0.2542491 0.152028052 0.1915395 0.1880040.04867586 0.3129237
0.2055123 0.3021658 0.1081114 0.1170336 0.3154806 0.204756613
0.1667430.04793226 0.1233618 0.07533077 0.6080031 0.0711667
0.4782345 0.1010838 0.07066752 0.04170945 0 0.1074445 0.04055525
0.1776291 0.09991926 0.1071691 0 0.6755824 0.077195332 0.1348283
0.03547515 0.09483311 0.2465079 0.08591797 0.3241736 0.035014699
0.04000756 0.0866283 0.02778914 0.1785887 0.080967438 0.1201974 0
0.1309878 0.1453044 0.2940318 0.4180354 0 0.06371962 0.1912642
0.0595403 0.2328180.2396480.16928 0.261822376 0.086418584 0.4273155
0.115744796 0 0 0.047717988 0.3120063 0.1096218 0.1792507
0.145951559 0.2250192 0.2199895 0.2245198 0.210400435 0 0.1371932
0.07524185 0.04787857 0.2258159 0.2034660.07858225 0.4192399 0
0.1218399 0.1371579 0.162143853 0.06773102 0.232990277 0.1387713
0.1314798 0.1130628 0.1520681 0.1455127 0.3773355 0.2027747 0
0.2634945 0.178507699 0.4696156 0.04894333 0.3841397 0.2991909
0.03310383 0 0.04509539 0.1710053 0.1349154 0.5162407 0.04472977
0.1637896 0 0.07976908 0.572187907 0 0.2377495 0.07137136
0.02602032 0.5187767 0 0.1576689 0.1816175 0.2233237 0.09008029
0.157111229 0.048873564 0.3578412 0 0.04363786 0.6407993 0
0.07646473 0 0.1212709 0.8397848 0 0.2463929 0.1401488
0.08625064 0.3867953 0.5082720.04339113 0.4482082 0.07415306
0.290099506 0.1922767 0.04087309 0.1629140 0 0 0.7895734
0.1208244 0.2413580.09898817 0.6386755 0.090118106 0 0.2586925
0.09115338 0.1071352 0.04016789 0.292525616 0.2251514 0.1148135
0.2781771 0.1000293 0.02898771 0.1165531 0.109968946 0.1727501
0.2478901 0.07387219 0.02752514 0.271435085 0.06078902 0 0.05050563
0 0.040461887 0.08160581 0.5363547 0 0.08476251 0.1305924 0
0.2193230.03676194 0.2724177 0.04662729 0.05138616 0.4197403
0.100115074 0.0425211 9.0127460 0.1479712 0 0.1486863 0.1560459
0 0.3025607 0.4536208 0.1019643 0.1086656 0.6404489 0.07452383
0.4003472 0.07159751 0.1257589 0.05228098 0.7483380.2940375 0.19522
0.1358057 0 0.08180144 0.1325842 0.16812 0.4068286 0 0.2030772
0.09432922 0.1733885 0.2929529 0.2097175 0 0.05946507 0
0.4908428 0.2411053 0 0.05272586 0.3474539 0 0.1210239
0.3365282 0.1399626 0.2833820.07259357 0.178092996 0.1635556 0
0.3641425 0.05954195 0.08838139 0.207909652 0 0 0.193844646
0.1531146 0.1786248 1.3806790.4355851 0.1092616 0.08142651
0.1711942 0 0.1130918 0.03404456 0.2518790.066111883 0.102739
0.6422931 0.09056053 0 0.2651614 0.09349343 0.1997345 0.4093587
0.04503564 0.3081135
AC108449.2 0.7778771 1.5990420.9309288 2.694595144 0.821461463 1.956318
1.9296391.0521192.6547392.7265810.6118332 1.051113391 4.2229250.538346835
2.018550417 1.5151211.8971010.4639694 1.4892761.1957420.569430351
2.8834160.6472292 0.7007708 0.9833838 1.0066750.6251285 2.648882

0.81071 0.29770344 0.9819359 1.384959256 0.4091745 2.295426 0.7236266
0.32114668 1.606429 0.2149542 1.799281 1.110316 0.04442036 0.4931385
1.088239 1.54413 1.879585 2.168877 0.4377505 0.298068 0.74098 0.7224006
0.3377997 0.9320739 0.7180817 0.711903535 0.5501601 3.056605
0.4810104 1.051189 0.9792964 0.6525154 1.604043 0.9794165 1.049697
0.5569014 0.2556135 0.58788081 0.1348336 3.305095 4.249461 0.483814779
0.5773446 0.3791718 1.054932 1.486420562 2.388757 0.9236191 0.7762396
0.7342383 1.358691 0.3065795 0.5735079 1.394508 0.3836742 1.890708
0.312576913 1.145406 0.5244042 1.853075 0.6212271 1.815596 0.8341518
0.554221929 0.2131762 0.3617534 1.907055 0.196608866 1.864819 0.3596944
1.175105 0.1589205 0.7170295 0.9356842 0.7337214 1.561653 0.7325539
0.5966213 0.767995167 22.76422 0.828340017 0.5305485 1.540912 0.8898967
0.9362976 2.282552 0.1559223 0.4218967 0.08748039 1.079839 0.5748094
0.4583819 0.6307823 1.752854 2.157465 2.806124 0.3887504 1.606949
0.2259365 0.569647 0.89288201 0.2370298 1.468396 0.5113988 0.2957909
0.345468662 1.663155 0.7947232 0.4110025 0.4058692 0.3631636
0.182897 0.1123405 0.5364672 0.7196989 0.6205576 0.5265042 1.46039
18.89047 0.5482067 0.3594036 0.9035217 0.4405055 0.08101532
0.6974438 2.346700051 2.799978 1.308997 0.408539502 0.5735138 0.8507421
0.2861108 1.503465 2.059442 0.7364934 0.770674815 1.10968 0.680748
0.329003 0.9888635 0.9128173 1.021178 1.461462 0.2260105 1.584033
0.494272077 0.9660219 0.5091066 0.5657992 0.6546408 0.7471869
0.2061514 2.237826 2.049694 0.6482336 0.6443808 0.3466873 1.556193
0.234956 0.3859089 1.25424 0.4139211 0.5633986 1.491037 0.596305972
0.9113126 0.411049 0.4120602 0.6460624 0.5392442 2.003299 0.135237931
0.5794573 0.2509396 0.9123093 0.9196884 0.390902575 0.7892093
0.5043438 0.7588747 0.7856969 1.206622 1.641496 0.6143495 1.35358
0.7848937 1.149818 2.742613 1.128071 0.24518 0.821633715 0.584108219
1.178878 1.639158796 0.480084739 0.4194147 0.276453126 0.4142421
2.857913 0.2307745 0.234879774 1.249325 0.5765615 0.7432862 0.660264771
0.8308246 0.4636483 1.017128 0.7859198 0.5451081 0.3143402
0.9105291 1.069854 0.4848266 0.8529344 1.562755 0.821954778 0.7193967
0.374951139 0.535979 0.812507 1.364639 0.3426124 0.7025203 0.8611849
0.1957952 0.1141069 0.4240416 1.447852661 0.4534513 0.897915
2.029137 1.123472 0.415537 0.4393584 0.6096047 0.5366375 2.136454
4.078403 0.08638031 0.6721458 0.133581 0.1925585 1.494981148 0.7036887
1.14783 0.5513179 0.7537409 3.460903 0.7688463 1.141814 0.9645139
0.8625472 0.6088579 1.02399805 0.660678508 0.5654032 0.5564276
0.5477658 1.662871 0.3008579 0.6275787 0.5023727 0.5074191
3.475197 0.8498273 1.379889 0.7036894 0.6940156 3.809513 5.316752
0.7960544 4.818292 0.7518073 2.065842199 1.188214 0.5919933 1.441976 0
0.2837086 0.3110058 4.846661 3.383301 2.63014 1.194762 1.045695 0.464086479
0.409311 0.7493647 1.05619 0.4482728 0.5429942 0.770336726 2.294795
1.302623 2.118973 0.1448794 0.8956782 0.6189769 0.283156756 0.9341025

0.9574308 0.7132949 1.461774 1.397825124 0.4695732 0.2821296
0.4389049 0.544034358 0.234415066 1.083458 1.916205 0.2747492 0.6956821
0.5043896 0.2879308 1.312998 0.425959 0.9864038 0.3151566 0.6450267
3.85028 0.451122393 0.4105751 16.31723 0.5638387 0.7715412 0.652598
1.507469 0.5650303 0.2947891 0.8764395 2.238703 0.5579098 0.2798007
4.355718 0.8635043 0.1449627 0.5530648 0.5160787 0.4543331
2.609317 1.7035 0.5183764 0.2950452 0.2363634 0.1974645 0.4267353
0.9739995 0.3928254 0.5548987 0.9150736 2.094895 0.8036178
0.9900428 1.128208 1.474856 0.6316009 1.040146 1.263861 1.202833 0.2979588
0.101822 4.622367 0.4987237 0.9640805 0.9748345 0.4504836 1.368139
0.8411385 1.261061877 0.6843457 0.7434 0.625082 0.22997 1.024071
0.803013365 0.565179074 0.4902369 1.3102072 0.7392219 0.6036678
5.332624 1.285143 0.6330044 0.8648616 0.5620255 0.3132821 0.4185967
0.5588366 2.397348 0.574526638 0.09920273 1.81921 0.3060519 0.4099319
1.735345 0.8726618 0.9367464 1.343913 0.2174275 0.7735212
SH3RF3-AS1 0.1569266 1.062636 0.2242587 0.53271404 0.243704708 0.8164905
0.7244349 0.3910421 0.8320295 0.5613907 0.105278 1.116644151
0.8438376 0.979265743 0.762435259 0.5810032 0.3046716 0.4790108
0.1127541 0.09840272 0.489908905 0.09602881 0.09684228 0.06029075
0.200546 0.1075149 0.09778705 0.1343389 0.325497 0.307354674 0.8389814
0.204265455 0.02166356 0.1215303 0.2801572 0.221038613 0.1633379
0.4808326 0.1667088 0.4776298 0.1070076 0.04848815 0.3745062
0.5579663 0.05390332 0.1492793 0.1970003 0.01465386 0.3494453
0.3390091 0.07750019 0.6962917 0.1482722 0.036749135 0.101948
0.1100825 0.1793293 0.01903978 0.4262245 0.2844382 0.1958763
0.5797364 0.4816563 0.1437389 0.01466112 0.101156548 0.8197613
0.05217496 0.06770407 0.37462466 0.09934358 0.1957321 0.06807074
0.201514282 1.472868 0.524459 0.4217918 0.03158506 0.1818364 0.1055062
0.1409763 0.1371159 0.1173667 0.3292536 0.197211868 0.04379775
0.1985151 0.5681476 0.05701037 0.8800259 0.02296518 0.200768149
0.04192137 0.3651815 0.03937759 0.135321813 0.5317424 3.249359
0.09628573 0.1777453 0.06643492 0.7130133 0.1178346 0.120921
0.05252101 0.3985644 0.660743972 0.2743448 0.161123495 0.08114791
0.03314305 0.4046858 0.09476975 0.147862 0.2325226 0.3339402 0
0.0512573 0.1163616 0.1577474 0.02170772 0.1262567 0.2427303
0.1668025 0.04180763 0.6522738 0.09071268 0.211118 0.556937937
0.03625393 0.6128508 0.7867905 0.1654142 0.190223143 0.487113
1.061807 0.04714742 0.2095135 0.04165966 0.02098071 0.05799124
0.1342688 0.1061473 0.2002111 0.2090665 0.111684 4.917934 0.06737845
0.123685 1.243751 0.09474713 0.08364175 0.06000452 0.032962961 0.2723171
0.508604 0.105445981 0.09210546 0.1330791 0.1828581 0.3184012
0.6299873 0.1408092 0.176813174 0.06364748 0.09370895 0.04852413
0.02835893 0.2458461 0.3388769 0.2566057 0.09074237 0.4927123
2.194272197 0.5725467 0.07963807 0.09223291 0.3629638 0.1653021

0.1891863	0.1711388	0.08397391	0.3170125	0.06929905	0.3698574
0.5474488	0.1212865	0.01475628	1.361306	0.1602525	0.1193156
0.06414062	0.487379757	0.1642765	0.04715275	0.2048312	0.1872301
0.3283256	0.09694893	0.116351836	0.05317713	0.1583235	0.2677909
0.118688	1.560490878	0.3275155	0.1041388	0.2031236	0	0.0366394
0.06482502	0.105711	0.4728785	0.015889	0.3363433	0.1083097	0.1791758
0.02812538	0.456760888	0.354168931	0.08113972	0.743585751	0.220288259
0.393647	0.063425655	0.1814363	1.019948	0.1687646	0.129330309	0.1869315
0.1148734	0.04263238	0.227223288	0.2199382	0.02279425	0.2125207
0.5886616	0	0.3380529	0.05222486	0.03980314	0.5839682	0.02024335
0.3463834	0.148168549	0.3150928	0.129035554	0.507242	0.1747599
0.1033178	0.08421893	0.1128237	0.05699382	0.1179165	0.2356124
0.05837181	0.640624512	0.3232476	0.09758152	0.09761272	0.2098855
0.2420047	0.02326164	0.2847139	0.09944213	0.7262723	0.04678468
0.07431722	0.5986899	0.4597053	0.132534	0.111843976	0.3497971
0.1843398	0.2134466	0.09511045	0.156715	0.01469947	0.02619623
0.1508761	0.6381989	0.3891311	0.143569691	0.178644456	0.1405283
0.1196805	0.9570403	0.0931586	0.02958204	0.4192451	0.4909972
0.5775992	0.5581114	0.132936	0.2128748	0.1490261	0.305713	0.5655313
0.04222397	0.1730235	0.9730551	0.1478438	0.373543807	0.5878105
0.3531292	0.3157898	0.06034703	0.1301805	0.1605441	0.5434822
0.1806716	0.2406057	0.0328932	0.08304582	0.109801009	0.4084944
0.04298102	2.33231	0.4390718	0.08008536	0.353471216	0.6151581
0.2289111	0.1335196	0.3157721	0.1541192	0.03872991	0.462865969
0.8151351	0.2608462	0.06136827	0.2743934	0.180392644	0.1615987
0.2330208	0.3524373	0.374447588	0.228569303	0.47455	0.1069366
0.1080595	0.07041524	0.2314406	0.0495442	0.07287978	0.4275523
0.6789213	0.1549398	0.008537664	0.5927784	0.210695211	0.0847771
1.073167	0.274889	0.04916998	0.04990774	0.654651	0.01296329	0.2130421
0.1407549	0.1842324	0.1129405	0.1323995	0.0277588	0.2971661
0.0498874	0.1903315	0.4492315	0.01737267	0.2210386	0.1052229
0.3567877	0.1692279	0.1138788	0.02718215	0.2202848	0.3240188
0.05069505	0.01591355	0.3149131	0.1044835	0.115232	0.08112214
0.1592867	0.02307075	0.1580792	0.9231485	0.2718404	0.04005896
0.03417981	0.05256151	0.05131419	0.5761885	0.2211856	0.139783	0
0.2017847	0.2050406	0.098632135	0.3894981	0.07675002	0.1882261
0.03957089	0.2447386	0.126659839	1.507379102	0.1124733	0.096620196
0.2204761	0.014839	6.652481	0.1769074	0.2723024	0.2705756	0.4664721
0.1976568	0.04384304	0.5543278	0.0239137	0.746932387	0.2560469
0.227659	0.2256956	0.201534	0.07832144	0.08284622	0.1611858	0.1496303
0.3741271	0.2866762
AP003171.1	0	0	0	0	0	0	0	0	0	0	0	0.094837298	0	0	0
0	0	0	0	0.05394325	0	0.6755711	1.576709	0	1.436052	0	0	0
0	0	0.07026557	0	0	0.1465674	0.09385382	0	0.1515292

0.1115176 0.1436095 0 0 0 0 0.6408722 0.108347 0 0.06987804
0.08836388 0 0 0 0 2.682278 0.369333413 0 0 0 0 0 0 0
0.1625979 0.09076324 0 0 2.744917167 0 0.314619 0 0 0 0 0
0.09347279 0 0.09583417 0.2543437 0.1904603 0 0 0 0
0.08846623 0.189088 0 1.901547 0 0 0 0 0 0 0 0.2001883 0
0 0 0 0.116122 0.08244748 0 0 0.1015073 0.08101809 0 0
0.061297474 2.20576 0 0 0.09992755 1.055259 0 0.1114523 0 0 0
0.07727135 0 0 0.5235967 0.1691861 0 0 0.1008413 0.513034 0
0 0 0 0 0 0.9974602 0 1.762397 0 0 0 0 0 0 0
0.2666983 7.485184 0 0 30.16462 0 0 0 0.3427994 0 0 0
0.8842037 0 0 0.3173731 0 0 0 0.6648024 0 0 0.9594977
0.3767147 0 0.2280086 0 0 0 0.07816904 0.06321473 0
0.2227419 0.1920894 0.3089841 0.4528342 0.2768845 0.7130047
0.2211385 0.2025476 0.1416007 0 0.5036089 0.03588215 0 0
1.001071 0 0 0 0 0 0 1.045125 0 0 0 0.070161032 0.08016552
0 0 0 0 0.4335241 0.627965 0 2.911551 0 0.05584282 0 0
0.2874356 0 0 0 0 0 0.057720683 0.9785572 0.077308241 0
0.07912403 0 0.0781482 0.1098279 0.1197251 0 0.5636054
0.1259446 0.1285382 0.421591849 3.050361 0 0 0 0 0 0
0.3600239 0 0 0 0 0 0 0.05561286 0 0.1132753 0
0.1943816 0 0 0.07893112 0 0 0 0.09807065 0 0.3996711 0
0.07013472 0.2710809 0 0.05406761 0.6582686 0 0 0 0
0.134885517 0 0 0 0 0 0 0 0.06065296 0.08949738 0
0.078703275 0 0 0 0 0 0 0.07660846 0 0.5669946 2.403895 0
0.0987424 0.3369895 0.230434 0.1550089 5.007403 0 0.1197468 0 0
0.5393867 0 0.05440676 0.2729229 2.551245 0 1.582115 0 0
0.09917424 0 0 0 0 0 0.3577886 0.08048679 0 0.2005108
1.207807 0.1238667 0 0 0 0 0 0 0 0 0 0.1218066
0.1170944 0 0 0 0.2452776 0 0 0 0.08722527 0 0 0 0
0.1868597 0 0.1401764 0.06686884 0 3.310885 0 0.237199 0
2.234488 0.07816961 0.6729171 0 0 0 0 0.1115918 0 0 0
1.196956 0 0 4.35087 0 0 0 0 0 0 0 0 0 0.1260087 0
0 0.06003191 0.2782367 0 0 0 0.322078 0 0.9508495 0.0773571
0.5913974 0.1818771 0 0 0 0 0 0 0 0 0 0 0 0 0
0 0.4090731 0 0 0 0 0.1631591 0 0 0 0 0.3605035
0.198708461 0 0 0 0 0.4722842 0 0 0 0 0
AC005785.1 0.6275905 0.2229812 0.3943903 0.04968157 0.294557502
0.6372294 0.278692 0.547036 0.095854 0.1278982 0.2945508 0.158409553
0.4485748 0.877484816 0.312864881 0.7377507 0.9376635 1.578451
0.4387869 0.9986417 0.411205591 0.3358412 1.089215 0.4723144
0.5260266 0.4512136 0.4842583 0.8537355 0.4033045 0.376218633
0.7042 0.636815918 1.972892 0.2754179 0.3265976 0.910778768 0.3058337
1.552259 1.039459 0.9888844 0.6543809 0.1933183 0.4807586 1.42729
0.3167067 1.033707 1.585438 0.3197931 0.2933073 0.6070351 1.116689

0.2101333 0.4978098 0.29817247 1.22243 0.4619892 1.4704670.2477064
0.4659096 0.7162305 2.2718330.2150104 0.1642382 0.6568581
0.7629616 0.693397879 0.2336833 0.4116553 0.4205234 0.267857685
0.5241289 0.7666771 1.1331820.28623902 0.5678143 0.4935635
0.4366388 0.4109198 0.5886948 1.4169080.7454702 1.0549770.1600818
0.1644996 0.406300917 0.1617514 0.1666239 0.2919645 1.10059
0.5392151 0.7517580.522396411 0.4134443 0.2298866 1.14579 1.628016066
0.8618565 0.8571680.3717607 4.2691490.3505054 0.8590919 1.737895
0.5751394 0.8023209 0.2432985 0.230370728 2.6097450.426523588
0.5789485 1.6969380.2019680.5090886 0.4343783 0.9458147 0.1462398
1.3518950.9895261 0.2246369 1.0857250.3279672 0.3650206 0.4074719
0.5010736 1.1229210.2380377 0.5982412 0.3797188 0.515825086
0.6770625 0.3790294 0.2519922 0.2136009 0.33928602 0.2657582
0.4996283 1.3257040.9378955 0.3613266 0.6163563 2.4256380.5494061
0.3192564 0.7170022 0.1871789 0.2421671 2.3916873.8230621.061222
1.2233710.2624362 0.5616384 3.6892260.179838738 0.2637342 1.731983
0.167178383 0.3423676 0.7372208 1.0743750.2449796 0.7006343
0.8036809 0.614834539 0.5475815 0.9569657 0.6380842 0.5316025
0.7795488 0.2844367 0.4695334 0.5657950.08799119 0.171326962
2.6353710.3342215 0.2494509 0.1996009 0.8478955 0.4168335
1.2620290.7894163 0.1773899 1.6402880.8311786 0.5593935 0.656057
0.6935993 0.2786864 1.9428951.7275471.7093090.23683738 1.103085
0.6629267 0.3967508 3.5359140.6350332 0.1537068 0.195320088
1.8857982.2711940.2867763 0.2656557 0.587151623 0.5095716 1.281999
0.54801 0.8672797 0.6765731 1.0804460.2440038 0.2784302 1.24029
0.9631758 0.4805236 0.7436018 1.2983890.273844832 0.257100187
4.9546180.408912455 0.208011598 1.6300130.292800171 0.8919773 0.6114959
1.7998240.705598015 2.6045590.4470342.3885510.32275713 0.2584475
1.5497230.3147874 0.6142788 0.2519306 1.9748660.5698550.9688556
0.7877481 0.4672601 0.3748981 1.537609059 0.8689844 0.162459206
1.2385570.4693918 0.2128581.9792990.7981749 1.0524321.49874 1.626039
0.9063972 0.497878616 0.9168685 0.4914310.3025157 2.6796020.4985842
0.9957588 0.9181667 0.9181355 0.2634063 0.8508234 0.3493182
0.1142071 0.5209031 0.4449704 1.17345459 0.2710177 0.7515256
0.7663924 0.1524047 1.5653130.2837744 0.4067750.3123739 0.7723657
0.5904242 0.372471178 0.681570114 0.9708406 1.26572 0.6572396
0.5026678 0.6207430.2452593 0.3714876 0.4509847 0.2453793
0.3570560.7696843 0.3283487 0.4650885 0.7551717 0.4548227
0.5688076 0.5750480.4860305 0.313534415 4.6120250.2165995 0.2877779
1.4309290.6282862 1.7180990.2044911 0.05054897 0.6731755 1.44947
0.7667506 0.284863129 0.6148018 0.4329140.6991526 1.0458440.6721974
0.289269301 0.1465271 0.3122223 1.3965630.4324396 0.3314853
0.4551119 0.500969645 0.2120008 1.1408011.2259270.2226353 0.395355562
2.8653551.1898160.6057240.32857905 0.090282307 0.1536354 0.2917113

0.3174497 1.193883 0.886311 0.4990198 1.85962 0.3281065 0.1804536
0.6502445 0.3726371 0.3219436 0.753927803 0.094877 2.848927 1.370798
0.5200131 0.3141759 0.425071 1.055433 0.4427853 1.147673 0.5623115
1.023805 0.8991397 0.5747181 0.8522079 1.158488 0.2928841 0.7453569
0.189563 1.75093 0.2775738 0.5581035 0.9942914 1.133357 1.608483
0.3574662 0.2719655 1.730405 0.45414 0.2265623 0.859444 0.8414648
1.675013 0.1671218 0.2130091 0.1741477 0.7906589 1.095214 1.591514
0.7841622 0.3823522 2.465793 0.607103 0.4894471 1.30233 8.471071
2.517941 0.3543257 0.662295948 0.3497345 0.7730426 0.5078178
2.690324 0.2218553 0.086982609 0.499966104 0.3776184 0.094614649
0.4982301 2.017732 0.1925437 0.8909246 0.1523716 0.1362648
0.4583812 1.478049 1.140791 0.4652757 0.5218717 0.295029901 1.103222
1.170401 0.5430551 0.4059788 0.7560007 0.4606831 0.3342495
1.061826 0.7536577 1.374983

LSAMP-AS1 0 0 0.1238994 0.057228137 0 0 0.08755226 0 0.09464066
0 0.4580451 0.045617941 0 0 0 0 0 0.4116715 0.05946331
2.439054 0.056840064 0.09284522 0.1685371 0.02914598 0.07271152 0
0.311999 0 0.08991599 0 0.1013959 3.18811007 0.03141804 0 0
2.739381709 1.020425 0 0 0.1847181 0.1330201 0.4570863 0.9584745
0.1541338 0 0 0 8.670845 0.1643645 0 0.4121197 0.0226924
0.04778554 0.035530809 0.08448697 0.5321647 1.64048 0.08283854
0.1725045 0.08684473 0.1549493 0 0.08731653 0 0 0.635719213 0
0.1210686 0 0 0.123493 0 0.2961631 0.629462332 0 0 0.04078084
2.656801 0.8664825 0 1.104051 0 0.2978736 2.114674 0 0 0.8113567
0.1345254 0 2.016517 0.03330574 0.145584137 0 0 0.2284327
1.439191123 0.1063684 0.1346415 0.05585617 0.03965828 0.3303382
0.1000706 0.2441315 0.07794145 0.6398256 0.2452233 0 0.08841654
0.179748286 0 0 0.06344911 0 1.0722 0.7263218 0.08422675
1.833766 1.040718 0.06750235 0 0.4407491 0.2848323 0 3.183012
0.2667826 0.08225861 0.413468 0.3061783 0 0.1840231 0 0.4504183
0 0.896594256 0 0 0 0.1688064 0 0.1217109 0 0 0.5387981
0.4258618 0.1212812 0 33.30466 0 3.786845 0.05636799 0.9343814
0.04043441 0.04351141 0.430247107 0 0 0 0.1526605 0.7720034 0
0 0.3197784 0 0 0.2307653 0.04530113 0.656816 0 0.07923188 0
0 0.0376003 0 0.049337828 0 0 0.2675255 0 0 0.1028893
1.418272 0 0 0 0 0.03451953 1.05539 1.284037 0 0 0 0.4185958
0.297613831 0 0.03419214 0 0 1.821834 0.09373483 0.371231788 0
2.254372 0 0 0 0.06951019 0.3524023 0 0.7002465 0 0.6983887
0.3449467 0.08188682 0 0 1.256628 1.299267 0.1223683 1.577207577 0
0.0588373 0 0.359412445 1.294028 0 0 0 0.02879465 0 0.2168811
0 0.1854855 0.050697754 0.127588 2.578512 0 0 0.3808857 0
1.363324 0.02886268 0.06049374 0 0 0.039069925 0 0 0.1605029
0.5068986 0.5176252 1.563396 0.32725 0.0330626 0 0 0 0.172051724
0.06466161 0.1415197 0 0.1602057 0.2871593 0.3036212 0 0

0.05201441 0 0 0.4735979 0.06666974 0 0.129763282 0.2341387 0
0.03439506 0.02507926 0.04545586 0 0.4938914 0 0.2152469 0
0.378572717 0 0.5016718 0.1388553 0 0.3474135 0.2145099 0
0.7221085 0 0.6166955 1.310995 0.7124452 0.6213688 0.02771042 0
0.5103017 1.463764 0.2015991 2.644435 0.034950958 0 0.5909228 0
1.312793 0 0 0.2717919 0.6987275 0 0.715561 0.6691054 0 0 0
0 0.03442017 0 0 0.409905 0.2489874 1.608699 0 0 0.449351 0
0.03330048 0.07964162 0.4984035 0 0.029068696 0.468724 0 0
0.345577636 0.038998528 0 1.809349 0.3526102 1.266303 0.08391292
0.07185253 0.2959472 0.2834591 0 0.94376 0.2724024 0.1348533
0.932777999 0.2458996 4.343394 0 0.08557178 0.1809494 0 0.1128017
0 0 0.9715888 0.1965533 0.03491184 0.1878697 0.1436571
0.9646702 0.9661132 0.4242374 0 0 0.1308015 0.2351995
5.137592 3.20873 0 0.04259636 0.03240794 0 0.6923691 0 0 0
0.1411789 0.6063982 1.773319 0 0 0 0.0387309 0 0.8131034
0.03720975 0.9245264 3.120305 0.6487144 0.5396027 0.7413612
0.1399362 0.05721734 0 0.07420557 0.1559879 0.05738853 0
0.033398386 0.282078525 0 5.231351713 0.6395004 4.777568 3.326863 0 0
0 0.06600108 0.5211926 0.07266778 0.2296931 0.2427694 0.031860428
0.04951167 0.2476255 0.1309279 0 0 0.03003737 0.02750154
0.8285627 0.04340687 0.1781821
CARMN 0.4634589 4.027011 0.1302715 3.669956642 0.082553859 0.602208
2.930546 4.145332 3.907951 2.210548 0.8738717 0.575568816 1.452862 0.832506468
3.622974781 0.2688445 0.3568919 0.1573977 0.1178091 0.02705639
0.045439898 0.1468337 0.02050305 0.04153531 0.005054616 0.2661659
0.02366066 0.02902213 0.05000482 0.082630949 0.6050082 0.075509317
0.02074855 0.03063084 0.1569146 0.014181027 0.1697394 0.06525635
0.1512639 0.5874696 0.053941 0.02688639 0.1465845 0.6080632
0.003622918 0.05894547 0.06892935 0.01181888 0.006665146 0.08430553
0.0325556 0.02129605 0.05481071 0.024699607 0.08516138 0.01726386
0.005562911 0.01631605 0.07195097 1.08215 0.01974768 0.1427203
0.04704171 0.07084654 0.02660566 0.052691266 0.1543764 0.008416205
0.009556019 0.43783565 0.0143079 0.006577713 0.02401943 0.042194945
0.08028837 0.05287444 0.181435 0.02069804 0.3051005 0.01418244
0.1691326 0.06220632 0.1005768 0.03240404 0.025304781 0.06535031
0.03638858 0.1905403 0.02155358 0.01419547 0.03588688 0.11132474
0.01373577 0.02175523 0.006616557 0.040928257 0.05176015 0.1497558
0.194145 0.04135329 0.02392063 0.04985499 0.04582185 0.02438181
0.02541608 0.06575253 0.126054731 15.95904 0.116207097 0.04908658
0.03174317 0.4090952 0.0210197 0.01956545 0.02344225 0.03659443
0.0166933 0.01808664 0.0539637 0.03498799 0.03282764 0.01272884
0.06765612 0.03363309 0.02444656 0.1887036 0.002612968 2.041773
0.305914509 0.01279256 0.201617 0.1064584 0.2976128 0.134244215
0.07120851 0.2189624 0.1473513 0.04224509 0.06860011 0.1057607

0.0107186	0.07106711	0.01248503	0.04171504	0.05480144	0.01032136	
7.820209	0.02445444	0.04295073	0.07053262	0.008596917	0.04918969	
0.07108141	0.02658584	0.03801353	0.2373345	0.015355539	0.08357225	
0.08318324	0.03261617	0.6366557	0.0508108	0.02555276	0.077669517	0
0.003149155	0.01956825	0.007624173	0.04681703	0.3834026	0.07191927	
0.02483132	0.8518532	0.109752633	0.119169	0.01445198	0.01756413	
0.02397469	0.02407207	0.1382808	0.07764172	0.01354561	0.439158	
0.03074073	0.4197901	2.925187	0.05298693	0.005950742	0.4686345	
0.03231238	0.01052542	0.05981485	0.043963979	0.3191926	0.06655325	
0.09848696	0.06724571	0.2058008	0.06081668	0.01524933	0.002680582	
0.05804259	0.01396443	0.07977184	0.811033164	0.1570423	0.2834723	
0.06874867	0.01362994	0.02955102	0.01867277	0.01332185	0.01138489	
0.02402825	0.3271236	0.0124794	0.01906753	0.0141776	0.07455602	
0.026055936	0.02454081	0.045238198	0.059686211	0.2658985	0.007992988	
0.01698532	0.9823763	0.01901605	0.068453208	0.03957636	0.04211346	
0.02793747	0.084583355	0.1042156	0.009192202	0.1575421	0.01844574	
0.009456306	0.0654365	0.03685611	0.04213479	0.1072347	0.001020438	
0.1792018	0.078763593	0.04084295	0.122284505	0.8144995	0.4290175	
0.01325699	0.05094425	0.0129995	0.02183462	0.2768207	0.254693	
0.01324097	0.417416299	0.1134992	0.04591013	0.05223317	0.01447792	
0.1064649	0.01289844	0.1042409	0.05155954	0.1120909	0.02515571	
0.004495468	0.09053727	0.1436731	0.09887656	0.010148206	0.03436128	
0.06106371	0.04303814	0.04445697	0.01737951	0.01037371	0.04093594	
0.03042179	0.07780815	0.02715997	0.035527776	0.009823863	0.1220598	
0.007239504	1.274785	0.2066235	0.0730682	0.3573489	0.05368409	
0.03520997	2.047053	0.12062	0.1700405	0.007512189	0.06067904	0.6349423
0.005675864	0.2907288	0.03703798	0.06334704	0.12634189	0.05539647	
0.01369287	0.3147316	0.01368903	0.01148387	0.1106015	1.936624	
0.02023865	0.0358082	0.008290492	0.01488433	0.033209457	1.238337	
0.02166611	0.06565507	0.1316014	0.0497895	0.163925331	0.1106275	
0.02500133	0.08180182	0.09215459	0.07380479	0.003904637	0.153502641	
0.2245469	0.09827055	0.01361133	0.07838004	0.018186663	0.03462032	
0.04502725	0.3570091	0.463304272	0.060998012	0.1175565	0.01078103	
0.05174769	0.05253304	0.02624982	0.03995922	0.01469506	0.07635669	
0.2030591	0.04217557	0.01377188	0.005859039	0.081612768	0.01424497	
10.22806	0.0766197	0.1150065	0.08427852	0.281434	0.0248315	0.01125053
0.02229935	0.04221312	0.05009987	0.04004438	0.02705277	0.04493913	
0.04526553	0.02158726	0.05792934	0.0140117	0.01114224	0.0181856	
0.1308011	0.04208396	0.0196816	0.006851063	0.05033921	0.06195396	
0.01703643	0.01604358	0.06576501	0.01896071	0.0360138	0.0228998	
0.01505514	0.01395556	0.04083879	2.820565	0.0566394	0.03365523	
0.02412136	0.2914504	0.01810672	0.0593256	0.05676182	0.06200707	
0.03282216	0.06509872	0.07174261	0.021876372	0.08675504	0.04900553	
0.02168732	0.0359048	0.3089164	0.051077909	0.278202383	0.09213126	

0.019481935 0.02222776 0.007480126 1.572638 0.07782673 0.07595257
0.08183594 0.2695528 0.1340556 0.01389183 0.2030134 0.01326
0.031007346 0.1479997 0.02582091 0.01820318 0.04876338 0.169767
0.06681852 0.02102978 0.0137139 0.2670463 0.1228329
AC020765.2 0.4455656 0.6341027 0.1982534 0.366286574 0.289557297
0.5318595 0.560375 0.9679498 0.302872 1.101766 0.5211913 0.817532637
0.6266275 0.442213472 0.943631292 0.2765697 0.7002879 2.898377
0.7992447 2.856495 2.546619069 1.36678 2.55296 0.4477147 2.187321
0.975819 1.633859 2.63649 1.035907 0.47549855 0.692246 1.48074442 1.689156
0.9024753 0.5778962 0.820708182 0.6997704 3.570627 1.237967 2.009878
1.305465 0.2250434 2.781055 0.9372014 0.3335684 2.771343 0.6023746
0.6529109 1.016942 0.6713218 0.7673475 1.220032 0.3058496 0.54579271
1.297814 0.5903908 1.075593 0.388817 2.723468 1.55637 1.190097 0.2502953
0.447093 0.4743975 4.518206 0.688582616 0.6891495 1.123598 1.055807
0.515173144 0.9484946 0.363373 1.642837 0.23022002 0.9856389 3.363513
1.305081 1.40729 3.95446 1.632252 0.1046879 1.527319 0.3268336 1.07697
0.266269223 0.2601911 0.167518 0.4305125 1.958016 3.104129 4.135539
0.857261404 1.712177 1.694882 1.462075 4.187040673 1.633936 0.9192189
0.7865118 4.162834 0.5990567 1.579895 3.562625 2.594079 0.7800342
0.5829731 0.377433522 0.7922695 1.196491135 1.129872 1.599766 0.8122073
0.3518766 4.632238 1.992341 0.3773631 4.079988 3.092643 1.598571 3.104264
0.4835997 0.5729613 1.060292 1.075681 2.390545 0.3685452 6.736256
0.6159004 2.495729228 3.735414 0.2794464 0.1921924 0.6614212
1.500869409 0.2712949 0.5376068 5.251699 0.777916 1.082766 2.181216
3.696315 1.121704 0.1313736 2.279687 0.05175041 0.5874615 0.5485869
1.701181 3.826983 1.515281 1.055378 0.9316762 0.8911782 0.795536639
1.399989 1.726562 3.610656986 1.270227 0.2964707 4.909298 0.4433295
2.105208 0.2091277 0.375143296 1.063443 2.319586 1.861739 0.8072667
1.064954 0.605818 0.5907158 1.636483 0.2724672 0.631569876 3.223055
0.5913864 2.473303 0.09294283 0.9547389 1.141468 0.8623719 3.11792
0.3051626 0.5660706 0.7973807 0.9500463 1.150849 0.4383162
0.4931201 3.834519 0.6091446 3.4532 0.285729945 1.552607 9.016435
0.7605308 2.911059 0.9010448 1.079903 1.166427151 1.579558 10.31408
0.3085752 0.7344734 0.699281273 0.3262581 0.7733272 1.292898
1.362498 1.496448 0.5501552 0.2453131 0.3930852 3.067754 2.718042
0.6893999 1.737102 1.566428 0.161517739 0.248786834 3.464591 0.952036674
0.562321477 3.800151 0.176622831 1.202976 0.946757 2.359028 0.900372467
3.747976 1.512209 2.176521 2.271423678 0.653298 2.369758 1.253247 0.5316516
2.995066 2.309527 0.2908635 1.773451 0.5420631 4.509768 0.3722947
2.300603586 4.345447 1.533133548 1.232752 2.238626 1.115972 4.002583 1.735485
4.867167 1.563642 2.235651 0.7043802 2.598857223 1.034661 2.053127 0.5854678
0.65625 2.368916 0.7773264 1.50224 2.901053 0.2330414 1.389678 2.483434
0.4546868 0.2560303 2.657308 0.996654099 0.7659493 0.9777815
0.5723751 0.3210378 2.152945 1.146151 4.522853 0.821623 7.494577 0.9446962

0.290764878 0.603000226 0.9632795 6.665539 2.153609 0.5435484 1.09837
0.99059 0.6419206 1.296738 0.6907489 1.184608 1.763192 0.4495793
0.3547191 1.049898 1.56776 1.070716 2.285723 0.9148977 0.715847391
2.182519 0.5547197 0.1005014 1.904563 0.3625166 7.500854 0.6262539
0.1490726 1.020983 1.160247 0.4453887 0.704186776 1.202919 0.1595869
0.7310205 1.233708 1.238975 0.524970213 0.185194 0.3187269 5.758366
0.9873263 0.9298882 1.222323 0.587943542 0.1705108 1.121435 1.276005
0.6452479 0.669791205 3.150053 0.8651974 1.308587 0.568763192 1.148199719
0.2265411 0.529402 0.9529001 0.8366373 1.074163 0.2759337 0.9200397
1.043205 0.3781214 1.323157 0.3170003 0.8199671 2.388084415 0.1573871
0.7413262 1.440921 0.9493449 0.463264 0.5044838 0.9626443 1.243027
2.31445 5.596756 1.803179 0.8938077 0.4466254 2.942311 0.6791769
0.2944558 1.241287 1.032065 2.667302 0.5581267 1.204309 3.309241 0.8456558
2.169916 2.780892 0.7467329 9.034984 0.8272102 1.503335 1.086242 1.112415
2.289147 0.2587486 0.08566085 0.9904651 1.259124 1.076622 1.041162
1.438295 0.5854768 1.00027 0.4096659 0.3359675 2.28364 8.404021 1.94797
0.7165254 0.732434929 0.5044856 4.84449 1.247994 2.571192 0.1453928
0.256518158 0.496494117 0.5742127 0.119582403 2.707742 2.258963 0
1.343558 0.3707179 0.5023186 0.7181437 1.534502 2.232516 0.2940284
0.8435114 0.774901669 5.450639 1.109442 2.178798 0.448973 0.3998563
0.5767593 0.2464316 1.060637 0.8890369 7.86909
GRK5-IT1 0.03701622 0.228277 0.08564545 0.0791179 0.117270705 0.2297633
0.242082 0.116154 0.06542035 0.06431934 0 0.18920041 0.1879883
0.424524933 0.135882906 0.05973905 0 0 0.2055201 0 0 0.06417926
0.03883375 0.08058864 0 0.04790384 0.03921261 0 0 0.102707687
0.233633 0 0 0 0 0 0 0 0.09550032 0 0 0 0.2650182 0
0.07205078 0 0.5111579 0 0.1514892 0.09062844 0 0 0.06606351
0 0 0.0980957 0 0.07634952 0.03974791 0 0.2856232 0 0 0
0 0.067606293 0.2480938 0 0 0.111277399 0.1138194 0.1308143
0.09098788 0.062159405 0.03433839 0 0 0 0.1041663 0 0.05653149
0 0 0 0.07189269 0.03512579 0 0.04649535 0 0.1058671
0.09209036 0 0.04202617 0 0 0 0.07352713 0 0 0 0 0
0.06750237 0.1616311 0 0 0.04076282 9.778869 0 0 0 0 0 0
0 0.05822174 0 0 0.233305 0 0 0 0 0.03352972 0 0
0.06047022 0 0 0 0.1245407 0 0 0 0 0 0.1400249 0 0
0 0 0 0.1070462 0 0.1119632 19.55164 0.1621126 0 0.07792875
0 0.05590057 0.06015453 0 0 0 0.046982043 0 0 0 0.05319954
0 0.7905029 0.050644345 0 0.1252576 0 0 0.05476904 0.100659
0.02057978 0 0.1261134 0.341047733 0.07406168 0.3832184 0.04109489
0 0.07365128 0 0.09803807 0 0.04708223 0.1111557 0 0.02386163
0 0 0.04438081 0 0.1196138 0 0 0.1197725 0.09454126 0 0
0 0 0 0.05331007 0 0.1110871 0 0.107889111 0.1281304 0 0
0 0 0.07427096 0 0.02830213 0 0 0 0.03991638 0 0.174439158
0.153537018 0 0.05140998 0 0.105235 0 0 0 0.0398086 0.064826818

0	0	0	0	0	0	0.05012989	0.09569729	0.07522492	0.05422368	0
0	0.1672652	0	0.03655257	0	0	0	0.03698255	0	0.03766405	0
0	0	0	0	0	0.190289207	0.08939468	0.06521698	0	0	0.04411086
0	0	0.05696613	0.1438199	0	0	0.05456242	0	0.05314615	0	0
0	0.09510233	0.1040162	0.06284272	0	0.05252345	0	0	0	0
0.195372073	0	0	0.2325898	0	0	0.05094463	0.02773097	0.05386449
0.9591542	0.1066147	0.06566369	0	0.03830966	0	0.02821968	0
0.03981582	0	0.193278796	0	0.05446339	0	0	0	0	0.9769561
0.08049923	0	0	0	0.080054918	0.05648491	0	0	0.04758589	0
0.035435489	0	0	0	0.2665781	0	0	0.04884454	0.1841516
0.05505227	0.9351296	0.1100317	0.080374945	0	0.07786777	0	0	0
0.1631096	0	0	0.05647302	0	0	0.1168992	0.09797056	0
0.06213086	0	0	0	0	9.4074290.06484146	0.03943433	0.05003251	0
0.05198279	0	0	0	0	0	0	0	0.1333657	0	0	0	0.0553978
0.06027768	0	0	0	0	0	0.04480398	0.06776238	0.1276267
0.09020011	0.0418979	0.04620803	0	0.03992122	0	0.1584744
0.2266424	0	0.05354547	0	0	0.05144247	0.2457996	0.04031609	0
0.4351671	0	0	0	0	0	0.05391332	0	0	0	0.146240086	0
0.064574498	0	0	2.7596330.06449079	0.1091933	0.05425041	0.1368697
0	0	0	0	0.044047042	0	0	0	0	0.03925862	0	0.07604176
0.05454705	0	0
AC024270.4	0.1055094	0.5060772	0.12206	0.150342803	0.037140366	0.272878
0.1150033	0.1655401	0.06215711	0.2138885	0	0.299604767	0.1071667
0.050418638	0.322762247	0.4540735	0.3316547	0	0.2733758	0.4089949
0	0.2439117	0.11069	0	0.2865283	0	0.3353097	0.07311829
0.05905409	0	0.04439582	0.148237435	0.08253766	0.09260554	0.415097
0.267957411	0.3350920.1409201	0	0	0	0.04618468	0.08393292
0.0506152	0	0.3791671	0	0.2233234	0.1439328	0.1722156
0.04921231	0.1788441	0.1255364	0.093342221	0.05548847	0.4194115
0.05255696	0	0.07553047	0.2851849	0.1356880.1541011	0.05734684
0.06084909	0	0.064234008	0	0.3578137	0.1547705	0.052863372
0.4325676	0	0.04322465	0.295294087	0.2610044	0	0.1607017	0
0.3463962	0	0.1074233	0	0	0	0	0.1001210	0	0.2172082
0.4694029	0.1749936	0	0.03992986	0.0316212	0.1500277	0.343715263
0.2794380	0.1467385	0	0	0	0	0.1535687	0.08004152	0.04601578
0.116188562	5.3423750.047221284	0.0772929	0.3788227	0.1666858	0
0.2112564	0.2044398	0	0	0.09764455	0.1330005	0.06010137
0.1654118	0.05344835	0.2175992	0.1337923	0.06371444	0.1080499	0
0.05745389	0.146339332	0.2417216	0.2867479	0.1183284	0	0.090593207
0.2319861	0.1225897	0.1796306	0	0.1058149	0.07993599	0.1104726
0.1278903	0.03370154	0	0	0.03545946	3.2971060	0.05235967	0
0.03609843	0.05311218	0.05715395	0.062794049	0.6384749	0	0.133915561
0.05013137	0	0.05359117	0.1010918	0.3600355	0.1072959	0.144354427
0.06062385	0	0	0.07203123	0	0.0956380.07821293	0	0	0.12961434

0.1407348 0 0 0 0.3498873 0.0450497 0.2794434 0.06398772
0.04473371 0.2112222 0.1363691 0.1360283 0.1026886 0 0.1265011
0 0 0.2443742 0 0.2275962 0.8982543 0 0 0.0543875
0.1231244 0 0.1519527 0.1096743 0.07036394 0.07536639 0
0.06086957 0.06612774 0.2211131 0.2943364 0.0930635 0.07056623 0
0 0 0.3769 0.2358042 0.1137759 0.05357852 0.165737917 0.255287203
0.3091404 0.097691174 0 0.1499786 0.06041256 0.0493763 0.1387849
0 0.061593176 0.3561022 0 0.08121417 0.199780461 0.08379594
0.08684556 0 0.09092379 0.2858904 0.1030379 0 0.3412103 0
0.07712668 0.1736464 0 0 0.147486719 0.1405513 0.1331662
0.03578532 0.3208721 0.06140795 0.1302871 0 0.09974194 0.1111976
0.180797346 0.04246778 0.495711 0.200255 0.3366984 0.04191055 0.1329394
0 0.1082492 0.06832297 0.2376647 0.05662937 0 0 0.05049516
0.085224579 0 0.1003329 0.1355378 0.06588519 0.2388322 0 0
0.2682561 0.3958293 0.171067 0.14918103 0.185626673 0.123556 0
0.2209879 0.3042275 0 0.09680689 0.02634772 0 1.366966 0
0.2495532 0.03548662 0.1455949 0.1958783 0.2144964 0.1098692
0.07565952 0 0 0.1460572 0 0.2062546 0 0.06199809 0
1.213832 0.1529677 0.08730494 0.1253223 0.3164026 0 0.1073348
0.245635 0 0.04521224 0 0.033667923 0.0950164 0 0.2347876 0
0.2201964 0.2951201 0.046408114 0.04374148 0.3138372 0 0.03484774
0.038182872 0.2308828 0 0 0.06484711 0.051226095 0.05165783
0.237665 0.05146304 0.05365608 0.2204458 0 0.05553404 0.04654183 0
0 0 0.08856752 0.211248355 0.05383312 9.603783 0.06160708 0.1124019
0 0 0 0 0.2298312 0.063811 0.2151503 0 0.2467742 0 0
0.1208598 0.1194112 0.06618946 0.4210759 0.08590644 0 0 0 0
0.167856 0 0.4506762 0.1212605 0.08570082 0.1592319 0.2634187
0.1854441 0.0758598 0 0 0 0 0.1017491 0 0 0.2443823
0.09341551 0.07661016 0.159771 0.05906578 0.153759 0.09190587 0.45094331
0.03451113 0 0.6659128 0 0.1118938 0.350960711 0.0926303 0
0.122706884 0.06461607 0.1696087 0 0.06127391 0.06916438 0.05154433
0 0.0684607 0.09545197 0.0862031 0.4099978 0.041849921 0.06503552
0.3794767 0 0.153568 0.07460071 0.1578211 0 0.1554785 0.1140332
0.07801639

AC010422.2 0.1632211 0 0.2157996 0.049838047 0.07387131 0.07236639
0.03812315 0.1097518 0 0.08103224 0.2216089 0.119181361 0.1657849
0.23399012 0.128393297 0.3386781 0.4031215 0.1344415 0.07767687
1.062045 0.34650061 0.08085575 0.6115552 0.3299692 0.506576 0.0603513
0.5928205 0.6059607 0.2740668 0.226442144 0.3826431 0.540541385
2.079429 0.1227935 0.4324666 0.583719148 0.253901 1.027718 0.3609461
0.4825941 0.4247566 0.03062008 0.834703 0.2013449 0.09077263
0.2199617 0.3805327 0.6662776 0.4771315 0.4567103 0.522038
0.07904817 0.04161481 0.154712894 0.441461 0.5252368 1.21957 0.1683296
0.475723 0.8697466 0.5847404 0.1702797 0.07604101 0.6454794

2.814567 0.766559545 0.351629 0.3426622 0.2394272 0.070095999 0.2867889
0.2472081 0.573152 0.078311062 0.5191315 0.6423153 0.3196319
0.1994584 0.4921241 1.332535 0.2848831 1.154509 0.0370583 0.3564383
0 0.2212648 0.159551 0.2635957 0.9360474 0.9780899 0.4930822
0.152141154 0.5029904 0.1257876 0.3978687 0.62667141 1.713703
0.0390849 0.583719 1.208797 0.1678136 0.7843328 1.403199 0.3393829
0.5572024 0.305081 0.154064203 0.5389928 0.187844035 0.2049783
0.6697506 0.2762784 0.383019 0.1167178 0.6325291 0.03667511
1.634988 0.2913191 0.2057493 2.191571 0.6031669 0.03543583 0.2524665
0.4213405 0.8448434 0.2149087 0.7856187 0.2666403 0.291065474
0.9157686 0.2172704 0.1307513 0.2892695 0.240250332 0.2153269
0.121914 0.5359214 0.2352124 0.3858494 1.37792 1.58692 0.4239512
0.2904701 0.404584 0.1760329 0.1645654 0.5864759 0.714827 0.694281
0.2945339 0.1914638 0.2464907 0.6441745 0.249791759 0.105826
0.7341289 0.295949875 0.2991304 0.06723116 0.2131831 0.2010691
0.7558856 0.2134091 0.319019496 0.6832833 1.183537 0.2042853
0.4059272 0.1380007 0.03170362 0.3629819 0.196469 0 0.128899931
0.4198772 0.402329 0.2070923 0.09484559 0.4175506 0.298676 0.3087813
0.1272701 0.1186324 0.07001933 0.7232934 0.2404952 0.8510216
0.2236448 0.08386924 2.833567 0.2260419 2.551786 0.032397726 0.7921961
0.6848659 0.4378004 1.034557 0.4327018 0.1360508 0.117561162
0.3358115 1.70876 0.1399522 0.2498366 0.203884934 0.1412462
1.841371 0.4031397 0.7805709 0.5553033 0.327494 0.1668902 0.5526716
0.4013537 0.1999054 0.6644295 0.3520184 1.136708 0.329648803
0.072537174 1.178506 0.323842396 0.313000129 1.822968 0.160212205 0.2946249
0.3680531 1.27889 0.449193697 1.794306 0.3429262 1.049965 0.309056702
0.2222242 1.093981 0.09473364 0.2813149 0.2369289 0.5806631
0.06595959 0.7540658 0.05268195 0.2301047 0.2072272 0.510370121
0.3979597 0.032594178 0.8153634 0.4414408 0.2372538 3.105941
0.2849909 0.575862 1.191421 0.6282172 0.5529235 0.149834021 0.3660255
2.752465 0.4741696 0.613878 0.527941 0.5875853 0.5677758 0.2511892
0.09059518 0.8272415 0.2628138 0.03437003 0.05806042 0.301301
0.565032245 0.3171832 0.06651994 0.4792558 0.1747254 0.7521333
0.1113919 0.5293702 0.1016295 0.149961 0.5292749 0.560466474
2.010127367 0.9283891 1.692942 0.5494247 0.4370176 0.2988951
0.2567288 0.06987331 0.2714431 0.1342648 0.4029532 0.08272591
0.2823282 0.1447924 0.1298657 0.4799568 0.2549485 0.5517783
0.435693 0.060875211 2.42087 0.2401535 0.09116345 0.9146139 0.4521467
2.26422 0.1893555 0 0.1736475 0.2492631 0.5593931 0.176499031
0.1423242 0.4885622 0.1147671 0.2697783 0.1685786 0.066964704
0.04199675 0.1686492 0.3632122 0.3358464 0.02433142 0.3913246
0.153841072 0.2320021 0.6242147 0.589058 0.04620756 0.177204602
0.6633183 0.6376573 0.3391433 0.257958739 0.237737484 0.06849747
0.2926301 0.3753155 1.138354 0.2923077 0.1251479 1.509564 0.3394256

0.2858241 0.2739629 0.2587920.4110370.812324777 0.1784547 1.071712
0.4901401 0.4222889 0.5672978 0.2808065 0.6876464 0.05125152
0.7618813 0.2961433 0.4279290.9425097 0.3038460.6255307 0.8821035
0.1602581 0.4486227 0.2194155 0.8026138 0.2468063 0.1638619
0.2849780.08218747 1.7165420.2596701 0.1128919 0.8110143 0.3617765
0.3409138 0.1847466 0.4657187 0.3688439 0.1257361 0 0.2246094
0.1903558 0.4119988 0.8432358 1.2375060.04425651 0.5184753
0.3096687 0.4571274 2.1538475.28662 1.4611540.8530601 0.548115084
0.1144030.7754774 0.7471453 0.2498886 0.2225541 0.116342094
0.122826319 0.2130793 0.122030547 0.8567989 1.6867390 0.5687377
0.1604940.1366939 0.6322592 0.4538890.4904503 0.1428799 0.2718253
0.110984673 1.5953670.2515901 0.3800690.1527215 0.1483790.5231706
0.1437010.2405224 0.1890078 1.137933
AC002398.2 0.4262474 3.3203920.4152507 3.116765757 0.260601568 0.9051281
1.9439922.9214482.8547063.1185130.7675726 0.917335323 0.6152343
1.768853886 3.541190887 0.5792877 0.6346665 0.6467452 0.3238498 0
0.047625084 0 0 0 0.03046172 0.1161305 0.04753043 0 0 0
1.6141920.094557515 0.05264902 0.02953555 0 0.097671056 0.1526772
0.08988992 0.1157580.5416997 0.1857579 0 0.1070781 0.4197227 0
0 0.4224445 0 0 0.05492633 0.03139149 0.01901348 0.08007699
0.029770511 0 0 0 0 0.04817929 0.7276536 0 0.06553187 0 0
0 0.122920533 0.8645693 0 0 0.370924664 0 0 0 0.037672367
0.1873003 0.3476167 0 0 0.1262621 0 0.4111381 0.1110777
0.4635094 0.01905205 0.043571327 0.04257672 0 0.0281790.1385525 0
0 0.073189116 0.1018816 0 0 0 0.0445619 0.7896926 0.0936014
0.03322883 0 0.11179600 0.06530549 0 0.02935249 0.02470474 0
0.210850186 0.1479105 0 0.1329067 0 0 0 0 0 0 0.2262352
0.03833739 0.15826900 0 0.06400747 0 0.27569100 1.7957820.186693511
0 0.1567803 0 0.7730898 0.173362456 0.0295958 0.3909868
0.05729126 0 0.06749707 0.05098947 0 0.04078924 0.0214975 0 0
0 0.05129644 0 0.1001974 0.09445909 0 0.2032748 0.1458292
0.040054992 0 0.1412642 0 0.03197774 0.1293691 0 0.6126008
0.0382765 0 0 0 0 0 0 0 0.1220109 0.06236296 0.03150449
0.4331166 0.041339119 0 0 0 0 0 0.08620875 0.02970851 0
0.3138816 0 0.4639306 2.7621640.09825431 0 0.4303593 0 0
0.1169108 0 0.3266523 0.2864887 0.03829246 0 0.1387705
0.02617947 0 0 0 0 0 6.604121322 0.1747233 0.2109074
0.1763042 0 0 0 0 0.03430562 0 0.1923332 0 0.02419174 0
0 0 0 0 0.100381355 0.9566815 0 0 1.37218300 0 0.04543001
0 0 0 0 0 0.36458100 0 0 0.06346112 0 1.16578800
0.2215307 0.196415168 0.05469793 0.03135955 0.5379280.3610116 0 0
0 0 0.2046951.39971200 0.922614336 0.0270893 0 0 0 0.0534677
0 0.1092538 0 0.2832815 0 0 0.1322725 0.2234446 0.03220979
0.027181475 0.02179777 0.06400024 0 0.02101338 0 0.03572417 0 0

0.03607016 0.10912 0 0.039469106 0.2627126 0 2.572584 0.09703012
0.03594666 0.3396309 0.01680665 0.03264514 0 0.4523048 0.07959236
0 0.04643595 0.7496795 0.05130852 0.1401665 0.07239241 0.02994211
0.29284666 0.0310556 0 0.4385514 0 0 0.130057 0.04554574 0 0
0 0 0.024259066 4.142227 0 0.03680663 0.08651979 0 0.064428163
0.04040596 0 0 0.04039062 0.02340977 0 0.029602752 0.05580353
0.03336501 0.0298287 0.02222863 0 0 0 0.6933811 0 0
0.01647572 0 0.06565436 0.06845215 0 0.1204074 0.03542399
0.02968805 0.05499948 0 0 0 0.053900338 0 0 0.0785957
0.2867951 0.1212909 0.09005662 0.03150472 0 0 0 0.1097919 0
0 0.06018364 0 0 0.1269498 0 0 0.01826596 0.1182412
0.02741834 0 0 0.03569059 0.02715392 0 0.03867476 0 0
0.02800486 0 0 0 0 3.983412 0 0 0.0276891 0.04258013 0 0
0.04886799 0 0.03767681 0 0 0 0 0 0 0 0.2617071 0
0.502238677 0 0.039136059 0.08243444 0 0 0 0.02205925 0.03287904
0.1106018 0.9170621 0 0.08248069 0 0.026695177 0.04148478 0 0
0.1469366 0.04758621 0.02516768 0 0 0.6546545 0.0746475
HAND2-AS1 0.1431088 5.487256 0.1864699 4.79424369 0.17339854 0.8742646
4.748541 4.268472 6.16598 4.851598 0.8876531 1.095921256 1.422965 1.19423282
5.272737856 0.4935203 0.05445472 0.217139 0.1304756 0.01313869
0.057562926 0.007835498 0.01422337 0.001639814 0.008181803 0.4971199
0.06861907 0.01879101 0.01264715 0.014629236 1.188488 0.082541813
0.003535289 0.02181586 0.2260551 0.047548683 0.496197 0.04526962
0.1204806 0.550809 0.08232386 0.007912808 0.05752082 0.6829113
0.005864342 0.008120325 0.04160406 0.004782743 0.001541248 0.09589336
0.01686306 0.04596198 0.0376392 0.011994224 0 0.02195651 0.02251142
0.004660668 0.0355867 0.9405676 0.02615331 0.2288182 0.01228152
0.05994523 0.009570228 0.112803257 0.0555302 0.001702892 0.008838932
0.434739254 0.004631981 0.005323605 0.01295992 0.010118532 0.002794863
0.1167092 0.1124263 0.002577192 0.1589678 0 0.2553666 0
0.2944794 0.01407242 0.03803459 0.06146746 0.1001324 0.02081387
0.01860711 1.496436 0.0187385 0.103204919 0.001710293 0.002708826
0.01071008 0.033124863 0.0508683 0.0580766 0.1602718 0.02900635
0.01858552 0.06005525 0.03021777 0.01096287 0.006856746 0.02759355
0.091238381 0.04477049 0.285186959 0.1158725 0.002704317 1.045945
0.003093106 0.007540523 0.005837768 0.04738772 0.01473879 0.002091177
0.1272271 0.01802002 0.02833996 0.004578646 0.08854288 0.01289397
0.006822611 0.3077647 0.002114779 2.190192 0.372950157 0.004437224
0.322844 0.01858374 0.4921221 0.205657364 0.01987306 0.3150489
0.2885255 0.02279375 0 0.09244404 0.007886353 0.04108388 0.002887038
0.04574158 0.02956865 0.01366933 0.1618899 0.006597316 0.006728077
0.1046556 0.001546184 0.02047432 0.1224021 0.01344811 0.05127644
0.1493989 0.021031742 0.06871196 0.04343452 0.00229544 1.292507
0.0154212 0.03676597 0.057708481 0 0 0.03167472 0 0.03120425

0.2478327 0.1842528 0.01692377 1.048699 0.061068675 0.07233616
0.01299618 0.006689574 0.008169967 0.01948248 0.173663 0.01595899
0.010963 0.8392316 0.02714148 0.4069246 2.279102 0.01979281 0.002408086
1.336526 0.02615169 0.00730169 0.02616786 0.043953981 0.7896285
0.09041488 0.1722752 0.01909636 0.1281252 0.0650425 0.017088769
0.004339002 0.01174404 0.004520788 0.01936876 2.700246336 0.2268257
0.7392596 0.1160175 0.01103125 0.00996534 0.004533785 0.0215638
0.01266959 0.007778805 0.6554279 0.01010007 0.006497731 0.006884695
0.007098959 0.003124164 0.02979278 0.046027859 0.125821374 0.4582416
0.005175234 0.01268944 1.590152 0.01458042 0.007914557 0.01220218
0.003408409 0.0104358 0.037080695 0.05742694 0.01487923 0.4243368
0.05062833 0.003061346 0.04634031 0.04261302 0.008119371 0.4016134 0
0.1085904 0.063746504 0 0.145295951 0.006020153 0.9111875 0.009196633
0.09345739 0.01841177 0.01860171 0.4700757 0.6664617 0 0.830542982
0.02364698 0.01061626 0.05514049 0 0.1148884 0.00189804 0.2029683
0.01622802 0.1419323 0.005089879 0.002425574 0.1931806 0.1012764
0.07353624 0.00912594 0.002927363 0.1418176 0 0.01975416 0.01790209
0 0.01709991 0.008207187 0.03875276 0.03175126 0.021299289 0.002650281
0.1887554 0.007812291 1.680118 0.2084928 0.06758514 0.6903881
0.02708488 0.02630476 0.2970894 0.03904903 0.3821308 0.001519978
0.07639326 0.3230128 0.006890553 0.06823652 0.03402716 0.03417952
0.176977227 0.02293862 0.008865746 1.102826 0.02215815 0.01062212
0.02619928 0.01529158 0.01637994 0.02430664 0 0.00150581 0.021176395
1.386119 0 0.0815595 0.06584272 0.01960376 0.191796524 0.5765527
0.02490409 0.01676088 0.0461067 0.08331202 0.003160178 0.161009575
0.6463762 0.1299433 0.01402061 0.06716761 0.009812792 0.003296424
0.02535119 0.4436829 0.00277756 0.004388277 0.02433894 0 0.04188146
0.03906975 0.009442231 0.08489403 0.04519451 0.023922 0.1680369
0.02781319 0.01114613 0.001896781 0.050670376 0.004611607 0.5701929
0.168882 0.1620864 0.17307 0.1874614 0.002115487 0.01489991 0
0.03006498 0.01474466 0.005892638 0.2400887 0.04041225 0.04070576
0.00129418 0.1125228 0.002835056 0.01127232 0.008585691 0.1587938
0.01656984 0.0053097 0.00443587 0.05032776 0.06564017 0.008272953
0.02337248 0.05873239 0.01705073 0.0244462 0 0.0601113 0
0.03224629 2.785472 0.04140434 0.004358162 0.01301492 0.06290197
0.002093498 0.008002427 0.1575072 0.1323053 0.01011972 0.0241482
0.1633669 0.03863 0.1093865 0.08767416 0.002194051 0.03228798
0.5942927 0.116501888 0.525704516 0.1399537 0.018395421 0.005535327
0.00484317 0 0.01574707 0.1570114 0.07727193 0.2859286 0.2785721
0.00408844 0.2344603 0 0.043020817 0.02228504 0.009287949 0
0.1249761 0.01118365 0.04055916 0.01083106 0.05105635 0.2222366
0.1152863
AC109460.3 0.07518811 0.125938 0.07087455 0.059520707 0.052933928 0.09074721
0.1365894 0.170397 0.06890239 0.05080708 0.1270385 0.047445404

0.1216249	0.167670182	0.117559031	0.1752735	0.1838229	0.1712652	
0.01855363	0.1646197	0.106410757	0.09173638	0.6047461	0.1060975	
0.2646852	0.2162295	0.0884994	0.8134255	0.1262494	0.115901095	
0.1792783	0.243551847	0.147045	0.2383064	0.2018996	0.100022363	
0.2008895	0.4797961	0.3592263	0.2593592	0.1429605	0.07679494	
0.2658323	0.2965706	0.09756735	0.09382015	0.1887772	0.0663103	
0.08832361	0.1909044	0.1285888	0.08260512	0.1093397	0.118253376	
0.2767953	0.4058878	0.19975	0.06318185	0.1674541	0.2619389	
0.4566104	0.06507584	0.09535517	0.2264474	0.2299897	0.259388449	
0.07465678	0.2455412	0.22467	0.129757358	0.08990809	0.1082533	
0.2395769	0.126259465	0.302245	0.1678044	0.0721047	0.1429258	
0.2860311	0.0742664	0.2041385	0.1792452	0.1106452	0.2506829	0
0.06342065	0.08166385	0.1713953	0.128989	0.1486693	0.1454879	
0.136274638	0.08852608	0.1427147	0.2534227	0.23133065	0.1604128	
0.1073603	0.2904689	0.3464748	0.1660596	0.1942816	0.2589897	
0.2512978	0.1457668	0.1202365	0.110397708	0.3034631	0.145820247	
0.09180076	0.2599582	0.1253827	0.2229985	0.08084854	0.1079169	
0.008760089	0.04541017	0.1082409	0.129882	0.1760766	0.130973	0.07194467
0.1206065	0.1880377	0.2724251	0.03422156	0.1368282	0.1410252	
0.324440106	0.2105346	0.06162703	0.1093078	0.1113179	0.057385353	
0.1653179	0.1019198	0.199124	0.2633532	0.07121658	0.2848199	
0.1720287	0.31898	0.09606556	0.1248235	0.02943262	0.07580735	
0.5030274	0.1260232	0.1368127	0.1582905	0.1028978	0.09251928	
0.2670015	0.079552513	0.06319304	0.2980975	0.381723574	0.3334297	
0.1766446	0.1867073	0.1640911	0.1757969	0.1954009	0.186689705	
0.172807	0.1319245	0.1903	0.0884032	0.1030074	0.1741702	0.1548225
0.07430249	0.1043631	0.123154344	0.323158	0.1489533	0.1669456	
0.03020598	0.1994693	0.1569495	0.132758	0.1266637	0.1239705	
0.07944171	0.1907595	0.2728582	0.06098161	0.1513539	0.1001636	
0.1880044	0.08548667	0.6723968	0.1044685	0.3063581	0.359175	
0.171117	0.3918468	0.2024009	0.1722321	0.126361113	0.3248535	
0.3473604	0.03899991	0.03580506	0.251612746	0.1229011	0.3246316	
0.2188467	0.300059	0.125269	0.1815916	0.155465	0.12988	0.5224693
0.1611519	0.1633711	0.1291258	0.330903	0.144354391	0.066416239	
0.5568684	0.085087047	0.120450074	0.1741711	0.062185055	0.1876615	
0.2362632	0.458207	0.317001553	0.6541511	0.2488809	0.2379299	
0.13709466	0.1127943	0.2750572	0.2300488	0.3695664	0.1584576	
0.2896281	0.1575488	0.1320833	0.1069591	0.1679395	0.1512425	
0.276318498	0.2036899	0.093423974	0.2643101	0.1845218	0.06800354	
0.2439039	0.1410062	0.4367161	0.3303146	0.1776952	0.1012532	
0.203996329	0.2521949	0.1864394	0.08607645	0.1566262	0.09955431	
0.1543834	0.1175347	0.2871342	0.04057359	0.2775697	0.08519435	
0.09030455	0.09014271	0.08796053	0.354274261	0.1488168	0.1509428	
0.1895965	0.0443427	0.4538575	0.0665167	0.2607897	0.06068718	

0.2283471 0.2663866 0.192931821 0.215569462 0.2021854 0.3177194
0.2449695 0.321183 0.1204757 0.06132135 0.1063968 0.0607837
0.1483239 0.04812399 0.2420554 0.1292522 0.1325743 0.2791735
0.08916507 0.1130928 0.2186613 0.1523855 0.152674618 0.4163331
0.1311137 0.07349056 0.04096153 0.04909008 0.5448596 0.06218959
0.01816797 0.2626866 0.2034219 0.2644457 0.087327162 0.1274815
0.1231794 0.1187891 0.2362737 0.2778361 0.106633032 0.05015598
0.05754709 0.5948954 0.130356 0.0697406 0.1226799 0.24987239
0.1489284 0.4307268 0.1592135 0.1434807 0.123956093 0.1401566
0.1288765 0.1468244 0.236191298 0.15007502 0.07566993 0.06720827
0.08964654 0.1402001 0.1701852 0.05231167 0.3253917 0.07738882
0.10582 0.0794602 0.1545354 0.06662136 0.123777154 0.0682001 1.039
0.2536585 0.157233 0.1355028 0.2533848 0.2150877 0.09487364
0.3275645 0.1970496 0.3134551 0.05446562 0.1646907 0.1195297
0.2257459 0.05981047 0.315166 0.06289057 0.4501006 0.07935749
0.2397292 0.1191205 0.1079706 0.1025018 0.3544222 0.04718869
1.473262 0.1344197 0.1492874 0.1512958 0.2398611 0.1517291
0.1111217 0.03827914 0.04768837 0.2159717 0.2569557 0.4632484
0.4846243 0.04228382 0.1393214 0.1886143 0.1031219 0.5439785
0.4817132 0.4869854 0.1309879 0.434419182 0.0901755 0.1543565
0.1906283 0.04775002 0.05315849 0.187576189 0.117351457 0.08906693
0.097159297 0.05116299 0.2909751 0 0.3735784 0.08762296 0.07754432
0.2196643 0.09757291 0.1435998 0.1604007 0.07935552 0.102723965
0.04119609 0.2918848 0.2723458 0.1276749 0.08269637 0.2218088
0.1172734 0.2831481 0.117379 0.8802705
AC008750.5 0.09391007 0 0.03621372 0 0.033057281 0.09715149 0 0 0
0 0.238007 0.053333449 0.2225654 0.134627357 0.114911549 0 0.2459948
0.180487 0 0.2123517 0.132907211 0.4884679 0.3940846 0.06815107
0.2550283 0.2835745 0.1658038 0.9111189 0.05256189 0.130284592
0.1185453 0.230896258 0.03673188 0.0824248 0 0 0.1704303
0.0627139 0.5653296 0.32394 0 0.08221458 0.2241169 0.180203
0.1218618 0.5062242 0.07859433 0 0.1921639 0.07664139 0
0.2387739 0.1117353 0.124620746 0.09877652 0.08295619 0.2338951
0.1291324 0.3025211 0.2538326 0.4226982 0 0 0.2707977
0.09943528 0.114344682 0.1049022 0.5661811 0.4132668 0.094103509
0.2887595 0.3318755 0.07694536 0.105132187 0.8130865 0.05389406
0.1907129 0.428435 0.2202247 0.1788921 0.09561351 0.2324883
0.1990023 0.1329212 0 0.1188188 0.06119897 0.6291125 0.193329
0.02984274 0.1168165 0.034041449 0.2487807 0.08443462 0.6231593 0
0.1865382 0 0.2612132 0.04636581 0.1287365 0.07799723 0.5137601
0.1822479 0.2137261 0.2866987 0 5.168536 0.084059875 0 0.4495683
0.2596316 0.06427513 0.4700791 0 0.1477084 0.2552282 0.08690985
0.03945962 0.2674702 0.147227 0.1427173 0.2420964 0.2977092
0.538744 0.04808563 0.08789075 0.2045504 0.19537693 0.1536768

0.1823027 0.1053198 0.08629839 0.0806337 0.1238894 0.3818941
0.0799413 0.03947142 0 0.0711481 0.1311035 0.1707457 0.1499826
0 0.1417939 0.1262447 5.797691 0.3198838 0.09320686 0.06590169
0.1285196 0.1418196 0.1017413 0.055890686 0.1420708 0.1478346
0.039731114 0.08924021 0.09025748 0 0.3149233 0.2670454 0.0955002
0.171312795 0.05395907 0.05296306 0.1097008 0.2885056 0.3242142
0.3404956 0.226247 0 0 0.057682492 0.1878943 0.1080249 0.1390102
0 0.1868532 0.08019419 0.9948895 0.1139063 0.1194475 0.1410009
0.04045906 0.1008948 0.04569968 0 0.07506267 0.1207635 0.05057668
0.1631314 0.130481328 0.1012875 0 0 0.1587299 0.1936333
0.7671195 0.03945631 0.04508251 0.09761709 0.1252567 0 0.273714446
0.1354444 0.4120052 0.2460059 0.1964835 0.3313297 0.59668 0
0.1196708 0.9159858 0.06709298 0.5247018 0.3375592 0.09537656
0.073758629 0.064920515 0.5503091 0.043475671 0.046689002 0.2669809
0.107542019 0.2636882 0.3088184 0.2693182 0.109643666 0.9508606
0.2124815 0.2168573 0.118544854 0.0745837 0.07729806 0.08478628
0.02697598 0.06361515 0.5961166 0.442752 0.3374431 0 0.03432382
0.123645 0.593813298 0.1526454 0.30630258 0.09382466 0.7704216
0.318512 0.3427159 0.05465697 0.03865458 0.2284967 0 0.2969187
0.160921105 0 0.2206071 0.2546281 0.07492072 0.0746061 0.1183245
0.05081574 0.04817432 0.1824353 0.5288416 0 0.09228316 0
0.04494389 0.341348761 0.06083097 0.1786053 0.2412744 0.234568
0.3188637 0.04984768 0 0.1364371 0.452974 0.3552744 0.088520386
0.275365854 0.1832879 0 0 0.3610423 0.1504744 0.1292464
0.09380455 0 0.0901249 0.1803208 0.3331773 0.09475602 0.1619859
0.1743441 0.2386443 0 0.3367089 0.04177969 0.040862325 0.6066675
0.1842313 0.03059661 0 0 0.0453687 0.06355219 0 0.1942673
0.2230896 0.3754912 0.101549578 0.1433021 0.3643845 0.1540743
0.1609671 0.09052637 0.119866349 0.08457062 0.03234438 0.3134638 0
0.0653296 0.1313378 0.247836991 0.03893269 0.1862233 0.3329715
0.1861002 0.13594071 0.1370002 0.06585012 0.1138245 0.115436081
0.319161315 0.02298937 0.09065869 0.04580537 0.04775731 0.1962108 0
0.3460018 0 0.07674346 0.05254196 0.3763792 0.3941535 0.188024435
0 2.285103 0.4386737 0.2000896 0.08462158 0.1675472 0.04396008
0.0688049 0.2045643 0 0.153198 0.04081659 0.1568891 0.1679543
0.6766967 0.05378643 0.2125672 0 1.264897 0.07646218 0 0.1147744
0.1103362 0.09217794 0.09960164 0.2273352 0.4011304 0 0.3051166
0.283453 0.4298421 0.3851329 0.10128 0.0782357 0.0335041 0.2555517
0.3072812 0.1358448 0.0772719 0 0.6090441 0.1662915 0.1363758
0.2370105 0.2628614 0.5930395 0.1227031 0.267578765 0.1228684
0.1735124 0.136778 0.1341897 0.09959256 0.078094323 0.082446842
0.04767632 0.054608455 0.1150248 0.1509625 0 0.2181507 0.1539017
0.04587773 0.1157461 0.1218687 0.06371873 0.1918156 0.5676596
0.074498169 0 0.4342607 0.2551203 0 0.6307939 0.1404708

0.03215297 0.6458001 0 0.6943953
AC012557.1 0.5573637 1.444633 0.7008629 0.569752471 0.255909886 0.3760447
0.9905155 0.6843769 0.556769 0.6947752 0.5527528 0.743176734
0.7876431 0.521102618 0.800619414 0.7821806 0.3427822 0.1862965
0.40364 0.9628561 0.257222221 0.336127 0.6864232 0.3956889 0.5922835
0.6272189 0.5647642 0.4282386 0.2034515 0.437053987 0.5200342
0.663914468 0.5687129 0.3509462 0.1634378 0.685775503 0.4287955
0.2427469 1.062851 0.2507752 0 0.4455196 0.693992 0.3836317
0.8962126 0.4898609 0.1521077 0.6924488 0.1983493 0.4153186
0.4408167 0.1437681 0.4324944 0.225105995 0.2294008 0.7064175 0
0.07497498 0.2341939 0.5109057 0.6077089 0.3893301 0.4346534
0.2515627 0.1539538 0.088518878 0.04060455 0.8766077 0.6754007
0.364246806 0.298054 0.1712789 0.2680494 0.24416133 0.5844832
0.8344317 0.2583676 0.7462552 0.3750668 0.4154629 0.5551374
0.479943 0.07702788 0.5144988 0.235327954 0.1379737 0.1421299
0.3043885 0.1496639 0.623767 1.23591 0.447998844 0.2751304 0.239669
0.6202453 0.710494889 0.240678 0.4062015 0.4549855 0.03589367
0.07474511 0.09057125 0.5302968 0.7406989 0.3860595 0.4121839
0.106743883 11.6034 0.357907961 0.6390896 0.8265693 0.2297045
0.8458859 0.6307728 1.079973 0.2668099 0 0.1682011 0.4887568
0.7454144 0.284936 0.4051052 0.2623833 0.7375001 0.702423 0.4094755
0.3061791 0.4750525 0.201665658 0.09517398 0.5927375 0.2989519
0.6346675 0.124843667 0.1918157 0.2956398 0.6188581 0.3666772
0.2916404 0.6058651 0.3044778 0.4846643 0.4412089 0.2452777
0.1829474 0.4397902 6.316766 0.1061293 0.1803881 0.1530516
0.8705571 0.07319224 0.1575241 0.129801773 0.274957 1.525931 0.153787374
0.2417957 0 0.4431139 0.9751798 0.1653846 0.369653 0.099465162
0.501263 0.08200173 0.1273858 0.8933759 1.183226 0.527183 0.2290384
0.4083725 0.3027271 0.178617471 0.1939422 0.1254397 0.4304538
0.5585697 1.133097 0.155204 0.4813653 0.8817948 0.5856383 0.1819242
0.4071731 0.6560971 0.2476459 0.5810739 0.3486544 0.2337198 0
0.7998156 0.202021827 0.3528486 0.4641961 0.3722704 0.522237
0.6745475 0.1696738 0.305447372 0.1047006 0.4156315 0.3393822
0.4154403 0.353155845 0.4613535 0.7290286 0.6475068 0.7351786
0.6091775 0.437603 0.3468913 0.3149828 0.5839663 0.3635759
0.4061931 0.5487685 0.6645139 0.228398243 0.201030465 0.585773
0.40387546 0.614445607 0.2411276 0.33301064 0.4763075 1.099717
0.5212255 0.127319445 0.6379533 0.1644906 0.5036342 0.091770403
0.1154766 0.6582354 0.4266373 0.6264962 0.09849416 0.2129899
0.1371009 0.1828599 0.1642539 0.398572 0.5503824 0.424333914
0.236338 0.169372364 0.7505461 0.1605732 0.3452024 0.3095287
0.1269366 0.7481022 0.4864437 0.4810794 0.5746423 0.467158446
0.5852352 0.8539049 0.3942359 0.2899959 0.4042893 0.4885325
0.4720624 0.3356433 0.1176922 1.228194 0.6243133 0.7858417

0.6034103 0.2783432 0.587226637 0.1883669 0.8987268 0.6537313
0.431273 0.3291272 0.1929456 0.137541 0.4224861 0.1168886 0.3143227
0.445427048 0.085268772 1.532418 0.816883 0.4187378 0.3843092 0.1941473
0.366868 0.5446344 0.5642107 0.976771 0.8375623 0.2149384 0.146709
0.953039 0.944768 0.4249118 0.1135555 0.7298482 0.2910902 0.474497862
0.3354615 0.1782762 0.5210938 0.5148757 0.2990317 0.1756088
1.328355 0.9485998 0.571483 0.3885812 0.3391302 0.288250113 0.4807227
0.8462547 0.2783087 0.4049863 0.3153607 0.417570415 0.2400551
0.1752737 0.3774791 0.1308894 0.3793073 0.1525109 0.703489289
0.8439028 0.8649784 0.4510919 0.2881355 0.368330352 0.1590862
0.3058636 0.0881162 0.089363775 0.176482701 0.3025492 0.2105478
0.3900578 0.628505 0.8354211 0.390191 0.3443838 0.09620677 0.3267563
0.4474236 0.02241311 0.6102607 0.64045246 0.296743 4.651791 0.212247
0.2839788 0.09826353 0.7133781 0.3062816 0.07989702 0.3167231
0.2638078 0.5336855 0.2211845 0.8501796 0.5200812 0.3928939
0.312287 0.6856538 0.1368204 0.4714707 0.1381158 0.8514916
0.08885157 0.1708316 0.5351903 0.1927642 0.3226473 0.8428709
0.08355267 0.4133557 0.438865 0.363009 0.5111093 0.20908 0.06056544
0.1037476 0.6429571 0.5709094 1.29701 0.4486449 0.2299745
1.044004 0.386199 0.2639352 0.366959 0.5697769 0.4944111 0.411621
0.517859066 0.4042491 0.1679038 0.4588368 0.1038817 0.2826951
0.181367994 0.414865491 0.1476327 0.295922412 0.7568858 0.5064204 0
0.2744289 0.5718796 0.2486107 0.4181509 0.1415153 0.2959636
0.1187938 0.7847294 0.432540513 0.3584936 0.8591239 0.2764986
0.4232545 0.7453356 0.4077905 0.4729275 0.1785501 0.1964321
0.2150239
AL136084.3 0.6408275 3.605286 0.7023317 4.109084555 0.142470105 0.5757167
2.481478 1.799196 4.351422 2.051186 0.4487706 2.470954485 1.610104 1.547243491
3.157188251 0.05443193 0.5300935 1.879834 0.4868812 0.1634272
0.429602124 1.75433 0.1415353 0.03671464 0.8243399 0.7856667
0.4644774 0.1753007 5.606641 0.374333255 2.043624 0.177699203 0
0.04440425 0.5686815 0.293680625 0.2754449 0.202713 0.5220974
1.745144 0.3351258 0.088582 1.207372 1.116417 0.06564991 0.3181678
0.127022 0.2141667 0.0690156 0.4128858 0.377556 0.5717038 0.7825291
0.313302421 0.2128533 0.04469052 0.25201 0.1043501 1.086503 0.8751733
0.6506223 0.2955651 1.594869 0.2334165 0.3214093 0.246401069
1.808429 0.03812694 0 0.557654392 0.2074157 0.3575789 0.2072617
0.906196342 0.03128782 0.4645447 0.3082251 0.2885105 0.1423685 0
0.05150933 0.2504942 0.5896405 0.8306518 0.32752934 0.3840633
0.3296938 0.9743899 0.2083021 0.2893862 0.0419546 0.440135003
0.07658528 0.1212986 0.04795873 0.082405539 1.004927 0.6784212
0.07036096 0.04995679 0.03467674 1.176532 0.1230111 0.3436348
0.1151395 0.3971613 1.225670227 1.33652 0.588706345 0.07412371
0.06054835 0.7193308 0.6232784 0.2363602 0.2614097 0.7957413 0

0.2809227 1.147924 0.3458225 0.2379443 0.05125684 0.5738621
1.090606 0.183306 1.191626 0 0.9917667 2.315594916 0 1.492806 3.366475
0.2789463 0.608150756 0.5784328 0.4114713 0.4306632 0.1701137
0.1522144 0.6899259 0 0.4905859 0.1292788 0.1463046 0.2037009
0.3060499 1.542399 0.04923692 0.1004256 3.905313 0.1038549 0.662148
0.1644315 0.06021935 0.535759 0.5840422 0.299657676 0.1923035
0.8752303 0.1541814 0.9694677 0.3452732 0.2057931 0.276871134
0.05813813 0.1711949 0.1772954 0 0.3992276 1.329891 0.150012
0.04736439 4.060142 0.621499286 0.3374108 0.2327826 0.1123323
0.2743825 0.4026494 0.9072538 0.1339928 0 1.329885 0.1012808
1.176999 2.239406 0.1477172 0 1.415333 0.2602329 0.1089876 0.05858858
1.218420176 1.473284 0.3445695 0.6332648 0.2137792 0.9388348
0.118076 0.212560756 0.04857409 0.1577661 0.2024366 0 4.128786013
0.9339805 0.5073308 1.431317 0.1058505 0.2231192 0.03383643
0.1931212 0.3094538 0.2322183 0.867471 0.1696018 0.3637027
0.05138168 1.033124854 0.27979411 0.1482325 0.843170521 1.106710151
0.6712025 0 0.6155728 2.395699 0.2539045 0 0.1366005 0.03815633
0.1168263 0 0.4821607 0.1665694 0.5481172 0.1453262 0.06854207
0 0.2862256 0.03635777 1.828868 0.03698215 0.6994113 0.295293874
1.397974 0.424318283 1.752248 0.8939428 0.2402263 0 0.1766703
0.1665933 0.1846451 1.24348 0 1.430420006 1.140342 1.48558 0.2743487
0.04036162 0.9244188 0 0.5475135 0.05190536 3.570926 0.1709399 0
0.5468671 0.6718609 0.3389732 0.081730177 0.5898803 0.2886571
0.2599608 0.6634293 0.6871186 0.1074166 0.1914294 0.183755
0.7591987 0.1640528 0.286128536 0.059338519 0.07899331 0.6121971
3.443813 0.1945023 0.1621284 1.392564 0.6064176 0.196317 0.9224971
0.3885729 0.239321 0.03403159 0.3839693 1.221004 0.3599777 0.7375504
0.1451146 1.035356 1.452892997 0.09337902 0.2977497 0.9889884
0.05512337 0 0.3910596 2.533547 1.026869 0.3767636 0.06009191
0.1348575 0.802372751 1.955742 0.2355634 6.69562 1.344112 0.4876876
0.226012233 0.2126145 1.707621 0.4503216 0.4857915 0.4575304
0.07075487 0.623073864 1.006752 0.7022613 0.08968993 2.038553 0.768963024
0.221416 0.1419003 0.4905604 0.746258767 0.049125709 1.684351 0
0.5922352 0.5145605 0.211407 0.3620451 0.05325702 0.4017016
0.7028408 0.05661127 0.1559728 0.08493605 0.364656045 0.05162584
0.1823764 0.2954053 0.3593105 0.319114 1.53445 0.3789178 0.5560032
0.1469384 0.3671676 0 0.3958001 0.2704639 0.09048109 0.06075884
0.4346409 0.2672019 0 0.05047636 0.08238408 0.4740423 0.1648848
0.2377633 0.2482925 0.4292626 0.9389444 0 0.05814429 0.9040558
0.152703 0.08420597 0 0.1091241 0.7586546 0.3609896 2.753438
0.1324319 0.09757717 0.1248848 0.1920471 0.09374483 1.388572
0.4040793 0.1021467 0.1132879 0.2457575 0.3525501 0 1.025979
0.140213 0.6877326 0.1445825 1.001522 0.631069729 2.398470651 0.4623191
1.0590806 0.1859 0.05421813 3.352629 0.2350462 0.6301206 0.7414635

0.7066927 0.9191511 0.06865367 0.9093543 0 0.280937855 0.3742135
0.4159055 0.5497581 0.1472713 0.3577096 0.4918876 0.3117886 0
1.531007 0.2618614
AC058791.1 0.06018898 0.009517489 0.03213713 0.03958369 0.058672023
0.08621512 0.07569793 0.1089625 0 0.03620225 0.04400302 0.047329684
0.05643175 0.053098803 0.008497993 0.08219256 0.03638384 0.08008477
0.05141215 0.004486837 0.039315266 0 0.004857255 0.01511982 0.01257332
0.02995863 0.009809283 0.02887692 0.04664498 0.019269735 0.02922239
0.004878671 0 0.006095518 0 0.025196567 0.006301872 0.009275695
0.01194501 0.07985388 0.09200765 0.1398393 0.02209866 0.01998968
0.01802396 0.01871826 0.1511184 0.02204952 0.004737 0.02267128 0
0.01961991 0.008263104 0.055296072 0.02921907 0 0.0276754 0.009549658
0.04474437 0.007508621 0.07145046 0.0067622 0.007549413 0.02403139
0.3970882 0.016912143 0.05430446 0.03663668 0.04074947 0.020877561
0.01423632 0 0.0113806 0.062198279 0.06871972 0.07174077 0.04231108
0.007920957 0.01954339 0.01763935 0.02121257 0.02292411 0.03679177
0.007863884 0.017984413 0.03514776 0.06336141 0.02907776 0.02859432
0.1412444 0.01151849 0.010069797 0.005256557 0.02497659 0.01975036
0.113120799 0.0459832 0.01552152 0.04829341 0.01371547 0.01428057
0.0461447 0.07598766 0.01347768 0.0210741 0.01817321 0.056083931
16.49687 0.006216435 0.06105124 0.01662334 0 0.02851983 0.0139054
0.01794228 0.01456454 0 0.006427198 0.01167255 0.007912032 0 0
0.03580725 0.008806531 0.008387673 0.01422421 0.006499738 0.02269051
0.028897213 0.02272956 0.03235617 0.03115463 0.006381979 0.023852245
0.01832386 0.008069146 0.02364743 0.08173226 0.04178993 0.01052316
0.004847715 0.02525413 0.01774653 0.02677828 0.03495337 0.004668053
22.52809 0.05407131 0.006892878 0 0.00475217 0.01398388 0.04514411
0.008266509 0.03151946 0.02915396 0.052887853 0.03299767 0.02669905
0.007054998 0 0.0473968 0.1694994 0.012669004 0 0.007833498
0.04056322 0.009482535 0.04110247 0.08813171 0.03603713 0.006501866
0.03680607 0.07678373 0.037054 0 0.01028015 0 0.04145476 0.06523614
0.01839363 0.0168473 0.0294448 0.01390315 0.02393638 0.002984569
0.01351842 0.01480242 0.03330642 0 0.1047276 0.0160853 0.006432944
0.03745232 0 0 0.005869235 0.02147949 0.04322315 0.052522048
0.08001511 0.007219025 0.009263045 0 0.026989146 0.04407239 0.0609376
0.01455419 0.009686962 0.006125662 0.01393451 0.02651039 0.01415992 0
0.06946369 0.0232818 0.01997067 0.02116 0.032727797 0.024005162
0.0508709 0.025721066 0.006905534 0 0.007952998 0 0.009135152
0.01493756 0.04865052 0.0281274 0.1414218 0.01603713 0.008766685
0.0110313 0.01143276 0.09405232 0.03989881 0.02822699 0.04747539
0.01309704 0.03992761 0.05230306 0.005076662 0.0320035 0.013511988
0.04515401 0.038831713 0.01850284 0.00438266 0.0188438 0.01689646
0.008084036 0.04573763 0.03379579 0.03282626 0.02195787 0.089253849
0.01677198 0 0.01129823 0.07202744 0.05517305 0.02333438 0.05261129

0.02137566 0.04047463 0.007821829 0 0.0204737 0.08069999 0.01994227
0.044877483 0 0.02641661 0.01784284 0.03903048 0.09432303 0.007372719
0 0.00504493 0.01488824 0.04504015 0.032731506 0.04072797 0.01626551
0 0.05091084 0.07342492 0.007418636 0.01274412 0 0.07410999 1.57293
0.04000552 0.09855714 0.01868651 0.02875021 0.0515727 0.01411867
0.02169557 0.009960181 0 0.024174959 0.006409223 0.01362436 0.05430468
0.007566967 0.02448517 0.02684102 1.456951 0.0100687 0.01149324
0.02474704 0.004628083 0.015019684 0.00706503 0.06467313 0.01519223
0.04166369 0.08033578 0.044322063 0.02084735 0.004783894 0 0.05001465
0.00966257 0.009712754 0.152734648 0.01727501 0.04131502 0.2093046
0.03670018 0.030159454 0 0.02921867 0.02525281 0.0512207 0.053949183
0.0408029 0.01340887 0.02709936 0.007063542 0.02902054 0 0.0219323
0.06126989 0.03972758 0.04662729 0.02569308 0.005829726 0.03893364
0.03543425 16.92393 0.04055125 0.009864747 0.01877393 0.01858579
0.02600765 0.02035317 0.01008536 0.01680077 0.02832342 0.03018488
0.03712747 0 0.1084274 0.01193292 0 0.01742699 0.04850339
0.143249 0.01626834 0.01131714 0.06527711 0.01363357 0.03682894
0.039228 0 0.03192663 0 0.02620256 0.005779615 0.04882548 0.01997309
0 0.03468796 0.03779735 0.009089682 0.02009211 0.03428669 0.02636293
0.01930299 0.02459533 0.02521332 0.05608804 0.1632897 0.01349438
0.02419786 0.019788111 0.02725926 0.006415835 0 0.009923658 0.03437054
0.017325804 0.018291443 0 0.056538022 0.008506368 0.03721351 1.265623
0.03226556 0.01365769 0.006785542 0.0627712 0.01802499 0.01884863
0.04539275 0.005997119 0.071621207 0.05136952 0.04281954 0.0679204
0.08086569 0.01473119 0.04674672 0.04755582 0.02729058 0.04503564
0.0205409
AC005070.3 0.167874 0.4716229 0.2395224 0.526256629 0.082730656 0.2344523
0.7319183 0.4477591 0.4549261 0.5833954 0.2340042 0.200212074
0.5172164 0.304836497 0.534082473 0.4425115 0.2286654 0.333394
0.1988403 0.3091034 0.130671999 0.1940416 0.1350229 0.1644666
0.3191228 0.2027676 0.1304123 0.2850259 0.1127518 0.0931589
0.2472307 0.194582642 0.3808399 0.2210144 0.08491536 0.060905988
0.1827972 0.04484304 0.3464864 0.09651274 0.1019352 0.124922
0.2804425 0.1530125 0.2723007 0.1583625 0.1053716 0.02664942
0.09160347 0.08220267 0.1957519 0.09485183 0.09986926 0.118812019
0.1236013 0.3336588 0.2174182 0.1385025 0.2403502 0.2994765
0.388603 0.179804 0.09124347 0.1258606 0.195526 0.091981351 0.1593951
0.4364714 0.3529618 0.050465941 0.2494907 0.0395508 0.123793
0.159744503 0.3270323 0.1926825 0.1193217 0.5265371 0.3070661
0.2878094 0.188011 0.540276 0.1067212 0.1853362 0.445593393 0.1274405
0.05469976 0.1757194 0.08639894 0.2720697 0.2018609 0.066937891
0.05717847 0.0855302 0.3182749 0.314455299 0.1667282 0.05627871
0.2217992 0.1989209 0.0632857 0.1394282 0.2347021 0.2443403
0.1719259 0.1537511 0.086270519 8.481672 0.112699322 0.0491917

0.3817338	0.1856474	0.2412868	0.1400525	0.5855043	0.07041178	
0.09124939	0.3340248	0.1340225	0.5833195	0.03947753	0.2040975	
0.2942852	0.1277247	0.4815304	0.2922574	0.08641262	0.2742414	
0.162986399	0.1538394	0.4627568	0.2761294	0.1311272	0.07207056	
0.16979	0.1560401	0.1143227	0.07055927	0.2441221	0.2034954	
0.1464757	0.05087094	0.1876766	0.3074645	0.1182867	0.197466	
5.693789	0.2450681	0.2082712	0.08246428	0.1608195	0.05070347	
0.1909668	0.119892492	0.3428545	0.3875957	0.085228195	0.1276209	
0.1774791	0.1449554	0.3216903	0.08592685	0.3755764	0.168431684	
0.1350404	0.1230801	0.08824569	0.2234847	0.1573109	0.1826014	
0.1835536	0.04714958	0.2033574	0.123736139	0.2015284	0.1158635	
0.2050085	0.2200279	0.3117515	0.07167772	0.363104	0.3054289	
0.1708199	0.1176251	0.2386718	0.3498984	0.2369095	0.4114806	
0.1945644	0.1187325	0.009041107	0.7484769	0.085524609	0.3168585	
0.1643575	0.1146169	0.2766526	0.08653492	0.1828408	0.077585479	
0.05641277	0.2268509	0.1007592	0.2038538	0.293575811	0.2179089	
0.2419936	0.1495188	0.2809878	0.2591252	0.2133255	0.07209203	
0.2267593	0.2889564	0.1559164	0.4314612	0.09654754	0.3580406	
0.17140658	0.092841734	0.4426806	0.093260735	0.050076866	0.1511311	
0.086509142	0.2749659	0.331227	0.09026894	0.058799835	0.5099287	
0.1329415	0.3488894	0.116551186	0.2666522	0.3592636	0.1515643	
0.448468	0.09097496	0.1885328	0.0158293	0.5368611	0.05057148	
0.1043076	0.2431312	0.212300597	0.1637217	0.078221099	0.2348099	
0.2171752	0.1537308	0.2858988	0.06839351	0.7393602	0.2246539	
0.253916	0.4423109	0.18698108	0.2432508	0.226756	0.141104	0.3013393
0.1000246	0.1551128	0.1635091	0.1205632	0.1521903	0.557762	
0.0991121	0.1237243	0.05573449	0.1205128	0.216958715	0.1794238	
0.1277102	0.1365793	0.1415187	0.3040011	0.1514835	0.1985013	
0.1158504	0.098968	0.127018	0.213623176	0.098449016	0.4521516	0.1305901
0.3076585	0.2984978	0.08966287	0.1694304	0.06707415	0.05699946	
1.79635	0.03223423	0.4169123	0.1806787	0.1853226	0.2025778	
0.3327491	0.113627	0.2166848	0.02240566	0.175309565	0.1549259	
0.1399664	0.3391067	0.1371834	0.1085084	0.129762	1.431438	0.2798915
0.2500366	0.3688862	0.4027377	0.217836511	0.2476285	0.2214676	
0.1468927	0.0431618	0.08900391	0.251771429	0.2267682	0.3700412	
0.6350624	0.09067282	0.07007006	0.05869499	0.177213752	0.1600713	
0.5076627	0.1860066	0.2328714	0.11542887	0.1959217	0.05885697	
0.06104195	0.092859297	0.163009721	0.160274	0.04321649	0.1228228	
0.1792794	0.0789181	0.1802016	0.3446007	0.1110777	0.1714836	
0.1033166	0.09833478	0.3100198	0.10755623	0.06852228	12.5269	
0.1470328	0.2682607	0.1663969	0.2396064	0.3064745	0.03074898	
0.3169226	0.3350439	0.3628609	0.0729639	0.3870286	0.1050825	
0.1411277	0.04807442	0.2026591	0.01053128	0.8123336	0.1776893	
0.3834126	0.1367808	0.04930938	0.1071055	0.05341448	0.06095779	

0.1536562 0.1543483 0.06817846 0.1646781 0.3073550.3442326
0.2595030.1328617 0.07785969 0.3426189 0.1428172 0.5180514
0.05525262 0.07434626 0.2099693 0.05945265 0.1340822 0.3474183
0.1973547 0.3914288 0.1608526 0.406576124 0.16473 0.2326286
0.1548531 0.1559207 0.2195729 0.153561891 0.140013183 0.1022716
0.156189261 0.09252845 0.5037411.8077710.2388548 0.1155484 0.0820112
0.1517327 0.1089265 0.1252942 0.1097249 0.3116734 0.06658661
0.06208606 0.4053939 0.07296865 0.1099526 0.5519353 0.144386
0.3161222 0.3545764 0.1451489 0.1489565
AC036108.3 0.43141815.713050.1663648.038875156 0.181067955 0.2574859
4.15075217.30681 7.2238548.5486810.8410707 0.932926939 3.3651091.038714348
10.243246341 0.4016820.5389647 0.0106301 0.05527622 0.03752048
0.023483418 0.2301535 0.1624722 0.1926662 0.1952645 0.4652596
0.1581980.2702263 0.08358459 0.053713396 0.9285974 0.209813836
0.02596067 0.3568098 0.2517961 0.084280964 0.4065308 0.06648563
0.3710136 0.7822399 0.05495713 0.0363163 0.1055981 0.6527224
0.1722544 0.0521762 0.04166057 0.1756055 0.04527135 0.1895851
0.007739403 0.09375344 0.08884148 0.02935904 0.1047171 0.2711648
0.1818388 0.1197863 0.08314842 0.8431757 0.1387038 0.2908176
0.02705606 0.1339726 0.0351385 0.111119795 0 0.2938637 0.2352875
0.764850259 0.08503501 0.0195464 0.03398875 0.111455041 0.3181143
0.06665791 0.1347885 0.1797883 0.5681089 0.0316085 0.4899255
0.2327778 0.2637133 0.1456126 0 0.0839765 0.1405725 0.2501057
0.008539843 0.04218339 0.06880116 0.042103563 0.03767756 0.02983756
0.3067245 0.094595525 0.1318380.1390675 0.1153846 0.1228859
0.06255284 0.1929390.1210353 0.1288057 0.1321717 0.08684039
0.176633813 1.84472550.148525666 0.07293308 0.3177375 0.5177253
0.05678396 0.2048775 0.02143423 0.1652915 0.03607708 0.1382052
0.06972133 0.05671127 0.09104754 0.07565028 0.1454387 0.1157251
0.1052109 0.4672947 0 2.4938040.172606066 0.04887577 0.2705735
0.08063895 0.4879389 0.128224752 0.1678238 0.2891871 0.2542474
0.02092266 0.09152573 0.1005695 0.02316472 0.05028184 0.111302
0.1199621 0.05010723 0.02788277 2.6431940.0403717 0.0658750.2911048
0.06244745 0.008352719 0.08089515 0.039501374 0.01255127 0.2612095 0
0.09460729 0.07973812 0.05899633 0.9220989 0.0849318 0.1181177
0.04540397 0.05720427 0.03743224 0.009691533 0.2548808 0.09002009
0.4060953 0.2060284 0.01553453 0.9296366 0.081535506 0.1217301
0.02863044 0.1596514 0.07499309 0.1980909 0.1416954 0.4687663
0.1207568 1.0974720.1494809 0.5218569 5.6975540.02422407 0.1503079
0.9947136 0.04267547 0 0.2401979 0.092219153 0.5011028 0.1695174
0.1510528 0.2454029 0.2651518 0.1678146 0.069715482 0.1035534
0.1207360.03319748 0.1896406 4.078587226 0.1196586 0.5095813
0.1130138 0.3008784 0.02927137 0.1276229 0.03958733 0.04228933
0.27609 0.3556404 0.2039614 0.09542951 0.1179648 0.156389234

0.080295788 0.7535658 0.122907686 0.131991919 0.3695213 0.06650572
0.1009475 2.1389570.2141367 0.106551363 0.2240105 0.03754343
0.1660385 0.115201508 0.1054257 0.09560473 0.2846374 0.157291
0.4383671 0.1296345 0.2033980.1430950.07497879 0.04851749 0.1146961
0.056495958 0.06742741 0.06958376 0.9007331 1.3036690.06753359 0
0.01931472 0.2253864 0.3229852 0.5097942 0.0786940.646855354
0.2938637 0.1169374 0.1664642 0.3640386 0.08568414 0.04878253
0.2244662 0.1957745 0.1772901 0.1308177 0.008905852 0.3424164
0.04131681 0.02382345 0.160834729 0.005374122 0.0946735 0.07105143
0.01554221 0.07512017 0.05284562 0.03139242 0.02410712 0.02667871
0.09864415 0.093844174 0.204348825 0.1943111 0.05736795 3.848983
0.6299528 0.1329368 0.8068929 0.1077333 0.08048485 0.159242
0.04779142 0.1569846 0.04464659 0.2003494 0.6931105 0.04216613
0.01727865 0.06544252 0.1033489 0.072199774 0.3981428 0.1139316
0.7082024 0.03615862 0.009750167 0.01603242 0.4491623 0 0.2540062
0.06898111 0.2709114 0.017942828 2.0002880.1287664 0.1814895
0.2417512 0.0239927 0.465942931 0.2590087 0.01714482 0.1538501
0.05974855 0.09234486 0.02320612 0.131371196 0.1857338 0.2961347
0.1838526 0.06028386 0.090072781 0.07261983 0.02327016 0.2715078
0.122378632 0.233626969 0.06093 0.1121296 0.07284029 0.2193946
0.1993441 0.04452871 0.1484711 0.05855543 0.1627179 0.02785097
0.05115582 0.06267881 0.132888394 0.0169322 1.1364970.1647075
0.2298003 0.3289399 0.4514605 0.1165098 0.02431432 0.1927709
0.1705997 0.2436174 0.1009666 0.04989741 0.05935181 0.1494572
0.06177302 0.1940526 0.0416373 0.3642142 0.03152363 0.2429312
0.08111813 0.07798139 0.0325739 0.02639797 0.04016793 0.1316266
0.09535055 0.1078223 0.1752919 0.2071335 0.1847051 0.04772054
0.02073524 0.05327866 4.5605040.1845983 0.2960303 0.06826598
0.05248951 0.1152988 0.9108448 0.07831292 0.2596403 0.0650231
0.3465943 0.08672179 0.082737513 0.2279514 0.03832245 0.06444626 0
0.9033149 0.103488835 0.211229609 0.05896761 0.231570855 0.01016188
0.2133890.9621431 0.1975437 0.2556137 0.0810615 0.2999511
0.1830307 0.04878676 0.1762382 0.1361214 0.04607087 0.03068355
0.3410207 0.0631082 0.02415096 0.4927492 0.0558446 0.1079412
0.2200628 0.8070077 0.2147126
AL731566.2 0.2140329 0.2042747 0.07958792 0.049014703 0.096867903
0.3321307 0.1249778 0.1199318 0.1350962 0.1195403 0.2179478
0.130236042 0.5745434 0.241082822 0.196423406 0.1973825 0.3003498
0.1762940.00848818 0.4222445 0.194729305 0.2120533 0.5533359
0.2080244 0.4151726 0.2670942 0.2348303 0.5085440.1668575
0.243910872 0.2798289 0.483283118 0.08969632 0.3220397 0.2448824
0.024959821 0.3745596 0.2756562 0.5916385 0.2373107 0.1898815
0.1505711 0.6202457 0.5060475 0.4017286 0.5253676 0.172729
0.2790966 0.3206535 0.2620131 0.0534806 0.3433617 0.1500668

0.304313953	0.1809033	0.3342445	0.354115	0.1261324	0.3857825	
0.1611582	0.545589	0.1116444	0.1246413	0.2512813	0.07284392	
0.153571342	0.1921222	0.8295415	0.3251753	0.26426228	0.364316	
0.02701379	0.3851836	0.37225044	0.319097	0.1710865	0.2212103	
0.3923266	0.3871952	0.1892974	0.5370054	0.3217068	0.07289216	
0.2531748	0.029692386	0.1305657	0.1195544	0.4416698	0.1770353	
0.5538394	0.1616454	0.241066887	0.3992161	0.2680374	0.4891196	
0.056028959	0.3036743	0.0384392	0.07973274	0.1018995	0.1807594	
0.09523152	0.1672748	0.5117901	0.3044429	0.2800382	0.244113948	
5.023219	0.123160519	0.06719734	0.5214598	0.1539714	0.5022552	
0.4132425	0.1925484	0.1322538	0.199439	0.2652837	0.1830788	
0.3135076	0.1438066	0.4414387	0.4138261	0.3998401	0.6508609	
0.1526477	0.2360844	0.4495463	0.159031643	0.1726226	0.3828451	
0.1457368	0.2423439	0.019690109	0.1714326	0.3064109	1.015093	
0.2313266	0.04599697	0.173738	0.3361516	0.3613544	0.1538231	
0.09947503	0.1154165	0.2157956	2.534367	0.04463605	0.1479424	
0.08046334	0.09415037	0.0808062	0.1614887	0.204720917	0.2601942	
0.3249004	0.203742467	0.1198546	0.3306024	0.1048306	0.1647895	
0.2738835	0.3964466	0.386957308	0.1317638	0.2327968	0.08036417	
0.3209425	0.2262015	0.5820242	0.3867341	0.07514237	0.1909822	
0.281712118	0.152941	0.3033565	0.3903696	0.2280146	0.4562805	
0.08812252	0.3644162	0.3198725	0.4083539	0.2984045	0.1679563	
0.2907251	0.133914	0.07331671	0.1466377	0.01474474	0.2593589	
0.5842525	0.201795852	0.3462705	0.204994	0.05219003	0.2907044	
0.1536721	0.2676064	0.269777679	0.1210967	0.2741291	0.1835202	
0.1801868	0.178237044	0.2315212	0.3449431	0.3243925	0.4478107	
0.2932917	0.5444747	0.08753766	0.1052015	0.8026038	0.4095893	
0.4868865	0.2885025	0.2212569	0.198123979	0.277428788	0.4871315	
0.201711849	0.091208665	0.2607784	0.420174498	0.3541481	0.3921371	
0.5179023	0.187418781	0.5263032	0.3113178	0.1235613	0.260529398	
0.145702	0.2076313	0.310562	0.7509545	0.5592353	0.246344	0.04324661
0.3708042	0.06908216	0.2346849	0.3245762	0.145004486	0.2050113	
0.192334255	0.2214753	0.6222789	0.1866674	0.1534289	0.4804847	
0.3020525	0.2231883	0.1300713	0.9304834	0.186654359	0.544583	
0.9292663	0.4290293	0.3567534	0.09109108	0.1733635	0.1985408	
0.6234807	0.04454926	0.7231779	0.1107737	0.2140807	0.07613499	
0.08779953	0.30563373	0.4827693	0.196263	0.4615188	0.2076387	
0.8824584	0.2556206	0.1735416	0.233218	0.3687089	0.5081375	
0.475551438	0.336210761	0.3222536	0.1982112	0.180116	0.4849001	
0.2694608	0.2209266	0.2519696	0.2224656	0.506179	0.3082304	
0.5152753	0.2468125	0.1977783	0.7876086	0.7983679	0.02387968	
0.6166622	0.1938431	0.239478115	0.4021035	0.2024452	0.1419575	
0.1873967	0.09432544	0.1107868	0.5742008	0.08311743	0.7400412	
0.2996219	0.4737418	0.165317331	0.06998647	0.2313475	0.3135309	

0.09826719 0.05526455 0.343927311 0.3097721 0.1026771 0.9355546
0.1651491 0.1435767 0.1443224 0.252165929 0.3232398 1.068645
0.2134361 0.2878132 0.232369484 0.2174533 0.08040038 0.1111802
0.267791446 0.222676159 0.09543498 0.1771051 0.1118531 0.1749293
0.3114352 0.08205358 0.4465946 0.0505785 0.1592912 0.3079279
0.09897872 0.4812458 0.146925031 0.09360349 4.257363 0.2410214
0.3175919 0.1653113 0.3477664 0.1717552 0.1092105 0.2664159
0.3467273 0.2899254 0.119605 0.3141507 0.1640525 0.2891769
0.09850664 0.2854897 0.1007023 0.4690366 0.2427289 0.1745844
0.4951438 0.06735809 0.180073 0.1945754 0.09252241 0.3218451
0.03953331 0.1490141 0.1903467 0.467567 0.3761856 0.3462449
0.05731361 0.2372616 0.202812 0.3901853 0.6413294 0.1792572
0.0725423 0.5630255 0.1624282 0.1665094 0.1736282 0.4621584
0.1782345 0.2796557 0.310367883 0.3825444 0.1482962 0.1558671
0.0655361 0.1053854 0.143025096 0.140930043 0.09313742 0.240029109
0.1544848 0.1843193 0 0.5393654 0.2555547 0.3472923 0.4522284
0.01487969 0.1763422 0.2060953 0.4950642 0.118247093 0.1837581
0.5302151 0.06229835 0.08344362 0.5269614 0.1372074 0.235545
0.4956262 0.07435415 0.2797836
AC024075.1 1.084724 3.288972 1.066647 2.086625933 0.801850977 2.146141 2.438555
2.680476 3.067328 1.390052 1.030928 1.478489165 4.021434 0.505386825 2.389149998
1.269181 2.514643 0.625424 0.3613539 1.366562 0.172706348 2.350888 1.251784
1.505502 1.730627 0.807171 0.8618156 2.790748 0.7285501 0.263353043
0.9584944 3.514734009 0.2227459 2.035032 0.8687495 0.265643807
1.660994 0.5976198 1.399272 0.748345 0.08981699 0.3561126 2.977005
1.678181 0.4222756 1.827259 0.680863 0.2582944 0.8046132 0.5311557
0.3794576 0.9652998 1.06476 1.439453302 0.2994955 5.461739 0.769969
0.3076354 0.7571031 0.4837697 1.88323 1.069394 1.901374 0.1876737
0.9906194 0.792454719 0.04543843 3.739932 7.160263 0.244565712 0.7087652
0.4791732 1.233169 0.910760517 1.282973 0.3735075 0.7021624 3.293986
0.9157474 0.5165809 1.40811 1.812642 0.08619787 0.9211978 0.158005912
1.363859 0.2915918 1.158126 0.1256108 0.6721722 0.4722582 0.26541108
0.5541913 0.3657287 1.812331 1.126359952 2.962632 0 1.074873 0.1205002
0.08364333 0.2027071 0.3956183 1.263051 1.542925 2.838483 0.418080209
8.059508 0.509747703 0.1787929 1.168384 1.156726 0.7238604 0.6515675
0.9983599 0.7251059 0.08844171 0.9411254 0.3418388 2.039044
0.7014853 1.071512 2.181173 2.553265 1.473834 1.95786 0 0.9303111
1.35404085 0.3993908 1.895147 1.155689 0.8971239 0.279412017 1.18058
0.3780971 0.6232786 0.4787175 0.7751038 0.3081781 0.3123313
0.4437511 1.611131 0.2744774 0.5322899 1.011634 8.43291 0.2771155
0.1211177 0.5138159 0.4731803 0.0409528 0.528831 1.162034921 1.53845
1.921038 0.653962499 0.5798162 0.07819009 0.4958655 2.221519 0.3701464
0.6618548 0.519429179 2.430728 0.6882288 0.1425507 0.2777028
0.4012384 0.8111714 0.8593753 0.03808236 1.570643 0.499703638

0.4340611 0.3275371 0.9633966 1.5075121.78058 0.4515697 1.903303
2.2202330.9657894 0.3664473 0.7360437 1.6606990.03958965 0.4768495
1.0079160.6800132 0.0438146 2.4966680.602858785 1.4916720.7272405
0.3703007 1.8907320.8806592 0.5379738 0.170905077 0.6639349
0.4651115 1.2207371.6852530.553277491 0.7744148 0.8158177 0.85246
1.4184481.7580652.3396711.28102 1.0367080.7001637 0.6393494 2.181837
0.7603119 0.9088722 0.76676553 0.815489654 1.4897910.828585765
0.202233498 0.2312856 0.465818454 0.4568664 3.0498362.7997260.284953044
1.5925460.4295033 1.5342190.616172703 0.5815070.4687434 1.4690081.33205
0.5510983 0.8342105 1.1506692.6309440.3676158 0.3568168 1.124694
0.712274785 0.2644735 0.41697863 1.5172331.2834930.7450031 0.2474124
0.4261442 2.8798370.8907605 0.3076291.5433250.975842086 1.375303
1.0033382.8455392.3040870.3554721 0.2391774 0.6163037 0.9598689
1.3960534.8104251.1789490.7195045 0.1350490.07786982 1.511409512
0.8431663 1.0830810.7663924 0.3556111 2.4860860.6909291 0.2308723
1.0342110.5232154 0.6595164 1.303649125 1.001908068 1.61958 0.2109533
0.4259887 2.9322290.3476161 0.7091194 0.5688404 0.03946116
1.2882410.9372721 0.8658949 0.8755966 0.3648539 3.1717213.121738
0.2541481 2.0418370.4705188 0.707980936 1.7643680.4388991 2.120468
0.1772832 0 0.5109382.91793 0.5307641 1.9522090.8696818 1.192723
0.439862185 0.4551898 1.7045991.46822 0.1743073 0.3136921 1.03840262
1.3431650.3082194 4.3750340.2441191 0.4810579 0.9102238 0.393619007
2.5295551.2906030.54085 1.0479210.677151548 0.5340750.1140920.3451218
0.700016237 0.552979129 0.3584826 0.9948104 0.1984060.6619548
0.6799098 0.1455474 2.2694710.6459597 0.9307605 0.5006883
0.4514641 2.6633520.293194146 0 7.9916590.7125435 1.0978050.6597694
1.9231871.1424790.1192114 1.1814282.1156970.6303984 0.6364697
4.9472361.5277381.5144080.3727616 0.9514262 0.0510362 2.394484
1.0377481.4769330.4640027 0 0.1996345 0.1294274 0.5579982
0.5957133 0.2337486 0.5286453 1.5654162.1326781.0485850.4386947
0.1694390.4643939 0.9408841 1.5439473.0989690.2008220.2573524
3.2787510.9723939 1.1518881.8068360.1366302 1.6993280.3897583
0.347705373 1.19746 0.1127354 1.1059140.05812428 1.0640840.744184547
0.607101698 0.4130201 0.662302541 1.0961060.4795226 12.130251.559118
0.5866305 0.8346217 0.8355906 0.1055749 1.1407910.4985097
2.4236930.35495785 0.3510251.7974010.2210108 0 4.4579390.3346471
1.2534362.4775950.3517069 1.413654
AL731567.1 1.1858010.0658808 1.55719 0.068500346 0.135377438 1.4919690
1.6593420.0566410.05568774 0.5482661 0.218413172 1.5625 1.608048988
0.235295076 4.4998243.9792580.9239218 0.5694062 4.1618042.517325863
11.502262.2526940.06977371 2.00177 0.9539293 2.7839255.0971521 1.35462
0.400159819 1.1327663.478365734 2.7828770.9282603 2.5937780.383707722
8.2445684.9439467.1935310.2211019 0 0.6312905 15.373351 2.6378
3.8052797.9901050.08046562 0.8140188 2.4920317.9250850.2242249

4.128642 1.31555 1.275879063 5.359802 2.463009 2.777783 0.3305174 3.510205
5.821229 2.225635 0.5148933 0.2612881 0.887185 1.628845 0.643869459
0.1610999 8.550008 1.410355 0.529892377 4.188158 0.3397773 3.072318
0.645812003 0.2973021 1.60014 0.6345746 4.715335 6.042546 3.479877
3.328261 5.077839 0.509351 0.2721721 0 0.03041194 1.12781 1.247927
0.8412115 0.3971927 7.255604 6.133944956 0.0363863 0.4898548
15.12963 2.192486752 3.564952 0.1074412 8.825275 11.06045 0 9.662371
8.532767 4.431444 4.084543 1.1741 0.705849704 28.15129 1.333950157 1.197371
0.5753404 1.746773 12.89788 1.732577 8.445456 0.4536759 0.5226101
1.423666 0.3231931 1.533496 1.431958 4.432164 0.7931538 0.06095949
0.5515712 1.181533 1.034809 2.146562 1.533553841 1.888029 4.14348 0.3594247
3.578301 1.733624558 0.7610349 1.675658 3.273786 1.576038 0 4.079158
3.456295 0.4078924 4.944425 1.621912 0.5322899 4.071391 2.711383 12.95965
4.055608 3.036185 2.072378 1.887552 2.135356 2.803849412 1.527261 13.36965
0.488125119 0.5938723 0.1848129 3.174291 1.796348 3.718289 0.5866441
0.789262519 3.038409 6.50689 9.153485 4.824455 5.785128 2.614519 1.175987
0.1350193 0.7643235 0.531502961 11.79857 0.2764923 2.526179 0.1303614
13.39114 0.7389322 9.591604 7.172026 1.549026 0.2405967 1.905429 2.003964
3.509082 0.7684765 2.036522 4.883731 1.915893 7.29301 1.603056315 6.429347
1.023174 1.312884 11.57878 1.883314 0.0747985 0.363561709 0.8308063 0
0.6411952 0.2747121 0.840694369 0.3882741 11.63004 4.130556 1.072863
3.985817 4.115447 0.7799047 1.029168 4.468317 3.297141 1.128109 2.350055
12.64534 1.208236593 0.199398724 2.676206 0.40059725 0.09560129 4.601182
3.79853776 17.50283 4.995505 6.479668 1.178669411 5.256901 0.6526219
4.033373 1.395724531 2.443504 5.302279 4.947885 0.3590374 0.651298 0.1408407
0.4532937 3.8348 0.7965009 4.67376 2.246954 3.507413712 4.922814
0.313595498 3.810324 0.9101134 0.03260956 1.169586 0.2238333 10.80396
7.485987 1.13613 9.018318 1.647525601 6.965819 3.33143 0.1042763 11.42898
1.26031 3.472731 5.462692 11.14662 2.054569 8.175639 0.7740573 0.1417206
0.5586116 0.9202797 4.931496254 1.463564 0.1828578 2.140832 1.500956
1.197004 4.797246 2.091843 4.714389 0.9275183 6.079542 1.042222161 0.620228804
2.02664 2.07757 0.6041294 0.8316868 0.3081142 0.2205395 0.576228
0.6529029 6.458951 0 2.103512 0.09701212 0.6302022 6.872062 2.712022
0.5005947 5.791393 0.5988422 2.384608517 1.286589 0.2829267 0.7518024
0.05237913 0.2824805 1.114774 7.352384 0.1393926 14.43961 6.623637
2.626948 0.831739404 0.8313797 1.193793 0.8938753 0.8651979 1.760953
0.245440619 0.8369807 0.2318014 2.924004 0.519308 0.4347529 3.496087
0.54976539 1.395088 2.144894 8.394648 2.318129 1.913689158 1.893539 0.7415978
1.981089 1.418214714 0.186720225 0.2353674 0.06187816 0.8441275
7.871997 7.081106 0.2580159 9.463266 0.9754644 0.7464216 12.04962
3.349499 0.6053072 0.654504103 0.1962229 29.1139 4.771882 2.731382
0.8230457 1.844016 3.150472 0.950982 5.654742 0.988518 8.78335 5.975743
0.8352476 3.525042 1.558819 0.4956035 3.663409 0.361893 6.762849 0.443602
1.069802 2.350143 1.299078 6.181409 0.6118386 2.05594 1.642724 0 0.234286

7.6177999 8.601494 4.281002 2.592287 0.08009846 0.3430182 1.569817 6.858224
3.894216 1.582234 0.6082876 11.49105 0.4681896 1.780191 1.310329 1.399424
12.18989 0.58624 815.409669927 1.415181 4.840789 3.827613 1.373847 0.577795
1.359213098 0.46425424 0.4881147 2.571798177 0.9421079 15.81632 0
2.456792 1.638687 4.039425 0.4740078 3.431185 5.43632 0.3142122 11.08384
0.953399183 0.5333757 5.631603 0.626867 0.5597586 3.976848 1.617922
0.6583702 5.478318 1.610658 1.883961
AC006160.1 0.05922595 0.08116515 0.04567757 0.042196213 0 0.09190531
0.0968328 0.1238976 0.1395634 0.1715182 0.1125773 0.134542514
0.04010416 0.056603324 0.036235442 0.1593041 0.09308442 0 0.153455
0.3443737 0.083820148 0.1369157 0.04142267 0.1289418 0 0.1532923
0.2091339 0.2052187 0.09944709 0.082166149 0.1246043 0.166421227
0.06949671 0.3378867 0.1331473 0.021487632 0.1074847 0.1186547
0.6621355 0.06809939 0.03269338 0.337025 0.04711435 0.454592 0.2305625
0.05320978 0.1239171 0.09401917 0.08079426 0.04833517 0.1381226
0.1338549 0.1409355 0.02619805 0.09344258 0.2092708 0.2065138
0.1832388 0.08479555 0.2241173 0.03808309 0.3171742 0.06438139
0.2049397 0.1567763 0.144226759 0 0.2231697 0.05791858 0.148369865
0.2428146 0 0.09705374 0.331516828 0.09156903 0.1699459 0.09020722
0.2701997 0.166666 0.1128213 0.1206005 0.0977484 0 0.0502974 0
0.05620127 0.0771923 0.07439255 0.335297 0.2634915 0.3192466
0.021468807 0.2241396 0.03550007 0.1965029 0.192938834 0.3529302 0
0.04118462 0.2924137 0.0811898 0.2705464 0.1800063 0.05746883
0.08985995 0.129151 0.130441025 2.477314 0 0.04338708 0.7442604
0.04678314 0.08107236 0.7708044 0.0765059 0.03105159 0 0.1370279
0.0746576 0.3036321 0.2785534 0.09000702 0.06107284 0.1126531
0.1788252 0 0.08314465 0 0.041072572 0.2326052 0.06898335
0.1549839 0.05442552 0.152558961 0.07813292 0.2408479 0.1512489
0.02489331 0 0.3140952 0.1860359 0.3230508 0.1135068 0.08563695
0.2086577 0.01990458 4.288383 0.02882001 0.1175649 0.20781 0.1013163
0 0.09624725 0.035248393 0.08959931 0.03107812 0.150342537 0.3376849
0.2846119 0.2105776 0.02837309 0.1684166 0 0.054020635 0.1020906
0.03340204 0.06918462 0.4043353 0.05842031 0.2147392 0.1536623 0
0.291462 0.436541098 0.2764969 0.06812771 0.1972555 0.1070701
0.2946051 0.0758637 0.3137218 0.1795922 0.1255526 0.2074906
0.1275809 0.190893 0.1152851 0 0.04733953 0 0.06379405 0.3772323
0.109720299 0.1596967 0.201688 0 0.2252378 0.1221181 0.1382276
0.174186455 0.2558883 0.1231277 0.1184929 0.08461133 0.345245154
0.0854203 0.1855985 0.4964727 0.08261042 0.1044793 0.2376671
0.05652015 0.04528341 0.1699064 0.04231331 0.1323648 0.2341761
0.150377 0.093034218 0.286602433 0.8242713 0.137093281 0.235561579
0.08418797 0.033911583 0.02771656 0.1168569 0.1061563 0.276594422
0.1599136 0.06700251 0.2279411 0.074762288 0.09407491 0.09749862
0.2673594 1.003758 0.1203599 0.2313544 0.3909205 0.2340955 0

0.06494066 0.1364629 0.086422674 0.3850734 0.027596404 0.2958604
0.3924409 0.08034996 0 0.2757626 0.1218908 0.07205262 0.08398271
0.2496757 0.152231365 0.1668701 0.2086943 0.1445269 0.378
0.1176289 0.22387 0.3204779 0.06076388 0.1150559 0.3335228
0.03178795 0.2036997 0 0.02834461 0.095678793 0.01918203 0
0.1521637 0.01849178 0.4021934 0 0.1400625 0.2366274 0.1904504
0.3520938 0.16748057 0.104198439 0.161831 0.2047653 0.06202395
0.4838569 0.06326612 0.0815114 0.2662173 0.02872773 0.2557745 0
0.7354334 0.09959911 0.2451818 0.3848355 0.1053535 0 0.08494042
0.1317453 0.103082024 0.2186314 0.6099899 0.09648131 0.06453109 0
0.2575128 0.5210433 0 0.3920574 0.0351738 0.4144171 0.170783824
0.09037586 0.5055714 0.2267288 0.4060662 0.1427299 0.170090349
0.03555725 0.04079705 0.351451 0.142175 0.0824024 0.1242456 0.208403372
0.1473213 0.4991406 0.3937388 0.07824479 0.214333186 0.08640146
0.1245884 0 0.691616042 0.143774574 0.08699179 0.1334093 0.1155517
0.1204758 0.1237436 0.05297926 0.1246924 0.05225097 0.07259932
0.1656823 0.01825922 0.0497159 0.118580743 0.09065498 1.120885
0.1383284 0.1051582 0.08005202 0.2641661 0.360414 0.04339296
0.1720159 0.07163848 0.04830842 0.07722499 0.1978894 0.0529616
0.07112835 0.1187246 0.1117159 0.03715435 0.3840914 0.2250367
0.1387364 0.07238442 0 0.02906678 0.2826695 0.09558181 0.1806997
0.06806758 0.7216009 0.06703663 0.1971542 0.06939729 0.2129132
0.02467032 0.02112992 0.2417519 0.2713088 0 0.1949312 0.03747051
0.08230796 0.1310931 0.06450575 0.3587391 0.3315559 0.1150801
0.1031797 0.295317763 0.07748899 0.05471424 0.2012764 0 0.1046829
0.197005946 0.207985899 0 0.172198661 0.07254231 0.1586784 0
0.1547779 0.09706068 0.05786711 0.1703268 0.03842927 0.05358037
0.04838867 0.1790002 0.023491756 0 0.1825825 0.1608959 0.08620282
0.2512552 0.8416071 0.1216668 0.116367 0.03200533 0.0437932
GATA6-AS1 0.4385904 1.619519 0.4322206 1.024236116 0.205850937 0.3781074
1.420887 1.185115 1.105292 1.241931 0.2470155 0.595035117 0.5197262
0.74518913 1.520576678 0.4587749 0.4084889 0.2965875 0.08117084
0.7398777 0.103453282 0.01408212 0.02556254 0.05304792 0.02205676
0.1786868 0.05162384 0.2617298 0.05455134 0.123947839 0.6869298
0.136934635 0.01906108 0.1390099 0.1917233 0.017680444 0.1105507
0.1464471 0.1047727 0.3642178 0.107603 0.0106658 0.05814991
0.3156023 0.03161855 0.04378205 0.2345112 0.05157387 0 0.07954224
0.1704749 0.137673 0.01449556 0.032334401 0 0.01076201 0.0121374
0.0167525 0.04360714 0.7508044 0.01566776 0.09490079 0 0.1264713
0.01289986 0.044502223 0.2177455 0.01836284 0.01191413 1.171983578
0.1123833 0.02870308 0.02994664 0.06819463 0.4445343 0.3216201
0.06185355 0.08337209 0.1371361 0.04641577 0.1116365 0.08042929
0.07745042 0 0 0.02312175 0.381092 0.0510097 0.1254041 0.5807302
0.05051583 0.114822201 0.1198772 0.007302537 0.2425296 0.09922115

0.04839965 0.02722865 0.1524938 0.1082717 0.01670114 0.1720175
0.08886763 0.01182162 0.009242315 0.08501434 0.089441186 0.1341041
0.806984021 0.1070992 0 0.2694586 0.01667698 0.1057055 0.01573764
0.05109972 0 0.01127492 0.2559572 0.04163908 0.0572998 0.03702977
0.08794087 0.08496877 0.03678521 0.1122877 0.4218804 0.7960973
0.304157832 0.07177204 0.2081231 0.1548503 0.1903251 0.481192554
0.06428929 0.2264849 0.1866759 0.1228962 0.02443668 0.3138244
0.2381151 0.1033715 0.007782967 0 0.0122634 0.0655115 0.01857139
0 0.04836736 0.2564852 0.0083365 0.06132811 0.1583882 0.014501533
0.01843101 0.166216 0.123704775 0.1505041 0.608879 0.4579208 0.3852079
0.0277153 0.346902 0.033336925 0.01400035 0.02748385 0.07115807
0.04990425 0.6729712 0.3202535 0.1761078 0.2851476 0.2674923
0.014966439 0.09750303 0.5045113 0.0901698 0 0.105043 0.135248
0.06453411 0.05910879 0.3822365 0.06097404 0.3884114 1.010487
0.1067161 0 0.4674226 0.04700037 0.01312275 0.08465295 0.033855002
0.1445417 0.09334848 0.194088 0.09266501 0.4019245 0.07582426
0.020474859 0.1169722 1.190414 0.008124854 0 1.633428116 0.1124568
0.5344993 0.089361 0 0.01074595 0.08148213 0.1046382 0 0.489308
0.4700189 0 0.3328189 0.0742399 0.019137593 0.008422217 0.0535442
0.045121212 0.181710625 0.4502648 0.111612452 0.01140286 0.9775465 0
0.071121029 0.08223754 0.01837699 0.009377719 0.046136903 0.1935167 0
0.1979897 0.06999253 0.08252871 0.297442 0.1608285 0.6741644
1.669899 0.03562296 0.1684265 0.142220527 0.05940861 0.06812058
0.1379492 0.2383369 0.00826419 0.2223047 0.5388944 0.07020588
0.07410788 0.783163 0 0.334023841 0.6865197 0.02861963 0.08588634
0.009719556 0.2322898 0 0.118663 0.0499977 0.2682323 0.06860726
0.07846725 0.1316921 0.2224641 0.05830626 0.03936319 0.1025918
0.3012189 0.06260165 0.05325388 1.227208 0 0.04609848 0.01770015
0.2481183 0.02633724 0.045935428 0.042868255 0.1236464 0.1263637
1.288622 0.2107731 0.0520566 0.2794549 0.3468273 0.0709132
0.1286121 0 0.2881566 0.0901473 0.168117 0.09047147 0.006191922
0.2029839 0.06115431 0.1951247 0.180238043 0.1349207 0.09560221
0.3413628 0.02654872 0.01431772 0.1883436 0.04946822 0 0.0705671
0.01447084 0.04871293 0.043913833 0.991507 0.03781764 0.1732314
0.06264736 0.1056969 0.108852847 0.1097145 0.0167843 0.0271107
0.2193457 0.08475288 0 0.107173978 0.4040628 0.1570334 0.4751648
0.120715 0.079360889 0.05331961 0.5638259 0.5168308 0.104829639
0.153790685 0.02982439 0 0.2020411 0.04956491 0.05090932 0.08718475
0.07694953 0.07523795 0.1692524 0.2999186 0 0.0102268 0.702507969
0 0.1537144 0.1991838 0.04326311 0.1097806 0.2390971 0.2281198
0.05355686 0.0353845 0.01473639 0.04968639 0 0.02442409 0
0.1463145 0.04884445 0.06434539 0 0.03646591 0.006613021 0.2140406
0.04963276 0.02862811 0 0.1550573 0.01966165 0.04460497 0.1820238
0.01979158 0.06435223 0.2129168 0.02855072 0.06131619 0.01014961

0.09562362 1.790264 0.01594559 0.01174887 0.01002458 0.07707868
0.02257487 0 0.1238454 0.03689719 0.5183403 0.02367253 0.1167351
0.156209929 0.1673686 0.1012949 0.04731833 0.05222577 0.1378162
0.010131271 0.246006361 0.1855532 0.056675368 0.02984461 0 0
0.02122571 0.03993171 0.130939 0.2302435 0.3003934 0.03306523
0.1692144 0 0.048323689 0.2553259 0 0.05295531 0.2305204
0.0861407 0.227793 0.4338093 0.0359059 0.3950191 0.2252119
MIR1-1HG-AS1 0.5797065 8.691146 0.05546658 5.533827799 0.147293203
0.4666961 3.484783 5.935923 3.890171 4.142794 1.267613 1.151057349 2.470355
2.161990384 3.096066215 0.3868881 0.5685904 0.1842948 0.2371618
0.01689597 0.018506067 0.01511434 0.004572712 0 0.01183676 0.2369104
0.01385197 0.1540499 0.007318744 0.006046964 1.166446 0.101043209
0.09206217 0.05164592 0.09553916 0.056929259 0.4093564 0.05239389
0.07871678 0.706654 0.02165443 0.1259236 0.06241229 0.6021972
0.008484048 0.01762173 0.0766046 0.01383856 0.004459501 0.08537269
0.01829707 0.007388223 0.03111617 0.028920426 0.03438423 0.01732629
0.01302707 0 0.009360709 1.25117 0.008408101 0.05729457 0 0.07541203
0.01384542 0.031842823 0.05112324 0 0 0.432399667 0.02010351
0.01540351 0 0.007319329 2.446244 0.02251271 0.1062199 0.01491388
0.4722286 0.01660602 0.2329823 0.04316238 0.4294922 0.05182246
0.008465433 0.08685801 0.04686757 0.04927384 0.06056827 0.2243872
0.005421864 0.066359281 0.004948622 0.007837813 0.006197787 0.010649406
0.05194734 0.4018369 0.05455719 0.025824 0.013444 0.005430187 0.07153622
0.01903222 0 0.02281147 0.057598333 0 0.046818158 0.03831653 0
0.6094065 0.008949701 0.008727207 0 0.03427833 0 0 0.02747189
0.02234561 0.04099992 0 0.07416117 0 0.007896314 0.2477323 0
3.261155 0.190430679 0.02139804 0.2081482 0.01466478 0.5287142
0.112274773 0.01725042 0.1975076 0.166966 0.02198408 0 0.04953349
0.009127461 0 0.01670692 0.03781437 0 0.004394593 0.04983169
0.006362969 0.03893451 0.1101142 0.03131648 0.01974704 0.1487483
0.007782247 0.03956402 0.1921226 0.022128722 0.07455511 0.02513499
0.01992512 0.7705085 0.05949365 0.132975 0.059634201 0 0 0.007637396
0 0.01289821 0.2666856 0.04361919 0.01224196 0.6335988 0.032126957
0.06976667 0 0.004838962 0.02363927 0.01300876 0.1563279 0.01154408
0.03965091 0.6153816 0.04581041 0.4056149 3.509352 0.08908545
0.006967641 0.5173623 0.008407582 0 0 0.006056095 0.3948932
0.03896314 0.1264767 0.01105082 0.4313856 0.1169869 0.027469583
0.01255462 0.1223303 0.008720406 0.04670187 2.655145615 0.04149058
0.2868391 0.1575683 0.01823898 0.02306725 0.004372738 0.02495738
0.003332603 0 0.3456563 0 0.01880077 0.0332007 0 0.009039565
0.1819851 0.006053574 0.039006002 0.9169723 0 0.006119347 2.150001 0
0.022900259 0.1059187 0.1923092 0 0.041265614 0.1973164 0
0.2951421 0 0 0.02553956 0.09863842 0.004698575 0.009847818
0.004779266 0.2496378 0.050881763 0.02125442 0.182784541 1.720119

0.6766507 0.008869955 0.03976661 0 0.005382284 0.8351699 0.5500781
0.02067156 0.711413873 0.1105262 0.007679362 0.04963636 0.005216
0.05194095 0.01647557 0.2901 0 0.06350597 0.01472723 0 0.1092212
0.0759725 0.03129005 0 0.004235068 0.03730364 0.05599195 0.01633068
0.02959916 0 0.01236937 0 0.02803215 0.06360247 0.012325623
0.046010497 0.2347941 0.01130218 3.108501 0.3204822 0.04888831
0.5878803 0.04244953 0.02537038 0.03137259 0.06276991 0.1701032 0
0.08570898 0.5462046 0.003322895 0.05446565 0.02813011 0.005817425
0.085345362 0.0663714 0.01923935 0.4856728 0.007123686 0.06915242
0.1326604 0.00884904 0 0.01081995 0.03882889 0 0.028279629
3.698041 0.04058967 0.04290676 0.2521478 0.02520987 0.154384823
0.08635481 0.009007296 0.04849652 0.02354237 0.06822394 0.02743131
0.069017896 0.09215713 0.1944737 0.01159078 0.01295634 0.018928453
0.01907598 0.04584502 0.3328287 0 0.044440183 0.003201052 0.008415575
0.03188981 0.08644678 0.01366024 0.05848462 0.006882494 0.02307225
0.06945769 0.01463194 0.08465783 0 0.12043084 0 0.08249081
0.03817572 0.1810938 0.3181343 0.2974493 0 0.004790215 0.02848364
0.01581656 0.07999261 0.02273329 0 0.03507907 0.1256315 0
0.2515827 0.0164061 0 0.02839102 0.05360362 0.005327084 0 0
0.006934291 0.03165427 0.007979085 0.05259859 0.03186345 0 0.01632312
0 0 0.005446789 0.009330257 3.691751 0.04278599 0 0.03227813
0.09100139 0 0 0.1186815 0.05940263 0.01464038 0.03175966
0.06264588 0.083830056 0.0598789 0.03019994 0 0.02802696 0.4714889
0.005436947 0 0.01991542 0.007603709 0 0 0 0.2809725 0.09428915
0.03194019 0.236376 0.4581647 0.008872228 0.2243514 0 0.020746325
0.09672047 0.04031112 0.01420923 0 0.2172688 0.04889805 0
0.0385378 0.7419546 0.05801277
AP001001.1 0.04749475 0 0.05494495 0.025378637 0.025077938 0.1105517
0.05823945 0.1304054 0.02098488 0.04126341 0.2708356 0.141609503
0.1326621 0.051065549 0.065380709 0.1341374 0.05598501 0.0228202 0
0.2991752 0.10082616 0.1029338 0.1619371 0.05170081 0.3869396
0.3073222 0.4025031 0.382625 0.1395609 0.082363823 0.1199079
0.212697538 0.1114622 0.2032198 0.06006043 0.103388769 0.3070685
0.1903042 0.1838017 0.04095793 0.03932644 0.1403318 0.2550295
0.5980874 0.06933515 0.2880241 0.01490582 0.05654721 0.1336313
0.2616378 0.1329167 0.2113287 0.04238237 0.094539876 0.1686013
0.1887969 0.1596939 0.06122656 0.1657491 0.1540502 0.3206676
0.2254464 0.2710524 0.04108655 0.03771683 0.130116362 0.07958101
0.4563615 0.1741737 0.035694434 0.2190589 0.1258838 0.1459308
0.119633178 0.1652208 0.1839832 0.3255272 0.1421961 0.7350948 0
0.3989391 0.411531 0.0188709 0.1008368 0 0.03380189 0.0464268
0.1342287 0.09166463 0.07923762 0.02953981 0.116210465 0.01348073
0.09608077 0.135069 0.290104502 0.1415117 0.05970869 0.07431066
0.3165666 0.04883107 0.1479258 0.2165272 0.2592319 0.1216028

0.0932124 0.169981288 2.744671 0.06376956 0.05218975 0.490262 0.1406871
0.1462813 0.3090635 0.1610489 0.1494062 0 0.08241451 0.1796093
0.385526 0.08376707 0.1082683 0.5877098 0.3952346 0.08604259
0.1276757 0.08334468 0.1939702 0.148216982 0.04663296 0.4840452
0.06658148 0.08183468 0.18351118 0.06265671 0.0620813 0.2425805
0.02994384 0.0178621 0.2158977 0.1864835 0.0647656 0.2730717
0.1201802 0.01792797 0.02394296 2.497771 0.08666803 0.2298031
0.124986 0.121872 0.07172487 0.03859152 0.31799873 0.1886112 0.1121504
0.180845072 0.06769947 0.06847119 0.2894869 0.1706481 0.04051723
0.2535695 0.146206609 0.1228034 0.04017887 0.1456372 0.2431848
0.3513651 0.177586 0.1980412 0.1000463 0.09439119 0.218795659
0.3325946 0.2253623 0.171366 0.08049579 0.1771883 0.07604621
0.1572383 0.02160291 0.03020512 0.1069662 0.07673271 0.09184909
0.3986903 0.2277698 0.1423602 0.2977444 0.03836852 0.5568969
0.115483474 0.4418232 0.1213039 0.02026706 0.2107276 0.05508534
0.1662722 0 0.08550131 0.1295951 0.1662891 0.1526668 0.034607574
0.1541264 0.06697622 0.1306376 0.07452824 0.1413864 0.1191194
0.101981 0.1180201 0.2861294 0.1781434 0.4179522 0.07682382
0.1627979 0.139887055 0.098500092 0.1826457 0.263852345 0.070838486
0.0337562 0.061187701 0.4000787 0.2108484 0.1660022 0.083177954
0.6251635 1.235812 0.3427342 0.292274381 0.1980326 0.2931995
0.2572825 0.1739486 0.217169 0.1217533 0 0.2815905 0 0.1822713
0.2579492 0.294543997 0.1447499 0.132781414 0.2609835 0.078677
0.06040745 0.06499783 0.04146391 0.2492554 0.2816813 0.05051085
0.1689365 0.06103904 0.2150634 0.3347143 0.222141 0.2841821
0.2122415 0.104724 0.1734744 0.2375492 0.1268659 0.2808331
0.07647463 0.2275257 0.02956565 0.06819073 0.604226543 0.05768454
0.2709874 0.04575894 0.04448703 0.2015805 0.03781548 0.2190232
0.06469001 0.4963624 0.2695185 0.285401933 0.250677881 0.1807598
0.1231548 0.1865195 0.4621964 0.07610199 0.163415 0.06226681
0.05183431 0.4102237 0 0.1895664 0.1557484 0.2580603 0.2645221
0.1086243 0.09273246 0.2043475 0.0475424 0.092997015 0.3451729
0.1397617 0.08123928 0.05821771 0.02093119 0.1548794 0.3374841
0.05164345 0.2505379 0.2961707 0.4866279 0.218273232 0.01811866
0.1105719 0.09740327 0.06105647 0.08584397 0.250066004 0.2245496
0.1472227 0.224589 0 0.1115109 0.02490889 0.062671423 0.1624433
0.6180688 0.03157488 0.1058846 0.232036729 0.2858108 0 0.06476223
0.153251349 0.069177822 0.07848096 0.4699663 0.0347489 0.3622968
0.3535177 0.03186403 0.1499909 0.141417 0.1601027 0.1594377
0.2635653 0.2541611 0.05705569 0.03634923 2.34347 0.1247951
0.1517921 0.1604892 0.1906572 0.3001412 0.1696397 0.284509
0.08617299 0.2324383 0.09289293 0.4403714 0.1274136 0.06416952
0.06120525 0.2418868 0.04469249 0.6930278 0.09667632 0.2503262
0.1160937 0.02092584 0.1223742 0.09444983 0.1293459 0.3043058

0.0614082 0.1446674 0.2284729 0.2371543 0.06260782 0.268916
0.02967561 0.08895918 0.1211667 0.4662198 0.1889335 0.1025851
0.0225364 0.3960287 0.1261521 0.2198472 0.1798011 0.1994121
0.3114637 0.1085993 0.101495393 0.0932105 0.09872257 0.2075252
0.1272488 0.05036865 0.236975877 0.07818235 0.01808412 0.082854207
0.1090752 0.3244827 0 0.206867 0.1634542 0.05220569 0.1170765
0.02311303 0.1208459 0.02910305 0.09227964 0.042386889 0.1097833
0.8602023 0.01935395 0.02592306 0.3274175 0.1465255 0.1097636
0.4024321 0.09624698 0.6321392
AC090825.1 0.1720351 0.872321 0.09287655 0.514787266 0.096892914 0.3737435
0.3375272 0.1799442 0.5675507 0.8967839 0.1526029 0.156323603
0.3261764 0.44392615 0.589422366 0.1665845 0.1982821 0.1763395
0.1273556 0 0.12173724 0.1988513 0.01203215 0 0.01557299
0.08905439 0.2186915 0.1311428 0.1925776 0.111379366 0.6225388
0.072511185 0.02691584 0.01509951 0.1160269 0.062415663 0.1092748
0.1148864 0.1479478 0.079124 0.05697917 0.225915 0.1094834 0.3631278
0.02232402 0.04636794 0.4751274 0.01820665 0 0.02808008 0.04814496
0.03888117 0.08187577 0.182635516 0.090475 0.07598428 0.03427805 0
0.07389232 0.1115999 0 0.2512647 0.03740205 0.1190587 0.01821568
0.251363445 0.1345203 0.01296493 0.1009425 0.103433678 0.03526549 0
0.1127658 0.115555827 0.2766222 0.07898339 0.08734238 0 0.0645492
0 0.0525467 0 0.145822 0.04870004 0.378675672 0.06529969 0.1009001
0.01440599 0 0.02186772 0.04279955 0.037416589 0 0.01031179
0.0163082 0.252195416 0.06834435 0.2306948 0.11963 0 0.2594174
0.1857494 0.06274426 0.06677246 0 0.1050414 0.164187968 0.1136197
0.153990398 0.07561652 0.02058925 0.3261412 0.02354929 0 0.15556
0.1262749 0 0.06368452 0.1879452 0.07839714 0.02697071 0.01742971
0 0.06544528 0.02077752 0.07047097 0 0.4496624 0.143165435 0
0.2137362 0.09003713 0.07904558 0.354513465 0.09078186 0.1199309
0.0292891 0.04338493 0 0.02606743 0.02401703 0.166822 0.0329706
0.0331669 0.1039017 0.1040712 0.8916283 0.03348568 0 0.1931619
0 0.01732008 0.1863812 0 0.0780784 0.07221872 0.029113582
0.09808819 0.09920632 0.06990514 0.08241602 0.07827263 0.06997923
0.078457544 0 0 0 0 0.08484746 0.2806912 0.1339040 0.1302488
0.190204778 0.09178829 0.1781031 0.08912911 0.03110093 0.06845975
0.08814528 0.01518793 0.02086664 0.3355202 0.01722009 0.1630585
0.5323121 0.06697428 0 0.2750166 0 0.01853042 0 0.015935365
0.14844 0.05858483 0.1370342 0.01453897 0.6562309 0.1739891
0.130104965 0.1321396 0.0178826 0.03441892 0.02457727 0.401136895
0.1687233 0.1940806 0.2703969 0 0 0 0.01641755 0.03507623 0
0.1229085 0.01922416 0.148411 0 0.351310465 0.083250126 0.1512178
0.095572388 0.051318121 0.0815143 0.03940153 0.0161018 0.1357748
0.02466838 0.200857684 0 0.0518997 0.01324212 0.065149166 0.02732617
0.02832067 0.8542661 0.16802 0.02330749 0 0.09732999 0.01236333

0.2850375 0 0.1698803 0.083678034 0.02796331 0.064127972 0.5041773
0.2931256 0.07001836 0.02092752 0.04005073 0.01416237 0.04185861
0.1626311 0.01813098 0.545367982 0.04154671 0.06061997 0.02798739
0.233322 0.02733438 0.01445069 0.07447202 0.03530047 0.3676263
0.116255 0.03693411 0.08452738 0.14279 0.08233331 0.027792059 0.04457483
0.1308758 0.1915306 0.04297082 0.03894204 0 0.0650949 0
0.09220102 0.07438079 0.129729334 0.020177854 0.05372279 0.02973935
0.2702438 0.09920973 0.01837707 0.06313819 0.1288814 0 0.0990606
0 0.2237957 0.1157232 0.1068279 0.7345814 0.008743512 0.1970583
0.02467286 0.09184411 0.089827481 0.06350642 0.06749916 0.325092
0.03748902 0.1617425 0.1496008 0.2794131 0.1247083 0.08541142 0
0.02292891 0 0.2975193 0 0.5268679 0.132695 0.04975094 0.197626277
0.4647781 0.2725597 0.02552171 0.1238938 0.08377471 0 0.060535449
0.08558558 0.1705725 0.04574812 0.2727355 0 0 0.04825268 0.12511
0.46523155 0 0.04211454 0.06643154 0.1678229 0.01749745 0.03594411
0 0.03621973 0.01517747 0.3233509 0.05775138 0.0636456 0.0144411
0.055111112 0.01755519 0.1550411 0.1406321 0.04887291 0.0775097
0.1227728 0.01610621 0.1008357 0.07494882 0 0 0 0.1034666
0.1538389 0.06198249 0.05911924 0.05192061 0.02158464 0.2403003 0
0.181346 0.01401712 0.04042529 0.0168862 0.05473847 0.05552778 0
0.03954352 0.2515263 0 0.04295091 0 0.03710725 0.07166051
0.09820258 0.7256302 0.045033 0 0.05662216 0.1523782 0.0159388
0.5331052 0.08744002 0.03473461 0 0.06685519 0.1198834 0.073527162
0.1800674 0.04767895 0 0.02458238 0.2189339 0.042918607 0.166139008
0.15721 0.200075903 0 0 0 0.09990828 0.01127738 0 0.2968515
0.04465059 0.02334544 0.07027789 0 0.040942255 0.1060416 0.0353568
0.1869433 0.02503955 0.08514651 0.02573302 0.03534087 0.05070214
0.09296668 0.1272072
AC025031.2 0.04722963 0 0 0.050473939 0 0 0 0.07410142 0
0.06154961 0.04488729 0.040234005 0.02398574 0.033853663 0.04334383
0.03811103 0.037115 0.09077126 0.02622265 0.1373101 0.100263335
0.0409437 0.1238716 0.07711831 0.4168446 0.1528033 0.02501602
0.04909538 0.03965195 0.065523245 0.05961927 0.049767113 0.1939701
0.06218012 0.03981677 0.128514548 0 0.04731048 0.06092524 0.3258344
0 0.2170753 0.05635687 0.03398565 0.09193082 0.0954721 0.02964523
0 0.07248289 0 1.25566 0.06004257 0 0.03133738 0 0.09387148
0.03528944 0 0.1267876 0.1148927 0.2733236 0.03449046 0
0.0408572 0.6751132 0.129390035 0.03956839 0.6406786 0.0346403 0
0.03630601 0 0.02902325 0.039655123 0.02190647 0.04065692 0.2158067
0 0 0.08996912 0.1081942 0.2338478 0.03753113 0.04010957
0.09172911 0.1792704 0.04616764 0.2669589 0.03646118 0.04502589
0.1468746 0.025680392 0.02681096 0.0212321 0.03357876 0.865455297
0.2814436 0.03958359 0.1970556 0.1049332 0.02427925 0.02942001
0.2153186 0 0.05374399 0 0.026004989 2.339442 0 0 0.6782961 0

0.1454648 0.1891312 0.2287856 0.03714306 0.346573 0.1311271 0
0.04035515 0 0.1794398 0.03652682 0.04491752 0.06417172 0.07255026
0 0.07715499 0.098259743 0.02318633 0.1100213 0.1059357 0.06510229
0.060828932 0.06230695 0.2880955 0.1809198 0 0.1420991 0.1073463
0.02472567 0.08587209 0.1131447 0.1365821 0.07131157 0.1428558
3.671745 0.03447369 0.03515697 0.04971531 0 0 0 0.168652596
0.0535881 0 0.029972595 0.2692862 0 0.1079516 0 0 0
0.226162944 0.04070596 0.03995459 0 0.0725482 0.03494038 0.06421627
0.07877426 0 0 0.087029725 0 0 0.02621683 0 0.09397293
0.03024868 0.1563606 0.08592928 0.03003651 0.07091272 0 0.01522273
0.1379008 0.07549945 0 0.2733068 0.114463 0.2461281 0.032811094
0.2292297 0.1206268 0.08061571 0.1197436 0 0.1929014 0.148826431
0.03400961 0.1104614 0.09449193 0.05060487 0.068828779 0.08174191
0.04440156 0.3711668 0.02470407 0.2499504 0.1184545 0 0.1083335
0.2032373 0.101228 0.1979139 0.05092998 0.03597537 0.055642475
0.048975125 0.1037864 0.098392307 0 0 0 0.03315378 0 0
0.124070464 0.1434632 0.2671551 0.02726568 0 0.2813245 0.1166251
0 0.0407006 0 0.2075548 0 0.1018249 0 0.02589341 0.09327609
0.068917603 0 0.033010052 0.07078 0 0.1201405 0 0.1649298
0.08748143 0 0.03348593 0.335987 0 0.0570301 0.1664229 0
0.1130383 0 0.02975412 0 0.03634203 0.04587555 0.03989507
0.03802387 0 0 0.1017151 0.085836238 0.09178007 0 0 0.02211935
0 0.03760439 0.2010467 0.02573156 0 0.07657543 0.133557073
0.041546427 0.02765396 0 0 0.4085479 0.1135158 0 0.05307363
0.03436331 1.903691 0 0.1256721 0.07148262 0 0 0.1800299 0
0.1016034 0.03151801 0.061651928 0.1634505 0.2779631 0.02308165
0.03859515 0.0416287 0.03422551 1.869772 0.05135517 0.2931051
0.1262218 0.09442176 0.102143436 0.03603503 0 0.07748764 0.09107347
0.2048755 0.090425491 0.1275978 0.1952012 0.210198 0.08503289
0.2217768 0.04953969 0.031160791 0.02937028 0.07024213 0.06279726
0.02339856 0.076913823 0.05167552 0.09935281 0 0.04354168 0.034395831
0.05202858 0.2507694 0 0.03602744 0.1850233 0.06337233 0.03728841
0.03125058 0.0289471 0 0.08736467 0.02973439 0.056737198 0
3.639237 0 0.02515747 0.03191867 0.1895929 0.03316287 0.28548
0.05144015 0.2570759 0.3178186 0.03079146 0.1183549 0.1267024 0
0.08115145 0.08017884 0.08888601 0.070683 0.2499553 0.04148815 0 0
0.03476887 0.07513808 0.08574924 0.08645918 0.08142055 0.05754393
0.1603747 0.147394 0 0.1018723 0.2360797 0.05055005 0.04819615
0.1390852 0 0 0.1344636 0.0984545 0 0.07715999 0.07151895
0.07931959 0.2408973 0.06171032 0.151393248 0.09269019 0.09817148 0
0.1012308 0.02504374 0.029456631 0 0.03596634 0 0.08677309
0.6073814 1.760531 0.1439986 0.2322026 0.03460951 0.1455287 0
0.03204568 0.05788119 0 0 0.3056773 0.03640002 0.1154755 0
0.02504537 0.02649229 0.02425575 0.27839 0.03828389 0.3928816

CRTC3-AS1 0.1856613 0.09541358 0.1431899 0.099207398 0.032677313
0.2160783 0.1517755 0.1456476 0.02734393 0.09409307 0.1176356
0.131801052 0.2200072 0.088719944 0 0.0749079 0.4133843 0.2973541
0.2577054 0.2998726 0.131379542 0.5096785 0.4220178 0.1010516
0.3991536 0.200225 0.2950165 0.3216594 0.4935984 0.128787068
0.1367132 0.114121139 0.3630967 0.5907111 0.7304318 0.101039024
0.526473 0.1859791 0.6386646 0.3202166 0.1537306 0.06095219
0.3323112 0.3117304 0.1505763 0.3961543 0.291341 0.1719264
0.4748879 0.3788023 0.04329861 0.3802696 0.331353 0.164251098
0.3905646 0.5740187 0.4624134 0.06382405 0.2990439 0.07527451
0.1790741 0.5875271 0.05045564 0.5353702 0.1720116 0.197803157
0.1036965 0.2798366 0.2950398 0.209299183 0.2854404 0.1640304
0.3993199 0.207847541 0.1865827 0.3995594 0.09426041 0.5029187
0.6095414 0.7662887 0.4489439 0.1532107 0.02458936 0.09197539
0.030049191 0.1761795 0.1663627 0.1943379 0.4777672 1.165239
2.059283 0.117775589 0.2283554 0.06955342 0.4399976 0.151205983
0.5224499 0 0.2259344 0.2520808 0.1908851 0.2505773 0.5078548
0.3152679 0.2288753 0.6477791 0.102226509 4.649306 0.498562016
0.4420314 0.4444009 0.3116432 0.2223772 1.657344 0.149894 0.3163564
0.7064247 0.4939876 0.2925455 0.2115166 0.3274531 0.1881024
0.2871764 0.1765723 0.4064209 0.2376646 0.4561227 0.4296735
0.193131218 0.3038208 0.198228 0.1908669 0.1919395 0.039853438
0.3673962 0.1617876 0.6716907 0.1170532 0.1163743 0.4923122
0.2591932 0.4219577 0.4002983 0.335568 0.04672137 0.3275832
1.415074 0.2032759 0.391576 0.2605768 0.4287679 0.2336492 0.1005718
0.331489586 0.2457661 0.2192031 0.333832696 0.08821446 0.5799302
0.2121807 0.4669552 0.5015542 0.04720125 0.02116796 0.2933637
0.4188343 0.3253195 1.077382 0.5265153 0.3155454 0.3440715
0.4345447 0.08785328 0.057019475 0.2166904 0.4538288 0.3607075
0.1678215 0.4309793 0.3963621 0.3483066 0.3659402 0.2755073
0.6969008 0.3799432 0.388967 0.451744 0.07419773 0.1112998 0.3879699
0.6249417 0.483769 0.042993848 0.4505549 0.9286185 0.1848602
0.2549713 0.1435557 0.2708221 0.253518127 0.1114108 0.1447426
0.1392941 0.1657746 0.135284151 0.1204989 0.1745441 0.2431782
0.5179336 0.1637606 0.1552162 0.110737 0.07097714 0.3728353
0.1989654 0.1556012 1.05109 0.2828408 0.072910829 0.256697208
0.2039939 0.193391776 0.253837908 0.5938023 0.13288238 0.2606573
0.03052687 0.6489176 0.108383394 0.4699656 0.07001307 0.321547
0.55661578 0.2211792 0.1146144 0.3981057 0.1333296 0.3773037
0.1586482 0.1750652 0.450312 0.2097369 0.5937626 0.3972275
0.225764561 0.8298996 0.519054618 0.3246117 1.17164 0.173168 0.4234707
0.4592442 0.1146308 0.1411689 0.08775623 0.6603882 0.238607156
0.616515 0.6814732 0.2391163 0.2592085 0.8296713 0.2924112
0.3265057 0.8095501 0.150282 0.9932496 0.1743853 0.1368337

0.1155748 0.2443501 0.281187677 0.4058894 0.2648286 0.4571272
0.08695193 0.1838658 0.3449231 0.6366479 0.2191619 0.5721473
0.2257655 0.109378638 0.272200729 1.2139130.8826092 0.1701284
0.2230578 0.0495816 0.3619890.1854527 0.1801111 1.2027010.3119343
0.4391302 0.2185560.3682851 0.2585102 0.7666790.1208332 0.1664193
0.08259892 0.363533785 0.4069354 0.02276422 0.2873268 0.2781513
0.1090959 0.1345417 1.3820780.2018789 0.4608825 0.2756567
0.4949002 0.184034294 0.3305282 0.2881570.1269194 0 0.06711438
0.16292179 0.1950633 0.04795891 0.4992202 0.4178340.3228934
0.7465119 0.122494145 0.1539408 0.4602071 0.1645721 0.2452815
0.10078363 0.1015691 0.06509322 0.2250324 0.313800382 0.225351996
0.1704385 0.07468053 0.1131972 0.1180209 0.2424443 0.0415198
0.3908854 0.1637961 0.2086187 0.3116282 0.2575752 0.6623592
0.631934996 0.1184104 1.33857 0.1084079 0.4450269 0.2300345
0.2898374 0.2824561 0.2550526 0.4381282 0.3368581 0.20822600
0.4652573 1.03764900.5295606 0.1196284 0.6653922 0.2038248
0.3241669 0.1637638 0.1359095 0.9076416 0.19086900.09111843
0.4922840.2809027 0.3398740.2400502 0.4147131 0.2451706 0.4055877
0.2175464 0.7508693 0.1160047 0.1159165 0 0.06074985 0.7161777
0.1336715 0.08809682 0.5375389 0.1438326 0.3033186 0.7497159
0.4936961 0.06764111 0.3638781 0.59513208 0.4858244 0.4931142
1.1717840.03316182 0.1476717 0.308786748 0.183373148 0.07069247
0.053980771 0.1989797 0.67152300 0.5121526 0.3042654 0.3174528
0.1716235 0.2710529 0.1469681 0.1706497 0.3607295 0.073641868
0.2002713 0.3338761 0.1513127 0.1351141 0.2789536 0.2603553
0.2065920.3191886 0.2508255 0.600612
DCUN1D2-AS 0.1587827 0 0 0 0 0.1847962 0.06490134 0
0.07015588 0.03448758 0.1509079 0.067631961 0.3225536 0.113813655
0.072859467 0.3843799 0.2495561 0.07629166 0 0.3077513 0.168539171
0.3441247 0.2915134 0.08642213 0.6468011 0.5650855 0.3364084
0.1650552 0.06665354 0.055071146 0.1503269 0.376455523 0.04657957
0.0522613 0.1338612 0 0.3241832 0.07952728 0.2048264 0 0 0
0 0.4570295 0.3090650.4279607 0.09966519 0.1260311 0.08122748
0.2915660.1110905 0.1009295 0.07084559 0.052677044 0.1252582
0.2629912 0.5932032 0.2046904 0.4688762 0.1287537 0.4594475 0
0.3883596 0.2060386 0.06304677 0.217500139 0.1330262 0.673099
0.5822913 0.119332331 0.3051457 0.1402834 0.1951483 0.133317759
0.3682401 0.06834286 0.0604606 0.3395606 0.2792661 0 0.3031179
0.1965450 0.1348456 0 0.1130052 0.1552124 0.5484706 0.06128997
0.1513739 0.4444039 0.043167843 0 0.03569041 0.2257789 0.096986679
0.2365484 0 0 0.1175927 0.04081257 0.24727 0.4343316 0.2311079
0.04517088 0.05193738 0.305994362 1.8351770 0 0.35631 0.04703399 0
0.1987019 0 0 0 0 0.2501930.0678356 0 0.06032642 0.4298022
0.5662859 0.2516977 0 0 0.1945423 0.082585601 0.1559016

0.09247098 0.1780742 0.05471734 0 0.1571037 0.06918265 0
0.05005357 0.2388636 0.09022266 0.2493779 0.288696 0.07607694 0
0.2397445 0.04002261 1.996848 0 0 0.4178485 0 0.05994699
0.06450888 0 0.09007973 0.2499581 0.100765775 0.2263304 0.4578208
0.06048762 0.3423027 0.474095 0.1211035 0.325861737 0.06842531
0.2014868 0.06955559 0.6097755 0.1174671 0.161918 0.3531114 0.3344713
0.04508074 0.146293934 0.07942271 0.136986 0.3966263 0.5382212
0.2764391 0.152541 0.5256733 0 0.05049033 0.1788027 0.051306
0.2558888 0 0.2538239 0.1903734 0.1531397 0 0.8274655
0.110308611 0.2568847 0.2027695 0.0677561 0.4025697 0.1841593
0.3242605 0.150103226 0.05716897 0 0.1191282 0.08506501 0.231397556
0.06870265 0 0.1247837 0.4567935 0.210079 0.5973535 0.2272928
0.06070163 0.1366539 0 0.3326863 0.1284173 0.1209467 0.187066121
0.246976436 0.6978451 0.220525385 0 0 0.06818683 0.2229214
0.2349669 0.2134509 0.208558126 0.4823132 0.2694471 0.137498
0.826794739 0.1891587 0.1960428 0.1075172 0.3762896 0.1613403
0.4651898 0 0.4279105 0.08968642 0.3482073 0.3135877 0.057924044
0.09678454 0.110977495 0.03965957 0.2630301 0.0403904 0 0.3465516
0.09803549 0 0 0.3765216 0.357111112 0.239664 0.2098134 0.1937358
0.04750335 0 0 0.1288785 0.1221794 0.192788 0.8718089 0
0.3510719 0 0 0.384767266 0.07713955 0.2264888 0.2039728
0.1115456 0.202175 0.06321167 0 0.1297616 0.06382387 0.06436032
0.112252393 0.139676192 0.139456 0 0.06235652 0.2861478 0.06360535
0.05463231 0.1486915 0 1.371445 0 0.9858356 0.2002663 0.5751584
0.4421702 0.1210496 0 0.3415835 0.3708647 0.310904229 1.044068
0.2920289 0.2327967 0 0 0 0.3223613 0.1726525 0.1970798
0.353624 1.388797 0.042924889 0 0.2772448 0.06512701 0.1020609
0.1147962 0.266003674 0.1072437 0 0.1325008 0.07146866 0.2071106
0.166549 0.104760408 0.1974816 1.062671 0.21112 0.5113182 0.25857891 0
0 0.07217024 0.365960231 0.231272772 0.08745823 0.1916066 0
0.3028044 0.2488142 0 0.125361 0 0.3406134 0.3331414 0.03671424
0.4998247 0.143059878 0 2.468437 0.1390701 0.1268665 0.05365417
0.212466 0.5017106 0 0.0864691 0.1440452 0.1942698 0 0.5172713
0.1064912 0 0.170516 0.04492595 0.07470714 0.2970392 0.4848071
0.2092204 0.04851502 0 0.05844526 0.1894567 0.04804716 0.3633372
0.06843255 0.3869174 0.2246536 0.1982114 0.2790776 0.1284329
0.04960521 0.04248644 0.1620321 0.3117298 0.7464784 0.04899411 0
0.05516619 0.263592 0.08646883 0.2404417 0 0.1156971 0.1555993
0.424144624 0.07790449 0.1100152 0.1734477 0 0.2104883 0.247577847
0.052275276 0 0 0.4375876 0.1276234 0 0.6570108 0.1561298
0.1163548 0.1957029 0.07727065 0.0808015 0.2432406 0.3085059
0.094470868 0.1468094 0.4894973 0.1941103 0 0.2947028 0.08906524
0.3261845 0.2924775 0 0.132084
AC006059.1 0.2640648 0.8142364 0.1527435 0.765982216 0.139430138

0.3512305	0.8018002	0.799021	0.466693	0.4260644	0.0717053	0.482039262
0.4214769	0.75711453	0.830876369	0.3348431	0.2371577	0.2900055	
0.3770049	0.2924618	0.560580605	0.2289196	0.0395758	0.1231928	
0.3585558	0.2196864	0.2397714	0.5293858	0.3167105	0.235508072	
0.357146	0.536628955	0.2655925	0.2979893	0.318027	0.020529585	1.335002
0.4534574	0.3893007	0.7156943	0.1561786	0.1733837	0.3601097	
0.1900166	0.2202826	0.1779308	0.3314979	0.05988482	0.09648996	
0.3232607	0.1583571	0.2078161	0.3029664	0.325389794	0.1785527	
0.4498656	0.2536794	0.01945211	0.5063428	0.4588389	0.4366215	
0.192839	0.1845327	0.3263371	0.2097008	0.068898133	0.8849206	
1.257995	0.5533622	0.255158367	0.4349784	0.1333139	0.185453	0.443430153
0.8398688	0.3896849	0.1149136	0.6131123	0.5573227	0.2515124	
0.8353698	0.09339019	0.1798626	0.112128	0	0.1252895	0.4793789
0.236919	0.6698174	0.4135786	0.2111626	0.266650792	0.08565844	
0.8309729	0.1877416	0.276504699	0.4870587	0.916877	0.2360902	
0.1676257	0.03878493	0.3054809	0.4127533	0.2745326	0.3219504	
0.1974282	0.207708639	8.533154	0.177275316	0.2487158	0.3047472	
0.3799268	0.2711018	0.3398941	0.6213059	0.1483356	0.1845446	
0.6545917	0.1902104	0.5479561	0.1330669	0.2579819	0.787723	
0.2331992	0.2050225	0.2028172	0.9002924	0.3389414	0.392413113	
0.3518709	0.2416614	0.2538408	0.4679901	0.583027877	0.2985971	
0.4602189	0.3853476	0.3329678	0.9363607	0.2572208	0.1974903	
0.3086473	0.3434123	0.5454583	0.1708752	0.4564107	7.97872	
0.1376752	0.140404	0.5559248	0.4259155	0.4272655	0.122608	0.303091274
0.599231	0.4156947	0.335158521	0.1882002	0.1631533	0.1724475	
0.3524046	0.06436304	0.2301737	0.232254321	0.1950775	0.2233894	
0.1982999	0.3090461	0	0.3590385	0.3250818	0.2913664	0.2998874
0.173782284	0.1509537	0.06509017	0.1884606	0.2557408	0.3189992	
0.289925	0.4995571	0.2059019	0.455828	0.3681589	1.097033	0.5593044
0.3855074	0.09045507	0.5653606	0.1455315	0.3656984	0.9174151	
0.052414156	0.4882446	0.1445217	0.1287797	0.3347483	0.35002	
0.1980969	0.142645868	0.8964241	0.2352758	0.1132098	0.1616777	
0.494778087	0.1142564	0.567435	0.3261067	0.2565132	0.1996419	
0.2838381	0.2970008	0.7499164	0.1623309	0.4851207	0.1896948	
0.1423769	0.3735481	0.266658586	0.176029702	0.04144844	0.209569347	
0.33758825	0.4289833	0.226797214	0.7944237	0.7815268	0.6288238	
0.09909832	0.4201552	0.1066919	0.1960003	0.178572344	0.1348207	
0.5589093	0.07663167	0.2438147	0.229987	0.1105196	0.2667792	
0.2643233	0.6818453	0.1654539	0.3725102	0.247708301	0.5058687	
0.2900259	0.3203584	0.6606148	0.5373724	0.1376684	0.2305341	
0.4425334	0.3442004	0.294207	0.5367232	0.436331939	0.2505329	
0.9969475	0.1227405	0.1128583	0.4270605	0.1663578	0.2449513	
0.1161093	0.238173	0.6373047	0.3644479	0.4170376	0.4696607	
0.2166467	0.297091795	0.4948232	0.1614274	0.2665288	0.936367	

0.1280871 0.4204986 0.4817437 0.1233148 0.181959 0.3975582
0.373364328 0.099552649 0.5521985 0 0.6222148 0.2991247 0.1511134
0.1557543 0.4521738 0.1372344 2.362248 0.4889337 0.5688065
0.1332217 0.1952085 0.5252532 0.8340085 0.117847 0.2231715
0.2265683 0.418565545 0.1827731 0.2220164 0.2949747 0.4007504
0.06649986 0.6560835 2.833699 0.04101872 0.2809328 0.2016332
0.3770856 0.142773102 0.6619888 0.5269418 0.5879683 0.2424759
0.1090929 0.216675604 0.2038314 0.1364232 0.4616992 0.4414669
0.2361852 0.2374119 0.398223004 0.5160938 0.6732507 0.2257101
0.3364028 0.184299236 0.2889221 0.1587114 0.5829701 0.521668151
0.219782788 0.3601571 0.273131 0.1103997 0.08632818 0.3546791 0
0.1786994 0.1996852 0.4161744 0.1266362 0.2093413 0.5937409
0.135952445 0.2598391 13.10589 0.2643219 0.2210332 0.1529656
0.6057311 0.2119044 0.5804154 0.2465195 0.2737776 0.2307727
0.2459395 0.1134398 0.202401 0.135914 0.2268623 0.4055926 0.1419912
0.5927918 0.3685769 0.6296157 0.04610472 0.6648286 0.138854
0.1800442 0.7533917 0.172643 0 0.1838474 0.2775402 0.4238188
0.4309704 0.2441043 0.1414222 0.06056347 0.3849552 0.2592122
0.7093923 0.2328 0.6801972 0.2359145 0.7765387 0.5546673
0.4855545 0.1583866 0.2748728 0.3696723 0.161228988 0.3516618
0.3397859 0.1648305 0.2425671 0.3800588 0.352916702 0.273229884
0.1723635 0.032904203 0.6584254 0.3941693 2.343637 0.3943386
0.7233184 0.4146528 0.1394851 0.7343173 0.2559572 0.4623122
0.5130631 0.134666116 0.2092735 0.6105466 0.1537222 0.4117969
0.3200703 0.4020416 0.387474 0.3335361 0.3975184 0.1673625
AP003900.1 0 0 0.2402138 0 0 0 0 0 0 0 0 0 0 0 0
0.02577737 0 0 0 0.01547886 0 0 0 0 0 0 0 0
0.0268196 0 0 0 0 0 0 0.817086346 0.1739235 0 0
0.2203864 0 0 0.8386049 0 0.03108987 0 0 0 0.06536753
0.1173184 0 0.08122264 0 0 0 0 0.04773782 0.2306134 0 0
0.3697388 0 0 0 0 0.525097423 0 0 0 0 0 0 0.01963061
2.01163124 0.3704249 0 0 0.1093041 0 0 0.2439331 0 0 0
0 0.1970379 0 0.1003136 0 0.3654528 0 0 0 0 0
0.15609938 0.06345384 0 0 0 0 0.3581815 0.1165089 0 0
0.3134732 0 0.05274471 0 0 0 0 0 0 0 0.4019624 0
1.086467 0.201342 0 0 0 0 0 0.04340417 0 0 0.1043715 0 0
0 0.1074784 0 0 0.08428578 0.1948607 0 0.02014022 0 0 0
0 0 0.06928555 0.3617504 0.04831208 16.36174 0 0 0 1.082018 0
0 1.083688446 0.4349481 0 0.304090891 0.09106929 0 0.0730158 0
0 0 0 0.1101301 0.0540486 0.2238985 0 0.07089843 0.5863631
0 0 0.2176714 0 0 0.1377988 0.3014512 0 0 0 0.3595787
0 0.08126383 0 0 0 0 0 0 0 0 1.304054 0.266311405 0 0
0.109053 0 0.02470026 1.081068 0 0 0.04980894 0 0 0 0.4146616
0.5105461 0 2.272455 0 0.1602394 0.9831579 0 0 0 0.7764116

0 0 0 0.612823048 0 0 0 0 0.027436556 0.0224244 0 0
0.727291158 0 0 0 0 0.03805619 0 0.1730481 0.3578755
0.3570546 0.07019247 1.084384 0.5681931 0 0.4553552 0 0.116535429
0 0 0.07978976 0.7710928 0.2112762 0.08743506 0.4741065 0 0
0.02264906 0 0.061582268 0.07714751 0.7035273 0 0 0 0 0 0
0 0 0.2314657 0.02354366 0 0 0 0.03103889 0 0.2462196 0
0.1898162 0 0.06799152 0 0.1027241 0 0 0 0 0.1242508
0.2007248 0.4835805 0.02559309 0 0.0119659 0 0 0 1.331691 0
0 0 0.06088389 0 0 0.08527202 0 0 0.2820111 0 0 0 0
0 0.1389415 0 0 0.04789842 0.017271827 0 0 0 0 0.02309545
0 0 0.01650366 0.03554318 0.5751415 0 0 0 0 0.02375502 0
0.01582621 0.034681745 0 0 0 0 0.558347858 0.1407634 0 0
0.02436808 0 0 0 0.9723076 1.194325 0.5361887 0.04431849 0
0.038375645 0.09779393 0.04318405 0 0.5955562 0 0 0 0.1228765
0 0 0 0 0.03202094 0 0.5179249 0 0.54231 0 0 0.3901468
0.1403078 0 0.02814943 0 0.4573939 0.1353302 0 0 0 0 0
0 1.033559 0.5987943 0 0 0 0 0 0 0 0.2757622 0.06958549
1.668891 0 0 0.1669574 0 0 0.02213359 0.32569 0 0.1355118
0.677407175 0.021034173 0 0.111455444 0 0.1540567 4.366193 0 0 0
0.5118466 0.03109164 0 0.09787353 0 0.057018825 0.1181443 0
0.07810479 0 0 0 0.01640599 0.4942775 0.1294714 0.1594413
AC004837.2 0.08102045 0 0.02343241 0 0.342240089 0.03143137 0.0662331
0 0 0.01759763 0.3465102 0.207059273 0.1645862 0.058074546
0.074354531 0.1307558 0 0.1167857 0 0.1962915 0.257996351
0.2107116 0.1912469 0.3748309 0.1925214 0.235915 0.2574836
0.1684421 0.2720851 0.056201197 0.1022744 0.170746812 0 0.5866707
0.06830401 0.022046134 0.1654177 0.04057958 0.1045147 0.1397388
0.1006294 0.02659883 0.09667787 0.2040533 0.2365552 0.1910748
0.07628273 0.0321543 0.04144713 0.2479574 0 0.2231678 0.07229933
0.215031875 0.06391422 0.348904 0.2118815 0.1044453 0.2827485
0.2299425 0.703313 0.1183339 0.1981643 0 0.09651071 0.110981603 0
0.6640138 0.05942398 0.152226264 0.06228145 0.071581 0.1991527
0.204080127 0.206688 0.03487262 0.246805 0.06930566 0.256497 0 0.03093378
0.2507226 0.06438309 0.3268299 0 0.05766204 1.069182 0.3561887
0.03127381 0.7337803 0.1511743 0.08810728 0.02299654 0.03642278
0.05760296 0.247442075 0.3218698 0.203712 0.2112754 0.1200056
0.1249501 0.1009376 0.073874 0.2653315 0.09219557 0.1590094
0.089220948 6.220485 0.027195842 0 0.254535 0.09599824 0.1663592
0.1419455 0.07849442 0.06371736 0.03302953 0.2811789 0.255327 0
0.09526452 0.1846929 0.1253205 0.0577906 0.1284312 0.06222847 0
0.1654452 0.126420367 0.1193255 0.07077635 0.5906169 0 0.052174747
0.1870487 0.2118068 0.2069069 0.1787823 0 0.09207402 0.06362377
0.07365499 0.116457 0.146438 0.3975791 0.1021097 3.612505 0.1182764 0
0.04264227 0.02078993 0 0.0329163 0.180822807 0.3217485 0.1275436

1.028334724 0.2309747 0.8760285 0.06172882 0.2328844 0.06911763
0.4943540.110849455 0.03491469 0.06854043 0.2129486 0.3941025
0.02996938 0.1101603 0.2477456 0.6826692 0.06900869 0.223943792
0.1621049 1.1183760.08994777 0.4119491 0.2821116 0.07783554
0.1877610.07370405 0.07728958 0.03041195 0.05235879 0.2089117
0.05914076 0.09713712 0.07285495 0 0.03272609 0.6333337 0.084429095
0.2949255 0.02586628 0 0.2310921 0.06264607 0.2600041 0.102122213
0 0.0947460.08104848 0.08681053 0 0.2629216 0.03808451 0.06367211
0.3390304 0.02679872 0.2438445 0.1159784 0.1548680.1045935
0.08682621 0.3055617 0.1310524 0.1542856 0.238630859 0.336059136
0.2225515 0.112525265 0.120842123 0.1151682 0.243551044 0.1421848
0.3996473 0.08713237 0.070945902 0.2871226 0.3895495 0.163706
0.268469228 0 0.1500492 0.1371543 0.2967358 0.2058137 0.6527612
0.05729732 0.2620147 0 0.06662859 0.1600112 0.118225272 0.04938527
0.028313684 0.08094676 0.1150403 0.1442672 0 0.1414651 0.02501179
0.1108881 0.02872186 0.2881858 0.104125421 0.1956655 0.2498051
0.2141871 0.07271717 0.09654907 0.05104196 0.06576154 0.1558581
0.3147904 0.3079725 0.06522836 0.1492816 0 0 0.245414142
0.2558479 0.1733522 0.07805937 0.05691723 0 0.06450877 0.0574812
0.04414142 0.2279673 0.03284049 0.17183369 0.035635581 0.118598
0.05252189 0.06363606 0.4964332 0.1622763 0.1393834 0.1213941
0.05894882 0.6414772 0.1750173 0.1796544 0.1635006 0.2305917
0.2820272 0.3088338 0.1581907 0.2396575 0.1622035 0.237962949
0.08411777 0.1788128 0.07919123 0.09931256 0.0714123 0.08806866
0.8224402 0.1321465 0.2765453 0.03608803 0.8098829 0.02190285 0
0.1886226 0.0332317 0.1301939 0.02928794 0.542924051 0.2371294
0.2511446 0.2253662 0.1823380.02113605 0.1274749 0.106910075
0.3274927 0.5723629 0.08079455 0.1605570.241894498 0.0443236
0.0426089 0.1104767 0.074693935 0.147511532 0.1636302 0.2737537
0.0889163 0.1236072 0.1269599 0.1630689 0.1279334 0.3216544
0.2482877 0.2379842 0.09366903 0.1785284 0.048665148 0.09301126
5.3667550.07096192 0.1510480.1095103 0.1355161 0.4551161 0
0.1323651 0.1470010.04956405 0.07923221 0.1015165 0.05433817
0.1459542 0.1566134 0.2292391 0.03812006 0.2728209 0.4947553
0.07117119 1.2872740 0.05964455 0.1288962 0.3922646 0.1483171
0.03491839 0.1974284 0.1604844 0.1769938 0.1780026 0.305826
0.1012462 0.2601495 0.08267848 0.1192974 0.2929985 0.04999946
0.1537778 0.2533419 0 0.1985471 0.3373914 0.06803473 0.3246958
0.02646537 0.0432848 0.3378881 0.02806818 0.2360091 0 0.2792497
0.075797431 0.160043869 0 0.636027884 0.0372139 0.06512109 0
0.2117345 0.1394168 0.2671703 0.2496484 0 0.1236893 0.8936349
0.1049455 0.096409395 0.03745548 0.2185495 0.1650778 0 0.08592859
0.1136161 0.2704632 0.5074144 0.06567441 0.584109
AC087854.1 0.2470170.3733676 0.3193844 0.302806487 0.26085731 0.1803833

0.4573188	0.319167	0.2568022	0.3692523	0.2002418	0.396101611	
0.3357592	0.572837863	0.300035857	0.2403632	0.4110696	0.3002066	
0.2097555	0.1689763	0.03855809	0.2519303	0.232469	0.0593145	
0.08385194	0.22095	0.2347369	0.1963574	0.1280906	0.120951152	
0.4126982	0.111005326	0.1065639	0.181735	0.06124906	0.0909376	
0.1334989	0.1164423	0.4498543	0.1879583	0.06015711	0.1574199	
0.4074536	0.1516097	0.07777807	0.09301269	0.1231266	0.05189969	
0.08919877	0.08893861	0.06607924	0.2001174	0.1361466	0.086769793	
0.06877521	0.2502932	0.04342783	0.1948074	0.08191425	0.1178242	
0.112119	0.1008059	0.2073125	0.1696937	0.08654236	0.119422304	
0.2434679	0.2012141	0.2877456	0.02184051	0.1005274	0.1925627	
0.09375591	0.170801114	0.2291472	0.09381215	0.08299241	0.1491534	
0.1891144	0.1176375	0.1331458	0.4136801	0.1039196	0.05244454	
0.105828297	0.06549461	0.06746751	0.2509562	0.1570443	0.2043232	
0.1039293	0.134311724	0.127852	0.07185367	0.1962825	0.097629133	
0.1443123	0.1887601	0.04546879	0.1452742	0.06349178	0.1719728	
0.1324875	0.1216066	0.198415	0.1473386	0.128008857	2.099242	0.092670031
0.1117675	0.1565107	0.1635574	0.1193411	0.1527411	0.2674703	
0.2399718	0.1007012	0.06555553	0.2014803	0.09932357	0.04271244	
0.09384932	0.06742586	0.6045848	0.1020041	0.2901654	0.1529894	
0.2611079	0.16626532	0.07133379	0.1142389	0.2240672	0.09513784	
0.290071626	0.06229931	0.1582748	0.09276799	0.1190921	0.07650551	
0.1155895	0.07606964	0.07265208	0.1705665	0.01575753	0.03290899	
0.1904511	2.741012	0.1113629	0.05408102	0.1988368	0.1640548	
0.1097165	0.5844258	0.084315954	0.370949	0.3431098	0.041495425	
0.08802509	0.3456401	0.1273119	0.4019985	0.1053637	0.299223	
0.059640053	0.5385051	0.04916885	0.07638145	0.08927914	0.2526148	
0.2518945	0.07068618	0.0918237	0.2557746	0.140569232	0.1817019	
0.05641095	0.1855118	0.07387997	0.3216371	0.1209799	0.2357147	
0.297411	0.2079196	0.2727078	0.2253637	0.369984	0.1803097	0.1103318
0.1437259	0.08408407	0.09390685	0.05679183	0.060566897	0.158678	
0.08350063	0.1426103	0.1335438	0.387611	0.08054235	0.05952327	
0.1412534	0.02265598	0.2034961	0.1868258	0.497623965	0.1948991	
0.157094	0.07422425	0.08360352	0.09131684	0.03644306	0.1663989	
0.1722014	0.1625699	0.2180028	0.1400449	0.2507015	0.2490297	
0.085593306	0.033901697	0.2634254	0.085767386	0.260065531	0.1652364	
0.205915528	0.1325989	0.344034	0.03515971	0.050894459	0.2059731	
0.0493149	0.08388411	0.061904495	0.08655076	0.134551	0.3443662	
0.1690433	0.06644006	0.01596379	0.2260686	0.1762139	0.1887684	
0.07567913	0.2152261	0.12721689	0.1151394	0.147257553	0.4355158	
0.09971949	0.09979678	0.1657105	0.04439873	0.1121419	0.02651593	
0.2524014	0.06316929	0.182072597	0.1579101	0.03200048	0.2275223	
0.08694186	0.1471805	0.1006939	0.1945983	0.07267515	0.3422602	
0.2270671	0.0701893	0.2141803	0.1628142	0.130388	0.184855373	

0.08823924 0.3627099 0.3546505 0.1122845 0.314522 0.2313833
0.08762499 0.1068718 0.09929027 0.08245574 0.344124086 0.153383374
0.4764405 0.06593585 0.2054277 0.1152173 0.05238547 0.1549837
0.1986615 0.1374364 0.1098148 0.1360148 0.08377115 0.0146613
0.04887395 0.6878806 0.04984831 0.09078498 0.4141783 0.0824216
0.13277248 0.1508589 0.2191363 0.3124518 0.07124385 0.08965082
0.1790038 0.3982459 0.4502901 0.1713327 0.0776654 0.09441017
0.023568671 0.2716154 0.1775972 0.3039527 0.05603834 0.06303087
0.097369353 0.09159743 0.367834 0.09700255 0.09156264 0.2274355
0.03810278 0.134214635 0.180718 0.1728825 0.1497289 0.2843476
0.15775259 0.07949104 0.09169904 0.1651098 0.140655863 0.058201189
0.1413937 0.2595057 0.1435182 0.2327642 0.09107722 0.1364775
0.08030364 0.1394085 0.1424913 0.09145858 0.08063442 0.05946149
0.11782434 0.09452494 0.5008862 0.1718076 0.1122272 0.1669387
0.2138734 0.04591218 0.03593014 0.2136483 0.164772 0.07555599
0.04736567 0.1784209 0.1851577 0.07852737 0.1654038 0.09866955
0.05469237 0.2065865 0.04732301 0.1531682 0.07547443 0.1088339
0.08022602 0.06934972 0.04396857 0.05319912 0.09393526 0.1947402
0.2014717 0.1042968 0.102155 0.07443603 0.1134859 0.07775977
0.4374191 0.1854242 0.05780179 0.06725271 0.06894722 0.07067661
0.1350812 0.07517231 0.1925274 0.3172394 0.2011642 0.1091664
0.194070099 0.1318891 0.05033817 0.07936211 0.1245765 0.3120453
0.077030968 0.181783554 0.182576 0.228133652 0.2402652 0.07007388
0.631908 0.09809689 0.1357325 0.1277733 0.1701358 0.2262764
0.1133785 0.1691704 0.07999003 0.047548328 0.1612166 0.2407706
0.1184219 0.1031008 0.3505922 0.17116 0.07089271 0.1498839
0.1236712 0.1813077
AC040904.1 0.1327936 0 0.2304362 0.141915516 0 0.1030329 0 0
0.1173459 0 0.1262078 0 0 0.095184962 0 0.2143105 0 0
0.2949167 0.06434487 0.422859489 0 0.1393139 0.2891072 0
0.08592617 0.140673 0.1380395 0.2229755 0 0.1676291 0 0.3116444
0 0.1119512 0.072267822 0 0.2660419 0.342602 0.4580677 0.1099553
2.441367 0.1584563 0.09555605 0.2584781 0 0 0.1054027 0.1358648
0.3251245 0.1858151 0.05627308 0 0.088110034 0 0 0.5953313
0.2054247 0.1425934 0.323039 0.3842465 0.09697541 0 0.2297531
0.1054549 0.242533787 0 0.1501142 0 0.099800358 0.2041603 0
0.163207 0.222993382 0.123187 0.3429402 0.2022583 0.2271858 0 0
0.2028036 0 0.1055247 0.05638722 0 0.06300591 0.2596154
0.08339972 0 0 0.2477767 0.072204509 0.1507665 0.05969742
0.09441203 0.648897424 0.1318872 0.1112956 0.2770266 0.09834542
0.1365299 0.4135952 0.3632414 0 0 0 0.804288833 5.700686
0.267446104 0.5836827 0.1191961 0 0 0.06647157 0.2573068
0.1044336 0 0 0.2510906 0.1134649 0.1561398 0.1009047 0
0.1894391 0.0601430 0.186423 0.3254003 0.138136454 0.06519204

0.1546712	0.1489275	0	0.171030225	0.5255578	0	0.5086847
0.3348876	0	0.6036422	0.5561612	0	0.06362489	0.2880166
0.2005038	0.06694364	17.155555	0	0.1976989	0	0.1362999	0.2005402
0	0.118548406	0	0.1045228	0	0.1892853	0	0.1011744	0.1908504
0	0.4051264	0.181683749	0	0	0.4653674	0.2039808	0	0.722217
0.07382879	0.0932420	0	0.122348963	0.3985382	0.3436936	0.9582664
0.09002534	0.1321099	0.340196	0.08792652	0.1208019	0	0.1993824
0.1716335	0	0	0.1061394	0.1592137	0.512297	0.536385	0.1153381
0.276760844	0.1074193	0	0.1133319	0	0	0.2324456	0.167379681	0
0.6211599	0	0.1422836	0	0.2298304	0.9987365	0.3130784
0.2778377	0.3513877	0	0.1900902	0	0.4571472	0	0	0.2147966
0	0.156447675	0	1.896777	0.092215211	0.297092798	0	0	0.1864343	0
0.1428111	0.348843862	0	0.07511492	0	0.377164009	0.1581977	0
0.2697572	0.2288724	0.4047978	0.3890488	0	0.07157426	0	0
0.06556514	0.581318434	0.8094312	0.092813017	0	0.06285088	0
0.2423088	0.1159316	0.1639786	0.3634942	0.188302	0.1049645
0.255994449	0	0.1169811	0.05400856	0.1589126	0	0.08365845	0	0
0.1289864	0.1121713	0.1069102	0.3914793	0	0.2859883	0.160894832
0.1935407	0	0.08529357	0.06219207	0.1127224	0	0.4710623
0.07234837	0.2135094	0.322956	0.28163773	0.116814394	0	0.1721682
0.3129011	0	0.2127784	0.0913805	0.1989666	0.4830896	0.3823236
0	0.3533472	0.06699492	0.549735	0	0.1518548	0.1037106	0.2856741
0.2658537	0	0.2757403	0.09769217	0.3244887	0	0.9363658
0.192461	0.8087943	0	0.2472335	0	0.06637045	0.430788795	0
0.4637324	0	0	0.1920133	0.190684248	0.05979358	0.0686049
0.2955025	0.5977087	0	0.4178662	0.087613525	0.08257921	0.09874847
0.08828224	0.3289439	0.360425761	0	0.5586925	0.482861	0.122424364
0.096709354	0.09752443	0.064098	0.3886267	0.1012969	0.1040445
0.1781814	0	0.08786597	0	1.2259	0.1842298	0.1672059	0.239288496
0.5081557	5.923961	0.3489226	0.2122027	0.3589777	0.1776902	0
0.1459404	0	0.1204683	0.08123614	0	0.1331095	0	0.5980522
0.1140851	0	0.2499172	0.09936826	0.1621821	0.4666021	0.08114845
0.1170158	0.09775822	0	0.08036588	0.4861875	1.144634	0.4853817
0.07515318	0	0	0.07160756	0	0.07106476	0.1355111	0.1303534
0.09604569	0.3277992	0	0.0922735	0.1763584	0.07231586	0.3016304
0	0	0.08675419	0	0.0651533	0.09200825	0.2901165	0	0.1408289
0.165644012	0	0.2022502	0.231657391	0.4879527	0.1067343	0
0.05783928	0.06528745	0.09731015	0.1636709	0.1292464	0.1351523
0.2441133	0.172007	0.079008148	0.4911202	0	0.1082259	0.5798396	0
0.2979492	0.2727956	0.6848957	0	0
AC008434.1	0.02696992	0	0.03120053	0	0	0	0.04409508	0.04231474	0
0	0.1537941	0	0.1095742	0.154653892	0.049501969	0	0.1695527	0
0	0.1306822	0	0.1402825	0.2546476	0	0.2197239	0.3490262
0.08571062	0.4766007	0.09057112	0	0.06808977	0.284189253	0.03164695

0.3905795 0.1364214 0.088064067 0.1468371 0 0 0.1860639 0
0.03541666 0.06436386 0.2716994 0.05249601 0.5088367 0 0.08562766
0.08278101 0 0 0.1828619 0 0 0 0.03573614 0.2015162
0.02781403 0.1158409 0 0.1040522 0 0.04397636 0 0.1285052
0.197031091 0 0.2743890.07912374 0 0.04146425 0 0.06629354
0.181156737 0.07505658 0 0.2053898 0.04614066 0.1138429 0.05137581
0.2471322 0 0.1714537 0.0458082 0 0.02559256 0.1054540.2032583
0 0.07713451 0.0335484 0.029328972 0.06124033 0 0.2300971
0.065894411 0.3214301 0 0.05626314 0 0.02772876 0.0671998 0
0.07850933 0 0.03528715 0.178198122 1.4249720 0 0.2420831
0.0319557 0.05537729 0.4860053 0.05225813 0.08484042 0.1759168
0.07487861 0 0 0.06342292 0.2049340 0.5642917 0.1954374
0.04142897 0 0 0.056110072 0 0.06282637 0.03024667 0.0371759 0
0.07115931 0.09400775 0 0.03400725 0 0 0.0282386 0 0.07753196
0 0.08144327 0.05438409 1.3566930.1574864 0.04015195 0.05677869
0.05536408 0 0.1753138 0.192614167 0.06120171 0.04245645 0.034230997
0.07688637 0.07776282 0.04109632 0.3100884 0.04601547 0.08227977 0
0 0.0456312 0 0.6904633 0.07980916 0.03666994 0.08996624 0
0.1531431 0.099394603 0.05396115 0 0 0.07313534 0.08049321 0
0.1428606 0.09813782 0.06860799 0 0 0.08692761 0.03937331 0 0
0 0.04357517 0.4216457 0 0.2181649 0.1377650 0.06837819
0.04170699 0.1258904 0.033994234 0.1165247 0.08410361 0.0539585
0.3467678 0.157215462 0.04667776 0 0.2119504 0.08464182 0.1070484
0.1623409 0.03860666 0.06186259 0 0 0.3164459 0 0.1643465
0.254191851 0.11186668 0.1777980.112371542 0.080451359 0 0.0926546
0.2271849 0.1064270.02900445 0 0.1638459 0.03051116 0.09341848
0.051067137 0 0.06659742 0.2556715 0.0697248 0.1644260.1185217
0.07629206 0.05814594 0 0.02957225 0.1597926 0 0.06575707
0.075400011 0.05389079 0.2042367 0 0 0 0.1332140.04921627
0.03824349 0.08527175 0 0.1302655 0.09503386 0.08775161 0.06454918
0.03213906 0 0.08756227 0.04150538 0.02619669 1.5491490.08685233
0.03975404 0 0 0.130708734 0.1310248 0.07694018 0.03464566
0.02526199 0.2289352 0.04294709 0.03826845 0.08816223 0.1734521
0.1311825 0.30506479 0 0.2210805 0 0 0.4665929 0.04321457
0.07423625 0.1616377 0 0.1164729 0 0 0.08163862 0.08373696 0
0.02056079 0.04212655 0.1740582 0 0.105616828 0.0373346 0.03968188
0.1054441 0 0 0 0.2737722 0.05865153 0.2008491 0.1441549
0.4583067 0.087491713 0.04115476 0.2511532 0.1769936 0 0.03899724
0.129091036 0.04857548 0 0.2400622 0 0 0.2263125 0.071176015
0.1341724 0.2005547 0.07171927 0.1068918 0.02928049 0 0.1134685
0 0.198911718 0.157130681 0.07922749 0.07810849 0 0.0411461
0.2535729 0 0.1703449 0 0.0661196 0.04526839 0.02494428
0.3395895 0.03239911 0 1.09376 0.04724332 0.02873175 0 0.144353
0.1514981 0 0.1174972 0.1468002 0.09899267 0.07033241 0.2433067

0 0.1457548 0.04634058 0.1526173 0 0.2421762 0.2195908
0.04738264 0.2307336 0.04753101 0 0.08581343 0.09793219 0.1481145
0 0 0.1221068 0.1346682 0.2370126 0.05817299 0.03370263
0.02886601 0.05504369 0 0.5851964 0 0.05118923 0.262366 0.2149067
0.05874841 0.1633602 0.04529452 0.4716396 0 0 0.1587889 0
0.07856222 0.05780666 0.1430094 0.067283451 0.142066871 0.04107632
0.047048814 0.1486523 0.04335475 0.6702204 0.1879513 0.05303862
0.1581068 0.06648197 0 0.07319723 0 0.4192084 0.096277688 0
0.207858 0.04396062 0.05888171 0.1430187 0.1210249 0.05540383
0.03974284 0 0.08974016
AP001469.2 0.1898268 0.2341302 0.2635245 0.162293128 0.040092549
0.176741 0.1862169 0.2382646 0.0670978 0.03298428 0.2164948
0.064683901 0.501302 0.217705094 0.278734166 0.2450833 0 0 0
0.3311286 0.080596296 0.5924239 0.4381244 0.4132751 0.3608542
0.4421893 0.5228348 0.6709072 0.2549925 0.052670609 0.1916989
0.48006123 0.08909835 0.3498827 0.3200656 0.08264474 0.6717796
0.0760607 0.4897452 0.1309604 0 0.04985576 0.8154406 0.4371077
0.1477965 0.613959 0.04766041 0.1205374 0.7380245 0 0.1062481
0.4182966 0.3387873 0.352666059 0.1197982 1.458859 0.6240802
0.195768 0.2446025 0.1231414 0.2197102 0.2772502 0.1238104
0.1970574 0.5426872 0.277359152 0 0.7295933 0.6682913 0.171195999
0.6420579 0.1341685 1.07319 0.382519417 0.5282829 0.1960914
0.5782514 0.2598074 0.4807674 0.07232133 0.8117342 0.1879777
0.06033851 0.3224192 0.147472185 0.396291 0.4082285 0.4768753
0.05861836 0.5429083 0.6139357 0.247717008 0.08620754 0.06826936
0.3239059 0 0.3016498 0 0.4752071 0.281167 0.3903356 0.6621765
0.2769328 0.221034 0.2592114 0.2483672 0.167232084 4.764065 0.05097477
0.2503101 0.4089343 0 0.1559084 0.1140243 0.3678168 0.1791438
0.0619092 0.4743272 0.1435723 0.5190293 0.3571198 0.1153936
0.8808583 0.2527474 0.7909576 0.233277 0 0.9923318 0.63188573
0.1118294 0.0442201 0.681248 0.680319 0.195588412 0.3005112 0.529336
0.3878178 0.2872305 0 0.3451595 0.3577614 0.3451397 0.3274235
0.1646864 0.8025294 0.3445023 4.514087 0 0.0565216 0 0.1169034
0.1146678 0.1233939 0.474497593 0.1723064 0.2988281 1.01192092
0.2705809 0.2189322 0.2314039 0.05456363 0.6477562 0 0.415543343
0.1308853 0.3211734 0.2660947 0.6998111 0.449387 0.36134 0.1899672
0 0.1293471 0.349792547 0.2278821 0.1965223 0.8851207 0
0.2266193 0.04863058 0.1508278 0 0.5794736 0.05700291 0.4906958
0.2202612 0.05542551 0.4855195 0.04551877 0.2196966 0.3680426
2.30824 0.211000575 0.4913744 0.1939308 0.06480263 0.1443832
0.1174212 0.3544298 0.095706843 0.5467699 0.177588 0.2278709
0.2440712 0.110655498 0.3613936 0.2141522 0.298361 0.5163151
0.5525347 0.1904384 0.2173852 0.3483339 0.6534861 0 1.527286
0.2046994 0.1156746 0.357823914 0.157473864 0.5839979 0.158184555

0.05662538 0.2158666 0.065214584 0.2665054 1.0487150.2858053
0.132978087 0.1537631 0.2147516 0.3068438 0.287547261 0.09045664
0.4687434 0.1542458 0.2290192 0.2314613 0.05561403 0.8591659
0.6138869 0.1715540.1665145 0.4123879 0.2215966 0.1851315
0.053070007 0.5689623 0.6109428 0.6953362 0.06927547 0.5303128
0.2344054 0.4849696 0 0.7802365 0.146376313 0.3667474 1.003338
0.4323454 0.4997596 0.09048381 0.04783547 0.4930430.8179752
0.1106307 0.5131120.1833920.05596146 0.09453427 0 0.2299971
0.1844426 0.3249243 0.04877043 0.4978555 1.0312650.1813689
0.1616106 0.5791580.3662508 0.1231097 0.16103901 0.400763227
0.2222953 0.09844488 0.1789152 0.6568193 0 0.0522509 0.1137681
0 1.14770600 0.4040843 0.5363029 0.4715035 0.3171721 0.6367518
0 0.2858572 0.2026850.346910659 0.4204450.5027390.0371082 0.06204913
0.1338523 0.05502409 0.5395418 0.4128166 0.5183451 0.2705677
0.9487571 0.164215216 0 0.4419330.1245763 0 0.1646884 0.181720459
0.2735173 0.2353676 1.1827680.2050601 0.07923307 0.2389338 0
0.2833102 0.8469580.4543140.2633238 0.329743363 0.3323133 0
0.06902436 0.350008118 0.552979129 0.2788198 0.6963672 0 0.5212894
0.3569526 0.1018832 0.2397932 0.2512066 0.2326901 0.06372396
0.4213665 0.4780375 0.136823935 0.1743365 8.3142580.1330081
0.2426728 0.0513154 0.2032047 0.2665784 0.1251720.4961996
0.4821821 0.2322520 0.4947236 0.2036985 0.4103558 0.09784993
0.3007735 0.1429014 1.8750030.7109675 0 0.5104029 0.06690903
0.05589765 0.1207989 0.3216696 0.1389998 0.0654496 0 0.1718888
0.6634999 0.6672816 0.2866139 0.1897717 0.2438068 0.2324537
0.2236062 0.7688581 0.4685847 0 0.6331382 0.1512613 0.3307987
0.2874512 0.06376075 0.5532696 0.6448728 0 0.2607803 0.1052197
0.3317743 0 0.2818384 0.094714397 0.349976273 0.05782281 0
0.2790089 0.3051508 0 0.5952996 0.03733103 0.05564145 0.1403792
0 0.12879990.2326379 0.2950582 0.135529361 0.07020501 1.111881
0.1856491 0.1657747 0.3623873 0.4259145 0.6239324 1.118914
0.06154871 0.547415
AC011510.1 0 0.1064228 0.1197838 0.331963217 0.109343315 0.5623577
0.2539322 0.2436797 0.0914970.08995712 0 0.352821278 0.3155048
0.148435291 0 1.5874710 0 0.05748813 0 0 0 0.1086259 0
0.07029628 0.4019903 0 0.05381609 0 0.143647114 0 0.054552413 0
0.06815897 0.1745812 0 0.07046639 0 0 0.7143293 0.0857344 0
0.1235516 0.07450699 0.7053922 0.06976807 0.2599659 0 0
0.1267531 0 0.04387726 0.1847930 0 0.06859839 0 0 0.1667745
0.08396003 0 0.07561369 0 0 0 0 0.4337304 0 0.2278266
0.155632726 0 0 0 0.347744925 1.0565660 0.1577049 0.0885707 0
0 0 0.1281666 0.2468393 0.04396626 0 0 0.05060684 0.1300569
0 0 0 0.05629932 0.05877787 0 0.07361497 0 0.1028352 0 0
0 0 0.1289954 0 0 0 0 0.285054688 3.2482260 0 0 0 0

0 0 0 0 0 0.5220811 0 0 0 0.08007803 0 0 0.2385787
0 0 0.107707795 0.05083156 0 0.05806091 0 0 0.136596 0.1804554
0 0 0 0 0 0 0.04960961 0 0.07816845 0 11.36413 0 0
0.5449563 0 0 0.08413221 0 0 0.08149858 0.525673205 0.07379479
0 0 0 0 0 0 0 0 0 0 0 0.1407818 0.1151316 0
0.05879412 0.095397967 0.1035828 0 0.1724261 0 0.05150439 0
0.06855809 0 0 0.3109250 0.06674581 0 0.08275901 0.1862132 0
0 0 0.071932014 0.0837570 0 0.06562872 0 0 0.130509332
0.3727977 0 0.05178884 0.1109414 0 0.1344026 0.2920257 0 0
0 0 0 0.1187502 0 0 0 0 0 0.24397085 0.107368544
0.1137658 0.07190207 0 0 0 0.1453666 0.4085905 0 0
0.4193539 0 0 0 0 0 0 0.9815107 0.1052097 0.1516746 0
0.1674237 0.3509060 0.05112247 0.075544295 0 0.144736384 0.05172385
0 0.05267699 0 0 0.06392873 0.09447459 0 0 0 0.06251376 0
0 0 0 0 0 0 0.2514333 0 0 0.07631108 0 0 0
0.1006051 0 0 0.04849242 0 0 0 0 0.08323882 0 0 0 0
0 0.08132511 0.07463856 0 0.1425025 0.3490612 0 0.3726318 0
0.09183734 0.0522373 0 0.1441691 0.03946809 0.0808653 0
0.2763887 0.540639988 0.07166677 0 0.05060209 0 0 0 0 0
0.1285153 0 0 0 0 0.1205272 0.3397535 0.1331074 0
0.09912025 0.6060895 0 0 0.2796274 0.2701127 0 0 0
0.1539924 0 0.05129685 0.11241251 0.3398659 0.2178119 0.09412412
0 0.226218735 0.07604177 0 0 0 0 0 0 0 0.5711485 0 0
0 0 0 3.0793550.09068735 0 0 0.2078230 0 0 0 0 0
0.05189408 0 0 0.1779090 0 0.3099178 0.08430445 0 0.06327309
0 0 0 0.0626629 0 0 0.126154 0.1171969 0 0.09099295
0.05583387 0 0 0.2113216 0.1016392 0 0 0 0 0.6875512 0
0 0 0 0.0676440 0.2032054 0 0 0 0.05490359 0 0.818126352
0.07884929 0.090313983 0 0 1.286542 0.09019691 0.1018119 0.0758747
0.1914262 0 0.03512699 0.3172334 0 0 0 0.07980005 0
0.1130282 0.2745358 0 0 0.07628958 0.08393006 0.1722635
AC012568.1 0.1882181 0 0.1814522 0 0.099381954 0.4381080.1025771
0.04921779 0 0 0.05962782 0 0.1911745 0.044970862 0 0.4556368
0.04930319 0 0.766346 0.03040022 0.066594397 0 0.03290996 0.06829546
0.08518955 0.04059648 0 0.0326089 0 0 0 0 0.1104291
0.1238991 0.1057844 0.034143484 0 0.06284677 0 0 0 0
0.0748639 0 0.06105998 0 0 0 0 0.07680376 0 0 0 0 0
0.04156597 0.1875125 0.03235149 0 0.4069927 0.1210268 0.09163354
0.1023009 0 0 0.057293469 0 0.248229 0.04601582 0 0 0 0 0
0 0 0.04777925 0 0.04413825 0 0.04790804 0 0 0 0 0 0
0 0.09686932 0 0 0.102340713 0 0 0 0.076644134 0.1246223 0
0 0.04646405 0.0322523 0 0.0572054 0 0 0 0 11.0841 0 0
0.1689453 0 0 0 0 0 0 0.04354699 0.03954322 0 0
0.04767321 0 0 0.02841502 0.3373126 0 0 0 0 0.2192267 0

0	0	0	0.05467188	0	0	0	0	0	0	0.03006006	0.3628684	0
0.03162804	27.973950	0	0.06604131	0	0	0	0	0	0	0.119445871
0	0	0.0956012	0	0.1070445	0.09570253	0	0.05407339	0	0	0
0	0	0	0	0	0	0.06276414	0	0.03482618	0	0.03120817	0
0.4569571	0	0	0.04709987	0	0	0	0	0.03761085	0	0
0.05449235	0.043585924	0	0	0	0	0	0.03660689	0	0	0.04891195
0	0	0	0	0	0.09861084	0.1640832	0.04150395	0.1258830.04490478
0.02398486	0	0	0.05258135	0.03382744	0	0	0	0.06893438	0	0
0	0.053884931	0	0	0.0337361	0.054937981	0.12705	0.07097724
0.1086584	0	0.1494834	0	0	0.02703313	0	0	0	0.06763161
0.07087508	0.03439654	0	0	0	0	0.06268229	0	0	0	0
0.07747296	0.1144904	0.04448237	0.1487739	0	0	0	0.05103351	0
0.1121462	0.03952507	0.0509234	0.04827638	0.06094061	0.1059924	0
0	0	0	0	0.03047993	0	0	0	0	0	0	0.1025446	0	0
0.044353964	0	0.03673524	0	0	0	0.0502644	0.04317341	0
0.09129574	1.6256850	0.1112944	0	0	0.1747135	0.02391499	0	0
0	0	0	0	0	0	0	0.09092965	1.4647970	0.1946789	0
0.03135723	0.033921575	0	0.0730313	0	0.04032702	0.04535908	0
0.1412497	0	0.03490311	0	0.16367	0	0	0	0.04665447	0	0
0.10217154	0.06864523	0	0	0	0.091382144	0	0.03028359	0.1377072
0	0.1474698	0	0.09906709	0	0.07690606	0.2106131	0.1160543
0.03949886	0	0	33.840280.1099008	0	0	0.08395109	0.08810643	0
0	0	0	0.08180613	0	0	0.1130217	0	0.03550292	0	0.1408419
0	0	0	0	0	0.04990633	0	0	0	0.1528815	0.1065201
0.1174780.2756778	0	0	0	0.06402327	0	0.04537752	0	0
0.04359532	0	0	0	0.31610200	0	0.067036417	0	0	0	0.13447400
0	0	0	0	0	0	1.55911500	0.06169111	0.09194985	0	0
0.04256915	0	0.1218991	0	0	0	0	0	0.03327002	0	0	0	0
0.06958665
MKNK1-AS1	0.1332281	0.03734593	0.0560461	0.012943624	0.025580522
0.1597536	0.01980221	0.07601078	0.03210815	0.01052259	0.1035988
0.020635355	0.22758500.043407457	0.055575831	0.1661454	0.04758917	0	0
0.1232421	0.038567553	0.1469954	0.1778888	0.1054739	0.1973471
0.09404435	0.0128303	0.1888515	0.1423578	0.033605787	0.08408878
0.114861276	0.007106003	0.1754013	0.1531602	0.013182597	0.107155
0.02426476	0.1718615	0.2088938	0.04011458	0.03180981	0.1300703
0.1045840.1768117	0.1550592	0.02280682	0.03845365	0.08674231
0.0741337	0.1440542	0.05132473	0	0.09643454	0.05732674	0.256774
0.3438880.08118967	0.1235518	0.03928437	0.1518651	0.09729271
0.03949779	0.1362074	0.1635090.121663677	0	0.3012107	0.1065986
0.054614674	0.1396557	0.1070055	0.1041988	0.122030734	0.1629142
0.02085226	0.05534185	0.1968479	0.1448529	0.0692155	0.1294791
0.08995252	0.07699638	0.005142883	0	0.0229862	0.09471448	0.1673452
0.009350167	0.04041281	0.07532954	0.046098666	0.06187903	0.01633439

0.2583299 0.029591846 0.08420285 0.01015088 0.2273997 0.02690924
0.01245242 0.0452671 0.08834666 0.2027275 0.0964754 0.09508046
0.14671281 1.739799 0.04878567 0 0.2500437 0.1219806 0.06217205
0.1879419 0.08213824 0.009525029 0.01975017 0.1260993 0.08397072
0.1034874 0.02848195 0.1012349 0.09367 0.1094279 0.2029611
0.03720982 0 0.04946439 0.251978972 0.02972971 0.1763378 0.08829058
0.05843231 0.077995379 0.159781 0.0949882 0.123721 0.08399583 0.04555017
0.08258418 0.06340692 0.07707415 0.1044541 0.01751267 0.07314905
0.03663419 4.583321 0 0.04507857 0.0637454 0.006215721 0.02743586 0
0.054061952 0.04122667 0.1525307 0.053803566 0.1035843 0.1920694
0.04613882 0.130551 0.08265846 0.09237545 0.149136721 0.08350966
0.09221421 0.04244455 0.1984468 0.04480085 0.06587092 0.3366835
0.008504281 0.1031602 0.078113182 0.1211643 0.04179614 0.08740017
0.0656872 0.1506161 0.02327107 0.168409 0.297484 0.0539183 0.02727747
0.07044345 0.121016 0.008840879 0.03872242 0.05808531 0.05840604
0.02935309 0.2945484 0.033656533 0.1665548 0.05413406 0.06201969
0.1458595 0.04682441 0.1978719 0.061064489 0.06977187 0.01888461
0.05452126 0.05190879 0.01765057 0.06812662 0.04554565 0.09518265
0.06335155 0.2003054 0.09113001 0.06934988 0.05093226 0.2084741
0.03893863 0.1725615 0.05877258 0.0645791 0.114152414 0.087914857
0.1863061 0.050463784 0.018064538 0.01721635 0.114425588 0.1105262
0.09558844 0.1042025 0.042422457 0.2452663 0.08906264 0.1538253
0.194932346 0.07214333 0.08972265 0.04920725 0.09915466 0.06153367
0.09758045 0.1199143 0.1697291 0 0.0398409 0.1076395 0.079530068
0.05906034 0.101581855 0.03025158 0.2522261 0.1170743 0.01105007
0.02114744 0.1121694 0.2210203 0.00858719 0.2776302 0.108959055
0.1608739 0.09602501 0.08866681 0.1014571 0.028866 0.03815099
0.05898367 0.08387652 0.1117618 0.3683074 0.05850544 0.0267791
0.01507909 0.008694667 0.080710639 0.100029 0.120933 0.1089106 0.08508486
0.1747773 0.04821668 0.03437117 0.01319729 0.9055158 0.1669157
0.145560823 0.031962711 0.04254977 0.1570286 0.02853863 0.3055754
0.03881357 0 0.04536764 0.05287312 0.3574217 0.01744208 0.1181678
0.0244415 0.1002789 0.2023675 0.1662018 0.04729545 0.06513836
0.04041266 0.102765822 0.1760452 0.07128132 0.0177573 0.01979481 0
0.02633055 0.3565421 0 0.08268082 0.1510531 0.2058163 0.032742298
0.02772266 0.09868934 0.07948425 0.03114005 0.05253862 0.150727644
0.09271062 0.01251443 0.2762556 0.04361196 0.03159601 0.1651526
1.094756979 0.09038117 0.1711236 0.03220767 0.03600221 0.085470289
0.02650352 0 0.03303006 0.200986257 0.149948942 0.01778973 0.05846157
0.04430662 0.02771682 0.1992803 0 0.219933 0.1282232 0.08907892
0.4065823 0.06161085 0.1601279 0.043649353 0.05561655 2.374063
0.1060801 0.1161256 0.04911167 0.07292929 0.1105564 0.01996608
0.131914 0.2197499 0.09632048 0.04737729 0.07891296 0.06498356
0.2181851 0.04162124 0.1439284 0.03419112 0.3987735 0.07396035

0.03191788 0.02960508 0.06403564 0.01783238 0.06743988 0.1099484
0.07760109 0.01043981 0.02951333 0.1439437 0.1436323 0.1383688
0.03265539 0.01513517 0.006481571 0.1977520.2615598 0.2014799
0.02989743 0.01149402 0.3366379 0.1045528 0.0659568 0.24759600
0.1412025 0.0949506 0.051764726 0.08319371 0.1007011 0.1234825 0
0.01284452 0.098200816 0.063799356 0.04611635 0.095079015 0.01112612
0.05840923 0.4514736 0.1899115 0.1071836 0.06212726 0.09703104
0.03536436 0.05341602 0.05195103 0.2745429 0.072060601 0 0.4013828
0.03948365 0.02644258 0.3275566 0.1902244 0.1119633 0.3123348
0.01963517 0.1746355
C1QTNF7-AS1 0.2775196 0.1677890.1321980.593166174 0.206872248 0.3039867
0.1067616 0.1024511 0.08655394 0.5814979 0.1861808 0.055626728
0.1160677 0.257429673 0.179778928 0.1053831 0.05131445 0.06274926
0.1087647 0.2689431 0.034655519 0 0.01712624 0.03554074 0 0
0.1037599 0.06787828 0.05482199 0 0.1236426 0 0.09577827
0.02149224 0 0.159913472 0.0666595 0 0 0.1689345 0 0.06431229
0.07791788 0.1644575 0 0 0.2664148 0.05182975 0.01670223 0
0.04568552 0.02767122 0.02913496 0.021663217 0.2575595 0.08652322
0.04879047 0.2020274 0.0876470.02647472 0.03149098 0.04768581
0.05323709 0.1129767 0 0.149076721 0.05470646 0.09226973 0.04789298
0.024537464 0.1505879 0 0 0 0.1211498 0.05621144 0.3232342
0.1396429 0.02296941 0 0.07479359 0 0.02594489 0.2911371
0.031705707 0.1084368 0.01595761 0.1025256 0 1.0115930 0.071010388
0.03706829 0.04403261 0.09285064 0.199426855 0.2594122 0.02736374 0
0.02417975 0 0.1220265 0.08930853 0.1900843 0.01857634 0.0640771
0.035953976 2.4797570.109592946 0 0 0.01934253 0.06703889 0.08171533
0.09489431 0.02567662 0 0 0.06173451 0.08369133 0.07677879
0.09923596 0.02525062 0.1242041 0.04436126 0.05015326 1.420884
0.02666823 0 0 0.07605662 0.03661614 0.02250228 0.126151291
0.1507526 0.02845108 0.04168934 0.02058433 0 0 0.1025556 0
0.06257266 0.0472089 0.2957818 0.03291826 18.327590.09532528
0.04860733 0 0.05026716 0 0 0 0.03704492 0.07709568 0
0.04653872 0.04706923 0.1243764 0.02346176 0.0278528 0 0.022334882
0.02813962 0 0.02860445 0.01671728 0.04830786 0.06658809 0.01815195
0.04584998 0.05561781 0.030081388 0.2286358 0 0 0.06640237 0
0.08364245 0.0216181 0.02970103 0.04152788 0.04902125 0.06329814
0.09470988 0.07149707 0 0.03914514 0 0 0.02835765 0.02268198
0.0264107 0.08338811 0 0.02069439 0.2272042 0.2286014 0.185187993
0.0235105 0 0.01633033 0 0 0.08476104 0 0.05131678 0.3073982
0.04319706 0.04913183 0.04673662 0.02496329 0.08429755 0.03498895
0.08208952 0.1408295 0 0 0.084640032 0.03587324 0.022672538
0.024348289 0.3944861 0 0.02291888 0.4831458 0.03511233 0.085768667
0 0.03693633 0 0.092731614 0.07779072 0.1612436 0.0221080 0
0.1673939 0 0.1407811 0.03688317 0.1610987 0.06448082 0.023821024

0.03980224	0	0.1794082	0.04635859	0.03322077	0.2383015	0.02850358
0.1007917	0.05958045	0.09259395	0	0.062940201	0.01971217	0.02876162
0.02655768	0.9181712	0	0	0.05300076	0	0.07928327	0.2482115	0
0.09625124	0.0406487	0.02343821	0	0	0.04657129	0.04194149
0.01529088	0.2217163	0	0.06949078	0	0.0262473	0	0.138489997
0.028720628	0.2485197	0.04233021	0.179507	0.1412125	0.05231488
0.1797385	0.09783804	0.07126503	0.3055006	0.3291303	0.08687589
0.1811891	0.152056	0.09092034	0.01244529	0.02549887	0.0702374
0.02178808	0.127858079	0.1129917	0.02401913	0.06382446	0.08004132	0
0.09463902	0.3645668	0.07100263	0.1013103	0.1745117	0.09790922
0.017652683	0.2740172	0	0.2678321	0.02098606	0.0944189	0.093765352
0.07350589	0.1012055	0.4722502	0	0.03406935	0.2739704	0.064623427
0.04060676	0.2913461	0.06511667	0.06470077	0.070893005	0.7859008
0.0686816	0.08903914	0.030099926	0	0.02397789	0.6461389	0.04777495
0.1743379	0.02558095	0	0.3351023	0.02160321	0.02001084	0.137003
0.03019716	0.06166526	0.039221856	0	2.118542	0	0.03478221	0.1765204
0.04368789	0.02292516	0	0	0.266571	0	0	0.03272703	0.04379405
0.02940808	0.05609919	0.01847561	0	0.02443122	0.4784998	0.05736068
0.1197096	0.02877015	0.02403537	0.1558266	0.0197592	0	0.0281426
0.1591182	0.05543272	0.0611352	0	0.08802914	0.1019997	0.05241712
0.06663503	0.1281976	0.07084296	0.02014863	0.03098444	0.2722424
0.195122	0.3911595	0.02472017	1.672402	0.09515992	0	0	0.0320379	0
0	0	0.06924996	0	0.107489956	0.02486317	0.113913116	0.05998537
0.07872688	2.839754	0.02844136	0.01605193	0	0.04024101	0	0.01107643
0.02000634	0.1268718	0.097126885	0	0.02516297	0.1862631	0.03564064
0	0.05494157	0	0.02405603	0.02646527	0.07242535
ADAMTS9-AS1	0.6988816	7.069392	0.1528283	6.544390402	0.733539597
0.6712037	5.302184	5.950614	10.53655	12.2104	0.9315291	1.364980175
1.759499	1.591433947	10.176063367	2.420854	1.326141	0.7698772	0.2105775
0.006194663	0.022616603	0.03694302	0.05588395	0	0.1764842	0.2095664
0.03160032	0.01328945	0.03577748	0.065032929	1.178083	0.118996326
0.01250121	0.008415657	0.1472974	0.020872286	0.2494159	0.03415015
0.08245818	0.3895454	0.03175714	0.002798064	0.1423804	0.5182358
0.01658962	0	0.01069941	0.01691236	0.002180015	0.2034543	0.01490746
0.012641	0.04943593	0.005655069	0.01008518	0.01693982	0.0127365
0.002197425	0.09609511	0.846608	0.1931831	0.1338174	0.07296064
0.02949197	0.003384148	0.023349418	0.1106765	0	0.006251105	0.233796234
0.003275849	0	0.002618733	0.064404622	0.01185957	0.05135796	0.01298132
0.007290609	0.3177903	0	0.1204009	0.03164972	0.1185236	0.2406661
0.012414913	0.0101096	0.01249695	0.01605826	0.01644924	0.07109607
0.01060185	0.03939089	0	0.01149448	0.009089315	0.020823734	0.04655625
0.1178621	0.07556539	0.03155998	0.002190687	0.5468336	0.05051264
0.02170898	0.009698512	0.08363496	0.009385597	1.794223	0.025747797
0.07024099	0.01912558	0.3433502	0.008750075	0.01706509	0.03715749

0.08043297 0 0.01774717 0.4028867 0.01092359 0.01002135 0.006476257
0.03295772 0.05876639 0.01351032 0.4058596 0 2.791603 0.031030509 0
0.06204431 0.1385975 0.4288095 0.07683931 0.008432823 0.2339507
0.03808974 0.002686715 0.05128573 0.04842863 0.004461935 0.02711848
0.0122507 0.01848545 0.006434358 0.04081742 1.978039 0.01244208
0.01268869 0.4710041 0.01093498 0.04504867 0.04501418 0.003804331
0.01450557 0.1341695 0.013521958 0.009111516 0.0491487 0.01298712
0.6645158 0.03635414 0.01300089 0.020406413 0 0.01081517 0.007467041
0.002181979 0.0220684 0.3157816 0.03080007 0.02094557 0.5638109
0.003926294 0.05968419 0.003676485 0.01419308 0.01155599 0.02755696
0.09006699 0.02821645 0.007753297 0.4336254 0.05438614 0.3029335
4.354086 0.0777664 0.006812225 0.3014497 0.01233007 0.00344262
0.03331177 0.032565566 0.3722962 0.01904702 0.05819087 0.01350541
0.2998474 0.03729699 0.013428432 0.009205941 0.01328907 0 0.05022618
4.719857484 0.09219344 0.1842898 0.07702686 0.01337411 0.008457274
0.01710081 0 0.09611892 0.007335144 0.2329089 0.01071452 0.04135817
0.01947609 0.025102771 0.004418967 0.00468226 0.035511283 0.025423972
0.5027775 0.01830025 0.005982853 1.89184 0.009165889 0.018657884
0.008629675 0.009642036 0.00246015 0.004034517 0.08630415 0.01052295
0.3058724 0.003672367 0 0.04057608 0.06027391 0.004593772 2.402226 0
0.06943357 0.059074356 0.07792625 0.047655324 0.7557235 0.4800327
0.002168027 0.0194398 0.01116107 0.002631115 0.2993984 0.1691982
0.02021047 1.065225233 0.07204061 0.02252421 0.02599779 0.005099655
0.07617359 0.002684679 0.2870881 0.003279098 0.4429047 0.007199369
0.01372339 0.1319109 0.07958347 0.0275329 0.010326535 0.01656241
0.1458863 0.03010867 0.08182791 0.07596472 0.006785999 0.03325704
0.004643456 0.07194306 0.01727328 0.039164761 0.007497369 0.08982661
0.03315024 2.316191 0.4423535 0.01365652 0.4310756 0.03990642
0.0155028 0.2116424 0.09205471 0.1474102 0 0.04851418 0.9256368
0.06010236 0.0366099 0.05500531 0.04550132 0.105692822 0.04129425
0.003135034 0.624789 0.003482395 0 0.03705753 0.3417405 0.310459
0.007933958 0.01518512 0.01490923 0.00691221 2.48732 0.04960538
0.06642037 0.1424358 0.04005228 0.101987306 0.1170488 0.04623352
0.01896591 0.007672416 0.1067235 0.004469908 0.022492809 0.09805167
0.1647845 0.01133224 0.1731204 0.043952337 0.00466262 0.004482243
0.1317115 0.007857428 0.043448929 0.07511163 0.004113931 0.0935355
0.05526213 0.01335555 0.0571801 0.01345796 0.01973792 0.1044745
0.07510423 0.00788281 0.005365797 0.058872293 0 2.672999 0.05971871
0.2338026 0.08351952 0.2394944 0.02393797 0.0140501 0.01392415
0.007731884 0.01564167 0.03333932 0.008543219 0.08574155 0.01535365
0.00183055 0.1133396 0 0.01275529 0.008674296 0.06363825 0.02343718
0.01126544 0.006274308 0.01016943 0.0154741 0.08971275 0.0183662
0.02076848 0.03858779 0.01861886 0.01497999 0.1057059 0.005325297
0.01596375 3.987741 0.02928214 0.003082197 0.005259693 0.004044163

0.002961144 0.05376534 0.1160342 0.04517149 0.02147074 0.02173587
0.04454439 0.050086786 0.09408741 0.008857896 0.01241349 0.009133935
0.5671765 0.031894041 0.252537203 0.06814921 0.104077475 0.007829433 0
0.2647511 0.01484894 0.02933191 0.07806938 0.0551497 0.161758
0.005782887 0.09400591 0.008279804 0.012677232 0.07486244 0.01313732
0.01389229 0.1535127 0.1536674 0.02390368 0.008754268 0.01569925
0.5561438 0.08271488
AL513327.1 0.1032582 0.09855046 0.1109231 0.110351243 0.202509811
0.3376347 0.1808832 0.150436 0.1107991 0.5510798 0.1892645
0.23247539 0.4157748 0.45465933 0.318131835 1.154608 0.3825387
0.2126286 0.04914054 0.6218454 0.234863427 0.3964248 0.6151499
0.2930496 0.3755554 0.3197567 0.371128 0.7091928 0.2353044
0.266042327 0.5632796 0.41967993 0.1557834 0.8302323 0.3544238
0.37329075 0.622421 0.2364228 0.9133779 0.6996513 0.1832132
0.2857238 0.7920843 0.9924739 0.2081666 0.7852253 1.087939
0.2341698 0.2716619 0.4153333 0.21157 0.2781698 0.1316334
0.406183965 0.2152787 0.3860304 0.5070077 0.2890442 0.609831
0.2212864 4.1687747 0.177744 0.1082377 0.4083481 0.1288572 0.484946761
0.04325421 1.125576 0.6437401 0.365843504 0.3458521 0.2606511
0.6118737 0.359177635 0.8552525 0.1333324 0.7301954 0.4921134
0.716062 0.3161244 0.5012506 0.28302 0.2637462 0.09082345 0.02148723
0.2134668 0.5659647 0.8337895 0.08540906 0.1582074 0.3027628
0.272704651 0.4480001 0.3448325 0.5820625 0.396449659 0.5347428
0.07417856 0.1692519 0.6063124 0.3677807 0.707531 0.369876 0.3810978
0.4825915 0.2846789 0.483265318 7.769513 0.227767716 0.2026171
1.012914 0.196629 0.2953134 0.1402939 0.3072622 0.2088152 0.04209517
0.9470792 0.3625961 0.163853 0.4943196 0.5660466 0.4221099
0.3682621 0.8351114 0.2379251 0.04659414 0.1445863 0.583097542
0.238978 0.4553073 0.3101884 0.1880832 0.161488315 0.6373233
0.784116 0.3861274 0.4603557 0.09431098 0.3268914 0.4131557
0.3553693 0.3145114 0.1493048 0.1113633 0.08179963 7.698682
0.2907124 0.3952992 0.5667545 0.1779028 0.09467594 0.2037613
0.829632155 0.1924767 0.6269808 0.53827496 1.414029 0.3402583
0.1910595 0.4664069 0.2957253 0.3712734 0.438959702 0.3623388
0.2121424 0.5169461 0.4796132 0.3055608 0.2707639 0.5535781
0.06732467 0.4774404 0.428114713 0.8485273 0.08272045 0.9007099
0.1700055 0.4952888 0.3117686 0.7618401 0.4965065 0.3987038
0.3322212 0.266919 0.4683179 0.06460557 0.4067662 0.3846705
0.2418578 0.1966255 0.8071653 0.163965577 0.4295701 0.5651284
0.1195984 0.8461609 0.2737391 0.344278 0.102262106 0.5682867
0.2645022 0.213966 0.1264428 0.333206718 0.6318762 0.4091026
0.3883523 0.5593949 0.6635671 0.6844397 0.1161373 0.2763247
1.047368 0.3319724 0.5192392 0.1749759 0.6797866 1.164375949
0.359464002 1.880097 0.317551062 0.236514972 0.3145254 0.291394677

0.3158238 0.4365761 0.3331430.303547739 1.1351280.2252886 0.4343087
0.188535035 0.1669449 0.47353 0.1947761 0.6896243 0.3072699
0.4105604 0.2086393 0.6201558 0.3499446 0.2790103 0.2986113
0.322874249 0.4225977 0.195889791 0.3831816 0.5410811 0.1313317
0.1076660.1931712 0.8014737 0.3903224 0.1882551 0.2973255
0.279627975 0.7258447 0.3638507 0.1679848 0.2074175 0.188968
0.1440426 0.3591907 0.2894419 0.3940270.7227019 0.2968987
0.3750742 0.0642786 0.05824236 0.808741981 0.2830724 0.3366588
0.3174026 0.1761670.5259067 0.2583885 0.1465162 0.1647525
0.5632874 0.4305008 0.656991028 0.194642165 0.2159282 0.2677505
0.5503370.3721703 0.2718158 0.1065840.4558516 0.2200193 0.737913
0.4142439 0.3663429 0.2753550.3320489 0.9037233 0.7534621
0.3801777 0.4482379 0.2313335 0.288835062 0.9444306 0.3798194
0.2415037 0.02410875 0.05850828 0.2351715 0.7337231 0.05613894
0.5538482 1.1366870.6930298 0.223316333 0.2419777 0.4378604
0.4779820.2607446 0.3039454 0.324788546 0.3420669 0.1714695
0.5334134 0.2987799 0.2462836 0.2630354 0.520684017 0.5962569
1.2505020.4363979 0.2375114 0.588548785 0.5810310.3103074 0.1542088
0.734363235 0.241713168 0.2383337 0.3880523 0.2428312 0.5007321
0.3582862 0.0593791.10057 0.1561673 0.5062966 0.6870761 0.2080596
0.9008298 0.27024005 0.1580535 8.0162930.8527096 0.2160785
0.1644905 0.4786571 0.7820076 0.2472264 0.4337884 0.5754274
0.4376635 0.2019583 0.7799751 0.2869028 0.5646864 0.2978147
0.2545953 0.1388083 1.1810840.5224566 0.44057 0.4507124 0.5134414
0.1683194 0.3344159 0.4820752 0.4050553 0.1652949 0.4942472
1.1938030.8562583 1.1213320.4374928 0.07373447 0.1263059 0.6397544
1.6652120.8855341 0.1228941 0.04899631 0.8456297 0.3134480.293208
0.4299955 0.5016693 1.1608330.3469303 0.299468289 0.4089161
0.2555148 0.4189529 0.04742594 0.2933206 0.322005359 0.48564578
0.2415165 0.115799846 0.1897121 0.4446157 1.2830171.4391970.2357015
0.3080721 0.7136090.1651071 0.4704130.4474295 0.6114283 0.162365187
0.02727766 1.1766680.3546522 0.0724619 1.0521120.4633611 0.2878792
0.7825429 0.1554431 0.6503535
AF001548.1 0.5384177 21.471870.2180066 16.456523195 0.369580405
0.2924261 0.563586.75804920.3159918.898191.4839742.109871242 2.242187
1.234981622 24.409511134 0.2606795 0.5500443 0.3104377 0.2391506
0.07826675 0.114300201 0.1400275 0.31067 0 0.03655406 0.9754964
0.2281461 0.5037186 0.09040644 0.186741249 0.6796597 0.056734509
0.03158941 0.53164 0.3177379 0.087903951 0.4763528 0.05393395
0.1389096 0.9286281 0.04458189 0 0 0.3099491 0.05240057 0
0.03379556 0.2991519 0.0550870.1977348 0 0 0.04804619 0.071449228
0.2973173 0.07134233 0 0.08329038 0.08672272 0.8731843 0.2596575
0.5111486 0 0.3726177 0.08551434 0.098336426 0.2255398 0.2434579
0.3159195 0.404645088 0.04138886 0 0 0.226034201 0.02497337

0.04634889 0.1640131 0.04605676 0.530301 0 0.5344795 0.06664664
0.2139274 0.04572491 0 0.1532762 0.07894667 0 0.08313149
0.02566476 0.0334874 0.204929525 0.09169347 0.02420459 0 0
0.1069486 0.1353759 0.7300909 0 0.05535668 0 0.2945558
0.03918329 0 0.03522299 0.148228438 1.777976 0.325311716 0 0
2.200934 0 0.3773168 0.1043262 0.04234308 0 0 0.03393527
0.04600487 0.1266152 0.08182456 0.04164057 0.1024119 0 1.157902 0
3.386332 0.280040267 0.07929723 0.2194925 0 0.5195163 0 0.03551496
0.04691842 0.137499 0 0.04049824 0 0.02818726 0.04894709 0.025797
0.03892588 0.08129519 0.0271426 0.8002245 0 0.04007895 0.2267018
0.08289512 0 0.04374875 0 0 0.5509304 0 0 0.4657286
0.08204321 0.5803586 0.09186361 0 0.220993505 0 0.04554823 0
0.08270495 0.1991601 1.207908 0.134704 0.1512216 2.29297 0 0.05386304
0 0.6575182 0.7300237 0.05356457 0.206901 0.07130042 0.09795938
0.6505909 0 0.6611011 4.477309 0 0 2.227109 0 0 0.04676434
0.074809295 0.3048755 0.3437864 0 0.03412693 0.249787 0.2199076 0
0.03877096 0.1679014 0 0.1730686 1.647760964 0.09318578 0.1518533
0.3808171 0.3379517 0 0.02700762 0 0 0.04633811 0.7500995
0.3158705 0.1451505 0 0.063432421 0.055831643 0.05915823 0.149556307
0.080305084 0.8036124 0 0.03779531 2.018437 0.1737103 0.612908094
0.109032 0 0 0.050974287 0 0 0.1458324 0.1391961 0.1094181 0
0 0.02902011 0 0.02951848 0.1329184 0.196415168 0 0 0.8606849
0.586113 0 0 0 0.09972883 0.3930143 0.2290438 0 1.107137204
0.422593 0.09486107 0.06569404 0.1288636 0.06416124 0.0678394 0
0.2900094 0.1307453 0.2274019 0 0.158727 0.06703339 0.07730349 0
0.02615732 0 0.06916533 0.02521606 0.0457038 0.08573802 0.1527955
0.02933398 0.3029893 0.261888 0.038063766 0.047362927 0.4413572
0.06980637 3.975171 0.1164361 0.04313599 0.6298608 0.2016798
0.1566967 0.4262908 0 0.2865325 0.02716339 0.02786157 0.8246475
0.02052341 0 0.02895696 0.07186106 0.070283198 0.1118002 0.03960974
1.36828 0.08799694 0 0.07803417 0 0 0.03341398 0 0.134551
0.116443517 1.725357 0.06267414 0.1766718 0.415295 0.03892634 0.25771265
0.6788202 0.2503455 0 0 0 0 0.106569906 0.1339285 0
0.03579444 0.1066974 0 0 0.1132622 0.1957782 0 0.039211247
0.01977086 0.07796648 0 0.08214257 0 0.07224445 0.08501757 0
0.2309978 0.09037217 0.04979786 0 0.161701014 0.04120681 0.8006322
0.04715742 0.3728336 0.1455491 0.4682944 0 0 0.1172835
0.2442221 0 0.03510227 0.1619095 0.1444407 0 0.02312816
0.1828078 0 0 0.06575747 0.5675579 0.131608 0 0 0.08565741
0.03258471 0.04928173 0 0 0.06094239 0.03360584 0 0.02903361 0
0.02881353 3.791109 0.05285237 0.1557686 0.03322692 0 0.03741271
0.07150533 0.8796239 0.2445948 0.04521217 0 0.2462242 0.230117738
0.07925011 0.07461033 0.03920969 0.05770156 1.141995 0.067161118
0.070904284 0 0.093926542 0.04946066 0.3894833 0 0.07035359

0.1852977	0.07890969	0.09954164	0.4192284	0.01826604	0.2309459	0
0	0.09956346	0.2074801	0.08776138	0	0.4853793	0.06040242	0	0
0.6110108	0
LINC02489	0.3346099	3.267241	0.3387108	3.93354531	0.353358058	0.2596195
0.6838474	1.246851	4.730964	2.834411	0.3975188	1.068928872	1.147047
0.719533784	3.608190162	0.1350035	0.7231134	0.3215456	0.3715617
0.04053363	0	0	0	0	0	0	0.04430803	0.7826136	0.070231	0
1.583953	0.308513926	0	0	0.4936605	0	0.3985133	0	0.2158199
0.5771135	0.1385313	0	0.09981853	0.7825339	0	0	0.1050145
0.5975794	0.04279357	0.102405	0.05852651	0.2835909	0.1492961
0.055504343	0.1979716	0	0	0	0.0449129	0.4069927	0	0.06108903
0	0.07236572	0	0.152782583	0.350415	0	0.06135442	0.565817284
0.1929142	0	0	0	0.1940022	0.07201098	0	0	0.529659	0.07967604
0.5110191	0	0	0.3196869	0	0.07938032	0.2044288	0	0.1291591
0.1196239	0	0.045484761	0.3324104	0.037606	0.2973712	0	0.08308151
0.2103298	0.08725555	0	0.04300307	0.05210832	0	0.060878	0.09519062
0.10945	0	0	0.336951871	0	0	1.585869	0	0	0	0.06578728	0	0
0.05272429	0	0.09835927	0	0	0	0	0.6425001	0	0.8199352	0
0.0410673	0.2435853	0	0.749504	0.538696896	0	0.07289584	0
0.05274006	0	0.09506512	0	0	0.08016017	0	0.06315304	0	0	0
0.1245391	0	0	0.06316449	0.2039137	0	0.189829	0.06584348	0	0
0.2411965	0.06373413	0.5410123	0.1427259	0	0	0.07209785	0	0
0	0	0.3412169	0.09301594	0.05873719	1.140008	0	0.08368551	0	0
0	0	0.4821845	0.05538874	0.07609839	0.2128011	0.06279982
0.919015	2.777116	0.122124	0.1337236	0.752217	0	0	0	0.11622913
0.2706723	0.05341314	0.1427854	0.05302207	0.06468118	0.04880919	0
0.5421363	0	0	0.08963065	2.681989193	0.1447802	0.2359303
0.1314811	0	0	0	0.05987304	0	0	0.537881	0.2103254	0
0.06371909	0.098553197	0	0	0	0.062383893	0.7134574	0	0
1.650521	0.08996294	0	0	0	0.09658521	0.079197339	0	0	0.2832197
0	0	0.0612697	0.1183173	0	0.6615007	0.04586205	0.2891164
0.183098885	0.1019792	0.116933915	0.6268229	0.47511	0	0.07632043
0.07303036	0	0.4579616	2.609632	0	0.591294146	0.2020219	0
0.06804468	0.05005297	0.2990566	0.0527001	0.06789786	0	0.2031354
0	0.3367368	0.3082623	0.4165917	0.1801564	0	0.0812798	0	0
0.03917749	0	0	0	0.09115078	0	0	0	0	0.3428622	0.1084562
2.496727	0.2412048	0	1.093726	0.03133443	0	0.06021056	0	0	0
0.173151	0.3494269	0	0	0.04498963	0	0.818977947	0.1158005	0
0.6541106	0	0	0.1212395	0	0	0.05191438	0	0	0.045228767
0.7658971	0	0	0	0.06047877	0.160160404	0.3389992	0	0
0.07530455	0.1745813	0	0.110383142	0.05202024	0.4354417	0
0.04144322	0.13622872	0	0	0.3041751	0	0.060921429	0	0
0.4284225	0.191434	0.1310843	0.1122442	0.1981342	0.0553506
0.0512707	0.1404087	0	0.05266515	0.050246078	0.06402188	0.2261673

0 0.08911712 0.5653391 0.3917717 0 1.838685 0.0911101 0
0.05117418 0 0 0.1122068 0.07534783 0.03593359 0 0 0.06259639
0 0.07348325 0 0.3685667 0 0 0 0 0 0 0.04734226 0 0
0 0 0 2.304838 0 0.06050336 0.1032475 0.07938668 0 0
0.4555491 0 0 0 0.05465024 0 0.0820858 0 0 0.3585973
0.2217857 0.052173185 0.716053149 0.1274062 1.386345149 0 0.06723661
0 0 0 0.1225998 0.1546551 0.5699256 0 0.05125919 0 0
0.309378 0.06447122 0.2727049 0 0.3105202 0 0.1718458 0
1.084926 0.0463911
MBNL1-AS1 1.627927 37.06973 0.6893255 27.797596565 0.774087572 1.015355
8.131896 13.53983 16.32876 12.47512 1.512998 3.401872222 5.48349 2.478813009
18.216769858 0.7039863 0.7774705 0.3586809 0.4569193 0.2462303
0.281628059 0.3703586 0.3656329 0.1346204 0.2320364 0.9835383
0.402547 0.4324084 0.2038779 0.171569409 2.418288 0.220347011 0.1108148
0.6867857 0.6937914 0.205576438 0.9365128 0.2837982 0.3828714
2.947354 0.3388494 0.0797238 0.7405216 2.355801 0.1838198 0.1151465
0.1100858 0.353375 0.1633369 0.7817305 0.3901412 0.2591724
0.4334007 0.465477883 0.7840095 0.4588227 0.4804991 0.2040625
0.06277575 1.309113 0.3296486 0.7192085 0.1393219 0.4162595
0.2892683 0.525655613 0.1469348 0.2770558 0.2935509 1.706760412
0.2696404 0.1589239 0.1105396 0.275634721 0.5944687 0.394863
0.5239827 0.7424336 0.8953396 0.1970303 1.016451 0.3228592
2.669464 0.8134659 0.209618777 0.345657 0.1802326 0.2146482 0.361057
0.2700938 0.08670618 0.63086017 0.145512 0.2931384 0.4667983
0.093392944 0.3037112 0.6784212 0.558197 0.2364621 0.1225245
0.1540697 0.2788253 0.2749078 0.2686587 0.3853935 0.344178104
2.01963 0.492098124 0.2322543 0.145316 1.721065 0.3231814 0.4434567
0.6055991 0.6118367 0.08066529 0.5399959 0.5101886 0.2343908
0.5076145 0.2289472 0.2260669 0.2010136 0.08961627 1.360871
0.2114917 3.88993 0.682983551 0.1435017 0.4426039 0.3454734
1.847244 0.306971334 0.175013 0.7484859 0.5225381 0.0878921 0.2672208
0.8074689 0.4779206 0.2371165 0.2994959 0.2796043 0.217281
0.2561751 2.015401 0.07877907 0.1472909 0.5822467 0.4638854
0.2988155 0.2777066 0.658398222 0.2245085 0.5061699 0.05422377
0.5640903 0.09724781 0.2912316 3.263875 0.2723822 0.1989334
0.809078981 0.2984424 0.1902166 0.5594656 0.4213745 0.3393435
1.660071 0.685055 0.06946777 7.839394 0.140873171 0.2069453 0.04267681
0.3444856 0.06402258 0.5569983 0.7459643 0.2828737 0.2945476
1.349905 0.2430739 2.031414 4.957131 0.2593257 0.3378729 2.356193
0.1561397 0.1743801 0.3827787 0.428009139 1.265933 0.2813984
0.8405151 0.1909761 1.491704 0.1889216 0.113365737 0.1101013
0.3996742 0.1462042 0.1108235 11.147722236 0.8794983 1.07385
0.7209598 0.1905309 0.4164892 0.3000163 0.7338607 0.3077347
0.2167371 0.7518082 0.4296579 0.5964725 0.2021013 0.121855752

0.165544848 0.3952866 0.218599765 0.482928066 1.412721 0.065660241
0.4640472 4.58732 0.3651389 0.181140994 0.377928 0.3459507 0.1817298
0.676947824 1.275047 0.3331388 0.9378894 0.1143233 0.6488649
0.3524333 0.9477246 0.06786783 0.4826178 0.4142001 0.4684946
0.446221854 0.4111689 0.691481646 2.720478 3.024506 0.3271653 0.1764236
0.3611925 0.1416043 1.177625 1.04261 0.2559318 1.340838147 0.3991197
0.2773084 3.672615 0.3363468 0.8038425 0.1728179 0.7336681
0.1522557 0.8932774 0.2507119 0.2570552 1.246194 0.5542852
0.2840918 0.242466191 0.1310845 0.4297784 0.3841643 0.1642777
0.797821 0.2040916 0.3350015 0.2009054 0.3940603 0.3171688
0.384683921 1.182814475 1.029546 0.1515917 9.589694 1.053554 0.5043996
4.388123 0.6771663 0.1766853 0.7638924 1.256386 0.8376234 0.1361263
0.3979318 1.108296 0.7970936 0.2212651 0.4788782 1.206415 0.21132989
0.3268266 0.2315831 2.964767 0.09187228 0.1308033 0.3421772
0.6756124 0.1760348 0.1367512 0.1922941 0.5484205 0.240711825
3.060564 0.03140845 0.3393923 0.7024067 0.09753752 1.373293378
0.6722669 0.19748 0.1476054 0.2671853 0.6123868 0.1509437
0.513294183 0.8389596 0.3778835 0.1195866 0.3230494 0.156233757
0.3493453 0.1513603 0.8707446 0.505797609 0.645184305 0.2658633
0.1736535 0.2467647 0.4836869 0.2008367 1.176647 0.1384683
0.1458028 0.5264415 0.3170231 0.3015474 0.1868593 0.856266416
0.2374789 1.0821 0.3544863 0.7258072 0.723325 1.287734 0.1641977
0.1878055 0.2840809 0.159106 0.1595609 0.2697304 0.2411636
0.3558923 0.2794907 0.2066959 0.4606052 0.2539022 0.3466043
0.1025224 0.6123046 0.1346559 0.1030308 0.1820812 0.3469873
0.4027935 1.39538 0.3837523 0.8657019 0.2774105 0.2049012
0.1146083 0.09942414 0.1657801 0.2623191 10.61909 0.3399085
0.4195818 0.2580952 1.015716 0.3468559 0.2090323 0.4065283
0.6877875 0.06797273 0.3047394 0.390743 0.999448336 0.5361568
0.3552063 0.3438663 0.057833 1.538051 0.260842154 1.305834021 0.2225981
0.294189056 0.1693755 0.8168865 0.6146486 0.2683444 0.6212768
0.4349919 0.684522 1.028574 0.1647688 0.5318345 0.1893122 0.139131128
0.3035287 0.3535197 0.2162382 0.1178171 0.5580269 0.2774751
0.4526708 0.6129812 1.738786 0.4314478
AC004918.5 0.8003507 0.5264766 1.351809 0.307918313 0.963521471 1.639392
0.4972495 0.4269443 0.7072471 0.3754859 2.525383 0.436354099
2.324957 0.43599858 0.734502195 0.8524923 0.9056862 0.5845178
0.8176365 1.59777 1.223321074 1.609685 1.494575 1.132597 1.4345 1.222195
2.136557 2.495903 0.4569191 0.421934281 0.7072135 2.108377026 0.3193092
1.706995 0.9716154 0.452982521 1.285459 0.4168949 3.303795 1.435609
1.590489 0.5255067 1.37523 1.082728 1.215127 2.610156 0.4621771 2.18531
1.719608 0.7446229 0.8287353 2.61831 0.8856082 1.741816304 0.3788213
3.117853 1.411311 0.6438121 1.667264 0.9605027 1.883569 0.9117785
0.835218 0.7754476 2.211817 0.935524922 0.4291352 4.270383 2.254128

1.178938931 2.042596 0.2262733 2.596843 1.102069456 2.479788 2.011792
0.4388459 2.902821 1.599093 0.8842748 0.2933526 1.109576 2.060642
2.745966 0.466330963 0.7594766 0.3129418 2.553474 1.359312 0.8240468
1.971231 1.253314159 0.4725105 0.7627717 3.072729 1.212385918 0.9538655
0.2414813 2.036969 1.043206 0.5430939 1.176578 1.080038 1.630872 1.402544
0.6492454 0.987121273 15.48761 1.439968375 1.547863 2.068986 0.7586455
1.413288 1.810829 1.922986 0.6797782 0.9396812 1.266582 0.7667537
0.6017934 0.6022777 2.213686 1.782667 1.065638 1.507931 0.9343689
0.5393166 1.045971 1.065666744 0.7072456 2.255942 1.023253 0.6839964
1.566821762 0.8657972 1.562256 0.6131713 1.372496 0.9150414 2.328427
1.273759 1.396976 1.089052 1.550723 0.7975717 1.178136 11.78543 0.2804109
0.8817369 0.8761719 0.6407569 1.692125 1.872919 1.114611334 2.252091
0.9323437 0.731396124 1.414626 1.523058 1.024431 0.7361666 0.5462161
4.199737 1.927222641 1.186458 0.6499856 1.963347 0.8359906 0.4026264
1.567016 2.732104 0.5170143 1.272495 0.973368665 0.8326935 0.1104774
2.221345 1.692866 1.289893 1.619793 2.79806 2.533706 0.9772745 1.586222
1.241328 1.052491 3.15476 1.202645 0.6717095 1.235051 0.6982862 2.864
1.668045084 1.605599 1.022068 0.7923435 0.5478756 1.559481 3.455691
0.766689414 0.5071661 0.4492497 1.377079 1.852302 0.606511757 0.4155582
0.6922324 1.962409 2.12666 0.9530205 2.055499 1.741432 1.28507 2.011324
1.097859 2.173287 0.897579 1.853295 0.867481218 1.112105966 0.3869268
1.511731316 0.811732467 1.023908 1.209818654 1.438265 3.44254 1.532093
2.971521154 1.426257 0.9416569 1.589427 1.272979497 1.220431 1.106741
1.409056 0.675916 1.07348 1.524125 1.403691 3.071426 0.2169927 1.544535 1.40678
2.452535339 1.170831 0.60413749 0.9115699 1.015195 1.107527 0.8762411
1.788731 1.304561 0.8178946 0.6809409 3.365561 0.740585021 1.700915
3.384233 1.640576 1.647365 1.335248 0.6655595 1.377189 0.9114581 0.839597
2.731281 0.8247685 1.415674 0.8370116 1.378927 1.454576252 1.586406
1.141626 0.246752 0.5247666 1.875091 1.809767 1.044791 1.203486 1.981714
1.375502 1.516378133 0.647720027 0.7872858 0.3735584 0.6537659
1.269259 0.8720466 0.7710538 0.6475556 0.9084173 4.70072 0.9220731
3.464204 0.856014 0.8448833 3.075712 2.196559 0.9000963 1.136365 1.516851
2.173080513 1.152247 0.447483 1.048256 0.7586764 1.01583 1.06717 4.842128
1.148746 3.83448 1.654119 1.568065 1.003931941 0.7816291 1.714222 0.7878608
0.8642626 0.7869433 0.245175278 1.095548 0.3142475 2.903033 0.5187466
0.9687849 0.839497 0.697024793 1.552846 2.142575 0.8513271 1.728787
1.320755847 1.015801 0.5050882 0.902167 1.298624716 1.328943355 1.398922
0.2472245 0.4216075 0.7814645 0.95316 0.816167 0.8088158 5.274523
0.6867503 2.283729 2.23552 1.269771 0.557649982 0.5635309 23.67275
1.990808 1.500636 1.644312 1.863442 1.88824 1.161055 1.464459 1.132662 1.17507
0.3130743 3.417604 0.7300112 2.162686 1.182662 1.050733 0.3615018
2.539315 2.710867 1.434234 0.4890839 1.833669 1.249086 0.9422315 0.6974889
1.289317 0.2483547 0.6630927 2.264751 1.61853 0.5908148 1.018531 1.08016
0.65103 0.6207143 1.162752 2.060777 0.3161047 0.303815 1.201251 0.5739752

7.217671 1.6725 0.4570095 1.282987 0.7320178 1.40247432 0.7853614
0.5767177 1.585343 0.4803265 0.5262435 1.33777686 1.264779118
0.8776566 1.563749999 0.5587718 0.7204857 3.977849 0.613534 0.3620101
0.9383855 0.5524112 0.9970838 0.5647661 0.7650642 2.011181
0.47618424 0.1479997 1.899845 1.669838 0.6989418 1.884414 0.6644267
1.660588 2.26444 0.8304085 1.029732
HMGA2-AS1 0.009816999 0 0.008517697 0.005245675 0.02591761 0.01142532
0.02006315 0.0192531 0.008675001 0.01066125 0.009330127 0.029270189
0.0024928 0.003518357 0.063065164 0.007921637 0 0 0.002725278
0.002378403 0.015630311 0.02127607 0.03089707 0.06144667 0.003332461
0.1524538 0.04419787 0.002551202 0.02884674 0.102145884 0.1022362
0.033619405 0.002879857 0.2229486 0.1613856 0.005342524 0.3908414
0.7768706 0.06331863 0.1523855 0.07722206 0.08057247 0.05857079
0.6287091 0.009554222 0.02315194 0.1294012 0 0.00251101 0
0.0137367 0.006240128 0 0.058623185 0.003872144 0.04227562 0.003667575
0.1670502 0.02108293 0.1552279 0.08995261 0.5018353 0.004001827
0.1401254 0.09744921 0.017929731 0.0205614 0.005548725 0.00360011
0.121735593 0 0 0.0150817 0.206064662 0.07968464 0.2239466
0.1420468 0.02099388 0.06215789 0 0.02248891 0.02430343 0.06630935
0 0.038133036 0.06753841 0.1295495 0.1202267 0 0.03041654
0.006105775 0.373649059 0.00278642 0.1522566 0 0 0.01462499
0.5389155 0.1638374 0 0.09588551 0.03669089 0.05370651 0.01071647
0.0223421 0.1027556 0.191888629 0.1620897 0.128514214 0.07011836 0
0.2064646 0.03023584 0.002457013 0.004755464 0.1428281 0.004002086
0.01362783 0.08353038 0.02935832 0.01731436 0 0.01898087 0.02100693
0.002223088 0.01131005 0.04823575 0.04811156 0.576976714 0.002409718
0.008575752 0.09358268 0.0879577 0.233908568 0.119796 1.082164 0.1065486
0.2413822 0.01476813 0.1617667 0 0 0.01175895 0.01064606
0.09634686 0.00989785 7.890112 0.003582796 0 0.0155005 0.01259526 0
0 0.048201432 0.09467853 0.0231811 0.074760088 0.4897635 0.04245827
0.01869873 0.03527236 0.004187385 0.1572358 0.130955044 0.008461011
0.008304832 0.004300387 0.002513273 0.01089389 0.06673893 0.1091584
0.05169808 0.0836157 0.927098097 0 0.008469383 0.1008129 0.04658696
0 0.09745461 0.02275042 0.004465246 0.09052775 0.2468898 0.02854869
0.1044169 0.1289863 0.0117698 1.053428 0 0.1387862 0.01278985
0.057970073 0.09926447 0.02820724 0.1089174 0.009333572 0.01518126
0.03723187 0.012373834 0.007069129 0.003826693 0.1153896 0.1577785
0.135912024 0.07433392 0.2953333 0.05400468 0.01026982 0 0
0.05621099 0.01125893 0.008448851 0.6838302 0.01645509 0.002646536 0
0.584066135 0.111977934 0.01078635 0.08521462 0.117136538 0.01744322
0.046373374 0.01378248 0.1888537 0.00263939 0.330957398 0.0298198
0.11106 0 0.413590479 0.134493 0.01212066 0.01994227 0.323591 0.06982588
0.06830764 0.06942539 0.005291251 0.08872014 0 0.0557407 0.150412411
0.01795158 0.205840904 0.007356052 0.06969541 0.1248601 0.0179131 0

0 0.004478656 0.04872195 0.01163953 0.1293197 0.05334342 0.06918427
0.09382789 0.002936972 0.3626551 0.003092298 0.003984062 0.0491006
0.1382654 0.008292461 0 0.1410863 0.3483337 0.1550426 0
0.03338494 0.1260273 0.006305475 0.0160918 0.1499975 0.003908164
0.1079547 0.005348479 0.1538945 0.007958362 0.069401861 0.030224974
0.01437016 0.01909175 0.03084234 0.03184479 0.05112255 0.40195
0.03125652 0.1678522 0.6041416 0.04948138 0.06965815 0.03466901
0.03048009 0.05467578 0.01309715 0.5865247 0.007919607 0.02292931
0.39085054 0.04416659 0.003611032 0.2278897 0.004011132 0.02163202
0.1671791 0.1345309 0 0.003046194 0.004372675 0.009813102 0.045116314
0.02247038 0.05142334 0.04831899 0.05679072 0.1490466 0.143316085
0.4486649 0.1622956 0.01365346 0.2430266 0.02817088 0.005148581
0.265556261 0.6898437 0.09490197 0.02610567 0.04620373 0.05062569
0.01074111 0.08776742 0.4149699 0.004525217 0.028597628 0.09372561
0.07107834 0.03232114 0.1123282 0 0.05927566 0 0.1883737
0.6979546 0.03707461 0.002269917 0 0.023586423 0.0638627 0.8692427
0.6921581 0.02353117 0.1492765 0.2692891 0 0.0107889 0.005346092
0.008905828 0.006005523 0 0.002460087 0.006583988 0.06631808 0.217174
0.04999715 0.01385667 0 0.04396184 0.3535673 0.03899376 0.06487947
0.003613473 0.2225559 0.3000299 0.004492782 0.02115477 0.09568717
0.01111166 0.00612737 0.004313606 0.00794057 0.03680307 0.1628612
0.03506264 0 0.04970241 0.02423312 0.2329097 0 0.03259401
0.1897857 0.003716425 0.03709601 0.01072974 0.2148507 0.047202171
0.03130771 0.09182524 0.04289474 0 0.02863032 0.293892509 0.084032095
0.2242755 0 0.01803638 0 1.036825 0.01068967 0.2944158 0.1115042
0.5777584 0.03344168 0.006660912 0.216558 0 0.131418326 0.04084528
0.003782996 0.03600355 0.09644771 0.007808776 0.02202641 0.007562582
0.02893263 0.3461541 0.1714925
AL445649.1 0.06428287 0 0 0.045799074 0.135769268 0.6317659 0.2802686
0.06723819 0.03786996 0.05584892 0 0.036507556 0.3917556 0.030718157
0.196646753 0.5878806 0.4378066 1.153097 0.2617332 0 0.045488503
0.2600607 0.2697569 0 0 0 0.06809715 0 0 0.029727261
0.08114606 0 0 0 0.2890317 0 0.2333243 0 0 0.07391396
0.03548486 0 0 0.4934066 0 0.02887651 0 0 0 0.0524622
0.02998319 0 0 0 0 0 0 0.02209827 0.02300892 0 0.04133476
0 0.1048177 0.1112191 0.03403248 0.039135336 0.2872287 0.02422247
0 0 0 0.07572462 0 0.03598229 0.03975501 2.951304 0.03263647
0.03665878 0 0 0 0 0.1021651 0.03639465 0 0 0 0 0
0.7558289 0 0.652452901 0 0 0.1828123 0.104706314 0.08512548
1.257108 0.0447011 0.1269524 0 0 0.1172255 0.03118786 0.02438313
0.3083923 0 0.7783471 0 0.04709162 0 0.07616647 0 0.1501623
0.04151912 0.6403549 0 0.1189822 0.1080428 0 0 0 4.242397
0.04075729 0.5240536 0.0658307 0.6617881 0.03500447 0.044579493 0
0.07487339 0.048062 0 0.165584979 0.08480418 0.03734461 0 0.02701879

0	0.04870196	0.04487118	3.4673810	0.1549149	0	0	9.652045
0.03128076	0	0.5864392	0	0.09707769	0.03482173	0	0	0.2361218
0	0.1221726	0	0	0.06159136	0.2559153	0	0.029316553	0
0.03625402	0	0.04388589	0.03170422	0.1456715	0.02382608	0.1203645
0.02433447	0.039484542	0.5573383	0	0	0	0.0213173	0.08234121	0
0	0.02725455	0	0.2215587	0.04143843	0	0	0	0.08266442	0
0.03722198	0.119088602	0.1386657	0	0	0.05432652	0.06627247	0	0
0	0.2004611	0.02143504	0.04591787	0.124907798	0.1112565	0.6849150
0.04483199	0	0	0	0.01638329	0.03688272	0	0	0.06931932
0.09793006	0	0	0	0.089279271	0	0.8528448	0	0	0.1691127
0.09217621	0	0	0	0	0.324583016	0.1021074	0	0.08705624
0.01846547	1.0886380	0	0	0.1452375	0	0.1057961	0	0.05224404
0	0.02140813	0.04056656	0.3270395	0.3909903	0	0	0.03910236
0.3038448	0	0	0.0517480	0	0	0	0.1349916	0.06956829
0.09892816	0.1873196	0	0.03450212	0.03158461	0.1600653	0	0	0
0.2445161	0	0.02007067	0.07275568	0	0.1520216	0	0.1033559
0.1042246	0	0	0.02509267	0	0.1346395	0	0	0.02949038	0	0
0.03084593	0	0	0	0.04435272	0.05967059	0	0.06693915	0.04609647
0	0.39159235	0.02966237	0	0.08377538	0	0	0.06211113	0.7395415
0.04659869	0.02659579	0	0.2784485	0	0.1634874	0	1.0195060	0
0	0.01929663	0	0.0238412	0	0.02235955	0	0	0	0	0	0
0.02326337	0.6095617	0.09015082	0.1168719	0	0.03121011	0.07868288
0	0.03135447	1.0460990	0	0.1015043	0.02835617	0	0.1798288	0
0	0	0	0.1158657	0	0.04565479	0.02896238	0.08601645	0
0.0235490	0.1166328	0.2883824	0	0.02147859	0.2299346	0	0
0.2182582	0.04032672	0	0	0.03764554	0	0	0	0	0.02593573	0
0.03693971	0	0	0	0.1506453	0	0.05355353	0	0.0874645
0.2103387	0.03099593	0	0.1626795	0	2.07738	0.04667565	0	0	0
0	0	0	0.3266226	0.09362661	0.09185487	0.1817937	0.026728375	0
0.06527033	0	0.3936811	0	0	0.4666483	0	0.0314040.02640999	0
0.1308493	0.05252027	0	0	0	0	0.03492674	0.7485050	0	0	0
0	0.07129862
AL355102.1	0.09656405	0.06616724	0	0.343990869	0.033991509	1.798147
0.2105061	0.1010035	0.1137745	0.02796493	0.1223666	0.274203493
0.7519530.092288029	0.118159049	1.8181450.05058936	0	0.2144557	0
0.136663284	0	0.1350739	0.03503854	0.04370595	0.2082776	0
0.1003787	0	0	0.04063183	0.101752109	0	0.6356565	0	0	0	0
0	0	0	0	0	0.04632391	0.1253057	0	0	0	0	0	0
0.02728021	0	0	0	0.08530061	0	0.06639088	0.0691268	0	0
0.04701199	0	0.05569014	0	0	0	0.03638637	0	0	0	0	0	0
0.02985947	0	0.04902566	0	0	0	0	0	0	0.08200663	0
0.09163251	0	0.2830151	0	0	0	0	0	0	0	0	0.1278733
0.05395415	0.06714883	0	0.1323747	0.3208060	0.04684959	0.07325539
0.04211444	0	2.1258410.04321774	0	0.1155684	0	0	0.2577941	0

0.1012552	0	0	0	0	0.07569387	0.09783372	0.09957529	0	0
0.09888915	0	0	0	0	0	0.07219748	0	0	0.2547813	0	0
0.04058692	0	0	0	0	0	0	0.09720077	0.2596249	28.8509	0	0
0.3388206	0	0	0	0	0	0	0	0	0	0	0	0.1098369	0	0
0.0554840	0	0.09888635	0	0	0.01789546	0.0452021	0.1096638	0
0.06440146	0	0.03573468	0.1309282	0	0	0.0852505	0	0.04094107
0	0	0.06224773	0	0	0.0385920.3725290.1560181	0.05591388
0.044722948	0	0	0	0	0	0.1502474	0	0	0	0	0	0
0.1114178	0	0.05059165	0	0	0	0.09215242	0.0492211	0	0
0.3237182	0.06941978	0	0	0	0	0.044704331	0.048008474	0.04575433
0	0.09038009	0.1905275	0	0	0	0.03641441	0.03716431	0.060947517
0	0	0	0	0	0.04715103	0	0	0	0.03529384	0.1271394
0.046968844	0	0	0.03215874	0	0.03275134	0	0	0	0	0	0
0.206836094	0.03886725	0	0.02618241	0	0	0	0	0	0.03126518
0.2718936	0	0	0	0	0.077999016	0	0	0	0	0	0	0	0
0	0.045511024	0.056629586	0	0	0	0.04640571	0	0.04429968
0.04822778	0	0.3706876	0	0	0.09743391	0.1665637	0.2689068
0.04907771	0	0.06924491	0	0	0	0.04735947	0.0314613	0	0	0
0.5881341	0	0	0	0.03217524	0.034806486	0	0.1498729	0	0
0.04654236	0.061626938	0.05797377	0	0	0	0.03358793	0	0	0	0
0.0427977	0.03189326	0.104836884	0	0.0677111	0	0	0.140649039	0
0.5282511	0	0	0	0	0.05082572	0	0	0.1080537	0.05954092
0.08105853	0	0	3.9161360	0	0.08701306	0.1292117	0	0	0	0
0	0	0	0	0	0	0	0	0	0.05241538	0	0	0	0	0.05120823
0.07791996	0.05892381	0	0	0	0	0	0.1041423	0.08044671
0.06890192	0	0	0	0	0	0	0.04274775	0	0	0	0	0	0
0.03158519	0	0	0	0	0.120452005	0.042388431	0	0	0	0
3.9994680.1401974	0	0.04717427	0	0	0	0.03944729	0	0	0	0
0	0	0	0	0	0	0.0521826	0.03570098
AC005180.1	5.19614475.858990.5252532	68.847399161	1.544963297	3.483634
35.2606763.6373452.7820837.189076.1370949.067867479	9.7100588.027643532
64.58489064	1.3026571.2289680.04847835	1.2884390.1466668	0	0.3061361
0.1323126	0.0274578	0.03424997	1.5668720.3206482	0	0.1694158
0.069988202	6.2089870.106316797	0.05919652	0.09962589	1.10578	0.082363157
2.7466290.2526718	0.3253844	6.8737460.4177178	0	0.3611832
3.3397410	0.5098893	0.2216574	0	0.1806516	0.8028414	0.1058861
0.1282681	0.2701064	0	0.1591867	0	0.03769417	0.02601346
0.1625128	5.1134090	2.3209670.08225902	0.4364134	0	0.414621219
0.0422647	0.05702804	0	1.364901659	0.07756004	0.0891409	0.09300295
0.127072038	0.2807909	0.3474193	0.4610250.0431537	3.65558	0.04804995
2.0802050.4371202	2.9665580.2142138	0.146969724	0.1914864	0.2712251
0.2534670.1557830.1202352	0.06275322	0.850340325	0.02863794	0.06803675
0.03586692	0.123257347	0.1503110.5073713	0.7366925	0.07472242
0.1556023	0.09427433	0.1839928	0.1101404	0	0.0330028	0.277770497

2.248973 0.812817461 0.05543494 0.2264116 4.423278 0.05179239 0.126262
0.0977503 0.1586964 0.2056609 0.03501564 0.03179625 0.08621015
0.355903 0.03833348 0.1560635 0.6237184 0.1370892 1.937351 0
19.49057 0.367344132 0.02476631 1.145805 0.08486586 2.955389 0.324870019
0.06655274 1.450714 0.3864964 0.06361153 0.1897277 0.8026282 0
0.04586184 0.02417095 0.03647229 0.1142564 0.07629523 1.384217
0.03682284 0.03755268 0.3186184 0.05178003 0 0.2049558 0
0.228959 1.072116 0.032015021 0.1438181 0.1454575 0.1153077 6.452823
0.3442929 0 0.276085015 0 0 0.04419801 0 0.1866066 3.086647
0.7713034 0.03542243 10.62761 0.418320899 0.1009359 0.08704563
0.02800333 0.3420043 0.02509413 1.777046 0.2004185 0 4.716244
0.3787246 3.87959 17.93484 0.1472978 0 4.506126 0 0 0 0.210281663
2.203651 0.6120218 1.4208 0.1279033 1.092197 0.323787 0.031793585
0.1089814 0.03932954 0.07569818 0.1081065 13.306936312 1.047745
1.138253 0.3171674 0.07916245 0 0.2277474 0.07221484 0.0771438 0
1.784079 0 0.08160077 0 0.237736502 0 0 0.105097064 0.902919169
2.366442 0 0.07082597 10.30212 0.02712681 0.044175003 0 0 0.1164946
0.191045029 0.9615834 0 2.254564 0.1521592 0 0.2586479 0.4281193
0.0271909 0.05698984 0.02765786 0.3487127 0.147227724 0.06150023
0.282075678 10.93726 4.226213 0.0513309 0 0 0.03114756 2.301511
1.538014 0.0398758 3.30655862 0.5482468 0.1777635 0.2667305
0.03018527 0.6612871 0 1.105567 0.1164555 0.5390182 0.08522728
0.04061493 2.528276 0.06280812 0.1810772 0 0.2695942 0 0.1296114
0.02362663 0.08564596 0 0.1431644 0.05496997 0.4055588 0.08179352
0.035664517 0.044377529 0.8566131 0.06540631 25.43827 2.618326 0.4445873
8.262229 0.5291088 0 1.561372 0.2179517 1.029141 0.02545122 0.3132647
3.020429 0 0.1575978 0.1085269 0.2693259 0.296338869 0.1396708
0.03711304 4.339194 0.08245029 0.04446541 0 0.6145183 0 0.1252313
0 0.07564196 0.081827855 7.42868 0.05872364 0.5793752 0.6809565 0
1.110754694 1.158488 0 0.05613038 0.4541365 0.6053839 0 0.46597858
0.8784064 0.2250859 0.03353823 0.1999441 0.082154969 0 0
0.6878918 0.046508745 0.293917356 0.1481973 0.02435069 0.03690961
0.1539299 0.03952627 0.3384536 0 0.03338009 0.772991 0.1693516
0.04665899 0.09528175 0.57573274 0.07721889 0.4773787 0.3534798
0.9405121 1.261467 3.408958 0.1416907 0.02772122 0 0.04576564
0.1851686 0.1973381 0.202272 0.1353363 0.181759 0.02167034 0.9135198
0 0.1132493 0.02053754 0.7090445 0.09248435 0.08890809 0
0.1203873 0.1831849 0.09235078 0.0434844 0.1843955 0.05710105
0.1574379 0.04433387 0.08161067 0.03152086 0.02699735 21.10696
0.1485629 0.03648755 0.03113254 0.1436263 0.0350545 0.4019891
0.4120895 0.7257285 0 0.4043486 0.7909854 0.053903218 0.7672995
0.1048612 0.2571675 0 9.202104 0.18878338 0.830437737 0.3073376
0.264018389 0 0.04054814 0 0 0.8928921 0.1109037 1.243564
0.5892052 0.05134405 1.020119 0 0 0.3265071 0.07776086 0.2055739

0 0.8828174 0.1980829 0 0.1858502 2.739809 0.4476306
LINC00685 1.107027 0.5689146 1.200637 0.073941963 0.47492809 1.503124
0.6221733 0.1628327 0.4891241 0.1803346 0.4603044 0.117881876
1.40552 1.53741506 1.015946962 1.563265 1.739896 7.912051 0.6914693
3.251971 1.982894848 1.199612 2.431646 2.485444 0.3288158 3.581596 0.623004
2.697091 0.5808825 1.487821632 0.9607339 3.535965021 5.03364 1.275273
0.4666377 0.866031914 1.553883 6.653534 5.265905 3.699324 1.661404 0.7268691
2.229125 4.431077 0.6733717 2.843864 1.606868 1.043437 0.8140777 1.439891
0.6292967 0.7329963 1.54354 0.596801375 1.910333 1.0543 5.169738
0.3924508 0.6315089 1.234291 1.06775 1.3137 0.2256357 1.07737
0.3846147 2.085053902 1.623044 1.525167 1.167168 0.571986631 0.9573591
1.711589 3.273863 0.929486435 1.412046 1.905936 0.421529 0.4734807 9.540462
1.054404 0.8981639 1.113373 1.20959 2.144691 0.13437886 0.623729 1.285035
2.867937 8.17233 0.4617258 0.688526 2.031510976 0.4713216 0.5287685
7.083549 5.324976586 0.7558864 1.21775 0.5051851 10.86303 0.4623835
4.008201 2.649626 2.014095 0.4330284 0.6336846 0.571441408 2.855978
1.11477535 5.626128 9.191466 0.4918788 1.917892 0.9351059 1.139545
0.2720642 3.892469 0.5282616 1.221036 0.9458945 1.057592 1.104058
0.3745706 2.86239 9.839563 0.1062827 0.8741844 3.842967 0.50381076
1.222808 3.102639 0.1163931 0.9537182 1.069338512 1.597344 0.7235083
2.120312 0.5670789 0.8847163 1.651201 8.765711 3.648159 0.9613601
0.7003021 0.1044681 0.2441566 1.97755 3.131146 1.339086 0.7283061
1.171765 2.507689 3.373151 1.111806776 2.11961 3.2131 0.307359158 4.092852
1.595955 3.004737 0.7955071 2.951226 0.7387877 1.325272578 1.133014
0.3511896 1.394199 2.656993 2.149813 1.693329 0.6539368 1.99185 0.6678902
0.191241719 2.837877 0.2984567 1.344225 1.078832 2.305905 0.2658775
3.756599 1.258824 1.232058 3.168456 1.967369 0.9812258 0.8585774 0.8295237
0.2488643 2.268829 0.2235773 3.785945 0.672934076 2.630518 3.666787
0.472393 12.19161 3.905353 0.3229617 0.436047533 2.39148 7.443759 0.3460656
0.3706688 2.823266448 1.736347 9.561794 1.141858 1.628564 2.013911 1.735303
0.5942539 0.3703083 5.83557 3.11418 1.855581 0.6341854 1.475663
0.570595377 1.542544804 11.70725 1.153121056 0.257989463 2.753812 0.415971526
6.216799 1.433408 4.836559 0.908787163 6.305006 3.130958 4.793154 1.113573798
0.4945527 3.929559 1.218109 3.517844 2.179413 2.432464 0.5871636 4.027557
0.3908065 1.327642 2.254644 3.028832028 2.530418 0.628656396 4.63146
0.458459 0.7392009 11.6781 1.630898 6.792279 6.691803 3.139541 0.6015842
0.800281711 0.7936911 0.3047523 1.013039 2.069948 1.731454 0.5230608
1.179329 0.6388725 0.436836 2.863776 1.281173 1.427802 1.205975 0.6953702
1.970021887 2.319323 1.480379 1.066568 0.9721144 3.171502 0.9365075
1.079921 0.4900419 3.337332 3.365383 1.418497817 0.852090039 2.795311
2.063202 1.412929 1.69576 1.330362 0.3332826 1.036672 2.718395 0.09960065
0.4981995 2.147878 0.9773744 1.109906 1.156048 1.397797 0.2161446
1.488442 0.4155517 0.903171942 6.848195 0.8144058 0.5410167 1.526583
1.219682 6.768735 0.2809363 0.07523292 1.545787 2.835271 6.501203 0.561132601

1.742058 0.4832352 0.7378525 0.6670919 0.2501108 0.894166555
0.4361578 0.1429803 3.964601 2.366815 0.7219836 0.65316 1.780315024
0.7744695 7.614707 3.863792 0.9255006 0.56337578 0.7570222 3.202039
2.390049 1.02058442 0.806212562 0.3302842 1.135493 0.1518641 2.586148
1.029992 0.8355375 3.386798 0.6409289 0.8057167 2.671047 1.535822
0.9583088 1.994816244 0.7942901 1.683572 11.3321 1.031926 0.7949091
2.175667 2.089028 0.6083125 3.391084 0.4393715 4.825199 3.157564 5.097502
1.020872 1.308728 0.8024608 1.370346 0.6510693 2.27804 1.57736 1.580233
0.549648 3.2923 1.578978 0.4402951 1.465551 7.916166 0.1789159 0.3371967
1.448806 1.943328 6.019566 0.3357859 1.21046 0.1481069 1.129682 2.852546
4.253612 3.543946 1.641524 1.057696 0.6891576 0.7158933 4.348035 10.10936
10.18378 1.31084 2.143912333 0.4413071 2.109311 2.317769 5.783638 0.3668792
1.078814684 0.592249567 0.9484022 0.362099987 1.144067 9.398358 0
2.862909 0.8163982 0.6591171 2.558313 2.222253 2.816725 1.187105 2.464564
0.740978281 5.949382 5.332433 1.296941 1.435035 3.59565 0.9314393 2.522881
2.141099 3.477214 4.604425
AL513320.1 0.1666325 0.2936038 0.1101549 0.101759357 0.075415245
0.07387887 0.3113595 0.2614398 0.1262129 0.04136292 0.1809925
0.202787149 0.1208928 0.511886977 0.393230275 0.07683479 0.6734403
1.738518 0 0.9227592 0.960158444 0.5778197 0.524443 0.3368657
0.6141318 0.369676 0.7312962 1.484701 0.1598828 0.561424655 0.4807882
0.903009067 1.480436 0.2193798 0.521779 0.621828591 1.03683 0.9538159
0.675565 0.5747939 0.5124767 0.5314208 1.022578 1.47313 0.1390047
1.924791 0.9562731 1.020304 0.7550107 0.5245376 0.6328767 0.2421009
0.5098149 0.315892887 1.051605 0.5046724 0.8181817 0.1718478
0.7412794 0.7335029 3.260329 0.3824448 0.271706 0.6177845 1.36108
0.391290443 0.598297 1.1302 0.7332939 0.500927199 0.4025765 0.9253743
1.521341 0.319791153 0.9716328 0.6557399 0.5801107 0.4887052
1.440241 0.6801926 1.199704 0.5303872 0.2648298 0.20216 0 0.1581225
0.791159 0.2093038 1.690697 1.429717 1.450941 0.854265244 0.3243177
0.192625 1.590888 1.0468948 0.851118 0.07980357 0.9435383 2.291828
0.5629116 1.809047 1.606166 0.4504189 0.5146721 0.7163518 0.471852584
1.021908 0.319616727 0.5754717 1.111091 0.5641054 0.7331705 0.476629
0.4151245 0.1497665 0.4269943 0.6278608 0.6001415 3.173005
0.4478351 0.07235291 0.1472818 0.7697361 2.760003 0.1097002
1.370149 0.8555273 0.99049612 0.2337271 0.7486135 0.293665 0.1640641
1.655583581 0.6908859 0.4563608 2.370863 0.3902086 0.03581034
1.352615 1.495465 0.6059367 0.3193519 0.6883999 0.1078272 0.7680223
0.1632909 0.7297671 1.488463 0.5011495 0.5130968 0.6830295
1.083168 0.170008324 0.1080377 2.435785 0.211494726 0.74649 0.2059089
1.088194 0.444755 1.096603 0.3631157 0.325687106 0.3692986 0.7249638
1.543306 0.6094282 0.5283187 0.8415222 0.8470133 0.6351549
0.2703395 0.438646602 1.047818 0.4518116 1.215669 0.1291039 0.4025954
0.3659021 0.8511344 0.6929603 0.1816678 0.3931551 0.8922459

0.4910432	0.7297989	0.6088509	0.7705992	1.744857	0.9230651
2.811875	0.396898187	2.503284	1.39836	0.3656868	0.7845902	0.8466788
0.8889236	0.120018228	1.062773	1.855823	0.1190644	0.3060699	0.624438583
0.1853978	3.625438	1.534017	0.3486372	2.173152	0.5015081	0.1703783
0.1092044	2.007736	0.5612296	0.7581182	0.3080363	0.9428786
0.560897614	0.197475264	2.353965	0.165305403	0.142018637	1.015128	0.449791903
0.568145	1.080268	4.224064	0.875474707	1.446164	0.8348384	2.253758	0.81132708
0.1701516	2.586378	1.192801	1.148778	1.064275	1.080987	0.7407198	1.411348
0.05378302	0.234914	0.6581813	1.354696254	0.8125544	0.266203253
1.212933	0.7210673	0.3148763	1.607146	0.08312781	2.733724	2.128564
1.215184	0.7150077	1.040166083	1.178515	2.22283	0.3291743	0.9115756
0.8510133	1.109752	0.6569282	1.904977	0.1618551	3.378125	0.3449657
0.8070326	0.1778217	0.3417759	1.470946081	0.6244955	0.7470125
0.7339087	0.847292	2.384386	0.2653468	0.7768742	0.07781521	0.8802974
1.428033	0.201946025	0.586325622	1.839833	4.19736	0.5609079	0.51479
0.6102842	0.36038	0.3745019	0.3810349	0.3426775	0.27425	0.8867766
0.3602862	0.5912745	0.19887	0.1451817	0.2974595	0.7681509
0.09531411	0.932212841	2.108985	0.2101481	0.3722752	0.583581	0.4196334
2.104539	0.1449848	0	1.004568	0.5513577	1.451507	0.386116966	0.7628188
0.443354	0.6639395	0.1224074	0.5507263	0.091152384	0.06431174
0.09838516	1.006466	0.6857314	0.2732395	0.3495655	0.18846768
0.5625211	2.903078	1.5509	0.542493	0.180908235	0.625091	0.4506816
1.298368	0.790050479	0.277378598	0.3146809	0.04596095	0.2438289
1.162146	0.8579489	0.1916452	0.9021157	0.3150179	0.6127756
0.6792442	0.4623515	0.4496012	0.343159707	0.4008058	0.7079546
1.417755	0.3550357	0.4182782	1.051143	0.4345828	0.4709043	0.9333658
0.8206176	2.329988	0.2172729	0.5249478	0.1277209	2.015494	0.2454114
1.91282	0.4032024	1.745654	0.3294922	2.091083	1.629231	0.755147	1.366886
0.6816787	0.2593156	1.917392	0.4514128	0.5220585	1.077759	0.6240312
0.5020704	0.2567281	0.1189887	0.2293038	0.4858358	1.215095
2.444842	1.057706	0.6325408	1.356361	0.3793691	0.4926082	3.604693
5.916834	2.636481	0.3421351	0.559570865	0.3270235	1.880252	0.9361156
1.428624	0.1767154	0.326627623	0.125393428	0.5438319	0.581378114
0.6122944	0.8035964	2.957806	1.078303	0.1404415	0.2791018	0.7921722
0.7877383	0.4199426	0.6418112	0.5550131	0.708151805	0.8363651
1.247549	0.3880126	0.5716827	0.9846214	1.148326	0.4156624	1.087433
1.543665	1.821783
AC079313.1	0.2982992	3.065991	0.06274392	3.1299389	0.171825209	1.178273
1.773494	3.148496	2.252569	5.253924	0.3092783	0.323419505	0.9915864
0.427635005	3.633498955	0.6127082	0.08524214	0	0.2409026	0.02628005
0.057568783	0.2350888	0.2275971	0.2066375	0.03682186	0.9475485
0.05745437	0.05637876	0.1366031	0	0.4107833	0.142875366	0.190925
0.03570232	0.5029602	0.088547936	0.3691097	0.05432907	0.349818
0.4677156	0	0.2492788	0.129435	0.5463846	0.1055689	0.03654518

0.03404315 0.04304907 0 0.1327889 0.03794576 0.04596666 0.09679636
0.107958998 0.08557013 0.03593249 0 0.195768 0.08735805 1.055498
0.4708075 0.3564646 0.04421799 0.1407553 0.08614082 1.040096818
0.2726306 0.03065518 0.3182339 0.366848568 0.3335366 0 0.06665779
0.227690129 0.2012506 0 0.1652147 0.1855767 0.267093 0 0.4141501
0 0.3016925 0.06908984 0 0.2573318 0.3976252 0.06812505 0
0.2068222 0.1686637 0.20643084 0 0.04876383 0.03856022 0.198769403
0.1615981 0 0.05657228 0.1606669 0.1115244 0.3716297 0.1978091
0.03947035 0 0.1064431 0.238902977 3.044703 0.109231651 0.05959764
0.0973653 0.5141005 0 0.1357432 0.1050905 0 0 0.112935
0.03418388 0.1390257 0.06377139 0.08242401 0.3355651 0.05158111 0
0.1666264 0.4187688 1.063213 0.394928581 0.1065042 0 0.06082571
0.2616611 0.139706008 0.3219763 0.2363107 0.06925317 0.03419411
0.08158987 0.1849069 0.02839376 0 0.02598599 0.1960553 0.08189076
0 4.278461 0.03958792 0 0.05709066 0 0.0409528 0.04406925 0 0
0.04268973 0.172095395 0.03865441 0.1563802 0 0.4676883 0
0.4136593 0.408122926 0.1402343 0 0 0.08331085 0.08024767 0.2581
0.3618422 0.03808236 1.817018 0.449733274 0 0.04679101 0.1806369
0.03676859 0.107914 0.1389445 0.1436455 0 0.3794173 0.08143273
0.2803976 2.394903 0.1187689 0.1300499 0.9428889 0 0 0.3768555
0 0.4387271 2.631918 0 0.3093925 0.0838723 0.7278469 0
0.117165 0 0.02712749 0.3486731 1.264634265 0.4693423 0.254943
0.213115 0.1134758 0.03587888 0.02720548 0.03881878 0.04146833
0.09335516 0.2324907 0.2727296 0 0.2065619 0.447279892 0.056240666
0.1191832 0 0 0.2698332 0 0.2665054 1.17713 0 0.332445219
0.05491539 0.153394 0.09393177 0.051347725 0 0 0.2938015 0.1402158
0.2204393 0.03972431 0.07671124 0.1461636 0.3676158 0.05946947
0.1606706 0.039570821 0.1983551 0.075814296 0.3793082 0.6417466
0.05518541 0 0 0.1004594 0.1484601 0.07690725 0.2143507
0.45306954 0.2292171 0.04777801 0 0.03245192 0 0.1025046
0.1320651 0.1252003 0.289747 0.04581357 0.04366477 0.07994494
0.135049 0 0.098570186 0.0526979 0.07736293 0 0.02540079 0.04603862
0 0.4617446 0.05909776 0.3924116 0.04396776 0.268398349 0.095419816
0.1587823 0 1.959548 0.4300602 0.08690402 0.6717973 0.223473 0
0.9759404 0 0.1924211 0.1094496 0.1122627 0.453103 0.2067376
0.08471602 0.2041837 0.2171625 0.318591421 0 0.03989992 0.3975878
0 0.04780439 0.07860585 0.4404423 0.05897379 0.06731755 0.04831565
0.1355367 0.175944874 1.779378 0.1262666 0.1334746 0.03486146
0.07842303 0.181720459 0.4884237 0.3362394 0.2413812 0.09764766
0.1414876 0 0.071567092 0.4721837 0.120994 0.03605667 0.05373956
0.35329646 0.1186833 0 0.04930311 0.05000116 0.039498509 0.1792413
0.235613 0.03968121 0.2068609 0.04249436 0.07277371 0 0.2153199
0.299173 0 0.1003254 0.1024366 0.065154255 0.04150869 5.05894
0.0950058 0.08666885 0.2199231 0.3265789 0.03808263 0.02980286

0.1181428 0.1476068 0.1658943 0.1767971 0.2174609 0 0.04885189
0.2096784 0.184147 0.0510362 0.08116894 0.1545582 0.1905719
0.4971457 0 0.03992689 0.08628494 0.09847028 0 0.04674971
0.1321613 0.1534721 0.06770407 0.04766297 0.05849263 0.169439
0.02902462 6.641535 0.1064791 0.03922745 0.03347034 0 0.03768679 0
0.1772136 0.2053223 0.1366302 0.03951925 0.2834606 0.057950895
0.1862716 0.03757846 0.03949694 0.5812428 0.2300722 0.169132852
0.357118646 0.123906 0.141921973 0.04982301 0.1743719 0 0.448837
0.05333004 0.07948778 0.3676599 0.3167248 0.07359941 0.1329359
0.3512598 0.290420059 0.351025 0.2926002 0.1326065 0 0.08628269 0
0 0.07992241 0.4396336 0.240622
GNG12-AS1 0.273482 0.6675921 0.1384169 0.840270915 0.03610065 0.3779656
0.5589195 0.6503282 0.7627655 0.5643024 0.1786942 0.269376246
0.7855902 0.220532433 0.737257903 0.4275695 0.2686419 0.01642528
0.1613318 0.02898769 0.163286002 0.2963544 0.174836 0.08838004
0.3017161 0.05530025 0.1629615 0.3775668 0.07175115 0.023713174
0.1941885 0.198120508 0.1103122 0.1293939 0.2017383 0.037208021
0.3373439 0.1027313 0.5512284 0.1768815 0.09907086 0.106618
0.336531 0.2090926 0.1497159 0.04606908 0.06973687 0.03391745
0.06120778 0.08369726 0.05979331 0.05794585 0.04575828 0.14176434
0.04719322 0.1811869 0.08301421 0.1366139 0.04129653 0.2148311
0.07418785 0.237163 0.01393537 0.05175246 0.2103924 0.039022391 0
0.2077121 0.8023352 0.147727572 0.1313932 0 0.1417993 0.0645812
0.4518991 0.07356966 0.03905074 0.2851133 0.3246741 0.08954069
0.08483802 0.07405182 0.1154529 0.1306426 0.082993004 0.1581421
0.1461975 0.0966137 0.06597737 0.1833197 0.1169401 0.176583264
0.1406937 0.01536799 0.2248178 0.083523305 0.1188317 0.05013921
0.1604595 0.06329301 0.03954049 0.1703559 0.1168872 0.1554893
0.1118387 0.09504616 0.089407629 2.060195 0.126223241 0.08452029
0.1841089 0.1063253 0.05264439 0.136895 0.0413993 0.2890083
0.07664948 0.1720263 0.1346638 0.00730236 0.1205859 0.2207964
0.5948654 0.1015992 0.1509563 0.2166143 0.05998892 0.2722469
0.480069029 0.1090861 0.3683094 0.06709261 0.2297181 0.02201428
0.07892214 0.1117105 0.1418641 0.07004611 0.01285659 0.01942456
0.04474168 0.07769379 0.1310324 0.1482891 0.09032799 0.0258501
6.947004 0.04366668 0.06997912 0.09895714 0.1008777 0.02581267
0.03472123 0.267033277 0.07757516 0.1950791 0.092201411 0.2619129
1.601712 0.01302273 0.2272304 0.08748915 0.1434019 0.052617501
0.1031218 0.1301378 0.1123127 0.2362999 0.1580636 0.4648035
0.1211623 0.2160308 0.1164683 0.070867061 0.1624441 0.4350146
0.04269599 0.1274644 0.1232835 0.1970486 0.06224639 0.2176875
0.2228423 0.3015479 0.2098734 0.6555922 0.01871511 0.1571108
0.2715362 0.02472775 0.05523295 0.3562997 0.112807667 0.08295931
0.05456927 0.02188141 0.1300074 0.05286497 0.09474476 0.026930497

0.06769533 0.03331377 0.1025912 0.1098849 0.211730434 0.09614406
0.03213827 0.2283559 0.03576208 0.1243801 0.1200339 0.1039873
0.05227522 0.1544604 0.4121426 0.3294794 0.1382385 0.1432162
0.251715957 0.310175793 0.2159745 0.02967387 0.082854451 0.109335
0.022020509 0.05999256 0.4215616 0.2022024 0.12722146 0.3807467
0.09185048 0.04440411 0.186096604 0.1119939 0.0211036 0.098379
0.1509799 0.1389436 0.4319116 0.1087905 0.3224457 0.07723645
0.2061608 0.3249117 0.031177011 0.08334923 0.125438199 0.2561562
0.2548317 0.2043533 0.1169586 0.08207222 0.3482594 0.07018113
0.05453423 0.1486165 0.1372938 0.1186769 0.007528656 0.1633661
0.05625 0.07129027 0.06999303 0.4439521 0.1775568 0.1950803
0.1443822 0.2476983 0.1826621 0.05320111 0.03681119 0.196742108
0.186838 0.07314313 0.04940381 0.04002549 0.558602 0.04763223 0.2000893
0.009312374 0.06870506 0.1247086 0.072502411 0.045107549 0.04503645
0.1662056 0.3624776 0.2464257 0.2738793 0.2293611 0.4641836
0.08705371 0.7074089 0.2830751 0.1212836 0.2414524 0.08402696
0.1308964 0.4560757 0.1802141 0.07354149 0.0969554 0.200809138
0.06506887 0.05658534 0.2464241 0.02793554 0.007532813 0.06812507
0.9716424 0.1300998 0.2704941 0.2131745 0.2861878 0.027724647
0.2151806 0.1492241 0.1892912 0.02197328 0.07414541 0.208624526
0.246284 0.1103816 0.1521433 0.09232142 0.1917372 0.1703222
0.045108955 0.2072702 0.1016838 0.05113491 0.04234026 0.148455886
0.1215605 0.03595626 0.1165346 0.157579413 0.143152173 0.05962641
0.1485076 0.05002237 0.3064048 0.3214119 0.04586949 0.1282011
0.04523893 0.1204751 0.04303437 0.01976106 0.2098398 0.133467503
0.07194839 2.35684 0.2619857 0.3141089 0.1559455 0.4402768
0.0540081 0.03756966 0.07446574 0.186074 0.1359328 0 0.2484326
0.05731775 0.2309362 0.106463 0.1209046 0.0241262 0.2366198
0.1148146 0.1201181 0.08356065 0.2711119 0.02516604 0.1291659
0.04137741 0.2894324 0.05156635 0.1041271 0.1837945 0.1280222
0.03004212 0.1751234 0.06407876 0.03201503 0.3401271 0.03355706
0.2657957 0.0369188 0.05677351 0.06532378 0.431302 0.1023901
0.245889 0.1435307 0.1432274 0.2568325 0.118711531 0.1509526
0.01776436 0.2116079 0.02747693 0.09516623 0.165237671 0.241974937
0.07809835 0.007454487 0.04710539 0.2610294 2.548579 0.6402549
0.210088 0.1002028 0.2686044 0.04159012 0.0695849 0.06284243
0.2103301 0.1169503 0.02370559 0.3029868 0.02089557 0.04664655
0.2447291 0.02876306 0.2896829 0.1826106 0.1316236 0.3033295
AC145285.6 0.192417 0.1384396 0.3339004 0.102817284 0.060959431 0.2239408
0.267407 0.0905684 0.05101002 0.1170202 0.219449 0.147524686 0.1270356
0.193091262 0.035317195 0.1863206 0.3477813 0.6656559 0.06409982
0.7738526 0.122544076 0.21685 0.5248487 0.3351314 0.2612701
0.09960513 0.5095855 1.43013 0.4846349 0.266946555 0.255038 0.760330896
0.4402813 0.3039917 0.3082113 0.261788894 0.4321389 1.310676

0.6701777 0.5973631 0.7010277 0.06317007 1.05617 0.3876881
0.2808997 0.7130941 0.6884281 0.2443643 0.1870238 0.4239927
0.1884713 0.4076965 0.3090691 0.217040376 0.2580448 0.3951874
0.5894644 0.4365664 0.1652935 0.374465 0.5010933 0.210775 0.1098123
0.282974 0.9015401 0.298715266 0.2095659 0.8591816 0.3387051
0.101227004 0.4585315 0.2379987 0.5202686 0.161557909 0.3480694
0.6128654 0.8205984 0.5596241 0.5414751 0.4765031 0.5289496
0.3572675 0.1529046 0.3676711 0 0.3012739 0.1504723 0.2175221
0.3862184 0.6695516 0.3350916 0.449882415 0.3932274 0.5709075
0.7797734 0.352592899 0.4013177 0.1128865 0.2207752 1.396518
0.2077224 0.335606 0.9824906 0.3220719 0.1751656 0.1762294
0.169514003 1.207268 0.439197826 0.4863074 0.6217714 0.284985 0.3555805
0.6260594 0.1118507 0.07566178 1.208014 0.2804664 0.3517008
0.7398444 0.2262455 0.3362835 0.1934569 0.7685887 0.6448862
0.07389378 0.5537574 0.3143351 0.40031747 0.538438 0.2353233
0.1510565 0.2254468 0.223039417 0.2919196 0.2850469 0.491387
0.5216434 0.3328803 0.3717361 1.017415 0.2099095 0.1106304
0.08346681 0.20337 0.2425022 1.077925 0.5758384 0.544282 0.4050877
0.325871 0.5666335 0.6879271 0.154598213 0.1964897 0.636102 1.50196004
0.3839822 0.1941797 1.099507 0.1797515 0.8864032 0.1467563
0.289584299 0.364846 0.3743893 0.337157 0.2758623 0.1565846 0.5624869
0.3476765 0.5944708 0.185742 0.230467604 0.6737255 0.3320064
0.630175 0.1174015 0.1914263 0.3820297 0.3822147 0.2100494
0.256979 0.158897 0.3606088 0.4093223 0.3511363 0.2768313 0.3114442
0.4082731 0.388609 0.9693193 0.240614691 0.8716388 0.761736 0.1642172
0.7927471 0.446338 0.3929473 0.097012784 0.8036346 2.130133 0.1924836
0.2474016 0.19628948 0.191488 0.37988 0.4687699 0.8957513 0.6237189
0.1833851 0.1928075 0.2059674 1.937529 0.1443436 0.6289262
0.1971179 0.4103857 0.362706501 0.119716973 2.114167 0.187066855
0.31568925 0.6290849 0.247891692 0.5943122 0.284739 0.4966375
0.286434282 1.188442 0.5333208 0.5887368 0.491857157 0.09169094
0.7602231 0.5472268 0.4725792 0.8211687 0.7610341 0.1905071
0.5392951 0.0652106 0.25318 0.1805065 0.842326256 0.7271733
0.121036859 0.2691388 0.2732114 0.2447306 1.088422 0.8735172
0.4395672 0.6320406 0.6821208 0.3193951 0.655316957 0.3717517
0.491564 0.2191224 0.5641448 0.4585924 0.4242717 0.2655032
0.3405383 0.1308303 0.4876064 0.3562977 0.6523382 0.1197803
0.248637 0.338046419 0.4860944 0.164679 0.3831283 0.2162781 1.257671
0.1685234 0.2730264 0.1048323 0.9435896 0.3275727 0.639342656
0.47393702 0.4619235 1.421982 0.332487 0.5132068 0.277483 0.3839881
0.2666786 0.1259988 0.4154881 0.3879417 0.2730654 0.3591781
0.1493554 0.3482917 0.2567093 0.1953588 0.1655759 0.2311321
0.276291976 0.6525913 0.3255771 0.07522909 0.3930988 0.2204779
0.6832427 0.5664363 0.2092248 0.7164792 0.4628131 0.3558301

0.27049095 0.2642569 0.2239816 0.3314749 0.4205121 0.2921372
0.257880105 0.1906090.24852 0.6529761 0.1558936 0.2710605 0.3632911
0.266597881 0.2273477 1.1446870.8186902 0.08579473 0.240237001
1.2842320.1011927 0.3323395 0.266088043 0.392367257 0.1978371
0.3993734 0.3801042 0.3669462 0.6331908 0.1549101 0.714004
0.2291709 0.2948315 0.3229675 0.4983022 0.2786223 0.34672732
0.2356205 1.4306290.5224393 0.07174536 0.3250975 0.2317246
0.1756404 0.2326133 0.3772278 0.9775231 1.2830450.2508934
0.3471744 0.4645754 0.3639608 0.2066356 0.3157661 0.1629577
1.0222860.4856681 0.4225645 0.4820924 0.8477753 0.6799246
0.5816244 0.2561891 3.0468860.4643987 0.7267585 0.5880406
0.8046622 0.4565611 0.2697730.1683161 0.2986198 0.2552611
0.6988601 2.1571280.6649701 0.2556468 0.4412220.2044337 0.28292
1.9522031.8742930.3785529 0.4274011 0.020559577 0.1699321.093218
0.5044522 1.5672030.2040601 0.216015291 0.215385008 0.08791773
0.285319418 0.26514 0.5876978 0.7172535 0.5447565 0.2081223
0.3807046 0.5454632 0.4681929 0.4700033 0.1886498 0.6106306
0.240412706 1.0496540.7118226 0.4861376 0.2310505 0.3163138
0.4856920.09881971 0.6805090.4055256 1.547274
AC008870.4 0.07084444 0 0.02731912 0.050473939 0 0.03664486 0.03860957
0 0 0.02051654 0.1795491 0.160936022 0.1439145 0 0 0.03811103
0.1113450.09077126 0 0.09154006 0 0.1228311 0.5945838 0.2827672
0.2565197 0.2444853 0.1250801 0.9082645 0.03965195 0 0.05961927
0.348369792 0 0.2487205 0.07963355 0.128514548 0.9642769 0
0.4264767 0.0814586 0.07821384 0.1240431 0.1690706 0.2039139
0.09193082 0.3182403 0 0.03748771 0.07248289 0 0 0.02001419
0.1685831 0.156686902 0.1490312 0.1251620.3528944 0.1217696
0.1014301 0.03829756 0.5922012 0.1379618 0.07701123 0.1225716
0.1500252 0 0 0.5872887 0.1039209 0 0.03630601 0 0.1451162
0 0.2190647 0.08131384 0.1079034 0.04040067 0.4984032 0.08996912
0.2163885 0.05846196 0.03753113 0.04010957 0 0.0448176 0.1154191
0.1483105 0 0.1575906 0.05874983 0.102721566 0.02681096 0.0212321
0.2014726 0.230788079 0.04690726 0 0.2463195 0.1748886 0.02427925
0 0.1291911 0.2062279 0.02687199 0.2162815 0.104019956 2.417424
0.06341359 0 0.3815415 0.1399017 0 0.1182070.09151423 0.03714306
0.07701623 0.1311271 0.2083745 0.1210654 0.05553298 0.07177593
0.3287414 0.1122938 0.1283434 0.07255026 0 0.03857749 0
0.1623043 0.1925373 0.1059357 0.1627557 0 0.2492278 0.1234695
0.06030659 0.1191067 0 0.1610194 0 0.1288081 0.1131447 0
0.03565578 0.0476186 2.2138460.1378948 0.03515697 0 0.04847668
0.03566225 0.0383761 0.042163149 0 0.2230487 0.05994519 0.1009823
0 0.03598386 0.1357564 0.4432016 0.2161320.129235968 0.1628239
0.3595913 0.1241351 0.09673093 0.1747019 0.1284325 0.1444195 0
0.05363674 0.174059449 0.3779862 0 0.1835178 0.09605574 0.09397293

0.1512434	0.1876327	0.1288939	0.06007302	0.1063691	0.03052175
0.4110137	0	0.03774972	0.1132525	0	0	1.107576	0.032811094
0.1146148	0.1507835	0.04030785	0.08980772	0.1825928	0.1102294
0.029765286	0.1360385	0.1472819	0.1181149	0.1518146	0	0.08174191
0.2220078	0.11135	0.1482244	0.06248761	0.2132181	0.1352157
0.1986115	0.3658272	0.101228	0.3562449	0.15279	0.07195074	0.278212373
0.048975125	0.4670387	0.065594871	0	0.03356777	0.08112819	0.1989227
0.0931873	0.2285661	0.082713643	0.6216738	0.1335776	0.2726568
0.313000009	0.0562649	0.1166251	0.03198079	0.0814012	0.1919615
0.3113321	0	0.05091247	0	0.1553604	0.1399141	0.103376404
0.3454606	0.231070367	0.21234	0.2682439	0.0480562	0.04309	0.1649298
0.1166419	0.129281	0	0.1493276	0.151745779	0.2851505	0.1664229
0.1728791	0.05651914	0	0.02975412	0.07666936	0.07268406	0.1146889
0.1595803	0.07604774	0.03480856	0	0.03390504	0.228896635	0.2523952
0.3368434	0.1516784	0.02211935	0.2004552	0	0.06701557	0.1286578
0.1139058	0.1531509	0.133557073	0	0.1106158	0	0.07419133
0.2383196	0	0.09750168	0.03538242	0	0.1019834	0	0.293235
0.09531015	0.2443997	0.06576136	0.09001494	0.1106578	0.1270042
0.03151801	0.092477893	0.2288307	0.1737269	0.02308165	0	0
0.03422551	0.5273717	0	0.1758631	0.08414784	0.3304761	0.051071718
0.03603503	0.3298639	0.03874382	0.1517891	0.1365836	0.090425491
0.1488641	0.07320044	0.1576485	0.08503289	0.02464186	0.09907939
0.124643165	0.08811083	0.526816	0.06279726	0.1169928	0.128189704
0.103351	0	0	0.130625039	0.240770817	0.05202858	0.2051749	0.1036648
0.1441098	0.03700466	0	0.2610189	0.03125058	0.05789419	0.07927383
0.131047	0.1189376	0.085105797	0	2.490005	0.08273232	0.05031493
0.095756	0.4423834	0.1989772	0.02595273	0.2057606	0.2570759
0.08667779	0.09237439	0.1893679	0.0633512	0.340327	0.1014393
0.187084	0.04444301	0.212049	0.115364	0.04148815	0.08658423	0	0.1390755
0.03756904	0.02858308	0.08645918	0.04071027	0.2301757	0.213833
0.2358304	0.2490333	0.4584255	0.1180398	0.1011001	0.0963923
0.4172555	0.170799	0.05829283	0	0.1312727	0.03136199	0.15432
0.2503163	0.2379588	0.2064834	0.1542758	0.100928832	0.06951764
0.06544766	0.3095502	0	0.07513123	0.147283153	0.06219674	0.03596634
0.164783407	0.1735462	0.07592268	0	0.1645698	0.06966077	0.06921903
0.08731722	0	0.09613703	0.1157624	0.2141172	0	0	0.4004002
0.3464265	0	0.07513612	0.07947687	0.1212787	0.2435913	0.07656777
0.2357289
AC139887.2	0.3948397	0.4831259	0.4132733	0.522429308	0.218408933
0.4668206	0.5533303	0.5309896	0.4153673	0.3593715	0.6969072
0.496525945	0.7638888	0.363878514	0.690198888	0.4551546	0.531911
0.2168136	0.1252694	0.920152	0.259443314	2.233031	0.4339518	0.5526078
0.7275999	0.7177972	3.196761	1.250857	0.6787658	0.260844919	0.48655
0.703327803	0.2426869	0.569333	0.2536139	0.22510853	2.008941	0.3578475

1.236957 1.426844 0.280229 0.25925 1.41343 2.042958 0.4940625 1.292238
0.7080975 0.4924813 0.4809182 0.3912847 0.3551723 0.6374043
0.6375653 0.187128929 0.2521467 1.880946 1.587487 0.5138444 0.827766
0.6403352 0.7435271 0.3569927 0.2605913 0.3253011 0.388208
1.219059509 0.3780477 2.603647 1.392804 0.678262242 0.9972744 0.1661134
0.9127673 1.010337001 1.901148 0.5503011 0.8304791 1.495748 1.706343
0.2148977 0.775289 0.4189217 0.1942325 0.9181271 0.054775383
0.5174085 1.203832 1.133601 0.3773906 2.563217 1.379894 0.439599389
0.3522194 0.3803578 1.149609 0.436409268 0.8776572 0.1733384
0.7648572 0.5291296 0.3479563 1.768507 1.817207 1.039912 0.5134854
0.5904044 0.745377288 11.30003 0.40391437 0.3099077 1.248872 0.5569422
1.061662 1.458787 0.3460981 0.3992348 0.6898454 0.6394633 0.4384653
0.8193248 0.3979335 1.514404 0.8870103 1.242761 1.013343 0.332142
0.937027 0.6757307 0.72365961 0.2584502 0.459889 0.2952075 0.4924214
0.750686951 1.004566 0.6717525 2.04066 0.3082029 0.5939742 0.7692126
0.9154148 0.4273159 0.2702543 0.7204378 0.4400263 0.4739185
4.729043 0.1784096 0.5458371 0.4552028 0.2991242 0.2697424
0.5347069 0.654613005 1.365323 0.5623661 0.656257105 0.9246135
1.328189 0.2865001 0.4863958 1.860603 0.3154841 0.797447464 0.5833746
0.3817376 0.8895166 2.012049 0.3755591 1.009786 0.7160456 0.343249
0.4804319 0.554337903 0.9028471 0.2433133 1.471588 0.8540118
0.5518001 0.337172 1.531261 0.7696809 0.944634 0.4516802 0.3159146
0.7332716 0.2058662 0.9016791 0.42831 0.4714758 0.501239 2.073961
0.496353733 0.8517156 1.152503 0.4172055 0.6912058 0.5669768 1.21772
0.497675584 1.150821 1.260593 0.432593 1.26917 0.383605727 0.366087 0.4242252
0.7979025 0.7867659 1.194049 1.084592 0.1749433 0.3450165 1.262162
1.047758 0.6145508 0.7907244 0.5585433 0.398718076 0.721380274
2.685596 0.48309061 0.252387406 0.3073529 0.242225596 0.7919018
0.8717893 0.4852858 0.971373267 1.561062 0.4147775 0.8466384
1.851256654 0.7167612 0.9285583 0.7129584 0.9802643 1.413751
1.225627 0.4786782 2.371358 0.08496009 0.7731031 0.677673 0.480125966
0.2292104 0.394234341 1.023771 0.9788775 0.9565472 0.4116942
0.9356233 0.9286917 0.977857 0.05332236 0.8322523 0.616174575
0.499475 1.043472 0.7876334 1.1925 0.4257048 0.3908842 0.625695 1.14294
0.5844108 2.620536 0.7265818 0.568142 0.3043103 0.5398974 0.956787935
2.073487 0.536383 0.2536062 0.854144 1.372565 0.5988052 1.627393 0.604373
1.98008 0.868803 0.917155502 0.628498521 0.8256682 0.9019426 0.7383803
2.195653 0.873675 0.8409907 0.5634229 0.3283169 1.407436 0.4603047
1.450855 0.9959911 1.303745 0.8377371 0.9245306 0.1762093 0.5359336
0.6022641 0.785386852 1.028088 0.7607584 0.3675479 0.1536455
0.1657219 0.3951254 4.103454 0.8382142 0.653429 0.8039725 2.236537
0.772593491 0.344289 0.525269 0.971695 0.3021326 0.2990531 1.034941278
0.770407 0.3788297 1.746795 0.7278005 0.5395395 1.853823 0.272909178
0.5027632 1.55195 1.337462 0.4843725 0.540936135 0.3085767 0.336191

0.2734679 0.936021711 1.916994314 0.2968767 0.9529236 0.2476107
0.6597483 0.42721 0.07568466 0.964882 0.5225097 0.6107562 0.3471439
0.9912146 0.5800188 0.485616378 0.5611975 7.980906 0.7081099
0.4807232 0.4955601 0.8679743 0.8845326 0.5165828 1.003426
1.074577 0.9201605 0.1593532 1.865815 0.3278575 0.5419303 0.2422951
2.053444 0.2476957 1.603898 2.319256 0.8588442 1.034063 0.5136065
0.2491438 0.7478028 0.3299848 0.6023323 0.1458591 0.4810672
0.8831811 0.8801529 0.7931119 1.003733 0.6931186 0.5835883
0.7482796 0.8859063 1.373484 0.2552671 0.2141172 1.89439 0.3620667
0.5119504 1.58016 1.120975 1.233001 0.7492808 0.924123613 0.8117895
0.8597952 1.944303 0.1007488 0.4287012 0.621506852 0.742806784
0.2577245 0.508396045 0.2763516 0.8160603 2.336197 0.9172024
0.6378273 0.4822259 0.7647326 0.2378955 0.3061736 0.2880278
0.8402135 0.503394768 0.1912251 1.564993 0.3064683 0.3078672
0.8574582 1.434318 0.994578 1.191377 0.3505346 1.199099
AC244093.5 0.2062185 0.04239127 0.07157001 0.044076824 0.04355458
0.1280018 0.1011485 0.03235487 0 0.0358325 0.1175947 0.035134709
0.1047288 0 0.075700783 0.3328081 0.06482202 0.03963341 0
0.3397373 0.175111729 0.07150892 0.2596128 0.08979236 0.3920143
0.2401864 0.283991 0.1929284 0.2770114 0 0.1041261 0.282487284 0
0.08144902 0.2086221 0.157117089 0.1122751 0.1239429 0.05320352 0
0 0.1354021 0.0984283 0.5342089 0.2006985 0.2501156 0
0.09820944 0.1054939 0.1009788 0.05771137 0.08738786 0.2576293
0.21892521 0.09760716 0.3005718 0.3081682 0.04253455 0.1992932
0.1003311 0.1193412 0.2409528 0.2017523 0.07135784 0.06550541
0.075327346 0 0.349674 0.1209998 0.03099649 0.1902273 0 0.02534481
0.173145976 0.01913002 0 0.5025472 0.0705605 0.1450784 0
0.3464325 0.2042097 0 0.08756511 0.080103276 0.1565495 0.2015816
0.2072216 0 0 0.05130382 0.089702538 0.04682582 0.05562336
0.1172918 0.403075559 0.2457732 0 0.2581209 0 0.1908186
0.1541477 0.07521155 0.2401205 0.1173311 0.08094419 0.090836369
4.017781 0.083064737 0.1359623 0.2221231 0.1954727 0.211714 0.1238704
0.1598313 0.06487102 0 0.114508 0.1819649 0.140962 0 0.125358
0.1594869 0.4510833 0.07471805 0.1267103 0 0.1010644 0.08580621
0.02024767 0.07205782 0.1618913 0 0 0.08161517 0.251582 0.05266328
0.02600276 0.06204466 0.2343527 0.04318382 0.1874714 0.0592828
0.08945363 0.1245469 0.04158338 2.970621 0 0.03070115 0.04341434 0
0 0 0.073638703 0.3275741 0.1623161 0.314086079 0.1763675
0.1783779 0.1256929 0.2667386 0.1055536 0.2516523 0.112856479
0.0710937 0.06978141 0.1084021 0.401238 0.061024 0.1401936 0.3439517
0 0.1405163 0.113999242 0.2475599 0.03558202 0.1144704 0.1957236
0.3077352 0.02641494 0.2457779 0.1125578 0.2622966 0.03096259
0.1066136 0.1994008 0 0.09889587 0.09889873 0.03977793 0
0.2865781 0.028652585 0.6005307 0.05266923 0.0351992 0.1568508

0.1594508 0.3128411 0.025992806 0 0.03215382 0.04125797 0.08838231
0 0.08922733 0.1550963 0.09723743 0 0.1091357 0.3517009
0.05903915 0.1261376 0.3194619 0.04419914 0.3802261 0.1334252
0.2827424 0.048590295 0.064151957 0.1812648 0.11456263 0.123030072
0.02931336 0 0.02895184 0.2034416 0.2883074 0.036115219 0.08352035
0.1399774 0.14286 0.078094312 0.09826766 0 0.02792751 0.07108434
0.2095402 0.2416654 0.1750042 0.1778391 0 0.0452233 0.1629084
0.210640222 0 0.028826327 0.2678402 0.2342465 0.08393102 0.03762874
0.1440265 0.1018586 0.1505278 0 0.3912049 0.1060107 0.1743072
0.1453303 0.05032274 0.07403378 0 0 0.03347611 0.2538881
0.1402143 0.1741936 0.06640937 0.1215876 0 0 0.074957272
0.1602955 0.2353212 0 0.1352115 0.1750494 0.03283838 0.05852195
0.06741095 0.2652513 0.0334351 0.145787404 0.072561587 0.2897888
0.05347284 0.1619706 0.2973068 0.09912868 0.05676282 0 0
0.3859178 0 0.6950475 0.2704999 0.1280546 0.2871335 0.3301467
0.1288439 0.3549043 0.1376169 0.080757157 0.6565794 0 0.1814064
0 0 0 0.5861318 0 0.153574 0.25719 0.3504323 0.08919768
0.03146792 0.1920377 0 0.05302049 0 0.217153417 0.09285493
0.06392295 0.137668 0.03712787 0.1506311 0.04326099 0.027211443
0.1282393 0.5213864 0.02741914 0.122598 0.11194282 0.04512611 0 0
0.152092664 0.090109412 0.01514481 0.1791709 0.0301754 0.09438388
0.09694396 0 0.09768732 0.1091594 0 0.1038399 0.05721899
0.05193165 0.148638815 0 3.958558 0.07224675 0.3515037 0.0836198
0.1931576 0.7819139 0 0.08984115 0.1496626 0.05046145 0
0.1446963 0.05532201 0.185746 0.1240159 0.1400338 0.03881026
0.1234491 0.2854375 0.1086897 0.1764244 0 0.03036223 0.1640375
0.0748813 0.07550126 0 0 0.1867316 0.3603969 0.2174706
0.1779219 0.05153967 0.08828658 0.04208772 0.2834006 0.4176249
0.1272619 0 0.08597628 0.1643228 0.04492044 0.1561364 0
0.1803135 0.1347227 0.088137016 0.06070691 0 0.3003528 0
0.04373935 0.077169808 0.054313866 0.03140794 0.107923952 0 0.06630017
2.049866 0.2694601 0.1419411 0.1208923 0.07625056 0 0.06996045
0.07581791 0.1869798 0.049077484 0.07626728 0.3496531 0.06722669
0.04502237 0.1749688 0.1619424 0.1694524 0.3950483 0 0.1829799
AC004637.1 0.1535943 1.641826 0.03553753 2.363688298 0.064880058 0.2383437
1.155163 1.15672 2.605956 1.708065 0.4087351 0.157012789 0.7488329
0.704605697 0.676595383 0.04957597 0.1448409 0.05903898 0 0.08930854
0 0.2130432 0.03222718 0 0 0.2385254 0.03254158 0 0
0.042617297 0.1163318 0.032369274 0 0 0 0.066870225 0.04181201
0.06154289 0 0 0.1017429 0.04033973 0.07331071 0.1768382 0
0.04139765 0.1156902 0.04876513 0 0 0.0429842 0.05207011 0 0
0 0 0 0.1267212 0.03298582 0.2989116 0.1185159 0.1794649 0
0.05314827 0.0487893 0 0.1029435 0.1389023 0.04506113 0.092346389
0.04722795 0 0.1510172 0.154753706 0.02849659 0.05288774 0

0.0525544	0.08644504	0.05851726	0.0469141	0	0.09764323	0.02608786
0	0	0	0.0771707	0	0	0	0.033405821	0	0	0.04368025	0
0.1830551	0.05149152	0.06408395	0	0.03158317	0.1530817	0.05601856
0.08942245	0.1048677	0	0.067656133	4.4633840	0.06751102	0
0.2183860	0.03075345	0	0	0	0.04264352	0.03872282	0.1049904	0
0	0	0.02921503	0	0.09437554	0	0.4516448	0.063909604	0
0.03577975	0.1722554	0.1270305	0.158256184	0.04052537	0.05353761	0
0	0.1848468	0	0	0	0	0	0.04638211	0.03097185	3.9334370	0
0.06467116	0	0	0.04992077	0	0	0	0	0.04378695	0	0.04680888
0.3973410.1572354	0	0.042028502	0.05295153	0	0	0	0	0.2506033
0.08539326	0.2588336	0.1395445	0.113210866	0.06146198	0	0	0	0
0.2360904	0.0813594	0.1117794	0.03907239	0	0.1191108	0.6930763
0	0	0.1104916	0	0.0496323	0.2134472	0	0.2484907	0.03922874
0.05243366	0.03894152	0.09500887	0	0.038719573	0	0	0	0.7899398
1.163948912	0.07974924	0.05775888	0.1448475	0.06427159	0.1219286
0.1540891	0	0.02348725	0	0	0.3604332	0.1325025	0	0	0	0
0	0	0.2183298	0	0.04312743	0.6667145	0.06607237	0.053798189
0.1244141	0.03475234	0	0	0	0	0.1248047	0.02647228	0
0.04499891	0	0	0	0	0	0.134475115	0	0.042940462	0.2455273
0.1453916	0	0.1121055	0	0.1137985	0.1681726	0.2613570
0.118437266	0.07418646	0	0.04997472	0.07352179	0	0	0.0498669
0.0472748	0.08951443	0	0.8903265	0.04528002	0	0	0	0.02984756
0	0	0.1150940	0	0.04358793	0	0.1481720	0.086867516	0
0.03597309	0	0.7238272	0.2657256	0	0.3804993	0	0	0.4422103
0	0	0	0.1271690.08554435	0.07025648	0	0.1321687	0.04099957	0
0	0	0.2402024	0	0	0.1335647	0.1870966	0	0.1525120
0.1535333	0	0.3281281	0.1430323	0	0.07898073	0	0	0.0829915
0	0.03417898	0	0	0.06444271	0	0.03820573	0	0.04084427
0.03043755	0.100051798	0	0.06462055	0.05584958	0	0.044743125
0.04512022	0.0296553	0	0	0	0	0	0.08130337	0.07531049	0	0
0	0.036902721	0	6.8103570.05381034	0.03272558	0.08304151	0.04110468
0.04313925	0	0.06691488	0.05573533	0.03758436	0	0	0	0	0
0.06953269	0	0	0	0.05396902	0.03754378	0.05413801	0.04522839
0.09774186	0	0	0	0	0	0	0.05399167	0.09938892	0	0	1.37929
0	0.1777443	0	0.05830474	0.2134543	0.1223898	0.1338294
0.09303399	0.2063626	0	0.04013731	0	0	0	0	0	0.1954659	0
0	0	0.053588795	0.05643852	0	0.7633839	0	0	0.1800844
0.03786162	0.05979658	0	0.1129404	0.07958002	0	0	0	0	0
0.1954786	0	0.0315526	0	0.1494025	0.03407147
AC008635.2	0.02634606	0	0.06095761	0	0.055644463	0.04088315	0
0.04133593	0	0.02288944	0.2003155	0.089774809	0	0	0	0.1275567
0.1656306	0	0	0.05106372	0.167789441	0.04567919	0.02763968
0.1433962	0.0715471	0.1022858	0.02790933	0.05477367	0.04423803
0.073101556	0	0.13880774	0	0.2428012	0.2221096	0.114702664

0.07172024 0.05278234 0 0 0 0 0.06287502 0.3791637 0.05128169
0.1420190.0661479 0.1254704 0.05391076 0 0.0737309 0.111645
0.04702029 0.069923621 0.04156699 0.2094570.4724515 0.1086826
0.2546130.04272699 0.3557584 0.07695913 0.04295911 0 0.1673768
0.096236716 0 0.4169541 0.07729347 0 0.04050511 0 0.1295201
0.176966278 0.1466408 0 0.2006388 0.13522 0.1112095 0 0.2011797
0 0.08374384 0 0 0.1000023 0.02575366 0.1985566 0 0.02511675
0.09831711 0.028650544 0.1794712 0.0710633 0.1498497 0 0.3139949
0.08832353 0.1099234 0.1170696 0.05417469 0.06564536 0.1441332
0.1533866 0.1498998 0.0344709 0.203088785 1.8270130 0 0.28378
0.06243302 0 0.1055029 0 0.04143896 0 0.1828664 0.09963203
0.04502256 0.06195584 0.1201161 0.1630058 0.07516891 0.1193229 0
0 0.08607861 0.328872911 0.02586802 0.06137309 0 0 0 0.03475664
0.0918332 0 0.06644122 0 0.3592853 0 0.2395098 0.07573852
0.03809473 0.07955935 0.05312609 2.4698960 0 0 0.08112512 0 0
0.04703967 0.05978601 0.1658975 0.066878353 0.1877696 0 0
0.03786444 0.08990211 0.2411295 0 0.04541395 0.04457567 0.04616412
0.1618780.1169446 0.1791085 0.2197129 0 0.05984027 0.14564316
0.1581388 0 0.02924903 0 0.07863126 0 0.5582239 0 0 0
0.1021555 0.06793346 0 0.1684632 0.06317552 0 0.04256721
0.6407214 0.036605972 0.2557420.06728916 0.1798792 0.1001947
0.1222267 0.2459566 0.099623671 0.03794311 0 0.05271035 0.05645774
0 0 0.04953698 0.2070477 0.1378065 0.1045722 0.2378785
0.07542725 0.0604316 0.1360460 0.1324825 0.0852307 0 0.186233977
0.163918524 0.1157901 0.073181467 0.235771153 0.03745016 0.135767016
0.07397659 0.05198259 0.08500057 0.092280167 0.2134079 0.2980539
0.1825151 0.199543473 0 0 0.03567963 0.1135199 0.2141635
0.3473403 0.2235819 0.1704028 0.05952498 0.05777639 0 0.26910975
0.2569440.036827941 0.0263221 0.1246952 0.1608429 0 0.09200266
0.2277319 0.09615564 0.03735886 0.1665985 0.101577868 0.1272522
0.04641778 0.08572177 0.1261121 0.09418688 0 0.1283052 0.4865435
0 0.1780371 0 0.03883446 0 0.03782644 0.063842611 0.1023952
0.2254813 0.06768849 0.04935527 0 0.2097683 0.03738324 0.02870763
0.2965198 0.2135801 0.111753049 0 0.03085237 0 0.04138609
0.03798332 0.08442988 0.1450381 0.05921204 0 0.3034098 0
0.1402072 0.02658339 0.1090666 0.2934689 0.2008518 0 0.2550480
0.103173734 0.2917679 0.1162919 0.02575124 0 0 0.03818398
0.4279030.05729483 0.1308021 0.09388024 0.2370205 0.028489295
0.1206084 0 0.1296746 0.06773792 0.07619034 0.151325933 0.09490369
0 0.1758818 0.04743383 0.08247571 0 0.069529595 0.06553439
0.3134649 0 0 0.114412733 0 0.0554219 0 0 0.422113961
0.01934871 0.1271695 0.07710298 0.04019432 0.04128456 0.1414037 0
0 0.1291803 0.04422126 0.04873456 0.1658671 0.094948994 0
4.3450680.0923010.08420141 0.03561033 0.1057604 0.4069827 0

0.1147793 0.2868089 0.03223427 0.06870551 0.05281746 0.2120349
0.1423833 0.06790297 0.0894522 0 0.1182871 0.2145113 0.0462866
0.06439895 0 0.03879018 0 0.03188895 0.09645891 0.09083752 0
0.1192823 0.1973296 0.2778361 0.1420684 0.06584606 0.02819829 0
0.05172385 0.114332 0.06503489 0 0.07322772 0 0.05738946 0.07979072
0.1327403 0.03839415 0 0.112602096 0.2326738 0 0.2686073 0
0.02794026 0.065727073 0 0 0.091920993 0.04840456 0.2964632 0
0.04590092 0.1036235 0.07722478 0.03247206 0 0.03575203 0.06457563
0.1023779 0.094050624 0 0.2436599 0.04294374 0 0.1117683
0.05911269 0.1082445 0.4270587 0.04271174 0.2337715
AC139530.1 1.340598 1.170059 1.273635 0.3521687 0.427086029 0.627578 1.763268
1.10455 0.8206499 0.3773922 1.238521 1.148409167 1.148659 2.362050713
0.989738922 1.244941 1.388968 4.203026 0.7152139 3.03381 1.542214401
4.025406 3.079986 1.067993 1.830477 2.083829 3.800271 2.312213 3.206754 1.340343106
2.31624 3.353993802 3.647083 1.469159 1.502706 2.070507827 2.579063 3.330974
3.99971 4.753524 4.179693 0.7769631 2.091191 2.080241 0.510223 2.523226
2.406887 1.272136 2.643591 0.8068086 1.08989 2.640537 2.138627 1.391398715
4.029327 2.480923 2.697259 0.7492062 1.415404 3.716665 2.138201 1.312626
0.7937766 3.083944 2.236263 3.446997649 2.309017 3.733606 1.318329 1.294577734
1.577466 0.7146152 2.632528 1.144462495 2.237119 2.359644 1.973425 1.60162
4.38373 2.282676 1.910118 3.800915 1.225998 2.251553 0.421828632 0.9594299
2.159686 1.458124 13.72595 4.441028 2.06819 1.376414432 2.431864 1.393876
5.015872 5.910398657 2.276106 1.205167 1.031178 5.180471 2.079026 2.416592
3.783137 2.725191 1.730043 1.156283 0.874225385 4.501149 2.815601878 4.542805
3.53603 1.11811 2.42971 2.091888 3.120024 0.9188256 5.117138 3.004632
1.982561 3.826758 3.046898 0.830869 1.841916 3.091266 4.782692 0.7593006
7.990634 2.251193 2.851396342 3.448781 1.107851 1.646263 3.117699 2.064214572
1.936527 4.346539 3.653079 2.710311 1.475922 4.000236 2.94847 2.791494 0.9832088
4.006787 1.503982 1.72164 0.9418618 4.220229 3.38958 1.2929 2.652057
2.793615 2.616737 1.056382024 2.583288 3.784552 2.157799433 2.530075 1.597973
5.044184 1.840591 4.587372 1.096731 2.151808284 0.8649554 2.661012 3.740064
2.814701 1.263262 2.209712 1.20335 5.479583 0.5613544 0.717632053 3.686226
4.419514 2.87685 0.4163387 2.011719 4.163486 1.933983 2.507205 1.257431
1.214448 4.443068 2.19667 3.542523 1.40075 1.373852 2.730372 6.171276 2.02953
1.07181248 3.756143 13.17935 1.482889 3.797635 1.760427 2.674358 1.227197003
2.858304 5.920499 0.7566882 1.428378 1.309731238 0.9721582 3.196576
1.730403 2.891051 1.416971 2.40432 0.5789241 0.841763 1.701642 4.639054
0.9038566 4.288436 3.616807 1.094104987 1.071730321 4.344853 1.258595373
0.94948474 3.076672 1.003453532 2.323733 1.108278 5.903835 1.901848974
6.187857 1.703023 3.891256 5.417146053 1.784425 3.587772 3.428205 0.9874682
1.978602 2.446511 2.351625 1.396694 0.7445289 2.085853 2.36658 3.518980903
4.674639 4.606380007 4.077995 2.339502 1.417403 5.657672 1.752285 0.9618089
2.81541 3.57893 3.741346 1.203143695 1.501216 11.29504 1.949444 2.957606 3.24863
3.444299 2.528809 2.31668 0.8220356 3.631299 3.967465 1.269535 2.181891

4.7850352.023555666 1.90656 1.9442875.7629031.6695913.1151171.681584
2.5823372.4318866.5867723.3149961.175413711 1.014577618 4.2887139.55468
2.3176632.8289411.5960544.1332923.4113162.8662341.0242193.3650772.311681
1.4509133.3252091.8144753.1060211.2283182.8598111.9591861.417573362
6.5004690.9586878 1.3615722.8275191.6635063.6471213.9076720.765496
2.8259072.1750114.8511861.976064736 1.9314011.1682051.0321491.617487
1.8842952.014642898 0.3507164 0.8976588 2.7248791.7259483.493349
3.2208321.353910376 1.2109112.6621133.2562621.7216231.08955413 6.129391
2.3947193.5130153.4660895 2.661684063 1.1330491.2797231.6986451.393972
1.6078221.4872782.2824011.0505691.2852692.7655621.8702541.8766062.555154106
1.63931 1.5996454.3686811.7233823.7353561.8439511.8195291.6708592.479743
1.4947333.4178761.5722342.26717 1.8082493.29198 0.5662132.4496121.888727
1.8493961.0915232.5920962.0137291.6498812.2935853.3242611.0696755.991333
0.7746692.4819861.2546061.4210661.6980750.4765507 0.5334631.234449
1.7119511.4262393.8784582.0151281.2509122.07123 1.1438321.1093554.366292
7.01852 3.06691 2.2800514.737375781 0.7863546 5.4901052.0943576.0197
1.3502340.69131449 1.370923749 1.1064431.110537644 4.4444555.116716
1.1166953.2229090.7879736 2.3379542.0215462.6387322.1495160.7801664
1.8140782.575537725 4.7364412.7590553.7477262.2073941.3106072.520587
1.5000651.7768492.4526092.408958
AC020978.1 0 0 0 0 0 0 0.05656121 0 0 0 0 0 0.3162419
0 0 0 0.05437174 0 0 0.1005764 0.07344055 0.1199612
0.03629323 0.03765824 0.1409210 0.0366473 0.3955734 0 0.047994246
0 0.255172733 0.1217816 0 0.1166594 0.075307123 0.04708736
0.06930765 0.08925263 0 0.05728981 0 0.4953611 0 0.1346743
0.1398622 0.1737155 0.05491774 0.03539468 0 0 0.05863971 0
0.091815596 0.1091619 0.04583911 0.1550921 0 0.03714758 0
0.5338751 0.1010538 0 0 0 0.063183452 0 0.3519616 0.1014928
0 0.1595598 0 0.1700708 0.116185804 0.1925517 0.05956049
0.05269113 0 0.5354341 0 0.1584995 0.1712881 0 0.1175173 0
0.03282784 0.1690836 0.1303608 0 0.03298041 0.08606575 0.188102868
0.0392768 0.03110403 0.09838263 0 0.1374339 0.05798811 0.1443386
0.2562036 0 0.1723957 0.1892590.1007047 0.03936621 0 0 3.88413
0 0.07602876 0.1863135 0 0.07103303 0.06926711 0.1340641
0.05441284 0 0.1440713 0.2180421 0.05911841 0.08135323 0.1051484
0.4280807 0.0987031 0.1253448 0 0 0.1130285 0 0.03396688
0.2014701 0.03879771 0.2384295 0 0.09127678 0.06029236 0 0 0
0.2358859 0 0.0628993 0.1326014 0 0 0 2.5312640.2020094 0
0 0.07101607 0 0 0.061767043 0.1570081 0.2722967 0 0.09862294
0 0.1581440 0.1180490 0.047331164 0 0.05853161 0.1212347
0.03542657 0.1023720.04703692 0.05770031 0 0.03928766 0 0.1384330
0.1536257 0 0.1032495 0 0.1832487 0.06294119 0 0.1558257
0.08942587 0.1115029 0 0.05530158 0 0.06673027 0.05589432
0.1201887 0 0.1679054 0 0 0.0438547 0 0.08074043 0.043604753

0 0.1078806 0.03460656 0.2965351 0.201661889 0 0.5854159 0
0.07238062 0 0.1041182 0.04952116 0.05290118 0 0 0.2319476
0.1119151 0 0.163027251 0 0.3801054 0 0 0.04917522 0 0
0.06825752 0.0744086 0.181757433 0.1401112 0.03913698 0.1198288 0
0.08242544 0 0 0.05962448 0.07030366 0 0 0.2610454 0
0.07586526 0.03416128 0.050480534 0.08434727 0 0.1382525 0.06549414
0.1760002 0 0.06040362 0.04271873 0.1262604 0.09811065 0.2187579
0.044460095 0.1670929 0.2438018 0.1125599 0.2069948 0.1649004
0.0435884 0 0.2661969 0 0.1168888 0 0.1019858 0 0 0 0
0.09869196 0 0.03240381 0.05873151 0 0 0.03769553 0.1112444
0.1121794 0.146741154 0.060863574 0.04051176 0 0 0.0498753 0 0
0 0 0.5478036 0 0.06136794 0.06981246 0 0 0.1582412 0
0.03721105 0.09234482 0.180634388 0.1915579 0 0.1014406 0 0 0
0.4214045 0 0.1717541 0 0.03458087 0 0.05278964 0 0.2270315
0 0 0.165586399 0.06230826 0.1072352 0.2309477 0 0.1443967 0
0.045649103 0 0.4116058 0.09199505 0.1028334 0 0.07570222 0 0
0.255146105 0.20155314 0.05081296 0.03339686 0.1518641 0.05277852
0.0542101 0 0 0.04578064 0.04240614 0 0.09598887 0 0.041558672
0 5.4248450 0.07370902 0 0.1388724 0 0 0.07535742 0
0.2539578 0 0.1733844 0 0.2492816 0 0.03915275 0 0.1553209
0.2535043 0.0607782 0.1268418 0 0 0.05503689 0.04187287
0.2533173 0.1192773 0.08429917 0.07831382 0 0.1216074 0.03730955
0.08646142 0.07405347 0.2118153 0.06791776 0.3002550.04269814 0 0
0.04594384 0 0.2095439 0.05809975 0.1008295 0.1356041 0.073928011
0 0.09587776 0.1007726 0 0 0.043152587 0 0 0 0.3177963
0.05561159 0 0.06027177 0.1360664 0 0.04263855 0.06734101 0
0.04239662 0.08962049 0.04116546 0 0.2666216 0 0 0.3302127
0.1940498 0.07106707 0.2039142 0.1121682 0.3069617
AC127024.6 0.2793677 0.2871409 0.1211964 0.074639528 0.147510321
0.1083789 0.2854740.2191584 0.1234346 0.06067862 0 0.178490953
0.3192253 0.40049522 0.384574263 0.2817880.3841928 0.6040356
0.3102190.4061011 0.444800312 0.4238253 0.2198137 0.1140405
0.6638356 0.0451923 1.7386720.5808076 0.3518175 0.484470221
0.3085719 0.551957193 0.3278147 0.1379255 0.23552 0.836193242
0.4277842 0.6996149 1.0811360.3613765 0.1734908 0.1375737
0.4166953 0.5528278 0.2039173 0.7059081 0.3945472 0.05543583
1.1075860 0.3420488 0.1479823 0.4985926 0.231704452 0.8815338
0.1850862 0.4696658 0.1800696 0.2624862 0.9061347 0.6062757
0.2040143 0.1708233 0.6646041 0.05546331 0.765354262 0.4095888
0.1973789 0.2049006 0.367425374 0.2147535 0.2468194 0.6008634
0.234563794 0.8746570.3006119 0.2127529 0.4182041 0.2948102
0.6652198 0.5866475 0.7780680.05550004 0.05931297 0.067823293
0.1988252 0.2389502 0.1754541 2.5880560.6658309 0.8253381
0.075950969 0.1585893 0.2197822 0.9931077 0.511924973 0.6936522

0.9365627 0.07285015 1.75862 0.5385526 0.9136161 1.910444 0.5591012
0.5165887 0.3655216 0.153821964 1.960387 0.234435854 0.3837301
0.8776655 0.3723894 0.2151094 0.2796823 0.1353288 0.2746308
0.6833374 0.1454305 0.1320594 0.8354658 0.4927243 0.3184211
0.2700745 2.424434 0.7275317 0.1072854 0.5882876 0.2852369
0.36325978 1.02862 0.3660673 0.07832745 0.2888147 0.539713306
0.6449651 0.3651669 0.3567192 0.3962968 0.8930627 2.222371
3.290729 0.3174634 0.3346309 0.8583892 0.1581805 0.3520857
1.756661 0.7646818 0.4159136 0.3675884 0.107529 0.6328367 1.021492
0.062349751 0.07924467 0.2198924 0.443226818 0.1991067 1.006882
0.6385438 0.1505647 0.7745576 0 0.143333052 0.1203898 0.4135865
0.6118923 1.072824 0.2583445 0.284884 0.6989358 0.3923201 0.1982915
0.064348629 0.279478 0.1205089 0.2326126 0.1893929 0.3821529
0.4025786 1.017376 1.461304 0.222086 0.734047 0.5416171 0.3151536
0.4588291 0.3907631 0.3349495 0.8756774 0.1692649 0.7279355
0.097040359 0.3954753 5.217608 0.05960619 0.7968317 0.3240161
0.4075107 0.044016119 0.5532177 1.687924 0.1397322 0.1496663
0.30534654 0.09065833 1.247537 0.329323 0.5845077 0.2772151 0.7006694
0 0.2670013 1.142059 0.22454 0.1756019 0.6401683 0.4787948
0.164565243 0 1.381289 0.242499908 0.052084665 0.744587 0.119970225
0.1961077 0.4134087 2.103096 0.061157375 0.9900313 0.3160496
0.9676745 1.256323824 0.4992183 0.6036176 0.5202158 0.1805609
0.2838676 0.5115442 0.5927029 0.7528802 0.1577973 0.2680334
0.2758684 0.458610887 0.170286 0.390515149 0.6280056 0.6611201
0.2131927 1.210687 0.3658408 1.207409 0.5098063 0.2475905 0.1656162
0.448795299 0.3373384 0.3691527 0.1988381 0.6268426 0.08322803
0.4399961 0.3401299 0.4836747 0.2713582 1.12092 0.7872017
0.6691618 0.2608611 0.4512409 0.888526531 0.7125402 0.6973611
0.8523322 0.4252236 2.489995 0.1112168 0.9910085 0.3805115
0.6176163 0.2264755 0.592502016 0.307188794 0.7769849 0.7244057
0.1097122 0.6041498 0 0.2403048 0.1308065 0.2540778 0.4524312
0.4023196 0.4955751 0.4228264 0.07228238 0.4862308 0.5324469
0.3818215 0.1878105 0.093216 0.182338486 1.933651 0.2055222 0.2730603
1.31269 0.6155942 1.771412 0.1417933 0.07594267 0.216718 0.4977424
0.8028633 0.302094029 0.3197259 0.325196 0.2864667 0.6284928
0.5554348 0.401156484 0.06289608 0.4329875 0.7382336 0.2514888
0.2186384 0.4395479 0.230398775 0.4777519 0.5712971 1.300081
0.1384047 0.113738129 0.6877475 0.5142211 0.5079151 0.386329716
0.254318232 0.2051693 0.2022715 0.562088 0.372935 0.2736076 0.3748533
1.268246 0.2772752 0.1712248 0.2930701 0.5167703 0.2638226
0.880965428 0.2672611 2.26594 0.7952254 0.186011 0.5192053 0.4205474
0.392323 0.3070256 0.1521367 0.5702356 0.5981586 0.3187352
0.8400966 0.4684104 0.3145416 0.1800069 0.7904424 0.3286057
1.045242 0.08529861 0.2454063 0.5975129 1.16933 0.3084917 0.1111122

0.3381432 1.470316 0.1204026 0.08509444 0.5928948 0.1307774
0.4296412 0.5272614 0.08727709 0.2616323 0.4276271 0.4799095
0.6566898 0.4310095 0.5965235 0.4853064 0.1855091 0.1901703
0.7932026 8.973122 0.5089036 0.1825112 0.597003564 0.7538729
0.5323025 0.2543081 1.272428 0.2962722 0.087119374 0.183949793
0.4786747 0.304596687 0.1924766 1.403406 0 0.9430257 0.2403625
0.3582574 0.2582448 0.4758341 0.5686596 0.4707624 0.2261649
0.207769067 0.2583014 0.9150656 0.2276828 0.9148885 0.6666559
0.391761 0.4304251 0.7204327 0.849198 0.6197151
STARD13-AS 0.04806101 0.03039893 0.03849234 0.047411476 0.042945577
0.09752749 0.1148454 0.07540583 0.02613547 0.2601681 0.03513649
0.094482103 0.08636664 0.164298039 0.027142653 0.280423 0.0464841
0.07815847 0.05747377 0.01074824 0.007848328 0.01922974 0.01163558
0.01609761 0.005019909 0.03349083 0.0900764 0.007686093 0.0372461
0.041031785 0.0513351 0.011686884 0.03470497 0.06327467 0.01870046
0.020119506 0.005032057 0 0.01907622 0.1147743 0 0.01456461
0.01764582 0.03724419 0.01439217 0 0.004641088 0.01173772 0.003782503
0.05430918 0.01034626 0.006266615 0.02639242 0.009812004 0.134156
0.04408795 0.005524714 0.03431439 0.0277888 0.4616649 0.01426333
0.00539963 0.00602822 0.01279274 0 0.006752189 0.006194602 0.004179208
0 0.011113848 0.01705159 0.02613019 0.004543715 0.00620818 0.02057731
0.01909505 0.02815456 0.0189747 0.0468163 0.007042526 0.01129218
0.01830494 0 0.006279326 0.007180294 0.007016388 0.08673292 0 0
0.03524498 0 0.040203759 0.008394741 0.01661988 0.0210276 0
0.01468707 0.08056076 0.007712475 0.02190365 0.0380102 0.04145246
0.08764352 0.03766684 0.004206926 0.01934846 0.028498351 4.993205
0.004963835 0.08937414 0 0 0.04554627 0.01850582 0.03581737
0.03488943 0.1507153 0.04105704 0.05126302 0.03790663 0.008693928
0.01123683 0 0.05274024 0 0.01135806 0 0.06039473 0.038457465
0.007259838 0.02153038 0.04975407 0.03567216 0.019046063 0 0.03865937
0.009441257 0.01864667 0.04449245 0 0.04645091 0.02688729 0.02479861
0.04276502 0.0167462 0.007454898 18.81715 0.01619102 0.03302385
0.03113258 0.01517847 0.03349847 0.01802383 0.013201645 0 0.005819873
0.009384678 0.00526974 0 0.02253372 0.01062662 0.01261548 0.0563940
0 0 0.006477961 0.03785911 0.03829046 0.09550667 0.002055408
0.06749277 0.01679412 0.027249756 0.007396922 0.06379 0.03693922
0.01503794 0 0.02841337 0.01958314 0.0067263 0.03291642 0.005550846
0.03822649 0.02859821 0.1025476 0 0.03989286 0.0570498 0.0238929
0.02568828 0.005136718 0.0119623 0.004721162 0.03786221 0.00937319
0.02286855 0.0215711 0.009319767 0.06921657 0.06917286 0.007396559
0.03168964 0.043101767 0.0671845 0.006951256 0.005810776 0 0
0.05934259 0.005292149 0.08197372 0.006363535 0.00792384 0.006196853
0.03189323 0.04505679 0.026133207 0.015334534 0.08124101 0.015403739 0
0.03678625 0.031752419 0.005190367 0.02188331 0.00397589 0.038847531

0.02994637 0.04600672 0.01707424 0.028000857 0.01761702 0.009129084
0.01502019 0.0700904 0.0150262 0.02707799 0.03137404 0.003985283
0.05846973 0.01216117 0.04380832 0 0 0.015503598 0.05540457
0.006999124 0 0.1214267 0.04518583 0 0.0067465 0.04193893 0
0.023756455 0.03571323 0.01302711 0.009021654 0.01769663 0.01762231
0.01397441 0.04201021 0.005689501 0.1687774 0.03122872 0.0059528
0.01089886 0.02761675 0.03715586 0.004479344 0.003592141 0.01054686 0
0.05540607 0 0.02354852 0.02098315 0 0.005944145 0.02397643
0.015681701 0.019512816 0 0.009586393 0.07549731 0.01598998 0.01184759
0.04070482 0.06093197 0.0215189 0.612028 0.0532408 0.01311634
0.01492122 0.01530473 0.0411809 0 0.0230986 0.04374273 0
0.077214999 0.04094221 0 0.02890826 0.01208447 0.01955146 0.0107163
0.8706571 0.04019937 0.01376606 0 0.1736901 0.019988743 0.01692432
0.02582081 0 0.009505296 0.04276552 0.03539125 0.009987991 0.003819948
0.004113425 0.03993679 0.1080181 0.007755653 0.043905205 0.01379413
0.03299012 0.009831181 0.01098944 0.040137301 0.008090025 0 0.02688589
0.02726655 0.021539262 0.03529629 0.01784501 0 0.005640252 0.02317296
0.02976363 0.05253895 0.01956965 0.0634451 0.00620533 0.006838658
0.004655047 0.013323679 0.02263545 4.857769 0.03238025 0.01575404
0.01998802 0.01978772 0.005191789 0.02031505 0 0.006707723 0.01809304
0 0.003705794 0 0.01331992 0.03493768 0.004184114 0.006957744
0.01106573 0.003010121 0.01948545 0.02259189 0.006515486 0 0.02352638
0.03132363 0.006767779 0.01274674 0.0180175 0.008369118 0 0.006497873
0.01196141 0.03233938 0.007913829 0.03018126 0 0 0.03650398 0 0
0.06873795 0.02013288 0.0223932 0 0.01077529 0.0579661 0.039502109
0.05804419 0.01024611 0.005384602 0.03169624 0.09801765 0.009223125
0.053554422 0.02252274 0.012898776 0 0.04754409 1.286221 0.1094975
0.007270463 0.02167307 0.03645303 0.007196493 0 0.07702317 0.01436613
0.017596821 0.01367289 0.01139716 0.01807819 0.07264283 0 0.05391727
0.01898671 0.005447895 0.04195455 0.02050243
AC015849.4 0 0 0 0.150342803 0 0 0 0 0 0.0305555 0
0.059920953 0.2500556 0 0 0.1135184 0.05527578 0.06759333
0.03905369 0.2044975 0.074661652 0.1219558 0.11069 0.1914219
0.1432641 0.3186004 0.1117699 0.4021506 0.05905409 0.04879225
0.08879165 0.111178076 0.04126883 0.04630277 0 0.07655926 0.335092
0.4227602 0.09073665 0 0 0.04618468 0.2517988 0.1012304
0.1369136 0.1895836 0 0.1116617 0 0 0.04921231 0.08942207
0.1255364 0.046671111 0 0.1398038 0.1051139 0 0.1132957 0
0.33922 0.102734 0.3440811 0.06084909 0.05585854 0.064234008 0
0.3975707 0.2063607 0 0.1622129 0 0.1296739 0.059058817
0.03262555 0 0 0.1203383 0.3958813 0.066996 0.1074233 0.1741361
0 0.02986781 0.204919782 0 0 0.0883522 0 0 0.04374839
0.038246094 0.03992986 0.0316212 0.1000184 0.257786447 0.3492976 0
0.3668464 0.05209271 0.2892748 0.1314466 0.06413527 0.2559479

0.04002076 0.04601578 0.077459041 6.2714840 0 0.1894114 0.04167144
0.2166423 0.3873035 0.1362932 0.05531757 0.05735056 0.2441114
0.08866698 0.1202027 0.08270589 0 0.2175992 0.06689617 0.1274289
0.1080499 0 0.05745389 0 0.06906331 0.1228920.2366568 0.09695757
0 0.09279444 0.06129484 0.08981528 0 0.1587223 0.9592319
0.2209453 0 0 0.05085329 0 0.03545946 3.6991920.7701309 0 0
0.07219686 0.05311218 0 0 0 0.1107297 0.04463852 0.05013137 0
0.05359117 0.2021835 0.1200118 0.2145919 0 0.1212477 0.05950482
0.1232505 0.2160937 0.05203709 0.0478190.2737452 0 0.07988179 0
0.07036739 0.06068383 0.03904502 0.09537126 0.4198648 0.0450497
0.3260173 0.1919632 0.04473371 0 0.04545638 0.04534276 0.05134429
0 0.1265011 0 0.05682368 0.4276549 0.048865929 0.1137981 0 0
0 0 0.1231244 0 0 0.1645115 0.03518197 0.2260992 0
0.1217391 0 0.05527828 0.0735841 0.0930635 0.07056623 0
0.1075616 0.1210734 0 0.1768532 0.0379253 0.3214711 0.082868959
0.145878402 0.0772851 0 0.104911629 0 0.120825119 0.0493763 0
0.2647603 0.061593176 0.2848818 0.1193631 0.2436425 0.066593487
0.1675919 0 0.2381467 0.1818476 0.3573630.1030379 0.09948774
0.1895613 0 0.07712668 0.03472928 0 0.08574972 0 0.03513782
0.09987467 0.03578532 0 0.06140795 0.3474322 0 0 0.277994
0.045199337 0.3397422 0 0.1716471 0 0.1257317 0 0.1141845
0.2706230.1024845 0.1188323 0 0 0 0.2019806 0.340898314
0.06834454 0.1003329 0.09035851 0.03294259 0.05970805 0.1120093
0.1497105 0.0383223 0.1696411 0 0.09945402 0.061875558 0.123556
0.09119597 0 0.4056366 0.05635344 0.04840345 0 0 1.518851
0.1012966 0.3119415 0.1064599 0.03639873 0.1958783 0.3753687
0.05493462 0.1891488 0 0.183637811 0.4381716 0 0 0 0
0.1019449 2.1420560 0.08730494 0.06266116 0.3515584 0.114092519 0
0 0.0577016 0.04521224 0 0.033667923 0.0950164 0.07267882
0.03913127 0 0.07339881 0 0.092816229 0.04374148 0.2615310
0.1045432 0.038182872 0.07696092 0 0.0639418 0 0 0.05165783
0.2037129 0.1029261 0 0 0 0 0.1396255 0 0 0.09758489
0.04428376 0 0.05383312 10.079220.06160708 0.1873365 0.1426105
0.09412093 0.04938983 0 0.07661039 0.2552440.1721203 0.3210065
0.1762673 0 0.0633566 0 0.1592150.06618946 0.2105380.02863548
0.1235775 0.1289509 0 0 0 0 0.1287646 0.1212605 0.08570082
0.1194239 0.1317093 0.06181469 0.1896495 0.04394951 0 0.1435581
0.1380941 0.2543728 0.04340808 0 0.04887646 0.04670776 0
0.1065141.0041180.0512530 0.075157218 0.1035334 0.04873596 0 0
0.03729793 0.043870089 0.0926303 0.1071302 0 0.06461607 0.1696087
5.2439580.03063696 0.1383288 0 0.04334751 0.0684607 0.09545197
0.0862031 0.1822212 0 0.3251776 0.1084219 0.05732632 0
0.07460071 0.1578211 0.1806218 0.4664356 0 0.3120655
AC093249.2 0.06143771 0.1262943 0 0 0 0.04766873 0.2008979

0.04819667 0.05429075 0 0 0.104675192 0.1872082 0.264227137
0.056382949 0 0.1931212 0.4132729 0.03411121 0.5060817 0.130425541
0.3195648 0.4189534 0.3343927 0.6673771 0.2385254 0.780998 1.21343
0.2063218 0.213086487 0.07755453 0.356062012 0.2883681 0.6875289
0.4661536 1.136793831 0.3344961 0.6769718 0.4755203 0.3178914
0.5087145 0.04033973 1.319593 0.7957718 0.05979318 0.7865554
0.5013242 0.2925908 0.6285861 0 0.214921 0.1041402 0.1644735
0.244587605 0.3392625 0.2442217 1.698506 0.1900818 0.4288157
0.09963721 0.4148055 0.04486622 0.200357 0.3720379 0.6830502
0.22441923 0.1544152 0.243079 0.09012226 0.277039168 0.1889118 0
0.6418232 0.154753706 0.3134625 0.2115509 1.169699 0.3678808
0.6483378 0.1755518 0.1407423 0.1520981 0.1464648 0.6000209 0
0.1457502 0.2101969 0 0.04742979 1.69856 0.7260236 0.133623282
0.6626534 0.3314321 0.2620815 0.825594078 0.5491653 0.1544745
0.2563358 0.3640005 0.4737475 0.07654085 0.6162042 0.2235561
0.06991178 0.2411532 0.1014842 0.4057622 0.371206315 0.2025331
0.4411739 0.2547837 0.06307497 1.107124 0.5357 0.09663359 0.7012951
0.4264352 0.07744564 1.049904 0.8668676 0.09336828 0.09503036
0.6135156 0.2782550 0.3881234 0.4014621 0.639096044 0.4825834
0.2146785 0.1033532 0.08468701 0.316512367 0.4863045 0.1070752
0.8629348 0.5035473 0.2772701 0.907655 0.7076055 0.111705 0.1766185
0.1332525 0 0.3097185 0.4214396 0.1793776 0.8689316 0.1293423
0.4414194 0.04639052 0.5990493 0.329082504 0.278836 0.4835808
0.194946235 0.8757389 0.2657165 0.3276622 0.132447 0.4717063
0.093717 0.42028502 0.1059031 0.05197412 0.3767835 0.1887457
0.1363545 0.1670689 0.1878652 0.3019725 0.06977227 0 0.06146198
0.2120157 0.3410364 0 0.305607 0.4328323 0.5695158 0.3353381
0.195362 0.1844908 0.4367396 0.2376262 0.3139246 0.09821194
0.1473222 0.2370171 1.141543 0.266809 0.170726606 0.3975851 1.059176
0.05243366 0.6620058 0.1425133 0.1075422 0.193597867 0.7078515
0.2394859 0.1229179 0.3291416 0.268603595 0.1860816 0.7508654
0.3379774 0.8033949 0.2845001 0.4314495 0.1319194 0.2113852
1.0046330 0.566395 0.3312562 0.6083719 0.144762787 0.127416612 1.28258
0.469302726 0.091634432 1.83397 0.105533973 0.301892 0 1.090194 0.376587322
2.177247 0.5212851 0.3901482 0.523491124 0.2927642 0.1517095
1.123242 0.6088624 1.123692 0.5399869 0.1737939 0.4967135 0
0.3031462 0.1213363 0.493075421 0.8238734 0.300583237 0.4603637
0.5234096 0.1562823 1.457372 1.072728 0.834522 0.728748 0.1306785
0.2428122 0 0.2967458 1.08244 0.3498231 0.6249352 0.4392782
0.3096404 0.0498669 0.472748 0.08951443 0.3113802 0.6924762
0.1811201 0.1529808 0.4410469 0.521072267 0.8655793 0 0.5919232
0.3452821 1.825307 0.2445846 0.4794672 0.2343067 1.135985 0.09961161
0.130301273 0.270224168 0.4676502 1.513437 0.2412757 0.3100133
0.3937726 0.0422777 0.1380795 0.2682049 0.4422103 0.2654308

0.3814488 0.2789602 0.09537675 0.08554435 0.1170941 0.04798231
0.1652109 0.2459974 0.240596011 1.148155 0.3163848 0.03002531
0.2008229 0.1624556 0.4897373 0.3741932 0.06680434 0.152512 0
0.4605999 0.298960273 0.04687545 0 0.5039912 0.3554133 0.04441802
0.088221135 0.1106553 0.03174044 1.367159 0.16592 0.1602744 0.7733126
0.202674441 0.1146172 1.461969 0.449287 0.1826253 0.166752997 0.6049895
0.1292411 0.05584958 0.28320159 0.402688121 0.06768033 0.2668977
0.3146506 0.09373115 0.1925471 0.1648732 0.8245999 0.2439101
0.4518863 0.1031218 0.1136466 0.2320763 0.258319047 0.1410606
0.4983188 0.2152413 0.1963535 0.2906453 0.2055234 0.1294177
0.405121 0.6691488 0.1114707 0.6013497 0.2002723 0.8313819
0.5768639 0.4427076 0.02639106 2.294579 0.05781279 0.8734923
0.1250574 0.05396902 0.9010508 0.2706901 0.7236542 0.3909674
0.1487269 1.068453 0.1059143 0.2994194 0.2433903 0.3067753
0.4319333 0.6957225 0 0.2959066 0.25078 0.06030872 2.48842
0.6066325 0.4081332 0.4695994 0 0.5018601 0.604721 2.631123
0.4476662 0.2408239 0.656456202 0.09043062 0.6810901 0.4026721 0
0.09773295 0.114954195 0.040453689 0 0.482299153 0.5643852 0.4444312
0.7633839 0.401395 0.1812336 0.1350633 0.3407546 0.1793897
0.2292729 0.3388211 0.2785301 0.511749868 0.2840244 0.3788019
0.9012839 0.134133 0.1954786 0.3446196 0.0946578 0.3621381
0.4482074 1.635431
DHDDS-AS1 0.2519062 0.1150735 0.09714039 0.179473497 0.059115667
0.08686703 0.1372866 0.2195727 0 0.07295199 0.1064058 0.190750256
0.3695799 0.12037569 0.359614496 0.3613704 0.1319722 0.1613807
0.09324161 0.2441213 0 0.1455862 0.3817307 0.2742146 0.5320724
0.3259997 0.2075526 0.9310488 0.09399536 0.11649265 0.1413281
0.501387202 0.4598081 0.2947972 0.5191234 0.060929014 0.2285829
0.5046750 0.07221196 0.1930985 0.09270336 0.07351134 0.2671891
0.8056333 0.1634422 0.2263176 0.1405486 0.1332975 0.1431845 0
0.07833037 0.04744385 0.09990702 0.111428379 0.2208001 0.2225233
0.3764429 0.1731936 0.2404411 0.1815695 0.6479165 0.12264 0
0.1452786 0.1778181 0.408960564 0.09379728 0.5695257 0.3284607
0.210354252 0.3012233 0 0.3095996 0.423012966 0.7270131 0.09637765
0.2984171 0.09577021 0.472588 0.2132727 0.3419674 0.3464617
0.3113878 0.07131011 0 0.2390413 0.2736022 0.1406286 0.3889422
1.520964 0.06963354 0.152189088 0.2224448 0.05033091 0.7561886
0.478699849 0.3891795 0.04691666 0.2919514 0.8291504 0.08633131
0.6625341 0.5614564 0.1222163 0.06370034 0.07324251 0.030822549
6.562379 0.187903217 0 0.5024712 0.1326554 0.1724129 0.6444853
0.05423386 0.132072 0.04564194 0.349693 0.3175418 0.334818 0.1974623
0.2552184 0.3030552 0.2661936 0.6845388 0.04299528 0.3143465
0.3657934 0.174694306 0.1374086 0.3912099 0.06278042 0.2314885
0.288391231 0.1846241 0.1463429 0.2859148 0.3529297 0.1263178

0.3816981	0.2930622	0.610682	0.08046323	0.1214134	0.1267836		
0.1128804	6.84793	0.4494614	0.1666799	0.176776	0.1723717	0.295882	
0.4548547	0.199896366	0.3175779	0.3524929	0.355251741	0.2792763		
0.2421084	0.1706002	0.160906	0.525307	0.2561716	0.306355206	0.04824696	
0.1894255	0.09808784	0.6019169	0.165653	0.342507	0.2178577	0.1965309	
0.1907197	0.103152433	0.2240051	0	0.5903991	0.07590038	0.4733731	
0.03585241	0.6301124	0.2036963	0.1424037	0.3361987	0.1085282		
0.2706423	0.1634476	0.1342292	0.2013496	0.2699485	0.2261131		
0.7779323	0.15555808	0.3169783	2.394807	0.09555018	0.5322253		
0.2164191	0.1633124	0.176397301	0.3627906	1.0474	0	0.1199594	
0.571057675	0.1453275	0.6315262	0.5279128	0.556332	0.5184464		
0.2527178	0.2403976	0.1498034	0.04817762	0.4799241	0.3284098		
0.1207301	0.08527999	0.197851597	0.087072033	0.9841067	0.272113318		
0.125239497	0.1193591	0.144236414	0.1964785	0.2761268	0.6020204		
0.147055163	1.417004	0.09499411	0.2262175	0.370984888	0.06668826		
0.1382306	0.4927701	0.1688421	0.2275234	0.1230027	0.5542351		
1.025853	0	0.2762126	0.4422239	0.081684947	0.2047295	0.195626681	
0.5033542	0.3709271	0.1708766	0.2042906	0.04887098	0.1036878		
0.51077	0.1190681	0.486726	0.323743452	0.4393689	0.246567	0.06830194	
0.3684428	0.5670205	0.2468636	0.2726183	0.516895	0.05437423		
1.182146	0.135204	0.1650281	0	0	0.135650463	0.4623269	0.1596981
0.3595551	0.1835195	0.7127719	0.08914161	0.3177222	0.1829908		
0.4050222	0.63533	0.118724412	0.098486237	0.426101	0.4354651	0.1758713	
0.3631751	0.08969677	0.1540858	0.08387439	0.2036464	0.4432135		
0.1612321	0.09930237	0.1412086	0.7531578	0.467664	0.4267627	0	
0.4214908	0.07471377	0.146146537	0.658684	0.1647286	0.2462189	0	
0.2960438	0.2028298	1.136491	0	0.1389617	0.3989466	1.231053	
0.090799529	0.2135535	0.3258107	0.09184263	0.07196353	0.2023581		
0.321531851	0.05041198	0.0289204	0.4982765	0.2015713	0.2044483		
0.2935859	0.295467919	0.1044338	1.998116	0.4093693	0.05546654		
0.243100022	0.2449947	0.1177584	0.6106503	0.361256016	0.244607025		
0.3494472	0.3242462	0.1228692	0.2562103	0.08771993	0.2253371		
0.4419628	0.07407982	0.2401679	0.09395972	0.362423	0.7400997		
0.336240293	0.08568523	8.62685	0.04902946	0.08945405	0.2269906		
0.4119792	0.2751452	0.1230424	0.1219394	0.1015668	0.4451863		
0.145983	0.2805616	0.1501747	0.8571705	0.07213889	0.2534197		
0.0526763	0.7121079	0.2506822	0.2458702	0.06841627	0.09865604		
0.3708899	0.1781156	0.1016347	0.8198095	0.1930083	0	0.5068933	
0.2445793	0.9346988	0.09055853	0.03497683	0.08987207	0.1713742		
0.5495048	1.25513	0.2418216	0	0.7001622	0.1115159	0.1829086	
0.5086093	0.2350349	0.489471	0.5119973	0.358879269	0.1098615		
0.3878609	0.3261298	0.05999217	0.1187329	0.244395372	0.07371901	0	
0.097655195	0.2056965	0.719902	2.086679	0.4144966	0.08256579	0.2461266	
0.6209592	0.4358707	0.1329381	0.137208	0.2900383	0.099917676		

0.2587897	0.5608631	0.2737359	0.1222157	0.564018	0.3768015
0.574985	0.6186811	0.3176332	1.055507
MSC-AS1	0.5325058	1.046209	0.5233586	0.694988555	0.547412586	1.308963
1.78364	1.046199	0.6746084	0.3787103	0.2732024	1.645919122	1.199008
1.2193945	1.055233262	0.8898114	0.336994	0.3894468	0.2930394
0.04795146	0.570227771	0.2001772	0.07168548	0.1718472	0.0479903
0.8568415	0.3694122	0.1347115	0.427287	0.323617473	1.635877	0.275592121
0.05529656	0.2016352	0.7786701	0.184648655	0.5067211	0.5617405
0.3221844	0.7396218	0.3550801	0.2104034	0.5229513	1.600549
0.4356985	0.1270125	0.1686013	2.749208	0.3712499	0.3518991
0.2274934	0.1757325	0.290158	0.171971605	0.07806709	0.08117404
0.0387319	0.06560907	0.4781894	1.184579	0.2318075	0.9773467
0.4034082	0.8357066	0.2731859	0.352878169	1.042278	0.02397192
0.04493206	0.747279797	0.03260262	0.05828771	0.0984592	2.290916338
0.6382437	1.046612	0.8433599	0.05240386	0.208863	0.03590747	0.4713956
0.1924947	1.670159	0.1640829	0.581182154	0.5097824	0.4606479	1.04178
0.09822586	0.8805413	0.09085936	0.597020064	0.01070049	0.158886
0.05695672	0.120893803	0.3088982	0.6753714	6.616148	0.05932965
0.2204489	0.4755423	0.08593554	0.08573684	0.0214497	0.6843932
1.066423882	0.9259114	0.540978267	0.4142624	0.02960934	0.8794173
0.2515772	0.1462502	0.1460966	0.4076634	0.07300241	0.1341059
1.315775	0.2778302	0.2160961	0.02864641	1.47604	0.2196055	0.05976017
0.6623559	0.04300129	0.6197139	1.745126805	0.0138808	0.9056535
0.7742502	0.4611965	1.97860524	0.9045465	1.839704	0.6318085
0.4813076	0.170139	0.407007	0.0296047	0.6554579	0.07676701	0.9573513
0.2810531	0.09740066	11.55639	0.01719845	0.1262831	0.501006	0.03143964
0.5301836	0.2756923	0.185104544	0.3528935	0.3931749	0.218312484
1.249391	0.6657854	0.1292533	1.544175	0.2613084	1.840218	0.551252896
0.004061528	0.6258895	0.03302894	0.07721234	0.1603676	1.486498
0.569841	0.09595735	3.5187759	0.87704191	0.202715	0.02032773	0.1778773
0.4312878	0.1195486	0.8088587	0.09984781	0.08145095	0.6803098
0.6261794	1.068927	0.8885457	0.3130253	0.01883281	2.971904	0.1090791
0.04949013	0.2087428	0.851187869	0.7090289	12.46609	0.9451242
0.1373984	1.180565	0.7286426	0.288080035	2.266781	0.08817228	0.2192043
0.4291831	1.950382396	1.080667	3.424594	0.5629166	0.02957884	0.02493936
0.007091434	0.168643	0.1657413	0.008111381	1.464536	0.04344404	0.05335734
0.03589522	1.415722002	0.527752957	0.1190884	0.471230883	0.8399191
0.2779918	0.113326425	0.1091637	1.636442	0.06588311	2.372719295
0.04771452	0.1705984	0.01632296	0.874447512	0.8084094	0.07563745
1.266809	0.1218298	0.05746015	0.13461	4.352399	0.05587895	1.911146
0.04133715	0.5258358	0.364449532	0.2412837	0.797063543	0.9934206
0.8810025	1.203523	0.2493652	0.05348277	0.04946237	0.4256769
0.7083202	0.0260741	1.21126166	0.4353083	0.2698348	3.258218
0.03101627	0.5840266	0.01484392	0.4475165	0.1450443	2.007161

0.05572866	0.01896958	1.50038	0.9856597	0.6664412	0.059951504
0.4006432	0.4839738	0.2270107	0.3840192	0.4200182	0.01125618
0.4346307	0.1951242	0.5303759	0.236855	0.253193096	0.23628704
0.3780152	0.01832917	2.961041	4.755773	0.1812206	1.29064	3.163202
1.176036	6.200346	0.1425149	0.6854771	0.02852934	0.348713	0.9973458
1.016699	1.383821	0.3522851	0.3899526	0.756629276	0.1239456	0.1317383
1.671995	0.02695642	0.2616765	1.434269	0.3205017	0.2664519	0.1052828
0.02938613	0.1036325	0.287912339	0.7766226	0.1535935	3.86575
0.2362635	1.526329	0.843594719	1.9967	2.500312	0.06816215	1.866556
0.1721088	0.4003775	1.175253741	2.748794	0.4520524	0.3414826
1.162652	1.460664971	0.06187247	0.441135	1.700675	1.146939398	0.168164086
0.4326056	0.1569503	0.3620184	0.3486876	0.1107667	0.442618
0.0334848	1.995581	2.460798	0.08305208	5.197513	0.0207677	0.070763493
1.197384	7.708188	1.803674	0.2911764	0.9012859	2.011513	0.03970678
1.393146	0.04619299	0.1453518	0.07207057	0.0460843	0.08266387
0.1769882	4.550222	0.5080909	0.7093352	0.7538479	0.07757808
0.3472394	1.630991	0.3225276	0.07059308	0.05203712	0.9633732
1.018143	0.05175994	0.06499132	0.4191348	0.08000871	0.04706101
0.05383701	0.2719014	1.301434	0.353062	1.8418	0.1989113	0.04090042
9.623063	2.039293	0.02292154	2.162285	0.3207837	0.4317257	0.03957144
0.123614	0.8928061	0.276935931	1.44737	0.4701733	0.175021	0.1666587
0.6271975	6.818713104	2.109977894	1.388797	0.168526697	0.06060584
0.01893838	1.229626	3.856722	2.117603	0.6526625	1.254567	0.8439273
0.03836914	1.715241	0.009156008	0.428975014	0.4052094	0.05811028
0.2112334	0.7819161	0.2174093	0.396499	0.06534461	0.06944245
0.8594684	0.4155266
FMR1-IT1	0.215838	0.1774748	0.1997561	0.092265773	0.18234512	0.6028774
0.07057784	0.4740979	0	0.1500159	0.4102672	0.294189171	0.8769132
0.185652303	0.15846403	0.4179992	0.5427663	0.0829644	0	0.4183354
0.824760928	0.8232907	2.128497	0.5168951	0.1172288	0.6703745
0.5487479	1.480805	0.724833	0	0.5994083	1.137171282	0.2532679
1.079813	0.4367076	0	0.4112936	0.08648301	1.67056	0.4467161
0.142974	0.1133746	0.8241577	1.428883	0.7562181	1.338001	0	0
0.5741575	0	0.3624207	0.6585427	0.462252	0.400990563	0.06810684
0.4575893	0.6450869	0.1780747	0.741652	0.28003	0.9992649	0.3152408
0.774266	0.2240595	0.4799274	0.394205791	0	1.561537	0.8231868
0.389308685	0.7300367	0.9153185	1.485516	0.144978205	0.8809849
0.3716019	0.7232357	0.3692598	1.396982	0.328925	0.3296297	0.6412067
0	0.07331983	0	0.2867412	0.2531817	0.3795538	0.06665061
0.205767	0.214788	0.046943456	0.1470303	0.03881203	1.780066	0.949225314
0.3429837	0	0.9905921	0.1918166	0.488204	0.1613383	0.2361599
1.256607	0.5403386	1.29904	0.332757718	6.557259	0.115919303	0	1.317412	0
0.3545438	0.4321621	0.5855043	0.2036912	0	1.378269	1.197134
0.07376874	1.11665	0.918439	0.8012472	0.4105435	0.9384412	0.3315526

0.1212021 0.2820768 0.179617664 0.127153 0.7039117 0.2420615
0.6545343 0 0.3986373 0.1504672 0.5511987 0.8164716 0.5844499
0.4905693 0.2711894 0 0.2481927 0.4369232 0.4562485 0.3046619
3.849476 0.4411226 0.2570662 0.181758 0.443074 0.2607607 0.3507553
0.077073745 0.4897921 0.06795508 0.602685096 0.3076576 0.1244659
0.5262247 0.3722417 0.4419095 0.3950868 0.236241842 0.2232301
0.3651826 0.453835 1.237761 0.1916118 0.4108531 0.6959924 0.1212418
0.3921893 0.318178643 1.036432 0.07448365 0.1437722 0.292648 0.7730164
0.1105885 0.9146409 0.7853887 0.4392512 0.324069 0.5021406
0.3617506 0.1890608 0.2070182 0.3622923 0.6661354 0.1394914
0.674879 0.119956595 0.7682202 0.3858827 0.2947291 1.039727 0.400533
0.3526215 0.108821192 0.1243384 0 0.3022777 0.1850102 0.125818205
0.3362023 0.1623311 0.6106397 0.6322226 0.9709263 2.468481
0.1235863 0.330054 0.6687278 0.09252181 0.5788547 0.5120468
0.591863 0.915424153 0.40286681 3.699545 0.29976665 0.257538169 0.245446 0
0.4242331 0.2555179 0.5570883 0.151199575 2.447658 0.830206 0.3987308
0.817371953 0.2057031 0.5329735 0.5261446 0.5952018 0.7018068
0.1897038 0 1.582146 0 0.5206613 0.5967766 0.188970861 0.8419973
0.422393937 1.03508 0.4494846 0.3074616 0.3150721 0.6029796
0.7462701 0.2363242 0.1224238 0.5459381 0.38834532 0.4691266
0.3042192 0.561815 0.7748725 0.1028825 0.2175608 0 0.5978953
0.1677199 2.260759 0.2085216 0.2545186 0.107488 0.123956 0.836840759
0.377489 0.7388949 0.1663598 0.485207 0.6595737 0.5499231 0.367511
0.3762959 0.4858429 0.419937 0.610351931 0.531624689 0.45496 0.4477376
0.2034313 0.7468208 0.4841795 0.2376426 0.6144472 0.06281573
0.4971321 0.497328 1.07206 0.3484517 1.206252 1.562743 0.9543683
0.2697082 0.7893516 0.2880728 0.394446522 1.195143 0.06351415
0.295351 0 0 0.2502554 0.4381952 0 1.393061 0.9998382 0.5609561
0.140037757 0.1317432 0.6029873 0.2124697 0.1109875 0.37451
0.495890231 0.3109963 0.04460319 1.777107 0.3108782 0.2702702
0.4527899 0.056961563 0.3221311 1.027214 0.2869816 0.427723 0.515524426
0 0 0.07848251 1.512279975 0.880252491 0.2219178 0.2083652
0.442162 0.3951465 0.2029323 0.1158439 0.8179533 0.3427541
0.3174897 0.07245582 0.1597016 0.5435412 0.363002276 0.06607506
6.652484 0.2268506 0.7817884 0.2917348 0.6353849 1.212427 0.1423238
0.658224 0.7048976 0.3697073 0.2814322 0.8221354 0.3474157
0.3888211 0.1112579 0.8305406 0.1624826 2.51955 0.5272101
0.4550391 0.4748249 0 0.06355709 0.2747031 0.3657467 1.501441
0.2232537 0.2103793 0.7329078 1.56272 2.124407 0.4655536 0.05394386
0.5544294 0.352408 1.186482 2.18553 0.1598379 0 0.9598596 0.1146587
0.09403171 0.8497828 0.2899906 1.509797 0.5076259 0.461241821
0.296514 0 1.446072 0.1850487 0.2288984 0.269231886 0 0.1972382
0.075305537 0.3965505 0.4857502 1.072744 1.692178 0.5518027 0.1897974
0.8512793 0.08402902 0.3514748 0.2116123 1.006467 0.308200879 0

1.197699 0.7036263 0 1.327697 1.065407 0.1773569 1.081399 0 1.005456
AC025280.1 0.05694803 0.5853256 0.1317622 2.069237384 0.481110586
0.530223 0.6517592 0.7147937 0.7045269 6.0855599 0 0.67918096
0.751953 0.408197051 0.940727812 1.838125 0 0.1094492 0 0 0 0
0.1194885 0.1859738 0 0.07369822 0.1206542 0.05919769 0
0.079005913 0.6469838 0.180022961 0 0.2998995 0.288059 0 0 0 0
0.1964406 0 0 0 0.4097885 0 0 0 0 0.05826509 0.2788567
0 0 0 0 0 0 0 0 0 0.2770681 0 0 0 0.09852871 0
0 0.09542069 0 0 0 0 0.4025055 0 0 0.1056566 0
0.7806394 0 0.2403837 0.108482 0 0 0.09050776 0 0 0.2161587
0 0 0 0 0 0.061929252 0 0 0 0 0.1131187 0 0 0 0
0.4966324 0 0 0 0 0 0.3761104 0.152924311 0 0.1022336
0.06747568 0 0 0 0.08957191 0.5571828 0 0.5025031 0 0 0
0 0 0.05158419 0.08747887 0 0.6512178 0 0 0.1326603
0.063867 0.07849834 0.146691309 0.07512781 0.198501 0 0.07180763
0.08566936 0.1294348 0.05962689 0 0.05457058 0 0 0 9.114983
0.08313464 0 0.1198904 0 0 0 0 0 0 0.08117426 0 0
0.08184545 0 0 0 0.098164 0 0 0 0.08426006 0.15486 0 0
0.3233676 0.104937764 0 0.09826113 0.0632229 0.1544281 0 0 0
0 0 0 0.07360437 0.550653 0 0 0.6145035 0 0.09201065 0 0
0.1842654 0 0 0 0.2641978 0.06645559 0 0 0.08879401 0 0
0.497949742 0.1478428 0.3212282 0 0 0 0 0 0 0 0 0 0
0 0 0 0.1251424 0 0 0.08094997 0 0 0.4494495 0.061244 0
0 0 0 0 0 0 0 0 0 0 0 0.06138869 0 0 0.2249389
0.083098725 0.1388486 0 0.2844812 0 0 0 0 0 0 0.1615052
0 1.24419866 0.06876514 0 0 0 0.1357257 0 0.09244555 0
0.05531533 0 0 0 0.1418014 0 0 0 0 0.07315564 0.3733916
0 0 0.08080531 0.1241053 0 0.0923323 0 0 0.06668858
0.1476673 0.8051186 0.08210241 0 0.3135054 0 0.2486053
1.967496 0.1640226 0 0.05746103 0 0 0.0434149 0 0 0.07600689
0.817717981 0 0.1675797 0.0556623 0 0.2007784 0.4952169 0
0.2476899 0.2827337 0 0.05692542 0 0.08689987 0 0.09343221
0.1464181 0 0.054516138 0 0.05884189 0 0 0.1782744 0 0 0
0.0846958 0 0.05642653 0 0 0 0 0 0.082946869 0 0 0 0
0 0.3056496 0.08992243 0 0.2792281 0.09558595 0 0 0.068411967
0 10.93171 0.09975609 0 0 0.1524035 0 0.125172 0 0 0 0
0.05708349 0 0 0 0.1289029 0 0.2556822 0 0.1000503
0.1392008 0 0 0.09059918 0.06892919 0 0 0 0 0 0 0 0
0 0.5811343 0 0 0.2811508 0.108088 0 0 0 0.1724707 0 0
0.1488168 0.12169688 0 0.07891477 0.08294358 0 0.06039395 0
0.149989831 0 0.099345381 0 0 0 0.0496083 0 0.08346217
0.07018961 0.1108537 0 0 0 0.06776468 0.210615 0 0 0
0.1811936 0.06388718 0.05849366 0 0.09232306 0
AC024267.6 0.04943736 0 0 0 0 0.03835781 0.08082871 0.03878263 0

0.04295114 0.04698552 0 0.07532084 0 0 0 0 0.09501432 0
0.1197738 0.052475051 0.214288 0.1037296 0.08072318 0.06712766
0.03198921 0.1309269 0.05139032 0.1245164 0.034293051 0.03120307
0.078140184 0 0.3254335 0.04167799 0 0.06729011 0.148566 0.2550927
0 0 0.09738104 0.05899125 0.03557429 0.1443421 0.1665582
0.03103098 0 0 0.06051983 0.1037649 0.06284924 0 0.065604466
0.03899941 0.06550631 0.03693903 0 0.05308569 0.08017552 0.3337834
0.2527189 0 0 0.03925951 0.045146106 0.08283599 0.3353134
0.5438931 0 0.1140094 0 0.09113978 0.041508785 0.02293048
0.08511482 0 0.08457836 0.2086803 0 0.1132517 0.06119474 0
0.02099224 0 0 0.09665146 0.1552432 0 0 0.03074803 0.053761621
0 0 0.07029677 0.060394042 0.1472998 0 0.05156672 0.07322547
0.1270708 0 0.1352301 0.03597798 0.1406406 0.09702493 0.027220581
5.55061 0.033188916 0 0.4881269 0.02928828 0.1522644 0.07423954 0
0 0 0.06862831 0.06231853 0 0 0.03756553 0.2676398 0.07052576
0.1119523 0 0 0.1211423 0.051426426 0.1213508 0.3742836
0.05544381 0 0 0.03260973 0 0 0 0 0.2247282 0.05176291 0
0.02368673 0 0.0373225 0 4.182501 0 0.03680037 0.05203923
0.0507427 0 0.1205099 0.088268095 0.05609306 0.03891251 0
0.1057027 0 0 0.07105114 0 0 0 0.04260875 0.04182225
0.08662515 0.2025052 0.0731473 0.1680451 0.08245653 0 0.05614396
0 0 0 0.5214042 0 0.1229571 0 0.8183478 0.269838 0.03144056
0.0742275 0 0.1274745 0 0 0.0296366 0.04768039 0 0.3864499
0 0.1599633 0.03156637 0.1265761 0.1880115 0.07645121 0
0.031156652 0.03559938 0.03854164 0.02472723 0.1059407 0 0.0855629
0.1859084 0 0.02585885 0.06540857 0.07439495 0.03538407 0.03779918
0 0.05297993 0.2071653 0.05331069 0.1506281 0 0.051264447
0.1086378 0.034330538 0 0 0.084920493 0.06940708 0.04877165
0.425334 0 0.3503951 0.05592864 0.08562061 0 0.05889497 0.06103837
0.03347572 0.02130157 0.1004673 0.03620947 0.06992377 0.02664618
0.05584814 0.08131135 0.04881812 0.072139127 0.06026817 0.069106187
0.123481 0.0935943 0.02515128 0.04510423 0.08631969 0.06104714
0.09021614 0.03505122 0.1953848 0.031767814 0.1790878 0.08710115 0
0.08874166 0 0.03114497 0.04012662 0.1521633 0.09603997 0.6263992
0 0 0 0.425879 0.179697233 0.02401757 0.0705178 0.0635074
0.04630662 0.08393018 0.07872439 0.07014818 0.05386874 0 0.1202324
0.069900071 0 0.1157865 0.064096 0 0.1425484 0 0.03401978 0 0
0.3914189 0 0.2192444 0.02494135 0.07674723 0 0.07537812 0
0.07976459 0 0.129067643 0.1026546 0.03636954 0.07248179 0.04039926
0 0 0.4014716 0 0.06136124 0.2642439 0.1235443 0.026729522 0
0.05754721 0.1216646 0 0.03574205 0.094652392 0 0.02554072
0.08250885 0.1780154 0.02579374 0 0 0 0.07352557 0 0
0.026836375 0.05409107 0 0 0.091154034 0 0.03630709 0.04772572 0
0.07542307 0.03873443 0.06633464 0 0 0.06060043 0.1244692

0.02286212 0.06224862 0.089084031 0 4.009844 0.0432998 0.02633344
0.1670535 0.03307589 0 0.02716587 0.3230682 0.2690928 0.06048634
0 0.09910989 0.06631252 0.1335883 0.06370863 0.1398779 0.04652048
0.1479741 0.04025221 0 0.03021052 0 0 0.03932519 0.02991918 0
0 0.0602338 0.1398928 0.1234271 0.08689143 0.05331715 0
0.05291299 0.1008981 0 0.1072697 0.03050885 0 0.1030567 0
0.02692227 0.2620173 0.2490821 0.07204512 0 0 0 0 0.03600222
0 0.05242881 0.030833568 0.03255205 0 0 0 0.1192075 1.228551 0
0.02430568 0.07245464 0.06093255 0 0 0 0.03201801 0.029413718 0
0.3048122 0.04029112 0 0.1048644 0.1109227 0.1015583 0.2549779
0.04007345 0.1644987
AC010976.1 0.1477962 0.03314366 0.0901531 0.040205103 0.068106399
0.1667973 0.1449856 0.08010619 0.009498418 0.07937777 0.1174809
0.114458808 0.2756706 0.115569401 0.054254518 0.3469418 0.1013623
0.05164572 0.06863102 0.2213533 0.182548416 0.167728 0.4567019
0.231089 0.3648772 0.2017004 0.1622591 0.2122952 0.1398757
0.048464607 0.1322931 0.336957679 0.03468529 0.2193457 0.1676423
0.099443671 0.1207009 0.2853312 0.3535765 0.2224663 0.01780039
0.07763384 0.1988037 0.3828659 0.2039913 0.2426308 0.07084187
0.05119011 0.1787078 0.2434303 0.1015238 0.09565403 0.1294893
0.124809222 0.1823063 0.2100779 0.3975538 0.1413367 0.3376048
0.2222579 0.7153539 0.1255928 0.1796486 0.1766722 0.08109119
0.211039608 0.00450261 0.4951451 0.1300803 0.076743034 0.1032844
0.05697899 0.1618296 0.194036619 0.3440071 0.0601442 0.180087
0.3953693 0.3554131 0.03071359 0.2790665 0.1397039 0.01281235
0.06389869 0.00521907 0.09179881 0.09719085 0.2497754 0.01659612
0.1485854 0.1437345 0.16072383 0.07017074 0.0217446 0.1566623
0.164138074 0.3042502 0.0225217 0.1962062 0.2786156 0.1326149
0.2879103 0.2401173 0.1760048 0.1773552 0.08438175 0.180510493
8.660777 0.08659235 0.04724546 0.3039176 0.07641529 0.1820818
0.129131 0.08330951 0.05071948 0.02629175 0.1044495 0.1456568
0.1515405 0.1074276 0.1756035 0.282642 0.1610061 0.3480763
0.09081291 0.05658665 0.09218691 0.139766013 0.08179176 0.1377163
0.06931482 0.06667373 0.041531478 0.1949778 0.1592331 0.08234969
0.2202448 0.09297691 0.1893366 0.1800711 0.2100905 0.1596511
0.1243368 0.1136067 0.04876802 2.72197 0.1608376 0.1280199
0.1640605 0.03861418 0.04869741 0.1397419 0.139138392 0.1646448
0.1395977 0.180765754 0.2757862 0.1782053 0.06142073 0.1737916
0.1237908 0.2049528 0.180150483 0.09727322 0.1682226 0.1600915
0.192628 0.2067506 0.27768 0.2435211 0.007547356 0.07324189 0.168357502
0.3440978 0.1390992 0.3430789 0.08380036 0.1550553 0.1204732
0.4377017 0.1515615 0.1333 0.1976998 0.2014435 0.2459783
0.06669167 0.2104873 0.1256516 0.1244017 0.04775871 0.5041383
0.097075582 0.1304238 0.08922225 0.06880134 0.2486745 0.1496002

0.3010402 0.128709203 0.1199718 0.09636809 0.0994609 0.1266867
0.117483605 0.1651046 0.2324192 0.2829827 0.2248922 0.1777665
0.3261991 0.06923975 0.1068393 0.4394131 0.1727857 0.3513312
0.09562544 0.2742812 0.208947086 0.15047185 0.6259392 0.208998831
0.056111538 0.05729671 0.0969342 0.09054412 0.3181221 0.3063312
0.2682489 0.3754778 0.2097629 0.2544170.22387981 0.1600639
0.2720583 0.1746813 0.3010441 0.2129780.7675948 0.1824363
0.2722939 0.07892731 0.06776929 0.1406378 0.180374002 0.1375886
0.060101061 0.4322462 0.1424468 0.08476119 0.08826022 0.2486743
0.2422331 0.2549957 0.08001983 0.2718791 0.151955084 0.2238923
0.08522001 0.1398929 0.3312217 0.04162912 0.07787372 0.1526778
0.3101604 0.1252877 0.2587677 0.06922966 0.1505170.04014704
0.0385816 0.38744702 0.1958238 0.1609879 0.1795035 0.1258515
0.2372284 0.1198154 0.1105757 0.1141949 0.2419515 0.3006246
0.273561729 0.127647816 0.1195795 0.1463273 0.1139732 0.3874161
0.03875194 0.1035535 0.1147488 0.02737215 0.4603321 0.08513697
0.2240431 0.1301477 0.3337321 0.4340250.3216327 0.0461710.1098368
0.09324983 0.210466928 0.5021876 0.1186135 0.07091639 0.008783724
0.04737059 0.04673553 0.9329024 0.07597023 0.2401440.2489615
0.4647016 0.113326381 0.07380969 0.2502416 0.1366722 0.1070898
0.09713923 0.192933699 0.1524573 0.05553137 0.2451706 0.1306281
0.1289874 0.07328458 0.148927092 0.1136326 0.3397055 0.1321991
0.1038412 0.195467197 0.2587340.02261133 0.05374129 0.222963429
0.156560334 0.06907243 0.2412574 0.08257436 0.1352893 0.2189655
0.07211334 0.2673193 0.0853464 0.1383472 0.1353122 0.1540932
0.09473992 0.109757131 0.05758483 6.2045370.1741658 0.2376079
0.1416528 0.2588923 0.6188877 0.0502051 0.2165808 0.25353
0.3222023 0.1681851 0.07542065 0.1009250.5228127 0.07156719
0.1459808 0.0657450.5791131 0.1531556 0.1416320.1609272 0.02841509
0.1424325 0.04275098 0.1853958 0.1574153 0.06485568 0.2946646
0.3041590.2381684 0.3022749 0.2811149 0.02686424 0.1092927
0.1261410.3851221 0.3926020.1392998 0.05610376 0.1493792 0.09278822
0.1463379 0.4842326 0.1940595 0.3524458 0.1018220.189502583
0.07119565 0.05957993 0.5870779 0.0345581 0.06554552 0.134078457
0.130934862 0.05320538 0.084380469 0.1036789 0.5486068 0.5342302
0.4073103 0.08191146 0.1220880.1291693 0.03138507 0.1221603
0.1580755 0.2262470.118311475 0.05466052 0.4721961 0.1095026
0.02346719 0.2536492 0.2532298 0.1987298 0.5108217 0.06534663
0.2384385
UBL7-AS1 1.5239430.2669906 2.4942360.592227555 0.658361855 1.370518
0.8494105 0.9917240.8569684 0.3987047 0.5925122 1.150692554
1.6534171.266126992 0.492674864 1.10395 1.8916281.4478021.95184 1.401326
0.753646064 2.3119541.1718261.0368132.2691312.3531711.80699 1.512138
1.5120611.117171331 0.9290671.277355906 0.8433081 2.92972 1.722739

0.942440018 1.319988 0.7632758 1.831819 0.8960446 0.6309249 0.909649
1.260515 1.009374 1.264049 2.567139 1.31526 1.704441 1.727509 1.674771 1.041977
1.695202 2.086216 1.263941013 0.8606554 2.673251 2.316164 0.9019066
1.199411 1.221692 1.803936 1.466994 2.583727 1.54304 1.127689 1.154446644
0.5368112 2.094663 1.803605 0.885013233 1.983519 1.315793 2.373134 1.512182024
1.622539 0.9540824 0.698974 2.207223 1.230502 1.830872 2.15547 1.050367
0.633025 1.580986 1.362177286 1.446115 0.8294787 2.077336 0.7353004
4.061335 2.358806 0.725043055 2.575639 1.775003 2.388569 1.18471214 1.685534
0.7111852 3.576559 0.4873562 1.22853 0.3883441 0.9631917 2.142478
0.9656003 1.223535 1.506555702 4.57491 1.081201706 0.6850592 2.518174
2.226303 1.493438 3.978848 2.063646 1.593437 0.9177767 2.692477 1.364357
1.154157 1.201364 1.736978 1.848257 1.894022 1.749036 1.409893 0.9116738
1.598394 1.54922861 1.725835 2.047313 0.8448372 1.157736 1.048285259 2.52447
1.373255 1.393082 1.299256 1.849657 1.594092 0.8975417 1.511349 1.476916
2.178484 1.098198 3.42219 2.078856 0.2528071 1.508234 1.075508 2.292947
0.8761025 1.083485 1.700580345 2.456121 1.881044 1.472653503 1.407021
2.970949 1.622872 1.455987 1.684166 0.9509809 0.758184346 1.776137 1.992402
2.397186 2.19015 1.39645 1.77772 1.261264 1.264601 0.9243398 0.973282419
0.9008672 3.09264 0.9612839 1.338377 1.576396 1.175666 1.318641 2.158257
1.288566 1.820093 1.219853 2.031727 1.883495 1.32879 0.5086923 1.252656
2.196418 1.68461 1.51587255 2.143298 1.238435 1.463175 0.7354254 1.365794
3.243499 1.484295603 1.683476 0.6345396 1.541793 1.874067 1.009488755
0.854203 1.400129 2.640233 2.06526 1.157062 1.667839 1.363425 0.9599554
2.041858 0.5196371 1.53845 3.118613 1.925881 1.774994942 1.768818274
0.8562375 1.443087169 1.511003548 1.944693 1.085765611 2.273244 1.178819
1.415417 1.379939276 1.753439 0.5779456 1.419633 1.62312862 1.34098
2.116746 3.224729 0.6790217 1.425314 1.445965 1.910514 2.249483 0.5086427
2.269125 1.590357 1.08660087 2.448932 1.404027565 1.574462 1.745821 1.136529
1.169175 2.056125 0.8660661 1.09027 1.00681 2.039566 1.70258764 2.049282
1.449266 1.535422 1.802961 1.145335 1.331001 2.263024 1.159311 0.9167464
3.540022 2.258618 0.8551303 0.3449672 2.038823 1.300895876 1.859311
1.630322 1.256908 1.265227 1.837506 2.743867 2.408093 3.056909 2.965346 1.431961
0.954933074 1.85851017 1.815021 1.234879 1.5642 1.884995 1.276422 1.418108
1.232488 0.8819916 5.459513 1.421529 2.07359 1.424093 1.245624 1.374412 1.50505
1.081987 1.275969 1.398349 1.254616743 1.414392 1.248517 1.946553 0.9198511
0.8242483 1.091794 3.216967 1.186304 1.977482 1.75869 1.609891 1.301477609
1.123092 1.370768 1.377989 0.6790033 0.7136495 0.936657381 0.9357171
0.8409918 2.620468 0.8418256 1.454691 2.179747 0.594132421 0.9369118
1.519571 1.38154 1.655838 1.522893687 0.4926399 0.7103726 0.8658319
1.708285238 1.185509642 1.348118 1.487898 0.7602084 0.7001333 1.424679
1.06888 2.064535 0.6531371 1.337356 0.5813414 1.137197 1.384631 1.52906748
0.8614874 9.41094 0.6370389 2.509038 1.697009 1.065933 1.653722 0.9706319
2.640594 1.084003 1.048801 0.7564436 0.8939741 3.832747 1.825004 1.093516
2.302915 1.531802 1.723487 0.7261522 0.9279516 2.306989 0.5493584

0.9051497 1.859667 1.110929 1.711892 0.9553344 1.054972 1.215284 1.902365
1.734932 1.213979 1.70961 0.6394582 0.6892049 0.9859594 3.118789
0.6732822 1.166845 1.588399 1.207436 1.942717 2.202784 0.203587 1.009474
1.640466 1.794851067 0.9261294 1.52384 3.821225 0.1484718 1.175386
1.080076456 1.345522814 0.8835732 0.860993303 1.861283 1.503269 2.366936
2.00638 0.8854658 1.535509 1.088555 0.7584725 1.351259 0.6260815
2.142192 0.906699349 1.184863 2.108775 1.002071 0.6238362 1.359129 1.078236
0.9516338 1.862893 0.9264701 1.478981
Z98885.3 0.06741713 0.06929296 0.009749055 0.018012043 0.071194509 0.06538511
0.02755629 0 0.01489365 0.007321495 0.0480552 0.028715676 0.1198332
0.04832384 0.015467605 0.06800119 0.05297918 0.01619624 0 0.1306673
0.035779801 0.1022777 0.114932 0.1742953 0.03432795 0.2181165
0.03570869 0.2102411 0.1981018 0 0.06382689 0.071039226 0.009888547
0.1331369 0.07104473 0.027516888 0.09176272 0.05064941 0.1956751
0.1453459 0 0.02213289 0.04022283 0.2183051 0.06561255 0.1249234
0.03173744 0.04013339 0.07759836 0.04126508 0.07075145 0.04999561
0.01504007 0.08946403 0.05318303 0.08933018 0.1259333 0.07821806
0.01809808 0.0410004 0.1950756 0.06154103 0.05496419 0.07290115
0.02676886 0.369391243 0 0.1905262 0.09889341 0.03800025 0.1036488
0.1191251 0.1035719 0.14151259 0.1016276 0.08705255 0.1155186
0.2450943 0.1304302 0.03210622 0.14157 0 0.02678659 0.150291
0.016367146 0.007996766 0.1070895 0.317555 0 0.04016965 0.08386145
0.073314083 0.1052448 0.151537 0.1437943 0.061768978 0.1506532 0
0.07032092 0.02496418 0.1472922 0.05249387 0.1844117 0.07359412
0.01917899 0.01102598 0.074240766 5.593491 0.045259329 0 0.257184 0
0.1557309 0.2024791 0.03265761 0.02650961 0 0.08188916 0.07436016
0.1008075 0.07926962 0.1024553 0.06517449 0.2324232 0.1984685
0.01294508 0.01183049 0.05506679 0.052597204 0.03309693 0.1276015
0.07560806 0 0.021707308 0.1334085 0.1028093 0 0.1275127
0.06338655 0.0574611 0.03529423 0.03064417 0.0888286 0.04874044
0.06362031 0.05097928 3.757457 0.07381334 0.05018423 0.05322396
0.06919722 0 0.01369483 0.090277613 0.07649343 0.1061291 0.021391937
0.1081092 0.09719246 0.02568228 0.03633435 0.0143782 0.051419
0.092237851 0.05810504 0.02851625 0.05906485 0.08629809 0.0997501
0.04583221 0.1311858 0.01183436 0.02871107 0.062114556 0.1011656
0.02908127 0.03742278 0.0114261 0.2179776 0 0.2566734 0.107326
0.06431267 0 0.02178388 0.04345886 0.01230276 0.06735651 0.04041508
0.03251059 0.01361569 0.1903045 0.070253464 0.1363375 0.09685502
0.02876838 0.2136575 0.1042556 0.157345 0.053109944 0.01213661
0.03941908 0.03372023 0.03611753 0.02456212 0.02917028 0.07922533
0.06622697 0.05289512 0.1895434 0.2705374 0.03618962 0.01288657
0.1450538 0.01806203 0.1977562 0.05452436 0.07702865 0.079425911
0.034954358 0.2222222 0.03511211 0.025138188 0.02395787 0.014475633
0.0118312 0.08313665 0.06344 0.162343766 0.1023921 0.1144038

0.04864992 0.191479963 0.08031438 0 0.04565044 0.1307191 0.01712576
0.08641224 0.09535422 0.1726011 0.03807972 0.01848055 0.02496476
0.049187635 0.06164024 0.023559821 0.1431311 0.06381671 0.1543433
0.09226215 0.02942829 0.1144677 0.1230267 0.01194973 0.06661097
0.064982085 0.1933408 0.1336261 0.06854814 0.07059263 0 0.05309003
0.0273601 0.03890686 0.05729875 0.498291 0.01356913 0.03726517 0
0.01209929 0.112315032 0.09006931 0.02404107 0.03247661 0.03946737
0.4005910 0 0.0459126 0.2167905 0.08197973 0.178728554 0.059304746
0.1480282 0.04370347 0.02647579 0.2551390.06751507 0.01159809
0.01893976 0 0.2790179 0 0.1943376 0.05952138 0.08721607
0.1408049 0.2762542 0 0.1903545 0.03374239 0.055002503 0.174986
0.02479835 0.09060567 0 0 0.04885463 0.3592851 0.03665304
0.1882746 0.09008656 0.2105949 0.027338048 0.0257188 0.07847644
0.02765211 0.01083344 0.0243705 0.088739985 0.06071243 0.06095176
0.1781509 0.01517234 0.01758731 0.05303595 0.055599932 0.06288616
0.3007978 0.04481944 0.05009983 0.064043781 0.03688167 0.01772744
0.03064257 0.108767463 0.08592106 0.111401 0.08135375 0.01233121
0.1414236 0.0660271 0 0.1596801 0.01115203 0.1342901 0.01414476
0.03897101 0.03183289 0.040494278 0.01289911 2.8935770.05904743
0.06284355 0.1366853 0.09021039 0.2721922 0 0.01835684 0.2140592
0.02062113 0.02197638 0.3547818 0.1356444 0.03036213 0 0.06676246
0 0.1135072 0.2470116 0.07402695 0.0308983 0.01485175 0.01240756
0.02681365 0 0.1234147 0.0290556 0.04107005 0.09538508 0.1157173
0.3851016 0.06361947 0 0.04509801 0.05159758 0.0496337 0.0121902
0.04160456 0.01599481 0.09369147 0.06715071 0.01835679 0.1148496
0.09907049 0.1350897 0.04404368 0.054025934 0.04134658 0 0.0736437
0.01806253 0.008937068 0.021023685 0.011097711 0.01283489 0.073505404 0
0.09482773 1.0471 0.7561243 0.05800439 0.03705207 0.03115987
0.01640407 0.005717883 0.04131076 0.1746503 0.040111137 0 0.2857723
0.09615291 0.03679688 0.1787530.1796259 0.06924691 0.2111098
0.01366192 0.09346869
AC026356.1 0.3382470.1121479 0.5806471 0.90953518 0.345676361 0.7449952
0.6243824 0.7532461 0.5592307 0.4360633 0.2281412 0.855143097
0.6206245 0.500545241 0.280377404 0.5634928 0.5144680.3564963
0.5209941 0.1797578 0.833877666 0.1324259 1.0416720.2494269
1.3778480.8895923 0.5316964 0.9527470.2198535 0.726598227 0.9641451
0.091979307 0.2816742 0.5889699 0.3679457 0.237519887 0.4009886
1.5301820.4504068 1.3926 0.5240096 1.3038920.7811885 0.8165571
0.9344832 0.5440573 1.0821050.5542766 1.2168260.4541440.3969624
0.3883962 0.1947340.796366666 0.5508774 0.5493937 0.7500501
0.5963927 1.1013421.0440250.2525776 0.6055782 0.4625782 0.3964383
0.4159135 0.757270196 0.3839329 0.4933745 0.4161431.754853604
0.2851775 0.1156795 1.3007851.39251726 0.2935337 0.7326335
1.2962720.1120024 0.5373352 0.1454954 1.0998020.3511595 0.7109895

0.7969006 0.635749654 0.8179625 0.7892728 0.9319627 0.3200899
1.393878 0.0678632 0.996709553 0.4211908 1.569642 0.4033905 0.613153075
0.6718765 1.18882 1.229165 0.517165 0.7852735 0.3534303 0.2188728
0.5558426 0.446982 0.8851171 1.658148627 1.729498 0.571353173 1.151021
0.6855751 0.2973505 0.2912511 0.8083374 0.4016983 1.115523
0.2846819 0.1817613 1.196612 0.1678144 0.7184503 0.4145496
0.8776127 0.9131795 0.6918439 0.3855001 0.6586625 0.3386689
0.771813263 0.3106831 0.7116926 0.6607905 0.7520107 0.843177751
1.669753 2.262941 1.811198 2.297632 1.116164 0.4215931 0.5026762 1.031604
0.6064291 0.1893227 0.8731595 0.01100106 11.40165 0.07964262
0.2274192 0.8269521 0.347178 1.021617 1.560383 0.545479915 0.321884
0.1717656 3.863814272 1.446421 2.23369 0.04987889 1.129069 1.154219
1.065211 1.030054474 0.6770929 1.310728 1.472149 0.4245968 0.5650453
1.157171 0.3761079 0.8274283 1.25153 0.743921363 0.4147891 0.4518419
0.6056726 0.5621774 0.0651301 1.593305 0.2745355 0.2382211
0.8743348 1.900377 1.452561 0.4782876 0.3982304 0.4534974 0.8895784
0.6734987 1.427962 0.1705847 0.45480964 0.8296695 1.142577 0.5587257
0.4564509 0.3880871 1.502474 0.123777089 3.205675 0.1531157 0.8186225
0.6546935 1.081276275 1.104736 0.5334077 0.4973416 1.32408 0.3175955
0.3064977 0.4217145 0.7258027 1.145647 2.408772 0.6035424 0.9177531
0.6150277 1.311186007 3.009642351 0.2637489 0.666776459 1.139184127
0.3101989 1.986713992 0.5974277 0.9041983 0.8566036 1.834450845
0.3756262 1.62939 0.6047074 1.012348593 0.883902 0.4580351 1.773202
0.8180461 1.219564 0.9909708 0.7716348 0.3999087 1.207958 0.5144526
0.4202064 0.748317183 0.5054621 1.082909732 1.896821 1.105147 1.054704
0.7764774 0.4381822 0.4446274 0.1194682 0.6188851 0.08624574
0.729184875 0.4743122 0.07689549 0.9230409 0.3917189 0.3770714
1.292298 0.7616369 0.8563811 0.7842792 0.2765019 3.513775 0.5629137
0.7607327 0.986944 1.123714643 2.385386 0.4669141 0.4485298 0.429249
1.333729 0.3822513 0.7276646 0.4517908 0.94734 0.4776513 0.478251443
0.518304689 0.4344341 1.188303 0.73702 0.8337297 0.5070148 0.8259262
1.593969 1.587752 1.052376 1.885595 0.425822 1.034891 0.7227173 0.9115484
1.43074 2.062214 0.6689763 0.7135798 0.56972379 0.4380281 0.4174055
0.9705011 0.1961612 0.5578001 0.6009264 0.2436714 0.8305005
0.2979434 0.6026461 0.5344362 1.321466585 0.8158469 1.320914
2.953752 1.052009 1.419936 1.044524375 0.6386941 1.66856 0.3763465
1.571573 0.1252431 0.4349052 2.217261377 1.343479 0.3732357 0.8704616
0.7351666 1.492592929 0.5491618 1.928045 0.6943128 0.42248585
1.573362135 1.730861 0.5266711 1.181488 0.3495751 0.5471345 1.317649
0.3101231 1.025189 1.618371 1.153794 0.7669678 0.4533782 0.445660863
0.651353 5.752516 1.20413 0.6393185 0.4719353 0.7884102 1.057277
0.8393995 1.283464 0.277157 0.4672425 0.04268146 0.7437259 0.6439693
0.4717429 0.6936745 0.3704652 0.5133708 0.8817926 1.545809
0.5367472 0.2533721 0.711494 0.2570386 1.892101 1.082955 0.3395609

0.7147848 1.80799 1.407918 0.8853417 0.55615 0.5883746 0.3545109
0.280279 0.7794138 1.413811 0.489288 1.481377 0.8283828 0.6520343
1.478058 0.4753556 0.9583118 0.6230417 0.302117 0.7698556 1.608874013
0.7601859 0.2268 0.8581635 0.04677356 0.3124286 1.483533166
1.681168264 1.728293 0.361655256 1.182755 0.4384996 0.813451 2.09108
0.7402933 1.599128 1.143103 0.7009023 0.6959131 1.885447 0.4946629
1.272397978 0.5447743 0.4204645 0.4979828 1.262551 0.5207481
0.5753142 1.17677 0.6592274 0.2653353 0.7382236
AL121906.1 0 0 0 0 0 0 0.2750932 0.05279726 0.1784191 0
0.06396438 0.114666915 0 0.04824147 0.061764957 0 0 0.2586981 0
0.0652223 0.071437626 0.1166896 0.1059102 0 0.1370777 0.4790384
0.1425913 3.7079280.1130081 0 0.04247873 0.709181363 0 0.2215167
0.0567389 0.109879938 0.5496379 0.06741744 0.2604554 0.1160785
0.1114547 0 0 0.3390068 0.06550071 0.4081432 0.04224445 0.10684
0.1032881 0.08238949 0.09417447 0.3707629 0.06005774 0.044655767 0
0.04458895 0.2011498 0.03470433 0 0.05457402 0.06491436 0.1474467
0 0.1164430.2672323 0.061460267 0.3383097 0.2282418 0 0.050580636
0.05173607 0 0.2481488 0 0.2809504 0 0 0.1151419 0.3314381
0 0.1541768 0 0 0.02857807 0 0.2554603 0.1315778 0 0
0.06416189 0.1255778 0.109783674 0.2674393 0 5.789818 0 0.1336857
0 0.07020105 0.04984325 0.03459792 0.2096176 0.2454632 0.1469374
0.03829259 0.2641724 0.185285547 1.777976 0.135546548 0.07395525
0.1208215 0 0.06909576 0.202134 0 0 0.2194962 0.04671404
0.08483818 0.1150122 0.3956725 0.05114035 0.05205072 0.4480523
0.3352972 0 0 0 0.070010067 0 0.07839017 0 0.7421662 0
0.0443937 0 0 0.1272953 0.0506228 0.07648421 0.03523407
0.06118386 0 0.1459721 0.101619 0.03392826 0.6155573 0.04912502 0
0 0.03453963 0.2540935 0.9296609 0.060082487 0.07636305 0
0.128132844 0.2398331 0 0.102554 0.09672644 0.2870738 0 0.092080627
0 0.1138706 0 0.1378416 0.09958007 0.09150818 0 0 0
0.124017357 0 0.05806339 0.2241539 0.1825059 0.03347786 0.04310438
0.08912552 0 0 0.05052531 0.1304805 0.1518467 0 0.1075867 0
0 0.1631098 1.286019 0.093511618 0.3266523 2.105692 0 0 0.2081558
0.03926921 0.042415533 0.0969274 0.2098767 0.1009882 0 0 0
0.1265445 0.05289127 0.1760165 0.1335673 0.2363167 0 0.1286461
0.1158453 0 0.2820272 0.07257523 0.1537947 0.079290526 0
0.1478956 0.046736346 0 0 0.346823013 0.04724414 0.1327919
0.6876031 0 0.13629 0.07613922 0.0388536 0.509742872 0 0.1661908
0.09114525 0.2029942 0.6154766 0.04929426 0 0.3627514 0
0.1106943 0.06645921 0.049103792 0.1640938 0.09407865 0.4370665
0.3503936 0 0 0.1762687 0.124661 0.06140849 0.0954349 0.3191877
0.086495094 0 0.05928817 0.1094901 0.4026989 0 0 0 0.6732361
0 1.19386 0.3792881 0 0 0.09662937 0.040772213 0.0653933 0
0.2161417 0 0.228519 0 0.1432458 0.03666747 0.2164209 0.49104 0

0.059203659 0.4334758 0 0.1057226 0.1940602 0.10784 0.1389399
0.1764698 0.04896771 0.2422107 0 0 0 0.1393079 0.1874199
0.1539256 0 0.1085886 0.04491316 0.395342991 0.1397502 0.1485365
0.03289136 0.05499809 0 0 0.341593 0.1463622 0.2506049 0.05995533
0.269102 0.072777198 0.05134992 0.5483987 0.2208398 0 0.04865792
0.161070406 0 0.03477021 0.3369736 0.06058593 0.03511466 0.4941585
0 0.1255579 0.1501426 0 0.0666859 0.073068131 0 0 0
0.062046894 0.343098414 0.04942715 0 0 0 0 0 0.106272 0
0.08249922 0.2259304 0 0.169486 0 0 2.274523 0.1768403 0.03584939
0 0.09005662 0.09451417 0 0 0.06105552 0.04117195 0 0.2023869
0.1805509 0.1818623 0.02891021 0.5331893 0.06333128 0 0.2465905
0.05912061 0 0 0 0.1070718 0.04073089 0.2464087 0 0.0820001
0 0.08401459 0.1774362 0.2177521 0 0.1080507 0 0.06606546
0.7788432 0.08306729 0 0.5144247 0 0.03665099 0.05095726
0.1130304 0 0.4836546 0.503382551 0.09906264 0.04663145 0.2940727
0.1442539 0 0.041975699 0 0 0 0.1236517 0 0 0.08794199
0.2977998 0.2959113 0 0.06550444 0 0.04124035 0 0.120128297
0.1244543 0.1556101 0 0.07346832 0.03568966 0.2265091 0.1382578
0.1487647 0 0.1119712
LINC-PINT 0.9411876 0.8494027 0.7777327 1.303736824 0.761891626
2.636044 1.999924 2.006647 0.4115035 1.817751 0.7667226 1.044827663
1.329033 2.378844031 1.089470919 4.519791 1.417398 1.934936 0.9868604
0.3909004 0.918958429 0.7164196 0.6743226 0.3105742 1.282428
0.8530385 1.063039 1.418122 0.4845705 0.696091964 1.639312 0.867353153
0.6195443 0.6130835 0.6192897 0.27484181 0.8882667 0.9066638
1.023746 1.244341 0.8526347 2.278127 0.6261043 1.198779 1.34048 0.6938485
0.3828667 0.7704782 0.3959187 2.296322 0.2202619 0.6892861
0.6672195 0.892126291 1.56255 0.5562015 1.181059 0.4295189 1.729012
0.7392574 0.9552404 0.795091 0.3636158 1.66244 1.697976 1.695023785
0.3956313 0.9156631 0.7263875 1.261883241 0.7613166 0.06953583
0.3063158 2.924176675 1.271623 1.592184 1.288675 0.3254066 0.3045393
0.9807829 0.811349 0.8930502 0.4065332 0.2924267 0.235661531
0.6348316 1.67656 0.589044 0.8911548 1.194278 1.346171 0.952187967
0.6515686 0.6014961 0.6062026 1.177824278 1.055273 0.4672442
2.209739 1.185198 0.6271305 2.823128 0.909002 0.4152648 0.4440753
0.5105968 1.859831895 14.9986 0.84540217 0.922516 0.4592019 0.5634182
0.7272271 1.158932 0.6672026 0.3713812 0.2566866 0.4188227
0.6944893 1.120827 0.6092393 0.7226477 0.5275395 0.1840126
0.3624032 0.5591672 0.2301901 1.323246 0.859658493 0.3091099
0.5653121 0.5700643 0.5243655 3.454962075 1.566119 0.7601511
1.013351 1.174369 0.8485346 1.870856 0.3055961 1.097109 0.4399488 1.10484
1.084386 0.6678944 20.85328 0.4978872 0.2245842 2.430203 0.3837227
1.327252 0.9113112 0.40986503 0.1637197 1.218345 1.356912153 1.795003
0.5389661 0.7295758 0.6127079 0.7385712 3.211535 0.883892444

0.1921971 0.5714966 1.206709 1.044421 1.251864 1.413462 2.136833 0.1473699
1.23646 1.553032423 1.148243 0.3168731 1.773042 0.3512683 2.410999
1.310602 0.590617 0.8949778 0.8968044 1.792278 1.089312 1.179605 0.8521902
0.6552901 0.7077338 0.1960968 1.31403 2.500831 0.829281329 1.856938
0.1507635 0.3862323 0.490551 1.592403 1.576683 2.323037196 1.738038
0.2761169 0.5150438 0.4357062 1.892539882 1.140829 0.6474367
1.536014 0.3087599 0.6508261 0.4342754 0.5492407 0.4488223
0.1693419 2.129723 0.6486314 1.400388 1.30893 1.406331197 1.047384307
1.736749 1.298059306 0.743466777 0.5360806 0.974535539 0.8885874
1.391153 1.56942 2.222633747 0.5976838 2.556187 0.7799978 0.788605529
0.5078795 0.9879428 5.080717 0.7997736 0.8663778 0.6869534
0.7421367 0.8307529 2.067198 1.528227 1.068646 1.373383577 1.695091
0.788468682 1.805306 0.7543358 0.6373274 1.28056 0.2977504 0.7491626
0.9335722 1.636872 2.913574 3.565551916 1.690876 0.4680023 0.8882882
0.6475291 0.8519306 0.7313583 2.203951 0.6813225 0.9301306
0.9917039 0.1372902 0.9571083 0.9962217 0.8098461 1.088703879
0.5608036 0.5332645 0.7709292 0.9215171 1.09679 0.5169917
0.9353013 0.2894416 0.9701793 0.9889679 1.446676019 0.525030894
0.6490136 0.3996666 1.777264 1.564947 0.6936153 0.762759 1.098183
2.242862 4.73027 0.6611764 2.041901 0.5757557 0.3088531 1.954314
1.252541 0.9630045 0.6807933 0.8184839 1.331333454 1.198478 0.9312455
1.269322 0.3376627 1.722737 1.725307 3.52866 0.3423223 1.00945 0.8705807
0.5015489 0.361709927 1.631369 1.343711 0.769393 1.252094 1.356171 1.274578844
0.6201846 0.4913266 0.4524494 2.113731 0.6228088 0.2958227
3.587342103 0.7953393 1.926525 2.363305 1.13078 1.135752263 0.3731625
0.5863797 1.484597 1.717249134 1.146375337 1.038025 0.3957324 0.9501342
0.3702329 1.526239 1.13527 0.4971127 2.126493 0.9527153 2.845773 1.08888
0.3138213 0.673664688 1.184639 21.9797 1.459112 0.7965472 1.351931
1.728928 1.671739 1.438022 0.5571942 0.5057534 1.873757 0.6071954
0.1610728 0.3958924 2.168115 0.4282424 0.6420878 0.820852 0.9030517
1.999911 1.457654 0.4729436 0.8958253 0.4731801 1.559951 0.6390645
0.7444107 0.8593249 1.73408 0.8240384 0.4093734 0.1383335
0.8558968 0.4835711 0.7441102 2.034678 1.255463 0.507585 1.060465
0.7033502 0.5149944 1.398036 1.003661 2.145284 0.1817499 0.9844889
0.8655436 1.555779389 1.38695 0.6498452 1.089013 1.321449 0.9146708
1.300828295 1.502888143 1.54335 3.340997268 0.5663602 0.2688571
4.237753 0.9884303 0.3901775 1.369778 1.358087 0.5553732 0.8321869
1.028862 0.4545139 0.971668972 0.9824036 0.9199894 2.261095 1.775606
0.5982266 1.754881 1.1621 0.8360256 0.7921585 0.8147572
AC023825.2 0.08813386 0 0.02548972 0 0.16287598 0.06838193 0
0.1382785 0 0.01914266 0 0.037539764 0.06713866 0.063173353 0
0.03555896 0 0.2117321 0 0.04270507 0.140323908 0 0.06934599
0.09593886 0.08975328 0.2566277 0.09336335 0.1374232 0.03699668
0.061135528 0.08344036 0.023217247 0 0.3480976 0.07430094 0

0.02999016 0.1324271 0.05684543 0.2280114 0.03648815 0.4918805 0
0.8878750.2144368 0 0.1106402 0.2448416 0.04508608 0.0539455 0
0.1867395 0.07864704 0.087716686 0 0.0583903 0.3951157 0.1136154
0.1419568 0.1071990.04250345 0.1609041 0.03592712 0.3049698
0.03499471 0.201209206 0.07383744 0 0 0.231827915 0.06774962 0
0.02707973 0.480995398 0.1430766 0.03793436 0.03355923 0.03769527
0.06200373 0 0 0 0.1050537 0.03742366 0 0.1254493 0.1507662
0.3044338 0 0.02100538 0.3288941 0.071882167 0.05003116 0.0198103
0.06266037 0.21533352 0.2188308 0 0 0.03263546 0.3851079
0.1921494 0.2008999 0 0.02507253 0.2017984 0.218372252 1.091392
0.118334288 0 0.1186640.02610666 0.09048254 0 0 0.0346558 0
0.03058658 0.1110976 0 0 0.06696951 0 0.12572900 0 0
0.1799709 0.091679849 0.08653468 0.02566345 0.09884179 0.03037138
0.454044527 0.08720192 0 0 0.02778271 0 0.05007895 0.09227972
0.7210955 0.04222723 0.03185898 0.2994131 0.02221493 5.642609
0.1286608 0 0.04638616 0.04523047 0.1330966 0.03580627 0.078679448
0.04999961 0.03468541 0.195758511 0.2512537 0.1270589 0.03357423
0.03166639 0.03759299 0.4705374 0.060290887 0 0.03727906 0.07721498
0.02256335 0.06520124 0.2097062 0.02449973 0 0.1501350.284206444
0.04408433 0.0380177 0.4158411 0.1792469 0.2849603 0.0282231
0.0583560.1603502 0.1401257 0.03308205 0.05695576 0.04261005
0.03216659 0.03522184 0.02641715 0 0 0.07654877 0.030613923
0.07129316 0 0.07521733 0 0.1022194 0.1285599 0.138860375
0.3490540.03435482 0 0.1416484 0.064219709 0.1144022 0.04142824
0.06926237 0 0.02915159 0 0.03154026 0.05053952 0 0.04722467
0.1477286 0 0 0.207665664 0.159934393 0.1936728 0.153005894
0.197177661 0.06263986 0.037847749 0.03093366 0 0.0236956 0.463048697
0.08923752 0.4486775 0.1017594 0.12516008 0 0.05440771 0.3282314
0.5506392 0 0.0322760.1246558 0.04750316 0.0497813 0 0.02175748
0.064302585 0.3223271 0.061599116 0.04402685 0.1459974 0.04483815
0.3618406 0.1538854 0 0.08041587 0.03124357 0.03483199 0.22653477
0.07981668 0 0.1254574 0.02636719 0 0 0.07153525 0.1695421
0.06420529 0 0.03547763 0.03247763 0.1097273 0 0.026696092
0.02140852 0 0.05660853 0.08255257 0.03740638 0 0.09379188
0.1680592 0.07085209 0.1786190.342687178 0.0387643 0.02580213 0
0.03461158 0.4447214 0.1765238 0 0.4456763 0.09618658 0.2537445
0 0.5862830.02223194 0 0.1840731 0.03359486 0 0.1895992
0.2940743 0.057523451 0.09150311 0.2593495 0.04307201 0 0.2718875
0.09580088 0.1341973 0 0.1640865 0.03925647 0 0.023825868
0.1344879 0.1538874 0.4699418 0.1416247 0.09557806 0.274203192
0.2381066 0.1138310 0.3570242 0.02299174 0.0462223 0.232593049
0.1644211 0.1310768 0 0.3056437 0.143526687 0.0482151 0
0.2002939 0.853144789 0.51348062 0.1132706 0.6168479 0.03224098
0.06722979 0 0.05912864 0.03479142 0.0291579 0.1350434 0

0.1222715 0.02774325 0.079406748 0.1011774 1.727555 0.1929805
0.09389125 0 0.1474141 0.06188428 0.04842964 0.1439865 0
0.02695783 0 0.1546011 0.1182179 0.1190765 0.1135758 0.02493657
0.1658676 0 0.2332172 0.1548397 0.1346436 0.07766227 0
0.1752663 0.0800071 0.04033475 0.265889 0.3758338 0.02493922
0.05500955 0 0.1425758 0.02753384 0.18866 0.04496873 0.04325715 0
0.0543893 0.2090988 0 0.1170474 0.04799535 0.06672974 0
0.03210939 0.02878897 0.282510615 0.1297249 0 0.09627379 0
0.1402002 0.027484088 0.17409534 0.1006736 0.076874402 0.0404812
0.03541929 1.095092 0.01919369 0.2166533 0 0.1629402 0 0.04484964
0.2160209 0.02853986 0.078655433 0 0.06792504 0.143657 0.04810425
0.02336823 0.7168294 0.09052591 0.06493696 0.07144047 0.09775267
AC005180.2 8.90497 116.8735 0.6705338 90.042364456 2.253600744 5.600991
47.8995 80.31572 78.69068 40.3312 10.4664913.107122138 12.33631
12.992578012 89.605346985 2.29602 1.739122 1.519046 2.164909 0.2042549
0.167789441 0.2740751 0.1934778 0 0.1073206 1.500191 0.3070026
0.02738683 0.6193324 0.073101556 10.11024 0.166569288 0.2164044
0.06937177 1.199392 0.14337833 2.97639 0.5278234 0.5437741 10.36032
0.2181498 0 0.8173752 5.990786 0.05128169 0.3905522 0.4299613 0
0.1886877 0.5805381 0.0737309 0.066987 0.5642435 0.069923621
0.2909689 0.069819 0.1181129 0.05434129 0.254613 7.989947 0.1524679
2.847488 0.08591823 1.549811 0.1255326 0.818012088 0.1324344 0.05956487
0 1.900823899 0.1620204 0.09310625 0.06476006 0.132724709 0.3177217
0.3175146 0.3611499 0.13522 4.96736 0.1505622 3.098167 0.5217886
4.940886 0.2461172 0 0.2750062 0.4120585 0.2316494 0.2033911
0.3767513 0.4915855 1.575779899 0.1196475 0.1421266 0.03746242
0.193110488 0.2616624 1.722309 1.209157 0.2341391 0.2437861 0.1969361
0.2402219 0.1533866 0.1199199 0.2412963 0.580253671 1.914014
1.768697545 1.042216 0.09459334 5.89992 0.162289 0.5011389 0.3062959
0.5801455 0.5155428 0.03657327 0.09963203 0.2251128 0.5576025 0
0.4075145 0.8519143 0.3102394 2.954357 0 29.18065 0.986618733
0.05173604 1.749133 0.118188 4.684759 0.746507372 0.1042699 2.02033
0.6728155 0.2325443 0.7530367 1.197618 0.08275619 0.3832157
0.1009847 0.3047578 0.1591187 0.05312609 1.626517 0 0.1176695
0.3882571 0.02704171 0 0.3853315 0 0.179358 1.824872 0.100317529
0.3755393 0 0.2007285 8.292313 0.4495105 0 0.576732754 0 0
0.2769847 0 0.5847228 3.188132 1.318278 0.1479926 12.74598 0.67966808
0.89612 0.1363767 0.05849806 0.178609 0.02621042 2.632281 0.209334
0.04793386 7.338796 0.07911436 7.423302 24.88063 0.500013 0 6.096438
0.304917 0.08513441 0.04576581 0.21963583 2.727915 1.480361 1.843762
0.3339824 2.526019 0.215212 0.066415781 0.1517724 0.1643163 0.2371966
0.1129155 27.567395548 1.139951 1.981479 0.5383239 0.05512261 0
0.2114476 0.07542725 0.1007193 0.136046 3.105737 0.0883217 0.2272819
0 0.248311969 0.054639508 0 0.036590733 1.060970189 3.557766 0.090511344

0 14.50314 0.1983347 0.046140084 0.1067039 0.1192216 0 0.798173893
1.945944 0.06505691 3.817721 0.3178558 0.1606226 0.03859337 0.8943275
0.08520139 0 0.02888819 0.9105622 0.2306655 0.064236 0.515591175
14.95095 7.332081 0.08042145 0.3365161 0.09200266 0.09759938 3.653914
4.184192 0 4.469426211 0.7953263 0.04641778 0.535761 0.03152803
0.8162863 0.06639087 1.625199 0.1216359 0.5885866 0.5341112
0.04242165 3.495102 0.2624083 0.340438 0.031921306 0.2047904 0
0.3384425 0.1727435 0.1341837 0.125861 0.03738324 0.1148305
0.9319193 0.04271601 0.074502033 0.185406475 1.604323 0.2732634
36.95777 3.684382 0.6332241 11.09541 0.8684432 0.03833771 1.554975
0.4552941 2.196579 0.1595004 0.6271332 3.741728 0.08034074 0.2880646
0.05667733 0.5626133 0.240738713 0.3647099 0.1162919 6.64382
0.08611801 0.3715473 0.5345757 0.427903 0.1145897 0.1635026
0.04694012 0.05267121 0.085467884 14.63381 0 0.9509471 1.219283
0.03809517 1.967237134 1.969252 0.05444446 0.2051955 0.5692059
0.5498381 0.05526937 0.591001556 1.671127 0.7444791 0.1401206
0.130524 0.085809549 0.05765222 0 2.68237 0.097155278 0.613983944
0.3482767 0.1017356 0.1156545 0.3215546 0.04128456 0.6363168
0.04160113 0.03486497 1.194918 0.2653275 0.1218364 0.06634684
0.569693963 0.1209808 0.4986144 0.7845585 2.329572 1.816127 3.419588
0.07399686 0.05790875 0.05738963 0.04780148 0.6769196 0.1717638
0.3697222 0.4240698 0.1898443 0 1.461053 0.2479161 0 0.08580453
0.694299 0.2897953 0.09286307 0.07758037 0.1257426 0.4145564
0.04822945 0.04541876 0.5135949 0.2385645 0.2302179 0.04630602
0.1420684 0.03292303 0.08459488 31.56325 0.3103431 0.07622131
0.03251744 0.1500154 0.03661386 0.4898497 0.8321471 1.675605
0.1327403 0.3455474 0.9294431 0.394107335 1.473601 0.3650861
0.3453523 0.0564695 11.34375 0.131454145 1.734757835 0.4012615
0.091920993 0.1452137 0.1270557 2.618868 0.4131083 1.528447 0.3088991
2.04574 1.743677 0.01787601 1.517527 0.06825191 0.125400832 1.364126
0.08121998 0.08588748 0 1.425046 0.4137889 0.216489 0.3105882
5.979643 0.8474218
AC023024.1 0.04001754 0.04700684 0.02645419 0.012218981 0.072445223
0.09758286 0.09346795 0.1166024 0.1414494 0.02980047 0.2390638
0.068180328 0.2322635 0.065563696 0.094435997 0.06458275 0.06289488
0.1538205 0.02539244 0.2603855 0.060680609 1.050657 0.1739272 0.30493
0.1785363 1.07275 0.3936401 0.6715167 0.2975733 0.118966433
0.1659787 0.13855049 0.1207472 0.1128965 0.1735027 0.149334897
0.8403729 0.4352199 0.176989 0.1380393 0.07573757 0.1351303
0.2182904 0.139866 0.2336782 0.7318913 0.1722399 0.03630084
1.041123 0.12597 0.4879619 0.3682302 0.14284 0.151726159 0.06313688
0.2499736 0.2221191 0.1179143 0.3683204 0.2595953 0.8270942
0.0417481 0.503368 0.05934548 0.2269927 0.271469285 0.009578911
0.09693665 0.03354358 0.842099237 0.4482461 0.06060893 0.7236874

0.038399633	0.1962194	0.2559031	0.06095082	0.2249491	0.4343613	
0.0653405	0.2531912	0.04245828	0.009085721	0.4078168	0.011103118	
0.2386927	0.1844121	0.1005305	0.1412272	0.6213051	0.7111225	
0.186505083	0.110339	0.2312987	0.7153443	0.027935159	0.4315106	
0.03833036	0.3816335	0.6604712	0.04114348	0.2920078	0.271052	
0.3827557	0.2797281	0.3291105	0.384020393	3.794503	0.54497737	
0.05025531	0.09236546	0.5215688	0.8451559	0.652448	0.7975518	
0.2517697	0.3449227	0.6110702	0.3819356	0.3223893	0.06721849	
0.08687936	0.2475926	0.1902925	0.8129987	0.1317249	0.2166897	
0.2334758	0.047574408	0.02245224	0.1931001	0.1987521	0.165483	
0.161983453	0.4072565	0.1295238	0.277387	0.4108838	0.3870006	
0.1819084	0.1795713	0.2806426	0.2355595	0.1818545	0.08631729	
0.2997214	0.2614336	0.3087858	0.2212853	0.2647772	0.09388379	
0.146766	0.1858055	0.489939048	0.713508	0.5219685	0.007255914	0.3096532
0.1153832	0.1306673	0.1971875	0.09753858	0.05232231	0.500577309	
0.2463575	0.1644309	0.09015371	0.07610558	0.3045074	0.09327475	
0.1303121	0.03211269	0.04544626	0.189617272	0.3202667	0.1282327	
0.1967477	0.07751216	0.1535587	0.04393651	0.8251853	0.1664175	
0.1090708	0.0343338	0.3916094	0.08475945	0.01669185	0.1553569	
0.2330421	0.363899	0.2586245	0.3277115	0.230349083	0.4069491	
0.2190145	0.3122536	0.3768457	0.1237683	0.2201502	0.014411455	
0.3293286	0.1069642	0.5833144	0.3062669	0.149961698	0.01978849	
0.4837027	0.3504302	1.841989	0.06050924	0.4358759	0.2782363	
0.4239856	0.1279218	0.01225289	0.1533183	0.08014099	0.03483638	
0.026940411	0.029640351	0.2638137	0.142915776	0.025579805	0.3331763	
1.139114966	0.1765727	0.1691943	0.3196984	0.090106774	0.335726	
0.07114166	0.1518141	0.357213634	0.2996594	0.2540987	0.1858096	
0.3990466	0.7435359	0.159112	0.04851468	1.380416	0.1420785	0.4826671
0.214517	0.233574794	0.2787694	0.135851023	0.3712534	0.09199524	
0.04653473	0.3442376	0.2096169	0.5153298	0.2295113	0.00810644	
0.4518744	0.16163562	0.3865716	0.08057697	0.0325511	0.2257601	
0.1566872	0.04321815	0.1392037	0.4222972	0.5386303	0.2317919	
0.01841001	0.04213314	0.02846978	0.0492474	0.166237286	0.4388168	
0.3587967	0.4406286	0.2516738	0.1649925	0.3095176	0.1541229	
0.5419424	0.5055392	0.5097884	0.129328625	0.241386345	0.2744789	
0.3409462	0.08980301	0.1236291	0.1282421	0.06294317	0.1199177	
0.07486956	0.4443958	0.08232795	0.09127	0.2884144	0.7514018	
0.1591984	0.1002398	0.01785905	0.5165296	0.03052014	0.858187702	
0.4036029	0.04205667	1.134307	0.09343305	0.1209322	0.1988516	
1.706119	0.1367554	0.04966943	0.07129824	0.2342954	0.049454775	
0.1831943	0.2528744	0.05627577	0.08084089	0.06612969	0.158707211	
0.1544475	0.04725527	0.9731902	0.3190703	0.05368882	0.131921	
0.0905227	0.2986243	0.6121642	0.1064159	0.8553304	0.099304951	
0.1125888	0.132285	0.1247236	0.231897271	0.099920553	0.2687005	

0.4415090.05855659 0.5320238 0.2329150.04602446 0.3430246 0.1361753
0.03503828 0.3358425 0.07402385 0.05038774 0.020602832 0.03500192
1.5765350.2703814 0.03045122 0.09272435 0.09179516 0.06422584
0.03769658 0.3735866 0.1140960.1538782 0.1565371 0.04011272
0.6134548 0.5973133 0.1031391 0.3623217 0.2151797 0.08555645
0.02327324 0.1506548 0.2934503 0.05037553 0.1094213 0.09094899
0.2906204 0.1779090.1478302 0.2507493 0.4594209 0.3853642
0.5827765 0.4377462 0.02143175 0.07954314 0.07000537 0.1683521
0.7690701 0.3598513 0.02170107 0.5402453 0.02277679 0.1307549
0.4934394 0.5088551 0.04165543 0.3884176 0.25655018 1.694137
0.2535023 0.3497081 0.2818242 0.2000695 0.02139302 0.135512048
0.02612073 0.019945791 0.3045937 0.3675947 0 0.07967974 0.3091701
0.1591904 0.06341456 0.1669225 0.08921442 0.1261095 0.2147432
0.108842112 0.2748567 0.1498022 0.1397744 0.1747355 0.1394515
0.1026142 0.2642378 0.2695761 0.03707181 0.4184871
AC104984.6 0.04112913 0.08454703 0 0 0 0 0 0.06452999 0 0 0
0 0 0 0 0 0 0 0 0.1195742 0 0.07131028 0 0.1343144
0.05584649 0 0.04356956 0.04275389 0.1381210 0 0.216694305
0.1930464 0.1082970.1386951 0.044765901 0 0.08239909 0.2122229
0.2837475 0 0.1620312 0.1963098 0 0.08005642 0 0.05163211 0
0 0 0 0 0.0734039 0 0 0.1089952 0 0.0424164 0.04416435
0.06670158 0 0.06007088 0.1341279 0.07115963 0.06532346 0.150236207
0 0 0.06033185 0 0.06323298 0 0.1010976 0.069066006 0.03815376
0 0 0 0.05787015 0 0.06281277 0 0 0 0 0.03902866 0
0.05166149 0.06350322 0.07842009 0 0 0 0.03697924 0.116966
0.200977952 0.2450904 0 0.08580129 0.06091953 0.1691454 0.1024797
0 0.05986337 0.04680205 0 0 11.408680 0.09038975 0.2215061 0
0 0.04117545 0 0 0.2012049 0 0.05184556 0.2108557 0 0
0.06361754 0.03911567 0.1117657 0 0 0 0 0 0 0 0 0 0
0 0 0.1555832 0 0 0.04306387 0 0.03941208 0 0 0 15.141
0 0.06123173 0 0 0.06211175 0 0 0.09333261 0.1942383 0 0
0 0.0626719 0.0591106 0 0.1254766 0 0.1417924 0 0 0
0.06085449 0.05592167 0.04573284 0 0 0 0 0 0.1369830
0.2455043 0 0.2723280.07483009 0 0 0 0.02651292 0 0 0 0
0 0.1428910 0 0 0 0.1042767 0 0.1439871 0 0.05923341
0.2565160 0.08813681 0 0 0.07733272 0 0.04302626 0 0.04126164
0 0 0.2123830 0.13788 0 0.0626571 0 0.042649172 0.3615225 0
0 0 0 0 0.0811506 0.1769271 0.072029797 0.08328835 0 0 0
0.2939841 0 0.1670996 0.03544344 0.08358324 0.1204971 0.1163454
0.04433628 0 0.1352930.04061396 0.120031491 0 0 0 0.1167979
0.04184894 0 0.1436264 0.3555148 0 0 0.1300394 0 0.04966371
0 0.0669106 0.04921875 0 0 0.06676623 0 0.03994996 0.06948391
0 0 0 0.1771538 0.149498115 0 0 0.05283463 0 0.06982525 0
0.1167188 0 0.1322572 0 0 0 0.04816398 0 0.1292166

0.1185924 0.1318044 0 0 0.05984943 1.3617620 0.1459193 0 0
0 0.06271041 0 0.1327194 0 0.053688554 0.1138705 0 0.04020055
0 0 0 1.085507 0.08944359 0.1020983 0.07327874 0.08222561 0 0
0 0 0.05287321 0 0 0.0370388 0 0.0915237 0 0 0.08628163
0.162815135 0.05115323 0 0 0.0407525 0.089305494 0.09000152 0 0
0.075835092 0 0.06041096 0 0 0 0 0 0 0.1632843 0
0.06903429 0.1521601 0 0 0 16.56864 0.07204606 0.08763184
0.05559168 0 0.05775866 0 0.08959159 0.07462342 0.2012851 0
0.08245393 0 0 0 0.04654827 0.0774049 0.1846593 0.0669752
0.07225853 0.05026696 0 0.06055579 0 0.04978219 0.07529154 0 0
0 0.1540268 0.2168665 2.306559 0 0.04402067 0 0 0.1189899
0.05076334 0 0.05715831 0.1092442 0 0.1868433 0 0 0.1074788
0.087892191 0.04035885 0 0.05990369 0 0 0 0.270814973 0.1252828
0.071749442 0 0 3.066259 0.1074847 0 0 0.20277 0 0.05581289 0
0 0.097882316 0.07605542 0 0.06703995 0 0 0.04614074 0
0.1818235 0.06667777 0.04561791
MIR762HG 0.8484158 0.3353932 0.7078136 0.435911295 0.249832908
0.3924332 0.5201762 0.5503715 0.2883542 0.2693262 0.8063389
0.667152962 0.7954544 0.783558416 0.344386429 0.3291408 0.7180065
0.9720777 1.277282 1.794601 0.623455644 0.6082001 1.052684 0.5416977
0.8529281 0.4750794 1.555541 2.993479 0.4109384 0.667741435 0.5457873
1.220651558 1.148706 1.181422 0.7565187 1.829112511 1.532324 1.291146
1.473283 1.238171 1.310437 0.5570661 1.907936 1.221012 0.5398846 1.209313
1.126519 0.5827634 1.402214 0.7390088 0.7191505 1.313659 0.6842942
1.407333269 0.8366072 1.383609 1.365381 0.5384429 1.051184 1.217166
0.771104 0.8102122 0.226133 1.63726 2.202642 0.864168596 0.5877704
1.447837 0.610299 0.882862009 0.5393093 0.3459551 1.022674 0.315077977
2.240036 1.1517 0.6709628 1.060701 2.226805 1.476311 0.971781 1.353129
0.7390221 1.579588 0.697141237 0.7121924 0.4864391 0.5123454
1.624843 4.401895 1.197428 0.789555312 0.9261967 0.9095058 1.125194
1.315492051 1.782476 0.8204599 1.174272 1.836648 0.5116299 0.5793067
1.72568 1.365478 0.9468713 0.9713006 0.817502294 0.9967443 1.80728731
0.6454277 1.581663 0.7056135 1.03853 0.8657053 0.6006661 0.6800556
0.6518373 1.585446 1.120892 1.491673 0.4987872 0.4215205 0.567826
0.9620516 1.839978 0.5263191 2.359201 0.6130314 1.731158012 1.818229
0.646125 0.4574496 1.0008 1.05068155 1.205356 0.5687081 2.270817
0.8434923 0.4295268 2.243537 1.622903 1.364586 0.6957373 1.309325
0.4064759 0.773153 1.212461 0.8931821 1.943222 0.8243702 1.523931
0.8007795 0.6098311 0.932188895 1.388419 1.232851 0.921521058 1.872151
1.693559 1.752741 0.9262289 1.64241 0.72175 1.138451391 1.040592 0.8557546
1.701027 0.7518632 0.3621093 0.5656869 0.4898327 0.8363011
0.4817554 0.330712953 1.289449 0.4926591 1.132091 0.4756215 0.8683953
1.61968 1.231553 1.884976 0.7989712 0.6736708 0.9594928 1.083305
0.9289498 0.5998774 0.5086065 1.038566 1.304878 0.8219187 0.83877088

1.09544 3.948335 0.7936983 1.251321 0.6433907 0.7425451 0.771191505
1.033892 3.752342 0.6202106 0.821639 0.737093649 0.4376908 0.935662
1.192458 1.237449 0.8310852 0.5565207 0.5838858 1.09778 2.330947
0.9441813 0.6153322 0.8005268 1.702616 0.499770591 0.346832933 1.75682
0.634481301 0.876055458 2.052517 0.82677001 0.91625 0.6438395 1.412493
1.228673568 2.16412 1.273601 1.375182 1.652802646 1.243966 1.571259 1.21527
1.181057 1.641271 0.7409076 1.361529 0.967337 0.5713742 1.690604 0.8216775
0.928508066 1.630993 1.619293119 1.165511 1.042494 1.419405 1.756505 1.253467
1.098024 1.295161 1.064244 0.683513 1.132299413 0.994916 1.135458 1.074993
1.239824 1.52626 1.767936 0.953488 1.029471 0.6973084 0.5788412 1.983463
1.046156 0.6906471 2.869599 1.344247512 1.458469 1.047277 1.236592 0.3591378
1.274166 1.026258 4.329206 0.5866795 3.161057 0.7406933 0.519051353
1.17689697 0.8884462 2.686487 0.9995595 1.117317 0.6535757 1.268704
1.057291 0.8072254 0.9394838 0.9868468 1.172178 0.6996632 1.401521
0.5225039 0.3793721 0.6243781 1.026656 0.7294986 0.692183015
1.321275 1.296319 0.3269202 0.9199681 0.4026631 2.707549 0.7121697
0.727376 0.7999105 1.075562 0.4811218 0.890969332 0.5975264 0.3988354
0.3747584 0.8494671 0.6251806 0.788756899 0.844852 1.045214 1.642888
0.5874999 1.242846 1.728485 0.516702576 0.953733 1.395264 1.062986 0.4203233
0.681969227 1.213905 0.6006329 0.6377623 0.962666957 1.675389659
0.4014084 0.9686744 0.5968578 1.331705 1.431744 0.3502761 1.842047
0.6801262 1.719984 0.6435594 1.410939 0.6368566 1.754210997 0.6742932
2.051207 1.371852 0.5475179 0.8049308 0.8296125 0.7790259 0.8875832
1.261687 1.332121 1.29754 0.6382231 1.373778 1.269328 1.01402 0.5536742
1.966568 0.6294746 1.599364 0.7439229 1.57655 1.734833 0.5175773
0.8167524 1.37571 0.6813167 2.210211 0.4640971 1.312002 0.7940979
1.283132 0.7455907 0.4399032 0.5403005 1.047765 0.7658806 0.8328252
1.203667 1.701621 0.4335431 0.9409863 0.8017264 1.066211 2.248296
3.192253 1.224509 0.6608614 2.196578404 0.5043189 2.147873 1.176291
1.731047 0.6748151 0.437564858 0.837959719 0.8448821 0.498095299 1.55876
1.324506 0 1.378646 1.114995 1.016261 0.8948692 1.556227 1.317369 0.5498713
0.8559127 0.582440228 1.01072 0.8173459 1.409501 0.997388 0.7613794
1.107378 0.3686873 0.9977752 0.96545 4.469802
AC011442.1 0.7281879 0.08316101 0 0 0.213607842 0.6277685 0
0.06347212 0 0.03514718 0.1537941 0.137850936 0.6985356 0.173985628
0 0.5223086 0.5722403 0.07775079 0.2695345 1.568186 0.601169092
1.12226 0.7639427 0.4844126 0.3295858 0 0.9856721 1.387749
1.018925 0 0.05106733 3.410271036 0.2373522 0.1065217 1.97811
0.044032034 2.367748 0.4862897 0.4174877 0.4186438 0.1339893 0.10625
5.020381 0.756877 0.3149761 0.6542186 0.2539284 0.1284415 0.2069525
0.6933323 0.1132152 0.2742928 2.238217 0.536845289 0.1914807
0.3216252 0.1209097 0.6675368 0.3475227 0 1.560783 0.1181722
0.4617518 0.06999308 0 0.591093274 0 1.097556 0.4747424 0.060807322
0.497571 0.4288993 0.1988806 0.067933776 0.2251698 0.2785999

0.1848509 0.2768439 0.5692146 0.07706371 1.482793 0.2003041 0
0.2748492 0.078571246 0.1151665 0.672269 0.3557021 0 0 0.6038712
0.307954204 0 0.03637302 0.4601942 0.988416159 1.125005 0 0.1687894
0.3595251 0.7486764 0.4535986 0.5164116 0.353292 1.104835 0.4763765
0 11.88961 1.520886588 0.2667239 0.5083746 1.198339 0 0.1215013
0.8622591 1.272606 0.3298441 0.8423844 0.8159301 0.414798 0
0.3688812 0.1877239 0.1538977 0.7328901 0.9321519 0 0.1321753
0 0.07944167 0.1413593 0.22685 1.059513 0.104206941 0.3202169
0.2820233 0.6198727 0.4590979 0.1825741 0.09194823 0.1270737
0.07355437 0.5039578 0.1754855 0.06108245 0.5302449 6.197619
1.240205 0.4215955 0.3406721 0.2491383 0.2443741 0.3944559 0
0.9180257 0.3821081 0.10269299 0.1153296 0.1166442 0.3082224
0.2907079 0.1380464 0.1234197 0.332094065 0.3486699 0.2737872
0.2126576 0.0828556 1.137281 0.1100098 0.4723227 0.2272455
0.09188589 0.149091905 0.7284755 0.139606 0.2245622 0.0548515
0.5634524 0.1554584 3.107218 0.4416202 0.154368 0.1214816 1.098032
0.2086263 0 0.1940088 1.164087 0.3901715 0.6536276 0.5621942
0.281045848 0.06544946 0.05166189 0.2762079 0.7692546 0.250242
0.9913866 0.050991351 0 0.3153885 0.6879709 0.08669194 0
0.3500832 0 0.6358513 1.777478 0.1605727 1.379898 0.05790999
0.2474503 0.6267039 0.5202456 0.2712393 0.2181223 0.4930395
0.095321944 0 0.9778888 0 0 0.05750544 0.625418548 0.3407774
0.1596405 1.43572 0.283395924 0.5734608 0.183067 0.2335462 0.306402819
0.4819411 0.6992729 0.05478676 0.2091744 0.164426 0.2963043
0.1144381 1.657159 0.1828035 0.4435838 0.4793779 0.472255048
0.4931781 0.056550008 1.091288 0.3446495 0.1234887 0 0.3531796
0.6993734 0.7382441 0 0.4476767 0.207966346 0.4884955 0.2851016
0.1974411 0.8230021 0.3374601 0.1529167 0.3940302 0.3112903
0.8251959 0.7517931 0.06513925 0.05963106 0 0 0.490157753
0.5109968 0.5770514 0.6236219 0.03789298 0.4120834 0.5153651
0.05740268 0.4408111 0.3902673 0.3279563 0.228798593 0.142347594
0.1894976 0.3147008 0.1906474 0.2332965 0.1944655 0.4454175
0.06061414 0 0.6406009 0.2330376 0.2152908 0.04081931 2.847057
0.1126568 0.5243001 0.06318982 0.3481165 0.4319517 2.481995463
1.008034 0.1190457 1.502578 0 0 0.1172645 2.792476 0 0.6025472
0.2883098 1.172726 0.174983427 0.86425 0.3767298 0.1991178 0
0.1754876 0.038727311 0.3278845 0.04180025 0.8102098 0 0.2110717
0.3394687 0.053382011 0.1006293 0.9024962 0.3227367 1.082279
0.307445143 0.4426304 0 0.2942022 0.149183788 0.117848011 0.5050753
0.2343255 0 0.5554723 0.2535729 0 1.980259 0.05353582 0.0495897
0.5432207 0.03741643 0.356569 0.242993327 0 8.750079 0.2834599
0.04309763 0 0.8119859 0.2272472 0 0.4406144 0.07340008
0.04949634 1.793477 1.013778 0.2170557 0.2915096 0.1737772 1.007274 0
0.5448964 0.1976317 0.1421479 0.3461003 0.142593 0.1786892

0.2574403 0.1958644 0.2221717 0.06974137 0.1971587 0.8242209
0.353504 0.7821416 0.174519 0.1011079 0.1298971 0.1651311 0.3176918
1.404471 0.1997246 0 2.305073 0.4298134 0.1321839 0.4900807
0.6794178 0.2947748 1.057169 1.123867366 0.1587889 0.168179 0.1178433
0 0.1716112 0.201850354 0.106550153 0 0.070573222 3.27035 0.520257 0
0.3171678 0.4375686 0.5336106 0.4986147 0.157497 0.2744896
0.04957856 1.519631 0.144416532 1.645789 0.5612167 0.1978228 0.2649677
0.3432448 0.04538434 0.4155288 0.5365283 0.06558469 0.8076614
AL021368.2 0.2268558 0.4890837 0.1280201 0.266091592 0.064273929
0.2618752 0.3482869 0.3863118 0.2151342 0.3509202 0.152501
0.146118902 0.3905885 0.241928446 0.375759905 0.07143683 0.421767
0.1701452 0.04300868 0.294914 0.117460969 0.2830027 0.1857519
0.0271038 0.2516848 0.1145682 0.1641185 0.4975172 0.08826115
0.072923993 0.2549359 0.212807412 0.0844037 0.1675447 0.1539329
0.009033478 0.16192 0.1551911 0.2212638 0.2767492 0.09621091
0.2034473 0.1650587 0.1353711 0.1615488 0.1118479 0.09377132
0.1624958 0.1245427 0.04741398 0.09677869 0.2415053 0.1234371
0.110137542 0.1134859 0.4838801 0.07855065 0.03138433 0.2435971
0.2288193 0.176113 0.157585 0.04059925 0.119663 0.1406065 0.242533787
0.05562641 0.5754376 0.7183007 0.09564201 0.250947 0.02933056
0.1088046 0.097559605 0.1257534 0.09526117 0.07163316 0.1325251
0.1829527 0.1686417 0.1901283 0.2054689 0.08793729 0.09632817
0.032238874 0.1076351 0.09194711 0.05559982 0.3417214 0.1424221
0.120447 0.051144861 0.2230088 0.0646722 0.4877955 0.243336534
0.2088215 0.03246121 0.1558274 0.2294727 0.06826496 0.1826712
0.1664857 0.318108 0.1416654 0.1665063 0.082256813 2.156029 0.14486664
0.1459207 0.2731577 0.05572543 0.1306519 0.3489758 0.1876196
0.07397381 0.0225566 0.1612996 0.1081084 0.321484 0.08457574
0.09670037 0.1882851 0.2499544 0.120286 0.06374581 0.07767624
0.2937642 0.178426254 0.1113697 0.2126729 0.05895048 0.2173665
0.078388853 0.1094912 0.1542908 0.1554314 0.1046524 0.03329452
0.2452296 0.1245569 0.1358117 0.1590622 0.2240129 0.04594879
0.07252228 0.7780727 0.05250282 0.1276805 0.1630796 0.07666869
0.03760128 0.1348756 0.069153237 0.06905776 0.3135685 0.017556817
0.2918148 0.1834662 0.1011744 0.1749462 0.09440393 0.1772428
0.075701562 0.09060728 0.09361557 0.07756124 0.3513003 0.1187072
0.1617465 0.2076435 0.1126674 0.1602337 0.035685114 0.1439166
0.06682932 0.1750679 0.03751056 0.2724767 0.07796158 0.435969
0.2969714 0.09149014 0.0539994 0.1787849 0.4208777 0.06058281
0.1724765 0.1857493 0.06937355 0.03128912 0.1153381 0.096097515
0.2327418 0.3921555 0.08499896 0.161325 0.1411819 0.06456821
0.034870767 0.23109 0.06039054 0.09409468 0.183783 0.298348064 0.06942793
0.2236754 0.1174044 0.1157657 0.1830144 0.0888143 0.04752255
0.04442039 0.2761931 0.2431118 0.1159305 0.2147966 0.1770133

0.189040941 0.14057014 0.2857325 0.088372911 0.115536088 0.09438113
0.104548043 0.1514778 0.2074253 0.5682693 0.101746126 0.4649955
0.1533596 0.3290069 0.20953556 0.1252399 0.266427 0.2322909
0.4958903 0.1293104 0.07699925 0.1173888 0.1729711 0.09375851
0.1213391 0.2731881 0.141292675 0.07419786 0.255235798 0.3814344
0.1859339 0.04785417 0.100962 0.01449145 0.1332326 0.2069897
0.192225 0.284279 0.18488488 0.1002182 0.2242138 0.4410699 0.2847183
0.01648388 0.1394308 0.1886221 0.1404994 0.123612 0.5094448
0.1024556 0.08155818 0.07577579 0.03972059 0.227934345 0.1075226
0.2052026 0.1705871 0.05960074 0.8078436 0.08810895 0.06280831
0.1386677 0.1467877 0.242217 0.13690723 0.06814173 0.2170619
0.1362998 0.1694881 0.3509909 0.1551509 0.06472785 0.1326444
0.0603862 0.2588649 0.1274914 0.3827928 0.1200326 0.1517498
0.2542355 0.134982 0.08642554 0.1755705 0.0886179 0.234736953
0.3868024 0.06512812 0.110867 0.08590883 0.03413834 0.1002401
0.4156304 0.1143113 0.1579547 0.1774463 0.4728895 0.038890655
0.1899717 0.2576291 0.1951742 0.06757338 0.072005 0.222464956
0.05730218 0.03716099 0.1754546 0.1942553 0.08083195 0.1218776
0.237286631 0.2615008 0.111092 0.06253325 0.1425424 0.0750887
0.1331861 0.05237742 0.1458643 0.198939592 0.072532016 0.03250814
0.1041593 0.08501209 0.227918 0.2861224 0.06681802 0.1354213
0.03294974 0.06443325 0.04643562 0.02558747 0.2159743 0.179466372
0.07622335 1.219199 0.1986921 0.3772492 0.1271379 0.1924977
0.1592896 0.02432341 0.1807902 0.1054098 0.2707871 0.06853862
0.2495803 0.1335915 0.34388 0.08794061 0.2129114 0.0676859
0.4388765 0.1396568 0.2673241 0.1487722 0.03412961 0.1425641
0.1232366 0.04353152 0.4102207 0.0524624 0.2494323 0.1784888
0.2486531 0.09724956 0.1581334 0.02765731 0.09771405 0.4065334
0.2878637 0.3041447 0.1195101 0.01575274 0.08458404 0.04776374
0.06026322 0.3896059 0.2137272 0.419294 0.2421888 0.14780077
0.1710274 0.1686818 0.09670551 0.08894579 0.08508416 0.110429342
0.054648761 0.06741673 0.053088152 0.07115977 0.1645488 0.2750008
0.1759278 0.3101154 0.0810918 0.231867 0.06462321 0.1651861
0.06441878 0.1110879 0.04279608 0.07673753 0.3923204 0.1262636
0.07247995 0.2875445 0.2203582 0.2301713 0.1793774 0.1076412
0.3037785
COL4A2-AS1 0.02741942 0.2818234 0.03172054 0.058605852 0.086867189 0
0.40347 0.04301999 0.2422976 0.4764395 0.05211913 0.093432301
0.08355034 0.314462913 0.503270023 0.2655069 0.08618927 0 0
0.1062882 0.174625308 0.2852411 0.1438287 0.1790859 0.186155
0.106453 0.2614174 0.08550778 0.2302016 0 0.0692246 0 0
0.03609901 0.0462317 0.268595405 0.07464218 0 0.2122229 0.189165 0
0 0.1963098 0.2367667 0 0.1108537 0.06884281 0 0.5891248
0.06713218 0 0 0.04893594 0 0.1297814 0 0.04097496 0.0282776

0.1766574 0.1334032 0 0.04004725 0 0 0.04354897 0.100157471 0
0 0 0 0.04215532 0.09689946 0 0.046044004 0.4578452 0 0
0.04690967 0.1929005 0 0.04187518 0.1357617 0 0.04657167 0 0
0.02680288 0.1722050 0.07842009 0.06821508 0.059635576 0.0311305
0.1479169 0.1169660 0.05446454 0 0 0 0.0563818 0.4099188 0
0.03990891 0 0.03587527 0.120778727 2.2636270.073630224 0 0
0.06497659 0 0.2196024 0.1062582 0.04312721 0 0 0.0345637
0.04685681 0 0.04166992 0.0424117 0.07823135 0.1738578 0.08423891
0 0.1791710.171135719 0.0269219 0.09581021 0.03075078 0 0
0.1446906 0.1433618 0.2100680.1037221 0 0.06232047 0.229674
0.04985352 0.02627472 0 0.04140033 0 1.6300870.08005558 0 0.2309
0 0 0 0.048956101 0 0.1726562 0.034801513 0.1172517 0.07905887
0 0.1182212 0.04678239 0.0836511 0.075028659 0 0.09278343 0
0.1123154 0 0.1118433 0.03048856 0 0.3425302 0.353679131
0.1097210.1892437 0 0 0.1091130.03512208 0.1815520 0 0.04116877
0.1417566 0.3004798 0.08005907 0.04383162 0.1314987 0 0.04430143
0 0.076194652 0.2661611 0 0 0.1042767 0.04240211 0.03199714
0.069121609 0.07897788 0.04275267 0 0 0.319671439 0.09491144
0.1031103 0.2585795 0.02868417 0.03627753 0.02750776 0.0392501
0.08385817 0.1415887 0.1763054 0 0.1774061 0 0.129214191 0
0.1807613 0.038081467 0.040896107 0.03897591 0 0 0.3787028
0.08846356 0.09603973 0.05552557 0.03101968 0 0.103836511 0
0.1354147 0.03713325 0.5670950 0 0.1551272 0.1182301 0.1858502
0 0 0 0 0.038328339 0.3835228 0.05191017 0.02789929 0.1000646
0.04787546 0 0.05003654 0.07776177 0 0.281909936 0.1324366
0.04830888 0 0.0328125 0.03267471 0.03454784 0.04451082 0.1687885
0.3728663 0 0 0 0 0 0.033221803 0.05328343 0 0.07044617
0.07704907 0.4655016 0 0.2723438 0.0298772 0.1322572 0.08891259
0.116305951 0.144720054 0.03210932 0 0.2584331 0 0 0.1132103 0
0.03989962 0 0.07897385 0.04863977 0.08299926 0.1986427 0.1527125
0.02090347 0.08565731 0.08847961 0.03659591 0.107377109 0.07591369 0
0.1340018 0 0.04833555 0 0.4453361 0.1192581 0.1361310
0.08222561 0 0.1255220.06383477 0.1349576 0.2467416 0.0396472
0.131242553 0.02469253 0 0.0610158 0.04936631 0.05722389 0.05752109
1.04925309 0.2387151 0.1631178 0.07291459 0.1086733 0 0.06000102
0 0.04985093 0.101113456 0 0.2416439 0 0.1203663 0 0 0
0.04329599 0 0.1680540.09204573 0 0 0.263512763 0.0419699
0.5930609 0.04803071 0.08763184 0 0.1467589 0.1925289 0
0.1791832 0.09949789 0.06709503 0.03575231 0 0 0.04939469
0.07066939 0.03103218 0 0.04103541 0.02232507 0 0 0.09664638 0
0 0.2323169 0.05019436 0 0.06681489 0.09310644 0.03422817 0
0.05914254 0 0 0.6715330.2691556 0.07932663 0 0 0.07621107
0.1092442 0.02986377 0.04152073 0.04604943 0 0.03582627 0.058594794
0.0538118 0.0379960.1198074 0 0.05815714 0.102607263 0.433303957 0

0.143498884	0.0503766	0	3.406954	0.2388548	0.0269613	0.04018549
0.2365650	0	0.2016195	0	0	0	0.08452894	0	0	0.1163218
0.09228148	0.02816362	0	0.04445185	0.1824717
AC138393.3	0.1033612	0.1062371	0.0597874	0.055230646	0.081864367
0.1202949	0.04224817	0.0405424	0.09133732	0.06735009	0.3438225
0.088051384	0.2362156	0.03704406	0.094857177	0.125108	0.1218382
0.04966281	0	0.5509178	0	0.3584182	0.4066362	0.253158	0.3859548
0.2675258	0.3284826	0.7252492	0	0	0.2283324	1.007458341
0.06064285	0.4422601	0.2178457	0.056250347	0.7386059	0.2070763
0.7333358	0.08913533	0	0.03393324	0.185004	0.7065811	0.05029723
0.2437621	0.03243902	0.3281646	0.1850654	0.1265319	0.07231547
0.2847045	0.4150587	0.377197056	0.04076902	0.410872	0.8109181
0.1065962	0.6381864	0.04190675	0.7975517	0.1509635	0.08426884
0.2682457	0.3693682	0.14158386	0	0.9055316	0.3790483	0.155361116
0.2780927	0.09131887	0.1270337	0.477314805	0.4554481	0.1334654
0.3148594	0.2210403	0.3272239	0	0.1183906	0.1279429	0.2464086
0.1316686	0	0.1226031	0.1768148	0.2596599	0.1196919	0.1970767
0.06428646	0.0843016	0.05867529	0	0.2204595	0.568209917	0.3079671
0	0.1617197	0.1530962	0.1062694	0.03219257	0.2827324	0.03761049
0.0882133	0.1014275	0.455291537	5.461149	0.104084609	0	0.2783322
0.2755552	0.1061157	0.2586939	0.1001386	0.2032172	0.2949601
0.1076135	0.2931581	0.529899	0.1215329	0.1963504	0.3597222
0.5160812	0.1404386	0.07938745	0	0.04221307	0.483839204	0.05074285
0.2106821	0.08693937	0.2493316	0.332807507	0.170447	0.1351054
0.06598993	0.03258287	0	0.1174627	0.0811675	0.2818942	0.1980921
0.5230878	0.03901602	0.1302656	6.853849	0.07544505	0.1538808	0
0.1060903	0.1170693	0.04199269	0.138409919	0.1172766	0.3661036
0.065594475	0.184165	0	0.07875003	0.1856877	0.1322644	0.157667
0.212122911	0.04454213	0.1748798	0.09055579	0.2116939	0.1529327
0.1756702	0.215495	0.1088637	0.05869151	0.380925915	0.361907	0.0445862
0.5737506	0.03503604	0.2827798	0.09929804	0.5475076	0.2350683
0.3944061	0.3879779	0.06679629	0.1166013	0.1508967	0.08261458
0.1239255	0	0.04175003	0.8977447	0.035903239	0.2926379	0.3959844
0.0441065	0.1637855	0.07992021	0.3618524	0.097711175	0.03721471
0.08058095	0.07754769	0.4429913	0.150630521	0.1565293	0.24293
0.4467604	0.135161	0.1709412	0.3888532	0.295917	0.3161146	0.1334343
0	0.5630701	0.2507835	0.1574629	0.243545072	0.401929366	0.3974855
0.251218054	0.038540834	0.07346245	0.088773779	0.1451129	0.3568927
0.3056856	0.362034584	0.5756053	0.2923321	0.537034	0.048928199
0.1231347	0.127616	0.4199362	0.3785582	0.1575391	0.7191972	0
0.4456841	0	0.1983354	0.1530998	0.339356049	0.3780171	0.28896758
0.129084	0.1467617	0.1051701	0	0.1353547	0.1595429	0.2357743
0.03664167	0.3676507	0.232465	0.280821	0.09105338	0.1891713	0.4638416
0.1231717	0.1953491	0.2516842	0.1590677	0.1756962	0.698477

0.2912509 0.1904448 0 0.03710028 0.563553103 0.2008859 0 0.03319453
0.1210195 0.1316078 0.04114826 0.1466623 0.08446957 0.4154678
0.2513759 0.40189491 0.227308986 0.1210404 0 0 0.8940996
0.04140453 0.03556344 0.2323013 0.1128052 1.1159440 0.2232769
0.2291927 0.1303653 0.4011483 0.3597938 0.3545929 0.04036209
0.3057411 0.2069298 0.269848231 0.8227295 0.1520793 0 0.08446478
0.04555183 0.1123529 0.9442991 0 0.4810914 0.184156 1.627292
0.167654278 0.039431 0.3609505 0.1695803 0 0.03736385 0.593683592
0.4886795 0.1067986 0.2587581 0.1860929 0.0808924 0.1626251
0.068194821 0.1285526 0.6148945 0.2748613 0.3328476 0.224432655
0.4523637 0.05435795 0.04697993 0.285870505 0.075274646 0.07590907
0.1496739 0.07562282 0.3548043 0.5263961 0.1386892 0.1224075
0.1367826 0.2534008 0.260234 0.09559799 0.1301463 0.155210397
0.07910556 7.265773 0.2715872 0.2752833 0.1397068 0.5878041
0.1814408 0 0.5628791 0.1406515 0.5374628 0.03369328 0.1554105
0.06932147 0.1396499 0.06659943 0.3509399 0.0972627 0.5027375
0.3366293 0.2723882 0.03158133 0 0.1902276 0.2466575 0.1876606
0.09460717 0 0.3148346 0.2632329 0.2903122 0.3633366 0.2508139
0.032291 0.1106279 0.2109528 0.6087707 0.4485485 0.2551456
0.1961807 0.1077329 0.2059054 0.2532948 0.7434601 0 0.3012567
0.1012889 0.165660675 0.2535635 0.1790387 0.1881791 0 0
0.193395889 0.10208732 0.1180675 0.360625467 0.1899013 0.0830777
0.6421484 0.2926284 0.05081711 0.03787114 0.1910921 0 0.05259853
0.1266719 0.3681791 0.215238601 0 1.2347420 0.08423868 0 0.1370284
0.3768563 0.5042909 0.1523129 0.08378358 0.3152652
AC007619.1 0.3593808 0.7387605 0.08315092 0.15362699 0.265661792
0.3346067 0.4113044 0.4510834 0.1905447 0.2810068 0.136623
0.122459812 0.8395592 0.051520016 0.395775455 0.1739972 0.1129665 0
0.1197204 0.2786195 0.07629261 0.3738597 0.3393241 0.0391207
0.1951916 0.2790515 0.1903525 0.6724398 0.06034411 0 0.2268282
0.151475631 0.126511 0.4731424 0.06059494 0.078231671 0.146748 0.1439984
0.3708750 0 0 0.08576641 0.3620461 0.2098566 0.1937249
0.04511543 0.05705046 0.07353847 0.1759776 0 0.1218340 0.238453126
0.0567006 0.2380964 0.05370505 0.2594401 0.1543608 0 0.6932602
0.2624456 0.2343983 0 0.05707875 0.065637178 0 0.3250044
0.2635858 0.162054465 0.1105042 0 0.2650133 0.120697874 0.1666912
0 0.2189496 0.2459342 0.4045292 0 0.1097699 0.2669101
0.1142331 0.03052027 0 0 0.07025998 0.2257056 0 0.06852241
0.04470406 0.156326267 0.1632084 0.06462391 0.153305 0.175611803
0.3569278 0.06024007 0 0.05323065 0 0.1790907 0.2621451
0.1046156 0.122685 0.2821259 0.158302215 6.171131 0 0 0.3225816
0.08516349 0.07379159 0.4677211 0.06963522 0.1130519 0 0.1995551
0.04530194 0.2456571 0.08451257 0.1638477 0.1667644 0.3759662
0.3255313 0.05520511 0 0.1761269 0.149536065 0.03528599 0.4185883

0.08060884 0.09907558 0 0.1422323 0.2505352 0.2753318 0
0.1621896 0.08168217 0.07525724 0.130684 0.1721887 0.05196417
0.05426257 0 4.355217 0.7344905 0.2140138 0.07565898 0.07377398 0
0 0.19249729 0.2446583 0.1697228 0.045613634 0 0.1036208
0.1095237 0.1033001 0.1226335 0 0.442523403 0.06194815 0.06080468
0.1889143 0.2576165 0.1595215 0.1954544 0.1198822 0 0.2040667 0
0.07190454 0 0.0797959 0.09745461 0.3575305 0.0920676 0.1427739
0.130771 0.09142181 0.05395907 0 0.4633326 0 0.05744922 0.08617632
0.1386435 0 0.1248562 0.049933388 0.465136 0.1376815 0.1226846
0.227789 0.2223023 0.08387599 0 0.05175735 0.05603505 0.1078515
0.07701274 0 0.3109962 0.2027168 0.3954007 0.1879788 0.1901929
0.07210773 0.05144431 0.0549556 0.3711547 0.4621599 0.3011941
0.1550151 0.2189957 0.254037609 0.335396398 0.1579467 0.099825205
0.214406777 0 0.185196754 0.05045491 0.2836332 0.1545965 0.062938658
0.7277623 0.1626275 0.04149413 0.068048199 0.4281322 0.1774853
0.04866979 0.18582 0.0730339 0 0 0.5036257 0.08119669 0.1970287
0.2129276 0 0.3504916 0.150708516 0.3590539 0.3061692 0.03656703
0 0.06274939 0.2662663 0.06558188 0.05096038 0.3976934 0.138560102
0.08679095 0.1899524 0.05846557 0.2580401 0 0 0.2916971
0.05530693 0.1745387 0.5464269 0.05786642 0.1589197 0 0.05159821
0.130629421 0.1745938 0.1025247 0.09233236 0.1009866 0.3050618
0.05722805 0.1529809 0.1174783 0.1155646 0.1748039 0.101626559 0
0.2525102 0.1863762 0.05645384 0.3108732 0.1151689 0 0.1615396
0.05229562 0.3104059 0.1035094 0.1912535 0.1813091 0.2603569
0.2001572 0.2465798 0 0.7344667 0.09593103 0.093824658 0.4477434
0.05287707 0.1053801 0 0 0.1562577 0 0 0.3122423 0.1920899
0.5029334 0.194308053 0 0 0.1768862 0.04619989 0 0.206420327
0.097092 0.1856662 0.03998608 0.2588137 0.07500218 0 0 0.223485
0.5344881 0.04778383 0.1068269 0.11705089 0 0 0 0.198791019
0.157035335 0.1319657 0.1387753 0 0.05482817 0.2815767 0
0.05674717 0.04755852 0 0.06032123 0.06647773 0.09050225 0 0
9.133426 0.06295287 0.153143 0.04857525 0.1442655 0.1514062 0
0.1565678 0.1956148 0.2638203 0.04685982 0.576377 0 0.3884436
0.1234999 0.1220197 0.06763535 0.2151371 0.2048271 0.2525541
0.08784516 0 0.1058256 0.3430455 0 0.1315774 0 0 0.04067757
0.269173 0.25266 0.1162754 0 0 0.4400823 0.4233321 0.1559577
0.04435632 0 0.1997766 0.04772807 0.03914184 0.2721018 0
0.2094904 0 0.153598004 0.03526502 0 0.1046861 0.07702878
0.2286761 0.044828416 0.141980666 0 0 0 0.1733138 0 0.06261242
0.1413505 0.0526703 0.2657665 0.0699562 0.1706899 0 0.0931009
0.085528237 0 0.609347 0.05857859 0.07846131 0.6098427 0.1612686
0.1845674 0.4236664 0 0.3986031
AL354993.2 0.109273 0 0.8848976 0.058389594 0.057697764 0.7206597
0.08932915 0.04286125 0 0.02373407 0.1038536 0.139631299 1.054399

0.156651267 0 0.4849664 0.6440342 0.2100132 0.3336857 0.5559539

0.231974578 1.468308 0.8311283 0.08921252 0.7789657 0.3535339

1.244385 0.4543587 0.4587043 0.075799031 0.1724229 1.007508579

0.1923341 0.359658 0.1842444 0.356805704 3.049037 0.7662204 2.466798

0.188467 0.2261996 0.07174815 2.086244 1.926459 0.9039581 1.141261

0.06858878 1.170903 1.034152 0 0.2293548 0.439906 0.2437768 0.326267229

0.5603107 2.678628 0.4898851 0.112693 0.4986823 0.3987327 0.8958661

0.3590953 0.4899875 0.3308529 0.1301648 2.394909279 0.4119639

0.4632213 1.322403 0.123185313 0.04199976 0.5792514 1.611594 0.0458741

0.4054717 0.517363 0.7489529 0.4673657 0.3075019 0.6765113

1.960871 0.6086732 0.08683401 0.997596 0.053057336 0.2073848 0.2136318

0.6176503 0.3796133 0.3125229 0.1699084 0.534739666 0 0.02456185

0.9711201 0.533963563 1.627907 0.137374 0.5698978 0.2832421 0.1685213

0.5105077 0.7970759 0.3180932 0.2486899 0.8578292 0.150416312

11.09595 0.293434102 0.3001874 0.8827548 0.161842 0.44874 1.996477

0.2646652 0.1289042 0.08909442 0.8343024 0.3443616 0.8403103

0.7066623 0.6227423 1.309911 1.376987 1.33624 1.426777 0.3451577

0.4909022 0.852521107 0.241403 0.3500078 0.2144611 0.07531206

0.070368524 0.4685092 0.5237203 0.3488213 0.3100182 0.328768

0.0620905 0.2860331 0.3476869 0.7067997 0.6715074 0.5774658

0.02754323 0.7495716 0.1595203 0.3253642 0 0.4486329 0

0.04439449 0.731631765 1.115859 0.2580287 0.069346189 0.6230349

0.3150685 0.2913896 0.4711398 1.118634 0 0.074751801 0.8476153

0.09244106 0.6701462 1.063059 0.4850395 0.6314402 0.3037605

0.4603609 0.0930726 0.100678298 0.2186323 0.141409 0.5459099

0.2222397 0.1630656 0.4549023 2.206761 1.292269 0.1042411 0.3691517

0.1412335 0.316981 0.4387001 0.4803687 0.5895605 0.5269475

0.2206898 0.9490918 0 0.9281264 1.360557 0.233146 0.3116759

0.5491934 0.478186 0.103299821 0.1180297 0.04259491 0.3006046 0 0

0.1182015 0.7704736 1.58869 2.486314 1.734896 1.09625 0.03910527

0.2506462 0.7993751 0 0.4121136 1.178343 0.8323453 0.064368693 0

1.140597 0.265586615 0.203725996 0.6989776 0 0.1534127 0.5390077

0.5288227 0.09568534 0.4978861 0.1854313 0.4415833 0.879353479

0.2603549 0.7420328 0 0 0.4996489 0.6402796 0.3863869

1.855252 0 0.8986254 0.5664974 0.119588571 0.5328507 0.420055963

0.6004547 0.2327338 0.7505013 0.8474103 0.5723856 0.6409386

0.548371 0.03873741 0.6046113 0.210652259 0.7587002 0.2406531

0.5555308 3.399908 0.2929872 0.8260886 0.9312781 0.2102071

0.2653503 0.3692134 0.3958832 0.4832096 0 0.1961113 0.496488204

0.6370417 0.1558677 0.03509311 0.204706 0 0.1305053 0.3876269

0.1786017 0.8784614 0 0.193127963 0.288372064 1.983432 0.07083672 0

0.4332341 0.2626361 0.15039 0.5525728 0.03975239 1.061794 0.3934122

0.9692058 0.5237223 0.6220019 0.5325214 0.8122271 0 0.3819968

0.07292174 0.071320589 0.5672517 0.08038876 0.08010441 0.08929579

0.1926288 0.1979649 3.993235 1.009953 0.5086077 0.5353945 0.4369184
0.50218952 0.04168628 1.144786 0.9860373 0.03511874 0.0395009
0.235364874 0.5658326 0.3387209 1.306999 0.1967366 0.2850636
0.9742502 0.144190525 0 1.340756 0.5448415 0.1624085 0.26692786
0.05977961 0.114934 0.1490009 0.604442064 0.119370033 0.2808775
0.3955864 0.1598962 0.2917425 0.7705435 0.07331079 0.7333158
0.72303 0.5692751 0.3209713 0.227398 0.5847581 0.459445685 0.08363005
5.613289 0.6220951 0.5529537 0.406168 0.5117609 0.5754553 0.0600456
0.8331026 0.941742 0.6684745 0.1424815 0.1369161 1.465727 0.8858235
0.3285736 0.9275301 0.05141285 3.025415 1.112134 1.295854 2.236972
0.3370141 0.3217724 0.304226 0.2645253 0.350064 0.2354736 0.06656835
0.711182 1.46638 0.3361031 0.3535458 0.341379 0.2923882 0.05575458
0.5363248 0.1975848 0 0.155551 1.594527 0.1451215 0.6843322
1.199658 0.5505541 1.074895 0.535411 0.642164351 0.348486 0 0.9151339
0.234213 0.2607414 0.034076213 0.143901683 0.1248205 1.239067853
0.4517164 1.361355 0 0.7615149 0.2417563 0.1601488 0.6060653
0.2658852 0.3521773 0.1004377 0.9554008 0.29256338 0.101033 1.13693
0.04452838 0.8349904 2.288879 0.09194096 0.8979101 0.8051225
0.1771513 0.2726975
AC034236.2 0.8276919 0.6427675 0.5319593 0.570042012 0.223372772
0.3853173 1.699085 0.9234602 0.5526191 0.2956293 0.4719856
0.407388047 0.896739 1.028352322 0.962152254 0.237472 2.066937 1.573077
0.3267897 1.844859 0.507606484 0.7813127 0.7814992 0.1501652
0.9115811 0.5950788 1.159329 1.338383 0.9574099 0.625177224 0.7545911
1.143499887 2.870516 1.259205 0.7443016 1.811767764 0.7510579 1.289725
0.9253447 1.713059 0.7919515 0.7970729 1.580233 1.29707 0.5907269
1.561591 1.166052 0.4525767 0.6304211 0.5178768 1.029485 0.8807611
0.5416387 0.61020303 1.915283 1.279509 1.594206 0.6544244 1.007276
1.088769 1.312802 0.5104159 0.2849176 0.5250785 2.599955 0.772643351
0.6009726 1.736149 1.322054 0.373228544 0.551423 0.6500088 1.096376
0.679506543 1.458848 0.6966727 0.5602933 0.6922818 1.721008 2.224892
0.9410211 1.297747 1.008515 0.7419648 0.107169227 0.2967148 0.6472646
0.5544787 1.022362 2.647774 0.7092673 1.120111688 0.3445624 0.4961189
1.516926 1.797566778 0.7855073 0.81702 0.4412638 3.541657 0.6713287
0.9967901 2.012493 1.177933 0.3348818 0.5896022 0.891211974 1.062921
0.703831766 0.6467639 1.370311 0.5666281 0.660916 4.087878 0.2851152
0.9836219 1.244721 1.148991 0.6607893 2.184517 0.6920582 0.4192874
0.7397021 1.049563 1.616091 0.66397 3.214754 0.8413248 1.281923457
1.607288 0.6319903 0.2681621 0.3929793 0.663299831 0.5823572
0.7693454 1.808411 0.8117384 0.4565485 1.212346 1.973983 0.4681896
0.4670699 1.741994 0.2221732 0.4450713 0.8201058 2.054097 2.149576
0.8712531 1.170486 0.6388637 1.360015 0.426921525 0.9391232 1.6649
0.87544179 1.1798 1.193249 1.63959 0.5419084 2.05552 1.206446 0.440385608
0.665808 0.6068382 1.208582 1.582182 0.9661122 0.8127745 0.7413833

1.4077230.5326528	0.457554723	1.76644	0.5712572	1.09246	0.1496322
0.7593859	0.8599457	1.8633321.3553080.6316623	0.8284896	0.8677087
1.1145310.8592675	0.8526748	0.6395246	1.8981241.2778621.59754
0.293893658	1.8003073.0535070.6906913	1.5388910.9528623	0.7941649
0.463672905	0.9801106	1.6490320.7175811	0.2956141	0.830947188
0.6764651	1.2450092.7174940.9043532	0.8395657	0.4244055	0.4081036
0.5554953	0.9814469	0.4927792	0.5241152	0.9421256	2.521851
0.541736516	0.438677193	2.3038640.702496627	0.397784498	1.0981040.458122323
1.2265871.2157471.17695	1.578392081	2.7004050.6970758	3.1748940.835851666
0.5916201	1.4079771.8931040.2932339	0.4485461	0.7409447	0.2081209
0.6146495	0.4155657	0.3226865	1.2895560.993055569	2.8925350.385664029
2.1500420.6529074	0.87025	2.6849620.6262467	0.5451017	1.426508
1.9561190.6106199	1.264654977	1.0771780.4536834	0.5460902	1.474728
1.4356581.9466960.6270220.7076538	0.5717062	1.3361630.4738577
0.6777941	1.2594781.3336052.039117143	0.6433726	0.6821392	1.039628
0.3014964	1.0148510.3807621.2788321.2926992.5137090.9542916	0.455110244
0.695734919	1.3785071.2161921.1557261.7103820.5894359	1.3163330.9025484
0.7092715	0.3839265	1.3244060.8972679	1.0485740.7328805	0.6402543
0.2594108	0.4740414	0.7122334	0.7610131	0.576234919	2.877176
0.8389391	0.5483255	0.7966183	0.6160531	1.3728680.3734185
0.4799954	0.9245919	1.2780541.1031510.477346093	1.75419	0.9634689
0.7845984	1.1113230.5452105	0.607465533	0.4058164	1.14029	1.852076
0.5795176	1.3243240.5980764	0.691711504	0.5833375	1.1078841.369526
0.4920674	0.339471816	2.2137020.8125332	1.1035321.390467034	0.388459252
0.4525194	1.2695780.2825992	1.2627512.3922480.7897531.0600790.8397477
0.6425716	0.5248322	1.0121960.4284523	0.905927418	0.6475355
0.6216089	1.6270780.4212860.6090915	1.1198340.8007287	0.6468515
1.9231590.4838935	4.2194861.1751630.6729469	1.6036510.8449252
0.5372630.7181733	0.7615488	3.37204	1.14565	1.43799	1.3600181.150752
1.2998811.0680570.4007312	1.4478420.7927125	1.0532681.6655061.308749
1.8588560.2578762	0.4826803	0.6201173	1.0886341.6069091.995483
0.9194156	0.3141937	0.7540636	0.8427414	0.9215107	3.760076
13.344612.4660010.6128295	0.668199021	0.7309715	1.4910481.928825
2.6413690.3803694	0.401506682	0.423884306	0.3921895	0.304823716
1.0813760.6504829	0	1.5862330.4792748	0.6065263	1.0314821.718576
2.1902230.7438631	1.3460890.251697384	2.7720081.5309711.9037681.566107
1.0046311.7848730.3589549	0.9621964	1.1927451.621844
AC019183.1	0	0	0.1356760	0	0	0	0	0	0	0	0	0	0	0	0
0	0	0	0.02841367	0	0.3558454	0.3383535	0	0.1194341	0
0.1863569	0.06095604	0.1476937	0	0	0	0	0	0.04943587	0
0.2793539	0	0.07564382	0	0	0.07700494	0.0699718	0.04219604	0
0.1185366	0	0.04654414	0.08999361	0.2871396	0	0	0	0	0	0
0.04381481	0.1814246	0.06296699	0.04754964	0.1696772	0	0	0
0.04656722	0.053549539	0	0	0	0	0	0.1036153	0.1801740.295411035

0.02719872 0.2523949 0 0.4514475 0 0 0.04477742 0 0 0 0
0 0 0 0 0.2515654 0.2553 0.031884367 0 0.1581686 0 0 0
0.04914636 0 0.04342778 0 0.07305483 0.1069344 0 0 0.07672334
0 9.97251 0.039366654 0 0.0526351 0 0 0 0 0.09223246 0 0
0.3695921 0.1503129 0.1378977 0 0 0 0.5311639 0 0
0.04789721 0 0 0.06830035 0.09864606 0 0 0 0.05109926
0.07487571 0.2218216 0 0.3331985 0.03069899 0 0 0 0.04426966
0.05912251 9.9220520 0 0 0.1805636 0 0 0 0 0 0.148853997
0.1253781 0 0 0 0.5502721 0 0 0.05053988 0.2480349
0.1027494 0 0.08676283 0.03986495 0 0 0.06659452 0.108054727 0
0.3035393 0 0 0 0.1126689 0.03882696 0 0.1118786 0 0 0
0.1712154 0.04686946 0 0 0 0.1527944 0 0.2371733 0
0.1501368 0 0 0 0.036956108 0.1689032 0.04571573 0 0 0
0.0761171 0.2205131 0.09216696 0.1840331 0.2715427 0.1764854
0.08394082 0 0 0 0.1474360 0 0 0.121613479 0 0 0 0 0
0 0 0 0 0.05937387 0 0 0 0.1397152 0 0 0.9348646
0.1787523 0.04294945 0 0.2212424 0 0 0 0.085567004 0.0714864
0 0.02929311 0.2497856 0.4176607 0.1069997 0.7167099 0.07241037
0 0.0415756 0.09270136 0.113043093 0.07080767 0.5682272 0.02384932
0.03508664 0.03493929 0 0 0.09024338 0 0 0 0 0 0 0
0.1139527 0 0.3013143 0 0 0 0.2080137 0 0.4714119
0.09507485 0.041455587 0 0 0.3041069 0.04605739 0 0.09395959 0
0 0 0 0 0.05201085 0 0 0 0.1117611 0 0 0.03913226 0
0 0 0 0 0 0 3.8096080 0.1091744 0 0.3223895 0.253639343
0 0 0.09620742 0.1884590.08479004 0 0 0.03029484 0 0.1055755
0.1223798 0 0 0.07293134 0.04360576 0.07796808 0 0.031831661
0.5774355 0 0 0 0.042705319 0.4306524 0 0 0.0447311 0 0
0 0 0.03594026 0.1476377 0.1627059 0 0.105666009 0 0 0 0
0 0 0.4117449 0.06444499 0 0.05319689 0 0 0.05877904 0 0
0 0.06636585 0.3310784 0 0.1193617 0.1030221 0 0.1033446 0
0.09329025 0.3193947 0 0.1516357 0 0 0.07320083 0 0.06324153
0 0 0 0 0 0 0.05564928 0 0.3893854 0 0.0887968
0.1969639 0 0.1149278 0 0.02877067 0.1625175 0.2562217 0
0.03109392 0 0 0 0.102296234 0 0.0471322 0 0 0 0.08594125
0.1806861 0 0 0 0 0 0 0 0 0 0.03109594 0.09867723 0
0.04320558 0.1901307 0.1625985
AL031429.2 0.3422147 0.5158802 0.1319653 0.487629584 0 0.07080533
0.4103085 0.3579475 0.9273764 0.9712319 0.08673137 0.116610422 0
0.130824324 0.711867305 0.1840957 0.07171373 0.5700127 0 0
0.048432289 0 0.07180355 0 0 0.08857413 0 0 0.07661563
0.126604236 0.2303931 0 0 0 0.03846705 0 0.1242119 0.04570674
0 0.1573946 0 0 0.1088929 0.3283359 0 0 0 0.1810847 0
0 0.03192355 0 0 0.030275096 0 0 0.03409319 0 0 0.07399867
0 0.1666064 0.07440068 0.1184166 0 0.041667977 0 0 0

0.10287587 0 0.2418754 0 0.268176171 0 0.1178362 0.03474854
0.03903116 0.03210055 0 0.06968443 0 0.1450356 0.03874992 0
0.3896852 0.02230132 0.143283 0.07045041 1.544235 0 0 0 0
0.0324405 0 0 0 0.09518787 0 0.02345622 1.449559 0.1664157 0
0 0.1194 0.025123464 0 0 0 0 0.08109558 0 0 0 0.2153038
0 0 0.2300696 0.03898718 0 0 0.03528862 0 0.02066547
0.1051364 0 0.4845072 0.284786712 0 0 0 0.06289544 0.058766934
0 0.1590455 0 0 0.06864109 0.0518537 0 0.1244418 0 0 0
0.02300221 2.608294 0 0 0 0.0234167 0.06890671 0.03707521 0
0.05177156 0 0.028956575 0 0 0.06952814 0.1311545 0.07785052
0.06960184 0 0.0393261 0.6176031 0 0 0 0.1550986 0.1395239
0.06407694 0.3109113 0 0 0.15746 0.05065626 0 0 0.02922331 0
0 0.05803665 0 0.1179485 0.07353352 0.1332262 0.03647007
0.8206004 0 0 0.0396308 0 0.07381973 0 0.2336489 0
0.2469645 0.02662319 0 0 0 0 0.04888945 0.997433997 0.1184565
0.3431714 0.1792924 0.04773329 0 0.02288781 0.06531604 0 0
0.2933897 0 0 0 0 0 0.1002682 0 0 0.2918689 0 0
0.8102556 0.02453535 0 0 0 0 0.172794194 0.2174304 0
0.03089669 0.01966046 0 0 0.06453673 0.02459331 1.49482 0.3252036
0.02252855 0.133162826 0.05562501 0.031891068 0.02279356 0.1295755
0.02321359 0 0 0 0.1248986 0.2911573 0 0.029320371 0 0 0
0 0.02718697 0 0.0740704 0.0351101 0.04432045 0 0 0.1008858
0.2840398 0.2620457 0.082926535 0.06650166 0.26034 1.172294 0.08547816
0.2323922 0 0.03237193 0 0.1100445 0 0 0 0.1068661 0
1.003469 0 0 0.408185 0.03418301 0.03319845 0.1313685 0.06571014
0.1618828 0 0.0236115 0.06353216 0 0.2138133 0 0 0 0
0.03356757 0.3344884 0 0 0 0 0.3473002 0 0 0.02280525 0
0.4177621 0 0 0.02932874 0.09896527 0.04368011 0.4109081 0 0
0 0 0.04786038 0.210731453 0.2837467 0 0.5763511 0.2034485
0.049537716 0.09984761 0 0.4562627 0.210328453 0 0.01675497
0.04404886 0 0.03480618 0 0.06122411 0 0.1509562 0.08389752
0.03829329 0.02110079 0 0 0 0.06168199 0.1598557 0 0
0.06105533 0 0 0.04969642 0 0 0 0 0 0.08219763 0
0.02582031 0 0 0 0.2004089 0.02788306 0 0 0.03629551 0 0
0.03933027 0 0.02582305 0 0 0.09841903 0 0 0.372499 0 0
0.0281584 0 0.03170569 1.06046 0.09939253 0.03454729 0 0
0.05961844 0 0.06716111 0.1580727 0 0 0.120974 0.056916202
0.270397693 0.1389886 0 0 0 1.133901 0.05962169 0.1570319 0
0.02811911 0.1776392 0 0.05591911 0 0.027147638 0 0 0
0.2988542 0 0 0.04686703 0 0.2219168 0.05060847
AC131097.3 0.0259308 0.9061783 0.02999841 0.498816883 0.136918512
0.2414325 0.5087538 0.9764257 0.1374859 0.4505733 0.2464477
0.441798973 0.3160573 0.743476239 0.523541845 0.2510923 0.285285
0.3488573 0.0287944 0 0.27524129 0.0899184 0 0.08468158

0.1408388 0.1006736 0 0.02695517 0.3047853 0.035974671 0.2945986
0.191267828 0 0 0.1748870 0.0352949 0.1039008 0 0.4472377 0
0.0340521 0.06188399 0.1865937 0 0 0.5208423 0 0.05306103
0.06348753 0.07256877 0.1318623 0 0.068821498 0.1636473 0
0.0775008 0.08022716 0.05568884 0 0 0.07574612 0.126846 0.1345926
0 0.09471985 0.086898 0.02931301 0.03803759 0.077952644 0.1993334 0
0 1.088606047 1.37113 0 0 0 0.1459422 0 0.03960174 0.06419553
0 0.02202163 0 0.09842604 0.1774341 0 0 0.2719296 0.06451163
0.084596877 0 0.0233144 0 0 0 0.04346569 0 0 0.05332079
0.03230533 0.09457425 0.07548445 0 0.1017827 0.256998518 0.1712587
0.104449179 0.05698829 0 0.2765203 0 0.88264 0 0.203929 0.08456948
0.07199362 0.09806165 0.04431292 0 0 0.04010914 0.07398411 0
0.1194983 0 0.5506920 0 0.06040574 0 0.1429742 0.400767849 0
0.4067358 0.06622107 0 0.07801763 0.05893705 0 0 0 0.1499771
0 0 0.5336267 0 0 0.4367285 0.02661548 0.1566391 0.08427955
0 0 0.08164131 0.032912114 0 0 0 0.4099439 0.04424254
0.07910962 0.03547765 0 0 0 0 0 0.2115426 0.1297499 0
0.4417281 0.525607715 0 0 0 0.07031752 0 0.1328611 0.03433908
0 0.1319292 0 0.2346059 0.4011762 0.1135689 0 0.4041685 0
0 0 0.036028995 0.04195184 0.09934284 0.04426099 0.03287183
0.08020014 0.03025999 0.065368947 0.07469011 0.0404316 0 0.1111357
0 0.08975863 0.04875618 0.08151369 0.02712689 0 0.05202869 0
0.01982636 0 0.0555779 0 0 0 0.12219906 0.026889145 0
0.072027994 0.116027485 0.03685988 0.04454236 0.07281058 0.3069795 0
0.045412832 0 0.0880068 0 0.049099576 0 0 0.2107035 0.2011151
0 0.3418656 0.07335261 0 0.2929338 0 0 0.037838299 0.1264471
0 0.5699588 0.02454596 0 0 0.04527627 0 0.04732002 0.07354003
0 0.466558476 0.1252465 0.04568615 0.08437064 0 0.03090077 0
0.0841886 0 0.3274357 0 0.0417530 0.07644472 0.1291361 0.03723023
0.031418168 0.1007813 0 0.0666216 0.07286602 0 0 0.03679401
0.05651029 0.1667692 0.1261282 0.109991618 0.273726197 0.06073216 0
0.5702727 0 0.1246487 0.214128 0.5827875 0.1509338 0 0.2240589
0.1379973 0 0 0.07221081 0.1581488 0.04050346 0.027892 0.1730455
0.101547528 0 0.07630597 0.2281082 0 0.1371341 0.1503285
0.1579344 0 0 0 0.05184102 0.028040251 0.1978456 0 0.5530663
0.9333834 0.1124842 0.02482346 0.02335196 0.05358631 0.05770321
0.2334309 0.08117574 0.05439823 0.37638525 0.06450145 0.1156965 0
0.1798534 0.028152345 0 0.05454835 0.1885779 0.286871803 0.037769152
0.1142624 0 0.2276631 0 0 0.06958748 0 0.06863086 0.1907162
0.04352425 0.02398321 0 0.031150808 0.2778392 0.2804316 0.1362693
0.1104990 0 0.2775826 0.03641527 0.1709880 0 0.09409608 0 0 0
0.2782571 0 0.02227757 0.02934742 0 0 0.08445209 0.1822282
0.1267678 0.04569969 0 0.2475214 0.03138632 0.04746927 0.1341086
0.1895624 0 0.0323699 0 0.05593165 0 0.08326151 0.8996896 0

0 0 0 0 0.3099399 0 0.07853307 0 0 0.03388124 0
0.1781161 0.07186634 0 0 0.2199990 0.170741489 0.1184811
0.090472151 0 0 1.2887950.02258872 0.02549755 0.03800379 0.06392049
0.05047628 0.05278277 0.0635578 0 0.030856072 0 0.0399699 0
0.1132261 0.02750166 0.08727145 0.07990378 0.03821159 0.2101926 0
LINC01081 0.1273676 2.0945840.5893880.816700903 0.067252017 0.6917604
2.1344862.5478940.9566848 2.2961310.3631526 1.03076926 1.908182
0.912956845 1.227329475 0.1541653 0.4003631 0 0.5303743 0.03085786
0.067596893 0 0 0 0 0.1648304 0.03373128 0 0.05346618
0.044175349 0.8440935 0.134210667 0.03736382 0 0.1073768 0
0.08668124 0 0.1643016 0.4393509 0.1054625 0 0.3039635
0.1374774 0.06197916 0 0.07994649 0 0 0.3118398 0 0.1079475
0 0 0 0.0843834 0.09516765 0 0.06838351 0.1032799 0.1228487
0 0.05192048 0 0 0.116311902 0.05335351 0.03599511 0.04670853
0.239306235 0 0 0.03913457 0.053470456 0 0 0.09699701 0
0.1344081 0 0.09725848 0.07882935 0 0.02704161 0 0 0
0.0399960 0.03035616 0 0 0 0 0.1358315 0.155595834 0.1264983
0 0 0.0471635 0 0 0.1161331 0.04634583 0 0 0 0.1051491
0.042753033 0 0 0.5659251 0.06538094 0.03187777 0 0.3004993
0.05192384 0 0 0 0 0 0 0.09084931 0.02884277 0.2445646
0.04470142 0.9363131 0.066246085 0.03126414 0.6675808 0.03571058
0.2633493 0 0 0.1109898 0.08131663 0 0 0 0 0 0
0.04604137 0 0 0.3640393 0 0.04740521 0.3351774 0.06536533 0
0.1034917 0 0 0.25063 0 0.04538776 0 0 0.3661044 0.05432794
0.09714321 0.043565028 0 0 0 0 0.0942263 0.04329419 0 0
0.4701 0 0.06370897 0 0 0 0.03167797 0 0 0 0.1620034 0
0 0.6568362 0 0 0.5344785 0 0 0 0.044242056 0.1030301 0
0 0.1614608 0.1969646 0.03715796 0 0 0 0 0 0.64965486
0.02755493 0.4190935 0.2001906 0 0 0 0 0.02434592 0
0.3412364 0 0.06867333 0.09701745 0 0 0 0 0.047492254
0.4978858 0 0 0.6910890 0.055765004 0.0644813 0 0.07352939
0.060292168 0.3793343 0 0.2156124 0 0 0 0 0 0.5755361 0
0.4402030.046463803 0 0 0.2226906 0.2109897 0 0.2324080.05559731
0.1572784 0.1743209 0.6321280 0.450146511 0.03844932 0 0.02590088
0 0.03794482 0 0.2584499 0 0.4330060 0 0 0.2378604 0 0
0.1856326 0 0 0 0.05405825 0 0 0 0.05119635 0 0 0
0.1118647 0 1.2004640 0 0.26294 0 0 0.09167544 0.2751347
0.3953943 0 0 0.3546871 0.0242750 0.2740014 0 0.374087991
0.1322367 0 0.2489840 0.0561316 0 0 0 0.07904383 0 0 0
1.3119080 0 0.1228023 0 0 0.1147008 0.03290084 0.07085706 0
0.2990410 0 0.15841 0.3314975 0 0.1577516 0.034569869 0 0
0.1736742 0 0 0 0 0.04659342 0 0 0 0 0 0.1170957
0.05344591 0 0 0.076503705 0 0.3443580 0.0678440.1291162
0.5112892 0 0.2449602 0.06936123 0.05777297 0.1168752 0.04151881

0.0638353 0.08542194 0 0.02735589 0.1441495 0 0 0 0
0.1167491 0 0 0.05065761 0.03854105 0 0 0 0.0720824
0.03974884 0.1119311 0.1717042 0.03979085 0 1.169767 0.06251355 0
0 0.06043631 0 0.2537286 0.03468051 0 0 0 0 0.544364541
0.03124556 0 0 0 0.06753732 0 0.461259051 0 0 0 0 0
0.02773798 0.06261979 0.1400011 0.03924581 0.1239654 0.04320998 0
0 0.037889929 0 0 0 0 0.06754172 0 0 0.09384438 0.103243
0.1059513
AL513327.2 0.1262813 0 0.07304516 0 0.100017659 0.1469701 0.1548499
0.09906522 0 0.02742829 0 0.107576637 0.4489272 0.045258522
0.057945802 0.1528504 0.4961856 0.06067546 0.07011345 0.2447574
0.067020374 0.1642113 0.2980843 0.1374646 0.3000707 0.9396916
0.3344359 0.7548022 1.060204 0.131395761 0 0.465731044 0.2222709
0.4156389 0.2129221 0.034361886 0.2578259 0.3162439 0.8959518
0.2178019 0.1045631 0 1.205484 0.9995693 0.1843517 1.616715
0.2377939 0.2505841 0.2261034 0.3091802 0.1767026 0.4281074
0.1690324 0.209472682 0 0.1673274 1.17945 0.1302337 0.6441026
0.4607956 0.5481042 0.3688787 0.1029553 0.2184858 0.1002833
0.461279612 0.1586954 1.213396 0.6020321 0.142359146 0.1941482 0
0.582012 0.212057673 0.4685835 0.2174148 0.384679 0.6481336 0.8439911
0.661532 0.4821449 0.3907852 0.1505246 0.4021647 0 0.2097062
0.4320465 0.1586195 0.2437224 0.1504863 0.1178128 0.446313159
0.07168644 0.3122341 0.8080381 0 0.1254194 0 0.3951616
0.8417026 0.162293 0.07866245 0.5757132 0.09190112 0.2155489
0.1652251 0.139062927 7.506168 0.169553607 1.318264 0.3400519 0.03740656
0.2592933 0.3792706 0.1835163 0.04965607 0.257405 0.3506043
0.1989808 0.1079005 0.1484826 0.1919126 0.1953289 0.4203475
0.7435162 0 0.1329605 0.1031475 0.262724344 0.0309975 0.9192877
0.03540601 0.5222065 0.081321408 0.8329736 0.3301296 0.1612462
0.07961613 0.3799409 0.1435098 0.396665 0 0.09075703 0.2282435
0.09533551 0.09549104 5.125269 0.1843497 0.0940018 0.1993913
0.09721176 0 0.102609 0 0.1432825 0.2484925 0.040069972 0.1800026
0.2730818 0.4329572 0.1361182 0.2154584 0.0963147 0.172773885
0.1088385 0.3204886 0.3319092 0.4202846 0.2335567 0.5580243
0.2808328 0.1773387 0.1434125 0.058174453 0.3158281 0.2723656
0.3504894 0.04280523 0.1884468 0.1213172 1.421448 0.2871944 0
0.09480229 0.04080413 0.2238618 0.09217888 0.05046712 0.1514057
0.5480704 0.1020161 1.041977 0.08772945 0.2043028 0.2822127
0.05388704 0.5602929 0.09764238 0.2210463 0 0.5910711 0.7875972
0.06316251 0.3382649 0.552097795 0.2185594 0.2967994 0.1984832
0.8917169 0.5430027 0.6967851 0.1355761 0.1206914 1.03248
0.2706608 0.8466831 0.06808763 0.19238 0.148775401 0.425582352
2.011884 0.745389908 0.047087203 0.1346289 0.054229611 0.7091658
0.06229044 0.5432304 0.110578793 0.5114508 0.2500094 0.2551579

0.47822359 0 0.1559146 0.0855094 0.4625029 0.1924731 0.647447
0.2679169 0.7487064 0 0.2423159 0.2805741 0.829215421 0.1539473
0.308914969 0.2207914 0.2988432 0.09636856 0.3456388 0.05512313
1.4424180 0.2880569 0.3581352 0.2994511 0.162293567 0.762428 0.2781108
0.3338397 0.6044776 0.1128636 0.1591116 0.2562457 0.3886815
0.09199564 2.2934140 0.1525008 0.04653511 0 0.04532721 0.191255584
0.1226996 0.2701929 0.3244429 0.3844239 1.33993 0 0.04479612
0.03440019 0.2030388 0.1535591 0.044637679 0.055542878 0.07394043
0.2455874 0.04959271 0.3186064 0.4046874 0.04344958 0.09460459
0.09187972 0.5908081 0.1818588 0.3920221 0.2229831 0.4247553
0.351662 1.5644170 0 0.3056225 0 0.206054153 0.7866536 0.0929013
0.1542878 0 0.2226115 0.3202895 0.7691306 0 0.3526636
0.2249923 1.3254280 0.273108461 0.1445243 0.2939938 0.1553883
0.08116996 0 0.090666497 0.2274451 0 1.896824 0.2841984 0.2635471
0.1324579 0.291609196 0.1963237 0.469529 0.3777878 0.3128123
0.102825089 0 0.265647 0.5165789 0.232841222 0.137950017 0.1391127 0
0.09239206 0.09632925 0.3462973 0 0.3489527 0.2506709 0.5804849
0.4239206 0.1459959 0.3577637 0.113776833 0.04832355 6.657709
0.3318113 0.4372249 0.128015 0.3379523 0.310345 0.03469586 0
0.4582419 0.1545046 0.2058235 1.075945 0.08469339 0.3981064
0.1898581 0.1429201 0.2970764 0.7559615 0.3598668 0.1109299
0.3086755 0.1669159 0.3718564 0.05022556 0.1910617 0.75131
0.05442504 0.5385081 0.2501367 0.3152786 0.1109765 0.1702397
0.03945148 0.1013696 0.1288656 0.3099019 2.603063 0.3506892
0.1198417 0.5703643 0.08385486 0.06876946 1.147353 0.9013513
0.5060824 0.6187479 0 0.2788117 0.04374806 0.3218706 0.2030012
0.100442 0.393801863 0.083150013 0.2404147 0.275370992 0.1740087
0.9135004 0.7845438 0.6875351 0.4035572 0.4164211 0.3891109
0.1229081 0.2784694 0.2708321 0.5316081 0.187833869 0.7589326
0.6326153 0.1029185 0 0.9375194 0.3187548 0.5512623 0.651308
0.3070874 1.505683
AP003419.3 0.7732595 0 0.6626340 0.48390233 0.7555079 0.374595
0.4942722 0.1012307 0.02488176 0.2177511 0.195177728 0.3490691
0.328452559 0.157697763 0.5546372 0.8102125 4.183203 0.572435 0.4440667
0.425585856 0.7944821 1.111682 0.4676323 0.388873 0.8895104 0.5157558
1.071742 0.3366197 0.516518348 0.3615211 0.603558607 2.419615 0.9803291
0.5311727 0.280544524 0.07796282 1.434414 0.5911045 1.580644 1.565109
0.1504352 0.7518247 1.071632 0.780434 1.080663 1.042629 0.09092763
0.4395239 0.7011872 1.04193 0.461178 1.022259 0.342044174 1.17481
0.2656363 1.840307 0.4725695 1.107099 2.600975 2.265097 0.2091443
0.0466983 0.2973013 0.8187543 0.889212367 0.479872 0.3884966
1.848465 0.301331448 0.440307 0.9108924 1.583928 0.288554299 0.5579158
0.5423806 0.8724102 0.1959862 0.8865215 3.600679 0.6998095
0.9926095 0.1365494 0.09728704 0 0.2989429 0.7278771 0.4316783

2.210944 0.3822411 0.7124979 0.872040531 0.6503083 1.338977 2.321221
1.749324536 0.4551003 0.09601126 0.4779646 2.842126 0.4711207
0.2140775 2.61131 0.7503184 0.5866099 0.1124138 0.252303724
1.702318 0.192264607 0.692347 1.336748 0.4072034 1.352513 3.497922 2.386185
0.1351375 5.417354 0.755376 0.2527095 2.349185 0.7408336 0.2176185
0.2214924 0.4085583 1.867807 0.3079527 1.12575 0.6082111 0.238332142
0.6467505 0.900653 0.1927128 0.03947692 1.696738929 0.6800738
0.3493925 0.5119645 0.8305795 0.3446659 0.5858365 2.548848
0.833142 0.2195489 1.076673 0.3891791 0.7507529 0.3929089 1.045213
1.577576 0.9646886 0.7054904 0.994749 1.256613 0.255670159 0.5849084
2.254216 0.654295372 0.5715171 0.330304 1.091 0.3704417 0.5374998
0.6116076 0.901214649 0.246834 0.2907334 1.354921 1.114464 0.593243
2.102741 0.7005881 1.809833 0.162622 0.263866718 0.9741188 0.04941565
1.939488 0.3494794 1.054196 0.5502686 2.5031 1.146333 0.2914181
0.5160032 0.6292676 0.3323089 2.132328 0.5035974 0.377709 2.485928
1.526985 1.591978 0.159168712 2.919021 1.133764 0.7332599 3.267472 0.5314616
0.2005236 0.613671538 0.4537027 6.56423 0.3724389 0.3682311
1.085151597 0.09913381 1.561612 0.1800554 0.7490064 1.023068 0.9481399
0.2459774 0.1970748 3.056343 0.5524465 1.104107 1.111791 0.4799267
0.202443897 0.267279141 4.4054 0.397756135 0.04271547 0.7327773
0.787116058 1.206233 1.017129 0.7391926 1.203747484 1.91386 0.7451923
2.347749 0.650735582 0.1364723 0.848634 0.4654225 0.1727611 0.4656088
1.132719 0.810142 0.4630869 0.06470607 0.5966502 0.5656103 3.385027361
1.256889 0.400334679 0.9442354 0.1897683 0.6410901 4.546454 0.4000425
4.385239 1.567876 1.665034 1.49407 0.220838538 0.3804029 1.160534 0.3727322
1.405162 0.6143098 1.010374 1.673676 0.1322231 0.1947271 3.338453
0.1383422 0.5065757 0.7131212 0.4111888 1.179791699 0.7791542
0.2451073 0.8093816 0.8047678 0.7293159 0.5472639 1.178476
1.310667 2.394444 0.7893787 0.40493368 1.05810794 1.240898 1.262456
0.7198137 0.6606306 0.780119 0.1576623 0.1501871 0.7084691
0.2061367 0 1.320894 0.2311778 0.8891991 0.8772846 0.6550023
0.134202 0.8009373 0.1146719 0.48600084 2.458018 0.4635182 0.6998161
3.089254 1.009717 14.61065 0.0581435 0 0.7109358 1.479749 0.7729526
0.278721183 0.4370206 0.2666985 0.09397437 0.3681693 0.41411
0.137081197 0.1547462 0.4734667 1.083414 0.7734374 0.4482722
0.4806404 0.188953735 0.1424771 1.831526 0.2665543 0.2837698
0.248742575 0.8773843 1.265163 1.197579 0.264029335 0.208570465 0.6940834
0.2764769 0.2095352 1.354479 0.9873158 0.768558 0.9948865 0.7579927
0.9478634 1.057547 1.880664 0.4687911 0.412853652 0.4822073 4.723266
1.354522 0.5796922 0.7354876 1.264625 0.5630631 0.09442375 1.185312
0.6235458 0.9811189 1.680428 0.4880252 0.6914716 0.9286583
0.2460443 0.5186036 0.1616969 3.043129 0.3264555 0.5031541
0.8050491 0.2523648 3.246821 0.3644996 0.2079875 2.673796 0.5924644
0.4885112 1.653225 1.430036 2.516826 0.06177364 0.07157734 0.1226108

0.3507039	0.9558407	3.852788	0.7069557	0.3805033	1.07462	0.3803475
0.6550391	2.688808	15.67996	5.00832	0.1871004	0.428410682	0.1686173
3.611457	1.459935	3.069232	0.151861	0.428687986	0.150860178	0.5670438
0.549570193	1.262825	1.473223	3.558521	0.3243251	0	0.3777591	0.6000737
1.170718	1.049325	0.3158835	2.29997	0.170394748	2.012453	2.118946	0.560179
0.562736	1.30609	0.9638685	0.6177474	1.266082	1.53217	1.747069
OPA1-AS1	0	0.06124131	0	0	0.157304769	0.09246007	0	0.04674204
0.0526522	0	0	0.050757991	0.1815581	0.042708746	0	0.04807972	0
0	0	0.2020973	0.126489156	0	0.1562727	0	0.2427131	0.0771088
0.2524755	0.09290584	0.2501184	0	0.03760692	0.031392334	0.0349581
0.1176668	0	0.032426005	0.04055008	0.05968546	0.3074457	0	0
0.03912223	0	0.04287525	0.05798855	0.08029645	0	0	0.03048073
0.0729404	0	0.02524929	0.05316983	0.039534281	0	0.07895026
0.04452008	0.2457932	0.06398054	0	0.4597557	0.2175605	0.09715502
0	0	0.217646014	0.04991827	0.3030977	0.2622068	0	0.2290128	0
0.03661484	0.150083071	0.1658192	0.1538746	0.2268793	0.3567777
0.08383603	0	0.04549818	0	0	0.2277045	0	0.02827026	0
0.1122624	0.04599831	0.1420082	0.03705849	0.064795193	0.03382388	0
0.1694477	0.072788796	0.2367071	0	0.1242997	0	0.03062995	0
0.1086557	0.216809	0.03390088	0.03897916	0.032807099	11.01844	0	0
0.160447	0	0.1835139	0.05965055	0	0.04685854	0	0.0413565
0.1502165	0.05091082	0.07005871	0.1811006	0.1382434	0.02833329
0.1619142	0.1830544	0	0.09733637	0.061980743	0	0.277599	0.2338792
0.1642621	0	0.1965114	0	0	0	0	0.1354248	0.03119314
0.1625004	0.02854799	0.08615387	0.04498225	0.09011126	7.697563
0.1304729	0.04435296	0.06271931	0.09173504	0	0.04841411	0.212766957
0	0.04689858	0.037812509	0.08493082	0	0	0.1712661	0	0.09088852
0.122280109	0	0.100811	0.3132101	0.1220328	0	0.08101328	0.2153216
0.04183695	0	0.109794039	0.178821	0.1028084	0.1322974	0.04039366
0.2963834	0.03816082	0.2761636	0	0	0.134192	0	0.03840906
0.04349285	0	0.1071569	0	0	0.3105077	0.041393473	0.2891892
0.03804477	0	0.03776622	0.0460707	0.486717	0.112652924	0.04290549	0
0.08940609	0.3192078	0	0.1804654	0	0.1873011	0.623318	0
0.3885402	0.127938	0.06833513	0.5127959	0	0.1498092	0.03212586
0.04538543	0.140393689	0.03089276	0.3928011	0.124128725	0.044434402
0.08469615	0.051174422	0	0.05878112	0.09611735	0.052174501	0.1206592
0.1685174	0	0.056410177	0	0	0	0.05134663	0.1816296	0.4364079
0	0.3853777	0	0.09799899	0.02941857	0.04347217	0.07263709	0	0
0.0282007	0	0	0.1560528	0.1103639	0	0	0.5651612	0	0
0.104977	0.1211661	0.2139085	0	0	0	0.22924	0.0289376	0.2013216
0	0	0.07418182	0.1283207	0.25267287	0.08684019	0.08499026	0
0.08371529	0.1011553	0.04744055	0	0.1298486	0.1437	0	0.08424576
0.052413702	0.2093243	0	0	0.4295096	0.04773601	0.04100171
0.06695607	0	2.701843	0	0.1056961	0.06012019	0.3083272	0

0.2952563 0.04653415 0.06408986 0 0.194445468 0.08248167 0
0.02911911 0.04869046 0 0 1.2096660 0.1848862 0.1061583
0.2382392 0 0 0 0.09775603 0.0382985 0.04307744 0.085558526
0.3219469 0 0.3646216 0.05363744 0.03108742 0 0.039311501 0
0.04430766 0.03961155 0.08855673 0.032344042 0 0 0.05416398 0
0.130178185 0.02187922 0.2588424 0 0.1363534 0.1400518 0
0.09408383 0.07884954 0.03651877 0.1000094 0.02755415 0 0.035788957
0.09120219 10.954320.1565588 0.1269516 0 0.1594564 0.1255118 0
0.1297906 0.1621595 0.2187001 0 0.1194504 0.07992193 0.2146731
0 0 0 0.2675145 0.1940529 0.05234018 0.1456427 0 0.04386335
0 0.03605954 0.1090743 0 0.07259566 0.06744128 0.1115687
0.05236214 0.2891678 0 0.0637724 0 0.1169771 0.2154747
0.03677023 0 0.1242072 0 0 0.2255653 0.05003359 0.1736621
0.1557037 0.445650548 0.02923378 0 0.04339101 0 0.09478327
0.185807921 0.039232753 0 0.15591428 0 0.2873452 0 0.1038081
0.05858793 0.0436623 0 0 0 0 0.3087128 0.070900672 0.05509045
0.3673692 0.09712024 0 0.1579824 0.06684373 0.1224012 0.1756042
0 0.1321726
AC007365.1 0.1180741 0.194175 0.08195737 0.252369697 0.099751796 0.1099346
0.1930478 0.2223043 0.1669419 0.1436157 0.314211 0.040234005
0.1679002 0.033853663 0.216719149 0.03811103 0.14846 0 0 0.1373101
0.100263335 0 0.07432298 0.2056488 0 0.3361673 0.02501602
0.02454769 0.1982597 0.065523245 0.08942891 0.099534226 0.08313004
0.6839813 0.1194503 0 0.09642769 0 0.06092524 0 0 0 0
0.06797129 0.04596541 0.06364807 0.05929046 0.1499508 0.04832193
0.1156344 0 0 0.08429156 0.125349522 0 0 0 0.07306174
0.05071504 0.03829756 0.1366618 0 0.1155169 0.0408572 0.03750629
0 0.1187052 0.1067798 0.1385612 0.141980733 0.03630601 0 0
0.158620492 0.328597 0.08131384 0.2877423 0 0.09968064 0.04498456
0.1442590 0.07506226 0.3008218 0.18345822 0.0448176 0.1846706 0
0 0.1350777 0.08812474 0.051360783 0 0.0212321 0.1678938 0
0.2814436 0 0.0492639 0.06995544 0 0.17652 0.2153186 0.06874262
0.107488 0.06179472 0.182034924 4.912829 0.06341359 0.05189842 0.2119675
0.1119214 0 0.6383179 0 0 0 0.03278178 0.05953557 0 0
0.03588797 0 0.04491752 0.06417172 0 0.1657589 0.03857749
0.098259743 0 0.1925373 0.1589035 0.06510229 0 0.06230695
0.0411565 0.06030659 0.05955337 0.03552478 0.05367313 0 0.04293604
0.02262895 0.1024365 0.2495905 0.07142791 1.72788 0 0 0.04971531
0 0 0.0383761 0.210815745 0.1071762 0.03717479 0.029972595
0.302947 0.2042669 0 0.0339391 0.2417463 0.288176 0.064617984
0.04070596 0.07990918 0 0.04836547 0 0.06421627 0.2757099 0
0.08045511 0.130544587 0 0.08149248 0.1835178 0.06403716 0.1174662
0.3932329 0.2189048 0.08592928 0.2402921 0.1772818 0.09156525
0.1065591 0 0.03774972 0.1698787 0.04555113 0.1526174 0.1230641

0.328110942 0.07640989 0.1206268 0.1209236 0 0.1460743 0.6613762
0 0.1360385 0 0 0.2530243 0 0.08174191 0.04440156 0.07423336
0.1729285 0 0.2369090.1352157 0.03611117 0.08129492 0 0.3166622
0.07639498 0 0.055642475 0.097950251 0.05189318 0.065594871 0.035221528
0.06713554 0.040564095 0 0.04659365 0 0.082713643 0.04782106
0.1068621 0 0.044714287 0.1125298 0 0.2878271 0.1424521
0.1439711 0.2075548 0.6012106 0.05091247 0 0.02589341 0.02331902
0.068917603 0 0.099030157 0.02359333 0.06706099 0.0961124 0.08618001
0 0.05832095 0 0.03348593 0 0.091047467 0.0570301 0.04160573
0.4610108 0.02825957 0 0.1190165 0.1533387 0 0.0917511
0.03989507 0 0.1044257 0.05880122 0 0.057224159 0.06883505 0
0.1516784 0.0884774 0.1202731 0 0 0 0 0.03828772 0.033389268
0.789382114 0 0 0.03709566 0.9192327 0 0.06500112 0.1415297
0.06872662 0.8838565 0 0.08378143 0.1667928 0.1221999 0.1315227
0.1260209 0.07377185 0.02540084 0.09455403 0 0.1307604 0.1042361
0.04616331 0 0 0.03422551 1.0068010.1027103 0.05862102 0
0.1180272 0.076607577 0.03603503 0 0.03874382 0.09107347 0.1024377
0.180850983 0.2551956 0.1464009 0.07882424 0 0.02464186 0.09907939
0 0.05874055 0 0.03139863 0.09359425 0.051275882 0.05167552 0 0
0.217708399 0.206374986 0.03468572 0.09118886 0.1036648 0.03602744
0.03700466 0.1901170.03728841 0.06250115 0.2315768 0.2774584
0.1092058 0.1189376 0.028368599 0.03614632 3.8946230.08273232 0
0.1915120.1579941 0.0994886 0.07785818 0.05144015 0.04284598
0.0577852 0 0.02367098 0.0633512 0.2127044 0.1014393 0.08017884
0 0.1767075 0.05768199 0.3319052 0.02886141 0.08323612 0 0 0
0.04322959 0 0.05754393 0 0 0.08301111 0.2037446 0 0.1011001
0.0963923 0 0.03415979 0.2040249 0.1344636 0.06563633 0.2822579
0.3857999 0.1787974 0.2379588 0 0.2159861 0 0.02317255 0
0.03439447 0 0 0.117826522 0.279885331 0.07193269 0.205979259 0 0
0 0.0822849 0.1625418 0.06921903 0.05821148 0 0.01602284
0.02894059 0 0.056200373 0.1310046 0.03640002 0.03849184 0.1546701
0.02504537 0.07947687 0.04851149 0.1739938 0 0.0261921
LINC00630 0.2433247 0.2911553 0.2142712 0.326019308 0.176419016
0.4283054 0.3028252 0.2564105 0.2567392 0.3502304 0.2899342
0.358879038 0.5348697 0.197840906 0.146648219 0.4923306 0.3139344
0.0977177 0.2661628 0.3237918 0.585939222 0.5226272 0.472444
0.5297463 0.3550505 0.4088922 0.8386901 1.0306240.7500608
0.216650995 0.2246361 0.386509931 0.1619374 0.8128254 0.4470085
0.122538007 0.9046039 0.2837585 0.4403743 0.6890115 0.1984698
0.3004553 0.2860142 0.3240515 0.42414 0.6656117 0.1458920.345913
0.5833655 0.2134003 0.2947428 0.7017779 0.2527817 0.404826381
0.1031375 0.7170105 0.4830161 0.3932646 0.3431755 0.2885984
0.5604576 0.3288649 0.5507238 0.3141705 0.4441418 0.577066226
0.01825561 0.4885430.6286232 0.300233355 0.3294257 0.1668468

0.3124431	0.451292224	0.5592516	0.2313465	0.8186576	0.4846297	
0.3730261	0.2490536	0.3993394	0.2697252	0.4271203	0.5736642	
0.543118679	0.5376133	0.5147573	0.6796965	0.1682204	1.367591	
0.8041248	0.35544383	0.2845039	0.5844842	0.4389453	0.221829969	
0.6636739	0.07305051	0.7273222	0.3442693	0.5824875	0.1131122	
0.6291611	0.5391667	0.3967327	0.5464444	0.547903497	5.192851	
0.585140851	0.08779579	0.2477478	0.4001869	0.581645	1.003481	0.1337024
0.3370206	0	0.7209339	0.5402009	0.2110108	0.2903733	0.4249779
0.3819861	0.4075629	0.5362124	0.4965075	0.1478535	0.2373126	
0.35511606	0.1711591	0.5541341	0.6190885	0.5556689	0.205806791	
0.4647347	0.2658365	0.3709809	0.467093	0.1966802	0.2228679	
0.2433631	0.6272959	0.7134196	0.3465807	0.5593157	0.300253	
3.687003	0.1696542	0.1081355	0.2370166	0.3280291	0.3235844	
0.2655829	1.076385793	0.5851316	0.3087231	0.470166138	0.4037807	
0.2094275	0.1272807	0.2139986	0.991415	0.9639266	0.660848455	
0.3818693	0.8663884	0.2545424	0.357029	0.231059	0.237019	0.3876691
0.07650098	0.4578067	0.762902949	0.2034562	0.2694529	0.354805	
0.1969649	0.3143322	0.274464	0.6011655	0.1982254	0.2633002	
0.2453768	0.3332688	0.3324357	0.3287193	0.2322205	0.2525471	
0.1611216	0.4283449	0.8895204	0.383496262	0.2761495	0.6771167	
0.2851506	0.4235523	0.4380615	0.4195651	0.24718986	0.6224085	
0.1925286	0.5231474	0.700425	0.243458381	0.2828487	0.2321689	
0.6792707	0.4179151	0.5189369	0.601163	0.7850021	0.2138106	
0.525097	0.3113562	0.4930807	0.3720437	0.6971121	0.367960942	
0.990439923	1.077385	0.489258582	0.243751634	0.4284769	0.299440031	
0.3773042	0.3081215	0.5038322	0.413416055	0.2868208	0.5382223	
0.08386354	0.563880391	0.4153417	0.197293	0.2459156	0.7511193	
0.5682922	1.106551	0.3287463	1.538557	0.106669	0.7964271	0.3514498
0.174880319	0.3541882	0.335055676	0.4499226	0.3781536	0.6318913	
0.07952151	0.7799578	0.4574268	0.1192924	0.1338944	0.4650416	
0.331384196	0.3552106	0.4670925	0.5996844	0.5475994	0.2336999	
0.251673	0.1886551	0.8048195	0.2857348	0.4478875	0.4151848	
0.7440938	0.09947315	0.3337114	0.281615286	0.6139945	0.2486544	
0.1819471	0.1700862	0.252789	0.4221709	0.2885762	0.4392542	
0.6189522	0.3120773	0.308095235	0.926463923	0.5528757	0.2825129	
0.2624266	0.5235866	0.2153097	0.2549106	0.4788473	0.169111	
2.289862	0.6171467	0.3350024	0.421409	0.4510335	0.4045364	0.7170859
0.2893062	0.4453278	0.1793442	0.265479827	0.3066712	0.1870217	
0.4614632	0.03561319	0.1600515	0.03684468	1.872771	0.3869965	
0.3155355	1.028814	0.6171459	0.683323801	0.1717962	0.4481116	
0.452838	0.312804	0.1732924	0.545829957	0.42844	0.4165261	0.9495837
0.425008	0.2311693	0.609495	0.498389227	0.5329873	0.3888902	0.1207195
0.4030269	0.417941826	0.2702033	0.07639712	0.1650693	0.187495151	
0.883383141	0.6401162	0.6836648	0.2231958	0.3823045	0.5577111	

0.282634 0.2637901 0.5046313 0.5698254 0.3047872 0.3896374
0.4618567 0.222502499 0.150091 4.585421 0.2544673 0.344337 0.667591
0.3304506 1.00472 0.4230733 0.3401712 0.3953559 0.2710462
0.06629567 0.8263594 0.3897101 0.3794559 0.3744074 0.4891165
0.3075691 0.3369795 1.020152 0.2105546 0.1242803 0.04480296
0.09624761 0.6355498 0.6417837 1.096963 0.3756489 0.6194758
0.4850578 0.5938927 0.4723509 0.3955892 0.1588413 0.6607959
0.2964826 0.2424183 1.003403 0.6096084 0.1930049 0.9841827
0.2266878 0.7554941 0.7534225 0.06099262 0.2434549 0.3511449
0.80713359 0.4312072 0.1107168 0.5025034 0.06227296 0.1232469
0.525494152 0.6552168 0.2212499 0.804607428 0.1334479 0.3736357
0.5415026 0.544146 0.6963514 0.8037098 0.8101848 0.09897163
0.2414862 0.3427086 2.305029 0.423508476 0.04029426 0.9180626
0.2959821 0.1268621 0.2465099 0.4359434 1.029557 0.4602449
0.2649448 0.3504423
AL161719.1 0.1153752 0.5533987 0.5338937 0.164400831 0.121839694
0.2387151 0.3143922 0.3620384 0.4757844 0.2673011 0.07310215
0.458667661 0.8203124 0.220532433 0.070588523 0.2482662 0 0
0.3416437 0.1118096 0 0.266719 0.08069352 0.1674569 0.05222009 0
0.04074037 0.599665 0 0.106709285 0.1941885 0.243147896 0 0.05063238
0.1296889 0.041859024 0.3140788 0.0770485 0.2976633 0 0 0 0
0.1106961 0.4491477 0 0 0.305257 0 0 0 0.09778362 0.06863742
0.102070325 0 0.1528764 0.05747137 0.03966209 0 0.1247406
0.1483757 1.404254 0.06270915 0.1330777 0.1221633 0 0 0
0.0564142 0.057806441 0 0.2718219 0.04726643 0.193743601 0.3567625
0.0662127 0.05857611 0.06579538 0.3246741 0 0.4111381 0 0
0.0653213 0 0.03649433 0.07518731 0.0966137 0 0.1833197
0.04783915 0.20911176 0 0 0.4374833 0 0 0.1289294 0 0 0
0.04791258 0.07013233 0 0.04376296 0.05031855 0.042350982 0.7619898
0 0.1690406 0.1380817 0.04556799 0.07896658 0.8855395 0
0.1814704 0 0.3203249 0.09695792 0 0 0.05844612 0 0 0
0.05907664 0.05399003 0.2513048 0.080011505 0.1888029 0.8510933
0.04313096 0 0.09906426 0 0.3351315 0 0.09698692 0 0.08741053
1.6107 0 0.25797 0.4448673 0 0 6.067636 0.05614287 0 0
0.2368432 0.05807852 0 0.2746628 0 0 0.048812512 0.4385519 0
0.1758069 0 0.1312337 0.3519864 0.052617501 0 0.0650689
0.1347752 0.3150665 0.1138058 0 0.256579 0 0 0 0 0
2.433671 0 0.2295624 0.344835 0.1018577 0.06997099 0.1956664
0.8661482 0.04970685 0.7685304 0.05614532 0 0.04610993 0 0
0.2004186 0.106870421 0 0.04911235 0.06564422 0.09750552 0.1189462
0 0 0.05538708 0.05996478 0 0 0.224185165 0.06656127 0 0 0
0.05088277 0.1543293 0 0.3528578 0.1985919 0.08242852 0
0.08294312 0.1757654 0.271853233 0.119639235 0.8451176 0.053412967
0.057360774 0.05466751 0.066061526 0 0.07588108 0.4135959 0.134705076

0 0.08701625 0.2220206 0.655383693 0.3665256 0.3798647 0.156249
0.4308449 0.4689345 0.1690089 0.435162 0.1658292 0 0.2530156
0.5696504 0 0 0 0.1536937 0.03640454 0.03913148 0.1403503
0.06715 0.09497983 0 0.1636027 0 0.098851536 0.7430207 0
0.06256576 0.2301136 0 0 0.2497231 0 0 0.06497197 0.2476983
1.700647 0.383048 0.1104336 0.27958089 0.0747352 0.1097147 0.04940381
0.07204588 0 0.1224829 0 0 0 0.1870628 0 0 0 0 0
0.1663374 0 0 0.144057 0.0559631 0 0.8861482 0.136444 0.03880485
0.1990112 0 0.02931915 0.06007136 0.1654684 0.153988 0.200809138
0.9050489 0 0 0 0 0.1114774 0.1561568 0.1672711 1.050154
0.4111223 0.07688629 0 0.1173712 0.2686034 0 0.9887977
0.2780453 0.699505765 0.2770695 0 0.0855806 0 0.4815724
0.2420368 0.101495149 0.09566319 0 0.05113491 0 0.041753218
0.08415727 0.08090158 0.1398416 0.709107357 0.336096406 0.02824409 0
0 0.4107129 0.1807942 0.3096191 0.1214537 0 0.1885697
0.06455155 0.6758281 0.04842458 0.184801158 0 0.8318257 1.279987
0.04097073 0.1039637 1.852593 0 1.521571 0.1675479 0.2093332
0.3764293 0.0501461 0.4625986 0 0.2078426 0.03304023 0.04352566
1.230436 0.4028931 0.3444439 0 0.2350143 0.2033339 0 0 0
0.3520124 0.4640971 0 0.04353028 0.144025 0.2027843 0 0 0
0.3924543 0.07550338 0 0.6170713 0 0.1068935 0.6129028
0.2094343 0.2911843 0 0.1681365 0.904497 0.082184906 0.03773815 0
0 0.1648616 0 0.047972227 0.050645917 0.1757213 0 0 0.06182275
0 0.5025257 0.1890792 0 0.5688093 0 0.2609434 0 0.09963005
0.091526322 0 0.05928003 0.2507468 0 1.305222 0.04314459 0.2370133
0.1700168 0.2493922 0
AP001432.1 0.1138961 0.1170651 0.3623461 0.060859923 0.060138823
0.176741 0.2793254 0.1786984 0.2012934 0.09895283 0.4329897
0.145538777 0.4048978 0 0.156787969 0.09190623 0.4027691 0
0.1580923 0.1655643 0.060447222 0.2468433 0.4182097 0.1549782
0.4252925 0.4421893 0.3619625 0.9175643 0.956222 0.197514782
0.2516048 0.240030615 0 0.9371859 0.09601968 0.340909553 0.2325391
0.4563642 0.8080796 0.09822028 0 0.5608774 0.7474872 0.9015346
0.332542 0.1534898 0.2502171 0.2260076 0.2330604 0.4879993
0.07968609 0.07239749 0.05081809 0.415642141 0.3593946 0.3018329
0.1276528 0.0587304 0.4280544 0.09235603 0.4394203 0.2495252
0.6964333 0.1970574 1.537614 0.208019364 0.9064966 1.062202 0.6265231
0.085597999 0.08755335 0.2012527 0.3499534 0.812853762 0.5811112
1.813846 0.3903197 0.6332805 0.801279 0.054241 0.2609146 0 0.3167772
0.1450887 0.221208277 0.1080794 0.2505039 0.107297 0.04396377
1.900179 0.4250324 0.495434016 0.3879339 0.1536061 0.04048824
0.208707874 0.339356 0.3818294 0.1782027 0.08435011 0.02927517
0.07094748 0.4673241 0.3315509 0.3564156 0.4843161 0.125424063
5.4536 0.076462155 0 0.6134014 0.3373784 0.1169313 0.7696641

0.110345 0.3582876 0.2321595 0.592909 0.1794654 0.194636 0 0.2596356
0.4844721 0.9207228 0.4384656 0.2186972 0.1598936 0.1395467
0.236957149 0.08387207 0.3979809 0.255468 1.255974 0.220036963 0.4507668
0.6451282 0.436295 0.4308458 0.2998428 0.06471741 0.5664555 0
0.190997 0.4528877 0.1289779 0.02870852 3.515779 0 0 0.1798356
0.02922584 0.08600088 0.5552726 0.711746389 0.581534 0.1792969
0.144560131 0.2841099 0.9851951 0.2169412 0.4910727 0.09716343
0.4343422 1.285587218 0.24541 0.3372321 0.399142 0.4082231 0
0.348435 0.3957649 0.3998647 0.2263573 0.104937764 0.2278821
0.2456528 0.7270634 0.03860702 0.4249113 0 0.754139 0.1036109
0.5070394 0.1710087 0.03680219 0.8076243 0.2078457 0.09103491
0.2389736 0.1647724 0.5980692 1.533331 0.276938254 0.4606635
0.3636202 0.4374177 0.2526706 0.1320989 0.5981003 0 0.4920929
0.177588 0.1139355 0.3050889 0 0.07392141 0.2141522 0.4922956
0.6851104 0.414401 0.6855781 0.1630389 0.1523961 0.4411031
0.3661729 0.8590984 0.1228196 0.173512 0.335459919 0.118105398
0.1251424 0.079092278 0.084938069 0.08094997 0.195643751 0.2798307
0.6741742 0.30622 0.199467131 0.2306447 0.128851 0.09862836 0.37740578
0.135685 0 0.3470531 0.4907553 0.173596 0.4171052 0.2416404
1.012913 0.06433276 0.1248859 0.1687042 0.207746812 0.2776972
0.676642595 0.6543067 0.6468806 0.4635575 0.259783 0.2983009
0.6328945 0.1039221 0.1211289 0.630191 0.256158548 1.134625 1.655508
0.3242591 1.022236 0.1696571 0.07175321 0.3235594 0.1752804
0.331892 0.384834 0 0.08394219 0.1418014 0 0.241496955 0.08299918
0.1624622 0.2194669 0.4534041 0.7251083 0.1813689 0.4848318
0.1551316 0.04578135 0.2308308 0.362337771 0.801526454 0.23341
0.3691683 0.2683729 0.5747169 0 0.4310699 0.3413043 0.1657369
1.147706 0 0.5556159 0.7757238 0.4125656 0.7136373 0.4992713
0.08895183 0.2143929 0.03800344 0.148675997 0.5124174 0.2513695
0.2783115 0 0.05019461 0.1238042 1.156161 1.300372 0.5301257
0.3043886 0.7684932 0.123161412 0.04344993 0.5303196 0.04671611
0.1830226 0.04117209 0.463387169 0.2564225 0.02942095 2.376096
0.4101202 0.5645356 0.1194669 0.300581787 0.3895515 0.9316538
0.227157 0.1410663 0.401874723 0.1246175 0.05989828 0.569451 0.105002436
0.290314043 0.1882034 0.1924173 0.1249958 0.3040855 0.3569526
0.1528248 0.3147285 0.07536197 0.5235528 0.5735157 0.6320498
0.3585281 0.307853853 0.2615047 9.16108 0.09975609 1.243698 0.6927579
0.5334123 0.9996691 0.031293 0.06202495 1.033247 0.1045134 0.0371274
0.4852096 0.4583215 0.05129448 0.2201623 0.2578058 0 0.5965917
0.5332257 0.2501257 0.8352048 0.1003636 0.04192324 0.1811984
0.1378584 0.1563747 0.245436 0.5550776 0.2578332 0.4976249
0.3002767 0.3070863 0.0355822 0.3352343 0.05811343 0.3354092
0.5354547 0.1054316 0.108088 0.9892784 0.2268919 0.1860743
0.6036475 0.4303851 0.2904665 0.1488168 0.304242201 0.5588149

0.1578295 0.08294358 0 0.1509849 0.106553696 0.449969494 0.08673422
0.546399596 0.1569425 0.2288631 2.830392 0.6697121 0.1679896
0.2503865 0.3509481 0.1108537 0.07727938 0.104687 0.5163519
0.135529361 0.3685763 0.6583504 0 0.124331 0.4831831 0.2236051
0.1462342 1.007022 0.1846461 0.6947959
AC009716.1 0.2604156 0.0764747 0.08607583 0 0.157146674 0.2309179
0.06082462 0.05836883 0.1314982 0.2908915 0.07071439 0.063383722
0.4912256 0 0.068282867 0.7805102 0.2923506 0.2859979 0.2478634
0.1802626 0 0.1935053 0 0 0 0 0.3940966 0.03867186 0 0
0.04696141 0.156803919 0 0 0.1254528 0 0 0.1490637 0.1919604
0.128328 0.4312569 0.2442682 0 0.5889422 0.434477 0.1504045
0.3269169 0.1181145 0.07612525 0.09108386 0 0.06305979 0 0 0
0.147883 0.2223767 0.1150998 0.2396859 0 0.1435292 0.1086709
0.06066086 0.06436549 0.05908654 0.067946023 0.1246703 0.2943822
0.2728576 0.503264619 0.171587 0 0 0.374830585 0.2070657 0
0.1133256 1.145633 0.05234486 0 0.05681557 0 0.05912567 0.3475323
0 0.1412092 0 0.186916 0 0.3191973 0 0.080912591 0.04223736 0
0 0.272683654 0 0.1247181 0.3104368 0 0.2677427 0.5561712
0.06784158 0 0 0.1946999 0.081935317 2.457002 0 0.3270383 0
0.04407959 0.07638727 0.4469295 0 0 0.06066479 0.154931 0.1406864
0 0.08748538 0.2261483 0 0.106143 0 0.228588 0.1566796 0 0
0.0365272 0.3033188 0.04172216 0.1025607 0 0.2453923 0 0
0.187638 0.2238596 0 0 0 0.07129824 0 0.0561713 0.03750862
7.910994 0 0 0 0 0.05618148 0.06045682 0.06642285 0 0
0.047218133 0 0.4290632 0 0.05346687 0.0634736 0.2269929
0.101797678 0 0.06294354 0 0.1142908 0 0.05058241 0.2275151
0.05224363 0.3379923 0.137104616 0.07443385 0 0.206507 0.2522067 0
0.04765308 0.2463268 0.270742 0.1419565 0 0.09616652 0.1678708
0.05431143 0.05947004 0.08920765 0 0 0 0.258449197 0.06018719
0.09501635 0.06350006 0.09432067 0.0575305 0.434132 0.281349263
0.1607339 0.05800613 0.2605057 0.2391652 0 0.03219358 0
0.1169455 0.1556729 0 0.3358988 0.1065078 0.2275548 0.06403507
0.07973613 1.309513 0 0 0.175315737 0.15430856 0 0.051668322
0.110974362 0 0.063903738 0 0.1468051 0.04000864 0.195457742 0
0.08417401 0.04295373 0.281767417 0.1772768 0.09186428 0 0.06411879
0.07560293 1.035427 0.4209481 0 0.08405285 0.2855431 0.1102087 0
0 0.156009821 0.03716839 0 0.3785331 0.203649 0 0.09187748 0
0.1582589 0.0588118 0.143434076 0.04492195 0.1310894 0.2420886 0
0 0 0.06039157 0.1145048 0.2168138 0.5656479 0 0.2193464 0
0.1602397 0.135224425 0 0.1061311 0.09558023 0 0 0.2369644
0.05278739 0 0 0 0.420805452 0.261805123 0.2613924 0 0.1753189
0.5363474 0.2384402 0 0.1393516 0.05413516 0.4819871 0.1071505
0.1319873 0 0.03850217 0.3107968 0 0.3486554 0.08003181 0 0
0.1029985 0.1094741 0 0 0.06558089 0 0.07552811 0.323615

0.1385253 0.2651291 0.1859373 0.201143009 0.1135375 0.4330499
0.06103612 0.1913 0.3227561 0.106840672 0.1340097 0.1921971
0.1241779 0 0.03882022 0 0.245449952 0.04626926 0 0.1978587
0.1105847 0.403894194 0.08140841 0 0.06763693 0 0.108372794
0.1912508 0.8260267 0.05443703 0 0.1165926 0 0.3524598
0.09846287 0.1368078 0.1248862 0.03440807 0.1873715 0.044691235 0
4.4256050.06516729 0 0.1005679 0.2489002 0 0 0.08103762
0.4049914 0.2275837 0 0.2610350 0 0.1340358 0.1917662 0.2105198
0.1400290.5567618 1.0904510.2614379 0.0454676 0 0 0.0591854 0
0.0681029 0.1282681 0.09065338 0.1263253 0.04644023 0.1307738 0
0 0.3185415 0.1518542 0 0 0.04591659 0.07061026 0.2585049
0.2964417 0.2836308 0.05633465 0.06247913 0.1626446 0.04860851
0.238501424 0.1095165 0 0.05418425 0.2392150.03945334 0.046405295
0.244958267 0 0 0.06835016 0 0 0.03240743 0.1829033 0.1090461
0.1375575 0.07241697 0 0.2279617 0.1927516 0.177073537 0.06879385
0.2867188 0.1819174 0 0.0394559 0.2504121 0.1910597 0.1096423
0.06031155 0.08252487
AC068790.3 0.05354969 0.2201586 0.06194969 0.171684412 0.056550069
0.2908396 0 0.08401734 0 0.02326197 0.1526817 0.045617941
0.1903679 0.038383809 0 0.08642177 0.04208156 0 0.05946331
0.3373160.113680128 0.1392678 0.1404476 0.1457299 0.2544903
0.5890527 0.1701813 0.5288185 0.2697480.111436912 0.06759726
0.141066817 0.1570902 0.1410016 0 0.466277738 0.2186624 0.2682068
0.1381560.5541542 0.2217002 0.1406419 0.2555932 0.8477360.1042326
0.3608258 0.1344489 0.08500829 0.1095763 0.3277701 0.1498617
0.2042316 0.09557109 0.142123237 0.4646783 0.3902541 0.2000586
0.3313541 0.1150030.6079131 0.1549493 0.07821163 0 0.2316227
0.4252522 0.146704434 0.1345898 0.4540071 0.3927571 0 0.123493
0.3784862 0.2303491 0.134884785 0.3228926 0.09219489 0.2039042
0.09161381 0.1506926 0.1530126 0.1635631 0.1325702 0.1702135
0.04547685 0.104003892 0 0.2355552 0.3026822 0.1240208 1.352853
0.2664459 0.553219719 0.1823921 0.1444397 0.2665048 0.130835557
0.2659209 0 0.2234247 0.6741908 0.30281 0.4336392 0.1953052
0.3897073 0.1523394 0.1050957 0.707636666 11.140480.071899314
0.1765297 0.3845313 0.09517366 0.05497674 0.6969298 0.1037603
0.08422675 0.4366110 0.4387653 0.1372659 0 0.04069033 0.2484881
0.7129946 0.3152885 0.08225861 0.2631160.08747953 0.167112637
0.1577341 0.1559298 0.09008365 0.1845350.206906367 0.07064459
0.04666388 0.2051297 0.3038514 0.04027854 0.2434217 0.2242748
0.3407709 0.07697116 0.1935736 0.08085417 0.08098607 7.591502
0.2345209 0.1195846 0.3945759 0.1099272 0.08086882 0.3916027
0.191220936 0 0.1264481 0.305850549 0.1908256 0.5404024 0.3671916
0.07696136 0.2284131 0.2450538 0.402956813 0.2307653 0.6342159
0.5160697 0.2741876 0.1584638 0.4732611 0.3721479 0.3384027

0.2736637	0.246689138	0.2142833	0.4157885	0.2675255	0.1089095
0.3196439	0.1028893	0.6382225	0.09742796	0.1362235	0.2814068
0.1730302	0.2071172	0.5472392	0.08560245	0	0.6714055	0.2595599
0.2790639	0.148806915	0.2599042	0.4786899	0.04570167	0.1018254
0.04140531	0.1874697	0	0.2699244	0.2922334	0.08035231	0.05737658
0.15607828	0.2085306	1.0572070.1262504	0.1680592	0.2833977
0.4297777	0.07665481	0.06141512	0.6913009	0.2869347	0.1346387
0.3175986	0.1223683	0.063088303	0.138821897	1.3532580.185931213
0.199673581	0.1522386	0.137976605	0.5638541	0.1056572	0.3455358
2.063204322	0.5964229	0.6058093	0.06182851	0.202791016	0.06379401	0
0.1450413	0.2307349	0.3264734	0.5491006	0.1514804	0.3463521
0.06049374	0.2055084	0.1057579	0.664188723	0.06528143	0	0.2140039
0.1267246	0.05448686	0.2931367	0.6545	0.1983756	0.04886028
0.07593374	0.04232748	0.06882069	0.2263156	0.7075984	0	0.2883703
0.03190659	0.1012071	0.2173223	0.1236155	0.05201441	0.5880374
0.1724482	0.07893298	0.06666974	0.1537683	0.291967385	0.2341387
0.1527673	0.06879011	0.3260304	1.2273080.1279093	0.1139749
0.02917484	0.4304937	0.2604673	0	0.329741896	0.4703173	0.208283
0.08411928	0.1930075	0.04290198	0.1842482	0.2005857	0.1168852.00426
0.3084693	0.4749634	0.1620962	0.2216834	0.2982450.7756586
0.08364367	0.2879987	0	0.13980383	0.2223873	0	0.05234068
0.1312793	0.1887971	0.1164163	1.7394680.1164546	0.1661636
0.1431122	0.2944064	0.086858862	0.1225712	0.3116707	0.2196417
0.2409412	0.07743033	0.230683114	0.04822412	0.05533053	0.1787443
0.3856464	0.1396966	0	0.176652949	0.2331033	0.2787457	0.6052043
0.07958896	0.174412176	0.7030860.1689716	0.1460371	0.148104701	0
0.09831803	0.07754351	0.1567157	0.2042424	0.1678258	0.2155576
0.2959472	0.07086478	0.0984620.3145867	0.02476386	0.1011399
0.128659034	0.1229498	11.509990.09380319	0.1426196	0.07237976
0.32244450.3760057	0	0.05832364	0.04857944	0.7534542	0	0.5367706
0.1436571	0.6752691	0.0230027	0.09090802	0.1007803	0.4808489
0.4142047	0.1411197	0.2290646	0.1415616	0.1182645	0.1703854
0.2592635	0	0.2769477	0.3914652	0.4242825	0.1336941	0.2823579
0.2310089	0	0.02865722	0.1639366	0.6307877	0.8133490.29742	0
0.3348877	0.1066761	0.2041321	0.7298037	0.1798676	0.07803802
0.06996812	0.457738719	0.1050936	0.1113084	0.3509728	0.1721656
0.02839499	0.500975788	0.317338341	0.2854544	0.420376477	0.09838468
0.2152058	0	0.8863111	0	0.1177224	0.09900163	0.2084771
0.1271686	0.06562659	0.2427694	0.063720857	0.2475583	0.1238127	0
0.1753674	0.3407620.2102616	0.5500308	0.4340091	0.1302206
0.3266672
AC037487.2	0	0	0	0	0	0	0	0	0	0.02395506	0	0.046977135
0.0280057	0	0	0	0.04333539	0	0	0	0	0.04780578	0.02892645
0.06002878	0	0.03568256	0.02920865	0.1433091	0	0	0.06961133

0.11621594 0.0647083 0 0.04648998 0.03001066 0 0 0.2134085 0
0 0 0 0.03968156 0.05366911 0.07431534 0.0346137 0 0 0 0
0 0 0 0.04350213 0.03653471 0 0 0.1480369 0.04471614
0.05318868 0.08054196 0 0.09540955 0 0.10071701 0.09239993
0.06233791 0.08089187 0 0 0 0.03388748 0.092602466 0.05115588 0
0.08399182 0.1415152 0.03879563 0 0 0 0.04382126 0 0
0.07849339 0 0 0 0.02628606 0 0.059968736 0 0 0.03920649 0
0 0 0.05752042 0 0 0.06870147 0.2011244 0.04013186 0 0 0
29.6826 0 0 0 0.06533958 0 0 0 0 0 0 0.06951359
0.04711858 0 0 0.04264863 0 0.07492675 0.08470952 0 0.045043
0.114727856 0 0.03211515 0 0.1520266 0 0.1818737 0 0.07041384
0.03476719 0.04147865 0 0.05773926 0.05013203 0.02642151 0.1993411
0.08326323 0 42.11467 0.08050282 0.0410492 0 0 0 0.08961569
0.049229599 0 0 0 0 0.07950053 0 0 0.04704375 0 0
0.04752819 0 0 0.02823571 0 0 0.07664721 0 0.03131307 0 0
0 0.03061072 0 0.02743065 0 0.146053 0.1003308 0 0 0.03563713
0.01777402 0 0 0.03305833 0 0.04454892 0.1915857 0 0 0 0
0.0349531 0 0.1287036 0 0 0 0 0.1181723 0 0 0 0 0
0.1094406 0.02766144 0.03946938 0.02108166 0.09491977 0 0.184867 0
0 0 0.057183247 0 0 0 0 0.047362547 0 0.1632079 0.05930518
0.048288132 0 0.09357893 0 0.052208302 0.06569477 0 0.07468139
0.02376097 0 0 0 0.02972264 0 0.06046617 0 0.040234019 0
0.038542463 0 0 0.02805515 0.1006236 0 0 0 0 0.08717726
0.106306819 0.06658821 0 0.04485627 0.03299581 0 0 0.2237974
0.2545972 0 0 0 0 0 0 0.1002222 0 0 0.03541986
0.0258265 0 0 0.03912361 0 0.04433203 0 0.038985235 0.048509515
0.0322887 0 0.08662563 0.1192549 0 0 0.04131243 0 2.024286 0
0.09782301 0 0 0.07678282 0.1261215 0 0.05931594 0.03680036
0.035992327 0 0 0.02695009 0 0 0 2.51901 0 0.1026687 0
0.05512332 0.029815612 0 0.06419139 0 0.03544573 0 0.079185451 0
0 0 0 0 0.05784244 0.072766541 0.03429267 0.04100728 0
0.02732011 0.029934802 0 0 0 0.101678336 0.080320991 0 0
0.08069251 0 0 0.07399339 0.08707573 0 0 0.04627997 0 0
0.033123113 0 25.71863 0.04829904 0 0 0 0.1548836 0 0
0.1500806 0.06746987 0.1078561 0.02763819 0 0 0.02368806 0
0.05189155 0.08252931 0.08979915 0.04844147 0 0 0 0.04386553 0
0.1009495 0.04753323 0 0 0 0.09692359 0.1784188 0 0.02951106
0 0.05413185 0.1196547 0 0.05233312 0.03831842 0 0.03003061
0.04175269 0 0.1607264 0.03602642 0 0.05411243 0 0.0401589 0
0 0 0.072620775 0 0.048100185 0 0.04432358 13.01875 0.07205675
0.05422384 0.04040999 0 0 0.01870823 0.06758195 0 0.032809715
0.1019738 0 0 0 0.02924292 0.06186468 0 0.04063095 0
0.1223274
AL117336.1 1.546947 0.8177086 2.556581 1.417037014 2.193720658 5.349712

1.300739 2.08037 0.3905693 1.91998 0.4200646 3.313360419 3.63631
3.991801606 0.892365354 8.202974 0.9030578 3.652663 2.306733 0.5139906
1.219770805 0.9962153 0.6491613 0.1443379 1.140269 0.2859931
1.123705 0.4594448 1.038999 0.551862198 2.008547 0.558877253 0.3111793
0.5237051 0.7452274 0.721599596 0.3609562 1.505321 0.7982116
0.7623066 0.512359 1.857313 0.4219195 0.6360895 3.097108 0.7147583
0.3883967 1.754089 0.3617654 0.8657045 0.06184592 0.374594 0.5521727
0.645175861 0.5578662 0.6442106 1.387033 0.5925634 0.6644427
0.5017552 1.023128 0.3227689 0.1441374 1.376461 0.9827768 0.726515389
0.3702893 0.2498168 0.06483423 0.730776949 0.951326 0.1561962
0.3802479 2.597706491 0.8200212 0.6848567 0.4039129 0.4536935
0.3109441 0.5893649 0.4050017 1.422458 0.2809793 0.6756367
0.343368072 0.2935887 1.123321 0.6106851 0.3412113 0.9691318
0.3848552 0.961289881 0.5519856 0.317911 0.4399319 0.539940767
0.9657295 0.6667767 1.198657 0.4582603 0.1817682 1.596848 1.853796
0.8363001 0.7544212 0.2891439 1.119456561 5.546224 0.652781386
0.8742173 0.3967273 0.6807994 0.8167738 0.4424824 0.8564093
0.4866295 0.2882936 0.7976248 1.44858 0.981895 0.4157514 0.4701859
0.1367302 0.3783128 1.481313 0.6110464 0.6825307 0.866439 0.827581684
0.2169825 0.6177613 0.8426631 0.7920131 0.910799768 0.4664652
0.3851512 0.677234 2.006327 0.2659586 0.7031981 1.110662 0.7232481
0.296473 0.383449 0.4004091 0.5347498 9.701918 0.1290448 0.1974038
2.698428 0.2268274 0.4672287 5.530626 0.236742935 1.002977 1.600291
0.841469422 1.890028 1.656696 0.6061401 0.8257839 1.206567 1.618087
0.423296018 0.7618698 0.7478068 0.6195638 0.8599669 1.046334
1.261993 0.8109046 0.6827541 1.154471 0.651553878 1.061182 1.525247
1.177645 0.958837 1.626924 1.188909 0.9364831 0.6433154 0.5621759
2.322656 1.08539 0.8547449 1.806706 0.2826158 0.5299201 0.08525539
0.4284675 0.84455 0.552695535 0.9295777 1.128851 0.4526512 0.3361757
0.6834967 0.2578874 1.615588651 1.273076 0.4134885 0.4421376
0.2841425 2.189987919 1.109189 0.914142 0.7641604 0.9247435 0.4678177
0.4434087 0.7592259 0.6082847 0.6086199 1.231507 0.7408477
1.668147 0.8753291 0.728999466 1.008302803 0.9712545 0.306925256
0.593298752 0.5026147 0.227764367 0.62052 1.482513 0.3327286 1.470697952
0.7160312 1.950073 0.5103159 0.334756513 0.5265387 0.2182805
1.616128 1.790158 0.9880288 0.7769363 0.5001115 0.7623193 3.495093
0.2907791 0.5673831 1.031912524 2.155262 0.432480956 1.103957 1.087789
0.134916 0.1612981 0.8488962 0.1637339 0.7259033 1.754863 1.048079
4.032995131 0.2668498 0.5450972 0.3954716 0.3702426 0.5266968
0.2784453 1.434976 0.2040578 1.674321 1.568055 0.6405036 0.8469391
1.100548 0.5711228 0.321309381 0.8159522 1.38699 0.6813301 0.413995
0.7503608 0.1407639 0.2508583 0.9632052 4.121688 1.218235 0.874898499
0.311040117 1.345716 0.3438224 0.9720171 1.593032 1.487226 0.5474647
1.953585 0.5788422 1.654263 1.14571 1.881706 0.3121763 0.3202001

7.631066 1.448891 0.4142234 0.3803303 1.297789 2.538587166 2.141446
0.5852782 0.4320059 0.3611815 0.5453981 1.088984 0.3589276
0.8650663 0.6034465 0.787473 1.89978 0.525733788 1.281449 0.2057957
1.522806 1.818207 0.9586337 0.59235445 0.9552694 0.3196784 0.6884768
1.591511 0.6918112 0.4636028 1.924620696 0.6596477 2.037756 0.5289029
1.182431 0.62380554 1.837643 0.4648822 0.8839239 1.629888551 0.836896771
0.7790309 0.3413458 1.487512 0.5394438 0.8311136 1.304713 0.2093716
0.5848989 0.6501431 2.819072 0.4496673 0.7791298 0.796437829
0.8794886 6.33336 1.47103 0.6592007 0.4779225 0.8279832 1.365518
1.360078 0.6739427 0.4009616 0.8111489 0.7491977 0.5316432
0.474283 0.1592426 0.4176877 0.7503304 0.4159069 0.9260528
0.3598668 0.7765095 0.9183097 0.3894705 0.3253744 1.546947
0.6419674 0.2427309 0.3047802 0.6462097 0.4002187 0.3862163
0.2330506 1.763684 0.05523207 1.040728 2.164942 0.7809529 1.342633
0.6000682 1.342227 0.5528147 2.758825 0.09627724 0.2677157 0.5938315
0.7085153 0.5197483 1.605672273 1.561345 0.9187092 3.025584 0.1894678
0.4218563 0.220529043 3.783325595 1.413638 0.385519389 0.5684285
0.6394503 0 0.2695138 0.34768 1.165979 1.361888 0.8603568 0.6897473
1.51666 0.2862505 0.262967416 1.062506 0.4768946 0.7204293 1.929914
0.4218837 0.2479204 0.8171652 0.7164388 0.6448835 0.5882668
LINC00641 1.199546 9.123091 1.058017 6.268518829 0.960327036 2.078523 5.549036
4.463239 5.526675 7.23888 0.9406066 1.522872944 3.37096 1.868206834
6.020219042 3.650197 2.101118 1.000842 0.8199225 2.242095 0.335500591
1.298184 1.668866 0.8488952 0.9885268 1.713306 1.07037 1.413908 2.022874
0.460072928 1.393213 0.565111448 0.6384212 0.8970746 0.3931514
0.707794716 0.4231683 0.3685251 1.336837 1.555026 0.283171 0.4116699
1.11912 1.827018 1.654079 1.218518 0.8000944 1.575212 0.4930359 0.7294678
0.7069265 0.6367769 0.6380938 0.422881911 0.4823371 1.071071
0.6581805 0.7908821 1.429951 1.331931 2.18403 0.5865188 2.3277
0.7889193 0.1604797 0.525235244 0.1779851 3.057601 1.744398 0.576344884
1.501659 0.5859747 0.8374388 0.426360225 1.055087 0.2274867 0.765538
2.016746 1.334203 0.7995208 1.764693 0.8081561 0.3252895 0.5038541
0.130828342 0.4916996 0.6154111 1.470929 0.5160262 1.699308 0.9152637
0.493050038 0.329444 0.1770344 0.950464 1.38627312 1.734288 0.1476541
0.5188613 0.3530454 0.3542731 1.120014 0.311822 0.905021 1.297191
0.8576166 0.396572671 10.78841 0.281766053 1.161544 1.637168 0.8748816
0.4734541 0.5784016 1.495981 0.1915256 0.0168991 1.010625 0.3331179
1.567305 0.8590559 0.5984719 1.46671 4.302108 1.997116 1.002907 0.6546823
2.069637 0.409647704 0.6232314 0.5612819 0.7002461 0.5356842
0.340354638 0.8442183 0.8940359 0.5954682 0.9800516 0.4248241
0.3533129 0.7487009 0.3627138 1.032783 0.7454835 0.3872724
1.094491 9.40731 0.4992442 0.4859967 1.22177 0.4573857 0.08607612
0.5978613 0.828013422 1.716732 2.173837 0.40446483 1.410714 0.8291843
0.4618968 1.440995 0.4641403 1.106566 0.935790521 0.6609537 0.1797222

0.5175228 1.231047 0.877838 1.04622 0.6193739 0.5057257 1.009201
0.606307081 1.03155 1.546732 0.417061 0.4074848 1.778456 0.4446954
1.856119 0.8767493 1.242345 0.5056951 0.5123328 3.548974 0.3101501
0.7206333 0.8169485 1.129429 0.21767 0.4995561 0.755946941 0.611961
4.314324 0.6987118 1.271027 1.145859 0.1602376 0.522494052 0.6678917
0.545349 0.5779508 0.5052252 1.872720872 1.47748 1.173996 0.9325151
0.85917 0.644426 3.451683 0.6564348 0.6319076 1.681226 0.5053164
1.220292 1.02253 1.203806 0.714239072 0.556118332 1.337918 0.636890046
0.622136059 0.5524145 0.458384921 0.6074365 1.819818 0.8720974
0.367522116 1.810046 0.5803367 0.9841551 0.63283039 0.3271634
0.8444762 0.7859384 1.259219 0.2843146 0.6262053 1.319192 0.8127154
0.8897395 0.1164728 1.179404 0.937568815 0.4105934 0.452696914
1.972403 1.243392 0.2029838 0.5483853 0.5926002 1.394866 0.4869698
0.5584152 1.384357 0.945619156 1.001095 0.8535836 1.036851 1.209154
0.1914172 0.6724588 0.4920724 0.4704815 0.7776086 4.245631
0.8134713 1.168581 0.2322417 0.2678231 1.261905347 0.9087551
0.9904079 1.660755 1.213371 1.781368 0.618844 0.2867422 0.6492998
0.7248113 0.953535 1.080637973 0.624461336 1.286395 0.7860087 2.161071
1.54637 0.6725139 0.5134577 2.397043 0.1885021 0.5930029 0.544733
1.709669 1.50575 1.276318 1.947986 0.5569869 0.3156506 1.379446 0.3907406
0.426126478 2.646817 0.4726831 1.144608 0.419198 0.05480566 0.4693656
1.404387 1.729712 1.164081 0.7477894 1.214608 0.282958764 1.154407 1.278706
0.5440812 0.3896797 0.5581826 0.910223945 1.077917 0.2998208
0.8676735 0.2285621 0.5920645 0.4837207 0.444429764 0.5638939
2.920708 1.377914 0.8753765 1.322002589 0.6576489 0.7085081 0.2072542
1.442658134 0.275473592 0.6469206 0.5177301 0.5989883 1.003965
1.209829 0.3337276 1.296834 0.2742833 0.6034064 0.9175582 0.5798859
1.934484 0.650482293 0.3212184 5.260497 0.8532069 0.6072127 0.8124264
0.9637555 0.6076029 0.1907696 1.29802 1.993092 0.6815165 0.4324059
4.962817 0.7019845 1.232145 0.5720326 1.073176 0.2535468 1.647879
0.5252543 0.5371027 0.509794 0.4246353 0.3967119 0.4492705
0.7149821 1.323234 0.236718 0.6376351 1.199387 2.118373 1.689396 0.4694148
0.388509 0.2662037 2.924077 1.322465 1.465356 0.4125021 0.2311173
0.7669114 1.0804 0.3527217 0.4550933 1.435872 1.87647 0.382523
0.370946572 1.083015 0.2154104 1.369766 0.5608612 0.9534107 0.46213665
0.644838172 0.3314568 0.320895142 1.242358 1.253601 0.4506837 1.085567
0.5324326 0.7328315 1.73073 0.5648397 1.001995 0.7048744 1.016828
0.521011383 0.4120168 2.148499 0.5405426 0.4525083 1.178789 0.3836584
0.7743886 2.756463 0.8106337 1.103451
AC015908.2 0.4208563 0.0393242 0.1549142 0.204439018 0.080806688
0.2968518 0.2814907 0.3601674 0.06761794 0.06647994 0.1454487
0.293333969 0.8355034 0.329089095 0.17555931 0.06174578 1.713763
0.4411905 0.8284529 0.01853864 0.12183161 1.393038 0.04013825 0
0.2857262 0.2228086 1.519869 2.247065 0.09636346 0 0.2414812

0.141103269 0.06734177 0.02518536 0.1612734 0.062464048 4.113999
0.1916258 0.2467709 0.06598778 0.03167963 0 0.3652275 0.3854341
0.4840621 0.6444983 0.02401493 0 0 0.6088733 0.02676794
0.01621305 0.03414135 0 0 0 0 0 0.5957051 0.09307197 0 0
0.1247701 0 0.030383 0 0.09616039 0.02162497 0.02806133 0.08626155
0.2941069 0 0.1410665 0 0.01774594 0.5598994 0 0.4254598
0.5652433 0.1822049 0 0.04735872 0.03040312 0.01624593 0.037153845
0 0 0 0.236291 0 0.4521232 0.020803108 0.02171895 0.03439929
1.904098 0.186956235 3.039882 0.03206578 0.07981515 0.02833466 0 0
0.2093097 0.3619645 0.1306104 1.026199 0.042132114 5.11685 2.054796941
0.2522505 0.03434202 0.090665 0 0.153211 0 0.7221301 0.06238911
0.05311157 1.639766 0.2288356 0.04498602 0 0 0 0.6931209
0.3232423 0.107422 0 0.596985065 0.5446989 0.3119402 0 0.6064859
0 0 0.03333996 1.709855 0 0 0.9130673 3.565288 0.03478152
0.0549936 0 0 0.01928738 0.04374115 0.02792637 0.02847987
0.04027326 0.01963493 0.4911161 0.03108761 0 0.1736421 0.2710302
0 0.05453568 0.05515735 0 0.2474397 0.4243054 0.05836123 0 0
0 0.2346378 0.05876967 2.490788 0.07803023 0.05317772 0 0.02172496
0 0.497572 0 0.04247534 0 0.3045014 0 0 0.2784375 0.07299571
0.0287224 0.04944997 0.2219686 0.1954931 0 0 0.7379989 0
0.06646095 0.186056708 0.2785407 0 0.06530497 0.09700162 0.02958287
0 0.024112189 0.02755042 0.02982744 0.09568223 0 0.278783232
0.03310864 0 0.03006739 0.02001221 0.278409 0.3262548 0 0.04387928
0.329276 1.558048 0 6.291728 0.08742851 0 0 0 0.026568465 0
0.1903475 0 0.05371427 0.1887223 0.8434897 0 0.1162163 0 0
0.253554271 0.04557893 0.0472377 0.4145107 0 0.1166278 0.08406772
0 2.742663 0.6915356 4.803428 0.2077924 0.167485802 0.792908 0.080222104
0.4204735 0.1991902 0 0.4886874 0 0.188978 0.1745461 0.7052812
0.06048345 0.688384728 0.1616958 0.06740773 0 0.4807413 0.02279631
0.02410315 0.3726487 0.02943986 0.1858138 0.2262267 0.03080228
0.2819763 0.04763355 0.05493142 0.069534007 0 0 2.727741 0
0.06495372 0.09138742 0.2985829 0.06253367 0.2153025 0.1240641 0
0.033655827 0.4032333 1.73614 1.051763 0.02757962 0 2.501157 0.04299375
0.05567389 0.08261449 0 0 0 0.2771758 0.266359 0 0 0.1028833
0.05106406 2.247427856 0.2648152 0.02814645 0.3178648 0 0.03372248
0.3881545 2.369085 0.08320334 0.332413 0 0.01912224 0.248232303
0.2919117 0.1336077 0.0313855 0 0.02766083 0.091564572 0.08613674
0.1185961 2.639288 0 0.0998091 0.4013099 0 0.0713766 0.398311 0
0.1326825 0.269993354 0.7535011 0 2.121563 0 0 0.02809812 0
0.02799217 0 0.02997664 0 0.60413 0 0.02344939 0.7706154 0 0
0.091923057 0 0.1034409 0.06701959 0.1018975 0 0.05119498
0.2149159 0.08409488 0.04167051 0.2776685 0.04681049 0 0
0.1539581 0.03446141 0.04930423 0.1299022 0 0.1145174 0.01557563
0 1.005339 0 0.3943168 0.03043383 0 1.575869 0 0 0.1948739

0.04776023 0.2689817 0.06189336 0 0 0.1952131 0.03755659
0.4427533 0.09444343 0.1089259 0.9038988 0.02540564 0.02083519 0
0.8995703 0 0.4499113 0 0.01877156 0.02650884 0.2507596
0.2870168 0 0 0.025192091 0.1456776 0.200231001 0.1757323
0.06150326 0 0 0.01881021 0.08410916 0.02357791 0.2606637
0.2076759 0.04688825 0.1982303 0.113816647 0.2829969 0.05897368 0
0.04176493 0.1623096 0.02146081 0.2947355 0 0.06202583 0.0636529
ITFG1-AS1 0.09461015 0.04862129 0.1368138 0 0.099911144 0.1101102
0.07734249 0.0371099 0.08360429 0.06164793 0.179836 0.080596554
0.1921925 0.067815484 0.086826138 0.07634383 0.1115229 0.04545813
0.02626454 0.3896667 0.200846999 0.1230273 0.4714641 0.07724151
0.3532781 0.1530474 0.1252799 0.2212821 0.07943058 0.098441872
0.2090008 0.07476992 0.05550856 0.560515 0.4386842 0.102975874
0.1287756 0.04738606 0.4881805 0.08158873 0 0.0931809 0.4515752
0.2042396 0.3222718 0.254999 0 0.1501904 0.1451974 0.1158191
0.03309646 0.1603693 0.04221311 0.06277488 0.111952 0.1567024
0.1767291 0.02439282 0.1523882 0.2685111 0.5018938 0.1036367
0.1157014 0.1636899 0.3005296 0.302392366 0 0.5614893 0.06939126
0.106655654 0.1818201 0 0.1744177 0.03971847 0.06582438 0.1221656
0.1080757 0.202326 0.2995196 0.09011284 0.3612236 0.05855535 0
0.1807814 0.045937821 0.04488919 0.1618449 0.2673854 0 0.2029402
0.05884368 0.180049902 0.1342689 0.04253203 0.1008972 0.173367563
0.04698219 0.07929364 0.1480278 0.07006719 0.07295409 0.206269 0.34506
0.06885243 0.05382984 0.216627 0 2.186968 0 0.2599066 0.382151
0.02802504 0.09713142 0.28415 0.3666417 0.3720239 0 0.1313366
0.08944601 0.04041961 0.1112434 0.2875624 0.07317034 0.247441
0.02142474 0.1816654 0 0.03863912 0.246041768 0.04644673 0.1928448
0 0.09780944 0.121852205 0.1872195 0.3710002 0.1208059 0.05964851
0.2134891 0.2687944 0.07429549 0 0.09066038 0.1026002 0.142851
0.1669314 1.29798 0.03452876 0.1760657 0.04979473 0.04855412
0.03571921 0.0384374 0.168922009 0.2146948 0.1861709 0.240163797
0.1685728 0.2045932 0.108124 0.1359733 0.04035542 0.2164773
0.097081811 0.2853969 0.04001841 0.1243334 0.1695495 0.06999238
0.09647827 0.1052001 0 0.1074448 0.043584375 0.2839425 0.04081133
0.2100697 0.06413946 0.2353076 0.151485 0.2505765 0.08606655
0.240676 0.106539 0.09171152 0.1372234 0.03453027 0.1512401 0.1417917
0.410615 0.1528611 0.698477 0.098590524 0.2295959 0.6343024 0.161489
0.329821 0.0365769 0.1932095 0.089438504 0.03406394 0.0368793
0.09464287 0.1520571 0.068938729 0.1023406 0.08894498 0.1858799
0.1732047 0.03129372 0.04745748 0.1015738 0.1808443 0.2442744
0.3041691 0.277522 0.07651701 0.3242955 0.111462721 0.171686761
0.2598804 0.065699655 0.035277792 0 0 0.2324472 0.2800085
0.3052417 0 0.1436924 0.2140655 0.08192771 0.447857156 0.2254191
0.1168114 0.1601594 0.2038281 0.09613408 0 0 0.3824535

0.05343935	0.07780431	0.2569190.069027695	0.05766874	0.099188353
0.2126792	0.2686724	0.1203324	0	0.08259664	0.1168282	0.1726501
0.03353942	0.2617407	0.091192911	0.3998484	0.2500332	0.1731552
0.1981330.02818585	0.1192066	0	0.1820004	0.1378465	0.1198764
0.3427615	0.1045925	0	0.06791841	0.085973357	0.1838534	0.06747629
0.1519207	0.1329281	0.2007755	0.07532893	0.06712262	0.1030907
0.3042339	0.03834888	0.234098241	0	0.0830944	0.06133147	0.03715492
0.2046003	0	0.09765743	0.1063168	0	0.3064391	0.06812441
0.2517458	0.2147904	0.1468741	0.1975992	0.1803175	0.1477794
0.02544142	0.1578418	0.092625621	0.3601656	0.1044027	0.0924741
0.0386568	0.1250856	0.03428019	0.5762336	0.1543116	0.176144
0.5478347	0.2364315	0.127883255	0.07218519	0	0.1552228	0.03040632
0.03420046	0.158497397	0.3195040.1221956	0.5789678	0	0.1480874
0.09923766	0	0	0.9146064	0.2201415	0.04687188	0.25678896
0.05175806	0.04975576	0	0.305278646	0.344507764	0.1215940.1826691
0.03461013	0.1804250.2223826	0	0.1867399	0.1878030.17396	0.1588009
0.04375212	0.1191276	0.056827832	0	3.32531	0.1242967	0.2015812
0.03196966	0.1265972	0.1660792	0.1299709	0.1545670.04291443	0.23151
0.2775659	0.2133792	0.0634524	0.3408707	0.1625622	0.3212277	0
0.7079591	0.2118385	0.1662177	0.02890751	0.04168454	0	0.2634034
0.05725748	0.2164932	0.1223259	0	0.1338591	0.2362072	0.2078593
0.2040701	0.08867129	0.0253154	0.09654628	0.2321789	0.1368574
0.2335438	0.1346784	0.1314824	0.06282417	0.2060887	0.5730656
0.3177852	0.1723443	0.09271335	0.202180121	0.09283825	0.1638805
0.3444941	0	0.07525125	0.236029487	0.124592192	0.0720476	0.08252332
0	0.1901099	0	0.3502695	0.3721176	0.1386592	0.1166089	0
0.09629061	0.1449341	0.2144593	0.05629015	0	0.3645817	0.11566
0.1032781	0.1003415	0.1592077	0.1700615	0.2091261	0.03834504
0.3672752
AC138932.6	0.4733868	0.1081241	0.1216987	0.168634956	0.194409695
0.2856737	0.04299858	0.1650501	0.04647983	0.1370927	0.2499496
0.134423027	0.1869865	0.527828513	0.434439131	0.6366506	0.2066706
0.05054492	0.02920356	0.2803516	1.004948484	0.3191863	0.4966306
0.3149112	0.9998802	0.2722776	0.1671585	0.8474839	0.08831891
0.145943427	0.2987847	0.526532522	0	0.6232369	0.5764592	0.228997859
0.2505750.5795744	1.0177650.2721557	0.08710493	0.1726798	0.3138167
0.7191314	0.1535718	0.6379503	0.2311064	0.3339935	0.161445
0.8370655	0.1471999	0.1783147	0.1408103	0.383896826	0.3319452
0.3833224	0.3930103	0.1084895	0.5648016	0.3838599	1.369774
0.1920561	0.1715312	0.1820069	0.5430085	0.672460465	0	1.27837
0.3086247	0.158120638	0.4043316	0	0.2585801	0.441629878	0.1219836
0.3622293	0.2002824	0.2699597	0.9251001	0	0.1204934	0.1302155
0.08359509	0.08933819	0	0.1746931	0.1542475	0	0	1.429112
0.3271416	0.257396891	0.05971748	0.07093708	0.1121877	0.064255828

0.3656767 0.04408332 0.3840484 0 0.1081569 0.950167 0 0.8038491
0.2394137 0.1376387 0.46337842 5.384457 0.423733437 0.1155961
0.4721266 0.4362549 0.3240014 0.6582221 0.1019173 0.1654614
0.04288559 0.1460332 0.4641236 1.39322 0 0.4396435 0.569507
0.2751298 1.238754 0.2423926 0.07384072 0.6874057 0.164144384
0.1032883 0.2450563 0.2064617 0.3625146 0.135487532 0.1734745
0.2750102 0.4701343 0.165808 0.2373787 0.2390981 0.7710191
0.2869012 0.1764093 0.2661894 0.07941804 0.2651587 5.23169 0
0.391535 0.05536678 0.2969304 0.1985811 0.5128628 0.281736708
0.2387193 0.4968084 0.534076507 0.5997956 0.682462 0.4408183
0.7559437 0.1346136 0.1604675 0.323835954 0.09066657 0.4894615
0.09216424 0.4578397 0.6225965 0.6078874 0.6725895 0.332392
0.2688029 0 0.05261931 0.2722688 0.7007299 0.0713167 0.9157353
0.2358108 0.5224054 0.1913949 0.1672548 0.1184608 0.4078963
0.4407832 0.07678845 0.2942869 0.2207216 0.2029169 0.2549496
1.187798 0.146163808 0.2552878 0.369433 0.1795597 0.1000168 0.1626795
0.3375897 0.132595626 0.53026 0.2870428 0.2104669 0.6199321
0.306612038 0.1593096 0.6922858 0.3720238 0.6052717 0.278364
0.685984 0 0.221189 0.09053627 0.4509411 0.6612362 0.1985184
0.08012986 0.371806376 0.136356144 1.098052 0.328731669 0.196126981
0.1495346 0.135525866 0.4799937 0.3632318 1.499009 0.322406908
1.384687 0.2082671 0.3340167 0.547769872 0 1.42871 0.2849301
0.2039729 0.7482407 0.847546 0.967134 0.1134001 0.3565155 0.6055746
0.519397 0.307007722 0.3206095 0.294100219 0.5517823 0.298737 0.2943549
0.57586 0.3214374 0.8118836 0.5759091 0.074585 0.2910296 0.371790635
0.349322 0.834036 0.1925314 1.132993 0.06267972 0.3976377 0.2561546
1.902244 0.2299074 0.6664531 1.058657 0.6202478 0.3929133 0.4153518
1.465771925 0.2810866 0.4501616 0.4054096 0.1724366 0.04464847
0.04187914 0.3731684 0.2865664 0.9725488 0.8101627 0.632142471
0.416423602 0.6159514 1.773057 0.2065629 0.8720647 0.1685598
0.2533658 0.09851144 0.2678873 1.32506 0.6817281 0.6997907
0.3184341 1.143166 0.1464738 0.4410892 0.246474 0.5374783 0.3510087
0.343301591 1.34703 0.1160854 0.308466 0.1719301 0.2781655 1.029136
1.441608 0.1715792 0.5222789 0.5154242 1.077842 0.170632151 0.4013138
0.3673617 0.2588885 0.1014264 0.4563301 0.302114297 0.5447269
0.1630433 0.7900625 0.1420487 0.5763045 0.4413696 0.069406096
0.3925079 1.016951 0.874198 0.1563506 0.285523782 0.1726495 0.1659704
0.3825151 0.484913557 0.68950506 0.1738291 0.7870506 0.1154491 0
0.08242246 0 0.2491634 0.1044091 0.225664 0.7504261 0.462156
0.4967175 0.473901727 0.04025532 4.266246 0.1842741 0 0.1777354
0.2111452 0.1846636 0.2023206 0.0572877 0.5725989 0.2574161 0
0.5535983 0.1411055 0.9475357 0.2937236 0.5059954 0.346466
1.220129 0.4496737 0.5082482 0.06428456 0.695236 0.1936064 0.5020772
0.1591615 0.4332941 0.2266904 0.06408533 0.2083732 0.1969791

0.1848951 0.7658067 0.09859366 0.08444463 0.3757245 0.2065279
0.2282578 1.233468 0.2994979 0.4751348 0.1746356 0.4010127
1.433682 0.4416819 1.149779 0.5154401 0.056201046 0.2064538 0.03644377
0.6128691 0.1127384 0.2510157 0.164025821 0.069267062 0.2803842
0.229394308 0 0.4650433 0 0.0229097 0.181019 0.3083505 0.421387
0.3071611 0.1427541 0.1933828 0.03406534 0.25035619 0.2917935
0.9323707 0.4286746 0.22967 0.7252037 0.6785885 0.2161045
0.5425639 0.5969022 0.758408
AC127024.4 0.4807301 0.0988212 0.4449114 0 0.253832696 0.6713862
0.7859805 0.5279726 0.339846 0.08353161 0.8223992 0.163809879
0.390625 0.551331081 0.176471307 0.6206654 0.4533332 1.29349 0.05338183
1.397621 0.408215005 0.4167484 1.563437 1.255927 1.305502 1.244256 2.291646
2.348688 1.856561 0.466853122 0.606839 1.418362726 0.338458 0.3164524
0.8105557 1.726684747 0.3271654 1.733591 2.604554 0.6633058 0
0.8206777 1.032538 2.283107 0.9357244 0.712631 0.6638414 0.07631426
1.131251 0.3530978 0.4036049 0.6518908 1.458545 0.318969766 0.6826163
1.146573 1.86782 0.247888 1.393758 1.247406 1.020083 0.5617017 0.3135457
1.247604 0.07635209 0.790203427 0.3221997 1.141209 0.7051775 1.011612719
0.7390867 0.509666 1.122578 0.887991504 1.382455 0.7448928 0.2928806
0.6579538 1.48809 0.9157571 1.248098 0.3570356 0.1528053 0.4490839
0.18673426 0.5930329 0.2349603 1.630356 0.1484491 1.008258 1.255778
0.83644704 0.3820561 0.3025574 1.708919 1.996729006 1.623326 0.08058088
0.902585 0.9256603 2.47128 0.7785795 1.227316 1.329433 0.4376296
0.2515928 0.370571095 14.76355 0.322729877 0.4226014 1.898623 0.5126399
0.2961247 1.876959 0.4657421 0.1512253 0 0.8008121 1.030178
1.314425 0.9043943 0.1461153 1.412805 2.926056 2.090165 0.4430748
0.2024626 0.7853276 0.400057524 1.038416 1.287839 0.3234822 0.5301187
0 0.2536783 0.5864802 0.6138349 0.6061683 1.229411 3.059368
2.214713 1.048866 0.5067268 0.7646156 0.362925 0.5331583 14.28972
0.4912502 0.7156955 0.5060308 0.296054 0.2903926 0.6249821
0.429160624 0.2181801 1.210836 0.488125119 0.4111424 0.8316582
0.3662643 0.9672644 1.066274 1.46661 0.460403136 0.3314628 0.8946974
0.2527036 2.559915 0.2133859 0.7843558 1.042352 0.6075867 0.2183781
0.354335307 0.48092 0.4147385 1.22751 0.2607227 1.769544 0.6157768
2.419121 1.311956 0.4891661 1.227043 0.7456027 0.960663 0.3509082
0.4610859 0.288187 1.390936 0.3883567 2.922771 0.667940131 1.322164
1.718932 0.6564422 1.096937 0.9664377 0.5609887 0.242374473 0.7615724
0.5996478 0.3847171 0.6181023 0.560462913 0.5824111 1.355833
0.3777948 1.005809 1.335673 2.556078 0.06881512 0.4043162 0.7447196
0.6182139 1.531005 1.399665 1.904125 0.226544361 0.747745216 2.429713
0.333831042 0.430205805 0.6833439 0.247730723 0.4049498 1.138216 1.96458
1.010288067 3.991351 1.957866 1.498639 1.001280642 0.458157 1.06837 0.52083
0.828548 0.3907788 1.267567 1.087905 2.487438 0.434455 0.4744042 0.9494173
1.192520662 0.7032591 0.268796142 1.440879 0.7735964 0.5380578

1.228065 0.5875625 2.849395 1.491349 0.06816779 1.899927 0.6178221
1.044873 0.5928817 0.7038648 1.955966 0.1718605 0.545138 0.5462692
2.145478 0.3268633 3.492243 0.5418401 0.6377426 0.239405 0.138042
1.223166394 1.261156 0.8228603 0.6175476 0.5853728 2.530032 0
0.8867596 0.7857315 1.468571 1.169143 0.679710105 0.930343206 0.9570245
0.7479254 0.3020647 1.594066 0.4621714 0.1323237 0.6842707
0.2098616 1.59167 1.246146 2.131938 1.067133 1.24382 0.9370994 1.7225
0.3003568 1.034177 0.1283233 0.941292836 3.859767 0.3536584 0.3289136
0.4714122 0.2542324 0.9754271 1.366372 0.2090889 0.2386713
1.284757 4.565123 0.571820841 0.5868562 1.343017 0.7887135 0.432599
0.2780453 0.782341974 0.432921 0.3477021 1.604636 0.778962 0.1003276
0.4033947 0.507475744 0.5978949 3.43183 1.086617 0.3810623 0.782872837
1.051966 0.2022539 0.4370049 1.240937874 1.050301268 0.317746 0.1392259
0.2110319 0.7334158 0.7533092 0.1290079 1.745897 0.3817035
0.3535681 0.4841366 1.333871 0.9079609 0.462002896 0.1471672
18.45613 0.5894678 1.075482 0.7147502 1.608154 1.957793 0.2113293
1.466044 1.918888 1.646878 0.8148741 1.83112 0.3868948 0.8660107
0.2478018 0.9793273 0.5428396 2.877808 0.2739895 0.5912061
1.351332 0.6777798 0.9201334 0.9942378 0.05818698 1.320046 0.5801214
0.468572 0.8161928 0.8401459 2.027843 2.125675 0.1802215 0.2058109
0.7849087 1.698826 4.589612 0.4746702 0.1824863 1.336168 0.5745964
0.3665099 2.03829 2.341344 1.611308 0.3768737 1.027311328 0.3302088
0.5995473 1.82045 0.1030385 0.4588372 0.599652837 0.759688756
0.5125204 0.503177904 0.7949035 2.395632 5.973231 3.182663 0.6145075
0.3522754 0.7702627 0.2807333 0.5545047 0.1767443 0.5604191
0.514835559 0.9778554 2.000701 0.7835838 0.5247737 2.039409 1.186476
0.641911 1.841848 0.7014155 1.49295
AL359220.1 0.3814532 0.05645762 0.2753646 0.039134988 0.138572144 0.43566
0.2145409 0.1675758 0.02157308 0.05302501 0.2842276 0.317153752
0.7655911 0.284358209 0.050409982 0.4580158 0.5995235 0.09970433
0.2473696 0.7777745 0.213782837 0.7989338 0.4706152 0.4816716
0.9033042 0.8214344 0.7208913 0.4250717 0.6302552 0.080438831
0.30432 0.344066968 0.1933647 0.6307655 0.2881382 0.225859285
0.5690466 0.2567754 0.5353686 0.1368444 0.1920366 0.120221
0.8666437 0.5314074 0.403912 0.7978178 0.02298544 0.1404862
0.7337175 0.7770287 0.2775546 0.470713 0.261422 0.340164294 0.06259035
0.760181 0.4104252 0.4531876 0.5963825 0.3018893 0.6593116
0.4412461 0.502565 0.1583932 0.1696363 0.334408705 0.04090582
1.045246 0.8012746 0.096324212 0.5864559 0.2372559 0.296292 0.174230815
0.06227902 0.3572647 0.1115506 0.6473766 1.726086 0.3255354
0.2842886 0.6950371 0.1115491 0.1347622 0.302269348 0.182434
0.06860919 0.1954874 0.1601977 0.01745542 0.7326232 0.398226023
0.353394 0.1536483 0.3818511 0.141662118 0.5758514 0.0613823
0.5474872 0.2711999 0.1913867 0.4524145 0.3450245 0.5774125

0.3889239	0.6787612	0.144501342	5.300583	0.090140876	0.1676644	
0.4930473	0.5423643	0.4950059	0.3146714	0.5026021	0.3983845	
0.1741675	0.7667573	0.2769655	0.4902003	0.2511689	0.4173862	
0.9298756	0.01741341	0.3731667	0.6375212	0.3384392	0.7128806	
0.272998858	0.2157303	0.7286473	0.2429895	0.555248	0.070745602	
0.3542713	0.4201577	0.4597943	0.08080578	0.1973997	0.4508344	
0.309932	0.2052913	0.4824975	0.3618214	0.3317487	0.7845734	
0.844298	1.051348	0.3452803	0.263403	0.2787659	0.1106029	0.09918307
0.435882843	0.3877958	1.056861	0.042605315	0.5741758	0.04399401	
0.3441013	0.403492	0.1457852	0.260677	0.371586703	0.7311721	0.113589
0.4277697	0.3250016	0.3115469	0.1701162	0.2222548	0.05999619	
0.1559522	0.089971372	0.2808605	0.09477784	0.2235996	0.3392835	
0.5221773	0.1837177	1.313371	0.7217741	0.3027547	0.215347	0.09860437
0.3088438	0.3831351	0.370744	0.1097629	0.153045	0.1331234	0.7103322
0.347681285	0.8491703	0.2766871	0.2812744	0.5067695	0.2689895	
0.217227	0.369256411	0.08350299	0.4758129	0.3083208	0.2681161	
0.168993661	0.258796	0.2582008	0.6331255	1.238645	0.8640213	1.02865
0.5460392	0.2379899	1.544285	0.1700555	0.4347823	0.3389437	
0.2928819	0.12942724	0.07911017	0.6772959	0.500113577	0.182060165	
0.4120907	0.199192112	0.4841258	0.5720014	0.2395736	0.064132054	
0.7539209	0.3210652	0.5038475	0.369805216	0.3053751	0.4144495	
0.1157161	0.2761257	0.3038764	0.2279809	0.3625602	0.3454063	
0.1585779	0.1806882	0.6237742	0.280535267	0.3571373	0.311398003	
0.487816	0.3148632	0.1366214	0.3229619	0.4262613	1.070187	0.6014285
0.194725	0.7622261	0.349046567	0.3795405	1.064548	0.4021255	0.5952507
0.3309223	0.3037533	0.7876544	0.295867	0.4535127	0.8609589	0.1081
0.3688474	0.08358451	0.3066967	0.56939925	0.1779043	0.8966893	
0.3057701	0.1229101	0.08289229	0.3644573	0.2987731	0.8711925	
0.1128493	0.1880138	0.146700829	0.563728159	0.4395509	0.3560815	
0.3259709	0.677531	0.6258809	0.6383828	0.06629828	0.09325261	
1.027948	0.1142614	0.3735198	0.1447184	0.1926537	0.7053353	
0.9282485	0.1048649	0.8993846	0.2036458	0.187223895	0.8068567	
0.166129	0.2326528	0.02992476	0.1075894	0.2034485	1.623201	0.08627288
0.6135997	0.4675824	0.927326	0.36958577	0.2235182	0.1705067	
0.03004003	0.1176897	0.2029753	0.233704794	0.258325	0.07567465	
1.089909	0.1538373	0.331172	0.1216336	0.056374573	0.04174913	0.6353931
0.07303481	0.2751549	0.32468032	0.3004997	0.05777493	0.08322187	
0.067520115	0.266688203	0.1277445	0.2238938	0.2500603	0.3398624	
0.4638472	0.1801644	0.9589008	0.05249865	0.3029959	0.1690287	
0.2765978	0.3073943	0.304272518	0.07473617	6.229156	0.1336386	
0.4063719	0.3464741	0.53492	0.488544	0.05701362	0.5051991	0.4041845
0.3770984	0.3819868	0.07647211	0.5812459	0.2418833	0.209736	
0.6838336	0.1493219	0.616546	0.2882198	0.209091	0.5930092	0.01075619
0.2336365	0.140791	0.2327	0.2178675	0.4050807	0.2082113	0.480116

0.8761621 0.7240803 0.2040491 0.09533565 0.3200844 0.06850965
0.3594658 1.032946 0.0715625 0.07529627 0.661585 0.08105509 0.3356905
0.2911243 0.2460019 0.6982006 0.3867642 0.828200947 0.1467292
0.3805865 0.3555702 0.03924467 0.1941768 0.098969908 0.116541975
0.07436405 0.191647314 0.4036773 0.4611223 0.2275048 0.9995275
0.2190467 0.4025175 0.4551041 0.1900871 0.2608896 0.100976
0.8182211 0.15251243 0.2200779 0.7761262 0.0994822 0.05329935
0.3592504 0.1129744 0.1755292 0.5216371 0.3166232 0.9849408
ACTA2-AS1 2.400298 8.300112 1.119337 2.077994483 0.201407432 3.659754 11.51468
10.08638 6.938709 3.02703 0.9107339 1.410730884 5.579997 3.314308696
9.562604766 0.3228123 1.250191 0.5989962 0.3977391 0.1217155
0.128376758 0.4113279 0.1220036 0.1367197 0.05053028 1.56519
0.1773992 0.125723 0.117162 0.24523356 3.875534 0.254886421 0.2620046
0.2204728 0.9882516 0.096198166 1.133351 0.456651 0.4920532 2.920379
0.6547921 0.06108623 0.6216785 2.523875 0.0362178 0.21314 0.2511043
0.08861373 0.04759323 0.2277812 0.1301815 0.07096455 0.7471839
0.111113313 0.3889771 0.07396471 0.03475727 0.05277064 0.199801
6.638725 0.2422817 0.4891729 0.1213598 0.9094482 0.1034339
0.178414346 1.231506 0.05784323 0.09553017 1.349452594 0.07866874
0.03287823 0.05145403 0.171851325 0.8371533 0.368403 0.6872526
0.111416 1.426846 0.1417798 0.9874806 0.1266768 0.9167359 0.1185141
0.099380407 0.2074662 0.2319043 0.4732797 0.294473 0.1463447
0.2546011 0.632328209 0.02640664 0.1129243 0.03968687 1.000154183
0.1478397 0.538016 0.3493511 0.2618219 0.2104354 0.2607872
0.1017943 0.1151002 0.1323338 0.5903696 0.276618574 3.533051
0.712013285 0.5929431 0.158666 2.110974 0.1814768 0.1164244 0.1442147
0.1097488 0.1137822 0.1420647 0.4866934 0.1589864 0.1203302
0.07069354 0.3453695 0.1681126 0.0926992 0.900348 0.05224295
3.845169 1.112946709 0.04567335 1.181148 0.3077976 0.9938684 1.306073418
0.1227347 2.002471 0.5702127 0.1583693 0.1819431 0.4229099
0.0730584 0.5920399 0.2629949 0.1008918 0.2317794 0.05159056
4.892743 0.115443 0.1523579 0.2056555 0.07639303 0.2669459 0.14363
0.107971042 0.126672 0.54189 0.070849452 0.4774057 0.2280114 0.1134119
3.175593 0.1190504 0.1561067 0.369132517 0.01603685 0.03148166
0.09781049 0.1095631 0.4817886 1.068889 0.5224147 0.1633126
1.664078 0.308582303 0.1675288 0.1525008 0.2065719 0.006307148
0.04165011 0.3217593 0.295685 0.02539004 1.875598 0.3841384 1.869824
6.546018 0.3191799 0.02230827 4.194077 0.1076741 0.03006317 0.2747387
0.116338677 1.708348 0.2138539 0.4367001 0.7489055 0.9279708
0.3799848 0.205214956 0.08039219 0.7035453 0.1721739 0.229272
7.307851797 1.191537 1.609337 0.4752404 0.1167914 0.05539076 0.07933436
0.05327064 0.1564931 0.1201035 1.674985 0.03898586 0.1203887
0.04251943 0.131528112 0.01929463 0.1328876 0.419937006 0.49954152
1.527444 0.055933338 0.05224611 4.836913 0.05502917 0.676170388 0.6782388

0.1157756 0.1503853 0.440399905 0.8755794 0.3445993 1.070949
0.2806078 0.1228934 0.381593 0.2500165 0.06017365 0.2522378
0.005100586 1.800642 0.549814372 0.0567085 0.31862012 4.219938 2.738861
0.189326 0.5007944 0.0243664 0.0746739 1.655309 1.741395 0.102953
1.141854807 0.747062 0.2212829 0.234597 0.07793356 0.9091021 0.1523882
0.6569646 0.2076051 0.7048664 0.0707282 0.007490093 0.8570911
1.447862 0.347295 0.101450182 0.135594 0.5971747 0.1971962 0.3485726
0.1263567 0.03703731 0.3432258 0.09123669 0.2916889 0.2639723
0.197314526 0.032735922 0.7844237 0.03618615 5.560818 0.7980674
0.4770301 1.4340068 0.2962152 0.2436848 0.5022276 0.3483484
0.618885 0.01877457 0.4092141 0.6995125 0.0248241 0.8864442
0.2101495 0.1179623 0.255033284 0.3670473 0.06159855 2.205157
0.04561576 0.1148026 1.779856 0.944398 0.1315099 0.2598165 0.07459099
0.1627463 0.065392016 6.722113 0.0649779 0.259485 0.5800605 0.1479763
0.449762417 0.6199897 0.07209657 0.1707982 0.7537552 0.08737295 0
0.847068372 2.400972 1.660389 0.1917358 0.2120205 0.040402109 0.1323302
0.1957091 1.30242 0.094347146 0.189711973 0.167397 0.04939755 0.3539519
0.2129049 0.1093399 0.11235 0.05141653 0.1846759 1.528167 0.1171176
0.0860472 0 0.206761899 0.1139239 1.735584 1.458576 0.4707837
0.8299455 2.060296 0.1045209 0.0357859 0.1519933 0.1097196
0.2674948 0.06065425 0.3217335 0.04991668 0.2178767 0.06793853
0.6054346 0.03501824 0.09746393 0.03787476 0.8335951 0.3126879
0.07378282 0.1095826 0.1554105 0.3828678 0.06812429 0.06415417
0.1926989 0.100039 0.3658317 0.06540744 0.2207394 0.06975587
0.2688539 15.67438 0.4840234 0.08074718 0.2353965 0.4326237
0.06464653 0.4756913 0.3090521 0.1901892 0.1484345 0.3931812
1.191283 0.228635672 0.2967003 0.5027934 0.0813019 0.329024 0.8633129
0.400371414 2.058295894 0.8076665 0.056804459 0.05982518 0.1346
0.3467964 0.4133246 0.4574019 0.2249781 1.112273 1.159036 0.1009998
1.533524 0.06627918 0.254623179 0.6795527 0.1577449 0.2274682
0.7515326 0.7054958 0.412266 0.04777993 0.09596716 1.900411 0.6500875
AC138956.2 0.02804259 0.0576457 0.1622073 0 0.207296701 0.3046104
0.320942 0.307984 0.04956087 0.02436339 0.3198219 0.286667288 0.9969074
0.160804899 0.051470798 1.086164 0.4407406 0.1077909 0.0311394
1.087038 0.178594065 0.5834478 0.8237463 0.122104 0.7234658 0.6532342
0.4158913 1.603271 0.3766935 0.038904427 0.2831915 0.561435246
0.03290564 0.7753083 0.2364121 0.06104441 0.4580316 0.280906
1.012882 0.09673209 0.09287893 0.4419034 0.6692379 0.6860852
0.6004231 0.8314029 0.3168334 0.13355 0.3156026 0.1373158
0.2354362 0.2376685 0.2502406 0.260491975 0.1769746 0.966094
0.8800304 0.1156811 0.6022411 0.2273917 0.2704765 0.4095742
0.1371763 0.1940717 0.1781549 0.153650666 0.04698746 2.123916 1.15179
0.29505371 0.9484946 0.2973052 0.2412558 0.65926642 0.9365015 0
0.3844057 0.8155885 0.4340261 0.1602575 0.2997882 0.2082707

0.1337046 0.3095957 0 0.2128836 0.3015324 0.4579087 0.04329765
0.2673412 0.1395308 0.24396372 0.1910281 0.2017049 0.9569947
0.68515211 0.5570237 0 0.2925044 0 0.3171476 0.3493626
0.2556908 1.714269 0.06382098 0.2935249 0.524975718 9.075087 0.150607276
0 1.258557 0.19936 0.0575798 0.8141509 0 0.08821476 0.09145677
0.4282121 0.2474447 0.5750609 0 0.7671053 0.5205072 1.1468
1.270065 0.2584603 0 0.3664862 0.175025167 0.05506752 0.6205889 0
0.07730898 0.144468713 0.3329528 0.3909868 0.1432282 0.2475187 0
0.1912105 0.2348938 0.4078924 0.1343594 0.08109559 0.04234124
0.08482064 4.103715 0.08187503 0.1669956 0.1771108 0.3741794
0.08469784 0.4557161 0.350481176 0.3818152 0.5297408 0.071184913
0.3597496 0.242567 0.04273084 0.6045402 0.3349194 0.5133136
0.19183464 0.1933533 0.3795686 0.1474104 1.234831 0.2489502
0.4194125 0.5768574 0 0.1910809 0.516738989 0.4488587 0.09677232
0.2801924 0.3041765 1.422809 0.1077609 0.705577 0.8673487 0.2140102
0.1684177 0.1449783 0.7773106 0.08187859 0.224139 0.2689746
0.1622759 0 1.461386 0.155852697 0.5444205 0.2864887 0 0.6043311
0.5203895 1.308974 0.247423941 0.4442506 0.04372432 0.2244183
0.7211193 0 0.3882741 0.1581806 0.6170648 0.6453939 0.9275505
1.15345 0.2007108 0.5145842 0.6757641 0.3005207 1.598154 0.8164713
0.4699283 0.528603509 0.203552864 1.047594 0.506310413 0.167302258
0.1195852 0.144509589 0.354331 1.051269 0.4222124 0.392889804 1.306113
0.2537974 0.32378 0.318589295 0.5345165 0.6232156 0.6456122
0.6524815 0.4559086 0.6161782 0.3173056 0.755732 0.06335802
0.5227231 0.7753575 0.040919827 0.06837241 0.235196624 0.67241
0.5308995 0.1141335 0 0.3427448 0.8657016 0.1023475 0.03976454
0.4433162 0.57663396 0.270893 0.6916953 0.7755547 0.6040483 0
0.1413321 0.1820897 0.3884055 0.4085791 3.316278 0.4063801
0.248011 0 0.08052447 1.325096927 0.2179777 0.3200012 0.2881889
0.4990678 0.7141218 0 0.03979049 0.4277872 0.58614 0.2728
0.594746342 0.049336382 0.7552987 0.2181449 0.0440511 1.091589
0.4043999 0.1543777 0.3151247 0.08161286 0.4036844 0.08076871
0.8456688 0.6507896 0.8416516 1.249466 0.6841136 0.08760407
1.297031 0.3368487 0.695510817 1.281043 0.6189021 0.7126461 0 0 0
0.7970504 0.06098426 0.6613184 0.7994045 0.8129123 0.151619162
0.1711664 0.2611422 0.7361326 0.2523494 0.08109654 0.483211219
0.555582 0.1448759 0.4368177 0.1514648 0.3511466 0.1176568
0.370034397 0.2092632 1.292894 0.1491435 0.4445727 0.63934615 0
0.1769722 0.0509839 0.620468937 0.653520789 0.3501090 0.6226489 0
0.3422607 0.4833734 0.07525464 0.6641998 0.1855503 0.2062481
0.04706884 0.1556183 0.9533589 0.370564823 0.1287713 7.051023
0.04912231 0.5676153 0.3032273 1.013137 0.9057608 0 0.8551925
0.8140736 0.3087896 0 1.068153 0.2256886 0.1515519 0.3131939
0.1904247 0 2.895794 0.8219684 0.2956031 0.445548 0.04942145

0.04128804 0.3122926 0.169712 0.5646865 0.04834345 0.4100005
1.364856 0.7701338 0.6900299 1.239977 0.1051292 0.3901832 0.4578634
1.541527 1.582025 0.06922274 0.1064503 0.545602 0.4096659 0.09162749
1.231467 0 1.675527 0.1099215 0.239705977 0.3852436 0.07771909
0.7351817 0 0.267655 0.384777237 0.258505202 0.1281301 0.48920074
0.1545646 0.2704745 2.090631 1.685555 0.3033146 0.4931856 0.5875722
0 0.152217 0.3093026 0.9807333 0.400427657 0 1.426426 0.1371272 0
0.9814656 0.4089748 0.6624851 1.033088 0.04546212 0.7775781
AC024075.3 0.6944882 2.169988 1.221211 2.196894784 0.498712193 1.336334
1.862169 2.440759 1.521974 1.086068 0.686447 0.804604622 2.539429 0.915954358
1.631614633 0.6276523 2.401333 1.975424 0.431862 0.8883937 0.294864497
1.926582 0.49544 1.270065 1.433358 0.9347091 0.6179848 2.396785 0.5597397
0.308315758 0.7013374 1.63923347 0.5215513 0.8411815 0.7962608
0.483774088 0.9830921 0.6121959 1.433401 0.4791233 0.04600382
0.3283184 2.055176 1.27934 0.5407188 1.235405 0.7323428 0.1322971
0.4547520.2720554 0.5830689 0.5415096 0.2478931 1.253377631
0.3506288 3.570463 1.245393 0.4870326 0.6860803 0.495569 1.446872
0.5680248 0.9965225 0.1922511 0.7059345 0.558100732 0.4189201
2.669244 5.541927 0.167020486 0.7687611 0.09817206 1.263246 0.373189675
1.005026 0.1913087 0.4231108 1.710927 1.289864 0.6879346 1.272754
0.6877233 0.08830026 0.4482414 0.215812953 1.001711 0.3530136
0.6280797 0.3860233 1.19175 0.4492213 0.302093912 0.6307869
0.4995319 1.382525 0.88234223 1.876115 0 0.4056646 0.7406351
0.1428057 0.588345 0.2532922 0.92996 1.517335 1.453857 0.367094818
7.889144 0.372986124 0.3663074 0.9475306 0.7570443 0.5703965
0.5284054 0.376788 0.568017 0.3170959 1.041206 0.4552293 1.424166
0.2613072 0.5910405 1.203124 2.113567 1.258151 1.79225 0.4289827
0.6807154 0.346766559 0.3273056 1.456028 0.7477112 0.8041294
0.787124096 1.136079 0.3389041 0.6384805 0.7005622 0.2507396
0.2525557 0.407208 0.3030495 1.011552 0.4820091 0.08388809 0.9522828
4.763928 1.824907 0.2067863 0.9942129 0.7698515 0.0839033 0.8577382
1.041580082 0.8825448 1.705507 0.493619961 0.5147636 0.4004858
0.8042697 1.796607 0.2843808 0.7627473 1.026189354 1.388661 0.3760079
0.1460276 0.5973997 0.7398444 1.133122 0.7104463 0.2340672
1.009538 0.358324072 0.389067 0.191729 0.6476493 1.129962 1.381825 0.3914168
1.140405 1.010838 0.7773427 0.5005134 0.8976143 0.8058336 0.0811105
0.5328873 0.9658862 0.4822608 0.04488325 1.447677 0.308781329
1.348283 0.7804532 0.5689987 1.338186 0.7303027 0.7131819 0.245102891
0.5601058 0.7363405 0.8614632 1.785887 0.323869751 0.6731055
0.7312513 0.4366258 1.162435 0.7718334 1.393451 0.9146084 1.125713
0.5737927 0.8931045 1.303781 0.359472 0.9733598 0.720011534 0.48970531
0.8546311 0.462979186 0.290032432 0.3553901 0.429461892 0.4680095
2.028004 1.643132 0 1.181351 0.4085519 1.122599 0.157800326 0.7280656
0.4801761 0.7147976 1.077268 1.129079 0.7731707 0.314329 1.766797

0.1255273 0.3045997 1.124694 0.72964734 0.7450412 0.232990277
1.137925 0.6836949 0.282657 0.6082724 0.4850422 3.739051 0.811099
0.9453964 0.9222307 1.142449272 1.375303 0.7830932 1.875505 1.196764
1.026219 0.3500157 0.8117171 0.9832803 0.8364757 2.862789 0.8051358
0.2866319 0.06917142 0.3988454 1.480956935 0.7557649 0.7132485
0.4995995 0.2602032 1.414845 0.9289626 0.3153378 0.72647 0.3573179
0.6756022 0.70700053 0.439862079 1.821737 0.2160985 0.4363786
1.842298 0.5341418 0.7646473 0.2913573 0.4850836 0.7997951
1.200165 0.8377357 0.4484763 0.5175039 2.243413 2.393114 0.3905202
1.135461 0.4819949 0.435149258 1.999678 0.3678578 1.330465 0.227009
0.0979407 1.368892 2.537915 0.3020609 0.9654321 0.593929 0.8608235
0.570748006 0.7630232 1.293463 0.9571104 0.03571174 0.6025184
0.638237708 0.8505721 0.315737 3.801754 0.5001465 0.4058279 0.5244887
0.329906839 1.382001 0.4957803 0.7017858 0.798229 0.422232354 0.1823671
0.4090614 0.6060675 0.102441401 0.3236951 0.408029 0.6972612
0.1219471 0.3814313 0.4788389 0.4472921 1.62299 0.3308574
0.6129398 0.326391 0.4367824 1.539048 0.23360184 0.08504219 6.384028
0.3406305 1.124581 0.750957 1.077976 0.7022066 0.1831785 0.9076822
1.411265 0.9176788 0.8331026 2.728869 0.9688098 2.101823 0.4534509
0.7231139 0.104562 2.078717 0.4523653 1.805785 0.5092712 0.1468735
0.3272058 0.486142 0.5379839 0.5085357 0.04788995 0.4738467
1.477824 1.491141 0.6347313 0.2097175 0.104143 0.5054531 0.9638325
0.7089952 1.567185 0.3085802 0.05272586 2.393571 0.4058229 0.7866555
1.430245 2.146094 1.254977 0.3992646 0.118728664 1.144889 0.5389301
0.4046028 0.2381678 1.620326 0.762335389 0.585326171 0.1692375
0.6299951 0.8166113 0.6251869 0 0.8953694 0.5463078 0.5292723
0.7190156 0.3244498 1.112069 0.2723565 1.655205 0.561951008 0.9760208
1.284586 0.1358408 0.1212985 2.946238 0.4363027 0.5421364 1.964922
0.4503564 1.324888
AL354726.1 0 0 0.127829 0 0 0.05715505 0 0.05778805 0 0
0.07001077 0 0.0374106 0.052801609 0.067603436 0 0 0 0
0.1070814 0 0 0 0.04009385 0.1500353 0 0.07803504 0.4977319
0.2473808 0.102196703 0 0.349298283 0 0.3879297 0.06210228
0.080177733 0.05013281 0.07379023 0.1900504 0.1270511 0 0.09673507
0.1757998 0 0 0.198544 0.04623771 0 0.1130517 0.09017755 0
0.03121616 0 0 0 0.04880383 0.05504099 0.1139545 0 0.05973276
0 0.05379482 0.1201145 0 0.05849862 0 0 0.3330891 0.108057
0.05536189 0.05662655 0 0.1810704 0 0.2733404 0.06341266
0.112198 0.2520519 0.3109441 0.07016248 0.1125005 0 0 0 0
0.1398042 0.07200774 0.2775842 0 0.1755674 0.0916322 0.040053745
0.04181709 0.0993472 0.3142371 0 0 0 0 0 0 0 0.1343331
0 0 0.04819066 0.202800102 7.297664 0.049453135 0 0.06612121 0
0.0756272 0.3318618 0 0 0 0.1533894 0.09285771 0.06294199 0
0 0 0.1751448 0.1000887 0.05657837 0 0.06016937 0 0 0 0

0	0	0.1457704	0	0	0	0	0	0	0	0	0	0.05561238
0.0371354	6.6532225	0.05376867	0	0.07754104	0	0.05562246	0
0.131523853	0.2507443	0.05798158	0	0	0.1061985	0.05612409
0.1588046	0.1256840	0.100784766	0.06348915	0	0	0.1885892
0.05449656	0.1502373	0.02047739	0.0517238	0	0.135740391	0	0
0.04089044	0	0.03664243	0.04717892	0.2438758	0.1340240	0
0.09520964	0	0	0.0588783	0	0	0.05950938	0.3199053	0.153526538
0.1191766	0.2351773	0	0	0.1708742	0.08596245	0.046424961
0.1060897	0.1148579	0.0736896	0.07892848	0	0.09561974	0.1385064
0.05789094	0.2311859	0.1949240.1108522	0.05272402	0.02816133
0.06339791	0	0.06173731	0.03971779	0	0.173571302	0.19096644
0.3237515	0.102308419	0	0.0523557	0.126535759	0	0.07267218
0.1584422	0	0.07458658	0.08333646	0.04252632	0.278963761	0.1755129
0.0909502	0.09976097	0.3174039	0.1497013	0	0	0.1588165	0	0
0.1454829	0	0.08980257	0	0	0.1045951	0	0	0.06431032
0.09096327	0	0	0	0.047335624	0	0.06489252	0.1497999	0.1322295
0	0	0	0.2267309	0.03577608	0.9333660.05930588	0.1085819	0
0.05288174	0.044626303	0.07157475	0	0	0.1034987	0.06253007
0.1759549	0.05226214	0.1204007	0.2368786	0	0.052077292	0.129600049
0.04313192	0.1910124	0	0.5310106	0.05901691	0	0	0	1.0604250
0.1960110.03716385	0.1524763	0	0.08423786	0.1725931	0.1980887
0.1474761	0.096158605	0	0.05419242	0.03600049	0	0	0	0.5982127
0	0.1371469	0	0.2577221	0	0.05620389	0	0.06042879	0.2367457
0	0.176295967	0.06633815	0.03805695	0.6147114	0.06631296	0	0
0.097203065	0.04580887	0.1095568	0	0.03649477	0.079975069	0
0.07748037	0	0.203736069	0.053647229	0.2163975	0.1066706	0	0
0.1154324	0.09884191	0.05815879	0	0.04514883	0.3091088	0.0340657
0.04637677	0	0	7.86690900	0	0.04978359	0.04928472	0.1551725	0
0.08023128	0.1336539	0.09012766	0	0.1476787	0.09880896	0	0
0.3334802	0	0.7165885	0.0299889	0	0.1800607	0	0	0.3515789
0.04458107	0.06742526	0.06349588	0.08975135	0.04168945	0.04597813
0.4531539	0.5958391	0.04602672	0	0.0751716	0.1446209	0.1065582
0.04545971	0	0	0	0	0.1115482	0.06185744	0.1610262	0.04812484
0	0.1084268	0.25519700	0	0	0	0.048504174	0.05609676	0.128506463
0.06767006	0.05920836	0	0.06416994	0.07243334	0.05398051	0.04539627
0	0	0.04513869	0	0	0	0.2270927	0	0	0.1171899	0.08264013
0.2648221	0.2171027	0.05971143	0.285963
AC067852.3	0.09381991	0.1577951	0.06906891	0.182299309	0.162125399
0.3705859	0.3625652	0.2141087	0.1507381	0.1259711	0.2756069
0.174378258	0.5804246	0.097816805	0.17220184	0.5230608	0.2278852
0.0819608	0.04735485	0.1405135	0.090531553	0.1478785	0.3937063
0.1764037	0.2316214	0.3200948	0.1264923	0.4698988	0.1002491
0.035498048	0.2368630.368480178	0.02001633	0.1122896	0.2300932
0.111399016	0.3250546	0.05126211	0.3740796	0.2647874	0	0.01120032

0.1017735 0.2700449 0.2158203 0.229881 0.05353559 0.08123776
0.07853705 0.3341141 0.01193455 0.05782902 0.09133205 0.16977423
0.05382637 0.2486297 0.1784393 0.02638808 0.1373776 0.2351461
0.3619649 0.2491419 0.08344362 0.1918359 0.05418535 0.311549738
0.04287335 0.298888 0.5004485 0.102559815 0.1966924 0.06028308
0.1362722 0.128901993 0.3639553 0.0440528 0.1818694 0.1459172
0.3360202 0 0.1563083 0.12669 0.09488717 0.1231359 0 0.16187
0.1083849 0.2571171 0 0.04878669 0.05304744 0.157677012 0.06778415
0.01533701 0.194045 0.145871094 0.3388347 0 0.2846863 0 0.05261436
0.03187732 0.06221416 0.1862109 0.03882198 0.1450716 0.112708259
2.591175 0.114517053 0.1874442 0.5052788 0.06063483 0.08756375
0.1536964 0.049579 0.02683029 0.05563269 0.08287975 0.1290166
0.1166022 0.08022853 0.1425802 0.3034293 0.2514579 0.1776921
0.1179151 0 0.1253991 0.159700382 0.03349729 0.1986848 0.1817413
0.10581 0.043939793 0.2587928 0.1932412 0.08712496 0.08603679
0.1667986 0.0387708 0.05358177 0.01550743 0.09807615 0.02466502
0.1931697 0.04299664 3.80291 0.03735312 0.01269782 0.0538678
0.02626286 0 0.1663259 0.045684841 0.01935469 0.1342661 0.043301422
0.09725949 0.09836817 0.0519859 0.2451592 0.2473862 0.2341845
0.081684427 0.05880792 0.1154448 0.05977934 0.3231655 0.06309797
0.440673 0.1991591 0.02395503 0.2615254 0.125731883 0.2047788 0
0.05681321 0.1040788 0.1357627 0.06555043 0.4404936 0.1241421
0.1518782 0.1920893 0.1433081 0.4563399 0.02490317 0.05453704
0.1636159 0.0493558 0.0137804 0.3555814 0.035551652 0.4553563
0.2614044 0.08734916 0.1729937 0.07913758 0.1194363 0.021500961
0.171968 0.01329864 0.05972423 0.03655444 0.049718484 0.1254734
0.09622044 0.09383934 0.2230624 0.06770691 0.2994797 0.0732548
0.05216983 0.1468085 0.09140259 0.3145189 0.1747491 0 0.341643512
0.035377193 0.2623954 0.118456176 0.012721139 0.06061922 0.073253709
0.1197432 0.2355985 0.1375873 0.119496438 0.1381742 0.0482449
0.1772583 0.080748439 0.02032148 0.0421221 0.103956 0.1911006
0.05199879 0.199903 0.1688885 0.1930773 0.09635091 0.07481643
0.370579 0.074673969 0 0.059612048 0.3749383 0.2099132 0.1041402
0.04668911 0.2829506 0.1158524 0.1089505 0.02418857 0.6337174
0.109613598 0.144185 0.1502695 0.1248792 0.265373 0.05081896 0.0214929
0.2492196 0.2100134 0.2568212 0.273773 0.06866638 0.1508638 0 0
0.124007653 0.2983381 0.04866378 0.02191298 0.1517902 0.2027185
0.04074531 0.02420435 0.05576159 0.1234198 0.05531428 0.156771847
0.105038749 0.1598067 0.02211607 0.1071842 0.4795613 0.06833179
0.03521518 0.051117 0.01241117 0.4174506 0.1965248 0.2118184
0.103271 0.1677151 0.7837951 0.4876674 0 0.2201797 0.07968464
0.18927071 0.3896261 0.1003933 0.1333843 0.01393961 0.01503525
0.02472281 0.4328944 0.1112893 0.3387593 0.04558816 0.5285932
0.064560417 0.05205983 0.2779901 0.1539263 0.08771592 0.08632857

0.269440357 0.06912771 0.07931454 0.132857 0.07677941 0.1068003
0.1073555 0.157563034 0.2651953 0.4059154 0.04536161 0.1352157
0.129637007 0.07465564 0.05382565 0.04651987 0.267344911 0.21118961
0.03131905 0.4775596 0.0624019 0.1040977 0.1069213 0.04577701
0.3501588 0.03386079 0.06272982 0.1861063 0.1104388 0.4080942
0.112706352 0.01305515 4.542758 0.104583 0.3089325 0.1037541 0.6619369
0.1317536 0 0.1486312 0.154749 0.06261173 0.06672666 0.6326534
0.02288088 0.07682353 0.05861977 0.05791721 0.03210342 0.3318762
0.1944441 0.1798138 0.04169609 0.07515704 0.01255765 0.04070701
0.1548524 0.2342018 0.02940708 0.1247006 0.1737701 0.2768223
0.08994464 0.1563734 0.1065826 0.06390097 0.487403 0.3181493 0.37013
0.01052696 0.0161883 0.3555931 0.07929018 0.1300519 0.2453932
0.2005378 0.07457666 0.18945 0.018226491 0.1004322 0.0354571
0.1366467 0 0.03618071 0.191502035 0.146015446 0.0259803 0.089273499
0.06268056 0.02742138 2.119534 0.6389641 0.07547921 0.2125016
0.2417822 0.04980752 0.02893525 0.08362098 0.2209538 0.111639969
0.1734905 0.1840549 0.09731605 0 0.4703798 0.08611521 0.2365354
0.2890742 0.06913594 0.2554183
HID1-AS1 0.1635072 0.1867296 0.2101729 0.038830871 0.13429774
0.3946854 0.7425828 0.3990566 0.1284326 0.06313555 0.03453292
0.433342453 0.4613209 0.312533693 0.366800483 0.05863955 0.114214
0.3142468 0.06052124 0.2288782 0.077135105 1.322959 0.07623805
0.01977635 0.02466839 0.2351109 0.07698181 0.03777031 0.2745472
0.02520434 0.5962659 0 0.02131801 0 0.06126409 0.158191159
0.1236404 0.03639715 0.04687132 0.3133408 0 0.02385736 0.08671352
0.1307301 0.07072469 0.07344909 0.04561364 0.02884023 0 0
0.05084266 0.04619226 0.06484762 0 0 0.1203628 0.05429811 0
0.03901636 0.2651695 0.1051374 0.07960313 0.2073634 0.09429742
0.05770906 0 0 0.1026855 0.1332483 0.081922012 0.02793113 0
0.08931326 0.122030734 0.03370639 0 0.05534185 0 0.1022491 0
0.1387276 0.1349288 0.02887364 0.0308573 0.176423779 0.05171896 0
0.09127921 0.112202 0.08659887 0.04519772 0.118539427 0 0.01633439
0.1033319 0 0.1804347 0.0609053 0.1515998 0.02690924 0.03735727
0.2716026 0.19878 0.07932814 0.0206733 0.1901609 0 1.859785
0.195142679 0.03992676 0 0.08610394 0 0.1273155 0.0704042
0.05715017 0 0.02521985 0.09160442 0.03104623 0.08544584 0.05521903
0.084303 0.1036685 0.213932 0.08372211 0.02550449 0.3561436 0.037796846
0.05351347 0.08464215 0.162998 0.05008483 0.046797227 0 0 0.1391861
0 0.0273301 0.08258418 0.05706623 0.06606356 0.06963607 0.07880701
0.05486178 0.03663419 0.08308135 0.3182578 0 0.3442251 0.111883 0
0.05904739 0 0 0.171597 0.046117343 0.1294804 0.3666779 0.08304987
0.07833061 0.09299077 0 0.099424481 0.03131612 0.03073807 0.1591671
0 0.2956856 0.09880638 0.04040202 0.02551284 0.1856884 0
0.254445 0 0 0 0.1084436 0.04654215 0.09623369 0.06610756

0.1386471 0 0.1174057 0.1639572 0.05304527 0.02904181 0.10891 0
0.1174124 0 0.2019392 0.02939203 0.06960093 0 0.1842436
0.2809465 0.02120056 0.06869755 0.02616445 0.08498077 0.05452126
0.1557264 0.370661975 0.1257722 0.2049554 0.1142192 0.01900546
0.02403665 0.1093560 0.08334371 0.09381334 0.1946931 0 0.3918172
0.1383838 0.085614311 0.037677796 0.07984546 0.22708703 0.027096807
0.07747359 0 0.3315785 0.1075370.1367657 0 0.07357989 0.06165875
0.04195235 0.034399826 0.0432860 0.3444507 0.1095920.0369202
0.02661285 0 0.09792061 0.49256 0.1195227 0.2511587 0.053020045 0
0.025395464 0.2359623 0.1547751 0 0.03315022 0 0.06730166
0.0663061 0.1545694 0.02872036 0.163438583 0.1754988 0.03200834
0.0147778 0.0434816 0.1298970.06867178 0.1769510.02795884 0.2999923
0.1227691 0.08775816 0.0535582 0 0.0260840.132071954 0.194174
0.1036568 0.11669 0.05105092 0 0 0.07733514 0.03959187 0.3797324
0.08836711 0.051374408 0.063925423 0.04254977 0.09421718 0.02853863
0.02619218 0 0.1750245 0.08166175 0.1586193 0.07845843 0.3662836
0.03222758 0 0.07520915 0.5059187 0 0.1986409 0.1954151
0.07274279 0.166006328 0.1257466 0 0.1065438 0.02969222 0
0.2369749 0.8483243 0.1580353 0.02254931 0 0.1089615 0.117872271
0.3326719 0 0.08941978 0.02335504 0.1050772 0 0 0.1501732
0.1212829 0.09812691 0.09478803 0 0 0.04519059 0.1891366
0.02415575 0.3600221 0.118343477 0.03975527 0 0.03303006 0.133990838
0 0.2134768 0 0.2392558 0.1385841 0.02846862 0 0.02868691
0.07212557 0.02226973 0.2439494 0.01680296 0.02287541 0.06547403 0
0.3438298 0.06364805 0.05806281 0.02455583 0.09723905 0.05102605 0
0.1978710.09888747 0 0 0.01821068 0.04873767 0.06545553 0.01560796
0.0205612 0.06838224 0.05437821 0.01479207 0.03191788 0.02220381
0.09605346 0.02674857 0.2023196 0.02198968 0.2328033 0.1252778 0
0.04112677 0.04535757 0.0957938 0.01959324 0 0.01944471 0.3337066
0 0.05255997 0 0.03448207 0.05049568 0.2412756 0.01978704
0.02751067 0 0.05295095 0.0474753 0.038823545 0 0.07552579
0.05292105 0.1168191 0.1734010.090646907 0.598118959 0 0 0.03337836
0 0 0.1266077 0.01786393 0.05325194 0.1791342 0.2121862
0.1109410.1113236 0 0.108090901 0 0.1680207 0.02961274 0.03966388
0.1156082 0.04076237 0.1119633 0.02677156 0.1767165 0.04030049
AC008764.6 0.1645960.1323984 0.3973883 0.137662852 0.15870371 0.0555252
0.08190304 0.2245606 0.07588635 0.06217432 0.3672774 0.195083394
0.1453752 0.235961176 0.19702693 0.1154936 0.3036828 0.6739404
0.1191996 1.3107510.334227268 1.00503 0.4204331 0.3661349 0.5150077
0.3148827 0.9779461 1.4357370.3244407 0.486483969 0.5149186
1.11604375 0.9824934 0.4805059 0.2051266 0.288198066 1.081211
0.6164997 0.9416175 0.7899398 0.2725765 0.1127719 0.8026973
1.0814140.09750709 1.4466170.8714322 1.0110810.8786230.1752121
0.5607682 0.3760424 0.4342503 0.398858665 0.3161421 0.5499807

1.219149 0.2878329 0.6147575 0.6151117 0.9525375 0.261304 0.4900952

0.4457365 0.9092873 0.967204581 0.47964 0.8898744 0.8188102

0.527075163 0.4620989 0.4552261 0.7476046 0.636916091 0.5510075

0.4558723 0.4141946 0.6856203 0.6645697 0.8997346 0.229514

0.3366154 0.2502196 0.182325 0.083394131 0.2648441 0.1888768

0.5752928 0.8287032 1.773833 0.3649787 0.303510205 1.243114 0.3024111

2.21834 0.734361198 0.6965354 0.335877 0.4777336 0.9221844 0.4561776

0.5438507 0.6655623 0.6666273 0.3745974 0.4119847 0.252181779

1.630599 0.40356053 0.5190093 0.8350639 0.1695861 0.367353 1.93439

0.3743945 0.1800962 1.225317 0.6059956 0.1804195 2.592638 0.6058436

0.228389 0.3763551 1.592607 2.126196 0.1758879 1.125205 0.2805772

0.297771187 0.5972524 0.2917372 0.1685422 0.2762049 0.460847514

0.8024768 0.137195 0.7675755 0.6226336 0.1614841 0.4066343

1.341245 0.494439 0.2125852 0.9416339 0.2377167 0.1731669 0.6218051

0.8357663 1.885783 0.6327709 0.4260278 0.3458324 1.639785 0.332210563

0.2435941 0.5294847 0.445069133 0.6630474 0.6190206 0.6760931

0.3188373 1.35531 0.5894795 0.55809138 0.3330649 0.4358893

0.7398307 0.4250504 0.6141332 0.3405573 0.5092724 0.7436827

0.1056532 0.329673981 0.4581873 0.5927012 1.024889 0.4366381

0.5268434 0.3941686 1.127746 1.380143 0.2730724 0.4512845 1.600157

0.3275352 0.7522225 0.2287973 0.291725 0.9524787 1.017498 0.2610576

0.268467382 0.8451806 2.193321 0.4153131 1.41522 0.4094703 0.3340445

0.24354593 1.339837 0.7476035 0.3006707 0.4600665 0.271156779

0.4644651 0.7804288 0.5848971 0.7561309 0.8900181 0.5528143

0.2458586 0.3064127 0.8745786 0.2914281 0.4318329 0.5016074

0.6541292 0.387829931 0.259729126 1.761308 1.0734225 0.224147829

1.047772 0.725271904 0.3114597 0.3106393 2.455088 0.626648793 1.333258

0.4857601 1.883902 0.325210532 0.4262698 0.7952099 1.250219 0.770882

0.4508405 0.6918827 0.5263378 1.334588 0.1131809 0.4472714

0.3957359 1.221780189 1.116694 0.290102361 0.4861892 0.3183857

0.1820399 1.60616 0.349868 1.290192 0.3003644 0.8321149 0.3620237

0.413872127 0.5098392 0.6052035 0.4540478 1.293152 0.187615 0.4508418

0.3717482 0.2202654 0.1668282 2.6598 0.6337618 0.1054856

0.2851106 0.5137376 1.430673114 0.4241557 0.2041574 1.011238

0.3619705 1.919604 0.4102496 0.6803424 0.4132844 1.162125 1.125481

0.394619516 1.05759653 1.089451 0.7793751 0.1911081 0.8460239

1.318681 0.22653 0.4825108 0.6039904 0.3708668 0.6389668 0.3554535

0.3610408 0.2962564 0.5380735 0.2618743 0.3018085 0.5696222

0.5348769 0.336299499 2.912532 0.4001177 0.3217598 0.5497151

0.5046147 1.472805 0.5375674 0.2178809 0.595121 0.752267 0.5651272

0.340494575 0.3822082 1.049618 0.786655 0.469189 0.3000846 0.506955286

0.3737893 0.1330981 1.242139 0.4123006 0.1269491 0.6155229

0.349395456 0.7120411 0.4683035 1.351158 0.503448 0.303008491 0.9865803

0.8731427 0.312261 1.042411806 0.208469657 0.2627833 0.2210746

0.4398112 0.4148815 0.5270615 0.3456839 0.7571043 0.5019275
0.4473858 0.4084001 0.3706559 0.4685646 0.438444991 0.3505267
0.9287179 1.291188 0.289706 0.3869116 0.4596413 0.2311463 0.1730265
1.480924 1.168583 0.8668198 0.4852223 0.8321112 1.20949 0.5156719
0.2028884 0.7289338 0.3771104 1.71361 0.2097628 1.081259 0.7959137
0.6306071 1.454041 0.2732426 0.3118305 1.388654 0.6168522 1.011427
1.004415 0.687872 1.144602 0.285565 0.07154275 0.1991462 0.4819848
0.674385 2.308485 0.7684427 0.09507984 1.273009 0.5892537 0.3741271
1.571326 5.144004 1.387051 0.243113 0.229394796 0.3300492 1.279266 0.3543843
1.380487 0.1821451 0.14282693 0.405240772 0.3596808 0.436946819
0.8151806 0.7132475 0 0.9351009 0.2181402 0.2517177 0.6879871
0.6965189 0.8108919 0.4735962 0.4727492 0.442812218 0.6749046
1.257516 0.5132493 0.3906 0.5161114 0.497758 0.2940233 0.6010984
0.8237234 0.9762983
AC104170.1 0.04367696 0.04489223 0.05052829 0.093354454 0.046124171
0.1016652 0.1071159 0.1027911 0 0.01897326 0.08302162 0.074415107
0.133089 0.031307148 0 0 0.03432316 0.04197167 0.1940012 0.2116358
0.278164207 0 0.1145538 0.09508984 0.207571 0.08478556 0.1388057
0.2043106 0.2200157 0.060594506 0.02756732 0.046023569 0 0.02875143
0.1104651 0.118847524 0.1783486 0.3500139 0.1126847 0.07533119
0.1808262 0.05735619 0.1042353 0.09428761 0 0.05886038 0.05483056
0.06933567 0.1564048 0.1604043 0.1222323 0.07403479 0.1559021
0.115920576 0.2756418 0.08681035 0.2610794 0.06756594 0.07035029
0.1062503 0.1263819 0.1594802 0.03560918 0.1511355 0.1387401
0.119657156 0 0.1481215 0 0.131300766 0.1007251 0.1543531
0.1342004 0.146688862 0.08103454 0.3007892 0.06652449 0 0.2150926
0.1664031 0 0 0.17354 0.07418496 0.127243699 0.08289273 0.1067371
0 0.1348741 0.2290144 0.1629918 0.047497361 0 0.07853997
0.06210585 0.320141871 0.04337884 0.07321213 0.04555821 0.4205065
0.02245293 0.163242 0.199122 0.03178586 0.02485065 0.05714644 0.168342031
4.182702 0 0 0.2352277 0.2070051 0.1345227 0.06558921 0.211576
0.03434911 0 0.1212636 0.1651717 0.1119588 0.1540672 0.1327537
0.1688961 0.02076938 0.1186893 0.1341859 0.03065806 0.1427026
0.045434262 0.06432666 0.1017454 0.09796708 0.150513 0.281266521
0.05762015 0.07612132 0.05577025 0.1101474 0 0.09927154 0.1143289
0.03970634 0.02092677 0.2210393 0.461632 0.02201834 6.491497 0.3188054
0.1300497 0.04597566 0.1344906 0.09893906 0.1419576 0.077983169
0.2477857 0.2406492 0.332616232 0.1245151 0.1259345 0.1996627
0.03138616 0 0.1332495 0.119514678 0.112932 0 0.1147975 0.06709104
0.09693635 0.2078504 0.04856584 0.1226724 0.09920423 0.160966482
0.3058594 0.07536252 0.6061193 0.08883031 0.1086302 0.08392002
0.1156791 0.03973279 0.05555426 0.1311571 0.02822587 0.07038828
0.1275277 0.03491014 0.1309168 0.04212471 0.105853 0.113807 0.030343003
0.07066225 0.05577656 0.1491034 0.1107364 0.06754318 0.1274222

0.165157827 0.0943541 0.4086096 0 0 0.063651393 0.07559318
0.1231849 0.1372989 0.0913832 0.05778722 0.08763535 0.1563057
0.05009227 0.07517982 0.3276473 0.07321061 0.1883959 0.133077
0.102913958 0.090582311 0.2879383 0.030330372 0.065144242 0.124171
0.075025627 0.3065991 0.04308882 0.3288026 0.267721371 0.3537912
0.1235297 0.1260736 0.165403292 0.1040652 0.1617787 0.2661764
0.1505562 0.08876096 0.3518941 0.06177631 0.1177069 0.04934076
0.4070764 0.1725195 0.223067374 0.2129831 0.152634977 0.1963675
0.1653775 0.1333241 0.1195462 0.07626179 0.7011417 0.03985211
0.1548354 0.2071424 0.028066255 0.1582207 0.0384761 0.05329163 0
0.3643375 0.05503196 0.1772555 0.20165 0.04242473 0.1106823
0.2109820.03219022 0.1087562 0.2508373 0.264598433 0.1485334
0.06230112 0.1122151 0.06136651 0.03707535 0.03477574 0.3718474
0.11898 0.03511254 0.1062230.123510745 0.11526376 0.02557379 0.2265103
0.06861056 0.1574235 0.1749616 0.06011165 0.08180227 0.2224492
0.3458111 0.2515981 0.07747929 0.0661056 0.2712188 0.1216294
0.3829220.2387792 0.1879213 0.08744155 0 0.2418489 0.03213179
0.1280725 0.2141519 0.4234707 0.1582551 0.4877022 0 0.08131721
0.1556363 0.04365962 0.07084506 0.03332443 0 0.07165892 0.08422281
0.06315483 0.146341254 0.07866648 0.1805179 0.14579 0.1179549 0
0.04581326 0.14408419 0.02716101 0.2598337 0.08711035 0.1081925
0.023709423 0 0.04593969 0.3573385 0.040266421 0.031808534 0.08019155
0.1475767 0.1917340.1665871 0.1026634 0.05860538 0.06896706
0.1444994 0.2141573 0.4765199 0.1413877 0.2199819 0.157408066
0.1002821 6.90811 0.3060364 0 0.05903541 0.08766573 0.1533416
0.1680037 0 0.07924611 0.2404734 0.1993270.06567127 0.2343434
0.1573636 0.05628536 0.07414769 0.0821999 0 0.1955913 0.07673472
0.1067617 0.07697499 0.3858422 0.1737152 0.02643302 0 0.150592
0.0532154 0.07415557 0.05452274 0.03838346 0.04710468 0.02729018
0.11686690.04457077 0.08574868 0.1895415 0.05390798 0.04144968
0.1213982 0.08700869 0.04757061 0.1653480.4034419 0.1591262
0.1426710.280010521 0.1071474 0.0605246 0 0.0936161 0.1621195
0.027240866 0.057518224 0.06652182 0.038097049 0.04012296 0.2457409
2.17080300.09511916 0.08589441 0 0.1614982 0.2975718 0.05927032
0.05352729 0.1131492 0.2338781 0.2826839 0.06732393 0.07119286 0
0.16213 0.1714966 0 0.03218115 0.03540413 0
AC068790.2 0.02658256 0.0546444 0 0.056817163 0.028071982 0.1650006 0
0.08341399 0 0.06928474 0 0.226451718 0.1080004 0.114324488
0.04879099 0.04290057 0.2088968 0.4087151 0 0.1288053 0.112863754
0.4608923 0.13943900.02893667 0.1443787 0.3440132 0.1689592
0.3315921 0.3570811 0.036878882 0.03355591 0.112043016 0.1247697 0
0.08964135 0.028933078 0.2170922 0.1597684 0.1371638 0 0 0
0.1903183 0.6503644 0.05174203 0.2149408 0.1668542 0.1265967
0.1087894 0.06508326 0 0.1126472 0.1423271 0.105826953 0.04194012

0.1761144 0.2383463 0.2193164 0 0 0.1025577 0.03882498
0.04334474 0.09198372 0.2955388 0.194201201 0 0.4807965 0.6238985
0.159823913 0.04086871 0 0.1960242 0.178554845 0.2219357 0.09153281
0 0.09095591 0.1122078 0 0.04059712 0.1974272 0.1689912
0.09030054 0.464656525 0.1008999 0.07795452 0.6677967 0 0.6588977
0.09919967 0.317985028 0.2112626 0.0239004 0.1889936 0.324739959
0.3168135 0.08911638 0 0.4724818 0.08199149 0.03311732 0.3878053
0.1160726 0.2117436 0.1391213 0.409823688 12.28942 0 0.175262
0.4294911 0 0.1091639 0.4258 0.1030151 0.08362189 0 0
0.2680704 0.04542671 0.06251199 0.2019906 0.08223453 0.6067494
0.3611819 0.04083394 0 0.08685131 0.055304182 0.05220045 0.2786581
0 0.07328391 0 0.1753432 0 0.135771 0.1340753 0.1999464
0.2416736 0.2226642 0.04833196 0.0764184 0.2306201 0.1605471
0.05360299 5.40961 0.07761223 0.07915053 0.05596319 0.0545689
0.08028807 0.1295968 0.047461929 0.06032269 0.04184667 0.303654136
0.07578208 0.6131675 0.08101214 0.03820434 0.4989002 0 0.072738736
0.09164323 0.2248791 0.04657852 0.1361093 0.03933145 0.4698625
0.2216852 0.07466056 0.1207549 0.195934066 0.1063723 0.1376009
0.08853477 0.03604246 0.264457 0.1362005 0.4928305 0 0 0.1197368
0 0.1713582 0.3104625 0.04249385 0.03187131 0.05127568 0.04294932
0.1385299 0.036934571 0 0.2036796 0.1814939 0.2021883 0.08221594
0.4032673 0 0.07656742 0 0.05318352 0.1708936 0.15495743
0.04600734 0.4498349 0.04178125 0.1668523 0.1406813 0.4800277
0.07610433 0.04064938 0.4118018 0.341849 0.3564581 0.02866526
0.3239721 0.062635245 0.110259977 0.8178068 0.110757588 0.039647932
0.03778634 0.13698575 0.4105236 0.05244922 0.1429393 0.931085352
0.2691544 0.2405835 0.03069225 0 0.06333589 0.1969227 0.1439997
0.1603545 0.108043 0.194699 0.1503926 0.2292432 0.1201186 0.1457376
0.0524992 0.271525456 0 0.074317066 0.07967516 0.1006517 0.08114336
0 0.3248999 0.1313007 0.09701879 0 0.168094 0 0.1604931
0.1873378 0.04324563 0.1272442 0 0 0.04315232 0.163637 0.1032818
0.5838146 0 0 0 0.038166 0.161039262 0.2066287 0.07583512
0.1024442 0.09959664 0.2256471 0.04233026 0 0.02896533 0.384662 0
0.037585406 0.327373911 0.2179052 0.2067873 0.08351519 0.1916214 0
0.2195101 0.1194871 0 1.875067 0.07656352 0.2829315 0.08046607
0.02751143 0.1480516 0.4863716 0 0.2573375 0.03547898 0.10409989
0.03679838 0.07822389 0.0779472 0 0.04686032 0.07705349 1.133328
0.2312366 0.1979643 0.1420845 0.4782962 0.057490067 0 0.1856595
0.1744515 0.102519 0.03843714 0.254473892 0.09575561 0.02746659
0.08873034 0.2871577 0.05547738 0 0.140307477 0.06612267 0.07906969
0.176723 0.2107131 0.057719888 0.05816975 0 0.09665889 0.147041113 0
0.1171344 0.1026489 0.07779511 0.2433308 0.2499309 0 0.2098728
0.1407118 0.1955098 0.1338546 0.04917204 0.03347121 0.127735092
0.0813779 11.78669 0.2793887 0.1415954 0.10779 0.2134196 0.335975

0.02921429 0.2316192 0.1929223 0.2276654 0 0.3197495 0.1426255
0.6225327 0 0.09025518 0.0500283 0.5171788 0.2597243 0.0934042
0.2274196 0 0.03913839 0.2960332 0.03217521 0.09732479 0.1833059
0.06477566 0.2106178 0.066367 0.4204952 0.2293499 0.09965571
0.08535426 0.05425311 0.2087526 0.8459608 0.2624747 0 0.3324828
0 0 0.4427884 0 0.0387388 0.06946566 0 0.1565083 0.07367267
0.1548677 0 0.05638215 0.29842687 0.21003962 0.1619454 0.370984547
0.04883907 0.3418565 0 0.5094425 0.07841527 0.155836 0.06552711 0
0.01803648 0.0651553 0.1377292 0.031631628 0 0.4097453 0
0.116072 0.2537362 0.1491083 0.3822566 0.6267524 0.04309515
0.1769025
PRR34 0.1051224 0.3889704 0.0851286 0.23592132 0.027753096 0.08156311
0.30937 0.346359 0.4366002 0.1552614 0.04995444 0.179103453 0.2295632
0.324007315 0.279773112 0.0169653 0.09087048 0.4444795 0.07003878
0.04074946 0.0334745 0.06379195 0.0661704 0.125875 0.06423238
0.1632506 0.1614719 0.07649258 0.02647686 0.204175735 0.3980967
0.160616865 0.04317332 0.2767975 0.1329346 0.034325291 0.1717009
0.06318141 0.1491663 0.1631775 0.1305646 0.07592518 0.10035
0.2571906 0.04092341 0.1558327 0.2177456 0.1251586 0.04302144
0.02573758 0.1176763 0.08018465 0.121949 0.146474721 0.0414637
0.04178731 0.0314185 0.1084125 0.05079605 0.2557249 0.1216712
0.06909111 0.02571142 0.1818776 0.3255738 0.047998788 0.2025615
0.06535854 0.06168112 0.079004188 0.06464713 0.01857498 0.1356582
0.132394899 0.5070916 0.1628875 0.184129 0.0269768 0.04437328 0
0.07224472 0.0520492 0.1837786 0.1249847 0.040833619 0.06483995
0.1644138 0.2244717 0.04057714 0.2304999 0.06538187 0.074306309
0.1133826 0.3733367 0.1046341 0.051368167 0.1252859 0.4228994
0.0548251 0.02335573 0.1134841 0.03274112 0.07668001 0.04590162
0.06579203 0.09627867 0.173643537 0.5380635 0.261116768 0.1039627
0.08492245 0.2242004 0.04316952 0.2262676 0.05092246 0.07440478
0.2057047 0.05837182 0.1192613 0.02694641 0.1359641 0.02396353
0.219511 0.1699595 0.01428316 0.08881418 0.007378829 0.2404212
0.131222276 0.02064299 0.1530515 0.3242095 0.08694172 0.270782679
0.07627459 0.219852 0.1476516 0.07953134 0.2213962 0.1314106
0.02201348 0.07645268 0.04533019 0.09120016 0.1031701 0.04239526
1.37009 0.007673059 0.007825141 0.06639297 0.03236941 0.1508145
0.264791 0.04692278 0.03578247 0.04137131 0.166780414 0.1947952
0.2273258 0.1041194 0.1435273 0.0627751 0.1122475 0.079103698
0.05436132 0.05335789 0.02762964 0.0215301 0.03888466 0.1929565
0.1198113 0.04428747 0.2626429 0.135595833 0.09464751 0.009069185
0.08752905 0.09977249 0.1202683 0.161584 0.08352551 0.1051925
0.1337089 0.2367533 0.1494558 0.2439527 0.09975411 0.008402228
0.3213945 0.09124778 0.1698457 0.109565 0.138757034 0.1275533
0.3557463 0.1345741 0.05996745 0.4226664 0.1533409 0.231877602

0.1438255 0.1721034 0.1261905 0.1802158 0.291074633 0.1410026
0.1087105 0.09087459 0.08797701 0.06954159 0.1265533 0.1053357
0.1004691 0.08142479 0.1351863 0.1057227 0.06801512 0.03202919
0.03715424 0.092656347 0.06930144 0.124099349 0.109753132 0.07471421
0.11737236 0.04427566 0.2281551 0.02826312 0.138076289 0.1064388
0.1248716 0.03641232 0.129380956 0.1001863 0.01297905 0.3915007
0.07700172 0.1281788 0.07699493 0.1189474 0.05665978 0.1781312
0.02881641 0.1505182 0.084367182 0.1025222 0.227765847 0.2835723
0.08955748 0.2085762 0.2109988 0 0.02596183 0.08632503 0.2385026
0.04154614 0.16887576 0.0571212 0.04630244 0.1752929 0.02515975
0.1064799 0.05960331 0.1365188 0.03235563 0.2961147 0.03551893
0.04231623 0.07747593 0.4711612 0.06791841 0.127367936 0.1174619
0.08996839 0.1485446 0.1181583 0.2230839 0.1339181 0.05966455
0.04009081 0.1521169 0.07669776 0.126338733 0.08322559 0.04308599
0.01362922 0.1568763 0.2803781 0.05053205 0.2097826 0.2598856
0.2600486 0.3253355 0.3936077 0.05594351 0.1060693 0.06527738
0.2634657 0.09616932 0.0738897 0.1074193 0.1052279 0.096056199
0.03638036 0.08506883 0.2106355 0.04295201 0.1111872 0.2056811
0.1280519 0.04572196 0.05871467 0.02809409 0.02627016 0.051153302
0.41707 0.04894679 0.1983403 0.1418961 0.1140015 0.251583169
0.1940691 0.1683584 0.07602608 0.1892638 0.1974498 0.06615844
0.235813188 0.1961146 0.07035434 0.4053399 0.1562396 0.051357792
0.04600717 0.2211367 0.2293461 0.319815725 0.137803106 0.185286
0.2587812 0.1153671 0.05613222 0.03294558 0.1269471 0.09129505
0.0834680 0.3543630 0.06175592 0.1458404 0.03309099 0.044199425 0.1689523
2.4300340 0.1565218 0.1735838 0.1634005 0.2320948 0.07381298
0.2946007 0.06869643 0.03814616 0.01286167 0.02741391 0.1211783
0.0846032 0.06628041 0.08579671 0.05948661 0.1187040 0.08652834
0.05991392 0.1108118 0.09635838 0.2871602 0 0.1421542 0.1717724
0.02886576 0.2627742 0.1793115 0.07734081 0.05249048 0.02771457
0.03401169 0.1510696 0.09563596 0.2360020 0.05159532 0.09884148
0.09730992 0.2294520 0.1533961 0.2303553 0.09159496 0.1830626
0.01765473 0.0153195 0.1030148 0.011232229 0.1340997 0.138388
0.06124339 0.01126583 0.2452633 0.268811361 0.145357557 0.2641745
0.137538866 0.1255391 0.02534799 0.3918541 0.1190458 0.1963954
0.2233954 0.3368703 0.1637030 0.02139791 0.3414004 0.006808231
0.075053533 0.04859771 0.1053236 0.1884829 0.3213098 0.06689435
0.2476564 0.07018409 0.06970869 0.3408448 0.05829765
AL139383.1 0.05787552 0.1388003 0.1785443 0.206170423 0.152795707
0.2394927 0.4258120 0.3480836 0.3239053 0.3184540 0.1100104 0.295818231
0.4506819 0.082969042 0.318683037 0.3424779 0.2425653 0.2039249
0.2784897 0.03739128 0 0.03344847 0.02023909 0.06300089 0.01309755
0.02496617 0.2452385 0.2807552 0 0.13382109 0.1339395 0.21344742
0.01131868 0.2412872 0.04879176 0.157482648 0.07877546 0 0.09954431

0.03327332 0.0479219 0.02533387 0.02302004 0.06941042 0.1314281

0.01299913 0 0.1531257 0.009869006 0.02361653 0 0.05722624

0.06886099 0.038401051 0.04565599 0.1150308 0.08648787 0.06963474

0.04143105 0.03128673 0.09303687 0.394472 0.01572835 0.03337781

0.04596048 0.03523455 0 0.09813652 0.09904642 0.014498684 0.04448965

0 0.0474204 0.080989453 0.04474057 0.01660709 0.04407519 0.06600969

0.176438 0.0551244 0.05892533 0.0238799 0 0.08191759 0 0

0.06600319 0.0242321 0.1042528 0.009195841 0.02399749 0.115386098

0.04380578 0.008672654 0.1783065 0.212107025 0.0574805 0.08084336 0

0.02857463 0.01983464 0.04806865 0.1407215 0 0.02195276 0.06310307

0.021244467 0.5733539 0.025902489 0.1271934 0.1558479 0.01142911

0.01980595 0.222106 0.07476146 0.04551537 0 0.09373241 0.02431844

0.01648383 0.06805052 0.02931825 0.01492008 0.03669483 0 0.0889036

0 0.06303085 0 0.1515343 0.3482873 0.02163573 0.02659227

0.124233682 0.03817569 0 0 0.02432571 0.01451077 0 0.7675749 0

0.1201619 0.4463163 0 0.02917609 4.455271 0.04224431 0 0.1015358

0.178211 0.0582677 0.09405269 0 0.04377816 0.121478 0 0.04124816 0

0.08818977 0 0.1810341 0 0.026394447 0.01662713 0.01632021

0.2704285 0.06914529 0.02854412 0.03934554 0.0643537 0.02709181

0.04381789 0.106646848 0.01929946 0.09986147 0.3962243 0.0261572

0.04798129 0.06177826 0.02554738 0.07019891 0.1226899 0.8400039

0.02493438 0.466351 0 0.04625878 0.04626012 0 0 0.06702381

0.1608278 0.01560554 0.02463616 0.01646451 0.06113945 0.02983341

0.04502528 0 0.111135 0.03008005 0.009649257 0.02067052 0.028114426

0.05843082 0.01813666 0.1212881 0.03027255 0.03828638 0.019354

0.04142356 0.545761 0.1162226 0.02067425 0.01616834 0.1144183

0.249812 0.090912916 0.080019293 0.6570996 0.026793475 0.057547617

0.06855698 0.033138355 0.01354229 0.1141924 0.05186788 0.016892982

0.136734 0 0.0890975 0.200908428 0.1378948 0.1429133 0.03918949

0.3324987 0.1176155 0.1412995 0.1364311 0.08318468 0 0.1057666

0.1714518 0.014075354 0 0.215737359 0.009637147 0.1187001 0.08833261

0.07040373 0.03368436 0 0.0528073 0.06838983 0 0.086776837

0.2096553 0.01699465 0.04707717 0.0577158 0.06896811 0.03646091

0.2035609 0 0.08432434 0.0488877 0.1242526 1.080585 0.09607392

0.0692458 0.11687149 0.0656063 0 0.01239118 0.03614028 0

0.01536023 0.01368689 0.03153164 0.03101798 0.01563935 0.013638483

0.03394086 0 0 0.07576215 0.06953299 0 0.05310189 0.03613156

0.01403635 0.04165708 0.1944763 0.1197774 0.02919844 0.07986379

0.02686148 0 0.04520028 0.03112638 0.1416154 0.10073162 0.4005869

0 0.05656885 0.01576492 0.01700404 0.01398006 0.2545814 0.06293099

0.4190353 0.1031154 0.1157051 0.083444865 0.1913495 0.336848

0.07912826 0.4092076 0.2510559 0.461700188 0.2866586 0.01993341

0.1180566 0.1215666 0.2617014 0.02023543 0.025456438 0 0.04303761

0.141079 0.04778794 0.178029193 0 0.1217477 0.0526114 0.035570858

0.252893387 0.03542011 0.01862391 0.04234385 0.02943219 0.04534578
0.0776569 0.1370805 0.02552979 0.02364799 0.01619045 0.2765647 0
0.115876948 0 0.3390299 0.4055231 0.03082814 0.1564535 0.6969854
0 0.4134345 0.08404684 0.1575113 0.1062156 0 0.2610593 0 0
0.03314786 0.03275058 0.4538398 0.1732309 0.1020990.01694662
0.1886239 0.1529972 0.02840402 0.07672895 0 0.1765795 0.04988666
0 0.04367207 0.0602059 0.01695374 0.05201462 0.09643124 0.02064813
0.05905991 0 0 0.05952705 0.01830807 0.06702603 0.2049664
0.08404658 0.0730332 0.2429970.02811402 0.7688061 0.144292067
0.1041179 0.02673334 0 0.0826993 0.09206635 0.036096366 0.050810887
0.05876455 0.050481692 0 0.2015784 0.7191225 0.4201355 0.08536281
0.08482149 0.05944397 0.05632955 0.08508284 0.08274936 0.1249432
0.06886834 0.07134841 0.02973656 0 0.04211864 0.4705920.01082128
0.2476930.1563564 0 0.02139733
AC090559.1 0.4789161 0.4648947 0.2462403 0.881457286 0.519798275
0.4128720.5655105 0.2713390.4467158 0.1733675 0.3034429 1.427927625
0.9188285 1.02984485 0.586017924 0.4293912 0.3345352 0.3068118
0.1034063 0.1160289 0.367136765 0.2306532 0.06978213 0.130332
0.07225421 0.4820512 0.8878326 0.5808076 0.4467524 0.719784328
1.6960960.686880062 0 0.3502869 0.4037485 0.04343861 1.3580450.39978
1.0639750.6424471 0.08812233 0.2096361 0.6667125 0.7466765
0.1812598 0.2330617 0.06680165 0.2745393 0.1497198 0.2931378
0.3164533 0.6201162 0.2611675 0.335419779 0.1259334 0.546445
0.09940018 0.08231754 0.1714196 0.2588956 0.2822871 0.582898
0.6941390.6904977 0.1479022 0.413048332 0.2229055 0.06015356
0.5073730.439910652 0.3476961 0 0.1308002 4.311505921 0.3208618
0.2290376 0.8510115 0.04551881 0.2058992 0.05068341 0.101584
0.1646705 0.5285718 0.1581679 0.284211893 0.0504953 0.3641146
1.3033740 0.10146 0.2316739 0.419538688 0.1057262 0.1076484
0.1324144 0.097509519 0.3699478 0.4905804 0.1942671 0 0.06838763
0.1325883 0.1698173 0.07745125 0.06055252 0.8876953 0.966880916
0.3953721 0.285788469 0.05847316 0.04776411 0.5674505 0.4097323
0.1198638 0.1546615 0.7951217 0 0.35088 0.3018501 0.3637402
0.03128408 0.1213033 0.08230841 0.4681229 0.0843515 0.5108829
0.0186758 0.5650407 0.44283097 0 0.3408881 1.3427560.293399
0.753885252 0.6318026 0.4173336 0.1019198 0.1845191 0.06003783
0.1209454 0 0.3628154 0.2294612 0.1154137 0.1807777 0.04023836
4.7148480.01942049 0.01980541 0.6721617 0.09558138 0.4821613
0.4107587 0.118761431 0.2113191 0.2094213 0.996205039 0.5878388
0.2301444 0.1013562 0.3823866 0.1134883 0.4058544 0.200211247
0.09172557 0.09003244 0.09324073 0.1362316 0.1377837 0.9405693
0.6952377 0.01868191 2.2964040.980550535 0.02661696 0.02295408
0.3396882 0.8477588 0.2249904 0.5623320.08808452 0.1452228
1.1167750.2596629 0.2751071 0.7460779 0.1942134 0 0.7974987

0.02566088 0.1074698 0.2310906 0.665419602 0.5165391 0.1528969
0.5903851 0.3372832 1.110912 0.06209687 0.670721812 1.743474 0.04148507
0.03992348 0.4846337 0.387741638 1.208778 0 0.6691007 0.09741794
0.08800479 0.1067687 0.533209 0.3458302 0.04579687 0.5132342
0.0891946 0.07172754 0.0405329 0.658260974 0.538000332 0.5262053
1.071618639 0.49604443 0.09455073 0.091405885 0.2241231 0.5249635
0.1001474 0.629047287 0.05387926 0.8578489 0.03071982 0.176326151
0.2852676 0.03284994 0.8467582 0.7107795 0.2703501 0.1558992
0.3763193 0.1147246 1.021926 0.1458685 0.5386003 0.271769415
0.9406275 0.520686865 1.143037 0.3274119 0.09475231 0.1213721
0.2090519 0.01642733 0.1456589 0.1509123 0.1261838 1.726793342
0.2088285 0.3046975 0.3354548 0.01591981 0.3012062 0.05028527
0.4103154 0.06141901 1.511853 0.2921695 0.06426136 0.4117919
0.3975026 0.4393033 0.274012717 0.2972957 0.1897581 0.3076086
0.1744507 1.106664 0.08473658 0.09438176 0.2754178 0.1925038
0.2803982 1.053336918 0.234048605 0.07789322 0.03449551 0.4179511
1.553528 0.277109 0.7323576 1.883613 0.3871661 0.2106558 0.3831615
0.377581 0.08053836 0.1101446 0.8520617 0.1825532 0.2493528
0.5151373 1.083081 0.659891665 0.2762358 0.2740296 0.507112 0
0.117256 0.539859 0.1620495 0.1157222 0.2146541 0.07110606 0.1329794
0.647344348 0.9541028 0.4026236 2.248081 0.4788517 0.4039526
0.496669932 0.6109904 0.2474214 0.08881006 1.029906 0.2498725
0.1674468 0.912818006 2.895466 0.3165629 0.1061291 1.950847 0.361073426
0.08733301 0.8115599 0.3869829 2.256656116 0.116259763 0.4884984
0.1797969 0.8370488 0.2029578 0.1876166 0.1428013 0.08402456
1.021077 1.973162 0.1786332 0.1230405 0.01675064 0.319624645 0.5497943
0.2877385 0.7923125 0.1700672 0.2517359 0.9612512 0.05604614
1.140381 0.1159137 0.1448217 0.113935 0.1214229 0.06667433 0.1427536
0.5272317 0.2628673 0.2408967 0.02503663 0.219003 0.3899365
0.5843007 0.3089182 0.6330208 0.176281 1.227525 1.111042 0 0
0.3241693 0.3011529 0.04981997 0 0.6025844 0.1496179 0.2847698
1.900565 0.2872927 0.03848731 0.1313553 0.4292444 0 3.657179
0.2463158 0.02014483 0 0.3101889 0.2259662 0.028428741 3.002437
0.1474777 0.135631 0 0.8888167 0.464636664 0.788356256 1.600648
0.858672566 0.04888295 0 4.6283150 0.162241 0.2223762 0.233964 0.9018073
0.2589574 0.04513172 0.6684419 0.06892645 0.221620338 0.07380041
0.184551 1.019152 0.3194849 0.2539641 0.328333 0.3416072 0.1960361
0.4097717 0.1623063
AC022973.4 0 0 0.110037 0 0.033482 0 0 0 0 0 0 0.054018675
0.06440711 0 0.058193964 0.1023367 0 0 0.03520686 0.2150799 0
0 0.03326232 0.10354 0.08610165 0.1230934 0.06717363 0.1977482
0.1064744 0.043986161 0.1200684 0 0 0.0834838 0.106917 0 0
0.06351964 0.245397 0 0 0 0 0 0.2468549 0.04272734 0.07960411
0 0 0.07762608 0.04436485 0 0.1131709 0.168295825 0 0.126033

0.04738004 0.03269787 0.06809064 0.1028376 0.1834839 0.04630732
0.1033962 0 0.05035641 0.057906889 0 0.5734554 0.09301698 0
0.04874491 0 0.1948349 0.212965843 0.05882379 0 0.0482908
0.1084849 0 0 0.04842098 0.07849176 0 0.0269258 0 0 0
0.2787729 0 0 0.1183174 0.034478813 0 0 0.1352498 0.154929471
0.3778696 0 0.06614232 0.04696152 0.03259762 0 0.2890894 0
0.07215734 0.04148318 0.034914621 28.16398 0.042569937 0 0.2845902 0
0.06510093 0.03174124 0 0 0 0 0 0.05418132 0.07455927 0
0.04904136 0 0.2297540 0.04870344 0 0 0.131924751 0.03113025
0.2215739 0.07111529 0 0 0.08365409 0 0 0 0 0 0.03319699
0.05764647 0 0 0.0957438 0 16.60159 0 0 0.06674839 0.0325427
0 0.1030484 0.056608768 0 0 0.040241578 0 0 0.04831238 0
0.1622858 0 0 0.05465233 0 0.0555551 0.1298722 0 0
0.05288165 0 0.10802 0.058423595 0.06343613 0 0.1407962 0.04298855
0 0.1218368 0.251918 0.05768486 0 0 0 0.04087645 0.04628683
0.1520497 0.03801354 0 0 0.1652273 0 0.1025889 0 0
0.08038464 0 0.03699883 0 0 0.09887127 0.06343302 0.1358854 0
0.05487385 0.1192282 0.04983331 0.2653439 0.08389664 0.6043461
0.04538556 0.04848331 0.5457379 0 0.3188659 0 0.1932039 0 0
0.4180346 0 0.047288861 0.04506851 0.108923716 0 0 0.0340973 0
0.2568206 0.03586858 0.1830362 0.060033957 0 0.07829118 0.0429378
0.1092903 0.06443248 0 0.2690643 0.3417786 0 0 0 0
0.07730328 0 0.06335341 0 0 0 0.2214368 0.1566049 0.0578581
0 0.05012226 0.040747154 0.114854 0.1117207 0.02578995 0.0758833
0.03778232 0 0.05146862 0.04879326 0 0 0 0.04673441 0.07894725
0 0.115244799 0.09241879 0 0 0.1781863 0.2691337 0.05048813 0
0 0.1529313 0.05140556 0.044828846 0.111561498 0.07425709 0.08221307
0 0.4113911 0 0 0 0 1.049752 0.0913188 0.168729 0 0.06562683
0 0.07251311 0 0.03410349 0 0 0 0 0 0 0 0.0919032
2.188536 0 0.2361159 0.05648897 0.1901577 0 0 0.07381322 0
0.04075879 0 0.030351597 0.05710479 0 0.3174912 0 0.03308447
0.06651261 0.041836865 0.0394329 0.1414619 0.04215619 0.0314152
0.068843636 0 0 0.05764347 0 0.13854081 0.02328474 0.06121565 0
0.0483709 0.09936583 0 0 0.04195741 0.03886473 0.106434 0.02932422
0.07984352 0.038088033 0 18.51569 0.05553872 0.06755345 0.0428544 0
0.08904975 0 0.1381284 0 0 0.041341 0.2224667 0 0.05711591 0
0.07176607 0 0.1423498 0.05162971 0.0557025 0 0 0 0.05044066
0.038376 0 0 0 0.0717737 0 0.1114517 0.1709688 0.03962044 0
0 0 0 0 0.06017748 0.2643725 0.126321 0.03453199 0.09602224 0
0.04620452 0 0 0.0622235 0.04393542 0.1385353 0 0.1008721
0.039548838 0 0.04828886 0.055310062 0.05825132 0.05096737 7.879037
0.1104767 0.03117581 0 0.03907773 0 0.1290748 0 0.2464084 0
0 0.04887112 0 0 0.2017574 0 0 0.7475398 0 0.3164926
AC136604.2 0.2786572 0.5108946 0.07841394 0.305847426 0.143158542

0.2687973 1.145149 0.6853439 0.4924828 0.5234534 0.243364 0.141146457
0.5431197 0.442663333 0.691164324 0.4618685 0.2485723 0.2894892
0.1672595 0.3649264 0 0.2219832 0.2370321 0.1147753 0.1329408
0.2241685 0.3271042 0.3757819 0.278209 0.062690348 0.2091527
0.246013067 0.2209331 0.4957647 0.1396827 0.090169363 0.2870269
0.2414134 0.1943038 0.2857681 0.03741613 0.02967001 0.2875749
0.314324 0.161253 0.5582156 0.1229086 0.09564513 0.06934909 0.2212698
0.0737684 0.05106367 0.1612944 0.039976679 0.1307055 0.1796259
0.1350548 0.06990291 0.258786 0.4885568 0.1888658 0.131997 0.07368165
0.5081796 0.04784627 0.275101905 0.01261922 0.4171662 0.1657131
0.056600915 0.1389453 0.1863072 0.2314036 0.113821902 0.2235663
0.1166974 0.1720636 0.1030772 0.3920806 0.129119 0.2415384
0.1118687 0.1795424 0.04477134 0 0.02143996 0.103067 0.1986576
0.04651304 0.05025906 0.1217879 0.139230566 0.1624613 0.162513
0.3641058 0.312814107 0.3291143 0.01262406 0.0628453 0.06693091
0.07743177 0.1219748 0.2197432 0.3507762 0.05999043 0.1478076
0.182458199 2.685956 0.070783888 0.09930919 0.2974446 0.2409356
0.03092791 0.2412722 0.1167435 0.07107435 0.171935 0.1463675
0.07594873 0.1415714 0.2833707 0.1144545 0.1863871 0.2292027
0.2319452 0.1388271 0.07400973 0.6028567 0.094011483 0.2588118
0.1228086 0.08446297 0.1453377 0.096998322 0.09935519 0.1575084
0.1346316 0.1804322 0.7590846 0.1540577 0.2996509 0.05477294
0.1732047 0.1851256 0.06822841 0.02277991 2.238675 0.05497204
0.02242464 0.1585529 0.04638079 0.07961424 0.1835845 0.080680451
0.05127122 0.2489728 0.15294256 0.1610273 0.1954354 0.1606645
0.2273022 0.06424845 0.1608348 0.092736339 0.07789208 0.1146815
0.1979468 0.2005223 0.1337189 0.3071994 0.1507369 0.02115256
0.2480358 0.041633497 0.361644 0.01299486 0.1086945 0.0510571
0.1723275 0.2315271 0.6582414 0.23294 0.1245308 0.0339234
0.1460107 0.7622127 0.03298466 0.09631364 0.243801 0.1016907
0.06084122 0.3270649 0.062785013 0.316794 0.4616461 0.03856514
0.2577746 0.1630519 0.1142522 0.094927947 0.1952352 0.1409142
0.1130082 0.04841696 0.329264834 0.1042771 0.1699274 0.2485836
0.1812093 0.2092509 0.2417773 0.05390401 0.09213309 0.2074137
0.2098447 0.2524761 0.3248535 0.2868331 0.248438272 0.078096143
0.4964958 0.156897194 0.14602781 0.1605823 0.038810307 0.1691754
0.6686876 0.1862864 0.065947932 0.3660281 0.07668141 0.1391299
0.099822485 0.08972047 0.1673743 0.2957816 0.1233129 0.1224414
0.09929057 0.3621738 0.1948451 0.1531421 0.06606375 0.2900407
0.120886243 0.05508744 0.168441936 0.3762213 0.2851627 0.1072831
0.206135 0.1972488 0.1115989 0.3435877 0.192229 0.07143569 0.193580068
0.136411 0.1326897 0.07963927 0.198277 0.1256464 0.1518278 0.3300955
0.2433955 0.1609378 0.6107233 0.3516724 0.03330361 0.1312709
0.0216261 0.146000193 0.1463533 0.02148533 0.16447 0.2327932

0.1534309 0.08394999 0.02137272 0.1230953 0.2179621 0.1343186
0.202322662 0.119250502 0.3351139 0.05858622 0.6861754 0.4777475
0.1810132 0.1451119 0.06770532 0.04383681 0.2276731 0.0433834
0.1469584 0.09118942 0.2805998 0.2307 0.2296617 0.09410977
0.1701185 0.06031066 0.137634951 0.3231931 0.08864844 0.1693085
0.04923532 0.06638147 0.1091526 0.3975406 0 0.2430417 0.2146925
0.2936032 0.06515151 0.7010335 0.2104015 0.0864937 0.125863
0.05444935 0.245128513 0.07460511 0.03890868 0.08379586 0.08135649
0.0943059 0.09479569 0.238508437 0.09366818 0.2800217 0.2002741
0.05223616 0.098117938 0.6756981 0.269329 0.1506177 0.124977466
0.153574061 0.1050893 0.1235989 0.1102031 0.1378792 0.07080953
0.06063242 0.2616258 0.0697654 0.2123327 0.2528214 0.1044843
0.2086247 0.144757774 0.06916707 1.914024 0.1846959 0.1765117
0.1628729 0.3426366 0.148069 0.04966132 0.2624861 0.1503096
0.1842895 0.1374809 0.2113773 0.3636732 0.09497045 0.04529168
0.1789954 0.1133907 0.4057621 0.1226402 0.2513979 0.2669313
0.2389122 0.08870838 0.1677421 0.0729261 0.1516554 0.01298339
0.1835201 0.2045879 0.1692257 0.3044515 0.1462018 0 0.08866829
0.9991017 0.2513579 0.2832517 0.1022497 0.01429445 0.2302613
0.1300262 0.1722559 0.410561 0.3541543 0.2853588 0.1869672 0
0.07390227 0.1043634 0.1425988 0.0322847 0.09584391 0.065760504
0.079343553 0.06882268 0.052552999 0.1106953 0.1937071 0.1871572
0.3673941 0.05924355 0.05518862 0.4455577 0.219903 0.1992912
0.1938254 0.2829017 0.080655825 0.4038752 0.522394 0.1595864
0.09865532 0.3035255 0.1858772 0.1315066 0.1886673 0.2564008
0.2088307
AL353804.1 1.181893 1.957142 0.3798024 2.17530368 0.728066464 1.426468
1.502948 3.348119 2.436944 5.077092 0.6240428 0.950896371 1.133765 1.223686059
1.325686889 0.3708854 1.031978 0.3154855 0.328103 0.5726836 0.209085734
0.2846087 0.9299436 0.142951 0.5349375 0.6373017 0.7303457
1.057945 0.4961329 0.273279876 0.4558693 0.48431898 0.07704735
0.9509008 0.05535503 0.285866507 0.9384061 0.5261849 0.9317104
0.679484 0.2718408 0.08622505 0.4700988 0.3779867 0.7668375
0.3539453 0.1648564 0.1042341 0.8061512 0.16076 0.4134489
0.4451937 0.6445221 0.522799226 0 0.3480114 0.4415484 0.06771576
0.4935439 0.3194577 0.5699798 0.4315513 0.1605966 0.2840074
0.2085716 0.239844943 0.1650291 0.5566872 0.5779015 0.444122657
0.2523711 0.9281722 0.4438433 0.220521172 0.6091066 0.2260921
0.600048 0.7301682 1.247224 0.06253951 0.9526371 0.6502111 0.4695968
0.2230483 0 0.2492296 0.09627643 0.4536131 0.1013799 1.43973
0.2450298 0.464126101 0.1118213 0.2361423 0.3267787 0.48127758
0.5869128 0.1100617 0.2054665 0.4862756 0.4050489 0.122703
0.2993453 0.9556901 0.1867931 0.8590973 0.723065551 14.63578
0.396721605 0.1443029 0.3536239 0.1944975 0.269642 0.9202849

0.5089084 0.4131033 0.05353567 1.048218 1.117381 0.336621 0.5404307
0.1496791 0.5078119 0.7493557 0.8029291 0.7060379 0 0.9653783
0.204907512 0.1289386 0.8412604 0.4786485 0.4977944 0.084567052
0.4331093 0.2288703 0.08384087 0.3311749 0.1481643 0.298475
0.3781218 0.2984579 0.3145976 0.4272353 0.2974214 0.1324029
18.46672 0.3834148 0.2443838 0.345582 0.5054581 0.09915844 0.6402256
0.175851183 1.937014 0.4651382 0.458361392 0.4679669 0.662622 0.7503952
0.4246526 0.7281871 0.8012699 0.583925929 0.3961385 0.3888264
0.5752602 0.5379184 0.6314834 0.5356576 0.4015566 0.1383124
0.9321018 0.423473904 0.1313733 0.1699416 0.2186868 0.3115955
0.4245972 0.1682122 0.7390897 0.2986567 0.2923065 0.3943439
0.2545961 1.290962 0.09585786 0.4198506 0.3936213 0.6965989
0.4773945 1.596831 0.273692542 0.9560555 0.5450272 0.5603775
0.208091 1.066164 0.7279171 0.248286045 0.4728166 0.3583261 0.1313668
0.9849435 0.57413274 0.5113854 0.308645 0.5160124 0.5151703
1.085913 0.5928502 0.4229612 0.3263217 0.6216087 0.07036581
1.210654 1.168284 0.4501308 0.232069833 0.068087369 1.442884 0.27357861
0.244832572 0.1866696 0.225575943 0.3687347 0.6477654 1.235744
0.919937101 0.5983465 0.5571162 0.4548714 0.12432753 0.3128877
0.9728243 0.4890721 0.707297 1.20093 0.1923678 0.1857399 0.07078075
0 0.8999537 0.9401547 0.287436831 0.640366 1.055516556 0.8200122
0.3729245 0 0 0.4585853 0.6081026 0.1198214 0.2327681
0.4671039 0.548505474 0.8325003 0.4048948 0.5608028 0.4714524
0.273859 0.6618478 0.2131782 0.4547186 0.5421147 0.4991749
0.3700372 0.241962 0 0.1414088 1.034221991 0.7336809 0.5619533
0.126522 0.3690155 3.34418 0.5227928 0.09316798 0.2861851 0.3694992
0.3193756 0.41777304 0.635356336 0.9611437 0.3405189 0.5672922
1.419953 0.2630243 0.3614696 0.6394725 0.4777338 1.370558 0.4727925
0.6406214 0.2318826 0.3397753 0.3656973 0.9761132 0.2051217
0.670954 0 0.428556088 0.7271555 0.1932182 0.7701391 0.05365667
0.1736221 0.1903272 1.799612 0.4283773 0.6927289 0.175479 0.4922598
0.177505361 0.6512673 0.4585913 0.5386336 0.2110239 0.2373557
0.754280928 0.4730454 0.1696108 0.9132077 0.2955411 0.5823894
0.2066168 0.433211001 0.3674866 0.8788832 0.4801693 0.1301188
0.35642991 0.1436831 0.207187 0.2387539 0.665869103 0.286911566
0.5786594 0.4437117 0.1441193 0.3005216 0.9774646 0.176206 0.15552
0.4779052 0.3621917 0.5510498 0.9716656 0.4547186 0.591589074
0.1507566 12.69295 0.5750905 0.5596002 1.109368 0.3514405 0.4149402
0.07216124 0.6436292 0.5360973 0.4016776 0.08561529 0.4936266
0.9688098 0.4139954 0.1974356 0.4830286 0.1235732 0.9826661
0.5613443 0.1153573 0.0802488 0.05785925 0.09667443 0.5223013
0.3576371 0.4206977 0.1131944 0.4000005 0.52024 1.516361 0.8655427
0.1770342 0.2461562 0.2459692 2.412158 0.6445411 1.139771 0.324165
0.06231238 0.365002 0.3052057 0.2860565 1.093717 0.2756839 0.334906

1.158197 1.192683396 0.3543704 0.0454941 0.5738003 0 0.3481691
0.286663308 0.475577514 0.200008 0.916356509 0.6634967 0.105551 0
0.4003863 0.2582546 0.721735 1.294851 0.1278135 0.4232374 0.3621104
0.8505005 0.507859467 0.3035471 1.467542 0.2675652 0.1433528
0.974937 0.4051382 0.337214 0.6289238 0.1064479 0.9103353
AC114271.1 0.2000165 0.09738087 0.4993194 0.078752264 0.111170239
0.1470224 0.2151457 0.1651674 0.08372317 0.05030307 0.210107
0.233165573 0.2993912 0.324468043 0.135254937 0.04247355 0.3143626
0.3136013 0.128587 0.209138 1.217969618 0.4289264 0.132529 0.1432432
0.3859423 0.2384123 0.2509161 0.4705512 0.1590871 0.430839213
0.2325533 0.843051006 0.8338123 0.2979805 0.7366174 0.183328538
0.4155338 0.4323536 0.2308575 0.3631322 0.6537514 0.1244179
0.1884239 0.2272556 0.204908 0.2979218 0.6805967 0.701885 0.2154131
0.1288709 0.2504177 0.2988895 0.2254567 0.50989806 0.141177
0.4463651 0.5112766 0.13028 0.1695609 0.2134071 0.5076843
0.3228836 0.1459052 0.4189134 0.7858321 0.490283818 0.5732704
0.44031 0.3860552 0.308554466 0.3884343 0.3720278 0.388146 0.371232851
0.3271485 0.4349842 0.3447307 0.2521415 0.2295879 0.3609639
0.4260461 0.3518317 0.3597145 0.2235043 0.265795939 0.2896973
0.2315357 0.3504093 0.983363 0.5620153 0.2618993 0.211787915
0.4422235 0.4448551 0.6885735 0.450110593 0.564588 0.4235008
0.4282439 0.14813 0.140704 0.2754165 0.1631767 0.3907186 0.4552095
0.1446234 0.318799164 0.2954866 0.515908891 0.3933063 0.4157668
0.3180688 0.3674627 0.4479092 0.3161682 0.6126426 1.235983
0.3068878 0.2654021 0.6206487 0.1856693 0.3439657 0.3500887
0.5556567 0.162106 0.2587359 0.43597 0.189171 0.448980307 0.5374809
0.4475457 0.3187674 0.406305 0.474543516 0.3402518 0.2385117
0.3763749 0.2654814 0.4355037 0.2512315 0.4023173 0.4880789
0.3278502 0.2892112 0.2145812 0.4988527 0.168496 0.1536794
0.3447958 0.2770307 0.4754265 0.2464156 0.7270722 0.507486634
0.2030557 0.4474454 0.387480765 0.1950722 0.4704747 0.2406173
0.3858054 0.5298568 0.2408723 0.172835305 0.4990207 0.7569782
0.1567906 0.1886563 0.1557598 0.286268 0.2019203 0.5395971
0.2988823 0.232780536 0.1369075 0.563089 0.3213962 0.192692 0.1361488
0.4921836 0.2509329 0.4979805 0.4217818 0.110642 0.8299789
0.3257329 0.5609542 0.4711939 0.3092299 0.4264285 0.1615829
0.4754568 0.372982708 0.4257821 0.3428087 0.6109369 0.3536438
0.4883855 0.6879441 0.311821265 0.3638654 0.5170444 0.4422948
0.4511802 0.09204901 0.150313 0.3760795 0.8355805 0.4570297
0.3203462 0.2323443 0.5952398 0.5875736 0.2989821 0.2481939
0.4146693 0.3292072 0.657532 0.434082512 0.327487418 0.3122999
0.263172313 0.28262365 0.6135277 0.226037039 0.2438624 0.2596358
0.5434235 0.129054454 0.3837245 0.1012302 0.5712708 0.199330665
0.2382807 0.5978852 0.4134424 0.3719482 0.2246318 0.3701012

0.3573495 0.5617295 0.1427076 0.4501752 0.4054177 0.2150582
0.25667 0.198658791 0.3102695 0.1494747 0.2356513 0.6627099
0.1562377 0.2794865 0.2305274 0.4776834 0.2413103 0.372054973
0.4449077 0.7604398 0.6165385 1.549525 0.3449836 0.4775045
0.2819704 0.137707 0.2198455 0.5068645 0.4915663 0.3801719
0.3276606 0.5063338 0.452799125 0.3477724 0.1501605 0.2501803
0.2760948 0.4289301 0.2263081 0.3510277 0.2179453 0.3131295
0.5291137 0.520958047 0.36115705 0.3883253 0.3139177 0.2315149
0.5463765 0.3457933 0.3911852 0.7255597 0.3370121 0.2348918
0.6367319 0.1680692 0.2655504 0.4031162 0.1905513 0.1043316
0.4357469 0.1868357 0.337208 0.309191106 0.6994962 0.3175256
0.1749217 0.6710041 0.287642 0.6942073 0.3526435 0.2976154
0.307057 0.3000964 0.5051044 0.37565763 0.3373432 0.2328278
0.3108871 0.6292908 0.3044363 0.196513912 0.1611642 0.1414046
0.368958 0.3032527 0.2801183 0.4306414 0.152801975 0.6153663
0.1409088 0.3709235 0.1981849 0.171436071 0.1958085 0.575773
0.4210658 0.388206658 0.306664572 0.2473993 0.2591491 0.3234872
0.192727 0.3794129 0.24013 0.8311352 0.3204156 0.5355264 0.194366
0.2969638 0.457305 0.60702549 0.3142147 0.2988496 0.3688103
0.3756985 0.4624406 0.5071087 0.3843733 0.2487421 0.3095735
0.3056032 0.296239 0.4186566 0.6384097 0.4800998 0.3698017
0.4024613 0.3395558 0.1882153 0.2993411 0.2957099 0.5548468
0.4181467 0.2226337 0.2247431 0.3182083 0.3695173 0.655221
0.3992588 0.4873948 0.2859721 0.3942384 0.3700531 0.1589469
0.4472757 0.4337906 0.2793081 0.3410133 0.5482082 0.4287725
0.2897205 1.382528 0.3075771 0.6076795 0.6695273 0.7867528
0.3221669 0.7565163 0.528665439 0.2737458 0.568927 0.3603166
0.5302472 0.4521495 0.347981974 0.221812193 0.464967 0.183645923
0.2707765 0.6684462 0 0.4218381 0.2484312 0.4551403 0.2789619
0.3483635 0.4499951 0.1612669 0.388621 0.294377673 0.8370693
0.1947201 0.2573876 0.6090583 0.2177158 0.4015376 0.2000388
0.4421161 0.6399928 0.2802266
WASHC5-AS1 0.025224 0 0.1459037 0.05391339 0.079911895 0 0.1237216
0.07915092 0.04457946 0.0219146 0 0.128927026 0.2049616 0
0.046297413 0.1221241 0.1189324 0.04847835 0.02800955 0.415556
0.267738972 0.3936036 0.1852377 0.1647468 0.1712499 0.4243611
0.1336034 0.3408658 0.08470791 0.104982303 0.2547277 0.345529591
0.1479913 0.3985036 0.17012 0.109817542 0.2746629 0.4548093
0.9761532 0.3480378 0.04177178 0.0993718 0.1203944 0.3267138
0.2454882 0.985786 0.06333069 0.1201267 0.02580737 0.4940562
0.1411815 0.1924022 0.1800709 0.234309647 0.03979667 0.3342273
0.4146359 0.07804039 0.5417092 0.4090727 0.2919488 0.2578852
0.5346836 0.261848 0.1602483 0.32248317 0 0.3421682 0.2590056
0.037913935 0.07756004 0.2674227 0.3410108 0.254144076 0.3041901

0.1302822 0.3457687 0.6904591 0.2484374 0.04804995 0.50079
0.06244574 0 0.1713710 0.04787161 0.4438229 0.1901002 0
0.2404705 0.1882597 0.137151665 0.1431897 0.1360735 0.5738707
0.246514694 0.2505183 0 0.1578627 0.5604181 0.2852708 0.1571239
0.09199641 0.4405617 0.2296251 0.1980168 0.388878696 10.49521
0.033867394 0.05543494 0.1811293 0.02988702 0.3107544 0.3787861
0.1466254 0.0793482 0 0.1750782 0.1907775 0.08621015 0.1186343
0.2300009 0.4291746 0.4797834 0.4341157 0.1549881 0 0.288444
0.314866399 0.1981305 0.1762777 0.1697317 0.2433850.064974004
0.2662110.04396104 0 0.3180576 0 0.1719918 0.5546215 0.3210329
0.09668381 0.1458892 0.6093677 0.07629523 4.9601120.1472914
0.1126580.05310307 0.1294501 0 0.2459470.225181384 0 0.158832
0.160075103 0.3954998 0 0.1537437 0.07250363 0.1721465 0.07695331
0.241574388 0.04347979 0.08535444 0.4419801 0.3099674 0.1866066
0.4801451 0.2103555 0.03542243 0.2005210.278880599 0.4542113
0.2176141 0.1400167 0.2736034 0.2509413 0.1615496 0.4342402
0.3671391 0.03208329 0.4165971 0.09780479 0.2276407 0.1472978
0.2822548 0.1512123 0.09731024 0.08150858 0.2190834 0.035046944
0.2448501 0.1610584 0.1722182 0.4156858 0.3120564 0.2649167
0.222555095 0.07265427 0.1573182 0.1009309 0.1621597 0.220556955
0.08731206 0.1422817 0.1982296 0.3694248 0.3337286 0.4301895
0.03610742 0.1542876 0.5644250.1081260.3382405 0.1904018 0.07685368
0.475473003 0.23540599 0.1662880.140129418 0 0.07171037 0.216641291
0.03541298 0.2986121 0.2441413 0.265050021 0.5107974 0.3138959
0.1164946 0.095522514 0.1802969 0.1868585 0.2049603 0.2173703
0.4613453 0.2586479 0.4281193 0.6525817 0 0.2489208 0.1743564
0.257648517 0.1230005 0.07051892 0.2268095 0.09550764 0.1283272 0
0.5285059 0.2180329 0.2301511 0.1430711 0.6778886 0.064834483
0.1218326 0.3555270.2256950.3622232 0.09017551 0 0.1637877
0.4270034 0.02450083 0.2556819 0.08122987 0.07436105 0 0.1810772
0.611235904 0.3431199 0.1439188 0.3888341 0.1417598 0.2569379
0.2811681 0.1431644 0.4947298 0.3650029 0.4498644 0.285316133
0.044377529 0.1476919 0.06540631 0.07924695 0.6545814 0.3637533
0.0694305 0.1511739 0.1101148 0.5446645 0.2179517 0.2237264
0.07635366 0.2610539 0.4916978 0.4230549 0 0.3527126 0.1009972
0.230485787 0.3142594 0.2226782 0.07396353 0.04122514 0.04446541
0.03655775 1.9971850.05485467 0.8766190.7190555 0.3025678 0.054551903
0 0.4697891 0.1241518 0.4215445 0.3647272 0.265615253 0.09086179
0.2085028 0.1683911 0.04541365 0.1316052 0.2116619 0.399410211
0.1568583 1.0504010.1006147 0.1499581 0.219079917 0.2207874
0.05306151 0.09171891 0 0.183698348 0.1667219 0.2191562 0.1107288
0.1539299 0.2766839 0.1353814 0.2389761 0.06676018 0.1545982
0.0846758 0.06998848 0.3811270.030301723 0.1544378 6.1377260.1767399
0.1612306 0.1704685 0.4725288 0.2833815 0.1386061 0.2197818

0.2745938 0.1851686 0.3617866 0.12642 0.06766814 0.3635180.04334068
0.3996649 0.1424145 0.3397480.2464505 0.1772611 0.1233125
0.1778162 0.1114144 0.2006456 0.2747774 0.3232277 0.0869688
0.2458606 0.2284042 0.2519006 0.1773355 0.4624604 0.1891251
0.08099204 0.4118431 0.1980838 0.07297509 0.3735905 0.04787544
0.4557085 0.06699818 0.1648358 0.6875323 0.2118117 0.3308307
0.3954927 0.215612872 0.1485096 0.06990746 0.4408585 0 0.1337515
0.18878338 0.066435019 0.0768344 0.132009195 0.1390291 0.4865777 0
0.3295952 0.1240128 0.1478716 0.3108910.04910043 0.1198028
0.2473016 0.1960353 0.120060081 0.1399316 0.5054456 0.2466887
0.2753497 0.4012806 0.3112731 0.3627205 0.6318908 0.2044633
0.5595383
PABPC4-AS1 0.3224644 0 0.1865237 0.057435862 0.141888331 0.4169933
0.08787005 0 0 0.1634248 0.3064718 0.091567047 0.6550589
0.038523134 0.049322289 0.7372514 0.3378745 0 0.02983957 0.2864573
0.11409276 0.2329556 0.8457442 0.4972802 2.2257510.1391037
0.2561985 1.4525460.4963330.037280467 0.1017639 0.537999655 0
0.3184051 0.1359262 0.058496277 0.4389123 0.8613771 0.6239585
0.3707771 0.08900195 0.1411524 1.4108650.5800998 0.3661383
0.7604847 0 0.04265842 0.1924546 0.9210876 0.2632099 0.2505225
0.04795899 0.356597778 0.2119841 0.9613735 0.3614125 0.2494177
0.4328266 0.08715996 0.5702097 0.1177433 0.1752669 0.1859707
0.1280387 1.177895489 0.0900522 1.5492270.5124375 0.282737493
0.4131374 0.2848950.5944762 0.406123156 0.4985610.04626477 0.286502
1.0573831.2855370.2047573 0.5745487 0.06652568 0 0.1369258 0
0.2039973 0.0525356 1.11386300 0.07685453 0.06685325 0.292225145
0.1525451 0.09664265 1.5284130.131310459 0.7472812 0 0.1681768
0.07960446 0.1381405 0.1673897 0.5880424 0.5866827 0.06115695
0.07031813 0.207143189 16.238960.108240438 0 0.9648177 0.2547176
0.2207052 0.08070686 0.3124107 0.5494611 0 1.1564060.03387368
0.2296069 0.3791562 0.3675423 0.74817 0.5878 0.8519349 0.04127859
0 0.6584779 0.279532027 0.07915332 0.18779500 0.7037783 0.207657388
0.07090102 0.4214994 0.4803732 0.5082572 0.08084949 1.465832
0.5064498 0 0.2832520.1165657 0.08114765 0.1354667 8.602162
0.03922869 0.04000621 0.1131452 0.08274468 0.04058118 0.1310081
0.143936267 0.06097956 0.3807211 0.068213492 0.2681255 0.1549611
0.2456829 0.4248239 0.1833938 0.1639622 0.330888642 0.2316029
0.1363967 0.09417145 0.1651097 0.1987987 1.13264200.5677180.03773678
0.3356920.148550737 0.2150611 0.2318321 0.1789977 0.1093048
0.5079399 0.1032628 1.6725190.5377988 0.2392564 0.5245096
0.3473165 0.3118035 0.0392304 0.1288697 0.2899653 0.2073362 0
1.4470640.112010287 0.7825427 0.03431625 0.1834702 0.34065 0.08311121
0.2508668 0 0.07740119 0.1675967 0.2419319 0.3455091 0.078322403
0.2325416 0.9599924 0.8024882 0.3092268 1.1021520.5930893

0.03846653 0.2670982 0.6013021 1.09431 0.9909342 0.5505707
0.5731248 0.379903792 0.278651575 1.5943730.11196366 0.04007967
0.07639562 0.138477428 0.6413542 0.2651018 0.5201851.270652687
0.3265024 1.1248150.1551323 0.2035271 0.2561023 0.3317784
0.07278386 0.2084151 0.5460974 0.1574553 0.3040605 1.274567
0.06071331 0.1767895 0.5307088 0.392117395 0.06551839 0.187815816
0.4832566 0.8902924 0.4648194 0.1471004 0.4691969 0.7963826
0.2942258 0.03810468 0.7221790.138140985 8.0795921.0889220.5245985
0.1607872 0.3202240.1354326 0.08724444 0.1654189 0.07830482
0.5901719 0.08653708 0.07921949 0 0.0385816 0.455820023 0.339428
0.07666091 0.1380796 0.2013624 0.6386919 0.3423296 0.2669068
0.1756844 0.3024394 1.3070630.379946846 0.094553937 0.3461513
0.6271171 0 0.5811241 0.08611541 0.1849170.06039413 0.1173092
0.6576173 0 0.6196937 0.3524832 0.4171651 0.5986552 1.270142
0.04197364 0.4335661 0.1075960.666478606 0.3347918 0.2372271
0.05253066 0 0.04737059 0 3.0551190 0.3668867 0.3351405 2.17577
0.029058046 0.08201076 0.1251208 0.3527024 0.1036353 0.03885569
0.154346959 0.1451975 0.08329706 1.9733240 0.3925704 0.3382365
0.070917663 0.2673708 2.7975750.2501063 0.4526412 0.233393669
0.5292286 0.05652833 0.1465671 0.099094857 0.156560334 0.3552296
0.2075333 0 0.4509642 0.0842175 0 0.2970215 0.0355610.2305786
0.1804163 0.04970749 1.5226060.161407545 0 11.769730.5177902
1.5172550.1452850.5034018 0.9056893 0.05906482 0.3512121 0.9263597
0.2301445 0 0.7003346 0.2162679 0.3388601 0.04617238 0.7603166
0 0.4825943 0.2187938 0.2360533 0.4926344 0.09471696 0.2769521
0.1282529 0.1626279 0.3935383 0 0.2619241 0.4562384 0.8386211
0.5195351 0.1159237 0.06716059 0.2300899 0 0.6858338 1.243888
0.09949984 0 0.5975169 0 0.1756055 0.2034591 0.1805205
0.8223734 0.3511104 0.114850051 0.1318438 0.03723746 0.3913853 0
0.05699611 0.067039228 0 0 2.765804258 0.1974836 0.08639477 0
0.8193023 0.2113844 0.3150659 0.4968049 0.05230844 0.07293154
0.1975944 0.2784579 0.031976074 0 1.15978 0.1752042 0 1.082997
0.1205856 0.3588178 0.6731759 0.04356442 1.043168
LINC01409 0.07246242 0.01103387 0.06209567 0.011472597 0.065185819
0.02082321 0.06143099 0.05895072 0.01897273 0.006995034 0.1020277
0.073160693 0.1144899 0.084643362 0.029555825 0.03031889 0.06327108
0.07221226 0.0208612 0.1976648 0.148132398 0.1628620.2280612
0.1080942 0.1639864 0.1319809 0.2871469 0.4101026 0.07210229
0.029786532 0.1727792 0.435509599 0.0409397 0.05653353 0.1357538
0.108080805 0.2484014 0.1290426 0.2215704 0.1296073 0.04888896
0.03524334 0.4227225 0.2935443 0.06268692 0.1916883 0.04042971
0.1618963 0.1290555 0.1051336 0.04506445 0.04549174 0.0670574
0.089036331 0.03810872 0.2098114 0.2085512 0.1079439 0.2478391
0.03917222 0.1553144 0.06663654 0.05688948 0.09751074 0.2173896

0.171558475	0.03597518	0.3337231	0.09842064	0.10891752	0.1939282	
0.06639115	0.2012058	0.130695868	0.2340263	0.1478594	0.3515416	
0.2479402	0.4267098	0.1073612	0.1311588	0.106306	0.02132682	
0.07977202	0.010424896	0.05602809	0.1101848	0.1719241	0.04143766	
0.1483969	0.1702595	0.265587475	0.05789359	0.08445502	0.1946253	
0.085243503	0.3838284	0.05848212	0.07278413	0.1470814	0.03311166	
0.2541095	0.1761889	0.08593762	0.06718729	0.158015	0.100484749	3.52727
0.104499665	0.04718551	0.2312624	0.07313841	0.06061691	0.1773298	
0.1352062	0.05487639	0.08315156	0.2980486	0.1217905	0.353146	
0.08204628	0.06933656	0.1411417	0.1735638	0.5810117	0.08245244	
0.07912079	0.07453281	0.156339319	0.0711476	0.09377835	0.1234044	
0.08508612	0.096783825	0.1416221	0.1870954	0.1713442	0.1252111	
0.01211204	0.1951962	0.2304233	0.2293424	0.1285875	0.1901491	
0.03647011	0.05952973	1.705976	0.01958946	0.1158708	0.1130016	
0.1432421	0.04458266	0.05669813	0.091043972	0.1035337	0.1309709	
0.088565025	0.03060404	0.1702409	0.09814848	0.09642838	0.2243723	
0.1228156	0.11015627	0.09252362	0.1362236	0.2351299	0.1841386	
0.2541395	0.2371881	0.2193386	0.01884445	0.09448407	0.103853578	
0.08591531	0.09261516	0.3188075	0.03274984	0.2109281	0.09969401	
0.3660657	0.3466842	0.0921675	0.1732715	0.09365646	0.08650219	
0.1723947	0.1072552	0.05148399	0.1449512	0.04769811	0.2470873	
0.041018328	0.3343296	0.2090635	0.08245685	0.08505443	0.1494104	
0.09708755	0.057507374	0.1855272	0.1590148	0.04832499	0.05751178	
0.109512239	0.09057617	0.1867087	0.2784054	0.6401297	0.3337769	
0.177701	0.06531012	0.08413177	0.3418454	0.01725665	0.4363575	
0.179432	0.1022139	0.063236961	0.072357485	0.4423196	0.07827512	
0.036025935	0.1106331	0.069150864	0.1808585	0.1270874	0.404075	
0.084602698	0.3369578	0.1032303	0.1983174	0.167696812	0.1214942	
0.178933	0.1090373	0.2960366	0.1799835	0.2358833	0.08351056	
0.2430178	0.0424454	0.1265384	0.09805655	0.148815552	0.1046964	
0.146310461	0.1233422	0.256587	0.0436922	0.1028396	0.1265224	
0.4871655	0.3330328	0.08752956	0.09758259	0.165558853	0.1912012	
0.3168054	0.07858994	0.1862765	0.1151344	0.04395973	0.1611974	
0.2271624	0.02867534	0.5531509	0.06914182	0.1265904	0.03341342	
0.04238591	0.263389827	0.1929677	0.1148455	0.1723805	0.0980396	
0.537643	0.06410537	0.2208708	0.04094102	0.1941787	0.1522973	
0.136607316	0.089712269	0.1508562	0.2366103	0.2318731	0.2708472	
0.1548111	0.062792	0.1025398	0.06248554	0.1699909	0.1082187	
0.138064	0.1462303	0.3333086	0.2092635	0.2046017	0.02096019	
0.2338287	0.07880359	0.108603275	0.7170314	0.09477044	0.1023048	
0.08772579	0.05677258	0.1594771	0.4031999	0.04085515	0.1665551	
0.09085129	0.5714211	0.078357179	0.07371604	0.2124355	0.07485415	
0.03105115	0.04268703	0.105336601	0.09425861	0.02495741	0.3822197	
0.05315133	0.0168031	0.09571209	0.063744914	0.1134884	0.3113342	

0.1355997	0.09041337	0.049533227	0.1174571	0.1693698	0.1171049
0.11876295	0.042999465	0.05321684	0.07254448	0.07068822	0.1678734
0.07990511	0.0432131	0.1949379	0.02486112	0.1217226	0.1441499
0.07446662	0.1284125	0.10961787	0.05751177	1.6616350.1598412
0.1887015	0.1233355	0.1939229	0.1055297	0.04424241	0.2572287
0.4236370.1214935	0.09098467	0.3873855	0.0431987	0.25624	0.03458535
0.5285089	0.07071247	0.3052552	0.1158136	0.3489156	0.4887293
0.1655444	0.1580575	0.08112379	0.2663713	0.2505624	0.03701337
0.03923876	0.1518865	0.1641612	0.1933992	0.1823481	0.02683015
0.04595959	0.07668403	0.2529096	0.5823325	0.09274869	0.03056322
0.2498928	0.07484924	0.09353743	0.6421150.3200172	0.3207098
0.1087061	0.108969112	0.1106083	0.2008271	0.1250844	0.07478089
0.1110014	0.06360646	0.091891541	0.07357552	0.149819389	0.09368568
0.3580834	0.2000821	0.2080725	0.05541801	0.09833317	0.1653915
0.07313891	0.06737608	0.1019609	0.1286234	0.11177426	0.1240708
0.3888607	0.06999277	0.1171871	0.2533273	0.1505408	0.1488583
0.2451997	0.06526372	0.3869709
H1FX-AS1	0.3723269	0.2491912	0.2654508	0.148056889	0.233169824
0.5979220.6087442	0.9713461	0.2678026	0.1767841	0.2715681
0.501583934	0.4617255	0.645476506	0.476782127	0.5589618	0.8777697
2.4046820.2980642	1.1244170.726073647	0.3678109	0.7494233	0.06126622
0.7818508	0.7619792	1.7703	0.5130467	0.4725191	0.504528988
0.8470905	0.506380377	2.3876790.4445878	0.7883721	0.603161612
1.2021321.7780860.6925169	0.7168357	0.7814866	0.3524892.242064
1.7695190.2443828	1.2018880.6304552	2.7559710.6289904	0.58299
0.2847263	0.4219658	0.3245225	0.511321592	1.2841520.6597079
2.2126480.1205519	0.7903094	0.6951007	1.0105380.3224858	0.3176713
0.8314441	0.7426245	0.624666335	0.7399289	0.5677124	0.6985793
0.331879962	0.5857370.2447986	2.0166320.327154765	1.3775521.42367
0.3297047	0.4221871.2365940.9814131	0.7537532	0.9431863	0.2958704
0.3713478	0.092493519	0.3779618	0.5332363	0.1522655	1.891727
2.5878632.4395870.546136327	0.4423808	0.2958339	1.7606461.375112305
1.54794	0.9724369	0.4335223	4.5401080.9436533	0.8144439	3.821187
1.7580930.4581675	0.9573032	0.371871344	0.7291262	0.418529693
1.5318680.6839485	1.0002971.0134042.6569	1.4596520.3813354	1.468443
0.5469094	0.3383605	1.5388761.8529510.3684498	1.3058341.651093
1.2588350.2660176	0.7840395	0.7921245	0.972771453	1.2029840.6051171
0.2233478	0.9190274	1.695099568	0.5083209	0.5055391	1.6363190.46402
0.4103112	1.8696142.2891850.6218570.3941208	0.6635611	0.6013942
0.4670591	1.4156022.4332680.7476716	0.8658751.2220160.8826406
0.7246686	0.525633925	0.4715753	1.44486	0.131879418	1.29594	0.8363596
1.8339780.4542183	2.3563550.4358662	0.231009293	1.0746370.4541505
1.6537551.86207	0.3651269	0.6121951	0.5632360.8876529	0.3392524
0.231354018	1.0827731.3894471.3409910.07044088	0.9690958	1.053663

1.129444 1.189405 0.3469217 0.5135263 1.219853 0.3739717 0.5372385
0.3737223 0.4412127 2.672333 1.056239 2.541956 0.252645425 2.899755
2.974456 0.554233 1.586104 0.8167985 0.7780356 0.267391487 1.558774
5.211325 0.3811174 0.3247146 0.630930472 0.2247903 2.726996 1.190827
1.499125 1.071142 0.7904862 0.1921189 0.1919911 1.311558 0.7237803
0.4716947 0.7376359 1.259738 0.550860499 0.457917421 6.107828 0.258553118
0.445552328 1.686221 0.364400787 0.7840869 0.862759 1.28505 0.454925036
1.385217 0.7640637 2.494355 1.082085746 1.041838 2.255725 1.04364 0.6230584
1.302139 0.253678 1.396145 0.7093804 0.6358033 0.5364251 0.5856961
1.244537043 1.2878 0.683858254 1.548509 1.036837 0.5462388 3.34163
0.2041006 1.678671 1.019165 1.399712 0.8418341 1.101674356 0.9671354
0.6712391 0.4261149 0.4248355 1.140175 0.9655213 1.047176 1.692327
0.2985734 1.433563 1.345411 0.6253938 0.9810004 0.491058 2.523108534
0.3996257 1.099232 1.484931 0.6488343 1.499405 0.248189 1.775354 0.7642273
1.078941 0.6879026 0.440738342 0.776918186 4.349968 3.16578 1.081339
0.9237722 0.5896514 0.423049 0.9859901 0.3212969 0.4175655
1.633512 0.6911968 0.4193647 0.7527512 0.5425312 0.6271041
0.9940757 1.387733 0.3235849 0.655565506 1.576208 0.2038396 0.2158135
2.108582 1.97667 2.785957 0.2461068 0.1694721 0.3062948 0.6479383
0.8265838 0.458794265 0.766345 0.8466506 0.845261 0.2281896 0.5508874
0.091179037 0.3820845 0.1476209 4.065579 0.7327 0.6505452 1.326012
1.096859854 0.4630714 4.004972 2.377399 0.4590018 0.192711856 1.335812
2.149333 3.345259 1.596528259 0.201788875 0.3624658 0.1379232 0.4624601
3.692213 0.8819444 0.3834026 1.292043 0.378132 0.5837664 0.2616036
0.9209692 0.3488835 2.080363919 0.3711022 0.8427708 1.304413
0.4197104 0.8016905 0.7878638 0.1459166 0.328302 1.235421 0.8247851
1.26068 0.1749981 0.4513268 0.6039481 0.631732 0.3049942 2.729199
0.5459083 1.140352 0.331351 1.224592 3.047765 1.381026 1.587199 0.8058559
0.3458552 1.57716 0.2239065 1.814552 0.3724257 0.5836803 1.468605
1.349384 0.3895314 0.08340759 0.5389936 0.4674807 2.392324 0.4435113
0.3369059 1.185283 0.9372007 0.8723365 1.914324 10.57925 2.189297
1.459449 1.563555827 0.2931327 1.385854 1.387243 3.006555 0.3535342
0.626444344 0.342082071 0.8308225 1.170648789 0.8670078 2.004359
0.107588 0.5883371 0.357592 0.2728383 0.6403263 2.309127 0.5669414
0.5942469 0.7402338 0.628507503 1.961429 1.001001 0.5645469 1.890413
0.8586389 0.8111057 0.7426301 0.28071 1.186162 1.344528
AC079921.2 0.1301669 1.204098 0.2635245 0.486879384 0.54984067 0.1514923
0.6916628 1.378531 0.9201984 1.328795 0.185567 0.443546749 0.1322115
0.559813098 0.836202499 1.155393 0.4091623 0.2501696 0.1445416
0.1892163 0.276330157 0.5077919 0.06827913 0.247965 0.1325587
0.3790194 0.7239251 0.4397543 0.7649776 0.270877416 0.862645
0.205740527 0.15274 0.2998995 0.2743419 0.035419174 0.265759 0.3259744
1.175389 0.5612587 0.3233412 0.08546703 0 0.4683297 0.3167067
0.04385422 0.735332 1.239813 0 0.3186934 0.09106982 0.1379 0.2323113

0.043183599 0.1540262 0.2155949 0.5349259 1.040367 0.06988644
0.2638744 0.4394203 0.3327003 0.4775543 1.632762 0 1.664154909
0.1090522 0 0.04773509 0.293478855 0.5003049 0.1150016 0.3199574
1.256849514 0.3622511 0.05602613 1.189546 0.3897111 0.1831495 0
0.3478861 0.08056187 0.4654685 0.05527187 0.25280946 0.1235193
0.5089602 0.4905003 0.1004886 0.8066066 0.1214378 0.212328864
0.07389217 0.4681327 0.1388168 0.079507761 0.3231962 0.1636412 0
0 0.2676587 1.256784 0.3560564 0.04736442 0.0370302 0.170309
0.71670893 1.611902 0.436926602 0.3575858 0.05841918 0.3470178
0.06681788 0.09773513 0.3783259 0.1023679 0.3183902 0.1355221
0.08204132 0.1112206 0.3826284 0.6923617 0.05033476 0.4332813
0.2063368 0.1999517 0.1827355 0.584767 0.406212255 0.03195127
0.07580588 0.4744406 0.2691372 0.67058884 1.373766 0 0.1662076
0.5334281 0.7343088 0 0.2044351 0.2958341 0.1247327 0.7999055
0.2948067 0.09842923 8.482515 0.04750551 0.04844708 1.027632 0.1002029
0.1965734 0.5817141 0.232406984 0.07384558 0.2049107 0.578240526
1.020476 0.1876562 0.04958655 0.1870753 0.2776098 0.5956694
0.267135006 0.392656 0.1101166 0.05702029 0.199946 0 0.9734057
0.1990132 0.2284941 1.256514 0.899466549 0.1953275 0.1122984
0.6502927 0.4853454 0.3884903 0.4585169 0.08618732 0.2960311
0.3725188 0.2442982 0.3785368 1.090817 0.04750758 0.1560598
0.1950805 0 0.4206201 0.169585 0.452144089 0.3158835 0.1246698
0.2221804 0.2062617 0.6038806 0.1898731 0.205086092 0.6561239
0.05073943 0.2929769 0.1394692 0.569085419 0.5632108 0.1223727
0.4091808 0 0 0.1632329 0.09316508 0.07464299 0.1680393
0.2092416 0.3272756 0.2105479 0.04957485 0.843442083 0.506165992
0.3575497 0.406760285 0.339752277 0.3237999 0.055898215 0.1370599
0.06420707 0.244976 0.398934262 0.06589847 0.4417748 0.03757271
0.739407243 0.5427398 0 0.7932641 1.233899 1.322636 0.3813534
0.184107 0.140317 0.07352316 0.1784084 0.1606706 0 0.1586841
0.4093972 0.6827548 0.369646 0.1655562 0.4750318 0.1136385
0.2411027 0.05938403 0.1845774 0.05144416 0.376396233 0.07858873 0
0.3970519 0 0.349009 0.3690165 0.2641302 0.3505608 0.6005664
0.2199051 0.4715795 0.1439009 0.4861763 0.2336095 0.27599652
0.2529499 0.185671 0.08360645 0.3962523 0.3314781 0 0.2308723
0.1418346 0.4185724 0.1055226 0.322078019 0.286259448 0.2286466
0.08438132 1.12461 1.266723 0.7299937 0.3135054 1.29208 1.894136
0.327916 0.468636 0.5772633 0.3283487 0.1683941 0.5437236 0.1736596
0.5082961 0.1400117 0.3040276 0.127436569 0.135143 0.0957598
0.1272281 0.2659248 0.2294611 0.2358175 0.4624644 0 0.2423432
0.05797878 0.2602305 0.387078723 0.1986283 0.3030398 0.8542374
0.7530074 0.4705382 0.498433258 0.8205519 0.6724788 0.1448287
0.7616517 0.1697852 0.06826679 0.472342808 1.173714 0.241988 0.043268
0.7416059 1.095219026 0.14242 1.300648 0.2958187 0.780018092 0.236991055

0.6691676 0 0.1428523 0.04964661 0.6119188 0.4366423 0.2569212
0.9904716 0.2792281 0.3823438 0.2708785 0.08194929 0.586388291
0.4482938 0.2639447 0.3420209 0.3466754 0.3958616 0.3918947
0.09139832 0.7867954 0.07088566 0.4132989 0.2388878 0.04243131
0.1630957 0.08729934 0.5862226 1.006456 0.368294 0.3674606 0.09740273
0.3444439 0.9147453 0.1988583 0.9749602 0 0.9318773 0.3938811
0 0.1121993 1.268749 0.4419998 0.04062244 0 0.2105735 0.3659883
0.2786363 1.461138 0 0.1882918 0.2409864 0.6176458 0 1.38296
0.2835418 0.7391602 0.1639562 0.0474231 0.2551145 0.486787522
0.1915937 0 0.3317743 0 0.9663032 0.081183769 1.0285017
0.7434362 0.965069416 0.2391505 0 4.043418 0.141738 0.3199803
1.240009 0.7620587 0.3800697 0.1103991 0.5583308 0.08430235
1.200402909 0.5415815 0.05016003 0.2121704 0.07104628 0.2415915
0.2920557 0.6350741 0.3356741 0.2637802 0.2165598
AL355073.2 0.1860779 0.6010884 0.2767716 0.397720144 0.084215946
0.2062507 0.5215411 0.6256049 0.4698051 0.5080881 0.1010568
0.271742061 0.4590018 0.876487743 1.170983751 0.8151109 0.2088968
0.4087151 0.5313265 0.3348937 0 0.1382677 0.08366339 0.1157467
0.03609468 0.2408093 0.02815986 0.02763268 0.04463514 0 0.2684473
0.084032262 0.1559621 0.1749862 0.08964135 0.057866156 0.07236405
0.1065123 0.2743277 0 0.1320648 0.104724 0 0.1530269 0.1552261
0 0 0.2109945 0.0543947 0.1301665 0 0.06758832 0.04744238 0
0.6710419 0.1408915 0.317795 0 0 0.4311054 0.05127885 0 0
0.04599186 0 0.145650901 0.2227054 0.06009956 0.2729556 0
0.04086871 0 0.09801208 0 0.3205738 0.04576641 0 0.3638236
0.2618183 0.3544654 0.1623885 0.1316181 0 0.2483265 0.103257006
0 0.05196968 0.1001695 0.3693904 0.7349244 0 0.05781546
0.241443 0 0.1133961 0 0 0.4010237 0.05545505 0.2756144 0
0.03311732 0.290854 0 0.09074725 0.06956066 0.087819362 0.6144712
0.178457454 0.175262 0.09544247 0.03149673 0 0 0.1030151 0.08362189
0.04334755 0 0.06701759 0 0 0 0 0.1516874 0.0240788
0.1225018 0.1865903 0.6513848 0.27652091 0.07830068 0.09288602
0.02981224 0.03664196 0.136947003 0.07013727 0.09265755 0 0 0
0.06041841 0 0 0 0.1537468 0 0 2.005811 0 0 0.05596319
0.02728445 0.08028807 0.08639789 0 0 0.04184667 0 0.1515642
0.07664594 0.08101214 0.114613 0.04535456 0 0.18184684 0 0.04497581
0.04657852 0.1361093 0.1966572 0.2168596 0.2216852 0 0.1207549
0 0 0 0.08853477 0.07208492 0.1057828 0.06810027 0.07040436
0.04836415 0.0676226 0.1197368 0.1374301 0.5312104 0 0.3399508
0.1912279 0 0 0.04617664 0.110803713 0.04300629 0.4413057 0
0.03369805 0.2055398 0.06204112 0 0 0.1243435 0.02659176
0.1139291 0.542351005 0.04600734 0.2998899 0.0835625 0.05561743 0
0.05333641 0.03805217 0.02032469 0.04575576 0.4557986 0.04455727
0.114661 0 0 0 0.1168295 0.036919196 0.039647932 0.1889317 0 0

0.2097969 0.08576359 0.046554268 0.3768162 0 0.122769 0.15100103 0
0.1969227 0.3239993 0.4810636 0.2160859 0.07787962 0.3007852
0.0286554 0.1201186 0 0.131248 0.349104158 0.06481263 0.074317066
0.1062335 0.2013034 0.05409558 0.09701053 0 0.1313007 0.09701879
0.3392479 0 0.341632328 0.03209863 0 0.1081141 0 0.06335491
0.1674671 0.2157616 0 0.1032818 0.08981762 0.04280245 0.1175492
0.1323819 0.114498 0.354286376 0.129143 0 0.1365922 0.02489916
0.2256471 0 0.1131564 0 0 0.04309946 0.112756219 0.374141612
0.3424225 0 0.4593335 0.2299457 0.1277817 0.2195101 0
0.03868186 0 0.07656352 0.4715526 0 0 0.2220774 0 0.249129
0.1429653 0.1064369 0.10409989 0 0 0.3377712 0.2172276 0
0.07705349 0.05396802 0 0.03299406 0.04736148 0 0 0.4867641 0
0.04361288 0.03417299 0.03843714 0.050894778 0.3112057 0.02746659
0.02957678 0 0.02773869 0.05576551 0.105230608 0.198368 0.07906969
0.4948243 0 0.028859944 0.232679 0 0.09665889 0 0 0.03904479
0.02566222 0.07779511 0.2027757 0.08331031 0.2140096 0.1259237
0.1055338 0.1303399 0 0.1229301 0 0.031933773 0.04068895
0.2156101 0.09312956 0 0.2155799 0.07113988 0 0 0 0.3858446
0.1626181 0.06932225 0.07993738 0 0.1436614 0 0.03008506 0
0.1989149 0.08657477 0.0934042 0 0 0.5479375 0.08458092
0.06435041 0 0 0.06477566 0.1504413 0.0331835 0 0.05733748 0
0.08535426 0.488278 0 0 0 0.05045401 0 0.03530335 0.1158092 0
0.4017957 0 0.03473283 0.51125799 0.02608472 0 0 0.6267404
0.08457322 0.066317082 0.070013207 0 0 0 0.08546413 0 0.04631296
0 0 0.2293449 0.2587249 0.1623283 0 0.06886458 0.031631628
0.04915611 0.1229236 0 0.116072 0 0 0.08191213 0.2350322
0.1723806 0.0589675
KLF7-IT1 0.1808426 0.3717488 0.1046051 0.724744122 0.190975195 0.8068024
0.5174272 0.2128012 0.2796595 1.217649 0.1289053 0.154056314
0.2525644 0.09721945 0.124472891 0.6566735 0.1065852 0 0.1757118
0 0.095977268 0.1175803 0.2134374 0.1968577 0 0.2047798
0.3591995 0.09399329 0.0379569 0.125444503 0.05707066 0.619315633
0.07957639 0.1785661 0.3049175 0 0 0.0905761 0.1166416
0.2339292 0 0.6827576 0.2157909 0.1626641 0.1760019 0.1218545
0.02837795 0 0 0.3320737 0.03163112 0.05747587 0 0.059995535
0.07133022 0.08985866 0.1689044 0.3030668 0.09709414 0.1466416
0.1308198 0.09904817 0.03685958 0.1173319 0 0.289004001 0
0.07666135 0.1657974 0.237844823 0.104262 0 0.1666954 0.113879827
0.2726101 0.3113513 0.3787326 0.03867362 0.09541949 0.04306155
0.1035691 0.05596282 0.2155605 0.1151849 0.482943262 0.2359595
0.1546792 0.1135764 0.03490253 0.02155056 0.05623839 0.147495623
0.07699452 0.1422713 0.09643 0 0.2245103 0 0.09431592 0
0.6507579 0.1689742 0.2061141 0.131608 0 0.05915311 0.049786651
10.82394 0.03035139 0.04967986 0.04058126 0.08035272 0.09283095

0.04526156 0.1314033 0 0 0.06276085 0.1424763 0 0 0.1030615
0.2097922 0.4299738 0.04095233 0.1736222 0 0.1846419 0.141088989
0.02219515 0.02632952 0.1014071 0.0623193 0.058228611 0.2982173
0.1181914 0 0.02850379 0.06801232 0.05137871 0.04733738 0.04110061
0.108308 0.09805757 0.06826314 0.0227915 17.884050 0.06730816
0.04759008 0.09280879 0.06827551 0.2204135 0.040360755 0.05129731 0
0 0.03222185 0.1955349 0 0.06497654 0.03856869 0 0.061855689
0.1168976 0.03824661 0.1188285 0.09259587 0 0.09220672 0.1382458
0.03174499 0.3337351 0.374892165 0.09045701 0 0.07528834 0.0612997
0.02248894 0.05791122 0.5088999 0 0.08625753 0 0.05843401
0.1165759 0.1320058 0 0.05420556 0.3488312 0 0.03926777
0.062816965 0.03657176 0 0.07716954 0.0286562 0.1048724 0.7386209
0.455886032 0.06511153 0 0.06783943 0.0484416 0.197659439 0.2151809
0 0.14212 0.2601282 0.1794492 0.2041034 0.03235886 0.13827
0.5836479 0 0.113672 0.02437641 0.1721875 0.42611092 0.023440766
0.1490245 0.062790816 0.033715875 0 0.116490172 0 0.2230093
0.1701742 0.197944481 0.0457768 0.3836022 0.02610013 0 0.2154387
0 0 0.1558429 0.04593888 0.1324548 0.06394556 0.3898885
0.4085867 0.1239326 0.1116109 0 0.3306928 0.094796807 0.4516953
0.08559234 0.04600189 0 0 0.02791392 0.0412515 0.03205447
0.07147204 0.087155362 0.1364804 0.1194815 0.1471012 0.08115458
0.02693793 0.02848219 0.03669595 0 0.570888 0 0.03639842 0.06664113
0.1125752 0.03245567 0.027388968 0.3074983 0.2579552 0.02903888 0
0.1151317 0.1439875 0.06415078 0.1231579 0 0 0.351581349
0.079540793 0.0264718 0 0.2130594 0.1303611 0.03622106 0.03111122
0.06773978 0.06578869 1.204027 0 0.2004998 0.3421343 0.2573473
0.5665517 0.1723339 0.3177821 0.121575 0 0.088524639 0.093878
0.3326008 0.06628487 0.07389056 0 0 1.927523 0 0.08417263
0.08055068 0.1355781 0.122221248 0.0344946 0 0.3708759 0.1453004
0.03268624 0.238039929 0.2850008 1.237926 0.2766671 0.08139789
0.04717694 0.1896879 0.178972362 0.08434426 0.03361971 0.0300564
0.04479664 0.319045582 0.1483995 0 0.1232954 0.125041068 0.164627374
0.2324208 0.676503 0.06615554 0.1379494 0.1062683 0.1213266 0
0.02991467 0.249387 0.03794251 0.0209075 0.0569266 0.081467686 0
8.984194 0.07919567 0.1204102 0.06110841 0.09074407 0.06349043 0
0.1477235 0.2871008 0.08297248 0 0.04531819 0.1212861 0.04072234
0.03884119 0.2046703 0 0.2706458 2.116621 0.1588584 0.05525528 0
0.03328257 0.1078891 0.2736121 0 0.4286699 0 0.1791055
0.08465592 0.3973127 0.3413112 0.2259877 0.169362 0.1845434
0.0887597 0.06539905 0.02790046 0.04290517 0.1570763 0.09006396
0.04924103 0.205385 0 0.03294277 0.2362893 0 0.08872786 0 0 0
0.143839 0.028197416 0.178613845 0.0688577 0.078869616 0.08306371
0.1090157 0 0.3347614 0.04445527 0.1325201 0.2228922 0
0.07668947 0.1662206 0.05856118 0.053797914 0 0.2090639 0.1105391

0.09870552 0.1678231 0.2028784 0.06965658 0.1998670 0.1002898
AL133371.2 0.4028976 0.5176349 0.9321954 1.399364217 1.861439768
0.1563016 0.4940449 1.027212 1.157095 0.2625279 0.478645 0.514831048
0.4092261 1.732754827 1.109248213 0.1625552 0.4749205 2.71017
0.3355429 0 0.427653815 0.08731871 0 0.1644666 0.06838345
0.2607012 0.5335049 0.4711653 0.1691277 1.886467718 3.687269
0.583747925 0.1772875 0.06630431 0.5094922 0.054815389 0.7540383
1.109865 1.039459 0.6948918 0.2502045 0.3968112 0.1201897 1.159673
0.3921131 0.1357392 0.189669 0.07994827 0.1030539 0.246608 0.2818828
0.08536665 0.269647 0.801981125 0 0.1334635 0.2257804 0.1038769
0.3244728 0.0816754 0.1943015 0.2206685 0.4105956 0.6970739
0.2399637 0.643869459 2.700341 0.05693105 0 0.454193466 0.3871407 0
0.06189652 2.114265487 0.2803134 0.4335355 0.07670682 0 0.2125842
0 0.1538272 0 1.200613 0.1283097 0.097813184 0.0477902 0.3446085
0.3162949 0.4665543 0 0.501172 0.38337156 0.4002493 0.1358421
0.1432237 0 0.3001108 3.798813 0.1050628 0 0.1035584 0
0.1836799 0 0.05730864 0.8566135 0.720975055 1.663073 0.338097966
0.8854506 0 1.074103 0.5170431 0.05041891 0 0.7129193 0
0.2097365 1.01575 0.08606353 0 0.1530732 0.1557981 0.2394837
0.136856 0.4641736 0.4949086 0.8227241 0.314330912 0.04944839
0.4106152 1.807393 0.4165218 1.037816062 0.3321978 0.1755451 0
0.3810201 0 0.1144662 0.2109251 0 0.09651938 0.1456411
0.2281243 0.05077698 10.8246 0.07352043 0 0.212051 0.1550759
0.6844968 0.2455287 0.179838738 0.3428545 0.2378428 0.511369172
1.220375 0 0.3837055 0.3619016 0 0.4609346 0.620134836 0 0
0.5294741 0.2062935 0.2235471 2.259692 0.7279921 0.2121731
2.173382 0.64961473 0.1007642 0 0.5032027 1.911967 0.1503087
0.3870597 0.06669257 0.09162868 1.281149 0.3024644 0.4556461
0.973944 0.2940945 0 1.026495 0 0.4882198 0 0.139949361 0.4073895
0.1929414 0.9455893 0 2.18068 0.1763107 0.634790286 1.885798
0.07852531 0 0.3237679 0.146787906 0.8280539 0.3787725 0.712413
0.05268521 0.0666322 0.1010489 0.5767362 0 0.08668693 0.4317684
0 0.108616 0 0.355998282 0.783352131 1.217372 0.489618861 0.300461197
0.07158841 0.259527425 0.141411 0.5962085 0 0.088199752 0 0.740674 0
0 0 0 5.72913 0.347201 0.1023468 0.2950949 0.1424637 0.162868
1.934359 0.1104433 0.5470452 0.440932009 1.105121 0.915186863 1.610125
1.048797 0.0512436 1.562233 0 0.06218918 0.2757116 1.999588 0.1592319
3.559832101 0.5473144 0.3549224 0.08193135 0.06026786 0.4201034
0.1903657 0.4087729 0 1.663223 0.08508234 0 0.890815 0.627013
0.2169231 0.122039277 0.4893376 0.287348 1.293909 0.1415187 1.197004 0
0.142921 0.8780238 0.6477905 0.979853 0.213623176 0.354416459 0.0589763
0.1305901 0.4746731 0.508253 0.8069659 0.831749 2.490128 0.7328501
0.07249843 0.435162 0.3573534 0.05081587 0.05212199 0.5609847
0.03839413 0.2359946 0.4333695 0.4705188 0.85463413 0.06971665

0.4445991 0.7876026 0 1.065355 3.138619 0.2044911 0 0.1875275
0.08972907 0.1006844 0.544591277 1.690705 0.2344951 1.900424 0.1942281
1.674892 0.916019454 0.5442436 0.2601852 0.05603492 0.9067282
0.4729729 0.211302 0.731006727 0.7516393 0.5243073 0.2678495
1.596833 0.437414665 0.3306178 1.483196 0.8240663 2.414341715 0
0.2589041 0.1944742 1.768648 0.3073362 0.2367543 0.6757559 0
0.6664664 0.8642776 0.08453179 0 0 0.605003793 0.3854378
0.9531336 0.9704163 0.375565 0.1361429 0.3369465 0 4.538549 0 0
0 0.131335 0 0.4053184 0.3628997 0.2163349 0.1709937 0.09478151
0 0.08201045 0.3539193 0.1846541 3.816546 0.07414994 2.003043
0.6705357 0.09219372 0.08682089 0.3681637 0.1710118 0.3772084
0.08851695 0.4888312 0.1888035 0.1078057 0.5139283 0 0
0.1864776 0.5735283 0.2099693 5.21697 0.6033701 0.07626256
0.2537418 0.1467858 0.1974101 0.215246183 2.174436 0.2093657 0
0.1079451 1.762517 0.251283094 1.061152548 1.303963 0.966421054 0
0.08095837 5.006136 0.04387129 0.2971245 0.07381008 0.6207245
0.7842708 0.1366846 1.419566 0 0.179783846 1.583195 0 0.4104486
1.099526 0.2670655 0.2824943 0.2586455 0.07421367 0.5715237
0.167576

NCBP2-AS1 0.07903819 0 0.152394 0 0.278222314 0.286182 0.04307509
0.04133593 0.04656253 0.06866833 0.1502366 0.224437023 0.08027968
0.075538244 0.145070718 0.2551134 0.1656306 0 0 1.046806 0.167789441
0.5938294 0.8291905 0.2581131 0.6439239 0.8864768 0.251184
0.6298972 0.3539042 0.036550778 0.266059 0.41642322 0.1545745
0.1387435 0.3553753 0.229405328 0.2510209 0.05278234 0.271887
0.2726399 0 0.1037923 1.38325 0.9479092 0.1025634 1.100647
0.03307395 0.3345878 0.2156431 0.3225211 0.2949236 0.312606
0.4231827 0.209770864 0 0.4538235 0.944903 0.2445358 0.5658066
0.2563619 0.4065811 0.1923978 0.08591823 0.09116536 0.1673768
0.288710149 0.1324344 1.250862 0.579701 0.237602987 0.2430307 0.1862125
0.4857004 0.353932557 0.6110033 0.09071846 0.4414054 1.983227
0.481908 0.3011244 0.3218875 0.3261179 0 0.1118714 0 0.2000045
0.4378122 0.3971133 0 0.5023351 0.2949513 0.200553805 0.2692068
0.02368777 0.7117861 0.064370163 0.5233248 0 0.3297701 0.1170696
0.3792228 0.2954041 0.2882663 0.5751996 0.1798798 0.1034127
0.290126836 6.003043 0.212243705 0.05790091 1.087823 0.2185156 0.1081927
0.2373816 0.1531479 0.1657558 0.0859238 0.3657327 0.3985281
0.1350677 0 0.3603484 0.6927747 0.4259572 0.5966143 0.121412 0
0.2582358 0.274060759 0.1034721 0.1227462 0.2068291 0.1452638 0
0.382323 0.459166 0.1345631 0.1661031 0.07926703 0.479047 0.4689517
0.1437059 0.2777079 0.07618945 0.3182374 0.1859413 2.710862
0.4615304 0.235339 0.4437224 0.08112512 0 0.2140731 0.376317359
0.05978601 0.7465386 0.100317529 0.2628775 0.1519281 0.0401457
0.1514578 0.3596084 0.2411295 0.216274783 0.3178976 0.133727

0.1846565 0.3507357 0.2728707 0.1791085 0.8349091 0 0.1196805
0.14564316 0.6325553 0.09091777 0.3217393 0.0714436 0.5242084 0
0.6628909 1.006611 0.1340419 0.07911436 0.1702592 0.1358669 0
0.4211158 0.2211143 0.4573755 0.1277016 1.3272090 0.292847773 0.767226
0.1345783 0.4047281 0.5343719 0.2037112 1.752441 0.265663123
0.2656018 0 0.1054207 0.2258310 0.1823921 0.6439807 0.6625525
0.1102452 0.6274334 0.7664974 0.03771362 0.1410071 0.6802302
0.3952756 1.236504 0.1136409 0.1605449 0.620779922 0.191238278
0.5210556 0.292725867 0.117885577 0.224701 0.045255672 0.2589181
0.5718085 1.076674 0.36912067 0.9603354 0.2980539 0.5779645
0.149657605 0.0627724 0.3252845 0.2140778 0.6584155 0.2677043
0.3087469 0.5216911 0.8520139 0 0.2311056 0.416257 0.153777
0.385416 0.147311764 0.4737978 0.1995124 0.08042145 0.04807372
0.414012 0.4879969 0.528856 0 0.7913431 0.270874316 0.572635 1.021191
0.4929002 1.450289 0.06279125 0.1327817 0.3849157 0.4459982
0.2559072 1.023713 0.2545299 0.07766893 0 0.03782644 0.287291751
0.3583832 0.3006417 0.135377 0.2220987 0.2683675 0.125861 0.1121497
0.3157839 0.3388798 1.0679 0.260757115 0.185406475 0.1851142 0
0.4138609 0.4937832 0.2110747 0.2538166 0.1381614 0 0.3034098
0.1517647 0.9814502 0.3455841 0.1363333 0.6603049 0.7431518
0.2880646 1.133547 0.21098 0.06878249 1.167072 0.07752795 0.1545074
0.043059 0.04644341 0 1.069758 0.1145897 0.6540103 0.6571617
0.6057189 0.170935767 0 0.3066795 0.2161243 0 0.1142855
0.4539778 0.2372592 0.1905556 0.9380365 0.1423015 0.3299028
0.2210775 0 0.4587407 0.8620284 0.1751507 0.1566288 0.54346048
0.1153044 0 0.09579893 0.582931671 0.383739965 0 0.3306408 0
0.4019432 0.371561 0 0.7488203 0.06972994 0.1614753 0.08844251
0.04873456 0.2985608 0.094948994 0.08065389 2.920456 0.4153545
0.3648728 0.213662 0.2115209 1.701928 0.02895438 0.2869482 0.5258163
0.2256399 0.2061165 0.4753572 0.07067829 0.569533 0.2489776
0.6261654 0.09916642 1.498304 0.7293385 0.2777196 0.4507926
0.09286307 0.3103215 0.08382843 0.1275558 0.6269829 0.09083752
0.3209968 0.8349759 0.9208717 0.6482843 0.3693778 0.03292303
0.08459488 0.1075409 0.5689623 0.9146558 0.09755233 0.05000513
1.061802 0.2799141 0.08608419 0.7181165 0 0.5375181 0.3098144
0.225204192 0.1034106 0.07301722 0.4604697 0 0.2235221 0.032863536
0.10408547 0.0802523 0.551525959 0.1936183 0.2541113 1.309434 1.49178
0.1554353 0.193062 0.2922486 0 0.2323882 0.1291513 0.9214007
0.094050624 0 1.66501 0.3864936 0 0.5029575 0.4137889 0.1353056
1.164706 0.2562704 0.6720931
AC108463.2 0.07187616 0.4432563 0.08315092 0 0.075903369 0 0.2937888
0.1691563 0 0.06244596 0 0.244919625 0.07300515 0 0.131925152
0.05799907 0 0.4144193 0 0.1393098 0.30517044 0.8100294
0.1885134 0.0391207 0.1463937 0.2325429 0.07614099 0.07471554

0.4827529 0.249290502 0.2268282 0.227213446 0.126511 0.3311997
0.3029747 0.078231671 0.24458 0.359996 0.370875 0.4958694 0 0.3775485
0.3430656 0.827534 0.06995221 0.09686247 1.127886 0.1141009 0.1103077
0 0.05028734 0.1522924 0.192418 0.143071876 0 0.1904771 0.2148202
0.1111886 0.5402629 0.4079805 0.2773041 0.6298694 0.410197
0.4352482 0.05707875 0 0.1806508 0.1625022 0.1581515 0.378127084
0 0 0.08833775 0.844885121 0.8667942 0.5568616 0.4378991
0.06148354 0.3033969 0.06845951 0.1097699 0 0.6853986 0.1220811
0.418792369 0.06820542 0.2459099 0.3611289 0.221953 0.06852241
0.1788162 0.351734101 0 0.09693586 0 0 0.6424701 0.783121 0
0.159692 0 0.4477268 0.06553628 0.1046156 0.204475 0.09404196
0.593633307 4.272322 0.193011266 0.473888 0.3225816 0.08516349 0.1475832
0.2518498 0.2785409 0.1695779 0 0.1496663 0.4077175 0.2456571
0 0.2730795 0.2779407 0.1025362 0.3255313 0.1104102 0
0.05870895 0.971984421 0.07057197 0.08371766 0.5642618 0.1486134
0.740577481 0.3318753 0.5637043 0.1835545 0.2265775 0 0.2450465
0.07525724 0.5227359 0.3443774 0.1039283 0.2713128 0.07246812
3.944348 0.5770997 0.1605104 0.6052718 0.110661 0.271362 0.3504147
0.19249729 0.1631055 0.3960198 0.136840901 0.4610383 0.6217251
0.1642856 0.3099002 0.3065836 0.1096398 0.344184869 0 0
0.3148572 0.03680236 0.05317382 0.1465908 0.3596466 0 0.7754544
0.331114304 0.5752363 0 0.03989795 0.1949092 0.1430122 0.230169
0.1427739 0.130771 0.3199763 0.05395907 0.1857974 0.3011662
0.1049318 0 1.206468 0 0 0.2497125 0.299600331 0.406994 0.1376815
0.06134229 0.5466936 0.2778779 0.4613179 0.045298142 0.5693308
0.1120701 0.2516536 0.07701274 0.418986838 0.2798966 0.2702891
0.2824291 0.03759576 0.04754822 0 0.1028886 0.412167 0.06185912
0.4621599 0.1807165 0.3875376 0.1094978 0.846792029 0.223597599
0.2369201 0.299475614 0.053601694 0.1532548 0.061732251 0.3531843
0.7090829 0.1159474 0.503509263 0.07277623 0.1219706 0.2489648
0.136096398 0.5137586 0.4437134 0.04866979 1.45559 0.1460678 0
0.101661 0.1162213 0.6495735 0.1970287 0.2839034 0.262204714
0.2628687 0.150708516 0.07181078 0.2041128 0.1097011 0 0.1254988
0.2662663 0 0.4586435 0.05681334 0.415680306 0.3905593 0.06331746
0.02923279 0.2150334 0.556739 0.1811246 0.2916971 0.05530693
0.3490773 0.4249987 0 0.1059464 0.7158906 0.257991 0.130629421
0.2095125 0.2050493 0.1846647 0.1346489 0.1830371 0.2289122
0.05099364 0.1566377 0.5778229 0.1748039 0.152439839 0.063227208
0.08417006 0 0.1129077 0.259061 0 0.2967648 0.3230793 0.05229562
0.5690775 1.035094 0.2550046 0.2175709 0.07438769 0.3002357
0.2465798 0.6736158 0.3865614 0.09593103 0.187649316 0.3482448
0.05287707 0.5620271 0.05873582 0.1900573 0.2083437 1.678117
0.07815459 0.1784242 0 0.2873905 0.155446442 0 0.2510008
1.179241 0.5543987 0.3117886 0.137613551 0.64728 0.2970659 0.07997217

0.3882205 0.07500218 0.07539172 0.521640723 0.849243 0.1603464
0.3822707 0.4629167 0.039016963 0.3932105 0.3023991 0.8494017
0.331318365 0.261725559 0.7126148 0.1040815 0.1051745 0.05482817
0.168946 0.2893285 0.05674717 0.2853511 0.8370067 0.1206425
0.03323886 0.1357534 0.086345201 0.1100182 1.068805 0.3147643
0.1148573 0.1457258 0.2885309 0.1514062 0.03949602 0.3131357 0
0 0.09371965 0.2521649 0.1928214 0 0.3704997 0.2440395 0
0.05378427 0.02926101 0.9470778 0.1756903 1.076716 0.3174769
0.3430455 0.5219881 0.1315774 0.3097736 0.1751458 0.1627103
0.08972432 0 0.1162754 0.1796383 0.346182 0.7334705 0.211666
0.1559577 0.04435632 0.3410544 0.04994415 0.9545614 0.1565674
0.1088407 0.4828484 0.1571178 0.1408703 0 0.3526502 0.3984047
0.2617152 0.1540576 0.495465 0.358627328 0.757230216 0.3284111 0
0.1320552 0 2.679255 0.3130621 0.6360773 0.7373842 0.6644163
0.1399124 0.1706899 0.6166032 0.0931009 0.384877068 0.5316496
0.4431614 0.3514716 0.4707678 0.1905758 0.2419029 0.1476539
0.1059166 0.2913106 0.2391619
SDCBP2-AS1 0.3393306 0.3450219 0.4221067 0.249558679 0.273573849
0.6964239 0.7695559 0.5953678 0.3740148 0.5008603 0.215001
0.640303672 1.260049 0.601532371 0.415216372 0.971606 0.7397665 0.5259387
0.3362871 0.6294 0.278849678 1.050148 1.393345 0.7546496 1.293084
0.9963244 1.043608 1.13027 1.02927 0.708677977 0.6954872 0.949652915
0.462398 0.9079017 0.4614056 0.329621764 2.095793 0.4605252 2.814647
0.5160316 0.4048404 0.3928998 2.394612 1.144742 0.5468615 1.140772
0.4397254 0.4633769 0.9071436 0.6074652 0.3012283 0.4112872
0.6446809 0.39703797 0.6102091 2.083748 0.899672 0.3725284 1.065691
0.9349398 0.8868537 0.3890248 0.2379795 0.5050264 0.5968935
0.559776109 0.7703258 1.311631 0.9152362 0.400357326 0.6619361
0.6318284 1.273561 1.256055011 0.5652549 0.7977994 1.217064 0.642956
1.36561 0.6603019 1.069891 0.280021 0.1913644 0.858328 0.092124689
0.1765811 1.062868 0.7745405 0.3211153 1.9062 0.9304349 0.694376778
0.3728295 0.4724011 0.5551418 0.374419251 1.406039 0.3669627
0.388199 0.4539691 0.2813533 0.7636732 0.7518746 0.4779628
0.4276541 0.6110642 0.75940592 10.42228 1.474600365 0.5372599
0.9235787 0.2161616 0.3596113 0.8547609 0.2333072 0.3099038
0.7139848 0.7547 0.7175077 1.016347 1.13261 0.4879631 1.456086
1.415798 1.807862 0.5941147 0.9783531 0.9179314 0.554145722 0.2973483
0.7862198 1.514048 0.2212965 0.535723311 0.6065025 0.7821663
0.8199794 0.2438416 1.317341 1.194194 0.7449688 0.3715058 0.492991
1.00768 0.4352238 0.9380853 13.1735 0.2077342 0.6572829 0.3763932
0.4906024 0.551016 0.4269223 0.247555246 1.473818 0.4939722 2.320158048
0.8945572 0.6733057 0.75614 1.01732 2.72048 0.6011005 1.168139647
0.503157 0.1790277 0.7672026 1.079838 0.415694 0.5605947 0.4442554
0.7788395 0.6464165 0.921114626 0.5037216 0.3336713 0.9357239

0.4699820.9038526 2.3508770.4396976 0.7700602 1.3597920.7176636
0.3772723 1.0372580.8682601 0.2566386 0.3762197 0.4645137
1.1495660.9190380.359943686 1.3872121.0716850.3238534 0.3515293
0.8463703 0.5279764 0.234550161 1.3242130.7452745 0.2992982
0.4769552 0.467927144 0.2368108 0.7615120.6939209 1.3626750.7723949
1.2225970.2507054 0.4882083 1.0299890.6256280.7277943 0.4918233
0.4446832 0.636199985 0.601586639 1.7639590.334456255 0.397270713
0.4460427 0.250701701 0.5481158 1.0942730.6474538 0.37062153
0.6945485 0.3880136 0.3117479 0.649425986 0.3042714 0.61267
0.8795584 1.8016941.1196610.2084498 0.5676783 0.9439760.3379927
0.4280835 0.8791352 0.409964631 0.6761085 1.14758333 1.403478
1.1432260.3452692 0.6591242 0.2994281 0.7163862 0.8056648
0.4708247 1.4016570.468923625 0.5022668 0.7135619 0.6054601
2.5455971.0783140.3769784 1.1905380.9714284 0.7478065 0.7150439
0.7872571 0.8013604 0.3815849 0.4348083 0.786909742 0.7338617
0.7702740.6280785 0.5126478 0.5203340.6972288 0.3106365 0.3776984
2.1412770.7808883 0.283742957 0.661190194 0.5298275 1.58948 0.5788953
0.6891099 0.4969462 0.9641566 0.8829082 0.6212071.1450390.2311999
0.7896472 0.8504458 0.6797181.3920240.9318476 0.1709769 0.7417635
0.3116695 0.614415097 0.6717744 0.3113727 0.3281030.1490830.2765775
0.3543074 2.1408071.4600161.8613190.5590709 1.05406 1.144205329
0.5178016 0.4671988 0.7363140.4409141 0.1952072 0.576328808
0.4304460.3770057 1.62794 0.7423195 1.39351 1.1175360.86182087
0.6806985 2.2031761.1255220.5892944 0.491202224 0.7185927 0.3377212
0.7031708 0.847678607 0.807801941 0.5895205 0.7749256 0.3203441
0.4341937 0.3945127 0.2447908 0.8296441 0.6856491 0.6172194
0.3368356 0.7593008 0.9004728 0.951158994 1.17284 11.384050.4729687
0.6297054 0.7594881 1.2059330.7634732 1.0305560.3735559 0.8473748
0.5848081 0.2236061 0.8704599 0.8907415 1.8535790.3322750.4542414
0.5150154 1.0102110.7040814 0.5897492 0.4905302 0.5080008
0.1826523 1.43378 0.7551976 0.4274808 0.3459571 0.5601387
0.4129906 0.7606447 0.4681495 0.8066880.5334699 0.4959648
0.5585076 0.8237832 1.3458940.3737823 0.3047134 1.1510540.7220135
0.5841760.7182738 0.9743233 0.6433906 0.4147654 0.951262949
0.3687793 0.7735903 0.6961701 0.2424375 0.8938543 0.263977287
0.562184656 0.3556573 0.241875891 0.3284783 1.4428861.2694220.9090373
0.5740405 1.3208331.4165920.1846651 0.2698494 0.3666712 0.5718662
0.542718608 0.5532667 1.3891660.3449468 0.5257565 0.6385089
0.9905819 0.8582338 1.0430881.2954351.112906
ANKRD44-IT1 0.06925392 0 0.08011735 0.118417811 0.029253684 0.04298658
0.02264565 0.04346267 0 0 0.05265543 0.094393719 0.04220503
0.099280854 0.0762673 0.02235324 0.1088452 0 0.0769018 0
0.0294037 0.02401469 0.08718523 0 0.01880705 0.07169892 0.1027084
0.1007856 0 0.057646971 0.05245269 0.029189878 0 0 0 0 0 0

0.03573445 0 0 0.01818873 0 0.07973433 0.02696007 0 0.0347756
0 0 0 0 0 0 0.036760594 0 0.0183528 0.04139659 0.01428429
0 0.06738794 0 0.02022967 0 0.02396395 0 0.025297023 0 0 0
0.020818971 0 0 0.03404598 0.023258898 0.02569758 0.09538592 0 0
0.01948854 0 0.04230607 0 0 0 0 0.01314342 0 0.1217839 0
0.01320451 0 0.120498451 0 0 0.0590848 0.033841002 0 0
0.02889473 0.02051546 0.01424049 0 0.07577441 0 0 0.01812221 0
3.1102211 0 0.03043995 0.02486504 0 0 0.08319828 0.0268379 0
0.06775843 0 0 0.02366948 0 0 0 0 0.0125462 0.04255286 0
0 0 0 0.1290615 0.06213441 0 0.035677961 0 0.0241395 0
0.0174649 0 0.03148087 0.02900466 0 0.05309018 0 0.04182634 0
25.33632 0.04043968 0 0.02915949 0 0.02091696 0.06752613 0 0 0
0.087899051 0 0 0.0211056 0.01990628 0.07089568 0.08451187 0 0
0.04686909 0 0 0.02049356 0.07532947 0.02310171 0 0.1101083
0.025522749 0.02771251 0 0.03075389 0 0.04133842 0 0 0.07560009
0.0176173 0 0.01790191 0.02678574 0.04044144 0 0.01660648 0
0.02237864 0 0 0 0 0.02364174 0.05267487 0.08567685 0.01616319
0.034916435 0.03989528 0.06478889 0 0.02968124 0.040370107 0.03595803
0.02604283 0 0.01448967 0 0.01389541 0.01982699 0 0.02384093 0
0 0.01493597 0 0 0 0.3652426 0.038473325 0 0.01968849 0 0
0.0546571 0 0 0.02804846 0.03133887 0 0 0.03300102 0
0.1875767 0.03580815 0.05629554 0.0202895 0.03918086 0 0.03129377
0.01518725 0.01367729 0.060633307 0 0 0.0968673 0.03933324 0 0
0.0483681 0.03420696 0 0.1571239 0 0.071202697 0.01672492
0.02440299 0 0 0.0495164 0 0.02248442 0.02131567 0.05381472
0.04679927 0 0 0 0 0.100690966 0.05383171 0 0 0.0259473 0
0 0.07861321 0 0 0.02245687 0.039167579 0.048736407 0 0
0.0217577 0.07987512 0 0.01906254 0.01037642 0.02015509 0.6779180 0
0.02457014 0 0.01433477 0.07714195 0 0 0 0.01848624 0.018080336
0.07669484 0 0 0 0 0.08029709 0.5905182 0.03012132 0.01719148
0.02467759 0 0.029955067 0 0.06449163 0.04544878 0.03561152
0.06008275 0.013259304 0.01247331 0.02862281 0.01541091 0 0 0
0.018276722 0 0 0 0.09606763 0 0 0 0 0.076615435 0
0.0305163 0 0.06080245 0 0.02170432 0 0.02187075 0.01832938 0
0 0.03843147 0 0.033278038 0 5.879292 0.02426247 0.02951119 0
0.03706727 0.038902 0.01522204 0.1206847 0.02513043 0.01694636
0.0180601 0.04165119 0 0.07485443 0 0 0 0 0.02255479
0.02433402 0 0 0 0 0.05029445 0.05071086 0 0 0.01567742
0.01729019 0.02434423 0.01493778 0.01730846 0.01482455 0.08480538 0
0 0 0 0.01924882 0.03678946 0 0 0.06978492 0 0.03619492 0
0.1087310 0 0.0605201 0.08906227 0.1028223 0.017277182 0.01824011 0
0.024162581 0 0 1.032603 0 0.01361937 0.0202995 0.01707137 0
0.0281936 0.03394902 0.03588173 0.032963175 0.1536761 0.06404905 0
0 0.1028289 0.03107702 0.02845342 0.1428735 0 0.0768122

MCM3AP-AS1 0.2881473 0.176098 0.2432533 0.099872694 0.127473745
0.3021213 0.2291901 0.2260459 0.1720456 0.1133306 0.2516521
0.301858204 0.4231899 0.262362549 0.164381688 0.2765042 0.4130965
0.2357367 0.1059354 0.2754688 0.239722316 0.5299747 0.7087607
0.6379272 0.61332 0.2670773 0.6022397 1.264908 0.4021036 0.194476093
0.435009 0.447236531 0.1667738 0.2486346 0.630283 0.250053316 0.6413044
0.362751 0.9694444 0.5574211 0.2482634 0.2658974 0.7527144
0.6388497 0.2880136 0.7949982 0.3641744 0.5068752 0.7669081
0.2764734 0.245188 0.3547683 0.201535 0.335872437 0.2549551 1.049966
0.8350164 0.1867325 0.3951272 0.2083931 0.8525505 0.2303309
0.2666685 0.3132708 0.6895683 0.469377026 0.09460513 0.8539433
0.7625375 0.301422015 0.3771529 0.2752174 1.09353 0.457715542
0.7224381 0.3754229 0.3350893 0.5362691 0.7533392 0.4821422
0.6809053 0.4482545 0.2258826 0.3158055 0.166378875 0.4138424
0.256927 0.8608211 0.1352731 1.167485 0.7410822 0.44673751 0.2475704
0.3238418 0.9163491 0.36152247 0.6999822 0.1338035 0.7676423
0.5479153 0.444382 0.8756253 0.6035715 0.4732394 0.2592114
0.450882 0.340896171 4.931225 0.216969535 0.1155277 0.828354 0.2468341
0.515697 0.3742336 0.2791635 0.2725436 0.1365177 0.6810852
0.2208805 0.4524871 0.4898951 0.2248696 0.7107096 0.5925215
1.372051 0.3229989 0.1421276 0.8110404 0.372650559 0.2810073
0.5646566 0.600459 0.8185297 0.381146648 0.3878393 0.4037883
0.5270344 0.6603847 0.2518826 0.615092 0.6727137 0.3469097
0.3526099 0.2561789 0.4409502 0.3062243 3.427857 0.1108462
0.3217383 0.2828183 0.3417176 0.1822925 0.6169696 1.136708519
0.2518324 0.4719952 0.973618663 0.3746504 0.3648871 0.2343706
0.3385743 0.5580669 0.3920217 0.538075355 0.4295724 0.3788199
0.7198203 0.8114619 0.2707845 0.4526677 0.5195683 0.1968565
0.2233171 0.480740526 0.3739604 0.6953864 0.6570859 0.2824582
0.5229677 0.3042528 0.876606 0.3400562 0.4729892 0.309862 0.2440897
0.4292269 0.3297107 0.541541 0.2100867 0.634679 0.2327791 1.785715
0.178538948 0.8000583 0.5370391 0.4120782 0.5257028 0.2197884
0.5543646 0.238040097 0.6028489 0.3612474 0.2220281 0.3713219
0.18158851 0.4532162 0.8419657 0.3947545 1.246489 0.862933 0.5996367
0.1923023 0.2500859 1.13606 0.463193 1.073669 0.4198961 0.4745627
0.311949053 0.405798035 0.8385611 0.351521234 0.188751265 0.3570101
0.551815708 0.429142 0.6146318 0.7453971 0.269365869 0.9107507
0.3590207 0.7170928 0.497677952 0.2876057 0.4663395 0.4113221
0.3875709 0.3917037 0.5618443 0.4681353 0.8374047 0.1275658
0.3629163 0.4191169 0.539786589 0.5459004 0.195950797 0.7547261
0.5952776 0.3625257 0.2238131 0.3263463 0.6034435 0.5329336
0.2153403 0.7017512 0.252718079 0.5736305 0.936449 0.4925254
1.092715 0.3271338 0.2722942 0.6605511 0.7250916 0.2515193
0.9867538 0.2915463 0.1521004 0.1308936 0.1425617 0.702965494

0.4540126 0.3887983 0.3901634 0.5087974 1.447738 0.3317345
0.3011206 0.3394333 0.6166787 0.464029 0.613875199 0.363084633
0.5722678 0.509894 0.2660791 0.5726117 0.2526901 0.1688106
0.2523318 0.1954845 1.810545 0.2803805 0.3626397 0.4282565
0.5138179 0.5313311 0.5061361 0.1490133 0.2827159 0.2754438
0.411718144 0.805853 0.3093778 0.2226492 0.1113702 0.2848651
0.3329663 0.8774966 0.6308684 0.659712 0.5758235 1.373995 0.400011423
0.2198492 0.4441993 0.1309648 0.2252586 0.2336604 0.320573527
0.3138436 0.1850754 1.655008 0.3084664 0.1889404 0.4942049
0.249200285 0.4019615 0.9121086 0.5306802 0.4109009 0.34031206
0.3919593 0.3112663 0.3433519 0.31949459 0.303429573 0.3088466
0.6108485 0.1310497 0.5613886 0.3447491 0.1462938 0.6640426
0.5797075 0.3197998 0.3071822 0.4051601 0.7305394 0.301714317
0.2980111 10.20135 0.3853825 0.4397148 0.1710513 0.3725419
0.6835344 0.250344 0.572538 0.6499744 0.4097164 0.2995578 0.944561
0.4805194 0.5155753 0.2023904 0.4054382 0.1722145 1.643359
0.5421425 0.4480884 0.6115317 0.2744986 0.3841172 0.3623967
0.2662906 0.4348198 0.12083 0.3842845 0.5509257 0.6780823
0.7528305 0.3695534 0.2457301 0.6855764 0.2821233 0.7033083
1.163144 0.4109127 0.09238292 1.355565 0.3594071 0.3795703 0.7075721
0.6801147 0.9476514 0.5342142 0.262116358 0.3419756 0.2886797
0.7769757 0.171094 0.2518996 0.167571625 0.36664181 0.1423331
0.305678096 0.1681207 0.3974784 0.3386794 0.6122597 0.1856979
0.231126 0.3887425 0.2122327 0.265524 0.1789522 0.5875946 0.333610734
0.1440103 1.011346 0.2602261 0.1700253 0.7970456 0.4739664
0.8339097 1.454588 0.1578172 0.4470016
AP000766.1 0.5936901 0.7278396 0.5171827 1.016684125 0.366352928
0.7825271 1.157783 0.8360842 0.4803813 0.6804799 0.3670999
0.664181795 0.9335842 0.369152115 0.912450432 0.5598732 0.989303
0.1787141 0.5520249 0.5753426 0.47072909 0.8247189 0.3864615
0.1012224 0.8449816 0.5368933 0.6668038 0.9851983 0.6065312
0.243124476 0.3747158 0.493686157 0.2559963 0.8004563 0.2954809
0.630615299 0.3991734 0.4084129 0.5259438 0.320758 0.2013723
0.371028 0.3755491 0.7720652 0.6126056 0.3518401 0.09877447
0.3065842 0.3476202 0.5341386 0.3403019 0.2182417 0.2680838
0.327475626 0.3724161 0.4454587 0.2886062 0.3430196 0.5491741
0.3480082 0.5312316 0.5902617 1.842801 0.2598873 0.8974875
0.339664468 0.1737855 0.4649369 0.9023546 0.209653071 0.5168608
0.0631953 0.5186749 0.33031568 0.1924277 0.2031962 0.2669175
0.6791704 0.6491521 0.2180121 0.5188881 0.424993 0.2330466
0.3189147 0.159761534 0.3427734 0.1643133 0.3009844 0.3147551
0.4125579 0.2446845 0.167238898 0.276114 0.1221923 0.595001 0.550504826
0.5683257 0.1498726 0.4401979 1.403798 0.1434059 1.25649 0.3587082
0.520551 0.3703467 0.3509537 0.307198067 5.987786 0.163267018 0.1964995

0.3595461 0.3940971 0.4112366 1.117108 0.235616 0.4500231 0.04665621
0.2581684 0.365173 0.580617 0.4709841 0.5109094 0.5753239 0.4387758
0.7191883 0.8954962 0.1506244 0.8179547 0.394356221 0.165043
0.3707439 0.1644498 0.3795983 0.239524939 0.330272 0.355288 0.210068
0.1488187 0.3981349 0.333279 0.4006812 0.1495606 0.8396487
0.3154501 0.2592021 0.1514479 2.314287 0.2192827 0.2662249
0.4818783 0.4001258 0.1944368 0.2789775 0.427833751 0.3408669
0.5179687 0.236045046 0.2294055 0.5568494 0.3215341 0.5705458
0.5003682 0.4037075 0.503996863 0.3020795 0.2420437 0.3196029
1.003513 0.8572545 0.4133341 0.4990844 0.2611677 0.3777309
0.237250597 0.4293431 0.3702593 0.4526393 1.052274 0.3486872
0.2382194 0.374155 0.2147298 0.4776458 0.2631221 0.2865948
0.6708922 0.3498233 0.6346057 0.351616 0.2690487 0.05778447 1.31087
0.327968285 0.7753389 0.5389309 0.3296481 0.3853705 0.5641324
0.3422302 0.099174482 0.2935917 0.217481 0.6046285 0.3985316
0.500355296 0.5261395 0.5715897 0.3710054 0.3030545 0.5299674
0.4843758 0.3020551 0.3636892 0.855688 0.5135854 0.4795826
0.636348 0.4685661 0.303372449 0.415365845 0.1336061 0.307963168
0.45874764 0.218604 0.35017396 0.5975128 0.9596941 0.6269374
0.162849977 0.8763383 1.262366 0.6070171 0.379228997 0.613532 0.2119535
0.5860595 1.263636 0.2471157 0.4086422 0.2023398 0.7016698
0.1050458 0.831366 0.6286335 0.511438529 0.3487984 0.314958957
1.021933 0.3994826 0.192869 0.2414601 0.4621023 0.9185972 0.5351735
0.2992138 0.4862343 0.464232123 0.414584 1.134208 0.6661969 1.001495
0.2557151 0.4956864 1.027619 0.3687663 0.4689778 0.5558713
0.1957954 0.2003258 0.1692027 0.1181026 0.79299 0.2710505
0.4183204 0.5283463 0.1875977 0.279301 0.2335015 0.431352 0.1792632
0.7877931 0.5856633 0.581529757 0.245394875 0.7161774 0.8995579
0.4943938 0.5362438 0.1203432 0.6448064 0.5171089 0.07806447
0.5714768 0.2987272 0.4123807 0.3356057 0.6440465 0.6573277
0.2699274 0.418976 0.700143 0.2911762 0.536885543 0.8168973 0.2473217
0.6152432 0.09352332 0.1008742 0.4405915 1.234356 0.8088811
0.2485871 0.7455315 0.6792551 0.317125761 0.523918 0.4079874
0.1936349 0.2574695 0.2327118 0.81484507 0.3317388 0.6023477
0.3740534 0.5537613 0.3097554 0.3676348 0.217086846 0.5248766
0.6436063 0.1854567 0.581166 0.213556616 0.6026189 0.165516 0.2535895
0.329717793 0.171904381 0.2022454 0.3590727 0.130833 0.4692444
0.8574623 0.2207469 0.1750664 0.3455 0.2893451 0.6183072
0.1190818 0.6034358 0.184745361 0.16423 1.82753 0.4197466 0.4381592
0.2175327 0.5359892 0.5574966 0.7507296 0.4440627 0.3374276
0.5994795 0.3264341 1.355112 0.3454017 0.2963681 0.5192664
0.6921526 0.06730861 0.5405969 0.3843794 0.5906349 0.3890225
0.1071514 0.4001948 0.3698372 0.1991288 1.414171 0.1603041
0.2353046 0.6436488 0.5000289 0.3080133 0.5939969 0.2324016

0.2526404	0.5693432	0.4915015	0.4759598	0.2118818	0.09503391
0.3031875	0.1947397	0.241507	0.3303606	0.402432	0.3231415	0.3458014
0.481496353	0.6457416	0.17346	0.5365287	0.05365966	0.4854857
0.218598083	0.188393025	0.212436	0.124781638	0.3154013	0.3679499	0
0.4797866	0.5943208	0.2096634	0.2292183	0.3132821	0.3615705
0.3024292	0.365972	0.276623937	0.1984055	0.5181991	0.1165912
0.2889044	0.6372414	0.5376399	0.6391916	0.6271593	0.3072975
0.4561791
AP003071.4	1.637478	6.708451	1.107254	4.387223114	0.426217517	5.636716	5.071655
3.401394	5.93063	2.184052	1.139801	6.581738053	2.307395	4.446931733	2.201218417
2.214625	1.051187	1.817334	2.407338	0.07822612	0.4406433	0.07997415
0.1330752	0.2259495	0.1096053	1.074479	0.1465892	0.09589658
0.1355393	0.047994246	3.813823	0.413136807	0.2976884	0.3036365
1.088821	0.414189176	0.5493525	0.5082561	0.1487544	1.19333	0.2673524
0	0.7155216	2.60554	0.04489145	0.01554024	0.3763836	0.01830591
0.1769734	0.53643	0.1774939	0.1563726	0.9878656	0.030605199
0.1091619	0.1222376	0.05169738	0.08324714	0.6934215	2.188061
0.3559168	0.6736921	0.2820446	2.593669	0.07325995	0.126366903
1.178639	0.01303561	0.06766189	0.883979339	0.01772887	0.08150422
0.1133805	0.116185804	1.005548	0.4566304	0.7728032	0.09864182
0.7463627	0.02196676	0.7572754	0.3140282	1.282898	0.08813797
0.156775337	0.1860245	0.1803558	0.1593298	0.4985299	0.3847715
0.08606575	0.401286119	0	0.08294408	0.06558842	0.225395834	0.2519621
0.3092699	0.168395	0.2391234	0.09484789	0.3016926	0.1682302
0.1678412	0.05248829	0.2414037	0.660332293	0.03807971	0.603836648
0.3801438	0.0621045	1.844546	0.1183884	0	0.08937605	0.3083394
0.07521678	0.1600793	0.843096	0.3744166	0.3525306	0.01752473
0.3745706	0.02193402	0.1566809	1.417103	0.1295088	2.354759	3.310756424
0.03396688	1.262546	0.4138422	0.4450685	1.009930817	0.5628735
1.688186	0.9129122	0.3053502	0.2255159	0.4193527	0.01207398
0.628993	0.1768019	0.06669544	0.295993	0.3255421	0.07910199	0.08417059
0.1888455	0.9953516	0.1183601	0.1567306	0.2810959	0.205890144
0.3401843	0.7442775	0.043908451	0.9369179	0.09974717	0.1230009
2.419668	0.1377239	0.1055411	0.678413344	0	0.01951054	0.1010289
0.03542657	0.2047441	1.254318	0.3590241	0.161939	1.479835	0.297487119
0.4845157	0.1193827	0.1536257	0.03127048	0.2409154	0.5465259
0.09162437	0.2727452	1.276061	0.3809073	1.713996	4.80206	0.4377061
0.03686772	3.442623	0	0.01863144	0.1602516	0.416578238	1.082057
0.1178084	1.495911	0.24851	0.6241432	0.1211107	0.116279342	0
0.3236417	0.1499618	0.2718238	4.940716285	1.10767	1.387653	0.8337377
0.03619031	0.01525691	0.03470605	0.1485635	0.1498867	0.158791
2.001973	0.05798691	0.1367851	0.08783706	0.027171208	0	0.1013615
0.416404826	0.825566281	0.8523704	0	0.09713748	2.593786	0.04960573
0.141366892	0.1634631	0.02609132	0.0798859	1.637608527	1.483658

0.1423753 1.0307080.02981224 0.02343455 0.06756845 0.2283414
0.07458439 5.4973450.01264421 1.2298060.538459027 0.2530418 0.644775791
0.9677677 1.3972080.2112003 0.2524994 0 0.1708749 1.3257353.2213
0.05468947 0.785461679 0.7519179 0.2844354 0.1406999 0.1793955
0.9344356 0.01452947 1.0295730.2484504 3.5842950.3506665 0.05570319
0.8328843 0.8614104 1.1092810.027943573 0.2801115 0.2302812
0.1481345 0.2916343 0.1174630.03672579 0.2454367 0.1382169
0.3708147 0.09348286 0.114132008 0 0.6616920.08970444 8.115283
0.3823773 0.2032498 1.9362130.4751412 0.4362856 0.2158014
0.0664266 0.2863837 0 0.3938376 1.57351 0.02637354 0.6124098
0.1860553 0.2154712 0.722537554 0.3352263 0.05090036 2.186609
0.1319269 0.1422962 0.8356463 0.07023408 0.1504658 0.1145027
0.02054544 0.03458087 0.249392267 3.3081510.0268464 0.1324351
0.4892007 0.1500665 1.114948421 1.0073170.2144705 0.6671821
0.4359922 0.1082975 0 0.33476009 1.7067010.9432632 0.07666254
0.2742224 0.13771408 0.1514044 0.3153523 1.9707420 0.050388285
0.1355012 0.0779260.2362330.2990783 0.1264902 0.1856750.1456687
0.2899441.5124860.3483974 0.01066543 0.07259915 0.180087578 0.1059053
0.4676591.0907910.5528177 0.5455258 2.0985160.3724624 0.2027708
0.1255957 0.8159757 0.3103929 0.1052521 0 0.4330972 0.3739224
0.04953462 1.1745830 0.08628941 0.09389047 2.02594 0.2677772
0.8129135 0.03395652 0.2568388 0.4885169 0.1055489 0.09939775
0.3652964 0.16968 0.1295552 0 0.2736034 0.1296921 0.2221604
3.64793 0.1131963 0 0.1423271 0.9411402 0.1442312 0 0.4270241
0.2444678 0 0.3529032 0.7382890.344997387 0.3960448 0.7670221
0.1175680.3954624 2.0789820.417141678 1.700819271 0.7552092 0.080466664
0.2966099 0.0185372 0 0.1406341 1.7348460.3380088 0.5969397
0.9876682 0.03912118 1.0175190.1792410.46654188 0.1705916 0.159973
0.3195362 1.1329230.4280535 0.1681765 0.02368902 0.0679714
2.4863950.6906638
AC007622.2 0.1382169 0.07103134 0.08994271 0.073855683 0.045612876
0.06702546 0.02824761 0.05421422 0.03053459 0.02251552 0.09852157
0.147180405 0.1140652 0.049536165 0.126845187 0.1254729 0.1221937 0
0.01918507 0.2511477 0.14670971 0.1797319 0.3534461 0.1974751
0.2815132 0.2683062 0.04575567 0.2963339 0.1305461 0.071907365
0.1526657 0.172950881 0.08109301 0.1137308 0.1747849 0.037609625
0.2116459 0.06922678 0.2451583 0.02979845 0.05722296 0.147473
0.2061596 0.2237818 0.1513319 0.3143227 0.09760095 0.1097073
0.1325752 0.08460065 0.06043869 0.1098212 0.1233391 0.057317788
0.02725863 0.1831425 0.1290926 0.1425429 0.1020366 0.02801933
0.3332826 0.1261699 0.1690293 0.07473006 0.05488085 0.094664588
0.04342365 0.5175610.1900770.064922637 0.1726548 0 0.08493618
0.2756193 0.1682861 0.04461824 0.1315741 0.2512431 0.0850833 0
0.2374718 0.4063345 0.1372930.1760702 0.100666544 0.08197385

0.06755452 0.3797753 0.0400137 0.2141225 0.064474 0.225460054 0
0.09320321 0.1351183 0.168849622 0.2573879 0.02896022 0.1441702
0.1279523 0.09769776 0.06457297 0.1102722 0.3394819 0.147451
0.05651296 0.095129102 6.161715 0.092789547 0 0.4187162 0.08188414
0.0532126 0.181614 0.2343384 0.06793669 0.09860683 0.08394355
0.228677 0.1623859 0 0.2231796 0.2806001 0.2711168 0.3208212
0.1061587 0 0.08467242 0.089861229 0.008481812 0.1006175 0.07750488
0.09526053 0.02225189 0.1253591 0.090333 0.3088514 0.07624847
0.05198141 0.07853688 0.1356738 0.2670101 0.2069479 0.08743562
0.09130294 0.1132262 2.548062 0.2396063 0.1414689 0.1273048
0.1329998 0.01304564 0.07019199 0.123389939 0.05880935 0.1223905
0.131571648 0.1477618 0.2739846 0.03948988 0.08690707 0.1326502
0.1317724 0.165465771 0.2978138 0.1461583 0.06054664 0.115002
0.1150342 0.1761826 0.3361924 0.0242625 0.2060195 0.031836428
0.3802467 0.05962169 0.1438561 0.05856374 0.3007929 0.0663918
0.4232682 0.07858469 0.06592611 0.1426736 0.04466076 0.1837652
0.02522281 0.1380926 0.1035725 0.06665241 0.02791455 0.5702303
0.108023923 0.195661 0.07722157 0.1032154 0.1423615 0.1469478
0.5746043 0.065330809 0.1119698 0.161632 0.2160386 0.09255907
0.025178322 0.08223074 0.09745547 0.3394422 0.1265183 0.2971625
0.3813211 0.1483901 0.1387036 0.2973857 0.03703032 0.4488742
0.1863075 0.1710824 0.203546275 0.098536009 0.3606788 0.059988309
0.051537685 0.03683838 0.133549118 0.4366086 0.1193113 0.1579338
0.121030231 0.262402 0.1856837 0.08976677 0.114498837 0.1029116
0.08532551 0.1169892 0.163776 0.1228878 0.5188264 0.0244366
0.6052911 0.01951752 0.1420814 0.1023642 0.189081113 0.06318664
0.120754393 0.1898754 0.04906329 0.04394871 0.07881398 0.2714991
0.1813428 0.04729242 0.0612476 0.1365641 0.099918487 0.1251734
0.1521983 0.2037767 0.08270129 0.04117699 0.04353754 0.04206974
0.5051839 0.151036 0.554574 0.08345729 0.1400669 0.04302026 0.1116255
0.251198699 0.08393539 0.2217978 0.1331655 0.032366 0.1026602
0.1650732 0.1838627 0.08471598 0.1527826 0.1120485 0.244283212
0.106386971 0.1315096 0 0.06785 0.2864483 0.04152531 0.05944531
0.09060294 0.03771143 0.4974221 0.1244046 0.1532408 0.1307456
0.1698678 0.5051808 1.066882 0.09445293 0.3902601 0.1037667
0.112764758 0.5381279 0.2796253 0.1266528 0.02823706 0.03045647
0.05008027 0.9470538 0.1690766 0.1608316 0.09234661 0.3454051
0.140119459 0.02636402 0.1407791 0.1559021 0.05552612 0.1249095
0.330786366 0.1789272 0.1160359 0.3075709 0.01555298 0.09915691
0.07248865 0.079792715 0.08595176 0.3597348 0.05742981 0.1027134
0.0937864 0.1134208 0.01817217 0 0.127424192 0.150988467 0.1712936
0.2752026 0.05056228 0.2504055 0.1895139 0.09272917 0.2591697
0.05715902 0.1376591 0.1304966 0.08788713 0.1414032 0.13490808
0.05289088 4.671133 0.09079317 0.1932604 0.1634668 0.1271511

0.3032835 0 0.1693563 0.2507765 0.06341538 0.236541 0.1818412
0.09269825 0.09337152 0.1261659 0.1075444 0.0812887 0.4912757
0.309477 0.2124755 0.1900408 0 0.03815651 0.082459 0.1463841
0.1423247 0.02978453 0.0631506 0.352001 0.3019428 0.2884806
0.2701786 0.0647704 0.1017047 0.0881534 0.2543944 0.3498887
0.04264832 0.1311687 0.3961731 0.1147256 0.1411299 0.5101672
0.2321278 0.3524915 0.1241587 0.23998568 0.1441051 0.1316781
0.2893831 0.1851567 0.07329021 0.15085782 0.079632874 0.01315688
0.316467783 0.1110989 0.1527534 0.6440216 0.2709065 0.05945958
0.0633027 0.2022968 0.01681561 0.08205862 0.09528108 0.2909267
0.020558713 0.0159743 0.479359 0.1548881 0 0.1740756 0.0678382
0.1774604 0.3309739 0.05601866 0.1437204
AC004884.2 0.1355906 0.05574529 0.03137196 0.057961831 0.200462744
0.04208119 0.1330121 0.04254724 0.095854 0.235602 0 0.046202786
0.02754407 0.116627729 0 0.2188243 0 0.05211866 0.1505641
0.1576803 0.172706348 0.1410533 0.1137985 0.2951965 0.07364372
0.2456607 0.2872719 0.1409469 0.3642751 0.11286559 0.2053917
0.257175659 0.06364168 0.1785116 0.3200656 0.029515979 0.1107329
0.05432907 0.0699636 0.09354312 0.179634 0.07122252 0.06471751
0.2341648 0.05278445 0.4750874 0.03404315 0.9040304 0.1664717
0.3319723 0.07589151 0.2757999 0.09679636 0.107958998 0.2139253 0
0.04052469 0.195768 0.08735805 0.1319372 0.3661836 0.07921435
0.08843598 0.1407553 0.3445633 1.139153658 0 0.09196554 0.1193377
0.040760952 0.1250762 0.09583463 0.06665779 0.182152103 0.3270323
0.4668844 0.2065183 0.1391825 0.1144684 0 0.124245 0 0.2154947
0.2072695 0 0.1286659 0 0.2725002 0.08374051 0.4394972 0.37106
0.05898024 0.09236522 0.09752765 0.03856022 0 0.05386603 0.04545588
0.1697168 0 0.1951678 0.1689226 0.2967137 0.0789407 0
0.1419241 0.059725744 23.46212 0.254873851 0 0.1947306 0.1285251
0.05568157 0.1357432 0.210181 0.4691862 0.04422086 0.4893852
0.4102066 0.0463419 0.1275428 0.123636 0 0.1289528 0.0736917
0.08331321 0 0.2215026 0.282091844 0 0.09475735 0.1520643
0.1869008 0 0.1431006 0 0.2077595 0.4787175 0.2855645
0.2465425 0.1703626 0.09861135 0.103944 0.8626432 0.1637815
0.3280974 15.37766 0.03958792 0.08074514 0.1141813 0 0.1228584
0.4847618 0.58101746 0.2461519 0.04268973 0.172095395 0.2319265 0
0.04132213 0.03897402 0.2313415 0.579123 0.296816674 0.04674476
0.1835277 0.7602706 0.1110811 0 0.1106143 0.3015352 0.07616471
0.06159384 0.099940728 0.05425764 0.2807461 0.3010614 0.07353718
0.08093548 0.03473613 0.5745821 0.09867704 0.1724624 0.2035818
0.0700994 0.1048863 0.1583586 0.1300499 0 0.1569261 0.1752584
0.3768555 0.113036022 0.1316181 0 0.6017387 0.1718847 0.0838723
0.2531641 0.136724062 0.15622 0.08456572 0.02712749 0.05811218
0.079039642 0.07040134 0.05098861 0.127869 0.1702138 0.1076366

0.1360274 0.1164564 0.08293665 0.09335516 0.3487361 0.4090945
0.1754566 0.3304990 0.063897127 0.028120333 0.4171414 0.112988968
0.161786799 0.2312856 0 0.1142166 0.1070118 0.2041467 0.617398263
0.05491539 0.2454304 0.09393177 0.462129527 0 0.1339267 0.183626
0.1635851 0.2204393 0 0.1534225 0.1753963 0.0612693 0.2378779
0 0.197854107 0.2644735 0.303257185 0.1625607 0.2310288 0.3035198
0 0.2367468 0.2009189 0.1484601 0.03845362 0.2572208 0.243960522
0.0327453 0.5255581 0.198526 0.06490385 0.1292626 0 0.2201085
0.08346686 0.1053625 0.09162714 0.08732954 0 0 0.428284 0.131426914
0.2898384 0.1547259 0.1741801 0.1778055 0.04603862 0.04318307
0.3463085 0.1181955 0.2616077 0.04396776 0.153370485 0.429389172
0.0952694 0 0.2555932 0.1172892 0 0.1119662 0 0.1578446
2.029956 0.07810601 0.1443158 0.02736239 0.1122627 0.07551717
0.2067376 0.04235801 0.1458455 0.03619376 0.070798094 0.03753974
0.03989992 0.2385527 0 0.5258483 0.1572117 2.037046 0.1769214
0.1682939 0.04831565 0.2168588 0.20526902 0.1655236 0.1893999
0.04449153 0.06972291 0.1568461 0.129800328 0.2442119 1.316938
0.3620718 0.292943 0.08489258 0.05688899 0.071567092 0.1349096
0.3226507 0.07211333 0.1880884 0.029441372 0 0.05704598 0.04930311
0.250005799 0.157994037 0.2788198 0.07853766 0.03968121 0.04137218
0.08498872 0.07277371 0.08564041 0.1435466 0.3656559 0.4551712
0.3511388 0.06829107 0.065154255 0.04150869 24.34157 0.1425087
0.2311169 0.3665386 0 0.72357 0.08940857 0 0.09840451 0 0
0.08154784 0 0.2442594 0.0465952 0.09207351 0.255181 0.08116894
0.3532758 0.09528597 0.3314305 0.1433765 0.1597076 0.1725699
0.229764 0.09928554 0.1402491 0 0.06138886 0.06770407 0.04766297
0.1754779 0.169439 0.4063447 0.1106923 0.1064791 0.1961373
0.2342924 0 0.188434 0.2521021 0.1181424 0.2874512 0 0.03951925
0 0.057950895 0.02661023 0.1503138 0.3554725 0.05812428 0.08627708
0.03382657 0.107135594 0.123906 0.33115127 0.2491151 0.08718593
0.673903 0.188984 0 0.1589756 0.1002709 0.1055749 0.07359941
0.1329359 0.03512598 0.096806686 0 0.2090001 0.08840432 0.05920524
0.1438045 0.2433797 0.1949789 0.3596509 0.08792673 0.06015549
AC061992.2 0.1246337 0.05124062 0.2739501 0.026639024 0.105293563
0.5995507 0 0.1173272 0 0.8012847 0.09476205 0.084938456
0.1898871 0.73255565 0.137255461 1.146507 0.1762962 2.323492 0.4567112
0.2053294 0.370417319 0.2593101 0.3661095 0.122104 0.2030781
1.177434 2.23129 0.1036458 1.653266 0.138326851 0.5506502 0.118196894
0.2632451 0.3445815 0.4413026 0.108523396 0.2883902 0.3495719
0.1929299 1.332753 0.350876 0.2455019 1.189756 0.4484217 0.6307475
0.8733929 0.2503375 0.6726961 0.2422808 0.2746316 0.122078 0.24295
0.06673082 0.38040098 0.25563 0.1321154 0.01862498 0.1542414
0.2676627 0.2627638 0.1923389 0.4004725 0.101612 0.7762868
0.9897493 1.434072886 1.169466 0.126801 0.09141189 0.749342755 0.2107766

0.2642713 0.3369922 0.272078205 1.4452180.2360361.0630480.2771935
1.0521850.0949674 0.1713075 0.1851295 0.1980811.0161090.290475516
0.1537493 0.5482408 1.1428151.92434 0.5228006 0.4495994 0.04066062
0.1698027 0.2689399 0.4784973 0.395865664 0.7426983 0.7729795
0.1040016 0.2399860.2178388 1.1645421.2955 0.2902466 0.2978313
0.1304555 0.301946818 0.2880980.251012126 0.6025983 0.4698613 0
0.1791372 0.1622063 1.5938730.3332558 1.8901070.6920599 0.628431
0.6389565 0.2051635 0.2083496 0.5590633 0.3318906 2.878815
0.1531617 0.5598966 0.4275672 0.751959975 0.03671168 0.5806679
1.3138970.3607752 1.284166339 0.7070109 0.4995942 1.46411 0.2514476
3.1873620.1982924 0.2609931 0.5891779 0.01194306 0.4685523 0.11291
0.1633583 0.2279842 0.0363889 0.1855507 0.8396363 0.06396229
0.6964044 0.0405081 0.111263866 0.9333261 0.1569602 1.17059635
0.8172089 0.0718717 0.8925997 0.3403338 0.3615003 0.1140697
0.221675584 0.0214837 0.1897843 0.3494173 0.7530235 0.3134928
0.9320278 0.2840979 0.8926273 0.4529324 0.551188255 0.3740489
0.4731091 0.3320799 0.5407583 0.3099802 1.2133080.2805803
0.8163282 0.2219365 0.3742616 1.2886960.4097451 2.29260100
0.4034619 0.2884905 1.0068510.1515511 0.554142924 0.3629470.03183208
0.2127359 0.4265866 0.3469263 0.5963102 0.267060762 2.4949830.64129
0.07480611 0.05341625 1.017136397 0.2804202 0.3280782 0.2350723
0.03911478 0.1484081 1.1878350.08920478 0.0762347 0.1072641
0.1869906 0.1462363 1.4649440.0189870.234934893 0.245555836 0.3012688
0.294265881 0.334604515 0.4783407 0.470994215 0.1224848 0.4426397
0.6165642 0.349235381 0.07571668 0.6908929 0.04317066 0.330388899
0.08908609 0.03077608 0.81018 1.3425550.6838629 0.6755139 0.2467932
0.9135961.6895470.6559663 0.9230446 0.381918382 0.2431019 0.156797749
0.3486571 0.2123598 0.2663114 0.5685487 0.4352315 0.2924148
0.4321338 0.9190028 0.3546529 0.46451069 0.2407937 0.4611302
0.1824835 0.07457387 0.2673385 0.04711069 0.4046438 0.9590258
0.5689843 1.5370680.02006815 0.1102271 0.6517136 0.7336674
0.105705738 0.5570540.8177809 0.1921259 1.4709371.3541870.4763223
0.2122160.2851914 0.7214031 0.4041481 0.370064391 0.131563686
0.7151621 0.9372151 1.4879480.2156225 1.8173030.3087553 0.7189511
1.9587090.08970765 0.2871776 0.3758528 0.5533284 0.7868314
0.3470739 0.4655773 0.1168054 0.13406 0.3992281 0.178961848
0.05175933 0.4951217 0.8161929 0.6110899 1.0326230.7044752
0.2024255 0.1084165 0.4640831 0.02220568 1.1586340.350408731
0.3613513 0.1450790.2453775 0.8972423 0.4865792 0.441452225
0.2244776 0.2446793 0.5546891 2.5805121.2485210.8628163 0.328919464
0.7440470.3336501 0.4142875 1.6918461.001304024 0.02727319 0.5767983
0.4985093 14.224824893 0.03630671 0.6041096 0.1684461 1.094239
0.05704345 0.7616793 0.2341255 0.3542399 1.1050550.3208303
1.7153980.4841459 0.3923288 0.494086431 0.4960079 0.2021799

0.2838178 0.385049 0.4379951 0.2334801 0.4550682 0.9451118
0.05429794 0.04522631 0.06099549 0.1300084 0.7121021 0.2674828
0.9878937 0.2141497 0.08463322 0.4925766 0.03730492 0.3145805
0.8539644 0.2894158 0.4393017 0.05505072 0.9914052 0.2111972
0.8898091 3.416271 0.3948153 0.394997 0.1711408 0.1095285 0.3629202
0.0778735 0.200094 1.144659 0.1957495 0.7031224 0.8768214 0.6150463
0.3290933 0.1820737 0.2579144 0.4529534 0.1046578 0.01816289
0.439686 0.452777956 0.3913586 0.5353982 0.1633737 0.5342737
0.4361785 0.15546555 0.24619543 1.233845 0.521814123 0.6411567
0.3005273 0 0.4451385 0.1960822 1.443024 1.566859 0.6793053
0.5073899 0.2596614 0.1775813 2.254259401 0.3918007 0.1344779
0.3859876 1.741471 0.03965517 0.7130842 0.3456444 0.5693463
0.6869831 0.8294166
AC234775.3 0.0677126 0.7887619 0.2350028 0.434183597 0.095342037
0.7004978 0.6642494 0.2833024 0.5584664 0.529458 0.08580588
0.461464415 0.5502095 0.226499583 0.579988014 0.4006887 0.1773712
0.4337925 0.1002537 0.6561999 0.191661923 0.5478717 0.1420747 0
0.1838848 0.2920966 1.721529 0.8211875 0.07579809 0 0.1424592
0.35675282 0.02648503 0.1782939 0.456679 0.122833265 0.3072163
0.4069711 0.6987828 0.1557151 0.07475621 0 0.4309239 0.4872485
0.219667 0.4258405 0.05666939 0.1791524 0.09237149 0.1657837
0.2526632 0.1530353 0 0.179712234 0 0.8374023 0.5396702 0
0.363548 0.2562317 0.3918611 0.1977944 0.662461 0.07810203 0.1792412
0.123670049 0.03781918 0.6889004 0.2648715 0.101778109 0.4164123
0.2392944 0.3883629 0.303216611 0.8375216 0.3497363 0.3093997
0.6178346 0.03175801 0.1289877 0.1723521 0.111755 0.1434879
0.2108504 0.131511018 0.1927635 0.1103167 0.08505246 0.104548
0.1721417 0.3649923 0.049090261 0 0.04058697 0.288849 0.110292779
0.4483362 0.1891685 0.4237746 0.4011774 0 0.1405971 0.2469599
0.3613703 0.07705216 0.3543776 0.14913227 3.801237 0.151525613
0.1488124 0.3646746 0.3209209 1.297652 0.4293294 0.08746863
0.2840085 0.2576404 0.532654 0.1991628 0.732852 0 0.3087131
0.8029775 0.257591 0.06133486 0.346715 0.1901173 0.03687209 0.422621744
0.1108066 0.3154726 0.6075154 0.1555607 0 0.02977627 0.1573483
1.095171 0.1423017 0.2376802 0.1026008 0.2363261 0.4924555
0.3028001 0.2284522 0.2385568 0.6371892 2.219197 0.09884912
0.1680139 0.09503506 0.3011688 0 0.183398 0.161196917 0.5634103
0.07106278 0.171885522 0.1930364 0.1301579 0.2751449 0.4541424
0.1155297 0.2065774 0.216164887 0.1556258 0.2291298 0.3954914
0.3004778 0.133583 0.214821 0.1003892 0.09508978 0.2050624 0.124773561
0.2257978 0.03894496 0.1002314 0.09180938 0.1571826 0.05782294
0.5380138 0.4106529 0.5167562 0.2711114 0.1166899 0.451043
0.1977068 0.1804046 0.1894303 0 0.1823382 0.5097028 0.188163622
0.4381921 0.05764711 0.03852595 0.05722504 0.2443292 0.1316955

0.056898885 0.2600491 0.2463492 0.04515735 0.04836776 0.131572086
0.1171925 0.04243869 0.3902343 0.07083591 0.0895878 0.2490794
0.2584763 0.3106332 0.4273560.2902590.1134988 0.1703748 0.03438499
0.15954801 0.280860398 0.09919825 0.062695098 0.201986872 0.5775089
0.232625192 0.2851933 0.8906774 0.1213677 0.079057095 0.04570702
0.07660348 0.2606034 0.299163119 0.5377758 0.3901431 0.09170101
0.4279147 0.1376066 0.3636954 0.1915442 1.1435520.05099548
0.1237436 0.2451697 0.329354702 0.05503145 0.1893046 0.2480537
0.1281928 0.39042 0.4530362 0.03940968 0.3065851 0.1647545
0.06401121 0 0.145037505 0.2997992 0.3976645 0.2386750.4321647
0.3227624 0.02843877 0.10992 0.5210317 0.3946276 0.3431827
0.07268587 0.1996186 0.1124036 0.1620310.109388864 0.1973761
0.3863429 0 0.1902736 0.1915937 0.2875360.1601325 0.1967523
0.0362901 0 0.319132183 0.039709771 0.6872177 0.1170534 0.1418231
0.1301624 0.0723317 0.3106380.5410921 0.0328442 2.2094340.06500896
0.6806603 0.1138709 0.2102359 1.0056680.7399064 0.4583189 0
0.09037405 0.324095541 0.3436946 0.06641876 0.3309191 0.1475559
0.03978841 0.2617 0.8706457 0 0.2801477 0.08042789 0.2933048
0.17084891 0.1377681 0.1576407 0.2962485 0.1160631 0.09790924
0.15124904 0.6504375 0.1399289 1.2305470.08127381 0.3532877
0.2840981 0.268049307 0.3930065 0.9399168 0.06002116 0.2460059
0.122522781 0.4939108 0.09496069 0.1641433 0.041616819 0.197251702
0.2983712 0.02178941 0.09908204 0.2754781 0.2122127 0 0.2138399
0.2090835 0.1106697 0 0.1252538 0.1989394 0.433831988 0
5.2480470.1581499 0.8175409 0.06101526 0.06040383 0.1901809
0.04961085 0.2949967 0.2457113 0.1656920.2648723 0.3393683
0.1211012 0.1219808 0.1939099 0.6641644 0 0.1688957 0.6615843
0.3965407 0.3034408 0.1193347 0.1661592 0.2154493 0.327834
0.1652741 0.03891058 0.1100001 0.2554750.1690538 0.1190121
0.4868441 0.1692324 0 0.2763932 0.2215610.2285477 0 0.1285193
0.5332452 0.1199022 0.1474979 0.5810370.03790654 0.2960329
0.3244025 0.192934079 0.3322223 0.06255439 0.3287398 0.09675566
0.2154296 0.168926592 0.029723595 0.03437637 0.039374694 0.1244056
0.3628317 0 0.1376328 0.5548438 0.06615904 0.2781905 0.1757436
0.1225161 0.1936285 0.2046517 0.10743181 0.1252132 0.3131179
0.1471609 0.1478326 0.1915055 0.2532114 0.1391008 0.4656455
0.2561402 0.225308
AC078795.1 0.02355471 0.01936808 0.04359934 0.020138186 0.004974895
0.09503413 0.06932033 0.02217384 0.0166517 0.02455718 0.03581843
0.040131596 0.1483331 0.033767494 0.008646701 0.09123367 0.05923285
0.03621609 0 0.2008755 0.060004878 0.1143506 0.3360732 0.1230752
0.1855034 0.1585110.1397331 0.3917633 0.07119184 0.026142587
0.07730778 0.134029185 0.01658369 0.0806284 0.1032601 0.051274974
0.1410673 0.08494211 0.3403129 0.03250051 0.03900738 0 0.1573976

0.2847528 0.04584841 0.419008 0.124193 0.07478457 0.0722984 0.06920403
0.09228678 0.09981624 0.08407701 0.118778942 0.01486519 0.2684133
0.1900779 0.1117429 0.06576174 0.06876014 0.1635768 0.05504427
0.08449674 0.08965705 0.1720898 0.146268786 0 0.2982223 0.1174772
0.09913354 0.1665826 0.06659333 0.3416026 0.126573399 0.2709488
0.129771 0.05022673 0.2659657 0.2319962 0.1346102 0.1151134
0.05831316 0.02246136 0.05601047 0 0.08493669 0.06447018 0.1538503
0.007273674 0.1167693 0.07618038 0.046107047 0.08557668 0.02117805
0.4488101 0.149630419 0.2058666 0.0236897 0.1277601 0.04884416
0.07265234 0.05869025 0.2663154 0.1714191 0.02144288 0.1047836
0.067440874 3.87359 0.063252181 0.04141306 0.3721133 0.06140008
0.07738373 0.1980823 0.02738439 0.05186792 0 0.1504124 0.09501445
0.04025243 0.03323498 0.1002305 0.1967428 0.224016 0.1621546
0.0361828 0 0.0769586 0.039203855 0.05550554 0.1426636 0.05283303
0.08441756 0.024269641 0.1553709 0.08210349 0.08421433 0.1069232 0
0.03212191 0.1726391 0.171307 0.1760565 0.1021758 0.07824306
0.0237487 2.455962 0.00687719 0.04208098 0.05950652 0.06769461
0.007114295 0.2220148 0.218690314 0.0534517 0.1557367 0.113605958
0.1007253 0.0814988 0.02871382 0.1150992 0.1044901 0.114977
0.109570966 0.1218071 0.207235 0.1155645 0.1977937 0.01394058
0.160132 0.1388136 0.006615643 0.03745015 0.112850666 0.05655362
0.03251402 0.251041 0.08303642 0.3561882 0.1146524 0.3244021
0.2657028 0.06591213 0.04951255 0.03044406 0.1305822 0.05501991
0.06777655 0.0621303 0.07269629 0.07611444 0.548287 0.065455158
0.1067016 0.09625582 0.04020526 0.1731863 0.1311322 0.2144001
0.029689523 0.1356922 0.05141746 0.08953884 0.08076169 0.068653586
0.158991 0.02657313 0.1777066 0.1330624 0.2617799 0.4158985
0.07417932 0.05402889 0.40544 0.06058221 0.6790907 0.02540018
0.1220049 0.144302199 0.063505607 0.18634 0.052342328 0.042158253
0.03348233 0.064737353 0.06613879 0.1766052 0.126658 0.057752176
0.1717176 0.1172491 0.1794955 0.178401896 0.06734602 0.03489849
0.1084579 0.5074626 0.134031 0.3036388 0.1998934 0.4722808
0.02128733 0.1601305 0.03721547 0.116861713 0.1378325 0.052681649
0.1035464 0.1426993 0.06231404 0.008596065 0.213863 0.2734108
0.05158078 0.00668014 0.3872634 0.060543814 0.1308354 0.1493994
0.2490786 0.3889894 0.05613854 0.0534211 0.07647421 0.2392469
0.07778993 0.5252745 0.03034167 0.04166396 0.03519093 0.006763748
0.188359063 0.1098557 0.04031832 0.1331372 0.03530088 0.1679538
0.03000694 0.05347599 0.1129307 0.1060414 0.1222088 0.146538838
0.116033896 0.1489513 0.04886224 0.09620323 0.1833786 0.04529073
0.02593427 0.05646777 0.006855168 0.4611482 0.04070558 0.1587795
0.1045743 0.07800885 0.1705443 0.2801318 0.07358407 0.4155135
0.06287557 0.116840506 0.2086841 0.1109022 0.06446413 0.01539877
0.008304548 0.04779376 0.392131 0.07171424 0.3800668 0.1510806

0.2119082 0.071318412 0.01437732 0.2632194 0.2009551 0.07267332
0.0272472 0.117253926 0.08060623 0.07788173 0.639472 0.08481645
0.04424246 0.06917904 0.043514067 0.1054639 0.2382154 0.04384619
0.07935262 0.086947124 0.0103088 0.04954996 0.02569472 0.121606384
0.178403066 0.04843641 0.1318873 0.01378679 0.143743 0.1919344 0
0.2157223 0.04987365 0.08662025 0.06325764 0.05664249 0.1127031
0.09620773 0.03605432 4.101234 0.1650435 0.1455419 0.02546994
0.1260735 0.535871 0.02588667 0.3181171 0.222232 0.2074972 0.04299832
0.4486039 0.07582794 0.0424326 0.04047245 0.1172963 0.008865977
1.177402 0.3106899 0.07448859 0.1669701 0.01660485 0.1734019
0.05995746 0.05131858 0.3277086 0.03248532 0.05739746 0.3412619
0.3763683 0.3394793 0.1930647 0.04709575 0.05042139 0.04807347
0.2959598 0.3748013 0.06977335 0.01788284 0.2618771 0.08133361
0.02565453 0.185476 0.03955885 0.4187809 0.05539792 0.010067193
0.129436 0.02611243 0.1509504 0 0.034972 0.064639638 0.0434269
0.0358748 0.057527392 0 0.1211671 0.46828 0.1969811 0.1250702
0.06904284 0.1741899 0.009170205 0.0575354 0.04618709 0.3173073
0.061663056 0.02613422 0.6099638 0.3609023 0.02057019 0.4596619
0.06870464 0.1548416 0.4095801 0.01527458 0.1672028
AC007390.1 0.1557348 0.2320986 0.1171066 0.066573042 0.156237595
0.2114571 0.184601 0.2504503 0.172023 0.236779 0.1998151 0.172467724
0.2926349 0.184187783 0.214382708 0.3644349 0.299838 0.1721026
0.0691732 0.4942694 0.429790397 0.371271 0.4370457 0.2330989
0.4810757 0.3526972 0.3175778 0.3885287 0.1699727 0.102626666
0.2015031 0.406152251 0.1964474 0.1947809 0.2363251 0.20340646
0.3815524 0.2262021 0.3716552 0.09401047 0.2256643 0.1993968
0.2508718 0.9637518 0.1667228 0.3095626 0.1319653 0.3152102
0.2629053 0.2287755 0.1797809 0.1418887 0.09727996 0.273829211
0.2211368 0.4900916 0.4770895 0.2047765 0.3971656 0.3030774
0.7360261 0.1649066 0.2285429 0.3098615 0.2411626 0.526202019
0.06523634 0.6205679 0.3883575 0.152154214 0.1735872 0.1788678
0.2583931 0.241903549 0.3864536 0.1675775 0.3320802 0.4262945
0.4108583 0.2224979 0.3686513 0.3084357 0.01856325 0.208305
0.0831785 0.2179776 0.1712622 0.2053962 0.1743291 0.289513
0.3002683 0.160889037 0.1768127 0.1365208 0.3875287 0.304399949
0.5645523 0.1174705 0.3167631 0.4844087 0.1921399 0.3783368
0.3691952 0.2890064 0.1373418 0.234326 0.287258372 4.564158 0.078412413
0.2053556 0.4403328 0.1107148 0.3357585 0.3235136 0.3545665
0.09798031 0.03809296 0.2648316 0.3828092 0.5588811 0.2472043
0.1834221 0.4335963 0.3295467 0.571319 0.2691304 0.1585061
0.2607709 0.226800952 0.1758454 0.2630183 0.1135264 0.1717345
0.140403987 0.1900419 0.2714187 0.1491411 0.1963711 0.1288532
0.3451141 0.4239577 0.2548389 0.2051957 0.2026643 0.1645998
0.1884209 2.857656 0.1364081 0.2376493 0.3770415 0.139866 0.09407406

0.5694355	0.25720297	0.1590311	0.4045141	0.143305731	0.3718264	
0.2806455	0.2610368	0.2294169	0.3122107	0.3325808	0.22372469	
0.3489817	0.5072226	0.1227968	0.5143234	0.2477064	0.1958655	
0.3030417	0.1257529	0.1591754	0.150660056	0.3115927	0.1477922	
0.2204406	0.04751009	0.4221925	0.314187	0.5980761	0.4250144	
0.1634198	0.2922842	0.1559956	0.2735648	0.1932533	0.2365041	
0.1447073	0.3004003	0.09435749	0.3516855	0.183925034	0.5165052	
0.5966317	0.1860754	0.4590041	0.1746035	0.327123	0.161944132	
0.3364296	0.2185411	0.1557887	0.1835508	0.090782318	0.1246602	
0.4172673	0.2692545	0.2728881	0.2318023	0.3554382	0.1616241	
0.333404	0.7103633	0.60082	0.4176643	0.3190791	0.3499439	0.275212976
0.109006034	0.5732266	0.156811931	0.121946321	0.1272892	0.147131509	
0.2678369	0.3533668	0.5778161	0.320469148	0.6386243	0.2334429	
0.1618304	0.626623173	0.3246735	0.4230153	0.347996	0.355646	0.3085745
0.5646221	0.22027	0.9988778	0.2199121	0.2262592	0.2806561	
0.460178582	0.2847802	0.136059051	0.365644	0.1474178	0.0911147	
0.2273357	0.2787175	0.6682679	0.8454768	0.121458	0.5108569	
0.265194004	0.3525954	0.672234	0.1963509	0.4193236	0.1577459	
0.2403723	0.2148879	0.7968967	0.2117778	0.6182844	0.1441867	
0.1779053	0.1454182	0.1117984	0.462292356	0.3631624	0.2332483	
0.3150906	0.1932811	0.5816628	0.2169941	0.2541235	0.343631	
0.4131517	0.2146247	0.352312532	0.239741347	0.2963547	0.2927719	
0.1284351	0.493953	0.1497226	0.1500341	0.3150087	0.03965831	
0.717397	0.2691299	0.4765496	0.2671341	0.2578825	0.3794723	
0.6915789	0.1641972	0.2805848	0.1662838	0.193126327	0.8677276	
0.1374832	0.2473555	0.08908447	0.1166764	0.3103515	0.9564246	
0.08466919	0.3431018	0.3676462	0.3424806	0.265235857	0.09505744	
0.2447307	0.2171814	0.1451476	0.1407408	0.324258488	0.2033579	
0.1448225	0.5068339	0.1331839	0.08531671	0.2368604	0.200361512	
0.1743218	0.8569799	0.165654	0.1697396	0.219800006	0.2641118	
0.2047535	0.2406687	0.258433552	0.2098209	0.145825	0.2480658	
0.1652149	0.2851123	0.4270667	0.1776191	0.393455	0.1597206	
0.1479476	0.2287226	0.2520665	0.3284544	0.130959538	0.1013105	
7.210532	0.3478216	0.2530101	0.1947098	0.2813233	0.3881965	
0.1155282	0.2289851	0.4026486	0.4144261	0.1269146	0.1678131	
0.2924516	0.4909595	0.163898	0.3304768	0.1465462	0.6292887	
0.3518708	0.47197	0.366395	0.1646775	0.2694199	0.08671602	0.09896224
0.5773077	0.1006784	0.256156	0.3128846	0.2673091	0.2326625	
0.5248648	0.04378772	0.2416912	0.3019515	0.5579865	0.416762	
0.3363756	0.06650696	0.5681262	0.1344368	0.1653776	0.6544182	
0.4184771	0.6354671	0.4069668	0.224641625	0.2712522	0.1834359	
0.3459072	0.05841467	0.1156108	0.223399458	0.112798032	0.1304548	
0.169798837	0.07868446	0.8824739	0.2902585	0.4171637	0.3407205	
0.1654759	0.1919464	0.09852364	0.2403931	0.1431429	0.4034454	

0.15751769	0.2159870.6901455	0.1903843	0.07650132	0.5161533
0.2576990.3519156	0.8319034	0.1893557	0.4706934
AL139021.3	0.1113270.07628303	0	0	0.156752823	0.1151696	0.1213444	0
0.06558432	0	0.07053716	0	0.07538376	0.053198613	0	0	0.1749707
0	0	0.1438487	0.078778334	0.06434011	0.3114487	0.04039531
0.1511634	0.1920956	0.1179327	0.5014742	0.06231021	0	0.09368743
0.234616391	0.04354431	0.3419906	0.06256922	0.040390286	0.5050974
0.07434505	0.7659173	0	0	0	0	0.2136241	0.1444627	0.1500276
0	0	0.1518689	0	0.05192577	0.03145087	0.06622909	0.344711185
0.1756440.2458539	0.1109097	0.07654087	0.03984753	0.1805456
0.2863391	0.2167972	0.2420353	0	0	0.067775733	0	0.2936444
0.4354780.055778145	0.1141046	0.1311421	0.09121592	0.062315193
0.03442445	0	0.1130416	0.06348677	0.1044273	0.07069002	0.05667317
0.0918688	0.05897749	0.03151466	0.072072872	0.1760691	0.07254916	0
0.05729614	0.07075497	0	0	0.0421315	0.03336472	0.2638331
0.090666745	0.07371141	0	0.1548294	0	0.0381531	0.04623144	0
0.2160482	0.1266823	0.04855299	0.163459932	3.0635560	0	0.2664735
0.04396912	0.1523917	0.03715078	0.1438081	0.05836766	0	0.05151423
0.1871118	0.06341523	0.1745322	0.1127907	0.2869964	0.3529234
0.06722751	0.05700377	0	0.06062178	0.077204084	0	0.1728906
0.08323519	0	0	0.1468664	0.06467451	0	0	0.1116493	0.08434349
0.03885462	0.2024128	0.1066793	0.1073145	0.05603052	0.03741462
0.6788101	0	0.05524667	0.07812406	0.03808882	0	0.06030529	0	0
0.05841753	0.188399169	0.2115820.3209909	0	0.05333287	0.126629
0.3396360.101542546	0.1279330.06278578	0	0.3800144	0.2196252
0.05045564	0.1031568	0	0.1685726	0.068380498	0.0742473	0
0.1235936	0.05031491	0.1107538	0.0950673	0.2948513	0.2025476
0.09440047	0.1114343	0.0959255	0.09568573	0	0.1779630.08898407	0
0	0.3223106	0.051560291	0.2401454	0.09477821	0	0.2352107
0.1147726	0	0.046774021	0.05344368	0.05786076	0.03712183	0	0
0.09633868	0	0.05832621	0.03882068	0.04909741	0.1861427	0.1593613
0.02837307	0.4471221	0	0.1866045	0.2000821	0	0.087438174
0.038480456	0.1630929	0.103077655	0	0.05274935	0.063743578	0.260494
0.2928744	0.1596335	0	0.1502948	0.04198152	0.1713843	0.351326541
0.08841627	0.2749021	0.1005110.09593713	0.07541345	0.1630787	0	0
0	0.08137928	0.2198651	0.10829909	0.1809556	0.155618819	0.2224514
0.1053815	0.03775844	0	0	0.09164721	0.2708745	0	0.1759932
0.143074592	0.1792375	0.06538043	0.0905557	0.4440790	0.04675648
0.1204804	0.05710891	0	0.5015380.1195036	0.1093983	0	0
0.044961839	0.03605645	0.6351904	0	0	0.3150011	0.3545557
0.05265509	0.2021765	0.2386597	0.1804992	0.262344251	0	0.1303687
0.09622432	0.1165864	0.05350032	0.2973032	0	0.05560094	0
0.2670995	0.1068819	0.3949696	0.1123298	0	0.1033393	0.1414521
0.1159272	0.1995781	0.0990566	0.145322403	0.3595912	0	0.1450847

0 0 0 0.3013553 0.08070098 0.1842375 0.1322323 0.4080369
0.040127778 0.05662648 0.08639292 0.06088314 0.04770515 0 0.1065729
0.1336739 0 0.2477333 0.1336231 0 0.1556962 0.048966958
0.1846132 0.2759512 0.0986814 0.03676917 0 0 0 0 0.136845279
0.054050592 0.05450613 0.1432968 0.1086012 0.05661456 0.05815018 0
0 0 0.04548829 0.06228658 0.03432184 0.1401764 0.13373768 0
0.7023090.1300079 0.1185995 0.2507895 0.2979317 0 0.04078285 0
0.4039764 0.1816106 0 0.1487890 0.06684995 0.1594046 0.1679938
0 0.4442932 0 0.2607826 0.09070729 0 0 0.05903706 0.04491627
0.4755255 0 0 0.4620319 0.1389715 0.1304460.04002127 0.04637279
0.1191537 0 0.2914166 0.1610390.04580151 0.1408666 0.5672854
0.1971325 0.2020857 0.05619346 0 0.05407898 0.1939467 0
0.0364140 0.05404845 0 0.03935446 0.092577982 0 0 0 0.1363577
0.05965353 0 0.4525670.03648898 0.1631591 0.1372128 0 0.1510725
0 0.2884028 0.044157436 0 0.5720003 0.3629230 0.5116412
0.3746767 0.07623234 0.2187350 0.2469541
LAMTOR5-AS1 0.07571891 0.05660052 0.0557432 0.044138298 0.027623039
0.04059045 0.1080422 0.03887999 0.0340636 0.03109815 0.06018778
0.056293939 0.0741116 0.067102965 0.042956939 0.03332722 0.0865499
0.09260694 0.03974725 0.08938881 0.099368376 0.2291477 0.1169887
0.05245193 0.1252457 0.1140250.1254220.1402471 0.2219182 0.064938379
0.07820352 0.181334147 0.07754166 0.1993753 0.04410388 0.061436048
0.1667737 0.06067883 0.2379738 0.1614630.09575468 0.05242852
0.1774180.08717768 0.03483627 0.1057517 0.07950048 0.1442414
0.1422633 0.04044784 0.05779186 0.09217693 0.06879696 0.076730691
0.1064316 0.1970123 0.1728149 0.07950856 0.07835014 0.1428921
0.1619992 0.1286874 0.05836528 0.1714977 0.1311936 0.213725148
0.03690842 0.2256598 0.07674009 0.055871497 0.08889657 0.07784392
0.1032127 0.161828298 0.1200487 0.05214519 0.1258120.2002002
0.1491549 0.1101463 0.1093310.03749067 0.05470018 0.1426379
0.016042999 0.04703035 0.05248431 0.1175893 0.1126584 0.3950535
0.07363804 0.05838797 0.1813124 0.1213043 0.2701475 0.127818614
0.1668119 0.02538429 0.08616029 0.08972248 0.07784935 0.07032079
0.1631856 0.08616317 0.06893022 0.08105709 0.09399516 3.255077
0.10351362 0.1422031 0.08897315 0.05219876 0.04240186 0.1516083
0.1093704 0.01948845 0.1122482 0.07453398 0.1318916 0.1152795
0.1035996 0.06904304 0.09369613 0.2042526 0.1982790.05286961
0.09276948 0.09220936 0.148938199 0.09597263 0.05612314 0.06484683
0.06452119 0.049647268 0.3886668 0.05758467 0.1371155 0.1666498
0.1429018 0.2503249 0.1600030.3454286 0.09498477 0.1592505
0.0644390.06940229 1.0828770.07034172 0.1250255 0.04637322 0.05652232
0.07484595 0.08725343 0.056535079 0.0749785 0.09319103 0.094357241
0.1197047 0.1548098 0.07761875 0.1009085 0.1714702 0.05460072
0.111130315 0.07119286 0.1304403 0.1905713 0.1466209 0.06722002

0.2040322	0.1706849	0.07926654	0.06097532	0.045663295	0.1156891	
0.1377757	0.1589537	0.03546605	0.08628602	0.09346295	0.2789368	
0.1152195	0.07704763	0.08268205	0.09430667	0.1135951	0.08039404	
0.07482553	0.04291589	0.07170014	0.1912932	0.1123996	0.107118841	
0.1447738	0.3990863	0.08224601	0.07853478	0.1277386	0.2345564	
0.045117033	0.1506858	0.0665439	0.05508734	0.1150573	0.040126122	
0.06195056	0.1087188	0.1038646	0.2822811	0.1475387	0.1091103	
0.07291651	0.03684146	0.1232236	0.02065503	0.1407645	0.106889	
0.08598966	0.068121289	0.112779399	0.1845429	0.172083616	0.094454608	
0.08610578	0.089863331	0.0657157	0.1032209	0.1362117	0.072331177	
0.1616979	0.07320126	0.09696239	0.135552265	0.09512456	0.1053855	
0.1528833	0.2443966	0.1930458	0.1371347	0.1207266	0.08310733	
0.05909881	0.1101968	0.1128354	0.086382496	0.1174823	0.128937173	
0.1430473	0.1159834	0.07144087	0.2386477	0.07692124	0.05270034	
0.04773361	0.05466099	0.08705567	0.074310987	0.07314488	0.07034096	
0.1153438	0.161454	0.05577942	0.1006079	0.09162897	0.1970377	
0.05616396	0.2395596	0.2039395	0.071025	0.0479923	0.07115803	
0.120098904	0.1029998	0.08640479	0.114954	0.1108991	0.2313874	
0.07672975	0.08985881	0.07500552	0.5223884	0.1874977	0.105113328	
0.06539651	0.3659656	0.1499328	0.06271599	0.2282531	0.1235322	
0.04547359	0.1144821	0.1302163	0.3745651	0.1229216	0.04395896	
0.1611366	0.1781016	0.0575068	0.07451781	0.03225589	0.0977348	
0.09003513	0.118609024	0.1867667	0.06076807	0.07266375	0.03150053	
0.0679529	0.06384944	1.014584	0.08981783	0.07860286	0.06622681	
0.1334876	0.041683639	0.07352755	0.06730695	0.03839799	0.07787184	
0.04976636	0.122566267	0.07934672	0.07396954	0.1302011	0.1511972	
0.1134901	0.1068593	0.128980469	0.06335296	0.1719904	0.1885407	
0.1009434	0.053807494	0.1265293	0.1534911	0.100119	0.21068829	
0.13435002	0.1021173	0.08372969	0.07856547	0.06301034	0.08844991	
0.04433411	0.1891256	0.143927	0.07594071	0.05083697	0.09804461	
0.2912229	0.160423042	0.07164735	1.012422	0.1109328	0.1129314	
0.1898026	0.1086876	0.1778677	0.06657218	0.1139575	0.06244637	
0.119592	0.04487738	0.1959574	0.07755883	0.4067311	0.03075153	
0.08413746	0.07772877	0.1957338	0.0751019	0.07739825	0.1447015	
0.07278807	0.06283613	0.1292228	0.07665208	0.1789355	0.09493386	
0.1341889	0.093496	0.1202998	0.06775189	0.1321428	0.07741734	
0.04420486	0.08710261	0.08108452	0.2608817	0.09685391	0.04442111	
0.1607129	0.07679094	0.09446449	0.323132	0.30751	0.1986215	0.07554996
0.161809892	0.1458998	0.0972952	0.1343447	0.029508	0.09928081	
0.039497357	0.092462351	0.07338739	0.052836409	0.06829296	0.2368004	
0.2736967	0.1427132	0.1069427	0.07263653	0.1136869	0.03751812	
0.04296892	0.06748769	0.08202921	0.104844657	0.1349268	0.09973707	
0.1952293	0.09017032	0.09490695	0.1374569	0.1597902	0.1298377	
0.1629281	0.1175759					

AC087286.1	0.05213552	0	0	0.055716831	0.027528335	0	0	0.04089929	0
0	0	0.044413242	0.02647722	0	0	0	0.08194051	0.05009998	0
0.02526216	0	0	0	0	0	0	0	0	0.04377073	0	0.03290606
0.027468292	0	0	0	0.028372754	0	0.05222478	0	0	0	0	0	0
0	0.0351297	0	0	0	0	0.03647603	0.04418626	0.0465236	0	0
0.1036222	0	0.02688363	0	0	0.05028578	0	0	0	0	0	0	0
0	0.039182183	0	0	0	0	0	0	0	0	0.07335653	0.04965725	0
0	0	0	0	0	0	0	0	0	0	0	0	0	0	0	0.05177967	0
0	0	0	0.03247596	0.09507376	0	0	0	0	11.10453	0	0
0.04679706	0	0	0	0	0	0	0	0	0	0	0	0	0	0	0	0
0	0	0	0.03036239	0	0	0	0	0	0	0	0	0	0
0.04995898	0	0	0	15.914554	0	0	0	0	0	0	0	0	0	0
0.03715723	0	0	0	0	0	0	0	0.0441048	0	0	0.03856975	0
0.0144928	0	0.02960408	0	0	0	0	0	0.02593355	0	0.03452045
0	0	0	0	0	0	0	0.03125409	0	0	0.04528237	0	0	0	0
0	0.04031186	0	0	0	0	0	0	0.075978247	0.02255818	0	0	0
0	0.02615175	0.03731524	0	0	0	0.04369436	0	0	0.061422239	0
0	0	0	0	0	0	0	0.02803423	0	0	0	0	0	0.06210931	0
0.03530274	0	0.05297529	0	0	0	0.1177924	0	0.02574124	0	0
0	0	0	0	0.0475659	0	0	0	0.03696422	0	0	0	0	0	0
0	0	0	0	0.05064079	0	0	0	0	0	0	0	0	0	0	0	0
0	0	0	0	0.03685752	0.045861989	0	0	0.08189782	0.03758209	0
0.0358765	0.01952885	0	1.1632293	0	0	0.02630258	0.02697863	0	0
0	0.02803931	0	0.034027957	0	0.0767709	0	0	0	0	1.799377	0	0
0	0.02605741	0	0	0	0	0	0	0.02495457	0.04695059	0	0	0
0	0	0	0	0.0387692	0	0	0	0	0	0	0	0	0.05743296	0
0.03814426	0	0	0	0	0	0.03195392	0	0	0	0	0	12.545512	0
0	0	0.03488108	0	0	0	0	0.03189377	0	0	0	0	0.02239523
0	0	0	0	0	0.03185934	0	0	0	0.0315521	0	0	0	0
0.06508173	0.09163374	0	0.03257525	0	0.05320244	0	0	0	0	0
0.1038559	0	0.03947393	0	0.03798858	0	0	0	0	0	0	0	0
0.068657317	0	0	0	0	2.591204	0	0.02563222	0.03820451	0	0	0
0	0	0	0	0	0.04249011	0	0	0	0	0	0	0
AC012645.4	0.125597	0.02868702	0.04843286	0	0.206319808	0.06496606
0.0912656	0.1532663	0	0	0.4244197	0.023776363	0.1275698
0.180052987	0.02561412	0.04504358	0.4167304	0.4291316	0.03099266
0.5544817	0.414755679	0.5565025	0.33673	0.4557322	0.7011076
0.9571738	0.5174142	3.292976	0.8904311	0.096802721	0.2113927
0.455854561	0.3111304	0.1469818	0.588245	0.258216129	3.969895	2.739906
0.9361011	0.1925525	0.1617722	0.1649328	1.065735	1.064446	0.05432669
1.861841	0.3854159	0.1772275	0.1142237	0.580842	0.5662895	0.2483759
0.3486858	0.129632199	0.6605272	0.1479294	0.6047765	0.01439199
2.187821	0.2942162	1.48061	0.1426754	0.1592848	0.6277607	0.1773153
0.280365401	0.1169151	0.441712	0.1023538	0.188783523	0.4291022

0.2959041 1.252048 0.164039901 0.6343378 1.273393 0.04251047 0.644621
4.555434 0.1063348 0.319688 0.3454821 0.04435819 0.04740566 0.027103747
0.2383654 0.05456572 0.3155198 0.2585616 0.5321627 0.9200356
0.257989909 0.2059718 0.08783004 0.7738939 0.272769323 0.2494794
0.11696 0.4366889 0.8681463 0.1434787 0.3303304 2.188578 0.7312245
0.3970014 0.620801 0.092206191 2.718913 2.791841565 1.502804 0.6012606
1.025173 0.1146169 0.1397093 0.7030467 0.2633971 0.3641031
0.1356072 1.161028 0.8585263 0.3281732 0.2757048 0.1726848
0.09290434 3.147559 0.1071843 0.2546844 0.7067208 0.406467494
0.2329341 0.1950519 0.1252058 0.3847233 0.431363698 0.441845
0.753967 0.4989362 0.3343352 0.5038424 0.3171824 1.022818 0.3805971
0.09360834 0.564994 0.08428342 0.7738584 0.159546 0.3870736 0.3116412
0.411311 0.2291791 1.285555 0.5442823 0.099665578 0.3166799 1.142363
0.106274178 0.6365409 0.1207119 0.4890889 0.08022551 1.071453 0
0.229116735 0.0721658 0.7083372 0.9781049 0.3143983 0.2064808
0.2276924 0.2172418 0.07839007 0.174332 0.154291152 3.685633 0.07223721
1.952104 0.07568577 1.180087 0.3575104 0.2402441 0.3046805 0.08875067
0.146671 0.2344795 0.5217623 0.2648514 0.3123158 0.2007803
0.565289 0.2705686 0.412108 0.155118236 2.054533 0.4098867 0.30966
2.317479 0.1942264 0.2768461 0.2638478 0.2612746 1.392585 0.2094007
0.1794303 1.016862495 0.1811458 1.049568 0.1754734 0.6423524
0.3323446 0.308004 0.07990596 0.09602986 1.128973 2.093731 0.2339151
0.3310689 0.3188957 0.131528112 0.159180697 7.697261 0.135671956
0.10407115 0.9124992 0.982828645 0.5093996 0.2202769 2.941559
0.537677658 0.9608383 0.5999282 0.2094652 0.36993592 0.2992486
0.8270382 0.340184 0.1322865 0.7657202 0.2044248 0.3552866
0.8875613 0.4414161 1.591383 0.4823147 0.488723886 0.8166025
0.234088251 2.913988 0.3302485 0.4685819 0.4328901 0.7066255
1.189038 3.794477 0.6332344 0.3529816 1.470661165 1.027912 0.9343055
0.06810881 1.102203 0.6651967 0.1230828 0.4304251 1.07382 0.3524332
1.579597 0.2921136 0.329123 0.3127383 0.1001812 0.456525817 0.3525444
0.8360446 1.362446 0.2352865 0.4738377 0.3555582 0.9504715
0.7907179 0.9872548 0.8824218 0.138120168 0.147311648 0.3595275
1.88168 0.2411393 0.3017902 0.06708236 0.3265066 0.05227327
0.3452201 0.4017821 0.2009702 0.1980432 0.197133 1.675371 0.2720326
0.1170279 0.1961803 0.4503203 0.03725126 1.912749627 0.9465957
0.1026643 0.1773219 0.2280788 0.5412124 1.496697 0.6516346 0
0.9006974 0.5718643 1.269421 0.392352094 0.4471944 0.3573784
0.1602701 0.08970007 0.2421431 0.187029916 0.175943 0.04325794
7.965409 0.1005007 0.189308 0.3805825 0.313047007 0.1735642 2.843416
0.2597711 0.1521017 0.257563442 5.160879 0.2642073 1.014873 0.128655199
0.325220525 0.4202007 0.09430441 0.2246233 0.4045193 0.3280196
0.11235 0.7271767 0.1292731 0.1368507 1.288293 0.3484912 0.1932876
0.737637046 0.08544296 2.452455 1.637843 0.1338017 0.0188624 0.5041813

0.2351719 0.2453889 0.7295677 1.012797 0.3927051 0.4731031 0.1538725
0.3743751 0.7039092 0.08392407 1.689952 0.1575821 0.8771754
0.159074 0.5639025 1.381512 0.9099881 0.616402 0.3774254 0.08445613
4.240737 0.09623126 0.5440911 0.2843213 0.5051961 0.7603614
0.255857 0.03487793 0.1194906 0.1708896 0.5205535 1.51401 1.222913
0.1059487 0.8921221 0.1668008 0.3039856 0.6128318 5.859258
0.2033696 1.221672 0.387686575 0.05477544 0.2513967 0.5487878
0.1495563 0.1479965 0.800742828 0.073510568 0.1912894 0.146068609
0.1794755 0.3813666 1.040389 0.2188189 0.1509426 0.1636205
0.189201 0.4346383 0.2367182 0.102615 0.3253705 0.332117189 0.6193392
0.4517239 0.1137341 0.1218702 0.9028373 0.5009815 0.2866796
0.3495946 0.4751027 3.219481

AC110609.1 0.1465989 0.3013557 0.1130633 0.313338218 0.103208542
0.1516589 0.2396851 0.2300079 0.690908 0.2971851 0.09288557 0
0.1985355 0 0.358766749 0.2365903 0.07680232 0 0.1085254 0 0
0.4236254 0 0 0 0 0.2070633 0.2031868 0 0.067793853
0.1850559 0.102983432 0.2867026 0.3216744 0.08239312 0 0.2660513
0 0 0.3371257 0.1618484 0.06417079 0.349859 0.1406535 0
0.2634148 0 0 0 0 0 0 0 0 0.07709784 0 0 0.05039572
0 0.4754964 0 0 0.1593599 0.08454609 0.07761203 0 0.2456374
0.1104801 0.2150442 0 0 0.1726921 0.120116 0.246175863 0.09066241
0 0 0 0.06875661 0.4654343 0.1492581 0.1209757 0 0.08299901
0.189815683 0.04637068 0.2388376 0 0.5281456 0.09317238 0.1823571
0.106281224 0.1664403 0 0.4863933 0 0.09706552 0 0.1019421
0.3618982 0 0 0.08911204 0 0 0 0 2.097777 0.131222181
0.5369688 0 0 0.2006742 0.2935279 0.09468552 0 0.07968511
0.06783557 0.184796 0 0 0 0.0755852 0.09294814 0 0.1501287
0.2744048 0.3991434 0.101664783 0.1919185 0.1138339 0 0.4041499
0.12587373 0.06446611 0 0.8735499 0 0 0 0.05116499 0.3553914
0.04682624 0.2826302 0 0.197075 3.798984 0 0.07275057 0 0.05015656
0.2951845 0.3176477 0 0.7762316 0.07692605 0 0.06965448
0.140897 0.9680016 0.2106912 0.8337456 0.1490812 0 0 0
0.3424981 0.200166 0.2892094 0.1328832 0.05433604 0.1372473 0 0
0.09777119 0 0.162752 0 0 0.1251876 0.1294232 0.4445352 0
0.6603308 0 0.126002 0.4993783 0 0.117177 0.2827778 0.2368591
0.08488577 0.067896225 0.5534043 0.2496139 0.4170466 0.06194658 0
0.1140492 0 0.633387 0.3809644 0.0488832 0.104717 0.712139345 0
0.09188046 0 0.2556015 0.06465302 0.04902373 0.06995068 0.03736255
0.08411207 0.1047359 0 0.3688642 0 0 0.152016849 0.6442976 0
0.291536607 0.2778481 0.083939563 0 0.3856662 0.262763 0 0.2968694
0.0552826 0 0.092527584 0.349288 0 0 0.08422204 0 0.07158242 0
0.05267677 0.4416242 0.1071628 0.09650843 0.142611673 0.119144
0.273231721 0.1464656 0.09251317 0.09944302 0.08916645 0 0
0.1783481 0.6929267 0 0.062801718 0 0.08609503 0 0 0.05823215

0.06157041 0.07932622 0.1504056 0.0474653 0.08255514 0.3147322
0.3601480 0.07015994 0 0 0.1394065 0.06277382 0.09154345 0 0
0.1386758 0 0.07856866 0.3169162 0.069092644 0 0.05722453 0
0.6908608 0 0 0.4035218 0.07321708 0 0.14069 0.2814910
0.09861298 0.05057381 0.1360804 0 0.0763283 0.1051243 0.06522043
0.191365144 0 0.1437977 0.1432891 0 0.1722851 0.2124693
0.2976256 0.1062696 0.4852195 0 0.1465407 0.10568306 0.1491351
0 0 0 0 0.093559048 0.0440065 0 0.2718526 0 0.1019832 0
0.064481241 0.06077612 0.2180288 0.4548138 0 0.053052769 0.2138650
0.1776865 0.270303299 0.142351063 0.0358877 0 0.07150475 0.07455184
0.1531480 0 0 0.05990043 0.08202094 0.09039216 0 0.117406677
0.1495957 0.1321175 0.5135957 0.05205852 0 0.3269382 0.06862415
0.3759291 0.4257818 0 0.2989383 0 0 0 0.08803013 0.04198182
0 0.1839324 0.07313241 0 0.08585171 0.05972312 0.1722411 0 0
0.1182943 0.08945529 0 0 0.2212430.1220014 0.1717755 0.05270128
0.06106516 0.05230179 0.1994652 0.1918733 0.3534355 0.1809387 0
0.1358217 0.3244879 0.2128903 0.1479947 0.3282732 0 0
0.208852732 0.04795111 0.1354313 0.07117271 0.4189552 0.1554696
0.06095481 0.064352073 0 0 0.08978008 0.2356610 0.1702727 0
0.3580885 0.1806861 0 0.1989370 0.4430743 0.058147911 0.4518144
0.07532282 0.07965142 0.1066867 0.2073063 0.1644620 0.1440186
0.1584422 0.216798

AC069549.1 0.3857402 0.06099585 0.2746147 0 0.062669716 0.1381342
0.1455403 0.1862188 0.05244117 0.02577929 0.1128029 0.050554552
0.1808304 0.085075137 0.381234606 0 0.2331774 0 0.3953893 0 0
0 0.03112926 0.1292002 0 0.1535995 0.03143295 0 0.04982319
0.041165406 0.1123686 0.093799539 0 0.07813012 0.1000606 0.129184163
0.0807751 0.05944624 0.3827668 0 0.1474151 0 0.07081315
0.2989238 0.05775613 0.03998731 0 0 0 0 0 0 0.1059134
0.11812748 0 0 0.04434164 0 0.06372411 0 0.1144782 0.08667542
0.04838281 0.05133761 0 0.216773686 0 0.06708508 0.04352598 0 0
0 0 0.298963072 0.1926803 0 0 0.05076397 0.5010001 0.2826184
0.04531582 0 0.09431671 0.0503982 0 0 0 0.1118125 0
0.02828781 0.07381993 0.290409719 0 0 0 0.724970569 0 0 0
0.2197498 0 0 0.1082202 0 0.1688250.07764587 0.032675608
0.4899233 0.039840001 0.06521104 0 0 0 0.02970573 0.1149888
0.1400122 0 0.1647630.07480722 0.1014135 0 0 0 0 0
0.04558017 0.1666225 0.09694625 0.18519697 0.1748035 0.2073648 0
0.04090094 0.229297036 0.03914475 0 0 0 0 0 0.03106812
0.0539497 0.02843357 0.2145214 0 0 7.8024050 0 0 0.06091158
0 0 0 0 0.2335530 0.08459042 0.08555468 0.04521419 0.1705797
0 0 0 0 0 0 0 0.04390304 0.2420657 0.04949044 0
0.1010929 0 0.1781042 0.05119818 0.06588359 0.1206953 0 0 0
0 0 0.04455138 0.03835098 0.01912756 0 0 0.1778790 0 0 0

0 0 0 0.4889931 0 0.03462616 0.224402819 0.04273352 0 0
0.1271713 0.518905542 0.462194 0.1673734 0 0.06208198 0 0.02976792
0.04247506 0 0.1532222 0.06359716 0 0 0.04520352 0 0
0.6520446 0.041210405 0 0.04217834 0 0 0 0 0.10393077
0.4206145 0.3356839 0.03425968 0.056184084 0.07069757 0 0.1205528
0.07671125 0 0 0 0 0.06704015 0 0.1172026 0.129893798 0
0.041477561 0.05929066 0.05617533 0.1509581 0.1082863 0 0.1099216
0.05414776 0.1262265 0 0 0.03582953 0 0 0.1420341 0.176797
0.07477288 0.04816802 0.1369927 0.2305729 0.05012867 0 0
0.0738845 0.04260213 0.323563054 0.08649214 0 0.03811717 0
0.1511248 0.1890016 0.08420593 0 0 0 0 0.15661088 0 0 0
0.2138941 0.04754468 0 0.08891694 0.2158897 0.1281435 0
0.05263623 0.1796377 0.03070915 0.08263001 0.2488309 0.04634764
0.03191649 0 0.116199677 0 0 0.1160096 0 0 0.3870432
0.7228903 0.06452844 0.07365808 0.1057329 0 0 0.2263924 0
0.2920927 0.07629 0.1716191 0 0.3473779 0.06131821 0 0.2136899
0.03096282 0 0.03915394 0.1845207 0.04413008 0 0.0588012
0.032214407 0 0 0.1078938 0.109421376 0.04321881 0.1307492
0.200515 0.04341871 0.04526895 0.09299367 0 0 0.1178003 0 0
0.1097748 0 0 0.09083665 3.850736 0.415817 0.06322137 0.1203187
0.07940864 0.04166957 0.06521988 0 0.05383653 0.3267353 0.1160696
0 0.0796016 0 0 0 0 0 0.1932751 0.1563912 0 0.05229363
0 0.2360299 0.107745 0 0 0 0.134342 0 0.05215227 0.1600049 0
0.0317584 0.1211182 0.05825411 0 0 0.1126368 0 0.07881349
0.03231751 0.04493225 0.3488314 1.902626 0.07753982 0 0.145583 0 0
0.06359891 0.1888067 0.03701264 0.039075507 0.1807687 0.207052498 0
0 1.474754 0 0.05835311 0 0.03657174 0.05775943 0.06039872
0.07272847 0.03843444 0.105924751 0 0.0457371 0.04836549 0.06478168
0 0 0.5181202 0.08745018 0.1443126 0.09873216
AL353804.2 0 0 0 0.09120219 0.135182369 0.1324284 0 0 0 0
0.2433227 0.072699485 0.2600414 0 0 0.1377269 0.0670637 0 0
0.2894592 0.271751199 0.2219456 0.3133559 0.1393463 0.1738162 0
0.316413 0.2217781 0.286591 0 0.2154541 0.179850029 0.1001394
0.5055942 0.2158367 0.232215048 0.2323157 0.4274305 0.2201736 0
0 0 0.2036643 0.3070461 0.3322226 0.2875168 0.1071329 0
0.08731369 0 0.1194143 0.1808198 0 0.169872083 0.06732175
0.1130786 0.06376507 0.1320165 0.2290946 0.2076015 0.4115608
0.2492855 0 0 0.2033122 0.077932326 0.1429935 0.09647106
0.3129606 0.128273659 0.06560193 0.3015891 0.1573278 0.214960479
0.3166652 0 0.1299816 0.2190019 0.5403438 0.08128334 0.1954979
0.4225435 0.2034468 0.108712 0 0.1214725 0.08342107 0.3215816 0
0.08135802 0.05307802 0.046402321 0.04844516 0.1150939 0.2426961
0.417014771 0.169515 0 0 0.2528073 0.1316117 0.318957 0.07781253
0.1863183 0.1456663 0.4466316 0.187955368 14.3724 0.057291529 0

0.1532030 0.08761422 0.08543609 0.08267929 0 0 0.05923394
0.0537879 0.07291838 0 0 0.1320018 0.1623244 0.2705568 0 0
0.1394126 0.088773572 0 0.09939964 0.04785424 0.1176344 0.329738389
0.05629173 0 0 0.3228239 0.4493321 0.09698286 0.1340316 0
0.1226658 0 0.1932811 0 13.85447 0.2491643 0.1905771 0
0.04379665 0 0.1386848 0 0.09682922 0.268687 0.054157974 0.1216444
0 0.2600793 0 0.4368154 0.260355 0.583796484 0 0.4331676
0.1495345 0.2621771 0.2525374 0.1160334 0.09489234 0.05992209 0
0 0.3414948 0.07362505 0.2368581 0.1157098 0.2547018 0.1093137
0.3390366 0.07763352 0.1085469 0.06406668 0.1103005 0.2200496 0
0 0.1023189 0.4115354 0.0689417 0.6670994 0.059286905 0.4832320
0 0.4868251 0.131972 0.09958762 0 0 0.06653153 0.04268475 0
0.124367851 0.1477008 0.08022991 0.1341335 0.3124674 0.2258198
0.1712299 0.1832426 0.1304998 0.4406794 0.1829106 0.3576138
0.1840525 0.5200359 0 0.132740937 1.594033 0.177786676 0 0.06065416
0.219887789 0 0.08419083 0.5506665 0.44836992 0.3456346 0.2413635
0.2956009 0.16158996 0.101666 0.2107319 0.05778661 0.4780268
0.2601438 0 0 0.2759837 0.09640644 0 0.08427105 0.249056697
0.2080728 0.178939218 0.2131559 0.0807824 0 0.1557201 0.2235108
0.2107622 0.07786667 0 0.4047336 0.054838388 0.1030486 0.1503562
0.03470867 0.2553134 0.05084825 0 0.2078027 0.3283351 0.08289329
0.5046094 0.06870595 0.06289616 0 0.06126358 0.82719418 0.2487584
0.2434591 0.1644421 0.07993563 0.5795294 0.067948 0.2421831
0.04649478 0.2058182 0.1383654 0.180994852 0.225212765 0.09993678
0.1106441 0.2010863 0.3075883 0.3418559 0 0.09589961 0
0.4299763 0 0.3784651 0.04305437 0 0.3564759 0.1951794 0
0.1376916 0 0.055699941 0.413478 0 0.04170662 0 0 0.06184264
0.8662878 0 0.3177699 0 0.1279592 0 0 0.3973576 0
0.1097081 0.2467952 0.408478264 0.07685284 0 0.1899051 0.3072946
0 0 0.112609891 0.05306964 0.4442258 0.1134694 0 0.277953699
0.1867467 0 0.1551556 0.157352353 0.124300784 0 0.2059633 0
0.1301972 0.3343216 0.1145085 0.3368852 0 0.2092199 0.358103
0.3551865 0.2149102 0.153779062 0 11.30576 0.07474519 0.2272872
0.2306975 0.2283857 0.05992253 0 0.1858961 0.3096764 0.3654456
0.1669128 0.3421719 0.2289406 0.5380747 0.03665847 0.1931686
0.1606096 0.7663097 0.2084531 0.07496562 0 0.07520035 0.06282445
0.06788411 0 0.3905613 0 0 0.1931891 0.2130629 0.2999883
0.1840748 0 0.09133972 0.1741728 0 0.6172389 0.1579954
0.2429644 0.1185994 0.2266739 0.04647388 0.5169151 0 0.3109152
0.3345162 1.276589755 0 0.4139046 0.3107396 0 0 0.05322567
0.056192156 0 0 0.1567917 0.4801508 1.060378 0.1486819 0.1678283
0.0625365 0.2629582 0.08306038 0.08685579 0.1045865 0.1105406
0.253873442 0 0.2630872 0.2086546 0 0.452549 0.1914774 0.08765621
0.3143922 0 0.2366347

MAGI2-AS3 0.7527427 2.770088 1.398323 3.688765745 0.668485053 2.116787
4.066978 2.809677 2.692223 3.865246 0.5084137 2.680000077 1.935349 2.116233966
3.188718863 1.765706 0.8167407 0.6462445 0.6788791 0.1036824
0.508328637 0.3268311 0.3567695 0.03050237 0.1642968 0.8604148
0.4627931 0.1059193 0.5646028 0.3162955 2.294327 0.460342586
0.2615468 0.2833889 0.579835 0.245374899 0.7749303 0.6711008
0.6374903 0.966572 0.2615471 0.3562596 0.6048874 1.677224 0.2256042
0.1373156 0.7387056 0.06470136 0.08730993 0.5238909 0.1211914
0.2677098 0.2773247 0.197753023 0.1687995 0.3831006 0.1142012
0.1234728 0.3528589 1.379819 0.2555253 0.5766804 0.4049797
0.6677068 0.1416044 0.188425282 1.047863 0.1065455 1.548855 0.821298997
0.2056092 0.03600909 0.1847152 0.603146746 3.311845 1.458246 0.3530691
0.08716119 0.4032243 0.7885358 0.3909791 0.145046 0.5020164
0.5073007 1.083492413 0.1389924 0.2278418 0.3647635 0.153391
8.371016 0.08397056 0.527717135 0.03615155 0.1248228 0.05795477
0.242729501 0.4882834 0.5316051 0.3905896 0.1811079 0.3221402
1.202781 0.1463277 0.1761142 0.08551188 0.6682521 0.561036668
1.017844 0.54210846 0.3974816 0.1760616 0.9084984 0.2432171
0.1364368 0.2616008 0.5308821 0.04153908 0.1821144 1.655312
0.3047204 0.09584635 0.1083955 0.3349156 0.2822385 0.1211396
0.7434763 0.155561 1.562603 1.303722945 0.1438151 1.12747 1.328434
0.5144089 1.174539958 0.3578992 1.642649 0.3057505 0.1959344
0.2280095 0.5587131 0.03200618 0.4191555 0.2758337 0.4088474
0.1750032 0.2106033 2.62982 0.1245768 0.1687625 1.225409 0.2627686
0.4327786 0.4429443 0.241053524 0.3612875 0.5674264 0.265120172
1.009424 0.6096204 0.1882583 1.032414 0.4520085 0.1787435 0.388600049
0.03951893 0.09266371 0.1562233 0.07564983 0.3316766 0.7654413
0.5048905 0.220003 1.915115 0.532767899 0.5631873 0.1164764 0.1428158
0.08461995 0.1621912 0.5905948 0.2513148 0.1065967 0.813257
0.3748217 0.8724888 1.876088 0.3123853 0.05497334 2.053925 0.09335924
0.04938894 2.51562 0.32385185 0.7995137 0.1691577 0.550028 0.3261507
1.461478 0.1456592 0.154118891 0.4549126 0.1509307 0.1350564
0.2729398 3.051521686 1.23887 0.7902905 1.635557 0.04929976 0.1364971
0.1290558 0.2060251 0.1782121 0.09646298 0.8517259 0.1216902
0.3021627 0.3764277 0.204075058 0.095093895 0.2435029 0.599672546
0.518615411 0.4779704 0.063447479 0.2610721 2.706556 0.1808081
0.289978023 0.1495966 0.1095095 0.125 0.819973481 0.5189293
0.1666912 1.10566 3.383911 0.03623738 0.1119457 0.4359574 0.0741417
3.254657 0.05306979 0.6980361 0.32338807 0.3447028 0.393471307
2.100927 1.467283 0.7218537 0.2045191 0.1578964 0.1038038 0.3370203
1.083648 0.05235135 1.126184725 0.4890754 0.354556 1.014278 0.1112661
0.8954978 0.2856549 0.4652099 0.1685708 1.696146 0.1570785
0.06357591 0.878631 0.919727 0.6162668 0.179011936 0.4009664 0.7775823
0.2879658 0.4915233 4.134375 0.02839496 0.4843453 0.1415601

2.7011119 0.2767189 0.273731684 0.076188106 0.2192549 0.1354093
2.1468290.4517222 0.1653083 1.630228 0.3921696 0.3243453 0.951591
1.250946 0.4744732 0.06040204 0.3045 1.230764 0.103897 0.6386167
1.028875 0.3654869 0.701622811 0.4584207 0.07683437 1.094285 0.1373888
0.09654636 0.5353312 0.4318324 0.4071703 0.3288223 0.1180024
0.2062537 0.373245294 1.852219 0.1275048 0.68959 0.5763521 0.4512107
0.49137212 0.6147963 0.7646156 0.5541022 0.807187 0.2937247
0.04809505 0.502521035 1.593605 0.5095591 0.159189 1.392 0.316659997
0.2006744 0.1714765 0.935525 0.852484289 0.100178413 0.4798579
0.0811522 0.4361144 0.6470715 0.7843746 0.3247116 0.1749717
0.3151913 1.208427 0.7525183 0.1460737 0.05933833 0.148417188
0.140369 2.100584 0.6559445 0.3324356 0.335702 1.04303 0.06618024
0.08398637 0.1831137 0.1871844 0.5999595 0.1395032 0.5489836
0.2015958 0.1101342 0.1181775 0.5203797 0.03116172 0.1200881
1.916441 0.8212298 0.2257148 0.08529823 0.09001325 0.5471022
0.4763674 0.05829022 0.1866369 0.4034758 0.229222 0.1192465
0.0805904 0.5989041 0.6095958 0.2917292 3.244154 0.2625569
0.0515879 0.242092 0.3118514 0.06903248 0.9963076 0.4272639
0.352953 0.1005363 0.3155421 0.557503 0.449100746 0.9586139 0.2523915
0.1892184 0.1883677 0.8361121 0.622792989 2.235848418 0.5412215
0.199972638 0.1287041 0.09623077 0.7279891 0.1630993 0.6662746
0.5282707 1.620067 0.6594959 0.4830876 0.7211473 0.1253839
0.274323085 0.3674213 0.2552229 0.2221401 0.748018 0.3633744
0.2972059 0.09288542 0.9440745 1.115024 0.5438833
AL731577.2 0.1600701 1.096826 0.3703587 0.855328648 0.535289706 0.7037794
0.8287492 0.9627176 0.8015467 0.6026316 0.405684 0.363628416
0.5961429 0.305963915 0.97933626 0.7319379 0.6708787 0 0.2369961
0.5687854 0 0.8325957 0.2518946 0.4646552 0.434697 0.3452529
0.2260907 0.5269128 0.5823478 0.333106011 0.2357378 0.787127423 0
0.4917271 0.04498219 0.058074682 0.617311 0.05344806 0.3441453
0.09202621 0.04418025 0.2102027 0.5730123 0.4607351 0.3634994
0.3595256 0.2009466 0.7199666 0.6277933 0.3919068 0.3733042
0.6557082 0.3332934 0.247819393 0.1262738 0.353498 0.07973506
0.4677268 0.3151186 0.08653178 0.3602455 0.5455085 0.4785103
0.1384728 0.2966038 0.243626282 0.2682095 0.2714226 1.643635
0.320799709 0.5332078 0.2828417 0.3934611 0.268797428 0.4207226
0.09186266 0.8126776 0.6846276 0.0375374 0 0.488921 0.1981386
0.2544002 0.4984427 0.36270186 0.4303701 0.4172557 0.201061 0
0.5849715 0.3318571 0.174071411 0.4240479 0.1918922 0.4172842
0.065182039 0.2119701 0.04471876 0.3339293 0.03951537 0.3017188
0.03323666 0 0.4659635 0.1214324 0.6981133 0.293786093 1.321469
0.107460327 0.05863119 0.0478932 0.821866 0.4930076 0.4807512
0.2067727 0.4196161 0 0.333311 0.3026659 0.2279521 0.250949
0.3243496 0.2475926 0.5074466 0.2416557 0.3278487 0 0.4358214

0.277517381 0.05238856 0.745766 0.6582329 0.478062 0.137440505 0.6687052
0.4184615 0.2725206 0.06727922 0.3210672 0 0 0 0.3067751
0.0385752 0.604221 0.349675 0.9150176 0 0.03971788 0.6178135
0.1642966 0.1611548 0.08670923 0.238164815 0.4237805 0.08399493
0.372470249 0.3042207 0.6153771 0.1626082 0.5751302 0.5462161
0.1627805 0.219002573 0.4138806 0.3159652 0.04674637 0.3551594
0.07894636 0.2901881 0.4004713 0.1123944 0.5150576 0.294960202
0.1067556 0.4603224 0.4146511 0.5787575 0.238869 0.205037 0.4592775
0.1456153 0.8483285 0.1201683 0.103444 1.221027 0.03894765 0
0.3198617 0 0.1724164 0.2780582 0.556015028 0.2589676 0.6813785
0.3642958 0.03381948 0.2062805 0.6537793 0.067253457 0.5379034
0.08319438 0.640502 0.2286793 0 0.6464239 0.05016177 0.5031818
0.5023606 0.5647529 0.5085219 0.3437036 0.305969 0.3214445
0.05718015 0.3130248 0.2013799 0.04064244 0.251443831 0.276643275 0
0.666940286 0.119372422 0.07584502 0.366611713 0.6741866 0.5790205
0.2295271 0.140166092 0 0.1509066 0.1232114 0.202060238 0.1271283
0.1976323 0.3974261 0.5977488 0.2710808 0.3517212 0.07546728
0.776484 0.1808272 0.4972934 0.6059164 0.077858265 0.06504619
0.447509252 0.3465032 0.3282968 0.4343241 0 0.5589783 0.09883037
0.09736841 0 0.4217495 0.308577092 0.3865716 0.7050485 0.6727228
0.03192568 0.2861245 0.1680706 0.4330783 0.3284534 0.6737507
0.4957771 0.7732205 0.5112155 0.06642949 0.03830353 0.129295667
0.2592167 0.1522168 0.1028133 0.5247666 0.7699649 0.4673108
0.07570948 0.3779062 0.5147309 0.1297643 0.264045944 0.187744935
0.687313 0 0.5028969 0 0.2564843 0.1835842 0.3997257 0.2717488
1.228946 1.075752 0.3312763 0.5114549 0.02761057 0.6686331 1.220311
0.1666845 0.4304413 0.2492478 0.278600066 0.1477239 0.353276
0.3650644 0 0 0 1.51655 0.8122445 0.728485 0.04753216 0.5066876
0.375032047 0.1221295 0.372657 0.3501604 0.03429613 0.2700296
0.357547281 0.6006292 0.385918 0.2968336 0.528353 0.5010957 0.3357988
0.176016362 0.7299705 0.1190319 0.2483037 0.2643405 0.202747608 0
0.05612091 0.1455108 0.787045282 0.194289964 0.5094114 0.5408485
0.1171132 0.2849089 0.4598579 0.1431872 0.4633841 0.1765235
0.8829647 0.268674 0.09869847 0.7390201 0.064097699 0.2450135
0.9376774 0 0.2557902 0.3605947 0.285585 0.2247905 0.1465978
0.05811347 0.3388307 0.1305633 0.1739302 1.149898 0.2862789
0.09611939 0.3896366 0.573676 0.3012515 0.119779 0.7168157 0.234352
0.2608447 0.04701716 0.03927943 0.1697714 0.4843673 0.195351
0.1379748 0.8451181 0.1509834 0.5328493 0 0.9782496 0.1666914
0.4283092 0.6533835 0.2618811 0.4245046 0.03292757 0.1012717
0.5561349 0.1417223 0.406793 0.1211956 0 0 0.6274438 0.057011151
0.4450381 0.03696908 0.1942823 0 0.2829266 0.232946219 0.526991299
0.1218967 0.418861607 0.1470452 0.04288605 2.651899 0.4415586
0.5771175 0.5864909 0.5589876 0 0.09050739 0.1961703 0.5874583

0.380947392 0.04933325 0.2056109 0.1739415 0.1747355 0.4810066
0.1496456 0.1370122 0.2751923 0.08650089 0.11836
AC010618.2 0.1931281 0 0.1489486 0 0.067983018 0.09989708 0.05262652
0.1010035 0 0.05592986 0.1223666 0.274203493 0.4250169 0.184576058
0.236318098 0.2077880.7588404 1.2991140.1072279 1.0917650.341658211
0.5022724 0.7766751 0.1401542 0.4807654 0.5415217 0.3409792
0.7026509 0.3783313 0.312588612 0.4875821.187107934 1.3597180.0847542
0.2170880.385376016 0.5257406 0.8383212 0.7473938 0.9992846
0.3731310.4226902 0.4609012 1.29707 0.1879585 0.9109284 0.5657083
0.4087790.1317298 0.5516514 0.3603197 0.3819230.4021257 0.341713697
0.8633282 0.3838527 1.4911320.4647362 0.4493242 0.3654086
1.6143920.3760959 0.2099393 0.3898310.7668406 0.881821215 0.4314675
0.9460455 0.2832974 0.387051823 1.18768 0.5687577 0.9098788
0.324309941 0.3583136 0.38792 0.3922053 0.2753393 1.0416630.9197386
0.7373673 0.9562343 0.3069394 0.2460199 0.125030765 0.06108834
0.5034281 0.2830151 0.6460762 0.6137224 0.3603536 0.490048863
0.1096335 0.2315222 1.0069251.101009652 0.6393664 0.3237249
0.1342977 3.9571210.1323747 0.5213097 1.29135 0.4684959 0.842437
0.2948011 0.106337792 1.8069650.12965322 1.2733170.6934103 0.3432459
0.5948244 0.5155882 0.6236894 0.3037656 0.5248823 0.268098
0.4057478 1.4301510.3027755 0.3913349 0.2489382 0.5816331 3.29466
0.04944458 0.4518731 0.5258280.535729206 0.5372675 0.2249457
0.1443950.3105804 0.331649915 0.2547813 0.3365886 0.8220051
0.4464561 0.3389527 0.5121117 1.8873210.6437606 0.7711060.4188764
0.1944015 0.06490623 1.6191832.6313920.8146482 0.4065848 0.363417
0.6319195 0.8892409 0.689642464 0.5113004 1.5707970.163415801
0.6423355 0.1856165 0.6376184 0.5088652 0.6590215 0.2945973
0.660578413 0.3329040.6535181 0.7896071 0.1648106 0.8572545
0.2625887 0.4652819 0.4068189 0.0365546 0.177937948 0.837219
0.0555389 0.7504284 0.04364272 1.0567360.2886119 1.3213830.5856268
0.1637643 0.7249284 0.3744222 0.3734864 1.0338060.9776358
0.1543681.2417630.2600301 1.73333 0.178891792 1.2498 0.4110490.6592963
2.5706480.1991056 0.1502474 0.162285517 0.7880619 2.1078920.2575932
0.2069299 0.93816618 0.4735256 1.2709460.6070998 0.4040727
0.5962133 0.2583338 0.09215242 0.2214951.1080850.4829236 0.9711547
0.6247781 0.9807198 0.303372449 0.13351045 3.7488310.625860631
0.096016948 0.6863150.276453126 0.4067104 0.3810552.1808190.789196041
2.3465590.3641441 2.2670230.365685104 0.4601490.9537908 0.435912
0.6657203 0.1308259 0.6601144 0.1821058 1.2491260 0.3882322
0.5403423 1.127252268 0.3139186 0.404947231 1.6400960.2132831
0.1637567 1.4096050.1686049 0.7154459 0.7636012 1.5062120.9159298
0.206836094 0.9716813 0.6805250.2094593 1.6563180.4602872 1.095016
0.6270220.7925723 0.3439170.7613020.3627973 0.1897823 0.4007431
0.1386421 1.052986722 0.1876503 0.2754793 0.4961861 0.1507482

0.710396 0.2562821 0.6850885 0.1402929 0.621034 1.148132 0.591643318
0.453036692 0.414629 0.9181055 0.252815 0.5568685 0.5673321 0.132899
0.3617083 0.1873547 0.1853438 0.3708337 0.6280875 0.1623899
0.2331892 0.7170848 0.1226943 0.1508314 0.5193368 0.3436833
0.588239813 4.233013 0.2367973 0.4404582 0.5260687 0.2837086
2.005987 0.5227859 0.06999933 0.719127 0.7455315 1.319185 0.452484317
0.3929385 0.3746823 0.3696666 0.3310323 0.1396271 0.400575098
0.260882 0.3991015 0.6088316 0.1738553 0.1007638 0.4726733
0.297314594 0.1200989 1.101045 0.427977 0.1594663 0.314510653 1.549591
0.1354222 0.4096446 0.178047608 0 0.330947 0.2485888 0.5651984
1.227674 0.5548286 0.2591377 0.7623858 0.3407672 0.3945615
0.5402684 0.4167865 0.3242341 0.348008703 0.09853802 2.175631
0.902142 0.3429072 0.4350653 0.6891289 0.632834 0.1768735 0.7011516
0.1752028 1.732802 0.8394021 0.613027 0.604453 0.9857461 0.02765324
0.5100072 0.1211555 1.541504 0.3144923 0.5089514 0.3540542
0.510545 1.563919 0.5632906 0.1947999 1.414171 0.3329392 0.5490441
0.5829273 0.7634369 2.376103 0.1388564 0 0.1378038 0.5255476
0.7583166 2.234941 0.6753732 0.3665594 0.5815236 0.4274775
0.8063219 1.852185 7.081601 2.908273 0.5046831 0.20635558 0.1579259
1.159704 0.7969796 0.6899099 0.06827142 0.160602673 0.211942153
0.1470711 0.224606949 0.473102 2.017975 0 0.9253027 0.1582511
0.1415228 0.5554135 1.441098 1.070151 0.1577892 0.6253952 0.268112431
0.654738 1.786133 0.5771265 0.3513702 1.331379 0.4333217 0.4628629
0.664051 0.4696434 1.713647
AC008115.3 0.4854586 0.1108814 0.3432058 0 0.370253593 0.5859173
0.5732361 0.2115737 0.1906604 0.09372581 0.4613824 0.459503122
0.5752646 0.579952094 0.099003939 0.5223086 0.7205989 0.6220063
0.1796897 0.02613644 0.687050391 0.888456 1.782533 0.3523001 0.6591716
1.256494 2.171336 2.663357 0.09057112 0.299330235 1.327751 0.824148834
0.1265878 1.455796 0.8185284 0.205482824 1.578499 0.270161 1.809114
0.5581917 0.1786524 1.416667 1.287277 1.125612 0.6824482 0.5815277
0.2031427 0.5565798 0.7726227 0.6603165 0.6792912 0.7543053
0.5294708 0.823162776 0.1702051 1.57239 1.652433 0.2781403 0.9267273
1.574595 1.560783 0.4726889 0.3957872 0.6066067 1.413557 0.689608819
0.09038026 1.615846 0.9494848 0.445920361 0.8707492 0.6671766
1.292724 1.177518789 0.4253206 0.2321666 0.4929356 1.061235 0.5692146
0.2055032 1.153284 0.8679844 0.4286342 0.1832328 0.209523323
0.409481 0.3954524 0.779157 0.1249244 0.4113841 0.6038712 0.293289718
0.6430234 0.1697408 3.259709 2.438093192 1.125005 0.2712449 1.181526
0.6391557 1.053693 0.4703986 1.229551 5.142361 0.4603481 0.7763172
0.772191862 7.21392 0.86907805 0.8298076 2.711331 0.4793355 0.941414
3.240035 0.3658069 0.5090425 1.011522 0.4492717 0.9859155 0.7835073
0.8244979 2.541182 0.6674628 1.564627 2.125381 0.3314318 0.1514474
0.6168183 0.224440287 0.1059222 1.350767 0.06049333 0.8922216

1.66731105 1.387607 1.034085 0.9642464 0.5781233 0.4057201 0.4290918
0.8753966 0.9316887 0.3876598 0.7799358 0.5701029 0.2447284
10.11353 0.1574864 0.2810637 1.078795 0.5813228 0.3258321 0.7889119
0.963070836 0.1836051 0.2547387 0.205385979 0.422875 1.010917 0.6164449
1.007787 1.058356 2.962072 0.184496703 0.4648932 0.3194184 0.7088588
2.623761 0.478855 1.760157 0.884668 0.302994 0.6738298 0.447275715 1.780718
0.4188179 1.047957 0.2925414 2.039161 0.3109168 3.285793 0.8832404
0.8232959 0.647902 0.6274471 0.834505 0.5118531 0.1724523 0.3880289
0.05202287 0.1307255 4.497554 0.262309458 1.527154 2.617536 0.5984505
1.059862 1.501452 1.195958 0.815861614 1.204089 0.5046217 0.4046888
0.1155893 0.471646386 0.7468441 0.2028399 0.974972 1.100344 1.462995
2.516284 0.5790999 0.3917964 1.160563 3.352694 3.300078 0.5234934
0.2054331 0.571931666 0.755100087 3.793023 0.299657445 0.362031115
0.1533478 0.694909498 0.9087397 1.170697 1.363209 0.897420426 3.60461
3.356228 0.1556975 1.48094696 0.06425882 0.3995845 1.095735 0.6042816
1.479834 1.896347 0.3051682 2.529348 0.243738 1.715191 1.01202 0.629673398
1.512413 0.301600042 2.020904 0.2297663 1.317213 0.1968483 1.789443
0.4329454 0.1968651 0.4206784 1.108533 2.045002405 0.4559291
0.04751693 0.8336403 4.453894 0.1928343 0.1699074 0.7442793
0.5395699 1.074064 4.647448 0.521114 0.2782783 0.268622 2.633106 0.718898038
0.5503043 0.07694018 1.004724 0.3536679 1.739908 0.8159948 0.2296107
0.8228474 1.951336 0.52473 0.838928173 0.332144387 1.326483 0.3496676
0.508393 1.671958 0.3889311 0.59389 0.9496216 0.4709463 2.368282
1.398226 0.717636 0.8708119 1.116493 3.079285 1.418695 0.9689106 0.9863301
0.7199195 1.056168282 0.933365 0.7936377 0.3954152 0.2644717
0.3328021 0.5863223 2.573459 0.3519092 2.309764 0.6727229 2.021941
0.670769802 0.2057738 1.255766 1.371701 0.4160515 1.091923 0.671273388
1.06866 0.8081382 0.4801243 0.6797984 0.6754295 0.452625 0.711760151
0.5031466 1.764882 0.5737542 2.271451 0.263524409 0.1770522 0.3971398
1.078741 1.094014447 1.885568178 0.2772962 1.01541 0.1973218 0.2468766
0.5916701 0.6513844 0.2981035 0.0713811 0.7603754 0.9506362
0.1995543 1.358358 0.583183984 0.2476912 9.625087 0.4724332 0.114927
0.2187214 0.685677 1.817977 0.2964 1.057475 1.125468 0.9239316 0.1406648
1.432806 1.012927 1.311793 0.5097464 0.4273284 0.2030293 2.09886
1.734768 0.521209 0.9229343 0.237655 0.3970871 0.9439478 0.522305
1.925488 0.0929885 2.694502 1.068435 1.077346 1.279868 0.8725949 0.4044315
0.4329902 0.6605243 1.429613 3.316113 0.8654734 0.5118923 1.799081
0.4656312 0.3818646 1.184362 0.3170616 0.8253693 0.4581064
1.729026717 0.5292964 0.1494925 1.099871 0 0.5434356 0.370058982
0.461717331 0.78045 0.75278103 0.2973045 1.604126 2.010661 1.527104
0.6099442 0.5929007 0.6315787 0.367493 0.3476868 0.3635761 2.34058
0.673943816 0.5485965 1.746008 0.7033699 0.6476988 1.172753 1.149737
1.357394 2.623027 0.2623388 1.346102
AC087294.1 0.07798019 0.04007496 0.05638278 0.104171034 0.092643085

0.07562978	0.06374773	0.09176088	0.03445443	0.1016237	0.185282	
0.132859625	0.06930443	0.013973829	0.089455501	0.1730427	0.1838402	
0.2060729	0.1190636	0.07557027	0	0.2704063	0.224975	0.127329
0.2779456	0.06307287	0.2581475	0.1317236	0.08183599	0.027046132	
0.1845685	0.123254694	0.09150324	0.1154978	0.2300932	0.212188602	
0.2388157	0.136699	0.2514821	0.03362379	0.09685334	0.2560072	
0.1628376	0.3927926	0.07589285	0.3940817	0.1468405	0.1547386	
0.1097025	0.1909223	0.02727898	0.4543709	0.2957419	0.155222153	
0.1076527	0.0516633	0.2476301	0.4222093	0.1674698	0.09484885	
0.09401685	0.8399641	0.09536414	0.1686469	0.1393338	0.320451159	
0.06533082	0.2975106	0.2573735	0.102559815	0.2098053	0.1377899	
0.2276195	0.196422084	0.1537202	0.0839101	0.0742324	0.3502012	
0.1782964	0.1671151	0.148865	0.2171829	0.0929507	0.09933654	0
0.02774915	0.1429252	0.2081424	0.105351	0.3159519	0.2182523	
0.127201624	0.0774676	0.07011205	0.6514368	0.071447067	0.2517058	
0.03267796	0.02033474	0.2598805	0.2605663	0.09714994	0.2133057	
0.3405	0.1109199	0.06376775	0.203948279	9.07716	0.065438316	0.08568877
0.314979	0.1154949	0.06004372	1.10271	0.07554895	0.04599479	0.286111
0.1894394	0.1474475	0.3164917	0.1375346	0.1629488	0.2110813	
0.713816	0.4856181	0.08984005	0.1094729	0.2229317	0.081117655	
0.1531305	0.1248876	0.07652267	0.08061713	0.125542266	0.3857781	
0.1189176	0.2240356	0.1720736	0.190627	0.1550832	0.2551513	
0.2126733	0.1401088	0.1832259	0.1471769	0.06879462	2.429405	
0.09960833	0.1306062	0.1231264	0.09004408	0.07360181	0.2376084	
0.243652483	0.2433161	0.153447	0.148462018	0.3612495	0.08431558	
0.05941246	0.09806367	0.232834	0.416328	0.066681165	0.1176158	0.06596847
0.3415962	0.1896569	0.1586463	0.09277327	0.08670874	0.1231973	
0.1217685	0.089808488	0.1755247	0.1009133	0.1839666	0.1585963	
0.2327361	0.1997727	0.4776048	0.6916487	0.01239822	0.1609891	
0.1133866	0.1068197	0.1707646	0.06232805	0.05843424	0.2068243	
0.3779766	0.4233112	0.094804406	0.2523186	0.3360914	0.1996552	
0.1359236	0.1658121	0.3412466	0.098290109	0.210573	0.09119068	
0.07800716	0.1879942	0.170463374	0.3627129	0.183277	0.06128284	
0.3467027	0.3740001	0.4987253	0.08371977	0.044717	0.721459	0.1462442
0.3921274	0.3573816	0.1484961	0.045935262	0.080862155	0.5783408	
0.243681276	0.08723067	0.05542328	0.200924458	0.1642193	0.1538603	
0.2306226	0.307276555	0.2960876	0.1654111	0.06752699	0.129197502	
0.06967365	0.02406977	0.2244129	0.1092003	0.1188544	0.4283635	
0.1378681	0.3677664	0.1321384	0.08550449	0.1636323	0.455155623	
0.3089587	0.16350733	0.1460799	0.09226956	0.09918116	0.160077	
0.1191371	0.1805492	0.03557569	0.2349747	0.1232763	0.112745416	
0.09416161	0.3434733	0.1585768	0.7115496	0.1045419	0.2210698	
0.03164693	0.1500096	0.1041487	1.531482	0.1412565	0.05747194	
0.04854295	0.1819355	0.236205053	0.2273052	0.05561575	0.1878255	

0.575205 1.307327 0.0620881 0.0691553 0.4460927 0.2350853 0.1106286
0.165385685 0.154342652 0.2739544 0.2274796 0.1071842 0.1686369
0.203043 0.1341531 0.1387461 0.1985788 0.294671 0.2245998 0.1210391
0.1967066 0.2219388 0.08143326 0.1634847 0.03045092 0.3250271
0.07805843 0.050896325 1.012016 0.2151286 0.0571647 0.09558588
0.1718314 0.08476391 0.6926311 0.3391673 0.266168 0.1910361
0.282566 0.11594524 0.07437118 0.2723169 0.3038546 0.1127776
0.0704723 0.055987607 0.04389061 0.07050181 0.325364 0.08774789
0.1424005 0.2044858 0.16720975 0.1091089 0.2174547 0.2592092
0.183507 0.158739193 0.02133018 0.12303 0.1594967 0.161754904 0.141976208
0.1503314 0.4046318 0.09984304 0.04461331 0.3207639 0.05231659
0.1693075 0.1160941 0.1194854 0.3599418 0.09015413 0.1718292
0.210775515 0.1492017 5.876925 0.08537387 0.1557643 0.329378 0.430422
0.1095095 0.09641292 0.1910973 0.1591704 0.1788906 0.1652279
0.3712869 0.1307479 0.263395 0.1674851 0.1544459 0.4402754
0.6710465 0.1031744 0.1027508 0.5956585 0.13743 0.143516 0.2326115
0.1061845 0.4282547 0.08402022 0.09500999 0.3530567 0.1946882
0.2398524 0.1366625 0.1218087 0.2399547 0.19894 0.2105048
0.817811 0.1203081 0.1110055 0.3251137 0.116508 0.0636989 0.8856297
0.09822262 0.4403574 0.06368066 0.104151379 0.1530395 0.175597
0.2129559 0.0626778 0.07236142 0.060794297 0.102692182 0.133613
0.08502238 0.07163493 0.1096855 0.9689296 0.4075784 0.0766773
0.157144 0.5766731 0.07589718 0.08597904 0.05972927 0.2272667
0.162385409 0.2162998 0.3004979 0.2224367 0.148968 0.3825067
0.1421584 0.120145 0.4309181 0.2370375 0.5513791
AC138207.4 0.4724527 0.1649202 0.4537507 0.190530765 0.301237212
0.4703162 0.3789363 0.3496507 0.2835801 0.07744652 0.5252704
0.303753418 0.5523075 0.677297454 0.212700515 0.4172023 0.3922883
0.4283081 0.2573641 0.9416199 0.359553614 1.313723 1.028709 2.503536
0.6415117 0.6921687 1.841411 0.6486442 1.39202 0.742018207 1.249043
1.089603154 0.3138021 0.8449907 0.435875 0.882920477 0.6915973
0.6964981 2.046846 0.6457348 0.5904886 1.311078 0.3616544 1.154612
0.2776189 0.4805218 0.2238117 0.8349083 0.7113884 0.4801507
0.2993625 0.7403925 0.5727361 0.579637773 0.4359904 1.015801
0.3330295 0.7538423 0.6987592 1.301102 1.048947 0.6249397 0.2907047
0.8945292 0.3256341 1.302469224 0.8961847 0.4333066 0.3269035
0.254578035 0.3974427 0.1890152 0.4163202 1.332254563 0.8021249
0.6906291 0.3937401 1.159044 0.3888205 0.4245231 0.3403461
0.5958474 2.734304 0.302814 0.121191771 1.167335 0.4531156 1.769119
0.2202159 1.240747 0.4546303 0.242348066 0.4554311 0.4408121
0.3042102 0.78406811 0.7968022 0.2689587 0.4463113 0.3300877
5.938929 0.510856 0.4714193 0.843349 0.2333058 0.5131826 0.971828831
8.742698 0.323157333 0.4114067 0.7361309 0.6126028 0.6955335
1.561742 0.2763609 0.2804178 0.3488682 0.5444815 0.8202897

1.340539 0.2515534 0.6231672 0.3447067 1.873596 0.4925498 0.1643191
0.3253704 0.3786215 0.241094269 0.5601571 1.017515 0.5698428
0.2580379 0.574047867 0.4703969 0.6991155 0.3870006 1.0903
0.898471 1.863986 0.9986876 0.7941746 0.5039811 0.6186896 0.8075681
0.5122942 21.17762 0.208212 0.4379488 0.8069684 0.4391795 0.3903953
0.2028088 0.175074798 0.9912024 0.6455119 0.871190196 0.8767407
0.6168591 0.7334989 0.2434175 1.064644 0.4079313 0.341491068
0.2919507 0.3468905 0.2030554 0.5385863 0.7781754 1.090826
0.7186195 0.2879219 0.5972859 0.772028583 0.481557 1.368912 1.74177
0.4592864 0.434547 0.9020518 1.345736 1.540752 0.8163566 0.7896671
0.3456436 0.6206035 0.5335665 0.2992478 0.3954461 0.2407271
0.936171 0.8826382 0.842223848 0.5191825 0.1138366 0.6694841
0.4633127 0.6892576 1.040244 0.865164509 0.9885311 1.028535 0.6509618
0.7449975 0.311780693 1.272819 0.4525431 0.3222514 0.5222212
0.3774086 0.8764062 0.3700522 0.3544156 2.102093 0.5731785
0.6424991 0.422955 0.5975247 0.525102824 0.757979257 1.900115 1.200907429
0.478639572 1.178429 0.260308663 0.4255101 0.3869433 0.7765194
2.497842461 1.588546 2.148034 0.689587 0.928339668 0.4672595 0.3081684
0.3742388 0.4301865 0.4891205 1.29275 1.462548 0.8648377 0.3625254
0.6646546 0.5897705 0.585343215 0.5868255 1.208692583 0.7837362
1.063205 2.467097 0.3741126 0.4202434 1.331919 0.2277401 0.6573
0.7327928 0.515533673 0.785769 0.9894449 0.6163349 0.7893965
0.3611726 0.4604992 0.6077697 0.6859257 0.4069556 1.611391
0.3301278 0.5518655 1.109824 0.691124 0.669636348 0.2165341 0.3814584
0.3893413 0.3089385 1.604173 0.2555106 0.3541617 0.3885295
0.8026207 0.6214781 0.466344152 0.784154418 0.2922895 0.7627848
0.5741229 0.7711003 0.971274 0.7606422 1.248812 1.089613 0.4106353
0.872944 2.103136 0.8274941 0.793409 0.8440101 0.9785995 0.1810097
0.6136575 0.5948763 0.383997607 0.9995378 0.7607168 0.3746565
0.2476735 0.3928536 0.6976565 1.484007 0.6203432 0.7081109
0.5399951 1.523723 0.424132014 0.5577078 0.4358135 2.135272 1.386609
0.5155806 1.954178705 0.3371623 2.044765 3.332543 1.492581 0.6697365
0.2618058 0.811625114 0.7206416 0.9147758 0.3792788 0.4769588
0.48389491 0.448653 1.087617 0.3565497 1.347773585 0.519354269 0.6153844
5.060076 1.056557 0.2039965 0.4190594 1.028646 0.4222727 0.6252185
1.486079 0.2992456 0.7667552 0.4377454 0.289134263 1.009703 4.217659
1.093053 0.4083506 1.036194 1.001954 0.6008848 1.10703 0.3883561
1.051287 0.708921 0.09298614 2.207045 0.5261086 1.397087 0.4748166
0.332928 0.503295 0.947199 1.05241 0.8613599 0.8715763 2.356519 0.4199896
1.687621 1.467391 0.538509 0.6454331 1.325035 0.8979922 0.4339827
0.7520477 0.3172547 1.303324 0.5915361 1.073402 0.8225368 1.379739
0.4070847 0.7782858 0.668969 3.764684 0.2718484 1.160881 0.6437494
0.6495338 0.4076559 0.304791705 0.691033 0.3088176 1.532034 0.1337453
0.3403296 0.478133786 1.209129344 0.8553321 0.839740608 0.6878635

0.6161804 11.51924 0.5280402 0.2892537 0.3658065 1.010798 0.2602825
0.4173366 0.9941381 0.2424771 0.466723626 0.3626484 0.7969435
0.929922 0.4281597 0.5577986 0.4300173 0.5035878 1.353003 0.4190945
0.7019831
PRC1-AS1 0.07407359 0.08459403 0.0190429 0.035183057 0.008691548
0.03831517 0.09419533 0.06456586 0.02909188 0.04290339 0.06257772
0.028045271 0.05015807 0.070793485 0.04531948 0.05313089 0.05174231
0.1581812 0.02741791 0.1914251 0.06988895 0.07134992 0.1467868
0.04479635 0.08940405 0.1171634 0.1830939 0.1197774 0.1105582
0.09134647 0.09350516 0.234159939 0.1738384 0.2492216 0.04163166
0.179163139 0.1120255 0.1978678 0.1274045 0.02839052 0.01362982
0.01080809 0.05892567 0.2013635 0.03204037 0.3105629 0.04132865
0.02613095 0.1599942 0.0806034 0.05758305 0.1046323 0.01468894
0.109219219 0.0519414 0.3380073 0.1721905 0.04243998 0.1325667
0.08008639 0.2381516 0.06010427 0.06710123 0.1139187 0.1176476
0.060127887 0.01379065 0.06512735 0.03621923 0.012371028 0.0506145
0.058172 0.1011538 0.069104397 0.1145249 0.05668013 0 0.07040361
0.09264371 0.04703499 0.1131258 0.04075114 0.05232244 0.02795853 0
0.03124028 0.08045334 0.1033804 0.02541541 0.1255419 0.2866626
0.017900618 0.1308208 0.02959983 0.2340621 0.221198636 0.1798329
0.01379595 0.03433959 0.1219068 0.04230986 0.153805 0.1500886
0.0838553 0.1592154 0.03230569 0.126888157 1.277395 0.044202691 0
0.103427 0.05851143 0 0.1895123 0.03189518 0.05178143 0.02684223
0.06855201 0.03112463 0.1265837 0.05806423 0.01250792 0.08911407
0.1330672 0.2087454 0.02528572 0.1039886 0.08067178 0.017123085
0.1454591 0.01917269 0.03692145 0.03403486 0.042401045 0.05428913
0.07172076 0 0.04151191 0.02476268 0.1122392 0.1464985 0.01496437
0.05520759 0.2142114 0.06213501 0.0497891 2.295936 0.0961201
0.159291 0.05198138 0.059134 0 0.05350042 0.029389988 0.1680921
0.1295642 0.052231287 0.1290486 0.2135776 0.1379548 0.09462953
0.08425512 0.02510928 0.022521109 0.0567485 0.111402 0.02884295
0.1432817 0.1339543 0.1342866 0.1235473 0.05779034 0.009346924
0.075830513 0.1482057 0.02840233 0.08223546 0.02231868 0.1064443
0.08433986 0.1525884 0.08984602 0.01046853 0.0123575 0.0212753
0.08488851 0.01201554 0.05262719 0.0592073 0 0.02659563 0.2001587
0.022871102 0.1065236 0.05255212 0.02809675 0.08346776 0.1145493
0.2593209 0.072618033 0.04741307 0.2438257 0.07409921 0.03527432 0
0.02848926 0.06190057 0.1811063 0.08610035 0 0.04954149 0.01178158
0.04405001 0.05666695 0.01764035 0.1793436 0.1775047 0.05015354
0.019392903 0.05974203 0.2893788 0.102877115 0.036826956 0.128692
0.028275361 0.06932992 0.04871743 0.2478356 0.115311738 0.2166701
0.09311077 0.2565763 0.093504807 0 0.1016175 0.1226078 0.1134821
0.2508891 0.08439484 0.04656402 0.2306768 0 0.1714665 0.02438192
0.036029464 0.3210729 0.092039146 0.2549103 0.1168628 0.1256166

0.04505408 0.08622373 0.06097927 0.04505792 0.0233415 0.1171005
0.031732497 0.08944437 0.2320115 0.08703213 0.1969844 0.09807861
0.03111034 0.06680335 0.2279912 0.0479666 0.2502811 0.02650469 0
0 0 0.199441621 0.06397565 0.0939192 0.211456 0.03854595 0.2095922
0.01310614 0.07007019 0.1345221 0.2514284 0.08006581 0.104733541
0.115840399 0.1734867 0.6189058 0.1163595 0.2254508 0.07912661
0.05663661 0.04316104 0.03592962 0.2725038 0.07111598 0.1022003
0.03321816 0.09369789 0.04583922 0.06901979 0.05142296 0.07082303
0.1318185 0.096693116 0.4785221 0.04843881 0.04826747 0.04035435
0.01450872 0.1312137 0.2840596 0 0.02043101 0.08798337 0.08227132
0.044499676 0.05023672 0.09580538 0.0405098 0.03174156 0.02380154
0.047273582 0.09635441 0.05102466 0.1739917 0.1333631 0.008588354
0.207191 0.097743383 0.02047267 0.269294 0.1422625 0.08970533 0.044677568
0.1080619 0.1038814 0.07481795 0.106228145 0.119878744 0.03022227
0.06356355 0.06021662 0.1130088 0.02579424 0.02208696 0.1429562
0.03267501 0.02017769 0.09670174 0.03044894 0.08290589 0.049436108
0.0377939 2.247467 0.05766889 0.0964486 0.1557432 0.05506519
0.1040234 0.01809045 0.01792828 0.1791958 0.01006985 0.08585324
0.1237496 0.1987164 0.1186131 0.01414173 0.2887597 0.01548959
0.2833017 0.0938174 0.05783895 0.1106488 0.01450502 0.02423578
0.03928147 0.09961973 0.1958668 0.113509 0.06016683 0 0.1130157
0.1735897 0.1597736 0.02057003 0.02642708 0.08398828 0.1131082
0.1309617 0.02031662 0.03124279 0.2516363 0.04372199 0.08964112
0.06231571 1.202558 0.04797668 0.07527699 0.105529257 0.06461006
0.1254565 0.179811 0 0.02618526 0.03079929 0.01083862 0.01253524
0.08614719 0.01512138 0.2778416 0 0.1218837 0.04046443 0.06031174
0.152162 0.0160211 0.02233757 0.07060604 0.0106608 0.07834938
0.1065368 0.1649231 0.08049265 0.03593781 0.0698319 0.0830995
0.05072269 0.1334113 0.06671483 0.1369298
AC005740.4 0.1547177 0.1590226 0.1073924 0.165345663 0.13070925
0.3121131 0.2276633 0.1699222 0.1093757 0.1478605 0.1764535
0.131801052 0.3142959 0.155259902 0.227181452 0.1248465 0.267484
0.7433853 0.03436072 0.3148663 0.098534656 0.429203 0.08115727 0
0.08403233 0.06006751 0.1475082 0.08041484 0.2597886 0.064393534
0.2929568 0.211939258 0.09077418 0.0407387 0.1304343 0.101039024
0.1684714 0.1549826 0.1596662 0.05336943 0.02562177 0.0203174
0.1107704 0.1558652 0.06023053 0.3544539 0.1748046 0 0.04748879
0.03788023 0.1515451 0.06556373 0.08283826 0.102656936 0.2441029
0.123004 0.3236893 0.06382405 0.04984064 0.100366 0 0.06779159
0.2018225 0.107074 0.07371926 0.113030375 0.07777235 0.1574081
0.1815629 0.116277324 0.0951468 0.05467681 0.1140914 0.181866599
0.2152877 0.186461 0.07069531 0.1852858 0.06530801 0.2652538
0.02362863 0.07660533 0 0.1576721 0 0.02936325 0.03024777
0.1554703 0.02388836 0.4424958 0.0577369 0.033650168 0.1053948

0.01391068 0.175999 0.340213462 0.1229294 0.1815385 0.06455269
0.1374986 0.01590709 0.2505773 0.1410708 0.1576339 0.2112695
0.06072929 0.170377515 1.379464 0.062320252 0.06800483 0.1666503
0.09165976 0.03176817 0.1084244 0 0.09734042 0 0 0.1950303
0.1850771 0.2183021 0.07053842 0.02393136 0.2648585 0.1401451
0.1901317 0.7384843 0.07582473 0.128754146 0.2278656 0.1441658
0.1041092 0 0.318827505 0.0408218 0.1617876 0.03951121 0.01950886
0.09309941 0.1758258 0.1943949 0.08439154 0.05930345 0.1789696 0
0.04679759 3.431555 0.4065519 0.09213552 0.325721 0.1270423 0.07009476
0.1005718 0 0.03510945 0.1704913 0.019637217 0.1543753 0.1784401
0.1414538 0.04447192 0.1319879 0.1416037 0.02116796 0.2133554 0
0 0.06337544 0.1144598 0.1893273 0.1806375 0.1303634 0.1757066
0.028509737 0.1238231 0.05339163 0.1030593 0.06293307 0.2308818
0.05945431 0.1229318 0.2814925 0.1771118 0.09292011 0.09998504
0.2992054 0.112936 0.09893031 0.3524494 0 0 0.08062817 0.128981543
0.4255241 0.4149146 0.05281719 0.3922636 0.1196298 0.01805481
0.058504183 0.133693 0.07237129 0.04643138 0.06630982 0.180378868
0.09372133 0.08727204 0.2431782 0.09711256 0.04094016 0.124173
0.04429479 0.1182952 0.1864177 0.1658045 0.1296677 0.1334717
0.1178503 0.145821658 0 0.6459807 0.04297595 0.230761735 0.1099634
0.053152952 0.06516433 0.3663225 0.3161394 0.027095849 0.1879862
0.07001307 0.2500921 0.146477837 0.03686321 0.1528192 0.2304822
0.05333182 0.1572099 0.1586482 0.1750652 0.2501734 0.1747808 0
0.2750036 0.203188105 0.4149498 0 0.3709848 0.02929101 0.0314851
0.367008 0.02701437 0.03821028 0.1694027 0.372964 0 0.059651789
0.07472909 0.1090357 0.1006805 0.1110893 0.1659343 0.1949408
0.02511582 0.0238103 0.2705076 0.2875196 0.02491219 0.09122244
0.1926247 0.0444273 0.168712606 0.1052306 0 0.2782513 0.02898398
0.1575993 0.04927472 0.1097669 0.01685861 0.04975194 0.1755954
0.196881548 0.027220073 0.2717717 0.1203558 0.2187365 0.1338347
0.0495816 0.1916412 0.1159079 0.2476528 0.1113612 0 0.02744564
0.01561115 0.06404959 0.2585102 0.04718025 0.1208332 0.1331355
0.1858476 0.14137425 1.070883 0.06829265 0.1209797 0.2275783
0.05454795 0.8296737 0.2826977 0.03364649 0.09601719 0.2205254
0.07732816 0.033460781 0.4013557 0.1080589 0.07615164 0.05966882
0.02237146 0.059244287 0.153264 0.1278904 0.2237884 0.139278 0
0.03245704 0.367482436 0.2501537 0.1380621 0.2057152 0.04599028
0.033594543 0.06771275 0.1627331 0.1687743 0.11410923 0.0225352
0.1022631 0.01493611 0.0679183 0.1888335 0.0727333 0.207599
0.219873 0.06142355 0.01896534 0.3375972 0.01430973 0.1363681
0.037172647 0.02368207 0.794776 0.1355098 0 0.1045612 0.186324
0.06518218 0.06801404 0.03370217 0.08421452 0.1892963 0.06052115
0.03101715 0.08301191 0.05574322 0.0265841 0.3502064 0.02911783
0.4399407 0.02519443 0.08154567 0.1323644 0.109068 0.09111843

0.0492284 0.05618053 0.05664567 0.0533445 0.1508048 0.07004873
0.193137 0.05438659 0 0.1546729 0.1159165 0.2210375 0.1822495
0.04476111 0.1527674 0.02936561 0.06450467 0.2876651 0.1179572
0.6560014 1.922816 0.3382055 0.1415081 0 0.1214561 0 0.06760291
0.03316182 0.1312638 0.038598344 0.14262356 0.09425663 0.107961543
0.170554 0.04974244 0 0.1078216 0.06085309 0.06802559 0.09534639
0.2108189 0.1049772 0.1516886 0.1002026 0.036820934 0.02861019
0.2384829 0.1260939 0.4728995 0.1312723 0.1735702 0.04767509
0.02279918 0.1003302 0.1544431
FENDRR 3.04628 31.35519 4.227444 24.581855839 1.495726436 13.47809 19.34162
21.81541 38.54169 19.15287 3.810564 19.969762218 23.50476 9.155868858
20.353439565 10.22641 3.626799 3.65185 6.30564 0.2660724 2.419312209
0.3438006 0.6103672 0.002371998 0.5148232 1.452272 0.3947229
0.7814618 0.3439303 0.051391631 7.390987 1.747331035 0.4270033
0.7458869 1.491661 1.32815388 2.070208 0.9167581 1.973252 2.923914
0.4294164 0.008584436 0.4628231 4.051689 0.3986916 0.3494461
1.039481 2.224221 1.707737 3.072964 0.240876 1.739671 1.921141 0.164822169
0.2784713 0.5428106 0.6284636 0.03146117 0.6715321 5.042816
0.3909192 0.9102131 1.183168 0.3091438 0.1384338 0.58502576
0.266532 0.7241931 0.3931559 3.26544715 0.1876051 0.3388273
0.385644 0.032932135 1.200707 0.5777411 0.2986994 0.3504245 1.720008
0.3694295 2.818671 0.9548271 0.7480377 0.680995 0.181980025 0.1550808
0.1831826 0.1532738 0.9622209 0.1537241 0.08402652 0.777237175
0.08658814 0.06465244 0.5019469 0.681461666 2.263705 0.9423516
1.168261 0.5551342 0.06048909 0.5103624 0.6516785 0.4503628
0.1115811 0.04846725 0.249556046 0.7411505 1.269755646 0.3495869
0.1369132 6.209352 0.5055837 1.044929 0.5193281 0.918524 0.1883242
0.1572947 0.09064389 0.4505701 0.2869572 0.2781674 1.715567
0.02072354 0.5408179 3.534687 0.6882835 11.03148 2.275765051 0.0385108
4.852692 0.02199393 3.856643 1.049614633 0.8767675 0.9759997 0.9237425
0.4066457 0.4163065 0.6141249 0.2372791 0.01584748 0.3716742
0.6900107 0.2204362 0.5096978 39.18686 0.2417572 0.5547351
6.119614 0.2952262 0.006581377 1.63245 0.105044879 1.562549 2.603565
0.024891148 0.5777171 0.08795953 0.4382882 4.47205 0.5651065
0.1595465 1.123939252 0.03380482 0.06636166 0.4734488 0.0290086
1.940895 1.410263 0.4639914 1.233196 4.951736 0.128488918 2.171168 0.09023538
0.1016033 0.0118179 1.142435 0.7787334 0.917619 0.4757409 4.08531
0.317354 2.196757 13.82464 0.6394122 0.04876631 4.595487 1.185295 0.01760322
0.007570386 2.001244966 2.129289 0.2532231 1.086052 0.8452631 1.101893
0.2263109 0.074156958 0.05962568 0.9615104 0.318248 0.2288055
8.650182121 0.6958077 1.884665 0.941846 0.03191352 0.1095533 0.2470233
0.1341262 2.903936 0.1575291 3.414021 0.1716651 0.8365109 0.5112156
0.364538049 0.01807647 0.3447632 0.323818567 0.572003834 3.007598
0.052402005 0.4650019 8.951288 0.05858514 0.022896889 0.9266518

0.00246514 0.2364952 0.226927474 2.995652 0.5057875 1.454836 0.01502239
0.08856502 0.1468309 0.4068236 0.1573791 0.4578561 0.1051284
3.799961 0.473766682 0.3347079 0.319825873 4.639283 3.826226 0.04434325
0.1789234 0.4489513 1.818944 2.155216 7.140694 0.1894614 6.684626001
1.328751 0.2879337 0.3509483 0.2060017 1.07502 0.2004232 4.173995
0.03353415 9.293789 0.0441752 0.185956 1.728011 0.4883227 0.7883933
0.158408598 1.069197 0.01243272 0.02799186 0.02041035 0.4439221
0.09368724 0.04328643 0.1733273 0.6376375 0.8373093 0.154047618
0.199349479 1.8525559 0.1243057 16.86832 1.090107 0.3700998 3.259856
0.297103 0.05073329 1.383328 0.2133863 1.932706 0.008794623 0.349551
6.165135 0.2259236 0.1974089 2.078978 0.2181213 3.012247896 1.088934
0.04167912 3.876291 0.4558488 0.1267608 0.1073759 0.4512346
0.0142162 1.246816 0.1048226 0.1633621 0.438269748 13.19727 0.03043777
0.6721069 0.7311209 0.04096002 0.667511795 1.04003 0.1238321
0.6036928 0.2550048 1.969108 0.1737061 0.991986251 2.268358 2.161581
0.2781378 0.118749 0.721541077 0.3719268 0.6921579 3.105944 0.028124273
0.285644848 0.2832514 0.0399681 0.8545212 0.5784433 0.8331522
0.3567037 0.7707259 0.08362461 0.5448955 0.4718105 0.01813829
0.1152355 0.366474704 0.02001214 3.546583 0.6756108 1.255863 1.142759
3.845862 0.09180184 4.399165 0.3987123 1.17816 0.383908 0.3352667
0.00218421 1.350345 0.1334638 0.2452368 2.984022 0.03280738 0.7174403
0.1011281 1.653811 0.8948184 0 0.5133206 0.1317321 0.4641942
0.3350723 0.04507787 0.2230113 1.230731 0.524983 0.3829872 1.34422
0.0435679 0.06763441 16.80611 1.903696 0.1607546 0.02689449 0.6865458
0.2574016 22.74595 0.7452099 0.7688209 8.175451 0.8224541 0.8142765
2.020938336 0.2480332 0.1932512 0.4125817 2.07836 2.662133 0.179392831
14.095291636 0.3318749 0.186263452 0.2121822 0.04553685 17.86959
0.5523715 0.4778042 0.8878066 1.07965 1.75604 0.3711003 0.9667053
0.1439467 0.261883813 0.9307975 0.7993862 0.7742892 0.09990393
1.132405 0.4229059 0.6804031 0.4752296 3.140479 1.322013
AC234772.2 3.000557 0.0576457 0.3892975 1.378569469 2.398718974 2.393368
0.09169773 1.011948 0 0.8039917 0.2132146 0.191111526 0.3702799
0.281408573 0.102941596 1.312449 0.04407406 0.5389544 1.338994 0
0.357188129 0 0.1765171 0.15263 0.2284629 0 0.1188261 0.08745114
0 0 0.3539894 0.147746117 0.03290564 0.2584361 0.1418473
0.06104441 0.07633859 0.1685436 0.1446975 0.3869284 0.04643946
0.8838067 0 0.04035795 1.25543 0.03779104 0.4928519 0 0
0.8925528 0.1961968 0.02376685 0.1501443 0.111639418 2.78735
0.07431492 0.04190621 0.2313622 0.3011205 0.04547835 0.1622859 0
0 0.1455538 0.04453872 0.307301333 0.7987869 0.03170024 0.0822707
0.54795689 0.08622679 0.09910172 0.03446511 0.188361834 0.07804179
0.09656018 0.04271175 0 0 0 0 0.06942358 0 0 0 0.07983135
0.05482408 0 0.04329765 0 0 0.67090023 0 0.2521311 0
0.274060844 0 0.6110717 0.05850088 0.08307208 0.08649481 0.3144263

0.05113816 0 0.03191049 0 0.061761849 0.8334264 0.075303638
0.06162938 0.1510269 0 0 0.05614834 0 0.04410738 0.04572839 0
0.2120955 0.04792174 0.4616179 0.2557018 0.216878 0.02666978 0
0.5599973 0 0.1374323 0 0 0 0.06289932 0.1159635 0.144468713
0.1109843 0 0 0.8486356 0 0.2549474 0.05872346 0.458879
0.1881031 0.08109559 0.169365 0.05654709 56.42609 0.04093751 0
0.3542216 0.08634909 0 0 0.150206218 0 0.6180309 0.10677737
0.07994435 0 0.04273084 0 0.04784563 0.1711045 0.153467712
0.04833833 0.04744607 0 0.08615099 0 0.4575409 0.5300852
0.03938062 0.1910809 0.723434585 0.05610733 0.04838616 0.3735899 0
0.05579643 0.1436813 0.1114069 0.05102051 0.03566836 4.084129
0.1449783 0.09038496 0 0 0.1008655 0.3245518 0.1359248 0
0.038963174 0 0 0.04786558 0.07109777 0.1300974 0.03272434
0.247423941 0.2019321 0 0.2805229 0.1802798 0.16346835 0.6066782
0 0.04407606 0.1466804 0.03710202 0.02813294 0.1204265 0.1715281
0.09653772 0.1202083 0.2350227 0.1209587 0.0854415 0.594678948
0.116315922 1.17084 0 0.125476693 0 0.144509589 0 0.1106599 0
0.049111225 0.3407251 1.0786390 0 0.06681456 0.06924618 0.03797719
0.3866557 0.2279543 2.053927 0.1586528 0 1.393877 0.03074842
0.05538268 0.12275948 0.1367448 0.117598312 0.2241367 0.02654497
0.2282669 0.05116938 0 0 0.3070424 0.43741 0.08866324 0.14415849
0 0 0.04562086 0.1006747 0.03341732 0.1766651 2.913436 0.04315616
0.3541019 0 0 0.1653407 0.2793058 0 0.033976844 0.08174162 0
0.1801181 0.02626673 0 0 0.03979049 0 0.04508769 0 0.832644879
0.098672764 0 0 0.2202555 0.1617169 0 0.07718883 0.02100831
0.1224193 1.170685 0.1615374 0.2487261 0.1697712 0.02902247
0.2342749 0.427571 0.08760407 0.09049051 0.03742764 0.256240827
0.0388195 0.3713413 0.02740946 0.2291587 0 0.0406428 0.1707965
0.1829528 0.1044187 0 0.05606292 0 0.1283748 0.1958567
0.4600829 0.07209983 0.446031 0.080535203 0.6565969 0.08692553 0 0
1.141226 0.05882839 0.444041276 0.1046316 0.1668251 0.03728587 0
0.213115383 0.06136468 0.5309166 0.1019678 0 0.081690099 0.06178394
0.2977886 0.6155096 0.08556518 0.04394303 0.3762732 0 0.2226604
0 0.1882753 0.02593639 0 0 0.1287713 17.89292 0.1964893
0.1493725 0.1516137 0.1125708 0 0.06163772 0.06108518 0
0.1029299 0 0 0.07522955 0.2525865 0.1927347 0 0.05277607
0.04196803 0.2054921 0.09853435 0.1370917 0 0.08257607 0
0.2375968 0 0.04834345 0 0.03174083 0 0.1971514 0.06048669 0
0.1200564 0.05723293 0.1651636 0 0.1730569 0.05322516 0
0.3724236 0.06108499 0.1273931 0 0.1225995 0.0366405 0.239705977
0.1926218 0 0.08168686 0.1803174 0 0.069959498 0.221575887
0.1281301 0.391360592 0.05152152 0 2.787508 0.09771332 0.02757406
0.205494 0.4147568 0 0 0.03436696 0.03632346 0.066737943 0
0.7780504 0.09141811 0.4897888 0 0.06291919 0.08641109 0.2479411

0.09092423 0.2799281
AL590729.1 0.07752088 0 0 0 0.027288122 0.1202949 0 0 0.04566866
0.08980012 0 0.132077075 0.2624618 0 0.047428589 0.2502159
0.1624510 0 0.2253755 0.054856118 0.3136159 0.2168726 0.08438601
0.5964756 0.1337629 0.2463619 0.4566384 0.4338878 0.035849105
0.06523784 0.299514642 0 0.3402001 0.1307074 0.056250347 0.3165454
0 0.3333344 0 0.08558477 0.06786649 0.185004 0.3346963 0.1005945
0.2785853 0.03243902 0.04102058 0.2115033 0.2530637 0.03615774
0.08760138 0.1844705 0.171453207 0.08153803 0.410872 0.1158454
0.2131924 0.1664834 0.167627 0.2492349 0 0.04213442 0.1341229
0.04104091 0.2359731 0 0.4089497 0.03790483 0 0.1191826
0.09131887 0.4446179 0.08678451 0.3835352 0.1334654 0.4329317
0.3094564 0.5453732 0.04922394 0.1183906 0.2558859 0.1232043
0.1097238 0 0.1716444 0.1262963 0.2596599 0.03989731 0.1970767
0 0.028100533 0.2640388 0.2090972 0.146973 0.126268871 0.4619506 0
0 0 0.1062694 0.3541183 0.1884883 0.150442 0.205831 0.1352366
0.056911442 5.546479 0 0.05678937 0.2783322 0.06123448 0.05305783
0.1810857 0.1502079 0.0812869 0.1264115 0.1793558 0.06514625 0
0.2430658 0.1178102 0.1598766 0.04915059 0.2106579 0.2778561 0
0 0.107519823 0.07611427 0.0601949 0 0.07123759 0 0.1022682
0.04503513 0.2639597 0.0977486 0.1554906 0.2936567 0.02705583 0
0.2723767 0.2241805 0.2731121 0.07815933 3.308755 0.03772252
0.0384702 0 0.07956774 0.07804617 0.1679708 0.092273279 0.2345532
0.1220345 0.098391712 0.405163 0 0.118125 0.0742751 0.1322644 0
0.247476729 0.04454213 0.08743989 0.8150021 0.2116939 0.07646637
0.2108042 0.2011287 0 0.05869151 0.095231479 0.103402 0.0891724
0.2581878 0.03503604 0.1542435 0 0.4448499 0.2350683 0.1643359
0.1551912 0.1001944 0.09994399 0 0.04130729 0.06196273 0.1495317
0.1252501 0.3590979 0 0.1254162 0.1649935 0.088213 0 0.07992021
0.2713893 0 0.1860735 0.08058095 0.05169846 0.1107478 0
0.04472267 0.09717201 0.3249167 0.2973542 0.2051295 0.5443944 0
0.07902864 0.04447811 0 0.5630701 0.0835945 0.3542915 0.121772536
0.160771746 0.1135673 0.10766488 0 0 0 0.07255644 0.2549234
0.416844 0.135762969 0.3662943 0.08769962 0.1491761 0.146784597 0
0.127616 0.03499468 0.3117538 0.1575391 0.03785248 0 0.111421 0
0.1700017 0.204133 0.263943594 0.2520114 0.072241895 0.2065343
0.3179838 0.1051701 0.3772067 0.04511823 0.1276343 0.4715486 0
0.1225502 0 0.374428 0.3186868 0.1471333 0.2473822 0.03079292
0.03255818 0.1258421 0.1193008 0.0501989 0.7857866 0.04160727
0.1523558 0 0 0.093925517 0.2259664 0 0.09958359 0.1210195
0.3948234 0 0.03666558 0.1970957 0.2492807 0.2932719 0.073071802
0.090923594 0.03026009 0.1340087 0.2435495 0.1862707 0.1242136
0.03556344 0.07743378 0 0.260387 0.07442562 0.04583853 0.0782192
0.240689 0.1439175 0.2560949 0 0.1389732 0.03448829 0.202386173

0.5007919 0.07603963 0.05051378 0 0 0 0.7344548 0 0.09621827
0.138117 0.4907707 0 0.07886201 0.1203168 0.1695803 0.06643754
0.03736385 0.247368163 0 0.05339928 0.4600144 0.09304646 0.1617848
0.2710418 0.204584462 0.09641447 0.4227399 0.06871532 0.153622
0.056108164 0.1696364 0.05435795 0.1409398 0.047645084 0.075274646 0
0.09978258 0 0.0394227 0.04049201 0 0.244815 0.03419566 0.1583755
0 0.1672965 0.1301463 0 0 3.49316 0.09052908 0.05505665
0.1397068 0.4149205 0.1814408 0.02839853 0.05628791 0.2344191
0.2213082 0.06738655 0.3108211 0.2079644 0.2327498 0.06659943
0.1462249 0 0.3093769 0.126236 0.1361941 0.284232 0.04554018
0.1521821 0.04110958 0 0.1419107 0 0.06296692 0.2632329
0.1935415 0.3179195 0.3344186 0 0.0829709 0.05273819 0.3043854
0.3737904 0 0.09809035 0.6823086 0.2059054 0.1125755 0.1956474
0.1301921 0.3765709 0.1350519 0.2208809 0.07606904 0.1790387
0.07527166 0 0.08221166 0 0.034029107 0 0.090156367 0 0.2076942
0 0.2926284 0.1016342 0.07574228 0.1592435 0 0.1577956
0.06333596 0.1673541 0.061496743 0 0.1991519 0.04211934 0.05641546
0.1918397 0.2898895 0.2388746 0.6473297 0.1675672 0.2292838
RASA3-IT1 0.03896444 0 0.5859952 0 0.041147616 1.813921 0.3822347
0.183401 0.06886353 0 0 5.244502602 2.849506 0.111717088 0 4.779124
0.1837192 0.2995452 0.7788128 0 0 0 0 0 0 0 0.04127643
0 0.06542572 0 0.0491859 0.041057868 0 0 0 0.169639203
0.05303523 0 0 0 0 0 0 0.1121526 0 0 0.391317 0 0
0.1907967 0 0 0.3477027 0 0 0 0 0 0 0 0.075164 0
0.1270685 0 0 0.071164519 0 0 0 0 0 0.1376992 0 0 0
0 0 0 0.05482435 0 0 0 0 0 0 0 0.1142649 0 0
0.1485854 0 0 0.04423808 0 0 0 0.232191 0 0.08128543 0 0
0 0 0 0 0 0.042908232 0.1286693 0 0 0.1398986 0
0.08000562 0 0.07549924 0 0 0 0 0.066586 0 0.1184303 0 0
0 0.05985396 0 0 0 0 0.04538378 0 0 0 0 0 0 0 0
0 0 0.07084447 0 0 0 0 8.820288 0 0 1.066393 0 0
0.3166028 0 0 0.4293688 0 0 0 0.05937337 0 0.06648024 0
0 0 0 0 0.03990151 0.05765162 0.05297842 0 0 0.04425031 0
0 0.2016939 0.04325778 0 0 0 0.05159899 0 0 0 0 0 0
0 0 0 0 0 0 0 0 0 0 0.1205113 0 0 0.05611586
0.06075379 0.03897792 0 0 0 0 0.06124252 0 0 0 0
0.08937516 0 0 0.06531157 0.1680689 0.05935936 0.091810083 0 0
0 0 0.05538682 0 0 0 0 0 0 0 0 0 0 0.2886472 0
0.033578 0 0 0 0 0 0 0.1539055 0.056857022 0 0 0 0 0
0.07109851 0 0 0.07110456 0.2762589 0 0.951446034 0 0 0 0
0 0 0 0 0.1892366 0 0 0 0 0 0.094419862 0 0 0 0
0 0 0 0 0 0 0 0 0 0 0.06120784 0.05617533 0 0
0.0291905 0 0.05609089 0 0 0 0 1.7361 0.02970493 0 0 0
0.508628409 0 0 0 0.063682 0.06868736 0 0.1582115 0 0 0 0

0 0 0 0 0 0 0 0 0 0 0 0.2439545 0 0.051415306 0
0.05794976 0 0 0 0 0 0 0.143687543 0 0.02861572 0
0.05701563 0 0.1221154 0 0.1230518 0 0 0.1308018 0 0 0
0 4.213854 0.06825416 0 0 0 0 0 0.08487625 0 0 0.05080591
0 0 0 0 0 0 0 0 0.06845545 0 0 0 0 0 0 0 0
0 0 0 0.08404467 0 0 0 0 0 0 0.07395496 0 0.05174728
0.04243799 0 0 0 0 0 0.1529388 0 0 0 0.04132218 0
0.051312311 0 0.067973155 0 0 0 0.03394252 0.03831343 0.1713171
0 0 0.02643768 0 0 0.046365308 0.07205251 0 0 0 0.04132487
0 0 0 0 0
LINC00702 0.2073127 20.384 0.1060312 6.548653298 0.357198906 0.2438166
2.847184 1.503068 1.292016 2.654298 0.0954045 0.371801347 2.688634 0.506802057
8.179009411 0.2676591 0.06516595 0.1006578 0.06058071 0.06978834
0.060224498 0.177829 0.01602572 0.1211502 0.03555738 0.8613513
0.01618206 0.01361067 0.1538975 0.442013921 0.7988631 0.080481894
0.0307281 0.03160326 0.3385101 0.052254375 0.4306914 0.1573902
0.09571145 1.94964 0.7444551 0.0716424 0.135406 1.152602 0.1529154
0.0529353 0.1177988 0.01385691 0.02232708 0.05877158 0.07328536
0.02959209 0.1908393 0.020271152 0.04820178 0.02313237 0.04891631
0.07201723 0.08201485 0.8352198 0.05051553 0.439841 0.06049099
0.4002139 0.05892108 0.127540244 0.1791687 0.03700308 0.0352121
0.478894628 0.04697041 0.1079676 0.008046091 0.076954901 0.1477791
0.1728264 0.425443 0.01493366 0.4175862 0.01247103 0.3032787
0.01080491 0.717926 0.1130487 0.313636496 0.4949191 0.5588905
0.1206069 0.02695494 0.03744747 0.05157603 0.598025732 0.02725352
0.1785468 0.01861802 0.085308254 0.07802437 0.6620815 0.4689037
0.02585825 0.08974554 0.2256517 0.0676516 0.03176243 0.02731557
0.1684578 0.305195472 0.389137 0.363322018 0.1055102 0.05484601
0.7860395 0.07617337 0.01966226 0.09725326 0.2745904 0.02135115
0.02423485 0.5419141 0.1230639 0.07697682 0.04642956 0.0506315
0.05396061 0.005930091 1.401209 0.006127093 0.3850131 0.513028816
0.02356906 0.04320991 0.3181584 0.3218606 0.427210061 0.1871275
0.6883903 0.3789583 0.4485199 0.2757581 0.1735972 0.009139568
0.1150635 0.05855177 0.02524302 3.456393 0.04620443 0.1496934
0.02867134 0.09421654 0.2894332 0.02015873 0.1548903 0.04610222
0.019481426 0.01485619 0.07557687 0.12463917 0.2052985 0.08179709
0.03990311 1.699876 0.05957257 0.0599181 0.289609519 0.01128488
0.02215316 0.04588516 0.008938879 0.08072076 1.118598 0.5119921
0.009193647 3.977636 0.273441366 0.1790142 0.01882675 0.0242269
0.03550594 0.01953903 0.5702356 0.09247512 0.0158814 1.612662
0.09501886 1.44692 1.678227 0.07964609 0 2.1847 0.04209367 0.5359265
0.03032617 0.130378763 0.423661 0.2285154 0.9535585 0.04149553
0.5028259 0.07639699 0.011002408 0.01257128 0.05103856 0.1899204
0.09820402 5.813450092 0.2133934 0.9067931 0.3155547 0.04337503

0.002887232 0.01094635 0.04060954 0.1868733 0.003756221 0.1356396
0.02926266 0.05177069 0.07313843 0.462771532 0.099567115 0.00959087
0.109108875 0.38081298 0.4497883 0.056227754 0.09191196 1.489774
0.1431584 0.156692676 0.004419131 0.2814402 0.0352746 0.549560665
0.1559827 0.06466379 0.4255684 0.1579675 0.004434778 0.0639336
0.1419808 0.02117163 0.2958264 0.02871363 0.08404127 0.121004481
0.03192392 0.183026997 0.1395362 0.9254317 0.1332258 0.05176516
0.01143084 0.005389423 0.2946883 0.6343573 0.006899659 0.10096406
0.2213457 0.06920594 0.1668574 0.002611459 0.2080394 0.03024527
0.3329947 0.03022521 0.207728 0.02580685 0.02108265 0.4342477
0.2282198 0.1033941 0.031728413 0.08057302 0.1245104 0.07008278
0.2493733 0.01852401 0.003475012 0.04644668 0.03804554 0.1017513
0.08845394 0.018512959 0.011517882 0.2964374 0.05092727 6.314376
0.251692 0.2587524 2.0513 3.531254 0.1428977 0.8419007 0.2325568
0.1277466 0.2356029 0.2303661 0.1215398 0.006654605 0.3442704
0.04929299 0.1368908 0.136733709 0.1208353 0.09311353 2.158565
0.06419821 0.2538952 0.1454874 0.3367095 0.04745717 0.02166861 0
0.06762254 0.05427452 0.5061581 0.08128702 0.7661854 0.3815287
0.2776765 0.480481212 0.988502 0.2570485 0.04127672 0.4203958
0.1024717 0.009155899 0.918579713 0.4423982 0.0811382 0.1537815
0.1751426 0.19901239 0.03820256 0.1560798 1.233893 0.016094699
0.181175034 0.1474437 0.02317353 0.05428459 0.2363793 0.04787427
0.3455169 0.02067487 0.2108136 0.3129742 0.1025594 0.03027505
0.01648648 0.039323017 0.1369511 0.5015014 0.3402146 0.1418125
0.3215059 0.4788862 0.03983943 0.02877941 0.03327499 0.01979693
0.05873906 0.06829034 0.06124802 0.09952254 0.05110548 0.1612325
0.07162328 0.1560647 0.009797695 0.06396453 0.2607058 0.06934395
0.09230192 0.01606491 0.1388698 0.2139497 0.03595351 0.8389317
0.4892209 0.03705046 0.01634477 0.007671034 0.007060495 0.08453722
0.1588248 6.71632 0.1885081 0.01578348 0.2908885 0.5964356
0.006065435 0.06955566 0.1782583 0.208185 0.02931961 0.1908107
0.1026474 0.023317015 0.4111428 0.127008 0.1144218 0.07483769 0.4374
0.209600099 0.296000566 0.2160366 0.057103461 0.03608405 0.02104798
0.05423007 0.08364318 0.4205724 0.6652372 0.7235168 0.6201924
0.03997799 0.9306886 0.01413322 0.132433259 0.1372024 0.1009115
0.04268424 0.3716187 0.164325 0.1395443 0.01793173 0.01607872
1.146248 0.1355427
AL133243.3 0.2760198 0.07092494 0.182467 0.12642008 0.176973102 0.344186
0.233701 0.2706651 0.08711099 0.2055478 0.1592722 0.268726726
0.3003807 0.360365705 0.117608374 0.4613668 0.1859209 0.284189
0.2353492 0.6066281 0.491787651 0.162371 0.4447008 0.09121217
0.3948666 0.2232536 0.3916039 0.1742036 0.3641538 0.246170221
0.2675425 0.212943272 0.1966452 0.2011643 0.2160762 0.316520551
0.2281009 0.3061165 0.2797613 0.2720349 0.1469243 0.27185

0.1999693	0.5745769	0.3645712	0.3121912	0.04331322	0.1643145	
0.2370171	0.3982342	0.1724233	0.1378541	0.202325	0.228927701	
0.4510378	0.2547092	0.4124776	0.2338267	0.5716078	0.2717801	
0.4563886	0.2951552	0.1768131	0.2217224	0.125254	0.648156206	
0.1156229	0.4234578	0.1807545	0.222258454	0.310692	0.08709339	
0.2544268	0.2731372	0.2423348	0.2036636	0.4128992	0.7420597	
0.2635365	0.1408388	0.2785173	0.3660684	0.2115062	0.2218511	
0.076583425	0.1122529	0.2987219	0.3219385	0.3424608	0.5403781	
0.1410172	0.2626427	0.1119206	0.9217727	0.182224	0.493747433	
0.3035075	0.1569771	0.3290382	0.5548463	0.2584479	0.7368713	
0.2606616	0.1650029	0.2243507	0.2386111	0.331096145	5.859509	
0.158829511	0.595778	0.2566049	0.3212058	0.3643398	0.3503477	
0.8786463	0.1472987	0.1366371	0.684227	0.4038569	0.3453428	
0.1159094	0.2471897	0.1982224	0.1640671	0.8438239	0.2271422	
0.04151697	0.611949	0.20508941	0.2564929	0.2009335	0.3150829	
0.1426768	0.190444775	0.4291588	0.2233467	0.339857	0.2672468	
0.2743473	0.4145017	0.1857882	0.3853524	0.4912078	0.4133608	
0.275359	0.2832625	7.10022	0.2086668	0.2641688	0.2594166	0.2175399
0.1042088	0.3524367	0.308012752	0.637545	0.9311023	0.300284694	
0.238875	0.3552912	0.2703821	0.1700118	0.4709386	0.2857058	
0.256256339	0.543758	0.4336483	0.5613766	0.4845563	0.3281767	
0.3149782	0.2027834	0.08998288	0.2462933	0.299722375	0.5818428	
0.1785971	0.5034261	0.253953	0.8090853	0.5808472	0.3459399	
0.2690296	0.1441933	0.4736327	0.2229693	0.1938162	0.2086759	
0.2678924	0.2659303	0.3517778	0.2707637	0.8133944	0.198603237	
0.3827617	0.1951251	0.3701774	0.2624275	0.2286665	0.3048462	
0.416248304	0.3407301	0.2075013	0.1972252	0.07393634	0.3591515	
0.2687156	0.3521677	0.2556529	0.1650008	0.2086803	0.2076818	
0.2469464	0.3994709	0.2375522	0.5810333	0.1734974	0.2444944	
0.5406365	0.32518618	0.306665149	0.4007557	0.260130394	0.213193423	
0.2592339	0.110065892	0.4151948	0.3209285	0.4611649	0.500659843	
0.5888963	0.6356763	0.278856	0.559970532	0.2348741	0.486844	0.3738047
0.7603114	0.3906487	0.476533	0.2509714	0.3666156	0.1781786	
0.2540122	0.2968983	0.215768993	0.3485081	0.186027689	0.4727463	
0.3452614	0.3059263	0.2338393	0.6282459	0.2799755	0.4497293	
0.1607525	0.5532303	0.380071652	0.1964064	0.7294576	0.1764088	
0.3008177	0.1468404	0.2422029	0.3920627	0.5916589	0.2728939	
0.3580596	0.1031733	0.2106938	0.135004	0.2830687	0.400121452	
0.1724084	0.3093487	0.5445273	0.152354	0.2175647	0.2590122	
0.4615909	0.7035663	0.8638106	0.183804	0.480865484	0.234134309	
0.2885991	0.5112317	0.2709934	0.2913488	0.1579547	0.2035072	
0.424642	0.07889605	1.667416	0.31232	0.6907366	0.3282394	0.2142484
0.5353071	0.6876434	0.2463646	0.1802581	0.2105118	0.257361912	
0.5049119	0.4133706	0.1637998	0.1691686	0.2259092	0.164303	

1.3208870.3322871 0.1896499 0.4303053 0.6109440.21319552 0.1955536
0.2983489 0.2992070.3041442 0.1710478 0.292543321 0.2929567
0.4023344 0.4551811 0.2484749 0.08743596 0.2274802 0.305684873
0.1287345 1.2388590.2031608 0.3418652 0.235452567 0.7657919
0.0933169 0.1613019 0.190849966 0.481003483 0.5610739 0.5472015
0.3678305 0.3910249 0.3707364 0.1058174 0.1712238 0.1826349
0.3866808 0.4550197 0.3692568 0.6268275 0.171713127 0.1659795
7.8624010.3367266 0.2257891 0.2531605 0.2572190.6229629 0.06500277
0.2147335 0.2951152 0.5487766 0.1863786 0.2075072 0.1851188
0.6126651 0.1778497 0.2956528 0.3061153 0.3688269 0.722369
0.5455471 0.4156562 0.05211955 0.1669114 0.5410617 0.1551136
0.1714361 0.1869366 0.2282027 0.2566316 0.1661274 0.3551875
0.7760982 0.1847811 0.1582634 0.3118470.1838573 0.1711174
0.3285085 0.2806546 0.1575469 0.2029237 0.2362061 0.9926826
0.3559480.3878781 0.5796127 0.631981137 0.1837913 0.2049052
0.2943337 0.2218552 0.2090869 0.239781288 0.408927003 0.2252087
0.249355584 0.2626157 0.6893319 0.2449741 0.3177309 0.305334
0.3322928 0.3888 0.1918905 0.2040032 0.4107560.3000675 0.152493089
0.2187480.3570813 0.3374314 0.3443522 0.2456931 0.3151824
0.2531350.2396874 0.1678042 0.8364297
AL606534.1 0.06400499 0 0.02468169 0 0.022530395 0.0993213 0.1046464
0.06694754 0 0 0.1622152 0.109049228 0.1083506 0.03058537
0.039159339 0 0.1341274 0 0 0.3101348 0.181167466 0.1479637
0.0671477 0 0.08690808 0.05522057 0.02260093 0.06653343 0.1074716
0.029598757 0.05386352 0.112406268 0 0.02808857 0.2158367 0.092886019
0.08711839 0.08548609 0.3302605 0.07359444 0.03533147 0.05603386
0.1527482 0.1535231 0.04152783 0.02875168 0.05356645 0.1016057
0.06548526 0 0.08956073 0.01808198 0.03807695 0.056624028 0.06732175
0.02826965 0.0956476 0.02200274 0.3894608 0.5882041 0.2469365
0.1246427 0.06957643 0 0.1016561 0.11689849 0 0.1205888
0.09388818 0 0.1968058 0.1507946 0.1048852 0.10748024 0.1979158
0 0.06499079 0.1095009 0.2101337 0 0.1954979 0.1584538
0.06781561 0.1630679 0 0.04049083 0.06256581 0.1875893 0
0.2033950.1326950.046402321 0 0 0.03033701 0.208507386 0.2542725 0
0.08901574 0.1264036 0.06580585 0.3721165 0.03890626 0.03105304
0.1213886 0.2233158 0.305427473 0.5636236 0 0.1406642 0.07660152
0 0.1314213 0.06407706 0.1240189 0.1006716 0 0 0.2420455 0
0 0.03242328 0.1980027 0.1014528 0.2125804 0 0 0.03485315
0.221933929 0 0.09939964 0.09570847 0.0882258 0.109912796 0.1407293
0.2230991 0.1634535 0.08070598 0.03209515 0.09698286 0.04467721
0.1163728 0.1431102 0.2159433 0.06442702 0.08604284 2.731868
0.03114554 0.03176286 0.1796628 0.04379665 0 0.0346712 0.152370573
0.09682922 0.03358587 0.027078987 0.1216444 0.1230311 0.03250992
0.1226502 0.1456051 0.1952663 0.175138945 0.07355227 0.0360973

0.03738362 0.1310885 0.09470151 0.1450418 0.1067539 0.02996105
0.0969171 0.353824737 0.1707474 0.03681253 0.1894865 0.0578549
0.1273509 0 0.2542774 0.07763352 0.0814102 0.03203334 0.05515025
0.0962717 0.0311469 0.0341053 0.1023189 0.1234606 0 0.8524048
0 0.2070994 0.3541892 0.07283292 0.1352292 0.06598599 0.1244845
0.053783384 0 0.03326577 0.06402713 0 0 0.0553878 0 0.3353337
0.06695729 0 0.1284224 0.03054043 0.1304998 0.1468931 0.04572764
0.2503297 0 0.1625112 0.201082603 0.066370469 0.4219499 0.059262225
0.063642357 0 0 0 0.08419083 0.06883332 0.03736416 0.3024303
0.1930908 0.2217006 0.12119247 0.1524989 0.05268297 0.2600398
0.7721971 0.1300719 0.1250114 0 0.1379919 0.04820322 0.07018083
0.06320329 0.031132087 0.2080728 0.029823203 0.1278936 0.1211736
0.04341677 0 0.1117554 0.1317264 0.5450667 0 0.1349112
0.109676776 0.1545729 0.1127671 0.1214803 0.2297821 0.02542412
0.02688161 0.06926756 0.2955016 0.1450632 0 0.06870595 0 0
0.03063179 0.051699636 0.02072986 0.1825943 0 0 0.1086618 0
0.09081865 0.1162369 0.06860606 0.4150962 0.361989704 0 0.1748894
0.1106441 0.1005431 0.1230353 0.1367423 0 0.03196654 0
0.3071259 0 0.03784651 0.06458156 0.220805 0.4158885 0.1301196
0.2332742 0.1606402 0.1139008 0.167099823 0.2362731 0.031391
0.1459732 0 0 0.03092132 0.08662878 0.0927945 0.1324041
0.07602405 0.1492857 0.023070581 0.06511229 0.09933941 0.2100205
0.02742702 0.06169881 0.081695653 0.1152793 0.1543116 0.02373814
0.03841183 0.0222629 0.04475704 0.168914837 0.291883 0.2855737
0.1134694 0.1268377 0.301116507 0 0.04488056 0 0.157352353
0.15537598 0.1410169 0.08238533 0.03121893 0.1627465 0.06686433 0
0.03368852 0.2258687 0.07845748 0 0.236791 0 0.051259687 0.1306268
1.211331 0 0.3182021 0.05767437 0.142741 0.2696514 0.04689441
0.1858961 0.1548382 0.02610326 0.0556376 0.3635577 0.1144703
0.1537356 0.05498771 0.2414608 0.0401524 0.2554366 0.1737109
0.1499312 0.07822523 0 0.03141222 0.1018262 0.05164723 0.1171684
0 0 0.09659457 0.1065315 0.4124839 0.1380561 0.02666101
0.02283493 0.08708641 0.2932012 0.2160336 0.05266514 0.04049407
0.02964984 0.05666849 0.02323694 0.09692158 0 0.2176406 0.0557527
0.045592491 0.08374171 0 0.3728875 0 0.04525195 0.106451339
0.168576467 0.03249409 0.186093653 0.03919793 0.1371859 0 0.3159491
0.1048927 0.3126825 0.05259164 0.04153019 0.01447597 0.02614662
0.08290541 0.203098754 0 0.09865769 0.06955153 0 0.203647 0.07180404
0.1095703 0.1571961 0.1037637 0.07099041
WWTR1-IT1 0.1313214 0 0.151921 0.631539778 0.173349158 0.1528359
0.2147069 0.5150952 0.1740675 0.1711379 0 0 0.03334603
0.188259394 0 0.1589509 0.0515989 0.2523884 0.1458235 0 0
0.05692173 0.1033271 0.1072133 0.08915625 0 0.06955673 0.03412727
0.1102518 0 0.248656 0.034594213 0.115571 0.08644553 0 0 0.044686

0.06577311 0.5082057 0.1132473 0.1087363 0.08622505 0 0.09449667
0 0.04424317 0.0412141 0 0.03358963 0.16076 0.04593876 0
0.1757788 0.043566602 0.05179744 0.04350142 0.09812186 0.1354315
0.1057594 0.1597288 0.1266622 0.04795015 0 0.05680148 0.05214289
0.059961236 0.2200388 0 0 0.049346962 0 0 0.04034939 0.110260586
0.1522767 0.2260921 0.050004 0.05616678 0.04619347 0.375237 0.05013879
0 0.2087097 0.1394052 0 0.06230739 0.03209214 0.08247511 0 0
0 0.071404016 0.07454754 0.1180712 0.04668267 0 0.1304251
0.05503084 0 0.04862756 0.03375407 0.1636039 0.05986906 0
0.03735862 0 0 2.601916 0.088160357 0.1443029 0.1178746 0
0.0674105 0 0.06361355 0.05163791 0.321214 0 0.04138448 0.0561035
0.2316132 0.04989303 0 0.06244631 0.05947623 0.05043128 0.04608905
0.05363213 0.136605008 0 0.1147173 0 0.1357621 0.084567052 0
0.05721758 0 0.08279372 0 0 0.1374988 0 0 0 0.09914047 0
20.49355 0.91061 0.09775354 0.2764656 0.0336972 0.04957922 0 0
0.07450053 0.05168203 0.041669217 0.09359339 0 0.3501844 0.2359181
0.0560144 0.1001587 0.089834758 0.1131824 0 0 0.0336199
0.1457269 0.08927627 0.1095154 0.09220827 0.1118522 0 0 0
0.1093434 0.08902728 0 0.04205305 0.2173793 0.1194627 0.08351616
0 0.127298 0.1693065 0 0.1049626 0.07872427 0 0 0.1140594 0
0.1062284 0 0.05603775 0.1664728 0.1015394 0.1915571 0.124143023
0 0.2047578 0.03284171 0.07035311 0 0.0852309 0 0.2064049
0.06868937 0 0 0.09399138 0.05020334 0.1130198 0.07036581
0.1650891 0 0 0.154713222 0.068087369 0.2164325 0.045596435
0.097933029 0.09333478 0.056393986 0.04609184 0.1943296 0.03530696
0.057496069 0.06648294 0.1857054 0.03790595 0 0.07822193 0
0.2223055 0.2546269 0.200155 0.1923678 0.09286993 0.03539038
0.2225257 0.1079944 0 0.143718415 0 0.091784048 0 0.03107704
0.1336197 0.05990562 0 0.243241 0 0.1396608 0 0.042192729 0 0
0.1068196 0.07857539 0.03912271 0 0.05329455 0.05052429 0.2232237
0.1663916 0 0 0.4087402 0.04713628 0.079555538 0.06379834 0
0.126522 0 0 0 0.09316798 0 0.1055712 0 0.092838453 0.057759667
0.03844575 0.08512972 0.2062881 0 0.05260487 0.1355511 0.09838038
0.04777338 0.6616487 0 0 0.03312609 0.03397753 0.09142433
0.05005709 0.3076826 0.03531337 0.1314532 0.128566826 0.1363417 0
0.2246239 0 0.05787405 0.3330727 0.2666092 0.07139621 0.04074876
0.116986 0.03281732 0.035501072 0.2504874 0 0 0.1266143 0
0.031428372 0.02956534 0.06784431 0.07305661 0 0.1370328 0
0.0433211 0.04083185 0.09765369 0.0873035 0.06505942 0 0.07184157
0.207187 0 0.060533555 0 0.02411081 0.09508107 0.1441193 0.1001739
0 0.08810299 0 0.04344592 0 0 0 0 0.078878543 0.1005044
5.14819 0.1150181 0 0.04437473 0.08786012 0.04610447 0.03608062 0
0.05956636 0 0 0 0.08807361 0.0591422 0.08461524 0 0.06178662
0 0.1603841 0.05767865 0.0401244 0.1735778 0 0.05223013 0

0.1802299 0 0.1600002 0.11148 0 0.2308114 0 0 0.03513845
0.3350220 0.04749044 0 0 0 0.1308024 0.1430283 0 0.1654104
0.04784371 0.0857924 0 0.03221549 0.0454941 0.1434501 0
0.03481691 0.081903802 0.259405917 0.05000199 0.057272282 0.06031788 0
4.07928 0.05719804 0.06456364 0.04811566 0 0.06390677 0 0
0.04252502 0.117198339 0 0.05060491 0 0.2867056 0.06963836
0.1104922 0.1011642 0.1451363 0 0.07282683
AC093423.2 0.03388212 0.1392995 0.195985 0.07241913 0.107341607 0.1051548
0.332378 0.2126389 0.05988133 0.0883103 0.1932105 0.346362307
0.3441433 0.048572647 0 0.2734052 0.1065039 0.1953555 0.2633667
0 0.215784133 0 0.07109154 0.1475307 0 0.04384791 0.2512478
0.2113236 0 0.188023225 0.08554069 0.071404989 0.07951569 0.08921495
0 0 0.1844704 0.06788026 0.08741448 0.7012523 0.4488794
0.08898741 0.08085986 0.04876201 0.1319007 0 0 0 0.1733287
0.0829551 0.04741049 0 0 0.044962329 0.05345686 0.08979011
0.05063268 0.03494257 0.07276506 0.1098973 0 0.1484589 0.05524719
0.0586212 0.05381337 0.123764381 0.2270881 0.03830144 0.1988052
0.050927871 0.1562737 0 0.1665682 0.113792962 0.2828792 0 0 0
0.1906934 0 0.05174507 0 0 0.05754851 0.065805666 0 0.1656013
0.127676 0 0.3230118 0 0.073691558 0.07693579 0.03046344 0.09635644
0.082782681 0.06730173 0 0 0 0.03483544 0.2110566 0.1235741 0
0.07711094 0.04433099 0.298492049 6.041864 0.22746179 0 0.06082546
0.04014572 0 0.3052825 0.1969545 0.05329221 0 0.1411042
0.3843927 0.05790087 0 0 0 0.03222344 0.03069082 0.3122815
0.1426968 0.166051 0.070490685 0.06653467 0.03946416 0.1899934 0
0.174552587 0.08939693 0.2952532 0.1730537 0.04272307 0 0 0
0.1232078 0.1623381 0 0.0511583 0.06832235 14.64238 0 0.05044261
0 0.03477675 0 0.1101227 0.060494953 0.1537746 0 0.172016632
0.0965918 0.09769288 0.2065161 0.1460857 0.2312356 0.206735 0
0.05840421 0.1719784 0.1781069 0.03469697 0.1002637 0.1382046
0.03767465 0.2379058 0.5002209 0 0.135582 0.116924 0 0.09187941
0.06741536 0.1302009 0.134606 0 0 0 0.04379207 0.06552392
0.1483932 0.05416265 0.04062316 0 0 0.2942836 0.047076787 0
0.8653663 0.05783301 0 0.261981 0.07907759 0 0.0975928 0.2113175
0.06778769 0.07260698 0.197508669 0.2052433 0.1274132 0.585798
0.03544497 0.1344842 0 0 0.05181169 0.05832027 0 0 0.1096102
0.4645515 0.31933942 0.210805974 0.07445544 0 0.050535236 0.1444874
0 0.04756847 0.06685176 0.03643808 0.059338048 0.1372257 0.07666191
0.1564813 0.128310563 0 0.08366586 0.1376564 0.2627843 0 0
0.4792259 0 0 0 0.1003732 0.049440889 0.08261015 0.094724498
0.06770261 0.09621794 0.0344751 0.3709487 0.05915961 0.08367789 0
0.04804503 0 0.304811087 0.08182579 0 0.02756043 0.08109268
0.1615043 0.08538139 0 0.05214291 0.230375 0.1144815 0.3273359
0.09988544 0.08436697 0.1459391 0 0.29629 0 0.435251 0 0.3451316

0 0.04807638 0.1476768 0.1089533 0.2746728 0.287438049 0.178830273
0.3174193 0 0.1064484 0.1465443 0.2171606 0.2797874 0.1776813
0.04930388 0.2438734 0.09758783 0.06010407 0.1367494 0 0.5661196
0 0.05292328 0.03644469 0.04522149 0 0.1876128 0.09970414
0.09935147 0 0.05972814 0.04910617 0.8942390 0.04205421 0
0.06773735 0.183192031 0 0 0.05558896 0.08711375 0.09798392
0.064870461 0.03051251 0.07001782 0.0753971 0.1830056 0 0.1421574
0.402380653 0.2106998 0 0.1351506 0.4364341 0.183924358 0.2224294
0.4276491 0.3080034 0.437309915 0 0 0.1635452 0 0 0.05309364
0 0 0.04483778 0 0.1137407 0 0 0.081405545 0 5.31312
0.1780543 0 0.1373890.04533743 0 0.03723652 0 0 0.2487276
0.08835811 0.1698136 0 0.3051846 0.2619781 0.0383464 0.06376605
0.7099032 0.05517408 0.05952647 0.04140985 0.2388515 0 0.0539034
0.0410105 0 0.05841039 0.1651261 0.3068038 0 0 0 0.04234037
0.1450567 0.0691510.1330380.09802375 0 0 0.1412608 0.3599811
0.1107078 0.2565354 0.2276127 0 0 0.289621867 0.2659805 0
0.1973943 0.5809768 0.2874586 0.042263861 0 0.05160388 0.709285101
0 0.1089325 0.8419932 0.02951524 0.03331602 0 0.1252812
0.06595413 0.09195716 0.08304692 0 0 0.06265435 0 0 0.1479453
0.1078040.03801068 0.03480172 0.09985729 0.1098581 0.1127399
AL133445.2 0.1404790.1155102 0.1950181 0.120103074 0.118680031 0.08719669
0.1837434 0.2644872 0.09930984 0.6590596 0.1602144 0.382948332
0.08561135 0.322220063 0.051568465 0.1360282 0.1324731 1.133952
0.06239698 0.05445505 0 0 0.02947534 0.3670071 0.0762988
0.1090790.02976289 0.05841139 0.04717604 0 0.1063983 0.029605294
0.03296808 0.0739790.04737214 0.091740366 0.2676921 0.05628781
0.4349161 0.2907469 0 0.1475806 0.06705078 0.04043453 0.10937500
0 0 0.08623676 0.06878819 0.03931382 0 0 0 0 0.03722797
0.08397146 0.2028260.03016919 0.09112929 0.1625939 0.1641405
0.04581219 0.14583 0 0.153942224 0.09415325 0.06352079 0.08242682
0.084461024 0.0431952 0.09928977 0 0.141539443 0.1824430.2902302
0.1283784 0 0.03953179 0.05352053 0.0858163 0 0.1339584
0.07158074 0.054567507 0.1599657 0.05492811 0 0.04337981 0.1339243
0.0349489 0.122213325 0.09569527 0.02526096 0.07990088 0 0.1674242
0.04709471 0 0.1248446 0.05777263 0.3150230.1024704 0.04089338 0
0.1102807 0.216576655 7.9789640.226339587 0.1852390 0.1331588 0
0.02812744 0.1088794 0.1325732 0 0.07800447 0.3187469 0 0
0.04269783 0 0.02672039 0.05089901 0 0.03944243 0.0917954
0.175357283 0.08275802 0.09817365 0 0 0.144742847 0.1111949
0.04896609 0.07174997 0.4959768 0.29586 0 0.02941744 0.2554165
0.08076859 0 0.08484318 0.1133088 16.959950 0.04182813 0.1182979
0 0.04242928 0.09131618 0 0.06375662 0.04422883 0.071319989
0.2002401 0.08100908 0.04281192 0.04037916 0 0 0.038439731
0.04843006 0.09507221 0.04923004 0.1150860.3325634 0.1146023

0.2030642 0.1183366 0.1595362 0.310631711 0.2248552 0 0.1247663 0
0.1397558 0.07197695 0.2232366 0.1022347 0.07147208 0.126553
0.1452534 0.1086678 0.08203396 0 0.1347425 0.1083892 0.1815770
0 0.09090892 0.1435162 0.0479564 0.07123269 0.04344808 0.06557288
0.035413348 0.1618522 0 0.05621104 0.06020731 0.737003413 0.09725271
0.05282691 0.04415969 0 0 0.02818632 0 0.04296339 0.1450814
0.3613091 0.09418746 0.09089118 0 0.066200819 0.087402477 0.7408811
0.078041716 0.251429579 0.03993737 0 0 0.2771747 0.09064577 0 0
0.1907092 0 0.159596914 0.06694135 0.1387551 0.456591 0.09684735
0.1712901 0.329252 0.07947693 0.03028664 0.3173912 0 0.1109755
0.163989893 0.06850215 0.353464376 0.2526322 0.02659534 0.08576253 0
0 0.03469377 0.1025417 0.31872 0.04441574 0.252756062 0.1017776 0
0.04570743 0.1344877 0.06696145 0.07080013 0.04560881 0.1729522
0.1910321 0.1423959 0.1809561 0.0414136 0.8395074 0.1210159 0
0.02729891 0.2404563 0.07218393 0.02631657 0.1430954 0.04473995 0
0.06122841 0.04517325 0 0.27807495 0.098859999 0.06580278 0
0.3089428 0.2430356 0.1350558 0.1160029 0.1683854 0 0.4044505
0.08092197 0.2491981 0.2267911 0.2326203 0.1564796 0.04283823
0.0877703 0.09066222 0.1499946 0.073350587 0.03889316 0.08267687
0.02746147 0.09183742 0.2476394 0.3257593 0.7415227 0 0
0.1001152 0.0561693 0 0.1286184 0.06540944 0 0.07223664
0.4875025 0.134480036 0.1265082 0.05806031 0 0.1011682 0.05863548
0.11788 0.556104824 0.2446037 0.08357081 0.03735662 0.05567704
0.030502825 0 0.177308 0.1532419 0.207215433 0.081845108 0.1444361
0.1627384 0.1644474 0 0 0.2261923 0.133092 0.07436096 0.1033197
0.0943163 0.07795681 0.03537659 0.067503269 0.3440417 7.216325
0.04921553 0.1197247 0.07595068 0.03759479 0.1972781 0.1543867
0.06120109 0 0 0.03663425 0.1126505 0.0753723 0 0.09655021
0.03179768 0.05287621 0 0.1143789 0.2468033 0 0.1485457
0.08273276 0.04469789 0.1700341 0.05143255 0.09687037 0 0.03180106
0 0.09876274 0.1212029 0.1053287 0.09021314 0.2867076 0.110318
0.04064173 0.1040311 0.1599785 0.03904552 0.2238782 0.09180135
0.04254496 0 0 0 0 0.1378481 0.07786657 0.08184186 0.1806595
0.08938763 0 0.480992059 0.08558215 0.049012901 0.2064771 0 0
0.2202722 0.05525276 0 0.03462865 0.05469061 0.0762529 0.3098895
0 0.03343229 0.1558631 0.04330705 0.1831832 0.1226795 0.1489891
0.3782315 0.1442918 0.04140193 0 0.09348643
AL157838.1 0.1183572 0.01216504 0.171154 0.27827239 0.062494381 0.2112132
0.1064309 0.2599769 0.06275334 0.2467888 0.2699696 0.221817694
0.2825083 0.186641657 0.173791087 0.3629231 0.409244 0.1137362
0.04599969 0.7684865 1.507556612 0.8208378 0.6270518 0.3027711
1.060681 0.7964827 0.4701752 0.7997131 3.388435 0.049260281 0.2689301
1.820855746 0.1458264 0.7012038 1.067653 0.244763199 1.022974 0.2015517
0.4427673 0.1428944 0.05880105 0.2409097 0.9744874 1.481921

0.2764538	1.371715	0.02971632	0.2254656	0.308791	0.3187571	0.1656146
0.6219278	0.4119068	0.722488192	0.1120415	1.184049	0.9108822	
0.5187617	1.340817	0.8541641	1.107332	0.6482469	1.109691	0.4095518
0.4699529	0.421526049	0.4858752	1.846368	0.2517444	0.2579572	
0.5276997	0.188222	0.2763821	0.387564637	0.4995673	0.2649037	
0.5137701	0.5264682	1.498795	0.05636554	0.939932	0.2344086	0.1504845
0.4523148	0.011493636	0.415557	0.1561895	0.215566	0.05482292	0.06205906
0.4784871	0.231677777	0.2351584	0.2819999	1.152831	0.549436129	
1.034435	0.05951777	1.901208	0.2980236	0.2555434	0.5308301	0.3021689
2.368694	0.2020233	0.3174574	0.221572485	17.21659	0.150968444	0.1040458
1.274855	0.4908303	0.911335	0.5095091	0.8829368	0.279241	0.1158014
1.051533	0.6340823	0.1516947	0.2504977	1.438961	1.308965	0.7710572
0.2733838	0.2181727	0.008307819	0.4640401	0.344733821	0.168504	
0.5031759	0.23229	0.3344494	0.015243701	0.7807046	0.7013384	
0.5289481	0.1865506	0.5964669	0.2959101	0.1982797	2.119671	
0.5160431	0.9583671	0.5450547	0.6503594	5.169008	0.2246164	
0.2378787	0.137045	0.4069658	0.05362165	0.1442555	1.024908298	
1.020616	0.6986988	0.120177887	0.3795919	0.273009	0.4959646	0.6804098
0.3130045	0.7402219	0.250995155	0.3264286	0.6207813	0.207388	
0.9756892	0.2714373	0.5551935	0.6382856	0.04986323	0.3427542	
0.403477734	0.7577854	0.1429538	0.7226919	0.152453	0.8713341	
0.1212849	1.692744	0.1722707	0.3010854	0.204363	0.1376772	0.637073
0.09503414	0.7095047	0.6243822	0.1826414	0.08605313	1.716748	
0.148004	1.857383	0.06045804	0.2626293	0.787702	0.3111522	0.6905856
0.111887496	0.1107963	0.1568623	0.4143936	0.5199438	0.103490754	
0.5018699	0.4784614	0.4836739	0.6314645	0.4854403	2.303528	
0.3134361	0.6741835	0.7435951	0.1902577	0.7836344	0.9572272	
0.3696318	0.278879549	0.190233715	0.8973041	0.254789671	0.105917976	
0.1430051	0.142314799	1.105007	0.6889044	1.02465	0.54929198	1.977349
0.95737	0.4646281	0.437009777	0.4934985	0.7014289	0.2564598	
0.3569843	0.26458	0.7368526	0.1674034	0.6124148	0.02674104	
0.4542213	0.53178	0.544027623	0.3751465	0.12408455	0.4138735	
0.4481454	0.4215001	0.226765	0.867958	0.3361497	0.4643679	0.05034935
0.7203622	0.441118065	0.8432112	0.4379078	0.6594786	1.260567	
0.01410419	0.1491274	0.2881996	0.7559055	0.7185244	1.359685	
0.2286903	0.2442443	0	0.2039181	1.061185659	0.8567515	0.9285407
0.2128589	0.620827	0.2913571	0.5088767	0.1343526	0.8253845	
0.3996262	0.5469084	0.334693385	0.17699575	0.2286923	0.1994866	
0.1301462	1.100605	0.2086113	0.3502189	0.2438375	0.1463943	
2.973131	0.1534024	3.201827	0.280645	0.9615694	0.9228669	1.930939
0.2218463	1.012105	0.1895615	1.398219904	0.5734487	0.2176794	
0.6998944	0.1160631	0.0312964	0.1114997	2.9075	0.5662616	
0.7198296	0.56936	2.129584	0.28796733	0.09030362	0.8679692	0.4757499
0.1445455	0.1540251	0.600505496	1.204427	0.18344	0.915237	0.1491644

0.3952153 0.3351948 0.390443968 0.4710514 1.742662 0.04721089
0.850232 0.308393073 0.2071978 0 0.08607354 0.403726391 1.060208186
0.4824185 0.4856026 0.02597834 0.4062807 0.8253282 0.1111675
0.8223123 0.2349414 0.3844688 0.2582578 0.2134621 0.1788342
0.270147976 0.09058251 23.51977 0.4457526 0.8511006 0.3439485
1.330333 2.011165 0.02601496 1.082834 0.3328527 0.6806043 0.3858163
0.1186387 0.6032796 0.7249292 0.5694224 0.2612061 0.03341219
1.523329 1.768338 0.4158764 0.5424492 0.05214733 0.139409 0.2447844
0.3867969 0.4983325 0.02040395 0.7642855 0.9846508 0.7608995
0.5304649 1.487073 0.1922747 0.2343547 0.2294807 1.463896 1.224141
0.1168653 0.06739301 0.9704576 0.1336081 0.2835984 0.8871695
0.327978 0.5346946 0.7036382 0.480561622 0.4761764 0.1148081
1.318743 0.3932101 0.5585596 0.664363575 0.194830916 0.1261841
0.361328125 0.1522171 0.3900368 1.764753 0.7887363 0.4364239
0.2601938 0.4230453 0.1267152 0.2369036 0.1958175 0.8355266
0.422513595 0.2298078 1.204081 0.2507969 0.06460044 1.625577 0.5510333
0.5531415 2.363269 0.1822845 1.299618
AC091057.1 0.2257087 0.08285314 0.3562343 0.06547213 0.197494219
0.2551813 0.2556833 0.2327127 0.7493708 0.08123958 0.3953199
0.552109211 0.3406059 0.242678125 0.053264205 0.08065812 0.1900406
0.2076007 0.1736537 0.209359 0.941196943 0.9587755 0.8490655
1.133716 1.628694 2.082231 1.721529 1.486517 1.090951 0.832039623 0.6939796
1.270719568 0.4937566 2.544933 0.5110455 0.319366488 1.123534 0.8462416
1.247826 0.8953617 0.04004797 0.9061329 0.5848253 1.125312 0.7249011
1.0885 0.8338495 1.58166 1.867224 0.323673 0.6677528 1.008394 0.391317
0.785171308 0.5646846 1.251831 2.214093 0.9992609 0.5799456 0.9674053
1.760265 0.3861699 1.569402 0.7112863 0.8526759 1.869773352 0.1512767
0.7672674 0.2317626 0.702753609 0.8080381 0.4159165 2.338103
1.207451863 1.425282 0.6189777 0.9576659 1.950041 0.8960296 0.2149796
1.098129 0.3911426 0.3202855 0.9009062 0.109592515 0.7220982
0.7911286 1.051005 0.346004 1.321802 1.098988 0.413760769 1.045163 0.5870615
0.9674149 1.181708343 1.729297 0.1648469 0.6491151 1.611873 2.511213
0.325382 1.361219 1.001981 0.5668838 0.9091474 0.788270569 3.960953
0.285734013 0.2125891 1.519478 0.9570321 1.433376 1.462302 1.411994
0.2130064 0.3917186 0.371515 0.3759706 1.113053 0.8113354 0.8648867
0.4563506 1.643676 1.604929 0.4606357 0.4458704 0.8559591 0.291810252
0.4922979 0.2328489 0.6527174 0.8155828 0.419437475 1.027281
0.4439471 0.3746639 1.116052 0.9094907 0.4250603 0.8372696
0.8061027 0.2780818 0.9254646 0.9517928 1.222363 4.434707 0.5436701
0.5352443 0.5328752 1.027614 0.3165102 1.199947 0.869311947 0.9621877
0.6700205 0.785762387 2.164765 1.171421 0.4274573 0.7993833 0.8334642
0.9345168 0.800692388 0.8559422 0.5755523 0.7514336 1.717016
0.2719369 0.9886152 0.4185869 0.1720672 0.6847619 0.888268921
0.9644792 1.733051 0.5530627 1.202266 0.8853552 0.510081 0.9948986

0.3695876	0.6213378	0.1694446	0.7376466	0.3408804	0.4895598
0.7499675	0.3498661	0.2985424	0.46366	1.543111	0.707853627	0.5816478
1.66559	0.8668339	0.5293316	1.047125	1.264277	0.497865247	1.133071
0.8194882	1.277308	0.8222519	0.375920246	0.9682331	0.5365463
0.9477122.995347	0.7764276	1.410372	0.4477179	0.2600936	0.8325117
0.1658623	1.945693	0.7788561	0.4470049	0.907904154	1.385912322
1.434832	1.330031723	0.990697515	0.3139632	0.900037944	0.6405532
0.4135288	1.447743	0.474342568	1.576892	0.7824498	0.5547129	1.074545079
0.5992358	0.843983	1.325298	1.775568	1.307262	1.10998	1.076296	1.332985
0.101991	0.5073489	0.4680512	0.712817677	0.8097485	0.574674678
0.591141	0.5707632	0.7234253	0.3618406	1.145696	0.3424197	0.6854963
0.2080364	0.8308703	0.499343411	0.6365867	2.03377	1.43205	3.465034
0.3477380.2925131	0.3873372	1.260401	0.5747156	2.108123	0.3348742
0.161596	0.1806486	0.4536867	0.521550479	1.425494	0.6715008	1.375173
1.212617	1.390423	0.6700616	0.5398752	0.8766017	1.993363	1.730426
1.032620565	1.387005572	1.933272	0.5685449	0.2380601	1.059893	1.028143
0.2107901	0.8985994	0.4058605	0.8587086	2.145296	1.358461	0.8784329
2.008921	0.3950837	1.041031	0.4986106	0.9867249	1.118917	0.98701824
1.50199	1.031863	0.3435256	0.2529529	0.3239913	0.7804267	1.610367
0.5329217	0.6303324	0.8042789	0.858964	0.66421872	0.2706159
1.936729	0.9046159	0.6777258	0.6783711	0.944534822	0.9045146
0.5913662	2.154355	0.510864	0.1766439	0.903026	0.770109914	0.07218488
1.577717	0.9003174	0.7683692	0.4883408	0.536246	0.5968958	0.3693224
1.248504569	1.315011343	0.6251588	0.994531	0.6180832	0.7772419
0.2677922	0.5235048	2.120579	0.3498949	0.5454435	0.395083	0.6888957
1.861504	0.408654239	0.2517097	1.813279	0.2880587	0.5959805	0.9195871
0.3386929	0.7086504	0.4925649	1.309926	0.6201284	0.3708345
0.4372495	1.163548	0.9082591	1.112349	0.5013956	0.9962466	0.794951
0.615263	1.604079	0.5636543	0.4354572	0.6733887	0.6408997	0.6283937
0.6771333	1.133308	0.6225693	0.7307152	0.4233585	0.539362	1.606664
1.277966	0.5560493	0.8248124	0.5231728	0.544407	1.359626	0.3203662
0.6150566	2.686391	0.9635	0.7796318	1.101041	0.9368331	0.587367
0.8152193	0.609809499	0.882762	0.3596877	1.631958	0.1796891	0.6462889
0.412261326	0.42462278	0.3879619	0.655307403	0.308052	1.798609	1.602574
0.6811419	1.234924	0.3969542	0.6219546	0.3420724	0.1629902
0.9049167	0.8541075	0.911251963	0.3219768	1.262412	1.056405	0.4118193
0.5693868	0.7487822	0.8991872	0.8101282	0.297959	0.3397501
AP003071.3	0.1213647	0.6652881	0	0.691741202	0.042721568	0.5649917
0.9259967	0.4443048	1.143962	0.5623549	0.2306912	0.620329214
0.6163549	0.753937722	1.262300221	0.2611543	0.254329	0.388754	0.1347672
0	0.085881299	0	0	0	0.05493097	0.1047079	0.1285659	0	0
0.056124419	0.4596059	0.085256776	0	0.05326084	0.2046321	0
0.0550639	0.2431449	0.1043719	0.1395479	0	0	0.09654579
0.1746639	0	0.05451822	0	0	0	0.09904748	0	0	0.2166017

0.053684529 0 0.05360421 0.06045486 0 0.08688068 0.5248649
0.3901956 0.1772583 0 0.1399862 0 0 0.3389260 0 0.182421966
0.06219637 0 0 0 0.1501132 0 0.06161695 0 0 0.07706371 0
0.3004561 0.3214757 0.0687123 0 0 0.07909047 0 0.1249244
0.03856726 0.0503226 0.175973831 0 0.03637302 0 0 0 0.1356225
0 0 0 0.05039985 0 0.1177640 0 0.133648592 0.1335911
0.108634756 0 0.07262494 0.4314019 0.08306594 0 0 0 0
0.05615896 0.2549781 0 0 0 0 0 0 0.2485738 0 0.4626137
0.420825537 0 0.09423955 0.4537 0.2230554 0 0.1601084 0.2820233
0 0.05101088 0 0.09194823 0 0 0 0 0.06108245 0.04078807 0
0.0590574 0.06022793 0.08516803 0.08304612 0.06109352 0.06574266 0
0 0.1273694 0.10269299 0.1153296 0 0 0.2907079 0.2070696 0
0.221396044 0 0 0.07088588 0 0.1197137 0.2200197 0.06747468 0
0.3216006 0 0.2428252 0 0 0 0 0.1036389 0 0.1472067
0.05145599 0 0.1568618 0.9127399 0.3543598 0 0.3395253 0 0
0.07027428 0.05620917 0.06544946 0 0.1381040 0.1251210 0 0
0.3153885 0 0 1.532850753 0.2100499 0.1521299 0.06358513
0.04232091 0 0 0.05790999 0.03093129 0.06963377 0.3468304
0.06780983 0 0 0 0 0 0.168557313 0.060338519 0.1150109 0 0
0.2394608 0 0 0 0 0 0.15320141 0.09638822 0 0.05478676 0
0 0 0.1144381 0 0.2742052 0 0.1597926 0 0.09863561
0.113100016 0.4850171 0.3829439 0 0.07381812 0 0.1998210 0.07382441
0.6883829 0 0.15597476 0.04884955 0.1425508 0 0.04841189
0.09641717 0.05097223 0.2626868 0.06225807 0.5501306 0 0
0.1192621 0.2014665 0.1161665 0 0.03930745 0.1154103 0
0.03789298 0 0 0 0 0.06504454 0.06559125 0 0.071173797
0.09474881 0.1049003 1.143884 0.1749724 0 0.3340631 0.2121495
0.1177366 0 0 0 0 0.04186848 0.3379703 0 0.06318982
0.04351456 0 0.158425242 0.1120038 0 0.2372491 0.06611792
0.07131474 0.1758967 0.08213166 0.0879773 0.1004245 0.07207745
0.04043883 0.087491713 0.6790536 0 0 0.2080257 0 0.077454622
0.2185896 0 0.1800466 0.2185066 0.04221434 0.1697344 0.053382011
0.2012586 0.3008321 0 0.04008442 0.08784147 0 0 0.5148538 0
0 0.02971031 0 0.05919655 0 0 0.1085641 0 0.1070716
0.1487691 0.06790258 0 0 0.048598665 0.0619228 0.109376 0.14173 0
0.1093607 0.2706620 0 0 0 0 0 0 0.1085279 0.2186322 0
0.2289259 0 0 0.03293862 0.07107396 0.04944291 0.2138895 0
0.06436008 0.09793219 0 0 0.1971587 0.04579005 0 0.07110378
0.04362975 0 0 0.9082209 0 0 0.04993116 0.2303515 0 0
0.04406131 0 0.1358836 0 0.1057169 0.172902672 0.03969723
0.1121193 0 0.17342 0.3861253 0.100925177 0.266375383 0 0
0.1486523 0 0 0 0.07955793 0.1185801 0.04986147 0.3937425
0.02744896 0.2974713 0 0.096277688 0.07480861 0 0.06594093
0.2649677 0.0429056 0.136153 0 0.05961426 0.5902622 0.1794803

AC073130.2 0.1937599 0.6970289 0.2801923 0.552185998 0.10230814
0.3507836 0.5015876 0.8106711 0.7134172 0.2525078 0.09207523
0.330120672 0.3608063 0.138884929 0.56309165 0.495111 0.1522646
0.03103249 0.03585955 0.1095336 0 0.2799531 0.1355158 0.08788292
0.1534713 0.3552302 0.1539426 0.1846297 0.4066811 0 0.1426767
0.323269149 0.01894676 0.4038995 0.05444955 0.035148799 0.1758202
0.06469722 0.2499463 0.7240666 0 0.02120365 0.423875 0.348566
0.7228649 0.2611167 0.0608099 0.07689681 0.1486808 0.2767281
0.06778097 0.1779015 0.08645171 0.12856186 0.2802259 0.256739
0.2171628 0.08326011 0.2253971 0.07855802 0.4672135 0.1650803
0.0789848 0.0838085 0.1025799 0.088470613 0 0.2190324 0.1184267
0.485397597 0.17377 0.2282474 0.07937874 0.298256689 0.2097001
0.02779922 0.1721512 0.2486161 0.3862217 0.123033 0 0.03997345 0
0.1508374 0 0.06128819 2.41489 0.162252 0 0.1077528 0.1606811
0.087795014 0.05499608 0.0580699 0.3214333 0.078900832 0.1924374 0
0.134737 0.07174819 0.06640387 0.1810437 0.05888974 0.2585157
0.1653639 0.1267567 0.195590414 7.944665 0.021679564 0 0.4347992
0.0765264 0.2652313 0.2263078 0.1877189 0.05079323 0 0.1569021
0.1221226 0.08277866 0 0.220846 0.3996042 0 0.4095233 0.3472444
0 0.1846419 0 0.04756104 0.1316476 0.2897347 0.06677067
0.041591865 0.08520493 0.1125633 0.2474083 0.4479167 0.3400616
0.2201945 0.05071862 0.02935758 0.216616 0.04669408 0.1462781 0
5.094962 0 0.09615452 0.2379504 0.06629199 0 0.1049588 0 0
0.1016733 0.163950422 0.2761872 0 0.1230201 0.3480886 0.05509813
0.1970408 0.176730539 0.111331 0.109276 0.02829251 0.04960493 0.09556212
0.08781592 0.197494 0 0.3117306 0.238026771 0.2584486 0.0557206
0.1613322 0.0437855 0.2570165 0.1240955 0.2565882 0 0.1848376
0.1212167 0 0.260214 0 0.07743428 0.1742322 0 0.4956735
0.3365809 0.112173152 0.3134722 0.02061969 0.08268165 0.2456246
0.07490885 0.358005 0.020352055 0.02325412 0.02517605 0.129218 0.1730057
0.094123543 0.3074013 0.09107889 0.05075716 0.1182401 0.192267
0.4049671 0.0924539 0.1604919 0.2223421 0.03460739 0.243583
0.1044703 0.04919642 0.380456179 0.200920852 0.1064461 0.089701165
0.096331071 0.06885603 0.249621798 0.1133447 0.3504432 0.06945885
0.113111132 0.2942794 0.2557348 0.1305006 0.214014183 0.1154136
0.2790991 0.08746763 0.4035218 0.3281349 0.02365264 0 0.1566516
0.03648096 0.01770465 0.191333 0 0.07873639 0.090282673 0.1774517
0.1681278 0.2464387 0 0.1973492 0.05981554 0 0 0.07657719
0.06225383 0.05849161 0.1991358 0.0788042 0.4057729 0.03848275
0.08137769 0.2096911 0.1242446 0.3136747 0.2182265 0.05199774
0.2142036 0 0.02318262 0.195635483 0.2823964 0.1381903 0.06222617
0.8620757 0.1370615 0.1028482 0.09164397 0.05278197 0.1817275
0.2356135 0.365279324 0.085222278 0.2079927 0.1256058 0.2029137
0.3259027 0.1034888 0.08888921 0.07257833 0.07048788 0.5810943

0.1395176 0.4582853 0.08146056 0.3175065 0.6295019 0.1600244
0.0756624 0.2084143 0.08620193 0.231850246 0.08940761 0.0237572
0.142039 0.05277897 0 0.1170087 0.9178683 0.07022834 0.1603288
0.3164491 0.2743843 0.087300892 0.197112 0.2631357 0.07947342
0.08302881 0.02334731 0.386428456 0.08724516 0.01668363 0.1976193
0.2034947 0.4043738 0.1354913 0.170449868 0.1807377 0.6483801
0.02146885 0.239982 0.227889701 0.07066641 0.1698316 0.02935605
1.042008902 0.164627374 0.07114923 0.03117525 0.04725396 0.02463381
0.1518119 0.1299927 0.12748 0.06410287 0.07917047 0.1084072
0.1045375 0.1016547 0.096985341 0.0247151 8.469065 0.0848525
0.2752232 0.1309466 0.2160573 0.2494267 0.01774521 0.07034454
0.205072 0.05926606 0.08421482 0.1132955 0.08663293 0.1745243
0.2358215 0.2558378 0 0.0724944 0.2366408 0.1134703 0.09867014
0.05691281 0 0.1027515 0.1563498 0 0.1391785 0.07869148
0.2010368 0.2821864 0.1702769 0.2437937 0 0.06912734 0.1318167
0.3169989 0.07007041 0.05978671 0.03064655 0.2019552 0.0857752
0.08793041 0.1956047 0 0.1411833 0.02109726 0.069010227 0.1109098
0.02237496 0.8701387 0 0.5308345 0.080564045 0.297689741 0.07377611
0.140838599 0 0.1816928 0.4012552 0.1687872 0.06350753 0.0709929
0.2587142 0.0314307 0.0876451 0.2176699 0.1464029 0.038427082 0
0.398217 0.1315942 0.1762599 0.1712481 0.1811414 0.165849 0.4758739
0.2355899 0.07163555
AL132642.1 0.08032453 2.091507 0.1858491 1.030106473 0.056550069 0.2908396
1.181955 1.008208 2.271376 0.6280731 0.2035756 0.319325587 0.7886668
0.345454285 2.16233196 0.04321088 0.2945709 0.05145893 0 0.07784216
0 0 0.02808952 0 0 0.2079009 0.0567271 0.3618232 0
0.074291274 0.6421739 0.056426727 0 0.07050078 0 0 0.03644374 0
0.06907799 0 0.04434003 0.03516048 0.1916949 0.07706691 0 0 0
0 0 0.06555403 0.03746543 0.1361544 0 0.035530809 0 0
0.04001172 0.02761285 0 0.08684473 0 0.1173174 0 0 0 0 0
0 0 0.201224953 0 0.09462154 0 0.044961595 0.07451368 0
0.04078084 0.04580691 0 0.05100419 0 0 0.08510676 0.04547685 0
0 0 0.06726271 0 0 0 0.087350482 0.06079736 0 0.03807212 0
0.1063684 0 0 0 0 0 0 0.07794145 0 0.03503191 0.088454583
0.7957489 0.035949657 0 0 0.4124192 0 0.05360998 0 0 0 0
0 0 0 0 0 0.02546409 0 0.2467758 0 0.4373976 0.055704212
0 0.3430456 0.1501394 0.036907 0.068968789 0.07064459 0 0
0.03376127 0 0 0.1121374 0.04868155 0.0513141 0 0 0
2.326428 0 0 0 0 0 2.915265 0 0 0.04214935 0 0.03816512 0
0 0.4232875 0 0 0.036632438 0 0 0.04691543 0.109675 0.03961594
0.2184282 0.01488591 0 0.5473275 0.049337828 0 0 0 0
0.07991098 0.03429643 0.07091361 0 0.2383911 0 0.1730302
1.121885 0 0 0.2247129 0 0.1297799 0 0 0.08663473 0.03419214
0 0 0.2898372 0.03124494 0.033748344 0.5398488 0 0 0

0.702352258 0.1621904 0 0 0 0.1062741 0 0.03832741 0.02047171
0.04608672 0 0 0 0 0.126176606 0.111057517 0.2353492 0.037186243
0.119804148 0.07611932 0.045992202 0 0.2641430 0 0.05422026
0.06058093 0 0 0 0 0.3626032 0.04614697 0 0 0.3029609 0
0 0 0.07931841 0 0 0.074854622 0.7757642 0.2281044 0.02724343
0 0 0.0330626 0.04886028 0.03796687 0 0.103231034 0.1939848 0
0.08711688 0.03204114 0 0 0.1303934 0 0.07802162 0.0904673 0
0.07893298 0 0 0 0 0 0.06879011 0 0 0 0 0.02917484
0.04304937 0 0.075714543 0.09421197 0 0 0.7991331 0.5018194
0.08580397 0.07369928 0.08023427 0 0.07708694 0 0.09499269
0.02701603 0.02771042 0.2982450.02041207 0.08364367 0.05759975 0 0
0.0741291 0.3939485 0.2355330 0.04719927 0 0 0 0.06646543
0.04770406 0 0 0.6945704 0.06233413 0.04392834 0.1721009
0.1161455 0.076894371 0.04822412 0 0.08937215 0.0482058 0.02793933
0.1685066 0.07066118 0.1332019 0.03982081 0.1424010.2122372
0.116274784 0.0585905 0.05632388 0.04867902 0.296209402 0 0.03932721
0.02584784 0 0 0 0 0.08455636 0 0.2953860 0 0 0.096494276
0 0.6515091 0 0.02852393 0 0.1074817 0 0 0 0.04857944 0
0 0 0 0 0 0 0.1007803 0.08014149 0.02180025 0 0 0 0
0.08519272 0 0 0 0.1304884 0.09091768 0.06684705 0 0.08662832
0 0.4012011 1.47543 0.1051313 0 0.09913998 0 0 0.2489109
0.02916173 0 0 0.03901901 0 0 0.02627339 0 0 0 0.1703699
0 0 0.0815584 0 0 0 0 0.1865918 0.05265498 0.1177224
0.03300054 0 0.03633389 0.06562659 0 0.031860428 0.04951167
0.04127091 0 0.0584558 0.3123652 0.03003737 0.02750154 0.1183661
0.2604412 0.02969701
AC087276.3 0.1316968 0 0.1741204 0 0.039735944 0.08758448 0.0307601
0 0.0332505 0.0326909 0.1072846 0.096162851 0.2102029 0.080913265
0 0.1821775 0.02956938 0 0 0.2734861 0 0.09785909 0.256589
0.1433597 0.3831906 0.07304271 0.2391620.3715840.06318112 0.078303192
0.04749845 0.237895654 0.06622939 0.1238467 0.09516563 0.12286448
0.3072944 0.1884604 0.1941557 0.1297955 0 0.04941232 0.2244966
0.5415248 0.1831024 0.7859768 0.04723649 0.02986632 0.1347427
0.1842510.1842804 0.1116166 0.1007322 0.249663756 0.1187326
0.2492902 0.1968048 0.07761069 0.1616179 0.09153457 0.2540486
0.08243526 0.1227091 0.1953047 0.1195245 0.171807607 0 0.2764809
0.2759780 0.1446243 0.2659502 0.2312271 0 0.1221696 0.1295649
0.1719324 0.1931224 0.3706043 0.03583903 0.1723959 0.2328821 0
0.1437982 0.073080244 0.08926504 0.1103448 0.2126852 0.05809697
0.07174392 0.1404173 0.163675787 0.08544076 0.1014932 0.3210249
0.413703028 0.1868542 0.0315361 0.5494771 0.2786662 0.01934319
0.3515822 0.3087783 0.05476699 0.1070441 0.1723107 0.145026553
2.1123350.101042747 0.04134728 0.3039727 0.04458368 0.07726083
0.6215557 0.1822726 0.1183669 0.4908684 0.3134055 0.1660112

0.0321508 0.04424292 0.2573263 0.2328063 0.1968209 0.647588
0.2601023 0.07923569 0.1844073 0.039141587 0.2770869 0.2191339
0.02109964 0.1815337 0 0.1240993 0.1311569 0.2882762 0.04744595
0.02830246 0.1710447 0.1969885 0.102621 0.270426 0.05440721 0.02840683
0.01896878 2.667154 0.02746507 0.1680566 0.19804 0.1158636 0.1136479
0 0.235138578 0.2134672 0.1777021 0.095516224 0.08045226 0.2169849
0.3726867 0.4326265 0.2246982 0.1721916 0.128702274 0.2918726
0.06366335 0.131864 0.2119292 0.0835106 0.05116086 0.1568979
0.3170465 0.06409829 0.242676913 0.07528506 0.03246238 0.3968502
0.1020363 0.2058867 0 0.2242294 0.6845954 0.1435799 0.08474385
0.04863313 0.02425578 0.08239879 0.1203003 0.1353417 0.1088712
0.09119226 0.6536312 0.026140478 0.1521887 0.09610294 0 0.143099
0.1163768 0.0658645 0.047427788 0.1083813 0.02933474 0.3199458
0.2419003 0 0 0.03537456 0.1182829 0.1377715 0.1493509
0.377489 0.1346573 0.05753928 0.2914531 0.08064798 0.1892127
0.1014393 0.2866144 0.132990464 0.117054905 0.2480587 0.156777577
0.056121723 0.1337166 0 0.3433754 0.03712099 0.222564 0.131795309
0.266692 0.1489891 0.1520573 0.106871123 0.04482604 0.04645741
0.05095795 0.2594082 0.1147013 0.1377984 0.1596608 0.2028089
0.04250703 0.4951003 0.2415163 0.219625601 0.229356 0.052597974
0.3195443 0.03561816 0.09571549 0.0686593 0.2299482 0.1393923
0.3089931 0.1867468 0.327164 0.193432485 0.1817427 0.06629427
0.1377321 0.1125715 0.1120987 0.28446 0.09162329 0.1737214
0.05482332 0.4767638 0.2726413 0.08319556 0.04684672 0.08103606
0.364722211 0.09140105 0.1073448 0.1450099 0.08811202 0.06388078
0.1497964 0.1334776 0.2050024 0.1814966 0.03050368 0.212808856
0.099299656 0.110159 0.1951385 0.2659858 0.2441164 0.06029173
0.1553585 0.07047261 0 0.8937481 0.1083758 0.4672385 0.07593325
0.1752411 0.1571755 0.4016016 0.02938688 0.2226044 0.07533084
0.049117864 0.2604409 0.05536304 0.07355627 0 0.1658272 0.2726734
0.5347429 0.1227434 0.04670315 0.2681611 0.1504509 0.142410273
0.1722539 0.1752009 0.06173412 0.1209298 0.02720392 0.180104139
0.05082834 0.116637 0.08373198 0.06774539 0.03926417 0.1578724
0.049651375 0.07019757 0.1399041 0.150091 0.2236986 0.020425653
0.08233939 0 0.06841042 0.104068488 0.054806064 0.04145098 0.1634621
0.02752978 0.3444352 0.08844443 0.0504885 0.08912261 0.07469166
0.02306204 0 0.01740078 0.1184464 0.090404633 0.05759528 4.730546
0.1647813 0.1202572 0.1780064 0.3020959 0.1585244 0.06202932
0.1639287 0.1706762 0.2301863 0.1717201 0.320596 0.151415 0.1016765
0.1777959 0.1916346 0.07081516 0.4786595 0.2604118 0.1983207
0.2759253 0.03315695 0.02770023 0.05986223 0.0683161 0.1377634 0
0 0.1916549 0.1878852 0.09920197 0.2637742 0.02351047 0.1409556
0.07679539 0.2954897 0.1360749 0.04644169 0.2142533 0.1045844 0
0.2254013 0.1424472 0.5687426 0.1096697 0.02458219 0 0.01846148

0.1042838	0.4110292	0.2016255	0.2194748	0.093871956	0.024775957
0.05730851	0.131282333	0.0691318	0.09073098	0.4675362	0.3113914
0.05549848	0.1378664	0.2086959	0.07324512	0.153184	0.06917059
0.1949559	0.067161945	0.1043708	0.4059967	0	0.1232251	0.1197213
0.2321694	0.1352712	0.1386202	0.3965082	0.1669372
AC068790.6	0	0	0	0	0	0.08905554	0.09383023	0	0	0.04985995	0
0.09777799	0.05829093	0	0	0.1852374	0	0	0	0.1668477	0
0.1990054	0.2408295	0.1249436	0.1558507	0.07426952	0.3039737
0.1193132	0.09636346	0	0	0.181418488	0	0	0	0.062464048
0.07811391	0	0.296125	0.1979634	0.0950389	0	0.1369603	0.2477791
0	0.3093592	0	0	0.05871676	0.5620369	0.2409114	0.04863914	0
0.076157122	0	0	0.2572846	0.1183713	0.1232493	0.09307197
0.2214133	0.1676397	0	0.0992925	0	0.104815958	0.1923208
0.2594997	0.08418398	0.08626155	0	0.2028128	0.07053324	0.096371171
0.05323781	0	0	0.196366	0	0	0.08764572	0.1420762	0	0.04873779
0.111461535	0	0.05609906	0.07208581	0.08860914	0.1094234	0
0.124818647	0.1954706	0.2063957	0.1632084	0.140217176	0.1139956	0
0.2394455	0	0.05900421	0.07149746	0	0	0	0	0.252792685
11.181270	0	0.3090782	0	0	0.2872706	0	0	0	0	0.07234264
0.1961448	0	0	0.1775373	0.2729001	0.1559522	0	0	0.09375228
0	0	0.06684433	0	0	0	0.3028408	0	0.146559	0.1447285	0
0.1304382	0	0	0.1649808	0	0.2599555	0	6.298726	0.0837791	0
0.1208198	0.1767144	0.08666755	0.09326284	0	0.3906947	0.09034338
0.072840376	0.163607	0.165472	0.1748983	0	0.1958333	0.1750837
0.078518364	0.09892496	0.09709894	0	0.1175393	0	0.1560605
0.1914398	0.1611858	0.1955246	0	0.1148243	0	0.063713	0
0.1712821	0.07351134	0.6839865	0	0.07299571	0.0861672	0
0.07398955	0	0	0	0.1106998	0	0.1993828	0	0.09284691	0
0.09795746	0	0	0.3348537	0	0	0	0	0.1229816	0	0.09932593
0.2158122	0	0.06003664	0.07592971	0.3454463	0	0.08775855
0.2963484	0.2460076	0.288586	0.3094293	0	0.405672461	0.059510472
0.1261125	0	0	0	0.098580185	0.6445712	0.1132334	0.06171876
1.00506694	0.1162163	0.2596997	0.06626195	0	0	0	0	0.2472798
0.3498833	0.2522032	0	0.1237291	0	0	0.05667065	0.083742901	0
0	0.05733729	0.2172984	0.2335755	0	0.3006134	0.1417335
0.314183	0	0.09072517	0.073755507	0	0.1011116	0	0.1373547	0
0.07230944	0	0	0.1114883	0	0	0	0	0	0	0.05576173	0
0.07372274	0.1612655	0.2922917	0.09138742	0	0.1250673	0	0
0.081143687	0.10096748	0	0.4464361	0	0	0	0.07898392	0	0
1.487061	0	0.2036084	0.05790646	0	0.1598154	0.1750058	0.1792828
0.3086498	0	0.074914262	0.1588891	0.1688787	0.1121876	0
0.1011674	0.08317596	0.8155865	0	0	0	0.4589337	0.062058076	0
0	0	0.07377657	0	0.16481623	0	0.05929803	0	0.3099745	0
0.120393	0	0	0.08535236	0.3815298	0.1705918	0	0	0	0.1043391

0.105816408 0 0.04214718 0.2216101 0 0.08755507 0.1798599
0.1540095 0.2718585 0 0 0 0 0 0 0 13.343870 0.06113849
0.07756979 0 0.4835609 0 0.1250115 0.1041257 0.3510787
0.1496608 0.05752599 0 0.2067685 0 0 0 0.1717761 0.2336344
0.1008258 0.07013994 0.3034247 0.2534893 0 0.2083906 0.1050580
0 0.1948739 0.1432807 0.5043408 0.4332535 0 0.06142419 0
0.4506791 0.5811137 0 0 0.1595115 0 0 0.2607115 0
0.08363377 0 0 0.2252587 0 0.08358655 0 0 0 0.151152543 0
0.1001155 0 0 0 0.3999429 0 0 0.07073372 0 0.03893922
0.07033237 0 0 0 0.3538421 0 0 0.2434643 0.1287649
0.1768413 0.2537072 0 0.1273058
GTF3C2-AS1 0.08648118 0.01871315 0.06318754 0 0.048066689 0.09182056
0.04465084 0.04284807 0.01608862 0.01186339 0.09516988 0.062039278
0.1386941 0.045675907 0.033417253 0.3158658 0.08584484 0.00874786
0.02527145 0.2117268 0.202915359 0.1025921 0.1623545 0.05945673
0.2224932 0.08246588 0.1735818 0.02838869 0.09171266 0.037887865
0.07469362 0.110312256 0.02136388 0.1378265 0.1688388 0.039632891
0.1486878 0.06383207 0.1526596 0.0314015 0.02261301 0.05977174
0.3476005 0.1572136 0.06201727 0.2698928 0.04571179 0.06503016
0.05588291 0.04457593 0.0764281 0.04629166 0.08935735 0.108722311
0.02872505 0.1206218 0.2448673 0.04694099 0.4398785 0.1181067
0.2546287 0.09307016 0.08906125 0.04725017 0.0650624 0.166262018
0.05338631 0.2984286 0.09347449 0.027366099 0.1049671 0.04825611
0.1062877 0.084076663 0.1055591 0.04701855 0.1178545 0.1012314
0.1472994 0.0780350.1737826 0.1126826 0.03616972 0.06571287
0.026520513 0.05183025 0.04894224 0.1029101 0.02811086 0.08678522
0.1811799 0.103945194 0.06717987 0.03273907 0.1294429 0.155691497
0.1898641 0.03814773 0.05697227 0.1213521 0.03743766 0.1417641
0.1992077 0.1192483 0.0776917 0.08337443 0.085209708 3.231566
0.067224643 0.03000951 0.1225671 0.1563992 0.1495340.08657856
0.1763893 0.05011401 0.02226676 0.08845942 0.09753913 0.2255695
0.05351857 0.07608955 0.1408074 0.0995628 0.2185153 0.04894299 0
0.2081975 0.047347724 0.04469052 0.02650759 0.01531394 0.05646668
0.011724482 0.07806086 0.1031253 0.1627332 0.03443587 0.1780279
0.08276189 0.03336027 0.2068929 0.08287078 0.07897659 0.04810736
0.09637168 1.1240120.03986783 0.09486871 0.03832954 0.03270276 0
0.05917445 0.081267428 0.1136173 0.2292883 0.057770726 0.05190362
0.07874292 0.08322859 0.05233278 0.09319087 0.1249752 0.112093232
0.1255340.1617221 0.1196322 0.1864442 0.1481609 0.1299624
0.1265280 0.06719844 0.117421906 0.09106877 0.1963410.07579752
0.01234285 0.2581078 0.0466423 0.1808264 0.08281227 0.06947271
0.1298468 0.07059503 0.08508911 0.08638405 0.07276077 0.04911494
0.02633928 0.02941626 0.4664933 0.069565975 0.2135507 0.03487535
0.06992229 0.05193001 0.02111633 0.2337079 0.097530951 0.02622075

0.02129091 0.03187251 0.04876921 0.079598483 0.05908259 0.1112564
0.1001568 0.1333245 0.09635348 0.2739783 0.08470204 0.1322448
0.1253536 0.1463341 0.2059935 0.09816508 0.1109453 0.139422629
0.061358169 0.2300497 0.107466298 0.040732674 0.0776403 0.031274132
0.1469753 0.1436912 0.10769 0.119569906 0.2027801 0.1132843
0.08408525 0.198224634 0.06506872 0.08991573 0.1109546 0.08629328
0.1202489 0.4800621 0.1158804 0.4023384 0.08226999 0.07486243
0.1528174 0.126193575 0.04439057 0.159063195 0.118235 0.1637253
0.1343077 0.1162755 0.1748418 0.1011697 0.3073256 0.03227125
0.2590395 0.116993057 0.2748069 0.2004826 0.07404801 0.08715033
0.06508826 0.0458797 0.1994982 0.2101425 0.03094802 0.1922395
0.0439735 0.08051018 0 0.07188536 0.143385834 0.106141 0.06492493
0.04677643 0.06821436 0.1622745 0.03624032 0.07104309 0.1091119
0.2415026 0.1180764 0.167326115 0.048047236 0.095943 0.1298274
0.03575005 0.1640534 0.02917282 0.06890758 0.02386926 0.03974017
0.242434 0.09176796 0.3956374 0.08726021 0.09892442 0.139427 0.3747588
0.01421917 0.09302191 0.06074944 0.065357109 0.1764241 0.06697004
0.07118203 0.02975611 0.04011865 0.03298401 0.304945 0.02969538
0.1807827 0.08109544 0.3457874 0.054141045 0.04861905 0.1377559
0.08214452 0.01755397 0.0329073 0.056644496 0.06968259 0.05643612
0.1114153 0.06555871 0.0522456 0.1336795 0.006006092 0.06793174
0.4603202 0.06051934 0.1488287 0.088948601 0.1693235 0.02872466
0.03310114 0.075532032 0.072925929 0.04345578 0.1406097 0.02664118
0.1111058 0.08558963 0.02442942 0.1149946 0.06023397 0.05579413
0.09167788 0.02946846 0.0859674 0.071082831 0.01393406 1.981276
0.07973127 0.1503184 0.08613036 0.1339914 0.3004231 0.03501584
0.1487226 0.1321337 0.08910253 0.02967453 0.05018714 0.06105318
0.2213878 0.04301426 0.09787586 0.0342647 0.08855474 0.1185912
0.04797984 0.1112579 0.01604336 0.04691072 0.028965 0.1046757
0.1333167 0.07062037 0.03327394 0.09788626 0.1306836 0.1519999
0.3436195 0.09100642 0.04384475 0.08360617 0.169784 0.2567812
0.0730318 0.01727813 0.0885576 0.01813461 0.03965924 0.1309569
0.05350964 0.2586914 0.1011021 0.087540947 0.02233198 0.05676623
0.2916921 0.01951175 0.06275179 0.045420983 0.065934663 0.006932339
0.047641806 0.05017528 0.2634071 0 0.08723009 0.07160948 0.06003734
0.09536984 0.04430057 0.04941319 0.07251607 0.1002273 0.070410273
0.01683366 0.1543504 0.08161026 0.009937307 0.038619 0.1582941
0.05610213 0.207926 0.07379034 0.2675653
AC106820.5 0.03368939 0 0.03897404 0.14401438 0 0.2091133 0.1377031
0.05285733 0.02977035 0.01463466 0.06403715 0.028699342 0.1368743 0
0.123670449 0.08155501 0.1323726 0 0 0.01632412 0 0.5257004
0.1060307 0.1650279 0.04574456 0.1307955 0.08922095 0.1926114
0.3111257 0 0.1063176 0.319494676 0.01976585 0.4878911 0.08520518
0.073336629 0.04585526 0.03374707 0.5215034 0.4648423 0.1115815

0.06636092	0.2009998	0.09696928	0.06557522	0.4086076	0.04229251	
0.1604423	0.1206399	0	0.09428161	0.171316	0.0901891	0.067059855
0.02657639	1.182952	0.226551	0.05211571	0.05426336	0.05463611	0.3899293
0.04920482	0.4394634	0.05828775	0.1872754	0.092295281	0.08467365	
0.1713761	0.172965	0.050638179	0.02589746	0.05952868	0.351944	0.169718513
0.3125223	0.02900101	0.3335307	0.2017276	0.1659076	0.03208796	
0.1800776	0.08340307	0	0.1573582	0.098147017	0.07992217	0.08232967
0.1269497	0.07802443	0.4496442	0.2095344	0.091590475	0.4398644	
0.1211606	0.3592813	0.164623579	0.3345945	0.1129416	0.03514046	
0.02494998	0.1385491	0.0209856	0.3378958	0.1716219	0.07667231	
0.2424335	0.055648902	2.002499	0.067850377	0	0.2116781	0.07983471
0.06917437	0.8094559	0.03263904	0.02649453	0.02746825	0.4910555	
0.02123367	0.02878575	0.1188368	0.0767978	0.0781649	0.4005018	
0.2746462	0.0776263	0	0.0825532	0.245313999	0.04961715	0
0.1133476	0.04643816	0	0.06666631	0.05871474	0.04301733	0.1486802
0.2280618	0.03828561	0.3174674	0	0.0484244	0.09742542	0.1017346
0.1698343	0.6932898	0	0.02507784	0	0.1383157	0.1271913
0.2463667	0.150377102	0.3057997	0.07955151	0.085519077	0.09604236	
0.7285288	0	0.09683648	0.2586603	0.1027795	0.092185383	0.116144
0.05700006	0.236125	0.1379984	0.04984668	0.2519338	0.09365086	
0.02365525	0.0765193	0.031039612	0.0674054	0.1453236	0.2618104	
0.1370352	0.06703189	0.2157671	0.245374	0	0.1285522	0.07587419
0.06531446	0.1845951	0.04918304	0.08078183	0.1009802	0.0974763	
0.05443179	0.2926096	0.070213502	0.13626	1.18311	0.08625605	0.106768
0.2344417	0.1179417	0.106159468	0.5094478	0.05252888	0.06740209	
0.1443879	0	0.1020379	0.1583605	0.05295144	0.1938384	0.1782923
0.3041814	0.09645077	0.06439621	0.1449713	0.03610351	0.2541133	
0.05449335	0	0.079380732	0.087336188	0.3701596	0.350921368	0.025123889
0.04788849	0.086804395	0.1655426	0.06647148	0.2536157	0.059000517	
0.1705563	0.05716938	0.0777956	0.15947587	0.04013435	0.04159497	
0.3193713	0.08709651	0.2396243	0.9129812	0.1906	0.2905312	0
0.1292903	0.2661393	0.049159655	0.1642805	0.070639259	0.1514644	
0.1753961	0.06855799	0	0.2647039	0.1040024	0.1229567	0.02388587
0.07988769	0.086593496	0.08136034	0.5045227	0.3288439	0.3225256	
0.1605856	0.02122393	0.2187563	0.07776947	0.06544703	0.2561182	
0.1084913	0.04965863	0	0.02418482	0.040818598	0.2455038	0.09610958
0.1081938	0.06311186	0.8579211	0.1072944	0.04780291	0.1284822	
0.9479188	0.2731103	0.190535347	0	0.2564362	0.08735723	0.05292146
0.4857025	0.02699067	0.02318299	0.07571596	0	0.1212431	0.1940654
0	0.08498218	0.3660991	0.1407248	0.05136689	0	0.01811869
0.1573749	0.175907891	0.3031366	0.099137	0.03292878	0.02753033	
0.08908258	0.07324026	1.094341	0.3663219	0.5435949	0.09003531	
0.4209502	0.145719987	0	0.1176477	0.1381819	0.06496367	0.04871328
0.258005839	0.03033895	0.06961953	0.2249047	0.03032743	0.05273191	

0.2826975 0 0.1047506 0.22547 0.2239697 0.1335235 0.018287814
0.1105821 0.03543471 0 0.279528668 0.343488742 0.1237085 0.130092
0.02464839 0.02569876 0.05279163 0.04520415 0.2127857 0.1337482
0.1445379 0.02827343 0.06231814 0.2120986 0.060706865 0.07735066
0.40988 0.02950692 0.1076705 0.2276792 0.02253977 0.165588 0.1666112
0.07338561 0.183375 0.0206094 0.1537471 0.6922753 0.09037816
0.4855177 0.1013008 0.2478338 0.095105 0.1008379 0.2057259
0.05918787 0.2264586 0.1187464 0 0.133992 0.1019431 0.06167225 0
0.08209339 0.1906616 0.08411017 0.08881906 0.05449996 0.1262986
0.01802894 0 0.09921093 0.2436654 0.2494854 0.03197143 0.7022863
0.1118542 0.1834635 0.2550761 0.02828975 0.07364339 0.08803725 0
0.04958767 0.1633958 0.392543 0 0.07145587 0.084046905 0.044365593
0.07696552 0.146927185 0.03094808 0.02707823 0.4186018 0.3961892
0.06625302 0.07406199 0.2076143 0.06557896 0.06857556 0 0.08727547
0.040088321 0.2180429 0.1038581 0.302023 0 0.08932565 0.2267668
0.2595282 0.1737562 0.0273083 0.1494648
AC063919.1 0 0.3594913 0.06743736 0 0.061559425 0.180916 0.1906157
0.2743795 0.4120967 0.2025806 0 0 0.2960445 0.501407401
0.534972071 0.2822317 0.3664741 0 0.5178458 0.2824588 0.123750218
22.13426 0 0.06345562 0 0 0.2470086 0.06059607 2.153382 0
0.0735852 0 0 0 0 0.063447733 0.07934405 0 0.3007884
0.4021618 0 0 0 0 0.1134658 0.628462 0.1463587 0 0.05964143
0 0 0.5928613 0.2080741 0 0 0.07724071 0.1742242 0 0 0
0 0.08513983 0.09505127 0 0 0.212933207 0 0.1317931 0 0 0
0 0.35822 0 0.3785334 0 0.08878663 0.09972921 0 0.1110446 0
0.1443136 0 0 0 0 0 0 0.1800091 2.000638 0.1450242
0.443745034 0.1323659 0.05241151 0.0828893 0 0.2315815 0 0 0
0 0.2178702 0.2126059 0.1696914 0.1326672 0.07627025 0
21.36721 0 0 0.1046485 0.4144176 0.1196934 0 0.2259032 0 0
0.08092196 0 0 0 0.08858958 0.1803332 0 0.2112109 0.1790906
0 0.0952287 0.848940966 0.1717066 0.067897 0 0.08035264 0.150156458
0 0.20319 0 0.07350387 0 0 0.3662124 0 0.05585964 0
0.08801644 0 0 0 0 0 0.05983244 0.0880324 0.3789262 0 0
0.1835322 1.701711783 0 0 0.4441315 0.0837788 0.3978345 0 0
0.1004828 0 0.1021427 0.1790855 0.2587514 0.07925905 0.06481819
0 0 0 0.1166326 0.2011645 0 0 0.1739794 0 0 0 0 0
0 0.1503095 0 0 0.1397821 0 0.09418413 0 0.080994315
0.09430906 0.07444194 0 0 0.2704386 0.1360508 0 0 0.0908915
0 0 0.169904111 0.1513352 0.4384217 0 0 0 0 0 0.08914058
0.2006768 0.1249409 0 0.440023 0.4440267 0 0.241790579 0 0 0
0.3314487 0 0.2455207 0.4600664 0.3134536 0.510447383 0.236093
0.06594735 0.06730544 0 0.1388901 0 0.0789447 0.0502348 0 0
0 0.06283882 0.2634097 0.1917539 0.2878155 0 0 0 0.232961
0.4966209 0 0 0.8142598 0 0 0.1653203 0 0 0 0 0 0

0.06946591 0.1468963 0.2838879 0.2691313 0.05662199 0 0
0.08592507 0 0 0 0.1699196 0 0.07488373 0 0 0.09282659 0
0 0.1874512 0 0 0.102557519 0.0682639 0.1511555 0.09157079 0
0 0 0.2183541 0.08482596 0 0 0 0.2352735 0 0 0.1777618
0 0.5643188 0 0 0.2420870 0.05697715 0 0 0 19.645630 0
0 0.4078908 0 0.1779052 0 0 0.0749384 0 0.111607841 0 0
0.5837338 0 0.1216571 0.1222889 0.076920536 0 0.3467860
0.1155189 0 0 0.1226264 0 11.070729225 0 0 0 0 0 0
0 0.1840932 0 0.1429120.09784388 0 0 0.140055996 0 0
0.1021125 0.1863039 0 0.1560036 0.08186266 0 0.1269802 0
0.1426430 0 0 0 0.05008067 0.06597393 0.1097077 0.1744813
0.04746274 0 0 0 0 0 0 0.5335621 0.2009869 0 0.1979428
0.2183056 0 0 0.07284545 0.0623915 0.4758895 0.1144441
0.08432358 0 0 0.1620235 0.07741718 0 0.2648172 0.09790036 0
0.07616608 0 0 0 0.08490287 0 0.06182058 0 0.153532898 0 0
0 0.9370772 0 0.05078015 0.1146386 0.1708674 0 0 0.3559720
0.1510141 0 0 0.0898536 0 0 0.1236492 0 0.0598754
0.08590086 0.09450392 0.1293106
AL596223.1 0.0153197 0 0 0 0.016178059 0 0 0 0 0 0.4513576
0 0 0.032942959 0 0 0 0 0 0.01484625 0 0.01328076
0.02410787 0.7087464 0 0.009912849 0 0.01592488 0.02572351
0.042507061 0 0 0 0.1210148 0.03874565 0 0.01042596 0
0.01976209 0.7398279 0 0 0.07312108 0 0 0.03096793 0 0 0
0 0 0.006491928 0.06835335 0 0.02417035 0 0.0114467 0.007899588
0.01645025 0 0 0.03356262 0.1498791 0 0.06082888 0.041969763 0
0.01731788 0.01123614 0.046053761 0.01177645 0.02706969 0 3.344323928
0.04974003 0 0 0.01310461 0 0 0 0.07585235 0.6817346 0 0
0.1235672 0.2396036 0 0 0 0 0 0.0869657 0 0.01089182 0
0.01521513 0 0 0 0 0 0.01396842 0 0.0261491 0.02004412
0.008435141 0.1011782 0 0.0336682 0.02750205 0.009075881 0.01572795
0 0 0 0.0374722 0.0106333 0.009655665 0 0.01801303 0
0.01184807 0 0.00693838 0 0.02150663 0 0.015936073 0.01504172
0.008921799 0.1718098 0.05279247 0.019730854 0 0 0.01956142
0.06760987 0 0 0 0.06963502 0 0.02215131 0 0.01544587
18.004970 0 0 0.0157242 0 0.01244791 0.013676303 0 0
0.262496669 0 0 0 0.0220174 0 0.07010594 0.136239314 0 0 0
0.01568813 0.02266696 0.05207398 1.7417750.2151367 0 0.056459014 0
0.02643341 0.008503857 0.04154299 0 0.009811653 0.02028724 0
0.02922849 0.05750423 0 0.02962644 0.01118259 0 0.01836764
0.01477525 0.04950392 0 0.319284367 0 0.009781817 0.05229803 0 0
1.3408010 0 0.01194332 0.06896249 1.3952330 0 0.01440237
0.0120394 0.03205267 0 0 0.2631564 0 0.01318467 0 0.03851795
0.008259985 0.02333839 0 0.071486454 0.1009944 0.010638382 0.034274027
0 0 0 0.01511341 0.008237683 1.462208613 0.01551154 0 0.008844068

0 0.0547513 0 0 0.0132019 0 0.01122063 0.02166805 0 0 0
0 0 0.05602788 0.021414695 0 0 0.01558781 0 0.01337442
0.009458675 0 0.02172341 0 0.019688485 0 0 9.464406 0.0183329 0
0 0 0 0.02976096 0 0.03700098 0 0 0 0 0 0 0
0.0502234 0 0 0.01086879 0.00834645 0.03694718 0 0 0.30995397
0 0 0 0.0883461 0 0 0.005738432 0.03343889 0.01102666 0 0
0.0154577 0.03963752 0 0.005839562 0 0 0 0 0.01060357 0
0.02246073 0 0.02700589 0 0 0 0.009507341 0 0.01531362 0
0.03506565 0 0.02513438 0.009847054 0 0.029330979 0 0.01582917 0
0.1103272 0.06394392 0 0 0 0.01139209 0.02036931 0.007589705
0.074844749 0.03352359 0 0 0.014123452 0.022313695 0.02250176
0.6433344 0.0224169 0 0.02400613 0.3083377 0 0 0.02816838 0
1.579852 0.009644833 0 0 0.02070958 0.01341779 0 0.6833204
0.03074877 0.02151383 0.008418189 0 0 0.01874357 0 0 0
0.02759765 0.04606489 0.01733821 0.08649487 0.04585436 0 0 0 0
0 0 0 0.01402222 0.05282016 0.09332655 0.008670025 0 0 0 0
0 0 0.01503818 0 0.009454115 0.392539 0 13.91635 0.5255901
0.01159917 0.0771858 0 0.1100921 0 0.1503278 0.02122901 0 0
0 0.152875798 0.020174525 0 0.02672509 0.08443879 0.01231338
23.60369 0 0 0.01122616 0.03776368 0 0 0 0.009921772 0.072918021
0.07082243 0.04722776 0 0.01672326 0.02437162 0.1117117 0.06294197
0 0.08692601 0.008495835
AL117329.1 0 0 0.1806231 0 0 0.01009505 0 0 0 0.005651963
0.0123657 0.166256951 0.006607672 0 0 0 0 0.01250298 0 0
0.220966997 0.5978033 0 0.2407745 0.5123351 0.2020549 0.2549853
0.02028743 0.2075456 0 0 0 0 0 0.3948788 0.127453004 0
0.156399 0 0 0 0.4015185 0.4191852 0.4962113 0.06331351
1.209845 0.383838 0.3098171 0.5590991 0.5733961 0.3823252 0
0.02322092 0.01726585 0 0.8361408 0.2041547 0.348873 0.4191344
0.01055034 0.8282571 0 0.4243062 0 0.3513001 0.118815981
0.08720345 0 0 0 0.0100017 0.2069119 0.5996565 0.07647027
0.5672774 0 0.792682 0.3895398 0.8970382 0 0.3278628 0 0 0
0.050539677 0.1790243 0 0.4003993 0 0.6884154 0.9144297
0.594259606 0 0 0 0.015894566 0.4005872 0.1962831 0.9499967
0.2119871 0.3745575 0.1945133 0.09490667 1.013153 0 0.1617224 0
16.09045 0 0 0 0.2004111 0.2805118 1.211383 0.3277384 0.2353427
0.1697335 0.487665 0.1886121 0.8337877 0.1376857 0 0.6037517
0.4083429 0.6128455 0.04996596 0 0.4038433 0 0.1213615
0.03788629 0.06566291 0 0.251360327 0 0.3968275 0 0.2296834
0.2055161 0.8280189 0.2724603 0 0.01246779 0.009406519 0.2848546
0.203331 4.819522 0.009496927 0.09685159 0 0.3672492 0 0.01057197 0
0.3690657 0 0.107340344 0 0.5439639 0.9813824 0 0.02219903 0
0 0.358842 0.4512797 0.2963752 0 0.2406372 0.1326788 0
0.3745657 0.1108202 0.011987618 0 0.2693978 0.3972265 0.008820585

0 0.04999805 0 0 0.08274558 0.4493112 0 0.4696841 0.2374335
0.4575744 0 0 0 0.4068251 0.280206168 0.7156881 0.1163074
0.1998745 0 0.3018076 0.9185821 0.008199839 0 0.01014343 0
0.2648751 0 0.1463705 0.4403480.01022503 0.2994446 0.008607147
0.7048562 0.2514352 0 0.4815010 0.5779328 0 0 0 0.215869269
0.01429571 0 0 0.3236576 0 0.01826663 0.4620878 0.2798496
0.47851078 1.02756500 0 0 0 0.03212828 0.008810173 0 0
1.8582810.3680522 0.8836088 0 0.05706561 0 0.465148398 0 0
0.1819880.1908999 0.8340369 0.04748231 0.7383254 0.01606645
0.8310111 0.03689926 0.01028431 0.200656457 0.8169637 0.2865421
0.0052917 0.1712709 0.2015610 0.01056056 0 0.09478461 0
0.01047494 0.06712424 0 0.009340273 0 0.4803936 0.4639736
0.1253546 0 0.71788700 0.4338492 0.4394941 0.5752841 0
0.47830567 0 0 0.2699016 0.07153465 0.3939184 0.3127170 0 0
3.66168 0.2623209 0.7847331 0 0 0.2898585 0.03471666 0
0.04198505 0.1215576 0 0.009005578 0 0 0.4890867 0 0
3.9226170.3395398 0 0.01159066 0.9884415 0.281388111 0 0 0 0
0.009406628 0 0.3339351 0.5579122 0.6152516 0.05856285 0.2443832
0.4094208 0 0.3074588 0.4547376 0.5708867 0.1160264 0.014125649
0.3416578 0 0 0 0.13265668 0.2197728 0.7347895 0.05711583
0.05954976 0 0 0.5855230.1721802 0.4226454 0.2183862 0.2406751
0 0.171931658 0.009957708 15.038240.1595396 0.6098806 0.2813779
0.01740989 0.3106177 0.07864497 0 0.6373810 0.1442031 0.2673594
0.5410181 0 0 0.3018684 0 0.009735993 0.2171692 0.2171566
0.2623776 0 0.2298779 0.02069929 0.2283507 0 0.5943951
0.2536383 0 0.5603434 0 0.5332184 0.2276260 0.02655447 0 0
0.3854089 0 0.4972471 0.2937499 0.4747238 1.8421630.3496196
0.009480454 0.2465024 0.06951052 0.0510692 0.3335501 0.7295828 0
0.1034870.210985058 0.008567082 0 0 0.3705203 1.1294330.4849970 0
0.02860303 0.04009073 0.0506537 0 0.1753983 0.008426531 0.185787173
0.3368360 0.4453620 0.4277741 0 0.01336411 0.7956775 0.3269437
0.8802895
AC015849.3 0.7281879 0.1871123 0.5967101 0.194552213 0.288370587
0.9887354 0.4960697 0.7616654 0.5898557 0.4217661 1.384147
0.87879972 2.6195090.695942513 0.501207441 2.4972881.4782871.166262
0.1010754 2.1464550.515287793 1.3151493.02394 1.42021 3.1310652.23813
0.8356785 5.2986791.22271 0.378839829 1.3022177.737052413 0.4628367
1.4380432.1486372.014465539 3.6342182.1275184.5401790.7326267 0.5024598
0.3984375 2.0998715.2835841.6536246.74663 0.4570711 0.8188145
1.4590150.9657129 0.7217469 1.2343181.6786631.248165296 1.005274
2.2513771.5415990.8135604 2.0525560.6888852 2.1070561.63964 3.957872
0.8399169 0.2891366 1.108299888 0.1525167 4.3559251.8692980.820898846
1.6793021.3939232.8340491.222807973 1.8013580.1567124 0.7394034
6.3847132.0918640.4045845 1.71448 0.6009123 0 1.2110540.353570607

0.4894578	2.194761	2.820209	0.5621596	0.2892544	1.396452	0.758887145	
0.8267444	0.4364762	4.530036	2.298067569	2.229921	0	1.772289	1.797625
1.653327	0.8693974	1.770554	2.782174	0.655996	0.7542628	1.369898063	
16.23132	0.977712807	0.2667239	6.154963	0.683053	1.308289	3.280536	0.5879039
0.5249501	0.5442427	0.8423844	1.338635	0.829596	2.069173	1.936626	
2.581204	3.231852	3.627806	0.6058987	0.08518919	1.090447	1.388724273	0.47665
1.342914	0.476385	1.547447	0.390776027	1.481003	1.427743	0.8523249	0.7651632
1.369305	3.310136	0.4765264	0.8826525	1.162979	0.6580708	0.6413657	
0.3670926	18.52348	1.816015	0.4065385	0.5748842	0.7785573	0.5040215	
0.6902979	1.137627425	0.6885193	1.432905	0.654667808	1.124463	0.524899	
0.6472671	1.657035	1.760092	2.036424	0.539652856	0.7845074	1.745393	
1.169617	3.821714	0.5387119	1.608894	2.142321	0.08521707	0.7236013	
0.782732502	2.003308	0.3141134	1.347373	1.028466	2.98831	0.3497814	
4.299211	2.152898	1.466496	1.184446	0.4705853	1.858078	0.08858995	0.4365199
0.873065	0.4096801	0.09804414	6.746331	0.632353157	2.35618	1.123646	
0.2071559	2.653928	0.8445666	1.628707	0.267704592	1.3546	0.8515491	
0.5766815	1.755512	0.707469578	1.076506	1.483267	3.433597	1.650515	3.29174
6.087783	1.085812	1.275916	3.603547	0.7153377	3.763445	1.701354	1.016894
3.789047285	1.352887656	2.333598	1.011343877	0.181015557	0.6469362		
1.459309945	0.8945407	1.077574	4.07875	0.478230622	2.21192	1.270027	
2.837586	0.86175793	2.024153	0.6742988	0.8628915	0.9412848	1.973113	
3.022304	1.287428	3.728608	0.2056539	1.430558	3.175879	0.885478216	0.5918137
0.975487637	2.031009	2.268942	0.4939547	1.162635	1.112516	2.060654	0.8858929
0.4732632	3.933159	0.467924279	6.008495	0.588022	2.048452	1.561283	0.325408
0.2676042	0.6402991	2.941694	1.355679	10.09795	0.9770888	0.4919563	0
0.5663115	2.242471722	2.653253	1.12525	1.169291	1.13679	3.245157	0.5797858
1.549872	0.5289734	1.951336	1.377416	2.273686016	0.533803479	1.741009	
0.6294017	0.619604	3.368218	0.7778622	0.751642	0.5682576	0.2207561	
2.839027	0.3495564	2.744958	1.683796	2.983129	3.126225	3.585288	0.4265313
2.186607	0.6884231	2.297166013	12.89444	1.517832	0.8896842	0.1983538	
0.1604582	1.363199	2.032759	0.7917957	2.033597	1.567685	11.61605	0.820234812
0.4629911	2.260379	0.7466918	0.3900483	0.438719	1.85891092	0.4098556	
0.4389027	1.789213	0.6008933	1.013144	1.336658	0.760693662	1.094344	
2.887988	0.8068418	0.9620262	1.581146451	0.5975511	0.1276521		
0.6619549	1.734261537	2.077071196	0.4456547	1.318081	0.1331922		
0.4166042	1.093533	0	0.9581899	0.4416706	0.5206918	0.4583424	
0.8418696	3.629363	1.858898948	0.1857684	34.12531	0.9566772	2.81212	
1.107277	1.948766	3.366099	0.166725	1.784488	4.404005	1.336401	0.8703636
3.315053	0.9767508	0.983845	0.5734647	2.575417	0.2855099	6.447941	1.43283
1.439248	0.6674792	0.2138895	0.6700845	1.255021	0.5875931	2.83269	
0.2092241	1.47869	2.472663	3.522415	2.133113	2.257839	0.7962246	0.4546397
0.9288622	2.918794	5.135099	0.8238641	0.05758788	5.144247	0.5641301	
0.4295977	2.343198	4.942765	2.741405	1.466821	1.03741603	1.042052	0.2943133
2.739857	0.390195	0.6757192	1.135408241	0.559388305	0.2310543	1.746687233	

1.059147 3.023994 5.277986 13.08317 2.923754 1.334027 0.8227143 0.2362455
0.8646423 0.446207 2.043641 0.541561995 0.7854904 4.209125 0.3956456
0.4636935 2.79959 1.36153 2.025703 2.190824 0.8362048 3.062383
AC022272.1 0.03811194 0.1175171 0.02204516 0.814598713 0.080494693
0.1182823 0.09346795 0.1195923 0.2694275 0.1986698 0 0.064933646
0.3290399 0.08195462 0.06995259 0.6150738 0.1796996 0 0
0.0923353 0.080907478 0.1321581 0.07996655 0 0.284623 0 0.24224
0.2773225 0.06399427 0.026436985 0.09621952 0.060239344 0 0
0.03213014 0.165927663 0.02593744 0.03817719 0.1474908 0 0
0.07507239 0.04547717 0.05484942 0.1112753 0.07704119 0 0.06050139
0.07798674 0 0.05332917 0.03230089 0.06801906 0 0.06013036
0.2019989 0.08543042 0.1179143 0.04092449 0 0.03675974 0.1113283
0.0932163 0.09890913 0.09079708 0.034803755 0 0.1723318 0.1956709
0.143214156 0.02929713 0.2020298 0.07026091 0 0.05303226 0.06561619
0.0580484 0.2282092 0.2413118 0 0.05820488 0 0.03028574
0.04854961 0.037010394 0.03616555 0.1117649 0.09574331 0.02942234
0.05450045 0.284449 0.020722787 0.06490529 0.01713324 0.08128912
0.093117198 0 0 0 0.02822526 0.01959213 0.04748094 0.2085015
0.1664155 0 0 0.020984721 5.789292 0.076757376 0.04187942 0.03420943
0.1128937 0.07825517 0.1144646 0 0 0 0.1322663 0.02402111
0.1628229 0 0.08687936 0.02947531 0.3262157 0.01726112 0 0
0.0622602 0.07929068 0 0.1331725 0.08548479 0 0 0.05027858
0.03321123 0.04866439 0.04805658 0 0.08662305 0.01995237 0.03464723
0.2921668 0 0.02877243 0.1152775 1.263596 0 0.02836991 0.2005888
0.03911825 0 0.03096758 0.06804709 0.08648582 0.1499909 0.02418638
0.08148768 0.05494439 0 0.1095486 0 0.0581359 0.130358674
0.06569534 0.03224135 0.133561 0.07805701 0.02819513 0.07772896
0.04237792 0 0.2164108 0.105342929 0 0.1315207 0 0.02583739
0.1516629 0.02440917 0.2775853 0.1386812 0.09695183 0.0286115 0
0.1719757 0.02781975 0.06092427 0.02284726 0.2205448 0.03078864
0.2317152 0.026476906 0.03082947 0.02433495 0 0.1449406 0.08840594
0.06671217 0.048038184 0 0 0.07625024 0.1633423 0.05554137
0.1484136 0 0.1497565 0.03986989 0.2016975 0.05735209 0.05455613
0.1311296 0.06560092 0 0.06388262 0.08219588 0.08709095 0.089801368
0 0.04187519 0.052931769 0.142110026 0 0.032733189 0 0.1503949
0.06148046 0 0.2701244 0.08623232 0.06600611 0.072164371 0 0
0.1290345 0.09853003 0.07745165 0.02791438 0.0539052 0.0205419
0.0430541 0.04178936 0.131721 0 0 0.053274911 0 0.07215313
0.07755788 0 0 0.02353104 0 0 0.1204998 0.122451227 0
0.1007212 0.1705058 0.1368243 0 0.07203025 0.06186832 0.05865239
0.1665867 0.03219332 0 0.02808876 0 0.05471933 0.023088512
0.09257738 0.1087263 0.02447936 0.08924602 0.09705439 0 0.0270391
0.02076408 0.2144712 0.03089627 0.053886927 0.067051763 0.06694607 0
0 0.2197851 0.06106769 0.07867896 0.02855184 0.02772947 0.4937731

0.0548853 0.4056444 0.1345934 0.0591655 0.2653306 0.1452751 0
0.3484525 0.02543345 0.174125041 0.1318964 0 0.09312868 0 0
0.05523654 0.6963750.08288209 0.07095633 0.03395154 0.1333388
0.041212313 0 0.04436393 0 0.04899448 0.02755404 0.164179874
0.1029650.1575176 0.2332264 0 0.07953899 0 0.075435584 0.09480136
0.2550684 0.1013485 0.0566444 0.103442657 0.0833991 0 0
0.03513595 0.111022837 0.05597927 0.1839621 0 0 0 0 0.06017975
0.02521765 0.04671771 0.0319850.03524945 0.1919533 0.022892035 0
2.83364100 0.1218049 0 0.1019946 0.2140861 0 0.08301924
0.2420219 0.06994463 0.02484717 0.1910130.1022425 0.03432835
0.06548515 0.0647003 0 0.3137070.4189183 0.1004366 0.1164485 0
0.02805674 0 0.02306511 0 0.0657023 0.04643506 0.08627623
0.04757583 0.2009574 0.1233088 0.02381305 0.06118703 0.1555675
0.07482317 0.1378262 0.04703939 0 0.1059304 0.02530755 0.02075475
0.2308488 0 0.1110811 0.0746957 0 0.01869908 0.02640649
0.02775461 0.08168818 0.08083618 0 0.100379295 0.02902303 0.099728954
0.03501076 0 0.9471069 0.03319989 0.1311631 0.02792814 0.07046062
0 0.01292963 0 0.1234156 0.09070176 0 0.1468649 0 0
0.1818932 0.02137795 0.1370122 0.1965659 0 0.02113571
AP001020.3 0.04319279 0.01775784 0.0299809 0.018463921 0.063858027
0.1206458 0.02824761 0.02710711 0 0.06004139 0.01642026 0.058872162
0.09651672 0.024768082 0.015855648 0.08364861 0.04073122 0.06641025 0
0.1925466 0.073354855 0.2995531 0.3715715 0.01880715 0.1290269
0.06707656 0.07320907 0.1526568 0.07252559 0.035953683 0.02180938
0.100129457 0 0.07961159 0.1019579 0.009402406 0.2116459 0.01730669
0.1560099 0.0595969 0 0.02268815 0.02061596 0.03729697 0.1008879
0.1862653 0.0650673 0.04114024 0.03535338 0.02115016 0.03626321
0.0732141 0.1387565 0.04585423 0.02725863 0.09157125 0.1032741
0.08018036 0.1020366 0.07004833 0.1833054 0.07570192 0 0.05978405
0.06860106 0.094664588 0 0.4101427 0.0633590.038953582 0.09296797 0
0.1274043 0.058025116 0.1682861 0 0.05262965 0.1477901 0.1580118
0.01645584 0.1319288 0.04277205 0.0137293 0.00733626 0 0.02459215
0.1435534 0.1302087 0 0.02470645 0.05372833 0.046970845 0.04903871
0.0233008 0.1596852 0.042212405 0.2917063 0 0.07208509 0.1023619
0.03552646 0.1183838 0.1260254 0.1257340.07864056 0.03390778
0.095129102 7.0460350.046394773 0.03797002 0.2636362 0 0.08868767
0.02594486 0.03347691 0 0.01408669 0.05995968 0.04355753 0.02952471
0.02031457 0.07876927 0.09353338 0.08215661 0.4616695 0.01326984 0
0.05644828 0.035944492 0.02544544 0.03018525 0.01937622 0.04763027 0
0.1025666 0.0903330.1323649 0.07624847 0 0.05890266 0.07235936 0
0.09105709 0.08743562 0.03912983 0 2.2912810.1261086 0.01286081
0.03637281 0.0531999 0 0.02807679 0.092542454 0.1764280.1495884
0.043857216 0.04925393 0.04981539 0.01316329 0.03724589 0.07369455 0
0.059094918 0.01489069 0.02923165 0.04540998 0.1415409 0.1661604

0.08221855	0.09125222	0	0.09810453	0.015918214	0.1382715	0.08943253
0.2589409	0.03513825	0.1890698	0.0221306	0.1944746	0.04715081
0.05493843	0.1297032	0	0.05568641	0.01261141	0.01380926	0.06214348
0.166631	0.04187183	0.1650667	0	0.1118063	0.02206331	0.01474505
0.1752141	0.06679445	0.07056544	0.043553873	0.08708763	0.04040801
0.03456618	0.01851181	0.025178322	0.02990209	0.03248516	0.1629323
0.0903702	0.1485812	0.2686581	0.06182922	0.0198148	0.20817
0.03703032	0.2027174	0.07452299	0.1974028	0.101773138	0.026873457
0.2467802	0.035992985	0	0	0	0.06064009	0.1022668	0.2322555
0.075643895	0.1749347	0.09772828	0.08976677	0.032713953	0.06174695
0.06399413	0	0.1935534	0.07022163	0.2151231	0.09774642	0.3631746
0.05855257	0.03788837	0.06824283	0.088237853	0.06318664	0.024150879
0.0345228	0.0572405	0.1406359	0	0.04524985	0.1386739	0.01576414
0	0.2185026	0.022204108	0.2294846	0.4413752	0.06324106	0.1654026
0.01029425	0.01088438	0.04206974	0.3057692	0.03356356	0.1167524
0.05563819	0.02546671	0.02151013	0	0.04186645	0.01678708	0.1478652
0.09987409	0.129464	0.07332873	0.0550244	0.02451503	0.04706443
0.0555573	0.0840364	0.073284963	0.060792555	0.08092897	0.08959977
0.05428	0.0996342	0.04152531	0	0.04530147	0.01257048	0.2611466
0.04976182	0.0919445	0.06101462	0.08940411	0.09622491	0.09219967	0
0.06504335	0.02305927	0.112764758	0.1435008	0.07626145	0.08443522
0.01411853	0.01522824	0	0.5962931	0.01878628	0.1715537	0.1077377
0.1727026	0.084071676	0	0.06033391	0.02834583	0.01110522	0.0249819
0.074426932	0.05445611	0.02677753	0.2306782	0.03110596	0.01802853
0.05436649	0.068393756	0.01074397	0.3340394	0.01148596	0.0342378
0.03751456	0.03780694	0	0.1256453	0.063712096	0.11324135	0.05075367
0.08339471	0.03792171	0.01317923	0.05414684	0.02318229	0.2728102
0.05715902	0.01058917	0.08699771	0.03994869	0.04350866	0.145285625
0.03966816	2.662546	0.04539659	0.1012316	0	0.02311839	0.3518089
0.01898758	0.1129042	0.1097147	0.03170769	0.01126385	0.04329553
0.02317456	0.04668576	0.02968609	0.01955353	0.04877322	0.2844228
0.1828728	0.1062377	0.02111564	0	0	0.06871583	0.02091201
0.09488315	0.01489226	0	0.1760005	0.3342938	0.1518319	0.09316503
0.04318026	0.01849176	0	0.05087888	0.1624483	0.03198624	0
0.1800787	0.01147256	0	0.1046497	0.05803195	0.1384788	0.07901009
0.110762622	0.06781417	0.01197074	0.1761462	0	0.1099353	0.043102234
0.0455045	0	0	0.03174255	0.1110934	1.073369	0.165554	0.0679538
0.03798162	0.1916496	0.01681561	0.04689064	0.03176036	0.08951592
0.082234851	0.1118201	0.1997329	0.02816147	0.01886001	0.05497123
0.1066029	0.2484445	0.1782167	0	0.1533017
LINC02595	0.3158717	0	0.04567757	0.084392427	1.334280026	0	0
0.06194879	0	0.03430365	0.825567	0.336356285	0	0.113206649	0	0
0.06205628	0.07588477	0	0	0.083820148	0	0.04142267	1.160476
0.3216758	0.357682	0.4600946	0.04104373	0.06629806	0.602551763

0.4984171 1.539396346 0 1.143617 0.1331473 0 0.8598779 1.186547
0.407468 1.225789 2.680857 5.02945 0 0.170472 0.5379791 0.3192587
0.3469678 0 1.252311 0.09667034 0.4419922 0 0.07046775 0.366772701
0.9967209 0.2615885 0.05900394 0.7329554 0.932751 0.4482346 0
0.05766804 0.5794325 0.6831324 0.3762631 0.360566897 0.8600585 0
0.05791858 4.629139803 0.182111 0.6976761 0.09705374 0.265213463
0.03662761 2.107329 5.8334 0 0.4444428 0 0.1206005 0.3909936
0.8157766 0.1005948 0.996911969 0.6744152 0.7333269 0.1983801 0
0.07528329 0.2455743 1.37400367 0.3586234 1.171502 0.1122874 0
0.2352868 0 0.1647385 0 1.339632 0 0.2160076 0 0 0.671585
1.478331621 0.1303849 0.212055044 0 0.07088194 0 0.08107236
0.4348127 0.2295177 0.8073415 0.06438557 0.3288669 1.642467 0
1.207065 0.2400187 1.526821 0.03755105 0 0.5458681 0.05542977
0.06450157 0.16429029 0.03876754 0.1839556 0.4428112 0.05442552
0.305117922 0.677152 0.206441 0 3.933143 0.7721665 2.961469 0 2.29725
0.1135068 0.2854565 9.657868 0 21.57734 0.05764002 0 0.083124
0.08105301 0.5366455 0 0 0 0 0.601370147 0.4502466 0.7969134
0 0.1702385 0.2694666 2.288694 1.51257777 0.06806037 0.2004122
0.06918462 0.2021677 0 0.8589568 1.097588 0.1108958 0.1793612
0.800325347 0 0.4087663 1.008195 0.8565611 0 0.252879 0.1568609 0
0 0.3556982 0.2041295 0.2036192 0 0.06311754 0.6627534
0.4569689 2.934526 0.4801139 0.658321793 0 0 0.2695789 0.05005283
0.1221181 0.5529105 0.64697826 0.8529611 0 0.5134691 1.353781
0.460326872 0.7175305 0.2969577 0.1861773 4.626183 0.2611982
0.2376671 1.639084 0.9056683 0 2.031039 4.301855 0.04257747 0.1203016
0.465171088 1.39206896 0 0.329023875 1.118917499 0 1.55993284 0
0.2337137 0 2.696795613 0.3198273 0.4913517 0 0 0.09407491 0
0.05347188 1.327002 0.5616794 1.272449 0.5584579 0.1276885 0
0.04329377 0 1.26753255 0.7701468 1.159048962 0.1183442 0.3737532
0.5624497 0.3602324 0.206822 0 0 0.1679654 0 0.202975154
0.4290945 0 0.7708101 0.14175 0.4705158 0.09949779 0.769147 0
0.2301118 0.2668182 0.6357591 0.1163998 0 0.1133785 0.191357587
1.611291 0.1126404 0.5579337 1.590293 0.4021934 0 0 0 0.5713513
0 3.461265111 1.111450017 0.3698993 0 0.1240479 0.7400164
1.202056 1.14116 0.08873911 0.9192872 1.648324 0.1137223 0.4202476
0.4780757 0.9807273 0 1.926464 0.6784059 0.04247021 0.474283
0.72157417 0 3.543751 0.3087402 0.1290622 0.6264287 0.1144501
0.0801605 0.2575975 0.294043 0.1406952 0.2368098 0.853919122
0.5422552 1.562675 0.5182373 0.7106159 2.226587 0.98274424 0.426687
0.3263764 0 0.9952249 0.3708108 0.3313215 0.625210117 0.8839279
1.350616 0.209994 0.5085912 1.114532565 0 0.4152948 1.363921 0.218405066
1.610275224 1.681841 2.439484 0.5777584 0.3011894 0 1.589378 0.4987698
0.7837645 0.8227923 0 0.1825922 0.5468749 0.142296892 0.18131
7.792821 0.1383284 1.093645 2.134721 0.1584996 0 0.4339296 0.08600793

0	0.04830842	0	0.03957788	0	1.209182	0.881954	0.4021771	4.086979
0.118182	0.2571848	0.4855773	0.530819	0.4870978	0.2325342	0.6281543
0.9080272	0	1.429419	1.731842	0	0	0.6245756	0.04258263	0.2960439
1.605874	0.08058396	0	0	2.144244	0.3747051	0.05487197	0.4194979
0.9890882	0.3587391	0	0.1150801	0.5158983	1.012518045	1.12359
0.2735712	0.2300302	0	0.5443508	0.443263377	0.311978849	2.164886
0.206638393	0.07254231	0	0.9812027	1.75415	0.3105942	0.1736013
1.897927	0	0	1.306494	0.1022869	0	0	0.06086084	1.673317	1.20684
0.1256276	0.7087218	0.6083341	0.116367	0.7681279	0
AL139353.2	0.1322746	0.05098313	0.1147678	0.035340212	0.034921483
0.1026302	0.05406633	0.1037668	0.08766547	0.09337257	0.2357146
0.070426358	0.1679404	0.094812939	0.060695882	0.02668411	0.07796015
0.01588877	0.06426088	0.1842685	0.087751411	0.3010082	0.2168272
0.3509723	0.1347051	0.2246746	0.1313655	0.3781249	0.166578
0.137631741	0.1669739	0.087113289	0.02910248	0.2394507	0.1672705
0.224954276	0.1575363	0.1490637	0.5972103	0.085552	0.2327418
0.09770728	0.157837	0.2974456	0.09655046	0.1336929	0.166053	0.1443622
0.1437921	0.2226494	0.1041125	0.1541461	0.07377277	0.120677785
0.1304335	0.2957659	0.2100224	0.1619923	0.2751949	0.2145176
0.06379078	0.1448946	0.350485	0.2002482	0.1838248	0.271784093
0.110818	0.2429821	0.2667941	0.086984008	0.1016812	0.2045114
0.2336931	0.124943528	0.2224038	0.2704332	0.2014678	0.2262979
0.4652876	0.01574836	0.239888	0.2046658	0.1182513	0.1474379
0.080282178	0.1255193	0.08081271	0.2076844	0.2042315	0.1655097
0.1953899	0.026970864	0.1783355	0.1635263	0.4231936	0.181789103
0.1642147	0.06928786	0.1552184	0.2693929	0.1274965	0.07209627
0.1959868	0.1082955	0.1222968	0.1298	0.182078483	2.347802	0.122100371
0.09084397	0.3265081	0.1175456	0.1018497	0.5379707	0.1441694
0.1300318	0.3639888	0.1032873	0.1563183	0.2401706	0.09720598
0.1758931	0.230174	0.1729738	0.1647468	0.1015947	0.1740885
0.1485589	0.257993545	0.08117156	0.2599875	0.08344432	0.1139563
0.085180883	0.1417822	0.1873069	0.04222472	0.1772137	0.1243665
0.09395046	0.3462421	0.2555306	0.1663626	0.3347061	0.07489507
0.100023	1.285418	0.1568928	0.07384731	0.08702261	0.1187963	0.07490864
0.1343485	0.191888234	0.1500826	0.1431572	0.167886696	0.2238977
0.1191842	0.2015579	0.09505222	0.09873671	0.1765501	0.135730238
0.2137574	0.09791217	0.1014012	0.2116496	0.1712488	0.146127
0.1424842	0.06965818	0.0751094	0.076169231	0.1654086	0.1711752
0.2019179	0.08967348	0.1727166	0.1270749	0.3612793	0.3459481
0.1892753	0.3847935	0.07479618	0.2398154	0.2534533	0.07929339
0.1189435	0.1913605	0.2938587	0.31594	0.11486631	0.1604992
0.1794753	0.08466675	0.1257609	0.1022764	0.3087161	0.156305146
0.1428746	0.2320245	0.06616018	0.1948754	0.096383348	0.164545
0.233164	0.1039516	0.1297274	0.218759	0.1327008	0.1656788	0.1390613

0.3272904 0.07087656 0.2494311 0.1693827 0.05037757 0.077918105
0.162881258 0.3633392 0.241118836 0.086313393 0.1292668 0.071004153
0.1624924 0.2283635 0.5512302 0.130305161 0.1841551 0.2712274
0.1813602 0.172191199 0.03939485 0.1020714 0.1119595 0.07836741
0.1176046 0.2300949 0.2338601 0.142589 0.05603524 0.1087783
0.1061269 0.132698131 0.1209401 0.150231679 0.2064911 0.1565131
0.2944146 0.09051066 0.230957 0.3470927 0.07543198 0.1172288
0.1045543 0.11687221 0.289497 0.3932682 0.3362342 0.2473304
0.1477751 0.08333148 0.1476238 0.1653958 0.06424114 0.6145311
0.09318077 0.0974873 0.2264389 0.08308723 0.280465475 0.1044248
0.09433871 0.1699204 0.061949 0.2666693 0.09215281 0.08211371
0.1171066 0.1329218 0.4021172 0.093512323 0.334528769 0.24203
0.2572429 0.1947988 0.4171591 0.2119468 0.1479129 0.1238681
0.06015018 0.3927302 0.07143363 0.3373009 0.1418078 0.1026725
0.09208794 0.2268919 0.06456581 0.29345 0.187577 0.194250046 0.1945527
0.1216379 0.1292882 0.04053461 0.1020147 0.1557634 0.5706568
0.1078717 0.3180951 0.1325646 0.3140275 0.169854097 0.1135375
0.23096 0.1898901 0.06376668 0.1912629 0.087055362 0.1191198
0.1281314 0.2483558 0.2232647 0.1897878 0.1040583 0.130906641
0.1439488 0.3073829 0.0879372 0.1064889 0.188483957 0.05427228
0.1043454 0.07515215 0.335351162 0.15653848 0.1275005 0.1995234
0.1088741 0.07567574 0.1165926 0.06655686 0.0783244 0.1203435
0.1520086 0.1942674 0.1682172 0.2290096 0.109245241 0.1012339
3.173919 0.304114 0.15853 0.2793552 0.2654935 0.08126846 0.0999419
0.198092 0.3449927 0.1719521 0.08623673 0.2900389 0.02217822
0.1787144 0.1136392 0.149703 0.0622351 0.4454095 0.2490535
0.1307189 0.2829095 0.08741884 0.1217202 0.1183708 0.2601683
0.3026795 0.09976404 0.08058078 0.2245783 0.1548008 0.1598346
0.1159075 0.04132383 0.141574 0.1518542 0.2921488 0.3348461
0.1836664 0.1412205 0.1608475 0.1537105 0.2070955 0.1251881
0.055537 0.09638197 0.07561323 0.212001266 0.1541343 0.1145608
0.1444913 0.1063178 0.1227437 0.123747453 0.119757375 0.163686
0.100953989 0.04556677 0.3986894 0 0.2088479 0.1138065 0.1332785
0.2547362 0.04827798 0.1458427 0.1215796 0.1927516 0.118049024
0.1987378 0.216632 0.148229 0.0541475 0.4559349 0.2225885 0.1188816
0.2923795 0.2680513 0.4951492
GSN-AS1 0.07771619 0.07668343 0.05754051 0.17275376 0.052525195
0.1447176 0.1219813 0.2389899 0.1648214 0.2781815 0.06499845
0.04766754 0.3252201 0.053477842 0.176878862 0.05518619 0.1368026
0.1015674 0.0483272 0.1265282 0.032996594 0.118576 0.05218056
0.09813439 0.1055256 0.06839096 0.09220685 0.1583411 0.1357141
0.081941887 0.07063438 0.117923922 0.02553417 0.1596148 0.1467611
0.040602287 0.1353996 0.07473527 0.176444 0.09650869 0.03603617
0.01632899 0.1335385 0.1252681 0.1089157 0.1340578 0.07804979

0.0345441	0.02862488	0.1369987	0.05654818	0.05005859	0.05548059	
0.049502929	0.01471382	0.06590515	0.01858197	0.1763268	0.1134939	
0.1512444	0.1139375	0.07264503	0.07603303	0.112947	0.05431049	
0.085164342	0.03125263	0.09136698	0.2188819	0.037380609	0.01911725	
0.2197174	0.08023286	0.093963409	0.3489351	0.05352066	0.0473479	
0.09572999	0.1574632	0.07106106	0.02848532	0.05387141	0.04940587	
0.005280013	0.042263181	0.09439642	0.1367437	0.1718073	0.0239987	
0.005927195	0.03093524	0.104797348	0.02823509	0.06987468	0.1237684	
0.022785659	0.08644803	0.06774005	0.0907912	0.05064903	0.02556891	
0.0464741	0.05668895	0.01809852	0.03891164	0.06914443	0.092428818	
5.184053	0.091825221	0.01366379	0.06138736	0.0589332	0.0319149	
0.03734578	0.02409382	0.05867402	0.01013839	0.03020769	0.07445383	
0.09562213	0.01462069	0.0519671	0.05289219	0.06208577	0.1323449	
0.06207822	0	0.08633162	0.045272099	0.01831345	0.1086238	0.06972673
0.05142029	0.032030015	0.09022277	0.1408636	0.04763246	0.1215136	
0.1356177	0.02119655	0.04556832	0.07912925	0.04766189	0.01348472	
0.09856807	0.07835625	6.646029	0.04991918	0.04165249	0.1047122	
0.01914437	0.08450223	0.07577728	0.016651036	0.08465188	0.05383047	
0.035510257	0.07089753	0.1523746	0.06157978	0.06254827	0.1272937	
0.04741927	0.072303495	0.04286817	0.06837494	0.07081147	0.09550225	
0.1287872	0.1141211	0.08641519	0.02182759	0.0847287	0.091652551	
0.03109875	0.01609147	0.08627942	0.03793425	0.1670027	0.1075121	
0.2387658	0.07918195	0.09094196	0.00933493	0.05223237	0.1522976	
0.04992136	0.02981618	0.1863565	0.02998169	0.2159729	0.1674012	
0.073427137	0.2162594	0.0198491	0.05836726	0.1024596	0.1201823	
0.0979463	0.031346352	0.08506331	0.05331742	0.1026209	0.1132474	
0.099666615	0.08070362	0.07598512	0.06840441	0.05853667	0.09048437	
0.1528145	0.03559951	0.05466715	0.09631477	0.07329095	0.08337064	
0.06369189	0.05209366	0.219742798	0.029011824	0.2937418	0.047491899	
0.027819313	0.07953934	0.234953234	0.06110097	0.09200362	0.0501473	
0.043553604	0.1007224	0.158257	0.05024946	0.047089411	0.03703348	
0.03838125	0.03367954	0.05625707	0.0315872	0.159381	0.08793689	
0.1474463	0.08428227	0.02726881	0.1074398	0.054433765	0.06063512	
0.047799776	0.1397619	0.06179523	0.1138699	0.04537885	0.04885046	
0.0499028	0.06807407	0.04848886	0.03440052	0.067917626	0.07507426	
0.1095393	0.03540095	0.02976066	0.06297582	0.05091872	0.1362519	
0.06697677	0.1690933	0.1470497	0.0100109	0.07789722	0.02322173	
0.00446325	0.048964321	0.0120819	0.008868394	0.04392717	0.203825	
0.3588751	0.04455209	0.03528764	0.02032377	0.02499087	0.04032148	
0.061534956	0.027345801	0.05460539	0.03224313	0.1220816	0.1478981	
0.03486743	0.0727322	0.04424845	0.04071219	0.756279	0.0358143	
0.04963034	0.07841626	0.1029527	0.1731362	0.04502826	0.0825461	
0.03009384	0.05808631	0.077100506	0.07745969	0.04116485	0.1731924	
0.01524196	0.01095998	0.02703262	0.839388	0.01352076	0.1196113	

0.08307899 0.1615855 0.060507601 0.1138474 0.1881672 0.05100226
0.1079 0.07641429 0.09820459 0.1063806 0.01284811 0.1763988
0.08395279 0.1038031 0.1108637 0.082039959 0.1005237 0.1849331
0.01653323 0.09240541 0.097874171 0.1428536 0.07847266 0.09042872
0.063049961 0.036222861 0.08675436 0.03601223 0.05913441 0.01897057
0.0292277 0.02502695 0.1178073 0.0246829 0.2019611 0.03130676
0.02875165 0.02739959 0.112033035 0.03806633 4.656207 0.04900887
0.0761696 0.004201765 0.1788653 0.1135043 0.04099692 0.02708627
0.1353656 0.09508534 0.06485407 0.03427646 0.0250186 0.1400017
0.1735948 0.08091952 0.02340186 0.1814415 0.02277972 0.1201527
0.01519723 0.02739295 0.01830786 0.128585 0.1091174 0.08536097
0.05894998 0.0681756 0.2146355 0.03492523 0.1584496 0.05028917
0.05050087 0.04658076 0.1459241 0.04882436 0.08543892 0.03069461
0.07080299 0.1209649 0.1032121 0.06432969 0.03765894 0.03132483
0.3941305 0.06905 0.232509261 0.06405898 0.07323185 0.07697052
0.04664098 0.1087928 0.065920266 0.085969397 0.0426114 0.10846021
0.03997975 0.00499722 1.081527 0.06769976 0.04585062 0.05922783
0.1570903 0.03630731 0.08436968 0.156199 0.05637266 0.136866728
0.08622702 0.1437507 0.07093876 0.03393456 0.08901808 0.167397
0.08940455 0.1053607 0.125992 0.1551565
FTX 0.3549501 0.4367987 0.2402318 0.42320077 0.181044142 0.4871042
0.4974288 0.3902107 0.2389785 0.3671195 0.2707951 0.312659952
0.6425691 0.245770305 0.531831335 0.5299746 0.4174529 0.009281406
0.115295 0.4703408 0.435708938 0.5358738 0.1595908 0.1235377
0.2524567 0.1499925 0.3197375 0.4593324 0.3527355 0.08709719
0.307853 0.312955768 0.1246679 0.4704881 0.3379166 0.173456915
0.5159951 0.4595644 0.5357487 0.3414965 0.02799091 0.1268346
0.2074506 0.6845846 0.4793985 0.5011195 0.1454995 0.1571586
0.2421061 0.325151 0.1148769 0.3253873 0.1379017 0.211481324
0.5066807 0.3743378 0.4510453 0.1543924 0.3552162 0.489493
0.3819488 0.4619933 0.1063049 0.1336854 0.1649066 0.220503239
0.07282597 0.4913228 0.6552676 0.181469992 0.3229708 0.1194651
0.2670877 0.450077886 0.3875108 0.02494314 0.5663695 1.119498
0.3023744 0.165589 0.3798267 0.1733552 0.1036146 0.2235167
0.22041464 0.2039267 0.1581423 0.1122197 0.07829164 0.1427215
0.15018 0.076149145 0.0904673 0.1150626 0.3193104 0.271379231
0.3980921 0.06475906 0.4180924 0.1323301 0.1340585 0.4271658
0.2377774 0.1792386 0.05495349 0.233786 0.194108669 22.54147 0.142649553
0.06898631 0.2037336 0.3919576 0.2131917 0.4496262 0.1543967
0.3570022 0.02362484 0.288268 0.2830706 0.2269484 0.1533134
0.1798085 0.4183071 0.2594954 0.1946605 0.1780392 0.02033871
0.2721752 0.246154116 0.1161698 0.3403044 0.1706036 0.3661208
0.130615549 0.3886259 0.1472895 0.09866206 0.2435745 0.2687995
0.1152502 0.08090276 0.114146 0.5761414 0.2199579 0.3901029

0.2312787 15.55306 0.148048 0.1150342 0.1575859 0.1759651 0.105748
0.1098713 0.258672157 0.1643823 0.3116935 0.25743585 0.1514406
0.2019018 0.110381 0.1978065 0.3707802 0.2946614 0.244467158
0.2122724 0.2737202 0.1480835 0.2967236 0.5787726 0.4005348
0.5302682 0.0610361 0.3318051 0.204673134 0.2270645 0.07499382
0.5817085 0.157148 0.1681536 0.0866024 0.7610261 0.2064783
0.1719899 0.1522681 0.1903727 0.4498376 0.0634519 0.158257
0.4197793 0.1816474 0.0546182 0.8682518 0.093938612 0.2461079
0.2713513 0.2060749 0.1928405 0.1157553 0.1944249 0.070000847
0.26429 0.2258947 0.2318843 0.3156367 0.218171065 0.1901479
0.1543628 0.3605439 0.5254087 0.7443638 0.1647236 0.1520846
0.1883117 0.4073098 0.1397333 0.2792677 0.4660816 0.2795659
0.500673383 0.24287493 0.4297944 0.33200183 0.154860994 0.1269959
0.141021751 0.2983192 0.4954792 0.2207261 0.164921454 0.4743035
0.1748272 0.3707946 0.274323464 0.2473839 0.3100494 0.2844945
0.3620641 0.2551661 0.1450209 0.1775918 0.1587777 0.1472981
0.2594661 0.4268043 0.20083547 0.2296029 0.135011761 0.3859888
0.1782825 0.2628867 0.167427 0.2866906 0.1967904 0.08812699
0.1129904 0.1908602 0.359973059 0.3090618 0.1829309 0.3928215
0.4796661 0.06905809 0.2099238 0.1763883 0.1672196 0.3049018
0.3834533 0.1166388 0.3167681 0.04809963 0.0970706 0.257452722
0.3237672 0.1515466 0.1737029 0.3596126 1.750414 0.1499576
0.3323402 0.07630087 0.4037599 0.1644274 0.238984831 0.123196132
0.2460039 0.3318421 0.3337884 0.4281858 0.2437479 0.2060383
0.2604866 0.03865032 3.747076 0.2016847 0.3598011 0.3021108
0.139944 0.2286205 0.2816446 0.1131481 0.2103772 0.1224638
0.283677293 0.7219971 0.1207929 0.2643324 0.08287383 0.01702622
0.06999151 3.294267 0.1207751 0.2307701 0.2108019 0.5768666
0.164496406 0.1731762 0.1461579 0.1703477 0.12106 0.111726 0.50853279
0.3500928 0.3393101 0.3008998 0.252145 0.2393661 0.4406948
0.127448246 0.3423563 0.466849 0.2439999 0.1866161 0.372251864
0.2166377 0.07111212 0.1097501 0.271581642 0.077373792 0.2252111
0.4335709 0.06006539 0.1805074 0.3178345 0.1036776 0.1486976
0.08308006 0.2279088 0.6808862 0.2411932 0.3618024 0.13053164
0.08500752 26.9554 0.3172283 0.2803876 0.261096 0.4200292 0.2339734
0.09287876 0.2261706 0.6308673 0.2895197 0.1259377 0.2323555
0.2008084 0.2566397 0.1763277 0.7023232 0.1226966 0.3685959
0.5426171 0.301195 0.1652612 0.05532107 0.08532322 0.322682 0.3740971
0.2165921 0.1040661 0.2059363 0.3088364 0.4310338 0.2843455
0.294266 0.2413926 0.07236275 0.4385991 0.270209 0.3981855 0.151992
0.2566474 0.4463046 0.08337624 0.3524042 0.4863044 0.6204507
0.3765156 0.5047928 0.12384027 0.3980599 0.1104189 0.1793594
0.1138599 0.1434012 0.352398722 0.193969237 0.1985891 0.227463984
0.06210814 0.2018414 3.180268 0.216652 0.2421768 0.2901848 0.3660574

0.0705038 0.1359825 0.2781638 0.3096379 0.152282664 0.2098594
0.4131331 0.247956 0.1739661 0.2202375 0.4307071 0.1810518
0.5977759 0.4775746 0.3187005
AC005519.1 0.1624706 0.1113275 0.1253042 0.023150812 0.057191273
0.2689256 0.247926 0.305892 0.09571376 0.1317441 0.1852955 0.036908151
0.6050829 0.295024642 0.159043636 0.5244101 0.2383288 0.1040848 0
0.7872479 0.18395058 0.4882694 0.5908867 0.282974 0.6324094
0.6027404 0.3212737 1.137185 0.1636839 0.120213825 0.3691641
0.273919356 0.2796137 0.5276212 0.4017795 0.141469416 0.4422836
0.4556965 1.11778 0.7472501 0.01793712 0.3698153 0.2843404 1.09117
0.3373262 0.6568508 0.3943227 0.2235276 0.3102925 0.1856325
0.1667171 0.4498142 0.367288 0.359336825 0.4272247 0.7893582
0.615074 0.2569186 0.5466428 0.7904649 0.6686132 0.2214757
0.08830659 0.4310181 0.08601479 0.197823828 0 1.310122 0.7785324
0.211646845 0.4995729 0.1913888 0.4659219 0.600222495 1.657888
0.2237766 0.2969513 1.167423 0.9296478 0.6602562 0.4466277
0.7776253 0.08607176 0.1379775 0.021036632 0.2158425 0.423513
0.7074639 0.2006832 0.3510834 0.4176739 0.318027322 0.6394619
0.07790797 0.9240914 0.502812434 0.6884765 0 0.3615329 0.6256841
0.0779529 0.6207256 0.2370237 0.7882521 0.4313869 0.2550897
0.489034568 3.970207 0.276315324 0.1428251 0.8750057 0.308009 0.578241
0.45543 0.2728356 0.01703635 0.08831232 0.1804317 0.2594174
0.666347 0.534896 0.625506 0.6533945 0.5047553 0.6279158 0.06655305
0.03041136 0.5308286 0.315480622 0.265871 0.5046331 0.1093261
0.2239528 0.083700966 0.3286498 0.6984563 0.3042681 0.3960713
0.0325882 0.2708 0.2381586 0.9255906 0.3217548 0.09396885
0.2126045 0.1419675 3.145331 0.15812 0.4998874 0.3192399 0.1667605
0.06542861 0.4752516 0.328761526 0.6882171 1.074207 0.192464842
0.756518 0.6870661 0.3135887 0.2023684 0.2772036 0.4295762
0.118552966 0.5974575 0.4214961 0.2846843 0.8651652 0.5609119
0.3681748 0.4697067 0.2129492 0.3075186 0.498972543 0.7368243
0.1308232 0.3246707 0.1321733 0.9590292 0.1803636 1.37698
0.6306091 0.1515449 0.3089917 0.1539931 0.6074504 0.1106888
0.5540676 0.2597267 0.334286 0.1400016 0.9407603 0.18059303
0.6133194 0.8852437 0.05546384 1.24949 0.3517483 0.5561463
0.423224482 0.5771683 0.1519956 0.09751608 0.2553195 0.063139202
0.3561783 0.7942585 0.4426306 0.3739224 0.415586 0.9127667
0.2325719 0.2236013 1.118623 0.2553656 0.4901951 0.5372797
0.511524 0.433864768 0.179706824 1.523314 0.376078862 0.290790171 0.1693613
0.093027387 0.5474376 0.470163 0.7571497 0.58804136 1.030899 1.421414
0.9879671 0.799852568 0.2580694 0.6151614 0.4840633 0.7280555
0.1320701 0.9202556 0.7353505 0.980782 0.02447186 0.1187649
0.577568 0.537375807 0.1848606 0.423938904 0.8116142 0.3280935
0.2975652 0.03952807 0.3404166 0.6286248 0.5534401 0.2150252

0.8047794 0.598568829 0.4577635 0.1717492 0.4405233 0.7388214
0.02581469 0.2047092 0.5626533 0.5667443 0.2525001 1.646875
0.3662475 0.1756215 0.02697028 0.04665354 1.08924742 0.2946765
0.4634985 0.4035066 0.05072726 1.342361 0.1379836 0.1844276
0.3304632 0.9926549 0.9658756 0.520696724 0.343008761 0.4312561
0.3089455 0.22119 1.04625 0.2429752 0.1192559 0.3894913 0.03152274
0.1871064 0.3431636 0.9222699 0.2404368 0.4259743 0.8445549
0.7844535 0.2537762 0.8504916 0.4336896 0.438305974 0.9596112
0.07968308 0.2964312 0.03540477 0.01909378 0.03139634 0.2418889 0
0.9007354 0.5596416 1.136843 0.327949919 0.3140346 0.7060583
0.3731821 0.2645594 0.4541881 0.207376675 0.3804131 0.05595791
1.735402 0.3315163 0.1695368 0.227223 0.228679633 0.3637231 1.111516
0.3744407 0.3756265 0.540928669 0.2607213 0.2278501 0.09846196
0.758905679 0.299749477 0.1034101 0.3555165 0.1901911 0.4792151
0.4582677 0.1744014 0.9919753 0.02866732 0.5178079 0.1272615
0.1602857 0.4636999 0.286259293 0.04973755 5.036896 0.5502274
0.3577071 0.2781617 0.565242 0.1825292 0.08332591 1.226887 0.7664322
1.152935 0.07061539 1.433142 0.3196293 0.8975596 0.297773 0.4167892
0.1426923 1.296803 0.6966989 0.5899087 0.3309456 0.1718001
0.4624743 0.4135617 0.2622032 0.9914028 0.09336263 0.4750847
0.3923139 1.054638 0.7234172 0.4906192 0.06767646 0.1391143
0.4200152 1.084498 1.488462 0.2005282 0.1027904 1.038634 0.4315428
0.2241421 0.65607 0.0181907 0.9944282 0.3821119 0.925857759
0.2763415 0.09005636 1.829977 0 0.1263546 0.486389953 0.370860007
0.01649663 0.491276354 0.1990005 0.9924655 0 0.7737008 0.1384552
0.1904916 0.5206457 0.04216818 0.5438388 0.2654828 0.5892531
0.14177541 0.02002923 1.268862 0.2824799 0.09458978 0.9419772
0.4981985 0.6452703 1.02151 0.1229173 0.9370527
AC016924.1 0.2048696 0.8039944 0.1939142 0.079615497 0.078672171
0.2312083 0.3349562 0.2045479 0.2633272 0.1456287 0.07080334
0.1269269 0.4350923 0.480594263 1.299006398 0.09017215 0.4390774
0.6443047 0.3309002 0.1082936 0.118613416 11.07597 0.4298579
0.02027387 0.2276008 0.02410256 0.5524292 0 1.344725 0.025838412
0.1410614 0 0.1092716 0.1471205 0.06280533 0.202713513 0.1014007
0.1119384 0.1441514 0.2569788 0.03084281 0.02445754 0.0444475
0.08041132 0.03625196 0.1003958 0.6078395 0 0.09527625 0 0.02606086
0.4577585 0.2326765 0.024715142 0.05876892 0.148069 0.4453128
0.03841485 0.05999685 0.06040898 0 0 0 0.09666968 0.02958043
0.170078725 0 0.06316124 0.3551611 0.1959602 0 0.0658185
0.1831203 0.031275172 1.209402 0 0.08510115 0.1274527 0 0.07095677
0.3413222 0.04610774 0.02960002 0.03163359 0 0.2651003 0.03641146
0.4210899 0.6901482 2.450258 0.6486868 2.025359184 0.866955 0.06698126
0.02648287 0.136513326 0.07398957 0 0 0.4965516 0.03829707
0.3480442 1.018904 0.2168635 0.4662544 0.1949448 0 8.364316 0 0

0.2340441 0.1324051 0.1912084 0 0.2165261 0.02929396 0.0607411
0.1034173 0.0704317 0.06365454 0.2189886 0.1415205 0.3745033
0.1948403 0.1687030.1144378 0.8366757 0.06085054 9.531936623
0.1097194 0.8460222 0.02088732 0.02567241 0 0.1474206 0.3895114
0.04756256 0.4227166 0.02801765 0.04233088 0.5265167 0 0.1606228
0 0.1124839 0.0187779 0.1277572 0.08156606 0.3604585 0
0.01911628 0.02812607 0 0.066506401 0 0.3225088 3.994951053
0.1858329 0.1074007 0.9365310.05353413 0.2224370 0 0.2247276
0.09453407 0.5221481 0.1716518 0.2204540.07596906 0.03106381
0.1830827 0.1057555 0.034319269 0.4099011 0.6427143 0.1033834
0.02525239 0.0741145 0.02385651 0.1233183 0.03388532 0.07106752
0.6431650.1203594 0.2040995 0.02718987 0.3274967 0.04465993
0.6466541 0.3310069 0 0.025877429 0.0602629 0.04756793 0.03178997
0.1888786 0.20161 0.1086695 0 0.1072907 0.2032768 0.09315478 0
0.217135317 0.03223407 0.4202231 0.02927315 0.07793435 0.02464134
0.01868452 0.07998134 0.1281606 0.7373296 0.3592639 0.03121811
0.2811720.05674605 0.043884065 0.212441157 0.3683437 0.155199941
0.027778488 0.4500615 0.09597618 0.0784431 0.07349489 0.1201769
0.619728068 0.2640083 0.06320992 0 0.141060921 0.1774998 0
0.1765581 0 0.03784901 0.02728236 0.0526847 0.2007681 0.1683172
0.1021080.2023035 0 0.1362288 0.026034343 0.2046833 1.145941
0.1705542 0.1699211.3658060.2759791 0 0.4225545 0 0.023935749
0.06746768 0 0.03029914 0.1114387 0.2441356 0.3050640.1511688
0.2866221 0.03618109 0.1573221 0 0 0.09275061 0 0.112828766
0.1266738 0.05313228 0.07177535 0 0 0.4745249 0.3699765
0.04058789 0.83846100 0 0.262134438 0.1090505 0.3863497 0.2340526
0 0.05968502 0.02563252 0.6278710.4065244 0 0.1072852 0.06607667
0.1691306 0.0192753 0.05186462 0.2981702 0 1.2620860 0.048623596
0.2062561 0.02740296 0.07281609 0.2739528 0 0.02699295 10.4359900
0.02311659 0.09954848 0.1117027 0.080558408 0.1989406 0 0.1527822
0.1915407 0 0.267437656 0.1006337 0.03848778 0.3730023 0.3688502
0.1749107 0.03907093 0.122879347 0 0.1938948 0.1981076 0
0.040440224 0.08151081 0.5485025 0 11.744423354 0.027127278 0
0.01797969 0 0.1704846 0.2042937 0.04998044 0.05881719 0.04929336
0.4794295 0.2813473 0 0.0234509 0.64883803 0.02850785 0.1007085
0.3914956 0.2380940.02517359 0.3488986 0.1046195 0 0.6896862 0
0.2734439 0.02428459 0.01866881 0 0 0.06400247 0.1264708
1.1917430.1951118 0.1971346 0 0.02276239 0 0.2193719 0.05925984
0 0.1704714 0.03210735 0 0.4005333 0.09299728 0.4910186
0.1205169 0.3956561 0.05980167 0.1900565 0.2193872 0.08082336
0.02298717 0 0.8800222 0 0 0.5640552 0.6568560.05428305
0.02433482 0.039800238 0 0.1032344 0.1627572 0.3991932 0.01975148
0 1.030118841 0 0 0 1.6466630 0.9734459 0.07325334 0.0272958
0.04591019 0.1087621 0.9098554 0.06847454 0.1447456 0.022162034

0.6888038 0.1148318 0.06071542 0.1626468 0.3555498 0.02089392
0.11478 0.2744506 0.1811622 0.3511719
AC099811.5 0 0.07432706 0.08365856 0.193206105 0.07636676 0.2805412
0.08867472 0.1701890.03195133 0.8952875 0.1374570.246414861 0.09181356
0.311007277 0.033182639 0.6418847 0.08524214 0.4864409 0.3011283
0.03504006 0 0.06269036 0.03793285 0 0.04909581 0.02339626
0.05745437 0.09396459 0 0 0.0228213 0.038100098 0 0.1428093
0.06096488 0.059031957 0.04921462 0.07243876 0.1399272 0.06236208 0
0.07122252 0.08629001 0.1040733 0 0.02436345 0.0680863 0
0.03699371 0.2655779 0 0.03064444 0.0645309 0 0.02852338
0.1197750.08104937 0.05593371 0.0582387 0.05863875 0 0.02640478
0.05895732 0 0 0.066037893 0 0.1226207 0.02651949 0.054347936 0
0 0.04443853 0.121434736 0.01677089 0.03112563 0 0 0.07631229 0
0.05522001 0 0.08619787 0.06141319 0 0.01715545 0.01767223 0 0
0.08617592 0 0.05898024 0.04105121 0.01625461 0.1285341 0
0.1077321 0 0.1508594 0 0.01858741 0.04504602 0.03296819 0 0
0.1182701 0.099542907 5.25360500 0 0.0649102 0.06426256 0
0.07239639 0 0.02843553 0 0 0.1823140.0308946 0 0 0.02796376
0.08596852 0 0.05554214 0 0.08860106 0 0.03550141 0.1474003 0
0.04984022 0 0.09540039 0.03150809 0 0 0.02719662 0.04109042 0
0 0.06929597 0.0261407 0.02729692 0 7.68882900 0 0.03806044 0
0.02730187 0 0.032278748 0.1230760.02845982 0 0 0 0 0.05196536
0.06169107 0.05515457 0.024734723 0.03116317 0 0.03167794 0.05554056
0 0.1229048 0.03015352 0.02538824 0.3285005 0 0 0 0.06021229
0.02451239 0.07194265 0.02315742 0.2154683 0 0.06898496 0
0.04673293 0.03496209 0.1055724 0 0.02167561 0 0 0.03140462
0.025119116 0.05849695 0.0461740.03085839 0.04583593 0.02795743
0.02109701 0 0.02603666 0.02818857 0 0 0 0.04693423 0.1019772
0.05683067 0 0 0.1813699 0.02587919 0.0552911 0.03111839 0
0.06060659 0.05848553 0.1377079 0 0 0.03972775 0.075325979
0.026964466 0 0 0.05076293 0.1426824 0.03888508 0.126645798
0.03661026 0 0.08349491 0.034231817 0 0 0 0.03115907 0.07347977
0 0 0.03897695 0 0.1982316 0.03570458 0.052761095 0 0
0.1083738 0.05133973 0.03679028 0 0.06313247 0.02232432 0 0
0.08574027 0.116171677 0.0436604 0.06370401 0.04411688 0.02163462
0.04308753 0 0.08804339 0.08346686 0.2282855 0.06108476 0 0 0
0 0.021904486 0.03513193 0 0.06967204 0.03386772 0 0 0 0
0.05813505 0 0.025561748 0.031806605 0.1270259 0.09375703 0.02839924
0.05212851 0 0.1244069 0.01354382 0 1.40535400 0.03207018 0
0.01871046 0.1006896 0.1929551 0 0.1166764 0.02412917 0.117996823
0.1751854 0.02659994 0.03534114 0 0 0.02620195 0.9175881
0.03931586 0.04487836 0 0.1084294 0 0.02758726 0 0 0.02324097
0 0.01730671 0 0 0.0201151 0 0.05659505 0 0.047711395
0.04496987 0.1075502 0 0.01791319 0.058882743 0 0 0.03286874

0.10000232	0.052664679	0.06638568	0.1047169	0	0.0551629	0	0
0.0285468	0	0	0	0	0.04552738	0	0.02767246	8.8470350	0
0.1155585	0.0244359	0.09676413	0.02538842	0	0.03938092	0.09840451
0.04423848	0.09429181	0.05436523	0	0.09770377	0.01553173	0.02046078
0	0.02705631	0.07359912	0.06352398	0.02209536	0.03186144	0
0.02876165	0	0	0	0.04405378	0.06138886	0.02256802	0	0.09748771
0	0.03869949	0.1106923	0	0.1830614	0.02231356	0	0.05024906
0.02400973	0.0590712	0.0273763	0	0	0.02362172	0	0.01774015
0.05010462	0.1053252	0	0.03834537	0	0.095231639	0	0.126152865
0.03321534	0.05812396	0.8985373	0	0.01777668	0	0.02228242	0	0
0.04431197	0	0.021512597	0	0.08360005	0	0	0.09586965	0.08112658
0.03713883	0.1065632	0	0.120311
SPAG5-AS1	0.2200704	0.07142956	0.2947901	0.033008772	0.150856709
0.3115434	0.1451861	0.2483605	0.06141155	0.06708666	0.1027433
0.124982453	0.3176438	0.293348389	0.070864619	0.6604786	0.358017
0.3932751	0.1243303	0.7745041	0.426204141	0.7765104	0.6359217
0.3278182	1.7352460.3047859	0.5276064	0.4575279	0.3046945
0.149977326	0.3070431	0.549222594	0.1540344	0.2338199	0.6184318
0.407620667	0.3205624	0.5105091	1.1455060.2130881	0.2046001
0.1825228	0.2119224	0.6223228	0.3006030.6815986	0.2471879
0.3064510.3041635	0.2174137	0.1998905	0.2715928	0.1998271
0.245926937	0.5421376	0.7059821	0.7500501	0.1791775	0.5845586
0.3318556	0.7149890.2086425	0.3714311	0.5277131	0.20849	0.662841362
0.08409959	0.8728933	0.2548568	0.197310642	0.2671119	0.163731
0.4555326	0.291752098	0.2972710.1794735	0.1470131	0.5152112
0.4834836	0.2647696	0.4304353	0.3345366	0.1104501	0.1606631
0.037492928	0.2308136	0.2792811	0.5431529	0.1967192	0.2870973
0.6435519	0.222525448	0.2016380.1457956	0.8179996	0.594286826
0.4754819	0.05824516	0.2255220.5947398	0.6827564	0.9138997
0.6054971	0.6237653	0.2899653	0.2071130.272106441	7.8664440.212538873
0.1866719	0.8247996	0.2607535	0.2378263	0.4445016	0.2842788
0.07287206	0.06925436	0.5359629	0.1898075	0.4948373	0.2814588
0.1760242	0.3702591	0.8812501	1.0281870.1126846	0.05420121
0.2018302	0.385557395	0.2388222	0.2428358	0.2468081	0.202233
0.198903469	0.1782694	0.2960693	0.6211658	0.2823622	0.3368694
0.9740519	0.4931851	0.3439698	0.2441802	0.3740336	0.1340788
0.3425559	1.8715510.3945373	0.2873979	0.4633052	0.2100298
0.09911966	0.3701817	0.144761977	0.0876134	0.6017081	0.200914081
0.5998641	0.2449073	0.2941575	0.2718933	0.2700814	0.2355755
0.121493605	0.3127938	0.4899256	0.3856118	0.5811991	0.2913399
0.4672047	0.4808209	0.1789225	0.2148476	0.21343274	0.6566094
0.2598091	0.3257587	0.2146289	0.7374731	0.1780374	1.600309
0.6181531	0.1571454	0.4869401	0.2594866	0.3086145	0.1521853
0.5554673	0.1573869	0.1414995	0.1309982	1.8711830.219941277

0.7058302 0.6606795 0.2240633 0.553061 0.376146 0.3018661 0.257921832
0.3503035 0.3431362 0.09269325 0.2813024 0.168796523 0.24724
0.7549771 0.3823066 0.5452611 0.4188707 0.8676242 0.1658023
0.1830229 0.544941 0.3227286 0.4983094 0.4288278 0.4999498
0.418472035 0.236210796 0.4666327 0.289558169 0.120928777 0.3951456
0.179063772 0.2005566 0.2970937 1.071251 0.31779509 0.7583909
0.2882768 0.4903557 0.372836703 0.2851684 0.4766882 0.4705804
0.3160797 0.1961536 0.5485994 0.2948832 0.6534254 0.1657386
0.2243713 0.3393146 0.478874008 0.385952 0.145717648 0.570891 0.3581599
0.2435641 0.4086079 0.485371 0.319427 0.612964 0.2463639 0.7690467
0.352295533 0.969705 0.4693582 0.336036 0.2310137 0.1932366 0.2870128
0.5390046 0.8793729 0.2025103 0.8479393 0.2735339 0.1650389
0.0865229 0.1884714 0.491180247 0.5852146 0.3084027 0.5257287
0.3290909 0.583364 0.1598505 0.2739163 0.3660054 0.9621843
0.4945256 0.371208564 0.135851941 0.5154236 0.290329 0.1940775
0.8293735 0.3526241 0.1275276 0.1359432 0.1292186 0.3056844
0.189043 1.013635 0.3622966 0.3316508 0.5375799 0.6239972 0.1929802
0.4734294 0.1700493 0.584624258 1.33616 0.306756 0.2000069 0.09465121
0.1565391 0.2350182 1.214949 0.08396269 0.5654674 0.4058515
0.737136 0.183698377 0.1885285 0.287631 0.2407067 0.2680196 0.2847155
0.280896709 0.121692 0.1436141 0.6787312 0.2710964 0.20144 0.323978
0.254730328 0.4033569 0.5053038 0.292609 0.2180551 0.377248762
0.616751 0.162436 0.280777 0.370178078 0.258681762 0.1049119 0.2758136
0.1468877 0.3180749 0.2904022 0.124332 0.8047296 0.1634972
0.1609111 0.4212262 0.1392652 0.4618328 0.375686058 0.1477428
3.507333 0.162315 0.4030836 0.2817998 0.2841421 1.062699 0.3394495
0.5466606 0.5744155 0.3448352 0.3523956 0.5263291 0.3107265
0.4312214 0.1359948 0.8477005 0.08719405 0.676041 0.4086623
0.5494286 0.6181459 0.1633034 0.3694929 0.2026965 0.1588876
0.4452704 0.1464296 0.2352024 0.642398 0.4530435 0.4546563
0.5871073 0.1688647 0.2768653 0.2521532 0.9020045 0.6255117
0.3430993 0.102592 0.6438693 0.1897175 0.2312788 0.6197253
0.6808351 0.7370676 0.2471872 0.437283706 0.1894288 0.2835582
0.93909 0.03310129 0.2047253 0.16855952 0.172869636 0.1587677
0.235734803 0.1702427 0.9806201 0.4797275 1.294861 0.1974115
0.2150212 0.199862 0.1954031 0.2829211 0.222386 0.3450681 0.215928134
0.06425543 0.6427285 0.2769003 0.1264383 0.3521504 0.4764468
0.3965672 1.080985 0.2315901 0.6851609
TGFB2-AS1 0.1329128 0.9835992 0 3.124943985 0.140359911 0.2062507
0.4780793 0.250242 0.4698051 2.032352 0 1.856904087 0.5670023
0.152432651 0.195163959 0.6435086 0.08355872 0 0.1475907 0.1288053
0.112863754 0.1843569 0.0278878 0.4051134 0.108284 0.03440132
0.05631972 0.05526535 0.3570811 1.36451864 0.4026709 0.42016131 0
1.819857 0 0.057866156 0.1085461 0 0 2.475786 0.5722809 0.9774236

0 0.2677971 0.2587102 0.2507642 7.908889 0.04219891 0 0.260333 0
0.360471 0.1423271 0.105826953 0.2097006 0.07044574 0 0.02741455
0.19981 0.7328791 0 0.2717749 0.6935159 0 0.04221983 0
0.4454108 0.1802987 0.03899365 0.599339672 0.08173742 0 0.1306828
1.785548447 2.391974 0.04576641 0.4048798 0.04547795 0.03740261 0 0
0 0.7182124 0.1580259 0.309771017 0.1513499 0.9094694 1.368983 0
2.939697 0 0.02890773 0.724329 0.0239004 0.07559742 0 0.4752203
0.08911638 0 0.03937348 0.327966 0.1324693 0.145427 0.07738173 0
0.03478033 0.526916171 0.08778159 0.107074473 0.05842067 0 0.1259869
0.4366554 0.2129 0 0.4181094 0 0.03690158 0.1340352 0 0
0.2019906 0.1233518 4.373652 0.09631518 0.3675055 0.03731806
0.08685131 0.442433455 0.02610023 0.3096201 0.5366204 0.03664196
0.068473501 0.2805491 0.09265755 0 1.039083 0.07997858 0 0
0.09666391 0.254728 0.03843669 0.2408206 0.08040448 10.45453 0
0.03957527 0.7834847 0 0.04014404 0 0.094923858 0.06032269 0.12554
0.539829575 0.3410193 4.215527 0 0.07640867 0.6349639 4.784783
1.672990929 0 0 0.09315703 0.05444371 0.03933145 0.1445731
0.2364642 0.1866514 2.083022 4.50648351 0.3191168 0.04586695
0.7673013 0.07208492 0.1586742 0.06810027 0.03520218 0.04836415
0.0676226 0 0.3092176 0.1713582 0 0 0.1274853 0.05127568
0.1717973 0 0.443214852 0.08601257 0.03394659 0.09074694 0.03369805
0.1233239 0 0.033505986 0.3445534 0 0.2659176 1.481078 0.077478715
0.8051285 0.0999633 0.04178125 0.02780871 0 0 0.1141565
0.1422728 0 0.2848742 0.08911453 0.114661 0.04049651 0.626352453
1.350684716 0.4089034 0.443030352 0.356831386 0.03778634 0 0.4105236
0.3671446 0.05717573 0 0.05383089 0.09021882 0.03069225 0.956339859
0.1266718 0.1969227 0 0.9163116 0.4861934 0.6619767 0
0.05731081 0 0 0.2362464 0.193946755 0 1.969402251 1.965321
0.1258146 1.487628 0.09701053 0 0 0 0 0 0.307469095 0.1604931
0 0.583816 0 0.03167745 0 0.8630465 0.04090925 1.006997 0
0.0856049 1.91997 0.7281006 0.038166 0 0.3357717 0 0.03414805
0.09959664 3.204189 0.04233026 0 0.2027573 0.1282207 0.2154973
0.375854062 0.60798012 0.06225864 0.1378582 0.5846063 0 0.1703756
0.4024351 0.9758115 0.270773 0.03826668 0.3062541 0.4243973
0.1072881 0.05502285 0.1480516 0.04053096 0.1245645 0.1143722
0.1773949 0.069399927 0.03679838 0 0.4417008 0 0 1.309909
0.7555523 0.05780914 0.2639525 0.09472297 0.2125761 0.057490067
0.1622547 0.06188649 0.1308386 1.469439 1.537486 0.890658621 0.5745336
0.1098664 0 2.105823 0.2219095 0 0.280614954 0.06612267 0.03953484
0.1413784 0.05267827 0.34631933 0 0.3355164 0.1449883 0.147041113
0.619495469 0.4880599 0.718542 0 0.04055513 0.04165516 0.4993557
0.04197456 0.3166014 0.6516994 0.1338546 0 0 0.127735092
0.1220668 0.07187004 0.2793887 0 0.2874399 0.5335491 0 2.045
0.0579048 0.09646115 0 0.03466113 0.2931037 0 0.04788713

0.5709376 0.1203402 0.6003396 0 0.3246554 0.7005315 0.1299541
0.1873933 0 0.2960332 1.2226580 0.687397 1.165962 0.06017651
0.0331835 0.09344339 0.1433437 3.853354 0.02845142 1.030809 0.1565645
0.07690553 0 0.2522701 0.03694253 3.177301 0.08685694 0 0
0.1162164 0.06946566 0.68167732 0.1304236 0.03683634 0.2710185 0
0.7047768 0.033158541 0.280052827 0.688268 0.092746137 0.04883907
0.04273206 15.85426 0.7641638 0.1306921 1.870032 1.605414 0 0.03607296
2.801678 0.1032969 0.094894885 0 0.08194906 0.1733169 0 0.02819291
1.043758 0.3003445 0.03917203 0.3878563 0.1474188
CDC42-IT1 0.02139666 0 0.1485181 0 0.045191023 0.1328111 0 0 0
0 0 0 0.2390602 0 0.039272516 0.03453124 0.1345151 0.04112253
0 0.2280895 0.090845536 0.1483913 0.1122363 0.1164576 0.2614778
0.1661405 0.09066499 0.2224191 0.03592742 0.059368605 0 0.157823598
0.02510719 0.2816975 0.07215352 0.232886189 0.05824679 0 0.22081 0
0 0.1123916 0.2553162 0.1231734 0.1249435 0.1730087 0.02686063
0 0.02189151 0 0.02993986 0.01813424 0.038187 0.056787681 0.06751632
0.1134054 0.1278987 0.066199 0.1608297 0 0.04127503 0.1875045
0.03488876 0.07403892 0.2038998 0.078157564 0.03585171 0.3386246
0.3766381 0.096483294 0.06579154 0.1512304 0.1314854 0.071860584
0.07939512 0.07367598 0 0.4392697 0.1806352 0.2037956 0.09803149
0.264853 0.03400581 0.05451308 0 0.02030393 0 0.02687592 0.06607271
0.04079658 0.07984713 0.162877512 0.02429259 0.0384755 0.4259456
0.156832506 0.2550074 0 0.178546 0.03169224 0 0.1066263 0.1170561
0.155714 0.09739156 0.08398545 0.047124648 17.16955 0.057457111 0.04702357
0.1920573 0.07605641 0.04393372 0.1285245 0 0 0 0.1485129
0.08091503 0 0.05031673 0.06503397 0.2978625 0.02034919 0.1550507
0 0 0.2097233 0.044515071 0.08403368 0.2242956 0.07198882
0.0294936 0 0.08468163 0.1118719 0 0.02697974 0 0.04863158
0.04480634 0.03890303 0.06151019 0.06187641 0.03230661 0.08629152
7.19188 0.06247112 0.06370931 0.09009104 0 0.09693741 0.1043142
0.152810951 0.1942182 0.2020976 0.08147175 0.30499 0 0.09781163 0
0.1095195 0 0.029274188 0.03688243 0.07240326 0.07498333 0.1314674
0.09497521 0 0.1308541 0 0 0.078854967 0.2568613 0 0.1187714
0.02901106 0.08514599 0.05481481 0.4250205 0.2335737 0.02721516 0
0.05530964 0.1241356 0.03123692 0.03420387 0.02565364 0.2476349
0.1382819 0.2973455 0.089187382 0.4846286 0.02732406 0.07304342
0.108496 0 0.1997509 0.026969414 0.03081507 0.2668953 0.06421218
0.1375546 0.187090943 0.05554788 0.1206927 0.06726057 0.1790688
0.08492717 0.2146559 0.06125739 0.03271923 0.2209765 0.0458598
0.07172947 0 0.1955771 0 0.044374861 0.5172071 0 0 0 0
0.09011888 0 0.2531183 0.074944298 0.4766212 0.2420611 0.2964552
0.081028491 0.2039196 0.05283523 0.05795362 0.1475103 0.1304478
0.06268634 0.0605265 0.1845209 0 0.04692244 0.1056433 0.031222064
0.1043371 0.059818795 0.0427544 0.1417778 0.1088556 0.03904253

0.03735946 0.2642141 0.3123669 0.06068115 0.3044275 0.054996881
0.2325295 0.1507907 0.03480898 0.1536308 0 0.0269593 0.06946776
0.2634272 0.02078322 0.2168862 0 0 0 0 0.440716986 0.06236933
0 0 0 0.07265054 0 0.03036038 0 0.03440217 0.06938265 0
0.150575779 0.07516921 0 0 0.3701727 0.06856878 0.02944776 0
0.03113554 0.8008353 0 0.1518236 0 0.1992988 0.05958436
0.09787174 0.06684241 0.09205971 0.08567251 0.055860924 0.3258145
0.09444518 0.02091358 0 0 0.06202138 1.737583 0 0.07967207
0.343097 0.3635988 0.138823557 0.03265024 0.04981326 0.03510459
0.1100252 0.1237543 0.040965884 0.03853748 0 0.7142023 0.07704569
0.04465448 0.0897728 0.056467677 0.02661151 0.1272885 0.08534801
0.02120072 0.162608269 0.1404648 0.04501027 0 0 0.124660035
0.01571383 0.1032793 0.09392748 0.06528673 0.2347015 0.1148394
0.2702871 0.05663038 0.02622808 0.1436552 0.05936884 0.05388284
0.051407837 0.06550215 13.01606 0.07496122 0.2279441 0.02892053
0.08589215 0.03004786 0 0.04660834 0.3105714 0.2356083 0.2231936
0.1286853 0 0.1541799 0.01838221 0.04843173 0.04026845 0.3202185
0.1045278 0 0.1569026 0 0.09450903 0 0.0517965 0.07833801 0
0 0.04843687 0.1335492 0.4888899 0.2538343 0.02673807 0 0
0.1260208 0.6809251 0 0 0.1784132 0 0.0699123 0.1620028
0.2515417 0.249451 0.08387075 0 0.0629878 0 0.06232754 0 0
0.026689751 0 0 0 0.1572449 0.171978 0 0.09319479 0.1472742
0.06271724 0.1582309 0 0.05807121 0.07866656 0.1940051 0.050921436
0.03956641 0.1649047 0.1046288 0 0.1361571 0.168027 0.04395478
0.3153009 0.1040636 0.4034417
SBF2-AS1 0.6589762 0.6275656 0.6589768 0.564985758 0.314530431
0.3813019 0.6634885 0.6717479 0.8751227 0.3913824 0.7359845
0.907070947 0.5710078 0.459003573 0.50567438 0.1081522 0.7840905
0.4293204 0.4258198 0.5267639 0.21339678 0.6777794 0.7850726
1.33335 1.253702 0.9877081 0.6783574 1.849909 2.644337 1.224124807
0.5592623 1.388761493 0.5373457 1.48517 0.4708031 0.737506116
1.454368 1.633479 6.137763 1.297091 1.07279 0.7089121 0.8529628 0.7876346
0.5000255 0.7576072 0.336511 1.95627 0.6323162 0.4922239 0.776223
0.4007317 2.272439 0.494054251 0.6989985 0.3551868 0.5841791
1.877542 0.3757912 0.5434074 0.3375482 1.957552 0.449229 0.605492
1.915848 1.427942828 1.179023 0.3408992 1.747606 0.279802361 0.7212089
0.657854 0.6405986 0.925279556 0.2935643 0.4679175 1.309899 0.535032
0.5867047 0.6666583 0.2558629 0.4608455 0.5443665 2.086767
0.433850783 0.7101116 2.078049 0.4162017 0.6840528 4.411796 0.4214344
1.186263619 1.779534 0.5790965 1.450532 0.55487501 0.6211998 0.4618052
0.5980417 1.34553 1.022018 0.598335 1.507219 0.2546865 0.4109449
0.5942828 0.578079726 2.434267 1.794563134 0.981855 0.5547398 0.5514105
0.37458 2.91096 0.7646742 0.5445936 1.001724 0.8889411 1.605035
1.164292 0.6303699 2.563061 0.4031084 1.547317 1.173582 0.9722318

0.3815413 0.6325273 0.642888518 0.4276906 1.738894 0.8434234

0.467003 0.824746709 1.144391 0.5061101 0.6940641 1.239345 1.613005

0.3723244 0.5418446 0.6972222 0.5494106 0.737507 0.7701273

1.009743 10.55595 0.3097952 0.5043881 0.5643319 0.2522079 0.8489803

3.279231 0.452016098 1.225035 0.5509197 3.510953758 1.130357 2.082526

1.265099 0.4066548 0.730497 1.669657 0.47881 0.26312 0.9448652 0.7306397

2.245601 0.352549 1.270576 0.8817687 2.263856 0.4820023 1.769983709

0.662961 0.3147716 0.8514505 1.075211 0.7666993 1.664347 0.4634437

1.158295 0.9470904 4.460761 1.645688 1.351179 0.7011459 0.2678171

0.6026059 0.5745146 1.515851 0.7243358 0.817315528 0.9938378

2.101445 0.4130617 0.4483611 0.8290641 0.6082433 0.427034934

2.241248 0.9752382 0.6070637 2.06635 0.900658405 0.3962797 0.5110143

0.5851687 1.324218 0.5073564 0.6648343 0.7407877 1.027616 1.057376

0.901698 1.347944 0.7266647 0.3346334 0.359668367 0.853197911 1.562628

1.535706732 0.349832933 0.6350614 0.581962679 1.233551 0.565626 0.5845668

0.423811229 0.7765481 2.4471 0.3352918 0.909385386 0.5056201

0.2666074 1.285705 0.968924 1.271087 1.090745 0.4949872 0.7545082

0.3953464 2.102366 0.6323401 0.92355133 0.4538677 1.483211296

1.394866 0.5392027 1.568309 0.4483657 0.8450735 0.3539951 0.4008459

0.4856933 0.8063294 0.559814866 0.7687448 1.062625 4.924163 0.8286869

0.62556 0.5019297 0.4774533 0.4812829 0.5677575 1.912074 0.2877461

1.300608 0.3800863 2.539042 0.897665321 1.47953 0.6585089 1.544785

0.5265762 1.181955 0.7173579 1.056544 0.4868098 2.454258 0.6519213

0.479027277 2.973729654 1.041999 1.467391 0.5672922 0.8641707 0.9902731

1.12214 0.4713637 1.121443 1.061171 0.4825408 0.6472258 1.100674

1.417949 0.5391208 0.4200667 0.2209817 0.4445116 1.252193 0.476271611

0.752456 0.8983651 0.7387116 0.3103236 0.7416231 0.8903195 1.16401

0.9068055 2.444505 0.8026206 0.7108163 0.36233053 0.7442317

1.100777 0.3787094 1.115163 0.446816 1.32225965 0.3989797 2.688943

0.9320367 1.206539 2.093988 1.421467 0.741818013 0.3472812 0.5260212

0.7078784 0.7304094 0.905405461 0.3666142 0.7753492 0.6904171

1.599458774 0.477199993 1.276878 1.976771 0.6700806 0.6702355 0.7117512

0.4695799 0.811269 1.064201 0.5476438 1.056084 0.7885392 1.092262

0.532226872 1.464567 2.164143 2.249968 0.2697042 0.6290233 1.72867

0.3293856 1.100645 1.443558 0.3309925 0.7014864 1.101966 2.25779

0.1797791 0.8987175 0.4190053 0.6025409 1.996918 0.7689109

0.6487012 0.5167293 0.6188258 0.4789792 0.3288924 1.131294 0.67144

0.6610964 1.777853 2.29526 0.5562507 0.3439169 0.8441272 0.5099316

0.8700058 0.3387057 0.5622844 0.6212844 0.8455257 1.116615

1.059953 0.9675376 8.4302239 2.213989 0.6257864 1.694462 0.2984066

0.413484 2.514110057 0.5553021 0.4385256 1.117037 0.6942468 0.7304373

1.119211235 0.642274787 0.4025933 0.766385791 0.5677343 0.5266671

4.348428 0.5578283 0.4502815 0.9876112 2.257645 0.4420767 0.5633214

0.5338328 0.6028032 1.337913679 0.9500713 0.6656893 0.3216299

1.853241 0.564645 1.298948 0.699806 0.4169551 0.7423917 0.5987566
AL133415.1 0.1048618 0.0431118 0.291146 0.134478017 0.022147442 0.8136094
0.2743139 0.3619529 0.1853268 0.5101817 0 0.0714638 0.08520714
0.180393031 0.307949929 0.06769297 0.09888571 0 0.06986517 0.02032423
0 0.03636218 0.04400425 0.06848893 0.02847696 0.1085639 0.0888671
0.06540255 0.07042995 0.640104564 0.6883239 0.022099136 0.02460932
0.05522228 0.601143 0.022826805 0.2283670 0 0.2893742 0.1389237
0.02754072 0.1001013 0.3018272 0.04082197 0.02826299 2.053583
0.06658581 0 0.05134756 0 0.01777464 0.1122893 0.139153949
0.06617747 0.0555783 0 0.02162876 0.1351204 0.1360485 0
0.03063104 0.06839383 0.1088561 0.1332376 0 0.07028153 0.04741566
0.06152823 0.283710083 0.06448689 0 0 0.21130676 0.3112828 0
0.1277723 0.03587991 0.08852658 0 0 0.1038404 0.1333259
0.05343208 0.081464805 0.0199013 0.1845071 0.1053719 0.03238124
0.1199627 0.05217584 0.022806807 0.02381086 0.01885627 0 0
0.1249753 0.3515426 0 0.03106378 0.06468734 0.1828958 0.03824497
0.06105046 0 0.10976 0.09238033 0.1385109 0.028158867 0.04609109
0.03764976 0.1739458 0.04306251 0.02099598 0 0 0 0.1746814
0.5287365 0.03583949 0 0.03187217 0.06487908 0.2592937 0.01899701
0.1288641 0 0.1713037 0.218161681 0.02059182 0.4152680 0.2352043
0.02890874 0.43217836 0.08300239 0.1462047 0 0.1322237 0.06309925
0.04766721 0.02195891 0.03813159 0.04019362 0.0303247 0.09499792
0.08458035 3.452701 0 0.06244596 0.5298272 0.04305224 0.03167171
0.1363275 0.037445176 0.1427751 0 0.133093605 0.0298942 0.1814099
0.03195734 0.2109897 0 0 0 0 0 0 0.04295347 0.1241225
0.5132753 0.1049394 0.08835538 0.1190872 0.42510199 0.04196129 0
0.02328322 0.1137431 0.08345755 0.1611835 0.05554565 0 0.1867284
0.03148886 0.2168514 0.1081547 0.03061749 0 0.8549277 0 0
0.03643114 0.029139598 0.03392989 0 0.03579748 0.1063445 0.2594577
0.02447373 0.052869219 0 0.03270034 0.1048981 0 0.2445079
0.1270415 0.1182993 0.2307438 0 0 0 0.03002133 0.03207041 0
0.1348512 0 0.1130776 0 0.444745729 0.021747453 0 0.116509871
0.062560618 0.05962321 0 0.05888788 0.1241397 0.09021779 0.146916301
0 0 0.02421471 0.119132541 0.1998759 0 0.1136088 0.1988046 0
0 0 0.02260773 0.6159907 0.02299599 0.06212901 0.275426369
0.05113404 0.029316293 0.3352526 0.01985233 0.0426788 0.03826832 0
0 0 0.08921676 0 0.242578309 0.3038913 0.1478005 0.05117808
0.05019476 0.3248958 0.1056988 0.06809021 0 0.3259373 0.1062927
0 0.03091355 0.05222148 0.1505557 0 0.04075503 0.05983025
0.3771764 0.1375097 0.1780247 0 0.05951666 0.09140899 0.03371998
0.0340034 0.088959226 0 0.04911907 0.1087635 0.1647237 0.3023602
0 0.05772762 0.8641379 0.09155437 0.4226679 0.3624296 0.1116097
0.04232257 0 0.2336112 0.06395396 0.1965508 0.02255854 0.139956
0.383272399 0.08709644 0.03085744 0.02049886 0.03427643 0 0.3039575

0.2128908 0.1824345 0.02603072 0 0.04192807 0 0.2240195
0.09765092 0.8258031 0.1617651 0.3032505 0.020076765 0.01888664
0.3033775 0.04666931 0.1510358 0.1094224 0 0.276739606 0.4434246
0.03119109 0.05577037 0.332485 0.045538212 0 0.04411772 0.0381296
0.386694522 0 0.3234468 0 0.0306883 0.03199605 0.03286391
0.1688433 0 0.0832611 0.1028319 0.03520162 0.03879437 0
0.025194209 0.03210162 0.170106 0 0.04468479 0 0.08418891 0.02945201
0.2996327 0 0 0 0.02734596 0 0.05626232 0 0.7747608
0.04747133 0 0 0.01707583 0.07369141 0.02563188 0.3326497
0.06175661 0.06673028 0.4569244 0.03839228 0 0.2044195 0.04747637
0.05236037 0.03686117 0.04523651 0.1310393 0.0673404 0.4280309 0
0 0.05176998 0.07961157 0 0.3063791 0.02284198 0 0.03522194
0.09168915 0.3836354 0 0.3292733 0.0290621 0.1221832 0
0.111207 0.026160492 0.497133435 0.287476 0.036586118 0.03853168 0
5.732948 0.09134673 0.1031098 0.09221033 0.1033955 0 0.01422991
0.4626396 0 0.174690819 0.0775636 0.09698079 0 0.2289381
0.1112142 0.09411143 0 0.09271453 0.06799999 0.1395676
AC106782.5 0.6940541 0.2377885 0.6356498 0.123621719 0.335931708
1.121891 0.4728164 0.7259623 0.1022193 0.2009979 0.2198776
0.197083761 0.9399413 0.746235233 0.424634082 2.333557 1.31809 0.6113763
1.2845 2.886596 0.306958549 0.9025208 3.519309 0.6610787 2.316757
0.9356219 1.133489 5.110426 0.971163 0.601802853 0.6570929 1.249378105
1.153754 2.055952 0.4875999 3.462362644 1.574483 2.143664 1.716021 1.695834
0.7183605 0.1898804 1.380303 1.872861 0.4503174 1.208132 1.270634
0.5968013 0.8876323 0.9204451 1.052105 1.004892 1.393527 0.959401249
1.916303 1.302834 3.673341 0.5368325 1.086857 2.344977 2.454574 1.90068
0.141463 1.150785 1.010472 0.369721916 3.391907 3.694069 1.739254 0.478145074
0.5779889 0.6131919 1.208433 0.485620354 1.985188 1.493665 0.7487904
0.9400257 3.458646 2.478983 2.164096 1.145489 0.1838439 0.8104562
0.112332328 0.4665144 0.593642 0.4358939 1.562775 4.493839 0.9712655
2.421530516 1.805812 0.3900154 1.356989 1.907720406 1.321191 0.04847443
0.7842774 3.041217 0.9217103 1.224953 1.318406 1.304834 1.447939 1.21079
0.605073116 1.527948 1.630794408 3.749762 1.349803 0.6167699 1.840754
1.592332 0.7844843 0.1364572 2.310712 1.405071 1.093617 0.9389666
0.952087 0.7031798 2.05763 0.4950578 2.200388 0.4886515 2.029899
0.7558778 1.504122526 2.669054 0.9094485 0.5513518 0.9168361
0.595933442 0.8393185 1.209615 0.7385202 1.093944 0.8700795 0.7230148
2.210388 1.261917 1.967525 0.5435939 0.3056509 0.2624139 0.7273675
0.6332522 1.291607 1.278519 0.9201574 1.13548 0.5639487 1.394101465
1.443739 2.412804 1.68841716 2.061065 0.7504416 0.8813235 0.8728049
2.615063 0.7940319 0.870449679 0.498489 0.8807178 1.469498 0.9772753
1.454803 0.8257183 0.8521314 0.8934478 0.5911565 0.746041916
1.504378 0.2993894 3.017906 0.1960513 1.812512 1.70397 1.531845 0.9996832
1.655235 1.867068 1.532466 1.509994 0.7599357 1.433089 1.24821 0.2231293

1.4484449 3.164813 0.723253923 1.637515 1.587998 0.1974455 3.079422 0.8049775
1.619855 0.400959333 1.457697 1.26254 0.376076 0.3718272 0.674306942
1.6516822 2.011859 0.8181618 0.8168266 1.147844 0.9864111 0.3311728
0.7738865 3.335077 1.30163 0.7271015 1.652787 2.467123 0.340701481
0.449815482 3.43164 1.00410118 0.345060906 1.233222 0.198700684 0.9744104
0.6276492 3.172248 1.519378538 2.635295 0.8506178 2.804744 1.478453448
1.240245 2.142304 0.7049515 1.221137 2.115701 1.016694 1.22708 1.527523
0.5227037 4.344175 1.313608 1.139361423 1.339672 3.274377997 1.386846
1.505598 0.7650509 1.055368 1.817771 4.535194 2.005371 1.02518 1.325792
0.854814796 1.606311 1.273769 0.7056977 1.488098 1.309541 1.311738 1.03279
1.557668 0.3932574 1.709956 1.117545 3.410151 1.440171 0.3321635 1.927124136
1.629724 1.402505 1.374526 0.4604886 1.66926 0.4144562 2.626173 0.9138221
1.906364 1.172187 0.81777622 1.475466178 0.9482283 3.824353 1.18112
1.459242 0.3706999 0.2786034 0.6716094 0.2524898 0.2914097
0.7496346 2.308489 0.5544091 2.06513 0.8053198 0.3086528 0.3161959
1.430881 0.3859725 0.528496707 2.76225 0.7233419 0.3674581 0.1890559
0.3058734 2.17947 0 0.2515601 1.112712 2.421633 2.283688 0.875600662
0.6178037 0.3366287 0.2846763 0.4832942 0.5017848 0.968939164
1.093802 0.5378517 2.445399 0.7809905 1.026006 2.426671 0.648716552
0.7553095 3.999892 2.230161 0.6017361 0.973290345 1.202364 0.3650052
2.103086 0.853144789 2.148194312 0.2548588 0.4746006 0.296214 1.720662
1.404804 0.6984571 2.100532 1.45425 1.5952 1.262033 3.316615 0.4733692
1.146434921 0.1770605 1.876482 3.49536 0.462121 1.328989 2.321772 2.233636
1.112368 1.259882 1.574088 2.547515 0.9426879 0.6667173 0.3879023
2.292222 0.2732918 0.8509606 0.3265519 2.640052 1.295028 0.6604881
1.590478 0.4586928 0.4257829 2.346377 1.225109 5.135118 0.8973753
0.7046883 1.145646 1.624501 1.626499 0.4678268 0.6143489 0.5571366
0.5902145 1.93035 2.384447 1.284947 0.1646653 1.687956 0.422468 0.9449085
2.277152 3.739717 2.317897 1.511421 3.398955841 0.4824144 1.282365 2.02175
3.843014 0.5520385 0.829675918 0.685500401 0.3523578 0.908078874
1.062631 4.602293 0 1.838995 0.5687149 0.4662145 1.675229 1.125858
1.491251 1.063228 2.959227 0.619411532 0.5882412 3.61064 1.885498 1.767831
0.7974408 1.362594 0.5940763 4.005799 0.7970077 6.447094
AC093010.2 7.478202 18.29116 11.09973 18.528384914 5.373452215 15.46352
26.80348 20.40823 19.66897 17.38423 4.123588 21.846130362 15.58545
15.967093211 20.184155321 10.36094 11.93354 6.16931 9.821876 3.281703
4.736412453 4.846891 5.319692 0.1535936 2.358924 3.423741 7.099841 6.51774
5.241851 2.495815142 12.55691 3.893528175 5.484404 2.14788 6.304473 3.906551396
5.101364 5.423912 7.508066 7.300709 1.942326 1.609697 3.30417 811.95538 4.754751
1.592491 3.675443 7.489615 0.8391006 5.009124 1.246311 3.266151 4.139298
4.060775781 3.047041 3.178324 3.465911 1.700698 3.26695 216.31825 3.232186
7.315839 2.18567 5.340151 2.29702 3.060201478 4.314675 4.824932 6.364489
4.188643016 2.996317 0.6544609 2.28689 9.418316087 6.086423 3.993071 3.02213
2.911808 4.504152 3.695753 4.956185 6.331232 3.251595 2.014592 0.993395122

0.7531439 3.051602 1.240614 8.591656 4.741636 0.4826654 5.821128377
1.842243 1.054536 7.937527 4.28768925 5.710505 4.227641 3.035495 5.316217
0.9701431 11.06341 5.741119 2.515759 3.783189 3.0807 3.942752851 9.289635
4.996681174 7.674784 10.16368 4.148207 4.599251 8.484251 2.238447 3.009915
1.078525 1.175224 8.759707 2.561915 5.931081 3.698919 2.809933 2.818006 2.412383
6.949334 1.47323 12.2309 9.191792195 1.497942 10.01471 3.916466 3.768295
9.177162042 3.17604 6.439755 5.067154 6.02688 1.552151 3.688006 2.031363
2.645592 4.723816 2.881767 1.890755 1.662666 22.14027 4.389945 2.560135 11.80767
3.168019 2.903234 7.982388 1.306856724 1.741023 9.634404 3.861537757 6.523916
7.526366 2.069404 9.809754 7.989508 1.18377 2.533725418 1.960941 2.96918
6.119069 5.996396 6.497911 6.211002 6.834594 0.8792737 8.382521 2.112505315
10.90414 0.4260518 4.885372 1.877235 8.457396 5.862606 4.22749 6.754763
6.079469 7.083775 5.847142 10.42578 2.278751 3.70754510.73175 3.657248 2.350957
4.013545 3.614594421 5.692583 9.358403 2.122392 10.85497 9.600706 2.315457
1.533931596 9.525325 4.79879 2.972908 3.21261 14.727923868 4.555919
6.483236 7.179856 2.011138 2.613538 1.282822 1.300233 1.941874 6.982454 13.79782
2.05465 5.287315 4.016921 2.410356442 1.911211239 2.926201 1.690191137
3.538158764 2.619906 0.742257709 1.696174 13.36302 9.42698 3.768372483
9.482669 3.172543 5.213185 12.055919868 7.417014 4.072121 10.16332 4.392538
2.544833 2.131485 5.513067 2.576204 11.25728 2.465734 7.663195 3.19130779
6.385877 2.674993882 21.40098 7.746642 1.597189 2.478073 1.047044 3.615343
3.282922 12.34231 2.314221 8.250776807 3.428837 1.087597 2.890825 2.321695
4.066921 6.166759 4.738454 5.577863 7.9405 3.813959 4.714235 9.515114 7.246314
3.329944 6.143758142 3.350287 3.849163 4.644664 3.048004 1.646861 1.432371
3.290927 1.777684 2.325333 4.103536 1.346627572 0.822292625 3.149735 1.120477
14.29617 4.322724 0.9043401 10.85039 13.10078 1.796552 3.833824 14.42696
5.741174 2.010965 2.646766 9.823079 18.51507 6.873502 2.807739 1.765497 4.696709268
8.31343 0.6487598 13.39476 3.372605 1.492385 6.582245 4.082027 3.356132
5.429026 6.001961 3.111742 1.554371838 15.17927 3.510722 5.888622 1.881896
3.315346 3.706058332 7.457174 2.487551 5.269018 4.366229 4.453852 2.478991
4.34044397 5.725262 4.026455 4.701884 5.137873 3.408395507 4.399836 4.730502
11.65603 17.056798251 2.774446803 3.361282 1.081191 3.677661 3.753656
9.535036 1.75125 3.564763 3.19761 3.934805 7.341729 7.642444 2.487276 3.411226864
1.309341 7.510398 9.577535 5.542883 3.349407 12.50816 4.730775 3.178877 5.436326
3.472056 7.822426 4.760445 7.062849 1.821639 6.068574 2.689562 3.632928 0.9128154
5.859827 2.233039 9.729725 4.343496 5.626097 2.986313 4.321133 2.049402 5.940812
3.116555 2.320813 6.927252 3.874979 2.882943 2.891257 1.961577 1.43467 15.96441
3.878149 3.852468 2.514282 3.514953 4.032061 12.33243 1.978586 7.157689 6.087082
5.654619 5.911014 5.06937452 5.356502 1.564195 5.07344 2.721836 5.583293
2.662044305 4.865959565 6.339502 4.784435685 1.83084 7.782708 4.602127
9.917524 4.803897 2.649514 5.065023 4.961015 4.9723 5.003861 6.819378 2.361068375
2.886392 6.864515 2.041145 8.413632 4.620312 5.144459 5.02718 3.365738 8.749521
5.624126

AC105339.2 0.08558664 0 0.0237629 0.007317262 0.075920919 0.05312446 0

0.01611385 0.01210087 0.008922914 0.07158095 0.034996608 0.06259031
0.044170224 0.018850808 0.3259749 0.1183732 0 0.007603054 0.2355543
0.043605857 0.09497046 0.3124658 0.1267058 0.1115638 0.09303868
0.07615859 0.2491094 0.06898064 0.042745396 0.06914457 0.126258879
0.008034302 0.09465036 0.0923565 0.055892685 0.1164936 0.07544518
0.2031452 0.1299006 0.01700812 0.08541762 0.08170118 0.2414199
0.03331828 0.212224 0.04727472 0.06521561 0.1436083 0.1592546
0.09580755 0.139271 0.07331904 0.199892637 0.04321044 0.3492887
0.2455655 0.09179595 0.1066071 0.04441631 0.3566163 0.1600038
0.05023981 0.07107736 0.07612259 0.068778657 0.005736273 0.7314291
0.1958518 0.025728878 0.1421097 0.04839372 0.1893389 0.149470015
0.2635918 0.0530467 0.1095001 0.1464232 0.2456638 0.05217169
0.135937 0.14408 0.02176372 0.05523992 0.006649035 0.0454808 0.03346487
0.1935066 0.02114327 0.1501314 0.1192384 0.055843719 0.06218902
0.03693648 0.1752462 0.133830405 0.1972057 0.005738476 0.585631
0.03549531 0.08799472 0.05118061 0.03121497 0.2939879 0.1402438
0.08062603 0.14325893 1.153115 0.059755395 0.02257131 0.4547916
0.05678878 0.1476173 0.3872872 0.09950189 0.02692335 0.01674769
0.1900964 0.06041656 0.08775495 0.08050678 0.2133114 0.2276993
0.175817 0.1922629 0.01051769 0.07689678 0.06711146 0.170937874
0.09075637 0.1076619 0.03071523 0.07078463 0.026455311 0.1264579
0.07756455 0.08742713 0.06906814 0.02060026 0.1011537 0.1182887
0.3236732 0.09513575 0.08910202 0.01550717 0.04487159 0.7436501
0.004997689 0.1223219 0.0864874 0.1264989 0 0.05563425 0.134473637
0.1242996 0.199403 0.09124836 0.1659146 0.1678059 0.05738282
0.1279249 0.09345662 0.2611075 0.131148362 0.1652333 0.08688391
0.07798267 0.1402319 0.08611086 0.09309503 0.2226898 0.02403811
0.02332733 0.094625961 0.1643912 0.04725622 0.07601366 0.04641769
0.2418146 0.08331852 0.3400164 0.236688 0.04789869 0.1336438
0.05752203 0.152273 0.03498535 0.1915417 0.04515041 0.2113151
0.04425021 0.3508677 0.047566604 0.2270831 0.07869331 0.02337389
0.09113666 0.1270593 0.2037459 0.038835956 0.1725644 0.03736534
0.09246554 0.1613973 0.029934551 0.1007268 0.1223019 0.1452828
0.1038599 0.1358835 0.1099038 0.0539065 0.05758584 0.2298156
0.0220127 0.2926562 0.1476675 0.1043078 0.193597216 0.113599643
0.2858745 0.123621687 0.066379347 0.03893085 0.094089943 0.1297712
0.1486041 0.1767226 0.095928701 0.2773069 0.166538 0.3162189
0.162056983 0.08156784 0.1690727 0.1483613 0.08850616 0.2852459
0.2306857 0.05810544 0.1845209 0.02320442 0.05630693 0.08451461
0.104906136 0.08346967 0.038284028 0.07866809 0.09397841 0.05921746
0.02498722 0.08966271 0.1817793 0.1249467 0.01941797 0.2760143
0.092394759 0.1942913 0.1809489 0.2060692 0.2580997 0.05303502
0.05607534 0.06113163 0.08956525 0.02660252 0.4222051 0.09371015
0.06560106 0 0.02949151 0.311094343 0.1496864 0.1367312 0.1539229

0.04168666 0.0929927 0.0654186 0.02914596 0.1007190.09357391
0.1665184 0.150054846 0.174667904 0.1403159 0.04438555 0.0107556
0.3010738 0.09325353 0.03298149 0.06411785 0.04483518 0.6948786
0.08874287 0.2672095 0.05181457 0.08857725 0.1620694 0.120056
0.01604218 0.2504024 0.0639688 0.165348334 0.4123035 0.09066737
0.07696197 0.01119036 0.01206992 0.01984684 0.3058146 0.01489003
0.1954621 0.1524876 0.2121706 0.0481255 0.04701634 0.2869244
0.1235681 0.03960906 0.07425255 0.147477181 0.1603159 0.03183585
0.3123445 0.03698193 0.03929594 0.05745459 0.07679604 0.08089899
0.2240277 0.1092454 0.06445019 0.126369855 0.05993165 0.0504115
0.03112081 0.164119414 0.154578443 0.04022742 0.1553321 0.02504733
0.08878995 0.1823966 0.09187156 0.1081148 0.04077387 0.07134037
0.0804469 0.07282578 0.1250082 0.074027285 0.04716155 3.396902
0.08995346 0.08388342 0.08329112 0.1603320.07211486 0.02257437
0.5816721 0.1801314 0.1926752 0.05356647 0.05147413 0.09184093
0.1295112 0.05882308 0.1666051 0.05798656 0.4918556 0.05574815
0.1744229 0.2133876 0.02413366 0.09576915 0.0653571 0.04972464
0.2130794 0.05311632 0.06673765 0.2131222 0.2649616 0.1444105
0.08491912 0.02566855 0.06595467 0.07685753 0.1949122 0.326844
0.1140855 0.03898666 0.2473996 0.1136645 0.1006737 0.4199113
0.1609867 0.4939130.0715697 0.102422346 0.08062439 0.04269608
0.3241032 0.01467554 0.03630619 0.046973961 0.072133838 0.03128448
0.071666495 0.05660816 0.2366418 0 0.4354060.09088919 0.03512165
0.1097068 0.01999211 0.09059109 0.0587377 0.2261545 0.081474298
0.0253225 0.5804646 0.08370305 0.0224227 0.1960662 0.1113779
0.05626211 0.1967477 0.01110011 0.2961737
SOCAR 0.0999313 0.03423727 0.03853563 0.106795815 0.052765222 0.05169028
0.08169246 0.1567883 0.02943548 0.05788017 0 0.283765145 0
1.146074059 0.030569833 0 0 0.9282867 0 0.0322810.459643667
0.2598935 0.1572570.09065089 0.0678450.1077702 0.9703907 0.1904448
0.3076260.55455219 0.9671198 0.07020018 0 0.2192741 0.224658
0.163151315 0.1360184 0.3670421 0.3007884 0.3447101 0.08274478
0.7217589 0.03974776 0.3116052 0.09725639 0.1571155 1.630854
0.1850771 0.05112123 0.1631108 0.09322107 0.3528936 0.1188995
0.22101842 0.1576647 0.06620632 0.2737810.08588247 0.1251903
0.2971184 0.3212859 0.2675823 0.2987326 0.8644814 0.343885
0.608380591 0.7534907 0.05648277 0.02443135 0.325445712 0.02560615 0
0.06140914 0.503428254 0.3244572 0.5734958 0.3297789 0.02849406
0.09373793 0 0 0.1236974 0.4235234 0.2828875 0.388172005
0.1264371 0.4395793 0.1464420.1285779 0.06351233 0.06215322
0.235456549 0.1134565 0.08984831 0 0.244157692 0.2977477 0.3629312
0.1737259 0.07400797 0.2911051 0.4979891 0.03037227 0.04848327
0.01895246 0.3268725 0.64193418 2.5299660.245986237 0.3660327
0.08969874 0.11840500 0.1500657 0.06454378 0.05239302 0.407389

0.09248224 0.1679586 0.1992337 0.2350001 0.07593393 0.07728566
0.1267189 0.03017298 0.1023375 0 0.1632492 0.346506517 0.01635301
0.7177683 0.6350779 0.06887369 1.587368268 0.2636656 0.1161086
0.1276003 1.428075 0.1002206 0.03785495 0.2441416 0.5450801
0.0319198 0.07224714 0.2766231 0.1679239 6.131338 0.02431384
0.099183 1.332415 0.03418996 0.9054761 0 0.118948456 0.03779498
0.02621889 0.549621197 0.5222911 0.2401113 0.1776526 0.2154312
0.02841675 0.2540585 0.296232389 0 0.02817945 0 0.03411153
0.09857195 0.407618 0.2314935 0.1871133 0.3026343 0.368285516
0.2665887 0 0.2958462 0.2484051 0.3976672 0.618686 0.2867255
0.151512 0.1271061 0.1250345 0.2152659 0.1395731 0.4133533 0
0.4792528 0.1285064 0.3498268 0.02893182 0.277694795 0.2155636
0.1276148 0.1137144 0.0633402 0.4636091 0.07774332 0.440854356
0.5037172 0.129845 0.1832708 0.1784547 0.242720159 0.3170832
0.2192109 0.1308895 0.05227037 0.1322151 0.05012663 0.1430487
0.07640621 0 0.5711583 0.139586 0.143681 0 0.274707336 0.39722738
0.4757945 0.346973996 0.347777921 0.1657243 0.085827967 0.1636805
0.1314475 0.05373489 0.291684219 0.1686378 0.4710525 0.03846025
0.220754787 0.4761947 0.08225418 0.6315576 0.2583504 0.1015409 0
0.141342 0 1.317049 0.05478684 0.1809126 0.243033391 0.4060814
0.186252445 0.08320034 0.1418917 0.08473351 0.5470346 0.08724212
0.082266 0.2127538 0.4014922 0 0.321072902 0.1810016 0.1173759
0.0135477 0.0199311 0.3374059 0 0.2703695 0.1025262 0.2750211
0.0844124 0.0804532 0.3682503 1.700341 0.7412962 0.121078338
0.2589251 0.190057 0.7702326 0.2028063 0.1413784 0.02652188
0.354489 0.145185 0.1874512 0.4320612 0.235490115 0.32232363 0.09751986
0.04318729 0.05232617 0.2401195 0.2134965 0.2292222 0.4866177
0.5574278 0.2397586 0 0.2363598 0.0336105 0.06894877 0.4174256
0.05078908 0.2341364 0.1433191 0.2889801 0.086964587 0.2075031
0.2450546 0.1465127 0.08166196 0.499123 0.7000253 0.8453371
0.03622013 0.1653785 0 0.03329721 0.162090947 0.3558105 0.3101982
0.5465101 1.09196 0.3371573 0.398599431 0.2249826 0.172091 0.01853123
0.419808 0.03475917 0.1048191 0.813159953 0.6007196 0.2229338
0.1328702 0.2970486 0.126574716 0.1822303 0.4905056 0.2725259
0.614187474 0.024258927 0.672743 0.09647146 0.3411959 0.05081937
0.0521978 0.4916523 0.05259805 0.2644874 0.2041601 0.02795539
0.2002557 0.02097127 0.160063996 0.3569094 1.125748 0.05835002 0
0.2251182 0.200576 0 1.262984 0.03628006 0.2115309 0.02037757
0.02171681 0.01669483 0.08936153 0.2100246 0.3863366 0.09424847
0.1567253 0.04985179 0.09492548 0.643743 0.2035557 0.7338167
0.1716542 0.4769451 0.3427076 0.09146778 0.3732615 0.3652648 0
0.02079101 0.02927332 0.1436984 0.08325194 0.3030444 0.1359684
0.6539663 0.02409245 0.2877922 1.390919 0 0.3317879 0.1269798
0.05044138 0 0 0.8051843 0.427102663 0.6047018 0.2769562

0.3638695 0.3212854 0.4769016 0.207753739 0.460598695 1.902494
0.261494029 0.1529998 0 0 0.1741034 0.3602927 0.1464578
0.2873905 0.1621032 0.0113007 0.5715198 0.02157344 0.158549533
0.8931593 0.1540347 0.5701035 1.490853 0.07065669 0.4671166
0.1539653 0.09817241 0.216009 0.1108377
RNF216-IT1 0.08787233 0.4515865 0 0.187816973 0.046397905 0.06817902
0.4310065 0.2068023 0.4659016 0.2290303 0 0 0.2677578 0
0.645141394 0.1418138 0 0.08444151 0 0 0 0 0 0.09565417
0.05965797 0.2274366 0.04654315 0.09134362 0 0 0.1109237 0
0.1031108 0.05784411 0.07408047 0.04782114 0 0 0.226707 0.1515565
0 0 0.1048539 0.1896944 0 0 0.05515596 0 0.0899046 0 0
0 0.07841367 0 0.1386388 0 0.2626288 0.1359339 0.1415356
0.2137617 0 0.06417067 0 0 0.06978173 0.160489717 0 0.1986674
0 0 0 0.3105383 0 0 0.1630309 0 0.1338386 0 0.06181975
0 0 0 0.06982795 0.1119378 0 0.08338467 0.04294824 0.05518735
0 0.0418861 0 0.09555849 0 0.03950304 0.06247443 0.107347274
0.2618177 0 0 0 0.04517236 0.1094739 0.0801215 0 0.199985
0.05748559 0.04838317 5.94857 0 0 0 0 0 0 0 0.2073178 0
0.06099163 0 0 0 0 0.0679594 0 0.1591916 0.2024733 0
0.07177474 0.091407802 0.0862779 0.2046984 0 0.06056252 0 0
0.07657308 0.1122025 0.1108011 0 0.09986069 0 0 0.08420386 0
0 0.1328941 7.936488 0.3206974 0 0 0.04509626 0 0.07140004 0
0 0 0.278825179 0.06262703 0 0 0.06314485 0.1499258 0 0 0
0.07433688 0.2309576 0 0 0.2986914 0.0732811 0 0.2494824
0.080960886 0 0 0.04877731 0 0.04370996 0.1688361 0.2327313 0
0 0 0.05678676 0.4248361 0 0 0 0.08474942 0 0.3816081 0
0 0 0.1499883 0.05569677 0.06794403 0 0 0.06327605 0.1370115
0 0.09415208 0.128058292 0.07604181 0 0 0.09192554 0.05813017
0.1322332 0 0 0.07562599 0.1883382 0 0 0 0.103524723 0 0
0.122041497 0 0 0.075470883 0 0.08668907 0 0.076945778 0
0.04970513 0 0 0.1046828 0.1084925 0.3570081 0.2271746
0.08928774 0.06436045 0 0.0473622 0 0.04817557 0 0.064111776
0.1071235 0.061416329 0.08779241 0.332718 0.0447051 0.2405113 0
0.05425406 0.08017725 0.1869051 0.06945725 0.05646564 0.1061064
0.1548178 0.0714772 0 0.0523571 0 0.07132298 0.1352312
0.08535303 0 0 0.06476252 0.1094017 0 0.05323375 0 0 0 0
0 0 0 0 0 0 0 0 0.05145113 0 0.276071 0 0 0.1209368
0.0987453 0 0.1897437 0 0 0 0 0.1223513 0.06699035
0.06862752 0.04725914 0 0.114705517 0.1216421 0 0.08588841 0 0
0.1273555 0.3567975 0 0.05453321 0 0.08783745 0 0.2681776
0.1022872 0 0.1129635 0 0.126179784 0 0.09079462 0 0.07910329
0.09169406 0 0.115951431 0 0.06534394 0 0.08706765 0 0.09614406
0.1848493 0 0.081010781 0.063994617 0.06453397 0.16966 0 0.06703029
0 0 0.06937636 0 0.05385706 0 0.08127248 0 0.052780746

0.06725147 6.4145610 0 0.05938577 0.176372 0.1851019 0 0 0 0
0.05728857 0.08808135 0.1178671 0.1582975 0 0 0 0 0.1073193
0 0.1073953 0.1548636 0 0.06989848 0.05317979 0 0 0 0 0
0 0.09476847 0 0.04702505 0.1793412 0 0 0 0 0.06105932 0
0.04785293 0.1330635 0 0.06402823 0.05740708 0.093890768 0.08622663
0.1217676 0.06399208 0 0.04659474 0 0.057859579 0 0 0 0 0
0.07654694 0.04320208 0 0.1083044 0 0 0.3230698 0 0.104562712
0.08124615 0 0 0 0.04659777 0.09857963 0.04512864 0.1942328
0.5698278 0.0487313
AC087301.1 0 0.0948191 0.1600849 0.098589283 0 0.07157735 0 0 0
0 0.08767704 0.078587917 0.04685066 0.066125379 0.169324494 0.07444118
0.07249565 0.3546017 0 0.3576051 0.391682933 0.3198966 0.09678194
0.100422 0.2505263 0.5969326 0.6352198 0.9589658 0.154902 0.191976985
0.1164526 0.340230311 0.1082503 0.1821819 0.1555459 0.401637989
0.1255663 0.2772306 1.071032 0.3182215 0.07638641 0.1211448
0.440321 0.5310654 0.0897829 0.3108048 0.2895258 0.1464473 0
0.1129326 0.06454325 0.1172794 0.08232214 0.183631192 0.1455492
0.6111882 0.6203686 0.04756979 0.1981204 0.2244165 0.08897919
0.2021076 0.2256357 0.2394156 0.2197799 0.168489204 0 1.147134
0.06766189 0.138663426 0.4254929 0 0.3401416 0.154914406 0.3423141
0 0.1405097 0.7102211 0.1298022 0.08786703 0.3522211 0.4567682
0 0.07834486 0 0.2188523 0.1352669 0.7531956 0 0.2638433 0
0.200643059 0.1571072 0 0.6558842 1.014281255 0.6413582 0
0.4811287 0.06832096 0 0.2873262 0.0841151 0.06713648 0.2624414
0 0.152384375 2.58942 0.061931964 0 0.496836 0.1093064 0.1894214
0.2308904 0.3575042 0 0.1504336 0.1280634 0.1162891 0.07882454
0 0.07009892 0.4994275 0.8773609 0.2924711 0 0 0.2260569
0.191927909 0.04528918 0.3760775 0.362112 0.2543249 0.11881539
0.3042559 0.1607796 0 0.05816194 0.06938951 0 0.04829593
0.08386573 0.3978042 0.1333909 0.06964541 0.2325301 2.636733 0 0
0.09710747 0.1893762 0 0.1499178 0.164712115 0 0.07261244
0.175633805 0.06574863 0.7979773 0.2108587 0.265169 0.3147974 0
0.252432872 0.07950978 0 0.2424695 0.4723543 0.136496 0.4390112
0.2820904 0.06477559 0 0.254988959 0.2768661 0.1591769 0.1024172
0.1876229 0.6424411 0.2954194 0.4275804 0.5035295 0.2933472
0.2770235 0.238469 0.267607 0.0673394 0 0.1106064 0.2669211
0.07452576 1.121761 0.128177919 1.119369 0.2356169 0.3149286 0.5262564
0.213992 0.4844426 0.116279342 0.13286 0.07192038 0.1845683 0.2965351
0.134441259 0.1995801 0.2601849 0.07249893 0.2412687 0.2441105
0.6015716 0.1320564 0.07053491 0.3969775 0.09886287 0.2319476
0.3979203 0.07026965 0 0.047830847 0.7095302 0.064062281 0 0
0.158465343 0.06475832 0.18202 0.6944803 0.242343243 0.186815 0.1565479
0.5858299 0.174678243 0.1099006 0 0.6246715 0.3577469 0.2812146
0.4729792 0.2609616 0.5469522 0 0 0.04554837 0 0 0.193432737

0.6451785 0.3929648 0.04693339 0.1683329 0.08053816 0.05695831
0.08417363 0.1308142 0.2187579 0.118560253 0.3341857 0.1625345
0.2626397 0.551986 0 0.1162357 0.4492681 0.2129575 0.1792148
0.8571847 0.1485418 0 0.1148547 0.06622573 0.335322874 0.1792713
0.1315893 0 0.04320508 1.018013 0 0.06544978 0.1507821 0.5933035
0.2991452 0.195654871 0.162302865 0.162047 0.3588178 0.07245788
0.3325502 0.07390902 0.0634824 0.1036672 0 0.2656017 0 0
0.1396249 0 0.5137991 0.3868119 0.07204821 0.2480737 0.1231264
0 0.7662316 0.47507 0.2705084 0.07538679 0 0.2674068 0.2809363
0.2006211 0.9160219 0.2465453 1.198803 0.049878453 0.07038618
0.3221568 0.07567718 0.1185941 0 0.397407358 0.04153884 0
0.2052868 0 0.09626448 0.1935289 0.243461884 0.1147362 0.2744038
0.1839901 0.09140747 0.200311388 0.3028089 0.09703149 0 0.170097403
0.537475041 0.03387531 0.04452914 0.06749514 0.1407427 0.1445603 0
0.2185031 0.3662451 0.113083 0 0.2559703 0.2903966 0.221646249 0
2.993018 0.2423980 0.2948361 0.3740749 0.9875368 0.1295521 0.1013854
0.5023828 0.4184491 0.2821754 0 0.6935377 0 0.1661877
0.0396277 0.469833 0 0.9664414 0.1502248 0.2431128 0.05637416 0
0 0.146765 0.223322 0.5066346 0 0.1123989 0.2088369 0.5758009 0
0.09949213 0.1152819 0.04936898 0.09414014 0.9961272 0.6672333
0.05693085 0 0.1923083 0 0.1004762 0.6984795 0.7746633
0.201659 0.1808055 0.098570682 0.2263113 0.1917555 0.3359086 0 0
0.057536783 0.242974182 0 0.160933328 0 0.1482976 0 0.7634424
0.1814218 0.06760176 0.0568514 0.08978802 0.1251878 0.05652882
0.418229 0.10977456 0.1705916 0.3554955 0.07518499 0 0.3424428
0.05174663 0.1895122 1.155514 0 0.5627631
LINC00092 0.2311507 0.06599508 0.3639746 0.082343001 0.176295943
0.7871335 0.5563897 0.6044439 0.1134785 0.1952452 0.2318917
0.503221675 0.7565182 0.902069458 0.047140644 0.2279721 0.8779645
0.5429743 0.377886 0.03111211 0.149938469 0.05566284 0.03368062
0.01397895 0.03487378 0.04985656 0.2040552 0.03337251 0.04312536
0.204880902 0.3323141 0.115019007 0.04520601 0.06762694 0.01082616
0.090851872 0.03495818 0.06431846 0.1159588 0.04429709 0 0.1264771
0.1072638 0.1386101 0.08748577 0.008652935 0.0403026 0.5708015
0.0131387 0.06288183 0.01796911 0.03265108 0.06875648 0.059644303
0.06078225 0.0510472 0.03838071 0.01324363 0.01378939 0.124957
0.03715827 0.04688967 0.06281792 0.08887239 0.01019794 0.105543216
0.1613793 0.02177502 0.1130241 0.048255594 0.01974317 0 0.04734842
0.291119504 0.452683 0.03316377 0 0 0.04517184 0 0.03922393
0.1589577 0 0.07634037 0.012470545 0.01218588 0.06904125 0.08065108
0.1784479 1.499708 0 0.034912414 0 0 0.1095605 0 0.08927839
0.06457652 0.01339482 0.01902084 0.02640605 0.03199713 0.105381
0.01869108 0 0.05040584 0.155556296 0.4876713 0.060347408 0.0846669
0.02305354 0.0380392 0.01318393 1.84486 0.037324 0.0403967 0

0.008913347	0.0566569	0.07680778	0.01509938	0.01951583	0.00993162	
0.07327828	0.02326433	0.157811	0.03605579	0.1992947	0.12022544	
0.01891304	0.2617538	0.1584212	0.01770127	0.529259188	0.0762355	
0.1119043	0.01639733	0.04857758	0.03863666	0.01459369	0.08739761	
0.1400914	0.01845839	0.009284144	0.02908436	0.01294747	0.3229941	
0.05624026	0.00955916	0.1216581	0	0.04848272	0.2504265	0
0.08742343	0.1415092	0.065196243	0.05491407	1.110801	0.04891995	
0.0461401	0.2738778	0.03917744	0	0.01106793	0.1955453	0.07875526
0.01315054	0.0855024	0.1658735	0.02855823	2.046836	0.08021088	
0.094653317	0.1541613	0	0.04989839	0.06964666	0.07665355	0.04934759
0.05101721	0.09345648	0.1715051	0.03856225	0.04149423	0.09105912	
0.04686892	0.01026413	0.02309495	0	0.04149656	0	0.02676395
0.04155161	0.9101544	0.03287904	0.02441866	0.3574575	0.04495695	
0.040465816	0.5363372	0.05005733	0.01284615	0.01375943	0.224574038	
0.1500226	0.01207276	0.20184	0.006717022	0.05097103	0.00644154	
0.07353012	0.009818607	0	0.01376192	0.01076253	0.1177066	0.009781681
0.015129155	0.03994892	0.1128778	0.071340823	0.086190409	0.164287	
0.033088058	0.01802899	0.2153693	0.04833655	0.044979579	0	0.05084753
0.03706761	0.060788937	0.07649196	0.04756546	0.4695601	0.07746528	
0.1957283	0.1034624	0.0363264	0.01384307	0.05802782	0	0.1394894
0.084323945	0.04696526	0.053852479	0.7441416	0.03646768	0.006533226	
0.07029688	0.03363324	0	0.02343429	0.5644979	0.04060207	0.346580516
0.02325967	0.01131257	0.01566857	0.00768376	0.05356045	0.01618026	
0.1563476	0.009881376	0.1184986	0.07593211	0.05169333	0.06625099	
0.1438922	0.1106251	0.031118428	0.04990989	0.05495251	0.2474475	
0.03007119	0.1308088	0	0	0.03498198	0.03097091	0.1249249
0.063549652	0.429265035	0.1428628	0	0.1210355	0.1295979	0.03086487
0.04418441	1.115973	0.1868673	0.009243078	2.607575	0.1366807	0
0.03322607	0.339729	0.03426501	0.04011704	0.06215827	0.05998809	
0.117341853	0.1155495	0.1417087	0.08786243	0.05246999	0.498028	
0.1582002	0.1042851	0.2653054	0.00796951	0.02287975	0.01925491	
0.041659107	0.3821182	0	0.08427537	0.08254274	0.05570551	0.030733295
0.01156459	0.0464407	0	0.1849631	0.05360086	0.08081888	0.093198603
0.1197864	0.03819759	0.221969	0.1399652	0.034854703	0.02810108	
0.1350699	0.02334735	6.973145263	0	0.01886205	0.01859565	0.09395463
0.03918336	0	0.05169269	0.0101387	0.01699403	0.03148279	0.04310901
0.0178158	0.01616953	0.061707239	0.117938	0.03471952	0.02249487	
0.0273612	0.1215013	0.03436679	0	0.1340741	0.02797309	0.06989878
0.0392794	0.05023308	0.03218063	0.06890061	0.06940103	0.02758129	
0.05813488	0.01208403	0.08648399	0.0575069	0.07896422	0.0706266	
0.3281618	0.01890727	0.07150499	0.09326066	0.0587705	0.0442764	
0.06258464	0.07267632	0.01603054	0	0.02769902	0	0.01374453
0.4062396	0.02521145	0.009288027	0.03169957	0.07312112	0.02676972	
0.1534915	0.04895276	0.07778384	0.01078347	0.09357117	0	0.178376173

0.2961283	0	0.009351834	0.137623	0.251947	0.01601848	0.186023815
0.2444807	0.033603381	0.03539033	0	0	0.1398326	0.03156787
0.03764122	0.237415	0.08749075	0.0566358	0.0786893	0.05821834	0
0.1187335	0.02969143	0.1883863	0.1682189	0.1293866	0.09364209
0.5408001	0.06623232	0.114503	0.07833779
AC087286.4	0.01984784	0.04080018	0.04592249	0.084844933	0.12575947
0.1539968	0.06490134	0.06228095	0.07015588	0.1379503	0	0
0.0604788	0	0.036429734	0	0.03119451	0	0.02203968	0.05770337	0
0.06882494	0.04164478	0.1512387	0	0.05137141	0	0.1237914	0
0.055071146	0.02505448	0.041828391	0	0.02613065	0.0334653	0.064808543
0	0.07952728	0.1024132	0	0	0.07819202	0	0	0.07726625	0	0
0.03150776	0	0.145783	0	0.06728631	0.07084559	0.026338522	0.03131454
0.07889735	0	0.04093808	0.02131255	0	0.03828729	0	0.0323633	0
0	0	0	0.02243663	0	0	0.03051457	0	0	0.333294398	0.07364802
0	0.0302303	0	0	0	0	0.04913626	0	0.05056709	0	0	0
0.06128997	0	0	0	0.02253414	0	0	0.04849334	0.07884947	0	0
0	0	0.148362	0	0	0.02258544	0	0.02185674	7.602874	0.026649009	0
0.106893	0.02351699	0	0.03974038	0	0	0	0.02755252	0.05003861	0
0.0466745	0.09048963	0.06140031	0.03775239	0	0.09146584	0	0	0
0	0.04623549	0.02225928	0	0	0.02618395	0	0.05068664	0	0	0
0	0	0.01901924	0	0	0	9.98424	0.02897454	0	0.04178485
0.0407438	0.0299735	0.03225444	0.035437392	0.09007973	0.03124476
0.050382888	0.0282913	0	0.06048762	0.02852522	0	0.1211035
0.05431029	0	0.06716227	0	0.04065033	0.05873355	0.08095898
0.08827786	0	0.1352422	0	0.03971136	0.03424651	0.02203479
0.02691106	0.07898261	0.07627048	0.05256733	0.03611103	0	0
0.025653	0.02558888	0	0.03172799	0	0.07656986	0	0	0	0.06422118
0	0	0.05032121	0.03069322	0.04632293	0.025017204	0	0.1237879	0
0.1275975	0.057849389	0.103054	0	0.03119592	0.04152668	0	0.03982357
0.0284116	0.04552622	0	0	0.09980589	0.04280576	0	0.04676653	0
0	0.027565673	0.02960308	0.02821313	0	0	0	0.02134509	0
0.08038554	0	0.02291633	0	0.09457933	0.0490107	0.08063789
0.03420815	0	0.02907436	0	0.06418657	0.04484321	0.04352591
0.03919846	0.028962022	0.04839227	0	0	0.01878786	0	0	0.03465516
0.2205798	0	0	0	0.025507937	0	0	0.04843395	0.02375168	0	0
0	0.06108969	0.096394	0.03353111	0	0.117024	0	0	0.024047954
0.03856977	0.0566222	0	0.01859093	0	0	0	0	0.03191193	0	0
0	0.02324267	0.05146582	0.06235652	0	0	0.1912131	0.02973831
0.05776352	1.600019	0	0	0.02002663	0.02054137	0.1658138	0.0302624
0	0	0.02649034	0	0.05495093	0	0.01939973	0	0	0	1.047674	0
0.02463497	0.07072479	0	0	0	0.04620747	0	0	0	0.019000262
0.05362186	0.0205079	0.1766677	0	0.02071106	0.04163725	0	0	0
0.02639	0.03933217	0	0	0	0	0.109788069	0.028909097	0.01457637
0.05748199	0.05808563	0.03028044	0.06220354	0.05326334	0	0.02626557

0 0 0 0 0 0 11.537260 0.02114441 0 0 0.02787281 0
0.04323455 0.0360113 0 0 0.01989505 0 0.03575487 0.0170516 0
0 0.1188157 0.01616024 0.03487007 0.02425751 0 0 0 0 0 0
0 0.02246536 0 0.06976939 0.06421644 0 0.04248644 0.08101605
0.03896622 0.05742142 0 0 0 0.0263592 0.02161721 0.06011044 0
0.05784856 0 0 0 0 0.02890795 0 0.1473418 0.024757785
0.026137638 0 0 0.03646564 0 0 0 0.05854867 0 0.02446287 0
0 0 0.1542530.047235434 0.03670235 0.03059358 0 0 0.0210502
0.04453262 0 0.02924775 0 0

AC092119.2 0.1886809 0.1633102 0.1263716 0.042450919 0.125843815
0.04623004 0.1948346 0.0779034 0.07020293 0.008627678 0.3586467
0.084596651 0.1613850.39861496 0.127589583 0.1762923 0.3589775
0.8779425 0.02205447 1.7418860.063244578 0.7231465 1.08349 0.4432105
0.2831653 0.3983955 0.7679464 3.0246010.3501658 0.399533591
0.4763544 1.935860596 0.7224675 0.8367417 0.2009265 0.497198738
0.5406677 0.6764352 1.3066440.5823389 0.03289073 0.07824446
0.8531773 0.9003803 0.1739657 0.8029645 0.1994641 0.4256401
0.4673712 0.1215673 0.3890776 0.2861586 0.4785285 0.408520902
0.4700331 0.3684346 0.7420013 0.3072415 0.4478638 0.1932601
1.2068590.3045847 0.1133475 0.3092652 0.3154453 0.108823007
0.2329519 0.7184538 0.8448887 0.507502558 0.5343632 0.5264156
0.5614276 0.650359975 0.4421845 0.7864701 0.4991345 0.6455978
1.4671311.2485250.2729891 0.6637844 0.1104790.05903451 0 0.05654051
0.3397548 0.3243136 0.3679865 0.7100410.2841151 0.496763148
0.7779493 0.2410719 1.2002551.358724184 0.2761583 0.1165207
0.2071661 1.1178790.1735697 0.4082692 0.4708415 0.6359730.2599068
0.2858472 0.196842594 2.4922670.453336991 0.2182449 1.1231290.1764958
0.2242948 1.0438850.1924193 0.01561951 0.08096776 0.2619245
0.1376985 2.1552250.1634703 0.1509172 0.3532886 0.8311097
1.3942610.1220362 0.3903505 0.5191273 0.475185697 0.7410294
0.4395317 0.1336452 0.04106553 0.844139121 0.4585265 0.1211508
0.4057651 0.3756536 0.2240849 0.1805663 1.1021580.0902780.2283839
0.6030771 0.05997633 0.1201484 0.8401470.4639036 1.1236080.3554095
0.4077113 0.3599232 1.3233190.070922319 0.2478854 0.9848701
0.176458376 0.3255681 0.4867608 0.5598857 0.4709818 1.236862
0.6362196 0.298906932 0.2225314 0.08400915 0.8874285 0.2745738
0.4554903 0.6346040.6790912 0.5020434 0.06766647 0.274485097
0.4172489 0.1884821 0.5402145 0.06732277 0.7113202 0.4324892
1.42027 0.9033813 0.5431351 0.3727555 0.3850531 0.7809842
0.2899524 0.2539941 0.1309695 0.6321250.6899256 0.2587564
0.400136905 0.8515014 2.1812340.1864542 1.0826320.5682053 0.139062
0.16272089 0.7007896 0.9445157 0.1291421 0.3617688 0.405217317
0.3695244 0.4294533 0.5462948 0.5402090.9459895 0.3088397
0.08529198 0.1290775 0.9059395 0.06385308 0.6991098 0.2891327

0.635396 0.257388429 0.09267828 3.076942 0.606851546 0.325852285 0.3246686
0.204697687 0.6552708 0.1959371 1.4737999 0.608702513 2.071316 0.2583933
1.513492 0.300854277 0.5205352 1.37322 1.291071 0.9327972 0.5045266
0.6109711 0.8989261 0.7172307 0.02243664 0.2395531 0.6079837
1.405600162 0.5084596 0.388681745 0.912782 0.5922146 0.06062623
1.141582 0.3121056 1.15269 0.2355845 0.7322436 0.3610752 0.293538012
0.4916408 0.8923048 0.4119647 1.853873 0.343183 0.675664 0.2901712
0.1069787 0.05787519 1.224706 0.7355362 0.4830476 0.02472727
0.1853521 1.335556599 0.7333172 0.3682911 1.148117 0.2511459
1.803936 0.2530163 0.4227239 0.1623107 0.2554667 1.014355 0.210614399
1.397698713 0.3953904 0.2575017 0.2963921 1.80394 0.5091841
0.1093379 0.2975825 0.4624181 0.1429547 0.2002154 0.5989445
0.3507011 0.33916 0.7466627 0.09841875 0.07755692 0.8438498
0.1457946 0.2074085 2.804377 0.2776135 0.184421 0.2921428 0.1575525
0.6476679 0.5846717 0.02159604 0.6039617 0.6546431 0.9231769
0.279198908 0.2879177 0.4623847 0.4887802 0.4085173 0.1723097
0.323221097 0.1699164 0.08208661 1.425339 0.4112204 0.0414499
0.5416473 0.131038338 0.2840703 1.211076 0.963881 0.432944 0.269533684
0.782307 0.1253405 0.1805466 1.025375896 0.188035157 0.1750338
0.2684292 0.1889049 0.5302631 0.9025563 0.6928878 0.9094771
0.3153982 0.3286689 0.2333554 0.8266245 0.06252 0.548764004
0.2584062 1.369291 1.809124 0.08463437 0.4295212 0.6776896 0.1952405
0.2401019 1.34117 0.2522482 1.312201 0.4531983 0.8560609 0.5594535
0.1610048 0.1279727 1.314965 0.2055824 2.704869 0.04851323 1.308504
1.104457 0.7175556 0.6871924 0.1737852 0.156258 0.5817294 0.222555
0.2661841 1.303865 0.8429633 0.9948806 0.128519 0.07445772 0.1275448
0.5472251 0.9553127 1.422133 0.906999 0.01884835 0.3174184 0.2637688
0.2812122 1.413543 5.653795 2.387853 0.09082716 0.084885819 0.214381
0.8944732 0.173564 0.6811183 0.200098 0.123871948 0.170008595 0.2722442
0.398447605 0.784537 0.6545085 0 1.211095 0.1367052 0.0727705
0.6487008 0.4446042 0.5053481 0.6693604 0.373028 0.307236244
0.4407236 1.821539 0.7284018 0.2168079 0.4739472 0.6572967
0.102001 0.8780209 0.3058859 1.365784
LINC01697 0.9973282 0.02252919 0.05494157 0.023424989 0.65970197
0.07936556 0.1015394 0.06304928 0.01936946 0.06982608 1.090219
0.00622421 0.06679079 0.382313349 0.0268212 0.1061241 0.2756015
0.0631905 0.336702 0.007080634 0.00775538 0.551058 0.10348 0.2863253
0.01984183 0 0 0.09114079 0.06134165 0.010136461 0.1429583
0.019247459 0 0.2068145 0.006159664 0.007952492 0.009944924 0
0.3675808 0.113415 0 0.004797372 0.08718421 0.005257587 0.02844344 0
0.004586124 0 0 0.107332 0 0.0030962 0.01303992 0.029087398 0
0.009681293 0.01091857 0.003767556 0.003922814 0.04739712 0.07751925
0.005335681 0.01191365 0 0 0 0.1224248 0.008259427 0.005358862
0.005491113 0.01684965 0 0.01346971 0 0.003388935 0 0 0

0.01542062 0.006959121 0.005579224 0.4341158 0.1161215 0.006204959
0.007095257 0 0.003571072 0.0137662 0 0.0243793 0 0.007945525 0
0 0.1662285 0.017851484 0.02902626 0.00612359 0.007621136 0.04869955
0.03004804 0.01365385 0.07328162 0.2818142 0.02078552 0.04301845 0
1.809561 0.039240386 0 0.1180491 0.008657132 0 2.05542 0 0
0.005957214 0 0.07828641 0 0.2749109 0.005551876 0.1751719
0.03126938 0.003309126 0.03928239 0 0.06564742 0.045602412 0.02510851
0.05106096 0 0 0.028230748 0 0.03820152 0.0653061 0.00921292 0
0.08303245 0.9027156 0.07306437 0.04900984 0.06338782 0.02757979
0.003683304 2.898572 0.03733162 0.005438792 0.01538194 0.003749677
0.03310174 0.005936791 0 0 0.09201517 0.03245737 0.00520733 0
0.01113342 0.01050077 0 0 0.074973124 0 0.006180984 0.08321614
0.05237504 0.3135065 0.06457276 0.4671452 0 0.05808331 0
0.1169491 0 0.00405575 0.009906563 0.02180645 0 0 0 0.05575986
0.3510468 0.02833033 0.08242359 0.01066664 0.005839891 0.004380045 0
0.06492733 0.03807607 0.101517671 0 0.004665249 0.006235634 0.09262182
0.03954601 0.9933085 0 0 0.005696137 0.01096344 0.1174288
0.021295655 0.003161373 0 0.01148392 0.07261278 0.004833424 0.01099496
1.140025 0 0.0440172 0.9787497 0.2388151 0.3781862 0.06678477
0.017215806 0.003788231 0.008027887 0 0 0 0 0.005128897 0.02162414
0.1178641 0.076774914 0.03698964 0.008265792 0.004218007 0 0.008704192
0.05412581 0.03957948 0.01574098 0 1.006075 1.746473 0.003938085
0.2063474 0.004005716 0.03246712 0.490432149 0.1425142 0.0408533
0.02189936 0.02766493 0.1635547 0.006666033 0.07654404 0.02706679 0
0.02072112 0.005775252 0 0 0.09010982 2.09498 0.03497409 0
0.05063266 0.1067469 0 0.01774239 0 0.04117611 0.08615828
0.009096562 0.1573535 0.00885259 0.1455336 0 0 0.03421868
0.006202094 0.01745222 0.005183662 0 0.0117475 0 0 0.192817245
0.2053475 0 0.01147741 4.118693 0 0.01005569 0 0 0.02629477 0
0.006480503 0.003686125 0.007561739 0.02034659 0.7742484 0.005706261
1.94904 0 0.009537567 0.0151715 0 0 0.02985339 0 0.1164833
0.5710916 0.02383397 0.0317404 0.0585796 0.1935436 0.031603224
0.01114925 0.008505003 0 0 0.03169428 0.003497211 0.0197394
0.1434389 0.1991707 0.006577303 0.01143631 0.06897422 0 0
0.1575639 0.04371637 0.06515573 0.051560537 0.03197686 0.007684951
0.01328374 0.006735908 0.292657348 0.008048833 0.007053469 0.03207393
0.03901418 0.04579703 0 0.005768525 0.004834471 0.004478122 0.07358208
1.040681 0.1149979 0 0 0.1284014 0.03199677 0.4125377 0.5036582
0.04888343 0.728503 0.008029785 0.007957803 0 0.0536363 0 0
0.009800444 0.006581083 0 0 0 0 0.220111 0 0 0.06438323
0.02689376 0 0.004421809 0.3343814 0.03148944 0 0.07029501
0.009120755 0.1027347 0 0.01369559 0.007820104 0.007455955 0.02151651
0 0.004508957 0.006933848 0.04061582 3.920179 0.4177834 0.05531999
0.03681228 0.005323837 0 0.515252521 0.01075439 0 0.05320831

0.06264171 0.007748546 0 0.024054624 0 0 0 0.03523577 1.180203
2.4376980.0179609 0.0107082 0.04052398 0.007111264 0.04213852 0
0.06624796 0.00869421 0.1688870 0 0.05583084 0.2247224 0.01229509
0.003752369 0.01076675 0 0.06483079
RORA-AS1 0.06545108 0.05979751 0.2523933 0.015543792 0.053758673
0.09028027 0.08323055 0.02282003 0.07711633 0.05686385 0.02764669
0.037171004 0.1255717 0.052127325 0.053392105 0.07041931 0.04571926
0.1537449 0.1049808 0.07752355 0.077191934 0.4665290.1220707
0.1345783 0.3159880.1317591 0.2234117 0.09827515 0.1343220.100891637
0.1468813 0.091956719 0.1877367 0.1819135 0.2207132 0.047492311
0.1088837 0.1165567 0.1876234 0.05017146 0.01204324 0.1814495
0.1214884 0.1255933 0.07077679 0.5390235 0.1460712 0.1154459
0.1264891 0.05341559 0.07123216 0.04930804 0.1168117 0.028951676
0.09179036 0.1349057 0.1086762 0.09749938 0.1717984 0.2358798
0.1402864 0.1805667 0.2490194 0.3019741 0.3234089 0.199232691
0.03655606 0.02466266 0.1280126 0.087447858 0.07826478 0.1542015
0.1251307 0.195393023 0.08095494 0.0500823 0.02215305 0.2488332
0.06139466 0.1662396 0.02221277 0.07201503 0 0.06176007 0.028248601
0.04830659 0.1066323 0.03653858 0 0.3258516 0.1809241 0.047450704
0.02476985 0.05230854 0.05170403 0.071072753 0.2166811 0.07314021
0.1668827 0.0430865 0.03738479 0.05436054 0.2121882 0.03175463
0.09102954 0.2664214 0.104109365 1.9452070.048821612 0.06392988
0.1174983 0.1292511 0.08959372 0.1383301 0.1831858 0.04575382
0.01185883 0.09085835 0.04583595 0.2485528 0 0.09946748 0.1912276
0.1106612 0.1119853 0.02234234 0.04083725 0.2494842 0.075649384
0.03570193 0.1270568 0.2691448 0.02004869 0.149861455 0.1151271
0.02534885 0.3900083 0.1742287 0.1641014 0.2148771 0.3274208
0.06611223 0.1184685 0.1472148 0.1317653 0.1613092 1.962164
0.01061641 0.05413414 0.03062033 0.05225053 0.06589458 0.3072727
0.168797558 0.0825141 0.1373787 0.092302638 0.1658570.1887162
0.05540737 0.08361421 0.1116711 0.2662373 0.019899546 0.1629638
0.03691286 0.3185687 0.1563920.07532088 0.1779825 0.1940725
0.05106328 0.1156246 0.04020209 0.07275214 0.08783657 0.06458921
0.0493017 0.1808725 0.3353504 0.2311310.2646251 0.05549969
0.01091903 0.02819814 0.0984468 0.1061687 0.0697517 0.02615765
0.3366667 0.1527485 0.06316401 0.161670381 0.1176547 0.1114435
0.1737831 0.06453275 0.08996911 0.2630806 0.137496324 0.06284095
0.1474085 0.09457315 0.1558411 0.063588867 0.02517298 0.02734752
0.3200493 0.2586647 0.1635696 0.1386197 0.08328116 0.08340511
0.08762363 0.03117385 0.09751826 0.0941054 0.1218674 0.068541909
0.030164444 0.1917703 0.050500963 0.162700624 0.1653988 0.12491988
0.02041986 0.05739532 0.08603038 0.216513929 0.02945364 0.0329089
0.2099163 0.137700678 0.1212901 0.1257043 0.3545532 0.3697538
0.2216844 0.09587684 0.02057188 0.1881461 0.03286153 0.0956886

0.03590625 0.07428275 0.1773116 0.132153702 0.1089859 0.08260754
0.07399616 0.07961915 0.06348907 0.04490083 0.01327099 0.07218554
0.1264627 0.214963249 0.08781403 0.05125107 0.2543654 0.4351363
0.1559912 0.0549779 0.1652759 0.246219 0.04238306 0.1474315
0.08196796 0.01071953 0.03621647 0 0.079301554 0.05652859 0.08298656
0.121447 0.07492984 0.1234631 0.1852884 0.3302064 0.05546946
0.3975512 0.2004464 0.061694709 0.140739542 0.2129056 0.07542928
0.1370863 0.07339214 0.1165265 0.1100964 0.03268877 0.2751428
0.492035 0.04189183 0.1161048 0.1834463 0.08279102 0.3037749
0.08316213 0 0.03128945 0.09706184 0.047465323 0.1006714 0.08560061
0.08529782 0.1426277 0.1153786 0.1580997 0.8858601 0.06326069
0.1173428 0.05182779 0.1090418 0.070775467 0.06658339 0.03386127
0.05965711 0.1308846 0.09463918 0.013923571 0.0851383 0.06762772
0.02427446 0.1702119 0.1973044 0.1067926 0.038384708 0.07235821
0.1838681 0.02900826 0.1441149 0.071058399 0 0.03059637 0.01322176
0.080453733 0.14829401 0.1014762 0.0982878 0.02128285 0.110949
0.09116669 0.07806375 0.2411476 0.06736679 0.08023009 0.09765165
0.03363068 0.03662763 0.131044535 0.07792054 2.339765 0.1019119
0.2711594 0.06880699 0.05838641 0.04085092 0.04795391 0.01584134
0.3298678 0.1156697 0 0.1385032 0.1950943 0.05240301 0.03748671
0.1563802 0.04105957 0.3047423 0.07697543 0.06388278 0.2844181
0.01281656 0.06424386 0.1041268 0.14964 0.03993854 0.03761101
0.1594894 0.1070084 0.2541895 0.03834575 0.109803 0.06361454
0.1245379 0.02968466 0.04283222 0.0525987 0.10771 0.09662092
0.2324513 0.09658136 0.1980162 0.1211361 0.08549452 0.1165779
0.1045226 0.186490308 0.1712674 0.1007752 0.3283523 0.04676207
0.1156859 0.090713691 0.038307815 0.03322824 0.101492334 0.04008354
0.3507136 0 0.126701 0.07865919 0.1278988 0.06274317 0.08493699
0.0592121 0.1693366 0.09419836 0.0605755 0.0941354 0.04483853
0.05926911 0.06350896 0.06941603 0.1060603 0.01493944 0.01071651
0.08252848 0.1613207
