# Supplementary material for: Ferroptosis-Related Long Noncoding RNAs Have Excellent Predictive Ability for Multiomic Characteristics of Bladder Cancer
Source: Oxid Med Cell Longev. 2022 Aug 29;2022:9316847. doi: 10.1155/2022/9316847 (PMC9444476; doi:10.1155/2022/9316847)
Supplement: Supplementary Materials — Supplementary Table 1: expression matrix of 60 ferroptosis-related genes. Supplementary Table 2: results for coexpression analysis of ferroptosis-related lncRNAs and mRNAs. Supplementary Table 3: expression of 263 ferroptosis-related lncRNAs. Supplementary Table 4: expression of lncRNAs and clinical information of 403 bladder cancer samples. Supplementary Table 5: cluster analysis based on 20 prognostic lncRNAs. Supplementary Table 6: detailed clinical information of bladder cancer samples. Supplementary Table 7: data of the high- and low-risk groups of the training cohort. Supplementary Table 8: data of the high- and low-risk groups of the validation cohort. Supplementary Table 9: markers of different immune cells. Supplementary Table 10: infiltration of 22 types of immune cells analysed using the CIBERSORT algorithm. Supplementary Table 11: immune scores evaluated using the ESTIMATE algorithm. Supplementary Table 12: immunotherapeutic response evaluated using the Tumour Immune Dysfunction and Exclusion (TIDE) algorithm. Supplementary Figure 1: gene set enrichment analysis. (A–F) Most significantly enriched biological pathways in cluster 1. (G–I) Most significantly enriched biological pathways in cluster 2. [file 9316847.f1.zip › s-table 4.pdf]

id futime fustat AC092802.3 AC009095.1 TXNDC12-AS1 AL450998.3
AC079313.2 ZFAS1 AC027449.1 AL035530.2 AC004846.2 AC018752.1
MIRLET7A1HG MIR100HG AC073487.1 AP001160.1 SSBP3-AS1 AL139220.2
AL137779.1 AC069277.1 AP001189.3 AC011405.1 AL356124.1 FO680682.1
THUMPD3-AS1 GAS5 AC073529.1 OSMR-AS1 AL162724.2 AL162727.2
AC053503.3 AC009065.5 BNC2-AS1 AC078778.1 AC087286.2 AL031670.1
AC022893.1 AC022154.1 AC011472.4 AP001107.1 MIR497HG MAP3K20-AS1
ENTPD1-AS1 ARAP1-AS2 AL589765.6 RBMS3-AS3 AL390719.2 AP001619.1
AC092171.1 AC129507.1 AC005387.1 AP005899.1 EPB41L4A-AS1 AL157871.5
AC108449.2 SH3RF3-AS1 AP003171.1 AC005785.1 LSAMP-AS1 CARMN
AC020765.2 GRK5-IT1 AC024270.4 AC010422.2 AC002398.2 HAND2-AS1
AC109460.3 AC008750.5 AC012557.1 AL136084.3 AC058791.1 AC005070.3
AC036108.3 AL731566.2 AC024075.1 AL731567.1 AC006160.1 GATA6-AS1
MIR1-1HG-AS1 AP001001.1 AC090825.1 AC025031.2 CRTC3-AS1 DCUN1D2-
AS AC006059.1 AP003900.1 AC004837.2 AC087854.1 AC040904.1 AC008434.1
AP001469.2 AC011510.1 AC012568.1 MKNK1-AS1 C1QTNF7-AS1 ADAMTS9-
AS1 AL513327.1 AF001548.1 LINC02489 MBNL1-AS1 AC004918.5 HMGA2-AS1
AL445649.1 AL355102.1 AC005180.1 LINC00685 AL513320.1 AC079313.1
GNG12-AS1 AC145285.6 AC008870.4 AC139887.2 AC244093.5 AC004637.1
AC008635.2 AC139530.1 AC020978.1 AC127024.6 STARD13-AS AC015849.4
AC093249.2 DHDDS-AS1 MSC-AS1 FMR1-IT1 AC025280.1 AC024267.6
AC010976.1 UBL7-AS1 Z98885.3AC026356.1 AL121906.1 LINC-PINT
AC023825.2 AC005180.2 AC023024.1 AC104984.6 MIR762HG AC011442.1
AL021368.2 COL4A2-AS1 AC138393.3 AC007619.1 AL354993.2 AC034236.2
AC019183.1 AL031429.2 AC131097.3 LINC01081 AL513327.2 AP003419.3
OPA1-AS1 AC007365.1 LINC00630 AL161719.1 AP001432.1 AC009716.1
AC068790.3 AC037487.2 AL117336.1 LINC00641 AC015908.2 ITFG1-AS1
AC138932.6 AC127024.4 AL359220.1 ACTA2-AS1 AC138956.2 AC024075.3
AL354726.1 AC067852.3 HID1-AS1 AC008764.6 AC104170.1 AC068790.2
PRR34 AL139383.1 AC090559.1 AC022973.4 AC136604.2 AL353804.1
AC114271.1 WASHC5-AS1 PABPC4-AS1 LINC01409 H1FX-AS1 AC079921.2
AL355073.2 KLF7-IT1 AL133371.2 NCBP2-AS1 AC108463.2 SDCBP2-AS1
ANKRD44-IT1 MCM3AP-AS1 AP000766.1 AP003071.4 AC007622.2
AC004884.2 AC061992.2 AC234775.3 AC078795.1 AC007390.1 AL139021.3
LAMTOR5-AS1 AC087286.1 AC012645.4 AC110609.1 AC069549.1 AL353804.2
MAGI2-AS3 AL731577.2 AC010618.2 AC008115.3 AC087294.1 AC138207.4
PRC1-AS1 AC005740.4 FENDRR AC234772.2 AL590729.1 RASA3-IT1
LINC00702 AL133243.3 AL606534.1 WWTR1-IT1 AC093423.2 AL133445.2
AL157838.1 AC091057.1 AP003071.3 AC073130.2 AL132642.1 AC087276.3
AC068790.6 GTF3C2-AS1 AC106820.5 AC063919.1 AL596223.1 AL117329.1
AC015849.3 AC022272.1 AP001020.3 LINC02595 AL139353.2 GSN-AS1
FTX AC005519.1 AC016924.1 AC099811.5 SPAG5-AS1 TGFB2-AS1 CDC42-IT1
SBF2-AS1 AL133415.1 AC106782.5 AC093010.2 AC105339.2 SOCAR
RNF216-IT1 AC087301.1 LINC00092 AC087286.4 AC092119.2 LINC01697

RORA-AS1
TCGA-CF-A47X 384 0 0.3238117 0.2597087 0.07339593 0.3650406
0.01552912 65.22578 0 0.1985753 0.4062753 0.4179293 0.2499809
0.05846821 0.8408621 0.9565937 0.4429349 0.03408291 0.04790953
0.7784388 0.5069506 0.09646323 0.03321506 0 3.542064 62.86093
0.02795241 0 0 0.07265268 0 1.449532 0 0.1012268 0.04348153
0.815035 0.7034966 0.1834071 0.5552529 0.1901538 0.02240849
0.05647322 0.06757546 0.1258676 0.3920466 0 20.1786 0.1284018
0.0762226 0.03141179 0.7337508 0.9495599 7.543473 0.2692102
1.195742 0.09840272 0.05394325 0.9986417 2.439054 0.02705639 2.856495 0
0.4089949 1.062045 0 0.01313869 0.1646197 0.2123517 0.9628561
0.1634272 0.004486837 0.3091034 0.03752048 0.4222445 1.366562
4.161804 0.3443737 0.7398777 0.01689597 0.2991752 0 0.1373101
0.2998726 0.3077513 0.2924618 0.01547886 0.1962915 0.1689763
0.06434487 0.1306822 0.3311286 0 0.03040022 0.1232421 0.2689431
0.006194663 0.6218454 0.07826675 0.04053363 0.2462303 1.59777
0.002378403 0 0 0.1466668 3.251971 0.9227592 0.02628005 0.02898769
0.7738526 0.09154006 0.920152 0.3397373 0.08930854 0.05106372 3.03381
0.1005764 0.4061011 0.01074824 0.2044975 0.5060817 0.2441213
0.04795146 0.4183354 0 0.1197738 0.2213533 1.401326 0.1306673
0.1797578 0.0652223 0.3909004 0.04270507 0.2042549 0.2603855
0.1195742 1.794601 1.568186 0.294914 0.1062882 0.5509178 0.2786195
0.5559539 1.844859 0.02841367 0 0 0.03085786 0.2447574 0.4440667
0.2020973 0.1373101 0.3237918 0.1118096 0.1655643 0.1802626
0.337316 0 0.5139906 2.242095 0.01853864 0.3896667 0.2803516
1.397621 0.7777745 0.1217155 1.087038 0.8883937 0.1070814 0.1405135
0.2288782 1.310751 0.2116358 0.1288053 0.04074946 0.03739128
0.1160289 0.2150799 0.3649264 0.5726836 0.209138 0.415556 0.2864573
0.1976648 1.124417 0.1892163 0.3348937 0 0 1.046806 0.1393098
0.6294 0 0.2754688 0.5753426 0.07822612 0.2511477 0.1576803
0.2053294 0.6561999 0.2008755 0.4942694 0.1438487 0.08938881
0.02526216 0.5544817 0 0 0.2894592 0.1036824 0.5687854
1.091765 0.02613644 0.07557027 0.9416199 0.1914251 0.3148663
0.2660724 0 0.2253755 0 0.06978834 0.6066281 0.3101348 0 0
0.05445505 0.7684865 0.209359 0 0.1095336 0.07784216 0.2734861
0.1668477 0.2117268 0.01632412 0.2824588 0.01484625 0 2.146455
0.0923353 0.1925466 0 0.1842685 0.1265282 0.4703408 0.7872479
0.1082936 0.03504006 0.7745041 0.1288053 0.2280895 0.5267639
0.02032423 2.886596 3.281703 0.2355543 0.032281 0 0.3576051 0.03111211
0.05770337 1.741886 0.007080634 0.07752355
TCGA-ZF-AA51 1714 0 0.155167763 0.047409586 0.080390167 0.22554345 0
42.419235446 0 0.177220968 0.242722435 0 0 1.404461516 0.178256458
1.109384307 0.143746442 0.165041546 0.052475051 0.322331861 0.531118447
0.026413912 0.14552109 0.091108856 3.386200387 65.574664536 0.132669877

0.166814168 0 0.159152179 0 0.930147047 0.2937622 0.110873211
0.047625084 1.174609692 0.115137565 0.094534001 0.640174244 0.191387336
0.155444704 0.072163951 0.067742576 0.034465521 0.057254199 0 6.847010542
0.140637832 0.041743101 0.055048258 2.411019872 0.693365192 4.333255989
0.058972899 0.569430351 0.489908905 0 0.411205591 0.056840064 0.045439898
2.546619069 0 0 0.34650061 0.047625084 0.057562926 0.106410757
0.132907211 0.257222221 0.429602124 0.039315266 0.130671999 0.023483418
0.194729305 0.172706348 2.517325863 0.083820148 0.103453282 0.018506067
0.10082616 0.12173724 0.100263335 0.131379542 0.168539171 0.560580605 0
0.257996351 0.03855809 0.422859489 0 0.080596296 0 0.066594397
0.038567553 0.034655519 0.022616603 0.234863427 0.114300201 0 0.281628059
1.223321074 0.015630311 0.045488503 0.136663284 0 1.982894848 0.960158444
0.057568783 0.163286002 0.122544076 0 0.259443314 0.175111729 0
0.167789441 1.542214401 0.07344055 0.444800312 0.007848328 0.074661652
0.130425541 0 0.570227771 0.824760928 0 0.052475051 0.182548416
0.753646064 0.035779801 0.833877666 0.071437626 0.918958429 0.140323908
0.167789441 0.060680609 0 0.623455644 0.601169092 0.117460969 0.174625308
0 0.07629261 0.231974578 0.507606484 0 0.048432289 0.27524129
0.067596893 0.067020374 0.425585856 0.126489156 0.100263335 0.585939222 0
0.060447222 0 0.113680128 0 1.219770805 0.335500591 0.12183161
0.200846999 1.004948484 0.408215005 0.213782837 0.128376758 0.178594065
0.294864497 0 0.090531553 0.077135105 0.334227268 0.278164207 0.112863754
0.0334745 0 0.367136765 0 0 0.209085734 1.217969618 0.267738972
0.11409276 0.148132398 0.726073647 0.276330157 0 0.095977268 0.427653815
0.167789441 0.30517044 0.278849678 0.0294037 0.239722316 0.47072909
0.4406433 0.14670971 0.172706348 0.370417319 0.191661923 0.060004878
0.429790397 0.078778334 0.099368376 0 0.414755679 0 0 0.271751199
0.508328637 0 0.341658211 0.687050391 0 0.359553614 0.06988895
0.098534656 2.419312209 0.357188129 0.054856118 0 0.060224498 0.491787651
0.181167466 0 0.215784133 0 1.507556612 0.941196943 0.085881299 0 0
0 0 0.202915359 0 0.123750218 0 0.220966997 0.515287793 0.080907478
0.073354855 0.083820148 0.087751411 0.032996594 0.435708938 0.18395058
0.118613416 0 0.426204141 0.112863754 0.090845536 0.21339678 0
0.306958549 4.736412453 0.043605857 0.459643667 0 0.391682933 0.149938469
0 0.063244578 0.00775538 0.077191934
TCGA-BL-A0C8 1219 0 2.10138696666667 1.61770573333333
2.05787444333333 1.48387426666667 0.160236866666667 15.53759
0.101114176666667 0.40107027 0.779594366666667 2.96199246666667
4.78778796666667 0.250871266666667 2.89185166666667
0.436099666666667 1.02155093333333 0.05223884 1.22175
0.410754566666667 0.830786866666667 1.80744763333333
2.77196096666667 3.09086316666667 5.52581533333333
20.3680466666667 0.8051801 0.17595565 1.39453016666667 1.5348319
0 1.14356693333333 0.06149106 2.66573031333333 3.4094962

2.57014723333333	2.86842033333333	0.505105866666667
27.2797166666667	0.7658587	0.296343633333333	0.526981893333333
0.57551027	1.82680779666667	0.4706807	0.489728633333333
4.62606933333333	1.85836736666667	2.3408421	1.22328983333333
0.3416985	3.61638096666667	3.936633	1.76304849666667
10.1647656666667	0.304618603333333	1.48781536666667
1.05002576666667	0.151017886666667	6.0141659	0.928434466666667
3.60666812	2.26670623333333	0.269734683333333	0.0151819133333333
0.0226325226666667	0.152463023333333	1.90685203	4.42262733333333
1.874799	5.984607	3.36905053333333	0.841346933333333	1.93649103333333
4.44277533333333	15.6686446666667	1.045091	0.0658848133333333
0.00798779333333333	1.0616963	0.1972947	1.41671923333333
2.18035416666667	0.833887666666667	3.86525753333333	0.01758157
2.41787893333333	0.916472733333333	2.16982676666667	0.6130089
1.96534356666667	1.082742	4.18296566666667	0.747778833333333
0.948107933333333	0.724302173333333	2.96655363333333	0.6393345	0
1.021867	7.313141	0.105965556666667	0.592649766666667	0.904658366666667
1.05654246666667	1.44550876666667	0.581570833333333
1.24007803333333	1.10939726666667	0.6635181	1.02254226666667
5.87883833333333	1.55847390666667	1.6211746	0.766865063333333
4.14474233333333	1.47516523333333	0.842001866666667
1.96103061333333	2.84516526666667	0.366506733333333
2.61481873333333	0.482196766666667	2.6062483	0.125370133333333
2.05545653333333	3.25380246666667	3.36794366666667
2.01835126666667	0.7018651	0.745419533333333	6.41455986666667
0.408529766666667	0.871997366666667	2.18375966666667
4.18849909333333	0.772371366666667	5.26811533333333
0.951554033333333	0.998068066666667	2.2546221	2.18166356666667
5.51916433333333	0.7392174	4.71265446666667	0	0.139703833333333
0.0350497	2.81316996666667	0.851647866666667	4.07603533333333
1.97321	2.5294164	0.3949552	2.28553476666667	0.90867847	4.3397386
10.74980526	2.30045563333333	4.49699366666667	2.95965766666667
0.9620763	2.38175043333333	5.5155568	2.57423926666667	1.6295923
3.48519506666667	4.11788033333333	2.6319589	1.05731596666667
1.34368943333333	1.05773213333333	1.5568014	4.6278801	0.24330245
0.287127923333333	0.2626916	10.1175053333333	1.1018266
5.57333356666667	0.359018833333333	4.29533286666667
6.50901153333333	1.36444396666667	0.4476813	0.860719433333333
0.268902306666667	4.2896811	0.651627603333333	2.55554346666667
2.25348946666667	4.537745	1.24158129666667	2.11289876666667
2.68228696666667	0.0627626466666667	2.42950023333333
8.54673976666667	0.249944533333333	1.73991813333333
1.46002386666667	1.9639512	1.14308380333333	1.4996029
4.30130233333333	1.3090167	0.939676	0.4042712	5.15354446666667

0.592169166666667 1.22353823333333 0.944007766666667
3.55861166666667 3.34673246666667 3.84680933333333
0.544268173333333 0.697121566666667 0.5120619 0.334740966666667
2.19818323333333 0.0956269333333333 0.301225166666667 2.447589
0.266072026666667 0.975148976666667 2.31203433333333 2.9068289
6.9816426 2.1766985 0.0719075866666667 3.04749546666667
0.265249633333333 0.914978996666667 4.0652873 1.21303436666667
1.20751346666667 21.0490333333333 0.0381529866666667 6.8702901
6.52640933333333 2.2059417 2.64729373333333 0.0701818
1.07315566666667 2.0473398.79053693333333 1.56678843333333
9.95875866666667 2.07796115333333 3.2859678 0.342563596666667
6.35184143333333 1.42535213333333 0.129254626666667
0.810156266666667 6.0728510.517966686666667 1.2117322
2.10178916666667 1.06341763333333 0.21587308 2.90645764666667
1.26669506666667 0.977236866666667 1.0991987
TCGA-BT-A42C 873 0 0.1643177 0.3631512 0.07945525 0.5471684
0.05043345 33.109970 0.1194272 0.5797575 0 0.1407216 0.1222251
0.5285502 1.27923 0.1065560.1184578 0.02593239 0.005138439 0.1193049
0.08484686 0.1258501 0.5853204 3.3121635.387810.2067771 0.0471069
0.05043345 0.07865064 0.06934599 0.6974225 0 0.4657311 0.02353561
0.7894455 0.3370188 0.3036624 0.6643657 0.3671960.07681846
0.1528387 0.06571485 0.2640014 0.5658835 0 3.2735240.03475056
0.02062882 0.07481109 0.7060683 3.2837373.6232450.2331482 0.6472292
0.09684228 1.5767091.0892150.1685371 0.02050305 2.55296 0.03883375
0.11069 0.6115552 0 0.01422337 0.6047461 0.3940846 0.6864232
0.1415353 0.004857255 0.1350229 0.1624722 0.5533359 1.251784
2.2526940.04142267 0.02556254 0.004572712 0.1619371 0.01203215
0.1238716 0.4220178 0.2915134 0.0395758 0 0.1912469 0.232469
0.1393139 0.2546476 0.4381244 0.1086259 0.03290996 0.1778888
0.01712624 0.05588395 0.6151499 0.31067 0 0.3656329 1.494575
0.03089707 0.2697569 0.1350739 0.1323126 2.43164 60.5244430.2275971
0.1748360.5248487 0.5945838 0.4339518 0.2596128 0.03222718
0.02763968 3.0799860.03629323 0.2198137 0.01163558 0.11069 0.4189534
0.3817307 0.07168548 2.1284970.1194885 0.1037296 0.4567019
1.1718260.1149321.0416720.1059102 0.6743226 0.06934599 0.1934778
0.1739272 0 1.0526840.7639427 0.1857519 0.1438287 0.4066362
0.3393241 0.8311283 0.7814992 0.3383535 0.07180355 0 0
0.2980843 1.1116820.1562727 0.07432298 0.4724440.08069352 0.4182097
0 0.1404476 0.02892645 0.6491613 1.6688660.04013825 0.4714641
0.4966306 1.5634370.4706152 0.1220036 0.8237463 0.49544 0
0.3937063 0.07623805 0.4204331 0.1145538 0.1394390.0661704
0.02023909 0.06978213 0.03326232 0.2370321 0.9299436 0.132529
0.1852377 0.8457442 0.2280612 0.7494233 0.06827913 0.08366339
0.2134374 0 0.8291905 0.1885134 1.3933450.08718523 0.7087607

0.3864615	0.1330752	0.3534461	0.1137985	0.3661095	0.1420747
0.3360732	0.4370457	0.3114487	0.1169887	0	0.33673	0	0.03112926
0.3133559	0.3567695	0.2518946	0.7766751	1.782533	0.224975	1.028709
0.1467868	0.08115727	0.6103672	0.1765171	0.2168726	0	0.01602572
0.4447008	0.0671477	0.1033271	0.07109154	0.02947534	0.6270518
0.8490655	0	0.1355158	0.02808952	0.256589	0.2408295	0.1623545
0.1060307	0	0.02410787	0	3.02394	0.07996655	0.3715715	0.04142267
0.2168272	0.05218056	0.1595908	0.5908867	0.4298579	0.03793285
0.6359217	0.0278878	0.1122363	0.7850726	0.04400425	3.519309
5.319692	0.3124658	0.157257	0	0.09678194	0.03368062	0.04164478	1.08349
0.10348	0.1220707
TCGA-G2-A3IB	220	1	0.1591313	0.2917236	0.04122181	0.2943877	0
23.29633	0.1584052	0.1652253	0.3733837	0.1564828	0.2246373
0.1154987	0.517961	1.011308	0.2272698	0.09268852	0.05381545	0
0.02475841	0.02031646	0.01865478	0.1401542	0.7525887	24.21876
0.04709726	0.4154683	0.05233028	0.08160875	0	0.6743131	0.03765824
0.3126896	0.0732624	0.9395985	0.140786	0.1454231	0.295437	0.3232782
0.03775626	0.06872104	0.06818643	0	0	0.1577592	8.83454	0.1081727
0.06421405	0.007056798	0.366312	0.5629353	0.8531214	0.1511982
0.7007708	0.06029075	0	0.4723144	0.02914598	0.04153531	0.4477147
0.08058864	0	0.3299692	0	0.001639814	0.1060975	0.06815107
0.3956889	0.03671464	0.01511982	0.1644666	0.1926662	0.2080244
1.505502	0.06977371	0.1289418	0.05304792	0	0.05170081	0	0.07711831
0.1010516	0.08642213	0.1231928	0	0.3748309	0.0593145	0.2891072
0	0.4132751	0	0.06829546	0.1054739	0.03554074	0	0.2930496	0
0	0.1346204	1.132597	0.06144667	0	0.03503854	0.0274578	2.485444
0.3368657	0.2066375	0.08838004	0.3351314	0.2827672	0.5526078
0.08979236	0	0.1433962	1.067993	0.03765824	0.1140405	0.01609761
0.1914219	0.3343927	0.2742146	0.1718472	0.5168951	0.1859738
0.08072318	0.231089	1.036813	0.1742953	0.2494269	0	0.3105742
0.09593886	0	0.30493	0.1343144	0.5416977	0.4844126	0.0271038
0.1790859	0.253158	0.0391207	0.08921252	0.1501652	0	0	0.08468158
0	0.1374646	0.4676323	0	0.2056488	0.5297463	0.1674569
0.1549782	0	0.1457299	0.06002878	0.1443379	0.8488952	0
0.07724151	0.3149112	1.255927	0.4816716	0.1367197	0.122104	1.270065
0.04009385	0.1764037	0.01977635	0.3661349	0.09508984	0.02893667
0.125875	0.06300089	0.130332	0.10354	0.1147753	0.142951	0.1432432
0.1647468	0.4972802	0.1080942	0.06126622	0.247965	0.1157467
0.1968577	0.1644666	0.2581131	0.0391207	0.7546496	0	0.6379272
0.1012224	0.2259495	0.1974751	0.2951965	0.122104	0	0.1230752
0.2330989	0.04039531	0.05245193	0	0.4557322	0	0.1292002
0.1393463	0.03050237	0.4646552	0.1401542	0.3523001	0.127329
2.503536	0.04479635	0	0.002371998	0.15263	0.08438601	0	0.1211502
0.09121217	0	0.1072133	0.1475307	0.3670071	0.3027711	1.133716	0

0.08788292	0	0.1433597	0.1249436	0.05945673	0.1650279	0.06345562
0.7087464	0.2407745	1.42021	0	0.01880715	1.160476	0.3509723
0.09813439	0.1235377	0.282974	0.02027387	0	0.3278182	0.4051134
0.1164576	1.33335	0.06848893	0.6610787	0.1535936	0.1267058
0.09065089	0.09565417	0.100422	0.01397895	0.1512387	0.4432105
0.2863253	0.1345783
TCGA-2F-A9KP	364	1	0.1559606	0.4093724	0.1028375	0.4721268
0.02175837	84.76615	0	0.3091451	0.1034993	0.7807664	0.02802054
0.5254293	0.6840931	1.024947	0.1302517	0.02764747	0.03356383
0.01330118	0.2779456	0.1098158	0.2326937	0.05827459	2.624028
90.77307	0.1305502	0.03048481	0.1958253	0.2544903	0	0.574421	0
0.1772904	0.06092344	0.8114019	0.2039364	0.2267451	0.163786
0.3456401	0.05232883	0.05275103	0.08665834	0.2755583	0.2929652	0
8.84038	0.4947474	0.02669952	0.008802423	0.628273	0.5543585	5.302744
0.3017596	0.9833838	0.200546	1.436052	0.5260266	0.07271152	0.005054616
2.187321	0	0.2865283	0.506576	0.03046172	0.008181803	0.2646852
0.2550283	0.5922835	0.8243399	0.01257332	0.3191228	0.1952645
0.4151726	1.730627	2.00177	0	0.02205676	0.01183676	0.3869396
0.01557299	0.4168446	0.3991536	0.6468011	0.3585558	0	0.1925214
0.08385194	0	0.2197239	0.3608542	0.07029628	0.08518955	0.1973471
0	0.1764842	0.3755554	0.03655406	0	0.2320364	1.4345	0.003332461
0	0.04370595	0.03424997	0.3288158	0.6141318	0.03682186	0.3017161
0.2612701	0.2565197	0.7275999	0.3920143	0	0.0715471	1.830477
0.140921	0.6638356	0.005019909	0.1432641	0.6673771	0.5320724
0.0479903	0.1172288	0	0.06712766	0.3648772	2.269131	0.03432795
1.377848	0.1370777	1.282428	0.08975328	0.1073206	0.1785363	0.05584649
0.8529281	0.3295858	0.2516848	0.186155	0.3859548	0.1951916
0.7789657	0.9115811	0.1194341	0	0.1408388	0	0.3000707
0.388873	0.2427131	0	0.3550505	0.05222009	0.4252925	0	0.2544903
0	1.140269	0.9885268	0.2857262	0.3532781	0.9998802	1.305502
0.9033042	0.05053028	0.7234658	1.433358	0.1500353	0.2316214
0.02466839	0.5150077	0.207571	0.1443787	0.06423238	0.01309755
0.07225421	0.08610165	0.1329408	0.5349375	0.3859423	0.1712499
2.225751	0.1639864	0.7818508	0.1325587	0.03609468	0	0.06838345
0.6439239	0.1463937	1.293084	0.01880705	0.61332	0.8449816	0.1096053
0.2815132	0.07364372	0.2030781	0.1838848	0.1855034	0.4810757
0.1511634	0.1252457	0	0.7011076	0	0	0.1738162	0.1642968
0.434697	0.4807654	0.6591716	0.2779456	0.6415117	0.08940405
0.08403233	0.5148232	0.2284629	0.5964756	0	0.03555738	0.3948666
0.08690808	0.08915625	0	0.0762988	1.060681	1.628694	0.05493097
0.1534713	0	0.3831906	0.1558507	0.2224932	0.04574456	0	0
0.5123351	3.131065	0.284623	0.1290269	0.3216758	0.1347051	0.1055256
0.2524567	0.6324094	0.2276008	0.04909581	1.735246	0.108284	0.2614778
1.253702	0.02847696	2.316757	2.358924	0.1115638	0.067845	0.05965797

0.2505263 0.03487378 0 0.2831653 0.01984183 0.315988
TCGA-DK-A3IT 648 0 0.1621569 0.2023088 0.09801298 0.6624667
0.767291 22.26258 0.1412398 0.1571423 0.3452529 0.558103 0.02670597
1.610032 0.3984441 0.6762895 0.04381449 0.4838893 0.2559137 0
0.323774 0.09661279 0.1108885 0.2221627 2.11922 23.88991 0.06843406
0.02905464 0.06221278 0.1940409 2.65182 1.58376 0.3581596 0.4731244
0.2322611 0.515557 0.5183171 0.172886 0.3512298 0.2402055 0.129672
0.1508288 0.1453022 0.3361673 0.3141236 0.03125862 2.698535
0.2572018 0.2544693 0.1426209 0.598798 0.2113405 1.959703 0.3595035
1.006675 0.1075149 0 0.4512136 0 0.2661659 0.975819 0.04790384 0
0.0603513 0.1161305 0.4971199 0.2162295 0.2835745 0.6272189
0.7856667 0.02995863 0.2027676 0.4652596 0.2670942 0.807171
0.9539293 0.1532923 0.1786868 0.2369104 0.3073222 0.08905439
0.1528033 0.200225 0.5650855 0.2196864 0 0.235915 0.22095 0.08592617
0.3490262 0.4421893 0.4019903 0.04059648 0.09404435 0 0.2095664
0.3197567 0.9754964 0 0.9835383 1.222195 0.1524538 0 0.2082776
1.566872 3.581596 0.369676 0.9475485 0.05530025 0.09960513 0.2444853
0.7177972 0.2401864 0.2385254 0.1022858 2.083829 0 0.0451923
0.03349083 0.3186004 0.2385254 0.3259997 0.8568415 0.6703745
0.07369822 0.03198921 0.2017004 2.353171 0.2181165 0.8895923
0.4790384 0.8530385 0.2566277 1.500191 1.07275 0 0.4750794 0
0.1145682 0.106453 0.2675258 0.2790515 0.3535339 0.5950788 0
0.08857413 0.1006736 0.1648304 0.9396916 0.8895104 0.0771088
0.3361673 0.4088922 0 0.4421893 0 0.5890527 0.03568256
0.2859931 1.713306 0.2228086 0.1530474 0.2722776 1.244256 0.8214344
1.56519 0.6532342 0.9347091 0 0.3200948 0.2351109 0.3148827
0.08478556 0.3440132 0.1632506 0.02496617 0.4820512 0.1230934
0.2241685 0.6373017 0.2384123 0.4243611 0.1391037 0.1319809
0.7619792 0.3790194 0.2408093 0.2047798 0.2607012 0.8864768
0.2325429 0.9963244 0.07169892 0.2670773 0.5368933 1.074479
0.2683062 0.2456607 1.177434 0.2920966 0.158511 0.3526972 0.1920956
0.114025 0 0.9571738 0 0.1535995 0 0.8604148 0.3452529
0.5415217 1.256494 0.06307287 0.6921687 0.1171634 0.06006751
1.452272 0 0.1337629 0 0.8613513 0.2232536 0.05522057 0
0.04384791 0.109079 0.7964827 2.082231 0.1047079 0.3552302 0.2079009
0.07304271 0.07426952 0.08246588 0.1307955 0 0.009912849 0.2020549
2.23813 0 0.06707656 0.357682 0.2246746 0.06839096 0.1499925
0.6027404 0.02410256 0.02339626 0.3047859 0.03440132 0.1661405
0.9877081 0.1085639 0.9356219 3.423741 0.09303868 0.1077702
0.2274366 0.5969326 0.04985656 0.05137141 0.3983955 0 0.1317591
TCGA-FD-A43P 832 0 0.04424554 0.1182884 0.0401152 0.1637056 0
35.95742 0.1541528 0.3858955 0.4239201 0.228423 0.03279103 0.6479419
0.2965036 1.025166 0.2331237 0.03725663 0.209483 0.3268798 0.2409377
0.2767949 0.09076993 0.09092779 8.441178 22.34179 0.5347174 0.08324132

0.1527764 0.07941794 0 1.808581 0.4764149 0.2213057 0.04753043
0.3047916 0.1281674 0.176899 0.2236157 0.1572999 0.1265581
0.06687621 0.1464839 0.08599256 0.2571319 0.1023494 6.531462
0.1754479 0.04166014 0.05493885 0.4901576 0.7496528 4.244905
0.1765671 0.6251285 0.09778705 0 0.4842583 0.311999 0.02366066
1.633859 0.03921261 0.3353097 0.5928205 0.04753043 0.06861907
0.0884994 0.1658038 0.5647642 0.4644774 0.009809283 0.1304123
0.158198 0.2348303 0.8618156 2.783925 0.2091339 0.05162384 0.01385197
0.4025031 0.2186915 0.02501602 0.2950165 0.3364084 0.2397714 0
0.2574836 0.2347369 0.140673 0.08571062 0.5228348 0 0 0.0128303
0.1037599 0.03160032 0.371128 0.2281461 0.04430803 0.402547 2.136557
0.04419787 0.06809715 0 0.3206482 0.623004 0.7312962 0.05745437
0.1629615 0.5095855 0.1250801 3.196761 0.283991 0.03254158 0.02790933
3.800271 0.0366473 1.738672 0.0900764 0.1117699 0.780998 0.2075526
0.3694122 0.5487479 0.1206542 0.1309269 0.1622591 1.80699
0.03570869 0.5316964 0.1425913 1.063039 0.09336335 0.3070026
0.3936401 0.04356956 1.555541 0.9856721 0.1641185 0.2614174
0.3284826 0.1903525 1.244385 1.159329 0.1863569 0 0 0.03373128
0.3344359 0.5157558 0.2524755 0.02501602 0.8386901 0.04074037
0.3619625 0.3940966 0.1701813 0.02920865 1.123705 1.07037 1.519869
0.1252799 0.1671585 2.291646 0.7208913 0.1773992 0.4158913
0.6179848 0.07803504 0.1264923 0.07698181 0.9779461 0.1388057
0.1689592 0.1614719 0.2452385 0.8878326 0.06717363 0.3271042
0.7303457 0.2509161 0.1336034 0.2561985 0.2871469 1.7703
0.7239251 0.02815986 0.3591995 0.5335049 0.251184 0.07614099
1.043608 0.1027084 0.6022397 0.6668038 0.1465892 0.04575567
0.2872719 2.23129 1.721529 0.1397331 0.3175778 0.1179327 0.125422 0
0.5174142 0.2070633 0.03143295 0.316413 0.4627931 0.2260907
0.3409792 2.171336 0.2581475 1.841411 0.1830939 0.1475082 0.3947229
0.1188261 0.2463619 0.04127643 0.01618206 0.3916039 0.02260093
0.06955673 0.2512478 0.02976289 0.4701752 1.721529 0.1285659
0.1539426 0.0567271 0.239162 0.3039737 0.1735818 0.08922095
0.2470086 0 0.2549853 0.8356785 0.24224 0.07320907 0.4600946
0.1313655 0.09220685 0.3197375 0.3212737 0.5524292 0.05745437
0.5276064 0.05631972 0.09066499 0.6783574 0.0888671 1.133489
7.099841 0.07615859 0.9703907 0.04654315 0.6352198 0.2040552 0
0.7679464 0 0.2234117
TCGA-DK-A3IV 294 1 0.3147748 0.3946513 0.0393642 0.4016021 0
37.37716 0.1890835 0.4260049 0.8319676 0.2241466 0.4933829
0.2097746 0.5528102 1.52908 0.7566657 0.08466329 0.1027806
1.807459 0.07092811 0.05820278 0.08907061 0.08922551 3.899972 22.75011
0.1099386 0.03500698 0.3997766 0.4286213 0 1.476318 0.03596122
0.2443079 0.09328122 1.173335 0.3252621 0.2661671 0.3134705
0.2646086 0.08012181 0.01514405 0.1265417 0.3290924 0.3924947

0.05021664 14.36219 0.5853553 0.5110027 0.03369396 0.8307858
1.188307 3.534867 1.010692 2.648882 0.1343389 0 0.8537355 0 0.02902213
2.63649 0 0.07311829 0.6059607 0 0.01879101 0.8134255 0.9111189
0.4282386 0.1753007 0.02887692 0.2850259 0.2702263 0.508544
2.790748 5.097152 0.2052187 0.2617298 0.1540499 0.382625 0.1311428
0.04909538 0.3216594 0.1650552 0.5293858 0 0.1684421 0.1963574
0.1380395 0.4766007 0.6709072 0.05381609 0.0326089 0.1888515
0.06787828 0.01328945 0.7091928 0.5037186 0.7826136 0.4324084
2.495903 0.002551202 0 0.1003787 0 2.697091 1.484701 0.05637876
0.3775668 1.43013 0.9082645 1.250857 0.1929284 0 0.05477367
2.312213 0.3955734 0.5808076 0.007686093 0.4021506 1.21343 0.9310488
0.1347115 1.480805 0.05919769 0.05139032 0.2122952 1.512138 0.2102411
0.952747 3.707928 1.418122 0.1374232 0.02738683 0.6715167 0.04275389
2.993479 1.387749 0.4975172 0.08550778 0.7252492 0.6724398 0.4543587
1.338383 0.06095604 0 0.02695517 0 0.7548022 1.071742 0.09290584
0.02454769 1.030624 0.599665 0.9175643 0.03867186 0.5288185 0.1433091
0.4594448 1.413908 2.247065 0.2212821 0.8474839 2.348688 0.4250717
0.125723 1.603271 2.396785 0.4977319 0.4698988 0.03777031 1.435737
0.2043106 0.3315921 0.07649258 0.2807552 0.5808076 0.1977482
0.3757819 1.057945 0.4705512 0.3408658 1.452546 0.4101026 0.5130467
0.4397543 0.02763268 0.09399329 0.4711653 0.6298972 0.07471554
1.13027 0.1007856 1.264908 0.9851983 0.09589658 0.2963339 0.1409469
0.1036458 0.8211875 0.3917633 0.3885287 0.5014742 0.1402471 0
3.292976 0.2031868 0 0.2217781 0.1059193 0.5269128 0.7026509
2.663357 0.1317236 0.6486442 0.1197774 0.08041484 0.7814618
0.08745114 0.4566384 0 0.01361067 0.1742036 0.06653343 0.03412727
0.2113236 0.05841139 0.7997131 1.486517 0 0.1846297 0.3618232
0.371584 0.1193132 0.02838869 0.1926114 0.06059607 0.01592488
0.02028743 5.298679 0.2773225 0.1526568 0.04104373 0.3781249
0.1583411 0.4593324 1.137185 0 0.09396459 0.4575279 0.05526535
0.2224191 1.849909 0.06540255 5.110426 6.51774 0.2491094 0.1904448
0.09134362 0.9589658 0.03337251 0.1237914 3.024601 0.09114079
0.09827515
TCGA-2F-A9KW 254 1 0.07013194 0.2624923 0.0635851 0.1946127
0.08072004 32.70936 0 0.140174 0.3199713 0.3620646 0.06930111
0.4611148 0.4229791 0.9099734 0.08527285 0.1056757 0.04150546 0
1.642175 0.1044612 0.0863256 0.1441262 1.90970 129.42878 0.02421601
0.03769791 0.3228802 0.0629412 0.1109901 0.5581214 0.1161765
0.04384792 0.1883468 0.4459511 0.1821375 0.1121584 0.3038098
0.1157604 0.1423631 0.07338667 0.1448682 0.1635643 0.09057112 0
0.6045416 0.05561918 0.03301696 0.02177038 0.1412601 0.9140357
3.479459 0.1399349 0.81071 0.325497 0 0.4033045 0.08991599 0.05000482
1.035907 0 0.05905409 0.2740668 0 0.01264715 0.1262494 0.05256189
0.2034515 5.606641 0.04664498 0.1127518 0.08358459 0.1668575

0.7285501 11.354620.09944709 0.05455134 0.007318744 0.1395609
0.1925776 0.03965195 0.4935984 0.06665354 0.3167105 0.0268196
0.2720851 0.1280906 0.2229755 0.09057112 0.2549925 0 0
0.1423578 0.05482199 0.03577748 0.2353044 0.09040644 0.070231
0.2038779 0.4569191 0.02884674 0 0 0.1694158 0.5808825
0.1598828 0.1366031 0.07175115 0.4846349 0.03965195 0.6787658
0.2770114 0 0.04423803 3.2067540 0.3518175 0.0372461 0.05905409
0.2063218 0.09399536 0.4272870.7248330 0.1245164 0.1398757
1.5120610.1981018 0.2198535 0.1130081 0.4845705 0.03699668
0.6193324 0.2975733 0.1381210.4109384 1.0189250.08826115 0.2302016
0 0.06034411 0.4587043 0.9574099 0.1476937 0.07661563 0.3047853
0.05346618 1.0602040.3366197 0.2501184 0.1982597 0.7500608 0
0.9562220 0.2697480 1.0389992.0228740.09636346 0.07943058 0.08831891
1.8565610.6302552 0.1171620.3766935 0.5597397 0.2473808 0.1002491
0.2745472 0.3244407 0.2200157 0.3570811 0.02647686 0 0.4467524
0.1064744 0.2782090.4961329 0.1590871 0.08470791 0.4963330.07210229
0.4725191 0.7649776 0.04463514 0.0379569 0.1691277 0.3539042
0.4827529 1.02927 0 0.4021036 0.6065312 0.1355393 0.1305461
0.3642751 1.6532660.07579809 0.07119184 0.1699727 0.06231021
0.2219182 0.04377073 0.8904311 0 0.04982319 0.2865910.5646028
0.5823478 0.3783313 0.09057112 0.08183599 1.39202 0.1105582
0.2597886 0.3439303 0 0.4338878 0.06542572 0.1538975 0.3641538
0.1074716 0.1102518 0 0.04717604 3.3884351.0909510 0.4066811 0
0.06318112 0.09636346 0.09171266 0.3111257 2.1533820.02572351
0.2075456 1.22271 0.06399427 0.07252559 0.06629806 0.1665780.1357141
0.3527355 0.1636839 1.3447250 0.3046945 0.3570811 0.03592742
2.6443370.07042995 0.9711635.2418510.06898064 0.3076260 0.154902
0.04312536 0 0.3501658 0.06134165 0.134322
TCGA-GU-A762 232 1 0 0.046474066 0 0.107196542 0.022231101
73.044717822 0.100941216 0.063172334 0.26436985 0 0.085887961
0.989988383 0.232985301 0.671292071 0.039141649 0.256801317 0.068586101
0.027180334 0.189322925 0.025892694 0.071324783 0.238162752 1.721888232
48.805244534 0.21341857 0.342618516 1.06709285 0.052003892 0
0.733626335 1.199856155 0.036228461 0 0.153524196 0.046303832 0.108113355
0.125508375 0.183932104 0.10693148 0.067371392 0.067225657 0.090094462
0.074832559 1.910061387 1.227913581 0.045954222 0.054559196 0.080943011
0.875349604 0.226561074 3.427684765 0 0.29770344 0.307354674 0
0.376218633 0 0.082630949 0.47549855 0.102707687 0 0.226442144 0
0.014629236 0.115901095 0.130284592 0.437053987 0.374333255 0.019269735
0.0931589 0.053713396 0.243910872 0.263353043 0.400159819 0.082166149
0.123947839 0.006046964 0.082363823 0.111379366 0.065523245 0.128787068
0.055071146 0.235508072 0 0.056201197 0.120951152 0 0 0.052670609
0.143647114 0 0.033605787 0 0.065032929 0.266042327 0.186741249 0
0.171569409 0.421934281 0.102145884 0.029727261 0 0.069988202 1.487821632

0.561424655 0 0.023713174 0.266946555 0 0.260844919 0 0.042617297
0.073101556 1.340343106 0.047994246 0.484470221 0.041031785 0.04879225
0.213086487 0.11649265 0.323617473 0 0.079005913 0.034293051 0.048464607
1.117171331 0 0.726598227 0 0.696091964 0.061135528 0.073101556
0.118966433 0 0.667741435 0 0.072923993 0 0 0 0.075799031
0.625177224 0 0.126604236 0.035974671 0.044175349 0.131395761 0.516518348
0 0.065523245 0.216650995 0.106709285 0.197514782 0 0.111436912 0
0.551862198 0.460072928 0 0.098441872 0.145943427 0.466853122 0.080438831
0.24523356 0.038904427 0.308315758 0.102196703 0.035498048 0.02520434
0.486483969 0.060594506 0.036878882 0.204175735 0.13382109 0.719784328
0.043986161 0.062690348 0.273279876 0.430839213 0.104982303 0.037280467
0.029786532 0.504528988 0.270877416 0 0.125444503 1.886467718 0.036550778
0.249290502 0.708677977 0.057646971 0.194476093 0.243124476 0.047994246
0.071907365 0.11286559 0.138326851 0 0.026142587 0.102626666 0
0.064938379 0 0.096802721 0.067793853 0.041165406 0 0.3162955
0.333106011 0.312588612 0.299330235 0.027046132 0.742018207 0.09134647
0.064393534 0.051391631 0 0.035849105 0 0.442013921 0.246170221
0.029598757 0 0.188023225 0 0.049260281 0.832039623 0.056124419 0
0.074291274 0.078303192 0 0.037887865 0 0 0.042507061 0 0.378839829
0.026436985 0.035953683 0.602551763 0.137631741 0.081941887 0.08709719
0.120213825 0.025838412 0 0.149977326 1.36451864 0.059368605 1.224124807
0.640104564 0.601802853 2.495815142 0.042745396 0.55455219 0 0.191976985
0.204880902 0.055071146 0.399533591 0.010136461 0.100891637
TCGA-XF-AAME 2828 1 0.09226692 0.08457304 0 0.1950752 0.7484348
45.5225 1.056228 0.3448811 0.2886586 0.5443876 0.1172235 4.364739
0.3533203 0.6596697 0.1851969 0.3318004 0.3120307 0.0494625
1.981036 0.02355963 0.02163269 0.05417577 1.858639 38.92039 0.09102585
0.3542578 0.4247873 0.09463616 4.005138 0.8582438 1.266422 0.1318564
0.2831915 0.6425781 0.8584318 0.08431868 0.7993961 0.1071097
0.7102638 0.134862 0.1104013 0.0819765 0.1361795 0.8537324
1.969429 0.04181352 0.173751 0.6955799 0.5309841 0.2748624 4.376427
0.07013374 0.9819359 0.8389814 0.07026557 0.7042 0.1013959
0.6050082 0.692246 0.233633 0.04439582 0.3826431 1.614192 1.188488
0.1792783 0.1185453 0.5200342 2.043624 0.02922239 0.2472307
0.9285974 0.2798289 0.9584944 1.132766 0.1246043 0.6869298
1.166446 0.1199079 0.6225388 0.05961927 0.1367132 0.1503269
0.357146 0 0.1022744 0.4126982 0.1676291 0.06808977 0.1916989 0
0 0.08408878 0.1236426 1.178083 0.5632796 0.6796597 1.583953
2.418288 0.7072135 0.1022362 0.08114606 0.04063183 6.208987 0.9607339
0.4807882 0.4107833 0.1941885 0.255038 0.05961927 0.48655 0.1041261
0.1163318 0 2.31624 0 0.3085719 0.0513351 0.08879165 0.07755453
0.1413281 1.635877 0.5994083 0.6469838 0.03120307 0.1322931
0.929067 0.06382689 0.9641451 0.04247873 1.639312 0.08344036 10.11024
0.1659787 0 0.5457873 0.05106733 0.2549359 0.0692246 0.2283324

0.2268282	0.1724229	0.7545911	0	0.2303931	0.2945986	0.8440935
0	0.3615211	0.03760692	0.08942891	0.2246361	0.1941885	0.2516048
0.04696141	0.06759726	0.06961133	2.008547	1.393213	0.2414812	0.2090008
0.2987847	0.606839	0.30432	3.875534	0.2831915	0.7013374	0	0.236863
0.5962659	0.5149186	0.02756732	0.03355591	0.3980967	0.1339395
1.696096	0.1200684	0.2091527	0.4558693	0.2325533	0.2547277
0.1017639	0.1727792	0.8470905	0.862645	0.2684473	0.05707066
3.687269	0.266059	0.2268282	0.6954872	0.05245269	0.435009	0.3747158
3.813823	0.1526657	0.2053917	0.5506502	0.1424592	0.07730778
0.2015031	0.09368743	0.07820352	0.03290606	0.2113927	0.1850559
0.1123686	0.2154541	2.294327	0.2357378	0.487582	1.327751	0.1845685
1.249043	0.09350516	0.2929568	7.390987	0.3539894	0.06523784	0.0491859
0.7988631	0.2675425	0.05386352	0.248656	0.08554069	0.1063983
0.2689301	0.6939796	0.4596059	0.1426767	0.6421739	0.04749845	0
0.07469362	0.1063176	0.0735852	0	0	1.302217	0.09621952	0.02180938
0.4984171	0.1669739	0.07063438	0.307853	0.3691641	0.1410614
0.0228213	0.3070431	0.4026709	0	0.5592623	0.6883239	0.6570929
12.55691	0.06914457	0.9671198	0.1109237	0.1164526	0.3323141
0.02505448	0.4763544	0.1429583	0.1468813
TCGA-XF-AAN0	1718	1	0.110028138	0.188259306	0.039902788	0.33585497
0.033770541	13.869981293	0.038334128	0.991618021	0.120478687	0.378689077
0.195704404	0.787004975	0.235946919	0.632237503	0.511345709	0.09167315
0.026046728	0.01032219	0.431391428	0.104887328	0.036115718	0.135669478
2.657932637	18.27894944	0.055721393	0.201087058	0.709181363	0.157994835	0
1.846766162	0.182266238	0.055033474	0.141836273	0.746283527	0.083527137
0.269808849	0.508415152	0.195583685	0.040609032	0.061405011	0.125782327
0.179628174	0.682054207	0.02545186	12.614374886	0.139615123	0.227917516
0.081971926	1.241067386	0.43020193	2.085553088	0.23417621	1.384959256
0.204265455	0	0.636815918	3.18811007	0.075509317	1.48074442	0
0.148237435	0.540541385	0.094557515	0.082541813	0.243551847	0.230896258
0.663914468	0.177699203	0.004878671	0.194582642	0.209813836	0.483283118
3.514734009	3.478365734	0.166421227	0.136934635	0.101043209	0.212697538
0.072511185	0.049767113	0.114121139	0.376455523	0.536628955	0	0.170746812
0.111005326	0	0.284189253	0.48006123	0.054552413	0	0.114861276	0
0.118996326	0.41967993	0.056734509	0.308513926	0.220347011	2.108377026
0.033619405	0	0.101752109	0.106316797	3.535965021	0.903009067	0.142875366
0.198120508	0.760330896	0.348369792	0.703327803	0.282487284	0.032369274
0.13880774	3.353993802	0.255172733	0.551957193	0.011686884	0.111178076
0.356062012	0.501387202	0.275592121	1.137171282	0.180022961	0.078140184
0.336957679	1.277355906	0.071039226	0.091979307	0.709181363	0.867353153
0.023217247	0.166569288	0.13855049	0.216694305	1.220651558	3.410271036
0.212807412	0	1.007458341	0.151475631	1.007508579	1.143499887	0	0
0.191267828	0.134210667	0.465731044	0.603558607	0.031392334	0.099534226
0.386509931	0.243147896	0.240030615	0.156803919	0.141066817	0.11621594

0.558877253 0.565111448 0.141103269 0.07476992 0.526532522 1.418362726
0.344066968 0.254886421 0.561435246 1.63923347 0.349298283 0.368480178 0
1.11604375 0.046023569 0.112043016 0.160616865 0.21344742 0.686880062 0
0.246013067 0.48431898 0.843051006 0.345529591 0.537999655 0.435509599
0.506380377 0.205740527 0.084032262 0.619315633 0.583747925 0.41642322
0.227213446 0.949652915 0.029189878 0.447236531 0.493686157 0.413136807
0.172950881 0.257175659 0.118196894 0.35675282 0.134029185 0.406152251
0.234616391 0.181334147 0.027468292 0.455854561 0.102983432 0.093799539
0.179850029 0.460342586 0.787127423 1.187107934 0.824148834 0.123254694
1.089603154 0.234159939 0.211939258 1.747331035 0.147746117 0.299514642
0.041057868 0.080481894 0.212943272 0.112406268 0.034594213 0.071404989
0.029605294 1.820855746 1.270719568 0.085256776 0.323269149 0.056426727
0.237895654 0.181418488 0.110312256 0.319494676 0 0 0 7.737052413
0.060239344 0.100129457 1.539396346 0.087113289 0.117923922 0.312955768
0.273919356 0 0.038100098 0.549222594 0.42016131 0.157823598 1.388761493
0.022099136 1.249378105 3.893528175 0.126258879 0.07020018 0 0.340230311
0.115019007 0.041828391 1.935860596 0.019247459 0.091956719
TCGA-CF-A47W 368 0 0.02450519 0.03930809 0.04443524 0.2493359
0.09401611 102.0452 0 0.133579 0.1118029 0 0.01210744 0.1049726
0.1313737 1.271835 0.0926976 0.2563012 0.05801061 0 0 0.0876009
0.08043601 0 1.608669 109.1449 0.07333257 0.05268894 0.5640967
0.04398526 0 1.187826 0.04059387 0 0 0.07791111 0.04405962
0.02612658 0.5661647 0.1306797 0.1808868 0.05128488 0.03744435
0.009525317 0.1898817 0.0850286 23.58804 0.03886841 0.2076595
0.1293174 1.92498 0.06387565 10.9368 0.03259696 0.4091745 0.02166356
0 1.972892 0.03141804 0.02074855 1.689156 0 0.08253766 2.079429
0.05264902 0.003535289 0.147045 0.03673188 0.5687129 0 0 0.3808399
0.02596067 0.08969632 0.2227459 2.782877 0.06949671 0.01906108
0.09206217 0.1114622 0.02691584 0.1939701 0.3630967 0.04657957
0.2655925 0 0 0.1065639 0.3116444 0.03164695 0.08909835 0
0.1104291 0.007106003 0.09577827 0.01250121 0.1557834 0.03158941 0
0.1108148 0.3193092 0.002879857 0 0 0.05919652 5.03364 1.480436
0.190925 0.1103122 0.4402813 0 0.2426869 0 0 0 3.647083
0.1217816 0.3278147 0.03470497 0.04126883 0.2883681 0.4598081
0.05529656 0.2532679 0 0 0.03468529 0.8433081 0.009888547
0.2816742 0 0.6195443 0 0.2164044 0.1207472 0.1930464
1.148706 0.2373522 0.0844037 0 0.06064285 0.126511 0.1923341
2.870516 0 0 0 0.03736382 0.2222709 2.419615 0.0349581 0.08313004
0.1619374 0 0 0 0.1570902 0.0647083 0.3111793 0.6384212
0.06734177 0.05550856 0 0.338458 0.1933647 0.2620046 0.03290564
0.5215513 0 0.02001633 0.02131801 0.9824934 0 0.1247697
0.04317332 0.01131868 0 0 0.2209331 0.07704735 0.8338123
0.1479913 0 0.0409397 2.387679 0.15274 0.1559621 0.07957639
0.1772875 0.1545745 0.126511 0.462398 0 0.1667738 0.2559963

0.2976884 0.08109301 0.06364168 0.2632451 0.02648503 0.01658369
0.1964474 0.04354431 0.07754166 0 0.3111304 0.2867026 0
0.1001394 0.2615468 0 1.359718 0.1265878 0.09150324 0.3138021
0.1738384 0.09077418 0.4270033 0.03290564 0 0 0.0307281
0.1966452 0 0.115571 0.07951569 0.03296808 0.1458264 0.4937566 0
0.01894676 0 0.06622939 0 0.02136388 0.01976585 0 0 0
0.4628367 0 0 0 0.02910248 0.02553417 0.1246679 0.2796137
0.1092716 0 0.1540344 0 0.02510719 0.5373457 0.02460932
1.153754 5.484404 0.008034302 0 0.1031108 0.1082503 0.04520601 0
0.7224675 0 0.1877367
TCGA-GC-A3I6 630 1 0.04124147 0.2058134 0.09971082 0.4196245
0.02109682 50.63261 0.2394775 0.3596949 0.2759693 0.4731424
0.1494273 0.3697651 0.4421958 0.4586698 0.2005806 0.08773156
0.3254335 0 0.5988776 0.1310485 0 0.1695084 1.901195 18.69891
0.4810076 0.7241697 0.3164524 0.1480516 0.0870244 0.8752168 0
0.5844602 0.2953555 1.456911 0.2032286 0.02931348 0.5558218
0.3141855 0.05073781 0.03836038 0.0949155 0.2885546 0.1420289 0
3.199535 0.2616573 0.1812142 0 0.2215167 0.931673 3.754124 0.2560118
2.295426 0.1215303 0.1465674 0.2754179 0 0.03063084 0.9024753 0
0.09260554 0.1227935 0.02953555 0.02181586 0.2383064 0.0824248
0.3509462 0.04440425 0.006095518 0.2210144 0.3568098 0.3220397
2.035032 0.9282603 0.3378867 0.1390099 0.05164592 0.2032198
0.01509951 0.06218012 0.5907111 0.0522613 0.2979893 0 0.5866707
0.181735 0 0.3905795 0.3498827 0.06815897 0.1238991 0.1754013
0.02149224 0.008415657 0.8302323 0.53164 0 0.6867857 1.706995
0.2229486 0 0.6356565 0.09962589 1.275273 0.2193798 0.03570232
0.1293939 0.3039917 0.2487205 0.569333 0.08144902 0 0.2428012
1.469159 0 0.1379255 0.06327467 0.04630277 0.6875289 0.2947972
0.2016352 1.079813 0.2998995 0.3254335 0.2193457 2.92972 0.1331369
0.5889699 0.2215167 0.6130835 0.3480976 0.06937177 0.1128965
0.108297 1.181422 0.1065217 0.1675447 0.03609901 0.4422601 0.4731424
0.359658 1.259205 0 0 0 0 0.4156389 0.9803291 0.1176668
0.6839813 0.8128254 0.05063238 0.9371859 0 0.1410016 0
0.5237051 0.8970746 0.02518536 0.560515 0.6232369 0.3164524
0.6307655 0.2204728 0.7753083 0.8411815 0.3879297 0.1122896 0
0.4805059 0.02875143 0 0.2767975 0.2412872 0.3502869 0.0834838
0.4957647 0.9509008 0.2979805 0.3985036 0.3184051 0.05653353
0.4445878 0.2998995 0.1749862 0.1785661 0.06630431 0.1387435
0.3311997 0.9079017 0 0.2486346 0.8004563 0.3036365 0.1137308
0.1785116 0.3445815 0.1782939 0.0806284 0.1947809 0.3419906
0.1993753 0 0.1469818 0.3216744 0.07813012 0.5055942 0.2833889
0.4917271 0.0847542 1.455796 0.1154978 0.8449907 0.2492216
0.0407387 0.7458869 0.2584361 0.3402001 0 0.03160326 0.2011643
0.02808857 0.08644553 0.08921495 0.073979 0.7012038 2.544933 0.05326084

0.4038995 0.07050078 0.1238467 0 0.1378265 0.4878911 0
0.1210148 0 1.4380430 0.07961159 1.1436170.2394507 0.1596148
0.4704881 0.5276212 0.1471205 0.1428093 0.2338199 1.819857
0.2816975 1.48517 0.05522228 2.0559522.14788 0.09465036 0.2192741
0.05784411 0.1821819 0.06762694 0.02613065 0.8367417 0.2068145
0.1819135
TCGA-FD-A6TA 1912 0 0.1408469 0.1694465 0.1276988 0.2117068
0.4052779 44.07573 0.06133935 0.1279606 0.1606507 0.1211899
0.03479459 1.676735 0.3775443 0.6200487 0.04757073 0.3870077
0.04167799 0.04955035 0.9970707 0.05244772 0.2022637 0.07236266
1.468068 36.94232 0.09726669 0.1324911 0 0.1264057 0.5572571
1.222781 0.1166594 0.04403019 0.03782593 0.3731707 0.1266194
0.1126246 0.4576091 0.2682498 0.129959 0.06550375 0.117572 0.08212209
0 0.04072613 2.099397 0 0.165771 0.1093044 0.8510835 0.4589176
2.530854 0.1873555 0.7236266 0.2801572 0.09385382 0.3265976 0
0.1569146 0.5778962 0 0.415097 0.4324666 0 0.2260551 0.2018996
0 0.1634378 0.5686815 0 0.08491536 0.2517961 0.2448824
0.8687495 2.593778 0.1331473 0.1917233 0.09553916 0.06006043
0.1160269 0.03981677 0.7304318 0.1338612 0.318027 0 0.06830401
0.06124906 0.1119512 0.1364214 0.3200656 0.1745812 0.1057844
0.1531602 0 0.1472974 0.3544238 0.3177379 0.4936605 0.6937914
0.9716154 0.1613856 0.2890317 0 1.10578 0.4666377 0.521779
0.5029602 0.2017383 0.3082113 0.07963355 0.2536139 0.2086221 0
0.2221096 1.502706 0.1166594 0.23552 0.01870046 0 0.4661536
0.5191234 0.7786701 0.4367076 0.288059 0.04167799 0.1676423
1.722739 0.07104473 0.3679457 0.0567389 0.6192897 0.07430094
1.199392 0.1735027 0.1386951 0.7565187 1.97811 0.1539329 0.0462317
0.2178457 0.06059494 0.1842444 0.7443016 0.04943587 0.03846705
0.174887 0.1073768 0.2129221 0.5311727 0 0.1194503 0.4470085
0.1296889 0.09601968 0.1254528 0 0.04648998 0.7452274 0.3931514
0.1612734 0.4386842 0.5764592 0.8105557 0.2881382 0.9882516
0.2364121 0.7962608 0.06210228 0.2300932 0.06126409 0.2051266
0.1104651 0.08964135 0.1329346 0.04879176 0.4037485 0.106917
0.1396827 0.05535503 0.7366174 0.17012 0.1359262 0.1357538
0.7883721 0.2743419 0.08964135 0.3049175 0.5094922 0.3553753
0.3029747 0.4614056 0 0.630283 0.2954809 1.088821 0.1747849
0.3200656 0.4413026 0.456679 0.1032601 0.2363251 0.06256922
0.04410388 0 0.588245 0.08239312 0.1000606 0.2158367 0.579835
0.04498219 0.217088 0.8185284 0.2300932 0.435875 0.04163166 0.1304343
1.491661 0.1418473 0.1307074 0 0.3385101 0.2160762 0.2158367 0
0 0.04737214 1.067653 0.5110455 0.2046321 0.05444955 0 0.09516563
0 0.1688388 0.08520518 0 0.03874565 0.3948788 2.148637 0.03213014
0.1019579 0.1331473 0.1672705 0.1467611 0.3379166 0.4017795
0.06280533 0.06096488 0.6184318 0 0.07215352 0.4708031 0.601143

0.4875999 6.3044730.0923565 0.2246580.07408047 0.1555459 0.01082616
0.0334653 0.2009265 0.006159664 0.2207132
TCGA-DK-A1A7 560 0 0.113651088 0.157997297 0.041216686 0.252300968
0.06976504 23.041965162 0.039596374 0.272587854 0.228150537 0.078231671
0.01123047 0.418913727 0.365574086 0.800519639 0.036849979 0.165206825
0.080713143 0.063972442 0.024755337 0.108341004 0.037304917 0 2.468081444
29.12458907 0.094182804 0.024436276 0 0.081598604 0 0.559116966 0 0
0.024417764 0.144535644 0.476796577 0.024234172 0.393866989 0.075037398
0.062919278 0.026427885 0.034732164 0.035341501 0.058709378 0.262899255
4.457540766 0.180265373 0.085608097 0.014111843 0.366266462 0.08887348
5.069898915 0.181415283 0.32114668 0.221038613 0 0.910778768 2.739381709
0.014181027 0.820708182 0 0.267957411 0.583719148 0.097671056 0.047548683
0.100022363 0 0.685775503 0.293680625 0.025196567 0.060905988 0.084280964
0.024959821 0.265643807 0.383707722 0.021487632 0.017680444 0.056929259
0.103388769 0.062415663 0.128514548 0.101039024 0 0.020529585 0.817086346
0.022046134 0.0909376 0.072267822 0.088064067 0.08264474 0 0.034143484
0.013182597 0.159913472 0.020872286 0.37329075 0.087903951 0 0.205576438
0.452982521 0.005342524 0 0 0.082363157 0.866031914 0.621828591
0.088547936 0.037208021 0.261788894 0.128514548 0.22510853 0.157117089
0.066870225 0.114702664 2.070507827 0.075307123 0.836193242 0.020119506
0.07655926 1.136793831 0.060929014 0.184648655 0 0 0 0.099443671
0.942440018 0.027516888 0.237519887 0.109879938 0.27484181 0 0.14337833
0.149334897 0.044765901 1.829112511 0.044032034 0.009033478 0.268595405
0.056250347 0.078231671 0.356805704 1.811767764 0 0 0 0 0.034361886
0.280544524 0.032426005 0 0.122538007 0.041859024 0.340909553 0
0.466277738 0.03001066 0.721599596 0.707794716 0.062464048 0.102975874
0.228997859 1.726684747 0.225859285 0.096198166 0.06104441 0.483774088
0.080177733 0.111399016 0.158191159 0.288198066 0.118847524 0.028933078
0.034325291 0.157482648 0.04343861 0 0.090169363 0.285866507 0.183328538
0.109817542 0.058496277 0.108080805 0.603161612 0.035419174 0.057866156 0
0.054815389 0.229405328 0.078231671 0.329621764 0 0.250053316 0.630615299
0.414189176 0.037609625 0.029515979 0.108523396 0.122833265 0.051274974
0.20340646 0.040390286 0.061436048 0.028372754 0.258216129 0 0.129184163
0.232215048 0.245374899 0.058074682 0.385376016 0.205482824 0.212188602
0.882920477 0.179163139 0.101039024 1.32815388 0.06104441 0.056250347
0.169639203 0.052254375 0.316520551 0.092886019 0 0 0.091740366
0.244763199 0.319366488 0 0.035148799 0 0.12286448 0.062464048
0.039632891 0.073336629 0.063447733 0 0.127453004 2.014465539 0.165927663
0.009402406 0 0.224954276 0.040602287 0.173456915 0.141469416 0.202713513
0.059031957 0.407620667 0.057866156 0.232886189 0.737506116 0.022826805
3.462362644 3.906551396 0.055892685 0.163151315 0.04782114 0.401637989
0.090851872 0.064808543 0.497198738 0.007952492 0.047492311
TCGA-FD-A3SR 602 1 0.3268885 0.1823836 0.05154319 0.3286593
0.80700826.90113 0.1485508 0.2375848 0.1296872 1.0761520 1.710362

0.26668 0.6322632 0.3532987 0.2469099 0.06729011 0.1400004
0.8358548 0.00846781 0.04665136 0.3504937 1.718257 56.61376 0.1112362
0.07639647 0 0 1.169616 0.9459754 0.4708736 0.1421756 0.09160631
0.3614958 0.5735396 0.09091754 0.5746384 0.3753496 0.1258931
0.03304918 0.2187788 0.1657351 0.6607668 0.09862996 4.145023
0.4959447 0.214113 0.1676508 0.4007776 2.593267 2.591494 0.4159236
1.606429 0.1633379 0.1515292 0.3058337 1.020425 0.1697394 0.6997704
0 0.335092 0.253901 0.1526772 0.496197 0.2008895 0.1704303 0.4287955
0.2754449 0.006301872 0.1827972 0.4065308 0.3745596 1.660994
8.244568 0.1074847 0.1105507 0.4093564 0.3070685 0.1092748 0
0.526473 0.3241832 1.335002 0.1739235 0.1654177 0.1334989 0
0.1468371 0.6717796 0.07046639 0 0.107155 0.0666595 0.2494159
0.622421 0.4763528 0.3985133 0.9365128 1.285459 0.3908414 0.2333243
0 2.746629 1.553883 1.03683 0.3691097 0.3373439 0.4321389 0.9642769
2.008941 0.1122751 0.04181201 0.07172024 2.579063 0.04708736 0.4277842
0.005032057 0.335092 0.3344961 0.2285829 0.5067211 0.4112936 0
0.06729011 0.1207009 1.319988 0.09176272 0.4009886 0.5496379
0.8882667 0.02999016 2.97639 0.8403729 0 1.532324 2.367748 0.16192
0.07464218 0.7386059 0.146748 3.049037 0.7510579 0.2793539 0.1242119
0.0352949 0.08668124 0.2578259 0.07796282 0.04055008 0.09642769
0.9046039 0.3140788 0.2325391 0 0.2186624 0 0.3609562
0.4231683 4.113999 0.1287756 0.250575 0.3271654 0.5690466 1.133351
0.4580316 0.9830921 0.05013281 0.3250546 0.1236404 1.081211
0.1783486 0.2170922 0.1717009 0.07877546 1.358045 0 0.2870269
0.9384061 0.4155338 0.2746629 0.4389123 0.2484014 1.202132
0.265759 0.07236405 0 0.7540383 0.2510209 0.24458 2.095793 0
0.6413044 0.3991734 0.5493525 0.2116459 0.1107329 0.2883902
0.3072163 0.1410673 0.3815524 0.5050974 0.1667737 0 3.969895
0.2660513 0.0807751 0.2323157 0.7749303 0.617311 0.5257406
1.578499 0.2388157 0.6915973 0.1120255 0.1684714 2.070208 0.07633859
0.3165454 0.05303523 0.4306914 0.2281009 0.08711839 0.044686
0.1844704 0.2676921 1.022974 1.123534 0.0550639 0.1758202 0.03644374
0.3072944 0.07811391 0.1486878 0.04585526 0.07934405 0.01042596 0
3.634218 0.02593744 0.2116459 0.8598779 0.1575363 0.1353996
0.5159951 0.4422836 0.1014007 0.04921462 0.3205624 0.1085461
0.05824679 1.454368 0.228367 1.574483 5.101364 0.1164936 0.1360184 0
0.1255663 0.03495818 0 0.5406677 0.009944924 0.1088837
TCGA-GU-AATP 1003 0 0.06275813 0.2237079 0.2275985 0.232201
0.03210354 25.3391 0 0.06081737 0.4199491 0 0.02067155 0.9878166
0.33645 1.589818 0.06782852 0.3003822 0.09904399 0.02943798 0.2278316
0.07478237 0.2403307 0.1719633 26.57203 27.32718 0.04815531 0.1574266
0.1926213 0 0.2648542 2.028027 0.5544612 0.2092675 0 0.3547226
0.1588081 0.06691054 0.664563 0.1806169 0.1389761 0.1070188
0.07813707 0.09757787 0.270161 0.1451732 0.16649 0.3318084 0.157576

0.0129876 0.6741744 0.4907593 2.10236 0.1669625 0.2149542
0.4808326 0.1115176 1.5522590 0.06525635 3.5706270 0.1409201
1.0277180.08988992 0.04526962 0.4797961 0.0627139 0.2427469
0.2027130.009275695 0.04484304 0.06648563 0.2756562 0.5976198
4.9439460.1186547 0.1464471 0.05239389 0.1903042 0.1148864
0.04731048 0.1859791 0.07952728 0.4534574 0 0.04057958 0.1164423
0.2660419 0 0.0760607 0 0.06284677 0.02426476 0 0.03415015
0.2364228 0.05393395 0 0.2837982 0.4168949 0.7768706 0 0
0.2526718 6.6535340.9538159 0.05432907 0.1027313 1.3106760
0.3578475 0.1239429 0.06154289 0.05278234 3.3309740.06930765
0.6996149 0 0.4227602 0.6769718 0.5046750.5617405 0.08648301 0
0.1485660.2853312 0.7632758 0.05064941 1.5301820.06741744 0.9066638
0.1324271 0.5278234 0.4352199 0.08239909 1.2911460.4862897
0.1551911 0 0.2070763 0.1439984 0.7662204 1.2897250 0.04570674
0.1039008 0 0.3162439 1.4344140.05968546 0 0.2837585 0.0770485
0.4563642 0.1490637 0.2682068 0 1.5053210.3685251 0.1916258
0.04738606 0.5795744 1.7335910.2567754 0.4566510.2809060.6121959
0.07379023 0.05126211 0.03639715 0.6164997 0.3500139 0.1597684
0.06318141 0 0.39978 0.06351964 0.2414134 0.5261849 0.4323536
0.4548093 0.8613771 0.1290426 1.7780860.3259744 0.1065123
0.0905761 1.1098650.05278234 0.3599960.4605252 0 0.3627510.4084129
0.5082561 0.06922678 0.05432907 0.3495719 0.4069711 0.08494211
0.2262021 0.07434505 0.06067883 0.05222478 2.7399060 0.05944624
0.4274305 0.6711008 0.05344806 0.8383212 0.2701610.1366990.6964981
0.1978678 0.1549826 0.9167581 0.1685436 0 0 0.1573902
0.3061165 0.08548609 0.06577311 0.06788026 0.05628781 0.2015517
0.8462416 0.2431449 0.06469722 0 0.1884604 0 0.06383207
0.03374707 0 0 0.1563992.1275180.03817719 0.01730669 1.186547
0.1490637 0.07473527 0.4595644 0.4556965 0.1119384 0.07243876
0.5105091 0 0 1.6334790 2.1436645.4239120.07544518 0.3670421 0
0.2772306 0.06431846 0.07952728 0.6764352 0 0.1165567
TCGA-DK-AA6X 467 0 0.4040916 0.3745111 0.2930956 0.5731265
0.08268426 54.072850 0.4699144 0.7374542 0.5563125 0.4259248
1.2076761.2276030.7490221 0.6259929 0.2196782 0.06377317 0
0.4694332 0.09630284 0.08842622 0.1107254.3648593 8.652510.1860396
0.20273 0.3720792 0.09670918 0 2.2218440.1785053 0.6063512
0.05787898 1.71301 0.2690908 0.3733847 1.0114090.3967778 0.1093705
0.03758622 0.1311147 0.1675444 0.2087439 0.1246334 8.39861
0.0854589 0.2029223 0 0.7596616 0.8426499 2.2504260.4300202
1.7992810.1667088 0.1436095 1.0394590 0.1512639 1.2379670.09550032
0 0.3609461 0.1157580.1204806 0.3592263 0.5653296 1.062851
0.5220974 0.01194501 0.3464864 0.3710136 0.5916385 1.399272
7.1935310.6621355 0.1047727 0.07871678 0.1838017 0.1479478
0.06092524 0.6386646 0.2048264 0.3893007 0 0.1045147 0.4498543

0.3426020 0.4897452 0 0 0.1718615 0 0.08245818 0.9133779
0.1389096 0.2158199 0.3828714 3.303795 0.06331863 0 0 0.3253844
5.265905 0.6755650.349818 0.5512284 0.6701777 0.4264767 1.236957
0.05320352 0 0 3.99971 0.08925263 1.081136 0.01907622 0.09073665
0.4755203 0.07221196 0.3221844 1.67056 0 0.2550927 0.3535765
1.831819 0.1956751 0.4504068 0.2604554 1.023746 0.05684543 0.5437741
0.176989 0.2122229 1.473283 0.4174877 0.2212638 0.2122229 0.7333358
0.370875 2.466798 0.9253447 0.07564382 0 0 0.1643016 0.8959518
0.5911045 0.3074457 0.06092524 0.4403743 0.2976633 0.8080796
0.1919604 0.138156 0.2134085 0.7982116 1.336837 0.2467709 0.4881805
1.017765 2.604554 0.5353686 0.4920532 1.012882 1.433401 0.1900504
0.3740796 0.04687132 0.9416175 0.1126847 0.1371638 0.1491663
0.09954431 1.063975 0.245397 0.1943038 0.9317104 0.2308575 0.9761532
0.6239585 0.2215704 0.6925169 1.175389 0.2743277 0.1166416
1.039459 0.271887 0.370875 2.814647 0.03573445 0.9694444 0.5259438
0.1487544 0.2451583 0.0699636 0.1929299 0.6987828 0.3403129
0.3716552 0.7659173 0.2379738 0 0.9361011 0 0.3827668
0.2201736 0.6374903 0.3441453 0.7473938 1.809114 0.2514821
2.046846 0.1274045 0.1596662 1.973252 0.1446975 0.3333344 0
0.09571145 0.2797613 0.3302605 0.5082057 0.08741448 0.4349161
0.4427673 1.247826 0.1043719 0.2499463 0.06907799 0.1941557
0.296125 0.1526596 0.5215034 0.3007884 0.01976209 0 4.540179
0.1474908 0.1560099 0.407468 0.5972103 0.176444 0.5357487 1.11778
0.1441514 0.1399272 1.145506 0 0.22081 6.137763 0 1.716021 7.508066
0.2031452 0.3007884 0.226707 1.071032 0.1159588 0.1024132 1.306644
0.3675808 0.1876234
TCGA-XF-A9T3 68 0 0.1080562 0.1925888 0 0.09995019 1.492438
41.76135 0.8784320.3926797 0.262932 0.2479347 0.1779601 4.406319
0.1930985 0.8011693 0.2141082 1.136551 0 0.1689532 1.176833
0.1072995 0.1182281 0 2.856919 37.75381 0.2155744 1.587609 2.653223
0.1293026 6.840341 1.250805 8.114647 0.1801571 0.1547713 0.6871015
0.69078 0.1536077 1.040215 0.3292771 0.3855184 0.3350249 0.1386123
0.08400419 0.09303196 0.166638 4.191528 0.1142607 0.2034842 0.2236188
0.8705888 0.8449833 3.451808 0.287474 1.110316 0.4776298 0 0.9888844
0.1847181 0.5874696 2.009878 0 0 0.4825941 0.5416997 0.550809
0.2593592 0.32394 0.2507752 1.745144 0.07985388 0.09651274 0.7822399
0.2373107 0.748345 0.2211019 0.06809939 0.3642178 0.706654 0.04095793
0.079124 0.3258344 0.3202166 0 0.7156943 0.2203864 0.1397388
0.1879583 0.4580677 0.1860639 0.1309604 0.7143293 0 0.2088938
0.1689345 0.3895454 0.6996513 0.9286281 0.5771135 2.947354
1.435609 0.1523855 0.07391396 0 6.873746 3.699324 0.5747939 0.4677156
0.1768815 0.5973631 0.0814586 1.426844 0 0 0 4.753524 0
0.3613765 0.1147743 0 0.3178914 0.1930985 0.7396218 0.4467161
0.1964406 0 0.2224663 0.8960446 0.1453459 1.3926 0.1160785

1.2443341 0.2280114 10.36032 0.1380393 0.2837475 1.238171 0.4186438
0.2767492 0.189165 0.08913533 0 0.188467 1.713059 0 0.1573946
0.4472377 0.4393509 0.2178019 1.580644 0 0 0.6890115 0
0.09822028 0.128328 0.5541542 0 0.7623066 1.555026 0.06598778
0.08158873 0.2721557 0.6633058 0.1368444 2.920379 0.09673209
0.4791233 0.1270511 0.2647874 0.3133408 0.7899398 0.07533119 0
0.1631775 0.03327332 0.6424471 0 0.2857681 0.679484 0.3631322
0.3480378 0.3707771 0.1296073 0.7168357 0.5612587 0 0.2339292
0.6948918 0.2726399 0.4958694 0.5160316 0 0.5574211 0.320758
1.19333 0.02979845 0.09354312 1.332753 0.1557151 0.03250051 0.09401047
0 0.161463 0 0.1925525 0.3371257 0 0 0.966572 0.09202621
0.9992846 0.5581917 0.03362379 0.6457348 0.02839052 0.05336943
2.923914 0.3869284 0 0 1.94964 0.2720349 0.07359444 0.1132473
0.7012523 0.2907469 0.1428944 0.8953617 0.1395479 0.7240666 0
0.1297955 0.1979634 0.0314015 0.4648423 0.4021618 0.7398279 0
0.7326267 0 0.0595969 1.225789 0.085552 0.09650869 0.3414965
0.7472501 0.2569788 0.06236208 0.2130881 2.475786 0 1.297091
0.2893742 1.695834 7.300709 0.1299006 0.3447101 0.1515565 0.3182215
0.04429709 0 0.5823389 0.113415 0.05017146
TCGA-PQ-A6FN 507 0 0.01729199 0.03698347 0 0.03198961 0.6103473
70.23684 0 0.2010869 0.1893439 0.1190293 0 0.4754482 0
1.891088 0.03737811 0.1256809 0 0 0.03766519 0.02060507 0.2270374
0.4264354 2.532109 61.8167 0.08757156 1.301291 0.7961051 0.06207604
0.5473223 1.175961 0.2291592 0 0 0.4031695 0.13818 0.03687224
0.04993898 0.06147574 0.1212599 0.2814698 0.0939465 0.13443
0.08932618 0.1600002 4.248152 0 0.1953788 0.02147114 0.2089776 0
3.922913 0.04600382 0.04442036 0.1070076 0 0.6543809 0.1330201
0.053941 1.305465 0 0 0.4247566 0.1857579 0.08232386 0.1429605 0
0 0.3351258 0.09200765 0.1019352 0.05495713 0.1898815 0.08981699
0 0.03269338 0.107603 0.02165443 0.03932644 0.05697917 0 0.1537306
0 0.1561786 0 0.1006294 0.06015711 0.1099553 0 0 0.0857344
0 0.04011458 0 0.03175714 0.1832132 0.04458189 0.1385313
0.3388494 1.590489 0.07722206 0.03548486 0 0.4177178 1.661404
0.5124767 0 0.09907086 0.7010277 0.07821384 0.280229 0 0.1017429
0 4.179693 0.05728981 0.1734908 0 0 0.5087145 0.09270336
0.3550801 0.142974 0 0 0.01780039 0.6309249 0 0.5240096
0.1114547 0.8526347 0.03648815 0.2181498 0.07573757 0 1.310437
0.1339893 0.09621091 0 0 0 0.2261996 0.7919515 0 0 0
0.1054625 0.1045631 1.565109 0 0 0.1984698 0 0 0.4312569
0.2217002 0 0.512359 0.283171 0.03167963 0 0.08710493 0 0.1920366
0.6547921 0.09287893 0.04600382 0 0 0 0.2725765 0.1808262 0
0.1305646 0.0479219 0.08812233 0 0.03741613 0.2718408 0.6537514
0.04177178 0.08900195 0.04888896 0.7814866 0.3233412 0.1320648 0
0.2502045 0 0 0.4048404 0 0.2482634 0.2013723 0.2673524

0.05722296	0.179634	0.350876	0.07475621	0.03900738	0.2256643	0
0.09575468	0	0.1617722	0.1618484	0.1474151	0	0.2615471
0.04418025	0.373131	0.1786524	0.09685334	0.5904886	0.01362982
0.02562177	0.4294164	0.04643946	0.08558477	0	0.7444551	0.1469243
0.03533147	0.1087363	0.4488794	0	0.05880105	0.04004797	0	0
0.04434003	0	0.0950389	0.02261301	0.1115815	0	0	0	0.5024598
0	0	2.680857	0.2327418	0.03603617	0.02799091	0.01793712	0.03084281
0	0.2046001	0.5722809	0	1.07279	0.1389237	0.7183605	1.942326
0.01700812	0.08274478	0	0.07638641	0	0	0.03289073	0	0.01204324
TCGA-DK-A3WW	633	0	0.06856047	0.1026442	0	0.1395181	0.02104302
81.38981	0	0.1694227	0.3002895	0.1887743	0.0270993	0.4125342
0.1470227	0.6354166	0.03704983	0.03889236	0.09738104	0.1286388
0.2090726	0.03267857	0.2475477	0.1127174	1.620571	126.9868	0.05050324
0.1326715	0.4419034	0	0	0.2976084	0.1817173	0	0	1.017236
0.1314877	0.04385808	0	0.146246	0.0506084	1.913127	0.06208093
0.07461965	0	1.205322	1.556288	0.08699664	0.05164342	0.01702605
0.718093	0.393164	7.36118	0.1823991	0.4931385	0.04848815	0	0.1933183
0.4570863	0.02688639	0.2250434	0	0.04618468	0.03062008	0
0.007912808	0.07679494	0.08221458	0.4455196	0.088582	0.1398393
0.124922	0.0363163	0.1505711	0.3561126	0.6312905	0.337025	0.0106658
0.1259236	0.1403318	0.225915	0.2170753	0.06095219	0	0.1733837	0
0.02659883	0.1574199	2.441367	0.03541666	0.04985576	0	0	0.03180981
0.06431229	0.002798064	0.2857238	0	0	0.0797238	0.5255067
0.08057247	0	0	0	0.7268691	0.5314208	0.2492788	0.106618
0.06317007	0.1240431	0.25925	0.1354021	0.04033973	0	0.7769631	0
0.1375737	0.01456461	0.04618468	0.04033973	0.07351134	0.2104034
0.1133746	0	0.09738104	0.07763384	0.909649	0.02213289	1.303892	0
2.278127	0.4918805	0	0.1351303	0.1620312	0.5570661	0.10625
0.2034473	0	0.03393324	0	0.07174815	0.7970729	0.07700494	0
0.0340521	0	0	0.1504352	0.03912223	0	0.3004553	0	0.5608774
0.2442682	0.1406419	0	1.857313	0.4116699	0	0.0931809	0.1726798
0.8206777	0.120221	0.06108623	0.4419034	0.3283184	0.09673507
0.01120032	0.02385736	0.1127719	0.05735619	0	0.07592518	0.02533387
0.2096361	0	0.02967001	0.08622505	0.1244179	0.0993718	0.1411524
0.03524334	0.352489	0.08546703	0.104724	0.6827576	0.3968112	0.1037923
0.3775485	0.3928998	0.01818873	0.2658974	0.371028	0	0.147473
0.07122252	0.2455019	0	0	0.1993968	0	0.05242852	0	0.1649328
0.06417079	0	0	0.3562596	0.2102027	0.4226902	1.416667	0.2560072
1.311078	0.01080809	0.0203174	0.008584436	0.8838067	0.06786649	0
0.0716424	0.27185	0.05603386	0.08622505	0.08898741	0.1475806
0.2409097	0.9061329	0	0.02120365	0.03516048	0.04941232	0
0.05977174	0.06636092	0	0	0.4015185	0.3984375	0.07507239
0.02268815	5.02945	0.09770728	0.01632899	0.1268346	0.3698153
0.02445754	0.07122252	0.1825228	0.9774236	0.1123916	0.7089121

0.02754072 0.1898804 1.609697 0.08541762 0.7217589 0 0.1211448
0.1264771 0.07819202 0.07824446 0.004797372 0.1814495
TCGA-XF-AAMY 3011 0 0.3737915 0.5329677 0.1807456 0.2996509
0.03824216 28.48286 0 0.2173394 0.272863 0.1715328 0.1477453
0.7844668 0.2671891 0.8314297 0.1885292 0.2252936 0.1179825
0.4324919 0.4885128 0.1336226 0.2044894 0 4.91603536.22134
0.05736325 0.1339491 0.229453 0.08945762 1.41974 0.8293087 0.4953611
0.06232056 0.3212342 1.056375 0.5675228 0.106273 0.3598346 0.2531215
0.1471557 0.02317859 0.1776942 0.1743541 0.2574554 0.05764398
19.58324 0.07905092 0.1877066 0.07735499 0.301157 0.4546881 3.677286
0.198888 1.088239 0.3745062 0 0.4807586 0.9584745 0.1465845
2.781055 0.2650182 0.08393292 0.834703 0.1070781 0.05752082 0.2658323
0.2241169 0.693992 1.207372 0.02209866 0.2804425 0.1055981 0.6202457
2.97700515.37335 0.04711435 0.05814991 0.06241229 0.2550295 0.1094834
0.05635687 0.3323112 0 0.3601097 0.8386049 0.09667787 0.4074536
0.1584563 0.06436386 0.8154406 0.1235516 0.0748639 0.1300703
0.07791788 0.1423804 0.7920843 0 0.09981853 0.7405216 1.37523
0.05857079 0 0 0.3611832 2.229125 1.022578 0.129435 0.336531 1.05617
0.1690706 1.41343 0.0984283 0.07331071 0.06287502 2.091191 0.4953611
0.4166953 0.01764582 0.2517988 1.319593 0.2671891 0.5229513
0.8241577 0 0.05899125 0.1988037 1.260515 0.04022283 0.7811885 0
0.6261043 0 0.8173752 0.2182904 0.1963098 1.907936 5.020381
0.1650587 0.1963098 0.185004 0.08576641 2.086244 1.580233 0.0699718
0.1088929 0.06188399 0.3039635 1.205484 0.7518247 0 0 0.2860142
0 0.7474872 0 0.2555932 0 0.4219195 1.11912 0.3652275
0.4515752 0.3138167 1.032538 0.8666437 0.6216785 0.6692379
2.055176 0.1757998 0.1017735 0.08671352 0.8026973 0.1042353
0.1903183 0.10035 0.02302004 0.6667125 0 0.2875749 0.4700988
0.1884239 0.1203944 1.410865 0.4227225 2.242064 0 0 0.2157909
0.1201897 1.38325 0.3430656 2.394612 0 0.7527144 0.3755491
0.7155216 0.2061596 0.06471751 1.189756 0.4309239 0.1573976
0.2508718 0 0.177418 0 1.065735 0.349859 0.07081315 0.2036643
0.6048874 0.5730123 0.4609012 1.287277 0.1628376 0.3616544
0.05892567 0.1107704 0.4628231 0 0.185004 0 0.135406 0.1999693
0.1527482 0 0.08085986 0.06705078 0.9744874 0.5848253 0.09654579
0.423875 0.1916949 0.2244966 0.1369603 0.3476005 0.2009998 0
0.07312108 0.4191852 2.099871 0.04547717 0.02061596 0 0.157837
0.1335385 0.2074506 0.2843404 0.0444475 0.08629001 0.2119224 0
0.2553162 0.8529628 0.1001013 1.380303 3.304178 0.08170118 0.03974776
0.1048539 0.440321 0.1072638 0 0.8531773 0.08718421 0.1214884
TCGA-K4-A3WS 761 0 0.18033 0.3856832 0.05449872 0.6672094 0.6687889
33.11778 0.2617813 0.5024162 0.191973 0.2068835 0.01484948 3.622859
0.8056333 2.005553 0.1542953 0.5274637 0.1067229 0.2114687
1.505705 0.1611605 0.4932639 0.06176522 3.436607 57.21328 0.05534805

0.193865 0.4151104 0.1618405 4.851603 1.239401 0.9459603 0.3382381
0.3228636 1.178525 0.8706128 0.3044143 1.345375 0.4045046 0.4048821
0.1537547 0.1292692 0.3037467 0.3881421 0.2433328 12.21975
0.3813691 0.05659761 0.1679343 1.331812 1.488496 3.995437 0.5197317
1.54413 0.5579663 0.6408722 1.42729 0.1541338 0.6080632 0.9372014
0 0.0506152 0.2013449 0.4197227 0.6829113 0.2965706 0.180203
0.3836317 1.116417 0.01998968 0.1530125 0.6527224 0.5060475
1.678181 12.6378 0.454592 0.3156023 0.6021972 0.5980874 0.3631278
0.03398565 0.3117304 0.4570295 0.1900166 0 0.2040533 0.1516097
0.09555605 0.2716994 0.4371077 0.07450699 0 0.104584 0.1644575
0.5182358 0.9924739 0.3099491 0.7825339 2.355801 1.082728 0.6287091
0.4934066 0.04632391 3.339741 4.431077 1.47313 0.5463846 0.2090926
0.3876881 0.2039139 2.042958 0.5342089 0.1768382 0.3791637
2.080241 0 0.5528278 0.03724419 0.1012304 0.7957718 0.8056333
1.600549 1.428883 0.4097885 0.03557429 0.3828659 1.009374 0.2183051
0.8165571 0.3390068 1.198779 0.887875 5.990786 0.139866 0 1.221012
0.756877 0.1353711 0.2367667 0.7065811 0.3620461 1.926459 1.29707
0.04219604 0.3283359 0.1865937 0.1374774 0.9995693 1.071632
0.04287525 0.06797129 0.3240515 0.1106961 0.9015346 0.5889422
0.847736 0.03968156 0.6360895 1.827018 0.3854341 0.2042396 0.7191314
2.283107 0.5314074 2.523875 0.6860852 1.27934 0 0.2700449 0.1307301
1.081414 0.09428761 0.6503644 0.2571906 0.06941042 0.7466765 0
0.314324 0.3779867 0.2272556 0.3267138 0.5800998 0.2935443
1.769519 0.4683297 0.1530269 0.1626641 1.159673 0.9479092 0.827534
1.144742 0.07973433 0.6388497 0.7720652 2.60554 0.2237818 0.2341648
0.4484217 0.4872485 0.2847528 0.9637518 0.2136241 0.08717768 0
1.064446 0.1406535 0.2989238 0.3070461 1.677224 0.4607351 1.29707
1.125612 0.3927926 1.154612 0.2013635 0.1558652 4.051689 0.04035795
0.3346963 0.1121526 1.152602 0.5745769 0.1535231 0.09449667
0.04876201 0.04043453 1.481921 1.125312 0.1746639 0.348566 0.07706691
0.5415248 0.2477791 0.1572136 0.09696928 0 0 0.4962113
5.283584 0.05484942 0.03729697 0.170472 0.2974456 0.1252681 0.6845846
1.09117 0.08041132 0.1040733 0.6223228 0.2677971 0.1231734
0.7876346 0.3018272 1.872861 11.95538 0.2414199 0.3116052 0.1896944
0.5310654 0.1386101 0 0.9003803 0.005257587 0.1255933
TCGA-UY-A9PA 1072 0 0.02032462 0.3042868 0.07370924 0.319599
0.03119081 26.99575 0 0.1477207 0.2596424 0.5596177 0.2209222
0.1498316 0.2724037 0.1130208 0.2086837 0.01801495 0.09622808
0.08580311 0 0.6660156 0.1667842 0.08353713 1.14822 18.85107
0.05614346 0.349602 0.09357244 0.2188884 0 0.3529018 0.06733717
0.05082947 0.1310014 0.9046734 0.09744822 0.1300164 0.05869717
0.1754818 0.04500829 0.02835714 0.07131463 0.1422055 0 0
7.007978 0 0.03827399 0.06309175 0.4093794 1.271484 1.956403 0.05407188
1.879585 0.05390332 0.108347 0.3167067 0 0.003622918 0.3335684

0.07205078	0	0.09077263	0	0.005864342	0.09756735	0.1218618
0.8962126	0.06564991	0.01802396	0.2723007	0.1722544	0.4017286
0.4222756	3.8052790.2305625	0.03161855	0.008484048	0.06933515
0.02232402	0.09193082	0.1505763	0.3090650.2202826	0.03108987
0.2365552	0.07777807	0.2584781	0.05249601	0.1477965	0.7053922
0.06105998	0.1768117	0	0.01658962	0.2081666	0.05240057	0
0.1838198	1.2151270.009554222	0	0.1253057	0	0.6733717	0.1390047
0.1055689	0.1497159	0.2808997	0.09193082	0.4940625	0.2006985	0
0.05128169	0.5102230.1346743	0.2039173	0.01439217	0.1369136
0.05979318	0.1634422	0.4356985	0.7562181	0	0.1443421	0.2039913
1.2640490.06561255	0.9344832	0.06550071	1.34048	0.2144368	0.05128169
0.2336782	0.08005642	0.5398846	0.3149761	0.1615488	0	0.05029723
0.2098566	0.9039581	0.5907269	0	0	0	0.06197916	0.1843517
0.7804340.05798855	0.04596541	0.42414	0.4491477	0.3325420.434477
0.1042326	0.05366911	3.0971081.6540790.4840621	0.3222718	0.1535718
0.9357244	0.4039120.0362178	0.6004231	0.5407188	0	0.2158203
0.07072469	0.09750709	0	0.05174203	0.04092341	0.1314281	0.1812598
0.2468549	0.1612530.7668375	0.2049080.2454882	0.3661383	0.06268692
0.2443828	0.3167067	0.1552261	0.1760019	0.3921131	0.1025634
0.06995221	0.5468615	0.02696007	0.2880136	0.6126056	0.04489145
0.1513319	0.05278445	0.6307475	0.2196670.04584841	0.1667228
0.1444627	0.03483627	0	0.05432669	0	0.05775613	0.3322226
0.2256042	0.3634994	0.1879585	0.6824482	0.07589285	0.2776189
0.03204037	0.06023053	0.3986916	1.25543	0.1005945	0	0.1529154
0.3645712	0.04152783	0	0.1319007	0.1093750.2764538	0.7249011	0
0.7228649	0	0.1831024	0	0.06201727	0.06557522	0.1134658	0
0.06331351	1.6536240.1112753	0.1008879	0.5379791	0.09655046
0.1089157	0.4793985	0.3373262	0.03625196	0	0.3006030.2587102
0.1249435	0.5000255	0.04082197	0.4503174	4.7547510.03331828
0.09725639	0	0.0897829	0.08748577	0.07726625	0.1739657	0.02844344
0.07077679
TCGA-G2-AA3F	893	0	0.1125736	0.2708643	0.0510324	0.3254023	0
36.348950	0.07159184	0.3081648	0.2905874	0.2641949	0.2607406
0.9807096	1.69541	0.1748988	0.03492329	0.03331163	0	0.06130159
0.1676779	0.06928357	0.2313469	6.8907737.923510.05182771	0.01512788
0.06478464	0.2020625	0	3.0541330.1398622	0.3167249	0.03023283
0.8053050.3485848	0.2250414	0.3251107	0.4573907	0.1090648
0.006544332	0.1226399	0.2953668	0.2907638	0	16.415780.580308
0.2914879	0.1223081.0203580.9536684.0515970.1871826	2.1688770.1492793
0	1.0337070	0.05894547	2.7713430	0.3791671	0.2199617	0
0.008120325	0.09382015	0.5062242	0.4898609	0.3181678	0.01871826
0.1583625	0.0521762	0.5253676	1.8272597.9901050.05320978	0.04378205
0.01762173	0.2880241	0.04636794	0.0954721	0.3961543	0.4279607
0.1779308	0	0.1910748	0.09301269	0	0.5088367	0.6139590.06976807

0	0.1550592	0	0	0.7852253	0	0	0.1151465	2.610156	0.02315194
0.02887651	0	0.5098893	2.843864	1.924791	0.03654518	0.04606908
0.7130941	0.3182403	1.292238	0.2501156	0.04139765	0.142019	2.523226
0.1398622	0.7059081	0	0.1895836	0.7865554	0.2263176	0.1270125
1.338001	0	0.1665582	0.2426308	2.567139	0.1249234	0.5440573
0.4081432	0.6938485	0	0.3905522	0.7318913	0	1.209313	0.6542186
0.1118479	0.1108537	0.2437621	0.1937249	1.141261	1.561591	0.1185366
0	0	0	1.616715	1.080663	0.08029645	0.06364807	0.6656117	0
0.1534898	0.1504045	0.3608258	0.07431534	0.7147583	1.218518
0.6444983	0.254999	0.6379503	0.712631	0.7978178	0.21314	0.8314029
1.235405	0.198544	0.229881	0.07344909	1.446617	0.05886038	0.2149408
0.1558327	0.01299913	0.2330617	0.04272734	0.5582156	0.3539453
0.2979218	0.985786	0.7604847	0.1916883	1.201888	0.04385422	0
0.1218545	0.1357392	1.100647	0.09686247	1.140772	0	0.7949982
0.3518401	0.01554024	0.3143227	0.4750874	0.8733929	0.4258405
0.419008	0.3095626	0.1500276	0.1057517	0.0351297	1.861841	0.2634148
0.03998731	0.2875168	0.1373156	0.3595256	0.9109284	0.5815277
0.3940817	0.4805218	0.3105629	0.3544539	0.3494461	0.03779104
0.2785853	0	0.0529353	0.3121912	0.02875168	0.04424317	0	0
1.371715	1.0885	0.05451822	0.2611167	0	0.7859768	0.3093592
0.2698928	0.4086076	0.628462	0.03096793	1.209845	6.74663	0.07704119
0.1862653	0.3192587	0.1336929	0.1340578	0.5011195	0.6568508
0.1003958	0.02436345	0.6815986	0.2507642	0.1730087	0.7576072
0.02826299	1.208132	1.592491	0.212224	0.1571155	0	0.3108048	0.008652935
0	0.8029645	0	0.5390235
TCGA-FJ-A871	272	1	0.09175805	0.07008883	0	0.266749	0.1408148	61.0663
0	0.4573054	0.4784442	0	0.142483	13.32757	0.4567831	1.62793
0.06375306	0.113863	0	0.1906101	0.1142093	0.05466929	0.2796739	0
1.43961	54.65182	0.05431429	0.3945805	0.3017461	0	0.3319207
1.631153	1.302866	0.03278229	0.02816297	0.305625	0.09951074	0.08385365
0.2271392	0.07323179	0.1403017	0.3413917	0.06379829	0.07133383
0.03385712	0.6670902	0.39548	0.1247487	0.2221621	0.02441448	1.42575
0.03416831	5.369492	0	0.4377505	0.1970003	0.06987804	1.585438	0
0.06892935	0.6023746	0.5111579	0	0.3805327	0.4224445	0.04160406
0.1887772	0.07859433	0.1521077	0.127022	0.1511184	0.1053716
0.04166057	0.172729	0.680863	0.08046562	0.1239171	0.2345112	0.0766046
0.01490582	0.4751274	0.02964523	0.291341	0.09966519	0.3314979	0
0.07628273	0.1231266	0	0	0.04766041	0.2599659	0	0.02280682
0.2664148	0.01069941	1.087939	0.03379556	0.1050145	0.1100858
0.4621771	0.1294012	0	0	0.2216574	1.606868	0.9562731	0.03404315
0.06973687	0.6884281	0	0.7080975	0	0.1156902	0.0661479
2.406887	0.1737155	0.3945472	0.004641088	0	0.5013242	0.1405486
0.1686013	0	0	0.03103098	0.07084187	1.31526	0.03173744	1.082105
0.04224445	0.3828667	0.1106402	0.4299613	0.1722399	0.05163211

1.1265190.2539284 0.09377132 0.06884281 0.03243902 0.04511543
0.06858878 1.1660520 0 0.5208423 0.07994649 0.2377939 1.0426290
0.05929046 0.1458920 0.2502171 0.3269169 0.1344489 0.0346137
0.3883967 0.8000944 0.02401493 0 0.2311064 0.6638414 0.02298544
0.2511043 0.3168334 0.7323428 0.04623771 0.05353559 0.04561364
0.8714322 0.05483056 0.1668542 0.2177456 0 0.06680165 0.07960411
0.1229086 0.1648564 0.6805967 0.06333069 0 0.04042971 0.6304552
0.7353320 0.02837795 0.189669 0.03307395 1.127886 0.4397254 0.0347756
0.3641744 0.09877447 0.3763836 0.09760095 0.03404315 0.2503375
0.05666939 0.124193 0.1319653 0 0.07950048 0 0.3854159 0 0
0.1071329 0.7387056 0.2009466 0.5657083 0.2031427 0.1468405
0.2238117 0.04132865 0.1748046 1.039481 0.4928519 0.03243902
0.391317 0.1177988 0.04331322 0.05356645 0.0412141 0 0 0.02971632
0.8338495 0 0.0608099 0 0.04723649 0 0.04571179 0.04229251
0.1463587 0 0.383838 0.4570711 0 0.0650673 0.3469678 0.166053
0.07804979 0.1454995 0.3943227 0.607895 0.0680863 0.2471879
7.908889 0.02686063 0.336511 2.053583 1.270634 3.675443 0.04727472 1.630854
0.05515596 0.2895258 0.0403026 0 0.1994641 0.004586124 0.1460712
TCGA-ZF-A9RN 615 1 0.04972805 0.05317826 0 0.1073278 0.05087617
66.4275 0 0.4578073 0.2420056 0.1141009 0.01637965 0.04293414
0.088865 0.5223287 0.1074914 0.08227705 0 0.2332596 0 0.02962789
0 0.06812983 2.172554 39.7141 0.2060485 0.1603814 0.2289428 0 0
0.4796896 0 0.04145466 0.03561332 0.8783554 0.08609814 0.1060367
0.09574253 0.1262793 0.4343676 0.03854508 0.1388374 0.06443199
0.4281383 0.03834387 6.810912 0.1051669 0.2809338 0.07203745
0.2670999 0.129622 3.472299 0.04409905 0.298068 0.01465386 0.08836388
0.3197931 8.670845 0.01181888 0.6529109 0 0.2233234 0.6662776 0
0.004782743 0.0663103 0 0.6924488 0.2141667 0.02204952 0.02664942
0.1756055 0.2790966 0.2582944 0.8140188 0.09401917 0.05157387
0.01383856 0.05654721 0.01820665 0 0.1719264 0.1260311 0.05988482
0 0.0321543 0.05189969 0.1054027 0.08562766 0.1205374 0 0
0.03845365 0.05182975 0.01691236 0.2341698 0.2991519 0.5975794
0.353375 2.18531 0 0 0 0 1.043437 1.020304 0.04304907 0.03391745
0.2443643 0.03748771 0.4924813 0.09820944 0.04876513 0.1254704
1.272136 0.05491774 0.05543583 0.01173772 0.1116617 0.2925908
0.1332975 2.749208 0 0 0 0.05119011 1.704441 0.04013339 0.5542766
0.10684 0.7704782 0.2448416 0 0.03630084 0 0.5827634 0.1284415
0.1624958 0 0.3281646 0.05705046 1.170903 0.4525767 0.04654414
0.1810847 0 0 0.2505841 0.09092763 0 0.1499508 0.345913
0.305257 0.2260076 0.1181145 0.08500829 0 1.754089 1.575212 0
0.1501904 0.3339935 0.07631426 0.1404862 0.08861373 0.13355
0.1322971 0 0.08123776 0.02884023 1.011081 0.06933567 0.1265967
0.1251586 0.1531257 0.2745393 0 0.09564513 0.1042341 0.701885
0.1201267 0.04265842 0.1618963 2.755971 1.239813 0.2109945 0

0.07994827 0.3345878 0.1141009 0.4633769 0 0.5068752 0.3065842
0.01830591 0.1097073 0.9040304 0.6726961 0.1791524 0.07478457
0.3152102 0 0.1442414 0 0.1772275 0 0 0 0.06470136
0.7199666 0.4087790.5565798 0.1547386 0.8349083 0.02613095 0
2.2242210 0.04102058 0 0.01385691 0.1643145 0.1016057 0 0 0
0.2254656 1.58166 0 0.07689681 0 0.02986632 0 0.06503016
0.1604423 0 0 0.3098171 0.8188145 0.06050139 0.04114024 0
0.1443622 0.0345441 0.1571586 0.2235276 0 0 0.3064510.04219891
0 1.95627 0.06658581 0.5968013 7.4896150.06521561 0.1850771 0
0.1464473 0.5708015 0.03150776 0.4256401 0 0.1154459
TCGA-GC-A3RB 582 0 0.1175163 0.2513396 0.03874405 0.1877557 0
60.3390500 0.01552939 0.2339602 0.2206154 0.01055674 0.1702844
0.3722798 0.7722981 0.1674233 0.1401451 0 0.2906509 0.1396214
0.05728585 0.2279352 0.3512794 3.49089253.019150.1426360.05742579
0.2459241 0 0 0.8656528 0 0.05343536 0 0.3170179 0.1664717
0.02278034 0.1234128 0.09766499 0.05914468 0.02484245 0.1729164
0.09966398 0.5242797 0.02471276 4.5124460.3389021 0.1207086
0.01326526 0.2582203 0.2227783 5.6899070.08526599 0.74098 0.3494453
0 0.2933073 0.1643645 0.006665146 1.0169420.1514892 0.1439328
0.4771315 0 0.001541248 0.08832361 0.1921639 0.1983493 0.0690156
0.0047370.09160347 0.04527135 0.3206535 0.8046132 2.4920310.08079426
0 0.004459501 0.1336313 0 0.07248289 0.4748879 0.08122748
0.09648996 0.06536753 0.04144713 0.08919877 0.1358648 0.08278101
0.7380245 0 0 0.08674231 0.01670223 0.002180015 0.2716619
0.0550870.04279357 0.1633369 1.7196080.00251101 0 0 0.1806516
0.8140777 0.7550107 0 0.06120778 0.1870238 0.07248289 0.4809182
0.1054939 0 0.05391076 2.6435910.03539468 1.1075860.003782503 0
0.6285861 0.1431845 0.3712499 0.5741575 0.05826509 0 0.1787078
1.7275090.07759836 1.2168260.1032881 0.3959187 0.04508608 0.1886877
1.0411230 1.4022140.2069525 0.1245427 0.5891248 0.1850654
0.07353847 1.0341520.6304211 0.08999361 0 0.05306103 0 0.2261034
0.4395239 0.03048073 0.04832193 0.5833655 0 0.2330604 0.07612525
0.1095763 0 0.3617654 0.4930359 0 0.1451974 0.1614451.131251
0.7337175 0.04759323 0.3156026 0.4547520.1130517 0.07853705 0
0.8786230.1564048 0.1087894 0.04302144 0.009869006 0.1497198 0
0.06934909 0.8061512 0.2154131 0.02580737 0.1924546 0.1290555
0.6289904 0 0.0543947 0 0.1030539 0.2156431 0.1103077
0.9071436 0 0.7669081 0.3476202 0.1769734 0.1325752 0.1664717
0.2422808 0.09237149 0.0722984 0.2629053 0.1518689 0.1422633 0
0.1142237 0 0 0.08731369 0.08730993 0.6277933 0.1317298
0.7726227 0.1097025 0.7113884 0.1599942 0.04748879 1.7077370
0.2115033 0 0.02232708 0.2370171 0.06548526 0.03358963 0.1733287
0.08623676 0.3087911.8672240 0.1486808 0 0.1347427 0.05871676
0.05588291 0.1206399 0.05964143 0 0.5590991 1.4590150.07798674

0.03535338 1.252311 0.1437921 0.02862488 0.2421061 0.3102925
0.09527625 0.03699371 0.3041635 0 0.02189151 0.6323162 0
0.8876323 0.8391006 0.1436083 0.05112123 0.0899046 0 0.0131387
0 0.4673712 0 0.1264891
TCGA-UY-A9PF 117 0 0.1278257 0.08201669 0 0.2601208 0.1176993
48.93855 0 0.1486474 0.1866223 0 0.05052457 0.4991759 0.2055844
1.089911 0.1243378 0.07704381 0 0.1439023 0.6682281 0.09139003
0.1258728 0.1050765 1.432724 44.93733 0.07061957 0.05496797 0 0
0.8091825 0.9987624 0.08469948 0 0.2197053 0.4334993 0.9295225
0.02725667 0.1476635 0.3375845 0.1604043 0.0118896 0.2112349
0.1391226 0.06603165 0 4.444191 0.2432978 0.240713 0.03174376
0.3089606 0.9995784 4.466378 0.2040415 0.7224006 0.3390091 0
0.6070351 0 0.08430553 0.6713218 0.09062844 0.1722156 0.4567103
0.05492633 0.09589336 0.1909044 0.07664139 0.4153186 0.4128858
0.02267128 0.08220267 0.1895851 0.2620131 0.5311557 7.925085
0.04833517 0.07954224 0.08537269 0.2616378 0.02808008 0 0.3788023
0.291566 0.3232607 0.1173184 0.2479574 0.08893861 0.3251245 0 0
0.1267531 0.07680376 0.0741337 0 0.2034543 0.4153333 0.1977348
0.102405 0.7817305 0.7446229 0 0.0524622 0 0.8028414 1.439891
0.5245376 0.1327889 0.08369726 0.4239927 0 0.3912847 0.1009788
0 0 0.8068086 0 0 0.05430918 0 0 0 0.3518991 0
0.2788567 0.06051983 0.2434303 1.674771 0.04126508 0.454144 0.08238949
2.296322 0.0539455 0.5805381 0.12597 0 0.7390088 0.6933323
0.04741398 0.06713218 0.1265319 0.1759776 0 0.5178768 0.2871396
0 0.06348753 0.3118398 0.3091802 0.7011872 0.0729404 0.1156344
0.2134003 0 0.4879993 0.09108386 0.3277701 0 0.8657045
0.7294678 0.6088733 0.1158191 0.8370655 0.3530978 0.7770287
0.2277812 0.1373158 0.2720554 0.09017755 0.3341141 0 0.1752121
0.1604043 0.06508326 0.02573758 0.02361653 0.2931378 0.07762608
0.2212698 0.16076 0.1288709 0.4940562 0.9210876 0.1051336 0.58299
0.3186934 0.1301665 0.3320737 0.246608 0.3225211 0 0.6074652 0
0.2764734 0.5341386 0.53643 0.08460065 0.3319723 0.2746316
0.1657837 0.06920403 0.2287755 0 0.04044784 0 0.580842 0 0 0
0.5238909 0.3919068 0.5516514 0.6603165 0.1909223 0.4801507
0.0806034 0.03788023 3.072964 0.8925528 0.2530637 0.1907967
0.05877158 0.3982342 0 0.16076 0.0829551 0.06878819 0.3187571
0.323673 0.09904748 0.2767281 0.06555403 0.184251 0.5620369 0.04457593
0 0 0 0.5733961 0.9657129 0 0.02115016 0.09667034 0.2226494
0.1369987 0.325151 0.1856325 0 0.2655779 0.2174137 0.260333 0
0.4922239 0.05134756 0.9204451 5.009124 0.1592546 0.1631108 0
0.1129326 0.06288183 0.145783 0.1215673 0.107332 0.05341559
TCGA-YC-A89H 573 0 0.08766594 0.03124945 0.1059764 0.2702985
0.1569574 38.00987 0.05090512 0.1380518 0.2399813 0 0.08662739
0.1979556 0.1958259 1.32706 0.08685298 0.06734316 0 0.246729

0.1909528	0.03482081	0.02397961	0	2.26499	46.40096	0.0134535
0.03141529	0.1345350	0.0524516	0	1.0993430	0.03654036	0.03139149
0.2787226	0.2335123	0.1713550	0.3797667	0.06678562	0.1024595
0.02038542	0.06615065	0.1590227	0.07547681	0	9.8449050	0.0926997	0
0.009071096	0.9417447	0.1142558	4.9503550	0.3377997	0.07750019	0
1.1166890	0.4121197	0.0325556	0.7673475	0	0.04921231	0.522038
0.03139149	0.01686306	0.1285888	0	0.4408167	0.3775560	0.1957519
0.007739403	0.0534806	0.3794576	0.2242249	0.1381226	0.1704749
0.01829707	0.1329167	0.04814496	1.25566	0.04329861	0.1110905
0.1583571	0	0	0.06607924	0.1858151	0	0.1062481	0	0
0.1440542	0.04568552	0.01490746	0.21157	0	0.05852651	0.3901412
0.8287353	0.0137367	0.02998319	0	0.1058861	0.6292967	0.6328767
0.03794576	0.05979331	0.1884713	0	0.3551723	0.05771137	0.0429842
0.0737309	1.08989	0	0.3420488	0.01034626	0.04921231	0.214921
0.07833037	0.2274934	0.3624207	0	0.1037649	0.1015238	1.041977
0.07075145	0.3969624	0.09417447	0.2202619	0	0.0737309	0.4879619
0	0.7191505	0.1132152	0.09677869	0	0.07231547	0	0.2293548
1.0294850	0.03192355	0.07256877	0	0.1767026	1.04193	0	0
0.2947428	0	0.07968609	0	0.1498617	0	0.06184592	0.7069265
0.02676794	0.03309646	0.1471999	0.4036049	0.2775546	0.1301815
0.2354362	0.5830689	0	0.01193455	0.05084266	0.5607682	0.1222323
0	0.1176763	0	0.3164533	0.04436485	0.0737684	0.4134489
0.2504177	0.1411815	0.2632099	0.04506445	0.2847263	0.09106982	0
0.03163112	0.2818828	0.2949236	0.05028734	0.3012283	0	0.245188
0.3403019	0.1774939	0.06043869	0.07589151	0.1220780	0.2526632
0.09228678	0.1797809	0.05192577	0.05779186	0.03647603	0.5662895	0
0	0.1194143	0.1211914	0.3733042	0.3603197	0.6792912	0.02727898
0.2993625	0.05758305	0.1515451	0.2408760	0.1961968	0.03615774	0
0.07328536	0.1724233	0.08956073	0.04593876	0.04741049	0.03931382
0.1656146	0.6677528	0	0.06778097	0.03746543	0.1842804	0.2409114
0.0764281	0.09428161	0	0	0.3823252	0.7217469	0.05332917
0.03626321	0.4419922	0.1041125	0.05654818	0.1148769	0.1667171
0.02606086	0	0.1998905	0	0.02993986	0.7762230	1.0521051	1.246311
0.09580755	0.09322107	0	0.06454325	0.01796911	0	0.3890776	0
0.07123216
TCGA-XF-AAMQ	2177	0	0.1946938	0.09463724	0.0962831	0.1882744
0.05432423	49.81515	0	0.1157766	0.1938054	0.2436679	0.0349795
0.3579354	0.3083850	0.3608833	0.07173531	0.0376514	0.06284924
0.0207557	0.3469741	0.03163588	0.01452419	0.07274723	2.077817
48.38808	0.2240875	0.02854184	0.4074317	0.09530808	0	0.6914699
0.02931985	0.1106605	0.1140809	0.4314270	0.1025410	0.1698347	0.1789049
0.1483215	0.1273834	0.02469445	0.07612677	0.2201561	0.06857321
0.02047128	4.04311	0.1684416	0.06666081	0.03845984	0.3565028
0.2998817	4.1204210	0.1177195	0.9320739	0.6962917	0	0.2101333

0.0226924 0.02129605 1.2200320 0.1788441 0.07904817 0.01901348
0.04596198 0.08260512 0.2387739 0.1437681 0.5717038 0.01961991
0.09485183 0.09375344 0.3433617 0.9652998 4.1286420.1338549
0.1376730.007388223 0.2113287 0.03888117 0.06004257 0.3802696
0.1009295 0.2078161 0.08122264 0.2231678 0.2001174 0.05627308
0.1828619 0.4182966 0.04387726 0 0.05132473 0.02767122 0.012641
0.2781698 0 0.2835909 0.2591724 2.61831 0.006240128 0 0.02728021
0.1282681 0.7329963 0.2421009 0.04596666 0.05794585 0.4076965
0.02001419 0.6374043 0.08738786 0.05207011 0.1116452.6405370.05863971
0.1479823 0.006266615 0.08942207 0.1041402 0.04744385 0.1757325
0.6585427 0 0.06284924 0.09565403 1.6952020.04999561 0.3883962
0.3707629 0.6892861 0.1867395 0.0669870.3682302 0 1.313659
0.2742928 0.2415053 0 0.2847045 0.1218340.4399060.8807611 0 0
0.1318623 0.1079475 0.4281074 0.4611780.02524929 0 0.7017779
0.09778362 0.07239749 0.06305979 0.2042316 0 0.3745940.6367769
0.01621305 0.1603693 0.1783147 0.6518908 0.4707130.07096455
0.2376685 0.5415096 0.03121616 0.05782902 0.04619226 0.3760424
0.07403479 0.1126472 0.08018465 0.05722624 0.6201162 0 0.05106367
0.4451937 0.2988895 0.1924022 0.2505225 0.04549174 0.4219658
0.1379 0.06758832 0.05747587 0.08536665 0.3126060.1522924 0.4112872
0 0.3547683 0.2182417 0.1563726 0.1098212 0.2757999 0.24295
0.1530353 0.09981624 0.1418887 0.03145087 0.09217693 0.04418626
0.2483759 0 0 0.1808198 0.2677098 0.6557082 0.3819230.7543053
0.4543709 0.7403925 0.1046323 0.06556373 1.7396710.02376685
0.08760138 0 0.02959209 0.1378541 0.01808198 0 0 0 0.6219278
1.0083940 0.1779015 0.1361544 0.1116166 0.04863914 0.04629166
0.1713160.5928613 0.006491928 0 1.2343180.03230089 0.0732141 0
0.1541461 0.05005859 0.3253873 0.4498142 0.4577585 0.03064444
0.2715928 0.3604710.01813424 0.4007317 0.01777464 1.0048923.266151
0.1392710.3528936 0 0.1172794 0.03265108 0.06728631 0.2861586
0.0030962 0.04930804
TCGA-CF-A1HR 389 0 0.05590706 0.2192153 0.1351683 0.1379017
0.02859892 30.0187 0 0.05417818 0.1360381 0.1282787 0.09207458
0.7240346 0.1498605 0.5526882 0.04028263 0.06607177 0.04411587
0.1136389 0.1623681 0.1221342 0.1835098 0 2.50146526.36718
0.06863742 0.1001721 0.1715935 0.06689976 0 0.8359057 0.4321912
0.09321131 0 0.6319977 0.1042422 0.1788185 0.4305565 0.1325056
0.1238046 0.008666909 0.1096840.05795045 0.1444011 0.08621666 3.56087
0.1182345 0.1052805 0.03470938 0.3002887 0.3886089 2.931766
0.1983145 0.7180817 0.1482722 2.6822780.4978098 0.04778554
0.05481071 0.3058496 0.06606351 0.1255364 0.04161481 0.08007699
0.0376392 0.1093397 0.1117353 0.4324944 0.7825291 0.008263104
0.09986926 0.08884148 0.1500668 1.06476 1.31555 0.1409355 0.01449556
0.03111617 0.04238237 0.08187577 0 0.3313530.07084559 0.3029664 0

0.07229933 0.1361466 0 0 0.3387873 0.1847930 0 0.02913496
0.04943593 0.1316334 0.04804619 0.1492961 0.4334007 0.8856082 0
0 0 0.2701064 1.54354 0.5098149 0.09679636 0.04575828 0.3090691
0.1685831 0.6375653 0.2576293 0 0.04702029 2.1386270 0.4985926
0.02639242 0.1255364 0.1644735 0.09990702 0.2901580.4622520 0
0.1294893 2.0862160.01504007 0.1947340.06005774 0.6672195 0.07864704
0.5642435 0.14284 0.0734039 0.6842942 2.2382170.1234371 0.04893594
0.4150587 0 0.2437768 0.5416387 0 0 0 0 0.1690324
1.0222590.05316983 0.08429156 0.2527817 0.06863742 0.05081809 0
0.09557109 0 0.5521727 0.6380938 0.03414135 0.04221311 0.1408103
1.4585450.2614220.7471839 0.2502406 0.2478931 0 0.09133205
0.06484762 0.4342503 0.1559021 0.1423271 0.1219490.06886099
0.2611675 0.1131709 0.1612944 0.6445221 0.2254567 0.1800709
0.04795899 0.0670574 0.3245225 0.2323113 0.04744238 0 0.269647
0.4231827 0.1924180.6446809 0 0.2015350.2680838 0.9878656
0.1233391 0.09679636 0.06673082 0 0.08407701 0.09727996 0.06622909
0.06879696 0.0465236 0.3486858 0 0.1059134 0 0.2773247
0.3332934 0.4021257 0.5294708 0.2957419 0.5727361 0.01468894
0.08283826 1.9211410.1501443 0.1844705 0.3477027 0.1908393
0.2023250.03807695 0.1757788 0 0 0.4119068 0.3913170.2166017
0.08645171 0 0.1007322 0 0.08935735 0.0901891 0.2080741
0.06835335 0.02322092 1.6786630.06801906 0.1387565 0.07046775
0.07377277 0.05548059 0.1379017 0.3672880.2326765 0.0645309
0.1998271 0.1423271 0.0381872.2724390.1122893 1.3935274.139298
0.07331904 0.1188995 0.07841367 0.08232214 0.06875648 0.07084559
0.4785285 0.01303992 0.1168117
TCGA-E7-A3X6 904 1 0.166278174 0.08890741 0 0.307609199 0.042529302
38.123243649 0 0.171207144 0.227589238 0.190762501 0.013692361
0.209819988 0.297142345 0.590740345 0.164736214 0.06386573 0 0.188490767
0.45273128 0.024767064 0.068224089 0.113904566 1.468552051 27.083704362
0.140346697 0.119172328 0.191381859 0.099486266 0 1.042575463 0
0.103960516 0 0.146850057 0.077509024 0.118186692 0.120052164 0.147786278
0.061369717 0.006444256 0.067439904 0.043088898 0.17894843 0 6.330531862
0.219782299 0.156562052 0.06021881 0.111639418 0.108355906 1.698094306
0.147456192 0.711903535 0.036749135 0.369333413 0.29817247 0.035530809
0.024699607 0.54579271 0 0.093342221 0.154712894 0.029770511 0.011994224
0.118253376 0.124620746 0.225105995 0.313302421 0.055296072 0.118812019
0.02935904 0.304313953 1.439453302 1.275879063 0.02619805 0.032334401
0.028920426 0.094539876 0.182635516 0.03133738 0.164251098 0.052677044
0.325389794 0 0.215031875 0.086769793 0.088110034 0 0.352666059 0 0
0.09643454 0.021663217 0.005655069 0.406183965 0.071449228 0.055504343
0.465477883 1.741816304 0.058623185 0 0 0 0.596801375 0.315892887
0.107958998 0.14176434 0.217040376 0.156686902 0.187128929 0.21892521 0
0.069923621 1.391398715 0.091815596 0.231704452 0.009812004 0.046671111

0.244587605 0.111428379 0.171971605 0.400990563 0 0.065604466 0.124809222
1.263941013 0.08946403 0.796366666 0.044655767 0.892126291 0.087716686
0.069923621 0.151726159 0 1.407333269 0.536845289 0.110137542 0
0.377197056 0.238453126 0.326267229 0.61020303 0 0.030275096 0.068821498
0 0.209472682 0.342044174 0.039534281 0.125349522 0.404826381 0.102070325
0.415642141 0 0.142123237 0 0.645175861 0.422881911 0 0.06277488
0.383896826 0.318969766 0.340164294 0.111113313 0.260491975 1.253377631 0
0.16977423 0 0.398858665 0.115920576 0.105826953 0.146474721 0.038401051
0.335419779 0.168295825 0.039976679 0.522799226 0.50989806 0.234309647
0.356597778 0.089036331 0.511321592 0.043183599 0 0.059995535 0.801981125
0.209770864 0.143071876 0.39703797 0.036760594 0.335872437 0.327475626
0.030605199 0.057317788 0.107958998 0.38040098 0.179712234 0.118778942
0.273829211 0.344711185 0.076730691 0 0.129632199 0 0.11812748
0.169872083 0.197753023 0.247819393 0.341713697 0.823162776 0.155222153
0.579637773 0.109219219 0.102656936 0.164822169 0.111639418 0.171453207 0
0.020271152 0.228927701 0.056624028 0.043566602 0.044962329 0 0.722488192
0.785171308 0.053684529 0.12856186 0.035530809 0.249663756 0.076157122
0.108722311 0.067059855 0 0 0.01726585 1.248165296 0 0.04585423
0.366772701 0.120677785 0.049502929 0.211481324 0.359336825 0.024715142 0
0.245926937 0.105826953 0.056787681 0.494054251 0.139153949 0.959401249
4.060775781 0.199892637 0.22101842 0 0.183631192 0.059644303 0.026338522
0.408520902 0.029087398 0.028951676
TCGA-DK-A6AW 1621 0 0.1482693 0.1233217 0 0.1676237 0.02528208
83.57966 0 0.2394736 0.2705866 0 0.1465128 0.07877694 0.17664
0.8550302 0.02670806 0.02920446 0.07799882 0 0.3588425 0.009815398
0.2974156 0 1.93214 147.0672 0.02275388 0.3719284 0.1516925
0.05914088 0 1.358731 0.2729047 0 0.1061848 0.2095125 0.03949391
0.1405152 0.7136654 0.2259088 0.145928 0.03064696 0.0372935
0.03842212 0.2127563 0.03810872 11.43181 0 0.3102343 0.07159568
1.592771 0.1717695 11.65937 0 0.5501601 0.101948 0 1.22243 0.08448697
0.08516138 1.297814 0 0.05548847 0.441461 0 0 0.2767953 0.09877652
0.2294008 0.2128533 0.02921907 0.1236013 0.1047171 0.1809033
0.2994955 5.359802 0.09344258 0 0.03438423 0.1686013 0.090475 0
0.3905646 0.1252582 0.1785527 0 0.06391422 0.06877521 0 0
0.1197982 0 0 0.05732674 0.2575595 0.01008518 0.2152787
0.2973173 0.1979716 0.7840095 0.3788213 0.003872144 0 0
0.1591867 1.910333 1.051605 0.08557013 0.04719322 0.2580448 0.1490312
0.2521467 0.09760716 0 0.04156699 4.029327 0.1091619 0.8815338
0.134156 0 0.3392625 0.2208001 0.07806709 0.06810684 0 0.03899941
0.1823063 0.8606554 0.05318303 0.5508774 0 1.56255 0 0.2909689
0.06313688 0 0.8366072 0.1914807 0.1134859 0.1297814 0.04076902
0.0567006 0.5603107 1.915283 0 0 0.1636473 0 0 1.17481 0 0
0.1031375 0 0.3593946 0 0.4646783 0.04350213 0.5578662
0.4823371 0 0.111952 0.3319452 0.6826163 0.06259035 0.3889771

0.1769746 0.3506288 0 0.05382637 0 0.3161421 0.2756418
0.04194012 0.0414637 0.04565599 0.1259334 0 0.1307055 0
0.141177 0.03979667 0.2119841 0.03810872 1.284152 0.1540262 0.6710419
0.07133022 0 0 0 0.6102091 0 0.2549551 0.3724161 0.1091619
0.02725863 0.2139253 0.25563 0 0.01486519 0.2211368 0.175644
0.1064316 0 0.6605272 0.07709784 0 0.06732175 0.1687995
0.1262738 0.8633282 0.1702051 0.1076527 0.4359904 0.0519414
0.2441029 0.2784713 2.78735 0.08153803 0 0.04820178 0.4510378
0.06732175 0.05179744 0.05345686 0 0.1120415 0.5646846 0
0.2802259 0 0.1187326 0 0.02872505 0.02657639 0 0.02417035 0
1.005274 0.06013036 0.02725863 0.9967209 0.1304335 0.01471382
0.5066807 0.4272247 0.05876892 0.02852338 0.5421376 0.2097006
0.06751632 0.6989985 0.06617747 1.916303 3.047041 0.04321044 0.1576647
0.1386388 0.1455492 0.06078225 0.03131454 0.4700331 0 0.09179036
TCGA-GC-A3BM 651 1 0.2628799 0.3255061 0.1003537 0.3967341 0
86.97848 0.09640855 0.2916223 0.2272487 1.142863 0.109375 0.121293
0.2596105 1.179713 0.5458069 0.02943239 0 0.1946987 0.1205477
0.1154067 0.204366 0.1706012 2.507498 57.69032 0.1910955 0.1338683
0.127397 0 0 1.301269 0 0.2422116 0.08917791 0.7918039 0.3372126
0.1032586 0.4395327 0.3091842 0.07659737 0.06434615 0.1644324
0.3549515 0.07147228 0.03200512 3.97551 0.04389069 0.1563278
0.03435926 0.4458895 0.3967105 5.984307 0.5153245 3.056605 0.1100825
0 0.4619892 0.5321647 0.01726386 0.5903908 0.0980957 0.4194115
0.5252368 0 0.02195651 0.4058878 0.08295619 0.7064175 0.04469052
0 0.3336588 0.2711648 0.3342445 5.461739 2.463009 0.2092708
0.01076201 0.01732629 0.1887969 0.07598428 0.09387148 0.5740187
0.2629912 0.4498656 0 0.348904 0.2502932 0 0.03573614 1.458859
0.06859839 0.04156597 0.256774 0.08652322 0.01693982 0.3860304
0.07134233 0 0.4588227 3.117853 0.04227562 0 0.08530061 0 1.0543
0.5046724 0.03593249 0.1811869 0.3951874 0.125162 1.880946 0.3005718
0 0.209457 2.480923 0.04583911 0.1850862 0.04408795 0.1398038
0.2442217 0.2225233 0.08117404 0.4575893 0 0.06550631 0.2100779
2.673251 0.08933018 0.5493937 0.04458895 0.5562015 0.0583903
0.069819 0.2499736 0.1089952 1.383609 0.3216252 0.4838801 0
0.410872 0.2380964 2.678628 1.279509 0 0 0 0.0843834 0.1673274
0.2656363 0.07895026 0 0.7170105 0.1528764 0.3018329 0.147883
0.3902541 0.03653471 0.6442106 1.071071 0 0.1567024 0.3833224
1.146573 0.760181 0.07396471 0.966094 3.570463 0.04880383 0.2486297
0.1203628 0.5499807 0.08681035 0.1761144 0.04178731 0.1150308
0.546445 0.126033 0.1796259 0.3480114 0.4463651 0.3342273 0.9613735
0.2098114 0.6597079 0.2155949 0.1408915 0.08985866 0.1334635
0.4538235 0.1904771 2.083748 0.0183528 1.049966 0.4454587 0.1222376
0.1831425 0 0.1321154 0.8374023 0.2684133 0.4900916 0.2458539
0.1970123 0.1036222 0.1479294 0 0 0.1130786 0.3831006

0.353498	0.3838527	1.57239	0.0516633	1.015801	0.338073	0.123004	0.5428106
0.07431492	0.4108720	0.02313237	0.2547092	0.02826965	0.04350142
0.08979011	0.03722797	1.184049	1.251831	0.05360421	0.256739	0	0.2492902
0	0.1206218	1.182952	0.07724071	0	0.8361408	2.251377	0.2019989
0.09157125	0.2615885	0.2957659	0.06590515	0.3743378	0.7893582
0.148069	0.119775	0.7059821	0.07044574	0.1134054	0.3551868	0.0555783
1.302834	3.178324	0.3492887	0.06620632	0	0.6111882	0.0510472
0.07889735	0.3684346	0.009681293	0.1349057
TCGA-GV-A6ZA	691	0	0.2652681	0.2502995	0.05658946	0.4618704
0.02394641	36.6177	0	0.1701164	0.7973506	0	0.01541915	0.07461508
0.9620208	3.008044	0.219241	0.1825663	0.03693903	0.2415395	0.1019653
0.1394526	0.2048748	0	6.403456	101.2967	0.1293106	0.03355039
0.1436784	0.1680492	0	2.348116	0.1033948	0.1951189	0	0.9591448
0.1246913	0.1164552	0.405577	0.6339965	0.1785322	0.06531266
0.09713892	0.09704596	0.6851551	0.0360954	18.33785	0.1979998
0.4407665	0.0581256	2.325795	0.5287578	4.704523	0.2905917	0.4810104
0.1793293	0	1.470467	1.64048	0.005562911	1.075593	0	0.05255696	1.21957
0	0.02251142	0.19975	0.2338951	0	0.25201	0.0276754	0.2174182
0.1818388	0.354115	0.769969	2.777783	0.2065138	0.0121374	0.01302707
0.1596939	0.03427805	0.03528944	0.4624134	0.5932032	0.2536794
0.04773782	0.2118815	0.04342783	0.5953313	0.2015162	0.6240802	0
0.1875125	0.343888	0.04879047	0.0127365	0.5070077	0	0	0.4804991
1.411311	0.003667575	0	0	0.03769417	5.169738	0.8181817	0	0.08301421
0.5894644	0.3528944	1.587487	0.3081682	0	0.4724515	2.697259
0.1550921	0.4696658	0.005524714	0.1051139	1.698506	0.3764429
0.0387319	0.6450869	0	0.03693903	0.3975538	2.316164	0.1259333
0.7500501	0.2011498	1.181059	0.3951157	0.1181129	0.2221191	0
1.365381	0.1209097	0.07855065	0.04097496	0.8109181	0.05370505
0.4898851	1.594206	0.04381481	0.03409319	0.0775008	0.09516765	1.17945
1.840307	0.04452008	0	0.4830161	0.05747137	0.1276528	0.2223767
0.2000586	0	1.387033	0.6581805	0	0.1767291	0.3930103	1.86782
0.4104252	0.03475727	0.8800304	1.245393	0.05504099	0.1784393
0.05429811	1.219149	0.2610794	0.2383463	0.0314185	0.08648787
0.09940018	0.04738004	0.1350548	0.4415484	0.5112766	0.4146359
0.3614125	0.2085512	2.212648	0.5349259	0.317795	0.1689044	0.2257804
0.944903	0.2148202	0.899672	0.04139659	0.8350164	0.2886062	0.05169738
0.1290926	0.04052469	0.01862498	0.5396702	0.1900779	0.4770895
0.1109097	0.1728149	0	0.6047765	0	0.04434164	0.06376507
0.1142012	0.07973506	1.491132	1.652433	0.2476301	0.3330295	0.1721905
0.3236893	0.6284636	0.04190621	0.1158454	0	0.04891631	0.4124776
0.0956476	0.09812186	0.05063268	0.08397146	0.9108822	2.214093
0.06045486	0.2171628	0.04001172	0.1968048	0.2572846	0.2448673
0.226551	0.1742242	0.0114467	0.2041547	1.541599	0.08543042	0.1032741
0.05900394	0.2100224	0.01858197	0.4510453	0.615074	0.4453128

0.08104937	0.7500501	0	0.1278987	0.5841791	0	3.673341 3.465911
0.2455655	0.273781 0.2626288	0.6203686	0.03838071	0	0.7420013
0.01091857	0.1086762
TCGA-G2-A2EJ	1460	0	0.1076862	0.1612207	0.1562138	0.3486279
0.04957761	41.18721 0	0.07044035	0.6485287	0.296503 0.1383336
0.5578439	0.1154624	0.4191738	0.1163865	0.03436158	0	0.05556366
0.09382429	0.05774333	0.0530205	0	0.987981 21.20376 0.07436641
0.382037 0.3966209	0.03865798	0	0.2493045	0	0	0.04627243
0.4793243	0.1204726	0.04592452	0.0932988	0.1421982	0.03577021
0.2353839	0.0865392	0.04185829	0.0834421	0	3.991525 0.03416086
0.02027876	0	0.1735216	0.1684181	2.392772 0.05729795	1.051189
0.01903978	0	0.2477064	0.08283854	0.01631605	0.388817 0.07634952	0
0.1683296	0	0.004660668	0.06318185	0.1291324	0.07497498	0.1043501
0.009549658	0.1385025	0.1197863	0.1261324	0.3076354	0.3305174
0.1832388	0.0167525	0	0.06122656	0	0	0.06382405	0.2046904
0.01945211	0.2306134	0.1044453	0.1948074	0.2054247	0.02781403
0.195768 0	0.03235149	0.08118967	0.2020274	0.002197425	0.2890442
0.08329038	0	0.2040625	0.6438121	0.1670502	0.02209827	0.06639088
0.02601346	0.3924508	0.1718478	0.195768 0.1366139	0.4365664
0.1217696	0.5138444	0.04253455	0.1267212	0.1086826	0.7492062	0
0.1800696	0.03431439	0	0.1900818	0.1731936	0.06560907	0.1780747
0	0	0.1413367	0.9019066	0.07821806	0.5963927	0.03470433
0.4295189	0.1136154	0.05434129	0.1179143	0.0424164	0.5384429
0.6675368	0.03138433	0.0282776	0.1065962	0.2594401	0.112693
0.6544244	0.1814246	0	0.08022716	0	0.1302337	0.4725695
0.2457932	0.07306174	0.3932646	0.03966209	0.0587304	0.1150998
0.3313541	0	0.5925634	0.7908821	0	0.02439282	0.1084895
0.247888 0.4531876	0.05277064	0.1156811	0.4870326	0.1139545
0.02638808	0	0.2878329	0.06756594	0.2193164	0.1084125	0.06963474
0.08231754	0.03269787	0.06990291	0.06771576	0.13028	0.07804039
0.2494177	0.1079439	0.1205519	1.040367 0	0.3030668	0.1038769
0.2445358	0.1111886	0.3725284	0.01428429	0.1867325	0.3430196
0.08324714	0.1425429	0.195768 0.1542414	0	0.1117429	0.2047765
0.07654087	0.07950856	0.02688363	0.01439199	0.05039572	0	0.1320165
0.1234728	0.4677268	0.4647362	0.2781403	0.4222093	0.7538423
0.04243998	0.06382405	0.03146117	0.2313622	0.2131924	0	0.07201723
0.2338267	0.02200274	0.1354315	0.03494257	0.202826 0.5187617
0.9992609	0	0.08326011	0.02761285	0.07761069	0.1183713	0.04694099
0.05211571	0	0.007899588	0.348873 0.8135604	0.1179143	0.08018036
0.7329554	0.1619923	0.1763268	0.1543924	0.2569186	0.03841485
0.05593371	0.1791775	0.02741455	0.066199 1.877542 0.02162876	0.5368325
1.700698 0.09179595	0.08588247	0.1359339	0.04756979	0.01324363
0.04093808	0.3072415	0.003767556	0.09749938
TCGA-FD-A43N	699	0	0.1569734	0.2637871	0.1626513	0.3318808

0.03441378	39.808160	0.2200294	0.2455469	0.1543608	0.04431823
0.5406208	0.3907167	1.4340420.1999514	0.07553047	0.1327142
0.2734888	0.3174948	0.1002048	0.09200906	0	3.55989355.90761
0.07226893	0.06026977	0.1032413	0.2012553	0.1419568	0.5191564
0.1857379	0.1121634	0.02408964	0.9030916	0.3180729	0.09563407
0.1619060.3986180.1158711	0.08343281	0.08629815	0.1568997	0.05792046
0	10.454470.2489802	0.1900299	0.06264995	1.1292020.7306602	5.42017
0.1193183	0.9792964	0.4262245	0	0.4659096	0.1725045	0.07195097
2.7234680.03974791	0.07553047	0.4757230.04817929	0.0355867	0.1674541
0.3025211	0.2341939	1.0865030.04474437	0.2403502	0.08314842
0.3857825	0.7571031	3.5102050.08479555	0.04360714	0.009360709
0.1657491	0.07389232	0.1267876	0.2990439	0.4688762	0.5063428	0
0.2827485	0.08191425	0.1425934	0.1158409	0.2446025	0.1667745	0
0.1235518	0.0876470.09609511	0.6098310.08672272	0.0449129	0.06277575
1.6672640.02108293	0.02300892	0.0691268	0.1625128	0.6315089
0.7412794	0.08735805	0.04129653	0.1652935	0.1014301	0.827766
0.1992932	0.03298582	0.2546131.4154040.03714758	0.2624862	0.0277888
0.1132957	0.4288157	0.2404411	0.4781894	0.7416520	0.05308569
0.3376048	1.1994110.01809808	1.1013420	1.7290120.1419568	0.254613
0.3683204	0.04416435	1.0511840.3475227	0.2435971	0.1766574
0.6381864	0.1543608	0.4986823	1.0072760.06296699	0	0.05568884
0.06838351	0.6441026	1.1070990.06398054	0.05071504	0.3431755	0
0.4280544	0.2396859	0.1150030.1480369	0.6644427	1.4299510.5957051
0.1523882	0.5648016	1.3937580.5963825	0.1998010.6022411	0.6860803
0	0.1373776	0.03901636	0.6147575	0.07035029	0	0.05079605
0.04143105	0.1714196	0.06809064	0.2587860.4935439	0.1695609
0.5417092	0.4328266	0.2478391	0.7903094	0.06988644	0	0.09709414
0.3244728	0.5658066	0.5402629	1.0656910	0.3951272	0.5491741
0.6934215	0.1020366	0.08735805	0.2676627	0.3635480.06576174
0.3971656	0.03984753	0.07835014	0	2.1878210	0.06372411	0.2290946
0.3528589	0.3151186	0.4493242	0.9267273	0.1674698	0.6987592
0.1325667	0.04984064	0.6715321	0.3011205	0.1664834	0	0.08201485
0.5716078	0.3894608	0.1057594	0.07276506	0.03016919	1.340817
0.5799456	0.08688068	0.2253971	0	0.1616179	0.1232493	0.4398785
0.05426336	0	0.01645025	0.4191344	2.0525560.04092449	0.1020366
0.9327510.2751949	0.1134939	0.3552162	0.5466428	0.05999685
0.0582387	0.5845586	0.19981	0.1608297	0.3757912	0.1351204
1.0868573.2669520.1066071	0.1251903	0.1415356	0.1981204	0.01378939
0.02131255	0.4478638	0.003922814	0.1717984
TCGA-UY-A8OD	3432	0	0.1016047	0.0724361	0.1228264	0.2976117
2.88462752.275240.1769968	0.1846174	0.3399463	0	0.08366749
4.7539280.4539238	0.9730583	0.4301032	0.4683036	0.2806143	0
0.5532850.02017863	0.08337697	0	2.09703497.320010.07796288	0.964872
0.7796288	0.3039566	11.363091.02911	1.1220830.1270506	0	0.8614374

0.676601 0.1263819 1.369352 0.1720098 0.362501 0.03150222 0.1111693
0.0921535 0 0.1566889 4.184561 0.1074388 0.1275568 0.3469417
0.7503928 0.9710965 8.178922 0.1351552 0.6525154 0.2844382 0
0.7162305 0.08684473 1.08215 1.55637 0 0.2851849 0.8697466
0.7276536 0.9405676 0.2619389 0.2538326 0.5109057 0.8751733
0.007508621 0.2994765 0.8431757 0.1611582 0.4837697 5.821229
0.2241173 0.7508044 1.25117 0.1540502 0.1115999 0.1148927
0.07527451 0.1287537 0.4588389 0 0.2299425 0.1178242 0.3230390 0
0.1231414 0.08396003 0.4069927 0.03928437 0.02647472 0.846608
0.2212864 0.8731843 0.4069927 1.309113 0.9605027 0.1552279 0 0
5.113409 1.234291 0.7335029 1.055498 0.2148311 0.374465 0.03829756
0.6403352 0.1003311 0.2989116 0.04272699 3.716665 0 0.9061347
0.4616649 0 0.09963721 0.1815695 1.184579 0.28003 0.2770681
0.08017552 0.2222579 1.221692 0.0410004 1.044025 0.05457402 0.7392574
0.107199 7.989947 0.2595953 0.06670158 1.217166 0 0.2288193 0.1334032
0.04190675 0 0.3987327 1.088769 0.04754964 0.07399867 0 0.1032799
0.4607956 2.600975 0 0.03829756 0.2885984 0.1247406 0.09235603 0
0.6079131 0.04471614 0.5017552 1.331931 0.09307197 0.2685111
0.3838599 1.247406 0.3018893 6.638725 0.2273917 0.495569 0.05973276
0.2351461 0.2651695 0.6151117 0.1062503 0 0.2557249 0.03128673
0.2588956 0.1028376 0.4885568 0.3194577 0.2134071 0.4090727
0.08715996 0.03917222 0.6951007 0.2638744 0.4311054 0.1466416
0.0816754 0.2563619 0.4079805 0.9349398 0.06738794 0.2083931
0.3480082 2.188061 0.02801933 0.1319372 0.2627638 0.2562317
0.06876014 0.3030774 0.1805456 0.1428921 0 0.2942162 0.4754964
0 0.2076015 1.379819 0.08653178 0.3654086 1.574595 0.09484885
1.301102 0.08008639 0.100366 5.042816 0.04547835 0.167627 0 0.8352198
0.2717801 0.5882041 0.1597288 0.1098973 0.09112929 0.8541641
0.9674053 0.5248649 0.07855802 0.08684473 0.09153457 0.09307197
0.1181067 0.05463611 0 0 0.01055034 0.6888852 0 0.07004833
0.4482346 0.2145176 0.1512444 0.489493 0.7904649 0.06040898
0.05863875 0.3318556 0.7328791 0 0.5434074 0.1360485 2.344977
16.318250 0.04441631 0.2971184 0.2137617 0.2244165 0.124957 0
0.1932601 0.04739712 0.2358798
TCGA-2F-A9KT 2352 0 0.1007134 0.2154021 0.2191482 0.7638977 0
54.208560 0.07319918 0.5513961 0.138652 0.01990406 0.3732325
1.025868 2.202846 0.2503557 0.04641957 0.04768334 0.08503503 0.3948708
0.02400195 0 0.9106827 3.686929 39.99197 0.1298287 0.6496358
0.1854696 0.1446193 0.1275104 2.215037 0.333672 0.2518723 0.04327624
0.8965751 0.2736317 0.2147543 1.105263 0.6240335 0.1115135
0.281033 0.09803478 0.2505467 0.3641826 0 10.07061 0.5750803
0.2655196 0.02501079 3.651433 0.2100171 2.19513 0.6430541 1.604043
0.1958763 0 2.271833 0.1549493 0.01974768 1.190097 0.2856232
0.135688 0.5847404 0 0.02615331 0.4566104 0.4226982 0.6077089

0.6506223	0.07145046	0.3886030.1387038	0.5455891.88323	2.225635
0.03808309	0.01566776	0.008408101	0.3206676	0	0.2733236	0.1790741
0.4594475	0.4366215	0.3697388	0.7033130.1121190.3842465	0.1040522
0.2197102	0	0.1210268	0.1518651	0.03149098	0.1931831	4.168747
0.2596575	0	0.3296486	1.8835690.08995261	0.04133476	0	0	1.06775
3.2603290.4708075	0.07418785	0.5010933	0.5922012	0.7435271
0.1193412	0.1185159	0.3557584	2.1382010.5338751	0.6062757
0.01426333	0.33922	0.4148055	0.6479165	0.2318075	0.9992649	0
0.3337834	0.7153539	1.8039360.1950756	0.2525776	0.06491436
0.9552404	0.04250345	0.1524679	0.8270942	0	0.7711041.560783
0.1761130	0.7975517	0.6932602	0.8958661	1.3128020.1696772	0	0
0.1228487	0.5481042	2.2650970.4597557	0.1366618	0.5604576
0.1483757	0.4394203	0.1435292	0.1549493	0.05318868	1.0231282.18403
0	0.5018938	1.3697741.0200830.6593116	0.2422817	0.2704765
1.4468720	0.3619649	0.1051374	0.9525375	0.1263819	0.1025577
0.1216712	0.09303687	0.2822871	0.1834839	0.1888658	0.5699798
0.5076843	0.2919488	0.5702097	0.1553144	1.0105380.4394203
0.05127885	0.1308198	0.1943015	0.4065811	0.2773041	0.8868537	0
0.8525505	0.5312316	0.3559168	0.3332826	0.3661836	0.1923389
0.3918611	0.1635768	0.7360261	0.2863391	0.1619992	0.05028578
1.48061	0	0.1144782	0.4115608	0.2555253	0.3602455	1.614392
1.5607830.09401685	1.0489470.2381516	0	0.3909192	0.1622859
0.2492349	0.0751640.05051553	0.4563886	0.2469365	0.1266622	0
0.1625939	1.1073321.7602650.3901956	0.4672135	0	0.2540486
0.2214133	0.2546287	0.3899293	0	0	0.8282571	2.1070560.03675974
0.1833054	0	0.06379078	0.1139375	0.3819488	0.6686132	0	0
0.7149890	0.04127503	0.3375482	0	2.4545743.2321860.3566163
0.3212859	0	0.08897919	0.03715827	0.03828729	1.2068590.07751925
0.1402864
TCGA-BT-A20Q	593	1	0.03050143	0.09785302	0.1106165	0.1974933
0.3276593	56.652490	0.1884335	0.05566414	0.5248912	0.04521014
1.5679030.3679199	0.1696119	0.04120716	0.2243928	0.0361027	0
0.9301297	0.05451811	0.1001181	0.2507306	1.04078221.57191 0.02106381
0.1311631	0.07021272	0.05474814	1.15851	0.6178719	0.05052691
0.1525610.1638297	0.6788279	0.3168574	0.1138185	0.2642629
0.1781472	0.1125743	0.04255595	0.1104752	0.2015536	0.1181722
0.03527817	1.2927110.2418962	0.1723149	0.02840479	0.1843084
0.2385167	4.1388550.0811464	0.9794165	0.5797364	0.1625979
0.2150104	0	0.1427203	0.2502953	0	0.1541011	0.1702797
0.06553187	0.2288182	0.06507584	0	0.3893301	0.2955651	0.0067622
0.1798040.2908176	0.1116444	1.0693940.5148933	0.3171742	0.09490079
0.05729457	0.2254464	0.2512647	0.03449046	0.5875271	0	0.1928390
0.1183339	0.1008059	0.09697541	0	0.2772502	0.07561369	0.09163354
0.09729271	0.04768581	0.1338174	0.1777440.5111486	0.06108903

0.7192085 0.9117785 0.5018353 0 0.04701199 2.320967 1.3137
0.3824448 0.3564646 0.237163 0.210775 0.1379618 0.3569927 0.2409528
0.1794649 0.07695913 1.312626 0.1010538 0.2040143 0.00539963
0.102734 0.04486622 0.12264 0.9773467 0.3152408 0 0.2527189
0.1255928 1.466994 0.06154103 0.6055782 0.1474467 0.795091 0.1609041
2.847488 0.0417481 0.06007088 0.8102122 0.1181722 0.157585 0.04004725
0.1509635 0.2624456 0.3590953 0.5104159 0 0.1666064 0.07574612
0 0.3688787 0.2091443 0.2175605 0 0.3288649 1.404254 0.2495252
0.1086709 0.07821163 0.08054196 0.3227689 0.5865188 0 0.1036367
0.1920561 0.5617017 0.4412461 0.4891729 0.4095742 0.5680248
0.05379482 0.2491419 0.07960313 0.261304 0.1594802 0.03882498
0.06909111 0.394472 0.582898 0.04630732 0.131997 0.4315513 0.3228836
0.2578852 0.1177433 0.06663654 0.3224858 0.3327003 0 0.09904817
0.2206685 0.1923978 0.6298694 0.3890248 0.02022967 0.2303309
0.5902617 0.6736921 0.1261699 0.07921435 0.4004725 0.1977944
0.05504427 0.1649066 0.2167972 0.1286874 0 0.1426754 0
0.08667542 0.2492855 0.5766804 0.5455085 0.3760959 0.4726889
0.8399641 0.6249397 0.06010427 0.06779159 0.9102131 0 0 0
0.439841 0.2951552 0.1246427 0.04795015 0.1484589 0.1641405
0.6482469 0.3861699 0.1772583 0.1650803 0.1173174 0.08243526
0.1676397 0.09307016 0.04920482 0.08513983 0.03356262 0 1.63964
0.1113283 0.07570192 0.05766804 0.1448946 0.07264503 0.4619933
0.2214757 0 0.02640478 0.2086425 0.2717749 0.1875045 1.957552
0.03063104 1.90068 7.315839 0.1600038 0.2675823 0.06417067 0.2021076
0.04688967 0 0.3045847 0.005335681 0.1805667
TCGA-CU-A0YR 460 1 0.01702611 0.3641481 0.06174686 0.1102421 0
28.9544 0 0.08662284 0 0.4687965 0.01682441 0.2374612 0.4107508
0.6311901 0.009200847 0.01207302 0 0.1118102 0.4450327 0.04057651
0.05588662 0 0.9965008 23.22544 0.2586752 0.1098242 0.1567729
0.1833647 0 0.8868865 0 0.2554817 0.2560624 0.2887058
0.1564637 0.01815265 0.1966845 0.1470026 0.04398781 0.1187751
0.08093925 0.05294522 0.04397636 0.03938503 1.589966 0 0 0.0317115
0.2743525 0.6213278 2.765185 0.2264824 1.049697 0.4816563 0.09076324
0.1642382 0.08731653 0.04704171 0.447093 0 0.05734684 0.07604101 0
0.01228152 0.09535517 0 0.4346534 1.594869 0.007549413 0.09124347
0.02705606 0.1246413 1.901374 0.2612881 0.06438139 0 0 0.2710524
0.03740205 0 0.05045564 0.3883596 0.1845327 0 0.1981643
0.2073125 0 0.04397636 0.1238104 0 0.1023009 0.03949779
0.05323709 0.07296064 0.1082377 0 0 0.1393219 0.835218 0.004001827
0.1048177 0 0.08225902 0.2256357 0.271706 0.04421799 0.01393537
0.1098123 0.07701123 0.2605913 0.2017523 0 0.04295911 0.7937766
0 0.1708233 0.00602822 0.3440811 0.200357 0 0.4034082 0.774266 0
0 0.1796486 2.583727 0.05496419 0.4625782 0 0.3636158 0.03592712
0.08591823 0.503368 0.1341279 0.226133 0.4617518 0.04059925 0

0.08426884 0.2343983 0.4899875 0.2849176 0 0.07440068 0.126846
0.05192048 0.1029553 0.0466983 0.09715502 0.1155169 0.5507238
0.06270915 0.6964333 0.06066086 0 0 0.1441374 2.3277 0.1247701
0.1157014 0.1715312 0.3135457 0.502565 0.1213598 0.1371763
0.9965225 0.1201145 0.08344362 0.2073634 0.4900952 0.03560918
0.04334474 0.02571142 0.01572835 0.694139 0.1033962 0.07368165
0.1605966 0.1459052 0.5346836 0.1752669 0.05688948 0.3176713
0.4775543 0 0.03685958 0.4105956 0.08591823 0.410197 0.2379795 0
0.2666685 1.842801 0.2820446 0.1690293 0.08843598 0.101612 0.662461
0.08449674 0.2285429 0.2420353 0.05836528 0 0.1592848 0.1593599
0.04838281 0 0.4049797 0.4785103 0.2099393 0.3957872 0.09536414
0.2907047 0.06710123 0.2018225 1.183168 0 0.04213442 0.1270685
0.06049099 0.1768131 0.06957643 0 0.05524719 0.04581219 1.109691
1.569402 0 0.0789848 0 0.1227091 0 0.08906125 0.4394634
0.09505127 0.1498791 0.4243062 3.957872 0.0932163 0 0.5794325
0.350485 0.07603303 0.1063049 0.08830659 0 0.05895732 0.3714311
0.6935159 0.03488876 0.449229 0.06839383 0.141463 2.18567 0.05023981
0.2987326 0 0.2256357 0.06281792 0.0323633 0.1133475 0.01191365
0.2490194
TCGA-SY-A9G5 1186 0 0.05419774 0.1159161 0.1965534 0.1503961
0.02772453 18.55924 0.06294218 0.1969564 0.8242428 0 0.1428151
1.252617 0.2905573 0.6027639 0.1952551 0.307448 0.04276705 0.01694837
0.1574038 0.03229092 0.1185994 0.07425352 1.83710 919.57115 0.06653887
0.4467033 0.1663472 0.194563 1.258001 1.150172 0.538685 0 0.03881434
0.4595061 0.2309831 0.0385225 0.4173925 0.1651556 0.1333548
0.07561731 0.06952377 0.02808933 0.0933241 0.04179032 6.021532
0.1146195 0.1360822 0.03364816 0.7277689 0.09418186 2.068396
0.04806279 0.5569014 0.1437389 0 0.6568581 0 0.07084654
0.4743975 0 0.06084909 0.6454794 0 0.05994523 0.2264474
0.2707977 0.2515627 0.2334165 0.02403139 0.1258606 0.1339726
0.2512813 0.1876737 0.887185 0.2049397 0.1264713 0.07541203
0.04108655 0.1190587 0.0408572 0.5353702 0.2060386 0.3263371 0
0 0.1696937 0.2297531 0 0.1970574 0 0 0.1362074 0.1129767
0.02949197 0.4083481 0.3726177 0.07236572 0.4162595 0.7754476
0.1401254 0.1112191 0.05569014 0.4364134 1.07737 0.6177845
0.1407553 0.05175246 0.282974 0.1225716 0.3253011 0.07135784
0.05314827 0 3.083944 0 0.6646041 0.01279274 0.06084909 0.3720379
0.1452786 0.8357066 0.2240595 0.09852871 0 0.1766722 1.54304
0.07290115 0.3964383 0.116443 1.66244 0.3049698 1.549811 0.05934548
0.07115963 1.63726 0.06999308 0.119663 0 0.2682457 0 0.3308529
0.5250785 0 0.1184166 0.1345926 0 0.2184858 0.2973013 0
0.0408572 0.3141705 0.1330777 0.1970574 0.06436549 0.2316227
0.09540955 1.376461 0.7889193 0 0.1636899 0.1820069 1.247604
0.1583932 0.9094482 0.1940717 0.1922511 0 0.1918359 0.09429742

0.4457365	0.1511355	0.09198372	0.1818776	0.03337781	0.6904977	0
0.5081796	0.2840074	0.4189134	0.261848	0.1859707	0.09751074
0.8314441	1.632762	0.04599186	0.1173319	0.6970739	0.09116536
0.4352482	0.5050264	0.02396395	0.3132708	0.2598873	2.593669
0.07473006	0.1407553	0.7762868	0.07810203	0.08965705	0.3098615	0
0.1714977	0	0.6277607	0.08454609	0.05133761	0	0.6677068
0.1384728	0.389831	0.6066067	0.1686469	0.8945292	0.1139187
0.107074	0.3091438	0.1455538	0.1341229	0	0.4002139	0.2217224	0
0.05680148	0.0586212	0.14583	0.4095518	0.7112863	0.1399862
0.0838085	0	0.1953047	0.0992925	0.04725017	0.05828775	0	0	0
0.8399169	0.09890913	0.05978405	0.6831324	0.2002482	0.112947
0.1336854	0.4310181	0.09666968	0	0.5277131	0	0.07403892
0.605492	0.1088561	1.150785	5.340151	0.07107736	0.8644814	0	0.2394156
0.08887239	0	0.3092652	0	0.3019741
TCGA-4Z-AA84	460	0	0.0497527	0.05320462	0.1804331	0.214762	0.0254507
41.20358	0	1.458478	0.1210628	0.228315	0.06555106	0.2610368	0.04445453
0.7685112	0.01792412	0.1205368	0	0	0.07224714	0.009880859
0.4627078	0.340818	4.42971	144.97334	0.03817605	0.160461	0.1527042
0.0595353	0	2.207666	0.1098899	0.04147521	0	0.4218196	0.4041992
0.1414523	0.28737	0.1010735	0.02448354	0.07712838	0.1332749
0.03867836	0.4711856	0	1.906095	0.420876	0.03123034	0.01029617
0.6680808	0.3458301	9.064802	0	0.2556135	0.01466112	0	0.7629616
0	0.02660566	4.518206	0	0	2.814567	0	0.009570228	0.2299897
0.09943528	0.1539538	0.3214093	0.3970882	0.195526	0.0351385
0.07284392	0.9906194	1.628845	0.1567763	0.01289986	0.01384542
0.03771683	0.01821568	0.6751132	0.1720116	0.06304677	0.2097008	0
0.09651071	0.08654236	0.1054549	0.1285052	0.5426872	0	0
0.163509	0	0.003384148	0.1288572	0.08551434	0	0.2892683	2.211817
0.09744921	0.03403248	0	0	0.3846147	1.36108	0.08614082	0.2103924
0.9015401	0.1500252	0.388208	0.06550541	0.0487893	0.1673768
2.236263	0	0.05546331	0	0.05585854	0.6830502	0.1778181	0.2731859
0.4799274	0	0.03925951	0.08109119	1.127689	0.02676886	0.4159135
0.2672323	1.697976	0.03499471	0.1255326	0.2269927	0.06532346
2.202642	0	0.1406065	0.04354897	0.3693682	0.05707875	0.1301648
2.599955	0.04656722	0	0	0	0.1002833	0.8187543	0	0.03750629
0.4441418	0.1221633	1.537614	0.05908654	0.4252522	0	0.9827768
0.1604797	0.030383	0.3005296	0.5430085	0.07635209	0.1696363
0.1034339	0.1781549	0.7059345	0.05849862	0.05418535	0.05770906
0.9092873	0.1387401	0.2955388	0.3255738	0.04596048	0.1479022
0.05035641	0.04784627	0.2085716	0.7858321	0.1602483	0.1280387
0.2173896	0.7426245	0	0	0	0.2399637	0.1673768	0.05707875
0.5968935	0	0.6895683	0.8974875	0.07325995	0.05488085	0.3445633
0.9897493	0.1792412	0.1720898	0.2411626	0	0.1311936	0
0.1773153	0.07761203	0	0.2033122	0.1416044	0.2966038	0.7668406

1.4135570.1393338	0.3256341	0.1176476	0.07371926	0.1384338
0.04453872	0.04104091	0	0.05892108	0.1252540.1016561	0.05214289
0.05381337	0	0.4699529	0.8526759	0	0.1025799	0	0.1195245	0
0.0650624	0.1872754	0	0.06082888	0.3513001	0.2891366	0.09079708
0.06860106	0.3762631	0.1838248	0.05431049	0.1649066	0.08601479
0.02958043	0	0.20849	0.04221983	0.2038998	1.9158480.1332376
1.0104722.29702	0.07612259	0.3438850.06978173	0.2197799	0.01019794	0
0.3154453	0	0.3234089
TCGA-4Z-AA7Q	510	1	0.17163798	0.163152442	0.138324897	0.105841555
0.234134349	18.325736182	0.132887063	0.304938688	0.556860073	0.131274356
0.01884496	0.577555519	0.869041198	0.67164422	0.123670049	0.101422118
0.090292211	1.574423765	0.290779756	0.034087207	0.312992098	0.235152324
3.141672525	11.804568956	0.1843808	0.266529738	0.175600762	0.136924138
0.482902094	1.79363635	0.063183452	0.047694034	0.040973511	0.282956085
0.297170519	0.142329038	0.936298969	0.164657161	0.161889094	0.124170299
0.064756986	0.118607532	0.246288864	0.573495472	2.054902528	0.241991206
0.179565188	0.011839981	1.152379997	0.347973568	2.482671293	0.15220929
0.58788081	0.101156548	2.744917167	0.693397879	0.635719213	0.052691266
0.688582616	0.067606293	0.064234008	0.766559545	0.122920533	0.112803257
0.259388449	0.114344682	0.088518878	0.246401069	0.016912143	0.091981351
0.111119795	0.153571342	0.792454719	0.643869459	0.144226759	0.044502223
0.031842823	0.130116362	0.251363445	0.129390035	0.197803157	0.217500139
0.068898133	0.525097423	0.110981603	0.119422304	0.242533787	0.197031091
0.277359152	0	0.057293469	0.121663677	0.149076721	0.023349418	0.484946761
0.098336426	0.152782583	0.525655613	0.935524922	0.017929731	0.039135336	0
0.414621219	2.085053902	0.391290443	1.040096818	0.039022391	0.298715266	0
1.219059509	0.075327346	0	0.096236716	3.446997649	0.063183452	0.765354262
0.006752189	0.064234008	0.22441923	0.408960564	0.352878169	0.394205791	0
0.045146106	0.211039608	1.154446644	0.369391243	0.757270196	0.061460267
1.695023785	0.201209206	0.818012088	0.271469285	0.150236207	0.864168596
0.591093274	0.242533787	0.100157471	0.14158386	0.065637178	2.394909279
0.772643351	0.053549539	0.041667977	0.09471985	0.116311902	0.461279612
0.889212367	0.217646014	0	0.577066226	0	0.208019364	0.067946023
0.146704434	0.10071701	0.726515389	0.525235244	0	0.302392366	0.672460465
0.790203427	0.334408705	0.178414346	0.153650666	0.558100732	0	0.311549738
0	0.967204581	0.119657156	0.194201201	0.047998788	0.03523455	0.413048332
0.057906889	0.275101905	0.239844943	0.490283818	0.32248317	1.177895489
0.171558475	0.624666335	1.664154909	0.145650901	0.289004001	0.643869459
0.288710149	0	0.559776109	0.025297023	0.469377026	0.339664468	0.126366903
0.094664588	1.139153658	1.434072886	0.123670049	0.146268786	0.526202019
0.067775733	0.213725148	0	0.280365401	0	0.216773686	0.077932326
0.188425282	0.243626282	0.881821215	0.689608819	0.320451159	1.302469224
0.060127887	0.113030375	0.58502576	0.307301333	0.2359731	0.071164519
0.127540244	0.648156206	0.11689849	0.059961236	0.123764381	0.153942224

0.421526049 1.869773352 0 0.088470613 0 0.171807607 0.104815958
0.166262018 0.092295281 0.212933207 0.041969763 0.118815981 1.108299888
0.034803755 0.094664588 0.360566897 0.271784093 0.085164342 0.220503239
0.197823828 0.170078725 0.066037893 0.662841362 0 0.078157564 1.427942828
0 0.369721916 3.060201478 0.068778657 0.608380591 0.160489717 0.168489204
0.105543216 0 0.108823007 0 0.199232691
TCGA-FD-A5BT 328 1 0 0 0 0.1618355 0.02684998 142.5848 0.3047835
0.1271624 0.1915782 0 0 2.380892 0.04689864 0.3567362 0
0.2636326 0.1242540 0.1143289 0.03127233 0.3158602 0 1.581474
39.97788 0.03221997 0.2821385 1.530449 0 0 1.670836 1.970839 0 0 0
0.06291474 0.01865367 1.566376 0.06220117 1.259196 0.06509513 0.03366535
0.02720327 0.09038026 0.4451928 0 0 0 0.3801788 0 0.09121096
5.126579 0.09309336 0.1348336 0.8197613 0 0.2336833 0 0.1543764
0.6891495 0.2480938 0 0.351629 0.8645693 0.0555302 0.07465678
0.1049022 0.04060455 1.808429 0.05430446 0.1593951 0 0.1921222
0.04543843 0.1610999 0 0.2177455 0.05112324 0.07958101 0.1345203
0.03956839 0.1036965 0.1330262 0.8849206 0 0 0.2434679 0 0
0 0.4337304 0 0 0.05470646 0.1106765 0.04325421 0.2255398
0.350415 0.1469348 0.4291352 0.0205614 0.2872287 0 0.0422647
1.623044 0.598297 0.2726306 0 0.2095659 0 0.3780477 0 0.1029435
0 2.309017 0 0.4095888 0.006194602 0 0.1544152 0.09379728
1.042278 0 0.09542069 0.08283599 0.00450261 0.5368112 0 0.3839329
0.3383097 0.3956313 0.07383744 0.1324344 0.009578911 0 0.5877704
0 0.05562641 0 0 0 0.4119639 0.6009726 0 0 0.086898
0.05335351 0.1586954 0.479872 0.04991827 0.1187052 0.01825561 0
0.9064966 0.1246703 0.1345898 0.09239993 0.3702893 0.1779851
0.09616039 0 0 0.3221997 0.04090582 1.231506 0.04698746 0.4189201
0 0.04287335 0 0.47964 0 0 0.2025615 0 0.2229055 0
0.01261922 0.1650291 0.5732704 0 0.0900522 0.03597518 0.7399289
0.1090522 0.2227054 0 2.700341 0.1324344 0.1806508 0.7703258 0
0.09460513 0.1737855 1.178639 0.04342365 0 1.169466 0.03781918 0
0.06523634 0 0.03690842 0 0.1169151 0.2456374 0 0.1429935
1.047863 0.2682095 0.4314675 0.09038026 0.06533082 0.8961847
0.01379065 0.07777235 0.266532 0.7987869 0 0 0.1791687 0.1156229
0 0.2200388 0.2270881 0.09415325 0.4858752 0.1512767 0.338926 0
0 0 0.1923208 0.05338631 0.08467365 0 0 0.08720345 0.1525167
0 0 0.8600585 0.110818 0.03125263 0.07282597 0 0 0 0.08409959
0.4454108 0.03585171 1.179023 0.07028153 3.391907 4.314675 0.005736273
0.7534907 0 0 0.1613793 0 0.2329519 0.1224248 0.03655606
TCGA-CF-A27C 425 0 0.4839541 0.6437588 0.04280749 1.179175 0.01811442
49.26548 0 0.5404802 0.3015807 0.9750133 0.1399671 0.04468411
0.5062451 1.334642 0.3954799 0.06277433 0 0.2491557 0.07713239
0.07032659 0.07748948 0 3.822337 55.25156 0.1304239 0.02537942
0.05434327 0.169496 0 1.588376 0 0.4132772 0.1014408 1.300989

0.1603502 0.2768647 0.3068015 0.6894122 0.01742606 0.03293748
0.1108901 0.4404665 0.2134137 0 16.53423 0.4867796 0.4001051
0.007328252 0.9510073 1.292251 5.620954 0.5966545 3.305095 0.05217496
0.3146190 0.4116553 0.1210686 0.008416205 1.1235980 0.3578137
0.3426622 0 0.001702892 0.2455412 0.5661811 0.8766077 0.03812694
0.03663668 0.4364714 0.2938637 0.8295415 3.739932 8.550008 0.2231697
0.01836284 0 0.4563615 0.01296493 0.6406786 0.2798366 0.673099
1.2579950 0.6640138 0.2012141 0.1501142 0.274389 0.7295933 0
0.2482290 0.3012107 0.09226973 0 1.125576 0.2434579 0 0.2770558
4.270383 0.005548725 0.02422247 0.03638637 0.05702804 1.525167 1.1302
0.03065518 0.2077121 0.8591816 0.5872887 2.603647 0.349674 0.1389023
0.4169541 3.733606 0.3519616 0.1973789 0.004179208 0.3975707
0.2430790 0.5695257 0.02397192 1.5615370 0.3353134 0.4951451
2.094663 0.1905262 0.4933745 0.2282418 0.9156631 0 0.05956487
0.09693665 0 1.447837 1.097556 0.5754376 0 0.9055316 0.3250044
0.4632213 1.7361490 0 0.02931301 0.03599511 1.213396 0.3884966
0.3030977 0.1067798 0.4885430 1.062202 0.2943822 0.4540071
0.06233791 0.2498168 3.057601 0.02162497 0.5614893 1.27837 1.141209
1.045246 0.05784323 2.123916 2.669244 0.3330891 0.298888 0.1026855
0.8898744 0.1481215 0.4807965 0.06535854 0.09813652 0.06015356
0.5734554 0.4171662 0.5566872 0.44031 0.3421682 1.549227 0.3337231
0.5677124 0 0.06009956 0.07666135 0.05693105 1.250862 0.1625022
1.3116310 0.8539433 0.4649369 0.01303561 0.517561 0.09196554
0.126801 0.6889004 0.2982223 0.6205679 0.2936444 0.2256598 0
0.4417120 0.1104801 0.06708508 0.09647106 0.1065455 0.2714226
0.9460455 1.615846 0.2975106 0.4333066 0.06512735 0.1574081
0.7241931 0.03170024 0.4089497 0 0.03700308 0.4234578 0.1205888
0 0.03830144 0.06352079 1.846368 0.7672674 0 0.2190324 0
0.2764809 0.2594997 0.2984286 0.1713761 0.1317931 0.01731788 0
4.355925 0.1723318 0.4101427 0 0.2429821 0.09136698 0.4913228
1.310122 0.06316124 0.1226207 0.8728933 0.1802987 0.3386246
0.3408992 0.04741566 3.694069 4.824932 0.7314291 0.05648277 0.1986674
1.147134 0.02177502 0.02243663 0.7184538 0.008259427 0.02466266
TCGA-E7-A6MF 750 0 0.6892637 0.2456953 0.05554851 0.3541987
0.04701183 43.90373 0.3735535 0.512094 0.1677179 0.8434744 0.6659627
0.07019077 0.6569215 0.2839146 0.6704561 0.01357636 0.1087786 0
0.1668162 0.07300661 0.2011062 0 1.914082 66.7256 0.07756952
0.1975994 0.4231065 0.6048459 0 1.1081360 0 0.06581656
0.3895868 0.1774766 0.228626 0.5750571 0.1789211 0.03957217 0
0.1040204 0.2143368 0.03956187 0.07086286 13.97352 0.2915365
0.3172830 0.04754699 0.7404363 0.3194039 15.03605 0.3259957 4.249461
0.06770407 0 0.4205234 0 0.009556019 1.0558070 0.1547705
0.2394272 0 0.008838932 0.22467 0.4132668 0.6754007 0 0.04074947
0.3529618 0.2352875 0.3251753 7.160263 1.410355 0.05791858 0.01191413

0 0.1741737 0.1009425 0.0346403 0.2950398 0.5822913 0.5533622
0 0.05942398 0.2877456 0 0.07912374 0.6682913 0.2278266
0.04601582 0.1065986 0.04789298 0.006251105 0.6437401 0.3159195
0.06135442 0.2935509 2.254128 0.00360011 0 0 0 1.167168 0.7332939
0.3182339 0.8023352 0.3387051 0.1039209 1.392804 0.1209998
0.04506113 0.07729347 1.318329 0.1014928 0.2049006 0 0.2063607
0.09012226 0.3284607 0.04493206 0.8231868 0 0.5438931 0.1300803
1.803605 0.09889341 0.416143 0 0.7263875 0 0 0.03354358 0.06033185
0.610299 0.4747424 0.7183007 0 0.3790483 0.2635858 1.322403
1.322054 0 0 0.03803759 0.04670853 0.6020321 1.848465 0.2622068
0.1385612 0.6286232 0.0564142 0.6265231 0.2728576 0.3927571
0.08089187 0.06483423 1.744398 0.02806133 0.06939126 0.3086247
0.7051775 0.8012746 0.09553017 1.15179 5.541927 0.108057 0.5004485
0.1332483 0.8188102 0 0.6238985 0.06168112 0.09904642 0.507373
0.09301698 0.1657131 0.5779015 0.3860552 0.2590056 0.5124375
0.09842064 0.6985793 0.04773509 0.2729556 0.1657974 0 0.579701
0.1581515 0.9152362 0 0.7625375 0.9023546 0.06766189 0.190077
0.1193377 0.09141189 0.2648715 0.1174772 0.3883575 0.435478
0.07674009 0 0.1023538 0.2150442 0.04352598 0.3129606 1.548855
1.643635 0.2832974 0.9494848 0.2573735 0.3269035 0.03621923
0.1815629 0.3931559 0.0822707 0.03790483 0 0.0352121 0.1807545
0.09388818 0 0.1988052 0.08242682 0.2517444 0.2317626 0
0.1184267 0 0.275978 0.08418398 0.09347449 0.172965 0 0.01123614 0
1.869298 0.1956709 0.063359 0.05791858 0.2667941 0.2188819 0.6552676
0.7785324 0.3551611 0.02651949 0.2548568 0.03899365 0.3766381
1.747606 0.06152823 1.739254 6.364489 0.1958518 0.02443135 0 0.06766189
0.1130241 0 0.8448887 0.005358862 0.1280126
TCGA-FT-A61P 337 0 0.12555983 0.033567843 0.113838771 0.130658394
0.457634325 29.20101521 0.492135917 0.159701034 0.085928494 0.21607262
0.062036181 5.897686517 0.210354252 0.378197696 0.050889054 0.539762856
0.03715439 0.051534356 0.615358889 0.009351042 0.051517314 0.387051823
1.276808404 28.826086919 0.130064492 0.219348929 0.361290257 0
9.041288678 1.158193338 0.207995139 0.039251287 0.067440848 0.731869292
0.501673256 0.100400661 0.135980528 0.103625085 0.405487659 0.145985437
0.046187937 0.097611754 0.283767502 0.145223359 3.697972331 0.149365636
0.02955575 0.087696724 0.758709539 0.286375659 2.921697261 0.041755122
0.483814779 0.37462466 0 0.267857685 0 0.43783565 0.515173144
0.111277399 0.052863372 0.070095999 0.370924664 0.434739254 0.129757358
0.094103509 0.364246806 0.557654392 0.020877561 0.050465941 0.764850259
0.26426228 0.244565712 0.529892377 0.148369865 1.171983578 0.432399667
0.035694434 0.103433678 0 0.209299183 0.119332331 0.255158367 0
0.152226264 0.02184051 0.099800358 0 0.171195999 0.155632726 0
0.054614674 0.024537464 0.233796234 0.365843504 0.404645088 0.565817284
1.706760412 1.178938931 0.121735593 0 0 1.364901659 0.571986631

0.500927199 0.366848568 0.147727572 0.101227004 0 0.678262242 0.03099649
0.092346389 0 1.294577734 0 0.367425374 0.011113848 0 0.277039168
0.210354252 0.747279797 0.389308685 0 0 0.076743034 0.885013233
0.03800025 1.754853604 0.050580636 1.261883241 0.231827915 1.900823899
0.842099237 0 0.882862009 0.060807322 0.09564201 0 0.155361116
0.162054465 0.123185313 0.373228544 0 0.10287587 0.077952644 0.239306235
0.142359146 0.301331448 0 0.141980733 0.300233355 0.057806441 0.085597999
0.503264619 0 0 0.730776949 0.576344884 0.08626155 0.106655654
0.158120638 1.011612719 0.096324212 1.349452594 0.29505371 0.167020486
0.05536189 0.102559815 0.081922012 0.527075163 0.131300766 0.159823913
0.079004188 0.014498684 0.439910652 0 0.056600915 0.444122657 0.308554466
0.037913935 0.282737493 0.10891752 0.331879962 0.293478855 0 0.237844823
0.454193466 0.237602987 0.378127084 0.400357326 0.020818971 0.301422015
0.209653071 0.883979339 0.064922637 0.040760952 0.749342755 0.101778109
0.09913354 0.152154214 0.055778145 0.055871497 0.039182183 0.188783523 0
0 0.128273659 0.821298997 0.320799709 0.387051823 0.445920361 0.102559815
0.254578035 0.012371028 0.116277324 3.26544715 0.54795689 0 0
0.478894628 0.222258454 0 0.049346962 0.050927871 0.084461024 0.2579572
0.702753609 0.182421966 0.485397597 0.201224953 0 0.08626155 0.027366099
0.050638179 0 0.046053761 0 0.820898846 0.143214156 0.038953582
4.629139803 0.086984008 0.037380609 0.181469992 0.211646845 0.1959602
0.054347936 0.197310642 0.599339672 0.096483294 0.279802361 0.283710083
0.478145074 4.188643016 0.025728878 0.325445712 0 0.138663426 0.048255594
0 0.507502558 0.005491113 0.087447858

TCGA-DK-AA77 618 0 0.08026753 0.2918445 0.05821962 0.3118339
0.04927245 75.63938 0.05593089 0.2566915 0.3222684 0.5525211 0
0.07036745 0.4303189 0.7141607 0.1995309 0.05976263 0 0.007530225
0.2447728 0.02869396 0.02634707 0 2.633716 73.57664 0.1478173
0.06903373 0 0.2305202 0 0.5110257 0 0.2007396 0.03449071
0.5444274 0.1218691 0.08557846 0.1854491 0.1386052 0.04740004
0.02239804 0.1108394 0.09984154 0.2073212 0 3.436491 0 0.06046181
0 0.6467009 0.3766082 5.833923 0.1281269 0.5773446 0.09934358 0
0.5241289 0.123493 0.0143079 0.9484946 0.1138194 0.4325676
0.2867889 0 0.004631981 0.08990809 0.2887595 0.298054 0.2074157
0.01423632 0.2494907 0.08503501 0.364316 0.7087652 4.188158 0.2428146
0.1123833 0.02010351 0.2190589 0.03526549 0.03630601 0.2854404
0.3051457 0.4349784 0 0.06228145 0.1005274 0.2041603 0.04146425
0.6420579 0 0 0.1396557 0.1505879 0.003275849 0.3458521
0.04138886 0.1929142 0.2696404 2.042596 0 0 0 0.07756004
0.9573591 0.4025765 0.3335366 0.1313932 0.4585315 0.03630601
0.9972744 0.1902273 0.04722795 0.04050511 1.577466 0.1595598
0.2147535 0.01705159 0.1622129 0.1889118 0.3012233 0.03260262
0.7300367 0 0.1140094 0.1032844 1.983519 0.1036488 0.2851775
0.05173607 0.7613166 0.06774962 0.1620204 0.4482461 0.06323298

0.5393093	0.4975710.2509470.04215532	0.2780927	0.1105042	0.04199976
0.55142300	0	0.1993334	0	0.1941482	0.4403070.2290128	0.03630601
0.3294257	0	0.08755335	0.1715870.12349300	0.9513261.5016590.2941069
0.1818201	0.4043316	0.7390867	0.5864559	0.07866874	0.9484946
0.7687611	0.05662655	0.1966924	0.02793113	0.4620989	0.1007251
0.04086871	0.06464713	0.04448965	0.3476961	0.04874491	0.1389453
0.2523711	0.3884343	0.07756004	0.4131374	0.1939282	0.585737
0.5003049	0.04086871	0.1042620.3871407	0.2430307	0	0.6619361	0
0.3771529	0.5168608	0.01772887	0.1726548	0.1250762	0.2107766
0.4164123	0.1665826	0.1735872	0.1141046	0.08889657	0	0.4291022
0	0	0.06560193	0.2056092	0.5332078	1.18768	0.8707492	0.2098053
0.3974427	0.0506145	0.0951468	0.1876051	0.08622679	0.1191826	0
0.04697041	0.3106920.1968058	0	0.1562737	0.0431952	0.5276997
0.8080381	0.06219637	0.17377	0	0.1446243	0	0.1049671	0.02589746
0	0.01177645	0.0100017	1.6793020.02929713	0.09296797	0.182111
0.1016812	0.01911725	0.3229708	0.4995729	0	0	0.2671119
0.08173742	0.06579154	0.7212089	0.06448689	0.5779889	2.996317
0.1421097	0.02560615	0	0.4254929	0.01974317	0.03051457	0.5343632
0.01684965	0.07826478
TCGA-ZF-AA5N	168	1	0	0	0.1338253	0.1365315	0.05662956	100.59230
0.1340997	0.1346865	0.2540083	0	0.5073028	0.1978288	0.8207955
0.0598236	0.05233225	0.1747102	0	0	0	0.4844973	0.3033375
1.61771982.37228 0.08494434	0	0	0	0	1.3882330.4890253	0	0
0.1564296	0.1179503	0.2360558	0.3197090.1499304	0.09533576
0.05148479	0.05847381	0.05737468	0.1906219	0	4.1351630.2341195
0.06948966	0.02290968	0.4459578	0.2885609	5.2732550.2945162
0.3791718	0.1957321	0	0.7666771	0	0.006577713	0.3637730.1308143
0	0.2472081	0	0.005323605	0.1082533	0.3318755	0.1712789
0.3575789	0	0.0395508	0.0195464	0.02701379	0.4791732	0.3397773
0	0.02870308	0.01540351	0.1258838	0	0	0.1640304	0.1402834
0.1333139	0	0.0715810.1925627	0	0	0.1341685	0	0	0.1070055
0	0	0.2606511	0	0	0.1589239	0.2262733	0	0.07572462	0
0.0891409	1.7115890.9253743	0	0	0.2379987	0	0.1661134	0	0
0	0.7146152	0	0.2468194	0.02613019	0	0	0	0.05828771
0.9153185	0.4025055	0	0.05697899	1.3157930.1191251	0.1156795	0
0.06953583	0	0.09310625	0.06060893	0	0.3459551	0.4288993
0.02933056	0.09689946	0.09131887	0	0.5792514	0.6500088	0.1036153
0.2418754	0	0	0	0.9108924	0	0	0.1668468	0.2718219
0.2012527	0	0.3784862	0	0.1561962	0.5859747	0	0	0
0.5096660.2372559	0.03287823	0.2973052	0.09817206	0	0.06028308	0
0.4552261	0.1543531	0	0.01857498	0	0	0	0.1863072	0.9281722
0.3720278	0.2674227	0.2848950.06639115	0.2447986	0.1150016	0	0
0	0.1862125	0	0.6318284	0	0.2752174	0.0631953	0.08150422	0
0.09583463	0.2642713	0.2392944	0.06659333	0.1788678	0.1311421

0.07784392 0 0.2959041 0.1726921 0 0.3015891 0.03600909
0.2828417 0.5687577 0.6671766 0.1377899 0.1890152 0.058172
0.05467681 0.3388273 0.09910172 0.09131887 0.1376992 0.1079676
0.08709339 0.1507946 0 0 0.09928977 0.188222 0.4159165 0
0.2282474 0.09462154 0.2659502 0.2028128 0.04825611 0.05952868 0
0.02706969 0.2069119 1.393923 0.2020298 0 0.6976761 0.2045114
0.2197174 0.1194651 0.1913888 0.0658185 0 0.163731 0 0.1512304
0.6578554 0 0.6131919 0.6544609 0.04839372 0 0.3105383 0 0 0
0.5264156 0 0.1542015
TCGA-DK-AA6U 578 0 0.08983289 0.2470259 0.1396234 0.2492819
0.03938869 40.23263 0 0.09327307 0.6557685 0.4416888 0.1521749
0.2275656 0.5847992 0.975295 0.1178959 0.02047483 0 0.04213795
0.02795327 0.09175249 0.02106201 0.1054932 3.555415 26.7576 0.08862456
0.2069474 0.1772491 0 0 3.101016 0.1275531 0.2888504 0
0.9792418 0.57941 0.4378364 0.2964982 0.2802635 0.08052025
0.05371538 0.1118465 0.2294651 0.4309080 0 12.97983 0.4478151
0.09666707 0.02390225 0.8788602 0.1338057 2.541348 0.1365672
1.054932 0.06807074 0 1.133182 0.2961631 0.02401943 1.642837 0.09098788
0.04322465 0.573152 0 0.01295992 0.2395769 0.07694536 0.2680494
0.2072617 0.0113806 0.123793 0.03398875 0.3851836 1.233169 3.072318
0.09705374 0.02994664 0 0.1459308 0.1127658 0.02902325 0.3993199
0.1951483 0.185453 0.01963061 0.1991527 0.09375591 0.163207 0.06629354
1.07319 0 0 0.1041988 0 0.002618733 0.6118737 0 0 0.1105396
2.596843 0.0150817 0 0 0.09300295 3.273863 1.521341 0.06665779
0.1417993 0.5202686 0.1451162 0.9127673 0.02534481 0.1510172
0.1295201 2.632528 0.1700708 0.6008634 0.004543715 0.1296739
0.6418232 0.3095996 0.0984592 1.485516 0 0.09113978 0.1618296
2.373134 0.1035719 1.300785 0.2481488 0.3063158 0.02707973 0.06476006
0.7236874 0.1010976 1.022674 0.1988806 0.1088046 0 0.1270337
0.2650133 1.611594 1.096376 0.180174 0 0 0.03913457 0.582012 1.583928
0.03661484 0 0.3124431 0.04726643 0.3499534 0 0.2303491
0.03388748 0.3802479 0.8374388 0.1410665 0.1744177 0.2585801
1.122578 0.296292 0.05145403 0.2412558 1.263246 0.1810704 0.1362722
0.08931326 0.7476046 0.1342004 0.1960242 0.1356582 0.0474204
0.1308002 0.1948349 0.2314036 0.4438433 0.388146 0.3410108
0.5944762 0.2012058 2.016632 0.3199574 0.09801208 0.1666954
0.06189652 0.4857004 0.08833775 1.273561 0.03404598 1.09353 0.5186749
0.1133805 0.08493618 0.06665779 0.3369922 0.3883629 0.3416026
0.2583931 0.09121592 0.1032127 0 1.252048 0.120116 0 0.1573278
0.1847152 0.3934611 0.9098788 1.292724 0.2276195 0.4163202
0.1011538 0.1140914 0.385644 0.03446511 0.4446179 0 0.008046091
0.2544268 0.1048852 0.04034939 0.1665682 0 0.2763821 2.338103 0
0.07937874 0 0.2312271 0.07053324 0.1062877 0.351944 0.35822 0
0.5996565 2.834049 0.07026091 0.1274043 0.09705374 0.2336931

0.08023286 0.2670877 0.4659219 0.1831203 0.04443853 0.4555326
0.1306828 0.1314854 0.6405986 0 1.2084332.28689 0.1893389
0.06140914 0 0.3401416 0.04734842 0 0.5614276 0.01346971
0.1251307
TCGA-BT-A20O 370 1 0.105206328 0.150007615 0.190770553 0.210847298 0
25.395844416 0.244361299 0.382323354 0.191998163 0.362093623 0
2.525853592 0.376011525 0.260013199 0.066328634 0.0528421 0.124526355
0.016449727 1.183988673 0.073128712 0.057555005 0.072068876 1.858343336
39.764215547 0.121089751 0.452411138 0.403632502 0.1888387 0
0.380567788 0.058092902 0.131554297 0.414396035 0.668980147 0.126105302
0.130861906 0.15191708 0.195917896 0.232976428 0.260951259 0.166711049
0.122683037 0.045289184 0.283925505 0 0.111247258 0.165098022 0.174176968
0.635721191 0.27423267 3.145146943 0.279892257 1.486420562 0.201514282
0.09347279 0.28623902 0.629462332 0.042194945 0.23022002 0.062159405
0.295294087 0.078311062 0.037672367 0.010118532 0.126259465 0.105132187
0.24416133 0.906196342 0.062198279 0.159744503 0.111455041 0.37225044
0.910760517 0.645812003 0.331516828 0.06819463 0.007319329 0.119633178
0.115555827 0.039655123 0.207847541 0.133317759 0.443430153 2.01163124
0.204080127 0.170801114 0.222993382 0.181156737 0.382519417 0.347744925 0
0.122030734 0 0.064404622 0.359177635 0.226034201 0 0.275634721
1.102069456 0.206064662 0.03598229 0 0.127072038 0.929486435 0.319791153
0.227690129 0.0645812 0.161557909 0 1.010337001 0.173145976 0.154753706
0.176966278 1.144462495 0.116185804 0.234563794 0.00620818 0.059058817
0.154753706 0.423012966 2.290916338 0.144978205 0 0.041508785 0.194036619
1.512182024 0.14151259 1.39251726 0 2.924176675 0.480995398 0.132724709
0.038399633 0.069066006 0.315077977 0.067933776 0.097559605 0.046044004
0.477314805 0.120697874 0.0458741 0.679506543 0.295411035 0.268176171
1.088606047 0.053470456 0.212057673 0.288554299 0.150083071 0.158620492
0.451292224 0.193743601 0.812853762 0.374830585 0.134884785 0.092602466
2.597706491 0.426360225 0 0.03971847 0.441629878 0.887991504 0.174230815
0.171851325 0.65926642 0.373189675 0 0.128901993 0.122030734 0.636916091
0.146688862 0.178554845 0.132394899 0.080989453 4.311505921 0.212965843
0.113821902 0.220521172 0.371232851 0.254144076 0.406123156 0.130695868
0.327154765 1.256849514 0 0.113879827 2.114265487 0.353932557 0.844885121
1.256055011 0.023258898 0.457715542 0.33031568 0.116185804 0.2756193
0.182152103 0.272078205 0.303216611 0.126573399 0.241903549 0.062315193
0.161828298 0 0.164039901 0.246175863 0.298963072 0.214960479 0.603146746
0.268797428 0.324309941 1.177518789 0.196422084 1.332254563 0.069104397
0.181866599 0.032932135 0.188361834 0.08678451 0 0.076954901 0.2731372
0.10748024 0.110260586 0.113792962 0.141539443 0.387564637 1.207451863 0
0.298256689 0.044961595 0 0.096371171 0.084076663 0.169718513 0
3.344323928 0.07647027 1.222807973 0 0.058025116 0.265213463 0.124943528
0.093963409 0.450077886 0.600222495 0.031275172 0.121434736 0.291752098
1.785548447 0.071860584 0.925279556 0.21130676 0.485620354 9.418316087

0.149470015 0.503428254 0 0.154914406 0.291119504 0.333294398 0.650359975 0 0.195393023
TCGA-GD-A3OS 638 0 0.4165164 0.1346603 0 0.5196677 0.1337859 33.35554 0.0337478 0.3660884 0.6187097 0.4000589 0.1244318 4.22849 0.311577 1.077283 0.512982 0.2764586 0.1605133 0.01817447 0.3797794 0.02308463 0.0158974 0.1592505 2.696726 59.94985 0.1114883 0.4790192 0.5351437 0.03477305 0 0.8829871 1.636689 0.2906953 0.1248669 1.067621 0.7740409 0.1962194 0.3916394 0.2508965 0.3932559 0.06757308 0.1074445 0.1280159 0.2251698 0.806644 0.7375621 0.3687344 0.237131 0.05412355 0.8584597 0.5807227 4.11157 0.4123183 2.388757 1.472868 0 0.5678143 0 0.08028837 0.9856389 0.03433839 0.2610044 0.5191315 0.1873003 0.002794863 0.302245 0.8130865 0.5844832 0.03128782 0.06871972 0.3270323 0.3181143 0.319097 1.282973 0.2973021 0.09156903 0.4445343 2.446244 0.1652208 0.2766222 0.02190647 0.1865827 0.3682401 0.8398688 0.3704249 0.206688 0.2291472 0.123187 0.07505658 0.5282829 1.056566 0 0.1629142 0.1211498 0.01185957 0.8552525 0.02497337 0.1940022 0.5944687 2.479788 0.07968464 0.03975501 0.02985947 0.2807909 1.412046 0.9716328 0.2012506 0.4518991 0.3480694 0.2190647 1.901148 0.01913002 0.02849659 0.1466408 2.237119 0.1925517 0.874657 0.02057731 0.03262555 0.3134625 0.7270131 0.6382437 0.8809849 0.1056566 0.02293048 0.3440071 1.622539 0.1016276 0.2935337 0.2809504 1.271623 0.1430766 0.3177217 0.1962194 0.03815376 2.240036 0.2251698 0.1257534 0.4578452 0.4554481 0.1666912 0.4054717 1.458848 0.02719872 0 1.37113 0 0.4685835 0.5579158 0.1658192 0.328597 0.5592516 0.3567625 0.5811112 0.2070657 0.3228926 0.05115588 0.8200212 1.055087 0.01774594 0.06582438 0.1219836 1.382455 0.06227902 0.8371533 0.9365015 1.005026 0.2733404 0.3639553 0.03370639 0.5510075 0.08103454 0.2219357 0.5070916 0.04474057 0.3208618 0.05882379 0.2235663 0.6091066 0.3271485 0.3041901 0.498561 0.2340263 1.377552 0.3622511 0.3205738 0.2726101 0.2803134 0.6110033 0.8667942 0.5652549 0.02569758 0.7224381 0.1924277 1.005548 0.1682861 0.3270323 1.445218 0.8375216 0.2709488 0.3864536 0.03442445 0.1200487 0 0.6343378 0.09066241 0.1926803 0.3166652 3.311845 0.4207226 0.3583136 0.4253206 0.1537202 0.8021249 0.1145249 0.2152877 1.200707 0.07804179 0.3835352 0 0.1477791 0.2423348 0.1979158 0.1522767 0.2828792 0.182443 0.4995673 1.425282 0.1501132 0.2097001 0.07451368 0.1221696 0.05323781 0.1055591 0.3125223 0.3785334 0.04974003 0.5672774 1.801358 0.05303226 0.1682861 0.03662761 0.2224038 0.3489351 0.3875108 1.657888 1.209402 0.01677089 0.297271 2.391974 0.07939512 0.2935643 0.3112828 1.985188 6.086423 0.2635918 0.3244572 0.1630309 0.3423141 0.452683 0.07364802 0.4421845 0.003388935 0.08095494
TCGA-XF-A9SZ 859 1 0.01797735 0.05767396 0.1955899 0.2328026

0.02758862 47.4606 0 0.1567928 0.1640405 0 0.03552876 1.242895
0.1445665 0.5664864 0.01942979 0.2900065 0.04255741 0.1011917
0.1566322 0.02142175 0.354054 0.07388953 2.16935 54.06583 0.0662127
0.07730671 0.3310635 0.06453643 0.2276061 0.9624488 0.7742863
0.04495924 0.03862407 0.07620893 0.1364739 0.03833367 0.2595915
0.109564 0.1857816 0.05852516 0.07528735 0.05590326 0.04643331
0.1663418 6.095325 0 0.06770754 0.05580536 0.7966215 0.3748807
2.027764 0.09565437 0.9236191 0.524459 0.09583417 0.4935635 0
0.05287444 3.363513 0 0 0.6423153 0.3476167 0.1167092 0.1678044
0.05389406 0.8344317 0.4645447 0.07174077 0.1926825 0.06665791
0.1710865 0.3735075 1.60014 0.1699459 0.3216201 0.02251271
0.1839832 0.07898339 0.04065692 0.3995594 0.06834286 0.3896849 0
0.03487262 0.09381215 0.3429402 0 0.1960914 0 0 0.02085226
0.05621144 0.05135796 0.1333324 0.04634889 0.07201098 0.394863
2.011792 0.2239466 2.951304 0 0.3474193 1.905936 0.6557399 0
0.07356966 0.6128654 0.08131384 0.5503011 0 0.05288774 0
2.359644 0.05956049 0.3006119 0.01909505 0 0.2115509 0.09637765
1.046612 0.3716019 0 0.08511482 0.0601442 0.9540824 0.08705255
0.7326335 0 1.592184 0.03793436 0.3175146 0.2559031 0 1.1517
0.2785999 0.09526117 0 0.1334654 0 0.517363 0.6966727 0.2523949
0.1178362 0 0 0.2174148 0.5423806 0.1538746 0.08131384
0.2313465 0.0662127 1.813846 0 0.09219489 0 0.6848567 0.2274867
0.5598994 0.1221656 0.3622293 0.7448928 0.3572647 0.368403 0
0.1913087 0.06341266 0.0440528 0 0.4558723 0.3007892 0.09153281
0.1628875 0.01660709 0.2290376 0 0.1166974 0.2260921 0.4349842
0.1302822 0.04626477 0.1478594 1.42367 0.05602613 0.04576641
0.3113513 0.4335355 0.09071846 0.5568616 0.7977994 0.09538592
0.3754229 0.2031962 0.4566304 0.04461824 0.4668844 0.236036
0.3497363 0.129771 0.1675775 0 0.05214519 0 1.273393 0 0 0
1.458246 0.09186266 0.38792 0.2321666 0.0839101 0.6906291 0.05668013
0.186461 0.5777411 0.09656018 0.1334654 0 0.1728264 0.2036636 0
0.2260921 0 0.2902302 0.2649037 0.6189777 0 0.02779922 0
0.1295649 0 0.04701855 0.02900101 0 0 0 0.1567124 0.06561619
0 2.107329 0.2704332 0.05352066 0.02494314 0.2237766 0 0.03112563
0.1794735 0.04576641 0.07367598 0.4679175 0 1.493665 3.993071
0.0530467 0.5734958 0 0 0.03316377 0 0.7864701 0 0.0500823
TCGA-4Z-AA82 1556 1 0.04771186 0.1530665 0.1153545 0.4119051
0.170847 27.41809 0.1108197 0.3236548 0.0870726 0.4378991 0.09429326
0.7541561 0.4689411 0.4421922 0.1461054 0.2142689 0 0
0.3810597 0.05685329 0.1044065 0 1.150052 40.62554 0.3002026
1.111347 1.610843 0.1141863 1.510165 1.058554 0.4742202 0.1193217
0.2733552 0.5056458 0.1779235 0.1017375 0.2755821 0.1130821
0.04108866 0.1849115 0.1494093 0.04945571 0.08215593 0 2.35343 0
0.05989855 0.02962146 0.3844057 0.7876549 4.720772 0.2538665

0.7762396 0.4217918 0.2543437 0.4366388 0.04078084 0.181435
1.3050810 0.1607017 0.3196319 0 0.1124263 0.0721047 0.1907129
0.2583676 0.3082251 0.04231108 0.1193217 0.1347885 0.2212103
0.7021624 0.6345746 0.09020722 0.06185355 0.1062199 0.3255272
0.08734238 0.2158067 0.09426041 0.0604606 0.1149136 0 0.246805
0.08299241 0.2022583 0.2053898 0.5782514 0.1577049 0.04777925
0.05534185 0.3232342 0.01298132 0.7301954 0.1640131 0 0.5239827
0.4388459 0.1420468 0.03263647 0.04902566 0.461025 0.421529 0.5801107
0.1652147 0.03905074 0.8205984 0.1079034 0.8304791 0.5025472 0
0.2006388 1.973425 0.05269113 0.2127529 0.02815456 0 1.169699
0.2984171 0.8433599 0.7232357 0.7806394 0 0.180087 0.698974
0.1155186 1.296272 0 1.288675 0.03355923 0.3611499 0.06095082 0
0.6709628 0.1848509 0.07163316 0 0.3148594 0.2189496 0.7489529
0.5602933 0 0.03474854 0 0.09699701 0.384679 0.8724102 0.2268793
0.2877423 0.8186576 0.05857611 0.3903197 0.1133256 0.2039042
0.08399182 0.4039129 0.765538 0 0.1080757 0.2002824 0.2928806
0.1115506 0.6872526 0.3844057 0.4231108 0.112198 0.1818694
0.05534185 0.4141946 0.06652449 0 0.184129 0.04407519 0.8510115
0.0482908 0.1720636 0.600048 0.3447307 0.3457687 0.286502 0.3515416
0.3297047 1.189546 0 0.3787326 0.07670682 0.4414054 0.4378991
1.217064 0 0.3350893 0.2669175 0.7728032 0.1315741 0.2065183
1.063048 0.3093997 0.05022673 0.3320802 0.1130416 0.125812 0
0.04251047 0 0 0.1299816 0.3530691 0.8126776 0.3922053
0.4929356 0.0742324 0.3937401 0 0.07069531 0.2986994 0.04271175
0.4329317 0 0.425443 0.4128992 0.06499079 0.050004 0 0.1283784
0.5137701 0.9576659 0.06161695 0.1721512 0.04078084 0.1719324 0
0.1178545 0.3335307 0.08878663 0 0.792682 0.7394034 0.0580484
0.05262965 5.8334 0.2014678 0.0473479 0.5663695 0.2969513
0.08510115 0 0.1470131 0.4048798 0 1.309899 0.1277723 0.7487904
3.02213 0.1095001 0.3297789 0.1338386 0.1405097 0 0.0302303
0.4991345 0 0.02215305
TCGA-DK-AA76 366 0 1.036115 0.1146209 0.3887144 0.4626707 0
80.46587 0 0.06491855 0.3260131 0.1229671 0.4942677 0.03915181
0.2394255 1.357621 0.2509953 0.09183715 0.04228918 0.6284623
0.1945562 0.08514696 0.02931854 0 2.680467 46.61614 0.0740198
0.2304584 0 0.2565187 0 0.982231 0 0.1340276 0.07676127
0.4543717 0.4853544 0.09523015 0.2063643 0.2358924 0.07252562
0.02492413 0.1556916 0.1388773 0.3691252 0 11.80584 0.4533552
0.1009212 0 0.2158911 0.6519064 6.04495 0.2376287 0.7342383
0.03158506 0.1904603 0.4109198 2.656801 0.02069804 1.40729 0 0
0.1994584 0 0.002577192 0.1429258 0.428435 0.7462552 0.2885105
0.007920957 0.5265371 0.1797883 0.3923266 3.293986 4.715335 0.2701997
0.08337209 0.01491388 0.1421961 0 0 0.5029187 0.3395606
0.6131123 0.1093041 0.06930566 0.1491534 0.2271858 0.04614066

0.2598074 0.0885707 0 0.1968479 0.1396429 0.007290609 0.4921134
0.04605676 0 0.7424336 2.902821 0.02099388 0.03665878 0 0.0431537
0.4734807 0.4887052 0.1855767 0.2851133 0.5596241 0.04040067
1.495748 0.0705605 0.0525544 0.13522 1.60162 0 0.4182041 0.0189747
0.1203383 0.3678808 0.09577021 0.05240386 0.3692598 0 0.08457836
0.3953693 2.207223 0.2450943 0.1120024 0.1151419 0.3254066
0.03769527 0.13522 0.2249491 0 1.060701 0.2768439 0.1325251
0.04690967 0.2210403 0.2459342 0.4673657 0.6922818 0.4514475
0.03903116 0 0 0.6481336 0.1959862 0.3567777 0 0.4846297
0.06579538 0.6332805 1.145633 0.09161381 0.1415152 0.4536935
2.016746 0.4254598 0.202326 0.2699597 0.6579538 0.6473766 0.111416
0.8155885 1.710927 0.2520519 0.1459172 0 0.6856203 0 0.09095591
0.0269768 0.06600969 0.04551881 0.1084849 0.1030772 0.7301682
0.2521415 0.6904591 1.057383 0.2479402 0.422187 0.3897111 0.3638236
0.03867362 0 1.983227 0.06148354 0.642956 0 0.5362691 0.6791704
0.09864182 0.2512431 0.1391825 0.2771935 0.6178346 0.2659657
0.4262945 0.06348677 0.2002002 0 0.644621 0 0.05076397 0.2190019
0.08716119 0.6846276 0.2753393 1.061235 0.3502012 1.159044 0.07040361
0.1852858 0.3504245 0 0.3094564 0 0.01493366 0.7420597
0.1095009 0.05616678 0 0 0.5264682 1.950041 0 0.2486161
0.04580691 0.1931224 0.196366 0.1012314 0.2017276 0.09972921
0.01310461 0.3895398 6.384713 0.2282092 0.1477901 0 0.2262979
0.09572999 1.119498 1.167423 0.1274527 0 0.5152112 0.04547795
0.4392697 0.535032 0.03587991 0.9400257 2.911808 0.1464232 0.02849406
0 0.7102211 0 0 0.6455978 0 0.2488332
TCGA-DK-A3IL 413 1 0.249764 0.2199589 0.2131279 0.4348755 0.4509362
32.90781 0.2047494 0.1388172 0.3485615 0 0.01451795 1.440208
0.7876467 1.633981 0.3255196 0.1953362 0.03478006 0.1516148
0.2240135 0.1225486 0.07233769 0 3.061077 27.93068 0.08116852
0.06317894 0.2705617 0.1054848 3.348202 4.740628 0.04867583 0.1469718
0.03156554 0.7162404 0.4167825 0.2506256 0.3818723 0.3357795
0.1518301 0.1298235 0.07815796 0.3654957 0.4933193 0.06797147
9.815059 0.4194615 0.249003 0.04560695 1.242893 0.8042249 3.850165
0.2736075 1.358691 0.1818364 0 0.5886948 0.8664825 0.3051005
3.95446 0.1041663 0.3463962 0.4921241 0.1262621 0.1589678
0.2860311 0.2202247 0.3750668 0.1423685 0.01954339 0.3070661
0.5681089 0.3871952 0.9157474 6.042546 0.166666 0.1371361 0.4722286
0.7350948 0.0645492 0 0.6095414 0.2792661 0.5573227 0
0.256497 0.1891144 0 0.1138429 0.4807674 0 0.04413825 0.1448529
0.02296941 0.3177903 0.716062 0.530301 0.529659 0.8953396 1.599093
0.06215789 0 0 3.65558 9.540462 1.440241 0.267093 0.3246741 0.5414751
0.4984032 1.706343 0.1450784 0.08644504 0.1112095 4.38373 0.5354341
0.2948102 0.0468163 0.3958813 0.6483378 0.472588 0.208863 1.396982
0.2403837 0.2086803 0.3554131 1.230502 0.1304302 0.5373352

0.3314381 0.3045393 0.06200373 4.96736 0.4343613 0.05787015
2.2268050.5692146 0.1829527 0.1929005 0.3272239 0.4045292
0.3075019 1.7210080 0.03210055 0.1459422 0.1344081 0.8439911
0.8865215 0.08383603 0.09968064 0.3730261 0.3246741 0.801279
0.05234486 0.1506926 0.03879563 0.3109441 1.3342030.5652433
0.2995196 0.9251001 1.48809 1.7260861.4268460.4340261 1.289864
0.3109441 0.3360202 0.1022491 0.6645697 0.2150926 0.1122078
0.04437328 0.1764380.2058992 0 0.3920806 1.2472240.2295879
0.2484374 1.2855370.4267098 1.2365940.1831495 0.2618183 0.09541949
0.2125842 0.4819080.3033969 1.36561 0.01948854 0.7533392 0.6491521
0.7463627 0.0850833 0.1144684 1.0521850.03175801 0.2319962
0.4108583 0.1044273 0.1491549 0.07335653 4.5554340.06875661
0.5010001 0.5403438 0.4032243 0.0375374 1.0416630.5692146
0.1782964 0.3888205 0.09264371 0.06530801 1.7200080 0.5453732
0.05482435 0.4175862 0.2635365 0.2101337 0.04619347 0.1906934
0.03953179 1.4987950.8960296 0 0.3862217 0 0.3706043 0
0.1472994 0.1659076 0 0 0.8970382 2.0918640.2413118 0.1580118
0.4444428 0.4652876 0.1574632 0.3023744 0.9296478 0 0.07631229
0.4834836 0.03740261 0.1806352 0.5867047 0.08852658 3.458646
4.5041520.2456638 0.09373793 0.06181975 0.1298022 0.04517184 0
1.4671310.01542062 0.06139466
TCGA-E7-A678 798 0 0.07956366 0.02127098 0 0.2207853 0 106.65960
0.3469644 0.6534050 0.2751738 0.1149296 0.6931364 0.8848727
0.04299591 0.1339920.09417468 0.009330241 0 0.1303607 0.09793513 0
2.126074141.70240.07326057 0.04276773 0 0 0 2.7054080 0
0.08547066 0.2529625 0.1430532 0.06362102 0.2872232 0.1515329
0.07341311 0.02775203 0.04727903 0.01546344 0.1027516 0 22.890030
0.2247436 0 0.5609012 0.20739213.9424 0.1058361 0.3065795
0.1055062 0 1.4169080 0.01418244 1.6322520 0 1.3325350 0
0.0742664 0.1788921 0.4154629 0 0.01763935 0.2878094 0.0316085
0.1892974 0.5165809 3.4798770.1128213 0.04641577 0.01660602 0 0
0.08996912 0.7662887 0 0.2515124 0 0 0.1176375 0 0.05137581
0.07232133 0 0 0.0692155 0 0 0.3161244 0 0.07967604
0.1970303 0.8842748 0 0 0 0.04804995 1.0544040.6801926 0
0.08954069 0.4765031 0.08996912 0.2148977 0 0.05851726 0
2.2826760 0.6652198 0.007042526 0.0669960.1755518 0.2132727
0.03590747 0.3289250.1084820 0.03071359 1.8308720.03210622 0.1454954
0 0.9807829 0 0.1505622 0.0653405 0 1.4763110.07706371
0.1686417 0 0 0 0.6765113 2.2248920 0 0 0 0.661532
3.6006790 0.04498456 0.2490536 0 0.0542410 0.1530126 0
0.5893649 0.7995208 0.1822049 0.09011284 0 0.9157571 0.3255354
0.1417798 0.1602575 0.6879346 0.07016248 0 0 0.8997346
0.1664031 0 0 0.0551244 0.05068341 0 0.1291190.06253951
0.3609639 0.04804995 0.2047573 0.1073612 0.9814131 0 0.3544654

0.04306155	0	0.3011244	0.06845951	0.6603019	0	0.4821422
0.2180121	0.02196676	0	0	0.0949674	0.1289877	0.1346102
0.2224979	0.07069002	0.1101463	0.04965725	0.1063348	0.4654343
0.2826184	0.08128334	0.7885358	0	0.9197386	0.2055032	0.1671151
0.4245231	0.04703499	0.2652538	0.3694295	0	0.04922394	0
0.01247103	0.1408388	0	0.3752370	0.05352053	0.05636554	0.2149796
0.07706371	0.1230330.05100419	0.03583903	0	0.0780350.03208796
0.1110446	0	0	0.4045845	0	0.01645584	0	0.01574836	0.07106106
0.1655890.6602562	0.07095677	0	0.2647696	0	0.2037956	0.6666583
0	2.4789833.6957530.05217169	0	0	0.08786703	0	0	1.248525
0.006959121	0.1662396
TCGA-ZF-A9R1	773 0	0.04784047	0.3410648	0.2313309	0.3097616
0.5383951	101.18710.0555592	0.2202149	0.2910244	0.1097699
0.01575791	1.66171	0.9831563	0.7389737	0.1464993	0.3703276
0.2642540.3141677	0.06947034	0.04750545	0.05234397	0	2.533331
36.002040.1174680.2400124	0.07341752	0.1144942	2.22088	2.561223
0.3169990.2791679	0.1027845	1.8590330.6371540.4420507	0.4605417
0.3644580.2471965	0.04449839	0.1570319	0.2107534	0.2471322
0.07377682	10.447160.4046997	0.2102101	0.01980087	1.2205660.9560473
2.5696130.3818262	0.5735079	0.1409763	0	0.7454702	1.104051
0.1691326	0.1046879	0.05653149	0.1074233	0.2848831	0.4111381
0.2553666	0.2041385	0.09561351	0.5551374	0.05150933	0.02121257
0.1880110.4899255	0.5370054	1.40811	3.3282610.1206005	0.1116365
0.2329823	0.3989391	0.0525467	0.1081942	0.4489439	0.3031179
0.8353698	0.2439331	0.03093378	0.1331458	0.2028036	0.2471322
0.8117342	0	0.04790804	0.1294791	0.07479359	0.1204009	0.5012506
0.5344795	0.5110191	1.0164510.2933526	0.02248891	0	0	2.080205
0.8981639	1.1997040.4141501	0.08483802	0.5289496	0.2163885
0.7752890.3464325	0.0469141	0.2011797	1.9101180.1584995	0.5866475
0.01129218	0.1074233	0.1407423	0.3419674	0.4713956	0.3296297	0
0.1132517	0.2790665	2.15547	0.14157	1.0998020.1541768	0.8113490
3.0981670.2531912	0.06281277	0.9717811.4827930.1901283	0.04187518
0.1183906	0.1097699	1.9608710.9410211	0.04477742	0.06968443
0.03960174	0.09725848	0.4821449	0.6998095	0.04549818	0.144259
0.3993394	0.4111381	0.2609146	0.05681557	0.1635631	0	0.4050017
1.7646930	0.3612236	0.1204934	1.2480980.2842886	0.9874806
0.2997882	1.2727540.1125005	0.1563083	0.1387276	0.2295140
0.04059712	0.07224472	0.05892533	0.1015840.04842098	0.2415384
0.9526371	0.4260461	0.50079	0.5745487	0.1311588	0.7537532
0.3478861	0.1623885	0.1035691	0.1538272	0.3218875	0.1097699
1.0698910.04230607	0.6809053	0.5188881	0.7572754	0.2374718
0.1242450.1713075	0.1723521	0.1151134	0.3686513	0.05667317
0.1093310	0.3196880.1492581	0.04531582	0.1954979	0.3909791
0.4889210.7373673	1.1532840.1488650.3403461	0.1131258	0.02362863

2.8186710	0.1183906	0	0.3032787	0.2785173	0.1954979	0.05013879
0.05174507	0.0858163	0.9399321.0981290	0	0	0.1723959	0.08764572
0.1737826	0.1800776	0	0	0.3278628	1.71448	0.05820488	0.1319288
0.1206005	0.2398880.02848532	0.3798267	0.4466277	0.3413222
0.05522001	0.4304353	0	0.09803149	0.2558629	0	2.1640964.956185
0.1359370	0	0.3522211	0.03922393	0	0.2729891	0.005579224
0.02221277
TCGA-XF-A9SH	1971	1	0.1292512	0.08293133	0.09374847	0.2869327	0
51.216590	0.3945508	0.1887035	0.17794	0	0.5150436	0.2771694
0.81457	0.08381627	0.01374759	0.1835842	0	0.3378401	0.06160613
0.2121276	0.1062483	1.91602489.564560.07140711	0.1111619	0.1190119
0.1855982	0	1.2343230.08564404	0.1939452	0	0.3835419	0.185912
0.02756064	0.5972407	0.0919017	0.12403	0	0.09362874	0.1406741
0.06676803	0.1195943	9.6931350.08200369	0.09735896	0.08024442
0.6248122	0.2021451	12.298940.2063171.3945080.1371159	0	1.0549770
0.06220632	1.5273190	0	1.1545090.1110777	0	0.1792452	0.2324883
0.4799430.2504942	0.02292411	0.5402760.2327778	0.3217068	1.812642
5.0778390.0977484	0.08042929	0.04316238	0.4115310	0.2338478
0.1532107	0.1965450.09339019	0	0.2507226	0.4136801	0	0
0.1879777	0.1281666	0	0.08995252	0	0.03164972	0.28302	0.06664664
0	0.3228592	1.1095760.02430343	0	0	0.4371202	1.1133730.5303872
0	0.07405182	0.3572675	0.05846196	0.4189217	0.2042097	0	0
3.8009150.1712881	0.7780680.01830494	0.1741361	0.1520981	0.3464617
0.1924947	0.6412067	0	0.06119474	0.1397039	1.0503670	0.3511595
0	0.8930502	0	0.5217886	0.04245828	0	1.3531290.2003041
0.2054689	0.1357617	0.1279429	0.2669101	0.6086732	1.2977470	0
0.06419553	0.07882935	0.3907852	0.9926095	0	0	0.2697252	0	0
0	0.1325702	0	1.4224580.8081561	0.04735872	0.05855535	0.1302155
0.3570356	0.6950371	0.1266768	0.2082707	0.6877233	0	0.12669
0.1349288	0.3366154	0	0.1974272	0.0520492	0.0238799	0.1646705
0.07849176	0.1118687	0.6502111	0.3518317	0.06244574	0.06652568
0.1063060.9431863	0.08056187	0.1316181	0.05596282	0	0.3261179	0
0.2800210	0.4482545	0.4249930.3140282	0.4063345	0	0.1851295
0.1117550.05831316	0.3084357	0.0918688	0.03749067	0	0.3454821
0.1209757	0	0.4225435	0.1450460.1981386	0.9562343	0.8679844
0.2171829	0.5958474	0.04075114	0.07660533	0.9548271	0.06942358
0.2558859	0	0.01080491	0.3660684	0.1584538	0	0	0	0.2344086
0.3911426	0.3004561	0.03997345	0	0.2328821	0.1420762	0.1126826
0.08340307	0.1443136	0.07585235	0	0.6009123	0	0.04277205
0.3909936	0.2046658	0.05387141	0.1733552	0.7776253	0.04610774	0
0.3345366	0	0.2648530.4608455	0.1038404	1.1454896.3312320.14408
0.1236974	0	0.4567682	0.1589577	0.04913626	0.6637844	0.4341158
0.07201503
TCGA-FD-A5BU	588	0	0.03319043	0.05323986	0.1203684	0.06140122

0.6876239 11.37258 0.1156365 0.06030758 0.1211429 0.1142331
0.01639862 1.157258 0 0.3383689 0.08071195 0.3647919 0.03928551
0.2335298 0.2530325 0.02966221 0 0 0.8062763 15.32076 0.03056106
0.3032936 0.4584159 0 3.466771 0.3841962 0.4948322 0 0.07130915
0.1406997 0.2320712 0 0.335487 0.1011405 0.2572475 0.2006666
0.1164579 0.03870398 0.08572684 0 0.2622616 0 0 0.03090896
0.1337046 0.1297722 1.963347 0.04415013 0.3836742 0.1173667
0.08846623 0.1600818 0.2978736 0.1005768 0.3268336 0 0
0.0370583 0.4635094 0.2944794 0.1106452 0.1990023 0.07702788
0.5896405 0.03679177 0.1067212 0.2637133 0.07289216 0.08619787
0.509351 0 0.07745042 0.4294922 0.0188709 0.145822 0.03753113
0.02458936 0 0.1798626 0 0.06438309 0.1039196 0.1055247
0.1714537 0.06033851 0.2468393 0 0.07699638 0.02594489 0.1185236
0.2637462 0.2139274 0 2.669464 2.060642 0.06630935 0.1021651 0
2.966558 1.20959 0.2648298 0.3016925 0.1154529 0.1529046 0.03753113
0.1942325 0 0.09764323 0.08374384 1.225998 0 0.05550004 0 0
0.1464648 0.3113878 1.670159 0 0.09050776 0 0.01281235 0.633025
0.02678659 0.7109895 0 0.4065332 0.1050537 4.940886 0.009085721 0
0.7390221 0 0.08793729 0 0.2464086 0.1142331 0.08683401
1.008515 0 0.1450356 0 0 0.1505246 0.1365494 0 0.07506226
0.4271203 0 0.3167772 0.05912567 0.1702135 0.04382126 0.2809793
0.3252895 0.03040312 0 0.08359509 0.1528053 0.1115491 0.9167359
0.1337046 0.08830026 0 0.09488717 0.02887364 0.2502196 0.17354
0.1689912 0.1837786 0 0.5285718 0 0.1795424 0.4695968
0.3597145 0 0 0.02132682 0.2958704 0.4654685 0 0.2155605
1.200613 0 0.6853986 0.1913644 0 0.2258826 0.2330466 1.282898
0.137293 0.2154947 0.198081 0.1434879 0.02246136 0.01856325 0.05897749
0.05470018 0 0.04435819 0 0.09431671 0.2034468 0.5020164
0.2544002 0.3069394 0.4286342 0.0929507 2.734304 0.05232244 0
0.7480377 0 0.1232043 0 0.717926 0.2115062 0.06781561 0.2087097
0 0.1339584 0.1504845 0.3202855 0.3214757 0 0.08510676 0 0
0.03616972 0 0 0.6817346 0 0 0.03028574 0.0137293 0.8157766
0.1182513 0.04940587 0.1036146 0.08607176 0.02960002 0.08619787
0.1104501 0.7182124 0.03400581 0.5443665 0.1333259 0.1838439
3.251595 0.02176372 0.4235234 0.06982795 0 0 0 0.110479 0.1161215
0
TCGA-C4-A0F7 62 1 0.04433833 0.09482919 0.09647839 0.04101223
0.04082581 126.164 0 0.6251727 0.09709923 0.1831216 0.03505045
0.7491253 0.1901603 0.3123041 0.0527126 0.05973563 0.06297671
0.3078074 0.7533021 0.1003835 0 0.182237 1.897675 182.87 0.1592207
0.2859973 0.04082581 0 0.3929484 0.1411407 0.1762759 0.08870794
0.3429368 0.3947105 0.1877826 0.06618079 0.05121935 0.06755561
0.05563872 0.2433213 0.2418916 0 0.0229041 2.010255 1.567016 0
0.100194 0.005505407 0.2500581 0.2311461 7.276127 0.2123249 1.890708

0.3292536 0.189088 0.1644996 2.114674 0.03240404 1.07697 0 0
0.3564383 0.01905205 0.01407242 0.2506829 0.1329212 0.5144988
0.8306518 0.007863884 0.1853362 0.1456126 0.2531748 0.9211978
0.2721721 0.0502974 0 0.05182246 0.1008368 0.04870004 0.04010957
0.09197539 0.1348456 0.112128 0 0.3268299 0.05244454 0.05638722
0.0458082 0.3224192 0.04396626 0 0.005142883 0.2911371 0.2406661
0.09082345 0.04572491 0.3196869 0.8134659 2.745966 0 0.03639465
0.08200663 0.2142138 2.144691 0.20216 0.06908984 0.1306426 0.3676711
0.04010957 0.9181271 0.08756511 0.02608786 0 2.251553 0.1175173
0.05931297 0.006279326 0.02986781 0.6000209 0.07131011 0.1640829
0.07331983 0 0.02099224 0.06389869 1.580986 0.150291 0.7969006
0.02857807 0.2924267 0.03742366 0.2461172 0.4078168 0 1.579588
0.2748492 0.09632817 0.04657167 0.1316686 0.03052027 0.997596
0.7419648 0 0.03874992 0.02202163 0.02704161 0.4021647 0.09728704
0.2277045 0.3008218 0.5736642 0.0653213 0.1450887 0.3475323
0.04547685 0 0.6756367 0.5038541 0.01624593 0.1807814 0.08933819
0.4490839 0.1347622 0.1185141 0.3095957 0.4482414 0 0.1231359
0.0308573 0.182325 0.07418496 0.09030054 0.1249847 0.08191759
0.1581679 0.0269258 0.04477134 0.2230483 0.2235043 0.171371
0.1369258 0.07977202 0.3713478 0.05527187 0.2483265 0.1151849
0.1283097 0.1118714 0.1220811 0.858328 0 0.3158055 0.3189147
0.08813797 0.1760702 0.2072695 1.016109 0.2108504 0.05601047
0.208305 0.03151466 0.1426379 0 0.04740566 0.08299901 0.0503982
0.108712 0.5073007 0.4984427 0.2460199 0.1832328 0.09933654
0.302814 0.02795853 0.1576721 0.680995 0 0.1097238 0 0.1130487
0.2218511 0.1630679 0.1394052 0.05754851 0.07158074 0.4523148
0.9009062 0.0687123 0.1508374 0.04547685 0.1437982 0.04873779
0.06571287 0.1573582 0 0 0 1.211054 0.04854961 0.00733626
0.1005948 0.1474379 0.005280013 0.2235167 0.1379775 0.03163359
0.06141319 0.1606631 0.1580259 0.05451308 2.086767 0.05343208
0.8104562 2.014592 0.05523992 0.2828875 0.1119378 0.07834486
0.07634037 0.05056709 0.05903451 0.006204959 0.06176007
TCGA-C4-A0F0 59 0 0 0 0 0.018758693 0.155611883 49.526787666 0
0.058958639 0.037010394 0.977182195 0 0.763676742 0 0.187954745
0.032877755 0.079091846 0 0.009512761 0.353389567 0 0.033283653
0.083353844 0.586598523 23.862591601 0.289438103 0.130813083 0
0.072802724 0 0.850974699 0 0 0.130713982 0 0.105337275 0.021621862
0 0.102998123 0.052394464 0.056589851 0.075749003 0 0 0.328384229 0
0 0 0.025181328 0 0 3.319447174 0.053953238 0.312576913 0.197211868
0 0.406300917 0 0.025304781 0.266269223 0.07189269 0 0 0.043571327
0.03803459 0 0 0.235327954 0.32752934 0.017984413 0.445593393 0
0.029692386 0.158005912 0 0 0 0.008465433 0 0.378675672 0.09172911
0.030049191 0 0 0 0 0.105828297 0 0 0.147472185 0 0 0
0.031705707 0.012414913 0.02148723 0 0 0.209618777 0.466330963

0.038133036 0 0 0.146969724 0.13437886 0 0 0.082993004 0 0
0.054775383 0.080103276 0 0 0.421828632 0 0.067823293 0.007180294
0.204919782 0 0 0.581182154 0 0 0 0.00521907 1.362177286
0.016367146 0.635749654 0 0.235661531 0 0 0.011103118 0 0.697141237
0.078571246 0.032238874 0 0 0 0.053057336 0.107169227 0 0 0 0
0 0 0 0.18345822 0.543118679 0 0.221208277 0 0.104003892 0
0.343368072 0.130828342 0.037153845 0.045937821 0 0.18673426 0.302269348
0.099380407 0 0.215812953 0 0 0.176423779 0.083394131 0.127243699
0.464656525 0.040833619 0 0.284211893 0 0 0 0.265795939 0 0
0.010424896 0.092493519 0.25280946 0.103257006 0.482943262 0.097813184 0
0.418792369 0.092124689 0 0.166378875 0.159761534 0.156775337 0.100666544
0 0.290475516 0.131511018 0 0.0831785 0.072072872 0.016042999 0
0.027103747 0.189815683 0 0 1.083492413 0.36270186 0.125030765
0.209523323 0 0.121191771 0 0 0.181980025 0 0 0 0.313636496
0.076583425 0 0 0.065805666 0.054567507 0.011493636 0.109592515 0 0
0 0.073080244 0.111461535 0.026520513 0.098147017 0 0 0.050539677
0.353570607 0.037010394 0 0.996911969 0.080282178 0.042263181 0.22041464
0.021036632 0 0 0.037492928 0.309771017 0 0.433850783 0.081464805
0.112332328 0.993395122 0.006649035 0.388172005 0 0 0.012470545 0 0
0.007095257 0.028248601
TCGA-FD-A3N5 685 1 0.04954273 0.09536415 0.07186863 0.2108006
0.1368537 16.29912 0 0.1656368 0.09041388 0.477438 0.03916465
0.8094183 0.2390413 0.25713 0.08031813 0.1387642 0.04691258
0.009295612 0.2374094 0.01180699 0.01626194 0.1221767 0.6000773
21.78554 0.06842687 0.2023927 0.09123583 0.07114085 1.379942 0.6308306
0.4595898 0.1734607 0.04257672 0.693065 0.1227275 0.09507734
0.08584715 0.1459381 0.05851251 0.2810985 0.128974 0.08473327
0.02559256 0.06876175 1.395067 0.1257299 0.3918415 0.04306139
0.119747 0.6198669 0.8865481 0.1581649 1.145406 0.04379775 1.901547
0.1617514 0 0.06535031 0.2601911 0.03512579 0.100121 0.2212648
0.04257672 0.06146746 0.06342065 0.1188188 0.1379737 0.3840633
0.03514776 0.1274405 0.0839765 0.1305657 1.363859 0.03041194
0.05620127 0.02312175 0.08685801 0.03380189 0.06529969 0.1792704
0.1761795 0.1130052 0.1252895 0.1970379 0.05766204 0.06549461
0.06300591 0.02559256 0.396291 0 0 0.0229862 0.1084368 0.0101096
0.2134668 0.1532762 0.07938032 0.345657 0.7594766 0.06753841 0
0.09163251 0.1914864 0.623729 0.1581225 0.2573318 0.1581421
0.3012739 0.0448176 0.5174085 0.1565495 0 0.1000023 0.9594299
0.03282784 0.1988252 0.007016388 0 0.1457502 0.2390413 0.5097824
0.2867412 0.2161587 0 0.09179881 1.446115 0.007996766 0.8179625
0.2554603 0.6348316 0.1254493 0.2750062 0.2386927 0.03902866
0.7121924 0.1151665 0.1076351 0 0.1226031 0 0.2073848
0.2967148 0 0.3896852 0.09842604 0 0.2097062 0.2989429
0.02827026 0.0448176 0.5376133 0.03649433 0.1080794 0.1412092 0

0.07849339 0.2935887 0.4916996 0 0.04488919 0.1746931 0.5930329
0.1824340.2074662 0.2128836 1.0017110.1398042 0.16187 0.05171896
0.2648441 0.08289273 0.1008999 0.06483995 0 0.0504953 0
0.02143996 0.2492296 0.2896973 0.04787161 0.2039973 0.05602809
0.3779618 0.1235193 0 0.2359595 0.0477902 0.2000045 0.06820542
0.1765811 0.01314342 0.4138424 0.3427734 0.1860245 0.08197385
0.1286659 0.1537493 0.1927635 0.08493669 0.2179776 0.1760691
0.04703035 0 0.2383654 0.04637068 0 0.1214725 0.1389924
0.4303701 0.06108834 0.4094810.02774915 1.1673350.03124028 0.02936325
0.1550808 0.07983135 0.1716444 0 0.4949191 0.1122529 0.04049083
0.06230739 0 0.1599657 0.4155570.7220982 0 0.06128819 0
0.08926504 0 0.05183025 0.07992217 0 0.1235672 0.1790243
0.4894578 0.03616555 0.02459215 0.6744152 0.1255193 0.09439642
0.2039267 0.2158425 0.2651003 0.01715545 0.2308136 0.1513499
0.02030393 0.7101116 0.0199013 0.4665144 0.7531439 0.0454808
0.1264371 0.08338467 0.2188523 0.01218588 0 0.05654051 0
0.04830659
TCGA-ZF-AA5H 897 0 0.05103511 0.1091520.07403354 0.2454745 0.2506244
44.872540.4623003 0.2077189 0.1490199 0.4215599 0.0201722
0.5084149 0.1368011 0.3027149 0.05515837 0.2732225 0.02416287
0.1005441 0.2223281 0.04256928 0.0335036 0.2936664 1.17403
48.722190.09868334 0.5157370.2349603 0 0.3876845 0.4282991
0.5072508 0.07657966 0.06578889 0.6490384 0.09379876 0.09794136
0.2947771 0.1503342 0.07157667 0.07595176 0.07162839 0.06348051
0.1581809 0 0.7918663 0.06475864 0.03844238 0.03168467 0.6578889
1.1174442.1381390.1629293 0.5244042 0.1985151 0 0.1666239
0.8113567 0.03638858 0.1675180 0 0.1595510 0.1001324 0.08166385
0.06119897 0.1421299 0.3296938 0.06336141 0.05469976 0.1405725
0.1195544 0.2915918 1.12781 0.0771923 0.3810920.04686757 0.0464268
0.1009001 0.04616764 0.1663627 0.1552124 0.4793789 0 1.069182
0.06746751 0.2596154 0.1054540.4082285 0.05060684 0 0.09471448
0.01595761 0.01249695 0.5659647 0.07894667 0.2044288 0.1802326
0.3129418 0.1295495 0 0 0.2712251 1.2850350.7911590.3976252
0.1461975 0.1504723 0.1154191 1.2038320.2015816 0 0.02575366
2.1596860.1690836 0.2389502 0.08673292 0 0.2101969 0.2736022
0.4606479 0.2531817 0 0.09665146 0.09719085 0.8294787 0.1070895
0.7892728 0.1315778 1.67656 0.1507662 0.4120585 0.1844121 0
0.4864391 0.6722690.09194711 0.02680288 0.1768148 0.07025998
0.2136318 0.6472646 0 0.02230132 0.1774341 0 0.4320465
0.7278771 0 0.1846706 0.5147573 0.07518731 0.2505039 0
0.2355552 0 1.1233210.6154111 0 0.1618449 0.1542475 0.2349603
0.06860919 0.2319043 0.3015324 0.3530136 0.07200774 0.1083849 0
0.1888768 0.1067371 0.07795452 0.1644138 0.06600319 0.3641146 0
0.1030670.09627643 0.2315357 0.4438229 0.0525356 0.1101848

0.5332363 0.5089602 0.05196968 0.1546792 0.3446085 0.4378122
0.2459099 1.0628680 0.2569270.1643133 0.1803558 0.06755452 0
0.5482408 0.1103167 0.06447018 0.1712622 0.07254916 0.05248431 0
0.05456572 0.2388376 0 0.08342107 0.2278418 0.4172557 0.5034281
0.3954524 0.1429252 0.4531156 0.08045334 0.03024777 0.1831826
0.05482408 0.1262963 0.1142649 0.5588905 0.2987219 0.06256581
0.03209214 0.1656013 0.05492811 0.1561895 0.7911286 0.07909047
2.41489 0 0.1103448 0.05609906 0.04894224 0.08232967 0 0.2396036
0 2.1947610.1117649 0.1435534 0.7333269 0.08081271 0.1367437
0.1581423 0.4235130.03641146 0.01767223 0.2792811 0.9094694 0
2.0780490.1845071 0.5936423.0516020.03346487 0.4395793 0.04294824
0.1352669 0.06904125 0 0.3397548 0.003571072 0.1066323
TCGA-DK-A3WX 321 1 0.2229678 0.3506436 0.09513114 0.3882195
0.02012786 24.692490.04569567 0.1620545 0.4308449 0 0.1684849
0.6768186 0.7031432 1.4586780.2055434 0.05812645 0.1862919
0.01845664 0.3142543 0.007814344 0.04305125 0.05390765 1.950211
15.811750.1207671 0.4653060.3623014 0.2825034 0.0830274 0.7780854
0.08690719 0.4920142 0.0281792.3907920.1624520.3216219 0.1893904
0.3264 0.1500631 0.2988878 0.1514216 0.1019635 0.4403931
0.06067908 3.9193640.3328526 0.1234936 0.008142793 0.8453699
0.5470041 1.4070960.4187198 1.8530750.5681476 0 0.2919645
0.1345254 0.1905403 0.4305125 0.04649535 0 0.2635957 0.028179
0.02081387 0.1713953 0.6291125 0.3043885 0.9743899 0.02907776
0.1757194 0.2501057 0.4416698 1.1581261.2479270.07439255 0.0510097
0.04927384 0.1342287 0.01440599 0.2669589 0.1943379 0.5484706
0.2369190.1003136 0.3561887 0.2509562 0.08339972 0.2032583
0.4768753 0.1300569 0 0.1673452 0.1025256 0.01605826 0.8337895
0 0 0.2146482 2.5534740.1202267 0 0.2830151 0.2534672.867937
0.2093038 0.06812505 0.0966137 0.2175221 0.1483105 1.133601
0.2072216 0.0771707 0.1985566 1.4581240.1303608 0.1754541 0
0.0883522 0 0.1406286 1.04178 0.3795538 0 0.1552432 0.2497754
2.0773360.3175550.9319627 0 0.5890440.3044338 0.2316494 0.1005305
0.05166149 0.5123454 0.3557021 0.05559982 0.1722050.2596599
0.2257056 0.6176503 0.5544787 0 0.1432830 0.0399960.1586195
0.4316783 0.1122624 0 0.6796965 0.0966137 0.1072970.186916
0.3026822 0 0.6106851 1.4709290 0.2673854 0 1.6303560.1954874
0.4732797 0.4579087 0.6280797 0.2775842 0.2571171 0.09127921
0.5752928 0 0.6677967 0.2244717 0.0242321 1.3033740.2787729
0.1986576 0.4536131 0.3504093 0.1901002 1.1138630.1719241
0.1522655 0.4905003 0.1001695 0.1135764 0.3162949 0.3971133
0.3611289 0.7745405 0.1217839 0.8608211 0.3009844 0.1593298
0.3797753 0.2725002 1.1428150.08505246 0.1538503 0.2053962 0
0.1175893 0 0.3155198 0 0.1118125 0.3215816 0.3647635
0.2010610.2830151 0.7791570.2081424 1.7691190.1033804 0.1554703

0.1532738	0	0.2596599	0	0.1206069	0.3219385	0.1875893
0.08247511	0.1276760	0.2155661.0510050	0.1622520.06726271	0.2126852
0.07208581	0.1029101	0.1269497	0	0	0.4003993	2.8202090.09574331
0.1302087	0.1983801	0.2076844	0.1718073	0.1122197	0.7074639
0.4210899	0	0.5431529	1.3689830.02687592	0.4162017	0.1053719
0.4358939	1.2406140.1935066	0.1464420.05518735	0.7531956	0.08065108
0	0.3243136	0.0137662	0.03653858
TCGA-YF-AA3L	364 0	0.3063202	0.03448139	0	0.08947616	0.04948303
74.0567	0.05616992	0.1054590.5001798	0	0	0.2569752	0.0432158
1.6137290.06098632	0.1028881	0.1144966	0.1361233	0.03511699
0.05763317	0.1058387	0	2.185928148.04030.1261817	0.01733219
0.1484491	0	0.3061762	1.9595280.1602418	0	0	0.03417214
0.08374051	0.03437768	0.9777685	0.09006903	0.2380131	0.04498752
0.05474415	0.01253353	0.3331316	0.07458779	14.128980	0.1214404
0.02001853	1.3638760.04202419	9.1266890.04289148	0.6212271	0.05701037
0	1.10059 0	0.02155358	1.9580160	0.2172082	0.9360474	0.1385525
0.01860711	0.1289890.1933290.1496639	0.2083021	0.02859432	0.08639894
0.008539843 0.1770353	0.1256108	0.8412115	0.3352970.1254041
0.06056827	0.09166463	0	0.03646118	0.4777672	0.06128997	0.6698174
0	0.03127381	0.1570443	0	0	0.05861836	0	0.09686932	0.009350167
0	0.01644924	0.08540906	0.08313149	0.1291591	0.3610571.3593120	0
0	0.1557838.17233	1.6906970	0.06597737	0.3862184	0	0.3773906	0
0	0	13.725950	2.5880560	0	0.04742979	0.3889422	0.09822586
0.06665061	0	0	0.01659612	0.7353004	0	0.3200899	0	0.8911548
0	0.2033911	0.1412272	0.06350322	1.6248430	0.3417214	0
0.1196919	0	0.3796133	1.0223620	0.07045041	0	0	0.2437224
2.2109440.04599831	0	0.1682204	0	0.04396377	0	0.1240208	0
0.3412113	0.5160262	0.2362910	0	0.1484491	0.1601977	0.294473
0.04329765	0.3860233	0	0	0.1122020.8287032	0.1348741	0
0.04057714	0.1042528	0	0	0.04651304	0.1013799	0.9833630	0
0.04143766	1.8917270.1004886	0.3693904	0.03490253	0.4665543	0
0.2219530.3211153	0	0.1352731	0.3147551	0.4985299	0.0400137
0.08374051	1.92434	0.1045480.007273674	0.1743291	0.05729614	0.1126584
0	0.2585616	0.5281456	0	0	0.1533910	0.6460762	0.1249244
0.1053510.2202159	0.02541541	0.02388836	0.9622209	0.04329765
0.03989731	0	0.02695494	0.3424608	0	0	0	0.04337981	0.05482292
0.3460040.1249244	0	0	0.05809697	0.08860914	0.02811086	0.07802443
0.1800091	0	0	0.5621596	0.02942234	0	0	0.2042315	0.0239987
0.07829164	0.2006832	0.6901482	0	0.1967192	0	0.06607271
0.6840528	0.03238124	1.5627758.5916560.02114327	0.1285779	0	0
0.1784479	0.06128997	0.3679865	0	0
TCGA-BT-A2LA	522 0	0.07963676	0.09580735	0.1805066	0.1933646
0.2444263	27.066990.2080926	1.2444320.4723372	0.2055672	0.05902
4.23647	0.4269373	0.4981982	0.4088364	0.2999943	0.1413918

0.1167352 0.1734638 0.3617868 0.04901256 0.2454894.39777491.3647
0.2016517 0.2568421 0.7791087 0.07147147 0 1.20991 0.2968237
0.09958107 0 0.5696885 0.2107996 0.2971709 2.4723890.171895
0.09552473 0.1435173 0.2377391 0.04643295 0.3342496 1.059247
0.1001108 0.09473568 0.3936626 0.01236043 0.7218213 0.05189565
8.4254690 1.8155960.8800259 0 0.5392151 2.0165170.01419547
3.1041290.1058671 0.4694029 0.9780899 0 1.4964360.1486693
0.02984274 0.6237670.2893862 0.1412444 0.2720697 0.04218339
0.5538394 0.6721722 0.3971927 0.2634915 0.5807302 0.2243872
0.07923762 0.02186772 0.04502589 1.1652390.1513739 0.4135786
0.3654528 0.7337803 0.2043232 0 0.07713451 0.5429083 0 0
0.04041281 1.0115930.07109607 0.1582074 0.02566476 0.1196239
0.2700938 0.8240468 0.03041654 0.7558289 0 0.1202352 0.4617258
1.4297170.2068222 0.1833197 0.6695516 0.1575906 2.5632170 0
0.02511675 4.4410280.03298041 0.6658309 0.03524498 0 1.69856
1.5209640.8805413 0.2057670 0 0.1485854 4.0613350.04016965
1.3938780.06416189 1.1942780.02100538 0.3767513 0.6213051 0.07842009
4.4018950 0.1424221 0.07842009 0.1970767 0.06852241 0.3125229
2.6477740.2515654 1.5442350.2719296 0.03035616 0.1504863 0.3822411
0.1420082 0.1350777 1.3675910.1833197 1.9001790.3191973 1.352853
0.02628606 0.9691318 1.6993080 0.2029402 1.4291121.0082580.01745542
0.1463447 0.2673412 1.19175 0.1755674 0.04878669 0.08659887
1.7738330.2290144 0.6588977 0.2304999 0.009195841 0.10146 0
0.05025906 1.43973 0.5620153 0.2404705 0.07685453 0.1483969
2.5878630.8066066 0.7349244 0.02155056 0 0.5023351 0.06852241
1.9062 0.01320451 1.1674850.4125579 0.3847715 0.2141225 0.4394972
0.5228006 0.1721417 0.1167693 0.2895130.07075497 0.3950535 0
0.5321627 0.09317238 0.02828781 0.08135802 8.3710160.5849715
0.6137224 0.4113841 0.3159519 1.2407470.1255419 0.4424958
0.1537241 0 0.1970767 0.1485854 0.03744747 0.5403781 0.2033950
0.3230118 0.1339243 0.06205906 1.3218020.03856726 0.1077528 0
0.07174392 0.1094234 0.08678522 0.4496442 2.0006380 0.6884154
0.2892544 0.05450045 0.02470645 0.07528329 0.1655097 0.005927195
0.1427215 0.3510834 2.4502580.08617592 0.2870973 2.9396970.04079658
4.4117960.1199627 4.4938394.7416360.1501314 0.06351233 0.0418861
0.2638433 1.4997080 0.7100410.0243793 0.3258516
TCGA-4Z-AA87 1454 0 0.02597753 0.1250095 0.3768403 0.1441728
0.1594638 77.457720.04525325 0.2265678 0.2133367 0 0.05133957
0.2743172 0.2089006 1.9260730.1123053 0.07828659 0.1537402
1.6754780 0.01547737 0.02131721 0.2135429 10.0793156.623780.2690952
0.01396366 0.2391957 0 0 0.8081399 0.04303288 0 0 0.6332072
0.9756544 0.08308904 0.2625794 0.1649183 0.05752651 0.01208139
0.1102614 0.05048813 0.0670968 0 17.932890.2060187 0.2690555
0.0887035 1.2034540.1354277.7164230.1727774 0.8341518 0.02296518 0

0.7517580.03330574 0.03588688 4.1355390.09209036 0.1749936 0.4930822
0 0.0187385 0.1454879 0.1168165 1.23591 0.0419546 0.01151849
0.2018609 0.06880116 0.1616454 0.4722582 7.2556040.3192466
0.05051583 0.005421864 0.02953981 0.04279955 0.1468746 2.059283
0.4444039 0.2111626 0 0.1511743 0.1039293 0.2477767 0.0335484
0.6139357 0 0 0.07532954 0 0.01060185 0.3027628 0.0334874 0
0.08670618 1.9712310.006105775 0 0 0.06275322 0.6885261.450941
0.1686637 0.1169401 0.3350916 0.05874983 1.3798940.05130382 0
0.09831711 2.06819 0.08606575 0.8253381 0 0.04374839 0.7260236
0.06963354 0.09085936 0.2147880 0.03074803 0.1437345 2.358806
0.08386145 0.0678632 0.1255778 1.3461710.3288941 0.4915855
0.7111225 0 1.1974280.6038712 0.1204470.06821508 0.06428646
0.04470406 0.1699084 0.7092673 0.2553 0 0.06451163 0 0.1178128
0.7124979 0.03705849 0.08812474 0.8041248 0.04783915 0.4250324 0
0.2664459 0 0.3848552 0.9152637 0.4521232 0.05884368 0.3271416
1.2557780.7326232 0.2546011 0.1395308 0.4492213 0.0916322
0.05304744 0.04519772 0.3649787 0.1629918 0.09919967 0.06538187
0.02399749 0.2316739 0.1183174 0.1217879 0.2450298 0.2618993
0.1882597 0.06685325 0.1702595 2.4395870.1214378 0 0.05623839
0.5011720.2949513 0.1788162 0.9304349 0 0.7410822 0.2446845
0.08606575 0.0644740.37106 0.4495994 0.3649923 0.07618038 0.3002683
0 0.07363804 0 0.9200356 0.1823571 0.07381993 0.05307802
0.08397056 0.3318571 0.3603536 0.6038712 0.2182523 0.4546303
0.2866626 0.0577369 0.08402652 0 0 0 0.05157603 0.1410172
0.1326950 0 0.0349489 0.4784871 1.0989880.0503226 0.1606811 0
0.1404173 0 0.1811799 0.2095344 0.1450242 0 0.9144297
1.3964520.2844490.05372833 0.2455743 0.1953899 0.03093524 0.15018
0.4176739 0.6486868 0 0.6435519 0 0.07984713 0.4214344
0.05217584 0.9712655 0.4826654 0.1192384 0.06215322 0 0 0 0
0.2841151 0 0.1809241
TCGA-BT-A2LB 492 1 0.045420608 0.255002802 0.041180577 0.199563278
0.15683382 26.288042523 0 0.115542018 0.082891148 0.625305068
0.056103155 1.251115336 0.304378176 0.968201116 0.067499108 0.136880757
0.161284863 0.314255615 0.964612312 0.060888424 0.14908894 0 1.916796704
34.327328079 0.162061614 0.073244604 0.15683382 0.040763558 0.934468177
0.920091743 0.225723442 0.141989467 0.146378232 0.409158884 0.158792954
0.121064703 0.065586988 0.207613825 0.121537366 0.095057035 0.086111715
0.132414519 0.117315887 0.026266893 5.187751319 0.3241934 0.192449468
0.028198959 1.143579937 0.503175172 5.031751722 0.120837565 0.554221929
0.200768149 0 0.522396411 0.145584137 0.11132474 0.857261404 0 0
0.152141154 0.073189116 0.103204919 0.136274638 0.034041449 0.447998844
0.440135003 0.010069797 0.066937891 0.042103563 0.241066887 0.26541108
6.133944956 0.021468807 0.114822201 0.066359281 0.116210465 0.037416589
0.025680392 0.117775589 0.043167843 0.266650792 0 0.08810728 0.134311724

0.072204509 0.029328972 0.247717008 0.05629932 0.102340713 0.046098666
0.071010388 0.03939089 0.272704651 0.204929525 0.045484761 0.63086017
1.253314159 0.373649059 0.652452901 0 0.850340325 2.031510976 0.854265244
0.20643084 0.176583264 0.449882415 0.102721566 0.439599389 0.089702538
0.033405821 0.028650544 1.376414432 0.188102868 0.075950969 0.040203759
0.038246094 0.133623282 0.152189088 0.597020064 0.046943456 0.061929252
0.053761621 0.16072383 0.725043055 0.073314083 0.996709553 0.109783674
0.952187967 0.071882167 1.575779899 0.186505083 0 0.789555312 0.307954204
0.051144861 0.059635576 0.0843016 0.156326267 0.534739666 1.120111688
0.031884367 0 0.084596877 0 0.446313159 0.872040531 0.064795193
0.051360783 0.35544383 0.20911176 0.495434016 0.080912591 0.553219719
0.059968736 0.961289881 0.493050038 0.020803108 0.180049902 0.257396891
0.83644704 0.398226023 0.632328209 0.24396372 0.302093912 0.040053745
0.157677012 0.118539427 0.303510205 0.047497361 0.317985028 0.074306309
0.115386098 0.419538688 0.034478813 0.139230566 0.464126101 0.211787915
0.137151665 0.292225145 0.265587475 0.546136327 0.212328864 0.05781546
0.147495623 0.38337156 0.200553805 0.351734101 0.694376778 0.120498451
0.44673751 0.167238898 0.401286119 0.225460054 0.05898024 0.04066062
0.049090261 0.046107047 0.160889037 0 0.05838797 0 0.257989909
0.106281224 0.290409719 0.046402321 0.527717135 0.174071411 0.490048863
0.293289718 0.127201624 0.242348066 0.017900618 0.033650168 0.777237175
0.67090023 0.028100533 0 0.598025732 0.2626427 0.046402321 0.071404016
0.073691558 0.122213325 0.231677777 0.413760769 0.175973831 0.087795014
0.087350482 0.163675787 0.124818647 0.103945194 0.091590475 0.443745034 0
0.594259606 0.758887145 0.020722787 0.046970845 1.37400367 0.026970864
0.104797348 0.076149145 0.318027322 2.025359184 0.05898024 0.222525448
0.02890773 0.162877512 1.186263619 0.022806807 2.421530516 5.821128377
0.055843719 0.235456549 0.09555849 0.200643059 0.034912414 0 0.496763148
0.007945525 0.047450704
TCGA-XF-A8HB 1370 0 0.08298539 0.1267758 0 0.2631776 0.01819315
68.52346 0.1239101 0.04308168 0.1947159 0 0.1288607 0.01889613
0.1588891 0.5273873 0.17938 0.08616443 0.08419267 0 0.0774676
0.09182189 0.03891312 0.194904 1.67435 56.80652 0.3493085 0.1401935
0.05457945 0 0 0.7547558 0 0 0 0.3266608 0.0426301
0.2275101 0.1369488 0.09633507 0.04375448 0.03859406 0.05769877
0.0552976 0.2755815 0 2.912452 0 0.08929864 0 0.6208412
1.452375 6.977551 0.09461803 0.2131762 0.04192137 0 0.4134443 0
0.01373577 1.712177 0.04202617 0.03992986 0.5029904 0.1018816
0.001710293 0.08852608 0.2487807 0.2751304 0.07658528 0.005256557
0.05717847 0.03767756 0.3992161 0.5541913 0.0363863 0.2241396
0.1198772 0.004948622 0.01348073 0 0.02681096 0.2283554 0
0.08565844 0 0.02299654 0.127852 0.1507665 0.06124033 0.08620754
0.05877787 0 0.06187903 0.03706829 0 0.4480001 0.09169347
0.3324104 0.145512 0.4725105 0.00278642 0 0 0.02863794 0.4713216

0.3243177 0 0.1406937 0.3932274 0.02681096 0.3522194 0.04682582
0 0.1794712 2.4318640.0392768 0.1585893 0.008394741 0.03992986
0.6626534 0.2224448 0.01070049 0.1470303 0 0 0.07017074
2.5756390.1052448 0.4211908 0.2674393 0.6515686 0.05003116
0.1196475 0.1103390 0.9261967 0 0.2230088 0.0311305 0.05867529
0.1632084 0 0.3445624 0 0 0 0 0.07168644 0.6503083
0.03382388 0 0.2845039 0 0.3879339 0.04223736 0.1823921 0
0.5519856 0.3294440.02171895 0.1342689 0.05971748 0.3820561
0.3533940.02640664 0.1910281 0.6307869 0.04181709 0.06778415 0
1.2431140 0.2112626 0.1133826 0.04380578 0.1057262 0 0.1624613
0.1118213 0.4422235 0.1431897 0.1525451 0.05789359 0.4423808
0.07389217 0.2414430.07699452 0.4002493 0.2692068 0 0.3728295 0
0.2475704 0.2761140 0 0.09236522 0.1698027 0 0.08557668
0.1768127 0.0421315 0.1813124 0 0.2059718 0.1664403 0
0.04844516 0.03615155 0.4240479 0.1096335 0.6430234 0.0774676
0.4554311 0.1308208 0.1053948 0.08658814 0 0.2640388 0.04423808
0.02725352 0.1119206 0 0.07454754 0.07693579 0.09569527 0.2351584
1.0451630 0.05499608 0.06079736 0.08544076 0.1954706 0.06717987
0.4398644 0.1323659 0.0869657 0 0.8267444 0.06490529 0.04903871
0.3586234 0.1783355 0.02823509 0.0904673 0.6394619 0.866955
0.04105121 0.2016380.7243290.02429259 1.7795340.02381086 1.805812
1.8422430.06218902 0.1134565 0 0.1571072 0 0.02253414 0.7779493
0 0.02476985
TCGA-DK-A2I6 2656 0 0.02816472 0.06023767 0 0.2692030.02881499
16.980440 0.2524672 0.1027994 0 0.009277021 0.4582786 0.1006618
0.4002458 0 0.1414633 0.1111229 0 0.1635948 0.03356099
0.1386721 0.07717406 2.88738615.343680.2204347 1.0193820.4754473
0.03370259 0 0.7742999 0.2177282 0 0.02017049 0.2785880.04876383
0.1401318 0.1084523 0.10013 0.03464999 0.06549291 0.04675535
0.0437912 0.02424868 0 2.1445710.1786916 0.07071726 0.0233144
0.3025574 0.04894311 2.4523070.09990638 0.3617534 0.3651815
0.2001883 0.2298866 0 0.02175523 1.6948820 0.0316212 0.1257876
0 0.002708826 0.1427147 0.08443462 0.2396690.1212986 0.02497659
0.0855302 0.02983756 0.2680374 0.3657287 0.4898548 0.03550007
0.007302537 0.007837813 0.09608077 0.01031179 0.0212321 0.06955342
0.03569041 0.8309729 0 0.03642278 0.07185367 0.05969742 0
0.06826936 0 0 0.01633439 0.04403261 0.01149448 0.3448325
0.02420459 0.0376060.2931384 0.7627717 0.1522566 0 0 0.06803675
0.5287685 0.1926250.04876383 0.01536799 0.5709075 0.0212321
0.3803578 0.05562336 0 0.0710633 1.3938760.03110403 0.2197822
0.01661988 0.0316212 0.3314321 0.05033091 0.1588860.03881203 0 0
0.0217446 1.7750030.1515371.5696420 0.6014961 0.0198103 0.1421266
0.2312987 0.03697924 0.9095058 0.03637302 0.0646722 0.1479169 0
0.06462391 0.02456185 0.4961189 0.1581686 0 0.0233144 0

0.3122341 1.3389770 0.0212321 0.5844842 0 0.1536061 0
0.1444397 0 0.3179110.1770344 0.03439929 0.04253203 0.07093708
0.3025574 0.1536483 0.1129243 0.2017049 0.4995319 0.0993472
0.01533701 0.01633439 0.3024111 0.07853997 0.0239004 0.3733367
0.008672654 0.1076484 0 0.1625130.2361423 0.4448551 0.1360735
0.09664265 0.08445502 0.2958339 0.4681327 0 0.1422713 0.1358421
0.02368777 0.09693586 0.4724011 0 0.3238418 0.1221923 0.08294408
0.09320321 0.09752765 0.2689399 0.04058697 0.02117805 0.1365208
0.03336472 0.1213043 0 0.08783004 0 0 0.1150939 0.1248228
0.1918922 0.2315222 0.1697408 0.07011205 0.4408121 0.02959983
0.01391068 0.06465244 0.2521311 0.2090972 0 0.1785468 0.9217727
0 0.1180712 0.03046344 0.02526096 0.2819999 0.5870615 0.03637302
0.0580699 0 0.1014932 0.2063957 0.03273907 0.1211606 0.05241151
0 0 0.4364762 0.01713324 0.0233008 1.1715020.1635263 0.06987468
0.1150626 0.07790797 0.06698126 0.01625461 0.1457956 0.0239004
0.0384755 0.5790965 0.01885627 0.3900154 1.0545360.03693648
0.08984831 0.03950304 0 0 0 0.2410719 0 0.05230854
TCGA-ZF-AA4X 2044 0 0.3711898 0.1428997 0.05384625 0.1098702 0
19.871190 0.2589919 0.2709637 0.2044066 0.6895697 0.1153718
0.4377934 1.5962420.3690848 0.09475432 0.1054452 0.1532205 0
0.01769234 0.07310376 0 5.15694324.32996 0.1230422 0.1117340.06835676
0.2132039 0 4.4900630.09838263 0.2227924 0 1.1958860.4567904
0.2532798 0.9433512 0.3091723 0.2575566 0.01381035 0.1193188
0.1962259 0.6135922 0 15.742421.0833090.16776 0.08296189 1.196243
1.0449542.8646880.3555065 1.9070550.03937759 0 1.14579 0.2284327
0.006616557 1.4620750 0.1500277 0.3978687 0 0.01071008 0.2534227
0.6231593 0.6202453 0.04795873 0.01975036 0.3182749 0.3067245
0.4891196 1.81233115.129630.1965029 0.2425296 0.006197787 0.135069
0.0163082 0.03357876 0.4399976 0.2257789 0.1877416 0 0.05760296
0.1962825 0.09441203 0.2300971 0.3239059 0.07361497 0 0.2583299
0.09285064 0.009089315 0.5820625 0 0.2973712 0.4667983 3.0727290
0.1828123 0 0.03586692 7.0835491.5908880.03856022 0.2248178
0.7797734 0.2014726 1.1496090.1172918 0.04368025 0.1498497
5.0158720.09838263 0.9931077 0.0210276 0.1000184 0.2620815
0.7561886 0.05695672 1.7800660 0.07029677 0.1566623 2.388569
0.1437943 0.4033905 5.7898180.6062026 0.06266037 0.03746242
0.7153443 0.1169661.1251940.4601942 0.4877955 0.1169660.2204595
0.1533050.9711201 1.5169260 0.0324405 0 0.1358315 0.8080381
2.3212210.1694477 0.1678938 0.4389453 0.4374833 0.04048824 0
0.2665048 0.03920649 0.4399319 0.9504641.9040980.1008972 0.1121877
1.7089190.3818511 0.03968687 0.9569947 1.3825250.3142371 0.194045
0.1033319 2.21834 0.06210585 0.1889936 0.1046341 0.1783065
0.1324144 0.1352498 0.3641058 0.3267787 0.6885735 0.5738707
1.5284130.1946253 1.7606460.1388168 0.1133961 0.09643 0.1432237

0.7117861	0	0.5551418	0.0590848	0.9163491	0.595001	0.06558842
0.1351183	0.03856022	0.4784973	0.288849	0.4488101	0.3875287
0.2638331	0.2701475	0	0.7738939	0.4863933	0	0.2426961
0.05795477	0.4172842	1.006925	3.259709	0.6514368	0.3042102	0.2340621
0.175999	0.5019469	0	0.146973	0	0.01861802	0.182224	0.03033701
0.04668267	0.09635644	0.07990088	1.152831	0.9674149	0	0.3214333
0.03807212	0.3210249	0.1632084	0.1294429	0.3592813	0.0828893
0.01089182	0	4.530036	0.08128912	0.1596852	0.1122874	0.4231936
0.1237684	0.3193104	0.9240914	0.02648287	0.1285341	0.8179996
0.07559742	0.4259456	1.450532	0	1.356989	7.937527	0.1752462	0
0.06247443	0.6558842	0.1095605	0	1.200255	0.1662285	0.05170403
TCGA-BT-A20P	544	1	0.17858408	0.13641038	0.09252182	0.3775711	0
58.23970104	0	0.16688072	0.60526179	0.1756118	0.02520978	0.23382007
0.61547123	1.93884613	0.17922577	0.06331597	0.12078809	0	0.2778497
0.12160011	0.16748163	0	3.07280076	56.35131745	0.10570918	0.27426862
0	0	0	2.06720179	0.16904688	0.12760505	0.05481217	0.48667307
0.17328615	0.02720002	0.73678272	0.2073125	0.0753275	0.11864884
0.0808532	0.1586668	0.13178882	0	12.16863468	0.24279216	0.28825523
0	1.95268351	0.79800069	9.04400785	0.20361744	0.19660887	0.13532181
0	1.62801607	1.43919112	0.04092826	4.18704067	0	0.34371526
0.62667141	0	0.03312486	0.23133065	0	0.71049489	0.08240554
0.1131208	0.3144553	0.09459552	0.05602896	1.12635995	2.19248675
0.19293883	0.09922115	0.01064941	0.2901045	0.25219542	0.8654553
0.15120598	0.09698668	0.2765047	0.15609938	0.24744208	0.09762913
0.64889742	0.06589441	0	0	0.07664413	0.02959185	0.19942685
0.02082373	0.39644966	0	0	0.09339294	1.21238592	0	0.10470631	0
0.12325735	5.32497659	1.0468948	0.1987694	0.08352331	0.3525929
0.23078808	0.43640927	0.40307556	0	0	5.91039866	0	0.51192497	0
0.25778645	0.82559408	0.47869985	0.1208938	0.94922531	0	0.06039404
0.16413807	1.18471214	0.06176898	0.61315308	0	1.17782428	0.21533352
0.19311049	0.02793516	0.20097795	1.31549205	0.98841616	0.24333653	0
0.56820992	0.1756118	0.53396356	1.79756678	0	0	0	0.15559583	0
1.74932454	0.0727888	0	0.22182997	0	0.20870787	0.27268365
0.13083556	0	0.53994077	1.38627312	0.18695623	0.17336756	0.06425583
1.99672901	0.14166212	1.00015418	0.68515211	0.88234223	0	0.14587109
0	0.7343612	0.32014187	0.32473996	0.05136817	0.21210703	0.09750952
0.15492947	0.31281411	0.48127758	0.45011059	0.24651469	0.13131046
0.0852435	1.37511231	0.07950776	0	0	0	0.06437016	0	0.37441925
0.033841	0.36152247	0.55050483	0.22539583	0.16884962	0	0.39586566
0.11029278	0.14963042	0.30439995	0.09066675	0.12781861	0	0.27276932
0	0.72497057	0.41701477	0.2427295	0.06518204	1.10100965	2.43809319
0.07144707	0.78406811	0.22119864	0.34021346	0.68146167	0.27406084
0.12626887	0	0.08530825	0.49374743	0.20850739	0	0.08278268	0
0.54943613	1.18170834	0	0.07890083	0	0.41370303	0.14021718

0.1556915	0.16462358	0	0	0.01589457	2.29806757	0.0931172
0.04221241	0	0.1817891	0.02278566	0.27137923	0.50281243	0.13651333
0	0.59428683	0	0.15683251	0.55487501	0	1.90772041	4.28768925
0.13383041	0.24415769	0.10734727	1.01428125	0	0.04849334	1.35872418
0.01785148	0.07107275
TCGA-BT-A20W	254	1	0.1659287	0.2883417	0.6769762	0.2494071
0.06365986	34.747550	0.3165699	0.2649626	0.2855423	0.02049537
0.4421753	0.8339562	0.6920201	0.3026269	0.1139813	0.09819985
0.1459354	0.5421356	0.2347925	0.03404034	0	3.993314	56.33503
0.2673714	0.02229784	0.3819591	0.1489157	0.1312985	0.7202658
0.2061508	0.103742	0.2228095	0.8792482	0.5469474	0.1769074
0.5989991	0.2633493	0.2985485	0.1639831	0.1103377	0.04837311
0.160715	0.1919142	13.14102	0.9211453	0.2734077	0.141646	1.086196	1.18941
5.655257	0.7725177	1.864819	0.5317424	0	0.8618565	0.1063684
0.05176015	1.633936	0.07352713	0.279438	1.713703	0.0445619	0.0508683
0.1604128	0.1865382	0.240678	1.004927	0.0459832	0.1667282	0.131838
0.3036743	2.962632	3.564952	0.3529302	0.04839965	0.05194734	0.1415117
0.06834435	0.2814436	0.5224499	0.2365484	0.4870587	0.06345384
0.3218698	0.1443123	0.1318872	0.3214301	0.3016498	0.1028352
0.1246223	0.08420285	0.2594122	0.04655625	0.5347428	0.1069486
0.08308151	0.3037112	0.9538655	0.01462499	0.08512548	0.1278733
0.150311	0.7558864	0.851118	0.1615981	0.1188317	0.4013177	0.04690726
0.8776572	0.2457732	0.1830551	0.3139949	2.276106	0.1374339
0.6936522	0.01468707	0.3492976	0.5491653	0.3891795	0.3088982
0.3429837	0.1131187	0.1472998	0.3042502	1.685534	0.1506532
0.6718765	0.1336857	1.055273	0.2188308	0.2616624	0.4315106
0.2450904	1.782476	1.125005	0.2088215	0.05446454	0.3079671	0.3569278
1.627907	0.7855073	0	0	0	0.1264983	0.1254194	0.4551003
0.2367071	0.2814436	0.6636739	0	0.339356	0	0.2659209	0
0.9657295	1.734288	3.039882	0.04698219	0.3656767	1.623326	0.5758514
0.1478397	0.5570237	1.876115	0	0.3388347	0.1804347	0.6965354
0.04337884	0.3168135	0.1252859	0.0574805	0.3699478	0.3778696
0.3291143	0.5869128	0.564588	0.2505183	0.7472812	0.3838284	1.54794
0.3231962	0	0.2245103	0.3001108	0.5233248	0.6424701	1.406039	0
0.6999822	0.5683257	0.2519621	0.2573879	0.05386603	0.7426983
0.4483362	0.2058666	0.5645523	0.07371141	0.1668119	0.05177967
0.2494794	0.09706552	0	0.169515	0.4882834	0.2119701	0.6393664
1.125005	0.2517058	0.7968022	0.1798329	0.1229294	2.263705	0
0.4619506	0.232191	0.07802437	0.3035075	0.2542725	0.1304251
0.06730173	0.1674242	1.034435	1.729297	0	0.1924374	0.1063684
0.1868542	0.1139956	0.1898641	0.3345945	0.2315815	0.01521513
0.4005872	2.229921	0	0.2917063	0.2352868	0.1642147	0.08644803
0.3980921	0.6884765	0.07398957	0.1077321	0.4754819	0.4752203
0.2550074	0.6211998	0.1249753	1.321191	5.710505	0.1972057	0.2977477

0.2618177 0.6413582 0.08927839 0.07884947 0.2761583 0.02902626
0.2166811
TCGA-FD-A5BX 173 1 0.05250827 0.1123028 0 0.09713859 0.5909264
54.99331 0 0.1526535 0.03194197 0 0 4.090569 0 0.5190883 0
0.0961853 0.08286782 0.2709309 0 0.03128434 0.143628 0.1438777
1.26566 53.21951 0.05640662 0.7150255 0.7252279 0 1.107987 0.6837863
2.087572 0.04377233 0 0 0.1958099 0 0.2527384 0.04444647
0.1744171 0.08140017 0.03565935 0.01360686 0 0.3643886 0.1760206
0.05552329 0 0.09779781 0.2820331 0.091246 7.236375 0 0.3596944
3.249359 0 0.857168 0.1346415 0.1497558 0.9192189 0 0 0.0390849
0.7896926 0.0580766 0.1073603 0 0.4062015 0.6784212 0.01552152
0.05627871 0.1390675 0.0384392 0 0.1074412 0 0.02722865
0.4018369 0.05970869 0.2306948 0.03958359 0 0 0.916877 0
0.203712 0.1887601 0.1112956 0 0 0 0 0.01015088 0.02736374
0.1178621 0.07417856 0.1353759 0.2103298 0.6784212 0.2414813
0.5389155 1.257108 0.05395415 0.5073713 1.21775 0.07980357 0
0.05013921 0.1128865 0 0.1733384 0 0.05149152 0.08832353
1.205167 0.05798811 0.9365627 0.08056076 0 0.1544745 0.04691666
0.6753714 0 0 0 0.0225217 0.7111852 0 1.18882 0 0.4672442
0 1.722309 0.03833036 0 0.8204599 0 0.03246121 0 0 0.06024007
0.137374 0.81702 0.04914636 0 0.04346569 0 0 0.09601126 0 0
0.07305051 0.1289294 0.3818294 0.1247181 0 0 0.6667767
0.1476541 0.03206578 0.07929364 0.04408332 0.08058088 0.0613823
0.538016 0 0 0 0 0.0609053 0.335877 0.07321213 0.08911638
0.4228994 0.08084336 0.4905804 0 0.01262406 0.1100617 0.4235008
0 0 0.05848212 0.9724369 0.1636412 0.4010237 0 3.798813 0
0.783121 0.3669627 0 0.1338035 0.1498726 0.3092699 0.02896022
0.04545588 0.7729795 0.1891685 0.0236897 0.1174705 0 0.02538429
0 0.11696 0 0 0 0.5316051 0.04471876 0.3237249 0.2712449
0.03267796 0.2689587 0.01379595 0.1815385 0.9423516 0.6110717 0
0 0.6620815 0.1569771 0 0.05503084 0 0.04709471 0.05951777
0.1648469 0.1356225 0 0 0.0315361 0 0.03814773 0.1129416 0
0 0.1962831 0 0 0 0 0.06928786 0.06774005 0.06475906 0 0
0 0.05824516 0.08911638 0 0.4618052 0.3515426 0.04847443
4.227641 0.005738476 0.3629312 0 0 0.06457652 0 0.1165207
0.00612359 0.07314021
TCGA-BL-A13J 81 1 0.780818133333333 1.81331476666667
1.47412776666667 2.06714873 0.3766872 47.5375933333333
0.100485066666667 0.9133516 0.46141346 2.011396 2.07743228666667
2.823884 2.5657283 1.2929668 0.910396333333333 0.2854126
1.40992110666667 0.0667845533333333 0.5635947 1.68139729666667
1.39786033333333 2.27896883333333 5.39830866666667
129.484766666667 0.845275866666667 0.437750826666667 1.8774646
1.27819044666667 1.17257676666667 0.351461833333333

1.35002093333333 0.919612593333333 5.29966796666667 3.0992845
0.4718677 0.274145566666667 1.94785116666667 1.0954134 0.08443505
0.661333 0.420888976666667 3.75191813333333 0.342876466666667
0.5354382 3.4986794 1.81928223333333 2.13142784666667
0.377643413333333 0.806274833333333 3.54400653333333
4.50119366666667 3.28417693333333 6.98917733333333 0.57585471
1.943634 1.15535566666667 1.67198189 4.15641933333333
0.739528966666667 3.45552773333333 3.70582916666667
0.596565266666667 0.0312004666666667 0.342518033333333 0.4925976
0.8788137 2.02784916666667 0.39174482 6.18171780333333
4.84834973333333 0.470374533333333 1.61442491333333 3.451591
14.7993753333333 0.472614706666667 0.1449401 0.0561401966666667
0.94266812 0.124577233333333 1.91332786666667 0.9224018
1.27996066666667 5.23474273333333 0.0143946833333333 2.0731692
0.218723263333333 2.19443706666667 0.422165346666667
3.31239036666667 1.15066226666667 11.8219883333333 0.9862948
0.8080142 0.986735596666667 2.97448596666667 0.652337966666667
0.12454447 0.801396466666667 10.136813 0.545740566666667
0.0638042433333333 1.45132414333333 0.925147733333333
0.762785916666667 0.664723466666667 2.03048422666667 1.0749028
0.6889641 0.946102633333333 3.38439973333333 1.5341989 2.43888375
1.58613373333333 1.21834733333333 2.03899573333333
0.930407116666667 1.82583649166667 3.9883058 0.398954966666667
3.12067163333333 6.84156066666667 2.71340273333333
4.29973533333333 1.48427654 2.2870251 6.149004 1.08097194 2.678019
0.862311583333333 9.639903 0.660433166666667 1.08758213333333
0.800854766666667 6.00540109666667 1.38832093333333
3.18648976666667 0.544630433333333 0.197686966666667
2.84781223333333 3.14794396666667 2.4149936 0.4822664 0
0.0960794533333333 0.0934772 0.14534448 2.5478929 1.80245576666667
4.59348756666667 1.6092478 2.53420173333333 0.277275233333333
3.4956629 1.73934296666667 4.5792249 9.26681214 3.06209333333333
2.1241204 0.0886235133333333 1.25992563333333 1.9917848
6.98346166666667 2.6015304 0.862387566666667 2.58240393333333
2.52982923333333 2.975778 1.7482983 0.19129601 0.592439433333333
2.43315977 4.553919 0.936738033333333 0.12689566 0.230887133333333
6.54386144 0.734847466666667 4.75632483333333 0.320661766666667
2.2800844 4.19850803333333 0.634803343333333 0.564619533333333
0.1972869 0.09035505 3.42751230666667 0.376898276666667 1.1845453
0.545960833333333 4.30576266666667 2.19536824333333
4.25984576666667 0.946401566666667 0.2839518 1.77090843333333
8.84708093333333 0.13196305 2.6270852 1.56338076666667 2.7482307
0.374745966666667 0.49104913 4.569471 1.09697533333333
0.124916533333333 1.32629316666667 3.9119121 1.1475882

0.833517566666667 0.8317575 4.3916316666667 2.06397691333333
1.6915352 0.851436796666667 0.323563296666667 2.032724
6.37403529333333 1.25118233333333 1.45041010666667
0.604101933333333 3.28628506666667 0.535824213333333 1.9366129
1.85233113333333 2.5402585 9.464703 1.10703423333333
0.0364586666666667 3.0616321 0.242865346666667 2.05792373333333
5.02345883333333 0.760227423333333 0.18875452 0 0.0105787466666667
6.54997223333333 12.9122086666667 1.10913803333333
0.998592563333333 3.20196116666667 1.24062253333333
1.83442573333333 10.3735228 1.8618451 0.0335695 3.46774946666667
1.3461798 0.03671224 4.6651471 1.2744519 0.1975913
0.984056366666667 4.793239 1.5591372 0.5130775 2.2014349 1.2465828
0.0191191393333333 4.379093 0.573137266666667 0.0541057686666667
0.9995609
TCGA-CF-A8HX 345 0 0.1546617 0.1819316 0.1121792 0.4005667
0.07120464 49.073749 0 0.1011683 1.1572236 0 0 0.4807139 0.7047779
3.61217 0.3928195 0.09870197 0.07322547 0 0 0.1382208 0.1269157
0.1271364 2.949767 68.85534 0.04984325 0 0.1424093 0.05552159
0.09790638 3.893877 0.05124072 0.07735813 0 0.06556362 0.2718978
0.0164895 1.429314 0.1099693 0.1883717 0.1007 0.05076627 0.04809436
0.6391557 0 35.50738 0.1471881 0.02912487 0.04801013 4.236676
0.6853447 7.063313 0.0411464 0.1589205 0.1777453 0.08244748
4.269149 0.03965828 0.04135329 4.162834 0 0 1.208797 0.03322883
0.02900635 0.3464748 0.04636581 0.03589367 0.04995679 0.01371547
0.1989209 0.1228859 0.1018995 0.1205002 11.06045 0.2924137
0.1082717 0.025824 0.3165666 0 0.1049332 0.2520808 0.1175927
0.1676257 0 0.1200056 0.1452742 0.09834542 0 0.281167 0
0.04646405 0.02690924 0.02417975 0.03155998 0.6063124 0 0
0.2364621 1.043206 0 0.1269524 0 0.07472242 10.86303 2.291828
0.1606669 0.06329301 1.396518 0.1748886 0.5291296 0 0 0.1170696
5.180471 0.2562036 1.75862 0.02190365 0.05209271 0.3640005 0.8291504
0.05932965 0.1918166 0 0.07322547 0.2786156 0.4873562 0.02496418
0.517165 0.04984325 1.185198 0.03263546 0.2341391 0.6604712 0.06091953
1.836648 0.3595251 0.2294727 0 0.1530962 0.05323065 0.2832421
3.541657 0.04342778 0 0 0.0471635 0.8417026 2.842126 0 0.06995544
0.3442693 0 0.08435011 0 0.6741908 0 0.4582603 0.3530454
0.02833466 0.07006719 0 0.9256603 0.2711999 0.2618219 0
0.7406351 0 0 0.02690924 0.9221844 0.4205065 0.4724818
0.02335573 0.02857463 0 0.04696152 0.06693091 0.4862756 0.14813
0.5604181 0.07960446 0.1470814 4.540108 0 0.2756144 0 0
0.1170696 0.159692 0.4539691 0.02051546 0.5479153 1.403798 0.2391234
0.1279523 0 0.239986 0.4011774 0.04884416 0.4844087 0 0.08972248
0 0.8681463 0.3618982 0.2197498 0.2528073 0.1811079 0.03951537
3.957121 0.6391557 0.2598805 0.3300877 0.1219068 0.1374986

0.5551342	0.08307208	0	0	0.02585825	0.5548463	0.1264036
0.04862756	0	0.1248446	0.2980236	1.611873	0	0.07174819	0
0.2786662	0	0.1213521	0.02494998	0	0	0.2119871	1.797625
0.02822526	0.1023619	0	0.2693929	0.05064903	0.1323301	0.6256841
0.4965516	0	0.5947398	0.03937348	0.03169224	1.34553	0.03106378
3.041217	5.316217	0.03549531	0.07400797	0	0.06832096	0.01902084	0
1.117879	0.04869955	0.0430865
TCGA-PQ-A6FI	372	0	0.05367802	0.05740229	0.03893373	0.1390236
0.1153264	53.08738	0	0.1092379	0.2155135	0.07389848	0.1166927
0.8855358	0.2014397	1.47256	0.01740444	0.02474061	0.07624251
0.1208581	0.07015247	0.04477379	0.03523863	0.3088742	1.797659
51.23694	0.1383917	0.5770693	0.6919585	0	0.4077613	0.5747492
0.1778398	0.1073939	0	1.297034	0.08149863	0.02289186	0	0.08178593
0.06735879	1.11839	0.08505876	0.0918059	0	0.4221737	0.9254156
0.2383929	0.1415161	0.0266604	0.5189689	1.119345	8.603958	0.05712228
0.7170295	0.06643492	0	0.3505054	0.3303382	0.02392063	0.5990567
0	0	0.1678136	0	0.01858552	0.1660596	0.1287365	0.07474511
0.03467674	0.01428057	0.0632857	0.06255284	0.1807594	0.08364333	0
0.0811898	0.01670114	0.013444	0.04883107	0.2594174	0.02427925
0.1908851	0.04081257	0.03878493	0	0.1249501	0.06349178	0.1365299
0.02772876	0.3903356	0	0.0322523	0.01245242	0	0.002190687
0.3677807	0.05535668	0.04300307	0.1225245	0.5430939	0.09588551	0
0.1323747	0.1556023	0.4623835	0.5629116	0.1115244	0.03954049
0.2077224	0.02427925	0.3479563	0.1908186	0.03158317	0.05417469
2.079026	0	0.5385526	0.0380102	0.2892748	0.4737475	0.08633131
0.2204489	0.488204	0	0.1270708	0.1326149	1.22853	0.1472922
0.7852735	0.03459792	0.6271305	0.3851079	0.2437861	0.04114348
0.1691454	0.5116299	0.7486764	0.06826496	0.0563818	0.1062694	0
0.1685213	0.6713287	0	0.02345622	0.05332079	0	0.162293	0.4711207
0.03062995	0	0.5824875	0	0.02927517	0.2677427	0.30281	0
0.1817682	0.3542731	0	0.07295409	0.1081569	2.47128	0.1913867
0.2104354	0.3171476	0.1428057	0	0.05261436	0.03735727	0.4561776
0.02245293	0.08199149	0.1134841	0.01983464	0.06838763	0.03259762
0.07743177	0.4050489	0.140704	0.2852708	0.1381405	0.03311166
0.9436533	0.2676587	0	0.6507579	0.1035584	0.3792228	0
0.2813533	0.01424049	0.444382	0.1434059	0.09484789	0.09769776
0.1951678	0.2178388	0	0.07265234	0.1921399	0.0381531	0.07784935
0	0.1434787	0	0	0.1316117	0.3221402	0.3017188	0.1323747
1.053693	0.2605663	5.938929	0.04230986	0.01590709	0.06048909	0.08649481
0.1062694	0	0.08974554	0.2584479	0.06580585	0.03375407	0.03483544
0.05777263	0.2555434	2.511213	0	0.06640387	0	0.01934319	0.05900421
0.03743766	0.1385491	0	0	0.3745575	1.653327	0.01959213	0.03552646
1.339632	0.1274965	0.02556891	0.1340585	0.0779529	0.03829707
0.01858741	0.6827564	0.327966	0	1.022018	0.06468734	0.9217103

0.9701431 0.08799472 0.2911051 0.04517236 0 0.02640605 0
0.1735697 0.03004804 0.03738479
TCGA-FJ-A3Z7 945 0 0.1430958 0.1391127 0.1887094 0.1323612
0.13974533.30704 0.1359681 0.25528 0.07122141 0.1790907 0.03856379
0.9149304 0.6974043 3.038221 1.110717 0.0991615 0.0923857 1.354644
2.040149 0.03100226 0.1067497 0.05346767 27.26825 165.1535 0.07785795
0.2657169 0.5390166 0.4669961 0.08234975 0.9223172 0.04309894
0.06506647 0.02794901 0.3584492 0.5821332 0.02773886 0.6386718
0.1519577 0.2112543 0.08469952 0.05006192 0.06067877 0.5039985 0
12.4097 0.8253398 0.2939657 0.01615267 1.414918 0.4747221 3.618661
0.03460853 0.9356842 0.7130133 0 0.8590919 0.1000706 0.04985499
1.579895 0 0 0.7843328 0.111796 0.06005525 0.1942816 0.07799723
0.09057125 1.176532 0.0461447 0.1394282 0.192939 0.09523152 0.2027071
9.662371 0.2705464 0.1720175 0.005430187 0.1479258 0.1857494
0.02942001 0.2505773 0.24727 0.3054809 0.3581815 0.1009376
0.1719728 0.4135952 0.0671998 0.6621765 0.1289954 0 0.0452671
0.1220265 0.5468336 0.707531 0 0.05210832 0.1540697 1.176578
0.03669089 0 0.320806 0.09427433 4.008201 1.809047 0.3716297 0.1703559
0.335606 0 1.768507 0.1541477 0.1530817 0.06564536 2.416592 0.1723957
0.9136161 0.04145246 0.1314466 0.07654085 0.6625341 0.4755423
0.1613383 0.4966324 0 0.2879103 0.3883441 0.05249387 0.3534303
0.2096176 2.823128 0.1921494 0.1969361 0.2920078 0.1024797
0.5793067 0.4535986 0.1826712 0.4099188 0.03219257 0.1790907
0.5105077 0.9967901 0.07305483 1.449559 0.03230533 0 0.07866245
0.2140775 0 0.17652 0.1131122 0.04791258 0.07094748 0.5561712
0.4336392 0.06870147 1.596848 1.120014 0 0.206269 0.950167 0.7785795
0.4524145 0.2607872 0.3493626 0.588345 0 0.03187732 0.2716026
0.5438507 0.163242 0.03311732 0.03274112 0.04806865 0.1325883 0
0.1219748 0.122703 0.2754165 0.1571239 0.1673897 0.2541095
0.8144439 1.256784 0.03311732 0.1689742 0 0.2954041 0.4477268
0.7636732 0 0.8756253 1.25649 0.3016926 0.06457297 0.1689226
1.164542 0.1405971 0.05869025 0.3783368 0.04623144 0.07032079
0.03247596 0.3303304 0 0 0.318957 1.202781 0.03323666 0.5213097
0.4703986 0.09714994 0.510856 0.153805 0.2505773 0.5103624 0.3144263
0.3541183 0 0.2256517 0.7368713 0.3721165 0.1636039 0.2110566
0.315023 0.5308301 0.325382 0.05039985 0.1810437 0 0.3515822
0.07149746 0.1417641 0.0209856 0.2178702 0 0.1945133 0.8693974
0.04748094 0.1183838 0 0.07209627 0.0464741 0.4271658 0.6207256
0.3480442 0.04504602 0.9138997 0.1324693 0.1066263 0.598335
0.1828958 1.224953 11.06341 0.05118061 0.4979891 0.1094739 0.2873262
0.03199713 0.148362 0.4082692 0.01365385 0.05436054
TCGA-E7-A7PW 416 0 0.1904157 0.1425389 0.1381123 0.3346497
0.08766541 31.82813 0.1326828 0.2352722 0.4517533 0.2621451
0.01881599 0.07967119 0.6124979 1.482405 0.1234799 0.1822792

0.2253835	0.2590233	0	0.3176582	0.09375329	0	3.859737	50.23173
0.210397	0.1023539	0	0.2050705	0.1205399	3.664414	0.189259	0.04762072
0.08182105	0.4843219	0.6086435	0.2436176	0.6049092	0.2707831
0.302197	0.0974124	0.08836517	0.0592126	0.6885487	0	32.06191
0.6644528	0.4661518	0.02364356	0.6903651	0.545975	4.093744	0.2026337
0.7337214	0.1178346	0.1015073	1.737895	0.2441315	0.04582185
3.562625	0.06750237	0	1.403199	0	0.03021777	0.2589897	0.5137601
0.5302968	0.1230111	0.07598766	0.2347021	0.1210353	0.1672748
0.3956183	8.532767	0.1800063	0.08886763	0.07153622	0.2165272
0.06274426	0.2153186	0.5078548	0.4343316	0.4127533	0.1165089
0.073874	0.1324875	0.3632414	0	0.2769328	0	0.0572054	0.08834666
0.08930853	0.05051264	0.369876	0.2945558	0	0.2788253	1.080038
0.05370651	0.1172255	0	0.1839928	2.649626	1.606166	0.1978091
0.1168872	0.9824906	0.1291911	1.817207	0.07521155	0.05601856
0.1441332	3.783137	0.189259	1.910444	0.08764352	0.06413527	0.6162042
0.5614564	0.08593554	0.2361599	0	0.1352301	0.2401173	0.9631917
0.1844117	0.2188728	0.2454632	0.909002	0.2008999	0.2402219
0.271052	0	1.72568	0.5164116	0.1664857	0	0.2827324	0.2621451
0.7970759	2.012493	0.1069344	0.1664157	0.09457425	0.1161331
0.5757132	2.61131	0.1086557	0.2153186	0.6291611	0.07013233
0.4673241	0.06784158	0.1953052	0.2011244	1.853796	0.311822	0.2093097
0.34506	0	1.227316	0.3450245	0.1017943	0.2556908	0.2532922
0.1343331	0.06221416	0.19878	0.6655623	0.199122	0.3878053	0.07668001
0.1407215	0.1698173	0.2890894	0.2197432	0.2993453	0.1631767
0.09199641	0.5880424	0.1761889	3.821187	0.3560564	0.290854	0.2061141
0.1836799	0.2882663	0.06553628	0.7518746	0.07577441	0.6035715
0.3587082	0.1682302	0.1102722	0.2967137	1.2955	0.2469599
0.2663154	0.3691952	0	0.1631856	0.09507376	2.188578	0.08911204
0.1082202	0.07781253	0.1463277	0	1.29135	1.229551	0.2133057
0.4714193	0.1500886	0.1410708	0.6516785	0.05113816	0.1884883	0
0.0676516	0.2606616	0.03890626	0.05986906	0.1235741	0.1024704
0.3021689	1.361219	0	0.05888974	0	0.3087783	0	0.1992077
0.3378958	0.2126059	0.01396842	0.09490667	1.770554	0.2085015
0.1260254	0.2160076	0.1959868	0.05668895	0.2377774	0.2370237
1.018904	0.03296819	0.6054971	0.145427	0.1170561	1.507219	0.03824497
1.318406	5.741119	0.03121497	0.03037227	0.0801215	0.0841151	0.105381	0
0.4708415	0.07328162	0.2121882
TCGA-4Z-AA7S	1064	1	0.3039607	0.2762925	0	0.2389849	0
72.68475	0	0.08836827	0.05547184	0.6276935	0.1051259	0.202881
0.5296041	0.9014719	0.1478333	0.07274308	0.03597798	0.08554738
0.2317292	0.0724397	0.1496584	0.06246612	2.040029	54.79402	0.1399403
0	0.2798807	0.2727951	0.09620898	1.099531	0.1007047	0.3420764
0.03265274	1.095258	0.2307497	0.2916651	0.6583751	0.2470007
0.1234039	0.02827263	0.1066531	0.2126725	0.3140373	0.1054689

4.759956 0.2410605 0.2289595 0.03774223 1.224478 0.5942319 8.023553
0.2425983 1.561653 0.120921 0.08101809 0.5751394 0.07794145 0.02438181
2.594079 0.1616311 0.1535687 0.3393829 0.06530549 0.01096287
0.2512978 0.1822479 0.7406989 0.3436348 0.01347768 0.2443403
0.1288057 0.5117901 1.263051 4.431444 0.05746883 0.01182162 0.01903222
0.2592319 0.06677246 0 0.3152679 0.2311079 0.2745326 0
0.2653315 0.1216066 0 0.07850933 0.221034 0 0 0.2027275
0.1900843 0.02170898 0.3810978 0.03918329 0.060878 0.2749078
1.630872 0.01071647 0.03118786 0.04684959 0.1101404 2.014095 0.4504189
0.03947035 0.1554893 0.3220719 0.2062279 1.039912 0.2401205
0.08942245 0.1533866 2.725191 0.1007047 0.5591012 0.03766684
0.2559479 0.2235561 0.1222163 0.08573684 1.256607 0 0.03597798
0.1760048 2.142478 0.07359412 0.5558426 0.1469374 0.4152648 0
0.1533866 0.3827557 0.05986337 1.365478 0.353292 0.318108 0.03990891
0.03761049 0.1046156 0.3180932 1.177933 0 0 0.07548445 0.04634583
0.09190112 0.7503184 0.216809 0.06874262 0.5391667 0 0.3315509 0
0.3897073 0.04013186 0.8363001 0.905021 0.3619645 0.06885243
0.8038491 1.329433 0.5774125 0.1151002 1.714269 0.92996 0 0.1862109
0.07932814 0.6666273 0.03178586 0.1160726 0.04590162 0 0.07745125
0 0.3507762 0.9556901 0.3907186 0.4405617 0.5866827 0.08593762
1.758093 0.04736442 0 0.131608 0 0.5751996 0.1046156 0.4779628 0
0.4732394 0.520551 0.1678412 0.3394819 0.0789407 0.2902466
0.3613703 0.1714191 0.2890064 0.2160482 0.08616317 0 0.7312245
0 0 0.1863183 0.1761142 0.4659635 0.4684959 5.142361 0.3405
0.843349 0.0838553 0.1576339 0.4503628 0 0.150442 0 0.03176243
0.1650029 0.03105304 0 0 0.04089338 2.368694 1.001981 0.117764
0.2585157 0.07794145 0.05476699 0 0.1192483 0.1716219 0.1696914
0 1.013153 2.782174 0.1664155 0.125734 0 0.1082955 0.01809852
0.1792386 0.7882521 0.2168635 0 0.6237653 0.07738173 0.155714
0.2546865 0.06105046 1.304834 2.515759 0.2939879 0.04848327 0
0.06713648 0.01869108 0 0.635973 0.2818142 0.03175463
TCGA-CF-A3MH 398 0 0.1425845 0.2922481 0 0.3407116 0 36.19888 0
0.1554471 0.3903183 0 0.02348256 0.02604133 0.2229512 1.123249
0.1990514 0.1664021 0 0.2786758 0.1811692 0.06371372 0.05850257
0 1.584184 45.64612 0.0547037 0.07664324 0 0.08531007 0 0.8940148
0 0.05943118 0.05105679 0.3777744 0.1091916 0.07600935 0.3088364
0.1629355 0.0526249 0.02762994 0.02958751 0.1385587 0.1227595
0.08245713 10.60088 0.03769293 0.08950194 0.02950743 0.8137175
0.3716634 3.058369 0.09483343 0.7325539 0.05252101 0 0.8023209
0.6398256 0.02541608 0.7800342 0 0.08004152 0.5572024 0
0.006856746 0.1457668 0.2137261 0.3860595 0.1151395 0.0210741
0.1719259 0.1321717 0.3044429 1.542925 4.084543 0.08985995 0.009242315
0 0.1216028 0 0.05374399 0.2288753 0.04517088 0.3219504 0
0.09219557 0.198415 0 0 0.2592114 0 0 0.0964754 0.01857634

0.009698512	0.4825915	0	0.09519062	0.2686587	1.402544	0.0223421
0.02438313	0.07325539	0	0.4330284	0.5146721	0	0.1118387
0.1751656	0.02687199	0.5134854	0.1173311	0.1048677	0.1498998
1.730043	0.03936621	0.5165887	0.004206926	0.04002076	0.06991178
0.06370034	0.0214497	0.5403386	0	0.1406406	0.1773552	0.9656003
0.01917899	0.446982	0.03829259	0.4440753	0.02507253	0.1199199
0.2797281	0.04680205	0.9468713	1.104835	0.1416654	0	0.0882133
0.122685	0.2486899	0.3348818	0	0	0	0	0.2155489	0.5866099
0.03390088	0.107488	0.3967327	0.04376296	0.3564156	0	0.1523394	0
0.7544212	1.297191	0.1306104	0.05382984	0.2394137	0.4376296
0.3889239	0.1323338	0.06382098	1.517335	0	0.03882198	0.0206733
0.3745974	0.02485065	0.2117436	0.06579203	0.02195276	0.06055252
0.07215734	0.05999043	0.1867931	0.4552095	0.2296251	0.06115695
0.06718729	0.4581675	0.0370302	0.09074725	0	0.05730864	0.1798798
0.204475	0.4276541	0	0.2592114	0.3703467	0.05248829	0.147451	0
0.2978313	0.07705216	0.02144288	0.1373418	0.1266823	0.06893022	0
0.3970014	0	0.168825	0.1456663	0.08551188	0.1214324	0.842437
0.4603481	0.1109199	0.2333058	0.1592154	0.2112695	0.1115811
0.03191049	0.205831	0	0.02731557	0.2243507	0.1213886	0.03735862
0.07711094	0	0.2020233	0.5668838	0	0.1653639	0	0.1070441	0
0.0776917	0.07667231	0.1326672	0.0261491	0	0.655996	0	0.07864056
0	0.1222968	0.03891164	0.05495349	0.4313869	0.4662544	0
0.2899653	0	0.09739156	0.4109449	0	1.447939	3.783189	0.1402438
0.01895246	0.199985	0.2624414	0	0.02258544	0.2599068	0.02078552
0.09102954
TCGA-DK-A6B2	477	0	0.06830974	0.1899279	0	0.1769191	0.02096606
42.27779	0.2379932	0.2184511	0.1246631	0.3761678	0.0540004
1.091532	0.146485	0.5824455	0.05168007	0.04843766	0	0.08971783
1.160573	0.05697836	0.1121102	0.05615259	1.929546	46.17209	0.04402874
0.01468737	0.1886946	0.04904467	0	1.304688	0.3621055	0.1025008
0.2054674	0.4343642	0.4967345	0.1019613	0.4340103	0.1318339
0.2218627	0.05083016	0.1175224	0.1168306	0.2117229	0.03160301
4.906951	0.173357	0.07718185	0.2883844	0.3302155	0.07122295	4.350734
0.07269285	0.5966213	0.3985644	0	0.2432985	0.2452233	0.06575253
0.5829731	0	0.04601578	0.305081	0.02935249	0.02759355	0.1202365
0.2866987	0.4121839	0.3971613	0.01817321	0.1537511	0.08684039
0.2800382	2.838483	1.1741	0.129151	0.08501434	0.02281147	0.0932124
0.1050414	0	0.6477791	0.05193738	0.1974282	0.3134732	0.1590094
0.1473386	0	0.03528715	0.2483672	0	0	0.09508046	0.0640771
0.08363496	0.2846789	0.03522299	0.10945	0.3853935	0.6492454
0.1027556	0.3083923	0.04211444	0.0330028	0.6336846	0.7163518
0.1064431	0.09504616	0.1762294	0.2162815	0.5904044	0.08094419	0
0.0344709	1.156283	0	0.3655216	0.01934846	0.04601578	0.2411532
0.07324251	0.6843932	1.29904	0	0.09702493	0.08438175	1.223535

0.01102598 0.8851171 0.2641724 0.5105968 0.2017984 0.2412963
0.3291105 0 0.9713006 0.4763765 0.1665063 0.03587527 0.1014275
0.2821259 0.8578292 0.5896022 0.07672334 0.1194 0.1017827 0
0.1652251 0.1124138 0.03897916 0.06179472 0.5464444 0.05031855
0.4843161 0.1946999 0.1050957 0 0.2891439 0.8576166 1.026199
0.216627 0.1376387 0.2515928 0.6787612 0.5903696 0.2935249
1.453857 0.04819066 0.1450716 0.1901609 0.4119847 0.05714644
0.1391213 0.09627867 0.06310307 0.8876953 0.04148318 0.1478076
0.8590973 0.1446234 0.1980168 0.07031813 0.158015 0.9573032
0.170309 0.06956066 0.05915311 0.8566135 0.1034127 0.09404196
0.6110642 0.01812221 0.450882 0.3509537 0.2414037 0.05651296
0.1419241 0.1304555 0.3543776 0.1047836 0.234326 0.04855299
0.08105709 0 0.6208801 0 0.07764587 0.4466316 0.6682521 0.6981133
0.2948011 0.7763172 0.06376775 0.5131826 0.03230569 0.06072929
0.04846725 0 0.1352366 0 0.1684578 0.2386111 0.2233158 0
0.04433099 0.1102807 0.3174574 0.9091474 0 0.1267567 0.03503191
0.1723107 0 0.08337443 0.2424335 0.07627025 0.02004412 0.1617224
0.7542628 0 0.03390778 0.671585 0.1298 0.06914443 0.233786 0.2550897
0.1949448 0.1182701 0.207113 0.03478033 0.08398545 0.5942828 0.10976
1.21079 3.0807 0.08062603 0.3268725 0.05748559 0 0.05040584 0
0.2858472 0.04301845 0.2664214
TCGA-4Z-AA86 311 1 0.06899208 0.07377886 0.125103285 0.255266194
0.088231213 20.935697336 0.08012348 0.183861004 0.272801372 0.31660443
0.034087376 1.33795066 0.21575784 0.660730684 0.06835229 0.256837873
0.054441162 0 0.275509078 0.047956259 0.113230057 0 1.447323727
58.47573093 0.100583583 0.655171943 0.529387278 0 0.727907508 0.632239664
0.571441408 0.02875684 0.247047396 0.536192556 0.087291472 0.073556969
0.033208 0.18687826 0.063658751 0.042781576 0.169194338 0.080452935
0.059399374 0 1.486789803 0 0.06496067 0.057110782 0.416892482
0.599453241 2.398960245 0.214138644 0.767995167 0.660743972 0.061297474
0.230370728 0 0.126054731 0.377433522 0.04076282 0.116188562 0.154064203
0.02470474 0.091238381 0.110397708 0 0.106743883 1.225670227 0.056083931
0.086270519 0.176633813 0.244113948 0.418080209 0.705849704 0.130441025
0.089441186 0.057598333 0.169981288 0.164187968 0.026004989 0.102226509
0.305994362 0.207708639 0 0.089220948 0.128008857 0.804288833 0.178198122
0.167232084 0.285054688 0 0.14671281 0.035953976 0.009385597 0.483265318
0.148228438 0 0.344178104 0.987121273 0.191888629 0 0 0.277770497
0.571441408 0.471852584 0.238902977 0.089407629 0.169514003 0.104019956
0.745377288 0.090836369 0.067656133 0.203088785 0.874225385 0 0.153821964
0.028498351 0.077459041 0.1014842 0.030822549 1.066423882 0.332757718 0
0.027220581 0.180510493 1.506555702 0.074240766 1.658148627 0.185285547
1.859831895 0.218372252 0.580253671 0.384020393 0 0.817502294 0
0.082256813 0.120778727 0.455291537 0.158302215 0.150416312 0.891211974 0
0.025123464 0.256998518 0 0.139062927 0.252303724 0.032807099 0.182034924

0.547903497 0.042350982 0.125424063 0.081935317 0.707636666 0 1.119456561
0.396572671 0.042132114 0 0.46337842 0.370571095 0.144501342 0.276618574
0.524975718 0.367094818 0.202800102 0.112708259 0 0.252181779 0.168342031
0.409823688 0.173643537 0.021244467 0.966880916 0.034914621 0.182458199
0.723065551 0.318799164 0.388878696 0.207143189 0.100484749 0.371871344
0.71670893 0.087819362 0.049786651 0.720975055 0.290126836 0.593633307
0.75940592 0 0.340896171 0.307198067 0.660332293 0.095129102 0.059725744
0.301946818 0.14913227 0.067440874 0.287258372 0.163459932 0.09399516 0
0.092206191 0 0.032675608 0.187955368 0.561036668 0.293786093 0.106337792
0.772191862 0.203948279 0.971828831 0.126888157 0.170377515 0.249556046
0.061761849 0.056911442 0.042908232 0.305195472 0.331096145 0.305427473 0
0.298492049 0.216576655 0.221572485 0.788270569 0.133648592 0.195590414
0.088454583 0.145026553 0.252792685 0.085209708 0.055648902 0 0.008435141
0 1.369898063 0.020984721 0.095129102 1.478331621 0.182078483 0.092428818
0.194108669 0.489034568 0 0.099542907 0.272106441 0.526916171 0.047124648
0.578079726 0.09238033 0.605073116 3.942752851 0.14325893 0.64193418
0.04838317 0.152384375 0.155556296 0.02185674 0.196842594 0 0.104109365
TCGA-5N-A9KI 76 1 0.042059578 0.044977738 0.3050664 0.207490259
0.430306502 23.212724075 0.244228015 0.448348056 0.12792896 0.096505633
0.027707541 1.494433865 0.338225791 0.597704169 0.037881403 0.27587171
0.099566747 0.605019768 0.335916689 0 0.023009445 0.115247307 2.22087529
27.342748847 0.096818963 0.331587209 0.258183901 0.10065904 1.242510025
0.750577485 0.510938702 0.105186034 0 0.208013637 0.207260055 0.074737445
0.769293376 0.085444815 0.160407333 0.345571827 0.077756055 0.043596843
0.144846342 0 3.122011006 0.088949264 0.29041403 0.121857376 0.508299556
0.255810888 2.544470291 0 0.828340017 0.161123495 0 0.426523588
0.179748286 0.116207097 1.196491135 0 0.047221284 0.187844035 0.210850186
0.285186959 0.145820247 0.084059875 0.357907961 0.588706345 0.006216435
0.112699322 0.148525666 0.123160519 0.509747703 1.333950157 0 0.806984021
0.046818158 0.06376956 0.153990398 0 0.498562016 0 0.177275316 0
0.027195842 0.092670031 0.267446104 0 0.05097477 0 0 0.04878567
0.109592946 0.025747797 0.227767716 0.325311716 0.336951871 0.492098124
1.439968375 0.128514214 0 0.04321774 0.812817461 1.11477535 0.319616727
0.109231651 0.126223241 0.439197826 0.06341359 0.40391437 0.083064737 0
0 2.815601878 0 0.234435854 0.004963835 0 0.371206315 0.187903217
0.540978267 0.115919303 0.152924311 0.033188916 0.08659235 1.081201706
0.045259329 0.571353173 0.135546548 0.84540217 0.118334288 1.768697545
0.54497737 0 1.80728731 1.520886588 0.14486664 0.073630224 0.104084609
0 0.293434102 0.703831766 0.039366654 0 0.104449179 0.042753033
0.169553607 0.192264607 0 0.06341359 0.585140851 0 0.076462155 0
0.071899314 0 0.652781386 0.281766053 2.054796941 0 0.423733437
0.322729877 0.090140876 0.712013285 0.150607276 0.372986124 0.049453135
0.114517053 0.195142679 0.40356053 0 0 0.261116768 0.025902489
0.285788469 0.042569937 0.070783888 0.396721605 0.515908891 0.033867394

0.108240438 0.104499665 0.418529693 0.436926602 0.178457454 0.03035139
0.338097966 0.212243705 0.193011266 1.474600365 0 0.216969535 0.163267018
0.603836648 0.092789547 0.254873851 0.251012126 0.151525613 0.063252181
0.078412413 0 0.10351362 0 2.791841565 0.131222181 0.039840001
0.057291529 0.54210846 0.107460327 0.12965322 0.86907805 0.065438316
0.323157333 0.044202691 0.062320252 1.269755646 0.075303638 0 0
0.363322018 0.158829511 0 0.088160357 0.22746179 0.226339587 0.150968444
0.285734013 0.108634756 0.021679564 0.035949657 0.101042747 0 0.067224643
0.067850377 0 0 0 0.977712807 0.076757376 0.046394773 0.212055044
0.122100371 0.091825221 0.142649553 0.276315324 0 0 0.212538873
0.107074473 0.057457111 1.794563134 0.028158867 1.630794408 4.996681174
0.059755395 0.245986237 0 0.061931964 0.060347408 0.026649009 0.453336991
0.039240386 0.048821612
TCGA-GU-AATO 324 1 0 0.07362061 0.2496699 0.08490623 0 50.25396
0.07995162 0.1834666 0.2093971 0 0.02267617 1.0058830.06151287
1.2760910.04960413 0.1952663 0.1629732 0 0 0.0410172 0.1129872
0 1.96538331.42905 0.1479105 0.02467044 0.1056504 0.08238053
0.4358077 1.4277890.4561726 0 0.0493035 0.2918414 0.1100264
0.1957312 0.1988206 0.0582742 0.2710281 0.05336227 0.08311701
0.05352025 0.4741757 0.2123348 4.9123670 0 0.1296427 0.0997295
1.8488810.2990837 2.5884340.1221025 0.5305485 0.08114791 0
0.5789485 0 0.04908658 1.1298720 0 0.0772929 0.2049783 0.1479105
0.1158725 0.09180076 0 0.6390896 0.07412371 0.06105124 0.0491917
0.07293308 0.06719734 0.1787929 1.1973710.04338708 0.1070992
0.03831653 0.05218975 0.07561652 0 0.4420314 0 0.2487158 0 0
0.1117675 0.5836827 0 0.2503101 0 0 0 0 0.07024099
0.2026171 0 0 0.2322543 1.5478630.07011836 0.04709162 0
0.05543494 5.6261280.5754717 0.05959764 0.08452029 0.4863074 0
0.3099077 0.1359623 0.06751102 0 4.5428050.07602876 0.3837301
0.08937414 0 0.2025331 0 0.4142624 0 0 0 0.04724546
0.6850592 0 1.1510210.07395525 0.92251600 1.0422160.05025531
0.09038975 0.6454277 0.2667239 0.1459207 0 0 0 0.3001874
0.6467639 0 0 0.05698829 0 1.3182640.69234700 0.05189842
0.08779579 0.1690406 0 0.3270383 0.1765297 0 0.8742173
1.1615440.2522505 0.2599066 0.1155961 0.4226014 0.1676644
0.5929431 0 0.3663074 0 0.1874442 0.03992676 0.5190093 0
0.1752620.1039627 0.1271934 0.05847316 0 0.09930919 0.1443029
0.3933063 0.05543494 0 0.04718551 1.5318680.3575858 0.175262
0.04967986 0.8854506 0.05790091 0.4738880.5372599 0.03043995
0.1155277 0.1964995 0.3801438 0 0 0.6025983 0.1488124
0.04141306 0.2053556 0 0.1422031 0 1.5028040.5369688 0.06521104
0 0.3974816 0.05863119 1.2733170.8298076 0.08568877 0.4114067 0
0.06800483 0.3495869 0.06162938 0.05678937 0 0.1055102 0.595778
0.1406642 0.1443029 0 0.1852390.1040458 0.2125891 0 0 0

0.04134728 0 0.03000951 0 0 0.0336682 0 0.2667239 0.04187942
0.03797002 0 0.09084397 0.01366379 0.06898631 0.1428251 0 0
0.1866719 0.05842067 0.04702357 0.981855 0.04609109 3.749762 7.674784
0.02257131 0.3660327 0 0 0.0846669 0 0.2182449 0 0.06392988
TCGA-FD-A6TG 93 1 0.02474426 0.1323053 0.04486874 0.2746564
1.461973 23.67575 0.3879437 0.1438743 0 0.8516349 0.01222556
3.271106 0.06632771 0.2981279 0.06685853 0.3794295 0.08786483
0.0638374 1.428287 0.04422776 0.08122073 0.1525537 2.108037 25.13418
0.08543999 0.05320297 0.22784 0.04441437 3.916 2.112402 0.4918788
0.03094123 0.159488 0.02622373 0.7217179 0.02638147 0.6074186
0.1445217 0.2191818 0.03452347 0.152639 0.1442737 0.09586709
0.08585813 1.635277 0.2747332 0.1630886 0.1152168 0.2492 0.09674822
3.011385 0.06582994 0.8898967 0.4046858 1.055259 0.201968 0.06344911
0.4090952 0.8122073 0 0.1666858 0.2762784 0.1329067 1.045945
0.1253827 0.2596316 0.2297045 0.7193308 0 0.1856474 0.5177253
0.1539714 1.156726 1.746773 0.04678314 0.2694586 0.6094065 0.1406871
0.3261412 0 0.3116432 0.04703399 0.3799268 0 0.09599824
0.1635574 0 0.0319557 0 0 0 0.1219806 0.01934253 0.3433502
0.196629 2.200934 1.585869 1.721065 0.7586455 0.2064646 0.07616647 0
4.423278 0.4918788 0.5641054 0.5141005 0.1063253 0.284985 0.1399017
0.5569422 0.1954727 0.218386 0.06243302 1.11811 0 0.3723894 0
0.04167144 0.2547837 0.1326554 0.8794173 0 0.06747568 0.02928828
0.07641529 2.226303 0 0.2973505 0 0.5634182 0.02610666 5.89992
0.5215688 0 0.7056135 1.198339 0.05572543 0.06497659 0.2755552
0.08516349 0.161842 0.5666281 0 0.08109558 0.2765203 0.5659251
0.03740656 0.4072034 0 0.1119214 0.4001869 0.04556799 0.3373784
0.04407959 0.09517366 0.06533958 0.6807994 0.8748816 0.090665
0.02802504 0.4362549 0.5126399 0.5423643 2.110974 0.19936 0.7570443
0 0.06063483 0.08610394 0.1695861 0.2070051 0 0.2242004
0.01142911 0.5674505 0 0.2409356 0.1944975 0.3180688 0.02988702
0.2547176 0.07313841 1.000297 0.3470178 0.03149673 0.08035272
1.074103 0.2185156 0.08516349 0.2161616 0 0.2468341 0.3940971
1.844546 0.08188414 0.1285251 0 0.3209209 0.06140008 0.1107148
0.04396912 0.05219876 0 1.025173 0 0 0 0.9084984 0.821866
0.3432459 0.4793355 0.1154949 0.6126028 0.05851143 0.09165976
6.209352 0 0.06123448 0 0.7860395 0.3212058 0 0 0.04014572
0.1331588 0.4908303 0.9570321 0.4314019 0.0765264 0.4124192
0.04458368 0 0.1563992 0.07983471 0.4144176 0.009075881 0.2004111
0.683053 0.1128937 0 0 0.1175456 0.0589332 0.3919576 0.308009
0.1324051 0.06426256 0.2607535 0.1259869 0.07605641 0.5514105
0.1739458 0.6167699 4.148207 0.05678878 0.118405 0 0.1093064
0.0380392 0.02351699 0.1764958 0.008657132 0.1292511
TCGA-4Z-AA7O 512 0 0.1072008 0.3668433 0 0.2379815 0 54.32535
0.07469812 0.1714113 0.2347655 0.1475832 0 0.6792103 0.1149419

1.073017 0.1042756 0.01520297 0.2030193 0.1206833 0.3736053
0.03832202 0.105563 0.3524885 1.177326 93.71829 0.1579332 0 0.09870823
0.07696743 0 0.9306776 0.2130991 0 0.09212768 0.3635532
0.1027967 0.0457175 0.1857564 0.1960023 0.06330481 0.02991355
0.03882776 0.3000211 0.1107546 0 6.468539 0.1360274 0.121124 0
1.122806 0.614749 3.682077 0.2281586 0.9362976 0.09476975 0 0.5090886
0 0.0210197 0.3518766 0 0 0.383019 0 0.003093106 0.2229985
0.06427513 0.8458859 0.6232784 0.02851983 0.2412868 0.05678396
0.5022552 0.7238604 12.89788 0.08107236 0.01667698 0.008949701
0.1462813 0.02354929 0.1454648 0.2223772 0 0.2711018 0
0.1663592 0.1193411 0 0.05537729 0.1559084 0 0 0.06217205
0.06703889 0.008750075 0.2953134 0 0 0.3231814 1.413288 0.03023584
0 0 0.05179239 1.917892 0.7331705 0 0.05264439 0.3555805 0
1.061662 0.211714 0 0 2.42971 0.07103303 0.2151094 0.04554627
0.2166423 0.06307497 0.1724129 0.2515772 0.3545438 0 0.1522644
0.1820818 1.493438 0.1557309 0.2912511 0.06909576 0.7272271
0.09048254 0.162289 0.8451559 0 1.03853 0 0.1306519 0 0.1061157
0.07379159 0.44874 0.660916 0 0 0 0.06538094 0.2592933 1.352513
0.1835139 0 0.581645 0.07896658 0.1169313 0.07638727 0.05497674 0
0.8167738 0.4734541 0 0.09713142 0.3240014 0.2961247 0.4950059
0.1814768 0.0575798 0.5703965 0.0756272 0.08756375 0 0.367353
0.1345227 0.1091639 0.04316952 0.01980595 0.4097323 0.06510093
0.03092791 0.269642 0.3674627 0.3107544 0.2207052 0.06061691
1.013404 0.06681788 0 0.09283095 0.5170431 0.1081927 0.1475832
0.3596113 0 0.515697 0.4112366 0.1183884 0.0532126 0.05568157
0.1791372 1.297652 0.07738373 0.3357585 0.1523917 0.04240186 0
0.1146169 0.2006742 0 0.08761422 0.2432171 0.4930076 0.5948244
0.941414 0.06004372 0.6955335 0 0.03176817 0.5055837 0 0.05305783
0.08000562 0.07617337 0.3643398 0.1314213 0.0674105 0 0
0.911335 1.433376 0.08306594 0.2652313 0 0.07726083 0 0.149534
0.06917437 0.1196934 0.01572795 0.2805118 1.308289 0.07825517
0.08868767 0.08107236 0.1018497 0.0319149 0.2131917 0.578241
0.1912084 0 0.2378263 0.4366554 0.04393372 0.37458 0.04306251
1.840754 4.599251 0.1476173 0 0 0.1894214 0.01318393 0 0.2242948
0 0.08959372
TCGA-GV-A3QI 1110 0 0.6481213 0.08943083 0 0.2320651 0
40.10779 0.03642054 0.1063681 0.09538714 0.5756567 0.1756054
0.03749008 0.1681266 0.6588071 0.02259628 0.04632817 0.04949302
0.01471038 0.1138492 1.650478 0.5833188 0.1718627 3.635513 36.73743
1.073235 0.37086 0.3850172 0.150108 0 2.737747 0.5195033 0.1307157
0.2245933 0.576087 0.1002412 0.1114523 0.5434153 0.2866944
1.014704 0.1312645 0.2472893 0.121901 0.4860053 0 2.102576 0.6963901
0.2362257 0.07139 0.3790013 1.335174 9.20685 0.3893513 2.282552
0.147862 0.1114523 0.4343783 1.0722 0.01956545 4.632238 0 0.2112564

0.1167178 0 0.007540523 0.08084854 0.4700791 0.6307728 0.2363602
0.0139054 0.1400525 0.2048775 0.4132425 0.6515675 1.732577
0.7708044 0.1057055 0.008727207 0.3090635 0 0.1891312 1.657344
0.1987019 0.3398941 0 0.1419455 0.1527411 0.06647157 0.4860053
0.1140243 0 0 0.1879419 0.08171533 0.01706509 0.1402939
0.3773168 0 0.4434567 1.810829 0.002457013 0.1501623 0.2577941
0.126262 0.9351059 0.476629 0.1357432 0.136895 0.6260594 0.118207
1.458787 0.1238704 0.03075345 0.1055029 2.091888 0.06926711 0.2796823
0.01850582 0.3873035 1.107124 0.6444853 0.1462502 0.4321621 0
0.07423954 0.129131 3.978848 0.2024791 0.8083374 0.202134 1.158932 0
0.5011389 0.652448 0.04117545 0.8657053 0.1215013 0.3489758
0.2196024 0.2586939 0.4677211 1.996477 4.087878 0 0 0.88264
0.03187777 0.3792706 3.497922 0.05965055 0.6383179 1.003481 0.8855395
0.7696641 0.4469295 0.6969298 0 0.4424824 0.5784016 0.153211
0.28415 0.6582221 1.876959 0.3146714 0.1164244 0.8141509 0.5284054
0.3318618 0.1536964 0.1273155 1.93439 0.06558921 0.4258 0.2262676
0.222106 0.1198638 0.03174124 0.2412722 0.9202849 0.4479092
0.3787861 0.08070686 0.1773298 2.6569 0.09773513 0 0.04526156
0.05041891 0.2373816 0.2518498 0.8547609 0.08319828 0.3742336
1.117108 0 0.181614 0.1357432 0.1622063 0.4293294 0.1980823
0.3235136 0.03715078 0.1516083 0 0.1397093 0.2935279 0.02970573
0.08543609 0.1364368 0.4807512 0.5155882 3.240035 1.10271 1.561742
0.1895123 0.1084244 1.044929 0.05614834 0.1810857 0 0.01966226
0.3503477 0.06407706 0 0.3052825 0.02812744 0.5095091 1.462302 0
0.2263078 0.05360998 0.6215557 0.2872706 0.08657856 0.8094559 0
0 1.211383 3.280536 0.1144646 0.02594486 0.4348127 0.5379707
0.03734578 0.4496262 0.45543 0 0.07239639 0.4445016 0.2129
0.1285245 2.91096 0.02099598 1.592332 8.484251 0.3872872 0.1500657 0
0.2308904 1.84486 0.03974038 1.043885 2.05542 0.1383301
TCGA-DK-A6AV 1952 0 0.1618602 0.04327257 0 0.1871475 0.03104947
39.868885 0 0.07352566 0.2584659 0.1392704 0.03998566 0.1934953
0.3254032 0.9375723 0.1093358 0.04303995 0.09579203 0.2942048
0.04407022 0.048218 0 0.5821101 3.264455 18.39429 0.1304078 0
0.09314842 0 0 1.141733 0.1340641 0.1517974 0 0.1286534
0.07275498 0.08628485 0.3505871 0.07192979 0.08214129 0.04704774
0.08473175 0.01572901 0 0 5.900721 0 0.1143016 0.03768351
0.570534 0.2109538 2.333613 0.1076537 0.1559223 0.2325226 0
0.9458147 0.7263218 0.02344225 1.992341 0 0.2044398 0.6325291 0
0.005837768 0.1079169 0 1.079973 0.2614097 0.01794228 0.5855043
0.02143423 0.1925484 0.9983599 8.445456 0.0765059 0.01573764 0
0.1610489 0.15556 0.2287856 0.149894 0 0.6213059 0 0.07849442
0.2674703 0.2573068 0.05225813 0.3678168 0 0 0.08213824
0.09489431 0.03715749 0.3072622 0.1043262 0 0.6055991 1.922986
0.004755464 0.04151912 0 0.0977503 1.139545 0.4151245 0.1050905

0.0413993	0.1118507	0.09151423	0.3460981	0.1598313	0	0
3.1200240.1340641	0.1353288	0.03581737	0.1362932	0.5357	0.05423386
0.1460966	0.5855043	0	0	0.08330951	2.0636460.03265761	0.4016983
0	0.6672026	0	0.3062959	0.7975518	0	0.6006661	0.8622591
0.1876196	0.1062582	0.1001386	0.06963522	0.2646652	0.2851152	0
0	0	0	0.1835163	2.3861850	0	0.1337024	0	0.1103450
0.1037603	0	0.8564093	1.4959810	0.3666417	0.1019173	0.4657421
0.5026021	0.1442147	0	0.3767880	0.0495790.0704042	0.3743945
0.2115760.1030151	0.05092246	0.07476146	0.1546615	0	0.1167435
0.5089084	0.3161682	0.1466254	0.3124107	0.1352062	1.459652
0.3783259	0.1030151	0.1314033	0	0.1531479	0.2785409	0.2333072
0.0268379	0.2791635	0.2356160.08937605	0.2343384	0.2101811.593873
0.08746863	0.02738439	0.3545665	0.1438081	0.1093704	0	0.7030467
0.09468552	0.1149888	0.08267929	0.2616008	0.2067727	0.6236894
0.3658069	0.07554895	0.2763609	0.03189518	0	0.5193281	0
0.1502079	0.07549924	0.09725326	0.8786463	0.1240189	0.06361355
0.1969545	0.1088794	0.8829368	1.4119940	0.1877189	0	0.1822726
0	0.1763893	0.03263904	0.2259032	0	0.3277384	0.5879039	0
0.03347691	0.2295177	0.1441694	0.02409382	0.1543967	0.2728356
0.2165261	0	0.2842788	0	0	0.7646742	0	0.7844843	2.238447
0.09950189	0.06454378	0	0.3575042	0.0373240	0.1924193	0
0.1831858
TCGA-DK-A1A3	665	1	0.04927087	0.0878156	0.1191238	0.1063411
0.05040843	40.565730.1144408	0.2029256	0.08991775	0.1130519	0
0.8540607	0.2641440.1826564	0.05325159	0.07860918	0.07775858
0.02311151	0.6439271	0	0	0.1350069	1.06515639.195050.09829644
0.01765633	0.3024506	0	0.1039674	0.7366832	0.2176514	0.04107354
0.2822872	0.1044336	0.2362336	0.08755149	0.1897246	0.1167771
0.04849286	0.0534670.1747396	0	0	0.03799135	0.7314585	0.1563
0.1855673	0.07137516	0.2646443	0.08562021	4.0253790.04369361
0.4218967	0.3339402	0	0.1462398	0.08422675	0.03659443	0.3773631
0.05822174	0	0.03667511	0	0.04738772	0.008760089	0.1477084
0.2668099	0.7957413	0.01456454	0.07041178	0.1652915	0.1322538
0.7251059	0.4536759	0.03105159	0.05109972	0.03427833	0.1494062
0.1262749	0.03714306	0.3163564	0	0.1483356	0.4019624	0.06371736
0.2399718	0.1044336	0.08484042	0.1791438	0	0	0.009525029
0.02567662	0.08043297	0.2088152	0.04234308	0.06578728	0.6118367
0.6797782	0.1428281	0.6403549	0.1012552	0.1586964	0.2720642
0.1497665	0	0.2890083	0.07566178	0.03714306	0.3992348	0.06487102
0	0.04143896	0.9188256	0.05441284	0.2746308	0.03488943	0.05531757
0.09663359	0.1320720.4076634	0.2036912	0.08957191	0	0.05071948
1.5934370.02650961	1.1155230	0.3713812	0.0346558	0.5801455
0.2517697	0	0.6800556	1.2726060.07397381	0.04312721	0.2032172
0.1130519	0.1289042	0.9836219	0.09223246	0.2153038	0.203929

0.3004993	0.04965607	0.1351375	0.04685854	0	0.3370206	0.1814704
0.3582876	0	0.08422675	0	0.4866295	0.1915256	0.7221301
0.3720239	0.1654614	0.1512253	0.3983845	0.1097488	0.08821476
0.5680170	0.02683029	0.05715017	0.1800962	0.03434911	0.08362189
0.07440478	0.04551537	0.7951217	0	0.07107435	0.4131033	0.6126426
0.0793482	0.5494611	0.05487639	0.3813354	0.1023679	0.08362189	0
0.7129193	0.1657558	0.1695779	0.3099038	0	0.2725436	0.4500231
0.3083394	0.06793669	0.4691862	0.3332558	0.2840085	0.05186792
0.09798031	0.05836766	0.01948845	0	0.2633971	0	0.1400122	0
0.5308821	0.4196161	0.3037656	0.5090425	0.04599479	0.2804178
0.05178143	0.09734042	0.9185240.04410738	0.0812869	0	0.2745904
0.1472987	0.1006716	0.05163791	0.05329221	0.1325732	0.279241
0.2130064	0	0.05079323	0	0.1183669	0	0.05011401	0.02649453	0
0	0.2353427	0.5249501	0	0	0.8073415	0.1300318	0.05867402
0.3570022	0.01703635	0.02929396	0.02843553	0.07287206	0.4181094	0
0.5445936	0	0.1364572	3.0099150.02692335	0.05239302	0.2073178	0
0.0403967	0	0.01561951	0	0.04575382
TCGA-GD-A6C6	67	0	0.1191905	0.09104294	0	0.09449936	0	171.165	0
0.1856324	0.2796670	0.0168255	0.05767289	0	3.0930320.009201443
1.36434	0	0.1517523	0	0.1623166	0.05589025	0.5598745	5.229083
93.546150.1254264	0.1830522	0.07839152	0.2445022	0	5.297027
0.4513007	0	0	0.1443623	0.1632770.05446148	0.09834863	0.1470122
0.2765127	0.3801050.03661775	0	0.2198961	0.03938758	19.790240
0.3527098	0.08456948	0.4801480.3550675	7.2780340.04529941	0.08748039
0	0	1.3518951.8337660.0166933	4.0799880	0	1.6349880	0.01473879
0.04541017	0.2552282	0	0	0	0.09124939	0.03607708	0.199439
0.08844171	0.5226101	0	0	0	0	0	0.3465730.7064247	0
0.1845446	0	0.03302953	0.1007012	0	0.1759168	0.0619092	0	0
0.01975017	0	0	0.04209517	0	0	0.08066529	0.9396812	0.004002086
0	0	0.2056609	3.8924690.4269943	0	0.07664948	1.2080140.07701623
0.6898454	0	0	0	5.1171380	0.6833374	0.1507153	0.05735056
0.7012951	0.04564194	0.07300241	0	0.5571828	0	0.02629175
0.9177767	0	0.2846819	0.2194962	0.2566866	0	0.5155428
0.3449227	0.2012049	0.6518373	0.3298441	0.0225566	0	0.2949601
0	0.08909442	1.2447210	0	0.08456948	0.05192384	0.2574055.4173540
0	0	0	0.2321595	0.06066479	0.4366110	0.2882936	0.0168991
0.06238911	0	0.04288559	0	0.1741675	0.1137822	0.09145677
0.3170959	0	0.05563269	0	1.2253170	0	0.2057047	0	0	0
0.1719350.05353567	1.2359830	0	0.08315156	1.4684430.3183902
0.04334755	0	0	0.0859238	0	0.7139848	0.06775843	0.1365177
0.04665621	0.07521678	0.09860683	0.04422086	1.8901070.2576404	0
0.03809296	0	0.1122482	0	0.3641031	0.07968511	0	0	0.04153908
0	0.5248823	1.0115220.2861110.3488682	0.02684223	0	0.1883242
0.04572839	0.1264115	0	0.02135115	0.1366371	0	0.3212140	0

0.1158014	0.3917186	0	0	0	0.4908684	0	0.02226676	0.02746825
0	0.0374722	0.1697335	0.5442427	0	0.01408669	0.06438557
0.3639888	0.01013839	0.02362484	0.08831232	0.0607411	0	0.06925436
0	0	1.0017240	2.3107121.0785250.01674769	0.4073890	0.1504336	0
0	0.08096776	0.005957214	0.01185883
TCGA-GC-A3RD	428	0	0.05798076	0.1705098	0.2102729	0.1877094
0.02224478	29.910320	0.2844504	0.1322663	0.5986654	0.04297041
0.2368193	0.1942739	1.3971470.1253304	0.1259101	0.03431415
0.1835802	0.1262930.1036345	0.02378956	0.1191547	3.7341463 3.3871
0.1334687	0.2181641	0.3336717	0.05203589	0.09175973	2.370023
0.09604756	0.1087523	0.03114269	1.1060540.1158308	0.1081799
0.2511712	0.3091961	0.06419837	0.03370643	0.1132055	0.2366435
0.3369537	0	6.28912	0.6897375	0.1091855	0.0179984	0.3503553
0.1133502	2.26809	0.03856319	1.0798390.0512573	0.07727135	0.9895261
1.0407180.01808664	3.0926430	0.09764455	0.2913191	0	0.002091177
0.1082409	0.08690985	0.1682011	0.2809227	0.006427198	0.3340248
0.1382052	0.2652837	0.9411254	1.4236660.1370279	0.01127492	0
0.08241451	0.06368452	0.1311271	0.4939876	0	0.6545917	1.086467
0.2811789	0.06555553	0	0.07487861	0.4743272	0	0.04354699
0.1260993	0	0.01774717	0.9470792	0	0	0.5399959	1.266582
0.01362783	0.1189822	0	0.03501564	0.5282616	0.6278608	0.112935
0.1720263	0.2804664	0.1311271	0.6394633	0.1145080.04264352
0.1828664	3.0046320.1440713	0.1454305	0.04105704	0.2441114
0.4264352	0.3496930.1341059	1.3782690	0.06862831	0.1044495
2.6924770.08188916	0.1817613	0.04671404	0.4188227	0.03058658
0.03657327	0.6110702	0	1.5854460.8423844	0.1612996	0	0.1076135
0.1995551	0.8343024	1.1489910	0	0.07199362	0	0.3506043
0.7553760.0413565	0.03278178	0.7209339	0.3203249	0.5929090.1549310
0	0.7976248	1.0106250.05311157	0.1313366	0.1460332	0.8008121
0.7667573	0.1420647	0.4282121	1.0412060.1533894	0.08287975
0.02521985	0.6059956	0.1212636	0	0.05837182	0.09373241	0.35088	0
0.1463675	1.0482180.3068878	0.1750782	1.1564060.2980486	0.5469094
0.1355221	0	0.06276085	0.2097365	0.3657327	0.1496663	0.7547	0
0.6810852	0.2581684	0.1600793	0.08394355	0.4893852	0.6920599
0.5326540.1504124	0.2648316	0.05151423	0.07453398	0	0.1356072
0.06783557	0.1647630.05923394	0.1821144	0.3333110.2680980.4492717
0.1894394	0.5444815	0.06855201	0	0.1572947	0	0.1793558	0
0.02423485	0.6842270	0	0.1411042	0.07800447	1.0515330.371515
0.05615896	0.1569021	0	0.3134055	0	0.08845942	0.4910555
0.08092196	0.0106333	0.4876650.8423844	0.1322663	0.05995968
0.3288669	0.1032873	0.03020769	0.2882680.1804317	0.1034173	0
0.5359629	0.03690158	0.1485129	0.8889411	0.1746814	1.405071
1.1752240.1900964	0.09248224	0.06099163	0.1280634	0.008913347
0.02755252	0.2619245	0	0.09085835

TCGA-BT-A20R 154 1 0.1447873 0.1407572 0.1432051 0.1704511
0.02019957 17.4079 0.5503017 0.5644283 0.2642322 0.5436233
0.0260131 3.605951 0.4586715 0.6831414 0.1493721 0.2450009
0.06231853 0.07408958 0.9461217 0.1019484 0.04320463 0.1622991
1.252225 15.4877 0.1939158 0.1132033 0.1817961 0.09450329 0
0.2856796 0.7849514 0.06583563 0.4524703 0.8090697 0.178808
0.05613353 0.5701955 0.2673983 0.1991776 0.1163082 0.08342963
0.0409307 0.1699854 0.1217905 1.248075 0.04175481 0.04957344
0.1144053 0.3181432 0.2058574 2.377702 0.1050529 0.5748094
0.1163616 0 0.2246369 0.06750235 0.0539637 1.598571 0.233305
0.1330005 0.2057493 0.2262352 0.1272271 0.129882 0.03945962
0.4887568 1.147924 0.01167255 0.1340225 0.06972133 0.1830788
0.3418388 0.3231931 0.0746576 0.2559572 0.02747189 0.1796093
0.1879452 0 0.2925455 0.250193 0.1902104 0.201342 0.255327 0.2014803
0.2510906 0 0.1435723 0.5220811 0.03954322 0.08397072 0.06173451
0.4028867 0.3625961 0.03393527 0.05272429 0.5101886 0.7667537
0.08353038 0.1080428 0 0.03179625 1.221036 0.6001415 0.03418388
0.1346638 0.3517008 0.2083745 0.4384653 0.1819649 0.03872282
0.09963203 1.982561 0.2180421 0.1320594 0.05126302 0.08866698
0.07744564 0.3175418 1.315775 1.197134 0.5025031 0.06231853 0.1456568
1.364357 0.07436016 1.196612 0.08483818 0.6944893 0.1110976 0.09963203
0.3819356 0.05184556 1.120892 0.8159301 0.1081084 0.0345637
0.2931581 0.04530194 0.3443616 0.6607893 0.3695921 0.2300696
0.09806165 0 0.1989808 0.2527095 0.1502165 0.05953557 0.5402009
0.09695792 0.1794654 0.1406864 0.4387653 0.06951359 1.44858
0.3331179 1.639766 0.08944601 0.4641236 1.030178 0.2769655 0.4866934
0.2474447 0.4552293 0.09285771 0.1290166 0.09160442 0.1804195
0.1651717 0.2680704 0.1192613 0.02431844 0.3018501 0 0.07594873
1.117381 0.2654021 0.1907775 0.03387368 0.1217905 0.3383605
0.08204132 0.06701759 0.1424763 1.01575 0.3985281 0.4077175
0.7175077 0 0.2208805 0.365173 0.843096 0.228677 0.4102066 0.628431
0.1991628 0.09501445 0.3828092 0.1871118 0.1318916 0 1.161028
0.184796 0.07480722 0.0537879 1.655312 0.3026659 0.4057478 0.9859155
0.1474475 0.8202897 0.03112463 0.1950303 0.09064389 0.2120955
0.06514625 0 0.5419141 0.4038569 0.2420455 0.04138448 0.3843927
0.3187469 0.6340823 0.3759706 0.2549781 0.1221226 0 0.1660112
0.07234264 0.09753913 0.02123367 0 0.009655665 0.1886121 1.338635
0.02402111 0.04355753 1.642467 0.1563183 0.07445383 0.2830706
0.2594174 0.0704317 0.182314 0.1898075 0.1340352 0.08091503
1.605035 0.5287365 1.093617 8.759707 0.06041656 0.1679586 0 0.1162891
0.0566569 0.05003861 0.1376985 0.07828641 0.04583595
TCGA-XF-A9T0 799 0 0.1249074 0.0954098 0.2588509 0.2145694
0.08215155 109.8066 0 0.155629 0.5535979 0 0.01763253 0.4764028
0.5261426 1.786071 0.2314269 0.09489691 0 0.2594717 0.2332044

0.04252551 0.08785652 0.4400466 4.479885 202.951 0.09036671 0.1150994
0.08215155 0.06405741 0 1.574963 0.295592 0.1785021 0.03833739
0.5673246 0.4420304 0.09512285 0.7729942 0.3171897 0.1053729
0.05809077 0.07876792 0.04161625 0.276532 0 6.357714 0.05660554
0.2352175 0.03323469 1.797065 0.4186105 11.97035 0 0.4583819
0.1577474 0 1.085725 0 0.03498799 3.104264 0 0.06010137 2.191571
0.03833739 0.01802002 0.1760766 0.2674702 0.7454144 0.3458225
0.007912032 0.5833195 0.05671127 0.3135076 2.039044 1.533496 0.3036321
0.04163908 0.02234561 0.385526 0.07839714 0.04035515 0.2115166
0.0678356 0.5479561 0 0 0.09932357 0.1134649 0 0.5190293 0
0 0.1034874 0.08369133 0.01092359 0.163853 0.04600487 0 0.2343908
0.6017934 0.02935832 0 0 0.08621015 0.9458945 3.173005 0.1390257
0.00730236 0.7398444 0.1210654 0.8193248 0.140962 0.1049904
0.04502256 3.826758 0.05911841 0.8354658 0.03790663 0.1202027
1.049904 0.334818 0.2778302 0.07376874 0 0 0.1515405 1.154157
0.1008075 0.1678144 0.1150122 1.120827 0 0.2251128 0.3223893
0.2108557 1.491673 0.414798 0.321484 0.04685681 0.529899 0.2456571
0.8403103 2.184517 0.1503129 0.03898718 0.04431292 0 0.1079005
2.349185 0.05091082 0 0.2110108 0 0.194636 0 0.1372659 0.04711858
0.981895 1.567305 0.2288356 0.04041961 1.39322 1.314425 0.4902003
0.1589864 0.5750609 1.424166 0.06294199 0.1166022 0.03104623
2.592638 0.1119588 0.04542671 0.02694641 0.01648383 0.3637402
0.05418132 0.1415714 0.336621 0.6206487 0.08621015 0.2296069
0.353146 1.538876 0.1112206 0 0 0.08606353 0.1350677 0.2456571
1.016347 0.02366948 0.4524871 0.580617 0.3744166 0.1623859 0.0463419
0.6389565 0.732852 0.04025243 0.5588811 0.06341523 0.1152795 0
0.8585263 0 0.1014135 0.07291838 0.3047204 0.2279521 1.430151
0.7835073 0.3164917 1.340539 0.1265837 0.1850771 0.4505701
0.04792174 0 0.066586 0.1230639 0.3453428 0 0.0561035 0.05790087
0 0.1516947 1.113053 0 0.08277866 0 0.0321508 0.1961448
0.2255695 0.02878575 0 0 0.8337877 0.829596 0.1628229 0.02952471
0 0.2401706 0.09562213 0.2269484 0.666347 0.06365454 0.0308946
0.4948373 0 0 1.164292 0.03583949 0.9389666 2.561915 0.08775495
0.1992337 0 0.07882454 0.07680778 0 2.155225 0 0.2485528
TCGA-YF-AA3M 415 0 0.02455513 0.02625881 0 0.2952703 0.07536619
53.44736 0 0.07138735 0.2240616 0 0.07279271 0.9344476 0.1974623
1.046851 0.132695 0.1088235 0.1162577 1.071182 0 0.04388972 0.04029998
0 2.186256 33.7164 0.3504528 0.4223706 0 0.08814982 1.243542
0.5329466 0.08135323 0 0 1.040932 0.1864087 0.0785395 0.1418297
0.1371816 0.09062775 0.06851921 0.06114479 0.07635785 0.3171146 0
1.869723 0 0.04624061 0 1.187018 0.6400585 4.248273 0.06532679
0.6307823 0.02170772 0.5235967 0.3279672 0.4407491 0.03282764
0.4835997 0 0.1654118 0.6031669 0.158269 0.02833996 0.130973
0.147227 0.284936 0.2379443 0 0.03947753 0.09104754 0.1438066

0.7014853 1.431958 0.2785534 0.0572998 0.04099992 0.08376707
0.02697071 0 0.3274531 0 0.1330669 0 0.09526452 0.04271244
0.1561398 0.06342292 0.3571198 0 0 0.02848195 0.07677879
0.01002135 0.4943196 0.1266152 0.09835927 0.5076145 0.6022777
0.01731436 0 0.07569387 0.355903 1.057592 0.4478351 0.06377139
0.1205859 0.2262455 0.05553298 0.3979335 0 0 0.06195584
3.046898 0.08135323 0.4927243 0.008693928 0.08270589 0.8668676
0.1974623 0.2160961 1.11665 0 0 0.1074276 1.201364 0.07926962
0.7184503 0.3956725 0.6092393 0 0.5576025 0.06721849 0
0.4987872 0 0.08457574 0 0.1215329 0.08451257 0.7066623
0.6920582 0.1378977 0 0 0 0.1484826 0.7408336 0.07005871 0
0.2903733 0 0 0.08748538 0 0 0.4157514 0.8590559 0.04498602
0.1112434 0 0.9043943 0.2511689 0.1203302 0 0.2613072 0
0.08022853 0.08544584 0.6058436 0.1540672 0.06251199 0.1359641
0.06805052 0.03128408 0.07455927 0.2833707 0.5404307 0.1856693
0.1186343 0.3791562 0.08204628 1.852951 0.3826284 0 0 0 0 0
1.13261 0 0.4898951 0.4709841 0.3525306 0 0.1275428 0.2051635
0 0.03323498 0.2472043 0.1745322 0.1035996 0 0.3281732 0 0
0 0.09584635 0.250949 0.3027755 0.8244979 0.1375346 0.2515534
0.05806423 0.2183021 0.2869572 0.4616179 0.2430658 0 0.07697682
0.1159094 0 0.2316132 0 0 0.2504977 0.8113354 0 0 0
0.04424292 0 0.05351857 0.1188368 0 0.01801303 0.1376857
2.069173 0 0.02031457 1.207065 0.09720598 0.01462069 0.1533134
0.534896 0.2189886 0 0.2814588 0 0.05031673 0.6303699 0
0.952087 5.931081 0.08050678 0.2350001 0 0 0.01509938 0.0466745
0.1634703 0.2749109 0
TCGA-4Z-AA7Y 1522 0 0.3173731 0.1357572 0 0.3963127 0 30.1361
0 0.1499348 0.1158391 0.1092318 0.07840333 0.05691044 0.212682
0.1176562 0.03430146 0.03938299 0 0.02977407 0.2765193 0.04727259
0.1823059 0 1.136742 36.12303 0.124198 0.1364777 0 0.05696647 0
0.1607268 0.05257419 0.07937127 0.06818713 0.7736029 0.05706278
0.1353489 0.2749705 0.306256 0.0644246 0.05904035 0.06645633
0.1480379 0.0409868 0 3.16894 0.100679 0.149414 0.009851907 0.1278509
0.7445433 4.131602 0.08443435 1.752854 0.1262567 0.1691861 0.3650206
0.2848323 0.01272884 0.5729613 0 0.05344835 0.03543583 0
0.004578646 0.07194467 0.1427173 0.4051052 0.05125684 0 0.2040975
0.07565028 0.4414387 1.071512 4.432164 0.09000702 0.03702977 0
0.1082683 0.01742971 0.1794398 0.1881024 0.06032642 0.2579819 0
0.1846929 0.09384932 0.1009047 0.204934 0.1153936 0 0.04767321
0.1012349 0.09923596 0.006476257 0.5660466 0.08182456 0 0.2289472
2.213686 0 0 0.09783372 0.03833348 1.104058 0.07235291 0.08242401
0.2207964 0.3362835 0.07177593 1.514404 0.125358 0 0.1201161
0.830869 0.1051484 0.3184211 0.01123683 0 0.09336828 0.2552184
0.02864641 0.918439 0 0.03756553 0.1756035 1.736978 0.1024553

0.4145496 0.05114035 0.7226477 0.06696951 0 0.08687936 0
0.4215205 0.3688812 0.09670037 0.04166992 0.1963504 0.1638477
0.6227423 0.4192874 0 0 0 0 0.1919126 0.2176185 0.1811006
0.03588797 0.4249779 0.05844612 0.2596356 0.2261483 0.04069033 0
0.4701859 0.5984719 0 0.2875624 0.4396435 0.1461153 0.4173862
0.07069354 0.7671053 0.5910405 0 0.1425802 0.05521903 0.228389
0.1327537 0.2019906 0.02396353 0.02931825 0.1213033 0 0.1144545
0.1496791 0.3439657 0.2300009 0.3675423 0.06933656 0.3684498
0.6923617 0 0.1030615 0.1530732 0.3603484 0.2730795 0.4879631
0 0.2248696 0.5109094 0.01752473 0.2231796 0.123636 0.2083496
0.3087131 0.1002305 0.1834221 0.1127907 0.06904304 0 0.2757048
0 0 0 0.1083955 0.3243496 0.3913349 2.541182 0.1629488
0.6231672 0.01250792 0.07053842 0.2781674 0.2557018 0.1178102
0.1184303 0.04642956 0.2471897 0.03242328 0.04989303 0 0.04269783
1.438961 0.8648867 0 0.220846 0 0.2573263 0 0.07608955 0.0767978
0.08858958 0 0 1.936626 0.08687936 0.07876927 0.2400187 0.1758931
0.0519671 0.1798085 0.625506 0.1415205 0 0.1760242 0.2019906
0.06503397 2.563061 0.03187217 0.7031798 3.698919 0.2133114 0.07593393
0 0.07009892 0.01951583 0.09048963 0.1509172 0.005551876 0.09946748
TCGA-FD-A62O 216 1 0.2584182 0.1554456 0.0585737 0.2689117
0.2726466 103.8211 0 0.2347752 0.05895062 0.5558815 0.1117186
0.5502734 0.3896424 0.5089404 0.06982413 0.0543998 0.03823425 0
0.4573427 0.105851 0.2650731 0.1327671 2.164315 73.72136 0.07435817 0
0.1487163 0.1159611 0.1022425 0.9114187 0 0.2827446 0
1.266645 0.3420182 0.06887915 0.2798654 0.2296794 0.1669091
0.03755709 0.1133416 0.263678 0.04171642 0 6.775559 0.3586492
0.1520738 0.03008185 0.2602536 0.4630983 9.581441 0.3867183
2.157465 0.2427303 0 0.4074719 0 0.06765612 1.060292 0 0.2175992
0.2524665 0 0.08854288 0.1206065 0.2420964 0.2623833 0.5738621
0.03580725 0.2942852 0.1454387 0.4138261 2.181173 0.7931538
0.06107284 0.08794087 0.07416117 0.5877098 0 0.03652682 0.2871764
0.4298022 0.787723 0 0.1253205 0.06742586 0 0 0.8808583
0.08007803 0 0.09367 0.02525062 0.03295772 0.4221099 0.04164057 0
0.2260669 1.782667 0.01898087 4.242397 0.09957529 0.1560635 0.3745706
0.1472818 0.3355651 0.5948654 0.1934569 0.3287414 0.8870103
0.1594869 0 0.1630058 1.841916 0.4280807 0.2700745 0 0.2175992
0.09503036 0.3030552 1.47604 0.8012472 0 0.2676398 0.282642
1.848257 0.06517449 0.8776127 0.05205072 0.5275395 0 0.4075145
0.2475926 0.06361754 0.567826 0.1877239 0.1882851 0.0424117
0.3597222 0.1667644 1.309911 0.7397021 0 0.03528862 0.04010914 0
0.1953289 0.2214924 0.1382434 0 0.3819861 0 0.4844721 0
0.2484881 0.04264863 0.1367302 1.46671 0 0.07317034 0.569507
1.412805 0.9298756 0.3453695 0.5205072 1.203124 0 0.3034293
0.084303 0.3763551 0.1688961 0.08223453 0.219511 0.01492008 0.08230841

0.04904136 0.1863871 0.5078119 0.3500887 0.4291746 0.74817
0.1411417 1.3058340.05033476 0 0.2097922 0.1557981 0.6927747
0.2779407 1.4560860 0.7107096 0.5753239 0.3745706 0.2806001 0
0.5590633 0.8029775 0.1967428 0.4335963 0.2869964 0.09369613 0
0.1726848 0.0755852 0 0.1320018 0.3349156 0.2475926 0.2489382
0.6674628 0.2110813 0.3447067 0.08911407 0.02393136 1.715567
0.2168780.1598766 0 0.0506315 0.1982224 0.1980027 0 0 0
1.3089650.4563506 0 0.3996042 0 0.2328063 0.1775373 0.1408074
0.0781649 0.1803332 0.01184807 0.6037517 2.5812040.02947531
0.09353338 1.5268210.2301740.05289219 0.4183071 0.6533945 0.3745033
0.02796376 0.3702591 0.1233518 0.2978625 0.4031084 0.06487908
2.05763 2.8099330.2276993 0.07728566 0.0679594 0.4994275 0.00993162
0.06140031 0.3532886 0.1751719 0.1912276
TCGA-BL-A3JM 205 1 0.1886821 0.1592946 0.1440577 0.4684695
0.01523987 81.269220 0.1660060.5255697 0.2050725 0.07850393
0.3145962 0.4791485 0.5338139 0.1878268 0.08626085 0.2350859
0.004658168 0.6921852 0.1479164 0.2281748 0.1632654 2.975659
51.498250.1325869 0 0.09143924 0.1782486 0 1.5518550.2961093
0.4222009 0.1280149 3.1994170.5634244 0.5611482 0.2007566
0.1664378 0.2052504 0.05542136 0.3023597 0.04632119 0.4360436
0.1378301 9.8728440.1890153 0.1122043 0.09248014 0.4400513
1.8378614.8679360.2906155 2.8061240.1668025 0 0.5010736 3.183012
0.03363309 1.0756810 0.1337923 0.4213405 0.06400747 0.01289397
0.1880377 0.2977092 0.7375001 1.0906060.008806531 0.1277247
0.1157251 0.3998401 2.5532650.06095949 0.1126531 0.08496877 0
0.3952346 0.06544528 0.04491752 0.1765723 0.5662859 0.2331992 0
0.0577906 0.6045848 0.1894391 0.5642917 0.2527474 0 0
0.1094279 0.1242041 0.05876639 0.3682621 0.1024119 0 0.2010136
1.0656380.02100693 0.04075729 0 0.6237184 2.86239 0.7697361
0.05158111 0.1015992 0.7685887 0.1122938 1.2427610.4510833
0.02921503 0.07516891 3.0912660.0987031 2.4244340.05274024 0.06689617
0.6135156 0.2661936 0.2196055 0.4105435 0 0.07052576 0.1610061
1.8940220.2324232 0.9131795 0.4480523 0.1840126 0.1257290.8519143
0.1902925 0.03911567 0.9620516 0.1538977 0.2499544 0.07823135
0.5160812 0.3759662 1.3769871.0495630 0 0.07398411 0.09084931
0.4203475 0.4085583 0.02833329 0.04491752 0.4075629 0 0.9207228
0.1061430.7129946 0 0.3783128 4.3021080 0.2474410.2751298
2.9260560.01741341 0.1681126 1.1468 2.1135670.1751448 0.2514579
0.1036685 1.5926070.02076938 0.6067494 0.1699595 0.03669483
0.4681229 0 0.2292027 0.7493557 0.5556567 0.4797834 0.5878
0.1735638 1.6510930.4332813 0.1516874 0.4299738 0.2394837
0.4259572 0.1025362 1.4157980 0.5925215 0.4387758 0.02193402
0.2711168 0.1289528 0.3318906 0.2575910.2240160.3295467 0.3529234
0.2042526 0 0.09290434 0.09294814 0 0.1623244 0.2822385

0.5074466	0.5816331	1.564627 0.713816 1.873596 0.1330672	0.2648585
0.02072354	0.02666978	0.04915059	0	0.05396061	0.1640671	0.1014528
0.06244631	0.03222344	0.02672039	0.7710572	1.643676 0	0	0.02546409
0.1968209	0.2729001	0.0995628	0.4005018	0	0	0.4083429
3.231852 0.3262157	0.08215661	0.03755105	0.1729738	0.06208577
0.2594954	0.5047553	0.1948403	0.08596852	0.8812501	4.373652
0.02034919	1.547317 0.2592937	0.4950578	2.818006 0.175817 0.1267189	0
0.8773609	0.07327828	0.03775239	0.8311097	0.03126938	0.1106612
TCGA-FJ-A3ZF	524 0	0.2837494	0.3742382	0.3087136	0.2712124	0
37.74224 0	0.1649848	2.433818 0.1302125	0.2990805	0.4654681
1.191605 1.472678 0.2760069	0.04191739	0.08956186	0.403732 0.1648158
0.1352459	0.1397072	0	7.083187 81.71981 0.1045082	0	0.5225411
0.237679 0	2.025469 0.03133619	0.3311578	0	0.6214777	0.2305227
0.1714302	0.5189937	0.2834174	0.1815248	0.08797566	0.1059848
0.2573553	0.5130231	0.0218791	24.97574 0.3300465	0.2849804
0.03523263	1.333569 1.158748 3.425165 0.3522823	0.3887504	0.04180763
0.1008413	1.122921 0.2667826	0.02444656	2.390545 0.03352972	0.06371444
0.8448434	0	0.006822611	0.2724251	0.538744 0.702423 0.183306 0.008387673
0.4815304	0.1052109	0.6508609	1.473834 0.5515712	0.1788252
0.03678521	0.007896314	0.08604259	0.02077752	0.06417172	0.4064209
0.2516977	0.2050225	0.04340417	0.1284312	0.1020041	0.060143
0.1954374	0.7909576	0	0.02841502	0.2029611	0.04436126	0.01351032
0.8351114	0	0	0.08961627	1.507931 0.002223088	0.5240536	0
0.1370892	9.839563 2.760003 0	0.1509563	0.6448862	0.1283434
1.013343 0.07471805	0	0.1193229	4.782692 0.1253448	0.7275317	0
0.1274289	0.278255 0.6845388	0.05976017	0.9384412	0.05158419
0.1119523	0.3480763	1.749036 0.1984685	0.6918439	0.3352972
0.3624032	0	0.3102394	0.8129987	0.1117657	1.839978 0.7328901
0.120286 0.1738578	0.1404386	0.3255313	1.33624	1.616091 0.5311639
0.02066547	0	0.02884277	0.7435162	1.867807 0.1619142	0.06417172
0.5362124	0	0.4384656	0	0.3152885	0.07492675	1.481313 1.997116
0.6931209	0.02142474	1.238754 2.090165 0.3731667	0.0926992	1.270065
1.258151 0.1000887	0.1776921	0.213932 2.126196 0.1186893	0.3611819
0.01428316	0	0.0843515	0.229754 0.2319452	0.8029291	0.162106
0.4341157	0.8519349	0.5810117	1.258835 0.2063368	0.0240788
0.04095233	0.136856 0.5966143	0.3255313	1.807862 0.0125462	1.372051
0.7191883	0.1566809	0.3208212	0.0736917	2.878815 0.06133486
0.1621546	0.571319 0.06722751	0.198279 0	3.147559 0	0	0.2705568
0.1211396	0.2416557	3.29466	2.125381 0.4856181	0.4925498	0.2087454
0.1401451	0.5408179	0	0.2106579	0	0.005930091	0.8438239
0.2125804	0.05947623	0.03069082	0.05089901	0.2733838	1.604929 0
0.4095233	0	0.647588 0.1559522	0.2185153	0.2746462	0.2112109
0.00693838	0.6128455	3.627806 0.01726112	0.4616695	0	0.1647468
0.1323449	0.1946605	0.6279158	0.168703 0	1.028187 0.09631518

0.1550507 1.1735820.01899701 2.2003882.4123830.1922629 0.03017298
0.1591916 0.2924711 0.02326433 0 1.3942610.003309126 0.1119853
TCGA-CU-A0YO 149 1 0.08019924 0.1543745 0 0.1483660.4430748
63.001160.05588331 0.1865259 0.08781662 0.5520511 0.03169966
2.3680870.04299528 0.3567766 0.05200725 0.1222670.07594159 0.09028583
0.3843158 0.05733909 0.07897398 0 1.40997832.0841 0.1255379
0.0689750.0738458 0 1.8276830.5570083 0.1062827 0.08022753
0.1723069 0.3399777 0.5126967 0.0513034 0.4632282 0.08960944
0.2604785 0.05221762 0.1198226 0.01246958 0.04142897 0.0742072
2.0278790.1526477 0.1208207 0.07966552 0.1938452 0.3344785.903777
0.04267262 1.6069490.6522738 0.5130340.2380377 0.08225861 0.1887036
0.3685452 0 0.1080499 0.2149087 0.2756910.3077647 0.03422156
0.04808563 0.4094755 1.1916260.01422421 0.2922574 0.4672947
0.1526477 1.95786 1.1815330 0.1122877 0.2477323 0.1276757
0.07047097 0.07255026 0.2376646 0 0.2028172 0 0.06222847
0.2901654 0 0.04142897 0.2332770.2385787 0.3373126 0.03720982
0.05015326 0.4058596 0.2379251 1.1579020.6425001 1.3608710.9343689
0.01131005 0.0658307 0.09888915 1.9373510.1062827 0.1097002
0.1666264 0.2166143 0.07389378 0.07255026 0.3321420.1267103
0.09437554 0 0.7593006 0 0.1072854 0.01135806 0.1080499 0
0.04299528 0.6623559 0.3315526 0.08747887 0 0.09081291 1.409893
0.01294508 0.3855001 0 0.5591672 0 2.9543570.1317249 0
0.5263191 0.9321519 0.06374581 0.08423891 0.07938745 0.05520511
1.4267770.66397 0 0.1051364 0.1194983 0.2445646 0 0.3079527
0.1830544 0 0.4965075 0.05907664 0.2186972 0.2285880.08225861
0.08470952 0.6110464 1.0029070.3232423 0.1816654 0.2423926
0.4430748 0.6375212 0.9003480.2584603 1.79225 0.05657837 0.1179151
0.08372211 0.1758879 0.1341859 0.04083394 0.08881418 0.0889036
0.5108829 0.04870344 0.1388271 0.7060379 0.2587359 0.1549881
0.04127859 0.08245244 0.2660176 0.1999517 0.1225018 0.1736222
0.4641736 0.1214120.1104102 0.5941147 0.04255286 0.3229989
0.8954962 1.4171030.1061587 0.08331321 0.1531617 0.3467150.0361828
0.2691304 0.05700377 0.05286961 0 0.1071843 0.1501287 0.04558017
0 0.7434763 0.3278487 0.04944458 0.3314318 0.08984005 0.1643191
0.02528572 0.1901317 3.5346870.5599973 0.2778561 0.05985396
1.4012090.2271422 0 0.05043128 0.3122815 0 0.2181727 0.4606357
0.2485738 0.3472444 0.2467758 0.2601023 0 0.04894299 0.0776263
0.1790906 0 0.04996596 0.6058987 0 0.01326984 0.5458681
0.1015947 0.06207822 0.1780392 0.06655305 0.1144378 0.05554214
0.1126846 0.3675055 0 0.9722318 0.1288641 0.4886515 6.949334
0.01051769 0.1023375 0.2024733 0 0.1578110.09146584 0.1220362
0.03928239 0.02234234
TCGA-CF-A5UA 365 0 0.08795273 0 0 0.0677957 0.3599335 119.305
0.1532150.2876609 0.3477736 0 0 0.07301589 0.1178799 0.3804004

0.06337245 0.397585 0.06940288 0.4400643 0 0.1834073 0 0
3.627227 75.27327 0.06073879 0.1103132 0.7423629 0.0526232 0
4.263284 0.1942632 0 0 0 0.07613979 0 0.3386747 0.0297796
0.1136151 0.02726948 0.04147941 0 0.1135856 0.1356356 7.392039 0
0 0 0.295258 0 8.988275 0 0.2259365 0.09071268 0 0.5982412
0.413468 0.002612968 6.736256 0 0 0.7856187 0 0.002114779 0.1368282
0.08789075 0.3061791 0 0.006499738 0.08641262 0 0.2360844 0
1.034809 0.08314465 0.4218804 0 0.08334468 0 0 0.4561227 0
0.9002924 0 0 0.1529894 0.186423 0 0 0 0 1.420884 0
0.04659414 0 0 0.2114917 0.5393166 0.04823575 0.6617881 0 0
0.8741844 1.370149 0.4187688 0.05998892 0.5537574 0 0.937027 0 0
0 7.990634 0 0.5882876 0 0 0.3881234 0.3143465 0.04300129
0.1212021 0 0 0.05658665 0.9116738 0.01183049 0.6586625 0
0.2301901 0 0 0.2166897 0 2.359201 0 0.07767624 0 0 0
0.3451577 3.214754 0 0 0 0.04470142 0.1329605 1.12575 0
0.1657589 0.1478535 0.05399003 0.1598936 0.1566796 0.263116 0
0.6825307 0.6546823 0.107422 0 0.07384072 0.2024626 0.3384392
0.05224295 0 0.4289827 0 0 0.02550449 1.125205 0.03065806 0
0.007378829 0 0.0186758 0 0.07400973 0 0.43597 0 0 0.07912079
0.7840395 0.1827355 0.1865903 0 0.4949086 0 0 0.9783531 0
0.1421276 0.1506244 0.1295088 0 0 0.5598966 0.1901173 0
0.1585061 0 0.09276948 0 0.2546844 0.2744048 0.1666225 0
0.155561 0 0.4518731 0.1514474 0.1094729 0.3253704 0.1039886
0.7384843 0.6882835 0 0 0 0.006127093 0.04151697 0 0.04608905
0.1426968 0.03944243 0.008307819 0.4458704 0 0 0 0.07923569 0
0 0 0 0.02150663 0 0.08518919 0 0 0.05542977 0.1740885 0
0.02033871 0.03041136 0.8366757 0 0.05420121 0.03731806 0
0.3815413 0 2.029899 1.47323 0.07689678 0 0 0 0.03605579 0
0.3903505 0 0.04083725
TCGA-K4-AAQO 359 0 0.06823156 0.1641725 0 0.1104479 3.403086
36.65417 1.188604 0.3347403 0.2490408 0.1174179 0.03371162 5.536377
0.3200692 0.3161842 0.1013982 0.6108256 0.2019039 0.2000338
0.4087081 0.162609 0.1399774 0 2.339788 49.66852 0.1492122 0.1100292
0.314131 0.2449427 22.56835 0.9132238 0.5651423 0.08531954 0.03664862
0.6869562 1.513033 0.2909845 3.54693 0.2512373 0.7932585 0.08726434
0.139012 0.09282709 0.1321753 0.1972927 2.597708 0.05411206 0.06424459
0.1270828 0.6871616 0.3557072 4.4774 0.1815241 0.569647 0.211118 0
0.3797188 0.3061783 2.041773 0.6159004 0.06047022 0.05745389
0.2666403 1.795782 2.190192 0.1410252 0.2045504 0.4750525 0.9917667
0.02269051 0.2742414 2.493804 0.4495463 0.9303111 2.146562 0
0.7960973 3.261155 0.1939702 0.4496624 0.07715499 0.4296735
0.1945423 0.3389414 0.1043715 0.1654452 0.2611079 0.3254003 0
0.9923318 0 0 0.04946439 0.02666823 2.791603 0.1445863 3.386332
0.8199352 3.88993 1.045971 0.04811156 0.03500447 0 19.49057 3.842967

0.8555273 1.063213 0.2722469 0.3143351 0.03857749 0.6757307
0.1010644 0.4516448 0.08607861 2.251193 0.1130285 0.2852369
0.06039473 0.05745389 0.4014621 0.3657934 0.6197139 0.2820768
0.6512178 0.1211423 0.09218691 1.598394 0.05506679 0.3386689 0
1.323246 0.1799709 29.18065 0.2334758 0 0.6130314 0.1321753
0.2937642 0.179171 0.04221307 0.1761269 0.4909022 0.8413248
0.04789721 0.4845072 0.550692 0.9363131 0.1031475 0.6082111
0.09733637 0.03857749 0.2373126 0.2513048 0.1395467 0 0.08747953
0.045043 0.866439 2.069637 0 0.03863912 0.6874057 0.7853276 0.7128806
3.845169 0.3664862 0.6807154 0.06016937 0.1253991 0.3561436
0.2805772 0.1427026 0.08685131 0.2404212 0.06303085 0.5650407 0
0.6028567 0.9653783 0.189171 0.288444 0.6584779 0.07453281 0.7921245
0.584767 0.6513848 0.1846419 0.8227241 0.2582358 0.05870895
0.9179314 0 0.8110404 0.8179547 2.354759 0.08467242 0.2215026
0.4275672 0.03687209 0.0769586 0.2607709 0.06062178 0.09220936 0
0.7067208 0.3991434 0.09694625 0.1394126 1.562603 0.4358214
0.525828 0.6168183 0.2229317 0.3786215 0.08067178 0.07582473
11.03148 0.1374323 0 0 0.3850131 0.611949 0.03485315 0.05363213
0.166051 0.0917954 0.4640401 0.8559591 0.4626137 0.1846419
0.4373976 0.1844073 0.09375228 0.2081975 0.0825532 0.0952287 0
0.4038433 1.090447 0.0622602 0.05644828 0.06450157 0.1485589
0.08633162 0.2721752 0.5308286 0.06085054 0.08860106 0.2018302
0.08685131 0.2097233 0.6325273 0.1713037 0.7558778 12.2309
0.06711146 0.1632492 0.07177474 0.2260569 0.1992947 0 0.5191273
0.06564742 0.2494842
TCGA-XF-AAMJ 1670 1 0.13034314 0.11615546 0.07878371 0.34160163
0.30004314 23.30589237 0 0.2368357 0.11893602 0 0.12879901
2.27235147 0.05823143 0.84561179 0.27000834 0.18484968 0 0.11209022
3.1230297 0.09060126 0.46349257 0.1785764 2.18453722 35.80134723
0.0800115 0.09341753 0.5000719 0 0.55007909 1.54022147 1.36748635
0.0543288 0.23336689 0.32231839 0.29511147 0.04632245 0.06273814
0.14343037 0.10423154 0.11113438 0.08114194 0.01688839 0 0.30151154
12.01591215 0.20674114 0.08181787 0.13487053 0.35005033 0.45300631
3.38996112 0 0.89288201 0.55693794 0 0.51582509 0 0.30591451
2.49572923 0 0.14633933 0.29106547 0.18669351 0.37295016 0.32444011
0.19537693 0.20166566 2.31559492 0.02889721 0.1629864 0.17260607
0.15903164 1.35404085 1.53355384 0.04107257 0.30415783 0.19043068
0.14821698 0.14316544 0.09825974 0.19313122 0.0825856 0.39241311 0
0.12642037 0.16626532 0.13813645 0.05611007 0.63188573 0.10770779 0
0.25197897 0 0.03103051 0.58309754 0.28004027 0 0.68298355
1.06566674 0.57697671 0.04457949 0 0.36734413 0.50381076 0.99049612
0.39492858 0.48006903 0.40031747 0 0.72365961 0.08580621 0.0639096
0.32887291 2.85139634 0 0.36325978 0.03845747 0 0.63909604
0.17469431 1.7451268 0.17961766 0 0.05142643 0.13976601 1.54922861

0.0525972 0.77181326 0.07001007 0.85965849 0.09167985 0.98661873
0.04757441 0 1.73115801 0 0.17842625 0.17113572 0.4838392
0.14953606 0.85252111 1.28192346 0 0.28478671 0 0.06624608
0.26272434 0.23833214 0.06198074 0.09825974 0.35511606 0.0800115
0.23695715 0 0.16711264 0.11472786 0.82758168 0.4096477 0.59698507
0.24604177 0.16414438 0.40005752 0.27299886 1.11294671 0.17502517
0.34676656 0 0.15970038 0.03779685 0.29777119 0.04543426 0.05530418
0.13122228 0 0.44283097 0.13192475 0.09401148 0.20490751 0.44898031
0.3148664 0.27953203 0.15633932 0.97277145 0.40621226 0.27652091
0.14108899 0.31433091 0.27406076 0.97198442 0.55414572 0 0.37265056
0.39435622 3.31075642 0.08986123 0.28209184 0.75195998 0.42262174
0.03920386 0.22680095 0.07720408 0.1489382 0 0.40646749 0.10166478
0.18519697 0.08877357 1.30372295 0.27751738 0.53572921 0.22444029
0.08111765 0.24109427 0.01712308 0.12875415 2.27576505 0 0.10751982
0 0.51302882 0.20508941 0.22193393 0.13660501 0.07049068 0.17535728
0.34473382 0.29181025 0.42082554 0 0.05570421 0.03914159 0
0.04734772 0.2453114 0.84894097 0.01593607 0 1.38872427 0.07929068
0.03594449 0.16429029 0.25799354 0.0452721 0.24615412 0.31548062
9.53193662 0 0.3855574 0.44243345 0.04451507 0.64288852 0.21816168
1.50412253 9.19179219 0.17093787 0.34650652 0.0914078 0.19192791
0.12022544 0 0.4751857 0.04560241 0.07564938
TCGA-GU-A763 997 0 0.1127758 0.02192734 0 0.1232823 0.01573358
135.2932 0.03571947 0.1788354 0.2993632 0 0.05065445 0.0408538
0.2748171 1.064207 0.1385083 0.1453964 0.07281048 0.1202268 0
0.1221666 0.1177833 0 1.44104 170.6022 0.05664088 0.1102185
0.09440147 0.03680462 0 2.551537 0.03396688 0.0256399 0 0.2390375
0.05325211 0.08744557 0.4737377 0.1093461 0.1135176 0.05721676
0.06266304 0.08767329 0.02648056 0.02371587 11.53305 0.0325231
0.1158393 0.006365073 0.9912154 0.05344789 13.3162 0.1091019
0.2370298 0.03625393 0 0.6770625 0.1840231 0.01279256 3.735414 0
0.2417216 0.9157686 0 0.004437224 0.2105346 0.1536768 0.09517398
0 0.02272956 0.1538394 0.04887577 0.1726226 0.3993908 1.888029
0.2326052 0.07177204 0.02139804 0.04663296 0 0.02318633 0.3038208
0.1559016 0.3518709 0 0.1193255 0.07133379 0.06519204 0
0.1118294 0.05083156 0 0.02972971 0 0 0.238978 0.07929723
0.0410673 0.1435017 0.7072456 0.002409718 0 0 0.02476631
1.222808 0.2337271 0.1065042 0.1090861 0.538438 0.1623043 0.2584502
0.02024767 0 0.02586802 3.448781 0.03396688 1.02862 0.007259838
0.06906331 0.4825834 0.1374086 0.0138808 0.127153 0 0.1213508
0.08179176 1.725835 0.03309693 0.3106831 0 0.3091099 0.08653468
0.05173604 0.02245224 0 1.818229 0.07944167 0.1113697 0.0269219
0.05074285 0.03528599 0.241403 1.607288 0 0 0 0.03126414 0.0309975
0.6467505 0 0 0.1711591 0.1888029 0.08387207 0.0365272
0.1577341 0 0.2169825 0.6232314 0.5446989 0.04644673 0.1032883

1.0384160.2157303 0.04567335 0.05506752 0.3273056 0 0.03349729
0.05351347 0.5972524 0.06432666 0.05220045 0.02064299 0.1515343 0
0.03113025 0.2588118 0.1289386 0.5374809 0.1981305 0.07915332
0.0711476 1.2029840.03195127 0.07830068 0.02219515 0.04944839
0.1034721 0.07057197 0.2973483 0 0.2810073 0.1650430.03396688
0.008481812 0 0.03671168 0.1108066 0.05550554 0.1758454 0
0.09597263 0 0.2329341 0.1919185 0.1748035 0 0.1438151
0.05238856 0.5372675 0.1059222 0.1531305 0.5601571 0.1454591
0.2278656 0.0385108 0 0.07611427 0 0.02356906 0.2564929 0 0
0.06653467 0.08275802 0.1685040.4922979 0 0.04756104 0 0.2770869
0 0.04469052 0.04961715 0.1717066 0.01504172 0.1213615 0.47665 0
0.02544544 0.03876754 0.08117156 0.01831345 0.1161698 0.265871
0.1097194 0.03550141 0.2388222 0.02610023 0.08403368 0.4276906
0.02059182 2.6690541.4979420.09075637 0.01635301 0.0862779 0.04528918
0.01891304 0 0.7410294 0.02510851 0.03570193
TCGA-G2-A2EC 696 1 0.2675659 0.2341064 0.0882140.1687460.03732865
65.923230.04237307 0.1149133 0.08878166 0.3348706 0.2643961
2.4353050.3586091 0.4734152 0.08544051 0.07545997 0.2015373
0.09698288 0.6622826 0.06521535 0.0998023 0.04998794 1.60228340.3052
0.06719158 0.0784496 0.3359579 0.1746414 0.5389324 1.003074
0.3223521 0.1216638 0.2351705 0.7733559 0.3158578 0.2723027
0.4566108 0.1791294 0.2603484 0.02828114 0.1156330.06618468
0.1884791 0.05626701 2.4811640.2700690.1145142 0.1208115 0.4899386
0.09510572 6.2144840.4206302 1.4683960.6128508 0 0.3790294 0
0.2016170.2794464 0 0.2867479 0.2172704 0.1567803 0.322844
0.06162703 0.1823027 0.5927375 1.4928060.03235617 0.4627568
0.2705735 0.3828451 1.8951474.14348 0.06898335 0.2081231 0.2081482
0.4840452 0.2137362 0.1100213 0.1982280.09247098 0.2416614 0
0.07077635 0.1142389 0.1546712 0.06282637 0.0442201 0 0.2192267
0.1763378 0.07605662 0.06204431 0.4553073 0.2194925 0.2435853
0.4426039 2.2559420.008575752 0.07487339 0 1.1458053.1026390.7486135
0 0.3683094 0.2353233 0.1925373 0.4598890.07205782 0.03577975
0.06137309 1.1078510.2014701 0.3660673 0.02153038 0.1228920.2146785
0.3912099 0.9056535 0.7039117 0.1326603 0.3742836 0.1377163
2.0473130.1276015 0.7116926 0.07839017 0.5653121 0.02566345
1.7491330.1931001 0 0.6461250.1413593 0.2126729 0.09581021
0.2106821 0.4185883 0.3500078 0.6319903 0.06830035 0 0.06040574
0.6675808 0.9192877 0.9006530.2775990.1925373 0.5541341 0.8510933
0.3979809 0.3033188 0.1559298 0.03211515 0.6177613 0.5612819
0.3119402 0.1928448 0.2450563 1.2878390.7286473 1.1811480.6205889
1.4560280 0.1986848 0.08464215 0.2917372 0.1017454 0.2786581
0.1530515 0.3482873 0.3408881 0.2215739 0.1228086 0.8412604
0.4475457 0.1762777 0.1877950.09377835 0.6051171 0.07580588
0.09288602 0.02632952 0.4106152 0.1227462 0.08371766 0.7862198

0.1290615 0.5646566 0.3707439 1.262546 0.1006175 0.09475735
0.5806679 0.3154726 0.1426636 0.2630183 0.1728906 0.05612314
0.03036239 0.1950519 0.1138339 0.2073648 0.09939964 1.12747
0.7457766 0.2249457 1.350767 0.1248876 1.017515 0.01917269 0.1441658
4.852692 0 0.0601949 0.04538378 0.04320991 0.2009335 0.09939964
0.1147173 0.03946416 0.09817365 0.5031759 0.2328489 0.09423955
0.1316476 0.3430456 0.2191339 0.06684433 0.02650759 0 0.067897
0.008921799 0.03788629 1.342914 0.1331725 0.03018525 0.1839556
0.2599875 0.1086238 0.3403044 0.5046331 0.8460222 0.1474003
0.2428358 0.3096201 0.2242956 1.738894 0.415268 0.9094485 10.01471
0.1076619 0.7177683 0.2046984 0.3760775 0.2617538 0.04623549
0.4395317 0.05106096 0.1270568
TCGA-FD-A3B7 122 1 0.1171045 0.03756882 0 0.2707994 0.1078274
22.89075 0.2039978 0.2468258 0.5342799 0.4030442 0 1.710239
0.156951 0.3473029 0.03164143 0.1536193 0.1663314 0.4778933
1.428423 0.06279361 0.2114116 0.144395 0.9447227 25.87994 0.102436
0.2140198 0.2156548 0.1681562 0 0.4236077 0.1939886 0
0.5786737 0.2730338 0.1310092 0.1373381 0.2029176 0.249795
0.2506805 0.1143699 0.06759869 0.05462309 0.03024667 0.08126633
0.1514172 0.1485944 0.0441047 0.08724389 0 0.09157401 1.544344
0.0934639 0.5113988 0.7867905 0 0.2519922 0.4504183 0.1064584
0.1921924 0.1245407 0.1183284 0.1307513 0 0.01858374 0.1093078
0.1053198 0.2989519 3.366475 0.03115463 0.2761294 0.08063895
0.1457368 1.155689 0.3594247 0.1549839 0.1548503 0.01466478
0.06658148 0.09003713 0.1059357 0.1908669 0.1780742 0.2538408
0.1074784 0.5906169 0.2240672 0.1489275 0.03024667 0.681248
0.05806091 0 0.08829058 0.03661614 0.1385975 0.3101884 0 0
0.3454734 1.023253 0.09358268 0.048062 0.07219748 0.08486586 0.1163931
0.293665 0.06082571 0.06709261 0.1510565 0.1059357 0.2952075
0.1618913 0.1722554 0 1.646263 0.03879771 0.07832745 0.04975407
0.2366568 0.1033532 0.06278042 0.7742502 0.2420615 0.063867
0.05544381 0.06931482 0.8448372 0.07560806 0.6607905 0 0.5700643
0.09884179 0.118188 0.1987521 0 0.4574496 0.22685 0.05895048
0.03075078 0.08693937 0.08060884 0.2144611 0.2681621 0.09864606 0
0 0.03571058 0.03540601 0.1927128 0.2338792 0.1589035 0.6190885
0.04313096 0.255468 0.04172216 0.09008365 0 0.8426631 0.7002461 0
0 0.2064617 0.3234822 0.2429895 0.3077976 0 0.7477112 0
0.1817413 0.162998 0.1685422 0.09796708 0 0.3242095 0.02163573
1.342756 0.07111529 0.08446297 0.4786485 0.3187674 0.1697317 0
0.1234044 0.2233478 0.4744406 0.02981224 0.1014071 1.807393
0.2068291 0.5642618 1.514048 0.06213441 0.600459 0.1644498 0.4138422
0.07750488 0.1520643 1.313897 0.6075154 0.05283303 0.1135264
0.08323519 0.06484683 0 0.1252058 0 0 0.04785424 1.328434
0.6582329 0.144395 0.06049333 0.07652267 0.5698428 0.03692145

0.1041092	0.02199393	0.06289932	0	0	0.3181584	0.3150829
0.09570847	0	0.1899934	0	0.23229	0.6527174	0.4537	0.2897347
0.1501394	0.02109964	0	0.01531394	0.1133476	0	0.1718098
0.06566291	0.4763850.08548479	0.01937622	0.4428112	0.08344432
0.06972673	0.1706036	0.1093261	0.02088732	0	0.2468081	0.5366204
0.07198882	0.8434234	0.2352043	0.5513518	3.9164660.03071523
0.6350779	0	0.3621120.1584212	0.02225928	0.1336452	0	0.2691448
TCGA-FD-A5C0	550 1	0.07196604	0.2462693	0	0.2662697	1.303208
36.096640	0.1150719	0.05253428	0.6935291	0.0142227	1.597321
0.1543257	0.1867542	0.02333411	0.2832182	0.06814547	0.33082
0.2194577	0.02572635	0.09448875	0.05915817	1.96452313.788120.1259032
0.1083146	0.1987945	0	4.2823651.6660870.5722309	0.1079871
0.06184718	0.2745682	0.5693286	0.06138216	0.5819429	0.08772021
0.2496746	0.06024471	0.1498787	0.03356837	0.0743518	0.03329457
1.6749490.3196129	0.1084174	0.04467944	0	0.3751759	2.789226
0.03829188	0.2957909	0.1654142	0.9974602	0.2136009	0	0.2976128
0.6614212	0	0	0.2892695	0.7730898	0.4921221	0.1113179
0.08629839	0.6346675	0.2789463	0.006381979 0.1311272	0.4879389
0.2423439	0.8971239	3.5783010.05442552	0.1903251	0.5287142
0.08183468	0.07904558	0.06510229	0.1919395	0.05471734	0.4679901	0
0	0.09513784	0	0.0371759	0.6803190	0	0.05843231	0.02250228
0.4288095	0.1880832	0.5195163	0.7495041.8472440.6839964	0.0879577
0	0	2.9553890.9537182	0.1640641	0.2616611	0.2297181	0.2254468
0.1627557	0.4924214	0	0.1270305	0	3.1176990.2384295	0.2888147
0.03567216	0.09695757	0.08468701	0.2314885	0.4611965	0.6545343
0.07849834	0	0.06667373	1.1577360	0.7520107	0.7421662	0.5243655
0.03037138	4.6847590.1654830	1.0008	1.0595130.2173665	0	0.2493316
0.09907558	0.07531206	0.3929793	0	0.06289544	0.1429742	0.2633493
0.5222065	0.03947692	0.1642621	0.06510229	0.5556689	0	1.255974
0.1025607	0.1845350.1520266	0.7920131	0.5356842	0.6064859
0.09780944	0.3625146	0.5301187	0.5552480.9938684	0.07730898
0.8041294	0	0.10581	0.05008483	0.2762049	0.1505130.07328391
0.08694172	0.02659227	0.2933990	0.1453377	0.4977944	0.406305
0.2433850.7037783	0.08508612	0.9190274	0.2691372	0.03664196
0.0623193	0.4165218	0.1452638	0.1486134	0.2212965	0	0.8185297
0.3795983	0.4450685	0.09526053	0.1869008	0.3607752	0.1555607
0.08441756	0.1717345	0	0.06452119	0	0.3847233	0.4041499
0.04090094	0.1176344	0.5144089	0.4780620.3105804	0.8922216
0.08061713	0.2580379	0.03403486	0	3.8566430.1159635	0.07123759	0
0.3218606	0.1426768	0.0882258	0.1357621	0	0	0.3344494
0.8155828	0.2230554	0.06677067	0.0369070.1815337	0	0.05646668
0.04643816	0.08035264	0.05279247	0	1.5474470	0.04763027	0.05442552
0.1139563	0.05142029	0.3661208	0.2239528	0.02567241	0.04984022
0.2022330.03664196	0.0294936	0.4670030.02890874	0.9168361	3.768295

0.07078463 0.06887369 0.06056252 0.2543249 0.01770127 0 0.04106553
0 0.02004869
TCGA-FD-A5BS 1639 0 0.13448427 0 0 0.12439576 0.1651071
44.6522327 0.18741887 0.4887204 0.14725768 0 0 2.45440509
0.07209781 0.69798218 0.08720977 0.29561994 0.25468942 0.34064604
0.23434556 0.0641004 0.08828644 0.11054997 1.16090684 46.39582285
0.06191516 0.31807213 0.4953213 0 0.51080009 1.6345603 0.71289234
0 0 0.51309068 0.08597293 0.0286765 0.31071072 0.05464146
0.30773866 0.0750536 0.04870976 0 0.34735647 0.12443635 1.89346228
0.25597141 0 0.40076785 1.08351535 0.21032945 5.2122791 0.07155674
0.34546866 0.19022314 0 0.33928602 0.89659426 0.13424422 1.50086941
0 0.09059321 0.24025033 0.17336246 0.20565736 0.05738535 0.0806337
0.12484367 0.60815076 0.02385225 0.07207056 0.12822475 0.01969011
0.27941202 1.73362456 0.15255896 0.48119255 0.11227477 0.18351118
0.35451347 0.06082893 0.03985344 0 0.58302788 0 0.05217475
0.29007163 0.17103023 0 0.19558841 0 0 0.07799538 0.12615129
0.07683931 0.16148831 0 0.5386969 0.30697133 1.56682176 0.23390857
0.16558498 0 0.32487002 1.06933851 1.65558358 0.13970601 0.02201428
0.22303942 0 0.75068695 0 0.15825618 0 2.06421457 0 0.53971331
0.01904606 0 0.31651237 0.28839123 1.97860524 0 0.14669131 0
0.04153148 1.04828526 0.02170731 0.84317775 0 3.45496208 0.45404453
0.74650737 0.16198345 0 1.05068155 0.10420694 0.07838885 0
0.33280751 0 0.07036852 0.66329983 0 0.05876693 0.40076785 0
0.08132141 1.69673893 0 0 0.20580679 0.09906426 0.22003696 0
0.20690637 0 0.91079977 0.34035464 0 0.12185221 0.13548753 0
0.0707456 1.30607342 0.14446871 0.7871241 0 0.04393979 0.04679723
0.46084751 0.28126652 0 0.27078268 0.12423368 0.75388525 0
0.09699832 0.08456705 0.47454352 0.0649740.20765739 0.09678383
1.69509957 0.67058884 0.1369470.05822861 1.03781606 0 0.74057748
0.53572331 0.03567796 0.38114665 0.23952494 1.00993082 0.02225189 0
1.28416634 0 0.02426964 0.14040399 0 0.04964727 0 0.4313637
0.12587373 0.22929704 0.32973839 1.17453996 0.1374405 0.33164992
1.66731105 0.12554227 0.57404787 0.04240104 0.31882751 1.04961463
0.14446871 0 0 0.42721006 0.19044478 0.1099128 0.08456705
0.17455259 0.14474285 0.0152437 0.41943748 0 0.04159186 0.06896879
0 0 0.01172448 0 0.15015646 0.01973085 0.25136033 0.39077603 0
0 0.30511792 0.08518088 0.03203002 0.13061555 0.08370097 0 0
0.19890347 0.0684735 0 0.82474671 0.43217836 0.59593344 9.17716204
0.02645531 1.58736827 0 0.11881539 0.52925919 0 0.84413912
0.02823075 0.14986146
TCGA-CU-A0YN 393 1 0.1377520.1031166 0.04995711 0.5478988 0.1056993
24.6103 0 0.09010716 0.2262536 0.0948215 0.1361201 0.4446232
0.5169475 0.9447445 0.1488813 0.06349093 0.1304389 0.006461538
0.9601588 0.05745068 0.02260791 0.05661806 1.8739452 3.94448 0.05073566

0.6664104 0.2536783 0.04945122 0 0.4982967 0.09127678 0.1722507
0.1775748 0.9927202 0.1706199 0.1174931 0.2784778 0.2658534
0.08134609 0.3587605 0.1387658 0.1606351 0.1778983 0.03186498
2.612347 0.3932865 0.2594054 0 0.3329528 0.4667867 2.038606
0.256534 1.663155 0.487113 1.762397 0.2657582 0 0.07120851 0.2712949 0
0.2319861 0.2153269 0.0295958 0.01987306 0.1653179 0.1238894
0.1918157 0.5784328 0.01832386 0.16979 0.1678238 0.1714326 1.18058
0.7610349 0.07813292 0.06428929 0.01725042 0.06265671 0.09078186
0.06230695 0.3673962 0.1571037 0.2985971 0.08428578 0.1870487
0.06229931 0.5255578 0.07115931 0.3005112 0.1365960 0.159781
0.1507526 0.008432823 0.6373233 0.03551496 0 0.175013 0.8657972
0.119796 0.08480418 0.2547813 0.06655274 1.597344 0.6908859 0.3219763
0.07892214 0.2919196 0.2492278 1.004566 0.08161517 0.04052537
0.03475664 1.936527 0.09127678 0.6449651 0 0.09279444 0.4863045
0.1846241 0.9045465 0.3986373 0.07512781 0.03260973 0.1949778
2.52447 0.1334085 1.669753 0.0443937 1.566119 0.08720192 0.1042699
0.4072565 0 1.205356 0.3202169 0.1094912 0.1446906 0.170447
0.1422323 0.4685092 0.5823572 0 0 0 0 0.8329736 0.6800738
0.1965114 0.06230695 0.4647347 0 0.4507668 0.2453923 0.07064459
0.1818737 0.4664652 0.8442183 0 0.1872195 0.1734745 0.2536783
0.3542713 0.1227347 0.3329528 1.136079 0.1457704 0.2587928 0
0.8024768 0.05762015 0.1753432 0.07627459 0.03817569 0.6318026
0.08365409 0.09935519 0.4331093 0.3402518 0.266211 0.07090102
0.1416221 0.5083209 1.373766 0.07013727 0.2982173 0.3321978
0.382323 0.3318753 0.6065025 0 0.3878393 0.330272 0.5628735
0.1253591 0.1431006 0.7070109 0.02977627 0.1553709 0.1900419
0.1468664 0.3886668 0 0.441845 0.06446611 0.03914475 0.05629173
0.3578992 0.6687052 0.2547813 1.387607 0.3857781 0.4703969
0.05428913 0.0408218 0.8767675 0.1109843 0.1022682 0 0.1871275
0.4291588 0.1407293 0 0.08939693 0.1111949 0.7807046 1.027281
0.1601084 0.08520493 0.07064459 0.1240993 0.3028408 0.07806086
0.06666631 0 0 0 1.481003 0.05027858 0.1025666 0.677152 0.1417822
0.09022277 0.3886259 0.3286498 0.1474206 0.09540039 0.1782694
0.2805491 0.08468163 1.144391 0.08300239 0.8393185 3.17604 0.1264579
0.2636656 0 0.3042559 0.0762355 0.02618395 0.4585265 0
0.1151271
TCGA-FD-A5BZ 835 1 0.09099128 0.1362262 0.06599777 0.1683309
0.3351315 37.51695 0.06340326 0.05290647 0.0996337 0.3758028
0.07193067 2.422063 0.2926858 0.5734473 0.08850844 0.2516315
0.04308035 0.05121759 1.149537 0.02168498 0.1194682 0 1.447555
23.55224 0.03351315 0.07825664 0.1675658 0.1959883 1.382418 1.053271
0.6632159 0.2730701 0.2736908 1.00289 0.1526931 0.1940235
0.4204502 0.1386379 0.228364 0.05924431 0.1277074 0.08488529 0
0.1683858 2.954387 0.3463776 0.1028093 0.101684 0.4398601 1.04359

3.1098880.2420744 0.7947232 1.0618070 0.4996283 0 0.2189624
0.5376068 0 0.1225897 0.1219140.3909868 0.3150489 0.1019198
0.3818941 0.2956398 0.4114713 0.008069146 0.1560401 0.2891871
0.3064109 0.3780971 1.6756580.2408479 0.2264849 0.1975076
0.0620813 0.1199309 0.2880955 0.1617876 0.06918265 0.4602189
0.1948607 0.2118068 0.1582748 0 0.09400775 0.5293360.1804554
0.05467188 0.0949882 0.02845108 0.2339507 0.7841160.04691842
0.07289584 0.7484859 1.5622561.0821640.03734461 0 1.4507140.7235083
0.4563608 0.2363107 0.1117105 0.2850469 0.1234695 0.6717525
0.2515820.05353761 0.0918332 4.3465390.06029236 0.3651669 0.03865937
0.06129484 0.1070752 0.1463429 1.8397040.1504672 0.1985010
0.1592331 1.3732550.1028093 2.2629410 0.7601511 0 2.02033
0.1295238 0 0.5687081 0.2820233 0.1542908 0.1433618 0.1351054
0.2505352 0.5237203 0.7693454 0.05109926 0.1590455 0.4067358
0.1109898 0.3301296 0.3493925 0 0.0411565 0.2658365 0.3351315
0.6451282 0 0.04666388 0 0.3851512 0.8940359 0.03333996
0.3710002 0.2750102 0.5864802 0.4201577 2.0024710.3909868
0.3389041 0 0.1932412 0 0.1371950.07612132 0 0.2198520
0.4173336 0 0.1575084 0.2288703 0.2385117 0.04396104 0.4214994
0.1870954 0.5055391 0 0.09265755 0.1181914 0.1755451 0.459166
0.5637043 0.7821663 0.0241395 0.4037883 0.3552881.6881860.09033300
0.4995942 0.1573483 0.08210349 0.2714187 0.06467451 0.05758467 0
0.7539670 0 0 1.6426490.4184615 0.3365886 1.0340850.1189176
0.6991155 0.07172076 0.1617876 0.9759997 0 0.04503513 0
0.6883903 0.2233467 0.2230991 0.05721758 0.2952532 0.04896609
0.7013384 0.4439471 0.2820233 0.1125633 0 0.1311569 0
0.1031253 0.05871474 0.20319 0 0.3968275 1.4277430.03321123
0.0903330.2064410.1873069 0.1408636 0.1472895 0.6984563 0.3895114
0.03150809 0.2960693 0.09265755 0.1118719 0.5061101 0.1462047
1.2096156.4397550.07756455 0.1161086 0.07657308 0.1607796 0.1119043
0 0.1211508 0.03820152 0.02534885
TCGA-UY-A9PE 189 0 0 0.1710961 0 0.09866206 0.12276722.960440
0.1938095 0.2919864 0.1835545 0.02634999 1.1475960.4288722
0.8402718 0.0864609 0.3592611 0.0631256 0.1000655 0.2323332
0.01588749 0.3938774 0.3288026.31509581.633180.06138349 0 0.368301
0.09572717 2.5320692.3922020.08834634 0.06668824 0.05729126 0.2826026
0.3622474 0.0568605 0.3080426 0.09480144 0.3149379 0.2604319
0.04527327 0.02073039 0.2066242 0 6.0913320.6762790.3515081
0.0496658 1.93358 0.1390156 3.7540850 0.4110025 0.04714742 0
1.3257040 0.1473513 5.2516990 0.1796306 0.5359214 0.05729126
0.2885255 0.1991240.0799413 0.6188581 0.4306632 0.02364743
0.1143227 0.2542474 1.0150930.6232786 3.2737860.1512489 0.1866759
0.1669660.2425805 0.0292891 0.1809198 0.6716907 0 0.3853476 0
0.2069069 0.09276799 0.5086847 0 0.3878178 0 0 0.123721

0.04168934 0.03808974 0.3861274 0.1374990 0.5225381 0.6131713
0.1065486 0 0 0.3864964 2.1203122.370863 0.06925317 0.1418641
0.4913870.06030659 2.04066 0.05266328 0 0 3.6530790 0.3567192
0.009441257 0.08981528 0.8629348 0.2859148 0.6318085 0.5511987 0
0 0.08234969 1.3930820 1.8111980 1.0133510 0.6728155 0.2773870
2.2708170.6198727 0.1554314 0.2100680.06598993 0.2753318 0.3488213
1.8084110.07487571 0 0.06622107 0.08131663 0.1612462 0.5119645 0
0.06030659 0.3709809 0 0.4362950 0.2051297 0.07041384 0.677234
0.5954682 1.7098550.1208059 0.4701343 0.6138349 0.4597943
0.5702127 0.1432282 0.6384805 0 0.08712496 0.1391861 0.7675755
0.05577025 0.1357710.1476516 0 0.1019198 0 0.1346316 0.08384087
0.3763749 0 0.4803732 0.1713442 1.6363190.1662076 0 0 0
0.1345631 0.1835545 0.8199794 0 0.5270344 0.2100680.9129122
0.3088514 0.2077595 1.46411 1.0951710.08421433 0.1491411 0
0.1371155 0 0.4989362 0.8735499 0 0 0.3057505 0.2725206
0.8220051 0.9642464 0.2240356 0.3870006 0 0.03951121 0.9237425
0 0.2639597 0 0.3789583 0.3398570.1634535 0 0.1730537
0.07174997 0.5289481 0.3746639 0 0.2474083 0 0.2882762
0.1465590.1627332 0.04301733 0 0.01956142 0 0.8523249 0.04866439
0.1323649 0 0.04222472 0.04763246 0.09866206 0.3042681 0.04756256
0 0.6211658 0 0 0.6940641 0 0.7385202 5.0671540.08742713
0.1276003 0.1122025 0 0.01639733 0.05068664 0.4057651 0.0653061
0.3900083
TCGA-GV-A40E 261 1 0.07899852 0.05631971 0 0.1096085 0.04041122
67.696380.04587219 0.2679444 2.7392250.09063098 0.2862297 0.8525705
0.3176368 1.0982340.05692068 0.03734463 0 0.08028772 0.02867893
0.03137812 0.1080439 0.1623477 3.76375 48.338340.1636654 0.6086499
0.6667851 0.1417973 0.08334814 1.4097740 0.3951319 0.02828785
0.8651263 0.1052126 0.07018791 0.2281464 0.1203652 0.06803221
0.08572639 0.08494485 0.3582508 0.2040435 0.03045674 6.639477
0.0835346 0.1239707 0.04904549 1.3259930.5147973 2.6575690.03502811
0.4058692 0.2095135 0 0.9378955 0.1688064 0.04224509 0.777916
0.1400249 0 0.2352124 0 0.02279375 0.2633532 0.03947142
0.3666772 0.1701137 0.08173226 0.07055927 0.02092266 0.2313266
0.4787175 1.5760380.02489331 0.1228962 0.02198408 0.02994384
0.04338493 0 0.1170532 0.05005357 0.3329678 0.02014022 0.1787823
0.1190921 0.3348876 0.03400725 0.2872305 0 0 0.08399583
0.02058433 0.002686715 0.4603557 0 0.05274006 0.0878921 1.372496
0.2413822 0.02701879 0.04058692 0.06361153 0.5670789 0.3902086
0.03419411 0.07004611 0.5216434 0.1191067 0.3082029 0.02600276 0
0.06644122 2.7103110 0.3962968 0.01864667 0 0.5035473 0.3529297
0.4813076 0.8164716 0.07180763 0 0.2202448 1.2992560.1275127
2.2976320.1272953 1.1743690.02778271 0.2325443 0.4108838 0.1555832
0.8434923 0.4590979 0.1046524 0.1037221 0.03258287 0 0.3100182

0.8117384	0.2218216	0	0	0	0.07961613	0.8305795	0	0.05955337
0.4670930.09698692	0.4308458	0.1876380.3038514	0.03476719	2.006327
0.9800516	0	0.05964851	0.1658080.6061683	0.08080578	0.1583693
0.2475187	0.7005622	0	0.08603679	0	0.6226336	0.1101474
0.1340753	0.07953134	0.02432571	0.1845191	0	0.1804322	0.3311749
0.2654814	0.3180576	0.5082572	0.1252111	0.46402	0.5334281	0
0.02850379	0.3810201	0.1661031	0.2265775	0.2438416	0.0174649
0.6603847	0.1488187	0.3053502	0.07624847	0.4787175	0.2514476
0.1423017	0.1069232	0.1963711	0	0.1666498	0	0.3343352	0	0
0.3228239	0.1959344	0.06727922	0.4464561	0.5781233	0.1720736
1.0903	0.04151191	0.01950886	0.4066457	0.8486356	0.0977486	0
0.4485199	0.2672468	0.08070598	0.08279372	0.04272307	0.4959768
0.1865506	1.1160520.05101088	0.4479167	0.03376127	0.04744595
0.1447285	0.03443587	0.1486802	0.07350387	0.06760987	0.2296834
0.7651632	0.04805658	0.07624847	3.9331430.1772137	0.1215136
0.2435745	0.3960713	0.4227166	0	0.2823622	1.0390830.02697974
1.2393450.1322237	1.0939446.02688	0.06906814	1.4280750.1108011
0.05816194	0.04857758	0	0.3756536	0.00921292	0.1742287
TCGA-BT-A2LD	623	1	0.04712419	0.1847771	0.2848342	0.04358911
0.0482122	58.728770	0	0.1433336	0.1081264	0.03104395	0.3912118
0.2947415	0.4658622	0.2122145	0.2339066	0.03718537	0.0663137
0.2395057	0.04679419	0.2062411	0	1.1713827.862480.1518684
0.1350965	0.1446366	0	0	1.0909730.05204214	0	0	0.1331781
0.07531372	0.05024218	0.5897402	0.06382245	0.1275452	0.1534125
0.05156026	0.02442328	0.6085802	0	0.8575616	0.04983005	0.1183216
0.009752204	0	0.2456695	2.3912610.1671597	0.3631636	0.04165966	0
0.3613266	0	0.06860011	1.0827660	0.1058149	0.3858494	0.06749707
0	0.07121658	0	0.2916404	0.1522144	0.04178993	0.2441221
0.09152573	0.04599697	0.7751038	0	0	0.02443668	0	0.0178621	0
0.1420991	0.1163743	0.2388636	0.9363607	0	0	0.07650551	0	0
0	0	0	0.04555017	0	0.05128573	0.09431098	0.04049824	0
0.2672208	0.9150414	0.01476813	0	0	0.1897277	0.8847163
0.03581034	0.08158987	0.01285659	0.3328803	0	0.5939742	0.06204466
0.1848468	0	1.4759220	0.8930627	0.04449245	0.1587223	0.2772701
0.1263178	0.1701390.5844499	0.08566936	0	0.09297691	1.849657
0.06338655	1.1161640.0506228	0.8485346	0	0.7530367	0.3870006	0
0.4295268	0.1825741	0.03329452	0	0	0.1621896	0.3287680.4565485
0	0.06864109	0.07801763	0	0.3799409	0.3446659	0	0.03552478
0.1966802	0	0.2998428	0.2238596	0.04027854	0.04147865	0.2659586
0.4248241	0	0.2134891	0.2373787	1.2294110.1973997	0.1819431	0
0.2507396	0	0.1667986	0.0273301	0.1614841	0	0.1999464
0.2213962	0.01451077	0.06003783	0	0.7590846	0.1481643	0.4355037
0	0.08084949	0.01211204	0.4103112	0.7343088	0	0.06801232	0
0.07926703	0	1.3173410	0.2518826	0.3981349	0.2255159	0.05198141

0.2855645 3.187362 0.2376802 0 0.1288532 0.1116493 0.1429018 0
0.5038424 0 0 0.4493321 0.2280095 0.3210672 0.3389527
0.4057201 0.190627 0.898471 0.02476268 0.09309941 0.4163065 0
0.1554906 0 0.2757581 0.2743473 0.03209515 0 0 0.29586
0.5964669 0.9094907 0 0.3400616 0 0.02830246 0 0.1780279
0.2280618 0 0 0.2055161 1.369305 0 0 0.7721665 0.1243665
0.1356177 0.2687995 0.0325882 0.02801765 0.02719662 0.3368694
0.07997858 0 1.613005 0.06309925 0.8700795 1.552151 0.02060026
0.1002206 0 0.06938951 0.03863666 0 0.2240849 0 0.1641014
TCGA-UY-A9PD 542 0 0.04746552 0.05075875 0.1721384 0.1536669
0.2185263 30.78737 0 0.2414878 0.2165576 0 0.0234516 0.7423815
0.4453145 1.407709 0.2052015 0.4207158 0.05618206 0.7347342
0.3618608 0.08483963 0.07790059 0.1950902 4.252888 64.80368 0.0655579
0.02551407 0.3277895 0.425988 2.103316 0.927176 0.4717718 0.05935283 0
0.5030352 0.2180953 0 0.8224779 0.1687474 0.1401481 0.04414963
0.04835209 0.1107008 0.06129882 0.1097979 9.171906 0.1505729
0.04469197 0.02946852 1.051658 0.3711734 3.421662 0 0.182897 0.02098071
0 0.6163563 0.1217109 0.1057607 2.181216 0 0.07993599 1.37792
0.05098947 0.09244404 0.2848199 0.0711481 0.6058651 0.6899259
0.01052316 0.2034954 0.1005695 0.173738 0.3081781 4.079158 0.3140952
0.3138244 0.04953349 0.2158977 0.02606743 0.1073463 0.4923122
0.09022266 0.2572208 0 0.09207402 0.1155895 0.6036422 0
0.3451595 0 0 0.08258418 0 0.04842863 0.3268914 0 0.09506512
0.8074689 2.328427 0.1617667 0.04870196 0 0.8026282 1.651201
1.352615 0.1849069 0.01942456 0.3717361 0.1610194 0.7692126
0.2343527 0 0.3592853 4.000236 0.2358859 2.222371 0 0.9592319
0.907655 0.3816981 0.407007 0.4905693 0.1294348 0.2247282 0.1893366
1.594092 0.0574611 0.4215931 0.07648421 1.870856 0.05007895 1.197618
0.1819084 0 2.243537 0.09194823 0.2452296 0.06232047 0.1174627
0.08168217 0.0620905 1.212346 0.3331985 0.0518537 0.05893705 0
0.1435098 0.5858365 0.1354248 0.05367313 0.2228679 0.08741053
0.06471741 0 0.2434217 0 0.7031981 0.3533129 0.9130673
0.2687944 0.2390981 3.059368 0.4508344 0.4229099 0.1912105
0.2525557 0 0.0387708 0.08258418 0.4066343 0.09927154 0.2416736
0.1314106 0 0.1209454 0 0.1540577 0.298475 0.2512315 0.1719918
1.465832 0.1951962 1.869614 0 0.06041841 0.05137871 0.1144662
0.479047 0.2450465 1.194194 0.03148087 0.615092 0.333279 0.4193527
0.07853688 0.2465425 0.1982924 0.1026008 0.03212191 0.3451141
0.08434349 0.2503249 0 0.3171824 0 0 0.09698286 0.5587131 0
0.5121117 0.4290918 0.1550832 1.863986 0.1122392 0.1758258
0.6141249 0.2549474 0.2936567 0 0.1735972 0.4145017 0.09698286
0 0 0 0.2959101 0.4250603 0.09194823 0.2201945 0 0.1710447
0.1304382 0.08276189 0.03828561 0 0 0.8280189 3.310136 0.08662305
0.05890266 2.961469 0.09395046 0.02119655 0.1152502 0.2708 0.04233088

0.04109042 0.9740519 0 0.04863158 0.3723244 0.04766721 0.7230148
3.688006 0.1011537 0.03785495 0.09986069 0 0.01459369 0 0.1805663
0.08303245 0.2148771
TCGA-G2-AA3B 2008 0 0.262392 0.07014929 0.0396496 0.1516927 0
40.93122 0 0.1112465 0.4189998 0 0.1296417 0.01742643 0.8791867
3.18166 0.1358875 0.08527712 0.02588146 0.2102622 0 0.08468021
0.1076597 0.1797448 3.938131 45.94539 0.1006688 0 0.2013376
0.1177442 0 3.037321 0.03622194 0.08202641 0 0.3476007 0.3494616
0 1.199824 0.1166056 0.3147406 0.08643846 0.04331136 0.05949613
0.423579 0 35.1841 0.2774585 0.08235321 0.0135753 1.981917 0.3704759
3.087617 0.05817258 0.1123405 0.05799124 0 2.425638 0 0.0107186
3.696315 0 0.1104726 1.58692 0 0.007886353 0.1720287 0.1311035
0.3044778 0 0.004847715 0.1464757 0.02316472 0.3361516 0.3123313
3.456295 0.1860359 0.2381151 0.009127461 0.1864835 0.02401703
0.02472567 0.2591932 0.2493779 0.1974903 0 0.06362377 0.07606964
0.5561612 0.0282386 0.3577614 0 0 0.06340692 0.1025556
0.004461935 0.4131557 0.02818726 0 0.4779206 1.273759 0 0.04487118
0 0 8.765711 1.495465 0.02839376 0.04474168 1.017415 0 0.9154148
0.04318382 0 0 2.94847 0 3.290729 0.04645091 0.2209453 0.7076055
0.2930622 0.0296047 0.2711894 0.05962689 0.05176291 0.1800711
0.8975417 0.03529423 0.5026762 0.03523407 0.3055961 0.09227972
0.08275619 0.1795713 0.04306387 1.622903 0.1270737 0.1245569
0.229674 0.0811675 0.07525724 0.2860331 1.973983 0.03069899 0 0 0
0.396665 2.548848 0.03119314 0 0.2433631 1.6107 0.5664555 0
0.2242748 0.05773926 1.110662 0.7487009 3.565288 0.07429549 0.7710191
2.214713 0.309932 0.0730584 0.2348938 0.407208 0 0.05358177 0.05706623
1.341245 0.1143289 0.2226642 0.02201348 0.7675749 0 0.03319699
0.2996509 0.3781218 0.4023173 0.5546215 0.5064498 0.2304233
2.289185 0.2044351 0 0.04733738 0.2109251 0.4689517 0.07525724
0.7449688 0.02900466 0.6727137 0.4006812 0.01207398 0.1356738
0.1703626 0.2609931 0.2363261 0.1726391 0.4239577 0.03885462
0.160003 0 1.022818 0.05116499 0.03106812 0.1340316 0.03200618 0
1.887321 0.8753966 0.2551513 0.9986876 0.1464985 0.1943949
0.2372791 0.05872346 0.02705583 0 0.009139568 0.1857882 0.04467721
0.1374988 0 0.02941744 0.1982797 0.8372696 0 0.05071862
0.1121374 0.1969885 0 0.03336027 0.3174674 0.3662124 0
0.2724603 0.4765264 0.01995237 0.07235936 0 0.3462421 0.04556832
0.08090276 0.2381586 0.5265167 0 0.4931851 0 0.04480634
0.5418446 0.02195891 2.210388 2.031363 0.1182887 0.2441416 0
0.04829593 0.08739761 0 1.102158 0.9027156 0.3274208
TCGA-K4-A54R 842 0 0.01898512 0.06090701 0 0.245853 0 36.05456 0
0.09658959 0.03464723 0 0.03752042 0.4841752 0.2035607 0.7390051
0.02051898 0.1043315 0.2696584 0.06233751 0.2067657 0.1018017 0
0.07803159 1.84477 927.55852 0.332141 0 0.08740552 0 0 0.2747031

0.5031944	0.04747954	0	2.293709	0.09102587	0.1214477	0.5482872
0.1542745	0.231231	0.05297652	0.04942375	0.08855559	0.2942175	0
4.036771	0.1806772	0.03575153	0.01178673	0.6118386	0.2474347
1.755011	0.05050825	0.5364672	0.1342688	0	0.5494061	0	0.07106711
1.121704	0	0.1278903	0.4239512	0.04078924	0.04108388	0.31898
0.1707457	0.4846643	0.4905859	0.02525413	0.05087094	0.05028184
0.3613544	0.4437511	0.4078924	0.3230508	0.1033715	0	0.0647656
0.166822	0.08587209	0.4219577	0.288696	0.3086473	0	0.07365499
0.07265208	0	0	0.3451397	0	0	0.07707415	0	0.02711848
0.3553693	0.04894709	0	0.2371165	1.396976	0	3.467381	0	0.04586184
3.648159	0.6059367	0	0.07769379	0.2099095	0.1288081	0.4273159
0.1874714	0	0.2395098	2.791494	0.0628993	0.3174634	0.02688729	0
0.111705	0.610682	0.6554579	0	0	0	0.2100905	1.511349	0.03064417
1.031604	0.06118386	1.097109	0.7210955	0.3832157	0.2806426	0
1.364586	0.07355437	0.1358117	0.04985352	0.2818942	0.130684	0.3476869
0.4681896	0	0.1244418	0	0	0	0.833142	0.1625004	0.04293604
0.6272959	0	0	0	0.3407709	0.05013203	0.7232481	0.3627138
0.03478152	0	0.2869012	1.048866	0.2052913	0.5920399	0.4078924
0.3030495	0	0.01550743	0.06606356	0.494439	0.03970634	0.04833196
0.07645268	0	0.3628154	0.05764647	0.05477294	0.2984579	0.4880789
0.3210329	0	0.2293424	0.621857	0.2958341	0	0.04110061	0
0.1437059	0.5227359	0.3715058	0	0.3469097	0.1495606	0.628993
0.2670101	0.09861135	0.5891779	0.4924555	0.171307	0.2548389
0.2024128	0.3454286	0	0.3805971	0.3553914	0.0539497	0
0.4191555	0	0.6437606	0.9316887	0.2126733	0.7941746	0.01496437
0.08439154	0.01584748	0.458879	0	0.07084447	0.1150635	0.3853524
0.1163728	0	0.1232078	0.2554165	2.119671	0.8061027	0	0.02935758
0.04868155	0.102621	0	0.2068929	0	0	0.06963502	0	0.8826525
0.03464723	0	2.29725	0.2555306	0.07912925	0.114146	0.9255906	0	0
0.3439698	0.09666391	0.03890303	0.6972222	0.03813159	1.261917
2.645592	0.3236732	0.5450801	0	0.08386573	0.1400914	0	0.090278
0.07306437	0.06611223
TCGA-DK-A1AA	578	0	0.1600942	0.1391015	0.03628734	0.1851057
0.06142143	71.78938	0	0.334528	0.5112919	0.2755019	0.02966205
0.1355638	0.1341054	0.4636716	0.07570004	0.01596389	0.02368673
0.06101507	0.4794834	0.05961491	0.0821085	0.04112565	1.168984
62.26306	0.05527928	0.05378449	0.04606607	0.2155192	0.06334085
0.3329919	0.0663007	0.07507063	0.1074875	0.3181244	0.09994611
0.05333966	0.2600716	0.1880261	0.05170143	0.004653444	0.1053254
0.1322379	0.1292199	0	3.406929	0.09522382	0	0	0.5240015
0.1825707	4.675706	0.3194375	0.7196989	0.1061473	0.2666983
0.3192564	0.5387981	0.01248503	0.1313736	0	0.03370154	0.2904701
0.0214975	0.002887038	0.09606556	0.1499826	0.4412089	0.1292788
0.01774653	0.1876766	0.111302	0.1538231	1.611131	4.944425	0.1135068

0.007782967 0.01670692 0.2730717 0.0329706 0.1131447 0.4002983
0.07607694 0.3434123 0 0.1164570.1705665 0.06362489 0.07753196
0.3274235 0.04960961 0.03006006 0.1044541 0.06257266 0.0122507
0.3145114 0.0257970.08016017 0.2994959 1.0890520.01175895 0 0
0.02417095 0.9613601 0.3193519 0.02598599 0.1310324 0.1106304
0.1131447 0.2702543 0.0592828 0 0.07573852 0.9832088 0.1326014
0.3346309 0.02479861 0 0.1766185 0.08046323 0.07676701 0.2481927
0.05457058 0.02368673 0.1596511 1.4769160.0888286 0.6064291 0
0.4399488 0.04222723 0.1009847 0.2355595 0.03941208 0.6957373
0.5039578 0.1590622 0.02627472 0.1980921 0.1721887 0.7067997
0.4670699 0 0 0 0 0.09075703 0.2195489 0.02854799 0.02262895
0.7134196 0.25797 0.1909970.07129824 0.07697116 0.02642151 0.296473
1.0327830.0549936 0.09066038 0.1764093 0.5067268 0.4824975
0.2629949 0.1343594 1.0115520 0.09807615 0.06963607 0.2125852
0.02092677 0.0764184 0.04533019 0.1201619 0.2294612 0 0.1732047
0.3145976 0.3278502 0.09668381 0.2832520.1285875 0.3941208
0.1247327 0 0.1083080.09651938 0.2777079 0.3443774 0.492991
0.05309018 0.3526099 0.8396487 0.1768019 0.2069479 0.103944
0.01194306 0.3028001 0.1760565 0.2051957 0.1066793 0.09498477
0.04995898 0.09360834 0.04682624 0.02843357 0.1226658 0.2758337
0.3067751 0.7711060.3876598 0.1401088 0.5039811 0.05520759
0.05930345 0.3716742 0.1881031 0.2723767 0 0.05855177 0.4912078
0.1431102 0 0.1623381 0.08076859 0.5160431 0.2780818 0
0.2166160.0513141 0.2704260.1649808 0.08287078 0.0484244 0.05585964
0 0.01246779 1.1629790.2921668 0.09105709 0.1135068 0.1663626
0.04766189 0.5761414 0.3217548 0.1606228 0.06929597 0.2441802
0.2547280.06151019 0.5494106 0.04019362 1.9675254.7238160.09513575
0.0319198 0.08420386 0.3978042 0.01845839 0.01901924 0.2283839
0.04900984 0.1184685
TCGA-CU-A5W6 56 1 0.07549096 0.03229146 0 0.1117247 0.139021
28.4724770 0.2304428 0.5786280 0.01491933 0.4361865 0.3237692
0.5317327 0.05711305 0.1070596 0 0.6940474 0.2630935 0.1349322
0.1486753 0.06205576 1.97886234.575530.13207 0.3570911 0 0.2168024
0.09557695 1.3981540 0.1510352 0.03243824 0.1280074 0.1146169
0.06438868 0.7412567 0.1380252 0.1281657 0.06319552 0.04272277
0.04695008 0.3899679 0.1746267 3.1018450.09579077 0.05686384
0.02812071 0.3649302 0.3148417 5.63401 0.08033485 0.6205576
0.2002111 7.4851840.7170022 0.4258618 0.04171504 2.2796870.1070462
0 0.4045840 0.04574158 0.1248235 0 0.2452777 0.1463046
0.02677828 0.3074645 0.1199621 0.09947503 0.2744774 1.621912
0.08563695 0 0.03781437 0.1201802 0.0331669 0.1365821 0.3355680
0.5454583 0.06928555 0.1464380.01575753 0.2880166 0 0.1646864 0
0.3628684 0.01751267 0.0472089 0.01848545 0.1493048 0.03892588 0
0.2796043 1.5507230.01064606 0.1549149 0 0.03647229 0.7003021

0.6883999 0.1960553 0.1482891 0.08346681 0 0.7204378 0.08945363
0 0.03809473 4.006787 0 0.8583892 0.04276502 0.05085329 0.1332525
0.1214134 0.9573513 0.4369232 0 0 0.1243368 2.178484 0.04874044
0.1893227 0.1459721 1.10484 0.03185898 0.3047578 0.1818545 0
1.309325 0.1754855 0.2240129 0 0.5230878 0.05196417 0.6715074
1.741994 0 0 0.1499771 0.04604137 0.2282435 1.076673 0.08615387
0.1024365 0.3465807 0.4448673 0.4528877 0 0.1935736 0.1993411
0.383449 0.7454835 0 0.1026002 0.2661894 0.7646156 0.3618214
0.1008918 0.08109559 0.4820091 0 0.02466502 0.07880701 0.9416339
0.2210393 0.2306201 0.09120016 0.4463163 0.1154137 0 0.1851256
0.4272353 0.2892112 0.1458892 0.1165657 0.1901491 0.6635611
0.7999055 0.1537468 0.09805757 0.1456411 0.07618945 0.1039283
1.00768 0 0.2561789 0.3154501 0.06669544 0.08743562 0.8626432
0.4685523 0.2284522 0.1021758 0.2026643 0.1073145 0.1592505 0
0.564994 0.2826302 0.2145214 0 0.4088474 0.0385752 0.4188764
0.7799358 0.1832259 0.6186896 0.2142114 0.1789696 0.6900107
0.08109559 0.2241805 0 0.02524302 0.4133608 0.2159433 0 0 0
0.9583671 0.9254646 0 0.04669408 0 0.05440721 0 0.07897659
0.09742542 0 0.02215131 0.009406519 0.6580708 0 0.08743562
0.2854565 0.3347061 0.01348472 0.2199579 0.09396885 0 0.0261407
0.3740336 0.03843669 0.06187641 0.737507 0.0303247 0.5435939
2.881767 0.08910202 0.07224714 0 0.1333909 0.009284144 0 0.6030771
0.06338782 0.1472148
TCGA-DK-AA6R 5041 0 0.03153198 0.1011592 0 0.05833314 0.09677999
24.94762 0.05492918 0.1260471 0.402814 0.1085251 0.01557922 0.5999761
0.0422612 0.2922376 0.04259942 0.07546153 0.0373225 1.220235
0.1373651 0.05636011 0.1552512 0.129601 1.34715 22.40543 0.3266325
0.1694934 0.217755 0.2263917 0.4990218 0.5703106 0.05223406 0
0.033873 1.203023 0.1196865 0.03361831 0.04553193 0.1121012 0.06982668
0.1759752 0.1070698 0.03677003 0 0.1458804 1.562894 0 0.1187579
0.04894084 0.6986305 0.2465755 1.81176 0.08388809 0.5265042
0.2090665 0 0.1871789 0.1212812 0.05480144 0.05175041 0 0
0.1760329 0 0.02956865 0.02943262 0.1417939 0.1829474 0.2037009
0.03495337 0.1182867 0.05010723 0.1154165 0.5322899 0.5322899
0.2086577 0.0122634 0 0.01792797 0.1039017 0.07131157 0.04672137
0.2397445 0.1708752 0.3617504 0.3975791 0.03290899 0.2005038
0.08144327 0.8025294 0.07816845 0 0.07314905 0.2957818 0.006434358
0.1113633 0.08129519 0.06315304 0.217281 0.7975717 0.09634686 0
0.09720077 0.1142564 0.1044681 0.1078272 0.08189076 0.09032799
0.20337 0.03565578 0.4400263 0.1245469 0.04638211 0.07955935
1.503982 0 0.1581805 0.0167462 0 0 0.1267836 0.2810531
0.4562485 0 0.0373225 0.1136067 1.098198 0.06362031 0.8731595
0.101619 1.084386 0.2994131 0.1591187 0.08631729 0 0.4064759
0.06108245 0.04594879 0.04140033 0.03901602 0.05426257 0.5774658

0.2221732	0.04426966	0	0	0	0.09533551	0.3891791	0.04498225
0.2495905	0.5593157	0	0.1289779	0.0561713	0.08085417	0.08326323
0.4004091	0.3872724	0	0.142851	0.07941804	0.362925	0.3317487
0.2317794	0.04234124	0.08388809	0.05561238	0.1931697	0.05486178
0.2377167	0.461632	0.1605471	0.1031701	0	0.1807777	0.0957438
0.06822841	0.2974214	0.2145812	0.6093677	0.08114765	0.03647011
0.6013942	0.2948067	0	0.06826314	0.2281243	0.3182374	0.2713128
0.4352238	0.04182634	0.4409502	0.2592021	0.295993	0.09130294
0.1637815	0.11291	0.2385568	0.07824306	0.1645998	0.05603052
0.064439	0	0.08428342	0	0	0.1932811	0.1750032	0.604221	0.1944015
0.5701029	0.1471769	0.8075681	0.06213501	0	0.2204362	0.169365
0.2731121	0	3.456393	0.275359	0.06442702	0.09914047	0.0511583
0.08484318	0.5450547	0.9517928	0.06108245	0.1462781	0	0.02840683
0.2599555	0.04810736	0.1017346	0.08801644	0	0.2848546	0.6413657
0.02877243	0.03912983	9.657868	0.07489507	0.09856807	0.3901029
0.2126045	0.1124839	0.02729692	0.1340788	0.2408206	0.03230661
0.7701273	0.09499792	0.3056509	1.890755	0.01550717	0.2766231	0
0.06964541	0.02908436	0	0.05997633	0.02757979	0.1317653
TCGA-BT-A3PH	142	1	0.01052781	0.1013242	0.03818013	0.2434513	0
39.26118	0	0.1071236	0.2497679	0.1449362	0.02080618	0.241221
0.1975407	0.3512572	0.03413514	0.03359317	0.02492226	0.004938284
0.2751785	0	0.05183484	0.08654164	2.224396	56.47396	0.1114786
0.01131799	0	0.1133805	0	0.4569928	0.06975903	0.07898642
0.06785651	0.7586946	0.06730203	0.08979508	0.3952538	0.3315053
0.08548296	0.01958469	0.2430875	0	0.2175364	0.02435307	2.465385
0.1001908	0.09912638	0	0.3816929	0.1372099	3.374969	0.02800832
1.46039	0.111684	0	0.2421671	0	0.01032136	0.5874615	0.1119632
0.03545946	0.1645654	0	0.01366933	0.07580735	0.1262447	0.4397902
0.3060499	0.004668053	0.197466	0.02788277	0.2157956	1.011634	4.071391
0.01990458	0.0655115	0.004394593	0.02394296	0.1040712	0.1428558
0.3275832	0.04002261	0.4564107	0.04831208	0.1021097	0.1904511
0.06694364	0.05438409	0.3445023	0	0.03162804	0.03663419	0.03291826
0.04081742	0.08179963	0.0271426	0	0.2561751	1.178136	0.00989785	0
0.2596249	0.07629523	0.2441566	0.7680223	0	0.0258501	0.2425022
0.0476186	0.4739185	0.04158338	0.03097185	0.05312609	1.72164	0
0.3520857	0.007454898	0.03545946	0.3097185	0.1128804	0.09740066
0.3046619	0	0	0.04876802	3.42219	0.05097928	0.01100106	0.03392826
0.6678944	0.02221493	0.05312609	0.2997214	0	0.773153	0.5302449
0.07252228	0	0.1302656	0	0.02754323	0.4450713	0.05912251
0.02300221	0	0	0.09549104	0.7507529	0.09011126	0.07142791
0.300253	0	0.02870852	0.03750862	0.08098607	0	0.5347498	1.094491
0.01928738	0.1669314	0.2651587	0.5331583	0.7845734	0.05159056
0.08482064	0.9522828	0.0371354	0.04299664	0.03663419	0.1731669
0.02201834	0.05360299	0.04239526	0.02917609	0.04023836	0	0.02277991

0.1324029 0.4988527 0.07629523 0.1354667 0.05952973 0.4670591
0.09842923 0 0.0227915 0.05077698 0.1859413 0.07246812 0.9380853
0 0.3062243 0.1514479 0.3255421 0.1132262 0.3280974 0.1633583
0.6371892 0.0237487 0.1884209 0.03741462 0.06940229 0 0.7738584
0.1970750 0 0.2106033 0.3496750.06490623 0.2447284 0.06879462
0.5122942 0.0497891 0.04679759 0.5096978 0.05654709 0.07815933 0
0.04620443 0.2832625 0.08604284 0 0.06832235 0.1133088 0.6503594
1.2223630.04078807 0 0 0.01896878 0 0.09637168 0.1698343 0
0.01544587 0.2033310.3670926 0.1152775 0 0 0.1000230.07835625
0.2312787 0.1419675 0.0187779 0 0.3425559 0.08040448 0.08629152
1.0097430.08458035 0.2624139 1.6626660.04487159 0.1679239 0.1328941
0.2325301 0.01294747 0 0.1201484 0.003683304 0.1613092
TCGA-BL-A13I 223 1 0.737283923333333 1.3333784 1.50084733333333
2.0735654 0.0437340833333333 20.8166933333333 0 0.946930066666667
3.2607808 2.61545613333333 1.66086545 1.53764356666667
3.34213993333333 0.7323771 0.71933466 0.07118295 1.52909299333333
3.17108106666667 0.304243766666667 1.19303093333333
3.72857056666667 2.98196766666667 3.92793766666667
20.6465633333333 0.207203283333333 1.88623186666667
7.56412783333333 0.530857063333333 0.100760866666667
0.383387433333333 2.81545066666667 2.14229147666667 4.59958
2.94435056666667 1.32781263333333 0.936816533333333
1.35165603333333 1.02603183333333 0.1089786 2.43328923333333
1.09404902666667 3.91237391 0.0444344733333333 8.35742033333333
0.142340643333333 0.833890883333333 2.25047377666667
1.47668866666667 0.4158158 3.56736963333333 2.146296
1.83647233333333 8.2406059 4.04886783333333 10.2520058 1.0637779
12.5186831333333 3.1507242 0.3194176 7.5190242 1.13017383666667
0.298714866666667 0.0170988133333333 0.0566307756666667
0.230547233333333 1.98799416666667 2.23432166666667
2.09453333333333 7.93943611 2.52033755 1.50539396666667
0.898931733333333 7.17851796666667 1.05985753333333 1.4731587
0.00619046333333333 0.0166105633333333 0.874641033333333
0.474911166666667 1.81075866666667 0.519635533333333 0.75348
3.6996319 7.00123173333333 1.20416833333333 1.16933373333333
5.7773028 0.747273366666667 1.5551161 4.4460744 9.8443551.7131158
7.1208220.76551848 3.09838233333333 0.29057661 0 0.946360633333333
5.4457514 2.98651033666667 3.90980833333333 10.9643719166667
0.595402033333333 0.888902533333333 1.1668221.42615366666667
3.30896166666667 0.6665374 0.748402663333333 2.4757689
1.72826993333333 1.60640356666667 0.823298666666667
1.06746293333333 0.85906928 0.647390033333333 6.72403631666667
2.99661925333333 0.394941166666667 3.01537496666667 4.982767
1.67895956666667 3.03832766666667 1.8036841.11632947333333

1.88440933333333	1.62390257	4.56438633333333	0.205185766666667
8.82968966666667	2.28491613333333	1.57841156666667
0.0947367966666667	6.10550106666667	0.6713958	2.20914413333333
0.366945746666667	1.7154284	2.56730093333333	1.46764835666667
0.298231033333333	0.5542824	3.4371458	1.600885 0.7107872
0.205922633333333	1.99788922	1.4439258	2.56585433333333
0.670045966666667	1.48506446666667	2.22684626666667
2.19102096666667	2.7358119	2.56605936666667	18.3778066666667
4.153581 3.64613123333333	0.02304893	0.45360139	1.80210853333333
6.94584913333333	0.384285963333333	1.9050769	2.2013373	1.7232503
2.21774166666667	2.00057806	0.108118983333333	0.403686266666667
2.91643623	1.80320333333333	0.6640998	1.79311996666667
4.33344733333333	8.202316 0.8519528	6.25730856666667
0.158691033333333	1.67570339333333	2.86738733333333
0.660302446666667	0.820130233333333	4.63629766666667	0.68037145
5.99137132	7.015902 1.43340336666667	2.5260548	5.0549785
8.80190415333333	1.3753145	0.836342233333333	0.02636733
1.10226973333333	5.43455503333333	0.136685966666667
0.779699733333333	1.00185853666667	1.0941171	0.291980866666667
0.477788213333333	6.203201 0.4555786	1.37449063333333
3.11865749666667	5.04717396666667	1.45137223333333
1.23621296666667	0.682220166666667	4.1966074	1.1716142	12.153848
0.779885996666667	1.23974003333333	26.6008	19.8620072
1.17155346666667	2.95734509333333	0.09115971	2.51603926666667
0.929512163333333	8.23454413333333	5.28145143333333
5.72794273333333	2.3557897	2.33359366666667	0	1.86066413333333
0.8584463	1.04489673333333	2.09957533333333	0.380715536666667
0.407915266666667	0.0258057266666667	18.5083366666667
1.86608963333333	8.12186536666667	0.745336816666667
1.12537418666667	7.65934686666667	0.4797095	2.61025603333333
6.27223474666667	1.19229126666667	0.0425857333333333
2.87045867666667	0.846998666666667	9.828697 2.39729333333333
7.77820566666667	3.16400936666667	0.3832785	13.0249423333333
0.285771533333333	2.1543753	2.645496 0.878911 0.158828533333333
3.3368664	0.367971933333333	2.666318 0.686248453333333
TCGA-CF-A3MF	383 0	0	0.2445137	0	0.3806948	0	13.74186 0
0.04431575	1.168379 0	0.03012544	0.07289029	0.2860209	1.66704
0.115324 0.01621328	0.1443406	0.007150184	0	0.1180652	0.200139
0.06265219	2.187335 34.03872 0.02807144	0	0	0.1641646	0	2.514399 0
0	0.03275001	0.03230943	0.0609045	0.1300151	1.496762 0.1780606
0.1575274	0.007089212	0.04658407	0	0.3149728	0	22.3815	0.1450672
0.2296415	0.03785465	1.719376 0.1192004	3.315939 0.1622139	0.5482067
0.06737845	0	3.823062 0	0.02445444	1.701181 0.1621126	0	0.714827 0
0.006597316	0.1260232	0.3198838	0.1061293	0.04923692	0.05407131

0.2450681	0.0403717	0.04463605	0.2771155	12.95965 0.02882001	0
0.006362969 0.08666803	0.03348568	0.03447369	0.2032759	0	0.1376752
0	0.1182764	0.1113629	0	0.1574864	0	0	0	0	0.09532528
0.01244208	0.2907124	0	0	0.07877907	0.2804109	0.003582796
0.03128076	0	0.03682284	3.131146 0.7297671	0.03958792	0.04366668
0.5758384	0.1378948	0.1784096	0	0	0	4.220229 0.2020094
0.7646818	0.01619102	0.7701309	0.1793776	0.4494614	0.01719845
0.4411226	0.08313464	0	0.1608376	0.2528071	0.07381334	0.07964262
0.04912502	0.4978872	0.1286608	0	0.3087858	0	0.8931821
1.240205 0.05250282	0.08005558	0.07544505	0.7344905	0.1595203
2.054097 0	0	0	0	0.1843497	1.045213 0.1304729	0	0.1696542
0.05614287	0	0	0.2345209	0.08050282	0.1290448	0.4992442
0.02792637	0.03452876	0	0.4912502	1.051348 0.115443 0.08187503
1.824907 0.05376867	0.03735312	0.3182578	0.8357663	0.3188054
0.07761223	0.007673059 0.04224431	0.01942049	0	0.05497204	0.3834148
0.1536794	0.1472914	0.03922869	0.01958946	2.433268 0.04750551	0	0
0.07352043	0.4615304	0.5770997	0.2077342	0.04043968	0.1108462
0.2192827	0.08417059	0.2396063	0.03958792	0.0363889	0.09884912
0.00687719	0.1364081	0	0.07034172	0	0.3870736	0	0	0.2491643
0.1245768	0	2.631392 0.1574864	0.09960833	0.208212 0.0961201
0.4065519	0.2417572	0.04093751	0.03772252	0	0.02867134	0.2086668
0.03114554	0.91061 0	0	0.2246164	0.5436701	0.0590574	0	0
0.02746507	0.0837791	0.03986783	0	0	0	0.009496927 1.816015 0
0.1261086	0.05764002	0.1568928	0.04991918	0.148048 0.15812 0.08156606
0	0.3945373	0	0.06247112	0.3097952	0	0.6332522	4.389945
0.004997689 0.02431384	0.3206974	0	0.05624026	0.02897454	0.4639036
0.03733162	0.01061641
TCGA-CF-A47T	385 1	0.0466363	0.016624 0.2818852	0.1150342	0.04771304
91.1613 0	0.05649263	0.7659877	0.1070069	0.1075289	0.1765456
0.4583698	1.181412 0.09240764	0.06889431	0.07360074	0.2041733
0.1354435	0.1018814	0.05102644	0.06389398	1.029279 123.1906 0.04294173
0	0	0.1116123	0	0.7872651	0.05150332	0.03887729	0.06679825
0.4942471	0.09316747	0.06629601	0.3142647	0.0947425	0.1262247
0.02168917	0.04574784	0.01208521	0.1204559	0	13.71293 0.09862829
0.1463707	0.01930247	0.876727 0.1215631 0.07906 0.04135727	0.3594036
0.123685 0	1.061222 3.786845 0.04295073	3.826983 0	0.05235967	0.694281
0.1001974	0.006728077 0.1368127	0.09320686	0.1803881	0.1004256
0.006892878 0.2082712	0.065875 0.1479424	0.1211177	4.055608 0.1175649
0.04836736	0.03893451	0.2298031	0	0.03515697	0.391576 0	0.140404 0
0	0.05408102	0.1976989	0.04015195	0.0565216	0	0	0.04507857
0.04860733	0.01268869	0.3952992	0.04007895	0.1245391	0.1472909
0.8817369	0	0	0	0.03755268	1.339086 1.488463 0	0.06997912
0.544282 0.03515697	0.5458371	0.03070115	0	0	3.38958 0	0.4159136
0.03302385	0	0.8689316	0.1666799	0.1262831	0.2570662	0

0.03680037 0.1280199 1.508234 0.05018423 0.2274192 0 0.2245842 0
0.1176695 0.2212853 0.06123173 1.943222 0.4215955 0.1276805 0
0.1538808 0.2140138 0.3253642 2.149576 0 0 0 0.04740521
0.0940018 1.577576 0.04435296 0 0.1081355 0 0 0 0.1195846
0.0410492 0.1974038 0.4859967 0.02847987 0.1760657 0.391535
0.7156955 0.3452803 0.1523579 0.1669956 0.2067863 0 0.01269782
0 1.885783 0.1300497 0.07915053 0.007825141 0 0.01980541 0
0.02242464 0.2443838 0.3447958 0.112658 0.04000621 0.1158708
0.7476716 0.04844708 0 0.06730816 0 0.235339 0.1605104 0.6572829
0 0.3217383 0.2662249 0.1888455 0.1414689 0.08074514 0.1855507
0.1680139 0.04208098 0.2376493 0.05524667 0.1250255 0 0.3116412
0.07275057 0 0.1905771 0.1687625 0.03971788 0.8146482 0.2810637
0.1306062 0.4379488 0.159291 0.09213552 0.5547351 0 0.0384702 0
0.09421654 0.2641688 0.03176286 0.09775354 0.05044261 0.04182813
0.2378787 0.5352443 0.06022793 0.09615452 0 0.1680566 0
0.09486871 0.02507784 0 0 0.09685159 0.4065385 0.02836991
0.01286081 0 0.07384731 0.04165249 0.1150342 0.4998874 0.3604585
0 0.2873979 0.03957527 0.06370931 0.5043881 0.06244596 1.291607
2.560135 0.1223219 0.099183 0 0 0.00955916 0 1.123608 0.005438792
0.05413414
TCGA-YC-A8S6 293 0 0.04396544 0.1175396 0.0797225 0.1830029
0.1349416 44.58233 0 0.1118403 0.3209421 0 0.0434446 2.146134
0.2946266 1.263159 0.1306732 0.01169077 0.05203923 0.01031145
2.058947 0.01309727 0.2164687 0 1.43066 42.7095 0.0607237 0.1417961
0 0.2367456 0 0.5725377 0.1456612 0.05497619 0.1889182
0.279565 0.1053981 0.04687444 0.3174287 0.1339749 0.3326474
0.06134109 0.07713254 0.08544819 0.05677869 0.2034029 14.93832 0
0.04139641 0.2593076 0.7969986 0.2865028 4.474378 0.1169662
0.9035217 1.243751 0 1.223371 0.05636799 0.07053262 1.515281 0.07792875
0 0.2945339 0.09445909 0.1046556 0.1582905 0.06590169 0.1530516
3.905313 0 0.08246428 0.2911048 0.08046334 0.5138159 3.036185 0.20781
0.2564852 0.1101142 0.124986 0.1931619 0.04971531 0.2605768
0.4178485 0.5559248 0 0.04264227 0.1988368 0 0.05677869 0
0.5449563 0.06604131 0.0637454 0 0.4710041 0.5667545 0.2267018
0 0.5822467 0.8761719 0.0155005 0.5864392 0.3388206 0.3186184
0.7283061 0.5011495 0.05709066 0.09895714 0.4050877 0 0.4552028
0.04341434 0.06467116 0 1.2929 0 0.3675884 0.03113258 0
0.1293423 0.176776 0.501006 0.181758 0.1198904 0.05203923 0.1640605
1.075508 0.05322396 0.8269521 0 2.430203 0.04638616 0.3882571
0.2647772 0 0.8243702 0.3406721 0.1630796 0.2309 0 0.07565898
0 0.8712531 0 0 0.4367285 0.3351774 0.1993913 0.9646886
0.06271931 0.04971531 0.2370166 0 0.1798356 0 0.3945759 0
2.698428 1.22177 0.04027326 0.04979473 0.05536678 0.5060308 0.263403
0.2056555 0.1771108 0.9942129 0.07754104 0.0538678 0.3442251

0.6327709 0.04597566 0.05596319 0.06639297 0.1015358 0.6721617
0.06674839 0.1585529 0.3455820.2770307 0.05310307 0.1131452
0.1130016 0.8658751.0276320.05596319 0.04759008 0.2120510.4437224
0.6052718 0.3763932 0.02915949 0.2828183 0.4818783 0.9953516
0.1273048 0.1141813 0.8396363 0.09503506 0.05950652 0.3770415
0.07812406 0.04637322 0 0.4113110 0 0 1.2254090.6178135
0.4065848 1.0787950.1231264 0.8069684 0.05198138 0.3257216.119614
0.3542216 0 1.0663930.2894332 0.2594166 0.1796628 0.2764656 0
0.1182979 0.1370450.5328752 0.08516803 0.2379504 0 0.19804
0.1208198 0.03832954 0 0 0 0 0.5748842 0.2005888 0.03637281
0.0831240.08702261 0.1047122 0.1575859 0.3192399 0 0.03806044
0.4633052 0.7834847 0.09009104 0.5643319 0.5298272 1.278519
11.807670.0864874 1.3324150 0.09710747 0.1216581 0.04178485
0.3554095 0.01538194 0.03062033
TCGA-DK-A3IS 1529 0 0.1178927 0.10315 0.07773626 0.1189623
0.01644744 90.317370 0.04673738 0.09779562 0.07377398 0 0.07260263
0.05745724 0.5165143 0.1042507 0.03039868 0.07611405 0.2211999
0.09337904 0.02554191 0.03517926 0 2.53088874.819450.1134874
0.1152194 0.04934233 0.1538981 0 1.2561150 0.1608195 0.02302642
0.5451996 0.1370296 0.05713323 0.1238081 0.1306370.05142288
0.0149532 0.05337542 0.01666386 0.1384102 0.09916763 6.836538
0.06799749 0.1210951 0 0.4317454 0.1117459 5.76721 0.05702604
0.4405055 0.09474713 0.3427994 0.2624362 0.9343814 0.008596917
1.0553780 0.03609843 0.1914638 0 0.001546184 0.1028978 0.1285196
0.8705571 0.1038549 0.00475217 0.1608195 0.06244745 0.09415037
0.4731803 2.0723780.1013163 0.0083365 0.03131648 0.1218720 0
0.4287679 0 0.4259155 1.0820180.02078993 0.1640548 0.1362999
0.05536408 0.1169034 0 0 0.006215721 0.05026716 0.01093498
0.1779028 0.08289512 0 0.4638854 0.6407569 0.01259526 0 0
0.05178003 1.1717650.5130968 0 0.1008777 0.3258710.04847668
0.2991242 0 0 0.08112512 2.6520570.07101607 0.1075290.01517847
0.07219686 0.4414194 0.1723717 0.03143964 0.4430740 0.0507427
0.03861418 2.2929470.06919722 0.3471780.03453963 0.3837227 0.04523047
0.02704171 0.09388379 0 1.5239310.2491383 0.07666869 0 0.1060903
0.07377398 0.4486329 1.1704860.1805636 0.0234167 0.02661548
0.06536533 0.09721176 0.7054904 0.09173504 0 0.3280291 0.2368432
0.02922584 0 0.1099272 0 0.2268274 0.4573857 0.01963493
0.04855412 0.2969304 0.2960540.2787659 0.07639303 0.3741794
0.7698515 0 0.02626286 0.1118830.4260278 0.1344906 0.0545689
0.03236941 0.1782110.09558138 0.0325427 0.04638079 0.5054581
0.4754265 0.1294501 0.08274468 0.1432421 1.2220160.1002029
0.02728445 0.09280879 0.1550759 0.08112512 0.1106610.4906024 0
0.3417176 0.4001258 0.1183601 0.1329998 0 0.06396229 0.3011688
0.06769461 0.1398660.03808882 0.05652232 0 0.2291791 0.05015656

0.06091158	0.04379665	0.2627686	0.1642966	0.363417	0.5813228
0.09004408	0.4391795	0.059134	0.1270423	0.2952262	0.08634909
0.07956774	0	0.02015873	0.2175399	0.04379665	0.0336972	0.03477675
0	0.4069658	1.027614	0.08304612	0.06629199	0	0.1158636	0.1767144
0.03270276	0.1383157	0.05983244	0.0157242	0.3672492	0.7785573
0.03911825	0.0531999	0.08105301	0.1187963	0.01914437	0.1759651
0.1667605	0.01911628	0	0.2100298	0	0	0.2522079	0.04305224
0.9201574	3.168019	0.1264989	0.03418996	0.04509626	0.1893762	0
0.0407438	0.4077113	0.003749677	0.05225053
TCGA-XF-A9SJ	98	1	0.0630754	0.01686292	0	0.07292964	0.07259814
40.97215	0	0.2177572	0.1438882	0.1085448	0.01558204	0.7603169
0.1690755	0.9353298	0.02556429	0.1146105	0.03732926	0	0.1717375
0.02818516	0.1035196	0.1296245	1.771001	39.49835	0.116157	0.3220958
0.2177944	0.05660817	0	1.026745	0.7314093	0	0	0.2673869
0.07560517	0.05043661	0.1821607	0.05606074	0.3317369	0.06600264
0.04105087	0	0.4072901	0.1459069	1.472558	0.05002288	0	0.2153787
0.6352338	0.08220672	3.236349	0.04195165	0.08101532	0.08364175	0
0.5616384	0.04043441	0.04918969	0.9316762	0.05590057	0.05311218
0.2464907	0.2032748	0.02047432	0.09251928	0.1418196	0.07319224
0.662148	0.01398388	0.05070347	0.008352719	0.0808062	0.0409528
1.887552	0	0.06132811	0.01974704	0.07172487	0.01732008	0	0.2336492
0.05994699	0.4272655	0	0	0.1097165	0.2005402	0	0.1146678	0
0	0.02743586	0	0.04504867	0.09467594	0	0.06316449	0.2988155
1.692125	0	0.09707769	0	0	2.507689	0.6830295	0.0409528	0.02581267
0.5666335	0.03566225	0.2697424	0	0	0	2.793615	0	0.6328367
0.03349847	0.05311218	0.04639052	0.295882	0.5301836	0.2607607	0	0
0.04869741	0.8761025	0	1.021617	0.2540935	1.327252	0.1330966	0
0.146766	0.06211175	0.8007795	0.2443741	0.03760128	0	0.1170693	0
0	0.6388637	0	0.06890671	0.1566391	0	0	0.994749	0	0
0.3235844	0.05807852	0.08600088	0.05618148	0.08086882	0	0.4672287
0.08607612	0.4911161	0.03571921	0.1985811	0.2903926	0.1106029
0.2669459	0.08469784	0.0839033	0.05562246	0	0	0.3458324
0.09893906	0.08028807	0.1508145	0.0582677	0.4821613	0	0.07961424
0.09915844	0.2464156	0	0.04058118	0.04458266	0.8826406	0.1965734
0.08028807	0.06827551	0.6844968	0	0.271362	0.551016	0.02091696
0.1822925	0.1944368	0.1567306	0.01304564	0.1228584	0.6964044	0
0.007114295	0.09407406	0	0.07484595	0	1.285555	0.2951845	0	0
0.4327786	0.1611548	0.6319195	0.3258321	0.07360181	0.3903953	0
0.07009476	0.006581377	0	0.07804617	0	0.1548903	0.1042088	0
0.04957922	0	0.04242928	0.05362165	0.3165102	0.06109352	0	0
0.1136479	0.08666755	0	0.1271913	0.0880324	0	0	0.5040215	0
0	0.5366455	0.07490864	0.08450223	0.105748	0.06542861	0.02812607
0.02730187	0.09911966	0.04014404	0.09693741	0.8489803	0.03167171
1.13548	2.903234	0	0.9054761	0	0	0.04848272	0.0299735	0.3599232

0.03310174	0.06589458
TCGA-DK-AA6T	572	0	0.06787535	0.108877	0.1230783	0.2668303	0.05208185
69.71811	0.05911993	0.1973291	0.1238703	0.2336098	0.1676781
1.132599	0.7277675	0.9436005	0.1650582	0.1082917	0.1205099	0
0.960994	0.01011001	0.08354796	0.2092332	2.143514	92.09889	0.06249821
0.0547274	2.81242	0	0	1.718701	0.05621919	0	0.03645729	0.6114362
0.08813851	0.05427477	0.6860802	0.06032689	0.1565709	0.02367512
0.09219085	0.1978767	0.6135981	0.03925255	4.339415	0.2153182	0
0.2212338	0.5468594	0.3980815	5.360661	0.1805765	0.6974438
0.06000452	0	3.689226	0.04351141	0.07108141	0.8911782	0.06015453
0.05715395	0.6441745	0.1458292	0.1224021	0.2670015	0.1017413
0.1575241	0.1644315	0.04514411	0.1909668	0.08089515	0.1614887
0.528831	2.135356	0.09624725	0.1583882	0.1487483	0.03859152	0.1863812
0	0.1005718	0.06450888	0.122608	0	0.0329163	0.5844258	0
0.1753138	0.1233939	0.08413221	0	0	0	0.04501418	0.2037613
0.04374875	0.2039137	0.2777066	1.872919	0	0.03482173	0	0.2049558
3.373151	1.083168	0.04406925	0.03472123	0.6879271	0.0383761	0.5347069
0	0.04992077	0	2.616737	0	1.021492	0.01802383	0	0.5990493
0.4548547	0.2756923	0.3507553	0	0.1205099	0.1397419	1.083485
0.01369483	1.560383	0.9296609	0.9113112	0.03580627	0.3853315
0.1858055	0	0.6098311	0.3944559	0.1348756	0	0.04199269	0
0.04439449	1.360015	0	0.03707521	0.08427955	0.1034917	0.102609
1.256613	0.04841411	0.0383761	0.2655829	0	0.5552726	0.06045682
0.3916027	0.08961569	5.530626	0.5978613	0.03108761	0.0384374
0.5128628	0.6249821	0.09918307	0.14363	0.4557161	0.8577382	0
0.1663259	0.05904739	1.639785	0.1419576	0.1295968	0.264791	0.09405269
0.4107587	0.1030484	0.1835845	0.6402256	0.7270722	0.245947
0.1310081	0.05669813	0.7246686	0.5817141	0.08639789	0.2204135
0.2455287	0.2140731	0.3504147	0.4269223	0.06752613	0.6169696
0.2789775	0.2810959	0.07019199	0.4847618	0.0405081	0.183398
0.2220148	0.5694355	0.06030529	0.08725343	0	0.5442823	0.3176477
0	0.1386848	0.4429443	0.08670923	0.8892409	0.7889119	0.2376084
0.2028088	0.05350042	0.1005718	1.63245	0	0.1679708	0.3166028
0.04610222	0.3524367	0.0346712	0	0.1101227	0.09131618	0.1442555
1.199947	0.06574266	0.1049588	2.915265	0	0.09326284	0.05917445
0.2463667	0.3789262	0.01244791	0.01057197	0.6902979	0.03096758
0.02807679	0	0.1343485	0.07577728	0.1098713	0.4752516	0	0
0.3701817	0	0.1043142	3.279231	0.1363275	0.5639487	7.982388
0.05563425	0	0.07140004	0.1499178	0.2504265	0.03225444	1.323319
0.005936791	0.3072727
TCGA-BT-A20N	795	1	0.093216835	0.119621242	0	0.206937726	0
52.707583054	0	0.149051378	0.170117725	0.256663053	0	0.234015252
0.29984455	0.691144953	0.010074807	0.185077196	0.088268095	0.09619558
0.081217494	0.088861494	0.091792689	0.459761643	2.182310841	76.331068861

0.24032995 0.200426796 0.085832125 0 0 0.377661349 0 0.186499432 0
0.316129082 0.104285185 0.099384566 0.161525221 0.293526192 0.027523472
0.104045764 0.137161489 0.17392299 0.385228334 0 0.589258843 0.295707992
0.105323882 0 0.600824874 0.437365166 2.102887059 0.049599052 2.346700051
0.032962961 0 0.179838738 0.430247107 0.02658584 0.795536639 0
0.062794049 0.249791759 0.040054992 0.01344811 0.079552513 0.055890686
0.129801773 0.06021935 0.008266509 0.119892492 0.039501374 0.204720917
1.162034921 2.803849412 0.035248393 0.014501533 0.007782247 0.31799873 0
0.168652596 0.331489586 0 0.303091274 1.083688446 0.180822807 0.084315954
0.118548406 0.192614167 0.474497593 0 0 0.054061952 0 0.003804331
0.829632155 0 0 0.658398222 1.114611334 0.048201432 0 0 0
1.111806776 0.170008324 0 0.267033277 0.154598213 0.042163149 0.654613005
0.073638703 0 0.04703967 1.056382024 0.061767043 0.062349751 0.013201645
0 0.329082504 0.199896366 0.185104544 0.077073745 0 0.088268095
0.139138392 1.700580345 0.090277613 0.545479915 0.060082487 0.40986503
0.078679448 0 0.489939048 0 0.932188895 0 0.069153237 0.048956101
0.138409919 0.19249729 0.731631765 0.426921525 0 0 0 0 0
0.255670159 0.212766957 0.210815745 1.076385793 0.2746628 0.711746389
0.06642285 0.191220936 0.049229599 0.236742935 0.828013422 0 0.168922009
0.281736708 0.429160624 0.435882843 0.107971042 0.350481176 1.041580082
0.131523853 0.045684841 0 0.332210563 0.077983169 0.047461929 0.04692278
0 0.118761431 0.056608768 0.080680451 0.175851183 0.507486634 0.225181384
0.143936267 0.091043972 0.525633925 0.232406984 0 0.040360755 0.179838738
0.376317359 0.19249729 0.247555246 0 1.136708519 0.427833751 0.205890144
0.123389939 0.58101746 0.111263866 0.161196917 0.218690314 0.25720297 0
0.056535079 0 0.099665578 0 0 0 0.241053524 0.238164815 0.689642464
0.963070836 0.243652483 0.175074798 0.029389988 0 0.105044879 0.150206218
0.092273279 0 0.019481426 0.308012752 0.152370573 0 0.060494953 0
1.024908298 0.869311947 0 0 0 0.235138578 0 0.081267428 0.150377102
0 0.013676303 0 1.137627425 0.06804709 0.092542454 0 0.191888234
0.016651036 0.258672157 0.328761526 0.066506401 0.032278748 0.144761977
0.094923858 0.152810951 0.452016098 0.037445176 1.394101465 1.306856724
0.134473637 0.118948456 0 0.164712115 0 0.035437392 0.070922319 0
0.168797558
TCGA-C4-A0F6 700 0 0.09478065 0.07601751 0 0.1534235 0 53.17837 0
0.1205526 0.08648582 0.4893166 0.04682891 0.3163106 0.06351558
0.4831335 0.06402389 0.03360394 0 0.1667205 0.4129 0.02823508
0.07777718 0 1.729517 39.04084 0.2618162 0 0 0.08506263 0
0.8571362 0.1570081 0.0592588 0.1527261 0.5524621 0.1704129
0.1768407 0.5474499 0.2166172 0.06996302 0.143259 0.04022957
0.3315764 0.2448068 0.1096239 3.914863 0.4510032 0.1338635
0.1176874 0.1909076 0.1852927 3.978917 0 2.799978 0.2723171
0.8842037 0.2637342 0 0.03801353 1.399989 0 0.6384749 0.105826 0
0.05127644 0.06319304 0.1420708 0.274957 0.535759 0.03151946 0.3428545

0.01255127 0.2601942 1.53845 1.527261 0.08959931 0.01843101 0.03956402
0.1886112 0.0780784 0.0535881 0.2457661 0.09007973 0.599231
0.4349481 0.3217485 0.3709490 0.06120171 0.1723064 0 0
0.04122667 0.03704492 0.01450557 0.1924767 0 0.1898290.2245085
2.252091 0.09467853 0 0 0.228959 2.11961 0.1080377 0 0.07757516
0.1964897 0 1.365323 0.3275741 0 0.05978601 2.583288 0.1570081
0.07924467 0 0 0.278836 0.3175779 0.3528935 0.4897921 0
0.05609306 0.1646448 2.456121 0.07649343 0.321884 0.07636305 0.1637197
0.04999961 0.1793580.713508 0.09333261 1.388419 0.9180257 0.06905776 0
0.1172766 0.2446583 1.115859 0.9391232 0 0.05177156 0 0
0.1432825 0.5849084 0 0.1071762 0.5851316 0 0.5815340 0 0
1.002977 1.716732 0.1736421 0.2146948 0.2387193 0.2181801 0.3877958
0.126672 0.3818152 0.8825448 0.2507443 0.01935469 0 0.2435941
0.2477857 0.06032269 0.03578247 0.04377816 0.2113191 0 0.05127122
1.937014 0.2030557 0 0.06097956 0.1035337 0.4715753 0.07384558 0
0.05129731 0.3428545 0.05978601 0.1631055 1.473818 0 0.2518324
0.3408669 0.3401843 0.05880935 0.2461519 0.9333261 0.5634103
0.0534517 0.1590311 0 0.0749785 0 0.3166799 0.7762316 0
0.09682922 0.3612875 0.4237805 0.5113004 0.1836051 0.2433161
0.9912024 0.1680921 0.03510945 1.562549 0 0.2345532 0 0.01485619
0.637545 0.09682922 0.07450053 0.1537746 0.06375662 1.020616 0.9621877
0 0 0 0.2134672 0.3906947 0.1136173 0.3057997 0 0
0.3690657 0.6885193 0.08648582 0.176428 0 0.1500826 0.08465188
0.1643823 0.6882171 0 0.123076 0.0876134 0.06032269 0.1942182
1.225035 0.1427751 1.443739 1.741023 0.1242996 0.03779498 0 0
0.08742343 0.09007973 0.2478854 0 0.0825141
TCGA-CF-A7I0 368 0 0.2958778 0.1054687 0 0.380114 0 59.79387
0.05726927 0.310622 0.1799891 0.3394455 0.01624292 0.9694174
0.3965545 1.157812 0.2043055 0.03205332 0.1167375 0.007710418
0.3938472 0.03917411 0.4046631 0.06756114 2.544444 68.44291 0.1135159
0.1060285 0.07567725 0.2950455 2.393293 2.735192 0.05445933 0.08221726
0.1412642 0.4877733 0.3349502 0.2628789 0.3323017 0.333934
0.2972725 0.05351268 0.06883924 0.05111534 0.6368468 0.190119
9.729626 0.208578 0.4333603 0.1530775 2.118963 0.4284668 8.392209
0.1749238 1.308997 0.508604 0 1.731983 0 0.2373345 1.726562 0 0
0.7341289 0.1412642 0.1493989 0.2980975 0.1478346 1.525931
0.5840422 0.02915396 0.3875957 0.2612095 0.3249004 1.921038
13.369650.03107812 0.166216 0.1921226 0.1121504 0.07221872 0
0.2192031 0.2499581 0.4156947 0 0.1275436 0.3431098 0.1045228
0.04245645 0.2988281 0.08149858 0 0.1525307 0.07709568 0.1341695
0.6269808 0.5509304 0.06584348 0.5061699 0.9323437 0.0231811
0.2361218 0 1.072116 3.2131 2.435785 0.04268973 0.1950791 0.636102
0.2230487 0.5623661 0.1623161 0 0.1658975 3.784552 0.2722967
0.2198924 0.005819873 0.1107297 0.4835808 0.3524929 0.3931749

0.06795508 0 0.03891251 0.1395977 1.881044 0.1061291 0.1717656 0
1.218345 0.03468541 1.824872 0.5219685 0.1942383 1.232851 0.3821081
0.3135685 0.1726562 0.3661036 0.1697228 0.2580287 1.6649 0 0
0.08164131 0.25063 0.2484925 2.254216 0.04689858 0.03717479 0.3087231
0 0.1792969 0 0.1264481 0 1.600291 2.173837 0.2710302 0.1861709
0.4968084 1.210836 1.056861 0.54189 0.5297408 1.705507 0.05798158
0.1342661 0.171597 0.5294847 0.2406492 0.04184667 0.04137131
0.121478 0.2094213 0 0.2489728 0.4651382 0.4474454 0.158832
0.3807211 0.1309709 1.44486 0.2049107 0.04184667 0 0.2378428
0.7465386 0.3960198 0.4939722 0 0.4719952 0.5179687 0.7442775
0.1223905 0.04268973 0.1569602 0.07106278 0.1557367 0.4045141
0.05841753 0.09319103 0 1.142363 0.07692605 0.233553 0.268687 0.5674264
0.08399493 1.570797 0.2547387 0.153447 0.6455119 0.1295642 0.1704913
2.603565 0.6180309 0.1220345 0.4293688 0.07557687 0.9311023
0.03358587 0.05168203 0 0.04422883 0.6986988 0.6700205 0.1273694
0.1016733 0.04214935 0.1777021 0.09034338 0.2292883 0.07955151
0.1835322 0 0 1.432905 0.1499909 0.1495884 0 0.1431572
0.05383047 0.3116935 1.074207 0.3225088 0.02845982 0.6017081 0.12554
0.2020976 0.5509197 0 2.412804 9.634404 0.199403 0.02621889 0
0.07261244 0.1415092 0.03124476 0.9848701 0.09201517 0.1373787
TCGA-FD-A3B6 1005 1 0.026506089 0.056690248 0 0.1225885 0
23.761225703 0.046173998 0.375663694 0.169304658 0 0.01309604
5.011769963 0.248676219 1.228288698 0.028647587 0.401746684 0.156868256
0.22379802 0.057735229 0.015792283 0.108754728 0 2.509702106
27.508626495 0.103726588 0.056991106 0.549140759 0.142730256 0
0.575290319 0.087816902 0 0.028473965 0.308999982 0.063542915 0.141299377
0.612393225 0.16154284 0.14185104 0.419124815 0.088503848 0.21636467
0.171154983 0.061314248 0.266564781 0 0 0.057596199 0.160166055
0.172728098 2.090588503 0.035258569 0.408539502 0.105445981 0 0.167178383
0 0.015355539 3.610656986 0.046982043 0.133915561 0.295949875 0
0.021031742 0.381723574 0.039731114 0.153787374 0.299657676 0.052887853
0.085228195 0 0.203742467 0.653962499 0.488125119 0.150342537 0.123704775
0.022128722 0.180845072 0.029113582 0.029972595 0.333832696 0.100765775
0.335158521 0.304090891 1.028334724 0.041495425 0 0.034230997 1.01192092
0.525673205 0.119445871 0.053803566 0 0.013521958 0.53827496 0 0
0.05422377 0.731396124 0.074760088 0 0 0.032015021 0.307359158
0.211494726 0.172095395 0.092201411 1.50196004 0.05994519 0.656257105
0.314086079 0 0.066878353 2.157799433 0 0.443226818 0.009384678
0.04463852 0.194946235 0.355251741 0.218312484 0.602685096 0 0
0.180765754 1.472653503 0.021391937 3.863814272 0.128132844 1.356912153
0.195758511 0.100317529 0.007255914 0 0.921521058 0.10269299 0.017556817
0.034801513 0.065594475 0.045613634 0.069346189 0.87544179 0.148853997
0.028956575 0.032912114 0 0.040069972 0.654295372 0.037812509 0.029972595
0.470166138 0.048812512 0.144560131 0.047218133 0.305850549 0 0.841469422

0.40446483 0 0.240163797 0.534076507 0.488125119 0.042605315 0.070849452
0.071184913 0.493619961 0 0.043301422 0.046117343 0.445069133 0.332616232
0.303654136 0.166780414 0 0.996205039 0.040241578 0.15294256 0.458361392
0.387480765 0.160075103 0.068213492 0.088565025 0.131879418 0.578240526 0
0 0.511369172 0.100317529 0.136840901 2.320158048 0.087899051 0.973618663
0.236045046 0.043908451 0.131571648 0.172095395 1.17059635 0.171885522
0.113605958 0.143305731 0.188399169 0.094357241 0 0.106274178 0 0
0.054157974 0.265120172 0.372470249 0.163415801 0.205385979 0.148462018
0.871190196 0.052231287 0.019637217 0.024891148 0.10677737 0.098391712 0
0.12463917 0.300284694 0.027078987 0.041669217 0.172016632 0.071319989
0.120177887 0.785762387 0.10269299 0.163950422 0 0.095516224 0.072840376
0.057770726 0.085519077 1.701711783 0.262496669 0.107340344 0.654667808
0.02418638 0.043857216 0.601370147 0.167886696 0.035510257 0.25743585
0.192464842 3.994951053 0 0.200914081 0.539829575 0.08147175 3.510953758
0.133093605 1.68841716 3.861537757 0.09124836 0.549621197 0.278825179
0.175633805 0.065196243 0.050382888 0.176458376 0.03245737 0.092302638
TCGA-GC-A3YS 758 0 0.05953543 0.04774957 0.1619333 0.2753465
0.04568249 46.12493 0.1037116 0.2271714 0.4074384 0.2049059
0.0441226 1.233639 0.518656 0.9380137 0.088475 0.1266477 0 0.1535947
0.1620991 0.1064133 0.04885488 0.1223496 1.658154 75.65593 0.3837329
0.9280575 1.09638 0.1068623 0 0.7968331 0.3944918 0.1861138
0.03197774 0.6624985 0.1486708 0.1586865 0.2149217 0.143625
0.1153596 0.2976478 0.09770976 0.03471268 0.1537727 0.5508727
2.929511 0.04721546 0.0560566 0.2587341 0.5995826 0.1551861
4.809284 0.0395972 0.5735138 0.09210546 0.3173731 0.3423676
0.1526605 0.08357225 1.270227 0 0.05013137 0.2991304 0.03197774
0.06871196 0.3334297 0.08924021 0.2417957 0.1923035 0.03299767
0.1276209 0.09460729 0.1198546 0.5798162 0.5938723 0.3376849
0.1505041 0.07455511 0.06769947 0.09808819 0.2692862 0.08821446
0.2263304 0.1882002 0.09106929 0.2309747 0.08802509 0.1892853
0.07688637 0.2705809 0.07379479 0 0.1035843 0.04653872 0.009111516
1.414029 0 0 0.5640903 1.414626 0.4897635 0.1221726 0 0.1438181
4.092852 0.74649 0.03865441 0.2619129 0.3839822 0.1009823 0.9246135
0.1763675 0.04378695 0.1877696 2.530075 0.09862294 0.1991067
0.00526974 0.05013137 0.8757389 0.2792763 1.249391 0.3076576
0.08117426 0.1057027 0.2757862 1.407021 0.1081092 1.446421 0.2398331
1.795003 0.2512537 0.3755393 0.3096532 0 1.872151 0.1153296
0.2918148 0.1172517 0.184165 0 0.6230349 1.1798 0.1253781 0 0
0.04538776 0.1800026 0.5715171 0.08493082 0.302947 0.4037807
0.4385519 0.2841099 0 0.1908256 0 1.890028 1.410714 0.05453568
0.1685728 0.5997956 0.4111424 0.5741758 0.4774057 0.3597496
0.5147636 0 0.09725949 0.1294804 0.6630474 0.1245151 0.07578208
0.1947952 0.04124816 0.5878388 0 0.1610273 0.4679669 0.1950722
0.3954998 0.2681255 0.03060404 1.29594 1.020476 0.1515642 0.03222185

1.220375 0.2628775 0.4610383 0.8945572 0 0.3746504 0.2294055
0.9369179 0.1477618 0.2319265 0.8172089 0.1930364 0.1007253
0.3718264 0.211582 0.1197047 0.03715723 0.6365409 0.06965448
0.08459042 0.1216444 1.009424 0.3042207 0.6423355 0.422875 0.3612495
0.8767407 0.1290486 0.1543753 0.5777171 0.07994435 0.405163 0
0.2052985 0.238875 0.1216444 0.09359339 0.0965918 0.2002401
0.3795919 2.164765 0.1153296 0.2761872 0.03816512 0.08045226
0.163607 0.05190362 0.09604236 0 0 0 1.124463 0.08148768 0.04925393
0.4502466 0.2238977 0.07089753 0.1514406 0.756518 0.1858329 0
0.5998641 0.3410193 0.30499 1.130357 0.0298942 2.061065 6.523916
0.1659146 0.5222911 0.06262703 0.06574863 0.05491407 0.0282913
0.3255681 0.00520733 0.165857
TCGA-XF-A9T2 575 1 0.1505352 0.09658775 0.2183723 0.3063338
0.1386097 94.65281 0.3146815 0.262584 0.1648332 0 0 1.08574
0.6456225 0.5022563 0.03253947 0.04269712 0.1425435 0 1.114839
0.08968863 0.04941179 0.1237443 1.476353 118.2578 0.1108878 0.06473357
0.9702679 0 0 0.6534457 0.4987358 0 0 0.3190717 0.2646434
0 0.4347433 0.1681983 0.3889153 0.1820247 0.04770794 0.09362234
0.07776282 0 7.482958 0.09550735 0.2267824 0.09345838 0.9702679
0.2354326 10.64741 0 0.8507421 0.1330791 0 0.7372208 0.7720034
0.08318324 0.2964707 0 0 0.06723116 0.1293691 0.04343452
0.1766446 0.09025748 0 0.8752303 0.02669905 0.1774791 0.07973812
0.3306024 0.07819009 0.1848129 0.2846119 0.608879 0.02513499
0.06847119 0.09920632 0 0.5799302 0.4578208 0.1631533 0
0.8760285 0.3456401 0 0.07776282 0.2189322 0 0 0.1920694
0.04706923 0.0491487 0.3402583 0.4657286 0.2411965 0.09724781
1.523058 0.04245827 0 0 0.1454575 1.595955 0.2059089 0.1563802
1.601712 0.1941797 0 1.328189 0.1783779 0 0 1.597973 0 1.006882 0
0 0.2657165 0.2421084 0.6657854 0.1244659 0 0 0.1782053
2.970949 0.09719246 2.23369 0 0.5389661 0.1270589 0 0.1153832 0
1.693559 0.1166442 0.1834662 0.07905887 0 0.1036208 0.3150685
1.193249 0 0 0 0 0.2730818 0.330304 0 0.2042669 0.2094275 0
0.9851951 0.4290632 0.5404024 0.07950053 1.656696 0.8291843
0.05515735 0.2045932 0.682462 0.8316582 0.04399401 0.2280114
0.242567 0.4004858 0.1061985 0.09836817 0.3666779 0.6190206
0.1259345 0.6131675 0.2273258 0 0.2301444 0 0.1954354
0.662622 0.4704747 0 0.1549611 0.1702409 0.8363596 0.1876562
0.07664594 0.1955349 0 0.1519281 0.6217251 0.6733057 0
0.3648871 0.5568494 0.09974717 0.2739846 0 0.0718717 0.1301579
0.0814988 0.2806455 0.3209909 0.1548098 0 0.1207119 0.140897
0.08555468 0 0.6096204 0.6153771 0.1856165 1.010917 0.08431558
0.6168591 0.2135776 0.1784401 0.08795953 0 0 0 0.08179709
0.3552912 0.1230311 0 0.09769288 0.08100908 0.273009 1.171421 0 0
0 0.2169849 0.165472 0.07874292 0.7285288 0 0 0.5439639

0.5248899 0.05494439 0.04981539 0.7969134 0.1191842 0.1523746
0.2019018 0.6870661 0.1074007 0 0.2449073 4.2155270 2.082526
0.1814099 0.7504416 7.5263660.1678059 0.2401113 0 0.7979773
1.1108010 0.4867608 0 0.1887162
TCGA-UY-A78O 2312 0 0.09546636 0.08507497 0.1154060.2060446
0.02441762 96.2124 0 0.1618997 0.4065204 0 0.01572257 0.3106737
0.1279502 1.3566620.02579483 0.08179705 0.1129977 0.1119511
0.1732863 0.1421967 0 0 2.80090964.87085 0.1245299 0.01710529 0
0 0 2.3022330.1054294 0.03979168 0 0.2360735 0.1716458
0.05089146 1.1487720.09697084 0.1115762 0.02219929 0.1008513
0.03710836 0.3698669 0.03680568 5.3490180.1514220.1498133 0.06914763
1.9228880.3317924 10.633230.12699 0.2861108 0.1828581 0 1.0743750
0.03261617 4.9092980 0.05359117 0.2131831 0 0.00229544 0.1867073
0 0.4431139 0.1541814 0.007054998 0.1449554 0.05899633 0.1048306
0.4958655 3.1742910.2105776 0.4579208 0.01992512 0.2894869
0.06990514 0.1079516 0.2121807 0.06048762 0.1724475 0.0730158
0.06172882 0.1273119 0.1011744 0.04109632 0.2314039 0 0.0956012
0.04613882 0.1243764 0.01298712 0.1910595 0.08204321 0.06373413
0.2912316 1.0244310.01869873 0 0 0.1153077 3.0047371.0881940
0.01302273 1.0995070.03598386 0.2865001 0.1256929 0.04680888 0
5.0441840.1581440.6385438 0.02253372 0.05359117 0.3276622 0.1706002
0.1292533 0.5262247 0 0 0.06142073 1.6228720.02568228 0.04987889
0.1025540.7295758 0.03357423 0.2007285 0.1306673 0.0626719
1.7527410.3082224 0.1011744 0 0.07875003 0.1095237 0.2913896
1.63959 0 0.06952814 0 0 0.4329572 1.091 0 0 0.1272807
0.1758069 0.2169412 0 0.3671916 0 0.6061401 0.4618968 0
0.1081240.4408183 0.3662643 0.3441013 0.1134119 0.04273084
0.8042697 0.05612409 0.0519859 0.08304987 0.6760931 0.1996627
0.08101214 0.1041194 0.08818977 0.1013562 0.04831238 0.1606645
0.7503952 0.2406173 0.1537437 0.2456829 0.09814848 1.833978
0.04958655 0.08101214 0 0.3837055 0.0401457 0.1642856 0.75614
0.0211056 0.2343706 0.3215341 0.1230009 0.03948988 0.04132213
0.8925997 0.2751449 0.02871382 0.2610368 0 0.07761875 0
0.4890889 0.9680016 0.04521419 0.2600793 0.1882583 0.1626082
0.6376184 0.6164449 0.05941246 0.7334989 0.1379548 0.1414538
0.4382882 0.04273084 0.1181250.05937337 0.03990311 0.2703821
0.03250992 0.3501844 0.2065161 0.04281192 0.4959646 0.4274573 0
0.1230201 0 0.3726867 0.1748983 0.08322859 0 0.4441315 0
0.9813824 0.6472671 0 0.01316329 0 0.2015579 0.06157978
0.1103810.3135887 0.9365310 0.2941575 0 0.09781163 1.265099
0.03195734 0.8813235 2.0694040.05738282 0.1776526 0 0.2108587
0.04891995 0.06048762 0.5598857 0.01113342 0.05540737
TCGA-GC-A3OO 481 0 0.1500692 0.1283850.2721204 0.2359797 1.128475
19.714430.3137074 0.1963284 0.3286458 0.3099002 0.05931656

4.4281870.2815855 0.5007015 0.3000584 0.5666459 0.1421023 0
0.5556941 0.0268233 0.04925883 0 3.27929621.6842 0.1105445
0.5001321 0.1381806 0 6.3649450.9771344 0.5469112 0.1876527
0.3224215 0.9224434 1.1452370.04799958 1.5602310.1981645 0.3212466
0.05583422 0.1002162 0.05833283 0.1938052 0.2082853 6.532709
0.0952117 0.08478014 0.02795072 0.2418161 0.1564692 2.685431
0.2794723 1.5034650.3184012 0 0.2449796 0 0.6366557 0.4433295
0.05319954 0.1010918 0.2010691 0.6126008 1.2925070.1640911
0.3149233 0.9751798 0.9694677 0 0.3216903 0.9220989 0.1647895
2.2215191.7963480.02837309 0.3852079 0.7705085 0.1706481 0.08241602
0 0.4669552 0.3423027 0.3524046 0 0.2328844 0.4019985
0.1908504 0.3100884 0.05456363 0 0 0.1305510.02346176 0.6645158
0.4664069 0.5803586 0.5410123 3.2638750.7361666 0.03527236
0.06159136 0 6.4528230.7955071 0.4447550.4676883 0.2272304
0.1797515 0.1357564 0.4863958 0.2667386 0.3973410.03786444
1.8405910 0.1505647 0.01062662 0.2021835 0.1324470.1609061.544175
0.3722417 0.08184545 0.07105114 0.1737916 1.4559870.03633435
1.1290690.09672644 0.6127079 0.03166639 8.2923130.1971875 0.0591106
0.9262289 0.2907079 0.1749462 0.1182212 0.1856877 0.1033001
0.4711398 0.5419084 0 0.1311545 0.4099439 0.3661044 0.1361182
0.3704417 0.1712661 0.0339391 0.2139986 0 0.4910727 0.05346687
0.07696136 0 0.8257839 1.4409950.2474397 0.1359733 0.7559437
0.9672644 0.4034923.1755930.6045402 1.7966070.1588046 0.2451592
0.07833061 0.3188373 0.03138616 0.03820434 0.1435273 0 0.3823866
0 0.2273022 0.4246526 0.3858054 0.07250363 0.4248239 0.09642838
0.4542183 0.1870753 0.1146130.06497654 0.3619016 0.1514578
0.3099002 1.01732 0.01990628 0.3385743 0.5705458 2.4196680.08690707
0.03897402 0.3403338 0.4541424 0.1150992 0.2294169 0.05333287
0.1009085 0 0.08022551 0.2106912 0.1705797 0 1.0324140.5751302
0.5088652 1.0077870.09806367 0.2434175 0.09462953 0.04447192 4.47205
0 0.0742751 0 1.6998760.1700118 0.1226502 0.2359181 0.1460857
0.04037916 0.6804098 0.7993833 0.2907079 0.3480886 0.4232875
0.4326265 0 0.05233278 0.09683648 0.0837788 0.0220174 0
1.6570350.1095486 0.03724589 0.1702385 0.09505222 0.06254827
0.1978065 0.2023684 0.05353413 0.05196536 0.2718933 0.07640867 0
0.4066548 0.2109897 0.8728049 9.8097540.1279249 0.2154312
0.06314485 0.2651690.0461401 0.02852522 0.4709818 0.01050077
0.08361421
TCGA-E7-A6ME 731 0 0.1068935 0.1524132 0.1938299 0.3295824
0.05468072 32.008920.06207001 0.2201242 0.2926157 0.6131673
0.1232317 0.5359894 0.2865311 0.8255716 0.3080792 0.06316413 0
0.1504217 0.1552227 0.09553043 0 0.2196738 3.95745533.59751
0.1558401 0.09576381 0 0.2558227 0.5638949 1.2115690.05902452
0.08910932 0.0382765 0.6419467 0.1637186 0.03798871 0.6688644

0.1357226	0.1775344	0.0414275	0.09679116	0.1108004	0.4601547
0.08242249	8.8303355	0.2825782	0.1341965	0.05530318	0.1435369
0.5108225	3.2862339	0.1421904	2.0594420	0.6299873	0.6648024	0.7006343
0.3197784	0.0508108	2.1052080	0.3600355	0.7558856	0.0382765
0.0154212	0.1757969	0.2670454	0.1653846	0.3452732	0.0473968
0.08592685	0.0849318	0.2738835	0.3701464	3.7182890	0.1684166
0.0277153	0.05949365	0.04051723	0.07827263	0	0.5015542	0.474095
0.06436304	0	0.06911763	0.1053637	0	0.04601547	0.6477562	0
0.1070445	0.08265846	0.0278528	0.03635414	0.2957253	0.09186361
0.1427259	0.2723822	0.5462161	0.004187385	0.2559153	0.1098369
0.3442929	2.951226	1.0966030	0.08748915	0.8864032	0.4432016
1.8606030	0.1055536	0.1572354	0.08990211	4.5873720	0.1180490	0.7745576
0.01261548	0.1200118	0.4717063	0.5253070	0.2613084	0.4419095	0	0
0.1237908	1.6841660	0.0143782	1.1542190	0.2870738	0.7385712	0.03759299
0.4495105	0.09753858	0	1.64241	0.1380464	0.09440393	0.04678239
0.1322644	0.1226335	1.1186342	0.05552	0.5502721	0.07785052	0.04424254
0.05432794	0.2154584	0.5374998	0	0.2417463	0.9914150	0.1312337
0.09716343	0.0634736	0.2284131	0.04704375	1.2065670	0.4641403
0.4243054	0.04035542	0.1346136	1.0662740	0.1457852	0.1190504
0.3349194	0.2843808	0.1256840	0.2473862	0.09299077	1.35531	0
0.4989002	0.0627751	0.1810341	0.1134883	0.1622858	0.06424845
0.7281871	0.5298568	0.1721465	0.1833938	0.2243723	2.356355
0.2776098	0.04535456	0.03856869	0	0.3596084	0.3065836	2.72048
0.07089568	0.5580669	0.5003682	0.1377239	0.1326502	0.2313415
0.3615003	0.1155297	0.1044901	0.3122107	0.1266290	0.1714702	0
1.0714530	0.8337456	0	0.4368154	0.4520085	0.5462161	0.6590215
1.0583560	0.2328341	1.0646440	0.08425512	0.1319879	0.5651065	0.04784563
0.1322644	0.06648024	0.05957257	0.4709386	0.1456051	0.0560144
0.2312356	0	0.3130045	0.8334642	0.2070696	0.05509813	0
0.2246982	0.1958333	0.09319087	0.2586603	0.3978345	0	0.02219903
1.7600920	0.07369455	0.2694666	0.09873671	0.1272937	0.3707802
0.2772036	0.2224370	0.06169107	0.2700814	0.6349639	0.1095195
0.7304970	2.6150637	7.9895080	0.09345662	0.02841675	0.1499258	0.3147974
0.2738778	0	1.2368620	0.1116711
TCGA-XF-AAN5	2293	0	0.09556754	0.06813212	0	0.2062630	0.09777401
71.352190	0.1109867	0.3009896	0.4069513	0.2192796	0.2833062
0.4823719	0.4269527	0.8857175	0.1032887	0.1355317	0.1508233
0.2390822	0	0.07591864	0.4182555	0	2.1092077	5.93704	0.07333051
0.5479496	1.6132710	0.1143585	0	0.5992150	0.1055411	0.4780063
0.4106508	2.1606740	0.3945673	0.1698180	0.5519950	0.1132526	0.1058158
0.2370435	0.09014127	0.1485908	0	0.07368938	1.2814640	0.3031651
0.05998883	0.0197774	0.5133136	0.8303602	7.2269140	0.2542491
0.7364934	0.1408092	0	0.8036809	0	0.02555276	0.2091277
0.7905029	0.1072959	0.2134091	0	0.03676597	0.1954009	0.0955002

0.369653 0.2057931 0.1694994 0.3755764 0.1181177 0.3964466
0.6618548 0.5866441 0 0.346902 0.132975 0.2535695 0.06997923 0
0.04720125 0.1211035 0.2301737 0 0.494354 0.299223 0.4051264
0.08227977 0 0 0.09570253 0.09237545 0 0.01300089 0.3712734 0
0 0.1989334 4.199737 0.1572358 0 0 0 0.7387877 0.3631157
0.4136593 0.1434019 0.1467563 0.216132 0.3154841 0.2516523 0
0.2411295 1.096731 0 0 0.056394 0.2145919 0.093717 0.2561716
1.840218 0.3950868 0 0 0.2049528 0.9509809 0.051419 1.065211 0
3.211535 0.4705374 0 0.05232231 0.1254766 0.72175 0.1234197
0.1772428 0.0836511 0.157667 0 0 1.206446 0 0.06960184 0.07910962
0.09714321 0.0963147 0.6116076 0.09088852 0.288176 0.9639266
0.3519864 0.4343422 0.2269929 0.2450538 0 1.618087 1.106566
0.05836123 0.2164773 0.1604675 1.46661 0.260677 0.1561067 0.5133136
0.7627473 0 0.2341845 0 0.5894795 0.1332495 0 0.1122475 0
0.4058544 0 0.1608348 0.8012699 0.2408723 0.07695331 0.1639622
0.1228156 0.4358662 0.5956694 0 0 0.4609346 0.2411295
0.1096398 0.6011005 0.08451187 0.3920217 0.4037075 0.1055411
0.1317724 0.579123 0.1140697 0.2065774 0.114977 0.3325808 0.339636
0.05460072 0 0 0.1490812 0 0.260355 0.1787435 0.1627805
0.2945973 2.962072 0.416328 0.4079313 0.02510928 0.1416037 0.1595465
0.1711045 0 0 0.0599181 0.2857058 0.1952663 0.1001587
0.206735 0 0.7402219 0.9345168 0 0.1970408 0 0.1721916
0.1750837 0.1249752 0.1027795 0 0.07010594 0 2.036424 0.0581359
0 2.288694 0.1765501 0.04741927 0.2946614 0.4295762 0 0.05515457
0.2355755 4.784783 0 1.669657 0 0.7940319 1.18377 0.2611075
0.2540585 0 0 0.03917744 0.1211035 0.6362196 0 0.2662373
TCGA-FD-A3SN 887 0 0.07143067 0.168050617 0 0.264288819 0.285011465
20.537715639 0 0.488013165 0.078215205 0 0.028233781 0.816913138
0.306355206 0.688498284 0.077201745 0.217797908 0.101457786 0 0.311178771
0.008511655 0.164125192 0.058718081 1.656130611 48.471282736 0.078926252
0.030716813 0.328859383 0.051285413 0.180872661 1.343625479 0.189324654
0.035727933 0.12277417 0.333086873 0.114160259 0.106619674 0.082516244
0.15962401 0.089636009 0.079728716 0.108338797 0.144380808 0.110698022
0.231328166 2.29875041 0.045319324 0.188318945 0.008869412 0.748155096
0.670292801 2.071814112 0.152028052 0.770674815 0.176813174 0 0.614834539
0 0.077669517 0.375143296 0.050644345 0.144354427 0.319019496 0
0.057708481 0.186689705 0.171312795 0.099465162 0.276871134 0.012669004
0.168431684 0.04540397 0.386957308 0.519429179 0.789262519 0.054020635
0.033336925 0.059634201 0.146206609 0.078457544 0.226162944 0.02116796
0.325861737 0.232254321 0 0.110849455 0.059640053 0.181683749 0
0.415543343 0 0 0.149136721 0.022334882 0.020406413 0.438959702
0.220993505 0 0.809078981 1.927222641 0.130955044 0.029316553 0
0.276085015 1.325272578 0.325687106 0.408122926 0.052617501 0.289584299
0.129235968 0.797447464 0.112856479 0.042028502 0 2.151808284 0.047331164

0.143333052 0 0 0.42028502 0.306355206 0.551252896 0.236241842 0 0
0.180150483 0.758184346 0.092237851 1.030054474 0.092080627 0.883892444
0.060290887 0.576732754 0.500577309 0 1.138451391 0.332094065 0.075701562
0.075028659 0.212122911 0.442523403 0.074751801 0.440385608 0 0
0.03547765 0.043565028 0.172773885 0.901214649 0.122280109 0.064617984
0.660848455 0.052617501 1.285587218 0.101797678 0.402956813 0 0.423296018
0.935790521 0 0.097081811 0.323835954 0.460403136 0.371586703 0.369132517
0.19183464 1.026189354 0.100784766 0.081684427 0.099424481 0.55809138
0.119514678 0.072738736 0.079103698 0.026394447 0.200211247 0 0.092736339
0.583925929 0.172835305 0.241574388 0.330888642 0.11015627 0.231009293
0.267135006 0.18184684 0.061855689 0.620134836 0.216274783 0.344184869
1.168139647 0 0.538075355 0.503996863 0.678413344 0.165465771 0.296816674
0.221675584 0.216164887 0.109570966 0.22372469 0.101542546 0.111130315 0
0.229116735 0 0 0.583796484 0.388600049 0.219002573 0.660578413
0.184496703 0.066681165 0.341491068 0.022521109 0.02116796 1.123939252
0.153467712 0.247476729 0 0.289609519 0.256256339 0.175138945 0.089834758
0 0.038439731 0.250995155 0.800692388 0.221396044 0.176730539 0.036632438
0.128702274 0.078518364 0.112093232 0.092185383 0 0.136239314 0
0.539652856 0.130358674 0.059094918 1.51257777 0.135730238 0.072303495
0.244467158 0.118552966 0 0.024734723 0.121493605 1.672990929 0.029274188
0.47881 0 0.870449679 2.533725418 0.131148362 0.296232389 0 0.252432872
0 0.05431029 0.298906932 0.074973124 0.019899546
TCGA-ZF-A9R2 642 0 0.1439923 0.1539827 0.06527529 0.2996787
0.0276219 49.793610 0.1962274 0.1642383 0.3716889 0.03557162
0.02868921 0 0.2001776 0.1361726 0.03828874 0.1704350.3968125 0
0.08579038 0.02954009 0 2.310256 23.65993 0.1408717 0.07739997 0
0.06461428 0 0.3646091 0 0.1350404 0 0.3815043 0.129447
0.2302795 0.1559428 0.1005548 0.04650142 0.01674165 0.1038996
0.08395605 0.2324466 0 8.507546 0.1141953 0.3050515 0.02234907
0.3625375 0.04691662 3.327713 0.1915395 1.10968 0.06364748 0.9594977
0.5475815 0.2307653 0 1.063443 0 0.06062385 0.6832833 0 0
0.172807 0.05395907 0.501263 0.05813813 0 0.1350404 0.05720427
0.1317638 2.430728 3.038409 0.1020906 0.01400035 0 0.1228034 0
0.04070596 0.2933637 0.06842531 0.1950775 0.1101301 0.03491469
0.5385051 0 0 0.1308853 0 0.05407339 0.08350966 0.02813962 0
0.3623388 0 0.07209785 0.2984424 1.186458 0.008461011 0 0.055484 0
1.133014 0.3692986 0.1402343 0.1031218 0.364846 0.1628239 0.5833746
0.0710937 0.05295153 0.04541395 0.8649554 0 0.1203898 0
0.1212477 0.1059031 0.04824696 0.004061528 0.2232301 0.098164
0.04260875 0.09727322 1.776137 0.05810504 0.6770929 0 0.1921971 0
0 0.2463575 0.1417924 1.040592 0.3486699 0.09060728 0 0.04454213
0.06194815 0.8476153 0.665808 0.05053988 0.0393261 0 0 0.1088385
0.246834 0 0.04070596 0.3818693 0 0.24541 0 0.2307653 0.04752819
0.7618698 0.6609537 0 0.2853969 0.09066657 0.3314628 0.7311721

0.01603685 0.1933533 1.388661 0.06348915 0.05880792 0.03131612
0.3330649 0.112932 0.09164323 0.05436132 0.01662713 0.09172557
0.05465233 0.07789208 0.3961385 0.4990207 0.04347979 0.2316029
0.09252362 1.074637 0.392656 0 0.1168976 0 0.3178976 0 0.503157 0
0.4295724 0.3020795 0 0.2978138 0.04674476 0.0214837 0.1556258
0.1218071 0.3489817 0.127933 0.07119286 0 0.0721658 0 0 0
0.03951893 0.4138806 0.332904 0.4648932 0.1176158 0.2919507
0.0567485 0.2133554 0.03380482 0.04833833 0.04454213 0 0.01128488
0.543758 0.07355227 0.1131824 0.05840421 0.04843006 0.3264286
0.8559422 0 0.111331 0 0.2918726 0.09892496 0.125534 0.116144
0.1004828 0 0.358842 0.7845074 0.06569534 0.01489069 0.06806037
0.2137574 0.04286817 0.2122724 0.5974575 0.2247276 0.03116317
0.3127938 0 0.03688243 0.26312 0 0.498489 1.960941 0.1652333 0 0
0.07950978 0.01106793 0 0.2225314 0 0.1629638
TCGA-ZF-AA4T 599 1 0.07066721 0.2456032 0.1922112 0.1960981
0.02711204 19.24377 0 0.0256807 0.1934481 0 0.01745751 0.09503881
0.4262075 0.8514245 0.05728246 0.01565916 0 0.02486093 0
0.01052585 0.2609534 0.07261312 3.10599 27.4111 0.1138706 0 0
0.1268432 0 0.4345673 0 0.04418259 0.03795686 0.823817 0.1552926
0.1318501 0.3061286 0.161507 0.07824527 0.05751417 0.07798608
0.09614073 0.1368936 0 5.736216 0 0.03326896 0 1.850397 1.381518
2.82429 0.188004 0.680748 0.09370895 0.3767147 0.9569657 0.04530113
0.003149155 2.319586 0.1252576 0 1.183537 0 0 0.1319245 0.05296306
0.08200173 0.1711949 0.007833498 0.1230801 0.03743224 0.2327968
0.6882288 6.50689 0.03340204 0.02748385 0 0.04017887 0 0.03995459
0.4188343 0.2014868 0.2233894 0.0540486 0.06854043 0.04916885 0
0.0456312 0.3211734 0 0 0.09221421 0 0.01081517 0.2121424
0.04554823 0 0.1902166 0.6499856 0.008304832 0.03625402 0 0
0.3511896 0.7249638 0 0.1301378 0.3743893 0.3595913 0.3817376
0.06978141 0 0.04457567 2.661012 0.05853161 0.4135865 0 0.05950482
0.05197412 0.1894255 0.6258895 0.3651826 0 0.04182225 0.1682226
1.992402 0.02851625 1.310728 0.1138706 0.5714966 0.03727906 0
0.1644309 0 0.8557546 0.2737872 0.09361557 0.09278343 0.1748798
0.06080468 0.09244106 0.6068382 0.2480349 0.6176031 0 0
0.3204886 0.2907334 0.100811 0.07990918 0.8663884 0.0650689
0.3372321 0.06294354 0.6342159 0 0.7478068 0.1797222 0
0.04001841 0.4894615 0.8946974 0.113589 0.03148166 0.3795686
0.3760079 0 0.1154448 0.03073807 0.4358893 0 0.2248791
0.05335789 0.01632021 0.09003244 0 0.1146815 0.3888264 0.7569782
0.08535444 0.1363967 0.1362236 0.4541505 0.1101166 0.04497581
0.03824661 0 0.133727 0 0.1790277 0.04686909 0.3788199 0.2420437
0.01951054 0.1461583 0.1835277 0.1897843 0.2291298 0.207235
0.5072226 0.06278578 0.1304403 0.0441048 0.7083372 0 0
0.4331676 0.09266371 0.3159652 0.6535181 0.3194184 0.06596847

0.3468905 0.1114020 0.06636166 0.04744607 0.08743989 0 0.02215316
0.4336483 0.0360973 0 0.1719784 0.09507221 0.6207813 0.5755523
0 0.1092760 0.06366335 0.09709894 0.1617221 0.05700006 0 0
0.4512797 1.745393 0.03224135 0.02923165 0.2004122 0.09791217
0.06837494 0.2737202 0.4214961 0.09453407 0 0.4899256 0
0.07240326 0.9448652 0 0.8807178 2.96918 0.08688391 0.02817945
0.07433688 0 0.1955453 0.06716227 0.08400915 0.006180984 0.03691286
TCGA-E7-A4IJ 674 1 0.3659271 0.2152237 0 0.2369336 0 27.14718 0
0.07978749 0.2671221 0 0.05423882 0.1202978 0.3923514 0.6782806
0.06921099 0.1556849 0.04331258 0.2832152 0.358676 0.1417122
0.03002805 0.3760034 3.084366 41.1135 0.1263518 0.09834812 0.4211726
0 0 1.932581 0 0.04575703 0.07861889 0.2326837 0.2119985
0.09753471 0 0.09292347 0.06077514 0.06807277 0.07041059 0.04267144
0.3308008 0.04232338 17.87445 0.3482447 0.2411815 0.04543649
0.8844625 0.4292244 2.312016 0.04867586 0.329003 0.04852413 0
0.6380842 0.656816 0.01956825 1.861739 0 0 0.2042853 0 0.03167472
0.1903 0.1097008 0.1273858 0.1772954 0.04056322 0.08824569
0.009691533 0.08036417 0.1425507 9.153485 0.06918462 0.07115807
0.007637396 0.1456372 0 0 0.3253195 0.06955559 0.1982999
0.2238985 0.2129486 0.07638145 0.4653674 0 0.2660947 0 0
0.04244455 0.02860445 0.007467041 0.5169461 0 0 0.5594656
1.963347 0.004300387 0 0 0.04419801 1.394199 1.543306 0 0.1123127
0.337157 0.1241351 0.8895166 0.1084021 0 0.04616412 3.740064
0.1212347 0.6118923 0.006477961 0.1232505 0.3767835 0.09808784
0.03302894 0.453835 0 0.08662515 0.1600915 2.397186 0.05906485
1.472149 0 1.206709 0.07721498 0.2769847 0.09015371 0 1.701027
0.2126576 0.07756124 0 0.09055579 0.1889143 0.6701462 1.208582
0.1027494 0 0 0 0.3319092 1.354921 0.3132101 0 0.2545424
0.1347752 0.399142 0 0.5160697 0 0.6195638 0.5175228 0.2346378
0.1243334 0.09216424 0.2527036 0.4277697 0.09781049 0.1474104
0.1460276 0 0.05977934 0.1591671 0.7398307 0.1147975 0.04657852
0.02762964 0.2704285 0.09324073 0.0555551 0.1979468 0.5752602
0.1567906 0.4419801 0.09417145 0.2351299 1.653755 0.05702029
0.04657852 0.1188285 0.5294741 0.1846565 0.3148572 0.7672026 0
0.7198203 0.3196029 0.1010289 0.06054664 0.7602706 0.3494173
0.3954914 0.1155645 0.1227968 0 0.1905713 0 0.9781049
0.3424981 0 0.1495345 0.1562233 0.04674637 0.7896071 0.7088588
0.3415962 0.2030554 0.02884295 0 0.4734488 0 0.8150021 0
0.04588516 0.5613766 0.03738362 0 0.1781069 0.04923004 0.207388
0.7514336 0.07088588 0.02829251 0.04691543 0.131864 0 0.1196322
0.236125 0.1021427 0 0.2963752 1.169617 0.133561 0.04540998 0.06918462
0.1014012 0.07081147 0.1480835 0.2846843 0.5221481 0.03167794
0.3856118 0.09315703 0.07498333 0.7306397 0 1.469498 6.119069
0.07798267 0 0.2309576 0.2424695 0.07875526 0 0.8874285

0.08321614 0.3185687
TCGA-FD-A6TE 376 0 0.1283152 0.194392 0.07755792 0.3758498 0
42.635020 0.1243471 0.4293135 0.4416284 0.07396378 0.0937408
0.6592423 0.4360479 0.09245472 0.01895559 0.02531315 0 0.09316482
0.1401582 0.03509855 0.04394949 4.243104 24.51652 0.2806061 0.3333697
0.2461457 0.3454764 0 0.8200168 0.2125594 0.2406758 0.0229736
1.677176 0.5084098 0.2622099 0.4632151 0.3855812 0.0749841
0.03481076 0.06535592 0.133005 0.5247521 0.02473503 3.103181 0.6105734
0.2214984 0.03319303 0.4738304 3.065962 6.325296 0.3129237 0.9888635
0.02835893 0.2280086 0.5316025 0 0.007624173 0.8072667 0
0.07203123 0.4059272 0 0 0.0884032 0.2885056 0.8933759 0
0.009482535 0.2234847 0.2548808 0.3209425 0.2777028 4.824455
0.4043353 0.04990425 0 0.2431848 0 0.0725482 1.077382 0.609755
0.3090461 0 0.3941025 0.08927914 0.2039808 0.6904633 0.6998111
0 0 0.1984468 0.01671728 0.002181979 0.4796132 0.08270495 0
0.4213745 0.8359906 0.002513273 0.04388589 0.09888635 0 2.656993
0.6094282 0.08331085 0.2362999 0.2758623 0.09673093 2.012049
0.4012380 0.161878 2.814701 0.03542657 1.072824 0.03785911 0.2160937
0.1887457 0.6019169 0.07721234 1.237761 0 0.2025052 0.192628 2.19015
0.08629809 0.4245968 0.1378416 1.044421 0.02256335 0 0.07610558 0
0.7518632 0.0828556 0.3513003 0.1123154 0.2116939 0.2576165
1.063059 1.582182 0 0 0 0 0.4202846 1.114464 0.1220328 0.04836547
0.357029 0.3150665 0.4082231 0.1142908 0.2741876 0.02823571
0.8599669 1.231047 0.05876967 0.1695495 0.4578397 2.559915 0.3250016
0.1095631 1.234831 0.5973997 0.1885892 0.3231655 0 0.4250504
0.06709104 0.1361093 0.0215301 0.06914529 0.1362316 0.1298722
0.2005223 0.5379184 0.1886563 0.3099674 0.1651097 0.1841386
1.86207 0.199946 0.1361093 0.09259587 0.2062935 0.3507357 0.03680236
1.0798380 0.8114619 1.003513 0.03542657 0.115002 0.1110811 0.7530235
0.3004778 0.1977937 0.5143234 0.3800144 0.1466209 0 0.3143983
0.2001660 0.2621771 0.07564983 0.3551594 0.1648106 2.623761
0.1896569 0.5385863 0.1432817 0.06337544 0.0290086 0.08615099
0.2116939 0.03990151 0.008938879 0.4845563 0.1310885 0.0336199
0.03469697 0.115086 0.9756892 1.717016 0 0.04960493 0.109675 0.2119292
0.1175393 0.1864442 0.1379984 0.1790855 0.01568813 0 3.821714
0.07805701 0.1415409 0.2021677 0.2116496 0.09550225 0.2967236
0.8651652 0.1716518 0.05554056 0.5811991 0.05444371 0.1314674
2.245601 0.04295347 0.9772753 5.996396 0.1402319 0.03411153 0
0.4723543 0.01315054 0.04065033 0.2745738 0.05237504 0.156392
TCGA-H4-A2HO 46 0 0.06179863 0.1652158 0 0.1714882 0.02370954
74.776950 0.03368675 0.5075123 0.2126953 0.122133 0.4432621
0.2898928 0.8018474 0.1085362 0.01643277 0.1462946 0.2536442
0.5720883 0.05522928 0.07606811 0.2540013 1.585029 46.6751 0.02133859
0 0.1422572 0.05546232 0 0.5812224 0 0.1159133 0.06638671

0.4912021 0.9753179 0.03294378 0.2677102 0.180471 0.3079155
0.05748144 0.03497384 0.02402151 0.3990458 0.1072151 26.30246 0
0.2327502 0.01918355 0.8713256 0.4429849 10.58693 0.2055123
0.9128173 0.2458461 0 0.7795488 0.07923188 0.04681703 1.064954
0.05476904 0 0.1380007 0 0.03120425 0.1030074 0.3242142
1.183226 0.3992276 0.04110247 0.1573109 0.09002009 0.2262015
0.4012384 5.785128 0.05842031 0.6729712 0.01289821 0.3513651
0.08484746 0.03494038 0.5265153 0.1174671 0 0.07089843 0.02996938
0.2526148 0 0.07980916 0.449387 0 0 0.04480085 0.04830786
0.0220684 0.3055608 0.1991601 0 0.3393435 0.4026264 0.01089389
0.03170422 0 0.1866066 2.149813 0.5283187 0.08024767 0.1580636
0.1565846 0.1747019 0.3755591 0.061024 0 0.1169446 1.263262
0.102372 0.2583445 0.03829046 0.05203709 0.1363545 0.165653 0.1603676
0.1916118 0.08426006 0.0731473 0.2067506 1.39645 0.0997501
0.5650453 0.09958007 1.251864 0.06520124 0.5847228 0.3045074
0.06085449 0.3621093 1.137281 0.1187072 0 0.1529327 0.1595215
0.4850395 0.9661122 0.08676283 0 0 0.0942263 0.2335567
0.593243 0 0 0.231059 0.1138058 0 0 0.1584638 0 1.046334
0.877838 2.490788 0.06999238 0.6225965 0.2133859 0.3115469 0.4817886
0.2489502 0.7398444 0.05449656 0.06309797 0.2956856 0.6141332
0.09693635 0.03933145 0.03888466 0.02854412 0.1377837 0 0.1337189
0.6314834 0.1557598 0.1866066 0.1987987 0.2541395 0.3651269 0
0.1966572 0 0.2235471 0.2728707 0.05317382 0.415694 0.02049356
0.2707845 0.8572545 0.2047441 0.1150342 0 0.3134928 0.133583
0.01394058 0.2477064 0.2196252 0.06722002 0.03856975 0.2064808
0.2892094 0.04390304 0.2525374 0.3316766 0.07894636 0.8572545
0.478855 0.1586463 0.7781754 0.1339543 0.1144598 1.940895 0
0.07646637 0.05765162 0.08072076 0.3281767 0.09470151 0.1457269
0.1002637 0.3325634 0.2714373 0.2719369 0.1197137 0.09556212
0.03961594 0.0835106 0 0.1481609 0.04984668 0.2587514 0.02266696
0.2406372 0.5387119 0.02819513 0.1661604 0 0.1712488 0.1287872
0.5787726 0.5609119 0.220454 0 0.2913399 0.03933145 0.09497521
0.352549 0.1241225 1.454803 6.497911 0.08611086 0.09857195 0 0.136496
0.0855024 0.05873355 0.4554903 0.3135065 0.07532088
TCGA-FD-A6TB 572 0 0.1561705 0.3188294 0 0.3808364 0.4575409
30.94714 0.2473194 0.2373303 0.5441027 0.2931815 0.1683495
2.472277 0.4186197 0.8157984 0.0767218 0.3523505 0.3360901
0.3995726 0.3710931 0.03383496 0.2330069 0 2.409399 36.18845
0.09804448 0.4426249 0.3268149 0.1528997 4.044335 1.047675 0.4703692
0.2485407 0.1830164 0.4513856 0.3460242 0.1210935 0.2870114
0.2235264 0.3720348 0.05942479 0.1462319 0.1434832 0.07333989
0.06568287 0.4691301 0.1351127 0.1336773 0.290871 0.2859631
0.7401397 4.196132 0.3021658 1.021178 0.3388769 0 0.2844367 0
0.3834026 0.605818 0.100659 0.095638 0.03170362 0.1220109 0.2478327

0.1741702 0.3404956 0.527183 1.329891 0.08813171 0.1826014 0.4060953
0.5820242 0.8111714 2.614519 0.2147392 0.3202535 0.2666856
0.177586 0.2806912 0.06421627 0.3155454 0.161918 0.3590385 0.5863631
0.1101603 0.2518945 0.722217 0.03666994 0.36134 0.1407818 0
0.06587092 0.06658809 0.3157816 0.2707639 1.207908 0.3412169
1.660071 1.567016 0.06673893 0.1456715 0 3.086647 1.693329 0.8415222
0.2581 0.4648035 0.5624869 0.1284325 1.009786 0.1401936 0.2506033
0.1791085 2.209712 0.04703692 0.284884 0.09550667 0.047819 0.1670689
0.342507 1.486498 0.4108531 0.15486 0.1680451 0.27768 1.77772 0.04583221
1.157171 0.09150818 1.413462 0.2097062 3.188132 0.09327475 0.05592167
0.5656869 0.1100098 0.1617465 0.1118433 0.1756702 0.1954544
0.6314402 0.8127745 0.03986495 0.1550986 0.2115426 0.04329419
0.5580243 2.102741 0.08101328 0.06421627 0.237019 0 0.348435 0.05058241
0.4732611 0 1.261993 1.04622 0.07803023 0.09647827 0.6078874
0.7843558 0.1701162 1.068889 0.4194125 1.133122 0.1502373 0.440673
0.09880638 0.3405573 0.2078504 0.4698625 0.1929565 0.03934554
0.9405693 0 0.3071994 0.5356576 0.286268 0.4801451 1.132642
0.2371881 0.6121951 0.9734057 0.2168596 0.09220672 2.259692
0.1791085 0.1465908 0.5605947 0.07532947 0.4526677 0.4133341
1.254318 0.1761826 0.1106143 0.9320278 0.214821 0.160132 0.1958655
0.05045564 0.2040322 0 0.2276924 0.1328832 0.2420657 0.1160334
0.7654413 0.2901881 0.2625887 1.760157 0.09277327 1.090826 0.1342866
0.1893273 1.410263 0.4575409 0.2108042 0.05297842 1.118598 0.3149782
0.1450418 0.08927627 0.1382046 0.1146023 0.5551935 0.9886152
0.2200197 0.08781592 0.2184282 0.05116086 0.1560605 0.1299624
0.2519338 0.07925905 0.05207398 0.1326788 1.608894 0.07772896
0.08221855 0.8589568 0.146127 0.1141211 0.4005348 0.3681748
0.07596906 0.1229048 0.4672047 0.1445731 0 1.270576 0.5132753
0.8257183 6.211002 0.09309503 0.407618 0.2986914 0.4390112 0.1658735
0.08095898 0.634604 0.06457276 0.1779825
TCGA-XF-AAN3 2625 0 0.1393271 0.09932924 0.06316044 0.2845997
0.3563598 48.23907 0.06067747 0.1814311 0.1059448 0.1198822
0.09178437 0.9496134 0.6535732 0.548794 0.2572472 0.1903867
0.05497102 0 0.6196062 0.06571693 0.09527674 0.04772122 1.549305
62.9103 0.2111432 0.4181486 0.2939968 0.1667222 1.102488 0.8399909
0.307735 0.2032571 0.08730814 0.8244215 0.2180331 0.08665169
0.5029681 0.3213747 0.1028453 0.02699872 0.07293599 0.08123596
0.23991 0.1880045 10.4421 0.09207954 0.05466076 0.08649994 0.7249694
0.5447588 5.949919 0.1081114 1.461462 0.2566057 0 0.4695334 0
0.07191927 0.5907158 0.02057978 0.07821293 0.3629819 0.06236296
0.1842528 0.1548225 0.226247 0.2290384 0.150012 0.03603713 0.1835536
0.2060284 0.3867341 0.8593753 1.175987 0.1536623 0.1761078
0.04361919 0.1980412 0.133904 0.07877426 0.3440715 0.3531114
0.3250818 0 0.2477456 0.07068618 0.07382879 0.08996624 0.1899672

0.1151316 0 0.3366835 0.01815195 0.03080007 0.5535781 0.134704
0.09301594 0.685055 2.732104 0.1091584 0.02382608 0.01789546 0.7713034
0.6539368 0.8470133 0.3618422 0.1211623 0.3476765 0.1444195
0.7160456 0.3439517 0.08539326 0.2197129 1.20335 0.05770031
0.6989358 0.002055408 0.2737452 0.1878652 0.2178577 0.569841
0.6959924 0 0.08245653 0.2435211 1.261264 0.1311858 0.3761079 0
2.136833 0.02449973 1.318278 0.1303121 0.04573284 0.4898327 0.4723227
0.2076435 0.03048856 0.215495 0.1198822 0.3037605 0.7413833 0
0.1395239 0.1297499 0 0.2808328 0.7005881 0.2153216 0.2757099
0.3876691 0.256579 0.3957649 0.2275151 0.3721479 0.07664721
0.8109046 0.6193739 0.05317772 0.1052001 0.6725895 1.042352
0.2222548 0.5224147 0.5768574 0.7104463 0.02047739 0.1991591
0.04040202 0.5092724 0.04856584 0.2216852 0.1198113 0.0643537
0.6952377 0.05288165 0.1507369 0.4015566 0.2019203 0.2103555
0.567718 0.2193386 0.563236 0.1990132 0.2216852 0.1382458 0.7279921
0.8349091 0.3596466 0.4442554 0.02310171 0.5195683 0.4990844
0.3590241 0.3361924 0.3015352 0.2840979 0.1003892 0.1388136
0.3030417 0.1031568 0.1706849 0.0144928 0.2172418 0.05433604
0.04949044 0.09489234 0.5048905 0.4004713 0.4652819 0.884668
0.08670874 0.7186195 0.1235473 0.1806375 0.4639914 0.5300852
0.2011287 0 0.5119921 0.2027834 0.1067539 0.1095154 0.03767465
0.2030642 0.6382856 0.4185869 0.06747468 0.197494 0.01488591
0.1568979 0.1914398 0.126528 0.09365086 0.06481819 1.741775 0
2.142321 0.04237792 0.09125222 1.097588 0.1424842 0.08641519 0.5302682
0.4697067 0.03106381 0.03015352 0.4808209 0.2364642 0.1308541
0.8817687 0.1049394 0.8521314 6.834594 0.2226898 0.2314935
0.0732811 0.2820904 0.02855823 0.08827786 0.6790912 0.4671452
0.1940725
TCGA-XF-AAMH 344 1 0.01466359 0.01568097 0.1063579 0.05425431
0.02250321 74.47645 0.05108837 0.1492063 0.1338029 0 0.02897974
1.148185 0.1179185 1.114394 0.02377245 0.2235523 0.0347128 0.1650781
0.03194004 0.03494616 0.0721978 0 1.52757 977.11894 0.06750963
0.09458514 0.2025289 0 0.09282574 1.845906 0.1943268 0 0
0.2486453 0.1347529 0.04690143 0.6775712 0.1340521 0.08118024
0.08865482 0.04481243 0.01139965 0.07574851 0.13568 10.80729 0
0.1380675 0.04551875 0.7088511 0 10.2286 0.1170336 0.2260105
0.09074237 0.07816904 0.565795 0.0376003 0.02483132 1.636483 0 0
0.196469 0.03150449 0.01692377 0.07430249 0 0.4083725 0.04736439
0.006501866 0.04714958 0.01553453 0.07514237 0.03808236 0.1350193 0
0.2851476 0.01224196 0.1000463 0 0 0.4345447 0.3344713
0.2913664 0 0.6826692 0.0918237 0.093242 0 0 0 0 0.008504281
0.04584998 0.02094557 0.06732467 0.1512216 0.05873719 0.06946777
0.5170143 0.05169808 0.1203645 0.0452021 0.03542243 1.99185
0.6351549 0.03808236 0.2160308 0.5944708 0 0.343249 0 0.2588336

0	5.4795830	0.3923201	0.06749277	0	0.3019725	0.1965309
0.09595735	0.1212418	0	0	0.007547356	1.2646010.01183436	0.8274283
0	0.1473699	0	0.1479926	0.03211269	0	0.8363011	0.2272455
0.1126674	0	0.1088637	0	0.4603609	1.4077230	0.06407694	0	0
0.1773387	1.8098330.04183695	0	0.07650098	0	0.3998647	0.05224363
0.3384027	0	0.6827541	0.5057257	0	0	0.3323920.6075867
0.05999619	0.1633126	0	0.2340672	0.0517238	0.02395503	0.02551284
0.7436827	0.1226724	0.07466056	0.04428747	0.02709181	0.01868191	0
0.02115256	0.1383124	0.5395971	0.03542243	0.03773678	0.01884445
0.8876529	0.2284941	0	0.03174499	0.2121731	0	0	0.7788395	0
0.1968565	0.2611677	0.1619390.0242625	0.07616471	0.8926273
0.09508978	0.006615643	0.1257529	0	0.07926654	0	0.07839007
0.1372473	0	0.05992209	0.2200030.1123944	0.4068189	0.302994
0.1231973	0.2879219	0.05779034	0.1303634	1.2331960.03938062	0	0
0.009193647	0.08998288	0.02996105	0.09220827	0.2379058	0.118366
0.04986323	0.1720672	0	0	0	0.3170465	0.1611858	0	0.02365525
0	0.2151367	0.3745657	0.08521707	0	0	0.1108958	0.06965818
0.02182759	0.0610361	0.2129492	0.1830827	0.02538824	0.1789225
0.1866514	0	2.2638560.08835538	0.8934478	0.8792737	0.02403811
0.1871133	0	0.06477559	2.0468360	0.5020434	0	0.05106328
TCGA-FD-A3B8	384	0	0.1067250.08876759	0	0.1755002	4.24017620.87833
0.08262957	0.1723743	0.2164108	0.9795214	0.01171785	4.014175
0.1589331	0.1758444	0.1153476	1.5408780.1122879	0.07231112
2.0663740.04945623	0.07784776	0.09747894	0.6886829	12.846360.1091891
0.4589418	0.3275672	0.04256992	9.7587730.2402160.4321642	0.08896887
0.8152784	0.42729	1.1560690.1264295	0.3767134	0.1385199	0.4114058
0.1489040.2080414	0.1290634	0	0.3566012	0.4429514	0	0.0669925
0.09570776	0.04777022	0.2163712.1873730.3154806	1.5840330.4927123
0.06321473	0.08799119	0	0.8518532	0.2724672	0.1261134	0	0
0.4331166	1.0486990.1043631	0	0.3027271	4.0601420.03680607
0.2033574	0.9296366	0.1909822	1.5706430.7643235	0.2914620.2674923
0.6335988	0.09439119	0.1302488	0	0.08785328	0.04508074	0.2998874
0.2176714	0.06900869	0.2557746	0	0.1531431	0.1293471	0.05879412
0	0.1031602	0.05561781	0.5638109	0.4774404	2.29297	1.140008
7.8393941.2724950.0836157	0.02433447	0.1096638	10.627610.6678902
0.2703395	1.8170180.1164683	0.1857420.05363674	0.4804319	0.1405163
0.1395445	0.05984027	0.5613544	0.03928766	0.1982915	0.01679412
0.07988179	0.06977227	0.1907197	3.5187590.3921893	0.3233676
0.05614396	0.07324189	0.9243398	0.02871107	1.25153	0	1.23646
0.150135 12.745980.04544626	0	0.4817554	0.09188589	0.1602337
0.3425302	0.05869151	0.2040670.0930726	0.5326528	0.06659452
0.3109113	0.4417281	0.4701	0.1434125	0.1626220	0.08045511
0.4578067	0	0.2263573	0.3379923	0.2736637	0.03131307	1.154471
1.0092010.02172496	0.1074448	0.2688029	0.2183781	0.1559522

1.664078 0.1910809 1.009538 0 0.2615254 0.1856884 0.1056532
0.09920423 0.1207549 0.2626429 0.04381789 2.296404 0.10802 0.2480358
0.9321018 0.2988823 0.200521 0.335692 0.09448407 0.3392524 1.256514
0.1207549 0.3337351 2.173382 0.1196805 0.7754544 0.6464165
0.1101083 0.2233171 0.3777309 1.479835 0.2060195 0.06159384
0.4529324 0.2050624 0.03745015 0.1591754 0.1685726 0.06097532
0.02960408 0.174332 0 0.1010929 0 1.915115 0.5150576 0.0365546
0.6738298 0.1217685 0.5972859 0.009346924 0.1757066 4.951736
0.1910809 0.05869151 0.04425031 3.977636 0.2462933 0.0969171
0.1118522 0.5002209 0.1595362 0.3427542 0.6847619 0.3216006
0.3117306 0.5473275 0.06409829 0.1955246 0.06719844 0.0765193 0
0 0.1108202 0.7236013 0.2164108 0.09810453 0.1793612 0.0751094
0.0847287 0.3318051 0.3075186 0.1057555 0.3285005 0.2148476
2.083022 0 0.4820023 0.1190872 0.5911565 8.382521 0.02332733
0.3026343 0.2494824 0 0.08021088 0.1352422 0.06766647 0.05808331
0.1156246
TCGA-XF-A9SY 640 0 0.096205284 0.144032225 0 0.391548604 0.029527942
31.261441778 0 0.083907182 0.351143097 0.132445722 0.038026228
0.590376292 0.257881084 0.713302448 0.093580171 0.15690157 0.091097892
0.009025411 0.461016905 0.022927579 0.031578494 0.316334129 1.560938788
20.844206255 0.097442209 0.206852302 0 0.069072959 0.243605524
0.306246944 0.318736199 0.192478438 0.206695596 1.3050556 0.069189735 0
0.222271435 0.21498711 0.049710237 0.626391376 0.165514864 0.179498807
0.049697302 0.089017353 0.63579314 0.122075251 0.036233491 0 0.542575939
0.601848941 1.803147105 0.204756613 0.494272077 2.194272197 0 0.171326962
0.049337828 0.109752633 0.631569876 0.341047733 0.12961434 0.128899931
0.041339119 0.061068675 0.123154344 0.057682492 0.178617471 0.621499286
0.07678373 0.123736139 0.081535506 0.281712118 0.499703638 0.531502961
0.436541098 0.014966439 0.032126957 0.218795659 0.190204778 0.087029725
0.057019475 0.146293934 0.173782284 0 0.223943792 0.140569232 0.122348963
0.099394603 0.349792547 0.095397967 0 0.078113182 0.030081388 0.003926294
0.428114713 0 0 0.140873171 0.973368665 0.927098097 0.039484542 0
0.418320899 0.191241719 0.438646602 0.449733274 0.070867061 0.230467604
0.174059449 0.554337903 0.113999242 0.113210866 0.14564316 0.717632053 0
0.064348629 0.027249756 0 0 0.103152433 0.87704191 0.318178643
0.104937764 0 0.168357502 0.973282419 0.062114556 0.743921363 0.124017357
1.553032423 0.284206444 0.67966808 0.189617272 0 0.330712953 0.149091905
0.035685114 0.353679131 0.380925915 0 0.100678298 0.457554723 0.108054727
0 0.525607715 0 0.058174453 0.263866718 0.109794039 0.130544587
0.762902949 0 0.104937764 0.137104616 0.246689138 0 0.651553878
0.606307081 0 0.043584375 0 0.354335307 0.089971372 0.308582303
0.516738989 0.358324072 0.135740391 0.125731883 0 0.329673981 0.160966482
0.195934066 0.135595833 0.106646848 0.980550535 0.058423595 0.041633497
0.423473904 0.232780536 0.278880599 0.148550737 0.103853578 0.231354018

0.899466549 0 0.374892165 0.64961473 0.14564316 0.331114304 0.921114626
0.025522749 0.480740526 0.237250597 0.297487119 0.031836428 0.099940728
0.551188255 0.124773561 0.112850666 0.150660056 0.068380498 0.045663295 0
0.154291152 0 0 0 0.532767899 0.294960202 0.177937948 0.447275715
0.089808488 0.772028583 0.075830513 0.028509737 0.128488918 0.723434585
0.095231479 0 0.273441366 0.299722375 0.353824737 0 0 0.310631711
0.403477734 0.888268921 0 0.238026771 0.049337828 0.242676913 0
0.117421906 0.031039612 0 0.056459014 0.011987618 0.782732502 0.105342929
0.015918214 0.800325347 0.076169231 0.091652551 0.204673134 0.498972543
0.034319269 0 0.21343274 4.50648351 0.078854967 1.769983709 0.42510199
0.746041916 2.112505315 0.094625961 0.368285516 0.080960886 0.254988959
0.094653317 0 0.274485097 0 0.04020209
TCGA-CF-A9FF 361 0 0.3133781 0.2680966 0.07576642 0.2512203
0.03206133 54.349320 0.1062905 0.3812699 0.2876182 0 0.5994032
0.6160142.6720290.1580585 0.08888513 0 0.06859831 0.6825961
0.09957873 0.1714391 0.08586861 2.488 105.2977 0.0288552 0
0.0961840 0.1322531.8742140 0.0522481 0.04488587 0.6642303
0.2003359 0.1113709 0.6033538 0.3289272 0.3084297 0.1263105
0.08749304 0.03248319 0.1618835 0 12.63626 0.1325489 0.1573688
0.01297052 2.1040250.7079425 6.3873770.1667430.9660219 0.5725467
0.2227419 2.6353710 0.1191693.2230550.07406168 0.1407348 0.4198772
0 0.07233616 0.3231580.1878943 0.1939422 0.3374108 0.037054
0.2015284 0.1217301 0.1529410.4340611 11.79857 0.2764969 0.09750303
0.06976667 0.3325946 0.09178829 0 0.2166904 0.07942271 0.1509537
0 0.1621049 0.1817019 0.3985382 0.05396115 0.2278821 0.1035828
0.06276414 0.1211643 0.2286358 0.05968419 0.8485273 0.05386304
0.08368551 0.2069453 0.8326935 0 0.5573383 0.06440146 0.1009359
2.8378771.0478180 0.1624441 0.6737255 0.3779862 0.9028471
0.2475599 0.06146198 0.1581388 3.6862260.1384330.2794780.007396922
0.07036739 0.06146198 0.2240051 0.2027151.0364320 0 0.3440978
0.9008672 0.1011656 0.4147891 0 1.1482430.04408433 0.89612
0.3202667 0 1.2894490.7284755 0.1439166 0.1097210.3619070.07190454
0.2186323 1.76644 0 0 0 0.06370897 0.3158281 0.9741188
0.1788210 0.2034562 0 0.2278821 0.07443385 0.2142833 0
1.0611821.03155 0.4975720.2839425 0.05261931 0.48092 0.2808605
0.1675288 0.4488587 0.3890670 0.2047788 0.2544450.4581873
0.3058594 0.1063723 0.09464751 0.01929946 0.02661696 0.06343613
0.3616440.1313733 0.1369075 0.4542113 0.2150611 0.08591531
1.0827730.1953275 0 0.09045701 0.1007642 0.6325553 0.5752363
0.5037216 0.02771251 0.3739604 0.4293431 0.4845157 0.3802467
0.05425764 0.3740489 0.2257978 0.05655362 0.3115927 0.0742473
0.1156891 0 3.6856330.09777119 0.1781042 0.3414948 0.5631873
0.1067556 0.8372191.7807180.1755247 0.4815570.1482057 0.1238231
2.1711680.05610733 0.1034020 0.1790142 0.5818428 0.1707474 0

0.1355820.2248552 0.7577854 0.9644792 0.2428252 0.2584486 0
0.07528506 0.1148243 0.09106877 0.0674054 0.1166326 0 0
2.0033080 0.1382715 0 0.1654086 0.03109875 0.2270645 0.7368243
0.4099011 0 0.6566094 0.3191168 0.2568613 0.6629610.04196129
1.50437810.904140.1643912 0.2665887 0 0.2768661 0.1541613
0.03971136 0.4172489 0.1169491 0.07275214
TCGA-XF-A9ST 128 1 0 0.1348682 0 0.1999835 0.3041416 37.427720
0.07856861 0.2959215 0.1240189 0.1246239 0.0682043 0.1448841
1.6364010.1265711 0.06387772 0 0 0.1569763 0.04293764 0.08870796
0.9626742 1.43719699.538980.05806339 0.1549531 0.3317908 0 0
0.7038705 0 0.1802321 0 0.42007 0.3599309 0.01920894 0
0.09150392 0.04654744 0.1005493 0.1835337 0.01400652 0.5118886 0
0.4918034 0.2286165 0.1696407 0 0.3628962 0.1408891 7.510042
0.04793226 0.5091066 0.07963807 0.1920894 0.3342215 0 0.01445198
0.5913864 0.3832184 0 0.4023290 0.01299618 0.1489533 0.1080249
0.1254397 0.2327826 0 0.1158635 0.02863044 0.3033565 0.3275371
0.2764923 0.06812771 0.5045113 0 0.2253623 0.1781031 0
0.4538288 0.1369860.06509017 0.1377988 1.1183760.05641095 0.3436936
0 0.1965223 0 0 0.04179614 0 0.003676485 0.08272045 0 0
0.04267681 0.1104774 0.008469383 0 0 0.08704563 0.2984567
0.4518116 0.04679101 0.4350146 0.3320064 0 0.2433133 0.03558202
0 0 4.4195140 0.1205089 0.06379 0.06068383 0.2120157 0
0.02032773 0.07448365 0.09826113 0 0.1390992 3.09264 0.02908127
0.4518419 0.05806339 0.3168731 0.0380177 0.1363767 0.1282327 0
0.4926591 0.1396060.06682932 0.1892437 0.0445862 0 0.141409
0.5712572 0.3035393 0.15746 0 0 0.2723656 0.04941565 0.1028084
0.08149248 0.2694529 0 0.2456528 0 0.4157885 0 1.525247
1.5467320 0.04081133 0.2722688 0.4147385 0.09477784 0.1525008
0.09677232 0.1917290 0 0 0.5927012 0.07536252 0.1376009
0.009069185 0.09986147 0.02295408 0 0.01299486 0.1699416 0.563089
0.2176141 0.2318321 0.09261516 1.3894470.1122984 0 0 0
0.09091777 0 0.3336713 0 0.6953864 0.3702593 0.1193827
0.05962169 0.2807461 0.4731091 0.03894496 0.03251402 0.1477922 0
0.1377757 0 0.07223721 0 0.05119818 0.07362505 0.1164764
0.4603224 0.0555389 0.4188179 0.1009133 1.3689120.02840233
0.05339163 0.09023538 0.04838616 0.0891724 0.2016939 0.01882675
0.1785971 0.03681253 0 0.1169240 0.1429538 1.7330510 0.0557206
0 0.03246238 0 0.1963410.1453236 0.2011645 0.02643341 0.2693978
0.3141134 0.1315207 0.08943253 0.4087663 0.1711752 0.01609147
0.07499382 0.1308232 0.6427143 0 0.2598091 0.04586695 0
0.3147716 0 0.2993894 0.4260518 0.04725622 0 0 0.1591769 0
0.03424651 0.1884821 0 0.08783657
TCGA-DK-A6B5 1542 0 0.39414 0.2479330.04204081 0.2359002 0
40.981290 0.1685080.3384907 0.0797959 0.08018515 0.2540642

0.3728837	0.9669385	0.2568430.1500151	0.05488466	0.1631289
0.2272528	0.07597374	0.1712287	0.09529249	11.0546929.693350.16011
0.7103589	0.2668499	0.04161508	0	2.6334270.07681287	0.08697331
0.04981198	0.6142734	0.1991637	0.07415619	0.5356564	0.2001757
0.09412676	0.2264330.1246492	0.1802407	0.5389493	0	5.962298
0.2206437	0.9605180.09356104	1.1674680.8158544	2.0891540.1233618
0.5657992	0.09223291	0.3089841	0.2494509	0.2675255	0.01756413
2.4733030.04109489	0	0.2070923	0	0.006689574	0.1669456	0.1390102
0.4304538	0.1123323	0.01028015	0.2050085	0.1596514	0.3903696
0.9633966	2.5261790.1972555	0.0901698	0.004838962	0.1713660.08912911
0.02621683	0.3607075	0.3966263	0.1884606	0.3014512	0.08994777
0.1855118	0.9582664	0	0.8851207	0.1724261	0.03482618	0.08740017
0	0.01419308	0.9007099	0.6575182	0	0.3444856	2.2213450.1008129
0	0.03573468	0.02800333	1.3442251.2156690.1806369	0.04269599
0.6301750.1835178	1.4715880.1144704	0	0.02924903	2.87685	0.1536257
0.2326126	0.03693922	0.03904502	0.3410364	0.5903991	0.1778773
0.1437722	0.0632229	0.5214042	0.3430789	0.9612839	0.03742278
0.6056726	0.2241539	1.7730420.4158411	0.05849806	0.1967477
0.1369831.1320910.2245622	0.1750679	0	0.5737506	0.0797959
0.5459099	1.09246	0	0.05065626	0	0	0.3504894	1.9394880.1322974
0.1835178	0.3548052.4336710.7270634	0.2065070.2675255	0.03061072
1.1776450.4170610.04247534	0.2100697	0.7007299	1.22751	0.2235996
0.2065719	0.2801924	0.6476493	0.04089044	0.05681321	0	1.024889
0.6061193	0.08853477	0.08752905	0.3962243	0.3396882	0.1407962
0.1086945	0.2186868	0.3213962	0.1400167	0.1789977	0.3188075
1.3409910.6502927	0.08853477	0.07528834	0.5032027	0.3217393
0.03989795	0.9357239	0.03075389	0.6570859	0.4526393	0.1536257
0.1438561	0.3010614	0.3320799	0.1002314	0.2510410.2204406
0.1235936	0.1589537	0	1.9521040.1627520.06588359	0.2368581
0.1428158	0.4146511	0.7504284	1.0479570.1839666	1.74177	0.08223546
0.1030593	0.1016033	0.3735899	0.2581878	0.04325778	0.0242269
0.5034261	0.1894865	0.1093434	0	0.1247663	0.7226919	0.5530627
0	0.1613322	0	0.3968502	0.0637130.07579752	0.2618104	0
0.008503857	0.3972265	1.3473730	0.2589409	1.0081950.2019179
0.08627942	0.5817085	0.3246707	0.1033834	0.06021229	0.3257587
0.7673013	0.1187714	0.8514505	0.02328322	3.0179064.8853720.07601366
0.2958462	0.04877731	0.1024172	0.04989839	0.02203479	0.5402145
0.00405575	0.06458921
TCGA-GC-A3RC	484	0	0.1132618	0.03028002	0	0.1309566	0	18.7868	0
0.4115971	0.1033496	0.5847277	0.01399	0.09590728	0.07590038
0.5773390.1147617	0.02007816	0.06703055	0.03320485	0.3700581
0.03374059	0.06970712	0	1.5996538.2823810.1694698	0.2283054
0.1303614	0	0	0.3687364	0	0.03540679	0.1825059	0.4801351
0.1923280.03018895	0	0.1294275	0.07837969	0.1119338	0.07211081

0.03301916 0.03656767 0.5894971 0.6508823 0.04491197 0.07998267
0.008789689 0.1140662 0.3321339 3.536052 0.07533077 0.6546408
0.3629638 0.4528342 0.1996009 0 0.02397469 0.09294283 0 0
0.09484559 0 0.008169967 0.03020598 0 0.5585697 0.2743825 0
0.2200279 0.07499309 0.2280146 1.507512 0.1303614 0.1070701 0
0.02363927 0.08049579 0.03110093 0 0.1678215 0.5382212 0.2557408
0 0.4119491 0.07387997 0.09002534 0.07313534 0 0 0 0.0656872
0.06640237 0.01155599 0.1700055 0.7300237 0 0.06402258 1.692866
0.04658696 0 0.1309282 0.3420043 1.078832 0.1291039 0.03676859
0.1274644 0.1174015 0.09605574 0.8540118 0.1957236 0 0
0.4163387 0 0.1893929 0.01503794 0.09537126 0 0.07590038
0.4312878 0.292648 0.1544281 0 0.08380036 1.338377 0.0114261
0.5621774 0.1825059 0.3512683 0.1792469 0.178609 0.07751216 0
0.4756215 0.0548515 0.03751056 0 0.03503604 0.09745461 0.2222397
0.1496322 0 0 0.07031752 0 0.04280523 0.3494794 0.04039366
0.06403716 0.1969649 0 0.03860702 0.2522067 0.1089095 0
0.958837 0.4074848 0 0.06413946 0.0713167 0.2607227 0.3392835
0.006307148 0.3041765 1.129962 0 0.1040788 0 0.4366381 0.08883031
0.03604246 0.09977249 0.0261572 0.8477588 0.04298855 0.0510571
0.3115955 0.192692 0.2736034 0.1093048 0.03274984 0.07044088
0.4853454 0.07208492 0.0612997 1.911967 0.0714436 0.1949092
0.469982 0 0.2824582 1.052274 0.03127048 0.05856374 0.07353718
0.5407583 0.09180938 0.08303642 0.04751009 0.05031491 0.03546605 0
0.07568577 0 0.1206953 0.1157098 0.08461995 0.5787575 0.04364272
0.2925414 0.1585963 0.4592864 0.02231868 0.06293307 0.0118179 0
0.03503604 0 0.03550594 0.253953 0.0578549 0.08902728 0.09187941 0
0.152453 1.202266 0 0.0437855 0 0.1020363 0 0.01234285 0.1370352
0 0.04154299 0.008820585 1.028466 0.02583739 0.03513825 0.8565611
0.08967348 0.03793425 0.157148 0.1321733 0.02525239 0.02451239
0.2146289 0.07208492 0.02901106 1.075211 0.1137431 0.1960513
1.877235 0.04641769 0.2484051 0 0.1876229 0.06964666 0.02691106
0.06732277 0.009906563 0.0493017
TCGA-GV-A3QK 832 0 0.1142686 0.3221549 0.263713 0.2882633 0.06376735
30.38649 0 0.2189531 0.4739465 0.2860244 0.09238489 0.1345323
0.5012186 0.8664857 0.2077056 0.1049662 0.04918283 0.009745456
0.1357627 0.0742702 0.03409782 0.08539279 3.584558 39.08325 0.06695571
0.02233549 0.04782551 0.1864589 0 0.8116672 0 0.3377308 0
0.7926596 0.3112903 0.2104322 0.3900068 0.4537257 0.0498421
0.07246765 0.09053558 0.2745772 0.2414796 0.09611913 11.3126
0.2636288 0.1760588 0.09029054 1.046183 1.028952 4.272832 0.6080031
0.7471869 0.1653021 0.2768845 0.8478955 0 0.02407207 0.9547389
0.07365128 0.3498873 0.4175506 0 0.01948248 0.1994693 0.1868532
1.133097 0.4026494 0.04145476 0.3117515 0.1980909 0.4562805 1.78058
13.39114 0.2946051 0.105043 0.01300876 0.1771883 0.06845975 0.09397293

0.4309793 0.2764391 0.3189992 0 0.2821116 0.3216371 0.1321099
0.08049321 0.2266193 0.05150439 0.03120817 0.1506161 0 0.02755696
0.4952888 0.05356457 0 0.5569983 1.2898930 0.0213173 0
0.02509413 2.3059050.4025954 0.1079140.1232835 0.1914263 0.09397293
0.5518001 0.3077352 0 0.07863126 2.0117190.1032495 0.3821529 0
0.4198648 0.3056070.4733731 0.1195486 0.7730164 0 0.1229571
0.1550553 1.5763960.2179776 0.0651301 0.03347786 2.4109990.2849603
0.02621042 0.1535587 0.2455043 0.8683953 0.5634524 0.2724767
0.1091130.2827798 0.3575305 0.1630656 0.7593859 0 0 0
0.03167797 0.1884468 1.0541960.2963834 0.1174662 0.3143322
0.2295624 0.4249113 0 0.3196439 0.02743065 1.6269241.778456
0.3045014 0.2353076 0.9157353 1.7695440.5221773 0.04165011
1.4228091.3818250.03664243 0.1357627 0.1084436 0.5268434 0.1086302
0.2644570.1202683 0.04798129 0.2249904 0 0.1723275 0.4245972
0.1361488 0.2509413 0.5079399 0.2109281 0.9690958 0.3884903
0.1057828 0.02248894 0.1503087 0.5242084 0.1430122 0.9038526
0.04133842 0.5229677 0.3486872 0.2409154 0.3007929 0.08093548
0.3099802 0.1571826 0.3561882 0.4221925 0.1107538 0.08628602
0.02593355 1.1800870 0 0.2547018 0.1621912 0.2388691.056736
2.0391610.2327361 0.4345470.1064443 0.2308818 1.1424350.05579643
0.1542435 0 0.01953903 0.8090853 0.1273509 0 0.06741536
0.1397558 0.8713341 0.8853552 0 0.2570165 0.07991098 0.2058867
0.1712821 0.2581078 0.06703189 0.1739794 0 0 2.98831 0.1516629
0.1890698 0 0.1727166 0.1670027 0.1681536 0.9590292 0.0741145
0.07194265 0.7374731 0.1586742 0.08514599 0.7666993 0.08345755
1.8125128.4573960.2418146 0.3976672 0.04370996 0.6424411 0.07665355
0.07898261 0.7113202 0.02180645 0.1808725
TCGA-GU-A764 610 0 0.02675025 0.01430311 0.2910372 0.1855766
0.2463107 24.504350.1397980.2527494 0.3905467 0 0.06608336
0.9460311 0.5019338 1.2643950.09396228 0.3011217 0.09498794
0.1317514 0.2039347 0.0318755 0.2853669 0 2.07945127.61577
0.1416287 0.02875804 0.8620875 0.0480151.0160320.9676493 1.06351 0
0 0.4819443 0.1710086 0.04278028 0.4635257 0.08151544 0.2073318
0.2052724 0.0711525 0.02079597 0.2418242 0.8353671.5180430.08485873
0.1007486 0.05812674 0.4849242 0.2789107 2.5579040.0711667
0.2061514 0.1891863 0.7130047 0.4168335 0.1028893 0.1382808
1.1414680 0.0450497 0.2986760.08620875 0.1736630.1569495 0.08019419
0.1552040.9072538 0.06523614 0.07167772 0.1416954 0.08812252
0.4515697 0.7389322 0.0758637 0.1352480.1563279 0.07604621
0.08814528 0.03024868 0.3963621 0.1525410.2899250 0.07783554
0.1209799 0.3401960 0.04863058 0 0 0.02327107 0.08364245
0.09006699 0.3117686 0.2069010.4821845 0.7459643 1.6197930.09745461
0.08234121 0 1.7770460.2658775 0.3659021 0.1389445 0.1970486
0.3820297 0.1512434 0.3371720.02641494 0.2360904 0 4.1634860

0.4025786 0.02841337 0.0450497 0.4328323 0.03585241 0.8088587
0.1105885 0 0 0.1204732 1.175666 0 1.593305 0.04310438 1.310602
0.0282231 2.632281 0.04393651 0 1.61968 0.1554584 0.07796158
0.03512208 0.09929804 0.0920676 0.4549023 0.8599457 0.1126689
0.02922331 0.1328611 0 0.1213172 0.5502686 0.03816082 0.3932329
0.274464 0.344835 0 0.04765308 0.1028893 0 1.188909 0.4446954 0
0.151485 0.2358108 0.6157768 0.1837177 0.3217593 0.1077609
0.3914168 0.04717892 0.06555043 0.04654215 0.3941686 0.08392002
0.1362005 0.161584 0.06177826 0.562332 0.1218368 0.2315271 0.1682122
0.4921836 0.1615496 0.1032628 0.09969401 1.053663 0.4585169
0.06810027 0.05791122 0.3870597 0 0.230169 2.350877 0 0.3042528
0.2382194 0.5465259 0.0663918 0.03473613 1.213308 0.05782294
0.1146524 0.314187 0.0950673 0.09346295 0 0.3575104 0.1251876 0
0.1093137 0.5905948 0.205037 0.2886119 0.3109168 0.1997727
0.9020518 0.08433986 0.05945431 0.7787334 0.1436813 0 0
0.5702356 0.5808472 0 0.04205305 0.1302009 0.07197695 0.1212849
0.510081 0.1036389 0.1240955 0.03429643 0 0.07351134 0.0466423
0.2157671 0 0.009811653 0.04999805 0.3497814 0.02440917 0.0221306
0.252879 0.1270749 0.1075121 0.0866024 0.1803636 0.02385651
0.02315742 0.1780374 0.06810027 0.05481481 1.664347 0.1611835 1.70397
5.862606 0.08331852 0.618686 0.1688361 0.2954194 0.04934759 0.07627048
0.4324892 0 0.3353504
TCGA-GU-A42R 577 1 0.2212425 0.5027593 0.2005894 0.6650985
0.02122036 48.8748 0.04817596 0.2311508 0.3028203 0.1903652
0.437243 0.1294868 1.186094 1.589114 0.4931793 0.06128146 0.2946052
1.303715 0.1807153 0.3048243 0.045388 0.170501 4.79956 933.84757 0.1400544
0.3419068 0.4456276 0.2978372 0 1.140443 0.09162437 0.8299519
0.05941701 0.4396326 0.2651917 0.4717622 0.998351 0.4915949
0.1224838 0.0192925 0.1612056 0.2579949 0.1428606 0 13.34987
0.7018385 0.5728652 0.111602 1.169772 0.1441736 5.376849 0.4782345
2.237826 0.1711388 0.2211385 1.262029 1.418272 0.07764172 0.8623719
0.09803807 0.2794434 0.3087813 0.02970851 0.01595899 0.132758
0.9948895 0.4813653 0.1339928 0.01839363 0.363104 0.4687663
0.3644162 1.903303 9.591604 0.3137218 0.06453411 0.01154408 0.1572383
0.01518793 0.1563606 0.3483066 0.5256733 0.4995571 0.3595787
0.187761 0.2357147 0.08792652 0.1428606 0.1508278 0.06855809
0.4569571 0.168409 0.0216181 0.02821645 0.7618401 0.07130042
0.05538874 0.2828737 2.79806 0.02275042 0 0.0852505 0.2004185
3.756599 0.8511344 0.1436455 0.06224639 0.3822147 0.1876327
1.531261 0.2457779 0.0813594 0.5582239 1.933983 0.1832487 1.017376
0.01958314 0.3260173 0.5695158 0.6301124 0.09984781 0.9146409 0
0.8183478 0.4377017 1.318641 0.2566734 0.2745355 0.08912552
0.590617 0.058356 0.209334 0.8251853 0.272328 1.231553 3.107218 0.435969
0.181552 0.5475076 0.1427739 2.206761 1.863332 0.03882696 0 0.03433908

0 1.4214482.5031 0.2761636 0.2189048 0.6011655 0.1018577
0.7541390.2463268 0.6382225 0.1460530.9364831 1.8561190 0.2505765
0.5224054 2.4191211.3133710.2956850.7055771.1404050.2438758 0.4404936
0.09623369 1.1277460.1156791 0.4928305 0.08352551 0.02554738
0.08808452 0.2519180.6582414 0.7390897 0.2509329 0.4342402
1.6725190.3660657 1.1294440.08618732 0.07040436 0.5088999 0.06669257
0.6628909 0.1427739 0.4396976 0 0.8766060.3741550.09162437
0.4232682 0.5745821 0.2805803 0.5380138 0.3244021 0.5980761
0.2948513 0.2789368 0.03452045 0.2402441 0.1294232 0 0.3390366
0.2513148 0.4592775 1.3213833.2857930.4776048 1.3457360.1525884
0.1229318 0.9176190.1114069 0.4448499 0.05159899 0.09247512
0.3459399 0.2542774 0.2173793 0.1346060.2232366 1.6927440.9948986
0 0.2565882 0.07091361 0.2242294 0.6839865 0.1808264 0.2453740
0.02028724 0 4.2992110.2775853 0.1944746 0.1568609 0.3612793
0.2387658 0.7610261 1.37698 0.1233183 0.2154683 1.6003090.03520218
0.4250205 0.4634437 0.05554565 1.5318454.22749 0.3400164 0.2867255
0.2327313 0.4275804 0.05101721 0.05256733 1.42027 0 0.231131
TCGA-DK-A6B6 1115 0 0.2849665 0.2437903 0.06889727 0.2635893 0
25.3655 0 0.1380770.2426922 0.1307710.5068630.1097690.3564685
0.7394973 0.1642612 0.09429769 0.2248650.00891129 0.08276138
0.2376956 0.0623584 0 3.61903321.916090.02623912 0.08169471
0.08746374 0.4091974 0 1.8692250 0.1425335 0 0.5637423
0.3567554 0.2228024 0.3840567 0.2605116 0.04908168 0.0265059
0.0838613 0.1476910.2453445 0 9.3617210.4218609 0.1431014
0.1297404 0.3826538 0.4951991 5.3732660.1010838 2.0496940.08397391
0.2025476 0.7894163 0 0.01354561 3.11792 0 0.06398772 0.1272701
0 0.0109630.1266637 0.1139063 0.8817948 0 0.0168473 0.3054289
0.1207568 0.3198725 2.2202337.1720260.1795922 0.05910879 0.03965091
0.02160291 0.02086664 0.08592928 0.3659402 0 0.2059019 0
0.07370405 0.2974110.1208019 0.09813782 0 0 0 0.2974840.02970103
0.007753297 0.4965065 0.09795938 0.07609839 0.2945476 2.533706
0.004465246 0 0 0 1.2588240.6929603 0 0.2176875 0.2100494
0.1288939 0.7696809 0.1125578 0.1117794 0 2.5072050.06294119
1.4613040.0067263 0.1919632 0.3353381 0.2036963 0.08145095
0.7853887 0 0.2698380.1515615 2.1582570.1073260.2382211 0
0.8949778 0.1603502 0.04793386 0.1664175 0.07483009 1.884976
0.4416202 0.2969714 0 0.2350683 0.1307711.2922691.3553080 0 0
0 0.2871944 1.1463330 0.08592928 0.1982254 0.06997099 0.1036109
0.2707420.09742796 0.1003308 0.6433154 0.8767493 0.2784375
0.08606655 0.1913949 1.3119560.7217741 0.02539004 0.8673487
1.0108380.1340240.1241421 0.06610756 1.3801430.03973279 0 0.1051925
0.07019891 0.1452228 0.05768486 0.23294 0.2986567 0.4979805
0.3671391 0.5377988 0.3466842 1.1894050.2960311 0.04836415 0
0.09162868 1.0066110.1307710.7700602 0.07560009 0.3400562 0.2147298

0.2727452 0.07858469 0.09867704 0.8163282 0.4106529 0.2657028
0.4250144 0.2025476 0.1152195 0 0.3046805 0.4445352 0
0.07763352 0.1065967 0.1456153 0.5856268 0.8832404 0.6916487
1.5407520.08984602 0.2814925 0.4757409 0.05102051 0.2350683 0
0.0158814 0.2690296 0.07763352 0.1194627 0 0.1022347 0.1722707
0.3695876 0.1472067 0 0 0.6845954 0 0.08281227 0 0 0 0
2.1528980.1386812 0.04715081 0 0.3459481 0.07918195 0.2064783
0.6306091 0.03388532 0 0.6181531 0.04836415 0.2335737 1.1582950
0.9996832 6.7547630.2366880.1515120 0.5035295 0.09345648 0.03611103
0.9033813 0 0.2646251
TCGA-R3-A69X 433 0 0.03984392 0.1988390 0.1474199 0.6726033
20.201490.3701797 0.2123648 0.2423796 0.2742654 0.05249587
1.8761450.2136056 0.4923632 0.1148347 0.2684023 0.1886433
0.03114934 1.4464590.03165192 0.08718932 0.1091762 2.21492815.73125
0.06114576 0.1856161 0 0.2383911 3.1948660.8647757 0.2640125
0.1328599 0.4280203 0.2815081 0.4669751 0.02832014 0.3068496
0.1551420.2745034 0.0555907 0.2059472 0.1961760.03430399 0.3379475
2.2515520.2106588 0.1750734 0.2721040.2140102 0.2769543 2.168261
0.07066752 0.6482336 0.3170125 0.1416007 0.1773899 0 0.439158
0.3051626 0.04708223 0.04473371 0.1186324 0.3138816 0.8392316
0.1239705 0.1194475 0.5856383 1.3298850.0294448 0.1708199
1.0974720.4083539 0.9657894 1.5490260.1255526 0.3822365 0.6153816
0.03020512 0.3355202 0.03003651 0.2755073 0.05049033 0.455828
0.08126383 0.07728958 0.2079196 0 0.06860799 0.5794736 0 0
0.0539183 0.04152788 0.4336254 0.3987038 0.6505909 0.2128011
1.3499050.9772745 0.09052775 0.02725455 0.04094107 4.7162441.232058
0.1816678 0.3794173 0.2228423 0.2569790.06007302 0.9446340.2622966
0.03907239 0 1.2574310 0.2220860.03291642 0.04473371 0.195362
0.1424037 0.6803098 0.4392512 0 0.03144056 0.1333 1.288566
0.06431267 0.8743348 0 0.8968044 0.1401257 7.3387960.1090708 0
0.7989712 0.1543680.09149014 0 0.3944061 0.09142181 0.1042411
0.6316623 0.1118786 0.05803665 0.1319292 0.1620034 0 0.2914181
0 0.2402921 0.2633002 0.1956664 0.5070394 0.1419565 0.1362235
0 0.5621759 1.2423450.07299571 0.2406760.1672548 0.4891661
0.3027547 1.8755980.2140102 0.7773427 0 0.1518782 0.1386471
0.2730724 0.05555426 0 0.1337089 0.1226899 1.1167750 0.1245308
0.2923065 0.4217818 0.03208329 0.2392564 0.0921675 0.3469217
0.3725188 0.0676226 0.08625753 1.2811490.1340419 0.3199763
1.3597920.0176173 0.4729892 0.4776458 1.2760610.06592611 0.1724624
0.2219365 0.5167562 0.06591213 0.1634198 0.09440047 0.07704763 0
0.08875067 0 0 0.1085469 0.8132570.8483285 0.1637643 0.8232959
0.01239822 0.8163566 0.01046853 0.1771118 4.08531 0.03566836
0.1643359 0 1.6126620.1441933 0.0814102 0.08351616 0 0.07147208
0.3010854 0.6213378 0.05145599 0.1848376 0.2383911 0.1435799

0.07299571 0.06947271 0.1285522 0 0.02922849 0.08274558 1.466496
0.09695183 0.05493843 0 0.1892753 0.09094196 0.1719899 0.1515449
0.07106752 0.06898496 0.1571454 0.0676226 0.02721516 0.9470904
0.1867284 1.655235 6.079469 0.04789869 0.1271061 0 0.2933472
0.1715051 0 0.5431351 0.05575986 0.05549969
TCGA-5N-A9KM 530 1 0.1254225 0.08382781 0.1705714 0.261031 0.1202984
30.45755 0 0.1367369 0.286115 0 0.09295249 0.9495947 0.3782235
0.6102665 0.01694446 0.1222865 0.148455 0 0.1365968 0.1587938
1.003489 0.06443808 3.082245 30.60684 0.08661482 0.1853999 0.2887161
0.05628136 0.3969846 0.9300782 0.2077676 0 0 0.6978384
0.1691295 0.0835757 1.041377 0.07166197 0.1446586 0.1093693
0.0780787 0.08531686 0.08098775 0 1.193769 0.1492023 0.206664
0.01946684 1.199976 0.3677945 2.891909 0.04170945 0.6443808 0.06929905
0 1.640288 0 0.03074073 0.5660706 0.1111557 0.2112222 0.07001933
0 0.02714148 0.07944171 0.1410009 0.1819242 0.1012808 0.01390315
0.1176251 0.1494809 0.2984045 0.3664473 0.2405967 0.2074906
0.06097404 0.04581041 0.1069662 0.01722009 0.07091272 0.6969008
0.1788027 0.3681589 0 0.03041195 0.2727078 0.1993824 0
0.05700291 0.310925 0.04709987 0.02727747 0.04902125 0.05438614
0.3322212 0 0.06279982 0.2430739 1.586222 0.2468898 0 0
0.3787246 3.168456 0.3931551 0.08143273 0.3015479 0.158897 0.1063691
0.4516802 0.03096259 0 0 1.214448 0.1558257 0.734047 0.005550846 0
0.1844908 0.3361987 0.6261794 0.324069 0 0.0742275 0.1976998
1.820093 0 1.900377 0.05052531 1.792278 0.03308205 0.07911436 0.0343338
0 0.6736708 0.1214816 0.0539994 0.04116877 0.3879779 0.05395907
0.3691517 0.8284896 0 0 0 0 0.09480229 0.5160032 0.134192
0.1772818 0.2453768 0.8661482 0.1710087 0 0.2814068 0
2.322656 0.5056951 0.0287224 0.106539 0.1184608 1.227043 0.215347
0.3841384 0.1684177 0.5005134 0 0.1920893 0 0.4512845
0.1311571 0.1197368 0.2367533 0.8400039 0.2596629 0 0.0339234
0.3943439 0.110642 0.4165971 0.5245096 0.1732715 0.5135263
0.2442982 0.1197368 0 0.3024644 0.07911436 0.05395907 0.7176636
0 0.309862 0.2631221 0.3809073 0.1426736 0.2035818 0.3742616
0.2711114 0.04951255 0.2922842 0.1114343 0.08268205 0 0.146671
0.6603308 0.04455138 0.06406668 0.3748217 0.1201683 0.7249284
0.647902 0.1609891 0.7896671 0.0123575 0.09292011 0.317354 4.084129
0.1551912 0 0.09501886 0.4736327 0.03203334 0 0 0.126553
0.204363 0.1694446 0 0.1212167 0 0.08474385 0.0861672 0.1298468
0.07587419 0 0.05750423 0.4493112 1.184446 0.0286115 0.1297032
0.3556982 0.3847935 0.00933493 0.1522681 0.3089917 0.643165 0
0.4869401 0 0 4.460761 0.03148886 1.867068 7.083775 0.1336438
0.1250345 0 0.2770235 0.03856225 0 0.3727555 0.3510468
0.01091903
TCGA-FD-A6TF 69 1 0.1214628 0.04329669 0 0.2122187 0.994137

29.96417 0.4231799 0.2452222 0.4187018 0 0.05334394 2.670111
0.2894085 0.9506029 0.07293116 0.8564939 0.09584543 0 0.3821548
0.008040814 0.1328968 0.05546996 1.936587 31.69144 0.0994137 0.1450882
0.3106678 0.04844845 4.698851 1.132606 0.715407 0.06750313 0.02899566
0.4290838 0.4799113 0.0575553 0.5456617 0.1507941 0.4034638
0.1380836 0.08096011 0.01049185 0.5228726 0.2497506 2.210368 0
0.4066324 0.05865147 0.3805681 0.5980355 3.62255 0 0.3466873
0.3698574 0.5036089 0.8311786 0 0.4197901 0.7973807 0
0.1363691 0.7232934 0.4639306 0.4069246 0.1907595 0.04045906
0.4071731 1.176999 0.02393638 0.2386718 0.5218569 0.1679563
0.7360437 1.905429 0.1275809 0.3884114 0.4056149 0.07673271
0.1630585 0 0.3799432 0.051306 1.097033 0 0.05235879 0.2253637
0.1716335 0 0.4906958 0 0 0.07044345 0.06329814 0.3029335
0.266919 0.6611011 0.919015 2.031414 1.241328 0.02854869 0.2215587 0
3.87959 1.967369 0.8922459 0.2803976 0.2098734 0.3606088 0.03052175
0.3159146 0.1066136 0.1191108 0.1021555 4.443068 0.08942587
0.5416171 0.03822649 0.04545638 0.4367396 0.1085282 1.068927
0.5021406 0.07360437 0 0.2014435 1.219853 0.02178388 1.452561
0.1304805 1.089312 0.05695576 7.423302 0.3916094 0 0.9594928
1.098032 0.1787849 0.1417566 0.06679629 0 0.1412335 0.8677087 0
0.1179485 0.2346059 0 0.04080413 0.6292676 0 0.09156525
0.3332688 0.04970685 0.03680219 0.09616652 0.1730302 0.03563713
1.08539 0.5123328 0.04944997 0.09171152 0.4078963 0.7456027
0.09860437 1.869824 0.1449783 0.8976143 0.09520964 0.1433081
0.1174057 1.600157 0.02822587 0 0.1494558 0.02493438 0.2751071 0
0.1460107 0.2545961 0.8299789 0.09780479 0.3473165 0.09365646
1.219853 0.3785368 0.1374301 0.05843401 0.4556461 0.1702592
0.1857974 0.3772723 0.01790191 0.2440897 0.2865948 1.713996
0.04466076 0.0700994 1.288696 0.1166899 0.03044406 0.1559956
0.0959255 0.09430667 0 0.2344795 0 0.03835098 0.1103005
0.8724888 0.103444 0.3744222 0.6274471 0.1133866 0.3456436
0.0212753 0.09998504 2.196757 0.1449783 0.1001944 0 1.44692
0.2229693 0.05515025 0.127298 0.04379207 0.1452534 0.1376772
0.7376466 0.1568618 0 0.1730302 0.04863313 0 0.07059503
0.06531446 0 0 0 0.4705853 0 0 0.2041295 0.07479618
0.05223237 0.1903727 0.1539931 0.1203594 0.04673293 0.2594866
0.3092176 0.05530964 1.645688 0.2168514 1.532466 5.847142 0.05752203
0.2152659 0.05678676 0.238469 0.04149423 0.025653 0.3850531 0.02833033
0.02819814
TCGA-XF-AAMR 2790 0 0.100966 0.1511596 0.1220544 0.2739495
4.369467 27.83821 1.383623 0.557709 0.1105558 0.6486656 0.1263752
6.242848 0.2525995 0.26201 0.1891471 1.024985 0.09560586 0.05998954
1.275552 0.008020715 0.1877999 0.08299697 1.875467 24.99589 0.1208576
0.2677423 0.5887934 0.1208184 19.5154 1.090817 0.8474223 0.0336672

0.2747703 0.9844259 2.425831 0.2224693 2.604854 0.1880214 0.6185888
0.2316511 0.2140842 0.1046563 0.1043131 0.2646967 1.373192
0.2775849 0.025351 0.2799876 0.3796168 0.280725 4.535422 0.1074445
1.556193 0.5474488 0.03588215 0.5593935 0.03451953 2.925187 0.9500463
0.02386163 0.1360283 0.2404952 2.762164 2.279102 0.2728582 0.1008948
0.6560971 2.239406 0.002984569 0.3498984 5.697554 0.2907251 1.660699
2.003964 0.190893 1.010487 3.509352 0.09184909 0.5323121 0.01522273
0.388967 0.2558888 0.5593044 0 0.2089117 0.369984 0 0.08692761
0.2202612 0.06674581 0 0.121016 0.09470988 4.354086 0.4683179
4.477309 2.777116 4.957131 1.052491 0.1044169 0.04143843 0.06224773
17.93484 0.9812258 0.4910432 2.394903 0.6555922 0.4093223 0.4110137
0.7332716 0.1994008 0.6930763 0.06793346 2.19667 0.1115029
0.3151536 0.02859821 0.04534276 0.2376262 0.2706423 0.8885457
0.3617506 0.550653 0.1274745 0.2459783 2.031727 0.04345886 0.4782876
0.1518467 1.179605 0.04261005 24.88063 0.08475945 0.02651292 1.083305
0.2086263 0.4208777 0.3004798 0.1166013 0.4633326 0.316981
1.114531 0 0.07353352 0.4011762 0.6568362 0.2238618 0.3323089
0.03840906 0.1065591 0.3324357 0.7685304 0.8076243 0.1678708
0.2071172 0.01777402 0.8547449 3.548974 0.2219686 0.1372234
0.4407832 0.960663 0.3088438 6.546018 0.7773106 0.8058336 0
0.4563399 0.1639572 0.3275352 0.07038828 0.1713582 0.2439527
0.466351 0.7460779 0.04087645 0.7622127 1.290962 0.3257329 0.2276407
0.3118035 0.08650219 0.3739717 1.090817 0.5312104 0.1165759
0.973944 0.1358669 0.3011662 1.037258 0.02678574 0.4292269 0.6708922
4.80206 0.1837652 0.1048863 0.4097451 0.451043 0.1305822 0.2735648
0.09568573 0.1135951 0 0.5217623 0.126002 0.01912756 0.2200496
1.876088 1.221027 0.3734864 0.834505 0.1068197 0.6206035 0.08488851
0.2992054 13.82464 0.09038496 0.09994399 0 1.678227 0.1938162
0.0962717 0.1693065 0.06552392 0.1086678 0.637073 0.3408804
0.9127399 0.260214 1.121885 0.02425578 0.07398955 0.08508911 0.1845951
0.1503095 0.02962644 0.4696841 1.858078 0.1719757 0.05568641
0.2036192 0.2398154 0.1522976 0.4498376 0.6074504 0.2040995
0.03496209 0.3086145 0.1713582 0.1241356 1.351179 0.1081547
1.509994 10.42578 0.152273 0.1395731 0.4248361 0.267607 0.09105912
0.02558888 0.7809842 0.08242359 0.0984468
TCGA-XF-A8HF 2954 1 0.01524397 0.04890486 0.05528375 0.05640169
0.04678777 145.5101 0.1593313 0.06647652 0.5285753 0.1049318
0.03012676 0.5497382 0.1225857 1.582344 0.04942671 0.1864609
0.07217345 0.6006416 0.03320422 0.1271526 0.1501108 0.1879648
4.564324 73.84308 0.168436 0.1311051 0.8421798 0.1641718 0 2.183652
0.8080728 0 0 0.2907975 0.1096329 0.03250519 0.6163407
0.06967892 0.4332192 0.02126856 0.06901644 0.08295594 0.3937331 0
9.920997 0 0.05741288 0.0378563 0.7369073 0 4.583046 0.04055525
0.234956 0.1212865 0 0.656057 1.05539 0.05298693 1.150849 0 0.1026886

0.8510216	0.09825431	0.01979281	0.06098161	0.04569968	0.2476459
0.1477172	0.01351842	0.2369095	0.02422407	0.1339140.03958965
3.5090820.1152851	0.1067161	0.08908545	0.3986903	0.06697428
0.1379008	0.45174400	0.3855074	0	0.05914076	0.1803097	0
0.03937331	0.05542551	0	0	0.008840879	0.07149707	0.0777664
0.06460557	0	0.1221240.2593257	3.15476	0.1289863	0	0	0.1472978
0.8585774	0.7297989	0.1187689	0.01871511	0.3511363	0	0.2058662
0	0	0	3.5425230	0.4588291	0.1025476	0.05134429	0.3139246
0.1634476	0.3130253	0.1890608	0	0	0.06669167	1.8834950.01230276
0.3982304	0	0.8521902	0.03216659	0.5000130.01669185	0	0.9289498
0	0.06058281	0.08005907	0.1508967	0	0.4387001	0.8592675
0.1712154	0.1332262	0.1135689	0	0.09217888	2.1323280.04349285	0
0.3287193	0.05614532	0.2078457	0.05431143	0.5472392	0	1.806706
0.3101501	0.1954931	0.03453027	0.07678845	0.3509082	0.3831351
0.3191799	0.08187859	0.0811105	0	0.02490317	0.05304527	0.7522225
0.1275277	0.3104625	0.09975411	0	0.1942134	0.04628683	0.03298466
0.09585786	0.5609542	0.1472978	0.0392304	0.1723947	0.5372385
0.04750758	0	0.1320058	0.2940945	0	0.1049318	0.8682601
0.04044144	0.3297107	0.3498233	0.4377061	0.02522281	0.1583586
2.2926010.1977068	0.05501991	0.1932533	0	0.08039404	0	0.2648514
0.4993783	0	0	0.3123853	0.03894765	1.0338060.5118531	0.1707646
0.5335665	0.01201554	0.1129360.6394122	0	0	0	0.07964609
0.2086759	0.0311469	0	0.1483932	0.08203396	0.09503414	0.4895598
0.3543598	0	0	0.08239879	0	0.08638405	0.04918304	0	0.01118259
0.2374335	0.08858995	0.02781975	0.01261141	0	0.2534533	0.04992136
0.0634519	0.1106888	0.02718987	0.1055724	0.1521853	0	0.03123692
0.7011459	0.03061749	0.7599357	2.2787510.03498535	0.4133533	0
0.0673394	0.04686892	0	0.2899524	0.01066664	0.1061687
TCGA-ZF-AA4U	262	1	0.2503781	0.1427999	0	0.2007162	0	69.561550
0.1698447	0.3046213	0.1148984	0.03298826	0.02327997	0.4474306
0.7425593	0.02706068	0.03254905	0.03951432	0.1565933	0.03635803
0.01988998	0	0.06860602	1.3654515 7.780970.06147812	0.1256130.23054300
0	0.8936284	0	0.1252332	0	0.3537979	0.3267920.0533889
0.3856469	0.09325209	0.04312429	0.06210319	0.03778588	0.06488234
0.04311307	0	6.95444	0.2647548	0.1257321	0.03108898	1.344834
0.3480747	5.3156330.1776291	0.3859089	0.01475628	0	0.6935993
1.2840370.005950742	0.4383162	0	0	0.2236448	0	0.002408086
0.1513539	0	0.5810739	0	0.01480242	0.4114806	0.1503079
0.07331671	0.4768495	0.7684765	0	0	0.006967641	0.2277698	0
0.07549945	0.07419773	0.2538239	0.09045507	0	0.09713712	0.1103318
0.1061394	0	0.4855195	0.08275901	0	0.03872242	0	0.006812225
0.4067662	0	0.1337236	0.3378729	1.2026450.0117698	0	0	0
0.8295237	0.6088509	0.1300499	0.1571108	0.2768313	0.03774972
0.9016791	0.09889587	0	0.1684632	1.40075	0.05530158	0.3907631	0

0	0.09821194	0.1342292	0.01883281	0.2070182	0	0	0.2104873
1.32879	0.06735651	0.4534974	0.1075867	0.6552901	0.03522184	0
0.1553569	0	0.5998774	0.1940088	0.1724765	0.04383162	0.08261458
0.05744922	0.4803687	0.8526748	0.04686946	0.03647007	0	0
0.05046712	0.5035974	0	0.03774972	0.2322205	0	0.09103491
0.05947004	0.08560245	0	0.2826158	0.7206333	0	0.1512401
0.2942869	0.4610859	0.370744	0.02230827	0.2241390	0.5328873	0.0588783
0.05453704	0.02904181	0.2287973	0.03491014	0.04249385	0.008402228
0.04625878	0	0.1520497	0.09631364	0.4198506	0.4711939	0.2822548
0.1288697	0.1072552	0.3737223	0.1560598	0.3399508	0	0
0.4211580	0.2566386	0	0.541541	0.6346057	0.03686772	0.1380926
0.1300499	0	0.1804046	0.06777655	0.2365041	0.1779630	0.07482553	0
0.3123158	0	0	0	0.05497334	0	0.9776358	0.1724523	0.06232805
0.2992478	0.05262719	0.09893031	0.04876631	0	0.04130729	0	0
0.2678924	0.0341053	0.1049626	0.05416265	0	0.7095047	0.7499675
0	0.07743428	0	0.1203003	0	0.07276077	0.08078183	0	0
0.4575744	0.4365199	0.06092427	0.01380926	0.06311754	0.07929339
0.02981618	0.1582570	0.5540676	0.3274967	0	0.5554673	0	0.03420387
0.2678171	0	1.4330893	3.7075450	0.1915417	0	0	0	0.01026413
0.03172799	0.2539941	0.005839891	0.0697517
TCGA-DK-A3IQ	539	1	0.1126734	0.0535515	0.04540236	0.1968621	1.248811
27.50304	0.1308525	0.2456754	0.1370836	0.4308816	0.06185478
6.066271	0.1006748	0.2552621	0.09471513	0.8255862	0.08890979
0.005872419	3.272317	0.02237688	0.12328	0.1543681	1.270246	19.55151	0.0288187
0.3499320	0.1729122	0.1797703	7.528887	0.6883553	1.990915	0.09392763
0.8069237	0.3449629	1.0504330	0.1067809	0.4700207	0.1653153
0.2125473	0.1397362	0.1742924	0.09732633	0	0.2606377	1.49285
0.03971437	0.07072638	0.05440729	0.6556255	0.06526589	3.158682
0.09991926	1.25424	1.361306	1.001071	0.2786864	0	0.4686345	0.4931201
0.04438081	0.1265011	0.08386924	0.4303593	1.336526	0.1001636
0.07506267	0.3486544	1.415333	0.03330642	0.1945644	0.9947136
0.1466377	1.007916	2.036522	0.04733953	0.4674226	0.5173623	0.1423602
0.2750166	0	0.1112998	0.1903734	0.5653606	0	0.07285495
0.1437259	0.1592137	0	0.04551877	0.1862132	0.03761085	0.05808531
0.03914514	0.3014497	0.3846705	2.227109	0.752217	2.356193	0.6717095
1.053428	0	0.038592	4.506126	0.2488643	0.7705992	0.9428889	0.2715362
0.3114442	0.1132525	0.42831	0.09889873	0.1104916	0.06317552
1.373852	0	0.3349495	0.03989286	0.1265011	0.1473222	0.2013496
2.971904	0.3622923	0.6145035	0.0296366	0.1256516	0.5086923
0.04041508	0.8895784	0	0.7077338	0.02641715	6.096438	0.2330421	0
0.5086065	1.164087	0.1857493	0.1314987	0.1239255	0.08617632
0.5895605	0.6395246	0	0.8206004	0.4041685	0.5344785	0.1514057
0.3777090	0.1071569	0.1698787	0.2525471	0.04610993	0.2389736
0.08920765	0	0.03305833	0.5299201	0.8169485	0	0.1417917

0.2207216 0.288187 0.1097629 4.194077 0.2689746 0.9658862 0
0.1636159 0.10891 0.291725 0.1309168 0.03187131 0.3213945 0.04626012
0.7974987 0.03801354 0.243801 0.3936213 0.3092299 0.1512123
0.2899653 0.05148399 0.4412127 0.1950805 0.1912279 0.05420556
1.0264495 0.2211143 1.206468 0.3762197 0.01660648 0.2100867 0.351616
3.4426230 0.1035725 0 0.4034619 0.1894303 0.0621303 0.1447073
0.08898407 0.04291589 0.03125409 0.2007803 0.117177 0.177879 0.1023189
2.0539250 0.3198617 0.154368 0.3880289 0.05843424 0.3954461 0.0592073
0.3524494 4.595487 0.1008655 0.06196273 0 2.1847 0.2659303
0.1023189 0.07872427 0.04062316 0.1347425 0.6243822 0.3498661
0.3395253 0.1742322 0.2247129 0.1353417 0 0.04911494 0.1009802
0.1397821 0.01836764 0 0.873065 0.02284726 0.06214348 0.6627534
0.1189435 0.1863565 0.4197793 0.2597267 0.04465993 0.02167561
0.1573869 0.1274853 0.02565364 0.6026059 0.8549277 1.24821
10.73175 0.04515041 0.4792528 0 0.1106064 0.02309495 0 0.1309695
0.004380045 0.02615765

TCGA-E7-A4XJ 68 1 0 0.2369277 0 0.1676745 0.06181939 72.04442 0
0.1903061 1.065967 0 0.09951413 0.1886112 0.2699485 1.306692
0.03265307 0.02856413 0.04768039 0 0 0.08400164 0.0661124 0
2.76275 138.0047 0.102002 0.02165319 0.2781872 0.4338312 0 4.488087 0
0.05037135 0.04327357 0 0.3138523 0.0429482 0.2908407 0.1841297
0.1709768 0.09367188 0.07295161 0.2192148 0.2080915 0 39.35396
0.2555754 0 0.02500924 1.054793 0 9.548655 0.1071691 0.4139211
0.1602525 0 1.942895 0 0.03231238 3.834519 0 0 2.833567 0
0.02615169 0.1880044 0.1207635 0.2337198 0.2602329 0 0.1187325
0.04267547 0.01474474 0.6800132 4.883731 0 0.04700037 0.008407582
0.2977444 0 0.2733068 0.3879699 0.1531397 0.1455315 0 0
0.08408407 0.512297 0 0.2196966 0 0 0.05840604 0 0.01233007
0.2418578 0 0 0.1561397 1.235051 0 0.08266442 0.372529 0
2.268829 1.744857 0 0.02472775 0.4082731 0 0.4714758 0.03977793 0
0 2.730372 0.06673027 0.8756774 0.0570498 0 0.2370171 0.2699485
0.1090791 0.6661354 0 0.04768039 0.1244017 1.252656 0.03251059
0.6734987 0 0.1960968 0 0.304917 0.363899 0 1.038566 0.3901715
0.06937355 0 0 0.1386435 0.5269475 1.898124 0 0 0 0
0.5480704 2.485928 0 0.04555113 0.1611216 0 0.1647724 0
0.6714055 0 0.08525539 1.129429 0.7379989 0.410615 0.2029169
1.390936 0.153045 0.1076741 0.1622759 0.4822608 0 0.0493558 0
0.9524787 0.04212471 0.05127568 0.09124778 0 0.02566088 0
0.1016907 0.6965989 0.4264285 0.09731024 0.2073362 0.1449512
2.672333 0 0 0.3488312 0 0.4573755 0 0.4645137 0 0.634679
0.2690487 0 0.06665241 0.1569261 0.2884905 0 0.07269629
0.3004003 0 0.07170014 0 0.565289 0.2827778 0 0.4115354
0.09335924 0 1.241763 0.05202287 0.2068243 0.2407271 0 0
1.185295 0.3245518 0.1495317 0 0.04209367 0.3517778 0.1234606 0

0	0.1083892	0.1826414	0.2985424	0	0	0	0.1088712	0.1106998
0.02633928	0.0974763	0	0.01477525	0	0.4096801	0.2205448
0.166631	0.4569689	0.1913605	0.02998169	0.1816474	0.334286	0.6466541
0	0.1414995	0.05127568	0.2476349	0.5745146	0	0.2231293
3.657248	0.2113151	0.1285064	0.08474942	0.2669211	0	0.07656986
0.6321125	0	0.3366667
TCGA-XF-A9T6	64	0	0	0.0180413	0.1223671	0.09363119	0.02589044
118.3553	0	0.6130899	0.4926182	0	0.01667092	0.1344542	0.2261131
1.56358	0	0.2691648	0.03993785	0.1820125	0	0.2211348	0.2215071
0.06934136	2.763985	101.3703	0.1553427	0.1269594	2.640825	0.06056397	0
1.098495	0	0	0	0.286072	0.03370354	0.1079223	0.9744509	0.1285249
0.03113322	0.4629201	0.0821104	0.01311555	0.3050262	0	1.623937
0.107037	0.09530984	0.05237034	0.1359248	0.4837325	8.176951	0
0.5633986	0.1193156	0	1.727547	0	0.01052542	0.6091446	0.1196138
0	0.2260419	0	0.00730169	0.08548667	0.05057668	0	0.1089876
0.1047276	0.009041107	0	0.2593589	0.0438146	1.915893	0.06379405
0.01312275	0	0.03836852	0.01853042	0.114463	0.6249417	0	0.3656984
0	0.03272609	0.09390685	0.536385	0.04357517	0.3680426	0	0
0.02935309	0	0.00344262	0.1966255	0	0	0.1743801	0.6982862
0.1387862	0	0.1560181	0	0.2235773	0.9230651	0	0.05523295
0.388609	0	0.501239	0	0.0496323	0.04256721	6.171276	0.05589432
0.1692649	0.0238929	0.05682368	1.141543	0.2261131	0.04949013
0.1394914	0.09201065	0	0.04775871	2.196418	0.01361569	1.427962
0.1631098	1.31403	0	0.08513441	0.2586245	0	1.304878	0.6536276
0.03128912	0.04430143	0.04175003	0	0.2206898	1.277862	0	0	0	0
0.1020161	1.526985	0	0.1526174	0.4283449	0	0.5980692	0
0.2595599	0.04454892	0.4284675	0.21767	0	0.1528611	0.2549496
0.3883567	0.1331234	0.03006317	0	0.04488325	0.05950938	0.0137804
0.1174124	1.017498	0.105853	0.04294932	0.1698457	0	0.1074698	0
0.06084122	0.4773945	0.1615829	0.08150858	0	0.04769811	1.056239
0.4206201	0	0	0.4882198	0.1277016	0	1.149566	0.02237864
0.2327791	0.05778447	0.01863144	0.02791455	0.1752584	1.006851
0.1823382	0.07611444	0.09435749	0	0.1912932	0	0.2705686
0.2368591	0	0.0689417	0.04938894	0.1724164	0.2600301	0.1307255
0.3779766	0.936171	0.02659563	0	0.01760322	0.1359248	0.1252501	0
0.5359265	0.2707637	0	0	0	0.181577	0.08605313	0.46366	0
0.4956735	0.1297799	0.09119226	0	0.02941626	0.05443179	0.09418413
0.04950392	0	0.09804414	0.03078864	0.04187183	2.934526	0.2938587
0.2159729	0.0546182	0.1400016	0.3310069	0	0.1309982	0.1717973
0.1382819	1.515851	0	1.448449	2.350957	0.04425021	0.3498268	0
0.07452576	0.04149656	0	0.6899256	0.06492733	0.1527485
TCGA-4Z-AA7M	495	0	0.145108	0.4073364	0.2631242	0.5872234	0.02783592
23.87421	0	0.2504804	0.6620434	0.2497125	0.07169446	0.3794633
0.6806908	1.781935	0.06861384	0.06430901	0.04293888	0.587068	0.1185271

0.06484131 0.4167655 0.1491037 11.0751463.42615 0.1252616 0.01949991
0.334031 0.1302298 0 1.259774 0.2403774 0.1814489 0.03897028
1.499395 0.3696083 0.5994977 1.361976 0.6632765 0.1338906 0.06748544
0.1621895 0.4653359 0.5153447 0.04195822 7.270504 1.4385 0.61483
0.1238723 1.315247 0.4728012 3.022761 0.6755824 1.491037 0.06414062 0
1.709309 0.4185958 0.05981485 3.4532 0 0.2443742 2.551786 0.1169108
0.02616786 0.6723968 0.1631314 0.7998156 0.05858858 0.0160853
0.7484769 0.2401979 0.5842525 2.496668 7.29301 0.3772323 0.08465295
0 0.5568969 0 0.2461281 0.483769 0.8274655 0.9174151 1.304054
0.6333337 0.05679183 0.1153381 0.4216457 2.30824 0 0.05449235
0.2945484 0.02835765 0.03331177 0.8071653 0.04676434 0 0.3827787
2.864 0.01278985 0.03722198 0.05591388 0 3.785945 2.811875 0.3768555
0.3562997 0.9693193 1.107576 2.073961 0.2865781 0.2134472 0.6407214
2.02953 0.1201887 0.7279355 0.02568828 0.4276549 0.266809 0.7779323
0.2087428 0.674879 0 0.3864499 0.5041383 1.68461 0.1903045
0.1705847 1.286019 2.500831 0.07654877 0.04576581 0.3277115 0.142891
0.8219187 0.5621942 0.1153381 0 0.8977447 0.1248562 0.9490918
1.59754 0.1527944 0.0396308 0 0 1.041977 1.591978 0.3105077
0.1230641 0.8895204 0.2004186 1.533331 0 0.2790639 0.1915857
0.84455 0.4995561 0.06646095 0.698477 1.187798 2.922771 0.7103322
0.2747387 1.461386 1.447677 0.3199053 0.3555814 0 0.2610576
0.113807 0.1385299 0.109565 0.06702381 0.2310906 0.1652273 0.3270649
1.596831 0.4754568 0.2190834 1.447064 0.2470873 2.541956 0.169585
0.04617664 0.03926777 0 1.327209 0.2497125 0.919038 0 1.785715 1.31087
0.1602516 0.5702303 0.3768555 0.1515511 0.5097028 0.548287
0.3516855 0.3223106 0.1123996 0.04528237 0.412108 0.08488577 0
0.6670994 2.51562 0.2780582 1.73333 4.497554 0.4233112 0.8826382
0.2001587 0.08062817 0.007570386 0 0.3590979 0 0.03032617
0.8133944 0.8524048 0.1140594 0.2942836 0 1.716748 1.543111
0.07027428 0.3365809 0 0.6536312 0.1993828 0.4664933 0.2926096
0 0 0.4068251 6.746331 0.2317152 0.1650667 0.4801139 0.31594
0.1674012 0.8682518 0.9407603 0 0.03140462 1.871183 0 0.2973455
0.7243358 0.03643114 3.164813 4.013545 0.3508677 0.02893182 0.3816081
1.121761 0 0 0.2587564 0.03807607 0.06316401
TCGA-ZF-AA56 259 1 0 0.046544244 0.052615233 0.093938612 0.044529342
46.052074848 0 0.147624697 0.185338343 0 0 0.589686867 0.1944476
0.403383452 0.070561358 0.059153493 0 0.210965676 0.505623497 0.051863587
0.047621658 0.178891792 1.13108077 33.343291739 0.180343835 0.421121314
0.267176052 0.05208242 0.091841768 0.776718953 0.04806672 0.072566335
0.124682158 0.430516868 0.06376391 0.046404262 0.125697898 0.07368394
0.053546476 0.047231188 0.096885441 0.045115254 0 0.302044053 3.043154186
0.046023615 0.136603958 0.036028995 0.116889523 0.226903192 3.303667044
0.077195332 0.596305972 0.487379757 0 0.23683738 0.297613831 0.043963979
0.285729945 0 0 0.032397726 0 0.043953981 0.1044685 0.130481328

0.202021827 1.218420176 0.006432944 0.085524609 0.092219153 0.201795852
0.602858785 1.603056315 0.109720299 0.033855002 0.006056095 0.115483474
0.015935365 0.032811094 0.042993848 0.110308611 0.052414156 0.266311405
0.084429095 0.060566897 0.276760844 0 0.211000575 0.071932014 0.043585924
0.033656533 0.02268198 0.032565566 0.163965577 0.074809295 0.11622913
0.428009139 1.668045084 0.057970073 0.119088602 0.044722948 0.210281663
0.672934076 0.396898187 0 0.112807667 0.240614691 0.032811094 0.496353733
0.028652585 0 0.036605972 1.07181248 0 0.097040359 0.005136718
0.048865929 0.170726606 0.15555808 0.851187869 0.119956595 0 0
0.097075582 1.51587255 0.070253464 0.45480964 0.093511618 0.829281329
0.030613923 0.21963583 0.230349083 0 0.83877088 0.281045848 0.096097515
0.076194652 0.035903239 0.049933388 0 0.293893658 0 0 0.036028995
0.044242056 0.08772945 0.159168712 0.041393473 0.328110942 0.383496262
0.106870421 0.276938254 0.258449197 0.148806915 0 0.552695535 0.755946941
0.186056708 0.098590524 0.146163808 0.667940131 0.347681285 0.116338677
0.155852697 0.308781329 0.153526538 0.035551652 0.2019392 0.268467382
0.030343003 0.036934571 0.138757034 0.1608278 0.665419602 0 0.062785013
0.273692542 0.372982708 0.035046944 0.112010287 0.041018328 0.252645425
0.452144089 0.110803713 0.062816965 0.139949361 0.292847773 0.299600331
0.359943686 0 0.178538948 0.327968285 0.416578238 0.108023923 0.113036022
0.554142924 0.188163622 0.065455158 0.183925034 0.051560291 0.107118841 0
0.155118236 0.067896225 0 0.059286905 0.32385185 0.556015028 0.178891792
0.262309458 0.094804406 0.842223848 0.022871102 0.128981543 2.001244966
0.038963174 0 0 0.130378763 0.198603237 0 0 0.047076787 0
0.1480040.707853627 0.05620917 0.112173152 0 0.026140478 0 0.069565975
0.070213502 0.080994315 0.319284367 0.280206168 0.632353157 0.026476906 0
0.658321793 0.11486631 0.073427137 0.093938612 0.18059303 0.025877429
0.025119116 0.219941277 0.443214852 0.089187382 0.817315528 0.029139598
0.723253923 3.614594421 0.047566604 0.277694795 0 0.128177919 0.02676395
0 0.400136905 0.101517671 0.161670381
TCGA-XF-A8HH 57 1 0.05067948 0.3613047 0.06126471 0.3593957
0.8555179 33.55553 0.05885627 0.2210051 0.2466358 0.232568 0.03338606
1.7031020.5886741.7222190.1551930.3234262 0.03999082 0.2456466
0.5151506 0.1308439 0.02772512 0.06943334 4.23737 60.734 0.124439
0.01816111 0.07777436 0.1212886 1.6040963.1531950.2798423 0.2534868
0.1451788 1.0383861.0056980.1620981 0.6342332 0.2402314 0.1496377
0.03928255 0.1453179 0.1969943 0.2181649 0.1953875 14.19796
0.6430739 0.2863088 0.08390368 0.8846833 0.6605102 4.706372
0.1348283 0.9113126 0.1642765 0 1.1030850 0.3191926 1.552607
0.1197725 0.2275962 0.7921961 0.3266523 0.7896285 0.3063581
0.1012875 0.3528486 1.4732840.03745232 0.3168585 0.5011028
0.3462705 1.4916726.4293470.1596967 0.1445417 0.3948932 0.4418232
0.14844 0.2292297 0.4505549 0.2568847 0.4882446 0 0.2949255
0.1586780.1074193 0.2181649 0.4913744 0.0837570 0.1665548

0.0264107 0.3722962 0.4295701 0.3048755 0.2706723 1.265933
1.605599 0.09926447 0.1386657 0 2.203651 2.630518 2.503284 0.4387271
0.08295931 0.8716388 0.1146148 0.8517156 0.6005307 0.2484907
0.255742 3.756143 0.1679054 0.3954753 0.0119623 0.1137981 0.3975851
0.3169783 0.7090289 0.7682202 0.1842654 0.1599633 0.1304238
2.143298 0.1363375 0.8296695 0.3266523 1.856938 0.07129316 2.727915
0.4069491 0 1.09544 0.06544946 0.2327418 0.2661611 0.2926379
0.465136 0.9281264 1.800307 0.2371733 0.07381973 0.04195184 0.1030301
0.2043028 2.919021 0.2891892 0.07640989 0.2761495 0 0.4606635
0.06018719 0.2599042 0 0.9295777 0.611961 0.2785407 0.2295959
0.2552878 1.322164 0.8491703 1.708348 0.5444205 1.348283 0.1191766
0.4553563 0.02939203 0.8451806 0.07066225 0 0.1275533 0.01560554
0.5165391 0.1025889 0.316794 0.9560555 0.4257821 0.2448501
0.7825427 0.3343296 2.899755 0.3158835 0.04300629 0.03657176
0.4073895 0.767226 0.406994 1.387212 0 0.8000583 0.7753389 1.082057
0.195661 0.1316181 0.362947 0.4381921 0.1067016 0.5165052 0.2401454
0.1447738 0 2.054533 0.5534043 0 0.483232 0.7995137 0.2589676
1.2498 1.527154 0.2523186 0.5191825 0.1065236 0.4255241 2.129289 0
0.1254162 0 0.423661 0.3827617 0.2070994 0.1062284 0 0.09090892
1.857383 0.5816478 0.06544946 0.3134722 0.08663473 0.1521887
0.09284691 0.2135507 0.13626 0.09430906 0 0.7156881 2.35618
0.03082947 0.1118063 0 0.1604992 0.2162594 0.2461079 0.6133194
0.0602629 0.05849695 0.7058302 0.08601257 0.4846286 0.9938378
0.03392989 1.637515 5.692583 0.2270831 0.2155636 0 1.119369 0.04155161
0.06422118 0.8515014 0 0.1176547
TCGA-C4-A0EZ 273 1 0.146679 0.1568558 0 0.1973464 1.493834 71.94915 0
0.5524195 0.9733978 0.2753629 0 0.2550502 0.5361508 1.161684
0.3026473 0.245838 0.1262655 0.006254798 0.1742696 0.02383393
0.08753821 0.2192261 3.480324 63.01536 0.3315083 0.01433529 0.1227809
0 0.4220592 2.952003 0.1325345 0.2334352 0.02864887 1.017484
0.2930273 0.2416844 0.3465871 0.2167135 0.3051306 0.1488351
0.140363 0.02073273 2.376447 0.09253631 17.03084 0.3384027 0.05022112
0.01655714 0.6983162 1.876922 8.430683 0.03547515 0.411049 0.04715275 0
0.6629267 0.03419214 0.06655325 9.016435 0.09454126 0.8982543
0.6848659 0.2864887 0.09041488 0.359175 0 0.4641961 0.3445695 0
0.1643575 0.1695174 0.204994 0.7272405 1.023174 0.201688 0.09334848
0.03896314 0.1213039 0.05858483 0.1206268 0.9286185 0.2027695
0.1445217 0 0.02586628 0.08350063 0 0.137765 0.1939308 0 0
0.05413406 0.08338811 0.01904702 0.5651284 0.3437864 0.05341314
0.2813984 1.022068 0.02820724 0 0 0.6120218 3.666787 1.39836
2.631918 0.05456927 0.761736 0.1507835 1.152503 0.05266923 0.03922874
0.06728916 13.17935 0 5.217608 0.004721162 0 1.059176 2.394807 12.46609
0.3858827 0 0.03156637 0.08922225 1.238435 0.09685502 1.142577
2.105692 0.1507635 0 1.480361 0.2190145 0 3.948335 0.05166189

0.3921555	0	0.3959844	0.1376815	1.3605573.0535070	0	0.09934284
0	0.2822127	1.1337640.03804477	0.1206268	0.6771167	0.04911235
0.3636202	0.09501635	0.4786899	0	1.1288514.3143240	0.6343024
0.3694331.7189320.2766871	0.2138539	0.2864887	0.7804532	0.2351773
0.2614044	0.06960093	2.1933210.05577656	0.2036796	0.3557463
0.02463616	0.1528969	0	0.4616461	0.5450272	0.3428087	0.1610584
0.03431625	0.2090635	2.9744560.1246698	0.4413057	0	0.1929414
0.1345783	0.1376815	1.0716850	0.5370391	0.5389309	0.1178084
0.07722157	0	0.03183208	0.05764711	0.09625582	0.5966317	0.09477821
0.3990863	0	0.4098867	0.2496139	0	0	0.1691577	0.6813785
0.4110492.6175360.3360914	0.1138366	0.05255212	0.4149146	0.2532231
0	0.1649935	0	0.2285154	0.1951251	0.3541892	0	0.8653663
0.1435162	0.06045804	1.66559	0	0.02061969	0.03419214	0.09610294	0
0.03487535	1.18311	0.07444194	0.009781817	0.1163074	1.1236460.02433495
0.02206331	0	0.1794753	0.0198491	0.2713513	0.8852437	0.04756793
0.0461740.6606795	0.03394659	0.02732406	2.1014450	1.5879989.358403
0.07869331	0.1276148	0	0.2356169	0.9101544	0	2.1812340.004665249
0.1114435
TCGA-FD-A62P	191	1	0.01782301	0.05717879	0.06463689	0.2802618
0.1367588	22.985720	0.1295388	0.2276849	0	0	1.3529610.2388755
0.2973297	0.03852595	0.1769333	0	0.008360246	0.3882185	0.01061892
0.5265213	0.1465103	2.07822823.010910.03282211	0.1724467	0.1641105
0.2559294	2.25652	0.6705088	0.1180982	0	0.1531698	0.03777732
0.2919679	0.01900227	0.4632530.1086233	0.1315618	0.04144478
0.1351606	0	0	0.04122842	1.4083250.0565392	0.1006893	0.04426099
0.5025885	0.09291553	2.6905490.09483311	0.4120602	0.2048312
1.0451250.3967508	0	0.09848696	0.7605308	0	0	0.4378004
0.03829246	0.1722752	0.1711170	0.3722704	0.6332648	0	0.1146169
0.1510528	0.05219003	0.3703007	1.3128840	0.1940880.1264767
0.02026706	0.1370342	0.08061571	0.1848602	0.0677561	0.1287797
0.1090530	0.1426103	0.1133319	0	0.06480263	0	0	0.06201969	0
0.05819087	0.1195984	0	0.1427854	0.8405151	0.7923435	0.1089174
0	0	1.4208	0.4723930.3656868	0	0.02188141	0.1642172	0.04030785
0.4172055	0.0351992	0.05243366	0.1798792	1.4828890	0.05960619
0.03786221	0	0.05243366	0.09555018	0.9451242	0.2947291	0
0.1265761	0.06880134	1.4631750.02876838	0.5587257	0	0.3862323
0.07521733	1.8437620.3122536	0	0.7936983	0.2762079	0.08499896	0
0.0441065	0.1226846	0.2331460.6906913	0.1501368	0.2336489
0.04426099	0	0.05388704	0.7332599	0	0.1209236	0.2851506
0.06564422	0.4374177	0.06350006	0.04570167	0	0.4526512	0.6987118
0.06530497	0.1614890.1795597	0.6564422	0.2812744	0.4367001	0
0.5689987	0	0.08734916	0	0.4153131	0.1491034	0.1814939
0.1345741	0.01646451	0.5903851	0	0.03856514	0.5603775	0.6109369
0.1722182	0.1834702	0.08245685	0.5542330.2221804	0	0.07716954

0.9455893 0.4047281 0.06134229 0.3238534 0.02364174 0.4120782
0.3296481 1.495911 0.1032154 0.6017387 0.2127359 0.03852595
0.04020526 0.1860754 0 0.08224601 0 0.30966 0.4170466 0 0
0.550028 0.3642958 0.6592963 0.5984505 0.1996552 0.6694841
0.02809675 0.05281719 1.086052 0.04786558 0.088213 0 0.9535585
0.3701774 0.07283292 0.05603775 0.05783301 0.0479564 0.2626293
0.8668339 0.138104 0.08268165 0 0 0.09795746 0.06992229 0.08625605
0 0.05229803 0.1998745 0.2071559 0 0.01474505 0.2695789
0.08466675 0.05836726 0.2060749 0.05546384 0.03178997 0.03085839
0.2240633 0.09074694 0.07304342 0.4130617 0.03579748 0.1974455
2.122392 0.02337389 0.1137144 0.1499883 0.3149286 0.03287904 0
0.1864542 0.006235634 0.1737831
TCGA-CF-A47V 379 0 0.1985523 0.2547938 0.04800464 0.2938523
0.1625092 26.85347 0 0.1924122 0.6763896 0.2733468 0.1308001
0.2083483 0.4967436 1.692963 0.1859814 0.05396996 0.1566762
0.130389 0.8649683 0.03943238 0.4344864 0 2.189562 64.44078 0.02437638
0.08538201 0.06094095 0.2851111 0 2.33665 0 0.2317261 0.05687822
0.6172435 0.1269303 0.1411264 0.5734159 0.2890766 0.2540422
0.04924844 0.05843093 0.1337761 0.1367564 0.0306196 38.71868
0.1259719 0.04985342 0.02465387 3.35937 0.5520533 3.771924 0.2465079
0.6460624 0.1872301 0 3.535914 0 0.06724571 2.911059 0 0
1.034557 0 0.01909636 0.3918468 0.1587299 0.522237 0.2137792
0.005869235 0.2766526 0.2454029 0.2907044 1.89073 211.57878 0.2252378
0.09266501 0.01105082 0.2107276 0.01453897 0.1197436 0.2549713
0.4025697 0.3347483 0 0.2310921 0.1335438 0 0.06837819
0.1443832 0.06562872 0 0.1458595 0.02069439 0.01350541 0.8461609
0.03412693 0.05302207 0.1909761 0.5478756 0.009333572 0.05432652 0
0.1279033 12.19161 0.7845902 0.3093925 0.1300074 0.7927471
0.08980772 0.6912058 0.1568508 0.03894152 0.1001947 3.797635
0.0438547 0.7968317 0.00937319 0 0.6620058 0.5322253 0.1373984
1.039727 0 0.1880115 0.2486745 0.7354254 0.2136575 0.4564509 0
0.490551 0 0.3339824 0.3768457 0.1042767 1.251321 0.7692546
0.161325 0.1042767 0.1637855 0.227789 0.3116759 1.538891 0 0
0.03287183 0.1614608 0.5602929 3.267472 0.03776622 0 0.4235523
0.09750552 0.2526706 0.09432067 0.1018254 0.0349531 0.3361757
1.271027 0.09700162 0.329821 0.1000168 1.096937 0.5067695 0.7489055
0.6043311 1.338186 0 0.1729937 0.1842436 1.41522 0.1107364
0.2021883 0.05996745 0.06113945 0.3372832 0.08038464 0.2577746
0.208091 0.3536438 0.4156858 0.34065 0.08505443 1.586104 0.2062617
0.03369805 0.0286562 0 0.5343719 0.5466936 0.3515293 0.05267487
0.5257028 0.3853705 0.24851 0.1423615 0.1718847 0.4265866
0.05722504 0.1731863 0.4590041 0.2352107 0.07853478 0 2.317479
0.06194658 0.4889931 0.4868251 0.3261507 0.03381948 2.570648
1.059862 0.1359236 0.4633127 0.08346776 0.3922636 0.8452631

0.07109777	0	0	0.04149553	0.2624275	0.1352292	0.1664728	0
0.07123269	0.7877020.5293316	0	0.2456246	0	0.1430990	0.05193001
0.1067680	0	0	2.6539280.1449406	0.1752141	0.05005283	0.1257609
0.1024596	0.1928405	1.24949	0.1888786	0.04583593	0.5530610.03369805
0.1084960.4483611	0.1063445	3.07942210.854970.09113666	0.0633402
0.05569677	0.5262564	0.02441866	0.05032121	1.0826320.09262182
0.06453275
TCGA-XF-A9SI	2423	0	0.1291799	0.05180348	0	0.2837871	0.09912181
78.154480.2250333	0.3403470.2357492	0.1111512	0.1595619	2.052603
0.2164191	0.4788944	0.08726044	0.12595	0.3822561	0.06816879
0.6682729	0.03848259	0	0	2.48493337.829780.1189462	0.3124704
0.5203895	0	1.7377291.5887810.6419759	0.3634466	0.03469263
0.9240998	0.3871029	0.1377272	0.6062379	0.1476177	0.5065739
0.03003888	0.09503921	0.07531954	0.1668280.2988207	4.2221670.05122402
0.09122366	0.3909757	0.5854382	0.6313549	4.6013390.08591797
0.5392442	0.3283256	0	0.6350332	1.8218340.2058008	0.9010448	0
0.0543875	0.4327018	0.1387705	0.1281252	0.2024009	0.1936333
0.6745475	0.9388348	0.02147949	0.08653492	0.2651518	0.1536721
0.8806592	1.8833140.1221181	0.4019245	0.4313856	0.05508534
0.6562309	0	0.1435557	0.1841593	0.35002	0.02470026	0.06264607
0.3876110	0.04170699	0.1174212	0	0	0.04682441	0.2272042
0.2998474	0.2737391	0.2497870.06468118	1.4917041.5594810.01518126
0.06627247	0	1.0921973.9053530.8466788	0.0838723	0.05286497
0.4463380.1825928	0.5669768	0.1594508	0.09500887	0.1222267
1.7604270	0.3240161	0.02286855	0	0.1425133	0.2164191	1.180565
0.4005330.2641978	0.07645121	0.1496002	1.3657940.1042556	0.3880871
0.2081558	1.5924030.1022194	2.5260190.1237683	0	0.6433907
0.2502420.1411819	0.04240211	0.07992021	0.2223023	0.5491934
0.9528623	0	0.2469645	0.08020014	0.1969646	0.09764238	0.5314616
0.0460707	0.1460743	0.4380615	0.1189462	0.1320989	0.0575305
0.04140531	0	0.6834967	1.1458590.02958287	0.0365769	0.1626795
0.9664377	0.2689895	0.9279708	0.5203895	0.7303027	0.1708742
0.07913758	0.2809465	0.4094703	0.06754318	0.08221594	0.4226664
0.02983341	1.1109120	0.1630519	1.0661640.4883855	0.3120564
0.08311121	0.1494104	0.8167985	0.6038806	0.2055398	0.1048724
2.18068	0.2037112	0.2778779	0.8463703	0.08567685	0.2197884
0.5641324	0.6241432	0.1469478	0.0838723	0.3469263	0.2443292
0.1311322	0.1746035	0.1147726	0.1277386	0.04031186	0.1942264	0
0	0.1319721.4614780.2062805	0.1991056	1.5014520.1658121	0.6892576
0.1145493	0.1196298	1.1018930.1300974	0.07992021	0.1205113
0.5028259	0.2286665	0.06598599	0.1015394	0.2619810.04344808
0.3111522	1.0471250.1251210.07490885	0.2898372	0.1163768	0
0.02111633	0.2344417	0.2704386	0	0.3018076	0.8445666	0.08840594
0.06679445	0.1221181	0.1022764	0.1201823	0.1157553	0.3517483

0.20161 0.02795743 0.376146 0.1233239 0 0.8290641 0.2594577
0.8049775 9.600706 0.1270593 0.4636091 0.06794403 0.2139920.3574575
0.03069322 0.5682053 0.03954601 0.08996911
TCGA-GU-A42Q 344 1 0.09748069 0.1303051 0.2209521 0.4395693
0.1121977 74.39826 0 0.2302613 0.2223739 0.5032559 0.1083666
0.2621994 0.2939623 0.5646553 0.1251107 0.1360848 0.08653649
0.1886169 0.0265414 0.02177956 0 0.1001649 1.33271359.10649
0.2131757 0.5239864 0.6170876 0 0.6942236 0.6699808 0.08074043
0.2133142 0.02617947 1.2655370.2726383 0.05196527 0.03519033
0.3341821 0.06296157 0.2890132 0.08688877 0.1515654 0.03147259
0.05637342 1.628061 0.07730851 0.4359769 0.01512999 0.8835573
1.58809311.87377 0.3241736 2.0032990.09694893 0 0.1537068 0.09373483
0.06081668 1.0799030 0.1231244 0.1360508 0.02617947 0.0650425
0.1722321 0.7671195 0.1696738 0.1180760.04322315 0.1828408
0.1678146 0.2676064 0.5379738 0.0747985 0.1382276 0.07582426
0.1169869 0.1662722 0.1739891 0.1929014 0.2708221 0.3242605
0.1980969 1.0810680.2600041 0.08054235 0.2324456 0.1258904
0.3544298 0 0.03660689 0.1978719 0.2286014 0.03729699 0.344278
0.2199076 0.04880919 0.1889216 3.4556910.03723187 0 0.1502474
0.3237870.3229617 0.8889236 0.7278469 0.09474476 0.3929473
0.1102294 1.21772 0.3128411 0 0.2459566 2.6743580.08074043
0.4075107 0.0215711 0.1231244 0.1075422 0.1633124 0.7286426
0.3526215 0.06645559 0 0.3010402 3.2434990.1573451.5024740.03926921
1.5766830.1285599 0.2152120.2201502 0.1439871 0.7425451 0.9913866
0.06456821 0.03199714 0.3618524 0.08387599 0.4781860.7941649 0
0.02662319 0.03025999 0.03715796 0.2210463 0.2005236 0.486717
0.6613762 0.4195651 0 0.5981003 0.4341320.1874697 0.1287036
0.2578874 0.1602376 0 0.1932095 0.3375897 0.5609887 0.217227
0.3799848 1.3089740.7131819 0.08596245 0.1194363 0.02120056
0.3340445 0.1274222 0.4032673 0.1533409 0.04502528 0.06209687
0.03699883 0.1142522 0.7279171 0.6879441 0.2649167 0.2508668
0.09708755 0.7780356 0.1898731 0.06204112 0.7386209 0.1763107
1.7524410.4613179 0.5279764 0.01616319 0.5543646 0.3422302
0.1211107 0.5746043 0.2531641 0.5963102 0.1316955 0.2144001
0.3271230 0.2345564 0 0.2768461 0.1140492 0.03462616 0.09958762
0.1456592 0.6537793 0.1502474 1.1959580.3412466 1.0402440.2593209
0.01805481 0.2263109 0.03272434 0.2713893 0 0.07639699 0.3048462
0.1244845 0.1915571 0.07907759 0.06557288 0.6905856 1.2642770
0.3580050.03124494 0.0658645 0.3348537 0.2337079 0.1179417
0.1360508 1.3408010.9185821 1.6287070.06671217 0.07056544 0.5529105
0.3087161 0.0979463 0.1944249 0.5561463 0.1086695 0.02109701
0.3018661 0 0.1997509 0.6082433 0.02447373 1.6198552.315457
0.2037459 0.07774332 0 0.4844426 0.04495695 0.04632293 0.139062
0.9933085 0.2630806

TCGA-BT-A42F 864 0 0.052645513 0.042223607 0.095462069 0.219133085
0.040395745 47.751229028 0 0.153052461 0.096076368 0.181192567
0.039016379 0.550680796 0.03527946 0.878251031 0.021337082 0.100325276
0.311566518 0.092604206 0.086003845 0.039207635 0.172804022 0.054095172
1.796390755 63.204867751 0.042415533 0.396178441 0.181780855 0 0
0.666529801 0.043604753 0 0.028277022 0.390552589 0.042068942 0.098225444
0.19004923 0.140372322 0.102009246 0.532524301 0.086402011 0.07162272
0.203965403 0.456676209 0.132360528 0 0.049569281 0.024513355 0.159058248
0.274453447 2.410988178 0.035014699 0.135237931 0.116351836 0.070161032
0.195320088 0.371231788 0.01524933 1.166427151 0 0 0.117561162 0
0.017088769 0.126361113 0.03945631 0.305447372 0.212560756 0.052522048
0.077585479 0.069715482 0.269777679 0.170905077 0.363561709 0.174186455
0.020474859 0.027469583 0 0.130104965 0.148826431 0.253518127 0.150103226
0.142645868 0 0.102122213 0.05952327 0.167379681 0.033994234 0.095706843
0.130509332 0 0.061064489 0.185187993 0.013428432 0.102262106 0 0
0.113365737 0.766689414 0.012373834 0 0 0.031793585 0.436047533
0.120018228 0 0.026930497 0.097012784 0.029765286 0.497675584 0.025992806
0.038719573 0.099623671 1.227197003 0.043604753 0.044016119 0.009319767 0
0.193597867 0.176397301 0.288080035 0.108821192 0 0.031156652 0.128709203
1.484295603 0.053109944 0.123777089 0.042415533 2.323037196 0.138860375
0.066415781 0.014411455 0 0.771191505 0.050991351 0.034870767 0.069121609
0.097711175 0 0.103299821 0.463672905 0.036956108 0 0.065368947 0 0
0.613671538 0.112652924 0 0.24718986 0 0 0.281349263 0 0
1.615588651 0.522494052 0.024112189 0.089438504 0.132595626 0.242374473
0.369256411 0.205214956 0.247423941 0.245102891 0.046424961 0.021500961
0.06869755 0.24354593 0.165157827 0 0.231877602 0 0.670721812 0
0.094927947 0.248286045 0.311821265 0.222555095 0 0.057507374 0.267391487
0.205086092 0 0.455886032 0.634790286 0.265663123 0.045298142 0.234550161
0.034916435 0.238040097 0.099174482 0.116279342 0.065330809 0.136724062
0.267060762 0.056898885 0.029689523 0.161944132 0.046774021 0.045117033 0
0.2638478 0 0.224402819 0 0.154118891 0.067253457 0.162285517
0.815861614 0.098290109 0.865164509 0.072618033 0.058504183 0.074156958
0.247423941 0 0 0.011002408 0.416248304 0.053783384 0.124143023 0
0.035413348 0.111887496 0.497865247 0 0.020352055 0.033748344 0.047427788
0 0.097530951 0.106159468 0 0 0.008199839 0.267704592 0.048038184
0.043553873 0.64697826 0.156305146 0.031346352 0.070000847 0.423224482 0
0 0.257921832 0.033505986 0.026969414 0.427034934 0.052869219 0.400959333
1.533931596 0.038835956 0.440854356 0 0.116279342 0.040465816 0.025017204
0.16272089 0 0.137496324
TCGA-ZF-A9RF 1949 0 0.3158001 0.06432588 0.05453716 0.4173
0.0461559 19.061380 0.5464897 0.2744405 0.2070294 0.1485995
9.94142500.1612403 0.6968636 0.1381511 0.1946061 0.1423975 0
0.06551161 0.0358387 0.04936118 0.2472351 7.08195645.1616 0.1523145
1.3095121.9385480.1079698 0 0.8703685 6.8256820.03760851 0.03230913

0.3824938	0.06609307	0.160331 2.17149	0.1374758	0.1054546	0.04895644
0.1140413	0.09352644	0.8933563	0	1.253084 0.2862286	0.05663752
0.2334066	1.151013 0.1567943	3.743183 0.04000756	0.5794573	0.05317713
0.08016552	1.885798 0	0.002680582	1.579558 0.05331007	0.1519527
0.3358115	0	0.004339002	0.3248535	0.04508251	0.1047006	0.04857409
0.08001511	0.05641277	0.1035534	0.1210967	0.6639349	0.8308063
0.2558883	0.1169722	0.01255462	0.08550131	0.1321396	0.03400961
0.1114108	0.05716897	0.8964241	0	0	0.1412534	0	0.1165247
0.5467699	0.3727977	0	0.06977187	0.0235105	0.009205941	0.5682867
0.03877096	0.5421363	0.1101013	0.5071661	0.007069129	0	0
0.1089814	2.39148	1.062773 0.117165 0.06769533	0.8036346	0.1360385
1.150821 0	0	0.03794311	2.858304 0	0.5532177	0.06921657	0
0.7078515	0.3627906	2.266781 0.1243384	0	0.03559938	0.1199718
1.683476 0.01213661	3.205675 0.0969274	1.738038 0.349054 0.1517724
0.3293286	0.05923341	1.033892 0	0.23109	0.07897788	0.03721471
0.05175735	0.1180297	0.9801106	0.1689032	0	0.07469011	0
0.5910711	0.4537027	0.04290549	0.1360385	0.6224085	0.05538708
0.4920929	0.1607339	0.2699244	0	1.273076 0.6678917	0.02755042
0.03406394	0.53026	0.7615724	0.08350299	0.08039219	0.4442506
0.5601058	0.1060897	0.171968 0.02616445	1.339837 0.0943541	0.07656742
0.1438255	0.111135 1.743474 0	0.1952352	0.4728166	0.3638654
0.07265427	0.07740119	0.1855272	1.558774 0.6561239	0	0.06511153
1.885798 0.2656018	0.5693308	1.324213 0.03989528	0.6028489	0.2935917
0	0.1119698	0.15622	2.494983 0.2600491	0.1356922	0.3364296
0.05344368	0.1506858	0	0.2612746	0.633387 0.04273352	0	0.4549126
0.5379034	0.7880619	1.204089 0.210573 0.9885311	0.04741307	0.133693
0.05962568	0.2019321	0.1860735	0.05611586	0.01257128	0.3407301	0
0	0.0975928	0.1618522	0.1107963	1.133071 0	0.02325412	0.5398488
0.1083813	0	0.02622075	0.5094478	0	0	0	1.3546	0	0.08708763
0.8529611	0.1428746	0.08506331	0.26429	0.5771683	0.1072907
0.02603666	0.3503035	0.3445534	0.03081507	2.241248 0	1.457697
9.525325 0.1725644	0.5037172	0.06327605	0.13286	0.5363372	0
0.7007896	0	0.06284095
TCGA-KQ-A41N	1604	0	0.2767768	0.05223177	0.1771338	0.1505965
0.02498533	37.5888	0.2268938	0.05916566	1.753025 0	0.01608811
0.230313 0.2182083	2.414268 0.4047166	0.5310545	0.1156249	0.1298278
0.1063891	0.07760148	0.05344084	0.1338344	3.270733 84.63333 0.07495598
0	0.8245158	0	0	4.428828 0.05394028	0	0.03497946	0.1380355
0.2797174	0.06943291	1.551635 0.2563323	0.1021524	0.1514362
0.0884538	0.02531408	0.08410361	0	11.92916 0.103295 0.1532964
0.0303237	2.623459 0.2546299	4.052554 0.0866283	0.2509396	0.1583235
0	2.271194 2.254372 0.05804259	10.31408 0	0.1096743	1.70876	0
0.01174404	0.3473604	0.09761709	0.4156315	0.1577661	0.007219025
0.2268509	0.120736 0.2741291	0.4651115	0	0.1231277	1.190414

0.1223303 0.1295951 0.0178826 0.1104614 0.1447426 0 0.2352758
0.04980894 0.094746 0.02265598 0.6211599 0.08410361 0.177588 0
0.04891195 0.01888461 0 0.01328907 0.2645022 0.1679014 0
0.3996742 0.4492497 0.003826693 0.2004611 0 0.03932954 7.443759
1.855823 0 0.03331377 2.130133 0.1472819 1.260593 0.03215382 0 0
5.920499 0.1078806 1.687924 0.06917286 0.1645115 0.2394859 1.0474
0.08817228 0 0.08879401 0.03854164 0.09636809 0.6345396 0.03941908
0.1531157 0.2098767 0.2761169 0.03435482 0.1643163 0.1069642
0.256516 3.752342 0.3153885 0.06039054 0.04275267 0.08058095 0.05603505
0.04259491 1.649032 0.04571573 0 0.0404316 0 0.7875972 6.56423 0
0 0.1925286 0.05996478 0.177588 0.05800613 0.2922334 0 0.4134885
0.545349 0.02982744 0.0368793 0.2870428 0.5996478 0.4758129
0.7035453 0.04372432 0.7363405 0.1148579 0.01329864 0.08498077
0.7476035 0.4086096 0 0.1721034 0.03008005 0.04148507 0.09887127
0.1409142 0.3583261 0.5170444 0.1573182 0.1675967 0.1590148
5.211325 0.05073943 0.1243435 0 0.07852531 0 0.1120701 0.7452745
0.06478889 0.3612474 0.217481 0.3236417 0.161632 0.08456572 0.64129
0.2463492 0.05141746 0.2185411 0.05786076 0.0665439 0 1.392585
0.3809644 0 0.06653153 0.1509307 0.08319438 2.107892 0.5046217
0.09119068 1.028535 0.2438257 0.07237129 0.9615104 0 0.08058095
0.06075379 0.05103856 0.2075013 0.03326577 0.2047578 0.2113175 0
0.1568623 0.8194882 0.3153885 0.02517605 0 0.02933474 0
0.02129091 0.05252888 0.0908915 0.01194332 0.01014343 0.8515491 0
0.04040801 0 0.2320245 0.05331742 0.2258947 0.1519956 0.2032768
0.02818857 0.3431362 0 0.2668953 0.9752382 0.03270034 1.26254
4.79879 0.03736534 0.129845 0.1370115 0.07192038 0.05005733 0.1237879
0.9445157 0.005696137 0.1474085
TCGA-GD-A3OQ 95 0 0.1253451 0.078191 0.03788135 0.1739132 0 29.3921
0 0.06832624 0.2859384 0.6471092 0.09289511 0.3204985 0.08399779
0.232339 0.03386801 0.03518197 0.07418168 0 0.2730251 0.03111683
0.1714307 0.0429322 1.295868 20.17577 0.05770757 0.05614712 0.1923586
0.1874887 0.06612326 0.7708083 0.03460656 0.0783683 0.04488367
0.3542388 0.1252038 0.06681929 0.1206648 0.1697606 0.02698627
0.1263043 0.07211911 0.081204 0.02697925 0.0241625 4.006788 0.1325424
0.1573611 0 0.2103922 0.3539539 2.267806 0.02778914 0.9123093
0.2677909 0 0.2867763 0 0.01396443 0.3085752 0.1110871
0.07036394 0.1399522 0 0.004520788 0.03899991 0.1252567 0.3393822
0.2024366 0.009263045 0.1007592 0.03319748 0.1835202 1.220737
0.6411952 0.1184929 0.008124854 0.008720406 0.1662891 0.03441892
0.09449193 0.1392941 0.1191282 0.1132098 0 0.08104848 0.2034961
0 0.0539585 0.2278709 0.05178884 0 0.05452126 0.01633033 0
0.213966 0 0 0.1462042 1.377079 0.1153896 0.02143504 0 0.07569818
0.3460656 0.1190644 0.02712749 0.1025912 0.1924836 0.1181149
0.432593 0.04125797 0 0.05271035 0.7566882 0.03460656 0.1397322

0.007396559 0.03518197 0.1229179 0 0.2192043 0.3022777 0
0.02472723 0.0994609 1.541793 0.03372023 0.8186225 0.1009882
0.5150438 0 0.2371966 0.5833144 0 0.6202106 0.6879709
0.09409468 0 0.07754769 0.1078515 0.3006046 0.7175811 0 0 0
0 0.06316251 0.3724389 0.08940609 0 0.5231474 0 0.1139355
0.2605057 0.08035231 0 0.4421376 0.5779508 0.09568223 0.09464287
0.2104669 0.3847171 0.3083208 0.1721739 0.2244183 0.8614632
0.0736896 0.05972423 0.05452126 0.3006707 0 0.05318352 0.1261905
0.009649257 0.03992348 0.06343302 0.1130082 0.1313668 0.4422948
0.1009309 0.2419319 0.04832499 0.3811174 0.2929769 0.02659176
0.06783943 0 0.1054207 0.2516536 0.2992982 0 0.2220281
0.6046285 0.1499618 0.2160386 0.02712749 0.07480611 0.04515735
0.08953884 0.1557887 0.03712183 0.05508734 0 0.2094007 0.0488832
0 0.04268475 0.1350564 0.640502 0.2575932 0.4046888 0.07800716
0.6509618 0.07409921 0.04643138 0.318248 0.2805229 0.05169846
0.03897792 0.1899204 0.1972252 0.06402713 0.03284171 0.06778769
0.05621104 0.4143936 1.277308 0 0.129218 0 0.3199458 0 0.03187251
0.06740209 0 0.06896249 0 0.5766815 0.07625024 0.03456618
0.5134691 0.06616018 0.1026209 0.2318843 0.09751608 0.09315478 0
0.09269325 0.2659176 0.06421218 0.6070637 0.1048981 0.376076
2.972908 0.09246554 0.1832708 0 0.1845683 0.01284615 0 0.1291421
0.01096344 0.09457315
TCGA-BT-A20U 455 1 0.06712817 0 0.2434469 0.1448824 0.2060341
52.00032 0.07795885 0.1626307 0.2450135 0.4620765 0.02211098
0.610778 0.2399187 0.2073807 0.08464358 0.05553313 0.1059407
0.08396758 0.6823495 0.01333162 0.03672367 0.09196884 1.663573
48.28858 0.1648273 0.2405553 0 0 0 0.7446661 0.4448026 0
0.1442239 0.2845673 0.1519857 0.09542632 0.3231085 0.1591008
0.1156193 0.07284511 0.09370867 0.06958169 0.1155893 0.0517606
2.507479 0.1419653 0.04213712 0.06945983 0.1802798 0.5832583
5.645605 0.1785887 0.9196884 0.118688 0 0.2656557 0 0.07977184
0.7344734 0 0.07536639 0.2498366 0 0.01936876 0.03580506 0
0.4154403 0 0 0.2038538 0.1896406 0.1801868 1.685253 0.2747121
0.08461133 0 0.04670187 0.1526668 0.02457727 0.05060487 0.1657746
0.08506501 0.1616777 0 0.08681053 0.1868258 0.1422836 0.3467678
0.2440712 0.1109414 0 0.05190879 0 0.05022618 0.1264428
0.1730686 0.08963065 0.1108235 1.852302 0.1577785 0.04591787 0
0.1081065 0.3706688 0.3060699 0.3486731 0.1098849 0.2474016
0.1518146 1.26917 0.08838231 0.7899398 0.05645774 1.428378 0.2965351
0.1496663 0.03168964 0.2260992 0.3291416 0.1199594 0.4291831
0.1850102 0 0.1059407 0.1266867 1.874067 0.03611753 0.6546935 0
0.4357062 0.1416484 0.1129155 0.3062669 0.08813681 0.821639
0.08669194 0.183783 0 0.4429913 0.07701274 0 0.2956141 0
0.04888945 0.1111357 0 0.3382649 0.3682311 0.3192078 0.2530243

0.7004250	0.3050889	0.2391652	0.05737658	0.1181723	0.2841425
0.5052252	0	0.1520571	0.6199321	0.6181023	0.2681161	0.229272
0.7211193	1.7858870.07892848	0.03655444	0.1557264	0.4600665	0
0.1708936	0.1802158	0.02067052	0.4846337	0.1358854	0.04841696
0.9849435	0.4511802	0.1621597	0.3455091	0.05751178	0.3247146
0.1394692	0.1139291	0.0484416	0.3237679	0.2258310.07701274
0.4769552	0.02968124	0.3713219	0.3985316	0.2718238	0.09255907
0.05811218	0.05341625	0.04836776	0.08076169	0.1835508	0	0.1150573
0	0.1794303	0.1047170.1271713	0	0.2729398	0.2286793	0.2069299
0.1155893	0.1879942	0.7449975	0.03527432	0.06630982	0.2288055
0.1802798	0.1107478	0	0.09820402	0.07393634	0	0.07035311
0.07260698	0.06020731	0.5199438	0.8222519	0	0.1730057	0
0.2419003	0.1229816	0.04876921	0.1443879	0	1.3952330.2648751
1.7555120.1633423	0.01851181	1.3537810.1948754	0.1132474	0.3156367
0.2553195	0	0	0.2813024	1.4810780.1375546	2.06635	0	0.3718272
3.21261	0.1613973	0.1784547	0.09415208	0.2965351	0.01375943
0.1275975	0.3617688	0.1174288	0.1558411
TCGA-XF-A9SW	362	1	0.12173665	0.06509147	0	0.05630221	7.05249165
46.0993864	2.01463696	0.5529939	0	0.41898684	0	7.89499071
0.40789834	0.45130086	0.08223255	1.41891917	0.21613845	0.01427577
0.72920443	0.03626525	0	0	2.3314364	59.77133988	0.14011573
0.19631074	0.28023146	0	19.45857175	0.79265469	1.8149570	0.06538734
0.38704614	2.50494864	0.06489571	2.81258782	0	1.60626162	0.29723468
0.07578418	0	0.15721546	0.42240435	0.39351651	0.19309013	0
0.32121101	0.73560757	0.31732091	6.83985989	0.08096744	0.39090257
1.56049088	0	0.58715162	0	0.81103316	0.69928127	0.10788911	0
0.20388493	6.60412132	2.70024634	0.25161275	0.27371445	0.35315585
4.12878601	0.02698915	0.29357581	4.07858723	0.17823704	0.55327749
0.84069437	0.34524515	1.63342812	2.65514561	0.03460757	0.40113689
0.06882878	0.13528415	0.23139756	0.49477809	0	0	0.49762397	0
0.15721546	0.1106555	0	0	0.01765057	0	4.71985748	0.33320672
1.64776096	2.68198919	11.14772224	0.60651176	0.13591202	0.1249078	0
13.30693631	2.82326645	0.62443858	1.26463426	0.21173043	0.19628948	0
0.38360573	0	1.16394891	0	1.30973124	0.20166189	0.30534654
0.04310177	0	0.26860359	0.57105768	1.9503824	0.1258182	0.49794974
0	0.11748361	1.00948876	0.02456212	1.08127628	0	1.89253988
0.06421971	27.56739555	0.1499617	0	0.73709365	0	0.29834806
0.31967144	0.15063052	0	0	0.83094719	0	0.9974340	0.64965486
0.5520978	1.0851516	0	0	0.24345838	0.22418516	0	0	0.15607828
0	2.18998792	1.87272087	0.27878323	0.06893873	0.30661204	0.56046291
0.16899366	7.3078518	0	0.32386975	0	0.04971848	0.37066197
0.27115678	0.06365139	0.15495743	0.29107463	0.02811443	0.38774164	0
0.32926483	0.57413274	0.09204901	0.22055695	0.0783224	0.10951224
0.63093047	0.56908542	0.5423510.19765944	0.14678791	0	0.41898684

0.46792714 0.04037011 0.18158851 0.5003553 4.94071628 0.02517832
0.07903964 1.0171364 0.13157209 0.06865359 0.09078232 0 0.04012612
0.07597825 1.0168625 0.71213934 0.51890554 0.12436785 3.05152169 0
0.93816618 0.47164639 0.17046337 0.31178069 0 0.18037887 8.65018212
0.16346835 0 0 5.81345009 0.3591515 0 0 0.19750867 0.73700341
0.10349075 0.37592025 1.53285075 0.09412354 0.70235226 0 0
0.07959848 0 0.16990411 0 0 0.70746958 0.05554137 0.02517832
0.46032687 0.09638335 0.09966662 0.21817106 0.0631392 0.21713532 0
0.16879652 0.07747872 0.18709094 0.90065841 0.2445079 0.67430694
14.72792387 0.02993455 0.24272016 0.12805829 0.13444126 0.22457404
0.05784939 0.40521732 0.02129566 0.06358887
TCGA-FD-A3B3 974 1 0.1174680 0.1449439 0.03276994 0.2841771 0.4576087
23.80953 0.09444505 0.1116462 0.527693 0.3731955 0.08036056 0.9775865
0.2422126 1.239432 0.1562566 0.237071 0.2566887 0.2034489 0.6691913
0.01076726 0.01482991 0 1.123056 17.03348 0.07904151 0.5439958
0.2496048 0.03243809 2.173641 0.3399379 0.2394962 0.1581857
0.3106193 1.091694 0.2960467 0.04816934 0.2087665 0.1789788
0.1700847 0.4286424 0.1442081 0.05619756 0.1166944 0.1881197
0.1687635 0.1146581 0.08507998 0.02243966 0.3640069 0.2355339
2.731968 0.1201974 0.7892093 0.3275155 0.4335241 0.5095716
0.06951019 0.1570423 0.3262581 0.1281304 0.06086957 0.1412462
0.1747233 0.2268257 0.1229011 0.1354444 0.4613535 0.9339805
0.04407239 0.2179089 0.1196586 0.2315212 0.7744148 0.3882741
0.0854203 0.1124568 0.04149058 0.1541264 0.1687233 0.08174191
0.1204989 0.06870265 0.1142564 0.4146616 0.2629216 0.1948991
0.2298304 0.04667776 0.3613936 0.1344026 0 0.06812662 0.08476104
0.09219344 0.6318762 0.09318578 0.1447802 0.8794983 0.4155582
0.07433392 0.1112565 0.1114178 1.047745 1.736347 0.1853978 0.4693423
0.09614406 0.191488 0.08174191 0.366087 0.08922733 0.07974924 0
0.9721582 0 0.09065833 0.0671845 0.1217391 0.1860816 0.1453275
1.080667 0.3362023 0.1478428 0.0855629 0.1651046 0.854203 0.02917028
1.104736 0 1.140829 0.1144022 1.139951 0.01978849 0 0.4376908
0.3500832 0.06942793 0.09491144 0.1565293 0.3109962 0.1182015
0.6764651 0.0761171 0.1184565 0.08975863 0.02755493 0.2185594
0.09913381 0.1804654 0.08174191 0.2828487 0.06656127 0.07392141
0.03219358 0.2085306 0 1.109189 1.47748 0.03310864 0.1023406
0.1593096 0.5824111 0.258796 1.191537 0.3882741 0.6731055 0.09561974
0.1254734 0.1257722 0.4644651 0.07559318 0.04600734 0.1410026
0.05843082 1.208778 0.05487385 0.1042771 0.5113854 0.150313 0.08731206
0.2325416 0.09057617 0.2247903 0.5632108 0.04600734 0.2151809
0.8280539 0.1823921 0.2798966 0.2368108 0.03595803 0.4532162
0.5261395 1.10767 0.08223074 0.07040134 0.2804202 0.1171925
0.158991 0.1246602 0.09633868 0.06195056 0.02255818 0.1811458 0
0.462194 0.1477008 1.23887 0.6464239 0.4735256 0.7468441 0.3627129

1.2728190.02848926 0.09372133 0.6958077 0.6066782 0.04472267 0
0.2133934 0.2687156 0.0553878 0.0852309 0.2052433 0.09725271
0.5018699 0.9682331 0.2100499 0.3074013 0.1621904 0 0.09932593
0.05908259 0.1020379 0.1513352 0 0.1463705 1.0765060.1484136
0.02990209 0.7175305 0.1645450.08070362 0.1901479 0.3561783
0.03223407 0.04693423 0.24724 0.8051285 0.05554788 0.3962797
0.1270415 1.6516824.5559190.1007268 0.3170832 0.07604181 0.1995801
0.1500226 0.1030540.3695244 0.003161373 0.02517298
TCGA-ZF-A9RC 2868 0 0.1177988 0.1049765 0.1424030.2905652
0.4820741 35.696480 0.2568509 0.5374475 0 0 3.1763240.7894078
2.32908 0.1697548 0.7065227 0.0464771 0.04604661 0.7269989
0.01169739 0 0.16139 4.9423934 4.792070.1265445 0.1688539 0.3615556
0.07048046 0.8699931 3.1248731.0407390.1964006 0.08436297 0.7906664
0.5334193 0.1674573 0.2268007 0.1695116 0.2173851 0.2100082
0.07111055 0.06105215 1.06491 0 9.81585 0.3736883 0.1848591
0.01218905 2.1354380.4605846 3.6714540 0.5043438 0.1041388
0.6279651.2819990.3524023 0.2834723 0.7733272 0 0.06612774
1.8413710.2109074 0.7392596 0.3246316 0.4120052 0.7290286
0.5073308 0.0609376 0.2419936 0.5095813 0.3449431 0.8158177
11.630040.1855985 0.5344993 0.2868391 0.06697622 0.1940806
0.04440156 0.1745441 0 0.5674350.5105461 0.03808451 0.157094
0.9987365 0 0.2141522 0.2920257 0 0.04554565 0 0.1842898
0.4091026 0.1518533 0.2359303 1.07385 0.6922324 0.2953333
0.6849150 1.1382539.5617943.6254380.2549430.03213827 0.37988 0.2220078
0.4242252 0.1550963 0.05775888 0.04953698 3.1965760.5854159
1.2475370.006951256 0 0.7508654 0.6315262 3.4245940.1623311
0.3212282 0.1859084 0.2324192 1.4001290.07922533 0.5334077
0.1265445 0.6474367 0.04142824 1.9814790.4837027 0.07733272
0.9356620 0.2236754 0.1031103 0.24293 0.2027168 0.7704736
1.2450090.2205131 0.3431714 0.04875618 0.4190935 0.2967994
1.5616120 0.04440156 0.2321689 0 0.2141522 0 1.0572070
0.9141421.1739960 0.08894498 0.6922858 1.3558330.2582008 1.609337
0.1581806 0.7312513 0.1385064 0.09622044 0.2049554 0.7804288
0.1231849 0.4498349 0.1087105 0.01813666 0 0.1192282 0.1699274
0.3086450.3760795 0.1422817 0.9599924 0.1867087 2.7269960.1223727
0.2998899 0 0.3787725 0.6439807 0.2702891 0.7615120.02604283
0.8419657 0.5715897 1.3876530.09745547 0.05098861 0.3280782
0.04243869 0.02657313 0.4172673 0 0.1087188 0 1.0495680.09188046
0.1673734 0.08022991 0.7902905 0.05016177 1.2709460.2028399
0.1832770.4525431 0.06190057 0.08727204 1.8846650 0.09717201 0
0.9067931 0.3521677 0 0 0.1274132 0.05282691 0.4784614
0.5365463 0.1521299 0.09107889 0 0.03537456 0.2158122 0.1112564
0.1583605 0.4384217 0.01440237 0.4403481.4832670 0.03248516
0.2969577 0.2331640.07598512 0.1543628 0.7942585 0.4202231

0.1019772 0.7549771 0.0999633 0.1206927 0.5110143 0.1182993
2.0118859 6.483236 0.1223019 0.2192109 0 0.2601849 0.01207276 0
0.4294533 0 0.02734752
TCGA-E7-A6MD 129 0 0.2954153 0.1579558 0 0.3643391 0.1511179
31.6244 0.1143595 0.2624231 0.1198052 0.4518866 0 1.111779
0.3519419 0.5780013 0.1152965 0.09310027 0.03885168 0.5773774
0.5362248 0.08800396 0.05387073 0.06745553 6.323351 33.918 0.1284502
0.3352320 0.3022358 0.1767506 0.2077871 1.614803 0.271871 0.1641775
0.1410434 0.3130784 0.2295085 0.122485 0.2843848 0.3750885
0.1574899 0.06869446 0.1839035 0.1658652 0.2119504 0.1518575
10.94051 0.05206299 0.1236237 0.1222705 1.123939 0.2566782 4.426164
0.1309878 0.7588747 0.2031236 0 0.54801 0 0.06874867 1.292898 0
0.2211131 0.4031397 0.1763042 0.1160175 0.2188467 0.2460059
0.6475068 1.431317 0.01455419 0.1495188 0.1130138 0.3243925 0.85246
4.130556 0.4964727 0.089361 0.1575683 0.1306376 0.2703969 0.3711668
0.2431782 0.1247837 0.3261067 0 0.06367211 0.07422425 0.3130784
0.2119504 0.298361 0 0.09861084 0.09518265 0.05131678 0.07702686
0.3883523 0.3808171 0.1314811 0.7209598 1.962409 0.05400468 0
0.05059165 0.3171674 1.141858 1.534017 0.213115 0.2283559 0.4687699
0.11135 0.7979025 0.09723743 0.1448475 0.2070477 1.730403 0
0.329323 0.005810776 0.05527828 0.3379774 0.5279128 0.5629166
0.6106397 0 0 0.2829827 2.640233 0.06622697 0.4973416 0.05289127
1.536014 0.06926237 0.5383239 0.3504302 0 1.192458 0.6358513
0.1174044 0.2585795 0.4467604 0.3954007 1.58869 2.717494 0.09216696
0.1792924 0.08151369 0.2001906 0.1984832 0.1800554 0.1873011
0.07423336 0.6792707 0 0.4922956 0.1169455 0.1262504 0
0.7641604 0.9325151 0.03006739 0.1858799 0.3720238 0.3777948
0.6331255 0.4752404 0.6170648 0.4366258 0.05789094 0.09383934
0.1142192 0.5848971 0.1372989 0.04178125 0.09087459 0.1212881
0.6691007 0.04983331 0.2485836 0.5160124 0.8355805 0.1982296
0.8024882 0.2784054 1.190827 0.4091808 0.0835625 0.14212 0.712413
0.6625525 0.2824291 0.6939209 0 0.3947545 0.3710054 0.8337377
0.3394422 0.127869 0.2350723 0.3902343 0.1777066 0.2692545
0.05832621 0.1038646 0 0.1754734 0 0 0.1341335 1.635557
0.5031818 0.6070998 0.974972 0.06128284 0.3222514 0.1811063
0.2431782 0.941846 0.04407606 0.3249167 0.06124252 0.3155547
0.2556529 0.3353337 0.2064049 0.585798 0.04415969 0.4836739
0.947712 0.06358513 0.05075716 0 0.1182829 0 0.1001568 0.05295144
0 0.0120394 0.01022503 3.433597 0.1497565 0.1629323 0.1861773
0.1039516 0.06840441 0.3605439 0.4426306 0.02927315 0.05683067
0.3823066 0.04178125 0.06726057 0.5851687 0.2307438 0.8181618
7.179856 0.1452828 0.1308895 0 0.07249893 0.20184 0.03119592
0.5462948 0.01148392 0.3200493
TCGA-E5-A4TZ 467 1 0.0655407 0.3270774 0.1584599 0.5052007 0

31.828310	0.1508455	0.2591543	0.3759576	0.2590571	0.05876285
0.3220869	0.5264389	0.3836945	0.02904636	0.02585885	0.2459457
0.04758665	0.2017534	0.05378282	0.1795879	4.349389	28.38098	0.2162489
0.2818399	0	0.2744966	0	1.027362	0	0.1365913	0.0704066
1.366035	0.1614849	0.1863393	0.2839207	0.166434	0.07660063	0.04572157
0.1446572	0.1868245	0.1692836	0.2274145	14.26536	0.2425641
0.2057032	0.01356344	0.5280495	0.6264117	1.90839	0.1453044
0.7856969	0	2.911551	0.8672797	0.7002465	0.01362994	1.362498	0
0.2943364	0.7805709	0	0.01103125	0.300059	0.1964835	0.7351786
0.1058505	0.009686962	0.2809878	0.3008784	0.4478107	1.418448
1.072863	0.08261042	0	0.01823898	0.07452824	0	0.02470407	0.5179336
0.4567935	0.2565132	2.272455	0.3390304	0.08360352	0.2778377
0.08464182	0.5163151	0	0.1640832	0.06335155	0.3073982	0.01337411
0.5593949	0.3379517	0	0.1905309	2.12666	0.01026982	0.04483199	0
0.07916245	1.628564	0.3486372	0.1134758	0.03576208	0.8957513
0.1482244	0.7867659	0	0.06427159	0.1378065	2.891051	0.07238062
0.5845077	0	0.0735841	0.8033949	0.556332	0.02957884	0.6322226	0
0.02585885	0.2248922	2.06526	0.05289512	1.32408	0.1760165	0.3087599
0	0.05512261	1.841989	0.04302626	1.237449	1.777478	0.1157657	0.02868417
0.135161	0.1879788	2.486314	0.9043532	0.1840331	0.04773329	0.02712689
0	0.8917169	0.7490064	0.623318	0.1729285	0.4179151	0	0.6851104
0.1556729	0.1680592	0	0.9247435	0.85917	0.02001221	0.1732047
0.6052717	1.005809	1.238645	0.1167914	0.6453939	1.162435	0.2311859
0.2230624	0.01900546	0.7561309	0.0913832	0.1668523	0.08797701
0.03027255	0.09741794	0.2653439	0.1812093	0.5151703	0.4570297
0.3694248	0.3092268	0.6401297	1.499125	0	0.05561743	0.2601282
0.05268521	0.1102452	0.03759576	1.362675	0.01448967	1.246489	0.3030545
0.03619031	0.1265183	0.1702138	0.03911478	0.07083591	0.1330624
0.2728881	0.03882068	0.2822811	0	0.6423524	0.2556015	0.06208198
0.3124674	0.04929976	0.5023606	0.4040727	1.100344	0.3467027
0.5222212	0.08610035	0.09711256	0.03191352	0.1466804	0.2973542	0
0.04337503	0.1650008	0.06695729	0.06868937	0.03544497	0	0.6314645
2.995347	0.04232091	0.1182401	0	0.1377715	0.06003664	0.1333245
0.1938384	0	0.03205267	0.2994446	1.650515	0.03986989	0.0903702
4.626183	0.1297274	0.05853667	0.5254087	0.3739224	0.07793435	0
0.5452611	0.02780871	0.1790688	1.324218	0	0.8168266	2.011138
0.1038599	0.05227037	0.09192554	0.2412687	0.006717022	0.04152668
0.540209	0.07261278	0.2586647
TCGA-ZF-A9R4	921	0	0.2901179	0.05909462	0.1002039	0.3066907	0
63.15396	0	0.150614	0.3529706	0.1901929	0.6689223	0.06606109
0.2962551	0.614584	0.1418474	0.03183751	0	0	0.06018392	0.05761725
0	0.05678222	6.231294	63.71158	0.2416932	0	0.4452242	0.2975676	0
1.639152	0	0.3109503	0	0.9077516	0.2759914	0.2356676	0.1994895
0.2104928	0.04588985	0.01285003	0.104767	0.1074006	0.2854625	0

29.771830.4820772 0.2341418 0.0171540.7791424 0.4321294 3.966011
0.2940318 1.2066220.0366394 0 0.6765731 0 0.02955102 1.4964480
0.0930635 0.5553033 0 0.00996534 0.1252690.3313297 0.6091775
0.2231192 0.006125662 0.2591252 0.02927137 0.2932917 1.758065
3.9858170.1044793 0.01074595 0.02306725 0.1413864 0 0.2499504
0.1637606 0.2100790.1996419 0 0.02679872 0.09131684 0.3513877
0.1070484 0.5525347 0 0.04150395 0.2003054 0.04319706 0.008457274
0.6635671 0 0 0.4164892 0.9530205 0 0 0 0 2.013911
2.1731520.03587888 0.1243801 0.6237189 0.06248761 1.1940490.1091357
0.1219286 0.1045722 1.4169710 0.2772151 0 0.0930635 0.2845001
0.5184464 0.02493936 0.9709263 0 0.06540857 0.1777665 1.157062
0.1895434 0.3175955 0.1335673 0.6508261 0.02915159 0 0.06050924
0 0.8310852 0.1605727 0.1830144 0.03627753 0.1709412 0.1901929
1.7348960.8395657 0.2715427 0 0 0 0.5430027 1.0230680 0
0.5189369 0.05088277 0.4144010 0.2833977 0.1094406 0.4678177
0.64442660.2784090.03129372 0.2783641.3356730.8640213 0.05539076
0.9275505 0.7718334 0.1949240.06770691 0.02403665 0.8900181
0.05778722 0.1406813 0.06954159 0.03828638 0.08800479 0.08389664
0.2092509 1.0859130.3203462 0.3337286 1.1021520.3337769 1.0711420
0 0.1794492 0.0666322 0.6274334 0.04754822 0.7723949 0
0.8629330.5299674 0.01525691 0.2971625 0.1076366 0.1484081
0.0895878 0.2617799 0.2318023 0.04909741 0.1475387 0 0.3323446
0.06465302 0 0.2258198 0.1364971 0.5647529 0.5962133 1.462995
0.3740001 0.3774086 0 0.04094016 0.1095533 0.03710202 0.2051295
0 0.002887232 0.2086803 0 0 0.1344842 0 0.4854403 0.7764276
0 0.1922670.1062741 0.1493509 0.07592971 0.09635348 0.1782923 0
0 0.008607147 3.29174 0.2016975 0.1485812 0.2611982 0.218759
0.09048437 0.7443638 0.4155860.02464134 0 0.4188707 0 0.08492717
0.5073564 0 1.1478442.6135380.1358835 0.1322151 0.05813017
0.2441105 0.05097103 0 0.9459895 0.004833424 0.1635696
TCGA-GV-A3QH 258 1 0.2199845 0.5153038 0.1139708 0.4844811 0
25.224970 0.06852267 0.2485257 0.1442155 0.2277298 0.02295856
0.5896749 1.4951280.4245672 0.04456815 0.04959663 0.06879215
0.0228175 0.02496503 0.08596176 0 3.1010341 8.851040.1687976 0
0.2893674 0.1128166 0 0.8488109 0 0.3929680 2.17595 0.4227313
0.1898656 0.5142999 0.5852296 0.1237205 0.004871824 0.07706916
0.3908998 0.1623409 0 24.200140.1993851 0.2564469 0.01951076
0.8861876 1.63833 2.5941530.4180354 1.6414960.06482502 0.05584282
1.0804460.6983887 0.01867277 0.5501552 0.07427096 0.07056623
0.3274940 0.004533785 0.1815916 0.59668 0.4376030.03383643 0.01393451
0.2133255 0.1276229 0.5444747 2.3396714.1154470.2376671 0.08148213
0.004372738 0.1191194 0 0.1184545 0.1552162 0.5973535 0.2838381
0.1602394 0.2438445 0.03644306 0 0.1623409 0.1904384 0
0.1258830.09113001 0.04913183 0.01710081 0.6844397 0.02700762 0

0.3000163 2.0554990 0 0 0.2277474 1.7353030.5015081 0.02720548
0.1200339 0.1833851 0.2132181 1.0845920.3517009 0.1540891
0.2378785 2.40432 0.1041182 0.7006694 0.05934259 0.07056623
0.4314495 0.2527178 0.007091434 2.4684810 0.07439495 0.3261991
1.6678390.2705374 0.3064977 0.2363167 0.4342754 0 0.2114476
0.4358759 0.04126164 0.5565207 1.3798980.0888143 0.02750776
0.3888532 0.07210773 1.09625 0.4244055 0.1764854 0.02288781
0.05202869 0 0.6967851 0.9481399 0.3885402 0.2369090.601163
0.1543293 0.6855781 0.3358988 0.4297777 0.02766144 0.4434087
3.4516830.3262548 0.04745748 0.6859842.5560781.02865 0.07933436 1.15345
1.3934510.1108522 0.2994797 0.1093560.5528143 0.08763535 0.4800277
0.1265533 0.0193540.1067687 0.6043461 0.2417773 0.5928502
0.2323443 0.4301895 0.5930893 0.1777010.7904862 0.1632329
0.05333641 0.2041034 0.1010489 0.7664974 0 1.2225970.01389541
0.5996367 0.4843758 0.03470605 0.3813211 0.1360274 1.187835
0.2490794 0.4158985 0.3554382 0.1861427 0.1091103 0.02615175
0.3080040.04902373 0.02976792 0.1712299 0.1290558 0.5085219
0.2583338 2.5162840.4987253 0.8764062 0.04954149 0.1241730.2470233
0.02813294 0.5443944 0 0.01094635 0.2076818 0.1284224 0 0
0.02818632 2.3035281.4103720 0.4049671 0 0.3774890.3454463
0.2739783 0.3041814 0 0 0.7048562 6.0877830.05735209 0.2686581
0.2376671 0.1327008 0.1528145 0.1647236 0.9127667 0.01868452
0.1813699 0.8676242 0 0.2146559 0.6648343 0 0.9864111
1.2828220.1099038 0.05012663 0.1322332 0.6015716 0.00644154
0.03982357 0.3088397 0.01099496 0.1386197
TCGA-BT-A0YX 400 1 0.0298943 0.03196841 0.0542073 0.1659104
0.09175349 40.963250.1041526 0.1303643 0.08183419 0.4115545
0.01477007 0.1667730.1201988 0.1662357 0.05654169 0.07419197
0.1415363 0.3926309 0.1953461 0.03562194 0.02453131 0 1.124773
29.5073 0.1376302 0.1606903 0.06881512 0.05365837 0 0.2811589
0.04952116 0.1121432 0.1605686 0.3484987 0.1134703 0.1434252 0
0.08350488 0.03861671 0.07646626 0.08628256 0.05810048 0 0
2.1474220.1896649 0.05629497 0.03711919 0.2408529 0.3896152.6240290
0.6143495 0.1057110 0.2440038 0.3449467 0.01332185 0.2453131 0
0 0.1668902 0 0.0215638 0.1554650 0.3468913 0.1931212
0.02651039 0.07209203 0.03958733 0.08753766 1.28102 0.7799047
0.05652015 0.1046382 0.02495738 0.1019810.01641755 0 0.110737
0.2272928 0.2970008 0.9831579 0.1159784 0.1663989 0.1900902
0.03860666 0.2173852 0 0.04490478 0.06934988 0.04673662 0
0.1161373 0 0.05987304 0.7338607 1.7414320.05621099 0 0.09215242
0.07221484 0.5942539 0.1703783 0.03881878 0.1039873 0.1928075
0.1352157 0.1749433 0.05903915 0 0.07542725 0.5789241 0.04952116
0 0.005292149 0 0.1319194 0.2403976 0.1686430.1235863 0
0.03538407 0.06923975 1.3634250.03618962 0.4217145 0 0.5492407

0.03154026 0.07542725 0.2782363 0 0.5838858 0.05790999 0.04752255
0.0392501 0.295917 0.05144431 0.03910527 0.4081036 0.08394082
0.06531604 0 0 0.1355761 0.2459774 0.127938 0.1352157 0.7850021
0 0.1630389 0.1065078 0.07665481 0.03946938 0.7592259 0.6564348
0 0.1015738 0 0.06881512 0.5460392 0.05327064 0.2007108
0.9146084 0.05272402 0.0732548 0 0.2458586 0.1563057 0.07610433
0.1053357 0.04142356 0.5332209 0.04538556 0.05390401 0.4229612
0.5952398 0.03610742 0.03846653 0.06531012 0.1921189 0.09316508
0.03805217 0.03235886 0.5767362 0.03771362 0.1028886 0.2507054
0.01982699 0.1923023 0.3020551 0.1485635 0.1483901 0.1164564
0.08920478 0.2584763 0.07417932 0.1616241 0.1593613 0.07291651
0.03731524 0.07990596 0.06995068 0.04247506 0.1832426 0.2060251
0.3437036 0.09215242 0.5790999 0.08371977 0.3700522 0.01178158
0.04429479 0.1341262 0.1204265 0 0 0.04060954 0.2469464
0.03054043 0.09399138 0 0 0.3134361 0.4477179 0.05790999
0.0924539 0.03832741 0.1346573 0 0.08470204 0.09645077 0
0.2631564 0.2514352 1.085812 0.05455613 0.06182922 1.639084 0.1656788
0.03559951 0.1520846 0.2325719 0.07998134 0.02587919 0.1658023
0.1141565 0.06125739 0.7407877 0.03002133 0.3311728 1.300233
0.0539065 0.1430487 0 0.1320564 0.07353012 0.0284116 0.08529198
1.140025 0.08328116
TCGA-FT-A3EE 99 1 0.03193471 0.06830077 0 0.09600202 0.04900802
51.23405 0 0.1508673 0.07284976 0.1099112 0.01577819 0.3292703
0.1926043 0.3699625 0.07334394 0.1033157 0.1133975 0.0561736
0.313019 0.03805329 0.4323937 0.03281407 1.759017 55.3509 0.02940481
0.05149744 0.1102681 0.02866039 0 0.8201843 0 0.09983116
0.08576404 0.2876749 0.08612653 0.1702384 0.09222691 0.1256972
0.04125246 0.02970378 0.08042433 0.08689251 0.06186259 0.05540385
1.273166 0.1013052 0.1653777 0.02973954 0.3537767 0.3953975 3.92709
0.06371962 1.35358 0.4728785 0 0.2784302 0.08188682 0.01138489
0.3930852 0.02830213 0 0.5526716 0.03430562 0.01266959 0.12988
0.1196708 0.3149828 0.3094538 0.01415992 0.2267593 0.04228933
0.1052015 1.036708 1.029168 0.04528341 0 0.003332603 0.1180201
0.03507623 0.1083335 0.07097714 0.06070163 0.7499164 0 0.154868
0.1722014 0 0.06186259 0.3483339 0.1187502 0.02398486 0.05093226
0.02496329 0.09611892 0.2763247 0 0 0.3077347 1.28507 0.01125893
0.01638329 0.0492211 0.0771438 0.3703083 0.1092044 0.04146833
0.05227522 0.2059674 0.1986115 0.3450165 0.1261376 0.02348725
0.0604316 0.8417630 0.05290118 0.2670013 0.08197372 0.1075616
0.2113852 0.1498034 0.1657413 0.330054 0 0.03779918 0.1068393
0.9599554 0.01288657 0.7258027 0.1286461 0.4488223 0.05053952
0.1007193 0.4239856 0 1.09778 0.2474503 0.04442039 0.08385817
0.3161146 0.0549556 0.2506462 0.5554953 0 0 0.01982636
0.02434592 0.1206914 0.1970748 0.06833513 0.03611117 0.2138106

0.3528578 0.1523961 0.2275548 0.06141512 0.02108166 0.6082847
0.6319076 0.04387928 0.1808443 0.221189 0.4043162 0.2379899
0.1564931 0.5145842 1.125713 0.02816133 0.05216983 0.08334371
0.3064127 0.05009227 0.04064938 0.1004691 0.545761 0.3458302
0.04848331 0.09213309 0.3263217 0.5875736 0.1542876 0.2670982
0.08413177 0.1919911 0.07464299 0.02032469 0.13827 0 0.1410071
0.412167 0.4882083 0 0.2500859 0.3636892 0.1498867 0.1387036
0.08293665 0.0762347 0.3106332 0.05402889 0.333404 0.02837307
0.03684146 0 0.09602986 0.03736255 0 0.1304998 0.1782121
0.305969 0.221495 0.3917964 0.044717 0.3544156 0.04405001 0.1182952
2.903936 0.1715281 0.07902864 0.08937516 0.1868733 0.3994709
0.1304998 0.05020334 0.05181169 0.04296339 0.6741835 0.2600936
0.03093129 0.1604919 0.02047171 0.05753928 0.08775855 0.1322448
0.06439621 0.08914058 0 0 1.275916 0.1311296 0.0198148 0.9056683
0.1390613 0.05466715 0.1883117 0.2236013 0.1281606 0.0552911
0.1830229 0.1422728 0.03271923 1.027616 0.03207041 0.7738865
1.941874 0.05758584 0.07640621 0 0.07053491 0.009818607 0.04552622
0.1290775 0 0.08340511
TCGA-ZF-A9RL 2703 0 0.2156781 0.5958256 0.130363 0.6982454 0
17.71405 0 0.1175672 0.4920069 0.1237182 0.6571292 0.08952494
1.686217 0.7662445 0.1942527 0.1051427 0.08509502 0.09273753 0
0.1820426 0.324474 0.07387234 5.239344 36.66019 0.1489439 0.01932218
0.08274662 0.06452141 0 1.19628 0 0.1348463 0 1.638111 0.1867103
0.1149742 0.4671561 0.1643078 0.03316757 0.008358793 0.1139219
0.1257531 0.2321126 0 15.28641 0.1710467 0.1353836 0.01115848
0.7964362 0.6090395 4.001235 0.1912642 0.7848937 0.015889 0.2874356
1.24029 0 0.02402825 3.067754 0 0 0.4013537 0 0.007778805
0.5224693 0.9159858 0.5839663 0.2322183 0 0.2889564 0.27609
0.8026038 0.7001637 4.468317 0.1699064 0.489308 0 0.2861294 0
0.2032373 0.3728353 0.1366539 0.1623309 0 0.1045935 0.1625699
0.4571472 0 0.6534861 0 0 0.2084741 0.08429755 0.007335144
1.047368 0.04633811 0 0.2167371 2.011324 0.008448851 0.03688272 0 0
5.83557 2.007736 0.09335516 0.1544604 1.937529 0.3658272 1.262162
0.3194619 0 0.136046 1.701642 0 1.142059 0.006363535 0.1210734
1.004633 0.04817762 0.008111381 0.6687278 0 0 0.4394131 2.041858
0.1450538 1.145647 0.1158453 0.1693419 0 0.136046 0.1279218
0.212383 2.330947 0.6267039 0.2761931 0.1415887 0.1334343 0.3711547
0.7993751 0.9814469 0 0 0 0 1.03248 3.056343 0.5127959
0.08129492 0.525097 0.1985919 0.4411031 0.06403507 0.6913009
0.09491977 0.6086199 1.681226 0.329276 0.2442744 0.09053627 0.7447196
1.544285 0.1201035 0.6757641 0.5737927 0.06339791 0.1468085
0.09381334 0.8745786 0.07517982 0.4118018 0.08142479 0.1162226
0.04579687 0.5457379 0.2074137 0.6216087 0.2989821 0.564425
0.6013021 0.3418454 1.311558 0.1680393 0.04575576 0.5836479

0.08668693	0.6802302	0.06185912	1.029989 0.02384093	1.13606	0.855688
0.158791 0.2973857	0.09335516	0.1072641	0.427356 0.40544	0.7103633
0.4471221	0.1232236	0	1.128973 0.08411207	0.1532222	0.4406794
0.09646298	0.3214445	1.108085 1.160563 0.721459 2.102093 0.05666695
0.1864177	0.1575291	0.09653772	0.04447811	0	0.003756221	0.2375522
0.1468931	0.1130198	0.05832027	0.1450814	0.7435951	0.8325117
0.06963377	0.2223421	0.04608672	0.2914531	0.2963484	0.1253536
0.1449713	0.2006768	0.01318467	0.481501 3.603547 0.06560092	0.20817	0
0.3272904	0.09631477	0.4073098	1.118623 0.7373296	0.03111839
0.544941 0	0.2209765	1.057376 0	3.335077 6.982454 0.2298156	0
0.07562599	0.3969775	0	0	0.9059395	0.0440172	0.08762363
TCGA-ZF-A9R9	864 1	0.1119005	0.1675301	0	0.2277135	0.6182139
63.31433 0	0.1789261	0.2042148	0.1540533	0.02211497	3.455755
0.6598956	1.866764 0.1209412	0.2539116	0.3178796	0	0.8287169
0.08000415	0.3305727	0.1839709	2.235356 82.89027 0.02060713	0.1202994
0.4121426	0.08034172	3.966873 1.003862 1.112207 0	0.1923332	0.5218009
0.6974721	0.02386089	0.4524335	0.1363968	0.2725805	0.08326659
0.1266562	0.01739856	0	0.4659296	4.98371	0	0.1264342	0.1945227
1.081874 0.8167091	6.178072 0.0595403	1.149818 0.3363433	0	0.9631758
0	0.3271236	2.718042 0	0.3769	0.1999054	0.1923332	0.6554279
0.1611519	0.06709298	0.3635759	0.867471 0.06946369	0.1559164
0.3556404	0.4095893	0.6393494	3.297141 0.04231331	0.4700189
0.3456563	0.1781434	0.1229085	0.101228 0.1989654	0	0.4851207	0
0.08682621	0.2180028	0	0	0	0	0	0.03893863	0.03498895
0.2329089	0.3319724	0.7500995	0.537881 0.7518082	1.097859 0.6838302
0	0	1.784079 3.11418	0.5612296	0.2324907	0.4121426	0.1443436
0.101228 1.047758 0.04419914	0	0	4.639054 0	0.22454	0.00792384	0	0
0.4799241	1.464536 0.09252181	0	0.05297993	0.1727857	0.5196371
0.01806203	2.408772 0	2.129723 0.04722467	3.105737 0.01225289	0
0.9441813	0.5202456	0.2431118	0.1763054	0	0.4621599	0
0.4927792	0	0.2933897	0.0555779	0.3412364	0.2706608	0.5524465
0	0	0.3113562	0.08242852	0.3661729	0.07973613	0.2869347	0
1.231507 0.5053164	1.558048 0.3041691	0.4509411	0.6182139	0.1700555
1.674985 0.3005207	0.8931045	0	0.09140259	0.1946931	0.2914281
0.3276473	0.341849 0.1351863	0.02067425	0.5132342	0	0.2098447
0.07036581	0.2481939	0.108126 1.09431	0.01725665	0.7237803	0.2092416
0.4557986	0	0.4317684	0.3952756	0.4621599	0.625628 0	0.463193
0.5135854	2.001973 0.03703032	0.3487361	0.1869906	0.290259 0.06058221
0.60082	0	0.02065503	0	2.093731 0.1047359	0.06359716	0.1829106
0.8517259	0.05718015	0.4829236	3.352694 0.1462442	0.5731785
0.01764035	0.1658045	3.414021 0.1202083	0	0	0.1356396	0.5810333
0.04572764	0.07036581	0	0.3613091	0.1902577	0.1658623	0.3468304
0.03460739	0	0.08064798	0.2460076	0.1463341	0.03610351	0.1249409
0	0	0.7153377	0	0.03703032	2.031039 0.07087656	0.07329095

0.1397333 0.2553656 0.3592639 0 0.3227286 0.2848742 0.0458598
0.9016980.1348512 1.30163 13.797820.0220127 0.5711583 0.1883382
0.09886287 0.01376192 0 0.06385308 0.9787497 0.03117385
TCGA-ZF-A9RE 106 1 0.1225168 0.2246009 0.1269483 0.5018723
0.02685974 40.751740 0.3053006 0.2555305 0.3614330.1902456
0.05928239 0.2345784 0.9083858 0.4729115 0.03102687 0.0828661
0.3612337 0.1143705 0.08342318 0 0.07193738 2.36120865.50116
0.1369847 0.1881607 0.4834753 0.06283138 0 1.3928690 0.4377142
0 1.81779 0.2587435 0.3732090.5054663 0.4622338 0.0322988
0.008139848 0.1030138 0.2313118 0.1356197 0.04048678 10.76218
0.3331328 0.4284717 0.03259859 0.4935477 0.4562205 5.469844
0.2328182.7426130.1083097 0 0.4805236 1.2566280.0124794 0.6893999
0 0.2358042 0.6644295 0 0.01010007 0.1633711 0.5247018
0.4061931 0.1696018 0.0232818 0.4314612 0.2039614 0.4868865
2.1818371.1281090.1323648 0 0 0.4179522 0.01922416 0.1979139
0.1556012 0.3326863 0.1896948 0.7764116 0.3055617 0.1400449 0
0.3164459 1.5272860 0.05258135 0.1725615 0.08208952 0.01071452
0.5192392 0.3158705 0.2103254 0.4296579 2.1732870.01645509 0
0.3237182 0 1.8555810.7581182 0.2727296 0.3294794 0.6289262
0.3562449 0.6145508 0.3802261 0.3604332 0.1324825 0.9038566
0.2319476 0.1756019 0.006196853 0.1768532 0.5663950.3284098
0.04344404 0.5788547 0 0.2071653 0.3513312 1.53845 0.1977562
0.6035424 0.2820272 0.6486314 0.1477286 0.0883217 0.1533183
0.13788 0.6153322 0.2712393 0.1159305 0 0.5630701 0.3011941
0.4121136 0.5241152 0.1474360 0 0 0.8466831 1.1041070.1498092
0.3166622 0.4930807 0 0.8590984 1.3095130.1346387 0.184867
0.7408477 1.2202920 0.2775220.6612362 1.5310050.4347823 0.03898586
1.5981541.3037810.06173731 0.3145189 0 0.4318329 0.07321061
0.3564581 0.1057227 0.01616834 0.0891946 0.3188659 0.2524761
1.2106540.4146693 0.3382405 0.9909342 0.4363575 0.4716947
0.3272756 0.04455727 0.1136720 1.2365040.1807165 0.7277943 0
1.0736690.4795826 0.05798691 0.4488742 0.4090945 0.1462363
0.1134988 0.6790907 0.4176643 0.1866045 0.1407645 0.04369436
0.2339151 0 0 0.3576138 0.1216902 0.3130248 0.9711547
3.3000780.3921274 0.6424991 0.1793436 0.1296677 0.1716651
0.2350227 0.5630701 0.06531157 0.02926266 0.1734974 0.2503297
0.1650891 0 0.09418746 0.7836344 1.9456930.06780983 0.2435830
0.1892127 0.2885860.2059935 0.2541133 0 0.03851795 0.5779328
3.7634450.06388262 0.2027174 4.3018550.2494311 0.08337064 0.2792677
0.4901951 0.03121811 0.06060659 0.4983094 0.08911453 0.07172947
1.3479440 0.7271015 2.05465 0.2926562 0.1395860 0.2319476
0.01076253 0.09980589 0.6991098 0.2388151 0.09751826
TCGA-DK-A6B1 2049 0 0.1013392 0.1685759 0.08167033 0.135398
0.01727982 57.274570 0.3191673 0.3082346 0.3875376 0.08901213

0.2221004 0.7847454 0.5217827 0.05476332 0.01596855 0.1599321 0
0.1962095 0.05366908 0.2587172 0.04627986 2.246767 43.7706 0.191806
0.08473529 0 0.04042165 0 1.107883 0 0.1126388 0.04838349
0.6682562 0.08097997 0.1320542 0.6828871 0.160123 0.6940171
0.03665656 0.08666391 0.1050431 0.1744978 0.02604657 6.082495
0.2857548 0.02120392 0.04893426 0.4989547 0.6750564 6.464925
0.239648 1.128071 0.1791758 0 0.7436018 1.299267 0.01906753 1.737102
0.03991638 0.1137759 0.3520184 0.02419174 0.006497731 0.1291258
0.3375592 0.5487685 0.3637027 0.01997067 0.09654754 0.09542951
0.2885025 0.7603119 2.350055 0.2341761 0.3328189 0.01880077
0.07682382 0.148411 0.05092998 1.05109 0.1284173 0.1423769 0
0.1310524 0.2507015 0.2147966 0 0.2046994 0 0.03382744
0.05877258 0.1408295 0.04135817 0.1749759 0.1451505 0 0.5964725
0.897579 0.002646536 0.06931932 0.06941978 0.08160077 0.6341854
0.3080363 0 0.1382385 0.1971179 0.15279 0.7907244 0.1334252
0.1325025 0.0852307 4.288436 0.1119151 0.6401683 0.03189323
0.0379253 0.3312562 0.1207301 0.05335734 0.5120468 0 0.05331069
0.09562544 3.118613 0.05452436 0.9177531 0.07257523 1.400388 0
0.2272819 0.08014099 0 0.8005268 0.2181223 0.2147966 0.1774061
0.2507835 0.1550151 1.178343 0.9421256 0 0 0 0.06867333
0.06808763 1.111791 0.03212586 0.07639498 0.3720437 0.08294312
0.1228196 0 0.3175986 0 1.668147 1.02253 6.291728 0.07651701
0.1985184 1.399665 0.3389437 0.1203887 0.8164713 0.359472 0.03971779
0.1747491 0.3918172 0.5016074 0.1883959 0.02866526 0.06801512
0.1144183 0.07172754 0 0.3248535 1.168284 0.3292072 0.1904018
0.5505707 0.179432 0.7376359 0.2105479 0.114661 0.02437641 0.108616
0.1136409 0.3875376 0.4918233 0.01493597 0.4198961 0.636348
0.1367851 0.1863075 0.1754566 1.464944 0.1703748 0.02540018
0.3190791 0.2000821 0.106889 0 0.3310689 0.3688642 0 0.1840525
0.3021627 0.2013799 0.6247781 0.5234934 0.3573816 0.422955
0.1775047 0.1334717 0.8365109 0.1209587 0.0835945 0.1680689
0.05177069 0.2444944 0 0 0.1096102 0.09089118 0.9572272
0.7788561 0 0.1044703 0 0.1014393 0.3094293 0.09816508
0.05449335 0.440023 0.008259985 0 1.701354 0.08219588 0.07452299
0.04257747 0.1693827 0.06369189 0.4660816 0.5372797 0.281172
0.05848553 0.4288278 0.114661 0 0.7266647 0.1130776 1.652787
5.287315 0.1476675 0.143681 0 0.3979203 0.1177066 0.04280576
0.2891327 0.3781862 0.0941054
TCGA-CF-A5U8 399 0 0.1113511 0.2381537 0 0.3237079 0.04882372
94.45998 0 0.5433922 0.4064244 0.1094978 0.09431313 0.1616389
0.29848 0.9435422 0.07736624 0.05921836 0.07531405 0 0.03464909
0.03791018 0.208857 0 2.476728 112.9754 0.06591202 0 0 0 0
1.726267 0 0.3182583 0 0.6069029 0.06991325 0.01695982 0.1837601
0.09694795 0.1056788 0.04438811 0.07382015 0.04946613 0.1232599

0.07359399	21.77953	0.1513863	0.08986676	0.0395036	1.666109	0.4975711
8.31031	0.16928	0.24518	0.02812538	0	1.298389	0.1223683	0.0141776
1.566428	0	0.05357852	1.136708	0	0.006884695	0.330903	0.09537656
0.6645139	0.05138168	0.02116	0.3580406	0.1179648	0.2212569
0.9088722	12.64534	0.150377	0.0742399	0.0332007	0.1627979	0
0.03597537	0.2828408	0.1209467	0.3735481	0	0.1542856	0.2490297
0	0.1643465	0.1156746	0	0	0.0645791	0	0.01947609	0.6797866
0	0.06371909	0.2021013	1.853295	0	0.09793006	0	0	1.475663
0.9428786	0.2065619	0.1432162	0.4103857	0.07195074	0.5585433
0.2827424	0	0	3.616807	0	0.4787948	0.04505679	0.3214711
0.6083719	0.08527999	0.03589522	0.591863	0	0.1506281	0.2742812
1.925881	0.07702865	0.6150277	0.1537947	1.30893	0	0	0.03483638
0.0626571	1.702616	0.4930395	0.1770133	0	0.1574629	0.2189957
0.8323453	2.521851	0	0	0	0.09701745	0.19238	0.4799267	0.04538543
0	0.6971121	0.1757654	0.173512	0	0.1223683	0	0.8753291
1.203806	0.08742851	0.3242955	0.08012986	1.904125	0.2928819	0.04251943
0.4699283	0.9733598	0	0	0.1383838	0.6541292	0.133077	0.3239721
0.03202919	0.249812	0.0405329	0.1932039	0.2868331	0.4501308
0.657532	0.07685368	0.5731248	0.1022139	1.259738	0.04957485	0
0.1721875	0	0.1605449	0.1094978	0.4446832	0	0.4745627
0.4685661	0.08783706	0.1710824	0.330499	0.018987	0.03438499	0.1220049
0.3499439	0	0.08598966	0	0.3188957	0	0.04520352	0.5200359
0.3764277	0.04064244	0.9807198	0.2054331	0.1484961	0.5975247
0.05015354	0.1178503	0.5112156	0.0854415	0.3542915	0.05935936
0.07313843	0.5406365	0.1625112	0	0.4645515	0	0.3696318
0.4470049	0	0.04919642	0	0.2866144	0	0.1109453	0	0.4440267
0.02333839	0	1.016894	0.08709095	0.1974028	0.1203016	0.05037757
0.05209366	0.2795659	0.511524	0.05674605	0.1377079	0.4999498
0.04049651	0.1955771	0.3346334	0	2.467123	4.016921	0.1043078	0	0
0.07026965	0.009781681	0	0.635396	0.06678477	0.1218674
TCGA-XF-AAMW	253	1	0.295242581	0.052621164	0.089227191	0.273094572	0
25.088752736	0	0.160938229	0.404106158	0.338716812	0.048624156
0.990212447	0.131901065	0.410445078	0.132956675	0.082868959	0.058243458
0.56549909	0.321546835	0.058635011	0.161517739	0.10112415	2.01821241
31.544134171	0.067963308	0.185151495	0.226544361	0.176647249	0
0.320398454	0	0.123061134	0.105720702	1.355879853	0.157285237	0.209851619
0.142109294	0.324886684	0.036322573	0.160193389	0.097467601	0.095635503	0
0.056913265	1.59063062	0.07804884	0.185327127	0.015274882	0.396452632
0.38479226	3.484609975	0.261822376	0.821633715	0.456760888	0	0.273844832
1.577207577	0.07455602	0.161517739	0.174439158	0.165737917	0.329648803	0
0.007098959	0.144354391	0.073758629	0.228398243	1.033124854	0.032727797
0.17140658	0.156389234	0.198123979	0.76676553	1.208236593	0.093034218
0.019137593	0	0.139887055	0.351310465	0.055642475	0.072910829	0.187066121
0.266658586	0	0.238630859	0.085593306	0.156447675	0.254191851	0.357823914

0.24397085 0 0.114152414 0 0.025102771 1.164375949 0.063432421
0.098553197 0.121855752 0.867481218 0.584066135 0 0 0.237736502
0.570595377 0.560897614 0.447279892 0.251715957 0.362706501 0.278212373
0.398718076 0.048590295 0 0.186233977 1.094104987 0.163027251 0.164565243
0.026133207 0.082868959 0.144762787 0.197851597 1.415722002 0.915424153 0
0 0.208947086 1.774994942 0.079425911 1.311186007 0.079290526 1.406331197
0.207665664 0.248311969 0.026940411 0 0.499770591 0.095321944 0.189040941
0.129214191 0.243545072 0.254037609 0.064368693 0.541736516 0 0
0.12219906 0 0.148775401 0.202443897 0.140393689 0.055642475 0.367960942
0.271853233 0.335459919 0.175315737 0.063088303 0 0.728999466 0.714239072
0 0.111462721 0.371806376 0.226544361 0.12942724 0.131528112 0.528603509
0.720011534 0.173571302 0.341643512 0.085614311 0.387829931 0.102913958
0.062635245 0.03715424 0.090912916 0.658260974 0 0.248438272 0.232069833
0.434082512 0.475473003 0.379903792 0.063236961 0.550860499 0.843442083 0
0.42611092 0.355998282 0.620779922 0.846792029 0.636199985 0 0.311949053
0.303372449 0.027171208 0.203546275 0.063897127 0.234934893 0.15954801
0.144302199 0.275212976 0.087438174 0.068121289 0.061422239 0.131528112 0
0 0 0.204075058 0.251443831 0.303372449 0.571931666 0.045935262
0.525102824 0.019392903 0.145821658 0.364538049 0.594678948 0.121772536
0.091810083 0.462771532 0.32518618 0.201082603 0.154713222 0.31933942
0.066200819 0.278879549 0.907904154 0 0.380456179 0.126176606 0.132990464
0.405672461 0.139422629 0.079380732 0 0 0 3.789047285 0.089801368
0.101773138 0.465171088 0.077918105 0.219742798 0.500673383 0.433864768
0.043884065 0 0.418472035 0.626352453 0 0.359668367 0.444745729
0.340701481 2.410356442 0.193597216 0.274707336 0.103524723 0 0.015129155
0.04676653 0.257388429 0.017215806 0.068541909
TCGA-BT-A3PJ 789 0 0.064966298 0.046315842 0.039267779 0.230355604 0
14.607578946 0.113172249 0.070826917 0.158081871 0.074532533 0
0.146698289 0.261216098 0.923229126 0.03510755 0.069100295 0.025632223
0.010157924 0.212263158 0.019353406 0.035540976 0.13351045 1.529253164
21.070640572 0.094714394 0.128044518 0.099699362 0.116610393 0
0.172337469 1.112067184 0.135394195 0.023263184 1.17046157 0.095176511
0.10389723 0.093810805 0.230965428 0.05195162 0.276960839 0.096818522
0.033670399 0.02796667 0.82654512 0.451121936 0.137393304 0.081560169
0.21511316 0.567040122 0.846711495 1.868707123 0.086418584 0.584108219
0.354168931 0.057720683 0.257100187 0 0.026055936 0.248786834 0.153537018
0.255287203 0.072537174 0 0.003124164 0.066416239 0.064920515 0.201030465
0.27979411 0.024005162 0.092841734 0.080295788 0.277428788 0.815489654
0.199398724 0.286602433 0.008422217 0.009039565 0.098500092 0.083250126
0.048975125 0.256697208 0.246976436 0.176029702 0.612823048 0.336059136
0.033901697 0 0.11186668 0.157473864 0.107368544 0 0.087914857
0.084640032 0.004418967 0.359464002 0.055831643 0 0.165544848 1.112105966
0.111977934 0 0 0 1.542544804 0.197475264 0.056240666 0.310175793
0.119716973 0.048975125 0.721380274 0.064151957 0 0.163918524 1.071730321

0 0 0.015334534 0.145878402 0.127416612 0.087072033 0.527752957
0.40286681 0 0.051264447 0.15047185 1.768818274 0.034954358 3.009642351
0 1.047384307 0.159934393 0.054639508 0.029640351 0.042649172 0.346832933
0 0.14057014 0 0.401929366 0.335396398 0 0.438677193 0.121613479 0
0.026889145 0 0.425582352 0.267279141 0.03089276 0.097950251 0.990439923
0.119639235 0.118105398 0.15430856 0.138821897 0.057183247 1.008302803
0.556118332 0 0.171686761 0.136356144 0.747745216 0.07911017 0.01929463
0.203552864 0.48970531 0.19096644 0.035377193 0.037677796 0.259729126
0.090582311 0.110259977 0.092656347 0.080019293 0.538000332 0 0.078096143
0.068087369 0.327487418 0.23540599 0.278651575 0.072357485 0.457917421
0.506165992 0 0.023440766 0.783352131 0.191238278 0.223597599 0.601586639
0 0.405798035 0.415365845 0 0.098536009 0.028120333 0.245555836
0.280860398 0.063505607 0.109006034 0.038480456 0.112779399 0 0.159180697
0.152016849 0 0.132740937 0.095093895 0.276643275 0.13351045 0.755100087
0.080862155 0.757979257 0.05974203 0 0.01807647 0.116315922 0.160771746
0 0.099567115 0.306665149 0.066370469 0.068087369 0.210805974 0.087402477
0.190233715 1.385912322 0 0.200920852 0.111057517 0.117054905 0.059510472
0.061358169 0.087336188 0.241790579 0.071486454 0.215869269 1.352887656 0
0.026873457 1.39206896 0.162881258 0.029011824 0.24287493 0.179706824
0.212441157 0 0.236210796 1.350684716 0.044374861 0.853197911 0.021747453
0.449815482 1.911211239 0.113599643 0.39722738 0 0.047830847 0.03994892
0 0.09267828 0.003788231 0.030164444
TCGA-ZF-A9R7 665 0 0.1376743 0.3926036 0.1664298 0.7216301
0.1056397 72.312256 0 0.5336673 1.172505 0.1579467 0.793586 0.4343147
1.968213 2.594456 0.6075893 0.0772851 0.6518269 0.1937368 0.04998007
0.3144334 0.2259516 1.131723 6.180739 80.54953 0.07394779 0.3206834
0.8451176 0.1647444 0 5.743781 0.07602109 0.3443072 0.1478956
1.994048 0.5134048 0.2446393 2.054273 0.5011075 0.0931565 0
0.07272005 0.5886633 1.422384 0 33.49303 0.2911591 0.3456789
0.0854738 4.344433 1.256025 4.281188 0.4273155 1.178878 0.08113972
0.9785572 4.954618 0.0588373 0.02454081 3.464591 0 0.3091404
1.178506 0 0.02979278 0.5568684 0.5503091 0.585773 0.1482325
0.0508709 0.4426806 0.7535658 0.4871315 1.489791 2.676206 0.8242713
0.0535442 0.1819851 0.1826457 0.1512178 0.1037864 0.2039939
0.6978451 0.04144844 0 0.2225515 0.2634254 1.896777 0.177798
0.5839979 0.1137658 0.06893438 0.1863061 0.03587324 0.00468226
1.880097 0.05915823 0 0.3952866 0.3869268 0.01078635 0 0 0
11.70725 2.353965 0.1191832 0.2159745 2.114167 0.4670387 2.685596
0.1812648 0 0.1157901 4.344853 0.3801054 1.381289 0.08124101
0.0772851 1.28258 0.9841067 0.1190884 3.699545 0.1251424 0.1086378
0.6259392 0.8562375 0.2222222 0.2637489 0.1478956 1.736749
0.1936728 0 0.2638137 0.3615225 1.75682 0.9778888 0.2857325
0.1807613 0.3974855 0.1579467 1.140597 2.303864 0 0.1002682 0 0
2.011884 4.4054 0.3928011 0.05189318 1.077385 0.8451176 0.1251424 0

1.3532580	0.9712545	1.3379180	0.2598804	1.0980522.4297130.6772959
0.1328876	1.0475940.8546311	0.3237515	0.2623954	0.07984546
1.7613080.2879383	0.8178068	0.06930144	0.6570996	0.5262053
0.4180346	0.4964958	1.4428840.3122999	0.1662881.5943730.4423196
6.1078280.3575497	0.1168295	0.1490245	1.2173720.5210556	0.2369201
1.7639590.3652426	0.8385611	0.1336061	0.1013615	0.3606788
0.4171414	0.3012688	0.09919825	0.18634 0.5732266	0.1630929
0.1845429	0	7.6972610.6442976	0.6520446	1.5940330.2435029	0
3.7488313.7930230.5783408	1.9001150.2893788	0.6459807	0.3447632
1.17084 0.1135673	0	0.00959087	0.4007557	0.4219499	0.2164325
0.07445544	0.7408811	0.8973041	1.4348320	0.1064461	0.2353492
0.2480587	0.1261125	0.2300497	0.3701596	0	0.1009944	0.01429571
2.3335980.04187519	0.2467802	0	0.3633392	0.2937418	0.4297944
1.5233140.3683437	0.03972775	0.4666327	0.4089034	0.5172071
1.5626280	3.43164 2.9262010.2858745	0.4757945	0	0.7095302
0.1128778	0	3.0769420.008027887	0.1917703
TCGA-FD-A3SO	168 1	0.159523211 0.077541449 0	0.147556369 0	12.98199416
0.101051559 0.158103476 0.264658844 0.299475614 0.014330308 1.285791276
0.038873331 0.618263817 0.054858211 0.092549533 0.034330538 0.006802511
0.189529882 0.077762996 0.095203667 0.178817323 1.736879019 31.885231581
0.08011945	0.685986092 0.200298625 0.104121479 0	0.965248612 0.192186843
0.036268064 0.311575639 0.368860846 0.115886121 0.015461648 0.083763716
0.220959801 0.096343534 0.276524657 0.11654242	0.101467067 0.299657445
0.167732399 1.145916483 0.046004457 0.081928256 0.045017496 0.525783891
0.340213106 2.312219214 0.115744796 1.639158796 0.743585751 0.077308241
0.408912455 0	0.045238198 0.952036674 0.05140998	0.097691174 0.323842396
0	0.046027859 0.085087047 0.043475671 0.40387546	0.843170521 0.025721066
0.093260735 0.122907686 0.201711849 0.828585765 0.40059725	0.137093281
0.045121212 0.006053574 0.263852345 0.095572388 0.098392307 0.193391776
0.220525385 0.209569347 0	0.112525265 0.085767386 0.092215211 0.112371542
0.158184555 0.07190207	0	0.050463784 0.022672538 0.035511283 0.317551062
0.149556307 0	0.218599765 1.511731316 0.08521462	0.089279271 0.044704331
0.105097064 1.153121056 0.165305403 0	0.02967387	0.187066855 0.065594871
0.48309061	0.11456263	0	0.073181467 1.258595373 0	0.242499908
0.015403739 0	0.469302726 0.272113318 0.471230883 0.29976665	0
0.034330538 0.208998831 1.443087169 0.03511211	0.666776459 0.046736346
1.298059306 0.153005894 0.036590733 0.142915776 0	0.634481301 0
0.088372911 0.038081467 0.251218054 0.099825205 0.265586615 0.702496627 0
0	0.072027994 0	0.745389908 0.397756135 0.124128725 0.065594871
0.489258582 0.053412967 0.079092278 0.051668322 0.185931213 0	0.306925256
0.636890046 0.026568465 0.065699655 0.328731669 0.333831042 0.500113577
0.419937006 0.506310413 0.462979186 0.102308419 0.118456176 0.22708703
1.0734225	0.030330372 0.110757588 0.124099349 0.026793475 1.071618639 0
0.156897194 0.27357861	0.263172313 0.140129418 0.11196366	0.07827512

0.258553118 0.406760285 0.036919196 0.062790816 0.489618861 0.292725867
0.299475614 0.334456255 0.038473325 0.351521234 0.307963168 0.416404826
0.059988309 0.112988968 0.294265881 0.062695098 0.052342328 0.156811931
0.103077655 0.172083616 0 0.135671956 0 0.041210405 0.177786676
0.599672546 0.666940286 0.625860631 0.299657445 0.243681276 1.200907429
0.102877115 0.04297595 0.323818567 0 0.10766488 0 0.109108875
0.260130394 0.059262225 0.045596435 0 0.078041716 0.254789671 1.330031723
0.168557313 0.089701165 0.037186243 0.156777577 0 0.107466298 0.350921368
0 0.010638382 0 1.011343877 0.052931769 0.035992985 0.329023875
0.241118836 0.047491899 0.33200183 0.376078862 0.155199941 0.075325979
0.289558169 0.443030352 0 1.535706732 0.116509871 1.00410118 1.690191137
0.123621687 0.346973996 0.122041497 0.064062281 0.071340823 0.027565673
0.606851546 0 0.050500963
TCGA-E7-A97P 437 1 0.093443856 0.09992714 0 0.072028369 0.119501613
33.485214949 0.054260192 0.135831254 0.170532031 0.214406777 0.015389476
2.116231339 0.45921149 0.057735681 0.050496718 0.14080245 0.073735887
0.094968797 0.203538231 0.046394744 0.127800336 0.256045194 0.992455656
21.092525006 0.078871064 0.334858196 0.215102903 0.055908603 0.887299474
0.585899335 0.20639157 0.038948674 0.100381355 0.231072176 0.143119091
0.066417738 0.089954778 0.07118735 0.057480213 0.057943976 0.074036057
0.048429601 0 0.07205187 2.103228382 0.049404694 0.175967315 0.019337914
0.18821504 0.121786202 2.436889463 0 0.480084739 0.220288259 0
0.208011598 0.359412445 0.059686211 0.562321477 0 0 0.313000129
0.100381355 0.125821374 0.120450074 0.046689002 0.614445607 1.106710151
0.006905534 0.050076866 0.131991919 0.091208665 0.202233498 0.09560129
0.235561579 0.181710625 0.039006002 0.070838486 0.051318121 0 0.253837908
0 0.33758825 0 0.120842123 0.260065531 0.297092798 0.080451359
0.05662538 0 0 0.018064538 0.024348289 0.025423972 0.236514972
0.080305084 0.062383893 0.482928066 0.811732467 0.117136538 0 0.048008474
0.902919169 0.257989463 0.142018637 0 0.082854451 0.31568925 0
0.252387406 0.123030072 0 0.235771153 0.94948474 0 0.052084665 0
0.104911629 0.091634432 0.125239497 0.8399191 0.257538169 0 0
0.056111538 1.511003548 0.025138188 1.139184127 0 0.743466777 0.197177661
1.060970189 0.025579805 0 0.876055458 0 0.115536088 0.040896107
0.038540834 0.214406777 0.203725996 0.397784498 0 0 0.116027485
0.047492254 0.047087203 0.04271547 0.044434402 0.035221528 0.243751634
0.057360774 0.084938069 0.110974362 0.199673581 0 0.593298752 0.622136059
0 0.035277792 0.196126981 0.430205805 0.182060165 0.49954152 0.167302258
0.290032432 0 0.012721139 0.027096807 0.224147829 0.065144242 0.039647932
0.109753132 0.057547617 0.49604443 0.047288861 0.14602781 0.244832572
0.28262365 0 0.04007967 0.036025935 0.445552328 0.339752277 0.039647932
0.033715875 0.300461197 0.117885577 0.053601694 0.397270713 0 0.188751265
0.45874764 0.825566281 0.051537685 0.161786799 0.334604515 0.201986872
0.042158253 0.121946321 0 0.094454608 0 0.10407115 0.291536607 0 0

0.518615411 0.119372422 0.096016948 0.362031115 0.08723067 0.478639572
0.036826956 0.230761735 0.572003834 0.125476693 0 0 0.38081298
0.213193423 0.063642357 0.097933029 0.050535236 0.251429579 0.105917976
0.990697515 0.060338519 0.096331071 0.119804148 0.056121723 0 0.040732674
0.025123889 0 0.034274027 0 0.181015557 0.142110026 0 1.118917499
0.086313393 0.027819313 0.154860994 0.290790171 0.027778488 0.026964466
0.120928777 0.356831386 0 0.349832933 0.062560618 0.345060906 3.538158764
0.066379347 0.347777921 0 0 0.086190409 0.02960308 0.325852285 0
0.162700624
TCGA-GU-A767 144 1 0.01484273 0.01587254 0 0.1784807 0.706122
78.46409 0.3102751 0.582541 0.7042752 0 0 1.245017 0.1989319
1.238058 0.0561467 0.3867868 0.03513688 0.6544549 0.2263117
0.1238058 0 0.1830173 1.073313 70.23586 0.1571691 0.01595678
0.6150095 0.05328352 5.449668 1.675169 0.4425769 0 0 0.1258415
0.3380328 0.06329923 0.9001769 0.1206131 0.4163382 0.0414175
0.07391983 0.01153892 0.1916848 0.1373376 11.62169 0.141255 0.05590171
0.04607485 1.016474 0 10.29062 0 0.4194147 0.393647 0.07912403
1.630013 1.294028 0.2658985 3.800151 0.105235 0.1499786 1.822968 0.9566815
0.4582416 0.1741711 0.2669809 0.2411276 0.6712025 0 0.1511311
0.3695213 0.2607784 0.2312856 4.601182 0.08418797 0.4502648
0.9169723 0.0337562 0.0815143 0 0.5938023 0 0.4289833 0
0.1151682 0.1652364 0 0 0.2158666 0 0 0.01721635 0.3944861
0.5027775 0.3145254 0.8036124 0.7134574 1.412721 1.023908 0.01744322
0.8528448 0.04575433 2.366442 2.753812 1.015128 0.2698332 0.109335
0.6290849 0.03356777 0.3073529 0.02931336 0.2183298 0.03745016
3.076672 0.04917522 0.744587 0.03678625 0 1.83397 0.1193591 0.2779918
0.245446 0.08094997 0 0.05729671 1.944693 0.02395787 0.3101989 0
0.5360806 0.06263986 3.557766 0.3331763 0 2.052517 0.05750544
0.09438113 0.03897591 0.07346245 0 0.6989776 1.098104 0 0.2918689
0.03685988 0.4978858 0.1346289 0.7327773 0.08469615 0.06713554
0.4284769 0.05466751 0.08094997 0 0.1522386 0 0.5026147
0.5524145 0.1903475 0 0.1495346 0.6833439 0.4120907 1.527444
0.1195852 0.3553901 0.0523557 0.06061922 0.07747359 1.047772
0.124171 0.03778634 0.07471421 0.06855698 0.09455073 0.04506851
0.1605823 0.1866696 0.6135277 0.07171037 0.07639562 0.1106331
1.686221 0.3237999 0.1889317 0 0.07158841 0.224701 0.1532548
0.4460427 0.01968849 0.3570101 0.218604 0.8523704 0.03683838
0.2312856 0.4783407 0.5775089 0.03348233 0.1272892 0.05274935
0.08610578 0 0.9124992 0.2778481 0.04217834 0.06065416 0.4779704
0.07584502 0.686315 0.1533478 0.05542328 1.178429 0.128692 0.1099634
3.007598 0 0 0.05538682 0.4497883 0.2592339 0 0.09333478
0.1444874 0.03993737 0.1430051 0.3139632 0.1150109 0.06885603
0.07611932 0.1337166 0 0.0776403 0.04788849 0.3314487 0
0.3236576 0.6469362 0 0 0 0.1292668 0.07953934 0.1269959

0.1693613 0.4500615 0 0.3951456 0.03778634 0 0.6350614
0.05962321 1.2332222.619906 0.03893085 0.1657243 0 0 0.164287
0.02821313 0.3246686 0 0.1653988
TCGA-4Z-AA81 1270 1 0.16142679 0.05754228 0 0.199089531 0
54.972143352 0 0.078217389 0.196399132 0.246929006 0.070895296
0.125077851 0.144236414 0.531946408 0.048463582 0.031796084 0 0
0.039068645 0.053432117 0 0.294883335 1.722600356 54.481746692
0.066061526 0.077130213 0 0.064389083 0 0.648818562 0 0.134569776 0
0.684314465 0.035832189 0.05736922 0.051799771 0.109313844 0.046339358
0.083416489 0.079175752 0.111551261 0.231636499 0 6.828700321 0.11379726
0.168882388 0.03340677 0.289019177 0.140259307 3.863730056 0.047717988
0.276453126 0.063425655 0 0.292800171 0 0.007992988 0.176622831 0
0.06041256 0.160212205 0 0.005175234 0.062185055 0.107542019 0.33301064
0 0.007952998 0.086509142 0.06650572 0.420174498 0.465818454 3.79853776
0.033911583 0.111612452 0 0.061187701 0.03940153 0 0.13288238
0.06818683 0.226797214 0.027436556 0.243551044 0.205915528 0 0.0926546
0.065214584 0 0.053884931 0.114425588 0 0.01830025 0.291394677 0 0
0.065660241 1.209818654 0.046373374 0 0 0 0.415971526 0.449791903 0
0.022020509 0.247891692 0.08112819 0.242225596 0 0 0.135767016
1.003453532 0 0.119970225 0.031752419 0.120825119 0.105533973 0.144236414
0.113326425 0 0 0.084920493 0.0969342 1.085765611 0.014475633
1.986713992 0.346823013 0.974535539 0.037847749 0.090511344 1.139114966 0
0.82677001 0.625418548 0.104548043 0 0.088773779 0.185196754 0
0.458122323 0 0 0.04454236 0 0.054229611 0.787116058 0.051174422
0.040564095 0.299440031 0.066061526 0.195643751 0.063903738 0.137976605
0.047362547 0.227764367 0.458384921 0 0 0.135525866 0.247730723
0.199192112 0.055933338 0.144509589 0.429461892 0.126535759 0.073253709 0
0.725271904 0.075025627 0.13698575 0.11737236 0.033138355 0.091405885
0.108923716 0.038810307 0.225575943 0.226037039 0.216641291 0.138477428
0.069150864 0.364400787 0.055898215 0 0.116490172 0.259527425 0.045255672
0.061732251 0.250701701 0 0.551815708 0.35017396 0 0.133549118 0
0.470994215 0.232625192 0.064737353 0.147131509 0.063743578 0.089863331 0
0.982828645 0.083939563 0 0.219887789 0.063447479 0.366611713 0.276453126
0.694909498 0.200924458 0.260308663 0.028275361 0.053152952 0.052402005
0.144509589 0 0 0.056227754 0.110065892 0 0.056393986 0 0
0.142314799 0.900037944 0 0.249621798 0.045992202 0 0.098580185
0.031274132 0.086804395 0 0 0 1.459309945 0.032733189 0 1.55993284
0.071004153 0.234953234 0.141021751 0.093027387 0.09597618 0 0.179063772
0 0 0.581962679 0 0.198700684 0.742257709 0.094089943 0.085827967
0.075470883 0.158465343 0.033088058 0 0.204697687 0 0.12491988
TCGA-2F-A9KQ 2886 0 0.1759161 0.2037982 0.1063295 0.2576393
0.06749163 36.829790 0.127857 0.5083153 0.4036392 0.04345802
0.1226741 0.1571828 0.6521544 0.2455831 0.1637214 0 0.01375284
0.3831783 0.05240526 0.07217855 0.0602534 2.06383534.58464 0.08098995

0 0.06749163 0.3157583 0 1.3363334 0 0 0.2519687 0.4039409
0.08200166 0.171926 0.2540214 0.1861338 0.05410573 0.02727113
0.1028752 0.2165356 0.1893208 0.03391096 9.266936 0.2790258
0.2208491 0.01820265 0.5314966 0.5731826 5.184423 0.3120063
0.4142421 0.1814363 0.0781482 0.8919773 0 0.01698532 1.202976 0
0.0493763 0.2946249 0 0.01268944 0.1876615 0.2636882 0.4763075
0.6155728 0 0.2749659 0.1009475 0.3541481 0.4568664 17.50283
0.02771656 0.01140286 0.006119347 0.4000787 0.0161018 0.03315378
0.2606573 0.2229214 0.7944237 0.0224244 0.1421848 0.1325989
0.1864343 0.2271849 0.2665054 0.1453666 0 0.1105262 0.02291888
0.005982853 0.3158238 0.03779531 0 0.4640472 1.438265 0.01378248 0
0.09038009 0.07082597 6.216799 0.568145 0.2665054 0.05999256 0.5943122
0.1989227 0.7919018 0.02895184 0.04312743 0.07397659 2.323733 0
0.1961077 0.005190367 0.0493763 0.301892 0.1964785 0.1091637
0.4242331 0 0.06940708 0.09054412 2.273244 0.0118312 0.5974277
0.04724414 0.8885874 0.03093366 0 0.1765727 0 0.91625 0.3407774
0.1514778 0 0.1451129 0.05045491 0.1534127 1.226587 0 0
0.07281058 0 0.7091658 1.206233 0 0 0.3773042 0 0.2798307 0
0.5638541 0 0.62052 0.6074365 0.05371427 0.2324472 0.4799937
0.4049498 0.4841258 0.05224611 0.354331 0.4680095 0 0.1197432
0.3315785 0.3114597 0.3065991 0.4105236 0.04427566 0.01354229
0.2241231 0 0.1691754 0.3687347 0.2438624 0.03541298 0.6413542
0.1808585 0.7840869 0.1370599 0 0 0.141411 0.2589181 0.3531843
0.5481158 0 0.429142 0.5975128 0.09713748 0.4366086 0.1142166
0.1224848 0.2851933 0.06613879 0.2678369 0.260494 0.0657157 0
0.5093996 0 0 0 0.2610721 0.6741866 0.4067104 0.9087397
0.1642193 0.4255101 0.06932992 0.06516433 0.4650019 0 0.07255644
0 0.09191196 0.4151948 0 0.04609184 0.04756847 0 1.105007
0.6405532 0 0.1133447 0 0.3433754 0.6445712 0.1469753
0.1655426 0.2455207 0 0.01826663 0.8945407 0 0.06064009 0
0.1624924 0.06110097 0.2983192 0.5474376 0.0784431 0.05076293
0.2005566 0.4105236 0.09011888 1.233551 0.05888788 0.9744104
1.696174 0.1297712 0.1636805 0 0.06475832 0.01802899 0 0.6552708
0.005128897 0.02041986
TCGA-SY-A9G0 1008 1 0.1854217 0.132191 0.1494333 0.2858534
1.833793 66.12598 0.6460146 0.4342452 0.1503949 0.7090829 0.1832251
8.365684 0.4970283 1.03109 0.2672032 1.132192 0.0975433 0.01932796
2.782306 0.04909952 0.2028765 0.08467889 3.021518 61.55824 0.04742568
0.3986777 1.138216 0 15.78089 0.9837441 1.63818 0 0.5311676
1.266386 2.527124 0.1317935 1.01149 0.1778803 0.6919557 0.1533052
0.1982127 0.3043148 0 0.42892 5.801405 0.1960686 0.2715797
0.1790714 0.4979696 0.3759182 5.70481 0.1096218 2.857913 1.019948
0.1098279 0.6114959 0 0.9823763 0.946757 0 0.1387849 0.3680531
1.372183 1.590152 0.2362632 0.3088184 1.099717 2.395699 0.009135152

0.3312272.1389570.3921371 3.0498364.9955050.1168569 0.9775465
2.1500010.2108484 0.1357748 0 0.03052687 0.2349669 0.7815268 0
0.3996473 0.3440340 0.1064271.0487150.4085905 0 0.09558844
0.4831458 1.89184 0.4365761 2.0184371.6505214.58732 3.44254 0.1888537
0.1691127 0.1905275 10.302121.4334081.0802681.17713 0.4215616
0.2847390.0931873 0.8717893 0.2034416 0.6667145 0.05198259
1.1082780.06825752 0.4134087 0.02188331 0 0 0.2761268 1.636442
0.2555179 0.4494495 0.04877165 0.3181221 1.1788190.08313665
0.9041983 0.1327919 1.3911530 14.503140.1691943 0.0811506
0.6438395 0.1596405 0.2074253 0.3787028 0.3568927 0.2836332
0.5390077 1.2157470 0.8102556 0.3069795 0.6910890.06229044
1.0171290.05878112 0.04659365 0.3081215 0.07588108 0.6741742
0.1468051 0.1056572 0.1632079 1.4825131.8198180.1887223 0.2800085
0.3632318 1.1382160.5720014 4.8369131.0512692.0280040.07267218
0.2355985 0.1075370.3106393 0.04308882 0.05244922 0.2281551
0.1141924 0.5249635 0 0.6686876 0.6477654 0.2596358 0.2986121
0.2651018 0.1270874 0.8627590.06420707 0.2097969 0.2230093
0.5962085 0.5718085 0.7090829 1.0942730.0546571 0.6146318
0.9596941 2.5937860.1193113 0.1070118 0.4426397 0.8906774
0.1766052 0.3533668 0.2928744 0.1032209 0 0.2202769 0.3856662
0 0.08419083 2.7065560.5790205 0.3810551.1706970.1538603 0.3869433
0.04871743 0.3663225 8.9512880.1106599 0.2549234 0 1.489774
0.3209285 0.08419083 0.1943296 0.06685176 0.2771747 0.6889044
0.4135288 0.2394608 0.3504432 0.2641430.03712099 0.1132334
0.1436912 0.06647148 0.4600664 0.01511341 0.4620878 1.077574
0.1503949 0.1022668 0.2337137 0.2283635 0.09200362 0.4954792
0.4701630.07349489 0.1426824 0.2970937 0.3671446 0 0.565626
0.1241397 0.6276492 13.363020.1486041 0.1314475 0.08668907 0.18202
0.2153693 0 0.1959371 0.02162414 0.05739532
TCGA-GV-A40G 580 0 0.08983605 0.1801294 0.04072491 0.3323875 0
28.020430 0.2203654 0.7992460.07729825 0.02219295 0.06488405
0.6923235 2.43535 0.3944450.05573901 0 0.00526743 0.2201397
0.1271199 0 0.1846196 4.78083929.698030.2223078 0.1327959 0
0.04031251 0 3.57465 0.4092473 0.1404183 0.02412642 0.3808297
0.1390889 0.1795877 2.4647280.1311745 0.1740720.09400522 0.08261683
0.1222194 3.7995820.02597625 19.004880.2137375 0.06344 0.0209152
2.4428010.4683365 2.2380430.1792507 0.2307745 0.1687646 0.1197251
1.7998240.02879465 0.01901605 2.3590280.0398086 0 1.27889 0
0.01458042 0.4582070.2693182 0.5212255 0.2539045 0.01493756
0.09026894 0.2141367 0.5179023 2.7997266.4796680.1061563 0 0
0.1660022 0.02466838 0 0.6489176 0.2134509 0.6288238 0
0.08713237 0.03515971 0.1428111 0.02900445 0.2858053 0 0.0337361
0.1042025 0.03511233 0.009165889 0.3331430.1737103 0.08996294
0.3651389 1.5320930.00263939 0.09217621 0 0.02712681 4.836559

4.2240640 0.2022024 0.4966375 0.2285661 0.4852858 0.2883074
0.06607237 0.08500057 5.903835 0.0744086 2.103096 0.00397589 0.2647603
1.090194 0.6020204 0.06588311 0.5570883 0.061244 0.425334 0.3063312
1.415417 0.06344 0.8566036 0.6876031 1.56942 0.0236956 0.1983347
0.3196984 0.1769271 1.412493 1.43572 0.5682693 0.08846356 0.3056856
0.1545965 0.5288227 1.17695 0 0.02453535 0 0 0.5432304
0.7391926 0.09611735 0 0.5038322 0.4135959 0.30622 0.04000864
0.3455358 0.05930518 0.3327286 0.8720974 0.8434897 0.3052417
1.499009 1.96458 0.2395736 0.05502917 0.4222124 1.643132 0.1584422
0.1375873 0.1367657 2.455088 0.3288026 0.1429393 0.02826312
0.05186788 0.1001474 0.0340973 0.1862864 1.235744 0.5434235
0.2441413 0.520185 0.404075 1.28505 0.244976 0.08576359 0.1701742 0
1.076674 0.1159474 0.6474538 0 0.7453971 0.6269374 0.04960573
0.1579338 0.2041467 0.6165642 0.1213677 0.126658 0.5778161
0.1596335 0.1362117 0.02803423 2.941559 0.262763 0 0.5506665
0.1808081 0.2295271 2.180819 1.363209 0.2306226 0.7765194 0.2478356
0.3161394 0.05858514 0 0.416844 0 0.1431584 0.4611649 0.06883332
0.03530696 0.03643808 0.09064577 1.02465 1.447743 0 0.06945885 0
0.222564 0.06171876 0.10769 0.2536157 0.3134536 0.008237683 0.2798496
4.07875 0.06148046 0.2322555 0 0.5512302 0.0501473 0.2207261
0.7571497 0.1201769 0.03888508 1.071251 0.05717573 0.2531183
0.5845668 0.09021779 3.172248 9.42698 0.1767226 0.05373489 0
0.6944803 0.04833655 0.02134509 1.473799 0.1178641 0.08603038
TCGA-FD-A43Y 474 1 0.018286831 0.078222404 0.198956985 0.202980251
0.140317787 26.994314604 0.06371186 0.106327943 0.233610154 0
0.072280772 0.553810666 0.392147101 1.254165883 0.474342568 0.051867938
0.216450142 0.360268123 0 0.065371581 0 0.075161528 2.487696842
57.388962423 0.084190672 1.730025762 0.673525378 0.13129482 0
0.71441799 0.302929054 0 0 0.503885578 0.058451907 0.116980724
0.105624143 0.157887665 0.11473773 0.26364465 0.089002336 0.042649222
0.23616327 0.338410793 2.548409852 0.2320423 0.10330967 0.102178872
0.884002058 0.762668443 6.630015438 0.145951559 0.234879774 0.129330309 0
0.705598015 0 0.068453208 0.900372467 0.064826818 0.061593176 0.449193697
0 0.007914557 0.317001553 0.109643666 0.127319445 0 0.04865052
0.058799835 0.106551363 0.187418781 0.284953044 1.178669411 0.276594422
0.071121029 0.022900259 0.083177954 0.200857684 0.124070464 0.108383394
0.208558126 0.09909832 0.727291158 0.070945902 0.050894459 0.348843862 0
0.132978087 0 0.054937981 0.042422457 0.085768667 0.018657884 0.303547739
0.612908094 0 0.181140994 2.971521154 0.330957398 0 0 0.044175003
0.908787163 0.875474707 0.332445219 0.12722146 0.286434282 0.082713643
0.971373267 0.036115219 0.053798189 0.092280167 1.901848974 0.181757433
0.061157375 0.038847531 0.061593176 0.376587322 0.147055163 2.372719295
0.151199575 0 0 0.2682489 1.379939276 0.162343766 1.834450845 0
2.222633747 0.463048697 0.046140084 0.090106774 0.072029797 1.228673568

0.283395924 0.101746126 0.09603973 0.362034584 0.062938658 0.09568534
1.578392081 0 0 0.045412832 0.055765004 0.110578793 1.203747484
0.052174501 0.082713643 0.413416055 0.134705076 0.199467131 0.195457742
2.063204322 0.048288132 1.470697952 0.367522116 0 0 0.322406908
1.010288067 0.064132054 0.676170388 0.392889804 0 0 0.119496438 0
0.626648793 0.267721371 0.931085352 0.138076289 0.016892982 0.629047287 0
0.065947932 0.919937101 0.129054454 0.265050021 1.270652687 0.084602698
0.454925036 0.398934262 0.046554268 0.197944481 0.088199752 0.36912067
0.503509263 0.37062153 0 0.269365869 0.162849977 0.141366892 0.121030231
0.617398263 0.349235381 0.079057095 0.057752176 0.320469148 0 0.072331177
0 0.537677658 0 0.10393077 0.44836992 0.289978023 0.140166092
0.789196041 0.897420426 0.307276555 2.497842461 0.115311738 0.027095849
0.022896889 0.049111225 0.135762969 0 0.156692676 0.500659843 0.03736416
0.057496069 0.059338048 0 0.54929198 0.474342568 0 0.113111132 0
0.131795309 1.00506694 0.119569906 0.059000517 0.510447383 1.462208613
0.47851078 0.478230622 0 0.075643895 2.696795613 0.130305161 0.043553604
0.164921454 0.58804136 0.619728068 0.126645798 0.31779509 0 0.074944298
0.423811229 0.146916301 1.519378538 3.768372483 0.095928701 0.291684219
0.076945778 0.242343243 0.044979579 0 0.608702513 0.076774914 0.216513929
TCGA-S5-AA26 503 0 0.3806125 0.5426933 0.2300548 0.6454440.03245001
54.018970 0.2151580.6174271 0 0.1462625 0.1516674 0.7935223
3.0571721.0169810.1386924 0.2002257 0.3173939 0 0.1385806
0.1735174 0 5.361248146.92150.07788001 0.1136611 0.09735002
0.2277251 0.4015688 3.12076300.07005562 0.2644075 0 1.658297
0.5407054 0.3156191.4656040.2362621 0.2497350.06883786 0.06222693
0.2137004 0.8192297 0.04891322 27.887670.6037008 0.2787339
0.07876656 2.4702570.5511729 9.1009460.2250192 1.2493250.1869315
0.5636054 2.6045590.2168811 0.03957636 3.7479760 0.3561022
1.7943060.04543001 0.01220218 0.6541511 0.9508606 0.6379533
0.1366005 0.0281274 0.5099287 0.2240105 0.5263032 1.592546
5.2569010.1599136 0.08223754 0.1059187 0.6251635 0 0.1434632
0.4699656 0.4823132 0.4201552 0 0.2871226 0.2059731 0
0.1638459 0.1537631 0.4193539 0.12705 0.2452663 0 0.008629675
1.1351280.10903200 0.3779281.4262570.0298198 0 0 0 6.305006
1.4461640.05491539 0.3807467 1.1884420.6216738 1.5610620.08352035
0.1244141 0.2134079 6.1878570.1401112 0.9900313 0.02994637
0.2848818 2.1772471.4170040.04771452 2.44765800 0.3503951 0.3754778
1.7534390.1023921 0.3756262 0.13629 0.5976838 0.08923752 0.1067039
0.3357260.08328835 2.16412 0.5734608 0.4649955 0.05552557 0.5756053
0.7277623 0.4978861 2.7004050.05937387 0 0 0.0644813 0.5114508
1.91386 0.1206592 0.04782106 0.2868208 0 0.2306447 0 0.5964229
0 0.7160312 1.8100460.1162163 0.1436924 1.3846873.9913510.7539209
0.6782388 1.3061131.1813510.07458658 0.1381742 0.07357989 1.333258
0.3537912 0.2691544 0.1064388 0.1367340.05387926 0.2568206

0.3660281 0.5983465 0.3837245 0.5107974 0.3265024 0.3369578
1.3852170.06589847 0.3768162 0.0457768 0 0.9603354 0.07277623
0.6945485 0.02804846 0.9107507 0.8763383 0.1634631 0.262402
0.05491539 0.07571668 0.04570702 0.1717176 0.6386243 0.1502948
0.1616979 0 0.9608383 0.2968694 0.4206145 0.3456346 0.1495966
0 2.3465593.60461 0.2960876 1.5885460.2166701 0.1879862 0.9266518
0.3407251 0.3662943 0 0.004419131 0.5888963 0.3024303 0.06648294
0.1372257 0 1.9773491.5768920 0.2942794 0.05422026 0.266692
0.1162163 0.2027801 0.1705563 0.2360930.01551154 1.0275652.21192
0.2701244 0.1749347 0.3198273 0.1841551 0.1007224 0.4743035
1.0308990.2640083 0.03661026 0.7583909 0.05383089 0.4766212
0.7765481 0 2.6352959.4826690.2773069 0.1686378 0 0.1868150
0.08038554 2.0713160.03698964 0.02945364
TCGA-BT-A42E 1108 0 0.01181285 0.1263245 0.08568096 0.218534
0.05438516 34.3302 0.04115633 0.2575699 2.6085280 0.07003747
0.1647526 0.2533176 1.6422180.1723578 0.5088639 0.05592864 0
0.07719183 0.04222847 0.0387746 0.04855254 2.14354647.054920.1631555
0.5968760.2175406 0.12722 0 0.6495141 0.07827396 0.1477128 0
1.3771070.1368747 0.1385390.1705767 0.2939759 0.07411782 0.5823439
0.04813399 0.1102015 0.1830670.08197697 1.9856370.03747344 0.1334711
0.0146678 0.9041532 1.4472054.64349 0.2199895 0.5765615 0.1148734
0.1259446 0.4470340 0.04211346 1.5122090 0 0.3429262 0
0.003408409 0.2488809 0.2124815 0.1644906 0.03815633 0.1414218
0.1329415 0.03754343 0.3113178 0.4295033 0.6526219 0.06700251
0.01837699 0.1923092 1.2358120.0518997 0.2671551 0.07001307
0.2694471 0.1066919 0 0.3895495 0.0493149 0.07511492 0.03051116
0.2147516 0 0.07097724 0.08906264 0.03693633 0.009642036 0.2252886
0 0 0.3459507 0.9416569 0.11106 0 0.03641441 0 3.130958
0.8348384 0.1533940.09185048 0.5333208 0.1335776 0.4147775
0.1399774 0.03475234 0.2980539 1.7030230.03913698 0.3160496
0.04600672 0.1193631 0.5212851 0.09499411 0.1705984 0.8302060
0.05592864 0.2097629 0.5779456 0.1144038 1.62939 0.07613922
2.5561870.4486775 0.1192216 0.07114166 0 1.2736010.1830670.1533596
0.03101968 0.2923321 0.1626275 0.1854313 0.6970758 0 0
0.0880068 0 0.2500094 0.7451923 0.1685174 0.1068621 0.5382223
0.08701625 0.1288510.08417401 0.6058093 0.09357893 1.9500730.5803367
0 0.2140655 0.2082671 1.9578660.3210652 0.1157756 0.2537974
0.4085519 0.08333646 0.0482449 0.06165875 0.4857601 0.1235297
0.2405835 0.1248716 0 0.8578489 0.03586858 0.07668141 0.5571162
0.1012302 0.3138959 1.1248150.1032303 0.7640637 0.4417748 0
0.3836022 0.7406740.2980539 0.1219706 0.3880136 0.03133887
0.3590207 1.2623660.02609132 0.1856837 0.2454304 0.6908929
0.07660348 0.1172491 0.2334429 0.04198152 0.07320126 0 0.5999282
0.0552826 0.3356839 0.2413635 0.1095095 0.1509066 0.3641441

3.356228 0.1654111 2.148034 0.09311077 0.07001307 0.00246514 1.078639
0.08769962 0 0.2814402 0.6356763 0.1930908 0.1857054 0.07666191
0.1907092 0.95737 0.7824498 0 0.2557348 0.06058093 0.1489891
0.2596997 0.1132843 0.05716938 0.06594735 0 0 1.270027 0.08623232
0.09772828 0.4913517 0.2712274 0.158257 0.1748272 1.421414 0.06320992
0 0.2882768 0.09021882 0.2420611 2.4471 0 0.8506178 3.172543
0.166538 0.4710525 0.04970513 0.1565479 0.05084753 0 0.2583933
0.008265792 0.0329089
TCGA-FD-A43X 110 0 0.2531786 0.1547112 0.08744544 0.3122481 0
81.40028 0 0.1401995 0.8800815 0 0.04765319 0.04323739 0.4201183
1.095011 0.149847 0.03419544 0.142701 0.005655171 0.07878149 0.1221113
0.1582924 0.09910483 2.860615 95.44994 0.02775257 0.05184407 0
0.2163998 0.07631957 2.721338 0.03994295 0.1206038 0.0259024
0.6388484 0.1252424 0.2956379 0.5570862 0.2632916 0.1067918
0.01682084 0.0818754 0.1124709 0.6539294 0 23.40034 0.1529806
0.06810989 0 2.816886 0.1885542 7.576451 0.2245198 0.7432862
0.04263238 0.1285382 2.388551 0.1854855 0.02793747 2.176521 0
0.08121417 1.049965 0 0.0104358 0.2379299 0.2168573 0.5036342
0.1168263 0.01603713 0.3488894 0.1660385 0.1235613 1.534219
4.033373 0.2279411 0.009377719 0 0.3427342 0.01324212 0.02726568
0.321547 0.137498 0.1960003 0 0.163706 0.08388411 0 0.09341848
0.3068438 0 0.1086584 0.1538253 0 0.00246015 0.4343087 0
0.09658521 0.1817298 1.589427 0 0 0.03716431 0.1164946 4.793154
2.253758 0.09393177 0.04440411 0.5887368 0.2726568 0.8466384 0.14286
0 0.1825151 3.891256 0.1198288 0.9676745 0.01707424 0.2436425
0.3901482 0.2262175 0.01632296 0.3987308 0 0.08562061 0.254417
1.419633 0.04864992 0.6047074 0.0388536 0.7799978 0.1017594 0
0.1518141 0 1.375182 0.2335462 0.3290069 0 0.537034 0.04149413
0.4415833 3.174894 0 0 0 0.07352939 0.2551579 2.347749 0 0
0.08386354 0.2220206 0.09862836 0.04295373 0.06182851 0 0.5103159
0.9841551 0 0.08192771 0.3340167 1.498639 0.5038475 0.1503853
0.32378 1.122599 0.04252632 0.1772583 0.04195235 1.883902 0.1260736
0.03069225 0.03641232 0.0890975 0.03071982 0.1830362 0.1391299
0.4548714 0.5712708 0.1164946 0.1551323 0.1983174 2.494355
0.03757271 0.122769 0.02610013 0 0.5779645 0.2489648 0.3117479 0
0.7170928 0.6070171 0.0798859 0.08976677 0.09393177 0.04317066
0.2606034 0.1794955 0.1618304 0.1713843 0.09696239 0 0.2094652
0 0.03425968 0.2956009 0.125 0.1232114 2.267023 0.1556975
0.06752699 0.689587 0.2565763 0.2500921 0.2364952 0 0.1491761 0
0.0352746 0.278856 0.2217006 0.03790595 0.1564813 0 0.4646281
0.5547129 0 0.1305006 0 0.1520573 0.06626195 0.08408525
0.0777956 0.06730544 0.008844068 0 2.837586 0.06600611 0.08976677 0
0.1813602 0.05024946 0.3707946 0.9879671 0 0.08349491 0.4903557
0.03069225 0.2964552 0.3352918 0.02421471 2.804744 5.213185 0.3162189

0.03846025	0	0.5858299	0.03706761	0.02291633	1.513492	0.004218007
0.2099163
TCGA-ZF-AA4R	1036	1	0.118628454	0.190288629	0.071702962	0.292611694
0.091025513	15.236607611	0.068884172	0.273029883	0.469068409	0.136096398
0.019537183	1.331476745	0.264989206	0.916204509	0.117528368	0.252354266
0.046804437	0.009274184	0.602921677	0.10601795	0.097346729	0.162526713
1.858632178	17.35736952	0.081922962	0.255064667	0.273076539	0.212930567
0.12516008	1.401792899	0.982565116	0.148337873	0	0.335256897	0.323885651
0.147557147	0.570995071	0.261078907	0.138646877	0.119536292	0.05370854
0.061482145	1.429879824	0.091470988	3.15296883	0.188160121	0.14892886
0.135023834	0.318589295	0.669974547	1.894767913	0.210400435	0.660264771
0.227223288	0.421591849	0.32275713	0.050697754	0.084583355	2.271423678	0
0.199780461	0.309056702	0	0.037080695	0.13709466	0.118544854	0.091770403
0	0.008766685	0.116551186	0.115201508	0.260529398	0.616172703	1.395724531
0.074762288	0.046136903	0.041265614	0.292274381	0.065149166	0	0.55661578
0.826794739	0.178572344	0	0.268469228	0.061904495	0.377164009	0.051067137
0.287547261	0	0	0.194932346	0.092731614	0.004034517	0.188535035
0.050974287	0.079197339	0.676947824	1.272979497	0.413590479	0.324583016
0.060947517	0.191045029	1.113573798	0.81132708	0.051347725	0.186096604
0.491857157	0.313000009	1.851256654	0.078094312	0	0.199543473	5.417146053
0	1.256323824	0.028000857	0.066593487	0.523491124	0.370984888	0.874447512
0.817371953	0	0	0.22387981	1.62312862	0.191479963	1.012348593
0.509742872	0.788605529	0.12516008	0.798173893	0.357213634	0	1.652802646
0.306402819	0.20953556	0.103836511	0.048928199	0.068048199	0.879353479
0.835851666	0	0.172794194	0.049099576	0.060292168	0.47822359	0.650735582
0.056410177	0.044714287	0.563880391	0.655383693	0.37740578	0.281767417
0.202791016	0.052208302	0.334756513	0.63283039	0.253554271	0.447857156
0.547769872	1.001280642	0.369805216	0.440399905	0.318589295	0.157800326
0.278963761	0.080748439	0.034399826	0.325210532	0.165403292	0	0.129380956
0.200908428	0.176326151	0.060033957	0.099822485	0.12432753	0.199330665
0.095522514	0.2035271	0.167696812	1.082085746	0.739407243	0.15100103	0
0	0.149657605	0.136096398	0.649425986	0	0.497677952	0.379228997
1.637608527	0.114498837	0.462129527	0.330388899	0.299163119	0.178401896
0.626623173	0.351326541	0.135552265	0	0.36993592	0.092527584	0.056184084
0.16158996	0.819973481	0.202060238	0.365685104	1.48094696	0.129197502
0.928339668	0.093504807	0.146477837	0.226927474	0	0.146784597	0
0.549560665	0.559970532	0.12119247	0	0.128310563	0.159596914	0.437009777
1.074545079	0.15320141	0.214014183	0	0.106871123	0	0.198224634
0.15947587	0	0	0	0.86175793	0.072164371	0.032713953	0	0.172191199
0.047089411	0.274323464	0.799852568	0.141060921	0.034231817	0.372836703
0.956339859	0.081028491	0.909385386	0.119132541	1.478453448	12.055919868
0.162056983	0.220754787	0	0.174678243	0.060788937	0	0.300854277	0
0.137700678
TCGA-BT-A20T	453	1	0.0746363	0.1064196	0.09022529	0.06903736

0.0763595 77.964110 0.1084923 0.1362088 0 0 1.3879250.3334413
0.7378424 0.02688879 0.1014372 0.05889497 0.1867183 0.1625718
0.02964545 0.08166224 0.1022553 2.25442552.14473 0.1030853 0.1872226
0.2290785 0.1786232 0.7874574 0.7559591 0 0.3110943 0.05345165
0.2109303 0.08946261 0.02652488 0.8621936 0.2274375 0.06427554
0.1272742 0.09011006 0.1160463 0.06425882 0.0575499 1.9301080
0.2811003 0 0.7015529 0.1945483 6.1293570 0.8308246 0.2199382
3.0503610.2584475 0.1275880.1042156 0.6532980 0.08379594 0.2222242
0 0.05742694 0.1127943 0.0745837 0.1154766 0.4821607 0.0110313
0.2666522 0.1054257 0.1457020.5815072.4435040.09407491 0.1935167
0.1973164 0.1980326 0.02732617 0.2813245 0.2211792 0.1891587
0.1348207 0.03805619 0 0.08655076 0.1581977 0 0.09045664 0
0.1494834 0.07214333 0.07779072 0.08630415 0.1669449 0 0
1.2750471.2204310.1344930.1021074 0 0.9615834 0.4945527 0.1701516
0 0.1119939 0.09169094 0.0562649 0.7167612 0.09826766 0 0
1.7844250.08242544 0.4992183 0.01761702 0.1675919 0.2927642
0.06668826 0.8084094 0.2057031 0 0.05889497 0.1600639 1.34098
0.08031438 0.8839020 0.5078795 0 1.9459440.2996594 0.2939841
1.2439660.4819411 0.1252399 0 0.1231347 0.4281322 0.2603549
0.5916201 0.1397152 0.2174304 0 0.3793343 0 0.1364723 0
0.1125298 0.4153417 0.3665256 0.1356850.1772768 0.06379401
0.06569477 0.5265387 0.3271634 0.04557893 0.2254191 0 0.458157
0.3053751 0.8755794 0.5345165 0.7280656 0.1755129 0.02032148
0.0432860.4262698 0.1040652 0.06333589 0.1001863 0.1378948
0.2852676 0 0.08972047 0.3128877 0.2382807 0.1802969 0.2561023
0.1214942 1.0418380.5427398 0 0.2154387 0 0.0627724 0.5137586
0.3042714 0.03300102 0.2876057 0.6135321.4836580.1029116 0
0.08908609 0.5377758 0.06734602 0.3246735 0.08841627 0.09512456
0.06210931 0.2992486 0.3492880.07069757 0.1016660.5189293 0.1271283
0.4601490.06425882 0.06967365 0.4672595 0 0.03686321 2.995652
0.06681456 0 0 0.1559827 0.2348741 0.1524989 0.07822193 0
0.06694135 0.4934985 0.5992358 0.09638822 0.1154136 0 0.04482604
0 0.06506872 0.04013435 0.1388901 0.0547513 0 2.0241530
0.06174695 0.09407491 0.03939485 0.03703348 0.2473839 0.2580694
0.1774998 0 0.2851684 0.1266718 0.2039196 0.5056201 0.1998759
1.2402457.4170140.08156784 0.4761947 0.1046828 0.1099006 0.07649196
0.09457933 0.5205352 0.008704192 0.1212901
TCGA-E7-A7DU 28 0 0.1289210.08271942 0 0.1669497 0 53.400380
0.3560624 0.7058298 0 0.02547873 0.07705912 0.4838069 1.003662
0.05573473 0.07770392 0.06103837 0.3265543 0.2246512 0.01536218
0.1692684 0.1059768 2.62197582.14614 0.07122464 0.05543894 0
0.09256198 0 3.1711920.08542519 0.06448321 0.05539694 0.8197754
0.1854369 0.3298825 0.07446432 0.1964290.09516393 0.08394027
0.03210263 0.08017978 0.06659742 0.1192887 18.52177 0.2453824

0.4369958	0	1.3503	0.4704667	6.144687	0.1371932	0.4636483
0.02279425	0	1.549723	2.578512	0.009192202	2.369758	0	0.08684556
1.093981	0	0.01487923	0.2750572	0.07729806	0.6582354	0.1665694
0.01143276	0.3592636	0.09560473	0.2076313	0.4687434	5.302279
0.09749862	0	0	0.2931995	0.02832067	0.1166251	0.1146144
0.1960428	0.5589093	0	0.1500492	0.134551	0	0.06659742	0.4687434
0	0	0.08972265	0.1612436	0.01052295	0.47353	0	0	0.3331388
1.106741	0.01212066	0	0	0	3.929559	2.586378	0	0.0211036	0.7602231
0.1166251	0.9285583	0	0	0	3.587772	0	0.6036176	0.009129084	0
0.1517095	0.1382306	0.07563745	0.5329735	0	0.06103837	0.2720583
2.116746	0	0.4580351	0.1661908	0.9879428	0.05440771	0.06505691
0.2540987	0	1.571259	0.6992729	0.266427	0.1354147	0.127616	0.1774853
0.7420328	1.407977	0	0	0	0	0.1559146	0.848634	0	0	0.197293
0.3798647	0	0.09186428	0	0	0.2182805	0.8444762	0.0472377
0.1168114	1.42871	1.06837	0.4144495	0.3445993	0.6232156	0.4801761
0.0909502	0.0421221	0	0.7952099	0.1617787	0.1969227	0.01297905
0.1429133	0.03284994	0.07829118	0.1673743	0.9728243	0.5978852
0.1868585	0.3317784	0.178933	2.255725	0	0.1969227	0	0	0.3252845
0.4437134	0.61267	0	0.4663395	0.2119535	0.1423753	0.08532551
0.1339267	0.03077608	0.3901431	0.03489849	0.4230153	0.2749021
0.1053855	0	0.8270382	0	0	0.2107319	0.1666912	0.1976323
0.9537908	0.3995845	0.02406977	0.3081684	0.1016175	0.1528192
0.5057875	0.06924618	0.127616	0.2886472	0.06466379	0.486844	0.05268297
0	0.08366586	0.1387551	0.7014289	0.843983	0	0.2790991	0
0.04645741	0	0.08991573	0.04159497	0	0	0.03212828	0.6742988	0
0.06399413	0	0.1020714	0.03838125	0.3100494	0.6151614	0	0
0.4766882	0.1969227	0.05283523	0.2666074	0	2.142304	4.072121
0.1690727	0.08225418	0.1084925	0	0.04756546	0.0490107	1.37322
0.05412581	0.1257043
TCGA-DK-A3WY	4967	0	0.1272691	0.1663435	0	0.2354437	1.475685
48.9543	0.5419447	0.5961114	0.3612965	0.1946792	0.05589395
2.600527	0.0379054	0.8125629	0.1069845	0.1504085	0.5356115
0.02653252	0.5852337	0.05055115	0.02320828	0.2324864	3.163575	65.0156
0.2343735	0.3344526	1.367179	0.05076444	0.9846942	0.920753	0.7027554
0.035365	0.1822905	0.7493256	0.2881515	0.1507666	0.5309071
0.1292748	1.064706	0.1841439	0.1472525	0.1758943	0.07304901
0.7850684	0.2641088	0.7626023	0.2662942	0.3862898	0.6266236
0.2580215	7.363576	0.07524185	1.017128	0.2125207	0	0.3147874	0
0.1575421	1.253247	0.05012989	0	0.09473364	0.364581	0.4243368
0.2300488	0.08478628	0.4266373	0.5481172	0.09405232	0.1515643
0.2846374	0.310562	1.469008	4.947885	0.2673594	0.1979897	0.2951421
0.2572825	0.8542661	0	0.3981057	0.1075172	0.07663167	0.1730481
0.1371543	0.3443662	0.2697572	0.2556715	0.1542458	0	0
0.04920725	0.022108	0.3058724	0.1947761	0.1458324	0.2832197

0.9378894	1.409056	0.01994227	0.08705624	0	2.254564	1.218109	1.192801
0.2938015	0.098379	0.5472268	0.03198079	0.7129584	0.02792751
0.1248047	0.03567963	3.428205	0	0.5202158	0.01502019	0.2381467
1.123242	0.4927701	1.266809	0.5261446	0	0.03347572	0.1746813
3.224729	0.04565044	1.773202	0.09114525	5.080717	0.3282314	3.817721
0.1858096	0.1670996	1.21527	0.05478676	0.2322909	0.03713325
0.4199362	0.04866979	0	1.893104	0	0.03089669	0.2107035	0.2156124
0.0855094	0.4654225	0	0.2878271	0.2459156	0.156249	0.3470531	0
0.1450413	0.07468139	1.616128	0.7859384	0.4145107	0.1601594
0.2849301	0.52083	0.1157161	1.070949	0.6456122	0.7147976	0.09976097
0.103956	0.3444507	1.250219	0.2661764	0.1439997	0.3915007	0.03918949
0.8467582	0.0429378	0.2957816	0.4890721	0.4134424	0.2049603
0.07278386	0.1090373	1.04364	0.7932641	0.3239993	0	5.72913
0.2140778	0.04866979	0.8795584	0.1875767	0.4113221	0.5860595
1.030708	0.1169892	0.183626	0.81018	0.09170101	0.1084579	0.347996
0.100511	0.1528833	0.03530274	0.340184	0	0.1205528	0.05778661	1.10566
0.3974261	0.435912	1.095735	0.2244129	0.3742388	0.1226078	0.2304822
1.454836	0.03797719	0.03499468	0	0.4255684	0.3738047	0.2600398
0.2223055	0.1376564	0.456591	0.2564598	1.325298	0.05478676	0.08746763
0.3626032	0.05095795	0	0.1109546	0.3193713	0.0789447	0
0.008810173	0.8628915	0.1290345	0	0.05347188	0.1119595	0.03367954
0.2844945	0.4840633	0.1765581	0	0.4705804	0	0.05795362
1.285705	0.1136088	0.7049515	10.16332	0.1483613	0.6315576	0.3570081
0.6246715	0.4695601	0.08063789	1.291071	0.03957948	0.3545532
TCGA-G2-A2EF	1884	0	0.3779301	0.221321	0	0.2829926	0.01380913
39.39389	0.06270093	0.2354418	0.6568668	0.24776	0.2311851	1.869932
0.5065263	1.034114	0.1118413	0.01914185	0.08520627	0	0.03920012
0.05897312	0.0443043	0.480798	4.609737	40.85474	0.2112797	0.6384656
0.6628384	0.1938173	0	0.651002	1.4608	0.2025339	0.09666393
1.525816	0.09707248	0.2110617	0.571715	0.2924846	0.08634841	0.07951234
0.1700878	0.1469037	0.116208	2.227213	0.2714817	0.3425406	0.03389014
0.005586532	0.7974774	1.806052	2.636636	0.04787857	0.7859198	0.5886616
0	0.6142788	0	0.01844574	0.5316516	0.09569729	0.09092379
0.2813149	0	0.05062833	0.3695664	0.02697598	0.6264962	0.1453262
0.03989881	0.448468	0.157291	0.7509545	1.33205	0.3590374	1.003758
0.06999253	0	0.1739486	0.16802	0.0407006	0.1333296	0.3762896
0.2438147	0.3578755	0.2967358	0.1690433	0.2288724	0.0697248
0.2290192	0.9815107	0.02703313	0.09915466	0	0.003672367	0.6896243
0.1391961	0	0.1143233	0.675916	0.323591	0.01846547	0	0.1521592
3.517844	1.148778	0.1402158	0.1509799	0.4725792	0.0814012	0.9802643
0.07108434	0.02647228	0.1135199	0.9874682	0.05962448	0.1805609
0.0700904	0.1818476	0.6088624	0.1688421	0.1218298	0.5952018	0
0.02130157	0.3010441	0.6790217	0.1307191	0.8180461	0.2029942
0.7997736	0.5506392	0.3178558	0.3990466	0.03544344	1.181057

0.2091744	0.4958903	0.567095	0.3785582	0.18582	0	0.2932339
0.9348646	0.01966046	0.2011151	0	0.4625029	0.1727611	0.05134663
0.1424521	0.7511193	0.4308449	0.4907553	0.06411879	0.2307349
0.02376097	1.790158	1.259219	0	0.2038281	0.2039729	0.828548	0.2761257
0.2806078	0.6524815	1.077268	0.3174039	0.1911006	0.109592	0.770882
0.1505562	0.1603545	0.07700172	0.3324987	0.7107795	0.1092903
0.1233129	0.707297	0.3719482	0.2173703	0.2084151	0.2960366
0.6230584	1.233899	0.4810636	0.1558429	0.347201	0.6584155	1.45559
1.801694	0.03580815	0.3875709	1.263636	0.02981224	0.163776	0.1635851
1.342555	0.4279147	0.5074626	0.355646	0.09593713	0.2443966	0
0.1322865	0.08422204	0.07671125	0.4780268	3.383911	0.5977488
0.6657203	0.6042816	0.1092003	0.4301865	0.1134821	0.05333182
0.01502239	0.3866557	0.3117538	0.033578	0.1579675	0.7603114
0.7721971	0.2546269	0.2627843	0.09684735	0.3569843	1.775568	0
0.4035218	0.04614697	0.2594082	0.2472798	0.08629328	0.08709651
0.0502348	0.0132019	0	0.9412848	0.09853003	0.1935534	1.327002
0.07836741	0.05625707	0.3620641	0.7280555	0	0.03115907	0.3160797
0.9163116	0.1475103	0.968924	0.1988046	1.221137	4.392538	0.08850616
0.2583504	0.2271746	0.3577469	0.07746528	0.03420815	0.9327972
0.01574098	0.3697538
TCGA-BT-A20V	154	1	0.16976	0.1815383	0.07695644	0.1766534	0.0325649
31.40464	0	0.07711422	0.232355	0.4382034	0.04193724	0.08033011
0.5119276	0.3933329	0.1720082	0.2746053	0.05023367	0.1791662
0.04622115	0.1264284	0.1044791	0.0872173	1.970833	27.94364	0.3907788
1.209074	0.2930841	0	0	1.258866	0	0.1061374	0.04559086	0.4497753
0.2119609	0.1357442	0.1838491	0.07544046	0.02349555	0.07895039
0.1032781	0.01649669	0.05480868	0.1472592	4.115649	0.1346307
0.1198803	0	0.4274143	3.595309	3.869224	0.2258159	0.5451081	0	0
0.2519306	0.3808857	0.009456306	2.995066	0.07522492	0.2858904
0.2369289	0	0.003061346	0.1584576	0.06361515	0.09849416	0.06854207
0.02822699	0.09097496	0.4383671	0.5592353	0.5510983	0.651298
0.1203599	0.08252871	0	0.217169	0.02330749	0	0.3773037	0.1613403
0.229987	0.3570546	0.2058137	0.06644006	0.4047978	0.164426	0.2314613
0.1052097	0	0.06153367	0	0	0.3072699	0.1094181	0	0.6488649
1.07348	0.06982588	1.088638	0	0	2.179413	1.064275	0.2204393	0.1389436
0.8211687	0.1919615	1.413751	0.2095402	0	0.2141635	1.978602
0.07030366	0.2838676	0.0150262	0.357363	1.123692	0.2275234	0.05746015
0.7018068	0	0.1004673	0.212978	1.425314	0.01712576	1.219564	0.6154766
0.8663778	0	0.1606226	0.7435359	0.08358324	1.641271	0.164426
0.1293104	0	0.1575391	0.0730339	0.4996489	0.4485461	0.1787523
0	0	0	0.1924731	0.4656088	0.1816296	0.1439711	0.5682922
0.4689345	0.173596	0.07560293	0.3264734	0	0.9880288	0.2843146
0.1166278	0.09613408	0.7482407	0.3907788	0.3038764	0.1228934
0.4559086	1.129079	0.1497013	0.05199879	0.0369202	0.4508405

0.08876096 0.1080430.1281788 0.1176155 0.2703501 0.06443248
0.1224414 1.20093 0.2246318 0.4613453 0.5460974 0.1799835
1.3021391.3226360.2160859 0.04593888 0.1023468 0.2677043 0.1460678
1.1196610.05629554 0.3917037 0.2471157 0.02343455 0.1228878
0.2204393 0.6838629 0.1376066 0.1340310.3085745 0.07541345
0.1930458 0.05297529 0.7657202 0 0 0.2601438 0.03623738
0.2710808 0.1308259 1.4798340.1188544 0.4891205 0.2508891
0.1572099 0.08856502 0.2279543 0.1575391 0 0.004434778 0.3906487
0.1300719 0.2001550 0.1712901 0.26458 1.3072620 0.3281349 0
0.1147013 0.3498833 0.1202489 0.2396243 0 0 0 1.973113
0.07745165 0.07022163 0.5616794 0.1176046 0.0315872 0.2551661
0.1320701 0.03784901 0.07347977 0.1961536 0.4861934 0.1304478
1.2710870 2.1157012.5448330.2852459 0.1015409 0.08928774 0.2812146
0.1957283 0 0.5045266 0 0.2216844
TCGA-K4-A3WV 646 0 0.1223666 0.3107843 0.2773590.6508257 0
36.784110.1065822 0.2112250.8095170.2105774 0.03022923 0.9081724
0.3690081.3609080.6860618 0.2087873 0.1086284 0.1004474 0.1332687
0.1458116 0.1506213 0.1257361 4.57293629.371290.1830930.1644388
0.5633629 0.05491006 0 0.8410204 0.05067634 0.1147591 0.03286284
0.6159940.1344515 0.1630787 0.3533926 0.4194955 0.1354886
0.1849545 0.1436962 0.8561634 0.4345796 0.0353825 11.2293
0.2426116 1.0945550.06647387 0.6777960.9170182 1.4139670.203466
0.3143402 0.3380529 0 1.9748660 0.0654365 2.3095270.05422368
0.1030379 0.5806631 0 0.04634031 0.2896281 0.5961166 0.2129899
0 0.04747539 0.1885328 0.1296345 0.2463440.8342105 0.1408407
0.2313544 0.2974420.02553956 0.1217533 0 0.2075548 0.1586482
0.4651898 0.1105196 0.07019247 0.6527612 0.01596379 0.3890488
0.1185217 0.05561403 0.1516746 0 0.09758045 0.1673939 0.04057608
0.4105604 0 0.0612697 0.3524333 1.5241250.06830764 0 0.04715103
0.2586479 2.4324641.0809870.03972431 0.4319116 0.7610341 0.3113321
1.2256270.2416654 0.04499891 0.3473403 2.4465110 0.5115442
0.02707799 0.1030379 0.5399869 0.1230027 0.13461 0.1897038 0
0.03620947 0.7675948 1.4459650.08641224 0.9909708 0.04929426
0.6869534 0.0322760.03859337 0.1591120.1204971 0.7409076 0.2963043
0.07699925 0 0.7191972 0 0.6402796 0.7409447 0.04294945 0
0.3418656 0 0.6474471.1327190.4364079 0.2075548 1.1065510.1690089
0.4171052 1.0354270.5491006 0 0.7769363 0.6262053 0.08406772 0
0.8475461.2675670.2279809 0.3815930.6161782 0.7731707 0 0.199903
0.02661285 0.6918827 0.3518941 0.1946990.07699493 0.1412995
0.1558992 0 0.09929057 0.1923678 0.3701012 0.2586479 0.1574553
0.2358833 0.2536780.3813534 0.07787962 0.1324548 0.2950949
0.3087469 0 0.2084498 0.0202895 0.5618443 0.4086422 0.06756845
0.5188264 0 0.6755139 0.3636954 0.3036388 0.5646221 0.1630787
0.1371347 0 0.2044248 0.07158242 0 0 0.1119457 0.3517212

0.6601144	1.896347	0.4283635	1.29275	0.08439484	0.1586482	0.1468309
2.053927	0.03785248	0	0.0639336	0.476533	0.1250114	0.1923678	0
0.329252	0.7368526	1.10998	0	0.02365264	0	0.1377984	0.2522032
0.4800621	0.9129812	0	0.01122063	1.858281	3.022304	0.02791438
0.2151231	1.272449	0.2300949	0.159381	0.1450209	0.9202556	0.02728236
0	0.5485994	0.6619767	0.06268634	1.090745	0	1.016694	2.131485
0.2306857	0	0.06436045	0.4729792	0.1034624	0.02907436	0.6109711
1.006075	0.09587684
TCGA-GU-AATQ	213	1	0.1181505	0.06317397	0	0.2732181	0.2266469
27.56187	0.1029099	0.2146814	0.5929572	0	0.1167508	0.2000938
0.6334116	0.7665108	0.1276962	0.07330676	0.06992377	0.08313134	0
0.07039385	0.04847724	0.1214039	2.796739	36.37051	0.2039822	0.6033377
1.359881	0	0.7479346	0.4273912	0.1957212	0.2216103	0	0.4382517
0.1770259	0.1889519	0.6824332	0.135014	0.04360681	0.09615952
0.06017865	0.1837032	0.07629206	0.9565755	13.11273	0.09370098
0.1112466	0.05501446	0.9519168	0.5389529	1.828682	0.07858225
0.9105291	0.05222486	0	0.569855	1.363324	0.03685611	0.2908635	0	0
0.06595959	0.06346112	0.04261302	0.1575488	0.442752	0.1371009
0.2862256	0.01309704	0.0158293	0.203398	0.04324661	1.150669	0.4532937
0.3909205	0.1608285	0.09863842	0	0.09732999	0	0.1750652	0
0.2667792	1.084384	0.05729732	0.2260686	0	0.07629206	0.8591659	0
0	0.1199143	0	0.06027391	0.2086393	0	0.1183173	0.9477246
1.403691	0.06942539	0	0	0.4281193	0.5871636	0.7407198	0.07671124
0.1087905	0.1905071	0	0.4786782	0.1750042	0	0.2235819
2.351625	0	0.5927029	0.03137404	0.09948774	0.1737939	0.5542351
4.352399	0	0	0.06992377	0.1824363	1.910514	0.09535422	0.7716348	0
0.7421367	0.1246558	0.8943275	0.04851468	0.1163454	1.361529
0.1144381	0.1173888	0.1551272	0	0	0.3863869	0.2081209	0
0.06453673	0.07335261	0	0.2679169	0.810142	0	0.6012106	0.3287463
0.435162	0.2416404	0.4209481	0.1514804	0	0.5001115	1.319192	0	0
0.967134	1.087905	0.3625602	0.2500165	0.3173056	0.314329	0	0.1688885
0	0.5263378	0.06177631	0.1503926	0.1189474	0.1364311	0.3763193
0.2690643	0.3621738	0.1857399	0.3573495	0.4281193	0.3040605
0.08351056	1.396145	0.184107	0.3007852	0.06394556	0.1424637	0.5216911
0.101661	0.5676783	0.03918086	0.4681353	0.2023398	0.2283414
0.0244366	0.1534225	0.2467932	0.1915442	0.1998934	0.22027	0
0.1207266	0	0.3552866	0	0	0	0.4359574	0.07546728	0.1821058
0.3051682	0.1378681	1.462548	0.04656402	0.1750652	0.4068236
0.1586528	0	0	0.1419808	0.2509714	0	0.09286993	0.4792259
0.07947693	0.1674034	1.076296	0.1144381	0	0.3029609	0.1596608	0
0.1158804	0.1906	0	0.02166805	0.3680522	1.287428	0.0539052
0.09774642	0.5584579	0.2338601	0.08793689	0.1775918	0.7353505
0.0526847	0	0.2948832	0	0.0605265	0.4949872	0	1.22708
5.513067	0.05810544	0.141342	0	0.2609616	0.0363264	0	0.8989261

1.7464730.02057188
TCGA-G2-A3VY 536 0 0.2701449 0.5898129 0.08164225 0.6351107 0
46.033040 0.3190576 0.1643352 0.6198470.2335765 0.07400801
0.9956805 0.7093804 0.4075423 0.06385222 0.05329235 0.4963082
0.04903551 0.1140076 0.1108407 0.09252788 4.3978763 1.799040.1399184
0.03630264 0.4145730.1616310 1.8566950.0372922 0.1689001 0.02418342
0.5487360.3148138 0.3600239 0.5201160.4287534 0.1578658 0.02617425
0.1223070.3325221 0.4360945 0 16.753450.6070202 0.3603427
0.02096461 0.6801588 0.8802055 3.4031810.4192399 1.0698540.03980314
0.3600239 0.9688556 0.02886268 0.04213479 1.7734510 0.3412103
0.7540658 0 0.008119371 0.1320833 0.3374431 0.1828599 0.03635777
0.03992761 0.5368611 0.1430950.3708042 2.6309443.8348 0.2340955
0.6741644 0.004698575 0.2815905 0.01236333 0.1018249 0.450312
0.4279105 0.2643233 0.5681931 0.2620147 0.1762139 0.07157426
0.05814594 0.6138869 0.1674237 0.06763161 0.1697291 0.1407811
0.004593772 0.6201558 0.02902011 0 0.06786783 3.0714260.005291251 0
0 0.0271909 4.0275571.4113480.1461636 0.3224457 0.5392951
0.05091247 2.3713580.1778391 0 0.1704028 1.3966940.2610454
0.7528802 0.003985283 0.1895613 0.4967135 1.0258530.05587895
1.5821460.06138869 0.02664618 0.2722939 2.2494830.1726011 0.3999087
0.3627514 0.8307529 0.04750316 0.08520139 1.3804160.04433628
0.9673371.6571590.1729711 0.1182301 0.4456841 0.5036257 1.855252
0.6146495 0.2212424 0.02459331 0 0 0.7487064 0.4630869
0.3853777 0.05091247 1.5385570.1658292 1.0129130 0.3463521
0.02972264 0.7623193 0.8127154 2.7426630.3824535 0.1134001
2.4874380.3454063 0.06017365 0.7557321.7667970.1588165 0.1930773
0.09792061 1.3345880.1177069 0.2292432 0.05665978 0.08318468
0.1147246 0.3417786 0.1948451 0.07078075 0.5617295 0.6525817
1.2745670.2430178 0.7093804 0.1403170.0286554 0.3898885 0.162868
0.8520139 0.1162213 0.9439760 0.8374047 0.7016698 0.07458439
0.6052911 0.1753963 0.9135961.1435520.4722808 0.9988778 0
0.08310733 0 0.8875613 0.05267677 0 0.2759837 0.0741417
0.7764841.2491262.5293480.3677664 0.8648377 0.2306768 0.2501734
0.1573791 0 0.1114210 0.02117163 0.3666156 0.1379919 0.03539038
0 0.03028664 0.6124148 1.3329850 0.1566516 0 0.2028089
0.1237291 0.4023384 0.2905312 0.06283882 0 0.8836088 3.728608
0.0205419 0.3631746 0.1276885 0.1425890.1474463 0.1587777
0.9807820.2007681 0.03897695 0.6534254 0.05731081 0.1845209
0.7545082 0.02260773 1.5275232.5762040.1845209 0 0.0473622
0.5469522 0.01384307 0.06418657 0.7172307 0.003938085 0.1881461
TCGA-XF-AAN8 118 1 0 0 0.08555764 0.2182194 0.07240917 62.1782
0.7397477 0.2914924 0.1722164 0.1623934 0.1165611 11.06954
0.06323825 1.0057780.08924209 0.3094793 0 0 2.3638090.04216769
0.07743758 0 0.95960961.497940 0.4818860.3258413 0.08469123

0.1493439 0.682715 2.032194 0 0.1520593 0.2500227 0.07540837 0
1.021987 0.04792699 1.758853 0.2304079 0.05073497 0.07336192 0
0.9277351 0.101681 0 0 0.4540473 0.3801481 0.1229891 7.913084 0
0.4848266 0.5839682 0 0.7877481 0.06049374 0.1072347 0.5420631
0.1672652 0 0.05268195 1.165788 0.4016134 0.1069591 0 0.1642539
1.828868 0.05230306 0.05057148 0.07497879 0.06908216 0.3676158
0.7965009 0 1.669899 0.009847818 0 0.2850375 0 0.2097369
0.08968642 0.6818453 0 0 0.1887684 0 0 0.171554 0.350906
0.07087508 0 0.03688317 2.402226 0.3499446 0 0.6615007 0.4826178
0.2169927 0.08872014 0.1452375 0 0.05698984 0.3908065 0.05378302
0.3676158 0.07723645 0.0652106 0 0.08496009 0 0 0.05952498
0.7445289 0 0.1577973 0.05846973 0 0 0 1.911146 0 0
0.05584814 0.07892731 0.5086427 0.03807972 1.207958 0 2.067198
0.0497813 0 0.1420785 0 0.5713742 0.1828035 0.09375851
0.1858502 0 0.08119669 0 0.4155657 0 1.49482 0.2929338
0.5755361 0 0.06470607 0 0 0.106669 0 0.06433276 0.08405285
0.06049374 0 3.495093 0.8897395 0.6915356 0.05343935 0.3565155
0.434455 0.1585779 0.2522378 0.06335802 0.1255273 0 0.09635091
0.49256 0.1131809 0.04934076 0.1201186 0.1781312 0 1.021926 0
0.1531421 0 0.1427076 0 0.06071331 0.0424454 0.6358033
0.07352316 0.1201186 0.4085867 1.934359 0 0.6495735 0.3379927
0.03129377 0.1275658 0.1050458 5.497345 0.01951752 0.0612693
1.689547 0.05099548 0.02128733 0.2199121 0 0.05909881 0.1177924
0.4414161 0.4416242 0.06704015 0.09640644 3.254657 0.1808272 0
0.243738 0.1321384 0.3625254 0 0.1747808 0.4578561 1.393877 0 0
0.2958264 0.1781786 0.04820322 0.2225257 0 0.3173912 0.02674104
0.101991 0.2742052 0.03648096 0 0.04250703 0 0.08226999 0
0.2634097 0 0 0.2056539 0.0430541 0.05855257 0 0.05603524
0.08428227 0.1472981 0.02447186 0.1683172 0 0.1657386 0 0
0.3953464 0.6159907 0.5227037 11.25728 0.02320442 1.317049 0 0
0.05802782 0.04484321 0.02243664 0.2063474 0.03286153
TCGA-CU-A3QU 158 0 0.1259428 0.03673114 0.04152216 0.1800377 0
33.36786 0 0.1581077 0.1462628 0.2364344 0.03394111 0.01596827
0.09207088 0.7427853 0.136118 0.03044474 0.05420756 0.005370548
0.1496329 0.05457198 0.0187907 0.1882338 1.655858 25.55328 0.1950328
0 0 0.04110168 0 0.7123593 0.07586526 0.02863345 0.04919747
0.2426781 0.08691692 0.09765513 0.3967861 0.1628168 0.101417
0.021299 0.07127507 0.1691163 0.08871675 0 6.809887 0.1452811
0.02156064 0.02132465 0.09224526 1.014698 2.859603 0.1218399 0.8529344
0.02024335 0 0.4672601 0 0.001020438 4.509768 0 0.07712668
0.2301047 0 0 0.1679395 0.03432382 0.398572 0.03698215 0.005076662
0.1043076 0.04851749 0.2346849 0.3568168 4.67376 0.06494066
0.03562296 0.004779266 0.1822713 0 0.02589341 0.5937626 0.3482073
0.1654539 0.4553552 0.06662859 0.07567913 0 0.02957225 0.1665145

0	0.03439654	0.0398409	0.1610987	0	0.2790103	0.02951848
0.04586205	0.4142001	1.544535	0	0	0.03529384	0.02765786	1.327642
0.234914	0.05946947	0.2061608	0.25318	0.1553604	0.7731031	0.0452233
0	0.05777639	2.085853	0.07586526	0.2680334	0.01216117	0.07712668
0.3031462	0.2762126	0.04133715	0.5206613	0	0.08131135	0.06776929
2.269125	0.01848055	0.5144526	0.1106943	1.528227	0	0.02888819
0.4826671	0.135293	1.690604	0.4435838	0.1213391	0	0.1983354
0.1970287	0.8986254	0.3226865	0	0.3252036	0	0	0.2423159
0.5966502	0.09799899	0.02589341	0.7964271	0.2530156	0.1248859
0.2855431	0.2055084	0.06046617	0.2907791	0.1164728	4.803428
0.07780431	0.6055746	0.4744042	0.1806882	0.005100586	0.5227231
0.3045997	0	0.07481643	0.1195227	0.4472714	0.4070764	0.1457376
0.02881641	0.1057666	0.1458685	0	0.06606375	0.8999537	0.4501752
0.2489208	0.1767895	0.1265384	0.5364251	0.1784084	0	0.1239326
0.1104433	0.2311056	0.1970287	0.4280835	0.01518725	0.3629163
0.831366	0.01264421	0.1420814	0.2378779	0.6559663	0.1237436
0.1601305	0.2262592	0.08137928	0.1101968	0	1.591383	0.1071628	0
0	0.05306979	0.4972934	0.3882322	1.715191	0.08550449	0.6646546
0.1714665	0	0.1051284	0.03074842	0.1700017	0	0.02871363
0.2540122	0.07018083	0.1079944	0	0	0.4542213	0.5073489	0
0.01770465	0	0.4951003	0	0.07486243	0.1292903	0.1917539	0
0.05706561	1.430558	0.04178936	0.03788837	0.04329377	0.1087783
0.02726881	0.2594661	0.1187649	0.102108	0.1982316	0.2243713	0
0.04692244	2.102366	0.02299599	4.344175	2.465734	0.05630693	0.05478684
0.04817557	0	0	0.04352591	0.2395531	0.004005716	0.0956886
TCGA-GD-A3OP	64	0	0.2783975	0.1653962	0	0.2765882	0.04747087
25.57865	0.2514673	0.2997648	0.1505382	0.3548793	0.2445329
1.514079	0.4422239	0.5160362	0.2061654	0.06945856	0.1464544
0.004836595	1.504775	0.0552896	0.1015349	0.2966585	1.710252	33.84057
0.1376655	0.08867947	0.2373543	0.148061	0.2610898	0.9100843
0.1024838	0.07735993	0.1993776	0.917912	0.2307065	0.2308583
0.506227	0.2408908	0.2207235	0.009590703	0.1283775	0.1923819
0.1331605	0.1431096	9.969555	0.2289646	0.0582511	0.102424	0.8722772
1.290091	6.073148	0.1371579	1.562755	0.3463834	0	0.3748981	0
0.1792018	0.3722947	0.03655257	0.1736464	0.2072272	0.2215307
0.1085904	0.1512425	0.123645	0.5503824	0.6994113	0.0320035
0.2431312	0.1146961	0.3245762	1.124694	2.246954	0.1364629	0.1684265
0.2496378	0.2579492	0.1698803	0.09327609	0.3972275	0.3135877
0.3725102	0	0.1600112	0.2152261	0.06556514	0.1597926	0.4123879
0.05112247	0	0.1076395	0.06448082	0.06943357	0.2986113	0.1329184
0.2891164	0.4684946	1.40678	0.0557407	0.1057961	0.1271394
0.3487127	2.254644	0.6581813	0.1606706	0.3249117	0.1805065
0.1399141	0.677673	0.1629084	0	0	2.36658	0.03416128	0.2758684
0.04380832	0.03472928	0.1213363	0.4422239	0.5258358	0.5967766

0.2249389 0.04881812 0.1406378 1.590357 0.02496476 0.4202064
0.06645921 1.068646 0.02175748 0.9105622 0.214517 0.04061396 0.8216775
0.4793779 0.2731881 0 0.1530998 0.2129276 0.5664974 1.289556 0
0.02252855 0 0.440203 0.2805741 0.5656103 0.02941857 0.02331902
0.3514498 0.5696504 0.1687042 0.1102087 0.1057579 0 0.5673831
1.179404 0.2077924 0.256919 0.519397 0.9494173 0.6237742 1.800642
0.7753575 1.124694 0.1454829 0.370579 0.2511587 0.3957359 0.1725195
0.0524992 0.1505182 0.1714518 0.5386003 0 0.2900407 0.9401547
0.4054177 0.1743564 0.5307088 0.09805655 0.5856961 0.1606706
0.131248 0.1116109 0.5470452 0.416257 0.2839034 0.8791352 0.01367729
0.4191169 0.6286335 1.229806 0.1023642 0 0.9230446 0.2451697
0.03721547 0.2806561 0.2198651 0.1128354 0.02574124 0.4823147
0.09650843 0.1172026 0.08427105 0.6980361 0.6059164 0.5403423
1.01202 0.1636323 0.5897705 0.02438192 0.2750036 3.799961 0.05538268
0.204133 0.1539055 0.08404127 0.2968983 0.06320329 0 0.1003732
0.1109755 0.53178 0.4680512 0.1597926 0.191333 0.07931841 0.2415163
0.05667065 0.1528174 0.2661393 0.2878155 0 0 3.175879 0.131721
0.06824283 0 0.1061269 0.1074398 0.4268043 0.577568 0.2023035
0.03570458 0.3393146 0.2362464 0.1056433 0.6323401 0.06212901
1.313608 7.663195 0.08451461 0.1809126 0 0.04554837 0.1394894
0.03919846 0.6079837 0.03246712 0.03590625
TCGA-KQ-A41P 1094 0 0.137130477 0.081469338 0.110514928 0.155030889
0.023382758 52.015430094 0.212340722 0.088593207 0.389291324 0.104881886
0.03011243 0.783232045 0.449267208 2.089960087 0.074104808 0.099938709
0.10820869 0.221559948 0.630580186 0.018156024 0.075019682 0.062625126
10.005748614 59.116583911 0.119252066 0.0819017 0.561186194
0.164093685 0.482269385 1.741681437 0.201922135 0.114315706 0.065471723
0.968864057 0.219161472 0.146203772 0.572044175 0.216675758 0.213694795
0.035430745 0.075881977 0.130297343 0.314836699 0.140983155 14.666461875
0.096669658 0.286927868 0.009459575 1.841392199 0.158865209 9.051896387
0.162143853 0.821954778 0.148168549 0 1.537609059 0.039069925 0.078763593
2.300603586 0 0 0.510370121 0.196415168 0.063746504 0.276318498
0.593813298 0.424333914 0.295293874 0.013511988 0.212300597 0.056495958
0.145004486 0.712274785 3.507413712 0.086422674 0.142220527 0.050881763
0.294543997 0.083678034 0.068917603 0.225764561 0.057924044 0.247708301
0.116535429 0.118225272 0.12721689 0.581318434 0 0.2215966 0.075544295
0 0.079530068 0.023821024 0.059074356 0.322874249 0.196415168 0.183098885
0.446221854 2.452535339 0.150412411 0 0.046968844 0.147227724 3.028832028
1.354696254 0.039570821 0.031177011 0.842326256 0.103376404 0.480125966
0.210640222 0.134475115 0.26910975 3.518980903 0.050480534 0.458610887 0
0 0.493075421 0.081684947 0.364449532 0.188970861 0.083098725 0.072139127
0.180374002 1.08660087 0.049187635 0.748317183 0.049103792 1.373383577
0.064302585 0.2306655 0.233574794 0.120031491 0.928508066 0.472255048
0.141292675 0 0.339356049 0 0.119588571 0.993055569 0.085567004

0.133162826 0.037838299 0.046463803 0.829215421 3.385027361 0.04347217
0.068917603 0.174880319 0 0.207746812 0 0.664188723 0.040234019
1.031912524 0.937568815 0.167485802 0.069027695 0.307007722 1.192520662
0.280535267 0.549814372 0.040919827 0.72964734 0 0.074673969 0.053020045
1.221780189 0.223067374 0.271525456 0.084367182 0.014075354 0.271769415 0
0.120886243 0.287436831 0.2150582 0.257648517 0.392117395 0.148815552
1.244537043 0 0.349104158 0 0.440932009 0.1537770.262204714 0.409964631
0.060633307 0.539786589 0.511438529 0.538459027 0.189081113 0.197854107
0.381918382 0.329354702 0.116861713 0.460178582 0.10829909 0.086382496 0
0.488723886 0.142611673 0.129893798 0.249056697 0.32338807 0.077858265
1.127252268 0.629673398 0.455155623 0.585343215 0.036029464 0.203188105
0.473766682 0.12275948 0.263943594 0.056857022 0.121004481 0.215768993
0.031132087 0.143718415 0.049440889 0.163989893 0.544027623 0.712817677 0
0 0 0.219625601 0.083742901 0.126193575 0.049159655 0 0 0.465148398
0.885478216 0 0.088237853 1.26753255 0.132698131 0.054433765 0.20083547
0.537375807 0 0.052761095 0.478874008 0.193946755 0.031222064 0.92355133
0.275426369 1.139361423 3.19130779 0.104906136 0.243033391 0.064111776 0
0.084323945 0.028962022 1.405600162 0.490432149 0.07428275
TCGA-UY-A9PH 1561 0 0.07637652 0.1633513 0.09232899 0.3532352
0.1172098 41.363630 0.3885768 0.09292312 0.1752458 0.02515724
0.5275349 0.3412158 1.3685210.01375786 0.15796 0.1808045 0.07165194
0.2218165 0.2275250.04178314 0 4.96491624.945330.1640938 0
0.2344197 0.2741820 2.7259660.1686945 0.3183477 0.05469793
0.8094312 0.2034412 0.1357167 0.1470494 0.07758016 0.1409447
0.1420819 0.09509266 0.1385441 0.1972712 0.1177835 24.57915
0.08076204 0.1917696 0 1.1281450.4645302 3.69211 0.06773102
0.7193967 0.3150928 0 0.8689844 0 0.04084295 4.3454470 0
0.3979597 0.05469793 0 0.2036899 0.1526454 0.2363381.397974
0.04515401 0.1637217 0.06742741 0.2050113 0.2644735 4.922814
0.3850734 0.05940861 0.02125442 0.1447499 0.02796331 0 0.8298996
0.09678454 0.5058687 0 0.04938527 0.1151394 0.8094312 0.06575707
0.1851315 0 0 0.05906034 0.03980224 0.07792625 0.4225977 0
0.1019792 0.4111689 1.1708310.01795158 0.05224404 0 0.06150023
2.5304180.8125544 0.1983551 0.08334923 0.7271733 0.3454606
0.2292104 0 0 0.2569444.6746390.08434727 0.1702860 0.08574972
0.8238734 0.2047295 0.2412837 0.8419973 0.1388486 0.06026817
0.1375886 2.4489320.06164024 0.5054621 0.1640938 1.6950910.3223271
0.0642360.2787694 0 1.6309930.4931781 0.07419786 0 0.3780171
0.3504916 0.5328507 2.8925350.0714864 0.05562501 0.1264471 0
0.1539473 1.2568890.07263709 0 0.3541882 0 0.2776972 0
0.06528143 0 2.1552620.4105934 0.7929080.05766874 0.3206095
0.7032591 0.3571373 0.0567085 0.06837241 0.7450412 0.08980257 0
0 1.1166940.2129831 0 0.1025222 0 0.9406275 0.07730328
0.05508744 0.6403660.25667 0.1230005 0.06551839 0.1046964 1.2878

0.1586841 0.06481263 0.3306928 1.105121 0.385416 0.2628687 0.6761085
0 0.5459004 0.3487984 0.2530418 0.06318664 0.2644735 0.2431019
0.05503145 0.1378325 0.2847802 0.1809556 0.1174823 0 0.8166025
0.119144 0 0.2080728 0.3447028 0.06504619 0.3139186 1.512413
0.3089587 0.5868255 0.3210729 0.4149498 0.3347079 0.1367448
0.2520114 0 0.03192392 0.3485081 0.2080728 0 0.08261015
0.06850215 0.3751465 0.8097485 0.09863561 0.07873639 0 0.229356 0
0.04439057 0.1642805 0 0.05602788 0 0.5918137 0 0.06318664
0.7701468 0.1209401 0.06063512 0.2296029 0.1848606 0.1362288 0
0.385952 0 0.1043371 0.4538677 0.05113404 1.339672 6.385877 0.08346967
0.4060814 0.1071235 0 0.04696526 0.04839227 0.5084596 0.1425142
0.1773116
TCGA-FD-A5BY 251 0 0.10217286 0.04682648 0 0.10800941 0.13439807
23.68701584 0 0.12730273 0.37292438 0 0.05769283 0.61652778
0.19562668 0.51405066 0.12620307 0.14748672 0.10365928 0 0.12717237
0.10435615 0.07186563 0.95987492 2.32865272 24.9268261 0.30239566
0.10984164 0.80638842 0 0.46199337 0.40127424 0.53194003 0.1825159
0 0.3403144 0.29742532 0.10893317 0.33722693 0.05189152 0.12390369
0.1018234 0.13381877 0.05673603 0.18850003 0.27011162 1.95020179 0
0.02748646 0.04530933 0.29399578 1.67404656 2.77903379 0.23299028
0.37495114 0.12903555 0 0.16245921 0 0.12228451 1.53313355 0
0.14748672 0.03259418 0.03135955 0.14529595 0.09342397 0.30630258
0.16937236 0.42431828 0.03883171 0.0782211 0.06958376 0.19233426
0.41697863 0.3135955 0.0275964 0.06812058 0.18278454 0.13278141
0.06412797 0.03301005 0.51905462 0.11097749 0.2900259 0 0.02831368
0.14725755 0.09281302 0.07540001 0.05307001 0.14473638 0 0.10158185
0 0.04765532 0.19588979 0 0.11693391 0.69148165 0.60413749
0.2058409 0 0 0.28207568 0.6286564 0.26620325 0.0758143
0.1254382 0.12103686 0.23107037 0.39423434 0.02882633 0.04294046
0.03682794 4.60638001 0 0.39051515 0.0155036 0 0.30058324
0.19562668 0.79706354 0.42239394 0 0.06910619 0.06010106 1.40402757
0.02355982 1.08290973 0.09407865 0.78846868 0.06159912 0.51559117
0.13585102 0 1.61929312 0.05655001 0.2552358 0.03832834 0.28896758
0.15070852 0.42005596 0.38566403 0 0.03189107 0 0 0.30891497
0.40033468 0 0.09903016 0.33505568 0 0.6766426 0.15600982 0
0.03854246 0.43248096 0.45269691 0.0802221 0.09918835 0.29410022
0.26879614 0.311398 0.31862012 0.23519662 0.23299028 0 0.05961205
0.02539546 0.29010236 0.15263498 0.07431707 0.22776585 0.21573736
0.52068687 0 0.16844194 1.05551656 0.19865879 0.07051892 0.18781582
0.14631046 0.68385825 0.4093972 0.07431707 0.09479681 0.91518686
0.14731176 0.15070852 1.14758333 0 0.1959508 0.31495896 0.64477579
0.12075439 0.30325718 0.15679775 0.1893046 0.05268165 0.13605905
0.15561882 0.12893717 0 0.23408825 0.27323172 0.04147756 0.17893922
0.39347131 0.44750925 0.40494723 0.30160004 0.16350733 1.20869258

0.09203915 0 0.31982587 0.11759831 0.0722419 0 0.183027 0.18602769
0.0298232 0.09178405 0.0947245 0.35346438 0.12408455 0.57467468
0.11310002 0.09028267 0.07485462 0.05259797 0 0.15906319 0.07063926
0 0.0214147 0 0.97548764 0.05327491 0.02415088 1.15904896
0.15023168 0.04779978 0.13501176 0.4239389 0.02603434 0 0.14571765
1.96940225 0.0598188 1.4832113 0.02931629 3.274378 2.67499388
0.03828403 0.18625244 0.06141633 0.19343274 0.05385248 0 0.38868175
0.0408533 0.1321537
TCGA-K4-A3WU 105 0 0.1251878 0.178498 0.03783381 0.2508927 0.4963026
33.4904 0.2544254 0.4321897 0.6663523 0.2154323 0.3092618 4.57904
0.4753901 0.9088523 0.2875168 0.09986537 0.246962 0.08318933
0.7726002 0.04972444 0.05136466 0.04287832 5.72154 63.49877 0.1008615
0.1794453 0.5763515 0.2996055 9.113557 1.977434 0.4838839 0.2087199
0.201723 0.9950462 0.4001493 0.233574 1.416033 0.1960393 0.604504
0.1407011 0.1369724 0.1378735 0.2155631 0.5067756 9.862014
0.3971281 0.176809 0.4728067 0.8405125 0.5982472 5.423518 0.1387713
0.535979 0.507242 0.05561286 1.238557 0.1605029 0.8144995 1.232752
0.03698255 0.1405513 0.8153634 0.537928 0.006020153 0.2643101
0.09382466 0.7505461 1.752248 0.01850284 0.2348099 0.9007331
0.2214753 1.517233 3.810324 0.2958604 0.1379492 1.720119 0.2609835
0.5041773 0.07078 0.3246117 0.03965957 0.3203584 0.07978976
0.08094676 0.4355158 0 0.05389079 0.5689623 0.05172385 0.06268229
0.03025158 0.1794082 0.7557235 0.3831816 0.8606849 0.6268229
2.720478 0.9115699 0.007356052 0.02140813 0.03215874 10.93726 4.63146
1.212933 0.3793082 0.2561562 0.2691388 0.21234 1.023771 0.2678402
0.2455273 0.0263221 4.077995 0.1382525 0.6280056 0.05540457
0.03513782 0.4603637 0.5033542 0.9934206 1.03508 0.2844812
0.123481 0.4322462 1.574462 0.1431311 1.896821 0.4370665 1.805306
0.04402685 14.95095 0.3712534 0 1.165511 1.091288 0.3814344 0.3835228
0.129084 0.3590539 0.6004547 2.150042 0.02929311 0.02279356 0.5699588
0.2226906 0.2207914 0.9442354 0 0.02359333 0.4499226 0.1536937
0.6543067 0.03716839 0.2140039 0 1.103957 1.972403 0.4204735
0.2126792 0.5517823 1.440879 0.487816 4.219938 0.67241 1.137925 0
0.3749383 0.2359623 0.4861892 0.1963675 0.07967516 0.2835723
0.009637147 1.143037 0.06335341 0.3762213 0.8200122 0.3102695
0.2268095 0.4832566 0.1233422 1.548509 0.6827548 0.1062335
0.4516953 1.610125 0.4737978 0.07181078 1.403478 0.0968673 0.7547261
1.021933 0.9677677 0.1898754 0.1625607 0.3486571 0.2480537
0.1035464 0.365644 0.2224514 0.1430473 0 2.913988 0.1464656
0.05929066 0.2131559 2.100927 0.3465032 1.640096 2.020904 0.1460799
0.7837362 0.2549103 0.3709848 4.639283 0.2241367 0.2065343 0
0.1395362 0.4727463 0.1278936 0 0.06770261 0.2526322 0.4138735
0.591141 0.4850171 0.1774517 0.7757642 0.3195443 0.05733729
0.118235 0.1514644 0.232961 0 0.181988 2.031009 0 0.0345228 0.1183442

0.2064911	0.1397619	0.3859888	0.8116142	0.2046833	0.1083738
0.570891 1.965321 0.0427544	1.394866 0.3352526	1.386846 21.40098 0.07866809
0.08320034	0.08779241	0.6451785	0.7441416	0	0.912782 0.02189936
0.1089859
TCGA-FD-A3SJ	739 1	0.1877991	0.1479792	0.0716918	0.2742807
1.183147 27.00046 0.03443672	0.2442519	0.126268 0.1360752	0.02930121
2.412433 0.4769063	1.429056 0.2136547	0.6027524	0.1403914	0.2457276
0.3660026	0.05888969	0.04866578	0.0812507	10.79715 98.92469 0.0546068
0.04250416	0.3185397	0.1419316	6.757592 1.001125 0.458459 0.04943826
0.08494392	0.4399562	1.35063 0.08430524	0.4567249	0.1706789
0.2225301	0.055162 0.2058507	0.06915664	0.2042367	0.02286419
7.994797 0.1567757	0.09306604	0.1165933	0.4379921	0.2576424
3.000979 0.1314798	0.812507 0.1747599	0	0.4693918	0.5068986
0.4290175	2.238626 0	0.1331662	0.4414408	0.3610116	0.9111875
0.1845218	0.7704216	0.1605732	0.8939428	0.00438266	0.2171752
1.303669 0.6222789	1.283493 0.9101134	0.3924409	0.2383369	0.6766507
0.078677 0.2931256	0	1.17164 0.2630301	0.6606148	0.7710928
0.1150403	0.09971949	0.06285088	0.2042367	0.6109428	0	0
0.2522261	0.04635859	0.4800327	0.5410811	0.586113 0.47511	3.024506
1.015195 0.06969541	0.04056656	0	4.226213 0.458459 0.7210673	0.6417466
0.2548317	0.2732114	0.2682439	0.9788775	0.2342465	0.1453916
0.1246952	2.339502 0.06549414	0.6611201	0.006999124	0.09987467
0.5234096	0.3709271	0.8810025	0.4494846	0	0.0935943	0.1424468
1.745821 0.06381671	1.105147 0.3503936	0.7543358	0.1459974	7.332081
0.09199524	0.1167979	1.042494 0.3446495	0.1859339	0.05191017
0.1467617	0.3061692	0.2327338	0.6529074	0.2497856	0.1295755
0.02454596	0.2109897	0.2988432	0.1897683	0.0282007	0.06706099
0.3781536	0.03640454	0.6468806	0	0.1267246	0	1.087789 1.243392
0.1991902	0.2686724	0.298737 0.7735964	0.3148632	2.738861 0.5308995
0.6836949	0.1045951	0.2099132	0.1547751	0.3183857	0.1653775
0.1006517	0.08955748	0.1187001	0.3274119	0	0.2851627	0.3729245
0.1494747	0.09550764	0.8902924	0.256587 1.036837 0.369646 0.2013034
0.08559234	1.048797 0.1995124	0.2041128	1.143226 0.03933324	0.5952776
0.3994826	1.397208 0.04906329	0.2310288	0.2123598	0.1281928
0.1426993	0.1474178	0.1053815	0.1159834	0	0.3302485	0.09251317
0.05617533	0.0807824	1.467283 0.3282968	0.2132831	0.2297663
0.09226956	1.063205 0.1168628	0.02929101	3.826226 0.02654497	0.3179838
0	0.9254317	0.3452614	0.1211736	0.03107704	0.09621794	0.02659534
0.4481454	0.5707632	0.3829439	0.1681278	0.2281044	0.03561816
0.2172984	0.1637253	0.1753961	0.4966209	0	0.1908999	2.268942
0.07215313	0.0572405	0.3737532	0.1565131	0.06179523	0.1782825
0.3280935	1.145941 0.05133973	0.3581599	0.1258146	0.1417778
0.5392027	0.01985233	1.505598 7.746642 0.09397841	0.1418917	0.332718
0.3929648	0.03646768	0.01878786	0.5922146	0.02766493	0.08260754

TCGA-FD-A3B5	272	1	0.1381192	0.05680851	0.2697169	0.1572406
0.01630478	35.99494	0	0.138996	0.1551158	0.4388044	0.01049869
0.1587637	0.08543829	0.5711149	0.09186352	0.137491	0.176059	0.3638075
0.2777072	0.04431064	0.08718529	0	1.411726	21.19393	0.1663088
0.708164	0.3424004	0.03814081	0.06725722	0.7840271	0.1760002
0.02657075	0.09130678	0.3828333	0.03820529	0.1019478	0.06136708
0.2320278	0.05097684	0.3359994	0.1382939	0.1238949	0	1.523766
1.144804	0.1348154	0.1400522	0.05276924	0.4280005	0.4708006
1.693981	0.1130628	1.364639	0.1033178	0.1132753	0.212858	0.5176252
0.01325699	1.115972	0.03766405	0.03578532	0.2372538	0	0.009196633
0.06800354	0.318512	0.3452024	0.2402263	0.0188438	0.1537308
0.06753359	0.1866674	0.7450031	0.03260956	0.08034996	0.00826419
0.008869955	0.06040745	0.07001836	0.1201405	0.173168	0.0403904
0.5373724	0.2112762	0.1442672	0.09979678	0	0	0.6953362
0.05267699	0	0.1170743	0.03322077	0.002168027	0.1313317	0	0
0.3271653	1.107527	0.1248601	0.3270395	0.03275134	0.0513309
0.7392009	0.3148763	0.05518541	0.2043533	0.2447306	0.0480562
0.9565472	0.08393102	0	0.1608429	1.417403	0.1760002	0.2131927	0
0.03578532	0.1562823	0.1708766	1.203523	0.3074616	0	0.02515128
0.08476119	1.136529	0.1543433	1.054704	0	0.6373274	0.04483815
0.08042145	0.04653473	0.04184894	1.419405	0.1234887	0.04785417
0.02789929	0.1051701	0.03656703	0.7505013	0.87025	0.4176607
0.02321359	0	0	0.09636856	0.6410901	0	0.0961124	0.6318913
0.03913148	0.4635575	0.3785331	0.05448686	0.02805515	0.134916
0.2029838	0	0.1203324	0.2943549	0.5380578	0.1366214	0.189326
0.1141335	0.282657	0	0.1041402	0	0.1820399	0.1333241	0.08114336
0.2085762	0.08833261	0.09475231	0	0.1072831	0	0.2356513
0.1283272	0.4648194	0.0436922	0.5462388	0.1655562	0.05409558
0.04600189	0.0512436	0.08042145	0.1097011	0.3452692	0	0.3625257
0.192869	0.2112003	0.04394871	0.3035198	0.2663114	0.39042	0.06231404
0.0911147	0.03775844	0.07144087	0	0.4685819	0.09944302	0.1509581
0	0.7218537	0.4343241	0.1637567	1.317213	0.09918116	2.467097
0.1256166	0.0314851	0.04434325	0.2282669	0.1051701	0	0.1332258
0.3059263	0.04341677	0.1336197	0.0344751	0.08576253	0.4215001
0.7234253	0	0.2464387	0.02724343	0.09571549	0.2335755	0.1343077
0.06855799	0	0.01558781	0.8340369	0.4939547	0.07755788	0.1406359
0.5624497	0.2944146	0.1138699	0.2628867	0.2975652	0.1705542
0.03679028	0.2435641	1.487628	0.1088556	1.568309	0.0426788	0.7650509
1.597189	0.05921746	0.08473351	0.0447051	0.04693339	0.006533226	0
0.06062623	0.1635547	0.07399616
TCGA-GC-A6I3	0	0	0.07621279	0.04075028	0.1381966	0.2114868
0.2631568	97.10858	0.1991457	0.2492639	0.6954294	0	0.01882748
0.478319	0.153218	3.284472	0.0926665	0.2803409	0.3608338	0.214495	0
0.05675931	0.03127018	0.0783114	3.560704	116.0834	0.1491222	0.04096654

0.2631568	0.1367971	0.9649083	5.3483490.2524994	0.04764979	0
0.08076961	0.1141903	0.04062772	0.6603039	0.1645044	0.3445742
0.06202756	0.04528784	0.01481219	0.7381812	0	20.88582 0.5439752
0.1793986	0.05914499	2.149114 0.248322 5.267753 0.1520681	0.3426124
0.08421893	0	1.979299 1.563396 0.05094425	4.002583 0	0.3208721
3.105941 0	0.09345739	0.2439039	0.3427159	0.3095287	0	0.01689646
0.2858988	0	0.1534289	0.2474124	1.169586 0	0.2223047	0.03976661
0.06499783	0.02092752	0	0.4234707	0	0.1376684	0.08743506	0
0.1657105	0.2423088	0	0.06927547	0	0	0.01105007	0.2383015
0.0194398	0.107666 0	0.07632043	0.1764236	0.8762411	0.0179131
0.3909903	0	0	11.6781	1.607146 0	0.1169586	1.088422 0.04309
0.4116942	0.03762874	0.1121055	0	5.657672 0	1.210687 0.1214267	0
1.457372 0.2042906	0.2493652	0.3150721	0	0.04510423	0.08826022
1.169175 0.09226215	0.7764774	0	1.28056	0.3618406	0.3365161
0.3442376	0	1.756505 0	0.100962 0.1000646	0	0	0.8474103
2.684962 0.1069997	0	0	0.232408 0.3456388	4.546454 0	0.08618001
0.07952151	0.1403503	0.259783 0.203649 0.2931367	0.1006236	0.1612981
0.5483853	0.4886874	0	0.57586	1.228065 0.3229619	0.5007944	0
0.6082724	0	0.04668911	0.03315022	1.60616	0.1195462	0	0.2109988
0.07040373	0.1213721	0	0.206135 0	0.6627099	0	0.1471004
0.1028396	3.34163	0.4750318	0.09701053	0	1.562233 0.04807372	0
0.6591242	0	0.2238131	0.2414601	0.2524994	0.07881398	0
0.5685487	0.4530362	0.008596065	0.2273357	0	0.2386477	0.0475659
0.4328901	0.08916645	0.1082863	0.1557201	0.2045191	0	1.409605
0.1968483	0.160077 0.3741126	0.04505408	0.367008 0.1789234	0.05116938
0.3772067	0.07109851	0.05176516	0.2338393	0	0.05990562	0.3709487
0	0.226765 0.3618406	0.07381812	0	0	0.0686593	0	0.1162755	0
0	0	0.04748231	1.162635 0	0	0.3602324	0.09051066	0.04537885
0.167427 0.03952807	0.169921 0	0.4086079	0.09701053	0.03904253
0.4483657	0.03826832	1.055368 2.478073 0.02498722	0.5470346	0.2405113
0.1683329	0.07029688	0	1.141582 0.006666033	0.07961915
TCGA-4Z-AA80	19	1	0.2552458	0.1364776	0.1322391	0.1686415	0
31.73099 0	0.03975307	0.09981756	0	0.09007926	0.02906028	0.3420969
1.419367 0.118229 0.1195839	0.04315985	0.05986412	0	0.065175 0.05984432
0.2248065	1.812782 34.56764 0.1846625	0.03920053	0.33575	0.1309	0
0.6858892	0	0.136787 0.07834166	0.309151 0.0874142	0.1166289
0.1579598	0.1851916	0.04710287	0.05087455	0.1960418	0.1275629
0.04709062	0	4.583821 0.05783613	0.274664 0	0.8078984	0.5702812
3.811425 0.1455127	0.7025203	0.1128237	0.1943816	0.7981749	0.32725
0.0129995	1.735485 0	0.06140795	0.2849909	0	0.01841177	0.1410062
0.05465697	0.1269366	0.1766703	0.008084036	0.06839351	0.01931472
0.4804847	0.4261442	0.2238333	0.2757626	0.5388944	0	0.04146391
0.04005073	0.1649298	0.4592442	0.3465516	0.2305341	0.4741065
0.1414651	0.04439873	0.1159316	0	0.5303128	0	0	0.02114744

0.02850358 0.01116107 0.1931712 0 0.07303036 0.3611925 1.788731 0
0 0 0 1.630898 0.08312781 0 0.08207222 0.8735172 0.1649298
0.9356233 0.1440265 0 0.09200266 1.752285 0.06040362 0.3658408
0.04518583 0.06140795 1.072728 0.04887098 0.05348277 0.6029796 0
0.08631969 0.2486743 2.056125 0.02942829 0.4381822 0.1762687
0.2977504 0.1538854 0.09200266 0.2096169 0.1436264 1.253467
0.3531796 0.01449145 0.04787546 0.1353547 0.06274939 0.5723856
0.6262467 0.7167099 0 0.04527627 0.05559731 0.05512313 0.4000425
0.1560528 0 0.7799578 0.06715 0.2983009 0 0.6545 0 0.8488962
0.5926002 0 0.08259664 0.3214374 0.5875625 0.4262613 0.0243664
0.3427448 0.4850422 0.06431032 0.2829506 0 0.349868 0.07626179
0.3248999 0 0.03368436 0.2090519 0.2214368 0.1972488 0.4585853
0.1562377 0.5285059 0.4691969 0.1265224 0.2041006 0.1136385 0
0 0 0.414012 0.1254988 0.2994281 0.0483681 0.3263463 0.4621023
0 0.2714991 0.2367468 0.4352315 0.03940968 0.213863 0.2787175 0
0.07692124 0 0.7066255 0 0 0.2235108 0.1578964 0.5589783
0.1686049 1.789443 0.1191371 0.4202434 0.08622373 0.02701437
0.4489513 0 0.04511823 0 0.01143084 0.6282459 0.1117554 0
0.05915961 0 0.867958 1.145696 0 0.1973492 0 0.2299482 0.3006134
0.1748418 0.2647039 0.8142598 0.01337442 0.7383254 1.112516 0
0.04524985 0.206822 0.230957 0.04885046 0.2866906 0.3404166 1.365806
0.06313247 0.485371 0 0.03735946 0.8450735 0 1.817771 1.047044
0.08966271 0.08724212 0 0.08053816 0.03363324 0.03465516 0.3121056
0.07654404 0.06348907
TCGA-G2-A3IE 612 1 0.5157579 0.3171367 0.09352234 0.465141 0.01978747
25.65249 0 0.1874282 0.6353381 0.4437771 0.3185299 0.2080895
1.278816 2.198814 0.35536 0.13943 0.03052357 1.070526 0.02808543 0.03072877
0.3809087 0.05299599 7.406686 31.43111 0.02374496 0.08317036 0.296812
0.09257528 0 1.361943 0.08543747 0.03224624 0.08310735 0.6285848
0.9942909 0.2886887 0.2606626 0.3798173 0.0713832 0 0.06567392
0.1002391 0.7659804 0 34.71479 0.6135442 0.1942483 0 0.5713631
0.2688767 3.373502 0.3773355 0.8611849 0.05699382 0 1.052432
0.0330626 0.02183462 4.867167 0 0.1302871 0.575862 0 0.01860171
0.4367161 0.03865458 0.7481022 0.1665933 0.04573763 0.7393602
0.2253864 0.3020525 2.879837 10.80396 0.1218908 0.07020588 0.005382284
0.2492554 0.01416237 0.08748143 0.1146308 0.09803549 0.4425334 0
0.02501179 0.1121419 0.1639786 0.133214 0.2344054 0.06392873
0.07747296 0.1121694 0.1007917 0.002631115 0.8014737 0.09972883 0
0.1416043 1.304561 0 0 0 0.03114756 6.792279 2.733724 0.1004594
0.3482594 0.4395672 0.1166419 0.9286917 0.1018586 0.1137985
0.2277319 0.9618089 0.04271873 1.207409 0 0.3474322 0.834522
0.1036878 0.04946237 0.7462701 0 0.06104714 0.2422331 0.8660661
0.1144677 0.4446274 0.124661 0.7491626 0 0.09759938 0.5153298
0.3555148 1.098024 0.6993734 0.1332326 0 0.1595429 0.2662663

0.6409386	0.5451017	0.07241037	0	0	0.1572784	1.4424184.385239
0.1103639	0.05832095	0.4574268	0.09497983	0.6328945	0.09187748
0.1983756	0	0.1637339	1.3948660.1889780.1168282	0.8118836
2.8493951.0701870.0746739	0.8657016	3.7390510.09096327	0.1158524
0.06730166	1.2901920.7011417	0.1313007	0.02596183	0	0.01642733
0.1566049	0.1115989	0.6081026	0.2794865	0.2180329	0.7963826
0.4871655	1.6786710.2411027	0.1313007	0.02791392	0.06218918
0.4879969	0.2662663	0.7163862	0.03420696	0.6034435	0.9185972
0.1708749	0.1813428	0.2009189	0.2924148	0.3065851	0.2734108
0.6682679	0.09164721	0.05270034	0	1.1890380	0.1099216	0.2107622
0.1038038	0.09883037	0.7154459	0.4329454	0.1805492	1.331919
0.06097927	0.03821028	1.8189440	0.1276343	0	0.005389423	0.2799755
0.1317264	0.2432410.08367789	0.03469377	0.3361497	0.3424197
0.1998210.05981554	0.0330626	0.1393923	0.1417335	0.1011697
0.1040024	0	0.009458675	0.01606645	2.0606540.02353104	0.1386739	0
0.3470927	0.0499028	0.1967904	0.6286248	0.2759791	0.02232432
0.3194270	0.2642141	0.3539951	0	4.5351943.6153430.1817793
0.0822660.05425406	0.05695831	0	0.2205798	1.15269	0.02706679
0.04490083
TCGA-ZF-A9R0	680	1	0.01905482	0.1426381	0	0.1233778	0.5556006
44.3061	0.1327751	0.1384917	0.4520676	0.3934913	0.07531633
1.2034920.3575392.0132350.1853488	0.2668532	0.1353242	0	0.1245149
0.1362340.1250914	0	2.87205865.804330	0.04097003	0	0.1368088
4.10121	2.5916890.2525209	0.04765385	0.04093899	0.9693178	0.7384937
0.08126236	0.8804801	0.2032287	0.1265891	0.01772367	0.07548617
0.01481345	0.1968651	0.04407787	20.860220.06044683	0.1435311
0.01183001	1.5352120.2483431	6.30245	0.2027747	0.1957952	0.1179165
0	1.49874	0	0.2768207	1.5636420	0	1.1914210.2046950.4700757
0.3303146	0.2284967	0.4864437	0.1846451	0.03379579	0.2246539
0.3229852	0.2231883	0.8907605	7.4859870.07205262	0.07410788
0.8351699	0.2816813	0.04185861	0	0.1411689	0	0.3442004	0
0.1108881	0.02651593	0.3634942	0.04921627	0.4849696	0.09447459
0.1144904	0.2210203	0.05958045	0.2993984	0.3903224	0.3930143
0.4579616	1.1776250.8178946	0.004478656	0.03910236	0	2.301511
6.6918032.1285640.1484601	0.07018113	0.6320406	0.1292810.977857
0.1505278	0.1681726	0.09615564	2.81541	0.1262604	0.5098063
0.0067465	0	0.7287480.51077	0.4256769	0.2363242	0	0.09021614
0.2549957	1.09027	0.1230267	0.1194682	0.06140849	0.9335722
0.08041587	3.6539140.2295113	0	1.2951610.7382441	0.2069897
0.05003654	0.2357743	0.06558188	0.5483711.4265080	0.1248986
0.04732002	0.1743209	0.2880569	1.5678760	0	0.1192924	0
0.1039221	0	0.04886028	0	0.7259033	0.4869698	0.1745461
0.1726501	0.5759091	1.4913490.6014285	1.6553090.1023475	0.8110990
0.1089505	0.0663061	0.3003644	0.03985211	0.09701879	0.08632503

0.0528073 0.1456589 0.0578581 0.3435877 0.1198214 0.2305274
0.2301511 0.2942258 0.3330328 1.019165 0.05938403 0.09701879
0.0412515 0.2757116 0.528856 0 0.8056648 0 0.5329336 0.5351735
1.325735 0.04729242 0.1484601 0.4321338 0.1647545 0.05158078
0.8454768 0.2708745 0.04773361 0 3.794477 0.1783481 0.05414776
0.07786667 0.3370203 0.09736841 0.7636012 0.1968651 0.03557569
0.2277401 0.04505792 0.1694027 2.155216 0.3070424 0.4715486
0.07110456 0.2946883 0.4497293 0.5450667 0 0 0.1025417
0.4643679 0.6854963 0.07382441 0 0.04886028 0.3089931 0.314183
0.3073256 0.1229567 0 0 0.8310111 0.8858929 0 0.01576414 0
0.07543198 0.06807407 0.08812699 0.5534401 0 0 0.612964 0
0.3123669 0.4008459 0 2.005371 3.282922 0.1249467 0.2127538
0.08017725 0.08417363 0.02343429 0 0.2355845 0 0.01327099
TCGA-FD-A43S 455 0 0.02961309 0.01583385 0 0.1643497 1.454246
60.56266 0.5158643 0.2690374 0.5404293 0 0.02926227 2.991375
0.1984469 1.399712 0.1440252 0.5407063 0.3154609 0.04861718
0.06450285 0.07057371 0.07290166 0 1.977442 67.60134 0.06135101
0.4934542 1.431524 0.05315362 7.404726 2.249537 1.618826 0.03702942 0
0.09415102 0.2484696 0.03157245 1.539398 0.1052792 1.10935 0.08263304
0.07373961 0.04604315 0.1147305 0.4452591 14.9331 0 0.02788271
0.5607427 1.670111 0.23157 10.14624 0 0.1141069 0.2356124 0.07893112
1.626039 0 0.254693 2.235651 0 0.09974194 0.6282172 1.399712 0.6664617
0.1776952 0 0.4810794 1.24348 0.03282626 0.253916 0.5097942
0.1300713 0.307629 1.13613 0.08398271 0.783163 0.5500781 0.05051085
0.1626311 0.03348593 0.08775623 0 0.294207 0.02264906 0.02872186
0.2524014 0.188302 0.03824349 0 0 0.04448237 0.00858719 0.09259395
0.1691982 0.1882551 0.2290438 2.609632 1.04261 0.6809409 0.04872195
0.3038448 0 1.538014 3.139541 1.215184 0.07690725 0.05453423 0.6821208
0 0.05332236 0 0.261357 0.03735886 3.57893 0.09811065 0.2475905
0.04193893 0 0.1306785 0.1190681 0.7083202 0.1224238 0.1615052
0.03505122 0.08001983 1.00681 0.01194973 0.6188851 0.0954349
1.636872 0.03124357 4.184192 0.00810644 0 1.064244 0 0.192225 0.07776177
0.03664167 0.05096038 0.03873741 1.956119 0.0415756 0.2911573
0.07354003 0.632128 0.3581352 1.665034 0 0.03348593 0.1338944
0.1636027 0.1211289 0.1582589 0.07593374 0 1.754863 0.5584152
0.7052812 0.03353942 0.074585 0.06816779 0.194725 1.741395 0.03976454
0.9453964 0 0.02418857 0.1545694 0.8321149 0.1548354 0
0.2385026 0.06838983 0.1509123 0 0.192229 0.2327681 0.4776834
0.1430711 0.03810468 0.08752956 1.399712 0.1845774 0.3392479
0.03205447 1.999588 0 0.4586435 0.4708247 0.1571239 0.2153403
0.2992138 3.2213 0.0612476 0.03845362 0.9190028 0.06401121
0.00668014 0.121458 0 0.05466099 0.03696422 0.6332344 0.6929267
0.1262265 0 1.083648 0 1.506212 0.4206784 0.2349747 0.6573
0.0233415 0.372964 7.140694 0.43741 0 0.2762589 0.6343573 0.1607525

0	0.1396608	0.04804503	0.31872	0.05034935	0.2080364	0.6883829	0
0.03796687	0.1867468	0	0.03227125	0.02388587	0.1653203	0.02172341
0.03689926	0.4732632	0	0	0.1679654	0.1172288	0.04848886
0.1129904	0.2150252	0.4225545	0	0.2463639	0	0.06068115
0.4856933	0.08921676	1.02518	12.34231	0.01941797	0.4014922	0.1869051
0.1308142	0.5644979	0	0.7322436	0.02072112	0.07218554
TCGA-XF-A8HG	467	1	0.1155498	0.2824385	0.1795941	0.3817204	0
38.31959	0	0.3239321	0.2108747	0.5681334	0.1468041	0.1677339
0.3097347	0.8261327	0.08920387	0.04974629	0.03907695	0	0.0359556
0.09834914	0.05418309	0	3.558624	116.9259	0.2203915	0	0.07599706	0
0	0.7165437	0	0.2064118	0.03546529	0.8397164	1.727996	0.3695857
0.333706	0.3185779	0.1218485	0.05373882	0.2821257	0.2438239
0.1705435	0	7.470349	0.3141892	0.06217024	0.01024829	0.8644666
0.9896382	6.19476	0.2634945	0.4240416	0.05837181	0	0.9063972	0
0.01324097	0.7043802	0	0.1111976	0.5529235	0	0	0.1012532
0.2969187	0.5746423	0	0.02195787	0.4423109	0.078694	0.9304834
1.543325	9.018318	0.2496757	0	0.02067156	0.1689365	0.01813098
0.335987	0.6603882	0.3765216	0.5367232	0	0.2881858	0.06316929
0.1049645	0.08527175	0.7802365	0	0.1487739	0.2776302	0
0.02021047	0.2973255	0	0	0.2559318	3.365561	0.01163953	0	0
0.0398758	0.6015842	0.7150077	0.2143507	0.1486165	0.3193951
0.1493276	0.8322523	0.3912049	0	0.1665985	3.741346	0.2187579
0.1656162	0	0.277994	0.2428122	0.486726	0.0260741	0.5459381	0
0.1953848	0.2718791	2.039566	0.06661097	0.08624574	0.3191877
2.913574	0.03483199	0	0.4518744	0.1300394	0.683513	0.4476767
0.284279	0	0.3676507	0.3976934	0.6046113	0.6106199	0.09270136	0
0	0	0.2994511	1.49407	0.5651612	0	0.4650416	0	0.630191
0.0588118	0.04232748	0.08717726	1.048079	1.384357	0.06048345	0.2617407
0.2910296	1.899927	0.7622261	0.102953	0.4433162	0.9222307	0
0.6337174	0.02872036	0.3620237	0.2071424	0.168094	0.04154614	0
0.1261838	0.05012226	0.07143569	0.4671039	0.2413103	0.6778886
0.722179	0.09758259	0.8418341	0.05144416	0	0.07147204	0.1592319
0.7913431	0.05681334	1.401657	0	0.7017512	0.4862343	0.05468947
0.1365641	0.2572208	0.3546529	0	0.3872634	0.5108569	0.1759932
0.08705567	0	0.3529816	0	0	0.4047336	0.05235135	0.4217495
0.9159298	1.108533	0.1232763	0.7327928	0.1171005	0	0.1894614
0.08866324	0.1225502	0	0.006899659	0.5532303	0.1349112	0	0
0.04441574	0.7203622	0.8308703	0	0.07657719	0	0.327164	0.09072517
0.2590395	0.07988769	0	0	0.01028431	3.933159	0.1204998	0.2185026
0	0.1045543	0.03440052	0.1908602	0.8047794	0	0.08574027
0.7690467	0	0.3044275	0.8063294	0	1.325792	2.314221	0.2760143	0
0.06945725	0.2187579	0.04060207	0	0.3610752	0.005775252	0.1264627
TCGA-K4-A83P	495	0	0.0536782	0.0717531	0.04866732	0.26067015
1.50336711	39.14375392	0.60780336	0.46816412	0.17143172	0.1847468

0.02652114 5.71108256 0.25180046 0.69648337 0.16679313 0.16652387
0.2223747 0.02517886 1.13998142 0.04797207 0.11012107 0.11031258
1.61632107 40.14887397 0.12356442 0.30296235 0.6178221 0.19269793
6.28633987 1.12620143 0.8892019 0.03356071 0.25948528 0.39821359
0.55226228 0.11445967 0.54257615 0.18401893 0.49528685 0.08113348
0.11391835 0.07302766 0.2426274 0.15521142 3.70167454 0.17028117
0.12635405 0.24161064 1.08118867 0.48971487 5.16125331 0.1785077
1.44785266 0.64062451 0 0.49787862 0.17205172 0.4174163 2.59885722
0.19028921 0.18079735 0.14983402 0.92261434 0.83054298 0.20399633
0.16092111 0.46715845 1.43042001 0.08925385 0.18698108 0.64685535
0.18665436 0.97584209 1.6475256 0.15223137 0.33402384 0.71141387
0.06103904 0.54536798 0 0.23860716 0.35711111 0.43633194 0.06158227
0.10412542 0.1820726 0.25599445 0 0.14637631 0 0 0.10895905
0.0629402 1.06522523 0.27962797 1.1071372 0.59129415 1.34083815
0.74058502 0.1293197 0 0.20683609 3.30655862 0.80028171 1.04016608
0.45306954 0.1372938 0.65531696 0.15174578 0.61617457 0.1060107
0.11843727 0.10157787 1.2031437 0.04446009 0.4487953 0.02375645
0.04519934 0 0.32374345 1.21126166 0.38834532 1.24419866 0.03176781
0.15195508 1.70258764 0.06498208 0.72918487 0.08649509 3.56555192
0.22653477 4.46942621 0.16163562 0 1.13229941 0.20796635 0.18488488
0.28190994 0.232465 0.1385601 0.21065226 1.26465498 0.11304309
0.02932037 0.46655848 0.45014651 0.16229357 0.22083854 0 0.09104747
0.3313842 0.09885154 0.25615855 0.14343408 0.06882069 0.10630682
4.03299513 0.94561916 0.68838473 0.09119291 0.37179063 0.6178221
0.34904657 1.14185481 0.57663396 1.14244927 0.04733562 0.1096136
0.16343858 0.41387213 0.02806625 0 0.16887576 0.08677684 1.72679334
0.04074715 0.19358007 0.54850547 0.37205497 0.06483448 0.13814098
0.16555885 1.10167436 0.37639623 0.34163233 0.08715536 3.5598321
0.27087432 0.41568031 0.46892363 0.0712027 0.25271808 0.46423212
0.78546168 0.09991849 0.24396052 0.46451069 0.14503751 0.06054381
0.265194 0.14307459 0.07431099 0 1.47066117 0.06280172 0 0.05483839
1.12618473 0.30857709 0.20683609 2.0450024 0.11274542 0.51553367
0.0317325 0.05965179 6.684626 0.14415849 0 0.95144603 0.10096406
0.38007165 0.10967678 0.04219273 0.30481109 0.25275606 0.44111807
0.49934341 0.15597476 0.06225383 0.10323103 0.19343248 0.07375551
0.11699306 0.0865935 0 0.01968848 0.20065646 0.46792428 0.12245123
0.02220411 0.20297515 0.11687221 0.06791763 0.35997306 0.59856883
0.02393575 0.11617168 0.35229553 0.3074691 0.05499688 0.55981487
0.24257831 0.8548148 8.25077681 0.09239476 0.3210729 0.05646564
0.11856025 0.34658052 0.02550794 0.29353801 0 0.21496325
TCGA-GD-A2C5 812 0 0.08825991 0.3910174 0.09145236 0.2332542
0.03869899 30.76006 0.08785718 0.09163985 0.4371941 0.1735819
0.1245919 1.431923 0.5069641 1.262043 0.3066128 0.07823012 0.1790878
0 0.3570294 0.1051702 0.1655457 0.103646 2.330672 49.56961 0.05804849

0.01355492 0.05804849 0.1357894 0.5587167 0.9486782 0.2506393
0.06306503 0.1083572 0.6413968 0.2871511 0.2285277 0.8739195
0.2497416 0.1489136 0.02345547 0.09989827 0.1372287 0.3907964
0.1166652 7.4812570.1199929 0.07123082 0.07827905 1.47298 0.3943883
2.5503150.4696156 0.4534513 0.3232476 0 0.9168685 0.06466161
0.1134992 1.0346610.08939468 0.04246778 0.3660255 0.0270893
0.02364698 0.2521949 0 0.5852352 1.1403420.01677198 0.2432508
0.2938637 0.5445831.3753036.9658190.1668701 0.6865197 0.1105262
0.2150634 0.04154671 0.0570301 0.6165150.2396640.2505329 0.07714751
0.1956655 0.1579101 0 0.1302655 0.3667474 0.06251376 0
0.1608739 0.01971217 0.07204061 0.7258447 0.4225930.2020219
0.3991197 1.7009150.05334342 0.0517480.03886725 0.5482468 0.7936911
1.1785150.2292171 0.1186769 0.3717517 0.2851505 0.4994750.1743072
0.07418646 0.1272522 1.5012160.1670929 0.3373384 0.03571323
0.3397422 0.2967458 0.4393689 0.4353083 0.4691266 0.06876514
0.1790878 0.2238923 2.0492820.1933408 0.4743122 0 1.690876
0.07981668 0.7953263 0.3865716 0.04966371 0.9949160.4884955
0.1002182 0.1324366 0.2808210.08679095 0.7587002 1.0771780.07080767
0 0.1252465 0.03844932 0.7624280.3804029 0 0.0570301 0.3552106
0.7430207 1.1346250.04492195 0.2263156 0.06658821 0.2668498
1.0010950.1616958 0.3998484 0.3493221.0448730.3795405 0.747062
0.2708931.3753030 0.1441850.1754988 0.5098392 0.1582207 0.1604931
0.0571212 0.2096553 0.2088285 0.1148540.1364110.8325003 0.4449077
0.1218326 8.0795920.1912012 0.9671354 0.07858873 0.03209863
0.1364804 0.5473144 0.5726350.3905593 0.5022668 0.01672492
0.5736305 0.4145840.7519179 0.1251734 0.0327453 0.2407937
0.2997992 0.1308354 0.3525954 0.1792375 0.07314488 0 1.0279120
0.03582953 0.1030486 0.4890754 0.3865716 0.9716813 0.4559291
0.09416161 0.7857690.08944437 0.07472909 1.3287510 0.3744280
0.2213457 0.1964064 0.1545729 0 0.08182579 0.1017776 0.8432112
0.6365867 0.04884955 0.05849161 0.1939848 0.1817427 0 0.2748069
0.08136034 0 0 0.8169637 6.0084950 0.2294846 0.4290945
0.2894970.07507426 0.3090618 0.4577635 0.06746768 0.0436604
0.9697050.1604931 0.2325295 0.7687448 0.3038913 1.6063113.428837
0.1942913 0.1810016 0.1061064 0.3341857 0.02325967 0 0.4916408
0 0.08781403
TCGA-XF-AAMX 206 1 0.2023658 0.4918325 0.06671814 0.6126057
0.02823246 20.5275 0 0.1470808 0.3693111 0.1266349 0.1090737
0.5754709 0.6903877 0.9548107 0.08947452 0.05218011 0.08710115
0.4314719 0.4007188 0.05480419 0.03019305 0.07561389 5.778589
23.565050.0677579 0.01977771 0 0.06604251 0 1.1978630.1219009
0.1380254 0.1581018 0.4289308 0.2572662 0.1176848 0.9032087
0.3737363 0.08147879 0.04277926 0.08537345 0.1001138 0.2851016 0
10.202130.4668777 0.3117943 0.03426461 0.8893225 0.8631661.248172

0.04894333 0.8979150.09758152 0.09807065 0.4914310.1415197 0.04591013
2.0531270.06521698 0.4957112.7524650 0.01061626 0.1864394 0.2206071
0.8539049 1.48558 0 0.2267560.1169374 0.9292663 1.0033383.33143
0.2086943 0.02861963 0.007679362 0.3347143 0.06061997 0.1664229
0.6814732 0.2098134 0.9969475 0.7035273 0.2498051 0.03200048
0.1169811 0.09503386 1.0033380 0 0.09602501 0.02876162 0.02252421
0.3638507 0.09486107 0 0.2773084 3.3842330.06918427 0 0
0.1777635 0.3047523 2.22283 0.04777801 0.007528656 0.4915640.1664229
1.0434720.1453303 0 0.04641778 11.295040.2438018 0.3691527
0.01302711 0 1.08244 0.2465670.2698348 0.3042192 0 0.08710115
0.08522001 1.4492660.1336261 0.07689549 0.05928817 0.4680023 0
0.04641778 0.08057697 0 1.1354580.2851016 0.2242138 0.04830888
0.09105338 0.1899524 0.2406531 0.4536834 0.5682272 0 0.04568615
0 0.2781108 1.1605340.1049770.04160573 0.4670925 0 1.655508
0.1310894 0.7075984 0 0.5450972 0.8535836 0.06740773 0.2500332
0.8340360.5928817 1.0645480.2212829 0.6916953 0.7830932 0.06489252
0.1502695 0.03200834 0.6052035 0.0384761 0.1873378 0.04630244
0.01699465 0.3046975 0.1117207 0.1326897 0.4048948 0.7604398
0.3555271.0889220.3168054 0.6712391 0 0 0.1194815 0.3549224
1.0211910.06331746 0.7135619 0.02440299 0.9364491.1342080.2844354
0.1521983 0.5255581 0.4611302 0.3976645 0.1493994 0.672234
0.06538043 0.07034096 0 0.9343055 0.08609503 0 0.1503562
0.3545560.7050485 0.6805250.04751693 0.3434733 0.9894449 0.2320115
0.1090357 0.2879337 0 0.3186868 0 0.06920594 0.7294576
0.1127671 0 0 0 0.4379078 2.03377 0.1425508 0.1991358 0
0.06629427 0.1011116 0.2004826 0.5045227 0 0 0.2865421
0.5880220.1007212 0.4413752 0 0.3932682 0.1095393 0.1829309
0.1717492 0 0.06370401 0.4693582 0 0.1507907 1.0626250.1478005
1.2737691.0875970.1809489 0.1173759 0.1548178 0.1625345 0.01131257
0 0.8923048 0.09010982 0.05125107
TCGA-DK-A1AE 491 0 0.1358974 0.2634037 0.0924085 0.2356929
0.0782072 90.949490 0.2160619 0.1705058 0.7015869 0.04196484
0.3858383 0.1138366 0.6769778 0.05048894 0.07076681 0 0.2111568
0.2775094 0.09108838 0.02787942 0.2792791.6717528 8.0489 0.152504
0.7122250.3519324 0.06098181 0.5914419 0.5161675 0.02813997
0.08496584 0.1459868 0.5580884 0.2409460.2445004 0.3188815
0.1380391 0.1912229 0.05530164 0.1788128 0.0660302 0.0219379
1.5521516.00365 0.2424948 0.07997283 0.01054633 0.2737252 0.3320931
10.138640.3841397 2.0291370.09761272 0 0.3025157 0 0.05223317
0.5854678 0 0.2002550.4741696 0 0.05514049 0.08607645 0.2546281
0.3942359 0.2743487 0.01129823 0.1411040.1664642 0.4290293
2.8455390.1042763 0.1445269 0.08588634 0.04963636 0.2221410.02798739
0 0.2391163 0.1937358 0.1227405 0 0.2141871 0.2275223
0.05400856 0.08775161 0.4323454 0 0.05103351 0.08866681 0.02655768

0.02599779	0.1679848	0.06569404	0.06804468	3.672615 1.640576 0.09382789
0	0.02618241	0.2667305	1.013039 0.3291743	0	0.1633661	0.2191224
0.1728791	0.7876334	0.05032274	0.04997472	0.08572177	1.949444
0.1125599	0.1988381	0.009021654	0.1716471	0.3498231	0.06830194
3.258218 0.5618150	0	0.1398929	1.535422 0.06854814	0.9230409
0.1094901	0.8882882	0.1254574	0.535761 0.0325511	0.0669106
1.074993 0.1974411	0.4410699	0	0.1891713	0.05846557	0.5555308
0.5460902	0.02384932	0	0.08437064	0.02590088	0.3338397	0.3727322
0.1211661	0.4610108	0.5996844	0.06256576	0.3242591	0.2420886	0
0.04485627	0.3954716	1.036851 0	0.1731552	0.1925314	0.7038648
0.4021255	0.234597 0.7755547	1.875505 0.1497999	0.1248792	0.0147778
0.4540478	0.05329163	0.04324563	0.1752929	0.04707717	0.3354548
0.02578995	0.07963927	0.5608028	0.6165385	0.225695 0.5245985
0.07858994	0.4261149	0.3970519	0.1081141	0.1471012	0.08193135
0.4929002	0.02923279	0.6054601	0	0.4925254	0.6661969	0.1406999
0.2037767	0.198526 0.1824835	0.238675 0.2490786	0.1963509	0.0905557
0.1153438	0	0.06810881	0	0	0.03470867	1.014278 0.6727228
0.2094593	0.8336403	0.1585768	0.6163349	0.08703213	0.1006805
0.3509483	0.04562086	0.1471333	0	0.1668574	0.1764088	0.1214803
0.1068196	0.02756043	0.04570743	0.6594786	1.43205	0	0.0788042
0.08711688	0.1377321	0	0.07404801	0.3288439	0	9.464406 0.0052917
2.048452 0.1705058	0.06324106	0.7708101	0.3362342	0.03540095
0.3928215	0.4405233	0.03029914	0.04411688	0.336036 0.583816 0.03480898
4.924163 0.05117808	0.7056977	2.890825 0.2060692	0.0135477	0.0714772
0.2626397	0.01566857	0.04843395	0.4119647	2.09498	0.2543654
TCGA-FJ-A3ZE	324	1	0.1999295	0.3207014	0.0453165	0.1271404
0.03835227	112.6401 0	0.4540941	0.4788852	0.3440534	0.7778964
0.2589223	0.9378545	1.204412 0.1350515	0.07309898	0	0.02930657	0
0.1861213	0.1230469	0.1027174	7.722834 129.2137 0.1208097	0.1612017
0.2301136	0.0448576	0	0.958259 0	0.09375	0	0.8210482	0.1148299
0.06661185	2.453921 0.3109666	0.07840155	0.01162266	0.08344559
0.3011411	1.097336 0	16.67753 0.2378356	0.3059014	0	1.459783
0.1954274	10.85697 0.2991909	1.123472 0.2098855	0.3996711	2.679602
0.1602057	0.01447792	0.65625	0	0.3366984	0.613878 0	0	0.1566262
0.07492072	0.2899959	0.04036162	0.07202744	0.3013393	0.3640386
0.3567534	2.304087 11.42898 0.378	0.009719556	0.005216 0.2841821
0.233322 0.1130383	0.2592085	0.04750335	0.1128583	0	0.07271717
0.08694186	0.1589126	0.06454918	0.4997596	0	0	0.1014571
0.9181712	0.005099655	0.2074175	0.1288636	0.05005297	0.3363468
1.647365 0.002936972	0	0	0.03018527	2.069948 0.9115756	0.03245192
0.05625	0.5641448	0.05651914	1.1925	0.07403378	0.07352179	0.1261121
2.957606 0.2069948	0.6268426	0.01769663	0	0.6249352	0.3684428
0.03101627	0.7748725	0	0.08874166	0.3312217	1.802961 0.07059263
0.3917189	0.4026989	0.6475291	0.02636719	0.03152803	0.2257601

0.04921875 1.2398240.8230021 0.2847183 0.0328125 0.4638416
0.2580401 3.3999081.4747280.03508664 0 0 0 0.6044776 1.405162
0.2139085 0.02825957 0.5475994 0.2301136 1.0222360 0.2883703
0.03299581 0.3702426 1.2091540.4807413 0.1981331.1329931.955966
0.5952507 0.07793356 0.6040483 1.1967640.1322295 0.2653730.0434816
1.2931520 0.1272442 0.02515975 0.0577158 0.01591981 0.0758833
0.1982770.4714524 1.5495250.3622232 0.1607872 0.1862765 0.4248355
0 0 0.08115458 0.06026786 1.4502890.2150334 2.5455970 1.092715
1.0014950.1793955 0.08270129 0.06490385 0.07457387 0.4321647
0.3889894 0.4193236 0.4440790.1614540 1.1022030 0.1420341
0.2553134 0.1112661 0.03192568 1.6563184.4538940.7115496 0.7893965
0.1969844 0.1110893 0.2060017 0.1006747 0.2473822 0 0.002611459
0.3008177 0.2297821 0.07857539 0.08109268 0.1344877 1.260567
3.4650340.04841189 0.4057729 0.03204114 0.1125715 0.1373547
0.08715033 0.3225256 0 0.0183329 0.1712709 1.5612830.1368243
0.1654026 0.14175 0.2473304 0.02976066 0.4796661 0.7388214
0.1114387 0.02163462 0.2310137 0 0.1536308 0.8286869 0.05019476
1.4880982.3216950.2580997 0.0199311 0 0.5519860.00768376 0.02375168
1.8538730.03497409 0.4351363
TCGA-DK-A2I1 546 0 0 0.1064515 0 0.1956646 0.4773902 53.53087
0.1734088 0.2351373 0.2270829 0 0 1.7007190.03335414 1.568386
0.08069059 0.1455840.02945633 0.09338719 0.2439310.09637666 0.1633735
0.05114302 3.06491433.048520.1260310.1070165 0.1718605 0.1786769
0.4726163 2.4666070.9069522 0 0.08020156 0.0263742 0.4872205
0.01326642 0.8624526 0.2148666 0.1974770.09837777 0.08168677
0.06771403 0.1285562 0.05756719 10.2255 0.0394728 0.1405924
0.05407636 0.2005039 0.1297378 4.8226460.03310383 0.4155370.2420047
0 0.4985842 0.2871593 0.1064649 2.3689160.04411086 0.04191055
0.5279410.0534677 0.1148884 0.09955431 0.0746061 0.4042893
0.9244188 0.05517305 0.1000246 0.08568414 0.09109108 0.3554721
1.26031 0.1176289 0.2322898 0.05194095 0.2122415 0.02733438 0
0.8296713 0 0.4270605 0 0.09654907 0.1471805 0 0.03213906
0.09048381 0 0.1121462 0.0288660 0.07617359 0.1889680.06416124
0.2990566 0.8038425 1.3352480.3626551 0 0 0.6612871 1.731454
0.8510133 0 0.07129027 0.4585924 0 0.4257048 0 0 0.09418688
3.24863 0.1649004 0.08322803 0.01762231 0.1257317 0.4392782
0.5670205 0.5840266 0.1028825 0.1357257 0 0.04162912 1.1453350
0.3770714 0 0.8519306 0 0.8162863 0.1566872 0 1.52626
0.3374601 0.01648388 0.03267471 0.1231717 0 0.2929872 1.435658
0.03493929 0.02718697 0.03090077 0.03794482 0.1128636 0.6143098 0
0 0.2336999 0 0.1696571 0 0.03190659 0 0.5266968 0.1914172
0.02279631 0.02818585 0.06267972 0.1718605 0.3309223 0.9091021 0
1.0262190 0.05081896 0.1298970.1876150.3643375 0 0.1064799
0.06896811 0.3012062 0.03778232 0.1256464 0.2738590.3449836

0.09017551 0.3202240.1151344 1.1401750.3490090.06335491 0.02693793
0.4201034 0.06279125 0.5567391.0783140.0495164 0.3271338 0.2557151
0.9344356 0.04117699 0.1292626 0.2673385 0.3227624 0.05613854
0.1577459 0 0.05577942 0 0.6651967 0.05823215 0.1767970.05084825
0.8954978 0.2861245 0.4602872 0.1928343 0.1045419 0.3611726
0.09807861 0.1659343 1.07502 0.03341732 0.03079292 0 0.2080394
0.1468404 0.02542412 0.03912271 0.1615043 0.06696145 0.01410419
0.3477380.09641717 0.03848275 0 0.1120987 0 0.06508826 0.1605856
0.06946591 0 0.2015610.3254080 0.01029425 0.4705158 0.1477751
0.06297582 0.06905809 0.02581469 0.2441356 0.04308753 0.1932366
0.03167745 0 0.62556 0.3248958 1.3095414.0669210.05303502 0.3374059
0.0523571 0 0.05356045 0 0.3431830 0.1559912
TCGA-DK-A6B0 2330 0 0 0.05627703 0 0.07301697 0.04038059
72.261210.04583743 0.2008060.3361412 0 0.1170052 0.02883437
0.1763311 0.9754685 0.03554847 0.04198088 0.03114497 0 0.05731439
0.0548701 0.1295544 0 1.881935182.60210.0545138 0.07071962
0.1211418 0.04722996 0 1.27545 0.0435884 0 0 0.1394308
0.08410633 0.04208083 0.3039647 0.2138205 0.09711523 0 0.06552177
0.03068394 0.2378704 0.03043366 13.495710 0.1982028 0.01633611
0.9539916 0.06858763 9.8802280 0.4393584 0.02326164 0.07013472
0.9957588 0.3036212 0.01289844 0.7773264 0 0.1329394 0.5875853
0 0.00189804 0.1543834 0.1183245 0.4885325 0 0.02333438
0.1551128 0.04878253 0.1733635 0.2391774 3.4727310.22387 0
0.01647557 0.1047240.01445069 0.02975412 0.2924112 0 0.1663578 0
0.05104196 0.1006939 0.08365845 0 0.04783547 0 0.03952507
0.03815099 0 0.002684679 0.1440426 0.0678394 0.0527001 0.1728179
0.6655595 0.003092298 0.1349916 0 0 0.5230608 1.1097520.1025046
0.06999303 0.4242717 0.02975412 0.3908842 0 0 0 3.444299
0.0435884 0.4399961 0.01397441 0 0.3096404 0.2468636 0.01484392
0.2175608 0 0.03114497 0.07787372 1.3310010.05309003 1.2922980
0.7313583 0 0.06639087 0.04321815 0 1.7679360.1529167 0.1394308
0.03454784 0.1953491 0 0.8260886 1.9466960 0 0 0 0.1591116
1.0103740 0.1190165 0.2516730 0.07175321 0 0.1012071 0
0.2784453 0.6724588 0.02410315 0.1192066 0.3976377 0.545138
0.3037533 0.1523882 0.1413321 0.3500157 0 0.0214929 0.06867178
0.4508418 0.05503196 0 0.05960331 0.03646091 0.05028527 0
0.1518278 0.6618478 0.4775045 0 0.1354326 0.04395973 0.9655213
0.3690165 0.1674671 0.02848219 0.1903657 0.1327817 0.1811246
0.3769784 0 0.2722942 0.4956864 0.01452947 0.04353754 0
0.04711069 0.02843877 0.0534211 0.2403723 0.04675648 0.1006079 0
0.1230828 0.06157041 0.07477288 0 0.2856549 0.1680706 1.095016
0.1699074 0.2210698 0.4604992 0.03111034 0.1949408 0.2004232
0.1766651 0.03255818 0 0.03024527 0.2422029 0.02688161 0
0.08538139 0.07080013 0.1491274 0.2925131 0.05097223 0.08137769 0

0.28446 0.07230944 0.0458797 0.02122393 0.1468963 0 0 0.2676042
0.07203025 0.01088438 0.09949779 0.08333148 0.05091872 0.2099238
0.2047092 0.3050640 0.2870128 0 0.0269593 0.5019297 0.1056988
1.3117386.1667590.05607534 0 0 0.1162357 0.01618026 0 0.675664
0.05063266 0.0549779
TCGA-FD-A5BR 813 0 0.08475262 0.1268861 0.06147275 0.1724685
0.2341154 47.4073 0.1181123 0.2587149 0.4021441 0.1166789
0.05024915 1.3272570.3180546 2.0108420.08244001 0.1502428 0.1605065
0.2385297 0.3692142 0.05049547 0.1947348 0.06966911 2.70427264.6737
0.03901923 0.2004506 0.6243076 0.06085024 0.9657259 0.9565285
0.3369511 0.08478252 0 0.6826323 0.4131267 0.1265044 0.4405756
0.3013089 0.2877795 0.04729914 0.1247070.2503734 0.1313434
0.03921019 11.129060.2688573 0.2872810.1157593 0.4097019 0.2209177
4.6936020.04509539 0.6096047 0.2847139 0.2710809 0.9181667 0
0.1042409 1.50224 0 0 0.5677758 0.1092538 0.2029683 0.1175347
0.05081574 0.4720624 0.5475135 0.05261129 0.1635091 0.2244662
0.1985408 0.6163037 5.4626920.3204779 0.1186630.2901 0.1734744
0.07447202 0 0.3265057 0.1288785 0.2449513 0 0.06576154
0.1945983 0 0.08756227 0.4930430 0.0509234 0.05898367 0.05300076
0.2870881 0.3591907 0 0.06789786 0.7336681 1.3771890.003984062
0.06956829 0 1.1055671.1793290.6569282 0.1320651 0.4439521
0.2655032 0.07666936 0.6256950.03347611 0.0498669 0.1283052
2.5288090 0.3401299 0.04201021 0.1141845 0.0498669 0.2726183
0.4475165 0 0.09244555 0.04012662 0.1526778 2.2630240.0273601
0.7616369 0 2.2039510.07153525 1.6251990.1392037 0.06676623
0.9534880.3940302 0.1886221 0.04451082 0.2516842 0.2916971
0.9312781 0.6270220 0.0740704 0.0841886 0.2584499 0.2562457
1.6736760 0.1533387 0.1886551 0.2497231 0.3235594 0.06039157
0.2173223 0.2237974 1.4349760.4920724 0.3726487 0 0.2561546
0.5462692 0.7876544 0.6569646 0.1820897 0.8117171 0 0.2492196
0.1769510.3717482 0.1772555 0.04315232 0.1365188 0.2035609
0.4103154 0.05146862 0.3300955 0.2131782 0.2819704 0.1637877
0.08724444 0.1611974 1.0471760.2641302 0.2157616 0.03669595
0.4087729 0.3849157 0.2916971 1.1905380.02248442 0.6605511
1.0276191.0295730.04206974 0.2201085 0.4046438 0.10992 0.07647421
0.2148879 0.1204804 0.09162897 0 0.4304251 0.07932622 0.04816802
0.2078027 0.4652099 0.4330783 0.6270220.7442793 0.03164693
0.6077697 0.06680335 0.02511582 4.1739952.9134360.1258421 0
0.3329947 0.3920627 0.06926756 0.05329455 0 0.04560881 0.2881996
0.3873372 0.2626868 0.2096911 0.1303934 0.09162329 0 0.1994982
0.2187563 0.2838879 0 0.01056056 0.6402991 0.06186832 0.04206974
0.7691470.1476238 0.1362519 0.1763883 0.5626533 0.1511688
0.08804339 0.5390046 0.8630465 0.06946776 0.4774533 0.06809021
1.03279 4.7384540.06113163 0.2703695 0.07132298 0.4492681 0.1563476

0	0.2901712	0.1067469	0.1652759
TCGA-KQ-A41Q	361	0	0.1767637	0.4983463	0.2331095	0.2972792
0.04932133	15.64473	0	0.2102287	0.2932619	0.2212277	0.03175812
0.2209169	0.516895	3.544559	0.442877	0.03418395	0.07608165	0.271357
0.03500223	0.1053155	0.3692249	0.1320954	3.268177	65.24042	0.110973	0
0	0.4038106	0	0.7440475	0.1597181	0.1205632	0.1035748
0.579028	0.1605132	0.1199287	0.371266	0.4243895	0.20165	0.1943089
0.09821747	0.1499109	0.1660215	0	4.571143	0.1529292	0.03026089
0.01995311	3.107244	1.130945	4.081664	0.1710053	0.5366375	0.09944213	0
0.9181355	0	0.05155954	2.901053	0.05696613	0.1082492	0.2511892	0
0.01622802	0.2871342	0.04817432	0.3356433	0.05190536	0.02137566
0.1205632	0.1957745	0.6234807	0.9598689	11.14662	0.06076388
0.0499977	0	0.2375492	0.03530047	0.03634203	0.8095501	0.1221794
0.1161093	0	0.1558581	0.07267515	0	0.04150538	0.8179752	0
0.04827638	0.08387652	0	0.003279098	0.2894419	0.2900094	0
0.1522557	0.9114581	0.0491006	0.09892816	0	0.1164555	0.6388725
1.904977	0.1252003	0.1775568	0.3405383	0.07268406	1.14294	0.2538881
0.0472748	0.4865435	2.31668	0.2661969	0.4836747	0.005689501
0.270623	0.472748	0.516895	0.1450443	0.5978953	0	0.1521633	0.3101604
1.159311	0.03890686	0.8563811	0.6732361	0.6813225	0.1695421
0.1216359	0.4222972	0	1.029471	0.3112903	0.1404994	0.1687885
0.1590677	0.05530693	0.2102071	0.7076538	0.09024338	0.0351101	0
0	0.3886815	0.1322231	0.22924	0	0.8048195	0	0.1752804
0.1145048	0.1236155	0.2545972	0.2040578	0.4704815	0.02943986
0.1820004	1.902244	2.145478	0.295867	0.2076051	0.3884055	0.9832803
0.2267309	0.2100134	0.02795884	0.2202654	0.20165	0.163637	0.03235563
0	0.06141901	0.04879326	0.2433955	0.4547186	0.137707	0.4270034
0.1654189	0.2271624	1.692327	0.3505608	0	0	0	0.4459982
0.05530693	0.9714284	0.02131567	0.7250916	0.3687663	0.2484504
0.5051839	0.08346686	0.9590258	0.5210317	0.2392469	0.7968967
0.05710891	0.1970377	0	1.07382	0.1504056	0.1369927	0.3283351
0.1685708	0.3284534	0.7925723	0.5395699	0.1500096	0.6859257
0.2279912	0.0238103	0.03353415	0.04315616	0.1193008	0	0.03022521
0.5916589	0.2955016	0.05052429	0.05214291	0.1729522	0.7559055
1.260401	0.06225807	0.1242446	0	0.1737214	0	0.2101425	0.07776947
0.2691313	0	0	2.941694	0.05865239	0.3057692	0	0.1653958
0.06697677	0.1672196	0.5667443	0.2866221	0.08346686	0.8793729
0.04090925	0.2634272	0.4812829	0	1.557668	5.577863	0.08956525
0.1025262	0.1352312	0.2129575	0.009881376	0.06108969	0.1069787	0
0.246219
TCGA-DK-A1AF	536	0	0.05071222	0.151846	0	0.1594871	0.1089544
27.74447	0.1766829	0.4054378	0.1480771	0.6283392	0.02004458
3.831424	0.2446841	0.3007997	0.1096188	0.1384439	0.09603997	0
2.231306	0.0241714	0.1331665	0	0.8388536	21.773	0.08872004

0.2616889	0.1400843	0.1092303	0.5136423	0.3081854	1.176097
0.07609516	0.8280537	0.4299547	0.1904630.07569466	0.3222033
0.1493831	0.1347609	0.08962208	0.1446464	0.08673346	0.02619669
0.32846311.9379980.03217446	0.07639833	0.2770610.3268633	0.4758745
2.9116640.1349154	2.1364540.7262723	0.05406761	0.2634063	0.05201441
0.1120909	0.2330414	0.1438199	0.06832297	0.09059518	0.2832815
0.1419323	0.04057359	0.1824353	0.1176922	3.5709260.04047463
0.1521903	0.1772901	0.04454926	1.3960532.0545690.1150559	0.2682323
0.06350597	0.1268659	0.3676263	0.04587555	0.1502820.1927880.2381730
0.3147904	0.3422602	0.1289864	0.02619669	0.1106307	0.2514333
0.06094061	0.1117618	0.07928327	0.4429047	0.3940270.1307453
0.2031354	0.8932774	0.8395970.1382654	0.1873196	0.03126518
0.5390182	0.43688360.1618551	0.2897470.1950803	0.1308303	0.1146889
0.5844108	0.1402143	0.08951443	0	0.8220356	0	0.2713582
0.1687774	0.1024845	0.08951443	0.05437423	2.0071610.1677199
0.05531533	0.09603997	0.1252877	0.9167464	0.05729875	0.7842792	0
0.9301306	0.06420529	0.5885866	0.5386303	0.03994996	0.6973084
0.8251959	0.1236120.3728663	0.1756962	0.1745387	0.2653503
0.5717062	0	0.04432045	0.3274357	0.4330060.09199564	0.1947271
0.0289376	0.0917511	0.2857348	0	0.3318920.2168138	0.05201441	0
1.6743210.7776086	0.1858138	0.1378465	0.2299074	0.3268633
0.4535127	0.7048664	0.4085791	0.8364757	0.03577608	0.2568212
0.2999923	0.1668282	0.04242473	0.1032818	0.2961147	0.08432434
1.5118530	0.1609378	0.5421147	0.2198455	0.02450083	0.07830482
0.02867534	0.2985734	0.6005664	0.1032818	0.5708881.6632230.2559072
0.3490773	0.7478065	0.05381472	0.2515193	0.4689778	3.584295
0.1510360.1053625	0.5689843	0.3946276	0.07778993	0.2117778	0
0.05616396	0.05064079	0.3524332	0.0474653	0.2305729	0.08289329
1.6961460.6737507	0.3439171.0740640.1041487	0.4069556	0.0479666
0.2705076	9.2937890.3541019	0.0501989	0.1892366	0.2077280.2728939
0.1450632	0.2232237	0.2303750.1910321	0.7185244	0.5747156
0.5501306	0.3136747	0.07802162	0.05482332	0.1114883	0.03094802
0.06544703	0.05662199	0.02976096	0.09478461	1.3556790.1665867
0.03356356	0.2301118	0.06424114	0.1690933	0.3049018	0.2525001
0.03618109	0.2282855	0.2025103	1.0069970.02078322	0.5677575
0.3259373	0.3932574	7.9405	0.02660252	0.2750211	0.08535303
0.1792148	0.1184986	0.0963940.05787519	0.01774239	0.04238306
TCGA-LT-A5Z6	474	0	0.7761814	0.2075085	0.1919249	0.7505894	0
35.285310	0.07692734	0.9014129	0.2428564	0.6798287	0.08786781
0.7565734	1.7657090.4671098	0.02501734	0.3758395	0.01654926	0
0.06306103	0.02895163	0.07250495	7.09566460.575580.2517664	0.01896452
0	0.8865795	0	3.9818540.1168888	0.7058683	0.07580063	1.121713
0.5850040.3009228	0.5094543	0.8242504	0.1823003	0	0.1078199
0.1508532	1.4580230	24.127180.8394030.1328776	0.1752309	0.3553155

5.195966 7.914184 0.5162407 4.078403 0.04678468 0.6582686 0.8508234 0
0.02515571 1.389678 0 0.2376647 0.8272415 0 0.005089879 0.2775697
0.5288416 1.228194 0.1709399 0.007821829 0.557762 0.1308177 0.7231779
4.810425 8.175639 0.3335228 0.06860726 0.01472723 0.2808331 0.116255
0.03989507 0.9932496 0.8718089 0.6373047 0 0.3079725 0.2270671
0.1121713 1.549149 0.513112 0 0.1059924 0.3683074 0.2482115
0.007199369 0.7227019 0.2274019 0 0.2507119 2.731281 0.008292461 0
0.2718936 0.08522728 2.863776 3.378125 0.04581357 0.1443822 0.4876064
0.1595803 2.620536 0.1741936 0 0.1780371 3.631299 0.1168888 1.12092
0.03122872 0.1188323 0.3113802 1.182146 0.05572866 2.260759 0
0.6263992 0.2587677 3.540022 0.498291 0.2765019 1.19386 0.9917039 0
0.5341112 0.2317919 0.06948391 0.5788412 0.7517931 0.5094448 0
0.698477 0.5464269 0.3692134 1.336163 0 0 0 0 2.293414 3.338453
0.2013216 0.03989507 0.4478875 0.06497197 0.384834 0.5656479
0.5880374 0 1.568055 4.245631 0.2262267 0.1198764 0.6664531
3.492243 0.8609589 0.0707282 3.316278 2.862789 0.933366 0.273773 0.1227691
2.6598 0.1106823 0.5838146 0.03551893 0.0488877 0.2921695 0
0.6107233 0.4991749 0.5068645 0.2556819 0.5901719 0.5531509
1.433563 0.2199051 0.08981762 0 0.08508234 1.023713 0.4249987
0.7150439 0.04679927 0.9867538 0.5558713 0.3506665 0.554574
0.09162714 1.537068 0.3431827 0.5252745 0.6182844 0.501538 0.2395596
0 1.579597 0.08255514 0.05012867 0.5046094 0.1570785 0.4957771
0.761302 4.647448 1.531482 1.611391 0.2502811 0.2875196 0.0441752 0
0.7857866 0 0.02580685 0.3580596 0 0.1663916 0.1144815
0.1423959 1.359685 2.108123 0 0.2182265 0.0904673 0.4767638 0
0.1922395 0.2561182 0 0 0 10.09795 0.03219332 0.1167524
0.2668182 0.6145311 0.1470497 0.3834533 1.646875 0.1573221
0.06108476 0.8479393 0 0.2168862 1.912074 0.1062927 1.709956
3.813959 0.4222051 0.0844124 0 0.8571847 0.07593211 0.03353111
1.224706 0 0.1474315
TCGA-GC-A4ZW 15 0 0.06725237 0.08989806 0 0.1088629 0 24.2555 0
0.09775896 0.1227334 0.3471985 0 0.08039668 0 0.3116466
0.2180576 0.0447074 0.1592051 0.1340709 0.1098662 0.02003442
0.02759371 0.06910425 2.469582 14.09053 0.1780332 0.03615006 0
0.06035687 0 1.848891 0.1671096 0 0 0.249457 0.1007649 0.07170215
0.3398916 0.09392931 0.08687494 0.04691564 0.1141809 0 0.1737047
0 7.125719 0.106671 0.4432583 0.01043825 0.4063801 0.08765061 1.90357
0.04472977 0.08638031 0.07431722 0 0.3493182 0 0.004495468
2.483434 0 0.05662937 0.2628138 0 0.002425574 0.08519435 0
0.6243133 0 0 0.0991121 0.008905852 0.1107737 1.178949 0.7740573
0.03178795 0.07846725 0 0.07647463 0.03693411 0.03802387 0.1743853
0 0.3644479 0.2314657 0.06522836 0.0701893 0.1069102 0.08685233
0.183392 0 0 0.05850544 0 0.01372339 0.2968987 0 0.3367368
0.2570552 0.8247685 0 0.03450212 0 0.04061493 1.281173 0.3449657

0.04366477 0.2476983 0.3562977 0.07604774 0.7265818 0.06640937
0.8903265 0 3.967465 0 0.7872017 0.0059528 0 0.6924762
0.135204 0.01896958 0.2085216 0 0 0.06922966 2.258618 0.01356913
3.513775 0.3792881 0.1372902 0.03547763 0.04242165 0.01841001 0
1.983463 0.06513925 0.1024556 0 0.2912509 0.05786642 0.3958832
0.4738577 0 0 0.041753 0 0.1525008 0.1383422 0 0 0.4151848
0.2476983 0 0 0.1724482 0 0.6405036 0.8134713 0.03080228
0.3427615 1.058657 0.5418401 0.1081 0.007490093 0.4063801 0.8051358
0.05930588 0.06866638 0.08775816 0.6337618 0.210982 0 0.04231623
0.1242526 0.06426136 0 0.3516724 0.3700372 0.4915663 0.08122987
0.08653708 0.06914182 1.345411 0.4715795 0.04280245 0.03639842 0
0.2545299 0 0.7872571 0 0.2915463 0.1957954 0.05570319
0.08345729 0.08732954 0.02006815 0.07268587 0.03034167 0.1441867
0.1195036 0.2039395 0 0.2921136 0.3147322 0 0.06870595
0.06357591 0.7732205 0.3627973 0.521114 0.1412565 0.3301278
0.02650469 0.02491219 0.185956 0 0.04160727 0 0.02108265 0.1031733
0.06870595 0 0.3273359 0.1809561 0.2286903 0.3348742 0
0.05199774 0 0.2726413 0 0.0439735 0.1084913 0 0.03700098
0.01047494 0.9770888 0 0.05563819 0.6357591 0.09318077 0.0100109
0.1166388 0.3662475 0 0 0.2735339 0.0856049 0 0.2877461 0
1.117545 4.714235 0.09371015 0.0804532 0 0.1485418 0.05169333 0
0.7355362 0.04117611 0.08196796
TCGA-K4-A4AB 76 0 0.2308706 0.08229626 0.05581833 0.09965739
0.3543014 21.10493 0.160872 0.2349177 0.05617753 0 0.01520904
1.355436 0.1237711 0.4279406 0.1330791 0.4065409 0.1457427
0.7508424 0.6034554 0.07336124 0.02526038 0.1265216 1.116465
21.60257 0.04251617 0.2647456 0.7086029 0.05525308 1.558926 1.291682
0.4589363 0.03849201 0.06613627 0.4567258 0.1844883 0.08204875
0.1333501 0.1876072 0.09657066 0.06442267 0.1271729 0.0837581
0.07950808 0.1780177 7.849913 0 0.1159361 0.01911118 0.3720165
0.4011943 3.198037 0.1637896 0.6721458 0.5986899 0 0.1142071
0.4735979 0.09053727 0.4546868 0.05456242 0 0.03437003 0.1322725
0.1931806 0.09030455 0.09228316 0.7858417 0.5468671 0.0204737
0.1237243 0.3424164 0.2140807 0.7195045 0.1417206 0.2036997
0.1316921 0.1092212 0.2275257 0.08452738 0 0.1368337 0.3510719
0.4170376 0.02354366 0.1492816 0.2141803 0.3914793 0.03975404
0.05596146 0.07631108 0 0.0267791 0.09625124 0.1319109 0.3750742
0.158727 0.3082623 1.246194 1.415674 0.1410863 0.03158461 0 2.528276
1.427802 0.8070326 0.07994494 0.1826621 0.6523382 0.03480856
0.568142 0.1215876 0.04528002 0.03883446 1.269535 0.1019858 0.6691618
0.01089886 0 0.1811201 0.1650281 1.50038 0.2545186 0 0
0.150517 0.8551303 0.03726517 0.5629137 0 0.9571083 0.03247763
3.495102 0.04213314 0 1.046156 0.05963106 0.08155818 0 0.1904448
0.1589197 0.4832096 0.6777941 0 0.1008858 0.07644472 0

0.04653511	0.5065757	0	0.1044257	0.7440938	1.700647	0.08394219
0.2193464	0.07893298	0	0.8469391	1.168581	0.2819763	0.1045925
0.6202478	0.6377426	0.3688474	0.8570911	0.248011	0.2866319
0.1085819	0.1508638	0.0535582	0.1054856	0.03219022	0	0.07747593
1.080585	0.4117919	0.04673441	0.03330361	0.241962	0.3801719	0.07436105
0.07921949	0.1265904	0.6253938	0.1439009	0.1175492	0.06664113
0.890815	0.07766893	0.1059464	0.8013604	0	0.1521004	0.2003258
0.8328843	0.1400669	0	0.1102271	0.1996186	0.04166396	0.1779053
0.1093983	0.0710250	0.329123	0.360148	0	0.06289616	0.878631	0.5112155
0.1897823	0.2782783	0.05747194	0.5518655	0	0.09122244	1.728011
0.1653407	0.1523558	0	0.4342477	0.2106938	0	0	0.09988544
0.0414136	0.2442443	0.161596	0.1192621	0.2142036	0.07893298
0.08319556	0	0.08051018	0.04965863	0.08592507	0	0.06712424
0.4919563	0.02808876	0.02546671	0.1163998	0.0974873	0.07789722
0.3167681	0.1756215	0	0	0.1650389	1.91997	0	1.300608	0.03091355
3.410151	9.515114	0.06560106	0.3682503	0.06476252	0	0.06625099
0.117024	0.4830476	0.08615828	0.01071953
TCGA-DK-AA74	1708	0	0.02600026	0	0	0.04809963	0.3990083
53.60131	0.09058567	0.09448582	0.521946	0	0	1.839015	0	0.9638789	0
0.4102119	0.1846496	0.02439191	0.2265337	0.06196364	0	0.5343242
0.7696822	26.13582	0.09576199	0.3354211	0.239405	0.09333764	0
0.8276572	1.808962	0	0.1117223	0.330658	0.05194191	0	0.6007066
0.02641001	0.4414224	0.2176552	0.04708604	0	0.0671555	0.3007208
0.4108937	0	0.09792395	0.2421303	0.4189587	0.06777273	3.5832	0
0.133581	0.4597053	0	0.5209031	0.06666974	0.1436731	0.2560303	0
0	0.05806042	0.2234446	0.1012764	0.09014271	0	0.6034103
0.6718609	0.08069999	0.05573449	0.04131681	0.07613499	0.135049
0.5586116	0	0.2224641	0.0759725	0.02956565	0.14279	0	0.1155748
0	0.4696607	0	0	0.1628142	0	0	0.09453427	0	0	0.01507909
0.0406487	0.07958347	0.0642786	0.06703339	0.4165917	0.5542852
0.8370116	0.3483337	0.1600653	0	0.06280812	1.205975	0.1778217
0.135049	0.05320111	0.1197803	0	0.3043103	0	0	0	2.181891	0
0.2608611	0.02761675	0	0.1529808	0	0.9856597	0.107488	0.1418014
0	0.04014704	0.3449672	0	0.7607327	0	0.9962217	0.1097273
0.2624083	0.02846978	0	0.6906471	0	0.07577579	0	0	0	0
1.259478	0	0.2840398	0.1291361	0.2378604	0	0.7131212	0.07418182
0.05880122	0.09947315	0.383048	0.1418014	0	0.06666974	0	1.100548
0.2322417	0.04763355	0	0.3929133	0.239405	0.08358451	1.447862	0
0.06917142	0	0	0	0.2851106	0.1087562	0	0.4711612	0.09607392
0.3975026	0.07894725	0.1312709	0	0.3276606	0	0	0.03341342
0.9810004	0.4861763	0.1323819	0.1125752	0.627013	0	0.7158906
0.3815849	0	0.1308936	0.1692027	0.8614104	0.04302026	0
0.6517136	0.1124036	0.03519093	0.1454182	0	0.0479923	0
0.3127383	0	0.0738845	0	0.919727	0.06642949	0.4007431	0.268622

0.04854295 1.1098240 0.1926247 0.4883227 0.2793058 0 0
0.2282198 0.1350040 0.4087402 0.08436697 0.8395074 0 0.1806486
0.2014665 0 0 0.04684672 0 0 0 0 0 0 0 0 0.02151013
0 0.2264389 0.02322173 0.04809963 0.02697028 0.09275061 0
0.0865229 0.7281006 0 0.3800863 0.05222148 1.440171 7.246314 0
1.700341 0.1094017 0.1148547 0.1438922 0 0.02472727 0.009096562
0.03621647
TCGA-G2-A2EO 1804 1 0.01499186 0.04809607 0.2174779 0.01386723
0.06902098 119.3336 0.05223209 0.1416504 0.246237 0 0 0.9827222 0
0.861454 0.04050774 0.3800425 0.07097984 0.1125158 0.3592059
0.2054389 0.0492094 0 2.672976 99.66477 0.213965 0.1128197 0.06902098
0.05381889 0.09490384 1.062923 1.291402 0.03749288 0.06441958 0.1906588
0.0419299 0.01598381 0.4329625 0.09898281 0.1770616 0.3974196
0.07635941 0.06992915 0.03872215 0.1040381 6.461538 0.2377904
0.2258535 0.01861511 0.3019668 0.2344683 13.52175 0.07976908
0.1925585 0.1325340 0.4449704 0 0.09887656 2.657308 0.05314615
0.05049516 0.301301 0.03220979 0.07353624 0.08796053 0.04494389
0.2783432 0.3389732 0.01994227 0.1205128 0.02382345 0.08779953
0.07786982 0.9202797 0.02834461 0.05830626 0.03129005 0.06819073
0.08233331 0.1017151 0.2443501 0 0.2166467 0 0 0.130388
0.2859883 0 0 0 0 0.008694667 0.02343821 0.0275329 0.05824236
0.07730349 0.1801564 0.2840918 1.378927 0.1550426 0 0 0.1810772
0.6953702 0.3417759 0 0.03681119 0.248637 0.03390504 0.5398974 0
0 0.03782644 4.785035 0 0.4512409 0.03715586 0.2019806 0.4410469
0 0.6664412 0.123956 0 0.425879 0.0385816 2.038823 0.01209929
0.986944 0.09662937 0.8098461 0 0.340438 0.0492474 0.1771538
2.869599 0 0.03972059 0 0.03710028 0.05159821 0.1961113 1.333605 0
0.2620457 0.03723023 0 0.04532721 0.4111888 0.1283207 0
0.3337114 0.1104336 0 0.1602397 0.1537683 0 0.5711228
0.2678231 0.05493142 0.06791841 0.4153518 0.138042 0.3066967
0.347295 0.08052447 0.3988454 0.05288174 0 0.026084 0.5137376
0.2508373 0.038166 0.06791841 0.0692458 0.4393033 0 0.0216261
0.1414088 0.5063338 0.1810772 0.0385816 0.04238591 0.491058
0.2336095 0.114498 0.03245567 0.2169231 0.03782644 0.257991 0.4348083
0 0.1425617 0.1181026 1.109281 0.1116255 0.428284 0.7336674
0.162031 0.006763748 0.1117984 0 0.07115803 0 0.1001812 0.07015994
0.04260213 0.06126358 0.6162668 0.03830353 0.1386421 2.633106
0.1819355 0.691124 0 0.0444273 0.7883933 0 0 0 0.1033941
0.2830687 0.03063179 0.04713628 0.1459391 0.1210159 0.2039181
0.4536867 0.1161665 0.02318262 0 0.08103606 0 0.07188536
0.02418482 0 0 0.009340273 0.5663115 0.05471933 0 0.1133785
0.08308723 0.00446325 0.0970706 0.04665354 0 0 0.1884714
0.038166 0 2.539042 0.1505557 0.3321635 3.329944 0.02949151 0.7412962
0 0.06622573 0.1106251 0 0.1853521 0.1573535 0

TCGA-DK-A1AG 475 0 0.215074862 0.257055582 0.045881774 0.36277429
0.01941534 53.283447567 0 0.468953594 0.438681727 0.435431402 0
0.040331092 0.983465859 1.313238342 0.218777729 0.038126785 0.029949539
0.302655793 0.413358843 0.052764041 0.083054508 0 2.595906707 74.42214607
0.046596815 0.231217706 0.116492038 0.090834298 0.080088276 1.409553654
0.083830719 0.063279625 0.027181475 0.697210939 0.217359896 0.202328276
0.43844661 0.3148456 0.107395835 0.058838222 0.063006868 0.147531034
0.653543671 0 16.685625457 0.120401166 0.166770805 0.007854542
1.936680123 0.395730304 7.210244005 0.572187907 1.494981148 0.111843976
0.134885517 1.17345459 0.129763282 0.010148206 0.996654099 0 0.085224579
0.565032245 0.027181475 0.00912594 0.354274261 0.341348761 0.587226637
0.081730177 0.044877483 0.216958715 0.160834729 0.30563373 1.511409512
4.931496254 0.095678793 0.03936319 0 0.604226543 0.027792059 0.085836238
0.281187677 0.384767266 0.297091795 0 0.245414142 0.184855373 0.160894832
0.130708734 0.2299971 0 0 0.080710639 0 0.010326535 0.808741981 0
0 0.242466191 1.454576252 0 0 0.077999016 0 1.970021887 1.470946081
0.098570186 0.196742108 0.338046419 0.228896635 0.956787935 0.074957272 0
0.063842611 2.023555666 0 0.888526531 0.004479344 0.340898314 0.521072267
0.135650463 0.059951504 0.836840759 0 0.179697233 0.38744702 1.300895876
0.112315032 1.123714643 0.040772213 1.088703879 0.026696092 0.031921306
0.166237286 0.149498115 1.344247512 0.490157753 0.227934345 0.033221803
0.563553103 0.130629421 0.496488204 2.039117143 0 0.082926535 0.031418168
0 0.191255584 1.179791699 0.25267287 0.057224159 0.281615286 0.27958089
0.241496955 0.135224425 0.291967385 0.1002222 0.321309381 1.261905347
0.069534007 0.085973357 1.465771925 1.223166394 0.56939925 0.101450182
1.325096927 1.480956935 0.044626303 0.124007653 0.132071954 1.430673114
0.264598433 0.161039262 0.127367936 0.11687149 0.274012717 0.115244799
0.146000193 1.034221991 0.452799125 0.611235904 0.455820023 0.263389827
2.523108534 0.27599652 0.354286376 0.027388968 0.122039277 0.287291751
0.130629421 0.786909742 0.100690966 0.702965494 0.79299 0.027943573
0.251198699 0.131426914 0.105705738 0.109388864 0.188359063 0.462292356
0.044961839 0.120098904 0 0.456525817 0 0.323563054 0.82719418
0.179011936 0.129295667 1.052986722 0.718898038 0.236205053 0.669636348
0.199441621 0.168712606 0.158408598 0.033976844 0.093925517 0.094419862
0.031728413 0.400121452 0.051699636 0.079555538 0 0 1.061185659
0.521550479 0 0.195635483 0 0.364722211 0 0.143385834 0.040818598 0
0 0 2.242471722 0.023088512 0.04186645 0.191357587 0.280465475
0.048964321 0.257452722 1.08924742 0.112828766 0.021904486 0.491180247 0
0.440716986 0.897665321 0 1.927124136 6.143758142 0.311094343 0.121078338
0.05323375 0.335322874 0.031118428 0.024047954 1.335556599 0.00885259
0.079301554
TCGA-GV-A3JZ 603 0 0.08116516 0.1410444 0.07358837 0.1876911 0
13.70023 0 0.09586101 0.2036702 0.0698375 0.01002545 0.8853868
0.3263484 1.297608 0.1315841 0.1187037 0.04803514 0.4521071

0.3535859	0.04835807	0.03330214	0	3.340425 24.6762	0.04203855
0.01090715	0.0934190.1456863	0	0.8514474	0.6722675	0.02537306
0.0653933	0.2150452	0.3202411	0.1406202	0.4102064	0.1751941
0.1610153	0.06133973	0.05856584	0.2129584	0.4978943	0.02346905
10.07203 0.2252923	0.1528449	0.01259766	0.4495789	0.4495789
2.1726060	0.7036887	0.3497971	0	0.2710177	0.2341387	0.03436128
0.7659493	0	0	0.3171832	0.02179777	0.002927363	0.1488168
0.06083097	0.1883669	0.5898803	0	0.1794238	0.005374122	0.4827693
0.8431663	1.4635640.01918203	0.1025918	0.004235068	0.05768454
0.04457483	0.09178007	0.4058894	0.07713955	0.4948232	0.03103889
0.2558479	0.08823924	0.1935407	0.1310248	0.1844426	0.1006051
0.03047993	0.1000290	0.01656241	0.2830724	0.02615732	0.0812798
0.1310845	1.5864060.03338494	0	0	0.2695942	2.3193230.6244955
0.0526979	0.1868380.4860944	0.2523952	2.0734870.1602955	0.02984756
0.1023952	1.90656	0	0.7125402	0.003592141	0.06834454	0.8655793
0.4623269	0.4006432	0.3774890	0.02401757	0.1958238	1.859311
0.09006931	2.3853860.0653933	0.5608036	0.02140852	0.2047904
0.4388168	0	1.4584690.5109968	0.1075226	0.05328343	0.200859
0.1745938	0.6370417	0.6433726	0.1139527	0.06650166	0.1007813
0.1856326	0.1226996	0.7791542	0.08684019	0.06883505	0.6139945
0.0747352	0.08299918	0	0.2341387	0	0.8159522	0.9087551	0
0.1838534	0.2810866	1.2611560.1779043	0.1355940.2179777	0.7557649
0.07157475	0.2983381	0.1941740.4241557	0.1485334	0.2066287
0.1174619	0.0656063	0.2972957	0.09241879	0.1463533	0.7336809
0.3477724	0.3431199	0.3394280.1929677	0.3996257	0.2529499
0.1291430.3074983	0.4893376	0.3583832	0.2095125	0.7338617
0.05383171	0.4540126	0.2710505	0.2801115	0.08393539	0.2898384
0.5570540.1973761	0.1098557	0.3631624	0.03605645	0.1029998	0
0.3525444	0	0.08649214	0.2487584	0.4009664	0.2592167	0.1876503
0.5503043	0.2273052	0.2165341	0.06397565	0.1052306	1.069197
0.08174162	0.2259664	0	0.08057302	0.1724084	0.02072986	0.06379834
0.29629	0.02729891	0.8567515	1.4254940.03930745	0.2823964	0
0.09140105	0.05576173	0.1061410.2455038	0.1699196	0	0.4803936
2.6532530.09257738	0.01678708	1.6112910.1044248	0.0120819	0.3237672
0.2946765	0.1266738	0.03513193	0.5852146	0.3357717	0.06236933
1.47953	0.04075503	1.6297243.3502870.1496864	0.2589251	0	0.1792713
0.04990989	0.03856977	0.7333172	0.1455336	0.05652859
TCGA-XF-A9SV	388	1	0.08936564	0.09556597	0	0.2479853	0.04571446
58.548670.3113525	0.1082526	0.05436314	0.4100986	0.02943565
1.0208380.7186417	0.4417272	0.08048811	0.02112272	0.1410356
0.08383756	0.5839653	0.03549593	0.04888907	0.1224352	3.056101
45.032130.1234290.03202438	0.4114301	0	0	0.8620441	0.2960759
0.07449764	0	0.8839495	0.3213537	0.2223166	0.4301442	0.166419
0.08795452	0.02770755	0.08766317	0.06947395	0	0.2756291	4.194058

0.0944970.1682875 0.4068667 0.8400032 0.2329421 6.2400240.2377495
1.14783 0.1843398 0 0.7515256 0 0.06106371 0.9777815 0
0.1003329 0.06651994 0.06400024 0.1418176 0.1509428 0.1786053
0.8987268 0.2886571 0.02641661 0.1277102 0.0946735 0.196263
1.0830810.1828578 0 0.3012189 0.03730364 0.2709874 0.1308758 0
0.2648286 0.2264888 0.1614274 0 0.1733522 0.3627099 0
0.07694018 0.3249243 0 0 0.1209330.04657129 0.1458863 0.3366588
0 0 0.4297784 1.1416260.1260273 0.2445161 0 0 1.480379
0.7470125 0.07736293 0.07314313 0.1646790.3368434 0.5363830.2353212
0 0.2254813 1.9442870.09869196 0.6973611 0.01054686 0.1003329 0
0.1596981 0.4839738 0.7388949 0 0.0705178 0.1609879 1.630322
0.02404107 0.4669141 0 0.5332645 0 0 0.3587967 0 1.047277
0.5770514 0.2052026 0 0 0.1025247 0.1558677 0.6821392 0
0.26034 0 0 0.2701929 0.2451073 0.08499026 0 0.2486544
0.1097147 0.1624622 0.1061311 0.1527673 0 1.38699 0.9904079 0
0.06747629 0.4501616 0.8228603 0.8966893 0.5971747 0.3200012
0.7132485 0 0.04866378 0.1036568 0.2041574 0.06230112 0.07583512
0.08996839 0 0.1897581 0 0.02148533 0.5619533 0.1501605
0.1439188 0.07666091 0.1148455 1.0992320.1856710 0.2579552
0.2873480.3006417 0.2050493 0.7702740 0.3887983 0.4183204
0.2302812 0.2217978 0.1547259 0.8177809 0.3863429 0.04031832
0.2332483 0.6351904 0.08640479 0 0.8360446 0.1394065 0
0.2434591 0.7775823 0.1522168 0.2754793 0.07694018 0.05561575
0.3814584 0.0939192 0 0.01243272 0 0 0 0.1245104 0.3093487
0.1825943 0 0 0.2404563 0.9285407 0.6715008 0.1154103
0.1381903 0 0.1073448 0 0.06492493 0.09610958 0 0 0.4639736
1.12525 0.1087263 0.1478652 0.1126404 0.09433871 0.008868394
0.1515466 0.4634985 0.05313228 0 0.3084027 0 0 0.6585089
0.05983025 1.4025053.8491630.1367312 0.1900570 0.1315893 0.05495251
0.0566222 0.3682911 0 0.08298656
TCGA-UY-A78K 536 1 0.05365435 0.1004097 0.04864569 0.2853690.06175476
46.317340.2336667 0.1754835 0.4651079 0 0.1325468 0.5799412
0.6831548 1.3177580.1449731 0.0856028 0.0952611 0.05033534
0.2629558 0.04795076 0.02201443 0.05513179 4.22542321.985380.04322833
0.1297833 0.1235095 0.09630616 0 1.8826380.2666421 0.1006374 0
0.3127431 0.2143755 0.1001077 0.4648587 0.2452496 0.1881254
0.07485917 0.1047583 0.06256732 0.5196849 0.09308548 15.12773
0.1276541 0.2525958 0.2664864 0.7024604 0.6643178 3.253501
0.07137136 0.5513179 0.2134466 0 0.7663924 0.03439506 0.04303814
0.5723751 0.09510233 0.1355378 0.4792558 0 0 0.1895965
0.2412744 0.6537313 0.2599608 0.01784284 0.1365793 0.07105143
0.4615188 0.7663924 2.1408320.1521637 0.06260165 0.05599195
0.04575894 0.1915306 0 0.4571272 0.2039728 0.2665288 0.2462196
0.07805937 0.3546505 0.08529357 0.03464566 0.04877043 0 0

0.1089106 0.04194149 0.03010867 0.3174026 0.06916533 0 0.3841643
0.2467520.006305475 0 0 0.1296114 1.0665680.7339087 0 0.04940381
0.3831283 0.1516784 0.2536062 0 0 0.06768849 5.7629030
0.8523322 0 0.09035851 0.5919232 0.3595551 0.2270107 0.1663598
0.07315564 0.0635074 0.1795035 1.2569080.03247661 0.4485298
0.2161417 0.7709292 0.05660853 0.3384425 0.4406286 0.05283463
1.2365920.6236219 0.1705871 0.07044617 0.03319453 0.09233236
0.03509311 1.0396280.3013143 1.1722940.0666216 0 0.3244429
0.8093816 0 0.1516784 0.1819471 0.04940381 0.2194669 0.09558023
0.06879011 0.03541986 0.6813301 1.6607552.7277410.1519207 0.4054096
0.6175476 0.3057701 0.1971962 0.2881889 0.4995995 0 0.02191298
0.11669 1.0112380.1122151 0.1024442 0.1485446 0.01239118 0.3076086
0 0.16447 0.1265220.2501803 0.3888341 0.1380796 0.1723805
1.4849310.08360645 0.1365922 0.02903888 1.2939090.1353770.1846647
0.6280785 0 0.3901634 0.5283463 0.1481345 0.1331655 0.1741801
0.1921259 0 0.1331372 0.3150906 0 0.1149540 1.3624460.06277382
0.03811717 0.1644421 0.2879658 0.1028133 0.4961861 1.004724
0.1878255 0.3893413 0.2114560.2782513 0.02799186 0.1801181
0.09958359 0 0.07008278 0.5445273 0 0.1265220.4352510.07218393
0.2128589 1.3751730 0.06222617 0.06879011 0.1450099 0.07372274
0.04677643 0.1081938 0.07488373 0 0.1253546 1.1692910.02447936
0.09987409 0.5579337 0.1699204 0.04392717 0.1737029 0.4035066
0.07177535 0.06967204 0.5257287 0.03414805 0 1.5447850.3771764
1.3745264.6446640.1539229 0.7702326 0 0 0.2474475 0 1.1481170
0.121447
TCGA-FD-A6TH 131 1 0.1075861 0.1882647 0 0.2894994 0.01500956
49.038660 0.09241149 0.1070952 0 0.04832346 0.6761957 0.1048683
0.2900671 0.06342454 0.03467642 0.02315331 0 0.5539011 0.0291362
0.4976086 0.1607981 3.01025565.001320.1576004 0.01051466 0.3602294
0.1053329 0 0.5943785 0.2592305 0.1467601 0.02101338 0.6841128
0.08987972 0.1981262 0.1694766 0.1490204 0.1985387 0.0181946
0.06310053 0.08363879 0.2778819 0.09049809 0.05620601 0.2171853
0.05525431 0.06072168 0.2758006 0.5098835 2.4884270.02602032
0.7537409 0.09511045 0 0.1524047 0.02507926 0.04445697 0.3210378
0.1040162 0.06588519 0.1747254 0.02101338 0.01975416 0.0443427
0.2345680.4312730.6634293 0.03903048 0.1415187 0.01554221 0.2076387
0.3556111 1.5009560.01849178 0.05325388 0.01633068 0.04448703
0.04297082 0.02211935 0.08695193 0.1115456 0.9363670 0.05691723
0.1122845 0.06219207 0.02526199 0.4978555 0.04849242 0 0.08508486
0.01529088 0.08182791 0.1761670.02521606 0.03917749 0.1642777
0.5247666 0.0160918 0.02007067 0 0.02362663 0.9721144 0.847292
0.02540079 0.04002549 0.2162781 0.02211935 0.8541440.1352115
0.1150940.04935527 1.6695910.03240381 0.4252236 0.05540607 0.03294259
0.3452821 0.1835195 0.3840192 0.4852070.3733916 0.04630662

0.1258515 1.265227 0.03946737 0.429249 0 0.9215171 0.08255257
0.1727435 0.2516738 0 0.3591378 0.03789298 0.05960074 0.07704907
0.1210195 0.1009866 0.204706 0.3014964 0 0.08547816 0.07286602 0
0.3844239 0.8047678 0.08371529 0.0884774 0.1700862 0.07204588
0.4534041 0 0.3260304 0.0258265 0.413995 1.213371 0 0.1329281
0.1724366 0.5853728 0.1229101 0.3485726 0.4990678 0.2602032
0.1034987 0.1517902 0.05105092 0.3619705 0.06136651 0.09959664
0.1181583 0.03614028 0.1744507 0.1781863 0.2327932 0.3690155
0.2760948 0.1417598 0.2013624 0.0980396 0.6488343 0.3962523
0.02489916 0 0.1415187 0.2220987 0.1346489 0.5126478 0.0259473
0.5087974 0.1875977 0.2916343 0.032366 0.1778055 1.470937 0.1902736
0.03530088 0.1932811 0 0.1108991 0 0.2352865 0.09154345 0
0.07993563 0.4915233 0.5247666 0.1507482 0.3536679 0.575205
0.3089385 0.03854595 0.02898398 0.02041035 0.02626673 0.1210195 0
0.2493733 0.152354 0 0 0 0.02631657 0.620827 1.212617 0.03789298
0.8620757 0 0.08811202 0.1612655 0.06821436 0.06311186 0
0.0502234 0 1.13679 0.08924602 0.129464 1.590293 0.061949 0.203825
0.3596126 0.05072726 0 0.03386772 0.3290909 0.09959664 0
0.5265762 0.1375097 0.4604886 3.048004 0.04168666 0.2028063 0
0.04320508 0.03007119 0.01859093 0.2511459 0.03421868 0.07492984
TCGA-DK-AA6W 415 1 0.49636 0.246442 0.06428923 0.4755222 0 79.99489 0
0.1932631 0.6146778 0.2440494 0.3328256 1.306832 1.282989 1.018627
0.3544483 0.1162736 0.04196509 0.008315279 0.1544522 0 0.08728156
0.07286113 3.264817 75.6328 0.1713892 0 0.1632278 0.0636382 0
2.077657 0 0.3546679 0.07617299 1.127224 0.5595463 0.3213011
0.3071742 0.1170423 0.157025 0.04946623 0.1665372 0.137813 1.23625 0
19.45769 0.4498808 0.2336781 0.03301719 0.6427096 0.6931182
7.043496 0.5187767 3.460903 0.156715 0 1.565313 0.04545586 0.01737951
2.152945 0.06284272 0.2388322 0.7521333 0 0.01790209 0.4538575
0.3188637 0.3291272 0.6871186 0.09432303 0.3040011 0.07512017
0.8824584 2.486086 1.197004 0.4021934 1.227208 0.02959916 0.2015805
0.03894204 0 0.1838658 0.202175 0.1280871 0.1898162 0 0.314522
0.1127224 0.2289352 1.031265 0 0 0.1747773 0.2217163 0.07596472
0.5259067 0.0457038 0 0.797821 1.875091 0.1499975 0.07275568 0
0.08564596 3.171502 2.384386 0.04603862 0.558602 1.257671 0.2004552
1.372565 0.1750494 0 0 3.115117 0.05873151 2.489995 0 0.05970805
1.825307 0.7127719 0.4200182 0.6595737 0 0.08393018 0.2372284
1.837506 0.400591 1.333729 0.228519 1.09679 0.03740638 0.1341837 0.1649925
0.06982525 1.274166 0.4120834 0.8078436 0.4655016 0.1316078
0.3050618 0 1.014851 0 0.2323922 0 0.05405825 1.33993 0.7293159
0.1011553 0.1202731 0.252789 0 0.7251083 0 1.227308 0 0.7503608
1.781368 0.06495372 0.2007755 0.04464847 2.530032 0.08289229 0.1263567
0.7141218 1.414845 0.06253007 0.2027185 0 1.919604 0.03707535
0.2256471 0.2230839 0 1.106664 0.2691337 0.1534309 3.34418

0.4289301 0.2569379 0.6386919 0.5376431.499405 0.3314781 0.2256471
0.1151317 1.1970040.2683675 0.1830371 0.5203340 1.4477380.279301
0.1174630.1026602 0.04603862 1.3541870.1915937 0.1679538 0.5816628
0.3150011 0.2313874 0 0.4738377 0 0.1511248 0.5795294
4.1343750.7699649 0.7103961.7399081.3073271.6041730.2095922 0.1575993
0.4439221 0 0.3948234 0 0.01852401 0.2175647 0.1086618 0
0.3451316 0.1430954 0.2913571 1.3904230 0.1370615 0 0.06388078
0.2922917 0.1622745 0.8579211 0 0 0.7178873.2451570.09705439
0.07332873 0.4021934 0.2666693 0.3588751 1.7504141.3423610 0
0.5833643.2041890.07265054 1.1819550.1780247 1.66926 1.6468610.0929927
0.1413784 0 1.0180130.1308088 0 1.8039360.006202094 0.1234631
TCGA-CF-A3MG 369 0 0.01662761 0.03556253 0 0.2153238 0 32.281720
0.1329357 0.2427589 0.2289122 0.01643063 0.02319034 0.1782832
0.3698503 0.0359420.07074271 0 0 0 0.03962681 0.2183144
0.0683419 1.83739 27.539 0.1224829 0 0 0 0 0.6977150
0.08316739 0 0.2114615 0.06643549 0.08863893 0.09604055 0.1351172
0.05523206 0.0618641 0.06022466 0.02585302 0.08589419 0 5.3271370
0.1252481 0 0.4688798 0.1300255 2.3549750 0.7688463 0.01469947
0 0.2837744 0 0.01037371 1.1461510 0 0.1113919 0.03572417 0
0.0665167 0.04984768 0.1929456 0.1074166 0.007372719 0.1514835
0.05284562 0.2556206 0.6909291 4.7972460 0 0 0.03781548 0
0.03760439 0.3449231 0.06321167 0.4204986 0 0.06450877 0.2313833
0 0.04294709 0.1813689 0 0 0.04821668 0 0.006785999 0.2583885
0.08573802 0 0.2040916 1.8097670.003908164 0 0 0 0.9365075
0.2653468 0 0.04763223 0.1685234 0 0.5988052 0.03283838 0
0.2097683 1.6815840 0.1112168 0.02354852 0.1120093 0.2445846
0.08914161 0.01125618 0.5499231 0 0.07872439 0.1198154 2.7438670
0.3822513 0 0.5169917 0 0.1258610.3095176 0 1.0262580.5153651
0.08810895 0 0.04114826 0.05722805 0.1305053 0.3807620 0 0 0
0 0.5472639 0.04744055 0 0.4221709 0.1224829 0.1813689
0.2369644 0.1279093 0 0.1407639 0.6188440.09138742 0.07532893
0.04187914 0 0.3644573 0.03703731 0 0.9289626 0.1759549
0.04074531 0 0.4102496 0.03477574 0.04233026 0.1339181 0.01536023
0.08473658 0.05048813 0.08394999 0.5227928 0.2263081 0.2811681
0.3423296 0.06410537 0.2481890 0 0.1439875 0 0.1258610.2289122
0.6972288 0 0.3317345 0.2335015 0.03672579 0.1650732 0.04318307
0.4763223 0.2875360.03000694 0.2169941 0.3545557 0.07672975 0
0.3555582 0 0.1890016 0.0679480.02839496 0.4673108 0.2562821
0.8159948 0.0620881 0.2555106 0.01310614 0.04927472 0.09368724 0
0 0 0.003475012 0.2590122 0 0 0 0.04473995 0.5088767
0.6700616 0 0.1028482 0 0.1497964 0.09138742 0.03624032
0.1072944 0.09282659 0 0 0.5797858 0 0.0550244 0 0.09215281
0.04455209 0.1499576 0.1379836 0.4745249 0 0.1598505 0.04233026
0 0.7173579 0 0.4144562 1.4323710.0654186 0.02652188 0 0 0

0	0.2530163	0.01745222	0.1852884
TCGA-DK-A3X1	2009	0	0.1185296	0.1109092	0.05373243	0.109638
0.04547485	31.57495	0	0.08614815	0.2433519	0.3059618	0.01464068
0.3985199	0.1191458	0.8238973	0.1040861	0.136578	0.2104445
0.2432446	0.3227247	0.1235846	0.02431641	0.06089675	4.131566
65.53353	0.1159609	0.5097044	0.1364245	0	0	2.293881	0.3436114
0.03705358	0	0.1256166	0.3137495	0.09477916	0.2995227	0.1354473
0.09296186	0.1584834	0.05869479	0.0345549	0.420953	0	6.109111
0.1880034	0.05580181	0	0.7162289	0.8882642	3.342401	0.1576689
1.141814	0.02619623	0	0.406775	0.4938914	0.04093594	4.522853	0.05252345
0	0.5293702	0	0.01709991	0.2607897	0	0.137541	0.1914294	0
0.1985013	0.03139242	0.1735416	0.2308723	2.091843	0.1400625
0.04609848	0.01236937	0.2190232	0.0650949	0.2010467	0.6366479	0
0.4817437	0.06799152	0.0574812	0.08762499	0.4710623	0.03826845
0.1616106	0	0	0.03437117	0.06949078	0.03325704	0.1465162
0.1527955	0	0.3350015	1.044791	0.1079547	0.1520216	0	0.1431644
1.079921	0.7768742	0.4617446	0.2000893	0.2730264	0.06701557
1.627393	0.05852195	0.04358793	0.03738324	2.582337	0	0.9910085
0.02098315	0.1497105	0.4794672	0.3177222	0.4346307	0.367511
0.08080531	0.07014818	0.1105757	2.408093	0	0.7276646	0.1432458
0.9353013	0.09379188	0.03738324	0.1541229	0.1167188	4.329206
0.05740268	0.06280831	0.2723438	0.1466623	0.1529809	0.3876269
1.278832	0.2080137	0.03237193	0.03679401	0	0.04479612	1.178476	0	0
0.2885762	0	0.4848318	0.05278739	0.1139749	0.03912361	0.2508583
0.2867422	0.2985829	0.06712262	0.3731684	0.8867596	0.2987731
0.3432258	0.03979049	0.3153378	0.05226214	0.02420435	0.07733514
0.6803424	0.3718474	0	0.05966455	0.01368689	0.09438176	0
0.02137272	0.09316798	0.3510277	0.1431644	0.2669068	0.2208708
1.775354	0.2308723	0.1131564	0.06415078	0.142921	0.1121497	0.05099364
0.3106365	0.07861321	0.3011206	0.431352	0.2454367	0.1838627
0.3463085	0.212216	0.1601325	0.05347599	0.2541235	0.05265509
0.08985881	0	0.9504715	0.1386758	0.08420593	0.2421831	0.4843453
0.07570948	0.6850885	0.2296107	0.0691553	0.3541617	0.07007019
0.1097669	0.04328643	0.03979049	0.03666558	0	0.04644668	0.4615909
0.09081865	0.09316798	0.04807638	0	0.1343526	0.5398752	0
0.09164397	0	0.1334776	0	0.07104309	0.04780291	0	0.01086879
0.4338492	1.549872	0.0270391	0.02451503	0	0.08211371	0.03528764
0.3323402	0.1844276	0.3699765	0	0.2739163	0	0.03036038
1.056544	0.05951666	2.626173	3.290927	0.02914596	0.354489	0	0.06544978	0
0	0.4227239	0.005183662	0.3302064
TCGA-DK-A1A6	2020	0	0.1024	0.1095047	0.1237879	0.06314555	0
75.46077	0	0.05788612	0.1661126	0.07831887	0.02248598	0.174553
0.3354831	0.6748732	0.01844553	0.0161357	0.05386874	0.2935338
0.3221781	0.05423088	0.074693	0.2338216	3.66896	821.16797	0.2671487	0

0	0	0	0.7572955	0.07539106	0.08536343	0.07333494	0.4340902
0.48642	0.1819589	0.2628707	0.1502421	0.05039153	0.03704028
0.1480982	0.1326783	0.146937	0.02631923	2.566351	0.03609326	0.02142588
0.007063786	0.2291717	0.1482876	2.257944	0.1816175	0.9645139
0.1508761	0.06065296	0.3123739	0	0.03042179	0.821623	0	0.2682561
0.1016295	0	0.008207187	0.06068718	0.1364371	0.4224861	0.183755
0.00504493	0.1158504	0.02410712	0.233218	1.034211	4.714389	0.2366274
0.01770015	0	0.06469001	0	0.02573156	0.2191619	0.1297616
0.1233148	0	0.04414142	0.1068718	0.07234837	0.08816223	0.579158	0
0.1025446	0.01319729	0	0.004643456	0.1647525	0.02933398	0.09115078
0.2009054	1.203486	0.005348479	0	0	0.05496997	0.4900419	0.07781521
0.05909776	0.009312374	0.1048323	0.1286578	0.604373	0.06741095	0
0.02870763	2.431886	0.03769553	0.3805115	0	0.0383223	0.2343067
0.1829908	0.1951242	0.3762959	0.1241053	0.05386874	0.1141949
3.056909	0.0459126	0.4517908	0.03666747	0.2894416	0.1680592
0.1148305	0.5419424	0	0.5866795	0.4408111	0.1386677	0.0298772
0.08446957	0.1174783	0.1786017	1.292699	0	0	0.05651029	0
0.03440019	1.310667	0.1298486	0	0.4392542	0	0.1551316	0
0.02917484	0	0.9632052	0.6492998	0.06253367	0.1030907	0.2865664
0.7857315	0.8711925	0.09123669	0.4277872	0.72647	0.1204007
0.05576159	0.03959187	0.4132844	0.11898	0.02896533	0.04009081
0.03153164	0.2754178	0	0.1230953	0.2861851	0.2179453	0.4947298
0.1756844	0.04094102	0.7642273	0.1418346	0	0.1231579	0.8780238
0.3157839	0.1566377	0.3776984	0	0.3394333	0.1792632	0.1382169
0.08471598	0.1181955	0.2851914	0.1967523	0.1129307	0.343631
0.2021765	0.07500552	0	0.7907179	0	0	0.04649478	0.1415601
0.3779062	0.1402929	0.8228474	0.4460927	0.3885295	0.1345221
0.01685861	0.1733273	0	0.1970957	0	0.03804554	0.7035663
0.1162369	0	0.1476768	0.06122841	0.8253845	0.8766017	0
0.05278197	0.02917484	0.2050024	0.1250673	0.1091119	0.1284822	0
0.00834645	0.4394941	0.5289734	0.02076408	0.04706443	0	0.1171066
0.02032377	0.07630087	0.3304632	0.04058789	0	0.3660054	0.2027573
0	0.4868098	0.09140899	0.9138221	1.777684	0.100719	0.145185	0
0.1507821	0.03498198	0	0.1623107	0	0.05546946
TCGA-XF-AAMG	3364	0	0.1175205	0.2154417	0.06088568	0.3105845
0.05152879	30.80066	0.1169843	0.3294568	2.267267	0	0.08294879
0.4716438	0.4050222	4.107754	0.3719735	0.2708305	0.07948682
0.03150023	1.279909	0.03000794	0.08266077	0.06900378	8.445072	23.05232
0.5410523	0	0.2318796	0.1205382	0.1062781	2.283462	3.504199
0.1679457	0	0.8184529	0.7110364	0.2863916	2.08487	0.2216919
0.2106752	0.07027115	0.2546332	0.3523959	0.9539866	0.03883573
8.152513	0.2130318	0.4426139	0.0416923	0.8115785	1.269086	3.089694
0.2233237	0.8625472	0.6381989	0.08949738	0.7723657	0.2152469
0.07780815	7.494577	0	0.3958293	0.149961	0.03607016	0.03875276

0.2283471	0.4529740.1168886	0.7591987	0.01488824	0.0989680.02667871
0.3687089	0.5232154	0.9275183	0.1904504	0.2481183	0.02803215
0.4963624	0.09220102	0	0.5721473	0.06382387	0.1819590.1027241
0.2279673	0.09929027	0.2135094	0.1734521	0.3662508	0.08323882	0
0.9055158	0.0262473	0.07194306	0.5632874	0.3029893	0	0.3940603
1.9817140.1538945	0.1033559	0	0.4055588	3.3373320.8802974
0.3924116	0.06870506	0.9435896	0.1139058	1.98008	0.2652513
0.1481720.2965198	6.5867720.1112444	0.6176163	0.005944145	0.1696411
1.1359850.4050222	0.5303759	0.4858429	0	0	0.2419515	2.965346
0.2167905	0.94734	0.2164209	0.9701793	0.07085209	0.9319193
0.5055392	0.1322572	3.1610570.3902673	0.1467877	0.1322572
0.4154678	0.1155646	0.8784614	2.5137090.4714119	0.1100445
0.1667692	0.05119635	0.2030388	2.3944440.1437	0	0.6189522	0
0.04578135	0	0.4304937	0.04433203	4.1216880.7248113	0.2153025
0.3042339	0.9725488	1.4685710.1128493	0.2916889	0.58614	0.3573179
0.2368786	0.1234198	0.3797324	1.1621250.03511254	0.3846620.1521169
0.03101798	0.1925038	0.1529313	0.2179621	0.3694992	0.3131295
0.3650029	0.3024394	0.1941787	1.0789410.4185724	0	0	0.6477905
0.3388798	0.5778229	2.1412770	0.6166787	0.7877931	0.3708147
0.1527826	0.2616077	0.7214031	0.0362901	0.1060414	0.4131517
0.2386597	0.5223884	0	0.9872548	0.07856866	0	0.2058182
2.7011190.5147309	0.6210341.9513360.2350853	0.8026207	0.2514284
0.04975194	0.6376375	0.04508769	0.2492807	0	0.1017513	0.8638106
0.06860606	0.1055712	0.1089533	0.04517325	0.3996262	1.993363
0.06504454	0.1817275	0.04304937	0.1814966	0	0.2415026	0.9479188
0.1874512	0.03694718	0.5752841	1.9513360.2144712	0.0555573
0.5713513	0.1329218	0.02499087	0.4037599	0.9926549	0.838461
0.05813505	0.9621843	0.1282207	0.03440217	2.4542580.03371998
1.9063642.3253330.09357391	0.1874512	0	0.5933035	0.03097091
0.03191193	0.2554667	0.0117475	0.3975512
TCGA-E7-A541	778 1	0.1692976	0.1991481	0	0.2975353	0.0519619
47.713120	0.18457	0.3089627	0.1165359	0	0.2698485	0.09076143
0.9414274	0.2195707	0.06902702	0	0.00794125	0.2581333	0.01008672
0.1944963	0	1.98937531.001380.2494171	0.01820045	0	0.1215514	0
1.8862170.2243589	0.3387146	0	0.5741441	0.1420497	0.1263495
0.2444643	0.2321536	0.1437135	0.06298825	0.07473256	0.06580701
0.52473	0.1566486	6.46981	0.05370559	0.1594050.04204273	1.023
0.8384588	2.9361890.09008029	0.6088579	0.3891311	0	0.5904242	0
0.02715997	0.9446962	0	0.1710670.5292749	0.10912	0.03175126
0.2663866	0.3552744	0.3143227	0.1640528	0.04504015	0.127018
0.09864415	0.5081375	0.6595164	6.0795420.3520938	0.02633724
0.06360247	0.2695185	0.07438079	0.07657543	0.2257655	0.06436032
0.3975582	0	0.03284049	0.08245574	0.3229560.1311825	0.1231097	0
0	0.1669157	0	0.01727328	0.4305008	0.2618880	0.3171688

1.3755020.007958362 0.1042246 0 0.08179352 3.3653831.4280330.04396776
0.1247086 0.3275727 0.1531509 0.8688030.0334351 0 0.2135801
3.3149960.1121794 0.2264755 0.02397643 0 0.09961161 0.63533
0.2368550.4199370.0923323 0.1202324 0.3006246 1.4319610.08197973
0.4776513 0.49104 0.9889679 0.1786190.04271601 0.5097884 0
0.7406933 0.3279563 0.2422170.08891259 0.2513759 0.1748039 0
0.9542916 0.09507485 0 0.1261282 0 0.1535591 0.7893787 0
0.03828772 0.3120773 0.1870628 0.2308308 0 0.2604673 0
1.2182350.9535350.1240641 0.03834888 0.8101627 1.1691430.1880138
0.2639723 0.2728 0.6756022 0 0.05531428 0.08836711 1.125481
0.1062230 0.07669776 0.01563935 0.2803982 0.05140556 0.1343186
0.3193756 0.5291137 0.4498644 1.3070630.1522973 0.6879026
0.1055226 0.04309946 0 0.9798531.0679 0.1748039 0.7808883
0.02245687 0.4640290.5856633 0.09348286 0.1120485 0.04396776
0.4041481 0 0.1222088 0.2146247 0.1804992 0.1874977 0
0.8824218 0.3169162 0 0.1383654 0.2767189 0.1297643 1.148132
0.52473 0.1106286 0.6214781 0.08006581 0.1755954 0.8373093 0
0.2932719 0 0.08845394 0.1838040.4150962 0 0.2746728 0
0.5469084 1.7304260.06559125 0.2356135 0 0.03050368 0 0.1180764
0.2731103 0 0 0 1.3774160.03089627 0.0840364 0 0.4021172
0.04032148 0.1644274 0.9658756 0 0 0.4945256 0.2154973
0.06938265 0.6519213 0.0340034 1.1721874.1035360.1665184 0.4320612
0 0.2991452 0.1249249 0 1.0143550 0.2004464
TCGA-XF-A8HE 3817 0 0.132874218 0.157881382 0.107084763 0.31409435
0.022657004 77.281493466 0 0.321912935 0.511925809 0.203253119
0.043766699 0.182376622 0.356173235 0.875715398 0.111696264 0.044492588
0.139800142 0.36703896 0.578849896 0.035184994 0.07269122 0 1.394559327
46.753647112 0.095159415 0.222206973 0.339855053 0.265000902 0.093460139
0.640869528 0.097827436 0.073845048 0.095159415 0.438103135 0.082584107
0.188887861 0.085275239 0.344918795 0.065388041 0.130457971 0.145382439
0.241028781 0.076266198 0.307366462 6.320821914 0.046834611 0.278022194
0.009165968 0.71369561 0.577253803 6.498386347 0.157111229 1.02399805
0.143569691 0.078703275 0.372471178 0.378572717 0.035527776 0.290764878 0
0.14918103 0.560466474 0 0.021299289 0.192931821 0.088520386 0.445427048
0.286128536 0.032731506 0.213623176 0.093844174 0.475551438 1.303649125
1.042222161 0.16748057 0.045935428 0.012325623 0.285401933 0.129729334
0.133557073 0.109378638 0.112252393 0.373364328 0 0.17183369 0.344124086
0.28163773 0.30506479 0.16103901 0 0.044353964 0.145560823 0.138489997
0.039164761 0.656991028 0.038063766 0 0.384683921 1.516378133 0.069401861
0 0.045511024 0.035664517 1.418497817 0.201946025 0.268398349 0.072502411
0.639342656 0.133557073 0.917155502 0.145787404 0.086867516 0.111753049
1.175413711 0.146741154 0.592502016 0.015681701 0.09945402 0.130301273
0.118724412 0.253193096 0.610351931 0 0.069900071 0.273561729 0.954933074
0.178728554 0.478251443 0 1.446676019 0.342687178 0.074502033 0.129328625

0 0.519051353 0.228798593 0.13690723 0.116305951 0.40189491 0.101626559
0.193127963 0.455110244 0.041455587 0 0.109991618 0 0.044637679
0.40493368 0.08424576 0.033389268 0.308095235 0 0.362337771 0.420805452
0 0.038985235 0.874898499 1.080637973 0 0.234098241 0.632142471
0.679710105 0.146700829 0.197314526 0.594746342 0.70700053 0.052077292
0.156771847 0.051374408 0.394619516 0.123510745 0.037585406 0.126338733
0.013638483 1.053336918 0.044828846 0.202322662 0.41777304 0.520958047
0.285316133 0.379946846 0.136607316 0.440738342 0.322078019 0.112756219
0.351581349 0.213623176 0.260757115 0.152439839 0.283742957 0.039167579
0.613875199 0.581529757 0.114132008 0.244283212 0.153370485 0.370064391
0.319132183 0.146538838 0.352312532 0.262344251 0.105113328 0.03685752
0.138120168 0.069092644 0 0.180994852 0.273731684 0.264045944 0.591643318
0.838928173 0.165385685 0.466344152 0.104733541 0.196881548 0.154047618
0.832644879 0.073071802 0 0.018512959 0.480865484 0.361989704 0.092838453
0.287438049 0.27807495 0.334693385 1.032620565 0 0.365279324 0.075714543
0.212808856 0.081143687 0.167326115 0.190535347 0 0 0.47830567
2.273686016 0.053886927 0.073284963 3.461265111 0.093512323 0.061534956
0.238984831 0.520696724 0 0.025561748 0.371208564 0.375854062 0
0.479027277 0.088959226 0.81777622 1.346627572 0.150054846 0.235490115 0
0.195654871 0.063549652 0 0.210614399 0 0.061694709
TCGA-DK-AA6Q 413 1 0.055112009 0.137516794 0 0.11894799 0.056384437
37.663345724 0.064003955 0.080111563 0.603465863 0 0.072612153
0.047582539 0.295458712 0.544828439 0.059564656 0.123751115 0 0.215428463
0 0.032835643 0 0.377530576 2.183600755 58.168613593 0.135322648
1.046724274 0.33830662 0.065948379 0 0.691112096 0 0.229714372 0
0.272566918 0.058719887 0.097930864 0.371379365 0.083970805 0.081362654
0.111067539 0.118520734 0.114252674 0.237245991 2.08226897 4.222834409
0.233106128 0.069188871 0 0.444027439 0.383082497 6.302073654 0.048873564
0.660678508 0.178644456 0 0.681570114 0 0.009823863 0.603000226
0.195372073 0.185626673 2.010127367 0.039469106 0.002650281 0.215569462
0.275365854 0.085268772 0.059338519 0.04072797 0.098449016 0.204348825
0.336210761 1.001908068 0.620228804 0.104198439 0.042868255 0.046010497
0.250677881 0.020177854 0.041546427 0.272200729 0.139676192 0.099552649 0
0.035635581 0.153383374 0.116814394 0 0.400763227 0 0 0.031962711
0.028720628 0.007497369 0.194642165 0.047362927 0 1.182814475 0.647720027
0.030224974 0 0.056629586 0.044377529 0.852090039 0.586325622 0.095419816
0.045107549 0.47393702 0 0.628498521 0.072561587 0 0 1.014577618
0.060863574 0.307188794 0.019512816 0.061875558 0.270224168 0.098486237
0.23628704 0.531624689 0 0 0.127647816 1.85851017 0.059304746
0.518304689 0.059203659 0.525030894 0.0387643 0.185406475 0.241386345 0
1.17689697 0.142347594 0.06814173 0.144720054 0.227308986 0 0.288372064
0.695734919 0 0 0.273726197 0 0.055542878 1.05810794 0.052413702
0.789382114 0.926463923 0 0.801526454 0.261805123 0.329741896 0.048509515
0.311040117 0.624461336 0.033655827 0 0.416423602 0.930343206 0.563728159

0.032735922 0.049336382 0.439862079 0.129600049 0.105038749 0.063925423
1.05759653 0.11526376 0.327373911 0.08322559 0.03394086 0.234048605
0.111561498 0.119250502 0.635356336 0.36115705 0.044377529 0.094553937
0.089712269 0.776918186 0.286259448 0.374141612 0.079540793 0.354416459
0.185406475 0.063227208 0.661190194 0.048736407 0.363084633 0.245394875 0
0.106386971 0.429389172 0.131563686 0.039709771 0.116033896 0.239741347 0
0.06539651 0.045861989 0.147311648 0 0.15661088 0.225212765 0.076188106
0.187744935 0.453036692 0.332144387 0.154342652 0.784154418 0.115840399
0.027220073 0.199349479 0.098672764 0.090923594 0 0.011517882 0.234134309
0 0.057759667 0.178830273 0.098859999 0.17699575 1.387005572 0.071173797
0.085222278 0.09421197 0.099299656 0.10096748 0.048047236 0 0.102557519
0.30995397 0 0.533803479 0.067051763 0.060792555 1.111450017 0.334528769
0.027345801 0.123196132 0.343008761 0.262134438 0.031806605 0.135851941
0.60798012 0.150575779 2.973729654 0 1.475466178 0.822292625 0.174667904
0.32232363 0 0.162302865 0.429265035 0 1.397698713 0.192817245
0.140739542

TCGA-UY-A78N 2641 1 0.09782246 0.07845716 0.133036 0.1809684
0.2064174 0.3289 0.04260204 0.4088137 0.7587221 0.1683401 0.01208295
0.9745135 0.262216 0.8612852 0.2510988 0.3663328 0.05789326
0.1835425 0.2663446 0.1748474 0.04013665 0.2512903 3.603203
93.08448 0.3602916 0.03943677 0.3377733 0.2194814 2.089973 1.645439
0.162047 0.2446424 0.05254252 0.3887675 0.6351294 0.2085899
0.8122153 0.2297794 0.1805209 0.0796151 0.1439383 0.08555443
0.3789952 0.02828553 4.848605 0.349108 0.1381596 0.03036608 1.034431
0.2549858 11.77022 0.3578412 0.5654032 0.1405283 0 0.9708406
0.5016718 0.1220598 0.9632795 0 0.123556 0.9283891 0.2627126
0.1887554 0.2021854 0.1832879 1.532418 0.07899331 0.01626551
0.4521516 0.1943111 0.3222536 1.61958 2.02664 0.161831 0.1236464
0.2347941 0.1807598 0.05372279 0.02765396 1.213913 0.139456 0.5521985
0 0.118598 0.4764405 0 0.2210805 0.2222953 0 0.03673524
0.04254977 0.2485197 0.08982661 0.2159282 0.4413572 0.3428622
1.029546 0.7872858 0.01437016 0.02509267 0 0.8566131 2.795311
1.839833 0.1587823 0.04503645 0.4619235 0.1106158 0.8256682
0.2897888 0.03597309 0.03085237 4.288713 0.04051176 0.7769849 0
0.123556 0.4676502 0.426101 0.3780152 0.45496 0.06668858 0.1157865
0.1195795 1.815021 0.1480282 0.4344341 0.4334758 0.6490136
0.02580213 1.604323 0.2744789 0.04816398 0.8884462 0.1894976
0.2170619 0.03210932 0.1210404 0.2525102 1.983432 1.378507 0
0.1068661 0.06073216 0.1118647 0.07394043 1.240898 0.2093243 0
0.5528757 0 0.23341 0.2613924 0.4703173 0.0322887 1.345716
1.286395 0.4032333 0.0830944 0.6159514 0.9570245 0.4395509
0.7844237 0.7552987 1.821737 0.04313192 0.1598067 0.04254977
1.089451 0.02557379 0.2179052 0.04308599 0 0.07789322 0.07425709
0.335139 0.9611437 0.3883253 0.1476919 0.3461513 0.1508562

4.3499680.2286466 0.3424225 0.0264718 0.0589763 0.1851142
0.08417006 0.5298275 0 0.5722678 0.7161774 0.6616920.1315096
0.0952694 0.7151621 0.6872177 0.1489513 0.2963547 0.1303687
0.3659656 0 0.3595275 0.05722453 0 0.09993678 0.2192549
0.6873130.4146291.3264830.2739544 0.2922895 0.1734867 0.2717717
1.8525590 0.03026009 0 0.2964374 0.2885991 0.1748894 0.03844575
0.3174193 0.06580278 0.2286923 1.9332720.09474881 0.2079927 0
0.1101590 0.0959430.2564362 0.0682639 0 0 1.7410090.06694607
0.08092897 0.3698993 0.24203 0.05460539 0.2460039 0.4312561
0.1090505 0.1270259 0.5154236 0.06225864 0.07516921 1.041999
0.04911907 0.9482283 3.1497350.1403159 0.09751986 0.05145113
0.1620470.1428628 0.02324267 0.3953904 0.2053475 0.2129056
TCGA-DK-AA75 340 1 0.08122744 0.1158175 0.0981931 0.1502681 0
56.588910 0.03935777 0.6917748 0 0 0.1078924 0.2177325
1.7565650.1170534 0.3167860.0640960.1778065 0.05897619 0.1935807 0
0.2225712.318906103.99340.1495851 0.05821608 0.1246542 0.09719874
0.1713996 2.3114460.1794089 0 0 0.2295576 0.4110885 0.1154692
0.3127780.06875627 0.1798759 0.1636981 0.07968 0.04209814 0.6993352
0 20.188680.0858915 0.2039495 0 0.8725796 0.2823052 5.4191790
0.5564276 0.1196805 0 1.26572 0.1388553 0.007239504 6.6655390 0
1.6929420 0.007812291 0.3177194 0 0.8168830.6121971 0 0.1305901
0.05736795 0.1982112 0.2109533 2.07757 0.2047653 0.1263637
0.01130218 0.1231548 0.02973935 0 0.8826092 0 0 0.1242508
0.05252189 0.06593585 0.1721682 0 0.09844488 0 0 0.1570286
0.04233021 0.03315024 0.2677505 0.06980637 0.1084562 0.1515917
0.3735584 0.01909175 0 0 0.06540631 2.0632024.19736 0 0.1662056
1.4219820 0.9019426 0.05347284 0 0 9.55468 0 0.7244057
0.009586393 0.09119597 1.5134370.4354651 0.01832917 0.4477376
0.1476673 0.0640960.1463273 1.2348790.04370347 1.1883030 0.3996666
0 0.2732634 0.3409462 0 2.6864870.3147008 0.1362998 0 0
0.1863762 0.07083672 1.2161920.3041069 0 0 0 0.2455874
1.2624560 0 0.2825129 0 0.3691683 0 0.2082830 0.3438224
0.7860087 1.73614 0.06133147 1.7730570.7479254 0.3560815 0.03618615
0.2181449 0.2160985 0.1910124 0.02211607 0.09421718 0.7793751
0.2265103 0.2067873 0.01362922 0 0.03449551 0.08221307 0.05858622
0.3405189 0.3139177 0.06540631 0.6271171 0.2366103 3.16578
0.08438132 0 0 0.1305901 0 0 1.58948 0 0.5098940.8995579
0.08970444 0 0 0.9372151 0.1170534 0.04886224 0.2927719
0.09622432 0.1499328 0 1.88168 0 0 0.1106441 0.1354093 0
0.9181055 0.3496676 0.2274796 0.7627848 0.6189058 0.1203558
0.1243057 0 0.1340087 0 0.05092727 0.5112317 0.1106441
0.08512972 0 0 0.1994866 0.5685449 0.1049003 0.1256058 0
0.1951385 0.4464361 0.1298274 0.08735723 0.1511555 0 0.2699016
0.6294017 0 0.08959977 0 0.2572429 0.03224313 0.3318421

0.3089455	0.3863497	0.09375703	0.2903290.1378582	0	1.467391
0.1087635	3.8243531.1204770.04438555	0.04318729	0	0.3588178	0
0.05146582	0.2575017	0	0.07542928
TCGA-ZF-AA5P	372	0	0.1476240.08770355	0.05948588	0.1365498	6.444047
26.318010.8572106	0.3934118	0.1197374	0.3387230.1296668	8.598339
0.1319035	0.6688857	0.06204758	1.6515940.1164891	0.007694006
1.21475500.009772681	0.1076805	0	2.43895	24.213420.06041294	0.4055774
0.3775809	0.05888349	38.211181.49522	1.9020190	0.1057226	0.5910354
2.4707340.05246387	3.2685740.1416199	0.9686197	0.1830815	0.1837994
0.02550327	0	0.2656001	2.87497	0.1040670.03088842	0.02036688
0.3964599	0.3847993	4.5766770.04363786	0.5477658	0.9570403	0
0.6572396	0	1.2747852.1536090.2325898	0.2209879	0.5494247
2.5725841.6801180.2449695	0	0.4187378	3.4438130.05091084	0.3076585
3.8489830.1801160.4259887	0.6041294	0.06202395	1.2886223.108501
0.1865195	0.2702438	0	0.1701284	0.06235652	0.6222148	0.2007248
0.06363606	0.2054277	0.3129011	0	0.1789152	0.08132511	0
0.02853863	0.1795072.3161910.5503373.9751712.4967279.5896940.6537659
0.03084234	0.1346395	0	25.438271.4129290.5609079	1.9595480.3624776
0.3324870.07419133	0.7383803	0.1619706	0.7238272	0.04138609
2.3176630	0.1097122	0.07549731	0	0.2412757	0.1758713	2.961041
0.2034313	0.8051186	0	0.1139732	1.5642	0.02647579	0.73702
0.1057226	1.7772640.03461158	36.957770.08980301	0.1292166	0.9995595
0.1906474	0.1694881	0.2584331	0	0.05645384	0	1.1557260.04605739
1.0034690.5702727	1.2004640.04959271	0.7198137	0	0.03709566
0.2624266	0	0.2683729	0.1753189	0.08411928	0.08662563	0.9720171
2.1610711.0517630.03715492	0.2065629	0.3020647	0.3259709	5.560818
0.0440511	0.4363786	0	0.1071842	0.02853863	0.1911081	0.06861056
0.08351519	0.1568763	0.07576215	0.4179511	0	0.6861754	0.5672922
0.2315149	0.07924695	0	0.2318731	1.0813391.12461	0.4593335
0.2130594	0.4746731	0.4138609	0.1129077	0.5788953	0.0217577
0.2660791	0.4943938	8.1152830.06785	0.2555932	1.4879480.1418231
0.09620323	0.1284351	0.1165864	0.06271599	0.08189782	0.2411393
0.6908608	0	0.2010863	2.1468290.5028969	0.2528150.5083930.1071842
0.5741229	0.1163595	0.2187365	16.868320.2202555	0.2435495
0.06120784	6.3143760.2709934	0.1005431	0.2062881	0.1064484
0.3089428	0.1301462	0.2380601	1.1438840.2029137	0.7991331
0.2659858	0	0.03575005	0.05292146	0.09157079	0	0.07153465
0.61960400	0.05428	0.1240479	0.1947988	0.1220816	0.3337884	0.22119
0.2340526	0.02839924	0.1940775	0.5846063	0	0.5672922	0.1647237
1.18112	14.296170.0107556	0.05232617	0.2760710.07245788	0.1210355
0.06235652	0.2963921	0.01147741	0.1370863
TCGA-GC-A3WC	540	0	0.2709727	0.3380687	0.1637849	0.5012906
0.8085843	55.921880.2622436	0.4485965	0.6318821	0.8289952
0.6694064	0.9478058	0.4842335	0.9208349	0.2440544	0.1147525

0.1069113 0.3742545 0.4262767 0.01793834 0.1235337 0.06187428
1.721338 70.58908 0.2079217 0.5340703 0.2079217 0.4863788 2.477733
1.677235 0.3491271 0.301187 0.09703012 1.021064 0.5714087 0.2568015
0.5651856 0.3134712 0.2000204 0.08401427 0.05793305 0.1404383
0.1166482 0.03482321 9.537855 0.1910213 0.02834877 0.09346159
0.6670821 0.9417629 5.285588 0.6407993 1.662871 0.0931586 0
0.5026678 0.3474135 0.2066235 0.5435484 0 0.3042275 0.4370176
0.09703012 0.2084928 0.321183 0.3610423 0.3843092 0.1945023
0.07342492 0.2984978 0.6299528 0.4849001 2.932229 0.8316868
0.4838569 0.2107731 0.3204822 0.4621964 0.09920973 0.4085479
0.2230578 0.2861478 0.2991247 0.4835805 0.4964332 0.1152173 0
0.4665929 0.6568193 0.07463856 0 0.3055754 0.1412125 0.4423535
0.3721703 0.1164361 0.2412048 1.053554 1.269259 0.03184479 0
0.04640571 2.618326 1.69576 0.51479 0.4300602 0.2464257 0.5132068
0.2383196 2.195653 0.2973068 0.2657256 0.03798332 2.828941 0.0498753
0.6041498 0.01598998 0.4056366 0.3100133 0.3631751 4.755773
0.7468208 0.08210241 0.1425484 0.3874161 1.884995 0.255139 0.8337297
0.1940602 1.564947 0.4447214 3.684382 0.1236291 0.1185924 1.117317
0.2332965 0.3509909 0 0.8940996 0.3108732 0.4332341 1.710382 0
0 0 0 0.3186064 0.6606306 0.4295096 0.9192327 0.5235866
0.1663374 0.5747169 0.5363474 0.1930075 0.1192549 1.593032 1.54637
0.02757962 0.2046003 0.8720647 1.594066 0.677531 0.7980674 1.091589
1.842298 0.5310106 0.4795613 0.02619218 0.8460239 0.1574235
0.1916214 0.2803781 0.06953299 1.553528 0.4113911 0.4777475
1.419953 0.5463765 0.6545814 0.5811241 0.2708472 0.9237722
1.266723 0.2299457 0.1303611 0.508253 0.4937832 0.259061 0.6891099
0.07987512 0.5726117 0.5362438 0.3823773 0.2864483 0.1172892
0.2156225 0.1301624 0.1833786 0.493953 0.05350032 0.2282531
0.03758209 0.3017902 0 0.2138941 0.3075883 0.4517222 0
0.5568685 1.671958 0.1686369 0.7711003 0.2254508 0.1338347
1.090107 0.1617169 0.1862707 0.05617533 0.251692 0.2913488 0.1230353
0 0.1465443 0.2430356 1.100605 1.059893 0.1749724 0.3259027
0.5018194 0.2441164 0 0.1640534 0.4857025 0 0.0883461
0.3939184 3.368218 0.2197851 0.0996342 0.7400164 0.4171591
0.1478981 0.4281858 1.04625 0 0.05212851 0.8293735 0 0.3701727
0.8641707 0.3023602 1.459242 4.322724 0.3010738 0.2401195 0
0.332502 0.1295979 0 1.80394 4.118693 0.07339214
TCGA-K4-A4AC 278 1 0.01673117 0.03578401 0 0.09285635 0.2310857
31.06542 0.05829188 0.1702446 0.2442707 0 0.04959888 0.5967035
0.08969677 2.046845 0.2260366 0.2372776 0.118822 0.1569619 0.03644362
0.0199368 0.05491851 0.4126052 1.141815 18.77206 0.1694628 0.5575957
0 0.06006278 1.4828 1.331494 0 0 0.03594666 0.9929661 0.1336985
0.05351458 0.09663868 0.2719174 0.1543779 0.2334352 0.07574967
0.06503508 0.3889311 0.1161083 2.163355 0.1061512 0.03150703 0

0.3369999	0.6977881	2.8095150	0.3008579	0.02958204	0	0.620743
0.2145099	0.0730682	1.09837	0	0	0.2988951	0.03594666	0.06758514
0.1204757	0.1504744	0.1941473	0.1621284	0.007418636	0.08966287
0.1329368	0.2694608	0.3476161	0.3081142	0.06326612	0.0520566
0.04888831	0.07610199	0.01837707	0.1135158	0.0495816	0.06360535
0.1511134	0.02559309	0.1622763	0.05238547	0.2127784	0.04321457	0
0	0.0502644	0.03881357	0.05231488	0.01365652	0.2718158	0.04313599
0	0.5043996	0.8720466	0.05112255	0	0	0.4445873	1.330362
0.6102842	0.08690402	0.2738793	0.2774830	0.8736750.09912868	0
0.08442988	1.5960540	0	0.01184759	0.05635344	0.3937726	0.08969677
0.1812206	0.4841795	0	0	0.03875194	1.2764220.06751507	0.5070148
0.10784	0.6936153	0.1765238	0.6332241	0.1282421	0.1318044
0.6535757	0.1944655	0.1551509	0	0.04140453	0.1151689	0.2626361
0.5894359	0.09395959	0	0.1246487	0	0.4046874	0.7801190.04773601
0	0.2153097	0	0	0.2384402	0.04290198	0	1.4872260.6725139	0
0	0.1685598	0.4621714	0.6258809	0.4770301	0.4043999	0.5341418
0.05901691	0.06833179	0	1.3186810.1749616	0	0.05053205	0
0.2771090	0.1810132	0.2630243	0.3457933	0.3637533	0.08611541
0.1548111	0.5896514	0.7299937	0.1277817	0.03622106	0.8069659
0.2110747	0	0.4969462	0	0.2526901	0.1203432	0.2032498
0.04152531	0	1.8173030.0723317	0.04529073	0.1497226	0.2973032
0.1235322	0	0.06708236	0	0.04754468	0.3418559	0.1653083
0.2564843	0.5673321	0.3889311	0.2030430.9712740.07912661	0.0495816
0.3700998	0	0.1242136	0	0.2587524	0.1579547	0.1367423
0.05260487	0.2171606	0.1350558	0.2086113	1.0281430	0.1034888
0.08580397	0.06029173	0	0.02917282	0.02699067	0	0	0.312717
0.7778622	0.06106769	0.04152531	1.2020560.2119468	0.03486743
0.2437479	0.2429752	0.05968502	0	0.3526241	0.1703756	0.06856878
0.9902731	0	0.3706999	0.9043401	0.09325353	0.2134965	0
0.07390902	0.03086487	0	0.5091841	0	0.1165265
TCGA-FD-A3SL	712	1	0.1293376	0	0.05211727	0.02658559	1.4996915.7808
0.1502053	0.3237895	0	0.1978432	0.02840119	4.5210940.1155644
0.1864640.06989354	0.7922880.1360791	0.02696375	1.4712120.01712424
0.07075643	0	8.99223417.285540.03308093	0.1699442	0	0	4.366682
1.2476240.4285062	0	0.4013819	0.2132212	0.9933413	0.04596508
0.2905193	0.08028523	0.2970223	0.06683454	0.1886838	0.02234413	0
0.09972847	2.0233330.04558803	0.08118666	0.05353201	0.1736749
0.1498371	2.9006750.07646473	0.6275787	0.4192451	0.07660846
0.2452593	0	0.3573489	0.99059	0.05094463	0.09680689	0.2567288
0.3396309	0.6903881	0.06132135	0.1292464	0.3668681.3925640.01274412
0.1694304	0.8068929	0.2209266	0.7091194	0.2205395	0.0815114
0.2794549	0.5878803	0.1634150.06313819	0	0.3619890.05463231
0.1557543	0	0.1393834	0.1549837	0.0913805	0.07423625	0.0522509
0.1425025	0.04317341	0	0.1797385	0.4310756	0.1065840.6298608

1.093726 4.388123 0.7710538 0.40195 0.02949038 0.04429968 8.262229
0.3332826 0.36038 0.6717973 0.2293611 0.3839881 0.09750168
0.8409907 0.05676282 0.3804993 0.1450381 4.133292 0 0.2403048
0.04070482 0.04840345 0.0422777 0.1540858 1.29064 0.2376426
0.3135054 0.03401978 0.1035535 1.418108 0.01159809 0.8259262
0.1389399 0.762759 0 11.09541 0.06294317 0 1.268704 0.4454175
0.06472785 0.1132103 0.03556344 0 0.15039 1.316333 0 0.408185
0.214128 0.26294 0.04344958 0.1576623 0.04100171 0.06500112 0.2549106
0 0.4310699 0 0.1842482 0 0.5474647 0.5134577 2.501157
0.09765743 0.2533658 0.1323237 0.6383828 1.434068 0.1543777
0.7646473 0 0.03521518 0.1750245 0.22653 0.06011165 0.2195101
0.2097826 0.05310189 0.7323576 0 0.1451119 0.3614696 0.3911852
0.0694305 0.184917 0.062792 0.423049 0.3135054 0.2195101 0.03111122
0.831749 0.2538166 0.2967648 0.9641566 0.01906254 0.1688106
0.6448064 1.936213 0.05944531 0.1119662 0.3087553 0.310638 0.02593427
0.1500341 0 0.04547359 0.0358765 0.3265066 0.4035218 0 0
1.630228 0.1835842 0.132899 0.59389 0.1341531 0.7606422 0.05663661
0.1916412 3.259856 0.07718883 0.03556344 0 2.0513 0.2035072 0
0.1355511 0.2797874 0.1160029 0.3502189 0.2107901 0.3340631
0.08888921 0.07369928 0.1553585 0.07898392 0.06890758 0.02318299 0
0 0 0.751642 0.07867896 0 1.14116 0.1479129 0.0727322 0.2060383
0.1192559 0.02563252 0.1244069 0.1275276 0.4024351 0.02944776
1.12214 0.05772762 0.2786034 10.85039 0.03298149 0.2292222 0.1209368
0.0634824 0.04418441 0.1912131 0.1093379 0.01005569 0.1100964
TCGA-K4-A5RH 276 0 0.1095158 0.1003836 0.05673856 0.08682887
0.8643419 14.24257 0.2725403 0.3354438 0.171311 0.2692327 0.02318966
1.867175 0.314529 0.4204958 0.08454564 0.174727 0.1851818 0.003669331
0.51117 0.1165167 0.1668994 0.4822778 3.40638 12.86118 0.1368541
0.6980047 0.5402137 0.08424599 0.5447155 0.7130821 3.343267
0.1173798 0.1176465 0.9119303 0.1375219 0.05004085 0.5647856
0.07548545 0.1299213 0.1382456 0.08942618 0.07297624 0.06061414
0.09047625 0.03371547 0.07444556 0.02946185 0.08741812 0.06302493
0.1427329 2.242361 0 0.5023727 0.4909972 0 0.3714876 0.7221085
0.05368409 0.6419206 0.02773097 0.02634772 0.06987331 0.01680665
0.02708488 0.1063968 0.09380455 0.5446344 0.6064176 0 0.06707415
0.1077333 0.2519696 0.5688404 0.576228 0.2662173 0.3468273
0.04244953 0.06226681 0.1288814 0.05307363 0.1854527 0.1486915
0.4521738 0.0119659 0.1213941 0.1986615 0.1989666 0.1616377
0.1137681 0.3490612 0 0.04536764 0.09783804 0.03990642 0.4558516
0.2016798 0.03133443 0.6771663 0.6475556 0.03125652 0 0.04822778
0.5291088 1.036672 0.3745019 0.223473 0.4641836 0.2666786 0.03538242
0.5634229 0 0 0.05921204 3.411316 0 0.1308065 0.06093197 0
0.1380795 0.08387439 3.163202 0.6144472 0 0 0.1147488 1.232488
0.01893976 1.593969 0.1764698 1.098183 0.4456763 0.8684432 0.1199177

0 1.057291 0.06061414 0.1326444 0 0.2323013 0.1615396 0.5525728
0.9025484 0 0.03418301 0.5827875 0 0.09460459 0.1501871
0.06695607 0.1415297 0.4788473 0.144057 0.3413043 0.1393516
0.2005857 0.04131243 1.953585 2.397043 0.04299375 0.1063168 0.09851144
0.6842707 0.06629828 0.2962152 0.3151247 0.2913573 0 0.051117
0.08166175 0.4825108 0.08180227 0.1194871 0.2598856 0.03613156
1.883613 0 0.06770532 0.6394725 0.7255597 0.1511739 0.06039413
0.1025398 0.9859901 1.29208 0 0.06773978 2.490128 0.1381614
0.3230793 0.8829082 0.01037642 0.2523318 0.5171089 0.4751412
0.09060294 0 0.7189511 0.5410921 0.05646777 0.3150087 0.05560094
0.1144821 0.01952885 0.05227327 0.07321708 0.08891694 0.09589961
0.3921696 0.3997257 0.3617083 0.9496216 0.1387461 1.248812
0.04316104 0.1159079 0.297103 0.02100831 0.07743378 0.0291905
3.531254 0.424642 0.03196654 0.09838038 0.1776813 0.1683854 0.2438375
0.8985994 0.2121495 0.07257833 0.08023427 0.07047261 0 0.02386926
0.07571596 0.2183541 0.005738432 0 0.5682576 0.02855184 0.04530147
0.08873911 0.1238681 0.04424845 0.2604866 0.3894913 0.627871
0.01354382 0.1359432 0.9758115 0 0.4713637 0.8641379 0.6716094
13.10078 0.06411785 0.4866177 0.0987453 0.1036672 1.115973 0.02973831
0.2975825 0 0.03268877
TCGA-FD-A5C1 1792 0 0 0.09749228 0 0.08432796 0.1165898
55.42543 0 0.09939116 0.3327536 0 0.06005796 0.6115288 0.08145858
0.6196176 0.0821105 0.2020171 0 0 0.06619292 0.02715856
0.07481179 0 1.795769 43.29927 0.06995388 0.1796844 0 0 0
0.9014057 0.7551096 0 0.03264514 0.1610299 0.03035474 0.03239969
0.04388145 0.06945278 0.1289832 0.9751786 0.05331415 0 0.1177366
0.1054443 0.08731832 0 0.08583982 0.08490024 0.06120964 0.1188187
4.123597 0.1212709 0.5074191 0.5775992 0.5669946 0.4509847 0
0.03520997 1.296738 0.05386449 0 0.2714431 0.03264514 0.02630476
0.0607837 0 0.5642107 0.196317 0.07410999 0.05699946 0.08048485
0.2224656 0.03946116 0.6529029 0.02872773 0.0709132 0.02537038
0.05183431 0 0.03436331 0.1801111 0 0.1372344 0 0.05894882
0.1374364 0.4830896 0 0 0 0.09129574 0.05287312 0.07126503
0.0155028 0.2200193 0.1566967 0 0.1766853 0.9084173 0.1678522
0 0 0 2.718395 0.3810349 0 0.08705371 0.1259988 0 0.3283169
0 0 0 2.866234 0 0.2540778 0.0215189 0 0.2682049 0.2036464
1.176036 0.06281573 0.2486053 0 0.02737215 0.8819916 0 1.587752
0.04896771 2.242862 0.09618658 0.03833771 0.07486956 0.05984943
0.8072254 0 0.0603862 0.03989962 0.1128052 0.05229562 0.03975239
0.7092715 0 0.03319845 0.1509338 0 0.09187972 0.7084691 0
0.06872662 0.169111 0.0559631 0.1657369 0.05413516 0.116885 0
0.5788422 0.1885021 0.05567389 0 0.2678873 0.2098616 0.09325261
0.2436848 0.08161286 0.4850836 0 0.01241117 0.1586193 0.6039904
0.2224492 0 0.2600486 0.01403635 0.3871661 0 0.04383681

0.4777338	0.3370121	0.1101148	0.1173092	0.06248554	0.3212969
1.894136 0.03868186	0.06578869	0.7328501	0	0.05229562	0.621207
0.02015509	0.1954845	0.07806447	0.4362856	0.03771143	0.1578446
1.958709 0.0328442	0.006855168	0.03965831	0	0.1302163	0	0.3452201
0	0.2158897	0	0.3243453	0.2717488	0.1873547	0.4709463
0.1985788	1.089613 0.03592962	0.2476528	0.05073329	0.1224193	0	0
0.1428977	0.07889605	0	0.04777338	0.04930388	0	0.1463943
0.4058605	0.1177366	0.07048788	0	0	0	0.03974017	0	0.08482596
0.03343889	0	0.2207561	0.02772947	0.01257048	0.9192872	0.06015018
0.04071219	0.03865032	0.03152274	0.4065244	0	0.1292186	0.270773
0.03113554	1.121443 0.09155437	0.2524898	1.796552 0.04483518	0.5574278
0	0	0.1868673	0.05776352	0.4624181	0	0.2751428
TCGA-XF-AAN7	565	1	0.2405973	0.0321613	0	0.1669115	0.04615355
20.87614 0	0.06557542	0.1097706	0	0.02971838	2.193114 0.2418481
0.5574625	0.2112791	0.04798261	0.07119513	0.2962499	0.7205909
0.05375531	0.1974346	0.1236112	1.590301 57.32706 0.4153819	0
0.5538426	0	0	0.7832917	3.885956 0.2256396	0.06461497	0.7012028
1.670267 0.09619371	0.1737103	0.07637156	0.7658943	0.05594737
0.09190922	0	0	1.460952 0	0.09540465	0.1132693	0.05601473
0.2423061	0.07839316	3.698357 0	0.8498273	0.132936 0	0.357056
1.310995 0.12062	1.184608 0.1066147	0	0.4029532	0.4523048	0.03904903
0.04812399	0.1803208	0.8375623	0.3885729	0.04000552	0.03223423
0.04779142	0.3082304	0.9372721	0	0	0	0.06276991	0	0	0
0.3119343	0	0.4889337	0	0.1750173	0.1360148	0	0	0	0	0
0.01744208	0.3291303	0.09205471	0.4142439	0	0	1.256386 0.9220731
0.04948138	0	0	0.2179517	0.4981995	0.27425	0	0.2830751
0.3879417	0	0.4603047	0	0	0	3.365077 0	0.4023196	0.0532408
0.1012966	0.2654308	0.1612321	0.1425149	0.497328 0.1640226	0
0.08513697	1.421529 0	1.885595 0	0.6611764	0	0.4552941	0.08232795
0	0.9868468	0.2330376	0.1274914	0.07897385	0.2232769	0.1035094
0.3934122	1.324406 0	0.06571014	0.2240589	0.2751347	0.1818588	0
0	0	0.6171467	0.8861482	0	0.1071505	0.3084693	0	1.14571
0.544733 0	0.06812441	0.6817281	1.246146 0.1142614	0.3483484
0.08076871	1.200165 0	0.1965248	0.3662836	0.6389668	0.2515981
0.07656352	0.3936077	0.1944763	0.3831615	0.0913188	0.0433834
0.4727925	0.6367319	0.2179517	0	0.1082187	1.633512 0.468636
0.07656352	0	0.435162 0.1517647	1.035094 0.2311999	0	0.2803805
0.2987272	0.0664266	0.1244046	0.07810601	0.2871776	0.06500896
0.04070558	0.2691299	0.1068819	0.1229216	0	0.2009702	0.281491 0
0	1.250946 1.075752 0.3708337	1.398226 0.2245998	0.872944 0.07111598	0
0.2133863	0.1615374	0.07442562	0	0.2325568	0.31232	0	0
0.09758783	0.08092197	0.1534024	2.145296 0	0.1395176	0	0.1083758
0	0.09176796	0.1940654	0	0	0.2623209	0.3495564	0.0548853
0.04976182	0.1137223	0.07143363	0.0358143	0.2016847	0.3431636

0.1072852 0 0.189043 0.3062541 0 0.4825408 0.3624296 0.7496346
14.42696 0.08874287 0 0 0 2.607575 0 0.2002154 0 0.04189183
TCGA-2F-A9KO 734 1 0.2222748 0.6338577 0.2015254 0.4112012
0.2558326 121.8891 0.06453434 0.094238 0.0338037 0.2550046 0.07321388
1.572164 0.6951166 1.545028 0.2102039 0.0689555 0.08769776 0.1303282
0.6051953 0.06621549 0.06079972 0 3.084886 108.4979 0.2558326
0.2986976 0.5116652 0.06649488 1.289823 0.7236407 0.06136794
0.3242652 0.07959236 1.648954 0.3700405 0.07899392 0.5884323
0.4045185 0.2734563 0.09475902 0.1027306 0.3887982 0.2870544
0.1713897 3.486097 0.2350378 0.1046433 0.05749886 0.895414 1.834721
6.896259 0.2463929 1.379889 0.2128748 0.0987424 0.7696843 0.7124452
0.1700405 1.763192 0.06566369 0.2495532 0.08272591 0.07959236
0.3821308 0.2420554 0.3331773 0.2149384 0.239321 0.09855714
0.4169123 0.1569846 0.5152753 0.8658949 2.103512 0.7354334
0.2881566 0.1701032 0.1895664 0.2237957 0.1256721 0.4391302
0.9858356 0.5688065 1.331691 0.1796544 0.08377115 0.3533472 0
0.4040843 0.09183734 0.1112944 0.1181678 0.08687589 0.1474102
0.3663429 0.2865325 0 0.8376234 3.464204 0.06965815 0 0
1.029141 2.147878 0.8867766 0.1924211 0.1212836 0.2730654 0.293235
1.450855 0.6950475 0 0.1402072 2.311681 0.06136794 0.4955751
0.01311634 0.3119415 0.3814488 0.09930237 0.6854771 1.07206 0
0.2192444 0.2240431 2.07359 0.1943376 0.425822 0 2.041901 0.586283
2.196579 0.09127 0.1459193 1.172178 0.2152908 0.3827928 0.04863977
0.2291927 0.1912535 0.9692058 0.8972679 0.05201085 0.1618828
0.1379973 0.3953943 0.3920221 1.320894 0.1056961 0.08378143
0.3350024 0.136444 0.5556159 0.1319873 0.4749634 0.09782301
1.881706 1.709669 0 0.2517458 0.6997907 2.131938 0.3735198 0.618885
0.8456688 0.8377357 0.196011 0.2118184 0.03222758 0.3554535
0.07747929 0.2829315 0.05594351 0.1197774 0.377581 0.168729 0.1469584
0.6406214 0.1680692 0.2237264 0.6196937 0.138064 0.6911968
0.5772633 0.4715526 0.2004998 0.3573534 0.9814502 0.2550046
0.7896472 0.02457014 0.3626397 0.4123807 0.2863837 0.1532408
0.1443158 0.3758528 0.6806603 0.1587795 0.4765496 0.3949696
0.04395896 0 0.1980432 0 0.05263623 0.3784651 0.4744732
0.3312763 0.6280875 0.717636 0.1210391 2.103136 0.1022003 0.02744564
1.932706 0.2487261 0.04583853 0 0.1277466 0.6907366 0.03784651 0
0.06010407 0.2491981 3.201827 1.358461 0 0.4582853 0.09499269
0.4672385 0.2036084 0.3956374 0 0 0 0.7847331 2.744958
0.4056444 0.0919445 0.4202476 0.3373009 0.04963034 0.3598011
0.9222699 0.06607667 0.03207018 1.013635 0.4243973 0.1518236
0.6472258 0.1116097 2.308489 5.741174 0.2672095 0.2363598 0 0
0.1366807 0 0.5989445 0.006480503 0.1161048
TCGA-DK-A3IM 248 1 0.08428698 0.05633434 0.03820938 0.3703294
0.04850606 32.3575 0.03670729 0.3369319 0.672967 0.07252362 0.0728774

0.0965623 0.3106589 0.6835233 0.1537258 0.02614804 0.09976539
0.4299598 0.04589821 0.1757631 0.08645756 0.1299119 2.176102 45.5273
0.2764845 0.4077598 0.6790848 0.03782245 0.06669583 0.2286714
0.1745312 0.1580938 0.02263616 0.4019695 0.1220784 0.1460288
0.2434194 0.1337739 0.01166569 0.3429946 0.1001715 0.04095358 0
0.02437172 0.7114222 0.1671126 0.03968092 0.02616439 0.5093136
0.7415007 4.592758 0.1401488 0.7036894 0.1490261 0.3369895
0.3283487 0.6213688 0.007512189 0.4495793 0 0.03548662 0.2823282
0 0.001519978 0.1292522 0.09475602 0.146709 0.03403159 0.01868651
0.1806787 0.04464659 0.2468125 0.8755966 0.09701212 0.09959911
0.0901473 0 0.1557484 0.1157232 0.07148262 0.218556 0.2002663
0.1332217 0 0.1635006 0.0146613 0.06699492 0.08163862 0.5363029
0.0522373 0 0.0244415 0.1811891 0 0.275355 0.02716339 0
0.1361263 0.856014 0.03466901 0 0.09743391 0.02545122 0.9773744
0.3602862 0.1094496 0.2414524 0.3591781 0.09531015 0.9959911
0.270499 0 0.02658339 1.450913 0.06981246 0.4228264 0.01492122
0.1064599 0.2789602 0.1412086 0.02852934 0.3484517 0.05746103
0.02494135 0.1301477 1.424093 0.05952138 1.034891 0 0.5757557
0.02223194 0.1595004 0.2884144 0 0.6996632 0.04081931 0.1200326
0.08299926 0.1303653 0.1813091 0.5237223 1.048574 0 0 0 0
0.2229831 0.2311778 0.06012019 0.1667928 0.421409 0.03880485
0.7757238 0 0.1620962 0 0.3121763 1.50575 0 0.2147904
0.3184341 1.067133 0.1447184 0.01877457 0.6507896 0.4484763
0.03716385 0.103271 0 0.3610408 0.0661056 0.08046607 0.1060693
0.02919844 0.08053836 0 0.09118942 0.2318826 0.2655504 0.07635366
0.3524832 0.1462303 0.4193647 0.3283487 0 0.3421343 0.05081587
0.3455841 0.2175709 0.8504458 0 0.4282565 0.3356057 0
0.1307456 0.02736239 0.5533284 0.1138709 0.1045743 0.2671341
0.1123298 0.1611366 0.02630258 0.197133 0.09861298 0.1796377
0.04305437 0.06040204 0.5114549 0.1623899 0.8708119 0.1967066
0.8274941 0.03321816 0.01561115 0.008794623 0.1697712 0.0782192 0
0.2356029 0.3282394 0.06458156 0.03312609 0.1367494 0.2267911
0.280645 0.8784329 0 0.08146056 0.02701603 0.07593325 0.05790646
0.08726021 0.08498218 0.2352735 0.0154577 0 1.683796 0.1345934
0.06101462 0.4780757 0.1418078 0.07841626 0.3021108 0.2404368
0.1691306 0 0.3622966 0.1072881 0 1.100674 0.04232257 0.5544091
2.010965 0.05181457 0.0336105 0 0.1396249 0 0.02002663 0.3507011
0.003686125 0.1834463
TCGA-FD-A3SS 391 1 0.1512934 0.1040081 0.1567659 0.2299079
0.09950561 36.88522 0.03765077 0.2356309 0.2761057 0.1487754
0.0427146 0.9344607 0.3765789 1.141778 0.1985562 0.1226062
0.05116482 0.005069092 0.07061689 0.03863159 0.4433989 0 3.011048
43.28749 0.2537393 0.08132453 0.5970337 0 0.4104607 1.110198
0.1074103 0.2702622 0.0928719 1.191093 0.3108814 0.2534775

1.0611230.2414936 0.08774727 0.1809312 0.07828283 0.1008149
0.1674739 0.02499815 2.7480490.1028447 0.1221025 0 0.2176685
0.8450661.3701660.08625064 0.6940156 0.3057130.2304340.4650885
0.02771042 0.06067904 0.3547191 0.03830966 0.1455949 0.1447924
0.04643595 0.07639326 0.1325743 0.1619859 0.9530390.3839693
0.02875021 0.1853226 0.2003494 0.1977783 0.3648539 0.6302022
0.2451818 0.1681170.08570898 0.2580603 0.1068279 0 0.3682851
0.5751584 0.1952085 0 0.2305917 0.04887395 0.5497350.08373696
0.4715035 0 0 0.1002789 0.1520560.04851418 0.3320489 0.02786157
0.1731510.3979318 0.8448833 0.03048009 0.04435272 0.1665637
0.3132647 1.1099060.5912745 0.1122627 0.08402696 0.1493554
0.2443997 1.3037450.1280546 0.1271690.1090666 3.3252090 0.07228238
0.01530473 0.03639873 0.09537675 0.7531578 0.3487131.2062520
0.07674723 0.3337321 1.2456240.08721607 0.7227173 0.1393079
0.3088531 0 0.6271332 0.7514018 0 1.4015212.8470570.1517498
0.1986427 0.4011483 0.2603569 0.6220019 0.7328805 0 0.0236115
0 0 0.4247553 0.8891991 0.3083272 0.1221999 0.4510335
0.1990112 0.4125656 0.03850217 0.2216834 0 0.3202001 1.276318
0.2771758 0.1468741 1.1431661.24382 0.1926537 0.4092141 0.8416516
0.5175039 0.1524763 0.1677151 0.07520915 0.2962564 0.2712188
0.02751143 0.06527738 0.07986379 0.1101446 0.06562683 0.2805998
0.3397753 0.4031162 0.2610539 0.4171651 0.3333086 0.7527512
0.1683941 0 0.2573473 0.05212199 0.1363333 0.07438769 0.679718
0.01433477 0.5138179 0.6440465 0.3938376 0.1698678 0.1122627
0.7868314 0.2102359 0.07800885 0.2578825 0 0.1781016 0.02697863
1.6753710.05057381 0.03070915 0 0.3045 0.02761057 0.2331892
1.1164930.2219388 0.7934090.09369789 0.06404959 0.3495510.02902247
0.2406890 0.2303661 0.2142484 0.2208050.03397753 0 0.2326203
0.9615694 2.0089210.04186848 0.3175065 0.02771042 0.1752411 0
0.09892442 0.3660991 0 0.03963752 0 2.9831290.0591655 0.08940411
0.9807273 0.1026725 0.1029527 0.1399440.4259743 0.0192753
0.01871046 0.3316508 0.05502285 0.1992988 1.4179490 2.06513
2.6467660.08857725 0.06894877 0 0 0.03322607 0.02054137 0.33916
0.007561739 0.08279102
TCGA-4Z-AA7N 1367 1 0.2617005 0.2176671 0 0.9951715 1.115595
103.035 0.3039241 0.3381435 0.2122645 0.8006286 0.5746672
3.6672891.3250481.0779710.2985576 0.1134032 0.4130121 0 1.5834250
0 0.2390283 4.04110171.8245 0.08032281 0.1563016 0.2677427
0.2087715 4.9699741.3884370.1926746 0.07272024 0.3123665 1.355923
0.4182489 0.2170125 0.2519290.2953608 0.2468362 0.05409298
0.1250663 0.3164766 0.07510451 0.1345265 6.5169710 0.1095149
0.03610541 0.7028246 0.9095377 4.7224880.3867953 3.8095130.5655313
0.1550089 0.7551717 0 0.6349423 1.0498980 0.1958783 0.1298657
0.7496795 0.3230128 0.2791735 0.1743441 0.9447681.2210040.0515727

0.2025778 0.6931105 0.7876086 3.171721 6.872062 0.3848355 0.09047147
0.5462046 0.2645221 0.7345814 0 0.2585102 0.4421702 0.5252532
0 0.2820272 0.6878806 0 0 0.3171721 0.1441691 0.1747135
0.2023675 0.09092034 0.9256368 0.9037233 0.8246475 0.3494269
1.108296 3.075712 0.05467578 0.05967059 0.2689068 3.020429 1.156048 0.19887
0.453103 0.1308964 0.3482917 0.06576136 0.8377371 0.2871335
0.08554435 0.2934689 1.814475 0 0.4862308 0.0411809 0.1958783
0.08554435 0.467664 0.9973458 1.562743 0 0 0.434025 1.374412 0.1408049
0.9115484 0.1874199 1.954314 0.1840731 3.741728 0.1591984 0
0.5225039 0.1126568 0.2542355 0.1527125 0.3597938 0.2001572
0.5325214 0.6402543 0 0.06353216 0.07221081 0.3546871 0.351662
0.8772846 0 0.1315227 0.4045364 0 0.7136373 0.3107968
0.298245 0.07678282 7.631066 1.947986 0.266359 0.1975992 0.1464738
0.9370994 0.7053353 0.6995125 1.249466 2.243413 0 0.7837951
0.5059187 0.5380735 0.1216294 0.1480516 0.2634657 0.02686148
0.8520617 0 0.2307 0.3656973 0.1905513 0.4916978 0.5986552
0.2092635 0.5425312 0.5437236 0.2220774 0.5665517 0.5609847
0.6603049 0.3002357 1.392024 0.07714195 0.5313311 0.6573277 1.57351
0.5051808 0.07551717 0.3470739 1.005668 0.1705443 0.3794723
0.1033393 0.0575068 0 0.2720326 0.1360804 0.08263001 0.3564759
1.230764 0.6686331 0.7170848 3.079285 0.08143326 0.8440101 0.04583922
0.2585102 6.165135 0.2342749 0.1439175 1.7361 0.1215398 0.5353071
0.4158885 0.09142433 0.5661196 0.1564796 0.9228669 0.3950837
0.3379703 0.6295019 0.298245 0.1571755 0.1598154 0.139427 0.1407248
0 0 0.2898585 3.126225 0.2653306 0.09622491 0 0.09208794
0.1731362 0.2286205 0.8445549 0.05186462 0.1006896 0.5375799
0.1480516 0.05958436 0.5391208 0.2336112 0.8053198 9.823079
0.1620694 0.4174256 0.1223513 0.5137991 0.339729 0.1658138
0.7466627 0.02034659 0.3037749
TCGA-HQ-A2OE 1174 0 0.1592084 0.4086108 0.3175617 0.3387098
0.1465958 48.70443 0 0.08099961 0.2760227 0.3835685 0.01573223
0.09357658 1.024231 0.5902156 0.1591659 0.1397049 0.05653359
0.01120199 0.121375 0.04742804 1.032109 0.09815542 2.836809 38.63555
0.3958086 0 0 0.1714614 0 0.4952843 0 0.1592645 0.08551419
0.7086557 0.1971959 0.1442811 0.5287599 0.3881217 0.06463644
0.1036604 0.2883237 0.1423361 0.08224316 0 1.521061 0.2777777
0.04497161 0.1828596 0.3527461 0.89224 3.493705 0.508272 5.316752
0.04222397 5.007403 0.4548227 0.5103017 0.005675864 1.56776 0.02821968
0.2144964 0.4799568 0.05130852 0.006890553 0.08916507 0.2386443
0.4249118 0.3599777 0.01411867 0.3327491 0.04216613 0.7983679
3.121738 2.712022 0.1053535 0.006191922 0.003322895 0.1086243 0.008743512
0.1800299 0.766679 0.1210496 0.8340085 0.06088389 0.3088338
0.04984831 0.1518548 0.02056079 0.6367518 0.03946809 0.02391499
0.1662018 0.01244529 0.06010236 0.7534621 0.02052341 0 0.7970936

2.1965559 0.01309715 0 0.04907771 0 1.397797 0.1451817 0.2067376
0.4560757 0.2567093 0.09001494 0.9245306 0.3301467 0.07025648
0.2008518 3.106021 0.1582412 0.5324469 0 0.3753687 0.1170941
0.4267627 1.016699 0.9543683 0.0434149 0.07537812 0.3216327 1.50505
0.2762542 1.43074 0.1539256 1.252541 0.03359486 0.08034074 0.1002398
0.06271041 0.3793721 0.5243001 0.134982 0.02090347 0.3545929
0.2465798 0.8122271 0.2594108 0.1117611 0 0.1581488 0.024275
1.564417 0.6550023 0.2952563 0.1260209 0.7170859 0.02931915
0.4992713 0 0.7756586 0.1261215 1.448891 0.5569869 0 0.1803175
0.4410892 1.7225 0.9282485 0.0248241 0.6841136 2.393114 0.08423786
0.4876674 0 0.2618743 0.382922 0.4863716 0.09616932 0 0.1825532
0.07251311 0.2296617 0.9761132 0.1043316 0.4230549 1.270142
0.2046017 0.6271041 0.1736596 0 0.1723339 0.03839413 0.7431518
0.2465798 0.9318476 0 0.5061361 0.2699274 0.02637354 1.066882
0.2067376 0.4655773 0.7399064 0.2801318 0.6915789 0.1414521
0.07451781 0 0.1170279 0 0.2488309 0.1951794 0.103897 1.220311
0.1226943 1.418695 0.1634847 0.9785995 0.06901979 0.04718025
0.2259236 0.427571 0.2560949 0.02970493 0.006654605 0.6876434
0.1301196 0.05005709 0 0.04283823 1.930939 1.041031 0 0.1600244
0.02041207 0.4016016 0.1750058 0.3747588 0.05136689 0.1777618
0.005839562 0.03471666 3.585288 0.1452751 0.09219967 1.926464 0.2268919
0.04502826 0.2816446 0.7844535 0.2981702 0.1929551 0.6239972
0.04053096 0.09787174 0.4200667 0.06395396 0.3086528 18.51507
0.120056 0.05078908 0.06699035 0.3868119 0.03426501 0.0302624
0.09841875 0.7742484 0.08316213
TCGA-UY-A8OC 0 1 0.01630993 0.01744153 0 0.09051849 0.3003568
87.88028 0.05682426 0.213375 0.1785905 0 0 0.9261389 0.1748769
0.6348719 0.008813824 0.1329996 0.03861014 0.09180605 0.8881519
0.02915228 0.1873754 0 1.146238 74.07034 0.02252676 0.0175341
0.4505352 0.1171011 0.309743 0.6371855 0.9726508 0 0.1401665
0.4148426 0.09123264 0.06955632 0 0.06626784 0.1805893 0.2199727
0.05722795 0.05071815 0 0.1509134 2.108888 0 0.3685653 0.02025173
1.51117 0.08502748 6.480396 0.04339113 0.7960544 0.1730235 0
0.5688076 1.463764 0.2907288 1.070716 0 0.1098692 0.2549485
0.1401665 0.06823652 0.1130928 0 0.1135555 0.7375504 0.02169557
0.113627 0.01727865 0.02387968 0.2541481 0.5005947 0 0.2029839
0.05446565 0.09273246 0.1970583 0 0.1208332 0 0.117847 0
0.1581907 0.09078498 0.1037106 0.04212655 0 0.0808653 0
0.04729545 0.02549887 0.0366099 0.3801777 0 0 0.2212651
0.9000963 0.5865247 0.06693915 0 0.1575978 0.2161446 0.2974595
0.08471602 0.1802141 0.1953588 0.1106578 0.1762093 0.1288439 0
0 1.228318 0 0.3818215 0.0230986 0.05493462 0.04798231 0
1.383821 0.2697082 0 0 0.046171 1.081987 0 2.062214 0 0.9630045 0
0.2880646 0.01785905 0 0.6243781 0.06318982 0.08642554 0.08565731

0.04036209	0	0	0.4740414	0	0.2138133	0.04050346	0	0
0.1342020.04653415	0.07377185	0.2893062	0.06007136	0.08895183
0.3486554	0.08364367	0	0.4142234	0.3156506	0	0.1477794
0.2464740.3003568	0.1048649	0.8864442	0.08760407	0.3905202
0.1725931	0	0.1986409	0.3018085	0.2387792	0	0.0738897
0.04520028	0.2493528	0	0.09410977	0.2051217	0.4357469	0
0.04197364	0.02096019	0.9940757	0.5082961	0.2491290.3177821
0.2359946	0.2880646	0.6736158	0.1709769	0	0.1490133	0.418976
0.6124098	0.09445293	0.04235801	0.1168054	0.4583189	0.07358407
0.1641972	0.1159272	0.03225589	0	0.1961803	0.0763283	0.04634764
0	0.6386167	0.1666845	0.1508314	0.9689106	0.03045092	0.1810097
0.05142296	0.1208332	0.1974089	0.08760407	0	0	0.3442704
0.2463646	0.2332742	0.3076826	0.05292328	0.0877703	0.2218463
0.4986106	0.06318982	0.0756624	0.08364367	0.02938688	0.1792828
0.01421917	0	0	0	0	0.4265313	0	0	0.6784059	0.06456581
0.0825461	0.1131481	0.2537762	0	0	0.1929802	0.1245645
0.06684241	0.2209817	0.1965508	0.3161959	6.8735020.01604218
0.2341364	0.06862752	0.07204821	0.04011704	0	0.07755692	0.005706261
0
TCGA-GV-A3JV	434	1	0.4604933	0.1561405	0.0814646	0.5298388
0.03447257	35.665610	0.08979479	0.1639777	0.1546246	0.01109849
0.5370689	0.3311713	0.8952055	0.1578066	0.04579392	0.07976459
0.01053677	0.5382170.05353388	0.0368665	0.04616327	2.2994834 9.55979
0.04136709	0.0241491	0.2585443	0.1612793	0	1.1213440.0744221
0.1404438	0.1447848	0.6665728	0.2243777	0.2634430.3243652
0.3251435	0.2528647	0.08357536	0.09025937	0.3405301	0.1160388
0.02598096	8.0035050.1781469	0.08460202	0.0278920.6786788	0.5855268
4.8293360.4482082	4.8182920.9730551	0.1197468	0.5750480.2015991
0.03703798	2.2857230.03981582	0.07565952	0.5517783	0.07239241
0.03402716	0.2186613	0.3367089	0.7298482	0.1451146	0.009960181
0.2166848	0.06544252	0.6166622	2.0418375.7913930.08494042	0.06115431
0.02813011	0.2043475	0.02467286	0.1016034	0.1664193	0.3415835
0.2231715	0	0.2396575	0.4141783	0.2856741	0.1740582	0.2858572
0	0	0.06513836	0.0702374	0.05500531	0.4482379	0.02895696
0.04498963	0.4788782	1.1363650.007919607	0.04609647	0.06924491
0.1085269	1.4884420.7681509	0.2041837	0.07354149	0.1655759
0.1270042	0.5359336	0.3549043	0.1321687	0.2550482.8598110.03721105
0.1878105	0.04374273	0.1891488	0.1652109	0.4214908	0.3522851
0.7893516	0	0.07976459	0.1098368	1.2759690.1903545	0.6689763
0.1085886	0.6807933	0.1895992	0.05667733	0.5165296	0.1327194
1.0266560.3481165	0.1755705	0.08847961	0.3057411	0.7344667
0.3819968	0.7122334	0	0	0.0278920.2740014	0.3056225	0.8009373
0.06408986	0.02540084	0.4453278	0.1654684	0.2143929	0.08003181
0.2879987	0.05931594	0.3803303	1.3794460.1028833	0.02544142

0.5374783 1.034177 0.8993846 0.2101495 1.297031 1.135461 0.1980887
0.2201797 0.1954151 0.5696222 0.1879213 0.2573375 0.1074193
0.03112638 0.5151373 0.03410349 0.1701185 0.670954 0.1868357
0.3527126 0.4335661 0.2338287 1.387733 0.1400117 0.1429653
0.121575 0.4333695 1.133547 0.3865614 0.7417635 0 0.2827159
0.700143 0.1860553 0.3902601 0.1458455 0.13406 0 0.4155135
0.2805848 0.1995781 0.0977348 0.02803931 0.4503203 0.1051243
0.03191649 0.1376916 1.028875 0.4304413 0.5193368 0.9863301
0.3250271 0.6136575 0.07082303 0.1331355 2.078978 0.09049051
0.1389732 0 0.04929299 0.1802581 0.1606402 0.03531337 0.03644469
0.09066222 1.012105 0.9867249 0.04351456 0.2084143 0.05759975
0.2226044 0.3086498 0.09302191 0.01811869 0.5643188 0 0.04198505
2.186607 0.3484525 0.06504335 0.04247021 0.29345 0.03009384 0.2103772
0.8504916 1.262086 0.1166764 0.4734294 0.1143722 0.09205971
0.4445116 0.02255854 1.430881 2.807739 0.2504024 0.1433191 0.04725914
0.2480737 0.06215827 0 0.8438498 1.94904 0.03128945
TCGA-XF-A9T8 418 1 0.05574553 0.1043232 0 0.09023649 0.192485
63.88442 0.04855477 0.1519359 0.279768 0 0 0.4137287 0.07471377
0.4391509 0.1054364 0.1432909 0 0.1045943 0.2124924 0.06642619
0.09148977 0.1718417 1.102251 86.93882 0.1283233 0.4344897 0.2566466
0.05002985 0.4411114 0.5041273 0.5078965 0.1045597 0 0.4135502
0.1503433 0.04457547 0.2817363 0.1415601 0.07715431 0.09722101
0.08044791 0.173349 0.07199195 0.7092323 2.182407 0.08841965 0.07873224
0.02595682 0.6736974 0.3632682 4.296299 0.07415306 0.7518073
0.1478438 0 0.4860305 2.644435 0.06334704 0.9148977 0 0
0.435693 0.02994211 0.03417952 0.1523855 0.04177969 0.2910902
1.035356 0 0.02240566 0.1033489 0.1938431 0.4705188 0.5988422
0.1317453 0.1951247 0.005817425 0.0475424 0.09184411 0.03151801
0.08259892 0.3708647 0.2265683 0.08527202 0.1622035 0.0824216
0.2658537 0 0.202685 0.2763887 0 0.04041266 0.02178808 0.04550132
0.2313335 0.07186106 0 1.206415 1.516851 0.02292931 0 0 0.2693259
0.4155517 0.09531411 0.2171625 0.0969554 0.2311321 0.03151801
0.6022641 0.1376169 0.04099957 0 1.959186 0.09234482 0.093216 0 0
0.2459974 0.07471377 0.3899526 0.2880728 0.07600689 0 0.09324983
1.398349 0.03374239 0.7135798 0.04491316 0.8184839 0.2940743
0.5626133 0.03052014 0 0.7294986 0.4319517 0.0886179 0.03659591
0.2069298 0.09593103 0.07292174 0.7610131 0.03913226 0 0.1730455
0 0 0.1146719 0 0.09455403 0.1793442 0.153988 0.03800344 0 0
0.03680036 1.297789 0.3907406 0.05106406 0.1578418 0.3510087
0.1283233 0.2036458 0.1179623 0.3368487 0.4819949 0.1474761
0.07968464 0.07274279 0.5348769 0.08744155 0.03547898 0.1052279
0.1416154 1.083081 0 0.06031066 0 0.337208 0.1009972 0.107596
0.07880359 0.3235849 0.3040276 0.1064369 0 0.4705188 0.21098
0.09593103 0.3116695 0.01848624 0.2754438 0.2911762 0.2154712

0.1037667	0.03619376	0.3992281	0.09037405	0.06287557	0.1662838
0.0990566	0.09003513	0	0.03725126	0.06522043	0	0	0.3654869
0.2492478	0.3436833	0.7199195	0.07805843	0.5948763	0.1318185
0.1858476	0.2181213	0.03742764	0.03448829	0	0.1368908	0.2105118
0.1139008	0.1314532	0.04522149	0.1499946	0.1895615	1.118917	0
0.08620193	0	0.07533084	0	0.06074944	0.1573749	0	0	0.1215576
0.6884231	0.02543345	0.02305927	0.474283	0.187577	0.05808631	0.1224638
0.4336896	0	0.02412917	0.1700493	0.1773949	0.08567251	1.252193
0.139956	0.3859725	1.765497	0.0639688	0.2889801	0	0.1231264
0.05998809	0.02649034	0.1457946	0	0.09706184
TCGA-KQ-A41S	35	0	0.136303805	0.131184703	0.247159585	0.214333955	0
26.350715869	0.047488648	0.158506444	0.223875053	0.093824658	0.013468906
2.123704063	0.365366343	0.555834445	0.169413578	0.096651479	0.064533822
0.006393609	1.068822833	0.024362873	0.156591617	0.056022839	2.18997966
38.155395396	0.100404569	0.380989286	0.125505711	0.097862681	0.431425883
1.084727934	0.451585971	0.204527826	0.29284666	0.46225123	0.17427223
0.174386883	0.511736119	0.173064824	0.186135227	0.158477202	0.092566473
0.052982126	0.176028047	0.094589949	3.740248223	0.432391042	0.256678347
0.186170469	0.384361241	1.030346521	3.427461896	0.290099506	2.065842199
0.373543807	0	0.313534415	0.034950958	0.12634189	0.715847391	0.193278796
0	0.060875211	0.29284666	0.176977227	0.152674618	0.040862325	0.474497862
1.452892997	0.024174959	0.175309565	0.072199774	0.239478115	0.707980936
2.384608517	0.103082024	0.180238043	0.085345362	0.092997015	0.089827481
0.061651928	0.363533785	0.310904229	0.418565545	0	0.237962949	0.13277248
0	0.105616828	0.346910659	0.540639988	0	0.102765822	0.127858079
0.105692822	0.288835062	0.070283198	0.818977947	0.21132989	2.173080513
0.39085054	0.39159235	0	0.296338869	0.903171942	0.932212841	0.318591421
0.200809138	0.276291976	0.092477893	0.785386852	0.080757157	0	0.103173734
1.417573362	0.180634388	0.182338486	0.077214999	0.183637811	0.240596011
0.146146537	0.756629276	0.394446522	0.817717981	0.129067643	0.210466928
1.254616743	0.055002503	0.56972379	0.395342991	1.331333454	0.057523451
0.240738713	0.858187702	0.053688554	0.692183015	2.481995463	0.234736953
0.107377109	0.269848231	0.093824658	0.071320589	0.576234919	0	0
0.101547528	0.374087991	0.206054153	0.48600084	0.194445468	0	0.265479827
0.200809138	0.148675997	0	0.13980383	0.035992327	2.538587166	0.426126478
2.247427856	0.092625621	0.343301591	0.941292836	0.187223895	0.255033284
0.695510817	0.435149258	0.096158605	0.18927071	0.166006328	0.336299499	0
0.10409989	0.096056199	0.10073162	0.659891665	0	0.137634951	0.428556088
0.309191106	0.230485787	0.666478606	0.108603275	0.655565506	0.127436569
0.10409989	0.088524639	0.85463413	0.06878249	0.187649316	0.614415097
0.018080336	0.411718144	0.536885543	0.722537554	0.112764758	0.070798094
0.178961848	0.324095541	0.116840506	0.193126327	0.145322403	0.118609024
0.034027957	1.912749627	0.191365144	0.116199677	0.055699941	0.701622811
0.278600066	0.588239813	1.056168282	0.050896325	0.383997607	0.096693116

0.14137425 3.012247896 0.256240827 0.202386173 0.508628409 0.136733709
0.257361912 0.167099823 0.128566826 0 0.073350587 1.398219904 0.98701824
0.158425242 0.231850246 0 0.049117864 0.074914262 0.065357109 0.175907891
0 0 0 2.297166013 0.174125041 0.112764758 0.72157417 0.194250046
0.077100506 0.283677293 0.438305974 0.048623596 0.117996823 0.584624258
0.069399927 0.055860924 0.476271611 0.383272399 0.528496707 4.696709268
0.165348334 0.086964587 0.114705517 0 0.117341853 0 0.2074085
0.009537567 0.047465323
TCGA-CU-A3YL 906 0 0.3469116 0.3400658 0.05242122 0.4947017
0.02218257 23.50312 0 0.2941607 0.6067233 0 0.01428341 0.388795
1.084891 2.464962 0.2812047 0.007687222 0.06843638 0.1559457 0.3463343
0.1291809 0.1186151 0 5.141323 44.56098 0.1131311 0.06215829
0.1330954 0.4670133 0.7320248 3.869274 0.04788948 0.1084481
0.1242224 1.011048 0.2656658 0.1849326 1.628047 0.491862 0.1813867
0.03361216 0.0670789 0.1348467 1.269376 0.0668734 18.0047 0.6419547
0.2722005 0.03589614 2.445628 1.431754 4.572528 0.1922767 1.188214
0.5878105 0.5393867 4.612025 0 0.05539647 2.182519 0 0.1460572
2.42087 0.0310556 0.02293862 0.4163331 0.6066675 0.3354615
0.09337902 0.006409223 0.1549259 0.3981428 0.4021035 1.764368
1.286589 0.2186314 0.1349207 0.0663714 0.3451729 0.06350642
0.1634505 0.4069354 1.044068 0.1827731 0 0.08411777 0.1508589
0.2757403 0.0373346 0.420445 0.07166677 0 0.1760452 0.1129917
0.04129425 0.9444306 0.1118002 0.1158005 0.3268266 1.152247
0.04416659 0.02966237 0 0.1396708 6.848195 2.108985 0 0.06506887
0.6525913 0.2288307 1.028088 0.6565794 0 0.2917679 6.500469
0.1915579 1.933651 0.04094221 0.4381716 1.148155 0.658684 0.1239456
1.195143 0 0.1026546 0.5021876 1.414392 0.174986 0.4380281 0.1397502
1.198478 0.09150311 0.3647099 0.4036029 0.1138705 1.321275 1.008034
0.3868024 0.07591369 0.8227295 0.4477434 0.5672517 2.877176 0 0
0 0.1322367 0.7866536 2.458018 0.08248167 0.1307604 0.3066712
0.9050489 0.5124174 0.1029985 0.2223873 0 2.141446 2.646817
0.2648152 0.3601656 1.34703 3.859767 0.8068567 0.3670473 1.281043
1.999678 0 0.3896261 0.1257466 2.912532 0.2418489 0.03679838
0.03638036 0.4005869 0.2762358 0 0.3231931 0.7271555 0.6994962
0.3142594 0.3347918 0.7170314 1.576208 0.135143 0 0.093878 0.06971665
1.167072 0.3482448 0.6717744 0.07669484 0.805853 0.8168973 0.3352263
0.5381279 0.03753974 0.05175933 0.3436946 0.2086841 0.8677276
0.3595912 0.1867667 0 0.9465957 0 0 0.413478 0.4584207
0.1477239 4.233013 0.933365 1.012016 0.9995378 0.4785221 1.070883
1.088934 0.0388195 0.5007919 0 0.1208353 0.5049119 0.2362731
0.1363417 0.1876128 0.03889316 0.5734487 1.50199 0.1120038
0.08940761 0.0741291 0.2604409 0.1588891 0.1764241 0.3031366
0.242087 0.01060357 0.009005578 12.89444 0.1318964 0.1435008 0
0.1945527 0.07745969 0.7219971 0.9596112 0.2062561 0.1751854

1.33616 0.03679838 0.3258145 0.752456 0.08709644 2.76225 8.31343
0.4123035 0.2075031 0.1216421 0.7662316 0.1155495 0.05495093
2.804377 0.0151715 0.1006714
TCGA-E7-A7XN 428 0 0.06145375 0.2135819 0.1671511 0.412117 0
45.05674 0 0.2009925 0.2803778 0.1057541 0.01518143 0.05815958
0.2059107 0.6549845 0.1162328 0.05991722 0 0.05765228 0.03346445
0.04576755 0.07564359 0 1.086664 30.59309 0.1343902 0.346848 0.07073167
0.1103056 0.2917681 1.822856 0 0.03842214 0 0.3582046 0.09207673
0.0982798 0.08873872 0.1482528 0.05670316 0.05001557 0.09216346
0.02388745 0.07936377 0.03553891 2.582458 0 0.05786283 0.009538246
0.2475608 0.6407457 3.693868 0.04087309 0.5919933 0.3531292 0
0.2165995 0.5909228 0.01369287 0.5547197 0.05446339 0 0.2401535
0 0.008865746 0.1311137 0.1842313 0.1782762 0.2977497 0.01362436
0.1399664 0.1139316 0.2024452 0.4388991 0.2829267 0.6099899
0.09560221 0.01923935 0.1397617 0.06749916 0.2779631 0.02276422
0.2920289 0.2220164 0.2820111 0.1788128 0.2191363 0.09769217
0.03968188 0.502739 0 0 0.07128132 0.02401913 0.003135034 0.3798194
0.03960974 0 0.2315831 0.447483 0.003611032 0 0.04735947 0.03711304
0.8144058 0.2101481 0.03989992 0.05658534 0.3255771 0.1737269
0.7607584 0 0 0.1162919 0.9586878 0 0.2055222 0 0
0.3163848 0.1647286 0.1317383 0.06351415 0.1675797 0.03636954
0.1186135 1.248517 0.02479835 0.4174055 0.1485365 0.9312455
0.2593495 0.1162919 0.04205667 0 1.296319 0.1190457 0.06512812 0
0.1520793 0.05287707 0.08038876 0.8389391 0 0.03356757 0.07630597
0 0.0929013 0.4635182 0 0.1042361 0.1870217 0 0.2513695
0.1094741 0 0 0.5852782 0.4726831 0.02814645 0.1044027
0.1160854 0.3536584 0.166129 0.06159855 0.6189021 0.3678578
0.05419242 0.1003933 0 0.4001177 0.03213179 0.07822389 0.08506883
0 0.2740296 0 0.08864844 0.1932182 0.3175256 0.2226782
0.2372271 0.09477044 0.2038396 0.0957598 0 0.3326008 0.4445991
0.07752795 0.05287707 0.3113727 0 0.3093778 0.2473217 0.05090036
0.2796253 0.03989992 0.4951217 0.06641876 0.1109022 0.1374832 0
0.06076807 0.076709 0.1026643 0.1437977 0 0 0.07683437 0.353276
0.2367973 0.7936377 0.2151286 0.7607168 0.04843881 0.06829265
0.04167912 0.3713413 0.07603963 0 0.09311353 0.4133706 0.031391 0
0.09970414 0.08267687 0.2176794 1.031863 0 0.0237572 0.3939485
0.05536304 0.1688787 0.06697004 0.099137 0 0 0 1.517832 0
0.07626145 3.543751 0.1216379 0.04116485 0.1207929 0.07968308
0.02740296 0.02659994 0.306756 0 0.09444518 0.8983651 0.03085744
0.7233419 0.6487598 0.09066737 0.2450546 0 0.47507 0.1417087 0
0.2776135 0 0.08560061
TCGA-DK-A2HX 1420 1 0.1326788 0.1091418 0.03701329 0.1321662
0.7987901 28.98956 0.5333734 0.1631924 0.1862574 1.405068 0.02017031
3.780218 0.4377224 0.4729476 0.06618383 0.7327466 0.1449636

0.01436209 1.24492 0.03648453 0.03350046 0 1.74588918.25067 0.1362642
0.1645815 0.2349383 0.3663847 2.002849 0.4873291 0.371949 0.1020966
0.2631309 0.6489774 1.186647 0.09793216 0.5894989 0.1399532
0.2260104 0.04271885 0.09241505 0.09521182 0.5799423 0.1416528
2.492678 0.06475256 0.07687754 0.1013814 0.08222839 0.1330165
4.648068 0.162914 1.441976 0.3157898 0.05440676 0.2877779 0 0.3147316
0.1005014 0 0.2062546 0.09116345 0.4385514 1.102826 0.07349056
0.03059661 0.5210938 0.9889884 0.05430468 0.3391067 0.7082024
0.1419575 2.120468 0.7518024 0.09648131 0.3413628 0.4856728
0.08123928 0.325092 0.02308165 0.2873268 0.2327967 0.2949747 0
0.07919123 0.3124518 0.3244887 0.1054441 0.0371082 0.05060209 0
0.0177573 0.06382446 0.6247890.2415037 1.36828 0.6541106 2.964767
1.0482560.2278897 0.08377538 0.0314613 4.339194 0.5410167 0.3722752
0.3975878 0.2464241 0.07522909 0.02308165 0.3675479 0.1814064
0.2402024 0.02575124 1.361572 0.1014406 0.2730603 0.02890826 0
0.03002531 0.2462189 1.671995 0.295351 0.0556623 0.07248179 0.07091639
1.946553 0.09060567 0.9705011 0.03289136 1.269322 0.04307201 6.64382
1.134307 0.04020055 0.3269202 1.502578 0.110867 0.1340018 0 0.1053801
0.08010441 0.5483255 0 0.3344884 0.2281082 0.248984 0.1542878
0.6998161 0.02911911 0.04616331 0.4614632 0 0.2783115 0
0.05234068 0.02695009 0.4320059 1.144608 0.3178648 0.0924741
0.308466 0.3289136 0.2326528 2.205157 0.7126461 1.330465 0.03600049
0.1333843 0.1065438 0.3217598 0.1280725 0.0779472 0.2106355
0.05656885 0.507112 0 0.1693085 0.7701391 0.1749217 0.07396353
0.05253066 0.1023048 0.2158135 0.1272281 0.3377712 0.06628487
0.7876026 0.1545074 0.5620271 0.328103 0 0.2226492 0.6152432
2.186609 0.1266528 0.2385527 0.8161929 0.3309191 0.06446413
0.2473555 0.1450847 0.07266375 0 0.1773219 0.1432891 0.1160096
0.04170662 1.094285 0.3650644 0.4404582 0.3954152 0.0571647
0.3746565 0.04826747 0.1209797 3.876291 0.02740946 0.05051378 0
2.158565 0.1637998 0.1459732 0.2246239 0.09935147 0.02746147
0.6998944 0.3435256 0.2372491 0.142039 0.235533 0.07355627 0.1121876
0.07118203 0.03292878 0.05697715 0.02246073 0 0.8896842 0.09312868
0.08443522 0.3087402 0.1292882 0.1731924 0.2643324 0.2964312
0.07281609 0.03534114 0.2000069 0.4417008 0.02091358 0.7387116
0.02049886 0.3674581 13.39476 0.07696197 0.1465127 0.08588841
0.2705084 0.08786243 0.01939973 0.184421 0 0.08529782
TCGA-CF-A8HY 345 0 0.1023942 0 0 0.07892746 0 149.4241 0
0.161245 0.4048765 0 0 0.1054059 0.0457451 1.803075 0.05533344
0.04235382 0 0.1921204 0.07434458 0.01016771 0.05601657 0
1.650556 173.5528 0.03928435 0.2751989 0 0.1225274 0 1.851977
0.05654009 0 0.03666539 0.07234427 0.08182303 0.01819486 0.3449982
0.03466928 0.1196731 0.06349402 0.03863212 0.01326708 0.1763145 0
12.11184 0 0.09641099 0.01059508 2.406166 0.04448375 8.155638 0 0

0.06034703	0.2729229	1.430929	1.312793	0.01368903	1.904563	0	0
0.9146139	0	0.02215815	0.04096153	0	0.5148757	0.05512337
0.007566967	0.1371834	0.03615862	0.1873967	0.1772832	0.05237913
0.06453109	0.02654872	0.007123686	0.05821771	0.03748902	0.03859515
0.2781513	0	0.4007504	0	0.09931256	0.07124385	0	0	0.06204913
0	0	0.01979481	0.08004132	0.003482395	0.02410875	0.08799694	0
0.09187228	0.7586764	0.004011132	0	0	0.08245029	1.526583	0.583581	0
0.02793554	0.3930988	0	0.1536455	0	0	0	2.827519	0	1.31269
0.01208447	0	0.2008229	0	0.02695642	0	0	0.04039926	0.008783724
0.9198511	0	0.1961612	0.05499809	0.3376627	0	0.08611801
0.09343305	0	0.9199681	0	0.08590883	0	0.08446478	0	0.08929579
0.7966183	0	0	0	0	0	3.089254	0.04869046	0	0.03561319	0	0
0	0.1312793	0	0.3611815	0.419198	0	0.0386568	0.1719301
0.4714122	0.02992476	0.04561576	0	0.227009	0	0.01393961	0.02969222
0.5497151	0.2141519	0	0.04295201	0.01576492	0	0	0.04923532
0.05365667	0.6710041	0.04122514	0	0.08772579	2.108582	0.2659248
0.2172276	0.07389056	0	0.043059	0.05873582	0.149083	0	0.1113702
0.09352332	0.1319269	0.02823706	0	0.6110899	0.1475559	0.01539877
0.08908447	0	0.03150053	0	0.2280788	0	0	0	0.1373888	0
0.5260687	0.2644717	0.09558588	0.2476735	0.04035435	0.2275783
0.4558488	0.2291587	0	0.063682	0.06419821	0.1691686	0	0	0
0.09183742	0.1160631	0.2529529	0.06611792	0.05277897	0	0	0
0.02975611	0.02753033	0	0	0.4890867	0.1983538	0	0.01411853
0.1290622	0.04053461	0.01524196	0.08287383	0.03540477	0.2739528	0
0.09465121	0	0	0.3103236	0.03427643	0.1890559	3.372605	0.01119036
0.08166196	0	0.07538679	0.05246999	0	0.2921428	0.02985339
0.1426277
TCGA-4Z-AA7W	840	0	0	0.01968416	0	0.05107866	0.02824805	70.37897	0
0.1872972	0.3023305	0	0.09094493	0.2237141	0	1.467126	0.009947102
0.09462866	0.2614477	0.03453681	0	0.05483444	0	0.1513113
2.757784	39.31353	0.1779627	0.6926019	0	0.06607898	0.6991392
1.917639	0	0	0	0	0.02941808	0.05887488	0.4252741	0.01869713
0.06114283	0.1540904	0.03125143	0	0	0.04257944	3.067618	0	0
0.01142784	0.4449067	0	3.652919	0	0.2837086	0.1301805	2.551245
0.6282862	0	0.01148387	0.3625166	0	0.06199809	0.4521467	0
0.01062212	0.04909008	0	0.2990317	0	0.02448517	0.1085084
0.009750167	0.09432544	0	0.2824805	0	0.01431772	0.06915242
0.02093119	0.1617425	0.0416287	0.1090959	0	0.06649986	0
0.0714123	0.08965082	0.9363658	0	0.1338523	0	0	0	0	0
0.05850828	0	0	0.1308033	1.01583	0.02163202	0	0	0.04446541
1.219682	0.4196334	0.04780439	0.007532813	0.2204779	0	0.1657219	0
0	0	1.663506	0	0.6155942	0.01955146	0	0.1624556	0.2960438
0.2616765	0	0.2007784	0	0.04737059	0.8242483	0	0.5578001	0
1.722737	0.2718875	0.3715473	0.1209322	0	0.4026631	0	0.03413834

0.04833555	0.04555183	0	0.1926288	0.6160531	0	0	0.1371341
0.0561316	0.2226115	1.0097170	0	0.1600515	0	0.05019461
0.06558089	0.1887971	0	0.5453981	0.05480566	0.03372248	0.1250856
0.2781655	0.2542324	0.1075894	0.1148026	0	0.0979407	0
0.01503525	0	0.5046147	0.4234707	0.04686032	0.1111872	0.01700404
0.1172560	0.06638147	0.1736221	0.2876420.04446541	0.04737059
0.05677258	1.97667	0.2294611	0	0	1.0653550.04644341	0.1900573
0.2765775	0	0.2848651	0.1008742	0.1422962	0.03045647	0.5258483
1.0326230.03978841	0.008304548	0.1166764	0	0.0679529	0	0.5412124
0.1722851	0	0	0.09654636	0	0.2837086	0.3328021	0.1718314
0.3928536	0.01450872	0.05454795	0.1267608	0	0	0.06868736
0.2538952	0.2259092	0	0.05787405	0.05972814	0.2476394	0.0312964
0.3239913	0.07131474	0	0.04719927	0.1658272	0.1011674	0.04011865
0.08908258	0	0.02700589	0	0.1604582	0	0.01522824	0.6264287
0.1020147	0.01095998	0.01702622	0.01909378	0	0	0.1565391	0	0
0.7416231	0	0.3058734	1.4923850.01206992	0.4991230	0	0.4980280
0.1575525	0	0.1153786
TCGA-FD-A43U	636	0	0.03026713	0.04855067	0.05488337	0.1399830.1625712
48.467810.05272579	0.4179688	0.3314193	0	0.05981713	1.495554
0.2028298	5.7225060.08178124	0.06975176	0.3224283	0.01419742
0.1977825	0.07213243	0.3477217	0.1866035	3.20095538.019360.05573869
0.1301556	0.3483668	0	0.4790044	1.9707611.1531920.03784726
0.06502848	0.2886914	0.1511651	0	0.5244659	0.05380221	0.3574704
0.04222905	0.04110996	0.02353004	0.3908815	0.03500717	22.00295
0.0480076	0.1424927	0.01879107	3.2920670.1183423	4.3830050
0.3110058	0.1605441	0	1.7180990	0.1106015	7.5008540	0	2.26422
0.1300570.02619928	0.5448596	0.0453687	0.1756088	0.3910596
0.02684102	0.12976200.01603242	0.1107868	0.5109381.1147740.2575128
0.1883436	0.1326604	0.1548794	0.1496008	0.03422551	0.1345417	0
0.6560835	0	0.08806866	0.1790038	0.19246100	0.05502409	0
0.09092965	0.02633055	0.09463902	0.03705753	0.2351715	0.07803417
0.1212395	0.3421772	1.06717	0.1671791	0.06211113	0	0	6.768735
2.1045390.07860585	0.06812507	0.6832427	0.03422551	0.3951254	0
0.1335647	0.03818398	3.6471210	1.7714120.0107163	0.1019449
0.4897373	0.2028298	1.4342690.2502554	0.4952169	0	0.04673553
1.0917940.04885463	0.6009264	0	1.7253070.09580088	0.5345757
0.1988516	0	2.7075490.1172645	0.1002401	0	0.1123529	0.1562577
0.1979649	1.3728680	0	0.1503285	0	0.3202895	14.610650
0.03422551	0.03684468	0.1114774	0.1238042	0	0.1164163	0
1.0889840.4693656	0.3881545	0.03428019	1.0291360.9754271	0.2034485
1.7798560	1.3688920	0.02472281	0.2369749	1.4728050.1582551
0.07705349	0.2056811	0.01398006	0.5398590.0919032	0.1091526
0.1903272	0.6942073	0.03655775	0	0.1594771	2.7859570.2358175
0.07705349	0	3.1386190	0.2083437	0.3543074	0.08029709	0.3329663

0.4405915	0.8356463	0.05008027	0.1572117	0.7044752	0.2617
0.04779376	0.3103515	0	0.06384944	0	1.496697	0.2124693	0.3870432
0.06184264	0.5353312	0	2.005987	0.5863223	0.08476391	0.6976565
0.1312137	0.8296737	0.1073759	0.0406428	0	0	0.1454874
0.164303	0.03092132	0.3330727	0.04910617	0.3257593	0.1114997
0.7804267	0.1758967	0.1170087	0	0.2726734	0.08317596	0.03298401
0.07324026	0	0	0	1.363199	0.05523654	0	0.1144501	0.1557634
0.02703262	0.06999151	0.03139634	0.02699295	0.02620195	0.2350182
1.309909	0.06202138	0.8903195	0.3039575	2.17947	6.582245	0.01984684
0.7000253	0.1273555	0.2674068	0.1582002	0	0.6476679	0.1164833
0.1580997
TCGA-BT-A0S7	200	1	0	0.04856665	0	0.04200873	0.1045445	137.326	0
0.165042	0.08288209	2.969874	0	0.6198339	0	0.0420911	0.0490849
0.02415279	0.05375575	0.149122	0.2473095	0.08117569	0.07453632
0.2799973	0.8672108	158.9854	0.3345423	0.0976488	0	0.407591	0
0.2628546	0.1504658	0	0.09757482	0.09626216	0.09072891	0.04842059
0.06557982	0.06919705	0.3603818	0.04224295	0.1901961	0.0882667
0.05865153	0	0.2283666	0.0720351	0	0.07048969	0	0.1183812
5.815973	0.1208244	3.383301	0.1806716	0	0.05054897	0.6987275
0.02023865	0.1490726	0.08049923	0.1529677	0	0	0.01637994
0.01816797	0	0.9485998	1.026869	0.0100687	0.2798915	0	0.08311743
0.5307641	0.1393926	0	0	0	0.05164345	0.1247083	0.05135517
0.2018789	0.1726525	0.04101872	0.1389415	0.1321465	0.4502901	0
0.05865153	0.4128166	0	0	0	0.07100263	0.310459	0.05613894	0	0
0.1760348	1.148746	0	0.04659869	0	0	0.07523292	0	0.05897379
0.1300998	0.2092248	0	0.8382142	0	0	0.05729483	0.765496	0
0.07594267	0.04019937	0	0.06680434	0	0.2664519	0	0.2476899	0
0.07597023	1.186304	0.03665304	0.8305005	0.1463622	0.3423223	0
0.1145897	0.1367554	0.08944359	0.727376	0	0.1143113	0.1192581	0
0	1.009953	0.4799954	0	0.3473002	0	0	0	0	0	0.1027103
0.3869965	0.1672711	1.300372	0.323615	0.1164546	0	0.8650663
1.729712	0.08320334	0.1543116	0.1715792	0.2090889	0.08627288
0.1315099	0.06098426	0.3020609	0	0.1112893	0.1580353	0.2178809
0	0.2312366	0.04572196	0.06293099	0.1157222	0	0	0.4283773
0.2976154	0.05485467	0	0.04085515	0.1694721	0	0	0	0
0.1145897	0.07815459	1.460016	0.03012132	0.6308684	0.8088811
0.1504658	0.1690766	0.1769214	0.1084165	0	0.07171424	0.08466919
0.08070098	0.08981783	0	0	0.1062696	0.06452844	0	0.4071703
0.8122445	0.06999933	0.3519092	0.3391673	0.6203432	0	0.03364649
0.0142162	0.1829528	0	0	0.04745717	0.3322871	0.0927945
0.07139621	0	0	0.5662616	0.5329217	0.0879773	0.07022834	0
0.1227434	0	0.02969538	0.3663219	0	0	0.3395398	0.7917957
0.08288209	0.01878628	0.2575975	0.1078717	0.01352076	0.1207751	0
0	0.03931586	0.08396269	0.05780914	0	0.9068055	0.1824345

0.2515601 3.3561320.01489003 0.03622013 0 0.2006211 0.2653054 0
0.02159604 0.02383397 0.06326069
TCGA-ZF-A9R3 949 1 0.2462455 0.2633304 0.1880071 0.5634386
0.01988928 46.98068 0.04515403 0.1601336 0.3311296 0.6244846
0.204908 0.4312315 0.5905874 0.2882775 0.07704062 0.04594997
0.1227225 0.01215858 0.1411497 0.1235475 0.06381142 0 2.43962
23.4947 0.0358007 0.01393305 0 0.1395774 0 1.537725 0 0.3241215
0.02784499 1.373519 0.2692702 0.3178097 0.6737239 0.394936 0.1243675
0.02410979 0.1525606 0.3224156 0.1004245 0 15.56611 0.3700206
0.1708417 0.008046274 1.044187 0.6080853 3.302928 0.241358 2.63014
0.2406057 0 0.6731755 0 0.0358082 1.020983 0 0.08730494
0.1736475 0 0.02430664 0.2626866 0.1942673 0.571483 0.3767636
0.01149324 0.2500366 0.2540062 0.7400412 1.952209 14.43961 0.3920574
0.0705671 0.01081995 0.2505379 0.08541142 0.2931051 0.4608825
0.1970798 0.2809328 0 0.2765453 0.1713327 0.2472335 0.2008491
0.5183451 0.1285153 0.1946789 0.08268082 0.1013103 0.007933958
0.5538482 0.03341398 0.05191438 0.1367512 3.83448 0.003046194
0.02659579 0 0.1252313 1.545787 1.004568 0.06731755 0.2704941
0.7164792 0.1758631 0.6534290 0.153574 0.152512 0.1308021 2.825907
0.1717541 0.216718 0.01376606 0.08730494 0.152512 0.1389617 0.1052828
1.393061 0.2827337 0.06136124 0.240144 1.977482 0.1882746 0.2979434
0.2506049 1.00945 0.1640865 0.1635026 0.04966943 0.1020983
0.7999105 0.6025472 0.1579547 0.136131 0.4810914 0.3122423
0.5086077 0.9245919 0.1091744 0 0 0.07904383 0.3526636
0.7109358 0.1848862 0.05862102 0.3155355 1.050154 0.5301257
0.1385253 0.1661636 0.1026687 0.6034465 1.164081 0.332413 0.176144
0.5222789 0.2386713 0.6135997 0.2598165 0.6613184 0.9654321
0.1371469 0.3387593 0.02254931 0.595121 0.08131721 0.1979643
0.05871467 0.4190353 0.2146541 0.2361159 0.2430417 0.6927289
0.307057 0.876619 0.3668867 0.1665551 0.3062948 0.2423432 0.03299406
0.08417263 0.1875275 0.6540103 0.1784242 1.861319 0.01719148
0.659712 0.2485871 0.1145027 0.1608316 0.1682939 0.4640831
0.2801477 0.3800668 0.3431018 0.1842375 0.07860286 0 0.9006974
0.4852195 0.07365808 0.3177699 0.3288223 0.728485 0.719127 2.309764
0.266168 0.7081109 0.02043101 0.09601719 1.246816 0.1044187 0.09621827
0 0.02166861 0.1896499 0.1324041 0.04074876 0.04205421 0
0.7198296 0.6303324 0.1004245 0.1603288 0.06646543 0.04670315 0
0.1807827 0.5435949 0 0.009507341 0 2.033597 0.07095633 0.1715537
0.294043 0.3180951 0.1196113 0.2307701 0.9007354 0.02311659
0.04487836 0.5654674 0.2639525 0.07967207 2.444505 0.02603072
1.112712 5.429026 0.1954621 0.1653785 0.05453321 0.9160219 0.00796951
0.02463497 0.6039617 0.0317404 0.1173428
TCGA-4Z-AA89 1029 0 0.09301956 0.3183149 0.06746892 0.2064998
0.1142006 99.95835 0 0.3109933 0.5092731 0.5122397 0.1654517

0.04448	0.7978933	1.413849	0.1306953	0.04287342	0.08808129	0.4101476
0	0.09975761	0.3969265	0.3058591	3.165642	112.1256	0.08565048
0.06000079	0.171301	0.1335714	0	1.076749	0	0.1395786	0	0.7886501
0.1189308	0	0.3223668	0.2078681	0.08926198	0.05191279	0.05053706
0.2458695	0.09610327	0	15.55558	0.2950822	0.2802693	0.01155006
1.498883	0.678906	12.02262	0.09898817	1.194762	0.0328932	0.09917424	1.44947
0.715561	0.008290492	1.160247	0	0.1253223	0.2492631	0	0	0.2034219
0.2230896	0.3885812	0.06009191	0.02474704	0.3688862	0.06898111
0.2996219	0.8696818	6.623637	0.0351738	0.01447084	0.03882889
0.2961707	0	0.1262218	0.2756567	0.353624	0.2016332	0	0.03608803
0.0776654	0	0.1441549	0.2705677	0	0	0.1510531	0.1745117
0.01518512	1.136687	0	0	0.1922941	1.654119	0.004372675	0	0	0
2.835271	0.5513577	0.04831565	0.2131745	0.4628131	0.08414784
0.8039725	0.25719	0	0.09388024	2.175011	0	0.4977424	0	0.06266116
0	0.3989466	0.02938613	0.9998382	0	0.2642439	0.2489615	1.75869
0.09008656	0.6026461	0.05995533	0.8705807	0.03925647	0.04694012
0.07129824	0.07327874	1.075562	0.2883098	0.1774463	0	0.184156
0.1920899	0.5353945	1.278054	0	0	0	0	0.2249923	1.479749
0.1061583	0	1.028814	0.4111223	0.3043886	0.2651291	0.1431122	0
0.787473	0.7477894	0	0.5478347	0.5154242	1.284757	0.4675824
0.07459099	0.7994045	0.593929	0	0.04558816	0	0.752267	0.1556363
0.1420845	0.02809409	0.1031154	0.07110606	0.05648897	0.2146925
0.175479	0.3000964	0.7190555	0.3351405	0.09085129	0.6479383
0.05797878	0.04736148	0.08055068	0.08972907	0.6571617	0	0.5590709
0.02467759	0.5758235	0.7455315	0.02054544	0.09234661	0.04831565
0.02220568	0.08042789	0.1510806	0.3676462	0.1322323	0.06622681	0
0.5718643	0	0.1057329	0	0.1180024	0.04753216	0.7455315
0.6727229	0.1910361	0.5399951	0.08798337	0.2205254	0.1048226	0
0.138117	0	0	0.4303053	0.07602405	0.116986	0	0.1001152	0.56936
0.8042789	0.07207745	0.3164491	0.04770406	0.2681611	0	0.08109544
0.09003531	0	0	0.01159066	1.567685	0.03395154	0.1077377	0.1406952
0.1325646	0.08307899	0.2108019	0.5596416	0.09954848	0	0.4058515
0.09472297	0.343097	0.8026206	0	2.421633	6.001961	0.1524876	0	0
0.2465453	0.02287975	0.07072479	0.6546431	0.0585796	0.05182779
TCGA-GU-A42P	332	1	0.1461273	0.4464739	0.1135597	0.3765322	0
19.65057	0.03636514	0.1289648	1.142904	0.2873905	0.2269083	0.05406996
0.8393541	0.9673602	0.603531	0.09436569	0.1976709	0.6903361
0.2955575	0.01243749	0.01713034	0.04290032	8.052476	55.96188	0.1922157
0.06732651	0.672755	0.1498796	0	1.495164	1.971109	0.5481708	0.04485033
1.039804	0.4003546	0.2225656	0.4220133	0.4664949	0.07704638
0.04368826	0.1193217	0.1217155	0.5661436	0.02414455	10.04702
0.3642208	0.1965553	0.0712814	1.513699	3.019977	4.594209	0.6386755
1.045695	0.08304582	0	0.7667506	0.6691054	0.01488433	0.4453887	0
0.3164026	0.5593931	0	0.00150581	0.2644457	0.3754912	0.3391302

0.1348575 0.004628083 0.4027377 0.2709114 0.4737418 1.192723
2.626948 0.4144171 0.04871293 0 0.4866279 0.02292891 0.09442176
0.4949002 1.388797 0.3770856 0.04789842 0.8098829 0.09441017
0.06637045 0.4583067 0.9487571 0 0.03135723 0.2058163 0.09790922
0.01490923 0.6930298 0.134551 0 0.5484205 1.568065 0.009813102
0.2784485 0.03217524 0.07564196 6.501203 1.451507 0.1355367 0.2861878
0.3558301 0.3304761 2.236537 0.3504323 0.1535333 0.2370205
4.851186 0.03458087 0.8028633 0.1736901 0.3515584 0.4605999
1.231053 0.1036325 0.5609561 0.05692542 0.1235443 0.4647016
1.609891 0.2105949 0.5344362 0.269102 0.5015489 0 0.05267121
0.2342954 0.08222561 0.4811218 1.172726 0.4728895 0.08222561
1.627292 0.5029334 0.4369184 1.103151 0.3223895 0.02280525 0.05184102
0 1.325428 0.7729526 0.2382392 0.1180272 0.6171459 0.07688629
0.7684932 0.1859373 0.2944064 0.05512332 1.89978 1.214608 0.01912224
0.2364315 1.077842 4.565123 0.927326 0.1627463 0.8129123 0.8608235
0.2577221 0.5285932 0.1089615 0.5651272 0.04365962 0.4782962
0.02627016 0.1157051 0.1329794 0.1901577 0.2936032 0.4922598
0.5051044 0.3025678 2.17577 0.5714211 0.8265838 0.2602305 0
0.1355781 0.1006844 0.6057189 0.2873905 1.05406 0 1.373995
0.6792551 0.03458087 0.3454051 0.2168588 1.158634 0.2933048
0.2119082 0.3424806 0.4080369 0.1334876 0.02605741 1.269421
0.1465407 0 0.1279592 0.2062537 0.5066876 1.319185 2.021941
0.282566 1.523723 0.08227132 0.07732816 0.1633621 0.05606292 0.4907707
0 0.06762254 0.610944 0.1492857 0.03281732 0.06773735 0.0561693
2.129584 0.858964 0.04043883 0.2743843 0 0.1504509 0.4589337
0.3457874 0.4209502 0.4078908 0.01531362 0.9884415 11.61605
0.1333388 0.1727026 0.2368098 0.3140275 0.1615855 0.5768666
1.136843 0.1117027 0.1084294 0.737136 0.2125761 0.3635988 0.7108163
0.04192807 2.283688 3.111742 0.2121706 0.03329721 0.08783745 1.198803
0.01925491 0 0.9231769 0.1935436 0.1090418
TCGA-FD-A3NA 1845 0 0.033873731 0.024149296 0 0.261105407 0.034655809
37.782494806 0 0.073858971 0.144243095 0.077723221 0 0.267712104
0.181599057 0.544164146 0.097627948 0.118095766 0.053459044 0 0.319727782
0.074000344 0.018531231 0.23204324 1.347219013 22.007936883 0.08317394
0.279190767 0.051983713 0.040534136 0.071477605 0.702522747 0 0.056476132
0.072777198 0.311125241 0.130830804 0.072230001 0.260871422 0.108957266
0.045840921 0.063014694 0.088183099 0.043889757 0.087491713 0 4.444791782
0.07163751 0.085051705 0.007010063 0.500343235 0.35318346 2.829692365
0.090118106 0.464086479 0.109801009 0 0.284863129 0 0.033209457
0.704186776 0.080054918 0 0.176499031 0.024259066 0.021176395 0.087327162
0.101549578 0.288250113 0.802372751 0.015019684 0.217836511 0.017942828
0.165317331 0.439862185 0.831739404 0.170783824 0.043913833 0.028279629
0.218273232 0 0.102143436 0.184034294 0.042924889 0.142773102 0.017271827
0.02190285 0.023568671 0.430788795 0.087491713 0.164215216 0 0.033921575

0.032742298 0.017652683 0.00691221 0.223316333 0.116443517 0.045228767
0.240711825 1.003931941 0.045116314 0 0.034806486 0.081827855 0.561132601
0.386116966 0.175944874 0.027724647 0.27049095 0.051071718 0.772593491
0.08919768 0 0.028489295 1.976064736 0 0.302094029 0.019988743
0.114092519 0.298960273 0.090799529 0.287912339 0.140037757 0 0.026729522
0.113326381 1.301477609 0.027338048 1.321466585 0.072777198 0.361709927
0.023825868 0.085467884 0.049454775 0 0.890969332 0.174983427 0.038890655
0 0.167654278 0.194308053 0.50218952 0.477346093 0.253639343 0
0.028040251 0 0.273108461 0.278721183 0 0.076607577 0.683323801 0
0.123161412 0.201143009 0.086858862 0.029815612 0.525733788 0.282958764
0.248232303 0.127883255 0.170632151 0.571820841 0.36958577 0.065392016
0.151619162 0.570748006 0 0.064560417 0.117872271 0.340494575 0.07084506
0.057490067 0.051153302 0.083444865 0.647344348 0 0.06515151 0.177505361
0.37565763 0.054551903 0.029058046 0.078357179 0.458794265 0.387078723 0
0.122221248 0.544591277 0.170935767 0.155446442 1.144205329 0.029955067
0.400011423 0.317125761 0.249392267 0.140119459 0.20526902 0.350408731
0.17084891 0.071318412 0.265235857 0.040127778 0.041683639 0 0.392352094
0.10568306 0 0 0.373245294 0.375032047 0.452484317 0.670769802
0.11594524 0.424132014 0.044499676 0.033460781 0.438269748 0 0 0
0.05427452 0.21319552 0.023070581 0.035501072 0.183192031 0 0.28796733
0.66421872 0.087491713 0.087300892 0 0.142410273 0.062058076 0.054141045
0.145719987 0 0 0.281388111 0.820234812 0.041212313 0.084071676
0.853919122 0.169854097 0.060507601 0.164496406 0.327949919 0.080558408 0
0.183698377 0.057490067 0.138823557 0.36233053 0 0.875600662 1.554371838
0.0481255 0.162090947 0 0.049878453 0.041659107 0 0.279198908
0.031603224 0.070775467
TCGA-XF-A9SL 2020 1 0.1115358 0.05111757 0 0.05895359 4.670398
31.9418 1.2768090.521132 0.2326277 0.1096794 0.03148985 6.03183
0.1281321 0.6497611 0.1722101 0.9829604 0.1508779 0.007474021
0.6247179 0.02847979 0.1046017 0 1.44745443.61363 0.08069273
0.4796293 1.0269980 19.76972 1.106643 2.639482 0 0.06846656 0.3715002
1.419683 0.1019277 2.622925 0.04855437 0.8879981 0.2148983 0.07033562
0.06193521 0.04115476 0.6265863 2.472288 0.05054578 0.0900158
0.6133213 1.026998 0.08306605 6.790351 0 0.409311 0.4084944 0
0.6148018 0 1.238337 1.202919 0.05648491 0.1073348 0.1423242
4.142227 1.386119 0.1274815 0.1433021 0.4807227 1.955742 0.00706503
0.2476285 2.000288 0.06998647 0.4551898 0.8313797 0.09037586
0.991507 3.698041 0.01811866 0.2975193 0.03603503 0.3305282 0
0.6619888 0 0 0.2716154 0 0.04115476 0 0 0 0.02772266
0.2740172 2.48732 0.2419777 1.725357 0.7658971 3.060564 0.7816291
0.02247038 0.1634874 0 7.42868 1.742058 0.7628188 1.779378 0.2151806
0.2642569 0.03603503 0.344289 0.03146792 0.3281281 0.1206084
1.931401 0.05278964 0.3197259 0.01692432 0 0.04687545 0.2135535
0.7766226 0.1317432 0.08689987 0 0.07380969 1.123092 0.0257188

0.8158469 0.05134992 1.6313690.1344879 14.633810.1831943 0
0.5975264 0.86425 0.1899717 0.1255220.0394310 0.04168628 1.75419 0
0.4177621 0.1978456 1.3119080.1445243 0.4370206 0 0.03603503
0.1717962 0.1173712 0.04344993 0.1135375 0.1225712 0 1.281449
1.1544070.2919117 0.07218519 0.4013138 0.5868562 0.2235182
6.7221130.1711664 0.7630232 0.05620389 0.05205983 0.3326719
0.3822082 0.03332443 0 0.41707 0.1913495 0.9541028 0 0.7010335
0.6512673 0.3373432 0 0.08201076 0.07371604 0.7663450.1986283
0.4867641 0.0344946 1.69070500 0 0.5178016 0 0.2198492
0.5239183.3081510.02636402 0.1655236 0.3613513 0.1377681 0.01437732
0.09505744 0.05662648 0.07352755 0 0.4471944 0.1491351 0.2263924
0 1.8522190.1221295 0.3929385 0.2057738 0.07437118 0.5577078
0.05023672 0.4013557 13.197270.1283748 0.07886201 0 0.5061581
0.1955536 0.06511229 0.2504874 0 0.1286184 0.09030362 0.2706159
0.6790536 0.1971120.6945704 0.1722539 0 0.04861905 0 0.1779052
0.03506565 0 0.4629911 0 0 0.5422552 0.1135375 0.1138474
0.1731762 0.3140346 0.1989406 0.02758726 0.1885285 0.1622547
0.03265024 0.7442317 0.2240195 0.6178037 15.179270.04701634
0.3558105 0.2681776 0.07038618 0.3821182 0 0.2879177 0.01114925
0.06658339
TCGA-XF-AAN2 1869 1 0.1701660.2079685 0.08816055 0.3597733 0
41.447920.08469478 0.1413461 0.3105475 0.6693355 0.1441287
0.0581213 0.5212972 0.9462566 0.03941019 0.0301657 0.1726416
0.5131277 0.1059010.04345056 0.1196902 0.09991529 2.68624927.80636
0.06715086 0.1568041 0.2238362 0.2618034 0 0.4924397 0.1610784
0.2431801 0.1044569 0.9789906 0.2816716 0.1814251 0.2106157
0.1851940.04486046 0.3843904 0.08529646 0.1700861 0.5023064 0
5.0640960.1542317 0.1831117 0.01509228 0.58757 1.4574044.731485
0.2586925 0.7493647 0.04298102 0 0.4329140 0.02166611 0.1595869
0 0.2456350.4885622 0 0 0.1231794 0.3643845 0.8462547
0.2355634 0.06467313 0.2214676 0.1287664 0.2313475 1.704599
1.1937930.5055714 0.03781764 0.04058967 0.1105719 0 0 0.288157
0.2772448 0.5269418 0 0.1886226 0.1775972 0.4637324 0.2511532
0.4419330.1205272 0.0730313 0.09868934 0 0.04960538 0.4378604
0.06267414 0 0.03140845 1.7142220.05142334 0 0.1498729 0.05872364
0.4832352 0.4433540.1262666 0.1492241 0.2239816 0.3298639
0.5252690.1920377 0.1430323 0 1.1682050 0.3251960.02582081 0 0
0.3258107 0.1535935 0.6029873 0 0.05754721 0.2502416 1.370768
0.07847644 1.3209140.5483987 1.3437110.1538874 0 0.2528744 0
0.3988354 0.3767298 0.2576291 0.06383477 0.3609505 0 1.144786
0.9634689 0 0 0 0 0.2939938 0.2666985 0 0 0.4481116
0.2686034 0.5303196 0.4330499 0.3116707 0.06419139 0.2057957
1.2787060.1336077 0 0.3673617 1.3430170.1705067 0.0649779
0.2611422 1.2934630 0.2779901 0 1.0496180 0.1856595 0.04894679

0.3368848 0.4026236 0.07381322 0.2104015 0.4585913 0.2328278
0.4697891 0.1251208 0.2124355 0.8466506 0.3030398 0 0
0.2344951 0.3066795 0.2510008 0.4671988 0.06449163 0.4441993
0.4079874 0.0268464 0.1407791 0.1893999 0.1450790 0.1576407
0.2632194 0.2447307 0.08639292 0.06730695 0 0.3573784 0 0
0.3973576 0.1275048 0.3726570 0.3746823 1.2557660 0.2723169 0.4358135
0.09580538 0.1080589 0.03043777 0.1958567 0.1203168 0 0.08128702
0.2983489 0.09933941 0 0 0.06540944 0.8679692 1.9367290 0
0.2631357 0.06233413 0.1752009 0 0.1377559 0.1176477 0 0 0
2.2603790 0.04436393 0.06033391 1.5626750 0.23096 0.1881672 0.1461579
0.7060583 0 0 0.2876310 0.06188649 0.04981326 1.1007770 0.09765092
0.3366287 3.5107220 0.2869244 0.3101982 0.1022872 0.3221568 0
0.04620747 0.4623847 0.008505003 0.03386127
TCGA-ZF-AA54 590 1 0.0513943 0.03664008 0.06212884 0.1426167
0.1051618 65.20927 0.1790593 0.1494149 0.5627577 0.3537724
0.01692848 1.7271240 0.3214492 0.5398305 0.1388665 0.4190958
0.1622195 0.08839435 0.3358393 0.06124128 0.2811617 0.2816507
2.96154 47.21324 0.1340813 0.1473381 0.4732281 0.1229994 0.2168962
1.0411020 0.6810946 0.04284369 0.07361326 1.3435240 0.2464146 0.1095897
0.09895061 0.08700707 0.0821970 0.1035752 0.1337942 0.1065455
0.08849681 0.1188860 0 0 0.09678237 0.2446255 0.5520994 0.1786204
4.0390430 0.09115338 1.05619 2.33231 0 0.6991526 0 0.06565507
0.7310205 0 0 0.1147671 0.03680663 0.0815595 0.1187891
0.1540743 0.2783087 6.69562 0.01519223 0.1468927 0.1814895
0.3135309 1.46822 0.8938753 0.2267288 0.1732314 0.04290676
0.09740327 0.5268679 0.07748764 0.1269194 0.06512701 0.5879683 0
0.0332317 0.3039527 0 0.1769936 0.1245763 0.3397535 0
0.07948425 0.2678321 0.06642037 0.4779820 0.1766718 0 0.3393923
0.7878608 0.04831899 1.0195060 0 0.5793752 0.7378525 0.6639395
0.1334746 0.1892912 0.3314749 0.03874382 0.9716950 0 0 0.1296746
1.0321490 0.2270315 0.2864667 0 0.0577016 0.5039912 0.09184263
3.86575 0.2124697 0.09343221 0.1216646 0.1366722 1.3779890 0.02765211
2.9537520 0.2208398 0.7693930 0.4699418 0.9509471 0.05627577 0
0.3747584 0.1991178 0.1951742 0.1349576 0.1695803 0.1768862
0.9860373 0.7845984 0.09620742 0 0.5530663 0 0.1553883
0.09397437 0.09775603 0.03874382 0.4528380 0 0.04671611 0.06103612
0.2196417 0 1.5228060 0.5440812 0.0313855 0.1552228 0.2588885
0.7887135 0.03004003 0.2594850 0.7361326 0.9571104 0.06042879
0.1539263 0.08941978 0.7866550 0.07165892 0.1744515 0.1983403
0.07912826 2.2480810 0 0.0864937 0.5386336 0.3108871 0.1241518
0.3527024 0.07485415 0.8452610 0.8542374 0.04361288 0.3708759
1.9004240 0.2161243 1.1792410 0.7363140 0.04544878 0.1309648 0.1936349
0.1324351 0.1559021 0.04449153 0.2453775 0.2962485 0.2009551
0.2171814 0.06088314 0.03839799 0 0.1602701 0 0.2920927 0

0.68959	0.3501604	0.3696666	1.371701	0.3038546	2.135272	0.0405098
0.07615164	0.6721069	0.4600829	0.1695803	0	0.7661854	0.299207
0.2100205	0	0.05558896	0	0.4757499	0.9046159	0	0.07947342
0.04392834	0.06173412	0	0.08214452	0.1381819	0	0.02513438	0
0.7466918	0	0.02834583	0.5182373	0.1898901	0.05100226	0.1703477
0.3731821	0.1527822	0	0.2407067	0.1308386	0.03510459	0.3787094
0.8258031	0.2846763	5.888622	0.1235681	0.5465101	0	0.07567718
0.08427537	0	0.4887802	0	0.05965711
TCGA-GU-A766	480	0	0.05369353	0.1148377	0.04868121	0.1614133
0.1441997	63.99379	0.1403024	0.09756204	0.4654475	0	0.1061149
0.8852566	0.07196353	1.368483	0.04352368	0.08328571	0.1271075
0.06296512	0.3508637	0.04798577	0.1542135	0.1103441	1.880685
30.91838	0.08033981	0.8802848	0.1235997	0.09637648	1.274622	0.5438387
0.08894558	0.06714058	0.02883993	0.2560676	0.09117611	0.05724618
0.3101321	0.09544447	0.1783542	0.4806971	0.05925446	0.09391951
0.03467096	0.1863069	0.5592668	0	0.02527803	0.05833646	0.6488985
0.3848859	4.149535	0.1071352	0.4482728	0.4390718	0.3577886
1.045844	0.03442017	0.1316014	1.233708	0.04758589	0.04521224	0.2697783
0.08651979	0.06584272	0.2362737	0.1609671	0.4049863	1.344112
0.04166369	0.0431618	0.2417512	0.09826719	0.1743073	0.8651979
0.4060662	0.06264736	0.2521478	0.06105647	0.132695	0.09107347	0
0.1020609	0.2424759	0	0.1301939	0.05603834	0	0	0	0.1331074
0.04032702	0.03114005	0.02098606	0.1424358	0.2607446	0.415295	0
0.7024067	0.8642626	0.05679072	0	0	0.6809565	0.6670919
0.1224074	0.03486146	0.02197328	0.4205121	0.1517891	0.3021326
0.05302049	0.07898073	0.06773792	1.617487	0	0.6284928	0.009505296
0.04521224	0.3554133	0.07196353	0.2362635	0.1109875	0.1464181	0
0.1070898	0.6790033	0.01083344	1.052009	0	1.252094	0.1416247
1.219283	0.08084089	0.05287321	0.8494671	0	0.06757338	0.2467416	0
0.04619989	0.03511874	1.111323	0.188459	0.02932874	0.9333834	0.1228023
0.08116996	0.3681693	0.0382985	0.09107347	0.312804	0.9887977
0.1830226	0.1913	0.2409412	0.03544573	1.818207	0.3896797	0
0.03040632	0.1014264	0.432599	0.1176897	0.5800605	0.2523494
0.03571174	0.2367457	0.08771592	0.02335504	0.469189	0.08422281
0.102519	0.1418961	0.4092076	0.4788517	0.04075879	0.125863	0.2110239
0.6292908	0.4215445	0.1036353	0.03105115	0.2281896	0.7530074
0.03417299	0.1453004	0.1942281	0	0.5543987	0.4409141	0.03561152
0.2252586	0.2574695	0.4892007	0.05552612	0.06972291	0.8972423
0.1160631	0.07267332	0.1451476	0.04770515	0.07787184	0	0.08970007
0	0.07629	0.1097081	0.5763521	0.03429613	0.3310323	0.4160515
0.1127776	1.386609	0.03174156	0.05966882	0.7311209	0.07209983
0.06643754	0	0.3815287	0.3041442	0.02742702	0.1266143	0.08711375
0.07223664	0.1445455	0.6777258	0.2080257	0.08302881	0.1721009
0.1209298	0.07377657	0.01755397	0.06496367	0.0749384	0.009847054	0

0.3900483	0.04899448	0.01110522	0.7106159	0.06376668	0.1079	0.12106
0.2645594	0.1915407	0.02324097	0.2680196	1.4694390.1100252
1.1151630.1617651	0.4832942	1.8818960.03960906	1.09196	0.1129635
0.1185941	0.08254274	0	0.4085173	0	0.1308846
TCGA-FD-A6TD	386	1	0	0.08072959	0.1095114	0.1536232	0.06951132
70.098650	0.1097360.2204323	0.1039295	0.02983901	0.5354628
0.1214149	0.9515327	0.06527282	0.1124139	0.07148409	0.3753565
0.1973225	0.02698675	0.1238975	0	0.8870863	32.4002	0.1112181
0.03246321	0.06951132	0	0	0.5243139	1.0504650.07551847	0.09731585
0.28802	0.06635795	0.09658415	0.3924346	0.2530491	0.1671745
0.09830528	0.08373761	0.1056389	0.03899724	0.5238863	1.930541
0.09579188	0.1137290.1499789	0.7906913	0.1180673.7517820.04016789
0.5429942	0.08008536	0.08048679	0.6721974	0	0.0497895	1.2389750
0	0.1685786	0	0.01960376	0.2778361	0.09052637	0.3153607
0.4876876	0.08033578	0.08900391	0.0239927	0.05526455	0.3136921
1.7609530.1427299	0.1056969	0.02520987	0.08584397	0.04975094
0.2048755	0.06711438	0.1147962	0.1090929	0.02309545	0.02928794
0.06303087	0.1920133	0.03899724	0.1646884	0	0.04535908	0.05253862
0.0944189	0.04005228	0.3039454	0.03892634	0.06047877	0.09753752
0.7869433	0.1490466	0	0.04654236	0	0.2501108	0.5507263
0.07842303	0.07414541	0.2921372	0.1365836	0.2990531	0	0
0.07619034	1.8842950	0.5554348	0.04276552	0	0.04441802	0.2023581
1.5263290.37451	0	0.03574205	0.09713923	0.7136495	0.0243705
1.4199360.04865792	1.3561710.09557806	0.03809517	0.06612969	0
0.6251806	0.1754876	0.0720050.0396472	0.03736385	0	0.0395009
0.5452105	0.08479004	0.09896527	0.1124842	0	0	0.41411	0.04307744
0.1024377	0.1732924	0.2780453	0.04117209	0.3227561	0.07743033	0
0.9586337	0.5581826	0.02766083	0.03420046	0.4563301	0.2780453
0.2029753	0.1479763	0.08109654	0.6025184	0	0.08632857	0.1050772
0.3000846	0.06315483	0.03843714	0.1140015	0.2510559	0.4039526	0
0.05444935	0.2373557	0.3044363	0.3647272	0.03885569	0.04268703
0.5508874	0.4705382	0.03843714	0.03268624	1.6748920.1142855
0.3117886	0.1952072	0.06008275	0.2336604	0.2327118	0.1500665
0.1249095	0.1568461	0.4865792	0.09790924	0.0272472	0.1407408	0
0.04976636	0	0.2421431	0	0.1716191	0.2467952	0.4512107
0.2700296	0.1396271	1.0919230.0704723	0.5155806	0.02380154
0.02237146	0.04096002	0.4460310.03736385	0	0.2776765	0.1710478
0.06169881	0	0.09798392	0.4875025	0.1540251	0.6783711	0
0.02334731	0.1161455	0.02720392	0	0.0329073	0.04871328	0	0
0.009406628	0.4387190.02755404	0.0249819	2.2265870.1912629	0.07641429
0.1117260.4541881	0	0	0.2847155	1.5374860.1237543	0.446816
0.3032505	0.5017848	3.3153460.07425255	0.3371573	0	0	0.05570551
0	0.1723097	0.03169428	0.09463918
TCGA-FD-A3B4	510	1	0.109954974	0.192409897	0.03625114	0.277381522

0.460201161 24.139735985 0.034826034 0.167097131 0.510781829 0.550454204
0.128407348 1.065504389 0.455503456 0.537322454 0.237677063 0.23744746
0.047326196 0 0.174183665 0.041688811 0.032810638 0.123253876 1.190406561
48.823097332 0.220896557 0.752231769 0.138060348 0.10765212 0.822609576
0.593002068 0.198703679 0.224987234 0.107380271 0.78392413 0.407373336
0.03197187 0.202076111 0.172608401 0.125435361 0.158059251 0.095037711
0.101022229 0.077454622 0.231226685 1.077066547 0.095128831 0.112941799
0.018617595 0.362408414 1.328830853 4.077624499 0.292525616 0.770336726
0.353471216 0 0.289269301 0 0.163925331 0.524970213 0.035435489
0.033667923 0.066964704 0.064428163 0.191796524 0.106633032 0.119866349
0.417570415 0.226012233 0.044322063 0.251771429 0.465942931 0.343927311
1.03840262 0.245440619 0.170090349 0.108852847 0.154384823 0.250066004
0.197626277 0.090425491 0.16292179 0.266003674 0.216675604 0 0.542924051
0.097369353 0.190684248 0.129091036 0.181720459 0.09912025 0 0.150727644
0.093765352 0.101987306 0.324788546 0.25771265 0.160160404 1.373293378
0.245175278 0.143316085 0 0.061626938 1.110754694 0.894166555 0.091152384
0.181720459 0.208624526 0.257880105 0.090425491 1.034941278 0.217153417 0
0.151325933 2.014642898 0.165586399 0.401156484 0.03539125 0.033667923
0.088221135 0.321531851 0.843594719 0.495890231 0.054516138 0.094652392
0.192933699 0.936657381 0.088739985 1.044524375 0.161070406 1.274578844
0.274203192 1.967237134 0.158707211 0 0.788756899 0.038727311 0.222464956
0.131242553 0.593683592 0.206420327 0.235364874 0.607465533 0 0.04368011
0.02482346 0 0.090666497 0.137081197 0.085558526 0.180850983 0.545829957
0.699505765 0.463387169 0.106840672 0.230683114 0.079185451 0.59235445
0.910223945 0.091564572 0.158497397 0.302114297 0.782341974 0.233704794
0.449762417 0.483211219 0.638237708 0.176295967 0.269440357 0 0.506955286
0.146341254 0.254473892 0.251583169 0.461700188 0.496669932 0.030351597
0.245128513 0.754280928 0.196513912 0.265615253 0.154346959 0.105336601
0.091179037 0.498433258 0.050894778 0.238039929 0.916019454 0.4539778
0.137613551 0.576328808 0.013259304 0.320573527 0.81484507 1.114948421
0.330786366 0.129800328 0.441452225 0.15124904 0.117253926 0.324258488
0.1065729 0.122566267 0.02495457 0.187029916 0.093559048 0 0.408478264
0.49137212 0.357547281 0.400575098 0.671273388 0.055987607 1.954178705
0.047273582 0.059244287 0.667511795 0.080535203 0.247368163 0 0.480481212
0.292543321 0.081695653 0.031428372 0.064870461 0.134480036 0.600505496
0.944534822 0.077454622 0.386428456 0.076894371 0.180104139 0.16481623
0.056644496 0.258005839 0.111607841 0.029330979 0 1.85891092 0.164179874
0.074426932 0.98274424 0.087055362 0.09820459 0.50853279 0.207376675
0.267437656 0.01730671 0.280896709 0.890658621 0.040965884 1.32225965
0.020076765 0.968939164 3.706058332 0.147477181 0.398599431 0.126179784
0.397407358 0.030733295 0.019000262 0.323221097 0.003497211 0.013923571
TCGA-DK-A1AB 508 1 0.1410504 0.1206694 0.1364089 0.1739591
0.6349509 34.800290 0.2323705 0.20593 0.64728 0.08362767 1.585013
0.2268539 0.1220102 0.3557080.3200552 0.08904152 0.3219913

1.0036320.08964012 0.1388955 0 0.4611109 27.9165 0.07792579
0.4549117 0.4329210.2025413 1.1310060.4490010.1869248 0.0235167
0.7273073 0.7374541 0.3719535 0.08020432 0.3258811 0.06686087
0.09023508 0.07871808 0.1192053 0.05117203 0.09715095 0.1087599
0.9997099 0.05965981 0.08853896 0.1050838 0.2651641 0.3431536
2.7553150.2251514 2.2947950.6151581 0.2005108 0.1465271 0.409905
0.1106275 0.1851940 0.0950164 0.04199675 0.04040596 0.5765527
0.05015598 0.08457062 0.2400551 0.2126145 0.02084735 0.2267682
0.2590087 0.3097721 1.3431650.8369807 0.03555725 0.1097145
0.08635481 0.2245496 0.4647781 0.1275978 0.1950633 0.1072437
0.2038314 0 0.2371294 0.09159743 0.05979358 0.04857548 0.2735173
0.6060895 0.1412497 0.09271062 0.07350589 0.1170488 0.3420669
0.6788202 0.3389992 0.6722669 1.0955480.4486649 0.01929663
0.05797377 1.1584880.4361578 0.06431174 0.4884237 0.2462840.190609
0.1488641 0.7704070.09285493 0.0829915 0.09490369 0.3507164
0.06230826 0.06289608 0.009987991 0.0950164 0.1106553 0.05041198
1.9967 0.3109963 0 0 0.1524573 0.9357171 0.06071243 0.6386941
0 0.6201846 0.2381066 1.9692520.1544475 0.0370388 0.844852
0.3278845 0.05730218 0.02469253 0.4886795 0.0970920.5658326
0.4058164 0 0.4109081 0.02335196 0.1147008 0.2274451 0.1547462
0.3219469 0.2551956 0.42844 0.2770695 0.2564225 0.1340097
0.04822412 0 0.9552694 1.0779170.08613674 0.3195040.5447269
0.4329210.2583250.6199897 0.5555820.8505721 0.06633815 0.06912771 0
0.3737893 0.07866648 0.09575561 0.1940691 0.2866586 0.6109904
0.05710479 0.07460511 0.4730454 0.1611642 0.09086179 0.1451975
0.09425861 0.3820845 0.8205519 0.3112057 0.2850008 0.5442436
0.2372592 0.64728 0.4304460.01247331 0.3138436 0.3317388 1.007317
0.1789272 0.2442119 0.2244776 0.6504375 0.08060623 0.2033579
0.1336739 0.07934672 0.04695059 0.1759430.0440065 0.3473779
0.07685284 0.6147963 0.6006292 0.2608821.06866 0.04389061 0.3371623
0.09635441 0.1532641.04003 0.6565969 0 0 0.9885020.2929567
0.1152793 0.02956534 0.03051251 0.1265082 1.2044270.9045146
0.2185896 0.08724516 0.04822412 0.05082834 0 0.06968259 0.03033895
0 0 0.3339351 0.4098556 0.1029650.05445611 0.4266870.1191198
0.1063806 0.3500928 0.3804131 0.1006337 0 0.1216920.5745336
0.03853748 0.3989797 0.01888664 1.0938027.4571740.1603159 0.2249826
0 0.04153884 0.01156459 0.05362186 0.1699164 0.0197394 0.0851383
TCGA-BT-A20X 251 1 0.05394532 0.04615051 0.0391276 0.09979708
0.04967173 70.785510 0.4626523 0.4134836 0.2227994 0.01066125
0.4084291 0.1446020.2799797 0.03498222 0.1128434 0.05108144
0.1518249 0.6815175 0.03214053 0.01770705 0.1330338 0.9849805
35.1272 0.1887526 0.3827623 0.1490152 0.03873137 0.06829863
0.6244446 0.03574508 0.02698217 0.04636028 0.1143415 0.08190447
0.02300585 0.03115864 0.2520591 0.03982013 1.2042420.1257811

0.07548796 0 0.1996593 0.5580143 0 0.06095176 0.006698289
0.3477021 0.08436889 5.677609 0.1148135 1.302623 0.2289111 1.207807
0.3122223 0.2489874 0.02500133 0.3187269 0 0 0.1686492 0
0.02490409 0.05754709 0.03234438 0.1752737 1.707621 0.004783894
0.3700412 0.01714482 0.1026771 0.3082194 0.2318014 0.04079705
0.0167843 0.009007296 0.1472227 0.2725597 0.1952012 0.04795891 0
0.1364232 0.01650366 0.2511446 0.367834 0.0686049 0 0.2353676 0
0 0.01251443 0.1012055 0.04623352 0.1714695 0.2503455 0 0.19748
0.3142475 0.1622956 0 0 0 0.1429803 0.09838516 0.3362394
0.1103816 0.24852 0.07320044 0.3788297 0.06392295 0 0 0.8976588
0.1072352 0.4329875 0.003819948 0.07267882 0.03174044 0.0289204
2.500312 0.04460319 0.05884189 0.02554072 0.05553137 0.8409918
0.06095176 1.66856 0.03477021 0.4913266 0.113831 0.05444446 0.04725527
0 1.045214 0.04180025 0.03716099 0 0.1067986 0.1856662 0.3387209
1.14029 0.03029484 0 0.05358631 0.03290084 0 0.4734667 0
0.1464009 0.4165261 0 0.02942095 0.1921971 0.05533053 0
0.3196784 0.2998208 0.1185961 0.1221956 0.1630433 0.3477021
0.07567465 0.07209657 0.1448759 0.315737 0.03805695 0.07931454
0.1501732 0.1330981 0.1805179 0.02746659 0.1683584 0.01993341
0.2474214 0 0.03890868 0.1696108 0.1414046 0.2085028 0.08329706
0.02495741 0.1476209 0.6724788 0.02746659 1.237926 0.2601852
0.1905556 0.2970659 0.3770057 0.02862281 0.1850754 0.6023477
0.2144705 0.1160359 1.316938 0.2446793 0.1399289 0.07788173
0.1448225 0 0.07396954 0 0.04325794 0 0.06131821 0 0.7646156
0.385918 0.3991015 0.8081382 0.07050181 2.044765 0.05102466 0.1278904
0.1238321 0.08692553 0.05339928 0 0.2570485 0.4023344 0.1543116
0.06784431 0.07001782 0.05806031 0.18344 0.5913662 0 0.01668363 0
0.116637 0.05929803 0.05643612 0.06961953 0 0.01582917 0.5579122
0.4389027 0.1575176 0.02677753 0.3263764 0.1281314 0.01284811
0.3393101 0.05595791 0.03848778 0 0.1436141 0.1098664 0
2.688943 0.3033775 0.5378517 2.487551 0.03183585 0.172091 0.09079462 0
0.0464407 0.0205079 0.08208661 0.1434389 0.06762772
TCGA-GV-A3JW 649 0 0.3950106 0.5218093 0.1685347 0.6125461 0
44.67075 0 0.2279883 0.5936672 1.279555 0.2755278 0.2268481
0.5294188 2.562664 0.3076368 0.2471449 0.08250885 0.3487771
0.278367 0.1522831 0.7436301 0.2387575 11.10274 43.60168 0.06953424
0.02497993 0.3209273 0.291949 0 1.7651 0.2309477 0.1162206
0.1497661 1.649889 0.8076986 0.1362533 0.4697347 0.5369478
0.2572764 0.04322536 0.1814694 0.2077347 0.5101321 0 24.6196
0.5528277 0.2625381 0.07212901 0.7020284 0.6662387 4.00033
0.2781771 2.118973 0.1335196 0.1238667 1.396563 1.608699 0.08180182
5.758366 0 0.2347876 0.3632122 0 0.01676088 0.5948954 0.3134638
0.3774791 0.4503216 0 0.6350624 0.1538501 0.9355546 4.375034
2.924004 0.351451 0.0271107 0.04849652 0.224589 0.02552171 0.210198

0.4992202 0.1325008 0.4616992 0.03554318 0.2253662 0.09700255
0.2955025 0.2400622 1.1827680 0.03490311 0.2762556 0.4722502
0.01896591 0.5334134 0 0 0.1476054 2.9030330.01365346 0.0238412
0 0.05613038 3.9646011.0064660.2413812 0.1521433 0.6529761
0.1576485 1.7467950.1376680.03417898 0.1758818 2.7248790.2309477
0.7382336 0.004113425 0.03913127 1.3671590.4982765 0.06816215
1.7771070 0.08250885 0.2451706 2.6204680.1781509 0.3763465
0.3369736 0.4524494 0 0.2051955 0.9731902 0.0915237 1.642888
0.8102098 0.1754546 0.0610158 0.2587581 0.03998608 1.306999
1.8520760 0 0.05770321 0.07085706 1.8968241.0834140.3646216
0.07882424 0.9495837 0.0855806 2.3760960.1241779 0.1787443 0
0.6884768 0.8676735 2.6392880.5789678 0.7900625 1.6046361.089909
0.1707982 0.4368177 3.8017540.6147114 0.1328570.1212829 1.242139
0.14579 0.08873034 0.07602608 0.1180566 0.08881006 0.3174912
0.08379586 0.9132077 0.3689580.1683911 1.9733240.3822197 4.065579
0.1448287 0.02957678 0.2766671 0.05603492 0.9380365 0.07997217
1.62794 0.01541091 1.6550080.3740534 0.6671821 0.3075709 0.3620718
0.5546891 1.2305470.6394720.5068339 0.2477333 0.1302011 0
7.9654090.2718526 0 0.1899051 0.5541022 0.2968336 0.6088316
0.4801243 0.3253643.3325430.1739917 0.2237884 0.6036928 0
0.4600144 0 0.04127672 0.4551811 0.02373814 0.07305661 0.0753971
0 0.9152372.1543550.1800466 0.1976193 0.08937215 0.08373198 0
0.1114153 0.2249047 0.5837338 0 0.6152516 1.7892130.2332264
0.2306782 0 0.2483558 0.1763988 0.3008998 1.7354020.3730023
0.0201151 0.6787312 0 0.7142023 0.9320367 0.04666931 2.445399
5.2690180.3123445 0.01853123 0 0.2052868 0 0.1766677 1.425339
0.1991707 0.02427446
TCGA-UY-A9PB 899 0 0.07519834 0 0 0.1391145 0.05770089 73.1515
0.1964949 0.1776276 0.2744691 0 0.03715374 1.7417280.05039284
0.8014766 0.1727068 0.4599047 0.08900771 0.1499116 0.2456941
0.01120076 0.1542697 0.1545384.83451649.8283 0.1644475 0.5456868
0.2596540 0.2380162 0.6528443 1.5571150.1410466 0 0.7172505
0.1051590.08017386 0.3800502 0.08593134 0.1595861 0.3934409
0.08724219 0.08769016 0.04855703 1.1306740.8912946 0.1192743
0.1062064 0.1517301 0.4543945 0.14701 4.0496910.1000293 0.1448794
0.3157721 0 0.4324396 0 0.09215459 0.9873263 0.2665781 0
0.3358464 0.04039062 0.0461067 0.1303560 0.1308894 0.4857915
0.05001465 0.09067282 0.05974855 0.1651491 0.2441191 0.519308
0.1421750.2193457 0.02354237 0 0.1238938 0.08503289 0.417834
0.07146866 0.4414669 0.5751415 0.182338 0.09156264 0.5977087 0
0.2050601 0.2796274 0 0.04361196 0 0.007672416 0.2987799 0
0.07530455 0.2671853 0.5187466 0.2430266 0 0 0.4541365
2.3668150.6857314 0.09764766 0.09232142 0.1558936 0.08503289
0.7278005 0.03712787 0 0.04743383 1.7259480 0.2514888 0.03993679

0	0.16592	0.2015713	1.866556	0.3108782	0	0.1780154	0.1306281
0.8418256	0.01517234	1.571573	0.06058593	2.113731	0.3570242	0.5692059
0.3190703	0	0.5874999	0	0.1942553	0.04936631	0.1860929
0.2588137	0.1967366	0.5795176	0.1055755	0	0.2334309	0
0.2841984	0.7734374	0.05363744	0	0.4250080	0.4101202	0
0.3856464	0	1.591511	0.2285621	0	0	0.1420487	0.778962	0.1538373
0.7537552	0.1514648	0.5001465	0.06631296	0.07677941	0.09812691
0.4123006	0.1179549	0.2871577	0.1892638	0.1215666	1.029906	0
0.08135649	0.2955411	0.3032527	0.04541365	0	0.05315133	0.7327
0.7616517	0	0.08139789	0.9067282	0.1423015	0.3882205	0.7423195
0	0.3084664	0.5537613	0.4359922	0.01555298	0.292943	2.580512
0.08127381	0.08481645	0.1331839	0.1336231	0.1511972	0	0.1005007
0	0.2136899	0.3072946	0.807187	0.528353	0.1738553	0.6797984
0.08774789	1.492581	0.1333631	0.139278	0.2550048	0	0.09304646	0
0.4203958	0.2484749	0.03841183	0	0.1830056	0.1011682	0.1491644
0.510864	0.2185066	0.2034947	0.0482058	0.06774539	0.3099745
0.06555871	0.03032743	0	0.1103272	0.05856285	0.6008933	0
0.03110596	0.9952249	0.2232647	0.08395279	0.252145	0.3315163
0.3688502	0	0.2710964	2.105823	0.07704569	1.206539	0.1510358
0.7809905	4.366229	0.03698193	0.419808	0.07910329	0	0.1849631	0
0.4112204	0.006577303	0.1702119
TCGA-FD-A6TI	294	1	0.01089594	0.1398231	0	0.1511786	0.1839339
20.52024	0.2657325	0.2850929	0.1789627	0.5250153	0.06460118
1.324262	0.1168276	0.2827533	0.07654567	0.16225	0.02579374	0.01022193
0.118667	0.1038686	0.1251773	0.08956782	2.107663	31.54768	0.06521294
0.2342752	0.3009828	0.1173452	0.4828265	0.5990991	0.2165951	0
0.04681954	0.3926123	0.1393109	0.06970127	0.503477	0.1272781
0.7680989	0.06587597	0.07522968	0.05929449	0	0.07561394	1.330586	0
0.1436297	0.03382323	0.2194666	0.5112281	3.687039	0.02898771
0.8956782	0.1541192	0	0.3314853	0	0.07380479	0.9298882	0
0.2201964	0.02433142	0.02340977	0.08331202	0.0697406	0.0653296
0.3793073	0.4575304	0.00966257	0.07007006	0.09234486	0.1435767
0.4810579	0.4347529	0.0824024	0.08475288	0.06822394	0.1115109
0.08377471	0.2217768	0.3228934	0.2071106	0.2361852	0	0.02113605
0.2274355	0	0	0.07923307	0.2701127	0.16367	0.03159601	0.03406935
0.1067235	0.2462836	0	0.1745813	0.6123868	0.9687849	0.02817088
0.02235955	0.03358793	0.6053839	0.7219836	0.2732395	0.1414876
0.1917372	0.2710605	0.02464186	0.5395395	0.1506311	0	0.08247571
3.493349	0.1443967	0.2186384	0.1080181	0.07339881	0.1602744
0.2044483	0.1721088	0.2702702	0.1782744	0.02579374	0.1289874
1.454691	0.01758731	0.1252431	0.03511466	0.6228088	0.02299174
0.5498381	0.05368882	0	1.242846	0.2110717	0.08083195	0.05722389
0.0808924	0.07500218	0.2850636	1.324324	0.1223798	0	0.08117574
0.299041	0.2635471	0.4482722	0.03108742	0.02464186	0.2311693

0.4815724 0.5645356 0.03882022 0.1396966 0 0.6918112 0.5920645
0.0998091 0.1480874 0.5763045 0.1003276 0.331172 0.08737295
0.3511466 0.4058279 0 0.1068003 0.09478803 0.1269491 0
0.05547738 0.1974498 0.2617014 0.2498725 0.03308447 0.0943059
0.5823894 0.2801183 0.1316052 0.3925704 0.0168031 0.6505452
0.1697852 0.02773869 0.04717694 0.4729729 0.3299028 0.07500218
1.39351 0 0.1889404 0.3097554 0.1082975 0.09915691 0.08489258
1.248521 0.3532877 0.04424246 0.08531671 0 0.1134901 0 0.189308
0.1019832 0.03096282 0 0.2937247 0.5010957 0.1007638 0.6754295
0.1424005 0.6697365 0.008588354 0 1.969108 1.141226 0.1617848
0.2439545 0.1024717 0.08743596 0.0222629 0.1370328 0 0.05863548
0.3952153 0.1766439 0.04221434 0.4043738 0.02793933 0.03926417 0
0.0522456 0.05273191 0.1216571 0.06394392 0.2443832 1.013144
0.07953899 0.01802853 0.3708108 0.1897878 0.1038031 0.2393661
0.1695368 0.1749107 0.05659505 0.20144 0.2219095 0.04465448
2.093988 0.1094224 1.026006 4.453852 0.03929594 0.03475917 0.09169406
0.09626448 0.05360086 0.02071106 0.0414499 0.01143631 0.1973044
TCGA-XF-AAML 232 1 0.1314304 0.04684976 0.07944089 0.2228802 0
22.79405 0 0.254732 0.1998802 0 0 0.02618636 0.2348687 2.436187
0.2959355 0.1747422 0.05185541 0.0513751 0 0.05220401 0.1797534
0.270099 2.65321 217.91312 0.1008487 0 0.1008487 0 0 0.8240777
0.07257334 0.05478199 0 0.3714366 0.288821 0.02335443 0.8223993
0.2781285 0.08893162 0.03056221 0.04462843 0.08514635 0.3960468
0.05067111 3.839402 0.1389771 0.08250037 0.01359956 1.147154 0.2854907
2.795897 0.1165531 0.6189769 0.03872991 0 0.4551119 0.449351
0.003904637 1.222323 0 0.2951201 0.3913246 0 0.003160178 0.1226799
0.1313378 0.1525109 0.07075487 0.009712754 0.05869499 0.02320612
0.1443224 0.9102238 3.496087 0.1242456 0 0.02743131 0.02490889 0
0.04953969 0.7465119 0.166549 0.2374119 0 0.1274749 0.03810278
0.4178662 0.2263125 0.2389338 0 0 0.1651526 0.2739704
0.004469908 0.2630354 0 0 0.1509437 0.839497 0.005148581 0 0 0
0.65316 0.3495655 0 0.1703222 0.3632911 0.09907939 1.853823
0.04326099 0.06444271 0 3.220832 0 0.4395479 0.007755653 0
0.7733126 0.2935859 0.4003775 0.4527899 0 0 0.07328458
2.179747 0.05303595 0.4349052 0.4941585 0.2958227 0.0462223
0.05526937 0.131921 0.08628163 1.728485 0.3394687 0.1218776 0.05752109
0.1626251 0 0.9742502 0.5980764 0 0.04786038 0.05439823 0
0.1324579 0.4806404 0 0.09907939 0.609495 0.2420368 0.1194669 0
0 0.05784244 0.4636028 0.4837207 0.4013099 0.09923766 0.4413696
0.4033947 0.1216336 0 0.1176568 0.5244887 0 0.107355 0
0.6155229 0.04581326 0 0.06615844 0.02023543 0.1674468 0.06651261
0.09479569 0.2066168 0.4306414 0.2116619 0.3382365 0.09571209
1.326012 0.06826679 0.05576551 0.1896879 0.211302 0.2210775 0.07539172
1.117536 0 0.4942049 0.3676348 0 0.07248865 0.05688899 0.8628163

0.2840981 0.06917904 0.2368604 0.1556962 0.1068593 0 0.3805825
0 0 0 0.04809505 0.3357988 0.4726733 0.452625 0.2044858
0.2618058 0.207191 0.03245704 0.1737061 0.05882839 0.2710418 0
0.009155899 0.2274802 0.04475704 0 0.1421574 0.11788 0.3351948
0.903026 0.1697344 0.1354913 0.1685066 0.1578724 0.120393 0.1336795
0.2826975 0.1222889 0 0.4094208 1.336658 0 0.05436649 0.3313215
0.1040583 0.1108637 0.4406948 0.227223 0.03907093 0 0.323978 0
0.0897728 1.421467 0 2.426671 2.478991 0.05745459 0.1048191 0
0.1935289 0.08081888 0.04163725 0.5416473 0.06897422 0.1067926
TCGA-ZF-AA58 1649 0 0.096449022 0.132609612 0.149906518 0.216662019
0.253737872 26.767592086 0 0.140199603 0.226306751 0 0 1.745967553
0.480135369 0.791728498 0.044674885 0.422558094 0.097852168 0.019389166
0.360144077 0.049254999 0.158292492 0.226525405 1.662660371 44.477991834
0.101495149 0.325877146 0.697779148 0 1.046668723 0.897144619 0.502140135
0.103374689 0.148013759 2.0735201 0.286268369 0.058760349 0.278543204
0.174944629 0.147474406 0.185830327 0.06394081 0.074980654 0.071176015
0.31872457 0.633444901 0 0.05189327 0.025662631 0.777072233 0.502811445
4.533895137 0.109968946 0.283156756 0.462865969 0 0.500969645 0
0.153502641 0.587943542 0.04884454 0.046408114 0.153841072 0.029602752
0.161009575 0.24987239 0.247836991 0.703489289 0.623073864 0.152734648
0.177213752 0.131371196 0.252165929 0.393619007 0.54976539 0.208403372
0.107173978 0.069017896 0.062671423 0.060535449 0.031160791 0.122494145
0.104760408 0.398223004 0 0.106910075 0.134214635 0.087613525 0.071176015
0 0 0 1.094756979 0.064623427 0.022492809 0.520684017 0.106569906
0.110383142 0.513294183 0.697024793 0.265556261 0 0 0.46597858
1.780315024 0.18846768 0.071567092 0.045108955 0.266597881 0.124643165
0.272909178 0.027211443 0 0.069529595 1.353910376 0.045649103 0.230398775
0.043905205 0.092816229 0.202674441 0.295467919 1.175253741 0.056961563 0
0 0.148927092 0.594132421 0.055599932 2.217261377 0 3.587342103
0.232593049 0.591001556 0.0905227 0.162815135 0.516702576 0.053382011
0.237286631 1.04925309 0.068194821 0 0.144190525 0.691711504 0
0.210731453 0.37638525 0 0.291609196 0.188953735 0.039311501 0
0.498389227 0.101495149 0.300581787 0.245449952 0.176652949 0.072766541
1.924620696 0.444429764 0 0 0.069406096 0.507475744 0.056374573
0.847068372 0.370034397 0.329906839 0.097203065 0.157563034 0 0.349395456
0.14408419 0.140307477 0.235813188 0.025456438 0.912818006 0.041836865
0.238508437 0.433211001 0.152801975 0.399410211 0.070917663 0.063744914
1.096859854 0.472342808 0.105230608 0.178972362 0.731006727 0 0.521640723
0.86182087 0.018276722 0.249200285 0.217086846 0.33476009 0.079792715
0.071567092 0.328919464 0.268049307 0.043514067 0.200361512 0.048966958
0.128980469 0 0.313047007 0.064481241 0.03915394 0.112609891 0.502521035
0.176016362 0.297314594 0.711760151 0.16720975 0.811625114 0.097743383
0.367482436 0.991986251 0.444041276 0.204584462 0.051415306 0.918579713
0.305684873 0.168914837 0.0433211 0.402380653 0.556104824 0.390443968

0.770109914 0.053382011 0.170449868 0.07066118 0.049651375 0 0.006006092
0 0.076920536 0 0 0.760693662 0.075435584 0.068393756 0.625210117
0.130906641 0.082039959 0.127448246 0.228679633 0.122879347 0.047711395
0.254730328 0.280614954 0.056467677 0.741818013 0.276739606 0.648716552
4.34044397 0.07679604 0.813159953 0.115951431 0.243461884 0.093198603 0
0.131038338 0 0.038384708
TCGA-FD-A3SP 783 0 0.181814 0.111102 0.1412928 0.2282375 0.3786668
27.98809 0.1357383 0.3114815 0.09480136 0.804546 0.0384986 3.1438
0.2436789 0.3851538 0.06316177 0.5318043 0.06148636 0.04264179
1.668963 0.03868732 0.08525539 0.2135091 1.159202 46.7099 0.119579
0.1954604 0.5978949 0.2331033 1.397579 0.5449385 1.850122 0
0.6417406 0.7982657 0.3684071 0.09692191 0.3000434 0.1253181
0.2252766 0.1207948 0.110244 0.1413445 0 0.5407389 1.473959
0.1235917 0.1222786 0.1451283 0.3662106 0.1354056 2.74245
0.1727501 0.9341025 0.8151351 0 0.2120008 0.03330048 0.2245469
0.1705108 0.1841516 0.04374148 0.2320021 0.05580353 0.6463762
0.1489284 0.03893269 0.8439028 1.006752 0.01727501 0.1600713
0.1857338 0.3232398 2.529555 1.395088 0.1473213 0.4040628 0.09215713
0.1624433 0.08558558 0.02937028 0.1539408 0.1974816 0.5160938 0
0.3274927 0.180718 0.08257921 0.1341724 0.2833102 0 0 0.09038117
0.04060676 0.09805167 0.5962569 0.1339285 0.05202024 0.8389596
1.552846 0.6898437 0 0 0.8784064 0.7744695 0.5625211 0.4721837
0.2072702 0.2273477 0.08811083 0.5027632 0.1282393 0.03820573
0.06553439 1.210911 0 0.4777519 0.01379413 0.04374148 0.1146172
0.1044338 2.748794 0.3221311 0 0 0.1136326 0.9369118 0.06288616
1.343479 0.1255579 0.7953393 0.1644211 1.671127 0.2986243 0.05115323
0.953733 0.1006293 0.2615008 0.2387151 0.1285526 0.223485 0
0.5833375 0.07293134 0.2837467 0.06450145 0.15841 0.1963237
0.1424771 0 0.05874055 0.5329873 0.09566319 0.3895515 0.04626926
0.2331033 0.03429267 0.6596477 0.5638939 0.0713766 0 0.3925079
0.5978949 0.04174913 2.400972 0.2092632 1.382001 0.04580887 0.2651953
0.04519059 0.7120411 0.02716101 0.06612267 0.1961146 0 2.895466
0.0394329 0.09366818 0.3674866 0.6153663 0.1568583 0.2673708
0.1134884 0.4630714 1.173714 0.198368 0.08434426 0.7516393 0.4587407
0.849243 0.6806985 0 0.4019615 0.5248766 1.706701 0.08595176
0.1349096 0.744047 0.3930065 0.1054639 0.1743218 0.1846132
0.06335296 0 0.1735642 0.06077612 0.1845207 0.05306964 1.593605
0.7299705 0.1200989 0.5031466 0.1091089 0.7206416 0.02047267
0.2501537 2.268358 0.1046316 0.09641447 0 0.4423982 0.1287345
0.291883 0.04083185 0.2106998 0.2446037 0.4710514 0.07218488
0.2012586 0.1807377 0.1332019 0.07019757 0 0.06793174 0.1047506
0 0 0.3074588 1.094344 0.09480136 0.01074397 0.8839279 0.1439488
0.1005237 0.3423563 0.3637231 0 0.04496987 0.4033569 0.06612267
0.02661151 0.3472812 0.4434246 0.7553095 5.725262 0.08089899

0.6007196	0	0.1147362	0.1197864	0	0.2840703	0	0.07235821
TCGA-E7-A97Q	246	1	0.6677708	0.7639229	0.3942362	0.416573	0.02383215
62.57747	0	0.3950445	1.161978	0.3206928	0.2608749	1.215992	0.8741759
2.360411	0.4951347	0.0908476	0.03676278	0.02185337	0.4735679
0.06476738	0.1274358	0	10.50687	69.55599	0.1072447	0.1168661
0.3574823	0.1672474	0.1966153	5.055821	0.1543522	0.2330255
0.2001901	2.172466	0.8066267	0.2649132	0.9866802	0.8675859
0.5903575	0.06500104	0.1300724	0.3863317	1.002774	0.07184636
74.65244	0.4926378	0.9650597	0.04820689	1.188629	0.8500718	2.541887
0.2478901	0.9574308	0.2608462	0	1.140801	0.07964162	0.09827055
1.121435	0.05505227	0.3138372	0.6242147	0.03336501	0.1299433
0.4307268	0.1862233	0.8649784	0.7022613	0.04131502	0.5076627
0.2961347	1.068645	1.290603	2.144894	0.4991406	0.1570334	0.1944737
0.6180688	0.1705725	0.07024213	0.4602071	1.062671	0.6732507
0.02375502	0.5723629	0.1728825	0.09874847	0.2005547	0.846958
0.1539924	0.04665447	0.1711236	0.2913461	0.1647845	1.250502	0
0.4354417	0.3778835	2.142575	0.09490197	0	0	0.2250859	7.614707
2.903078	0.120994	0.1016838	1.144687	0.526816	1.55195	0.5213864	0
0.3134649	2.662113	0.4116058	0.5712971	0.03299012	0.261531	1.461969
1.998116	0.4520524	1.027214	0.0846958	0.07352557	0.3397055	1.519571
0.3007978	0.3732357	0.1501426	1.926525	0.1310768	0.7444791
0.6121642	0	1.395264	0.9024962	0.111092	0.1631178	0.6148945
0.5344881	1.340756	1.107884	0.04360576	0	0.1156965	0.3314975
0.469529	1.831526	0.04430766	0	0.3888902	0	0.9316538	0	0.2787457
0.04100728	2.037756	2.920708	0.398311	0.9146064	1.016951	3.43183	0.6353931
1.660389	1.292894	0.4957803	0.1095568	0.4059154	0.1891366	0.4683035
0.2598337	0.07906969	0.07035434	0.04303761	0.3165629	0.1414619
0.2800217	0.8788832	0.1409088	1.050401	2.797575	0.3113342	4.004972
0.241988	0.07906969	0.03361971	0.5243073	0.8620284	0.1603464
2.203176	0	0.9121086	0.6436063	0.9432632	0.3597348	0.3226507
0.3336501	0.9399168	0.2382154	0.8569799	0.2759512	0.1719904
0.0387692	2.843416	0.2180288	0.04413008	0.4442258	0.5095591
0.1190319	1.101045	1.764882	0.2174547	0.9147758	0.269294	0.1380621
2.161581	0.1668251	0.4227399	0.05794976	0.0811382	1.238859	0.2855737
0.09765369	0	0.08357081	1.742662	1.577717	0.3008321	0.6483801
0.03982081	0.1399041	0.08535236	0.4603202	0.22547	0.346786	0.01139209
0.4547376	2.887988	0.2550684	0.3340394	1.350616	0.3073829	0.1849331
0.466849	1.111516	0.1938948	0.1075502	0.5053038	0.03953484	0.1272885
0.5260212	0.03119109	3.999892	4.026455	0.2240277	0.2229338	0.06534394
0.2744038	0.03819759	0	1.211076	0.1575639	0.1838681
TCGA-YC-A9TC	20	1	0.1249524	0.1336218	0.05035023	0.2054736
0.06391864	78.04559	0.2902252	0.3229021	0.9374733	0	0.1234721
0.1881006	0.4465846	1.389666	0.1650582	0.3076469	0.09859903
0.3321316	0.1512054	0.07444641	0.02278581	0	2.054971	231.6455

0.08309423 0.2089592 1.470129 0.1495211 0 2.711976 0.5059728
0.06944247 0.0298287 0.500266 0.1886041 0.05920884 0.400956 0.1339727
0.1229793 0.03874111 0.04714306 0.04317311 0.3585964 0.4817359
13.40387 0.1761695 0.2353021 0 1.39822 0.2533246 11.7215 0.07387219
0.7132949 0.06136827 0 1.225927 0.4984035 0.01361133 1.276005
0.9351296 0 0.589058 0.0298287 0.01402061 0.1592135 0.3329715
0.4510919 0.08968993 0.2093046 0.1860066 0.1838526 0.2134361
0.54085 8.394648 0.3937388 0.4751648 0.01159078 0.03157488 0.04574812
0.06279726 0.1645721 0.21112 0.2257101 0 0.08079455 0.1497289
0.08828224 0.07171927 0.454314 0 0 0.03220767 0.06511667 0.01133224
0.4363979 0.03579444 0 0.1195866 0.8513271 0.02610567 0
0.0427977 0.03353823 3.863792 1.5509 0.03605667 0.05113491 0.8186902
0.06279726 1.337462 0.02741914 0.04084427 0 3.256262 0.09199505
1.300081 0.009831181 0 0.449287 0.4093693 0.3414826 0.2869816 0 0
0.1321991 1.38154 0.04481944 0.8704616 0 2.363305 0 0.1401206
0.1064159 0 1.062986 0.3227367 0.06253325 0.07291459 0.2748613
0.04778383 0.5448415 1.369526 0.07796808 0.5763511 0 0 0.3777878
0.2665543 0.03961155 0.03139863 0.1207195 0.05113491 0.227157
0.1978587 0.6052043 0 0.5289029 1.377914 0 0.2201415 0.874198
1.086617 0.07303481 0.1917358 0.1491435 0.7017858 0 0.04536161
0.02415575 1.351158 0.08711035 0.176723 0.4053399 0.141079 0.1061291
0.04215619 0.2002741 0.4801693 0.3709235 0.1006147 0.2501063
0.1355997 2.377399 0.043268 0.4948243 0.0300564 0.2678495 0.1751507
0.3822707 1.125522 0 0.5306802 0.1854567 0.07666254 0.05742981
0.07211333 0.4142875 0.06002116 0.04384619 0.165654 0.0986814
0.1885407 0 0.2597711 0.4548138 0 0.1134694 0.159189 0.2483037
0.427977 0.5737542 0.2592092 0.3792788 0.1422625 0.2057152
0.2781378 0.03728587 0.06871532 0 0.1537815 0.2031608 0.1134694
0.0873035 0.1351506 0.03735662 0.04721089 0.9003174 0 0.02146885
0.142401 0.150091 0.3815298 0.06051934 0.2239697 0 0.02036931
0.5708867 0.8068418 0.1013485 0.01148596 0.209994 0.0879372
0.01653323 0.2439999 0.3744407 0.1981076 0 0.292609 0.1413784
0.08534801 0.7078784 0.05577037 2.230161 4.701884 0.1092454 0.1328702
0 0.1839901 0.221969 0.02639 0.963881 0.04371637 0.02900826
TCGA-K4-A6FZ 55 0 0.068018289 0.084860424 0 0.188747424 0.104383045
29.046781144 0 0.06591487 0.517213286 0.156067854 0.15682916
0.53078859 0.273487524 0.504313378 0.049009113 0.128615988 0.026836375
0.010635123 0.44446974 0.027016788 0.111631868 0.046594171 1.15278805
32.296073862 0.130478806 0.536239206 0.41753218 0.244177047 0
0.442882348 0.713609321 0.085052851 0.048712088 0.672794753 0.108706603
0.060432289 0.163696425 0.207270631 0.062760238 0.115988769 0.089818744
0.12338259 0.175682939 0.209787784 3.159622949 0.179809719 0.042695854
0.014076172 0.685013733 0.768289912 3.264030347 0.271435085 1.397825124
0.180392644 0 0.395355562 0.029068696 0.018186663 0.669791205 0.080374945

0.038182872 0.177204602 0 0.009812792 0.123956093 0.13594071 0.368330352
0.768963024 0.030159454 0.11542887 0.090072781 0.232369484 0.677151548
1.913689158 0.214333186 0.079360889 0.018928453 0.232036729 0 0.076913823
0.10078363 0.25857891 0.184299236 0.034681745 0.241894498 0.15775259
0.360425761 0.02928049 0.329743363 0.11241251 0.10217154 0.085470289
0.070893005 0.043952337 0.588548785 0 0.13622872 0.156233757 1.320755847
0.05062569 0.02326337 0.104836884 0.082154969 0.56337578 0.180908235
0.35329646 0.148455886 0.240237001 0.128189704 0.540936135 0.11194282
0.100051798 0.114412733 1.08955413 0 0.113738129 0.040137301 0.038182872
0.166752997 0.243100022 1.460664971 0.515524426 0 0.026836375 0.195467197
1.522893687 0.064043781 1.492592929 0.073068131 1.135752263 0.143526687
0.085809549 0.099304951 0.089305494 0.681969227 0.307445143 0.0750887 0
0.224432655 0.11705089 0.26692786 0.339471816 0.031831661 0.049537716
0.028152345 0.034569869 0.102825089 0.248742575 0.032344042 0.051275882
0.417941826 0.041753218 0.401874723 0.403894194 0.174412176 0.029934802
0.62380554 1.322002589 0.269993354 0.25678896 0.285523782 0.782872837
0.32468032 0.040402109 0.63934615 0.422232354 0.079975069 0.129637007
0.118343477 0.303008491 0.023709423 0.057719888 0.051357792 0.178029193
0.361073426 0.068843636 0.098117938 0.35642991 0.171436071 0.219079917
0.233393669 0.049533227 0.192711856 1.095219026 0.028859944 0.319045582
0.437414665 0.54346048 0.039016963 0.491202224 0 0.34031206 0.213556616
0.13771408 0.0937864 0.029441372 1.001304024 0.122522781 0.086947124
0.219800006 0 0.053807494 0 0.257563442 0.053052769 0.032214407
0.277953699 0.316659997 0.202747608 0.314510653 0.263524409 0.158739193
0.48389491 0.044677568 0.033594543 0.721541077 0.213115383 0.056108164 0
0.19901239 0.235452567 0.301116507 0 0.183924358 0.030502825 0.308393073
0.4883408 0.08784147 0.227889701 0.116274784 0.020425653 0 0.088948601
0.018287814 0 0.074844749 0.014125649 1.581146451 0.103442657 0.03751456
1.114532565 0.188483957 0.097874171 0.372251864 0.540928669 0.040440224
0.058882743 0.377248762 0.34631933 0.162608269 0.905405461 0.045538212
0.973290345 3.408395507 0.126369855 0.126574716 0 0.200311388 0.034854703
0 0.269533684 0.051560537 0.071058399

TCGA-KQ-A41R 1350 0 0.1142474 0.0244348 0.08286585 0.1268123
0.2103932 50.74593 0 0.2158929 3.377663 0 0.02257878 0.04097301
0.367492 1.694146 0.07408662 0.05670805 0 0.3751304 0 0.1089094
0.1125019 0 5.550692 123.1953 0.1157162 0 0 0.1640534 0
1.124101 0.07570222 0 0 0 0.06390641 0.09744526 0.3959334
0.0580239 0.3879295 0.02125323 0.07500127 0.1598711 0.1180348
0.1057114 35.48632 0.07248445 0.1290859 0.01418588 1.104564 0.2382393
6.181684 0.06078902 0.4695732 0.1615987 0.1218066 2.865355 0.468724
0.03462032 3.150053 0 0.2308828 0.6633183 0 0.003296424 0.1401566
0.1370002 0.1590862 0.221416 0 0.1959217 0.07261983 0.2174533
0.534075 1.893539 0.08640146 0.05331961 0.01907598 0.2858108 0
0.05167552 0.1015691 0 0.2889221 0 0.0443236 0.07949104 0 0

0.3323133 0.3398659 0.06864523 0.02650352 0.7859008 0.00466262
0.5810031 0 0 0.3493453 1.015801 0.01074111 0.6095617 0 0
0.7570222 0.625091 0.1186833 0.1215605 1.284232 0.103351 0.3085767
0.04512611 0 0 6.129391 0.07570222 0.6877475 0.008090025 0.07696092
0.6049895 0.2449947 0.06187247 0 0 0.05409107 0.258734 0.4926399
0.03688167 0.5491618 0 0.3731625 0.0482151 0.05765222 0.1125888
0.09000152 1.213905 0.4426304 0.1331861 0.06000102 0.4523637 0
0.05977961 2.213702 0.5774355 0.09984761 0 0 0 0.8773843 0
0.05167552 0.2702033 0.08415727 0.1246175 0.08140841 0.703086 0
1.837643 0.6576489 0.7535011 0.05175806 0.1726495 1.051966 0.3004997
0.1323302 0 0.1823671 0 0.07465564 0.03975527 0.9865803 0
0.05816975 0.04600717 0 0.08733301 0 0.6756981 0.1436831
0.1958085 0.2207874 0.5292286 0.1174571 1.335812 0.14242 0.232679
0.1483995 0.3306178 0.1153044 0.3932105 0.7185927 0 0.3919593
0.6026189 0.1514044 0.1134208 0 0.02727319 0.4939108 0.0103088
0.2641118 0 0.1265293 0 5.160879 0.213865 0 0.1867467 0.2006744
0 1.549591 0.1770522 0.02133018 0.448653 0.1080619 0.06771275
0.3719268 0.06136468 0.1696364 0 0.03820256 0.7657919 0
0.07184157 0.2224294 0 0.2071978 0.536246 0 0.07066641 0.0585905
0.08233939 0 0.1693235 0.1105821 0 0.03352359 0.3416578
0.5975511 0.0833991 0.03780694 0 0.05427228 0.1428536 0.2166377
0.2607213 0.08151081 0 0.616751 0 0.1404648 0.3666142 0
1.202364 4.399836 0.05993165 0.1822303 0.09614406 0.3028089 0.02810108
0 0.782307 0.03197686 0
TCGA-DK-AA6M 1582 0 0.04393104 0.04697905 0.07966012 0.2234952
0.03370899 178.4536 0.07652852 0.1596469 0.6012955 0 0.0651159
0.3238564 0.05887922 0.6921579 0.07122052 0.124604 0.103997 0.875787
0.09569004 0.03926106 0.1081497 0.09028147 1.747216 45.54975 0.06067618
0.3542127 0.3033809 0.1577069 0 1.620921 1.164378 0 0 0.325904
0.1316446 0.1170944 0.1268721 0.07809089 0.1297121 0.1021552
0.04475159 0.01707627 0.1134685 0.1016219 3.913112 0 0.08272804
0.02727417 0.1769722 0.1145114 4.927279 0 0.2821296 0.2330208
0.1170944 1.189816 0 0.04502725 0.8651974 0.07786777 0 0.6376573
0 0.02535119 0.1288765 0.06585012 0.3058636 0.1419003 0.02921867
0.05885697 0.02327016 0.08040038 0.114092 0.7415978 0.1245884
0.5638259 0.04584502 0 0.04825268 0.09935281 0.06509322 0
0.1587114 0 0.0426089 0.09169904 0.5586925 0.1134685 0
0.2178119 0 0 0.0686816 0.004482243 0.3103074 0.1132622 0
0.1513603 0.5050882 0.08776742 0.09015082 0.0677111 0 3.202039
0.4506816 0 0.03595626 0.1011927 0 0.336191 0 0.06462055
0.0554219 2.394719 0 0.5142211 0 0 0.1292411 0.1177584
0.441135 0 0 0 0.02261133 0.7103726 0.01772744 1.928045 0
0.5863797 0 0 0.132285 0 0.6006329 0 0.05237742 0 0.05435795
0 0.114934 0.8125332 0 0 0.05454835 0 0.265647 1.265163 0 0

0.07639712 0.08090158 0.05989828 0 0.1689716 0 0.4648822
0.7085081 0 0.04975576 0.1659704 0.2022539 0.05777493 0.1957091
0.1769722 0.4090614 0.07748037 0.05382565 0 0.8731427 0.04593969
0 0.2211367 0.1217477 0.8115599 0 0.269329 0.207187 0.575773
0.05306151 0.05652833 0.1693698 2.149333 1.300648 0 0 1.483196 0
0.3023991 0.3377212 0 0.3112663 0.165516 0.3153523 0.01817217
0.05704598 0.5767983 0.09496069 0.04954996 0.2047535 0 0.1534911
0 0.2642073 0 0 0 0.1714765 0.05612091 0.1354222 0.3971398
0.12303 1.087617 0.1038814 0.1627331 0.6921579 0.5309166 0.05435795
0 0.1560798 0.0933169 0.04488056 0.207187 0.4276491 0.177308 0
0.5968958 0 0.1698316 0.05632388 0 0 0.02872466 0.03543471
0.1226264 0 0 0.1276521 0 0 0.4152948 0.1043454 0.07847266
0.07111212 0.2278501 0.5485025 0 0.162436 0.3355164 0.04501027
0.7753492 0.04411772 0.3650052 4.730502 0.0504115 0.4905056
0.1848493 0.09703149 0.1350699 0 0.1253405 0.007684951 0.03059637
TCGA-ZF-AA52 1077 1 0.0569524 0.1015064 0 0.1931601 0.2330693
39.37712 0.2645651 0.1655735 0.2078726 0 0.0562777 3.771075
0.101775 0.8093444 0.03077687 0.4374966 0.08988147 0.008904896
0.248106 0.01131071 0.1557841 0.07802754 3.19173 33.38433 0.03496039
0.3877714 0.6118068 0 8.412344 2.362323 1.383713 0 0.08157424
0.4023841 0.3868398 0.1012011 0.822388 0.09641655 0.3713518
0.1765792 0.07735488 0.04427549 0.1961348 0.1317431 11.07327
0.06022259 0.1787483 0.1414334 0.8412344 0.4948437 3.799642
0.05050563 0.4389049 0.3524373 0 0.605724 0 0.3570091 1.308587 0
0 0.3391433 0.6933811 0.4436829 0.1468244 0.1138245 0.0881162
0.4905604 0.02525281 0.06104195 0.2715078 0.1111802 0.3451218
1.981089 0 0.5168308 0.3328287 0.06476223 0.12511 0 0.2250324
0.07217024 0.5829701 0 0.1104767 0.1651098 0.482861 0 0.06902436
0.09412412 0 0.03303006 0.08903914 0.1317115 0.1542088 0.1957782
0.3041751 0.8707446 0.902167 0.4149699 0.1168719 0 0.6878918
2.390049 1.298368 0.04930311 0.1165346 0.3323395 0 0.2734679 0
0.05584958 0 3.513015 0 0.5079151 0.02688589 0.0639418 0.05584958
0.6106503 1.700675 0.07848251 0 0 0.05374129 0.8658319 0.03064257
0.6943128 0 1.484597 0.2002939 2.68237 0.1247236 0 0.6377623
0.2942022 0.1458643 0.04985093 0.04697993 0 0.1490009 1.103532 0
0.4562627 0.1885779 0.1736742 0.5165789 1.197579 0.05416398 0
0.1650693 0.1398416 0.569451 0.06763693 0.1460371 0 0.8839239
0.2072542 2.121563 0 0.3825151 0.4370049 0.08322187 1.30242
0.0509839 0.6060675 0 0.04651987 0.03303006 0.312261 0.3573385
0.09665889 0.2293461 0.0526114 0.3869829 0.05764347 0.1506177
0.2387539 0.4210658 0.09171891 0.1465671 0.1171049 3.345259
0.2958187 0.09665889 0.1232954 0.8240663 0.09579893 0.8494017
0.7031708 0 0.3433519 0.2535895 1.970742 0 0.04930311 0.4985093
0.1641433 0.02569472 0.2406687 0 0.100119 0 1.014873 0.1776865

0.1078938	0.1551556	0.935525 0.1455108	0.4096446	1.078741 0.1594967
0.3565497	0.07481795	0.1687743	3.105944 0.1019678	0.1409398	0
1.233893 0.1613019	0	0	0.3080034	0.1532419	0.08607354	0.3693224
0.5148538	0.02935605	0.04867902	0.06841042	0.1043391	0.03310114	0
0	0	0	0.6619549	0	0.1256453	1.363921 0.07515215	0.09042872
0.1097501	0.09846196	0	0.03286874	0.280777 0.1449883	0	0.6904171
0.0381296	2.103086 11.65603 0.03112081	0.2725259	0	0	0.02334735	0
0.1805466	0.01328374	0.01322176
TCGA-K4-A5RJ	539 0	0.173276234 0.102943564 0.069822591 0.409598542 0
19.766486123	0.067077723 0.181910934 0.175679751 0.132527346 0.095124158
0.395105413 0.464472021 0.249809716 0.052021024 0.017065029 0.045577017
0.090309736 0.083872913 0.195004523 0.189587731 0	2.101387387
30.781821114	0.168412997 0.186281804 0.886384196 0.414693163 0
1.309316084 3.06175326	0	0.041364596 0.816162429 0.10000232	0.041053584
0.444816835 0.293344913 0.113693423 0.080585667 0.102420958 0.119739619
0.8453748	1.024330448 0.055320432 0	0.326302389 0.047811967 0.310234469
0.150554963 2.465547619 0	0.544034358 0.374447588 0	0.32857905
0.345577636 0.463304272 0.568763192 0	0.06484711	0.257958739 0
0.00277756	0.236191298 0.115436081 0.089363775 0.746258767 0.0512207
0.092859297 0.122378632 0.267791446 0.700016237 1.418214714 0.691616042
0.104829639 0	0.153251349 0.46523155	0.04354168	0.313800382 0.365960231
0.521668151 0	0.074693935 0.140655863 0.122424364 0.198911718 0.350008118
0	0	0.200986257 0.030099926 0.007857428 0.734363235 0	0	0.505797609
1.298624716 0.004525217 0	0	0.046508745 1.02058442	0.790050479
0.05000116	0.157579413 0.266088043 0.130625039 0.936021711 0.152092664 0
0	3.4660895	0.255146105 0.386329716 0.02726655	0	0.28320159
0.361256016 1.146939398 1.512279975 0	0.091154034 0.222963429 1.708285238
0.108767463 0.42248585	0.062046894 1.717249134 0.853144789 0.097155278
0.231897271 0.075835092 0.962666957 0.149183788 0.198939592 0.101113456
0.285870505 0.198791019 0.604442064 1.390467034 0	0.210328453 0.286871803
0	0.232841222 0.264029335 0	0.217708399 0.187495151 0.709107357
0.105002436 0	0.148104701 0.101678336 1.629888551 1.442658134 0
0.305278646 0.484913557 1.240937874 0.067520115 0.094347146 0.620468937
0.102441401 0.203736069 0.267344911 0.133990838 1.042411806 0.040266421
0.147041113 0.319815725 0.035570858 2.256656116 0	0.124977466 0.665869103
0.388206658 0	0.099094857 0.11876295	1.596528259 0.780018092 0
0.125041068 2.414341715 0.582931671 0.331318365 0.847678607 0.076615435
0.31949459	0.329717793 0	0.127424192 0.250005799 14.224824893
0.041616819 0.121606384 0.258433552 0.136845279 0.21068829	0	0.128655199
0.270303299 0.109421376 0.157352353 0.852484289 0.787045282 0.178047608
1.094014447 0.161754904 1.347773585 0.106228145 0.11410923	0.028124273 0
0.047645084 0.143687543 0.016094699 0.190849966 0.157352353 0.060533555
0.437309915 0.207215433 0.403726391 1.248504569 0	1.042008902 0.296209402
0.104068488 0.105816408 0.075532032 0.279528668 11.070729225	0.014123452

0 1.734261537 0.03513595 0.063712096 0.218405066 0.335351162 0.063049961
0.271581642 0.758905679 11.744423354 0.10000232 0.370178078 0.147041113
0 1.599458774 0.386694522 0.853144789 17.056798251 0.164119414
0.614187474 0.081010781 0.170097403 6.973145263 0.109788069 1.025375896
0.006735908 0.080453733
TCGA-HQ-A5ND 274 1 0.045626628 0.146376828 0 0.253223319 0
12.528194561 0.105976344 0.077377397 0.305312801 0.523451117 0.015028701
0.206056014 0.122303512 0.507439671 0.147938776 0.072794977 0.072007299
0.185484895 0.033127782 0.072491382 0.07488259 0.125021368 0.649563213
23.745968536 0.063018076 0.277956909 0.420120507 0.109195879 0.962776162
0.748214617 0.050388285 0.076071203 0.032676039 0.805911287 0.206607587
0.162151775 0.087845972 0.146761295 0.028066354 0.091951597 0.125665037
0.059117835 0.117848011 0.07036276 1.332864588 0.048246501 0.286403533 0
0.673943313 0.911805659 1.380132719 0.040461887 0.234415066 0.228569303 0
0.090282307 0.038998528 0.060998012 1.148199719 0 0.051226095 0.237737484
0 0.004388277 0.15007502 0.319161315 0.176482701 0.049125709 0.053949183
0.163009721 0.233626969 0.222676159 0.552979129 0.186720225 0.143774574
0.153790685 0.044440183 0.069177822 0 0.034395831 0.225351996 0.231272772
0.219782788 0.558347858 0.147511532 0.058201189 0.096709354 0.157130681
0.552979129 0.226218735 0.091382144 0.149948942 0 0.043448929 0.241713168
0.039211247 0.060921429 0.645184305 1.328943355 0.028597628 0.03121011
0.140649039 0.293917356 0.806212562 0.277378598 0.039498509 0.143152173
0.392367257 0.240770817 1.916994314 0.090109412 0.044743125 0.422113961
2.661684063 0.20155314 0.254318232 0.021539262 0 0.402688121 0.244607025
0.168164086 0.880252491 0.082946869 0 0.156560334 1.185509642 0.08592106
1.573362135 0.343098414 1.146375337 0.51348062 0.613983944 0.099920553 0
1.675389659 0.117848011 0.072532016 0 0.075274646 0.157035335 0.119370033
0.388459252 0.042705319 0 0.037769152 0 0.137950017 0.208570465
0.130178185 0.206374986 0.883383141 0.336096406 0.290314043 0.108372794 0
0.080320991 0.836896771 0.275473592 0 0.344507764 0.68950506 1.050301268
0.266688203 0.189711973 0.653520789 0.3236951 0.053647229 0.21118961 0
0.208469657 0.031808534 0 0.137803106 0.252893387 0.116259763 0.13854081
0.153574061 0.286911566 0.306664572 0.183698348 0.156560334 0.042999465
0.201788875 0.236991055 0 0.164627374 0 0.383739965 0.261725559
0.807801941 0 0.303429573 0.171904381 0.050388285 0.150988467 0.157994037
0.03630671 0.197251702 0.178403066 0.2098209 0.054050592 0.13435002 0
0.325220525 0.142351063 0.04321881 0.124300784 0.100178413 0.194289964 0
1.885568178 0.141976208 0.519354269 0.119878744 0.0225352 0.285644848
0.081690099 0.075274646 0 0.181175034 0.481003483 0.15537598 0 0
0.081845108 1.060208186 1.315011343 0 0.164627374 0 0.054806064 0
0.072925929 0.343488742 0 0.022313695 0.13265668 2.077071196 0.111022837
0.11324135 1.610275224 0.15653848 0.036222861 0.077373792 0.299749477
0.027127278 0.052664679 0.258681762 0.619495469 0.124660035 0.477199993 0
2.148194312 2.774446803 0.154578443 0.024258927 0.063994617 0.537475041 0

0.028909097 0.188035157 0.292657348 0.14829401
TCGA-BT-A3PK 303 1 0.06134823 0.0492035 0.08343202 0.1702383
0.1294521 44.47033 0.05343476 0.1839268 0.2519067 0 0.03031073
0.6829656 0.1233343 0.3980017 0.02486427 0.1740053 0.05446064
0.06834453 0.5512153 0.1142224 0.03775685 0.03151876 2.594158 55.4929
0.04942715 0.4451816 0.07061022 0.08258714 0 0.5991781 0.1016259
0.07671234 0.4118929 0.3900977 0.05515118 0.0817592 0.1550261
0.112946 0.1358539 0.3138438 0.07290979 0.05961608 0.01980687
0.03547789 0.5398427 0.07297969 0.1155269 0.0142828 0.1235679
0.2998338 2.481113 0.08160581 1.083458 0.47455 0.2452776 0.1536354 0
0.1175565 0.2265411 0.1631096 0.05165783 0.06849747 0.01647572
0.02433894 0.07566993 0.02298937 0.3025492 1.684351 0.0408029
0.160274 0.06093 0.09543498 0.3584826 0.2353674 0.08699179 0.02982439
0.003201052 0.07848096 0.04211454 0.05202858 0.1704385 0.08745823
0.3601571 0.1407634 0.1636302 0.1413937 0.09752443 0.07922749
0.2788198 0.07604177 0 0.01778973 0.02397789 0.07511163 0.2383337
0.01977086 0 0.2658633 1.398922 0.09372561 0.07868288 0 0.1481973
0.3302842 0.3146809 0.1792413 0.05962641 0.1978371 0.05202858
0.2968767 0.01514481 0.04512022 0.01934871 1.133049 0.05081296
0.2051693 0.03529629 0.05165783 0.06768033 0.3494472 0.4326056
0.2219178 0 0.03630709 0.06907243 1.348118 0.111401 1.730861 0.04942715
1.038025 0.1132706 0.3482767 0.2687005 0.06041096 0.4014084
0.5050753 0.03250814 0.2416439 0.07590907 0.1319657 0.2808775
0.4525194 0.4306524 0.01675497 0.1142624 0 0.1391127 0.6940834
0.02187922 0.03468572 0.6401162 0.02824409 0.1882034 0.1912508
0.09831803 0 0.7790309 0.6469206 0.02809812 0.121594 0.1738291
0.317746 0.1277445 0.167397 0.350109 0.408029 0.2163975 0.03131905
0.2134768 0.2627833 0.08019155 0.1171344 0.185286 0.03542011
0.4884984 0.02328474 0.1050893 0.5786594 0.2473993 0.1667219
0.3552296 0.05321684 0.3624658 0.6691676 0.03904479 0.2324208
0.2589041 0 0.7126148 0.5895205 0.0305163 0.3088466 0.2022454
0.1355012 0.1712936 0.2788198 0.6041096 0.2983712 0.04843641
0.145825 0.05450613 0.1021173 0.05743296 0.4202007 0.0358877
0.1307492 0 0.4798579 0.5094114 0.330947 0.2772962 0.1503314
0.6153844 0.03022227 0.1022631 0.2832514 0.06178394 0 0.02861572
0.1474437 0.5610739 0.1410169 0.02411081 0 0.1444361 0.4824185
0.6251588 0.02971031 0.07114923 0.03932721 0.04145098 0.04214718
0.04345578 0.1237085 0 0.02250176 0.2197728 0.4456547 0.05597927
0.05075367 1.681841 0.1275005 0.08675436 0.2252111 0.1034101 0
0.06638568 0.1049119 0.4880599 0.01571383 1.276878 0.3234468
0.2548588 3.361282 0.04022742 0.672743 0.06453397 0.03387531 0.01886205
0.01457637 0.1750338 0.008048833 0.1014762
TCGA-FD-A3N6 851 0 0.02016058 0.1832545 0.1462287 0.3636401
0.03093908 29.499 0 0.293057 0.53349 0.2081629 0.07968699 0.0803364

0.2702052 0.672652 0.2560256 0.02501737 0.1431772 0.4019112
0.1317406 0.06005821 0 0.08286292 1.512529 50.51466 0.167071 0.6177026
0.5104948 0.03618697 0 0.4521525 0.03339686 0.1008385 0.04331471
0.726444 0.1288823 0.08597807 0.02911172 0.2355005 0.04836546
0.5297493 0.09812192 0.1097116 0 0.1165893 3.779834 0.09593191
0.7403191 0.01251651 0.3248603 1.10357 4.898552 0.5363547 1.916205
0.1069366 0 0.2917113 1.809349 0.01078103 0.529402 0 0.237665
0.2926301 0 0 0.06720827 0.09065869 0.2105478 0 0.01340887
0.04321649 0.1121296 0.1771051 0.9948104 0.06187816 0.1334093 0
0.008415575 0.4699663 0.06643154 0.2507694 0.07468053 0.1916066
0.273131 0 0.2737537 0.2595057 0.064098 0.07810849 0.6963672 0
0.03028359 0.05846157 0.6461389 0.004113931 0.3880523 0.07796648 0
0.1736535 0.247245 0.07107834 0 0.5282511 0.02435069 1.135493
0.04596095 0.235613 0.1485076 0.3993734 0.2051749 0.9529236
0.1791709 0.0296553 0.1271695 1.279723 0.03339686 0.2022715
0.01784501 0.2037129 0.2668977 0.3242462 0.1569503 0.2083652 0
0.04772572 0.2412574 1.487898 0.08135375 0.5266711 0 0.3957324
0.6168479 0.1017356 0.441509 0 0.9686744 0.2343255 0.1041593 0
0.1496739 0.1387753 0.3955864 1.269578 0 0.04404886 0 0 0
0.2764769 0.2588424 0.09118886 0.6836648 0 0.1924173 0.8260267
0.07754351 0 0.3413458 0.5177301 0 0.1826691 0.7870506
0.1392259 0.2238938 0.04939755 0.6226489 0.6972612 0.1066706
0.4775596 0 0.2210746 0.1475767 0.1026489 0.2587812 0.01862391
0.1797969 0.06121565 0.1235989 0.4437117 0.2591491 0.2191562
0.2075333 0.07254448 0.1379232 0 0.02566222 0.676503 0.1944742
0.3306408 0.1040815 0.7749256 0 0.6108485 0.3590727 0.077926
0.2752026 0.07853766 0.1684461 0.02178941 0.1318873 0.2480658
0.1432968 0.08372969 0 0.09430441 0 0.200515 0.2059633 0.0811522
0.5408485 0.2485888 1.01541 0.4046318 5.060076 0.06356355 0.01493611
0.0399681 0.2977886 0.09978258 0 0.02317353 0.5472015 0.08238533
0.09508107 0.1635452 0.1627384 0.4856026 0.994531 0 0.03117525
0.02584784 0.1634621 0.2216101 0.1406097 0.130092 0 0.6433344
0.7347895 1.318081 0.1839621 0.08339471 2.439484 0.1995234 0.03601223
0.4335709 0.3555165 0.01797969 0.1047169 0.2758136 0.718542
0.1032793 1.976771 0 0.4746006 1.081191 0.1553321 0.09647146 0.16966
0.04452914 0.01859565 0.05748199 0.2684292 0.007053469 0.0982878
TCGA-UY-A78P 2380 0 0.04583767 0.01633932 0.05541161 0.1413303
0.07034396 29.49002 0.05323326 0.3442563 0.6413341 0.1051745
0.04529465 0.5662308 0.04095641 0.8496447 0.02477051 0.05958878
0.1446807 0.2365121 0.09984304 0.01820667 0.05015264 0.1255996
1.384548 26.79154 0.03517198 0.1971124 0.6330956 0.05485048 0
0.7074592 0.3037281 0.1528461 0.06565436 0.2266989 0.04883841
0.01629018 0.04412615 0.07760007 0.1071455 0.03552958 0.05361144
0.07126954 0.03946437 0 5.202459 0 0.1150913 0.009485963 0.1231019

0.07965419	2.3324580	0.2747492	0.1080595	0	0.3174497	0.3526102
0.05174769	0.9529001	0	0.05146304	0.3753155	0.06565436	0.04188146
0.08964654	0.04580537	0.3900578	0.5922352	0.02709936	0.1228228
0.07284029	0.1118531	0.1984060.8441275	0.1155517	0.2020411
0.03188981	0.0347489	0.1678229	0	0.1131972	0	0.1103997	0
0.0889163	0.1435182	0.3886267	0	0	0	0.1377072	0.04430662
0.04777495	0.0935355	0.2428312	0	0.4284225	0.2467647	0.4216075
0.03232114	0.03135447	0	0.03690961	0.1518641	0.2438289	0.03968121
0.05002237	0.3801042	0.1036648	0.2476107	0.0301754	0	0.07710298
1.6986450.1518641	0.5620880	0.1029261	0.3146506	0.1228692
0.3620184	0.4421620	0	0.08257436	0.7602084	0.01233121	1.1814880
0.9501342	0.03224098	0.1156545	0.05855659	0	0.5968578	0
0.08501209	0.1203663	0.07562282	0	0.1598962	0.2825992	0	0
0.2276631	0.04659342	0.09239206	0.2095352	0	0.1036648	0.2231958
0	0.1249958	0.05443703	0.1567157	0.08069251	1.4875120.5989883
0.02799217	0.03461013	0.1154491	0.2110319	0.2500603	0.3539519	0
0.1219471	0	0.0624019	0.2392558	0.4398112	0.1917340.07779511
0.1153671	0.04234385	0.8370488	0	0.1102031	0.1441193	0.3234872
0.1107288	0	0.07068822	0.4624601	0.1428523	0.07779511	0.06615554
1.7686480	0.1051745	0.3203441	0.06080245	0.1310497	0.130833
0.2362330.05056228	0.03968121	1.0942390.09908204	0.01378679	0.1652149
0.1086012	0.07856547	0.03814426	0.2246233	0.07150475	0.04341871	0
0.4361144	0.1171132	0.5651984	0.1973218	0.09984304	1.056557
0.06021662	0.0679183	0.8545212	0.6155096	0	0.05701563	0.05428459
0.3678305	0.03121893	0.1441193	0	0.1644474	0.02597834	0.6180832
0.05919655	0.04725396	0	0.02752978	0	0.02664118	0.02464839	0
0.0224169	0.05711583	0.1331922	0	0.03792171	0.5777584	0.1088741
0.05913441	0.06006539	0.1901911	0	0	0.1468877	0	0.09392748
0.6700806	0.0306883	0.2962143.6776610.02504733	0.3411959	0
0.06749514	0.09395463	0.05808563	0.1889049	0.03207393	0.02128285
TCGA-CF-A9FL	565	1	0.2548853	0.0170356	0.05777291	0.1915588
0.09778878	48.139280.05550174	0.04631309	0.7849532	0	0.01574161
0.7554070.1708069	1.4764190.07747823	0.03953606	0.03771153	0.1419765
0.2775940.07593011	0.2614492	0.3273798	2.5307118 9.093930.07334158	0
0.07334158	0	0.1008447	0.9681089	0.1055570.1195196	0.06845215
0.2701251	0.1527588	0.1188906	0.6900980.1779952	0.1469886
0.07408727	0.04688054	0.02476887	0.3703149	0	13.960910.3032108
0.08999684	0.03956078	1.15513	0.8304856	4.2017010.08476251	0.6956821
0.07041524	0	1.1938831.2663030.05253304	0.8366373	0.05647302
0.05365608	1.1383540.06845215	0.03906975	0.1402001	0.04775731
0.6285050.5145605	0.007063542	0.1792794	0.2193946	0.1749293
0.6619548	7.8719970.1204758	0.04956491	0.08644678	0.3622968
0.01749745	0.03602744	0.1180209	0.3028044	0.08632818	0.02436808
0.1236072	0.2327642	0.1012969	0.0411461	0.5212894	0	0

0.02771682 0.1743379 0.05526213 0.5007321 0.08214257 0.191434
0.4836869 0.7814645 0.1123282 1.0460990 0.1539299 2.586148
1.1621460.2068609 0.3064048 0.3669462 0.1441098 0.6597483
0.09438388 0 0.04019432 1.3939720.05277852 0.3729350.005640252 0
0.09373115 0.2562103 0.3486876 0.3951465 0 0.07542307 0.1352893
0.7001333 0.1414236 0.3495751 0 0.3702329 0.06722979 0.3215546
0.5320238 0 1.3317050.5554723 0.2279180 0.3548043 0.05482817
0.2917425 1.2627510.0447311 0.03480618 0 0 0.09632925 1.354479
0.1363534 0.03602744 0.3823045 0.4107129 0.3040855 0 0.2042424
0 0.5394438 1.0039650 0.1804250 0.7334158 0.3398624 0.2129049
0.3422607 0.3814313 0 0.1040977 0.1385841 0.4148815 0.1665871
0.2433308 0.05613222 0.02943219 0.2029578 0.0483709 0.1378792
0.3005216 0.1927270.1539299 0.4509642 0.1678734 3.6922130.04964661
0.2027757 0.1379494 0.3073362 0.4019432 0.05482817 0.4341937 0
0.5613886 0.4692444 0.2990783 0.2504055 0.04137218 0.05704345
0.2754781 0.1437430.2851123 0.05661456 0.06301034 0 0.4045193
0.07455184 0.04526895 0.1301972 0.6470715 0.2849089 1.227674
0.2468766 0.04461331 0.2039965 0.1130088 0.1888335 0.5784433
0.08556518 0.0394227 0 0.2363793 0.3910249 0.1627465 0.1001739
0 0 0.4062807 0.7772419 0 0.02463381 0 0.3444352 0.08755507
0.1111058 0.02569876 0 0 0.05954976 0.4166042 0 0.01317923
0.3011894 0.07567574 0.01897057 0.1805074 0.4792151 0.1704846
0.0551629 0.3180749 0.04055513 0.06528673 0.6702355 0.03199605
1.7206623.7536560.08878995 0.05081937 0.06703029 0.1407427 0.03918336
0.03028044 0.5302631 0.03901418 0.110949
TCGA-CF-A9FM 398 0 0.1145370.06999071 0.1186799 0.1816197 0.1004412
56.158370 0.2021695 0.2687481 0.2252614 0.01616859 0.1662636
0.3508797 0.6369155 0.08842196 0.07251507 0.1162033 0.007675131
0.1425617 0.1754767 0.2148326 0 1.99665960.940990.07533092
0.1407243 0 0.05873904 0 1.1127450 0.0409205 0.03515443
0.5895855 0.05230075 0.1918956 0.2835265 0.1329623 0.04831217
0.05326778 0.1055643 0.2035256 0.1267865 0 11.377640.1557176
0.1540632 0.02031692 0.5932310.1279518 5.3425480.1305924 0.5043896
0.2314406 0.08722527 0.8863110.08391292 0.02624982 1.0741630
0.2204458 0.2923077 0 0.009442231 0.1701852 0.1962108 0.8354211
0.2114070.02902054 0.0789181 0.1993441 0.3114352 0.6799098
7.0811060.1237436 0.05090932 0.01366024 0.3535177 0.03594411
0.1850233 0.2424443 0.2488142 0.3546791 0 0.1269599 0.09107722
0.1040445 0.2535729 0.3569526 0 0.1474698 0.1992803 0.02558095
0.01335555 0.3582862 0 0.1310843 0.2008367 0.95316 0 0 0
0.03952627 1.0299920.8579489 0.04249436 0.3214119 0.6331908
0.03700466 0.42721 0.09694396 0 0.04128456 1.6078220.0542101
0.2736076 0.02317296 0 0.1925471 0.08771993 0.1107667 0.2029323
0 0.03873443 0.2189655 1.4246790.0660271 0.5471345 0 1.5262390

0.04128456	0.2329150	1.4317440.2535729	0.2861224	0	0.5263961
0.2815767	0.7705435	2.3922480	0	0	0	0.3462973	0.9873158
0.1400518	0.03700466	0.5577111	0.1807942	0.3569526	0.1165926
0.1678258	0	0.8311136	1.2098290.02997664	0.2223826	0.08242246
0.7533092	0.4638472	0.1093399	0.4833734	0.4788389	0.1154324
0.1069213	0.02846862	0.5270615	0.1026634	0.2499309	0.03294558
0.04534578	0.1876166	0.09936583	0.07080953	0.9774646	0.3794129
0.2766839	0.0842175	0.07990511	0.8819444	0.6119188	0.08331031
0.1062683	0.2367543	0.3715610.1689460.3945127	0.02170432	0.3447491
0.8574623	0.1264902	0.1895139	0.08498872	0.7616793	0.2122127
0.1919344	0.4270667	0.05815018	0.08844991	0	0.3280196	0.153148
0.09299367	0.3343216	0.7843746	0.4598579	0.5548286	0.5916701
0.3207639	0.4190594	0.02579424	0.0727333	0.8331522	0.04394303
0.04049201	0.1221154	0.04787427	0.3707364	0.06686433	0	0.05309364
0	0.8253282	0.2677922	0	0.1518119	0	0.08844443	0.1798599
0.08558963	0.05279163	0	0.02400613	0	1.0935330	0.05414684	0
0.1165926	0.0292277	0.3178345	0.4582677	0.2042937	0	0.2904022
0.04165516	0.2347015	0.7117512	0.03286391	1.4048049.5350360.1823966
0.0521978	0	0.1445603	0	0.06220354	0.9025563	0.04579703
0.09116669
TCGA-ZF-A9RD	408	1	0.05604295	0	0	0.1814358	0.2580159	49.477630
0.1018310.3068297	0	0.05537902	0.7090452	0.07511238	0.4674641
0.0454281	0.0745114	0.06633464	0	0.1220720.01669515	0.1839558
0.3455169	1.22844438.880890.09030556	0.5422442	0.3870238	0.1005935
0	0.6081803	0.1856750.07007839	0.1204074	0.1781814	0.1343515
0.02987553	0.3237022	0.2703992	0.09307913	0.2997351	0.06343303	0
0	0.5833769	1.9726320.08889138	0.2638410.01739687	0.2257639
0.2921651	4.7885710	0.2879308	0.0495442	0	0.4990198	0.07185253
0.03995922	0.2759337	0	0	0.1251479	0.1204074	0.08489403
0.05231167	0	0.3901910.3620451	0	0.1802016	0.04452871	0.08205358
0.1455474	0.2580159	0.05297926	0.08718475	0.05848462	0.03186403	0
0.06337233	0.0415198	0	0	0	0.1630689	0.1364775	0.1781814	0
0.1018832	0	0	0	0	0.0571801	0.0593790.07224445	0.1122442
1.1766470.8161670.05927566	0	0	0.3384536	0.8355375	0.1916452
0.07277371	0.04586949	0.1549101	0	0.07568466	0	0	0.1414037
1.4872780	0.3748533	0.02976363	0	0.1648732	0.2253371	0.442618
0.1158439	0.3056496	0.06633464	0.07211334	1.06888	0	1.3176490
1.13527	0.05912864	0.6363168	0.04602446	0	0.3502761	0	0.06681802
0	0.1386892	0	0.07331079	0.7897530	0.06122411	0.06958748	0	0
0.7685580	0.1901170.2826340.3096191	0.1528248	0	0.2155576
0.07399339	1.3047130.3337276	0	0	0	0.1290079	0.1801644	0.11235
0.07525464	0.4472921	0.09884191	0.04577701	0	0.3456839	0.05860538
0	0.1269471	0.0776569	0.1428013	0	0.06063242	0.1762060.24013
0.1353814	0	0.0432131	0.3834026	0.4366423	0.2140096	0.1213266

0.6757559	0	0.2893285	0.2447908	0	0.1462938	0.2207469
0.185675	0.09272917	0.07277371	0.2341255	0	0	0.1776191	0
0.04433411	0	0.11235	0	0	0.1145085	0.3247116	0.1431872
0.2591377	0.6513844	0.05231659	1.028646	0.02208696	0.207599	0.3567037
0.3762732	0	0	0.3455169	0.1058174	0	0.08810299	0	0.2261923
0.1111675	0.5235048	0.1085641	0.1299927	0	0.0504885	0.1540095
0.02442942	0.04520415	0	0.3083377	0	0	0	0.02318229	1.589378
0.06655686	0.02502695	0.1036776	0.1744014	0.04998044	0	0.124332
0.4993557	0.1148394	0.4695799	0.1688433	0.6984571	1.75125
0.09187156	0.4916523	0	0	0.05169269	0.05326334	0.6928878	0
0.07806375
TCGA-G2-AA3D	2139	0	0.1154153	0.2821096	0.1793849	0.2745186
0.02530285	35.83587	0	0.1078516	0.7522469	0.1134943	0.3095588
0.190534	2.033029	1.253037	0.14256	0.1812164	0.03903144	0.2010836
0.1077412	0.2062926	0.05411998	0.2710705	3.096198	24.41006	0.07590855
0.1063525	0.07590855	0.1775683	0	3.14912	0.05462578	0.1237028	0
0.8736858	0.1054036	0.193367	0.9047186	0.2930853	0.03651197
0.03067213	0.07651446	0.230722	0.4684484	0	15.58656	0.2615194
0.2794402	0.01023635	1.12914	1.418262	4.579483	0.219323	1.312998	0.07287978
0	1.85962	0.2959472	0.01469506	0.9200397	0.1168992	0.05553404
1.509564	0.03542399	0.04519451	0.3253917	0.3460018	0.3443838
0.05325702	0.0219323	0.3446007	0.1484711	0.4465946	2.269471
9.463266	0.1246924	0.07694953	0.006882494	0.1499909	0.03621973
0.03728841	0.3908854	0.125361	0.1786994	0	0.1279334	0.08030364	0
0.1703449	0.2397932	0	0.09906709	0.219933	0.3351023	0.01345796
1.10057	0.08501757	0.1981342	0.1384683	0.8088158	0	0.1015043
0.05082572	0	3.386798	0.9021157	0	0.1282011	0.714004	0.2610189
0.964882	0.09768732	0	0	2.282401	0	1.268246	0.05253895	0	0.8245999
0.4419628	0.0334848	0.8179533	0.08992243	0	0.2673193	2.064535
0.1596801	0.3101231	0.106272	0.4971127	0.03479142	0.04160113
0.3430246	0	1.842047	1.980259	0.1354213	0.04329599	0.1224075
0.05674717	0.7333158	1.060079	0	0	0	0	0.3489527	0.9948865
0.09408383	0.03728841	0.2637901	0.1214537	0.3147285	0.3524598
0.2959472	0.08707573	0.2093716	1.296834	0.60413	0.1867399	0.2491634
1.745897	0.9589008	0.05141653	0.6641998	1.62299	0.05815879	0.3501588
0.02868691	0.7571043	0.06896706	0.2098728	0.09129505	0.1370805
0.08402456	0	0.2616258	0.15552	0.8311352	0.2389761	0.2970215
0.1949379	1.292043	0.2569212	0.1259237	0	0	0.7488203	0.05674717
0.8296441	0.02187075	0.6640426	0.1750664	0.1456687	0.2591697
0.08564041	0.3542399	0.2138399	0.2157223	0.393455	0	0.1891256	0
0.7271767	0	0	0.3368852	0.1749717	0.4633841	0.7623858
0.2981035	0.1693075	0.4222727	0.1429562	0.219873	0.7707259	0
0.244815	0.1230518	0.02067487	0.1712238	0.03368852	0	0	0.133092
0.8223123	2.120579	0	0.12748	0.08455636	0.08912261	0.2718585

0.1149946 0.2127857 0.1840932 0 0.5855230.9581899 0.06017975
0.2728102 0.4987698 0.0783244 0.1178073 0.1486976 0.9919753
0.05881719 0.0285468 0.8047296 0.04197456 0.2702871 0.8112690
2.1005323.5647630.1081148 0.05259805 0.06937636 0.2185031 0.0101387
0 0.9094771 0.005768525 0.2411476
TCGA-DK-A2I2 237 1 0.05527253 0.1477686 0 0.06390774 0.1060287
79.057830.1444283 0.1606898 0.2017412 0.09511705 0.02730887
0.6442352 0.07407982 1.0501420.01493454 0.2057639 0.09813412
0.2398221 0.3611818 0.01646564 0.06803511 0 1.23029252.57654
0.1526814 0.3713820.1272345 0 0 0.7197837 0.1831225 0.06911503
0.02968805 0.1757319 0.07729433 0.02946483 0.1995327 0.133341
0.08159963 0.4884069 0.09853361 0.02148477 0.2141433 0.6712502
1.0323140.0876694 0.1561284 0.02573657 0.4453207 0.1440744 6.24035
0.03676194 0.4259590.4275523 0 0.3281065 0.2834591 0.07635669
1.0432050.09797056 0.04654183 0.3394256 0.02968805 0.0239220.07738882
0 0.09620677 0.4017016 0.06126989 0.1110777 0.05855543 0.0505785
0.6459597 0.9754644 0.05225097 0.07523795 0.02307225 0.141417
0.01517747 0.03125058 0.1637961 0 0.1996852 0.9723076 0.3216544
0.1394085 0.08786597 0 0.2512066 0 0 0.1282232 0.02160321
0.01973792 0.1561673 0 0.0553506 0.1458028 5.2745230.1883737
0.02835617 0 0.03338009 0.6409289 0.3150179 0.2153199 0.04523893
0.2291709 0.03125058 0.5225097 0.1091594 0.08130337 0 1.050569
0.04578064 0.2772752 0.01956965 0.1396255 0.2439101 0.07407982
1.9955810.3427541 0 0 0.0853464 0.6531371 0.01115203 1.0251890
2.1264930.0291579 0.03486497 0.1361753 0.1632843 0.6801262
0.05353582 0.03294974 0 0.1367826 0.04755852 0.72303 0.8397477 0
0.1509562 0.06863086 0 0.2506709 0.7579927 0.07884954 0.06250115
0.5046313 0 0.07536197 0.09846287 0.07086478 0 0.5848989
0.2742833 0 0.1878030.1044091 0.3817035 0.05249865 0.1846759
0.1855503 0.3308574 0 0.03386079 0.07212557 0.5019275 0.1444994
0.1407118 0.0834680.02552979 1.0210770.04195741 0.0697654 0.4779052
0.3204156 0.06676018 0.0355610.02486112 0.3781320.9904716 0.1055338
0.02991467 0.6664664 0.06972994 0.2853511 0.6856491 0.01832938
0.5797075 0.3455 0.2899440.05715902 0.1435466 1.1050550.2090835
0.04987365 0.1597206 0 0.1439270 0.1292731 0 0.1178003 0
0.3151913 0.1765235 0.3407672 0.0713811 0.1160941 0.6252185
0.03267501 0.06142355 0.08362461 0.2226604 0.03419566 0 0.2108136
0.1826349 0.2258687 0.04344592 0.04483778 0.07436096 0.2349414
0.3498949 0.1070716 0.06410287 0 0.07469166 0 0.06023397
0.1337482 0 0 0.1721802 0.4416706 0.02521765 0.05715902
0.7837645 0.1203435 0.0246829 0.08308006 0.02866732 0.04929336 0
0.1634972 0.3166014 0.05663038 1.0642010.0832611 1.45425 3.19761
0.04077387 0.2644874 0 0.3662451 0.01699403 0.02626557 0.3153982
0.004834471 0.06736679

TCGA-FD-A62S 406 1 0 0.1231889 0.046419 0.224949 0.1767841 28.41019
0.1337825 0.1767538 0.09343541 0.1762119 0.07588779 1.372012
0.2401679 0.4033295 0.2005888 0.2246322 0.1515011 0.1681096
1.672795 0.09913772 0.08402699 0.3156492 1.07886 17.86491 0.1060704
0.2889662 0.4714241 0.09189787 0.9723123 0.6852487 0.5088737
0.1600514 0.6049943 0.7325043 0.1994487 0.08187893 0.4435803
0.2275229 0.160618 0.4643118 0.1216943 0.1293573 0.1322392
0.08882461 2.335388 0.1218109 0.1205168 0.2145558 0.154686 0.100091
2.192743 0.2724177 0.9864038 0.6789213 0 0.1804536 0 0.2030591
0.3781214 0 0 0.2858241 0.05499948 0.1680369 0.10582 0.07674346
0.3267563 0.7028408 0.03972758 0.1714836 0.1627179 0.1592912
0.9307605 0.7464216 0.07259932 0.1692524 0.06945769 0.1601027
0.3233509 0.0289471 0.2086187 0.3406134 0.4161744 1.194325
0.2482877 0.1424913 0 0.0661196 0.2326901 0.5711485 0.07690606
0.08907892 0.02001084 0.1044745 0.5062966 0.2309978 0.0512707
0.5264415 0.6867503 0.6979546 0 0 0.772991 0.8057167 0.6127756
0.299173 0.1204751 0.2948315 0.05789419 0.6107562 0 0.07531049
0.1291803 1.285269 0.04240614 0.1712248 0.0634451 0 0.451863
0.2401679 2.460798 0.3174897 0.2792281 0.06060043 0.1383472
1.337356 0.1342901 1.618371 0.08249922 0.9527153 0.1350434 1.194918
0.03503828 0 1.719984 0.0495897 0.06443325 0.168054 0.2534008 0
0.5692751 0.6425716 0.03594026 0.08389752 0.1907162 0.1170957
0.5804849 0.9478634 0.03651877 0.2315768 0.5698254 0.1885697
0.5235528 0.1368078 0.098462 0 0.6501431 0.6034064 0.02344939
0.17396 0.225664 0.3535681 0.3029959 1.528167 0.2062481 0.6129398
0.04514883 0.06272982 0.02226973 0.4473858 0.2141573 0.1955098
0.354363 0.02364799 1.973162 0.03886473 0.2123327 0.3621917 0.5355264
0.1545982 0.2305786 0.1217226 0.5837664 0.2792281 0.1303399
0.249387 0.8642776 0.1614753 0.8370067 0.6172194 0 0.3197998
0.2893451 1.512486 0.1376591 0.3656559 0.3208303 0.1106697
0.08662025 0.1479476 0.04548829 0.07594071 0.03195392 0.1368507
0.05990043 0 0.2092199 1.208427 0.8829647 0.3945615 0.7603754
0.1194854 1.486079 0.02017769 0.01896534 0.5448955 0 0.1583755 0
0.3129742 0.3866808 0.07845748 0 0 0.1033197 0.3844688
0.5454435 0.1487691 0.07917047 0.295386 0.02306204 0 0.05579413
0.1445379 0.142912 0.02816838 0.4226454 0.5206918 0.04671771
0.01058917 0.8227923 0.1520086 0.2019611 0.2279088 0.5178079
0.4794295 0 0.1609111 0.6516994 0.02622808 0.5476438 0.1028319
1.5952 3.934805 0.07134037 0.2041601 0.05385706 0.113083 0.03148279 0
0.3286689 0.004478122 0.08023009
TCGA-MV-A51V 410 0 0.1226844 0.1874234 0 0.2755968 0 14.74742
0.06106228 0.1783356 0.415805 0 0.08659354 0.2165021 0.1879194
0.9421151 0.1894234 0.1491327 0.04148973 0.6823502 0.648986
0.09397946 0.02876429 0.2881431 2.15849 125.56417 0.1613789 0.03768362

0.08068944 0.566256 0 1.521572 0.1161325 0.1314939 0.1129652
0.4086335 0.09803687 0.1308018 0.5567735 0.1602229 0.1293719
0.04075491 0.03570739 0.05450076 0.3168787 0.08108433 13.9999
0.1667943 0.06600889 0.03264319 0.6354293 1.279165 2.24444 0.04662729
0.3151566 0.1549398 0.1868597 0.6502445 0.94376 0.04217557
1.323157 0.06213086 0 0.2739629 0 0.02781319 0.0794602 0.05254196
0.4474236 0.05661127 0.04662729 0.1033166 0.02785097 0.3079279
0.5006883 12.04962 0.1656823 0.2999186 0.01463194 0.1594377
0.05775138 0 0.3116282 0.3331414 0.1266362 0.5361887 0.2379842
0.09145858 1.2259 0.04526839 0.06372396 0 0.2106131 0.4065823
0.137003 0.07510423 0.6870761 0.09037217 0.1404087 0.3170231
2.283729 0.03707461 0.1798288 0.1080537 0.1693516 2.671047 0.6792442
0 0.04303437 0.3229675 0.07927383 0.3471439 0.1038399 0
0.04422126 2.765562 0 0.2930701 0.00620533 0 0.1031218 0.09395972
0.08305208 0.07245582 0.09558595 0.1244692 0.1353122 0.5813414
0.01414476 1.153794 0.2259304 2.845773 0 0.2653275 0.3358425
0.06903429 0.6435594 0.5432207 0.04643562 0.09204573 0.260234
0.06032123 0.3209713 0.5248322 0.1476377 0.03829329 0.04352425
0.05344591 0.4239206 1.057547 0.1000094 0.2774584 0.3047872
0.06455155 0.5735157 0.1248862 0.3145867 0.04627997 2.819072
0.9175582 0.7706154 0.1588009 0.7504261 0.4841366 0.1690287
0.1171176 0.04706884 0.326391 0.3091088 0.1861063 0.2439494
0.4084001 0.4765199 0.1338546 0.06175592 0.01619045 0.1786332
0.106434 0.2528214 0.5510498 0.194366 0.0846758 0.1804163 0.1441499
0.2616036 0.3823438 0 0.03794251 0.08453179 0.08844251 0.1206425
0.3368356 0 0.3071822 0.6183072 0.3483974 0.1304966 0.4551712
1.715398 0 0.06325764 0.2287226 0.06228658 0.05083697 0 1.288293
0.08202094 0 0.358103 0.7525183 0.268674 0.5402684 0.9506362
0.3599418 0.2992456 0.09670174 0.3375972 0.4718105 0.1882753 0
0.1308018 0.1025594 0.4550197 0 0 0.1137407 0.0943163
0.2582578 0.395083 0.06790258 0.1084072 0 0 0 0.09167788
0.02827343 0.09784388 0 0.2183862 0.4583424 0.031985 0.08699771 0
0.1942674 0.03130676 0.6808862 0.1272615 0.2813473 0 0.4212262
0.1338546 0.1436552 1.056084 0.03520162 1.262033 7.341729 0.0804469
0.02795539 0 0 0.04310901 0 0.2333554 0.07358208 0.09765165
TCGA-E5-A2PC 1326 0 0.02897266 0.08262089 0 0.11613 0 23.20052
0.06729441 0.04913423 0.2291214 0.1329555 0.02862943 0.03848367
0.1035494 0.6444429 0.06262689 0.1369612 0.02286212 0.4212968
0.04207193 0.0632935 0.06340006 0 1.433821 27.78106 0.1333871
0.09344178 0.2667743 0.0693388 0 1.215728 0.03199629 0.120762 0
0.4503394 0.1196187 0.07207585 0.05578172 0.09319263 0.03920836
0.02694866 0.03935176 0.03753951 0.6236071 0.02233999 3.177326
0.09190881 0.2000512 0 0.3890458 0.1762149 2.797035 0.05138616
0.6450267 0.008537664 0 0.3726371 0.2724024 0.01377188 0.3170003

0	0	0.2587920	0.01114613	0.1545354	0.3763792	0.02241311
0.1559728	0.02569308	0.09833478	0.05115582	0.09897872	0.4514641
3.3494990.01825922	0	0.08465783	0.2635653	0.0636456	0.08736467
0.2575752	0.03671424	0.2093413	0.04431849	0.09366903	0.08063442
0.1842298	0.02494428	0.4213665	0	0.1160543	0.06161085	0.03019716
0.00788281	0.2080596	0.04979786	0	0.3015474	2.23552	0.002269917	0
0.05954092	0.04665899	1.5358220.4623515	0.1003254	0.01976106
0.4983022	0.1310470.9912146	0.05721899	0	0.04873456	1.870254
0.09598887	0.5167703	0.006838658	0.09758489	0.1136466	0.362423
5.1975130.1597016	0	0.02286212	0.1540932	1.1371970.03897101
0.7669678	0	1.08888	0.1222715	0.1218364	0.07402385	0.1521601
1.4109390.03741643	0.02558747	0	0.09559799	0.06647773	0.227398
1.0121960.1627059	0.02110079	0.02398321	0	0.1459959	1.880664
0.02755415	0.1092058	0.3896374	0.6758281	0.6320498	0.03440807
0.02476386	0	0.4496673	0.5798859	0	0.04375212	0.4621561.333871
0.2765978	0.0860472	0.1556183	0.4367824	0.0340657	0.1104388
0.01680296	0.3706559	0.1413877	0.04917204	0.1458404	0.2765647
0.1230405	0.02932422	0.1044843	0.9716656	0.2969638	0.06998848
0.04970749	0.07446662	0.9209692	0.2708785	0.1229301	0.0209075	0
0.04873456	0.03323886	0.7593008	0.03843147	0.4051601	0.1190818
0.01066543	0.08788713	0.3511388	0.4841459	0.1252538	0.05664249
0.2520665	0.03432184	0.09804461	0	0.3484912	0.09039216	0.1097748
0.3551865	0.1460737	0.09869847	0.4167865	0.1995543	0.09015413
0.7667552	0.03044894	0.01430973	0.01813829	0.02593639	0.1672965	0
0.03027505	0.3692568	0.2367910	0	0.07795681	0.2134621	0.6888957
0	0.1045375	0	0.01740078	0	0.02946846	0.06231814	0	1.579852
0.2406751	0.8418696	0.03524945	0.03994869	0.1825922	0.1682172
0.02875165	0.2411932	0.1602857	0	0	0.1392652	0	0.05936884
0.7885392	0.03879437	3.3166157.6424440.07282578	0.2002557	0.08127248
0.2559703	0.0178158	0	0.8266245	1.0406810.03363068
TCGA-DK-A3X2	547	1	0.1314772	0.1687191	0.1430445	0.3283584
0.04035382	72.462640	0.0573351	0.1919533	0.1810045	0.05196784
0.04191308	0.2114570.5117813	0.1420996	0.07458317	0	0.1480129
0.08591458	0.04700033	0.02157808	0.1080782.19562165.177670.3813436
0.05653817	0.06053072	0.09439728	0.08322974	0.8180295	0	0
0.02824767	1.0311030.3256959	0.2382999	0.2657927	0.3071631
0.1843966	0.04280231	0.2366148	0.09199077	0.0679179	0	2.720019
0.2919558	0.1733124	0.008162637	0.3177863	0.6168793	8.541204
0.4197403	3.85028	0.5927784	0.1401764	0.3219436	0.1348533
0.005859039	0.8199671	0	0.08856752	0.4110370	0.001896781	0.06662136
0.3941535	0.6102607	0.08493605	0.005829726	0.3100198	0.06267881
0.4812458	2.6633520.6053072	0.0497159	0.0102268	0	0.2541611
0.0144411	0.02973439	0.6623592	0.4998247	0.5937409	0	0.1785284
0.05946149	0.1672059	0.3395895	0.4780375	0	0.03949886	0.1601279

0.06166526 0.005365797 0.9008298 0 0.05266515 0.1868593 1.269771 0
0 0.08105853 0.09528175 0.9583088 0.4496012 0.1024366 0.2098398
0.2786223 0.1189376 0.5800188 0.05193165 0 0.1658671 1.876606 0
0.2638226 0.004655047 0.04428376 0.2320763 0.7400997 0.0207677
0.5435412 0 0.06224862 0.09473992 1.384631 0.03183289 0.4533782
0.169486 0.3138213 0.02774325 0.06634684 0.05038774 0 0.6368566
0.356569 0.2159743 0 0.1301463 0.09050225 0.5847581 0.4284523 0
0 0 0 0.3577637 0.4687911 0 0.1189376 0.4618567 0.04842458
0.3585281 0.1873715 0.1011399 0 0.7791298 1.934484 0 0.1191276
0.4967175 0.9079609 0.3073943 0 0.9533589 1.539048 0.04637677
0.4080942 0.02287541 0.4685646 0.2199819 0.03347121 0.03309099 0
0.01675064 0.07984352 0.2086247 0.4547186 0.457305 0.381127 1.522606
0.1284125 0.3488835 0.08194929 0 0.0569266 0 0.2985608
0.1357534 0.9004728 0 0.7305394 0.6034358 0.07259915 0.1414032
0.06829107 0.3923288 0.1989394 0.1127031 0.3284544 0.1401764
0.2912229 0 0.1932876 0 0 0.2149102 0.05933833 0.7390201
0.3242341 1.358358 0.1718292 0.4377454 0.08290589 0.1363681
0.1152355 0 0.1301463 0 0.01648648 0.6268275 0 0 0
0.03537659 0.1788342 1.861504 0 0.1016547 0 0.1184464 0
0.0859674 0.2120986 0 0.009644833 0 3.629363 0.1919533 0.04350866
0.5468749 0.2290096 0.02739959 0.3618024 0.4636999 0.0234509
0.04552738 0.4618328 0 0.05388284 1.092262 0 0.4733692 2.487276
0.1250082 0.02097127 0 0.2903966 0.01616953 0 0.06252 0.1149979
0.03662763
TCGA-GD-A76B 224 0 0.13798182 0.06707056 0.18196534 0.08121969
0.11550072 17.86641205 0.39332679 0.10940304 0.16024425 0 0.061976
0.19494094 0.06724806 0.32551577 0.0813435 0.09784134 0.02969468
0.20005352 0.24590477 0.0298943 0.02058694 0.10311369 4.47866712
23.47937908 0.10972569 0.0404559 0.11550072 0.13509199 0.31762699
1.85131161 0 0.06274113 0 0.42540177 0.45607978 0.20060652
0.21735778 0.0764489 0.10648193 0.02333501 0.05395197 0.01950341
0.09719733 0.05803299 10.23614928 0.19896098 0.04724332 0.04672621
0.20212627 0.16348448 1.60637191 0.10011507 0.45112239 0.21069521
0.06686884 0.7539278 0.932778 0.08161277 2.38808441 0 0.21124835
0.81232478 0.05390034 0.05067038 0.12377715 0.18802443 0.64045246
0.36465604 0.03893364 0.10755623 0.13288839 0.14692503 0.29319415
0.6545041 0.11858074 0.70250797 0.12043084 0.05705569 0.05511111
0.0567372 0.631935 0.14305988 0.13595245 0.03837564 0.04866515
0.11782434 0.2392885 0.03239911 0.13682394 0 0 0.04364935
0.03922186 0.05887229 0.27024005 0.16170101 0.05024608 0.85626642
0.55764998 0.02358642 0 0 0.57573274 1.99481624 0.34315971
0.06515425 0.1334675 0.34672732 0.0851058 0.48561638 0.14863882
0.03690272 0.09494899 2.55515411 0.04155867 0.88096543 0.01332368 0
0.25831905 0.33624029 0.07076349 0.36300228 0.06841197 0.08908403

0.10975713 1.52906748 0.04049428 0.44566086 0 0.67366469 0.07940675
0.56969396 0.02060283 0 1.754211 0.24299333 0.17946637 0.26351276
0.1552104 0 0.45944568 0.90592742 0.10566601 0 0.03115081
0.07650371 0.11377683 0.41285365 0.03578896 0.0283686 0.2225025
0.18480116 0.30785385 0.04469124 0.12865903 0.03312311 0.79643783
0.65048229 0.09192306 0.05682783 0.47390173 0.4620029 0.30427252
0.2067619 0.37056482 0.23360184 0 0.11270635 0.06547403 0.43844499
0.15740807 0.12773509 0.04419943 0.11587695 0.31962464 0.03808803
0.14475777 0.59158907 0.60702549 0.03030172 0.16140755 0.10961787
2.08036392 0.58638829 0.03193377 0.08146769 0.60500379 0.09494899
0.0863452 0.95115899 0.03327804 0.30171432 0.18474536 0.18008758
0.13490808 0.06515425 0.49408643 0.43383199 0.09620773 0.13095954
0.13373768 0.16042304 0 0.73763705 0.11740668 0 0.15377906
0.14841719 0.0640977 0.3480087 0.58318398 0.21077551 0.28913426
0.04943611 0.03717265 0.3664747 0 0 0 0.03932302 0.17171313
0.05125969 0.07887854 0.08140554 0.06750327 0.27014798 0.40865424
0.04859866 0.09698534 0.09649428 0.09040463 0 0.07108283 0.06070686
0.1400056 0 0.17193166 1.85889895 0.02289204 0.14528563 0.14229689
0.10924524 0.11203304 0.13053164 0.28625929 0.64883803 0 0.37568606
0.12773509 0.05140784 0.53222687 0.02519421 1.14643492 3.41122686
0.07402728 0.160064 0.05278075 0.22164625 0.06170724 0 0.5487664 0
0.13104453
TCGA-GC-A6I1 0 0 0.01598289 0.01709181 0.1738906 0.02956783
0.2943343 49.81376 0 0.1510142 0.1458413 0 0.04738065 1.461663
0.1713705 0.5332646 0.03454839 0.1218329 0.2270158 0.3523639 0
0.03809033 0.05246237 0.197076 2.026193 35.7277 0.1398088 0.4467455
0.5886687 0 0 0.4625254 0.2118107 0 0 0.3387705 0.08940333
0.03408082 0.04615834 0.1298782 0.08258522 1.21904 0.1013066
0.06212649 0 0.1109156 1.722169 0 0 0 0.3863138 0.1666452
3.93769 0.0425211 0.4105751 0.0847771 0 0.094877 0.2458996
0.01424497 0.1573871 0 0.05383312 0.1784547 0 0.004611607
0.0682001 0 0.296743 0.05162584 0.03543425 0.06852228 0.0169322
0.09360349 0 0.1962229 0.09065498 0 0 0.03634923 0.01755519 0
0.1184104 0 0.2598391 0.09779393 0.09301126 0.09452494 0.5081557
0 0.1743365 0 0 0.05561655 0 0 0.1580535 0.04120681
0.06402188 0.2374789 0.5635309 0.0638627 0 0 0.07721889
0.7942901 0.4008058 0.04150869 0.07194839 0.2356205 0 0.5611975
0 0 0 1.63931 0 0.2672611 0.02263545 0.05383312 0.1410606
0.08568523 1.197384 0.06607506 0 0 0.05758483 0.8614874 0.01289911
0.651353 0 1.184639 0.1011774 0.1209808 0.03500192 0 0.6742932 0
0.07622335 0.0419699 0.07910556 0 0.08363005 0.6475355 0 0
0.2778392 0 0.04832355 0.4822073 0.09120219 0.03614632 0.150091 0
0.2615047 0 0.1229498 0 0.8794886 0.3212184 0 0 0.04025532
0.1471672 0.07473617 0.1139239 0.1287713 0.08504219 0 0.01305515

0 0.3505267 0.1002821 0.0813779 0.1689523 0 0.5497943 0
0.06916707 0.1507566 0.3142147 0.1544378 0 0.05751177 0.3711022
0.4482938 0.04068895 0 0.3854378 0.08065389 0.1100182 1.17284 0
0.2980111 0.16423 0.1059053 0.05289088 0.04150869 0.4960079 0
0.03605432 0.1013105 0 0.07164735 0 0.08544296 0.1495957
0.09083665 0 0.1403690 0.2450135 0.09853802 0.2476912 0.1492017
1.0097030 0.0377939 0.02368207 0.02001214 0.1287713 0 0 0.1369511
0.1659795 0.1306268 0.1005044 0 0.3440417 0.09058251 0.2517097
0.0619228 0.0247151 0 0.05759528 0 0.01393406 0.07735066 0 0
0.009957708 0.1857684 0 0.03966816 0.18131 0.1012339 0.03806633
0.08500752 0.04973755 0.02850785 0.02767246 0.1477428 0.1220668
0.06550215 1.4645670 0.03210162 0.1770605 1.3093410 0.04716155 0.3569094
0.06725147 0 0.1179380 0 0.2584062 0 0.07792054
TCGA-XF-A9SK 486 1 0.1097458 0.1369201 0 0.1861072 0.05613979
56.671950 0.4254078 0.2002825 0 0.05422282 1.51239 0.2941767
0.8476007 0.05930621 0.2788531 0.1731992 0 0.3585703 0.02179545
0.2701727 0 2.670125 29.4867 0.06736775 0.3932765 0.4210484
0.1313245 1.042095 2.328999 0.9695919 0.09148706 0 0.8916912
0.1461628 0.09750595 1.05648 0.1950817 0.1890225 0.144612 0.08281157
0.05687847 1.13384 0.0423109 7.016279 0.05802367 0.1722217 0.1703366
1.7684030 0.1907102 2.9938760 0.5638387 0.2748890 1.3707980
0.0766197 1.440921 0.06484146 0.06160708 0.4901401 0.0785957
0.168882 0.2536585 0.4386737 0.212247 0.2954053 0.04055125 0.1470328
0.1647075 0.2410214 0.7125435 4.771882 0.1383284 0.1991838
0.03817572 0.1247951 0.1406321 0 0.1084079 0.1390701 0.2643219
0 0.07096192 0.1718076 0.3489226 0.04724332 0.1330081 0.09068735
0.1099008 0.1060801 0 0.05971871 0.8527096 0.04715742 0
0.3544863 1.990808 0.6921581 0 0 0.3534798 11.3321 1.417755
0.0950058 0.2619857 0.5224393 0.08273232 0.7081099 0.07224675
0.05381034 0.092301 4.3686810 0.7952254 0.03238025 0.06160708
0.2152413 0.04902946 1.803674 0.2268506 0.09975609 0.0432998
0.1741658 0.6370389 0.05904743 1.20413 0.1768403 1.459112 0.1929805
0.7845585 0.2703814 0.07204606 1.371852 0.2834599 0.1986921
0.04803071 0.2715872 0.06295287 0.6220951 1.6270780 0.1598557
0.1362693 0 0.3318113 1.354522 0.1565588 0.08273232 0.2544673
1.279987 0.09975609 0.06516729 0.09380319 0.04829904 1.47103 0.8532069
0.06701959 0.1242967 0.1842741 0.5894678 0.1336386 1.458576
0.04912231 0.3406305 0 0.104583 0.06364805 1.291188 0.3060364
0.2793887 0.1565218 0.4055231 0.7923125 0.05553872 0.1846959
0.5750905 0.3688103 0.1767399 0.5177902 0.1598412 1.304413
0.3420209 0.09312956 0.07919567 0.9704163 0.4153545 0.3147643
0.4729687 0.02426247 0.3853825 0.4197466 1.090791 0.09079317
0.1425087 0.2838178 0.1581499 0.1650435 0.3478216 0.1300079
0.1109328 0 1.637843 0.5135957 0.415817 0.07474519 0.6559445 0

0.9021142 0.4724332 0.08537387 1.0930530 0.05766889 0.1355098 0.6756108
0.1964893 0.09052908 0.06825416 0.3402146 0.3367266 0 0.1150181
0.1780543 0.04921553 0.4457526 0.2880587 0.14173 0.0848525 0
0.1647813 0 0.07973127 0.02950692 0.1021125 0.01341779 0.1595396
0.9566772 0 0.04539659 0.1383284 0.3041140 0.04900887 0.3172283
0.5502274 0.3914956 0 0.1623150 0.2793887 0.07496122 2.2499680 0
3.49536 9.5775350 0.08995346 0.05835002 0 0.2423980 0.02249487 0
1.8091240 0.03199677 0.1019119
TCGA-DK-A1AD 3420 0 0.1891068 0.1427487 0.04034202 0.1337629
0.1194982 8.40397 0.1550244 0.1131893 0.1827073 0.7657151 0.03297644
0.9242161 0.2683621 0.8041510 0.1623059 0.1400094 0.2370010 0.1617551
0.12115 0.01988285 0.2921061 0.1828838 3.1421775 2.2475 0.2304604
0.04783542 0.1024268 0.1198005 0.7041844 1.1427910 0.3685451
0.1669178 0.07169878 0.4951395 0.5022349 0.1185995 0.2891315
0.1412404 0.09032329 0.04138729 0.08813547 0.03459151 0.2298540 0
2.9565760 0.1411520 0 0.05524949 0.5377408 0.4639333 3.8915450 0.1479712
0.7715412 0.04916998 0.2371990 0.5200131 0.08557178 0.1150065
0.9493449 0.03943433 0.1124019 0.4222889 0.2867951 0.1620864
0.1572330 0.2000896 0.2839788 0.3593105 0.009864747 0.2682607
0.2298003 0.3175919 1.0978052 0.7313820 0.1051582 0.04326311 0.1810938
0.1517921 0.04887291 0.02515747 0.4450269 0.1268665 0.2210332
0.5955562 0.1510480 0.1122272 0.2122027 0.02873175 0.2426728 0 0
0.1161256 0.03478221 0.2338026 0.2160785 0.3728336 0.08911712
0.7258072 1.5006360 0.02353117 0.04565479 0 0.9405121 1.031926
0.3550357 0.08666885 0.3141089 0.07174536 0.05031493 0.4807232
0.3515037 0.03272558 0.08420141 1.7233820 0.07370902 0.1860110 0.01575404
0.1873365 0.1963535 0.08945405 0.2911764 0.7817884 0 0.02633344
0.2376079 2.5090380 0.06284355 0.6393185 0.03584939 0.7965472
0.09389125 2.3295720 0.03045122 0.08763184 0.5475179 0.04309763
0.3772492 0.08763184 0.2752833 0.1531430 0.5529537 0.4212860 0 0
0.1104990 0.0678440 0.4372249 0.5796922 0.1269516 0 0.3443370 0.04097073
1.2436980 0 0.1426196 0 0.6592007 0.6072127 0.1018975 0.2015812
0 1.0754820 0.4063719 0.4707837 0.5676153 1.1245810 0 0.3089325
0.05806281 0.2897060 0 0.1415954 0.1735838 0.03082814 0.1700672
0.06755345 0.1765117 0.5596002 0.3756985 0.1612306 1.517255
0.1887015 0.4197104 0.3466754 0 0.1204102 0.3756650 0.3648728
0.1148573 0.6297054 0.02951119 0.4397148 0.4381592 0.5528177
0.1932604 0.2311169 0.3850490 0.8175409 0.1455419 0.2530101
0.1185995 0.1129314 0 0.1338017 0.05205852 0.06322137 0.2272872
0.3324356 0.2557902 0.3429072 0.1149270 0.1557643 0.4083506
0.0964486 0 1.2558630 0.1493725 0.05505665 0 0.1418125 0.2257891
0.3182021 0 0 0.1197247 0.8511006 0.5959805 0 0.2752232
0.02852393 0.1202572 0.06113849 0.1503184 0.1076705 0.1863039 0
0.6098806 2.81212 0.1218049 0.1012316 1.0936450 0.15853 0.0761696

0.2803876	0.3577071	0.238094	0.1155585	0.4030836	0	0.2279441
0.2697042	0.04468479	0.462121	5.542883	0.08388342	0	0	0.2948361
0.0273612	0.02114441	0.08463437	0.4125377	0.2711594
TCGA-K4-A5RI	356	1	0.08468126	0.1207421	0	0.195822	0.3682046	58.9594
0.049172	0.1333516	0.7984597	0.4857525	0.06973172	1.15292	0.1891588
0.3924119	0.04576144	0.2226725	0.2672855	0.05296197	0.2151936
0.1345412	0.0463264	0	1.486225	42.7047	0.240416	0.6979516	0.2599092
0	1.161469	1.082336	0.3740749	0.1764815	0.0909682	0.4786369
0.3045089	0.1354263	0.3668372	0.1146877	0.1510612	0.1050207
0.1261995	0.03291613	0.1093607	0.9467816	9.327236	0.08954365
0.2126216	0.01752452	0.5116961	0.478252	5.033175	0	0.652598	0.04990774
0	0.3141759	0.1809494	0.08427852	0.463264	0.05003251	0	0.5672978
0.1212909	0.17307	0.1355028	0.08462158	0.09826353	0.319114	0.01877393
0.1663969	0.3289399	0.1653113	0.6597694	0.8230457	0.08005202
0.1097806	0.3181343	0.1604892	0.0775097	0.03191867	0.2300345
0.05365417	0.1529656	0	0.1095103	0.1669387	0.3589777	0
0.0513154	0	0	0.04911167	0.1765204	0.08351952	0.1644905
0.1455491	0.5653391	0.723325	1.644312	0.1492765	0.02896238	0.08701306
1.261467	0.7949091	0.4182782	0.2199231	0.1559455	0.3250975
0.095756	0.4955601	0.0836198	0.08304151	0.03561033	3.735356	0
0.5192053	0.01998802	0.1426105	0.2906453	0.2269906	0.9012859
0.2917348	0	0.1670535	0.1416528	1.697009	0.1366853	0.4719353	0
1.351931	0	1.816127	0.09272435	0.05559168	0.8049308	0	0.1271379	0
0.1397068	0.04857525	0.406168	0.6090915	0	0	0	0.1291162
0.128015	0.7354876	0	0.191512	0.667591	0.1039637	0.6927579	0.1005679
0.07237976	0	0.4779225	0.8124264	0	0.03196966	0.1777354
0.7147502	0.3464741	0.8299455	0.3032273	0.750957	0.04978359
0.1037541	0.02455583	0.3869116	0.05903541	0.10779	0.1634005
0.1564535	0.2517359	0.0428544	0.1628729	1.109368	0.4624406
0.1704685	0.145285	0.1233355	0.8016905	0.3958616	0.2155799
0.06110841	0.1361429	0.213662	0.1457258	0.7594881	0	0.1710513
0.2175327	0.5455258	0.1634668	0.3665386	0.4379951	0.06101526
0.02546994	0.1947098	0.2507895	0.1898026	0	0.0188624	0
0.1203187	0.2306975	0.335702	0.3605947	0.4350653	0.2187214
0.329378	1.036194	0.1557432	0.1045612	1.142759	0.1516137	0.1397068	0
0.3215059	0.2531605	0.05767437	0.04437473	0.137389	0.07595068
0.3439485	0.9195871	0.1093607	0.1309466	0	0.1780064	0.07756979
0.08613036	0.2276792	0	0.6833204	0.2813779	1.107277	0	0
2.134721	0.2793552	0.004201765	0.261096	0.2781617	0.02517359	0.0244359
0.2817998	0.2874399	0.02892053	0.6290233	0	1.328989	3.349407
0.08329112	0.2251182	0.05938577	0.3740749	0.1215013	0	0.4295212
0.5036582	0.06880699
TCGA-FD-A3SQ	1423	1	0.1117769	0.1344737	0.05067124	0.4523392
0.1500944	26.21639	0.04867925	0.2031005	0.1784906	0.2885309

0.09664613 3.454722 0.2247159 0.3884795 0.1585601 0.2724553
0.2646071 0 1.308657 0.09989474 0.2522419 0.1148548 2.004926
23.62653 0.09648923 0.1802498 0.2573046 0.3511068 2.034315 1.394959
0.3703263 0.1397704 0.480302 0.8292209 0.5191768 0.3873112
0.3631611 0.2128846 0.3558197 0.05848216 0.1850301 0.1737935
0.2165296 0.06464097 3.733654 0.6648475 0.1841795 0.06939564
0.5628539 0.3641996 4.194742 0.1486863 1.507469 0.654651 2.234488
0.425071 0 0.281434 0.5044838 0 0 0.2808065 0.09005662 0.1874614
0.2533848 0.1675472 0.7133781 1.53445 0.01858579 0.2396064
0.4514605 0.3477664 1.923187 1.844016 0.2641661 0.2390971 0.2974493
0.1906572 0.1227728 0.1895929 0.2898374 0.212466 0.6057311 0
0.1355161 0.2138734 0.1776902 0.144353 0.2032047 0.207823 0.08395109
0.07292929 0.04368789 0.2394944 0.4786571 0.4682944 0.3917717
1.287734 1.863442 0.2692891 0.08601645 0.1292117 3.408958 2.175667
1.051143 0.3265789 0.4402768 0.2317246 0.4423834 0.8679743
0.1931576 0.04110468 0.1057604 1.843951 0.1388724 0.4205474
0.01978772 0.09412093 0.2055234 0.4119792 2.011513 0.6353849
0.1524035 0.03307589 0.2588923 1.065933 0.09021039 0.7884102
0.09005662 1.728928 0.1474141 3.419588 0.09179516 0 0.8296125
0.8119859 0.1924977 0.1467589 0.5878041 0.1442655 0.5117609
1.119834 0 0.06105533 0.2775826 0.5112892 0.3379523 1.264625
0.1594564 0.1579941 0.3304506 1.852593 0.5334123 0.2489002
0.322445 0 0.8279832 0.9637555 0.05119498 0.1265972 0.2111452
1.608154 0.53492 2.060296 1.013137 1.077976 0.04928472 0.6619369 0.09723905
0.4596413 0.08766573 0.2134196 0.2320948 0.6969854 0.9612512 0
0.3426366 0.3514405 0.5071087 0.4725288 0.5034018 0.1939229
0.7878638 0.3918947 0.07113988 0.09074407 0.3369465 0.2115209
0.2885309 1.205933 0.03706727 0.3725419 0.5359892 2.098516 0.1271511
0 0.2334801 0.06040383 0.1260735 0.2813233 0.2979317 0.1086876
0.03488108 0.5041813 0.3269382 0.07940864 0.2283857 1.04303
0.285585 0.6891289 0.685677 0.430422 1.001954 0.05506519 0.186324 3.845862
0.1125708 0.4149205 0 0.4788862 0.257219 0.142741 0.08786012
0.04533743 0.03759479 1.330333 0.3386929 0.270662 0.2160573 0.1074817
0.3020959 0 0.1339914 0.02253977 0.1560036 0.03074877 0.01740989
1.948766 0.1019946 0.02311839 0.1584996 0.2654935 0.1788653
0.4200292 0.565242 0.3488986 0.09676413 0.2841421 0.5335491
0.08589215 1.72867 0.08418891 2.321772 12.50816 0.160332 0.200576 0.176372
0.9875368 0.03436679 0 0.6776896 0.04888343 0.05838641
TCGA-GV-A3QF 617 1 0.3226012 0.2979406 0.159538 0.1898915 0
15.16609 0 0.04263069 0.2676077 0 0.02897995 0.03213774 0.7861292
1.467749 0.1505599 0.1325727 0.0694261 0.05502644 0.4152235
0.04368302 0 0.0602699 3.933296 32.22904 0.1485223 0 0 0.3684856
0 0.4667843 0 0.07334433 0 0.6837796 0.2753667 0.2657767
0.2964395 0.3872644 0.1190652 0.0136393 0.05975034 0.07979815

0.2272472	0.03392026	3.28645	0.04651704	0.0276137	0.01820763
0.826999	0.8791207	2.219839	0.1560459	0.5650303	0.01296329	0.07816961
1.055433	0.1128017	0.0248315	0.9626443	0.05198279	0	0.6876464
0.03150472	0.002115487	0.2150877	0.04396008	0.3062816	0.3789178
0.02600765	0.3064745	0.1165098	0.1717552	1.142479	3.150472	0.360414
0.2281198	0	0.3001412	0.01610621	0.03316287	0.2824561	0.5017106
0.2119044	0	0.4551161	0.04591218	0	0.1514981	0.2665784	0
0.08810643	0.1105564	0.02292516	0.02393797	0.7820076	0	0
0.1641977	1.88824	0	0	0	0.1416907	2.089028	0.4345828	0.03808263
0.0540081	0.1756404	0.1989772	0.8845326	0.7819139	0.04313925
0.4069827	1.819529	0	0.392323	0.005191789	0.04938983	0.1294177
0.2751452	0.03970678	1.212427	0	0	0.6188877	1.653722	0.2721922
1.057277	0.09451417	1.671739	0.06188428	0.07399686	0.06422584	0.05775866
0.7790259	0.2272472	0.1592896	0.1925289	0.1814408	0.1514062
0.5754553	0.8007287	0.4117449	0	0.03641527	0	0.310345	0.5630631
0.1255118	0.0994886	1.00472	0	0.9996691	0	0.3760057	0.1548836
1.365518	0.6076029	0.2149159	0.1660792	0.1846636	1.957793	0.488544
0.1045209	0.9057608	0.7022066	0.1551725	0.1317536	0.05102605
0.2311463	0.1533416	0.335975	0.07381298	0	0.05604614	0.08904975
0.148069	0.4149402	0.3843733	0.2833815	0.9056893	0.1055297
0.1459166	0.09139832	0	0.06349043	0	1.701928	0.1514062	0.7634732
0.038902	0.6835344	0.5574966	0.3724624	0.3032835	0.72357	0.4550682
0.1901809	0.535871	0.3881965	0	0.1778677	0	0.2351719	0.06862415
0.04166957	0.05992253	0.06618024	0.2247905	0.632834	1.817977	0.1095095
0.6008848	0.1040234	0.06518218	0.09180184	0	0.1814408	0
0.03983943	0.6229629	0.2696514	0.04610447	0	0.1972781	2.011165
0.7086504	0	0.2494267	0	0.1585244	0.4835609	0.3004231
0.165588	0.08186266	0.02151383	0.3106177	3.366099	0.2140861	0.3518089
0	0.08126846	0.1135043	0.2339734	0.1825292	0.1046195	0.02538842
1.062699	0	0.03004786	0.3293856	0.02945201	2.233636	4.730775	0.07211486
0	0.1851019	0.1295521	0	0.02787281	0.1952405	0.728503	0.04085092
TCGA-E7-A7DV	37	0	0.01147557	0	0	0.148606	0.1408862	54.85301
0.1999061	0.1501294	0.2722531	0	0.01133962	2.700247	0.06152121
0.9146555	0.01860407	1.735257	0	0.0107657	0	0.0273485	0.1130025
0.09433252	2.964033	52.5188	0.0792485	0.5921708	0.475491	0.04119585	0
2.822756	1.710879	0.05739809	0	0.1459404	0.04126549	0.01223486
0.4639776	0.1748463	0.2287109	0.213478	0.0623462	0.0178425	0.38532
0.05309089	0.1318935	0.03640349	0.1080502	0.1781125	0.2311415	0
2.710438	0	0.2947891	0.2130421	0.6729171	0.4427853	0	0.01125053
1.243027	0	0	0.05125152	0	0.01489991	0.09487364	0.0688049
0.07989702	0.5560032	0.02035317	0.03074898	0.02431432	0.1092105
0.1192114	0.950982	0.04339296	0.05355686	0.004790215	0.1696397
0.1008357	0.28548	0.2550526	0	0.5804154	0.1228765	0	0.03593014
0.1459404	0	0.125172	0	0	0.01996608	0	0.0140501	0.2472264	0

1.838685 0.1878055 1.161055 0.0107889 0.023549 0 0.02772122 0.6083125
0.4709043 0.02980286 0.03756966 0.2326133 0.02595273 0.5165828 0
0 0 1.670859 0 0.3070256 0.02031505 0 0.405121 0.1230424
1.393146 0.1423238 0.125172 0.02716587 0.0502051 0.9706319 0
0.8393995 0 1.438022 0.04842964 0.05790875 0.03769658 0 0.8875832
0 0.02432341 0 0 0 0.0600456 0.6468515 0.06444499 0
0.170988 0.2449602 0.03469586 0.09442375 0 0.07785818 0.4230733
1.521571 0.031293 0 0 0 1.360078 0.1907696 0.08409488 0.1299709
0.2023206 0.2113293 0.05701362 0.0357859 0 0.1831785 0 0 0
0.1730265 0.1680037 0.02921429 0.2946007 0.4134345 1.140381 0
0.04966132 0.07216124 0.2487421 0.1386061 0.05906482 0.04424241
0.328302 0.7867954 0 0 4.538549 0.02895438 0.03949602 1.030556
0.01522204 0.250344 0.7507296 0.2027708 0 0.08940857 0.9451118
0.04961085 0.02588667 0.1155282 0.04078285 0.06657218 0 0.245389
0.3759291 0.06521988 0 0.08398637 0.1465978 0.1768735 0.2964
0.09641292 1.10703 0.01809045 0.06801404 4.399165 0.06163772 0.02839853
0 0.02877941 0.06500277 0.04689441 0.03608062 0.03723652 0.1543867
0.02601496 0.4925649 0 0.01774521 0 0.06202932 0 0.03501584
0.1666112 0 0.008418189 0.07864497 0.166725 0 0.01898758 0.4339296
0.0999419 0.04099692 0.09287876 0.08332591 0 0 0.3394495 2.045
0 1.100645 0.2996327 1.112368 3.178877 0.02257437 1.262984 0 0.1013854
0.1340741 0 0.2401019 0.008029785 0.04795391
TCGA-XF-AAN1 941 1 0.2047086 0.4378232 0.08248842 0.2103912 0
120.3699 0 0.2314411 0.2490577 0.1565678 0.1573316 0.1450183
0.3048485 1.180501 0.04916612 0.06854614 0 0.1173611 0.1486312
0.16262 0.1493193 0 2.333522 85.87765 0.1047174 0.04890519 0
0.1633062 0.1439865 1.875938 0 0.2275342 0.04886814 0.9160037
0.08179114 0.07275107 0.3284417 0.2772462 0.05036904 0.03173464
0.08753202 0.3006034 0.4699887 0 13.95341 0.1443086 0.2141632
0.01412127 0.7330221 0.7114627 8.246499 0.3025607 0.8764395
0.1407549 0 1.147673 0 0.02229935 2.31445 0 0.2298312 0.7618813
0 0 0.3275645 0.2045643 0.3167231 0.1469384 0.01008536
0.3169226 0.1927709 0.2664159 1.181428 5.654742 0.1720159 0.0353845
0.02848364 0.284509 0.07494882 0.05144015 0.4381282 0.0864691
0.2465195 0 0.1323651 0.2136483 0 0.1174972 0.4961996 0 0
0.131914 0 0.01392415 0.4337884 0.1172835 0.0911101 0.2840809
1.464459 0.005346092 0 0 0 3.391084 0.9333658 0.1181428 0.07446574
0.3772278 0.2057606 1.003426 0.08984115 0.06691488 0.1147793
2.479743 0.07535742 0.1521367 0 0.07661039 0.6691488 0.1219394
0.04619299 0.658224 0 0.3230682 0.2165808 2.640594 0.01835684
1.283464 0 0.5571942 0.1439865 0.05738963 0.3735866 0.08959159
1.261687 0.4406144 0.1807902 0.1791832 0.5628791 0.1565678
0.8331026 1.923159 0 0.04969642 0 0.06936123 0 1.185312 0.1297906
0.05144015 0.3401712 0.1675479 0.06202495 0.08103762 0.05832364 0

0.6739427	1.29802	0.04167051	0.154567	0.0572877	1.466044	0.5051991
0.1519933	0.8551925	0.9076822	0.08023128	0.1486312	0.197871
1.480924	0	0.2316192	0.06869643	0.08404684	0.1159137	0.1381284
0.2624861	0.6436292	0.3095735	0.2197818	0.3512121	0.2572287
1.235421	0.07088566	0	0.1477235	0	0.2869482	0.3131357	0.3735559
0.1206847	0.572538	0.4440627	0.1255957	0.1693563	0	0.05429794
0.2949967	0.3181171	0.2289851	0	0.1139575	0	0.7295677
0.4257818	0	0.1858961	0.1831137	0.05811347	0.7011516	1.057475
0.1910973	0.3883561	0.01792828	0.03370217	0.3987123	0.06108518
0.05628791	0.08487625	0.03327499	0.2147335	0.1858961	0	0
0.06120109	1.082834	1.309926	0	0.07034454	0	0.1639287	0.1250115
0.1487226	0.07338561	0.1269802	0	0	1.784488	0.08301924	0.1129042
0.08600793	0.198092	0.02708627	0.2261706	1.226887	0.6896862	0.03938092
0.5466606	0.0579048	0.04660834	1.443558	0	1.259882	5.436326	0.5816721
0.03628006	0	0.5023828	0.02797309	0.04323455	1.34117	0.007957803
0.01584134
TCGA-DK-AA6P	457	0	0.4546871	0.2633768	0.06870698	0.578295	0.05814812
50.95019	0	0.05507828	0	0	0.4305797	0.1585368	0.355484	0.6321042
0.1740455	0.1175466	0	0.4887673	0.4126641	0.05643788	0.2176516
0	2.500212	40.27075	0.08722218	0.04073454	0.1744444	0.1360224	0
1.83665	0	0.2368997	0	0.401561	0.8856406	0.04039764	0.4377097
0.2020605	0.09089995	0.05286539	0.06861923	0.1325548	0.4404005	0
23.51658	0.3605964	0.1070296	0.01176201	0.6868746	0.2962989
3.454457	0.4536208	2.238703	0.1842324	0	0.5623115	0.9715888
0.04221312	5.596756	0	0.063811	0.2961433	0	0.03006498	0.1970496	0
0.2638078	0.3671676	0.01680077	0.3350439	0.1705997	0.3467273
2.115697	0.988518	0.07163848	0.01473639	0.01581656	0.08617299	0
0.2570759	0.3368581	0.1440452	0.2737776	0	0.147001	0.164772
0.1204683	0.1468002	0.4821821	0	0	0.2197499	0.266571	0.007731884
0.5754274	0.2442221	0	0.159106	1.132662	0.008905828	0.1166328	0
0.04576564	0.4393715	0.8206176	0.1476068	0.186074	0.9775231
0.2570759	1.074577	0.1496626	0.05573533	0.2868089	1.494733	0
0.5702356	0.006707723	0.255244	0.1114707	0.1015668	0.1453518
0.7048976	0	0.2690928	0.25353	1.084003	0.2140592	0.277157	0.06105552
0.5057534	0	0.04780148	0.114096	0.07462342	1.332121	0.07340008
0.1054098	0.09949789	0.1406515	0.1956148	0.941742	0.4838935
0.05319689	0	0.09409608	0.05777297	0.4582419	0.6235458	0.1621595
0.04284598	0.3953559	0.2093332	1.033247	0.4049914	0.04857944
0.1500806	0.4009616	1.993092	0.2776685	0.04291443	0.5725989
1.918888	0.4041845	0.1097196	0.8140736	1.411265	0.1336539	0.154749
0.09888747	1.168583	0.07924611	0.1929223	0.03814616	0.1575113
0.1448217	0	0.1503096	0.5360973	0.3056032	0.2745938	0.9263597
0.423637	0.8247851	0.4132989	0.3858446	0.2871008	0	0.5258163	0
0.8473748	0.02513043	0.6499744	0.3374276	0.8159757	0.2507765

0.09840451	0.04522631	0.2457113	0.222232 0.4026486	0.4039764
0.06244637	0	1.012797 0	0.05383653	0.3096764	0.1871844	0.3388307
0.1752028	1.125468 0.1591704	1.051287 0.1791958	0.08421452	1.17816	0
0.2344191	0	0.01979693	0.2951152	0.1548382	0.05956636	0	0
0.3328527	0.6201284	0	0.205072 0.04857944	0.1706762	0.1041257
0.1321337	0.183375 0	0	0.637381 4.404005 0.2420219	0.1097147	0
0.3449927	0.1353656	0.6308673	0.7664322	0	0.09840451	0.5744155
0.09646115	0.3105714	0.3309925	0	1.574088 3.472056 0.1801314
0.2115309	0	0.4184491	0.06989878	0.0360113	0.2522482	0
0.3298678
TCGA-CF-A47Y	373	0	0.204408 0.1502807	0	0.2599768	0	70.45966 0
0.2507036	0.4896124	0	0.1262415	0.2545407	0.2397157	1.18403
0.082846 0.05888325	0.09072951	0.2337118	0.1391372	0.1522324
0.1258032	0	3.161219 121.4313 0.04117195	0.1648127	0.1176342
0.1834497	0.161747 1.571256 0.04232631	0.03195002	0.1097919	0.2978658
0.1020888	0.1089664	0.5534315	0.2011409	0.1556004	0.01188302
0.05350251	0.1092501	0.06599512	0.02955246	12.86634 0.405272 0.1443479
0.03965775	2.573247 0.1998051	9.707913 0.1019643	0.5579098	0.1129405
0	1.023805 0.1965533	0.05009987	1.803179 0	0.2151503	0.427929
0.1097919	0.01474466	0.3134551	0.153198 0.5336855	0	0.02832342
0.3628609	0.2436174	0.2899254	0.6303984	8.78335	0.04830842
0.04968639	0.07999261	0.2324383	0	0.3178186	0.208226 0.1942698
0.2307727	0	0.04956405	0.07555599	0.08123614	0.09899267	0.232252 0
0	0.09632048	0	0.01564167	0.4376635	0	0.05117418	0.1595609
1.17507	0.006005523	0.2883824	0	0.1851686	4.825199 2.329988 0.1658943
0.1359328	1.283045 0.08667779	0.9201605	0.05046145	0.03758436
0.03223427	3.417876 0.2539578	0.5981586	0.01809304	0.1721203
0.6013497	0.4451863	0.07207057	0.3697073	0	0.06048634	0.3222023
1.048801 0.02062113	0.4672425	0.04117195	1.873757 0.02695783	0.6769196
0.1538782	0.2012851	1.29754	0.04949634	0.2707871	0.06709503
0.5374628	0.2638203	0.6684745	4.219486 0	0	0	0.1168752
0.1545046	0.9811189	0.2187001	0.0577852	0.2710462	0.3764293
0.1045134	0.2275837	0.7534542	0.06746987	0.8111489	0.6815165
0.04681049	0.23151	0.2574161	1.646878 0.3770984	0.2674948	0.3087896
0.9176788	0.09012766	0.06261173	0	0.8668198	0.2404734	0.2276654
0.01286167	0.1062156	0.113935 0	0.1842895	0.4016776	0.296239
0.1851686	0.2301445	0.1214935	1.26068	0.2388878	0.1626181
0.08297248	0	0.2256399	0	0.5848081	0.01694636	0.4097164
0.5994795	0.3103929	0.06341538	0	0.06099549	0.165692 0.2074972
0.4144261	0.1816106	0.119592 0.03189377	0.3927051	0.2989383
0.3267353	0.3654456	0.5999595	0.1305633	1.732802 0.9239316
0.1788906	0.708921 0.01006985	0.1892963	0.383908 0.1029299	0.2213082
0	0.05873906	0.5487766	0.02610326	0	0.2487276	0	0.6806043
0.3708345	0	0.05926606	0	0.2301863	0.3510787	0.08910253

0.0206094	0.1426430.01874357	0	1.3364010.06994463	0.03170769
0.04830842	0.1719521	0.09508534	0.2895197	1.1529350.2734439
0.04423848	0.3448352	0	0.2356083	0.7014864	0	2.5475157.822426
0.1926752	0.02037757	0	0.2821754	0.0392794	0	1.3122010.0536363
0.1156697
TCGA-CF-A3MI	370	0	0.01361513	0.1747172	0	0.1259377	0	54.062660
0.04947783	0.4969433	0	0	0.1356347	0.1459831.2113720.1103635
0.02172224	0.06446159	0	0.2075941	0.05678308	0.02234519	0.05596014
2.0798446.707840.06268262	0.0439111	0.1253652	0.09775315	0
1.4184180.04510805	0.1702491	0	0.2308669	0.1142382	0.1306438
0.7470849	0.1936157	0.1256263	0.006331993	0.03082096	0.05292283	0
0	11.090820.1295721	0	0.04226411	0.7130148	0.1064683	3.683429
0.1086656	0.2798007	0.1323995	0	0.8991397	0.03491184	0.04004438
0.8938077	0	0	0.9425097	0	0.005892638	0.05446562	0.04081659
0.2211845	0.3958001	0.03018488	0.0729639	0.1009666	0.119605
0.6364697	5.9757430.07722499	0	0.02273329	0.09289293	0	0.03079146
0	0	0.2459395	0	0.07923221	0.04736567	0	0.07033241	0	0
0.08180613	0.04737729	0	0.03333932	0.2019583	0.03510227	0
0.2697304	0.3130743	0	0	0	0.1973381	3.1575640.2172729
0.1767971	0	0.2508934	0.09237439	0.1593532	0	0	0.06870551
1.5722340	0.3187352	0	0.3210065	0.2002723	0.1459830.0460843
0.2814322	0	0	0.1681851	0.7564436	0.02197638	0.04268146	0
0.6071954	0	0.1717638	0.1565371	0	0.6382231	1.7934770.06853862
0.03575231	0.03369328	0.04685982	0.1424815	1.1751630	0	0
0.04151881	0.2058235	1.6804280	0	0.06629567	0.0501461	0.0371274
0	0	0.1078561	0.7491977	0.4324059	0	0.2775659	0	0.8148741
0.3819868	0.06065425	0	0.8331026	0	0.06672666	0	0.4852223
0.1993270	0.02741391	0	0.1214229	0.0413410.1374809	0.08561529
0.4186566	0.3617866	0	0.09098467	0.1749981	0.04243131	0.06932225
0	0.1313350.2061165	0.09371965	0.2236061	0.0180601	0.2995578
0.3264341	0.1052521	0.2365410	0.1300084	0.2648723	0.04299832
0.1269146	0	0.04487738	0	0.4731031	0	0.1160696	0.1669128
0.1395032	0.1739302	0.8394021	0.1406648	0.1652279	0.09298614
0.08585324	0.06052115	0.3352667	0	0.06738655	0.05080591	0.06829034
0.1863786	0.0556376	0	0.08835811	0.03663425	0.3858163	0.4372495
0	0.08421482	0	0.1717201	0.1496608	0.02967453	0.1537471	0	0
0.1442031	0.8703636	0.02484717	0.01126385	0	0.08623673	0.06485407
0.1259377	0.07061539	0.02428459	0.09429181	0.3523956	0.03466113
0.2231936	1.1019660.02734596	0.9426879	4.7604450.05356647	0.02171681
0.05728857	0	0.05023308	0	0.4531983	0	0
TCGA-G2-A2EL	819	1	0.2407329	0.3805566	0.1138750.3872591	0.2730617
58.916880	0.4792555	0.4584311	1.9452720.1034265	0.05839087
0.5050108	1.04765	0.3676490.1410138	0.09910989	0.05891514	0.1139916
0.0561241	0	0.1290583	2.30371736.570850.3373115	0	0	0.1127218

0 0.9389651 0 0.2879343 0.08994974 0.6655474 0.4600134
0.1897060 0.2711057 0.04249317 0.03894183 0.3009094 0.0488214
0.2433067 0 31.4578 0.3652322 0.0197101 0.01299624 0.3794755
0.4638033 10.98482 0.6404489 4.355718 0.0277588 0.1115918 0.5747181
0.1878697 0.02705277 0.4466254 0 0.2467742 0.303846 0 0.2400887
0.1646907 0.1568891 0.8501796 0.2704639 0.03712747 0.3870286
0.04989741 0.3141507 4.947236 0.8352476 0.1978894 0.02442409 0
0.4403714 0.1034666 0.1183549 0.4652573 0.5172713 0.1134398
0.03202094 0.1015165 0.1784209 0.1331095 0.2433067 0.4947236
0.05189408 0 0.07891296 0.03272703 0.008543219 0.7799751 0.1619095
0 0.2411636 3.417604 0.002460087 0.02147859 0 0.202272 5.097502
0.5249478 0.2174609 0.2484326 0.3471744 0.1893679 1.865815
0.1446963 0 0.05281746 2.26717 0.1733844 0.8400966 0.003705794
0.1762673 0.8313819 0.2805616 0.08266387 0.8221354 0.05708349
0.09910989 0.07542065 0.8939741 0.3547818 0.7437259 0.2023869
0.1610728 0.1546011 0.3697222 0.04011272 0.08245393 1.373778
1.013778 0.2495803 0 0.1554105 0.576377 0.1369161 0.6729469
0.05877904 0 0 0.0638353 1.075945 0.4880252 0.1194504 0.02367098
0.8263594 0.4625986 0.4852096 0.261035 0.5367706 0.02763819
0.5316432 4.962817 0 0.2133792 0.5535983 1.83112 0.07647211
0.3217335 1.068153 2.728869 0.1476787 0.6326534 0.01821068 0.8321112
0.06567127 0.3197495 0.1211783 0.2610593 0.06667433 0.2224667
0.2113773 0.4936266 0.6384097 0.12642 0.7003346 0.3873855
0.4513268 0.1630957 0.07993738 0.04531819 0 0.4753572 0.2521649
0.8704599 0.04165119 0.944561 1.355112 0 0.1818412 0.08154784
0.7121021 0.3393683 0.4486039 0.1678131 0.148789 0.1959574 0
0.1538725 0 0 0.3421719 0.5489836 1.149898 0.613027 1.432806
0.3712869 2.207045 0.1237496 0.03101715 0.00218421 0 0.3108211 0
0.06124802 0.2075072 0.3635577 0 0.1698136 0.1126505 0.1186387
1.163548 0 0.1132955 0 0.320596 0.05752599 0.05018714 0.6922753 0
0 0.2673594 3.315053 0.191013 0.04329553 0.03957788 0.2900389
0.03427646 0.2323555 1.433142 0.01866881 0.05436523 0.5263291
0.2931037 0.1286853 2.25779 0 0.6667173 7.062849 0.05147413
0.01669483 0.08808135 0.6935377 0.03218063 0.01989505 0.8560609 0
0.1385032
TCGA-XF-AAMZ 1348 1 0.1120485 0.1198226 0 0.440483 0.08597662
102.3224 0 0.1628752 0.1022425 0.1928214 0.05536056 0.4967243
0.3003493 0.7269239 0.0302753 0.04469194 0.06631252 0.1313966
0.4271097 0.1001375 0 0.2302678 2.513038 53.04547 0.2192404
0.06022926 0 0 0.7093071 1.134891 0.3712262 0.07005503 0.06018364
0.29687 0.1343067 0.1194623 0.08089857 0.1422679 0.1447415
0.01302762 0.1521885 0.08710789 0.2170557 0.06479804 6.922033
0.08886175 0 0.01739107 1.015599 0.1460338 5.784756 0.07452383
0.8635043 0.2971661 0 0.8522079 0.1436571 0.04493913 2.942311 0

0 0.6255307 0.06018364 0.04041225 0.1195297 0.1679543 0.5200812
0.09048109 0 0.1050825 0.05935181 0.1640525 1.527738 3.525042
0.0529616 0 0.03507907 0.1274136 0.1538389 0.1267024 1.037649
0.1064912 0.2024010 0 0.05433817 0.1851577 0 0 0.2036985 0 0
0.06498356 0.04379405 0.08574155 0.2869028 0.1444407 0.1122068
0.3558923 0.7300112 0.006583988 0.2299346 0 0.1353363 1.020872
0.1277209 0 0.05731775 0.4645754 0.0633512 0.3278575 0.05532201
0 0.2120349 1.808249 0 0.4684104 0 0 0.5768639 0.1501747
0.1769882 0.3474157 0 0.06631252 0.100925 3.832747 0.1356444
0.6439693 0.1805509 0.3958924 0.1182179 0.4240698 0.6134548 0
1.269328 0.2170557 0.1335915 0 0.06932147 0 1.465727 1.603651 0 0
0.2782571 0.08542194 0.08469339 0.6914716 0.07992193 0.0633512
0.3897101 0 0.4583215 0 0.1436571 0 0.474283 0.7019845
0.1539581 0.0634524 0.1411055 0.3868948 0.5812459 0.04991668
0.2256886 0.9688098 0.09880896 0.02288088 0.04873767 1.20949
0.2343434 0.1426255 0.0846032 0 0.1427536 0 0.3636732
0.9688098 0.4800998 0.06766814 0.2162679 0.0431987 0.6039481
0.08729934 0 0.1212861 0.4053184 0.07067829 0.1928214 0.8907415
0 0.4805194 0.3454017 0.4330972 0.09269825 0 0.2674828
0.1211012 0.07582794 0.2924516 0 0.07755883 0 0.3743751 0
0.0796016 0.2289406 0.2015958 0.2862789 0.604453 1.012927 0.1307479
0.5261086 0.1987164 0.08301191 1.350345 0.07522955 0.2079644 0
0.09952254 0.1851188 0.1144703 0.08807361 0 0.0753723 0.6032796
0.9082591 0.1085279 0.08663293 0 0.151415 0 0.06105318 0.09037816
0 0 0.5410181 0.9767508 0.1022425 0.02317456 0 0.02217822
0.0250186 0.2008084 0.3196293 0 0 0.3107265 0 0 0.1797791
0.05626232 0.3879023 1.821639 0.09184093 0.08936153 0.1178671 0
0.06890061 0 0.5594535 0.009800444 0.1950943
TCGA-DK-AA6L 1163 1 0.05643116 0.4023097 0.2728709 0.3305867
0.1154681 17.17165 0.06553595 0.1777297 0.7552238 0.1294812
0.1115253 0.3035722 1.058858 1.708475 0.2439616 0.1667279 0.04452943
0 0.04097255 0.1232792 0.06174336 0.3092537 3.22628 34.7904
0.02598032 0.3033335 0.6062075 0.2025807 0.2381529 3.837665
0.1246408 0.3292979 0.08082767 1.554936 0.323174 0.1403849 3.042149
0.1719614 0.08331014 0.1574668 0.08090509 0.07311713 0.5344343 0
7.701968 0.2386857 0.07084497 0.05839127 1.439743 0.1470945 2.472688
0.4003472 0.1449627 0.0498874 0 1.158488 0.9646702 0.04526553
0.6791769 0.1333657 0 0.8821035 0 0.04070576 0.2257459
0.6766967 0.3928939 0.06075884 0.1084274 0.1411277 0.1494572
0.2891769 1.514408 1.558819 0.07112835 0.1463145 0.1256315 0.06416952
0.06198249 0 0.5295606 0 0.135914 0.5179249 0.1459542 0.07852737
0.5980522 0.1457548 0.4103558 0 0.1130217 0.2181851 0.02940808
0.01535365 0.5646864 0 0.07534783 0.2794907 2.162686 0.06631808 0
0 0.181759 1.308728 2.015494 0.04885189 0.2309362 0.3639608 0.340327

0.5419303 0.1857460 0.1423833 3.29198 0.2492816 0.3145416
0.01331992 0.0633566 0.4427076 0.8571705 4.550222 0.3888211 0
0.1335883 0.5228127 1.825004 0.03036213 0.4717429 0.1818623
2.168115 0.1190765 0.1898443 0.5973133 0 1.01402 0.2915096 0.34388
0.04939469 0.1396499 0.3884436 0.8858235 0.8449252 0 0.08219763
0 0 0.3981064 0.9286583 0.2146731 0.2127044 0.3794559
0.2078426 0.05129448 0.1340358 0.6752691 0 0.1592426 1.232145
0.03446141 0.3408707 0.9475357 0.8660107 0.2418833 0.2178767
0.1515519 2.101823 0 0.07682353 0.06545553 0.5156719 0.1573636
0.6225327 0.06628041 0 0.5272317 0.05711591 0.09497045 0.4139954
0.3698017 0.363518 0.3388601 0.25624 0.631732 0.5862226 0.1436614
0.04072234 0.3628997 0.569533 0 1.853579 0.07485443 0.5155753
0.2963681 0.3739224 0.09337152 0.2442594 0.9878937 0.1219808
0.0424326 0.4909595 0.06684995 0.4067311 0 0.7039092 0.08803013
0 0.5380747 0.1101342 0.09611939 0.9857461 1.311793 0.263395
1.397087 0.1186131 0.05574322 0.1334638 0.2525865 0.2327498 0
0.05110548 0.6126651 0.1537356 0.0591422 0.3051846 0 0.7249292
1.112349 0.2186322 0.1745243 0 0.1016765 0.2067685 0.2213878
0.4855177 0 0.02759765 0 0.983845 0.03432835 0.04668576 1.209182
0.1787144 0.1400017 0.2566397 0.8975596 0 0.09770377 0.4312214
0.04788713 0.1541799 0.8987175 0 2.292222 6.068574 0.1295112
0.2100246 0.1582975 0.1661877 0.06940103 0.03575487 0.1610048
0.006581083 0.05240301
TCGA-XF-A9T4 495 1 0.08073654 0.04796565 0 0.1078711 0.04130029
53.71756 0 0.1043198 0.1473416 0.1234999 0.03545781 5.717697
0.1202315 0.3325618 0.03393424 0.05406884 0.02123621 0 0.2149392
0.0481027 0.4564065 0.07374197 1.161568 30.80804 0.06608047 0.1832368
0.04130029 0.09661132 0 0.3504625 0.5349739 0.04486945 0.09636735
0.9126809 0.02508972 0.07651423 0.2072585 0.1731299 0.03641994
0.1335047 0.06599871 0.01394791 0.06951088 0.394273 2.139414 0.1422874
0.1689308 0.01113878 0.144551 0.2338325 2.883413 0.07159751 0.5530648
0.1903315 0 0.2928841 0.9661132 0.02158726 0.2944558 0
0.1208598 0.1602581 0 0.00129418 0.05981047 0.05378643 0.312287
0.4346409 0.01193292 0.04807442 0.06177302 0.09850664 0.3727616
0.4956035 0.1187246 0.04884445 0 0.06120525 0.05911924 0.08115145
0.1196284 0.170516 0.2268623 0 0.1566134 0.1654038 0.1140851
0.04634058 0.09784993 0.177909 0 0.04162124 0.05609919 0.00183055
0.2978147 0.02312816 0.03593359 0.2066959 1.182662 0.217174 0 0
0.02167034 0.8024608 0.2454114 0.2096784 0.106463 0.2066356
0.1014393 0.2422951 0.1240159 0 0.06790297 0.566213 0 0.1800069
0.03493768 0 0.02639106 0.07213889 0.5080909 0.1112579 0
0.06370863 0.07156719 1.093516 0 0.6936745 0.02891021 0.4282424
0.1135758 0 0.1031391 0 0.5536742 0.1737772 0.08794061
0.07066939 0.06659943 0.1234999 0.3285736 0.537263 0 0 0.02227757

0.02735589 0.1898581 0.2460443 0 0.1014393 0.3744074 0.03304023
0.2201623 0.1917662 0.0230027 0.02368806 0.4176877 0.5720326
0.04930423 0.1625622 0.2937236 0.2478018 0.209736 0.06793853
0.3131939 0.4534509 0 0.05861977 0.01560796 0.2028884 0.05628536
0 0.08579671 0.03314786 0.2628673 0 0.04529168 0.1974356
0.4024613 0.04334068 0.04617238 0.03458535 0.3049942 1.006456 0
0.03884119 0.2163349 0.2489776 0.3704997 0.332275 0 0.2023904
0.5192664 0.04953462 0.1261659 0.0465952 0.2141497 0.1939099
0.04047245 0.163898 0.1594046 0.03075153 0.02239523 0.08392407
0.04198182 0 0.03665847 0.1181775 0.3896366 0.02765324 0.5097464
0.1674851 0.4748166 0.01414173 0.0265841 0.2452368 0.1927347
0.06659943 0 0.1612325 0.1778497 0.05498771 0.08461524 0.2619781
0.09655021 0.5694224 0.5013956 0 0.2358215 0 0.1777959 0
0.04301426 0.1013008 0.05008067 0.04606489 0 0.5734647 0.06548515
0.02968609 0.881954 0.1136392 0.1735948 0.1763277 0.297773 0.06400247
0.01553173 0.1359948 0.5709376 0.01838221 0.4190053 0.7747608
0.2732918 2.689562 0.05882308 0.3863366 0 0.0396277 0.02758129
0.0170516 0.1279727 0 0.03748671
TCGA-K4-A6MB 469 0 0.02363523 0.2148382 0.04285774 0.2404842
0.01813569 21.28478 0 0.1975498 0.1725341 0.2440395 0.2452299
1.165505 0.2534197 0.7666774 0.555599 0.1445505 0.02797559 0.3048812
0.5405606 0.09857281 0.174556 0.09714422 4.753487 23.15389 0.07072919
0.1143415 0.1632212 0.2121187 0.5236681 2.205818 0.1957638
0.08866338 0.05077993 0.4759196 0.2266425 0.08819673 0.5801943
0.2040655 0.1003175 0.08244036 0.1939511 0.06431011 2.106119
0.02733667 25.29736 0.412374 0.1335249 0.02201057 0.5712742 0.400452
3.177087 0.1257589 0.5160787 0.4492315 1.196956 0.7453569 0.4242374
0.05792934 1.241287 0 0.1194112 0.4486227 0.1269498 0.1125228
0.315166 0.2125672 0.6856538 0.2672019 0 0.2026591 0.1940526
0.2854897 0.9514262 3.663409 0.1117159 0.06434539 0.2515827
0.2418868 0.05192061 0.08017884 0.6653922 0.04492595 0.4055926
0.54231 0.2292391 0.09866955 0 0.1526173 0.3007735 0 0.03550292
0.1439284 0.01847561 0.1133396 0.2545953 0.1828078 0 0.4606052
1.050733 0.04999715 0.2182582 0 0.9135198 1.370346 1.91282 0.184147
0.1209046 0.3157661 0.187084 2.053444 0.1400338 0.06953269 0.0894522
2.449612 0.03915275 0.7904424 0.004184114 0.159215 2.294579 0.2534197
0.7093352 0.8305406 0.1289029 0.1398779 0.1459808 2.302915
0.06676246 0.3704652 0.5331893 0.6420878 0.02493657 1.461053
0.3623217 0.04654827 1.966568 1.007274 0.2129114 0.03103218 0.3509399
0.1220197 0.9275301 0.7181733 0.06636585 0.02582031 0.02934742
0.1441495 0.1429201 0.5186036 0 0.08017884 0.4891165 0.04352566
0.2578058 0.2105198 0.09090802 0 0.7503304 1.073176 0.1299022
0.3212277 0.5059954 0.9793273 0.6838336 0.6054346 0.1904247
0.7231139 0.3334802 0.05791721 0.0205612 0.7289338 0.07414769

0.09025518 0.05948661 0.03275058 0.2408967 0.07176607 0.1789954
0.4830286 0.3395558 0.3996649 0.7603166 0.5285089 2.729199
0.368294 0.03008506 0.2046703 0.1709937 0.6261654 0.2440395
0.4542414 0 0.4054382 0.6921526 1.174583 0.1075444 0.09207351
0.08463322 0.6641644 0.1172963 0.3304768 0.1679938 0.08413746 0
1.689952 0 0 0.1931686 0.5203797 0.573676 0.5100072 0.4273284
0.1544459 0.332928 0.2887597 0.3502064 2.984022 0 0.1462249 0
0.07162328 0.2956528 0.2414608 0 0.0383464 0.03179768 0.2612061
0.9962466 0.2289259 0.2558378 0 0.1916346 0 0.09787586
0.2478338 0.06597393 0.01733821 0.3018684 2.575417 0.0647003
0.01955353 0.4021771 0.149703 0.08091952 0.7023232 0.4167892
0.1264708 0.02046078 0.8477005 0.1203402 0.04843173 0.6025409
0.04747133 0.8509606 3.632928 0.1666051 0.09424847 0 0.469833
0.05813488 0 1.314965 0 0.1563802
TCGA-UY-A8OB 2109 0 0.01965146 0.02101491 0 0.1272409 0
33.58054 0 0.02856562 0.2510429 0 0 0.6264609 0.1053526
0.3278325 0.0424783 0.1149606 0.09304095 0.01843584 0.04280455 0
0 0.3230813 1.087986 24.99554 0.09952059 0.4436551 0.4523663 0 0
0.5402546 0.06510693 0 0 0.4581815 0.05496206 0.209517 0.3972716
0.0798446 0.06527639 0.1827863 0.05783125 0 0.1522719 0
0.7622854 0 0.07401266 0 0.3166564 0.6659098 3.641549 0.05228098
0.4543331 0.01737267 0 0.189563 0 0.0140117 1.032065 0 0.06618946
0.2194155 0 0.002835056 0.06289057 0 0.1368204 0 0.01742699
0.01053128 0.0416373 0.1007023 0.0510362 0.361893 0.03715435 0
0.0164061 0.04469249 0.02158464 0.08888601 0.2038248 0.07470714
0.1419912 0 0.03812006 0.05469237 0.2499172 0 0.1429014 0 0
0.03419112 0 0 0.1388083 0 0 0.2539022 0.3615018 0.01385667
0.04032672 0 0 0.6510693 0.4032024 0.0510362 0.0241262
0.1629577 0.04444301 0.2476957 0.03881026 0 0 1.888727 0
0.3286057 0.006957744 0.06618946 0.05781279 0.0526763 0.7538479
0.1624826 0 0.04652048 0.065745 1.531802 0 0.5133708 0.06333128
0.820852 0.1658676 0.2479161 0.2151797 0.0774049 0.6294746 0
0.0676859 0 0.0972627 0.06763535 0.05141285 0.7615488 0.3310784
0 0 0 0.2970764 0.1616969 0 0 0.3075691 1.230436 0
0.140029 0.1007803 0.05189155 0.4159069 0.2535468 0 0 0.346466
0.5428396 0.1493219 0.03501824 0 0.104562 0 0.03210342 0.06838224
0.3771104 0.0821999 0.0500283 0.118704 0.4538398 0.02503663 0
0.1133907 0.1235732 0.1882153 0.1424145 0 0.07071247 0.5459083
0.3674606 0 0 0.09478151 0.09916642 0 0.5150154 0 0.1722145
0.06730861 0 0.0812887 0.255181 0.4925766 0 0.008865977 0.1465462
0 0.07772877 0 0.1575821 0.1839324 0 0.1606096 0.03116172
0.3012515 0.1211555 0.2030293 0.4402754 0.503295 0.01548959
0.02911783 0.03280738 0.05277607 0 0 0.1560647 0.3061153
0.0401524 0.06178662 0.06376605 0.05287621 0.03341219 0.794951 0 0

0.1007803	0.07081516	0	0.0342647	0.095105 0.1097077	0.08649487	0
0.2855099	0	0.04877322	4.086979 0.0622351	0.02340186	0.1226966
0.1426923	1.191743 0	0.08719405	0.6003396	0.04026845	1.996918 0
0.3265519	0.9128154	0.05798656	0.1567253	0	0	0.01208403	0
0.2055824	0	0.04105957
TCGA-ZF-A9R5	1090	0	0.9688755	0.3676476	0.05667294	0.9251035	0
94.31704 0	0.3520926	0.7129704	0.4302742	1.111817 0.2023806
2.178212 1.535207 0.1942301	0.1218904	0.1479741	0	0.03403859
0.1582794	0.4360012	0	4.382386 147.583	0.07913972	0.1343995
0.2877808	0.05609904	0	3.233423 0.05177365	0.3517321	0	2.28547
0.2185318	0.3498809	0.2707836	0.563502 0.1326548	0.02180301
0.1149699	0.3523106	0.4439897	0.03614865	43.82432 0.9418866
0.3531334	0.04850946	1.510849 0.6517389	13.35246 0.748338 2.609317
0.2210386	0	1.75093	0	0.01114224	2.667302 0.0553978	0.4210759
0.8026138	0	0.01127232	0.4501006	1.264897 0.4714707	0.05047636
0.04850339	0.8123336	0.3642142	0.4690366	2.394484 6.762849 0.3840914
0.03646591	0	0.6930278	0.2403003	0.070683 0.3241669	0.2970392
0.5927918	0	0.2728209	0.2065865	0.09936826	0.2421762	1.875003
0.3099178	0.1408419	0.3987735	0.02443122	0.01275529	1.181084 0
0.06259639	0.3466043	2.539315 0	0	0	0.1132493	2.27804	1.745654
0.08116894	0.2366198	1.022286 0.212049 1.603898 0.1234491	0	0.1182871
1.849396 0.1553209	1.045242 0.01106573	0.210538 0.8734923	0.7121079
0.07757808	2.51955	0.2556822	0.1479741	0.5791131	1.723487 0.1135072
0.8817926	0	0.9030517	0	0	0.08555645	0.1846593	1.599364
0.5448964	0.4388765	0.04103541	0.5027375	0.2151371	3.025415 3.37204
0	0	0	0	0.7559615	3.043129 0.2675145	0.1767075	0.3369795
0.4028931	0.5965917	0.5567618	0.4808489	0.08252931	0.9260528
1.647879 0.1145174	0.7079591	1.220129 2.877808 0.616546 0.09746393
2.895794 2.078717 0.7165885	0.3318762	0.05437821	1.71361	0	0.5171788
0.08652834	0.1732309	0.219003 0.1423498	0.4057621	0.9826661
0.2993411	0.339748 0.4825943	0.3052552	1.140352 0.09740273	0.1989149
0.2706458	0	1.498304 0.05378427	1.010211 0	1.643359 0.5405969
0.08628941	0.4912757	0.08116894	0.03730492	0.1688957	1.177402
0.6292887	0.4442932	0.1957338	0	0.8771754	0.07313241	0
0.7663097	0.1200881	0.119779 1.541504 2.09886	0.6710465	0.947199
0.2833017	0.4399407	0.7174403	0.04196803	0.3093769	0	0.009797695
0.3688269	0.2554366	0	0.7099032	0	1.523329 0.615263 0	0.0724944
0.08014149	0.4786595	0.1717761	0.08855474	0.1008379	0.1744813
0.04585436	0.009735993	6.447941 0.313707 0.2844228	0.118182 0.4454095
0.1814415	0.3685959	1.296803 0.1951118	0.02705631	0.676041 0
0.3202185	0.7689109	0	2.640052 5.859827 0.4918556	0.04985179	0
0.9664414	0.08648399	0.1188157	2.704869 0	0.3047423
TCGA-CU-A3KJ	562	0	0.1445304	0.2181998	0.1233303	0.4167928
0.01304712	31.72708 0	0.4201827	0.3568563	0.4096542	0.05040642

0.06775628 0.04557859 0.8037024 0.05972636 0.04370678 0.06037831
0.3270109 0.07407396 0.1063724 0.0418595 0.1747179 1.008631
23.30095 0.2074492 0.4752751 0.0782827 0.03052035 0 0.2460314 0
0.04252394 0.01826596 1.333497 0.09511271 0.2538008 0.2946363
0.4490601 0.1160996 0.3400381 0.08468129 0.1454067 0 0.1573316
0.6351448 0.1887892 0.06404003 0.01055651 0.6849737 0.6648274
2.851654 0.2940375 1.7035 0.1052229 4.35087 0.2775738 0.1308015
0.0181856 0.5581267 0.06027768 0.08590644 0.2468063 0.01826596
0.008585691 0.07935749 0.07646218 0.1381158 0.08238408 0.143249
0.1776893 0.03152363 0.2427289 1.037748 0.443602 0.2250367 0.006613021
0.02839102 0.09667632 0 0.2499553 0.1637638 0.4848071 0.3685769
0.3901468 0.4947553 0.04732301 0.1621821 0.2195908 0.7109675
0.08430445 0 0.07396035 0.4784998 0.008674296 0.5224566 0.06575747
0 0.1025224 2.710867 0.04396184 0 0.05241538 0.02053754 1.57736
0.3294922 0.1545582 0.1148146 0.4856681 0.115364 2.319256 0.2854375
0 0.2145113 1.091523 0.2535043 0.08529861 0.003010121 0.02863548
0.1250574 0.2506822 0.3472394 0.5272101 0 0.04025221 0.1531556
0.7261522 0.2470116 1.545809 0.2465905 1.999911 0.2332172 0.08580453
0.02327324 0.0669752 0.7439229 0.1976317 0.1396568 0.02232507
0.3366293 0.2048271 1.112134 1.14565 0.1193617 0 0.08445209 0
0.3598668 0.3264555 0.1940529 0.05768199 1.020152 0.3444439
0.5332257 1.090451 0.4142047 0.08979915 0.3598668 0.5252543
0.01557563 0.2118385 0.4496737 0.2739895 0.2882198 0.03787476
0.8219684 0.4523653 0.0299889 0.1944441 0.01479207 0.2097628
0.1955913 0.2597243 0.05991392 0.102099 0.3899365 0.05162971
0.1226402 0.5613443 0.2957099 0.2464505 0.2187938 0.1158136
0.331351 0.3444439 0.08657477 2.116621 0.08201045 0.7293385 0.02926101
0.7040814 0.02255479 0.5421425 0.3843794 0.09389047 0.309477
0.3532758 0.3145805 0.6615843 0.3106899 0.3518708 0 0.0751019
0 0.159074 0 0.1932751 0.2084531 1.916441 0.7168157 0.3144923
1.734768 0.1031744 1.05241 0.0938174 0.02519443 0.1011281 0.2054921
0.126236 0 0.06396453 0.722369 0.1737109 0.1603841 0.05517408
0.1143789 1.768338 1.604079 0.03293862 0.2366408 0.02180025 0.2604118
0.2336344 0.1185912 0.2057259 0.04746274 0 0.2171692 1.43283
0.4189183 0.1828728 0.2571848 0.2490535 0.02277972 0.5426171
0.6966989 0.1971346 0.07359912 0.4086623 0.3246554 0.1045278
0.6487012 0.01707583 1.295028 2.233039 0.05574815 0.09492548 0.1073193
0.1502248 0.0575069 0.01616024 0.04851323 0.220111 0.07697543
TCGA-DK-AA6S 5050 0 0.2568286 0.1569416 0 0.2714997 0.3096794
29.28355 0.2556567 0.1599984 0.7365348 0.2525541 0.1450206
2.313654 0.2458702 0.2040241 0.1288757 0.243903 0.34742 0.4216475
0.8790899 0.02185972 0.1204309 0.0754002 5.111413 38.82259 0.0168916
0.3944362 0.3378321 0 1.393557 1.778445 0.5470038 0.1376353
0.1182412 0.7387867 0.2565391 0.0391174 0.1059595 0.121121

0.1624970.1109117 0.07890299 0.1426155 0.7107396 0.04243568
11.227530.3491687 0.06909182 0.0569463 1.6258170.4303633 4.115106
0.19522 0.5183764 0.3567877 0 0.5581035 0.2351995 0.1308011
1.2043090 0 0.1638619 0.1182412 0.1587938 0.2397292 0
0.8514916 0.4740423 0.01626834 0.3834126 0.2429312 0.1745844
1.4769331.0698020.1387364 0.2140406 0.05360362 0.2503262 0.181346
0.04148815 0.1359095 0.2092204 0.6296157 0.1403078 0.07117119
0.1531682 0.4666021 0.04738264 0 0 0 0.03191788 0.05736068
0.06363825 0.44057 0.5675579 0.07348325 0.6123046 1.4342340.3535673
0.03764554 0 0.7090445 1.5802332.0910830.1905719 0.1201181
0.4225645 0.04148815 0.8588442 0.1086897 0.05396902 0.0462866
2.5920960.0607782 0.2454063 0.01948545 0.1235775 0.05396902
0.2458702 1.6309910.4550391 0.1000503 0 0.1416320.9279516
0.07402695 0.5367472 0.05912061 1.4576540.1548397 0.6942990.1506548
0.07225853 1.57655 0.1421479 0.2673241 0 0.2723882 0.2525541
1.2958541.43799 0.1030221 0.2004089 0.1822282 0 0.1109299
0.5031541 0.05234018 0.3319052 0.2105546 0 0.2501257 0.2614379
0.1411197 0.04844147 0.7765095 0.5371027 0 0.1662177 0.5082482
0.5912061 0.2090910.8335951 0.2956031 1.8057850 0.1798138
0.03191788 1.0812590.07673472 0.0934042 0.1108118 0.01694662
0.5843007 0.0557025 0.2513979 0.1153573 0.5548468 0.1772611
0.2360533 0.3489156 1.2245920.9147453 0.0934042 0.1588584
0.3539193 0.2777196 0.9470778 0.5897492 0.02433402 0.4480884
0.5906349 2.02594 0.2124755 0.09528597 0.8539644 0.3965407
0.07448859 0.47197 0.2607826 0.07739825 0 0.5639025 0.08585171
0.1563912 0.07496562 0.8212298 0.2343520.5089514 0.5212090.1027508
0.8613599 0.05783895 0.08154567 1.6538110.09853435 0.1361941
0.06845545 0.2607058 0.5455471 0.1499312 0.05767865 0.05952647
0.2468033 0.4158764 0.5636543 0.07107396 0.1134703 0 0.1983207
0.1008258 0.04797984 0.05918787 0 0 0.2171566 1.4392480.1004366
0.1062377 0.4855773 0.1307189 0.1201527 0.3011950.5899087 0
0.06352398 0.5494286 0.7005315 0 0.5167293 0.07369141 0.6604881
9.7297250.1744229 0.6437430 0.2431128 0.07896422 0.03487007
1.3085040 0.06388278
TCGA-E7-A85H 394 0 0.2169491 0.1637656 0.09256319 0.3069137
0.2741834 89.3833 0 0.2597083 0.1397383 0 0.0504421 0.4983612
0.5131221.8687480.1172385 0.09501642 0.06042105 0.5626972 0.138987
0.3801702 0.2094456 0.1049049 4.7295 91.337730.08813038 0.02743913
0.1175072 0.09162584 0.1615724 1.4033710.04228062 0.3510708
0.02741834 0.02704948 0.3875187 0.08163656 0.3685561 0.2138866
0.08478133 0.01780528 0.07366709 0.08928999 0.2636955 0.02952056
18.022770.08096691 0.02403201 0.01584598 0.3598657 0.2328543
9.5350880.1358057 0.2950452 0.1692279 0 0.9942914 5.137592
0.04208396 3.3092410 0 0.2849780.02741834 0.01656984 0.1191205

0.1147744 0.08885157 0.1648848 0.01131714 0.1367808 0.08111813
0.4951438 0.4640027 2.350143 0.07238442 0.04963276 0.005327084
0.1160937 0.01401712 0 0.9076416 0.04851502 0.04610472 0
1.287274 0.07547443 0.08114845 0.2307336 0.5104029 0.06327309 0
0.02960508 0.1197096 0.02343718 0.4507124 0.131608 0 0.1346559
0.4890839 0.03899376 0 0 0.09248435 0.549648 1.629231 0.4971457
0.08356065 0.4820924 0.08658423 1.034063 0.1764244 0.03754378
0.06439895 2.013729 0.1268418 0.5975129 0.02259189 0.1289509
0.9010508 0.06841627 0.3225276 0.4748249 0.1392008 0.03021052
0.1609272 2.306989 0.0308983 0.2533721 0 0.4729436 0.1346436
0.2897953 0.2934503 0.05026696 1.734833 0.3461003 0.1487722 0
0.03158133 0.08784516 2.236972 1.360018 0 0.02788306 0.1267678
0.1167491 0.3086755 0.8050491 0.1456427 0.02886141 0.1242803
0.2350143 0.8352048 0.0454676 0.2290646 0 0.9183097 0.509794
1.005339 0.02890751 0.06428456 1.351332 0.5930092 0.3126879 0.445548
0.5092712 0.1800607 0.04169609 0.02220381 0.7959137 0.1067617
0.2274196 0.09635838 0.1886239 0.3089182 0 0.2669313 0.0802488
0.4181467 0.1233125 0.4926344 0.4887293 3.047765 0.1988583 0
0.05525528 0.1846541 0.4507926 0.1756903 0.4905302 0 0.6115317
0.3890225 0.2677772 0.1900408 0.3314305 0.2894158 0.3034408
0.1669701 0.366395 0.09070729 0.1447015 0.03185934 1.381512 0.05972312
0 0 0.2257148 0.2608447 0.3540542 0.9229343 0.5956585
0.8715763 0.1106488 0.1323644 0.8948184 0.1370917 0.284232 0
0.06934395 0.4156562 0.07822523 0.0401244 0.04140985 0 0.5424492
0.4354572 0.04944291 0.09867014 0 0.2759253 0.07013994 0.1112579
0.2264586 0 0 0.2623776 0.6674792 0.1164485 0.02111564
0.530819 0.2829095 0.01519723 0.1652612 0.3309456 0.02276239
0.02209536 0.6181459 0.1299541 0.1569026 0.6188258 0.02563188
1.590478 4.343496 0.2133876 0.2035557 0.1073953 0.05637416 0.0706266
0.02425751 1.104457 0 0.2844181
TCGA-G2-AA3C 211 1 0 0.01967913 0.06673791 0.1872405 0.5648165
20.83606 0 0.08024969 0.63809 0.1266724 0.1272904 4.740792 0.3452961
1.296199 0.02983368 1.3212 0.04356348 1.337959 0.2405025 0.01096409
0.06040399 0.0756363 1.37646 27.74744 0.04236124 0.3363206 0.7625023
0.1321242 0 1.251472 6.096851 0 0 0.1950263 0.1470528 0
0.2657283 0.05607706 0.09508676 0.1283758 0.02499475 0 0.190124
0.08513711 0 0.05837701 0.06930816 0.3084729 0.8154538 0.09593575
2.262982 0 0.2363634 0.1138788 0 1.133357 3.20873 0.0196816
0.8456558 0 0 0.08218747 0 0.0053097 0.1079706 0.1103362
0.1708316 0.2377633 0.06527711 0.04930938 0.07798139 0.06735809 0
1.299078 0 0.02862811 0 0.02092584 0.04042529 0 0.190869 0
0.6648286 0.02814943 0 0.1088339 0.1170158 0.04753101 0.06690903
0 0 0.06403564 0.02877015 0.01126544 0.5134414 0 0.3685667
0.1030308 1.833669 0.06487947 0 0 0.08890809 3.2923 0.755147 0

0.2711119	0.8477753	0	0.5136065	0	0.05413801	0	1.6498810
1.16933	0.006515486	0	0.2706901	0.09865604	0.07059308	0	0	0
0.02841509	0.5493584	0.01485175	0.7114940	0.8958253	0.07766227
0.09286307	0.05037553	0	0.5175773	0.1425930.03412961	0.09664638	0
0	0.3370141	1.1507520.1033446	0	0.04569969	0	0.1669159
0.2523648	0	0.08323612	0.04480296	0.2033339	0.1003636	0
0.1415616	0	0.3894705	0.4246353	0	0.04168454	0.6952360.6777798
0.01075619	0.07378282	0.04942145	0.1468735	0	0.07515704	0.09605346
0.6306071	0.07697499	0	0.2871602	0.1529972	0.6330208	0
0.2389122	0.05785925	0.2226337	0.1778162	0.09471696	0.1655444
1.3810260.9749602	0	0	3.8165460.09286307	1.0767160.5080008	0
0.2744986	0.1071514	0.8129135	0	0.1433765	0.4393017	0.1193347
0.01660485	0.1646775	0	0.07278807	0	0.9099881	0.1722411
0.05229363	0.07520035	0.08529823	0.04701716	0.5105450.2376550.13743
2.3565190.01450502	0.1090680	0	0.04554018	0	0.09230192	0.05211955
0	0.1735778	0.2388515	0.1485457	0.05214733	0.6733887	0.2138895
0.05691281	0	0.03315695	0.3034247	0.01604336	0.1187464	0	0	0
0.2138895	0	0	0.4870978	0.08741884	0.02739295	0.05532107
0.1718001	0	0.03186144	0.1633034	0.1873933	0	0.4789792
0.3326497	0.4586928	5.6260970.02413366	0.7338167	0.1548636	0
0.3281618	0	0.7175556	0.06438323	0.01281656
TCGA-E7-A8O8	13	0	0.2152335	0.09864291	0.1115094	0.1279848	0
118.52520	0.0893904	0.9539290	0.0151917	0.0275679	0.4945199
1.5103330.09138755	0.06813378	0	0	0.2344095	0.05495819	0.1513895
0.06318865	3.842373263.15	0.1557149	0	0.07077949	0.1103802	0
3.0253180	0.03844812	0	0.2606886	0.07985379	0.04917312	0.8879871
0.06246442	0.1021347	0	0.03132196	0.0358554	0.1588349	0	28.3379
0.1950790.02895097	0.009544695	0.8670488	0.1202218.2076270.08180144
0.1974645	0.02718215	0	1.6084830	0.006851063	2.1699160	0
1.7165420	0.00443587	0.1025018	0.09217794	0.5351903	0.2482925
0.01363357	0.1071055	0.0325739	0.1800730.1996345	6.1814090.02906678
0	0	0.1223742	0.0168862	0.03476887	0.09111843	0.05844526
0.1388540	0.05964455	0.08022602	0.09775822	0	0.05589765	0	0
0.01783238	0.02403537	0.006274308	0.1683194	0	0	0.1820812
1.2490860.003613473	0	0	0	1.5789781.3668860.03992689	0.02516604
0.6799246	0.1390755	0.2491438	0.03036223	0.04522839	0.03879018
2.2935850	0.3084917	0	0	0.7236542	0.3708899	0.05203712
0.06355709	0	0	0.1424325	0.9051497	0.01240756	0.2570386	0
0.4731801	0	0.07758037	0.1094213	0.06055579	0.8167524	0.1786892
0.1425641	0	0.1902276	0.1058256	0.3217724	1.2998810	0	0	0
0.3718564	3.2468210.04386335	0	0.09624761	0	0.04192324	0
0.1182645	0	0.3253744	0.3967119	0.3943168	0	0.1936064
0.9201334	0.2336365	0.1095826	0.04128804	0.3272058	0	0.01255765
0.02674857	1.4540410.3858422	0.03913839	0	0.02840402	0.1762810

0.08870838 0.09667443 0.2247431 0.1114144 0.2769521 0.1580575
1.5871990 0.5479375 0.03328257 0.07414994 0.3103215 0.3174769
0.1826523 0 0.3841172 0.4001948 0.03395652 0.03815651 0.1597076
0.05505072 0.1661592 0.1734019 0.2694199 0 0.06283613 0
0.6164020 0 0.06282445 0.09001325 0.03927943 1.5639190.3970871
0.1435160.4199896 0.02423578 0.09111843 0.5133206 0.08257607
0.1521821 0 0.01606491 0.1669114 0.03141222 0 0 0.08273276
0.1394090.6408997 0 0 0 0.02770023 0.2534893 0.04691072 0 0
0 0.2298779 0.6700845 0.02805674 0 0.2325342 0.1217202
0.01830786 0.08532322 0.4624743 0.2193719 0 0.3694929 0
0.09450903 0.3288924 0.06175661 0.4257829 2.9863130.09576915
0.1716542 0 0 0.01890727 0 0.6871924 0.02689376 0.06424386
TCGA-XF-A9SM 1048 0 0.01661198 0.05329364 0 0.1997555 0.07647983
33.081020.1736299 0.2294003 0.3637959 0 0.04924555 0.3938652
0.2226445 0.8005889 0.01795411 0.1884699 0.1573008 0.07792189
0.1447360 0.08179093 0.1365553 2.90177323.224930.1147197 0.9643764
1.6825560.1192698 0 0.5288034 0.2751844 0.0830892 0 1.056313
0.1128341 0.1239778 0.1439254 0.08436887 0.06744239 0.3322069
0.1090546 0.06457179 0.04290672 0.4995518 0.07159814 0.105395
0.03128259 0.08250714 0.4684390.6062151 3.0078710.1325842 0.4267353
0.2202848 0 0.3574662 0.04259636 0.05033921 2.7808920 0.167856
0.2596701 0.03569059 0.05032776 0.3544222 0.09960164 0.1927642
0.4292626 0.03682894 0.05341448 0.02639797 0.1945754 0.1294274
0.6118386 0.2826695 0.1550573 0.006934291 0.09444983 0.05473847
0.07513808 0.4922840.1894567 0.1800442 0.4573939 0.1288962
0.06934972 0 0.08581343 0.1207989 0 0.04990633 0.06743988
0.1558266 0.01016943 0.3344159 0.08565741 0 0.3469873 0.9422315
0.2225559 0 0.05120823 0.1203873 0.4402951 0.6816787 0.08628494
0.1291659 0.5816244 0.03756904 0.7478028 0.1640375 0.09774186 0
3.3242610.05503689 0.1111122 0.02352638 0 0.3909674 0.1781156
0.9633732 0.2747031 0.09059918 0.03932519 0.04275098 1.859667
0.02681365 1.8921010.1070718 1.5599510.1752663 0.1257426 0.09094899
0 1.37571 0.2574403 0.1232366 0 0.2466575 0.3430455 0.304226
1.0680570.09329025 0.03629551 0.2475214 0.05065761 0.05022556
0.3644996 0 0 0.6355498 0 0.1811984 0.0591854 0.1703854
0.04386553 1.5469470.4492705 0.03043383 0.2634034 0.5020772
0.9942378 0.1407910.1554105 0.3122926 0.4861420.3515789 0.04070701
0.2023196 0.2732426 0.1737152 0.2960332 0.1421542 0.07672895
1.2275250.05044066 0.1677421 0.5223013 0.3182083 0.2006456
0.1282529 0.08112379 0.8058559 0.9318773 0.08458092 0.1078891
2.0030430.08382843 0.3430455 1.43378 0 0.3623967 0.3698372
0.2568388 0.0824590.1725699 0.9914052 0.2154493 0.05995746
0.08671602 0.05903706 0.1292228 0 0.3774254 0 0.2360299
0.06788411 0.5471022 0.1697714 0.5632906 0.9439478 0.2326115

1.687621 0.03928147 0.0492284 0.1317321 0 0.04110958 0 0.1388698
0.5410617 0.1018262 0.05223013 0.0539034 0.04469789 0.2447844
0.6283937 0.06436008 0.1027515 0.08519272 0.05986223 0 0.028965
0.1339920 0 0.02069929 1.2550210 0.06871583 0.6281543 0.1183708
0.1285850.322682 0.4135617 0.05925984 0.02876165 0.2026965 0.2960332
0 1.131294 0.06673028 2.346377 4.321133 0.0653571 0.4769451 0.06989848
0.146765 0.07150499 0 0.1737852 0 0.1041268
TCGA-DK-A3IN 250 1 0.1263864 0.1081242 0.04583527 0.151977 0.0969783
19.47964 0 0.06430094 0.1845209 0.08699801 0 0.727742 0.2371477
0.4685383 0.1024481 0.1277073 0.02991918 0.2074944 0.7708211
0.06777072 0.08297032 0.1558399 11.64118 18.33125 0.06400568 0.2989196
0.2909349 0.09074223 0.5600497 0.1828734 0.3768559 0.2528619
0.1900775 0.5893498 0.1009952 0.02694976 0.1460007 0.1925672
0.09795768 0.09404573 0.1630796 0.1375561 0 0 1.906552 0.04009304
0.04760051 0.01569316 0.254568 0.2306087 1.779297 0.16812 0.9739995
0.3240188 0 0.2719655 0.03240794 0.06195396 0.7467329 0.04480398
0 0.1128919 0.02715392 0.06564017 0.04718869 0.2273352 0.3226473
0.9389444 0.039228 0.06095779 0.04016793 0.09252241 0.5579982 2.05594
0.09558181 0.01966165 0.03165427 0.1293459 0.05552778 0.08574924
0.2809027 0.04804716 0.7533917 0.1353302 0.3922646 0.04396857
0.08036588 0.09793219 0.3216696 0.0626629 0 0.1099484 0.0197592
0.0154741 0.4820752 0.03258471 0 0.4027935 0.6974889 0.3000299
0.02593573 0.07791996 0.1831849 1.465551 0.2593156 0.09847028
0.04137741 0.2561891 0.02858308 0.3299848 0.0748813 0 0.03188895
1.069675 0.04187287 0.3381432 0.03132363 0 0.1487269 0.1016347
1.018143 0.3657467 0.06892919 0.02991918 0.1853958 1.110929 0
1.082955 0.04073089 0.6390645 0.0800071 0.4145564 0.2906204
0.04978219 0.6813167 0.1958644 0.04353152 0.2323169 0.1876606 0
0.2645253 0.4007312 0.3193947 0 0.03138632 0.03854105 0.1910617
0.2079875 0.03605954 0 0.6417837 0 0.1378584 0 0.2592635 0
0.6419674 0.7149821 0 0.05725748 0.1591615 0.05818698 0.2327
0.3828678 0.169712 0.5379839 0.04458107 0.1548524 0.02198968
0.3118305 0.02643302 0.03217521 0.1717724 0 1.111042 0.038376
0.0729261 0.3576371 0.3695173 0.2747774 0.1626279 0.2663713
0.3458552 0.3938811 0.06435041 0.2736121 0.6705357 0.1275558
0.5219881 0.7551976 0.05029445 0.2662906 0.1991288 0.4885169
0.1463841 0.229764 0.2111972 0.327834 0.05131858 0.09896224 0.04491627
0.07665208 0.0315521 0.08445613 0.1182943 0.107745 0 0.4763674
0.4843673 0.1947999 0.522305 0.1061845 1.467391 0.09961973 0.05618053
0.4641942 0.2375968 0 0 0.2139497 0.1551136 0.05164723 0
0.0410105 0.1700341 0.3867969 0.6771333 0.09793219 0.1563498 0
0.0683161 0.2083906 0.1046757 0.1019431 0 0 0.2283507
0.5875931 0.02306511 0.02091201 0.9080272 0.2601683 0.1091174
0.3740971 0.2622032 0 0 0.1588876 1.222658 0.0517965 0.67144

0.4569244 1.225109 2.049402 0.04972464 0.3427076 0.05317979 0.223322
0.09326066 0 0.156258 0.004421809 0.14964
TCGA-XF-A8HC 200 1 0.1338044 0.143088 0 0.3005768 0.02933436
21.95707 0 0.1250357 0.4186093 0.1315774 0.05666541 0.0571272
0.5636191 1.558978 0.2272519 0.01355417 0.04525034 0.01793249
0.04163587 0.04555454 0.1254859 0.1571302 4.302236 24.97746 0.1848065
0 0 0.5489611 0 3.180683 0 0.3824332 0 0.6887656 0.152747
0.305695 0.7176464 0.1359131 1.389817 0.1066774 0.05408875 0.04458051
1.530516 0.04421689 16.36733 0.2425499 0.1079879 0.01186732 1.386049
0.7972045 4.239202 0.4068286 0.3928254 0.05069505 0 1.730405 0
0.01703643 9.034984 0.06776238 0.4506762 0.8110143 0 0.008272953
1.473262 0.4011304 0.8428709 0 0 0.1536562 0.1316266 0.3218451
0.5957133 1.642724 0.1806997 0.04460497 0.007979085 0.3043058 0
0.08645918 0.339874 0.3633372 0.172643 0 0.1483171 0.05319912
0.4861875 0.1481145 0.1389998 0 0 0.07760109 0 0.08971275
0.4050553 0.04928173 0 1.39538 1.289317 0.004492782 0 0.05892381
0.09235078 7.916166 1.917392 0 0.2894324 3.046886 0.08645918 0.6023323
0.07550126 0 0.09645891 5.991333 0.2533173 1.470316 0.006767779
0.1287646 1.068453 0.8198095 0.05175994 1.501441 0 0 0.1574153
1.711892 0.1234147 0.3395609 0.2464087 0.7444107 0.04033475
0.04822945 0.177909 0.07529154 2.210211 0.2221717 0.4102207 0.05019436
0.09460717 0.1315774 0.350064 1.447842 0 0 0.04746927 0 0.75131
2.673796 0.1090743 0.04322959 1.096963 0.3520124 0.1563747 0.0681029
0 0.1009495 0.2427309 1.323234 1.575869 0.2164932 0.4332941
1.320046 0.2178675 0.06812429 0.5646865 0.5085357 0.06742526
0.2342018 0.2328033 1.388654 0 0.09732479 0.02886576 0.1765795 0
0 0.1516554 0.4206977 0.655221 0.3232277 0.3935383 0.2505624
1.57716 0 0 0 0.09219372 0.6269829 0.1315774 0.4274808
0.05071086 0.4348198 1.414171 0.1055489 0.1423247 0.09928554
0.8898091 0.1652741 0.3277086 0.5773077 0.4755255 0.1789355 0
4.240737 0.08945529 0 0.3905613 0.05829022 0.195351 1.414171 1.925488
0.4282547 0.538509 0.1958668 0.05664567 0.3350723 0 0.1419107 0
0.03595351 0.1714361 0.1171684 0.180299 0 0.05143255 0.4983325
1.133308 0 0 0 0.1377634 0.105058 0.1333167 0.06167225 0.5335621
0.01402222 0 2.83269 0 0.09488315 0 0.3026795 0.08536097
0.2165921 0.9914028 0.1704714 0 0.4452704 0 0.07833801
0.6610964 0.03839228 5.135118 5.940812 0.2130794 0.09146778 0
0.5066346 0.0587705 0 0.5817294 0.3343814 0.03993854
TCGA-ZF-AA4W 1830 0 0 0.01924988 0 0.08325291 0.1381241
79.23895 0.06271583 0.2878306 0.1642557 0 0.01778769 0.1362882
0.04825206 0.8341615 0.03891058 0.04467493 0.04261326 0.2955299
0.07841887 0.0643496 0 0.2219595 0.5938964 76.0277 0.04143724
0.5031531 1.325992 0.1292422 0 0.8595268 0.1192773 0 0
0.3815447 0.05034584 0.03838397 0.9357559 0.04571157 0.01328753

0.4102138 0.07131122 0.02798831 0.04649425 0.1249201 3.827508
0.05710367 0.06779639 0 0.4350911 0.1876863 9.0758470 0.5548987
0.01591355 0 0.45414 0.6923691 0.01604358 0.8272102 0.1276267
0.1212605 0.3617765 0.03867476 0.02337248 0.1344197 0 0.08355267
0.05814429 0.03192663 0.1543483 0.09535055 0.03953331 0.2337486 0
0.06806758 0.1820238 0.05259859 0.0614082 0.03954352 0.08142055
0.2400502 0.06843255 0 0 0.03491839 0.09393526 1.1446340
0.0654496 0 0 0.01043981 0.0281426 0.0183662 0.1652949 0 0
0.3837523 0.2483547 0.02115477 0.03693971 0 0.0434844 0.1789159
0.4514128 0.04674971 0.05156635 0.4643987 0.04071027 0.1458591 0
0 0.09083752 0.7746690.1192773 0.1204026 0.01274674 0.1212605
0.1059143 0.1930083 0.06499132 0.2232537 0 0 0.06485568
0.9553344 0.0290556 0.7147848 0 0.8593249 0.2658890.04541876
0.1478302 0 0.4640971 0.06974137 0.0524624 0 0 0 0.2354736
0.7927125 0.1516357 0.03933027 0.1341086 0 0.05442504 0.5924644
0 0 0.3756489 0.4640971 0.2454360.1282681 0.2769477 0.04753323
0.3047802 0.2367180 0.1223259 0.2266904 0.5801214 0.4050807
0.06415417 0.04834345 0.04788995 0.06349588 0.02940708 0.1252778
0.6168522 0.1505920.1833059 0.2627742 0.04988666 0 0 0.01298339
0.1131944 0.3992588 0.0869688 0 0.03701337 0.2239065 0.1121993
0 0.4286699 0.08682089 0.09083752 0.3097736 0.3459571 0 0.12083
0.1603041 0.09939775 0.02978453 0.1402491 3.4162710.03891058
0.03248532 0.1006784 0 0.09493386 0 0.09623126 0 0 0
0.1866369 0.1379748 0.3329392 0.0929885 0.08402022 0.6454331
0.1135090.0533445 0.04507787 0.04834345 0 0 0.8389317 0.1869366
0 0 0.05841039 0.09687037 0.02040395 0.6225693 0 0.1391785 0
0 0 0.07062037 0 0.2009869 0.05282016 0.5943951 0.2092241
0.0657023 0.01489226 1.4294190.09976404 0.05894998 0.1040661
0.09336263 0.03210735 0 0.1464296 0.6873970 1.7778530 0.8973753
3.1165550.05311632 0.3732615 0 0 0.0442764 0 0.2225550.03148944
0.03761101
TCGA-LC-A66R 466 0 0.1017773 0.1360484 0 0.2118202 0 35.03448
0.08864876 0.3328758 0.4179156 0 0.1257144 0.8668944 0.3410212
0.6131249 0.1237501 0.08570082 0.2409352 0 0.1662675 0.06063873
0.2923152 0.1045798 4.39285328.945520.1640002 0.5744359 0.585715
0.09134188 0 0.2577146 2.9504710 0.1640002 1.0246950.05083128
0.0542557 0.07348278 0.2326077 0.06573667 0.1301674 0.08927852
0.1780265 0.1314391 0.05885815 1.4256550.2421479 0.1916603
0.03159373 0.5125006 1.0611784.1431630.2030772 0.9150736 0.3149131
0 0.2265623 0 0.06576501 1.5033350.09020011 0.08570082 0.3409138
0 0.05873239 0.1492874 0.3051166 0.4133557 0.9040558 0
0.06817846 0.1078223 0.1490141 0.5286453 0.2342860.7216009
0.01979158 0.03186345 0.1446674 0.2515263 0.05754393 0.4147131
0.3869174 0.1838474 0 0.1974284 0.1947402 0.4853817 0 0

0.1261154 0.1528815 0.02951333 0.1591182 0.02076848 0.4942472 0 0
0.8657019 0.6630927 0.09568717 0 0 0.1843955 0.3371967
0.5220585 0.1321613 0.1041271 0.7267585 0.2301757 0.4810672 0
0 0 2.481986 0.08429917 0.08509444 0.0180175 0.08570082 0.2994194
0 0.4191348 0.2103793 0 0.0602338 0.2946646 1.054972 0.04107005
1.80799 0.0820001 1.73408 0.3758338 0.5135949 0.2507493 0
1.312002 0.1971587 0.2494323 0.06681489 0.3148346 0 0.06656835
1.053268 0 0 0.1895624 0 0.5385081 0.4885112 0.07259566
0.05754393 0.6194758 0 0.5550776 0.09065338 0.3914652 0
0.6462097 0.6376351 0 0 0.06408533 0.468572 0.2082113 0.1926989
0.4100005 0.4738467 0.08975135 0.1247006 0 1.011427 0.0532154
0.06477566 0.1793115 0 0.3241693 0 0.1835201 0.4000005
0.4873948 0.2458606 0.2619241 0.03923876 1.814552 1.268749 0.06477566
0 0.3681637 0.3209968 0.1751458 0.5601387 0 0.3842845
0.2353046 0.3652964 0.0631506 0 0.3948153 0.1100001 0.05739746
0.256156 0 0.1341889 0 0.5440911 0 0 0 0.4034758 0.8451181
0.5490441 2.694502 0.09500999 1.325035 0.06016683 0.1508048 0.2230113
0 0.06296692 0 0.4892209 0.2282027 0 0.1600002 0.1651261 0
0.7642855 0.7307152 0.1971587 0.07869148 0.1304884 0 0
0.03327394 0.08209339 0 0.09332655 0.2536383 1.47869 0.04643506 0
1.731842 0.08058078 0.0681756 0.2059363 0.4750847 0 0.04405378
0.2352024 1.165962 0 2.29526 0.2044195 0.7046883 2.320813 0.06673765
0.3652648 0 0.1123989 0.06258464 0 0.2661841 0 0.1594894
TCGA-E7-A8O7 466 0 0.4254793 0.1516666 0.0428623 0.5247486 0
25.78505 0 0.1632107 0.1294144 0.2440654 0.5021908 0.1813201
1.045467 0.7667589 0.2107669 0.03352251 0.05595712 0.09424601
0.05148743 0.05633331 0.3685463 0 2.913823 39.50934 0.04353028
0.1524715 0 0.2121412 0 2.000838 0 0.0295576 0.02539266
0.8266849 0.1416666 0.05040348 0.2047962 0.2941256 0.09160394
0.03847626 0.04146982 0.09188135 0.2136869 0 15.36681 0.1499701
0.2670782 0.007337635 0.9046137 0.6469528 3.623466 0.09432922
2.094895 0.1044835 0.1260087 0.859444 0 0.01896071 1.086242 0.0418979
0.1592319 0.1847466 0 0.01705073 0.1512958 0.283453 0.438865
0.152703 0.02620256 0.1646781 0.1752919 0.1903467 1.565416 7.617799
0.06703663 0.06435223 0 0.2284729 0 0.1603747 0.2451706
0.2246536 0.2775402 0 0.1604844 0.2014717 0.07515318 0.1221068
0.1718888 0.1171969 0.1065201 0.1439437 0.05543272 0.03858779
1.193803 0.06094239 0.04734226 0.2774105 2.264751 0.01111166 0 0
0.05710105 1.448806 1.077759 0.1534721 0.1837945 0.5880406 0.213833
0.8831811 0.1867316 0 0.1192823 1.254606 0.07831382 0.5928948
0.008369118 0.1194239 0.2433903 0.5068933 0.08000871 0.7329078 0
0.1398928 0.304159 1.215284 0.09538508 1.407918 0 0.8240384 0.02493922
0.2385645 0.4594209 0 0.7940979 0.8242209 0.1784888 0.09310644
0.2632329 0.04067757 0.711182 1.665506 0 0.02582305 0 0.0720824

0.2501367 1.6532250.06744128 0 0.4850578 0.04353028 0.2578332
0.1263253 0.4242825 0 0.4002187 1.1993870.1948739 0.1338591
0.2083732 0.8161928 0.4801160.1000391.3648561.4778240.04168945
0.1737701 0.04112677 1.0044150.07415557 0.2106178 0.07734081
0.04367207 0.3011529 0.0717737 0.2045879 0.52024 0.2859721
0.2284042 0.4562384 0.1518865 0.3724257 0.4419998 0.1504413
0.1791055 0.1710118 0.8349759 0.1627103 0.4129906 0.01567742
0.5509257 0.6436488 0.16968 0.3520010.06138886 0.3949970.255475
0.3412619 0.3128846 0.4620319 0.0934960 0.2843213 0.221243
0.1343420.1931891 0.2292220.1509834 0.5829273 1.0684350.3530567
0.8979922 0 0.07004873 1.2307310.03174083 0.2632329 0 0.03705046
0.2566316 0.09659457 0.11148 0.3068038 0.03180106 0.9846508
0.4233585 0.04579005 0.2010368 0.09091768 0.1916549 0.1948739
0.09788626 0.1906616 0.1979428 0.008670025 0 2.4726630.08627623
0.1760005 0 0.2245783 0.2146355 0.3088364 0.3923139 0.4005333
0.06138886 0.6423980.06017651 0.04843687 0.5562507 0.04747637
1.1456466.9272520.2131222 0 0 0.2088369 0.07267632 0.02246536
1.3038650.07029501 0.1070084
TCGA-LT-A8JT 641 0 0.2997982 0.3624159 0.04727164 0.3375929
0.04000695 85.222180 0.3221062 0.5233341 0.3588973 0.180324
0.1869877 0.7686779 0.7731539 0.1479220.0231069 0.06171356 0
0.05678406 0.1553211 0.04278521 0 2.8866216.518760.04800834 0 0
0.04679294 0 1.4899730 0.4237773 0.05600973 1.5471750.1979042
0.2223544 0.2635081 0.4303040.07697329 0.006062056 0.05606338
0.5573336 0.1683352 0 13.445740.2480968 0.1472766 0.01618495
0.8401459 1.8007547.1965130.1733885 0.8036178 0.1152320 0.8414648
0 0.0360138 1.1124150.04620803 0.2634187 0.4657187 0.02800486
0.0244462 0.2398611 0.4298421 0.3630090.08420597 0.005779615
0.3073550.2071335 0.4675672.1326788.6014940.1971542 0.2129168
0.01632312 0.2371543 0.04295091 0.1473940.4055877 0.1982114
0.4238188 0 0.1769938 0.1042968 0 0.1346682 0.6634999 0
0.1174780.1436323 0.0611352 0.01861886 0.8562583 0.03360584 0
0.2049012 1.61853 0.00612737 0 0 0.1574379 1.9433280.6240312
0.06770407 0.1280222 0.8046622 0.2358304 0.8801529 0.3603969 0
0.1973296 1.4210660 0.1307774 0 0.1317093 0.3067753 0.2445793
0.04706101 1.56272 0 0.1234271 0.2381684 1.9023650.1157173
0.8853417 0.08401459 0.4093734 0.05500955 0.2302179 0.3853642
0.1540268 1.2831320.3535040.2486531 0.03422817 0.2903122 0.269173
1.46638 1.3087490.07320083 0 0.0323699 0.03974884 0.3152786
1.4300360.1115687 0 0.5938927 0.1440250.4976249 0.04644023
0.1336941 0 0.3862163 2.1183730.04776023 0.2362072 0.1969791
0.8401459 0.8761621 0.3658317 0.7701338 1.4911410.04597813
0.2768223 0.04535757 0.6878720.05452274 0.0663670.05249048 0.0602059
0.04981997 0 0.1692257 1.5163610.3942384 0.2519006 0.8386211

0.1641612 0.5836803 0.04062244 0.0331835 0.08465592 0.3772084
0.9208717 0.08972432 0.7606447 0.01729019 0.6780823 0.5000289
0.1295552 0.3019428 0.06770407 0.1711408 0.1690538 0.3763683
0.2673091 0.1389715 0.1202998 0.06508173 0.5051961 0.1220014 0
0.2130629 0.1192465 0.5328493 0.7634369 1.077346 0.1946882
0.4339827 0.1130157 0.193137 0.524983 0 0.1935415 0 0.01634477
0.1661274 0.1065315 0 0 0 0.7608995 0.539362 0 0.2821864
0.06684705 0.1878852 0.1432807 0.1306836 0.08411017 0.2183056 0
0.5603434 3.522415 0.04757583 0.3342938 0 0.1548008 0.03492523
0.4310338 1.054638 0.09299728 0.02256802 0.4530435 0.0331835
0.1335492 0.3439169 0.05236037 1.624501 3.874979 0.2649616 0.02079101
0 0.5758009 0.01603054 0 0.8429633 0.009120755 0.2541895
TCGA-FJ-A3Z9 385 1 0.2018786 0.3728927 0.0665575 0.2206861 0
53.45185 0 0.2134207 1.172251 0.25266 0.7435424 0.2157391 1.131478
0.7824204 0.07934141 0.01952043 0.1737829 0.008608661 0 0.03280334
0.03012035 0 4.201163 64.09597 0.08449345 0 0.2534804 0.131767 0
2.310292 0.0608037 0.2294884 0 1.517093 0.2126502 0.07826762
0.318012 0.2889482 0.08805616 0.03414101 0.08724515 0.1284078
0.04740252 0.04245348 26.47222 0.3493152 0.06912081 0.02278808
0.7393177 1.195955 8.070236 0.2929529 0.9900428 0.08112214 0
1.675013 0.1411789 0.0228998 2.289147 0 0.1854441 0.3688439 0 0
0.1517291 0.3851329 0.5111093 0 0.04882548 0.3442326 0.1847051
0.3761856 1.048585 4.281002 0.06939729 0.02855072 0 0.06260782 0 0
0.2175464 0.2790776 0.4309704 0 0.1780026 0.102155 0 0.2370126
0.6672816 0.09099295 0.2756778 0.1383688 0 0.01497999 1.121332 0
0 0.1146083 0.5908148 0.004313606 0.1506453 0 0.04433387
6.019566 0.5020704 0.04766297 0.03004212 0.4565611 0.2490333
0.7931119 0.2174706 0.05399167 0.2778361 1.698075 0.1216074
0.4296412 0.006497873 0.06181469 0.4319333 0.9346988 0.05383701
2.124407 0 0.08689143 0.3022749 1.734932 0.3851016 0.55615 0.1774362
0.1383335 0 0.04630602 0.5827765 0.2168665 0.7455907 0.7821416
0.09724956 0 0.3633366 0.25266 0.3361031 1.858856 0 0 0
0.1119311 0.1109765 2.516826 0.05236214 0.08301111 0.4723509
0.2027843 0.3002767 0.1307738 0.2823579 0.09692359 0.2330506
1.689396 0.2689817 0.2078593 0.1848951 2.027843 0.7240803 0.06540744
0.6900299 0.6347313 0.4531539 0.08994464 0.0957938 1.144602
0.03838346 0.4204952 0.02771457 0.01695374 0 0.1114517 0.3044515
0.8655427 0.3700531 0.1773355 0.5195351 0.1933992 1.468605 0 0
0.3973127 0.08851695 0.6482843 0 0.4681495 0.02434423 0.7528305
0.3080133 0 0.2884806 0.04766297 0.1095285 0.1190121 0.3394793
0.2326625 0.130446 0.06775189 0.09163374 0.7603614 0.1717755
0.05215227 0.2999883 0.0805904 0 2.376103 1.279868 0.2398524
0.7520477 0.1735897 0.05438659 0.3829872 0.1971514 0.3179195 0
0.007671034 0.3551875 0.4124839 0.2308114 0 0.09876274 0.5304649

1.606664 0.07110378 0.1702769 0 0.09920197 0.5043408 0.1519999
0.08881906 0 0 0 2.133113 0.2009574 0.1518319 0.6245756
0.1598346 0.1584496 0.2843455 0.7234172 0.4910186 0 0.4546563
0.09344339 0.4888899 0.8441272 0.03686117 1.626499 2.882943 0.1444105
0.02927332 0 0 0 0.06976939 0.9948806 0.1027347 0.03834575
TCGA-DK-A1AC 3981 0 0.2027029 0.1083834 0.08168024 0.2916619 0
76.18314 0.03923461 0.2291736 0.1438602 0.1550339 0.1112787
0.8997279 0.3924203 1.544664 0.352962 0.09183028 0.1599515 0.9508196
0.7849333 0.03354724 0.01848205 0.1388564 4.305766 46.88517 0.2021984
0.02421302 0.05184574 0 0 0.3258875 0.1119286 0.253468 0.09677871
0.5489913 0.4724404 0.1200638 0.2927014 0.4175142 0.1288452
0.03142372 0.169525 0.1138105 0.174519 0 4.950716 0.2143421 0
0.01398291 0.2268251 0.4990152 3.595775 0.2097175 1.128208 0.1592867
0.06003191 0.1671218 0.6063982 0.01505514 0.2587486 0.03992122
0.0758598 0.1257361 0 0.0601113 0.1111217 0.10128 0.20908
0.1091241 0.01997309 0.259503 0.04772054 0.3462449 0.4386947
2.592287 0.2129132 0.06131619 0 0.268916 0.03710725 0.1018723
0.7508693 0.1284329 0.2441043 1.033559 0.305826 0.07443603 0.07160756
0.05817299 0.2866139 0.05583387 0 0.03265539 0.08802914 0.1057059
0.4374928 0.02903361 0 0.09942414 1.018531 0.00794057 0 0.1041423
0.08161067 0.3357859 0.2567281 0.05849263 0.1751234 0.269773
0.4584255 1.003733 0.1779219 0.09938892 0.1420684 0.4765507
0.03730955 0.5272614 0.01196141 0.1896495 0.6957225 0.09055853
0.2719014 0.4655536 0 0.05331715 0.2811149 1.213979 0.06361947
0.5883746 0.2177521 0.8558968 0.1425758 0.1420684 0.4377462
2.306559 0.4399032 0.174519 0.1581334 0.05914254 0.2508139 0.1162754
0.3535458 0.2578762 0.06324153 0.09841903 0.05593165 0.1717042
0.1702397 0.06177364 0.2891678 0.2037446 0.3955892 0 0.3070863
0 0.2310089 0.1784188 1.763684 0.4694148 0.06189336 0.2040701
0.7658067 2.125675 0.2040491 0.2207394 1.239977 0.2097175 0.5958391
0.1563734 0.01959324 0.285565 0.04710468 0.2293499 0.03401169
0.05201462 0.6025844 0.1709688 0.1462018 0.1770342 0.1589469
0.4624604 0.1159237 0.1823481 1.349384 0.2105735 0.05733748
0.3413112 0.4888312 0.3693778 0.1162754 0.806688 0.01493778
0.3695534 0.5939969 0.2736034 0.2701786 0.1754779 0.3629202
0.4868441 0.1930647 0.5248648 0.04002127 0.1321428 0 0.255857
0.05270128 0.1600049 0.1840748 0.5989041 0.9782496 0.1388564
0.8725949 0.1366625 0.3172547 0.1597736 0 1.34422 0.06048669
0.3344186 0.08404467 0.007060495 0.7760982 0.1380561 0 0
0.1212029 1.487073 1.277966 0.04362975 0.2437937 0.08662832 0.2637742
0.4332535 0.3436195 0.05449996 0 0 0.5332184 2.257839 0.1233088
0.09316503 0.04258263 0.1159075 0.05028917 0.294266 0.4906192
0.1205169 0.09748771 0.5871073 0.1433437 0.2538343 0.5099316
0.04523651 0.4678268 2.891257 0.08491912 0.1436984 0.09476847

0.09949213 0.02769902 0.06421644 0.128519 0 0.109803
TCGA-C4-A0F1 89 0 0.1435333 0.09767662 0 0.1569052 0.02002461
71.98994 0 0.2181256 0.1428783 0.1796383 0.07736339 0.1793859
0.1748842 0.2418659 0.3243621 0.04394951 0 0.2264643 0.2273763
0.05441983 0 0.05363114 0.9347203 120.5659 0.1441772 0.08416714
0.2402954 0.04684239 0 0.2265642 0.04323071 0.06526541 0.1401723
0.1382866 0.1042702 0.04173551 0 0.1855576 0.05297506 0.1153008
0.1092914 0.01014405 0 1.297909 0.8623366 0 0.09828814 0.05670718
0.1051292 0.1360496 6.899797 0 1.474856 0.02307075 0.2782367
0.2130091 1.773319 0.01395556 0.08566085 0 0 0 0 0 0.03827914
0.0782357 0.06056544 0.7586546 0 0.1328617 0.02073524 0.05731361
0.169439 0.08009846 0.02467032 0.01014961 0.005446789 0.02967561
0.07166051 0.2360797 0.1160047 0.04960521 0.1414222 0.5987943
0.1012462 0.1134859 0 0.03370263 0.1897717 0 0 0.01513517
0.1019997 0.005325297 0.07373447 0 0 0.1657801 1.08016 0.03680307
0.05355353 0.08044671 0.03152086 1.21046 0.1189887 0.169439 0.06407876
0.1683161 0.1180398 0.6931186 0.05153967 0 0.06584606 0.533463
0.08646142 0.08727709 0.03233938 0.04394951 0 0.03497683 1.301434
0.05394386 0 0 0.02686424 1.70961 0 0.3545109 0 0.4835711
0.02753384 0.03292303 0.02143175 0 0.5403005 0.1011079 0.02765731
0 0.032291 0 0.341379 0.4826803 0 0 0 0.03979085 0.03945148
0.07157734 0 0 0.1588413 0 0.0355822 0 0 0 0.05523207
0.388509 0 0.08867129 0.09859366 0.1802215 0.09533565 0.06975587
0.1051292 0.104143 0.04602672 0.1065826 0 0.07154275 0.02729018
0.09965571 0.1510696 0.09643124 0.1496179 0.03962044 0 0.2461562
0.4472757 0.1891251 0.06716059 0.02683015 0.3895314 0.3659883 0
0.2259877 0.1888035 0.03292303 0.1796383 0.5334699 0.01730846
0.2457301 0.2324016 0.1296921 0.0647704 0.169439 0.0778735
0.1692324 0.04709575 0.04378772 0.04637279 0.07741734 0.03257525
0.03487793 0.06106516 0 0 0.6095958 0.1666914 0 0.4044315
0.1218087 1.303324 0.02057003 0.1546729 0.0435679 0 0 0
0.08453722 0.1847811 0.02666101 0 0.04234037 0.1053287 0.1922747
0.5560493 0 0 0 0.02351047 0 0.09100642 0.1262986 0.07284545
0 0.227626 0.7962246 0.02381305 0.04318026 0.2960439 0.04132383
0.05050087 0.2413926 0.06767646 0.3956561 0 0.1688647 3.853354
0.02673807 0.8700058 0.1310393 0.6143489 1.961577 0.02566855
0.08325194 0 0.1152819 0 0 0.07445772 0.01369559 0.06361454
TCGA-G2-A2ES 1004 1 0 0.1195131 0.04053054 0.1447255 0.06860364
76.11435 0 0.3898909 0.2651438 0 0.01104351 0.3874462 0.05991471
0.5800371 0.114749 0.05943534 0.2910215 0.09436121 0.4138349
0.0599273 0.01834195 0.2756077 2.48003 72.02272 0.0771791 0.3604422
0.2058109 0.04012011 0.07074751 0.5336383 0.2591871 0.05589926 0
0.3790121 0.09377184 0.05957685 0.1936551 0.1248724 0.02887353
0.1663228 0.1277611 0.0868829 0 0 2.954336 0.2836231 0.1894117

0.0346923 0.7653594 0.2330506 6.511479 0.05946507 0.6316009
0.1580792 0 0.1741477 0 0.04083879 0.9904651 0.1584744 0
0.2246094 0 0.03224629 0.04768837 0.0335041 0.1037476 0.3609896
0.03468796 0.07785969 0.05327866 0.2372616 0.4643939 0.3430182
0.02112992 0.09562362 0.009330257 0.08895918 0.09820258 0.05055005
0.1159165 0.04248644 0.06056347 0 0.2601495 0.07775977 0.07106476
0.02886601 0.2438068 0 0 0.006481571 0.05241712 0.01596375
0.1263059 0.02881353 0 0.2623191 0.65103 0.1628612 0 0.06890192
0.02699735 0.1481069 0.2293038 0.02902462 0.03201503 0.2986198
0.1011001 0.5835883 0.08828658 0 0.02819829 1.234449 0.07405347
0.2616323 0.007913829 0 0.2959066 0.08987207 0.353062 0.5544294 0
0.05291299 0.1092927 0.6394582 0.04509801 0.280279 0.1080507
0.7441102 0.18866 0.08459488 0.07954314 0.04402067 1.047765 0.1298971
0.09771405 0 0.1106279 0 0.2923882 0.6201173 0 0 0.08326151
0 0.1013696 0.1226108 0.0637724 0.1011001 0.6607959 0
0.3352343 0.3185415 0.02865722 0.02951106 1.040728 0.2662037 0
0.0253154 0.08444463 0.2058109 0.3200844 0.2688539 0.3901832
0.5054531 0 0.06390097 0.01944471 0.1991462 0.116869 0.08535426
0.09563596 0.02064813 0.2847698 0 0.08866829 0.2459692 0.4337906
0.08099204 0.2300899 0.04595959 0.08340759 0.2786363 0.08535426
0.169362 0.1078057 0.08459488 0.346182 0.4959648 0.01482455 0.6855764
0.2526404 0.2221604 0.1017047 0.4063447 0.200094 0 0.05042139
0.2416912 0.1191537 0.04420486 0 0.1194906 0.05230179 0.0317584
0.09133972 0.2917292 0.4283092 0.1378038 0.4329902 0.2399547
0.5915361 0.02642708 0.1159165 0.06763441 0.1200564 0.0829709 0
0.1588248 0.1582634 0.02283493 0.03513845 0.1450567 0.09021314
0.2343547 0.8248124 0 0.06912734 0.4012011 0.1409556 0.06142419
0.04384475 0.01802894 0.0623915 0 0 0.4546397 0.06118703
0.01849176 1.605874 0.141574 0.04658076 0.07236275 0.1391143 0.05980167
0.03869949 0.2768653 0.02845142 0 0.3387057 0.0673404 0.5571366
1.43467 0.06595467 0.3030444 0.04702505 0.04936898 0.01374453
0.04248644 0.1275448 0.007820104 0.1245379
TCGA-XF-AAN4 823 1 0.106555 0.09115833 0 0.3351094 13.40886 25.36886
1.113722 0.4027133 0.2333512 1.320247 0.2948194 12.36019 0.3998731
0.671533 0.1842621 3.252725 0.4540415 0.009996356 1.067645 0.03809116 0
0 1.7096 32.61586 0 0.5269421 2.256613 0 44.51904 0.8942353
2.259363 0.05329627 0.4578634 0.7227261 5.125903 0.2272104 2.831107
0.1515279 0.7629468 0.1982223 0.2050298 0.08283713 0.1100874
0.5915625 0.2755531 0.33802 0.04013145 0.1587687 0.2575482
0.0555496 4.500315 0 1.040146 0.9231485 0 0.7906589 0 2.820565
1.259124 0.2266424 0 0.1903558 3.983412 2.785472 0.2159717 0.2555517
0.6429571 2.753438 0.03779735 0.3426189 4.560504 0.202812 0.9408841
1.569817 0.2417519 1.790264 3.691751 0.1211667 0.7256302 0.04819615 0
0.1620321 0.3849552 0 0.08267848 0.4374191 0.1355111 0.05504369

0.2324537 0.2113216 0.06402327 0.1977520.06663503 3.9877410.6397544
3.7911092.30483810.619090.6207143 0.03506264 0.0874645 0 21.10696
1.1296820.4858358 6.6415350.3401271 0.2552611 0.0963923 0.7482796
0.04208772 1.37929 0 1.7119510.2118153 0.4276271 0.03018126
0.1435581 0.25078 0.1713742 1.8418 0.3524080.5811343 0.1008981
0.1261410.6892049 0.05159758 0.7794138 0 2.0346780.04496873
31.563250.07000537 0 0.7658806 0.1651311 0.4065334 0.671533
0.2109528 0.4400823 0.05575458 1.0886340 0.3724990.8996896
1.1697670.1288656 0.3507039 0 0.0963923 0.2964826 0.3924543
0.05811343 0.1518542 0.1639366 0 2.1649422.9240770.1952131
0.09654628 0.3757245 0.7849087 0.06850965 15.674380.4578634
0.9638325 0.0751716 0.4874030.3337066 0.4819848 0.04457077
0.05425311 0.2360020.05905991 1.9005650 0.9991017 2.4121580.2793081
0.4118431 0 0.07668403 0.5389936 1.4611380.4882780.1845434
0.5139283 0.1075409 0.7334705 0.5585076 0.08480538 0.2821233
0.5693432 3.64793 0.0881534 0.1106923 1.1446590.2763932 0.04807347
0.3019515 0 0.08710261 0.05320244 0.1708896 0.1994652 0.1211182
0.1741728 3.2441540.6533835 0.5255476 0.6605243 0.19894 1.073402
0.08398828 0.2210375 16.806110.05723293 0.05273819 0 6.71632
0.3118470.08708641 0.3350220.0691510.2867076 0.2294807 0.5231728
0.9082209 0.1318167 1.47543 0.07679539 0 0.08360617 0 0.4758895
0 0.02655447 0.9288622 0.1555675 0 0.08058396 0.1518542
0.1459241 0.4385991 0.4200152 0.1900565 0.1106923 0.2521532
1.0308090 0.5622844 0.4280309 0.5902145 15.964410.07685753
0.1359684 0.1793412 0.09414014 0.4062396 0.08101605 0.5472251
0.007455955 0.02968466
TCGA-4Z-AA83 2024 0 0.5124965 0.1534552 0 0.7774435 0.06291948
73.935580 0.08939662 0.3367043 0.2822214 0.2430841 0.469708
0.6044552 0.9879593 0.1550927 0.01453622 0 0.1442382 0.3572203
0.03664135 0.3028 0 3.3261196 2.7489 0.07550338 0.02203852 0
0.5151433 0 1.7203980 0.1025355 0 1.5207890.1556233 0.2841311
0.1184065 0.5413960.1815854 0.05720328 0.05336705 0.3187369
0.5824350.04742056 12.693670.1950926 0.2702279 0.03818144 1.569055
0.2137412 6.0849760.4908428 1.2638610.2718404 0 1.0952140
0.0566394 1.0766220 0 0.4119988 0 0.04140434 0.2569557
0.3072812 0.5709094 0.1324319 0.009089682 0.1428172 0.1845983
0.3901853 1.5439476.8582240.2713088 0.01594559 0.04278599 0.4662198
0.0450330.1390852 0.06074985 0.3117298 0.2592122 0 0.1192974
0.1854242 0.1303534 0 0.2236062 0.1016392 0 0.2615598
0.1281976 0.02928214 1.6652120.05285237 0 0.3399085 1.1627520
0.2103387 0 0.1485629 2.8525461.2150950.1064791 0.03355706
0.6988601 0.4172555 0.8859063 0.2834006 0 0.05172385 1.426239
0.06791776 0.4799095 0 0.1380941 0.06030872 0.5495048 0.1989113
1.1864820 0 0.3851221 0.9859594 0.0496337 1.4138110.06606546

1.2554630.04325715 0.3103431 0.1683521 0 0.8328252 0.3176918
0.2878637 0.2691556 0.6087707 0.4233321 0.5363248 1.6069090 0
0 0.06251355 0.3099019 0.9558407 0.1169771 0 0.2424183
0.07550338 0.3354092 0 0.6307877 0.05413185 0.7809529 1.322465
0.03755659 0.2321789 0.2065279 1.6988260.3594658 0.4840234
1.5415270.7089952 0.1446209 0.3181493 0 0.6743850.08574868
0.2087526 0.05159532 0 0.2872927 0 0.2513579 0.6445411
0.3410133 0.1980838 0.6858338 0.2529096 0.4674807 0 0
0.0887597 0 0.5689623 0.2116660.8237832 0 0.7033083 0.4915015
0.1131963 0.2543944 0.1064791 0.1957495 0.2215610.2959598
0.5579865 0.2914166 0.08108452 0 0.5205535 0.1918733 0.05825411
0 0.2625569 0.2618811 0.7583166 1.4296130.2105048 0.8225368
0.1131082 0.1822495 1.9036960.1651636 0.3043854 0 0.1885081
0.1838573 0.2932012 0 0.1330380.1103181.4638960.5444070 0.3169989
0.1051313 0.2954897 0.4506791 0.1697840.09921093 0.1144441
0.01503818 0 2.9187940.07482317 0.05087888 0 0.2921488 0.04882436
0.2702091.0844980.2193872 0 0.9020045 0.1565645 0.1260208
0.6212844 0 1.93035 3.87814900.1949122 0.6539663 0 0.9961272
0.02521145 0.03896622 0.9553127 0.02151651 0.04283222
TCGA-E7-A5KE 17 0 0.5135532 0.5814893 0.4382238 0.4610569 0
20.9312 0 0.1207586 0.2205219 0 0.3432880.02708497 0.6478091
1.2598930.5305573 0.0321313 0 0.8431242 0.03290044 0.2429795
0.02478957 0.1862451 9.08267777.438170.2851123 0.0162382 0.06953958
0.1626698 0 1.8358450 1.0199140.0649036 1.34464 0.09655988
0.2737663 0.5234588 0.5369890.03902325 0.02809864 0.1230930.1761364
1.9116420.0698799 34.546870.5270695 0.2844381 0.05626495 1.095248
0.9449201 4.85496 0.2411053 1.2028330.04005896 0.3220781.591514
0.0387309 0.03365523 1.0411620.05354547 0.1017491 0.8432358 0
0.004358162 0.4632484 0.1358448 1.29701 0.09757717 0.02009211
0.5180514 0.2960303 0.6413294 3.0989693.8942160 0.01174887 0
0.1889335 0 0 0.7161777 0.7464784 0.7093923 0 0.2929985
0.05780179 0.09604569 0.5851964 0.7688581 0 0.04537752 0.2014799
0.07084296 0.003082197 0.8855341 0.1557686 0.06050336 0.4195818
2.0607770.04970241 0.03099593 0 0.03648755 4.2536122.4448420.03922745
0.2657957 2.1571280.1707991.3734840.4176249 0.1777443 0.114332
3.8784580.3002550.6566898 0 0.2543728 2.48842 1.25513 0.04090042
2.18553 0 0.1072697 0.3926023.1187890.0121902 0.4892880.7788432
0.5075850 0.07622131 0.7690701 0.1189899 1.2036671.4044710.3041447
0.07932663 0.4485485 0.1559577 0.1975848 1.9954830 0 0 0
2.6030633.8527880.2154747 0.03415979 1.0034030 0.5354547 0
0.8133490.1196547 1.3426331.4653560.4427533 0.1368574 0.2282578
4.5896121.0329460.08074718 1.5820251.5671850.1065582 0.37013 0.05255997
2.3084850.1895415 0.8459608 0.09884148 0 0.03848731 0 0.2832517
1.1397710.5482082 0.07297509 1.2438880.5823325 2.3923240.1882918 0

0.06539905 0 0.9146558 0.1559577 1.3458940 1.163144 0.4759598 0
0.3498887 0.1961373 0.7031224 0.2285477 0.3748013 0.416762
0.161039 0.2608817 0 1.51401 0.3534355 0 0.6172389 0.0515879
0.4245046 2.234941 3.316113 0.817811 1.379739 0.1309617 0.04476111
0.1607546 0 0.3737904 0 0.01578348 0.1711174 0.2160336
0.04749044 0.09802375 0.04064173 1.224141 1.359626 0 0.07007041 0
0.1360749 0.5811137 0.2567812 0.2436654 0.08432358 0 0
5.135099 0.1378262 0.1624483 0 0.3348461 0.08543892 0.3981855
1.488462 0.08082336 0.1830614 0.6255117 0.07690553 0.6809251
0.8455257 0 2.384447 3.852468 0.326844 0.02409245 0 0.6672333
0.009288027 0.05742142 1.422133 0 0.0525987
TCGA-ZF-AA4V 1806 0 0.01288773 0.08269142 0.09347726 0.1192094
0.05933378 26.38155 0 0.2622729 0.3527954 0.08871264 0.08914538
0.1412272 0.2418216 0.5494385 0.1671476 0.3838189 0.1525443
0.006045254 0.08421569 0 0.08460557 0.1589113 1.843498 28.93469
0.1008674 0.1939706 0 0.04626533 0 1.715594 0.3842833 0.03223069
0 0.1092664 0.05664211 0.1099236 0.5582934 0.04581792 0.06183562
0.1798111 0.05688995 0.03005724 1.631084 0 1.184992 0.2861826
0.09707731 0.008001228 0.6749217 0.2687471 3.470245 0 0.2979588
0.03417981 0 0.7841622 0 0.02412136 1.438295 0 0 1.237506
0.0276891 0.01301492 0.4846243 0.0772719 0.4486449 0.1248848
0.03428669 0.05525262 0.06826598 0.1792572 0.200822 1.582234 0.1949312
0.01002458 0.03227813 0.1025851 0.05662216 0 0.1336715 0.04899411
0.2328 0 0.04999946 0.06725271 0.3277992 0 0.4685847 0 0
0.02989743 0.02014863 0.005259693 0.1228941 0.03322692 0.1032475
0.2580952 0.3161047 0.02423312 0 0 0.03113254 3.543946 1.057706
0.03347034 0.0369188 0.6649701 0.05829283 0.2552671 0.1272619 0
0.06503489 2.015128 0.04269814 0.4310095 0.03650398 0.04340808
0.6066325 0.2418216 9.623063 0.1598379 0.2811508 0.03050885
0.1392998 0.6732822 0.04160456 1.481377 0.08306729 1.060465 0.0543893
0.03251744 0.3598513 0.05076334 1.701621 0.1997246 0.1195101 0
0.2551456 0.04435632 0 0.9194156 0 0.0281584 0 0 0.3506892
0.7069557 0.03677023 0.2040249 0.6096084 0.6170713 0.1054316
0.04591659 0.29742 0 0.6000682 0.4125021 0.09444343 0.2335438
1.233468 0.4746702 0.0715625 0.2353965 0.06922274 0.3085802
0.04545971 0.01052696 0 0.7684427 0.05390798 0.2624747 0.09730992
0.05952705 0.1313553 0 0.1022497 0.324165 0.4287725 0.3735905
0.09949984 0.09274869 0.4435113 0.2409864 0 0.02790046 0.1864776
0.09755233 0.04435632 0.3737823 0 0.4109127 0.2118818 0.1423271
0.04264832 0.2342924 0.8768214 0 0.06977335 0.3363756 0.04580151
0.09685391 0 1.222913 0.1809387 0 0.1579954 0.242092 0.03292757
0.6753732 0.8654734 0.1203081 0.4070847 0.02031662 0.1527674
0.02689449 0.1730569 0 0 0.2908885 0.3285085 0.05266514 0 0
0.1040311 0.1168653 0.3203662 0.04993116 0.05978671 0.09913998

0.04644169 0 0.0730318 0.2494854 0 0.009454115 0.3854089
0.8238641 0.04703939 0.03198624 2.144244 0.1836664 0.03069461
0.151992 0.2005282 0.02298717 0.02231356 0.3430993 0 0 1.116615
0.05176998 1.284947 2.514282 0.1140855 0.2877922 0 0.05693085
0.03169957 0 0.906999 0.004508957 0.10771
TCGA-ZF-AA53 1761 0 0 0.04238746 0.07187439 0.403303 0.1216575
48.33746 0 0.2160652 0.3978529 0 0 0.6238943 0.05312455
0.5143012 0 0.175665 0.2345817 0.07437085 0.08633759 0.04723174
0.4553708 0.1629153 0.66282 548.42736 0.03649725 0.3835117 0.3649725
0 0.1254593 0.3154405 2.232472 0.09912834 0.04258013 0.3360584
0.04751122 0 0.05723602 0.06039302 0.1097197 0.1659075 0.05608015
0 0 0.1833793 0.1993112 0.12574 0.1119637 0.08612968 0.319351
0.1033194 3.412013 0.05272586 0.101822 0.05256151 0.9508495 0.3823522
0.8131034 0.2914504 0.5854768 0 0 0.04425651 0.04258013
0.06290197 0.04228382 0 0.2299745 0.1920471 0.02636293 0.07434626
0.05248951 0.0725423 0.2573524 0.6082876 0.03747051 0.07707868
0.09100139 0.0225364 0.1523782 0.1344636 0.08809682 0 0.6801972
0 0.1537778 0.06894722 0 0.05118923 0 0 0 0.01149402
0.03098444 0.004044163 0.04899631 0 0.07938668 1.015716 0.303815
0.2329097 0.1626795 0 0.1436263 1.641524 0.6325408 0 0.05677351
0.2556468 0 0.2141172 0 0.05830474 0 1.250912 0 0.5965235 0
0 0.4081332 0 2.039293 0 0.108088 0 0.05610376 1.166845 0.01599481
0.8283828 0 0.7033502 0.2090988 0.1500154 0.02170107 0
0.4335431 0 0.01575274 0 0.1961807 0 0.155551 0.3141937
0.05564928 0 0 0.06043631 0.1198417 0.3805033 0 0.1344636
0.1930049 0 0.108088 0.07061026 0 0.05233312 1.342227 0.2311173
0.1089259 0.1346784 0.2994979 0.1824863 0.07529627 0.4326237
0.1064503 0.05272586 0 0.0161883 0.03448207 0.09507984 0.04144968
0 0.229452 0.01830807 0.4292444 0.06017748 0.01429445 0.06231238
0.2897205 0.04787544 0 0.03056322 0.3369059 0.6176458 0.05045401
0.04290517 0.5735283 0.05000513 0.3410544 0.3047134 0 0.09238292
0.09503391 0.9411402 0.1311687 0 0.6150463 0.1285193 0.01788284
0.06650696 0.1408666 0.04442111 0 0.1059487 0 0.1126368
0.2429644 0.3118514 0.1012717 0.3665594 0.5118923 0.1110055
0.7782858 0.03124279 0.02936561 0.6865458 0.05322516 0.09809035
0.07395496 0.5964356 0.2806546 0.04049407 0 0 0.1599785
0.06739301 0.6150566 0.2303515 0.03064655 0 0.2142533 0
0.01727813 0.03197143 0 0.392539 0 0.05758788 0 0 0.3747051
0.1412205 0.07080299 0.2566474 0.1027904 0 0 0.102592 0.2522701
0 1.059953 0.07961157 0.1646653 3.514953 0.03898666 1.390919 0 0
0.07312112 0 0.01884835 0.006933848 0.09662092
TCGA-ZF-A9RM 1455 0 0.1306015 0.2793257 0.05262657 0.308722 0
22.70533 0 0.4218758 0.1324131 0.3995532 0.3584841 0.0838461
0.6612643 1.049023 0.2823063 0.08489069 0.03435224 0.2110111

0.03160828 2.783946 0.1190798 0.4175041 5.48610437.7991 0.253872
0.03120089 0 0.6251237 0 2.12069 0 0.2540369 0.03117726
0.553641 0.3478781 0.01547142 0.7124416 0.390609 0.02677901 0.03374383
0.1691754 0.4512498 0.1874043 0.03356768 20.63953 1.381006 0.08198004
0.00900919 0.7599457 0.1891267 3.944333 0.3474539 4.622367 0.05131419
0.0773571 2.465793 0.03720975 0.01810672 1.00027 0.05144247 0.2443823
0.5184753 0 0.002093498 0.1393214 0.6090441 1.044004 0.09374483
0.01930299 0.2099693 0.1152988 0.5630255 3.278751 11.49105 0.08230796
0.02257487 0 0.3960287 0.0159388 0.0984545 0.5375389 0.05516619
0.2359145 0 0.2533419 0.07067661 0.0922735 0.262366 0.6331382 0
0.04359532 0.3366379 0.2722424 0.002961144 0.8456297 0.03741271 0
0.3468559 1.201251 0 0 0 0.0350545 1.057696 1.356361 0.03768679
0.06532378 0.441222 0.1312727 1.89439 0.08597628 0.2134543 0.07322772
2.07123 0 0.4853064 0 0.04887646 0.4695994 0.7001622 0.02292154
0.9598596 0 0.1030567 0.1493792 1.588399 0.09369147 0.6520343
0.5144247 0.5149944 0 0.03661386 0.5402453 0.05715831 0.9409863
2.305073 0.08458404 0.07621107 0.1077329 0.1997766 1.594527 0.7540636
0 0.03170569 0 0 0.5703643 1.07462 0.1242072 0.06563633
0.9841827 0.1068935 0.9892784 0.2585049 0.3348877 0.03831842
0.5528147 0.7669114 0.9038988 0.1314824 0.4751348 1.336168
0.661585 0.06464653 0.545602 2.393571 0 0.3555931 0.05049568 1.273009
0.1213982 0.3324828 0.1533961 0.06702603 0 0.2643725 0.2302613
0.365002 1.382528 0.4557085 0.5975169 0.2498928 1.185283 0 0
0.1570763 0.2099693 1.061802 0.04994415 1.151054 0.01924882 1.355565
0.3031875 0.1442312 0.3961731 0.188434 0.3290933 0.5332452
0.2618771 0.5681262 0.5672854 0.1607129 0 0.8921221 0.1358217
0 0.1185994 0.06903248 0.5561349 0.5815236 1.799081 0.3251137
0.6689690 0.2516363 0.06450467 0.2574016 0 0.6823086 0 0.006065435
0.1575469 0.02964984 0 0.1412608 0.03904552 0.9704576 2.686391 0
0.2019552 0 0.1045844 0.1595115 0.0885576 0.7022863 0.1620235
0 0.4972471 5.144247 0.1059304 0.1800787 0.05487197 0.1608475
0.1209649 0.4463046 1.038634 0.8800222 0.05024906 0.6438693
0.03694253 0.1784132 0.9675376 0 1.687956 4.032061 0.2473996 0
0.06105932 0.1923083 0.02676972 0 0.3174184 0.04061582 0.2324513
TCGA-CU-A72E 413 1 0.04549066 0.1216172 0.08248817 0.178832 0.1396228
32.54046 0 0.4628808 0.1245285 0.07828368 0.02247587 0.6956325
0.1524238 0.5691684 0.05531171 0.1834612 0.02692227 0.2133832
0.3220333 0.2439293 0.09332429 0.1402299 2.07652 49.26314 0.09948127
0.330109 0.1570757 0 0.1439861 1.102522 0.1507144 0.05688338
0.219906 0.2169476 0.1317742 0.07275085 0.2627525 0.1328467
0.09654036 0.04760182 0.1506061 0.05304749 0.1174968 0.0526148
5.832967 0.07215408 0.06424876 0.01412122 0.8704611 0.02964419
4.750162 0.1210239 0.9640805 0.2211856 0.1818771 0.4894471
3.120305 0.05676182 0.3359675 0.04031609 0.07661016 0.4571274

0.04886799 0.1575072 0.1031219 0.1363758 0.2639352 0.4040793
0.02521332 0.1340822 0.07831292 0.1665094 1.151888 1.780191 0.06450575
0.1238454 0.1186815 0.2198472 0.08744002 0.07715999 0.3033186
0.08646883 0.5546673 0.06958549 0.1985471 0.07517231 0.07231586
0.05874841 0.3307987 0 0 0.0659568 0.3911595 0.1160342
0.293208 0.8796239 0.4555491 0.4065283 7.217671 0.1897857 0.04667565
0 0.4120895 0.7158933 0.4926082 0.1772136 0.1023901 0.28292
0.15432 0.5119504 0.04492044 0.1338294 0.05738946 1.109355 0
0.1901703 0.02013288 0 0.5018601 0.1829086 0.3207837 0.09403171
0 0.02692227 0.1463379 1.942717 0.01835679 0.4753556 0.03665099
1.003661 0.04799535 0.8321471 0.1307549 0 1.066211 0.1321839
0.06026322 0.02986377 0.2532948 0.03914184 0.6843322 0.9215107 0
0.09939253 0 0.03468051 0.06876946 0.6550391 0 0.3857999
0.7554941 0.2094343 0.1860743 0.2836308 0.2041321 0.03003061
0.09627724 0.3527217 0.02083519 0.2060887 0.4010127 0.3665099
0.3356905 0.3090521 0.09162749 0.7866555 0 0.1300519 0.01978704
0.3741271 0.04757061 0 0.09159496 0.08404658 0.2463158 0.03453199
0.1722559 0.2860565 0.6076795 0.1648358 0.1756055 0.09353743
0.8723365 0.2835418 0.1158092 0.04924103 0.6033701 0.08608419
0.1565674 0.584176 0 0.3795703 0.241507 0.4270241 0.1411299
0.1181424 0.2579144 0.1474979 0.02565453 0.1653776 0.2020857
0.09446449 0 0.3039856 0.2128903 0.03231751 0.04647388 0.4272639
0.406793 0.8063219 0.3818646 0.0636989 0.2718484 0.08964112
0.1179572 0.7452099 0.06108499 0.1125755 0.04243799 0.1782583
0.2362061 0.02323694 0.1430283 0.1107078 0.09180135 0.2835984
0.7796318 0.04406131 0.08793041 0.02916173 0.2254013 0 0.03965924
0.1834635 0 0.5255901 0.4747238 0.4295977 0.02075475 0
0.9890882 0.2070955 0.06432969 0.3524042 0.2241421 0 0.0590712
0.2312788 0.08685694 0.0699123 2.213989 0.02284198 0.9449085
1.978586 0.1006737 0.1269798 0.04785293 0.1004762 0.04895276
0.02161721 0.2812122 0.4177834 0.1980162
TCGA-2F-A9KR 3183 1 0.2371776 0.2874512 0.1146864 0.4533966
0.1213268 55.4882 0 0.5401312 1.096532 0.1088407 0.09374715
0.5670668 0.9324503 0.5568662 0.2990632 0.04484799 0.07486208
0.8158555 0.1377646 0.09420669 0.1038018 0.3249448 5.652642
52.61045 0.2256678 0.9349246 1.237533 0.22705 0.6005677 4.072421
0.1571579 0.1581742 0 1.273551 0.3285156 0.3203027 0.2283217
0.1606103 0.08753719 0.08824346 0.1216987 0.09833856 0.5717608 0
14.06152 0.6019112 0.5359648 0 3.184828 0.2060771 7.066967 0.3365282
0.9748345 0.139783 0 1.30233 0.6487144 0.06200707 2.28364 0
0.159771 2.153847 0 0.1323053 0.5439785 0.2370105 0.366959 0.1021467
0.05608804 0.3474183 0.2596403 0.1736282 1.806836 1.310329 0.3587391
0.03689719 0.05940263 0.1798011 0.03473461 0.07151895 0.7497159
0.2404417 0.4855545 1.668891 0.3373914 0.1925274 0.3016304

0.1633602	0.2874512	0	0	0.2475960.02472017	0.04517149	0.4299955
0.2445948	0	0.6877875	1.6725	0.003716425	0	0	0.7257285
4.34803553.6046930.2053223	0.2458891.9522030.2503163	1.58016	0.1561364
0.09303399	0.07979072	4.3662920.2095439	0.7932026	0.0223932
0.1065140.6047210.5086093	0.4317257	0.8497828	0.1724707	0.2620173
0.4842326	2.2027840.1148496	0.9583118	0.05095726	2.1452840.06672974
1.6756050.4934394	0.1868433	2.2482960.4900807	0.3896059	0.04152073
0.7434601	0.2721018	1.1996583.7600760.0887968	0.03454729	0.07853307
0	1.14735З2.6888080.2255653	0.1787974	0.7534225	0.2911843
0.6036475	0.05633465	0.7298037	0.04175269	0.2677157	0.4550933	0
0.5730656	1.4336822.03829	0.2911243	0.1901892	1.2314671.430245
0.1115482	0.2453932	0.02751067	1.5713260.1653480.4427884	0.1830626
0.0730332	0.02014483	0.09602224	0.4105611.0937170.6695273	0.6875323
0.2034591	0.6421151.9143240.7391602	0	0.2053850.07626256	0.7181165
0.1088407	0.7182738	0	0.7075721	0.3303606	0.2444678	0.5101672
0.2874512	0.4529534	0.5810370.1854760.6544182	0.05619346	0.323132
0.03947393	0.6128318	0.1479947	0.04493225	0.5169151	0.352953
0.1211956	1.8521851.1843620.8856297	1.1608810.06231571	0.6560014
0.7688209	0.1273931	0.1956474	0	0.2081850.9926826	0.09692158	0
0.2565354	0.04254496	0.8871695	1.1010410	0.1956047	0	0.1424472
0.2607115	0.1309569	0.2550761	0.2648172	0.01159917	1.842163
2.3431980.2308488	0.1046497	0.3587391	0.1251881	0.03765894
0.4863044	0.65607	0.5640552	0.0273763	0.6197253	0	0.1620028
0.6257864	0	2.2771527.1576890.4199113	0.05044138	0.1330635
0.6984795	0.07778384	0.06011044	1.4135430.05531999	0.1211361
TCGA-E5-A4U1	1181	0	0.2455104	0.2250379	0.1907930.4541862
0.0269128 4.4447 60	0.2804035	0.4800520.1207121	0.1906158	0.1677113
0.3290489	9.13404	0.5780748	0.1896322	0	0.9953258	0	0.01044819
0	0	4.62461	128.148	0.09688322	0.05655806	0	0	0	6.800279
0.1161995	0	0	0.4832093	0.2312203	0.01869676	0.6583849
0.6412614	0.3365612	0.01631138	0.07741068	1.4314712.9441440	176.158
0	0.0330235	0	7.7001970.1828433	23.052260.1399626	0.4504836	0
0	8.4710710.5396027	0.03282216	8.4040210.4351671	0.05906578	5.28662
0.03767681	0.01011972	0.4817132	0.2628614	0.5697769	0.1132879
0.1632897	0.1973547	0.0650231	0.4621584	0.1366302	1.399424
0.3315559	0.5183403	0.01464038	0.1994121	0	0.07931959	0.4936961
0	0.1583866	0	0.06803473	0.3172394	0	0.04529452	0.06376075	0
0.3161020	1.6724020.02147074	0.5016693	0.04521217	0	0.06797273
0.4570095	0.03709601	0	0	0	10.109365.9168340.1366302	0.1435307
1.8742930.2379588	1.1209750	0.2063626	0.1327403	7.01852	0.05809975
8.9731220	1.0041182.6311230.2350349	0.03957144	0.2899906	0
0.2490821	0.1940595	0.2035870.09907049	0.6230417	0.1130304
0.1817499	0	0.1327403	0.5088551	0	3.1922530.6794178	0.2137272
0.04604943	0	0	0.5505541	13.344610.1969639	0	0	0	0.9013513

15.67996 0.05003359 0.2379588 0.06099262 0 0.4303851 0.06247913
0.1798676 0 0.5938315 1.435872 0.8995703 0.3177852 0.4416819
2.3413344 0.2460019 0.1484345 0 2.146094 0.06185744 0.2005378 0
5.144004 0.4034419 0 0.01765473 0.242997 0 0 0.3541543 0.2756839
0.7867528 0.2118117 0.1805205 0.3200172 10.57925 0.1639562
0.4017957 0 0.2537418 0 0.4828484 0.9743233 0.06978492
0.6801147 0.402432 0 0.2321278 0 0.1046578 0.03790654 0.03955885
0.4184771 0 0.30751 0 5.859258 0.3282732 0.3488314 0 0.1005363
0 7.081601 0.3170616 0.09822262 0.6437494 1.202558 1.922816 8.175451 0
0.1301921 0 0.02931961 0.355948 0 0.1654104 0.2276127 0
0.327978 0.9368331 0.1358836 0 0 0.5687426 0 0.05350964
0.02828975 0.09790036 0.0771858 0.3496196 4.942765 0 0.05803195 0
0.055537 0.03132483 0.6204507 0.0181907 0.656856 0 0.6808351 0
0.2515417 1.694462 0.03522194 3.739717 6.087082 0.1609867 0 0
0.7746633 0.01078347 0 5.653795 0.03681228 0.08549452
TCGA-E7-A3Y1 163 0 0.3195541 0.6346327 0 0.5067126 0.02335229
86.79105 0.053016 0.353911 0.4720949 0 0.7367931 0.2304188 2.284198
2.143648 0.3042561 0.04046289 0.07204512 0.5924358 0.09943554
0.05439709 0.07492192 0 4.666245 262.775 0.0980796 0.06543597 0
0.1638798 0.09632818 3.41277 0 0.07611116 0.0326932 0.9676015
0.2553552 0.2595791 0.5712987 0.2936767 0.308892 0.007076915
0.04478092 0.1537871 0.4716396 0.07039971 41.49353 0.09654368
0.1146216 0.02834174 1.042096 0.7536264 11.65986 0.283382 1.368139
0.2017847 0 2.517941 0.7413612 0.06509872 1.94797 0 0.153759
1.461154 0 0.0241482 0.4869854 0.5930395 0.4944111 0.2457575
0.01349438 0.3914288 0.3465943 0.1782345 1.699328 12.18989 0.1150801
0.02367253 0.03175966 0.3114637 0.06685519 0.2408973 0.06764111
0.1156971 0.2748728 0 0.3246958 0.2011642 0 0.4716396
0.5532696 0 0 0.1412025 0.09515992 0.02173587 1.160833 0 0
0.3047394 1.282987 0.01072974 0 0 0.4043486 10.18378 2.636481
0.03951925 0.1432274 0.3785529 0.2064834 1.233001 0.1803135 0
0.03839415 3.06691 0.1008295 0.5089036 0.01077529 0.051253 0.4476662
0.489471 0.123614 1.509797 0 0.07204512 0.3524458 1.009474 0.1350897
0.302117 0 0.9844889 0.03210939 0.3455474 0.04165543 0 1.224509
0.2947748 0.419294 0 0.3012567 0.2094904 1.074895 2.466001 0 0 0
0 0.5060824 5.00832 0.1736621 0 0.2434549 0.1681365 0.2904665
0.1626446 0.07803802 0.1607264 0.7085153 1.87647 0 0.1723443
1.149779 1.611308 0.6982006 0.3931812 1.675527 1.254977 0.1610262
0.07457666 0.05295095 1.387051 0.1591262 0.0387388 0.0153195
0.02811402 0.3101889 0.04620452 0.2853588 0.334906 0.3221669
0.3308307 0.8223734 0.3207098 2.189297 0.0474231 0 0.03294277
0.1467858 0.5375181 0.1571178 0.6433906 0 0.9476514 0.3231415
0.3529032 0.3524915 0.03951925 0.01816289 0.2960329 0.4187809
0.6354671 0.05407898 0.1986215 0.03798858 0.2033696 0 1.902626

0.3109152	0.3155421	0	2.908273	0.8253693	0.4403574	0.6495338
0.04797668	0.3382055	0.8224541	0.1225995	0.3765709	0	0.1908107
0.3878781	0.2176406	0.04784371	0	0	0.5346946	0.5873670
0.1411833	0.03901901	0.1096697	0.08363377	0.2586914	0.07364339	0
0	0.009480454	2.741405	0.1110811	0.1384788	0.1150801	0.09638197
0.3941305	0.3765156	0.9944282	0.05428305	0	0.7370676	0.1162164
0.249451	0.2984066	0.09168915	2.317897	5.654619	0.493913	0	0.06402823
0.201659	0.09357117	0.05784856	2.387853	0.005323837	0.1165779
TCGA-FD-A6TC	187	0	0.09550303	0.2772078	0	0.3281153	0.3559363
52.75997	0.04753362	0.07932828	0.3236813	0.09391352	0.04044499
1.274886	0.1828562	0.5057826	0.08110062	0.1838117	0.1291899
0.1599916	0.1485882	0.0325146	0.1119571	0.2243036	2.713091
33.38562	0.2009993	0.1466731	0.06281229	0.09795537	0.6909352
1.006791	0.6780205	0.06824051	0.1172496	0.5494432	0.1417303
0.116368	0.7092288	0.1940162	0.09063804	0.03807055	0.111185	0.07424523
0.1761948	0.03155985	1.999302	0.04328006	0.1027686	0.1185843
0.8244113	0.213377	3.083588	0.07259357	0.8411385	0.2050406	0
0.3543257	0.1399362	0.07174261	0.7165254	0	0.09190587	0.8530601
0	0.1633669	0.1309879	0.1227031	0.411621	0.3525501	0.02419786
0.1608526	0.08672179	0.2796557	0.3897583	0.586248	0.1031797
0.1167351	0.06264588	0.1085993	0.1198834	0.06171032	0.3638781
0.1555993	0.3696723	0.1669574	0.02646537	0.1091664	0.08675419	0
0.6448728	0.067644	0	0.0949506	0	0.04454439	0.3469303	0.2462242
0.05465024	0.390743	0.7320178	0.2148507	0	0	0.7909854	1.31084
0.3421351	0.2834606	0.2568325	0.4274011	0.1542758	0.7492808
0.1347227	0.04013731	0	2.280051	0.1356041	0.1825112	0.0579661	0
0.2408239	0.5119973	0.8928061	0.5076259	0.1488168	0	0.101822
1.640466	0.04404368	0.7698556	0.4836546	0.8655436	0.02878897
0.9294431	0.3884176	0.1074788	0.6608614	1.057169	0.2421888
0.03582627	0.1012889	0	0.535411	0.6128295	0.1149278	0.05961844
0.03388124	0	0.6187479	0.1871004	0.1557037	0.2159861	0.3511449
0.904497	0.1488168	0.04860851	0.06996812	0.03602642	0.5197483
0.382523	0.4499113	0.09271335	0.5154401	0.3768737	0.3867642
1.191283	0.1099215	0.3992646	0.04812484	0.18945	0.0474753	0.243113
0.142671	0.06946566	0.1030148	0.7688061	0.2259662	0	0.1869672
1.158197	0.7565163	0.3954927	0.3511104	0.1087061	1.459449	0.2551145
0.03473283	0.2362893	0.1974101	0.3098144	0.1408703	0.4147654
0.03619492	0.5342142	0.3458014	0.738289	0.1241587	0	0.439686
0.3244025	0.05539792	0.4069668	0.1939467	0.07554996	0	1.221672	0
0.07753982	0.3345162	0.557503	0.6274438	0.5046831	0.4581064
0.06368066	0.4076559	0.07527699	0.1415081	0.8142765	0.0366405
0.1350519	0	0.1026474	0.5796127	0.0557527	0.0857924	0	0
0.7036382	0.8152193	0.1057169	0.02109726	0	0.02458219	0
0.1011021	0.08803725	0.07616608	0.1100921	0.2465024	1.466821

0.0746957 0.07901009 0.5158983 0.07561323 0.06905 0.5047928
0.3821119 0.02433482 0.02362172 0.2471872 0.06946566 0.08387075
0.4134840.3836354 1.5114215.9110140.0715697 0.8051843 0.05740708
0.1808055 0 0 0.09082716 0 0.1045226
TCGA-FD-A5BV 163 1 0.17851164 0.11931067 0 0.2270405 0 47.94846239
0 0.29192281 0.20361125 0 0 0.73356698 0.11962642 2.39893981
0.32557626 0.15822572 0.36976348 0.70127676 0.29162386 0.10635694 0
0 3.15151284 71.47370738 0.26710094 0.02398877 0.41092453 0
0.14125531 0.61345162 0 0 0 0.33107373 0.22288806 0.02379037 0
0.19265767 0.16471207 0.18679613 0.12880752 0.12143 0.11526845
0.05161695 5.96277724 0.14157132 0 0.06926705 0.62922819 1.163279
6.26389565 0.1780931.26106188 0.09863213 0 0.66229595 0.05721734
0.02187637 0.73243493 0 0.45094331 0.54811508 0 0.03863 0.43441918
0.26757876 0.51785907 0 0.01978811 0.40657612 0.08273751 0.31036788
0.34770537 15.40966993 0.29531776 0.15620993 0.08383006 0.10149539
0.07352716 0.15139325 0.59513208 0.42414462 0.16122899 0 0.0432848
0.1940701 0 0 0 0 0.06703642 0.05176473 0 0.05008679
0.29946829 0.23011774 0 0.99944834 1.40247432 0.04720217 0 0
0.05390322 2.14391233 0.55957086 0.0579509 0.11871153 0.02055958
0.10092883 0.92412361 0.08813702 0 0.1126021 4.73737578 0.07392801
0.59700356 0.03950211 0.07515722 0.6564562 0.35887927 0.27693593
0.46124182 0.12169688 0 0.18950258 1.79485107 0.05402593 1.60887401
0.50338255 1.55577939 0.28251062 0.39410734 0.25655018 0.08789219
2.1965784 1.12386737 0.14780077 0.05859479 0.16566068 0.153598
0.64216435 0.66819902 0 0 0 0.54436454 0 0.42841068 0.44565055
0 0.80713359 0.08218491 0.3042422 0.23850142 0.45773872 0
1.60567227 0.37094657 0 0.20218012 0.05620105 1.02731133 0.82820095
0.22863567 0.23970598 0.11872866 0 0.01822649 0.03882355 0.2293948
0.28001052 0 0.01123223 0.14429207 0.02842874 0 0 1.1926834
0.52866544 0.21561287 0.11485005 0.10896911 1.56355583 0.48678752
0.51125799 0 0.21524618 0.22520419 0 0.95126295 0 0.26211636
0.48149635 0.34499739 0.23998568 0.0579509 0.45277796 0.19293408
0.01006719 0.22464163 0 0.16180989 0 0.38768657 0.20885273 0
1.27658976 0.44910075 0.05701115 0.20635558 1.72902672 0.10415138
0.30479171 0.10552926 0 2.02093834 0.23970598 0.2208809 0
0.02331701 0.63198114 0.04559249 0 0.28962187 0 0.48056162
0.6098095 0.17290267 0.06901023 0 0 0 0.08754095 0 0 0
0.06951052 1.03741603 0 0.11076262 1.01251804 0.21200127 0.23250926
0.12384027 0.92585776 0.03980024 0 0.43728371 0.68167732 0
2.51411006 0 3.39895584 5.06937452 0.10242235 0.42710266 0.09389077
0.09857068 0.17837617 0 0.08488582 0.51525252 0.18649031
TCGA-DK-A2I4 3835 0 0.09221628 0.06574293 0 0.1232090.1257938
25.890840.1784912 0.2308584 0.2056899 0.5642403 0 1.34125
0.1098615 0.3988404 0.1384259 0.1998013 0.2183016 0.009612429

1.004322 0.0305235 0.4035885 0.04211358 1.606893 30.18277 0.1651044
0.2974132 0.2830361 0.03678275 0.4540371 0.6523308 1.289975
0.076874 0.4843062 0.4995087 0.2169757 0.0873936 0.2663191
0.09366954 0.196647 0.1191308 0.1217724 0.1194834 0.2381834
0.09480709 1.118762 0.06500755 0.05788521 0.1335871 0.3302088
0.400621 2.072495 0.1635556 0.6843457 0.3894981 0 0.3497345 0
0.08675504 0.5044856 0 0.03451113 0.114403 0 0.1093865 0.0901755
0.1228684 0.4042491 1.025979 0.02725926 0.16473 0.2279514 0.3825444
1.19746 1.415181 0.07748899 0.1673686 0.0598789 0.0932105 0.1800674
0.09269019 0.4858244 0.07790449 0.3516618 0 0.3378881 0.1318891
0.0651533 0.1587889 0.2607803 0.2032054 0 0.08319371 0.0320379
0.09408741 0.4089161 0.07925011 0.0820858 0.5361568 0.7853614
0.03130771 0 0.03158519 0.7672995 0.4413071 0.3270235 0.1862716
0.1509526 0.169932 0.06951764 0.8117895 0.06070691 0 0.2326738
0.7863546 0 0.7538729 0.05804419 0.1035334 0.09043062 0.1098615
1.44737 0.296514 0 0 0.07119565 0.9261294 0.04134658 0.7601859
0.09906264 1.38695 0.1297249 1.473601 1.694137 0.04035885 0.5043189
0.1587889 0.1710274 0.0538118 0.2535635 0.03526502 0.348486
0.7309715 0.02877067 0.06716111 0.1781161 0.03124556 0.2788117
0.1686173 0.02923378 0.02317255 0.4312072 0.03773815 0.5588149
0.1095165 0.1050936 0.05411243 1.561345 1.083015 0.01877156 0.09283825
0.2064538 0.3302088 0.1467292 0.2967003 0.3852436 1.144889
0.1084268 0.1004322 0 0.3300492 0.1071474 0.1565083 0.1340997
0.1041179 3.002437 0.0622235 0.07390227 0.3543704 0.2737458
0.1485096 0.1318438 0.1106083 0.2931327 0.1915937 0.02608472
0.08872786 2.174436 0.1034106 0.3526502 0.3687793 0.108731 0.3419756
0.6457416 0.3960448 0.1441051 0.02661023 0.3913586 0.3322223
0.129436 0.2712522 0.036414 0.1458998 0 0.05477544 0.04795111
0.145583 0 0.9586139 0.4450381 0.1579259 0.5292964 0.1530395
0.691033 0.06461006 0.1214561 0.2480332 0.1926218 0.07606904
0.1529388 0.4111428 0.1837913 0.08374171 0.03221549 0.2659805
0.1378481 0.4761764 0.882762 0.03969723 0.1109098 0.02627339
0.01846148 0.2252587 0.02233198 0.04958767 0 0.1503278 0.0510692
1.042052 0.01869908 0.06781417 1.12359 0.1541343 0.06405898 0.3980599
0.2763415 0 0.01774015 0.1894288 0.1304236 0.0629878 0.5553021
0.3292733 0.4824144 5.356502 0.08062439 0.6047018 0.08622663
0.2263113 0.2961283 0 0.214381 0.01075439 0.1712674
TCGA-UY-A78M 690 1 0.07234781 0.0928409 0.1049506 0.1338411
0.1332327 126.7569 0 0.1577486 0.1584389 0 0.2144722 0.5621721
0.07757217 1.287394 0.03909649 0.3847576 0.137014 0.1968301 0.09455226
0.04310471 0 0.237888 3.943349 109.0819 0.01332327 0.07777801 0.1998491
0 0.2747925 5.087587 0.5273277 0.03618667 0 0.5213801 0.3757846
0 0.08357572 0.1763711 0.1495314 0.07402304 0.06223483 0.4049574
1.08382 0.1004136 15.57028 0.1836048 0.3542257 0.1167828 1.340654

0.03771662	5.8902890	0.7434	0.07675002	0	0.7730426	0.07420557
0.04900553	4.84449	0	0	0.7754774	0	0.08767416	0.1543565
0.1735124	0.1679038	0.1402130.006415835	0.2326286	0.03832245
0.1482962	0.1127354	4.8407890.05471424	0.1012949	0.03019994
0.09872257	0.04767895	0.09817148	0.4931142	0.1100152	0.3397859
0.02213359	0.02806818	0.05033817	0.09200825	0	0.1052197	0	0
0.1007011	0	0.008857896	0.2555148	0.07461033	0	0.3552063
0.5767177	0.09182524	0.3266226	0	0.1048612	2.1093111.880252
0.03757846	0.01776436	1.0932180.06544766	0.8597952	0	0	0
5.4901050.09587776	0.5323025	0.01024611	0.04873596	0.6810901
0.3878609	0.4701733	0	0.07891477	0	0.05957993	1.52384	0	0.2268
0.04663145	0.6498452	0	0.3650861	0.2535023	0	2.1478730.168179
0.1686818	0.0379960.1790387	0	0	1.4910480.1625175	0.1580727
0.07186634	0	0.04374806	3.6114570	0	0.1107168	0	0.1578295	0
0.1113084	0	0.9187092	0.2154104	0.02650884	0.1638805	0.03644377
0.5995473	0.3805865	0.5027934	0.07771909	0.5389301	0.255197
0.0354571	0.07552579	1.2792660.0605246	0.07367267	0.1383880.02673334
0.1474777	0.04393542	0.1043634	0.0454941	0.5689270.06990746
0.03723746	0.2008271	1.3858540	0	0	0.2093657	0.07301722
0.3984047	0.7735903	0	0.2886797	0.17346	0.7670221	0.1316781
0.1503138	0.5353982	0.06255439	0.02611243	0.1834359	0	0.0972952
0	0.2513967	0.1354313	0	0.4139046	0.2523915	0.03696908
1.1597040.1494925	0.1755970.3088176	0.1254565	0	0.1932512	0
0.1790387	0	0.1270080.2049052	0	0.0454941	0	0.07786657
0.1148081	0.3596877	0.1121193	0.02237496	0	0.1042838	0
0.05676623	0.1633958	0	0.02122901	0.3335501	0.2943133	0.02640649
0.01197074	0.2735712	0.1145608	0.07323185	0.1104189	0.09005636
0.1032344	0.05010462	0.2835582	0.03683634	0	0.4385256	0.0290621
1.2823651.5641950.04269608	0.2769562	0.1217676	0.1917555	0	0
0.8944732	0	0.1007752
TCGA-4Z-AA7R	522	1	0.1520827	0.3090055	0.1103086	0.2813481
0.0233391	23.344010	0.1547487	0.2775461	0.1046861	0.04508431
0.2787706	0.5707271	0.9584591	0.2794288	0.2480323	0	0.6420376
0.5300235	0.0724885	0.07487962	0	2.67032143.148480.1680415	0
0.2100519	0.1091915	0	0.9682393	0.05038628	0.3423068	0.03267474
0.9025848	0.4921927	0.0972872	0.3513699	0.3398548	0.2469741
0.07072919	0.06541208	0.1655234	0.3142489	0.03517998	8.084268
0.1447338	0.2291137	0	0.4288560.3171372	1.7863630.3641425
0.6250820.1882261	0	0.5078178	0.1559879	0.02168732	1.247994
0.05391332	0.6659128	0.7471453	0	0.002194051	0.1906283	0.136778
0.4588368	0.6877326	0	0.1548531	0.06444626	0.1558671	1.105914
3.8276130.2012764	0.04731833	0	0.2075252	0	0	1.1717840.1734477
0.1648305	0.32569	0.2360091	0.07936211	0.2901165	0.07856222
0.3317743	0	0	0.1234825	0	0.01241349	0.4189529	0.03920969	0

0.3438663	1.5853430.04289474	0.09362661	0	0.2571675	2.317769
0.9361156	0.03949694	0.2116079	0.5044522	0.3095502	1.944303
0.3003528	0	0.2686073	2.0943570.1007726	0.2543081	0.005384602	0
0.4026721	0.3261298	0.1750211.4460720.08294358	0.03600222	0.5870779
3.8212250.0736437	0.8581635	0.2940727	1.0890130.09627379	0.3453523
0.3497081	0.05990369	1.1762910.1178433	0.09670551	0.1198074
0.1881791	0.1046861	0.9151339	1.9288250.2562217	0	0	0
0.3218706	1.4599350.04339101	0.03439447	0.5025034	0	0.08294358
0.05418425	0.3509728	0.0401589	3.0255841.3697660.2507596	0.3444941
0.6128691	1.82045	0.3555702	0.0813019	0.7351817	0.4046028	0
0.1366467	0.05292105	0.3543843	0	0.1548677	0.06124339	0
0.1356310.1385353	0.1425988	0.5738003	0.3603166	0.4408585
0.3913853	0.1250844	1.3872430.3317743	0	0	0	0.4604697
0.2617152	0.6961701	0.0605201	0.7769757	0.5365287	0.117568
0.2893831	0.3554725	0.1633737	0.3287398	0.1509504	0.3459072
0.05404845	0.1343447	0	0.5487878	0.07117271	0	0.3107396
0.1892184	0.1942823	0.7969796	1.0998710.2129559	1.5320340.179811
0.06760291	0.4125817	0.08168686	0.07527166	0	0.1144218	0.2943337
0.3728875	0.1434501	0.1973943	0.08184186	1.3187431.6319580
0.8701387	0	0.4110292	0.08358655	0.2916921	0.3925430.08490287	0
0.7295828	2.7398570.02775461	0.1761462	0.2300302	0.1444913
0.07697052	0.1793594	1.8299770.1627572	0.1053252	0.93909	0.2710185
0.06232754	1.1170370.1221832	2.02175	5.07344	0.3241032	0.3638695
0.06399208	0.3359086	0.009351834	0.02890795	0.1735640.05320831
0.3283523
TCGA-E7-A519	508	0	0	0	0.08116588	0.08280719	0.1030385	100.72560
0.01626646	1.9605160	0	0.4905078	0	2.4061170.01209446	0.05951217
0	0.01049813	0	0.1600127	0.03673132	0	2.240476183.17820.01030385
0.09624217	0.1030385	0	0	5.3432820	0	0	0.04743775	0.09836417
0	0.6463515	0.06820027	0.4047520.03122584	0.02279875	0.01739904	0
0.1553141	33.954470.07099744	0.1685836	0	1.0819040.05833797
10.486880.05954195	0.22997	0.03957089	0	2.6903240.05738853	0.0359048
2.5711920	0	0.2498886	0	0.03228798	0.04775002	0.1341897
0.1038817	0.1445825	0.009923658	0.1559207	0	0.0655361	0.05812428
1.3738470	0.05222577	0.02802696	0.1272488	0.02458238	0.1012308
0.03316182	0	0.2425671	0	0	0.1245765	0	0.05780666	0	0
0.1344740	0	0.009133935	0.04742594	0.05770156	0.3585973	0.057833
0.4803265	0	0.09185487	0	0	5.7836381.4286240.5812428	0.02747693
1.5672030	0.1007488	0	0	0	6.0197	0	1.2724280.03169624	0	0
0.05999217	0.1666587	0.1850487	0	0	0.0345581	0.1484718
0.01806253	0.04677356	0.1442539	1.3214490	0.0564695	0.2818242	0
1.7310470	0.08894579	0	0	0.07702878	0.2342132.6413690	0	0	0
0.2030012	3.0692320	0	0.06227296	0.1648616	0	0.2392150.1721656
0	0.1894678	0.5608612	0.2870168	0	0.1127384	0.1030385

0.03924467	0.3290240	0.2381678	0	0	0.1168191	1.3804870.0936161
0	0.01126583	0.0826993	0	0	0.0322847	0	0.5302472	0	0
0.07478089	3.0065550	0.6267404	0	0.1079451	0	0.1540576
0.2424375	0.08906227	0.1710940.05365966	0.3954624	0.1851567
0.05812428	0.5342737	0.09675566	0	0.05841467	0	0.0295080
0.1495563	0.4189552	0.06359891	0	0.1883677	0	0.6899099	0
0.0626778	0.1337453	0	0.03316182	2.07836	0.1803174	0	0
0.07483769	0.2218552	0	0	0.5809768	0.1806595	0.3932101
0.1796891	0.17342	0	0	0.2016255	0	0.01951175	0	0	0	0
0.3901950.08168818	0	0	0.1063178	0.04664098	0.1138599	0
0.3991932	0	0.03310129	0	0	0.6942468	0	3.8430142.721836
0.01467554	0.3212854	0	0	0.1376230	0.6811183	0.06264171
0.04676207
TCGA-DK-A3IU	706	1	0.04429458	0.1302615	0.08031932	0.1434012
0.9176743	50.467590.1157427	0.20121	0.1818814	0.07622538	0.02188492
1.9724560.2077826	0.2463126	0.07180988	0.3454966	0.1835008
0.1246637	0.5306504	0.01979298	0.1635670	0.8958517	25.60564
0.02039276	0.1666672	0.3568734	0.1192589	2.8741050.4005721
0.6236946	0.05538775	0.1427493	0.2816579	0.4203242	0.03541901
0.5436686	0.1181057	0.2247875	0.07725038	0.1027777	0.04304394
0.02860187	0.07684712	0.5568237	0	0.08341263	0.1581243	0.267655
0.1731885	2.8268130.08838139	1.0240710.2447386	0	0.2218553	0
0.3089164	0.1453928	0	0.1118938	0.2225541	0.2617071	0.5942927
0.05315849	0.09959256	0.2826951	1.0015220.03437054	0.2195729
0.9033149	0.1053854	1.0640840.5777950.1046829	0.1378162	0.4714889
0.05036865	0.2189339	0.02504374	0.1476717	0.2104883	0.3800588
0.1355118	0.2792497	0.3120453	0.1408289	0.1430094	0.2818384
0.05490359	0	0.01284452	0.06924996	0.5671765	0.2933206	1.141995
0.2217857	1.5380510.5262435	0.02863032	0.1817937	0	9.202104
0.3668792	0.1767154	0.2300722	0.09516623	0.2040601	0.07513123
0.4287012	0.04373935	0.1954659	0.02794026	1.3502340	0.2962722
0.09801765	0.03729793	0.09773295	0.1187329	0.6271975	0.2288984
0.06039395	0.05242881	0.06554552	1.1753860.008937068	0.3124286	0
0.9146708	0.1402002	11.343750.2000695	0	0.6748151	0.1716112
0.08508416	0.05815714	0	0.2286761	0.2607414	0.3803694	0.03109392
0.1209740.2199990.06753732	0.1004420.1518610.09478327	0	0.1232469	0
0.1509849	0.03945334	0.02839499	0	0.4218563	0.9534107	0
0.07525125	0.2510157	0.4588372	0.1941768	0.8633129	0.267655
1.6203260	0.03618071	0.1734010.1821451	0.1621195	0.05638215
0.2452633	0.09206635	0.8888167	0.1008721	0.09584391	0.3481691
0.4521495	0.1337515	0.05699611	0.1110014	0.3535342	0.9663032
0.08457322	0.1438391.7625170.2235221	0.4954650.8938543	0.1028223
0.2518996	0.4854857	2.0789820.07329021	0.08627708	0.4361785
0.2154296	0.0349720.1156108	0.03935446	0.09928081	0	0.1479965

0.1554696	0.1888067	0	0.8361121	0.2829266	0.06827142	0.5434356
0.07236142	0.3403296	0.02618526	0.1312638	2.6621330	0.08221166
0.04132218	0.4374	0.2090869	0.04525195	0.03481691	0.2874586
0.08938763	0.5585596	0.6462889	0.3861253	0.5308345	0.1703699
0.2194748	0	0.06275179	0.07145587	0.06182058	0	0.1034870.6757192
0.08083618	0.1099353	0.5443508	0.1227437	0.1087928	0.1434012
0.1263546	0.01975148	0.03834537	0.2047253	0.7047768	0	0.7304373
0.1112070.5520385	5.5832930.03630619	0.4769016	0.04659474	0
0.2519470.1473418	0.2000980.007748546	0.1156859
TCGA-FD-A3SM	547 1	0.065124497 0.097500114 0.04723608	0.337338948 0
34.88006514 0	0.170398531 0.142620134 0.717254656 0.038611792 0.986136969
0.558617993 0.26557174	0.07038608	0.110829698 0	0.030547978 0.794378813
0.031040853 0.021376513 0.160602673 1.303888952 31.002596681	0.053968755
0.084015045 0.239861135 0.233788701 0.247356795 0.565386961 0.215762937
0.065147469 0.279837991 0.30368069	0.16132672	0.111093578 0.112847092
0.191837362 0.120180293 0.03634498	0.10319696	0.101257168 0.033641726 0
1.403442811 0	0.024527633 0.032345547 0.367287363 0.339509327 1.513334397
0.207909652 0.803013365 0.126659839 0	0.086982609 0.033398386 0.051077909
0.256518158 0	0.350960711 0.116342094 0	0.116501888 0.187576189
0.078094323 0.181367994 0.631069729 0.017325804 0.153561891 0.103488835
0.143025096 0.744184547 1.359213098 0.197005946 0.010131271 0.005436947
0.236975877 0.042918607 0.029456631 0.308786748 0.247577847 0.352916702
0.677407175 0.075797431 0.077030968 0.165644012 0.067283451 0.094714397 0
0	0.098200816 0	0.031894041 0.322005359 0.067161118 0.052173185
0.260842154 1.33777686	0.293892509 0.026728375 0.120452005 0.18878338
1.078814684 0.326627623 0.169132852 0.165237671 0.216015291 0.147283153
0.621506852 0.077169808 0	0.065727073 0.69131449	0.043152587 0.087119374
0.009223125 0.043870089 0.114954195 0.244395372 6.818713104 0.269231886 0
0.030833568 0.134078457 1.080076456 0.021023685 1.483533166 0.041975699
1.300828295 0.027484088 0.131454145 0.02139302	0	0.437564858 0.201850354
0.110429342 0.102607263 0.193395889 0.044828416 0.034076213 0.401506682 0
0.056916202 0	0	0.393801863 0.428687986 0.185807921 0.117826522
0.525494152 0.047972227 0.106553696 0.046405295 0.500975788 0	0.220529043
0.46213665	0	0.236029487 0.164025821 0.599652837 0.098969908 0.400371414
0.384777237 0.762335389 0	0.191502035 0.090646907 0.14282693	0.027240866
0.29842687	0.268811361 0.036096366 0.464636664 0.039548838 0.065760504
0.286663308 0.347981974 0.18878338	0.067039228 0.06360646	0.626444344
0.081183769 0.066317082 0.028197416 0.251283094 0.032863536 0.358627328
0.263977287 0.017277182 0.167571625 0.218598083 0.417141678 0.15085782
0.03382657	0.15546555	0.168926592 0.064639638 0.223399458 0.092577982
0.039497357 0	0.800742828 0.06095481	0.03701264	0.05322567	0.622792989
0.232946219 0.160602673 0.370058982 0.060794297 0.478133786 0.03079929
0.038598344 0.179392831 0.069959498 0	0	0.209600099 0.239781288
0.106451339 0.081903802 0.042263861 0	0.664363575 0.412261326 0.100925177

0.080564045 0 0.093871956 0 0.045420983 0.084046905 0 0.152875798
0.210985058 1.135408241 0 0.043102234 0.443263377 0.123747453 0.065920266
0.352398722 0.486389953 0 0 0.16855952 0.033158541 0.026689751
1.119211235 0.026160492 0.829675918 2.662044305 0.046973961 0.207753739 0
0.057536783 0.01601848 0.024757785 0.123871948 0 0.090713691
TCGA-BL-A5ZZ 377 0 0.055003323 0.088229313 0 0.254385885 0.147717258
18.942318105 0 0.149912953 0.326232709 0.662576439 0.149467219
7.189058768 0.589752078 0.280373149 0.074308987 0.231575749 0.195312301
0.051600868 0.898556593 0.057349053 0.112839572 0 1.339271753
33.079552987 0.044315177 0.399138975 0.569766567 0.148091226 0.34819068
0.656588139 2.186767634 0.034389203 0.295434516 0.437190091 0.197788789
0.146606602 0.277985227 0.090788974 0.162404398 0.409286781 0.099610105
0.096210583 0 0.159043051 0.809975482 0.261727222 0 0.845170372
0.166181915 0.358431582 3.072907698 0 0.565179074 1.507379102 0
0.499966104 0.282078525 0.278202383 0.496494117 0.146240086 0.0926303
0.122826319 0.502238677 0.525704516 0.117351457 0.082446842 0.414865491
2.398470651 0.018291443 0.140013183 0.211229609 0.140930043 0.607101698
0.46425424 0.207985899 0.246006361 0 0.07818235 0.166139008 0
0.183373148 0.052275276 0.273229884 0.021034173 0.160043869 0.181783554 0
0.142066871 0.349976273 0.818126352 0 0.063799356 0.107489956 0.252537203
0.48564578 0.070904284 0.716053149 1.305834021 1.264779118 0.084032095 0
0.042388431 0.830437737 0.592249567 0.125393428 0.357118646 0.241974937
0.215385008 0.06219674 0.742806784 0.054313866 0 0 1.370923749 0
0.183949793 0.053554422 0.0926303 0.040453689 0.07371901 2.109977894 0
0.149989831 0.03255205 0.130934862 1.345522814 0.011097711 1.681168264 0
1.502888143 0.17409534 1.734757835 0.135512048 0.270814973 0.837959719
0.106550153 0.054648761 0.433303957 0.10208732 0.141980666 0.143901683
0.423884306 0 0.270397693 0.170741489 0.461259051 0.083150013 0.150860178
0.039232753 0.279885331 0.6552168 0.050645917 0.449969494 0.244958267
0.317338341 0.072620775 3.783325595 0.644838172 0.025192091 0.124592192
0.069267062 0.759688756 0.116541975 2.058295894 0.258505202 0.585326171
0.048504174 0.146015446 0.598118959 0.405240772 0.057518224 0.21003962
0.145357557 0.050810887 0.788356256 0 0.079343553 0.475577514 0.221812193
0.066435019 0 0.091891541 0.342082071 1.0285017 0.070013207 0.178613845
1.061152548 0.10408547 0.757230216 0.562184656 0.01824011 0.36664181
0.188393025 1.700819271 0.079632874 0.107135594 0.24619543 0.029723595
0.0434269 0.112798032 0 0.092462351 0.068657317 0.073510568 0.064352073
0.039075507 0.056192156 2.235848418 0.526991299 0.211942153 0.461717331
0.102692182 1.209129344 0.01083862 0.14262356 14.095291636 0.221575887
0.034029107 0.051312311 0.296000566 0.408927003 0.168576467 0.259405917 0
0.480992059 0.194830916 0.42462278 0.266375383 0.297689741 0 0.024775957
0.151152543 0.065934663 0.044365593 0.153532898 0.020174525 0.008567082
0.559388305 0.100379295 0.0455045 0.311978849 0.119757375 0.085969397
0.193969237 0.370860007 1.030118841 0.095231639 0.172869636 0.280052827 0

0.642274787 0.497133435 0.685500401 4.865959565 0.072133838 0.460598695
0.057859579 0.242974182 0.186023815 0.026137638 0.170008595 0.024054624
0.038307815
TCGA-FD-A62N 82 0 0.03180663 0 0 0.1323927 0.04881147 24.39076
0.1662228 0.1964968 0 0 0.03142982 1.051974 0.1278879 1.061219
0.06875274 0.2255372 0.1882379 0 0.3810444 0.09475167 0.07830173
0.06536492 1.132037 34.66168 0.08053892 0.478715 0.8786064 0 0.5033682
0.4372113 0.2634451 0 0 0.3370837 0.07624986 0.01695556
0.2296425 0.1050006 0.1936959 0.04437697 0.05400119 0.04945372
0.04107632 0.5150287 1.302331 0 0.08984421 0.2270887 0.2562602
0.08290771 3.156047 0 0.4902369 0.1124733 0 0.3776184 0
0.09213126 0.5742127 0 0 0.2130793 0 0.1399537 0.08906693
0.04767632 0.1476327 0.4623191 0 0.1022716 0.05896761 0.09313742
0.4130201 0.4881147 0 0.1855532 0.01991542 0.01808412 0.15721
0.03596634 0.07069247 0 0.1723635 0 0 0.182576 0.2022502
0.04107632 0.05782281 0.07884929 0 0.04611635 0.02486317 0.06814921
0.2415165 0 0.1274062 0.2225981 0.8776566 0.2242755 0.06527033
0 0.3073376 0.9484022 0.5438319 0.123906 0.07809835 0.08791773
0.03596634 0.2577245 0.03140794 0 0 1.106443 0 0.4786747
0.02252274 0.1071302 0 0 1.388797 0.1972382 0 0 0.05320538
0.8835732 0.01283489 1.728293 0 1.54335 0.1006736 0.4012615
0.02612073 0.1252828 0.8448821 0 0.06741673 0 0.1180675 0
0.1248205 0.3921895 0 0.1389886 0.1184811 0 0.2404147
0.5670438 0 0.07193269 0.2212499 0.1757213 0.08673422 0
0.2854544 0 1.413638 0.3314568 0.1456776 0.0720476 0.2803842
0.5125204 0.07436405 0.8076665 0.1281301 0.1692375 0.05609676
0.0259803 0 0.3596808 0.06652182 0.1619454 0.2641745 0.05876455
1.600648 0.04828886 0.06882268 0.200008 0.464967 0.0768344 0 0.07357552
0.8308225 0.7434362 0 0.0688577 1.303963 0.0802523 0.3284111
0.3556573 0 0.1423331 0.212436 0.7552092 0.01315688 0.123906
1.233845 0.03437637 0.0358748 0.1304548 0 0.07338739 0 0.1912894
0 0.1807687 0 0.5412215 0.1218967 0.1470711 0.78045 0.133613
0.8553321 0.01253524 0.09425663 0.3318749 0.1281301 0 0
0.2160366 0.2252087 0.03249409 0.05000199 0.05160388 0.08558215
0.1261841 0.3879619 0 0.07377611 0.0815584 0.05730851 0
0.006932339 0.07696552 0 0 0 0.2310543 0.02902303 0 2.164886
0.163686 0.0426114 0.1985891 0.01649663 0 0 0.1587677 0.688268 0
0.4025933 0.287476 0.3523578 6.339502 0.03128448 1.902494 0 0
0.2444807 0 0.2722442 0 0.03322824
TCGA-BT-A20J 579 1 0.145725242 0.077917946 0 0.134793472 0.027954328
53.548179111 0 0.211828183 0.398915816 0.626936871 0.03599972
0.264939628 0.488275975 0.337644432 0.078749387 0.051666056 0.043121534
0.145255238 0.238062664 0.03255857 0.269060407 0.299475931 2.350408809
43.656699803 0.109021879 0.254577246 1.006355809 0.065391896 0

0.711637322 0.120699996 0.045555201 0 1.042458259 0.101892699 0.213629917
0.105213031 0.092513607 0.053784069 0.059300949 0.105150044 0.07080537
0.376390515 0.463503736 0 0.057784785 0.274420176 0.033927057 0.366900555
0.28488749 1.714777332 0.193844646 1.3102072 0.096620196 0 0.094614649
5.231351713 0.019481935 0.119582403 0.064574498 0.122706884 0.122030547
0.039136059 0.018395421 0.097159297 0.054608455 0.295922412 1.0590806
0.056538022 0.156189261 0.231570855 0.240029109 0.662302541 2.571798177
0.172198661 0.056675368 0.007603709 0.082854207 0.200075903 0 0.053980771
0 0.032904203 0.111455444 0.636027884 0.228133652 0.231657391 0.047048814
0 0.090313983 0 0.095079015 0.113913116 0.104077475 0.115799846
0.093926542 1.386345149 0.294189056 1.563749999 0 0 0 0.264018389
0.362099987 0.581378114 0.141921973 0.007454487 0.285319418 0.164783407
0.508396045 0.107923952 0.053588795 0.091920993 1.110537644 0 0.304596687
0.012898776 0 0.482299153 0.097655195 0.168526697 0.075305537 0.099345381
0 0.084380469 0.860993303 0.073505404 0.361655256 0 3.340997268
0.076874402 0.091920993 0.019945791 0.071749442 0.498095299 0.070573222
0.053088152 0.143498884 0.360625467 0 1.239067853 0.304823716 0.102296234
0 0.090472151 0 0.275370992 0.549570193 0.15591428 0.205979259
0.804607428 0 0.546399596 0 0.420376477 0.048100185 0.385519389
0.320895142 0.200231001 0.08252332 0.229394308 0.503177904 0.191647314
0.056804459 0.48920074 0.6299951 0.128506463 0.089273499 0 0.436946819
0.038097049 0.370984547 0.137538866 0.050481692 0.858672566 0.055310062
0.052552999 0.916356509 0.183645923 0.132009195 2.765804258 0.149819389
1.170648789 0.965069416 0 0.078869616 0.966421054 0.551525959 0
0.241875891 0.024162581 0.305678096 0.124781638 0.080466664 0.316467783
0.33115127 0.521814123 0.039374694 0.057527392 0.169798837 0 0.052836409
0 0.146068609 0 0.207052498 0 0.199972638 0.418861607 0.224606949
0.75278103 0.08502238 0.839740608 0.08614719 0.107961543 0.186263452
0.391360592 0.090156367 0.067973155 0.057103461 0.249355584 0.186093653
0.057272282 0.709285101 0.049012901 0.361328125 0.655307403 0 0.140838599
0 0.131282333 0.1001155 0.047641806 0.146927185 0 0.02672509 0
1.746687233 0.099728954 0 0.206638393 0.100953989 0.10846021 0.227463984
0.491276354 0 0.126152865 0.235734803 0.092746137 0 0.766385791
0.036586118 0.908078874 4.784435685 0.071666495 0.261494029 0 0.160933328
0.033603381 0 0.398447605 0 0.101492334
TCGA-DK-AA71 415 0 0.07673728 0.08206143 0 0.1064711 0.08832261
160.04260 0.1394329 0.1400431 0 0.0379141 0.14907 0.411393
0.8178789 0.03110141 0.09182283 0.04541463 0.1709771 0.1253611
0.1257298 0 0 2.55776769.68287 0.1059871 0.02062423 0.5299357
0.06886928 0 1.027066 0.1906778 0.04797771 0 0.44729 0.2759428
0.06136097 0.387828 0.1364066 0.08496626 0.03568824 0.09554183
0.01491413 0.198203 0 11.16247 0.1217153 0.1806332 0 0.6182583
0.2500309 11.16932 0.1531146 0.7392219 0.2204761 0.4090731
0.4982301 0.6395004 0.02222776 2.707742 0 0.06461607 0.8567989

0.08243444	0.005535327	0.05116299	0.1150248	0.7568858	0.1859
0.008506368	0.09252845	0.01016188	0.1544848	1.096106	0.9421079
0.07254231	0.02984461	0	0.1090752	0	0.08677309	0.1989797
0.4375876	0.6584254	0	0.0372139	0.2402652	0.4879527	0.1486523
0.2790089	0	0	0.01112612	0.05998537	0.007829433	0.1897121
0.04946066	0	0.1693755	0.5587718	0.01803638	0.3936811	0	0
1.144067	0.6122944	0.04982301	0.04710539	0.26514	0.1735462	0.2763516
0	0.05643852	0.04840456	4.444455	0.3177963	0.1924766	0	0.06461607
0.5643852	0.2056965	0.06060584	0.3965505	0	0	0.1036789
1.861283	0	1.182755	0.1236517	0.5663602	0.0404812	0.1452137
0.3045937	0	1.55876	3.27035	0.07115977	0.0503766	0.1899013	0
0.4517164	1.081376	0	0	0	0	0.1740087	1.262825	0	0	0.1334479
0	0.1569425	0.06835016	0.09838468	0	0.5684285	1.242358	0.1757323
0	0	0.7949035	0.4036773	0.05982518	0.1545646	0.8166113
0.06767006	0.06268056	0.03337836	0.8151806	0.04012296	0.04883907
0.1255391	0	0.04888295	0.05825132	0.1106953	0.6634967	0.2707765
0.1390291	0.1974836	0.09368568	0.8670078	0.2391505	0	0.08306371
0	0.1936183	0.1320552	0.3284783	0	0.1681207	0.3154013
0.2966099	0.1110989	0.2491151	0.6411567	0.1244056	0	0.07868446
0.1363577	0.06829296	0	0.1794755	0.08978008	0	0.1567917
0.1287041	0.1470452	0.473102	0.2973045	0.07163493	0.6878635
0.01512138	0.170554	0.2121822	0.05152152	0	0	0.03608405	0.2626157
0.03919793	0.06031788	0	0.2064771	0.1522171	0.308052	0.1486523	0
0	0.0691318	0	0.05017528	0.03094808	0	0.08443879	0.3705203
1.059147	0.03501076	0.03174255	0.07254231	0.04556677	0.03997975
0.06210814	0.1990005	0	0.03321534	0.1702427	0.04883907	0.1572449
0.5677343	0.03853168	1.062631	1.83084	0.05660816	0.1529998	0	0
0.03539033	0.03646564	0.784537	0	0.04008354
TCGA-KQ-A41O	1538	0	0.2685671	0.2513006	0.06087407	0.2173677
0.1030379	88.94621	0	0.3415937	0.3982276	0	0	0.03009918
0.5849204	1.804578	0.2267698	0.04463388	0.1192075	0.2913216	0
0.1300096	0.02754833	0.1379812	3.542764	152.25	0.2627467	0
0.3863922	0	0	1.967288	0	0.2098921	0.03606327	0.4980936
0.2011983	0.07158424	0.6301892	0.5114991	0.2973671	0.03903208
0.1082934	0.1565905	0.346838	0.03882832	14.49327	0	0.4109202
0.01042109	1.758085	0.3937791	3.630053	0.1786248	0.6036678	0.0148839	0
2.017732	4.777568	0.007480126	2.258963	0	0.1696087	1.686739	0	0.00484317
0.2909751	0.1509625	0.5064204	0.05421813	0.03721351	0.503741
0.213389	0.1843193	0.4795226	15.81632	0.1586784	0	0	0.3244827	0
0.6073814	0.671523	0.1276234	0.3941693	0.1540567	0.06512109
0.07007388	0.1067343	0.04335475	0.3051508	0	0	0.05840923
0.07872688	0	0.4446157	0.3894833	0.06723661	0.8168865	0.7204857
0	0	0	0.04054814	9.398358	0.8035964	0.1743719	0.2610294
0.5876978	0.07592268	0.8160603	0.06630017	0	0.2964632	5.116716

0.05561159	1.4034060.04754409	0.1696087	0.4444312	0.7199020.01893838
0.4857502	0	0.1192075	0.5486068	1.5032690.09482773	0.4384996	0
0.2688571	0.03541929	0.1270557	0.3675947	0	1.3245060.520257
0.1645488	0	0.0830777	0.1733138	1.3613550.6504829	0.0471322	0
0	0	0.9135004	1.4732230.2873452	0	0.3736357	0.06182275
0.2288631	0	0.2152058	0.04432358	0.6394503	1.2536010.06150326
0.1901099	0.4650433	2.3956320.4611223	0.1346	0.2704745	0.6251869
0.05920836	0.02742138	0	0.7132475	0.2457409	0.3418565	0.02534799
0.2015784	0	0.05096737	0.1937071	0.1055510.6684462	0.4865777
0.08639477	0.3580834	2.0043590	0.08546413	0.1090157	0.08095837
0.2541113	0	1.4428860	0.3974784	0.3679499	0.0185372	0.1527534
0.08718593	0.3005273	0.3628317	0.1211671	0.8824739	0.05965353
0.2368004	0	0.3813666	0.2356610	0.4801508	0.09623077	0.04288605
2.0179751.6041260.1096855	0.6161804	0.2778416	0.04974244	0.04553685
0	0.2076942	0	0.02104798	0.6893319	0.1371859	0	0.1089325	0
0.3900368	1.7986090	0.1816928	0	0.09073098	0	0.2634071
0.02707823	0.9370772	0.01231338	1.1294333.0239940	0.1110934	0
0.3986894	0.00499722	0.2018414	0.9924655	1.6466630.05812396
0.9806201	0.04273206	0.1719780.5266671	0	4.6022937.7827080.2366418
0	0	0.1482976	0	0	0.6545085	0.03523577	0.3507136
TCGA-E7-A677	820	0	0.3365528	0.4766288	0.1319505	0.3533742
0.3210581	18.602560.03169081	0.1189989	0.9295970.5008994	0.7010846
0.3352761.1459610.5732515	0.2506883	0.1048106	0.1076641	0.00853335
0.4160696	0.17884	0.8807770	3.38538144.949960.1340068	0.02933622
0.1675086	0.6857249	0.05758107	1.23717	0.3616306	0.6141980.03908533
1.3495830.2980132	0.1260722	0.4203066	0.2633220.1275716	0.03384233
0.06383188	0.07778495	0.1409635	0.04208208	10.650460.1731296
0.01712903	0.1129436	1.5023421.0195245.7278010.4355851	1.285143
0.1769074	0	0.8909246	0	0.07782673	1.3435580.06449079	0.06127391
0.5687377	0	0.01574707	0.3735784	0.2181507	0.2744289	0.2350462
0.03226556	0.2388548	0.1975437	0.5393654	1.5591182.4567920.1547779
0.02122571	0.2809725	0.2068670.09990828	0.1439986	0.5121526
0.6570108	0.3943386	0	0.2117345	0.09809689	0.05783928	0.1879513
0.5952996	0.09019691	0	0.1899115	0.02844136	0.01484894	1.439197
0.07035359	0	0.2683444	0.6135340.01068967	0.4666483	0.1401974	0
2.8629091.0783030.4488370.6402549	0.5447565	0.1645698	0.9172024
0.2694601	0	0.04590092	3.2229090.06027177	0.9430257	0.1094975
0.03063696	0.4013950.4144966	3.8567221.6921780.0496083	0	0.4073103
2.00638	0.7561243	2.09108	0.08794199	0.9884303	0.01919369	0.4131083
0.07967974	0.1074847	1.3786460.3171678	0.1759278	0.2388548
0.2926284	0.06261242	0.7615149	1.5862330	0.05962169	0.02258872
0.02773798	0.6875351	0.3243251	0.1038081	0.0822849	0.544146
0.5025257	0.6697121	0.03240743	0.8863111	0.07205675	0.2695138
1.0855670	0.3502695	0.0229097	3.1826630.9995275	0.4133246

1.6855550.8953694	0.06416994	0.6389641	0.1266077	0.9351009
0.09511916	0.5094425	0.1190458	0.4201355	0.1622410.1104767
0.3673941	0.4003863	0.4218381	0.3295952	0.8193023	0.2080725
0.5883371	0.1417380.04631296	0.3347614	0.04387129	1.49178	0.3130621
0.9090373	0	0.6122597	0.4797866	0.1406341	0.2709065	0.188984
0.4451385	0.1376328	0.1969811	0.4171637	0.4525670.1427132	0
0.2188189	0.1702727	0	0.1486819	0.1630993	0.4415586	0.9253027
1.5271040.4075784	0.5280402	0.1218837	0.1078216	0.5523715
0.09771332	0.2926284	0.03394252	0.08364318	0.3177309	0.3159491
0.05719804	0.02951524	0.2202722	0.7887363	0.6811419	0	0.1687872
0.1865918	0.3113914	0.3999429	0.08723009	0.3961892	0.05078015	0
0	13.083170.03319989	0.1655541.75415	0.2088479	0.06769976	0.216652
0.7737008	0.9734459	0	1.2948610.7641638	0.09319479	0.5578283
0.09134673	1.8389959.9175240.4354060.1741034	0.07654694	0.7634424
0.1398326	0	1.2110952.4376980.126701
TCGA-DK-A1A5	65	1	0.1232082	0.0768580.03723555	0.2279311	0.1418094
32.986780.03577175	0.1268604	0.01873758	0.1413505	0.01014572
1.0453420.3027412	0.1332205	0.1220656	0.1237678	0.04861136
0.06260944	0.5591053	0.02446908	0.1011049	0.08440059	1.257535
13.532630.1039936	0.08830388	0.09453962	0.03685849	0.5849639
0.4605430.2040996	0.05135486	0.2867702	0.1740999	0.3076733
0.1204136	0.4151271	0.07300409	0.1250521	0.1432512	0.08483512
0.07183767	0.05303862	0	1.0620620	0.1546784	0.02549755	0.3722498
0.2141044	2.1377150.1092616	0.6330044	0.2723024	0	0.1523716	0
0.07595257	0.3707179	0.1091933	0.06916438	0.1604940.02205925
0.1570114	0.08762296	0.1539017	0.5718796	0.6301206	0.01365769
0.1155484	0.2556137	0.2555547	0.5866305	1.6386870.09706068
0.03993171	0.09428915	0.1634542	0.01127738	0.2322026	0.3042654
0.1561298	0.7233184	0	0.1394168	0.1357325	0.06528745	0.05303862
0.03733103	0.1018119	0.06169111	0.1071836	0.01605193	0.02933191
0.2357015	0.1852977	0	0.6212768	0.3620101	0.2944158	0	0
0.8928921	0.8163982	0.1404415	0.05333004	0.2100880.2081223
0.06966077	0.6378273	0.1419411	0	0.1036235	0.7879736	0.1360664
0.2403625	0.007270463	0.1383288	0.1812336	0.08256579	2.117603
0.5518027	0	0.02430568	0.08191146	0.8854658	0.05800439	0.7402933
0.2977998	0.3901775	0.2166533	1.5284470.3091701	0	1.114995
0.4375686	0.3101154	0.0269613	0.05081711	0.1413505	0.2417563
0.4792748	0	0.1570319	0.02549755	0.06261979	0.4035572	0
0.05858793	0.1625418	0.6963514	0.1890792	0.1679896	0.1829033	0
0.05422384	0.34768	0.5324326	0.01881021	0.3721176	0.1810190.6145075
0.2190467	0.4574019	0.3033146	0.5463078	0.07243334	0.07547921
0.01786393	0.2181402	0.08589441	0.07841527	0.1963954	0.08536281
0.2223762	0.03117581	0.05924355	0.2582546	0.2484312	0.1240128
0.2113844	0.05541801	0.3575920.3199803	0	0.04445527	0.2971245

0.1554353 0.6360773 0.5740405 0.01361937 0.1856979 0.5943208
1.734846 0.05945958 0 0.1960822 0.5548438 0.1250702 0.3407205
0.03648898 0.1069427 0.02563222 0.1509426 0 0.05835311 0.1678283
0.6662746 0.5771175 0.1582511 0.6099442 0.0766773 0.2892537
0.04046443 0.06085309 0.4778042 0.02757406 0.1016342 0.03831343
0.4205724 0.305334 0.1048927 0.06456364 0.03331602 0.05525276
0.4364239 1.234924 0.07955793 0.06350753 0.05265498 0.05549848 0
0.07160948 0.06625302 0.1146386 0 0 2.923754 0.1311631 0.0679538
0.3105942 0.1138065 0.04585062 0.2421768 0.1384552 0.07325334
0.01777668 0.1974115 0.1306921 0.1472742 0.4502815 0.1031098
0.5687149 4.803897 0.09088919 0.3602927 0.04320208 0.1814218
0.03156787 0.05854867 0.1367052 0.0179609 0.07865919
TCGA-HQ-A5NE 370 1 0.04591008 0.08182566 0 0.2831072 0.1174251
21.5357 0.3199041 0.456026 0.08378442 0.2106812 0.01512207 1.027533
0.08204221 0.2552961 0.03307952 0.2441574 0.03622732 0.8183324
0.6666717 0.02735315 0.05023186 0.1886971 1.13774 621.89738 0.1902287
0.6745313 0.2818203 0.1098743 0.1937515 1.107151 0.2535066
0.1148157 0.1972742 0.9406648 0.07337334 0.09789548 0.1767834
0.116584 0.06212957 0.1067571 0.1403028 0.04758808 0.03952671
0.03539994 2.836192 0.2427311 0.08645484 0.03800379 0.4931856 1.03714
3.160281 0.08142651 0.8648616 0.2705756 0.1631591 0.1362648 0
0.08183594 0.5023186 0.05425041 0.05154433 0.1366939 0.03287904
0.07727193 0.07754432 0.04587773 0.2486107 0.7414635 0.006785542
0.0820112 0.0810615 0.3472923 0.8346217 4.039425 0.05786711
0.130939 0.03194019 0.05220569 0 0.03460951 0.3174528 0.1163548
0.4146528 0 0.2671703 0.1277733 0.09731015 0.1581068 0.05564145
0.0758747 0.09194985 0.06212726 0 0.07806938 0.3080721 0.07890969
0.1225998 0.4349919 0.9383855 0.1115042 0.031404 0.04717427
0.1109037 0.6591171 0.2791018 0.07948778 0.1002028 0.3807046
0.06921903 0.4822259 0.1208923 0.1800844 0.07722478 2.337954 0
0.3582574 0.02167307 0 0.1350633 0.2461266 0.6526625 0.1897974
0.08346217 0.07245464 0.122088 1.535509 0.03705207 1.599128 0.2959113
1.369778 0 0.3088991 0.1591904 0 1.016261 0.5336106 0.0810918
0.04018549 0.03787114 0.0526703 0.1601488 0.6065263 0.08594125 0
0.03800379 0.1400011 0.4164211 0.3777591 0.0436623 0.06921903
0.8037098 0 0.2503865 0.1090461 0.1177224 0.04040999 1.165979
0.7328315 0.08410916 0.1386592 0.3083505 0.3522754 0.4025175
0.2249781 0.4931856 0.5292723 0.05398051 0.2125016 0.05325194
0.2517177 0 0.155836 0.2233954 0.08482149 0.233964 0 0.05518862
0.721735 0.4551403 0.1478716 0.3150659 0.09833317 0.2728383
1.240009 0 0.1325201 0.07381008 0.193062 0.7373842 1.320833 0.0202995
0.231126 0.2096634 0.3380088 0.0633027 0.1589756 1.443024 0.06615904
0.06904284 0.1654759 0.1631591 0.07263653 0.03820451 0.1636205
0.3580885 0 0.0625365 0.5282707 0.5864909 0.1415228 0.5929007

0.1571440.3658065 0.06031174 0.06802559 0.8878066 0.2054940.07574228
0.1713171 0.6652372 0.3322928 0.3126825 0.04811566 0 0
0.2601938 0.3969542 0.1185801 0.0709929 0.1177224 0.1378664 0
0.06003734 0.07406199 0.1708674 0.01122616 0.02860303 1.334027
0.02792814 0.03798162 0.1736013 0.1332785 0.05922783 0.2901848
0.1904916 0.0272958 0 0.2150212 1.8700320.06271724 0.9876112
0.09221033 0.4662145 2.6495140.03512165 0.1464578 0 0.06760176
0.03764122 0 0.0727705 0.0107082 0.1278988
TCGA-XF-AAMT 90 1 0.1544370.1101014 0.0933468 0.2857033 0.2962549
30.331430.1345157 0.3273836 0.3288162 0.4429442 0.3052148 1.88724
0.3794751.2643310.1669143 0.2007674 0.09139883 0.2475095 0.4765562
0.0843455 0.1267312 0.1586896 2.0939435.734650.1007267 0.4704141
0.8887646 0.09240152 0.8147009 1.0614390.6822168 0.2253 0.2488541
1.1456960.4833571 0.04116383 1.1150280.3268141 0.1044989 0.1436481
0.08011746 0.06003059 0 0.5656395.6578380 0.3150610.08789067
0.6739798 0.7715697 3.5581770.1711942 0.5620255 0.4664721 0
0.4583812 0.06600108 0.2695528 0.7181437 0.1368697 0 0.6322592
0.1106018 0.2859286 0.2196643 0.1157461 0.4181509 0.7066927
0.0627712 0.1517327 0.2999511 0.4522284 0.8355906 0.4740078
0.1703268 0.2302435 0.2363760.1170765 0.2968515 0.1455287
0.1716235 0.1957029 0.1394851 0.5118466 0.2496484 0.1701358
0.1636709 0.06648197 0.1403792 0.1914262 0 0.09703104 0.04024101
0.0551497 0.7136090.09954164 0.1546551 0.6845220.5524112 0.5777584
0.02640999 0 1.2435642.5583130.7921722 0.3676599 0.2686044
0.5454632 0.08731722 0.7647326 0.07625056 0.03786162 0.03247206
2.0215460.04263855 0.2582448 0.03645303 0.04334751 0.3407546
0.6209592 1.2545670.8512793 0.07018961 0.06093255 0.1291693
1.0885550.03115987 1.1431030 1.3580870.1629402 2.04574 0.06341456
0.20277 0.8948692 0.4986147 0.2318670.2365650.1910921 0.2657665
0.6060653 1.0314820.1806861 0.02811911 0.06392049 0.03924581
0.3891109 0.6000737 0 0.05821148 0.8101848 0.5688093 0.3509481
0.1375575 0.09900163 0 1.3618881.73073 0.02357791 0.1166089
0.4213870.7702627 0.4551041 1.1122730.5875722 0.7190156 0.04539627
0.2417822 0.1791342 0.6879871 0.1614982 0.06552711 0.3368703
0.05944397 0.9018073 0.03907773 0.4455577 1.2948510.2789619
0.3108910.4968049 0.1653915 0.6403263 0.7620587 0.2293449
0.2228922 0.6207245 0.2922486 0.6644163 1.4165920.01707137
0.3887425 0.2292183 0.5969397 0.2022968 0.1002709 1.566859
0.2781905 0.1741899 0.1919464 0.1372128 0.1136869 0 0.189201
0.1806861 0.03657174 0.2629582 1.6200670.5589876 0.5554135
0.6315787 0.5766731 1.0107980.1521620.09534639 1.07965 0.4147568
0.1592435 0 0.7235168 0.3888 0.05259164 0 0.1252812 0.03462865
0.4230453 0.6219546 0.04986147 0.2587142 0.03300054 0.2086959
0.07073372 0.09536984 0.2076143 0 0.03776368 0.04009073 0.8227143

0.07046062	0.1916496	1.897927	0.2547362	0.1570903	0.3660574
0.5206457	0.04591019	0.02228242	0.199862	1.605414	0.1582309	2.257645
0.1033955	1.675229	5.065023	0.1097068	0.2873905	0.1083044	0.0568514
0.2374150.02446287	0.6487008	0.04052398	0.06274317
TCGA-XF-A9SU	182	1	0	0.02173601	0.07371344	0.09400507	1.933941
117.0225	0.2124468	0.1329562	0.2225633	0	0.04016997	1.67254
0.1089677	1.130273	0.01098398	0.5693047	0.09623357	0.1430133
0.1328201	0.09688051	0.03335874	0.08354189	2.094472	58.50118	0.07486222
0.1311082	0.09357777	0	4.374761	1.529328	1.212138	0.1016647	0
0.1292464	0.3086036	0.02167064	0.5870052	0.05161525	0.4276032
0.1606996	0.04601206	0.01580151	0.367493	0	11.94344	0	0.1913808
0.1261907	0.900686	0	5.972971	0	0.3132821	0.1976568	0	1.478049
0.5211926	0.1340556	1.534502	0	0.0684607	0.453889	0.9170621
0.2785721	0.09757291	0.1218687	0.1415153	0.9191511	0.01802499
0.1089265	0.1830307	0.01487969	0.1055749	3.431185	0.03842927
0.3003934	0.4581647	0.02311303	0.04465059	0	0.2710529	0.07727065
0.7343173	0.03109164	0	0.2262764	0.1292464	0	0	0	0
0.03536436	0	0.161758	0.1651071	0.4192284	0.5699256	1.028574
0.9970838	0.03344168	0	0	0.5892052	2.222253	0.7877383	0.3167248
0.04159012	0.4681929	0	0.2378955	0	0.05979658	0	2.638732
0.06734101	0.4758341	0.007196493	0.0684607	0.1793897	0.4358707
0.8439273	0.08402902	0.1108537	0	0.03138507	0.7584725	0.01640407
0.7009023	0.06550444	0.5553732	0	1.743677	0.1669225	0	1.556227
0.157497	0.06462321	0	0	0.0699562	0.2658852	1.718576	0	0.1776392
0.05047628	0.1239654	0.1229081	1.170718	0	0	0.09897163	0
0.1108537	0.07241697	0.2084771	0	0.8603568	0.5648397	0.2606637
0	0.3071611	0.2807333	0.1900871	1.159036	0	0.3244498	0
0.04980752	0.2121862	0.6965189	0.2975718	0	0.163703	0.05632955
0.2589574	0	0.219903	0.1278135	0.3483635	0.04910043	0.05230844
0.07313891	2.309127	0.3800697	0.2587249	0	0.7842708	0	0.1399124
0.1846651	0	0.2122327	0.3132821	0.9876682	0.01681561	0.1055749
0.6793053	0.1757436	0.009170205	0.09852364	0	0.03751812	0
0.4346383	0	0.05775943	0.08306038	0.6594959	0	1.441098	0.367493
0.07589718	0.2602825	0.0160211	0.2108189	1.75604	0	0	0
0.6201924	0.1918905	0.04153019	0.06390677	0.06595413	0.05469061
0.1267152	0.3420724	0.3937425	0.0314307	0	0.07324512	0
0.04430057	0.06557896	0	0	0.0506537	0.2362455	0	0.01681561	0
0.04827798	0.03630731	0.0705038	0.04216818	0.1087621	0	0.1954031
0	0	0.4420767	0	1.125858	4.961015	0.01999211	0.1621032	0
0.08978802	0.08749075	0	0.4446042	0.007111264	0.08493699
TCGA-CF-A47S	333	0	0.2338001	0.03788205	0	0.2031545	0.07610848
52.43077	0	0.08753841	0.4654666	0.04876854	0.08401104	0.06069858
0.246885	0.8930062	0.04977216	0.04395814	0.01677182	0	0.07716068
0.08020171	0.1162769	0.1164791	1.83155	266.6001	0.03914151	0.03808309

0.09785377 0.07630116 0 1.886248 0 0.01771838 0.0152217 0.2703046
0.1019069 0.06798262 0.3069141 0.1259388 0.1359731 0.04612938
0.03688783 0.04406281 0.237891 0 22.00622 0.06742497 0.08005036
0.03518851 0.8562205 0.2031425 7.733023 0.1130918 0.4185967
0.04384304 0 1.140791 0.07266778 0.01389183 2.232516 0 0.09545197
0.4904503 0 0.00408844 0.1435998 0.06371873 0.2959636 0.06865367
0.01884863 0.1252942 0.04878676 0.1763422 1.140791 5.43632 0.05358037
0.03306523 0.008872228 0.1208459 0.02334544 0.03204568 0.1469681
0.0808015 0.2559572 0 0.1236893 0.1133785 0.1351523 0.07319723
0.128799 0.03512699 0.04256915 0.05341602 0.01107643 0.005782887
0.470413 0.01826604 0 0.1647688 0.5647661 0.006660912 0.1308493 0
0.05134405 2.816725 0.4199426 0.07359941 0.0695849 0.4700033
0.09613703 0.3061736 0.06996045 0 0.03575203 2.149516 0 0.5686596
0 0.09545197 0.2292729 0.1329381 0.03836914 0.3514748 0 0
0.1221603 1.351259 0.005717883 0.6959131 0 0.8321869 0.04484964
0.01787601 0.08921442 0.05581289 1.317369 0.2744896 0.1651861 0
0.05259853 0.1706899 0.3521773 2.190223 0 0 0.05278277 0.04320998
0.2784694 1.049325 0 0.01602284 0.2414862 0.2609434 0.07727938 0
0.1271686 0.01870823 0.6897473 1.001995 0.2076759 0.09629061
0.1427541 0.5545047 0.2608896 0.1009998 0.152217 1.112069 0
0.02893525 0.110941 0.8108919 0.05927032 0.01803648 0.02139791
0.08508284 0.04513172 0.1290748 0.1992912 0.4232374 0.4499951
0.1198028 0.07293154 0.06737608 0.5669414 0.1103991 0.1623283
0.07668947 0.1366846 0.2323882 0.1706899 0.2698494 0.0281936
0.265524 0.3615705 0.03912118 0.08205862 0.07359941 0.5073899
0.1225161 0.0575354 0.2403931 0.1510725 0.04296892 0 0.2367182
0.198937 0.06039872 0.08685579 0.4830876 0.09050739 1.070151 0.3476868
0.08597904 0.4173366 0.02233757 0.1049772 0.3711003 0 0.1577956
0.02643768 0.03997799 0.2040032 0.01447597 0 0.09195716 0.0762529
0.2369036 0.1629902 0.02744896 0.0876451 0.03633389 0.153184
0.03893922 0.04941319 0.06857556 0.355972 0 0 0.8646423 0.01292963
0.04689064 0 0.1458427 0.08436968 0.1359825 0.5438388 0.9098554
0 0.2829211 0.03607296 0.05807121 0.5633214 0.01422991 1.491251
4.9723 0.09059109 0.0113007 0 0.1251878 0.0566358 0 0.5053481
0.04213852 0.0592121
TCGA-FD-A6TK 330 0 0.03839017 0.1094766 0.09281715 0.3077557
0.4909565 21.09049 0.1337525 0.1581127 0.116768 0.3523447 0.101161
6.919709 0.3773219 0.8776377 0.138306 0.3334699 0.03029341 0.09604103
0.6968415 0.0228728 0.08400811 0.05259638 1.578897 14.38767 0.02945739
0.3852017 0.05891478 0.09187723 1.782172 0.6850947 0.08479324
0.1920185 0.1649614 0.4611028 0.1738393 0.05457369 0.2956538
0.1949758 0.1889199 0.1190276 0.09704385 0.05968997 0.1322095
0.02960155 1.323702 0.04059452 0.04819589 0.08739198 0.3093026
0.1334246 1.703877 0.03404456 0.5588366 0.5543278 0 0.4652757

0.2296931 0.2030134 0.2940284 0 0.0862031 0.1428799 0.08248069
0.2344603 0.1604007 0.1918156 0.1187938 0.9093543 0.04539275
0.1097249 0.1762382 0.2060953 0.4985097 0.3142122 0.04838867
0.1692144 0.2243514 0.02910305 0.07027789 0.05788119 0.1706497
0.2432406 0.4623122 0.09787353 0.8936349 0.1691704 0.2441133 0
0.2326379 0.3172334 0 0.05195103 0.02000634 0.09400591 0.4474295
0.2309459 0.05125919 0.5318345 0.7650642 0.216558 0.05252027
0.03944729 1.020119 1.187105 0.6418112 0.1329359 0.06284243 0.1886498
0.1157624 0.2880278 0.07581791 0.1129404 0.06457563 0.7801664
0.04239662 0.4707624 0.07702317 0.0862031 0.3388211 0.137208
1.715241 0.2116123 0 0 0.1580755 0.6260815 0.04131076 1.885447
0.04124035 1.028862 0.2160209 1.517527 0.1261095 0 0.5498713
0.04957856 0.06441878 0.2016195 0.1266719 0 0.1004377 0.7438631
0 0.05591911 0.0635578 0 0.2708321 0.3158835 0 0.02894059
0.3427086 0 0.104687 0.2279617 0.06562659 0.06758195 1.51666
0.7048744 0.04688825 0.1449341 0.1933828 0.1767443 0.100976
1.533524 0.3093026 0.2723565 0.04513869 0.08362098 0.1113236
0.4735962 0.05352729 0.0651553 0.3414004 0.08274936 0.6684419 0
0.1938254 0.3621104 0.1612669 0.2473016 0.1975944 0.1019609
0.5942469 0.5583308 0 0.1662206 1.419566 0.1291513 0.6166032
0.3666712 0.03394902 0.1789522 0.3024292 1.017519 0.09528108
0.1329359 0.2596614 0.1936285 0.04618709 0.1431429 0 0.06748769
0 0.102615 0 0.07272847 0.1045865 0.7211473 0.1961703 0.1577892
0.3635761 0.05972927 0.9941381 0.07060604 0.1516886 0.9667053
0.03436696 0.06333596 0 0.9306886 0.410756 0.02614662 0 0.08304692
0.3098895 0.1958175 0.9049167 0.2974713 0.2176699 0.06562659
0.06917059 0.07033237 0.07251607 0 0 0 0.1753983 0.446207 0
0.03176036 1.306494 0.1215796 0.156199 0.2781638 0.2654828 0.06847454
0.04431197 0.222386 2.801678 0.07866656 0.5338328 0.4626396 1.063228
5.003861 0.0587377 0.5715198 0.3230698 0.05652882 0.0786893 0
0.6693604 0 0.1693366
TCGA-E7-A5KF 20 0 0.4463328 0.4483728 0.0490506 0.3127656
0.04151252 47.99399 0 0.3440582 0.197465 0.4655045 0.1737451
0.01347393 0.5800767 0.5264765 0.07308988 0.004795296 0.09605402
0.5519531 0.117842 0.04834988 0.2441744 0 2.298398 72.4790 1 0.09340318
0.05816159 0.06226878 0.1456617 0 1.467764 0 0.06765004 0
0.6880282 0.07565596 0.2451424 0.234364 0.2198142 0.04492688
0.01887057 0.08113631 0.4626461 0.2794723 0 15.64227 0.04290556
0.4075174 0.0503821 0.3269111 0.9166331 4.679991 0.251879 2.397348
0.0239137 0.3605035 0.5218717 0.2427694 0.01326 0.8435114 0
0.4099978 0.2718253 0 0 0.07935552 0.5676596 0.7847294 0
0.005997119 0.3116734 0.1361214 0.4950642 2.423693 11.08384 0.179002 0
0 0.09227964 0 0 0.3607295 0.3085059 0.5130631 0 0.1049455
0.07999003 0.172007 0.4192084 0.2950582 0 0.1218991 0.2745429

0.1268718	0.008279804	0.6114283	0	0	0.1893122	2.011181	0	0	0
0	2.464564	0.5550131	0.3512598	0.2103301	0.6106306	0.2141172
0.8402135	0.1869798	0.07958002	0.1023779	1.814078	0.08962049
0.2261649	0.01436613	0.1822212	0.2785301	0.2900383	0.009156008
1.006467	0	0.03201801	0.226247	2.142192	0.1746503	0.4946629	0
0.4545139	0.02853986	0.06825191	0.2147432	0	0.8559127	1.519631
0.1110879	0	0.3681791	0.0931009	0.9554008	1.346089	0	0	0	0
0.5316081	2.29997	0.3087128	0	2.305029	0.09963005	0.5163519
0.1927516	0.2427694	0	0.2862505	1.016828	0.1982303	0.2144593
0.03406534	0.5604191	0.8182211	0.06627918	0.9807333	1.655205	0
0.2209538	0	0.4727492	0.1131492	0.1377292	0.006808231	0.1249432
0.06892645	0.2464084	0.2829017	0.8505005	0.388621	0.1960353
0.2784579	0.1286234	0.7402338	0.08430235	0.06886458	0.05856118	0
0.9214007	0.0931009	0.5718662	0.03588173	0.5875946	0.365972
0.179241	0.2909267	0.03512598	0.1775813	0.2046517	0.3173073
0.4034454	0.2884028	0.08202921	0	0.3253705	0.4430743	0.03843444
0.1105406	0.1253839	0.5874583	0.6253952	2.34058	0.2272667
0.2424771	0.0106608	0.1002026	0.1439467	0.03632346	0.1673541	0
0.01413322	0.3000675	0.08290541	0.04252502	0	0	0.8355266
0.8541075	0	0.1464029	0	0.1949559	0	0.1002273	0.08727547
0.1510141	0.009921772	0.008426531	2.043641	0.1234156	0.08951592
0.1022869	0.1927516	0.05637266	0.3096379	0.5892531	0.1447456	0
0.3450681	0.1032969	0.1940051	0.6028032	0	2.959227	6.819378
0.2261545	0.02157344	0	0.418229	0.05821834	0.154253	0.373028	0.06624796
0.09419836
TCGA-UY-A78L	1127	0	0.037275353	0.079723153	0	0.206874562	0.019067984
35.096243376	0	0.189643852	0.1133772	0	0.012277921	0.425802516
0.066611784	0.391529264	0.040286929	0.147576039	0.088241153	0.104908602
0.216513879	0.088834371	0.040784298	0.102138069	2.507347169	23.087394876
0.137289482	1.309055387	0.114407902	0.089209199	0	0.791048922	0.12349638
0.031073751	0.053390354	0.447712836	0.104253354	0.119225077	0.574136599
0.113588361	0.082545212	0.242699347	0.071724111	0.077275513	1.508350445	0
1.26741236	0.039415698	0.023398163	0.007714018	0.400427657	0.485812966
1.412034369	0.066111883	0.574526638	0.746932387	0.198708461	0.295029901
0.031860428	0.031007346	0.774901669	0.044047042	0.041849921	0.110984673
0.026695177	0.043020817	0.102723965	0.074498169	0.432540513	0.280937855
0.071621207	0.06658661	0.04607087	0.118247093	0.35495785	0.953399183
0.023491756	0.048323689	0.020746325	0.042386889	0.040942255	0	0.073641868
0.094470868	0.134666116	0.057018825	0.096409395	0.047548328	0.079008148
0.096277688	0.135529361	0	0	0.072060601	0.097126885	0.012677232
0.162365187	0	0	0.139131128	0.47618424	0.131418326	0	0	0
0.740978281	0.708151805	0.290420059	0.1169503	0.240412706	0	0.503394768
0.049077484	0	0.094050624	2.575537725	0.04116546	0.207769067	0.017596821
0	0.511749868	0.099917676	0.428975014	0.308200879	0.06776468	0.029413718

0.118311475 0.906699349 0.040111137 1.272397978 0.120128297 0.971668972
0.078655433 0.125400832 0.108842112 0.097882316 0.582440228 0.144416532
0.04279608 0 0.215238601 0.085528237 0.29256338 0.251697384 0
0.027147638 0.030856072 0.037889929 0.187833869 0.170394748 0.070900672
0.056200373 0.423508476 0.091526322 0.135529361 0.177073537 0.063720857
0.032809715 0.262967416 0.521011383 0.113816647 0.05629015 0.25035619
0.514835559 0.15251243 0.254623179 0.400427657 0.561951008 0 0.111639969
0.108090901 0.442812218 0.2338781 0.031631628 0.075053533 0.06886834
0.221620338 0 0.080655825 0.507859467 0.294377673 0.120060081 0.031976074
0.11177426 0.628507503 1.200402909 0.031631628 0.053797914 0.179783846
0.094050624 0.384877068 0.542718608 0.032963175 0.333610734 0.276623937
0.46654188 0.020558713 0.096806686 2.254259401 0.10743181 0.061663056
0.15751769 0.044157436 0.104844657 0 0.332117189 0.058147911 0.105924751
0.253873442 0.274323085 0.380947392 0.268112431 0.673943816 0.162385409
0.466723626 0.07834938 0.036820934 0.261883813 0.066737943 0.061496743
0.046365308 0.132433259 0.152493089 0.203098754 0.117198339 0 0.03343229
0.422513595 0.911251963 0.096277688 0.038427082 0.031860428 0.067161945 0
0.070410273 0.040088321 0 0.072918021 0.185787173 0.541561995 0.09070176
0.082234851 0 0.118049024 0.136866728 0.152282664 0.14177541 0.022162034
0.021512597 0.215928134 0.094894885 0.050921436 1.337913679 0.174690819
0.619411532 2.361068375 0.081474298 0.158549533 0.104562712 0.10977456 0
0.047235434 0.307236244 0.00869421 0.0605755
TCGA-S5-A6DX 56 1 0.05792656 0.1445397 0.1400509 0.1607434 0
95.4209 0.4709083 0.2385747 0.1057141 0.1329124 0 1.1733720
1.6463760 0.2053753 0.3199661 0.02717164 0.2102915 0.2300836
0.9506928 0 2.69218 72.857370.05333757 0.1660649 0.1777919 0 0
3.3805861.2154650.04828916 0 0.04092669 0.1465819 0.04117286 1.2268
0.4314892 0.3848301 0.06285984 0.04371001 0.1350984 0.2493620
15.229580.1225054 0.07272232 0.09590176 1.4778950.3019848 7.385382
0.1027390.09920273 0.2560469 0 1.1032220.04951167 0.1479997
5.4506390 0.06503552 1.5953670.04148478 0.02228504 0.04119609 0
0.3584936 0.3742135 0.05136952 0.06208606 0.03068355 0.1837581
0.3510250.5333757 0 0.2553259 0.09672047 0.1097833 0.1060416
0.3056773 0.2002713 0.1468094 0.2092735 0.1181443 0.03745548
0.1612166 0.4911202 0 0.07020501 0 0 0 0 0.07486244
0.02727766 0.09956346 0.3093780.3035287 0.1479997 0.04084528 0 0
0.3265071 5.9493820.8363651 0.3510250.02370559 1.0496540 0.1912251
0.07626728 0 0 4.7364410 0.2583014 0.01367289 0.3251776
0.2840244 0.2587897 0.4052094 0 0.2106150 0.05466052 1.1848630
0.5447743 0.1244543 0.9824036 0 1.3641260.2748567 0.07605542
1.01072 1.6457890.07673753 0 0 0 0.1010332.7720080 0 0 0
0.7589326 2.0124530.05509045 0.1310046 0.04029426 0 0.3685763
0.06879385 0.2475583 0.1019738 1.0625060.4120168 0.2829969 0
0.2917935 0.9778554 0.2200779 0.6795527 0 0.9760208 0

0.1734905 0 0.6749046 0.2826839 0 0.04859771 0.07134841
0.07380041 0 0.4038752 0.3035471 0.8370693 0.1399316 0
0.1240708 1.961429 0.5415815 0.04915611 0 1.583195 0 0.5316496
0.5532667 0.1536761 0.1440103 0.1984055 0.1705916 0.0159743 0
0.3918007 0.1252132 0.02613422 0.215987 0 0.1349268 0 0.6193392
0.4518144 0 0 0.3674213 0.04933325 0.654738 0.5485965 0.2162998
0.3626484 0.1065368 0.02861019 0.9307975 0 0 0.07205251
0.1372024 0.218748 0 0 0.06265435 0.1558631 0.2298078 0.3219768
0.07480861 0 0.04951167 0.1043708 0 0.01683366 0.2180429 0
0.07082243 0.336836 0.7854904 0 0.1118201 0 0.1987378 0.08622702
0.2098594 0.02002923 0.6888038 0 0.06425543 0 0.03956641
0.9500713 0.0775636 0.5882412 2.886392 0.0253225 0.8931593
0.08124615 0.1705916 0.1187335 0.03670235 0.4407236 0.168887
0.0941354
TCGA-XF-A8HI 544 1 0.5955177 0.3442355 0.2918518 0.5955072
0.09880006 61.7325 0 0.2924502 0.2056109 0.8863228 0.3021836
0.1859945 0.819723 1.253017 0.3566065 0.09415587 0.07620305 0.007549723
0.2804647 0.1054836 0.2113223 0.06615308 4.564563 86.37596 0.07410004
0.2249405 0 0.3466756 0 2.095973 0 0.322015 0.06916004 1.125789
0.1286155 0.4118402 0.6507523 0.4822872 0.07128416 0.02245603
0.0819785 0.2502501 0.2494297 0 19.7474 0.408462 0.2424734
0.01998495 1.296751 0.7551666 5.111928 0.6422931 1.81921 0.227659 0
1.170401 0.2476255 0.02582091 1.109442 0 0.3794767 0.2515901 0
0.009287949 0.2918848 0.4342607 0.8591239 0.4159055 0.04281954
0.4053939 0.3410207 0.5302151 1.797401 5.631603 0.1825825 0
0.04031112 0.8602023 0.0353568 0.03640002 0.3338761 0.4894973
0.6105466 0 0.2185495 0.2407706 0 0.207858 1.111881 0.07980005 0
0.4013828 0.02516297 0.01313732 1.176668 0.2074801 0.06447122
0.3535197 1.899845 0.003782996 0 0 0.07776086 5.332433 1.247549
0.2926002 0.3029868 0.7118226 0.4004002 1.564993 0.3496531 0
0.2436599 2.759055 0.2666216 0.9150656 0.01139716 0.1084219
0.3788019 0.5608631 0.05811028 1.197699 0 0.3048122 0.4721961
2.108775 0.2857723 0.4204645 0.1556101 0.9199894 0.06792504
0.08121998 0.1498022 0 0.8173459 0.5612167 0.3923204 0.08452894
1.234742 0.609347 1.13693 1.530971 0 0 0.0399699 0 0.6326153
2.118946 0.3673692 0.03640002 0.9180626 0.05928003 0.6583504
0.2867188 0.1238127 0 0.4768946 2.148499 0.05897368 0.3645817
0.9323707 2.000701 0.7761262 0.1577449 1.426426 1.284586 0.2270927
0.1840549 0.1680207 1.257516 0.06732393 0.4097453 0.1053236
0.02973656 0.184551 0.04887112 0.522394 1.467542 0.1947201 0.5054456
1.15978 0.3888607 1.001001 0.05016003 0.1229236 0.2090639 0 1.66501
0.4431614 1.389166 0.06404905 1.011346 0.5181991 0.159973 0.479359
0.2090001 0.1344779 0.3131179 0.6099638 0.6901455 0.5720003
0.09973707 0 0.4517239 0.07532282 0.0457371 0.2630872 0.2552229

0.2056109	1.786133	1.746008	0.3004979	0.7969435	0.1649231	0.2384829
0.7993862	0.7780504	0.1991519	0	0.1009115	0.3570813	0.09865769
0.05060491	0	0.04330705	1.204081	1.262412	0	0.398217	0.04127091
0.4059967	0.3538421	0.1543504	0.1038581	0.0898536	0.04722776	0
4.209125	0.1468649	0.1997329	0.06086084	0.216632	0.1437507	0.4131331
1.268862	0.1148318	0.08360005	0.6427285	0.08194906	0.1649047
0.6656893	0.09698079	3.61064	6.864515	0.5804646	0.1540347	0
0.3554955	0.02969143	0.03059358	1.821539	0	0.04483853
TCGA-XF-A9T5	2027	0	0.05106004	0.2002098	0.06172476	0.2046621
0.02611946	80.27936	0	0.1855539	0.1863659	0	0.1177287	0.2984158
0.1368679	0.8518017	0.1839511	0.06939502	0.2014556	0.06386869
0.1112183	0.09126446	0.05586662	0.06995473	1.134604	67.61748	0.1567168
0.4574371	1.332092	0	0	2.733588	0.3947212	0.08513009	0	0.8297321
0.1156057	0.1088769	0.2949211	0.06915295	0.1381979	0.07123956
0.06935167	0.01323157	0.04396062	0	3.325507	0	0.3525607	0.02113343
0.3428179	0.3549174	7.238871	0.09056053	0.3060519	0.2256956	0
0.5430551	0.1309279	0.01820318	2.178798	0	0	0.380069	0	0
0.2723458	0.2551203	0.2764986	0.5497581	0.0679204	0.07296865
0.0631082	0.06229835	0.2210108	0.626867	0.1608959	0.05295531
0.01420923	0.01935395	0.1869433	0.1154755	0.1513127	0.1941103
0.1537222	0.07810479	0.1650778	0.1184219	0.1082259	0.04396062
0.1856491	0	0	0.03948365	0.1862631	0.01389229	0.3546522
0.08776138	0.2727049	0.2162382	1.669838	0.03600355	0.03492674	0
0.2055739	1.296941	0.3880126	0.1326065	0.02089557	0.4861376
0.3464265	0.3064683	0.06722669	0	0.04294374	3.747726	0	0.2276828
0.01807819	0.05732632	0.9012839	0.2737359	0.2112334	0.7036263	0
0.04029112	0.1095026	1.002071	0.09615291	0.4979828	0	2.261095
0.143657	0.08588748	0.1397744	0.06703995	1.409501	0.1978228	0.1262636
0	0.08423868	0.05857859	0.04452838	1.903768	0	0	0	0	0.1029185
0.560179	0.09712024	0.03849184	0.2959821	0.2507468	0	0.1819174	0
0	0.7204293	0.5405426	0	0.11566	0.4286746	0.7835838	0.0994822
0.2274682	0.1371272	0.1358408	0	0.09731605	0.02961274	0.5132493
0.07119286	0	0.1884829	0	1.019152	0	0.1595864	0.2675652
0.2573876	0.2466887	0.1752042	0.06999277	0.5645469	0.2121704	0
0.1105391	0.4104486	0.3864936	0.3514716	0.3449468	0	0.2602261
0.1165912	0.3195362	0.1548881	0.08840432	0.3859876	0.1471609
0.3609023	0.1903843	0.362923	0.1952293	0.04249011	0.1137341
0.07965142	0.04836549	0.2086546	0.2221401	0.1739415	0.5771265
0.7033699	0.2224367	0.929922	0.08049265	0.1260939	0.7742892
0.09141811	0.04211934	0	0.04268424	0.3374314	0.06955153	0	0
0.1831832	0.2507969	1.056405	0.06594093	0.1315942	0	0	0
0.08161026	0.302023	0	0	0.445362	0.3956456	0	0.02816147	1.673317
0.148229	0.07093876	0.247956	0.2824799	0.06071542	0	0.2769003
0.1733169	0.1046288	0.3216299	0	1.885498	2.041145	0.08370305

0.5701035	0	0.07518499	0.1883863	0	0.7284018	0	0.05926911
TCGA-XF-A9SX	719	1	0.04559388	0.02437863	0.248026	0.105434	0	87.7401
0.397126	0.2485345	0.2496221	0	0.02252687	2.102988	0.2444314
1.309945	0.03695815	0.1616505	0.05396671	0.06416022	0.347592	0.05432951
0.2244865	0.09369872	1.493953	62.45313	0.04198189	0.196064	0.7346832
0.08183812	0	0.5277724	0.8308099	0	0	0.09663994	0.07286798	0
1.185069	0.05789051	0.3617961	0.1802371	0.03870457	0.01772262
0.2944085	0.1582026	2.489139	0	0.08585939	0.2972186	0.3673416
0.2971145	7.491835	0	0.4099319	0.201534	0	0.4059788	0	0.04876338
0.448973	0	0.153568	0.1527215	0.1469366	0.1249761	0.1276749	0
0.4232545	0.1472713	0.08086569	0.1099526	0.02415096	0.08344362	0
0.5597586	0.08620282	0.2305204	0	0.02592306	0.02503955	0
0.1351141	0	0.4117969	0	0	0.1031008	0.5798396	0.05888171
0.1657747	0.1130282	0	0.02644258	0.03564064	0.1535127	0.0724619
0	0	0.1178171	0.6989418	0.09644771	0.748505	0	0	1.435035
0.5716827	0	0.04664655	0.2310505	0	0.3078672	0.04502237	0	0
2.207394	0	0.9148885	0.07264283	0	0.134133	0.1222157	0.7819161	0
0	0	0.02346719	0.6238362	0.03679688	1.262551	0.07346832	1.775606
0.04810425	0	0.1747355	0	0.997388	0.2649677	0.07247995	0	0
0.07846131	0.8349904	1.566107	0	0.2988542	0.1132261	0	0
0.562736	0	0.1546701	0.1268621	0	0.124331	0	0.1753674	0
1.929914	0.4525083	0.04176493	0.1032781	0.22967	0.5247737	0.05329935
0.7515326	0	0.1212985	0	0	0.03966388	0.3906	0	0.116072
0.3213098	0.04211864	0.3194849	0	0.09865532	0.1433528	0.6090583
0.2753497	0	0.1171871	1.890413	0.07104628	0.116072	0.09870552
1.099526	0	0.4707678	0.5257565	0	0.1700253	0.2889044	1.132923	0
0.05920524	1.741471	0.1478326	0.02057019	0.07650132	0	0.09017032	0
0.1218702	0.1066867	0.06478168	0	0.748018	0.1747355	0.3513702
0.6476988	0.148968	0.4281597	0.03593781	0.4728995	0.09990393
0.4897888	0.05641546	0	0.3716187	0.3443522	0	0.2867056
0.1479453	0.1226795	0.06460044	0.4118193	0.2649677	0.1762599
0.0584558	0.1232251	0	0.009937307	0	0	0.01672326	0	0.4636935
0	0.01886001	1.20684	0.0541475	0.03393456	0.1739661	0.09458978
0.1626468	0	0.1264383	0	0	1.853241	0.2289381	1.767831	8.413632
0.0224227	1.490853	0	0	0.1682189	0	0.2168079	0.05583084
0.06350896
TCGA-G2-A2EK	485	0	0.3433053	0.2723826	0	0.5326676	0.01699507
38.26797	0	0.2656145	0.282945	0.8385337	0.3392383	0.9907052
0.8311845	0.8416228	0.4129337	0.05104259	0.02621611	0.181813
0.2170984	0.03299044	0.1454023	0.04551724	2.429968	28.67793	0.01529557
0.1309613	0.1529557	0.2385334	0.8412562	2.69202	0.1467612
0.1661741	0.07137931	1.00934	0.7831813	0.2479492	0.7995646	0.35434
0.2084529	0.07725541	0.07646156	0.1119215	0.7436971	0	15.38524
0.2459153	0.1668361	0.09625582	0.4907328	0.5195994	3.827246

0.2651614 1.735345 0.07832144 0.4722842 0.7560007 0 0.169767
0.3998563 0.03925862 0.07460071 0.148379 0.04758621 0.01118365
0.08269637 0.6307939 0.7453356 0.3577096 0.01473119 0.5519353
0.4927492 0.5269614 4.457939 3.976848 0.2512552 0.0861407 0.2172688
0.3274175 0.08514651 0.02504537 0.2789536 0.2947028 0.3200703 0
0.08592859 0.3505922 0 0.1430187 0.3623873 0.2745358 0.03327002
0.3275566 0 0.1536674 1.052112 0.4853793 0.3105202 0.5580269
1.884414 0.007808776 0 0 0.8828174 3.59565 0.9846214 0.08628269
0.2447291 0.3163138 0.07513612 0.8574582 0.1749688 0.1954786
0.1117683 1.310607 0.3302127 0.6666559 0 0.07460071 0.1954786
0.564018 0.2174093 1.327697 0.1811936 0.1048644 0.2536492 1.359129
0.178753 0.5207481 0.03568966 0.5982266 0.02336823 1.425046 0.1394515
0 0.7613794 0.3432448 0.2875445 0.1163218 0.1370284 0.6098427
2.288879 1.004631 0.03109594 0 0.02750166 0.06754172 0.9375194 1.30609
0.1579824 0.02504537 0.2465099 1.305222 0.4831831 0.0394559
0.340762 0.02924292 0.4218837 1.178789 0.1623096 0.1003415 0.7252037
2.039409 0.3592504 0.7054958 0.9814656 2.946238 0.1171899 0.4703798
0.1156082 0.5161114 0.16213 0.2537362 0.06689435 0.470592 0.2539641
0.2017574 0.3035255 0.974937 0.2177158 0.4012806 1.082997 0.2533273
0.8586389 0.2415915 0 0.1678231 0.2670655 0.5029575 0.1905758
0.6385089 0.1028289 0.7970456 0.6372414 0.4280535 0.1740756
0.1438045 0.03965517 0.1915055 0.4596619 0.5161533 0.5116412
0.09490695 0 0.9028373 0.2073063 0 0.452549 0.3633744 0.4810066
1.331379 1.172753 0.3825067 0.5577986 0.0698319 0.1312723 1.132405 0
0.1918397 0.04132487 0.164325 0.2456931 0.203647 0.06963836 0.107804
0.1489891 1.625577 0.5693868 0.0429056 0.1712481 0.3123652
0.1197213 0.2434643 0.038619 0.08932565 0.1236492 0.02437162
0.4277741 2.79959 0.1818932 0.05497123 0.1256276 0.4559349
0.08901808 0.2202375 0.9419772 0.3555498 0.09586965 0.3521504
0.02819291 0.1361571 0.564645 0.1112142 0.7974408 4.620312 0.1960662
0.07065669 0.04659777 0.3424428 0.1293866 0.0210502 0.4739472
0.2247224 0.06941603
TCGA-XF-A8HD 2964 0 0.05857076 0.1127421 0.1274476 0.08668319
0.05393074 36.82043 0 0.3150152 0.3634251 0.4838058 0.02315076
0.686179 0.3768015 0.6513987 0.02532114 0.1723572 0.166384 0.07143191
0.2296406 0.03489636 0.07690124 0.2407343 1.972103 32.08681 0.2534745
0.1889007 0.5393074 0.126157 0.2224643 1.389872 0.03880997 0.08788713
0.05033535 1.762866 0.135731 0.1748491 0.7442661 0.297469 0.05188131
0.09806232 0.04640592 0.1912412 0.272306 0.1083893 3.954921 0.03716033
0.1544152 0.02909048 1.368492 1.648853 2.746919 0.09349343 0.8726618
0.08284622 0 0.4606831 0.03003737 0.06681852 0.5767593 0
0.1578211 0.5231706 0.02516768 0.04055916 0.2218088 0.1404708
0.4077905 0.4918876 0.04674672 0.144386 0.0558446 0.1372074
0.3346471 1.617922 0.8416071 0.227793 0.04889805 0.1465255 0.02573302

0.02649229	0.2603553	0.08906524	0.4020416	0	0.1136161	0.17116
0.2979492	0.1210249	0.4259145	0	0	0.1902244	0.05494157
0.02390368	0.4633611	0.06040242	0	0.2774751	0.6644267	0.02202641
0	0	0.1980829	0.9314393	1.1483260	0.02876306	0.4856920.07947687
1.4343180.1619424	0	0.05911269	2.5205870.1940498	0.3917610.05391727
0.1578211	0.3446196	0.3768015	0.3964991.0654070.06388718	0.1109227
0.2532298	1.0782360.1796259	0.5753142	0.2265091	1.7548810.7168294
0.4137889	0.1026142	0.04614074	1.1073780.04538434	0.2203582
0.09228148	0.3768563	0.1612686	0.09194096	1.7848730.09867723	0
0.08727145	0	0.3187548	0.9638685	0.06684373	0.07947687	0.4359434
0.04314459	0.2236051	0.2504121	0.2102616	0.06186468	0.2479204
0.3836584	0.02146081	0.1592077	0.6785885	1.1864760.1129744
0.4122660.4089748	0.4363027	0.08264013	0.08611521	0.04076237
0.4977580.1714966	0.1491083	0.2476564	0.01082128	0.3283330
0.1858772	0.4051382	0.4015376	0.3112731	0.1205856	0.1505408
0.8111057	0.2920557	0	0.2028784	0.2824943	0.4137889	0.2419029
0.9905819	0.03107702	0.4739664	0.5376399	0.1681765	0.0678382
0.2433797	0.7130842	0.2532114	0.06870464	0.2576990.3746767
0.1374569	0	0.5009815	0.1644620	0.1914774	0.2972059	0.1496456
0.4333217	1.1497370.1421584	0.4300173	0.0830995	0.1735702
0.4229059	0.06291919	0.2898895	0	0.1395443	0.3151824	0.07180404
0.1104922	0.03801068	0.3782315	0.5510333	0.7487822	0.136153
0.1811414	0.03003737	0.2321694	0.1287649	0.1582941	0.2267668	0
0.1117117	0	1.36153	0.02137795	0.1066029	0.7087218	0.2225885
0.1673970.4307071	0.4981985	0.02089392	0.08112658	0.4764468
1.0437580.1680271.2989480.09411143	1.3625945.1444590.1113779	0.4671166
0.09857963	0.05174663	0.09364209	0.04453262	0.6572967	0.01229509
0.1060603
TCGA-GV-A3JX	581	0	0.1179773	0.1835095	0.4278565	0.3769846	0
30.165830	0.09354189	0.3523171	0.2214809	0.06358893	0.0427381
0.2299940.7952080.09273386	0.04563076	0.07616871	0.07546321	0.02336152
0.006390064	0.1056136	0.1322465	2.19582	27.153880.2617022	0
0.09875554	0.1925108	0.06789443	1.10183	0.07106707	0.2145799	0
0.9547845	0.2571150.1829576	0.2168197	0.2396732	0.07125204
0.1047476	0.1068275	0.1000550.2216153	0.04961942	6.9338990.06804632
0.04039403	0.2996392	0.9073165	2.3483491.4507970.1997345	0.9367464
0.1611858	0	0.3342495	0.02750154	0.02102978	0.2464316	0.07604176
0	0.1437010	0.01083106	0.1172734	0.03215297	0.4729275	0.3117886
0.04755582	0.3161222	0.1079412	0.2355451.2534360.6583702	0.1216668
0.4338093	0	0.1097636	0.03534087	0.02425575	0.2065920.3261845
0.3874740.01640599	0.2704632	0.07089271	0.2727956	0.05540383
0.6239324	0	0	0.1119633	0	0.008754268	0.2878792	0	0.1718458
0.4526708	1.6605880.007562582	0	0	0	2.5228810.4156624	0
0.2896829	0.09881971	0.1212787	0.9945780.1694524	0.0315526

0.1082445 1.500065 0.07106707 0.4304251 0.01898671 0.1806218
0.0946578 0.574985 0.06534461 0.1773569 0.05849366 0.1015583
0.1987298 0.9516338 0.06924691 1.17677 0.1382578 1.1621 0.09052591
0.216489 0.2642378 0 0.3686873 0.4155288 0.2301713 0.02816362
0.5042909 0.1845674 0.8979101 0.3589549 0 0.04686703 0.07990378
0 0.5512623 0.6177474 0.1224012 0.04851149 1.029557 0.2370133
0.1462342 0.1910597 0.5500308 0 0.8171652 0.7743886 0.2947355
0.1700615 0.2161045 0.641911 0.1755292 0.04777993 0.6624851
0.5421364 0.2648221 0.2365354 0.1119633 0.2940233 0 0.3822566
0.07018409 0.247693 0.3416072 0 0.1315066 0.337214 0.2000388
0.3627205 0.3588178 0.1488583 0.7426301 0.6350741 0.08191213
0.06965658 0.2586455 0.1353056 0.1476539 0.8582338 0.02845342
0.8339097 0.6391916 0.02368902 0.1774604 0.1949789 0.3456444
0.1391008 0.1548416 0.3519156 0.07623234 0.1597902 0 0.2866796
0 0.5181202 0.08765621 0.09288542 0.1370122 0.4628629 1.357394
0.120145 0.5035878 0.05072269 0.04767509 0.6804031 0.08641109
0.2388746 0 0.01793173 0.253135 0.1095703 0.1011642 0.03480172
0.1442918 0.5531415 0.8991872 0 0.165849 0.02750154 0.1352712
0.1768413 0.05610213 0.2595282 0.0598754 0.06294197 0.01336411
2.025703 0.1370122 0.2484445 0.6083341 0.1188816 0.08940455
0.1810518 0.6452703 0.11478 0.03713883 0.3965672 0.3003445
0.04395478 0.699806 0 0.5940763 5.02718 0.05626211 0.1539653
0.04512864 0.1895122 0.5408001 0 0.102001 0.003752369 0.01493944
TCGA-H4-A2HQ 590 0 0.5077722 0.8720946 0.05580261 0.6120086 0
45.96251 0 0.1453842 0.1123234 0.4236664 0.182457 0.1318265
0.8249081 1.682759 0.1413567 0.0409154 0.03642541 0.2093105 0
0.03667028 0.02525326 0.2529718 3.275431 130.288 0.1912689 0.4962582
0.7792435 0.110475 0 1.24679 0.05097855 0.4232928 0 2.3156
0.4426472 0.09843076 0.2666251 0.5235891 0.07382734 0.0500924
0.1027547 0.3110139 0.2781999 0 10.25728 0.3416817 0.202831
0.009552898 0.7438234 1.804866 2.65558 0.4093587 1.343913 0.1496303 0
1.061826 0.8285627 0.0137139 1.060637 0.05454705 0.1554785 0.2405224
0 0.05105635 0.2831481 0.6458001 0.1785501 0 0.02729058
0.3545764 0.2200628 0.4956262 2.477595 5.478318 0.116367 0.0359059
0.0385378 0.4024321 0.05070214 0.27839 0.3191886 0.2924775
0.3335361 0.4942775 0.5074144 0.1498839 0.6848957 0.03974284
1.118914 0.07628958 0 0.3123348 0.02405603 0.01569925 0.7825429 0
0 0.6129812 2.26444 0.02893263 0 0 0.1858502 2.141099 1.087433
0.07992241 0.1826106 0.680509 0.2435913 1.191377 0.3950483 0
0.4270587 1.776849 0.2039142 0.7204327 0.005447895 0.4664356
0.3621381 0.6186811 0.06944245 1.081399 0 0.2549779 0.5108217
1.862893 0.2111098 0.6592274 0.1487647 0.8360256 0.06493696
0.3105882 0.2695761 0.1818235 0.9977752 0.5365283 0.1793774 0
0.1523129 0.4236664 0.8051225 0.9621964 0.04320558 0 0.03821159

0.09384438 0.651308 1.266082 0.1756042 0.1739938 0.4602449 0.1700168
1.007022 0.1096423 0.4340091 0.04063095 0.7164388 2.756463 0
0.2091261 0.5425639 1.841848 0.5216371 0.09596716 1.033088 1.964922
0.2171027 0.2890742 0.02677156 0.6010984 0.03218115 0.6267524
0.06970869 0.1563564 0.1960361 0.7475398 0.1886673 0.6289238
0.4421161 0.6318908 0.6731759 0.2451997 0.28071 0.3356741
0.2350322 0.199867 0.07421367 1.164706 0.1059166 1.043088 0.1428735
1.454588 0.6271593 0.0679714 0.3309739 0.3596509 0.5693463
0.4656455 0.4095801 0.8319034 0.218735 0.1298377 0 0.3495946
0.1440186 0.08745018 0.3143922 0.9440745 0.2751923 0.664051
2.623027 0.4309181 1.353003 0.1334113 0.02279918 0.4752296 0.2479411
0.6473297 0 0.01607872 0.2396874 0.1571961 0.1451363 0.09985729
0.04140193 2.363269 0.8101282 0.05961426 0.4758739 0.1183661
0.1386202 0.2537072 0.207926 0.1737562 0.08590086 0 0.7956775
2.190824 0.1965659 0.1782167 0.116367 0.2923795 0.1053607 0.5977759
1.02151 0.2744506 0.1065632 1.080985 0.03917203 0.3153009 0.4169551
0.09271453 4.005799 3.365738 0.1967477 0.09817241 0.1942328 1.155514
0.06623232 0.02924775 0.8780209 0.01076675 0.01071651
TCGA-XF-A9SP 454 1 0.0507842 0.07241025 0.0613913 0.17224 0.961199
59.18061 0.3538673 0.2952824 0.525184 0 0.01672752 3.622354 0.3176332
0.9413332 0.07318292 0.408119 0.1202204 0.2382137 1.17992 0.01008571
0.1944768 0.2783072 1.553087 48.84757 0.07793505 0.1637877 0.1558701
0.06076961 5.465196 1.151212 1.23385 0.04233509 0 0.4664454 0.6898866
0.1082887 1.222199 0.0859742 0.6872565 0.1102184 0.08622125
0.05264034 0.2186156 0.2741076 5.253154 0 0.03187782 0.483443
1.091091 0.1765 6.352732 0.04503564 0.2174275 0.3741271 0 0.7536577
0.04340687 0.2670463 0.8890369 0 0.1140332 0.1890078 0.6546545
0.2222366 0.117379 0 0.1964321 1.531007 0.04503564 0.1451489
0.8070077 0.07435415 0.3517069 1.610658 0.03200533 0.3950191
0.7419546 0.09624698 0.09296668 0.03828389 0.2508255 0 0.3975184
0.1294714 0.06567441 0.1236712 0 0 0.06154871 0.08393006 0
0.01963517 0.02646527 0.5561438 0.1554431 0.6110108 1.084926
1.738786 0.8304085 0.3461541 0 0.0521826 2.739809 3.477214 1.543665
0.4396336 0.1316236 0.4055256 0.07656777 0.3505346 0 0.1494025
0.04271174 2.452609 0.1121682 0.849198 0.04195455 0 0.4482074
0.3176332 0.8594684 0 0.09232306 0.04007345 0.06534663 0.9264701
0.01366192 0.2653353 0 0.7921585 0.07144047 5.979643 0.03707181
0.06667777 0.96545 0.06558469 0.1076412 0.04445185 0.08378358 0
0.1771513 1.192745 0.1901307 0.2219168 0.2101926 0.103243 0.3070874
1.53217 0 0 0.2649448 0.2493922 0.1846461 0.06031155 0.1302206
0 0.6448835 0.8106337 0.06202583 0.03834504 0.5969022 0.7014155
0.3166232 1.900411 0.04546212 0.4503564 0.05971143 0.06913594
0.1767165 0.8237234 0.03540413 0.04309515 0.3408448 0 0.4097717
0 0.2564008 0.1064479 0.6399928 0.2044633 0.04356442 0.06526372

1.1861620.2637802	0.1723806	0	0.5715237	0.2562704	0.2913106
1.2954350	0.1578172	0.3072975	2.4863950.05601866	0.08792673
0.6869831	0.2561402	0.01527458	0.1893557	0	0.1629281	0
0.4751027	0.1584422	0.1443126	0	1.1150240.08650089	0.4696434
0.2623388	0.2370375	0.4190945	0.06671483	0.1003302	3.140479
0.09092423	0.1675672	0	1.1462480.1678042	0.1037637	0	0.1098581
0	0.1822845	0.2979590.5902622	0.2355899	0.2604412	0.3965082	0
0.07379034	0.0273083	0.09450392	0.08692601	0.3269437	0.8362048	0
0	0.7681279	0.2680513	0.1259920.4775746	0.1229173	0.1811622	0
0.2315901	0.3878563	0.1040636	0.7423917	0.06799999	0.7970077
8.7495210.01110011	0.2160090.5698278	0	0.1145030	0.3058859	0
0.08252848
TCGA-DK-A3IK	146	1	0.3590239	0.2972389	0.1260034	0.5034932
0.1244125	23.275510.04035	0.4545424	0.3170357	0.1594413	0.1487748
1.0960610.9002855	1.3095020.3004107	0.1847757	0.1644987	0.08148751
0.6306624	0.1656045	0.2851120.2380065	3.001841114.05260.1119712
0.09960545	0.2132786	0.1663033	0	1.8768510.1151106	0.4344563
0.19906	1.1782920.4210884	0.5432991	0.4682572	0.2529245	0.1538803
0.02693088	0.09831447	0.1080424	0.4187874	0	17.836740.6613067
0.2399030.04314129	0.6998203	0.5132015	5.2744350.3081135	0.7735212
0.2866762	0	1.3749830.1781821	0.1228329	7.86909	0	0.07801639
1.1379330.0746475	0.1152863	0.8802705	0.6943953	0.2150239
0.2618614	0.0205409	0.1489565	0.2147126	0.2797836	1.413654
1.8839610.0437932	0.2252119	0.05801277	0.6321392	0.1272072
0.3928816	0.6006120.1320840.1673625	0.1594413	0.5841090.1813077	0
0.08974016	0.5474150.1722635	0.06958665	0.1746355	0.07242535
0.08271488	0.6503535	0	0.0463911	0.4314478	1.0297320.1714925
0.07129862	0.03570098	0.4476306	4.6044251.8217830.2406220.3033295
1.5472740.2357289	1.1990990.1829799	0.03407147	0.2337715	2.408958
0.3069617	0.6197151	0.02050243	0.3120655	1.6354311.0555070.4155266
1.0054560	0.1644987	0.2384385	1.4789810.09346869	0.7382236
0.1119712	0.8147572	0.09775267	0.8474218	0.4184871	0.04561791
4.4698020.8076614	0.3037785	0.1824717	0.3152652	0.3986031
0.2726975	1.6218440.1625985	0.05060847	0	0.1059513	1.505683
1.7470690.1321726	0.0261921	0.3504423	0	0.6947959	0.08252487
0.3266672	0.1223274	0.5882668	1.1034510.0636529	0.3672752
0.7584081.49295	0.9849408	0.6500875	0.7775781	1.3248880.285963
0.2554183	0.04030049	0.9762983	0	0.1769025	0.05829765	0.02139733
0.1623063	0.3164926	0.2088307	0.9103353	0.2802266	0.5595383
1.0431680.3869709	1.3445280.2165598	0.0589675	0.1002898	0.167576
0.6720931	0.2391619	1.1129060.0768122	0.4470016	0.4561791
0.6906638	0.1437204	0.06015549	0.8294166	0.2253080.1672028
0.4706934	0.2469541	0.1175759	0	3.2194810.2167980.09873216
0.2366347	0.5438833	0.11836	1.7136471.3461020.5513791	0.7019831

0.1369298 0.1544431 1.322013 0.2799281 0.2292838 0 0.1355427
0.8364297 0.07099041 0.07282683 0.1127399 0.09348643 1.299618
0.3397501 0.1794803 0.07163555 0.02969701 0.1669372 0.1273058
0.2675653 0.1494648 0.1293106 0.008495835 0.8802895 3.062383
0.02113571 0.1533017 0 0.4951492 0.1551565 0.3187005 0.9370527
0.3511719 0.120311 0.6851609 0.1474188 0.4034417 0.5987566
0.1395676 6.447094 5.624126 0.2961737 0.1108377 0.0487313 0.5627631
0.07833779 0 1.365784 0.06483079 0.1613207
