# Supplementary material for: Ferroptosis-Related Long Noncoding RNAs Have Excellent Predictive Ability for Multiomic Characteristics of Bladder Cancer
Source: Oxid Med Cell Longev. 2022 Aug 29;2022:9316847. doi: 10.1155/2022/9316847 (PMC9444476; doi:10.1155/2022/9316847)
Supplement: Supplementary Materials — Supplementary Table 1: expression matrix of 60 ferroptosis-related genes. Supplementary Table 2: results for coexpression analysis of ferroptosis-related lncRNAs and mRNAs. Supplementary Table 3: expression of 263 ferroptosis-related lncRNAs. Supplementary Table 4: expression of lncRNAs and clinical information of 403 bladder cancer samples. Supplementary Table 5: cluster analysis based on 20 prognostic lncRNAs. Supplementary Table 6: detailed clinical information of bladder cancer samples. Supplementary Table 7: data of the high- and low-risk groups of the training cohort. Supplementary Table 8: data of the high- and low-risk groups of the validation cohort. Supplementary Table 9: markers of different immune cells. Supplementary Table 10: infiltration of 22 types of immune cells analysed using the CIBERSORT algorithm. Supplementary Table 11: immune scores evaluated using the ESTIMATE algorithm. Supplementary Table 12: immunotherapeutic response evaluated using the Tumour Immune Dysfunction and Exclusion (TIDE) algorithm. Supplementary Figure 1: gene set enrichment analysis. (A–F) Most significantly enriched biological pathways in cluster 1. (G–I) Most significantly enriched biological pathways in cluster 2. [file 9316847.f1.zip › s-table 5.pdf]

TCGA-CF-A47X	1
TCGA-ZF-AA51	1
TCGA-BL-A0C8	2
TCGA-BT-A42C	1
TCGA-G2-A3IB	1
TCGA-2F-A9KP	1
TCGA-DK-A3IT	1
TCGA-FD-A43P	1
TCGA-DK-A3IV	1
TCGA-2F-A9KW	2
TCGA-GU-A762	1
TCGA-XF-AAME	1
TCGA-XF-AAN0	1
TCGA-CF-A47W	1
TCGA-GC-A3I6	1
TCGA-FD-A6TA	1
TCGA-DK-A1A7	1
TCGA-FD-A3SR	2
TCGA-GU-AATP	2
TCGA-DK-AA6X	2
TCGA-XF-A9T3	1
TCGA-PQ-A6FN	1
TCGA-DK-A3WW	1
TCGA-XF-AAMY	2
TCGA-K4-A3WS	2
TCGA-UY-A9PA	1
TCGA-G2-AA3F	2
TCGA-FJ-A871	1
TCGA-ZF-A9RN	1
TCGA-GC-A3RB	1
TCGA-UY-A9PF	2
TCGA-YC-A89H	1
TCGA-XF-AAMQ	1
TCGA-CF-A1HR	1
TCGA-E7-A3X6	1
TCGA-DK-A6AW	1
TCGA-GC-A3BM	1
TCGA-GV-A6ZA	1
TCGA-G2-A2EJ	1
TCGA-FD-A43N	1
TCGA-UY-A8OD	1
TCGA-2F-A9KT	1
TCGA-BT-A20Q	1
TCGA-CU-A0YR	1

TCGA-SY-A9G5	1
TCGA-4Z-AA84	1
TCGA-4Z-AA7Q	1
TCGA-FD-A5BT	1
TCGA-CF-A27C	2
TCGA-E7-A6MF	1
TCGA-FT-A61P	1
TCGA-DK-AA77	1
TCGA-ZF-AA5N	1
TCGA-DK-AA6U	1
TCGA-BT-A20O	1
TCGA-GD-A3OS	1
TCGA-XF-A9SZ	1
TCGA-4Z-AA82	1
TCGA-DK-AA76	1
TCGA-DK-A3IL	2
TCGA-E7-A678	1
TCGA-ZF-A9R1	1
TCGA-XF-A9SH	1
TCGA-FD-A5BU	1
TCGA-C4-A0F7	1
TCGA-C4-A0F0	1
TCGA-FD-A3N5	1
TCGA-ZF-AA5H	1
TCGA-DK-A3WX	1
TCGA-YF-AA3L	1
TCGA-BT-A2LA	1
TCGA-4Z-AA87	2
TCGA-BT-A2LB	1
TCGA-XF-A8HB	1
TCGA-DK-A2I6	1
TCGA-ZF-AA4X	2
TCGA-BT-A20P	1
TCGA-BT-A20W	1
TCGA-FD-A5BX	1
TCGA-BL-A13J	2
TCGA-CF-A8HX	2
TCGA-PQ-A6FI	1
TCGA-FJ-A3Z7	2
TCGA-E7-A7PW	2
TCGA-4Z-AA7S	1
TCGA-CF-A3MH	1
TCGA-DK-A6B2	1
TCGA-4Z-AA86	1

TCGA-5N-A9KI	1
TCGA-GU-AATO	1
TCGA-FD-A6TG	1
TCGA-4Z-AA7O	2
TCGA-GV-A3QI	1
TCGA-DK-A6AV	2
TCGA-DK-A1A3	1
TCGA-GD-A6C6	1
TCGA-GC-A3RD	1
TCGA-BT-A20R	1
TCGA-XF-A9T0	1
TCGA-YF-AA3M	1
TCGA-4Z-AA7Y	1
TCGA-FD-A62O	1
TCGA-BL-A3JM	1
TCGA-FJ-A3ZF	1
TCGA-CU-A0YO	1
TCGA-CF-A5UA	1
TCGA-K4-AAQO	1
TCGA-XF-AAMJ	1
TCGA-GU-A763	1
TCGA-G2-A2EC	1
TCGA-FD-A3B7	1
TCGA-FD-A5C0	1
TCGA-FD-A5BS	1
TCGA-CU-A0YN	1
TCGA-FD-A5BZ	1
TCGA-UY-A9PE	1
TCGA-GV-A40E	1
TCGA-BT-A2LD	1
TCGA-UY-A9PD	1
TCGA-G2-AA3B	1
TCGA-K4-A54R	1
TCGA-DK-A1AA	1
TCGA-CU-A5W6	1
TCGA-DK-AA6R	1
TCGA-BT-A3PH	1
TCGA-BL-A13I	1
TCGA-CF-A3MF	2
TCGA-CF-A47T	1
TCGA-YC-A8S6	1
TCGA-DK-A3IS	1
TCGA-XF-A9SJ	1
TCGA-DK-AA6T	1

TCGA-BT-A20N	1
TCGA-C4-A0F6	1
TCGA-CF-A7I0	2
TCGA-FD-A3B6	1
TCGA-GC-A3YS	1
TCGA-XF-A9T2	1
TCGA-UY-A78O	1
TCGA-GC-A3OO	1
TCGA-E7-A6ME	1
TCGA-XF-AAN5	1
TCGA-FD-A3SN	1
TCGA-ZF-A9R2	1
TCGA-ZF-AA4T	2
TCGA-E7-A4IJ	2
TCGA-FD-A6TE	1
TCGA-H4-A2HO	1
TCGA-FD-A6TB	1
TCGA-XF-AAN3	1
TCGA-XF-AAMH	1
TCGA-FD-A3B8	1
TCGA-XF-A9SY	1
TCGA-CF-A9FF	2
TCGA-XF-A9ST	1
TCGA-DK-A6B5	1
TCGA-GC-A3RC	1
TCGA-GV-A3QK	2
TCGA-GU-A764	1
TCGA-GU-A42R	2
TCGA-DK-A6B6	2
TCGA-R3-A69X	1
TCGA-5N-A9KM	1
TCGA-FD-A6TF	1
TCGA-XF-AAMR	1
TCGA-XF-A8HF	1
TCGA-ZF-AA4U	1
TCGA-DK-A3IQ	1
TCGA-E7-A4XJ	1
TCGA-XF-A9T6	1
TCGA-4Z-AA7M	2
TCGA-ZF-AA56	1
TCGA-XF-A8HH	2
TCGA-C4-A0EZ	1
TCGA-FD-A62P	1
TCGA-CF-A47V	2

TCGA-XF-A9SI	1
TCGA-GU-A42Q	1
TCGA-BT-A42F	1
TCGA-ZF-A9RF	1
TCGA-KQ-A41N	1
TCGA-GD-A3OQ	1
TCGA-BT-A20U	1
TCGA-XF-A9SW	1
TCGA-FD-A3B3	1
TCGA-ZF-A9RC	2
TCGA-E7-A6MD	1
TCGA-E5-A4TZ	1
TCGA-ZF-A9R4	1
TCGA-GV-A3QH	1
TCGA-BT-A0YX	1
TCGA-FT-A3EE	1
TCGA-ZF-A9RL	1
TCGA-ZF-A9R9	1
TCGA-ZF-A9RE	1
TCGA-DK-A6B1	1
TCGA-CF-A5U8	2
TCGA-XF-AAMW	1
TCGA-BT-A3PJ	1
TCGA-ZF-A9R7	1
TCGA-FD-A3SO	1
TCGA-E7-A97P	1
TCGA-GU-A767	1
TCGA-4Z-AA81	1
TCGA-2F-A9KQ	2
TCGA-SY-A9G0	1
TCGA-GV-A40G	2
TCGA-FD-A43Y	1
TCGA-S5-AA26	2
TCGA-BT-A42E	1
TCGA-FD-A43X	1
TCGA-ZF-AA4R	1
TCGA-BT-A20T	1
TCGA-E7-A7DU	1
TCGA-DK-A3WY	1
TCGA-G2-A2EF	1
TCGA-BT-A20V	1
TCGA-K4-A3WV	1
TCGA-GU-AATQ	1
TCGA-G2-A3VY	1

TCGA-XF-AAN8	1
TCGA-CU-A3QU	1
TCGA-GD-A3OP	1
TCGA-KQ-A41P	1
TCGA-UY-A9PH	1
TCGA-FD-A5BY	1
TCGA-K4-A3WU	1
TCGA-FD-A3SJ	1
TCGA-FD-A3B5	1
TCGA-GC-A6I3	1
TCGA-4Z-AA80	1
TCGA-G2-A3IE	2
TCGA-ZF-A9R0	2
TCGA-FD-A43S	1
TCGA-XF-A8HG	2
TCGA-K4-A83P	1
TCGA-GD-A2C5	2
TCGA-XF-AAMX	1
TCGA-DK-A1AE	1
TCGA-FJ-A3ZE	2
TCGA-DK-A2I1	1
TCGA-DK-A6B0	1
TCGA-FD-A5BR	1
TCGA-KQ-A41Q	2
TCGA-DK-A1AF	1
TCGA-LT-A5Z6	2
TCGA-GC-A4ZW	1
TCGA-K4-A4AB	1
TCGA-DK-AA74	1
TCGA-G2-A2EO	1
TCGA-DK-A1AG	1
TCGA-GV-A3JZ	1
TCGA-XF-A9SV	1
TCGA-UY-A78K	1
TCGA-FD-A6TH	1
TCGA-DK-AA6W	1
TCGA-CF-A3MG	1
TCGA-DK-A3X1	1
TCGA-DK-A1A6	1
TCGA-XF-AAMG	1
TCGA-E7-A541	1
TCGA-XF-A8HE	1
TCGA-DK-AA6Q	1
TCGA-UY-A78N	1

TCGA-DK-AA75	1
TCGA-ZF-AA5P	1
TCGA-GC-A3WC	1
TCGA-K4-A4AC	1
TCGA-FD-A3SL	1
TCGA-K4-A5RH	1
TCGA-FD-A5C1	1
TCGA-XF-AAN7	1
TCGA-2F-A9KO	1
TCGA-DK-A3IM	1
TCGA-FD-A3SS	1
TCGA-4Z-AA7N	2
TCGA-HQ-A2OE	1
TCGA-UY-A8OC	1
TCGA-GV-A3JV	1
TCGA-XF-A9T8	1
TCGA-KQ-A41S	1
TCGA-CU-A3YL	1
TCGA-E7-A7XN	1
TCGA-DK-A2HX	1
TCGA-CF-A8HY	1
TCGA-4Z-AA7W	1
TCGA-FD-A43U	1
TCGA-BT-A0S7	1
TCGA-ZF-A9R3	2
TCGA-4Z-AA89	2
TCGA-GU-A42P	1
TCGA-FD-A3NA	1
TCGA-XF-A9SL	1
TCGA-XF-AAN2	1
TCGA-ZF-AA54	1
TCGA-GU-A766	1
TCGA-FD-A6TD	1
TCGA-FD-A3B4	1
TCGA-DK-A1AB	1
TCGA-BT-A20X	1
TCGA-GV-A3JW	1
TCGA-UY-A9PB	1
TCGA-FD-A6TI	1
TCGA-XF-AAML	1
TCGA-ZF-AA58	1
TCGA-FD-A3SP	1
TCGA-E7-A97Q	1
TCGA-YC-A9TC	2

TCGA-K4-A6FZ	1
TCGA-KQ-A41R	1
TCGA-DK-AA6M	1
TCGA-ZF-AA52	1
TCGA-K4-A5RJ	1
TCGA-HQ-A5ND	1
TCGA-BT-A3PK	1
TCGA-FD-A3N6	1
TCGA-UY-A78P	1
TCGA-CF-A9FL	2
TCGA-CF-A9FM	2
TCGA-ZF-A9RD	1
TCGA-G2-AA3D	2
TCGA-DK-A2I2	1
TCGA-FD-A62S	1
TCGA-MV-A51V	2
TCGA-E5-A2PC	1
TCGA-DK-A3X2	1
TCGA-GD-A76B	1
TCGA-GC-A6I1	1
TCGA-XF-A9SK	1
TCGA-DK-A1AD	1
TCGA-K4-A5RI	1
TCGA-FD-A3SQ	1
TCGA-GV-A3QF	1
TCGA-E7-A7DV	1
TCGA-XF-AAN1	1
TCGA-DK-AA6P	1
TCGA-CF-A47Y	2
TCGA-CF-A3MI	1
TCGA-G2-A2EL	1
TCGA-XF-AAMZ	1
TCGA-DK-AA6L	1
TCGA-XF-A9T4	1
TCGA-K4-A6MB	1
TCGA-UY-A8OB	1
TCGA-ZF-A9R5	2
TCGA-CU-A3KJ	1
TCGA-DK-AA6S	1
TCGA-E7-A85H	1
TCGA-G2-AA3C	1
TCGA-E7-A8O8	2
TCGA-XF-A9SM	1
TCGA-DK-A3IN	1

TCGA-XF-A8HC	1
TCGA-ZF-AA4W	1
TCGA-LC-A66R	1
TCGA-E7-A8O7	2
TCGA-LT-A8JT	2
TCGA-FJ-A3Z9	1
TCGA-DK-A1AC	1
TCGA-C4-A0F1	1
TCGA-G2-A2ES	1
TCGA-XF-AAN4	1
TCGA-4Z-AA83	2
TCGA-E7-A5KE	2
TCGA-ZF-AA4V	1
TCGA-ZF-AA53	1
TCGA-ZF-A9RM	2
TCGA-CU-A72E	1
TCGA-2F-A9KR	1
TCGA-E5-A4U1	2
TCGA-E7-A3Y1	2
TCGA-FD-A6TC	1
TCGA-FD-A5BV	2
TCGA-DK-A2I4	1
TCGA-UY-A78M	1
TCGA-4Z-AA7R	1
TCGA-E7-A519	1
TCGA-DK-A3IU	1
TCGA-FD-A3SM	1
TCGA-BL-A5ZZ	1
TCGA-FD-A62N	1
TCGA-BT-A20J	1
TCGA-DK-AA71	1
TCGA-KQ-A41O	2
TCGA-E7-A677	1
TCGA-DK-A1A5	1
TCGA-HQ-A5NE	1
TCGA-XF-AAMT	1
TCGA-XF-A9SU	1
TCGA-CF-A47S	1
TCGA-FD-A6TK	1
TCGA-E7-A5KF	2
TCGA-UY-A78L	1
TCGA-S5-A6DX	1
TCGA-XF-A8HI	2
TCGA-XF-A9T5	1

TCGA-XF-A9SX	1
TCGA-G2-A2EK	1
TCGA-XF-A8HD	1
TCGA-GV-A3JX	1
TCGA-H4-A2HQ	1
TCGA-XF-A9SP	1
TCGA-DK-A3IK	1
