# Supplementary material for: Ferroptosis-Related Long Noncoding RNAs Have Excellent Predictive Ability for Multiomic Characteristics of Bladder Cancer
Source: Oxid Med Cell Longev. 2022 Aug 29;2022:9316847. doi: 10.1155/2022/9316847 (PMC9444476; doi:10.1155/2022/9316847)
Supplement: Supplementary Materials — Supplementary Table 1: expression matrix of 60 ferroptosis-related genes. Supplementary Table 2: results for coexpression analysis of ferroptosis-related lncRNAs and mRNAs. Supplementary Table 3: expression of 263 ferroptosis-related lncRNAs. Supplementary Table 4: expression of lncRNAs and clinical information of 403 bladder cancer samples. Supplementary Table 5: cluster analysis based on 20 prognostic lncRNAs. Supplementary Table 6: detailed clinical information of bladder cancer samples. Supplementary Table 7: data of the high- and low-risk groups of the training cohort. Supplementary Table 8: data of the high- and low-risk groups of the validation cohort. Supplementary Table 9: markers of different immune cells. Supplementary Table 10: infiltration of 22 types of immune cells analysed using the CIBERSORT algorithm. Supplementary Table 11: immune scores evaluated using the ESTIMATE algorithm. Supplementary Table 12: immunotherapeutic response evaluated using the Tumour Immune Dysfunction and Exclusion (TIDE) algorithm. Supplementary Figure 1: gene set enrichment analysis. (A–F) Most significantly enriched biological pathways in cluster 1. (G–I) Most significantly enriched biological pathways in cluster 2. [file 9316847.f1.zip › s-table 6.pdf]

Id	Age	Gender	Grade	Stage	T	M	N
TCGA-ZF-A9RM	>65	MALE	High Grade	Stage I-II	T1-T2	unknow	N0
TCGA-CF-A9FM	<=65	MALE	High Grade	Stage I-II	T1-T2	M0	N0
TCGA-CF-A8HX	<=65	FEMALE	High Grade	Stage I-II	T1-T2	M0	N0
TCGA-DK-AA6M	<=65	FEMALE	High Grade	Stage I-II	T1-T2	M0	N0
TCGA-G2-AA3B	>65	FEMALE	High Grade	Stage I-II	T1-T2	M0	N0
TCGA-CF-A47V	<=65	MALE	High Grade	Stage I-II	T1-T2	M0	N0
TCGA-CF-A9FF	<=65	MALE	High Grade	Stage I-II	T1-T2	M0	N0
TCGA-CF-A8HY	<=65	MALE	High Grade	Stage I-II	T1-T2	M0	N0
TCGA-E7-A8O8	<=65	MALE	High Grade	Stage I-II	T1-T2	M0	N0
TCGA-E7-A4XJ	>65	MALE	High Grade	Stage I-II	T1-T2	M0	N0
TCGA-DK-AA6W	>65	MALE	High Grade	Stage I-II	T1-T2	M0	N0
TCGA-CF-A47T	<=65	FEMALE	Low Grade	Stage I-II	T1-T2	M0	N0
TCGA-CF-A47S	<=65	MALE	Low Grade	Stage I-II	T1-T2	M0	N0
TCGA-CF-A47W	<=65	MALE	Low Grade	Stage I-II	T1-T2	M0	N0
TCGA-CF-A3MG	<=65	MALE	Low Grade	Stage I-II	T1-T2	M0	N0
TCGA-E7-A8O7	<=65	MALE	Low Grade	Stage I-II	T1-T2	M0	N0
TCGA-CF-A7I0	<=65	MALE	Low Grade	Stage I-II	T1-T2	M0	N0
TCGA-CF-A47Y	<=65	MALE	Low Grade	Stage I-II	T1-T2	M0	N0
TCGA-CF-A5U8	<=65	MALE	Low Grade	Stage I-II	T1-T2	M0	N0
TCGA-CF-A47X	<=65	MALE	Low Grade	Stage I-II	T1-T2	M0	N0
TCGA-CF-A3MI	<=65	MALE	Low Grade	Stage I-II	T1-T2	M0	N0
TCGA-CF-A5UA	>65	MALE	Low Grade	Stage I-II	T1-T2	M0	N0
TCGA-CF-A3MH	>65	MALE	Low Grade	Stage I-II	T1-T2	M0	N0
TCGA-CF-A9FH	>65	MALE	Low Grade	Stage I-II	T1-T2	M0	N0
TCGA-LT-A8JT	>65	FEMALE	High Grade	Stage I-II	T1-T2	M0	N0
TCGA-E7-A5KE	>65	FEMALE	High Grade	Stage I-II	T1-T2	M0	N0
TCGA-DK-A6AV	>65	FEMALE	High Grade	Stage I-II	T1-T2	M0	N0
TCGA-4Z-AA83	<=65	MALE	High Grade	Stage I-II	T1-T2	M0	N0
TCGA-4Z-AA7W	<=65	MALE	High Grade	Stage I-II	T1-T2	M0	N0
TCGA-4Z-AA7Y	<=65	MALE	High Grade	Stage I-II	T1-T2	M0	N0
TCGA-DK-AA77	<=65	MALE	High Grade	Stage I-II	T1-T2	M0	N0
TCGA-4Z-AA7O	<=65	MALE	High Grade	Stage I-II	T1-T2	M0	N0
TCGA-DK-A6B1	>65	MALE	High Grade	Stage I-II	T1-T2	M0	N0
TCGA-DK-A3IS	>65	MALE	High Grade	Stage I-II	T1-T2	M0	N0
TCGA-DK-A6AW	>65	MALE	High Grade	Stage I-II	T1-T2	M0	N0
TCGA-4Z-AA80	>65	MALE	High Grade	Stage I-II	T1-T2	M0	N0
TCGA-E7-A5KF	>65	MALE	Low Grade	Stage I-II	T1-T2	M0	N0
TCGA-XF-A9SH	<=65	FEMALE	High Grade	Stage I-II	T1-T2	M0	N0
TCGA-DK-A2I1	>65	FEMALE	High Grade	Stage I-II	T1-T2	M0	N0
TCGA-DK-A3IU	<=65	MALE	High Grade	Stage I-II	T1-T2	M0	N0
TCGA-GC-A3RC	<=65	MALE	High Grade	Stage I-II	T1-T2	M0	N0
TCGA-C4-A0F0	<=65	MALE	High Grade	Stage I-II	T1-T2	M0	N0
TCGA-DK-A6B0	<=65	MALE	High Grade	Stage I-II	T1-T2	M0	N0

TCGA-GC-A3BM	>65	MALE	High Grade	Stage I-II	T1-T2	M0	N0
TCGA-DK-AA71	>65	MALE	High Grade	Stage I-II	T1-T2	M0	N0
TCGA-BT-A20W	>65	MALE	High Grade	Stage I-II	T1-T2	M0	N0
TCGA-E5-A4U1	>65	MALE	High Grade	Stage I-II	T1-T2	M0	N0
TCGA-ZF-A9R2	>65	MALE	High Grade	Stage I-II	T1-T2	M0	N0
TCGA-DK-A1A5	>65	MALE	High Grade	Stage I-II	T1-T2	M0	N0
TCGA-GC-A3OO	>65	MALE	High Grade	Stage I-II	T1-T2	M0	N0
TCGA-4Z-AA81	>65	MALE	High Grade	Stage I-II	T1-T2	M0	N0
TCGA-2F-A9KT	>65	MALE	High Grade	Stage I-II	T1-T2	M0	N0
TCGA-E7-A677	>65	MALE	Low Grade	Stage I-II	T1-T2	M0	N0
TCGA-UY-A78O	>65	FEMALE	High Grade	Stage I-II	T1-T2	unknow	N0
TCGA-UY-A78P	>65	FEMALE	High Grade	Stage I-II	T1-T2	unknow	N0
TCGA-KQ-A41R	>65	FEMALE	High Grade	Stage I-II	T1-T2	unknow	N0
TCGA-XF-AAMQ	<=65	FEMALE	High Grade	Stage I-II	T1-T2	unknow	N0
TCGA-GU-A766	<=65	MALE	High Grade	Stage I-II	T1-T2	unknow	N0
TCGA-PQ-A6FI	>65	MALE	High Grade	Stage I-II	T1-T2	unknow	N0
TCGA-YC-A8S6	>65	MALE	High Grade	Stage I-II	T1-T2	unknow	N0
TCGA-FD-A43P	>65	MALE	High Grade	Stage I-II	T1-T2	unknow	N0
TCGA-GV-A40G	>65	MALE	High Grade	Stage I-II	T1-T2	unknow	N0
TCGA-XF-A9SG	>65	MALE	High Grade	Stage I-II	T1-T2	unknow	N0
TCGA-FD-A43X	>65	MALE	High Grade	Stage I-II	T1-T2	unknow	N0
TCGA-FD-A3N6	<=65	FEMALE	High Grade	Stage I-II	T1-T2	unknow	N0
TCGA-YF-AA3L	<=65	FEMALE	High Grade	Stage I-II	T1-T2	unknow	N0
TCGA-XF-A8HB	<=65	FEMALE	High Grade	Stage I-II	T1-T2	unknow	N0
TCGA-XF-AAME	<=65	FEMALE	High Grade	Stage I-II	T1-T2	unknow	N0
TCGA-UY-A8OD	>65	FEMALE	High Grade	Stage I-II	T1-T2	unknow	N0
TCGA-XF-A9SI	>65	FEMALE	High Grade	Stage I-II	T1-T2	unknow	N0
TCGA-FD-A5BU	>65	FEMALE	High Grade	Stage I-II	T1-T2	unknow	N0
TCGA-K4-A3WV	>65	FEMALE	High Grade	Stage I-II	T1-T2	unknow	N0
TCGA-GD-A76B	>65	FEMALE	High Grade	Stage I-II	T1-T2	unknow	N0
TCGA-GC-A6I1	>65	FEMALE	High Grade	Stage I-II	T1-T2	unknow	N0
TCGA-GV-A6ZA	<=65	MALE	High Grade	Stage I-II	T1-T2	unknow	N0
TCGA-FD-A3B8	<=65	MALE	High Grade	Stage I-II	T1-T2	unknow	N0
TCGA-FD-A5BR	<=65	MALE	High Grade	Stage I-II	T1-T2	unknow	N0
TCGA-K4-A54R	<=65	MALE	High Grade	Stage I-II	T1-T2	unknow	N0
TCGA-FD-A3NA	<=65	MALE	High Grade	Stage I-II	T1-T2	unknow	N0
TCGA-E7-A541	>65	MALE	High Grade	Stage I-II	T1-T2	unknow	N0
TCGA-GU-A764	>65	MALE	High Grade	Stage I-II	T1-T2	unknow	N0
TCGA-FD-A3N5	>65	MALE	High Grade	Stage I-II	T1-T2	unknow	N0
TCGA-XF-AAN2	>65	MALE	High Grade	Stage I-II	T1-T2	unknow	N0
TCGA-UY-A9PH	>65	MALE	High Grade	Stage I-II	T1-T2	unknow	N0
TCGA-FD-A3B6	>65	MALE	High Grade	Stage I-II	T1-T2	unknow	N0
TCGA-K4-A5RJ	>65	MALE	High Grade	Stage I-II	T1-T2	unknow	N0
TCGA-XF-AAML	>65	MALE	High Grade	Stage I-II	T1-T2	unknow	N0

TCGA-BT-A20J	>65	MALE	High Grade	Stage I-II	T1-T2	unknow	N0
TCGA-FD-A62P	>65	MALE	High Grade	Stage I-II	T1-T2	unknow	N0
TCGA-BT-A3PK	>65	MALE	High Grade	Stage I-II	T1-T2	unknow	N0
TCGA-K4-A4AC	>65	MALE	High Grade	Stage I-II	T1-T2	unknow	N0
TCGA-BL-A0C8	>65	MALE	High Grade	Stage I-II	T1-T2	M0	unknow
TCGA-ZF-A9RF	>65	MALE	High Grade	Stage I-II	T1-T2	M0	unknow
TCGA-ZF-A9R3	<=65	FEMALE	High Grade	Stage I-II	T1-T2	M0	unknow
TCGA-ZF-AA51	>65	FEMALE	High Grade	Stage I-II	T1-T2	M0	unknow
TCGA-ZF-AA4X	<=65	MALE	High Grade	Stage I-II	T1-T2	M0	unknow
TCGA-ZF-AA53	<=65	MALE	High Grade	Stage I-II	T1-T2	M0	unknow
TCGA-E7-A97P	>65	MALE	High Grade	Stage I-II	T1-T2	M0	unknow
TCGA-ZF-A9R4	>65	MALE	High Grade	Stage I-II	T1-T2	M0	unknow
TCGA-E7-A3X6	>65	MALE	Low Grade	Stage I-II	T1-T2	M0	unknow
TCGA-E7-A6MF	<=65	MALE	Low Grade	Stage I-II	T1-T2	M0	unknow
TCGA-E7-A4IJ	<=65	MALE	High Grade	Stage I-II	T1-T2	M0	unknow
TCGA-E7-A519	>65	MALE	High Grade	Stage I-II	T1-T2	M0	unknow
TCGA-E7-A6ME	>65	MALE	High Grade	Stage I-II	T1-T2	M0	unknow
TCGA-GV-A3JW	>65	MALE	High Grade	Stage I-II	T1-T2	unknow	unknow
TCGA-YF-AA3M	<=65	MALE	High Grade	Stage I-II	T1-T2	unknow	unknow
TCGA-XF-AAMW	>65	FEMALE	High Grade	Stage III-IV	T1-T2	unknow	N0
TCGA-DK-A1A6	<=65	MALE	High Grade	Stage III-IV	T1-T2	M0	N1-3
TCGA-4Z-AA82	<=65	MALE	High Grade	Stage III-IV	T1-T2	M0	N1-3
TCGA-CU-A3QU	<=65	MALE	High Grade	Stage III-IV	T1-T2	M0	N1-3
TCGA-DK-A2I6	>65	MALE	High Grade	Stage III-IV	T1-T2	M0	N1-3
TCGA-UY-A78N	>65	MALE	High Grade	Stage III-IV	T1-T2	unknow	N1-3
TCGA-E5-A2PC	<=65	FEMALE	High Grade	Stage III-IV	T1-T2	unknow	N1-3
TCGA-XF-A9T4	<=65	MALE	High Grade	Stage III-IV	T1-T2	unknow	N1-3
TCGA-FD-A3B5	>65	MALE	High Grade	Stage III-IV	T1-T2	unknow	N1-3
TCGA-CU-A0YR	>65	MALE	High Grade	Stage III-IV	T1-T2	M0	N1-3
TCGA-ZF-AA5P	<=65	MALE	High Grade	Stage III-IV	T1-T2	M0	N1-3
TCGA-UY-A78M	>65	FEMALE	High Grade	Stage III-IV	T1-T2	unknow	N1-3
TCGA-GU-AATP	>65	MALE	High Grade	Stage III-IV	T1-T2	unknow	N1-3
TCGA-XF-A8HI	<=65	FEMALE	High Grade	Stage III-IV	T1-T2	unknow	N1-3
TCGA-FD-A3SJ	<=65	MALE	High Grade	Stage III-IV	T1-T2	unknow	N1-3
TCGA-UY-A9PE	>65	MALE	High Grade	Stage III-IV	T1-T2	unknow	N1-3
TCGA-ZF-AA5N	<=65	FEMALE	High Grade	Stage III-IV	T1-T2	M1	unknow
TCGA-HQ-A2OE	>65	MALE	unknow	unknow	T1-T2	unknow	N1-3
TCGA-G2-A2ES	>65	MALE	High Grade	Stage I-II	T3-T4	M0	N0
TCGA-CF-A3MF	<=65	MALE	Low Grade	Stage III-IV	T3-T4	M0	N0
TCGA-BL-A13I	<=65	FEMALE	High Grade	Stage III-IV	T3-T4	M0	N0
TCGA-DK-A3WX	>65	FEMALE	High Grade	Stage III-IV	T3-T4	M0	N0
TCGA-DK-A3WY	>65	FEMALE	High Grade	Stage III-IV	T3-T4	M0	N0
TCGA-DK-A3X1	>65	FEMALE	High Grade	Stage III-IV	T3-T4	M0	N0
TCGA-CF-A27C	<=65	MALE	High Grade	Stage III-IV	T3-T4	M0	N0

TCGA-DK-A3WW	<=65	MALE	High Grade	Stage III-IV	T3-T4	M0	N0
TCGA-DK-A1AA	<=65	MALE	High Grade	Stage III-IV	T3-T4	M0	N0
TCGA-HQ-A5NE	<=65	MALE	High Grade	Stage III-IV	T3-T4	M0	N0
TCGA-ZF-A9R5	<=65	MALE	High Grade	Stage III-IV	T3-T4	M0	N0
TCGA-CF-A1HR	<=65	MALE	High Grade	Stage III-IV	T3-T4	M0	N0
TCGA-BL-A3JM	<=65	MALE	High Grade	Stage III-IV	T3-T4	M0	N0
TCGA-DK-A3IT	<=65	MALE	High Grade	Stage III-IV	T3-T4	M0	N0
TCGA-E7-A85H	<=65	MALE	High Grade	Stage III-IV	T3-T4	M0	N0
TCGA-DK-A1AG	<=65	MALE	High Grade	Stage III-IV	T3-T4	M0	N0
TCGA-E7-A7XN	>65	MALE	High Grade	Stage III-IV	T3-T4	M0	N0
TCGA-DK-A3IQ	>65	MALE	High Grade	Stage III-IV	T3-T4	M0	N0
TCGA-DK-AA74	>65	MALE	High Grade	Stage III-IV	T3-T4	M0	N0
TCGA-DK-A3IM	>65	MALE	High Grade	Stage III-IV	T3-T4	M0	N0
TCGA-DK-AA75	>65	MALE	High Grade	Stage III-IV	T3-T4	M0	N0
TCGA-DK-A1AE	>65	MALE	High Grade	Stage III-IV	T3-T4	M0	N0
TCGA-E7-A678	<=65	MALE	Low Grade	Stage III-IV	T3-T4	M0	N0
TCGA-E7-A7DU	>65	MALE	Low Grade	Stage III-IV	T3-T4	M0	N0
TCGA-2F-A9KR	<=65	FEMALE	High Grade	Stage III-IV	T3-T4	M0	N0
TCGA-BT-A20U	>65	FEMALE	High Grade	Stage III-IV	T3-T4	M0	N0
TCGA-BT-A2LB	>65	FEMALE	High Grade	Stage III-IV	T3-T4	M0	N0
TCGA-BT-A20P	>65	FEMALE	High Grade	Stage III-IV	T3-T4	M0	N0
TCGA-GC-A3RD	>65	FEMALE	High Grade	Stage III-IV	T3-T4	M0	N0
TCGA-GC-A3I6	<=65	MALE	High Grade	Stage III-IV	T3-T4	M0	N0
TCGA-BT-A2LA	<=65	MALE	High Grade	Stage III-IV	T3-T4	M0	N0
TCGA-CU-A0YN	<=65	MALE	High Grade	Stage III-IV	T3-T4	M0	N0
TCGA-4Z-AA7N	<=65	MALE	High Grade	Stage III-IV	T3-T4	M0	N0
TCGA-4Z-AA7M	<=65	MALE	High Grade	Stage III-IV	T3-T4	M0	N0
TCGA-2F-A9KQ	>65	MALE	High Grade	Stage III-IV	T3-T4	M0	N0
TCGA-G2-A2EO	>65	MALE	High Grade	Stage III-IV	T3-T4	M0	N0
TCGA-R3-A69X	>65	MALE	High Grade	Stage III-IV	T3-T4	M0	N0
TCGA-GC-A4ZW	>65	MALE	High Grade	Stage III-IV	T3-T4	M0	N0
TCGA-BT-A42E	>65	MALE	High Grade	Stage III-IV	T3-T4	M0	N0
TCGA-MV-A51V	>65	MALE	High Grade	Stage III-IV	T3-T4	M0	N0
TCGA-XF-A8HF	>65	MALE	High Grade	Stage III-IV	T3-T4	M0	N0
TCGA-BT-A0YX	>65	FEMALE	High Grade	Stage III-IV	T3-T4	M0	N0
TCGA-C4-A0F6	>65	FEMALE	High Grade	Stage III-IV	T3-T4	M0	N0
TCGA-GC-A3RB	<=65	MALE	High Grade	Stage III-IV	T3-T4	M0	N0
TCGA-DK-AA6S	<=65	MALE	High Grade	Stage III-IV	T3-T4	M0	N0
TCGA-ZF-AA4V	>65	MALE	High Grade	Stage III-IV	T3-T4	M0	N0
TCGA-GU-A42Q	>65	MALE	High Grade	Stage III-IV	T3-T4	M0	N0
TCGA-XF-AAMJ	>65	MALE	High Grade	Stage III-IV	T3-T4	M0	N0
TCGA-C4-A0F1	>65	MALE	High Grade	Stage III-IV	T3-T4	M0	N0
TCGA-DK-A1AC	>65	MALE	High Grade	Stage III-IV	T3-T4	M0	N0
TCGA-KQ-A41N	>65	MALE	High Grade	Stage III-IV	T3-T4	M0	N0

TCGA-CU-A3KJ	>65	MALE	High Grade	Stage III-IV	T3-T4	M0	N0
TCGA-BT-A3PJ	>65	MALE	High Grade	Stage III-IV	T3-T4	M0	N0
TCGA-DK-A2I4	>65	MALE	High Grade	Stage III-IV	T3-T4	M0	N0
TCGA-CF-A9FL	>65	MALE	High Grade	Stage III-IV	T3-T4	M0	N0
TCGA-GU-A763	>65	MALE	High Grade	Stage III-IV	T3-T4	M0	N0
TCGA-XF-AAN1	>65	FEMALE	High Grade	Stage III-IV	T3-T4	M0	N0
TCGA-4Z-AA7S	>65	MALE	High Grade	Stage III-IV	T3-T4	M0	N0
TCGA-SY-A9G5	>65	MALE	High Grade	Stage III-IV	T3-T4	M0	N0
TCGA-CU-A3YL	>65	MALE	High Grade	Stage III-IV	T3-T4	M0	N0
TCGA-CU-A5W6	>65	MALE	High Grade	Stage III-IV	T3-T4	M0	N0
TCGA-4Z-AA87	>65	MALE	High Grade	Stage III-IV	T3-T4	M0	N0
TCGA-DK-A3IN	>65	MALE	High Grade	Stage III-IV	T3-T4	M0	N0
TCGA-4Z-AA89	<=65	MALE	High Grade	Stage III-IV	T3-T4	M0	N0
TCGA-4Z-AA7R	>65	MALE	High Grade	Stage III-IV	T3-T4	M0	N0
TCGA-FD-A3B3	>65	FEMALE	High Grade	Stage III-IV	T3-T4	unknow	N0
TCGA-GC-A3WC	>65	FEMALE	High Grade	Stage III-IV	T3-T4	unknow	N0
TCGA-FD-A5BY	<=65	FEMALE	High Grade	Stage III-IV	T3-T4	unknow	N0
TCGA-K4-A5RI	>65	FEMALE	High Grade	Stage III-IV	T3-T4	unknow	N0
TCGA-K4-A6FZ	>65	FEMALE	High Grade	Stage III-IV	T3-T4	unknow	N0
TCGA-PQ-A6FN	>65	FEMALE	High Grade	Stage III-IV	T3-T4	unknow	N0
TCGA-S5-AA26	<=65	MALE	High Grade	Stage III-IV	T3-T4	unknow	N0
TCGA-UY-A9PA	<=65	MALE	High Grade	Stage III-IV	T3-T4	unknow	N0
TCGA-UY-A9PB	<=65	MALE	High Grade	Stage III-IV	T3-T4	unknow	N0
TCGA-K4-AAQO	<=65	MALE	High Grade	Stage III-IV	T3-T4	unknow	N0
TCGA-FD-A6TK	<=65	MALE	High Grade	Stage III-IV	T3-T4	unknow	N0
TCGA-GD-A6C6	<=65	MALE	High Grade	Stage III-IV	T3-T4	unknow	N0
TCGA-FD-A3B7	>65	MALE	High Grade	Stage III-IV	T3-T4	unknow	N0
TCGA-K4-A3WS	>65	MALE	High Grade	Stage III-IV	T3-T4	unknow	N0
TCGA-K4-A5RH	>65	MALE	High Grade	Stage III-IV	T3-T4	unknow	N0
TCGA-BT-A20N	>65	MALE	High Grade	Stage III-IV	T3-T4	unknow	N0
TCGA-BT-A20O	>65	MALE	High Grade	Stage III-IV	T3-T4	unknow	N0
TCGA-FD-A43N	>65	MALE	High Grade	Stage III-IV	T3-T4	unknow	N0
TCGA-ZF-A9RC	>65	MALE	High Grade	Stage III-IV	T3-T4	unknow	N0
TCGA-XF-A8HD	>65	MALE	High Grade	Stage III-IV	T3-T4	unknow	N0
TCGA-FD-A6TD	>65	MALE	High Grade	Stage III-IV	T3-T4	unknow	N0
TCGA-UY-A9PD	>65	MALE	High Grade	Stage III-IV	T3-T4	unknow	N0
TCGA-FD-A6TB	>65	MALE	High Grade	Stage III-IV	T3-T4	unknow	N0
TCGA-FD-A5BV	<=65	FEMALE	High Grade	Stage III-IV	T3-T4	unknow	N0
TCGA-FD-A62S	<=65	FEMALE	High Grade	Stage III-IV	T3-T4	unknow	N0
TCGA-XF-AAN5	<=65	FEMALE	High Grade	Stage III-IV	T3-T4	unknow	N0
TCGA-FD-A5C1	<=65	FEMALE	High Grade	Stage III-IV	T3-T4	unknow	N0
TCGA-2F-A9KW	>65	FEMALE	High Grade	Stage III-IV	T3-T4	unknow	N0
TCGA-ZF-A9RG	>65	FEMALE	High Grade	Stage III-IV	T3-T4	unknow	N0
TCGA-FD-A43S	>65	FEMALE	High Grade	Stage III-IV	T3-T4	unknow	N0

TCGA-XF-AAN8	>65	FEMALE	High Grade	Stage III-IV	T3-T4	unknow	N0
TCGA-XF-AAN4	>65	FEMALE	High Grade	Stage III-IV	T3-T4	unknow	N0
TCGA-XF-AAMX	>65	FEMALE	High Grade	Stage III-IV	T3-T4	unknow	N0
TCGA-XF-A9T6	>65	FEMALE	High Grade	Stage III-IV	T3-T4	unknow	N0
TCGA-GV-A3QI	<=65	MALE	High Grade	Stage III-IV	T3-T4	unknow	N0
TCGA-XF-A8HE	<=65	MALE	High Grade	Stage III-IV	T3-T4	unknow	N0
TCGA-XF-AAMR	<=65	MALE	High Grade	Stage III-IV	T3-T4	unknow	N0
TCGA-XF-A9T2	<=65	MALE	High Grade	Stage III-IV	T3-T4	unknow	N0
TCGA-ZF-AA4W	<=65	MALE	High Grade	Stage III-IV	T3-T4	unknow	N0
TCGA-GV-A3JX	<=65	MALE	High Grade	Stage III-IV	T3-T4	unknow	N0
TCGA-XF-A9SP	<=65	MALE	High Grade	Stage III-IV	T3-T4	unknow	N0
TCGA-FD-A3SP	<=65	MALE	High Grade	Stage III-IV	T3-T4	unknow	N0
TCGA-XF-A9T8	<=65	MALE	High Grade	Stage III-IV	T3-T4	unknow	N0
TCGA-FD-A5BS	>65	MALE	High Grade	Stage III-IV	T3-T4	unknow	N0
TCGA-XF-A9ST	>65	MALE	High Grade	Stage III-IV	T3-T4	unknow	N0
TCGA-GU-AATQ	>65	MALE	High Grade	Stage III-IV	T3-T4	unknow	N0
TCGA-XF-A9T0	>65	MALE	High Grade	Stage III-IV	T3-T4	unknow	N0
TCGA-XF-A8HG	>65	MALE	High Grade	Stage III-IV	T3-T4	unknow	N0
TCGA-FD-A62N	>65	MALE	High Grade	Stage III-IV	T3-T4	unknow	N0
TCGA-XF-A9SM	>65	MALE	High Grade	Stage III-IV	T3-T4	unknow	N0
TCGA-XF-AAMY	>65	MALE	High Grade	Stage III-IV	T3-T4	unknow	N0
TCGA-FD-A3SN	>65	MALE	High Grade	Stage III-IV	T3-T4	unknow	N0
TCGA-FD-A5BT	>65	MALE	High Grade	Stage III-IV	T3-T4	unknow	N0
TCGA-KQ-A41Q	>65	MALE	High Grade	Stage III-IV	T3-T4	unknow	N0
TCGA-FD-A43Y	<=65	MALE	High Grade	Stage III-IV	T3-T4	unknow	N0
TCGA-FD-A3B4	<=65	FEMALE	High Grade	Stage III-IV	T3-T4	unknow	N0
TCGA-5N-A9KM	>65	FEMALE	High Grade	Stage III-IV	T3-T4	unknow	N0
TCGA-ZF-AA56	>65	FEMALE	High Grade	Stage III-IV	T3-T4	unknow	N0
TCGA-FD-A6TC	>65	FEMALE	High Grade	Stage III-IV	T3-T4	unknow	N0
TCGA-BL-A5ZZ	>65	FEMALE	High Grade	Stage III-IV	T3-T4	unknow	N0
TCGA-FT-A3EE	>65	FEMALE	High Grade	Stage III-IV	T3-T4	unknow	N0
TCGA-XF-AAMG	<=65	MALE	High Grade	Stage III-IV	T3-T4	unknow	N0
TCGA-H4-A2HO	<=65	MALE	High Grade	Stage III-IV	T3-T4	unknow	N0
TCGA-BT-A0S7	>65	MALE	High Grade	Stage III-IV	T3-T4	unknow	N0
TCGA-K4-A4AB	>65	MALE	High Grade	Stage III-IV	T3-T4	unknow	N0
TCGA-K4-A3WU	>65	MALE	High Grade	Stage III-IV	T3-T4	unknow	N0
TCGA-DK-AA6Q	<=65	FEMALE	High Grade	Stage III-IV	T3-T4	M0	N1-3
TCGA-2F-A9KO	<=65	MALE	High Grade	Stage III-IV	T3-T4	M0	N1-3
TCGA-DK-A6B2	>65	MALE	High Grade	Stage III-IV	T3-T4	M0	N1-3
TCGA-G2-AA3F	>65	MALE	High Grade	Stage III-IV	T3-T4	M0	N1-3
TCGA-KQ-A41O	>65	MALE	High Grade	Stage III-IV	T3-T4	M0	N1-3
TCGA-BT-A2LD	>65	FEMALE	High Grade	Stage III-IV	T3-T4	M0	N1-3
TCGA-4Z-AA86	>65	MALE	High Grade	Stage III-IV	T3-T4	M0	N1-3
TCGA-GU-A42P	>65	MALE	High Grade	Stage III-IV	T3-T4	M0	N1-3

TCGA-BT-A20R	>65	FEMALE	High Grade	Stage III-IV	T3-T4	M0	N1-3
TCGA-BT-A20T	<=65	MALE	High Grade	Stage III-IV	T3-T4	M0	N1-3
TCGA-G2-AA3C	>65	MALE	High Grade	Stage III-IV	T3-T4	M0	N1-3
TCGA-HQ-A5ND	>65	MALE	High Grade	Stage III-IV	T3-T4	M0	N1-3
TCGA-ZF-A9R1	>65	MALE	High Grade	Stage III-IV	T3-T4	M0	N1-3
TCGA-DK-AA6R	>65	MALE	High Grade	Stage III-IV	T3-T4	M0	N1-3
TCGA-SY-A9G0	>65	MALE	High Grade	Stage III-IV	T3-T4	M0	N1-3
TCGA-E7-A6MD	>65	MALE	High Grade	Stage III-IV	T3-T4	M0	N1-3
TCGA-C4-A0EZ	>65	FEMALE	High Grade	Stage III-IV	T3-T4	M1	N1-3
TCGA-DK-AA6L	<=65	MALE	High Grade	Stage III-IV	T3-T4	unknow	N1-3
TCGA-FD-A5BZ	>65	FEMALE	High Grade	Stage III-IV	T3-T4	unknow	N1-3
TCGA-XF-A9T5	>65	FEMALE	High Grade	Stage III-IV	T3-T4	unknow	N1-3
TCGA-GC-A6I3	>65	FEMALE	High Grade	Stage III-IV	T3-T4	unknow	N1-3
TCGA-BT-A42B	<=65	MALE	High Grade	Stage III-IV	T3-T4	unknow	N1-3
TCGA-XF-AAN7	<=65	MALE	High Grade	Stage III-IV	T3-T4	unknow	N1-3
TCGA-GC-A3YS	<=65	MALE	High Grade	Stage III-IV	T3-T4	unknow	N1-3
TCGA-UY-A8OB	<=65	MALE	High Grade	Stage III-IV	T3-T4	unknow	N1-3
TCGA-ZF-AA4R	>65	MALE	High Grade	Stage III-IV	T3-T4	unknow	N1-3
TCGA-FD-A3SO	>65	MALE	High Grade	Stage III-IV	T3-T4	unknow	N1-3
TCGA-XF-A9SU	>65	FEMALE	High Grade	Stage III-IV	T3-T4	unknow	N1-3
TCGA-K4-A6MB	<=65	MALE	High Grade	Stage III-IV	T3-T4	unknow	N1-3
TCGA-XF-A9SK	<=65	MALE	High Grade	Stage III-IV	T3-T4	unknow	N1-3
TCGA-GV-A3JV	>65	MALE	High Grade	Stage III-IV	T3-T4	unknow	N1-3
TCGA-XF-AAMH	>65	MALE	High Grade	Stage III-IV	T3-T4	unknow	N1-3
TCGA-FD-A5BX	>65	MALE	High Grade	Stage III-IV	T3-T4	unknow	N1-3
TCGA-UY-A8OC	>65	MALE	High Grade	Stage III-IV	T3-T4	unknow	N1-3
TCGA-GD-A3OQ	<=65	MALE	High Grade	Stage III-IV	T3-T4	unknow	N1-3
TCGA-BT-A42F	<=65	MALE	High Grade	Stage III-IV	T3-T4	unknow	N1-3
TCGA-K4-A83P	>65	MALE	unknow	Stage III-IV	T3-T4	unknow	N1-3
TCGA-FD-A6TI	>65	MALE	High Grade	Stage III-IV	T3-T4	unknow	N1-3
TCGA-DK-A1A7	>65	FEMALE	High Grade	Stage III-IV	T3-T4	M0	N1-3
TCGA-DK-A3IL	>65	FEMALE	High Grade	Stage III-IV	T3-T4	M0	N1-3
TCGA-DK-A2HX	>65	FEMALE	High Grade	Stage III-IV	T3-T4	M0	N1-3
TCGA-DK-A1AF	>65	FEMALE	High Grade	Stage III-IV	T3-T4	M0	N1-3
TCGA-DK-A1A3	<=65	MALE	High Grade	Stage III-IV	T3-T4	M0	N1-3
TCGA-G2-AA3D	<=65	MALE	High Grade	Stage III-IV	T3-T4	M0	N1-3
TCGA-DK-A3X2	>65	MALE	High Grade	Stage III-IV	T3-T4	M0	N1-3
TCGA-DK-A3IK	>65	MALE	High Grade	Stage III-IV	T3-T4	M0	N1-3
TCGA-ZF-AA5H	<=65	FEMALE	High Grade	Stage III-IV	T3-T4	M0	N1-3
TCGA-XF-A8HH	<=65	FEMALE	High Grade	Stage III-IV	T3-T4	M0	N1-3
TCGA-DK-A1AD	>65	MALE	High Grade	Stage III-IV	T3-T4	M0	N1-3
TCGA-BT-A20Q	>65	MALE	High Grade	Stage III-IV	T3-T4	M0	N1-3
TCGA-CU-A72E	>65	MALE	High Grade	Stage III-IV	T3-T4	M0	N1-3
TCGA-BL-A13J	<=65	MALE	High Grade	Stage III-IV	T3-T4	M0	N1-3

TCGA-GV-A3QK	<=65	FEMALE	High Grade	Stage III-IV	T3-T4	M0	N1-3
TCGA-BT-A20V	<=65	FEMALE	High Grade	Stage III-IV	T3-T4	M0	N1-3
TCGA-DK-A1AB	>65	FEMALE	High Grade	Stage III-IV	T3-T4	M0	N1-3
TCGA-DK-A6B5	<=65	MALE	High Grade	Stage III-IV	T3-T4	M0	N1-3
TCGA-BT-A20X	<=65	MALE	High Grade	Stage III-IV	T3-T4	M0	N1-3
TCGA-XF-AAN0	>65	MALE	High Grade	Stage III-IV	T3-T4	M0	N1-3
TCGA-C4-A0F7	>65	MALE	High Grade	Stage III-IV	T3-T4	M0	N1-3
TCGA-4Z-AA84	<=65	MALE	High Grade	Stage III-IV	T3-T4	M1	N1-3
TCGA-FD-A3SM	>65	MALE	High Grade	Stage III-IV	T3-T4	M1	N1-3
TCGA-FD-A6TF	>65	FEMALE	High Grade	Stage III-IV	T3-T4	M1	N1-3
TCGA-FD-A3SL	<=65	MALE	High Grade	Stage III-IV	T3-T4	M1	N1-3
TCGA-XF-A9SV	>65	MALE	High Grade	Stage III-IV	T3-T4	M1	N1-3
TCGA-FD-A6TH	<=65	MALE	High Grade	Stage III-IV	T3-T4	unknow	N1-3
TCGA-GD-A2C5	<=65	FEMALE	High Grade	Stage III-IV	T3-T4	unknow	N1-3
TCGA-ZF-AA58	<=65	FEMALE	High Grade	Stage III-IV	T3-T4	unknow	N1-3
TCGA-ZF-A9RD	>65	FEMALE	High Grade	Stage III-IV	T3-T4	unknow	N1-3
TCGA-XF-AAMZ	>65	FEMALE	High Grade	Stage III-IV	T3-T4	unknow	N1-3
TCGA-FD-A6TE	<=65	MALE	High Grade	Stage III-IV	T3-T4	unknow	N1-3
TCGA-FD-A5C0	<=65	MALE	High Grade	Stage III-IV	T3-T4	unknow	N1-3
TCGA-FD-A3SQ	<=65	MALE	High Grade	Stage III-IV	T3-T4	unknow	N1-3
TCGA-2F-A9KP	>65	MALE	High Grade	Stage III-IV	T3-T4	unknow	N1-3
TCGA-XF-A9SL	>65	MALE	High Grade	Stage III-IV	T3-T4	unknow	N1-3
TCGA-FD-A6TG	>65	MALE	High Grade	Stage III-IV	T3-T4	unknow	N1-3
TCGA-FD-A62O	>65	MALE	High Grade	Stage III-IV	T3-T4	unknow	N1-3
TCGA-UY-A9PF	>65	MALE	High Grade	Stage III-IV	T3-T4	unknow	N1-3
TCGA-XF-A8HC	>65	MALE	High Grade	Stage III-IV	T3-T4	unknow	N1-3
TCGA-CU-A0YO	>65	MALE	High Grade	Stage III-IV	T3-T4	unknow	N1-3
TCGA-XF-A9SY	<=65	FEMALE	High Grade	Stage III-IV	T3-T4	unknow	N1-3
TCGA-XF-A9SX	<=65	FEMALE	High Grade	Stage III-IV	T3-T4	unknow	N1-3
TCGA-XF-A9T3	>65	FEMALE	High Grade	Stage III-IV	T3-T4	unknow	N1-3
TCGA-XF-AAMT	>65	FEMALE	High Grade	Stage III-IV	T3-T4	unknow	N1-3
TCGA-ZF-A9R9	<=65	MALE	High Grade	Stage III-IV	T3-T4	unknow	N1-3
TCGA-FD-A6TA	<=65	MALE	High Grade	Stage III-IV	T3-T4	unknow	N1-3
TCGA-BT-A3PH	>65	MALE	High Grade	Stage III-IV	T3-T4	unknow	N1-3
TCGA-FT-A61P	>65	MALE	High Grade	Stage III-IV	T3-T4	unknow	N1-3
TCGA-XF-AAN3	>65	MALE	High Grade	Stage III-IV	T3-T4	unknow	N1-3
TCGA-GV-A3QF	>65	MALE	High Grade	Stage III-IV	T3-T4	unknow	N1-3
TCGA-XF-A9SZ	>65	MALE	High Grade	Stage III-IV	T3-T4	unknow	N1-3
TCGA-GU-A767	>65	MALE	High Grade	Stage III-IV	T3-T4	unknow	N1-3
TCGA-XF-A9SJ	>65	MALE	High Grade	Stage III-IV	T3-T4	unknow	N1-3
TCGA-XF-A9SW	>65	MALE	High Grade	Stage III-IV	T3-T4	unknow	N1-3
TCGA-ZF-AA4T	<=65	MALE	High Grade	Stage III-IV	T3-T4	unknow	N1-3
TCGA-XF-AAMF	>65	MALE	High Grade	Stage III-IV	T3-T4	unknow	N1-3
TCGA-GD-A3OP	>65	FEMALE	High Grade	Stage III-IV	T3-T4	unknow	N1-3

TCGA-FD-A3SR	>65	MALE	High Grade	Stage III-IV	T3-T4	unknow	N1-3
TCGA-GU-A42R	>65	MALE	High Grade	Stage III-IV	T3-T4	unknow	N1-3
TCGA-FD-A43U	>65	MALE	High Grade	Stage III-IV	T3-T4	unknow	N1-3
TCGA-GU-AATO	>65	MALE	High Grade	Stage III-IV	T3-T4	unknow	N1-3
TCGA-FJ-A3Z7	>65	MALE	High Grade	Stage III-IV	T3-T4	unknow	N1-3
TCGA-LC-A66R	>65	MALE	High Grade	Stage III-IV	T3-T4	unknow	N1-3
TCGA-S5-A6DX	>65	MALE	High Grade	Stage III-IV	T3-T4	unknow	N1-3
TCGA-GU-A762	>65	MALE	High Grade	Stage III-IV	T3-T4	unknow	N1-3
TCGA-E5-A4TZ	<=65	MALE	High Grade	Stage III-IV	T3-T4	unknow	N1-3
TCGA-DK-A2I2	<=65	FEMALE	High Grade	Stage III-IV	T3-T4	M0	N1-3
TCGA-KQ-A41P	>65	MALE	High Grade	Stage III-IV	T3-T4	M1	N1-3
TCGA-GV-A3QG	<=65	MALE	High Grade	Stage III-IV	T3-T4	unknow	N1-3
TCGA-E7-A7DV	<=65	MALE	High Grade	Stage III-IV	T3-T4	unknow	N1-3
TCGA-FD-A3SS	>65	MALE	High Grade	Stage III-IV	T3-T4	unknow	N1-3
TCGA-E7-A97Q	<=65	FEMALE	High Grade	Stage III-IV	T3-T4	unknow	N1-3
TCGA-GV-A3JZ	<=65	MALE	High Grade	Stage III-IV	T3-T4	unknow	N1-3
TCGA-E7-A7PW	<=65	MALE	High Grade	Stage III-IV	T3-T4	M0	unknow
TCGA-4Z-AA7Q	>65	MALE	High Grade	Stage III-IV	T3-T4	M0	unknow
TCGA-ZF-A9RN	>65	FEMALE	High Grade	Stage III-IV	T3-T4	M0	unknow
TCGA-ZF-A9R0	>65	MALE	High Grade	Stage III-IV	T3-T4	M0	unknow
TCGA-ZF-AA54	>65	MALE	High Grade	Stage III-IV	T3-T4	unknow	unknow
TCGA-ZF-AA52	>65	MALE	High Grade	Stage III-IV	T3-T4	unknow	unknow
TCGA-FJ-A871	<=65	MALE	High Grade	Stage III-IV	T3-T4	unknow	unknow
TCGA-5N-A9KI	>65	FEMALE	High Grade	Stage III-IV	T3-T4	unknow	unknow
TCGA-KQ-A41S	>65	FEMALE	High Grade	Stage III-IV	T3-T4	unknow	unknow
TCGA-ZF-AA4U	>65	MALE	High Grade	Stage III-IV	T3-T4	unknow	unknow
TCGA-G2-A2EJ	<=65	FEMALE	High Grade	Stage I-II	unknow	M0	N0
TCGA-G2-A2EC	<=65	FEMALE	High Grade	Stage I-II	unknow	M0	N0
TCGA-DK-AA76	<=65	FEMALE	High Grade	Stage I-II	unknow	M0	N0
TCGA-DK-AA6P	>65	FEMALE	High Grade	Stage I-II	unknow	M0	N0
TCGA-G2-A2EF	<=65	MALE	High Grade	Stage I-II	unknow	M0	N0
TCGA-G2-A2EK	<=65	MALE	High Grade	Stage I-II	unknow	M0	N0
TCGA-DK-AA6T	<=65	MALE	High Grade	Stage I-II	unknow	M0	N0
TCGA-DK-AA6X	<=65	MALE	High Grade	Stage I-II	unknow	M0	N0
TCGA-BT-A42C	<=65	MALE	High Grade	Stage I-II	unknow	M0	N0
TCGA-G2-A2EL	>65	MALE	High Grade	Stage I-II	unknow	M0	N0
TCGA-E7-A3Y1	<=65	MALE	Low Grade	Stage I-II	unknow	M0	N0
TCGA-DK-AA6U	<=65	MALE	unknow	Stage I-II	unknow	M0	N0
TCGA-ZF-A9R7	>65	FEMALE	High Grade	Stage I-II	unknow	M0	unknow
TCGA-DK-A3IV	<=65	MALE	High Grade	Stage I-II	unknow	M0	unknow
TCGA-GD-A3OS	<=65	FEMALE	High Grade	Stage I-II	unknow	unknow	unknow
TCGA-G2-A3IE	<=65	MALE	High Grade	Stage I-II	unknow	unknow	unknow
TCGA-LT-A5Z6	<=65	MALE	High Grade	Stage I-II	unknow	unknow	unknow
TCGA-DK-A6B6	<=65	MALE	High Grade	Stage I-II	unknow	unknow	unknow

TCGA-G2-A3IB	>65	MALE	High Grade	Stage I-II	unknow	unknow	unknow
TCGA-GV-A3QH	>65	MALE	High Grade	Stage I-II	unknow	unknow	unknow
TCGA-GV-A40E	>65	MALE	High Grade	Stage I-II	unknow	unknow	unknow
TCGA-ZF-A9RE	>65	FEMALE	High Grade	Stage I-II	unknow	unknow	unknow
TCGA-YC-A89H	>65	FEMALE	High Grade	Stage I-II	unknow	unknow	unknow
TCGA-ZF-A9RL	<=65	MALE	High Grade	Stage I-II	unknow	unknow	unknow
TCGA-G2-A3VY	>65	MALE	High Grade	Stage I-II	unknow	unknow	unknow
TCGA-FJ-A3ZF	>65	MALE	High Grade	Stage III-IV	unknow	M0	N0
TCGA-UY-A78L	<=65	MALE	High Grade	Stage III-IV	unknow	unknow	N1-3
TCGA-UY-A78K	<=65	MALE	High Grade	Stage III-IV	unknow	unknow	N1-3
TCGA-FJ-A3ZE	<=65	MALE	High Grade	Stage III-IV	unknow	M1	N1-3
TCGA-H4-A2HQ	<=65	FEMALE	High Grade	Stage III-IV	unknow	M1	unknow
TCGA-YC-A9TC	>65	MALE	High Grade	Stage III-IV	unknow	M1	unknow
TCGA-FJ-A3Z9	>65	MALE	High Grade	unknow	unknow	M0	N0
