# Supplementary material for: Ferroptosis-Related Long Noncoding RNAs Have Excellent Predictive Ability for Multiomic Characteristics of Bladder Cancer
Source: Oxid Med Cell Longev. 2022 Aug 29;2022:9316847. doi: 10.1155/2022/9316847 (PMC9444476; doi:10.1155/2022/9316847)
Supplement: Supplementary Materials — Supplementary Table 1: expression matrix of 60 ferroptosis-related genes. Supplementary Table 2: results for coexpression analysis of ferroptosis-related lncRNAs and mRNAs. Supplementary Table 3: expression of 263 ferroptosis-related lncRNAs. Supplementary Table 4: expression of lncRNAs and clinical information of 403 bladder cancer samples. Supplementary Table 5: cluster analysis based on 20 prognostic lncRNAs. Supplementary Table 6: detailed clinical information of bladder cancer samples. Supplementary Table 7: data of the high- and low-risk groups of the training cohort. Supplementary Table 8: data of the high- and low-risk groups of the validation cohort. Supplementary Table 9: markers of different immune cells. Supplementary Table 10: infiltration of 22 types of immune cells analysed using the CIBERSORT algorithm. Supplementary Table 11: immune scores evaluated using the ESTIMATE algorithm. Supplementary Table 12: immunotherapeutic response evaluated using the Tumour Immune Dysfunction and Exclusion (TIDE) algorithm. Supplementary Figure 1: gene set enrichment analysis. (A–F) Most significantly enriched biological pathways in cluster 1. (G–I) Most significantly enriched biological pathways in cluster 2. [file 9316847.f1.zip › s-table 7.pdf]

id	futime	fustat	AL136084.3	AL731567.1	AC006160.1	AC012568.1	AC034236.2	riskScore	risk
TCGA-CF-A47X	1.05205479452055	0	0.1634272	4.161804	0.3443737	0.03040022	1.844859	0.304943347874197	low
TCGA-BL-A0C8	3.33972602739726	0	1.874799	15.6686446666667	1.045091	4.18296566666667	0.7392174	0.422489915703895	low
TCGA-G2-A3IB	0.602739726027397	1	0.03671464	0.06977371	0.1289418	0.06829546	0.1501652	0.850740684333934	high
TCGA-2F-A9KP	0.997260273972603	1	0.8243399	2.00177	0	0.08518955	0.9115811	0.733241497242039	high
TCGA-FD-A43P	2.27945205479452	0	0.4644774	2.783925	0.2091339	0	1.159329	0.491643163410303	low
TCGA-CF-A47W	1.00821917808219	0	0	2.782877	0.06949671	0.1104291	2.870516	0.300307185218852	low
TCGA-GC-A3I6	1.72602739726027	1	0.04440425	0.9282603	0.3378867	0.1238991	1.259205	0.454217517071441	low
TCGA-FD-A6TA	5.23835616438356	0	0.5686815	2.593778	0.1331473	0.1057844	0.7443016	0.644203189599722	high
TCGA-PQ-A6FN	1.38904109589041	0	0.3351258	0	0.03269338	0	0.7919515	0.76267139720969	high
TCGA-XF-AAMY	8.24931506849315	0	1.207372	15.37335	0.04711435	0.0748639	1.580233	0.278418906243116	low
TCGA-K4-A3WS	2.08493150684932	0	1.116417	12.6378	0.454592	0	1.29707	0.232366421445017	low
TCGA-ZF-A9RN	1.68493150684932	1	0.2141667	0.8140188	0.09401917	0	0.4525767	0.762988178584598	high
TCGA-UY-A9PF	0.320547945205479	0	0.4128858	7.925085	0.04833517	0.07680376	0.5178768	0.552313125325991	low
TCGA-XF-AAMQ	5.96438356164384	0	0.5717038	4.128642	0.1338549	0	0.8807611	0.549581560981729	low
TCGA-CF-A1HR	1.06575342465753	0	0.7825291	1.31555	0.1409355	0	0.5416387	0.73532904164239	high
TCGA-G2-A2EJ	4	0	0.1043501	0.3305174	0.1832388	0.03235149	0.6544244	0.665155090649587	high
TCGA-FD-A43N	1.91506849315068	0	1.086503	3.510205	0.08479555	0	1.007276	0.606680057481719	high
TCGA-CU-A0YR	1.26027397260274	1	1.594869	0.2612881	0.06438139	0.1023009	0.2849176	1.03923160607369	high
TCGA-SY-A9G5	3.24931506849315	0	0.2334165	0.887185	0.2049397	0	0.5250785	0.665887372045942	high
TCGA-4Z-AA84	1.26027397260274	0	0.3214093	1.628845	0.1567763	0	2.599955	0.32631624944813	low
TCGA-FD-A5BT	0.898630136986301	1	1.808429	0.1610999	0	0	0.6009726	0.995558624649893	high

TCGA-E7-A6MF	2.05479452054795	0	0	1.410355	0.05791858	0.04601582	1.322054	0.555593675251966	low
TCGA-FT-A61P	0.923287671232877	0	0.557654392	0.529892377	0.148369865	0	0.373228544	0.787090932838872	high
TCGA-DK-AA6U	1.58356164383562	0	0.2072617	3.072318	0.09705374	0	1.096376	0.535661744224967	low
TCGA-BT-A20O	1.01369863013699	1	0.906196342	0.645812003	0.331516828	0	0.679506543	0.611543347474527	high
TCGA-GD-A3OS	1.74794520547945	0	0.03128782	0.2973021	0.09156903	0	1.458848	0.540071391062874	low
TCGA-E7-A678	2.18630136986301	0	0	3.479877	0.1128213	0	2.224892	0.33821336225344	low
TCGA-XF-A9SH	5.4	1	0.2504942	5.077839	0.0977484	0	1.297747	0.449269729873914	low
TCGA-FD-A5BU	1.61095890410959	0	0.5896405	0.509351	0	0	1.008515	0.731622666555142	high
TCGA-C4-A0F7	0.16986301369863	1	0.8306518	0.2721721	0.0502974	0	0.7419648	0.797483229719269	high
TCGA-ZF-AA5H	2.45753424657534	0	0.3296938	1.12781	0.0771923	0	0.6472646	0.721896576871893	high
TCGA-DK-A3WX	0.879452054794521	1	0.9743899	1.247927	0.07439255	0	0.5544787	0.802497176600151	high
TCGA-YF-AA3L	0.997260273972603	0	0.2083021	0.8412115	0.335297	0.09686932	1.022362	0.503714548777701	low
TCGA-4Z-AA87	3.98356164383562	0	0.0419546	7.255604	0.3192466	0	0.7092673	0.385140122759084	low
TCGA-DK-A2I6	7.27671232876712	0	0.1212986	0.4898548	0.03550007	0	0.4961189	0.801084726831554	high
TCGA-ZF-AA4X	5.6	0	0.04795873	15.12963	0.1965029	0	1.516926	0.212875353536435	low
TCGA-BT-A20P	1.49041095890411	1	0.08240554	2.19248675	0.19293883	0.07664413	1.79756678	0.401390093561478	low
TCGA-BT-A20W	0.695890410958904	1	1.004927	3.564952	0.3529302	0.1246223	0.7855073	0.513664897632209	low
TCGA-BL-A13J	0.221917808219178	1	0.39174482	14.7993753333333	0.472614706666667	11.8219883333333	0.4822664	4.90284652098401	high
TCGA-CF-A8HX	0.945205479452055	0	0.04995679	11.06045	0.2924137	0.04646405	3.541657	0.120023409766426	low
TCGA-FJ-A3Z7	2.58904109589041	0	1.176532	9.662371	0.2705464	0	0.9967901	0.366613077096111	low
TCGA-E7-A7PW	1.13972602739726	0	0.1230111	8.532767	0.1800063	0.0572054	2.012493	0.266628978232219	low
TCGA-CF-A3MH	1.09041095890411	0	0.1151395	4.084543	0.08985995	0			

0.3348818 0.660134833454102 high

TCGA-DK-A6B2 1.30684931506849 0 0.3971613 1.1741 0.129151 0 0.5896022 0.703946908527415 high

TCGA-GU-AATO 0.887671232876712 1 0.07412371 1.197371 0.04338708 0 0.6467639 0.721212078499695 high

TCGA-4Z-AA7O 1.4027397260274 0 0.6232784 12.89788 0.08107236 0 0.660916 0.389919270197883 low

TCGA-GD-A6C6 0.183561643835616 0 0 0.5226101 0 0 1.244721 0.627010631525902 high

TCGA-YF-AA3M 1.13698630136986 0 0.2379443 1.431958 0.2785534 0 0.6920582 0.567285408350302 low

TCGA-FD-A62O 0.591780821917808 1 0.5738621 0.7931538 0.06107284 0 0.7397021 0.744401936265708 high

TCGA-CU-A0YO 0.408219178082192 1 1.191626 1.181533 0 0.3373126 0.66397 0.931628848305914 high

TCGA-K4-AAQO 0.983561643835616 0 0.9917667 2.146562 0 0 0.8413248 0.744561705838062 high

TCGA-XF-AAMJ 4.57534246575342 1 2.31559492 1.53355384 0.04107257 0 1.28192346 0.741645269926123 high

TCGA-GU-A763 2.73150684931507 0 0 1.888029 0.2326052 0 1.607288 0.407652385609391 low

TCGA-G2-A2EC 1.90684931506849 1 1.492806 4.14348 0.06898335 0.2192267 0.6319903 0.753677277209956 high

TCGA-FD-A3B7 0.334246575342466 1 3.366475 0.3594247 0.1549839 0 0.2681621 1.14544679462498 high

TCGA-CU-A0YN 1.07671232876712 1 0.5784328 0.7610349 0.07813292 0 0.5823572 0.775518746058729 high

TCGA-UY-A9PD 1.48493150684931 0 0.6899259 4.079158 0.3140952 0 1.212346 0.416658577387681 low

TCGA-K4-A54R 2.30684931506849 0 0.4905859 0.4078924 0.3230508 0 0.4681896 0.640378692285211 high

TCGA-DK-A1AA 1.58356164383562 0 0.1292788 4.944425 0.1135068 0.03006006 0.4670699 0.592903331606097 high

TCGA-BT-A3PH 0.389041095890411 1 0.3060499 4.071391 0.01990458 0.03162804 0.4450713 0.701924622926615 high

TCGA-CF-A3MF 1.04931506849315 0 0.04923692 12.95965 0.02882001 0 2.054097 0.233873463294738 low

TCGA-YC-A8S6 0.802739726027397 0 3.905313 3.036185 0.20781 0.06604131 0.8712531 0.824074564998589 high

TCGA-C4-A0F6 1.91780821917808 0 0.535759 1.527261 0.08959931 0 0.9391232 0.645317277858606 high

TCGA-FD-A3B6 2.75342465753425 1 0.299657676 0.488125119 0.150342537 0.119445871 0.87544179 0.659316669867655 high

TCGA-E7-A6ME 2.0027397260274 0 0.3452732 3.718289 0.1684166 0.1070445

2.05552 0.3592334429712 low
TCGA-ZF-A9R2 1.75890410958904 0 0.05813813 3.038409 0.1020906
0.05407339 0.665808 0.618846223075809 high
TCGA-ZF-AA4T 1.64109589041096 1 0.1711949 6.50689 0.03340204 0
0.6068382 0.559254314508975 low
TCGA-FD-A6TE 1.03013698630137 0 0 4.824455 0.4043353 0 1.582182
0.296009200812073 low
TCGA-H4-A2HO 0.126027397260274 0 0.3992276 5.785128 0.05842031 0
0.9661122 0.513968673327618 low
TCGA-FD-A6TB 1.56712328767123 0 1.329891 2.614519 0.2147392 0
0.8127745 0.618228189111424 high
TCGA-XF-AAMH 0.942465753424658 1 0.04736439 0.1350193 0 0
1.407723 0.608124568122603 high
TCGA-XF-A9SY 1.75342465753425 0 0.621499286 0.531502961 0.436541098 0
0.457554723 0.580139165352952 low
TCGA-CF-A9FF 0.989041095890411 0 0.3374108 11.79857 0.2764969
0.06276414 1.76644 0.227358133627294 low
TCGA-GC-A3RC 1.32602739726027 0 0.2743825 0.1303614 0.1070701 0
0.1496322 0.87620462069355 high
TCGA-GU-A764 1.67123287671233 0 0.9072538 0.7389322 0.0758637 0
0.8599457 0.733987079963274 high
TCGA-GU-A42R 1.58082191780822 1 0.1339928 9.591604 0.3137218
0.4569571 1.863332 0.257634598421811 low
TCGA-DK-A6B6 3.05479452054795 0 0 7.172026 0.1795922 0 1.355308
0.351706213846733 low
TCGA-R3-A69X 1.18630136986301 0 1.329885 1.549026 0.1255526 0
0.6316623 0.762300231809235 high
TCGA-5N-A9KM 1.45205479452055 1 0.1012808 0.2405967 0.2074906
0.04709987 0.8284896 0.615962528828036 high
TCGA-FD-A6TF 0.189041095890411 1 1.176999 1.905429 0.1275809 0
0.8677087 0.674201411419768 high
TCGA-XF-A8HF 8.09315068493151 1 0.1477172 3.509082 0.1152851 0
0.8592675 0.554541897073125 low
TCGA-ZF-AA4U 0.717808219178082 1 0 0.7684765 0 0 0.8526748
0.710227248371722 high
TCGA-XF-A9T6 0.175342465753425 0 0.1089876 1.915893 0.06379405 0
1.277862 0.546555015078812 low
TCGA-ZF-AA56 0.70958904109589 1 1.218420176 1.603056315 0.109720299
0.043585924 0.293893658 0.867450199877218 high
TCGA-XF-A8HH 0.156164383561644 1 1.473284 6.429347 0.1596967 0
1.800307 0.380734756720067 low
TCGA-BT-A42F 2.36712328767123 0 0.212560756 0.363561709 0.174186455 0
0.463672905 0.719835131431323 high
TCGA-ZF-A9RF 5.33972602739726 0 0.04857409 0.8308063 0.2558883 0

							0.9801106	0.529475818413498	low
TCGA-ZF-A9RC	7.85753424657534	0	0.5073308	11.630004	0.1855985	0	1.245009	0.302780552200875	low
TCGA-E7-A6MD	0.353424657534247	0	1.431317	4.130556	0.4964727	0.09861084	2.717494	0.229033145054197	low
TCGA-E5-A4TZ	1.27945205479452	1	0.1058505	1.072863	0.08261042	0.1640832	0.9043532	0.667630557422683	high
TCGA-ZF-A9R4	2.52328767123288	0	0.2231192	3.985817	0.1044793	0.04150395	0.8395657	0.560680382482718	low
TCGA-GV-A3QH	0.706849315068493	1	0.03383643	4.115447	0.2376671	0.125883	0.4244055	0.564643171031619	low
TCGA-BT-A0YX	1.0958904109589	1	0.1931212	0.7799047	0.05652015	0.04490478	0.4081036	0.812733082410719	high
TCGA-ZF-A9RL	7.40547945205479	0	0.2322183	4.468317	0.1699064	0	0.9814469	0.482622231202275	low
TCGA-ZF-A9R9	2.36712328767123	1	0.867471	3.297141	0.04231331	0	0.4927792	0.7472045644361	high
TCGA-CF-A5U8	1.09315068493151	0	0.05138168	12.64534	0.150377	0	2.521851	0.179137105808068	low
TCGA-XF-AAMW	0.693150684931507	1	1.033124854	1.208236593	0.093034218	0	0.541736516	0.798785678673166	high
TCGA-BT-A3PJ	2.16164383561644	0	0.27979411	0.199398724	0.286602433	0	0.438677193	0.661456153791696	high
TCGA-ZF-A9R7	1.82191780821918	0	0.1482325	2.676206	0.8242713	0.06893438	2.303864	0.177035599736738	low
TCGA-FD-A3SO	0.46027397260274	1	0.843170521	0.40059725	0.137093281	0	0.702496627	0.73850588094118	high
TCGA-E7-A97P	1.1972602739726	1	1.106710151	0.09560129	0.235561579	0	0.397784498	0.783178119677522	high
TCGA-2F-A9KQ	7.90684931506849	0	0.6155728	17.50283	0.02771656	0	1.226587	0.261622251543173	low
TCGA-S5-AA26	1.37808219178082	0	0.1366005	5.256901	0.1599136	0.12705	2.700405	0.26019051998653	low
TCGA-E7-A7DU	0.0767123287671233	0	0.1665694	5.302279	0.09749862	0	1.407977	0.422751931278718	low
TCGA-DK-A3WY	13.6082191780822	0	0.5481172	4.947885	0.2673594	0	1.893104	0.321857351277502	low
TCGA-G2-A2EF	5.16164383561644	0	0.1453262	0.3590374	1.003758	0.02703313	0.2932339	0.338194859469244	low
TCGA-BT-A20V	0.421917808219178	1	0.06854207	0.651298	0.1203599	0	0.4485461	0.738257606739779	high
TCGA-K4-A3WV	1.76986301369863	0	0	0.1408407	0.2313544	0	0.7409447	0.609136051230414	high
TCGA-GU-AATQ	0.583561643835616	1	0.2862256	0.4532937	0.3909205	0			

0.2081209 0.639135747028854 high
TCGA-G2-A3VY 1.46849315068493 0 0.03635777 3.8348 0.2340955
0.06763161 0.6146495 0.530397774965411 low
TCGA-CU-A3QU 0.432876712328767 0 0.03698215 4.67376 0.06494066
0.03439654 0.3226865 0.657468022039173 high
TCGA-GD-A3OP 0.175342465753425 0 0.6994113 2.246954 0.1364629 0
1.289556 0.534112719688837 low
TCGA-4Z-AA80 0.0520547945205479 1 0.1766703 0.2238333 0.2757626 0
0.6262467 0.617324465034347 high
TCGA-G2-A3IE 1.67671232876712 1 0.1665933 10.80396 0.1218908
0.07747296 0.5451017 0.422399131146494 low
TCGA-FD-A43S 1.24657534246575 0 1.24348 1.13613 0.08398271 0.04448237
1.956119 0.509921128099847 low
TCGA-XF-A8HG 1.27945205479452 1 0 9.018318 0.2496757 0.1487739
0.6106199 0.40061023268362 low
TCGA-GD-A2C5 2.22465753424658 0 1.140342 6.965819 0.1668701 0
1.077178 0.45515565618374 low
TCGA-XF-AAMX 0.564383561643836 1 1.48558 3.33143 0.2086943 0
0.4536834 0.691382104550717 high
TCGA-DK-A1AE 1.34520547945205 0 0.2743487 0.1042763 0.1445269
0.05103351 0.5460902 0.745112454603334 high
TCGA-KQ-A41Q 0.989041095890411 0 0.05190536 11.14662 0.06076388
0.04827638 0.7076538 0.407060314018876 low
TCGA-DK-A1AF 1.46849315068493 0 3.570926 2.054569 0.1150559 0.06094061
0.5717062 1.01551731956036 high
TCGA-K4-A4AB 0.208219178082192 0 0.5468671 0.1417206 0.2036997 0
0.6777941 0.68312741066513 high
TCGA-DK-A1AG 1.3013698630137 0 0.081730177 4.931496254 0.095678793 0
2.039117143 0.342561846269707 low
TCGA-GV-A3JZ 1.65205479452055 0 0.5898803 1.463564 0.01918203
0.03047993 0.6433726 0.781054280728537 high
TCGA-XF-A9SV 1.06301369863014 1 0.2886571 0.1828578 0 0
0.6821392 0.805981879841308 high
TCGA-FD-A6TH 0.358904109589041 1 0.6634293 1.500956 0.01849178 0
0.3014964 0.880724160147312 high
TCGA-DK-AA6W 1.13698630136986 1 0.6871186 1.197004 0.4021934 0
1.014851 0.479277004739433 low
TCGA-DK-A3X1 5.5041095890411 0 0.1914294 2.091843 0.1400625 0
1.278832 0.507073618814003 low
TCGA-E7-A541 2.13150684931507 1 0.1640528 6.079542 0.3520938 0
0.9542916 0.370128963589957 low
TCGA-DK-AA6Q 1.13150684931507 1 0.059338519 0.620228804 0.104198439 0
0.695734919 0.687953166660672 high
TCGA-UY-A78N 7.23561643835616 1 0.07899331 2.02664 0.161831 0.03673524

1.3785070.478949574334953 low
TCGA-DK-AA75 0.931506849315068 1 0.6121971 2.07757 0.2047653 0
1.2161920.511941852304286 low
TCGA-ZF-AA5P 1.01917808219178 0 3.4438130.6041294 0.06202395 0
1.1557260.914321688565968 high
TCGA-GC-A3WC 1.47945205479452 0 0.1945023 0.8316868 0.4838569 0
1.7103820.333059143051597 low
TCGA-K4-A5RH 0.756164383561644 0 0.6064176 0.5762280.2662173 0
0.9025484 0.583802678139559 low
TCGA-FD-A5C1 4.90958904109589 0 0.1963170.6529029 0.02872773
0.09129574 0.7092715 0.765609109985741 high
TCGA-XF-AAN7 1.54794520547945 1 0.3885729 0 0 0 1.324406
0.657095040411403 high
TCGA-FD-A3SS 1.07123287671233 1 0.3839693 0.6302022 0.2451818 0
0.7328805 0.614277805479199 high
TCGA-HQ-A2OE 3.21643835616438 0 0.3599777 2.7120220.1053535
0.02391499 0.2594108 0.745450794088147 high
TCGA-GV-A3JV 1.18904109589041 1 0.1451146 5.7913930.08494042 0
0.7122334 0.531036684370399 low
TCGA-CU-A3YL 2.48219178082192 0 0.09337902 1.2865890.2186314 0
2.8771760.276159695733328 low
TCGA-FD-A43U 1.74246575342466 0 0.3910596 1.1147740.2575128
0.09092965 1.3728680.483163847683273 low
TCGA-GU-A42P 0.90958904109589 1 0.1348575 2.6269480.4144171
0.03135723 1.1031510.400660684851423 low
TCGA-XF-A9SL 5.53424657534247 1 1.9557420.8313797 0.09037586 0
1.75419 0.595578393441225 high
TCGA-XF-AAN2 5.12054794520548 1 0.2355634 1.1937930.5055714
0.0730313 0.9634689 0.425629201727032 low
TCGA-GU-A766 1.31506849315068 0 1.3441120.8651979 0.4060662
0.04032702 1.1113230.51350016399905 low
TCGA-FD-A6TD 1.05753424657534 1 0.4876876 1.7609530.1427299
0.04535908 0.5452105 0.698708666368553 high
TCGA-FD-A3B4 1.3972602739726 1 0.226012233 0.245440619 0.170090349 0
0.607465533 0.692609947725306 high
TCGA-BT-A20X 0.687671232876712 1 1.7076210.2318014 0.04079705 0
1.14029 0.778570324836112 high
TCGA-GV-A3JW 1.77808219178082 0 0.4503216 2.9240040.3514510.03490311
1.8520760.334818211120196 low
TCGA-FD-A6TI 0.805479452054795 1 0.4575304 0.4347529 0.0824024
0.16367 1.3243240.621902299626516 high
TCGA-FD-A3SP 2.14520547945205 0 1.0067521.3950880.1473213 0
0.5833375 0.736419485634181 high
TCGA-E7-A97Q 0.673972602739726 1 0.7022613 2.1448940.4991406

0.04665447 1.107884 0.405935329920668 low
TCGA-YC-A9TC 0.0547945205479452 1 0.08968993 8.394648 0.3937388 0
1.369526 0.268144931307371 low
TCGA-ZF-AA52 2.95068493150685 1 0.4905604 1.981089 0 0 1.103532
0.645260417469705 high
TCGA-K4-A5RJ 1.47671232876712 0 0.746258767 1.418214714 0.691616042 0
1.390467034 0.314462084452864 low
TCGA-BT-A3PK 0.83013698630137 1 1.684351 0.2353674 0.08699179 0
0.4525194 0.945107375442638 high
TCGA-FD-A3N6 2.33150684931507 0 0 0.06187816 0.1334093 0.03028359
1.269578 0.563419245146901 low
TCGA-CF-A9FL 1.54794520547945 1 0.5145605 7.871997 0.1204758 0
1.262751 0.39412412692667 low
TCGA-CF-A9FM 1.09041095890411 0 0.211407 7.081106 0.1237436 0.1474698
2.392248 0.275876891368207 low
TCGA-G2-AA3D 5.86027397260274 0 0.05325702 9.463266 0.1246924
0.09906709 1.060079 0.375014839037211 low
TCGA-DK-A2I2 0.649315068493151 1 0.4017016 0.9754644 0.05225097 0
0.8397477 0.703106347851577 high
TCGA-MV-A51V 1.12328767123288 0 0.05661127 12.04962 0.1656823
0.2106131 0.5248322 0.388614652385996 low
TCGA-DK-A3X2 1.4986301369863 1 0.08493605 0.6053072 0.0497159
0.03949886 0.4284523 0.808486617742408 high
TCGA-GC-A6I1 0.003 0 0.05162584 0.1962229 0.09065498 0 0.6475355
0.725026958922457 high
TCGA-K4-A5RI 0.975342465753425 1 0.319114 0.8230457 0.08005202 0
0.6090915 0.740914397681467 high
TCGA-GV-A3QF 1.69041095890411 1 0.3789178 3.150472 0.360414 0.08810643
0.8007287 0.476999236182209 low
TCGA-E7-A7DV 0.101369863013699 0 0.5560032 0.950982 0.04339296 0
0.6468515 0.774242634536955 high
TCGA-DK-AA6P 1.25205479452055 0 0.3671676 0.988518 0.07163848 0
0.4838935 0.778162060168524 high
TCGA-CF-A47Y 1.02191780821918 0 0 8.78335 0.04830842 0 4.219486
0.133655044495265 low
TCGA-CF-A3MI 1.01369863013699 0 0.3958001 5.975743 0.07722499
0.08180613 1.175163 0.473396413346554 low
TCGA-DK-AA6L 3.18630136986301 1 0.06075884 1.558819 0.07112835
0.1130217 0.8449252 0.659271118685691 high
TCGA-XF-A9T4 1.35616438356164 1 0.4346409 0.4956035 0.1187246 0
0.537263 0.755118210662254 high
TCGA-K4-A6MB 1.28493150684932 0 0.2672019 3.663409 0.1117159
0.03550292 0.7181733 0.593557445059435 high
TCGA-ZF-A9R5 2.98630136986301 0 0.05047636 6.762849 0.3840914

0.1408419 3.37204 0.150783315284307 low
TCGA-E7-A85H 1.07945205479452 0 0.1648848 2.350143 0.07238442 0
1.360018 0.517623513627617 low
TCGA-G2-AA3C 0.578082191780822 1 0.2377633 1.299078 0 0 1.150752
0.639101790034986 high
TCGA-XF-A9SM 2.87123287671233 0 0.4292626 0.6118386 0.2826695
0.04990633 1.068057 0.536269226978696 low
TCGA-DK-A3IN 0.684931506849315 1 0.9389444 2.05594 0.09558181 0
0.4007312 0.790549268922898 high
TCGA-XF-A8HC 0.547945205479452 1 0 1.642724 0.1806997 0 1.447842
0.459851655486775 low
TCGA-ZF-AA4W 5.01369863013699 0 0.05814429 0 0.06806758 0
0.7927125 0.712482448627342 high
TCGA-LC-A66R 1.27671232876712 0 0.9040558 0.234286 0.7216009
0.1528815 1.053268 0.3884670912256 low
TCGA-E7-A8O7 1.27671232876712 0 0.152703 7.617799 0.06703663 0.1065201
1.665506 0.359558313079187 low
TCGA-FJ-A3Z9 1.05479452054795 1 0 4.281002 0.06939729 0.2756778
1.858856 0.412017340996568 low
TCGA-G2-A2ES 2.75068493150685 1 0.3609896 0.3430182 0.02112992 0
0.6201173 0.806779560740708 high
TCGA-XF-AAN4 2.25479452054795 1 2.753438 1.569817 0.2417519 0.06402327
1.088634 0.696712716031512 high
TCGA-E7-A5KE 0.0465753424657534 0 0.09757717 3.894216 0 0.04537752
1.995483 0.409817448644292 low
TCGA-ZF-AA53 4.82465753424658 0 0.1920471 0.6082876 0.03747051 0
0.3141937 0.854120892385117 high
TCGA-CU-A72E 1.13150684931507 1 0.4040793 1.780191 0.06450575 0
0.9215107 0.646162853053611 high
TCGA-2F-A9KR 8.72054794520548 1 0.1021467 1.310329 0.3587391 0
3.760076 0.176320778680768 low
TCGA-E5-A4U1 3.23561643835616 0 0.1132879 1.399424 0.3315559
0.3161021 3.34461 0.00668942736251224 low
TCGA-FD-A5BV 0.446575342465753 1 0 15.40966993 0.29531776 0.06703642
0.66819902 0.259418961144343 low
TCGA-DK-A3IU 1.93424657534247 1 1.001522 0.577795 0.1046829 0
0.3803694 0.861873317997061 high
TCGA-FD-A3SM 1.4986301369863 1 0.631069729 1.359213098 0.197005946 0
0.401506682 0.716330246953883 high
TCGA-BT-A20J 1.58630136986301 1 1.0590806 2.571798177 0.172198661 0
0.304823716 0.748048355958394 high
TCGA-E7-A677 2.24657534246575 0 0.2350462 2.456792 0.1547779 0
1.586233 0.441979107353728 low
TCGA-HQ-A5NE 1.01369863013699 1 0.7414635 4.039425 0.05786711

						0.09194985	0.6065263	0.684489173134084	high
TCGA-CF-A47S	0.912328767123288	0	0.06865367	5.43632	0.05358037	0.04256915	2.190223	0.332357831447028	low
TCGA-E7-A5KF	0.0547945205479452	0	0	11.08384	0.179002	0.1218991	1.346089	0.294093700291729	low
TCGA-XF-A9SX	1.96986301369863	1	0.1472713	0.5597586	0.08620282	0	1.566107	0.522554344401004	low
TCGA-G2-A2EK	1.32876712328767	0	0.3577096	3.976848	0.2512552	0.03327002	1.004631	0.464709311076784	low
TCGA-XF-A8HD	8.12054794520548	0	0.4918876	1.617922	0.8416071	0	1.784873	0.226662091068943	low
TCGA-GV-A3JX	1.59178082191781	0	0.3117886	0.6583702	0.1216668	0	0.3589549	0.783064551356029	high
