# Supplementary material for: Ferroptosis-Related Long Noncoding RNAs Have Excellent Predictive Ability for Multiomic Characteristics of Bladder Cancer
Source: Oxid Med Cell Longev. 2022 Aug 29;2022:9316847. doi: 10.1155/2022/9316847 (PMC9444476; doi:10.1155/2022/9316847)
Supplement: Supplementary Materials — Supplementary Table 1: expression matrix of 60 ferroptosis-related genes. Supplementary Table 2: results for coexpression analysis of ferroptosis-related lncRNAs and mRNAs. Supplementary Table 3: expression of 263 ferroptosis-related lncRNAs. Supplementary Table 4: expression of lncRNAs and clinical information of 403 bladder cancer samples. Supplementary Table 5: cluster analysis based on 20 prognostic lncRNAs. Supplementary Table 6: detailed clinical information of bladder cancer samples. Supplementary Table 7: data of the high- and low-risk groups of the training cohort. Supplementary Table 8: data of the high- and low-risk groups of the validation cohort. Supplementary Table 9: markers of different immune cells. Supplementary Table 10: infiltration of 22 types of immune cells analysed using the CIBERSORT algorithm. Supplementary Table 11: immune scores evaluated using the ESTIMATE algorithm. Supplementary Table 12: immunotherapeutic response evaluated using the Tumour Immune Dysfunction and Exclusion (TIDE) algorithm. Supplementary Figure 1: gene set enrichment analysis. (A–F) Most significantly enriched biological pathways in cluster 1. (G–I) Most significantly enriched biological pathways in cluster 2. [file 9316847.f1.zip › s-table 8.pdf]

id	futime	fustat	AL136084.3	AL731567.1	AC006160.1	AC012568.1	AC034236.2	riskScore	risk
TCGA-ZF-AA51	4.6958904109589	0	0.429602124	2.517325863	0.083820148	0.066594397	0.507606484	0.718707588702786	high
TCGA-BT-A42C	2.39178082191781	0	0.1415353	2.252694	0.04142267	0.03290996	0.7814992	0.661283220147806	high
TCGA-DK-A3IT	1.77534246575342	0	0.7856667	0.9539293	0.1532923	0.04059648	0.5950788	0.734896798418092	high
TCGA-DK-A3IV	0.805479452054795	1	0.1753007	5.097152	0.2052187	0.0326089	1.338383	0.397821300230122	low
TCGA-2F-A9KW	0.695890410958904	1	5.606641	1.35462	0.09944709	0	0.9574099	0.68047816154325	high
TCGA-GU-A762	0.635616438356164	1	0.374333255	0.400159819	0.082166149	0	0.625177224	0.757345493557312	high
TCGA-XF-AAME	7.74794520547945	1	2.043624	1.132766	0.1246043	0	0.7545911	0.813978747341553	high
TCGA-XF-AAN0	4.70684931506849	1	0.177699203	3.478365734	0.166421227	0	1.143499887	0.479678355006248	low
TCGA-DK-A1A7	1.53424657534247	0	0.293680625	0.383707722	0.021487632	0.034143484	1.811767764	0.529259198278955	low
TCGA-FD-A3SR	1.64931506849315	1	0.2754449	8.244568	0.1074847	0	0.7510579	0.455183942161924	low
TCGA-GU-AATP	2.74794520547945	0	0.202713	4.943946	0.1186547	0.06284677	1.289725	0.4491652565566	low
TCGA-DK-AA6X	1.27945205479452	0	0.5220974	7.193531	0.6621355	0	0.9253447	0.270861304324913	low
TCGA-XF-A9T3	0.186301369863014	0	1.745144	0.2211019	0.06809939	0	1.713059	0.622688176831978	high
TCGA-DK-A3WW	1.73424657534247	0	0.088582	0.6312905	0.337025	0	0.7970729	0.529717422723838	low
TCGA-UY-A9PA	2.93698630136986	0	0.06564991	3.805279	0.2305625	0.06105998	0.5907269	0.538597731569975	low
TCGA-G2-AA3F	2.44657534246575	0	0.3181678	7.990105	0.05320978	0	1.561591	0.367817406075917	low
TCGA-FJ-A871	0.745205479452055	1	0.127022	0.08046562	0.1239171	0	1.166052	0.593657697545079	high
TCGA-GC-A3RB	1.59452054794521	0	0.0690156	2.492031	0.08079426	0	0.6304211	0.651119068726288	high
TCGA-YC-A89H	1.56986301369863	0	0.377556	0.2242249	0.1381226	0	1.029485	0.627982342415597	high
TCGA-E7-A3X6	2.47671232876712	1	0.313302421	1.275879063	0.02619805	0	0.61020303	0.761246947620839	high
TCGA-DK-A6AW	4.44109589041096	0	0.2128533	5.359802	0.09344258	0	1.915283	0.35585788626034	low

TCGA-GC-A3BM	1.78356164383562	1	0.04469052	2.463009	0.2092708	0.04156597	1.279509	0.460901716042963	low
TCGA-GV-A6ZA	1.89315068493151	0	0.25201	2.777783	0.2065138	0.1875125	1.594206	0.432467850572748	low
TCGA-UY-A8OD	9.4027397260274	0	0.8751733	5.821229	0.2241173	0.4069927	1.088769	0.48975645391547	low
TCGA-2F-A9KT	6.44383561643836	0	0.6506223	2.225635	0.03808309	0.1210268	1.312802	0.598784018681502	high
TCGA-BT-A20Q	1.62465753424658	1	0.2955651	0.5148933	0.3171742	0.09163354	0.5104159	0.630786586647795	high
TCGA-4Z-AA7Q	1.3972602739726	1	0.246401069	0.643869459	0.144226759	0.057293469	0.772643351	0.667042243911873	high
TCGA-CF-A27C	1.16438356164384	0	0.03812694	8.550008	0.2231697	0.248229	1.736149	0.292343967910717	low
TCGA-DK-AA77	1.69315068493151	0	0.2074157	4.188158	0.2428146	0	0.551423	0.529215453671437	low
TCGA-ZF-AA5N	0.46027397260274	1	0.3575789	0.3397773	0	0	0.6500088	0.814879385810466	high
TCGA-XF-A9SZ	2.35342465753425	1	0.4645447	1.60014	0.1699459	0	0.6966727	0.641459240376475	high
TCGA-4Z-AA82	4.26301369863014	1	0.3082251	0.6345746	0.09020722	0.04777925	0.5602933	0.762151418884154	high
TCGA-DK-AA76	1.0027397260274	0	0.2885105	4.715335	0.2701997	0	0.6922818	0.480979336941529	low
TCGA-DK-A3IL	1.13150684931507	1	0.1423685	6.042546	0.166666	0.04413825	1.721008	0.342581932723559	low
TCGA-ZF-A9R1	2.11780821917808	0	0.05150933	3.328261	0.1206005	0.04790804	0.9410211	0.541646506154689	low
TCGA-C4-A0F0	0.161643835616438	0	0.32752934	0	0	0	0.107169227	1.00137678785437	high
TCGA-FD-A3N5	1.87671232876712	1	0.3840633	0.03041194	0.05620127	0	0.2967148	0.890965287293286	high
TCGA-BT-A2LA	1.43013698630137	0	0.2893862	0.3971927	0.2634915	0	2.647774	0.307863355938074	low
TCGA-BT-A2LB	1.34794520547945	1	0.440135003	6.133944956	0.021468807	0.102340713	1.120111688	0.510760543660896	low
TCGA-XF-A8HB	3.75342465753425	0	0.07658528	0.0363863	0.2241396	0	0.3445624	0.715898200852212	high
TCGA-FD-A5BX	0.473972602739726	1	0.6784212	0.1074412	0	0	0.81702	0.808537716432847	high
TCGA-PQ-A6FI	1.01917808219178	0	0.03467674	0	0.0811898	0.0322523	0.6713287	0.738005866484698	high
TCGA-4Z-AA7S	2.91506849315068	1	0.3436348	4.431444	0.05746883	0	1.177933	0.510686799243884	low

TCGA-4Z-AA86	0.852054794520548	1	1.225670227	0.705849704	0.130441025	0	0.891211974	0.716033844163389	high
TCGA-5N-A9KI	0.208219178082192	1	0.588706345	1.333950157	0	0	0.703831766	0.778546143000379	high
TCGA-FD-A6TG	0.254794520547945	1	0.7193308	1.746773	0.04678314	0	0.5666281	0.775008729211867	high
TCGA-GV-A3QI	3.04109589041096	0	0.2363602	1.732577	0.7708044	0	4.087878	0.104095595245908	low
TCGA-DK-A6AV	5.34794520547945	0	0.2614097	8.445456	0.0765059	0	0.2851152	0.545954362147736	low
TCGA-DK-A1A3	1.82191780821918	1	0.7957413	0.4536759	0.03105159	0	0.9836219	0.735972833854692	high
TCGA-GC-A3RD	1.17260273972603	0	0.2809227	1.423666	0.1370279	0.04354699	1.148991	0.56425178355351	low
TCGA-BT-A20R	0.421917808219178	1	1.147924	0.3231931	0.0746576	0.03954322	0.6607893	0.838161273851625	high
TCGA-XF-A9T0	2.18904109589041	0	0.3458225	1.533496	0.3036321	0	2.184517	0.329666960393473	low
TCGA-4Z-AA7Y	4.16986301369863	0	0.05125684	4.432164	0.09000702	0.04767321	0.4192874	0.631447779722039	high
TCGA-BL-A3JM	0.561643835616438	1	1.090606	0.06095949	0.1126531	0	1.049563	0.702400709663092	high
TCGA-FJ-A3ZF	1.43561643835616	0	0.183306	0.5515712	0.1788252	0.02841502	1.616091	0.474431989784243	low
TCGA-CF-A5UA	1	0	0	1.034809	0.08314465	0	3.214754	0.28082352150536	low
TCGA-FD-A5C0	1.50684931506849	1	0.2789463	3.578301	0.05442552	0	0.3929793	0.702023106927056	high
TCGA-FD-A5BS	4.49041095890411	0	0.60815076	1.73362456	0.15255896	0	0.66329983	0.666759341805199	high
TCGA-FD-A5BZ	2.28767123287671	1	0.4114713	1.675658	0.2408479	0.05467188	0.7693454	0.585163146191091	high
TCGA-UY-A9PE	0.517808219178082	0	0.4306632	3.273786	0.1512489	0	1.808411	0.401523872801239	low
TCGA-GV-A40E	0.715068493150685	1	0.1701137	1.576038	0.02489331	0	0.8117384	0.686631364201497	high
TCGA-BT-A2LD	1.70684931506849	1	0.1522144	0	0	0	0.4565485	0.867121868119438	high
TCGA-G2-AA3B	5.5013698630137	0	0	3.456295	0.1860359	0	1.973983	0.344270990725829	low
TCGA-CU-A5W6	0.153424657534247	1	0.1463046	1.621912	0.08563695	0.3628684	1.741994	0.508201646369691	low
TCGA-DK-AA6R	13.8109589041096	0	0.2037009	0.5322899	0.2086577	0	0.2221732	0.749903780398246	high

TCGA-BL-A13I 0.610958904109589 1 2.09453333333333 1.05985753333333
1.4731587 9.844355 0.5542824 2.8181417152013 high
TCGA-CF-A47T 1.05479452054795 1 0.1004256 4.055608 0.1175649 0
2.149576 0.339036743840254 low
TCGA-DK-A3IS 4.18904109589041 0 0.1038549 2.072378 0.1013163 0
1.170486 0.54209989148118 low
TCGA-XF-A9SJ 0.268493150684932 1 0.662148 1.887552 0 0 0.6388637
0.779660865372619 high
TCGA-DK-AA6T 1.56712328767123 0 0.1644315 2.135356 0.09624725 0
1.360015 0.511572683033715 low
TCGA-BT-A20N 2.17808219178082 1 0.06021935 2.803849412 0.035248393 0
0.426921525 0.718375494624064 high
TCGA-CF-A7I0 1.00821917808219 0 0.5840422 13.36965 0.03107812 0
1.6649 0.278964553521669 low
TCGA-GC-A3YS 2.07671232876712 0 0.1923035 0.5938723 0.3376849 0
1.1798 0.469357340613281 low
TCGA-XF-A9T2 1.57534246575342 1 0.8752303 0.1848129 0.2846119 0
1.193249 0.545994467990134 low
TCGA-UY-A78O 6.33424657534247 0 0.1541814 3.174291 0.2105776
0.0956012 1.63959 0.40061962555441 low
TCGA-GC-A3OO 1.31780821917808 0 0.9694677 1.796348 0.02837309 0
0.5419084 0.817962058074444 high
TCGA-XF-AAN5 6.28219178082192 0 0.2057931 0.5866441 0 0.09570253
1.206446 0.664928895646788 high
TCGA-FD-A3SN 2.43013698630137 0 0.276871134 0.789262519 0.054020635 0
0.440385608 0.804035498371061 high
TCGA-E7-A4IJ 1.84657534246575 1 0.1772954 9.153485 0.06918462 0
1.208582 0.378373546854732 low
TCGA-XF-AAN3 7.19178082191781 0 0.150012 1.175987 0.1536623 0
0.7413833 0.632437049511428 high
TCGA-FD-A3B8 1.05205479452055 0 4.060142 0.7643235 0.291462 0
0.5326528 0.969871529024179 high
TCGA-XF-A9ST 0.350684931506849 1 0.2327826 0.2764923 0.06812771 0
0.5712572 0.774667901680331 high
TCGA-DK-A6B5 4.22465753424658 0 0.1123323 2.526179 0.1972555
0.03482618 1.09246 0.499580859040383 low
TCGA-GV-A3QK 2.27945205479452 0 0.4026494 13.39114 0.2946051
0.03120817 0.7593859 0.291849697424195 low
TCGA-XF-AAMR 7.64383561643836 0 2.239406 2.003964 0.190893 0 1.114531
0.655860600561125 high
TCGA-DK-A3IQ 1.47671232876712 1 1.415333 2.036522 0.04733953 0.03761085
0.6395246 0.81521523401679 high
TCGA-E7-A4XJ 0.186301369863014 1 0.2602329 4.883731 0 0 1.898124
0.405026908116262 low

TCGA-4Z-AA7M	1.35616438356164	0	0.05858858	7.29301	0.3772323	0.05449235	1.59754	0.269782806342414	low
TCGA-C4-A0EZ	0.747945205479452	1	0.3445695	1.023174	0.201688	0	3.053507	0.275861446478909	low
TCGA-FD-A62P	0.523287671232877	1	0.6332648	1.312884	0	0	0.6906913	0.787233991517779	high
TCGA-CF-A47V	1.03835616438356	0	0.2137792	11.57878	0.2252378	0	1.538891	0.254260518317085	low
TCGA-XF-A9SI	6.63835616438356	0	0.9388348	1.883314	0.1221181	0	0.9528623	0.640133744254448	high
TCGA-GU-A42Q	0.942465753424658	1	0.118076	0.0747985	0.1382276	0.03660689	0.7941649	0.672954808454003	high
TCGA-KQ-A41N	4.39452054794521	0	0.1577661	0	0.1231277	0.04891195	1.649032	0.511542483512615	low
TCGA-GD-A3OQ	0.26027397260274	0	0.2024366	0.6411952	0.1184929	0	0.7175811	0.683986217690773	high
TCGA-BT-A20U	1.24657534246575	1	0	0.2747121	0.08461133	0	0.2956141	0.81697314923374	high
TCGA-XF-A9SW	0.991780821917808	1	4.12878601	0.84069437	0.34524515	0	0.83094719	0.83160624303636	high
TCGA-FD-A3B3	2.66849315068493	1	0.9339805	0.3882741	0.0854203	0	0.6764651	0.793173849033195	high
TCGA-FT-A3EE	0.271232876712329	1	0.3094538	1.029168	0.04528341	0.02398486	0.5554953	0.776303436291715	high
TCGA-ZF-A9RE	0.29041095890411	1	0.1696018	1.128109	0.1323648	0.05258135	0.5241152	0.709966352775871	high
TCGA-DK-A6B1	5.61369863013699	0	0.3637027	2.350055	0.2341761	0.03382744	0.9421256	0.528388175812686	low
TCGA-GU-A767	0.394520547945205	1	0.6712025	4.601182	0.08418797	0	1.098104	0.527148985484497	low
TCGA-4Z-AA81	3.47945205479452	1	0	3.79853776	0.033911583	0.053884931	0.458122323	0.6782012776746	high
TCGA-SY-A9G0	2.76164383561644	1	2.395699	4.995505	0.1168569	0	1.215747	0.588977818409157	high
TCGA-GV-A40G	1.58904109589041	0	0.2539045	6.479668	0.1061563	0.0337361	1.17695	0.434552711202644	low
TCGA-FD-A43Y	1.2986301369863	1	0	1.178669411	0.276594422	0.054937981	1.578392081	0.415709312957121	low
TCGA-BT-A42E	3.03561643835616	0	0.03815633	0.6526219	0.06700251	0.07097724	0.6970758	0.722984864467625	high
TCGA-FD-A43X	0.301369863013699	0	0.1168263	4.033373	0.2279411	0.1086584	3.174894	0.218619023376753	low
TCGA-ZF-AA4R	2.83835616438356	1	0	1.395724531	0.074762288	0	0.835851666	0.641542053436944	high

TCGA-BT-A20T	1.24109589041096	1	0.4821607	2.443504	0.09407491	0.1494834	0.5916201	0.712682779086665	high
TCGA-XF-AAN8	0.323287671232877	1	1.828868	0.7965009	0	0.07087508	0.4155657	1.04758324298903	high
TCGA-KQ-A41P	2.9972602739726	0	0.295293874	3.507413712	0.086422674	0	0.993055569	0.553955859378021	low
TCGA-UY-A9PH	4.27671232876712	0	1.397974	4.922814	0.3850734	0	2.892535	0.223517015478403	low
TCGA-FD-A5BY	0.687671232876712	0	0.42431828	0.3135955	0.0275964	0	0.38566403	0.878680402205266	high
TCGA-K4-A3WU	0.287671232876712	0	1.752248	3.810324	0.2958604	0.06268229	2.150042	0.356863741534973	low
TCGA-FD-A3SJ	2.02465753424658	1	0.8939428	0.9101134	0.3924409	0	0.6529074	0.572292020504036	low
TCGA-FD-A3B5	0.745205479452055	1	0.2402263	0.03260956	0.08034996	0	0.87025	0.698795605400625	high
TCGA-GC-A6I3	0.003	0	0	1.169586	0	0	2.684962	0.364505305119887	low
TCGA-ZF-A9R0	1.86301369863014	1	0.1846451	7.485987	0.07205262	0.1144904	1.426508	0.394304663505043	low
TCGA-K4-A83P	1.35616438356164	0	1.43042001	1.6475256	0.15223137	0	1.26465498	0.598184241441121	high
TCGA-FJ-A3ZE	0.887671232876712	1	0.04036162	11.42898	0.378	0	1.474728	0.221061439789339	low
TCGA-DK-A2I1	1.4958904109589	0	0.9244188	1.26031	0.1176289	0.1121462	1.435658	0.576332123139901	low
TCGA-DK-A6B0	6.38356164383562	0	0	3.472731	0.22387	0.03952507	1.946696	0.337975579135924	low
TCGA-FD-A5BR	2.22739726027397	0	0.5475135	5.462692	0.3204779	0.0509234	0.627022	0.469860125537579	low
TCGA-LT-A5Z6	1.2986301369863	0	0.1709399	8.175639	0.3335228	0.1059924	1.336163	0.302114875473059	low
TCGA-GC-A4ZW	0.0410958904109589	0	0	0.7740573	0.03178795	0	0.4738577	0.786390905429094	high
TCGA-DK-AA74	4.67945205479452	0	0.6718609	0.5586116	0	0	1.259478	0.674539148978428	high
TCGA-G2-A2EO	4.94246575342466	1	0.3389732	0.9202797	0.02834461	0	1.333605	0.602213552328668	high
TCGA-UY-A78K	1.46849315068493	1	0.2599608	2.140832	0.1521637	0	1.039628	0.548128304748415	low
TCGA-CF-A3MG	1.01095890410959	0	0.1074166	4.797246	0	0	0.380762	0.681769920586151	high
TCGA-DK-A1A6	5.53424657534247	0	0.183755	4.714389	0.2366274	0.1025446	1.292699	0.407812574211814	low
TCGA-XF-AAMG	9.21643835616438	0	0.7591987	0.9275183	0.1904504	0			

2.5137090.356283667864571 low
TCGA-XF-A8HE 10.4575342465753 0 0.286128536 1.042222161 0.16748057
0.044353964 0.455110244 0.714485995259846 high
TCGA-K4-A4AC 0.761643835616438 1 0.1621284 0.3081142 0.06326612
0.0502644 0.5894359 0.775364827025879 high
TCGA-FD-A3SL 1.95068493150685 1 1.3925640.2205395 0.0815114
0.04317341 1.3163330.68498358043671 high
TCGA-2F-A9KO 2.01095890410959 1 0.2393212.1035120.7354334 0.1112944
0.8972679 0.334174330001603 low
TCGA-DK-A3IM 0.679452054794521 1 0.03403159 0.09701212 0.09959911 0
1.0485740.626115546606368 high
TCGA-4Z-AA7N 3.74520547945205 1 1.2210046.8720620.3848355 0.1747135
0.6402543 0.454566601441347 low
TCGA-UY-A8OC 0.003 1 0.7375504 0.5005947 0 0 0.4740414
0.899279650220717 high
TCGA-XF-A9T8 1.14520547945205 1 1.0353560.5988422 0.1317453 0
0.7610131 0.736128711610721 high
TCGA-KQ-A41S 0.0958904109589041 0 1.452892997 2.384608517 0.103082024 0
0.576234919 0.770470954885053 high
TCGA-E7-A7XN 1.17260273972603 0 0.2977497 0.2829267 0.6099899 0
0.8389391 0.417214819130064 low
TCGA-DK-A2HX 3.89041095890411 1 0.9889884 0.7518024 0.09648131 0
0.5483255 0.809995890185174 high
TCGA-CF-A8HY 0.945205479452055 0 0.05512337 0.05237913 0.06453109 0
0.7966183 0.711683027073551 high
TCGA-4Z-AA7W 2.3013698630137 0 0 0.2824805 0 0 0.6160531
0.792683900506312 high
TCGA-BT-A0S7 0.547945205479452 1 1.0268690.1393926 0 0 0.4799954
0.947435455033982 high
TCGA-ZF-A9R3 2.6 1 0.3767636 14.439610.3920574 0.1946789 0.9245919
0.245512663927465 low
TCGA-4Z-AA89 2.81917808219178 0 0.06009191 6.6236370.0351738 0
1.2780540.432200543666718 low
TCGA-FD-A3NA 5.05479452054795 0 0.802372751 0.831739404 0.170783824
0.033921575 0.477346093 0.758232299067108 high
TCGA-ZF-AA54 1.61643835616438 1 6.69562 0.8938753 0.2267288 0
0.7845984 1.28644653929049 high
TCGA-DK-A1AB 1.39178082191781 1 0.2126145 0.8369807 0.03555725
0.1412497 0.4058164 0.850075114610904 high
TCGA-UY-A9PB 2.46301369863014 0 0.4857915 0.5193080.1421750
0.5795176 0.730542533065174 high
TCGA-XF-AAML 0.635616438356164 1 0.07075487 3.4960870.1242456 0
0.5980764 0.597545382145561 high
TCGA-ZF-AA58 4.51780821917808 0 0.623073864 0.54976539 0.208403372 0

0.691711504 0.66780272469167 high
TCGA-K4-A6FZ 0.150684931506849 0 0.768963024 1.913689158 0.214333186
0.10217154 0.339471816 0.728487411962227 high
TCGA-KQ-A41R 3.6986301369863 0 0.221416 1.893539 0.08640146 0.06864523
2.213702 0.396986967546678 low
TCGA-DK-AA6M 4.33424657534247 0 0.1419003 0.7415978 0.1245884 0
0.8125332 0.649246271026389 high
TCGA-HQ-A5ND 0.750684931506849 1 0.049125709 0.186720225 0.143774574
0.091382144 0.388459252 0.771679105347788 high
TCGA-UY-A78P 6.52054794520548 0 0.5922352 0.8441275 0.1155517
0.1377072 0.2825992 0.857669796464118 high
TCGA-ZF-A9RD 1.11780821917808 1 0.3620451 0.2580159 0.05297926 0
0.7897531 0.740123943210056 high
TCGA-FD-A62S 1.11232876712329 1 0.7028408 0.7464216 0.07259932
0.07690606 0.6425716 0.790631544470491 high
TCGA-E5-A2PC 3.63287671232877 0 0.1559728 3.349499 0.01825922
0.1160543 1.012196 0.600971892277555 high
TCGA-GD-A76B 0.613698630136986 0 0.36465604 0.6545041 0.11858074 0
0.90592742 0.652087449375005 high
TCGA-XF-A9SK 1.33150684931507 1 0.2954053 4.771882 0.1383284
0.1099008 1.627078 0.40408748974807 low
TCGA-DK-A1AD 9.36986301369863 0 0.3593105 2.731382 0.1051582 0
0.421286 0.699251462858791 high
TCGA-FD-A3SQ 3.8986301369863 1 1.53445 1.844016 0.2641661 0.08395109
1.119834 0.577026364866409 low
TCGA-XF-AAN1 2.57808219178082 1 0.1469384 5.654742 0.1720159 0
1.923159 0.320745728963001 low
TCGA-G2-A2EL 2.24383561643836 1 0.2704639 0.8352476 0.1978894 0
0.6729469 0.6411324432114 high
TCGA-XF-AAMZ 3.69315068493151 1 0.09048109 3.525042 0.0529616 0
1.603651 0.450125097220473 low
TCGA-UY-A8OB 5.77808219178082 0 0 0.361893 0.03715435 0 0.7615488
0.722981553964644 high
TCGA-CU-A3KJ 1.53972602739726 0 0.08238408 0.443602 0.2250367 0
1.14565 0.527980901063932 low
TCGA-DK-AA6S 13.8356164383562 0 0.4740423 1.069802 0.1387364 0
1.43799 0.525024937296474 low
TCGA-E7-A8O8 0.0356164383561644 0 0.2482925 6.181409 0.02906678 0
1.299881 0.452057309101351 low
TCGA-LT-A8JT 1.75616438356164 0 0.08420597 8.601494 0.1971542
0.117478 1.308749 0.338202249199795 low
TCGA-DK-A1AC 10.9068493150685 0 0.1091241 2.592287 0.2129132 0
0.2578762 0.651627150714644 high
TCGA-C4-A0F1 0.243835616438356 0 0.7586546 0.08009846 0.02467032 0

0.4826803 0.897668879277434 high
TCGA-4Z-AA83 5.54520547945206 0 0.1324319 6.858224 0.2713088 0
1.606909 0.304022565158099 low
TCGA-ZF-AA4V 4.94794520547945 0 0.1248848 1.582234 0.1949312 0
0.9194156 0.556243291176431 low
TCGA-ZF-A9RM 3.98630136986301 0 0.09374483 11.49105 0.08230796
0.04359532 0.7540636 0.386250054283651 low
TCGA-E7-A3Y1 0.446575342465753 0 0.2457575 12.18989 0.1150801 0
2.466001 0.198450154663189 low
TCGA-FD-A6TC 0.512328767123288 0 0.3525501 0.586248 0.1031797 0
0.6128295 0.735668178673947 high
TCGA-DK-A2I4 10.5068493150685 0 1.025979 1.415181 0.07748899 0
0.7309715 0.749627755744176 high
TCGA-UY-A78M 1.89041095890411 1 0.140213 4.840789 0.05471424 0
1.491048 0.43772054054378 low
TCGA-4Z-AA7R 1.43013698630137 1 0.6877326 3.827613 0.2012764 0
1.928825 0.366582349887397 low
TCGA-E7-A519 1.39178082191781 0 0.1445825 1.373847 0 0.134474
2.641369 0.385274456736236 low
TCGA-BL-A5ZZ 1.03287671232877 0 2.398470651 0.46425424 0.207985899 0
0.423884306 0.911756313109545 high
TCGA-FD-A62N 0.224657534246575 0 0.4623191 0.4881147 0 0
0.3921895 0.896259488580041 high
TCGA-DK-AA71 1.13698630136986 0 0.1859 0.9421079 0.07254231 0
1.081376 0.618044415194885 high
TCGA-KQ-A41O 4.21369863013699 0 0.05421813 15.81632 0.1586784 0
0.6504829 0.288931034472181 low
TCGA-DK-A1A5 0.178082191780822 1 0.6301206 1.638687 0.09706068
0.06169111 0.4792748 0.76900011591428 high
TCGA-XF-AAMT 0.246575342465753 1 0.7066927 0.4740078 0.1703268 0
1.031482 0.623819613539527 high
TCGA-XF-A9SU 0.498630136986301 1 0.9191511 3.431185 0.03842927 0
1.718576 0.486580278003087 low
TCGA-FD-A6TK 0.904109589041096 0 0.9093543 0.3142122 0.04838867 0
0.7438631 0.804137112349486 high
TCGA-UY-A78L 3.08767123287671 0 0.280937855 0.953399183 0.023491756 0
0.251697384 0.877942358005164 high
TCGA-S5-A6DX 0.153424657534247 1 0.3742135 0.5333757 0 0
2.772008 0.38271853659473 low
TCGA-XF-A8HI 1.49041095890411 1 0.4159055 5.631603 0.1825825 0
1.530971 0.376811539210453 low
TCGA-XF-A9T5 5.55342465753425 0 0.5497581 0.626867 0.1608959 0
1.903768 0.450746106935621 low
TCGA-H4-A2HQ 1.61643835616438 0 0 5.478318 0.116367 0 0.9621964

0.471407740221611	low
TCGA-XF-A9SP	1.24383561643836	1	1.531007	1.610658	0.03200533	0
1.192745	0.700137682460827	high
TCGA-DK-A3IK	0.4	1	0.2618614	1.883961	0.0437932	0.06958665	1.621844
0.512714820835897	low
