# Supplementary material for: Ferroptosis-Related Long Noncoding RNAs Have Excellent Predictive Ability for Multiomic Characteristics of Bladder Cancer
Source: Oxid Med Cell Longev. 2022 Aug 29;2022:9316847. doi: 10.1155/2022/9316847 (PMC9444476; doi:10.1155/2022/9316847)
Supplement: Supplementary Materials — Supplementary Table 1: expression matrix of 60 ferroptosis-related genes. Supplementary Table 2: results for coexpression analysis of ferroptosis-related lncRNAs and mRNAs. Supplementary Table 3: expression of 263 ferroptosis-related lncRNAs. Supplementary Table 4: expression of lncRNAs and clinical information of 403 bladder cancer samples. Supplementary Table 5: cluster analysis based on 20 prognostic lncRNAs. Supplementary Table 6: detailed clinical information of bladder cancer samples. Supplementary Table 7: data of the high- and low-risk groups of the training cohort. Supplementary Table 8: data of the high- and low-risk groups of the validation cohort. Supplementary Table 9: markers of different immune cells. Supplementary Table 10: infiltration of 22 types of immune cells analysed using the CIBERSORT algorithm. Supplementary Table 11: immune scores evaluated using the ESTIMATE algorithm. Supplementary Table 12: immunotherapeutic response evaluated using the Tumour Immune Dysfunction and Exclusion (TIDE) algorithm. Supplementary Figure 1: gene set enrichment analysis. (A–F) Most significantly enriched biological pathways in cluster 1. (G–I) Most significantly enriched biological pathways in cluster 2. [file 9316847.f1.zip › s-table 9.pdf]

Gene symbol	B cells naive	B cells memory	Plasma cells	T cells CD8	T cells CD4 naive	T cells CD4 memory resting	T cells CD4 memory activated	T cells follicular helper	T cells regulatory (Tregs)	T cells gamma delta	NK cells resting	NK cells activated	Monocytes	Macrophages M0	Macrophages M1	Macrophages M2	Dendritic cells resting	Dendritic cells activated	Mast cells resting	Mast cells activated	Eosinophils	Neutrophils
ABCB4	555.7134486	10.744235	7.225818568	4.311280198	4.605860048	7.406441697	8.043975587	6.469992914	7.833081747	9.312294624	13.68283856	4.556712661	6.202495893	7.951360487	27.55771004	121.4322774	114.6367466	4.872184408	4.72798535	34.11365858	15.03053048	14.90688846
ABCB9	15.6035437	22.09478724	653.3923279	24.22372293	35.67150717	30.04819243	38.45542286	17.60479495	46.07365684	19.71572161	21.8637228	16.39614273	20.30942573	56.25188749	29.38087813	40.39026017	123.9933055	23.38460308	53.46675672	23.61574571	29.78644179	33.67914681
ACAP1	215.3059512	321.6210208	38.61687228	1055.613378	1790.09717	922.1527467	340.8834245	1107.798265	1995.482601	280.0757192	512.636909	387.5817422	190.8790244	48.97221651	30.97425375	23.05515036	61.38654966	26.41292251	104.2001984	73.60793243	696.4428018	596.0259609
ACHE	15.11794931	16.64884729	22.12373672	13.42828841	27.18773248	18.44492663	13.44126834	14.80554182	24.65271196	33.65844838	16.65373849	13.44126834	16.37526833	13.6929629	1373.06962	48.58576071	16.71547355	193.1599772	13.9487779	20.50124852	40.41492672	22.76649356
ACP5	605.8973843	1935.201479	1120.104684	306.3125193	744.6565995	557.8198203	248.5469318	711.9497439	958.9160181	493.96912	340.8201368	239.2770679	512.9313823	13644.1723	1062.475909	7633.960182	13825.05363	3989.865169	263.4979304	323.3812772	860.5633739	307.1427978
ADAM28	1943.742699	1148.120138	324.7808001	22.6897181	40.06171311	21.23331069	15.20961988	24.28140623	74.76633501	24.37075223	193.6093922	94.40581808	58.3112027	54.96648703	274.639997	360.6820442	37.29172579	53.21964861	137.8536284	116.8795038	546.0061574	128.6860657
ADAMDEC1	371.0335929	318.478799	127.9674477	44.61628687	80.9645004	47.43351387	30.55297649	68.73789331	156.7076551	231.8272543	68.27758592	48.39924584	106.301336	4464.262133	8524.022801	5004.969419	164.8412494	1784.613941	730.1921708	1569.761722	175.1699108	185.5059228
ADAMTS3	146.1955683	106.052311	74.3391695	42.39041586	79.24179515	57.38590645	53.41302407	35.41107579	76.28061758	65.26798809	31.43526856	50.81967419	54.79707074	45.56049098	32.34016681	33.35142773	48.69796983	44.22693487	650.1825865	406.2334876	100.7360208	124.3571431
ADRB2	486.3438163	510.0813396	289.7984504	899.6485015	77.15395496	595.2105488	142.8319114	97.17422647	217.0627702	2378.452129	1957.53925	2360.471299	664.8839501	143.0178779	787.6239741	363.1850058	175.6334734	82.9116614	4628.681578	2887.874236	1969.028818	735.6634863
AIF1	24.0742984	20.32185897	21.96957308	742.8158187	718.6147868	35.1722477	39.50876033	19.07768435	29.36703511	1086.959659	105.7345527	23.17042572										

8655.680002 1841.269508 1611.228571 11610.29035 3285.640815 26.9238589
1851.124125 1672.630327 51.17646344 6260.640469
AIM2 327.9580556 4538.991046 1429.674088 294.4846139 57.08079673 250.2995803
3311.978393 1115.681612 315.6458748 407.592905 87.22742351 134.4958651
536.1118999 181.9571 2965.888162 148.2845223 176.9615884 907.0399616
93.47144882 62.12545422 117.5077056 794.2145572
ALOX15 12.23359461 13.75323798 9.073509293 9.774971658 18.5565618 12.00966261
5.239540575 13.15225266 22.38142497 14.4527592 30.17685432 9.776317635
9.800855372 6.621226619 5.412216318 430.3353048 2907.327994 30.34226562
11.13947086 10.6548502 355.2522822 30.06854621
ALOX5 1799.683549 2425.680554 190.3314674 78.68443963 74.93090365 132.0101224
59.08735916 35.00087493 143.0134329 441.5556233 157.0365044 121.9977952
1979.301155 788.5397751 83.30089783 248.3724724 118.4834954 73.10596198
641.1396907 590.9257381 1460.758519 2917.27138
AMPD1 263.7450621 217.7617272 3012.768061 66.1074899 68.66194527 85.31836726
68.46140597 53.95513652 79.26421174 114.0495383 59.1404365 60.67685001
98.2326067 76.54456664 62.39764428 59.95269372 89.21099812 60.64349611
56.2085768 75.44109945 118.498452 173.7766404
ANGPT4 23.12217296 21.31255675 230.6806713 44.68465936 22.06014827 44.47513375
99.59399622 101.445586 29.46507768 29.2585845 9.380755775 26.17664299
16.91869965 39.33257003 21.218127 11.8097012 17.89428495 12.01870358
33.41141421 31.32955998 14.81234386 34.7186708
ANKRD55 284.4126543 218.9158033 117.2003668 141.6981114 1853.335599
436.4203479 79.77497227 1077.33032 123.8806106 176.2438812 83.38527977
88.45957062 126.1078201 123.600429 96.20392388 48.55417359 108.0271283
133.5596277 153.2289329 104.3009962 1068.295022 415.2425186
APOBEC3A 102.4291739 89.60068607 99.59399622 64.35533705 107.2355131
52.73721382 57.78231649 71.37789026 36.39006843 723.1437605 253.3068151
132.2193618 2415.197908 85.2400852 4501.140479 148.7993403 107.913789
2637.278954 71.9207859 33.35485521 1137.484256 5584.333309
APOBEC3G 1283.451966 1135.293933 2034.562362 2090.85254 336.6456997
2340.674986 1540.772502 386.0535874 979.7203756 5415.054458 6314.770723
8895.302228 865.7405246 278.7681406 1036.993881 258.3004281 203.4477818
1393.358139 627.1314036 327.1943796 306.3062145 464.4412542
APOL3 222.6275107 530.3981335 754.8680347 1209.122376 950.7902718 1615.032254
1119.486881 351.1290483 993.4624966 1173.270474 1172.693097 1002.837652
1000.826958 238.5980956 11009.29392 437.5272083 219.6004846 861.4587391
264.675663 98.21606371 107.8051428 106.4245095
APOL6 17.31182151 11.60650829 7.831610308 8.448971352 11.96227918 8.439383107
57.5171862 7.177587337 13.36764224 9.718189532 12.85527177 370.3966282
11.82068745 13.10943681 967.6451456 7.784612131 9.135536319 159.5346364
23.12992518 8.195434224 5.952127004 19.07117143
AQP9 21.34280658 15.11459289 15.66433549 13.67548268 14.1921014 14.8941249
13.11596785 18.66670647 64.26406026 188.8727485 93.46925864 12.57775597

2044.034835 4583.500675 4309.341662 399.4746192 1132.650087 1454.899429 576.553304 1876.266127 128.8196425 13705.68672
ARHGAP22 34.63556475 48.31289928 23.83351793 45.01409912 71.86055345 22.63123607 15.45266056 25.40966715 32.56780804 12.49456627 82.42753503 27.39864055 54.80800174 175.4574354 351.5922058 272.9890572 319.7744126 2985.731104 46.74782224 52.54547172 81.64009182 20.41036041
ARRB1 15.93115384 15.19815814 18.99250539 19.09130442 18.71219759 25.18421938 23.05515036 18.44492663 7.896579688 15.11426723 57.39670667 138.9893405 440.2370047 333.3962724 946.0138184 395.5562302 43.10758498 32.66449377 44.46607563 31.29032739 26.07419288 32.74962107
ASGR1 40.87013431 42.49782741 42.61290526 12.18880923 10.8262442 13.27420938 8.195551244 5.122171371 19.55662923 304.7934605 10.82390469 15.2477554 976.7071792 108.7218916 25.23626149 155.4913385 76.17616916 33.71862306 55.01698406 65.87597482 40.3944073 75.07267777
ASGR2 30.26095627 33.90854185 33.91363549 29.78023761 36.87140683 41.82249473 34.46381595 31.38273532 113.2301347 167.1042382 29.55398187 29.87461252 1484.961048 36.11978652 26.3738691 760.7257707 48.75909319 23.49623112 23.98629172 20.52967572 51.1819622 49.90264894
ATHL1 367.9599908 535.7827614 782.0675728 806.0999282 5333.944658 794.9480224 231.1788185 1765.552386 250.5151658 216.9823771 570.547141 277.0846656 183.2808703 52.25820981 167.5032595 132.631945 31.12354897 26.36096779 72.34940321 60.48932464 329.5023981 1241.008114
ATP8B4 123.6149862 86.80594392 83.72565704 69.79360213 59.88926338 51.01417813 77.34829047 29.05340287 58.6310728 106.4715425 612.7580118 1035.862627 219.3440174 106.0706443 33.35142773 1163.254876 641.207958 109.3567651 1798.044318 2105.047172 386.1946805 95.69691227
ATXN8OS 19.03486997 17.5814772 131.4519579 12.10850879 11.61080204 12.65987353 12.98839722 12.42360351 16.7813537 24.88673795 10.34693112 9.398323168 10.41544387 10.99877982 13.34400281 11.40281159 10.34858245 12.37331355 22.20849492 8.571375787 19.43850977 15.56008373
AZU1 26.08448842 37.31588708 54.50569955 75.49418368 9.996682874 45.14159359 20.30359339 12.10187282 11.81131187 32.59050723 411.6451897 118.8706557 96.50450872 51.60165293 163.9010445 315.8328244 63.667765 58.98475492 600.0026635 1034.586818 114.3003623 82.37125482
BACH2 4442.146224 1237.156184 145.9248694 716.5762166 1423.65694 449.1787857 442.4757485 245.0583665 444.6850323 597.1505699 478.0988813 511.1200359 52.1091524 60.73069122 78.3338478 56.28089968 58.86433429 82.81441868 78.94686859 68.30005918 187.9346031 132.7648842
BANK1 7633.960182 9177.359918 108.7748971 27.08870144 39.56449682 35.51276327 4.929861418 18.99250539 33.46526866 55.75942073 33.00482498 37.1948578 86.58724759 7.014183016 16.02027146 29.19263501 49.22937803 19.41137259 79.8154876 92.53939613 993.0314299 39.18510516
BARX2 16.2562128 22.62509146 15.03185841 14.68073716 13.89894647 10.89927471 17.9020085 19.74347261 400.0920776 134.5869269 19.02444933 26.10193827

18.15622799 17.14187893 15.56608274 28.07283413 22.65017098 21.75934628
48.17655596 26.33223631 65.61241103 61.55854792
BCL11B 166.5812991 133.7136259 149.8210195 4183.738789 7177.230404 4962.937555
842.2142194 3186.537019 2748.114787 2581.692496 818.2107999 512.27635
88.02675109 78.00838097 68.0390322 51.07857308 95.81819269 76.4604343
53.86480939 52.15823403 306.9880201 166.8927472
BCL2A1 112.6417383 314.019133 76.39326014 575.0352025 452.9144897 755.8584033
1206.5058 698.6513948 171.062582 431.9749142 539.9592883 526.5774707
2261.391134 5560.455558 7038.536622 195.959802 135.5924775 5551.253657
869.1471248 3291.227822 13257.93204 8790.33201
BCL7A 1097.312303 437.9146743 201.8089576 119.2680253 118.1838416 78.52160611
52.25679554 70.53816317 182.5834686 53.79298261 31.50174882 60.22893457
53.33648066 55.79800075 131.3538432 185.7375226 281.3217082 211.8047471
156.3160764 190.530536 79.74570454 121.2861905
BEND5 571.2009408 267.7407888 197.8545424 159.6750731 219.5683455 64.9845649
51.46605096 54.2462042 28.02242234 56.17742997 74.64354417 37.21954547
57.98412082 67.04718628 57.11383215 51.61492393 66.09074337 79.33688539
54.23882638 63.18438861 82.16727965 102.1569818
BFSP1 8.615516504 12.0925165 9.834897324 19.66358081 130.3828712 8.910362721
12.08950821 59.32684348 14.5326211 111.388406 34.61450761 19.45168576
21.37855994 15.68206397 36.95107529 30.8024485 19.39633132 12.20057862
34.02291075 49.68496671 13.21796321 19.08652196
BHLHE41227.283923 379.2503375 981.1066215 45.2704192 17.67634993 26.70594032
66.17589119 22.22269743 114.5180865 88.6299037 51.53421812 51.58608454
42.84587819 5455.337361 143.8681079 325.12977 328.1155298 552.0522552
257.3486083 252.5016943 54.15551876 77.67945327
BIRC3 1762.323129 1072.239215 363.8164437 370.9307246 677.9807091 1121.424012
2888.707681 402.5623243 2449.370773 238.9246256 283.6871924 1173.96483
159.952492 230.7650134 1971.112539 237.5017836 442.7298273 10553.57544
71.25617244 1951.560424 1582.230325 132.8542597
BLK 1788.424777 3284.235489 42.03948876 136.3936481 67.99945866 19.16528476
10.42128191 89.85602787 9.580065844 20.71481403 10.05664906 12.25757399
34.13408456 15.52409402 11.8204986 14.41666747 15.2857867 16.7439792
10.55696945 8.164952674 38.700173 9.863599005
BMP2K 282.3095234 216.4337119 104.8531332 117.76525 109.3081052 42.06887765
146.6778838 224.3125249 77.32098601 118.8177698 107.0425872 117.2003668
350.5910579 323.5873864 655.4319287 971.8036512 427.6761572 620.4619299
2822.782124 2472.95602 900.2382074 588.9037939
BPI 10.46971467 14.03167905 15.2477554 64.13890515 41.29948278 12.50772288
6.382539853 15.19584325 10.77959704 19.43743846 837.2008291 166.0621042
280.0017084 15.02182134 16.53368601 25.96026018 13.48246933 14.91304029
973.6001576 829.760687 390.5882271 60.80053179
BRAF 2571.803933 974.3131867 492.0692023 178.6931052 101.0267209 155.551421
178.3523031 102.1260256 593.3232903 441.0280541 298.7194597 257.1799046

264.8782577 312.9311159 369.770593 191.008753 230.0949786 360.063401 153.4806628 301.8437696 925.5224912 721.2799495
BRSK2 10.44585498 4.456868898 34.32244633 10.46220047 7.924164041 16.19041478 40.50367377 54.73054355 14.7642389 61.61611091 18.27029466 17.22013091 11.75864418 9.848570784 10.52617889 8.886630463 7.49854918 28.80995339 46.23597972 15.48193395 8.252301573 11.86171476
BST1 65.21842011 67.5879307 33.76856098 68.85559845 129.3703853 67.69994038 71.82322708 62.2914124 44.30333794 322.4608558 151.4101449 86.13707715 1567.219569 390.5390601 73.76833139 209.4648222 154.2773488 113.982438 427.4998589 444.1675314 10.16106693 1750.231329
BTNL8 15.22236138 13.26757452 9.870549848 13.49547351 23.17042572 17.46905843 21.218127 16.64319163 35.14611792 44.08665866 11.54872001 13.71677829 20.1092024 25.72341908 18.63943005 10.71025113 16.44465145 18.98239767 13.13000302 16.68244608 99.58265357 1062.582186
C11orf80531.8492615 393.7715656 2192.7069 162.5847884 91.4854186 158.5148937 138.9893405 136.961868 92.03471807 149.3466966 100.49005 141.0093392 85.67243058 99.33436102 34.48872876 88.34055902 103.7648187 141.8654708 147.6024091 142.9001411 157.069336 101.6588003
C1orf54 41.71079514 44.30946814 113.0970394 244.0295762 292.5617552 288.2644361 109.8410784 87.19841264 51.31778403 125.6817604 162.6593193 73.10888641 395.0230177 1418.001252 1157.753062 2036.475768 4773.895505 1134.906101 277.2757208 305.2108103 422.5487977 49.30939393
C3AR1 131.0995558 97.68302989 235.8730787 218.067024 100.57262 198.3433582 109.0856459 110.3968405 521.2960334 667.738611 753.7180287 473.1714371 1337.368233 799.6447121 4247.947251 2754.457112 879.0878468 259.2776033 4587.545998 4865.330497 9437.290602 746.04456
C5AR1 92.39233333 64.88366132 25.374709 71.29814039 39.1758312 24.57422621 23.7998802 27.36083709 42.31424562 422.4027943 335.1748215 170.9594157 4237.561451 4989.998907 262.8037637 1920.668117 1195.953955 889.9578222 536.888673 756.9007984 2647.728393 12587.17469
C5AR2 8.032047496 16.66742886 15.10593558 5.512627066 50.36671815 6.818438323 16.3457722 8.182355675 46.59340554 21.2390901 35.45497764 28.87408345 56.84374943 41.54578326 16.69585423 59.05366978 24.61393679 18.84768096 3.57062816 40.86964441 286.0155699 383.2737567
CA8 17.99617948 18.54386568 16.86720259 13.31718235 19.53973873 16.42178108 15.32647154 200.6236992 43.54005187 62.32018113 14.15699793 14.46846851 14.00907196 12.5219856 31.46897563 13.25016669 11.51310402 32.21701547 72.2168953 144.6321597 15.50755364 25.59129039
CAMP 32.24981999 29.66511957 52.127423 76.14506063 148.5655127 101.7397638 32.63242659 121.3399664 33.09276144 40.18619522 1655.267194 130.333791 126.178855 187.3573662 81.65650077 105.4403669 94.82321767 41.52861367 223.2341403 175.1542212 529.4562817 1507.37221
CASP5 16.22879037 25.41117138 7.489070848 20.72194816 11.62316713 9.073509293 7.44133492 44.6725684 117.8506598 13.78588342 35.16192527 57.15128061

120.1766089 50.41398411 518.0757148 31.73041766 21.43059083 52.52248298
9.129396815 70.37132603 59.19731483 483.5047319
CCDC102B 7.52422977 6.165616203 6.184617618 17.7200176 9.251047619
13.31038127 1.761755136 6.133755741 5.824258226 17.08721371 21.31213885
3.261428192 5.674857963 207.1228817 1.538367713 2.556420269 146.6828382
8.571628691 36.9435694 6.50271849 30.7604336 6.416534838
CCL1 9.151450435 10.41823708 10.05715446 10.7845404 22.5839555 9.669430796
15.97030854 12.61652384 9.104707458 11.24956199 11.53343942 29.60547378
11.12331494 12.30972478 13.39276407 10.20717886 10.50372943 366.165883
7.259316561 1266.591321 9.884652732 9.407691897
CCL13 195.3507704 171.4302952 101.3596853 120.948249 41.65976896 118.4029297
66.04578922 12.70708557 75.51129905 72.33766521 95.02050924 96.66643251
87.08769892 166.9319287 182.2839096 6581.432651 3717.503489 5513.790278
101.0268889 675.8697907 121.9773443 105.3162645
CCL14 70.01715851 58.91915912 153.5523731 71.06446098 31.21088513 91.76959128
77.60907212 33.08789345 152.0417291 152.9884125 67.54459099 72.99488239
105.0702474 166.0197886 1233.172359 2459.096418 283.0117609 172.6730315
98.30151884 149.3226672 119.0063797 248.3181959
CCL17 42.21616032 58.99587921 82.14621562 78.50428281 19.48358652 30.30478142
170.3383554 38.03375116 393.9264602 59.77162048 75.01179342 38.77174192
67.76049878 114.8422294 55.41158071 620.2134606 2541.410819 6414.906541
26.61741896 45.95170735 91.92866442 34.28591541
CCL18 19.93877192 21.68816978 56.60919591 35.31761702 24.35123147 12.82579482
81.202364 31.27670734 20.56828687 109.4946381 39.1384328 79.51319871
40.68134871 2820.01871 1252.527933 12334.37123 1697.959294 1749.786143
423.1104795 930.7824996 47.81945962 49.52876721
CCL19 78.52160611 139.7443318 258.1016824 77.62297831 36.87140683 60.92923205
89.77834495 48.98562369 34.99339888 21.17848138 109.9231796 146.4600699
115.5525527 114.3667988 32553.88074 137.0742038 170.8471166 3048.871233
38.04867174 19.15243809 152.8012995 117.2636391
CCL20 39.50876033 26.99246182 13.71677829 61.54110043 13.08202394 29.40136468
1562.138826 188.6755023 12.36926288 18.13033499 18.43517451 20.31818041
18.85113165 11.31047112 724.6148681 12.8049967 14.52087814 1509.776445
13.96528518 5473.438695 31.12895874 22.44468073
CCL22 46.07125476 31.32507211 19.84751256 24.03529684 39.42959137 27.87853733
28.17031886 24.33306321 560.1844434 23.02227816 26.9167755 76.9655571
33.117333 2604.46642 464.9729614 880.2022045 4123.086134 30950.32751
24.52312784 113.920916 94.52151623 59.92678374
CCL23 19.74347261 16.59253631 53.41302407 15.31837372 19.69815613 27.6569304
5.971548735 13.44126834 70.65994811 22.99098158 64.61571582 43.88212709
37.98614378 244.4793596 615.574234 5505.223986 321.7810006 377.5397731
662.6987872 545.134546 534.1983137 58.68966344
CCL4 1661.464071 817.4816647 307.8066133 2564.294556 270.8722361 442.4757485
11191.41447 356.4726389 481.2791164 8310.145807 11202.80591 38944.01868

3000.888772 1968.542066 11885.1113 3139.008389 2072.163482 4282.933369
1133.500818 35339.68536 6568.915331 2680.048326
CCL5 19.56849893 88.23956549 30.32542187 11434.94887 189.4932768 3143.971755
1850.010235 69.75841522 171.0009164 19080.91475 17238.65692 11744.53738
102.8425968 100.2336827 16862.16904 250.750217 59.29347738 6507.212063
46.19150568 660.0913807 300.4379114 133.3630669
CCL7 80.54919503 45.94840622 58.10858778 54.29720304 48.24358597 38.5055913
22.95842907 53.34937988 55.4673111 74.50448787 33.53994623 21.32839265
36.20654238 651.3648029 241.0650179 85.74723008 24.2434917 72.95235312
137.7438512 956.4362847 108.0688126 97.336807
CCL8 229.3383995 97.02939896 105.7382506 35.05420149 31.42579269 64.22389989
82.98777026 40.64411705 77.90022347 105.8701548 49.61858818 49.04713211
126.7672449 152.3193862 4535.517152 2829.09296 85.43977671 1523.493213
59.52076258 1333.111492 24.19311858 94.60984879
CCND2 299.0433892 447.6145479 677.7138981 682.2085787 363.696915 1162.572673
2369.155331 460.6314841 1118.95465 330.7988708 1065.420393 6016.733404
196.775837 378.8110795 81.43053168 108.189616 428.1711403 308.3936468
337.1012133 466.6854945 449.4451946 314.0695212
CCR10 52.98873326 92.31546536 773.8184665 130.744128 64.30250203 282.9223926
67.60022415 100.7324553 54.44144843 50.76403215 81.81890669 50.05941379
68.90758909 63.12794941 57.58401637 73.7311384 54.79311967 76.23824701
50.87012491 73.11439148 139.3579335 97.53796277
CCR2 420.0370653 360.0216463 1710.33313 312.6203444 64.9467235 756.5516447
297.1447653 95.47693449 258.8984705 1151.152529 92.11909435 97.00535628
2908.726283 122.7713689 80.9645004 74.52120484 115.7528109 117.1398876
115.0116104 63.82847845 213.9862512 243.2288166
CCR3 135.0222303 31.14530202 52.48031743 51.21539926 12.07805481 88.58205857
53.98997768 20.4848163 260.9680321 37.49630312 128.2007913 81.07845472
83.4012701 79.25178432 59.88926338 39.09280883 83.69246929 66.10168276
33.17881936 72.43377329 6575.502616 1435.2148
CCR5 98.76355416 93.00951267 46.81934 992.0442583 75.4038863 462.1524294
423.3973006 530.4856724 503.0168012 2444.179396 578.4832233 1425.773385
489.987509 2191.941547 1493.347557 787.3000077 1515.597414 624.5662045
149.3747509 260.5538316 130.089752 85.86154472
CCR6 327.4987971 331.0843447 40.64411705 173.0896162 69.04044011 486.3438163
155.6717837 151.0907582 101.9853805 262.8582877 46.43838291 122.9424087
29.59035049 17.97363923 25.58895596 29.92011687 30.03002814 42.18974701
27.61848758 31.69929888 34.98004676 45.63378677
CCR7 3811.326733 1939.045683 202.9261759 5074.201816 8788.597127 2693.556404
2885.120974 1858.531026 6339.949771 490.8051753 672.4665585 482.4429323
165.2353496 328.1949289 17366.91051 123.7463123 290.530233 14223.15516
66.65895255 81.24584846 647.9636327 376.1557339
CD160 446.1294222 281.8251718 129.5641036 1145.34982 114.9678358 125.3243228
159.1785523 73.65633284 249.1195624 3283.208487 2499.533496 688.496341

93.85626167 89.41070681 67.99945866 36.95107529 71.95840084 78.86266197 64.10120829 95.57948479 170.0131077 231.9423322
CD180 1502.166724 2247.885466 1123.318028 78.77964471 23.15489547 37.96319899 83.10064157 48.86157308 179.2163466 131.4034549 37.04952547 30.94941671 576.4469199 201.3638474 120.0804247 2787.643912 108.5104996 93.2676663 64.1551368 52.74308227 108.1883623 110.5409754
CD19 7315.689158 5295.93686 1743.474914 132.2416591 128.2039752 55.61361754 93.42592839 148.8556127 217.5476871 147.7927672 90.4860579 98.40190703 164.9026221 98.83889476 106.4449014 146.6235694 122.9982229 161.3344231 121.1411991 130.640189 393.1656925 257.8963108
CD1A 1294.174017 818.7834717 504.0040168 208.3949257 135.3722907 191.4053509 139.7165139 156.4816454 511.8243369 265.2998497 152.5357864 145.9248694 312.0225877 224.9858071 160.945247 156.9391502 14772.22773 1315.229715 110.8168315 174.7119173 260.6061886 419.7944138
CD1B 1351.157456 338.3307439 343.6898904 55.67758898 22.28653167 192.5532686 45.50400457 21.53089091 52.94106332 252.4165956 152.9052072 109.5758377 198.2817057 165.9539997 76.0826143 132.2193618 15969.53236 2349.595156 124.0396525 158.8890881 118.1030265 356.8836932
CD1C 1349.225244 2899.133023 31.29737364 44.14189025 77.23468675 204.5100438 33.58018677 40.92391452 16.8510481 50.88172305 36.32319474 30.67802465 526.834127 29.85115497 22.85969301 167.5032595 9523.260691 1002.723527 32.85124884 34.92829111 24.63751709 258.2129965
CD1D 243.5748238 416.3030749 201.5321093 16.92273192 30.19763591 10.79075018 6.357029724 48.61705515 52.42348265 288.6345968 16.24955986 17.37783232 2785.253606 49.35527967 373.0594532 39.50876033 90.82975345 51.31081795 69.97612949 23.92167028 51.54152805 136.9215217
CD1E 53.25152761 49.96271254 49.27253115 52.56295023 80.73544092 55.87769713 33.11414254 61.98706623 75.82638966 32.86570534 53.71078497 34.07711005 91.10615096 48.57029757 40.47880872 112.6050172 8828.20894 1421.6131 49.95483864 51.76582224 49.63979787 41.44795696
CD2 419.4350192 409.5822139 377.8722518 8182.951228 8002.722844 9578.936337 7866.426688 14057.43026 11226.69889 5897.393241 6247.906848 3097.47128 222.357789 149.914948 112.6050172 188.1947985 187.6124847 146.9688776 332.6238386 138.5888054 614.9120433 576.8272959
CD209 68.81156687 26.73032883 23.37353986 21.6089747 99.59399622 16.53368601 21.60591559 24.14453092 154.8153429 116.6935326 42.60499233 31.86953296 84.17376676 315.2176797 190.945084 2758.297802 2461.153302 228.3870623 66.9779578 173.3203491 91.6159891 130.8839822
CD22 3293.629651 1803.150148 19.03486997 37.47048645 53.38200923 54.37437693 40.69767509 45.41225697 127.573896 80.6118876 41.94230156 32.52035914 31.15232509 153.7017175 62.36131967 221.7347708 154.8175221 36.801049 287.7290641 205.9169697 104.7638451 72.3215622
CD244 59.70898915 38.51177638 28.83176888 623.6947469 174.4389829 69.11006059 23.25507848 123.3772483 85.18075466 1115.507714 1823.542464 1702.765135

828.4298346 22.32433652 46.7586588 96.28143387 23.85077614 18.38716012
105.2985222 38.33766231 2465.483607 51.86506899
CD247 23.66779296 53.69027623 65.55259868 5508.526368 7038.536622 5318.708728
2156.371127 5302.661793 9743.994501 9129.432322 16099.47966 15108.26954
35.27406041 23.92868112 26.9337182 81.83870096 43.67897681 32.48553108
128.4661867 105.4058403 459.1326292 59.83784952
CD27 388.8197482 4138.587476 7350.586942 5506.313911 4042.377012 4188.175594
1125.960048 5710.976341 6979.725316 2288.252945 196.4705551 127.3316351
136.4091916 90.85495783 137.4401366 136.436149 125.1576644 111.9872056
123.9116687 89.47064836 468.4975465 300.1045211
CD28 170.6541081 183.9284292 145.7577865 527.4027958 462.9998637 961.774233
2294.871098 1326.705724 1614.199583 524.1543544 77.96041968 71.78378935
79.14901833 62.22720273 64.48080982 207.2614073 87.37183838 66.03630917
62.4258293 75.1664189.18080942 177.5405702
CD300A 30.69853755 77.94519325 103.7065116 738.0313132 193.2963626 414.3723032
54.40588795 46.8193484.09971859 2268.609727 5340.892712 4668.361682
2187.807203 691.3037154 211.1048289 1737.241257 59.48756952 26.19487356
777.7278706 1075.456564 2406.92936 4130.857899
CD33 25.374709 16.71748672 29.48171985 21.33635265 17.83717204 11.69136762
10.49185361 16.00738743 55.45160433 146.4620047 13.328498 14.20413975
1494.400184 388.0381883 241.8450557 562.1160422 731.6958076 15.47840391
811.3522325 870.835795 36.39003364 36.74188801
CD37 13247.62405 11610.29035 802.2248179 4472.430176 2997.964479 4287.536344
865.4159386 3602.317619 4831.809557 1823.236151 2090.502205 1604.568691
4508.112105 1453.500603 340.0169777 2337.797784 408.0617171 99.31070429
783.7092702 1104.720671 1908.23192 3204.567177
CD38 588.2648306 659.9477137 12117.94539 318.883456 296.2994306 205.305051
492.6276527 1145.360549 696.5074435 224.0112245 1418.265485 867.2194289
237.7018615 90.8542014 12009.36242 642.6104871 65.77761797 3371.628895
814.6736615 1366.953817 298.7045221 400.4888321
CD3D 103.2248459 174.4025802 88.37677893 14396.85632 14916.803 12009.36242
7456.754789 18921.47848 12721.06851 11894.34724 250.8695535 243.8532814
79.18206065 74.30949816 26.01377304 12.22050099 106.1967153 132.5505719
40.77673955 13.20972496 795.027696 250.9974436
CD3E 1233.172359 756.7993492 1163.254876 5787.182458 3554.437369 6370.192958
2850.795927 2993.594839 5302.45403 1748.883874 461.7145711 342.1231155
225.2808846 188.3816952 172.5351641 175.317922 195.4486787 276.20651
144.3993653 129.5942842 425.8648102 809.110134
CD3G 55.98119171 87.6081271 31.36207011 3348.560467 2859.409338 3066.253514
1149.980649 2416.241107 1298.021187 1121.515185 252.9163802 117.4404361
31.97464945 31.47187386 22.5839555 15.49172903 29.83579519 38.2153575
19.63068007 17.90169501 114.9902097 160.0909817
CD4 138.4573773 118.3512664 351.0163026 164.9509326 2495.269425 3710.379943
1725.414846 4363.016183 5524.681644 399.5375472 81.07568775 75.96955929

3343.906153 552.3052303 1574.336844 10105.34674 1340.751384 701.0986434
213.8663803 287.7837723 163.6390935 364.5571609
CD40 1493.347557 1328.267122 838.3890443 203.0571538 119.8051169 168.9573287
169.3131278 129.5124355 338.6601465 269.4014427 164.1619074 204.8063535
298.5733076 1146.591678 12966.60573 643.639203 1598.408075 4943.303284
111.7010328 416.0597275 204.0560124 275.5927662
CD40LG 65.37231327 51.83259142 34.65893963 186.4853205 464.1789801 852.1721059
660.7418675 809.450384 31.04806788 449.7694466 29.30316525 52.25679554
24.75213495 29.86059304 26.39060217 32.06615205 20.16451827 16.89038862
15.73172412 21.43431914 36.42693635 51.29818379
CD5 482.7709118 212.2007839 459.3251879 947.6570017 1101.084704 1734.374565
918.9690411 1464.692495 3513.782725 508.249512 283.2620394 201.6701633
312.9288878 259.243454 285.0923469 188.25878 270.5730739 259.6191632
182.0748079 219.5267689 296.9402652 223.7346793
CD6 75.85653173 91.34978305 295.0724091 1720.140098 738.2321164 3148.85189
2731.999076 1835.581311 2092.360886 342.3979908 623.8033823 348.4243243
90.96996824 94.71792103 137.1320082 88.93386715 96.80906697 90.20058102
41.95697606 41.71625038 329.7270655 155.6335945
CD68 3.286119074 8.41213334 9.182953598 51.10845475 21.15896542 15.76945271
30.90820518 26.97070262 23.8360063 97.22500992 93.83368418 13.92293323
806.3047213 1705.45606 748.5959447 2770.911453 1137.825995 687.7634179
172.4465903 226.1109905 45.59685457 36.20155225
CD69 8246.962733 5037.059721 131.1507128 3798.407191 4397.041606 5489.202469
1071.887678 6081.160743 539.3938343 4572.324665 1781.265844 5471.704926
66.10288756 31.65121441 59.02137051 28.81247015 39.31238934 69.45698718
440.3983738 1484.634024 6363.149838 436.4221698
CD7 15.62904812 17.28012062 18.02258618 2234.707961 2032.402798 1507.773715
1271.833268 2132.53501 7917.977191 415.8325753 1666.682281 2106.83981
20.48373388 16.0113349 13.36812571 80.61769372 27.07256267 22.00857195
12.17345488 20.04765141 48.57212298 25.19240764
CD70 294.6566184 647.9228093 528.1256069 247.6214563 116.5526077 266.2959935
388.2749858 214.2359544 1999.95277 301.4293391 226.6449338 186.4718703
189.6644237 162.091307 365.3486293 116.8363113 163.5181238 705.6011675
205.9889771 115.0885118 280.4419984 309.3411047
CD72 1434.004736 194.0589782 8.359413914 18.8127059 43.04528298 8.195551244
15.72935292 10.23080265 37.64694269 11.4839684 67.34451995 21.03489045
9.096324111 9.476286725 9.588069329 123.9520803 24.27133459 8.05889839
6.665661843 15.55213132 13.37742609 9.272238244
CD79A 12117.94539 9835.923 3860.541888 425.4456431 183.3771612 161.6033691
97.84605147 437.0541054 424.1217962 196.7666923 149.4947867 139.2030869
174.974517 124.5658582 108.8170218 63.57950923 138.9136781 189.639752
61.91581828 118.775806 511.4149111 261.960019
CD79B 5975.189254 4719.84198 900.5698225 229.2346632 284.1304512 341.3861217
192.0177626 425.1070045 863.4669544 277.8145759 364.2599841 254.4761746

158.7986952 68.04622904 64.07909887 79.32420695 61.59710526 72.14957289
38.49728027 61.7152637 209.7392117 232.5557133
CD80 131.2576257 345.1713796 367.1492638 73.52859919 102.0859478 92.39233333
195.416557 109.7080589 392.7464198 196.5982704 163.1921911 139.2568175
148.4294518 350.482739 3650.811207 160.4810794 596.4318172 4526.24129
120.790446 177.1857239 109.740993 132.6404432
CD86 354.7433616 599.8609321 309.4183226 108.8922088 35.145174 95.47693449
90.30112485 29.58459024 176.4166456 341.3536821 110.1384066 87.77041198
1398.4001 764.4811798 1444.502959 2791.659069 1729.206247 4490.947531
161.7655034 203.6194781 107.8833958 161.5053365
CD8A 564.4780916 348.1087905 44.21285974 9990.737125 41.07506248 147.1712529
161.1817391 172.4731305 80.70305906 1127.953469 959.5951565 1190.400365
203.576442 135.9847653 45.89341104 63.47614462 176.6671569 163.4447696
87.086495 102.8346227 176.3456177 623.2298556
CD8B 21.53089091 18.55930203 22.19126923 3697.963718 143.5770588 16.54639136
12.46145751 118.4547967 12.43551104 108.6995574 20.09303314 16.68319539
14.49443263 14.7839247 9.306658239 9.973398059 14.20160728 13.49794899
9.973784848 10.95016398 33.0054369 31.43329135
CD96 140.0696821 182.4215585 83.87781162 1718.974851 1432.94769 1571.924274
253.7390252 473.0050907 2933.320651 3005.007369 1629.78438 2127.768209
35.69766875 37.23265829 64.30250203 32.94807285 50.26149649 56.78650106
103.3665611 42.58554439 433.031001 90.84379933
CDA7.453474496 8.282139574 18.8378526 10.06514533 9.6818797 14.73210587
10.15946955 10.55001689 11.57836151 167.1780478 98.13957615 8.147551977
1040.419935 346.9958858 8.817496805 28.81247015 343.4091893 36.03732383
88.0510502 82.11698268 346.4833211 3592.43191
CDC25A 14.44417944 13.22104832 18.49909288 9.90420085 13.45315049 9.040054131
226.961818 31.42579269 29.61784034 25.27567657 13.26647127 142.2923317
14.74858062 18.97174653 22.0447246 14.74324112 14.79591225 12.77212168
27.83891458 74.24230416 14.87374833 17.78100586
CDH12 10.62580967 18.14764196 6.994769751 7.907398998 7.285693652 8.59290329
20.54473142 9.729238863 130.5611509 197.4542946 27.0789387 9.729238863
9.440421022 12.70746733 12.51970773 9.085608856 33.16857129 23.20574621
84.74034858 38.3494807 62.3285341 24.73256754
CDHR1 15.70334805 21.35717484 8.220261044 32.70000097 86.84214478 12.73061175
27.50039747 22.14183418 7.934015528 130.6180895 237.0997067 29.5032353
10.5027912 9.74632817 9.294987078 23.8492483 6.785695372 8.710620771
19.2193508 11.06215587 8.510053764 27.59515717
CDK6 30.69853755 23.04874713 15.94453415 65.1412171 25.78013421 99.98686977
45.35113413 24.21507209 69.53996611 24.45714861 27.05878945 289.5847031
21.06647043 23.49923645 16.71107549 12.82579482 75.85179982 67.41087913
47.96802384 14.10751406 64.38971117 19.40986991
CEACAM3 68.46140597 61.8011542 104.89154 69.08981156 59.64008096
55.44751849 46.69869297 65.12057481 133.397401 120.6353537 189.912654

109.5758377 242.1017704 156.08054 123.6149862 113.8352122 147.5042141
210.180427 186.2935686 166.5230648 132.0579064 2096.525977
CEACAM8 77.91715796 86.88656507 22.74378501 143.8250498 7.285693652
73.7311384 35.28849776 4.767545237 39.35635057 15.77579613 956.067675
162.1045877 6.650645086 5.093941607 14.10673265 11.65755142 14.18862492
19.05023187 2385.025776 1835.208959 235.2654646 149.8673485
CEMP1 28.85299608 26.05680615 5.783504523 9.51802003 44.87716404 8.245074698
8.750532337 66.92570007 210.0815605 135.6592715 24.7914552 47.37880726
12.41773777 13.43483203 19.10532833 44.64572595 6.045328615 7.792685483
11.22269837 40.31486949 54.48869834 30.29430793
CFP 4.573719127 4.646460043 5.382740888 14.38688428 371.8319741 50.46125007
5.783504523 13.9469182 47.25871943 471.1297374 33.99443636 6.982740503
3702.502521 27.13104189 219.1912001 1971.112539 7.548560149 451.2500358
47.82909649 214.9062825 14.58477024 2060.180409
CHI3L1 9.040054131 10.11185342 10.0823446 13.16060514 12.37533181 7.285693652
12.94364358 9.949791059 19.88900634 16.32499594 344.721706 59.67623931
12.16796964 8959.873277 3395.663462 2050.816822 89.88071419 473.9150298
477.0548529 636.7916524 89.33007873 3791.328311
CHI3L2 574.7325605 445.1279343 320.5064848 362.8779127 662.9063033 312.1882039
166.1811986 4770.057676 515.3473004 404.1369074 322.0390633 170.2202856
200.9927361 181.8142129 1765.552386 175.3835932 184.1091199 210.5699575
148.963857 171.1295117 317.4730696 356.9094548
CHST15 375.0617962 380.4544171 2248.878473 37.3682688 56.31496953 41.68602369
34.12601784 41.65976896 82.84147995 339.1261865 43.29669296 21.63834323
3334.89402 1452.251166 103.4871553 1051.6856 317.7544695 99.79101855
1121.27006 895.2054973 1106.193591 4602.158626
CHST7 15.2998514 17.07503774 105.3543147 26.28908826 70.20092794 367.4048158
234.0049091 401.8384715 78.75058598 56.03427065 29.82523398 23.03809769
291.0641772 314.6462296 350.8883739 421.2349567 193.2489244 3085.840231
152.218479 368.050649 27.24137463 793.1481128
CLC 317.0133169 263.8099578 158.2726851 91.38509289 21.54663623 110.2519917
184.5831263 91.98053835 38.52410842 337.9147534 509.9159527 237.9275689
86.56595204 69.27115976 226.7134742 172.4089256 98.81299309 102.2341069
3917.54615 3098.629915 26617.23891 4605.247829
CLCA3P 17.20651635 25.26232098 11.94994798 10.28448645 11.3209152 2.307902038
0.972464723 4.366178616 10.45160006 25.67823744 4.29075067 1.761755136
2.691185739 2.022563272 0.251594145 9.029403736 2.311940973 0.758567527
5.985711194 5.420513422 18.88573028 6.90793389
CLEC10A 110.2519917 95.48720378 67.3906821 80.05432796 56.97214117 77.30955315
36.92529104 66.46616213 123.3530513 154.0857258 56.08638386 61.70274209
481.4264713 159.5970517 69.39655423 5770.127086 5129.9883 145.4750722
107.2146881 150.2976889 35.23355032 145.6285415
CLEC2D 1012.819733 595.0445595 134.5503286 1510.850313 1443.461253 1711.865828
1875.9525 1689.599596 2808.690677 332.2360807 979.2239401 344.4100434

93.13451585 78.64035879 1465.741277 187.9865712 66.49062417 2002.179445
45.51634127 41.11000417 92.59123087 68.77784236
CLEC4A 270.684059 105.2173251 19.48358652 43.67169279 94.87431986 19.995556
35.566978 67.3906821 75.54705953 326.2228204 66.20219764 39.06798479
2802.254283 1137.937654 51.67871576 6742.94324 4209.628791 558.9934716
321.2172134 548.5828994 234.9146008 2814.377261
CLEC7A 148.8556127 142.0062175 58.64250526 86.04241069 106.6702025 89.98404196
50.52462737 936.3792246 94.16503856 628.3365071 143.1492688 89.37299011
6165.953009 3760.306028 283.2038076 3071.447657 5359.771755 1178.503785
634.4501909 1330.094243 159.5272392 5247.49398
CLIC2 8.195551244 7.671472425 116.2438106 6.339831804 11.06762282 4.799625554
7.65617701 5.412216318 6.36193044 14.6317676 24.68882499 30.3854209
103.5590934 437.0461122 1393.382865 2233.164343 2244.054642 5239.88349
8.029450285 36.79183022 8.446042556 10.41872533
CMA1 77.95639785 68.10609278 75.6389405 25.22954469 42.09518393 46.6365232
76.31621718 29.75313496 40.08632954 77.40398982 57.33259038 53.020637
58.76829357 46.37300953 65.76280884 64.11513732 44.7774243 53.12239382
1378.879998 1622.195863 164.6813549 48.25538871
COL8A2 135.2230196 73.45098986 23.48774462 60.53487072 79.43023238 61.77215124
55.34420893 62.18440951 122.1817822 76.59825958 88.02552084 68.46140597
275.8747733 1233.715268 33.84353695 151.5623553 129.2643777 109.1988225
70.07666952 59.73326622 89.9998803 186.4210163
COLQ 28.85299608 27.39812397 23.55278029 152.7740895 66.85032911 135.3256446
36.18719498 48.33458991 163.7532278 410.2594987 406.5670516 351.0163026
33.83180651 24.84089345 86.1812316 128.1562926 31.5991765 20.18679185
51.67336008 36.89214997 27.50524235 40.46415035
CPA3 786.5967435 653.570348 431.8870773 147.6958112 25.20265438 243.2991012
226.7134742 148.1833551 145.5265348 271.5067499 458.1363071 308.1273629
135.1465191 139.386735 247.6828366 206.9088618 132.4987072 149.4195584
28290.30984 22328.97153 1091.417527 522.5988941
CR2 1471.268235 559.6347831 139.9135207 245.2703384 285.69747 67.24739132
17.8883765 25.49933683 25.46161624 22.26236223 25.27012551 64.40632604
35.1557773 27.25115703 55.67688175 39.48271435 16.28183461 51.3593367
16.71082041 16.32654871 122.0795542 33.0622167
CREB5 30.97425375 24.5582079 32.02053189 12.41011504 43.39553244 7.370460489
10.97804588 9.555358782 9.368720456 15.69291933 13.03890087 14.41666747
360.9106341 22.86410106 11.76274946 59.21766228 227.5494335 257.4524083
23.96095789 23.33830393 15.15801623 1385.315828
CRISP3 27.37818895 16.54772888 6.508150101 3.235495107 4.203532154 7.582193721
10.20717886 5.210162458 647.7895425 34.44926679 164.0262277 41.73838224
5.032005451 11.29862608 10.66334011 5.556158206 5.950386602 5.355044803
140.2065177 33.50781403 75.7877931 15.93701737
CRTAM 54.88908823 66.10701896 54.0562838 1443.48038 308.5611006 114.0594238
661.2766113 42.15294578 47.76902833 829.6692685 261.1619294 652.8575264

212.0416867 761.03216 38.22269719 85.70898291 128.6403474 153.9609051
15.54962061 106.1592663 78.33058859 83.96491114
CRYBB1 8.51087882 9.160277762 16.77479438 8.78944775 20.22798354 10.6390921
8.277095789 10.18494196 10.55258875 7.618702951 9.40235754 7.972776848
9.396148118 9.70690203 12.12718124 166.9553981 9.016747265 8.796010004
38.37689323 16.78915846 9.497994228 9.876168345
CSF1 22.1078749 21.81521667 14.62640697 22.99418539 21.94024148 32.27097928
235.5590589 24.17762134 159.729662 39.12354971 21.72047615 24.48518696
24.56893179 687.8844582 35.28849776 23.12217296 112.5821168 93.53462038
28.21718786 61.84446433 73.59342702 22.5275051
CSF2 24.24874392 18.24411695 43.36581215 24.4197256 40.95236426 25.87142267
1283.451966 22.7587994 16.26218479 62.26959997 47.27814858 741.3146239
27.09459243 28.52989192 18.82453343 16.29360796 34.06423073 38.03034172
29.73723499 1722.887037 20.96429003 19.5960694
CSF3R 99.76628743 29.61360061 22.72781743 24.3859179 107.0724606 36.16508067
25.6594246 38.93185048 85.4433645 338.8415636 115.6437107 45.2333023
2331.948628 214.3427639 860.3372222 737.8599039 92.48429351 67.25193329
186.0953351 212.2561883 248.3966271 9175.361959
CST7 29.71165699 21.33475082 14.02145998 5181.597893 197.8545424 1470.064999
746.2695012 322.0620463 1323.231227 12102.63842 11514.68497 10179.78364
63.6732263 254.880064 186.8061087 292.5617552 1490.444911 4059.440795
2285.360966 2487.597945 616.2101884 2347.728878
CTLA4 19.39158332 12.05483904 12.39999002 19.09265901 282.3095234 606.1036832
1865.797683 2019.936149 4077.44587 22.98413575 11.01469755 18.71219759
18.619765 14.31666435 8.580759955 18.03666634 16.48246747 12.49357301
18.08044096 14.80064982 62.02243666 38.69364838
CTSG 247.7712501 204.7033855 205.3757047 113.3980465 134.4958651 81.23896111
73.69460204 38.09184417 224.9735167 156.8702985 309.0786617 105.3144058
88.12615549 65.31852439 88.66013895 141.0660169 69.98276175 42.00016699
5638.069687 5475.90797 137.176414 185.0082007
CTSW 16.65790643 14.10507421 16.31812595 4894.215252 487.0683652 963.2767736
23.08754551 14.6382422 12.83317361 4144.004572 6980.778276 10811.41236
16.93731154 15.94992913 13.08202394 26.76355546 16.23954329 15.8711173
119.4688824 272.4656742 41.78979254 20.49140221
CXCL10 761.3944185 395.6381026 246.2438258 157.22658 120.4987696 152.9758784
529.512254 127.9224652 256.6183684 488.5453368 201.1109308 1510.914551
500.9300164 163.1981892 31273.67963 278.3434035 163.7176364 11302.3948
112.8085953 313.9187307 332.722032 486.9671286
CXCL11 90.26229276 71.79483122 44.79050636 22.77124293 6.633637977 14.33061258
52.00544678 23.48774462 42.51314662 42.34729139 28.53859717 183.3079432
38.41873667 16.90510228 9059.841342 29.35940998 21.12034731 3956.496269
27.84781811 33.7735536 33.86659948 74.22060498
CXCL13 50.1800947 22.77083572 17.24705732 22.35743472 6.024967349 8.95713845
280.5297599 9578.936337 13.50429071 7.577261926 10.02644064 16.12512522

10.31783604 11.75346746 627.930517 16.76076868 12.71410384 20.93152388
25.70939324 10.94557061 11.1092851 29.79046571
CXCL3 73.07039801 28.30659504 56.44753053 26.71170482 17.02062149 30.02788375
26.84183085 27.69750654 136.6242482 45.90656617 17.38441257 30.21807353
175.8826199 1496.816 271.246673 233.3303549 81.03679349 157.0218318
149.9605608 7936.428599 44.00159828 75.7691719
CXCL5 94.16490321 83.04902879 43.36581215 18.42018765 7.759898576 41.21600403
24.94052208 18.93654262 73.85830951 78.88888206 25.08008077 29.07388553
32.93471403 5315.265079 91.98053835 46.38135018 37.39284051 387.4036234
49.57406448 621.7471315 29.25747912 69.6139163
CXCL9 597.5841917 438.2088306 268.3992061 125.8362376 77.45884479 151.5042347
259.4612394 143.5770588 159.2182742 220.9114526 186.9242102 590.3615841
265.0825127 168.4971354 38944.01868 199.4359175 179.9813131 2918.819703
123.5555835 99.76021313 193.9209434 403.0543959
CXCR1 260.5780432 187.0592932 146.6778838 214.6841388 320.9597732 253.8205315
76.35489937 71.48534098 216.8895181 691.7310759 515.9159844 417.1870896
82.78141165 72.0058534 61.26982624 53.22101207 65.52991342 64.12271586
263.7886674 267.9679786 239.4764818 5613.081304
CXCR2 26.76355546 12.64597482 20.8510013 73.61765642 5.107669662 20.88194891
7.644655016 11.15831089 62.58074509 341.3628029 963.1701029 280.0023863
408.3528365 115.5505074 174.1206652 64.07909887 176.8360878 48.42702155
126.093962 56.93465721 442.7589038 17610.41012
CXCR5 368.7814351 298.694373 10.14786303 32.70801147 29.79215867 181.8245372
207.1265648 2508.080521 32.4851772 31.93919539 16.03380756 15.13101608
14.61246054 17.36373676 212.2750073 14.43114488 17.53626408 26.90915848
13.73208623 18.88504854 55.85644925 19.29227122
CXCR6 143.966553 137.922284 129.4184927 239.577985 92.27701669 190.1248095
1140.294509 187.5232686 914.3089198 1037.795776 175.2851029 139.1507517
89.88376184 60.50454252 68.56018671 56.08489855 61.54906754 93.76432338
58.16905397 53.14506673 86.73337152 166.6014244
CXorf57 39.26542516 19.44447612 10.62580967 102.1724937 236.7644719 111.2439257
152.5108229 14.24242632 12.49889885 284.2716475 54.61125396 77.38738398
8.024895821 11.29730888 6.694465385 10.44585498 7.429375053 5.419912679
7.400067061 6.598985716 73.86823463 12.15912559
CYP27A1 28.17031886 29.93010894 40.80982734 35.11559608 57.08079673 18.92107732
20.98904773 60.30106853 55.89770913 88.19698455 36.91521067 30.40510025
761.5963848 9212.241151 72.62100796 75.71085354 807.5821247 1970.12216
731.4259977 853.0000301 71.57804711 52.96843829
CYP27B1 839.568697 393.1234511 544.7418911 123.385104 89.21509024 112.6050172
253.1007469 62.71521001 451.157092 198.9157769 95.8119075 117.9119847
96.6530619 1182.186949 6662.564771 145.7011205 203.4468878 2253.07628
88.73893452 231.5401669 179.3795601 291.0919657
DACH1 8.110268958 6.614252509 7.019305551 6.072925338 26.64846634 6.332890006
3.990985982 4.929861418 16.74879697 14.24749768 34.41652414 18.5565618

63.04081707 7.182118842 4.273621867 8.23248634 5.418450145 6.024269222
30.55282309 11.69596161 232.9448016 80.62449793
DAPK2 50.49233083 52.66107689 24.63126285 36.778245 35.93880485 53.08765217
21.06633463 20.19963341 71.83050785 55.81143763 75.19081972 65.98252315
50.3687963 36.29552204 35.145174 76.65867431 50.65432393 57.82502739
260.2593867 126.5670604 1104.766554 494.7641499
DCSTAMP 38.69538004 19.46232551 134.084158 45.95452314 33.84353695
19.29191355 61.87372226 68.52517722 60.49713226 77.66579469 39.23336722
85.31836726 100.3959971 3167.902414 171.5562529 278.806442 919.9504445
632.4755841 126.0151417 250.4705436 145.246652 90.65655649
DEFA4 364.8313155 262.8085775 213.2412028 580.4081606 61.9081487 252.1933458
165.640875 89.90011254 42.80616145 290.2323357 3153.504694 224.7841847
108.489886 59.8115536 76.11959313 88.37677893 79.53417974 104.1710485
246.9127303 174.0874482 885.3172017 453.7054493
DENND5B 47.963256 82.49743428 338.5948695 4.937003728 5.354735415
6.171513508 8.639430617 2.140728959 35.50090535 5.928172884 28.75548047
4.929861418 5.019653713 14.58676995 7.808323788 36.71555117 12.87739467
28.44840466 6.111045892 12.88881259 9.439454235 11.06939913
DEPDC5 23.00370479 33.44683221 30.00802014 42.10826584 192.0177626 41.85091261
38.53463959 136.4948062 91.68554625 53.64503254 74.53481103 143.2597854
40.17793365 96.61739385 168.2830482 190.192431 106.0520414 38.1781776
110.3343475 32.01670875 572.5456158 27.33916292
DGKA 426.967722 460.0096029 200.6236992 2223.016764 3066.253514 2690.021108
845.8491856 3860.541888 2953.650091 550.2709334 393.4296648 301.113803
300.9545855 170.0857145 169.0248855 215.9013618 198.328799 236.2697561
148.5136694 129.2895441 429.5993645 314.1795669
DHRS11 53.98997768 69.55072395 70.16756594 88.61812105 123.9520803 27.7175438
149.3668195 72.38497496 59.33234796 34.77276169 61.62409019 85.55342451
46.95962914 266.5013357 18.60026599 242.6906605 1090.33834 299.290358
69.86702041 69.59671194 27.38267333 54.33030883
DHX58 13.86362993 16.88361737 12.83845224 67.97505932 298.2898502 30.99587635
14.48068577 86.56913311 85.15077625 19.01547885 126.714319 29.64538132
66.42778065 21.95980512 1394.307066 233.5831274 34.01497516 1538.10279
27.39483764 21.85490233 18.05914553 20.09780832
DPEP2 695.4566346 459.3705381 11.18070884 469.0254782 1220.279156 631.2800075
9.029403736 327.6079697 341.7222111 496.445567 636.0698166 22.1565212
1471.477094 116.2735817 16.21638188 2410.189476 502.9110289 37.70324907
158.0635767 131.4440963 4131.564833 4347.71622
DPP4 19.45168576 16.56719599 15.7816761 686.98687 689.9495562 1042.227242
892.3150761 22.53108052 866.5973448 383.7055093 43.07303297 391.9641115
19.65506451 17.52701962 175.6975199 32.80967907 11.79647193 138.2117037
68.77332318 25.76939706 18.57167619 19.5766283
DSC1 82.26251271 48.0259129 44.79050636 408.664347 788.0190869 15.6035437
10.21829304 7.631831021 23.3327929 82.60097115 21.21931052 23.81586063

18.85006161 14.93885569 11.66918119 6.707735929 45.60303808 14.70793626
34.51256333 35.70681106 71.06536769 42.13322455
DUSP2 13.29827708 139.2748932 32.34016681 4083.939908 318.0170106 2005.427058
2312.349458 2300.259226 319.7380921 4596.059629 494.2129873 307.9165172
404.8953793 490.424434 567.6636334 261.4949956 292.3186781 536.6914932
39.90444703 758.6700823 1013.317761 88.7690473
EAF2 3181.18246 2020.339327 4989.763973 219.0176307 240.986393 138.142275
265.1751702 197.1649083 208.0767508 181.1618028 198.0166857 184.1015768
683.9617628 281.0119542 284.1304512 281.4216469 230.8171913 468.0738156
401.7261393 399.8816761 742.5341803 240.8539709
EBI3 10.50267123 16.93649086 8.897758981 12.21399038 15.05714204 11.53252065
119.0986128 11.63480358 164.024998 8.368172831 10.01431702 16.85549775
12.4947299 13.18836854 20437.69231 311.2058679 16.81680477 6496.243408
8.959876311 134.9737807 9.497299095 11.62603766
EFNA5 5.427660306 9.117018877 8.933068014 12.03201722 116.9246518 24.29954135
13.95930187 76.65867431 240.3423442 77.73188438 29.85222409 43.79639678
16.87392513 14.68073282 64.26624737 56.80709441 19.84796253 35.5318796
59.94184526 12.07275719 81.94476603 38.58771285
EGR2 122.3401907 98.24319728 54.47162284 35.39583074 35.24465136 36.23835539
2100.112826 1667.329269 131.119 151.2415422 155.2354783 1331.912221
466.5929482 3919.367365 23.08754551 2175.756551 4531.291492 1121.590168
293.4561431 1906.729264 1430.716563 198.0753053
ELANE 11.04729816 8.908551164 9.3286347 103.4864778 12.73061175 12.43682411
6.90617027 9.857216379 16.19604013 18.50447818 734.2988939 167.9222444
28.96336488 15.08601415 103.3533021 206.3156576 12.68932527 13.01751849
4503.039803 4464.6663 119.9375404 31.87881829
EMR1 499.0111023 383.0612119 194.3869003 195.8964573 428.0646447 312.812165
400.2184096 182.9389251 335.8350828 447.9667425 348.0789413 188.5213167
1815.513906 203.312067 3528.214438 404.3935503 178.6781861 237.420847
287.3791829 248.1729828 16330.70798 1253.613443
EMR2 145.9766252 106.053844 67.17486231 43.05953599 56.18573504 83.26351882
72.53685465 21.24976442 96.27230625 165.095489 174.2285945 87.43886769
1319.424101 1045.041824 1516.793563 363.8164437 863.0024859 357.6021098
829.2405253 1558.475521 3871.759806 3956.252694
EMR3 55.17004911 124.3420312 135.3256446 104.1704046 89.37299011 63.19960659
89.53763691 139.7591552 112.6019046 136.763141 63.86912529 64.8769987
379.6236682 38.87513434 13.53604074 5.690710112 17.83104509 19.31544328
185.9826158 322.1691705 4792.729376 3015.993844
EPB41 21.98416943 67.00924563 15.20961988 235.3351386 12.29355102 385.6302551
240.8141851 89.94208868 45.47875651 9.639752894 104.9505198 97.80292842
25.10456886 51.91551646 7.165488052 25.55686935 17.91341178 73.20980687
9.708024058 7.155722446 104.6317907 16.47488638
EPHA1 47.02936831 46.76894576 52.03367718 400.0482873 763.4010715 292.8838925
35.76999194 57.88335321 43.93245365 61.83016944 155.7830488 99.21173158

63.80169822 58.11820163 45.61964119 38.90350151 60.8481688 58.64760753
39.16381447 38.69471267 56.73556026 71.4205995
EPN2 46.90834881 42.21678294 109.4328043 31.50834858 64.62199466 96.20392388
50.11843442 55.41158071 17.84892932 62.36421567 31.97660478 27.97705381
32.43586908 32.22402237 30.74111032 22.48126594 59.13787146 31.56870199
43.97341673 70.25798406 554.8063768 51.26114266
ETS1 988.8726693 792.2524045 410.3171187 613.4935899 287.8283832 1902.189462
1261.517205 701.5416085 1031.179862 99.66586714 759.7185652 696.2669173
85.66933558 96.62991012 130.1202875 75.4038863 120.6221577 115.7021417
147.1300736 156.3004608 230.763415 243.6131706
ETV3 37.98662268 36.84623894 103.0952183 49.08454024 20.64868683 455.2117491
21.48427937 32.1556293 21.61892339 14.30737014 33.20716288 38.00908858
40.95675962 24.47191439 90.34410993 33.76856098 40.52550185 2091.702661
17.83028298 22.50028072 60.82035259 40.75588852
FAIM3 11191.41447 8362.288551 254.0902643 5723.984822 8841.004838 5134.615329
1145.360549 7380.970003 4680.731681 1866.749257 2548.903709 478.4201622
117.871065 315.0175529 53.69761011 157.4129632 147.2831703 49.08029322
58.68422735 45.15325264 488.4465999 735.0353714
FAM124B 21.67016354 15.40803255 18.92107732 14.04816696 17.18073516
20.50088961 16.26784814 16.06928085 13.18060161 10.75869747 20.02563485
18.188607 29.15950369 19.3314478 9.129359328 22.79147187 16.61570142
11.26463001 473.0941252 126.8779546 96.55065376 19.72983414
FAM174B 117.7226808 92.46718738 82.29386042 94.16937409 137.0201675
104.0881916 92.90928381 163.1153582 160.6169251 69.86697577 40.33600095
37.77736037 39.85052889 53.40511981 57.84876862 68.59561619 42.79765197
40.87114944 866.8101902 390.2498203 73.78985768 103.0050021
FAM198B 9.800664188 16.43893928 9.140836633 4.526894401 30.53349636
8.45170015 5.598819437 13.63143065 20.58243315 54.27641657 23.76109791
10.27433459 515.249292 519.5115003 36.63587146 398.2258384 563.731154
45.53321991 34.9096904 48.35776342 58.16319768 8.88467942
FAM212B 49.97014577 48.44610942 115.1532722 23.00981627 12.55533168
25.23626149 70.72102235 17.60479495 98.45119026 31.95594959 13.78507742
20.4848163 95.87273144 44.3949005 15.81931361 15.45266056 19.30988108
12.50084037 52.82638983 87.80595326 113.7106812 569.9349175
FAM65B 4719.699579 5267.741473 237.5875518 3376.093302 4454.485846 2808.159439
256.5379971 665.2076215 910.8875823 2376.662113 1586.716996 696.5310083
2428.861545 92.82935445 193.0268711 86.87890188 136.2081634 126.2864186
187.4701407 170.8249765 2755.053484 8329.197426
FASLG 342.8390554 274.6088916 188.93844 169.2167794 144.0787779 140.8491762
478.9874938 126.7421568 171.4953465 600.2580034 1537.723722 2218.049204
108.383093 101.1502198 80.50952615 85.78259883 70.64960056 101.0667448
128.362777 147.2678931 115.7068375 250.192653
FBXL8 7.713639432 3.515112115 15.05714204 91.19310135 53.47478762 313.0593206
66.28866703 53.28186176 99.56256588 47.42553518 12.81344642 32.60848091

43.1055926 14.5008585 148.2319802 114.7348137 67.36258689 55.64683088
7.765887386 6.318548034 4.601188064 8.576738645
FCER1A 106.1351171 80.99986492 88.81589691 111.3010937 70.33336588 129.667324
64.11513732 29.85402332 73.23250104 286.9014407 168.922198 98.18855945
622.2119019 45.65742898 56.54869661 801.8866109 2215.373887 88.29115522
4443.124585 4222.609307 2525.841586 106.8980281
FCER2 990.3077473 210.1913338 26.3738691 8.626141867 19.37674145 10.14786303
17.85021806 10.37726055 30.97494747 9.772859254 12.55432737 13.36812571
12.66527557 212.7516179 10.52617889 352.9280319 1104.166801 98.25529772
13.539828 16.26399175 31.52912247 11.25081202
FCGR2B 3389.8606 4533.908058 2656.037745 65.97309417 8.255798098 30.69853755
59.92126523 108.5578105 127.1097924 121.0118537 119.9451162 181.4820226
287.6388369 991.9681697 311.314882 1548.943526 1529.233028 137.2036108
626.9589967 1610.39688 645.9279905 1302.127371
FCGR3B 434.6883898 191.9423831 121.1090782 178.1993614 61.46749025 133.7448215
106.4982845 64.11513732 203.850527 446.2450155 494.7560439 369.6606901
402.4458452 256.3156774 154.0822062 155.6717837 158.6201963 125.0983352
290.4715044 123.6149676 321.0470841 27546.9664
FCN1 160.5487443 92.91123613 109.9311142 130.0754644 144.5883358 98.71765801
63.37271678 116.5979064 122.2714734 5094.70768 465.2336051 121.8089713
29800.42108 1401.079575 306.8296547 593.0958939 121.7194027 120.9633626
483.4804269 562.6779332 168.3568238 5370.355855
FCRL2 4384.737162 3466.953007 714.6372738 73.7221496 34.17485561 55.77947477
79.24179515 46.90834881 24.53453756 157.2790481 54.32604608 49.01458544
47.6563456 36.19137437 30.94941671 17.75900367 61.14907238 38.40731991
42.54800493 30.86969384 82.33991761 116.1844543
FES 8.544430362 8.676013229 10.34392287 9.520155138 8.032047496 6.024967349
7.843078446 7.262313101 8.504060287 97.09901245 211.838797 258.841965
826.5409485 41.65766393 20.94215824 982.2000845 345.415859 10.72101379
121.9591412 76.89948356 389.5499618 534.5825131
FFAR2 12.83845224 15.54382491 20.15690171 11.77579288 11.92870205 15.89364427
16.94262088 14.98197508 28.272854 24.4582214 73.96615142 13.44126834
101.05938 31.01207332 454.5513273 87.81654144 17.74165652 16.51972226
76.33948281 89.36683692 1048.091741 4025.755477
FLJ13197 129.5124355 51.64103294 16.11115902 167.3200623 260.3922902 110.1171051
19.8911471 41.76287856 30.5070053 30.25891777 147.0679625 36.95107529
15.24807194 7.575081726 15.19584325 10.88684503 10.0917531 8.549571152
22.40135257 16.54334331 76.45802449 11.65518546
FLT3LG 26.22845437 56.33557728 33.23725547 1267.031752 2489.205564 1471.268235
234.4917711 309.7698509 644.6131105 222.022152 226.7845957 367.4048158
69.27835254 18.86233643 18.188607 60.37056445 18.33062213 62.75049101
22.87589336 47.4891672 156.402118 26.35814025
FLVCR2 69.18049192 86.59482486 45.35113413 83.37381583 90.1471958 145.8719106
242.0193671 256.9226064 217.1777617 144.8106524 140.2531417 88.34055902

566.9712028 1142.352389 1989.419532 951.2228505 2317.021706 1056.886238
266.9231614 198.7626785 75.80729715 108.4609673
FOSB 200.0093028 243.4082578 2911.316126 41.65285438 401.5583963 59.70898915
32.1556293 5833.248925 24.99410874 37.53954222 32.22859594 31.75264764
3422.450435 29.34004788 23.17042572 22.49743226 28.8254167 45.83078441
92.82531833 644.5586469 7325.609904 80.15125244
FOXP3 135.9782583 93.5353139 41.24636078 65.14694151 129.5124355 190.192431
478.810999 146.8412284 2001.919746 103.9721613 31.98561018 56.57949837
76.15295297 62.1654919 171.731211 63.79022174 63.27839509 64.02824977
69.49716875 23.27253143 140.8020324 164.6742962
FPR1 361.3212533 387.2639146 343.9217634 210.2034137 161.9808797 240.3083941
161.2444569 248.213136 222.4633603 911.9483813 397.2639649 303.3669097
9800.634834 880.9211063 801.8866109 408.5415267 281.0707287 222.4014754
660.8588777 716.9951177 3809.534017 27644.58995
FPR2 227.4506603 162.9187996 101.1071972 54.91749032 60.78015047 57.00754952
24.31747659 56.44753053 97.93080766 168.8394702 88.1553815 69.04044011
964.2419055 182.3163119 274.9365739 95.43864594 62.80252929 48.59895914
195.1670966 344.4167653 150.7924613 3797.403194
FPR3 325.6674246 243.6605005 84.16107071 105.807962 97.29879579 116.5979064
99.98686977 128.4835934 153.046445 196.9377727 112.657984 94.70041249
203.421307 3004.448975 608.2692535 2357.542622 5751.146697 4194.211448
200.0838912 328.0502272 211.1145595 277.4149037
FRK 138.3048999 125.3630578 59.60491785 26.38936082 11.24943836 5.639101241
6.171513508 3.210667699 6.588217809 57.57067756 3.655442581 6.694465385
7.43265118 10.88453313 3.762990897 3.129845825 13.42127435 18.05689621
2.434618453 5.775390014 6.41696694 61.16828044
FRMD4A 57.81750686 73.55800845 17.18073516 49.31923782 24.03797283 71.89460431
48.33458991 211.2458646 38.98612196 23.15443689 46.14200777 43.21756185
52.74025927 161.857003 256.1732314 1094.097795 229.1834068 57.27849364
43.95612501 57.91160526 76.09249167 21.62064394
FRMD8 197.3732828 150.7395951 80.20078747 28.9702418 14.61435957 141.1237411
249.2964218 228.186585 1019.519496 70.74900337 249.1822219 162.813157
247.2516889 125.8231332 271.9483575 170.7725366 241.4254373 290.6920702
31.1452567 134.4864268 132.1534127 172.2630314
FZD2 40.20305932 10.76683514 25.20265438 13.86980966 23.74932601 6.482478073
10.89927471 9.463569762 14.54663645 68.17805047 21.52230454 11.61080204
455.2912189 98.02418421 409.5554813 777.4188883 831.7517546 236.3498633
210.0179936 171.534545 40.47220877 16.40047837
FZD3 121.5674974 138.3505755 181.1627863 50.26515479 96.62044786 39.04159069
136.5432862 449.1787857 63.60598821 59.00916719 29.43502605 38.82769283
39.70835546 31.33678222 32.63242659 195.3507704 77.88233881 24.30648198
58.84581105 68.23533874 28.00346067 73.00874476
GAL3ST4 69.54706802 72.93354863 48.67912609 335.5930908 942.3193748 238.0119755
123.3772483 256.2684015 169.488552 50.30538263 35.6643216 36.41518352

44.09898382 84.21537991 40.44995594 141.0660169 141.2340099 57.36368731
198.8061414 98.79983558 310.5298451 77.37112075
GALR1 2.661554274 4.348941107 5.90308276 3.908160923 41.01608039 6.212037366
3.847184414 6.994769751 4.835860747 18.63540007 4.227180464 3.69898121
4.179834102 2.442423079 4.897631255 8.968861076 2.493139629 3.475112044
4.223909018 18.24601309 15.43906987 6.071751695
GFI1 27.63680315 25.20303785 40.28604041 293.640883 48.08423235 132.9727705
700.6608098 561.1786244 587.5134761 337.3673177 508.7800779 394.3207649
19.38352568 29.03365269 24.66700128 26.60841604 34.30719036 53.2689152
1114.642442 628.8432206 40.27670531 41.43881952
GGT5 11.49823256 9.101161188 16.62951352 12.99967839 24.53939902 9.261514296
11.57823288 10.13745863 6.912133983 14.80259974 16.95386801 13.68021641
11.13018839 10.60327972 290.2150742 407.7940357 21.9710094 12.62902183
15.14646617 52.37771168 99.69997101 16.79237197
GIPR 8.245074698 8.69890136 5.570829264 10.90883943 20.2876468 5.19508538
9.251047619 30.90820518 32.74751321 67.5773091 13.45361226 11.72814867
8.506723418 7.247773205 31.73041766 23.65144355 8.587227324 11.2653381
17.06083294 25.50899867 110.9133339 10.05277019
GNG7 768.0078466 1494.305763 2218.049204 133.5514698 99.42210121 104.2562884
75.96955929 63.64624142 214.2818797 51.82499254 60.45334031 53.08765217
112.449475 82.41009583 94.49057817 243.4861493 187.5124462 70.79123977
170.2524464 115.3507975 197.7715925 455.4134725
GNLY 9.016559093 14.52025513 19.25302109 3732.536058 134.9186388 1198.37505
233.4211369 12.65987353 24.36657201 8201.567151 21760.60028 23235.35012
19.94743339 16.57744727 20.40693277 15.74284151 22.37056888 21.45038814
26.17106781 18.37211337 24.52606794 118.2602821
GPC4 126.6902267 73.41170643 105.523008 43.02443597 59.39944184 77.95639785
53.75861921 60.05830259 60.95504449 92.09947118 77.48575035 42.58445228
56.14456485 1153.718547 129.3188315 145.8719106 161.2550359 86.47333389
72.23389989 79.65437831 31.55476171 111.2722902
GPR1 3.129845825 4.79850122 2.493139629 1.559283973 44.35621868 1.244201526
0.453354164 17.15481458 52.08421325 14.99847133 17.25032467 1.863695454
5.245664557 1.064159643 0.972464723 0.453354164 1.025582915 2.979127268
37.47986528 12.27884037 1.869736619 4.064088815
GPR171 33.51495052 22.98219258 13.87497835 924.3717963 899.2846127 969.236538
2693.556404 227.8557261 6933.009161 4853.059657 443.0770732 1041.132076
14.67922106 12.06520012 25.51768362 54.79422344 19.22969007 560.4038043
84.11631212 46.41606111 1086.272212 30.67179408
GPR18 3056.84082 1822.014185 15.28674853 1037.066376 1241.790015 678.2573057
431.4075402 1521.532591 215.7526442 840.8407261 1204.171373 2025.829576
109.6660347 23.11613842 51.61492393 54.63497421 21.83449399 33.94734448
52.30100217 39.9013615 266.0015774 156.3072275
GPR183 275.8348106 2308.149818 296.6071048 630.5280037 854.0807595 3012.768061
2265.096641 965.2070375 454.3725611 475.9389405 216.2351844 201.5996553

196.979149 437.1733508 1631.804333 2474.434424 437.8462334 1945.433337 303.5641536 795.5512127 5121.174533 144.0671927
GPR19 27.02537152 36.08351025 78.06875407 40.61246787 73.33196188 71.86055345 373.0594532 336.9689608 397.8626539 29.76682384 42.35493987 65.65533364 19.81202335 19.66810706 10.56154776 11.00115106 19.86290214 14.74706676 17.35908885 16.0100968 237.9715496 49.98684446
GPR25 4.292572754 29.42735453 141.0660169 28.7191378 10.77863651 138.1919025 12.75467855 21.82778451 41.74363822 10.93345478 11.1673751 21.43533197 14.04592183 8.494154594 27.36083709 14.41666747 11.76229619 4.701007424 22.81784638 15.84215873 18.26386402 16.9118569
GPR65 507.5709281 490.2704567 162.813157 1518.573945 572.3060778 1499.931247 850.211608 252.2799806 608.4463585 2511.23381 2138.331722 2137.237334 1473.160908 702.0291957 263.7450621 993.5543835 607.4918095 155.0751578 1700.97754 1507.86671 8034.40209 2530.19965
GPR97 154.4101049 141.428644 207.6511007 104.9389313 57.15128061 138.4573773 94.40581808 39.81497642 136.0103845 96.9370486 295.1646205 418.1169425 71.26388228 98.36835876 137.5495819 92.19065542 119.263697 138.6725511 261.4611106 460.8781982 4581.696353 2246.982554
GRAP2 12.62708927 15.00203046 11.63480358 280.9234111 318.9107126 269.2219489 151.959726 201.2672093 189.8106758 166.772216 200.763896 307.2558216 11.82509864 9.44203317 15.99426095 20.12785138 7.973524377 10.11500312 234.8866301 140.1321556 13.68032754 10.85570109
GSTT1 6.958858985 12.48982331 12.11447678 18.64410933 43.10259613 14.99403456 14.09510035 83.68091706 24.66756578 14.72558502 15.93181405 15.76945271 17.31196185 51.03653105 82.52506061 520.5704059 634.2257819 64.6589982 349.3679467 391.8491675 40.40866355 23.00205986
GUSBP11 8048.07448 10388.03123 30254.0679 521.3361484 587.5664324 151.2654723 9.073509293 447.7619209 268.2718711 15.07275333 311.6532884 98.43888337 43.51505491 6.401562035 14.61435957 286.719961 9.045254731 9.611149339 51.74630466 22.89358188 371.5811685 277.7647569
GYPE 38.71816582 61.40275529 35.67150717 26.87927605 8.008517109 3.69898121 4.833493917 6.90617027 75.98982389 61.8922118 15.90605862 24.75298907 6.627731906 7.19407517 7.071638638 13.09271261 5.369616159 8.386579586 9.235760341 3.745156913 8.022460261 14.23666653
GZMA 204.958358 217.1903236 91.85142742 6301.839315 440.9534741 4034.52042 1173.96483 130.7405303 318.1893547 14188.10221 13660.47953 22380.29444 132.0433731 95.37711154 109.7080589 68.0390322 93.29389844 128.6984558 50.07477091 68.27005989 150.5888289 334.8696127
GZMB 102.255299 68.32230989 62.71521001 2080.697741 85.36127082 58.9177582 21960.79819 64.03542312 53.64876826 6018.578754 15276.85279 23235.35012 61.71915165 136.87295 159.2974346 111.0281937 30.67820522 82.17563279 947.0769465 5600.547397 226.9706864 154.330993
GZMH 21.35853068 33.95743123 41.24636078 8555.821981 198.9335618 630.7749174 896.7120375 26.30390688 79.31202778 10428.44501 11889.00904 7419.827913

36.90093477 24.82219085 15.89364427 14.88173325 17.75887232 26.34630303
17.9815414 19.61378309 52.48247611 41.85793547
GZMK 184.0395826 278.1808222 100.8148697 8814.387272 1423.65694 3724.329794
187.7181578 119.8476496 51.85294841 15903.70006 1967.19369 233.6749633
67.13748264 69.54165218 107.0724606 24.66700128 79.45607344 81.98912286
38.19756342 76.14236186 151.2474716 421.2121304
GZMM 6.305414565 16.45527256 12.3063871 1048.989355 1215.533292 884.0128037
529.132872 1030.215674 644.0425249 893.7767856 3771.290343 2616.387026
13.20630847 17.49904782 29.38087813 10.33284771 10.30937689 20.75077043
10.50550296 31.99969933 137.5324623 65.82671625
HAL 380.2322774 308.5027329 108.9091177 84.17530879 61.60426486 75.48182942
78.63381891 548.1212227 79.74092409 191.4998166 99.60146076 96.12281291
877.1793038 102.784423 90.95985764 76.24018935 74.77632532 54.11520645
130.3179977 91.4599882 192.0721184 3105.095231
HCK 273.5706061 489.3443473 130.5834263 106.4569423 107.2851313 80.31784648
81.79892574 43.76861448 75.08844559 737.406205 391.7033029 184.1658664
7368.002228 3576.179245 8146.586422 4811.755806 4370.59343 3772.369126
616.1411859 834.9287387 3730.856433 7443.161769
HDC 147.9736788 177.8451002 310.1156544 123.8596636 18.24228689 118.9595662
82.7123624 23.22265447 91.91206074 90.33473739 200.3498771 150.8083027
129.5522336 114.2614669 200.9810494 172.2889652 149.3724413 109.8186109
9299.383668 9389.503009 1119.899701 150.6140872
HESX1 6.869435614 5.810228378 9.073509293 7.046990167 8.172396024 5.971548735
7.370460489 8.369699084 37.84864451 7.64453315 8.440480409 9.195676707
8.365084929 7.285721879 2324.301051 100.3133583 12.06326019 1074.504512
6.47248879 11.36628394 10.3966513 7.283926945
HHEX 4489.488986 2773.84053 93.42592839 139.0353705 83.41360206 105.0214682
88.61808798 78.89317933 55.90922856 263.6249346 245.8936654 202.8459866
1914.137567 489.3080097 66.50542817 566.3249387 929.3017456 94.43877672
1317.400196 636.7392929 999.3650819 946.2641209
HIC1 5.092351833 4.093578948 6.795317032 8.159927666 18.79636185 5.971548735
7.057961151 6.633637977 88.0877177 11.44970382 15.68354842 41.82249473
7.017818913 6.432515224 12.57775597 6.223850888 7.589776753 12.15568636
8.937587114 8.052876552 12.829329 7.476611925
HIST1H2AE 35.26722995 21.8243194 277.7164299 29.91298037 30.34545814
45.64822436 11.24943836 110.4902286 64.89000999 15.85352471 14.84822972
6.198523194 8.28188422 6.633607497 6.332890006 9.272327825 17.89759376
41.34339654 16.93785386 29.36019802 79.29384956 22.11671753
HIST1H2BG 17.44318854 42.30455978 689.6460957 78.31604621 138.8837705
190.054614 52.48031743 182.147041 117.0121723 72.04696546 68.37610587
11.21409988 23.39236935 11.49352112 10.13745863 8.737345423 38.3658796
137.8269375 49.45247756 29.77503454 62.63375304 45.19348156
HK3 12.94364358 9.44765425 13.29827708 21.81413027 25.374709 12.81515335
21.8935711 15.36690486 19.81013313 140.6970049 222.5053519 32.40772779

3103.136582 3713.557866 31.97610013 159.1217051 1093.669785 294.1705912
37.91335842 234.2311504 1199.688932 1255.80611
HLA-DOB 6607.334733 4832.779431 4287.536344 212.2757519 54.34069564
47.963256 44.00072194 118.2666793 117.0529938 44.15872342 230.4248523
188.3202183 102.1825691 49.6673024 1573.187324 41.5765162 37.2824505
2006.948626 63.49779998 48.71801042 227.8449074 96.88647987
HLA-DQA1 942.7749076 1379.121703 161.7246112 173.4117383 122.6942485
106.9349988 167.0688665 89.17794598 621.2518682 356.7324528 219.2102706
214.9325691 275.3534268 874.2342868 4169.716387 5225.867112 7712.247914
6417.316621 190.2949934 390.0099509 68.2343922 218.2099668
HMGB3P30 30.97425375 30.88530653 36.51263796 42.79987867 3.547906137
52.31915781 40.80982734 5.822857646 359.7407068 101.6070301 38.72002482
62.1534728 27.19577484 39.38790361 55.10360905 37.55103381 46.97065338
53.61427074 36.03095911 16.65415201 69.89874699 76.9105534
HNMT 61.73602193 55.47571769 28.54732378 19.61866274 10.96771886 11.06762282
31.71073352 8.992612544 24.60168339 79.57214932 26.91293797 13.5847049
815.4265044 774.9294257 161.3031858 948.8385009 636.1739965 279.1068543
131.7566915 122.4906095 25.91241295 29.96288574
HOXA1 12.90856012 10.23319877 9.656741815 12.85536224 13.26159685 6.91970575
11.04729816 18.89413582 11.40467609 14.51961531 11.76733746 9.61033962
10.68723722 12.91531933 6.869435614 9.891781855 11.52610311 8.30653615
760.2924663 450.3900541 8.713825016 17.61748588
HPGDS 203.7910498 224.547027 125.9523542 83.72110944 88.13751751 182.147041
92.78922471 56.70706623 219.6942345 118.9847774 177.6752993 148.3391263
36.75265198 239.7846459 64.8769987 138.6741034 195.4447153 125.1233721
21760.60028 17743.45795 256.5193159 219.1322864
HPSE 30.8024485 29.58277474 32.74521328 55.34751097 132.3282146 207.2614073
494.245822 234.9215764 157.5455198 302.9255809 29.9724331 54.88908823
1285.833086 61.93696691 841.0904631 894.528267 27.69056049 30.84139727
164.6993882 103.8988856 39.27972971 973.4037642
HRH1 206.5394742 165.4581351 161.4809975 75.97436695 26.48229454 101.1491714
284.8186666 78.2920509 108.9553664 212.4319062 54.07639831 42.47010982
72.1745748 217.6585736 145.2834807 2195.073255 202.5321442 128.2673018
119.1113422 116.6680467 81.41713941 227.8748322
HSPA6 70.64960056 148.2879125 17.74602894 106.7169109 89.65418124 21.31200497
20.34672736 259.1902042 358.1515065 78.69909351 184.8377232 284.0317902
641.2142131 22.41022369 139.8627137 207.4981375 65.31326001 20.58127818
69.23074494 129.0246893 727.0675325 3303.08264
HTR2B 6.171513508 13.11478166 26.40774882 22.71063607 11.3103513 5.822857646
4.605860048 9.317338154 26.43901488 77.03535453 30.97175272 6.958858985
13.85052231 6.900538825 31.86953296 216.0502815 19.64801361 382.9194372
52.69279218 4.888042211 8.275869344 7.446654092
ICA1 191.4053509 115.3270172 209.3903648 134.2533776 149.651559 289.2721081
160.7794316 2419.169387 216.2736493 194.6366579 133.7203845 135.275358

183.2555042 198.8629383 192.8882167 157.9232363 204.0001741 190.5740318 218.8477587 198.9860864 165.6905432 309.3789708
ICOS 6.458825767 8.407302028 6.147205936 194.1905981 1018.135876 786.5967435 1804.928958 7744.01596 738.9703291 36.42210641 12.554434 22.53108052 5.362446048 4.485992218 18.58723205 5.065226719 6.702810599 6.159261216 5.202255893 5.256582916 13.9959794 6.199101199
IDO1 241.0650179 312.4524995 345.5621343 241.3331484 192.0869901 248.9097098 201.7392421 241.4925332 91.27779079 183.5919996 667.1972433 671.6285143 269.4662793 338.8974249 26473.42255 91.36792646 131.5732395 20536.85787 207.8915127 164.8463693 1389.417624 527.4082419
IFI44L 24.57422621 37.30993947 52.98873326 84.26221988 147.4365503 127.385545 18.32015721 132.631945 3323.895627 492.7938791 302.4006942 311.314882 307.8493777 12.5901686 6016.733404 984.7302507 11.03225551 4019.777684 81.33879659 95.98817333 165.2059275 74.97678161
IFNA10 63.51120873 158.4224613 31.21088513 12.57137446 14.25566764 13.99751273 9.372947039 15.27320006 155.2422393 10.459288 8.565645005 13.16479841 13.10429637 10.54848621 8.485896389 7.997120003 21.86200716 8.554973126 31.31689645 22.44617229 11.35404201 20.7783228
IFNG 233.2462403 168.0836515 137.3910104 249.0786281 107.3272359 142.1822906 6344.879706 208.3394234 174.2692818 269.3932002 247.3486217 3575.236664 108.1486375 105.0542732 96.36432219 71.1513012 75.83739524 92.78571972 109.1827541 70.32219401 157.0213934 187.1620872
IGHD 10811.41236 2526.481303 21173.52695 62.3476822 125.466199 26.14159933 21.20380232 135.5693196 28.36731347 27.10540257 32.58210537 21.68739568 50.4303708 44.70050298 97.72702921 106.3962528 78.42668997 56.63839916 32.60266038 30.03853946 257.5071029 79.25564732
IGHE 18.17410575 73.92464991 4949.619115 15.63289317 46.81934 54.27863199 23.03809769 51.74744263 18.57562555 28.07995577 19.3650878 15.52894714 25.291882 23.51249171 11.92870205 16.09778833 36.23816826 14.94842 67.3916899 25.52891416 62.78628164 45.89295939
IGHM 21960.79819 14250.20741 31273.67963 361.7379219 46.93569691 156.9391502 260.5780432 2116.005563 593.8569709 226.0772361 162.309451 134.8648186 127.0436672 32.10709446 32.67724104 26.51832445 44.41793335 41.25586808 70.02577331 41.64994169 834.002996 98.13122154
IGKC 27164.52012 30254.0679 36524.44074 3097.474216 489.1798399 40.36292074 67.88787751 329.5978842 351.6837529 2059.744494 324.3571791 121.6124805 318.0695055 78.07530888 235.9541063 170.8361038 64.18688874 223.333702 100.706419 162.9166765 1108.467068 119.9968023
IGLL3P 6633.535586 8962.420222 42851.28444 217.4652812 18.00926412 41.51758679 96.40860336 50.27476208 551.6984117 36.19278033 33.45486904 62.78986393 70.0394246 45.93797103 157.7551766 102.1260256 159.0326323 142.2968228 37.36510829 56.06416798 80.27971882 83.43489613
IGSF6 46.07125476 70.4016677 65.01794569 27.09871228 40.17569562 48.71083673 35.64452519 27.93727573 56.76437031 137.7959697 100.2893009 74.41451203

2662.140929 6076.582495 295.0724091 1626.552661 3679.737192 1231.557711 156.3302861 366.6351782 246.6944983 4222.728231
IL12B 105.275436 89.59925365 56.97214117 41.94288686 28.4476789 59.255418 77.91715796 42.09518393 43.47558877 84.12179397 85.58316556 56.90002211 42.42610973 43.55770236 513.0848293 18.65300514 73.47854562 2407.546497 38.07655929 72.40950258 68.62419013 127.5501333
IL12RB2 13.44126834 18.33818525 13.07042258 95.11508424 33.86467066 106.8888316 726.4496109 117.5335217 219.4383155 386.7126926 594.0469774 1531.023729 20.50390251 19.79170172 14.52740162 43.64768257 14.03060068 10.65230466 39.54913244 31.27438783 11.68834565 14.16736964
IL17A 24.10976356 31.66676526 13.1991983 7.018701499 6.547727883 6.560415317 294.032794 69.2200367 11.18736285 18.32724833 9.511607877 8.087608443 7.929351943 7.622591789 11.84351258 5.797213647 7.653355249 8.556203816 12.06834847 20.20321364 8.063350097 39.5476383
IL18R1 32.74521328 56.21530515 34.90083093 197.574303 73.22426678 243.8532814 400.3612339 119.2372786 512.4917243 1022.471148 829.4466231 1084.441173 19.50301241 19.54166867 7.9840431 32.56301861 22.9410599 21.814318 1442.746463 2220.298354 1494.967961 516.0935366
IL18RAP 63.16533501 45.38600163 22.92310159 541.6070596 83.75974388 275.2312246 367.9599908 101.4843406 113.0686831 4488.262918 6172.262928 5732.166309 92.02184739 93.75421468 24.97523206 38.64095647 88.07756471 89.09515062 239.2744548 362.4767389 1797.696657 2653.493356
IL1A 207.8094975 114.0328396 101.4843406 65.8236721 98.02150804 89.41077945 147.6537197 52.93205499 47.70844227 104.0011372 81.80415638 75.92997592 61.10993853 627.6075867 90.02342615 27.04752387 107.4684033 387.9719904 140.5103893 2906.366436 2009.795325 204.892697
IL1B 508.1423339 364.9197528 198.7896427 144.2186891 136.961868 251.1017935 200.4208119 204.2875485 284.0853434 352.1592413 250.0432905 241.5910262 1257.182131 1273.463059 675.1218227 291.824541 437.0700597 2781.903216 3282.430504 17770.60586 1137.887994 3099.346146
IL1RL1 161.2444569 100.3685885 109.8410784 34.56285806 19.32045331 70.12950502 95.88469778 69.00006431 60.40587603 98.27886377 65.350782 125.0829545 37.42366918 98.08944243 93.26068194 76.11959313 100.3189187 44.282995 730.4759326 1305.536765 971.1736917 102.9096081
IL21 346.0439646 299.7702438 161.7246112 59.42001123 21.34280658 81.58442785 333.1909722 1466.934174 42.0591226 87.99639559 38.68027919 68.0390322 103.5720427 83.58872184 59.64008096 42.67287335 78.1233779 62.52092002 96.13005298 60.01519603 93.38643807 201.0983497
IL26 6.120665994 7.623841163 26.26628742 7.410920044 7.902366544 9.532387256 273.5706061 116.5979064 20.82075804 10.73746674 7.691378335 5.136655175 3.424557859 2.491129956 4.330193687 4.366178616 4.355733897 4.531899492 3.032229715 38.01550446 4.776302225 4.557666573
IL2RA 151.2654723 216.016679 79.81202979 85.8239546 87.16147336 360.4452087 4978.13072 184.4383466 3823.559035 31.18038026 42.04435301 628.1600217

25.41115885 33.9871692 3963.774053 146.4600699 44.95064885 2221.900378
193.2645954 310.0050923 133.8083659 31.22286334
IL2RB 2118.459057 1945.518804 848.6873784 4000.216538 818.7927424 2988.605281
7562.491571 7279.249666 12662.47545 10958.76994 18026.39249 19190.61594
321.9612928 294.5506048 221.9788101 172.9682935 246.8795571 366.1434094
341.9687806 442.6048403 381.1943018 1044.851269
IL3 20.12785138 12.58545616 13.81492461 9.157134905 9.485254596 8.603842518
1689.599596 15.86814667 37.29223107 5.976390119 13.59206642 28.81247015
11.52632712 9.762406535 10.04573553 7.30958941 9.891831901 12.94335151
8.370755211 411.2800676 112.5425353 12.25400672
IL4 10.0823446 28.84260472 39.12138574 13.17067743 10.44585498 10.77863651
232.713076 118.0039289 31.86954261 32.63339605 12.25198156 9.624468251
8.872339575 7.664565106 8.673663183 7.429093546 9.549519912 9.284199738
7.282627513 10.14540159 52.56882157 34.58584621
IL4R 11009.29392 1883.66674 291.1304361 884.659355 2125.328314 1148.814515
864.1960215 414.2300341 3102.322518 913.5881164 851.30019 1609.901147
1192.215763 1054.288345 3007.592556 846.6140012 1093.752458 2410.484546
3106.475575 4652.787414 1117.612583 2809.100785
IL5 6.618252721 11.61192289 9.694581053 18.71157669 11.36906118 35.07363094
159.9554562 28.07283413 86.16972173 34.7038725 8.169496573 13.81492461
11.47899088 11.32827552 48.58576071 18.8378526 15.96329864 22.53916948
12.570626 317.6908754 22.81507783 13.25903543
IL5RA 122.2890422 101.5434264 197.2344989 42.9451164 26.5887412 63.93112851
43.62068846 32.20308963 103.1997215 118.4320183 45.67953351 44.27072755
41.02366335 36.13968537 43.73965746 22.0447246 51.58293868 55.86616787
154.5243618 105.6851394 713.1604697 256.0574097
IL7 250.9247833 611.4516464 40.0906413 41.57938135 34.7070222 69.25499148
30.32542187 68.38795936 43.15687677 27.39425876 24.76130902 26.22845437
51.3943268 93.50347161 133.4873015 79.20083093 68.88084979 143.5066148
55.59054639 25.03009419 18.13188477 14.89186794
IL7R 277.7164299 454.5504216 126.0983758 15195.16858 19513.31337 18085.9446
1965.113683 3066.253514 507.1604755 2118.827609 1159.340489 341.6294609
163.3050166 5078.293455 2163.365124 85.59144108 2156.179359 7799.647099
36.56114051 325.7294175 358.0842541 742.6718185
IL9 60.05830259 42.70618828 16.73521702 43.06562332 31.36207011 89.90011254
6525.444386 46.35002341 15.37426695 47.46140523 45.8168679 31.82797542
15.74553195 22.74633599 28.83176888 10.07084824 31.51470707 34.55584304
4.125140757 15.58698236 56.79210664 24.3295572
IRF8 9506.386392 7886.092193 218.3135972 350.3493603 279.6879535 45.41225697
487.5663213 610.5494813 196.6158219 992.3568261 1625.377439 2594.872072
2458.705658 1929.656915 12117.94539 3495.416283 1652.355711 2798.258545
363.7262259 798.9447795 519.9851623 281.068165
ITK 468.9985735 409.7558423 192.2179775 6471.889098 9578.936337 7704.627853
3954.854034 9006.839826 7176.363772 5787.063934 3056.935046 3981.741268

124.1014103 115.0222464 90.2244571 71.26574474 102.079759 166.1778128
1344.476541 1083.589895 654.6984165 380.4695205
KCNA3 306.2764477 411.3873405 1214.683767 573.9051164 465.479642 975.6549806
324.103216 263.9154835 74.99289378 440.6509136 411.3943392 577.1730417
143.5528595 102.9419725 129.9134995 20.61657512 23.5447124.00174244
13.81687679 18.00422957 120.5070417 65.0351555
KCNG2 12.5310046 21.62331457 203.7216331 18.15238454 7.924164041 12.07805481
12.5310046 13.09271261 7.933580511 7.281883852 17.88167287 15.86814667
18.84293691 21.28655105 32.65230838 15.93115384 16.35770749 15.52250872
18.17240921 21.55778412 26.69902616 11.70521171
KIAA0226L 7209.224512 5267.694585 147.3808749 41.69784069 82.79229745
64.8769987 50.36671815 26.53483439 87.2937927 97.05270244 54.34410784
30.36307028 176.3964323 104.5999421 210.4700584 711.6687161 61.81257943
43.42734643 108.4065512 90.21510801 435.4505242 2046.296729
KIAA0754 9.870549848 7.165981269 4.818030863 23.11849958 80.9645004
7.879203828 4.366178616 76.11959313 56.16728339 60.22816379 151.0455484
150.2587239 44.79047534 7.819073969 414.8050146 157.7551766 7.952601123
4.64920776 7.690245228 54.64334078 85.39781903 7.870074424
KIR2DL1 7.406441697 5.157127328 4.493181638 8.01647279 12.79241876 6.332890006
4.126234993 6.198523194 24.55389477 24.17768826 281.9844186 532.2521388
5.116435124 4.751833847 8.603842518 8.522115249 4.531899492 7.023482621
30.90286056 25.65281368 7.453176714 12.34421311
KIR2DL4 331.7763894 242.5966898 326.5656738 122.2246008 125.3243228 79.00529624
62.54142777 71.00293957 119.6110333 247.0446849 602.9726262 1692.521387
128.8246048 99.74347628 300.0125058 75.07815363 110.773487 151.8311567
97.22199641 116.9912535 187.147452 282.8093
KIR2DS4 621.6999538 437.1452806 512.4557638 280.1613018 113.6054513 120.4987696
87.81654144 129.4623262 189.8263758 544.8216867 1172.82887 3017.546724
129.0849772 114.1247729 136.0850535 62.96408983 137.3858092 106.7559176
147.351626 135.0551191 121.4465272 344.8539572
KIR3DL2 12.24576038 10.57912504 7.59573833 22.06039056 14.16687271 9.251047619
13.16479841 9.926447424 30.03124066 214.4909511 511.2935608 1163.915182
11.38893073 9.60888568 28.01546378 13.76775417 10.64138208 9.637544809
17.20022019 14.72443266 13.64241979 20.01964437
KIRREL 15.52894714 13.70655219 28.13135229 34.23347857 26.78347553 9.694581053
12.95560951 23.95290683 171.4659907 30.4285789 18.20868192 12.13897809
13.64075973 10.87526239 22.74378501 37.96319899 14.30574871 11.31192453
42.11575303 19.78886308 34.10168949 15.74318836
KLRB1 119.5727193 241.3252476 39.37347577 8818.269216 317.5656356 10026.23944
500.6894521 4477.729529 1259.996581 12634.61161 12957.39238 5576.724303
90.24623781 26.68511703 33.3990692 13.5124231 94.79879985 37.00213293
37.48705237 72.13784357 276.4397193 322.6159656
KLRC3 13.84066629 44.61637486 9.097003233 724.7758509 13.18858231 32.02053189
12.41173255 5.4425507 47.20920823 586.9379733 1485.991589 662.1427421

12.58558886 6.245979354 14.44417944 9.753147704 18.67641809 9.447726589
8.281151752 15.21430708 34.50130668 8.77366203
KLRC4 68.25016134 85.07512829 24.46628657 218.1751146 48.80110093 17.67634993
22.20688826 9.624468251 33.76338165 124.8024312 205.1777143 119.9854466
20.05492885 24.93153862 17.06104789 6.458825767 10.4089605 5.35636677
14.14835289 12.25029921 4.900978773 44.4640693
KLRD1 204.0068731 170.2059521 244.5489843 1670.271427 75.78362334 207.8812187
155.9617882 149.0261337 59.999115 3580.074313 7278.146564 6195.927488
81.02147756 53.17243141 62.89115552 75.6389405 88.95467091 61.79015112
69.9817193 47.96373123 58.50047382 195.370024
KLRF1 153.1504496 198.6098814 112.8639054 851.1875307 21.90774996 127.2371142
37.05749544 13.02444905 8.539704435 4962.361286 10475.7852 5911.213148
100.1145382 41.2903796 76.15970242 182.5359056 75.15348707 65.02829076
69.58226846 59.59794077 107.0253016 121.9079756
KLRG1 1351.157456 1076.359373 482.7709118 3859.14824 467.4716087 1939.944493
275.5197677 283.2038076 373.3248469 4631.811312 1700.856514 912.2671752
305.4474128 270.2038954 253.1007469 148.5655127 250.0803963 224.2019093
1310.19144 1313.328247 448.570662 605.3949976
KLRK1 670.2708554 280.4713279 77.84292347 8228.460511 353.8455751 276.2455792
69.14462123 105.1489388 44.32349787 5199.517616 8790.503534 5454.73273
78.91616578 44.14759366 38.56086836 62.47198947 71.21203215 47.2591819
88.01964485 77.98871131 441.4201255 246.4439838
KRT18P50 23.97041806 28.15146632 87.81654144 23.80795592 44.1209146
22.87490438 18.71219759 30.47049066 73.21035599 162.0879363 23.74696304
16.91790381 58.20626127 20.18111153 56.97214117 20.17213484 10.05531958
51.62553838 14.97730628 18.08634139 163.0384385 68.38796964
KYNU 296.2994306 573.2552275 97.42434599 45.94858696 41.3563524 39.20610994
27.37818895 22.74378501 128.3281017 117.085962 70.07148215 66.70647411
858.7450615 3089.346164 6316.573655 1059.119128 1973.598471 7308.577846
132.0617598 327.6913744 62.48037948 100.1093538
LAG3 36.29565926 8.732532802 10.14786303 521.3214165 6.264629561 175.1856105
999.6943311 1296.73277 656.8213603 884.9362538 139.8539928 23.50429099
7.92284356 6.759088639 1427.702674 97.67723625 20.86020976 215.1741661
12.7599446 22.32766244 5.070710836 8.170942175
LAIR2 424.4791722 238.1828119 166.9553981 1078.390893 212.5228127 371.0335929
81.43053168 234.346304 3022.383345 1320.824363 1596.837227 1637.522871
103.0030242 85.54718583 207.8812187 88.73958185 96.22686841 78.2599751
85.27872952 114.86678 376.2549058 260.8513758
LAMP3 47.28278554 46.66817965 128.4317353 134.7841361 120.9265077 160.3048593
368.6319956 165.5835013 264.0396917 132.2010638 210.8261404 418.7096295
38.76231871 316.7767691 17116.19562 113.1413857 1014.136741 23356.30643
81.94628982 130.0600242 540.5707204 320.9232259
LAT 157.4129632 133.1507052 111.4231016 3224.978584 5669.605245 3282.260737
1447.742457 8480.619457 3200.697142 2738.316414 1722.578313 1262.245177

239.5833162 603.6863585 162.813157 181.9526719 205.2650782 218.7467931 3738.758621 2477.465096 405.7028156 209.6546017
LCK 337.4400897 327.1096376 40.80982734 8763.864592 8948.349603 7786.120969 4042.377012 9578.936337 6736.744097 4299.610023 3604.565205 2808.159439 80.75884433 64.45522652 25.99553349 22.85969301 43.79168499 57.6156067 33.89599017 20.84968098 245.2328838 186.8691266
LEF130.90820518 28.4865918 56.48122719 1204.840659 2704.715795 1154.223661 412.9279657 495.8728917 364.8725487 144.3431401 115.1927029 115.8526336 12.54164279 11.51825528 11.83156209 15.80624293 11.61178237 16.8510481 20.33091749 18.49440463 107.1460636 31.2755148
LHCGR 154.6890469 195.3792191 59.39944184 9.897454754 24.46628657 8.255798098 5.598819437 9.419742658 139.0898849 126.933563 21.7148195 26.06781415 11.47012114 31.7588928 23.03809769 22.03161941 20.63736847 21.31678215 31.75279947 22.23624099 51.25024965 82.91574196
LILRA2 20.88194891 19.97153628 13.23730918 20.16252339 27.39864055 18.02258618 13.56995609 14.6382422 11.58792629 151.8492133 101.3950793 38.77174192 1764.823942 852.0801094 317.6856228 220.1788316 135.3143975 67.56872756 133.8752859 107.1102323 283.3313914 2439.781118
LILRA3 7.153043448 6.787328787 5.58378914 9.02114446 28.11270143 6.052620848 7.382940272 5.625148525 40.72087151 19.25297574 20.37949338 12.10187282 880.456908 53.4640133 973.1747582 154.5203653 27.05609175 394.9475489 37.57554618 18.25338212 81.14096204 117.4599802
LILRA4 33.02123349 111.7082404 65.08699055 24.77824314 17.45746015 57.84876862 18.43170071 9.669430796 500.1910895 40.01532931 19.49828414 18.12117817 65.68769791 44.41590756 92.50411622 38.58613078 34.16139145 31.18408975 15.20381774 8.331049833 15.63266643 63.10838368
LILRB2 30.88657745 28.11397271 15.56608274 25.57422148 6.646385161 9.996682874 9.085608856 12.43682411 61.89822691 357.618743 201.7195756 36.09030097 3344.118855 541.4074958 4796.504314 1185.826811 250.1929104 291.7908682 334.3576063 328.4510229 1013.349786 2905.942642
LIME1 23.92087663 43.82308141 1021.385341 1287.731562 995.0546469 1225.59738 171.9065402 801.8866109 383.1735043 666.8094476 181.4780792 47.46263396 78.16252525 41.8287759 34.53702578 50.33543999 42.42770527 48.5118084 45.04108146 104.2880897 52.56517195 79.53831265
LINC00597 15.98296208 18.32668535 7.214039616 6.841729202 60.43870697 3.681017104 3.720542522 33.95721509 12.93495387 65.41694292 128.013785 72.72761985 23.18626902 11.37053999 87.56074506 126.3468697 7.572513244 6.369784788 68.98009213 313.742119 142.3813574 20.71458679
LINC00921 675.1218227 227.5869807 145.5963793 132.8112012 40.64411705 146.1436716 22.79147187 103.3123673 71.61705409 70.88276404 116.2458441 70.47336347 69.03803764 21.48566946 43.47348792 70.79707101 21.81066603 30.69076634 24.93138681 87.94657299 427.5881941 287.709765
LOC100130100 19.84751256 28.75087131 7525.221652 23.05076042 19.12046512 16.09778833 15.86814667 17.16741792 12.23607915 17.14185375 10.35285767

14.70906515 16.13831431 13.76101418 15.93115384 14.61435957 16.1139255
15.35656506 18.81189118 13.06014039 15.57160381 17.23014635
LOC126987 135.5693196 103.5430592 195.6949202 72.45319298 106.3115117
91.28169833 101.1966631 23.60168909 749.8002383 23.76710847 86.37729281
76.62216013 68.19210687 49.24995457 120.1283625 101.4058634 85.34834978
91.89975985 52.82210885 25.0176638 72.26321114 91.27327826
LRMP 2106.83981 2947.585258 1042.751166 631.9125478 861.6605651 327.4987971
185.1858088 2003.326231 135.9692767 613.0798899 1245.630394 436.5800269
1048.681062 155.3040258 38.79936244 773.4916653 535.9059585 114.6747087
727.1797268 621.2830904 6331.426168 3231.634624
LST1 29.98420127 20.94422774 39.94821809 520.3780954 264.6015763 332.3730932
131.0995558 36.87140683 71.2981542 1394.203591 672.9594285 291.3221307
5975.189254 2191.478522 2243.545374 2286.079952 3691.087351 946.6004764
1224.997584 1641.972335 4186.037105 9953.015759
LTA 11.68110017 12.01527664 12.60267124 21.16573991 258.3876509 27.69750654
2867.607058 524.4770391 138.2988262 13.28187351 24.18839952 2880.862643
12.51621864 12.5287015 114.1041953 14.98197508 14.60038983 12.82463268
12.95614608 17.78813205 26.31845898 16.83523098
LTB 10351.64143 12117.94539 106.6290931 9829.724672 13247.62405 12009.36242
5257.453469 9725.14216 17485.2086 3372.608476 1009.906661 16862.16904
321.3564756 127.317149 116.2863022 161.002021 184.5481051 112.0607213
222.2586327 400.9057785 2136.992363 5458.249488
LTC4S 61.1295924 25.94383276 66.32500458 132.3582101 77.11890893 78.40840372
50.09224489 112.5148376 13.48887099 117.9338674 162.8566162 97.97552647
91.87551209 110.0952748 145.9248694 581.6689523 224.3289545 67.02300291
1156.117714 1035.91691 595.9161078 114.1929024
LY86 6947.982466 7763.972248 815.0088739 382.951912 398.8717851 198.5520941
176.7444504 208.4980914 379.3366843 1584.314572 243.1158973 224.0756082
5218.063407 1832.781159 1246.621776 6395.572444 1997.214217 601.1020453
297.4214468 221.121219 654.4709644 691.7851206
LY9 289.0732036 607.833791 552.0344178 1310.964334 1851.721168 1298.433446
51.04583679 979.0134027 199.2633251 1132.316757 271.972779 28.5057754
30.00087721 34.01455014 28.29412112 252.2799806 74.17751535 32.7996725
53.62595267 25.98366937 71.60927294 59.93806713
MAGEA11 21.99996581 32.72734275 33.69520426 20.72781035 9.272327825
46.81934 31.49065027 14.26798853 78.55197164 84.08511613 19.87971163
10.00970252 5.93274616 8.165451989 6.881696305 5.473565526 12.44081637
7.145470373 5.96527055 28.6908932 54.72137242 18.05763943
MAK 20.11347878 20.28901775 15.05714204 8.152190939 13.44126834 9.822978292
40.92391452 13.84066629 9.960118091 22.0750038 11.47152467 14.95790508
24.81417634 11.76696799 40.72370334 106.2299542 13.83006736 54.01241952
27.90947126 58.25891278 356.3243879 1120.128556
MAN1A1 293.5258353 262.0187671 4141.039248 239.4182791 96.36432219 219.4246857
337.6772681 280.6334531 242.0517762 294.362322 381.9911766 308.6636084

205.5255231 541.5412754 493.8657698 534.8061268 366.5485771 444.6270407
641.7452468 519.5952215 169.353094 662.1081563
MANEA 3.436589508 17.60224736 658.3410173 52.68877507 48.36415338 63.61546587
343.1966006 85.74723008 33.23629102 24.98200909 72.1721013 132.9727705
67.09972733 120.265675 81.69270078 117.0081644 111.351923 130.2198315
87.12900391 34.12837499 4.4501978 3.047541504
MAP3K13 5.152513024 4.887907798 10.45807901 7.381955365 8.886630463
6.382539853 7.500776659 4.945111836 9.244897824 6.502030229 7.176074961
6.292627292 7.051783275 7.239320879 37.08407242 7.843078446 7.964226977
195.7507332 5.545198276 6.098718067 7.766884131 7.993696005
MAP4K1 48.65001463 923.4585215 201.4645711 1721.22046 2431.498593 1387.561664
1231.701055 4278.451524 2031.177327 1550.102008 747.3149188 451.4493332
317.9668785 25.60814698 31.1038533 367.2710553 58.98386899 23.10683533
361.741886 310.297677 272.9729346 35.18234699
MAP4K2 10.65044684 12.63242373 8.427360618 17.70603378 144.6964726 20.0115018
85.19343142 16.98151569 32.11507998 10.77970448 11.12538801 10.43288028
12.04202684 15.05384266 9.050871942 11.5997819 11.28413666 11.22679921
8.807533308 10.13352773 10.82778318 12.24076681
MAP9 10.29840785 13.08373168 8.288872254 93.40076641 96.15733766 25.30523597
55.74523407 64.48080982 22.3116708 3.353565773 37.63679768 43.71021332
1.421006596 2.140728959 2.387820415 3.785240835 2.574327617 2.772555856
2.778249883 2.815665601 8.739231654 2.738399982
MARCH3611.9846331 264.9416398 286.0949186 78.87501105 110.8989432 78.59525306
79.73662851 183.64099 205.2571817 85.76984856 190.5710992 194.2572943
103.5658121 175.8754876 328.0740952 56.54869661 138.0560759 125.2511774
1744.531054 2814.293695 1152.378442 205.0752959
MARCO 9.029403736 10.74173048 14.46846851 14.45792523 38.56086836 10.71025113
14.49169961 24.0051092 27.25608933 12.81976422 16.1160018 13.72935017
236.7362055 2418.052513 48.77266347 117.1511345 17.52051452 15.15488638
14.22873553 26.61544932 21.0620827 14.64273507
MAST1 9.251047619 14.90583928 845.8491856 6.640168162 10.18494196 8.220261044
11.14631073 19.18028488 18.85451819 8.103482987 10.71480628 7.644655016
8.492607794 7.33322736 9.151450435 8.533286435 7.34003434 7.654391457
28.7744498 20.84717616 15.59997418 23.9863503
MBL2 59.05366978 96.12663648 15.69138202 2.661464134 21.37467901 6.171513508
3.5940133.740216058 133.631472 38.13537967 14.15214366 6.332890006
4.939369175 9.464931333 5.398099627 9.195676707 8.24077408 23.80513621
4.872899118 5.575591779 2.332247338 21.75446848
MEFV 27.28348256 27.22990284 14.24242632 10.29578093 7.225818568 12.67206304
8.23248634 11.65755142 14.7398934 44.28123628 19.76334445 14.76826697
552.7124986 12.49592782 10.0823446 9.173433177 11.06899545 10.86423794
18.06194726 16.27540589 21.01530154 871.8164344
MEP1A 939.6261506 391.2004498 222.3862967 54.17625589 22.42983417 141.2290524
43.13043305 54.92226784 169.5472888 136.1749837 85.62849683 89.41077945

98.26125945 171.9329356 25.48235387 66.46616213 186.6180666 321.8834531
25.29772111 96.70274469 257.5044339 165.3704727
MGAM 8.043975587 5.311140732 5.166817607 3.964324624 4.929861418 6.159428432
5.092351833 5.783504523 9.150127078 48.51372535 314.5412721 47.87522011
159.713285 14.13313291 5.166817607 5.665802146 8.88168871 6.625392634
234.352858 137.7165021 609.1830453 5009.465768
MICAL3 328.9820884 319.7728118 89.29505406 100.6010965 19.30656418 126.6902267
131.4519579 93.58629209 14.43318731 38.76021394 40.04773519 117.9119847
15.57018571 19.84503831 25.35785089 17.81194748 21.72023967 22.58563747
150.5320494 127.6291786 47.68240621 10.63927101
MMP12 746.9258791 488.893271 163.9010445 79.27159307 52.70903607 114.8747346
105.1489388 81.69270078 265.1392895 233.2582309 98.1637984.9536497
95.85109514 243.058639 399.1895721 221.5740634 15992.46965 3929.353984
207.7939338 2823.785573 241.2406344 296.4122687
MMP25 136.2875659 125.5332669 196.2250897 211.5319494 232.4626329 168.1540221
115.6721617 162.7569122 370.1045701 134.7329729 149.937847 457.3283307
446.4796965 119.3129296 1099.921767 583.9384858 563.3057559 1130.6098
147.1246374 181.1486053 1253.630786 4281.638501
MMP9 338.94428 388.1056494 357.9745698 286.9137339 359.6153524 395.4202673
258.4783862 424.4791722 396.8938938 338.8946519 688.2490954 284.1304512
335.4295326 38944.01868 2727.981464 16386.05747 3188.623948 2899.726139
1191.482432 12655.47888 635.0862911 6429.776491
MNDA 29.5032353 276.0791648 5.02270779 50.81863936 9.217386051 8.750532337
4.525700455 13.50066477 38.28622427 2157.226727 790.3716005 301.3206314
11447.56145 2609.874357 4016.523813 1867.617958 4559.644609 2133.559627
2154.019115 1279.186504 3154.581987 22598.07416
MROH7 23.05515036 26.76307736 330.0530055 28.67423007 29.60547378 31.49065027
27.55771004 33.16402421 19.72411436 52.90645478 19.11115142 24.83406901
31.92868262 30.49050205 26.72336531 24.0216752 27.38987274 29.81710869
39.08973983 67.89494755 37.2994064 35.65136128
MS4A1 18085.9446 17478.14564 396.3913895 343.9223112 52.22897356 220.8581711
125.466199 112.2871276 272.6999955 505.1556973 193.3316222 177.3861387
128.6338935 31.97803261 94.49057817 64.19016147 64.56613288 122.1899811
80.29122433 52.09389632 704.4005013 274.6589928
MS4A2 151.2024037 187.5914373 119.1973269 108.6218788 99.13536849 111.3833723
94.92027298 79.20083093 67.67598186 124.4244268 93.27967612 69.18049192
52.17720971 70.5034722 67.69994038 68.77420162 73.06314632 95.08035664
4441.180768 2673.250779 321.6586355 126.264012
MS4A3 121.2914634 62.7546875 60.99801415 80.86216165 8.750532337 40.41954215
20.88194891 35.12086261 36.53908237 105.015473 592.6171613 191.1348695
23.55316735 7.818215887 20.77904136 38.22269719 15.87378625 21.23223044
1963.692404 893.55158 1384.096932 174.6897078
MS4A6A 1102.971199 616.0957235 326.3520803 221.1962643 167.8644908 216.3694922
177.7168106 149.2510114 453.0762101 2149.718655 222.7095319 191.822788

8814.800982 268.5081463 402.5623243 16167.182 3743.66762 167.4361623
704.4355853 501.7726181 408.7375549 1136.619238
MSC28.93411812 44.52165409 115.1532722 78.78518268 42.41248076 85.70898291
243.5748238 31.95509978 172.576955 132.0717019 60.98475714 42.30368151
47.46789206 631.8453475 1562.138826 101.4843406 573.3664602 1844.921425
63.76316724 148.3606244 75.28238775 111.4753412
MXD1 22.77650595 22.40684859 25.96026018 67.17780431 255.8877786 110.942725
65.29264421 148.9108567 94.33865234 100.6229174 201.8229525 62.75327788
250.0158171 83.19369257 135.7286165 26.56895465 18.2441129 247.6648431
27.88347076 51.87740674 1552.584099 2389.810861
MYB365.4798555 297.3542162 123.9035186 647.9999569 590.5897003 269.6240292
838.0011211 1793.189396 735.501768 250.0613928 615.5214621 235.7125916
300.7619354 95.31275979 78.10250073 118.9099184 97.44739467 366.9910132
4336.138621 5021.57244 3653.922546 505.0813817
MZB1 46.81934122.2593141 20117.66469 42.83903749 70.2363913 9.914966708
21.78305128 18.16093232 65.46672008 62.51579389 21.76048739 26.15989089
24.85648896 22.27069193 30.4274162 33.81911592 23.21322482 22.41174764
30.79487517 29.49339673 52.52655798 29.79626914
NAALADL1 11.62316713 15.79395952 10.56154776 70.98305911 207.9599013
147.3280124 20.45437223 150.7507919 48.57025297 62.46833869 344.9584692
228.0189635 31.52382891 14.37732844 8.288872254 10.286083 7.320372505
17.6608551 8.867943028 11.21471067 15.86794224 15.89251626
NCF2 153.4955828 345.1359814 194.190923 62.34284219 225.0278308 88.17850315
60.47608557 68.81156687 425.6902381 1620.910475 483.3850653 168.835817
12280.85608 15650.12064 762.0351121 11487.88902 11149.65069 3147.592954
956.1678627 1619.076321 20110.62767 17226.82018
NCR3 152.0118385 792.8512873 42.58445228 1756.075472 250.1023415 411.610723
407.2225704 201.4043697 669.2181531 1859.555439 1482.733424 3218.191907
56.94572009 30.83325447 105.9964013 55.94701428 86.33580407 33.09868234
96.4754312 83.51655348 146.729649 223.2727212
NFE2 6.094544149 6.768979235 11.86713177 107.3563954 48.98562369 33.47001344
7.618893885 56.38068068 67.68355 79.04536219 134.3635052 82.87062985
1517.644033 30.55010836 32.42880369 110.0759892 12.40993727 11.95985661
233.7156172 148.1955068 272.8867403 2938.86897
NIPSNAP3B 1577.975004 744.7742002 349.550369 128.0373349 41.71079514
137.6053305 86.05619964 79.24179515 106.126089 497.4339321 152.7729655
105.1928183 89.57172242 79.70267094 105.2363956 106.9349988 69.48205189
124.3434718 207.1530615 142.5503686 153.9906407 331.760106
NKG7 6.994769751 10.33850341 7.726182177 8640.630717 106.5387015 679.6910465
947.3726372 7.582193721 18.51226305 10086.41757 13911.17937 14574.73469
34.65463899 4.93466715 217.2245766 6.547727883 4.961302934 6.557717872
429.3526592 573.0034371 24.2643021 13.31565465
NLRP3 22.14183418 20.53269856 15.37842878 15.30576169 18.45676256 48.05321317
14.17835244 11.65755142 28.04117893 51.58523231 44.60257144 88.89574256

636.6292677 32.50574568 37.67910545 322.0620463 310.2780778 19.68892684
26.84988325 423.0729418 68.36800786 478.8774821
NMBR 507.384036 573.6463176 242.5274059 45.63830073 8.77366203 31.0595517
92.99539565 20.04059624 117.8521836 183.1728448 29.15508056 48.05321317
77.85728222 49.44515642 16.42178108 35.1722477 81.80338925 17.42986379
11.1988627 24.63797792 103.947176 201.7522159
NME8 4.031081011 3.928660473 3.866557468 4.66238798 6.421545431 3.210667699
3.69898121 3.740216058 11.91914197 34.0487377 253.5462988 13.4174629
14.82529394 5.97286223 97.09045136 422.959594 24.14230852 7.978826221
58.47502369 23.33658092 117.6088791 10.36459567
NOD2 6.932520098 10.18850108 224.3125249 8.398706067 8.568369563 124.1410057
147.9736788 116.1121546 42.78540525 146.6310156 178.2578801 166.0621042
1302.129171 91.56365214 2182.907505 811.2539416 31.53202085 249.2072515
31.6866415 7.939244863 617.7232142 689.3755544
NOX3 12.00966261 6.682825624 6.670735069 7.382208399 8.615516504 9.107287855
7.489070848 8.533286435 5.486578437 66.24889709 7.475615219 7.106612265
11.17305851 7.877966901 9.70690203 8.992612544 6.581035841 7.139098203
133.3117235 215.5792618 6.271681559 15.12937746
NPAS1 18.22767575 16.19541233 27.89933234 24.54202898 10.25273904 20.72030953
8.311325156 8.59290329 159.7592111 12.08977117 12.18029946 12.95560951
14.37797149 15.36265788 12.49569028 17.39161784 15.60612542 15.18946538
22.04957549 13.78738275 19.27166644 23.07570972
NPIPB15 1624.147916 2800.237224 1335.65936 315.43784 11.1028997 753.235961
217.6161372 262.1479746 1476.413161 908.3810389 288.973686 230.6806713
176.7823779 212.5601012 282.2159665 370.1582078 246.2668474 154.0423997
82.55559576 173.8764126 399.8752628 1079.514585
NPL 18.58723205 15.43171438 51.43557494 84.14120251 16.80025027 77.75838355
115.4350344 26.32189818 67.97537441 151.1552852 98.03373398 113.5081582
856.4061947 1653.078338 501.9616375 4287.536344 2046.531199 469.3198548
552.9189042 434.39859 1095.722226 2221.125735
NR4A3 136.961868 146.6985292 133.9396044 83.36646929 117.4897219 96.79674591
315.2674688 165.5835013 462.3914642 158.621241 81.37480444 142.7268857
91.30097224 198.7422467 448.7091592 100.4851433 479.801155 2205.779288
126.340664 1237.55158 4000.433332 289.5950348
NTN3 6.560415317 15.17015534 21.48427937 8.668709617 23.29056908 11.48733325
28.15146387 7.668026751 180.3938042 39.58499503 5.960532656 6.670735069
8.728208573 6.387053062 11.62316713 15.7165782 5.550003213 7.932018996
3.851996371 5.968588791 12.89560396 14.63118719
NTRK1 13.56995609 13.73659599 28.05481762 10.62433837 22.8422122 15.01956589
21.08132558 9.834897324 11.27070692 17.70640663 9.510068915 13.60956204
11.91460935 8.11603997 8.77366203 8.95713845 14.87419345 8.220089782
353.8858453 1165.767806 24.83620744 16.05784624
ORC1 23.68477152 41.00915539 30.47049066 48.78745757 40.53053139 49.69010165
571.8507896 149.8210195 27.67048227 17.56601887 28.16743979 199.5193663

42.26892867 50.27542876 21.53089091 49.33707158 43.52073776 20.24691855
138.2764869 141.3227716 139.6579855 15.70455066
OSM 2.05303015 2.708641993 2.05303015 3.823397028 2.307902038 4.818030863
45.01752131 12.94364358 142.6507045 20.55380014 2.264192614 172.7170749
4.071675747 3.540709893 3.129845825 6.319098182 10.91034349 3.537000212
13.94683468 8.408928144 1657.870692 188.2377246
P2RX1 161.7246112 37.90926885 36.61226829 180.7978777 168.4091537 119.891114
110.7662487 142.8858929 70.45015588 184.552453 133.7653785 137.761379
686.8592062 198.6785267 134.6490049 356.2137024 162.158759 57.63014286
2865.596717 2193.457858 1302.716482 1169.401245
P2RX5 9725.14216 9116.674392 2524.75311 784.9884834 670.8311858 770.0178375
524.8550881 2876.602126 725.3438736 1108.125761 507.7460936 492.8199974
175.5409499 33.05027296 21.68739568 20.0115018 37.21278271 35.5352843
174.3664719 298.9924978 355.9021695 35.46460502
P2RY10 436.5800269 348.9025998 408.3799473 319.1050423 340.8834245 516.3687437
293.2102273 313.2896621 931.7085834 107.4410957 236.9914349 207.4981375
20.95780349 11.36968834 20.4848163 16.64319163 5.340759621 142.7074755
27.33260384 59.53712782 1812.068581 350.0244998
P2RY13 9.195676707 35.59674022 8.020483187 7.470609935 7.357242825 6.469992914
7.274663001 6.982740503 10.2025435 233.2139703 76.92819841 13.81492461
2786.279688 120.6493256 473.3335035 2965.888162 25.78957652 8.194297426
43.78982454 60.88154521 1447.836993 4885.264485
P2RY14 1638.857158 196.0477382 30.69853755 154.8611784 63.19960659 75.4038863
76.77594259 33.74435445 102.9533339 512.624542 214.6448472 215.2283477
51.7107481 7.681017154 21.35853068 852.5563739 214.7529288 24.7275648
2202.531209 1476.439279 9767.658851 1604.23718
P2RY2 11.65755142 13.6018949 9.903062667 14.97257129 11.66918119 11.41545947
13.35591205 20.63255455 10.1169561 12.42139783 14.83438983 11.91498962
172.1808803 86.58027686 11.44146222 9.463569762 10.69402254 14.59353766
14.46354526 89.76543076 944.4078498 20.46490608
PADI4 10.86384541 14.72490782 16.99336407 54.8131472 22.06014827 15.94453415
24.61338222 19.92287086 9.858987446 140.6718371 117.7115047 26.4439513
765.8740271 15.82068937 17.75900367 12.5310046 21.13767299 36.58022188
110.3487033 145.7041526 609.7225739 834.1791302
PAQR5 23.55278029 20.52510491 15.54095466 14.17914437 25.79852882 15.95742958
14.88173325 13.05907552 36.93744762 28.61895 16.92537277 12.69616964
14.23047428 301.5136307 18.28085747 17.46905843 19.08196331 15.76945271
367.6396247 291.658959 16.33922468 21.35009578
PASK 513.8685117 628.0406815 674.8353905 1441.389436 1036.993881 2312.349458
982.6694359 7489.424599 1422.863691 232.6670491 194.6949707 256.2684015
146.7089795 114.1939024 240.986393 221.4207731 159.7552188 188.6396292
189.825041 170.7980542 412.5430957 260.1254538
PAX7 67.60022415 81.26725901 281.5200465 23.31147251 25.73025482 42.55469556
34.32244633 37.98662268 107.1993296 47.57678524 13.99767158 21.90774996

38.03678229 20.55062936 24.24874392 19.33487809 32.20861554 32.68914412
22.88582143 40.26434744 82.85959644 43.89458066
PBXIP1 399.906182 918.0224791 803.5855937 1520.324793 1070.73357 3186.537019
192.0869901 1612.60149 3045.590782 570.9583968 527.8700969 195.0766104
253.4748335 80.09674175 197.5776496 410.8735418 290.7321071 562.838958
173.2265498 203.1679252 835.0912646 980.0231145
PCDHA5 5.122171371 7.929425728 11.42909979 15.43591938 25.92410763 33.86467066
7.73695061 11.02174746 107.6797892 43.8130913 10.21122017 11.39071505
10.28963858 10.55544651 85.93860365 8.255798098 13.64170835 6.045929382
10.74438293 6.114001431 10.27064822 12.67803161
PDCD1 103.8732743 124.685259 105.4403669 153.8622347 130.6987203 149.0261337
234.7672258 2598.761593 263.9482706 208.0893621 128.5338061 122.4494002
153.2622842 114.2409952 134.5949207 133.0710845 111.798525 122.1858671
130.332615 95.2768144 223.7380224 277.5988266
PDCD1LG2 17.24705732 11.81520376 12.20918656 12.8049967 15.10593558
11.03474465 14.41666747 8.147551977 75.96003159 15.66812358 22.01108215
12.92033658 14.01790012 55.0450855 448.8743703 343.4394084 116.3739956
813.7897771 9.979746792 18.43935219 72.1116222 22.62515879
PDE6C 0.972464723 3.751282949 1.479085222 1.934003663 21.41944377 6.212037366
1.658857567 6.606824787 1.646250183 6.317187616 14.89875645 2.387820415
4.556478651 3.190692616 2.992189132 3.505149256 1.993538338 10.57243953
8.281616068 1.119174869 46.47392838 2.18143109
PDK1 32.80967907 24.86674344 1399.416902 231.2949243 518.4525497 121.1522918
170.8986502 117.2961766 486.7488725 23.37025579 32.92518869 37.96319899
25.63097887 126.8741645 131.2576257 121.9445976 96.83583568 22.47364617
23.67746813 30.15639337 571.5173059 43.65251141
PGLYRP1 18.90822468 23.3446173 20.11347878 9.957040446 9.789026033 15.62904812
16.08395924 19.77039029 12.62509239 16.07793448 99.27421716 30.4274162
13.97992365 13.38044445 12.64904623 15.47869427 11.05128893 16.05671329
237.8880314 262.4990034 752.182952 1587.726613
PIK3IP1 2462.087898 1132.15121 728.3720212 5245.606657 5090.528151 4353.539533
461.309405 2315.263539 4814.01712 1330.849657 1203.06945 322.6103849
647.7544918 249.271088 209.4648222 1631.804333 295.0789727 259.7420759
557.4000388 310.2471426 780.1455706 545.6500777
PKD2L2 9.70690203 6.77668278 24.81718933 18.90534167 52.06339581 11.46263134
23.12217296 6.039291579 47.37634103 47.67860232 10.97872887 7.924164041
13.39656756 9.864099442 12.7431858 20.11347878 5.885376139 14.50853995
160.8584776 48.32066723 259.9512623 62.5622821
PLA1A 35.62073897 28.11928425 15.15649598 16.1139255 16.45891984 7.843078446
14.26798853 19.93877192 16.46804088 31.59116116 25.1394749 12.84867382
59.35576681 26.2500019 2799.769624 14.40398388 38.04099365 647.1074354
48.99703525 43.37084455 53.1551885 26.03523647
PLA2G7 100.7324553 79.06118962 69.94065389 24.31875779 12.76669965 36.51263796
39.26542516 37.60063067 108.8451808 178.7417662 73.09936856 50.30565877

1290.202807 10066.60469 1679.064856 1569.463396 5706.822398 5086.100024
1067.947829 980.107544 43.5153618 59.09237491
PLCH2 31.91347153 14.44424591 32.27097928 26.92691404 169.1927281 136.8549573
42.93384847 176.0842582 981.1482044 71.66407227 152.093623 132.3765006
47.06577352 31.4082709 44.50406981 29.6256784 18.7750035 30.87091818
36.2777495 74.7391603 102.882811 101.4466056
PLEKHF1 39.92144865 32.66376393 301.5187063 884.5112502 341.0076265 508.7385617
485.6449165 919.8846228 373.2850334 1042.95957 1513.840196 982.2000845
45.53332862 292.5202514 73.43952307 33.49248832 40.58425981 34.5707663
73.36688598 281.0035199 61.66664344 50.11176168
PLEKHG311.39071505 10.80558704 11.07886417 293.5247521 76.73435744 559.7419793
106.6290931 26.01377304 17.7528531 927.8029843 855.4915686 317.7910043
417.6498993 137.0428414 590.7969437 180.2721835 25.67875771 21.42126529
204.7763582 37.68321735 1449.679742 1603.250854
PMCH 12.39999002 10.5949733 16.56020185 7.056096164 12.96669349 14.81798486
138.142275 9.800664188 9232.536023 19.44909386 6.727160344 11.09025115
9.49164333 8.822055734 6.066370195 7.417755579 9.763369734 11.88490948
10.02000176 32.33716932 10.81046055 13.42857679
PNOC 4198.527432 4255.796399 3031.838783 88.67789621 64.69325183 65.69416471
83.02319643 22.1078749 82.8028802 192.0353994 65.36645851 78.13755037
230.8474814 93.57509016 42.98994774 92.99539565 68.46161307 98.82686845
62.52678004 105.3515894 156.274342 269.3613294
PPBP 112.7304414 89.83852158 166.6443042 61.67249965 65.62156196 58.2388091
20.89581929 63.64624142 117.7291809 476.6620695 37.90535113 17.49572851
80.46672814 3754.147483 230.1459177 292.9918215 91.22599046 211.9393609
78.2133217 1402.0838 52.02064599 564.5799005
PPFIBP1 68.77420162 10.81398033 17.04707726 40.76620059 45.86348711 26.78347553
19.75629479 5.459102119 41.78961635 11.9742 21.69814253 9.753147704
24.45704984 36.42488944 6.90617027 11.4758086 218.6205764 76.72712856
44.20420842 30.45109048 8.539653683 12.05579356
PRF1 201.9573668 236.1351207 213.4124783 8182.518825 847.8104385 2130.073632
1288.368436 953.5011061 1643.00791 15440.12701 26164.60132 27164.52012
142.7487889 88.71604457 55.13574502 23.15489547 67.86490889 103.9210768
346.3646077 285.2205902 268.3717602 422.6697907
PRG2 98.84281244 125.3109437 157.5298559 126.6467707 107.0290453 139.1010495
165.4537351 35.31282708 68.52536924 16.54700608 70.81916153 82.7123624
117.086355 103.3752118 295.678865 263.6529152 85.47893602 89.83062765
19061.38442 20880.60329 189.2703998 59.65270386
PRR5L 114.4648121 191.9719939 14.28013525 302.0175264 88.78052856 230.07536
127.9674477 56.48122719 70.06950907 758.3736527 1048.389642 1138.959696
109.1297143 36.07946691 74.93090365 109.0856459 12.1948795 77.23080779
335.8228197 374.2790764 432.931894 183.3709267
PSG2 1541.92252 1234.841312 423.0944463 157.1462452 109.5312437 214.8615441
99.13536849 80.92660206 294.2433074 352.7444825 148.4491223 136.1349156

170.0696655 156.9681652 95.30143834 126.992246 116.270557 146.2605667
80.50482289 42.86988631 61.87660914 553.4992818
PTGDR 84.74897834 75.93103964 34.61104034 549.7297474 59.43850829 135.8294143
56.51547019 38.93185048 36.94548042 407.8586616 1627.918557 2044.535858
36.25887091 25.25504861 40.98318506 26.78347553 32.57760806 29.80079173
21.23770371 29.11895968 137.7868425 79.20265559
PTGER2 47.37880726 20.49640288 21.50099156 2260.804067 258.841965 4236.414392
1056.864994 14.85589132 2608.201318 2957.932915 2983.001501 5770.127086
1154.310555 619.1628375 170.3953887 1754.337137 584.1241872 826.8557439
460.4294121 892.0718245 501.0228289 183.6977894
PTGIR 32.06615205 21.65542661 30.36307028 46.51032228 233.2462403 59.60491785
161.002021 24.85041048 1296.620335 31.97751482 31.06375629 30.86641388
482.8617503 37.79124289 1832.0701 1096.655917 27.40247852 1333.258674
196.2521481 113.9045242 26.61063961 47.10622736
PTPRCAP 4113.169696 4036.430692 905.8167534 4378.93295 1887.920817
4607.979618 1493.347557 5576.724303 3413.915859 1703.230685 3602.345527
3305.985414 35.76573661 26.30209546 18.44492663 30.51218687 29.6087852
51.69649463 42.2402032 16.45954984 351.6052781 40.98388057
PTPRG 106.5825021 96.88881184 84.32615296 3.798829584 4.273621867 3.949271467
12.60267124 12.98839722 154.7918901 67.01744049 8.39063172 5.598819437
6.373268195 9.600273699 31.75264764 4.706761986 8.931399029 18.24345684
9.872674744 2.978201507 2.992900342 109.6278029
PVRIG 874.708657 1156.813644 303.2499758 3978.913368 2515.245009 1786.799569
818.4743673 3927.763769 2477.585452 4095.753219 4533.058778 4025.406714
99.88281165 68.65638518 20.76445065 113.3694758 57.36224685 96.69210326
87.81532271 17.22725976 164.3468784 357.6545327
QPCT 15.58990295 25.78610834 2273.276543 24.03521349 3.411622987 22.22269743
65.08699055 2.011800929 12.20302577 22.4979037 69.39293926 48.52460385
1499.926054 2646.756425 113.8352122 1278.360026 1578.799624 420.5800847
235.9062116 247.8655529 38.67780446 3236.166445
RAB27B 7.831610308 10.49747881 11.65755142 25.25617138 23.27195892 35.43354856
10.79075018 22.51380495 68.21956759 40.13993426 91.19546276 82.18072015
6.931516995 6.062575043 13.50066477 31.34081799 13.5363234 29.73319865
907.9390023 649.7572478 28.80815534 8.590150366
RALGPS21597.105013 1305.477004 112.819143 57.02675161 40.25707294 23.42229647
36.80214677 28.21199417 98.90129916 85.48154309 54.56356691 32.54129812
44.85995177 27.2457713 22.03161941 63.44359344 37.70351551 39.5414463
44.08881457 47.56182701 56.92763722 109.4299419
RASA3 10.56154776 24.23500243 13.14017301 270.9813064 156.4816454 327.8466714
24.57422621 71.22892237 79.60826141 68.96792103 212.7904928 65.33110512
93.34350756 21.36443032 14.43114488 115.9404818 17.29897036 12.32264713
15.37212197 19.09834122 26.66449652 19.42649153
RASGRP2 5911.213148 5135.507838 68.81156687 2427.952654 4016.523813
2855.39049 121.3399664 169.5987981 1173.438292 997.1942031 1363.762146

256.5379971 759.9791832 41.20902217 120.6402118 102.0421756 65.88033568
130.5735306 281.323554 209.5925636 1547.788877 1260.173261
RASGRP3 458.3600431 575.4533742 3254.10232 16.2266408 28.79489939
13.66874699 13.74179096 15.84342427 93.14886581 16.02504949 15.51147933
19.07768435 11.43917669 735.0103013 552.0344178 1320.702754 986.0715173
1037.512659 57.22500728 90.18575257 77.00158662 15.89265608
RASSF4 22.67712362 18.66101906 15.11794931 22.98193781 29.25535072 19.66949919
23.48774462 25.7630517 67.82146858 20.64721465 228.5281547 146.1955683
672.1035142 591.6261482 3036.440707 560.785394 657.8411955 2481.685967
39.53020938 30.91340781 15.57730018 33.77387846
RCAN3 46.66611859 40.66450557 41.29948278 155.029818 219.6437293 566.9862725
193.0935915 28.81247015 332.6704532 28.35364012 7.23560514 25.01169532
41.68337282 54.98818208 37.60063067 28.67150516 30.55512091 106.6270408
6.230147517 35.07280711 10.99141575 5.269930927
REN 63.75570668 51.28033369 391.9641115 30.74953796 76.81339366 42.47010982
37.29867542 35.79486574 74.05101213 25.63374355 26.14898837 26.42631865
31.44891199 30.59695762 29.94374704 26.03111393 25.36954972 25.18446444
87.85793968 51.61065942 33.52436889 43.29342529
RENBP 53.60553688 41.69053434 23.15489547 45.59103269 37.11302941 34.34525827
40.61494394 18.33363903 33.37503878 37.66459052 20.43861955 34.34525827
215.0882638 433.0934113 777.7696598 1584.310866 205.9372141 113.6596094
101.6442499 192.9259608 306.3902752 77.08759041
REPS2 40.36292074 27.95271373 23.92087663 13.88756297 12.08950821 18.95216894
8.288872254 18.57128353 22.28002508 19.43901612 16.00616409 21.71773363
153.2877211 14.49580783 9.789026033 171.2308983 46.40358284 43.11555986
108.2496101 19.0519557 735.5721017 1345.638366
RGS1 180.1991847 246.271589 742.0017818 646.7216655 215.6731441 1496.611591
1321.498692 1981.049393 1384.645326 110.3073506 147.0419785 255.510178
64.44174947 147.1485549 184.9939733 63.8234625 62.10361148 3515.825678
507.2404796 474.484624 6082.807012 111.3503483
RGS13 71.26574474 70.49121711 269.9126854 26.92097022 11.20330491 22.22269743
7.843078446 7.972776848 27.72115697 60.00992468 36.27118978 29.94374704
18.73599553 9.041316875 5.136655175 3.828607749 4.083493393 34.67367816
2908.942653 3857.995451 1591.017083 19.83442204
RNASE2 186.4718703 164.907335 350.7677573 216.8731189 156.1334939 98.76355416
102.5188931 83.75974388 107.0530669 600.512299 663.0545505 230.07536
3614.28076 144.2292522 407.2225704 677.7138981 143.2632325 145.7209604
1975.141115 2222.694963 6527.495415 672.1905923
RNASE6 1799.683549 1157.232625 2281.114952 17.05134667 28.13135229 64.4438491
8.863552717 22.17275389 11.37316837 777.2554078 84.58792101 14.36766267
4038.448716 2527.65046 306.1693936 5075.103934 8418.335804 853.6151668
681.3654348 506.4674568 144.0092188 2002.128163
RPL10L 36.038732 70.34302342 311.314882 321.3503467 328.0740952 588.7015627
346.9414841 160.244989 144.4448434 184.4753417 91.27669248 98.92836489

95.70491339 57.31550918 53.34937988 70.33336588 26.98167014 75.52316136 182.7339103 140.7121505 24.82149691 13.6945175
RPL3P7 76.85048537 379.4864795 1151.460639 1290.949662 4477.729529 3370.859953 1067.780098 2289.139001 1429.669295 649.1975723 256.6761145 55.10360905 109.4607889 25.67577369 39.64729475 31.93512985 20.79837075 74.47742389 459.1784166 252.4467177 266.1820622 142.5383492
RRP12 12.81515335 12.57253314 546.1354514 10.83222373 10.26293695 14.31729413 378.7293568 17.27184239 28.96853799 9.389333103 20.59701131 75.44491958 288.3451691 98.84973377 11.72814867 14.51466222 35.49710568 11.84870047 26.91471649 173.3527607 930.2305857 274.0937737
RRP9 9.544532505 16.3321486 17.54854083 92.36151715 116.4244851 49.53085908 524.2815539 82.56301801 21.61705767 72.63989178 53.29234769 186.6021403 36.13230183 164.9509697 183.1808863 91.16319084 29.29745433 16.43308988 110.6197466 173.3669546 45.90579476 9.945088387
RSAD2 215.2283477 211.2148732 215.5173489 164.1021811 230.6806713 222.8577454 149.4255108 101.6083258 852.5910812 390.7076696 300.3481477 666.8036659 334.986335 78.8723585 6976.481671 140.1673526 115.74094 7545.527873 399.4351209 312.6203522 1070.378854 713.3593658
RYR1 108.5578105 115.9141217 61.53204161 134.6786684 13.31038127 134.9186388 161.9808797 214.1715785 1289.511214 142.5812776 38.11141231 76.73435744 128.5919034 205.7970663 72.99488239 102.3003102 117.1694825 105.5402507 15.79705542 55.48825404 75.75195307 199.9238732
S100A12 190.663133 205.6096122 206.614297 103.0892269 122.0464109 78.96882194 77.60907212 114.3269206 93.856191 2375.556565 1088.208915 220.6263312 11149.65069 143.0161359 258.3004281 123.701236 132.8315959 138.8702312 1054.174516 976.5083429 374.4516108 8781.768452
S1PR5 7.997120003 7.027494673 7.65617701 41.04900098 8.45170015 6.547727883 6.843334165 8.886630463 5.615635204 13.06706142 587.6581174 175.6975199 6.788452669 9.800628668 6.633637977 6.433693872 6.920113656 7.668039292 8.974214161 9.124929522 8.09184729 9.352910216
SAMSN1 197.0884279 441.9005895 574.9216874 794.816235 609.2761383 1660.128114 2859.409338 577.4007393 1973.849465 1571.696585 1266.566015 2876.602126 1412.29406 645.6886884 5090.528151 3133.89778 2070.552906 7507.176097 6327.078194 4360.895256 11409.47833 1654.100485
SCN9A 7.11928632 7.905938062 8.439383107 5.599396915 20.4393522 6.409332909 6.276977095 22.14183418 39.74988474 56.53456409 11.24582219 10.59412632 6.128985193 6.720861612 111.6046467 88.10035668 304.8088511 6.084883035 61.22511339 108.6710445 17.76010304 15.23819177
SEC31B 64.80136266 51.08175979 101.2389769 423.4721951 539.2656032 63.79022174 23.57209837 163.9010445 1073.503244 58.50379417 248.3195373 30.97425375 136.5478618 33.18258928 58.5085105 492.0692023 38.33774434 29.75143595 207.472637 231.1663064 179.5015829 40.56722012
SELL11102.0298 8002.61479 376.9049927 7418.862396 9805.46284 7104.510327 2531.49842 4188.175594 5165.445456 10585.56882 5741.494585 1590.659482

7242.740165 103.9204555 234.1716363 287.2191393 121.953751 137.3301011
353.9899758 288.7165574 3001.389383 13724.93946
SEPT5 10.89927471 18.06590222 52.73721382 13.1121548 56.7692406 37.98662268
43.47348792 15.39081454 256.754317 93.46696057 19.49414625 22.41199796
16.50408571 14.40167057 27.46127525 14.79336806 14.32019206 25.86887464
23.98393978 23.25211628 35.94016466 25.73609401
SEPT8 27.7175438 22.87983558 37.65356333 31.02782537 35.00087493 35.93880485
272.6170427 126.6447075 33.16765019 30.19637375 26.97577912 67.28417387
15.62108755 27.18926965 183.0663448 72.01025469 34.66782519 19.83223519
417.7817187 267.4681455 24.51115472 21.77777366
SERGEF 15.69138202 11.98596801 11.09025115 63.60787074 264.2358173 28.58789254
16.22879037 189.1431751 15.82118936 17.27020744 64.18194259 41.5765162
20.42165917 24.06055832 113.6054513 103.0111836 16.8397593 19.0610534
88.98322459 39.40617951 19.49842933 16.74391215
SH2D1A 30.86641388 23.74579306 17.02062149 1249.210018 910.0374967 1354.892078
1484.478664 2984.358981 2057.444392 1522.337958 1296.002366 844.7081417
18.41801556 17.33796088 17.53465103 11.39071505 16.51251838 27.6285163
22.17158328 43.95158903 29.31150533 41.8750989
SIGLEC1 258.3004281 149.3986543 98.52007343 81.50052589 55.54431252 91.07262834
46.5723539 55.13574502 132.2882244 181.1221996 98.8058291 88.81589691
279.3221994 116.4299735 6220.794765 3457.591061 447.6079282 2742.909524
185.6630518 145.6660089 126.3742217 245.9321054
SIK1 5471.704926 5620.453994 7279.249666 1219.200313 494.4265452 410.006561
162.0409056 4922.268476 942.2614617 393.1870068 457.6523281 248.2954165
544.8091055 146.1531348 292.9918215 111.2439257 104.8119808 228.1768122
59.70681574 326.9960366 2371.944991 401.5465651
SIRPG 74.41451203 127.7355428 55.74523407 1310.139566 1078.535981 1148.814515
293.2102273 3092.334477 2278.399895 713.8212187 98.34637628 57.88335321
54.8626275 31.73696814 80.84943542 39.50876033 30.07003805 83.9411784
13.59528084 25.50236675 81.76982471 125.1446974
SIT1 759.1593175 1505.448039 424.4791722 1457.873439 1418.51616 1612.60149
927.3906158 1676.239317 4164.941026 1324.14278 115.1265171 107.7569918
99.63796028 121.9743848 60.40293882 107.7569918 411.7305737 77.55169941
76.7881106 51.40969236 76.98880064 286.5258188
SKA1 4.914356682 2.770472888 3.236183763 2.220631652 15.89364427 1.658857567
353.1886192 53.82754533 32.1969624 20.96016433 17.19149842 41.3563524
3.198489691 4.375489179 6.57228761 15.6035437 2.81889331 4.751056832
30.76766673 18.08123801 5.435240974 2.093797389
SKAP1 1378.742233 556.9267569 875.1463025 2273.257742 2670.874802 2395.678989
814.2486746 2389.171966 5831.851105 2886.347943 2037.376281 1513.442454
155.1060459 188.0291943 115.4350344 100.357131 168.3391756 153.5290423
498.3461422 480.9894709 445.0168928 388.8474803
SLAMF1 762.0351121 515.4872027 1019.767442 496.4217166 449.8358705 1081.526073
2005.427058 1074.944299 1504.845258 720.6522405 114.1243297 115.6197705

111.9089299 131.7093126 6057.915205 845.5065088 126.4411906 801.5218011
58.05601406 157.5576331 243.9828796 260.858135
SLAMF8 156.6501475 135.5923959 79.43023238 107.6408152 72.69074036 101.4058634
85.97854784 48.49238871 37.4347836 218.2134567 170.4258874 95.17401569
166.4059352 2483.002598 663.4733228 1407.517431 2859.321256 358.4664294
237.642389 389.3018874 111.1519762 120.6524521
SLC12A1 89.4899425 48.93661716 9.107287855 10.77936011 13.59723596 11.20330491
1.863695454 1.863695454 20.23145003 37.92005759 3.639923264 3.866557468
3.335133412 3.699265138 5.19508538 1.712065726 2.708807426 1.786805461
5.250153157 18.19115834 24.7590821 8.293088602
SLC12A8 7.225818568 8.434874182 52.06339581 14.91996119 11.19206299 3.740216058
9.306658239 34.82375423 48.42252291 7.503468941 10.14791696 18.06584929
11.98809147 466.0589582 24.5564624 25.3393543 37.95714189 52.24193196
447.9225018 581.4055879 107.0092016 13.16312363
SLC15A3 9.050871942 202.4492561 10.26293695 18.5710453 27.12597858 19.36453188
13.09271261 18.79636185 9.872890222 74.5094252 11.74522644 8.498902895
1682.54452 4564.069594 3860.541888 5890.338133 3688.669477 4650.121848
321.2954407 438.8157376 20.39019926 1431.042545
SLC2A6 112.56368 313.2302692 42.90224117 43.07557759 8.055918939 15.58990295
24.8858211.07886417 17.54138316 213.1524507 70.73672817 125.466199
623.8936693 328.7462744 6057.915205 579.7336116 500.2261802 1981.204438
552.6577118 528.6359134 14.41361211 12.17469756
SLC7A10 66.78073716 84.61698111 110.6318514 33.20097043 19.66949919 53.12123129
44.81896685 626.6515191 23.56884003 14.77666766 31.85028897 24.36857837
34.30894984 52.92098872 34.7291372 26.32189818 37.79824063 53.05335639
47.67700578 11.7742041 34.6614461 50.97452513
SLCO5A1 118.7296898 108.1291054 82.29386042 30.06870751 55.91267865
70.96791001 41.79365254 15.57807487 112.9325737 35.53050559 73.09344194
49.59274467 40.69340961 42.48673066 954.0382072 39.75712842 42.93594304
1056.080519 14.98054654 26.31428316 29.11032035 131.4344977
SMPD3 70.72102235 140.540597 328.7366695 70.12682543 148.9719357 47.62155017
17.258479 516.1544785 27.3493063 99.22270606 89.68398839 154.573264
35.63627466 44.17497653 66.63623186 116.4670669 23.5940494 53.33653667
15.1723682 30.90533172 1685.7279 246.5509419
SMPDL3B 2.724372492 3.034400379 6.771782908 3.73037929 5.877194227
3.847184414 4.624418991 4.914356682 6.387050179 9.073790284 4.588512766
4.182493252 4.661740608 4.125782205 4.751056832 4.031081011 3.678959719
4.248305374 4.795918331 5.282496017 155.7849254 6.39158455
SOCS1 27.14793524 25.09172222 16.85549775 160.8027185 64.4438491 117.8166952
1034.179076 255.1479692 604.6602261 80.88085602 49.70265419 1230.201795
20.71591309 82.36571476 2300.259226 705.1354113 103.7199643 398.8444411
50.34356835 59.60816677 52.27131121 31.69383195
SP140 881.125548 2397.760361 1001.792706 422.1251624 367.6750359 549.3361922
376.9049927 529.7231104 659.0216126 475.9591297 411.7724421 385.2475659

107.9949811 76.35053504 170.8986502 30.82412721 75.79589461 212.573622
54.88954044 26.60936953 654.2750566 218.1702966
SPAG4 16.39614273 24.95714972 3657.844908 60.34817877 79.5891519 64.66029759
120.1755798 87.31662416 50.08813703 83.87858963 8.399871249 50.78803941
16.94563165 34.0400967 21.82778451 11.61080204 11.74593292 50.70004672
41.63965234 106.8030875 52.47902692 22.84191822
SPIB 3945.300733 5363.4372 128.9672433 67.02484651 111.2439257 74.71026363
74.10530922 88.49719564 12.73818081 98.69157038 106.9259138 78.25416453
116.3915428 57.81514413 1435.057428 31.21088513 57.53286072 40.31588104
75.17714713 73.12638767 83.38153908 272.8204886
SPOCK2 1531.023729 1120.73105 433.012581 3283.92381 2040.442671 3212.756292
1022.450995 3128.50636 4263.9774 1757.141517 995.6524365 650.4634003
263.011432 175.9842183 132.2193618 118.3071595 202.7159891 217.0461442
211.7278211 172.4173117 437.5889734 594.0103963
SSX1 1.658857567 3.900272217 3.286119074 7.384988622 40.80982734 2.493139629
9.812231302 25.44533792 117.0747287 58.08304612 2.161370142 1.479085222
5.167103334 9.739377312 7.46633806 23.76513175 14.99089205 26.61872738
26.03413905 18.90635556 43.12335361 14.77955057
ST3GAL6 66.60162565 34.3893867 66.14136755 7.158395479 12.96669349 6.482478073
6.982740503 20.88194891 20.23462109 45.97919713 27.1082918.38880596
357.9772296 524.564052 41.07506248 192.2179775 398.9464519 1251.448838
742.8329889 710.1274498 1574.930342 466.5209967
ST6GALNAC4 264.3270269 441.0048109 2093.524176 408.1100167 297.0293513
398.8717851 276.2455792 263.2789632 799.8515516 351.5954793 240.2635016
227.0422204 195.8463286 688.7351586 133.9832531 243.2991012 349.1318417
182.6270057 113.4914762 129.7475492 134.8323638 181.6890388
ST8SIA1 50.56172691 44.96471016 9.741848192 75.93922657 9.129359328 278.4319646
97.33937156 2924.590009 32.76973467 78.19146733 6.718345522 14.10673265
8.218778885 5.382915835 13.60956204 3.807870165 6.257607885 14.96016422
313.2838676 62.58965914 5.018635304 15.88264599
STAP1 3534.504408 1961.960801 983.2371048 112.0597302 432.5194514 110.856908
108.0607513 92.39233333 615.5083627 68.95913217 299.8706305 435.480525
21.15804952 25.9443561 372.9209445 43.53483002 47.63471034 120.7564143
3297.989161 2140.766305 831.8707238 48.56097434
STEAP4 4.42067119 8.353897982 7.819769102 4.832288844 10.49185361 2.346954138
3.411622987 3.59401371.18929282 7.879999289 8.756230575 17.27184239
111.873397 5.310285765 22.35085197 26.46384319 48.28814175 4.304290172
33.18345138 43.22283208 25.0235825 800.5703388
STXBP6 16.73521702 15.04379369 32.06615205 16.13910899 18.76887513 21.45135324
14.46846851 17.85021806 8.586358526 14.44502275 10.5690911.99719249
12.89610083 12.90257104 24.24874392 13.12815713 12.40900014 14.61020736
315.8071292 239.9762281 16.29439284 14.80593608
TARDBPP1 42.03948876 48.24713311 11.19206299 20.13104889 56.90002211
38.53463959 63.68405364 29.13250856 4.882051391 104.0260167 13.59143299

31.08142239 9.112054424 40.62396736 4.672469314 6.606824787 30.78464147
22.03505447 18.59746591 23.04525243 47.62167267 62.01117545
TBX21 504.1990666 543.923946 450.3185771 1658.322072 162.9916872 522.1073862
1173.96483 445.5205989 334.0503226 1805.133857 4208.888552 3311.978393
273.2767975 315.8607585 526.5774707 86.6079297 245.4943449 178.261254
141.1042313 151.2597324 242.2058318 542.1588248
TCF7 32.1556293 157.1737661 24.80221189 3170.404097 5181.114553 3207.171426
351.3659373 4668.361682 754.808733 910.6990599 415.1493446 151.5623553
18.65570294 24.98661146 284.0317902 43.91152808 32.56860072 55.65274077
22.35563875 21.57656523 451.3650271 55.11585006
TCL1A 12117.94539 763.1449829 493.5179123 106.739179 133.6927662 105.523008
62.11998297 138.6741034 108.7230911 106.6455477 104.9994128 78.2920509
161.4604075 108.830925 109.218595 72.95993424 90.24888922 122.3968151
50.03226763 93.0399196 259.7561099 261.7385971
TEC 11.26237461 11.13599083 9.61033962 11.99089142 8.992612544 9.646923918
9.195676707 12.7431858 8.92091341 14.80669355 20.22252914 16.45891984
16.29861436 19.98621302 66.07482414 50.36671815 14.62208914 21.16569802
23.41596618 225.4910315 80.57831321 12.87726499
TEP1 13.00060521 15.87747831 6.805956619 98.35605225 13.17735831 95.04661844
15.47869427 8.393614631 9.251815429 9.550904671 181.4004368 91.56873261
16.10571842 8.991936007 69.86793771 112.1957614 48.0734321 12.2359858
13.60384434 5.707248958 8.370970376 11.99606546
TGM5 60.12655552 71.99773897 785.5941764 47.8969765 41.46606342 47.09380347
40.98318506 33.23725547 71.18774803 82.63306347 56.85336746 60.67685001
64.53380501 55.85317965 69.97949464 126.5453946 58.75579949 77.88602952
118.4957283 160.108741 152.1582745 82.75554452
TLR2 81.54272739 113.709418 115.9850691 120.5282543 135.7286165 126.8421582
102.0859478 151.8480214 81.98559599 438.5864476 138.3345347 140.2158809
4560.185304 793.699493 4668.361682 3071.447657 1202.759716 487.1255361
680.7876421 1591.232654 118.7000496 5374.798483
TLR7 49.81426014 102.4990755 37.62419139 7.997221294 58.36525286 9.70690203
7.262313101 36.29565926 49.5799296 84.85721753 31.8438018 10.83895443
570.6516022 34.35052469 966.7052764 546.9250913 35.19591687 60.11520175
33.4472936 22.98087009 158.4344162 20.0870008
TLR8 7.582193721 6.714925465 19.45168576 5.70074897 5.570829264 6.830950912
5.730458788 39.48271435 5.723066082 91.59801705 5.825720142 6.107123818
1522.070253 646.6061428 2380.195799 1082.104258 553.7198288 667.192622
19.05833985 9.50032415 7.370219005 1719.870237
TMEM156 574.4981382 577.0327019 771.380008 168.0820865 114.8275013
152.9758784 334.197775 276.9063479 221.7312302 207.1593629 138.8828404
141.9594113 23.45170307 26.25281018 17.46905843 21.15896542 30.88767375
23.225842 23.73534036 29.2534512 288.5489965 51.44861036
TMEM255A 64.48080982 43.77493033 47.15884594 38.72072177 5.079112797
22.20688826 16.47169916 4.573719127 52.65374427 63.73438687 49.63841616

29.92011687 66.84987758 354.6259629 289.1639534 167.0152257 926.137278
979.6564849 43.72927667 12.85862889 3.957441844 37.11078102
TNFAIP6 267.9179363 259.8916347 141.1803804 81.29435266 36.71555117 94.24800971
79.12333165 63.61546587 131.8853818 147.6840939 123.7740746 145.8185128
233.4391136 347.1638911 19190.61594 325.3643379 245.9512171 8826.486407
253.2624558 2285.704444 203.2806125 3460.952883
TNFRSF10C 44.84631996 28.85121323 27.82084 27.03922751 25.78013421 57.28934706
44.90546301 21.95552174 33.97430589 39.13114743 20.06638784 37.57632728
237.9058901 48.17879865 42.18070928 75.92997592 53.99033577 48.90501431
79.22645394 82.62208068 288.9786687 3919.76984
TNFRSF11A 50.02783296 32.36541213 28.03580356 9.855680429 6.818438323
22.07591774 45.77175188 14.45611207 930.5271046 87.70315573 86.12792797
68.56018671 17.83610862 37.89907656 74.82023836 396.3913895 281.4799185
708.1258587 24.86947846 80.59359524 12.25490482 67.7636041
TNFRSF13B 502.6927279 1498.256342 1292.53498 181.9551888 307.6962383
120.0804247 138.9893405 181.9526719 379.5542581 372.4166229 227.5044918
167.0688665 188.0078881 158.639318 240.986393 171.9065402 167.5646975
186.0239287 163.0912348 187.8628997 290.7927064 342.7765361
TNFRSF17 22.5997525 247.609295 7744.01596 13.81523144 4.347662735
13.44126834 7.947610014 7.855308418 41.18335555 10.04246187 12.50971798
8.910362721 10.1098504 10.59143865 9.409205288 13.52425156 10.59179267
17.5240948 7.227678736 6.534182401 20.98966863 9.35211403
TNFRSF4 15.01956589 14.8278152 142.8319114 18.71047795 22.20688826 524.4770391
1997.155963 1178.444669 2852.970487 21.35202747 195.89287 697.7043267
17.76981387 20.73872674 1462.50333 441.126154 203.4808519 1646.191207
76.94630793 347.3968639 25.15229317 28.18616394
TNFSF14 10.0823446 9.719004464 9.555358782 92.31649554 28.21199417 59.18404696
428.3545003 168.835817 258.1350672 17.77460958 362.1020637 2050.816822
35.07227527 936.6552461 20.45437223 16.04544391 17.95365709 8.691520443
13.49997975 66.46487192 291.7775578 614.120037
TNIP3 14.12983303 31.867553 12.33024148 70.50041584 64.72785062 30.8024485
446.3091826 257.640918 48.14764737 43.91377403 18.25984636 11.86713177
14.52350675 241.1663751 1192.706587 17.36410025 23.11775258 47.4835514
42.84667423 106.0845587 41.53015417 15.74452103
TPSAB1 1102.29779 794.7203184 151.7313169 376.0368021 43.36581215 284.8186666
147.2741095 481.0725006 28.78893808 262.6432093 465.2204213 311.7403372
543.6290931 358.1005731 368.9130231 365.4798555 397.8866549 396.8599394
27467.08759 29437.28907 918.5119625 1033.132677
TRAC 572.5113556 1006.350088 215.8269579 13721.45552 14057.43026 15532.16188
8731.942274 20117.66469 19538.4969 3189.238319 815.6846136 506.0537577
208.9784165 252.2397003 151.3173183 142.6133419 215.6237237 255.9384554
159.567675 187.1669874 880.0902068 790.0055576
TRAF4 194.7986362 450.3805458 103.831718 27.41737791 35.38777983 88.21790387
248.213136 107.5017488 48.57230164 40.01034813 54.5480586 70.6081762

28.30245079 30.40539832 23.05515036 23.70038398 39.85889969 30.04309979
166.0286165 185.660216 80.71335948 48.24388624
TRAT1 13.5847049 15.98508028 17.40314684 816.7413233 2327.160635 2578.359018
1616.337428 1427.702674 448.2961522 1435.573943 25.38745132 41.18448661
6.845897848 13.33976249 6.319098182 4.273621867 10.65205619 29.29706627
33.59153566 35.53946833 193.0502207 10.38229418
TRAV12-2 13.14017301 12.20201385 9.555358782 225.3780296 99.38436963
100.6104917 12.96669349 85.70898291 9.535532796 11.61297377 11.08896426
9.776317635 10.63253287 10.29206918 10.57301826 8.463162241 12.87147163
11.68801662 7.555697662 9.149337627 11.60323881 15.47264026
TRAV13-1 88.73958185 72.48117143 40.55965894 463.1984082 537.6097159
881.5627754 411.162234 707.2496886 138.6945511 76.99469029 42.45202479
28.3148996 29.72104173 23.48775343 37.77736037 12.25757399 32.45267986
36.23428561 66.13800202 35.7585025 29.26235006 74.99855252
TRAV13-2 72.69074036 67.18924471 68.66194527 369.2792078 411.162234
639.8040504 201.5321093 381.3344477 191.405895 38.54870306 44.35066443
55.64727919 56.85541485 51.02429694 35.79486574 39.12138574 41.21658712
67.13601356 25.93332534 33.83672753 55.62918464 88.04801861
TRAV21 119.891114 65.25320664 125.1327277 439.3576224 417.5047308 765.2981535
157.5889451 463.357295 2763.362726 49.08129044 55.8763643 46.6365232
51.31601583 42.96072752 31.46897563 36.23835539 52.02734337 17.85858588
79.46582934 72.06010035 196.2354888 88.25003141
TRAV8-6 24.5564624 22.1433316 18.81035485 244.1129158 317.7910043 396.1200681
172.8402474 439.2288461 676.1350103 22.66646971 18.30217166 15.40291048
34.39428346 25.30927131 19.19569957 13.1991983 24.16870446 22.76541274
22.73939893 36.64150351 23.43793277 65.39316048
TRAV9-2 60.43870697 91.39228534 39.20610994 220.3744213 498.4569511 911.8379139
448.8743703 656.9831726 1958.896333 113.9953881 56.17040361 22.09272622
47.77691068 27.28669595 23.52091869 17.66320162 27.7387923 33.54720142
14.88500975 38.63800306 102.811815 130.9175576
TRBC1 575.8478308 930.328823 525.0492335 8923.922332 12460.73946 10179.78364
3287.836318 16623.7078 19876.20631 5338.319292 4520.50562 2812.593654
78.94320693 56.8335872 223.4955364 131.0995558 189.1209091 145.715446
114.6022938 47.87130436 551.5102039 181.0122765
TRDC 88.17850315 81.15497901 101.0670278 3014.575466 282.0101621 781.3316356
486.8794819 370.2752613 124.6527289 17980.35398 10797.21174 11382.00872
61.01801703 30.12404697 22.31868643 24.26650252 46.6420448 30.42258616
247.3533379 209.0182876 165.8509884 162.1231973
TREM1 415.2301184 236.6369111 211.1048289 102.2864477 116.8363113 67.49889477
71.26574474 101.4843406 66.50026811 574.8694989 79.85743138 95.75746526
1775.188919 603.8863807 194.7254774 184.6497911 222.6374113 317.7853076
285.3975429 685.7072276 377.9594713 8433.289331
TREM2 17.00788201 11.99552131 19.32045331 16.18514962 14.88173325 16.93010618
12.76669965 14.95790508 101.0745387 14.19593878 13.80468131 14.68548476

24.23855513 2158.627387 160.8316593 3999.017687 6016.863497 928.15125
391.2846329 611.704972 16.04376238 24.6518745
TREML2 70.53816317 20.8510013 42.35560264 23.09902116 50.87816452 18.783374
52.41361977 57.35568408 59.68920701 35.87929016 55.01920603 123.3281827
40.72889444 27.33242059 32.02053189 32.60848091 32.7416014 27.32785335
101.9682716 38.88556376 577.2525196 1082.741035
TRIB2 1848.219528 915.3236717 262.8037637 1138.062023 2278.453629 2377.362513
275.8348106 5890.338133 839.9304115 754.1562238 1307.778436 1049.5197
123.0323791 152.1388719 71.30464757 22.3637411 255.2096152 126.8179678
1949.185675 1394.871276 311.6530554 155.8993479
TRPM4 7.691250656 6.199378707 63.7178043 7.796755019 17.98120025 9.463569762
9.520155138 11.62316713 6.76116021 8.48672507 7.443654876 7.702553769
18.36114786 14.40413982 248.6383328 237.2475823 28.55293563 34.88330592
16.83475086 16.02878528 22.68943065 10.22097545
TRPM6 45.92245257 35.63102651 64.55045854 25.76719358 16.86720259 31.1038533
63.79022174 32.92379314 19.946392 59.15056029 22.10779339 30.99587635
35.69642216 27.7197468 22.77650595 16.04544391 21.83288519 33.4402678
20.64070077 17.97104758 515.1758508 220.431397
TSHR 19.81562324 17.26446055 95.35040447 11.64652927 11.34522644 13.71677829
7.644655016 236.3005486 11.89081812 15.28593998 20.15779147 12.75467855
14.29339998 15.60070061 9.442115885 7.808323788 19.79865996 22.62901303
20.59108368 9.980145399 17.61165132 18.38429613
TTC38 24.85041048 25.03368325 39.64729475 374.209498 118.0039289 26.49834722
57.41736661 172.2330604 43.28600708 427.6618803 2894.462666 662.1427421
105.6462644 91.99909893 437.3613074 328.9820884 95.294272 143.9588074
458.4207043 370.5910615 155.2070688 47.0570675
TXK 309.7698509 195.384424 85.82317784 676.0594416 1384.385645 426.3148568
175.3835932 206.762571 166.3675052 319.0374935 1564.533681 2015.56144
62.96991111 64.00962037 86.84214478 154.3565359 82.39792906 85.15990882
173.7054259 201.212217 201.2173366 173.9732072
TYR 15.49172903 15.24296718 15.0813304 10.627147 44.38209766 197.0884279
37.45017394 6.818438323 1778.505105 304.5548851 71.99864479 51.17855685
14.06800259 36.31423107 25.02917588 31.80183546 32.73142982 12.31353089
142.7673868 104.4652597 13.13836219 150.834091
UBASH3A 10.12600478 12.70263006 8.087608443 876.3804334 2177.794994
714.9395195 203.0783034 935.0097588 1376.318036 319.0941238 6.038374156
8.533286435 6.105136566 4.993003471 8.795522384 8.195551244 4.841232059
5.677166893 13.69796446 17.94318894 59.1375484 10.09959562
UGT1A8 680.5299008 471.1925779 163.9010445 82.13823081 98.52007343 123.3281827
101.445586 93.50382185 450.365002 462.5102909 78.13378227 85.31836726
91.50678971 96.13395165 55.51233152 55.04284584 85.82524233 54.24689175
63.86973215 59.03064075 97.15471911 336.4203983
UGT2B17 29.46022982 12.69818111 309.8818349 3.976053906 5.049784849
5.598819437 4.541614018 7.819769102 8.39783357 10.13915266 4.743273044

6.482478073 7.244351309 6.408970503 7.748155333 7.395068314 6.871949567
9.056114065 5.248499675 5.642280383 9.001148941 8.791555748
UPK3A 15.1818271 14.61683336 17.258479 10.97861145 13.15225266 13.64441885
20.18467535 10.91012024 20.9331605 16.61685711 10.8839878 9.961241592
215.8718015 12.12418525 6.818438323 7.6068206 10.60159881 15.10114829
10.7435039 15.4131337 25.62748279 18.54314925
VILL 29.52223049 34.59261182 53.86489096 434.2235247 693.1658136 500.1222764
32.85440556 445.3565484 38.85635157 209.2510089 215.5413192 39.83941898
97.78124023 43.06693373 305.4093323 129.9134995 21.08681353 20.96596084
102.4969037 138.1446793 23.66126097 20.88408586
VNN1 22.95842907 21.92466999 16.71107549 12.26655159 29.07388553 11.49823256
6.881696305 4.556712661 50.60131657 119.8944937 62.57962649 27.08777116
1024.700138 924.7365315 12.28113965 120.6402118 158.589962 37.60366073
68.02099242 40.75362109 64.48867363 894.8557203
VNN2 64.9845649 79.68108191 51.04583679 445.4839483 574.7325605 87.43886769
7.680556652 183.1808863 81.25132533 1027.324135 600.7634958 133.9832531
3300.115435 462.8123954 6.198523194 8.255798098 6.33332463 7.339096499
242.3176356 17.98841772 9.753158043 18515.3558
VNN3 30.92999926 32.29662574 10.57301826 13.07856517 58.33320422 28.54732378
26.89503329 36.41518352 85.4261616 38.72160006 22.08322245 44.53194026
187.5997533 28.65180995 70.96791001 60.50951351 19.03566519 20.43299213
47.36063389 42.19644363 79.94263155 2232.466996
VPREB3 3778.568055 2591.601205 404.2572521 89.69557529 38.11603592 166.1221545
154.18198 86.99944061 335.2264761 155.1026171 126.7365483 114.2787683
136.159858 88.97247865 156.4260981 111.6979924 91.99785705 129.4706451
73.26322789 68.47189386 89.86960473 157.9569892
WNT5B 11.75096777 11.14541251 48.95771465 10.61879975 6.818438323 8.393614631
9.272327825 17.98120025 11.6468939 10.98399056 6.098592957 9.789026033
15.97840064 11.93775629 11.1694943 533.28442 99.77388102 132.7650466
23.59480205 13.46299768 12.38883289 15.95349649
WNT7A 12.86019319 17.53933245 20.33283198 101.4088521 230.7618491 87.39996412
46.54002774 100.096916 51.70572802 34.57067229 11.09709075 13.79321282
56.25300857 20.27568968 23.34134802 10.34392287 17.52213467 28.44004811
21.12626859 16.1427751 57.16727492 21.34233197
ZAP70 13.7050941 12.81144157 13.36812571 3230.539852 4287.536344 2667.26621
772.7826189 7380.970003 1758.634939 1422.384713 4781.948859 1772.000761
38.05970681 18.46666377 25.55686935 32.52035914 19.14980044 26.83408716
24.08409421 38.3203582 185.4848873 70.04945364
ZBP1 35.84871042 74.5068251 450.6459844 149.2911945 184.7781077 162.5267903
27.55771004 71.30464757 144.075716 106.9247563 499.9477503 127.0420484
24.54237397 14.89516342 16.16358042 22.09272622 10.54629785 25.50635842
13.08437525 28.70340729 143.7111215 269.6190106
ZBTB10 152.6895498 99.22415511 95.56043531 99.88073058 109.5758377 91.36792646
83.06394836 1221.65228 4.246104982 179.029292 122.4353858 106.5387015

62.01375558 65.65921306 222.3082742 74.03494821 76.59712517 773.9507313
117.0610741 216.3804759 144.6296186 20.31580867
ZBTB32 9.857216379 193.1962311 12.93200847 27.83511 29.13250856 7.771095444
187.3923151 24.71873759 112.5857434 29.81729559 16.3475771 12.38711595
10.52648555 10.28530289 223.0243338 14.98197508 10.56712858 71.90536722
11.02017204 8.852017078 57.7649518 12.27627866
ZFP36L2 923.602815 661.8816941 485.6449165 3227.833526 357.0079722 3731.327234
366.0960572 641.8482849 2943.240501 154.9349031 915.0156182 263.103889
1341.516598 297.4815667 23.57209837 303.5769537 532.3725666 124.3001947
34.42499963 54.25282854 311.9773023 517.338976
ZNF135 94.28786122 16.31493562 11.40281159 64.5605419 96.20392388 15.80624293
3.236183763 31.1690733 3.228407856 42.87083843 151.1023562 79.04663522
3.860059178 8.519318271 4.99336445 39.75712842 8.279532213 2.420046884
24.37863287 14.23180261 99.10011749 1.926397343
ZNF165 6.421545431 4.431621524 74.30319945 3.190692616 33.67116463 2.724372492
45.2032616 6.147205936 5.925447852 4.898005565 7.023083197 4.897631255
2.66873461 2.372973625 4.23966252 6.080237169 2.415107373 1.83777562
4.450391447 3.139406165 5690.382767 5.357879252
ZNF204P 77.87689746 42.94621317 41.07506248 151.9597341 400.6631845 168.8951305
40.0906413 52.22897356 83.76317704 129.5986466 37.01330204 32.9745764
29.90974572 26.84363388 33.02123349 27.46127525 25.55386032 36.16594047
38.88746337 25.20670047 65.48783698 27.48554596
ZNF222 9.800664188 7.20321789 11.69136762 71.91125741 56.64251081 15.0813304
15.58990295 182.9389251 10.02779726 13.84326232 92.27882159 91.80829026
19.8606097 70.82485981 47.02936831 148.6222178 86.41818993 39.91834899
222.5736107 211.4937423 346.5564511 26.82559709
ZNF286A 737.8599039 331.9087607 73.88202193 218.2270364 5.598819437 169.3131278
190.8748431 118.138051 136.8685992 246.5038172 225.4077006 199.2998879
11.04393742 13.50584518 36.038732 21.87563888 15.08086286 280.7699313
77.84039621 25.83329275 7.473171396 441.3134689
ZNF324 28.21199417 23.46442356 21.1733262 32.01125825 348.190389 27.16912681
24.0557628 179.5864792 63.46545944 45.38499365 44.94117978 42.90224117
28.71444884 26.20331776 37.88295257 112.914142 25.24781608 23.83651935
30.19634282 45.77517474 35.29328382 35.42469786
ZNF442 19.71319664 22.44753254 19.39158332 32.11029942 8.980486469 9.182953598
3.103039674 44.70063347 47.38616861 180.8575692 21.77015125 40.95236426
12.30022875 31.35256239 71.64242608 36.61226829 48.30333449 96.00867374
79.06662655 3.646156403 40.29650854 20.15752219
