# Supplementary figures and images for: Ferroptosis-Related Long Noncoding RNAs Have Excellent Predictive Ability for Multiomic Characteristics of Bladder Cancer
Source: Oxid Med Cell Longev. 2022 Aug 29;2022:9316847. doi: 10.1155/2022/9316847 (PMC9444476; doi:10.1155/2022/9316847)

A

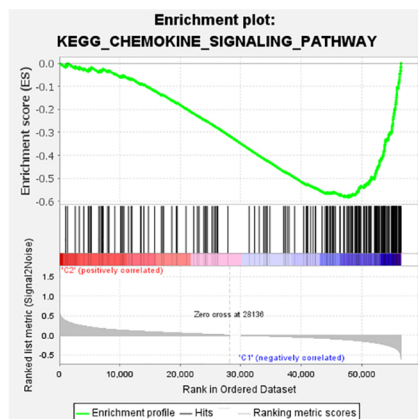

B

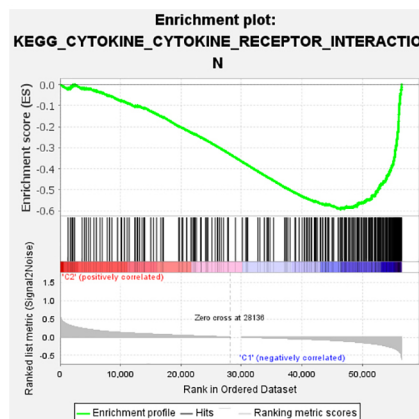

C

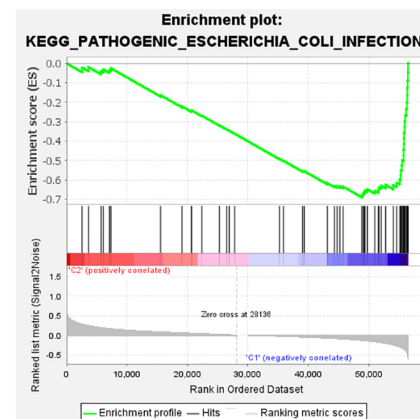

D

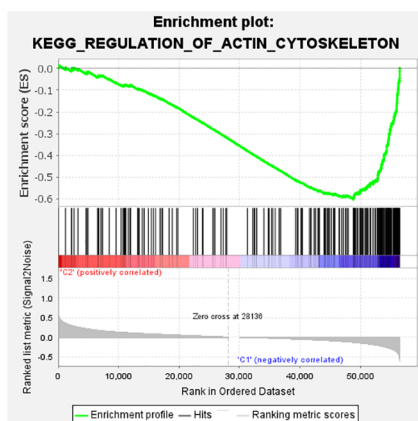

E

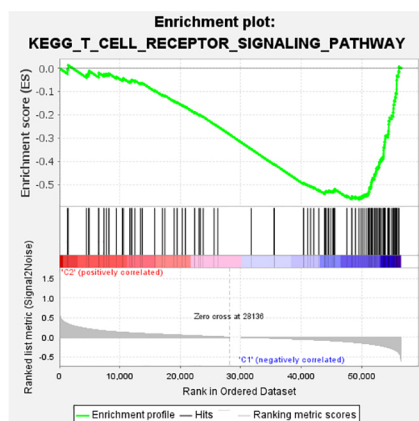

F

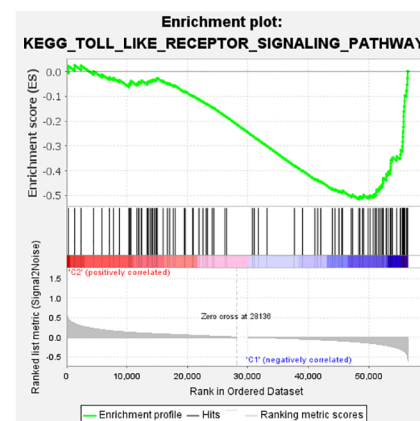

G

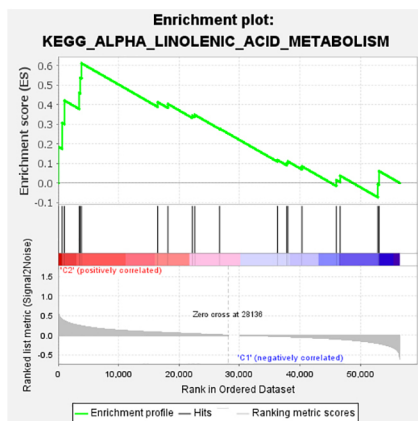

H

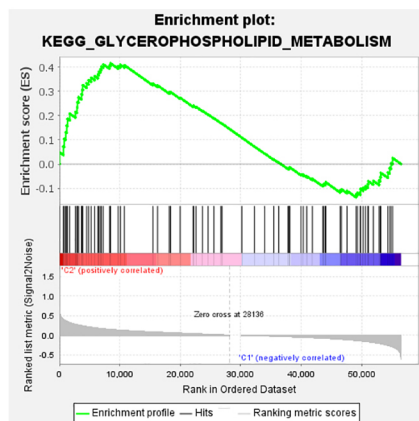

I

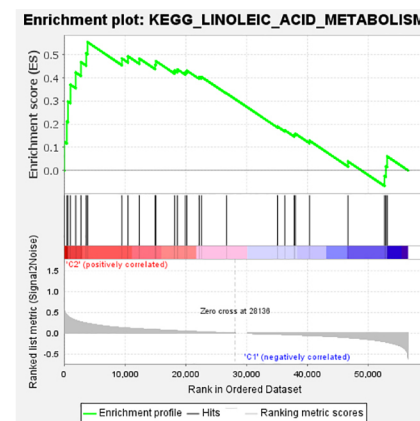

Supplement: Supplementary Materials — Supplementary Table 1: expression matrix of 60 ferroptosis-related genes. Supplementary Table 2: results for coexpression analysis of ferroptosis-related lncRNAs and mRNAs. Supplementary Table 3: expression of 263 ferroptosis-related lncRNAs. Supplementary Table 4: expression of lncRNAs and clinical information of 403 bladder cancer samples. Supplementary Table 5: cluster analysis based on 20 prognostic lncRNAs. Supplementary Table 6: detailed clinical information of bladder cancer samples. Supplementary Table 7: data of the high- and low-risk groups of the training cohort. Supplementary Table 8: data of the high- and low-risk groups of the validation cohort. Supplementary Table 9: markers of different immune cells. Supplementary Table 10: infiltration of 22 types of immune cells analysed using the CIBERSORT algorithm. Supplementary Table 11: immune scores evaluated using the ESTIMATE algorithm. Supplementary Table 12: immunotherapeutic response evaluated using the Tumour Immune Dysfunction and Exclusion (TIDE) algorithm. Supplementary Figure 1: gene set enrichment analysis. (A–F) Most significantly enriched biological pathways in cluster 1. (G–I) Most significantly enriched biological pathways in cluster 2. [file 9316847.f1.zip › Supplementary Figure 1 (1).pdf]
